# A Systematic Evaluation of Cohort Selection Criteria and Their Impact on Machine Learning Model Performance and Demographic Disparities in COVID-19 Outcomes: Cohort Study

**DOI:** 10.2196/88406

**Published:** 2026-08-20

**Authors:** Atefehsadat Haghighathoseini, Janusz Wojtusiak, Hua Min, Nirup M Menon

**Affiliations:** 1 Department of Health Administration, Policy and Informatics, George Mason University, 4400 University Dr, Fairfax, VA, 22030, United States, +1 (703) 993-1929; 2 Information Systems and Operations Management, Costello College of Business, George Mason University, Fairfax, VA, United States

**Keywords:** Cohort selection, machine learning, COVID-19, model performance, demographic disparities, data processing decisions

## Abstract

**Background:**

Cohort selection criteria play a critical role in shaping machine learning (ML) model performance and the equity of clinical outcome predictions across demographic groups. In practice, cohort definitions are often influenced by variable and sometimes inconsistent data processing decisions, which may introduce bias and limit the generalizability of ML models. During the COVID-19 pandemic, rapid cohort construction further increased concerns about transparency and fairness in ML-based analyses.

**Objective:**

This study aimed to systematically examine how cohort selection and data processing decisions influence ML performance and demographic equity in predicting COVID-19–related in-hospital mortality.

**Methods:**

Using data from the National COVID Cohort Collaborative (N3C), we evaluated 2 sets of cohorts. Set 1 consisted of 16 cohorts derived from 4 primary data processing decisions, including COVID-19 case identification, inpatient inclusion, diagnosis date selection, and admission timestamp availability. Set 2 expanded this design to 64 cohorts by additionally applying provider ID and location identifier filtering. Model performance was assessed using the area under the receiver operating characteristic curve (AUC) across multiple training-testing cohort combinations. Three ML models—logistic regression, random forest, and gradient boosting—were evaluated using 3 analytical approaches: maximum AUC classification, direct AUC regression, and AUC gap analysis. Performance was further examined across demographic subgroups defined by gender, race, and ethnicity.

**Results:**

This study analyzed data from the N3C, including patients with a first positive COVID-19 diagnosis between August 1, 2020, and December 31, 2021. Data preprocessing, cohort construction, and model development were completed prior to analysis. Cohort selection decisions had a substantial impact on ML model performance. Admission time inclusion or exclusion emerged as the most influential factor in Set 1 and consistently affected model accuracy across analytical approaches. In Set 2, this decision remained important, while additional criteria, particularly provider ID filtering, also significantly influenced results. The importance of specific decisions varied across models and evaluation strategies. Analyses across demographic subgroups showed that data processing decisions affected predictive performance differently by gender, race, and ethnicity.

**Conclusions:**

Seemingly minor cohort selection and data processing decisions can meaningfully affect both predictive accuracy and demographic equity in ML-based COVID-19 outcome prediction. These findings highlight the risk of bias introduced by differences in cohort definitions and underscore the need for transparent, standardized, and equity-aware cohort selection practices to support fair and reproducible ML research in health care.

## Introduction

The integration of machine learning (ML) in health care, particularly for clinical decision-making in diagnosis, prognosis, and risk prediction, is advancing rapidly [1]. ML’s ability to efficiently analyze large, complex datasets has enhanced traditional clinical reasoning and enabled precision medicine, where treatments are tailored to individual patient characteristics [2,3]. However, the performance and generalizability of ML models depend on the quality and structure of the data [4], underscoring the importance of thoughtful cohort selection [5,6]. Most ML studies evaluate generalizability using a test set; yet, the test set is selected after data processing decisions have been made, thus potentially limiting real generalizability. Cohort selection—defining which patients are included in a study based on diagnosis codes, treatment timelines, and other criteria—is a foundational step in ML model development. Poorly defined or nontransparent criteria can introduce bias [7], produce heterogeneous cohorts, and compromise model validity [5,6,8]. Real-world data sources like electronic health records (EHRs) offer rich clinical information but also present challenges, including missing data, inconsistent coding, and variation across institutions, all of which affect model development and reproducibility [9,10].

Inconsistencies in cohort construction practices and the absence of standardized guidelines make it difficult to compare studies or validate ML models across settings [6,11]. The use of proxy definitions without adequate clinical validation can distort clinical characteristics, while underrepresentation of racial and ethnic minority groups [12] can skew outcomes and reinforce health disparities [13,14]. Such limitations can impair a model’s ability to generalize to diverse patient populations, reducing its clinical utility [15,16].

Selection bias in ML carries significant implications. Inadequately defined cohorts can lead to overfitting, limited real-world applicability, and unequal model performance across subpopulations. For example, disparities in the allocation of advanced heart failure therapies by race and gender reveal how implicit bias in data and decision-making can affect outcomes [17,18]. Addressing these challenges requires a structured and transparent approach to cohort definition that accounts for equity, clinical relevance, and methodological rigor [8,19].

Several strategies have been proposed to improve ML practice in this context. These include advanced data preprocessing, interpretability frameworks, and performance metrics that reflect the specific characteristics of the cohort under study [20,21]. Collaboration between clinicians and data scientists is essential to ensure that cohort definitions are clinically appropriate and that ML models align with patient-centered goals and ethical standards [22,23].

The COVID-19 pandemic provided a critical test case for ML deployment in health care, particularly through large-scale data initiatives like the National COVID Cohort Collaborative (N3C). These resources enabled broad analysis of patient outcomes and risk factors [24]. However, the urgency of pandemic response often led to expedited cohort construction with less rigorous inclusion criteria [25], raising concerns about the generalizability and fairness of resulting models [24,26,27]. Vulnerable populations were disproportionately affected, and inconsistent or arbitrary cohort definitions sometimes reduced predictive accuracy for marginalized groups [28,29]. This context highlights the urgent need for standardized, explicit cohort selection criteria to ensure ML models are valid, equitable, and reproducible [30,31]. Moving forward, the development of comprehensive guidelines for cohort construction, along with validation of existing models against diverse benchmarks, will be critical for achieving trustworthy and impactful ML applications in health care [32,33].

To address these gaps—including the lack of standardized cohort construction criteria [30,31] and the limited validation of models against diverse benchmarks [32,34]—this study presents a comprehensive examination of how varying cohort construction decisions influence the performance and fairness of ML models in predicting COVID-19 outcomes. By systematically analyzing combinations of data processing criteria across multiple modeling approaches and evaluating their impact on both overall accuracy and subgroup consistency, this study offers insights into the unintended consequences of cohort definition choices. The findings aim to inform more transparent and equitable practices in ML-based health care research, emphasizing the importance of deliberate, well-documented cohort selection processes to support model reproducibility, reliability, and inclusivity.

## Methods

### Data Source

The N3C offers the most extensive harmonized repository of COVID-19–related patient data in the United States, integrating EHRs from more than 70 health care institutions nationwide [35,36]. This centralized initiative provides a unified data platform through the N3C Data Enclave, enabling researchers to conduct large-scale, high-quality analyses focused on COVID-19 outcomes, treatment effectiveness, and health care disparities [36,37]. This study used the limited dataset (LDS) available through the N3C, which contains individual-level patient data with direct identifiers removed. This approach maintains patients’ privacy while preserving the detailed clinical information necessary for research [38]. The dataset conforms to the Observational Medical Outcomes Partnership (OMOP) Common Data Model (CDM), which standardizes clinical data structures across contributing institutions to support consistency and reproducibility [36,39]. For inpatient data analysis, the N3C employs the concept of a “Macro-visit,” encompassing hospital admissions, observation periods, extended multiday stays following outpatient procedures, emergency department visits, and overlapping outpatient or telehealth encounters that occurred during the COVID-19 pandemic [27,40].

The primary objective of this research is to predict outcomes for patients hospitalized with COVID-19, with a specific focus on in-hospital mortality. The unit of analysis is the patient hospitalization. To support this objective, the data must be transformed so that each hospital stay is represented by a single, consolidated record—commonly referred to as a flat table or analytic file—which is a typical structure for applying ML methods.

### Data Preprocessing Decisions

#### Overview

One of the most critical steps in any data analysis, including building ML models, is cohort construction. In this study, a series of reasonable but variable data processing decisions were applied to approximately 22 million patients from the N3C dataset.

The study distinguishes between 4 sets of cohort-defining decisions. The first set comprises 4 primary data processing decisions (decisions 1-4) that result in 16 distinct cohorts (referred to as Set 1). These decisions include the identification of COVID-19 cases, identification of inpatient hospitalization records, identification of COVID-19–related hospitalizations, and potential exclusion of records with missing data on the specific time of admission. These different but plausible design choices can lead to the creation of up to 16 different datasets, each varying in size and characteristics. By incorporating 4 additional decisions—filtering by provider IDs and location identifiers (decisions 5 and 6)—into the primary set of decisions, the number of possible cohorts expands to 64 (referred to as Set 2). The details of these decisions are provided below for both Set 1 and Set 2.

The analysis of these cohorts revealed statistically significant differences in both demographic and outcome variables, underscoring the impact of these preprocessing decisions on cohort composition. Specifically, significant variations were observed in age, gender, race, ethnicity, and state, as well as in length of stay and survival (expiry flag). Consistently small *P* values (<.001) indicate that these differences are unlikely to be due to chance, indicating that cohort membership is strongly associated with survival outcomes and demographic characteristics. To evaluate these differences, multiple statistical tests were applied, including the chi-square test for categorical variables (gender, race, ethnicity, state, and expiry flag), with df reported for each; the independent 2-sample *t* test (Welch *t* test) for continuous variables (age and length of stay), as a parametric test assuming unequal variances; and the Mann-Whitney *U* test as a nonparametric alternative for the same continuous variables. Together, these results demonstrate that the seemingly minor preprocessing choices substantially influence the resulting cohorts, ultimately shaping both the composition and the outcomes observed in the study.

#### Set 1: Main Decisions

##### Decision A

Identifying patients with COVID-19: there have been a variety of tests and options for identifying patients with COVID-19. This study considers that some patients have a confirmed positive COVID-19 laboratory test result, while others are identified based on diagnostic codes [36].

##### Decision B

Identifying hospitalization records among patient encounters: 2 approaches were considered for identifying hospitalization records. One approach involved performing a wildcard text search for visit concepts containing terms such as “inpatient,” “observ,” and “hospital.” The other relied on an N3C-defined variable for hospitalization status, which is calculated by the N3C team based on clinical data.

##### Decision C

A time window is considered for a COVID-19 positive test to be related to the macro-visit. This study compared a 7-day versus a 10-day window prior to the start of the inpatient record, extending through the second day of hospitalization.

##### Decision D

The final decision relates to how admission timing is handled during data processing. One approach was to include only records with an exact admission time, thus allowing an exact 48-hour observation window used in subsequent analyses. However, excluding records without an exact time stamp will result in less generalizable results. The other extreme is including all records, which would require estimating the 2-day hospitalization period based on the admission and discharge date alone. Moreover, this approach does not capture the 48-hour window, as it is often calculated from midnight to midnight.

### Set 2: Additional Decisions

#### Decision E

Keep only records with provider ID (all encounters have a known provider) or include all records (with or without provider ID) to preserve the entire cohort.

#### Decision F

One approach is to drop the records without a location identifier. The other approach is to keep all the records, whether they have a location identifier, which preserves cohort size but introduces uncertainty to any analysis at the location level.

### Other Decisions

Data preprocessing contains other decisions that were not investigated for the simplicity of the presented work.

After decision C, data were filtered to include only patients whose first positive COVID-19 diagnosis occurred between August 1, 2020, and December 31, 2021. This period was selected to avoid the earliest phase of the COVID-19 pandemic, when testing availability, diagnostic coding practices, and clinical management protocols were rapidly evolving [25]. Restricting the analysis to this timeframe provides a more consistent basis for cohort comparison while maintaining a large and diverse patient population. Similar periods have been used in prior N3C studies of COVID-19 outcomes [36]. Then, hospitalization data were merged with other core data to be used in the final analysis with a unique identifier and variables including gender, date of birth, race, ethnicity, and age at death. Patients younger than 18 years were excluded from all cohorts. The analysis was restricted to adults aged 18 years and older, consistent with the standard definition of adulthood used in clinical research and prior N3C studies of COVID-19 outcomes [36]. Pediatric patients differ from adults in disease presentation, hospitalization patterns, and clinical outcomes. Restricting the study population to adults therefore provides a more clinically comparable population for evaluating the impact of cohort construction decisions [36,39].

### Study Design

#### Study Design Overview

**Figure 1.**
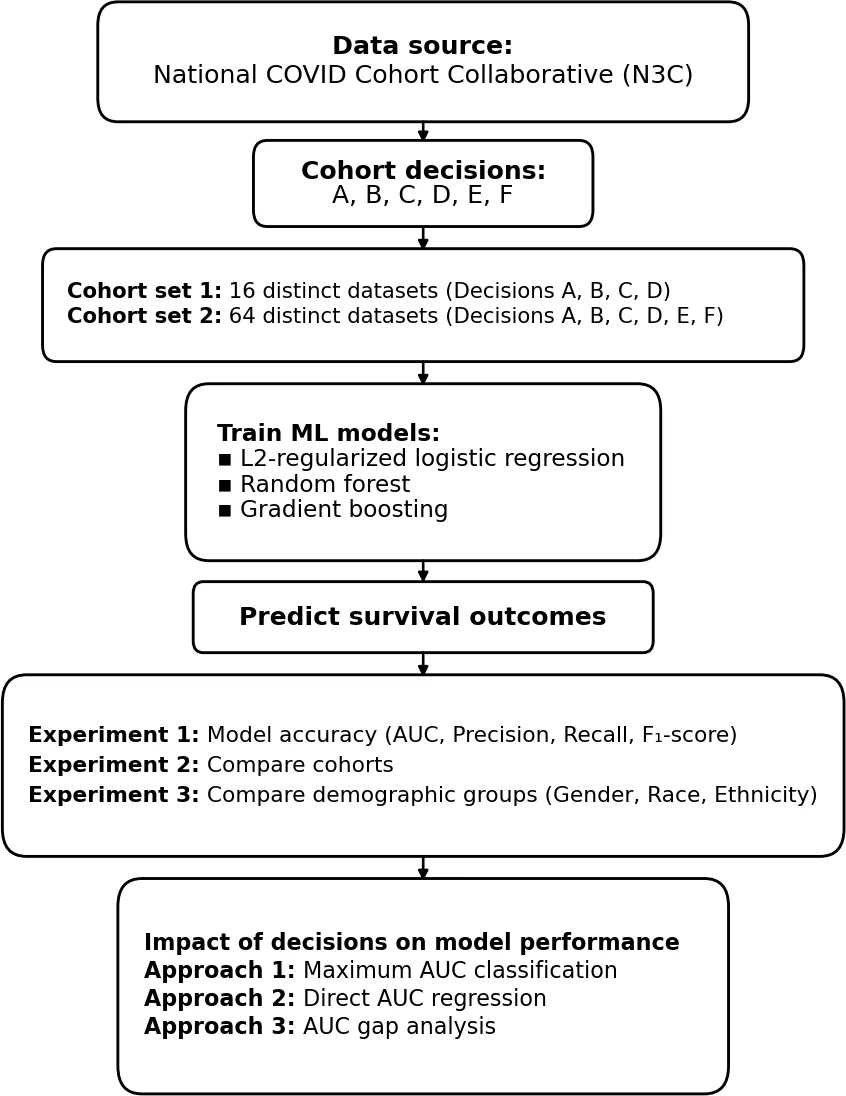
Flowchart illustrating experimental design for analyzing the impact of data processing decisions on machine learning (ML) model performance in COVID-19 outcomes. AUC: area under the receiver operating characteristic curve; ML: machine learning.

The study is structured to evaluate the impact of cohort selection criteria on ML model performance and demographic equity in predicting COVID-19 outcomes. The experiment is divided into 3 main approaches, each focusing on different aspects of model evaluation and data processing decisions. As shown in Figure 1, the flowchart illustrates the experimental design of this study for analyzing the impact of data processing decisions on ML model performance in COVID-19 outcomes.

Race, ethnicity, and gender were harmonized using rule-based recoding of the original N3C concept labels into analytically consistent categories. Sex was categorized as female, male, or unknown, with unmatched or ambiguous values (eg, no matching concept, sex unknown, other) grouped as unknown. Race categories were aggregated into Black, White, Asian, Multi_Race, Native Hawaiian, and unknown. Specifically, Black or African American and Black were grouped as Black, while multiple Asian subcategories (eg, Asian, Asian Indian, Filipino, Chinese, Korean, Japanese, and Vietnamese) were consolidated into Asian. Categories such as multiple race, multiple races, and more than one race were grouped as Multi_Race, and Native Hawaiian or Other Pacific Islander was retained as Native Hawaiian. Race values that were missing, unmapped, or ambiguous (eg, no matching concept, unknown, and no information) were assigned to unknown. Ethnicity was harmonized into Hispanic, non-Hispanic, and unknown, with Hispanic or Latino mapped to Hispanic, Not Hispanic or Latino mapped to non-Hispanic, and missing or ambiguous values grouped as unknown. These harmonized categories were used for all descriptive summaries and subgroup analyses.

The study involves the systematic extraction of relevant clinical measurements and medical device usage data for patients within defined cohorts from the N3C dataset. Measurements are organized based on their unique concept identifiers, selected from the concept set member’s table. These measurements cover a wide range of clinically important indicators, including, but not limited to, respiratory rate, glomerular filtration rate (GFR), cardiac troponin T, sodium, ABG indices, prothrombin time, alanine transaminase, calcium, systolic blood pressure (BP), heart rate, CD3+ CD8+ T cells, albumin, erythrocyte sedimentation rate, venous lactate, BMI, complete blood count (CBC) with platelets, CD3+ CD4+ T cells, cardiac troponin I, interleukin 6, fraction of inspired oxygen, blood urea nitrogen, temperature, bilirubin, D-dimer, alkaline phosphatase, fibrinogen, creatinine, chloride, glucose, C-reactive protein, urine output, N-terminal pro–B-type natriuretic peptide (NT-proBNP), diastolic BP, weight, ferritin, and interleukin 10.

In addition, the study incorporates data on a broad range of medical devices used during hospitalization, represented as binary indicators reflecting their presence or absence. These devices include endotracheal tube, peritoneal hemodialysis, imaging-related, medical supplies, oxygen delivery systems, respiratory therapy equipment, room air, skin care, surgical related, transfusion related, and ventilator, and categories labeled other or unknown. Device categories were defined through clinical review and inventory-based grouping of concept names according to their functional and clinical relevance.

This combined extraction and categorization focuses on the first 48 hours of hospital admission, capturing the acute phase of patient care. The resulting structured dataset of clinical measurements and device use provides a comprehensive and organized representation of patient status during this critical early period of hospitalization. This structured dataset served as the basis for subsequent ML analyses, including data partitioning, missing data handling, and model development as described below.

For each cohort, the dataset was partitioned into training and testing subsets using a deterministic hash-based approach applied to the patient identifier, resulting in approximately 80% of observations assigned to the training set and 20% assigned to the testing set. The outcome variable was defined as in-hospital mortality, and all remaining variables after cohort construction and preprocessing were used as input features. Missing data were addressed using mode imputation. Specifically, for each variable, the most frequently observed value in the training set was used to replace missing observations, and the same training-derived imputation value was subsequently applied to the corresponding testing set. This approach ensured that no information from the testing data was used during preprocessing or imputation. No additional feature-selection procedure was performed, as the primary objective was to evaluate the impact of cohort construction decisions rather than optimize model performance. Logistic regression (LR) and random forest (RF) models incorporated class-weight balancing to address class imbalance. Model hyperparameters were predefined and held constant across all cohorts to ensure comparability. LR was implemented with a maximum of 1000 iterations and balanced class weights; RF used 1000 trees with a maximum depth of 12 and balanced class weights; and gradient boosting (GB) used 150 estimators, a learning rate of 0.5, and a maximum depth of 5. No information from the testing datasets was used during feature construction, model training, or model selection.

Three ML models were implemented, including RF, GB, and L2-regularized LR [41,42]. Standard implementations of these models were used, with class balancing applied where appropriate. Hyperparameters were specified a priori and were not further tuned, as the primary objective of this study was to evaluate the impact of cohort construction decisions rather than to optimize model performance.

Cross-validation was not applied in this study. While cross-validation is commonly used to estimate model performance within a single dataset, the primary objective of this work is to evaluate the impact of cohort construction decisions across different cohort definitions. Therefore, a cross-cohort training-testing framework was used, in which models trained on one cohort were evaluated on multiple alternative cohorts. This design enables assessment of generalizability and the influence of data processing decisions, rather than model optimization. All analyses were conducted using standard ML libraries, and the deterministic data partitioning approach ensured reproducibility of results across experiments.

To provide a more comprehensive evaluation beyond discrimination performance, fairness, and calibration analyses were conducted. Fairness was assessed using demographic parity and equality of opportunity, which quantify disparities in model predictions across demographic subgroups. These metrics were computed for gender, race, ethnicity, and age groups across all cohorts and models. In addition to fairness metrics, model calibration was evaluated to assess the agreement between predicted probabilities and observed outcomes. Calibration curves were generated for each model across all training-testing cohort combinations. This analysis was performed separately for Set 1 (16 cohorts) and Set 2 (64 cohorts), allowing for assessment of prediction reliability and generalizability under different cohort construction scenarios.

To systematically investigate these effects, the study is organized into three complementary experimental components: (1) model performance evaluation, which examines predictive accuracy across cohorts; (2) cross-cohort analysis of data processing decisions, which identifies the influence of cohort construction choices on model performance; and (3) stratified analysis across demographic groups, which evaluates how these decisions affect performance across subpopulations. Each component is described in detail below.

To ensure full transparency and reproducibility, detailed numerical results are provided in the supplementary materials. Multimedia Appendix 1 presents cohort-level summaries, including sample sizes, training, and testing splits, and demographic distributions at both the row and patient levels across the 16- and 64-cohort configurations. Multimedia Appendices 2 and 3 report complete model performance metrics (area under the receiver operating characteristic curve [AUC], precision, recall, and *F*_1_-score) for all models across the 16 and 64 cohorts, respectively. Multimedia Appendix 4 provides subgroup-specific AUC results stratified by gender, race, and ethnicity across all cohorts. Due to space constraints, the main manuscript presents representative results and key trends, while the appendices provide the full set of numerical results for comprehensive evaluation.

#### Experiment 1: Model Accuracy

Standard supervised ML models were trained on the training set, and the predicted outcome was survival at the end of hospitalization. RFs [43], GB [43], and L2-regularized LR [44] models were trained [41]. Metrics are important for evaluating the performance of ML models [45]. They provide insights into how well a model’s predictions match the data. This study uses several metrics, including AUC, precision, recall, and *F*_1_-score. For each cohort in Set 1, metrics are computed across all training-testing combinations (eg, training on Cohort 1 and testing on Cohorts 1‐16, training on Cohort 2 and testing on Cohorts 1‐16, etc). The same procedure is applied to all cohort pairs in Set 2. It is important to evaluate ML models across subpopulations defined by sex, race, and ethnicity to test fairness and reliability. Subpopulations based on sex include males and females, while subpopulations based on race include White, Black, Asian, Native Hawaiian, and multiple races. Subpopulations based on ethnicity include Hispanic and non-Hispanic.

To assess model discrimination overall and across demographic subgroups, the performance of the ML models in predicting survival at the end of hospitalization was evaluated using the AUC.

This evaluation also allows for auditing fairness both in the overall population and within subgroups. AUC is a common evaluation metric for binary classification models that measures the probability of a randomly selected positive instance being ranked higher than a random negative instance across all possible thresholds. The best-performing model was identified by comparing the AUC scores of the 3 models.

#### Experiment 2: Across Cohorts

This experiment aims to address which data processing decisions most strongly impact model performance. For the purposes of analysis, the experiment is divided into 2 subsets: Set 1, which includes 16 cohorts derived from 4 key decisions (A, B, C, and D), and Set 2, which expands the design to 64 cohorts by incorporating 2 additional decisions, E and F. Each decision was implemented during both the training and testing phases. To clearly distinguish between the phases, a prime notation (eg, A′, B′, ..., F′) is used to represent decisions applied during testing. To determine the influence of these data processing decisions, models from each cohort were compared using the following 3 analyses.

##### Approach 1: Maximum AUC Classification

For each test cohort, the model—LR, RF, or GB—that achieves the highest AUC is identified. A new binary column, “max AUC,” is added, where a value of 1 indicates that the model achieved the highest AUC for that cohort, and 0 otherwise. A “DecisionTreeClassifier” is then trained to determine which data cohort and decisions are most strongly associated with a model achieving the top AUC performance.

##### Approach 2: Direct AUC Regression

This approach leverages the actual AUC values obtained by each model for every test cohort. A “DecisionTreeRegressor” is applied to examine how AUC values change across cohorts and to identify which data processing decisions most significantly influence model performance, either positively or negatively.

##### Approach 3: AUC Gap Analysis

For each test cohort, the highest AUC achieved among the 3 models is identified. The AUC gap is then calculated as the difference between this maximum AUC and the AUC of each individual model. A “DecisionTreeRegressor” is used to analyze these gaps and determine which data processing decisions are most associated with performance loss relative to the best-performing model. These approaches provide complementary insights into how data processing decisions affect model accuracy and consistency across varying cohort definitions.

### Experiment 3: Across Demographic Groups

This experiment identifies how data processing decisions can impact model performance across demographic subgroups. Similar to Experiment 2, the same 3 analytic approaches—maximum AUC classification, direct AUC regression, and AUC gap analysis—are applied across the 3 models—LR, RF, and GB.

However, in this experiment, the analysis is performed within stratified demographic groups. The test data are stratified by sex (male and female), race (Black, White, and Asian), and ethnicity (Hispanic or non-Hispanic). For each subgroup, shifts in model performance due to different data processing decisions are examined. This helps to determine whether certain decisions disproportionately impact certain demographic groups. The experiment is designed to help identify potential sources of bias and guides efforts toward more equitable predictive modeling.

### Ethical Considerations

The study was reviewed by the George Mason University Institutional Review Board (IRB), which determined that the proposed activity is not research involving human subjects as defined by the US Department of Health and Human Services (DHHS) and Food and Drug Administration (FDA) regulations. Therefore, the need for ethics approval was waived. The IRB reference number is #1706572. This study complies with the principles of the Declaration of Helsinki. The need for informed consent to participate was waived by the George Mason University IRB, as the study does not involve research on human subjects according to the relevant regulations.

## Results

### Overview

Consistent with the analytical framework outlined in the Methods, results are organized into 3 components reflecting key aspects of the study design. We first assess predictive performance across cohorts and demographic subpopulations to evaluate model accuracy and fairness. We then investigate the impact of data processing decisions on model performance across alternative cohort constructions. Finally, we examine the extent to which these effects vary across demographic subgroups. These findings collectively highlight the role of cohort selection and preprocessing decisions in shaping both predictive performance and equity.

### Model Performance Across Cohorts

Predictive performance across cohorts and demographic subpopulations demonstrated notable variability, reflecting the impact of cohort construction and data processing decisions on model outcomes. Across both cohort sets (Set 1 and Set 2), the models achieved moderate to high discriminative performance, with AUC values generally ranging from approximately 0.59 to 0.86 across different models and evaluation settings. However, performance was not consistent across demographic groups, with some subpopulations exhibiting wider variability in AUC ranges. These findings highlight the sensitivity of model performance to both cohort definition and population characteristics, underscoring the importance of evaluating predictive models across diverse groups.

It is important to note that for Native Hawaiian and multirace subgroups, some models show an AUC of zero due to the absence of data, leaving no cases for training or evaluation. Table 1 summarizes the AUC ranges for LR, RF, and GB models across Cohorts 1‐16 and 1‐64, based on the entire dataset and stratified by demographic groups.

**Table 1.** Range of area under the receiver-operator curve (AUC) values for logistic regression (LR), random forest (RF), and gradient boosting (GB) models across Cohorts 1‐16 and 1‐64, calculated using the entire dataset and stratified by demographic groups.

Cohort set	Entire datasets^a^	Demographic group						
		Sex		Race			Ethnicity	
		Male	Female	Black	White	Asian	Hispanic	Non-Hispanic
AUC^b^ range (LR^c^, RF^d^, and GB^e^ models)								
Cohorts 1-16	0.70‐0.77^a^ 0.70‐0.84 0.63‐0.86	0.70‐0.74 0.69‐0.75 0.65‐0.75	0.70‐0.76 0.70‐0.76 0.66‐0.77	0.68‐0.74 0.68‐0.75 0.63‐0.75	0.70‐0.73 0.68‐0.75 0.64‐0.75	0.74‐0.80 0.70‐0.84 0.65‐0.82	0.70‐0.77 0.69‐0.77 0.67‐0.78	0.70‐0.74 0.68‐0.75 0.64‐0.75
Cohorts 1-64	0.69‐0.79 0.66‐0.78 0.59‐0.78	0.68‐0.78 0.67‐0.85 0.59‐0.90	0.70‐0.80 0.69‐0.87 0.60‐0.91	0.69‐0.78 0.68‐0.86 0.61‐0.90	0.68‐0.75 0.66‐0.82 0.57‐0.86	0.71‐0.81 0.66‐0.93 0.50‐0.97	0.71‐0.81 0.70‐0.91 0.62‐0.93	0.68‐0.77 0.66‐0.83 0.59‐0.88

a The sequence of numbers in each cell, listed from top to bottom, represents the range of AUC values for the LR, RF, and GB models, respectively.

b AUC: area under the receiver operating characteristic curve.

c LR: logistic regression.

d RF: random forest.

e GB: gradient boosting.

### Influence of Data Processing Decisions

Model performance varied substantially across cohort definitions, indicating that data processing decisions play a critical role in shaping predictive outcomes. The importance of specific decisions differed depending on the cohort set and modeling context, suggesting that no single configuration consistently yields optimal performance. To better understand these effects, we further analyze how individual data processing decisions influence model accuracy across multiple evaluation settings.

To further interpret the findings, results are compared across multiple dimensions: within a single cohort set, across different cohort sets, across different models, and across the analytical approaches themselves. Due to page limitations, results are presented for a subset of approaches as representative examples.

#### Comparison 1: Within Cohort Set 1 (Cohorts 1-16)

**Figure 2.**
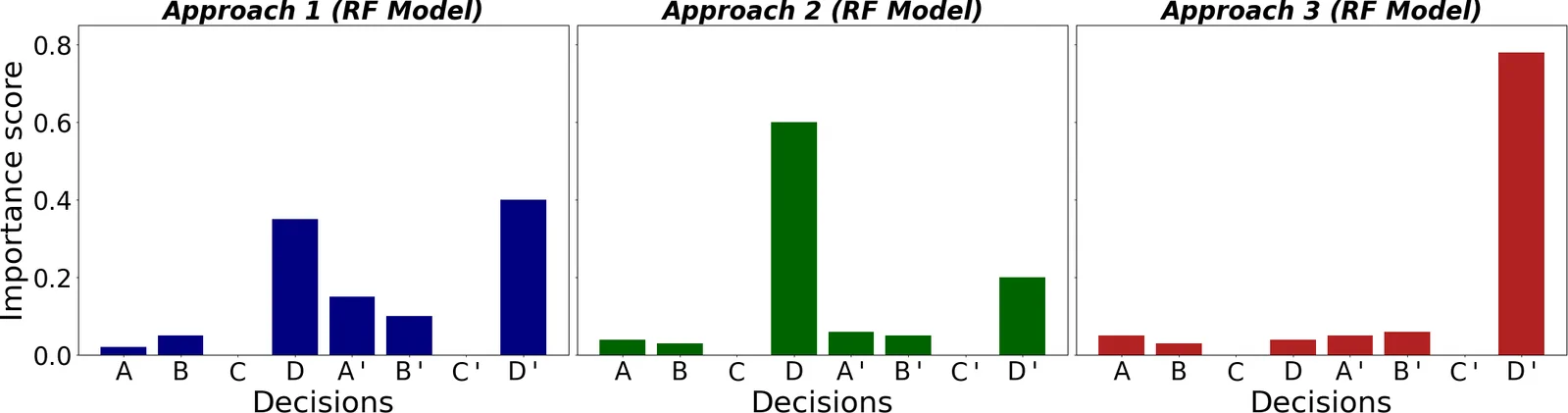
Feature importance for approaches 1, 2, and 3 using random forest (RF) model across cohorts 1-16 for 4 main decisions. RF: random forest. A, B, C, and D correspond to decisions applied to the training data, while A′, B′, C′, and D′ correspond to decisions applied to the testing data.

This comparison focuses on the impact of the initial 4 data processing decisions—A, B, C, and D—across 16 distinct cohorts. The objective is to assess how variations in these decisions affect model performance when applied in both the training and testing sets. Due to space limitations, only a few illustrative plots are shown in this paper. For instance, feature importance of Approaches 1, 2, and 3 using the RF model comparing cohorts 1-16 for 4 main decisions is shown in Figure 2.

Based on these results, decision D is concluded to play a crucial role across all 3 approaches. Specifically, including or excluding time-related information has a noticeable impact on model accuracy. Moreover, when considering A=A′, B=B′, C=C′, and D=D′, a more detailed understanding is gained. This alignment between decisions made during training (A, B, C, and D) and testing (A′, B′, C′, and D′) ensures that D=D′ remains consistent.

**Figure 3.**
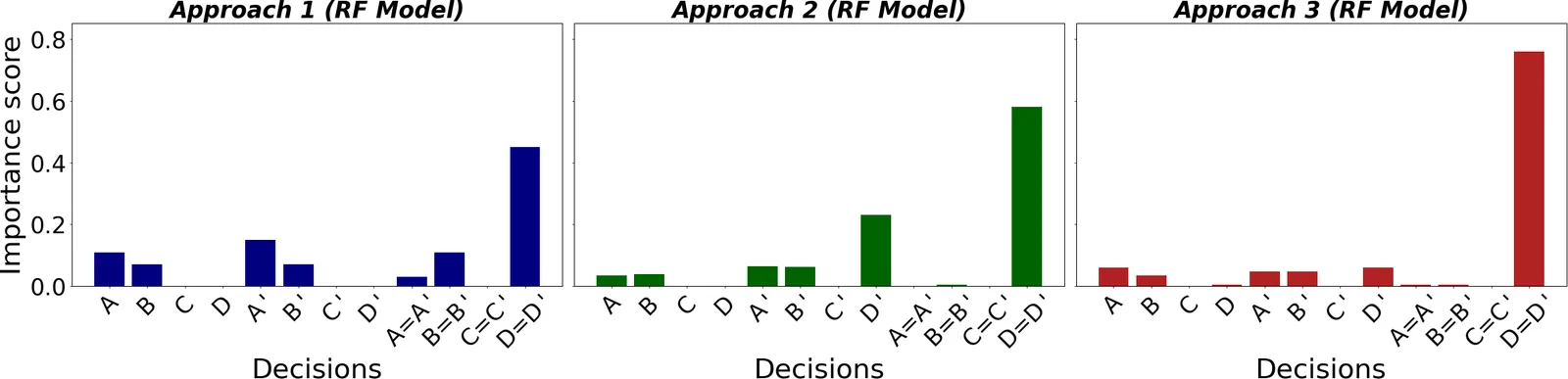
Feature importance for approaches 1, 2, and 3 using random forest (RF) model across cohorts 1-16 for 4 main decisions, offering deeper insights. RF: random forest.

As shown in Figure 3, the feature importance of D=D′ is significant across all approaches, highlighting its stability throughout both training and testing phases. Taken together, the results demonstrate that decision D is important and consistently influences model performance.

#### Comparison 2: Between Cohort Set 1 and Cohort Set 2

**Figure 4.**
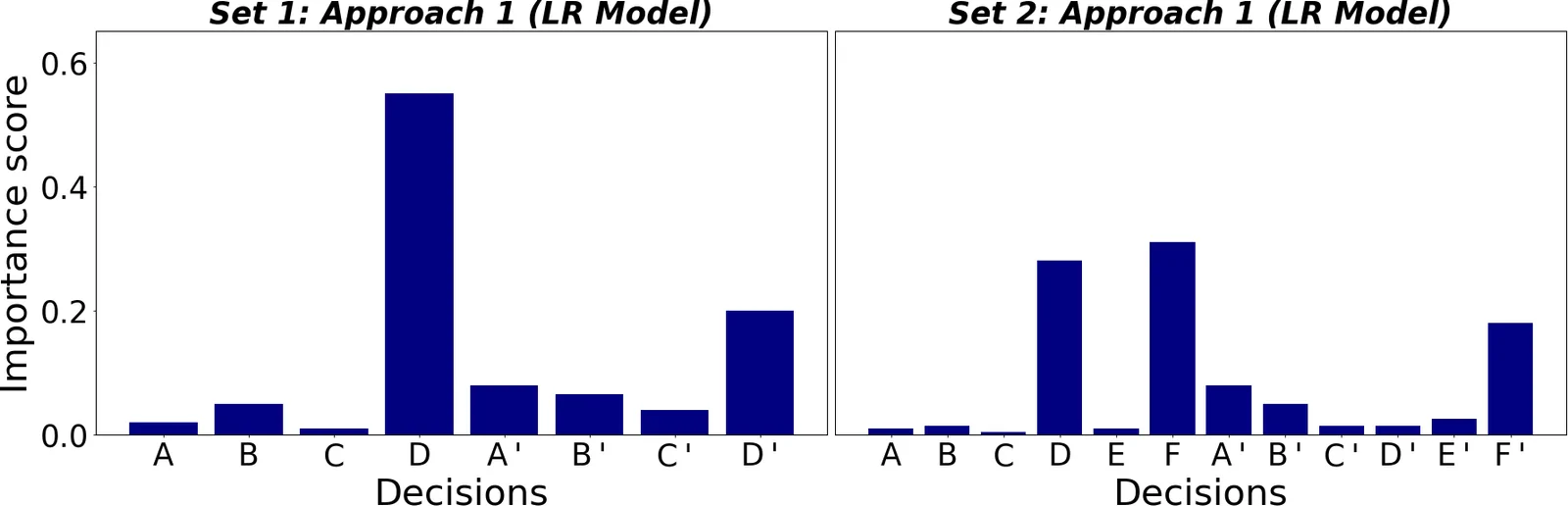
Feature importance comparing Set 1 (cohorts 1 to 16) and Set 2 (cohorts 1 to 64) using the logistic regression (LR) model. LR: logistic regression.

When comparing Set 1 (Cohorts 1-16) with Set 2 (Cohorts 1-64), decision D (time) remains highly important in Set 1. However, in Set 2, although decision D is not the most dominant factor, it still holds significant importance. This suggests that additional decisions—such as F (Provider IDs)—also play a crucial role in influencing model performance. Due to page limitations, only selected feature importance results comparing Set 1 (cohorts 1-16) and Set 2 (cohorts 1-64) using the LR model are shown in Figure 4.

#### Comparison 3: Across 3 Different Models

**Figure 5.**
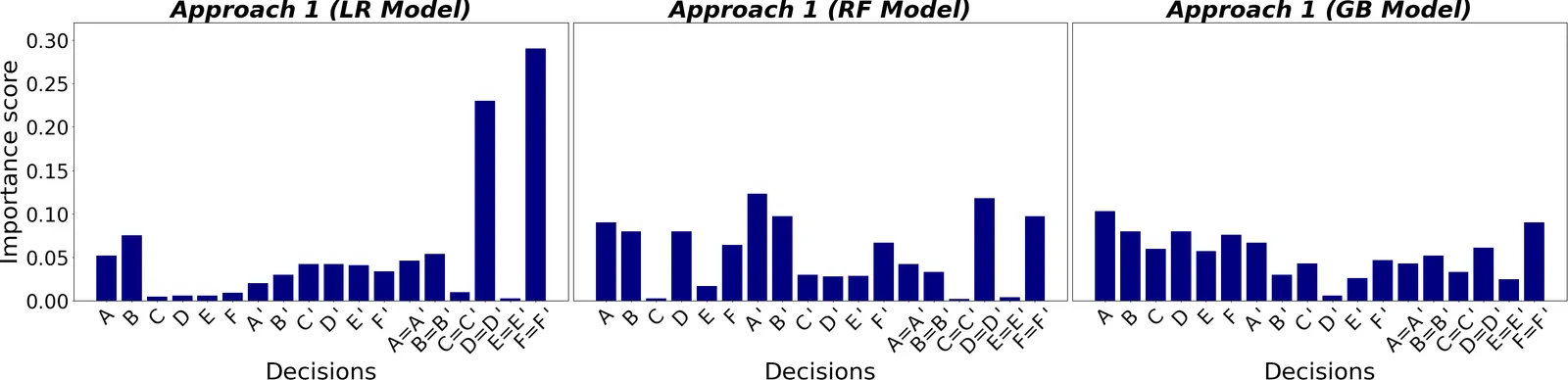
Feature importance for Approach 1 compared across logistic regression (LR), random forest (RF), and gradient boosting (GB) models. GB: gradient boosting; LR: logistic regression; RF: random forest.

This comparison examines how different ML models respond to the same data processing decisions. By analyzing 3 models, the study aims to understand whether the perceived importance of decisions remains stable across modeling approaches. The results reveal that the importance of decisions is not consistent across models. Each model assigns different levels of importance to the same decisions. Due to page limitations, only selected feature importance results for Approach 1 across the LR, RF, and GB models are shown in Figure 5.

#### Comparison 4: Among Different Analytical Approaches

**Figure 6.**
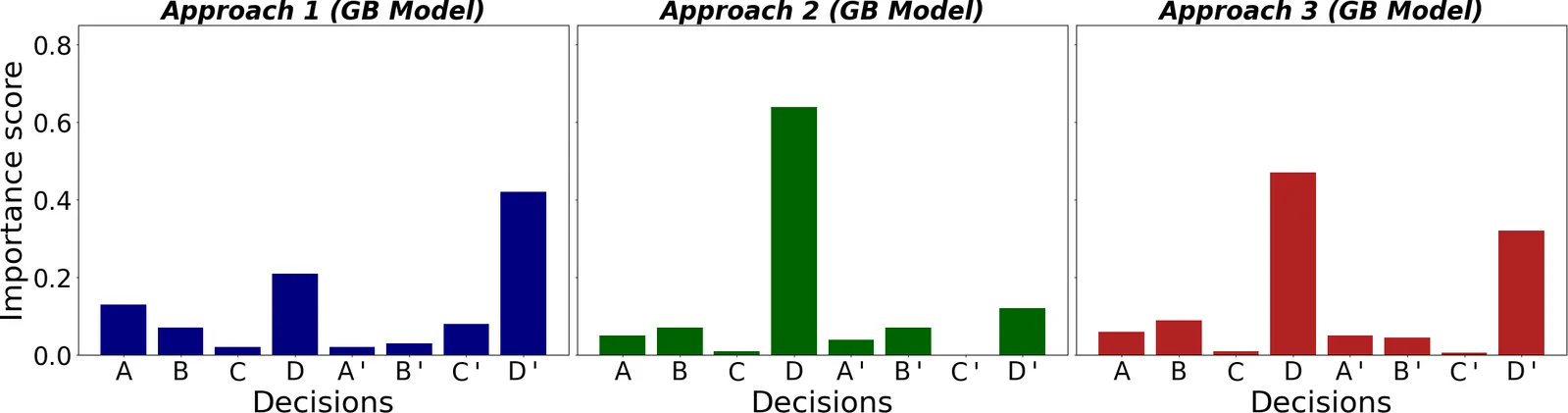
Feature importance comparing Approaches 1, 2, and 3 using the gradient boosting (GB) model. GB: gradient boosting.

This comparison assesses how the importance of data processing decisions varies across different analytical approaches. Decision D (time) consistently holds significant importance across all 3 approaches. However, the measurement ranges differ, indicating that changing the measurement scale can lead to different results. Due to page limitations, only selected feature importance comparisons of Approaches 1, 2, and 3 using the GB model are presented in Figure 6.

### Differential Effects Across Demographic Subgroups

Model performance varied across demographic subgroups, indicating that the impact of data processing decisions is not uniform across populations. The importance of specific decisions differed by subgroup, suggesting potential disparities in model behavior and performance.

**Figure 7.**
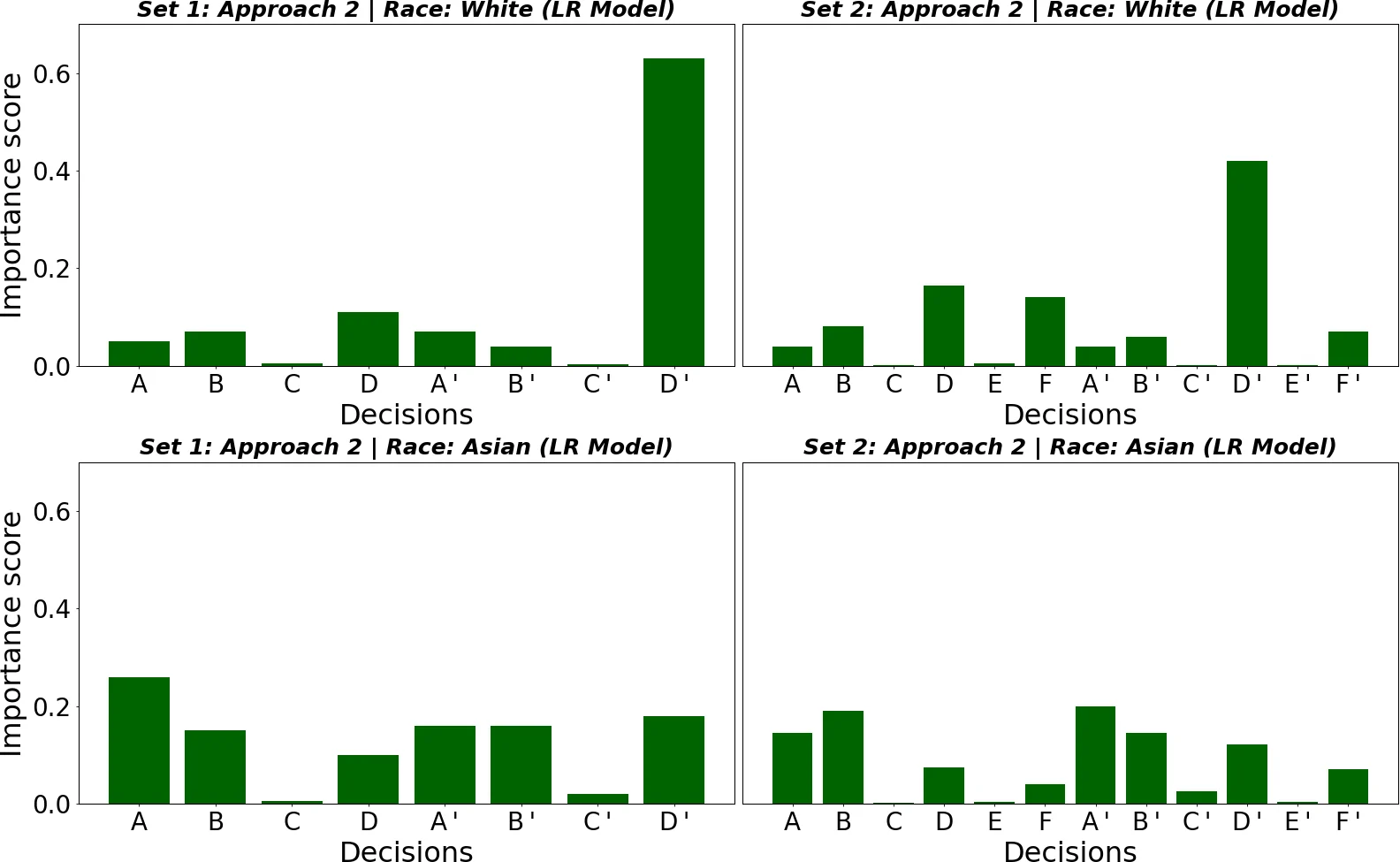
Feature importance comparing race groups White and Asian for Approach 2 using the logistic regression (LR) model. LR: logistic regression.

**Figure 8.**
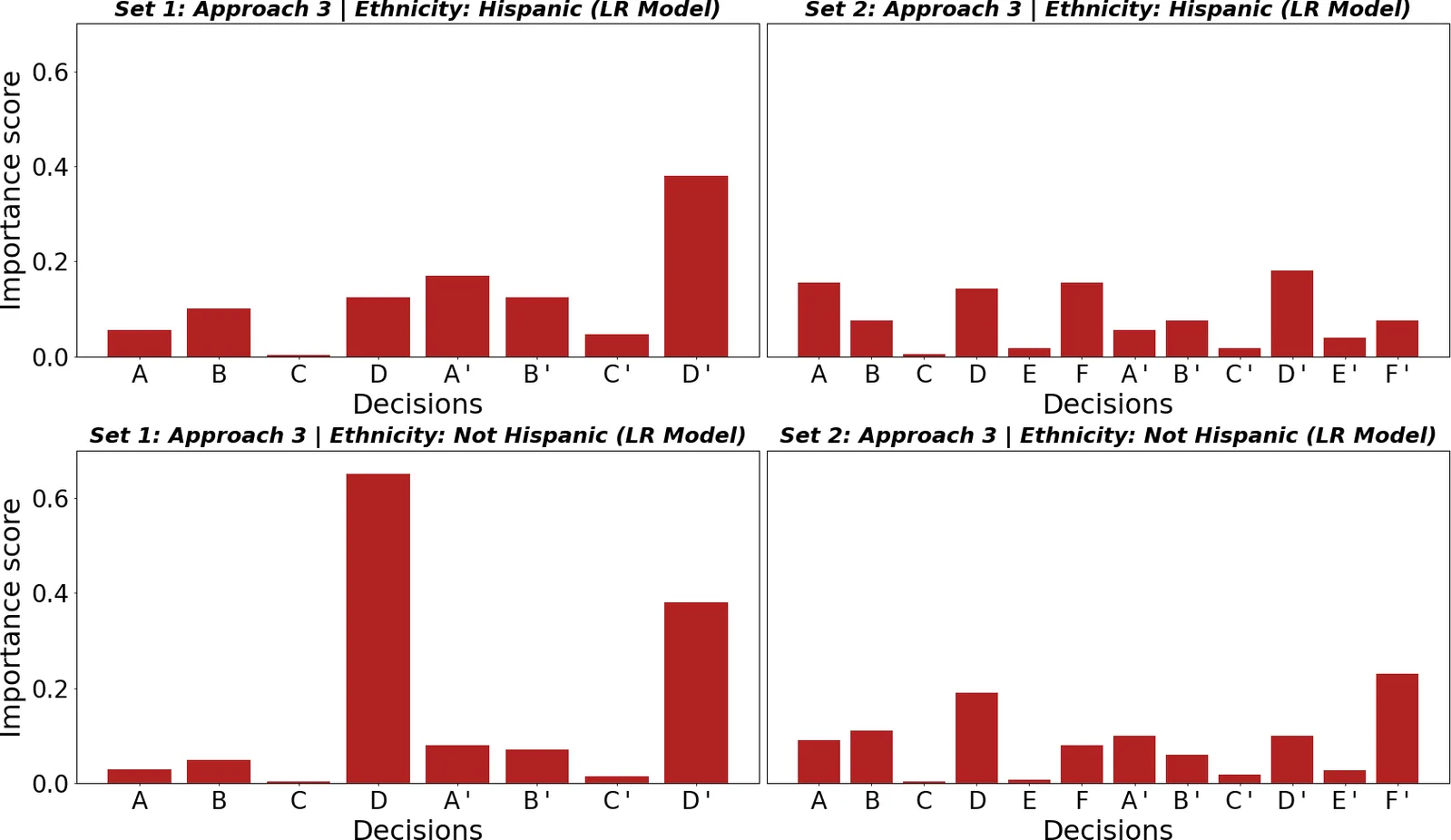
Feature importance comparing ethnicity groups Hispanic and non-Hispanic for Approach 3 using the logistic regression (LR) model. LR: logistic regression.

The impact of different decisions on cohort selection and cohort formation can be observed. These decisions affect subpopulations differently. Across different demographic groups, the quality of the model is influenced by different decisions to varying degrees. Certain decisions become more important depending on the subgroup, and the range of influential decisions varies across demographics. For illustration, a subset of representative plots is presented. Sex-specific differences (female and male) are provided in Multimedia Appendix 5, while Figure 7 presents differences by race (White and Asian) and Figure 8 presents differences by ethnicity (Hispanic and non-Hispanic).

Fairness analysis using demographic parity and equality of opportunity revealed substantial variation across cohorts and demographic subgroups [46-48]. While sex groups (female and male) exhibited relatively similar fairness patterns, notable disparities were observed across racial, ethnic, and age groups. In particular, certain racial categories and age groups showed consistently lower fairness metric values across multiple cohorts, indicating potential inequities in model performance (Figures S2 and S3 in Multimedia Appendix 5).

Because subgroup-level fairness metrics depend on both subgroup size and the distribution of outcome cases within each subgroup, these results should be interpreted cautiously. In this study, demographic parity was calculated as the proportion of predicted positive cases within each subgroup, while equality of opportunity and equalized odds were calculated among observed positive and negative cases, respectively. Therefore, when a subgroup contained very few patients, or when positive or negative outcome cases were absent, the corresponding fairness estimates could be unstable or not estimable. For this reason, fairness results for small demographic groups, including Native Hawaiian and multirace patients in some cohorts, should be interpreted as descriptive indicators of potential subgroup differences rather than definitive evidence of bias.

Calibration analysis further demonstrated variability in the alignment between predicted probabilities and observed outcomes. Across both Set 1 and Set 2, calibration curves showed that model reliability differs depending on cohort construction and training-testing combinations (Figures S4 and S5 in Multimedia Appendix 5). These findings highlight that models with similar AUC values may exhibit different levels of calibration and fairness, reinforcing the importance of multi-dimensional evaluation. Due to space limitations, representative calibration plots for Set 1 and Set 2 are presented in Figures S4 and S5 in Multimedia Appendix 5, while the complete set of calibration results is provided in Multimedia Appendices 6 and 7.

## Discussion

### Principal Findings

The study systematically explores the impact of cohort selection criteria on ML model performance and demographic disparities in predicting COVID-19 outcomes. Overall, the findings address the study objective by showing that model performance and subgroup results are sensitive to cohort construction decisions. The findings underscore the critical role of data processing decisions in shaping model accuracy. Notably, decision D (time inclusion/exclusion) was consistently associated with variation in model performance, particularly in Set 1, which comprises 16 cohorts based on 4 primary decisions.

The analysis reveals that different ML models—LR, RF, and GB—respond variably to the same data processing decisions, indicating that the perceived importance of these decisions is not consistent across models. This variability highlights the need for careful consideration of model choice in conjunction with cohort selection criteria to ensure robust and reliable predictions. These findings reinforce prior concerns in the literature that differences in data preprocessing—rather than model selection alone—can substantially influence observed performance, thereby affecting reproducibility and generalizability.

Furthermore, the study delves into the impact of data processing decisions on model performance across demographic subgroups, including gender, race, and ethnicity. The results indicate that certain decisions disproportionately affect specific demographic groups, potentially introducing bias and exacerbating health disparities. This finding emphasizes the importance of fair and transparent cohort selection practices to promote equity in ML-based health care research. Importantly, these results suggest that fairness is not solely a property of the algorithm but is strongly influenced by upstream data construction choices.

Although the AUC is widely used to evaluate model discrimination, it does not capture several critical dimensions of model performance, including calibration, subgroup-specific error patterns, and fairness across populations. As a result, models with similar AUC values may differ substantially in terms of reliability and equity. To address this limitation, this study incorporates additional evaluation dimensions, including fairness metrics and calibration analyses, to provide a more comprehensive assessment of model performance.

Furthermore, although confidence intervals and formal statistical tests are important for quantifying uncertainty, the large sample size of the N3C dataset inherently provides high statistical power to detect differences in AUC, as demonstrated in prior methodological studies. However, due to the computational complexity associated with large-scale data, resampling-based approaches such as bootstrapping were not feasible within the study environment [49]. Future work should explore scalable and computationally efficient methods for uncertainty estimation in large clinical datasets. So, these findings underscore that fairness assessment should not rely solely on discrimination metrics such as AUC, but instead should integrate multiple complementary measures to ensure a robust, reliable, and equitable evaluation of model performance.

Threshold-dependent metrics, such as false positive and false negative rate parity, provide important insights into subgroup-specific error disparities but require the selection of operating thresholds, which may vary across settings. In this study, we focused on threshold-independent metrics and calibration for consistency across cohorts. Future work will extend this analysis to include threshold-dependent fairness measures.

Importantly, this study was designed to evaluate how alternative cohort construction decisions affect model performance and subgroup estimates, rather than to identify a single optimal cohort definition. Therefore, the findings should be interpreted as demonstrating sensitivity to cohort construction choices, not as establishing a validated guideline for fair or standardized cohort selection. Development of such guidelines would require additional clinical validation, external replication, and evaluation across multiple datasets, outcomes, and health care settings.

The study also highlights the challenges posed by inconsistencies in cohort construction practices and the absence of standardized guidelines, which can hinder the comparability and validation of ML models across different settings. Addressing these challenges requires a structured and transparent approach to cohort definition, accounting for equity, clinical relevance, and methodological rigor. From a broader perspective, these findings suggest the need for future work to develop and validate standardized cohort design frameworks that may improve consistency across studies and support more reliable cross-study comparisons.

This research underscores the importance of considering cohort definition and data processing decisions when designing clinical ML models, especially in the context of equity and performance consistency. In addition to these contributions, several limitations should be considered when interpreting the findings. The study shows that cohort selection criteria can influence ML model performance and demographic subgroup results in clinical outcome prediction. Decision D (time inclusion/exclusion) was consistently associated with variation in model performance, particularly in Set 1, while other decisions, such as provider filtering, also contributed to performance differences in Set 2. The findings suggest that certain data processing decisions may affect demographic groups differently, highlighting the need for transparent reporting and sensitivity analysis in cohort construction. By systematically analyzing combinations of data processing criteria across multiple modeling approaches, this work provides insight into the potential consequences of cohort definition choices. This study relied on traditional ML methods; future work should examine whether similar patterns are observed with deep learning–based approaches.

Feature importance derived from tree-based models may be sensitive to data structure and model initialization and may exhibit variability under different sampling or training conditions. In addition, such measures can be influenced by correlated or structured features, which may introduce bias in the relative importance rankings. Therefore, the reported importance values should be interpreted with caution and should not be considered as evidence of causal relationships. Although consistent patterns were observed across multiple models and analytical approaches in this study, future work should further assess the stability and robustness of these findings using repeated resampling strategies, multiple random seeds, and complementary importance measures such as permutation importance or Shapley Additive Explanations (SHAP) values.

Some demographic subgroups, particularly Native Hawaiian and multirace, had very small or zero sample sizes in certain cohorts. In several cases, these groups were absent from either the training or testing sets, making it impossible to compute reliable performance metrics such as AUC. Consequently, reported values of zero or undefined AUC reflect data limitations rather than true model performance. Small sample sizes increase variability and reduce the statistical reliability of subgroup-level estimates, which may lead to unstable or misleading comparisons. Therefore, results for underrepresented subgroups should be interpreted with caution, and fairness assessments are more reliable for groups with sufficient representation. This limitation highlights the importance of adequate subgroup sample sizes when evaluating model performance across demographic populations.

Subgroup fairness analyses were affected by small sample sizes and limited outcome variation in some demographic groups. Although Multimedia Appendix 1 reports cohort-level sample sizes, train/test splits, and demographic distributions across the 16- and 64-cohort configurations, some subgroups, particularly multirace and Native Hawaiian patients, had very small counts in several cohorts. Because subgroup AUC requires both positive and negative outcome cases, and threshold-based fairness metrics depend on the distribution of observed outcomes and predicted classifications within each subgroup, estimates for sparsely represented groups may be unstable. Therefore, subgroup fairness results for small demographic groups should be interpreted cautiously as descriptive indicators rather than definitive evidence of model fairness or unfairness. Future studies should build on these descriptive summaries by reporting subgroup-specific outcome counts and uncertainty measures alongside fairness metrics.

This study has several limitations that should be acknowledged. First, use of data from a specific time period (August 1, 2020-December 31, 2021) may not capture the changing nature of COVID-19, including changes in virus variant, vaccination rates, and health care practices. Therefore, our findings may not be generalizable to current or future scenarios. Second, the study is limited to adult patients (18 years and older), thereby excluding pediatric populations. Accordingly, the results cannot be generalized to younger populations, and differences in the presentation and outcome of COVID-19 among children and adolescents were not examined.

Although 18 years is widely used as the standard threshold for defining adult populations in clinical research and public health reporting, alternative age thresholds could result in modest differences in cohort composition. Future studies could evaluate the sensitivity of the findings to alternative age cutoffs to further assess the robustness of the observed results.

Finally, while the study simplifies data processing decisions to maintain analytic feasibility, limiting the study to only including other possibly influential factors such as socioeconomic status, comorbidities, or geographic variations would give a more complete picture of model performance and demographic disparities.

### Conclusions

In conclusion, this study demonstrates that cohort selection is not merely a preprocessing step but a fundamental driver of ML performance and fairness in clinical research. Overall, the study calls for the development of comprehensive guidelines for cohort construction and the validation of existing models against diverse benchmarks to ensure ML models are valid, equitable, and reproducible. These efforts are essential for achieving trustworthy and impactful ML applications in health care, particularly in the context of the COVID-19 pandemic and beyond. More broadly, these findings highlight that improving equity and reliability in clinical ML requires careful attention to upstream data decisions, emphasizing transparency, standardization, and reproducibility as core principles for future research.

## Supplementary material

10.2196/88406 Multimedia Appendix 1 Sample sizes and demographic characteristics across the 16- and 64-cohort configurations.

10.2196/88406 Multimedia Appendix 2 Performance metrics for logistic regression, random forest, and gradient boosting models across 16 cohorts, overall and by demographic subgroup.

10.2196/88406 Multimedia Appendix 3 Performance metrics for logistic regression, random forest, and gradient boosting models across 64 cohorts.

10.2196/88406 Multimedia Appendix 4 Demographic subgroup-specific AUC values for logistic regression, random forest, and gradient boosting models across 64 cohorts.

10.2196/88406 Multimedia Appendix 5 Supplementary analyses of feature importance, demographic fairness, and model calibration across the 16- and 64-cohort configurations.

10.2196/88406 Multimedia Appendix 6 Calibration plots for logistic regression, random forest, and gradient boosting models across 16 cohorts.

10.2196/88406 Multimedia Appendix 7 Calibration plots for logistic regression, random forest, and gradient boosting models across 64 cohorts.

## Abbreviations

AUC: area under the receiver operating characteristic curve
BP: blood pressure
CBC: complete blood count
CDM: Common Data Model
DHHS: Department of Health and Human Services
EHR: electronic health record
FDA: Food and Drug Administration
GB: gradient boosting
GFR: glomerular filtration rate
IRB: Institutional Review Board
LDS: limited dataset
LR: logistic regression
ML: machine learning
N3C: National COVID Cohort Collaborative
NT-proBNP: N-terminal pro–B-type natriuretic peptide
OMOP: Observational Medical Outcomes Partnership
RF: random forest
SHAP: Shapley Additive Explanations
