# Supplementary material for: A Systematic Evaluation of Cohort Selection Criteria and Their Impact on Machine Learning Model Performance and Demographic Disparities in COVID-19 Outcomes: Cohort Study
Source: JMIR Form Res. 2026 Aug 20;10:e88406. doi: 10.2196/88406 (PMC13492188; doi:10.2196/88406)
Supplement: Supplementary file 1 [file formative-v10-e88406-s001.docx]

Appendix 1

Appendix 1 provides a structured summary of sample size, data partitioning, and demographic composition across both cohort configurations (set 1 and set 2). Specifically, it reports the distribution of total observations and their allocation into training and testing sets at the row level, alongside corresponding demographic characteristics. In addition, it presents the counts of unique patients across data splits and summarizes their demographic distribution based on patient identifiers. These summaries facilitate direct comparison of population structure and representativeness under the 16-cohort and 64-cohort construction strategies.

- ***Cohorts 1 to 16***

**Table 1. Summary of Row and Unique Patient Counts Across Cohorts 1–16**

|  | ***Rows*** | | | ***Patients*** | | |
| --- | --- | --- | --- | --- | --- | --- |
| ***Dataset*** | ***Total*** | ***Train*** | ***Test*** | ***Total*** | ***Train*** | ***Test*** |
| Cohort 1 | 123,367 | 98,821 | 24,546 | 114,648 | 91,481 | 23,167 |
| Cohort 2 | 313,743 | 250,901 | 62,842 | 289,119 | 230,766 | 58,353 |
| Cohort 3 | 127,925 | 102,487 | 25,438 | 117,951 | 94,085 | 23,866 |
| Cohort 4 | 329,997 | 263,873 | 66,124 | 301,369 | 240,588 | 60,781 |
| Cohort 5 | 126,056 | 101,013 | 25,043 | 117,270 | 93,611 | 23,659 |
| Cohort 6 | 321,467 | 257,140 | 64,327 | 296,420 | 236,666 | 59,754 |
| Cohort 7 | 130,680 | 104,732 | 25,948 | 120,614 | 96,246 | 24,368 |
| Cohort 8 | 338,242 | 270,534 | 67,708 | 309,057 | 246,801 | 62,256 |
| Cohort 9 | 125,347 | 100,393 | 24,954 | 116,543 | 93,001 | 23,542 |
| Cohort 10 | 316,573 | 253,161 | 63,412 | 291,787 | 232,907 | 58,880 |
| Cohort 11 | 129,996 | 104,130 | 25,866 | 119,917 | 95,666 | 24,251 |
| Cohort 12 | 333,000 | 266,272 | 66,728 | 304,183 | 242,851 | 61,332 |
| Cohort 13 | 123,299 | 98,726 | 24,573 | 115,423 | 92,069 | 23,354 |
| Cohort 14 | 313,710 | 250,862 | 62,848 | 290,410 | 231,871 | 58,539 |
| Cohort 15 | 127,910 | 102,429 | 25,481 | 118,747 | 94,694 | 24,053 |
| Cohort 16 | 330,617 | 264,370 | 66,247 | 303,245 | 242,183 | 61,062 |

**Table 2. Distribution of Demographic Characteristics Across Cohorts 1 to 16 (Rows)**

| ***Dataset*** | ***Female*** | ***Male*** | ***Black*** | ***White*** | ***Asian*** | ***Multi Race*** | ***Native Hawaiian*** | ***Hispanic*** | ***Non-Hispanic*** |
| --- | --- | --- | --- | --- | --- | --- | --- | --- | --- |
| Cohort 1 | 10,432 | 11,217 | 4,361 | 12,763 | 740 | 28 | 70 | 3,967 | 16,804 |
| Cohort 2 | 22,992 | 24,514 | 9,896 | 30,076 | 1,129 | 554 | 104 | 6,034 | 39,661 |
| Cohort 3 | 10,843 | 11,631 | 4,506 | 13,301 | 765 | 28 | 70 | 4,106 | 17,471 |
| Cohort 4 | 24,267 | 25,752 | 10,293 | 31,875 | 1,192 | 604 | 106 | 6,279 | 41,864 |
| Cohort 5 | 10,453 | 11,232 | 4,369 | 12,780 | 741 | 28 | 70 | 3,974 | 16,831 |
| Cohort 6 | 23,170 | 24,631 | 9,930 | 30,308 | 1,132 | 558 | 104 | 6,058 | 39,908 |
| Cohort 7 | 10,864 | 11,646 | 4,514 | 13,318 | 766 | 28 | 70 | 4,113 | 17,498 |
| Cohort 8 | 24,460 | 25,888 | 10,327 | 32,138 | 1,196 | 609 | 106 | 6,305 | 42,142 |
| Cohort 9 | 10,688 | 11,312 | 4,435 | 12,918 | 752 | 28 | 70 | 4,065 | 17,032 |
| Cohort 10 | 23,302 | 24,661 | 9,986 | 30,296 | 1,146 | 558 | 105 | 6,142 | 39,983 |
| Cohort 11 | 11,108 | 11,731 | 4,579 | 13,466 | 780 | 28 | 70 | 4,205 | 17,712 |
| Cohort 12 | 24,591 | 25,905 | 10,383 | 32,108 | 1,212 | 608 | 107 | 6,388 | 42,205 |
| Cohort 13 | 4,835 | 5,028 | 1,772 | 5,612 | 253 | <20 | <20 | 1,894 | 7,636 |
| Cohort 14 | 12,174 | 12,619 | 5,902 | 14,502 | 553 | <20 | 46 | 3,285 | 20,789 |
| Cohort 15 | 5,051 | 5,258 | 1,835 | 5,916 | 263 | <20 | <20 | 1,969 | 7,995 |
| Cohort 16 | 12,720 | 13,146 | 6,113 | 15,209 | 584 | <20 | 48 | 3,410 | 21,717 |

***Table 3. Demographic Distribution of Unique Patients Across Cohorts 1 to 16 (Patient IDs)***

| ***Dataset*** | ***Female*** | ***Male*** | ***Black*** | ***White*** | ***Asian*** | ***Multi Race*** | ***Native Hawaiian*** | ***Hispanic*** | ***Non-Hispanic*** |
| --- | --- | --- | --- | --- | --- | --- | --- | --- | --- |
| Cohort 1 | 10,672 | 9,918 | 4,087 | 12,029 | 753 | 27 | 63 | 3,876 | 15,850 |
| Cohort 2 | 22,935 | 21,950 | 9,315 | 28,250 | 1,106 | 513 | 91 | 5,904 | 37,207 |
| Cohort 3 | 10,980 | 10,213 | 4,174 | 12,441 | 780 | 29 | 64 | 3,983 | 16,329 |
| Cohort 4 | 23,924 | 22,963 | 9,605 | 29,700 | 1,158 | 563 | 92 | 6,121 | 38,946 |
| Cohort 5 | 10,678 | 9,943 | 4,092 | 12,050 | 754 | 28 | 63 | 3,880 | 15,873 |
| Cohort 6 | 23,031 | 22,145 | 9,336 | 28,497 | 1,110 | 514 | 91 | 5,928 | 37,445 |
| Cohort 7 | 10,987 | 10,239 | 4,179 | 12,464 | 781 | 30 | 64 | 3,987 | 16,354 |
| Cohort 8 | 24,039 | 23,178 | 9,628 | 29,981 | 1,162 | 565 | 92 | 6,147 | 39,220 |
| Cohort 9 | 10,786 | 10,157 | 4,150 | 12,176 | 773 | 27 | 63 | 4,007 | 16,048 |
| Cohort 10 | 23,100 | 22,231 | 9,398 | 28,455 | 1,130 | 517 | 91 | 6,044 | 37,486 |
| Cohort 11 | 11,104 | 10,459 | 4,241 | 12,598 | 800 | 29 | 64 | 4,117 | 16,541 |
| Cohort 12 | 24,099 | 23,257 | 9,692 | 29,920 | 1,182 | 567 | 92 | 6,264 | 39,245 |
| Cohort 13 | 4,866 | 4,780 | 1,679 | 5,398 | 280 | <20 | <20 | 1,981 | 7,352 |
| Cohort 14 | 11,745 | 11,734 | 5,550 | 13,524 | 554 | <20 | 36 | 3,337 | 19,433 |
| Cohort 15 | 5,037 | 4,936 | 1,720 | 5,629 | 289 | <20 | <20 | 2,034 | 7,619 |
| Cohort 16 | 12,135 | 12,128 | 5,691 | 14,038 | 576 | <20 | 36 | 3,445 | 20,096 |

- ***Cohorts 1 to 64***

**Table 4. Summary of Row and Unique Patient Counts Across Cohorts 1–64**

|  | ***Rows*** | | | ***Patients*** | | |
| --- | --- | --- | --- | --- | --- | --- |
| ***Dataset*** | ***Total*** | ***Train*** | ***Test*** | ***Total*** | ***Train*** | ***Test*** |
| Cohort 1 | 71,720 | 57,506 | 14,214 | 66,389 | 53,199 | 13,190 |
| Cohort 2 | 125,540 | 100,606 | 24,934 | 116,266 | 93,029 | 23,237 |
| Cohort 3 | 72,265 | 57,934 | 14,331 | 66,905 | 53,613 | 13,292 |
| Cohort 4 | 134,284 | 107,628 | 26,656 | 124,841 | 99,862 | 24,979 |
| Cohort 5 | 120,901 | 96,927 | 23,974 | 112,294 | 89,819 | 22,475 |
| Cohort 6 | 293,192 | 234,671 | 58,521 | 269,777 | 215,600 | 54,177 |
| Cohort 7 | 123,364 | 98,856 | 24,508 | 114,645 | 91,673 | 22,972 |
| Cohort 8 | 313,741 | 251,089 | 62,652 | 289,114 | 231,017 | 58,097 |
| Cohort 9 | 74,308 | 59,545 | 14,763 | 68,232 | 54,651 | 13,581 |
| Cohort 10 | 130,799 | 104,790 | 26,009 | 120,241 | 96,174 | 24,067 |
| Cohort 11 | 74,861 | 59,979 | 14,882 | 68,752 | 55,068 | 13,684 |
| Cohort 12 | 139,954 | 112,129 | 27,825 | 129,122 | 103,264 | 25,858 |
| Cohort 13 | 125,386 | 100,485 | 24,901 | 115,550 | 92,398 | 23,152 |
| Cohort 14 | 308,521 | 246,922 | 61,599 | 281,338 | 224,867 | 56,471 |
| Cohort 15 | 127,922 | 102,476 | 25,446 | 117,948 | 94,289 | 23,659 |
| Cohort 16 | 329,995 | 264,064 | 65,931 | 301,364 | 240,842 | 60,522 |
| Cohort 17 | 72,055 | 57,784 | 14,271 | 66,705 | 53,454 | 13,251 |
| Cohort 18 | 126,716 | 101,564 | 25,152 | 117,381 | 93,907 | 23,474 |
| Cohort 19 | 72,600 | 58,212 | 14,388 | 67,221 | 53,868 | 13,353 |
| Cohort 20 | 135,501 | 108,612 | 26,889 | 125,997 | 100,772 | 25,225 |
| Cohort 21 | 123,452 | 98,957 | 24,495 | 114,778 | 91,778 | 23,000 |
| Cohort 22 | 300,165 | 240,288 | 59,877 | 276,354 | 220,771 | 55,583 |
| Cohort 23 | 126,053 | 100,992 | 25,061 | 117,267 | 93,743 | 23,524 |
| Cohort 24 | 321,465 | 257,323 | 64,142 | 296,415 | 236,769 | 59,646 |
| Cohort 25 | 74,667 | 59,843 | 14,824 | 68,564 | 54,921 | 13,643 |
| Cohort 26 | 132,012 | 105,779 | 26,233 | 121,384 | 97,075 | 24,309 |
| Cohort 27 | 75,220 | 60,277 | 14,943 | 69,084 | 55,338 | 13,746 |
| Cohort 28 | 141,211 | 113,145 | 28,066 | 130,307 | 104,197 | 26,110 |
| Cohort 29 | 128,000 | 102,563 | 25,437 | 118,073 | 94,390 | 23,683 |
| Cohort 30 | 315,988 | 252,938 | 63,050 | 288,287 | 230,352 | 57,935 |
| Cohort 31 | 130,677 | 104,662 | 26,015 | 120,611 | 96,393 | 24,218 |
| Cohort 32 | 338,240 | 270,714 | 67,526 | 309,052 | 246,922 | 62,130 |
| Cohort 33 | 73,123 | 58,655 | 14,468 | 67,743 | 54,279 | 13,464 |
| Cohort 34 | 127,193 | 101,949 | 25,244 | 117,862 | 94,301 | 23,561 |
| Cohort 35 | 73,723 | 59,123 | 14,600 | 68,313 | 54,732 | 13,581 |
| Cohort 36 | 136,101 | 109,101 | 27,000 | 126,598 | 101,258 | 25,340 |
| Cohort 37 | 122,777 | 98,454 | 24,323 | 114,089 | 91,251 | 22,838 |
| Cohort 38 | 295,723 | 236,732 | 58,991 | 272,167 | 217,498 | 54,669 |
| Cohort 39 | 125,344 | 100,460 | 24,884 | 116,540 | 93,181 | 23,359 |
| Cohort 40 | 316,571 | 253,385 | 63,186 | 291,782 | 233,132 | 58,650 |
| Cohort 41 | 75,753 | 60,728 | 15,025 | 69,619 | 55,753 | 13,866 |
| Cohort 42 | 132,527 | 106,192 | 26,335 | 121,899 | 97,490 | 24,409 |
| Cohort 43 | 76,361 | 61,202 | 15,159 | 70,193 | 56,209 | 13,984 |
| Cohort 44 | 141,853 | 113,667 | 28,186 | 130,949 | 104,711 | 26,238 |
| Cohort 45 | 127,352 | 102,084 | 25,268 | 117,414 | 93,880 | 23,534 |
| Cohort 46 | 311,176 | 249,082 | 62,094 | 283,825 | 226,837 | 56,988 |
| Cohort 47 | 129,993 | 104,152 | 25,841 | 119,914 | 95,848 | 24,066 |
| Cohort 48 | 332,998 | 266,496 | 66,502 | 304,178 | 243,072 | 61,106 |
| Cohort 49 | 66,872 | 53,680 | 13,192 | 62,036 | 49,683 | 12,353 |
| Cohort 50 | 85,090 | 68,288 | 16,802 | 77,509 | 62,024 | 15,485 |
| Cohort 51 | 67,473 | 54,149 | 13,324 | 62,607 | 50,137 | 12,470 |
| Cohort 52 | 86,157 | 69,107 | 17,050 | 78,527 | 62,826 | 15,701 |
| Cohort 53 | 120,556 | 96,729 | 23,827 | 112,797 | 90,216 | 22,581 |
| Cohort 54 | 292,132 | 233,945 | 58,187 | 270,073 | 215,756 | 54,317 |
| Cohort 55 | 123,296 | 98,871 | 24,425 | 115,420 | 92,287 | 23,133 |
| Cohort 56 | 313,708 | 251,198 | 62,510 | 290,405 | 231,973 | 58,432 |
| Cohort 57 | 69,417 | 55,681 | 13,736 | 63,864 | 51,117 | 12,747 |
| Cohort 58 | 89,006 | 71,363 | 17,643 | 80,497 | 64,385 | 16,112 |
| Cohort 59 | 70,026 | 56,156 | 13,870 | 64,439 | 51,574 | 12,865 |
| Cohort 60 | 90,093 | 72,197 | 17,896 | 81,528 | 65,198 | 16,330 |
| Cohort 61 | 125,088 | 100,334 | 24,754 | 116,070 | 92,806 | 23,264 |
| Cohort 62 | 308,035 | 246,656 | 61,379 | 282,153 | 225,433 | 56,720 |
| Cohort 63 | 127,907 | 102,540 | 25,367 | 118,744 | 94,917 | 23,827 |
| Cohort 64 | 330,615 | 264,688 | 65,927 | 303,240 | 242,268 | 60,972 |

**Table 5. Distribution of Demographic Characteristics Across Cohorts 1 to 64 (Rows)**

| ***Dataset*** | ***Female*** | ***Male*** | ***Black*** | ***White*** | ***Asian*** | ***Multi Race*** | ***Native Hawaiian*** | ***Hispanic*** | ***Non-Hispanic*** |
| --- | --- | --- | --- | --- | --- | --- | --- | --- | --- |
| Cohort 1 | 26,220 | 25,214 | 14,040 | 28,518 | 1,249 | 124 | 61 | 8,231 | 41,287 |
| Cohort 2 | 32,018 | 30,925 | 15,842 | 37,032 | 1,386 | 761 | 71 | 9,169 | 51,811 |
| Cohort 3 | 26,436 | 25,410 | 14,126 | 28,624 | 1,249 | 124 | 61 | 8,423 | 41,444 |
| Cohort 4 | 32,239 | 31,126 | 15,929 | 37,142 | 1,387 | 761 | 71 | 9,365 | 51,974 |
| Cohort 5 | 45,963 | 48,745 | 19,961 | 55,633 | 3,648 | 129 | 264 | 16,740 | 74,018 |
| Cohort 6 | 103,588 | 108,152 | 45,558 | 133,554 | 5,337 | 2,744 | 378 | 25,432 | 179,230 |
| Cohort 7 | 47,190 | 49,882 | 20,777 | 56,323 | 3,741 | 129 | 279 | 17,437 | 75,529 |
| Cohort 8 | 109,583 | 114,196 | 48,307 | 140,418 | 5,731 | 2,744 | 421 | 27,907 | 187,041 |
| Cohort 9 | 27,136 | 26,057 | 14,456 | 29,627 | 1,289 | 130 | 66 | 8,469 | 42,758 |
| Cohort 10 | 33,494 | 32,352 | 16,387 | 39,063 | 1,444 | 818 | 76 | 9,469 | 54,362 |
| Cohort 11 | 27,356 | 26,254 | 14,542 | 29,734 | 1,289 | 130 | 66 | 8,665 | 42,916 |
| Cohort 12 | 33,720 | 32,554 | 16,474 | 39,175 | 1,445 | 818 | 76 | 9,669 | 54,527 |
| Cohort 13 | 47,772 | 50,527 | 20,611 | 57,982 | 3,782 | 135 | 273 | 17,306 | 76,935 |
| Cohort 14 | 109,638 | 114,014 | 47,494 | 142,132 | 5,601 | 3,002 | 390 | 26,513 | 189,804 |
| Cohort 15 | 49,045 | 51,689 | 21,454 | 58,693 | 3,882 | 135 | 288 | 18,023 | 78,496 |
| Cohort 16 | 115,908 | 120,340 | 50,348 | 149,348 | 6,021 | 3,002 | 436 | 29,081 | 198,007 |
| Cohort 17 | 26,255 | 25,229 | 14,049 | 28,553 | 1,250 | 126 | 61 | 8,236 | 41,326 |
| Cohort 18 | 32,057 | 30,943 | 15,852 | 37,071 | 1,387 | 763 | 71 | 9,177 | 51,854 |
| Cohort 19 | 26,471 | 25,425 | 14,135 | 28,659 | 1,250 | 126 | 61 | 8,428 | 41,483 |
| Cohort 20 | 32,278 | 31,144 | 15,939 | 37,181 | 1,388 | 763 | 71 | 9,373 | 52,017 |
| Cohort 21 | 46,079 | 48,803 | 19,985 | 55,732 | 3,654 | 131 | 264 | 16,768 | 74,150 |
| Cohort 22 | 104,491 | 108,683 | 45,681 | 134,714 | 5,355 | 2,769 | 378 | 25,543 | 180,429 |
| Cohort 23 | 47,306 | 49,940 | 20,801 | 56,422 | 3,747 | 131 | 279 | 17,465 | 75,661 |
| Cohort 24 | 110,494 | 114,733 | 48,433 | 141,587 | 5,750 | 2,769 | 421 | 28,019 | 188,249 |
| Cohort 25 | 27,176 | 26,074 | 14,466 | 29,668 | 1,290 | 132 | 66 | 8,475 | 42,803 |
| Cohort 26 | 33,538 | 32,373 | 16,398 | 39,108 | 1,446 | 820 | 76 | 9,478 | 54,412 |
| Cohort 27 | 27,396 | 26,271 | 14,552 | 29,775 | 1,290 | 132 | 66 | 8,671 | 42,961 |
| Cohort 28 | 33,764 | 32,575 | 16,485 | 39,220 | 1,447 | 820 | 76 | 9,678 | 54,577 |
| Cohort 29 | 47,898 | 50,588 | 20,637 | 58,091 | 3,788 | 137 | 273 | 17,335 | 77,079 |
| Cohort 30 | 110,653 | 114,622 | 47,630 | 143,456 | 5,622 | 3,029 | 390 | 26,639 | 191,165 |
| Cohort 31 | 49,171 | 51,750 | 21,480 | 58,802 | 3,888 | 137 | 288 | 18,052 | 78,640 |
| Cohort 32 | 116,931 | 120,954 | 50,487 | 150,681 | 6,043 | 3,029 | 436 | 29,208 | 199,377 |
| Cohort 33 | 27,149 | 25,480 | 14,319 | 28,941 | 1,267 | 124 | 61 | 8,692 | 41,903 |
| Cohort 34 | 32,990 | 31,226 | 16,127 | 37,473 | 1,421 | 760 | 72 | 9,664 | 52,471 |
| Cohort 35 | 27,414 | 25,681 | 14,413 | 29,063 | 1,267 | 124 | 61 | 8,915 | 42,075 |
| Cohort 36 | 33,260 | 31,430 | 16,221 | 37,599 | 1,421 | 760 | 72 | 9,890 | 52,648 |
| Cohort 37 | 47,172 | 49,226 | 20,299 | 56,304 | 3,711 | 129 | 265 | 17,307 | 75,008 |
| Cohort 38 | 104,959 | 108,809 | 45,953 | 134,462 | 5,412 | 2,750 | 380 | 26,019 | 180,534 |
| Cohort 39 | 48,487 | 50,378 | 21,130 | 57,034 | 3,809 | 129 | 280 | 18,040 | 76,578 |
| Cohort 40 | 111,080 | 114,914 | 48,723 | 141,422 | 5,816 | 2,750 | 423 | 28,546 | 188,468 |
| Cohort 41 | 28,085 | 26,325 | 14,741 | 30,059 | 1,307 | 130 | 66 | 8,937 | 43,388 |
| Cohort 42 | 34,490 | 32,661 | 16,678 | 39,520 | 1,481 | 817 | 77 | 9,972 | 55,045 |
| Cohort 43 | 28,354 | 26,527 | 14,835 | 30,182 | 1,307 | 130 | 66 | 9,164 | 43,561 |
| Cohort 44 | 34,765 | 32,866 | 16,772 | 39,648 | 1,481 | 817 | 77 | 10,202 | 55,224 |
| Cohort 45 | 49,022 | 51,047 | 20,958 | 58,704 | 3,852 | 135 | 274 | 17,882 | 77,993 |
| Cohort 46 | 111,066 | 114,714 | 47,899 | 143,108 | 5,683 | 3,008 | 392 | 27,110 | 191,195 |
| Cohort 47 | 50,383 | 52,225 | 21,817 | 59,455 | 3,957 | 135 | 289 | 18,635 | 79,614 |
| Cohort 48 | 117,467 | 121,104 | 50,777 | 150,424 | 6,114 | 3,008 | 438 | 29,730 | 199,529 |
| Cohort 49 | 10,148 | 9,004 | 5,193 | 7,902 | 386 | <20 | <20 | 4,924 | 13,352 |
| Cohort 50 | 10,205 | 9,078 | 5,237 | 7,925 | 393 | <20 | <20 | 4,966 | 13,441 |
| Cohort 51 | 10,403 | 9,203 | 5,286 | 8,016 | 386 | <20 | <20 | 5,144 | 13,515 |
| Cohort 52 | 10,462 | 9,279 | 5,330 | 8,040 | 393 | <20 | <20 | 5,188 | 13,606 |
| Cohort 53 | 18,848 | 18,999 | 7,571 | 20,625 | 1,307 | <20 | 36 | 6,899 | 29,602 |
| Cohort 54 | 50,185 | 50,812 | 26,905 | 56,440 | 2,529 | <20 | 138 | 11,933 | 85,938 |
| Cohort 55 | 19,359 | 19,409 | 7,749 | 20,904 | 1,363 | <20 | 36 | 7,210 | 30,138 |
| Cohort 56 | 54,811 | 55,467 | 28,217 | 62,463 | 2,884 | <20 | 166 | 14,034 | 92,817 |
| Cohort 57 | 10,468 | 9,295 | 5,345 | 8,209 | 397 | <20 | <20 | 5,056 | 13,805 |
| Cohort 58 | 10,529 | 9,371 | 5,389 | 8,237 | 405 | <20 | <20 | 5,098 | 13,900 |
| Cohort 59 | 10,727 | 9,495 | 5,438 | 8,324 | 397 | <20 | <20 | 5,280 | 13,969 |
| Cohort 60 | 10,791 | 9,573 | 5,482 | 8,354 | 405 | <20 | <20 | 5,324 | 14,067 |
| Cohort 61 | 19,647 | 19,836 | 7,835 | 21,718 | 1,354 | <20 | 37 | 7,132 | 30,971 |
| Cohort 62 | 52,475 | 52,983 | 27,892 | 59,298 | 2,640 | <20 | 142 | 12,373 | 89,887 |
| Cohort 63 | 20,172 | 20,252 | 8,013 | 22,005 | 1,415 | <20 | 37 | 7,449 | 31,521 |
| Cohort 64 | 57,318 | 57,873 | 29,251 | 65,638 | 3,020 | <20 | 173 | 14,553 | 97,130 |

**Table 6. Demographic Distribution of Unique Patients Across Cohorts 1 to 64 (Patient IDs)**

| ***Dataset*** | ***Male*** | ***Female*** | ***Black*** | ***White*** | ***Asian*** | ***Multi Race*** | ***Native Hawaiian*** | ***Hispanic*** | ***Non-Hispanic*** |
| --- | --- | --- | --- | --- | --- | --- | --- | --- | --- |
| Cohort 1 | 4,790 | 4,899 | 2,579 | 5,435 | 247 | <20 | <20 | 1,534 | 7,797 |
| Cohort 2 | 5,787 | 5,951 | 2,853 | 6,992 | 273 | 151 | <20 | 1,715 | 9,663 |
| Cohort 3 | 4,826 | 4,926 | 2,592 | 5,452 | 247 | <20 | <20 | 1,558 | 7,824 |
| Cohort 4 | 5,823 | 5,979 | 2,866 | 7,009 | 273 | 151 | <20 | 1,739 | 9,691 |
| Cohort 5 | 9,329 | 8,807 | 3,741 | 10,746 | 654 | <20 | 46 | 3,165 | 14,194 |
| Cohort 6 | 20,404 | 19,780 | 8,503 | 25,455 | 963 | 545 | 71 | 4,787 | 34,019 |
| Cohort 7 | 9,566 | 9,016 | 3,885 | 10,880 | 673 | <20 | 48 | 3,291 | 14,486 |
| Cohort 8 | 21,577 | 20,919 | 9,023 | 26,749 | 1,050 | 545 | 78 | 5,244 | 35,507 |
| Cohort 9 | 4,898 | 5,039 | 2,633 | 5,596 | 255 | 23 | <20 | 1,566 | 8,005 |
| Cohort 10 | 6,000 | 6,200 | 2,936 | 7,318 | 286 | 162 | <20 | 1,761 | 10,070 |
| Cohort 11 | 4,934 | 5,066 | 2,646 | 5,613 | 255 | 23 | <20 | 1,590 | 8,032 |
| Cohort 12 | 6,036 | 6,228 | 2,949 | 7,335 | 286 | 162 | <20 | 1,785 | 10,098 |
| Cohort 13 | 9,571 | 9,082 | 3,818 | 11,091 | 673 | 23 | 46 | 3,252 | 14,608 |
| Cohort 14 | 21,299 | 20,792 | 8,788 | 26,840 | 1,016 | 594 | 72 | 4,960 | 35,714 |
| Cohort 15 | 9,811 | 9,304 | 3,967 | 11,233 | 693 | 23 | 48 | 3,381 | 14,912 |
| Cohort 16 | 22,514 | 21,982 | 9,318 | 28,208 | 1,105 | 594 | 80 | 5,429 | 37,274 |
| Cohort 17 | 4,791 | 4,909 | 2,581 | 5,443 | 247 | 21 | <20 | 1,535 | 7,806 |
| Cohort 18 | 5,789 | 5,961 | 2,855 | 7,001 | 273 | 152 | <20 | 1,716 | 9,673 |
| Cohort 19 | 4,827 | 4,936 | 2,594 | 5,460 | 247 | 21 | <20 | 1,559 | 7,833 |
| Cohort 20 | 5,825 | 5,989 | 2,868 | 7,018 | 273 | 152 | <20 | 1,740 | 9,701 |
| Cohort 21 | 9,337 | 8,829 | 3,746 | 10,764 | 655 | 21 | 46 | 3,171 | 14,217 |
| Cohort 22 | 20,519 | 19,932 | 8,524 | 25,680 | 965 | 550 | 71 | 4,800 | 34,247 |
| Cohort 23 | 9,574 | 9,038 | 3,890 | 10,898 | 674 | 21 | 48 | 3,297 | 14,509 |
| Cohort 24 | 21,693 | 21,073 | 9,045 | 26,975 | 1,052 | 550 | 78 | 5,257 | 35,736 |
| Cohort 25 | 4,899 | 5,049 | 2,635 | 5,604 | 255 | 24 | <20 | 1,567 | 8,014 |
| Cohort 26 | 6,002 | 6,210 | 2,938 | 7,327 | 286 | 163 | <20 | 1,762 | 10,080 |
| Cohort 27 | 4,935 | 5,076 | 2,648 | 5,621 | 255 | 24 | <20 | 1,591 | 8,041 |
| Cohort 28 | 6,038 | 6,238 | 2,951 | 7,344 | 286 | 163 | <20 | 1,786 | 10,108 |
| Cohort 29 | 9,579 | 9,104 | 3,823 | 11,109 | 674 | 24 | 46 | 3,257 | 14,632 |
| Cohort 30 | 21,424 | 20,962 | 8,813 | 27,088 | 1,018 | 599 | 72 | 4,977 | 35,965 |
| Cohort 31 | 9,819 | 9,326 | 3,972 | 11,251 | 694 | 24 | 48 | 3,386 | 14,936 |
| Cohort 32 | 22,640 | 22,154 | 9,344 | 28,457 | 1,107 | 599 | 80 | 5,446 | 37,526 |
| Cohort 33 | 4,850 | 5,073 | 2,637 | 5,514 | 252 | <20 | <20 | 1,624 | 7,915 |
| Cohort 34 | 5,856 | 6,131 | 2,912 | 7,075 | 281 | 151 | <20 | 1,812 | 9,789 |
| Cohort 35 | 4,886 | 5,108 | 2,651 | 5,533 | 252 | <20 | <20 | 1,653 | 7,943 |
| Cohort 36 | 5,892 | 6,167 | 2,926 | 7,094 | 281 | 151 | <20 | 1,841 | 9,818 |
| Cohort 37 | 9,425 | 9,040 | 3,815 | 10,869 | 671 | <20 | 46 | 3,270 | 14,387 |
| Cohort 38 | 20,538 | 20,047 | 8,588 | 25,628 | 983 | 546 | 71 | 4,897 | 34,278 |
| Cohort 39 | 9,663 | 9,265 | 3,961 | 11,006 | 692 | <20 | 48 | 3,402 | 14,688 |
| Cohort 40 | 21,718 | 21,215 | 9,111 | 26,938 | 1,073 | 546 | 78 | 5,366 | 35,788 |
| Cohort 41 | 4,959 | 5,216 | 2,691 | 5,677 | 260 | 23 | <20 | 1,658 | 8,125 |
| Cohort 42 | 6,072 | 6,383 | 2,995 | 7,404 | 295 | 162 | <20 | 1,860 | 10,200 |
| Cohort 43 | 4,995 | 5,251 | 2,705 | 5,696 | 260 | 23 | <20 | 1,687 | 8,153 |
| Cohort 44 | 6,108 | 6,419 | 3,009 | 7,423 | 295 | 162 | <20 | 1,889 | 10,229 |
| Cohort 45 | 9,679 | 9,322 | 3,893 | 11,227 | 691 | 23 | 46 | 3,359 | 14,817 |
| Cohort 46 | 21,447 | 21,069 | 8,874 | 27,031 | 1,037 | 595 | 72 | 5,073 | 35,993 |
| Cohort 47 | 9,920 | 9,560 | 4,044 | 11,372 | 713 | 23 | 48 | 3,494 | 15,130 |
| Cohort 48 | 22,669 | 22,290 | 9,408 | 28,416 | 1,129 | 595 | 80 | 5,554 | 37,577 |
| Cohort 49 | 1,764 | 1,972 | 1,054 | 1,507 | 83 | <20 | <20 | 930 | 2,644 |
| Cohort 50 | 1,771 | 1,976 | 1,056 | 1,510 | 86 | <20 | <20 | 932 | 2,653 |
| Cohort 51 | 1,799 | 2,007 | 1,068 | 1,525 | 83 | <20 | <20 | 959 | 2,671 |
| Cohort 52 | 1,806 | 2,011 | 1,070 | 1,528 | 86 | <20 | <20 | 961 | 2,680 |
| Cohort 53 | 3,654 | 3,734 | 1,520 | 4,002 | 234 | <20 | <20 | 1,321 | 5,815 |
| Cohort 54 | 9,497 | 9,669 | 5,163 | 10,632 | 449 | <20 | 23 | 2,256 | 16,314 |
| Cohort 55 | 3,731 | 3,825 | 1,552 | 4,052 | 247 | <20 | <20 | 1,366 | 5,923 |
| Cohort 56 | 10,381 | 10,553 | 5,397 | 11,764 | 530 | <20 | 28 | 2,638 | 17,630 |
| Cohort 57 | 1,805 | 2,012 | 1,071 | 1,554 | 85 | <20 | <20 | 944 | 2,706 |
| Cohort 58 | 1,813 | 2,016 | 1,073 | 1,557 | 89 | <20 | <20 | 946 | 2,716 |
| Cohort 59 | 1,840 | 2,047 | 1,085 | 1,572 | 85 | <20 | <20 | 973 | 2,733 |
| Cohort 60 | 1,848 | 2,051 | 1,087 | 1,575 | 89 | <20 | <20 | 975 | 2,743 |
| Cohort 61 | 3,772 | 3,849 | 1,553 | 4,160 | 240 | <20 | <20 | 1,351 | 6,012 |
| Cohort 62 | 9,803 | 9,991 | 5,301 | 11,023 | 471 | <20 | 24 | 2,312 | 16,876 |
| Cohort 63 | 3,849 | 3,944 | 1,585 | 4,213 | 253 | <20 | <20 | 1,396 | 6,124 |
| Cohort 64 | 10,721 | 10,917 | 5,539 | 12,220 | 553 | <20 | 30 | 2,703 | 18,259 |
