# Supplementary material for: A Systematic Evaluation of Cohort Selection Criteria and Their Impact on Machine Learning Model Performance and Demographic Disparities in COVID-19 Outcomes: Cohort Study
Source: JMIR Form Res. 2026 Aug 20;10:e88406. doi: 10.2196/88406 (PMC13492188; doi:10.2196/88406)
Supplement: Supplementary file 2 [file formative-v10-e88406-s002.docx]

Appendix 2

Appendix 2 presents four performance metrics—AUC, Precision, Recall, and F1 Score—for the LR, RF, and GB models across the entire dataset for 16 cohorts. In addition, it provides AUC values stratified by demographic groups, including Male, Female, Black, White, Asian, Hispanic, and Non-Hispanic, across these 16 cohorts, offering a detailed view of model performance across subpopulations.

Table 1. Logistic Regression (LR) Performance Metrics Across 16 Test Cohorts Using a Model Trained on Cohorts 1–8.

| Original Decisions | Train Model | Test Dataset | AUC  (LR) | Precision (LR) | Recall (LR) | F1 (LR) | Entire Dataset | Train Model | Test Dataset | AUC  (LR) | Precision (LR) | Recall (LR) | F1 (LR) |
| --- | --- | --- | --- | --- | --- | --- | --- | --- | --- | --- | --- | --- | --- |
| ***0*** | Train1 | Test 1 | 0.73 | 0.22 | 0.69 | 0.33 | ***64*** | Train5 | Test 1 | 0.73 | 0.22 | 0.69 | 0.33 |
| ***1*** | Train1 | Test 2 | 0.71 | 0.28 | 0.65 | 0.39 | ***65*** | Train5 | Test 2 | 0.71 | 0.28 | 0.65 | 0.39 |
| ***2*** | Train1 | Test 3 | 0.73 | 0.22 | 0.69 | 0.33 | ***66*** | Train5 | Test 3 | 0.73 | 0.22 | 0.69 | 0.33 |
| ***3*** | Train1 | Test 4 | 0.71 | 0.28 | 0.65 | 0.39 | ***67*** | Train5 | Test 4 | 0.71 | 0.28 | 0.65 | 0.39 |
| ***4*** | Train1 | Test 5 | 0.73 | 0.22 | 0.69 | 0.33 | ***68*** | Train5 | Test 5 | 0.73 | 0.22 | 0.69 | 0.33 |
| ***5*** | Train1 | Test 6 | 0.71 | 0.28 | 0.65 | 0.39 | ***69*** | Train5 | Test 6 | 0.71 | 0.28 | 0.65 | 0.39 |
| ***6*** | Train1 | Test 7 | 0.73 | 0.22 | 0.69 | 0.33 | ***70*** | Train5 | Test 7 | 0.73 | 0.22 | 0.69 | 0.33 |
| ***7*** | Train1 | Test 8 | 0.72 | 0.28 | 0.65 | 0.39 | ***71*** | Train5 | Test 8 | 0.72 | 0.28 | 0.65 | 0.39 |
| ***8*** | Train1 | Test 9 | 0.73 | 0.22 | 0.69 | 0.33 | ***72*** | Train5 | Test 9 | 0.73 | 0.22 | 0.69 | 0.33 |
| ***9*** | Train1 | Test 10 | 0.71 | 0.27 | 0.65 | 0.39 | ***73*** | Train5 | Test 10 | 0.71 | 0.28 | 0.65 | 0.39 |
| ***10*** | Train1 | Test 11 | 0.73 | 0.22 | 0.69 | 0.33 | ***74*** | Train5 | Test 11 | 0.73 | 0.22 | 0.69 | 0.33 |
| ***11*** | Train1 | Test 12 | 0.72 | 0.28 | 0.65 | 0.39 | ***75*** | Train5 | Test 12 | 0.72 | 0.28 | 0.65 | 0.39 |
| ***12*** | Train1 | Test 13 | 0.74 | 0.25 | 0.71 | 0.37 | ***76*** | Train5 | Test 13 | 0.74 | 0.25 | 0.71 | 0.37 |
| ***13*** | Train1 | Test 14 | 0.72 | 0.31 | 0.65 | 0.42 | ***77*** | Train5 | Test 14 | 0.72 | 0.31 | 0.65 | 0.42 |
| ***14*** | Train1 | Test 15 | 0.74 | 0.25 | 0.71 | 0.37 | ***78*** | Train5 | Test 15 | 0.74 | 0.25 | 0.71 | 0.37 |
| ***15*** | Train1 | Test 16 | 0.72 | 0.31 | 0.65 | 0.42 | ***79*** | Train5 | Test 16 | 0.72 | 0.31 | 0.65 | 0.42 |
| ***16*** | Train2 | Test 1 | 0.72 | 0.22 | 0.66 | 0.33 | ***80*** | Train6 | Test 1 | 0.72 | 0.22 | 0.66 | 0.33 |
| ***17*** | Train2 | Test 2 | 0.74 | 0.28 | 0.71 | 0.4 | ***81*** | Train6 | Test 2 | 0.74 | 0.28 | 0.71 | 0.4 |
| ***18*** | Train2 | Test 3 | 0.72 | 0.22 | 0.66 | 0.33 | ***82*** | Train6 | Test 3 | 0.72 | 0.22 | 0.66 | 0.33 |
| ***19*** | Train2 | Test 4 | 0.74 | 0.28 | 0.71 | 0.4 | ***83*** | Train6 | Test 4 | 0.74 | 0.28 | 0.71 | 0.4 |
| ***20*** | Train2 | Test 5 | 0.72 | 0.22 | 0.66 | 0.33 | ***84*** | Train6 | Test 5 | 0.72 | 0.22 | 0.66 | 0.33 |
| ***21*** | Train2 | Test 6 | 0.74 | 0.28 | 0.71 | 0.4 | ***85*** | Train6 | Test 6 | 0.74 | 0.28 | 0.71 | 0.4 |
| ***22*** | Train2 | Test 7 | 0.72 | 0.22 | 0.66 | 0.33 | ***86*** | Train6 | Test 7 | 0.72 | 0.22 | 0.66 | 0.33 |
| ***23*** | Train2 | Test 8 | 0.74 | 0.28 | 0.71 | 0.4 | ***87*** | Train6 | Test 8 | 0.74 | 0.28 | 0.71 | 0.4 |
| ***24*** | Train2 | Test 9 | 0.72 | 0.22 | 0.65 | 0.33 | ***88*** | Train6 | Test 9 | 0.72 | 0.22 | 0.65 | 0.33 |
| ***25*** | Train2 | Test 10 | 0.74 | 0.28 | 0.71 | 0.4 | ***89*** | Train6 | Test 10 | 0.74 | 0.28 | 0.71 | 0.4 |
| ***26*** | Train2 | Test 11 | 0.72 | 0.22 | 0.65 | 0.33 | ***90*** | Train6 | Test 11 | 0.72 | 0.22 | 0.65 | 0.33 |
| ***27*** | Train2 | Test 12 | 0.74 | 0.28 | 0.71 | 0.4 | ***91*** | Train6 | Test 12 | 0.74 | 0.28 | 0.71 | 0.4 |
| ***28*** | Train2 | Test 13 | 0.74 | 0.26 | 0.65 | 0.38 | ***92*** | Train6 | Test 13 | 0.74 | 0.26 | 0.65 | 0.38 |
| ***29*** | Train2 | Test 14 | 0.74 | 0.31 | 0.7 | 0.43 | ***93*** | Train6 | Test 14 | 0.74 | 0.31 | 0.7 | 0.43 |
| ***30*** | Train2 | Test 15 | 0.74 | 0.26 | 0.65 | 0.38 | ***94*** | Train6 | Test 15 | 0.74 | 0.26 | 0.65 | 0.38 |
| ***31*** | Train2 | Test 16 | 0.74 | 0.31 | 0.71 | 0.43 | ***95*** | Train6 | Test 16 | 0.74 | 0.31 | 0.71 | 0.43 |
| ***32*** | Train3 | Test 1 | 0.73 | 0.22 | 0.69 | 0.33 | ***96*** | Train7 | Test 1 | 0.73 | 0.22 | 0.7 | 0.33 |
| ***33*** | Train3 | Test 2 | 0.71 | 0.28 | 0.65 | 0.39 | ***97*** | Train7 | Test 2 | 0.71 | 0.28 | 0.65 | 0.39 |
| ***34*** | Train3 | Test 3 | 0.73 | 0.22 | 0.69 | 0.33 | ***98*** | Train7 | Test 3 | 0.73 | 0.22 | 0.7 | 0.33 |
| ***35*** | Train3 | Test 4 | 0.71 | 0.28 | 0.65 | 0.39 | ***99*** | Train7 | Test 4 | 0.71 | 0.28 | 0.65 | 0.39 |
| ***36*** | Train3 | Test 5 | 0.73 | 0.22 | 0.69 | 0.33 | ***100*** | Train7 | Test 5 | 0.73 | 0.22 | 0.7 | 0.33 |
| ***37*** | Train3 | Test 6 | 0.71 | 0.28 | 0.65 | 0.39 | ***101*** | Train7 | Test 6 | 0.71 | 0.28 | 0.65 | 0.39 |
| ***38*** | Train3 | Test 7 | 0.73 | 0.22 | 0.69 | 0.33 | ***102*** | Train7 | Test 7 | 0.73 | 0.22 | 0.7 | 0.33 |
| ***39*** | Train3 | Test 8 | 0.71 | 0.28 | 0.65 | 0.39 | ***103*** | Train7 | Test 8 | 0.71 | 0.28 | 0.65 | 0.39 |
| ***40*** | Train3 | Test 9 | 0.73 | 0.22 | 0.69 | 0.33 | ***104*** | Train7 | Test 9 | 0.73 | 0.22 | 0.69 | 0.33 |
| ***41*** | Train3 | Test 10 | 0.71 | 0.28 | 0.65 | 0.39 | ***105*** | Train7 | Test 10 | 0.71 | 0.28 | 0.65 | 0.39 |
| ***42*** | Train3 | Test 11 | 0.73 | 0.22 | 0.69 | 0.33 | ***106*** | Train7 | Test 11 | 0.73 | 0.22 | 0.69 | 0.33 |
| ***43*** | Train3 | Test 12 | 0.71 | 0.28 | 0.65 | 0.39 | ***107*** | Train7 | Test 12 | 0.71 | 0.28 | 0.65 | 0.39 |
| ***44*** | Train3 | Test 13 | 0.74 | 0.25 | 0.71 | 0.37 | ***108*** | Train7 | Test 13 | 0.74 | 0.25 | 0.71 | 0.37 |
| ***45*** | Train3 | Test 14 | 0.72 | 0.31 | 0.64 | 0.42 | ***109*** | Train7 | Test 14 | 0.72 | 0.31 | 0.64 | 0.42 |
| ***46*** | Train3 | Test 15 | 0.74 | 0.25 | 0.71 | 0.37 | ***110*** | Train7 | Test 15 | 0.74 | 0.25 | 0.71 | 0.37 |
| ***47*** | Train3 | Test 16 | 0.72 | 0.31 | 0.65 | 0.42 | ***111*** | Train7 | Test 16 | 0.72 | 0.31 | 0.65 | 0.42 |
| ***48*** | Train4 | Test 1 | 0.72 | 0.22 | 0.65 | 0.33 | ***112*** | Train8 | Test 1 | 0.72 | 0.22 | 0.66 | 0.33 |
| ***49*** | Train4 | Test 2 | 0.74 | 0.28 | 0.71 | 0.4 | ***113*** | Train8 | Test 2 | 0.74 | 0.28 | 0.71 | 0.4 |
| ***50*** | Train4 | Test 3 | 0.72 | 0.22 | 0.65 | 0.33 | ***114*** | Train8 | Test 3 | 0.72 | 0.22 | 0.66 | 0.33 |
| ***51*** | Train4 | Test 4 | 0.74 | 0.28 | 0.71 | 0.4 | ***115*** | Train8 | Test 4 | 0.74 | 0.28 | 0.71 | 0.4 |
| ***52*** | Train4 | Test 5 | 0.72 | 0.22 | 0.65 | 0.33 | ***116*** | Train8 | Test 5 | 0.72 | 0.22 | 0.66 | 0.33 |
| ***53*** | Train4 | Test 6 | 0.74 | 0.28 | 0.71 | 0.4 | ***117*** | Train8 | Test 6 | 0.74 | 0.28 | 0.71 | 0.4 |
| ***54*** | Train4 | Test 7 | 0.72 | 0.22 | 0.65 | 0.33 | ***118*** | Train8 | Test 7 | 0.72 | 0.22 | 0.66 | 0.33 |
| ***55*** | Train4 | Test 8 | 0.74 | 0.28 | 0.71 | 0.4 | ***119*** | Train8 | Test 8 | 0.74 | 0.28 | 0.71 | 0.4 |
| ***56*** | Train4 | Test 9 | 0.72 | 0.22 | 0.65 | 0.33 | ***120*** | Train8 | Test 9 | 0.72 | 0.22 | 0.65 | 0.33 |
| ***57*** | Train4 | Test 10 | 0.74 | 0.28 | 0.71 | 0.4 | ***121*** | Train8 | Test 10 | 0.74 | 0.28 | 0.71 | 0.4 |
| ***58*** | Train4 | Test 11 | 0.72 | 0.22 | 0.65 | 0.33 | ***122*** | Train8 | Test 11 | 0.72 | 0.22 | 0.65 | 0.33 |
| ***59*** | Train4 | Test 12 | 0.74 | 0.28 | 0.71 | 0.4 | ***123*** | Train8 | Test 12 | 0.74 | 0.28 | 0.71 | 0.4 |
| ***60*** | Train4 | Test 13 | 0.74 | 0.27 | 0.65 | 0.38 | ***124*** | Train8 | Test 13 | 0.74 | 0.26 | 0.65 | 0.38 |
| ***61*** | Train4 | Test 14 | 0.74 | 0.31 | 0.7 | 0.43 | ***125*** | Train8 | Test 14 | 0.74 | 0.31 | 0.7 | 0.43 |
| ***62*** | Train4 | Test 15 | 0.74 | 0.26 | 0.65 | 0.38 | ***126*** | Train8 | Test 15 | 0.74 | 0.26 | 0.65 | 0.38 |
| ***63*** | Train4 | Test 16 | 0.74 | 0.31 | 0.71 | 0.43 | ***127*** | Train8 | Test 16 | 0.74 | 0.31 | 0.71 | 0.43 |

Table 2. Logistic Regression (LR) Performance Metrics Across 16 Test Cohorts Using a Model Trained on Cohorts 9–16.

| Original Decisions | Train Model | Test Dataset | AUC  (LR) | Precision (LR) | Recall (LR) | F1 (LR) | ***Entire Dataset*** | Train Model | Test Dataset | AUC  (LR) | Precision (LR) | Recall (LR) | F1 (LR) |
| --- | --- | --- | --- | --- | --- | --- | --- | --- | --- | --- | --- | --- | --- |
| ***128*** | Train 9 | Test 1 | 0.73 | 0.22 | 0.7 | 0.33 | ***192*** | Train13 | Test 1 | 0.71 | 0.21 | 0.7 | 0.32 |
| ***129*** | Train 9 | Test 2 | 0.71 | 0.27 | 0.66 | 0.39 | ***193*** | Train13 | Test 2 | 0.71 | 0.27 | 0.65 | 0.38 |
| ***130*** | Train 9 | Test 3 | 0.73 | 0.22 | 0.7 | 0.33 | ***194*** | Train13 | Test 3 | 0.72 | 0.21 | 0.7 | 0.32 |
| ***131*** | Train 9 | Test 4 | 0.71 | 0.27 | 0.66 | 0.39 | ***195*** | Train13 | Test 4 | 0.71 | 0.27 | 0.66 | 0.38 |
| ***132*** | Train 9 | Test 5 | 0.73 | 0.22 | 0.7 | 0.33 | ***196*** | Train13 | Test 5 | 0.72 | 0.21 | 0.7 | 0.32 |
| ***133*** | Train 9 | Test 6 | 0.71 | 0.27 | 0.65 | 0.39 | ***197*** | Train13 | Test 6 | 0.71 | 0.27 | 0.65 | 0.38 |
| ***134*** | Train 9 | Test 7 | 0.73 | 0.22 | 0.7 | 0.33 | ***198*** | Train13 | Test 7 | 0.72 | 0.21 | 0.7 | 0.32 |
| ***135*** | Train 9 | Test 8 | 0.71 | 0.27 | 0.66 | 0.39 | ***199*** | Train13 | Test 8 | 0.71 | 0.27 | 0.65 | 0.38 |
| ***136*** | Train 9 | Test 9 | 0.73 | 0.22 | 0.7 | 0.33 | ***200*** | Train13 | Test 9 | 0.72 | 0.21 | 0.7 | 0.32 |
| ***137*** | Train 9 | Test 10 | 0.71 | 0.27 | 0.65 | 0.39 | ***201*** | Train13 | Test 10 | 0.71 | 0.27 | 0.65 | 0.38 |
| ***138*** | Train 9 | Test 11 | 0.73 | 0.22 | 0.7 | 0.33 | ***202*** | Train13 | Test 11 | 0.72 | 0.21 | 0.7 | 0.32 |
| ***139*** | Train 9 | Test 12 | 0.71 | 0.27 | 0.66 | 0.39 | ***203*** | Train13 | Test 12 | 0.71 | 0.27 | 0.65 | 0.38 |
| ***140*** | Train 9 | Test 13 | 0.74 | 0.25 | 0.71 | 0.37 | ***204*** | Train13 | Test 13 | 0.75 | 0.26 | 0.7 | 0.38 |
| ***141*** | Train 9 | Test 14 | 0.72 | 0.31 | 0.65 | 0.42 | ***205*** | Train13 | Test 14 | 0.72 | 0.31 | 0.66 | 0.42 |
| ***142*** | Train 9 | Test 15 | 0.74 | 0.25 | 0.71 | 0.37 | ***206*** | Train13 | Test 15 | 0.75 | 0.26 | 0.7 | 0.38 |
| ***143*** | Train 9 | Test 16 | 0.72 | 0.31 | 0.66 | 0.42 | ***207*** | Train13 | Test 16 | 0.72 | 0.3 | 0.66 | 0.42 |
| ***144*** | Train 10 | Test 1 | 0.72 | 0.22 | 0.66 | 0.33 | ***208*** | Train14 | Test 1 | 0.7 | 0.2 | 0.68 | 0.32 |
| ***145*** | Train 10 | Test 2 | 0.74 | 0.28 | 0.71 | 0.4 | ***209*** | Train14 | Test 2 | 0.72 | 0.26 | 0.73 | 0.39 |
| ***146*** | Train 10 | Test 3 | 0.72 | 0.22 | 0.66 | 0.33 | ***210*** | Train14 | Test 3 | 0.7 | 0.21 | 0.68 | 0.32 |
| ***147*** | Train 10 | Test 4 | 0.74 | 0.28 | 0.71 | 0.4 | ***211*** | Train14 | Test 4 | 0.72 | 0.26 | 0.73 | 0.39 |
| ***148*** | Train 10 | Test 5 | 0.72 | 0.22 | 0.66 | 0.33 | ***212*** | Train14 | Test 5 | 0.7 | 0.2 | 0.68 | 0.32 |
| ***149*** | Train 10 | Test 6 | 0.74 | 0.28 | 0.71 | 0.4 | ***213*** | Train14 | Test 6 | 0.72 | 0.26 | 0.73 | 0.39 |
| ***150*** | Train 10 | Test 7 | 0.72 | 0.22 | 0.66 | 0.33 | ***214*** | Train14 | Test 7 | 0.7 | 0.21 | 0.68 | 0.32 |
| ***151*** | Train 10 | Test 8 | 0.74 | 0.28 | 0.71 | 0.4 | ***215*** | Train14 | Test 8 | 0.72 | 0.26 | 0.73 | 0.39 |
| ***152*** | Train 10 | Test 9 | 0.72 | 0.22 | 0.65 | 0.33 | ***216*** | Train14 | Test 9 | 0.7 | 0.2 | 0.68 | 0.31 |
| ***153*** | Train 10 | Test 10 | 0.74 | 0.28 | 0.71 | 0.4 | ***217*** | Train14 | Test 10 | 0.72 | 0.26 | 0.72 | 0.39 |
| ***154*** | Train 10 | Test 11 | 0.72 | 0.22 | 0.65 | 0.33 | ***218*** | Train14 | Test 11 | 0.71 | 0.2 | 0.68 | 0.31 |
| ***155*** | Train 10 | Test 12 | 0.74 | 0.28 | 0.71 | 0.4 | ***219*** | Train14 | Test 12 | 0.72 | 0.26 | 0.73 | 0.39 |
| ***156*** | Train 10 | Test 13 | 0.74 | 0.26 | 0.65 | 0.38 | ***220*** | Train14 | Test 13 | 0.74 | 0.27 | 0.64 | 0.38 |
| ***157*** | Train 10 | Test 14 | 0.74 | 0.31 | 0.71 | 0.43 | ***221*** | Train14 | Test 14 | 0.74 | 0.31 | 0.72 | 0.44 |
| ***158*** | Train 10 | Test 15 | 0.74 | 0.26 | 0.65 | 0.38 | ***222*** | Train14 | Test 15 | 0.74 | 0.27 | 0.65 | 0.38 |
| ***159*** | Train 10 | Test 16 | 0.74 | 0.31 | 0.71 | 0.43 | ***223*** | Train14 | Test 16 | 0.74 | 0.31 | 0.72 | 0.44 |
| ***160*** | Train 11 | Test 1 | 0.73 | 0.22 | 0.7 | 0.33 | ***224*** | Train15 | Test 1 | 0.72 | 0.21 | 0.7 | 0.32 |
| ***161*** | Train 11 | Test 2 | 0.71 | 0.28 | 0.66 | 0.39 | ***225*** | Train15 | Test 2 | 0.71 | 0.27 | 0.66 | 0.38 |
| ***162*** | Train 11 | Test 3 | 0.73 | 0.22 | 0.7 | 0.33 | ***226*** | Train15 | Test 3 | 0.72 | 0.21 | 0.7 | 0.32 |
| ***163*** | Train 11 | Test 4 | 0.71 | 0.28 | 0.66 | 0.39 | ***227*** | Train15 | Test 4 | 0.71 | 0.27 | 0.66 | 0.38 |
| ***164*** | Train 11 | Test 5 | 0.73 | 0.22 | 0.7 | 0.33 | ***228*** | Train15 | Test 5 | 0.72 | 0.21 | 0.7 | 0.32 |
| ***165*** | Train 11 | Test 6 | 0.71 | 0.28 | 0.66 | 0.39 | ***229*** | Train15 | Test 6 | 0.71 | 0.27 | 0.65 | 0.38 |
| ***166*** | Train 11 | Test 7 | 0.73 | 0.22 | 0.7 | 0.33 | ***230*** | Train15 | Test 7 | 0.72 | 0.21 | 0.7 | 0.32 |
| ***167*** | Train 11 | Test 8 | 0.71 | 0.28 | 0.66 | 0.39 | ***231*** | Train15 | Test 8 | 0.71 | 0.27 | 0.66 | 0.38 |
| ***168*** | Train 11 | Test 9 | 0.73 | 0.22 | 0.7 | 0.33 | ***232*** | Train15 | Test 9 | 0.72 | 0.21 | 0.7 | 0.32 |
| ***169*** | Train 11 | Test 10 | 0.71 | 0.27 | 0.65 | 0.39 | ***233*** | Train15 | Test 10 | 0.71 | 0.27 | 0.65 | 0.38 |
| ***170*** | Train 11 | Test 11 | 0.73 | 0.22 | 0.7 | 0.33 | ***234*** | Train15 | Test 11 | 0.72 | 0.21 | 0.7 | 0.32 |
| ***171*** | Train 11 | Test 12 | 0.71 | 0.27 | 0.66 | 0.39 | ***235*** | Train15 | Test 12 | 0.71 | 0.27 | 0.66 | 0.38 |
| ***172*** | Train 11 | Test 13 | 0.74 | 0.25 | 0.71 | 0.37 | ***236*** | Train15 | Test 13 | 0.75 | 0.26 | 0.7 | 0.38 |
| ***173*** | Train 11 | Test 14 | 0.72 | 0.31 | 0.65 | 0.42 | ***237*** | Train15 | Test 14 | 0.72 | 0.3 | 0.66 | 0.42 |
| ***174*** | Train 11 | Test 15 | 0.74 | 0.25 | 0.71 | 0.37 | ***238*** | Train15 | Test 15 | 0.75 | 0.26 | 0.7 | 0.38 |
| ***175*** | Train 11 | Test 16 | 0.72 | 0.31 | 0.65 | 0.42 | ***239*** | Train15 | Test 16 | 0.72 | 0.3 | 0.66 | 0.42 |
| ***176*** | Train 12 | Test 1 | 0.72 | 0.22 | 0.66 | 0.33 | ***240*** | Train16 | Test 1 | 0.7 | 0.2 | 0.69 | 0.32 |
| ***177*** | Train 12 | Test 2 | 0.74 | 0.28 | 0.71 | 0.4 | ***241*** | Train16 | Test 2 | 0.72 | 0.26 | 0.73 | 0.39 |
| ***178*** | Train 12 | Test 3 | 0.72 | 0.22 | 0.66 | 0.33 | ***242*** | Train16 | Test 3 | 0.7 | 0.21 | 0.69 | 0.32 |
| ***179*** | Train 12 | Test 4 | 0.74 | 0.28 | 0.71 | 0.4 | ***243*** | Train16 | Test 4 | 0.72 | 0.26 | 0.73 | 0.39 |
| ***180*** | Train 12 | Test 5 | 0.72 | 0.22 | 0.66 | 0.33 | ***244*** | Train16 | Test 5 | 0.7 | 0.2 | 0.69 | 0.31 |
| ***181*** | Train 12 | Test 6 | 0.74 | 0.28 | 0.71 | 0.4 | ***245*** | Train16 | Test 6 | 0.72 | 0.26 | 0.73 | 0.39 |
| ***182*** | Train 12 | Test 7 | 0.72 | 0.22 | 0.66 | 0.33 | ***246*** | Train16 | Test 7 | 0.7 | 0.21 | 0.69 | 0.32 |
| ***183*** | Train 12 | Test 8 | 0.74 | 0.28 | 0.71 | 0.4 | ***247*** | Train16 | Test 8 | 0.72 | 0.26 | 0.73 | 0.39 |
| ***184*** | Train 12 | Test 9 | 0.72 | 0.22 | 0.65 | 0.33 | ***248*** | Train16 | Test 9 | 0.71 | 0.2 | 0.68 | 0.31 |
| ***185*** | Train 12 | Test 10 | 0.74 | 0.28 | 0.71 | 0.4 | ***249*** | Train16 | Test 10 | 0.72 | 0.26 | 0.73 | 0.39 |
| ***186*** | Train 12 | Test 11 | 0.72 | 0.22 | 0.65 | 0.33 | ***250*** | Train16 | Test 11 | 0.71 | 0.2 | 0.68 | 0.31 |
| ***187*** | Train 12 | Test 12 | 0.74 | 0.28 | 0.71 | 0.4 | ***251*** | Train16 | Test 12 | 0.72 | 0.26 | 0.73 | 0.39 |
| ***188*** | Train 12 | Test 13 | 0.74 | 0.26 | 0.65 | 0.38 | ***252*** | Train16 | Test 13 | 0.74 | 0.27 | 0.65 | 0.38 |
| ***189*** | Train 12 | Test 14 | 0.74 | 0.31 | 0.7 | 0.43 | ***253*** | Train16 | Test 14 | 0.74 | 0.32 | 0.72 | 0.44 |
| ***190*** | Train 12 | Test 15 | 0.74 | 0.26 | 0.65 | 0.38 | ***254*** | Train16 | Test 15 | 0.74 | 0.27 | 0.65 | 0.38 |
| ***191*** | Train 12 | Test 16 | 0.74 | 0.31 | 0.71 | 0.43 | ***255*** | Train16 | Test 16 | 0.74 | 0.31 | 0.72 | 0.44 |

Table 3. Random Forest (RF) Performance Metrics Across 16 Test Cohorts Using a Model Trained on Cohorts 1–8.

| Original Decisions | Train Model | Test Dataset | AUC  (RF) | Precision (RF) | Recall (RF) | F1 (RF) | ***Entire Dataset*** | Train Model | Test Dataset | AUC  (RF) | Precision (RF) | Recall (RF) | F1 (RF) |
| --- | --- | --- | --- | --- | --- | --- | --- | --- | --- | --- | --- | --- | --- |
| ***0*** | Train1 | Test 1 | 0.75 | 0.24 | 0.67 | 0.36 | ***64*** | Train5 | Test 1 | 0.75 | 0.24 | 0.67 | 0.35 |
| ***1*** | Train1 | Test 2 | 0.71 | 0.29 | 0.57 | 0.38 | ***65*** | Train5 | Test 2 | 0.71 | 0.29 | 0.57 | 0.38 |
| ***2*** | Train1 | Test 3 | 0.75 | 0.24 | 0.67 | 0.36 | ***66*** | Train5 | Test 3 | 0.75 | 0.24 | 0.67 | 0.35 |
| ***3*** | Train1 | Test 4 | 0.71 | 0.29 | 0.57 | 0.38 | ***67*** | Train5 | Test 4 | 0.71 | 0.29 | 0.57 | 0.38 |
| ***4*** | Train1 | Test 5 | 0.75 | 0.24 | 0.67 | 0.36 | ***68*** | Train5 | Test 5 | 0.75 | 0.24 | 0.67 | 0.35 |
| ***5*** | Train1 | Test 6 | 0.71 | 0.29 | 0.57 | 0.38 | ***69*** | Train5 | Test 6 | 0.71 | 0.29 | 0.57 | 0.38 |
| ***6*** | Train1 | Test 7 | 0.75 | 0.24 | 0.67 | 0.36 | ***70*** | Train5 | Test 7 | 0.75 | 0.24 | 0.67 | 0.35 |
| ***7*** | Train1 | Test 8 | 0.71 | 0.29 | 0.57 | 0.38 | ***71*** | Train5 | Test 8 | 0.71 | 0.29 | 0.57 | 0.38 |
| ***8*** | Train1 | Test 9 | 0.75 | 0.24 | 0.67 | 0.35 | ***72*** | Train5 | Test 9 | 0.75 | 0.24 | 0.67 | 0.35 |
| ***9*** | Train1 | Test 10 | 0.71 | 0.29 | 0.57 | 0.38 | ***73*** | Train5 | Test 10 | 0.71 | 0.29 | 0.56 | 0.38 |
| ***10*** | Train1 | Test 11 | 0.75 | 0.24 | 0.67 | 0.35 | ***74*** | Train5 | Test 11 | 0.75 | 0.24 | 0.67 | 0.35 |
| ***11*** | Train1 | Test 12 | 0.71 | 0.29 | 0.57 | 0.38 | ***75*** | Train5 | Test 12 | 0.71 | 0.29 | 0.57 | 0.38 |
| ***12*** | Train1 | Test 13 | 0.75 | 0.26 | 0.69 | 0.38 | ***76*** | Train5 | Test 13 | 0.75 | 0.26 | 0.69 | 0.38 |
| ***13*** | Train1 | Test 14 | 0.7 | 0.31 | 0.58 | 0.4 | ***77*** | Train5 | Test 14 | 0.7 | 0.31 | 0.58 | 0.4 |
| ***14*** | Train1 | Test 15 | 0.75 | 0.26 | 0.7 | 0.38 | ***78*** | Train5 | Test 15 | 0.75 | 0.26 | 0.69 | 0.38 |
| ***15*** | Train1 | Test 16 | 0.7 | 0.31 | 0.58 | 0.4 | ***79*** | Train5 | Test 16 | 0.7 | 0.31 | 0.58 | 0.4 |
| ***16*** | Train2 | Test 1 | 0.74 | 0.25 | 0.61 | 0.35 | ***80*** | Train6 | Test 1 | 0.74 | 0.25 | 0.62 | 0.35 |
| ***17*** | Train2 | Test 2 | 0.75 | 0.3 | 0.7 | 0.42 | ***81*** | Train6 | Test 2 | 0.75 | 0.3 | 0.71 | 0.42 |
| ***18*** | Train2 | Test 3 | 0.74 | 0.25 | 0.61 | 0.35 | ***82*** | Train6 | Test 3 | 0.74 | 0.25 | 0.62 | 0.35 |
| ***19*** | Train2 | Test 4 | 0.75 | 0.3 | 0.7 | 0.42 | ***83*** | Train6 | Test 4 | 0.75 | 0.3 | 0.71 | 0.42 |
| ***20*** | Train2 | Test 5 | 0.74 | 0.25 | 0.61 | 0.35 | ***84*** | Train6 | Test 5 | 0.74 | 0.25 | 0.62 | 0.35 |
| ***21*** | Train2 | Test 6 | 0.75 | 0.3 | 0.7 | 0.42 | ***85*** | Train6 | Test 6 | 0.75 | 0.3 | 0.71 | 0.42 |
| ***22*** | Train2 | Test 7 | 0.74 | 0.25 | 0.61 | 0.35 | ***86*** | Train6 | Test 7 | 0.74 | 0.25 | 0.62 | 0.35 |
| ***23*** | Train2 | Test 8 | 0.75 | 0.3 | 0.71 | 0.42 | ***87*** | Train6 | Test 8 | 0.75 | 0.3 | 0.71 | 0.42 |
| ***24*** | Train2 | Test 9 | 0.74 | 0.25 | 0.61 | 0.35 | ***88*** | Train6 | Test 9 | 0.74 | 0.25 | 0.62 | 0.35 |
| ***25*** | Train2 | Test 10 | 0.75 | 0.3 | 0.7 | 0.42 | ***89*** | Train6 | Test 10 | 0.75 | 0.3 | 0.71 | 0.42 |
| ***26*** | Train2 | Test 11 | 0.74 | 0.25 | 0.61 | 0.35 | ***90*** | Train6 | Test 11 | 0.74 | 0.25 | 0.62 | 0.35 |
| ***27*** | Train2 | Test 12 | 0.75 | 0.3 | 0.7 | 0.42 | ***91*** | Train6 | Test 12 | 0.75 | 0.3 | 0.71 | 0.42 |
| ***28*** | Train2 | Test 13 | 0.75 | 0.28 | 0.65 | 0.39 | ***92*** | Train6 | Test 13 | 0.75 | 0.28 | 0.65 | 0.39 |
| ***29*** | Train2 | Test 14 | 0.75 | 0.32 | 0.73 | 0.45 | ***93*** | Train6 | Test 14 | 0.75 | 0.32 | 0.73 | 0.45 |
| ***30*** | Train2 | Test 15 | 0.75 | 0.28 | 0.65 | 0.39 | ***94*** | Train6 | Test 15 | 0.75 | 0.28 | 0.65 | 0.39 |
| ***31*** | Train2 | Test 16 | 0.75 | 0.32 | 0.73 | 0.44 | ***95*** | Train6 | Test 16 | 0.75 | 0.32 | 0.73 | 0.44 |
| ***32*** | Train3 | Test 1 | 0.75 | 0.24 | 0.67 | 0.35 | ***96*** | Train7 | Test 1 | 0.75 | 0.24 | 0.67 | 0.36 |
| ***33*** | Train3 | Test 2 | 0.71 | 0.29 | 0.56 | 0.38 | ***97*** | Train7 | Test 2 | 0.71 | 0.29 | 0.57 | 0.38 |
| ***34*** | Train3 | Test 3 | 0.75 | 0.24 | 0.67 | 0.35 | ***98*** | Train7 | Test 3 | 0.75 | 0.24 | 0.67 | 0.36 |
| ***35*** | Train3 | Test 4 | 0.71 | 0.29 | 0.57 | 0.38 | ***99*** | Train7 | Test 4 | 0.71 | 0.29 | 0.57 | 0.39 |
| ***36*** | Train3 | Test 5 | 0.75 | 0.24 | 0.67 | 0.35 | ***100*** | Train7 | Test 5 | 0.75 | 0.24 | 0.67 | 0.36 |
| ***37*** | Train3 | Test 6 | 0.71 | 0.29 | 0.56 | 0.38 | ***101*** | Train7 | Test 6 | 0.71 | 0.29 | 0.57 | 0.38 |
| ***38*** | Train3 | Test 7 | 0.75 | 0.24 | 0.67 | 0.35 | ***102*** | Train7 | Test 7 | 0.75 | 0.24 | 0.67 | 0.36 |
| ***39*** | Train3 | Test 8 | 0.71 | 0.29 | 0.57 | 0.38 | ***103*** | Train7 | Test 8 | 0.71 | 0.29 | 0.57 | 0.39 |
| ***40*** | Train3 | Test 9 | 0.75 | 0.24 | 0.67 | 0.35 | ***104*** | Train7 | Test 9 | 0.75 | 0.24 | 0.67 | 0.36 |
| ***41*** | Train3 | Test 10 | 0.71 | 0.29 | 0.56 | 0.38 | ***105*** | Train7 | Test 10 | 0.71 | 0.29 | 0.57 | 0.38 |
| ***42*** | Train3 | Test 11 | 0.75 | 0.24 | 0.67 | 0.35 | ***106*** | Train7 | Test 11 | 0.75 | 0.24 | 0.67 | 0.36 |
| ***43*** | Train3 | Test 12 | 0.71 | 0.29 | 0.57 | 0.38 | ***107*** | Train7 | Test 12 | 0.71 | 0.29 | 0.57 | 0.39 |
| ***44*** | Train3 | Test 13 | 0.75 | 0.26 | 0.69 | 0.38 | ***108*** | Train7 | Test 13 | 0.75 | 0.26 | 0.69 | 0.38 |
| ***45*** | Train3 | Test 14 | 0.7 | 0.31 | 0.58 | 0.4 | ***109*** | Train7 | Test 14 | 0.7 | 0.31 | 0.58 | 0.4 |
| ***46*** | Train3 | Test 15 | 0.75 | 0.26 | 0.7 | 0.38 | ***110*** | Train7 | Test 15 | 0.75 | 0.26 | 0.7 | 0.38 |
| ***47*** | Train3 | Test 16 | 0.7 | 0.31 | 0.58 | 0.4 | ***111*** | Train7 | Test 16 | 0.7 | 0.31 | 0.58 | 0.4 |
| ***48*** | Train4 | Test 1 | 0.74 | 0.25 | 0.61 | 0.35 | ***112*** | Train8 | Test 1 | 0.74 | 0.25 | 0.61 | 0.35 |
| ***49*** | Train4 | Test 2 | 0.75 | 0.3 | 0.7 | 0.42 | ***113*** | Train8 | Test 2 | 0.75 | 0.3 | 0.7 | 0.42 |
| ***50*** | Train4 | Test 3 | 0.74 | 0.25 | 0.62 | 0.35 | ***114*** | Train8 | Test 3 | 0.74 | 0.25 | 0.61 | 0.35 |
| ***51*** | Train4 | Test 4 | 0.75 | 0.3 | 0.7 | 0.42 | ***115*** | Train8 | Test 4 | 0.75 | 0.3 | 0.7 | 0.42 |
| ***52*** | Train4 | Test 5 | 0.74 | 0.25 | 0.61 | 0.35 | ***116*** | Train8 | Test 5 | 0.74 | 0.25 | 0.61 | 0.35 |
| ***53*** | Train4 | Test 6 | 0.75 | 0.3 | 0.7 | 0.42 | ***117*** | Train8 | Test 6 | 0.75 | 0.3 | 0.7 | 0.42 |
| ***54*** | Train4 | Test 7 | 0.74 | 0.25 | 0.62 | 0.35 | ***118*** | Train8 | Test 7 | 0.74 | 0.25 | 0.61 | 0.35 |
| ***55*** | Train4 | Test 8 | 0.75 | 0.3 | 0.7 | 0.42 | ***119*** | Train8 | Test 8 | 0.75 | 0.29 | 0.7 | 0.42 |
| ***56*** | Train4 | Test 9 | 0.74 | 0.25 | 0.61 | 0.35 | ***120*** | Train8 | Test 9 | 0.74 | 0.25 | 0.61 | 0.35 |
| ***57*** | Train4 | Test 10 | 0.75 | 0.3 | 0.7 | 0.42 | ***121*** | Train8 | Test 10 | 0.75 | 0.3 | 0.7 | 0.42 |
| ***58*** | Train4 | Test 11 | 0.74 | 0.25 | 0.61 | 0.35 | ***122*** | Train8 | Test 11 | 0.74 | 0.25 | 0.61 | 0.35 |
| ***59*** | Train4 | Test 12 | 0.75 | 0.3 | 0.7 | 0.42 | ***123*** | Train8 | Test 12 | 0.76 | 0.3 | 0.7 | 0.42 |
| ***60*** | Train4 | Test 13 | 0.75 | 0.28 | 0.64 | 0.39 | ***124*** | Train8 | Test 13 | 0.75 | 0.28 | 0.65 | 0.39 |
| ***61*** | Train4 | Test 14 | 0.75 | 0.32 | 0.72 | 0.44 | ***125*** | Train8 | Test 14 | 0.75 | 0.32 | 0.72 | 0.44 |
| ***62*** | Train4 | Test 15 | 0.75 | 0.28 | 0.65 | 0.39 | ***126*** | Train8 | Test 15 | 0.75 | 0.28 | 0.65 | 0.39 |
| ***63*** | Train4 | Test 16 | 0.75 | 0.32 | 0.72 | 0.44 | ***127*** | Train8 | Test 16 | 0.75 | 0.32 | 0.72 | 0.44 |

Table 4. Random Forest (RF) Performance Metrics Across 16 Test Cohorts Using a Model Trained on Cohorts 9–16.

| Original Decisions | Train Model | Test Dataset | AUC  (RF) | Precision (RF) | Recall (RF) | F1 (RF) | ***Entire Dataset*** | Train Model | Test Dataset | AUC  (RF) | Precision (RF) | Recall (RF) | F1 (RF) |
| --- | --- | --- | --- | --- | --- | --- | --- | --- | --- | --- | --- | --- | --- |
| ***128*** | Train 9 | Test 1 | 0.75 | 0.24 | 0.68 | 0.35 | ***192*** | Train13 | Test 1 | 0.72 | 0.24 | 0.58 | 0.34 |
| ***129*** | Train 9 | Test 2 | 0.71 | 0.29 | 0.57 | 0.38 | ***193*** | Train13 | Test 2 | 0.7 | 0.29 | 0.5 | 0.36 |
| ***130*** | Train 9 | Test 3 | 0.75 | 0.24 | 0.68 | 0.35 | ***194*** | Train13 | Test 3 | 0.72 | 0.24 | 0.58 | 0.34 |
| ***131*** | Train 9 | Test 4 | 0.71 | 0.29 | 0.57 | 0.38 | ***195*** | Train13 | Test 4 | 0.7 | 0.29 | 0.5 | 0.36 |
| ***132*** | Train 9 | Test 5 | 0.75 | 0.24 | 0.68 | 0.35 | ***196*** | Train13 | Test 5 | 0.72 | 0.24 | 0.58 | 0.34 |
| ***133*** | Train 9 | Test 6 | 0.71 | 0.29 | 0.57 | 0.38 | ***197*** | Train13 | Test 6 | 0.7 | 0.29 | 0.5 | 0.36 |
| ***134*** | Train 9 | Test 7 | 0.75 | 0.24 | 0.68 | 0.35 | ***198*** | Train13 | Test 7 | 0.72 | 0.24 | 0.58 | 0.34 |
| ***135*** | Train 9 | Test 8 | 0.71 | 0.29 | 0.57 | 0.38 | ***199*** | Train13 | Test 8 | 0.7 | 0.29 | 0.5 | 0.36 |
| ***136*** | Train 9 | Test 9 | 0.75 | 0.24 | 0.67 | 0.35 | ***200*** | Train13 | Test 9 | 0.72 | 0.24 | 0.58 | 0.34 |
| ***137*** | Train 9 | Test 10 | 0.71 | 0.29 | 0.57 | 0.38 | ***201*** | Train13 | Test 10 | 0.7 | 0.29 | 0.5 | 0.36 |
| ***138*** | Train 9 | Test 11 | 0.75 | 0.24 | 0.67 | 0.35 | ***202*** | Train13 | Test 11 | 0.72 | 0.24 | 0.58 | 0.34 |
| ***139*** | Train 9 | Test 12 | 0.71 | 0.29 | 0.57 | 0.38 | ***203*** | Train13 | Test 12 | 0.7 | 0.29 | 0.5 | 0.36 |
| ***140*** | Train 9 | Test 13 | 0.75 | 0.26 | 0.69 | 0.38 | ***204*** | Train13 | Test 13 | 0.75 | 0.28 | 0.66 | 0.39 |
| ***141*** | Train 9 | Test 14 | 0.7 | 0.31 | 0.58 | 0.4 | ***205*** | Train13 | Test 14 | 0.7 | 0.32 | 0.52 | 0.39 |
| ***142*** | Train 9 | Test 15 | 0.75 | 0.26 | 0.69 | 0.38 | ***206*** | Train13 | Test 15 | 0.75 | 0.28 | 0.66 | 0.39 |
| ***143*** | Train 9 | Test 16 | 0.7 | 0.31 | 0.59 | 0.4 | ***207*** | Train13 | Test 16 | 0.7 | 0.32 | 0.52 | 0.39 |
| ***144*** | Train 10 | Test 1 | 0.74 | 0.25 | 0.62 | 0.35 | ***208*** | Train14 | Test 1 | 0.72 | 0.22 | 0.62 | 0.33 |
| ***145*** | Train 10 | Test 2 | 0.75 | 0.3 | 0.71 | 0.42 | ***209*** | Train14 | Test 2 | 0.73 | 0.28 | 0.67 | 0.4 |
| ***146*** | Train 10 | Test 3 | 0.74 | 0.25 | 0.62 | 0.35 | ***210*** | Train14 | Test 3 | 0.72 | 0.22 | 0.62 | 0.33 |
| ***147*** | Train 10 | Test 4 | 0.75 | 0.3 | 0.71 | 0.42 | ***211*** | Train14 | Test 4 | 0.73 | 0.28 | 0.67 | 0.4 |
| ***148*** | Train 10 | Test 5 | 0.74 | 0.25 | 0.62 | 0.35 | ***212*** | Train14 | Test 5 | 0.72 | 0.22 | 0.62 | 0.33 |
| ***149*** | Train 10 | Test 6 | 0.75 | 0.3 | 0.71 | 0.42 | ***213*** | Train14 | Test 6 | 0.73 | 0.28 | 0.67 | 0.4 |
| ***150*** | Train 10 | Test 7 | 0.74 | 0.25 | 0.62 | 0.35 | ***214*** | Train14 | Test 7 | 0.72 | 0.22 | 0.62 | 0.33 |
| ***151*** | Train 10 | Test 8 | 0.75 | 0.29 | 0.71 | 0.42 | ***215*** | Train14 | Test 8 | 0.73 | 0.28 | 0.67 | 0.4 |
| ***152*** | Train 10 | Test 9 | 0.74 | 0.25 | 0.62 | 0.35 | ***216*** | Train14 | Test 9 | 0.72 | 0.22 | 0.62 | 0.33 |
| ***153*** | Train 10 | Test 10 | 0.75 | 0.3 | 0.71 | 0.42 | ***217*** | Train14 | Test 10 | 0.73 | 0.28 | 0.67 | 0.4 |
| ***154*** | Train 10 | Test 11 | 0.74 | 0.25 | 0.62 | 0.35 | ***218*** | Train14 | Test 11 | 0.72 | 0.22 | 0.62 | 0.33 |
| ***155*** | Train 10 | Test 12 | 0.76 | 0.3 | 0.71 | 0.42 | ***219*** | Train14 | Test 12 | 0.73 | 0.28 | 0.67 | 0.4 |
| ***156*** | Train 10 | Test 13 | 0.75 | 0.28 | 0.65 | 0.39 | ***220*** | Train14 | Test 13 | 0.75 | 0.29 | 0.62 | 0.39 |
| ***157*** | Train 10 | Test 14 | 0.75 | 0.32 | 0.73 | 0.45 | ***221*** | Train14 | Test 14 | 0.76 | 0.33 | 0.71 | 0.45 |
| ***158*** | Train 10 | Test 15 | 0.75 | 0.28 | 0.65 | 0.39 | ***222*** | Train14 | Test 15 | 0.75 | 0.29 | 0.62 | 0.39 |
| ***159*** | Train 10 | Test 16 | 0.75 | 0.32 | 0.73 | 0.44 | ***223*** | Train14 | Test 16 | 0.76 | 0.33 | 0.71 | 0.45 |
| ***160*** | Train 11 | Test 1 | 0.75 | 0.24 | 0.67 | 0.35 | ***224*** | Train15 | Test 1 | 0.72 | 0.23 | 0.59 | 0.33 |
| ***161*** | Train 11 | Test 2 | 0.71 | 0.29 | 0.58 | 0.38 | ***225*** | Train15 | Test 2 | 0.7 | 0.28 | 0.51 | 0.36 |
| ***162*** | Train 11 | Test 3 | 0.75 | 0.24 | 0.67 | 0.35 | ***226*** | Train15 | Test 3 | 0.72 | 0.23 | 0.59 | 0.34 |
| ***163*** | Train 11 | Test 4 | 0.71 | 0.29 | 0.58 | 0.38 | ***227*** | Train15 | Test 4 | 0.7 | 0.28 | 0.51 | 0.36 |
| ***164*** | Train 11 | Test 5 | 0.75 | 0.24 | 0.67 | 0.35 | ***228*** | Train15 | Test 5 | 0.72 | 0.23 | 0.59 | 0.33 |
| ***165*** | Train 11 | Test 6 | 0.71 | 0.29 | 0.58 | 0.38 | ***229*** | Train15 | Test 6 | 0.7 | 0.28 | 0.51 | 0.36 |
| ***166*** | Train 11 | Test 7 | 0.75 | 0.24 | 0.67 | 0.35 | ***230*** | Train15 | Test 7 | 0.72 | 0.23 | 0.59 | 0.34 |
| ***167*** | Train 11 | Test 8 | 0.71 | 0.29 | 0.58 | 0.38 | ***231*** | Train15 | Test 8 | 0.7 | 0.28 | 0.51 | 0.36 |
| ***168*** | Train 11 | Test 9 | 0.75 | 0.24 | 0.67 | 0.35 | ***232*** | Train15 | Test 9 | 0.72 | 0.23 | 0.59 | 0.33 |
| ***169*** | Train 11 | Test 10 | 0.71 | 0.29 | 0.58 | 0.38 | ***233*** | Train15 | Test 10 | 0.7 | 0.28 | 0.51 | 0.36 |
| ***170*** | Train 11 | Test 11 | 0.75 | 0.24 | 0.67 | 0.35 | ***234*** | Train15 | Test 11 | 0.72 | 0.23 | 0.59 | 0.33 |
| ***171*** | Train 11 | Test 12 | 0.71 | 0.29 | 0.58 | 0.38 | ***235*** | Train15 | Test 12 | 0.7 | 0.28 | 0.51 | 0.36 |
| ***172*** | Train 11 | Test 13 | 0.75 | 0.26 | 0.69 | 0.38 | ***236*** | Train15 | Test 13 | 0.75 | 0.28 | 0.66 | 0.39 |
| ***173*** | Train 11 | Test 14 | 0.7 | 0.31 | 0.59 | 0.4 | ***237*** | Train15 | Test 14 | 0.7 | 0.31 | 0.52 | 0.39 |
| ***174*** | Train 11 | Test 15 | 0.75 | 0.26 | 0.69 | 0.38 | ***238*** | Train15 | Test 15 | 0.75 | 0.27 | 0.66 | 0.39 |
| ***175*** | Train 11 | Test 16 | 0.7 | 0.31 | 0.59 | 0.4 | ***239*** | Train15 | Test 16 | 0.7 | 0.31 | 0.53 | 0.39 |
| ***176*** | Train 12 | Test 1 | 0.74 | 0.25 | 0.61 | 0.35 | ***240*** | Train16 | Test 1 | 0.72 | 0.22 | 0.62 | 0.33 |
| ***177*** | Train 12 | Test 2 | 0.75 | 0.3 | 0.7 | 0.42 | ***241*** | Train16 | Test 2 | 0.73 | 0.28 | 0.67 | 0.4 |
| ***178*** | Train 12 | Test 3 | 0.74 | 0.25 | 0.61 | 0.35 | ***242*** | Train16 | Test 3 | 0.72 | 0.22 | 0.62 | 0.33 |
| ***179*** | Train 12 | Test 4 | 0.75 | 0.3 | 0.7 | 0.42 | ***243*** | Train16 | Test 4 | 0.73 | 0.28 | 0.67 | 0.4 |
| ***180*** | Train 12 | Test 5 | 0.74 | 0.25 | 0.61 | 0.35 | ***244*** | Train16 | Test 5 | 0.72 | 0.22 | 0.62 | 0.33 |
| ***181*** | Train 12 | Test 6 | 0.75 | 0.3 | 0.7 | 0.42 | ***245*** | Train16 | Test 6 | 0.73 | 0.28 | 0.67 | 0.4 |
| ***182*** | Train 12 | Test 7 | 0.74 | 0.25 | 0.61 | 0.35 | ***246*** | Train16 | Test 7 | 0.72 | 0.22 | 0.62 | 0.33 |
| ***183*** | Train 12 | Test 8 | 0.75 | 0.3 | 0.7 | 0.42 | ***247*** | Train16 | Test 8 | 0.73 | 0.28 | 0.67 | 0.4 |
| ***184*** | Train 12 | Test 9 | 0.74 | 0.25 | 0.61 | 0.35 | ***248*** | Train16 | Test 9 | 0.72 | 0.22 | 0.62 | 0.33 |
| ***185*** | Train 12 | Test 10 | 0.75 | 0.3 | 0.7 | 0.42 | ***249*** | Train16 | Test 10 | 0.73 | 0.28 | 0.67 | 0.4 |
| ***186*** | Train 12 | Test 11 | 0.74 | 0.25 | 0.61 | 0.35 | ***250*** | Train16 | Test 11 | 0.72 | 0.22 | 0.62 | 0.33 |
| ***187*** | Train 12 | Test 12 | 0.75 | 0.3 | 0.7 | 0.42 | ***251*** | Train16 | Test 12 | 0.73 | 0.28 | 0.67 | 0.4 |
| ***188*** | Train 12 | Test 13 | 0.75 | 0.28 | 0.65 | 0.39 | ***252*** | Train16 | Test 13 | 0.75 | 0.29 | 0.61 | 0.39 |
| ***189*** | Train 12 | Test 14 | 0.75 | 0.32 | 0.72 | 0.44 | ***253*** | Train16 | Test 14 | 0.76 | 0.33 | 0.71 | 0.45 |
| ***190*** | Train 12 | Test 15 | 0.75 | 0.28 | 0.65 | 0.39 | ***254*** | Train16 | Test 15 | 0.75 | 0.28 | 0.61 | 0.39 |
| ***191*** | Train 12 | Test 16 | 0.75 | 0.32 | 0.72 | 0.44 | ***255*** | Train16 | Test 16 | 0.76 | 0.33 | 0.71 | 0.45 |

Table 5. Gradient Boosting (GB) Performance Metrics Across 16 Test Cohorts Using a Model Trained on Cohorts 1–8.

| Original Decisions | Train Model | Test Dataset | AUC  (GB) | Precision (GB) | Recall (GB) | F1 (GB) | ***Entire Dataset*** | Train Model | Test Dataset | AUC  (GB) | Precision (GB) | Recall (GB) | F1 (GB) |
| --- | --- | --- | --- | --- | --- | --- | --- | --- | --- | --- | --- | --- | --- |
| ***0*** | Train1 | Test 1 | 0.74 | 0.39 | 0.08 | 0.13 | ***64*** | Train5 | Test 1 | 0.74 | 0.41 | 0.08 | 0.14 |
| ***1*** | Train1 | Test 2 | 0.69 | 0.37 | 0.07 | 0.11 | ***65*** | Train5 | Test 2 | 0.69 | 0.32 | 0.1 | 0.15 |
| ***2*** | Train1 | Test 3 | 0.74 | 0.4 | 0.08 | 0.13 | ***66*** | Train5 | Test 3 | 0.74 | 0.4 | 0.08 | 0.13 |
| ***3*** | Train1 | Test 4 | 0.69 | 0.36 | 0.07 | 0.11 | ***67*** | Train5 | Test 4 | 0.69 | 0.31 | 0.1 | 0.15 |
| ***4*** | Train1 | Test 5 | 0.74 | 0.39 | 0.08 | 0.13 | ***68*** | Train5 | Test 5 | 0.74 | 0.41 | 0.08 | 0.14 |
| ***5*** | Train1 | Test 6 | 0.69 | 0.36 | 0.07 | 0.11 | ***69*** | Train5 | Test 6 | 0.69 | 0.31 | 0.1 | 0.15 |
| ***6*** | Train1 | Test 7 | 0.74 | 0.4 | 0.08 | 0.13 | ***70*** | Train5 | Test 7 | 0.74 | 0.4 | 0.08 | 0.13 |
| ***7*** | Train1 | Test 8 | 0.69 | 0.36 | 0.07 | 0.11 | ***71*** | Train5 | Test 8 | 0.69 | 0.31 | 0.1 | 0.15 |
| ***8*** | Train1 | Test 9 | 0.74 | 0.39 | 0.08 | 0.13 | ***72*** | Train5 | Test 9 | 0.75 | 0.41 | 0.08 | 0.13 |
| ***9*** | Train1 | Test 10 | 0.69 | 0.36 | 0.07 | 0.11 | ***73*** | Train5 | Test 10 | 0.69 | 0.32 | 0.1 | 0.15 |
| ***10*** | Train1 | Test 11 | 0.74 | 0.39 | 0.08 | 0.13 | ***74*** | Train5 | Test 11 | 0.75 | 0.4 | 0.08 | 0.13 |
| ***11*** | Train1 | Test 12 | 0.69 | 0.36 | 0.07 | 0.11 | ***75*** | Train5 | Test 12 | 0.69 | 0.31 | 0.1 | 0.15 |
| ***12*** | Train1 | Test 13 | 0.75 | 0.42 | 0.11 | 0.17 | ***76*** | Train5 | Test 13 | 0.74 | 0.44 | 0.11 | 0.18 |
| ***13*** | Train1 | Test 14 | 0.69 | 0.45 | 0.07 | 0.12 | ***77*** | Train5 | Test 14 | 0.69 | 0.33 | 0.11 | 0.17 |
| ***14*** | Train1 | Test 15 | 0.75 | 0.43 | 0.11 | 0.17 | ***78*** | Train5 | Test 15 | 0.74 | 0.44 | 0.11 | 0.18 |
| ***15*** | Train1 | Test 16 | 0.69 | 0.45 | 0.07 | 0.12 | ***79*** | Train5 | Test 16 | 0.69 | 0.33 | 0.11 | 0.17 |
| ***16*** | Train2 | Test 1 | 0.74 | 0.41 | 0.08 | 0.13 | ***80*** | Train6 | Test 1 | 0.75 | 0.43 | 0.07 | 0.13 |
| ***17*** | Train2 | Test 2 | 0.76 | 0.51 | 0.12 | 0.2 | ***81*** | Train6 | Test 2 | 0.76 | 0.52 | 0.12 | 0.2 |
| ***18*** | Train2 | Test 3 | 0.74 | 0.41 | 0.07 | 0.13 | ***82*** | Train6 | Test 3 | 0.74 | 0.42 | 0.07 | 0.12 |
| ***19*** | Train2 | Test 4 | 0.76 | 0.51 | 0.12 | 0.19 | ***83*** | Train6 | Test 4 | 0.76 | 0.52 | 0.12 | 0.2 |
| ***20*** | Train2 | Test 5 | 0.74 | 0.41 | 0.08 | 0.13 | ***84*** | Train6 | Test 5 | 0.75 | 0.43 | 0.07 | 0.13 |
| ***21*** | Train2 | Test 6 | 0.76 | 0.51 | 0.12 | 0.2 | ***85*** | Train6 | Test 6 | 0.76 | 0.52 | 0.12 | 0.2 |
| ***22*** | Train2 | Test 7 | 0.74 | 0.41 | 0.07 | 0.13 | ***86*** | Train6 | Test 7 | 0.74 | 0.42 | 0.07 | 0.12 |
| ***23*** | Train2 | Test 8 | 0.76 | 0.51 | 0.12 | 0.19 | ***87*** | Train6 | Test 8 | 0.76 | 0.52 | 0.12 | 0.2 |
| ***24*** | Train2 | Test 9 | 0.75 | 0.41 | 0.07 | 0.13 | ***88*** | Train6 | Test 9 | 0.75 | 0.43 | 0.07 | 0.13 |
| ***25*** | Train2 | Test 10 | 0.76 | 0.51 | 0.12 | 0.2 | ***89*** | Train6 | Test 10 | 0.76 | 0.52 | 0.12 | 0.2 |
| ***26*** | Train2 | Test 11 | 0.75 | 0.41 | 0.07 | 0.13 | ***90*** | Train6 | Test 11 | 0.75 | 0.42 | 0.07 | 0.12 |
| ***27*** | Train2 | Test 12 | 0.76 | 0.51 | 0.12 | 0.19 | ***91*** | Train6 | Test 12 | 0.76 | 0.52 | 0.12 | 0.2 |
| ***28*** | Train2 | Test 13 | 0.75 | 0.45 | 0.11 | 0.18 | ***92*** | Train6 | Test 13 | 0.75 | 0.48 | 0.11 | 0.18 |
| ***29*** | Train2 | Test 14 | 0.75 | 0.52 | 0.14 | 0.22 | ***93*** | Train6 | Test 14 | 0.75 | 0.53 | 0.13 | 0.21 |
| ***30*** | Train2 | Test 15 | 0.75 | 0.45 | 0.11 | 0.17 | ***94*** | Train6 | Test 15 | 0.75 | 0.48 | 0.11 | 0.17 |
| ***31*** | Train2 | Test 16 | 0.75 | 0.52 | 0.14 | 0.22 | ***95*** | Train6 | Test 16 | 0.75 | 0.53 | 0.13 | 0.21 |
| ***32*** | Train3 | Test 1 | 0.75 | 0.4 | 0.08 | 0.13 | ***96*** | Train7 | Test 1 | 0.75 | 0.41 | 0.09 | 0.14 |
| ***33*** | Train3 | Test 2 | 0.68 | 0.3 | 0.14 | 0.19 | ***97*** | Train7 | Test 2 | 0.7 | 0.37 | 0.12 | 0.18 |
| ***34*** | Train3 | Test 3 | 0.75 | 0.4 | 0.08 | 0.13 | ***98*** | Train7 | Test 3 | 0.75 | 0.41 | 0.08 | 0.14 |
| ***35*** | Train3 | Test 4 | 0.68 | 0.29 | 0.14 | 0.19 | ***99*** | Train7 | Test 4 | 0.7 | 0.37 | 0.12 | 0.18 |
| ***36*** | Train3 | Test 5 | 0.75 | 0.4 | 0.08 | 0.13 | ***100*** | Train7 | Test 5 | 0.75 | 0.41 | 0.09 | 0.14 |
| ***37*** | Train3 | Test 6 | 0.68 | 0.29 | 0.14 | 0.19 | ***101*** | Train7 | Test 6 | 0.7 | 0.37 | 0.12 | 0.18 |
| ***38*** | Train3 | Test 7 | 0.75 | 0.4 | 0.08 | 0.13 | ***102*** | Train7 | Test 7 | 0.75 | 0.41 | 0.08 | 0.14 |
| ***39*** | Train3 | Test 8 | 0.68 | 0.29 | 0.14 | 0.19 | ***103*** | Train7 | Test 8 | 0.7 | 0.37 | 0.12 | 0.18 |
| ***40*** | Train3 | Test 9 | 0.75 | 0.4 | 0.08 | 0.13 | ***104*** | Train7 | Test 9 | 0.75 | 0.41 | 0.08 | 0.14 |
| ***41*** | Train3 | Test 10 | 0.68 | 0.3 | 0.14 | 0.19 | ***105*** | Train7 | Test 10 | 0.7 | 0.37 | 0.12 | 0.18 |
| ***42*** | Train3 | Test 11 | 0.75 | 0.4 | 0.08 | 0.13 | ***106*** | Train7 | Test 11 | 0.75 | 0.41 | 0.08 | 0.14 |
| ***43*** | Train3 | Test 12 | 0.68 | 0.29 | 0.14 | 0.19 | ***107*** | Train7 | Test 12 | 0.7 | 0.37 | 0.12 | 0.18 |
| ***44*** | Train3 | Test 13 | 0.75 | 0.44 | 0.11 | 0.17 | ***108*** | Train7 | Test 13 | 0.74 | 0.45 | 0.12 | 0.19 |
| ***45*** | Train3 | Test 14 | 0.67 | 0.29 | 0.18 | 0.22 | ***109*** | Train7 | Test 14 | 0.69 | 0.42 | 0.13 | 0.2 |
| ***46*** | Train3 | Test 15 | 0.75 | 0.44 | 0.11 | 0.17 | ***110*** | Train7 | Test 15 | 0.75 | 0.45 | 0.12 | 0.19 |
| ***47*** | Train3 | Test 16 | 0.67 | 0.29 | 0.18 | 0.23 | ***111*** | Train7 | Test 16 | 0.7 | 0.42 | 0.13 | 0.2 |
| ***48*** | Train4 | Test 1 | 0.75 | 0.42 | 0.07 | 0.12 | ***112*** | Train8 | Test 1 | 0.75 | 0.43 | 0.08 | 0.13 |
| ***49*** | Train4 | Test 2 | 0.76 | 0.52 | 0.12 | 0.2 | ***113*** | Train8 | Test 2 | 0.76 | 0.53 | 0.12 | 0.2 |
| ***50*** | Train4 | Test 3 | 0.75 | 0.42 | 0.07 | 0.12 | ***114*** | Train8 | Test 3 | 0.74 | 0.43 | 0.08 | 0.13 |
| ***51*** | Train4 | Test 4 | 0.76 | 0.52 | 0.12 | 0.2 | ***115*** | Train8 | Test 4 | 0.76 | 0.53 | 0.12 | 0.2 |
| ***52*** | Train4 | Test 5 | 0.75 | 0.42 | 0.07 | 0.12 | ***116*** | Train8 | Test 5 | 0.75 | 0.43 | 0.08 | 0.13 |
| ***53*** | Train4 | Test 6 | 0.76 | 0.52 | 0.12 | 0.2 | ***117*** | Train8 | Test 6 | 0.76 | 0.53 | 0.12 | 0.2 |
| ***54*** | Train4 | Test 7 | 0.75 | 0.42 | 0.07 | 0.12 | ***118*** | Train8 | Test 7 | 0.74 | 0.43 | 0.08 | 0.13 |
| ***55*** | Train4 | Test 8 | 0.76 | 0.51 | 0.12 | 0.19 | ***119*** | Train8 | Test 8 | 0.76 | 0.53 | 0.12 | 0.2 |
| ***56*** | Train4 | Test 9 | 0.75 | 0.42 | 0.07 | 0.12 | ***120*** | Train8 | Test 9 | 0.75 | 0.43 | 0.08 | 0.13 |
| ***57*** | Train4 | Test 10 | 0.76 | 0.52 | 0.12 | 0.2 | ***121*** | Train8 | Test 10 | 0.76 | 0.53 | 0.12 | 0.2 |
| ***58*** | Train4 | Test 11 | 0.75 | 0.42 | 0.07 | 0.12 | ***122*** | Train8 | Test 11 | 0.75 | 0.43 | 0.08 | 0.13 |
| ***59*** | Train4 | Test 12 | 0.76 | 0.51 | 0.12 | 0.19 | ***123*** | Train8 | Test 12 | 0.76 | 0.53 | 0.12 | 0.2 |
| ***60*** | Train4 | Test 13 | 0.75 | 0.48 | 0.1 | 0.17 | ***124*** | Train8 | Test 13 | 0.75 | 0.48 | 0.11 | 0.18 |
| ***61*** | Train4 | Test 14 | 0.75 | 0.53 | 0.13 | 0.22 | ***125*** | Train8 | Test 14 | 0.75 | 0.54 | 0.14 | 0.22 |
| ***62*** | Train4 | Test 15 | 0.75 | 0.47 | 0.1 | 0.17 | ***126*** | Train8 | Test 15 | 0.75 | 0.49 | 0.11 | 0.18 |
| ***63*** | Train4 | Test 16 | 0.75 | 0.53 | 0.13 | 0.21 | ***127*** | Train8 | Test 16 | 0.75 | 0.54 | 0.14 | 0.22 |

Table 6. Gradient Boosting (GB) Performance Metrics Across 16 Test Cohorts Using a Model Trained on Cohorts 9–16.

| Original Decisions | Train Model | Test Dataset | AUC  (GB) | Precision (GB) | Recall (GB) | F1 (GB) | ***Entire Dataset*** | Train Model | Test Dataset | AUC  (GB) | Precision (GB) | Recall (GB) | F1 (GB) |
| --- | --- | --- | --- | --- | --- | --- | --- | --- | --- | --- | --- | --- | --- |
| ***128*** | Train 9 | Test 1 | 0.74 | 0.39 | 0.08 | 0.13 | ***192*** | Train13 | Test 1 | 0.68 | 0.25 | 0.13 | 0.17 |
| ***129*** | Train 9 | Test 2 | 0.7 | 0.39 | 0.09 | 0.14 | ***193*** | Train13 | Test 2 | 0.66 | 0.31 | 0.14 | 0.19 |
| ***130*** | Train 9 | Test 3 | 0.74 | 0.39 | 0.08 | 0.13 | ***194*** | Train13 | Test 3 | 0.68 | 0.25 | 0.13 | 0.17 |
| ***131*** | Train 9 | Test 4 | 0.7 | 0.39 | 0.09 | 0.14 | ***195*** | Train13 | Test 4 | 0.66 | 0.31 | 0.14 | 0.19 |
| ***132*** | Train 9 | Test 5 | 0.74 | 0.39 | 0.08 | 0.13 | ***196*** | Train13 | Test 5 | 0.68 | 0.25 | 0.13 | 0.17 |
| ***133*** | Train 9 | Test 6 | 0.7 | 0.39 | 0.09 | 0.14 | ***197*** | Train13 | Test 6 | 0.66 | 0.31 | 0.14 | 0.19 |
| ***134*** | Train 9 | Test 7 | 0.74 | 0.39 | 0.08 | 0.13 | ***198*** | Train13 | Test 7 | 0.68 | 0.25 | 0.13 | 0.17 |
| ***135*** | Train 9 | Test 8 | 0.7 | 0.39 | 0.09 | 0.14 | ***199*** | Train13 | Test 8 | 0.66 | 0.31 | 0.14 | 0.19 |
| ***136*** | Train 9 | Test 9 | 0.74 | 0.38 | 0.08 | 0.13 | ***200*** | Train13 | Test 9 | 0.68 | 0.25 | 0.13 | 0.17 |
| ***137*** | Train 9 | Test 10 | 0.7 | 0.39 | 0.09 | 0.14 | ***201*** | Train13 | Test 10 | 0.66 | 0.31 | 0.14 | 0.19 |
| ***138*** | Train 9 | Test 11 | 0.74 | 0.38 | 0.08 | 0.13 | ***202*** | Train13 | Test 11 | 0.68 | 0.25 | 0.13 | 0.17 |
| ***139*** | Train 9 | Test 12 | 0.7 | 0.39 | 0.09 | 0.14 | ***203*** | Train13 | Test 12 | 0.66 | 0.31 | 0.14 | 0.19 |
| ***140*** | Train 9 | Test 13 | 0.74 | 0.43 | 0.11 | 0.17 | ***204*** | Train13 | Test 13 | 0.74 | 0.41 | 0.14 | 0.21 |
| ***141*** | Train 9 | Test 14 | 0.7 | 0.45 | 0.11 | 0.18 | ***205*** | Train13 | Test 14 | 0.68 | 0.38 | 0.14 | 0.21 |
| ***142*** | Train 9 | Test 15 | 0.74 | 0.43 | 0.11 | 0.17 | ***206*** | Train13 | Test 15 | 0.74 | 0.41 | 0.14 | 0.21 |
| ***143*** | Train 9 | Test 16 | 0.7 | 0.45 | 0.11 | 0.17 | ***207*** | Train13 | Test 16 | 0.69 | 0.38 | 0.14 | 0.21 |
| ***144*** | Train 10 | Test 1 | 0.74 | 0.43 | 0.08 | 0.13 | ***208*** | Train14 | Test 1 | 0.69 | 0.26 | 0.15 | 0.19 |
| ***145*** | Train 10 | Test 2 | 0.76 | 0.51 | 0.12 | 0.2 | ***209*** | Train14 | Test 2 | 0.71 | 0.33 | 0.19 | 0.24 |
| ***146*** | Train 10 | Test 3 | 0.74 | 0.43 | 0.08 | 0.13 | ***210*** | Train14 | Test 3 | 0.69 | 0.26 | 0.15 | 0.19 |
| ***147*** | Train 10 | Test 4 | 0.76 | 0.51 | 0.12 | 0.2 | ***211*** | Train14 | Test 4 | 0.71 | 0.33 | 0.19 | 0.24 |
| ***148*** | Train 10 | Test 5 | 0.74 | 0.43 | 0.08 | 0.13 | ***212*** | Train14 | Test 5 | 0.69 | 0.26 | 0.15 | 0.19 |
| ***149*** | Train 10 | Test 6 | 0.76 | 0.51 | 0.12 | 0.2 | ***213*** | Train14 | Test 6 | 0.71 | 0.33 | 0.19 | 0.24 |
| ***150*** | Train 10 | Test 7 | 0.74 | 0.43 | 0.08 | 0.13 | ***214*** | Train14 | Test 7 | 0.69 | 0.26 | 0.15 | 0.19 |
| ***151*** | Train 10 | Test 8 | 0.76 | 0.51 | 0.12 | 0.2 | ***215*** | Train14 | Test 8 | 0.71 | 0.32 | 0.19 | 0.24 |
| ***152*** | Train 10 | Test 9 | 0.74 | 0.43 | 0.08 | 0.13 | ***216*** | Train14 | Test 9 | 0.69 | 0.26 | 0.15 | 0.19 |
| ***153*** | Train 10 | Test 10 | 0.76 | 0.51 | 0.12 | 0.2 | ***217*** | Train14 | Test 10 | 0.71 | 0.33 | 0.19 | 0.24 |
| ***154*** | Train 10 | Test 11 | 0.74 | 0.43 | 0.07 | 0.13 | ***218*** | Train14 | Test 11 | 0.69 | 0.26 | 0.15 | 0.19 |
| ***155*** | Train 10 | Test 12 | 0.76 | 0.51 | 0.12 | 0.2 | ***219*** | Train14 | Test 12 | 0.71 | 0.33 | 0.19 | 0.24 |
| ***156*** | Train 10 | Test 13 | 0.75 | 0.48 | 0.11 | 0.18 | ***220*** | Train14 | Test 13 | 0.75 | 0.42 | 0.12 | 0.19 |
| ***157*** | Train 10 | Test 14 | 0.75 | 0.53 | 0.14 | 0.22 | ***221*** | Train14 | Test 14 | 0.76 | 0.52 | 0.16 | 0.25 |
| ***158*** | Train 10 | Test 15 | 0.75 | 0.47 | 0.11 | 0.18 | ***222*** | Train14 | Test 15 | 0.75 | 0.43 | 0.12 | 0.19 |
| ***159*** | Train 10 | Test 16 | 0.75 | 0.52 | 0.14 | 0.22 | ***223*** | Train14 | Test 16 | 0.76 | 0.52 | 0.16 | 0.25 |
| ***160*** | Train 11 | Test 1 | 0.75 | 0.41 | 0.08 | 0.13 | ***224*** | Train15 | Test 1 | 0.68 | 0.22 | 0.18 | 0.19 |
| ***161*** | Train 11 | Test 2 | 0.69 | 0.33 | 0.13 | 0.18 | ***225*** | Train15 | Test 2 | 0.66 | 0.28 | 0.17 | 0.21 |
| ***162*** | Train 11 | Test 3 | 0.74 | 0.4 | 0.08 | 0.13 | ***226*** | Train15 | Test 3 | 0.68 | 0.22 | 0.18 | 0.2 |
| ***163*** | Train 11 | Test 4 | 0.69 | 0.33 | 0.13 | 0.18 | ***227*** | Train15 | Test 4 | 0.66 | 0.29 | 0.17 | 0.21 |
| ***164*** | Train 11 | Test 5 | 0.75 | 0.41 | 0.08 | 0.13 | ***228*** | Train15 | Test 5 | 0.68 | 0.22 | 0.18 | 0.19 |
| ***165*** | Train 11 | Test 6 | 0.69 | 0.33 | 0.13 | 0.18 | ***229*** | Train15 | Test 6 | 0.66 | 0.28 | 0.17 | 0.21 |
| ***166*** | Train 11 | Test 7 | 0.74 | 0.4 | 0.08 | 0.13 | ***230*** | Train15 | Test 7 | 0.68 | 0.22 | 0.18 | 0.2 |
| ***167*** | Train 11 | Test 8 | 0.69 | 0.33 | 0.13 | 0.18 | ***231*** | Train15 | Test 8 | 0.66 | 0.29 | 0.17 | 0.21 |
| ***168*** | Train 11 | Test 9 | 0.75 | 0.41 | 0.08 | 0.13 | ***232*** | Train15 | Test 9 | 0.68 | 0.22 | 0.18 | 0.2 |
| ***169*** | Train 11 | Test 10 | 0.69 | 0.33 | 0.13 | 0.18 | ***233*** | Train15 | Test 10 | 0.66 | 0.28 | 0.17 | 0.21 |
| ***170*** | Train 11 | Test 11 | 0.75 | 0.4 | 0.08 | 0.13 | ***234*** | Train15 | Test 11 | 0.68 | 0.22 | 0.18 | 0.2 |
| ***171*** | Train 11 | Test 12 | 0.69 | 0.33 | 0.13 | 0.18 | ***235*** | Train15 | Test 12 | 0.66 | 0.29 | 0.17 | 0.21 |
| ***172*** | Train 11 | Test 13 | 0.74 | 0.45 | 0.11 | 0.17 | ***236*** | Train15 | Test 13 | 0.74 | 0.42 | 0.14 | 0.21 |
| ***173*** | Train 11 | Test 14 | 0.68 | 0.33 | 0.17 | 0.22 | ***237*** | Train15 | Test 14 | 0.69 | 0.36 | 0.19 | 0.25 |
| ***174*** | Train 11 | Test 15 | 0.74 | 0.45 | 0.11 | 0.17 | ***238*** | Train15 | Test 15 | 0.74 | 0.42 | 0.14 | 0.21 |
| ***175*** | Train 11 | Test 16 | 0.68 | 0.33 | 0.17 | 0.23 | ***239*** | Train15 | Test 16 | 0.69 | 0.36 | 0.19 | 0.24 |
| ***176*** | Train 12 | Test 1 | 0.75 | 0.42 | 0.07 | 0.12 | ***240*** | Train16 | Test 1 | 0.66 | 0.19 | 0.22 | 0.21 |
| ***177*** | Train 12 | Test 2 | 0.76 | 0.52 | 0.12 | 0.19 | ***241*** | Train16 | Test 2 | 0.68 | 0.28 | 0.19 | 0.22 |
| ***178*** | Train 12 | Test 3 | 0.74 | 0.42 | 0.07 | 0.12 | ***242*** | Train16 | Test 3 | 0.66 | 0.19 | 0.22 | 0.2 |
| ***179*** | Train 12 | Test 4 | 0.76 | 0.51 | 0.12 | 0.19 | ***243*** | Train16 | Test 4 | 0.69 | 0.28 | 0.18 | 0.22 |
| ***180*** | Train 12 | Test 5 | 0.75 | 0.42 | 0.07 | 0.12 | ***244*** | Train16 | Test 5 | 0.66 | 0.19 | 0.22 | 0.21 |
| ***181*** | Train 12 | Test 6 | 0.76 | 0.52 | 0.12 | 0.19 | ***245*** | Train16 | Test 6 | 0.69 | 0.28 | 0.19 | 0.22 |
| ***182*** | Train 12 | Test 7 | 0.75 | 0.42 | 0.07 | 0.12 | ***246*** | Train16 | Test 7 | 0.66 | 0.19 | 0.22 | 0.21 |
| ***183*** | Train 12 | Test 8 | 0.76 | 0.51 | 0.12 | 0.19 | ***247*** | Train16 | Test 8 | 0.69 | 0.28 | 0.19 | 0.22 |
| ***184*** | Train 12 | Test 9 | 0.75 | 0.42 | 0.07 | 0.12 | ***248*** | Train16 | Test 9 | 0.66 | 0.19 | 0.22 | 0.21 |
| ***185*** | Train 12 | Test 10 | 0.76 | 0.52 | 0.12 | 0.19 | ***249*** | Train16 | Test 10 | 0.69 | 0.28 | 0.19 | 0.22 |
| ***186*** | Train 12 | Test 11 | 0.75 | 0.42 | 0.07 | 0.12 | ***250*** | Train16 | Test 11 | 0.66 | 0.19 | 0.22 | 0.2 |
| ***187*** | Train 12 | Test 12 | 0.76 | 0.51 | 0.12 | 0.19 | ***251*** | Train16 | Test 12 | 0.69 | 0.28 | 0.18 | 0.22 |
| ***188*** | Train 12 | Test 13 | 0.75 | 0.46 | 0.1 | 0.16 | ***252*** | Train16 | Test 13 | 0.75 | 0.45 | 0.12 | 0.19 |
| ***189*** | Train 12 | Test 14 | 0.75 | 0.53 | 0.13 | 0.21 | ***253*** | Train16 | Test 14 | 0.76 | 0.52 | 0.16 | 0.25 |
| ***190*** | Train 12 | Test 15 | 0.75 | 0.46 | 0.1 | 0.16 | ***254*** | Train16 | Test 15 | 0.75 | 0.44 | 0.12 | 0.19 |
| ***191*** | Train 12 | Test 16 | 0.75 | 0.52 | 0.13 | 0.2 | ***255*** | Train16 | Test 16 | 0.76 | 0.52 | 0.16 | 0.25 |

Table 7. Logistic Regression (LR) AUC Performance for Male Patients Across 16 Cohorts.

| Original Decisions | Train Model | Test Dataset | AUC (LR) | ***Male*** | Train Model | Test Dataset | AUC (LR) | Male | Train Model | Test Dataset | AUC (LR) | Male | Train Model | Test Dataset | AUC (LR) |
| --- | --- | --- | --- | --- | --- | --- | --- | --- | --- | --- | --- | --- | --- | --- | --- |
| ***1*** | Train 1 | Test Male 1 | 0.73 | ***65*** | Train 5 | Test Male 1 | 0.73 | ***129*** | Train 9 | Test Male 1 | 0.73 | ***193*** | Train 13 | Test Male 1 | 0.71 |
| ***2*** | Train 1 | Test Male 2 | 0.71 | ***66*** | Train 5 | Test Male 2 | 0.71 | ***130*** | Train 9 | Test Male 2 | 0.71 | ***194*** | Train 13 | Test Male 2 | 0.7 |
| ***3*** | Train 1 | Test Male 3 | 0.73 | ***67*** | Train 5 | Test Male 3 | 0.73 | ***131*** | Train 9 | Test Male 3 | 0.73 | ***195*** | Train 13 | Test Male 3 | 0.71 |
| ***4*** | Train 1 | Test Male 4 | 0.71 | ***68*** | Train 5 | Test Male 4 | 0.71 | ***132*** | Train 9 | Test Male 4 | 0.71 | ***196*** | Train 13 | Test Male 4 | 0.7 |
| ***5*** | Train 1 | Test Male 5 | 0.73 | ***69*** | Train 5 | Test Male 5 | 0.73 | ***133*** | Train 9 | Test Male 5 | 0.73 | ***197*** | Train 13 | Test Male 5 | 0.71 |
| ***6*** | Train 1 | Test Male 6 | 0.71 | ***70*** | Train 5 | Test Male 6 | 0.71 | ***134*** | Train 9 | Test Male 6 | 0.71 | ***198*** | Train 13 | Test Male 6 | 0.7 |
| ***7*** | Train 1 | Test Male 7 | 0.73 | ***71*** | Train 5 | Test Male 7 | 0.73 | ***135*** | Train 9 | Test Male 7 | 0.73 | ***199*** | Train 13 | Test Male 7 | 0.71 |
| ***8*** | Train 1 | Test Male 8 | 0.71 | ***72*** | Train 5 | Test Male 8 | 0.71 | ***136*** | Train 9 | Test Male 8 | 0.71 | ***200*** | Train 13 | Test Male 8 | 0.7 |
| ***9*** | Train 1 | Test Male 9 | 0.73 | ***73*** | Train 5 | Test Male 9 | 0.73 | ***137*** | Train 9 | Test Male 9 | 0.73 | ***201*** | Train 13 | Test Male 9 | 0.71 |
| ***10*** | Train 1 | Test Male 10 | 0.71 | ***74*** | Train 5 | Test Male 10 | 0.71 | ***138*** | Train 9 | Test Male 10 | 0.71 | ***202*** | Train 13 | Test Male 10 | 0.7 |
| ***11*** | Train 1 | Test Male 11 | 0.73 | ***75*** | Train 5 | Test Male 11 | 0.73 | ***139*** | Train 9 | Test Male 11 | 0.73 | ***203*** | Train 13 | Test Male 11 | 0.71 |
| ***12*** | Train 1 | Test Male 12 | 0.71 | ***76*** | Train 5 | Test Male 12 | 0.71 | ***140*** | Train 9 | Test Male 12 | 0.71 | ***204*** | Train 13 | Test Male 12 | 0.7 |
| ***13*** | Train 1 | Test Male 13 | 0.74 | ***77*** | Train 5 | Test Male 13 | 0.74 | ***141*** | Train 9 | Test Male 13 | 0.74 | ***205*** | Train 13 | Test Male 13 | 0.74 |
| ***14*** | Train 1 | Test Male 14 | 0.71 | ***78*** | Train 5 | Test Male 14 | 0.71 | ***142*** | Train 9 | Test Male 14 | 0.71 | ***206*** | Train 13 | Test Male 14 | 0.71 |
| ***15*** | Train 1 | Test Male 15 | 0.74 | ***79*** | Train 5 | Test Male 15 | 0.74 | ***143*** | Train 9 | Test Male 15 | 0.74 | ***207*** | Train 13 | Test Male 15 | 0.74 |
| ***16*** | Train 1 | Test Male 16 | 0.71 | ***80*** | Train 5 | Test Male 16 | 0.71 | ***144*** | Train 9 | Test Male 16 | 0.71 | ***208*** | Train 13 | Test Male 16 | 0.71 |
| ***17*** | Train 2 | Test Male 1 | 0.72 | ***81*** | Train 6 | Test Male 1 | 0.72 | ***145*** | Train 10 | Test Male 1 | 0.72 | ***209*** | Train 14 | Test Male 1 | 0.7 |
| ***18*** | Train 2 | Test Male 2 | 0.73 | ***82*** | Train 6 | Test Male 2 | 0.73 | ***146*** | Train 10 | Test Male 2 | 0.73 | ***210*** | Train 14 | Test Male 2 | 0.71 |
| ***19*** | Train 2 | Test Male 3 | 0.72 | ***83*** | Train 6 | Test Male 3 | 0.72 | ***147*** | Train 10 | Test Male 3 | 0.72 | ***211*** | Train 14 | Test Male 3 | 0.7 |
| ***20*** | Train 2 | Test Male 4 | 0.73 | ***84*** | Train 6 | Test Male 4 | 0.73 | ***148*** | Train 10 | Test Male 4 | 0.73 | ***212*** | Train 14 | Test Male 4 | 0.72 |
| ***21*** | Train 2 | Test Male 5 | 0.72 | ***85*** | Train 6 | Test Male 5 | 0.72 | ***149*** | Train 10 | Test Male 5 | 0.72 | ***213*** | Train 14 | Test Male 5 | 0.7 |
| ***22*** | Train 2 | Test Male 6 | 0.73 | ***86*** | Train 6 | Test Male 6 | 0.73 | ***150*** | Train 10 | Test Male 6 | 0.73 | ***214*** | Train 14 | Test Male 6 | 0.71 |
| ***23*** | Train 2 | Test Male 7 | 0.72 | ***87*** | Train 6 | Test Male 7 | 0.72 | ***151*** | Train 10 | Test Male 7 | 0.72 | ***215*** | Train 14 | Test Male 7 | 0.7 |
| ***24*** | Train 2 | Test Male 8 | 0.73 | ***88*** | Train 6 | Test Male 8 | 0.73 | ***152*** | Train 10 | Test Male 8 | 0.73 | ***216*** | Train 14 | Test Male 8 | 0.72 |
| ***25*** | Train 2 | Test Male 9 | 0.71 | ***89*** | Train 6 | Test Male 9 | 0.71 | ***153*** | Train 10 | Test Male 9 | 0.71 | ***217*** | Train 14 | Test Male 9 | 0.7 |
| ***26*** | Train 2 | Test Male 10 | 0.73 | ***90*** | Train 6 | Test Male 10 | 0.73 | ***154*** | Train 10 | Test Male 10 | 0.73 | ***218*** | Train 14 | Test Male 10 | 0.71 |
| ***27*** | Train 2 | Test Male 11 | 0.72 | ***91*** | Train 6 | Test Male 11 | 0.72 | ***155*** | Train 10 | Test Male 11 | 0.72 | ***219*** | Train 14 | Test Male 11 | 0.7 |
| ***28*** | Train 2 | Test Male 12 | 0.73 | ***92*** | Train 6 | Test Male 12 | 0.73 | ***156*** | Train 10 | Test Male 12 | 0.73 | ***220*** | Train 14 | Test Male 12 | 0.71 |
| ***29*** | Train 2 | Test Male 13 | 0.73 | ***93*** | Train 6 | Test Male 13 | 0.73 | ***157*** | Train 10 | Test Male 13 | 0.73 | ***221*** | Train 14 | Test Male 13 | 0.74 |
| ***30*** | Train 2 | Test Male 14 | 0.73 | ***94*** | Train 6 | Test Male 14 | 0.73 | ***158*** | Train 10 | Test Male 14 | 0.73 | ***222*** | Train 14 | Test Male 14 | 0.73 |
| ***31*** | Train 2 | Test Male 15 | 0.73 | ***95*** | Train 6 | Test Male 15 | 0.73 | ***159*** | Train 10 | Test Male 15 | 0.73 | ***223*** | Train 14 | Test Male 15 | 0.74 |
| ***32*** | Train 2 | Test Male 16 | 0.73 | ***96*** | Train 6 | Test Male 16 | 0.73 | ***160*** | Train 10 | Test Male 16 | 0.73 | ***224*** | Train 14 | Test Male 16 | 0.73 |
| ***33*** | Train 3 | Test Male 1 | 0.73 | ***97*** | Train 7 | Test Male 1 | 0.73 | ***161*** | Train 11 | Test Male 1 | 0.73 | ***225*** | Train 15 | Test Male 1 | 0.71 |
| ***34*** | Train 3 | Test Male 2 | 0.71 | ***98*** | Train 7 | Test Male 2 | 0.71 | ***162*** | Train 11 | Test Male 2 | 0.7 | ***226*** | Train 15 | Test Male 2 | 0.7 |
| ***35*** | Train 3 | Test Male 3 | 0.73 | ***99*** | Train 7 | Test Male 3 | 0.73 | ***163*** | Train 11 | Test Male 3 | 0.73 | ***227*** | Train 15 | Test Male 3 | 0.71 |
| ***36*** | Train 3 | Test Male 4 | 0.71 | ***100*** | Train 7 | Test Male 4 | 0.71 | ***164*** | Train 11 | Test Male 4 | 0.71 | ***228*** | Train 15 | Test Male 4 | 0.7 |
| ***37*** | Train 3 | Test Male 5 | 0.73 | ***101*** | Train 7 | Test Male 5 | 0.73 | ***165*** | Train 11 | Test Male 5 | 0.73 | ***229*** | Train 15 | Test Male 5 | 0.72 |
| ***38*** | Train 3 | Test Male 6 | 0.71 | ***102*** | Train 7 | Test Male 6 | 0.71 | ***166*** | Train 11 | Test Male 6 | 0.71 | ***230*** | Train 15 | Test Male 6 | 0.7 |
| ***39*** | Train 3 | Test Male 7 | 0.73 | ***103*** | Train 7 | Test Male 7 | 0.73 | ***167*** | Train 11 | Test Male 7 | 0.73 | ***231*** | Train 15 | Test Male 7 | 0.71 |
| ***40*** | Train 3 | Test Male 8 | 0.71 | ***104*** | Train 7 | Test Male 8 | 0.71 | ***168*** | Train 11 | Test Male 8 | 0.71 | ***232*** | Train 15 | Test Male 8 | 0.7 |
| ***41*** | Train 3 | Test Male 9 | 0.73 | ***105*** | Train 7 | Test Male 9 | 0.73 | ***169*** | Train 11 | Test Male 9 | 0.73 | ***233*** | Train 15 | Test Male 9 | 0.71 |
| ***42*** | Train 3 | Test Male 10 | 0.7 | ***106*** | Train 7 | Test Male 10 | 0.7 | ***170*** | Train 11 | Test Male 10 | 0.7 | ***234*** | Train 15 | Test Male 10 | 0.7 |
| ***43*** | Train 3 | Test Male 11 | 0.73 | ***107*** | Train 7 | Test Male 11 | 0.73 | ***171*** | Train 11 | Test Male 11 | 0.73 | ***235*** | Train 15 | Test Male 11 | 0.71 |
| ***44*** | Train 3 | Test Male 12 | 0.71 | ***108*** | Train 7 | Test Male 12 | 0.71 | ***172*** | Train 11 | Test Male 12 | 0.71 | ***236*** | Train 15 | Test Male 12 | 0.7 |
| ***45*** | Train 3 | Test Male 13 | 0.74 | ***109*** | Train 7 | Test Male 13 | 0.74 | ***173*** | Train 11 | Test Male 13 | 0.74 | ***237*** | Train 15 | Test Male 13 | 0.74 |
| ***46*** | Train 3 | Test Male 14 | 0.71 | ***110*** | Train 7 | Test Male 14 | 0.71 | ***174*** | Train 11 | Test Male 14 | 0.71 | ***238*** | Train 15 | Test Male 14 | 0.71 |
| ***47*** | Train 3 | Test Male 15 | 0.74 | ***111*** | Train 7 | Test Male 15 | 0.74 | ***175*** | Train 11 | Test Male 15 | 0.74 | ***239*** | Train 15 | Test Male 15 | 0.74 |
| ***48*** | Train 3 | Test Male 16 | 0.71 | ***112*** | Train 7 | Test Male 16 | 0.71 | ***176*** | Train 11 | Test Male 16 | 0.71 | ***240*** | Train 15 | Test Male 16 | 0.71 |
| ***49*** | Train 4 | Test Male 1 | 0.72 | ***113*** | Train 8 | Test Male 1 | 0.72 | ***177*** | Train 12 | Test Male 1 | 0.72 | ***241*** | Train 16 | Test Male 1 | 0.7 |
| ***50*** | Train 4 | Test Male 2 | 0.73 | ***114*** | Train 8 | Test Male 2 | 0.73 | ***178*** | Train 12 | Test Male 2 | 0.73 | ***242*** | Train 16 | Test Male 2 | 0.71 |
| ***51*** | Train 4 | Test Male 3 | 0.72 | ***115*** | Train 8 | Test Male 3 | 0.72 | ***179*** | Train 12 | Test Male 3 | 0.72 | ***243*** | Train 16 | Test Male 3 | 0.7 |
| ***52*** | Train 4 | Test Male 4 | 0.73 | ***116*** | Train 8 | Test Male 4 | 0.73 | ***180*** | Train 12 | Test Male 4 | 0.73 | ***244*** | Train 16 | Test Male 4 | 0.72 |
| ***53*** | Train 4 | Test Male 5 | 0.72 | ***117*** | Train 8 | Test Male 5 | 0.72 | ***181*** | Train 12 | Test Male 5 | 0.72 | ***245*** | Train 16 | Test Male 5 | 0.7 |
| ***54*** | Train 4 | Test Male 6 | 0.73 | ***118*** | Train 8 | Test Male 6 | 0.73 | ***182*** | Train 12 | Test Male 6 | 0.73 | ***246*** | Train 16 | Test Male 6 | 0.71 |
| ***55*** | Train 4 | Test Male 7 | 0.72 | ***119*** | Train 8 | Test Male 7 | 0.72 | ***183*** | Train 12 | Test Male 7 | 0.72 | ***247*** | Train 16 | Test Male 7 | 0.7 |
| ***56*** | Train 4 | Test Male 8 | 0.73 | ***120*** | Train 8 | Test Male 8 | 0.73 | ***184*** | Train 12 | Test Male 8 | 0.73 | ***248*** | Train 16 | Test Male 8 | 0.72 |
| ***57*** | Train 4 | Test Male 9 | 0.72 | ***121*** | Train 8 | Test Male 9 | 0.72 | ***185*** | Train 12 | Test Male 9 | 0.72 | ***249*** | Train 16 | Test Male 9 | 0.7 |
| ***58*** | Train 4 | Test Male 10 | 0.73 | ***122*** | Train 8 | Test Male 10 | 0.73 | ***186*** | Train 12 | Test Male 10 | 0.73 | ***250*** | Train 16 | Test Male 10 | 0.71 |
| ***59*** | Train 4 | Test Male 11 | 0.72 | ***123*** | Train 8 | Test Male 11 | 0.72 | ***187*** | Train 12 | Test Male 11 | 0.72 | ***251*** | Train 16 | Test Male 11 | 0.7 |
| ***60*** | Train 4 | Test Male 12 | 0.73 | ***124*** | Train 8 | Test Male 12 | 0.73 | ***188*** | Train 12 | Test Male 12 | 0.73 | ***252*** | Train 16 | Test Male 12 | 0.71 |
| ***61*** | Train 4 | Test Male 13 | 0.73 | ***125*** | Train 8 | Test Male 13 | 0.73 | ***189*** | Train 12 | Test Male 13 | 0.73 | ***253*** | Train 16 | Test Male 13 | 0.74 |
| ***62*** | Train 4 | Test Male 14 | 0.73 | ***126*** | Train 8 | Test Male 14 | 0.73 | ***190*** | Train 12 | Test Male 14 | 0.73 | ***254*** | Train 16 | Test Male 14 | 0.73 |
| ***63*** | Train 4 | Test Male 15 | 0.73 | ***127*** | Train 8 | Test Male 15 | 0.73 | ***191*** | Train 12 | Test Male 15 | 0.73 | ***255*** | Train 16 | Test Male 15 | 0.74 |
| ***64*** | Train 4 | Test Male 16 | 0.73 | ***128*** | Train 8 | Test Male 16 | 0.73 | ***192*** | Train 12 | Test Male 16 | 0.73 | ***256*** | Train 16 | Test Male 16 | 0.73 |

Table 8. Random Forest (RF) AUC Performance for Male Patients Across 16 Cohorts.

| Original Decisions | Train Model | Test Dataset | AUC (RF) | ***Male*** | Train Model | Test Dataset | AUC (RF) | Male | Train Model | Test Dataset | AUC (RF) | Male | Train Model | Test Dataset | AUC (RF) |
| --- | --- | --- | --- | --- | --- | --- | --- | --- | --- | --- | --- | --- | --- | --- | --- |
| ***1*** | Train 1 | Test Male 1 | 0.74 | ***65*** | Train 5 | Test Male 1 | 0.74 | ***129*** | Train 9 | Test Male 1 | 0.74 | ***193*** | Train 13 | Test Male 1 | 0.72 |
| ***2*** | Train 1 | Test Male 2 | 0.7 | ***66*** | Train 5 | Test Male 2 | 0.7 | ***130*** | Train 9 | Test Male 2 | 0.7 | ***194*** | Train 13 | Test Male 2 | 0.69 |
| ***3*** | Train 1 | Test Male 3 | 0.74 | ***67*** | Train 5 | Test Male 3 | 0.74 | ***131*** | Train 9 | Test Male 3 | 0.74 | ***195*** | Train 13 | Test Male 3 | 0.72 |
| ***4*** | Train 1 | Test Male 4 | 0.7 | ***68*** | Train 5 | Test Male 4 | 0.7 | ***132*** | Train 9 | Test Male 4 | 0.7 | ***196*** | Train 13 | Test Male 4 | 0.69 |
| ***5*** | Train 1 | Test Male 5 | 0.74 | ***69*** | Train 5 | Test Male 5 | 0.75 | ***133*** | Train 9 | Test Male 5 | 0.74 | ***197*** | Train 13 | Test Male 5 | 0.72 |
| ***6*** | Train 1 | Test Male 6 | 0.7 | ***70*** | Train 5 | Test Male 6 | 0.7 | ***134*** | Train 9 | Test Male 6 | 0.7 | ***198*** | Train 13 | Test Male 6 | 0.69 |
| ***7*** | Train 1 | Test Male 7 | 0.74 | ***71*** | Train 5 | Test Male 7 | 0.74 | ***135*** | Train 9 | Test Male 7 | 0.74 | ***199*** | Train 13 | Test Male 7 | 0.72 |
| ***8*** | Train 1 | Test Male 8 | 0.7 | ***72*** | Train 5 | Test Male 8 | 0.7 | ***136*** | Train 9 | Test Male 8 | 0.7 | ***200*** | Train 13 | Test Male 8 | 0.69 |
| ***9*** | Train 1 | Test Male 9 | 0.74 | ***73*** | Train 5 | Test Male 9 | 0.74 | ***137*** | Train 9 | Test Male 9 | 0.74 | ***201*** | Train 13 | Test Male 9 | 0.72 |
| ***10*** | Train 1 | Test Male 10 | 0.7 | ***74*** | Train 5 | Test Male 10 | 0.7 | ***138*** | Train 9 | Test Male 10 | 0.7 | ***202*** | Train 13 | Test Male 10 | 0.69 |
| ***11*** | Train 1 | Test Male 11 | 0.74 | ***75*** | Train 5 | Test Male 11 | 0.74 | ***139*** | Train 9 | Test Male 11 | 0.74 | ***203*** | Train 13 | Test Male 11 | 0.72 |
| ***12*** | Train 1 | Test Male 12 | 0.7 | ***76*** | Train 5 | Test Male 12 | 0.7 | ***140*** | Train 9 | Test Male 12 | 0.7 | ***204*** | Train 13 | Test Male 12 | 0.69 |
| ***13*** | Train 1 | Test Male 13 | 0.74 | ***77*** | Train 5 | Test Male 13 | 0.74 | ***141*** | Train 9 | Test Male 13 | 0.74 | ***205*** | Train 13 | Test Male 13 | 0.74 |
| ***14*** | Train 1 | Test Male 14 | 0.69 | ***78*** | Train 5 | Test Male 14 | 0.69 | ***142*** | Train 9 | Test Male 14 | 0.69 | ***206*** | Train 13 | Test Male 14 | 0.69 |
| ***15*** | Train 1 | Test Male 15 | 0.74 | ***79*** | Train 5 | Test Male 15 | 0.74 | ***143*** | Train 9 | Test Male 15 | 0.74 | ***207*** | Train 13 | Test Male 15 | 0.74 |
| ***16*** | Train 1 | Test Male 16 | 0.69 | ***80*** | Train 5 | Test Male 16 | 0.69 | ***144*** | Train 9 | Test Male 16 | 0.69 | ***208*** | Train 13 | Test Male 16 | 0.69 |
| ***17*** | Train 2 | Test Male 1 | 0.74 | ***81*** | Train 6 | Test Male 1 | 0.74 | ***145*** | Train 10 | Test Male 1 | 0.74 | ***209*** | Train 14 | Test Male 1 | 0.71 |
| ***18*** | Train 2 | Test Male 2 | 0.75 | ***82*** | Train 6 | Test Male 2 | 0.75 | ***146*** | Train 10 | Test Male 2 | 0.75 | ***210*** | Train 14 | Test Male 2 | 0.73 |
| ***19*** | Train 2 | Test Male 3 | 0.74 | ***83*** | Train 6 | Test Male 3 | 0.74 | ***147*** | Train 10 | Test Male 3 | 0.74 | ***211*** | Train 14 | Test Male 3 | 0.71 |
| ***20*** | Train 2 | Test Male 4 | 0.75 | ***84*** | Train 6 | Test Male 4 | 0.75 | ***148*** | Train 10 | Test Male 4 | 0.75 | ***212*** | Train 14 | Test Male 4 | 0.73 |
| ***21*** | Train 2 | Test Male 5 | 0.74 | ***85*** | Train 6 | Test Male 5 | 0.74 | ***149*** | Train 10 | Test Male 5 | 0.74 | ***213*** | Train 14 | Test Male 5 | 0.71 |
| ***22*** | Train 2 | Test Male 6 | 0.75 | ***86*** | Train 6 | Test Male 6 | 0.75 | ***150*** | Train 10 | Test Male 6 | 0.75 | ***214*** | Train 14 | Test Male 6 | 0.73 |
| ***23*** | Train 2 | Test Male 7 | 0.74 | ***87*** | Train 6 | Test Male 7 | 0.74 | ***151*** | Train 10 | Test Male 7 | 0.74 | ***215*** | Train 14 | Test Male 7 | 0.71 |
| ***24*** | Train 2 | Test Male 8 | 0.75 | ***88*** | Train 6 | Test Male 8 | 0.75 | ***152*** | Train 10 | Test Male 8 | 0.75 | ***216*** | Train 14 | Test Male 8 | 0.73 |
| ***25*** | Train 2 | Test Male 9 | 0.74 | ***89*** | Train 6 | Test Male 9 | 0.74 | ***153*** | Train 10 | Test Male 9 | 0.74 | ***217*** | Train 14 | Test Male 9 | 0.71 |
| ***26*** | Train 2 | Test Male 10 | 0.75 | ***90*** | Train 6 | Test Male 10 | 0.75 | ***154*** | Train 10 | Test Male 10 | 0.75 | ***218*** | Train 14 | Test Male 10 | 0.72 |
| ***27*** | Train 2 | Test Male 11 | 0.74 | ***91*** | Train 6 | Test Male 11 | 0.74 | ***155*** | Train 10 | Test Male 11 | 0.74 | ***219*** | Train 14 | Test Male 11 | 0.71 |
| ***28*** | Train 2 | Test Male 12 | 0.75 | ***92*** | Train 6 | Test Male 12 | 0.75 | ***156*** | Train 10 | Test Male 12 | 0.75 | ***220*** | Train 14 | Test Male 12 | 0.73 |
| ***29*** | Train 2 | Test Male 13 | 0.74 | ***93*** | Train 6 | Test Male 13 | 0.74 | ***157*** | Train 10 | Test Male 13 | 0.74 | ***221*** | Train 14 | Test Male 13 | 0.74 |
| ***30*** | Train 2 | Test Male 14 | 0.74 | ***94*** | Train 6 | Test Male 14 | 0.74 | ***158*** | Train 10 | Test Male 14 | 0.74 | ***222*** | Train 14 | Test Male 14 | 0.75 |
| ***31*** | Train 2 | Test Male 15 | 0.74 | ***95*** | Train 6 | Test Male 15 | 0.74 | ***159*** | Train 10 | Test Male 15 | 0.74 | ***223*** | Train 14 | Test Male 15 | 0.74 |
| ***32*** | Train 2 | Test Male 16 | 0.74 | ***96*** | Train 6 | Test Male 16 | 0.74 | ***160*** | Train 10 | Test Male 16 | 0.74 | ***224*** | Train 14 | Test Male 16 | 0.75 |
| ***33*** | Train 3 | Test Male 1 | 0.75 | ***97*** | Train 7 | Test Male 1 | 0.75 | ***161*** | Train 11 | Test Male 1 | 0.74 | ***225*** | Train 15 | Test Male 1 | 0.72 |
| ***34*** | Train 3 | Test Male 2 | 0.7 | ***98*** | Train 7 | Test Male 2 | 0.7 | ***162*** | Train 11 | Test Male 2 | 0.7 | ***226*** | Train 15 | Test Male 2 | 0.69 |
| ***35*** | Train 3 | Test Male 3 | 0.74 | ***99*** | Train 7 | Test Male 3 | 0.74 | ***163*** | Train 11 | Test Male 3 | 0.74 | ***227*** | Train 15 | Test Male 3 | 0.72 |
| ***36*** | Train 3 | Test Male 4 | 0.7 | ***100*** | Train 7 | Test Male 4 | 0.7 | ***164*** | Train 11 | Test Male 4 | 0.7 | ***228*** | Train 15 | Test Male 4 | 0.69 |
| ***37*** | Train 3 | Test Male 5 | 0.75 | ***101*** | Train 7 | Test Male 5 | 0.75 | ***165*** | Train 11 | Test Male 5 | 0.74 | ***229*** | Train 15 | Test Male 5 | 0.72 |
| ***38*** | Train 3 | Test Male 6 | 0.7 | ***102*** | Train 7 | Test Male 6 | 0.7 | ***166*** | Train 11 | Test Male 6 | 0.7 | ***230*** | Train 15 | Test Male 6 | 0.69 |
| ***39*** | Train 3 | Test Male 7 | 0.74 | ***103*** | Train 7 | Test Male 7 | 0.74 | ***167*** | Train 11 | Test Male 7 | 0.74 | ***231*** | Train 15 | Test Male 7 | 0.72 |
| ***40*** | Train 3 | Test Male 8 | 0.7 | ***104*** | Train 7 | Test Male 8 | 0.7 | ***168*** | Train 11 | Test Male 8 | 0.7 | ***232*** | Train 15 | Test Male 8 | 0.69 |
| ***41*** | Train 3 | Test Male 9 | 0.74 | ***105*** | Train 7 | Test Male 9 | 0.74 | ***169*** | Train 11 | Test Male 9 | 0.74 | ***233*** | Train 15 | Test Male 9 | 0.72 |
| ***42*** | Train 3 | Test Male 10 | 0.7 | ***106*** | Train 7 | Test Male 10 | 0.7 | ***170*** | Train 11 | Test Male 10 | 0.7 | ***234*** | Train 15 | Test Male 10 | 0.69 |
| ***43*** | Train 3 | Test Male 11 | 0.74 | ***107*** | Train 7 | Test Male 11 | 0.74 | ***171*** | Train 11 | Test Male 11 | 0.74 | ***235*** | Train 15 | Test Male 11 | 0.72 |
| ***44*** | Train 3 | Test Male 12 | 0.7 | ***108*** | Train 7 | Test Male 12 | 0.7 | ***172*** | Train 11 | Test Male 12 | 0.7 | ***236*** | Train 15 | Test Male 12 | 0.69 |
| ***45*** | Train 3 | Test Male 13 | 0.74 | ***109*** | Train 7 | Test Male 13 | 0.74 | ***173*** | Train 11 | Test Male 13 | 0.74 | ***237*** | Train 15 | Test Male 13 | 0.74 |
| ***46*** | Train 3 | Test Male 14 | 0.69 | ***110*** | Train 7 | Test Male 14 | 0.69 | ***174*** | Train 11 | Test Male 14 | 0.69 | ***238*** | Train 15 | Test Male 14 | 0.69 |
| ***47*** | Train 3 | Test Male 15 | 0.74 | ***111*** | Train 7 | Test Male 15 | 0.74 | ***175*** | Train 11 | Test Male 15 | 0.74 | ***239*** | Train 15 | Test Male 15 | 0.74 |
| ***48*** | Train 3 | Test Male 16 | 0.69 | ***112*** | Train 7 | Test Male 16 | 0.69 | ***176*** | Train 11 | Test Male 16 | 0.69 | ***240*** | Train 15 | Test Male 16 | 0.69 |
| ***49*** | Train 4 | Test Male 1 | 0.74 | ***113*** | Train 8 | Test Male 1 | 0.74 | ***177*** | Train 12 | Test Male 1 | 0.74 | ***241*** | Train 16 | Test Male 1 | 0.71 |
| ***50*** | Train 4 | Test Male 2 | 0.75 | ***114*** | Train 8 | Test Male 2 | 0.75 | ***178*** | Train 12 | Test Male 2 | 0.75 | ***242*** | Train 16 | Test Male 2 | 0.73 |
| ***51*** | Train 4 | Test Male 3 | 0.74 | ***115*** | Train 8 | Test Male 3 | 0.74 | ***179*** | Train 12 | Test Male 3 | 0.74 | ***243*** | Train 16 | Test Male 3 | 0.71 |
| ***52*** | Train 4 | Test Male 4 | 0.75 | ***116*** | Train 8 | Test Male 4 | 0.75 | ***180*** | Train 12 | Test Male 4 | 0.75 | ***244*** | Train 16 | Test Male 4 | 0.73 |
| ***53*** | Train 4 | Test Male 5 | 0.74 | ***117*** | Train 8 | Test Male 5 | 0.74 | ***181*** | Train 12 | Test Male 5 | 0.74 | ***245*** | Train 16 | Test Male 5 | 0.71 |
| ***54*** | Train 4 | Test Male 6 | 0.75 | ***118*** | Train 8 | Test Male 6 | 0.75 | ***182*** | Train 12 | Test Male 6 | 0.75 | ***246*** | Train 16 | Test Male 6 | 0.73 |
| ***55*** | Train 4 | Test Male 7 | 0.74 | ***119*** | Train 8 | Test Male 7 | 0.74 | ***183*** | Train 12 | Test Male 7 | 0.74 | ***247*** | Train 16 | Test Male 7 | 0.71 |
| ***56*** | Train 4 | Test Male 8 | 0.75 | ***120*** | Train 8 | Test Male 8 | 0.75 | ***184*** | Train 12 | Test Male 8 | 0.75 | ***248*** | Train 16 | Test Male 8 | 0.73 |
| ***57*** | Train 4 | Test Male 9 | 0.74 | ***121*** | Train 8 | Test Male 9 | 0.74 | ***185*** | Train 12 | Test Male 9 | 0.74 | ***249*** | Train 16 | Test Male 9 | 0.71 |
| ***58*** | Train 4 | Test Male 10 | 0.75 | ***122*** | Train 8 | Test Male 10 | 0.75 | ***186*** | Train 12 | Test Male 10 | 0.75 | ***250*** | Train 16 | Test Male 10 | 0.73 |
| ***59*** | Train 4 | Test Male 11 | 0.74 | ***123*** | Train 8 | Test Male 11 | 0.74 | ***187*** | Train 12 | Test Male 11 | 0.74 | ***251*** | Train 16 | Test Male 11 | 0.71 |
| ***60*** | Train 4 | Test Male 12 | 0.75 | ***124*** | Train 8 | Test Male 12 | 0.75 | ***188*** | Train 12 | Test Male 12 | 0.75 | ***252*** | Train 16 | Test Male 12 | 0.73 |
| ***61*** | Train 4 | Test Male 13 | 0.74 | ***125*** | Train 8 | Test Male 13 | 0.74 | ***189*** | Train 12 | Test Male 13 | 0.74 | ***253*** | Train 16 | Test Male 13 | 0.74 |
| ***62*** | Train 4 | Test Male 14 | 0.74 | ***126*** | Train 8 | Test Male 14 | 0.74 | ***190*** | Train 12 | Test Male 14 | 0.74 | ***254*** | Train 16 | Test Male 14 | 0.75 |
| ***63*** | Train 4 | Test Male 15 | 0.74 | ***127*** | Train 8 | Test Male 15 | 0.74 | ***191*** | Train 12 | Test Male 15 | 0.74 | ***255*** | Train 16 | Test Male 15 | 0.74 |
| ***64*** | Train 4 | Test Male 16 | 0.74 | ***128*** | Train 8 | Test Male 16 | 0.74 | ***192*** | Train 12 | Test Male 16 | 0.74 | ***256*** | Train 16 | Test Male 16 | 0.75 |

Table 9. Gradient Boosting (GB) AUC Performance for Male Patients Across 16 Cohorts.

| Original Decisions | Train Model | Test Dataset | AUC (GB) | ***Male*** | Train Model | Test Dataset | AUC (GB) | Male | Train Model | Test Dataset | AUC (GB) | Male | Train Model | Test Dataset | AUC (GB) |
| --- | --- | --- | --- | --- | --- | --- | --- | --- | --- | --- | --- | --- | --- | --- | --- |
| ***1*** | Train 1 | Test Male 1 | 0.74 | ***65*** | Train 5 | Test Male 1 | 0.74 | ***129*** | Train 9 | Test Male 1 | 0.74 | ***193*** | Train 13 | Test Male 1 | 0.67 |
| ***2*** | Train 1 | Test Male 2 | 0.68 | ***66*** | Train 5 | Test Male 2 | 0.69 | ***130*** | Train 9 | Test Male 2 | 0.69 | ***194*** | Train 13 | Test Male 2 | 0.65 |
| ***3*** | Train 1 | Test Male 3 | 0.74 | ***67*** | Train 5 | Test Male 3 | 0.74 | ***131*** | Train 9 | Test Male 3 | 0.74 | ***195*** | Train 13 | Test Male 3 | 0.67 |
| ***4*** | Train 1 | Test Male 4 | 0.68 | ***68*** | Train 5 | Test Male 4 | 0.69 | ***132*** | Train 9 | Test Male 4 | 0.69 | ***196*** | Train 13 | Test Male 4 | 0.65 |
| ***5*** | Train 1 | Test Male 5 | 0.74 | ***69*** | Train 5 | Test Male 5 | 0.74 | ***133*** | Train 9 | Test Male 5 | 0.74 | ***197*** | Train 13 | Test Male 5 | 0.67 |
| ***6*** | Train 1 | Test Male 6 | 0.68 | ***70*** | Train 5 | Test Male 6 | 0.69 | ***134*** | Train 9 | Test Male 6 | 0.69 | ***198*** | Train 13 | Test Male 6 | 0.65 |
| ***7*** | Train 1 | Test Male 7 | 0.74 | ***71*** | Train 5 | Test Male 7 | 0.74 | ***135*** | Train 9 | Test Male 7 | 0.74 | ***199*** | Train 13 | Test Male 7 | 0.67 |
| ***8*** | Train 1 | Test Male 8 | 0.68 | ***72*** | Train 5 | Test Male 8 | 0.69 | ***136*** | Train 9 | Test Male 8 | 0.69 | ***200*** | Train 13 | Test Male 8 | 0.65 |
| ***9*** | Train 1 | Test Male 9 | 0.74 | ***73*** | Train 5 | Test Male 9 | 0.74 | ***137*** | Train 9 | Test Male 9 | 0.74 | ***201*** | Train 13 | Test Male 9 | 0.67 |
| ***10*** | Train 1 | Test Male 10 | 0.68 | ***74*** | Train 5 | Test Male 10 | 0.69 | ***138*** | Train 9 | Test Male 10 | 0.69 | ***202*** | Train 13 | Test Male 10 | 0.65 |
| ***11*** | Train 1 | Test Male 11 | 0.74 | ***75*** | Train 5 | Test Male 11 | 0.74 | ***139*** | Train 9 | Test Male 11 | 0.74 | ***203*** | Train 13 | Test Male 11 | 0.67 |
| ***12*** | Train 1 | Test Male 12 | 0.68 | ***76*** | Train 5 | Test Male 12 | 0.69 | ***140*** | Train 9 | Test Male 12 | 0.69 | ***204*** | Train 13 | Test Male 12 | 0.65 |
| ***13*** | Train 1 | Test Male 13 | 0.74 | ***77*** | Train 5 | Test Male 13 | 0.74 | ***141*** | Train 9 | Test Male 13 | 0.73 | ***205*** | Train 13 | Test Male 13 | 0.73 |
| ***14*** | Train 1 | Test Male 14 | 0.68 | ***78*** | Train 5 | Test Male 14 | 0.69 | ***142*** | Train 9 | Test Male 14 | 0.69 | ***206*** | Train 13 | Test Male 14 | 0.67 |
| ***15*** | Train 1 | Test Male 15 | 0.74 | ***79*** | Train 5 | Test Male 15 | 0.74 | ***143*** | Train 9 | Test Male 15 | 0.73 | ***207*** | Train 13 | Test Male 15 | 0.73 |
| ***16*** | Train 1 | Test Male 16 | 0.68 | ***80*** | Train 5 | Test Male 16 | 0.69 | ***144*** | Train 9 | Test Male 16 | 0.69 | ***208*** | Train 13 | Test Male 16 | 0.67 |
| ***17*** | Train 2 | Test Male 1 | 0.74 | ***81*** | Train 6 | Test Male 1 | 0.74 | ***145*** | Train 10 | Test Male 1 | 0.74 | ***209*** | Train 14 | Test Male 1 | 0.68 |
| ***18*** | Train 2 | Test Male 2 | 0.75 | ***82*** | Train 6 | Test Male 2 | 0.75 | ***146*** | Train 10 | Test Male 2 | 0.75 | ***210*** | Train 14 | Test Male 2 | 0.7 |
| ***19*** | Train 2 | Test Male 3 | 0.74 | ***83*** | Train 6 | Test Male 3 | 0.74 | ***147*** | Train 10 | Test Male 3 | 0.74 | ***211*** | Train 14 | Test Male 3 | 0.69 |
| ***20*** | Train 2 | Test Male 4 | 0.75 | ***84*** | Train 6 | Test Male 4 | 0.75 | ***148*** | Train 10 | Test Male 4 | 0.75 | ***212*** | Train 14 | Test Male 4 | 0.7 |
| ***21*** | Train 2 | Test Male 5 | 0.74 | ***85*** | Train 6 | Test Male 5 | 0.74 | ***149*** | Train 10 | Test Male 5 | 0.74 | ***213*** | Train 14 | Test Male 5 | 0.69 |
| ***22*** | Train 2 | Test Male 6 | 0.75 | ***86*** | Train 6 | Test Male 6 | 0.75 | ***150*** | Train 10 | Test Male 6 | 0.75 | ***214*** | Train 14 | Test Male 6 | 0.7 |
| ***23*** | Train 2 | Test Male 7 | 0.74 | ***87*** | Train 6 | Test Male 7 | 0.74 | ***151*** | Train 10 | Test Male 7 | 0.74 | ***215*** | Train 14 | Test Male 7 | 0.69 |
| ***24*** | Train 2 | Test Male 8 | 0.75 | ***88*** | Train 6 | Test Male 8 | 0.75 | ***152*** | Train 10 | Test Male 8 | 0.75 | ***216*** | Train 14 | Test Male 8 | 0.7 |
| ***25*** | Train 2 | Test Male 9 | 0.74 | ***89*** | Train 6 | Test Male 9 | 0.74 | ***153*** | Train 10 | Test Male 9 | 0.74 | ***217*** | Train 14 | Test Male 9 | 0.68 |
| ***26*** | Train 2 | Test Male 10 | 0.75 | ***90*** | Train 6 | Test Male 10 | 0.75 | ***154*** | Train 10 | Test Male 10 | 0.75 | ***218*** | Train 14 | Test Male 10 | 0.7 |
| ***27*** | Train 2 | Test Male 11 | 0.74 | ***91*** | Train 6 | Test Male 11 | 0.74 | ***155*** | Train 10 | Test Male 11 | 0.74 | ***219*** | Train 14 | Test Male 11 | 0.69 |
| ***28*** | Train 2 | Test Male 12 | 0.75 | ***92*** | Train 6 | Test Male 12 | 0.75 | ***156*** | Train 10 | Test Male 12 | 0.75 | ***220*** | Train 14 | Test Male 12 | 0.7 |
| ***29*** | Train 2 | Test Male 13 | 0.74 | ***93*** | Train 6 | Test Male 13 | 0.74 | ***157*** | Train 10 | Test Male 13 | 0.74 | ***221*** | Train 14 | Test Male 13 | 0.74 |
| ***30*** | Train 2 | Test Male 14 | 0.74 | ***94*** | Train 6 | Test Male 14 | 0.75 | ***158*** | Train 10 | Test Male 14 | 0.75 | ***222*** | Train 14 | Test Male 14 | 0.75 |
| ***31*** | Train 2 | Test Male 15 | 0.74 | ***95*** | Train 6 | Test Male 15 | 0.74 | ***159*** | Train 10 | Test Male 15 | 0.74 | ***223*** | Train 14 | Test Male 15 | 0.74 |
| ***32*** | Train 2 | Test Male 16 | 0.74 | ***96*** | Train 6 | Test Male 16 | 0.75 | ***160*** | Train 10 | Test Male 16 | 0.75 | ***224*** | Train 14 | Test Male 16 | 0.75 |
| ***33*** | Train 3 | Test Male 1 | 0.75 | ***97*** | Train 7 | Test Male 1 | 0.74 | ***161*** | Train 11 | Test Male 1 | 0.74 | ***225*** | Train 15 | Test Male 1 | 0.68 |
| ***34*** | Train 3 | Test Male 2 | 0.68 | ***98*** | Train 7 | Test Male 2 | 0.69 | ***162*** | Train 11 | Test Male 2 | 0.68 | ***226*** | Train 15 | Test Male 2 | 0.65 |
| ***35*** | Train 3 | Test Male 3 | 0.75 | ***99*** | Train 7 | Test Male 3 | 0.74 | ***163*** | Train 11 | Test Male 3 | 0.74 | ***227*** | Train 15 | Test Male 3 | 0.68 |
| ***36*** | Train 3 | Test Male 4 | 0.68 | ***100*** | Train 7 | Test Male 4 | 0.69 | ***164*** | Train 11 | Test Male 4 | 0.68 | ***228*** | Train 15 | Test Male 4 | 0.66 |
| ***37*** | Train 3 | Test Male 5 | 0.75 | ***101*** | Train 7 | Test Male 5 | 0.74 | ***165*** | Train 11 | Test Male 5 | 0.74 | ***229*** | Train 15 | Test Male 5 | 0.68 |
| ***38*** | Train 3 | Test Male 6 | 0.68 | ***102*** | Train 7 | Test Male 6 | 0.69 | ***166*** | Train 11 | Test Male 6 | 0.68 | ***230*** | Train 15 | Test Male 6 | 0.66 |
| ***39*** | Train 3 | Test Male 7 | 0.75 | ***103*** | Train 7 | Test Male 7 | 0.74 | ***167*** | Train 11 | Test Male 7 | 0.74 | ***231*** | Train 15 | Test Male 7 | 0.68 |
| ***40*** | Train 3 | Test Male 8 | 0.68 | ***104*** | Train 7 | Test Male 8 | 0.69 | ***168*** | Train 11 | Test Male 8 | 0.68 | ***232*** | Train 15 | Test Male 8 | 0.66 |
| ***41*** | Train 3 | Test Male 9 | 0.74 | ***105*** | Train 7 | Test Male 9 | 0.74 | ***169*** | Train 11 | Test Male 9 | 0.74 | ***233*** | Train 15 | Test Male 9 | 0.68 |
| ***42*** | Train 3 | Test Male 10 | 0.68 | ***106*** | Train 7 | Test Male 10 | 0.69 | ***170*** | Train 11 | Test Male 10 | 0.68 | ***234*** | Train 15 | Test Male 10 | 0.65 |
| ***43*** | Train 3 | Test Male 11 | 0.74 | ***107*** | Train 7 | Test Male 11 | 0.74 | ***171*** | Train 11 | Test Male 11 | 0.74 | ***235*** | Train 15 | Test Male 11 | 0.68 |
| ***44*** | Train 3 | Test Male 12 | 0.68 | ***108*** | Train 7 | Test Male 12 | 0.69 | ***172*** | Train 11 | Test Male 12 | 0.68 | ***236*** | Train 15 | Test Male 12 | 0.66 |
| ***45*** | Train 3 | Test Male 13 | 0.74 | ***109*** | Train 7 | Test Male 13 | 0.74 | ***173*** | Train 11 | Test Male 13 | 0.73 | ***237*** | Train 15 | Test Male 13 | 0.73 |
| ***46*** | Train 3 | Test Male 14 | 0.66 | ***110*** | Train 7 | Test Male 14 | 0.68 | ***174*** | Train 11 | Test Male 14 | 0.66 | ***238*** | Train 15 | Test Male 14 | 0.68 |
| ***47*** | Train 3 | Test Male 15 | 0.74 | ***111*** | Train 7 | Test Male 15 | 0.74 | ***175*** | Train 11 | Test Male 15 | 0.73 | ***239*** | Train 15 | Test Male 15 | 0.73 |
| ***48*** | Train 3 | Test Male 16 | 0.67 | ***112*** | Train 7 | Test Male 16 | 0.68 | ***176*** | Train 11 | Test Male 16 | 0.66 | ***240*** | Train 15 | Test Male 16 | 0.68 |
| ***49*** | Train 4 | Test Male 1 | 0.75 | ***113*** | Train 8 | Test Male 1 | 0.74 | ***177*** | Train 12 | Test Male 1 | 0.74 | ***241*** | Train 16 | Test Male 1 | 0.65 |
| ***50*** | Train 4 | Test Male 2 | 0.75 | ***114*** | Train 8 | Test Male 2 | 0.75 | ***178*** | Train 12 | Test Male 2 | 0.75 | ***242*** | Train 16 | Test Male 2 | 0.68 |
| ***51*** | Train 4 | Test Male 3 | 0.75 | ***115*** | Train 8 | Test Male 3 | 0.74 | ***179*** | Train 12 | Test Male 3 | 0.74 | ***243*** | Train 16 | Test Male 3 | 0.65 |
| ***52*** | Train 4 | Test Male 4 | 0.75 | ***116*** | Train 8 | Test Male 4 | 0.75 | ***180*** | Train 12 | Test Male 4 | 0.75 | ***244*** | Train 16 | Test Male 4 | 0.68 |
| ***53*** | Train 4 | Test Male 5 | 0.75 | ***117*** | Train 8 | Test Male 5 | 0.74 | ***181*** | Train 12 | Test Male 5 | 0.74 | ***245*** | Train 16 | Test Male 5 | 0.65 |
| ***54*** | Train 4 | Test Male 6 | 0.75 | ***118*** | Train 8 | Test Male 6 | 0.75 | ***182*** | Train 12 | Test Male 6 | 0.75 | ***246*** | Train 16 | Test Male 6 | 0.68 |
| ***55*** | Train 4 | Test Male 7 | 0.75 | ***119*** | Train 8 | Test Male 7 | 0.74 | ***183*** | Train 12 | Test Male 7 | 0.74 | ***247*** | Train 16 | Test Male 7 | 0.65 |
| ***56*** | Train 4 | Test Male 8 | 0.75 | ***120*** | Train 8 | Test Male 8 | 0.75 | ***184*** | Train 12 | Test Male 8 | 0.75 | ***248*** | Train 16 | Test Male 8 | 0.68 |
| ***57*** | Train 4 | Test Male 9 | 0.74 | ***121*** | Train 8 | Test Male 9 | 0.74 | ***185*** | Train 12 | Test Male 9 | 0.74 | ***249*** | Train 16 | Test Male 9 | 0.65 |
| ***58*** | Train 4 | Test Male 10 | 0.75 | ***122*** | Train 8 | Test Male 10 | 0.75 | ***186*** | Train 12 | Test Male 10 | 0.75 | ***250*** | Train 16 | Test Male 10 | 0.68 |
| ***59*** | Train 4 | Test Male 11 | 0.74 | ***123*** | Train 8 | Test Male 11 | 0.74 | ***187*** | Train 12 | Test Male 11 | 0.74 | ***251*** | Train 16 | Test Male 11 | 0.65 |
| ***60*** | Train 4 | Test Male 12 | 0.75 | ***124*** | Train 8 | Test Male 12 | 0.75 | ***188*** | Train 12 | Test Male 12 | 0.75 | ***252*** | Train 16 | Test Male 12 | 0.68 |
| ***61*** | Train 4 | Test Male 13 | 0.74 | ***125*** | Train 8 | Test Male 13 | 0.74 | ***189*** | Train 12 | Test Male 13 | 0.74 | ***253*** | Train 16 | Test Male 13 | 0.74 |
| ***62*** | Train 4 | Test Male 14 | 0.74 | ***126*** | Train 8 | Test Male 14 | 0.74 | ***190*** | Train 12 | Test Male 14 | 0.75 | ***254*** | Train 16 | Test Male 14 | 0.75 |
| ***63*** | Train 4 | Test Male 15 | 0.74 | ***127*** | Train 8 | Test Male 15 | 0.74 | ***191*** | Train 12 | Test Male 15 | 0.74 | ***255*** | Train 16 | Test Male 15 | 0.74 |
| ***64*** | Train 4 | Test Male 16 | 0.74 | ***128*** | Train 8 | Test Male 16 | 0.74 | ***192*** | Train 12 | Test Male 16 | 0.75 | ***256*** | Train 16 | Test Male 16 | 0.75 |

Table 10. Logistic Regression (LR) AUC Performance for Female Patients Across 16 Cohorts.

| Original Decisions | Train Model | Test Dataset | AUC (LR) | ***Female*** | Train Model | Test Dataset | AUC (LR) | Female | Train Model | Test Dataset | AUC (LR) | Female | Train Model | Test Dataset | AUC (LR) |
| --- | --- | --- | --- | --- | --- | --- | --- | --- | --- | --- | --- | --- | --- | --- | --- |
| ***1*** | Train 1 | Test Female 1 | 0.73 | ***65*** | Train 5 | Test Female 1 | 0.73 | ***129*** | Train 9 | Test Female 1 | 0.73 | ***193*** | Train 13 | Test Female 1 | 0.71 |
| ***2*** | Train 1 | Test Female 2 | 0.72 | ***66*** | Train 5 | Test Female 2 | 0.72 | ***130*** | Train 9 | Test Female 2 | 0.72 | ***194*** | Train 13 | Test Female 2 | 0.71 |
| ***3*** | Train 1 | Test Female 3 | 0.73 | ***67*** | Train 5 | Test Female 3 | 0.73 | ***131*** | Train 9 | Test Female 3 | 0.73 | ***195*** | Train 13 | Test Female 3 | 0.71 |
| ***4*** | Train 1 | Test Female 4 | 0.72 | ***68*** | Train 5 | Test Female 4 | 0.72 | ***132*** | Train 9 | Test Female 4 | 0.72 | ***196*** | Train 13 | Test Female 4 | 0.71 |
| ***5*** | Train 1 | Test Female 5 | 0.73 | ***69*** | Train 5 | Test Female 5 | 0.73 | ***133*** | Train 9 | Test Female 5 | 0.73 | ***197*** | Train 13 | Test Female 5 | 0.71 |
| ***6*** | Train 1 | Test Female 6 | 0.72 | ***70*** | Train 5 | Test Female 6 | 0.72 | ***134*** | Train 9 | Test Female 6 | 0.72 | ***198*** | Train 13 | Test Female 6 | 0.71 |
| ***7*** | Train 1 | Test Female 7 | 0.73 | ***71*** | Train 5 | Test Female 7 | 0.73 | ***135*** | Train 9 | Test Female 7 | 0.73 | ***199*** | Train 13 | Test Female 7 | 0.72 |
| ***8*** | Train 1 | Test Female 8 | 0.72 | ***72*** | Train 5 | Test Female 8 | 0.72 | ***136*** | Train 9 | Test Female 8 | 0.72 | ***200*** | Train 13 | Test Female 8 | 0.71 |
| ***9*** | Train 1 | Test Female 9 | 0.74 | ***73*** | Train 5 | Test Female 9 | 0.73 | ***137*** | Train 9 | Test Female 9 | 0.73 | ***201*** | Train 13 | Test Female 9 | 0.72 |
| ***10*** | Train 1 | Test Female 10 | 0.72 | ***74*** | Train 5 | Test Female 10 | 0.72 | ***138*** | Train 9 | Test Female 10 | 0.72 | ***202*** | Train 13 | Test Female 10 | 0.71 |
| ***11*** | Train 1 | Test Female 11 | 0.73 | ***75*** | Train 5 | Test Female 11 | 0.73 | ***139*** | Train 9 | Test Female 11 | 0.73 | ***203*** | Train 13 | Test Female 11 | 0.72 |
| ***12*** | Train 1 | Test Female 12 | 0.72 | ***76*** | Train 5 | Test Female 12 | 0.72 | ***140*** | Train 9 | Test Female 12 | 0.72 | ***204*** | Train 13 | Test Female 12 | 0.72 |
| ***13*** | Train 1 | Test Female 13 | 0.75 | ***77*** | Train 5 | Test Female 13 | 0.75 | ***141*** | Train 9 | Test Female 13 | 0.75 | ***205*** | Train 13 | Test Female 13 | 0.75 |
| ***14*** | Train 1 | Test Female 14 | 0.73 | ***78*** | Train 5 | Test Female 14 | 0.73 | ***142*** | Train 9 | Test Female 14 | 0.73 | ***206*** | Train 13 | Test Female 14 | 0.73 |
| ***15*** | Train 1 | Test Female 15 | 0.75 | ***79*** | Train 5 | Test Female 15 | 0.75 | ***143*** | Train 9 | Test Female 15 | 0.75 | ***207*** | Train 13 | Test Female 15 | 0.75 |
| ***16*** | Train 1 | Test Female 16 | 0.73 | ***80*** | Train 5 | Test Female 16 | 0.73 | ***144*** | Train 9 | Test Female 16 | 0.73 | ***208*** | Train 13 | Test Female 16 | 0.73 |
| ***17*** | Train 2 | Test Female 1 | 0.72 | ***81*** | Train 6 | Test Female 1 | 0.72 | ***145*** | Train 10 | Test Female 1 | 0.72 | ***209*** | Train 14 | Test Female 1 | 0.7 |
| ***18*** | Train 2 | Test Female 2 | 0.74 | ***82*** | Train 6 | Test Female 2 | 0.74 | ***146*** | Train 10 | Test Female 2 | 0.74 | ***210*** | Train 14 | Test Female 2 | 0.73 |
| ***19*** | Train 2 | Test Female 3 | 0.72 | ***83*** | Train 6 | Test Female 3 | 0.72 | ***147*** | Train 10 | Test Female 3 | 0.72 | ***211*** | Train 14 | Test Female 3 | 0.7 |
| ***20*** | Train 2 | Test Female 4 | 0.74 | ***84*** | Train 6 | Test Female 4 | 0.74 | ***148*** | Train 10 | Test Female 4 | 0.74 | ***212*** | Train 14 | Test Female 4 | 0.73 |
| ***21*** | Train 2 | Test Female 5 | 0.72 | ***85*** | Train 6 | Test Female 5 | 0.72 | ***149*** | Train 10 | Test Female 5 | 0.72 | ***213*** | Train 14 | Test Female 5 | 0.71 |
| ***22*** | Train 2 | Test Female 6 | 0.74 | ***86*** | Train 6 | Test Female 6 | 0.74 | ***150*** | Train 10 | Test Female 6 | 0.74 | ***214*** | Train 14 | Test Female 6 | 0.73 |
| ***23*** | Train 2 | Test Female 7 | 0.72 | ***87*** | Train 6 | Test Female 7 | 0.72 | ***151*** | Train 10 | Test Female 7 | 0.72 | ***215*** | Train 14 | Test Female 7 | 0.71 |
| ***24*** | Train 2 | Test Female 8 | 0.74 | ***88*** | Train 6 | Test Female 8 | 0.74 | ***152*** | Train 10 | Test Female 8 | 0.74 | ***216*** | Train 14 | Test Female 8 | 0.73 |
| ***25*** | Train 2 | Test Female 9 | 0.73 | ***89*** | Train 6 | Test Female 9 | 0.73 | ***153*** | Train 10 | Test Female 9 | 0.73 | ***217*** | Train 14 | Test Female 9 | 0.71 |
| ***26*** | Train 2 | Test Female 10 | 0.74 | ***90*** | Train 6 | Test Female 10 | 0.74 | ***154*** | Train 10 | Test Female 10 | 0.74 | ***218*** | Train 14 | Test Female 10 | 0.73 |
| ***27*** | Train 2 | Test Female 11 | 0.73 | ***91*** | Train 6 | Test Female 11 | 0.73 | ***155*** | Train 10 | Test Female 11 | 0.73 | ***219*** | Train 14 | Test Female 11 | 0.71 |
| ***28*** | Train 2 | Test Female 12 | 0.74 | ***92*** | Train 6 | Test Female 12 | 0.74 | ***156*** | Train 10 | Test Female 12 | 0.74 | ***220*** | Train 14 | Test Female 12 | 0.73 |
| ***29*** | Train 2 | Test Female 13 | 0.75 | ***93*** | Train 6 | Test Female 13 | 0.75 | ***157*** | Train 10 | Test Female 13 | 0.75 | ***221*** | Train 14 | Test Female 13 | 0.75 |
| ***30*** | Train 2 | Test Female 14 | 0.75 | ***94*** | Train 6 | Test Female 14 | 0.75 | ***158*** | Train 10 | Test Female 14 | 0.75 | ***222*** | Train 14 | Test Female 14 | 0.75 |
| ***31*** | Train 2 | Test Female 15 | 0.75 | ***95*** | Train 6 | Test Female 15 | 0.75 | ***159*** | Train 10 | Test Female 15 | 0.75 | ***223*** | Train 14 | Test Female 15 | 0.75 |
| ***32*** | Train 2 | Test Female 16 | 0.75 | ***96*** | Train 6 | Test Female 16 | 0.75 | ***160*** | Train 10 | Test Female 16 | 0.75 | ***224*** | Train 14 | Test Female 16 | 0.75 |
| ***33*** | Train 3 | Test Female 1 | 0.73 | ***97*** | Train 7 | Test Female 1 | 0.73 | ***161*** | Train 11 | Test Female 1 | 0.73 | ***225*** | Train 15 | Test Female 1 | 0.71 |
| ***34*** | Train 3 | Test Female 2 | 0.72 | ***98*** | Train 7 | Test Female 2 | 0.72 | ***162*** | Train 11 | Test Female 2 | 0.72 | ***226*** | Train 15 | Test Female 2 | 0.71 |
| ***35*** | Train 3 | Test Female 3 | 0.73 | ***99*** | Train 7 | Test Female 3 | 0.73 | ***163*** | Train 11 | Test Female 3 | 0.73 | ***227*** | Train 15 | Test Female 3 | 0.72 |
| ***36*** | Train 3 | Test Female 4 | 0.72 | ***100*** | Train 7 | Test Female 4 | 0.72 | ***164*** | Train 11 | Test Female 4 | 0.72 | ***228*** | Train 15 | Test Female 4 | 0.71 |
| ***37*** | Train 3 | Test Female 5 | 0.73 | ***101*** | Train 7 | Test Female 5 | 0.73 | ***165*** | Train 11 | Test Female 5 | 0.73 | ***229*** | Train 15 | Test Female 5 | 0.72 |
| ***38*** | Train 3 | Test Female 6 | 0.72 | ***102*** | Train 7 | Test Female 6 | 0.72 | ***166*** | Train 11 | Test Female 6 | 0.72 | ***230*** | Train 15 | Test Female 6 | 0.71 |
| ***39*** | Train 3 | Test Female 7 | 0.73 | ***103*** | Train 7 | Test Female 7 | 0.73 | ***167*** | Train 11 | Test Female 7 | 0.73 | ***231*** | Train 15 | Test Female 7 | 0.72 |
| ***40*** | Train 3 | Test Female 8 | 0.72 | ***104*** | Train 7 | Test Female 8 | 0.72 | ***168*** | Train 11 | Test Female 8 | 0.72 | ***232*** | Train 15 | Test Female 8 | 0.72 |
| ***41*** | Train 3 | Test Female 9 | 0.74 | ***105*** | Train 7 | Test Female 9 | 0.74 | ***169*** | Train 11 | Test Female 9 | 0.74 | ***233*** | Train 15 | Test Female 9 | 0.72 |
| ***42*** | Train 3 | Test Female 10 | 0.72 | ***106*** | Train 7 | Test Female 10 | 0.72 | ***170*** | Train 11 | Test Female 10 | 0.72 | ***234*** | Train 15 | Test Female 10 | 0.71 |
| ***43*** | Train 3 | Test Female 11 | 0.74 | ***107*** | Train 7 | Test Female 11 | 0.74 | ***171*** | Train 11 | Test Female 11 | 0.74 | ***235*** | Train 15 | Test Female 11 | 0.72 |
| ***44*** | Train 3 | Test Female 12 | 0.72 | ***108*** | Train 7 | Test Female 12 | 0.72 | ***172*** | Train 11 | Test Female 12 | 0.72 | ***236*** | Train 15 | Test Female 12 | 0.72 |
| ***45*** | Train 3 | Test Female 13 | 0.75 | ***109*** | Train 7 | Test Female 13 | 0.75 | ***173*** | Train 11 | Test Female 13 | 0.75 | ***237*** | Train 15 | Test Female 13 | 0.76 |
| ***46*** | Train 3 | Test Female 14 | 0.73 | ***110*** | Train 7 | Test Female 14 | 0.73 | ***174*** | Train 11 | Test Female 14 | 0.73 | ***238*** | Train 15 | Test Female 14 | 0.73 |
| ***47*** | Train 3 | Test Female 15 | 0.75 | ***111*** | Train 7 | Test Female 15 | 0.75 | ***175*** | Train 11 | Test Female 15 | 0.75 | ***239*** | Train 15 | Test Female 15 | 0.76 |
| ***48*** | Train 3 | Test Female 16 | 0.73 | ***112*** | Train 7 | Test Female 16 | 0.73 | ***176*** | Train 11 | Test Female 16 | 0.73 | ***240*** | Train 15 | Test Female 16 | 0.73 |
| ***49*** | Train 4 | Test Female 1 | 0.72 | ***113*** | Train 8 | Test Female 1 | 0.72 | ***177*** | Train 12 | Test Female 1 | 0.72 | ***241*** | Train 16 | Test Female 1 | 0.7 |
| ***50*** | Train 4 | Test Female 2 | 0.74 | ***114*** | Train 8 | Test Female 2 | 0.74 | ***178*** | Train 12 | Test Female 2 | 0.74 | ***242*** | Train 16 | Test Female 2 | 0.73 |
| ***51*** | Train 4 | Test Female 3 | 0.72 | ***115*** | Train 8 | Test Female 3 | 0.72 | ***179*** | Train 12 | Test Female 3 | 0.72 | ***243*** | Train 16 | Test Female 3 | 0.71 |
| ***52*** | Train 4 | Test Female 4 | 0.74 | ***116*** | Train 8 | Test Female 4 | 0.74 | ***180*** | Train 12 | Test Female 4 | 0.74 | ***244*** | Train 16 | Test Female 4 | 0.73 |
| ***53*** | Train 4 | Test Female 5 | 0.72 | ***117*** | Train 8 | Test Female 5 | 0.73 | ***181*** | Train 12 | Test Female 5 | 0.72 | ***245*** | Train 16 | Test Female 5 | 0.71 |
| ***54*** | Train 4 | Test Female 6 | 0.74 | ***118*** | Train 8 | Test Female 6 | 0.74 | ***182*** | Train 12 | Test Female 6 | 0.74 | ***246*** | Train 16 | Test Female 6 | 0.73 |
| ***55*** | Train 4 | Test Female 7 | 0.72 | ***119*** | Train 8 | Test Female 7 | 0.72 | ***183*** | Train 12 | Test Female 7 | 0.72 | ***247*** | Train 16 | Test Female 7 | 0.71 |
| ***56*** | Train 4 | Test Female 8 | 0.74 | ***120*** | Train 8 | Test Female 8 | 0.74 | ***184*** | Train 12 | Test Female 8 | 0.74 | ***248*** | Train 16 | Test Female 8 | 0.73 |
| ***57*** | Train 4 | Test Female 9 | 0.73 | ***121*** | Train 8 | Test Female 9 | 0.73 | ***185*** | Train 12 | Test Female 9 | 0.73 | ***249*** | Train 16 | Test Female 9 | 0.71 |
| ***58*** | Train 4 | Test Female 10 | 0.74 | ***122*** | Train 8 | Test Female 10 | 0.74 | ***186*** | Train 12 | Test Female 10 | 0.74 | ***250*** | Train 16 | Test Female 10 | 0.73 |
| ***59*** | Train 4 | Test Female 11 | 0.73 | ***123*** | Train 8 | Test Female 11 | 0.73 | ***187*** | Train 12 | Test Female 11 | 0.73 | ***251*** | Train 16 | Test Female 11 | 0.71 |
| ***60*** | Train 4 | Test Female 12 | 0.74 | ***124*** | Train 8 | Test Female 12 | 0.74 | ***188*** | Train 12 | Test Female 12 | 0.74 | ***252*** | Train 16 | Test Female 12 | 0.73 |
| ***61*** | Train 4 | Test Female 13 | 0.75 | ***125*** | Train 8 | Test Female 13 | 0.75 | ***189*** | Train 12 | Test Female 13 | 0.75 | ***253*** | Train 16 | Test Female 13 | 0.75 |
| ***62*** | Train 4 | Test Female 14 | 0.75 | ***126*** | Train 8 | Test Female 14 | 0.75 | ***190*** | Train 12 | Test Female 14 | 0.75 | ***254*** | Train 16 | Test Female 14 | 0.75 |
| ***63*** | Train 4 | Test Female 15 | 0.75 | ***127*** | Train 8 | Test Female 15 | 0.75 | ***191*** | Train 12 | Test Female 15 | 0.75 | ***255*** | Train 16 | Test Female 15 | 0.75 |
| ***64*** | Train 4 | Test Female 16 | 0.75 | ***128*** | Train 8 | Test Female 16 | 0.75 | ***192*** | Train 12 | Test Female 16 | 0.75 | ***256*** | Train 16 | Test Female 16 | 0.75 |

Table 11. Random Forest (RF) AUC Performance for Female Patients Across 16 Cohorts.

| Original Decisions | Train Model | Test Dataset | AUC (RF) | ***Female*** | Train Model | Test Dataset | AUC (RF) | Female | Train Model | Test Dataset | AUC (RF) | Female | Train Model | Test Dataset | AUC (RF) |
| --- | --- | --- | --- | --- | --- | --- | --- | --- | --- | --- | --- | --- | --- | --- | --- |
| ***1*** | Train 1 | Test Female 1 | 0.75 | ***65*** | Train 5 | Test Female 1 | 0.75 | ***129*** | Train 9 | Test Female 1 | 0.75 | ***193*** | Train 13 | Test Female 1 | 0.72 |
| ***2*** | Train 1 | Test Female 2 | 0.72 | ***66*** | Train 5 | Test Female 2 | 0.72 | ***130*** | Train 9 | Test Female 2 | 0.72 | ***194*** | Train 13 | Test Female 2 | 0.7 |
| ***3*** | Train 1 | Test Female 3 | 0.75 | ***67*** | Train 5 | Test Female 3 | 0.75 | ***131*** | Train 9 | Test Female 3 | 0.75 | ***195*** | Train 13 | Test Female 3 | 0.72 |
| ***4*** | Train 1 | Test Female 4 | 0.72 | ***68*** | Train 5 | Test Female 4 | 0.72 | ***132*** | Train 9 | Test Female 4 | 0.72 | ***196*** | Train 13 | Test Female 4 | 0.7 |
| ***5*** | Train 1 | Test Female 5 | 0.75 | ***69*** | Train 5 | Test Female 5 | 0.75 | ***133*** | Train 9 | Test Female 5 | 0.75 | ***197*** | Train 13 | Test Female 5 | 0.72 |
| ***6*** | Train 1 | Test Female 6 | 0.72 | ***70*** | Train 5 | Test Female 6 | 0.72 | ***134*** | Train 9 | Test Female 6 | 0.72 | ***198*** | Train 13 | Test Female 6 | 0.7 |
| ***7*** | Train 1 | Test Female 7 | 0.75 | ***71*** | Train 5 | Test Female 7 | 0.75 | ***135*** | Train 9 | Test Female 7 | 0.75 | ***199*** | Train 13 | Test Female 7 | 0.72 |
| ***8*** | Train 1 | Test Female 8 | 0.72 | ***72*** | Train 5 | Test Female 8 | 0.72 | ***136*** | Train 9 | Test Female 8 | 0.72 | ***200*** | Train 13 | Test Female 8 | 0.7 |
| ***9*** | Train 1 | Test Female 9 | 0.75 | ***73*** | Train 5 | Test Female 9 | 0.75 | ***137*** | Train 9 | Test Female 9 | 0.75 | ***201*** | Train 13 | Test Female 9 | 0.72 |
| ***10*** | Train 1 | Test Female 10 | 0.72 | ***74*** | Train 5 | Test Female 10 | 0.72 | ***138*** | Train 9 | Test Female 10 | 0.72 | ***202*** | Train 13 | Test Female 10 | 0.7 |
| ***11*** | Train 1 | Test Female 11 | 0.75 | ***75*** | Train 5 | Test Female 11 | 0.75 | ***139*** | Train 9 | Test Female 11 | 0.75 | ***203*** | Train 13 | Test Female 11 | 0.72 |
| ***12*** | Train 1 | Test Female 12 | 0.72 | ***76*** | Train 5 | Test Female 12 | 0.72 | ***140*** | Train 9 | Test Female 12 | 0.72 | ***204*** | Train 13 | Test Female 12 | 0.7 |
| ***13*** | Train 1 | Test Female 13 | 0.76 | ***77*** | Train 5 | Test Female 13 | 0.76 | ***141*** | Train 9 | Test Female 13 | 0.76 | ***205*** | Train 13 | Test Female 13 | 0.76 |
| ***14*** | Train 1 | Test Female 14 | 0.71 | ***78*** | Train 5 | Test Female 14 | 0.71 | ***142*** | Train 9 | Test Female 14 | 0.71 | ***206*** | Train 13 | Test Female 14 | 0.71 |
| ***15*** | Train 1 | Test Female 15 | 0.76 | ***79*** | Train 5 | Test Female 15 | 0.76 | ***143*** | Train 9 | Test Female 15 | 0.76 | ***207*** | Train 13 | Test Female 15 | 0.76 |
| ***16*** | Train 1 | Test Female 16 | 0.71 | ***80*** | Train 5 | Test Female 16 | 0.72 | ***144*** | Train 9 | Test Female 16 | 0.72 | ***208*** | Train 13 | Test Female 16 | 0.71 |
| ***17*** | Train 2 | Test Female 1 | 0.74 | ***81*** | Train 6 | Test Female 1 | 0.74 | ***145*** | Train 10 | Test Female 1 | 0.74 | ***209*** | Train 14 | Test Female 1 | 0.72 |
| ***18*** | Train 2 | Test Female 2 | 0.76 | ***82*** | Train 6 | Test Female 2 | 0.76 | ***146*** | Train 10 | Test Female 2 | 0.76 | ***210*** | Train 14 | Test Female 2 | 0.74 |
| ***19*** | Train 2 | Test Female 3 | 0.74 | ***83*** | Train 6 | Test Female 3 | 0.74 | ***147*** | Train 10 | Test Female 3 | 0.74 | ***211*** | Train 14 | Test Female 3 | 0.72 |
| ***20*** | Train 2 | Test Female 4 | 0.76 | ***84*** | Train 6 | Test Female 4 | 0.76 | ***148*** | Train 10 | Test Female 4 | 0.76 | ***212*** | Train 14 | Test Female 4 | 0.74 |
| ***21*** | Train 2 | Test Female 5 | 0.74 | ***85*** | Train 6 | Test Female 5 | 0.74 | ***149*** | Train 10 | Test Female 5 | 0.74 | ***213*** | Train 14 | Test Female 5 | 0.72 |
| ***22*** | Train 2 | Test Female 6 | 0.76 | ***86*** | Train 6 | Test Female 6 | 0.76 | ***150*** | Train 10 | Test Female 6 | 0.76 | ***214*** | Train 14 | Test Female 6 | 0.74 |
| ***23*** | Train 2 | Test Female 7 | 0.74 | ***87*** | Train 6 | Test Female 7 | 0.74 | ***151*** | Train 10 | Test Female 7 | 0.74 | ***215*** | Train 14 | Test Female 7 | 0.72 |
| ***24*** | Train 2 | Test Female 8 | 0.76 | ***88*** | Train 6 | Test Female 8 | 0.76 | ***152*** | Train 10 | Test Female 8 | 0.76 | ***216*** | Train 14 | Test Female 8 | 0.74 |
| ***25*** | Train 2 | Test Female 9 | 0.75 | ***89*** | Train 6 | Test Female 9 | 0.75 | ***153*** | Train 10 | Test Female 9 | 0.75 | ***217*** | Train 14 | Test Female 9 | 0.72 |
| ***26*** | Train 2 | Test Female 10 | 0.76 | ***90*** | Train 6 | Test Female 10 | 0.76 | ***154*** | Train 10 | Test Female 10 | 0.76 | ***218*** | Train 14 | Test Female 10 | 0.74 |
| ***27*** | Train 2 | Test Female 11 | 0.75 | ***91*** | Train 6 | Test Female 11 | 0.75 | ***155*** | Train 10 | Test Female 11 | 0.75 | ***219*** | Train 14 | Test Female 11 | 0.72 |
| ***28*** | Train 2 | Test Female 12 | 0.76 | ***92*** | Train 6 | Test Female 12 | 0.76 | ***156*** | Train 10 | Test Female 12 | 0.76 | ***220*** | Train 14 | Test Female 12 | 0.74 |
| ***29*** | Train 2 | Test Female 13 | 0.76 | ***93*** | Train 6 | Test Female 13 | 0.76 | ***157*** | Train 10 | Test Female 13 | 0.76 | ***221*** | Train 14 | Test Female 13 | 0.76 |
| ***30*** | Train 2 | Test Female 14 | 0.76 | ***94*** | Train 6 | Test Female 14 | 0.75 | ***158*** | Train 10 | Test Female 14 | 0.76 | ***222*** | Train 14 | Test Female 14 | 0.76 |
| ***31*** | Train 2 | Test Female 15 | 0.76 | ***95*** | Train 6 | Test Female 15 | 0.76 | ***159*** | Train 10 | Test Female 15 | 0.76 | ***223*** | Train 14 | Test Female 15 | 0.76 |
| ***32*** | Train 2 | Test Female 16 | 0.76 | ***96*** | Train 6 | Test Female 16 | 0.76 | ***160*** | Train 10 | Test Female 16 | 0.76 | ***224*** | Train 14 | Test Female 16 | 0.76 |
| ***33*** | Train 3 | Test Female 1 | 0.75 | ***97*** | Train 7 | Test Female 1 | 0.75 | ***161*** | Train 11 | Test Female 1 | 0.75 | ***225*** | Train 15 | Test Female 1 | 0.72 |
| ***34*** | Train 3 | Test Female 2 | 0.72 | ***98*** | Train 7 | Test Female 2 | 0.72 | ***162*** | Train 11 | Test Female 2 | 0.72 | ***226*** | Train 15 | Test Female 2 | 0.7 |
| ***35*** | Train 3 | Test Female 3 | 0.75 | ***99*** | Train 7 | Test Female 3 | 0.75 | ***163*** | Train 11 | Test Female 3 | 0.75 | ***227*** | Train 15 | Test Female 3 | 0.72 |
| ***36*** | Train 3 | Test Female 4 | 0.72 | ***100*** | Train 7 | Test Female 4 | 0.72 | ***164*** | Train 11 | Test Female 4 | 0.72 | ***228*** | Train 15 | Test Female 4 | 0.7 |
| ***37*** | Train 3 | Test Female 5 | 0.75 | ***101*** | Train 7 | Test Female 5 | 0.75 | ***165*** | Train 11 | Test Female 5 | 0.75 | ***229*** | Train 15 | Test Female 5 | 0.72 |
| ***38*** | Train 3 | Test Female 6 | 0.72 | ***102*** | Train 7 | Test Female 6 | 0.72 | ***166*** | Train 11 | Test Female 6 | 0.72 | ***230*** | Train 15 | Test Female 6 | 0.7 |
| ***39*** | Train 3 | Test Female 7 | 0.75 | ***103*** | Train 7 | Test Female 7 | 0.75 | ***167*** | Train 11 | Test Female 7 | 0.75 | ***231*** | Train 15 | Test Female 7 | 0.72 |
| ***40*** | Train 3 | Test Female 8 | 0.72 | ***104*** | Train 7 | Test Female 8 | 0.72 | ***168*** | Train 11 | Test Female 8 | 0.72 | ***232*** | Train 15 | Test Female 8 | 0.7 |
| ***41*** | Train 3 | Test Female 9 | 0.75 | ***105*** | Train 7 | Test Female 9 | 0.75 | ***169*** | Train 11 | Test Female 9 | 0.75 | ***233*** | Train 15 | Test Female 9 | 0.72 |
| ***42*** | Train 3 | Test Female 10 | 0.72 | ***106*** | Train 7 | Test Female 10 | 0.72 | ***170*** | Train 11 | Test Female 10 | 0.72 | ***234*** | Train 15 | Test Female 10 | 0.7 |
| ***43*** | Train 3 | Test Female 11 | 0.75 | ***107*** | Train 7 | Test Female 11 | 0.75 | ***171*** | Train 11 | Test Female 11 | 0.75 | ***235*** | Train 15 | Test Female 11 | 0.72 |
| ***44*** | Train 3 | Test Female 12 | 0.72 | ***108*** | Train 7 | Test Female 12 | 0.72 | ***172*** | Train 11 | Test Female 12 | 0.72 | ***236*** | Train 15 | Test Female 12 | 0.7 |
| ***45*** | Train 3 | Test Female 13 | 0.75 | ***109*** | Train 7 | Test Female 13 | 0.76 | ***173*** | Train 11 | Test Female 13 | 0.76 | ***237*** | Train 15 | Test Female 13 | 0.76 |
| ***46*** | Train 3 | Test Female 14 | 0.71 | ***110*** | Train 7 | Test Female 14 | 0.71 | ***174*** | Train 11 | Test Female 14 | 0.71 | ***238*** | Train 15 | Test Female 14 | 0.71 |
| ***47*** | Train 3 | Test Female 15 | 0.75 | ***111*** | Train 7 | Test Female 15 | 0.76 | ***175*** | Train 11 | Test Female 15 | 0.76 | ***239*** | Train 15 | Test Female 15 | 0.76 |
| ***48*** | Train 3 | Test Female 16 | 0.71 | ***112*** | Train 7 | Test Female 16 | 0.71 | ***176*** | Train 11 | Test Female 16 | 0.72 | ***240*** | Train 15 | Test Female 16 | 0.71 |
| ***49*** | Train 4 | Test Female 1 | 0.74 | ***113*** | Train 8 | Test Female 1 | 0.74 | ***177*** | Train 12 | Test Female 1 | 0.74 | ***241*** | Train 16 | Test Female 1 | 0.72 |
| ***50*** | Train 4 | Test Female 2 | 0.76 | ***114*** | Train 8 | Test Female 2 | 0.76 | ***178*** | Train 12 | Test Female 2 | 0.76 | ***242*** | Train 16 | Test Female 2 | 0.74 |
| ***51*** | Train 4 | Test Female 3 | 0.74 | ***115*** | Train 8 | Test Female 3 | 0.74 | ***179*** | Train 12 | Test Female 3 | 0.74 | ***243*** | Train 16 | Test Female 3 | 0.72 |
| ***52*** | Train 4 | Test Female 4 | 0.76 | ***116*** | Train 8 | Test Female 4 | 0.76 | ***180*** | Train 12 | Test Female 4 | 0.76 | ***244*** | Train 16 | Test Female 4 | 0.74 |
| ***53*** | Train 4 | Test Female 5 | 0.74 | ***117*** | Train 8 | Test Female 5 | 0.74 | ***181*** | Train 12 | Test Female 5 | 0.74 | ***245*** | Train 16 | Test Female 5 | 0.72 |
| ***54*** | Train 4 | Test Female 6 | 0.76 | ***118*** | Train 8 | Test Female 6 | 0.76 | ***182*** | Train 12 | Test Female 6 | 0.76 | ***246*** | Train 16 | Test Female 6 | 0.74 |
| ***55*** | Train 4 | Test Female 7 | 0.74 | ***119*** | Train 8 | Test Female 7 | 0.74 | ***183*** | Train 12 | Test Female 7 | 0.74 | ***247*** | Train 16 | Test Female 7 | 0.72 |
| ***56*** | Train 4 | Test Female 8 | 0.76 | ***120*** | Train 8 | Test Female 8 | 0.76 | ***184*** | Train 12 | Test Female 8 | 0.76 | ***248*** | Train 16 | Test Female 8 | 0.74 |
| ***57*** | Train 4 | Test Female 9 | 0.75 | ***121*** | Train 8 | Test Female 9 | 0.75 | ***185*** | Train 12 | Test Female 9 | 0.75 | ***249*** | Train 16 | Test Female 9 | 0.72 |
| ***58*** | Train 4 | Test Female 10 | 0.76 | ***122*** | Train 8 | Test Female 10 | 0.76 | ***186*** | Train 12 | Test Female 10 | 0.76 | ***250*** | Train 16 | Test Female 10 | 0.74 |
| ***59*** | Train 4 | Test Female 11 | 0.75 | ***123*** | Train 8 | Test Female 11 | 0.75 | ***187*** | Train 12 | Test Female 11 | 0.75 | ***251*** | Train 16 | Test Female 11 | 0.72 |
| ***60*** | Train 4 | Test Female 12 | 0.76 | ***124*** | Train 8 | Test Female 12 | 0.76 | ***188*** | Train 12 | Test Female 12 | 0.76 | ***252*** | Train 16 | Test Female 12 | 0.74 |
| ***61*** | Train 4 | Test Female 13 | 0.76 | ***125*** | Train 8 | Test Female 13 | 0.76 | ***189*** | Train 12 | Test Female 13 | 0.76 | ***253*** | Train 16 | Test Female 13 | 0.76 |
| ***62*** | Train 4 | Test Female 14 | 0.76 | ***126*** | Train 8 | Test Female 14 | 0.75 | ***190*** | Train 12 | Test Female 14 | 0.75 | ***254*** | Train 16 | Test Female 14 | 0.76 |
| ***63*** | Train 4 | Test Female 15 | 0.76 | ***127*** | Train 8 | Test Female 15 | 0.76 | ***191*** | Train 12 | Test Female 15 | 0.76 | ***255*** | Train 16 | Test Female 15 | 0.76 |
| ***64*** | Train 4 | Test Female 16 | 0.76 | ***128*** | Train 8 | Test Female 16 | 0.76 | ***192*** | Train 12 | Test Female 16 | 0.76 | ***256*** | Train 16 | Test Female 16 | 0.76 |

Table 12. Gradient Boosting (GB) AUC Performance for Female Patients Across 16 Cohorts.

| Original Decisions | Train Model | Test Dataset | AUC (GB) | ***Female*** | Train Model | Test Dataset | AUC (GB) | Female | Train Model | Test Dataset | AUC (GB) | Female | Train Model | Test Dataset | AUC (GB) |
| --- | --- | --- | --- | --- | --- | --- | --- | --- | --- | --- | --- | --- | --- | --- | --- |
| ***1*** | Train 1 | Test Female 1 | 0.75 | ***65*** | Train 5 | Test Female 1 | 0.75 | ***129*** | Train 9 | Test Female 1 | 0.75 | ***193*** | Train 13 | Test Female 1 | 0.69 |
| ***2*** | Train 1 | Test Female 2 | 0.7 | ***66*** | Train 5 | Test Female 2 | 0.69 | ***130*** | Train 9 | Test Female 2 | 0.7 | ***194*** | Train 13 | Test Female 2 | 0.67 |
| ***3*** | Train 1 | Test Female 3 | 0.75 | ***67*** | Train 5 | Test Female 3 | 0.75 | ***131*** | Train 9 | Test Female 3 | 0.75 | ***195*** | Train 13 | Test Female 3 | 0.69 |
| ***4*** | Train 1 | Test Female 4 | 0.7 | ***68*** | Train 5 | Test Female 4 | 0.69 | ***132*** | Train 9 | Test Female 4 | 0.7 | ***196*** | Train 13 | Test Female 4 | 0.67 |
| ***5*** | Train 1 | Test Female 5 | 0.75 | ***69*** | Train 5 | Test Female 5 | 0.75 | ***133*** | Train 9 | Test Female 5 | 0.75 | ***197*** | Train 13 | Test Female 5 | 0.69 |
| ***6*** | Train 1 | Test Female 6 | 0.7 | ***70*** | Train 5 | Test Female 6 | 0.69 | ***134*** | Train 9 | Test Female 6 | 0.7 | ***198*** | Train 13 | Test Female 6 | 0.67 |
| ***7*** | Train 1 | Test Female 7 | 0.75 | ***71*** | Train 5 | Test Female 7 | 0.75 | ***135*** | Train 9 | Test Female 7 | 0.75 | ***199*** | Train 13 | Test Female 7 | 0.69 |
| ***8*** | Train 1 | Test Female 8 | 0.7 | ***72*** | Train 5 | Test Female 8 | 0.69 | ***136*** | Train 9 | Test Female 8 | 0.7 | ***200*** | Train 13 | Test Female 8 | 0.67 |
| ***9*** | Train 1 | Test Female 9 | 0.75 | ***73*** | Train 5 | Test Female 9 | 0.75 | ***137*** | Train 9 | Test Female 9 | 0.75 | ***201*** | Train 13 | Test Female 9 | 0.69 |
| ***10*** | Train 1 | Test Female 10 | 0.7 | ***74*** | Train 5 | Test Female 10 | 0.69 | ***138*** | Train 9 | Test Female 10 | 0.7 | ***202*** | Train 13 | Test Female 10 | 0.67 |
| ***11*** | Train 1 | Test Female 11 | 0.75 | ***75*** | Train 5 | Test Female 11 | 0.75 | ***139*** | Train 9 | Test Female 11 | 0.75 | ***203*** | Train 13 | Test Female 11 | 0.69 |
| ***12*** | Train 1 | Test Female 12 | 0.7 | ***76*** | Train 5 | Test Female 12 | 0.69 | ***140*** | Train 9 | Test Female 12 | 0.7 | ***204*** | Train 13 | Test Female 12 | 0.67 |
| ***13*** | Train 1 | Test Female 13 | 0.75 | ***77*** | Train 5 | Test Female 13 | 0.75 | ***141*** | Train 9 | Test Female 13 | 0.75 | ***205*** | Train 13 | Test Female 13 | 0.75 |
| ***14*** | Train 1 | Test Female 14 | 0.71 | ***78*** | Train 5 | Test Female 14 | 0.69 | ***142*** | Train 9 | Test Female 14 | 0.7 | ***206*** | Train 13 | Test Female 14 | 0.7 |
| ***15*** | Train 1 | Test Female 15 | 0.75 | ***79*** | Train 5 | Test Female 15 | 0.75 | ***143*** | Train 9 | Test Female 15 | 0.75 | ***207*** | Train 13 | Test Female 15 | 0.75 |
| ***16*** | Train 1 | Test Female 16 | 0.71 | ***80*** | Train 5 | Test Female 16 | 0.69 | ***144*** | Train 9 | Test Female 16 | 0.71 | ***208*** | Train 13 | Test Female 16 | 0.7 |
| ***17*** | Train 2 | Test Female 1 | 0.75 | ***81*** | Train 6 | Test Female 1 | 0.75 | ***145*** | Train 10 | Test Female 1 | 0.74 | ***209*** | Train 14 | Test Female 1 | 0.69 |
| ***18*** | Train 2 | Test Female 2 | 0.76 | ***82*** | Train 6 | Test Female 2 | 0.76 | ***146*** | Train 10 | Test Female 2 | 0.76 | ***210*** | Train 14 | Test Female 2 | 0.71 |
| ***19*** | Train 2 | Test Female 3 | 0.74 | ***83*** | Train 6 | Test Female 3 | 0.75 | ***147*** | Train 10 | Test Female 3 | 0.74 | ***211*** | Train 14 | Test Female 3 | 0.69 |
| ***20*** | Train 2 | Test Female 4 | 0.76 | ***84*** | Train 6 | Test Female 4 | 0.76 | ***148*** | Train 10 | Test Female 4 | 0.76 | ***212*** | Train 14 | Test Female 4 | 0.71 |
| ***21*** | Train 2 | Test Female 5 | 0.75 | ***85*** | Train 6 | Test Female 5 | 0.75 | ***149*** | Train 10 | Test Female 5 | 0.75 | ***213*** | Train 14 | Test Female 5 | 0.69 |
| ***22*** | Train 2 | Test Female 6 | 0.76 | ***86*** | Train 6 | Test Female 6 | 0.76 | ***150*** | Train 10 | Test Female 6 | 0.76 | ***214*** | Train 14 | Test Female 6 | 0.71 |
| ***23*** | Train 2 | Test Female 7 | 0.74 | ***87*** | Train 6 | Test Female 7 | 0.75 | ***151*** | Train 10 | Test Female 7 | 0.74 | ***215*** | Train 14 | Test Female 7 | 0.69 |
| ***24*** | Train 2 | Test Female 8 | 0.76 | ***88*** | Train 6 | Test Female 8 | 0.76 | ***152*** | Train 10 | Test Female 8 | 0.76 | ***216*** | Train 14 | Test Female 8 | 0.71 |
| ***25*** | Train 2 | Test Female 9 | 0.75 | ***89*** | Train 6 | Test Female 9 | 0.75 | ***153*** | Train 10 | Test Female 9 | 0.75 | ***217*** | Train 14 | Test Female 9 | 0.69 |
| ***26*** | Train 2 | Test Female 10 | 0.76 | ***90*** | Train 6 | Test Female 10 | 0.76 | ***154*** | Train 10 | Test Female 10 | 0.76 | ***218*** | Train 14 | Test Female 10 | 0.71 |
| ***27*** | Train 2 | Test Female 11 | 0.75 | ***91*** | Train 6 | Test Female 11 | 0.75 | ***155*** | Train 10 | Test Female 11 | 0.75 | ***219*** | Train 14 | Test Female 11 | 0.7 |
| ***28*** | Train 2 | Test Female 12 | 0.76 | ***92*** | Train 6 | Test Female 12 | 0.76 | ***156*** | Train 10 | Test Female 12 | 0.76 | ***220*** | Train 14 | Test Female 12 | 0.71 |
| ***29*** | Train 2 | Test Female 13 | 0.76 | ***93*** | Train 6 | Test Female 13 | 0.76 | ***157*** | Train 10 | Test Female 13 | 0.76 | ***221*** | Train 14 | Test Female 13 | 0.76 |
| ***30*** | Train 2 | Test Female 14 | 0.76 | ***94*** | Train 6 | Test Female 14 | 0.76 | ***158*** | Train 10 | Test Female 14 | 0.76 | ***222*** | Train 14 | Test Female 14 | 0.77 |
| ***31*** | Train 2 | Test Female 15 | 0.76 | ***95*** | Train 6 | Test Female 15 | 0.76 | ***159*** | Train 10 | Test Female 15 | 0.76 | ***223*** | Train 14 | Test Female 15 | 0.76 |
| ***32*** | Train 2 | Test Female 16 | 0.76 | ***96*** | Train 6 | Test Female 16 | 0.76 | ***160*** | Train 10 | Test Female 16 | 0.76 | ***224*** | Train 14 | Test Female 16 | 0.77 |
| ***33*** | Train 3 | Test Female 1 | 0.75 | ***97*** | Train 7 | Test Female 1 | 0.75 | ***161*** | Train 11 | Test Female 1 | 0.75 | ***225*** | Train 15 | Test Female 1 | 0.68 |
| ***34*** | Train 3 | Test Female 2 | 0.68 | ***98*** | Train 7 | Test Female 2 | 0.7 | ***162*** | Train 11 | Test Female 2 | 0.69 | ***226*** | Train 15 | Test Female 2 | 0.67 |
| ***35*** | Train 3 | Test Female 3 | 0.75 | ***99*** | Train 7 | Test Female 3 | 0.75 | ***163*** | Train 11 | Test Female 3 | 0.75 | ***227*** | Train 15 | Test Female 3 | 0.69 |
| ***36*** | Train 3 | Test Female 4 | 0.68 | ***100*** | Train 7 | Test Female 4 | 0.7 | ***164*** | Train 11 | Test Female 4 | 0.69 | ***228*** | Train 15 | Test Female 4 | 0.67 |
| ***37*** | Train 3 | Test Female 5 | 0.75 | ***101*** | Train 7 | Test Female 5 | 0.75 | ***165*** | Train 11 | Test Female 5 | 0.75 | ***229*** | Train 15 | Test Female 5 | 0.68 |
| ***38*** | Train 3 | Test Female 6 | 0.68 | ***102*** | Train 7 | Test Female 6 | 0.7 | ***166*** | Train 11 | Test Female 6 | 0.69 | ***230*** | Train 15 | Test Female 6 | 0.67 |
| ***39*** | Train 3 | Test Female 7 | 0.75 | ***103*** | Train 7 | Test Female 7 | 0.75 | ***167*** | Train 11 | Test Female 7 | 0.75 | ***231*** | Train 15 | Test Female 7 | 0.69 |
| ***40*** | Train 3 | Test Female 8 | 0.68 | ***104*** | Train 7 | Test Female 8 | 0.7 | ***168*** | Train 11 | Test Female 8 | 0.69 | ***232*** | Train 15 | Test Female 8 | 0.67 |
| ***41*** | Train 3 | Test Female 9 | 0.75 | ***105*** | Train 7 | Test Female 9 | 0.75 | ***169*** | Train 11 | Test Female 9 | 0.75 | ***233*** | Train 15 | Test Female 9 | 0.69 |
| ***42*** | Train 3 | Test Female 10 | 0.68 | ***106*** | Train 7 | Test Female 10 | 0.7 | ***170*** | Train 11 | Test Female 10 | 0.69 | ***234*** | Train 15 | Test Female 10 | 0.67 |
| ***43*** | Train 3 | Test Female 11 | 0.75 | ***107*** | Train 7 | Test Female 11 | 0.75 | ***171*** | Train 11 | Test Female 11 | 0.75 | ***235*** | Train 15 | Test Female 11 | 0.69 |
| ***44*** | Train 3 | Test Female 12 | 0.68 | ***108*** | Train 7 | Test Female 12 | 0.7 | ***172*** | Train 11 | Test Female 12 | 0.69 | ***236*** | Train 15 | Test Female 12 | 0.67 |
| ***45*** | Train 3 | Test Female 13 | 0.75 | ***109*** | Train 7 | Test Female 13 | 0.75 | ***173*** | Train 11 | Test Female 13 | 0.75 | ***237*** | Train 15 | Test Female 13 | 0.75 |
| ***46*** | Train 3 | Test Female 14 | 0.68 | ***110*** | Train 7 | Test Female 14 | 0.7 | ***174*** | Train 11 | Test Female 14 | 0.69 | ***238*** | Train 15 | Test Female 14 | 0.7 |
| ***47*** | Train 3 | Test Female 15 | 0.75 | ***111*** | Train 7 | Test Female 15 | 0.75 | ***175*** | Train 11 | Test Female 15 | 0.75 | ***239*** | Train 15 | Test Female 15 | 0.75 |
| ***48*** | Train 3 | Test Female 16 | 0.68 | ***112*** | Train 7 | Test Female 16 | 0.71 | ***176*** | Train 11 | Test Female 16 | 0.69 | ***240*** | Train 15 | Test Female 16 | 0.7 |
| ***49*** | Train 4 | Test Female 1 | 0.75 | ***113*** | Train 8 | Test Female 1 | 0.75 | ***177*** | Train 12 | Test Female 1 | 0.75 | ***241*** | Train 16 | Test Female 1 | 0.66 |
| ***50*** | Train 4 | Test Female 2 | 0.76 | ***114*** | Train 8 | Test Female 2 | 0.76 | ***178*** | Train 12 | Test Female 2 | 0.76 | ***242*** | Train 16 | Test Female 2 | 0.69 |
| ***51*** | Train 4 | Test Female 3 | 0.75 | ***115*** | Train 8 | Test Female 3 | 0.75 | ***179*** | Train 12 | Test Female 3 | 0.75 | ***243*** | Train 16 | Test Female 3 | 0.67 |
| ***52*** | Train 4 | Test Female 4 | 0.76 | ***116*** | Train 8 | Test Female 4 | 0.76 | ***180*** | Train 12 | Test Female 4 | 0.76 | ***244*** | Train 16 | Test Female 4 | 0.69 |
| ***53*** | Train 4 | Test Female 5 | 0.75 | ***117*** | Train 8 | Test Female 5 | 0.75 | ***181*** | Train 12 | Test Female 5 | 0.75 | ***245*** | Train 16 | Test Female 5 | 0.66 |
| ***54*** | Train 4 | Test Female 6 | 0.76 | ***118*** | Train 8 | Test Female 6 | 0.76 | ***182*** | Train 12 | Test Female 6 | 0.76 | ***246*** | Train 16 | Test Female 6 | 0.69 |
| ***55*** | Train 4 | Test Female 7 | 0.75 | ***119*** | Train 8 | Test Female 7 | 0.75 | ***183*** | Train 12 | Test Female 7 | 0.75 | ***247*** | Train 16 | Test Female 7 | 0.67 |
| ***56*** | Train 4 | Test Female 8 | 0.76 | ***120*** | Train 8 | Test Female 8 | 0.76 | ***184*** | Train 12 | Test Female 8 | 0.76 | ***248*** | Train 16 | Test Female 8 | 0.69 |
| ***57*** | Train 4 | Test Female 9 | 0.75 | ***121*** | Train 8 | Test Female 9 | 0.75 | ***185*** | Train 12 | Test Female 9 | 0.75 | ***249*** | Train 16 | Test Female 9 | 0.67 |
| ***58*** | Train 4 | Test Female 10 | 0.76 | ***122*** | Train 8 | Test Female 10 | 0.76 | ***186*** | Train 12 | Test Female 10 | 0.76 | ***250*** | Train 16 | Test Female 10 | 0.69 |
| ***59*** | Train 4 | Test Female 11 | 0.75 | ***123*** | Train 8 | Test Female 11 | 0.75 | ***187*** | Train 12 | Test Female 11 | 0.75 | ***251*** | Train 16 | Test Female 11 | 0.67 |
| ***60*** | Train 4 | Test Female 12 | 0.76 | ***124*** | Train 8 | Test Female 12 | 0.76 | ***188*** | Train 12 | Test Female 12 | 0.76 | ***252*** | Train 16 | Test Female 12 | 0.7 |
| ***61*** | Train 4 | Test Female 13 | 0.76 | ***125*** | Train 8 | Test Female 13 | 0.76 | ***189*** | Train 12 | Test Female 13 | 0.76 | ***253*** | Train 16 | Test Female 13 | 0.76 |
| ***62*** | Train 4 | Test Female 14 | 0.76 | ***126*** | Train 8 | Test Female 14 | 0.76 | ***190*** | Train 12 | Test Female 14 | 0.76 | ***254*** | Train 16 | Test Female 14 | 0.76 |
| ***63*** | Train 4 | Test Female 15 | 0.76 | ***127*** | Train 8 | Test Female 15 | 0.76 | ***191*** | Train 12 | Test Female 15 | 0.76 | ***255*** | Train 16 | Test Female 15 | 0.76 |
| ***64*** | Train 4 | Test Female 16 | 0.76 | ***128*** | Train 8 | Test Female 16 | 0.76 | ***192*** | Train 12 | Test Female 16 | 0.76 | ***256*** | Train 16 | Test Female 16 | 0.77 |

Table 13. Logistic Regression (LR) AUC Performance for Black Patients Across 16 Cohorts.

| Original Decisions | Train Model | Test Dataset | AUC (LR) | ***Black*** | Train Model | Test Dataset | AUC (LR) | Black | Train Model | Test Dataset | AUC (LR) | Black | Train Model | Test Dataset | AUC (LR) |
| --- | --- | --- | --- | --- | --- | --- | --- | --- | --- | --- | --- | --- | --- | --- | --- |
| ***1*** | Train 1 | Test Black 1 | 0.72 | ***65*** | Train 5 | Test Black 1 | 0.72 | ***129*** | Train 9 | Test Black 1 | 0.72 | ***193*** | Train 13 | Test Black 1 | 0.69 |
| ***2*** | Train 1 | Test Black 2 | 0.7 | ***66*** | Train 5 | Test Black 2 | 0.7 | ***130*** | Train 9 | Test Black 2 | 0.7 | ***194*** | Train 13 | Test Black 2 | 0.69 |
| ***3*** | Train 1 | Test Black 3 | 0.72 | ***67*** | Train 5 | Test Black 3 | 0.72 | ***131*** | Train 9 | Test Black 3 | 0.72 | ***195*** | Train 13 | Test Black 3 | 0.69 |
| ***4*** | Train 1 | Test Black 4 | 0.7 | ***68*** | Train 5 | Test Black 4 | 0.7 | ***132*** | Train 9 | Test Black 4 | 0.7 | ***196*** | Train 13 | Test Black 4 | 0.69 |
| ***5*** | Train 1 | Test Black 5 | 0.72 | ***69*** | Train 5 | Test Black 5 | 0.72 | ***133*** | Train 9 | Test Black 5 | 0.72 | ***197*** | Train 13 | Test Black 5 | 0.69 |
| ***6*** | Train 1 | Test Black 6 | 0.7 | ***70*** | Train 5 | Test Black 6 | 0.7 | ***134*** | Train 9 | Test Black 6 | 0.7 | ***198*** | Train 13 | Test Black 6 | 0.69 |
| ***7*** | Train 1 | Test Black 7 | 0.72 | ***71*** | Train 5 | Test Black 7 | 0.72 | ***135*** | Train 9 | Test Black 7 | 0.72 | ***199*** | Train 13 | Test Black 7 | 0.69 |
| ***8*** | Train 1 | Test Black 8 | 0.7 | ***72*** | Train 5 | Test Black 8 | 0.7 | ***136*** | Train 9 | Test Black 8 | 0.7 | ***200*** | Train 13 | Test Black 8 | 0.69 |
| ***9*** | Train 1 | Test Black 9 | 0.72 | ***73*** | Train 5 | Test Black 9 | 0.72 | ***137*** | Train 9 | Test Black 9 | 0.72 | ***201*** | Train 13 | Test Black 9 | 0.69 |
| ***10*** | Train 1 | Test Black 10 | 0.7 | ***74*** | Train 5 | Test Black 10 | 0.7 | ***138*** | Train 9 | Test Black 10 | 0.7 | ***202*** | Train 13 | Test Black 10 | 0.69 |
| ***11*** | Train 1 | Test Black 11 | 0.72 | ***75*** | Train 5 | Test Black 11 | 0.72 | ***139*** | Train 9 | Test Black 11 | 0.72 | ***203*** | Train 13 | Test Black 11 | 0.69 |
| ***12*** | Train 1 | Test Black 12 | 0.7 | ***76*** | Train 5 | Test Black 12 | 0.7 | ***140*** | Train 9 | Test Black 12 | 0.7 | ***204*** | Train 13 | Test Black 12 | 0.69 |
| ***13*** | Train 1 | Test Black 13 | 0.73 | ***77*** | Train 5 | Test Black 13 | 0.73 | ***141*** | Train 9 | Test Black 13 | 0.73 | ***205*** | Train 13 | Test Black 13 | 0.74 |
| ***14*** | Train 1 | Test Black 14 | 0.71 | ***78*** | Train 5 | Test Black 14 | 0.71 | ***142*** | Train 9 | Test Black 14 | 0.71 | ***206*** | Train 13 | Test Black 14 | 0.71 |
| ***15*** | Train 1 | Test Black 15 | 0.73 | ***79*** | Train 5 | Test Black 15 | 0.73 | ***143*** | Train 9 | Test Black 15 | 0.73 | ***207*** | Train 13 | Test Black 15 | 0.74 |
| ***16*** | Train 1 | Test Black 16 | 0.71 | ***80*** | Train 5 | Test Black 16 | 0.71 | ***144*** | Train 9 | Test Black 16 | 0.71 | ***208*** | Train 13 | Test Black 16 | 0.71 |
| ***17*** | Train 2 | Test Black 1 | 0.71 | ***81*** | Train 6 | Test Black 1 | 0.71 | ***145*** | Train 10 | Test Black 1 | 0.71 | ***209*** | Train 14 | Test Black 1 | 0.68 |
| ***18*** | Train 2 | Test Black 2 | 0.72 | ***82*** | Train 6 | Test Black 2 | 0.72 | ***146*** | Train 10 | Test Black 2 | 0.72 | ***210*** | Train 14 | Test Black 2 | 0.71 |
| ***19*** | Train 2 | Test Black 3 | 0.71 | ***83*** | Train 6 | Test Black 3 | 0.71 | ***147*** | Train 10 | Test Black 3 | 0.71 | ***211*** | Train 14 | Test Black 3 | 0.68 |
| ***20*** | Train 2 | Test Black 4 | 0.72 | ***84*** | Train 6 | Test Black 4 | 0.72 | ***148*** | Train 10 | Test Black 4 | 0.72 | ***212*** | Train 14 | Test Black 4 | 0.71 |
| ***21*** | Train 2 | Test Black 5 | 0.71 | ***85*** | Train 6 | Test Black 5 | 0.71 | ***149*** | Train 10 | Test Black 5 | 0.71 | ***213*** | Train 14 | Test Black 5 | 0.68 |
| ***22*** | Train 2 | Test Black 6 | 0.72 | ***86*** | Train 6 | Test Black 6 | 0.72 | ***150*** | Train 10 | Test Black 6 | 0.72 | ***214*** | Train 14 | Test Black 6 | 0.71 |
| ***23*** | Train 2 | Test Black 7 | 0.71 | ***87*** | Train 6 | Test Black 7 | 0.71 | ***151*** | Train 10 | Test Black 7 | 0.71 | ***215*** | Train 14 | Test Black 7 | 0.68 |
| ***24*** | Train 2 | Test Black 8 | 0.72 | ***88*** | Train 6 | Test Black 8 | 0.72 | ***152*** | Train 10 | Test Black 8 | 0.72 | ***216*** | Train 14 | Test Black 8 | 0.71 |
| ***25*** | Train 2 | Test Black 9 | 0.71 | ***89*** | Train 6 | Test Black 9 | 0.71 | ***153*** | Train 10 | Test Black 9 | 0.71 | ***217*** | Train 14 | Test Black 9 | 0.68 |
| ***26*** | Train 2 | Test Black 10 | 0.72 | ***90*** | Train 6 | Test Black 10 | 0.72 | ***154*** | Train 10 | Test Black 10 | 0.72 | ***218*** | Train 14 | Test Black 10 | 0.71 |
| ***27*** | Train 2 | Test Black 11 | 0.71 | ***91*** | Train 6 | Test Black 11 | 0.71 | ***155*** | Train 10 | Test Black 11 | 0.71 | ***219*** | Train 14 | Test Black 11 | 0.68 |
| ***28*** | Train 2 | Test Black 12 | 0.72 | ***92*** | Train 6 | Test Black 12 | 0.72 | ***156*** | Train 10 | Test Black 12 | 0.72 | ***220*** | Train 14 | Test Black 12 | 0.71 |
| ***29*** | Train 2 | Test Black 13 | 0.73 | ***93*** | Train 6 | Test Black 13 | 0.73 | ***157*** | Train 10 | Test Black 13 | 0.73 | ***221*** | Train 14 | Test Black 13 | 0.74 |
| ***30*** | Train 2 | Test Black 14 | 0.73 | ***94*** | Train 6 | Test Black 14 | 0.73 | ***158*** | Train 10 | Test Black 14 | 0.73 | ***222*** | Train 14 | Test Black 14 | 0.74 |
| ***31*** | Train 2 | Test Black 15 | 0.73 | ***95*** | Train 6 | Test Black 15 | 0.73 | ***159*** | Train 10 | Test Black 15 | 0.73 | ***223*** | Train 14 | Test Black 15 | 0.73 |
| ***32*** | Train 2 | Test Black 16 | 0.73 | ***96*** | Train 6 | Test Black 16 | 0.73 | ***160*** | Train 10 | Test Black 16 | 0.73 | ***224*** | Train 14 | Test Black 16 | 0.74 |
| ***33*** | Train 3 | Test Black 1 | 0.72 | ***97*** | Train 7 | Test Black 1 | 0.72 | ***161*** | Train 11 | Test Black 1 | 0.72 | ***225*** | Train 15 | Test Black 1 | 0.69 |
| ***34*** | Train 3 | Test Black 2 | 0.7 | ***98*** | Train 7 | Test Black 2 | 0.7 | ***162*** | Train 11 | Test Black 2 | 0.7 | ***226*** | Train 15 | Test Black 2 | 0.69 |
| ***35*** | Train 3 | Test Black 3 | 0.72 | ***99*** | Train 7 | Test Black 3 | 0.72 | ***163*** | Train 11 | Test Black 3 | 0.72 | ***227*** | Train 15 | Test Black 3 | 0.69 |
| ***36*** | Train 3 | Test Black 4 | 0.7 | ***100*** | Train 7 | Test Black 4 | 0.7 | ***164*** | Train 11 | Test Black 4 | 0.7 | ***228*** | Train 15 | Test Black 4 | 0.69 |
| ***37*** | Train 3 | Test Black 5 | 0.72 | ***101*** | Train 7 | Test Black 5 | 0.72 | ***165*** | Train 11 | Test Black 5 | 0.72 | ***229*** | Train 15 | Test Black 5 | 0.69 |
| ***38*** | Train 3 | Test Black 6 | 0.7 | ***102*** | Train 7 | Test Black 6 | 0.7 | ***166*** | Train 11 | Test Black 6 | 0.7 | ***230*** | Train 15 | Test Black 6 | 0.69 |
| ***39*** | Train 3 | Test Black 7 | 0.72 | ***103*** | Train 7 | Test Black 7 | 0.72 | ***167*** | Train 11 | Test Black 7 | 0.72 | ***231*** | Train 15 | Test Black 7 | 0.69 |
| ***40*** | Train 3 | Test Black 8 | 0.7 | ***104*** | Train 7 | Test Black 8 | 0.7 | ***168*** | Train 11 | Test Black 8 | 0.7 | ***232*** | Train 15 | Test Black 8 | 0.7 |
| ***41*** | Train 3 | Test Black 9 | 0.72 | ***105*** | Train 7 | Test Black 9 | 0.72 | ***169*** | Train 11 | Test Black 9 | 0.72 | ***233*** | Train 15 | Test Black 9 | 0.69 |
| ***42*** | Train 3 | Test Black 10 | 0.7 | ***106*** | Train 7 | Test Black 10 | 0.7 | ***170*** | Train 11 | Test Black 10 | 0.7 | ***234*** | Train 15 | Test Black 10 | 0.69 |
| ***43*** | Train 3 | Test Black 11 | 0.72 | ***107*** | Train 7 | Test Black 11 | 0.72 | ***171*** | Train 11 | Test Black 11 | 0.72 | ***235*** | Train 15 | Test Black 11 | 0.69 |
| ***44*** | Train 3 | Test Black 12 | 0.7 | ***108*** | Train 7 | Test Black 12 | 0.7 | ***172*** | Train 11 | Test Black 12 | 0.7 | ***236*** | Train 15 | Test Black 12 | 0.7 |
| ***45*** | Train 3 | Test Black 13 | 0.73 | ***109*** | Train 7 | Test Black 13 | 0.73 | ***173*** | Train 11 | Test Black 13 | 0.73 | ***237*** | Train 15 | Test Black 13 | 0.74 |
| ***46*** | Train 3 | Test Black 14 | 0.7 | ***110*** | Train 7 | Test Black 14 | 0.71 | ***174*** | Train 11 | Test Black 14 | 0.71 | ***238*** | Train 15 | Test Black 14 | 0.71 |
| ***47*** | Train 3 | Test Black 15 | 0.73 | ***111*** | Train 7 | Test Black 15 | 0.73 | ***175*** | Train 11 | Test Black 15 | 0.73 | ***239*** | Train 15 | Test Black 15 | 0.74 |
| ***48*** | Train 3 | Test Black 16 | 0.71 | ***112*** | Train 7 | Test Black 16 | 0.71 | ***176*** | Train 11 | Test Black 16 | 0.71 | ***240*** | Train 15 | Test Black 16 | 0.71 |
| ***49*** | Train 4 | Test Black 1 | 0.71 | ***113*** | Train 8 | Test Black 1 | 0.71 | ***177*** | Train 12 | Test Black 1 | 0.71 | ***241*** | Train 16 | Test Black 1 | 0.68 |
| ***50*** | Train 4 | Test Black 2 | 0.72 | ***114*** | Train 8 | Test Black 2 | 0.72 | ***178*** | Train 12 | Test Black 2 | 0.72 | ***242*** | Train 16 | Test Black 2 | 0.71 |
| ***51*** | Train 4 | Test Black 3 | 0.71 | ***115*** | Train 8 | Test Black 3 | 0.71 | ***179*** | Train 12 | Test Black 3 | 0.71 | ***243*** | Train 16 | Test Black 3 | 0.68 |
| ***52*** | Train 4 | Test Black 4 | 0.72 | ***116*** | Train 8 | Test Black 4 | 0.72 | ***180*** | Train 12 | Test Black 4 | 0.72 | ***244*** | Train 16 | Test Black 4 | 0.71 |
| ***53*** | Train 4 | Test Black 5 | 0.71 | ***117*** | Train 8 | Test Black 5 | 0.71 | ***181*** | Train 12 | Test Black 5 | 0.71 | ***245*** | Train 16 | Test Black 5 | 0.68 |
| ***54*** | Train 4 | Test Black 6 | 0.72 | ***118*** | Train 8 | Test Black 6 | 0.72 | ***182*** | Train 12 | Test Black 6 | 0.72 | ***246*** | Train 16 | Test Black 6 | 0.71 |
| ***55*** | Train 4 | Test Black 7 | 0.71 | ***119*** | Train 8 | Test Black 7 | 0.71 | ***183*** | Train 12 | Test Black 7 | 0.71 | ***247*** | Train 16 | Test Black 7 | 0.68 |
| ***56*** | Train 4 | Test Black 8 | 0.72 | ***120*** | Train 8 | Test Black 8 | 0.72 | ***184*** | Train 12 | Test Black 8 | 0.72 | ***248*** | Train 16 | Test Black 8 | 0.71 |
| ***57*** | Train 4 | Test Black 9 | 0.71 | ***121*** | Train 8 | Test Black 9 | 0.71 | ***185*** | Train 12 | Test Black 9 | 0.71 | ***249*** | Train 16 | Test Black 9 | 0.69 |
| ***58*** | Train 4 | Test Black 10 | 0.72 | ***122*** | Train 8 | Test Black 10 | 0.72 | ***186*** | Train 12 | Test Black 10 | 0.72 | ***250*** | Train 16 | Test Black 10 | 0.71 |
| ***59*** | Train 4 | Test Black 11 | 0.71 | ***123*** | Train 8 | Test Black 11 | 0.71 | ***187*** | Train 12 | Test Black 11 | 0.71 | ***251*** | Train 16 | Test Black 11 | 0.68 |
| ***60*** | Train 4 | Test Black 12 | 0.72 | ***124*** | Train 8 | Test Black 12 | 0.72 | ***188*** | Train 12 | Test Black 12 | 0.72 | ***252*** | Train 16 | Test Black 12 | 0.71 |
| ***61*** | Train 4 | Test Black 13 | 0.73 | ***125*** | Train 8 | Test Black 13 | 0.73 | ***189*** | Train 12 | Test Black 13 | 0.73 | ***253*** | Train 16 | Test Black 13 | 0.74 |
| ***62*** | Train 4 | Test Black 14 | 0.73 | ***126*** | Train 8 | Test Black 14 | 0.73 | ***190*** | Train 12 | Test Black 14 | 0.73 | ***254*** | Train 16 | Test Black 14 | 0.74 |
| ***63*** | Train 4 | Test Black 15 | 0.73 | ***127*** | Train 8 | Test Black 15 | 0.73 | ***191*** | Train 12 | Test Black 15 | 0.73 | ***255*** | Train 16 | Test Black 15 | 0.73 |
| ***64*** | Train 4 | Test Black 16 | 0.73 | ***128*** | Train 8 | Test Black 16 | 0.73 | ***192*** | Train 12 | Test Black 16 | 0.73 | ***256*** | Train 16 | Test Black 16 | 0.74 |

Table 14. Random Forest (RF) AUC Performance for Black Patients Across 16 Cohorts.

| Original Decisions | Train Model | Test Dataset | AUC (RF) | ***Black*** | Train Model | Test Dataset | AUC (RF) | Black | Train Model | Test Dataset | AUC (RF) | Black | Train Model | Test Dataset | AUC (RF) |
| --- | --- | --- | --- | --- | --- | --- | --- | --- | --- | --- | --- | --- | --- | --- | --- |
| ***1*** | Train 1 | Test Black 1 | 0.74 | ***65*** | Train 5 | Test Black 1 | 0.74 | ***129*** | Train 9 | Test Black 1 | 0.74 | ***193*** | Train 13 | Test Black 1 | 0.69 |
| ***2*** | Train 1 | Test Black 2 | 0.7 | ***66*** | Train 5 | Test Black 2 | 0.7 | ***130*** | Train 9 | Test Black 2 | 0.7 | ***194*** | Train 13 | Test Black 2 | 0.69 |
| ***3*** | Train 1 | Test Black 3 | 0.73 | ***67*** | Train 5 | Test Black 3 | 0.73 | ***131*** | Train 9 | Test Black 3 | 0.73 | ***195*** | Train 13 | Test Black 3 | 0.68 |
| ***4*** | Train 1 | Test Black 4 | 0.7 | ***68*** | Train 5 | Test Black 4 | 0.7 | ***132*** | Train 9 | Test Black 4 | 0.7 | ***196*** | Train 13 | Test Black 4 | 0.69 |
| ***5*** | Train 1 | Test Black 5 | 0.74 | ***69*** | Train 5 | Test Black 5 | 0.74 | ***133*** | Train 9 | Test Black 5 | 0.74 | ***197*** | Train 13 | Test Black 5 | 0.69 |
| ***6*** | Train 1 | Test Black 6 | 0.7 | ***70*** | Train 5 | Test Black 6 | 0.7 | ***134*** | Train 9 | Test Black 6 | 0.7 | ***198*** | Train 13 | Test Black 6 | 0.69 |
| ***7*** | Train 1 | Test Black 7 | 0.74 | ***71*** | Train 5 | Test Black 7 | 0.73 | ***135*** | Train 9 | Test Black 7 | 0.74 | ***199*** | Train 13 | Test Black 7 | 0.68 |
| ***8*** | Train 1 | Test Black 8 | 0.7 | ***72*** | Train 5 | Test Black 8 | 0.7 | ***136*** | Train 9 | Test Black 8 | 0.7 | ***200*** | Train 13 | Test Black 8 | 0.69 |
| ***9*** | Train 1 | Test Black 9 | 0.74 | ***73*** | Train 5 | Test Black 9 | 0.74 | ***137*** | Train 9 | Test Black 9 | 0.74 | ***201*** | Train 13 | Test Black 9 | 0.69 |
| ***10*** | Train 1 | Test Black 10 | 0.7 | ***74*** | Train 5 | Test Black 10 | 0.7 | ***138*** | Train 9 | Test Black 10 | 0.7 | ***202*** | Train 13 | Test Black 10 | 0.69 |
| ***11*** | Train 1 | Test Black 11 | 0.74 | ***75*** | Train 5 | Test Black 11 | 0.74 | ***139*** | Train 9 | Test Black 11 | 0.74 | ***203*** | Train 13 | Test Black 11 | 0.69 |
| ***12*** | Train 1 | Test Black 12 | 0.7 | ***76*** | Train 5 | Test Black 12 | 0.7 | ***140*** | Train 9 | Test Black 12 | 0.7 | ***204*** | Train 13 | Test Black 12 | 0.69 |
| ***13*** | Train 1 | Test Black 13 | 0.74 | ***77*** | Train 5 | Test Black 13 | 0.74 | ***141*** | Train 9 | Test Black 13 | 0.74 | ***205*** | Train 13 | Test Black 13 | 0.74 |
| ***14*** | Train 1 | Test Black 14 | 0.69 | ***78*** | Train 5 | Test Black 14 | 0.69 | ***142*** | Train 9 | Test Black 14 | 0.69 | ***206*** | Train 13 | Test Black 14 | 0.7 |
| ***15*** | Train 1 | Test Black 15 | 0.74 | ***79*** | Train 5 | Test Black 15 | 0.74 | ***143*** | Train 9 | Test Black 15 | 0.74 | ***207*** | Train 13 | Test Black 15 | 0.74 |
| ***16*** | Train 1 | Test Black 16 | 0.69 | ***80*** | Train 5 | Test Black 16 | 0.69 | ***144*** | Train 9 | Test Black 16 | 0.69 | ***208*** | Train 13 | Test Black 16 | 0.7 |
| ***17*** | Train 2 | Test Black 1 | 0.74 | ***81*** | Train 6 | Test Black 1 | 0.74 | ***145*** | Train 10 | Test Black 1 | 0.74 | ***209*** | Train 14 | Test Black 1 | 0.7 |
| ***18*** | Train 2 | Test Black 2 | 0.75 | ***82*** | Train 6 | Test Black 2 | 0.75 | ***146*** | Train 10 | Test Black 2 | 0.75 | ***210*** | Train 14 | Test Black 2 | 0.73 |
| ***19*** | Train 2 | Test Black 3 | 0.74 | ***83*** | Train 6 | Test Black 3 | 0.74 | ***147*** | Train 10 | Test Black 3 | 0.74 | ***211*** | Train 14 | Test Black 3 | 0.7 |
| ***20*** | Train 2 | Test Black 4 | 0.74 | ***84*** | Train 6 | Test Black 4 | 0.74 | ***148*** | Train 10 | Test Black 4 | 0.74 | ***212*** | Train 14 | Test Black 4 | 0.73 |
| ***21*** | Train 2 | Test Black 5 | 0.74 | ***85*** | Train 6 | Test Black 5 | 0.74 | ***149*** | Train 10 | Test Black 5 | 0.74 | ***213*** | Train 14 | Test Black 5 | 0.7 |
| ***22*** | Train 2 | Test Black 6 | 0.75 | ***86*** | Train 6 | Test Black 6 | 0.75 | ***150*** | Train 10 | Test Black 6 | 0.75 | ***214*** | Train 14 | Test Black 6 | 0.73 |
| ***23*** | Train 2 | Test Black 7 | 0.74 | ***87*** | Train 6 | Test Black 7 | 0.74 | ***151*** | Train 10 | Test Black 7 | 0.74 | ***215*** | Train 14 | Test Black 7 | 0.7 |
| ***24*** | Train 2 | Test Black 8 | 0.75 | ***88*** | Train 6 | Test Black 8 | 0.75 | ***152*** | Train 10 | Test Black 8 | 0.75 | ***216*** | Train 14 | Test Black 8 | 0.73 |
| ***25*** | Train 2 | Test Black 9 | 0.74 | ***89*** | Train 6 | Test Black 9 | 0.74 | ***153*** | Train 10 | Test Black 9 | 0.74 | ***217*** | Train 14 | Test Black 9 | 0.7 |
| ***26*** | Train 2 | Test Black 10 | 0.75 | ***90*** | Train 6 | Test Black 10 | 0.75 | ***154*** | Train 10 | Test Black 10 | 0.75 | ***218*** | Train 14 | Test Black 10 | 0.73 |
| ***27*** | Train 2 | Test Black 11 | 0.74 | ***91*** | Train 6 | Test Black 11 | 0.74 | ***155*** | Train 10 | Test Black 11 | 0.74 | ***219*** | Train 14 | Test Black 11 | 0.7 |
| ***28*** | Train 2 | Test Black 12 | 0.75 | ***92*** | Train 6 | Test Black 12 | 0.74 | ***156*** | Train 10 | Test Black 12 | 0.75 | ***220*** | Train 14 | Test Black 12 | 0.73 |
| ***29*** | Train 2 | Test Black 13 | 0.74 | ***93*** | Train 6 | Test Black 13 | 0.74 | ***157*** | Train 10 | Test Black 13 | 0.74 | ***221*** | Train 14 | Test Black 13 | 0.74 |
| ***30*** | Train 2 | Test Black 14 | 0.74 | ***94*** | Train 6 | Test Black 14 | 0.74 | ***158*** | Train 10 | Test Black 14 | 0.74 | ***222*** | Train 14 | Test Black 14 | 0.75 |
| ***31*** | Train 2 | Test Black 15 | 0.74 | ***95*** | Train 6 | Test Black 15 | 0.74 | ***159*** | Train 10 | Test Black 15 | 0.74 | ***223*** | Train 14 | Test Black 15 | 0.74 |
| ***32*** | Train 2 | Test Black 16 | 0.74 | ***96*** | Train 6 | Test Black 16 | 0.74 | ***160*** | Train 10 | Test Black 16 | 0.74 | ***224*** | Train 14 | Test Black 16 | 0.75 |
| ***33*** | Train 3 | Test Black 1 | 0.74 | ***97*** | Train 7 | Test Black 1 | 0.74 | ***161*** | Train 11 | Test Black 1 | 0.74 | ***225*** | Train 15 | Test Black 1 | 0.68 |
| ***34*** | Train 3 | Test Black 2 | 0.7 | ***98*** | Train 7 | Test Black 2 | 0.7 | ***162*** | Train 11 | Test Black 2 | 0.7 | ***226*** | Train 15 | Test Black 2 | 0.69 |
| ***35*** | Train 3 | Test Black 3 | 0.74 | ***99*** | Train 7 | Test Black 3 | 0.74 | ***163*** | Train 11 | Test Black 3 | 0.73 | ***227*** | Train 15 | Test Black 3 | 0.68 |
| ***36*** | Train 3 | Test Black 4 | 0.7 | ***100*** | Train 7 | Test Black 4 | 0.7 | ***164*** | Train 11 | Test Black 4 | 0.7 | ***228*** | Train 15 | Test Black 4 | 0.69 |
| ***37*** | Train 3 | Test Black 5 | 0.74 | ***101*** | Train 7 | Test Black 5 | 0.74 | ***165*** | Train 11 | Test Black 5 | 0.74 | ***229*** | Train 15 | Test Black 5 | 0.69 |
| ***38*** | Train 3 | Test Black 6 | 0.7 | ***102*** | Train 7 | Test Black 6 | 0.7 | ***166*** | Train 11 | Test Black 6 | 0.7 | ***230*** | Train 15 | Test Black 6 | 0.69 |
| ***39*** | Train 3 | Test Black 7 | 0.74 | ***103*** | Train 7 | Test Black 7 | 0.74 | ***167*** | Train 11 | Test Black 7 | 0.73 | ***231*** | Train 15 | Test Black 7 | 0.68 |
| ***40*** | Train 3 | Test Black 8 | 0.7 | ***104*** | Train 7 | Test Black 8 | 0.7 | ***168*** | Train 11 | Test Black 8 | 0.7 | ***232*** | Train 15 | Test Black 8 | 0.69 |
| ***41*** | Train 3 | Test Black 9 | 0.74 | ***105*** | Train 7 | Test Black 9 | 0.74 | ***169*** | Train 11 | Test Black 9 | 0.74 | ***233*** | Train 15 | Test Black 9 | 0.69 |
| ***42*** | Train 3 | Test Black 10 | 0.7 | ***106*** | Train 7 | Test Black 10 | 0.7 | ***170*** | Train 11 | Test Black 10 | 0.7 | ***234*** | Train 15 | Test Black 10 | 0.69 |
| ***43*** | Train 3 | Test Black 11 | 0.74 | ***107*** | Train 7 | Test Black 11 | 0.74 | ***171*** | Train 11 | Test Black 11 | 0.74 | ***235*** | Train 15 | Test Black 11 | 0.69 |
| ***44*** | Train 3 | Test Black 12 | 0.7 | ***108*** | Train 7 | Test Black 12 | 0.7 | ***172*** | Train 11 | Test Black 12 | 0.7 | ***236*** | Train 15 | Test Black 12 | 0.69 |
| ***45*** | Train 3 | Test Black 13 | 0.74 | ***109*** | Train 7 | Test Black 13 | 0.74 | ***173*** | Train 11 | Test Black 13 | 0.74 | ***237*** | Train 15 | Test Black 13 | 0.74 |
| ***46*** | Train 3 | Test Black 14 | 0.69 | ***110*** | Train 7 | Test Black 14 | 0.69 | ***174*** | Train 11 | Test Black 14 | 0.69 | ***238*** | Train 15 | Test Black 14 | 0.7 |
| ***47*** | Train 3 | Test Black 15 | 0.74 | ***111*** | Train 7 | Test Black 15 | 0.74 | ***175*** | Train 11 | Test Black 15 | 0.74 | ***239*** | Train 15 | Test Black 15 | 0.74 |
| ***48*** | Train 3 | Test Black 16 | 0.69 | ***112*** | Train 7 | Test Black 16 | 0.69 | ***176*** | Train 11 | Test Black 16 | 0.69 | ***240*** | Train 15 | Test Black 16 | 0.7 |
| ***49*** | Train 4 | Test Black 1 | 0.74 | ***113*** | Train 8 | Test Black 1 | 0.74 | ***177*** | Train 12 | Test Black 1 | 0.74 | ***241*** | Train 16 | Test Black 1 | 0.7 |
| ***50*** | Train 4 | Test Black 2 | 0.75 | ***114*** | Train 8 | Test Black 2 | 0.75 | ***178*** | Train 12 | Test Black 2 | 0.75 | ***242*** | Train 16 | Test Black 2 | 0.73 |
| ***51*** | Train 4 | Test Black 3 | 0.74 | ***115*** | Train 8 | Test Black 3 | 0.74 | ***179*** | Train 12 | Test Black 3 | 0.74 | ***243*** | Train 16 | Test Black 3 | 0.7 |
| ***52*** | Train 4 | Test Black 4 | 0.74 | ***116*** | Train 8 | Test Black 4 | 0.74 | ***180*** | Train 12 | Test Black 4 | 0.74 | ***244*** | Train 16 | Test Black 4 | 0.73 |
| ***53*** | Train 4 | Test Black 5 | 0.74 | ***117*** | Train 8 | Test Black 5 | 0.74 | ***181*** | Train 12 | Test Black 5 | 0.74 | ***245*** | Train 16 | Test Black 5 | 0.7 |
| ***54*** | Train 4 | Test Black 6 | 0.75 | ***118*** | Train 8 | Test Black 6 | 0.75 | ***182*** | Train 12 | Test Black 6 | 0.75 | ***246*** | Train 16 | Test Black 6 | 0.73 |
| ***55*** | Train 4 | Test Black 7 | 0.74 | ***119*** | Train 8 | Test Black 7 | 0.74 | ***183*** | Train 12 | Test Black 7 | 0.74 | ***247*** | Train 16 | Test Black 7 | 0.7 |
| ***56*** | Train 4 | Test Black 8 | 0.74 | ***120*** | Train 8 | Test Black 8 | 0.74 | ***184*** | Train 12 | Test Black 8 | 0.74 | ***248*** | Train 16 | Test Black 8 | 0.73 |
| ***57*** | Train 4 | Test Black 9 | 0.74 | ***121*** | Train 8 | Test Black 9 | 0.74 | ***185*** | Train 12 | Test Black 9 | 0.74 | ***249*** | Train 16 | Test Black 9 | 0.7 |
| ***58*** | Train 4 | Test Black 10 | 0.75 | ***122*** | Train 8 | Test Black 10 | 0.75 | ***186*** | Train 12 | Test Black 10 | 0.75 | ***250*** | Train 16 | Test Black 10 | 0.73 |
| ***59*** | Train 4 | Test Black 11 | 0.74 | ***123*** | Train 8 | Test Black 11 | 0.74 | ***187*** | Train 12 | Test Black 11 | 0.74 | ***251*** | Train 16 | Test Black 11 | 0.7 |
| ***60*** | Train 4 | Test Black 12 | 0.74 | ***124*** | Train 8 | Test Black 12 | 0.74 | ***188*** | Train 12 | Test Black 12 | 0.74 | ***252*** | Train 16 | Test Black 12 | 0.73 |
| ***61*** | Train 4 | Test Black 13 | 0.74 | ***125*** | Train 8 | Test Black 13 | 0.74 | ***189*** | Train 12 | Test Black 13 | 0.74 | ***253*** | Train 16 | Test Black 13 | 0.74 |
| ***62*** | Train 4 | Test Black 14 | 0.74 | ***126*** | Train 8 | Test Black 14 | 0.74 | ***190*** | Train 12 | Test Black 14 | 0.74 | ***254*** | Train 16 | Test Black 14 | 0.75 |
| ***63*** | Train 4 | Test Black 15 | 0.74 | ***127*** | Train 8 | Test Black 15 | 0.74 | ***191*** | Train 12 | Test Black 15 | 0.74 | ***255*** | Train 16 | Test Black 15 | 0.74 |
| ***64*** | Train 4 | Test Black 16 | 0.74 | ***128*** | Train 8 | Test Black 16 | 0.74 | ***192*** | Train 12 | Test Black 16 | 0.74 | ***256*** | Train 16 | Test Black 16 | 0.75 |

Table 15. Gradient Boosting (GB) AUC Performance for Black Patients Across 16 Cohorts.

| Original Decisions | Train Model | Test Dataset | AUC (GB) | ***Black*** | Train Model | Test Dataset | AUC (GB) | Black | Train Model | Test Dataset | AUC (GB) | Black | Train Model | Test Dataset | AUC (GB) |
| --- | --- | --- | --- | --- | --- | --- | --- | --- | --- | --- | --- | --- | --- | --- | --- |
| ***1*** | Train 1 | Test Black 1 | 0.72 | ***65*** | Train 5 | Test Black 1 | 0.73 | ***129*** | Train 9 | Test Black 1 | 0.73 | ***193*** | Train 13 | Test Black 1 | 0.64 |
| ***2*** | Train 1 | Test Black 2 | 0.67 | ***66*** | Train 5 | Test Black 2 | 0.68 | ***130*** | Train 9 | Test Black 2 | 0.68 | ***194*** | Train 13 | Test Black 2 | 0.64 |
| ***3*** | Train 1 | Test Black 3 | 0.72 | ***67*** | Train 5 | Test Black 3 | 0.73 | ***131*** | Train 9 | Test Black 3 | 0.73 | ***195*** | Train 13 | Test Black 3 | 0.64 |
| ***4*** | Train 1 | Test Black 4 | 0.67 | ***68*** | Train 5 | Test Black 4 | 0.68 | ***132*** | Train 9 | Test Black 4 | 0.68 | ***196*** | Train 13 | Test Black 4 | 0.64 |
| ***5*** | Train 1 | Test Black 5 | 0.72 | ***69*** | Train 5 | Test Black 5 | 0.73 | ***133*** | Train 9 | Test Black 5 | 0.73 | ***197*** | Train 13 | Test Black 5 | 0.64 |
| ***6*** | Train 1 | Test Black 6 | 0.67 | ***70*** | Train 5 | Test Black 6 | 0.68 | ***134*** | Train 9 | Test Black 6 | 0.68 | ***198*** | Train 13 | Test Black 6 | 0.64 |
| ***7*** | Train 1 | Test Black 7 | 0.72 | ***71*** | Train 5 | Test Black 7 | 0.73 | ***135*** | Train 9 | Test Black 7 | 0.73 | ***199*** | Train 13 | Test Black 7 | 0.64 |
| ***8*** | Train 1 | Test Black 8 | 0.67 | ***72*** | Train 5 | Test Black 8 | 0.68 | ***136*** | Train 9 | Test Black 8 | 0.68 | ***200*** | Train 13 | Test Black 8 | 0.64 |
| ***9*** | Train 1 | Test Black 9 | 0.72 | ***73*** | Train 5 | Test Black 9 | 0.73 | ***137*** | Train 9 | Test Black 9 | 0.73 | ***201*** | Train 13 | Test Black 9 | 0.64 |
| ***10*** | Train 1 | Test Black 10 | 0.67 | ***74*** | Train 5 | Test Black 10 | 0.68 | ***138*** | Train 9 | Test Black 10 | 0.68 | ***202*** | Train 13 | Test Black 10 | 0.64 |
| ***11*** | Train 1 | Test Black 11 | 0.72 | ***75*** | Train 5 | Test Black 11 | 0.73 | ***139*** | Train 9 | Test Black 11 | 0.73 | ***203*** | Train 13 | Test Black 11 | 0.64 |
| ***12*** | Train 1 | Test Black 12 | 0.67 | ***76*** | Train 5 | Test Black 12 | 0.68 | ***140*** | Train 9 | Test Black 12 | 0.68 | ***204*** | Train 13 | Test Black 12 | 0.64 |
| ***13*** | Train 1 | Test Black 13 | 0.72 | ***77*** | Train 5 | Test Black 13 | 0.73 | ***141*** | Train 9 | Test Black 13 | 0.73 | ***205*** | Train 13 | Test Black 13 | 0.72 |
| ***14*** | Train 1 | Test Black 14 | 0.66 | ***78*** | Train 5 | Test Black 14 | 0.66 | ***142*** | Train 9 | Test Black 14 | 0.66 | ***206*** | Train 13 | Test Black 14 | 0.67 |
| ***15*** | Train 1 | Test Black 15 | 0.72 | ***79*** | Train 5 | Test Black 15 | 0.73 | ***143*** | Train 9 | Test Black 15 | 0.73 | ***207*** | Train 13 | Test Black 15 | 0.72 |
| ***16*** | Train 1 | Test Black 16 | 0.67 | ***80*** | Train 5 | Test Black 16 | 0.66 | ***144*** | Train 9 | Test Black 16 | 0.67 | ***208*** | Train 13 | Test Black 16 | 0.67 |
| ***17*** | Train 2 | Test Black 1 | 0.74 | ***81*** | Train 6 | Test Black 1 | 0.74 | ***145*** | Train 10 | Test Black 1 | 0.73 | ***209*** | Train 14 | Test Black 1 | 0.66 |
| ***18*** | Train 2 | Test Black 2 | 0.75 | ***82*** | Train 6 | Test Black 2 | 0.75 | ***146*** | Train 10 | Test Black 2 | 0.74 | ***210*** | Train 14 | Test Black 2 | 0.69 |
| ***19*** | Train 2 | Test Black 3 | 0.74 | ***83*** | Train 6 | Test Black 3 | 0.74 | ***147*** | Train 10 | Test Black 3 | 0.73 | ***211*** | Train 14 | Test Black 3 | 0.66 |
| ***20*** | Train 2 | Test Black 4 | 0.74 | ***84*** | Train 6 | Test Black 4 | 0.74 | ***148*** | Train 10 | Test Black 4 | 0.74 | ***212*** | Train 14 | Test Black 4 | 0.7 |
| ***21*** | Train 2 | Test Black 5 | 0.74 | ***85*** | Train 6 | Test Black 5 | 0.74 | ***149*** | Train 10 | Test Black 5 | 0.73 | ***213*** | Train 14 | Test Black 5 | 0.66 |
| ***22*** | Train 2 | Test Black 6 | 0.75 | ***86*** | Train 6 | Test Black 6 | 0.75 | ***150*** | Train 10 | Test Black 6 | 0.74 | ***214*** | Train 14 | Test Black 6 | 0.69 |
| ***23*** | Train 2 | Test Black 7 | 0.74 | ***87*** | Train 6 | Test Black 7 | 0.74 | ***151*** | Train 10 | Test Black 7 | 0.73 | ***215*** | Train 14 | Test Black 7 | 0.66 |
| ***24*** | Train 2 | Test Black 8 | 0.74 | ***88*** | Train 6 | Test Black 8 | 0.74 | ***152*** | Train 10 | Test Black 8 | 0.74 | ***216*** | Train 14 | Test Black 8 | 0.7 |
| ***25*** | Train 2 | Test Black 9 | 0.74 | ***89*** | Train 6 | Test Black 9 | 0.74 | ***153*** | Train 10 | Test Black 9 | 0.73 | ***217*** | Train 14 | Test Black 9 | 0.66 |
| ***26*** | Train 2 | Test Black 10 | 0.75 | ***90*** | Train 6 | Test Black 10 | 0.74 | ***154*** | Train 10 | Test Black 10 | 0.74 | ***218*** | Train 14 | Test Black 10 | 0.7 |
| ***27*** | Train 2 | Test Black 11 | 0.74 | ***91*** | Train 6 | Test Black 11 | 0.74 | ***155*** | Train 10 | Test Black 11 | 0.73 | ***219*** | Train 14 | Test Black 11 | 0.66 |
| ***28*** | Train 2 | Test Black 12 | 0.74 | ***92*** | Train 6 | Test Black 12 | 0.74 | ***156*** | Train 10 | Test Black 12 | 0.74 | ***220*** | Train 14 | Test Black 12 | 0.7 |
| ***29*** | Train 2 | Test Black 13 | 0.74 | ***93*** | Train 6 | Test Black 13 | 0.74 | ***157*** | Train 10 | Test Black 13 | 0.73 | ***221*** | Train 14 | Test Black 13 | 0.73 |
| ***30*** | Train 2 | Test Black 14 | 0.74 | ***94*** | Train 6 | Test Black 14 | 0.74 | ***158*** | Train 10 | Test Black 14 | 0.74 | ***222*** | Train 14 | Test Black 14 | 0.74 |
| ***31*** | Train 2 | Test Black 15 | 0.74 | ***95*** | Train 6 | Test Black 15 | 0.74 | ***159*** | Train 10 | Test Black 15 | 0.73 | ***223*** | Train 14 | Test Black 15 | 0.73 |
| ***32*** | Train 2 | Test Black 16 | 0.74 | ***96*** | Train 6 | Test Black 16 | 0.74 | ***160*** | Train 10 | Test Black 16 | 0.74 | ***224*** | Train 14 | Test Black 16 | 0.75 |
| ***33*** | Train 3 | Test Black 1 | 0.73 | ***97*** | Train 7 | Test Black 1 | 0.74 | ***161*** | Train 11 | Test Black 1 | 0.73 | ***225*** | Train 15 | Test Black 1 | 0.65 |
| ***34*** | Train 3 | Test Black 2 | 0.66 | ***98*** | Train 7 | Test Black 2 | 0.67 | ***162*** | Train 11 | Test Black 2 | 0.66 | ***226*** | Train 15 | Test Black 2 | 0.65 |
| ***35*** | Train 3 | Test Black 3 | 0.72 | ***99*** | Train 7 | Test Black 3 | 0.74 | ***163*** | Train 11 | Test Black 3 | 0.73 | ***227*** | Train 15 | Test Black 3 | 0.65 |
| ***36*** | Train 3 | Test Black 4 | 0.66 | ***100*** | Train 7 | Test Black 4 | 0.67 | ***164*** | Train 11 | Test Black 4 | 0.66 | ***228*** | Train 15 | Test Black 4 | 0.65 |
| ***37*** | Train 3 | Test Black 5 | 0.73 | ***101*** | Train 7 | Test Black 5 | 0.74 | ***165*** | Train 11 | Test Black 5 | 0.73 | ***229*** | Train 15 | Test Black 5 | 0.65 |
| ***38*** | Train 3 | Test Black 6 | 0.66 | ***102*** | Train 7 | Test Black 6 | 0.67 | ***166*** | Train 11 | Test Black 6 | 0.67 | ***230*** | Train 15 | Test Black 6 | 0.65 |
| ***39*** | Train 3 | Test Black 7 | 0.72 | ***103*** | Train 7 | Test Black 7 | 0.74 | ***167*** | Train 11 | Test Black 7 | 0.73 | ***231*** | Train 15 | Test Black 7 | 0.65 |
| ***40*** | Train 3 | Test Black 8 | 0.66 | ***104*** | Train 7 | Test Black 8 | 0.67 | ***168*** | Train 11 | Test Black 8 | 0.66 | ***232*** | Train 15 | Test Black 8 | 0.65 |
| ***41*** | Train 3 | Test Black 9 | 0.73 | ***105*** | Train 7 | Test Black 9 | 0.74 | ***169*** | Train 11 | Test Black 9 | 0.73 | ***233*** | Train 15 | Test Black 9 | 0.65 |
| ***42*** | Train 3 | Test Black 10 | 0.66 | ***106*** | Train 7 | Test Black 10 | 0.67 | ***170*** | Train 11 | Test Black 10 | 0.66 | ***234*** | Train 15 | Test Black 10 | 0.65 |
| ***43*** | Train 3 | Test Black 11 | 0.73 | ***107*** | Train 7 | Test Black 11 | 0.74 | ***171*** | Train 11 | Test Black 11 | 0.73 | ***235*** | Train 15 | Test Black 11 | 0.65 |
| ***44*** | Train 3 | Test Black 12 | 0.66 | ***108*** | Train 7 | Test Black 12 | 0.67 | ***172*** | Train 11 | Test Black 12 | 0.66 | ***236*** | Train 15 | Test Black 12 | 0.65 |
| ***45*** | Train 3 | Test Black 13 | 0.72 | ***109*** | Train 7 | Test Black 13 | 0.73 | ***173*** | Train 11 | Test Black 13 | 0.72 | ***237*** | Train 15 | Test Black 13 | 0.72 |
| ***46*** | Train 3 | Test Black 14 | 0.64 | ***110*** | Train 7 | Test Black 14 | 0.65 | ***174*** | Train 11 | Test Black 14 | 0.64 | ***238*** | Train 15 | Test Black 14 | 0.66 |
| ***47*** | Train 3 | Test Black 15 | 0.72 | ***111*** | Train 7 | Test Black 15 | 0.73 | ***175*** | Train 11 | Test Black 15 | 0.72 | ***239*** | Train 15 | Test Black 15 | 0.72 |
| ***48*** | Train 3 | Test Black 16 | 0.64 | ***112*** | Train 7 | Test Black 16 | 0.66 | ***176*** | Train 11 | Test Black 16 | 0.64 | ***240*** | Train 15 | Test Black 16 | 0.66 |
| ***49*** | Train 4 | Test Black 1 | 0.74 | ***113*** | Train 8 | Test Black 1 | 0.74 | ***177*** | Train 12 | Test Black 1 | 0.74 | ***241*** | Train 16 | Test Black 1 | 0.63 |
| ***50*** | Train 4 | Test Black 2 | 0.74 | ***114*** | Train 8 | Test Black 2 | 0.74 | ***178*** | Train 12 | Test Black 2 | 0.75 | ***242*** | Train 16 | Test Black 2 | 0.68 |
| ***51*** | Train 4 | Test Black 3 | 0.74 | ***115*** | Train 8 | Test Black 3 | 0.74 | ***179*** | Train 12 | Test Black 3 | 0.74 | ***243*** | Train 16 | Test Black 3 | 0.63 |
| ***52*** | Train 4 | Test Black 4 | 0.74 | ***116*** | Train 8 | Test Black 4 | 0.74 | ***180*** | Train 12 | Test Black 4 | 0.74 | ***244*** | Train 16 | Test Black 4 | 0.68 |
| ***53*** | Train 4 | Test Black 5 | 0.74 | ***117*** | Train 8 | Test Black 5 | 0.74 | ***181*** | Train 12 | Test Black 5 | 0.74 | ***245*** | Train 16 | Test Black 5 | 0.63 |
| ***54*** | Train 4 | Test Black 6 | 0.74 | ***118*** | Train 8 | Test Black 6 | 0.74 | ***182*** | Train 12 | Test Black 6 | 0.75 | ***246*** | Train 16 | Test Black 6 | 0.68 |
| ***55*** | Train 4 | Test Black 7 | 0.74 | ***119*** | Train 8 | Test Black 7 | 0.74 | ***183*** | Train 12 | Test Black 7 | 0.74 | ***247*** | Train 16 | Test Black 7 | 0.63 |
| ***56*** | Train 4 | Test Black 8 | 0.74 | ***120*** | Train 8 | Test Black 8 | 0.74 | ***184*** | Train 12 | Test Black 8 | 0.74 | ***248*** | Train 16 | Test Black 8 | 0.68 |
| ***57*** | Train 4 | Test Black 9 | 0.74 | ***121*** | Train 8 | Test Black 9 | 0.74 | ***185*** | Train 12 | Test Black 9 | 0.74 | ***249*** | Train 16 | Test Black 9 | 0.64 |
| ***58*** | Train 4 | Test Black 10 | 0.74 | ***122*** | Train 8 | Test Black 10 | 0.74 | ***186*** | Train 12 | Test Black 10 | 0.74 | ***250*** | Train 16 | Test Black 10 | 0.68 |
| ***59*** | Train 4 | Test Black 11 | 0.74 | ***123*** | Train 8 | Test Black 11 | 0.74 | ***187*** | Train 12 | Test Black 11 | 0.74 | ***251*** | Train 16 | Test Black 11 | 0.64 |
| ***60*** | Train 4 | Test Black 12 | 0.74 | ***124*** | Train 8 | Test Black 12 | 0.74 | ***188*** | Train 12 | Test Black 12 | 0.74 | ***252*** | Train 16 | Test Black 12 | 0.68 |
| ***61*** | Train 4 | Test Black 13 | 0.74 | ***125*** | Train 8 | Test Black 13 | 0.73 | ***189*** | Train 12 | Test Black 13 | 0.74 | ***253*** | Train 16 | Test Black 13 | 0.74 |
| ***62*** | Train 4 | Test Black 14 | 0.73 | ***126*** | Train 8 | Test Black 14 | 0.74 | ***190*** | Train 12 | Test Black 14 | 0.74 | ***254*** | Train 16 | Test Black 14 | 0.74 |
| ***63*** | Train 4 | Test Black 15 | 0.74 | ***127*** | Train 8 | Test Black 15 | 0.73 | ***191*** | Train 12 | Test Black 15 | 0.74 | ***255*** | Train 16 | Test Black 15 | 0.74 |
| ***64*** | Train 4 | Test Black 16 | 0.74 | ***128*** | Train 8 | Test Black 16 | 0.74 | ***192*** | Train 12 | Test Black 16 | 0.74 | ***256*** | Train 16 | Test Black 16 | 0.75 |

Table 16. Logistic Regression (LR) AUC Performance for White Patients Across 16 Cohorts.

| Original Decisions | Train Model | Test Dataset | AUC (LR) | ***White*** | Train Model | Test Dataset | AUC (LR) | White | Train Model | Test Dataset | AUC (LR) | White | Train Model | Test Dataset | AUC (LR) |
| --- | --- | --- | --- | --- | --- | --- | --- | --- | --- | --- | --- | --- | --- | --- | --- |
| ***1*** | Train 1 | Test White 1 | 0.72 | ***65*** | Train 5 | Test White 1 | 0.72 | ***129*** | Train 9 | Test White 1 | 0.72 | ***193*** | Train 13 | Test White 1 | 0.71 |
| ***2*** | Train 1 | Test White 2 | 0.7 | ***66*** | Train 5 | Test White 2 | 0.7 | ***130*** | Train 9 | Test White 2 | 0.7 | ***194*** | Train 13 | Test White 2 | 0.7 |
| ***3*** | Train 1 | Test White 3 | 0.72 | ***67*** | Train 5 | Test White 3 | 0.72 | ***131*** | Train 9 | Test White 3 | 0.72 | ***195*** | Train 13 | Test White 3 | 0.71 |
| ***4*** | Train 1 | Test White 4 | 0.7 | ***68*** | Train 5 | Test White 4 | 0.7 | ***132*** | Train 9 | Test White 4 | 0.7 | ***196*** | Train 13 | Test White 4 | 0.7 |
| ***5*** | Train 1 | Test White 5 | 0.72 | ***69*** | Train 5 | Test White 5 | 0.72 | ***133*** | Train 9 | Test White 5 | 0.72 | ***197*** | Train 13 | Test White 5 | 0.71 |
| ***6*** | Train 1 | Test White 6 | 0.7 | ***70*** | Train 5 | Test White 6 | 0.7 | ***134*** | Train 9 | Test White 6 | 0.7 | ***198*** | Train 13 | Test White 6 | 0.7 |
| ***7*** | Train 1 | Test White 7 | 0.72 | ***71*** | Train 5 | Test White 7 | 0.72 | ***135*** | Train 9 | Test White 7 | 0.72 | ***199*** | Train 13 | Test White 7 | 0.71 |
| ***8*** | Train 1 | Test White 8 | 0.71 | ***72*** | Train 5 | Test White 8 | 0.71 | ***136*** | Train 9 | Test White 8 | 0.71 | ***200*** | Train 13 | Test White 8 | 0.7 |
| ***9*** | Train 1 | Test White 9 | 0.72 | ***73*** | Train 5 | Test White 9 | 0.72 | ***137*** | Train 9 | Test White 9 | 0.72 | ***201*** | Train 13 | Test White 9 | 0.71 |
| ***10*** | Train 1 | Test White 10 | 0.7 | ***74*** | Train 5 | Test White 10 | 0.7 | ***138*** | Train 9 | Test White 10 | 0.7 | ***202*** | Train 13 | Test White 10 | 0.7 |
| ***11*** | Train 1 | Test White 11 | 0.72 | ***75*** | Train 5 | Test White 11 | 0.72 | ***139*** | Train 9 | Test White 11 | 0.72 | ***203*** | Train 13 | Test White 11 | 0.71 |
| ***12*** | Train 1 | Test White 12 | 0.71 | ***76*** | Train 5 | Test White 12 | 0.71 | ***140*** | Train 9 | Test White 12 | 0.7 | ***204*** | Train 13 | Test White 12 | 0.7 |
| ***13*** | Train 1 | Test White 13 | 0.72 | ***77*** | Train 5 | Test White 13 | 0.72 | ***141*** | Train 9 | Test White 13 | 0.72 | ***205*** | Train 13 | Test White 13 | 0.72 |
| ***14*** | Train 1 | Test White 14 | 0.7 | ***78*** | Train 5 | Test White 14 | 0.7 | ***142*** | Train 9 | Test White 14 | 0.7 | ***206*** | Train 13 | Test White 14 | 0.7 |
| ***15*** | Train 1 | Test White 15 | 0.72 | ***79*** | Train 5 | Test White 15 | 0.72 | ***143*** | Train 9 | Test White 15 | 0.72 | ***207*** | Train 13 | Test White 15 | 0.72 |
| ***16*** | Train 1 | Test White 16 | 0.7 | ***80*** | Train 5 | Test White 16 | 0.7 | ***144*** | Train 9 | Test White 16 | 0.7 | ***208*** | Train 13 | Test White 16 | 0.7 |
| ***17*** | Train 2 | Test White 1 | 0.71 | ***81*** | Train 6 | Test White 1 | 0.71 | ***145*** | Train 10 | Test White 1 | 0.71 | ***209*** | Train 14 | Test White 1 | 0.7 |
| ***18*** | Train 2 | Test White 2 | 0.73 | ***82*** | Train 6 | Test White 2 | 0.73 | ***146*** | Train 10 | Test White 2 | 0.73 | ***210*** | Train 14 | Test White 2 | 0.71 |
| ***19*** | Train 2 | Test White 3 | 0.71 | ***83*** | Train 6 | Test White 3 | 0.71 | ***147*** | Train 10 | Test White 3 | 0.71 | ***211*** | Train 14 | Test White 3 | 0.7 |
| ***20*** | Train 2 | Test White 4 | 0.73 | ***84*** | Train 6 | Test White 4 | 0.73 | ***148*** | Train 10 | Test White 4 | 0.73 | ***212*** | Train 14 | Test White 4 | 0.71 |
| ***21*** | Train 2 | Test White 5 | 0.71 | ***85*** | Train 6 | Test White 5 | 0.71 | ***149*** | Train 10 | Test White 5 | 0.71 | ***213*** | Train 14 | Test White 5 | 0.7 |
| ***22*** | Train 2 | Test White 6 | 0.73 | ***86*** | Train 6 | Test White 6 | 0.73 | ***150*** | Train 10 | Test White 6 | 0.73 | ***214*** | Train 14 | Test White 6 | 0.71 |
| ***23*** | Train 2 | Test White 7 | 0.71 | ***87*** | Train 6 | Test White 7 | 0.71 | ***151*** | Train 10 | Test White 7 | 0.71 | ***215*** | Train 14 | Test White 7 | 0.7 |
| ***24*** | Train 2 | Test White 8 | 0.73 | ***88*** | Train 6 | Test White 8 | 0.73 | ***152*** | Train 10 | Test White 8 | 0.73 | ***216*** | Train 14 | Test White 8 | 0.71 |
| ***25*** | Train 2 | Test White 9 | 0.71 | ***89*** | Train 6 | Test White 9 | 0.71 | ***153*** | Train 10 | Test White 9 | 0.71 | ***217*** | Train 14 | Test White 9 | 0.7 |
| ***26*** | Train 2 | Test White 10 | 0.73 | ***90*** | Train 6 | Test White 10 | 0.73 | ***154*** | Train 10 | Test White 10 | 0.73 | ***218*** | Train 14 | Test White 10 | 0.71 |
| ***27*** | Train 2 | Test White 11 | 0.71 | ***91*** | Train 6 | Test White 11 | 0.71 | ***155*** | Train 10 | Test White 11 | 0.71 | ***219*** | Train 14 | Test White 11 | 0.7 |
| ***28*** | Train 2 | Test White 12 | 0.73 | ***92*** | Train 6 | Test White 12 | 0.73 | ***156*** | Train 10 | Test White 12 | 0.73 | ***220*** | Train 14 | Test White 12 | 0.71 |
| ***29*** | Train 2 | Test White 13 | 0.71 | ***93*** | Train 6 | Test White 13 | 0.71 | ***157*** | Train 10 | Test White 13 | 0.71 | ***221*** | Train 14 | Test White 13 | 0.72 |
| ***30*** | Train 2 | Test White 14 | 0.72 | ***94*** | Train 6 | Test White 14 | 0.72 | ***158*** | Train 10 | Test White 14 | 0.72 | ***222*** | Train 14 | Test White 14 | 0.73 |
| ***31*** | Train 2 | Test White 15 | 0.71 | ***95*** | Train 6 | Test White 15 | 0.71 | ***159*** | Train 10 | Test White 15 | 0.71 | ***223*** | Train 14 | Test White 15 | 0.72 |
| ***32*** | Train 2 | Test White 16 | 0.72 | ***96*** | Train 6 | Test White 16 | 0.72 | ***160*** | Train 10 | Test White 16 | 0.72 | ***224*** | Train 14 | Test White 16 | 0.73 |
| ***33*** | Train 3 | Test White 1 | 0.72 | ***97*** | Train 7 | Test White 1 | 0.72 | ***161*** | Train 11 | Test White 1 | 0.72 | ***225*** | Train 15 | Test White 1 | 0.71 |
| ***34*** | Train 3 | Test White 2 | 0.7 | ***98*** | Train 7 | Test White 2 | 0.7 | ***162*** | Train 11 | Test White 2 | 0.7 | ***226*** | Train 15 | Test White 2 | 0.7 |
| ***35*** | Train 3 | Test White 3 | 0.72 | ***99*** | Train 7 | Test White 3 | 0.72 | ***163*** | Train 11 | Test White 3 | 0.72 | ***227*** | Train 15 | Test White 3 | 0.71 |
| ***36*** | Train 3 | Test White 4 | 0.7 | ***100*** | Train 7 | Test White 4 | 0.7 | ***164*** | Train 11 | Test White 4 | 0.7 | ***228*** | Train 15 | Test White 4 | 0.7 |
| ***37*** | Train 3 | Test White 5 | 0.72 | ***101*** | Train 7 | Test White 5 | 0.72 | ***165*** | Train 11 | Test White 5 | 0.72 | ***229*** | Train 15 | Test White 5 | 0.71 |
| ***38*** | Train 3 | Test White 6 | 0.7 | ***102*** | Train 7 | Test White 6 | 0.7 | ***166*** | Train 11 | Test White 6 | 0.7 | ***230*** | Train 15 | Test White 6 | 0.7 |
| ***39*** | Train 3 | Test White 7 | 0.72 | ***103*** | Train 7 | Test White 7 | 0.72 | ***167*** | Train 11 | Test White 7 | 0.72 | ***231*** | Train 15 | Test White 7 | 0.71 |
| ***40*** | Train 3 | Test White 8 | 0.7 | ***104*** | Train 7 | Test White 8 | 0.7 | ***168*** | Train 11 | Test White 8 | 0.7 | ***232*** | Train 15 | Test White 8 | 0.7 |
| ***41*** | Train 3 | Test White 9 | 0.72 | ***105*** | Train 7 | Test White 9 | 0.72 | ***169*** | Train 11 | Test White 9 | 0.72 | ***233*** | Train 15 | Test White 9 | 0.71 |
| ***42*** | Train 3 | Test White 10 | 0.7 | ***106*** | Train 7 | Test White 10 | 0.7 | ***170*** | Train 11 | Test White 10 | 0.7 | ***234*** | Train 15 | Test White 10 | 0.7 |
| ***43*** | Train 3 | Test White 11 | 0.72 | ***107*** | Train 7 | Test White 11 | 0.72 | ***171*** | Train 11 | Test White 11 | 0.72 | ***235*** | Train 15 | Test White 11 | 0.71 |
| ***44*** | Train 3 | Test White 12 | 0.7 | ***108*** | Train 7 | Test White 12 | 0.7 | ***172*** | Train 11 | Test White 12 | 0.7 | ***236*** | Train 15 | Test White 12 | 0.7 |
| ***45*** | Train 3 | Test White 13 | 0.72 | ***109*** | Train 7 | Test White 13 | 0.72 | ***173*** | Train 11 | Test White 13 | 0.72 | ***237*** | Train 15 | Test White 13 | 0.73 |
| ***46*** | Train 3 | Test White 14 | 0.7 | ***110*** | Train 7 | Test White 14 | 0.7 | ***174*** | Train 11 | Test White 14 | 0.7 | ***238*** | Train 15 | Test White 14 | 0.7 |
| ***47*** | Train 3 | Test White 15 | 0.72 | ***111*** | Train 7 | Test White 15 | 0.72 | ***175*** | Train 11 | Test White 15 | 0.72 | ***239*** | Train 15 | Test White 15 | 0.73 |
| ***48*** | Train 3 | Test White 16 | 0.7 | ***112*** | Train 7 | Test White 16 | 0.7 | ***176*** | Train 11 | Test White 16 | 0.7 | ***240*** | Train 15 | Test White 16 | 0.7 |
| ***49*** | Train 4 | Test White 1 | 0.71 | ***113*** | Train 8 | Test White 1 | 0.71 | ***177*** | Train 12 | Test White 1 | 0.71 | ***241*** | Train 16 | Test White 1 | 0.7 |
| ***50*** | Train 4 | Test White 2 | 0.73 | ***114*** | Train 8 | Test White 2 | 0.73 | ***178*** | Train 12 | Test White 2 | 0.73 | ***242*** | Train 16 | Test White 2 | 0.71 |
| ***51*** | Train 4 | Test White 3 | 0.71 | ***115*** | Train 8 | Test White 3 | 0.71 | ***179*** | Train 12 | Test White 3 | 0.71 | ***243*** | Train 16 | Test White 3 | 0.7 |
| ***52*** | Train 4 | Test White 4 | 0.73 | ***116*** | Train 8 | Test White 4 | 0.73 | ***180*** | Train 12 | Test White 4 | 0.73 | ***244*** | Train 16 | Test White 4 | 0.71 |
| ***53*** | Train 4 | Test White 5 | 0.71 | ***117*** | Train 8 | Test White 5 | 0.71 | ***181*** | Train 12 | Test White 5 | 0.71 | ***245*** | Train 16 | Test White 5 | 0.7 |
| ***54*** | Train 4 | Test White 6 | 0.73 | ***118*** | Train 8 | Test White 6 | 0.73 | ***182*** | Train 12 | Test White 6 | 0.73 | ***246*** | Train 16 | Test White 6 | 0.71 |
| ***55*** | Train 4 | Test White 7 | 0.71 | ***119*** | Train 8 | Test White 7 | 0.71 | ***183*** | Train 12 | Test White 7 | 0.71 | ***247*** | Train 16 | Test White 7 | 0.7 |
| ***56*** | Train 4 | Test White 8 | 0.73 | ***120*** | Train 8 | Test White 8 | 0.73 | ***184*** | Train 12 | Test White 8 | 0.73 | ***248*** | Train 16 | Test White 8 | 0.71 |
| ***57*** | Train 4 | Test White 9 | 0.71 | ***121*** | Train 8 | Test White 9 | 0.71 | ***185*** | Train 12 | Test White 9 | 0.71 | ***249*** | Train 16 | Test White 9 | 0.7 |
| ***58*** | Train 4 | Test White 10 | 0.73 | ***122*** | Train 8 | Test White 10 | 0.73 | ***186*** | Train 12 | Test White 10 | 0.73 | ***250*** | Train 16 | Test White 10 | 0.71 |
| ***59*** | Train 4 | Test White 11 | 0.71 | ***123*** | Train 8 | Test White 11 | 0.71 | ***187*** | Train 12 | Test White 11 | 0.71 | ***251*** | Train 16 | Test White 11 | 0.7 |
| ***60*** | Train 4 | Test White 12 | 0.73 | ***124*** | Train 8 | Test White 12 | 0.73 | ***188*** | Train 12 | Test White 12 | 0.73 | ***252*** | Train 16 | Test White 12 | 0.71 |
| ***61*** | Train 4 | Test White 13 | 0.71 | ***125*** | Train 8 | Test White 13 | 0.71 | ***189*** | Train 12 | Test White 13 | 0.71 | ***253*** | Train 16 | Test White 13 | 0.72 |
| ***62*** | Train 4 | Test White 14 | 0.72 | ***126*** | Train 8 | Test White 14 | 0.72 | ***190*** | Train 12 | Test White 14 | 0.72 | ***254*** | Train 16 | Test White 14 | 0.73 |
| ***63*** | Train 4 | Test White 15 | 0.71 | ***127*** | Train 8 | Test White 15 | 0.71 | ***191*** | Train 12 | Test White 15 | 0.71 | ***255*** | Train 16 | Test White 15 | 0.72 |
| ***64*** | Train 4 | Test White 16 | 0.72 | ***128*** | Train 8 | Test White 16 | 0.72 | ***192*** | Train 12 | Test White 16 | 0.72 | ***256*** | Train 16 | Test White 16 | 0.73 |

Table 17. Random Forest (RF) AUC Performance for White Patients Across 16 Cohorts.

| Original Decisions | Train Model | Test Dataset | AUC (RF) | ***White*** | Train Model | Test Dataset | AUC (RF) | White | Train Model | Test Dataset | AUC (RF) | White | Train Model | Test Dataset | AUC (RF) |
| --- | --- | --- | --- | --- | --- | --- | --- | --- | --- | --- | --- | --- | --- | --- | --- |
| ***1*** | Train 1 | Test White 1 | 0.74 | ***65*** | Train 5 | Test White 1 | 0.74 | ***129*** | Train 9 | Test White 1 | 0.74 | ***193*** | Train 13 | Test White 1 | 0.72 |
| ***2*** | Train 1 | Test White 2 | 0.7 | ***66*** | Train 5 | Test White 2 | 0.7 | ***130*** | Train 9 | Test White 2 | 0.7 | ***194*** | Train 13 | Test White 2 | 0.69 |
| ***3*** | Train 1 | Test White 3 | 0.74 | ***67*** | Train 5 | Test White 3 | 0.74 | ***131*** | Train 9 | Test White 3 | 0.74 | ***195*** | Train 13 | Test White 3 | 0.72 |
| ***4*** | Train 1 | Test White 4 | 0.71 | ***68*** | Train 5 | Test White 4 | 0.71 | ***132*** | Train 9 | Test White 4 | 0.71 | ***196*** | Train 13 | Test White 4 | 0.69 |
| ***5*** | Train 1 | Test White 5 | 0.74 | ***69*** | Train 5 | Test White 5 | 0.74 | ***133*** | Train 9 | Test White 5 | 0.74 | ***197*** | Train 13 | Test White 5 | 0.72 |
| ***6*** | Train 1 | Test White 6 | 0.7 | ***70*** | Train 5 | Test White 6 | 0.7 | ***134*** | Train 9 | Test White 6 | 0.7 | ***198*** | Train 13 | Test White 6 | 0.69 |
| ***7*** | Train 1 | Test White 7 | 0.74 | ***71*** | Train 5 | Test White 7 | 0.74 | ***135*** | Train 9 | Test White 7 | 0.74 | ***199*** | Train 13 | Test White 7 | 0.72 |
| ***8*** | Train 1 | Test White 8 | 0.71 | ***72*** | Train 5 | Test White 8 | 0.71 | ***136*** | Train 9 | Test White 8 | 0.71 | ***200*** | Train 13 | Test White 8 | 0.69 |
| ***9*** | Train 1 | Test White 9 | 0.74 | ***73*** | Train 5 | Test White 9 | 0.74 | ***137*** | Train 9 | Test White 9 | 0.74 | ***201*** | Train 13 | Test White 9 | 0.72 |
| ***10*** | Train 1 | Test White 10 | 0.7 | ***74*** | Train 5 | Test White 10 | 0.7 | ***138*** | Train 9 | Test White 10 | 0.7 | ***202*** | Train 13 | Test White 10 | 0.69 |
| ***11*** | Train 1 | Test White 11 | 0.74 | ***75*** | Train 5 | Test White 11 | 0.74 | ***139*** | Train 9 | Test White 11 | 0.74 | ***203*** | Train 13 | Test White 11 | 0.72 |
| ***12*** | Train 1 | Test White 12 | 0.71 | ***76*** | Train 5 | Test White 12 | 0.71 | ***140*** | Train 9 | Test White 12 | 0.71 | ***204*** | Train 13 | Test White 12 | 0.69 |
| ***13*** | Train 1 | Test White 13 | 0.73 | ***77*** | Train 5 | Test White 13 | 0.73 | ***141*** | Train 9 | Test White 13 | 0.73 | ***205*** | Train 13 | Test White 13 | 0.73 |
| ***14*** | Train 1 | Test White 14 | 0.68 | ***78*** | Train 5 | Test White 14 | 0.69 | ***142*** | Train 9 | Test White 14 | 0.68 | ***206*** | Train 13 | Test White 14 | 0.68 |
| ***15*** | Train 1 | Test White 15 | 0.73 | ***79*** | Train 5 | Test White 15 | 0.73 | ***143*** | Train 9 | Test White 15 | 0.73 | ***207*** | Train 13 | Test White 15 | 0.73 |
| ***16*** | Train 1 | Test White 16 | 0.69 | ***80*** | Train 5 | Test White 16 | 0.69 | ***144*** | Train 9 | Test White 16 | 0.69 | ***208*** | Train 13 | Test White 16 | 0.68 |
| ***17*** | Train 2 | Test White 1 | 0.73 | ***81*** | Train 6 | Test White 1 | 0.73 | ***145*** | Train 10 | Test White 1 | 0.73 | ***209*** | Train 14 | Test White 1 | 0.71 |
| ***18*** | Train 2 | Test White 2 | 0.75 | ***82*** | Train 6 | Test White 2 | 0.75 | ***146*** | Train 10 | Test White 2 | 0.75 | ***210*** | Train 14 | Test White 2 | 0.72 |
| ***19*** | Train 2 | Test White 3 | 0.73 | ***83*** | Train 6 | Test White 3 | 0.73 | ***147*** | Train 10 | Test White 3 | 0.73 | ***211*** | Train 14 | Test White 3 | 0.71 |
| ***20*** | Train 2 | Test White 4 | 0.75 | ***84*** | Train 6 | Test White 4 | 0.75 | ***148*** | Train 10 | Test White 4 | 0.75 | ***212*** | Train 14 | Test White 4 | 0.72 |
| ***21*** | Train 2 | Test White 5 | 0.73 | ***85*** | Train 6 | Test White 5 | 0.73 | ***149*** | Train 10 | Test White 5 | 0.73 | ***213*** | Train 14 | Test White 5 | 0.71 |
| ***22*** | Train 2 | Test White 6 | 0.75 | ***86*** | Train 6 | Test White 6 | 0.75 | ***150*** | Train 10 | Test White 6 | 0.75 | ***214*** | Train 14 | Test White 6 | 0.72 |
| ***23*** | Train 2 | Test White 7 | 0.73 | ***87*** | Train 6 | Test White 7 | 0.73 | ***151*** | Train 10 | Test White 7 | 0.73 | ***215*** | Train 14 | Test White 7 | 0.71 |
| ***24*** | Train 2 | Test White 8 | 0.75 | ***88*** | Train 6 | Test White 8 | 0.75 | ***152*** | Train 10 | Test White 8 | 0.75 | ***216*** | Train 14 | Test White 8 | 0.73 |
| ***25*** | Train 2 | Test White 9 | 0.73 | ***89*** | Train 6 | Test White 9 | 0.73 | ***153*** | Train 10 | Test White 9 | 0.73 | ***217*** | Train 14 | Test White 9 | 0.71 |
| ***26*** | Train 2 | Test White 10 | 0.75 | ***90*** | Train 6 | Test White 10 | 0.75 | ***154*** | Train 10 | Test White 10 | 0.75 | ***218*** | Train 14 | Test White 10 | 0.72 |
| ***27*** | Train 2 | Test White 11 | 0.73 | ***91*** | Train 6 | Test White 11 | 0.73 | ***155*** | Train 10 | Test White 11 | 0.73 | ***219*** | Train 14 | Test White 11 | 0.71 |
| ***28*** | Train 2 | Test White 12 | 0.75 | ***92*** | Train 6 | Test White 12 | 0.75 | ***156*** | Train 10 | Test White 12 | 0.75 | ***220*** | Train 14 | Test White 12 | 0.73 |
| ***29*** | Train 2 | Test White 13 | 0.73 | ***93*** | Train 6 | Test White 13 | 0.73 | ***157*** | Train 10 | Test White 13 | 0.73 | ***221*** | Train 14 | Test White 13 | 0.73 |
| ***30*** | Train 2 | Test White 14 | 0.74 | ***94*** | Train 6 | Test White 14 | 0.74 | ***158*** | Train 10 | Test White 14 | 0.74 | ***222*** | Train 14 | Test White 14 | 0.74 |
| ***31*** | Train 2 | Test White 15 | 0.73 | ***95*** | Train 6 | Test White 15 | 0.73 | ***159*** | Train 10 | Test White 15 | 0.73 | ***223*** | Train 14 | Test White 15 | 0.73 |
| ***32*** | Train 2 | Test White 16 | 0.74 | ***96*** | Train 6 | Test White 16 | 0.74 | ***160*** | Train 10 | Test White 16 | 0.74 | ***224*** | Train 14 | Test White 16 | 0.74 |
| ***33*** | Train 3 | Test White 1 | 0.74 | ***97*** | Train 7 | Test White 1 | 0.74 | ***161*** | Train 11 | Test White 1 | 0.74 | ***225*** | Train 15 | Test White 1 | 0.72 |
| ***34*** | Train 3 | Test White 2 | 0.7 | ***98*** | Train 7 | Test White 2 | 0.7 | ***162*** | Train 11 | Test White 2 | 0.7 | ***226*** | Train 15 | Test White 2 | 0.69 |
| ***35*** | Train 3 | Test White 3 | 0.74 | ***99*** | Train 7 | Test White 3 | 0.74 | ***163*** | Train 11 | Test White 3 | 0.74 | ***227*** | Train 15 | Test White 3 | 0.72 |
| ***36*** | Train 3 | Test White 4 | 0.71 | ***100*** | Train 7 | Test White 4 | 0.71 | ***164*** | Train 11 | Test White 4 | 0.71 | ***228*** | Train 15 | Test White 4 | 0.69 |
| ***37*** | Train 3 | Test White 5 | 0.74 | ***101*** | Train 7 | Test White 5 | 0.74 | ***165*** | Train 11 | Test White 5 | 0.74 | ***229*** | Train 15 | Test White 5 | 0.72 |
| ***38*** | Train 3 | Test White 6 | 0.7 | ***102*** | Train 7 | Test White 6 | 0.7 | ***166*** | Train 11 | Test White 6 | 0.7 | ***230*** | Train 15 | Test White 6 | 0.69 |
| ***39*** | Train 3 | Test White 7 | 0.74 | ***103*** | Train 7 | Test White 7 | 0.74 | ***167*** | Train 11 | Test White 7 | 0.74 | ***231*** | Train 15 | Test White 7 | 0.72 |
| ***40*** | Train 3 | Test White 8 | 0.71 | ***104*** | Train 7 | Test White 8 | 0.71 | ***168*** | Train 11 | Test White 8 | 0.71 | ***232*** | Train 15 | Test White 8 | 0.69 |
| ***41*** | Train 3 | Test White 9 | 0.74 | ***105*** | Train 7 | Test White 9 | 0.74 | ***169*** | Train 11 | Test White 9 | 0.74 | ***233*** | Train 15 | Test White 9 | 0.72 |
| ***42*** | Train 3 | Test White 10 | 0.7 | ***106*** | Train 7 | Test White 10 | 0.7 | ***170*** | Train 11 | Test White 10 | 0.7 | ***234*** | Train 15 | Test White 10 | 0.69 |
| ***43*** | Train 3 | Test White 11 | 0.74 | ***107*** | Train 7 | Test White 11 | 0.74 | ***171*** | Train 11 | Test White 11 | 0.74 | ***235*** | Train 15 | Test White 11 | 0.72 |
| ***44*** | Train 3 | Test White 12 | 0.71 | ***108*** | Train 7 | Test White 12 | 0.71 | ***172*** | Train 11 | Test White 12 | 0.71 | ***236*** | Train 15 | Test White 12 | 0.69 |
| ***45*** | Train 3 | Test White 13 | 0.73 | ***109*** | Train 7 | Test White 13 | 0.73 | ***173*** | Train 11 | Test White 13 | 0.73 | ***237*** | Train 15 | Test White 13 | 0.73 |
| ***46*** | Train 3 | Test White 14 | 0.68 | ***110*** | Train 7 | Test White 14 | 0.68 | ***174*** | Train 11 | Test White 14 | 0.69 | ***238*** | Train 15 | Test White 14 | 0.68 |
| ***47*** | Train 3 | Test White 15 | 0.73 | ***111*** | Train 7 | Test White 15 | 0.73 | ***175*** | Train 11 | Test White 15 | 0.73 | ***239*** | Train 15 | Test White 15 | 0.73 |
| ***48*** | Train 3 | Test White 16 | 0.69 | ***112*** | Train 7 | Test White 16 | 0.69 | ***176*** | Train 11 | Test White 16 | 0.69 | ***240*** | Train 15 | Test White 16 | 0.68 |
| ***49*** | Train 4 | Test White 1 | 0.73 | ***113*** | Train 8 | Test White 1 | 0.73 | ***177*** | Train 12 | Test White 1 | 0.73 | ***241*** | Train 16 | Test White 1 | 0.71 |
| ***50*** | Train 4 | Test White 2 | 0.75 | ***114*** | Train 8 | Test White 2 | 0.75 | ***178*** | Train 12 | Test White 2 | 0.75 | ***242*** | Train 16 | Test White 2 | 0.73 |
| ***51*** | Train 4 | Test White 3 | 0.73 | ***115*** | Train 8 | Test White 3 | 0.73 | ***179*** | Train 12 | Test White 3 | 0.73 | ***243*** | Train 16 | Test White 3 | 0.71 |
| ***52*** | Train 4 | Test White 4 | 0.75 | ***116*** | Train 8 | Test White 4 | 0.75 | ***180*** | Train 12 | Test White 4 | 0.75 | ***244*** | Train 16 | Test White 4 | 0.73 |
| ***53*** | Train 4 | Test White 5 | 0.73 | ***117*** | Train 8 | Test White 5 | 0.73 | ***181*** | Train 12 | Test White 5 | 0.73 | ***245*** | Train 16 | Test White 5 | 0.71 |
| ***54*** | Train 4 | Test White 6 | 0.75 | ***118*** | Train 8 | Test White 6 | 0.75 | ***182*** | Train 12 | Test White 6 | 0.75 | ***246*** | Train 16 | Test White 6 | 0.73 |
| ***55*** | Train 4 | Test White 7 | 0.73 | ***119*** | Train 8 | Test White 7 | 0.73 | ***183*** | Train 12 | Test White 7 | 0.73 | ***247*** | Train 16 | Test White 7 | 0.71 |
| ***56*** | Train 4 | Test White 8 | 0.75 | ***120*** | Train 8 | Test White 8 | 0.75 | ***184*** | Train 12 | Test White 8 | 0.75 | ***248*** | Train 16 | Test White 8 | 0.73 |
| ***57*** | Train 4 | Test White 9 | 0.73 | ***121*** | Train 8 | Test White 9 | 0.73 | ***185*** | Train 12 | Test White 9 | 0.73 | ***249*** | Train 16 | Test White 9 | 0.71 |
| ***58*** | Train 4 | Test White 10 | 0.75 | ***122*** | Train 8 | Test White 10 | 0.75 | ***186*** | Train 12 | Test White 10 | 0.75 | ***250*** | Train 16 | Test White 10 | 0.73 |
| ***59*** | Train 4 | Test White 11 | 0.73 | ***123*** | Train 8 | Test White 11 | 0.73 | ***187*** | Train 12 | Test White 11 | 0.73 | ***251*** | Train 16 | Test White 11 | 0.71 |
| ***60*** | Train 4 | Test White 12 | 0.75 | ***124*** | Train 8 | Test White 12 | 0.75 | ***188*** | Train 12 | Test White 12 | 0.75 | ***252*** | Train 16 | Test White 12 | 0.73 |
| ***61*** | Train 4 | Test White 13 | 0.73 | ***125*** | Train 8 | Test White 13 | 0.73 | ***189*** | Train 12 | Test White 13 | 0.73 | ***253*** | Train 16 | Test White 13 | 0.73 |
| ***62*** | Train 4 | Test White 14 | 0.74 | ***126*** | Train 8 | Test White 14 | 0.74 | ***190*** | Train 12 | Test White 14 | 0.74 | ***254*** | Train 16 | Test White 14 | 0.74 |
| ***63*** | Train 4 | Test White 15 | 0.73 | ***127*** | Train 8 | Test White 15 | 0.73 | ***191*** | Train 12 | Test White 15 | 0.73 | ***255*** | Train 16 | Test White 15 | 0.73 |
| ***64*** | Train 4 | Test White 16 | 0.74 | ***128*** | Train 8 | Test White 16 | 0.74 | ***192*** | Train 12 | Test White 16 | 0.74 | ***256*** | Train 16 | Test White 16 | 0.74 |

Table 18. Gradient Boosting (GB) AUC Performance for White Patients Across 16 Cohorts.

| Original Decisions | Train Model | Test Dataset | AUC (GB) | ***White*** | Train Model | Test Dataset | AUC (GB) | White | Train Model | Test Dataset | AUC (GB) | White | Train Model | Test Dataset | AUC (GB) |
| --- | --- | --- | --- | --- | --- | --- | --- | --- | --- | --- | --- | --- | --- | --- | --- |
| ***1*** | Train 1 | Test White 1 | 0.74 | ***65*** | Train 5 | Test White 1 | 0.74 | ***129*** | Train 9 | Test White 1 | 0.74 | ***193*** | Train 13 | Test White 1 | 0.68 |
| ***2*** | Train 1 | Test White 2 | 0.69 | ***66*** | Train 5 | Test White 2 | 0.69 | ***130*** | Train 9 | Test White 2 | 0.7 | ***194*** | Train 13 | Test White 2 | 0.65 |
| ***3*** | Train 1 | Test White 3 | 0.74 | ***67*** | Train 5 | Test White 3 | 0.74 | ***131*** | Train 9 | Test White 3 | 0.74 | ***195*** | Train 13 | Test White 3 | 0.68 |
| ***4*** | Train 1 | Test White 4 | 0.69 | ***68*** | Train 5 | Test White 4 | 0.69 | ***132*** | Train 9 | Test White 4 | 0.7 | ***196*** | Train 13 | Test White 4 | 0.65 |
| ***5*** | Train 1 | Test White 5 | 0.74 | ***69*** | Train 5 | Test White 5 | 0.74 | ***133*** | Train 9 | Test White 5 | 0.74 | ***197*** | Train 13 | Test White 5 | 0.68 |
| ***6*** | Train 1 | Test White 6 | 0.69 | ***70*** | Train 5 | Test White 6 | 0.69 | ***134*** | Train 9 | Test White 6 | 0.7 | ***198*** | Train 13 | Test White 6 | 0.65 |
| ***7*** | Train 1 | Test White 7 | 0.74 | ***71*** | Train 5 | Test White 7 | 0.74 | ***135*** | Train 9 | Test White 7 | 0.74 | ***199*** | Train 13 | Test White 7 | 0.68 |
| ***8*** | Train 1 | Test White 8 | 0.69 | ***72*** | Train 5 | Test White 8 | 0.69 | ***136*** | Train 9 | Test White 8 | 0.7 | ***200*** | Train 13 | Test White 8 | 0.65 |
| ***9*** | Train 1 | Test White 9 | 0.74 | ***73*** | Train 5 | Test White 9 | 0.74 | ***137*** | Train 9 | Test White 9 | 0.74 | ***201*** | Train 13 | Test White 9 | 0.68 |
| ***10*** | Train 1 | Test White 10 | 0.69 | ***74*** | Train 5 | Test White 10 | 0.69 | ***138*** | Train 9 | Test White 10 | 0.7 | ***202*** | Train 13 | Test White 10 | 0.65 |
| ***11*** | Train 1 | Test White 11 | 0.74 | ***75*** | Train 5 | Test White 11 | 0.74 | ***139*** | Train 9 | Test White 11 | 0.74 | ***203*** | Train 13 | Test White 11 | 0.68 |
| ***12*** | Train 1 | Test White 12 | 0.69 | ***76*** | Train 5 | Test White 12 | 0.69 | ***140*** | Train 9 | Test White 12 | 0.7 | ***204*** | Train 13 | Test White 12 | 0.65 |
| ***13*** | Train 1 | Test White 13 | 0.72 | ***77*** | Train 5 | Test White 13 | 0.72 | ***141*** | Train 9 | Test White 13 | 0.72 | ***205*** | Train 13 | Test White 13 | 0.72 |
| ***14*** | Train 1 | Test White 14 | 0.68 | ***78*** | Train 5 | Test White 14 | 0.68 | ***142*** | Train 9 | Test White 14 | 0.69 | ***206*** | Train 13 | Test White 14 | 0.67 |
| ***15*** | Train 1 | Test White 15 | 0.72 | ***79*** | Train 5 | Test White 15 | 0.72 | ***143*** | Train 9 | Test White 15 | 0.72 | ***207*** | Train 13 | Test White 15 | 0.72 |
| ***16*** | Train 1 | Test White 16 | 0.68 | ***80*** | Train 5 | Test White 16 | 0.68 | ***144*** | Train 9 | Test White 16 | 0.69 | ***208*** | Train 13 | Test White 16 | 0.67 |
| ***17*** | Train 2 | Test White 1 | 0.73 | ***81*** | Train 6 | Test White 1 | 0.74 | ***145*** | Train 10 | Test White 1 | 0.74 | ***209*** | Train 14 | Test White 1 | 0.68 |
| ***18*** | Train 2 | Test White 2 | 0.75 | ***82*** | Train 6 | Test White 2 | 0.75 | ***146*** | Train 10 | Test White 2 | 0.75 | ***210*** | Train 14 | Test White 2 | 0.69 |
| ***19*** | Train 2 | Test White 3 | 0.73 | ***83*** | Train 6 | Test White 3 | 0.74 | ***147*** | Train 10 | Test White 3 | 0.73 | ***211*** | Train 14 | Test White 3 | 0.68 |
| ***20*** | Train 2 | Test White 4 | 0.75 | ***84*** | Train 6 | Test White 4 | 0.75 | ***148*** | Train 10 | Test White 4 | 0.75 | ***212*** | Train 14 | Test White 4 | 0.69 |
| ***21*** | Train 2 | Test White 5 | 0.73 | ***85*** | Train 6 | Test White 5 | 0.74 | ***149*** | Train 10 | Test White 5 | 0.74 | ***213*** | Train 14 | Test White 5 | 0.68 |
| ***22*** | Train 2 | Test White 6 | 0.75 | ***86*** | Train 6 | Test White 6 | 0.75 | ***150*** | Train 10 | Test White 6 | 0.75 | ***214*** | Train 14 | Test White 6 | 0.69 |
| ***23*** | Train 2 | Test White 7 | 0.73 | ***87*** | Train 6 | Test White 7 | 0.74 | ***151*** | Train 10 | Test White 7 | 0.74 | ***215*** | Train 14 | Test White 7 | 0.68 |
| ***24*** | Train 2 | Test White 8 | 0.75 | ***88*** | Train 6 | Test White 8 | 0.75 | ***152*** | Train 10 | Test White 8 | 0.75 | ***216*** | Train 14 | Test White 8 | 0.69 |
| ***25*** | Train 2 | Test White 9 | 0.73 | ***89*** | Train 6 | Test White 9 | 0.74 | ***153*** | Train 10 | Test White 9 | 0.74 | ***217*** | Train 14 | Test White 9 | 0.68 |
| ***26*** | Train 2 | Test White 10 | 0.75 | ***90*** | Train 6 | Test White 10 | 0.75 | ***154*** | Train 10 | Test White 10 | 0.75 | ***218*** | Train 14 | Test White 10 | 0.69 |
| ***27*** | Train 2 | Test White 11 | 0.73 | ***91*** | Train 6 | Test White 11 | 0.74 | ***155*** | Train 10 | Test White 11 | 0.74 | ***219*** | Train 14 | Test White 11 | 0.68 |
| ***28*** | Train 2 | Test White 12 | 0.75 | ***92*** | Train 6 | Test White 12 | 0.75 | ***156*** | Train 10 | Test White 12 | 0.75 | ***220*** | Train 14 | Test White 12 | 0.69 |
| ***29*** | Train 2 | Test White 13 | 0.73 | ***93*** | Train 6 | Test White 13 | 0.73 | ***157*** | Train 10 | Test White 13 | 0.73 | ***221*** | Train 14 | Test White 13 | 0.72 |
| ***30*** | Train 2 | Test White 14 | 0.74 | ***94*** | Train 6 | Test White 14 | 0.74 | ***158*** | Train 10 | Test White 14 | 0.74 | ***222*** | Train 14 | Test White 14 | 0.74 |
| ***31*** | Train 2 | Test White 15 | 0.73 | ***95*** | Train 6 | Test White 15 | 0.73 | ***159*** | Train 10 | Test White 15 | 0.73 | ***223*** | Train 14 | Test White 15 | 0.73 |
| ***32*** | Train 2 | Test White 16 | 0.74 | ***96*** | Train 6 | Test White 16 | 0.74 | ***160*** | Train 10 | Test White 16 | 0.74 | ***224*** | Train 14 | Test White 16 | 0.74 |
| ***33*** | Train 3 | Test White 1 | 0.75 | ***97*** | Train 7 | Test White 1 | 0.74 | ***161*** | Train 11 | Test White 1 | 0.74 | ***225*** | Train 15 | Test White 1 | 0.68 |
| ***34*** | Train 3 | Test White 2 | 0.69 | ***98*** | Train 7 | Test White 2 | 0.7 | ***162*** | Train 11 | Test White 2 | 0.69 | ***226*** | Train 15 | Test White 2 | 0.66 |
| ***35*** | Train 3 | Test White 3 | 0.74 | ***99*** | Train 7 | Test White 3 | 0.74 | ***163*** | Train 11 | Test White 3 | 0.74 | ***227*** | Train 15 | Test White 3 | 0.68 |
| ***36*** | Train 3 | Test White 4 | 0.69 | ***100*** | Train 7 | Test White 4 | 0.7 | ***164*** | Train 11 | Test White 4 | 0.69 | ***228*** | Train 15 | Test White 4 | 0.66 |
| ***37*** | Train 3 | Test White 5 | 0.75 | ***101*** | Train 7 | Test White 5 | 0.74 | ***165*** | Train 11 | Test White 5 | 0.74 | ***229*** | Train 15 | Test White 5 | 0.68 |
| ***38*** | Train 3 | Test White 6 | 0.69 | ***102*** | Train 7 | Test White 6 | 0.7 | ***166*** | Train 11 | Test White 6 | 0.69 | ***230*** | Train 15 | Test White 6 | 0.66 |
| ***39*** | Train 3 | Test White 7 | 0.74 | ***103*** | Train 7 | Test White 7 | 0.74 | ***167*** | Train 11 | Test White 7 | 0.74 | ***231*** | Train 15 | Test White 7 | 0.68 |
| ***40*** | Train 3 | Test White 8 | 0.69 | ***104*** | Train 7 | Test White 8 | 0.7 | ***168*** | Train 11 | Test White 8 | 0.69 | ***232*** | Train 15 | Test White 8 | 0.66 |
| ***41*** | Train 3 | Test White 9 | 0.74 | ***105*** | Train 7 | Test White 9 | 0.74 | ***169*** | Train 11 | Test White 9 | 0.74 | ***233*** | Train 15 | Test White 9 | 0.68 |
| ***42*** | Train 3 | Test White 10 | 0.69 | ***106*** | Train 7 | Test White 10 | 0.7 | ***170*** | Train 11 | Test White 10 | 0.69 | ***234*** | Train 15 | Test White 10 | 0.66 |
| ***43*** | Train 3 | Test White 11 | 0.74 | ***107*** | Train 7 | Test White 11 | 0.74 | ***171*** | Train 11 | Test White 11 | 0.74 | ***235*** | Train 15 | Test White 11 | 0.68 |
| ***44*** | Train 3 | Test White 12 | 0.69 | ***108*** | Train 7 | Test White 12 | 0.7 | ***172*** | Train 11 | Test White 12 | 0.69 | ***236*** | Train 15 | Test White 12 | 0.66 |
| ***45*** | Train 3 | Test White 13 | 0.73 | ***109*** | Train 7 | Test White 13 | 0.72 | ***173*** | Train 11 | Test White 13 | 0.72 | ***237*** | Train 15 | Test White 13 | 0.72 |
| ***46*** | Train 3 | Test White 14 | 0.67 | ***110*** | Train 7 | Test White 14 | 0.68 | ***174*** | Train 11 | Test White 14 | 0.67 | ***238*** | Train 15 | Test White 14 | 0.68 |
| ***47*** | Train 3 | Test White 15 | 0.73 | ***111*** | Train 7 | Test White 15 | 0.72 | ***175*** | Train 11 | Test White 15 | 0.72 | ***239*** | Train 15 | Test White 15 | 0.72 |
| ***48*** | Train 3 | Test White 16 | 0.67 | ***112*** | Train 7 | Test White 16 | 0.69 | ***176*** | Train 11 | Test White 16 | 0.67 | ***240*** | Train 15 | Test White 16 | 0.68 |
| ***49*** | Train 4 | Test White 1 | 0.74 | ***113*** | Train 8 | Test White 1 | 0.74 | ***177*** | Train 12 | Test White 1 | 0.74 | ***241*** | Train 16 | Test White 1 | 0.64 |
| ***50*** | Train 4 | Test White 2 | 0.75 | ***114*** | Train 8 | Test White 2 | 0.75 | ***178*** | Train 12 | Test White 2 | 0.75 | ***242*** | Train 16 | Test White 2 | 0.67 |
| ***51*** | Train 4 | Test White 3 | 0.74 | ***115*** | Train 8 | Test White 3 | 0.74 | ***179*** | Train 12 | Test White 3 | 0.74 | ***243*** | Train 16 | Test White 3 | 0.64 |
| ***52*** | Train 4 | Test White 4 | 0.75 | ***116*** | Train 8 | Test White 4 | 0.75 | ***180*** | Train 12 | Test White 4 | 0.75 | ***244*** | Train 16 | Test White 4 | 0.67 |
| ***53*** | Train 4 | Test White 5 | 0.74 | ***117*** | Train 8 | Test White 5 | 0.74 | ***181*** | Train 12 | Test White 5 | 0.74 | ***245*** | Train 16 | Test White 5 | 0.64 |
| ***54*** | Train 4 | Test White 6 | 0.75 | ***118*** | Train 8 | Test White 6 | 0.75 | ***182*** | Train 12 | Test White 6 | 0.75 | ***246*** | Train 16 | Test White 6 | 0.67 |
| ***55*** | Train 4 | Test White 7 | 0.74 | ***119*** | Train 8 | Test White 7 | 0.74 | ***183*** | Train 12 | Test White 7 | 0.74 | ***247*** | Train 16 | Test White 7 | 0.64 |
| ***56*** | Train 4 | Test White 8 | 0.75 | ***120*** | Train 8 | Test White 8 | 0.75 | ***184*** | Train 12 | Test White 8 | 0.75 | ***248*** | Train 16 | Test White 8 | 0.67 |
| ***57*** | Train 4 | Test White 9 | 0.74 | ***121*** | Train 8 | Test White 9 | 0.74 | ***185*** | Train 12 | Test White 9 | 0.74 | ***249*** | Train 16 | Test White 9 | 0.64 |
| ***58*** | Train 4 | Test White 10 | 0.75 | ***122*** | Train 8 | Test White 10 | 0.75 | ***186*** | Train 12 | Test White 10 | 0.75 | ***250*** | Train 16 | Test White 10 | 0.67 |
| ***59*** | Train 4 | Test White 11 | 0.74 | ***123*** | Train 8 | Test White 11 | 0.74 | ***187*** | Train 12 | Test White 11 | 0.74 | ***251*** | Train 16 | Test White 11 | 0.64 |
| ***60*** | Train 4 | Test White 12 | 0.75 | ***124*** | Train 8 | Test White 12 | 0.75 | ***188*** | Train 12 | Test White 12 | 0.75 | ***252*** | Train 16 | Test White 12 | 0.67 |
| ***61*** | Train 4 | Test White 13 | 0.73 | ***125*** | Train 8 | Test White 13 | 0.73 | ***189*** | Train 12 | Test White 13 | 0.73 | ***253*** | Train 16 | Test White 13 | 0.73 |
| ***62*** | Train 4 | Test White 14 | 0.74 | ***126*** | Train 8 | Test White 14 | 0.74 | ***190*** | Train 12 | Test White 14 | 0.74 | ***254*** | Train 16 | Test White 14 | 0.74 |
| ***63*** | Train 4 | Test White 15 | 0.73 | ***127*** | Train 8 | Test White 15 | 0.73 | ***191*** | Train 12 | Test White 15 | 0.73 | ***255*** | Train 16 | Test White 15 | 0.73 |
| ***64*** | Train 4 | Test White 16 | 0.74 | ***128*** | Train 8 | Test White 16 | 0.74 | ***192*** | Train 12 | Test White 16 | 0.74 | ***256*** | Train 16 | Test White 16 | 0.74 |

Table 19. Logistic Regression (LR) AUC Performance for Asian Patients Across 16 Cohorts.

| Original Decisions | Train Model | Test Dataset | AUC (LR) | ***Asian*** | ***Train Model*** | ***Test Dataset*** | ***AUC (LR)*** | ***Asian*** | ***Train Model*** | ***Test Dataset*** | ***AUC (LR)*** | ***Asian*** | ***Train Model*** | Test Dataset | AUC (LR) |
| --- | --- | --- | --- | --- | --- | --- | --- | --- | --- | --- | --- | --- | --- | --- | --- |
| ***1*** | Train 1 | Test Asian 1 | 0.79 | ***65*** | Train 5 | Test Asian 1 | 0.79 | ***129*** | Train 9 | Test Asian 1 | 0.79 | ***193*** | Train 13 | Test Asian 1 | 0.76 |
| ***2*** | Train 1 | Test Asian 2 | 0.77 | ***66*** | Train 5 | Test Asian 2 | 0.77 | ***130*** | Train 9 | Test Asian 2 | 0.77 | ***194*** | Train 13 | Test Asian 2 | 0.74 |
| ***3*** | Train 1 | Test Asian 3 | 0.79 | ***67*** | Train 5 | Test Asian 3 | 0.79 | ***131*** | Train 9 | Test Asian 3 | 0.79 | ***195*** | Train 13 | Test Asian 3 | 0.76 |
| ***4*** | Train 1 | Test Asian 4 | 0.77 | ***68*** | Train 5 | Test Asian 4 | 0.77 | ***132*** | Train 9 | Test Asian 4 | 0.77 | ***196*** | Train 13 | Test Asian 4 | 0.74 |
| ***5*** | Train 1 | Test Asian 5 | 0.79 | ***69*** | Train 5 | Test Asian 5 | 0.79 | ***133*** | Train 9 | Test Asian 5 | 0.79 | ***197*** | Train 13 | Test Asian 5 | 0.76 |
| ***6*** | Train 1 | Test Asian 6 | 0.77 | ***70*** | Train 5 | Test Asian 6 | 0.77 | ***134*** | Train 9 | Test Asian 6 | 0.77 | ***198*** | Train 13 | Test Asian 6 | 0.74 |
| ***7*** | Train 1 | Test Asian 7 | 0.79 | ***71*** | Train 5 | Test Asian 7 | 0.79 | ***135*** | Train 9 | Test Asian 7 | 0.79 | ***199*** | Train 13 | Test Asian 7 | 0.76 |
| ***8*** | Train 1 | Test Asian 8 | 0.77 | ***72*** | Train 5 | Test Asian 8 | 0.77 | ***136*** | Train 9 | Test Asian 8 | 0.77 | ***200*** | Train 13 | Test Asian 8 | 0.74 |
| ***9*** | Train 1 | Test Asian 9 | 0.78 | ***73*** | Train 5 | Test Asian 9 | 0.78 | ***137*** | Train 9 | Test Asian 9 | 0.78 | ***201*** | Train 13 | Test Asian 9 | 0.75 |
| ***10*** | Train 1 | Test Asian 10 | 0.76 | ***74*** | Train 5 | Test Asian 10 | 0.76 | ***138*** | Train 9 | Test Asian 10 | 0.76 | ***202*** | Train 13 | Test Asian 10 | 0.74 |
| ***11*** | Train 1 | Test Asian 11 | 0.78 | ***75*** | Train 5 | Test Asian 11 | 0.78 | ***139*** | Train 9 | Test Asian 11 | 0.78 | ***203*** | Train 13 | Test Asian 11 | 0.75 |
| ***12*** | Train 1 | Test Asian 12 | 0.76 | ***76*** | Train 5 | Test Asian 12 | 0.76 | ***140*** | Train 9 | Test Asian 12 | 0.76 | ***204*** | Train 13 | Test Asian 12 | 0.74 |
| ***13*** | Train 1 | Test Asian 13 | 0.79 | ***77*** | Train 5 | Test Asian 13 | 0.79 | ***141*** | Train 9 | Test Asian 13 | 0.79 | ***205*** | Train 13 | Test Asian 13 | 0.79 |
| ***14*** | Train 1 | Test Asian 14 | 0.78 | ***78*** | Train 5 | Test Asian 14 | 0.78 | ***142*** | Train 9 | Test Asian 14 | 0.78 | ***206*** | Train 13 | Test Asian 14 | 0.77 |
| ***15*** | Train 1 | Test Asian 15 | 0.8 | ***79*** | Train 5 | Test Asian 15 | 0.8 | ***143*** | Train 9 | Test Asian 15 | 0.8 | ***207*** | Train 13 | Test Asian 15 | 0.79 |
| ***16*** | Train 1 | Test Asian 16 | 0.78 | ***80*** | Train 5 | Test Asian 16 | 0.78 | ***144*** | Train 9 | Test Asian 16 | 0.78 | ***208*** | Train 13 | Test Asian 16 | 0.77 |
| ***17*** | Train 2 | Test Asian 1 | 0.78 | ***81*** | Train 6 | Test Asian 1 | 0.78 | ***145*** | Train 10 | Test Asian 1 | 0.78 | ***209*** | Train 14 | Test Asian 1 | 0.76 |
| ***18*** | Train 2 | Test Asian 2 | 0.78 | ***82*** | Train 6 | Test Asian 2 | 0.78 | ***146*** | Train 10 | Test Asian 2 | 0.78 | ***210*** | Train 14 | Test Asian 2 | 0.76 |
| ***19*** | Train 2 | Test Asian 3 | 0.78 | ***83*** | Train 6 | Test Asian 3 | 0.78 | ***147*** | Train 10 | Test Asian 3 | 0.78 | ***211*** | Train 14 | Test Asian 3 | 0.76 |
| ***20*** | Train 2 | Test Asian 4 | 0.77 | ***84*** | Train 6 | Test Asian 4 | 0.77 | ***148*** | Train 10 | Test Asian 4 | 0.77 | ***212*** | Train 14 | Test Asian 4 | 0.76 |
| ***21*** | Train 2 | Test Asian 5 | 0.78 | ***85*** | Train 6 | Test Asian 5 | 0.78 | ***149*** | Train 10 | Test Asian 5 | 0.78 | ***213*** | Train 14 | Test Asian 5 | 0.76 |
| ***22*** | Train 2 | Test Asian 6 | 0.78 | ***86*** | Train 6 | Test Asian 6 | 0.78 | ***150*** | Train 10 | Test Asian 6 | 0.78 | ***214*** | Train 14 | Test Asian 6 | 0.76 |
| ***23*** | Train 2 | Test Asian 7 | 0.78 | ***87*** | Train 6 | Test Asian 7 | 0.78 | ***151*** | Train 10 | Test Asian 7 | 0.78 | ***215*** | Train 14 | Test Asian 7 | 0.76 |
| ***24*** | Train 2 | Test Asian 8 | 0.78 | ***88*** | Train 6 | Test Asian 8 | 0.77 | ***152*** | Train 10 | Test Asian 8 | 0.77 | ***216*** | Train 14 | Test Asian 8 | 0.76 |
| ***25*** | Train 2 | Test Asian 9 | 0.78 | ***89*** | Train 6 | Test Asian 9 | 0.78 | ***153*** | Train 10 | Test Asian 9 | 0.78 | ***217*** | Train 14 | Test Asian 9 | 0.75 |
| ***26*** | Train 2 | Test Asian 10 | 0.77 | ***90*** | Train 6 | Test Asian 10 | 0.77 | ***154*** | Train 10 | Test Asian 10 | 0.77 | ***218*** | Train 14 | Test Asian 10 | 0.76 |
| ***27*** | Train 2 | Test Asian 11 | 0.77 | ***91*** | Train 6 | Test Asian 11 | 0.77 | ***155*** | Train 10 | Test Asian 11 | 0.77 | ***219*** | Train 14 | Test Asian 11 | 0.75 |
| ***28*** | Train 2 | Test Asian 12 | 0.77 | ***92*** | Train 6 | Test Asian 12 | 0.77 | ***156*** | Train 10 | Test Asian 12 | 0.77 | ***220*** | Train 14 | Test Asian 12 | 0.75 |
| ***29*** | Train 2 | Test Asian 13 | 0.79 | ***93*** | Train 6 | Test Asian 13 | 0.79 | ***157*** | Train 10 | Test Asian 13 | 0.79 | ***221*** | Train 14 | Test Asian 13 | 0.78 |
| ***30*** | Train 2 | Test Asian 14 | 0.79 | ***94*** | Train 6 | Test Asian 14 | 0.79 | ***158*** | Train 10 | Test Asian 14 | 0.79 | ***222*** | Train 14 | Test Asian 14 | 0.79 |
| ***31*** | Train 2 | Test Asian 15 | 0.79 | ***95*** | Train 6 | Test Asian 15 | 0.79 | ***159*** | Train 10 | Test Asian 15 | 0.79 | ***223*** | Train 14 | Test Asian 15 | 0.78 |
| ***32*** | Train 2 | Test Asian 16 | 0.79 | ***96*** | Train 6 | Test Asian 16 | 0.79 | ***160*** | Train 10 | Test Asian 16 | 0.79 | ***224*** | Train 14 | Test Asian 16 | 0.79 |
| ***33*** | Train 3 | Test Asian 1 | 0.79 | ***97*** | Train 7 | Test Asian 1 | 0.79 | ***161*** | Train 11 | Test Asian 1 | 0.79 | ***225*** | Train 15 | Test Asian 1 | 0.76 |
| ***34*** | Train 3 | Test Asian 2 | 0.77 | ***98*** | Train 7 | Test Asian 2 | 0.77 | ***162*** | Train 11 | Test Asian 2 | 0.77 | ***226*** | Train 15 | Test Asian 2 | 0.74 |
| ***35*** | Train 3 | Test Asian 3 | 0.79 | ***99*** | Train 7 | Test Asian 3 | 0.79 | ***163*** | Train 11 | Test Asian 3 | 0.79 | ***227*** | Train 15 | Test Asian 3 | 0.76 |
| ***36*** | Train 3 | Test Asian 4 | 0.77 | ***100*** | Train 7 | Test Asian 4 | 0.76 | ***164*** | Train 11 | Test Asian 4 | 0.76 | ***228*** | Train 15 | Test Asian 4 | 0.74 |
| ***37*** | Train 3 | Test Asian 5 | 0.79 | ***101*** | Train 7 | Test Asian 5 | 0.79 | ***165*** | Train 11 | Test Asian 5 | 0.79 | ***229*** | Train 15 | Test Asian 5 | 0.76 |
| ***38*** | Train 3 | Test Asian 6 | 0.77 | ***102*** | Train 7 | Test Asian 6 | 0.77 | ***166*** | Train 11 | Test Asian 6 | 0.77 | ***230*** | Train 15 | Test Asian 6 | 0.74 |
| ***39*** | Train 3 | Test Asian 7 | 0.79 | ***103*** | Train 7 | Test Asian 7 | 0.79 | ***167*** | Train 11 | Test Asian 7 | 0.79 | ***231*** | Train 15 | Test Asian 7 | 0.76 |
| ***40*** | Train 3 | Test Asian 8 | 0.77 | ***104*** | Train 7 | Test Asian 8 | 0.77 | ***168*** | Train 11 | Test Asian 8 | 0.77 | ***232*** | Train 15 | Test Asian 8 | 0.74 |
| ***41*** | Train 3 | Test Asian 9 | 0.78 | ***105*** | Train 7 | Test Asian 9 | 0.78 | ***169*** | Train 11 | Test Asian 9 | 0.78 | ***233*** | Train 15 | Test Asian 9 | 0.75 |
| ***42*** | Train 3 | Test Asian 10 | 0.76 | ***106*** | Train 7 | Test Asian 10 | 0.76 | ***170*** | Train 11 | Test Asian 10 | 0.76 | ***234*** | Train 15 | Test Asian 10 | 0.74 |
| ***43*** | Train 3 | Test Asian 11 | 0.78 | ***107*** | Train 7 | Test Asian 11 | 0.78 | ***171*** | Train 11 | Test Asian 11 | 0.78 | ***235*** | Train 15 | Test Asian 11 | 0.75 |
| ***44*** | Train 3 | Test Asian 12 | 0.76 | ***108*** | Train 7 | Test Asian 12 | 0.76 | ***172*** | Train 11 | Test Asian 12 | 0.76 | ***236*** | Train 15 | Test Asian 12 | 0.74 |
| ***45*** | Train 3 | Test Asian 13 | 0.79 | ***109*** | Train 7 | Test Asian 13 | 0.79 | ***173*** | Train 11 | Test Asian 13 | 0.8 | ***237*** | Train 15 | Test Asian 13 | 0.79 |
| ***46*** | Train 3 | Test Asian 14 | 0.77 | ***110*** | Train 7 | Test Asian 14 | 0.77 | ***174*** | Train 11 | Test Asian 14 | 0.77 | ***238*** | Train 15 | Test Asian 14 | 0.77 |
| ***47*** | Train 3 | Test Asian 15 | 0.8 | ***111*** | Train 7 | Test Asian 15 | 0.8 | ***175*** | Train 11 | Test Asian 15 | 0.8 | ***239*** | Train 15 | Test Asian 15 | 0.78 |
| ***48*** | Train 3 | Test Asian 16 | 0.78 | ***112*** | Train 7 | Test Asian 16 | 0.78 | ***176*** | Train 11 | Test Asian 16 | 0.78 | ***240*** | Train 15 | Test Asian 16 | 0.77 |
| ***49*** | Train 4 | Test Asian 1 | 0.78 | ***113*** | Train 8 | Test Asian 1 | 0.78 | ***177*** | Train 12 | Test Asian 1 | 0.78 | ***241*** | Train 16 | Test Asian 1 | 0.76 |
| ***50*** | Train 4 | Test Asian 2 | 0.78 | ***114*** | Train 8 | Test Asian 2 | 0.78 | ***178*** | Train 12 | Test Asian 2 | 0.78 | ***242*** | Train 16 | Test Asian 2 | 0.76 |
| ***51*** | Train 4 | Test Asian 3 | 0.78 | ***115*** | Train 8 | Test Asian 3 | 0.78 | ***179*** | Train 12 | Test Asian 3 | 0.78 | ***243*** | Train 16 | Test Asian 3 | 0.76 |
| ***52*** | Train 4 | Test Asian 4 | 0.77 | ***116*** | Train 8 | Test Asian 4 | 0.77 | ***180*** | Train 12 | Test Asian 4 | 0.77 | ***244*** | Train 16 | Test Asian 4 | 0.76 |
| ***53*** | Train 4 | Test Asian 5 | 0.78 | ***117*** | Train 8 | Test Asian 5 | 0.78 | ***181*** | Train 12 | Test Asian 5 | 0.78 | ***245*** | Train 16 | Test Asian 5 | 0.76 |
| ***54*** | Train 4 | Test Asian 6 | 0.78 | ***118*** | Train 8 | Test Asian 6 | 0.78 | ***182*** | Train 12 | Test Asian 6 | 0.78 | ***246*** | Train 16 | Test Asian 6 | 0.76 |
| ***55*** | Train 4 | Test Asian 7 | 0.78 | ***119*** | Train 8 | Test Asian 7 | 0.78 | ***183*** | Train 12 | Test Asian 7 | 0.78 | ***247*** | Train 16 | Test Asian 7 | 0.76 |
| ***56*** | Train 4 | Test Asian 8 | 0.77 | ***120*** | Train 8 | Test Asian 8 | 0.77 | ***184*** | Train 12 | Test Asian 8 | 0.78 | ***248*** | Train 16 | Test Asian 8 | 0.76 |
| ***57*** | Train 4 | Test Asian 9 | 0.78 | ***121*** | Train 8 | Test Asian 9 | 0.78 | ***185*** | Train 12 | Test Asian 9 | 0.78 | ***249*** | Train 16 | Test Asian 9 | 0.75 |
| ***58*** | Train 4 | Test Asian 10 | 0.77 | ***122*** | Train 8 | Test Asian 10 | 0.77 | ***186*** | Train 12 | Test Asian 10 | 0.77 | ***250*** | Train 16 | Test Asian 10 | 0.75 |
| ***59*** | Train 4 | Test Asian 11 | 0.77 | ***123*** | Train 8 | Test Asian 11 | 0.77 | ***187*** | Train 12 | Test Asian 11 | 0.77 | ***251*** | Train 16 | Test Asian 11 | 0.75 |
| ***60*** | Train 4 | Test Asian 12 | 0.77 | ***124*** | Train 8 | Test Asian 12 | 0.77 | ***188*** | Train 12 | Test Asian 12 | 0.77 | ***252*** | Train 16 | Test Asian 12 | 0.75 |
| ***61*** | Train 4 | Test Asian 13 | 0.79 | ***125*** | Train 8 | Test Asian 13 | 0.79 | ***189*** | Train 12 | Test Asian 13 | 0.79 | ***253*** | Train 16 | Test Asian 13 | 0.78 |
| ***62*** | Train 4 | Test Asian 14 | 0.79 | ***126*** | Train 8 | Test Asian 14 | 0.79 | ***190*** | Train 12 | Test Asian 14 | 0.79 | ***254*** | Train 16 | Test Asian 14 | 0.79 |
| ***63*** | Train 4 | Test Asian 15 | 0.79 | ***127*** | Train 8 | Test Asian 15 | 0.79 | ***191*** | Train 12 | Test Asian 15 | 0.79 | ***255*** | Train 16 | Test Asian 15 | 0.78 |
| ***64*** | Train 4 | Test Asian 16 | 0.79 | ***128*** | Train 8 | Test Asian 16 | 0.79 | ***192*** | Train 12 | Test Asian 16 | 0.79 | ***256*** | Train 16 | Test Asian 16 | 0.79 |

Table 20. Random Forest (RF) AUC Performance for Asian Patients Across 16 Cohorts.

| Original Decisions | Train Model | Test Dataset | AUC (RF) | ***Asian*** | ***Train Model*** | ***Test Dataset*** | ***AUC (RF)*** | ***Asian*** | ***Train Model*** | ***Test Dataset*** | ***AUC (RF)*** | ***Asian*** | ***Train Model*** | Test Dataset | AUC (RF) |
| --- | --- | --- | --- | --- | --- | --- | --- | --- | --- | --- | --- | --- | --- | --- | --- |
| ***1*** | Train 1 | Test Asian 1 | 0.82 | ***65*** | Train 5 | Test Asian 1 | 0.82 | ***129*** | Train 9 | Test Asian 1 | 0.82 | ***193*** | Train 13 | Test Asian 1 | 0.78 |
| ***2*** | Train 1 | Test Asian 2 | 0.75 | ***66*** | Train 5 | Test Asian 2 | 0.75 | ***130*** | Train 9 | Test Asian 2 | 0.75 | ***194*** | Train 13 | Test Asian 2 | 0.7 |
| ***3*** | Train 1 | Test Asian 3 | 0.81 | ***67*** | Train 5 | Test Asian 3 | 0.81 | ***131*** | Train 9 | Test Asian 3 | 0.81 | ***195*** | Train 13 | Test Asian 3 | 0.77 |
| ***4*** | Train 1 | Test Asian 4 | 0.75 | ***68*** | Train 5 | Test Asian 4 | 0.75 | ***132*** | Train 9 | Test Asian 4 | 0.75 | ***196*** | Train 13 | Test Asian 4 | 0.7 |
| ***5*** | Train 1 | Test Asian 5 | 0.82 | ***69*** | Train 5 | Test Asian 5 | 0.82 | ***133*** | Train 9 | Test Asian 5 | 0.82 | ***197*** | Train 13 | Test Asian 5 | 0.78 |
| ***6*** | Train 1 | Test Asian 6 | 0.75 | ***70*** | Train 5 | Test Asian 6 | 0.75 | ***134*** | Train 9 | Test Asian 6 | 0.75 | ***198*** | Train 13 | Test Asian 6 | 0.7 |
| ***7*** | Train 1 | Test Asian 7 | 0.81 | ***71*** | Train 5 | Test Asian 7 | 0.81 | ***135*** | Train 9 | Test Asian 7 | 0.81 | ***199*** | Train 13 | Test Asian 7 | 0.77 |
| ***8*** | Train 1 | Test Asian 8 | 0.75 | ***72*** | Train 5 | Test Asian 8 | 0.75 | ***136*** | Train 9 | Test Asian 8 | 0.75 | ***200*** | Train 13 | Test Asian 8 | 0.7 |
| ***9*** | Train 1 | Test Asian 9 | 0.81 | ***73*** | Train 5 | Test Asian 9 | 0.81 | ***137*** | Train 9 | Test Asian 9 | 0.81 | ***201*** | Train 13 | Test Asian 9 | 0.77 |
| ***10*** | Train 1 | Test Asian 10 | 0.75 | ***74*** | Train 5 | Test Asian 10 | 0.75 | ***138*** | Train 9 | Test Asian 10 | 0.75 | ***202*** | Train 13 | Test Asian 10 | 0.7 |
| ***11*** | Train 1 | Test Asian 11 | 0.8 | ***75*** | Train 5 | Test Asian 11 | 0.8 | ***139*** | Train 9 | Test Asian 11 | 0.8 | ***203*** | Train 13 | Test Asian 11 | 0.76 |
| ***12*** | Train 1 | Test Asian 12 | 0.75 | ***76*** | Train 5 | Test Asian 12 | 0.74 | ***140*** | Train 9 | Test Asian 12 | 0.75 | ***204*** | Train 13 | Test Asian 12 | 0.7 |
| ***13*** | Train 1 | Test Asian 13 | 0.83 | ***77*** | Train 5 | Test Asian 13 | 0.83 | ***141*** | Train 9 | Test Asian 13 | 0.83 | ***205*** | Train 13 | Test Asian 13 | 0.83 |
| ***14*** | Train 1 | Test Asian 14 | 0.75 | ***78*** | Train 5 | Test Asian 14 | 0.75 | ***142*** | Train 9 | Test Asian 14 | 0.75 | ***206*** | Train 13 | Test Asian 14 | 0.73 |
| ***15*** | Train 1 | Test Asian 15 | 0.83 | ***79*** | Train 5 | Test Asian 15 | 0.83 | ***143*** | Train 9 | Test Asian 15 | 0.83 | ***207*** | Train 13 | Test Asian 15 | 0.82 |
| ***16*** | Train 1 | Test Asian 16 | 0.75 | ***80*** | Train 5 | Test Asian 16 | 0.75 | ***144*** | Train 9 | Test Asian 16 | 0.75 | ***208*** | Train 13 | Test Asian 16 | 0.73 |
| ***17*** | Train 2 | Test Asian 1 | 0.81 | ***81*** | Train 6 | Test Asian 1 | 0.81 | ***145*** | Train 10 | Test Asian 1 | 0.81 | ***209*** | Train 14 | Test Asian 1 | 0.78 |
| ***18*** | Train 2 | Test Asian 2 | 0.81 | ***82*** | Train 6 | Test Asian 2 | 0.81 | ***146*** | Train 10 | Test Asian 2 | 0.81 | ***210*** | Train 14 | Test Asian 2 | 0.78 |
| ***19*** | Train 2 | Test Asian 3 | 0.8 | ***83*** | Train 6 | Test Asian 3 | 0.81 | ***147*** | Train 10 | Test Asian 3 | 0.81 | ***211*** | Train 14 | Test Asian 3 | 0.77 |
| ***20*** | Train 2 | Test Asian 4 | 0.81 | ***84*** | Train 6 | Test Asian 4 | 0.81 | ***148*** | Train 10 | Test Asian 4 | 0.81 | ***212*** | Train 14 | Test Asian 4 | 0.78 |
| ***21*** | Train 2 | Test Asian 5 | 0.81 | ***85*** | Train 6 | Test Asian 5 | 0.81 | ***149*** | Train 10 | Test Asian 5 | 0.81 | ***213*** | Train 14 | Test Asian 5 | 0.78 |
| ***22*** | Train 2 | Test Asian 6 | 0.81 | ***86*** | Train 6 | Test Asian 6 | 0.81 | ***150*** | Train 10 | Test Asian 6 | 0.81 | ***214*** | Train 14 | Test Asian 6 | 0.78 |
| ***23*** | Train 2 | Test Asian 7 | 0.8 | ***87*** | Train 6 | Test Asian 7 | 0.81 | ***151*** | Train 10 | Test Asian 7 | 0.81 | ***215*** | Train 14 | Test Asian 7 | 0.77 |
| ***24*** | Train 2 | Test Asian 8 | 0.81 | ***88*** | Train 6 | Test Asian 8 | 0.81 | ***152*** | Train 10 | Test Asian 8 | 0.81 | ***216*** | Train 14 | Test Asian 8 | 0.78 |
| ***25*** | Train 2 | Test Asian 9 | 0.8 | ***89*** | Train 6 | Test Asian 9 | 0.8 | ***153*** | Train 10 | Test Asian 9 | 0.8 | ***217*** | Train 14 | Test Asian 9 | 0.77 |
| ***26*** | Train 2 | Test Asian 10 | 0.81 | ***90*** | Train 6 | Test Asian 10 | 0.81 | ***154*** | Train 10 | Test Asian 10 | 0.81 | ***218*** | Train 14 | Test Asian 10 | 0.78 |
| ***27*** | Train 2 | Test Asian 11 | 0.8 | ***91*** | Train 6 | Test Asian 11 | 0.8 | ***155*** | Train 10 | Test Asian 11 | 0.8 | ***219*** | Train 14 | Test Asian 11 | 0.77 |
| ***28*** | Train 2 | Test Asian 12 | 0.81 | ***92*** | Train 6 | Test Asian 12 | 0.81 | ***156*** | Train 10 | Test Asian 12 | 0.81 | ***220*** | Train 14 | Test Asian 12 | 0.78 |
| ***29*** | Train 2 | Test Asian 13 | 0.82 | ***93*** | Train 6 | Test Asian 13 | 0.82 | ***157*** | Train 10 | Test Asian 13 | 0.83 | ***221*** | Train 14 | Test Asian 13 | 0.82 |
| ***30*** | Train 2 | Test Asian 14 | 0.83 | ***94*** | Train 6 | Test Asian 14 | 0.83 | ***158*** | Train 10 | Test Asian 14 | 0.83 | ***222*** | Train 14 | Test Asian 14 | 0.83 |
| ***31*** | Train 2 | Test Asian 15 | 0.83 | ***95*** | Train 6 | Test Asian 15 | 0.83 | ***159*** | Train 10 | Test Asian 15 | 0.83 | ***223*** | Train 14 | Test Asian 15 | 0.82 |
| ***32*** | Train 2 | Test Asian 16 | 0.83 | ***96*** | Train 6 | Test Asian 16 | 0.83 | ***160*** | Train 10 | Test Asian 16 | 0.83 | ***224*** | Train 14 | Test Asian 16 | 0.83 |
| ***33*** | Train 3 | Test Asian 1 | 0.82 | ***97*** | Train 7 | Test Asian 1 | 0.82 | ***161*** | Train 11 | Test Asian 1 | 0.82 | ***225*** | Train 15 | Test Asian 1 | 0.78 |
| ***34*** | Train 3 | Test Asian 2 | 0.75 | ***98*** | Train 7 | Test Asian 2 | 0.75 | ***162*** | Train 11 | Test Asian 2 | 0.75 | ***226*** | Train 15 | Test Asian 2 | 0.7 |
| ***35*** | Train 3 | Test Asian 3 | 0.81 | ***99*** | Train 7 | Test Asian 3 | 0.81 | ***163*** | Train 11 | Test Asian 3 | 0.81 | ***227*** | Train 15 | Test Asian 3 | 0.77 |
| ***36*** | Train 3 | Test Asian 4 | 0.74 | ***100*** | Train 7 | Test Asian 4 | 0.75 | ***164*** | Train 11 | Test Asian 4 | 0.75 | ***228*** | Train 15 | Test Asian 4 | 0.7 |
| ***37*** | Train 3 | Test Asian 5 | 0.82 | ***101*** | Train 7 | Test Asian 5 | 0.82 | ***165*** | Train 11 | Test Asian 5 | 0.82 | ***229*** | Train 15 | Test Asian 5 | 0.78 |
| ***38*** | Train 3 | Test Asian 6 | 0.75 | ***102*** | Train 7 | Test Asian 6 | 0.75 | ***166*** | Train 11 | Test Asian 6 | 0.75 | ***230*** | Train 15 | Test Asian 6 | 0.7 |
| ***39*** | Train 3 | Test Asian 7 | 0.81 | ***103*** | Train 7 | Test Asian 7 | 0.81 | ***167*** | Train 11 | Test Asian 7 | 0.81 | ***231*** | Train 15 | Test Asian 7 | 0.77 |
| ***40*** | Train 3 | Test Asian 8 | 0.74 | ***104*** | Train 7 | Test Asian 8 | 0.75 | ***168*** | Train 11 | Test Asian 8 | 0.75 | ***232*** | Train 15 | Test Asian 8 | 0.7 |
| ***41*** | Train 3 | Test Asian 9 | 0.81 | ***105*** | Train 7 | Test Asian 9 | 0.81 | ***169*** | Train 11 | Test Asian 9 | 0.81 | ***233*** | Train 15 | Test Asian 9 | 0.77 |
| ***42*** | Train 3 | Test Asian 10 | 0.74 | ***106*** | Train 7 | Test Asian 10 | 0.75 | ***170*** | Train 11 | Test Asian 10 | 0.75 | ***234*** | Train 15 | Test Asian 10 | 0.7 |
| ***43*** | Train 3 | Test Asian 11 | 0.8 | ***107*** | Train 7 | Test Asian 11 | 0.8 | ***171*** | Train 11 | Test Asian 11 | 0.8 | ***235*** | Train 15 | Test Asian 11 | 0.76 |
| ***44*** | Train 3 | Test Asian 12 | 0.74 | ***108*** | Train 7 | Test Asian 12 | 0.74 | ***172*** | Train 11 | Test Asian 12 | 0.75 | ***236*** | Train 15 | Test Asian 12 | 0.7 |
| ***45*** | Train 3 | Test Asian 13 | 0.83 | ***109*** | Train 7 | Test Asian 13 | 0.82 | ***173*** | Train 11 | Test Asian 13 | 0.83 | ***237*** | Train 15 | Test Asian 13 | 0.82 |
| ***46*** | Train 3 | Test Asian 14 | 0.74 | ***110*** | Train 7 | Test Asian 14 | 0.75 | ***174*** | Train 11 | Test Asian 14 | 0.75 | ***238*** | Train 15 | Test Asian 14 | 0.72 |
| ***47*** | Train 3 | Test Asian 15 | 0.82 | ***111*** | Train 7 | Test Asian 15 | 0.82 | ***175*** | Train 11 | Test Asian 15 | 0.83 | ***239*** | Train 15 | Test Asian 15 | 0.82 |
| ***48*** | Train 3 | Test Asian 16 | 0.75 | ***112*** | Train 7 | Test Asian 16 | 0.75 | ***176*** | Train 11 | Test Asian 16 | 0.75 | ***240*** | Train 15 | Test Asian 16 | 0.72 |
| ***49*** | Train 4 | Test Asian 1 | 0.81 | ***113*** | Train 8 | Test Asian 1 | 0.81 | ***177*** | Train 12 | Test Asian 1 | 0.81 | ***241*** | Train 16 | Test Asian 1 | 0.78 |
| ***50*** | Train 4 | Test Asian 2 | 0.81 | ***114*** | Train 8 | Test Asian 2 | 0.81 | ***178*** | Train 12 | Test Asian 2 | 0.81 | ***242*** | Train 16 | Test Asian 2 | 0.79 |
| ***51*** | Train 4 | Test Asian 3 | 0.8 | ***115*** | Train 8 | Test Asian 3 | 0.8 | ***179*** | Train 12 | Test Asian 3 | 0.8 | ***243*** | Train 16 | Test Asian 3 | 0.78 |
| ***52*** | Train 4 | Test Asian 4 | 0.81 | ***116*** | Train 8 | Test Asian 4 | 0.81 | ***180*** | Train 12 | Test Asian 4 | 0.81 | ***244*** | Train 16 | Test Asian 4 | 0.78 |
| ***53*** | Train 4 | Test Asian 5 | 0.81 | ***117*** | Train 8 | Test Asian 5 | 0.81 | ***181*** | Train 12 | Test Asian 5 | 0.81 | ***245*** | Train 16 | Test Asian 5 | 0.78 |
| ***54*** | Train 4 | Test Asian 6 | 0.81 | ***118*** | Train 8 | Test Asian 6 | 0.81 | ***182*** | Train 12 | Test Asian 6 | 0.81 | ***246*** | Train 16 | Test Asian 6 | 0.79 |
| ***55*** | Train 4 | Test Asian 7 | 0.8 | ***119*** | Train 8 | Test Asian 7 | 0.8 | ***183*** | Train 12 | Test Asian 7 | 0.8 | ***247*** | Train 16 | Test Asian 7 | 0.78 |
| ***56*** | Train 4 | Test Asian 8 | 0.81 | ***120*** | Train 8 | Test Asian 8 | 0.81 | ***184*** | Train 12 | Test Asian 8 | 0.81 | ***248*** | Train 16 | Test Asian 8 | 0.78 |
| ***57*** | Train 4 | Test Asian 9 | 0.8 | ***121*** | Train 8 | Test Asian 9 | 0.8 | ***185*** | Train 12 | Test Asian 9 | 0.8 | ***249*** | Train 16 | Test Asian 9 | 0.77 |
| ***58*** | Train 4 | Test Asian 10 | 0.81 | ***122*** | Train 8 | Test Asian 10 | 0.81 | ***186*** | Train 12 | Test Asian 10 | 0.81 | ***250*** | Train 16 | Test Asian 10 | 0.78 |
| ***59*** | Train 4 | Test Asian 11 | 0.8 | ***123*** | Train 8 | Test Asian 11 | 0.8 | ***187*** | Train 12 | Test Asian 11 | 0.8 | ***251*** | Train 16 | Test Asian 11 | 0.77 |
| ***60*** | Train 4 | Test Asian 12 | 0.8 | ***124*** | Train 8 | Test Asian 12 | 0.8 | ***188*** | Train 12 | Test Asian 12 | 0.81 | ***252*** | Train 16 | Test Asian 12 | 0.78 |
| ***61*** | Train 4 | Test Asian 13 | 0.82 | ***125*** | Train 8 | Test Asian 13 | 0.82 | ***189*** | Train 12 | Test Asian 13 | 0.82 | ***253*** | Train 16 | Test Asian 13 | 0.81 |
| ***62*** | Train 4 | Test Asian 14 | 0.83 | ***126*** | Train 8 | Test Asian 14 | 0.83 | ***190*** | Train 12 | Test Asian 14 | 0.83 | ***254*** | Train 16 | Test Asian 14 | 0.83 |
| ***63*** | Train 4 | Test Asian 15 | 0.82 | ***127*** | Train 8 | Test Asian 15 | 0.83 | ***191*** | Train 12 | Test Asian 15 | 0.83 | ***255*** | Train 16 | Test Asian 15 | 0.81 |
| ***64*** | Train 4 | Test Asian 16 | 0.83 | ***128*** | Train 8 | Test Asian 16 | 0.83 | ***192*** | Train 12 | Test Asian 16 | 0.83 | ***256*** | Train 16 | Test Asian 16 | 0.84 |

Table 21. Gradient Boosting (GB) AUC Performance for Asian Patients Across 16 Cohorts.

| Original Decisions | Train Model | Test Dataset | AUC (GB) | ***Asian*** | ***Train Model*** | ***Test Dataset*** | ***AUC (GB)*** | ***Asian*** | ***Train Model*** | ***Test Dataset*** | ***AUC (GB)*** | ***Asian*** | ***Train Model*** | Test Dataset | AUC (GB) |
| --- | --- | --- | --- | --- | --- | --- | --- | --- | --- | --- | --- | --- | --- | --- | --- |
| ***1*** | Train 1 | Test Asian 1 | 0.77 | ***65*** | Train 5 | Test Asian 1 | 0.75 | ***129*** | Train 9 | Test Asian 1 | 0.78 | ***193*** | Train 13 | Test Asian 1 | 0.67 |
| ***2*** | Train 1 | Test Asian 2 | 0.74 | ***66*** | Train 5 | Test Asian 2 | 0.72 | ***130*** | Train 9 | Test Asian 2 | 0.76 | ***194*** | Train 13 | Test Asian 2 | 0.67 |
| ***3*** | Train 1 | Test Asian 3 | 0.77 | ***67*** | Train 5 | Test Asian 3 | 0.75 | ***131*** | Train 9 | Test Asian 3 | 0.78 | ***195*** | Train 13 | Test Asian 3 | 0.67 |
| ***4*** | Train 1 | Test Asian 4 | 0.73 | ***68*** | Train 5 | Test Asian 4 | 0.72 | ***132*** | Train 9 | Test Asian 4 | 0.75 | ***196*** | Train 13 | Test Asian 4 | 0.67 |
| ***5*** | Train 1 | Test Asian 5 | 0.77 | ***69*** | Train 5 | Test Asian 5 | 0.75 | ***133*** | Train 9 | Test Asian 5 | 0.78 | ***197*** | Train 13 | Test Asian 5 | 0.67 |
| ***6*** | Train 1 | Test Asian 6 | 0.74 | ***70*** | Train 5 | Test Asian 6 | 0.72 | ***134*** | Train 9 | Test Asian 6 | 0.76 | ***198*** | Train 13 | Test Asian 6 | 0.67 |
| ***7*** | Train 1 | Test Asian 7 | 0.77 | ***71*** | Train 5 | Test Asian 7 | 0.75 | ***135*** | Train 9 | Test Asian 7 | 0.78 | ***199*** | Train 13 | Test Asian 7 | 0.68 |
| ***8*** | Train 1 | Test Asian 8 | 0.73 | ***72*** | Train 5 | Test Asian 8 | 0.72 | ***136*** | Train 9 | Test Asian 8 | 0.75 | ***200*** | Train 13 | Test Asian 8 | 0.67 |
| ***9*** | Train 1 | Test Asian 9 | 0.77 | ***73*** | Train 5 | Test Asian 9 | 0.74 | ***137*** | Train 9 | Test Asian 9 | 0.78 | ***201*** | Train 13 | Test Asian 9 | 0.67 |
| ***10*** | Train 1 | Test Asian 10 | 0.73 | ***74*** | Train 5 | Test Asian 10 | 0.72 | ***138*** | Train 9 | Test Asian 10 | 0.75 | ***202*** | Train 13 | Test Asian 10 | 0.67 |
| ***11*** | Train 1 | Test Asian 11 | 0.76 | ***75*** | Train 5 | Test Asian 11 | 0.74 | ***139*** | Train 9 | Test Asian 11 | 0.77 | ***203*** | Train 13 | Test Asian 11 | 0.67 |
| ***12*** | Train 1 | Test Asian 12 | 0.73 | ***76*** | Train 5 | Test Asian 12 | 0.72 | ***140*** | Train 9 | Test Asian 12 | 0.75 | ***204*** | Train 13 | Test Asian 12 | 0.67 |
| ***13*** | Train 1 | Test Asian 13 | 0.75 | ***77*** | Train 5 | Test Asian 13 | 0.75 | ***141*** | Train 9 | Test Asian 13 | 0.75 | ***205*** | Train 13 | Test Asian 13 | 0.76 |
| ***14*** | Train 1 | Test Asian 14 | 0.72 | ***78*** | Train 5 | Test Asian 14 | 0.71 | ***142*** | Train 9 | Test Asian 14 | 0.71 | ***206*** | Train 13 | Test Asian 14 | 0.73 |
| ***15*** | Train 1 | Test Asian 15 | 0.75 | ***79*** | Train 5 | Test Asian 15 | 0.75 | ***143*** | Train 9 | Test Asian 15 | 0.74 | ***207*** | Train 13 | Test Asian 15 | 0.76 |
| ***16*** | Train 1 | Test Asian 16 | 0.72 | ***80*** | Train 5 | Test Asian 16 | 0.71 | ***144*** | Train 9 | Test Asian 16 | 0.71 | ***208*** | Train 13 | Test Asian 16 | 0.73 |
| ***17*** | Train 2 | Test Asian 1 | 0.79 | ***81*** | Train 6 | Test Asian 1 | 0.79 | ***145*** | Train 10 | Test Asian 1 | 0.79 | ***209*** | Train 14 | Test Asian 1 | 0.77 |
| ***18*** | Train 2 | Test Asian 2 | 0.79 | ***82*** | Train 6 | Test Asian 2 | 0.81 | ***146*** | Train 10 | Test Asian 2 | 0.8 | ***210*** | Train 14 | Test Asian 2 | 0.77 |
| ***19*** | Train 2 | Test Asian 3 | 0.79 | ***83*** | Train 6 | Test Asian 3 | 0.79 | ***147*** | Train 10 | Test Asian 3 | 0.78 | ***211*** | Train 14 | Test Asian 3 | 0.76 |
| ***20*** | Train 2 | Test Asian 4 | 0.79 | ***84*** | Train 6 | Test Asian 4 | 0.8 | ***148*** | Train 10 | Test Asian 4 | 0.79 | ***212*** | Train 14 | Test Asian 4 | 0.77 |
| ***21*** | Train 2 | Test Asian 5 | 0.79 | ***85*** | Train 6 | Test Asian 5 | 0.8 | ***149*** | Train 10 | Test Asian 5 | 0.79 | ***213*** | Train 14 | Test Asian 5 | 0.77 |
| ***22*** | Train 2 | Test Asian 6 | 0.79 | ***86*** | Train 6 | Test Asian 6 | 0.81 | ***150*** | Train 10 | Test Asian 6 | 0.8 | ***214*** | Train 14 | Test Asian 6 | 0.77 |
| ***23*** | Train 2 | Test Asian 7 | 0.79 | ***87*** | Train 6 | Test Asian 7 | 0.79 | ***151*** | Train 10 | Test Asian 7 | 0.78 | ***215*** | Train 14 | Test Asian 7 | 0.76 |
| ***24*** | Train 2 | Test Asian 8 | 0.79 | ***88*** | Train 6 | Test Asian 8 | 0.8 | ***152*** | Train 10 | Test Asian 8 | 0.8 | ***216*** | Train 14 | Test Asian 8 | 0.77 |
| ***25*** | Train 2 | Test Asian 9 | 0.79 | ***89*** | Train 6 | Test Asian 9 | 0.79 | ***153*** | Train 10 | Test Asian 9 | 0.78 | ***217*** | Train 14 | Test Asian 9 | 0.76 |
| ***26*** | Train 2 | Test Asian 10 | 0.79 | ***90*** | Train 6 | Test Asian 10 | 0.8 | ***154*** | Train 10 | Test Asian 10 | 0.8 | ***218*** | Train 14 | Test Asian 10 | 0.77 |
| ***27*** | Train 2 | Test Asian 11 | 0.78 | ***91*** | Train 6 | Test Asian 11 | 0.78 | ***155*** | Train 10 | Test Asian 11 | 0.77 | ***219*** | Train 14 | Test Asian 11 | 0.76 |
| ***28*** | Train 2 | Test Asian 12 | 0.79 | ***92*** | Train 6 | Test Asian 12 | 0.8 | ***156*** | Train 10 | Test Asian 12 | 0.79 | ***220*** | Train 14 | Test Asian 12 | 0.76 |
| ***29*** | Train 2 | Test Asian 13 | 0.77 | ***93*** | Train 6 | Test Asian 13 | 0.79 | ***157*** | Train 10 | Test Asian 13 | 0.78 | ***221*** | Train 14 | Test Asian 13 | 0.79 |
| ***30*** | Train 2 | Test Asian 14 | 0.8 | ***94*** | Train 6 | Test Asian 14 | 0.82 | ***158*** | Train 10 | Test Asian 14 | 0.81 | ***222*** | Train 14 | Test Asian 14 | 0.81 |
| ***31*** | Train 2 | Test Asian 15 | 0.77 | ***95*** | Train 6 | Test Asian 15 | 0.79 | ***159*** | Train 10 | Test Asian 15 | 0.78 | ***223*** | Train 14 | Test Asian 15 | 0.78 |
| ***32*** | Train 2 | Test Asian 16 | 0.8 | ***96*** | Train 6 | Test Asian 16 | 0.81 | ***160*** | Train 10 | Test Asian 16 | 0.81 | ***224*** | Train 14 | Test Asian 16 | 0.81 |
| ***33*** | Train 3 | Test Asian 1 | 0.77 | ***97*** | Train 7 | Test Asian 1 | 0.79 | ***161*** | Train 11 | Test Asian 1 | 0.78 | ***225*** | Train 15 | Test Asian 1 | 0.7 |
| ***34*** | Train 3 | Test Asian 2 | 0.73 | ***98*** | Train 7 | Test Asian 2 | 0.76 | ***162*** | Train 11 | Test Asian 2 | 0.74 | ***226*** | Train 15 | Test Asian 2 | 0.66 |
| ***35*** | Train 3 | Test Asian 3 | 0.77 | ***99*** | Train 7 | Test Asian 3 | 0.78 | ***163*** | Train 11 | Test Asian 3 | 0.78 | ***227*** | Train 15 | Test Asian 3 | 0.7 |
| ***36*** | Train 3 | Test Asian 4 | 0.72 | ***100*** | Train 7 | Test Asian 4 | 0.76 | ***164*** | Train 11 | Test Asian 4 | 0.73 | ***228*** | Train 15 | Test Asian 4 | 0.66 |
| ***37*** | Train 3 | Test Asian 5 | 0.77 | ***101*** | Train 7 | Test Asian 5 | 0.79 | ***165*** | Train 11 | Test Asian 5 | 0.78 | ***229*** | Train 15 | Test Asian 5 | 0.7 |
| ***38*** | Train 3 | Test Asian 6 | 0.73 | ***102*** | Train 7 | Test Asian 6 | 0.76 | ***166*** | Train 11 | Test Asian 6 | 0.74 | ***230*** | Train 15 | Test Asian 6 | 0.66 |
| ***39*** | Train 3 | Test Asian 7 | 0.77 | ***103*** | Train 7 | Test Asian 7 | 0.78 | ***167*** | Train 11 | Test Asian 7 | 0.78 | ***231*** | Train 15 | Test Asian 7 | 0.7 |
| ***40*** | Train 3 | Test Asian 8 | 0.73 | ***104*** | Train 7 | Test Asian 8 | 0.76 | ***168*** | Train 11 | Test Asian 8 | 0.73 | ***232*** | Train 15 | Test Asian 8 | 0.66 |
| ***41*** | Train 3 | Test Asian 9 | 0.76 | ***105*** | Train 7 | Test Asian 9 | 0.78 | ***169*** | Train 11 | Test Asian 9 | 0.77 | ***233*** | Train 15 | Test Asian 9 | 0.7 |
| ***42*** | Train 3 | Test Asian 10 | 0.73 | ***106*** | Train 7 | Test Asian 10 | 0.76 | ***170*** | Train 11 | Test Asian 10 | 0.73 | ***234*** | Train 15 | Test Asian 10 | 0.65 |
| ***43*** | Train 3 | Test Asian 11 | 0.76 | ***107*** | Train 7 | Test Asian 11 | 0.78 | ***171*** | Train 11 | Test Asian 11 | 0.77 | ***235*** | Train 15 | Test Asian 11 | 0.7 |
| ***44*** | Train 3 | Test Asian 12 | 0.72 | ***108*** | Train 7 | Test Asian 12 | 0.76 | ***172*** | Train 11 | Test Asian 12 | 0.73 | ***236*** | Train 15 | Test Asian 12 | 0.66 |
| ***45*** | Train 3 | Test Asian 13 | 0.72 | ***109*** | Train 7 | Test Asian 13 | 0.77 | ***173*** | Train 11 | Test Asian 13 | 0.75 | ***237*** | Train 15 | Test Asian 13 | 0.79 |
| ***46*** | Train 3 | Test Asian 14 | 0.7 | ***110*** | Train 7 | Test Asian 14 | 0.75 | ***174*** | Train 11 | Test Asian 14 | 0.7 | ***238*** | Train 15 | Test Asian 14 | 0.68 |
| ***47*** | Train 3 | Test Asian 15 | 0.72 | ***111*** | Train 7 | Test Asian 15 | 0.76 | ***175*** | Train 11 | Test Asian 15 | 0.74 | ***239*** | Train 15 | Test Asian 15 | 0.78 |
| ***48*** | Train 3 | Test Asian 16 | 0.7 | ***112*** | Train 7 | Test Asian 16 | 0.75 | ***176*** | Train 11 | Test Asian 16 | 0.7 | ***240*** | Train 15 | Test Asian 16 | 0.68 |
| ***49*** | Train 4 | Test Asian 1 | 0.82 | ***113*** | Train 8 | Test Asian 1 | 0.79 | ***177*** | Train 12 | Test Asian 1 | 0.78 | ***241*** | Train 16 | Test Asian 1 | 0.7 |
| ***50*** | Train 4 | Test Asian 2 | 0.81 | ***114*** | Train 8 | Test Asian 2 | 0.8 | ***178*** | Train 12 | Test Asian 2 | 0.79 | ***242*** | Train 16 | Test Asian 2 | 0.73 |
| ***51*** | Train 4 | Test Asian 3 | 0.81 | ***115*** | Train 8 | Test Asian 3 | 0.79 | ***179*** | Train 12 | Test Asian 3 | 0.78 | ***243*** | Train 16 | Test Asian 3 | 0.7 |
| ***52*** | Train 4 | Test Asian 4 | 0.81 | ***116*** | Train 8 | Test Asian 4 | 0.8 | ***180*** | Train 12 | Test Asian 4 | 0.79 | ***244*** | Train 16 | Test Asian 4 | 0.73 |
| ***53*** | Train 4 | Test Asian 5 | 0.82 | ***117*** | Train 8 | Test Asian 5 | 0.79 | ***181*** | Train 12 | Test Asian 5 | 0.78 | ***245*** | Train 16 | Test Asian 5 | 0.7 |
| ***54*** | Train 4 | Test Asian 6 | 0.81 | ***118*** | Train 8 | Test Asian 6 | 0.8 | ***182*** | Train 12 | Test Asian 6 | 0.79 | ***246*** | Train 16 | Test Asian 6 | 0.73 |
| ***55*** | Train 4 | Test Asian 7 | 0.81 | ***119*** | Train 8 | Test Asian 7 | 0.79 | ***183*** | Train 12 | Test Asian 7 | 0.78 | ***247*** | Train 16 | Test Asian 7 | 0.7 |
| ***56*** | Train 4 | Test Asian 8 | 0.81 | ***120*** | Train 8 | Test Asian 8 | 0.8 | ***184*** | Train 12 | Test Asian 8 | 0.79 | ***248*** | Train 16 | Test Asian 8 | 0.73 |
| ***57*** | Train 4 | Test Asian 9 | 0.81 | ***121*** | Train 8 | Test Asian 9 | 0.78 | ***185*** | Train 12 | Test Asian 9 | 0.78 | ***249*** | Train 16 | Test Asian 9 | 0.69 |
| ***58*** | Train 4 | Test Asian 10 | 0.81 | ***122*** | Train 8 | Test Asian 10 | 0.8 | ***186*** | Train 12 | Test Asian 10 | 0.79 | ***250*** | Train 16 | Test Asian 10 | 0.73 |
| ***59*** | Train 4 | Test Asian 11 | 0.8 | ***123*** | Train 8 | Test Asian 11 | 0.78 | ***187*** | Train 12 | Test Asian 11 | 0.77 | ***251*** | Train 16 | Test Asian 11 | 0.7 |
| ***60*** | Train 4 | Test Asian 12 | 0.81 | ***124*** | Train 8 | Test Asian 12 | 0.79 | ***188*** | Train 12 | Test Asian 12 | 0.79 | ***252*** | Train 16 | Test Asian 12 | 0.73 |
| ***61*** | Train 4 | Test Asian 13 | 0.8 | ***125*** | Train 8 | Test Asian 13 | 0.75 | ***189*** | Train 12 | Test Asian 13 | 0.74 | ***253*** | Train 16 | Test Asian 13 | 0.77 |
| ***62*** | Train 4 | Test Asian 14 | 0.81 | ***126*** | Train 8 | Test Asian 14 | 0.8 | ***190*** | Train 12 | Test Asian 14 | 0.79 | ***254*** | Train 16 | Test Asian 14 | 0.82 |
| ***63*** | Train 4 | Test Asian 15 | 0.8 | ***127*** | Train 8 | Test Asian 15 | 0.75 | ***191*** | Train 12 | Test Asian 15 | 0.74 | ***255*** | Train 16 | Test Asian 15 | 0.77 |
| ***64*** | Train 4 | Test Asian 16 | 0.81 | ***128*** | Train 8 | Test Asian 16 | 0.8 | ***192*** | Train 12 | Test Asian 16 | 0.79 | ***256*** | Train 16 | Test Asian 16 | 0.82 |

Table 22. Logistic Regression (LR) AUC Performance for Hispanic Patients Across 16 Cohorts.

| Original Decisions | Train Model | Test Dataset | AUC (LR) | ***Hispanic*** | Train Model | Test Dataset | AUC (LR) | Hispanic | Train Model | Test Dataset | AUC (LR) | Hispanic | Train Model | Test Dataset | AUC (LR) |
| --- | --- | --- | --- | --- | --- | --- | --- | --- | --- | --- | --- | --- | --- | --- | --- |
| ***1*** | Train 1 | Test Hispanic 1 | 0.73 | ***65*** | Train 5 | Test Hispanic 1 | 0.73 | ***129*** | Train 9 | Test Hispanic 1 | 0.73 | ***193*** | Train 13 | Test Hispanic 1 | 0.73 |
| ***2*** | Train 1 | Test Hispanic 2 | 0.72 | ***66*** | Train 5 | Test Hispanic 2 | 0.72 | ***130*** | Train 9 | Test Hispanic 2 | 0.72 | ***194*** | Train 13 | Test Hispanic 2 | 0.71 |
| ***3*** | Train 1 | Test Hispanic 3 | 0.73 | ***67*** | Train 5 | Test Hispanic 3 | 0.73 | ***131*** | Train 9 | Test Hispanic 3 | 0.73 | ***195*** | Train 13 | Test Hispanic 3 | 0.73 |
| ***4*** | Train 1 | Test Hispanic 4 | 0.72 | ***68*** | Train 5 | Test Hispanic 4 | 0.72 | ***132*** | Train 9 | Test Hispanic 4 | 0.72 | ***196*** | Train 13 | Test Hispanic 4 | 0.71 |
| ***5*** | Train 1 | Test Hispanic 5 | 0.73 | ***69*** | Train 5 | Test Hispanic 5 | 0.73 | ***133*** | Train 9 | Test Hispanic 5 | 0.73 | ***197*** | Train 13 | Test Hispanic 5 | 0.73 |
| ***6*** | Train 1 | Test Hispanic 6 | 0.72 | ***70*** | Train 5 | Test Hispanic 6 | 0.72 | ***134*** | Train 9 | Test Hispanic 6 | 0.72 | ***198*** | Train 13 | Test Hispanic 6 | 0.71 |
| ***7*** | Train 1 | Test Hispanic 7 | 0.73 | ***71*** | Train 5 | Test Hispanic 7 | 0.73 | ***135*** | Train 9 | Test Hispanic 7 | 0.73 | ***199*** | Train 13 | Test Hispanic 7 | 0.73 |
| ***8*** | Train 1 | Test Hispanic 8 | 0.72 | ***72*** | Train 5 | Test Hispanic 8 | 0.72 | ***136*** | Train 9 | Test Hispanic 8 | 0.72 | ***200*** | Train 13 | Test Hispanic 8 | 0.71 |
| ***9*** | Train 1 | Test Hispanic 9 | 0.74 | ***73*** | Train 5 | Test Hispanic 9 | 0.74 | ***137*** | Train 9 | Test Hispanic 9 | 0.74 | ***201*** | Train 13 | Test Hispanic 9 | 0.73 |
| ***10*** | Train 1 | Test Hispanic 10 | 0.72 | ***74*** | Train 5 | Test Hispanic 10 | 0.72 | ***138*** | Train 9 | Test Hispanic 10 | 0.72 | ***202*** | Train 13 | Test Hispanic 10 | 0.71 |
| ***11*** | Train 1 | Test Hispanic 11 | 0.73 | ***75*** | Train 5 | Test Hispanic 11 | 0.73 | ***139*** | Train 9 | Test Hispanic 11 | 0.73 | ***203*** | Train 13 | Test Hispanic 11 | 0.73 |
| ***12*** | Train 1 | Test Hispanic 12 | 0.72 | ***76*** | Train 5 | Test Hispanic 12 | 0.72 | ***140*** | Train 9 | Test Hispanic 12 | 0.72 | ***204*** | Train 13 | Test Hispanic 12 | 0.72 |
| ***13*** | Train 1 | Test Hispanic 13 | 0.75 | ***77*** | Train 5 | Test Hispanic 13 | 0.75 | ***141*** | Train 9 | Test Hispanic 13 | 0.75 | ***205*** | Train 13 | Test Hispanic 13 | 0.77 |
| ***14*** | Train 1 | Test Hispanic 14 | 0.74 | ***78*** | Train 5 | Test Hispanic 14 | 0.74 | ***142*** | Train 9 | Test Hispanic 14 | 0.74 | ***206*** | Train 13 | Test Hispanic 14 | 0.74 |
| ***15*** | Train 1 | Test Hispanic 15 | 0.76 | ***79*** | Train 5 | Test Hispanic 15 | 0.76 | ***143*** | Train 9 | Test Hispanic 15 | 0.76 | ***207*** | Train 13 | Test Hispanic 15 | 0.77 |
| ***16*** | Train 1 | Test Hispanic 16 | 0.74 | ***80*** | Train 5 | Test Hispanic 16 | 0.74 | ***144*** | Train 9 | Test Hispanic 16 | 0.74 | ***208*** | Train 13 | Test Hispanic 16 | 0.74 |
| ***17*** | Train 2 | Test Hispanic 1 | 0.72 | ***81*** | Train 6 | Test Hispanic 1 | 0.72 | ***145*** | Train 10 | Test Hispanic 1 | 0.72 | ***209*** | Train 14 | Test Hispanic 1 | 0.7 |
| ***18*** | Train 2 | Test Hispanic 2 | 0.73 | ***82*** | Train 6 | Test Hispanic 2 | 0.73 | ***146*** | Train 10 | Test Hispanic 2 | 0.73 | ***210*** | Train 14 | Test Hispanic 2 | 0.71 |
| ***19*** | Train 2 | Test Hispanic 3 | 0.72 | ***83*** | Train 6 | Test Hispanic 3 | 0.72 | ***147*** | Train 10 | Test Hispanic 3 | 0.72 | ***211*** | Train 14 | Test Hispanic 3 | 0.7 |
| ***20*** | Train 2 | Test Hispanic 4 | 0.73 | ***84*** | Train 6 | Test Hispanic 4 | 0.73 | ***148*** | Train 10 | Test Hispanic 4 | 0.73 | ***212*** | Train 14 | Test Hispanic 4 | 0.71 |
| ***21*** | Train 2 | Test Hispanic 5 | 0.72 | ***85*** | Train 6 | Test Hispanic 5 | 0.72 | ***149*** | Train 10 | Test Hispanic 5 | 0.72 | ***213*** | Train 14 | Test Hispanic 5 | 0.7 |
| ***22*** | Train 2 | Test Hispanic 6 | 0.73 | ***86*** | Train 6 | Test Hispanic 6 | 0.73 | ***150*** | Train 10 | Test Hispanic 6 | 0.73 | ***214*** | Train 14 | Test Hispanic 6 | 0.71 |
| ***23*** | Train 2 | Test Hispanic 7 | 0.72 | ***87*** | Train 6 | Test Hispanic 7 | 0.72 | ***151*** | Train 10 | Test Hispanic 7 | 0.72 | ***215*** | Train 14 | Test Hispanic 7 | 0.7 |
| ***24*** | Train 2 | Test Hispanic 8 | 0.73 | ***88*** | Train 6 | Test Hispanic 8 | 0.73 | ***152*** | Train 10 | Test Hispanic 8 | 0.73 | ***216*** | Train 14 | Test Hispanic 8 | 0.71 |
| ***25*** | Train 2 | Test Hispanic 9 | 0.73 | ***89*** | Train 6 | Test Hispanic 9 | 0.73 | ***153*** | Train 10 | Test Hispanic 9 | 0.73 | ***217*** | Train 14 | Test Hispanic 9 | 0.71 |
| ***26*** | Train 2 | Test Hispanic 10 | 0.73 | ***90*** | Train 6 | Test Hispanic 10 | 0.73 | ***154*** | Train 10 | Test Hispanic 10 | 0.73 | ***218*** | Train 14 | Test Hispanic 10 | 0.71 |
| ***27*** | Train 2 | Test Hispanic 11 | 0.73 | ***91*** | Train 6 | Test Hispanic 11 | 0.73 | ***155*** | Train 10 | Test Hispanic 11 | 0.73 | ***219*** | Train 14 | Test Hispanic 11 | 0.71 |
| ***28*** | Train 2 | Test Hispanic 12 | 0.73 | ***92*** | Train 6 | Test Hispanic 12 | 0.73 | ***156*** | Train 10 | Test Hispanic 12 | 0.73 | ***220*** | Train 14 | Test Hispanic 12 | 0.72 |
| ***29*** | Train 2 | Test Hispanic 13 | 0.76 | ***93*** | Train 6 | Test Hispanic 13 | 0.76 | ***157*** | Train 10 | Test Hispanic 13 | 0.76 | ***221*** | Train 14 | Test Hispanic 13 | 0.76 |
| ***30*** | Train 2 | Test Hispanic 14 | 0.76 | ***94*** | Train 6 | Test Hispanic 14 | 0.76 | ***158*** | Train 10 | Test Hispanic 14 | 0.76 | ***222*** | Train 14 | Test Hispanic 14 | 0.76 |
| ***31*** | Train 2 | Test Hispanic 15 | 0.76 | ***95*** | Train 6 | Test Hispanic 15 | 0.76 | ***159*** | Train 10 | Test Hispanic 15 | 0.76 | ***223*** | Train 14 | Test Hispanic 15 | 0.76 |
| ***32*** | Train 2 | Test Hispanic 16 | 0.76 | ***96*** | Train 6 | Test Hispanic 16 | 0.76 | ***160*** | Train 10 | Test Hispanic 16 | 0.76 | ***224*** | Train 14 | Test Hispanic 16 | 0.76 |
| ***33*** | Train 3 | Test Hispanic 1 | 0.73 | ***97*** | Train 7 | Test Hispanic 1 | 0.73 | ***161*** | Train 11 | Test Hispanic 1 | 0.73 | ***225*** | Train 15 | Test Hispanic 1 | 0.73 |
| ***34*** | Train 3 | Test Hispanic 2 | 0.72 | ***98*** | Train 7 | Test Hispanic 2 | 0.72 | ***162*** | Train 11 | Test Hispanic 2 | 0.71 | ***226*** | Train 15 | Test Hispanic 2 | 0.71 |
| ***35*** | Train 3 | Test Hispanic 3 | 0.73 | ***99*** | Train 7 | Test Hispanic 3 | 0.73 | ***163*** | Train 11 | Test Hispanic 3 | 0.73 | ***227*** | Train 15 | Test Hispanic 3 | 0.73 |
| ***36*** | Train 3 | Test Hispanic 4 | 0.72 | ***100*** | Train 7 | Test Hispanic 4 | 0.72 | ***164*** | Train 11 | Test Hispanic 4 | 0.72 | ***228*** | Train 15 | Test Hispanic 4 | 0.72 |
| ***37*** | Train 3 | Test Hispanic 5 | 0.73 | ***101*** | Train 7 | Test Hispanic 5 | 0.73 | ***165*** | Train 11 | Test Hispanic 5 | 0.73 | ***229*** | Train 15 | Test Hispanic 5 | 0.73 |
| ***38*** | Train 3 | Test Hispanic 6 | 0.72 | ***102*** | Train 7 | Test Hispanic 6 | 0.72 | ***166*** | Train 11 | Test Hispanic 6 | 0.72 | ***230*** | Train 15 | Test Hispanic 6 | 0.71 |
| ***39*** | Train 3 | Test Hispanic 7 | 0.73 | ***103*** | Train 7 | Test Hispanic 7 | 0.73 | ***167*** | Train 11 | Test Hispanic 7 | 0.73 | ***231*** | Train 15 | Test Hispanic 7 | 0.73 |
| ***40*** | Train 3 | Test Hispanic 8 | 0.72 | ***104*** | Train 7 | Test Hispanic 8 | 0.72 | ***168*** | Train 11 | Test Hispanic 8 | 0.72 | ***232*** | Train 15 | Test Hispanic 8 | 0.72 |
| ***41*** | Train 3 | Test Hispanic 9 | 0.74 | ***105*** | Train 7 | Test Hispanic 9 | 0.74 | ***169*** | Train 11 | Test Hispanic 9 | 0.74 | ***233*** | Train 15 | Test Hispanic 9 | 0.73 |
| ***42*** | Train 3 | Test Hispanic 10 | 0.72 | ***106*** | Train 7 | Test Hispanic 10 | 0.72 | ***170*** | Train 11 | Test Hispanic 10 | 0.72 | ***234*** | Train 15 | Test Hispanic 10 | 0.71 |
| ***43*** | Train 3 | Test Hispanic 11 | 0.73 | ***107*** | Train 7 | Test Hispanic 11 | 0.73 | ***171*** | Train 11 | Test Hispanic 11 | 0.73 | ***235*** | Train 15 | Test Hispanic 11 | 0.73 |
| ***44*** | Train 3 | Test Hispanic 12 | 0.72 | ***108*** | Train 7 | Test Hispanic 12 | 0.72 | ***172*** | Train 11 | Test Hispanic 12 | 0.72 | ***236*** | Train 15 | Test Hispanic 12 | 0.72 |
| ***45*** | Train 3 | Test Hispanic 13 | 0.75 | ***109*** | Train 7 | Test Hispanic 13 | 0.75 | ***173*** | Train 11 | Test Hispanic 13 | 0.75 | ***237*** | Train 15 | Test Hispanic 13 | 0.77 |
| ***46*** | Train 3 | Test Hispanic 14 | 0.74 | ***110*** | Train 7 | Test Hispanic 14 | 0.74 | ***174*** | Train 11 | Test Hispanic 14 | 0.74 | ***238*** | Train 15 | Test Hispanic 14 | 0.74 |
| ***47*** | Train 3 | Test Hispanic 15 | 0.76 | ***111*** | Train 7 | Test Hispanic 15 | 0.76 | ***175*** | Train 11 | Test Hispanic 15 | 0.76 | ***239*** | Train 15 | Test Hispanic 15 | 0.77 |
| ***48*** | Train 3 | Test Hispanic 16 | 0.74 | ***112*** | Train 7 | Test Hispanic 16 | 0.74 | ***176*** | Train 11 | Test Hispanic 16 | 0.74 | ***240*** | Train 15 | Test Hispanic 16 | 0.74 |
| ***49*** | Train 4 | Test Hispanic 1 | 0.72 | ***113*** | Train 8 | Test Hispanic 1 | 0.72 | ***177*** | Train 12 | Test Hispanic 1 | 0.72 | ***241*** | Train 16 | Test Hispanic 1 | 0.7 |
| ***50*** | Train 4 | Test Hispanic 2 | 0.73 | ***114*** | Train 8 | Test Hispanic 2 | 0.73 | ***178*** | Train 12 | Test Hispanic 2 | 0.73 | ***242*** | Train 16 | Test Hispanic 2 | 0.71 |
| ***51*** | Train 4 | Test Hispanic 3 | 0.72 | ***115*** | Train 8 | Test Hispanic 3 | 0.72 | ***179*** | Train 12 | Test Hispanic 3 | 0.72 | ***243*** | Train 16 | Test Hispanic 3 | 0.7 |
| ***52*** | Train 4 | Test Hispanic 4 | 0.73 | ***116*** | Train 8 | Test Hispanic 4 | 0.73 | ***180*** | Train 12 | Test Hispanic 4 | 0.73 | ***244*** | Train 16 | Test Hispanic 4 | 0.71 |
| ***53*** | Train 4 | Test Hispanic 5 | 0.72 | ***117*** | Train 8 | Test Hispanic 5 | 0.72 | ***181*** | Train 12 | Test Hispanic 5 | 0.72 | ***245*** | Train 16 | Test Hispanic 5 | 0.7 |
| ***54*** | Train 4 | Test Hispanic 6 | 0.73 | ***118*** | Train 8 | Test Hispanic 6 | 0.73 | ***182*** | Train 12 | Test Hispanic 6 | 0.73 | ***246*** | Train 16 | Test Hispanic 6 | 0.71 |
| ***55*** | Train 4 | Test Hispanic 7 | 0.72 | ***119*** | Train 8 | Test Hispanic 7 | 0.72 | ***183*** | Train 12 | Test Hispanic 7 | 0.72 | ***247*** | Train 16 | Test Hispanic 7 | 0.7 |
| ***56*** | Train 4 | Test Hispanic 8 | 0.73 | ***120*** | Train 8 | Test Hispanic 8 | 0.73 | ***184*** | Train 12 | Test Hispanic 8 | 0.73 | ***248*** | Train 16 | Test Hispanic 8 | 0.71 |
| ***57*** | Train 4 | Test Hispanic 9 | 0.73 | ***121*** | Train 8 | Test Hispanic 9 | 0.73 | ***185*** | Train 12 | Test Hispanic 9 | 0.73 | ***249*** | Train 16 | Test Hispanic 9 | 0.71 |
| ***58*** | Train 4 | Test Hispanic 10 | 0.73 | ***122*** | Train 8 | Test Hispanic 10 | 0.73 | ***186*** | Train 12 | Test Hispanic 10 | 0.73 | ***250*** | Train 16 | Test Hispanic 10 | 0.71 |
| ***59*** | Train 4 | Test Hispanic 11 | 0.73 | ***123*** | Train 8 | Test Hispanic 11 | 0.73 | ***187*** | Train 12 | Test Hispanic 11 | 0.73 | ***251*** | Train 16 | Test Hispanic 11 | 0.71 |
| ***60*** | Train 4 | Test Hispanic 12 | 0.73 | ***124*** | Train 8 | Test Hispanic 12 | 0.73 | ***188*** | Train 12 | Test Hispanic 12 | 0.73 | ***252*** | Train 16 | Test Hispanic 12 | 0.72 |
| ***61*** | Train 4 | Test Hispanic 13 | 0.76 | ***125*** | Train 8 | Test Hispanic 13 | 0.76 | ***189*** | Train 12 | Test Hispanic 13 | 0.76 | ***253*** | Train 16 | Test Hispanic 13 | 0.76 |
| ***62*** | Train 4 | Test Hispanic 14 | 0.76 | ***126*** | Train 8 | Test Hispanic 14 | 0.76 | ***190*** | Train 12 | Test Hispanic 14 | 0.76 | ***254*** | Train 16 | Test Hispanic 14 | 0.76 |
| ***63*** | Train 4 | Test Hispanic 15 | 0.76 | ***127*** | Train 8 | Test Hispanic 15 | 0.76 | ***191*** | Train 12 | Test Hispanic 15 | 0.76 | ***255*** | Train 16 | Test Hispanic 15 | 0.76 |
| ***64*** | Train 4 | Test Hispanic 16 | 0.76 | ***128*** | Train 8 | Test Hispanic 16 | 0.76 | ***192*** | Train 12 | Test Hispanic 16 | 0.76 | ***256*** | Train 16 | Test Hispanic 16 | 0.76 |

Table 23. Random Forest (RF) AUC Performance for Hispanic Patients Across 16 Cohorts.

| Original Decisions | Train Model | Test Dataset | AUC (RF) | ***Hispanic*** | Train Model | Test Dataset | AUC (RF) | Hispanic | Train Model | Test Dataset | AUC (RF) | Hispanic | Train Model | Test Dataset | AUC (RF) |
| --- | --- | --- | --- | --- | --- | --- | --- | --- | --- | --- | --- | --- | --- | --- | --- |
| ***1*** | Train 1 | Test Hispanic 1 | 0.75 | ***65*** | Train 5 | Test Hispanic 1 | 0.75 | ***129*** | Train 9 | Test Hispanic 1 | 0.75 | ***193*** | Train 13 | Test Hispanic 1 | 0.72 |
| ***2*** | Train 1 | Test Hispanic 2 | 0.71 | ***66*** | Train 5 | Test Hispanic 2 | 0.71 | ***130*** | Train 9 | Test Hispanic 2 | 0.71 | ***194*** | Train 13 | Test Hispanic 2 | 0.69 |
| ***3*** | Train 1 | Test Hispanic 3 | 0.75 | ***67*** | Train 5 | Test Hispanic 3 | 0.75 | ***131*** | Train 9 | Test Hispanic 3 | 0.75 | ***195*** | Train 13 | Test Hispanic 3 | 0.72 |
| ***4*** | Train 1 | Test Hispanic 4 | 0.71 | ***68*** | Train 5 | Test Hispanic 4 | 0.71 | ***132*** | Train 9 | Test Hispanic 4 | 0.71 | ***196*** | Train 13 | Test Hispanic 4 | 0.7 |
| ***5*** | Train 1 | Test Hispanic 5 | 0.75 | ***69*** | Train 5 | Test Hispanic 5 | 0.75 | ***133*** | Train 9 | Test Hispanic 5 | 0.75 | ***197*** | Train 13 | Test Hispanic 5 | 0.72 |
| ***6*** | Train 1 | Test Hispanic 6 | 0.71 | ***70*** | Train 5 | Test Hispanic 6 | 0.71 | ***134*** | Train 9 | Test Hispanic 6 | 0.71 | ***198*** | Train 13 | Test Hispanic 6 | 0.7 |
| ***7*** | Train 1 | Test Hispanic 7 | 0.75 | ***71*** | Train 5 | Test Hispanic 7 | 0.75 | ***135*** | Train 9 | Test Hispanic 7 | 0.75 | ***199*** | Train 13 | Test Hispanic 7 | 0.72 |
| ***8*** | Train 1 | Test Hispanic 8 | 0.72 | ***72*** | Train 5 | Test Hispanic 8 | 0.72 | ***136*** | Train 9 | Test Hispanic 8 | 0.72 | ***200*** | Train 13 | Test Hispanic 8 | 0.7 |
| ***9*** | Train 1 | Test Hispanic 9 | 0.75 | ***73*** | Train 5 | Test Hispanic 9 | 0.75 | ***137*** | Train 9 | Test Hispanic 9 | 0.76 | ***201*** | Train 13 | Test Hispanic 9 | 0.73 |
| ***10*** | Train 1 | Test Hispanic 10 | 0.71 | ***74*** | Train 5 | Test Hispanic 10 | 0.71 | ***138*** | Train 9 | Test Hispanic 10 | 0.71 | ***202*** | Train 13 | Test Hispanic 10 | 0.69 |
| ***11*** | Train 1 | Test Hispanic 11 | 0.75 | ***75*** | Train 5 | Test Hispanic 11 | 0.75 | ***139*** | Train 9 | Test Hispanic 11 | 0.75 | ***203*** | Train 13 | Test Hispanic 11 | 0.73 |
| ***12*** | Train 1 | Test Hispanic 12 | 0.71 | ***76*** | Train 5 | Test Hispanic 12 | 0.71 | ***140*** | Train 9 | Test Hispanic 12 | 0.71 | ***204*** | Train 13 | Test Hispanic 12 | 0.7 |
| ***13*** | Train 1 | Test Hispanic 13 | 0.75 | ***77*** | Train 5 | Test Hispanic 13 | 0.76 | ***141*** | Train 9 | Test Hispanic 13 | 0.76 | ***205*** | Train 13 | Test Hispanic 13 | 0.76 |
| ***14*** | Train 1 | Test Hispanic 14 | 0.71 | ***78*** | Train 5 | Test Hispanic 14 | 0.71 | ***142*** | Train 9 | Test Hispanic 14 | 0.71 | ***206*** | Train 13 | Test Hispanic 14 | 0.71 |
| ***15*** | Train 1 | Test Hispanic 15 | 0.76 | ***79*** | Train 5 | Test Hispanic 15 | 0.76 | ***143*** | Train 9 | Test Hispanic 15 | 0.76 | ***207*** | Train 13 | Test Hispanic 15 | 0.77 |
| ***16*** | Train 1 | Test Hispanic 16 | 0.71 | ***80*** | Train 5 | Test Hispanic 16 | 0.71 | ***144*** | Train 9 | Test Hispanic 16 | 0.71 | ***208*** | Train 13 | Test Hispanic 16 | 0.72 |
| ***17*** | Train 2 | Test Hispanic 1 | 0.75 | ***81*** | Train 6 | Test Hispanic 1 | 0.75 | ***145*** | Train 10 | Test Hispanic 1 | 0.75 | ***209*** | Train 14 | Test Hispanic 1 | 0.71 |
| ***18*** | Train 2 | Test Hispanic 2 | 0.75 | ***82*** | Train 6 | Test Hispanic 2 | 0.75 | ***146*** | Train 10 | Test Hispanic 2 | 0.75 | ***210*** | Train 14 | Test Hispanic 2 | 0.72 |
| ***19*** | Train 2 | Test Hispanic 3 | 0.75 | ***83*** | Train 6 | Test Hispanic 3 | 0.75 | ***147*** | Train 10 | Test Hispanic 3 | 0.75 | ***211*** | Train 14 | Test Hispanic 3 | 0.71 |
| ***20*** | Train 2 | Test Hispanic 4 | 0.75 | ***84*** | Train 6 | Test Hispanic 4 | 0.75 | ***148*** | Train 10 | Test Hispanic 4 | 0.75 | ***212*** | Train 14 | Test Hispanic 4 | 0.73 |
| ***21*** | Train 2 | Test Hispanic 5 | 0.75 | ***85*** | Train 6 | Test Hispanic 5 | 0.75 | ***149*** | Train 10 | Test Hispanic 5 | 0.75 | ***213*** | Train 14 | Test Hispanic 5 | 0.71 |
| ***22*** | Train 2 | Test Hispanic 6 | 0.75 | ***86*** | Train 6 | Test Hispanic 6 | 0.75 | ***150*** | Train 10 | Test Hispanic 6 | 0.75 | ***214*** | Train 14 | Test Hispanic 6 | 0.72 |
| ***23*** | Train 2 | Test Hispanic 7 | 0.75 | ***87*** | Train 6 | Test Hispanic 7 | 0.75 | ***151*** | Train 10 | Test Hispanic 7 | 0.75 | ***215*** | Train 14 | Test Hispanic 7 | 0.71 |
| ***24*** | Train 2 | Test Hispanic 8 | 0.75 | ***88*** | Train 6 | Test Hispanic 8 | 0.75 | ***152*** | Train 10 | Test Hispanic 8 | 0.75 | ***216*** | Train 14 | Test Hispanic 8 | 0.73 |
| ***25*** | Train 2 | Test Hispanic 9 | 0.75 | ***89*** | Train 6 | Test Hispanic 9 | 0.75 | ***153*** | Train 10 | Test Hispanic 9 | 0.75 | ***217*** | Train 14 | Test Hispanic 9 | 0.72 |
| ***26*** | Train 2 | Test Hispanic 10 | 0.75 | ***90*** | Train 6 | Test Hispanic 10 | 0.75 | ***154*** | Train 10 | Test Hispanic 10 | 0.75 | ***218*** | Train 14 | Test Hispanic 10 | 0.72 |
| ***27*** | Train 2 | Test Hispanic 11 | 0.75 | ***91*** | Train 6 | Test Hispanic 11 | 0.75 | ***155*** | Train 10 | Test Hispanic 11 | 0.75 | ***219*** | Train 14 | Test Hispanic 11 | 0.72 |
| ***28*** | Train 2 | Test Hispanic 12 | 0.75 | ***92*** | Train 6 | Test Hispanic 12 | 0.75 | ***156*** | Train 10 | Test Hispanic 12 | 0.75 | ***220*** | Train 14 | Test Hispanic 12 | 0.73 |
| ***29*** | Train 2 | Test Hispanic 13 | 0.76 | ***93*** | Train 6 | Test Hispanic 13 | 0.76 | ***157*** | Train 10 | Test Hispanic 13 | 0.76 | ***221*** | Train 14 | Test Hispanic 13 | 0.76 |
| ***30*** | Train 2 | Test Hispanic 14 | 0.76 | ***94*** | Train 6 | Test Hispanic 14 | 0.76 | ***158*** | Train 10 | Test Hispanic 14 | 0.76 | ***222*** | Train 14 | Test Hispanic 14 | 0.76 |
| ***31*** | Train 2 | Test Hispanic 15 | 0.76 | ***95*** | Train 6 | Test Hispanic 15 | 0.76 | ***159*** | Train 10 | Test Hispanic 15 | 0.76 | ***223*** | Train 14 | Test Hispanic 15 | 0.76 |
| ***32*** | Train 2 | Test Hispanic 16 | 0.76 | ***96*** | Train 6 | Test Hispanic 16 | 0.76 | ***160*** | Train 10 | Test Hispanic 16 | 0.76 | ***224*** | Train 14 | Test Hispanic 16 | 0.76 |
| ***33*** | Train 3 | Test Hispanic 1 | 0.75 | ***97*** | Train 7 | Test Hispanic 1 | 0.75 | ***161*** | Train 11 | Test Hispanic 1 | 0.75 | ***225*** | Train 15 | Test Hispanic 1 | 0.72 |
| ***34*** | Train 3 | Test Hispanic 2 | 0.71 | ***98*** | Train 7 | Test Hispanic 2 | 0.71 | ***162*** | Train 11 | Test Hispanic 2 | 0.71 | ***226*** | Train 15 | Test Hispanic 2 | 0.69 |
| ***35*** | Train 3 | Test Hispanic 3 | 0.75 | ***99*** | Train 7 | Test Hispanic 3 | 0.75 | ***163*** | Train 11 | Test Hispanic 3 | 0.75 | ***227*** | Train 15 | Test Hispanic 3 | 0.72 |
| ***36*** | Train 3 | Test Hispanic 4 | 0.71 | ***100*** | Train 7 | Test Hispanic 4 | 0.71 | ***164*** | Train 11 | Test Hispanic 4 | 0.71 | ***228*** | Train 15 | Test Hispanic 4 | 0.7 |
| ***37*** | Train 3 | Test Hispanic 5 | 0.75 | ***101*** | Train 7 | Test Hispanic 5 | 0.75 | ***165*** | Train 11 | Test Hispanic 5 | 0.75 | ***229*** | Train 15 | Test Hispanic 5 | 0.72 |
| ***38*** | Train 3 | Test Hispanic 6 | 0.71 | ***102*** | Train 7 | Test Hispanic 6 | 0.71 | ***166*** | Train 11 | Test Hispanic 6 | 0.71 | ***230*** | Train 15 | Test Hispanic 6 | 0.7 |
| ***39*** | Train 3 | Test Hispanic 7 | 0.75 | ***103*** | Train 7 | Test Hispanic 7 | 0.75 | ***167*** | Train 11 | Test Hispanic 7 | 0.75 | ***231*** | Train 15 | Test Hispanic 7 | 0.72 |
| ***40*** | Train 3 | Test Hispanic 8 | 0.71 | ***104*** | Train 7 | Test Hispanic 8 | 0.71 | ***168*** | Train 11 | Test Hispanic 8 | 0.71 | ***232*** | Train 15 | Test Hispanic 8 | 0.7 |
| ***41*** | Train 3 | Test Hispanic 9 | 0.75 | ***105*** | Train 7 | Test Hispanic 9 | 0.75 | ***169*** | Train 11 | Test Hispanic 9 | 0.75 | ***233*** | Train 15 | Test Hispanic 9 | 0.73 |
| ***42*** | Train 3 | Test Hispanic 10 | 0.71 | ***106*** | Train 7 | Test Hispanic 10 | 0.71 | ***170*** | Train 11 | Test Hispanic 10 | 0.71 | ***234*** | Train 15 | Test Hispanic 10 | 0.69 |
| ***43*** | Train 3 | Test Hispanic 11 | 0.75 | ***107*** | Train 7 | Test Hispanic 11 | 0.75 | ***171*** | Train 11 | Test Hispanic 11 | 0.75 | ***235*** | Train 15 | Test Hispanic 11 | 0.73 |
| ***44*** | Train 3 | Test Hispanic 12 | 0.71 | ***108*** | Train 7 | Test Hispanic 12 | 0.71 | ***172*** | Train 11 | Test Hispanic 12 | 0.71 | ***236*** | Train 15 | Test Hispanic 12 | 0.7 |
| ***45*** | Train 3 | Test Hispanic 13 | 0.75 | ***109*** | Train 7 | Test Hispanic 13 | 0.75 | ***173*** | Train 11 | Test Hispanic 13 | 0.75 | ***237*** | Train 15 | Test Hispanic 13 | 0.76 |
| ***46*** | Train 3 | Test Hispanic 14 | 0.71 | ***110*** | Train 7 | Test Hispanic 14 | 0.7 | ***174*** | Train 11 | Test Hispanic 14 | 0.71 | ***238*** | Train 15 | Test Hispanic 14 | 0.71 |
| ***47*** | Train 3 | Test Hispanic 15 | 0.76 | ***111*** | Train 7 | Test Hispanic 15 | 0.76 | ***175*** | Train 11 | Test Hispanic 15 | 0.76 | ***239*** | Train 15 | Test Hispanic 15 | 0.77 |
| ***48*** | Train 3 | Test Hispanic 16 | 0.71 | ***112*** | Train 7 | Test Hispanic 16 | 0.71 | ***176*** | Train 11 | Test Hispanic 16 | 0.71 | ***240*** | Train 15 | Test Hispanic 16 | 0.72 |
| ***49*** | Train 4 | Test Hispanic 1 | 0.75 | ***113*** | Train 8 | Test Hispanic 1 | 0.75 | ***177*** | Train 12 | Test Hispanic 1 | 0.75 | ***241*** | Train 16 | Test Hispanic 1 | 0.71 |
| ***50*** | Train 4 | Test Hispanic 2 | 0.75 | ***114*** | Train 8 | Test Hispanic 2 | 0.75 | ***178*** | Train 12 | Test Hispanic 2 | 0.75 | ***242*** | Train 16 | Test Hispanic 2 | 0.72 |
| ***51*** | Train 4 | Test Hispanic 3 | 0.75 | ***115*** | Train 8 | Test Hispanic 3 | 0.75 | ***179*** | Train 12 | Test Hispanic 3 | 0.75 | ***243*** | Train 16 | Test Hispanic 3 | 0.71 |
| ***52*** | Train 4 | Test Hispanic 4 | 0.75 | ***116*** | Train 8 | Test Hispanic 4 | 0.75 | ***180*** | Train 12 | Test Hispanic 4 | 0.75 | ***244*** | Train 16 | Test Hispanic 4 | 0.73 |
| ***53*** | Train 4 | Test Hispanic 5 | 0.75 | ***117*** | Train 8 | Test Hispanic 5 | 0.75 | ***181*** | Train 12 | Test Hispanic 5 | 0.75 | ***245*** | Train 16 | Test Hispanic 5 | 0.71 |
| ***54*** | Train 4 | Test Hispanic 6 | 0.75 | ***118*** | Train 8 | Test Hispanic 6 | 0.75 | ***182*** | Train 12 | Test Hispanic 6 | 0.75 | ***246*** | Train 16 | Test Hispanic 6 | 0.73 |
| ***55*** | Train 4 | Test Hispanic 7 | 0.75 | ***119*** | Train 8 | Test Hispanic 7 | 0.75 | ***183*** | Train 12 | Test Hispanic 7 | 0.75 | ***247*** | Train 16 | Test Hispanic 7 | 0.72 |
| ***56*** | Train 4 | Test Hispanic 8 | 0.75 | ***120*** | Train 8 | Test Hispanic 8 | 0.75 | ***184*** | Train 12 | Test Hispanic 8 | 0.75 | ***248*** | Train 16 | Test Hispanic 8 | 0.73 |
| ***57*** | Train 4 | Test Hispanic 9 | 0.75 | ***121*** | Train 8 | Test Hispanic 9 | 0.75 | ***185*** | Train 12 | Test Hispanic 9 | 0.75 | ***249*** | Train 16 | Test Hispanic 9 | 0.72 |
| ***58*** | Train 4 | Test Hispanic 10 | 0.75 | ***122*** | Train 8 | Test Hispanic 10 | 0.75 | ***186*** | Train 12 | Test Hispanic 10 | 0.75 | ***250*** | Train 16 | Test Hispanic 10 | 0.73 |
| ***59*** | Train 4 | Test Hispanic 11 | 0.75 | ***123*** | Train 8 | Test Hispanic 11 | 0.75 | ***187*** | Train 12 | Test Hispanic 11 | 0.75 | ***251*** | Train 16 | Test Hispanic 11 | 0.72 |
| ***60*** | Train 4 | Test Hispanic 12 | 0.75 | ***124*** | Train 8 | Test Hispanic 12 | 0.75 | ***188*** | Train 12 | Test Hispanic 12 | 0.75 | ***252*** | Train 16 | Test Hispanic 12 | 0.73 |
| ***61*** | Train 4 | Test Hispanic 13 | 0.75 | ***125*** | Train 8 | Test Hispanic 13 | 0.76 | ***189*** | Train 12 | Test Hispanic 13 | 0.76 | ***253*** | Train 16 | Test Hispanic 13 | 0.76 |
| ***62*** | Train 4 | Test Hispanic 14 | 0.76 | ***126*** | Train 8 | Test Hispanic 14 | 0.76 | ***190*** | Train 12 | Test Hispanic 14 | 0.76 | ***254*** | Train 16 | Test Hispanic 14 | 0.76 |
| ***63*** | Train 4 | Test Hispanic 15 | 0.76 | ***127*** | Train 8 | Test Hispanic 15 | 0.76 | ***191*** | Train 12 | Test Hispanic 15 | 0.76 | ***255*** | Train 16 | Test Hispanic 15 | 0.76 |
| ***64*** | Train 4 | Test Hispanic 16 | 0.76 | ***128*** | Train 8 | Test Hispanic 16 | 0.76 | ***192*** | Train 12 | Test Hispanic 16 | 0.76 | ***256*** | Train 16 | Test Hispanic 16 | 0.76 |

Table 24. Gradient Boosting (GB) AUC Performance for Hispanic Patients Across 16 Cohorts.

| Original Decisions | Train Model | Test Dataset | AUC (GB) | ***Hispanic*** | Train Model | Test Dataset | AUC (GB) | Hispanic | Train Model | Test Dataset | AUC (GB) | Hispanic | Train Model | Test Dataset | AUC (GB) |
| --- | --- | --- | --- | --- | --- | --- | --- | --- | --- | --- | --- | --- | --- | --- | --- |
| ***1*** | Train 1 | Test Hispanic 1 | 0.76 | ***65*** | Train 5 | Test Hispanic 1 | 0.76 | ***129*** | Train 9 | Test Hispanic 1 | 0.75 | ***193*** | Train 13 | Test Hispanic 1 | 0.7 |
| ***2*** | Train 1 | Test Hispanic 2 | 0.73 | ***66*** | Train 5 | Test Hispanic 2 | 0.72 | ***130*** | Train 9 | Test Hispanic 2 | 0.73 | ***194*** | Train 13 | Test Hispanic 2 | 0.69 |
| ***3*** | Train 1 | Test Hispanic 3 | 0.76 | ***67*** | Train 5 | Test Hispanic 3 | 0.77 | ***131*** | Train 9 | Test Hispanic 3 | 0.75 | ***195*** | Train 13 | Test Hispanic 3 | 0.7 |
| ***4*** | Train 1 | Test Hispanic 4 | 0.73 | ***68*** | Train 5 | Test Hispanic 4 | 0.72 | ***132*** | Train 9 | Test Hispanic 4 | 0.73 | ***196*** | Train 13 | Test Hispanic 4 | 0.69 |
| ***5*** | Train 1 | Test Hispanic 5 | 0.76 | ***69*** | Train 5 | Test Hispanic 5 | 0.76 | ***133*** | Train 9 | Test Hispanic 5 | 0.75 | ***197*** | Train 13 | Test Hispanic 5 | 0.7 |
| ***6*** | Train 1 | Test Hispanic 6 | 0.73 | ***70*** | Train 5 | Test Hispanic 6 | 0.72 | ***134*** | Train 9 | Test Hispanic 6 | 0.73 | ***198*** | Train 13 | Test Hispanic 6 | 0.69 |
| ***7*** | Train 1 | Test Hispanic 7 | 0.76 | ***71*** | Train 5 | Test Hispanic 7 | 0.77 | ***135*** | Train 9 | Test Hispanic 7 | 0.75 | ***199*** | Train 13 | Test Hispanic 7 | 0.7 |
| ***8*** | Train 1 | Test Hispanic 8 | 0.73 | ***72*** | Train 5 | Test Hispanic 8 | 0.72 | ***136*** | Train 9 | Test Hispanic 8 | 0.73 | ***200*** | Train 13 | Test Hispanic 8 | 0.69 |
| ***9*** | Train 1 | Test Hispanic 9 | 0.77 | ***73*** | Train 5 | Test Hispanic 9 | 0.77 | ***137*** | Train 9 | Test Hispanic 9 | 0.76 | ***201*** | Train 13 | Test Hispanic 9 | 0.71 |
| ***10*** | Train 1 | Test Hispanic 10 | 0.73 | ***74*** | Train 5 | Test Hispanic 10 | 0.72 | ***138*** | Train 9 | Test Hispanic 10 | 0.73 | ***202*** | Train 13 | Test Hispanic 10 | 0.69 |
| ***11*** | Train 1 | Test Hispanic 11 | 0.77 | ***75*** | Train 5 | Test Hispanic 11 | 0.77 | ***139*** | Train 9 | Test Hispanic 11 | 0.76 | ***203*** | Train 13 | Test Hispanic 11 | 0.71 |
| ***12*** | Train 1 | Test Hispanic 12 | 0.73 | ***76*** | Train 5 | Test Hispanic 12 | 0.73 | ***140*** | Train 9 | Test Hispanic 12 | 0.73 | ***204*** | Train 13 | Test Hispanic 12 | 0.7 |
| ***13*** | Train 1 | Test Hispanic 13 | 0.78 | ***77*** | Train 5 | Test Hispanic 13 | 0.77 | ***141*** | Train 9 | Test Hispanic 13 | 0.76 | ***205*** | Train 13 | Test Hispanic 13 | 0.78 |
| ***14*** | Train 1 | Test Hispanic 14 | 0.75 | ***78*** | Train 5 | Test Hispanic 14 | 0.72 | ***142*** | Train 9 | Test Hispanic 14 | 0.73 | ***206*** | Train 13 | Test Hispanic 14 | 0.73 |
| ***15*** | Train 1 | Test Hispanic 15 | 0.78 | ***79*** | Train 5 | Test Hispanic 15 | 0.77 | ***143*** | Train 9 | Test Hispanic 15 | 0.76 | ***207*** | Train 13 | Test Hispanic 15 | 0.78 |
| ***16*** | Train 1 | Test Hispanic 16 | 0.75 | ***80*** | Train 5 | Test Hispanic 16 | 0.72 | ***144*** | Train 9 | Test Hispanic 16 | 0.74 | ***208*** | Train 13 | Test Hispanic 16 | 0.73 |
| ***17*** | Train 2 | Test Hispanic 1 | 0.75 | ***81*** | Train 6 | Test Hispanic 1 | 0.75 | ***145*** | Train 10 | Test Hispanic 1 | 0.75 | ***209*** | Train 14 | Test Hispanic 1 | 0.7 |
| ***18*** | Train 2 | Test Hispanic 2 | 0.76 | ***82*** | Train 6 | Test Hispanic 2 | 0.75 | ***146*** | Train 10 | Test Hispanic 2 | 0.75 | ***210*** | Train 14 | Test Hispanic 2 | 0.71 |
| ***19*** | Train 2 | Test Hispanic 3 | 0.75 | ***83*** | Train 6 | Test Hispanic 3 | 0.75 | ***147*** | Train 10 | Test Hispanic 3 | 0.75 | ***211*** | Train 14 | Test Hispanic 3 | 0.7 |
| ***20*** | Train 2 | Test Hispanic 4 | 0.76 | ***84*** | Train 6 | Test Hispanic 4 | 0.75 | ***148*** | Train 10 | Test Hispanic 4 | 0.75 | ***212*** | Train 14 | Test Hispanic 4 | 0.71 |
| ***21*** | Train 2 | Test Hispanic 5 | 0.75 | ***85*** | Train 6 | Test Hispanic 5 | 0.75 | ***149*** | Train 10 | Test Hispanic 5 | 0.75 | ***213*** | Train 14 | Test Hispanic 5 | 0.7 |
| ***22*** | Train 2 | Test Hispanic 6 | 0.76 | ***86*** | Train 6 | Test Hispanic 6 | 0.75 | ***150*** | Train 10 | Test Hispanic 6 | 0.75 | ***214*** | Train 14 | Test Hispanic 6 | 0.71 |
| ***23*** | Train 2 | Test Hispanic 7 | 0.75 | ***87*** | Train 6 | Test Hispanic 7 | 0.75 | ***151*** | Train 10 | Test Hispanic 7 | 0.75 | ***215*** | Train 14 | Test Hispanic 7 | 0.7 |
| ***24*** | Train 2 | Test Hispanic 8 | 0.76 | ***88*** | Train 6 | Test Hispanic 8 | 0.76 | ***152*** | Train 10 | Test Hispanic 8 | 0.75 | ***216*** | Train 14 | Test Hispanic 8 | 0.71 |
| ***25*** | Train 2 | Test Hispanic 9 | 0.76 | ***89*** | Train 6 | Test Hispanic 9 | 0.76 | ***153*** | Train 10 | Test Hispanic 9 | 0.75 | ***217*** | Train 14 | Test Hispanic 9 | 0.7 |
| ***26*** | Train 2 | Test Hispanic 10 | 0.76 | ***90*** | Train 6 | Test Hispanic 10 | 0.76 | ***154*** | Train 10 | Test Hispanic 10 | 0.76 | ***218*** | Train 14 | Test Hispanic 10 | 0.71 |
| ***27*** | Train 2 | Test Hispanic 11 | 0.76 | ***91*** | Train 6 | Test Hispanic 11 | 0.75 | ***155*** | Train 10 | Test Hispanic 11 | 0.75 | ***219*** | Train 14 | Test Hispanic 11 | 0.71 |
| ***28*** | Train 2 | Test Hispanic 12 | 0.76 | ***92*** | Train 6 | Test Hispanic 12 | 0.76 | ***156*** | Train 10 | Test Hispanic 12 | 0.76 | ***220*** | Train 14 | Test Hispanic 12 | 0.71 |
| ***29*** | Train 2 | Test Hispanic 13 | 0.77 | ***93*** | Train 6 | Test Hispanic 13 | 0.77 | ***157*** | Train 10 | Test Hispanic 13 | 0.77 | ***221*** | Train 14 | Test Hispanic 13 | 0.77 |
| ***30*** | Train 2 | Test Hispanic 14 | 0.78 | ***94*** | Train 6 | Test Hispanic 14 | 0.78 | ***158*** | Train 10 | Test Hispanic 14 | 0.77 | ***222*** | Train 14 | Test Hispanic 14 | 0.77 |
| ***31*** | Train 2 | Test Hispanic 15 | 0.77 | ***95*** | Train 6 | Test Hispanic 15 | 0.78 | ***159*** | Train 10 | Test Hispanic 15 | 0.77 | ***223*** | Train 14 | Test Hispanic 15 | 0.78 |
| ***32*** | Train 2 | Test Hispanic 16 | 0.78 | ***96*** | Train 6 | Test Hispanic 16 | 0.78 | ***160*** | Train 10 | Test Hispanic 16 | 0.77 | ***224*** | Train 14 | Test Hispanic 16 | 0.77 |
| ***33*** | Train 3 | Test Hispanic 1 | 0.76 | ***97*** | Train 7 | Test Hispanic 1 | 0.76 | ***161*** | Train 11 | Test Hispanic 1 | 0.75 | ***225*** | Train 15 | Test Hispanic 1 | 0.69 |
| ***34*** | Train 3 | Test Hispanic 2 | 0.72 | ***98*** | Train 7 | Test Hispanic 2 | 0.73 | ***162*** | Train 11 | Test Hispanic 2 | 0.71 | ***226*** | Train 15 | Test Hispanic 2 | 0.69 |
| ***35*** | Train 3 | Test Hispanic 3 | 0.76 | ***99*** | Train 7 | Test Hispanic 3 | 0.76 | ***163*** | Train 11 | Test Hispanic 3 | 0.75 | ***227*** | Train 15 | Test Hispanic 3 | 0.69 |
| ***36*** | Train 3 | Test Hispanic 4 | 0.72 | ***100*** | Train 7 | Test Hispanic 4 | 0.73 | ***164*** | Train 11 | Test Hispanic 4 | 0.72 | ***228*** | Train 15 | Test Hispanic 4 | 0.69 |
| ***37*** | Train 3 | Test Hispanic 5 | 0.77 | ***101*** | Train 7 | Test Hispanic 5 | 0.76 | ***165*** | Train 11 | Test Hispanic 5 | 0.76 | ***229*** | Train 15 | Test Hispanic 5 | 0.69 |
| ***38*** | Train 3 | Test Hispanic 6 | 0.72 | ***102*** | Train 7 | Test Hispanic 6 | 0.73 | ***166*** | Train 11 | Test Hispanic 6 | 0.72 | ***230*** | Train 15 | Test Hispanic 6 | 0.69 |
| ***39*** | Train 3 | Test Hispanic 7 | 0.76 | ***103*** | Train 7 | Test Hispanic 7 | 0.76 | ***167*** | Train 11 | Test Hispanic 7 | 0.76 | ***231*** | Train 15 | Test Hispanic 7 | 0.69 |
| ***40*** | Train 3 | Test Hispanic 8 | 0.72 | ***104*** | Train 7 | Test Hispanic 8 | 0.73 | ***168*** | Train 11 | Test Hispanic 8 | 0.72 | ***232*** | Train 15 | Test Hispanic 8 | 0.69 |
| ***41*** | Train 3 | Test Hispanic 9 | 0.77 | ***105*** | Train 7 | Test Hispanic 9 | 0.77 | ***169*** | Train 11 | Test Hispanic 9 | 0.76 | ***233*** | Train 15 | Test Hispanic 9 | 0.69 |
| ***42*** | Train 3 | Test Hispanic 10 | 0.72 | ***106*** | Train 7 | Test Hispanic 10 | 0.73 | ***170*** | Train 11 | Test Hispanic 10 | 0.72 | ***234*** | Train 15 | Test Hispanic 10 | 0.69 |
| ***43*** | Train 3 | Test Hispanic 11 | 0.77 | ***107*** | Train 7 | Test Hispanic 11 | 0.77 | ***171*** | Train 11 | Test Hispanic 11 | 0.76 | ***235*** | Train 15 | Test Hispanic 11 | 0.7 |
| ***44*** | Train 3 | Test Hispanic 12 | 0.72 | ***108*** | Train 7 | Test Hispanic 12 | 0.73 | ***172*** | Train 11 | Test Hispanic 12 | 0.72 | ***236*** | Train 15 | Test Hispanic 12 | 0.69 |
| ***45*** | Train 3 | Test Hispanic 13 | 0.77 | ***109*** | Train 7 | Test Hispanic 13 | 0.77 | ***173*** | Train 11 | Test Hispanic 13 | 0.76 | ***237*** | Train 15 | Test Hispanic 13 | 0.77 |
| ***46*** | Train 3 | Test Hispanic 14 | 0.71 | ***110*** | Train 7 | Test Hispanic 14 | 0.74 | ***174*** | Train 11 | Test Hispanic 14 | 0.71 | ***238*** | Train 15 | Test Hispanic 14 | 0.74 |
| ***47*** | Train 3 | Test Hispanic 15 | 0.78 | ***111*** | Train 7 | Test Hispanic 15 | 0.77 | ***175*** | Train 11 | Test Hispanic 15 | 0.77 | ***239*** | Train 15 | Test Hispanic 15 | 0.77 |
| ***48*** | Train 3 | Test Hispanic 16 | 0.72 | ***112*** | Train 7 | Test Hispanic 16 | 0.74 | ***176*** | Train 11 | Test Hispanic 16 | 0.72 | ***240*** | Train 15 | Test Hispanic 16 | 0.74 |
| ***49*** | Train 4 | Test Hispanic 1 | 0.75 | ***113*** | Train 8 | Test Hispanic 1 | 0.76 | ***177*** | Train 12 | Test Hispanic 1 | 0.76 | ***241*** | Train 16 | Test Hispanic 1 | 0.67 |
| ***50*** | Train 4 | Test Hispanic 2 | 0.76 | ***114*** | Train 8 | Test Hispanic 2 | 0.76 | ***178*** | Train 12 | Test Hispanic 2 | 0.76 | ***242*** | Train 16 | Test Hispanic 2 | 0.69 |
| ***51*** | Train 4 | Test Hispanic 3 | 0.75 | ***115*** | Train 8 | Test Hispanic 3 | 0.76 | ***179*** | Train 12 | Test Hispanic 3 | 0.76 | ***243*** | Train 16 | Test Hispanic 3 | 0.68 |
| ***52*** | Train 4 | Test Hispanic 4 | 0.76 | ***116*** | Train 8 | Test Hispanic 4 | 0.76 | ***180*** | Train 12 | Test Hispanic 4 | 0.76 | ***244*** | Train 16 | Test Hispanic 4 | 0.69 |
| ***53*** | Train 4 | Test Hispanic 5 | 0.75 | ***117*** | Train 8 | Test Hispanic 5 | 0.76 | ***181*** | Train 12 | Test Hispanic 5 | 0.76 | ***245*** | Train 16 | Test Hispanic 5 | 0.67 |
| ***54*** | Train 4 | Test Hispanic 6 | 0.76 | ***118*** | Train 8 | Test Hispanic 6 | 0.76 | ***182*** | Train 12 | Test Hispanic 6 | 0.76 | ***246*** | Train 16 | Test Hispanic 6 | 0.69 |
| ***55*** | Train 4 | Test Hispanic 7 | 0.75 | ***119*** | Train 8 | Test Hispanic 7 | 0.76 | ***183*** | Train 12 | Test Hispanic 7 | 0.76 | ***247*** | Train 16 | Test Hispanic 7 | 0.68 |
| ***56*** | Train 4 | Test Hispanic 8 | 0.76 | ***120*** | Train 8 | Test Hispanic 8 | 0.76 | ***184*** | Train 12 | Test Hispanic 8 | 0.76 | ***248*** | Train 16 | Test Hispanic 8 | 0.69 |
| ***57*** | Train 4 | Test Hispanic 9 | 0.76 | ***121*** | Train 8 | Test Hispanic 9 | 0.76 | ***185*** | Train 12 | Test Hispanic 9 | 0.76 | ***249*** | Train 16 | Test Hispanic 9 | 0.68 |
| ***58*** | Train 4 | Test Hispanic 10 | 0.76 | ***122*** | Train 8 | Test Hispanic 10 | 0.76 | ***186*** | Train 12 | Test Hispanic 10 | 0.76 | ***250*** | Train 16 | Test Hispanic 10 | 0.69 |
| ***59*** | Train 4 | Test Hispanic 11 | 0.76 | ***123*** | Train 8 | Test Hispanic 11 | 0.76 | ***187*** | Train 12 | Test Hispanic 11 | 0.76 | ***251*** | Train 16 | Test Hispanic 11 | 0.69 |
| ***60*** | Train 4 | Test Hispanic 12 | 0.76 | ***124*** | Train 8 | Test Hispanic 12 | 0.76 | ***188*** | Train 12 | Test Hispanic 12 | 0.76 | ***252*** | Train 16 | Test Hispanic 12 | 0.69 |
| ***61*** | Train 4 | Test Hispanic 13 | 0.77 | ***125*** | Train 8 | Test Hispanic 13 | 0.78 | ***189*** | Train 12 | Test Hispanic 13 | 0.78 | ***253*** | Train 16 | Test Hispanic 13 | 0.78 |
| ***62*** | Train 4 | Test Hispanic 14 | 0.77 | ***126*** | Train 8 | Test Hispanic 14 | 0.78 | ***190*** | Train 12 | Test Hispanic 14 | 0.78 | ***254*** | Train 16 | Test Hispanic 14 | 0.77 |
| ***63*** | Train 4 | Test Hispanic 15 | 0.77 | ***127*** | Train 8 | Test Hispanic 15 | 0.78 | ***191*** | Train 12 | Test Hispanic 15 | 0.78 | ***255*** | Train 16 | Test Hispanic 15 | 0.78 |
| ***64*** | Train 4 | Test Hispanic 16 | 0.78 | ***128*** | Train 8 | Test Hispanic 16 | 0.78 | ***192*** | Train 12 | Test Hispanic 16 | 0.78 | ***256*** | Train 16 | Test Hispanic 16 | 0.78 |

Table 25. Logistic Regression (LR) AUC Performance for Non-Hispanic Patients Across 16 Cohorts.

| Original Decisions | ***Train Model*** | ***Test Dataset*** | ***AUC (LR)*** |  | ***Train Model*** | ***Test Dataset*** | ***AUC (LR)*** |  | ***Train Model*** | ***Test Dataset*** | ***AUC (LR)*** |  | ***Train Model*** | ***Test Dataset*** | AUC (LR) |
| --- | --- | --- | --- | --- | --- | --- | --- | --- | --- | --- | --- | --- | --- | --- | --- |
| ***1*** | Train 1 | Test Not Hispanic 1 | 0.72 | ***65*** | Train 5 | Test Not Hispanic 1 | 0.72 | ***129*** | Train 9 | Test Not Hispanic 1 | 0.72 | ***193*** | Train 13 | Test Not Hispanic 1 | 0.71 |
| ***2*** | Train 1 | Test Not Hispanic 2 | 0.7 | ***66*** | Train 5 | Test Not Hispanic 2 | 0.7 | ***130*** | Train 9 | Test Not Hispanic 2 | 0.7 | ***194*** | Train 13 | Test Not Hispanic 2 | 0.7 |
| ***3*** | Train 1 | Test Not Hispanic 3 | 0.72 | ***67*** | Train 5 | Test Not Hispanic 3 | 0.72 | ***131*** | Train 9 | Test Not Hispanic 3 | 0.72 | ***195*** | Train 13 | Test Not Hispanic 3 | 0.71 |
| ***4*** | Train 1 | Test Not Hispanic 4 | 0.71 | ***68*** | Train 5 | Test Not Hispanic 4 | 0.71 | ***132*** | Train 9 | Test Not Hispanic 4 | 0.71 | ***196*** | Train 13 | Test Not Hispanic 4 | 0.7 |
| ***5*** | Train 1 | Test Not Hispanic 5 | 0.72 | ***69*** | Train 5 | Test Not Hispanic 5 | 0.72 | ***133*** | Train 9 | Test Not Hispanic 5 | 0.72 | ***197*** | Train 13 | Test Not Hispanic 5 | 0.71 |
| ***6*** | Train 1 | Test Not Hispanic 6 | 0.7 | ***70*** | Train 5 | Test Not Hispanic 6 | 0.7 | ***134*** | Train 9 | Test Not Hispanic 6 | 0.7 | ***198*** | Train 13 | Test Not Hispanic 6 | 0.7 |
| ***7*** | Train 1 | Test Not Hispanic 7 | 0.72 | ***71*** | Train 5 | Test Not Hispanic 7 | 0.72 | ***135*** | Train 9 | Test Not Hispanic 7 | 0.72 | ***199*** | Train 13 | Test Not Hispanic 7 | 0.71 |
| ***8*** | Train 1 | Test Not Hispanic 8 | 0.71 | ***72*** | Train 5 | Test Not Hispanic 8 | 0.71 | ***136*** | Train 9 | Test Not Hispanic 8 | 0.71 | ***200*** | Train 13 | Test Not Hispanic 8 | 0.7 |
| ***9*** | Train 1 | Test Not Hispanic 9 | 0.72 | ***73*** | Train 5 | Test Not Hispanic 9 | 0.72 | ***137*** | Train 9 | Test Not Hispanic 9 | 0.72 | ***201*** | Train 13 | Test Not Hispanic 9 | 0.71 |
| ***10*** | Train 1 | Test Not Hispanic 10 | 0.7 | ***74*** | Train 5 | Test Not Hispanic 10 | 0.7 | ***138*** | Train 9 | Test Not Hispanic 10 | 0.7 | ***202*** | Train 13 | Test Not Hispanic 10 | 0.7 |
| ***11*** | Train 1 | Test Not Hispanic 11 | 0.72 | ***75*** | Train 5 | Test Not Hispanic 11 | 0.72 | ***139*** | Train 9 | Test Not Hispanic 11 | 0.72 | ***203*** | Train 13 | Test Not Hispanic 11 | 0.71 |
| ***12*** | Train 1 | Test Not Hispanic 12 | 0.71 | ***76*** | Train 5 | Test Not Hispanic 12 | 0.71 | ***140*** | Train 9 | Test Not Hispanic 12 | 0.71 | ***204*** | Train 13 | Test Not Hispanic 12 | 0.7 |
| ***13*** | Train 1 | Test Not Hispanic 13 | 0.73 | ***77*** | Train 5 | Test Not Hispanic 13 | 0.73 | ***141*** | Train 9 | Test Not Hispanic 13 | 0.73 | ***205*** | Train 13 | Test Not Hispanic 13 | 0.74 |
| ***14*** | Train 1 | Test Not Hispanic 14 | 0.71 | ***78*** | Train 5 | Test Not Hispanic 14 | 0.71 | ***142*** | Train 9 | Test Not Hispanic 14 | 0.71 | ***206*** | Train 13 | Test Not Hispanic 14 | 0.71 |
| ***15*** | Train 1 | Test Not Hispanic 15 | 0.73 | ***79*** | Train 5 | Test Not Hispanic 15 | 0.73 | ***143*** | Train 9 | Test Not Hispanic 15 | 0.73 | ***207*** | Train 13 | Test Not Hispanic 15 | 0.74 |
| ***16*** | Train 1 | Test Not Hispanic 16 | 0.71 | ***80*** | Train 5 | Test Not Hispanic 16 | 0.71 | ***144*** | Train 9 | Test Not Hispanic 16 | 0.71 | ***208*** | Train 13 | Test Not Hispanic 16 | 0.71 |
| ***17*** | Train 2 | Test Not Hispanic 1 | 0.71 | ***81*** | Train 6 | Test Not Hispanic 1 | 0.71 | ***145*** | Train 10 | Test Not Hispanic 1 | 0.71 | ***209*** | Train 14 | Test Not Hispanic 1 | 0.7 |
| ***18*** | Train 2 | Test Not Hispanic 2 | 0.73 | ***82*** | Train 6 | Test Not Hispanic 2 | 0.73 | ***146*** | Train 10 | Test Not Hispanic 2 | 0.73 | ***210*** | Train 14 | Test Not Hispanic 2 | 0.71 |
| ***19*** | Train 2 | Test Not Hispanic 3 | 0.71 | ***83*** | Train 6 | Test Not Hispanic 3 | 0.71 | ***147*** | Train 10 | Test Not Hispanic 3 | 0.71 | ***211*** | Train 14 | Test Not Hispanic 3 | 0.7 |
| ***20*** | Train 2 | Test Not Hispanic 4 | 0.73 | ***84*** | Train 6 | Test Not Hispanic 4 | 0.73 | ***148*** | Train 10 | Test Not Hispanic 4 | 0.73 | ***212*** | Train 14 | Test Not Hispanic 4 | 0.72 |
| ***21*** | Train 2 | Test Not Hispanic 5 | 0.71 | ***85*** | Train 6 | Test Not Hispanic 5 | 0.71 | ***149*** | Train 10 | Test Not Hispanic 5 | 0.71 | ***213*** | Train 14 | Test Not Hispanic 5 | 0.7 |
| ***22*** | Train 2 | Test Not Hispanic 6 | 0.73 | ***86*** | Train 6 | Test Not Hispanic 6 | 0.73 | ***150*** | Train 10 | Test Not Hispanic 6 | 0.73 | ***214*** | Train 14 | Test Not Hispanic 6 | 0.71 |
| ***23*** | Train 2 | Test Not Hispanic 7 | 0.71 | ***87*** | Train 6 | Test Not Hispanic 7 | 0.71 | ***151*** | Train 10 | Test Not Hispanic 7 | 0.71 | ***215*** | Train 14 | Test Not Hispanic 7 | 0.7 |
| ***24*** | Train 2 | Test Not Hispanic 8 | 0.73 | ***88*** | Train 6 | Test Not Hispanic 8 | 0.73 | ***152*** | Train 10 | Test Not Hispanic 8 | 0.73 | ***216*** | Train 14 | Test Not Hispanic 8 | 0.72 |
| ***25*** | Train 2 | Test Not Hispanic 9 | 0.71 | ***89*** | Train 6 | Test Not Hispanic 9 | 0.71 | ***153*** | Train 10 | Test Not Hispanic 9 | 0.71 | ***217*** | Train 14 | Test Not Hispanic 9 | 0.7 |
| ***26*** | Train 2 | Test Not Hispanic 10 | 0.73 | ***90*** | Train 6 | Test Not Hispanic 10 | 0.73 | ***154*** | Train 10 | Test Not Hispanic 10 | 0.73 | ***218*** | Train 14 | Test Not Hispanic 10 | 0.71 |
| ***27*** | Train 2 | Test Not Hispanic 11 | 0.71 | ***91*** | Train 6 | Test Not Hispanic 11 | 0.71 | ***155*** | Train 10 | Test Not Hispanic 11 | 0.71 | ***219*** | Train 14 | Test Not Hispanic 11 | 0.7 |
| ***28*** | Train 2 | Test Not Hispanic 12 | 0.73 | ***92*** | Train 6 | Test Not Hispanic 12 | 0.73 | ***156*** | Train 10 | Test Not Hispanic 12 | 0.73 | ***220*** | Train 14 | Test Not Hispanic 12 | 0.72 |
| ***29*** | Train 2 | Test Not Hispanic 13 | 0.73 | ***93*** | Train 6 | Test Not Hispanic 13 | 0.73 | ***157*** | Train 10 | Test Not Hispanic 13 | 0.73 | ***221*** | Train 14 | Test Not Hispanic 13 | 0.73 |
| ***30*** | Train 2 | Test Not Hispanic 14 | 0.73 | ***94*** | Train 6 | Test Not Hispanic 14 | 0.73 | ***158*** | Train 10 | Test Not Hispanic 14 | 0.73 | ***222*** | Train 14 | Test Not Hispanic 14 | 0.73 |
| ***31*** | Train 2 | Test Not Hispanic 15 | 0.73 | ***95*** | Train 6 | Test Not Hispanic 15 | 0.73 | ***159*** | Train 10 | Test Not Hispanic 15 | 0.73 | ***223*** | Train 14 | Test Not Hispanic 15 | 0.73 |
| ***32*** | Train 2 | Test Not Hispanic 16 | 0.73 | ***96*** | Train 6 | Test Not Hispanic 16 | 0.73 | ***160*** | Train 10 | Test Not Hispanic 16 | 0.73 | ***224*** | Train 14 | Test Not Hispanic 16 | 0.74 |
| ***33*** | Train 3 | Test Not Hispanic 1 | 0.72 | ***97*** | Train 7 | Test Not Hispanic 1 | 0.72 | ***161*** | Train 11 | Test Not Hispanic 1 | 0.72 | ***225*** | Train 15 | Test Not Hispanic 1 | 0.71 |
| ***34*** | Train 3 | Test Not Hispanic 2 | 0.7 | ***98*** | Train 7 | Test Not Hispanic 2 | 0.7 | ***162*** | Train 11 | Test Not Hispanic 2 | 0.7 | ***226*** | Train 15 | Test Not Hispanic 2 | 0.7 |
| ***35*** | Train 3 | Test Not Hispanic 3 | 0.72 | ***99*** | Train 7 | Test Not Hispanic 3 | 0.72 | ***163*** | Train 11 | Test Not Hispanic 3 | 0.72 | ***227*** | Train 15 | Test Not Hispanic 3 | 0.71 |
| ***36*** | Train 3 | Test Not Hispanic 4 | 0.7 | ***100*** | Train 7 | Test Not Hispanic 4 | 0.7 | ***164*** | Train 11 | Test Not Hispanic 4 | 0.7 | ***228*** | Train 15 | Test Not Hispanic 4 | 0.7 |
| ***37*** | Train 3 | Test Not Hispanic 5 | 0.72 | ***101*** | Train 7 | Test Not Hispanic 5 | 0.72 | ***165*** | Train 11 | Test Not Hispanic 5 | 0.72 | ***229*** | Train 15 | Test Not Hispanic 5 | 0.71 |
| ***38*** | Train 3 | Test Not Hispanic 6 | 0.7 | ***102*** | Train 7 | Test Not Hispanic 6 | 0.7 | ***166*** | Train 11 | Test Not Hispanic 6 | 0.7 | ***230*** | Train 15 | Test Not Hispanic 6 | 0.7 |
| ***39*** | Train 3 | Test Not Hispanic 7 | 0.72 | ***103*** | Train 7 | Test Not Hispanic 7 | 0.72 | ***167*** | Train 11 | Test Not Hispanic 7 | 0.72 | ***231*** | Train 15 | Test Not Hispanic 7 | 0.71 |
| ***40*** | Train 3 | Test Not Hispanic 8 | 0.71 | ***104*** | Train 7 | Test Not Hispanic 8 | 0.71 | ***168*** | Train 11 | Test Not Hispanic 8 | 0.71 | ***232*** | Train 15 | Test Not Hispanic 8 | 0.7 |
| ***41*** | Train 3 | Test Not Hispanic 9 | 0.72 | ***105*** | Train 7 | Test Not Hispanic 9 | 0.72 | ***169*** | Train 11 | Test Not Hispanic 9 | 0.72 | ***233*** | Train 15 | Test Not Hispanic 9 | 0.71 |
| ***42*** | Train 3 | Test Not Hispanic 10 | 0.7 | ***106*** | Train 7 | Test Not Hispanic 10 | 0.7 | ***170*** | Train 11 | Test Not Hispanic 10 | 0.7 | ***234*** | Train 15 | Test Not Hispanic 10 | 0.7 |
| ***43*** | Train 3 | Test Not Hispanic 11 | 0.72 | ***107*** | Train 7 | Test Not Hispanic 11 | 0.72 | ***171*** | Train 11 | Test Not Hispanic 11 | 0.72 | ***235*** | Train 15 | Test Not Hispanic 11 | 0.71 |
| ***44*** | Train 3 | Test Not Hispanic 12 | 0.71 | ***108*** | Train 7 | Test Not Hispanic 12 | 0.71 | ***172*** | Train 11 | Test Not Hispanic 12 | 0.7 | ***236*** | Train 15 | Test Not Hispanic 12 | 0.7 |
| ***45*** | Train 3 | Test Not Hispanic 13 | 0.73 | ***109*** | Train 7 | Test Not Hispanic 13 | 0.73 | ***173*** | Train 11 | Test Not Hispanic 13 | 0.73 | ***237*** | Train 15 | Test Not Hispanic 13 | 0.74 |
| ***46*** | Train 3 | Test Not Hispanic 14 | 0.71 | ***110*** | Train 7 | Test Not Hispanic 14 | 0.71 | ***174*** | Train 11 | Test Not Hispanic 14 | 0.71 | ***238*** | Train 15 | Test Not Hispanic 14 | 0.71 |
| ***47*** | Train 3 | Test Not Hispanic 15 | 0.73 | ***111*** | Train 7 | Test Not Hispanic 15 | 0.73 | ***175*** | Train 11 | Test Not Hispanic 15 | 0.73 | ***239*** | Train 15 | Test Not Hispanic 15 | 0.74 |
| ***48*** | Train 3 | Test Not Hispanic 16 | 0.71 | ***112*** | Train 7 | Test Not Hispanic 16 | 0.71 | ***176*** | Train 11 | Test Not Hispanic 16 | 0.71 | ***240*** | Train 15 | Test Not Hispanic 16 | 0.71 |
| ***49*** | Train 4 | Test Not Hispanic 1 | 0.71 | ***113*** | Train 8 | Test Not Hispanic 1 | 0.71 | ***177*** | Train 12 | Test Not Hispanic 1 | 0.71 | ***241*** | Train 16 | Test Not Hispanic 1 | 0.7 |
| ***50*** | Train 4 | Test Not Hispanic 2 | 0.73 | ***114*** | Train 8 | Test Not Hispanic 2 | 0.73 | ***178*** | Train 12 | Test Not Hispanic 2 | 0.73 | ***242*** | Train 16 | Test Not Hispanic 2 | 0.71 |
| ***51*** | Train 4 | Test Not Hispanic 3 | 0.71 | ***115*** | Train 8 | Test Not Hispanic 3 | 0.71 | ***179*** | Train 12 | Test Not Hispanic 3 | 0.71 | ***243*** | Train 16 | Test Not Hispanic 3 | 0.7 |
| ***52*** | Train 4 | Test Not Hispanic 4 | 0.73 | ***116*** | Train 8 | Test Not Hispanic 4 | 0.73 | ***180*** | Train 12 | Test Not Hispanic 4 | 0.73 | ***244*** | Train 16 | Test Not Hispanic 4 | 0.71 |
| ***53*** | Train 4 | Test Not Hispanic 5 | 0.71 | ***117*** | Train 8 | Test Not Hispanic 5 | 0.71 | ***181*** | Train 12 | Test Not Hispanic 5 | 0.71 | ***245*** | Train 16 | Test Not Hispanic 5 | 0.7 |
| ***54*** | Train 4 | Test Not Hispanic 6 | 0.73 | ***118*** | Train 8 | Test Not Hispanic 6 | 0.73 | ***182*** | Train 12 | Test Not Hispanic 6 | 0.73 | ***246*** | Train 16 | Test Not Hispanic 6 | 0.71 |
| ***55*** | Train 4 | Test Not Hispanic 7 | 0.71 | ***119*** | Train 8 | Test Not Hispanic 7 | 0.71 | ***183*** | Train 12 | Test Not Hispanic 7 | 0.71 | ***247*** | Train 16 | Test Not Hispanic 7 | 0.7 |
| ***56*** | Train 4 | Test Not Hispanic 8 | 0.73 | ***120*** | Train 8 | Test Not Hispanic 8 | 0.73 | ***184*** | Train 12 | Test Not Hispanic 8 | 0.73 | ***248*** | Train 16 | Test Not Hispanic 8 | 0.72 |
| ***57*** | Train 4 | Test Not Hispanic 9 | 0.71 | ***121*** | Train 8 | Test Not Hispanic 9 | 0.71 | ***185*** | Train 12 | Test Not Hispanic 9 | 0.71 | ***249*** | Train 16 | Test Not Hispanic 9 | 0.7 |
| ***58*** | Train 4 | Test Not Hispanic 10 | 0.73 | ***122*** | Train 8 | Test Not Hispanic 10 | 0.73 | ***186*** | Train 12 | Test Not Hispanic 10 | 0.73 | ***250*** | Train 16 | Test Not Hispanic 10 | 0.71 |
| ***59*** | Train 4 | Test Not Hispanic 11 | 0.71 | ***123*** | Train 8 | Test Not Hispanic 11 | 0.71 | ***187*** | Train 12 | Test Not Hispanic 11 | 0.71 | ***251*** | Train 16 | Test Not Hispanic 11 | 0.7 |
| ***60*** | Train 4 | Test Not Hispanic 12 | 0.73 | ***124*** | Train 8 | Test Not Hispanic 12 | 0.73 | ***188*** | Train 12 | Test Not Hispanic 12 | 0.73 | ***252*** | Train 16 | Test Not Hispanic 12 | 0.72 |
| ***61*** | Train 4 | Test Not Hispanic 13 | 0.73 | ***125*** | Train 8 | Test Not Hispanic 13 | 0.73 | ***189*** | Train 12 | Test Not Hispanic 13 | 0.73 | ***253*** | Train 16 | Test Not Hispanic 13 | 0.73 |
| ***62*** | Train 4 | Test Not Hispanic 14 | 0.73 | ***126*** | Train 8 | Test Not Hispanic 14 | 0.73 | ***190*** | Train 12 | Test Not Hispanic 14 | 0.73 | ***254*** | Train 16 | Test Not Hispanic 14 | 0.73 |
| ***63*** | Train 4 | Test Not Hispanic 15 | 0.73 | ***127*** | Train 8 | Test Not Hispanic 15 | 0.73 | ***191*** | Train 12 | Test Not Hispanic 15 | 0.73 | ***255*** | Train 16 | Test Not Hispanic 15 | 0.73 |
| ***64*** | Train 4 | Test Not Hispanic 16 | 0.73 | ***128*** | Train 8 | Test Not Hispanic 16 | 0.73 | ***192*** | Train 12 | Test Not Hispanic 16 | 0.73 | ***256*** | Train 16 | Test Not Hispanic 16 | 0.74 |

Table 26. Random Forest (RF) AUC Performance for Non-Hispanic Patients Across 16 Cohorts.

| Original Decisions | ***Train Model*** | ***Test Dataset*** | ***AUC (RF)*** |  | ***Train Model*** | ***Test Dataset*** | ***AUC (RF)*** |  | ***Train Model*** | ***Test Dataset*** | ***AUC (RF)*** |  | ***Train Model*** | ***Test Dataset*** | AUC (RF) |
| --- | --- | --- | --- | --- | --- | --- | --- | --- | --- | --- | --- | --- | --- | --- | --- |
| ***1*** | Train 1 | Test Not Hispanic 1 | 0.74 | ***65*** | Train 5 | Test Not Hispanic 1 | 0.74 | ***129*** | Train 9 | Test Not Hispanic 1 | 0.74 | ***193*** | Train 13 | Test Not Hispanic 1 | 0.71 |
| ***2*** | Train 1 | Test Not Hispanic 2 | 0.7 | ***66*** | Train 5 | Test Not Hispanic 2 | 0.7 | ***130*** | Train 9 | Test Not Hispanic 2 | 0.7 | ***194*** | Train 13 | Test Not Hispanic 2 | 0.68 |
| ***3*** | Train 1 | Test Not Hispanic 3 | 0.74 | ***67*** | Train 5 | Test Not Hispanic 3 | 0.74 | ***131*** | Train 9 | Test Not Hispanic 3 | 0.74 | ***195*** | Train 13 | Test Not Hispanic 3 | 0.71 |
| ***4*** | Train 1 | Test Not Hispanic 4 | 0.7 | ***68*** | Train 5 | Test Not Hispanic 4 | 0.7 | ***132*** | Train 9 | Test Not Hispanic 4 | 0.7 | ***196*** | Train 13 | Test Not Hispanic 4 | 0.69 |
| ***5*** | Train 1 | Test Not Hispanic 5 | 0.74 | ***69*** | Train 5 | Test Not Hispanic 5 | 0.74 | ***133*** | Train 9 | Test Not Hispanic 5 | 0.74 | ***197*** | Train 13 | Test Not Hispanic 5 | 0.71 |
| ***6*** | Train 1 | Test Not Hispanic 6 | 0.7 | ***70*** | Train 5 | Test Not Hispanic 6 | 0.7 | ***134*** | Train 9 | Test Not Hispanic 6 | 0.7 | ***198*** | Train 13 | Test Not Hispanic 6 | 0.69 |
| ***7*** | Train 1 | Test Not Hispanic 7 | 0.74 | ***71*** | Train 5 | Test Not Hispanic 7 | 0.74 | ***135*** | Train 9 | Test Not Hispanic 7 | 0.74 | ***199*** | Train 13 | Test Not Hispanic 7 | 0.71 |
| ***8*** | Train 1 | Test Not Hispanic 8 | 0.7 | ***72*** | Train 5 | Test Not Hispanic 8 | 0.7 | ***136*** | Train 9 | Test Not Hispanic 8 | 0.7 | ***200*** | Train 13 | Test Not Hispanic 8 | 0.69 |
| ***9*** | Train 1 | Test Not Hispanic 9 | 0.74 | ***73*** | Train 5 | Test Not Hispanic 9 | 0.74 | ***137*** | Train 9 | Test Not Hispanic 9 | 0.74 | ***201*** | Train 13 | Test Not Hispanic 9 | 0.71 |
| ***10*** | Train 1 | Test Not Hispanic 10 | 0.7 | ***74*** | Train 5 | Test Not Hispanic 10 | 0.7 | ***138*** | Train 9 | Test Not Hispanic 10 | 0.7 | ***202*** | Train 13 | Test Not Hispanic 10 | 0.68 |
| ***11*** | Train 1 | Test Not Hispanic 11 | 0.74 | ***75*** | Train 5 | Test Not Hispanic 11 | 0.74 | ***139*** | Train 9 | Test Not Hispanic 11 | 0.74 | ***203*** | Train 13 | Test Not Hispanic 11 | 0.71 |
| ***12*** | Train 1 | Test Not Hispanic 12 | 0.7 | ***76*** | Train 5 | Test Not Hispanic 12 | 0.7 | ***140*** | Train 9 | Test Not Hispanic 12 | 0.7 | ***204*** | Train 13 | Test Not Hispanic 12 | 0.69 |
| ***13*** | Train 1 | Test Not Hispanic 13 | 0.74 | ***77*** | Train 5 | Test Not Hispanic 13 | 0.74 | ***141*** | Train 9 | Test Not Hispanic 13 | 0.74 | ***205*** | Train 13 | Test Not Hispanic 13 | 0.74 |
| ***14*** | Train 1 | Test Not Hispanic 14 | 0.69 | ***78*** | Train 5 | Test Not Hispanic 14 | 0.69 | ***142*** | Train 9 | Test Not Hispanic 14 | 0.69 | ***206*** | Train 13 | Test Not Hispanic 14 | 0.69 |
| ***15*** | Train 1 | Test Not Hispanic 15 | 0.74 | ***79*** | Train 5 | Test Not Hispanic 15 | 0.74 | ***143*** | Train 9 | Test Not Hispanic 15 | 0.74 | ***207*** | Train 13 | Test Not Hispanic 15 | 0.74 |
| ***16*** | Train 1 | Test Not Hispanic 16 | 0.69 | ***80*** | Train 5 | Test Not Hispanic 16 | 0.69 | ***144*** | Train 9 | Test Not Hispanic 16 | 0.69 | ***208*** | Train 13 | Test Not Hispanic 16 | 0.69 |
| ***17*** | Train 2 | Test Not Hispanic 1 | 0.73 | ***81*** | Train 6 | Test Not Hispanic 1 | 0.73 | ***145*** | Train 10 | Test Not Hispanic 1 | 0.74 | ***209*** | Train 14 | Test Not Hispanic 1 | 0.71 |
| ***18*** | Train 2 | Test Not Hispanic 2 | 0.75 | ***82*** | Train 6 | Test Not Hispanic 2 | 0.75 | ***146*** | Train 10 | Test Not Hispanic 2 | 0.75 | ***210*** | Train 14 | Test Not Hispanic 2 | 0.72 |
| ***19*** | Train 2 | Test Not Hispanic 3 | 0.73 | ***83*** | Train 6 | Test Not Hispanic 3 | 0.73 | ***147*** | Train 10 | Test Not Hispanic 3 | 0.73 | ***211*** | Train 14 | Test Not Hispanic 3 | 0.71 |
| ***20*** | Train 2 | Test Not Hispanic 4 | 0.75 | ***84*** | Train 6 | Test Not Hispanic 4 | 0.75 | ***148*** | Train 10 | Test Not Hispanic 4 | 0.75 | ***212*** | Train 14 | Test Not Hispanic 4 | 0.72 |
| ***21*** | Train 2 | Test Not Hispanic 5 | 0.73 | ***85*** | Train 6 | Test Not Hispanic 5 | 0.73 | ***149*** | Train 10 | Test Not Hispanic 5 | 0.74 | ***213*** | Train 14 | Test Not Hispanic 5 | 0.71 |
| ***22*** | Train 2 | Test Not Hispanic 6 | 0.75 | ***86*** | Train 6 | Test Not Hispanic 6 | 0.75 | ***150*** | Train 10 | Test Not Hispanic 6 | 0.75 | ***214*** | Train 14 | Test Not Hispanic 6 | 0.72 |
| ***23*** | Train 2 | Test Not Hispanic 7 | 0.73 | ***87*** | Train 6 | Test Not Hispanic 7 | 0.73 | ***151*** | Train 10 | Test Not Hispanic 7 | 0.74 | ***215*** | Train 14 | Test Not Hispanic 7 | 0.71 |
| ***24*** | Train 2 | Test Not Hispanic 8 | 0.75 | ***88*** | Train 6 | Test Not Hispanic 8 | 0.75 | ***152*** | Train 10 | Test Not Hispanic 8 | 0.75 | ***216*** | Train 14 | Test Not Hispanic 8 | 0.73 |
| ***25*** | Train 2 | Test Not Hispanic 9 | 0.73 | ***89*** | Train 6 | Test Not Hispanic 9 | 0.73 | ***153*** | Train 10 | Test Not Hispanic 9 | 0.74 | ***217*** | Train 14 | Test Not Hispanic 9 | 0.71 |
| ***26*** | Train 2 | Test Not Hispanic 10 | 0.75 | ***90*** | Train 6 | Test Not Hispanic 10 | 0.75 | ***154*** | Train 10 | Test Not Hispanic 10 | 0.75 | ***218*** | Train 14 | Test Not Hispanic 10 | 0.72 |
| ***27*** | Train 2 | Test Not Hispanic 11 | 0.73 | ***91*** | Train 6 | Test Not Hispanic 11 | 0.73 | ***155*** | Train 10 | Test Not Hispanic 11 | 0.74 | ***219*** | Train 14 | Test Not Hispanic 11 | 0.71 |
| ***28*** | Train 2 | Test Not Hispanic 12 | 0.75 | ***92*** | Train 6 | Test Not Hispanic 12 | 0.75 | ***156*** | Train 10 | Test Not Hispanic 12 | 0.75 | ***220*** | Train 14 | Test Not Hispanic 12 | 0.73 |
| ***29*** | Train 2 | Test Not Hispanic 13 | 0.74 | ***93*** | Train 6 | Test Not Hispanic 13 | 0.74 | ***157*** | Train 10 | Test Not Hispanic 13 | 0.74 | ***221*** | Train 14 | Test Not Hispanic 13 | 0.74 |
| ***30*** | Train 2 | Test Not Hispanic 14 | 0.74 | ***94*** | Train 6 | Test Not Hispanic 14 | 0.74 | ***158*** | Train 10 | Test Not Hispanic 14 | 0.74 | ***222*** | Train 14 | Test Not Hispanic 14 | 0.75 |
| ***31*** | Train 2 | Test Not Hispanic 15 | 0.74 | ***95*** | Train 6 | Test Not Hispanic 15 | 0.74 | ***159*** | Train 10 | Test Not Hispanic 15 | 0.74 | ***223*** | Train 14 | Test Not Hispanic 15 | 0.74 |
| ***32*** | Train 2 | Test Not Hispanic 16 | 0.74 | ***96*** | Train 6 | Test Not Hispanic 16 | 0.74 | ***160*** | Train 10 | Test Not Hispanic 16 | 0.74 | ***224*** | Train 14 | Test Not Hispanic 16 | 0.75 |
| ***33*** | Train 3 | Test Not Hispanic 1 | 0.74 | ***97*** | Train 7 | Test Not Hispanic 1 | 0.74 | ***161*** | Train 11 | Test Not Hispanic 1 | 0.74 | ***225*** | Train 15 | Test Not Hispanic 1 | 0.71 |
| ***34*** | Train 3 | Test Not Hispanic 2 | 0.7 | ***98*** | Train 7 | Test Not Hispanic 2 | 0.7 | ***162*** | Train 11 | Test Not Hispanic 2 | 0.7 | ***226*** | Train 15 | Test Not Hispanic 2 | 0.68 |
| ***35*** | Train 3 | Test Not Hispanic 3 | 0.74 | ***99*** | Train 7 | Test Not Hispanic 3 | 0.74 | ***163*** | Train 11 | Test Not Hispanic 3 | 0.74 | ***227*** | Train 15 | Test Not Hispanic 3 | 0.71 |
| ***36*** | Train 3 | Test Not Hispanic 4 | 0.7 | ***100*** | Train 7 | Test Not Hispanic 4 | 0.7 | ***164*** | Train 11 | Test Not Hispanic 4 | 0.7 | ***228*** | Train 15 | Test Not Hispanic 4 | 0.69 |
| ***37*** | Train 3 | Test Not Hispanic 5 | 0.74 | ***101*** | Train 7 | Test Not Hispanic 5 | 0.74 | ***165*** | Train 11 | Test Not Hispanic 5 | 0.74 | ***229*** | Train 15 | Test Not Hispanic 5 | 0.71 |
| ***38*** | Train 3 | Test Not Hispanic 6 | 0.7 | ***102*** | Train 7 | Test Not Hispanic 6 | 0.7 | ***166*** | Train 11 | Test Not Hispanic 6 | 0.7 | ***230*** | Train 15 | Test Not Hispanic 6 | 0.69 |
| ***39*** | Train 3 | Test Not Hispanic 7 | 0.74 | ***103*** | Train 7 | Test Not Hispanic 7 | 0.74 | ***167*** | Train 11 | Test Not Hispanic 7 | 0.74 | ***231*** | Train 15 | Test Not Hispanic 7 | 0.71 |
| ***40*** | Train 3 | Test Not Hispanic 8 | 0.7 | ***104*** | Train 7 | Test Not Hispanic 8 | 0.7 | ***168*** | Train 11 | Test Not Hispanic 8 | 0.7 | ***232*** | Train 15 | Test Not Hispanic 8 | 0.69 |
| ***41*** | Train 3 | Test Not Hispanic 9 | 0.74 | ***105*** | Train 7 | Test Not Hispanic 9 | 0.74 | ***169*** | Train 11 | Test Not Hispanic 9 | 0.74 | ***233*** | Train 15 | Test Not Hispanic 9 | 0.71 |
| ***42*** | Train 3 | Test Not Hispanic 10 | 0.7 | ***106*** | Train 7 | Test Not Hispanic 10 | 0.7 | ***170*** | Train 11 | Test Not Hispanic 10 | 0.7 | ***234*** | Train 15 | Test Not Hispanic 10 | 0.68 |
| ***43*** | Train 3 | Test Not Hispanic 11 | 0.74 | ***107*** | Train 7 | Test Not Hispanic 11 | 0.74 | ***171*** | Train 11 | Test Not Hispanic 11 | 0.74 | ***235*** | Train 15 | Test Not Hispanic 11 | 0.71 |
| ***44*** | Train 3 | Test Not Hispanic 12 | 0.7 | ***108*** | Train 7 | Test Not Hispanic 12 | 0.7 | ***172*** | Train 11 | Test Not Hispanic 12 | 0.7 | ***236*** | Train 15 | Test Not Hispanic 12 | 0.69 |
| ***45*** | Train 3 | Test Not Hispanic 13 | 0.74 | ***109*** | Train 7 | Test Not Hispanic 13 | 0.74 | ***173*** | Train 11 | Test Not Hispanic 13 | 0.74 | ***237*** | Train 15 | Test Not Hispanic 13 | 0.74 |
| ***46*** | Train 3 | Test Not Hispanic 14 | 0.69 | ***110*** | Train 7 | Test Not Hispanic 14 | 0.69 | ***174*** | Train 11 | Test Not Hispanic 14 | 0.69 | ***238*** | Train 15 | Test Not Hispanic 14 | 0.69 |
| ***47*** | Train 3 | Test Not Hispanic 15 | 0.74 | ***111*** | Train 7 | Test Not Hispanic 15 | 0.74 | ***175*** | Train 11 | Test Not Hispanic 15 | 0.74 | ***239*** | Train 15 | Test Not Hispanic 15 | 0.74 |
| ***48*** | Train 3 | Test Not Hispanic 16 | 0.69 | ***112*** | Train 7 | Test Not Hispanic 16 | 0.69 | ***176*** | Train 11 | Test Not Hispanic 16 | 0.69 | ***240*** | Train 15 | Test Not Hispanic 16 | 0.69 |
| ***49*** | Train 4 | Test Not Hispanic 1 | 0.73 | ***113*** | Train 8 | Test Not Hispanic 1 | 0.74 | ***177*** | Train 12 | Test Not Hispanic 1 | 0.73 | ***241*** | Train 16 | Test Not Hispanic 1 | 0.71 |
| ***50*** | Train 4 | Test Not Hispanic 2 | 0.75 | ***114*** | Train 8 | Test Not Hispanic 2 | 0.75 | ***178*** | Train 12 | Test Not Hispanic 2 | 0.75 | ***242*** | Train 16 | Test Not Hispanic 2 | 0.72 |
| ***51*** | Train 4 | Test Not Hispanic 3 | 0.74 | ***115*** | Train 8 | Test Not Hispanic 3 | 0.74 | ***179*** | Train 12 | Test Not Hispanic 3 | 0.73 | ***243*** | Train 16 | Test Not Hispanic 3 | 0.71 |
| ***52*** | Train 4 | Test Not Hispanic 4 | 0.75 | ***116*** | Train 8 | Test Not Hispanic 4 | 0.75 | ***180*** | Train 12 | Test Not Hispanic 4 | 0.75 | ***244*** | Train 16 | Test Not Hispanic 4 | 0.73 |
| ***53*** | Train 4 | Test Not Hispanic 5 | 0.74 | ***117*** | Train 8 | Test Not Hispanic 5 | 0.74 | ***181*** | Train 12 | Test Not Hispanic 5 | 0.74 | ***245*** | Train 16 | Test Not Hispanic 5 | 0.71 |
| ***54*** | Train 4 | Test Not Hispanic 6 | 0.75 | ***118*** | Train 8 | Test Not Hispanic 6 | 0.75 | ***182*** | Train 12 | Test Not Hispanic 6 | 0.75 | ***246*** | Train 16 | Test Not Hispanic 6 | 0.72 |
| ***55*** | Train 4 | Test Not Hispanic 7 | 0.74 | ***119*** | Train 8 | Test Not Hispanic 7 | 0.74 | ***183*** | Train 12 | Test Not Hispanic 7 | 0.74 | ***247*** | Train 16 | Test Not Hispanic 7 | 0.71 |
| ***56*** | Train 4 | Test Not Hispanic 8 | 0.75 | ***120*** | Train 8 | Test Not Hispanic 8 | 0.75 | ***184*** | Train 12 | Test Not Hispanic 8 | 0.75 | ***248*** | Train 16 | Test Not Hispanic 8 | 0.73 |
| ***57*** | Train 4 | Test Not Hispanic 9 | 0.73 | ***121*** | Train 8 | Test Not Hispanic 9 | 0.73 | ***185*** | Train 12 | Test Not Hispanic 9 | 0.73 | ***249*** | Train 16 | Test Not Hispanic 9 | 0.71 |
| ***58*** | Train 4 | Test Not Hispanic 10 | 0.75 | ***122*** | Train 8 | Test Not Hispanic 10 | 0.75 | ***186*** | Train 12 | Test Not Hispanic 10 | 0.75 | ***250*** | Train 16 | Test Not Hispanic 10 | 0.72 |
| ***59*** | Train 4 | Test Not Hispanic 11 | 0.73 | ***123*** | Train 8 | Test Not Hispanic 11 | 0.73 | ***187*** | Train 12 | Test Not Hispanic 11 | 0.73 | ***251*** | Train 16 | Test Not Hispanic 11 | 0.71 |
| ***60*** | Train 4 | Test Not Hispanic 12 | 0.75 | ***124*** | Train 8 | Test Not Hispanic 12 | 0.75 | ***188*** | Train 12 | Test Not Hispanic 12 | 0.75 | ***252*** | Train 16 | Test Not Hispanic 12 | 0.73 |
| ***61*** | Train 4 | Test Not Hispanic 13 | 0.74 | ***125*** | Train 8 | Test Not Hispanic 13 | 0.74 | ***189*** | Train 12 | Test Not Hispanic 13 | 0.74 | ***253*** | Train 16 | Test Not Hispanic 13 | 0.74 |
| ***62*** | Train 4 | Test Not Hispanic 14 | 0.74 | ***126*** | Train 8 | Test Not Hispanic 14 | 0.74 | ***190*** | Train 12 | Test Not Hispanic 14 | 0.74 | ***254*** | Train 16 | Test Not Hispanic 14 | 0.75 |
| ***63*** | Train 4 | Test Not Hispanic 15 | 0.74 | ***127*** | Train 8 | Test Not Hispanic 15 | 0.74 | ***191*** | Train 12 | Test Not Hispanic 15 | 0.74 | ***255*** | Train 16 | Test Not Hispanic 15 | 0.74 |
| ***64*** | Train 4 | Test Not Hispanic 16 | 0.74 | ***128*** | Train 8 | Test Not Hispanic 16 | 0.74 | ***192*** | Train 12 | Test Not Hispanic 16 | 0.74 | ***256*** | Train 16 | Test Not Hispanic 16 | 0.75 |

Table 27. Gradient Boosting (GB) AUC Performance for Non-Hispanic Patients Across 16 Cohorts.

| Original Decisions | ***Train Model*** | ***Test Dataset*** | ***AUC (GB)*** |  | ***Train Model*** | ***Test Dataset*** | ***AUC (GB)*** |  | ***Train Model*** | ***Test Dataset*** | ***AUC (GB)*** |  | ***Train Model*** | ***Test Dataset*** | AUC (GB) |
| --- | --- | --- | --- | --- | --- | --- | --- | --- | --- | --- | --- | --- | --- | --- | --- |
| ***1*** | Train 1 | Test Not Hispanic 1 | 0.73 | ***65*** | Train 5 | Test Not Hispanic 1 | 0.73 | ***129*** | Train 9 | Test Not Hispanic 1 | 0.74 | ***225*** | Train 15 | Test Not Hispanic 1 | 0.67 |
| ***2*** | Train 1 | Test Not Hispanic 2 | 0.68 | ***66*** | Train 5 | Test Not Hispanic 2 | 0.68 | ***130*** | Train 9 | Test Not Hispanic 2 | 0.69 | ***226*** | Train 15 | Test Not Hispanic 2 | 0.65 |
| ***3*** | Train 1 | Test Not Hispanic 3 | 0.73 | ***67*** | Train 5 | Test Not Hispanic 3 | 0.73 | ***131*** | Train 9 | Test Not Hispanic 3 | 0.73 | ***227*** | Train 15 | Test Not Hispanic 3 | 0.67 |
| ***4*** | Train 1 | Test Not Hispanic 4 | 0.68 | ***68*** | Train 5 | Test Not Hispanic 4 | 0.68 | ***132*** | Train 9 | Test Not Hispanic 4 | 0.69 | ***228*** | Train 15 | Test Not Hispanic 4 | 0.65 |
| ***5*** | Train 1 | Test Not Hispanic 5 | 0.73 | ***69*** | Train 5 | Test Not Hispanic 5 | 0.74 | ***133*** | Train 9 | Test Not Hispanic 5 | 0.74 | ***229*** | Train 15 | Test Not Hispanic 5 | 0.67 |
| ***6*** | Train 1 | Test Not Hispanic 6 | 0.68 | ***70*** | Train 5 | Test Not Hispanic 6 | 0.68 | ***134*** | Train 9 | Test Not Hispanic 6 | 0.69 | ***230*** | Train 15 | Test Not Hispanic 6 | 0.65 |
| ***7*** | Train 1 | Test Not Hispanic 7 | 0.73 | ***71*** | Train 5 | Test Not Hispanic 7 | 0.73 | ***135*** | Train 9 | Test Not Hispanic 7 | 0.73 | ***231*** | Train 15 | Test Not Hispanic 7 | 0.67 |
| ***8*** | Train 1 | Test Not Hispanic 8 | 0.68 | ***72*** | Train 5 | Test Not Hispanic 8 | 0.68 | ***136*** | Train 9 | Test Not Hispanic 8 | 0.69 | ***232*** | Train 15 | Test Not Hispanic 8 | 0.65 |
| ***9*** | Train 1 | Test Not Hispanic 9 | 0.73 | ***73*** | Train 5 | Test Not Hispanic 9 | 0.73 | ***137*** | Train 9 | Test Not Hispanic 9 | 0.74 | ***233*** | Train 15 | Test Not Hispanic 9 | 0.67 |
| ***10*** | Train 1 | Test Not Hispanic 10 | 0.68 | ***74*** | Train 5 | Test Not Hispanic 10 | 0.68 | ***138*** | Train 9 | Test Not Hispanic 10 | 0.69 | ***234*** | Train 15 | Test Not Hispanic 10 | 0.65 |
| ***11*** | Train 1 | Test Not Hispanic 11 | 0.73 | ***75*** | Train 5 | Test Not Hispanic 11 | 0.73 | ***139*** | Train 9 | Test Not Hispanic 11 | 0.73 | ***235*** | Train 15 | Test Not Hispanic 11 | 0.67 |
| ***12*** | Train 1 | Test Not Hispanic 12 | 0.68 | ***76*** | Train 5 | Test Not Hispanic 12 | 0.68 | ***140*** | Train 9 | Test Not Hispanic 12 | 0.69 | ***236*** | Train 15 | Test Not Hispanic 12 | 0.65 |
| ***13*** | Train 1 | Test Not Hispanic 13 | 0.73 | ***77*** | Train 5 | Test Not Hispanic 13 | 0.73 | ***141*** | Train 9 | Test Not Hispanic 13 | 0.73 | ***237*** | Train 15 | Test Not Hispanic 13 | 0.73 |
| ***14*** | Train 1 | Test Not Hispanic 14 | 0.68 | ***78*** | Train 5 | Test Not Hispanic 14 | 0.68 | ***142*** | Train 9 | Test Not Hispanic 14 | 0.69 | ***238*** | Train 15 | Test Not Hispanic 14 | 0.67 |
| ***15*** | Train 1 | Test Not Hispanic 15 | 0.73 | ***79*** | Train 5 | Test Not Hispanic 15 | 0.73 | ***143*** | Train 9 | Test Not Hispanic 15 | 0.73 | ***239*** | Train 15 | Test Not Hispanic 15 | 0.73 |
| ***16*** | Train 1 | Test Not Hispanic 16 | 0.68 | ***80*** | Train 5 | Test Not Hispanic 16 | 0.68 | ***144*** | Train 9 | Test Not Hispanic 16 | 0.69 | ***240*** | Train 15 | Test Not Hispanic 16 | 0.68 |
| ***17*** | Train 2 | Test Not Hispanic 1 | 0.74 | ***81*** | Train 6 | Test Not Hispanic 1 | 0.74 | ***145*** | Train 10 | Test Not Hispanic 1 | 0.74 | ***241*** | Train 16 | Test Not Hispanic 1 | 0.64 |
| ***18*** | Train 2 | Test Not Hispanic 2 | 0.75 | ***82*** | Train 6 | Test Not Hispanic 2 | 0.75 | ***146*** | Train 10 | Test Not Hispanic 2 | 0.75 | ***242*** | Train 16 | Test Not Hispanic 2 | 0.68 |
| ***19*** | Train 2 | Test Not Hispanic 3 | 0.74 | ***83*** | Train 6 | Test Not Hispanic 3 | 0.74 | ***147*** | Train 10 | Test Not Hispanic 3 | 0.73 | ***243*** | Train 16 | Test Not Hispanic 3 | 0.64 |
| ***20*** | Train 2 | Test Not Hispanic 4 | 0.75 | ***84*** | Train 6 | Test Not Hispanic 4 | 0.75 | ***148*** | Train 10 | Test Not Hispanic 4 | 0.75 | ***244*** | Train 16 | Test Not Hispanic 4 | 0.68 |
| ***21*** | Train 2 | Test Not Hispanic 5 | 0.74 | ***85*** | Train 6 | Test Not Hispanic 5 | 0.74 | ***149*** | Train 10 | Test Not Hispanic 5 | 0.74 | ***245*** | Train 16 | Test Not Hispanic 5 | 0.64 |
| ***22*** | Train 2 | Test Not Hispanic 6 | 0.75 | ***86*** | Train 6 | Test Not Hispanic 6 | 0.75 | ***150*** | Train 10 | Test Not Hispanic 6 | 0.75 | ***246*** | Train 16 | Test Not Hispanic 6 | 0.68 |
| ***23*** | Train 2 | Test Not Hispanic 7 | 0.74 | ***87*** | Train 6 | Test Not Hispanic 7 | 0.74 | ***151*** | Train 10 | Test Not Hispanic 7 | 0.74 | ***247*** | Train 16 | Test Not Hispanic 7 | 0.64 |
| ***24*** | Train 2 | Test Not Hispanic 8 | 0.75 | ***88*** | Train 6 | Test Not Hispanic 8 | 0.75 | ***152*** | Train 10 | Test Not Hispanic 8 | 0.75 | ***248*** | Train 16 | Test Not Hispanic 8 | 0.68 |
| ***25*** | Train 2 | Test Not Hispanic 9 | 0.74 | ***89*** | Train 6 | Test Not Hispanic 9 | 0.74 | ***153*** | Train 10 | Test Not Hispanic 9 | 0.74 | ***249*** | Train 16 | Test Not Hispanic 9 | 0.64 |
| ***26*** | Train 2 | Test Not Hispanic 10 | 0.75 | ***90*** | Train 6 | Test Not Hispanic 10 | 0.75 | ***154*** | Train 10 | Test Not Hispanic 10 | 0.75 | ***250*** | Train 16 | Test Not Hispanic 10 | 0.68 |
| ***27*** | Train 2 | Test Not Hispanic 11 | 0.74 | ***91*** | Train 6 | Test Not Hispanic 11 | 0.74 | ***155*** | Train 10 | Test Not Hispanic 11 | 0.74 | ***251*** | Train 16 | Test Not Hispanic 11 | 0.64 |
| ***28*** | Train 2 | Test Not Hispanic 12 | 0.75 | ***92*** | Train 6 | Test Not Hispanic 12 | 0.75 | ***156*** | Train 10 | Test Not Hispanic 12 | 0.75 | ***252*** | Train 16 | Test Not Hispanic 12 | 0.68 |
| ***29*** | Train 2 | Test Not Hispanic 13 | 0.74 | ***93*** | Train 6 | Test Not Hispanic 13 | 0.74 | ***157*** | Train 10 | Test Not Hispanic 13 | 0.74 | ***253*** | Train 16 | Test Not Hispanic 13 | 0.74 |
| ***30*** | Train 2 | Test Not Hispanic 14 | 0.74 | ***94*** | Train 6 | Test Not Hispanic 14 | 0.74 | ***158*** | Train 10 | Test Not Hispanic 14 | 0.74 | ***254*** | Train 16 | Test Not Hispanic 14 | 0.75 |
| ***31*** | Train 2 | Test Not Hispanic 15 | 0.74 | ***95*** | Train 6 | Test Not Hispanic 15 | 0.74 | ***159*** | Train 10 | Test Not Hispanic 15 | 0.74 | ***255*** | Train 16 | Test Not Hispanic 15 | 0.74 |
| ***32*** | Train 2 | Test Not Hispanic 16 | 0.74 | ***96*** | Train 6 | Test Not Hispanic 16 | 0.75 | ***160*** | Train 10 | Test Not Hispanic 16 | 0.75 | ***256*** | Train 16 | Test Not Hispanic 16 | 0.75 |
| ***33*** | Train 3 | Test Not Hispanic 1 | 0.74 | ***97*** | Train 7 | Test Not Hispanic 1 | 0.74 | ***161*** | Train 11 | Test Not Hispanic 1 | 0.74 | ***225*** | Train 15 | Test Not Hispanic 1 | 0.67 |
| ***34*** | Train 3 | Test Not Hispanic 2 | 0.67 | ***98*** | Train 7 | Test Not Hispanic 2 | 0.68 | ***162*** | Train 11 | Test Not Hispanic 2 | 0.68 | ***226*** | Train 15 | Test Not Hispanic 2 | 0.65 |
| ***35*** | Train 3 | Test Not Hispanic 3 | 0.74 | ***99*** | Train 7 | Test Not Hispanic 3 | 0.74 | ***163*** | Train 11 | Test Not Hispanic 3 | 0.74 | ***227*** | Train 15 | Test Not Hispanic 3 | 0.67 |
| ***36*** | Train 3 | Test Not Hispanic 4 | 0.67 | ***100*** | Train 7 | Test Not Hispanic 4 | 0.68 | ***164*** | Train 11 | Test Not Hispanic 4 | 0.68 | ***228*** | Train 15 | Test Not Hispanic 4 | 0.65 |
| ***37*** | Train 3 | Test Not Hispanic 5 | 0.74 | ***101*** | Train 7 | Test Not Hispanic 5 | 0.74 | ***165*** | Train 11 | Test Not Hispanic 5 | 0.74 | ***229*** | Train 15 | Test Not Hispanic 5 | 0.67 |
| ***38*** | Train 3 | Test Not Hispanic 6 | 0.67 | ***102*** | Train 7 | Test Not Hispanic 6 | 0.68 | ***166*** | Train 11 | Test Not Hispanic 6 | 0.68 | ***230*** | Train 15 | Test Not Hispanic 6 | 0.65 |
| ***39*** | Train 3 | Test Not Hispanic 7 | 0.74 | ***103*** | Train 7 | Test Not Hispanic 7 | 0.74 | ***167*** | Train 11 | Test Not Hispanic 7 | 0.74 | ***231*** | Train 15 | Test Not Hispanic 7 | 0.67 |
| ***40*** | Train 3 | Test Not Hispanic 8 | 0.67 | ***104*** | Train 7 | Test Not Hispanic 8 | 0.69 | ***168*** | Train 11 | Test Not Hispanic 8 | 0.68 | ***232*** | Train 15 | Test Not Hispanic 8 | 0.65 |
| ***41*** | Train 3 | Test Not Hispanic 9 | 0.74 | ***105*** | Train 7 | Test Not Hispanic 9 | 0.74 | ***169*** | Train 11 | Test Not Hispanic 9 | 0.74 | ***233*** | Train 15 | Test Not Hispanic 9 | 0.67 |
| ***42*** | Train 3 | Test Not Hispanic 10 | 0.67 | ***106*** | Train 7 | Test Not Hispanic 10 | 0.68 | ***170*** | Train 11 | Test Not Hispanic 10 | 0.68 | ***234*** | Train 15 | Test Not Hispanic 10 | 0.65 |
| ***43*** | Train 3 | Test Not Hispanic 11 | 0.74 | ***107*** | Train 7 | Test Not Hispanic 11 | 0.74 | ***171*** | Train 11 | Test Not Hispanic 11 | 0.74 | ***235*** | Train 15 | Test Not Hispanic 11 | 0.67 |
| ***44*** | Train 3 | Test Not Hispanic 12 | 0.67 | ***108*** | Train 7 | Test Not Hispanic 12 | 0.68 | ***172*** | Train 11 | Test Not Hispanic 12 | 0.68 | ***236*** | Train 15 | Test Not Hispanic 12 | 0.65 |
| ***45*** | Train 3 | Test Not Hispanic 13 | 0.74 | ***109*** | Train 7 | Test Not Hispanic 13 | 0.73 | ***173*** | Train 11 | Test Not Hispanic 13 | 0.73 | ***237*** | Train 15 | Test Not Hispanic 13 | 0.73 |
| ***46*** | Train 3 | Test Not Hispanic 14 | 0.66 | ***110*** | Train 7 | Test Not Hispanic 14 | 0.68 | ***174*** | Train 11 | Test Not Hispanic 14 | 0.66 | ***238*** | Train 15 | Test Not Hispanic 14 | 0.67 |
| ***47*** | Train 3 | Test Not Hispanic 15 | 0.74 | ***111*** | Train 7 | Test Not Hispanic 15 | 0.73 | ***175*** | Train 11 | Test Not Hispanic 15 | 0.73 | ***239*** | Train 15 | Test Not Hispanic 15 | 0.73 |
| ***48*** | Train 3 | Test Not Hispanic 16 | 0.66 | ***112*** | Train 7 | Test Not Hispanic 16 | 0.68 | ***176*** | Train 11 | Test Not Hispanic 16 | 0.67 | ***240*** | Train 15 | Test Not Hispanic 16 | 0.68 |
| ***49*** | Train 4 | Test Not Hispanic 1 | 0.74 | ***113*** | Train 8 | Test Not Hispanic 1 | 0.74 | ***177*** | Train 12 | Test Not Hispanic 1 | 0.74 | ***241*** | Train 16 | Test Not Hispanic 1 | 0.64 |
| ***50*** | Train 4 | Test Not Hispanic 2 | 0.75 | ***114*** | Train 8 | Test Not Hispanic 2 | 0.75 | ***178*** | Train 12 | Test Not Hispanic 2 | 0.75 | ***242*** | Train 16 | Test Not Hispanic 2 | 0.68 |
| ***51*** | Train 4 | Test Not Hispanic 3 | 0.74 | ***115*** | Train 8 | Test Not Hispanic 3 | 0.74 | ***179*** | Train 12 | Test Not Hispanic 3 | 0.74 | ***243*** | Train 16 | Test Not Hispanic 3 | 0.64 |
| ***52*** | Train 4 | Test Not Hispanic 4 | 0.75 | ***116*** | Train 8 | Test Not Hispanic 4 | 0.75 | ***180*** | Train 12 | Test Not Hispanic 4 | 0.75 | ***244*** | Train 16 | Test Not Hispanic 4 | 0.68 |
| ***53*** | Train 4 | Test Not Hispanic 5 | 0.74 | ***117*** | Train 8 | Test Not Hispanic 5 | 0.74 | ***181*** | Train 12 | Test Not Hispanic 5 | 0.74 | ***245*** | Train 16 | Test Not Hispanic 5 | 0.64 |
| ***54*** | Train 4 | Test Not Hispanic 6 | 0.75 | ***118*** | Train 8 | Test Not Hispanic 6 | 0.75 | ***182*** | Train 12 | Test Not Hispanic 6 | 0.75 | ***246*** | Train 16 | Test Not Hispanic 6 | 0.68 |
| ***55*** | Train 4 | Test Not Hispanic 7 | 0.74 | ***119*** | Train 8 | Test Not Hispanic 7 | 0.74 | ***183*** | Train 12 | Test Not Hispanic 7 | 0.74 | ***247*** | Train 16 | Test Not Hispanic 7 | 0.64 |
| ***56*** | Train 4 | Test Not Hispanic 8 | 0.75 | ***120*** | Train 8 | Test Not Hispanic 8 | 0.75 | ***184*** | Train 12 | Test Not Hispanic 8 | 0.75 | ***248*** | Train 16 | Test Not Hispanic 8 | 0.68 |
| ***57*** | Train 4 | Test Not Hispanic 9 | 0.74 | ***121*** | Train 8 | Test Not Hispanic 9 | 0.74 | ***185*** | Train 12 | Test Not Hispanic 9 | 0.74 | ***249*** | Train 16 | Test Not Hispanic 9 | 0.64 |
| ***58*** | Train 4 | Test Not Hispanic 10 | 0.75 | ***122*** | Train 8 | Test Not Hispanic 10 | 0.75 | ***186*** | Train 12 | Test Not Hispanic 10 | 0.75 | ***250*** | Train 16 | Test Not Hispanic 10 | 0.68 |
| ***59*** | Train 4 | Test Not Hispanic 11 | 0.74 | ***123*** | Train 8 | Test Not Hispanic 11 | 0.74 | ***187*** | Train 12 | Test Not Hispanic 11 | 0.74 | ***251*** | Train 16 | Test Not Hispanic 11 | 0.64 |
| ***60*** | Train 4 | Test Not Hispanic 12 | 0.75 | ***124*** | Train 8 | Test Not Hispanic 12 | 0.75 | ***188*** | Train 12 | Test Not Hispanic 12 | 0.75 | ***252*** | Train 16 | Test Not Hispanic 12 | 0.68 |
| ***61*** | Train 4 | Test Not Hispanic 13 | 0.74 | ***125*** | Train 8 | Test Not Hispanic 13 | 0.74 | ***189*** | Train 12 | Test Not Hispanic 13 | 0.74 | ***253*** | Train 16 | Test Not Hispanic 13 | 0.74 |
| ***62*** | Train 4 | Test Not Hispanic 14 | 0.74 | ***126*** | Train 8 | Test Not Hispanic 14 | 0.74 | ***190*** | Train 12 | Test Not Hispanic 14 | 0.74 | ***254*** | Train 16 | Test Not Hispanic 14 | 0.75 |
| ***63*** | Train 4 | Test Not Hispanic 15 | 0.74 | ***127*** | Train 8 | Test Not Hispanic 15 | 0.74 | ***191*** | Train 12 | Test Not Hispanic 15 | 0.74 | ***255*** | Train 16 | Test Not Hispanic 15 | 0.74 |
| ***64*** | Train 4 | Test Not Hispanic 16 | 0.74 | ***128*** | Train 8 | Test Not Hispanic 16 | 0.74 | ***192*** | Train 12 | Test Not Hispanic 16 | 0.75 | ***256*** | Train 16 | Test Not Hispanic 16 | 0.75 |
