# Supplementary material for: A Systematic Evaluation of Cohort Selection Criteria and Their Impact on Machine Learning Model Performance and Demographic Disparities in COVID-19 Outcomes: Cohort Study
Source: JMIR Form Res. 2026 Aug 20;10:e88406. doi: 10.2196/88406 (PMC13492188; doi:10.2196/88406)
Supplement: Supplementary file 3 [file formative-v10-e88406-s003.docx]

Appendix 3

Appendix 3 reports the four-performance metrics (AUC, Precision, Recall, and F1 Score) for the three models using the entire dataset across 64 cohorts.

Table 1. Logistic Regression (LR) Performance Metrics Across 64 Cohorts.

| Add Decisions | Train  Model | Test  Dataset | AUC (LR) | Precision (LR) | Recall (LR) | F1 (LR) | Add Decisions | Train  Model | Test  Dataset | AUC (LR) | Precision (LR) | Recall (LR) | F1 (LR) |
| --- | --- | --- | --- | --- | --- | --- | --- | --- | --- | --- | --- | --- | --- |
| 1 | Train 1 | Test 1 | 0.76 | 0.22 | 0.71 | 0.33 | 2049 | Train 33 | Test 1 | 0.76 | 0.22 | 0.71 | 0.33 |
| 2 | Train 1 | Test 2 | 0.75 | 0.25 | 0.69 | 0.36 | 2050 | Train 33 | Test 2 | 0.75 | 0.25 | 0.7 | 0.36 |
| 3 | Train 1 | Test 3 | 0.76 | 0.22 | 0.71 | 0.33 | 2051 | Train 33 | Test 3 | 0.76 | 0.22 | 0.71 | 0.33 |
| 4 | Train 1 | Test 4 | 0.75 | 0.25 | 0.7 | 0.36 | 2052 | Train 33 | Test 4 | 0.75 | 0.25 | 0.7 | 0.36 |
| 5 | Train 1 | Test 5 | 0.72 | 0.2 | 0.62 | 0.31 | 2053 | Train 33 | Test 5 | 0.71 | 0.2 | 0.63 | 0.31 |
| 6 | Train 1 | Test 6 | 0.7 | 0.3 | 0.47 | 0.37 | 2054 | Train 33 | Test 6 | 0.7 | 0.3 | 0.48 | 0.37 |
| 7 | Train 1 | Test 7 | 0.71 | 0.2 | 0.62 | 0.31 | 2055 | Train 33 | Test 7 | 0.71 | 0.2 | 0.62 | 0.31 |
| 8 | Train 1 | Test 8 | 0.7 | 0.3 | 0.47 | 0.36 | 2056 | Train 33 | Test 8 | 0.7 | 0.3 | 0.48 | 0.37 |
| 9 | Train 1 | Test 9 | 0.76 | 0.22 | 0.69 | 0.34 | 2057 | Train 33 | Test 9 | 0.76 | 0.22 | 0.69 | 0.33 |
| 10 | Train 1 | Test 10 | 0.75 | 0.25 | 0.67 | 0.37 | 2058 | Train 33 | Test 10 | 0.75 | 0.25 | 0.67 | 0.37 |
| 11 | Train 1 | Test 11 | 0.76 | 0.22 | 0.69 | 0.34 | 2059 | Train 33 | Test 11 | 0.76 | 0.22 | 0.69 | 0.34 |
| 12 | Train 1 | Test 12 | 0.75 | 0.25 | 0.67 | 0.37 | 2060 | Train 33 | Test 12 | 0.75 | 0.25 | 0.67 | 0.37 |
| 13 | Train 1 | Test 13 | 0.71 | 0.21 | 0.6 | 0.31 | 2061 | Train 33 | Test 13 | 0.71 | 0.21 | 0.6 | 0.31 |
| 14 | Train 1 | Test 14 | 0.7 | 0.3 | 0.47 | 0.37 | 2062 | Train 33 | Test 14 | 0.7 | 0.3 | 0.48 | 0.37 |
| 15 | Train 1 | Test 15 | 0.71 | 0.21 | 0.6 | 0.31 | 2063 | Train 33 | Test 15 | 0.71 | 0.21 | 0.6 | 0.31 |
| 16 | Train 1 | Test 16 | 0.7 | 0.3 | 0.47 | 0.36 | 2064 | Train 33 | Test 16 | 0.7 | 0.3 | 0.48 | 0.37 |
| 17 | Train 1 | Test 17 | 0.76 | 0.22 | 0.71 | 0.33 | 2065 | Train 33 | Test 17 | 0.76 | 0.22 | 0.71 | 0.33 |
| 18 | Train 1 | Test 18 | 0.75 | 0.25 | 0.69 | 0.36 | 2066 | Train 33 | Test 18 | 0.75 | 0.25 | 0.7 | 0.36 |
| 19 | Train 1 | Test 19 | 0.76 | 0.22 | 0.71 | 0.33 | 2067 | Train 33 | Test 19 | 0.76 | 0.22 | 0.71 | 0.33 |
| 20 | Train 1 | Test 20 | 0.75 | 0.25 | 0.7 | 0.36 | 2068 | Train 33 | Test 20 | 0.75 | 0.25 | 0.7 | 0.36 |
| 21 | Train 1 | Test 21 | 0.72 | 0.2 | 0.62 | 0.31 | 2069 | Train 33 | Test 21 | 0.72 | 0.2 | 0.63 | 0.31 |
| 22 | Train 1 | Test 22 | 0.7 | 0.3 | 0.47 | 0.37 | 2070 | Train 33 | Test 22 | 0.7 | 0.3 | 0.48 | 0.37 |
| 23 | Train 1 | Test 23 | 0.71 | 0.2 | 0.62 | 0.31 | 2071 | Train 33 | Test 23 | 0.71 | 0.2 | 0.62 | 0.31 |
| 24 | Train 1 | Test 24 | 0.7 | 0.3 | 0.47 | 0.36 | 2072 | Train 33 | Test 24 | 0.7 | 0.3 | 0.48 | 0.37 |
| 25 | Train 1 | Test 25 | 0.76 | 0.22 | 0.69 | 0.34 | 2073 | Train 33 | Test 25 | 0.76 | 0.22 | 0.69 | 0.33 |
| 26 | Train 1 | Test 26 | 0.75 | 0.25 | 0.67 | 0.37 | 2074 | Train 33 | Test 26 | 0.75 | 0.25 | 0.67 | 0.37 |
| 27 | Train 1 | Test 27 | 0.76 | 0.22 | 0.69 | 0.34 | 2075 | Train 33 | Test 27 | 0.76 | 0.22 | 0.69 | 0.34 |
| 28 | Train 1 | Test 28 | 0.75 | 0.25 | 0.67 | 0.37 | 2076 | Train 33 | Test 28 | 0.75 | 0.25 | 0.68 | 0.37 |
| 29 | Train 1 | Test 29 | 0.71 | 0.21 | 0.6 | 0.31 | 2077 | Train 33 | Test 29 | 0.71 | 0.21 | 0.6 | 0.31 |
| 30 | Train 1 | Test 30 | 0.7 | 0.3 | 0.47 | 0.37 | 2078 | Train 33 | Test 30 | 0.7 | 0.3 | 0.48 | 0.37 |
| 31 | Train 1 | Test 31 | 0.71 | 0.21 | 0.6 | 0.31 | 2079 | Train 33 | Test 31 | 0.71 | 0.21 | 0.6 | 0.31 |
| 32 | Train 1 | Test 32 | 0.7 | 0.29 | 0.47 | 0.36 | 2080 | Train 33 | Test 32 | 0.7 | 0.29 | 0.48 | 0.37 |
| 33 | Train 1 | Test 33 | 0.76 | 0.22 | 0.71 | 0.33 | 2081 | Train 33 | Test 33 | 0.76 | 0.22 | 0.71 | 0.33 |
| 34 | Train 1 | Test 34 | 0.75 | 0.25 | 0.69 | 0.36 | 2082 | Train 33 | Test 34 | 0.75 | 0.24 | 0.7 | 0.36 |
| 35 | Train 1 | Test 35 | 0.76 | 0.22 | 0.71 | 0.33 | 2083 | Train 33 | Test 35 | 0.76 | 0.22 | 0.71 | 0.33 |
| 36 | Train 1 | Test 36 | 0.75 | 0.25 | 0.69 | 0.36 | 2084 | Train 33 | Test 36 | 0.75 | 0.25 | 0.7 | 0.36 |
| 37 | Train 1 | Test 37 | 0.72 | 0.2 | 0.62 | 0.31 | 2085 | Train 33 | Test 37 | 0.72 | 0.2 | 0.62 | 0.31 |
| 38 | Train 1 | Test 38 | 0.7 | 0.3 | 0.47 | 0.37 | 2086 | Train 33 | Test 38 | 0.7 | 0.3 | 0.48 | 0.37 |
| 39 | Train 1 | Test 39 | 0.72 | 0.2 | 0.62 | 0.31 | 2087 | Train 33 | Test 39 | 0.72 | 0.2 | 0.62 | 0.31 |
| 40 | Train 1 | Test 40 | 0.7 | 0.3 | 0.47 | 0.36 | 2088 | Train 33 | Test 40 | 0.7 | 0.3 | 0.48 | 0.37 |
| 41 | Train 1 | Test 41 | 0.76 | 0.22 | 0.69 | 0.33 | 2089 | Train 33 | Test 41 | 0.76 | 0.22 | 0.69 | 0.33 |
| 42 | Train 1 | Test 42 | 0.75 | 0.25 | 0.67 | 0.37 | 2090 | Train 33 | Test 42 | 0.75 | 0.25 | 0.67 | 0.36 |
| 43 | Train 1 | Test 43 | 0.76 | 0.22 | 0.69 | 0.34 | 2091 | Train 33 | Test 43 | 0.76 | 0.22 | 0.69 | 0.33 |
| 44 | Train 1 | Test 44 | 0.75 | 0.25 | 0.67 | 0.37 | 2092 | Train 33 | Test 44 | 0.75 | 0.25 | 0.67 | 0.36 |
| 45 | Train 1 | Test 45 | 0.72 | 0.21 | 0.6 | 0.31 | 2093 | Train 33 | Test 45 | 0.72 | 0.21 | 0.6 | 0.31 |
| 46 | Train 1 | Test 46 | 0.7 | 0.3 | 0.47 | 0.37 | 2094 | Train 33 | Test 46 | 0.7 | 0.3 | 0.48 | 0.37 |
| 47 | Train 1 | Test 47 | 0.71 | 0.21 | 0.59 | 0.31 | 2095 | Train 33 | Test 47 | 0.71 | 0.21 | 0.6 | 0.31 |
| 48 | Train 1 | Test 48 | 0.7 | 0.3 | 0.47 | 0.36 | 2096 | Train 33 | Test 48 | 0.7 | 0.3 | 0.48 | 0.37 |
| 49 | Train 1 | Test 49 | 0.78 | 0.25 | 0.77 | 0.38 | 2097 | Train 33 | Test 49 | 0.78 | 0.25 | 0.77 | 0.37 |
| 50 | Train 1 | Test 50 | 0.78 | 0.25 | 0.77 | 0.38 | 2098 | Train 33 | Test 50 | 0.78 | 0.25 | 0.77 | 0.38 |
| 51 | Train 1 | Test 51 | 0.78 | 0.25 | 0.77 | 0.38 | 2099 | Train 33 | Test 51 | 0.78 | 0.25 | 0.77 | 0.38 |
| 52 | Train 1 | Test 52 | 0.78 | 0.25 | 0.77 | 0.38 | 2100 | Train 33 | Test 52 | 0.77 | 0.25 | 0.77 | 0.38 |
| 53 | Train 1 | Test 53 | 0.74 | 0.26 | 0.59 | 0.36 | 2101 | Train 33 | Test 53 | 0.74 | 0.26 | 0.6 | 0.36 |
| 54 | Train 1 | Test 54 | 0.72 | 0.35 | 0.49 | 0.41 | 2102 | Train 33 | Test 54 | 0.72 | 0.34 | 0.51 | 0.41 |
| 55 | Train 1 | Test 55 | 0.74 | 0.24 | 0.63 | 0.34 | 2103 | Train 33 | Test 55 | 0.74 | 0.24 | 0.64 | 0.35 |
| 56 | Train 1 | Test 56 | 0.72 | 0.34 | 0.49 | 0.4 | 2104 | Train 33 | Test 56 | 0.72 | 0.34 | 0.5 | 0.4 |
| 57 | Train 1 | Test 57 | 0.78 | 0.25 | 0.76 | 0.38 | 2105 | Train 33 | Test 57 | 0.78 | 0.25 | 0.77 | 0.38 |
| 58 | Train 1 | Test 58 | 0.78 | 0.25 | 0.76 | 0.38 | 2106 | Train 33 | Test 58 | 0.78 | 0.25 | 0.76 | 0.38 |
| 59 | Train 1 | Test 59 | 0.78 | 0.25 | 0.76 | 0.38 | 2107 | Train 33 | Test 59 | 0.78 | 0.25 | 0.77 | 0.38 |
| 60 | Train 1 | Test 60 | 0.78 | 0.25 | 0.76 | 0.38 | 2108 | Train 33 | Test 60 | 0.78 | 0.25 | 0.76 | 0.38 |
| 61 | Train 1 | Test 61 | 0.74 | 0.26 | 0.58 | 0.36 | 2109 | Train 33 | Test 61 | 0.74 | 0.26 | 0.59 | 0.36 |
| 62 | Train 1 | Test 62 | 0.72 | 0.34 | 0.5 | 0.41 | 2110 | Train 33 | Test 62 | 0.72 | 0.34 | 0.51 | 0.41 |
| 63 | Train 1 | Test 63 | 0.75 | 0.26 | 0.58 | 0.36 | 2111 | Train 33 | Test 63 | 0.75 | 0.26 | 0.59 | 0.36 |
| 64 | Train 1 | Test 64 | 0.72 | 0.33 | 0.5 | 0.4 | 2112 | Train 33 | Test 64 | 0.72 | 0.33 | 0.51 | 0.4 |
| 65 | Train 2 | Test 1 | 0.75 | 0.23 | 0.66 | 0.34 | 2113 | Train 34 | Test 1 | 0.75 | 0.23 | 0.67 | 0.34 |
| 66 | Train 2 | Test 2 | 0.77 | 0.26 | 0.72 | 0.38 | 2114 | Train 34 | Test 2 | 0.77 | 0.25 | 0.73 | 0.38 |
| 67 | Train 2 | Test 3 | 0.75 | 0.23 | 0.67 | 0.34 | 2115 | Train 34 | Test 3 | 0.75 | 0.23 | 0.67 | 0.34 |
| 68 | Train 2 | Test 4 | 0.77 | 0.26 | 0.72 | 0.38 | 2116 | Train 34 | Test 4 | 0.77 | 0.26 | 0.73 | 0.38 |
| 69 | Train 2 | Test 5 | 0.71 | 0.21 | 0.59 | 0.31 | 2117 | Train 34 | Test 5 | 0.71 | 0.21 | 0.59 | 0.31 |
| 70 | Train 2 | Test 6 | 0.71 | 0.34 | 0.4 | 0.37 | 2118 | Train 34 | Test 6 | 0.71 | 0.33 | 0.42 | 0.37 |
| 71 | Train 2 | Test 7 | 0.71 | 0.21 | 0.59 | 0.31 | 2119 | Train 34 | Test 7 | 0.71 | 0.21 | 0.59 | 0.31 |
| 72 | Train 2 | Test 8 | 0.71 | 0.33 | 0.41 | 0.36 | 2120 | Train 34 | Test 8 | 0.71 | 0.32 | 0.42 | 0.37 |
| 73 | Train 2 | Test 9 | 0.75 | 0.23 | 0.65 | 0.34 | 2121 | Train 34 | Test 9 | 0.75 | 0.23 | 0.65 | 0.34 |
| 74 | Train 2 | Test 10 | 0.77 | 0.26 | 0.71 | 0.38 | 2122 | Train 34 | Test 10 | 0.77 | 0.26 | 0.71 | 0.38 |
| 75 | Train 2 | Test 11 | 0.75 | 0.23 | 0.65 | 0.34 | 2123 | Train 34 | Test 11 | 0.75 | 0.23 | 0.65 | 0.34 |
| 76 | Train 2 | Test 12 | 0.77 | 0.26 | 0.71 | 0.38 | 2124 | Train 34 | Test 12 | 0.77 | 0.26 | 0.72 | 0.38 |
| 77 | Train 2 | Test 13 | 0.71 | 0.21 | 0.57 | 0.31 | 2125 | Train 34 | Test 13 | 0.71 | 0.21 | 0.57 | 0.31 |
| 78 | Train 2 | Test 14 | 0.71 | 0.33 | 0.41 | 0.37 | 2126 | Train 34 | Test 14 | 0.71 | 0.33 | 0.42 | 0.37 |
| 79 | Train 2 | Test 15 | 0.71 | 0.21 | 0.57 | 0.31 | 2127 | Train 34 | Test 15 | 0.71 | 0.21 | 0.57 | 0.31 |
| 80 | Train 2 | Test 16 | 0.71 | 0.33 | 0.41 | 0.36 | 2128 | Train 34 | Test 16 | 0.71 | 0.32 | 0.43 | 0.37 |
| 81 | Train 2 | Test 17 | 0.75 | 0.23 | 0.66 | 0.34 | 2129 | Train 34 | Test 17 | 0.75 | 0.23 | 0.67 | 0.34 |
| 82 | Train 2 | Test 18 | 0.77 | 0.26 | 0.72 | 0.38 | 2130 | Train 34 | Test 18 | 0.77 | 0.25 | 0.73 | 0.38 |
| 83 | Train 2 | Test 19 | 0.75 | 0.23 | 0.67 | 0.34 | 2131 | Train 34 | Test 19 | 0.75 | 0.23 | 0.67 | 0.34 |
| 84 | Train 2 | Test 20 | 0.77 | 0.26 | 0.72 | 0.38 | 2132 | Train 34 | Test 20 | 0.77 | 0.26 | 0.73 | 0.38 |
| 85 | Train 2 | Test 21 | 0.71 | 0.21 | 0.59 | 0.31 | 2133 | Train 34 | Test 21 | 0.71 | 0.21 | 0.59 | 0.31 |
| 86 | Train 2 | Test 22 | 0.71 | 0.34 | 0.4 | 0.37 | 2134 | Train 34 | Test 22 | 0.71 | 0.33 | 0.42 | 0.37 |
| 87 | Train 2 | Test 23 | 0.71 | 0.21 | 0.59 | 0.31 | 2135 | Train 34 | Test 23 | 0.71 | 0.21 | 0.59 | 0.31 |
| 88 | Train 2 | Test 24 | 0.71 | 0.33 | 0.41 | 0.36 | 2136 | Train 34 | Test 24 | 0.71 | 0.32 | 0.42 | 0.37 |
| 89 | Train 2 | Test 25 | 0.75 | 0.23 | 0.65 | 0.34 | 2137 | Train 34 | Test 25 | 0.75 | 0.23 | 0.65 | 0.34 |
| 90 | Train 2 | Test 26 | 0.77 | 0.26 | 0.71 | 0.38 | 2138 | Train 34 | Test 26 | 0.77 | 0.26 | 0.72 | 0.38 |
| 91 | Train 2 | Test 27 | 0.76 | 0.23 | 0.65 | 0.34 | 2139 | Train 34 | Test 27 | 0.75 | 0.23 | 0.65 | 0.34 |
| 92 | Train 2 | Test 28 | 0.77 | 0.26 | 0.71 | 0.38 | 2140 | Train 34 | Test 28 | 0.77 | 0.26 | 0.72 | 0.38 |
| 93 | Train 2 | Test 29 | 0.71 | 0.21 | 0.57 | 0.31 | 2141 | Train 34 | Test 29 | 0.71 | 0.21 | 0.57 | 0.31 |
| 94 | Train 2 | Test 30 | 0.71 | 0.33 | 0.41 | 0.37 | 2142 | Train 34 | Test 30 | 0.71 | 0.33 | 0.42 | 0.37 |
| 95 | Train 2 | Test 31 | 0.71 | 0.21 | 0.57 | 0.31 | 2143 | Train 34 | Test 31 | 0.71 | 0.21 | 0.57 | 0.31 |
| 96 | Train 2 | Test 32 | 0.71 | 0.33 | 0.41 | 0.36 | 2144 | Train 34 | Test 32 | 0.71 | 0.32 | 0.43 | 0.37 |
| 97 | Train 2 | Test 33 | 0.76 | 0.22 | 0.66 | 0.34 | 2145 | Train 34 | Test 33 | 0.75 | 0.22 | 0.67 | 0.34 |
| 98 | Train 2 | Test 34 | 0.77 | 0.26 | 0.72 | 0.38 | 2146 | Train 34 | Test 34 | 0.77 | 0.25 | 0.73 | 0.38 |
| 99 | Train 2 | Test 35 | 0.76 | 0.23 | 0.66 | 0.34 | 2147 | Train 34 | Test 35 | 0.76 | 0.23 | 0.67 | 0.34 |
| 100 | Train 2 | Test 36 | 0.77 | 0.26 | 0.72 | 0.38 | 2148 | Train 34 | Test 36 | 0.77 | 0.25 | 0.73 | 0.38 |
| 101 | Train 2 | Test 37 | 0.71 | 0.21 | 0.59 | 0.31 | 2149 | Train 34 | Test 37 | 0.71 | 0.21 | 0.59 | 0.31 |
| 102 | Train 2 | Test 38 | 0.71 | 0.34 | 0.4 | 0.37 | 2150 | Train 34 | Test 38 | 0.71 | 0.33 | 0.42 | 0.37 |
| 103 | Train 2 | Test 39 | 0.71 | 0.21 | 0.59 | 0.31 | 2151 | Train 34 | Test 39 | 0.71 | 0.21 | 0.59 | 0.31 |
| 104 | Train 2 | Test 40 | 0.71 | 0.33 | 0.41 | 0.36 | 2152 | Train 34 | Test 40 | 0.71 | 0.32 | 0.42 | 0.37 |
| 105 | Train 2 | Test 41 | 0.76 | 0.23 | 0.65 | 0.34 | 2153 | Train 34 | Test 41 | 0.76 | 0.23 | 0.65 | 0.34 |
| 106 | Train 2 | Test 42 | 0.77 | 0.26 | 0.71 | 0.38 | 2154 | Train 34 | Test 42 | 0.77 | 0.26 | 0.71 | 0.38 |
| 107 | Train 2 | Test 43 | 0.76 | 0.23 | 0.65 | 0.34 | 2155 | Train 34 | Test 43 | 0.76 | 0.23 | 0.65 | 0.34 |
| 108 | Train 2 | Test 44 | 0.77 | 0.26 | 0.71 | 0.38 | 2156 | Train 34 | Test 44 | 0.77 | 0.26 | 0.71 | 0.38 |
| 109 | Train 2 | Test 45 | 0.71 | 0.21 | 0.57 | 0.31 | 2157 | Train 34 | Test 45 | 0.71 | 0.21 | 0.57 | 0.31 |
| 110 | Train 2 | Test 46 | 0.71 | 0.33 | 0.41 | 0.37 | 2158 | Train 34 | Test 46 | 0.71 | 0.33 | 0.42 | 0.37 |
| 111 | Train 2 | Test 47 | 0.71 | 0.21 | 0.56 | 0.31 | 2159 | Train 34 | Test 47 | 0.71 | 0.21 | 0.56 | 0.31 |
| 112 | Train 2 | Test 48 | 0.71 | 0.33 | 0.41 | 0.36 | 2160 | Train 34 | Test 48 | 0.71 | 0.32 | 0.43 | 0.37 |
| 113 | Train 2 | Test 49 | 0.77 | 0.25 | 0.72 | 0.38 | 2161 | Train 34 | Test 49 | 0.77 | 0.25 | 0.73 | 0.38 |
| 114 | Train 2 | Test 50 | 0.77 | 0.25 | 0.72 | 0.38 | 2162 | Train 34 | Test 50 | 0.77 | 0.25 | 0.73 | 0.38 |
| 115 | Train 2 | Test 51 | 0.77 | 0.25 | 0.73 | 0.38 | 2163 | Train 34 | Test 51 | 0.77 | 0.26 | 0.73 | 0.38 |
| 116 | Train 2 | Test 52 | 0.77 | 0.25 | 0.72 | 0.38 | 2164 | Train 34 | Test 52 | 0.77 | 0.26 | 0.73 | 0.38 |
| 117 | Train 2 | Test 53 | 0.74 | 0.28 | 0.45 | 0.34 | 2165 | Train 34 | Test 53 | 0.74 | 0.28 | 0.46 | 0.35 |
| 118 | Train 2 | Test 54 | 0.72 | 0.38 | 0.4 | 0.39 | 2166 | Train 34 | Test 54 | 0.72 | 0.37 | 0.42 | 0.4 |
| 119 | Train 2 | Test 55 | 0.73 | 0.24 | 0.57 | 0.34 | 2167 | Train 34 | Test 55 | 0.73 | 0.24 | 0.57 | 0.34 |
| 120 | Train 2 | Test 56 | 0.72 | 0.36 | 0.41 | 0.38 | 2168 | Train 34 | Test 56 | 0.72 | 0.36 | 0.43 | 0.39 |
| 121 | Train 2 | Test 57 | 0.78 | 0.26 | 0.73 | 0.38 | 2169 | Train 34 | Test 57 | 0.78 | 0.26 | 0.73 | 0.38 |
| 122 | Train 2 | Test 58 | 0.78 | 0.26 | 0.72 | 0.38 | 2170 | Train 34 | Test 58 | 0.77 | 0.26 | 0.72 | 0.38 |
| 123 | Train 2 | Test 59 | 0.78 | 0.26 | 0.73 | 0.39 | 2171 | Train 34 | Test 59 | 0.78 | 0.26 | 0.73 | 0.39 |
| 124 | Train 2 | Test 60 | 0.78 | 0.26 | 0.72 | 0.39 | 2172 | Train 34 | Test 60 | 0.77 | 0.26 | 0.72 | 0.39 |
| 125 | Train 2 | Test 61 | 0.75 | 0.28 | 0.43 | 0.34 | 2173 | Train 34 | Test 61 | 0.75 | 0.28 | 0.45 | 0.34 |
| 126 | Train 2 | Test 62 | 0.72 | 0.37 | 0.4 | 0.39 | 2174 | Train 34 | Test 62 | 0.72 | 0.37 | 0.42 | 0.39 |
| 127 | Train 2 | Test 63 | 0.75 | 0.29 | 0.44 | 0.35 | 2175 | Train 34 | Test 63 | 0.75 | 0.28 | 0.46 | 0.35 |
| 128 | Train 2 | Test 64 | 0.72 | 0.36 | 0.41 | 0.38 | 2176 | Train 34 | Test 64 | 0.72 | 0.35 | 0.43 | 0.39 |
| 129 | Train 3 | Test 1 | 0.76 | 0.22 | 0.71 | 0.33 | 2177 | Train 35 | Test 1 | 0.76 | 0.22 | 0.71 | 0.33 |
| 130 | Train 3 | Test 2 | 0.75 | 0.25 | 0.69 | 0.36 | 2178 | Train 35 | Test 2 | 0.75 | 0.25 | 0.7 | 0.36 |
| 131 | Train 3 | Test 3 | 0.76 | 0.22 | 0.71 | 0.33 | 2179 | Train 35 | Test 3 | 0.76 | 0.22 | 0.71 | 0.33 |
| 132 | Train 3 | Test 4 | 0.75 | 0.25 | 0.69 | 0.36 | 2180 | Train 35 | Test 4 | 0.75 | 0.25 | 0.7 | 0.36 |
| 133 | Train 3 | Test 5 | 0.72 | 0.2 | 0.63 | 0.31 | 2181 | Train 35 | Test 5 | 0.71 | 0.2 | 0.63 | 0.31 |
| 134 | Train 3 | Test 6 | 0.7 | 0.3 | 0.46 | 0.36 | 2182 | Train 35 | Test 6 | 0.7 | 0.3 | 0.47 | 0.37 |
| 135 | Train 3 | Test 7 | 0.71 | 0.2 | 0.62 | 0.31 | 2183 | Train 35 | Test 7 | 0.71 | 0.2 | 0.62 | 0.31 |
| 136 | Train 3 | Test 8 | 0.7 | 0.3 | 0.46 | 0.36 | 2184 | Train 35 | Test 8 | 0.7 | 0.3 | 0.47 | 0.36 |
| 137 | Train 3 | Test 9 | 0.76 | 0.22 | 0.69 | 0.34 | 2185 | Train 35 | Test 9 | 0.76 | 0.22 | 0.69 | 0.34 |
| 138 | Train 3 | Test 10 | 0.75 | 0.25 | 0.67 | 0.37 | 2186 | Train 35 | Test 10 | 0.75 | 0.25 | 0.68 | 0.37 |
| 139 | Train 3 | Test 11 | 0.76 | 0.22 | 0.69 | 0.34 | 2187 | Train 35 | Test 11 | 0.76 | 0.22 | 0.69 | 0.34 |
| 140 | Train 3 | Test 12 | 0.75 | 0.25 | 0.67 | 0.37 | 2188 | Train 35 | Test 12 | 0.75 | 0.25 | 0.68 | 0.37 |
| 141 | Train 3 | Test 13 | 0.71 | 0.21 | 0.6 | 0.31 | 2189 | Train 35 | Test 13 | 0.71 | 0.21 | 0.6 | 0.31 |
| 142 | Train 3 | Test 14 | 0.7 | 0.3 | 0.46 | 0.36 | 2190 | Train 35 | Test 14 | 0.7 | 0.3 | 0.48 | 0.37 |
| 143 | Train 3 | Test 15 | 0.71 | 0.21 | 0.6 | 0.31 | 2191 | Train 35 | Test 15 | 0.71 | 0.21 | 0.6 | 0.31 |
| 144 | Train 3 | Test 16 | 0.7 | 0.3 | 0.46 | 0.36 | 2192 | Train 35 | Test 16 | 0.7 | 0.3 | 0.47 | 0.36 |
| 145 | Train 3 | Test 17 | 0.76 | 0.22 | 0.71 | 0.33 | 2193 | Train 35 | Test 17 | 0.76 | 0.22 | 0.71 | 0.33 |
| 146 | Train 3 | Test 18 | 0.75 | 0.25 | 0.69 | 0.36 | 2194 | Train 35 | Test 18 | 0.75 | 0.25 | 0.7 | 0.36 |
| 147 | Train 3 | Test 19 | 0.76 | 0.22 | 0.71 | 0.33 | 2195 | Train 35 | Test 19 | 0.76 | 0.22 | 0.71 | 0.33 |
| 148 | Train 3 | Test 20 | 0.75 | 0.25 | 0.69 | 0.36 | 2196 | Train 35 | Test 20 | 0.75 | 0.25 | 0.7 | 0.36 |
| 149 | Train 3 | Test 21 | 0.72 | 0.2 | 0.63 | 0.31 | 2197 | Train 35 | Test 21 | 0.72 | 0.2 | 0.63 | 0.31 |
| 150 | Train 3 | Test 22 | 0.7 | 0.3 | 0.46 | 0.36 | 2198 | Train 35 | Test 22 | 0.7 | 0.3 | 0.47 | 0.37 |
| 151 | Train 3 | Test 23 | 0.71 | 0.2 | 0.62 | 0.31 | 2199 | Train 35 | Test 23 | 0.71 | 0.2 | 0.62 | 0.31 |
| 152 | Train 3 | Test 24 | 0.7 | 0.3 | 0.46 | 0.36 | 2200 | Train 35 | Test 24 | 0.7 | 0.3 | 0.47 | 0.36 |
| 153 | Train 3 | Test 25 | 0.76 | 0.22 | 0.69 | 0.34 | 2201 | Train 35 | Test 25 | 0.76 | 0.22 | 0.69 | 0.34 |
| 154 | Train 3 | Test 26 | 0.75 | 0.25 | 0.67 | 0.37 | 2202 | Train 35 | Test 26 | 0.75 | 0.25 | 0.68 | 0.37 |
| 155 | Train 3 | Test 27 | 0.76 | 0.22 | 0.69 | 0.34 | 2203 | Train 35 | Test 27 | 0.76 | 0.22 | 0.69 | 0.34 |
| 156 | Train 3 | Test 28 | 0.75 | 0.25 | 0.67 | 0.37 | 2204 | Train 35 | Test 28 | 0.75 | 0.25 | 0.68 | 0.37 |
| 157 | Train 3 | Test 29 | 0.71 | 0.21 | 0.6 | 0.31 | 2205 | Train 35 | Test 29 | 0.71 | 0.21 | 0.6 | 0.31 |
| 158 | Train 3 | Test 30 | 0.7 | 0.3 | 0.46 | 0.36 | 2206 | Train 35 | Test 30 | 0.7 | 0.3 | 0.48 | 0.37 |
| 159 | Train 3 | Test 31 | 0.71 | 0.21 | 0.6 | 0.31 | 2207 | Train 35 | Test 31 | 0.71 | 0.21 | 0.6 | 0.31 |
| 160 | Train 3 | Test 32 | 0.7 | 0.3 | 0.46 | 0.36 | 2208 | Train 35 | Test 32 | 0.7 | 0.29 | 0.47 | 0.36 |
| 161 | Train 3 | Test 33 | 0.76 | 0.22 | 0.7 | 0.33 | 2209 | Train 35 | Test 33 | 0.76 | 0.22 | 0.71 | 0.33 |
| 162 | Train 3 | Test 34 | 0.75 | 0.25 | 0.69 | 0.36 | 2210 | Train 35 | Test 34 | 0.75 | 0.24 | 0.69 | 0.36 |
| 163 | Train 3 | Test 35 | 0.76 | 0.22 | 0.71 | 0.33 | 2211 | Train 35 | Test 35 | 0.76 | 0.22 | 0.71 | 0.33 |
| 164 | Train 3 | Test 36 | 0.75 | 0.25 | 0.69 | 0.36 | 2212 | Train 35 | Test 36 | 0.75 | 0.24 | 0.69 | 0.36 |
| 165 | Train 3 | Test 37 | 0.72 | 0.2 | 0.62 | 0.31 | 2213 | Train 35 | Test 37 | 0.72 | 0.2 | 0.62 | 0.31 |
| 166 | Train 3 | Test 38 | 0.7 | 0.3 | 0.46 | 0.36 | 2214 | Train 35 | Test 38 | 0.7 | 0.3 | 0.47 | 0.37 |
| 167 | Train 3 | Test 39 | 0.72 | 0.2 | 0.62 | 0.31 | 2215 | Train 35 | Test 39 | 0.72 | 0.2 | 0.62 | 0.31 |
| 168 | Train 3 | Test 40 | 0.7 | 0.3 | 0.46 | 0.36 | 2216 | Train 35 | Test 40 | 0.7 | 0.3 | 0.47 | 0.36 |
| 169 | Train 3 | Test 41 | 0.76 | 0.22 | 0.68 | 0.33 | 2217 | Train 35 | Test 41 | 0.76 | 0.22 | 0.69 | 0.33 |
| 170 | Train 3 | Test 42 | 0.75 | 0.25 | 0.67 | 0.36 | 2218 | Train 35 | Test 42 | 0.75 | 0.25 | 0.68 | 0.37 |
| 171 | Train 3 | Test 43 | 0.76 | 0.22 | 0.69 | 0.33 | 2219 | Train 35 | Test 43 | 0.76 | 0.22 | 0.69 | 0.34 |
| 172 | Train 3 | Test 44 | 0.75 | 0.25 | 0.67 | 0.36 | 2220 | Train 35 | Test 44 | 0.75 | 0.25 | 0.68 | 0.37 |
| 173 | Train 3 | Test 45 | 0.72 | 0.21 | 0.6 | 0.31 | 2221 | Train 35 | Test 45 | 0.72 | 0.21 | 0.6 | 0.31 |
| 174 | Train 3 | Test 46 | 0.7 | 0.3 | 0.46 | 0.36 | 2222 | Train 35 | Test 46 | 0.7 | 0.3 | 0.48 | 0.37 |
| 175 | Train 3 | Test 47 | 0.71 | 0.21 | 0.59 | 0.31 | 2223 | Train 35 | Test 47 | 0.71 | 0.21 | 0.6 | 0.31 |
| 176 | Train 3 | Test 48 | 0.7 | 0.3 | 0.46 | 0.36 | 2224 | Train 35 | Test 48 | 0.7 | 0.3 | 0.47 | 0.36 |
| 177 | Train 3 | Test 49 | 0.78 | 0.25 | 0.77 | 0.37 | 2225 | Train 35 | Test 49 | 0.78 | 0.25 | 0.77 | 0.37 |
| 178 | Train 3 | Test 50 | 0.78 | 0.25 | 0.77 | 0.37 | 2226 | Train 35 | Test 50 | 0.78 | 0.25 | 0.77 | 0.37 |
| 179 | Train 3 | Test 51 | 0.78 | 0.25 | 0.77 | 0.37 | 2227 | Train 35 | Test 51 | 0.78 | 0.25 | 0.77 | 0.37 |
| 180 | Train 3 | Test 52 | 0.78 | 0.25 | 0.77 | 0.37 | 2228 | Train 35 | Test 52 | 0.78 | 0.25 | 0.77 | 0.37 |
| 181 | Train 3 | Test 53 | 0.74 | 0.26 | 0.58 | 0.36 | 2229 | Train 35 | Test 53 | 0.74 | 0.26 | 0.6 | 0.36 |
| 182 | Train 3 | Test 54 | 0.71 | 0.35 | 0.49 | 0.4 | 2230 | Train 35 | Test 54 | 0.72 | 0.34 | 0.5 | 0.41 |
| 183 | Train 3 | Test 55 | 0.74 | 0.23 | 0.63 | 0.34 | 2231 | Train 35 | Test 55 | 0.74 | 0.24 | 0.64 | 0.34 |
| 184 | Train 3 | Test 56 | 0.72 | 0.34 | 0.48 | 0.4 | 2232 | Train 35 | Test 56 | 0.72 | 0.34 | 0.5 | 0.4 |
| 185 | Train 3 | Test 57 | 0.78 | 0.25 | 0.77 | 0.38 | 2233 | Train 35 | Test 57 | 0.78 | 0.25 | 0.77 | 0.38 |
| 186 | Train 3 | Test 58 | 0.78 | 0.25 | 0.76 | 0.38 | 2234 | Train 35 | Test 58 | 0.78 | 0.25 | 0.77 | 0.38 |
| 187 | Train 3 | Test 59 | 0.78 | 0.25 | 0.77 | 0.38 | 2235 | Train 35 | Test 59 | 0.78 | 0.25 | 0.77 | 0.38 |
| 188 | Train 3 | Test 60 | 0.78 | 0.25 | 0.76 | 0.38 | 2236 | Train 35 | Test 60 | 0.78 | 0.25 | 0.77 | 0.38 |
| 189 | Train 3 | Test 61 | 0.74 | 0.26 | 0.57 | 0.36 | 2237 | Train 35 | Test 61 | 0.74 | 0.26 | 0.58 | 0.36 |
| 190 | Train 3 | Test 62 | 0.72 | 0.34 | 0.49 | 0.4 | 2238 | Train 35 | Test 62 | 0.72 | 0.34 | 0.5 | 0.41 |
| 191 | Train 3 | Test 63 | 0.75 | 0.26 | 0.57 | 0.36 | 2239 | Train 35 | Test 63 | 0.75 | 0.26 | 0.59 | 0.36 |
| 192 | Train 3 | Test 64 | 0.72 | 0.34 | 0.49 | 0.4 | 2240 | Train 35 | Test 64 | 0.72 | 0.33 | 0.5 | 0.4 |
| 193 | Train 4 | Test 1 | 0.75 | 0.23 | 0.67 | 0.34 | 2241 | Train 36 | Test 1 | 0.75 | 0.22 | 0.67 | 0.34 |
| 194 | Train 4 | Test 2 | 0.77 | 0.26 | 0.72 | 0.38 | 2242 | Train 36 | Test 2 | 0.77 | 0.25 | 0.73 | 0.38 |
| 195 | Train 4 | Test 3 | 0.75 | 0.23 | 0.67 | 0.34 | 2243 | Train 36 | Test 3 | 0.75 | 0.23 | 0.67 | 0.34 |
| 196 | Train 4 | Test 4 | 0.77 | 0.26 | 0.72 | 0.38 | 2244 | Train 36 | Test 4 | 0.77 | 0.25 | 0.73 | 0.38 |
| 197 | Train 4 | Test 5 | 0.71 | 0.21 | 0.6 | 0.31 | 2245 | Train 36 | Test 5 | 0.71 | 0.21 | 0.6 | 0.31 |
| 198 | Train 4 | Test 6 | 0.71 | 0.34 | 0.39 | 0.36 | 2246 | Train 36 | Test 6 | 0.71 | 0.33 | 0.41 | 0.37 |
| 199 | Train 4 | Test 7 | 0.71 | 0.21 | 0.59 | 0.31 | 2247 | Train 36 | Test 7 | 0.71 | 0.21 | 0.6 | 0.31 |
| 200 | Train 4 | Test 8 | 0.71 | 0.33 | 0.39 | 0.36 | 2248 | Train 36 | Test 8 | 0.71 | 0.33 | 0.42 | 0.37 |
| 201 | Train 4 | Test 9 | 0.75 | 0.23 | 0.65 | 0.34 | 2249 | Train 36 | Test 9 | 0.75 | 0.23 | 0.65 | 0.34 |
| 202 | Train 4 | Test 10 | 0.77 | 0.26 | 0.71 | 0.38 | 2250 | Train 36 | Test 10 | 0.77 | 0.26 | 0.72 | 0.38 |
| 203 | Train 4 | Test 11 | 0.76 | 0.23 | 0.65 | 0.34 | 2251 | Train 36 | Test 11 | 0.75 | 0.23 | 0.66 | 0.34 |
| 204 | Train 4 | Test 12 | 0.77 | 0.26 | 0.71 | 0.38 | 2252 | Train 36 | Test 12 | 0.77 | 0.26 | 0.72 | 0.38 |
| 205 | Train 4 | Test 13 | 0.71 | 0.21 | 0.57 | 0.31 | 2253 | Train 36 | Test 13 | 0.71 | 0.21 | 0.57 | 0.31 |
| 206 | Train 4 | Test 14 | 0.71 | 0.33 | 0.39 | 0.36 | 2254 | Train 36 | Test 14 | 0.71 | 0.33 | 0.42 | 0.37 |
| 207 | Train 4 | Test 15 | 0.71 | 0.21 | 0.57 | 0.31 | 2255 | Train 36 | Test 15 | 0.71 | 0.21 | 0.57 | 0.31 |
| 208 | Train 4 | Test 16 | 0.71 | 0.33 | 0.4 | 0.36 | 2256 | Train 36 | Test 16 | 0.71 | 0.32 | 0.42 | 0.37 |
| 209 | Train 4 | Test 17 | 0.75 | 0.23 | 0.67 | 0.34 | 2257 | Train 36 | Test 17 | 0.75 | 0.22 | 0.67 | 0.34 |
| 210 | Train 4 | Test 18 | 0.77 | 0.26 | 0.72 | 0.38 | 2258 | Train 36 | Test 18 | 0.77 | 0.25 | 0.73 | 0.38 |
| 211 | Train 4 | Test 19 | 0.75 | 0.23 | 0.67 | 0.34 | 2259 | Train 36 | Test 19 | 0.75 | 0.23 | 0.67 | 0.34 |
| 212 | Train 4 | Test 20 | 0.77 | 0.26 | 0.73 | 0.38 | 2260 | Train 36 | Test 20 | 0.77 | 0.25 | 0.73 | 0.38 |
| 213 | Train 4 | Test 21 | 0.71 | 0.21 | 0.6 | 0.31 | 2261 | Train 36 | Test 21 | 0.71 | 0.21 | 0.6 | 0.31 |
| 214 | Train 4 | Test 22 | 0.71 | 0.34 | 0.39 | 0.36 | 2262 | Train 36 | Test 22 | 0.71 | 0.33 | 0.41 | 0.37 |
| 215 | Train 4 | Test 23 | 0.71 | 0.21 | 0.59 | 0.31 | 2263 | Train 36 | Test 23 | 0.71 | 0.21 | 0.6 | 0.31 |
| 216 | Train 4 | Test 24 | 0.71 | 0.33 | 0.39 | 0.36 | 2264 | Train 36 | Test 24 | 0.71 | 0.33 | 0.42 | 0.37 |
| 217 | Train 4 | Test 25 | 0.76 | 0.23 | 0.65 | 0.34 | 2265 | Train 36 | Test 25 | 0.75 | 0.23 | 0.66 | 0.34 |
| 218 | Train 4 | Test 26 | 0.77 | 0.26 | 0.71 | 0.38 | 2266 | Train 36 | Test 26 | 0.77 | 0.26 | 0.72 | 0.38 |
| 219 | Train 4 | Test 27 | 0.76 | 0.23 | 0.65 | 0.34 | 2267 | Train 36 | Test 27 | 0.75 | 0.23 | 0.66 | 0.34 |
| 220 | Train 4 | Test 28 | 0.77 | 0.26 | 0.71 | 0.38 | 2268 | Train 36 | Test 28 | 0.77 | 0.26 | 0.72 | 0.38 |
| 221 | Train 4 | Test 29 | 0.71 | 0.21 | 0.57 | 0.31 | 2269 | Train 36 | Test 29 | 0.71 | 0.21 | 0.57 | 0.31 |
| 222 | Train 4 | Test 30 | 0.71 | 0.33 | 0.39 | 0.36 | 2270 | Train 36 | Test 30 | 0.71 | 0.33 | 0.42 | 0.37 |
| 223 | Train 4 | Test 31 | 0.71 | 0.21 | 0.57 | 0.31 | 2271 | Train 36 | Test 31 | 0.71 | 0.21 | 0.57 | 0.31 |
| 224 | Train 4 | Test 32 | 0.71 | 0.33 | 0.4 | 0.36 | 2272 | Train 36 | Test 32 | 0.71 | 0.32 | 0.42 | 0.37 |
| 225 | Train 4 | Test 33 | 0.76 | 0.23 | 0.66 | 0.34 | 2273 | Train 36 | Test 33 | 0.75 | 0.22 | 0.67 | 0.34 |
| 226 | Train 4 | Test 34 | 0.77 | 0.26 | 0.72 | 0.38 | 2274 | Train 36 | Test 34 | 0.77 | 0.25 | 0.73 | 0.38 |
| 227 | Train 4 | Test 35 | 0.76 | 0.23 | 0.67 | 0.34 | 2275 | Train 36 | Test 35 | 0.76 | 0.22 | 0.67 | 0.34 |
| 228 | Train 4 | Test 36 | 0.77 | 0.26 | 0.72 | 0.38 | 2276 | Train 36 | Test 36 | 0.77 | 0.25 | 0.73 | 0.38 |
| 229 | Train 4 | Test 37 | 0.71 | 0.21 | 0.59 | 0.31 | 2277 | Train 36 | Test 37 | 0.71 | 0.21 | 0.6 | 0.31 |
| 230 | Train 4 | Test 38 | 0.71 | 0.34 | 0.39 | 0.36 | 2278 | Train 36 | Test 38 | 0.71 | 0.33 | 0.41 | 0.37 |
| 231 | Train 4 | Test 39 | 0.71 | 0.21 | 0.59 | 0.31 | 2279 | Train 36 | Test 39 | 0.71 | 0.21 | 0.59 | 0.31 |
| 232 | Train 4 | Test 40 | 0.71 | 0.33 | 0.39 | 0.36 | 2280 | Train 36 | Test 40 | 0.71 | 0.32 | 0.42 | 0.37 |
| 233 | Train 4 | Test 41 | 0.76 | 0.23 | 0.64 | 0.34 | 2281 | Train 36 | Test 41 | 0.76 | 0.23 | 0.65 | 0.34 |
| 234 | Train 4 | Test 42 | 0.77 | 0.26 | 0.71 | 0.38 | 2282 | Train 36 | Test 42 | 0.77 | 0.26 | 0.72 | 0.38 |
| 235 | Train 4 | Test 43 | 0.76 | 0.23 | 0.65 | 0.34 | 2283 | Train 36 | Test 43 | 0.76 | 0.23 | 0.65 | 0.34 |
| 236 | Train 4 | Test 44 | 0.77 | 0.26 | 0.71 | 0.38 | 2284 | Train 36 | Test 44 | 0.77 | 0.26 | 0.72 | 0.38 |
| 237 | Train 4 | Test 45 | 0.71 | 0.21 | 0.57 | 0.31 | 2285 | Train 36 | Test 45 | 0.71 | 0.21 | 0.57 | 0.31 |
| 238 | Train 4 | Test 46 | 0.71 | 0.33 | 0.39 | 0.36 | 2286 | Train 36 | Test 46 | 0.71 | 0.33 | 0.42 | 0.37 |
| 239 | Train 4 | Test 47 | 0.71 | 0.21 | 0.57 | 0.31 | 2287 | Train 36 | Test 47 | 0.71 | 0.21 | 0.57 | 0.31 |
| 240 | Train 4 | Test 48 | 0.71 | 0.33 | 0.4 | 0.36 | 2288 | Train 36 | Test 48 | 0.71 | 0.32 | 0.42 | 0.36 |
| 241 | Train 4 | Test 49 | 0.77 | 0.25 | 0.73 | 0.38 | 2289 | Train 36 | Test 49 | 0.77 | 0.25 | 0.73 | 0.38 |
| 242 | Train 4 | Test 50 | 0.77 | 0.25 | 0.72 | 0.38 | 2290 | Train 36 | Test 50 | 0.77 | 0.25 | 0.73 | 0.38 |
| 243 | Train 4 | Test 51 | 0.77 | 0.25 | 0.73 | 0.38 | 2291 | Train 36 | Test 51 | 0.77 | 0.26 | 0.74 | 0.38 |
| 244 | Train 4 | Test 52 | 0.77 | 0.25 | 0.73 | 0.38 | 2292 | Train 36 | Test 52 | 0.77 | 0.26 | 0.73 | 0.38 |
| 245 | Train 4 | Test 53 | 0.74 | 0.28 | 0.45 | 0.35 | 2293 | Train 36 | Test 53 | 0.74 | 0.28 | 0.46 | 0.35 |
| 246 | Train 4 | Test 54 | 0.72 | 0.38 | 0.39 | 0.38 | 2294 | Train 36 | Test 54 | 0.72 | 0.38 | 0.41 | 0.39 |
| 247 | Train 4 | Test 55 | 0.73 | 0.24 | 0.57 | 0.34 | 2295 | Train 36 | Test 55 | 0.73 | 0.24 | 0.57 | 0.34 |
| 248 | Train 4 | Test 56 | 0.72 | 0.36 | 0.4 | 0.38 | 2296 | Train 36 | Test 56 | 0.72 | 0.36 | 0.42 | 0.39 |
| 249 | Train 4 | Test 57 | 0.78 | 0.26 | 0.73 | 0.38 | 2297 | Train 36 | Test 57 | 0.78 | 0.26 | 0.73 | 0.38 |
| 250 | Train 4 | Test 58 | 0.78 | 0.26 | 0.72 | 0.38 | 2298 | Train 36 | Test 58 | 0.77 | 0.26 | 0.72 | 0.38 |
| 251 | Train 4 | Test 59 | 0.78 | 0.26 | 0.73 | 0.39 | 2299 | Train 36 | Test 59 | 0.78 | 0.26 | 0.73 | 0.39 |
| 252 | Train 4 | Test 60 | 0.78 | 0.26 | 0.72 | 0.39 | 2300 | Train 36 | Test 60 | 0.77 | 0.26 | 0.72 | 0.38 |
| 253 | Train 4 | Test 61 | 0.75 | 0.28 | 0.43 | 0.34 | 2301 | Train 36 | Test 61 | 0.75 | 0.28 | 0.44 | 0.34 |
| 254 | Train 4 | Test 62 | 0.72 | 0.37 | 0.39 | 0.38 | 2302 | Train 36 | Test 62 | 0.72 | 0.37 | 0.41 | 0.39 |
| 255 | Train 4 | Test 63 | 0.75 | 0.29 | 0.43 | 0.34 | 2303 | Train 36 | Test 63 | 0.75 | 0.28 | 0.45 | 0.35 |
| 256 | Train 4 | Test 64 | 0.72 | 0.36 | 0.4 | 0.38 | 2304 | Train 36 | Test 64 | 0.72 | 0.35 | 0.42 | 0.39 |
| 257 | Train 5 | Test 1 | 0.75 | 0.21 | 0.74 | 0.33 | 2305 | Train 37 | Test 1 | 0.75 | 0.21 | 0.74 | 0.33 |
| 258 | Train 5 | Test 2 | 0.75 | 0.25 | 0.75 | 0.37 | 2306 | Train 37 | Test 2 | 0.75 | 0.24 | 0.76 | 0.37 |
| 259 | Train 5 | Test 3 | 0.75 | 0.22 | 0.74 | 0.33 | 2307 | Train 37 | Test 3 | 0.75 | 0.21 | 0.74 | 0.33 |
| 260 | Train 5 | Test 4 | 0.75 | 0.25 | 0.76 | 0.37 | 2308 | Train 37 | Test 4 | 0.75 | 0.24 | 0.76 | 0.37 |
| 261 | Train 5 | Test 5 | 0.73 | 0.21 | 0.71 | 0.32 | 2309 | Train 37 | Test 5 | 0.73 | 0.21 | 0.71 | 0.32 |
| 262 | Train 5 | Test 6 | 0.7 | 0.29 | 0.59 | 0.39 | 2310 | Train 37 | Test 6 | 0.7 | 0.29 | 0.6 | 0.39 |
| 263 | Train 5 | Test 7 | 0.73 | 0.21 | 0.7 | 0.32 | 2311 | Train 37 | Test 7 | 0.73 | 0.21 | 0.71 | 0.32 |
| 264 | Train 5 | Test 8 | 0.7 | 0.29 | 0.59 | 0.38 | 2312 | Train 37 | Test 8 | 0.7 | 0.28 | 0.6 | 0.38 |
| 265 | Train 5 | Test 9 | 0.75 | 0.22 | 0.71 | 0.33 | 2313 | Train 37 | Test 9 | 0.75 | 0.22 | 0.72 | 0.33 |
| 266 | Train 5 | Test 10 | 0.75 | 0.25 | 0.73 | 0.37 | 2314 | Train 37 | Test 10 | 0.75 | 0.25 | 0.73 | 0.37 |
| 267 | Train 5 | Test 11 | 0.75 | 0.22 | 0.71 | 0.34 | 2315 | Train 37 | Test 11 | 0.75 | 0.22 | 0.72 | 0.33 |
| 268 | Train 5 | Test 12 | 0.75 | 0.25 | 0.73 | 0.37 | 2316 | Train 37 | Test 12 | 0.75 | 0.25 | 0.73 | 0.37 |
| 269 | Train 5 | Test 13 | 0.73 | 0.22 | 0.69 | 0.33 | 2317 | Train 37 | Test 13 | 0.73 | 0.21 | 0.69 | 0.33 |
| 270 | Train 5 | Test 14 | 0.7 | 0.29 | 0.59 | 0.39 | 2318 | Train 37 | Test 14 | 0.7 | 0.29 | 0.6 | 0.39 |
| 271 | Train 5 | Test 15 | 0.73 | 0.22 | 0.68 | 0.33 | 2319 | Train 37 | Test 15 | 0.73 | 0.21 | 0.68 | 0.33 |
| 272 | Train 5 | Test 16 | 0.7 | 0.28 | 0.59 | 0.38 | 2320 | Train 37 | Test 16 | 0.7 | 0.28 | 0.6 | 0.38 |
| 273 | Train 5 | Test 17 | 0.75 | 0.21 | 0.74 | 0.33 | 2321 | Train 37 | Test 17 | 0.75 | 0.21 | 0.74 | 0.33 |
| 274 | Train 5 | Test 18 | 0.75 | 0.25 | 0.75 | 0.37 | 2322 | Train 37 | Test 18 | 0.75 | 0.24 | 0.76 | 0.37 |
| 275 | Train 5 | Test 19 | 0.75 | 0.22 | 0.74 | 0.33 | 2323 | Train 37 | Test 19 | 0.75 | 0.22 | 0.74 | 0.33 |
| 276 | Train 5 | Test 20 | 0.75 | 0.25 | 0.76 | 0.37 | 2324 | Train 37 | Test 20 | 0.75 | 0.24 | 0.76 | 0.37 |
| 277 | Train 5 | Test 21 | 0.73 | 0.21 | 0.71 | 0.32 | 2325 | Train 37 | Test 21 | 0.73 | 0.21 | 0.71 | 0.32 |
| 278 | Train 5 | Test 22 | 0.7 | 0.29 | 0.59 | 0.39 | 2326 | Train 37 | Test 22 | 0.7 | 0.29 | 0.6 | 0.39 |
| 279 | Train 5 | Test 23 | 0.73 | 0.21 | 0.71 | 0.32 | 2327 | Train 37 | Test 23 | 0.73 | 0.21 | 0.71 | 0.32 |
| 280 | Train 5 | Test 24 | 0.7 | 0.28 | 0.59 | 0.38 | 2328 | Train 37 | Test 24 | 0.7 | 0.28 | 0.6 | 0.38 |
| 281 | Train 5 | Test 25 | 0.75 | 0.22 | 0.71 | 0.33 | 2329 | Train 37 | Test 25 | 0.75 | 0.22 | 0.72 | 0.33 |
| 282 | Train 5 | Test 26 | 0.75 | 0.25 | 0.73 | 0.37 | 2330 | Train 37 | Test 26 | 0.75 | 0.25 | 0.73 | 0.37 |
| 283 | Train 5 | Test 27 | 0.75 | 0.22 | 0.71 | 0.34 | 2331 | Train 37 | Test 27 | 0.75 | 0.22 | 0.72 | 0.33 |
| 284 | Train 5 | Test 28 | 0.75 | 0.25 | 0.73 | 0.37 | 2332 | Train 37 | Test 28 | 0.75 | 0.25 | 0.74 | 0.37 |
| 285 | Train 5 | Test 29 | 0.73 | 0.22 | 0.69 | 0.33 | 2333 | Train 37 | Test 29 | 0.73 | 0.21 | 0.69 | 0.33 |
| 286 | Train 5 | Test 30 | 0.71 | 0.29 | 0.59 | 0.39 | 2334 | Train 37 | Test 30 | 0.7 | 0.29 | 0.6 | 0.39 |
| 287 | Train 5 | Test 31 | 0.73 | 0.22 | 0.68 | 0.33 | 2335 | Train 37 | Test 31 | 0.73 | 0.21 | 0.68 | 0.33 |
| 288 | Train 5 | Test 32 | 0.71 | 0.28 | 0.59 | 0.38 | 2336 | Train 37 | Test 32 | 0.71 | 0.28 | 0.6 | 0.38 |
| 289 | Train 5 | Test 33 | 0.75 | 0.21 | 0.74 | 0.33 | 2337 | Train 37 | Test 33 | 0.75 | 0.21 | 0.74 | 0.33 |
| 290 | Train 5 | Test 34 | 0.75 | 0.24 | 0.75 | 0.37 | 2338 | Train 37 | Test 34 | 0.75 | 0.24 | 0.76 | 0.37 |
| 291 | Train 5 | Test 35 | 0.75 | 0.21 | 0.74 | 0.33 | 2339 | Train 37 | Test 35 | 0.75 | 0.21 | 0.74 | 0.33 |
| 292 | Train 5 | Test 36 | 0.75 | 0.24 | 0.75 | 0.37 | 2340 | Train 37 | Test 36 | 0.75 | 0.24 | 0.76 | 0.37 |
| 293 | Train 5 | Test 37 | 0.73 | 0.21 | 0.71 | 0.32 | 2341 | Train 37 | Test 37 | 0.73 | 0.21 | 0.71 | 0.32 |
| 294 | Train 5 | Test 38 | 0.7 | 0.29 | 0.59 | 0.39 | 2342 | Train 37 | Test 38 | 0.7 | 0.29 | 0.6 | 0.39 |
| 295 | Train 5 | Test 39 | 0.73 | 0.21 | 0.7 | 0.32 | 2343 | Train 37 | Test 39 | 0.73 | 0.21 | 0.7 | 0.32 |
| 296 | Train 5 | Test 40 | 0.7 | 0.28 | 0.59 | 0.38 | 2344 | Train 37 | Test 40 | 0.7 | 0.28 | 0.6 | 0.38 |
| 297 | Train 5 | Test 41 | 0.75 | 0.22 | 0.71 | 0.33 | 2345 | Train 37 | Test 41 | 0.75 | 0.22 | 0.71 | 0.33 |
| 298 | Train 5 | Test 42 | 0.75 | 0.25 | 0.73 | 0.37 | 2346 | Train 37 | Test 42 | 0.75 | 0.25 | 0.73 | 0.37 |
| 299 | Train 5 | Test 43 | 0.75 | 0.22 | 0.71 | 0.33 | 2347 | Train 37 | Test 43 | 0.75 | 0.22 | 0.71 | 0.33 |
| 300 | Train 5 | Test 44 | 0.75 | 0.25 | 0.73 | 0.37 | 2348 | Train 37 | Test 44 | 0.75 | 0.25 | 0.73 | 0.37 |
| 301 | Train 5 | Test 45 | 0.74 | 0.22 | 0.69 | 0.33 | 2349 | Train 37 | Test 45 | 0.73 | 0.21 | 0.69 | 0.33 |
| 302 | Train 5 | Test 46 | 0.71 | 0.29 | 0.59 | 0.39 | 2350 | Train 37 | Test 46 | 0.7 | 0.29 | 0.6 | 0.39 |
| 303 | Train 5 | Test 47 | 0.73 | 0.22 | 0.68 | 0.33 | 2351 | Train 37 | Test 47 | 0.73 | 0.21 | 0.68 | 0.33 |
| 304 | Train 5 | Test 48 | 0.71 | 0.28 | 0.59 | 0.38 | 2352 | Train 37 | Test 48 | 0.71 | 0.28 | 0.6 | 0.38 |
| 305 | Train 5 | Test 49 | 0.77 | 0.25 | 0.77 | 0.37 | 2353 | Train 37 | Test 49 | 0.77 | 0.25 | 0.77 | 0.37 |
| 306 | Train 5 | Test 50 | 0.76 | 0.25 | 0.77 | 0.37 | 2354 | Train 37 | Test 50 | 0.76 | 0.25 | 0.77 | 0.37 |
| 307 | Train 5 | Test 51 | 0.77 | 0.25 | 0.77 | 0.37 | 2355 | Train 37 | Test 51 | 0.77 | 0.25 | 0.77 | 0.37 |
| 308 | Train 5 | Test 52 | 0.76 | 0.25 | 0.77 | 0.38 | 2356 | Train 37 | Test 52 | 0.76 | 0.25 | 0.77 | 0.38 |
| 309 | Train 5 | Test 53 | 0.76 | 0.25 | 0.73 | 0.38 | 2357 | Train 37 | Test 53 | 0.76 | 0.25 | 0.73 | 0.37 |
| 310 | Train 5 | Test 54 | 0.72 | 0.33 | 0.59 | 0.43 | 2358 | Train 37 | Test 54 | 0.72 | 0.33 | 0.6 | 0.43 |
| 311 | Train 5 | Test 55 | 0.76 | 0.25 | 0.74 | 0.37 | 2359 | Train 37 | Test 55 | 0.76 | 0.24 | 0.74 | 0.37 |
| 312 | Train 5 | Test 56 | 0.71 | 0.32 | 0.59 | 0.42 | 2360 | Train 37 | Test 56 | 0.72 | 0.32 | 0.6 | 0.42 |
| 313 | Train 5 | Test 57 | 0.77 | 0.25 | 0.76 | 0.38 | 2361 | Train 37 | Test 57 | 0.77 | 0.25 | 0.76 | 0.38 |
| 314 | Train 5 | Test 58 | 0.77 | 0.25 | 0.76 | 0.38 | 2362 | Train 37 | Test 58 | 0.77 | 0.25 | 0.76 | 0.38 |
| 315 | Train 5 | Test 59 | 0.77 | 0.25 | 0.76 | 0.38 | 2363 | Train 37 | Test 59 | 0.77 | 0.25 | 0.76 | 0.38 |
| 316 | Train 5 | Test 60 | 0.77 | 0.25 | 0.76 | 0.38 | 2364 | Train 37 | Test 60 | 0.77 | 0.25 | 0.76 | 0.38 |
| 317 | Train 5 | Test 61 | 0.76 | 0.26 | 0.72 | 0.38 | 2365 | Train 37 | Test 61 | 0.76 | 0.25 | 0.73 | 0.38 |
| 318 | Train 5 | Test 62 | 0.72 | 0.33 | 0.59 | 0.42 | 2366 | Train 37 | Test 62 | 0.72 | 0.33 | 0.6 | 0.42 |
| 319 | Train 5 | Test 63 | 0.77 | 0.26 | 0.73 | 0.38 | 2367 | Train 37 | Test 63 | 0.76 | 0.25 | 0.73 | 0.38 |
| 320 | Train 5 | Test 64 | 0.72 | 0.32 | 0.6 | 0.41 | 2368 | Train 37 | Test 64 | 0.72 | 0.31 | 0.6 | 0.41 |
| 321 | Train 6 | Test 1 | 0.73 | 0.21 | 0.74 | 0.32 | 2369 | Train 38 | Test 1 | 0.73 | 0.21 | 0.74 | 0.32 |
| 322 | Train 6 | Test 2 | 0.75 | 0.23 | 0.78 | 0.36 | 2370 | Train 38 | Test 2 | 0.75 | 0.23 | 0.78 | 0.36 |
| 323 | Train 6 | Test 3 | 0.73 | 0.21 | 0.74 | 0.32 | 2371 | Train 38 | Test 3 | 0.73 | 0.21 | 0.74 | 0.32 |
| 324 | Train 6 | Test 4 | 0.75 | 0.24 | 0.78 | 0.36 | 2372 | Train 38 | Test 4 | 0.75 | 0.23 | 0.78 | 0.36 |
| 325 | Train 6 | Test 5 | 0.72 | 0.2 | 0.71 | 0.31 | 2373 | Train 38 | Test 5 | 0.72 | 0.2 | 0.71 | 0.31 |
| 326 | Train 6 | Test 6 | 0.74 | 0.29 | 0.7 | 0.41 | 2374 | Train 38 | Test 6 | 0.74 | 0.29 | 0.7 | 0.41 |
| 327 | Train 6 | Test 7 | 0.72 | 0.2 | 0.7 | 0.31 | 2375 | Train 38 | Test 7 | 0.72 | 0.2 | 0.7 | 0.31 |
| 328 | Train 6 | Test 8 | 0.74 | 0.28 | 0.7 | 0.4 | 2376 | Train 38 | Test 8 | 0.74 | 0.28 | 0.7 | 0.4 |
| 329 | Train 6 | Test 9 | 0.73 | 0.21 | 0.71 | 0.32 | 2377 | Train 38 | Test 9 | 0.73 | 0.21 | 0.71 | 0.32 |
| 330 | Train 6 | Test 10 | 0.75 | 0.24 | 0.76 | 0.36 | 2378 | Train 38 | Test 10 | 0.75 | 0.24 | 0.76 | 0.36 |
| 331 | Train 6 | Test 11 | 0.73 | 0.21 | 0.72 | 0.32 | 2379 | Train 38 | Test 11 | 0.73 | 0.21 | 0.72 | 0.32 |
| 332 | Train 6 | Test 12 | 0.75 | 0.24 | 0.76 | 0.36 | 2380 | Train 38 | Test 12 | 0.75 | 0.24 | 0.76 | 0.36 |
| 333 | Train 6 | Test 13 | 0.72 | 0.2 | 0.68 | 0.31 | 2381 | Train 38 | Test 13 | 0.72 | 0.2 | 0.68 | 0.31 |
| 334 | Train 6 | Test 14 | 0.74 | 0.29 | 0.7 | 0.41 | 2382 | Train 38 | Test 14 | 0.74 | 0.28 | 0.71 | 0.41 |
| 335 | Train 6 | Test 15 | 0.72 | 0.2 | 0.68 | 0.31 | 2383 | Train 38 | Test 15 | 0.72 | 0.2 | 0.68 | 0.31 |
| 336 | Train 6 | Test 16 | 0.74 | 0.28 | 0.7 | 0.4 | 2384 | Train 38 | Test 16 | 0.74 | 0.28 | 0.71 | 0.4 |
| 337 | Train 6 | Test 17 | 0.73 | 0.21 | 0.74 | 0.32 | 2385 | Train 38 | Test 17 | 0.73 | 0.21 | 0.74 | 0.32 |
| 338 | Train 6 | Test 18 | 0.75 | 0.23 | 0.78 | 0.36 | 2386 | Train 38 | Test 18 | 0.75 | 0.23 | 0.78 | 0.36 |
| 339 | Train 6 | Test 19 | 0.73 | 0.21 | 0.74 | 0.32 | 2387 | Train 38 | Test 19 | 0.73 | 0.21 | 0.74 | 0.32 |
| 340 | Train 6 | Test 20 | 0.75 | 0.24 | 0.78 | 0.36 | 2388 | Train 38 | Test 20 | 0.75 | 0.23 | 0.78 | 0.36 |
| 341 | Train 6 | Test 21 | 0.72 | 0.2 | 0.71 | 0.31 | 2389 | Train 38 | Test 21 | 0.72 | 0.2 | 0.71 | 0.31 |
| 342 | Train 6 | Test 22 | 0.74 | 0.29 | 0.7 | 0.41 | 2390 | Train 38 | Test 22 | 0.74 | 0.29 | 0.7 | 0.41 |
| 343 | Train 6 | Test 23 | 0.72 | 0.2 | 0.71 | 0.31 | 2391 | Train 38 | Test 23 | 0.72 | 0.2 | 0.71 | 0.31 |
| 344 | Train 6 | Test 24 | 0.74 | 0.28 | 0.7 | 0.4 | 2392 | Train 38 | Test 24 | 0.74 | 0.28 | 0.7 | 0.4 |
| 345 | Train 6 | Test 25 | 0.73 | 0.21 | 0.72 | 0.32 | 2393 | Train 38 | Test 25 | 0.73 | 0.21 | 0.71 | 0.32 |
| 346 | Train 6 | Test 26 | 0.75 | 0.24 | 0.76 | 0.36 | 2394 | Train 38 | Test 26 | 0.75 | 0.24 | 0.76 | 0.36 |
| 347 | Train 6 | Test 27 | 0.73 | 0.21 | 0.72 | 0.32 | 2395 | Train 38 | Test 27 | 0.73 | 0.21 | 0.72 | 0.32 |
| 348 | Train 6 | Test 28 | 0.75 | 0.24 | 0.76 | 0.36 | 2396 | Train 38 | Test 28 | 0.75 | 0.24 | 0.76 | 0.36 |
| 349 | Train 6 | Test 29 | 0.72 | 0.2 | 0.68 | 0.31 | 2397 | Train 38 | Test 29 | 0.72 | 0.2 | 0.68 | 0.31 |
| 350 | Train 6 | Test 30 | 0.74 | 0.28 | 0.7 | 0.41 | 2398 | Train 38 | Test 30 | 0.74 | 0.28 | 0.71 | 0.4 |
| 351 | Train 6 | Test 31 | 0.72 | 0.2 | 0.68 | 0.31 | 2399 | Train 38 | Test 31 | 0.72 | 0.2 | 0.68 | 0.31 |
| 352 | Train 6 | Test 32 | 0.74 | 0.28 | 0.7 | 0.4 | 2400 | Train 38 | Test 32 | 0.74 | 0.28 | 0.71 | 0.4 |
| 353 | Train 6 | Test 33 | 0.74 | 0.21 | 0.74 | 0.32 | 2401 | Train 38 | Test 33 | 0.73 | 0.2 | 0.73 | 0.32 |
| 354 | Train 6 | Test 34 | 0.75 | 0.23 | 0.78 | 0.36 | 2402 | Train 38 | Test 34 | 0.75 | 0.23 | 0.78 | 0.36 |
| 355 | Train 6 | Test 35 | 0.74 | 0.21 | 0.74 | 0.32 | 2403 | Train 38 | Test 35 | 0.74 | 0.21 | 0.74 | 0.32 |
| 356 | Train 6 | Test 36 | 0.75 | 0.23 | 0.78 | 0.36 | 2404 | Train 38 | Test 36 | 0.75 | 0.23 | 0.78 | 0.36 |
| 357 | Train 6 | Test 37 | 0.72 | 0.2 | 0.71 | 0.31 | 2405 | Train 38 | Test 37 | 0.72 | 0.2 | 0.7 | 0.31 |
| 358 | Train 6 | Test 38 | 0.74 | 0.29 | 0.7 | 0.41 | 2406 | Train 38 | Test 38 | 0.74 | 0.29 | 0.7 | 0.41 |
| 359 | Train 6 | Test 39 | 0.72 | 0.2 | 0.7 | 0.31 | 2407 | Train 38 | Test 39 | 0.72 | 0.2 | 0.7 | 0.31 |
| 360 | Train 6 | Test 40 | 0.74 | 0.28 | 0.7 | 0.4 | 2408 | Train 38 | Test 40 | 0.74 | 0.28 | 0.7 | 0.4 |
| 361 | Train 6 | Test 41 | 0.74 | 0.21 | 0.71 | 0.32 | 2409 | Train 38 | Test 41 | 0.74 | 0.21 | 0.71 | 0.32 |
| 362 | Train 6 | Test 42 | 0.75 | 0.24 | 0.76 | 0.36 | 2410 | Train 38 | Test 42 | 0.75 | 0.24 | 0.76 | 0.36 |
| 363 | Train 6 | Test 43 | 0.74 | 0.21 | 0.71 | 0.32 | 2411 | Train 38 | Test 43 | 0.74 | 0.21 | 0.71 | 0.32 |
| 364 | Train 6 | Test 44 | 0.75 | 0.24 | 0.76 | 0.36 | 2412 | Train 38 | Test 44 | 0.75 | 0.24 | 0.76 | 0.36 |
| 365 | Train 6 | Test 45 | 0.72 | 0.2 | 0.68 | 0.31 | 2413 | Train 38 | Test 45 | 0.72 | 0.2 | 0.68 | 0.31 |
| 366 | Train 6 | Test 46 | 0.74 | 0.28 | 0.7 | 0.41 | 2414 | Train 38 | Test 46 | 0.74 | 0.28 | 0.7 | 0.4 |
| 367 | Train 6 | Test 47 | 0.72 | 0.2 | 0.68 | 0.31 | 2415 | Train 38 | Test 47 | 0.72 | 0.2 | 0.68 | 0.31 |
| 368 | Train 6 | Test 48 | 0.74 | 0.28 | 0.7 | 0.4 | 2416 | Train 38 | Test 48 | 0.74 | 0.28 | 0.7 | 0.4 |
| 369 | Train 6 | Test 49 | 0.77 | 0.26 | 0.74 | 0.39 | 2417 | Train 38 | Test 49 | 0.77 | 0.26 | 0.74 | 0.39 |
| 370 | Train 6 | Test 50 | 0.77 | 0.26 | 0.74 | 0.39 | 2418 | Train 38 | Test 50 | 0.77 | 0.26 | 0.74 | 0.39 |
| 371 | Train 6 | Test 51 | 0.77 | 0.26 | 0.75 | 0.39 | 2419 | Train 38 | Test 51 | 0.77 | 0.26 | 0.75 | 0.39 |
| 372 | Train 6 | Test 52 | 0.76 | 0.26 | 0.75 | 0.39 | 2420 | Train 38 | Test 52 | 0.76 | 0.26 | 0.74 | 0.39 |
| 373 | Train 6 | Test 53 | 0.76 | 0.26 | 0.66 | 0.38 | 2421 | Train 38 | Test 53 | 0.76 | 0.26 | 0.66 | 0.37 |
| 374 | Train 6 | Test 54 | 0.75 | 0.33 | 0.7 | 0.45 | 2422 | Train 38 | Test 54 | 0.75 | 0.33 | 0.7 | 0.45 |
| 375 | Train 6 | Test 55 | 0.75 | 0.25 | 0.7 | 0.37 | 2423 | Train 38 | Test 55 | 0.75 | 0.25 | 0.7 | 0.37 |
| 376 | Train 6 | Test 56 | 0.74 | 0.32 | 0.71 | 0.44 | 2424 | Train 38 | Test 56 | 0.74 | 0.32 | 0.71 | 0.44 |
| 377 | Train 6 | Test 57 | 0.77 | 0.27 | 0.74 | 0.39 | 2425 | Train 38 | Test 57 | 0.77 | 0.26 | 0.73 | 0.39 |
| 378 | Train 6 | Test 58 | 0.77 | 0.27 | 0.73 | 0.39 | 2426 | Train 38 | Test 58 | 0.77 | 0.27 | 0.73 | 0.39 |
| 379 | Train 6 | Test 59 | 0.77 | 0.27 | 0.74 | 0.39 | 2427 | Train 38 | Test 59 | 0.77 | 0.27 | 0.74 | 0.39 |
| 380 | Train 6 | Test 60 | 0.77 | 0.27 | 0.74 | 0.39 | 2428 | Train 38 | Test 60 | 0.77 | 0.27 | 0.73 | 0.39 |
| 381 | Train 6 | Test 61 | 0.76 | 0.27 | 0.65 | 0.38 | 2429 | Train 38 | Test 61 | 0.76 | 0.26 | 0.65 | 0.38 |
| 382 | Train 6 | Test 62 | 0.75 | 0.33 | 0.7 | 0.45 | 2430 | Train 38 | Test 62 | 0.75 | 0.33 | 0.7 | 0.45 |
| 383 | Train 6 | Test 63 | 0.76 | 0.27 | 0.65 | 0.38 | 2431 | Train 38 | Test 63 | 0.76 | 0.26 | 0.65 | 0.38 |
| 384 | Train 6 | Test 64 | 0.74 | 0.31 | 0.71 | 0.43 | 2432 | Train 38 | Test 64 | 0.74 | 0.31 | 0.71 | 0.43 |
| 385 | Train 7 | Test 1 | 0.75 | 0.22 | 0.74 | 0.33 | 2433 | Train 39 | Test 1 | 0.75 | 0.21 | 0.74 | 0.33 |
| 386 | Train 7 | Test 2 | 0.75 | 0.25 | 0.76 | 0.37 | 2434 | Train 39 | Test 2 | 0.75 | 0.24 | 0.76 | 0.37 |
| 387 | Train 7 | Test 3 | 0.75 | 0.22 | 0.74 | 0.34 | 2435 | Train 39 | Test 3 | 0.75 | 0.22 | 0.74 | 0.33 |
| 388 | Train 7 | Test 4 | 0.75 | 0.25 | 0.76 | 0.37 | 2436 | Train 39 | Test 4 | 0.75 | 0.25 | 0.76 | 0.37 |
| 389 | Train 7 | Test 5 | 0.73 | 0.21 | 0.71 | 0.32 | 2437 | Train 39 | Test 5 | 0.73 | 0.21 | 0.71 | 0.32 |
| 390 | Train 7 | Test 6 | 0.7 | 0.29 | 0.57 | 0.39 | 2438 | Train 39 | Test 6 | 0.7 | 0.29 | 0.58 | 0.39 |
| 391 | Train 7 | Test 7 | 0.73 | 0.21 | 0.71 | 0.32 | 2439 | Train 39 | Test 7 | 0.73 | 0.21 | 0.71 | 0.32 |
| 392 | Train 7 | Test 8 | 0.7 | 0.29 | 0.57 | 0.38 | 2440 | Train 39 | Test 8 | 0.7 | 0.29 | 0.58 | 0.38 |
| 393 | Train 7 | Test 9 | 0.75 | 0.22 | 0.71 | 0.33 | 2441 | Train 39 | Test 9 | 0.75 | 0.22 | 0.72 | 0.33 |
| 394 | Train 7 | Test 10 | 0.75 | 0.25 | 0.73 | 0.37 | 2442 | Train 39 | Test 10 | 0.75 | 0.25 | 0.74 | 0.37 |
| 395 | Train 7 | Test 11 | 0.75 | 0.22 | 0.72 | 0.34 | 2443 | Train 39 | Test 11 | 0.75 | 0.22 | 0.72 | 0.34 |
| 396 | Train 7 | Test 12 | 0.75 | 0.25 | 0.73 | 0.37 | 2444 | Train 39 | Test 12 | 0.75 | 0.25 | 0.74 | 0.37 |
| 397 | Train 7 | Test 13 | 0.73 | 0.21 | 0.69 | 0.33 | 2445 | Train 39 | Test 13 | 0.73 | 0.21 | 0.69 | 0.33 |
| 398 | Train 7 | Test 14 | 0.7 | 0.29 | 0.57 | 0.39 | 2446 | Train 39 | Test 14 | 0.7 | 0.29 | 0.58 | 0.39 |
| 399 | Train 7 | Test 15 | 0.73 | 0.21 | 0.68 | 0.33 | 2447 | Train 39 | Test 15 | 0.73 | 0.21 | 0.69 | 0.33 |
| 400 | Train 7 | Test 16 | 0.7 | 0.29 | 0.57 | 0.38 | 2448 | Train 39 | Test 16 | 0.7 | 0.28 | 0.59 | 0.38 |
| 401 | Train 7 | Test 17 | 0.75 | 0.22 | 0.74 | 0.33 | 2449 | Train 39 | Test 17 | 0.75 | 0.21 | 0.74 | 0.33 |
| 402 | Train 7 | Test 18 | 0.75 | 0.25 | 0.76 | 0.37 | 2450 | Train 39 | Test 18 | 0.75 | 0.24 | 0.76 | 0.37 |
| 403 | Train 7 | Test 19 | 0.75 | 0.22 | 0.74 | 0.34 | 2451 | Train 39 | Test 19 | 0.75 | 0.22 | 0.74 | 0.33 |
| 404 | Train 7 | Test 20 | 0.75 | 0.25 | 0.76 | 0.37 | 2452 | Train 39 | Test 20 | 0.75 | 0.25 | 0.76 | 0.37 |
| 405 | Train 7 | Test 21 | 0.73 | 0.21 | 0.71 | 0.32 | 2453 | Train 39 | Test 21 | 0.73 | 0.21 | 0.71 | 0.32 |
| 406 | Train 7 | Test 22 | 0.7 | 0.29 | 0.57 | 0.39 | 2454 | Train 39 | Test 22 | 0.7 | 0.29 | 0.58 | 0.39 |
| 407 | Train 7 | Test 23 | 0.73 | 0.21 | 0.71 | 0.32 | 2455 | Train 39 | Test 23 | 0.73 | 0.21 | 0.71 | 0.32 |
| 408 | Train 7 | Test 24 | 0.7 | 0.29 | 0.57 | 0.38 | 2456 | Train 39 | Test 24 | 0.7 | 0.29 | 0.58 | 0.38 |
| 409 | Train 7 | Test 25 | 0.75 | 0.22 | 0.71 | 0.33 | 2457 | Train 39 | Test 25 | 0.75 | 0.22 | 0.72 | 0.33 |
| 410 | Train 7 | Test 26 | 0.75 | 0.25 | 0.73 | 0.37 | 2458 | Train 39 | Test 26 | 0.75 | 0.25 | 0.74 | 0.37 |
| 411 | Train 7 | Test 27 | 0.75 | 0.22 | 0.72 | 0.34 | 2459 | Train 39 | Test 27 | 0.75 | 0.22 | 0.72 | 0.34 |
| 412 | Train 7 | Test 28 | 0.75 | 0.25 | 0.73 | 0.37 | 2460 | Train 39 | Test 28 | 0.75 | 0.25 | 0.74 | 0.37 |
| 413 | Train 7 | Test 29 | 0.73 | 0.21 | 0.69 | 0.33 | 2461 | Train 39 | Test 29 | 0.73 | 0.21 | 0.69 | 0.33 |
| 414 | Train 7 | Test 30 | 0.7 | 0.29 | 0.57 | 0.39 | 2462 | Train 39 | Test 30 | 0.7 | 0.29 | 0.58 | 0.39 |
| 415 | Train 7 | Test 31 | 0.73 | 0.21 | 0.68 | 0.33 | 2463 | Train 39 | Test 31 | 0.73 | 0.21 | 0.69 | 0.33 |
| 416 | Train 7 | Test 32 | 0.7 | 0.29 | 0.57 | 0.38 | 2464 | Train 39 | Test 32 | 0.7 | 0.28 | 0.58 | 0.38 |
| 417 | Train 7 | Test 33 | 0.75 | 0.21 | 0.73 | 0.33 | 2465 | Train 39 | Test 33 | 0.75 | 0.21 | 0.74 | 0.33 |
| 418 | Train 7 | Test 34 | 0.75 | 0.25 | 0.75 | 0.37 | 2466 | Train 39 | Test 34 | 0.75 | 0.24 | 0.76 | 0.37 |
| 419 | Train 7 | Test 35 | 0.75 | 0.22 | 0.74 | 0.33 | 2467 | Train 39 | Test 35 | 0.75 | 0.21 | 0.74 | 0.33 |
| 420 | Train 7 | Test 36 | 0.75 | 0.25 | 0.75 | 0.37 | 2468 | Train 39 | Test 36 | 0.75 | 0.24 | 0.76 | 0.37 |
| 421 | Train 7 | Test 37 | 0.73 | 0.21 | 0.71 | 0.32 | 2469 | Train 39 | Test 37 | 0.73 | 0.21 | 0.71 | 0.32 |
| 422 | Train 7 | Test 38 | 0.7 | 0.29 | 0.57 | 0.39 | 2470 | Train 39 | Test 38 | 0.7 | 0.29 | 0.58 | 0.39 |
| 423 | Train 7 | Test 39 | 0.73 | 0.21 | 0.7 | 0.32 | 2471 | Train 39 | Test 39 | 0.73 | 0.21 | 0.71 | 0.32 |
| 424 | Train 7 | Test 40 | 0.7 | 0.29 | 0.57 | 0.38 | 2472 | Train 39 | Test 40 | 0.7 | 0.29 | 0.58 | 0.38 |
| 425 | Train 7 | Test 41 | 0.75 | 0.22 | 0.71 | 0.33 | 2473 | Train 39 | Test 41 | 0.75 | 0.22 | 0.72 | 0.33 |
| 426 | Train 7 | Test 42 | 0.75 | 0.25 | 0.73 | 0.37 | 2474 | Train 39 | Test 42 | 0.75 | 0.25 | 0.73 | 0.37 |
| 427 | Train 7 | Test 43 | 0.75 | 0.22 | 0.71 | 0.33 | 2475 | Train 39 | Test 43 | 0.75 | 0.22 | 0.72 | 0.33 |
| 428 | Train 7 | Test 44 | 0.75 | 0.25 | 0.73 | 0.37 | 2476 | Train 39 | Test 44 | 0.75 | 0.25 | 0.73 | 0.37 |
| 429 | Train 7 | Test 45 | 0.74 | 0.21 | 0.69 | 0.33 | 2477 | Train 39 | Test 45 | 0.73 | 0.21 | 0.69 | 0.33 |
| 430 | Train 7 | Test 46 | 0.7 | 0.29 | 0.57 | 0.39 | 2478 | Train 39 | Test 46 | 0.7 | 0.29 | 0.58 | 0.39 |
| 431 | Train 7 | Test 47 | 0.73 | 0.21 | 0.68 | 0.33 | 2479 | Train 39 | Test 47 | 0.73 | 0.21 | 0.68 | 0.33 |
| 432 | Train 7 | Test 48 | 0.7 | 0.29 | 0.57 | 0.38 | 2480 | Train 39 | Test 48 | 0.7 | 0.28 | 0.58 | 0.38 |
| 433 | Train 7 | Test 49 | 0.77 | 0.25 | 0.77 | 0.37 | 2481 | Train 39 | Test 49 | 0.77 | 0.24 | 0.77 | 0.37 |
| 434 | Train 7 | Test 50 | 0.76 | 0.25 | 0.77 | 0.37 | 2482 | Train 39 | Test 50 | 0.76 | 0.24 | 0.77 | 0.37 |
| 435 | Train 7 | Test 51 | 0.77 | 0.25 | 0.78 | 0.38 | 2483 | Train 39 | Test 51 | 0.77 | 0.25 | 0.78 | 0.37 |
| 436 | Train 7 | Test 52 | 0.76 | 0.25 | 0.78 | 0.38 | 2484 | Train 39 | Test 52 | 0.76 | 0.25 | 0.77 | 0.37 |
| 437 | Train 7 | Test 53 | 0.76 | 0.26 | 0.72 | 0.38 | 2485 | Train 39 | Test 53 | 0.76 | 0.25 | 0.73 | 0.38 |
| 438 | Train 7 | Test 54 | 0.72 | 0.34 | 0.57 | 0.42 | 2486 | Train 39 | Test 54 | 0.72 | 0.33 | 0.58 | 0.42 |
| 439 | Train 7 | Test 55 | 0.76 | 0.24 | 0.74 | 0.37 | 2487 | Train 39 | Test 55 | 0.76 | 0.24 | 0.74 | 0.36 |
| 440 | Train 7 | Test 56 | 0.71 | 0.32 | 0.58 | 0.41 | 2488 | Train 39 | Test 56 | 0.72 | 0.32 | 0.59 | 0.41 |
| 441 | Train 7 | Test 57 | 0.77 | 0.25 | 0.76 | 0.38 | 2489 | Train 39 | Test 57 | 0.77 | 0.25 | 0.76 | 0.38 |
| 442 | Train 7 | Test 58 | 0.77 | 0.25 | 0.76 | 0.38 | 2490 | Train 39 | Test 58 | 0.77 | 0.25 | 0.76 | 0.38 |
| 443 | Train 7 | Test 59 | 0.77 | 0.25 | 0.76 | 0.38 | 2491 | Train 39 | Test 59 | 0.77 | 0.25 | 0.77 | 0.38 |
| 444 | Train 7 | Test 60 | 0.77 | 0.25 | 0.76 | 0.38 | 2492 | Train 39 | Test 60 | 0.77 | 0.25 | 0.77 | 0.38 |
| 445 | Train 7 | Test 61 | 0.76 | 0.26 | 0.71 | 0.38 | 2493 | Train 39 | Test 61 | 0.76 | 0.26 | 0.72 | 0.38 |
| 446 | Train 7 | Test 62 | 0.72 | 0.33 | 0.57 | 0.42 | 2494 | Train 39 | Test 62 | 0.72 | 0.33 | 0.58 | 0.42 |
| 447 | Train 7 | Test 63 | 0.77 | 0.26 | 0.72 | 0.38 | 2495 | Train 39 | Test 63 | 0.77 | 0.26 | 0.72 | 0.38 |
| 448 | Train 7 | Test 64 | 0.72 | 0.32 | 0.58 | 0.41 | 2496 | Train 39 | Test 64 | 0.72 | 0.32 | 0.59 | 0.41 |
| 449 | Train 8 | Test 1 | 0.74 | 0.2 | 0.77 | 0.32 | 2497 | Train 40 | Test 1 | 0.74 | 0.2 | 0.77 | 0.32 |
| 450 | Train 8 | Test 2 | 0.75 | 0.23 | 0.8 | 0.36 | 2498 | Train 40 | Test 2 | 0.75 | 0.23 | 0.8 | 0.36 |
| 451 | Train 8 | Test 3 | 0.74 | 0.2 | 0.77 | 0.32 | 2499 | Train 40 | Test 3 | 0.74 | 0.2 | 0.77 | 0.32 |
| 452 | Train 8 | Test 4 | 0.75 | 0.23 | 0.8 | 0.36 | 2500 | Train 40 | Test 4 | 0.75 | 0.23 | 0.8 | 0.36 |
| 453 | Train 8 | Test 5 | 0.72 | 0.2 | 0.74 | 0.31 | 2501 | Train 40 | Test 5 | 0.72 | 0.2 | 0.74 | 0.31 |
| 454 | Train 8 | Test 6 | 0.74 | 0.29 | 0.71 | 0.41 | 2502 | Train 40 | Test 6 | 0.74 | 0.29 | 0.71 | 0.41 |
| 455 | Train 8 | Test 7 | 0.72 | 0.2 | 0.73 | 0.31 | 2503 | Train 40 | Test 7 | 0.72 | 0.2 | 0.74 | 0.31 |
| 456 | Train 8 | Test 8 | 0.74 | 0.28 | 0.71 | 0.4 | 2504 | Train 40 | Test 8 | 0.74 | 0.28 | 0.71 | 0.4 |
| 457 | Train 8 | Test 9 | 0.74 | 0.2 | 0.74 | 0.32 | 2505 | Train 40 | Test 9 | 0.74 | 0.2 | 0.75 | 0.32 |
| 458 | Train 8 | Test 10 | 0.75 | 0.23 | 0.78 | 0.36 | 2506 | Train 40 | Test 10 | 0.75 | 0.23 | 0.79 | 0.36 |
| 459 | Train 8 | Test 11 | 0.74 | 0.2 | 0.75 | 0.32 | 2507 | Train 40 | Test 11 | 0.74 | 0.21 | 0.75 | 0.32 |
| 460 | Train 8 | Test 12 | 0.75 | 0.23 | 0.79 | 0.36 | 2508 | Train 40 | Test 12 | 0.75 | 0.23 | 0.79 | 0.36 |
| 461 | Train 8 | Test 13 | 0.72 | 0.2 | 0.71 | 0.31 | 2509 | Train 40 | Test 13 | 0.72 | 0.2 | 0.71 | 0.31 |
| 462 | Train 8 | Test 14 | 0.74 | 0.29 | 0.71 | 0.41 | 2510 | Train 40 | Test 14 | 0.74 | 0.28 | 0.71 | 0.41 |
| 463 | Train 8 | Test 15 | 0.72 | 0.2 | 0.71 | 0.31 | 2511 | Train 40 | Test 15 | 0.72 | 0.2 | 0.71 | 0.31 |
| 464 | Train 8 | Test 16 | 0.74 | 0.28 | 0.71 | 0.4 | 2512 | Train 40 | Test 16 | 0.74 | 0.28 | 0.71 | 0.4 |
| 465 | Train 8 | Test 17 | 0.74 | 0.2 | 0.77 | 0.32 | 2513 | Train 40 | Test 17 | 0.74 | 0.2 | 0.77 | 0.32 |
| 466 | Train 8 | Test 18 | 0.75 | 0.23 | 0.8 | 0.36 | 2514 | Train 40 | Test 18 | 0.75 | 0.23 | 0.8 | 0.36 |
| 467 | Train 8 | Test 19 | 0.74 | 0.2 | 0.77 | 0.32 | 2515 | Train 40 | Test 19 | 0.74 | 0.2 | 0.77 | 0.32 |
| 468 | Train 8 | Test 20 | 0.75 | 0.23 | 0.8 | 0.36 | 2516 | Train 40 | Test 20 | 0.75 | 0.23 | 0.8 | 0.36 |
| 469 | Train 8 | Test 21 | 0.72 | 0.2 | 0.74 | 0.31 | 2517 | Train 40 | Test 21 | 0.72 | 0.2 | 0.74 | 0.31 |
| 470 | Train 8 | Test 22 | 0.74 | 0.29 | 0.71 | 0.41 | 2518 | Train 40 | Test 22 | 0.74 | 0.29 | 0.71 | 0.41 |
| 471 | Train 8 | Test 23 | 0.72 | 0.2 | 0.73 | 0.31 | 2519 | Train 40 | Test 23 | 0.72 | 0.2 | 0.74 | 0.31 |
| 472 | Train 8 | Test 24 | 0.74 | 0.28 | 0.71 | 0.4 | 2520 | Train 40 | Test 24 | 0.74 | 0.28 | 0.71 | 0.4 |
| 473 | Train 8 | Test 25 | 0.74 | 0.2 | 0.74 | 0.32 | 2521 | Train 40 | Test 25 | 0.74 | 0.2 | 0.75 | 0.32 |
| 474 | Train 8 | Test 26 | 0.75 | 0.23 | 0.78 | 0.36 | 2522 | Train 40 | Test 26 | 0.75 | 0.23 | 0.79 | 0.36 |
| 475 | Train 8 | Test 27 | 0.74 | 0.2 | 0.75 | 0.32 | 2523 | Train 40 | Test 27 | 0.74 | 0.21 | 0.75 | 0.32 |
| 476 | Train 8 | Test 28 | 0.75 | 0.23 | 0.79 | 0.36 | 2524 | Train 40 | Test 28 | 0.75 | 0.23 | 0.79 | 0.36 |
| 477 | Train 8 | Test 29 | 0.72 | 0.2 | 0.71 | 0.31 | 2525 | Train 40 | Test 29 | 0.72 | 0.2 | 0.71 | 0.31 |
| 478 | Train 8 | Test 30 | 0.74 | 0.28 | 0.71 | 0.41 | 2526 | Train 40 | Test 30 | 0.74 | 0.28 | 0.71 | 0.41 |
| 479 | Train 8 | Test 31 | 0.72 | 0.2 | 0.71 | 0.31 | 2527 | Train 40 | Test 31 | 0.72 | 0.2 | 0.71 | 0.31 |
| 480 | Train 8 | Test 32 | 0.74 | 0.28 | 0.71 | 0.4 | 2528 | Train 40 | Test 32 | 0.74 | 0.28 | 0.71 | 0.4 |
| 481 | Train 8 | Test 33 | 0.74 | 0.2 | 0.76 | 0.32 | 2529 | Train 40 | Test 33 | 0.74 | 0.2 | 0.76 | 0.32 |
| 482 | Train 8 | Test 34 | 0.75 | 0.23 | 0.8 | 0.35 | 2530 | Train 40 | Test 34 | 0.75 | 0.23 | 0.8 | 0.36 |
| 483 | Train 8 | Test 35 | 0.74 | 0.2 | 0.76 | 0.32 | 2531 | Train 40 | Test 35 | 0.74 | 0.2 | 0.76 | 0.32 |
| 484 | Train 8 | Test 36 | 0.75 | 0.23 | 0.8 | 0.36 | 2532 | Train 40 | Test 36 | 0.75 | 0.23 | 0.8 | 0.36 |
| 485 | Train 8 | Test 37 | 0.72 | 0.2 | 0.73 | 0.31 | 2533 | Train 40 | Test 37 | 0.72 | 0.2 | 0.74 | 0.31 |
| 486 | Train 8 | Test 38 | 0.74 | 0.29 | 0.71 | 0.41 | 2534 | Train 40 | Test 38 | 0.74 | 0.29 | 0.71 | 0.41 |
| 487 | Train 8 | Test 39 | 0.72 | 0.2 | 0.73 | 0.31 | 2535 | Train 40 | Test 39 | 0.72 | 0.2 | 0.73 | 0.31 |
| 488 | Train 8 | Test 40 | 0.74 | 0.28 | 0.71 | 0.4 | 2536 | Train 40 | Test 40 | 0.74 | 0.28 | 0.71 | 0.4 |
| 489 | Train 8 | Test 41 | 0.74 | 0.2 | 0.74 | 0.32 | 2537 | Train 40 | Test 41 | 0.74 | 0.2 | 0.74 | 0.32 |
| 490 | Train 8 | Test 42 | 0.75 | 0.23 | 0.78 | 0.36 | 2538 | Train 40 | Test 42 | 0.75 | 0.23 | 0.78 | 0.36 |
| 491 | Train 8 | Test 43 | 0.74 | 0.2 | 0.74 | 0.32 | 2539 | Train 40 | Test 43 | 0.74 | 0.2 | 0.74 | 0.32 |
| 492 | Train 8 | Test 44 | 0.75 | 0.23 | 0.78 | 0.36 | 2540 | Train 40 | Test 44 | 0.75 | 0.23 | 0.78 | 0.36 |
| 493 | Train 8 | Test 45 | 0.72 | 0.2 | 0.71 | 0.31 | 2541 | Train 40 | Test 45 | 0.72 | 0.2 | 0.71 | 0.31 |
| 494 | Train 8 | Test 46 | 0.74 | 0.28 | 0.71 | 0.41 | 2542 | Train 40 | Test 46 | 0.74 | 0.28 | 0.71 | 0.41 |
| 495 | Train 8 | Test 47 | 0.72 | 0.2 | 0.71 | 0.31 | 2543 | Train 40 | Test 47 | 0.72 | 0.2 | 0.71 | 0.31 |
| 496 | Train 8 | Test 48 | 0.74 | 0.28 | 0.71 | 0.4 | 2544 | Train 40 | Test 48 | 0.74 | 0.28 | 0.71 | 0.4 |
| 497 | Train 8 | Test 49 | 0.77 | 0.26 | 0.77 | 0.38 | 2545 | Train 40 | Test 49 | 0.77 | 0.26 | 0.77 | 0.39 |
| 498 | Train 8 | Test 50 | 0.77 | 0.26 | 0.77 | 0.39 | 2546 | Train 40 | Test 50 | 0.77 | 0.26 | 0.77 | 0.39 |
| 499 | Train 8 | Test 51 | 0.77 | 0.26 | 0.77 | 0.39 | 2547 | Train 40 | Test 51 | 0.77 | 0.26 | 0.77 | 0.39 |
| 500 | Train 8 | Test 52 | 0.77 | 0.26 | 0.77 | 0.39 | 2548 | Train 40 | Test 52 | 0.77 | 0.26 | 0.77 | 0.39 |
| 501 | Train 8 | Test 53 | 0.76 | 0.26 | 0.66 | 0.37 | 2549 | Train 40 | Test 53 | 0.76 | 0.26 | 0.67 | 0.37 |
| 502 | Train 8 | Test 54 | 0.75 | 0.33 | 0.71 | 0.45 | 2550 | Train 40 | Test 54 | 0.75 | 0.33 | 0.71 | 0.45 |
| 503 | Train 8 | Test 55 | 0.75 | 0.24 | 0.72 | 0.36 | 2551 | Train 40 | Test 55 | 0.75 | 0.24 | 0.72 | 0.36 |
| 504 | Train 8 | Test 56 | 0.74 | 0.32 | 0.71 | 0.44 | 2552 | Train 40 | Test 56 | 0.74 | 0.32 | 0.71 | 0.44 |
| 505 | Train 8 | Test 57 | 0.77 | 0.26 | 0.75 | 0.39 | 2553 | Train 40 | Test 57 | 0.77 | 0.26 | 0.76 | 0.39 |
| 506 | Train 8 | Test 58 | 0.77 | 0.26 | 0.75 | 0.39 | 2554 | Train 40 | Test 58 | 0.77 | 0.26 | 0.75 | 0.39 |
| 507 | Train 8 | Test 59 | 0.77 | 0.26 | 0.76 | 0.39 | 2555 | Train 40 | Test 59 | 0.77 | 0.26 | 0.76 | 0.39 |
| 508 | Train 8 | Test 60 | 0.77 | 0.26 | 0.76 | 0.39 | 2556 | Train 40 | Test 60 | 0.77 | 0.27 | 0.76 | 0.39 |
| 509 | Train 8 | Test 61 | 0.76 | 0.27 | 0.66 | 0.38 | 2557 | Train 40 | Test 61 | 0.76 | 0.26 | 0.66 | 0.38 |
| 510 | Train 8 | Test 62 | 0.75 | 0.33 | 0.71 | 0.45 | 2558 | Train 40 | Test 62 | 0.75 | 0.33 | 0.71 | 0.45 |
| 511 | Train 8 | Test 63 | 0.76 | 0.27 | 0.66 | 0.38 | 2559 | Train 40 | Test 63 | 0.76 | 0.26 | 0.66 | 0.38 |
| 512 | Train 8 | Test 64 | 0.74 | 0.32 | 0.71 | 0.44 | 2560 | Train 40 | Test 64 | 0.74 | 0.31 | 0.71 | 0.44 |
| 513 | Train 9 | Test 1 | 0.76 | 0.21 | 0.73 | 0.33 | 2561 | Train 41 | Test 1 | 0.76 | 0.21 | 0.73 | 0.33 |
| 514 | Train 9 | Test 2 | 0.75 | 0.24 | 0.72 | 0.36 | 2562 | Train 41 | Test 2 | 0.75 | 0.24 | 0.72 | 0.36 |
| 515 | Train 9 | Test 3 | 0.76 | 0.22 | 0.73 | 0.33 | 2563 | Train 41 | Test 3 | 0.76 | 0.21 | 0.74 | 0.33 |
| 516 | Train 9 | Test 4 | 0.75 | 0.24 | 0.72 | 0.37 | 2564 | Train 41 | Test 4 | 0.75 | 0.24 | 0.72 | 0.36 |
| 517 | Train 9 | Test 5 | 0.72 | 0.2 | 0.65 | 0.31 | 2565 | Train 41 | Test 5 | 0.72 | 0.2 | 0.65 | 0.31 |
| 518 | Train 9 | Test 6 | 0.7 | 0.3 | 0.48 | 0.37 | 2566 | Train 41 | Test 6 | 0.7 | 0.3 | 0.5 | 0.37 |
| 519 | Train 9 | Test 7 | 0.71 | 0.2 | 0.65 | 0.31 | 2567 | Train 41 | Test 7 | 0.71 | 0.2 | 0.65 | 0.31 |
| 520 | Train 9 | Test 8 | 0.7 | 0.3 | 0.48 | 0.37 | 2568 | Train 41 | Test 8 | 0.7 | 0.29 | 0.5 | 0.37 |
| 521 | Train 9 | Test 9 | 0.76 | 0.22 | 0.71 | 0.33 | 2569 | Train 41 | Test 9 | 0.76 | 0.22 | 0.71 | 0.33 |
| 522 | Train 9 | Test 10 | 0.75 | 0.25 | 0.7 | 0.37 | 2570 | Train 41 | Test 10 | 0.75 | 0.25 | 0.7 | 0.37 |
| 523 | Train 9 | Test 11 | 0.76 | 0.22 | 0.71 | 0.33 | 2571 | Train 41 | Test 11 | 0.76 | 0.22 | 0.72 | 0.33 |
| 524 | Train 9 | Test 12 | 0.75 | 0.25 | 0.7 | 0.37 | 2572 | Train 41 | Test 12 | 0.75 | 0.25 | 0.7 | 0.37 |
| 525 | Train 9 | Test 13 | 0.71 | 0.2 | 0.62 | 0.31 | 2573 | Train 41 | Test 13 | 0.71 | 0.2 | 0.62 | 0.31 |
| 526 | Train 9 | Test 14 | 0.7 | 0.3 | 0.49 | 0.37 | 2574 | Train 41 | Test 14 | 0.7 | 0.3 | 0.5 | 0.37 |
| 527 | Train 9 | Test 15 | 0.71 | 0.2 | 0.62 | 0.31 | 2575 | Train 41 | Test 15 | 0.71 | 0.2 | 0.62 | 0.31 |
| 528 | Train 9 | Test 16 | 0.7 | 0.29 | 0.48 | 0.37 | 2576 | Train 41 | Test 16 | 0.7 | 0.29 | 0.5 | 0.37 |
| 529 | Train 9 | Test 17 | 0.76 | 0.21 | 0.73 | 0.33 | 2577 | Train 41 | Test 17 | 0.76 | 0.21 | 0.73 | 0.33 |
| 530 | Train 9 | Test 18 | 0.75 | 0.24 | 0.72 | 0.36 | 2578 | Train 41 | Test 18 | 0.75 | 0.24 | 0.72 | 0.36 |
| 531 | Train 9 | Test 19 | 0.76 | 0.22 | 0.73 | 0.33 | 2579 | Train 41 | Test 19 | 0.76 | 0.21 | 0.74 | 0.33 |
| 532 | Train 9 | Test 20 | 0.75 | 0.24 | 0.72 | 0.37 | 2580 | Train 41 | Test 20 | 0.75 | 0.24 | 0.72 | 0.36 |
| 533 | Train 9 | Test 21 | 0.72 | 0.2 | 0.65 | 0.31 | 2581 | Train 41 | Test 21 | 0.72 | 0.2 | 0.65 | 0.31 |
| 534 | Train 9 | Test 22 | 0.7 | 0.3 | 0.48 | 0.37 | 2582 | Train 41 | Test 22 | 0.7 | 0.3 | 0.5 | 0.37 |
| 535 | Train 9 | Test 23 | 0.71 | 0.2 | 0.65 | 0.31 | 2583 | Train 41 | Test 23 | 0.71 | 0.2 | 0.65 | 0.31 |
| 536 | Train 9 | Test 24 | 0.7 | 0.29 | 0.48 | 0.37 | 2584 | Train 41 | Test 24 | 0.7 | 0.29 | 0.5 | 0.37 |
| 537 | Train 9 | Test 25 | 0.76 | 0.22 | 0.71 | 0.33 | 2585 | Train 41 | Test 25 | 0.76 | 0.22 | 0.72 | 0.33 |
| 538 | Train 9 | Test 26 | 0.75 | 0.25 | 0.7 | 0.37 | 2586 | Train 41 | Test 26 | 0.75 | 0.25 | 0.7 | 0.37 |
| 539 | Train 9 | Test 27 | 0.76 | 0.22 | 0.71 | 0.33 | 2587 | Train 41 | Test 27 | 0.76 | 0.22 | 0.72 | 0.33 |
| 540 | Train 9 | Test 28 | 0.75 | 0.25 | 0.7 | 0.37 | 2588 | Train 41 | Test 28 | 0.75 | 0.25 | 0.7 | 0.37 |
| 541 | Train 9 | Test 29 | 0.72 | 0.2 | 0.62 | 0.31 | 2589 | Train 41 | Test 29 | 0.71 | 0.2 | 0.63 | 0.31 |
| 542 | Train 9 | Test 30 | 0.7 | 0.3 | 0.49 | 0.37 | 2590 | Train 41 | Test 30 | 0.7 | 0.3 | 0.5 | 0.37 |
| 543 | Train 9 | Test 31 | 0.71 | 0.2 | 0.62 | 0.31 | 2591 | Train 41 | Test 31 | 0.71 | 0.2 | 0.62 | 0.31 |
| 544 | Train 9 | Test 32 | 0.7 | 0.29 | 0.48 | 0.37 | 2592 | Train 41 | Test 32 | 0.7 | 0.29 | 0.5 | 0.37 |
| 545 | Train 9 | Test 33 | 0.76 | 0.21 | 0.73 | 0.33 | 2593 | Train 41 | Test 33 | 0.76 | 0.21 | 0.73 | 0.33 |
| 546 | Train 9 | Test 34 | 0.75 | 0.24 | 0.72 | 0.36 | 2594 | Train 41 | Test 34 | 0.75 | 0.24 | 0.72 | 0.36 |
| 547 | Train 9 | Test 35 | 0.76 | 0.21 | 0.73 | 0.33 | 2595 | Train 41 | Test 35 | 0.76 | 0.21 | 0.73 | 0.33 |
| 548 | Train 9 | Test 36 | 0.75 | 0.24 | 0.72 | 0.36 | 2596 | Train 41 | Test 36 | 0.75 | 0.24 | 0.72 | 0.36 |
| 549 | Train 9 | Test 37 | 0.72 | 0.2 | 0.65 | 0.31 | 2597 | Train 41 | Test 37 | 0.72 | 0.2 | 0.65 | 0.31 |
| 550 | Train 9 | Test 38 | 0.7 | 0.3 | 0.48 | 0.37 | 2598 | Train 41 | Test 38 | 0.7 | 0.3 | 0.5 | 0.37 |
| 551 | Train 9 | Test 39 | 0.72 | 0.2 | 0.64 | 0.31 | 2599 | Train 41 | Test 39 | 0.72 | 0.2 | 0.65 | 0.31 |
| 552 | Train 9 | Test 40 | 0.7 | 0.29 | 0.48 | 0.37 | 2600 | Train 41 | Test 40 | 0.7 | 0.29 | 0.5 | 0.37 |
| 553 | Train 9 | Test 41 | 0.76 | 0.22 | 0.71 | 0.33 | 2601 | Train 41 | Test 41 | 0.76 | 0.22 | 0.71 | 0.33 |
| 554 | Train 9 | Test 42 | 0.75 | 0.25 | 0.7 | 0.36 | 2602 | Train 41 | Test 42 | 0.75 | 0.25 | 0.7 | 0.36 |
| 555 | Train 9 | Test 43 | 0.76 | 0.22 | 0.71 | 0.33 | 2603 | Train 41 | Test 43 | 0.76 | 0.22 | 0.71 | 0.33 |
| 556 | Train 9 | Test 44 | 0.75 | 0.25 | 0.7 | 0.36 | 2604 | Train 41 | Test 44 | 0.75 | 0.25 | 0.7 | 0.37 |
| 557 | Train 9 | Test 45 | 0.72 | 0.2 | 0.62 | 0.31 | 2605 | Train 41 | Test 45 | 0.72 | 0.2 | 0.62 | 0.31 |
| 558 | Train 9 | Test 46 | 0.7 | 0.3 | 0.49 | 0.37 | 2606 | Train 41 | Test 46 | 0.7 | 0.3 | 0.5 | 0.37 |
| 559 | Train 9 | Test 47 | 0.71 | 0.2 | 0.62 | 0.31 | 2607 | Train 41 | Test 47 | 0.71 | 0.2 | 0.62 | 0.31 |
| 560 | Train 9 | Test 48 | 0.7 | 0.29 | 0.48 | 0.37 | 2608 | Train 41 | Test 48 | 0.7 | 0.29 | 0.5 | 0.37 |
| 561 | Train 9 | Test 49 | 0.78 | 0.24 | 0.79 | 0.37 | 2609 | Train 41 | Test 49 | 0.78 | 0.24 | 0.79 | 0.37 |
| 562 | Train 9 | Test 50 | 0.78 | 0.24 | 0.79 | 0.37 | 2610 | Train 41 | Test 50 | 0.78 | 0.24 | 0.79 | 0.37 |
| 563 | Train 9 | Test 51 | 0.78 | 0.24 | 0.79 | 0.37 | 2611 | Train 41 | Test 51 | 0.78 | 0.24 | 0.79 | 0.37 |
| 564 | Train 9 | Test 52 | 0.78 | 0.24 | 0.79 | 0.37 | 2612 | Train 41 | Test 52 | 0.77 | 0.24 | 0.79 | 0.37 |
| 565 | Train 9 | Test 53 | 0.74 | 0.26 | 0.6 | 0.36 | 2613 | Train 41 | Test 53 | 0.74 | 0.25 | 0.62 | 0.36 |
| 566 | Train 9 | Test 54 | 0.72 | 0.34 | 0.51 | 0.41 | 2614 | Train 41 | Test 54 | 0.72 | 0.34 | 0.52 | 0.41 |
| 567 | Train 9 | Test 55 | 0.74 | 0.23 | 0.65 | 0.34 | 2615 | Train 41 | Test 55 | 0.74 | 0.23 | 0.66 | 0.35 |
| 568 | Train 9 | Test 56 | 0.72 | 0.33 | 0.51 | 0.4 | 2616 | Train 41 | Test 56 | 0.72 | 0.33 | 0.52 | 0.41 |
| 569 | Train 9 | Test 57 | 0.78 | 0.25 | 0.78 | 0.38 | 2617 | Train 41 | Test 57 | 0.78 | 0.25 | 0.78 | 0.38 |
| 570 | Train 9 | Test 58 | 0.78 | 0.25 | 0.78 | 0.38 | 2618 | Train 41 | Test 58 | 0.78 | 0.25 | 0.78 | 0.38 |
| 571 | Train 9 | Test 59 | 0.78 | 0.25 | 0.78 | 0.38 | 2619 | Train 41 | Test 59 | 0.78 | 0.25 | 0.78 | 0.38 |
| 572 | Train 9 | Test 60 | 0.78 | 0.25 | 0.78 | 0.38 | 2620 | Train 41 | Test 60 | 0.78 | 0.25 | 0.78 | 0.38 |
| 573 | Train 9 | Test 61 | 0.74 | 0.26 | 0.59 | 0.36 | 2621 | Train 41 | Test 61 | 0.75 | 0.26 | 0.61 | 0.36 |
| 574 | Train 9 | Test 62 | 0.72 | 0.34 | 0.51 | 0.41 | 2622 | Train 41 | Test 62 | 0.72 | 0.34 | 0.53 | 0.41 |
| 575 | Train 9 | Test 63 | 0.75 | 0.26 | 0.6 | 0.36 | 2623 | Train 41 | Test 63 | 0.75 | 0.26 | 0.61 | 0.36 |
| 576 | Train 9 | Test 64 | 0.72 | 0.33 | 0.51 | 0.4 | 2624 | Train 41 | Test 64 | 0.72 | 0.33 | 0.53 | 0.41 |
| 577 | Train 10 | Test 1 | 0.75 | 0.22 | 0.69 | 0.34 | 2625 | Train 42 | Test 1 | 0.75 | 0.22 | 0.7 | 0.34 |
| 578 | Train 10 | Test 2 | 0.77 | 0.25 | 0.74 | 0.38 | 2626 | Train 42 | Test 2 | 0.77 | 0.25 | 0.75 | 0.38 |
| 579 | Train 10 | Test 3 | 0.75 | 0.22 | 0.69 | 0.34 | 2627 | Train 42 | Test 3 | 0.75 | 0.22 | 0.7 | 0.34 |
| 580 | Train 10 | Test 4 | 0.77 | 0.25 | 0.75 | 0.38 | 2628 | Train 42 | Test 4 | 0.77 | 0.25 | 0.75 | 0.38 |
| 581 | Train 10 | Test 5 | 0.71 | 0.21 | 0.62 | 0.31 | 2629 | Train 42 | Test 5 | 0.71 | 0.2 | 0.62 | 0.31 |
| 582 | Train 10 | Test 6 | 0.71 | 0.33 | 0.43 | 0.37 | 2630 | Train 42 | Test 6 | 0.71 | 0.33 | 0.44 | 0.37 |
| 583 | Train 10 | Test 7 | 0.71 | 0.21 | 0.62 | 0.31 | 2631 | Train 42 | Test 7 | 0.71 | 0.2 | 0.62 | 0.31 |
| 584 | Train 10 | Test 8 | 0.71 | 0.32 | 0.43 | 0.37 | 2632 | Train 42 | Test 8 | 0.71 | 0.32 | 0.44 | 0.37 |
| 585 | Train 10 | Test 9 | 0.75 | 0.23 | 0.66 | 0.34 | 2633 | Train 42 | Test 9 | 0.75 | 0.23 | 0.67 | 0.34 |
| 586 | Train 10 | Test 10 | 0.77 | 0.26 | 0.73 | 0.38 | 2634 | Train 42 | Test 10 | 0.77 | 0.26 | 0.73 | 0.38 |
| 587 | Train 10 | Test 11 | 0.76 | 0.23 | 0.67 | 0.34 | 2635 | Train 42 | Test 11 | 0.75 | 0.23 | 0.67 | 0.34 |
| 588 | Train 10 | Test 12 | 0.77 | 0.26 | 0.73 | 0.38 | 2636 | Train 42 | Test 12 | 0.77 | 0.26 | 0.73 | 0.38 |
| 589 | Train 10 | Test 13 | 0.71 | 0.21 | 0.59 | 0.31 | 2637 | Train 42 | Test 13 | 0.71 | 0.21 | 0.59 | 0.31 |
| 590 | Train 10 | Test 14 | 0.71 | 0.33 | 0.43 | 0.37 | 2638 | Train 42 | Test 14 | 0.71 | 0.32 | 0.44 | 0.37 |
| 591 | Train 10 | Test 15 | 0.71 | 0.21 | 0.59 | 0.31 | 2639 | Train 42 | Test 15 | 0.71 | 0.21 | 0.59 | 0.31 |
| 592 | Train 10 | Test 16 | 0.71 | 0.32 | 0.43 | 0.37 | 2640 | Train 42 | Test 16 | 0.71 | 0.32 | 0.45 | 0.37 |
| 593 | Train 10 | Test 17 | 0.75 | 0.22 | 0.69 | 0.34 | 2641 | Train 42 | Test 17 | 0.75 | 0.22 | 0.7 | 0.34 |
| 594 | Train 10 | Test 18 | 0.77 | 0.25 | 0.74 | 0.38 | 2642 | Train 42 | Test 18 | 0.77 | 0.25 | 0.75 | 0.38 |
| 595 | Train 10 | Test 19 | 0.75 | 0.23 | 0.69 | 0.34 | 2643 | Train 42 | Test 19 | 0.75 | 0.22 | 0.7 | 0.34 |
| 596 | Train 10 | Test 20 | 0.77 | 0.25 | 0.75 | 0.38 | 2644 | Train 42 | Test 20 | 0.77 | 0.25 | 0.75 | 0.38 |
| 597 | Train 10 | Test 21 | 0.71 | 0.21 | 0.62 | 0.31 | 2645 | Train 42 | Test 21 | 0.71 | 0.2 | 0.62 | 0.31 |
| 598 | Train 10 | Test 22 | 0.71 | 0.33 | 0.43 | 0.37 | 2646 | Train 42 | Test 22 | 0.71 | 0.33 | 0.44 | 0.37 |
| 599 | Train 10 | Test 23 | 0.71 | 0.21 | 0.62 | 0.31 | 2647 | Train 42 | Test 23 | 0.71 | 0.21 | 0.62 | 0.31 |
| 600 | Train 10 | Test 24 | 0.71 | 0.32 | 0.43 | 0.37 | 2648 | Train 42 | Test 24 | 0.71 | 0.32 | 0.44 | 0.37 |
| 601 | Train 10 | Test 25 | 0.76 | 0.23 | 0.66 | 0.34 | 2649 | Train 42 | Test 25 | 0.75 | 0.23 | 0.67 | 0.34 |
| 602 | Train 10 | Test 26 | 0.77 | 0.26 | 0.73 | 0.38 | 2650 | Train 42 | Test 26 | 0.77 | 0.26 | 0.73 | 0.38 |
| 603 | Train 10 | Test 27 | 0.76 | 0.23 | 0.67 | 0.34 | 2651 | Train 42 | Test 27 | 0.75 | 0.23 | 0.67 | 0.34 |
| 604 | Train 10 | Test 28 | 0.77 | 0.26 | 0.73 | 0.38 | 2652 | Train 42 | Test 28 | 0.77 | 0.26 | 0.73 | 0.38 |
| 605 | Train 10 | Test 29 | 0.71 | 0.21 | 0.59 | 0.31 | 2653 | Train 42 | Test 29 | 0.71 | 0.21 | 0.59 | 0.31 |
| 606 | Train 10 | Test 30 | 0.71 | 0.33 | 0.43 | 0.37 | 2654 | Train 42 | Test 30 | 0.71 | 0.32 | 0.44 | 0.37 |
| 607 | Train 10 | Test 31 | 0.71 | 0.21 | 0.59 | 0.31 | 2655 | Train 42 | Test 31 | 0.71 | 0.21 | 0.59 | 0.31 |
| 608 | Train 10 | Test 32 | 0.71 | 0.32 | 0.43 | 0.37 | 2656 | Train 42 | Test 32 | 0.71 | 0.32 | 0.45 | 0.37 |
| 609 | Train 10 | Test 33 | 0.76 | 0.22 | 0.69 | 0.34 | 2657 | Train 42 | Test 33 | 0.75 | 0.22 | 0.69 | 0.34 |
| 610 | Train 10 | Test 34 | 0.77 | 0.25 | 0.74 | 0.38 | 2658 | Train 42 | Test 34 | 0.77 | 0.25 | 0.75 | 0.38 |
| 611 | Train 10 | Test 35 | 0.76 | 0.22 | 0.69 | 0.34 | 2659 | Train 42 | Test 35 | 0.76 | 0.22 | 0.7 | 0.34 |
| 612 | Train 10 | Test 36 | 0.77 | 0.25 | 0.74 | 0.38 | 2660 | Train 42 | Test 36 | 0.77 | 0.25 | 0.75 | 0.38 |
| 613 | Train 10 | Test 37 | 0.71 | 0.2 | 0.62 | 0.31 | 2661 | Train 42 | Test 37 | 0.71 | 0.2 | 0.62 | 0.31 |
| 614 | Train 10 | Test 38 | 0.71 | 0.33 | 0.43 | 0.37 | 2662 | Train 42 | Test 38 | 0.71 | 0.33 | 0.44 | 0.37 |
| 615 | Train 10 | Test 39 | 0.71 | 0.21 | 0.62 | 0.31 | 2663 | Train 42 | Test 39 | 0.71 | 0.2 | 0.62 | 0.31 |
| 616 | Train 10 | Test 40 | 0.71 | 0.32 | 0.43 | 0.37 | 2664 | Train 42 | Test 40 | 0.71 | 0.32 | 0.44 | 0.37 |
| 617 | Train 10 | Test 41 | 0.76 | 0.23 | 0.66 | 0.34 | 2665 | Train 42 | Test 41 | 0.76 | 0.22 | 0.67 | 0.34 |
| 618 | Train 10 | Test 42 | 0.77 | 0.26 | 0.72 | 0.38 | 2666 | Train 42 | Test 42 | 0.77 | 0.26 | 0.73 | 0.38 |
| 619 | Train 10 | Test 43 | 0.76 | 0.23 | 0.66 | 0.34 | 2667 | Train 42 | Test 43 | 0.76 | 0.23 | 0.67 | 0.34 |
| 620 | Train 10 | Test 44 | 0.77 | 0.26 | 0.72 | 0.38 | 2668 | Train 42 | Test 44 | 0.77 | 0.26 | 0.73 | 0.38 |
| 621 | Train 10 | Test 45 | 0.71 | 0.21 | 0.59 | 0.31 | 2669 | Train 42 | Test 45 | 0.71 | 0.21 | 0.59 | 0.3 |
| 622 | Train 10 | Test 46 | 0.71 | 0.33 | 0.43 | 0.37 | 2670 | Train 42 | Test 46 | 0.71 | 0.32 | 0.44 | 0.37 |
| 623 | Train 10 | Test 47 | 0.71 | 0.21 | 0.59 | 0.31 | 2671 | Train 42 | Test 47 | 0.71 | 0.21 | 0.59 | 0.31 |
| 624 | Train 10 | Test 48 | 0.71 | 0.32 | 0.43 | 0.37 | 2672 | Train 42 | Test 48 | 0.71 | 0.32 | 0.45 | 0.37 |
| 625 | Train 10 | Test 49 | 0.77 | 0.25 | 0.74 | 0.37 | 2673 | Train 42 | Test 49 | 0.77 | 0.25 | 0.74 | 0.37 |
| 626 | Train 10 | Test 50 | 0.77 | 0.25 | 0.74 | 0.37 | 2674 | Train 42 | Test 50 | 0.77 | 0.25 | 0.74 | 0.37 |
| 627 | Train 10 | Test 51 | 0.77 | 0.25 | 0.74 | 0.38 | 2675 | Train 42 | Test 51 | 0.77 | 0.25 | 0.75 | 0.38 |
| 628 | Train 10 | Test 52 | 0.77 | 0.25 | 0.74 | 0.38 | 2676 | Train 42 | Test 52 | 0.77 | 0.25 | 0.75 | 0.38 |
| 629 | Train 10 | Test 53 | 0.74 | 0.28 | 0.47 | 0.35 | 2677 | Train 42 | Test 53 | 0.74 | 0.27 | 0.48 | 0.35 |
| 630 | Train 10 | Test 54 | 0.72 | 0.37 | 0.42 | 0.39 | 2678 | Train 42 | Test 54 | 0.72 | 0.37 | 0.44 | 0.4 |
| 631 | Train 10 | Test 55 | 0.73 | 0.23 | 0.58 | 0.33 | 2679 | Train 42 | Test 55 | 0.73 | 0.23 | 0.59 | 0.33 |
| 632 | Train 10 | Test 56 | 0.72 | 0.35 | 0.43 | 0.39 | 2680 | Train 42 | Test 56 | 0.72 | 0.35 | 0.45 | 0.39 |
| 633 | Train 10 | Test 57 | 0.78 | 0.25 | 0.73 | 0.38 | 2681 | Train 42 | Test 57 | 0.78 | 0.26 | 0.73 | 0.38 |
| 634 | Train 10 | Test 58 | 0.77 | 0.26 | 0.73 | 0.38 | 2682 | Train 42 | Test 58 | 0.77 | 0.26 | 0.73 | 0.38 |
| 635 | Train 10 | Test 59 | 0.78 | 0.26 | 0.73 | 0.38 | 2683 | Train 42 | Test 59 | 0.78 | 0.26 | 0.74 | 0.38 |
| 636 | Train 10 | Test 60 | 0.77 | 0.26 | 0.73 | 0.38 | 2684 | Train 42 | Test 60 | 0.77 | 0.26 | 0.73 | 0.38 |
| 637 | Train 10 | Test 61 | 0.75 | 0.28 | 0.46 | 0.35 | 2685 | Train 42 | Test 61 | 0.75 | 0.28 | 0.47 | 0.35 |
| 638 | Train 10 | Test 62 | 0.72 | 0.37 | 0.42 | 0.39 | 2686 | Train 42 | Test 62 | 0.72 | 0.37 | 0.44 | 0.4 |
| 639 | Train 10 | Test 63 | 0.75 | 0.28 | 0.46 | 0.35 | 2687 | Train 42 | Test 63 | 0.75 | 0.28 | 0.48 | 0.35 |
| 640 | Train 10 | Test 64 | 0.72 | 0.35 | 0.43 | 0.39 | 2688 | Train 42 | Test 64 | 0.72 | 0.35 | 0.45 | 0.39 |
| 641 | Train 11 | Test 1 | 0.76 | 0.22 | 0.73 | 0.33 | 2689 | Train 43 | Test 1 | 0.76 | 0.21 | 0.74 | 0.33 |
| 642 | Train 11 | Test 2 | 0.75 | 0.24 | 0.72 | 0.37 | 2690 | Train 43 | Test 2 | 0.75 | 0.24 | 0.72 | 0.36 |
| 643 | Train 11 | Test 3 | 0.76 | 0.22 | 0.73 | 0.33 | 2691 | Train 43 | Test 3 | 0.76 | 0.22 | 0.74 | 0.33 |
| 644 | Train 11 | Test 4 | 0.75 | 0.25 | 0.72 | 0.37 | 2692 | Train 43 | Test 4 | 0.75 | 0.24 | 0.72 | 0.36 |
| 645 | Train 11 | Test 5 | 0.71 | 0.2 | 0.65 | 0.31 | 2693 | Train 43 | Test 5 | 0.72 | 0.2 | 0.65 | 0.31 |
| 646 | Train 11 | Test 6 | 0.7 | 0.3 | 0.47 | 0.37 | 2694 | Train 43 | Test 6 | 0.7 | 0.3 | 0.49 | 0.37 |
| 647 | Train 11 | Test 7 | 0.71 | 0.2 | 0.64 | 0.31 | 2695 | Train 43 | Test 7 | 0.71 | 0.2 | 0.65 | 0.31 |
| 648 | Train 11 | Test 8 | 0.7 | 0.3 | 0.47 | 0.36 | 2696 | Train 43 | Test 8 | 0.7 | 0.3 | 0.49 | 0.37 |
| 649 | Train 11 | Test 9 | 0.76 | 0.22 | 0.71 | 0.33 | 2697 | Train 43 | Test 9 | 0.76 | 0.22 | 0.71 | 0.33 |
| 650 | Train 11 | Test 10 | 0.75 | 0.25 | 0.69 | 0.36 | 2698 | Train 43 | Test 10 | 0.75 | 0.25 | 0.7 | 0.37 |
| 651 | Train 11 | Test 11 | 0.76 | 0.22 | 0.71 | 0.33 | 2699 | Train 43 | Test 11 | 0.76 | 0.22 | 0.71 | 0.33 |
| 652 | Train 11 | Test 12 | 0.75 | 0.25 | 0.7 | 0.37 | 2700 | Train 43 | Test 12 | 0.75 | 0.25 | 0.7 | 0.37 |
| 653 | Train 11 | Test 13 | 0.71 | 0.2 | 0.62 | 0.31 | 2701 | Train 43 | Test 13 | 0.71 | 0.2 | 0.62 | 0.31 |
| 654 | Train 11 | Test 14 | 0.7 | 0.3 | 0.47 | 0.37 | 2702 | Train 43 | Test 14 | 0.7 | 0.3 | 0.49 | 0.37 |
| 655 | Train 11 | Test 15 | 0.71 | 0.2 | 0.62 | 0.31 | 2703 | Train 43 | Test 15 | 0.71 | 0.2 | 0.62 | 0.31 |
| 656 | Train 11 | Test 16 | 0.7 | 0.3 | 0.47 | 0.36 | 2704 | Train 43 | Test 16 | 0.7 | 0.29 | 0.49 | 0.37 |
| 657 | Train 11 | Test 17 | 0.76 | 0.22 | 0.73 | 0.33 | 2705 | Train 43 | Test 17 | 0.76 | 0.21 | 0.74 | 0.33 |
| 658 | Train 11 | Test 18 | 0.75 | 0.24 | 0.72 | 0.37 | 2706 | Train 43 | Test 18 | 0.75 | 0.24 | 0.72 | 0.36 |
| 659 | Train 11 | Test 19 | 0.76 | 0.22 | 0.73 | 0.33 | 2707 | Train 43 | Test 19 | 0.76 | 0.22 | 0.74 | 0.33 |
| 660 | Train 11 | Test 20 | 0.75 | 0.25 | 0.72 | 0.37 | 2708 | Train 43 | Test 20 | 0.75 | 0.24 | 0.72 | 0.37 |
| 661 | Train 11 | Test 21 | 0.72 | 0.2 | 0.65 | 0.31 | 2709 | Train 43 | Test 21 | 0.72 | 0.2 | 0.65 | 0.31 |
| 662 | Train 11 | Test 22 | 0.7 | 0.3 | 0.47 | 0.37 | 2710 | Train 43 | Test 22 | 0.7 | 0.3 | 0.49 | 0.37 |
| 663 | Train 11 | Test 23 | 0.71 | 0.2 | 0.65 | 0.31 | 2711 | Train 43 | Test 23 | 0.71 | 0.2 | 0.65 | 0.31 |
| 664 | Train 11 | Test 24 | 0.7 | 0.3 | 0.47 | 0.36 | 2712 | Train 43 | Test 24 | 0.7 | 0.29 | 0.49 | 0.37 |
| 665 | Train 11 | Test 25 | 0.76 | 0.22 | 0.71 | 0.33 | 2713 | Train 43 | Test 25 | 0.76 | 0.22 | 0.71 | 0.33 |
| 666 | Train 11 | Test 26 | 0.75 | 0.25 | 0.69 | 0.36 | 2714 | Train 43 | Test 26 | 0.75 | 0.25 | 0.7 | 0.37 |
| 667 | Train 11 | Test 27 | 0.76 | 0.22 | 0.71 | 0.33 | 2715 | Train 43 | Test 27 | 0.76 | 0.22 | 0.71 | 0.33 |
| 668 | Train 11 | Test 28 | 0.75 | 0.25 | 0.7 | 0.37 | 2716 | Train 43 | Test 28 | 0.75 | 0.25 | 0.7 | 0.37 |
| 669 | Train 11 | Test 29 | 0.71 | 0.2 | 0.62 | 0.31 | 2717 | Train 43 | Test 29 | 0.71 | 0.2 | 0.62 | 0.31 |
| 670 | Train 11 | Test 30 | 0.7 | 0.3 | 0.47 | 0.37 | 2718 | Train 43 | Test 30 | 0.7 | 0.3 | 0.49 | 0.37 |
| 671 | Train 11 | Test 31 | 0.71 | 0.2 | 0.62 | 0.31 | 2719 | Train 43 | Test 31 | 0.71 | 0.2 | 0.62 | 0.31 |
| 672 | Train 11 | Test 32 | 0.7 | 0.3 | 0.47 | 0.36 | 2720 | Train 43 | Test 32 | 0.7 | 0.29 | 0.49 | 0.37 |
| 673 | Train 11 | Test 33 | 0.76 | 0.21 | 0.73 | 0.33 | 2721 | Train 43 | Test 33 | 0.76 | 0.21 | 0.73 | 0.33 |
| 674 | Train 11 | Test 34 | 0.75 | 0.24 | 0.72 | 0.36 | 2722 | Train 43 | Test 34 | 0.75 | 0.24 | 0.72 | 0.36 |
| 675 | Train 11 | Test 35 | 0.76 | 0.22 | 0.73 | 0.33 | 2723 | Train 43 | Test 35 | 0.76 | 0.21 | 0.73 | 0.33 |
| 676 | Train 11 | Test 36 | 0.75 | 0.24 | 0.72 | 0.36 | 2724 | Train 43 | Test 36 | 0.75 | 0.24 | 0.72 | 0.36 |
| 677 | Train 11 | Test 37 | 0.72 | 0.2 | 0.64 | 0.31 | 2725 | Train 43 | Test 37 | 0.72 | 0.2 | 0.65 | 0.31 |
| 678 | Train 11 | Test 38 | 0.7 | 0.3 | 0.47 | 0.37 | 2726 | Train 43 | Test 38 | 0.7 | 0.3 | 0.49 | 0.37 |
| 679 | Train 11 | Test 39 | 0.72 | 0.2 | 0.64 | 0.31 | 2727 | Train 43 | Test 39 | 0.72 | 0.2 | 0.65 | 0.31 |
| 680 | Train 11 | Test 40 | 0.7 | 0.3 | 0.47 | 0.36 | 2728 | Train 43 | Test 40 | 0.7 | 0.3 | 0.49 | 0.37 |
| 681 | Train 11 | Test 41 | 0.76 | 0.22 | 0.7 | 0.33 | 2729 | Train 43 | Test 41 | 0.76 | 0.22 | 0.71 | 0.33 |
| 682 | Train 11 | Test 42 | 0.75 | 0.25 | 0.69 | 0.36 | 2730 | Train 43 | Test 42 | 0.75 | 0.25 | 0.7 | 0.36 |
| 683 | Train 11 | Test 43 | 0.76 | 0.22 | 0.71 | 0.33 | 2731 | Train 43 | Test 43 | 0.76 | 0.22 | 0.71 | 0.33 |
| 684 | Train 11 | Test 44 | 0.75 | 0.25 | 0.69 | 0.36 | 2732 | Train 43 | Test 44 | 0.75 | 0.25 | 0.7 | 0.36 |
| 685 | Train 11 | Test 45 | 0.72 | 0.2 | 0.62 | 0.31 | 2733 | Train 43 | Test 45 | 0.72 | 0.2 | 0.62 | 0.31 |
| 686 | Train 11 | Test 46 | 0.7 | 0.3 | 0.47 | 0.37 | 2734 | Train 43 | Test 46 | 0.7 | 0.3 | 0.49 | 0.37 |
| 687 | Train 11 | Test 47 | 0.71 | 0.2 | 0.61 | 0.31 | 2735 | Train 43 | Test 47 | 0.71 | 0.2 | 0.62 | 0.31 |
| 688 | Train 11 | Test 48 | 0.7 | 0.3 | 0.47 | 0.36 | 2736 | Train 43 | Test 48 | 0.7 | 0.29 | 0.49 | 0.37 |
| 689 | Train 11 | Test 49 | 0.78 | 0.24 | 0.8 | 0.37 | 2737 | Train 43 | Test 49 | 0.78 | 0.24 | 0.8 | 0.37 |
| 690 | Train 11 | Test 50 | 0.78 | 0.25 | 0.8 | 0.38 | 2738 | Train 43 | Test 50 | 0.78 | 0.24 | 0.79 | 0.37 |
| 691 | Train 11 | Test 51 | 0.78 | 0.25 | 0.8 | 0.38 | 2739 | Train 43 | Test 51 | 0.78 | 0.25 | 0.8 | 0.38 |
| 692 | Train 11 | Test 52 | 0.78 | 0.25 | 0.8 | 0.38 | 2740 | Train 43 | Test 52 | 0.78 | 0.25 | 0.8 | 0.38 |
| 693 | Train 11 | Test 53 | 0.74 | 0.26 | 0.59 | 0.36 | 2741 | Train 43 | Test 53 | 0.74 | 0.26 | 0.61 | 0.36 |
| 694 | Train 11 | Test 54 | 0.71 | 0.34 | 0.5 | 0.41 | 2742 | Train 43 | Test 54 | 0.72 | 0.34 | 0.51 | 0.41 |
| 695 | Train 11 | Test 55 | 0.73 | 0.23 | 0.65 | 0.34 | 2743 | Train 43 | Test 55 | 0.74 | 0.23 | 0.66 | 0.35 |
| 696 | Train 11 | Test 56 | 0.72 | 0.33 | 0.5 | 0.4 | 2744 | Train 43 | Test 56 | 0.72 | 0.33 | 0.51 | 0.4 |
| 697 | Train 11 | Test 57 | 0.78 | 0.25 | 0.78 | 0.38 | 2745 | Train 43 | Test 57 | 0.78 | 0.25 | 0.78 | 0.37 |
| 698 | Train 11 | Test 58 | 0.78 | 0.25 | 0.78 | 0.38 | 2746 | Train 43 | Test 58 | 0.78 | 0.25 | 0.78 | 0.38 |
| 699 | Train 11 | Test 59 | 0.78 | 0.25 | 0.79 | 0.38 | 2747 | Train 43 | Test 59 | 0.78 | 0.25 | 0.78 | 0.38 |
| 700 | Train 11 | Test 60 | 0.78 | 0.25 | 0.78 | 0.38 | 2748 | Train 43 | Test 60 | 0.78 | 0.25 | 0.78 | 0.38 |
| 701 | Train 11 | Test 61 | 0.74 | 0.26 | 0.58 | 0.36 | 2749 | Train 43 | Test 61 | 0.74 | 0.26 | 0.6 | 0.36 |
| 702 | Train 11 | Test 62 | 0.72 | 0.34 | 0.5 | 0.41 | 2750 | Train 43 | Test 62 | 0.72 | 0.34 | 0.52 | 0.41 |
| 703 | Train 11 | Test 63 | 0.75 | 0.26 | 0.59 | 0.36 | 2751 | Train 43 | Test 63 | 0.75 | 0.26 | 0.6 | 0.36 |
| 704 | Train 11 | Test 64 | 0.72 | 0.33 | 0.5 | 0.4 | 2752 | Train 43 | Test 64 | 0.72 | 0.33 | 0.52 | 0.4 |
| 705 | Train 12 | Test 1 | 0.75 | 0.22 | 0.69 | 0.34 | 2753 | Train 44 | Test 1 | 0.75 | 0.22 | 0.69 | 0.34 |
| 706 | Train 12 | Test 2 | 0.77 | 0.25 | 0.74 | 0.38 | 2754 | Train 44 | Test 2 | 0.77 | 0.25 | 0.75 | 0.38 |
| 707 | Train 12 | Test 3 | 0.75 | 0.22 | 0.69 | 0.34 | 2755 | Train 44 | Test 3 | 0.75 | 0.22 | 0.7 | 0.34 |
| 708 | Train 12 | Test 4 | 0.77 | 0.25 | 0.74 | 0.38 | 2756 | Train 44 | Test 4 | 0.77 | 0.25 | 0.75 | 0.38 |
| 709 | Train 12 | Test 5 | 0.71 | 0.2 | 0.62 | 0.31 | 2757 | Train 44 | Test 5 | 0.71 | 0.2 | 0.62 | 0.31 |
| 710 | Train 12 | Test 6 | 0.71 | 0.33 | 0.41 | 0.37 | 2758 | Train 44 | Test 6 | 0.71 | 0.33 | 0.43 | 0.37 |
| 711 | Train 12 | Test 7 | 0.71 | 0.2 | 0.62 | 0.31 | 2759 | Train 44 | Test 7 | 0.71 | 0.2 | 0.62 | 0.31 |
| 712 | Train 12 | Test 8 | 0.71 | 0.33 | 0.42 | 0.37 | 2760 | Train 44 | Test 8 | 0.71 | 0.32 | 0.44 | 0.37 |
| 713 | Train 12 | Test 9 | 0.75 | 0.23 | 0.67 | 0.34 | 2761 | Train 44 | Test 9 | 0.75 | 0.23 | 0.67 | 0.34 |
| 714 | Train 12 | Test 10 | 0.77 | 0.26 | 0.73 | 0.38 | 2762 | Train 44 | Test 10 | 0.77 | 0.26 | 0.73 | 0.38 |
| 715 | Train 12 | Test 11 | 0.76 | 0.23 | 0.67 | 0.34 | 2763 | Train 44 | Test 11 | 0.75 | 0.23 | 0.67 | 0.34 |
| 716 | Train 12 | Test 12 | 0.77 | 0.26 | 0.73 | 0.38 | 2764 | Train 44 | Test 12 | 0.77 | 0.26 | 0.73 | 0.38 |
| 717 | Train 12 | Test 13 | 0.71 | 0.21 | 0.59 | 0.31 | 2765 | Train 44 | Test 13 | 0.71 | 0.21 | 0.59 | 0.31 |
| 718 | Train 12 | Test 14 | 0.71 | 0.33 | 0.41 | 0.37 | 2766 | Train 44 | Test 14 | 0.71 | 0.32 | 0.43 | 0.37 |
| 719 | Train 12 | Test 15 | 0.71 | 0.21 | 0.59 | 0.31 | 2767 | Train 44 | Test 15 | 0.71 | 0.21 | 0.59 | 0.31 |
| 720 | Train 12 | Test 16 | 0.71 | 0.32 | 0.42 | 0.37 | 2768 | Train 44 | Test 16 | 0.71 | 0.32 | 0.44 | 0.37 |
| 721 | Train 12 | Test 17 | 0.75 | 0.22 | 0.69 | 0.34 | 2769 | Train 44 | Test 17 | 0.75 | 0.22 | 0.69 | 0.34 |
| 722 | Train 12 | Test 18 | 0.77 | 0.25 | 0.74 | 0.38 | 2770 | Train 44 | Test 18 | 0.77 | 0.25 | 0.75 | 0.38 |
| 723 | Train 12 | Test 19 | 0.75 | 0.22 | 0.69 | 0.34 | 2771 | Train 44 | Test 19 | 0.75 | 0.22 | 0.7 | 0.34 |
| 724 | Train 12 | Test 20 | 0.77 | 0.25 | 0.74 | 0.38 | 2772 | Train 44 | Test 20 | 0.77 | 0.25 | 0.75 | 0.38 |
| 725 | Train 12 | Test 21 | 0.71 | 0.2 | 0.62 | 0.31 | 2773 | Train 44 | Test 21 | 0.71 | 0.2 | 0.62 | 0.31 |
| 726 | Train 12 | Test 22 | 0.71 | 0.33 | 0.41 | 0.37 | 2774 | Train 44 | Test 22 | 0.71 | 0.33 | 0.43 | 0.37 |
| 727 | Train 12 | Test 23 | 0.71 | 0.2 | 0.62 | 0.31 | 2775 | Train 44 | Test 23 | 0.71 | 0.2 | 0.62 | 0.31 |
| 728 | Train 12 | Test 24 | 0.71 | 0.33 | 0.41 | 0.37 | 2776 | Train 44 | Test 24 | 0.71 | 0.32 | 0.43 | 0.37 |
| 729 | Train 12 | Test 25 | 0.76 | 0.23 | 0.67 | 0.34 | 2777 | Train 44 | Test 25 | 0.75 | 0.23 | 0.67 | 0.34 |
| 730 | Train 12 | Test 26 | 0.77 | 0.26 | 0.73 | 0.38 | 2778 | Train 44 | Test 26 | 0.77 | 0.26 | 0.73 | 0.38 |
| 731 | Train 12 | Test 27 | 0.76 | 0.23 | 0.67 | 0.34 | 2779 | Train 44 | Test 27 | 0.75 | 0.23 | 0.67 | 0.34 |
| 732 | Train 12 | Test 28 | 0.77 | 0.26 | 0.73 | 0.38 | 2780 | Train 44 | Test 28 | 0.77 | 0.26 | 0.73 | 0.38 |
| 733 | Train 12 | Test 29 | 0.71 | 0.21 | 0.59 | 0.31 | 2781 | Train 44 | Test 29 | 0.71 | 0.21 | 0.59 | 0.31 |
| 734 | Train 12 | Test 30 | 0.71 | 0.33 | 0.41 | 0.37 | 2782 | Train 44 | Test 30 | 0.71 | 0.32 | 0.43 | 0.37 |
| 735 | Train 12 | Test 31 | 0.71 | 0.21 | 0.59 | 0.31 | 2783 | Train 44 | Test 31 | 0.71 | 0.21 | 0.59 | 0.31 |
| 736 | Train 12 | Test 32 | 0.71 | 0.32 | 0.42 | 0.37 | 2784 | Train 44 | Test 32 | 0.71 | 0.32 | 0.44 | 0.37 |
| 737 | Train 12 | Test 33 | 0.76 | 0.22 | 0.69 | 0.34 | 2785 | Train 44 | Test 33 | 0.76 | 0.22 | 0.69 | 0.34 |
| 738 | Train 12 | Test 34 | 0.77 | 0.25 | 0.74 | 0.38 | 2786 | Train 44 | Test 34 | 0.77 | 0.25 | 0.74 | 0.38 |
| 739 | Train 12 | Test 35 | 0.76 | 0.22 | 0.69 | 0.34 | 2787 | Train 44 | Test 35 | 0.76 | 0.22 | 0.69 | 0.34 |
| 740 | Train 12 | Test 36 | 0.77 | 0.25 | 0.74 | 0.38 | 2788 | Train 44 | Test 36 | 0.77 | 0.25 | 0.74 | 0.38 |
| 741 | Train 12 | Test 37 | 0.71 | 0.2 | 0.62 | 0.31 | 2789 | Train 44 | Test 37 | 0.71 | 0.2 | 0.62 | 0.31 |
| 742 | Train 12 | Test 38 | 0.71 | 0.33 | 0.41 | 0.37 | 2790 | Train 44 | Test 38 | 0.71 | 0.33 | 0.43 | 0.37 |
| 743 | Train 12 | Test 39 | 0.71 | 0.2 | 0.61 | 0.31 | 2791 | Train 44 | Test 39 | 0.71 | 0.2 | 0.62 | 0.31 |
| 744 | Train 12 | Test 40 | 0.71 | 0.33 | 0.41 | 0.37 | 2792 | Train 44 | Test 40 | 0.71 | 0.32 | 0.43 | 0.37 |
| 745 | Train 12 | Test 41 | 0.76 | 0.23 | 0.66 | 0.34 | 2793 | Train 44 | Test 41 | 0.76 | 0.22 | 0.67 | 0.34 |
| 746 | Train 12 | Test 42 | 0.77 | 0.26 | 0.73 | 0.38 | 2794 | Train 44 | Test 42 | 0.77 | 0.26 | 0.73 | 0.38 |
| 747 | Train 12 | Test 43 | 0.76 | 0.23 | 0.67 | 0.34 | 2795 | Train 44 | Test 43 | 0.76 | 0.23 | 0.67 | 0.34 |
| 748 | Train 12 | Test 44 | 0.77 | 0.26 | 0.73 | 0.38 | 2796 | Train 44 | Test 44 | 0.77 | 0.26 | 0.73 | 0.38 |
| 749 | Train 12 | Test 45 | 0.71 | 0.21 | 0.59 | 0.31 | 2797 | Train 44 | Test 45 | 0.71 | 0.21 | 0.59 | 0.31 |
| 750 | Train 12 | Test 46 | 0.71 | 0.33 | 0.41 | 0.37 | 2798 | Train 44 | Test 46 | 0.71 | 0.32 | 0.43 | 0.37 |
| 751 | Train 12 | Test 47 | 0.71 | 0.21 | 0.59 | 0.31 | 2799 | Train 44 | Test 47 | 0.71 | 0.21 | 0.59 | 0.31 |
| 752 | Train 12 | Test 48 | 0.71 | 0.32 | 0.42 | 0.36 | 2800 | Train 44 | Test 48 | 0.71 | 0.32 | 0.44 | 0.37 |
| 753 | Train 12 | Test 49 | 0.77 | 0.25 | 0.74 | 0.37 | 2801 | Train 44 | Test 49 | 0.77 | 0.25 | 0.74 | 0.37 |
| 754 | Train 12 | Test 50 | 0.77 | 0.25 | 0.74 | 0.37 | 2802 | Train 44 | Test 50 | 0.77 | 0.25 | 0.74 | 0.37 |
| 755 | Train 12 | Test 51 | 0.77 | 0.25 | 0.75 | 0.38 | 2803 | Train 44 | Test 51 | 0.77 | 0.25 | 0.75 | 0.38 |
| 756 | Train 12 | Test 52 | 0.77 | 0.25 | 0.74 | 0.38 | 2804 | Train 44 | Test 52 | 0.77 | 0.25 | 0.75 | 0.38 |
| 757 | Train 12 | Test 53 | 0.74 | 0.28 | 0.46 | 0.35 | 2805 | Train 44 | Test 53 | 0.74 | 0.27 | 0.48 | 0.35 |
| 758 | Train 12 | Test 54 | 0.72 | 0.37 | 0.41 | 0.39 | 2806 | Train 44 | Test 54 | 0.72 | 0.37 | 0.43 | 0.4 |
| 759 | Train 12 | Test 55 | 0.73 | 0.23 | 0.58 | 0.33 | 2807 | Train 44 | Test 55 | 0.73 | 0.23 | 0.59 | 0.33 |
| 760 | Train 12 | Test 56 | 0.72 | 0.36 | 0.42 | 0.39 | 2808 | Train 44 | Test 56 | 0.72 | 0.35 | 0.44 | 0.39 |
| 761 | Train 12 | Test 57 | 0.78 | 0.26 | 0.74 | 0.38 | 2809 | Train 44 | Test 57 | 0.78 | 0.25 | 0.73 | 0.38 |
| 762 | Train 12 | Test 58 | 0.77 | 0.26 | 0.73 | 0.38 | 2810 | Train 44 | Test 58 | 0.77 | 0.25 | 0.73 | 0.38 |
| 763 | Train 12 | Test 59 | 0.78 | 0.26 | 0.74 | 0.38 | 2811 | Train 44 | Test 59 | 0.78 | 0.26 | 0.74 | 0.38 |
| 764 | Train 12 | Test 60 | 0.77 | 0.26 | 0.74 | 0.38 | 2812 | Train 44 | Test 60 | 0.77 | 0.26 | 0.73 | 0.38 |
| 765 | Train 12 | Test 61 | 0.75 | 0.28 | 0.45 | 0.35 | 2813 | Train 44 | Test 61 | 0.75 | 0.28 | 0.47 | 0.35 |
| 766 | Train 12 | Test 62 | 0.72 | 0.37 | 0.41 | 0.39 | 2814 | Train 44 | Test 62 | 0.72 | 0.37 | 0.43 | 0.4 |
| 767 | Train 12 | Test 63 | 0.75 | 0.28 | 0.46 | 0.35 | 2815 | Train 44 | Test 63 | 0.75 | 0.28 | 0.47 | 0.35 |
| 768 | Train 12 | Test 64 | 0.72 | 0.35 | 0.42 | 0.38 | 2816 | Train 44 | Test 64 | 0.72 | 0.35 | 0.44 | 0.39 |
| 769 | Train 13 | Test 1 | 0.75 | 0.21 | 0.76 | 0.33 | 2817 | Train 45 | Test 1 | 0.75 | 0.21 | 0.76 | 0.33 |
| 770 | Train 13 | Test 2 | 0.75 | 0.24 | 0.77 | 0.37 | 2818 | Train 45 | Test 2 | 0.75 | 0.24 | 0.78 | 0.37 |
| 771 | Train 13 | Test 3 | 0.75 | 0.21 | 0.76 | 0.33 | 2819 | Train 45 | Test 3 | 0.75 | 0.21 | 0.76 | 0.33 |
| 772 | Train 13 | Test 4 | 0.75 | 0.24 | 0.78 | 0.37 | 2820 | Train 45 | Test 4 | 0.75 | 0.24 | 0.78 | 0.37 |
| 773 | Train 13 | Test 5 | 0.73 | 0.21 | 0.73 | 0.32 | 2821 | Train 45 | Test 5 | 0.73 | 0.2 | 0.73 | 0.32 |
| 774 | Train 13 | Test 6 | 0.7 | 0.29 | 0.6 | 0.39 | 2822 | Train 45 | Test 6 | 0.7 | 0.28 | 0.61 | 0.39 |
| 775 | Train 13 | Test 7 | 0.73 | 0.21 | 0.73 | 0.32 | 2823 | Train 45 | Test 7 | 0.73 | 0.2 | 0.73 | 0.32 |
| 776 | Train 13 | Test 8 | 0.7 | 0.28 | 0.61 | 0.38 | 2824 | Train 45 | Test 8 | 0.7 | 0.28 | 0.62 | 0.38 |
| 777 | Train 13 | Test 9 | 0.75 | 0.21 | 0.74 | 0.33 | 2825 | Train 45 | Test 9 | 0.75 | 0.21 | 0.74 | 0.33 |
| 778 | Train 13 | Test 10 | 0.75 | 0.25 | 0.76 | 0.37 | 2826 | Train 45 | Test 10 | 0.75 | 0.24 | 0.76 | 0.37 |
| 779 | Train 13 | Test 11 | 0.75 | 0.22 | 0.75 | 0.33 | 2827 | Train 45 | Test 11 | 0.75 | 0.21 | 0.74 | 0.33 |
| 780 | Train 13 | Test 12 | 0.75 | 0.25 | 0.76 | 0.37 | 2828 | Train 45 | Test 12 | 0.75 | 0.25 | 0.76 | 0.37 |
| 781 | Train 13 | Test 13 | 0.73 | 0.21 | 0.71 | 0.32 | 2829 | Train 45 | Test 13 | 0.73 | 0.21 | 0.71 | 0.32 |
| 782 | Train 13 | Test 14 | 0.7 | 0.28 | 0.61 | 0.39 | 2830 | Train 45 | Test 14 | 0.7 | 0.28 | 0.62 | 0.39 |
| 783 | Train 13 | Test 15 | 0.73 | 0.21 | 0.71 | 0.32 | 2831 | Train 45 | Test 15 | 0.73 | 0.21 | 0.71 | 0.32 |
| 784 | Train 13 | Test 16 | 0.71 | 0.28 | 0.61 | 0.38 | 2832 | Train 45 | Test 16 | 0.7 | 0.28 | 0.62 | 0.38 |
| 785 | Train 13 | Test 17 | 0.75 | 0.21 | 0.76 | 0.33 | 2833 | Train 45 | Test 17 | 0.75 | 0.21 | 0.76 | 0.33 |
| 786 | Train 13 | Test 18 | 0.75 | 0.24 | 0.77 | 0.37 | 2834 | Train 45 | Test 18 | 0.75 | 0.24 | 0.78 | 0.37 |
| 787 | Train 13 | Test 19 | 0.75 | 0.21 | 0.76 | 0.33 | 2835 | Train 45 | Test 19 | 0.75 | 0.21 | 0.76 | 0.33 |
| 788 | Train 13 | Test 20 | 0.75 | 0.24 | 0.78 | 0.37 | 2836 | Train 45 | Test 20 | 0.75 | 0.24 | 0.78 | 0.37 |
| 789 | Train 13 | Test 21 | 0.73 | 0.21 | 0.73 | 0.32 | 2837 | Train 45 | Test 21 | 0.73 | 0.2 | 0.73 | 0.32 |
| 790 | Train 13 | Test 22 | 0.7 | 0.29 | 0.6 | 0.39 | 2838 | Train 45 | Test 22 | 0.7 | 0.28 | 0.61 | 0.39 |
| 791 | Train 13 | Test 23 | 0.73 | 0.21 | 0.73 | 0.32 | 2839 | Train 45 | Test 23 | 0.73 | 0.2 | 0.73 | 0.32 |
| 792 | Train 13 | Test 24 | 0.7 | 0.28 | 0.61 | 0.38 | 2840 | Train 45 | Test 24 | 0.7 | 0.28 | 0.62 | 0.38 |
| 793 | Train 13 | Test 25 | 0.75 | 0.21 | 0.74 | 0.33 | 2841 | Train 45 | Test 25 | 0.75 | 0.21 | 0.74 | 0.33 |
| 794 | Train 13 | Test 26 | 0.75 | 0.25 | 0.76 | 0.37 | 2842 | Train 45 | Test 26 | 0.75 | 0.24 | 0.76 | 0.37 |
| 795 | Train 13 | Test 27 | 0.75 | 0.22 | 0.75 | 0.33 | 2843 | Train 45 | Test 27 | 0.75 | 0.21 | 0.74 | 0.33 |
| 796 | Train 13 | Test 28 | 0.75 | 0.25 | 0.76 | 0.37 | 2844 | Train 45 | Test 28 | 0.75 | 0.25 | 0.76 | 0.37 |
| 797 | Train 13 | Test 29 | 0.73 | 0.21 | 0.71 | 0.33 | 2845 | Train 45 | Test 29 | 0.73 | 0.21 | 0.71 | 0.32 |
| 798 | Train 13 | Test 30 | 0.71 | 0.28 | 0.61 | 0.39 | 2846 | Train 45 | Test 30 | 0.7 | 0.28 | 0.62 | 0.39 |
| 799 | Train 13 | Test 31 | 0.73 | 0.21 | 0.71 | 0.32 | 2847 | Train 45 | Test 31 | 0.73 | 0.21 | 0.71 | 0.32 |
| 800 | Train 13 | Test 32 | 0.71 | 0.28 | 0.61 | 0.38 | 2848 | Train 45 | Test 32 | 0.71 | 0.28 | 0.62 | 0.38 |
| 801 | Train 13 | Test 33 | 0.75 | 0.21 | 0.75 | 0.33 | 2849 | Train 45 | Test 33 | 0.75 | 0.21 | 0.76 | 0.33 |
| 802 | Train 13 | Test 34 | 0.75 | 0.24 | 0.77 | 0.37 | 2850 | Train 45 | Test 34 | 0.75 | 0.24 | 0.77 | 0.37 |
| 803 | Train 13 | Test 35 | 0.75 | 0.21 | 0.76 | 0.33 | 2851 | Train 45 | Test 35 | 0.75 | 0.21 | 0.76 | 0.33 |
| 804 | Train 13 | Test 36 | 0.75 | 0.24 | 0.77 | 0.37 | 2852 | Train 45 | Test 36 | 0.75 | 0.24 | 0.78 | 0.37 |
| 805 | Train 13 | Test 37 | 0.73 | 0.2 | 0.73 | 0.32 | 2853 | Train 45 | Test 37 | 0.73 | 0.2 | 0.73 | 0.32 |
| 806 | Train 13 | Test 38 | 0.7 | 0.29 | 0.6 | 0.39 | 2854 | Train 45 | Test 38 | 0.7 | 0.28 | 0.61 | 0.39 |
| 807 | Train 13 | Test 39 | 0.73 | 0.2 | 0.72 | 0.32 | 2855 | Train 45 | Test 39 | 0.73 | 0.2 | 0.73 | 0.32 |
| 808 | Train 13 | Test 40 | 0.7 | 0.28 | 0.61 | 0.38 | 2856 | Train 45 | Test 40 | 0.7 | 0.28 | 0.62 | 0.38 |
| 809 | Train 13 | Test 41 | 0.75 | 0.21 | 0.74 | 0.33 | 2857 | Train 45 | Test 41 | 0.75 | 0.21 | 0.74 | 0.33 |
| 810 | Train 13 | Test 42 | 0.75 | 0.25 | 0.76 | 0.37 | 2858 | Train 45 | Test 42 | 0.75 | 0.24 | 0.76 | 0.37 |
| 811 | Train 13 | Test 43 | 0.75 | 0.21 | 0.74 | 0.33 | 2859 | Train 45 | Test 43 | 0.75 | 0.21 | 0.74 | 0.33 |
| 812 | Train 13 | Test 44 | 0.75 | 0.25 | 0.76 | 0.37 | 2860 | Train 45 | Test 44 | 0.75 | 0.24 | 0.76 | 0.37 |
| 813 | Train 13 | Test 45 | 0.74 | 0.21 | 0.71 | 0.32 | 2861 | Train 45 | Test 45 | 0.74 | 0.21 | 0.71 | 0.32 |
| 814 | Train 13 | Test 46 | 0.71 | 0.28 | 0.61 | 0.39 | 2862 | Train 45 | Test 46 | 0.7 | 0.28 | 0.62 | 0.39 |
| 815 | Train 13 | Test 47 | 0.73 | 0.21 | 0.7 | 0.32 | 2863 | Train 45 | Test 47 | 0.73 | 0.21 | 0.7 | 0.32 |
| 816 | Train 13 | Test 48 | 0.71 | 0.28 | 0.61 | 0.38 | 2864 | Train 45 | Test 48 | 0.71 | 0.28 | 0.62 | 0.38 |
| 817 | Train 13 | Test 49 | 0.77 | 0.24 | 0.78 | 0.37 | 2865 | Train 45 | Test 49 | 0.77 | 0.24 | 0.79 | 0.37 |
| 818 | Train 13 | Test 50 | 0.76 | 0.24 | 0.78 | 0.37 | 2866 | Train 45 | Test 50 | 0.76 | 0.24 | 0.78 | 0.37 |
| 819 | Train 13 | Test 51 | 0.77 | 0.24 | 0.79 | 0.37 | 2867 | Train 45 | Test 51 | 0.77 | 0.24 | 0.79 | 0.37 |
| 820 | Train 13 | Test 52 | 0.76 | 0.24 | 0.79 | 0.37 | 2868 | Train 45 | Test 52 | 0.76 | 0.24 | 0.79 | 0.37 |
| 821 | Train 13 | Test 53 | 0.76 | 0.25 | 0.74 | 0.37 | 2869 | Train 45 | Test 53 | 0.76 | 0.24 | 0.74 | 0.37 |
| 822 | Train 13 | Test 54 | 0.72 | 0.33 | 0.6 | 0.43 | 2870 | Train 45 | Test 54 | 0.72 | 0.33 | 0.61 | 0.43 |
| 823 | Train 13 | Test 55 | 0.76 | 0.24 | 0.76 | 0.36 | 2871 | Train 45 | Test 55 | 0.76 | 0.24 | 0.77 | 0.36 |
| 824 | Train 13 | Test 56 | 0.71 | 0.31 | 0.61 | 0.42 | 2872 | Train 45 | Test 56 | 0.71 | 0.31 | 0.62 | 0.42 |
| 825 | Train 13 | Test 57 | 0.77 | 0.25 | 0.78 | 0.38 | 2873 | Train 45 | Test 57 | 0.77 | 0.25 | 0.78 | 0.37 |
| 826 | Train 13 | Test 58 | 0.77 | 0.25 | 0.78 | 0.38 | 2874 | Train 45 | Test 58 | 0.77 | 0.25 | 0.78 | 0.37 |
| 827 | Train 13 | Test 59 | 0.77 | 0.25 | 0.78 | 0.38 | 2875 | Train 45 | Test 59 | 0.77 | 0.25 | 0.78 | 0.38 |
| 828 | Train 13 | Test 60 | 0.77 | 0.25 | 0.78 | 0.38 | 2876 | Train 45 | Test 60 | 0.77 | 0.25 | 0.78 | 0.38 |
| 829 | Train 13 | Test 61 | 0.76 | 0.25 | 0.74 | 0.38 | 2877 | Train 45 | Test 61 | 0.76 | 0.25 | 0.74 | 0.37 |
| 830 | Train 13 | Test 62 | 0.72 | 0.33 | 0.61 | 0.43 | 2878 | Train 45 | Test 62 | 0.72 | 0.33 | 0.62 | 0.43 |
| 831 | Train 13 | Test 63 | 0.77 | 0.25 | 0.74 | 0.38 | 2879 | Train 45 | Test 63 | 0.77 | 0.25 | 0.74 | 0.37 |
| 832 | Train 13 | Test 64 | 0.72 | 0.31 | 0.61 | 0.41 | 2880 | Train 45 | Test 64 | 0.72 | 0.31 | 0.62 | 0.41 |
| 833 | Train 14 | Test 1 | 0.73 | 0.21 | 0.74 | 0.32 | 2881 | Train 46 | Test 1 | 0.73 | 0.21 | 0.74 | 0.32 |
| 834 | Train 14 | Test 2 | 0.75 | 0.23 | 0.78 | 0.36 | 2882 | Train 46 | Test 2 | 0.75 | 0.23 | 0.78 | 0.36 |
| 835 | Train 14 | Test 3 | 0.73 | 0.21 | 0.74 | 0.32 | 2883 | Train 46 | Test 3 | 0.73 | 0.21 | 0.74 | 0.32 |
| 836 | Train 14 | Test 4 | 0.75 | 0.23 | 0.79 | 0.36 | 2884 | Train 46 | Test 4 | 0.75 | 0.23 | 0.79 | 0.36 |
| 837 | Train 14 | Test 5 | 0.72 | 0.2 | 0.71 | 0.31 | 2885 | Train 46 | Test 5 | 0.72 | 0.2 | 0.71 | 0.31 |
| 838 | Train 14 | Test 6 | 0.74 | 0.29 | 0.7 | 0.41 | 2886 | Train 46 | Test 6 | 0.74 | 0.29 | 0.7 | 0.41 |
| 839 | Train 14 | Test 7 | 0.72 | 0.2 | 0.71 | 0.31 | 2887 | Train 46 | Test 7 | 0.72 | 0.2 | 0.71 | 0.31 |
| 840 | Train 14 | Test 8 | 0.74 | 0.28 | 0.7 | 0.4 | 2888 | Train 46 | Test 8 | 0.74 | 0.28 | 0.7 | 0.4 |
| 841 | Train 14 | Test 9 | 0.73 | 0.21 | 0.72 | 0.32 | 2889 | Train 46 | Test 9 | 0.73 | 0.21 | 0.71 | 0.32 |
| 842 | Train 14 | Test 10 | 0.75 | 0.24 | 0.76 | 0.36 | 2890 | Train 46 | Test 10 | 0.75 | 0.24 | 0.76 | 0.36 |
| 843 | Train 14 | Test 11 | 0.73 | 0.21 | 0.72 | 0.32 | 2891 | Train 46 | Test 11 | 0.73 | 0.21 | 0.72 | 0.32 |
| 844 | Train 14 | Test 12 | 0.75 | 0.24 | 0.77 | 0.36 | 2892 | Train 46 | Test 12 | 0.75 | 0.24 | 0.76 | 0.36 |
| 845 | Train 14 | Test 13 | 0.72 | 0.2 | 0.68 | 0.31 | 2893 | Train 46 | Test 13 | 0.72 | 0.2 | 0.68 | 0.31 |
| 846 | Train 14 | Test 14 | 0.74 | 0.29 | 0.7 | 0.41 | 2894 | Train 46 | Test 14 | 0.74 | 0.29 | 0.7 | 0.41 |
| 847 | Train 14 | Test 15 | 0.72 | 0.2 | 0.68 | 0.31 | 2895 | Train 46 | Test 15 | 0.72 | 0.2 | 0.68 | 0.31 |
| 848 | Train 14 | Test 16 | 0.74 | 0.28 | 0.7 | 0.4 | 2896 | Train 46 | Test 16 | 0.74 | 0.28 | 0.7 | 0.4 |
| 849 | Train 14 | Test 17 | 0.73 | 0.21 | 0.74 | 0.32 | 2897 | Train 46 | Test 17 | 0.73 | 0.21 | 0.74 | 0.32 |
| 850 | Train 14 | Test 18 | 0.75 | 0.23 | 0.78 | 0.36 | 2898 | Train 46 | Test 18 | 0.75 | 0.23 | 0.78 | 0.36 |
| 851 | Train 14 | Test 19 | 0.73 | 0.21 | 0.74 | 0.32 | 2899 | Train 46 | Test 19 | 0.73 | 0.21 | 0.74 | 0.32 |
| 852 | Train 14 | Test 20 | 0.75 | 0.23 | 0.79 | 0.36 | 2900 | Train 46 | Test 20 | 0.75 | 0.23 | 0.79 | 0.36 |
| 853 | Train 14 | Test 21 | 0.72 | 0.2 | 0.71 | 0.31 | 2901 | Train 46 | Test 21 | 0.72 | 0.2 | 0.71 | 0.31 |
| 854 | Train 14 | Test 22 | 0.74 | 0.29 | 0.7 | 0.41 | 2902 | Train 46 | Test 22 | 0.74 | 0.29 | 0.7 | 0.41 |
| 855 | Train 14 | Test 23 | 0.72 | 0.2 | 0.71 | 0.31 | 2903 | Train 46 | Test 23 | 0.72 | 0.2 | 0.71 | 0.31 |
| 856 | Train 14 | Test 24 | 0.74 | 0.28 | 0.7 | 0.4 | 2904 | Train 46 | Test 24 | 0.74 | 0.28 | 0.7 | 0.4 |
| 857 | Train 14 | Test 25 | 0.73 | 0.21 | 0.72 | 0.32 | 2905 | Train 46 | Test 25 | 0.73 | 0.21 | 0.72 | 0.32 |
| 858 | Train 14 | Test 26 | 0.75 | 0.24 | 0.77 | 0.36 | 2906 | Train 46 | Test 26 | 0.75 | 0.24 | 0.76 | 0.36 |
| 859 | Train 14 | Test 27 | 0.73 | 0.21 | 0.72 | 0.32 | 2907 | Train 46 | Test 27 | 0.73 | 0.21 | 0.72 | 0.32 |
| 860 | Train 14 | Test 28 | 0.75 | 0.24 | 0.77 | 0.36 | 2908 | Train 46 | Test 28 | 0.75 | 0.24 | 0.76 | 0.36 |
| 861 | Train 14 | Test 29 | 0.72 | 0.2 | 0.68 | 0.31 | 2909 | Train 46 | Test 29 | 0.72 | 0.2 | 0.68 | 0.31 |
| 862 | Train 14 | Test 30 | 0.74 | 0.29 | 0.7 | 0.41 | 2910 | Train 46 | Test 30 | 0.74 | 0.28 | 0.7 | 0.41 |
| 863 | Train 14 | Test 31 | 0.72 | 0.2 | 0.68 | 0.31 | 2911 | Train 46 | Test 31 | 0.72 | 0.2 | 0.68 | 0.31 |
| 864 | Train 14 | Test 32 | 0.74 | 0.28 | 0.7 | 0.4 | 2912 | Train 46 | Test 32 | 0.74 | 0.28 | 0.7 | 0.4 |
| 865 | Train 14 | Test 33 | 0.74 | 0.2 | 0.74 | 0.32 | 2913 | Train 46 | Test 33 | 0.74 | 0.2 | 0.74 | 0.32 |
| 866 | Train 14 | Test 34 | 0.75 | 0.23 | 0.78 | 0.36 | 2914 | Train 46 | Test 34 | 0.75 | 0.23 | 0.78 | 0.36 |
| 867 | Train 14 | Test 35 | 0.74 | 0.21 | 0.74 | 0.32 | 2915 | Train 46 | Test 35 | 0.74 | 0.21 | 0.74 | 0.32 |
| 868 | Train 14 | Test 36 | 0.75 | 0.23 | 0.78 | 0.36 | 2916 | Train 46 | Test 36 | 0.75 | 0.23 | 0.78 | 0.36 |
| 869 | Train 14 | Test 37 | 0.72 | 0.2 | 0.71 | 0.31 | 2917 | Train 46 | Test 37 | 0.72 | 0.2 | 0.71 | 0.31 |
| 870 | Train 14 | Test 38 | 0.74 | 0.29 | 0.7 | 0.41 | 2918 | Train 46 | Test 38 | 0.74 | 0.29 | 0.7 | 0.41 |
| 871 | Train 14 | Test 39 | 0.72 | 0.2 | 0.7 | 0.31 | 2919 | Train 46 | Test 39 | 0.72 | 0.2 | 0.7 | 0.31 |
| 872 | Train 14 | Test 40 | 0.74 | 0.28 | 0.7 | 0.4 | 2920 | Train 46 | Test 40 | 0.74 | 0.28 | 0.7 | 0.4 |
| 873 | Train 14 | Test 41 | 0.74 | 0.21 | 0.71 | 0.32 | 2921 | Train 46 | Test 41 | 0.74 | 0.21 | 0.71 | 0.32 |
| 874 | Train 14 | Test 42 | 0.75 | 0.24 | 0.76 | 0.36 | 2922 | Train 46 | Test 42 | 0.75 | 0.24 | 0.76 | 0.36 |
| 875 | Train 14 | Test 43 | 0.74 | 0.21 | 0.72 | 0.32 | 2923 | Train 46 | Test 43 | 0.74 | 0.21 | 0.71 | 0.32 |
| 876 | Train 14 | Test 44 | 0.75 | 0.24 | 0.76 | 0.36 | 2924 | Train 46 | Test 44 | 0.75 | 0.24 | 0.76 | 0.36 |
| 877 | Train 14 | Test 45 | 0.72 | 0.2 | 0.68 | 0.31 | 2925 | Train 46 | Test 45 | 0.72 | 0.2 | 0.68 | 0.31 |
| 878 | Train 14 | Test 46 | 0.74 | 0.29 | 0.7 | 0.41 | 2926 | Train 46 | Test 46 | 0.74 | 0.28 | 0.7 | 0.4 |
| 879 | Train 14 | Test 47 | 0.72 | 0.2 | 0.68 | 0.31 | 2927 | Train 46 | Test 47 | 0.72 | 0.2 | 0.68 | 0.31 |
| 880 | Train 14 | Test 48 | 0.74 | 0.28 | 0.7 | 0.4 | 2928 | Train 46 | Test 48 | 0.74 | 0.28 | 0.7 | 0.4 |
| 881 | Train 14 | Test 49 | 0.77 | 0.26 | 0.74 | 0.38 | 2929 | Train 46 | Test 49 | 0.77 | 0.26 | 0.74 | 0.39 |
| 882 | Train 14 | Test 50 | 0.76 | 0.26 | 0.74 | 0.39 | 2930 | Train 46 | Test 50 | 0.77 | 0.26 | 0.74 | 0.39 |
| 883 | Train 14 | Test 51 | 0.77 | 0.26 | 0.75 | 0.39 | 2931 | Train 46 | Test 51 | 0.77 | 0.26 | 0.75 | 0.39 |
| 884 | Train 14 | Test 52 | 0.76 | 0.26 | 0.75 | 0.39 | 2932 | Train 46 | Test 52 | 0.76 | 0.26 | 0.75 | 0.39 |
| 885 | Train 14 | Test 53 | 0.76 | 0.26 | 0.66 | 0.37 | 2933 | Train 46 | Test 53 | 0.76 | 0.26 | 0.66 | 0.37 |
| 886 | Train 14 | Test 54 | 0.75 | 0.33 | 0.7 | 0.45 | 2934 | Train 46 | Test 54 | 0.75 | 0.33 | 0.7 | 0.45 |
| 887 | Train 14 | Test 55 | 0.75 | 0.25 | 0.7 | 0.37 | 2935 | Train 46 | Test 55 | 0.75 | 0.25 | 0.7 | 0.37 |
| 888 | Train 14 | Test 56 | 0.74 | 0.32 | 0.71 | 0.44 | 2936 | Train 46 | Test 56 | 0.74 | 0.32 | 0.71 | 0.44 |
| 889 | Train 14 | Test 57 | 0.77 | 0.26 | 0.74 | 0.39 | 2937 | Train 46 | Test 57 | 0.77 | 0.27 | 0.74 | 0.39 |
| 890 | Train 14 | Test 58 | 0.77 | 0.27 | 0.74 | 0.39 | 2938 | Train 46 | Test 58 | 0.77 | 0.27 | 0.74 | 0.39 |
| 891 | Train 14 | Test 59 | 0.77 | 0.27 | 0.74 | 0.39 | 2939 | Train 46 | Test 59 | 0.77 | 0.27 | 0.74 | 0.39 |
| 892 | Train 14 | Test 60 | 0.77 | 0.27 | 0.74 | 0.39 | 2940 | Train 46 | Test 60 | 0.77 | 0.27 | 0.74 | 0.39 |
| 893 | Train 14 | Test 61 | 0.76 | 0.27 | 0.65 | 0.38 | 2941 | Train 46 | Test 61 | 0.76 | 0.27 | 0.65 | 0.38 |
| 894 | Train 14 | Test 62 | 0.75 | 0.33 | 0.7 | 0.45 | 2942 | Train 46 | Test 62 | 0.75 | 0.33 | 0.7 | 0.45 |
| 895 | Train 14 | Test 63 | 0.76 | 0.27 | 0.65 | 0.38 | 2943 | Train 46 | Test 63 | 0.76 | 0.26 | 0.65 | 0.38 |
| 896 | Train 14 | Test 64 | 0.74 | 0.31 | 0.71 | 0.43 | 2944 | Train 46 | Test 64 | 0.74 | 0.31 | 0.71 | 0.43 |
| 897 | Train 15 | Test 1 | 0.75 | 0.21 | 0.76 | 0.33 | 2945 | Train 47 | Test 1 | 0.75 | 0.21 | 0.76 | 0.33 |
| 898 | Train 15 | Test 2 | 0.75 | 0.24 | 0.78 | 0.37 | 2946 | Train 47 | Test 2 | 0.75 | 0.24 | 0.78 | 0.37 |
| 899 | Train 15 | Test 3 | 0.75 | 0.21 | 0.76 | 0.33 | 2947 | Train 47 | Test 3 | 0.75 | 0.21 | 0.76 | 0.33 |
| 900 | Train 15 | Test 4 | 0.75 | 0.24 | 0.78 | 0.37 | 2948 | Train 47 | Test 4 | 0.75 | 0.24 | 0.78 | 0.37 |
| 901 | Train 15 | Test 5 | 0.73 | 0.2 | 0.73 | 0.32 | 2949 | Train 47 | Test 5 | 0.73 | 0.2 | 0.74 | 0.32 |
| 902 | Train 15 | Test 6 | 0.7 | 0.29 | 0.59 | 0.39 | 2950 | Train 47 | Test 6 | 0.7 | 0.29 | 0.6 | 0.39 |
| 903 | Train 15 | Test 7 | 0.73 | 0.2 | 0.73 | 0.32 | 2951 | Train 47 | Test 7 | 0.73 | 0.2 | 0.73 | 0.32 |
| 904 | Train 15 | Test 8 | 0.7 | 0.28 | 0.59 | 0.38 | 2952 | Train 47 | Test 8 | 0.7 | 0.28 | 0.6 | 0.38 |
| 905 | Train 15 | Test 9 | 0.75 | 0.21 | 0.74 | 0.33 | 2953 | Train 47 | Test 9 | 0.75 | 0.21 | 0.74 | 0.33 |
| 906 | Train 15 | Test 10 | 0.75 | 0.25 | 0.76 | 0.37 | 2954 | Train 47 | Test 10 | 0.75 | 0.24 | 0.76 | 0.37 |
| 907 | Train 15 | Test 11 | 0.75 | 0.22 | 0.74 | 0.33 | 2955 | Train 47 | Test 11 | 0.75 | 0.21 | 0.74 | 0.33 |
| 908 | Train 15 | Test 12 | 0.75 | 0.25 | 0.76 | 0.37 | 2956 | Train 47 | Test 12 | 0.75 | 0.25 | 0.76 | 0.37 |
| 909 | Train 15 | Test 13 | 0.73 | 0.21 | 0.71 | 0.32 | 2957 | Train 47 | Test 13 | 0.73 | 0.21 | 0.71 | 0.32 |
| 910 | Train 15 | Test 14 | 0.7 | 0.29 | 0.59 | 0.39 | 2958 | Train 47 | Test 14 | 0.7 | 0.29 | 0.6 | 0.39 |
| 911 | Train 15 | Test 15 | 0.73 | 0.21 | 0.7 | 0.32 | 2959 | Train 47 | Test 15 | 0.73 | 0.21 | 0.71 | 0.32 |
| 912 | Train 15 | Test 16 | 0.7 | 0.28 | 0.59 | 0.38 | 2960 | Train 47 | Test 16 | 0.7 | 0.28 | 0.6 | 0.38 |
| 913 | Train 15 | Test 17 | 0.75 | 0.21 | 0.76 | 0.33 | 2961 | Train 47 | Test 17 | 0.75 | 0.21 | 0.76 | 0.33 |
| 914 | Train 15 | Test 18 | 0.75 | 0.24 | 0.78 | 0.37 | 2962 | Train 47 | Test 18 | 0.75 | 0.24 | 0.78 | 0.37 |
| 915 | Train 15 | Test 19 | 0.75 | 0.21 | 0.76 | 0.33 | 2963 | Train 47 | Test 19 | 0.75 | 0.21 | 0.76 | 0.33 |
| 916 | Train 15 | Test 20 | 0.75 | 0.24 | 0.78 | 0.37 | 2964 | Train 47 | Test 20 | 0.75 | 0.24 | 0.78 | 0.37 |
| 917 | Train 15 | Test 21 | 0.73 | 0.2 | 0.73 | 0.32 | 2965 | Train 47 | Test 21 | 0.73 | 0.2 | 0.74 | 0.32 |
| 918 | Train 15 | Test 22 | 0.7 | 0.29 | 0.59 | 0.39 | 2966 | Train 47 | Test 22 | 0.7 | 0.29 | 0.6 | 0.39 |
| 919 | Train 15 | Test 23 | 0.73 | 0.2 | 0.73 | 0.32 | 2967 | Train 47 | Test 23 | 0.73 | 0.2 | 0.73 | 0.32 |
| 920 | Train 15 | Test 24 | 0.7 | 0.28 | 0.59 | 0.38 | 2968 | Train 47 | Test 24 | 0.7 | 0.28 | 0.6 | 0.38 |
| 921 | Train 15 | Test 25 | 0.75 | 0.21 | 0.74 | 0.33 | 2969 | Train 47 | Test 25 | 0.75 | 0.21 | 0.74 | 0.33 |
| 922 | Train 15 | Test 26 | 0.75 | 0.25 | 0.76 | 0.37 | 2970 | Train 47 | Test 26 | 0.75 | 0.24 | 0.76 | 0.37 |
| 923 | Train 15 | Test 27 | 0.75 | 0.22 | 0.74 | 0.33 | 2971 | Train 47 | Test 27 | 0.75 | 0.21 | 0.74 | 0.33 |
| 924 | Train 15 | Test 28 | 0.75 | 0.25 | 0.76 | 0.37 | 2972 | Train 47 | Test 28 | 0.75 | 0.25 | 0.76 | 0.37 |
| 925 | Train 15 | Test 29 | 0.73 | 0.21 | 0.71 | 0.32 | 2973 | Train 47 | Test 29 | 0.73 | 0.21 | 0.71 | 0.32 |
| 926 | Train 15 | Test 30 | 0.7 | 0.29 | 0.59 | 0.39 | 2974 | Train 47 | Test 30 | 0.7 | 0.29 | 0.6 | 0.39 |
| 927 | Train 15 | Test 31 | 0.73 | 0.21 | 0.7 | 0.32 | 2975 | Train 47 | Test 31 | 0.73 | 0.21 | 0.71 | 0.32 |
| 928 | Train 15 | Test 32 | 0.7 | 0.28 | 0.59 | 0.38 | 2976 | Train 47 | Test 32 | 0.7 | 0.28 | 0.6 | 0.38 |
| 929 | Train 15 | Test 33 | 0.75 | 0.21 | 0.75 | 0.33 | 2977 | Train 47 | Test 33 | 0.75 | 0.21 | 0.76 | 0.33 |
| 930 | Train 15 | Test 34 | 0.75 | 0.24 | 0.77 | 0.36 | 2978 | Train 47 | Test 34 | 0.75 | 0.24 | 0.77 | 0.36 |
| 931 | Train 15 | Test 35 | 0.75 | 0.21 | 0.76 | 0.33 | 2979 | Train 47 | Test 35 | 0.75 | 0.21 | 0.76 | 0.33 |
| 932 | Train 15 | Test 36 | 0.75 | 0.24 | 0.77 | 0.37 | 2980 | Train 47 | Test 36 | 0.75 | 0.24 | 0.78 | 0.37 |
| 933 | Train 15 | Test 37 | 0.74 | 0.2 | 0.73 | 0.32 | 2981 | Train 47 | Test 37 | 0.73 | 0.2 | 0.73 | 0.32 |
| 934 | Train 15 | Test 38 | 0.7 | 0.29 | 0.59 | 0.39 | 2982 | Train 47 | Test 38 | 0.7 | 0.29 | 0.6 | 0.39 |
| 935 | Train 15 | Test 39 | 0.73 | 0.2 | 0.73 | 0.32 | 2983 | Train 47 | Test 39 | 0.73 | 0.2 | 0.73 | 0.32 |
| 936 | Train 15 | Test 40 | 0.7 | 0.28 | 0.59 | 0.38 | 2984 | Train 47 | Test 40 | 0.7 | 0.28 | 0.6 | 0.38 |
| 937 | Train 15 | Test 41 | 0.75 | 0.21 | 0.74 | 0.33 | 2985 | Train 47 | Test 41 | 0.75 | 0.21 | 0.74 | 0.33 |
| 938 | Train 15 | Test 42 | 0.75 | 0.24 | 0.76 | 0.37 | 2986 | Train 47 | Test 42 | 0.75 | 0.24 | 0.76 | 0.37 |
| 939 | Train 15 | Test 43 | 0.75 | 0.21 | 0.74 | 0.33 | 2987 | Train 47 | Test 43 | 0.75 | 0.21 | 0.74 | 0.33 |
| 940 | Train 15 | Test 44 | 0.75 | 0.25 | 0.76 | 0.37 | 2988 | Train 47 | Test 44 | 0.75 | 0.24 | 0.76 | 0.37 |
| 941 | Train 15 | Test 45 | 0.74 | 0.21 | 0.71 | 0.32 | 2989 | Train 47 | Test 45 | 0.74 | 0.21 | 0.71 | 0.32 |
| 942 | Train 15 | Test 46 | 0.7 | 0.29 | 0.59 | 0.39 | 2990 | Train 47 | Test 46 | 0.7 | 0.29 | 0.6 | 0.39 |
| 943 | Train 15 | Test 47 | 0.73 | 0.21 | 0.7 | 0.32 | 2991 | Train 47 | Test 47 | 0.73 | 0.21 | 0.71 | 0.32 |
| 944 | Train 15 | Test 48 | 0.7 | 0.28 | 0.59 | 0.38 | 2992 | Train 47 | Test 48 | 0.7 | 0.28 | 0.6 | 0.38 |
| 945 | Train 15 | Test 49 | 0.77 | 0.24 | 0.79 | 0.37 | 2993 | Train 47 | Test 49 | 0.77 | 0.24 | 0.79 | 0.37 |
| 946 | Train 15 | Test 50 | 0.76 | 0.24 | 0.78 | 0.37 | 2994 | Train 47 | Test 50 | 0.76 | 0.24 | 0.79 | 0.37 |
| 947 | Train 15 | Test 51 | 0.77 | 0.24 | 0.79 | 0.37 | 2995 | Train 47 | Test 51 | 0.77 | 0.24 | 0.79 | 0.37 |
| 948 | Train 15 | Test 52 | 0.76 | 0.24 | 0.79 | 0.37 | 2996 | Train 47 | Test 52 | 0.76 | 0.24 | 0.79 | 0.37 |
| 949 | Train 15 | Test 53 | 0.76 | 0.25 | 0.74 | 0.38 | 2997 | Train 47 | Test 53 | 0.76 | 0.25 | 0.74 | 0.37 |
| 950 | Train 15 | Test 54 | 0.72 | 0.33 | 0.59 | 0.42 | 2998 | Train 47 | Test 54 | 0.72 | 0.33 | 0.6 | 0.43 |
| 951 | Train 15 | Test 55 | 0.76 | 0.24 | 0.76 | 0.36 | 2999 | Train 47 | Test 55 | 0.76 | 0.24 | 0.77 | 0.36 |
| 952 | Train 15 | Test 56 | 0.71 | 0.32 | 0.6 | 0.41 | 3000 | Train 47 | Test 56 | 0.71 | 0.31 | 0.61 | 0.41 |
| 953 | Train 15 | Test 57 | 0.77 | 0.25 | 0.78 | 0.37 | 3001 | Train 47 | Test 57 | 0.77 | 0.24 | 0.78 | 0.37 |
| 954 | Train 15 | Test 58 | 0.77 | 0.25 | 0.78 | 0.37 | 3002 | Train 47 | Test 58 | 0.77 | 0.24 | 0.78 | 0.37 |
| 955 | Train 15 | Test 59 | 0.77 | 0.25 | 0.78 | 0.38 | 3003 | Train 47 | Test 59 | 0.77 | 0.25 | 0.78 | 0.38 |
| 956 | Train 15 | Test 60 | 0.77 | 0.25 | 0.78 | 0.38 | 3004 | Train 47 | Test 60 | 0.77 | 0.25 | 0.78 | 0.38 |
| 957 | Train 15 | Test 61 | 0.76 | 0.26 | 0.73 | 0.38 | 3005 | Train 47 | Test 61 | 0.76 | 0.25 | 0.73 | 0.37 |
| 958 | Train 15 | Test 62 | 0.72 | 0.33 | 0.59 | 0.42 | 3006 | Train 47 | Test 62 | 0.72 | 0.33 | 0.6 | 0.42 |
| 959 | Train 15 | Test 63 | 0.77 | 0.26 | 0.73 | 0.38 | 3007 | Train 47 | Test 63 | 0.77 | 0.25 | 0.74 | 0.38 |
| 960 | Train 15 | Test 64 | 0.72 | 0.32 | 0.6 | 0.41 | 3008 | Train 47 | Test 64 | 0.72 | 0.31 | 0.61 | 0.41 |
| 961 | Train 16 | Test 1 | 0.74 | 0.2 | 0.77 | 0.32 | 3009 | Train 48 | Test 1 | 0.74 | 0.2 | 0.77 | 0.32 |
| 962 | Train 16 | Test 2 | 0.75 | 0.23 | 0.8 | 0.36 | 3010 | Train 48 | Test 2 | 0.75 | 0.23 | 0.8 | 0.36 |
| 963 | Train 16 | Test 3 | 0.74 | 0.2 | 0.77 | 0.32 | 3011 | Train 48 | Test 3 | 0.74 | 0.2 | 0.77 | 0.32 |
| 964 | Train 16 | Test 4 | 0.75 | 0.23 | 0.8 | 0.36 | 3012 | Train 48 | Test 4 | 0.75 | 0.23 | 0.8 | 0.36 |
| 965 | Train 16 | Test 5 | 0.72 | 0.2 | 0.74 | 0.31 | 3013 | Train 48 | Test 5 | 0.72 | 0.2 | 0.74 | 0.31 |
| 966 | Train 16 | Test 6 | 0.74 | 0.29 | 0.71 | 0.41 | 3014 | Train 48 | Test 6 | 0.74 | 0.29 | 0.71 | 0.41 |
| 967 | Train 16 | Test 7 | 0.72 | 0.2 | 0.73 | 0.31 | 3015 | Train 48 | Test 7 | 0.72 | 0.2 | 0.73 | 0.31 |
| 968 | Train 16 | Test 8 | 0.74 | 0.28 | 0.71 | 0.4 | 3016 | Train 48 | Test 8 | 0.74 | 0.28 | 0.71 | 0.4 |
| 969 | Train 16 | Test 9 | 0.74 | 0.2 | 0.75 | 0.32 | 3017 | Train 48 | Test 9 | 0.74 | 0.2 | 0.75 | 0.32 |
| 970 | Train 16 | Test 10 | 0.75 | 0.23 | 0.79 | 0.36 | 3018 | Train 48 | Test 10 | 0.75 | 0.23 | 0.79 | 0.36 |
| 971 | Train 16 | Test 11 | 0.74 | 0.21 | 0.75 | 0.32 | 3019 | Train 48 | Test 11 | 0.74 | 0.21 | 0.75 | 0.32 |
| 972 | Train 16 | Test 12 | 0.75 | 0.23 | 0.79 | 0.36 | 3020 | Train 48 | Test 12 | 0.75 | 0.23 | 0.79 | 0.36 |
| 973 | Train 16 | Test 13 | 0.72 | 0.2 | 0.71 | 0.31 | 3021 | Train 48 | Test 13 | 0.72 | 0.2 | 0.71 | 0.31 |
| 974 | Train 16 | Test 14 | 0.74 | 0.29 | 0.71 | 0.41 | 3022 | Train 48 | Test 14 | 0.74 | 0.29 | 0.71 | 0.41 |
| 975 | Train 16 | Test 15 | 0.72 | 0.2 | 0.71 | 0.31 | 3023 | Train 48 | Test 15 | 0.72 | 0.2 | 0.71 | 0.31 |
| 976 | Train 16 | Test 16 | 0.74 | 0.28 | 0.71 | 0.4 | 3024 | Train 48 | Test 16 | 0.74 | 0.28 | 0.71 | 0.4 |
| 977 | Train 16 | Test 17 | 0.74 | 0.2 | 0.77 | 0.32 | 3025 | Train 48 | Test 17 | 0.74 | 0.2 | 0.77 | 0.32 |
| 978 | Train 16 | Test 18 | 0.75 | 0.23 | 0.8 | 0.36 | 3026 | Train 48 | Test 18 | 0.75 | 0.23 | 0.8 | 0.36 |
| 979 | Train 16 | Test 19 | 0.74 | 0.2 | 0.77 | 0.32 | 3027 | Train 48 | Test 19 | 0.74 | 0.2 | 0.77 | 0.32 |
| 980 | Train 16 | Test 20 | 0.75 | 0.23 | 0.8 | 0.36 | 3028 | Train 48 | Test 20 | 0.75 | 0.23 | 0.8 | 0.36 |
| 981 | Train 16 | Test 21 | 0.72 | 0.2 | 0.74 | 0.31 | 3029 | Train 48 | Test 21 | 0.72 | 0.2 | 0.74 | 0.31 |
| 982 | Train 16 | Test 22 | 0.74 | 0.29 | 0.71 | 0.41 | 3030 | Train 48 | Test 22 | 0.74 | 0.29 | 0.71 | 0.41 |
| 983 | Train 16 | Test 23 | 0.72 | 0.2 | 0.73 | 0.31 | 3031 | Train 48 | Test 23 | 0.72 | 0.2 | 0.73 | 0.31 |
| 984 | Train 16 | Test 24 | 0.74 | 0.28 | 0.71 | 0.4 | 3032 | Train 48 | Test 24 | 0.74 | 0.28 | 0.71 | 0.4 |
| 985 | Train 16 | Test 25 | 0.74 | 0.2 | 0.75 | 0.32 | 3033 | Train 48 | Test 25 | 0.74 | 0.2 | 0.75 | 0.32 |
| 986 | Train 16 | Test 26 | 0.75 | 0.23 | 0.79 | 0.36 | 3034 | Train 48 | Test 26 | 0.75 | 0.23 | 0.79 | 0.36 |
| 987 | Train 16 | Test 27 | 0.74 | 0.21 | 0.75 | 0.32 | 3035 | Train 48 | Test 27 | 0.74 | 0.21 | 0.75 | 0.32 |
| 988 | Train 16 | Test 28 | 0.75 | 0.23 | 0.79 | 0.36 | 3036 | Train 48 | Test 28 | 0.75 | 0.23 | 0.79 | 0.36 |
| 989 | Train 16 | Test 29 | 0.72 | 0.2 | 0.71 | 0.31 | 3037 | Train 48 | Test 29 | 0.72 | 0.2 | 0.71 | 0.31 |
| 990 | Train 16 | Test 30 | 0.74 | 0.29 | 0.71 | 0.41 | 3038 | Train 48 | Test 30 | 0.74 | 0.28 | 0.71 | 0.41 |
| 991 | Train 16 | Test 31 | 0.72 | 0.2 | 0.71 | 0.31 | 3039 | Train 48 | Test 31 | 0.72 | 0.2 | 0.71 | 0.31 |
| 992 | Train 16 | Test 32 | 0.74 | 0.28 | 0.71 | 0.4 | 3040 | Train 48 | Test 32 | 0.74 | 0.28 | 0.71 | 0.4 |
| 993 | Train 16 | Test 33 | 0.74 | 0.2 | 0.76 | 0.32 | 3041 | Train 48 | Test 33 | 0.74 | 0.2 | 0.76 | 0.32 |
| 994 | Train 16 | Test 34 | 0.75 | 0.23 | 0.8 | 0.35 | 3042 | Train 48 | Test 34 | 0.75 | 0.23 | 0.8 | 0.35 |
| 995 | Train 16 | Test 35 | 0.74 | 0.2 | 0.76 | 0.32 | 3043 | Train 48 | Test 35 | 0.74 | 0.2 | 0.76 | 0.32 |
| 996 | Train 16 | Test 36 | 0.75 | 0.23 | 0.8 | 0.36 | 3044 | Train 48 | Test 36 | 0.75 | 0.23 | 0.8 | 0.36 |
| 997 | Train 16 | Test 37 | 0.72 | 0.2 | 0.73 | 0.31 | 3045 | Train 48 | Test 37 | 0.72 | 0.19 | 0.73 | 0.31 |
| 998 | Train 16 | Test 38 | 0.74 | 0.29 | 0.71 | 0.41 | 3046 | Train 48 | Test 38 | 0.74 | 0.29 | 0.71 | 0.41 |
| 999 | Train 16 | Test 39 | 0.72 | 0.2 | 0.73 | 0.31 | 3047 | Train 48 | Test 39 | 0.72 | 0.19 | 0.73 | 0.31 |
| 1000 | Train 16 | Test 40 | 0.74 | 0.28 | 0.71 | 0.4 | 3048 | Train 48 | Test 40 | 0.74 | 0.28 | 0.71 | 0.4 |
| 1001 | Train 16 | Test 41 | 0.74 | 0.2 | 0.74 | 0.32 | 3049 | Train 48 | Test 41 | 0.74 | 0.2 | 0.74 | 0.32 |
| 1002 | Train 16 | Test 42 | 0.76 | 0.23 | 0.79 | 0.36 | 3050 | Train 48 | Test 42 | 0.75 | 0.23 | 0.78 | 0.36 |
| 1003 | Train 16 | Test 43 | 0.74 | 0.21 | 0.75 | 0.32 | 3051 | Train 48 | Test 43 | 0.74 | 0.2 | 0.74 | 0.32 |
| 1004 | Train 16 | Test 44 | 0.76 | 0.23 | 0.79 | 0.36 | 3052 | Train 48 | Test 44 | 0.75 | 0.23 | 0.79 | 0.36 |
| 1005 | Train 16 | Test 45 | 0.72 | 0.2 | 0.71 | 0.31 | 3053 | Train 48 | Test 45 | 0.72 | 0.2 | 0.71 | 0.31 |
| 1006 | Train 16 | Test 46 | 0.74 | 0.29 | 0.71 | 0.41 | 3054 | Train 48 | Test 46 | 0.74 | 0.28 | 0.71 | 0.41 |
| 1007 | Train 16 | Test 47 | 0.72 | 0.2 | 0.71 | 0.31 | 3055 | Train 48 | Test 47 | 0.72 | 0.2 | 0.71 | 0.31 |
| 1008 | Train 16 | Test 48 | 0.74 | 0.28 | 0.71 | 0.4 | 3056 | Train 48 | Test 48 | 0.74 | 0.28 | 0.71 | 0.4 |
| 1009 | Train 16 | Test 49 | 0.77 | 0.25 | 0.77 | 0.38 | 3057 | Train 48 | Test 49 | 0.77 | 0.25 | 0.77 | 0.38 |
| 1010 | Train 16 | Test 50 | 0.77 | 0.26 | 0.77 | 0.38 | 3058 | Train 48 | Test 50 | 0.77 | 0.26 | 0.77 | 0.38 |
| 1011 | Train 16 | Test 51 | 0.77 | 0.26 | 0.78 | 0.38 | 3059 | Train 48 | Test 51 | 0.77 | 0.26 | 0.77 | 0.38 |
| 1012 | Train 16 | Test 52 | 0.77 | 0.26 | 0.77 | 0.39 | 3060 | Train 48 | Test 52 | 0.77 | 0.26 | 0.77 | 0.39 |
| 1013 | Train 16 | Test 53 | 0.76 | 0.26 | 0.66 | 0.37 | 3061 | Train 48 | Test 53 | 0.76 | 0.26 | 0.67 | 0.37 |
| 1014 | Train 16 | Test 54 | 0.75 | 0.33 | 0.71 | 0.45 | 3062 | Train 48 | Test 54 | 0.75 | 0.33 | 0.71 | 0.45 |
| 1015 | Train 16 | Test 55 | 0.75 | 0.24 | 0.72 | 0.36 | 3063 | Train 48 | Test 55 | 0.75 | 0.24 | 0.72 | 0.36 |
| 1016 | Train 16 | Test 56 | 0.74 | 0.32 | 0.71 | 0.44 | 3064 | Train 48 | Test 56 | 0.74 | 0.32 | 0.71 | 0.44 |
| 1017 | Train 16 | Test 57 | 0.77 | 0.26 | 0.76 | 0.39 | 3065 | Train 48 | Test 57 | 0.77 | 0.26 | 0.76 | 0.39 |
| 1018 | Train 16 | Test 58 | 0.77 | 0.26 | 0.76 | 0.39 | 3066 | Train 48 | Test 58 | 0.77 | 0.26 | 0.76 | 0.39 |
| 1019 | Train 16 | Test 59 | 0.77 | 0.26 | 0.77 | 0.39 | 3067 | Train 48 | Test 59 | 0.77 | 0.26 | 0.77 | 0.39 |
| 1020 | Train 16 | Test 60 | 0.77 | 0.26 | 0.77 | 0.39 | 3068 | Train 48 | Test 60 | 0.77 | 0.26 | 0.76 | 0.39 |
| 1021 | Train 16 | Test 61 | 0.76 | 0.27 | 0.66 | 0.38 | 3069 | Train 48 | Test 61 | 0.76 | 0.26 | 0.66 | 0.38 |
| 1022 | Train 16 | Test 62 | 0.75 | 0.33 | 0.71 | 0.45 | 3070 | Train 48 | Test 62 | 0.75 | 0.33 | 0.71 | 0.45 |
| 1023 | Train 16 | Test 63 | 0.76 | 0.27 | 0.66 | 0.38 | 3071 | Train 48 | Test 63 | 0.76 | 0.26 | 0.67 | 0.38 |
| 1024 | Train 16 | Test 64 | 0.74 | 0.31 | 0.71 | 0.44 | 3072 | Train 48 | Test 64 | 0.74 | 0.31 | 0.71 | 0.44 |
| 1025 | Train 17 | Test 1 | 0.76 | 0.22 | 0.71 | 0.33 | 3073 | Train 49 | Test 1 | 0.74 | 0.22 | 0.68 | 0.33 |
| 1026 | Train 17 | Test 2 | 0.75 | 0.25 | 0.69 | 0.36 | 3074 | Train 49 | Test 2 | 0.72 | 0.24 | 0.64 | 0.35 |
| 1027 | Train 17 | Test 3 | 0.76 | 0.22 | 0.71 | 0.33 | 3075 | Train 49 | Test 3 | 0.74 | 0.22 | 0.68 | 0.33 |
| 1028 | Train 17 | Test 4 | 0.75 | 0.25 | 0.7 | 0.36 | 3076 | Train 49 | Test 4 | 0.72 | 0.24 | 0.64 | 0.35 |
| 1029 | Train 17 | Test 5 | 0.72 | 0.2 | 0.63 | 0.31 | 3077 | Train 49 | Test 5 | 0.7 | 0.2 | 0.65 | 0.31 |
| 1030 | Train 17 | Test 6 | 0.7 | 0.3 | 0.47 | 0.37 | 3078 | Train 49 | Test 6 | 0.69 | 0.28 | 0.54 | 0.37 |
| 1031 | Train 17 | Test 7 | 0.71 | 0.21 | 0.62 | 0.31 | 3079 | Train 49 | Test 7 | 0.7 | 0.2 | 0.65 | 0.31 |
| 1032 | Train 17 | Test 8 | 0.7 | 0.3 | 0.47 | 0.36 | 3080 | Train 49 | Test 8 | 0.69 | 0.28 | 0.54 | 0.37 |
| 1033 | Train 17 | Test 9 | 0.76 | 0.22 | 0.69 | 0.34 | 3081 | Train 49 | Test 9 | 0.74 | 0.22 | 0.66 | 0.33 |
| 1034 | Train 17 | Test 10 | 0.75 | 0.25 | 0.67 | 0.37 | 3082 | Train 49 | Test 10 | 0.72 | 0.25 | 0.61 | 0.35 |
| 1035 | Train 17 | Test 11 | 0.76 | 0.22 | 0.69 | 0.34 | 3083 | Train 49 | Test 11 | 0.74 | 0.22 | 0.66 | 0.33 |
| 1036 | Train 17 | Test 12 | 0.75 | 0.25 | 0.67 | 0.37 | 3084 | Train 49 | Test 12 | 0.72 | 0.25 | 0.62 | 0.35 |
| 1037 | Train 17 | Test 13 | 0.71 | 0.21 | 0.6 | 0.31 | 3085 | Train 49 | Test 13 | 0.7 | 0.2 | 0.63 | 0.31 |
| 1038 | Train 17 | Test 14 | 0.7 | 0.3 | 0.47 | 0.37 | 3086 | Train 49 | Test 14 | 0.69 | 0.28 | 0.54 | 0.37 |
| 1039 | Train 17 | Test 15 | 0.71 | 0.21 | 0.6 | 0.31 | 3087 | Train 49 | Test 15 | 0.7 | 0.2 | 0.62 | 0.31 |
| 1040 | Train 17 | Test 16 | 0.7 | 0.3 | 0.47 | 0.36 | 3088 | Train 49 | Test 16 | 0.69 | 0.28 | 0.54 | 0.37 |
| 1041 | Train 17 | Test 17 | 0.76 | 0.22 | 0.71 | 0.33 | 3089 | Train 49 | Test 17 | 0.74 | 0.22 | 0.68 | 0.33 |
| 1042 | Train 17 | Test 18 | 0.75 | 0.25 | 0.69 | 0.36 | 3090 | Train 49 | Test 18 | 0.72 | 0.24 | 0.64 | 0.35 |
| 1043 | Train 17 | Test 19 | 0.76 | 0.22 | 0.71 | 0.33 | 3091 | Train 49 | Test 19 | 0.74 | 0.22 | 0.68 | 0.33 |
| 1044 | Train 17 | Test 20 | 0.75 | 0.25 | 0.7 | 0.36 | 3092 | Train 49 | Test 20 | 0.72 | 0.24 | 0.64 | 0.35 |
| 1045 | Train 17 | Test 21 | 0.72 | 0.2 | 0.63 | 0.31 | 3093 | Train 49 | Test 21 | 0.7 | 0.2 | 0.65 | 0.31 |
| 1046 | Train 17 | Test 22 | 0.7 | 0.3 | 0.47 | 0.37 | 3094 | Train 49 | Test 22 | 0.69 | 0.28 | 0.54 | 0.37 |
| 1047 | Train 17 | Test 23 | 0.71 | 0.21 | 0.62 | 0.31 | 3095 | Train 49 | Test 23 | 0.7 | 0.2 | 0.65 | 0.31 |
| 1048 | Train 17 | Test 24 | 0.7 | 0.3 | 0.47 | 0.36 | 3096 | Train 49 | Test 24 | 0.69 | 0.28 | 0.54 | 0.37 |
| 1049 | Train 17 | Test 25 | 0.76 | 0.22 | 0.69 | 0.34 | 3097 | Train 49 | Test 25 | 0.74 | 0.22 | 0.66 | 0.33 |
| 1050 | Train 17 | Test 26 | 0.75 | 0.25 | 0.67 | 0.37 | 3098 | Train 49 | Test 26 | 0.72 | 0.25 | 0.61 | 0.35 |
| 1051 | Train 17 | Test 27 | 0.76 | 0.22 | 0.69 | 0.34 | 3099 | Train 49 | Test 27 | 0.74 | 0.22 | 0.66 | 0.33 |
| 1052 | Train 17 | Test 28 | 0.75 | 0.25 | 0.67 | 0.37 | 3100 | Train 49 | Test 28 | 0.72 | 0.25 | 0.62 | 0.35 |
| 1053 | Train 17 | Test 29 | 0.71 | 0.21 | 0.6 | 0.31 | 3101 | Train 49 | Test 29 | 0.7 | 0.2 | 0.63 | 0.31 |
| 1054 | Train 17 | Test 30 | 0.7 | 0.3 | 0.47 | 0.37 | 3102 | Train 49 | Test 30 | 0.69 | 0.28 | 0.54 | 0.37 |
| 1055 | Train 17 | Test 31 | 0.71 | 0.21 | 0.6 | 0.31 | 3103 | Train 49 | Test 31 | 0.7 | 0.2 | 0.62 | 0.31 |
| 1056 | Train 17 | Test 32 | 0.7 | 0.3 | 0.47 | 0.36 | 3104 | Train 49 | Test 32 | 0.7 | 0.28 | 0.54 | 0.37 |
| 1057 | Train 17 | Test 33 | 0.76 | 0.22 | 0.71 | 0.33 | 3105 | Train 49 | Test 33 | 0.74 | 0.21 | 0.68 | 0.33 |
| 1058 | Train 17 | Test 34 | 0.75 | 0.25 | 0.69 | 0.36 | 3106 | Train 49 | Test 34 | 0.72 | 0.24 | 0.64 | 0.35 |
| 1059 | Train 17 | Test 35 | 0.76 | 0.22 | 0.71 | 0.33 | 3107 | Train 49 | Test 35 | 0.74 | 0.22 | 0.68 | 0.33 |
| 1060 | Train 17 | Test 36 | 0.75 | 0.25 | 0.69 | 0.36 | 3108 | Train 49 | Test 36 | 0.72 | 0.24 | 0.64 | 0.35 |
| 1061 | Train 17 | Test 37 | 0.72 | 0.2 | 0.63 | 0.31 | 3109 | Train 49 | Test 37 | 0.71 | 0.2 | 0.65 | 0.31 |
| 1062 | Train 17 | Test 38 | 0.7 | 0.3 | 0.47 | 0.37 | 3110 | Train 49 | Test 38 | 0.69 | 0.28 | 0.54 | 0.37 |
| 1063 | Train 17 | Test 39 | 0.72 | 0.21 | 0.62 | 0.31 | 3111 | Train 49 | Test 39 | 0.71 | 0.2 | 0.65 | 0.31 |
| 1064 | Train 17 | Test 40 | 0.7 | 0.3 | 0.47 | 0.36 | 3112 | Train 49 | Test 40 | 0.69 | 0.28 | 0.54 | 0.37 |
| 1065 | Train 17 | Test 41 | 0.76 | 0.22 | 0.69 | 0.33 | 3113 | Train 49 | Test 41 | 0.74 | 0.22 | 0.65 | 0.33 |
| 1066 | Train 17 | Test 42 | 0.75 | 0.25 | 0.67 | 0.36 | 3114 | Train 49 | Test 42 | 0.72 | 0.24 | 0.61 | 0.35 |
| 1067 | Train 17 | Test 43 | 0.76 | 0.22 | 0.69 | 0.34 | 3115 | Train 49 | Test 43 | 0.74 | 0.22 | 0.66 | 0.33 |
| 1068 | Train 17 | Test 44 | 0.75 | 0.25 | 0.67 | 0.37 | 3116 | Train 49 | Test 44 | 0.72 | 0.25 | 0.61 | 0.35 |
| 1069 | Train 17 | Test 45 | 0.72 | 0.21 | 0.6 | 0.31 | 3117 | Train 49 | Test 45 | 0.71 | 0.2 | 0.62 | 0.31 |
| 1070 | Train 17 | Test 46 | 0.7 | 0.3 | 0.47 | 0.37 | 3118 | Train 49 | Test 46 | 0.69 | 0.28 | 0.54 | 0.37 |
| 1071 | Train 17 | Test 47 | 0.71 | 0.21 | 0.6 | 0.31 | 3119 | Train 49 | Test 47 | 0.7 | 0.2 | 0.62 | 0.31 |
| 1072 | Train 17 | Test 48 | 0.7 | 0.3 | 0.47 | 0.36 | 3120 | Train 49 | Test 48 | 0.7 | 0.28 | 0.54 | 0.37 |
| 1073 | Train 17 | Test 49 | 0.78 | 0.25 | 0.77 | 0.37 | 3121 | Train 49 | Test 49 | 0.78 | 0.27 | 0.74 | 0.4 |
| 1074 | Train 17 | Test 50 | 0.78 | 0.25 | 0.77 | 0.38 | 3122 | Train 49 | Test 50 | 0.78 | 0.27 | 0.74 | 0.4 |
| 1075 | Train 17 | Test 51 | 0.78 | 0.25 | 0.77 | 0.38 | 3123 | Train 49 | Test 51 | 0.78 | 0.27 | 0.74 | 0.4 |
| 1076 | Train 17 | Test 52 | 0.78 | 0.25 | 0.77 | 0.38 | 3124 | Train 49 | Test 52 | 0.78 | 0.27 | 0.74 | 0.4 |
| 1077 | Train 17 | Test 53 | 0.74 | 0.26 | 0.59 | 0.36 | 3125 | Train 49 | Test 53 | 0.73 | 0.23 | 0.69 | 0.35 |
| 1078 | Train 17 | Test 54 | 0.71 | 0.35 | 0.49 | 0.41 | 3126 | Train 49 | Test 54 | 0.71 | 0.33 | 0.58 | 0.42 |
| 1079 | Train 17 | Test 55 | 0.74 | 0.24 | 0.63 | 0.34 | 3127 | Train 49 | Test 55 | 0.73 | 0.23 | 0.69 | 0.35 |
| 1080 | Train 17 | Test 56 | 0.72 | 0.34 | 0.49 | 0.4 | 3128 | Train 49 | Test 56 | 0.72 | 0.32 | 0.58 | 0.41 |
| 1081 | Train 17 | Test 57 | 0.78 | 0.25 | 0.77 | 0.38 | 3129 | Train 49 | Test 57 | 0.79 | 0.28 | 0.72 | 0.4 |
| 1082 | Train 17 | Test 58 | 0.78 | 0.25 | 0.76 | 0.38 | 3130 | Train 49 | Test 58 | 0.79 | 0.28 | 0.72 | 0.4 |
| 1083 | Train 17 | Test 59 | 0.78 | 0.25 | 0.77 | 0.38 | 3131 | Train 49 | Test 59 | 0.79 | 0.28 | 0.72 | 0.4 |
| 1084 | Train 17 | Test 60 | 0.78 | 0.25 | 0.76 | 0.38 | 3132 | Train 49 | Test 60 | 0.79 | 0.28 | 0.72 | 0.4 |
| 1085 | Train 17 | Test 61 | 0.74 | 0.26 | 0.58 | 0.36 | 3133 | Train 49 | Test 61 | 0.73 | 0.24 | 0.68 | 0.35 |
| 1086 | Train 17 | Test 62 | 0.72 | 0.34 | 0.5 | 0.41 | 3134 | Train 49 | Test 62 | 0.71 | 0.32 | 0.58 | 0.41 |
| 1087 | Train 17 | Test 63 | 0.75 | 0.26 | 0.58 | 0.36 | 3135 | Train 49 | Test 63 | 0.73 | 0.24 | 0.68 | 0.35 |
| 1088 | Train 17 | Test 64 | 0.72 | 0.33 | 0.49 | 0.4 | 3136 | Train 49 | Test 64 | 0.72 | 0.32 | 0.58 | 0.41 |
| 1089 | Train 18 | Test 1 | 0.75 | 0.23 | 0.66 | 0.34 | 3137 | Train 50 | Test 1 | 0.73 | 0.23 | 0.64 | 0.33 |
| 1090 | Train 18 | Test 2 | 0.77 | 0.26 | 0.72 | 0.38 | 3138 | Train 50 | Test 2 | 0.72 | 0.25 | 0.6 | 0.35 |
| 1091 | Train 18 | Test 3 | 0.75 | 0.23 | 0.67 | 0.34 | 3139 | Train 50 | Test 3 | 0.73 | 0.23 | 0.64 | 0.34 |
| 1092 | Train 18 | Test 4 | 0.77 | 0.26 | 0.72 | 0.38 | 3140 | Train 50 | Test 4 | 0.72 | 0.25 | 0.61 | 0.36 |
| 1093 | Train 18 | Test 5 | 0.71 | 0.21 | 0.59 | 0.31 | 3141 | Train 50 | Test 5 | 0.7 | 0.21 | 0.59 | 0.31 |
| 1094 | Train 18 | Test 6 | 0.71 | 0.34 | 0.4 | 0.37 | 3142 | Train 50 | Test 6 | 0.69 | 0.29 | 0.48 | 0.36 |
| 1095 | Train 18 | Test 7 | 0.71 | 0.21 | 0.59 | 0.31 | 3143 | Train 50 | Test 7 | 0.7 | 0.21 | 0.58 | 0.31 |
| 1096 | Train 18 | Test 8 | 0.71 | 0.33 | 0.41 | 0.36 | 3144 | Train 50 | Test 8 | 0.69 | 0.29 | 0.48 | 0.36 |
| 1097 | Train 18 | Test 9 | 0.75 | 0.23 | 0.65 | 0.34 | 3145 | Train 50 | Test 9 | 0.73 | 0.23 | 0.61 | 0.33 |
| 1098 | Train 18 | Test 10 | 0.77 | 0.26 | 0.71 | 0.38 | 3146 | Train 50 | Test 10 | 0.72 | 0.25 | 0.57 | 0.35 |
| 1099 | Train 18 | Test 11 | 0.75 | 0.23 | 0.65 | 0.34 | 3147 | Train 50 | Test 11 | 0.73 | 0.23 | 0.61 | 0.33 |
| 1100 | Train 18 | Test 12 | 0.77 | 0.26 | 0.71 | 0.38 | 3148 | Train 50 | Test 12 | 0.72 | 0.26 | 0.57 | 0.35 |
| 1101 | Train 18 | Test 13 | 0.71 | 0.21 | 0.57 | 0.31 | 3149 | Train 50 | Test 13 | 0.7 | 0.21 | 0.56 | 0.31 |
| 1102 | Train 18 | Test 14 | 0.71 | 0.33 | 0.41 | 0.37 | 3150 | Train 50 | Test 14 | 0.69 | 0.29 | 0.48 | 0.36 |
| 1103 | Train 18 | Test 15 | 0.71 | 0.21 | 0.56 | 0.31 | 3151 | Train 50 | Test 15 | 0.7 | 0.21 | 0.55 | 0.31 |
| 1104 | Train 18 | Test 16 | 0.71 | 0.33 | 0.41 | 0.36 | 3152 | Train 50 | Test 16 | 0.69 | 0.29 | 0.48 | 0.36 |
| 1105 | Train 18 | Test 17 | 0.75 | 0.23 | 0.66 | 0.34 | 3153 | Train 50 | Test 17 | 0.73 | 0.23 | 0.64 | 0.33 |
| 1106 | Train 18 | Test 18 | 0.77 | 0.26 | 0.72 | 0.38 | 3154 | Train 50 | Test 18 | 0.72 | 0.25 | 0.6 | 0.35 |
| 1107 | Train 18 | Test 19 | 0.75 | 0.23 | 0.67 | 0.34 | 3155 | Train 50 | Test 19 | 0.73 | 0.23 | 0.64 | 0.34 |
| 1108 | Train 18 | Test 20 | 0.77 | 0.26 | 0.72 | 0.38 | 3156 | Train 50 | Test 20 | 0.72 | 0.25 | 0.61 | 0.36 |
| 1109 | Train 18 | Test 21 | 0.71 | 0.21 | 0.59 | 0.31 | 3157 | Train 50 | Test 21 | 0.7 | 0.21 | 0.59 | 0.31 |
| 1110 | Train 18 | Test 22 | 0.71 | 0.34 | 0.4 | 0.37 | 3158 | Train 50 | Test 22 | 0.69 | 0.29 | 0.48 | 0.36 |
| 1111 | Train 18 | Test 23 | 0.71 | 0.21 | 0.59 | 0.31 | 3159 | Train 50 | Test 23 | 0.7 | 0.21 | 0.58 | 0.31 |
| 1112 | Train 18 | Test 24 | 0.71 | 0.33 | 0.41 | 0.36 | 3160 | Train 50 | Test 24 | 0.69 | 0.29 | 0.48 | 0.36 |
| 1113 | Train 18 | Test 25 | 0.75 | 0.23 | 0.65 | 0.34 | 3161 | Train 50 | Test 25 | 0.73 | 0.23 | 0.61 | 0.33 |
| 1114 | Train 18 | Test 26 | 0.77 | 0.26 | 0.71 | 0.38 | 3162 | Train 50 | Test 26 | 0.72 | 0.25 | 0.57 | 0.35 |
| 1115 | Train 18 | Test 27 | 0.75 | 0.23 | 0.65 | 0.34 | 3163 | Train 50 | Test 27 | 0.73 | 0.23 | 0.61 | 0.33 |
| 1116 | Train 18 | Test 28 | 0.77 | 0.26 | 0.71 | 0.38 | 3164 | Train 50 | Test 28 | 0.72 | 0.26 | 0.57 | 0.35 |
| 1117 | Train 18 | Test 29 | 0.71 | 0.21 | 0.57 | 0.31 | 3165 | Train 50 | Test 29 | 0.7 | 0.21 | 0.56 | 0.31 |
| 1118 | Train 18 | Test 30 | 0.71 | 0.33 | 0.41 | 0.37 | 3166 | Train 50 | Test 30 | 0.69 | 0.29 | 0.48 | 0.36 |
| 1119 | Train 18 | Test 31 | 0.71 | 0.21 | 0.56 | 0.31 | 3167 | Train 50 | Test 31 | 0.7 | 0.21 | 0.55 | 0.31 |
| 1120 | Train 18 | Test 32 | 0.71 | 0.33 | 0.41 | 0.36 | 3168 | Train 50 | Test 32 | 0.69 | 0.29 | 0.48 | 0.36 |
| 1121 | Train 18 | Test 33 | 0.76 | 0.22 | 0.66 | 0.34 | 3169 | Train 50 | Test 33 | 0.73 | 0.23 | 0.63 | 0.33 |
| 1122 | Train 18 | Test 34 | 0.77 | 0.25 | 0.72 | 0.38 | 3170 | Train 50 | Test 34 | 0.72 | 0.25 | 0.6 | 0.35 |
| 1123 | Train 18 | Test 35 | 0.76 | 0.23 | 0.66 | 0.34 | 3171 | Train 50 | Test 35 | 0.74 | 0.23 | 0.64 | 0.33 |
| 1124 | Train 18 | Test 36 | 0.77 | 0.25 | 0.72 | 0.38 | 3172 | Train 50 | Test 36 | 0.72 | 0.25 | 0.6 | 0.35 |
| 1125 | Train 18 | Test 37 | 0.71 | 0.21 | 0.59 | 0.31 | 3173 | Train 50 | Test 37 | 0.7 | 0.21 | 0.58 | 0.31 |
| 1126 | Train 18 | Test 38 | 0.71 | 0.34 | 0.4 | 0.37 | 3174 | Train 50 | Test 38 | 0.69 | 0.29 | 0.48 | 0.36 |
| 1127 | Train 18 | Test 39 | 0.71 | 0.21 | 0.59 | 0.31 | 3175 | Train 50 | Test 39 | 0.7 | 0.21 | 0.58 | 0.31 |
| 1128 | Train 18 | Test 40 | 0.71 | 0.33 | 0.41 | 0.36 | 3176 | Train 50 | Test 40 | 0.69 | 0.29 | 0.48 | 0.36 |
| 1129 | Train 18 | Test 41 | 0.76 | 0.23 | 0.65 | 0.34 | 3177 | Train 50 | Test 41 | 0.74 | 0.23 | 0.6 | 0.33 |
| 1130 | Train 18 | Test 42 | 0.77 | 0.26 | 0.71 | 0.38 | 3178 | Train 50 | Test 42 | 0.72 | 0.25 | 0.57 | 0.35 |
| 1131 | Train 18 | Test 43 | 0.76 | 0.23 | 0.65 | 0.34 | 3179 | Train 50 | Test 43 | 0.74 | 0.23 | 0.61 | 0.33 |
| 1132 | Train 18 | Test 44 | 0.77 | 0.26 | 0.71 | 0.38 | 3180 | Train 50 | Test 44 | 0.72 | 0.25 | 0.57 | 0.35 |
| 1133 | Train 18 | Test 45 | 0.71 | 0.21 | 0.57 | 0.31 | 3181 | Train 50 | Test 45 | 0.7 | 0.21 | 0.56 | 0.31 |
| 1134 | Train 18 | Test 46 | 0.71 | 0.33 | 0.41 | 0.37 | 3182 | Train 50 | Test 46 | 0.69 | 0.29 | 0.48 | 0.36 |
| 1135 | Train 18 | Test 47 | 0.71 | 0.21 | 0.56 | 0.31 | 3183 | Train 50 | Test 47 | 0.7 | 0.21 | 0.55 | 0.31 |
| 1136 | Train 18 | Test 48 | 0.71 | 0.33 | 0.41 | 0.36 | 3184 | Train 50 | Test 48 | 0.69 | 0.29 | 0.48 | 0.36 |
| 1137 | Train 18 | Test 49 | 0.77 | 0.25 | 0.72 | 0.38 | 3185 | Train 50 | Test 49 | 0.78 | 0.27 | 0.75 | 0.4 |
| 1138 | Train 18 | Test 50 | 0.77 | 0.25 | 0.72 | 0.38 | 3186 | Train 50 | Test 50 | 0.78 | 0.27 | 0.74 | 0.4 |
| 1139 | Train 18 | Test 51 | 0.77 | 0.25 | 0.73 | 0.38 | 3187 | Train 50 | Test 51 | 0.78 | 0.27 | 0.75 | 0.4 |
| 1140 | Train 18 | Test 52 | 0.77 | 0.25 | 0.72 | 0.38 | 3188 | Train 50 | Test 52 | 0.78 | 0.27 | 0.74 | 0.4 |
| 1141 | Train 18 | Test 53 | 0.74 | 0.28 | 0.45 | 0.35 | 3189 | Train 50 | Test 53 | 0.73 | 0.24 | 0.63 | 0.35 |
| 1142 | Train 18 | Test 54 | 0.72 | 0.38 | 0.4 | 0.39 | 3190 | Train 50 | Test 54 | 0.71 | 0.33 | 0.53 | 0.41 |
| 1143 | Train 18 | Test 55 | 0.73 | 0.24 | 0.57 | 0.34 | 3191 | Train 50 | Test 55 | 0.73 | 0.24 | 0.63 | 0.35 |
| 1144 | Train 18 | Test 56 | 0.72 | 0.36 | 0.41 | 0.38 | 3192 | Train 50 | Test 56 | 0.71 | 0.33 | 0.53 | 0.4 |
| 1145 | Train 18 | Test 57 | 0.78 | 0.26 | 0.73 | 0.38 | 3193 | Train 50 | Test 57 | 0.79 | 0.28 | 0.72 | 0.4 |
| 1146 | Train 18 | Test 58 | 0.77 | 0.26 | 0.72 | 0.38 | 3194 | Train 50 | Test 58 | 0.79 | 0.28 | 0.72 | 0.4 |
| 1147 | Train 18 | Test 59 | 0.78 | 0.26 | 0.73 | 0.39 | 3195 | Train 50 | Test 59 | 0.79 | 0.28 | 0.73 | 0.4 |
| 1148 | Train 18 | Test 60 | 0.77 | 0.26 | 0.72 | 0.39 | 3196 | Train 50 | Test 60 | 0.79 | 0.28 | 0.72 | 0.4 |
| 1149 | Train 18 | Test 61 | 0.75 | 0.28 | 0.43 | 0.34 | 3197 | Train 50 | Test 61 | 0.73 | 0.24 | 0.62 | 0.35 |
| 1150 | Train 18 | Test 62 | 0.72 | 0.37 | 0.4 | 0.39 | 3198 | Train 50 | Test 62 | 0.71 | 0.33 | 0.53 | 0.41 |
| 1151 | Train 18 | Test 63 | 0.75 | 0.28 | 0.44 | 0.34 | 3199 | Train 50 | Test 63 | 0.73 | 0.24 | 0.62 | 0.35 |
| 1152 | Train 18 | Test 64 | 0.72 | 0.36 | 0.41 | 0.38 | 3200 | Train 50 | Test 64 | 0.71 | 0.32 | 0.53 | 0.4 |
| 1153 | Train 19 | Test 1 | 0.76 | 0.22 | 0.71 | 0.33 | 3201 | Train 51 | Test 1 | 0.74 | 0.21 | 0.67 | 0.33 |
| 1154 | Train 19 | Test 2 | 0.75 | 0.25 | 0.69 | 0.36 | 3202 | Train 51 | Test 2 | 0.72 | 0.24 | 0.62 | 0.34 |
| 1155 | Train 19 | Test 3 | 0.76 | 0.22 | 0.71 | 0.33 | 3203 | Train 51 | Test 3 | 0.74 | 0.22 | 0.67 | 0.33 |
| 1156 | Train 19 | Test 4 | 0.75 | 0.25 | 0.69 | 0.36 | 3204 | Train 51 | Test 4 | 0.72 | 0.24 | 0.62 | 0.34 |
| 1157 | Train 19 | Test 5 | 0.71 | 0.2 | 0.62 | 0.31 | 3205 | Train 51 | Test 5 | 0.7 | 0.2 | 0.64 | 0.31 |
| 1158 | Train 19 | Test 6 | 0.7 | 0.3 | 0.45 | 0.36 | 3206 | Train 51 | Test 6 | 0.69 | 0.28 | 0.53 | 0.37 |
| 1159 | Train 19 | Test 7 | 0.71 | 0.2 | 0.62 | 0.31 | 3207 | Train 51 | Test 7 | 0.7 | 0.2 | 0.64 | 0.31 |
| 1160 | Train 19 | Test 8 | 0.7 | 0.3 | 0.45 | 0.36 | 3208 | Train 51 | Test 8 | 0.69 | 0.28 | 0.52 | 0.37 |
| 1161 | Train 19 | Test 9 | 0.76 | 0.22 | 0.69 | 0.34 | 3209 | Train 51 | Test 9 | 0.74 | 0.22 | 0.65 | 0.33 |
| 1162 | Train 19 | Test 10 | 0.75 | 0.25 | 0.67 | 0.37 | 3210 | Train 51 | Test 10 | 0.72 | 0.25 | 0.6 | 0.35 |
| 1163 | Train 19 | Test 11 | 0.76 | 0.22 | 0.69 | 0.34 | 3211 | Train 51 | Test 11 | 0.74 | 0.22 | 0.66 | 0.33 |
| 1164 | Train 19 | Test 12 | 0.75 | 0.25 | 0.67 | 0.37 | 3212 | Train 51 | Test 12 | 0.72 | 0.25 | 0.6 | 0.35 |
| 1165 | Train 19 | Test 13 | 0.71 | 0.21 | 0.6 | 0.31 | 3213 | Train 51 | Test 13 | 0.7 | 0.21 | 0.62 | 0.31 |
| 1166 | Train 19 | Test 14 | 0.7 | 0.3 | 0.46 | 0.36 | 3214 | Train 51 | Test 14 | 0.69 | 0.28 | 0.53 | 0.37 |
| 1167 | Train 19 | Test 15 | 0.71 | 0.21 | 0.6 | 0.31 | 3215 | Train 51 | Test 15 | 0.7 | 0.21 | 0.61 | 0.31 |
| 1168 | Train 19 | Test 16 | 0.7 | 0.3 | 0.45 | 0.36 | 3216 | Train 51 | Test 16 | 0.69 | 0.28 | 0.52 | 0.36 |
| 1169 | Train 19 | Test 17 | 0.76 | 0.22 | 0.71 | 0.33 | 3217 | Train 51 | Test 17 | 0.74 | 0.21 | 0.67 | 0.33 |
| 1170 | Train 19 | Test 18 | 0.75 | 0.25 | 0.69 | 0.36 | 3218 | Train 51 | Test 18 | 0.72 | 0.24 | 0.62 | 0.34 |
| 1171 | Train 19 | Test 19 | 0.76 | 0.22 | 0.71 | 0.33 | 3219 | Train 51 | Test 19 | 0.74 | 0.22 | 0.67 | 0.33 |
| 1172 | Train 19 | Test 20 | 0.75 | 0.25 | 0.69 | 0.36 | 3220 | Train 51 | Test 20 | 0.72 | 0.24 | 0.62 | 0.34 |
| 1173 | Train 19 | Test 21 | 0.72 | 0.2 | 0.62 | 0.31 | 3221 | Train 51 | Test 21 | 0.7 | 0.2 | 0.64 | 0.31 |
| 1174 | Train 19 | Test 22 | 0.7 | 0.3 | 0.45 | 0.36 | 3222 | Train 51 | Test 22 | 0.69 | 0.28 | 0.53 | 0.37 |
| 1175 | Train 19 | Test 23 | 0.71 | 0.2 | 0.62 | 0.31 | 3223 | Train 51 | Test 23 | 0.7 | 0.2 | 0.64 | 0.31 |
| 1176 | Train 19 | Test 24 | 0.7 | 0.3 | 0.45 | 0.36 | 3224 | Train 51 | Test 24 | 0.69 | 0.28 | 0.52 | 0.37 |
| 1177 | Train 19 | Test 25 | 0.76 | 0.22 | 0.69 | 0.34 | 3225 | Train 51 | Test 25 | 0.74 | 0.22 | 0.65 | 0.33 |
| 1178 | Train 19 | Test 26 | 0.75 | 0.25 | 0.67 | 0.37 | 3226 | Train 51 | Test 26 | 0.72 | 0.25 | 0.6 | 0.35 |
| 1179 | Train 19 | Test 27 | 0.76 | 0.22 | 0.69 | 0.34 | 3227 | Train 51 | Test 27 | 0.74 | 0.22 | 0.66 | 0.33 |
| 1180 | Train 19 | Test 28 | 0.75 | 0.25 | 0.67 | 0.37 | 3228 | Train 51 | Test 28 | 0.72 | 0.25 | 0.6 | 0.35 |
| 1181 | Train 19 | Test 29 | 0.71 | 0.21 | 0.6 | 0.31 | 3229 | Train 51 | Test 29 | 0.7 | 0.21 | 0.62 | 0.31 |
| 1182 | Train 19 | Test 30 | 0.7 | 0.3 | 0.46 | 0.36 | 3230 | Train 51 | Test 30 | 0.69 | 0.28 | 0.53 | 0.37 |
| 1183 | Train 19 | Test 31 | 0.71 | 0.21 | 0.59 | 0.31 | 3231 | Train 51 | Test 31 | 0.7 | 0.21 | 0.61 | 0.31 |
| 1184 | Train 19 | Test 32 | 0.7 | 0.3 | 0.45 | 0.36 | 3232 | Train 51 | Test 32 | 0.69 | 0.28 | 0.52 | 0.36 |
| 1185 | Train 19 | Test 33 | 0.76 | 0.22 | 0.7 | 0.33 | 3233 | Train 51 | Test 33 | 0.74 | 0.21 | 0.67 | 0.32 |
| 1186 | Train 19 | Test 34 | 0.75 | 0.25 | 0.69 | 0.36 | 3234 | Train 51 | Test 34 | 0.72 | 0.24 | 0.62 | 0.34 |
| 1187 | Train 19 | Test 35 | 0.76 | 0.22 | 0.71 | 0.33 | 3235 | Train 51 | Test 35 | 0.74 | 0.21 | 0.67 | 0.32 |
| 1188 | Train 19 | Test 36 | 0.75 | 0.25 | 0.69 | 0.36 | 3236 | Train 51 | Test 36 | 0.72 | 0.24 | 0.62 | 0.34 |
| 1189 | Train 19 | Test 37 | 0.72 | 0.2 | 0.62 | 0.31 | 3237 | Train 51 | Test 37 | 0.71 | 0.2 | 0.64 | 0.31 |
| 1190 | Train 19 | Test 38 | 0.7 | 0.3 | 0.45 | 0.36 | 3238 | Train 51 | Test 38 | 0.69 | 0.28 | 0.53 | 0.37 |
| 1191 | Train 19 | Test 39 | 0.72 | 0.2 | 0.62 | 0.31 | 3239 | Train 51 | Test 39 | 0.71 | 0.2 | 0.64 | 0.31 |
| 1192 | Train 19 | Test 40 | 0.7 | 0.3 | 0.45 | 0.36 | 3240 | Train 51 | Test 40 | 0.69 | 0.28 | 0.53 | 0.37 |
| 1193 | Train 19 | Test 41 | 0.76 | 0.22 | 0.68 | 0.33 | 3241 | Train 51 | Test 41 | 0.74 | 0.22 | 0.65 | 0.33 |
| 1194 | Train 19 | Test 42 | 0.75 | 0.25 | 0.67 | 0.36 | 3242 | Train 51 | Test 42 | 0.72 | 0.25 | 0.6 | 0.35 |
| 1195 | Train 19 | Test 43 | 0.76 | 0.22 | 0.69 | 0.33 | 3243 | Train 51 | Test 43 | 0.74 | 0.22 | 0.65 | 0.33 |
| 1196 | Train 19 | Test 44 | 0.75 | 0.25 | 0.67 | 0.37 | 3244 | Train 51 | Test 44 | 0.72 | 0.25 | 0.6 | 0.35 |
| 1197 | Train 19 | Test 45 | 0.72 | 0.21 | 0.6 | 0.31 | 3245 | Train 51 | Test 45 | 0.71 | 0.21 | 0.62 | 0.31 |
| 1198 | Train 19 | Test 46 | 0.7 | 0.3 | 0.46 | 0.36 | 3246 | Train 51 | Test 46 | 0.69 | 0.28 | 0.53 | 0.37 |
| 1199 | Train 19 | Test 47 | 0.71 | 0.21 | 0.59 | 0.31 | 3247 | Train 51 | Test 47 | 0.7 | 0.21 | 0.61 | 0.31 |
| 1200 | Train 19 | Test 48 | 0.7 | 0.3 | 0.45 | 0.36 | 3248 | Train 51 | Test 48 | 0.69 | 0.28 | 0.52 | 0.36 |
| 1201 | Train 19 | Test 49 | 0.78 | 0.25 | 0.77 | 0.37 | 3249 | Train 51 | Test 49 | 0.78 | 0.27 | 0.74 | 0.4 |
| 1202 | Train 19 | Test 50 | 0.78 | 0.25 | 0.77 | 0.37 | 3250 | Train 51 | Test 50 | 0.78 | 0.27 | 0.74 | 0.4 |
| 1203 | Train 19 | Test 51 | 0.78 | 0.25 | 0.77 | 0.37 | 3251 | Train 51 | Test 51 | 0.78 | 0.27 | 0.74 | 0.4 |
| 1204 | Train 19 | Test 52 | 0.78 | 0.25 | 0.77 | 0.37 | 3252 | Train 51 | Test 52 | 0.78 | 0.27 | 0.74 | 0.4 |
| 1205 | Train 19 | Test 53 | 0.74 | 0.26 | 0.58 | 0.36 | 3253 | Train 51 | Test 53 | 0.73 | 0.23 | 0.69 | 0.35 |
| 1206 | Train 19 | Test 54 | 0.71 | 0.35 | 0.48 | 0.4 | 3254 | Train 51 | Test 54 | 0.71 | 0.33 | 0.57 | 0.42 |
| 1207 | Train 19 | Test 55 | 0.74 | 0.23 | 0.63 | 0.34 | 3255 | Train 51 | Test 55 | 0.73 | 0.24 | 0.69 | 0.35 |
| 1208 | Train 19 | Test 56 | 0.72 | 0.34 | 0.48 | 0.4 | 3256 | Train 51 | Test 56 | 0.71 | 0.32 | 0.57 | 0.41 |
| 1209 | Train 19 | Test 57 | 0.78 | 0.25 | 0.77 | 0.38 | 3257 | Train 51 | Test 57 | 0.79 | 0.28 | 0.73 | 0.41 |
| 1210 | Train 19 | Test 58 | 0.78 | 0.25 | 0.76 | 0.38 | 3258 | Train 51 | Test 58 | 0.79 | 0.28 | 0.73 | 0.41 |
| 1211 | Train 19 | Test 59 | 0.78 | 0.25 | 0.77 | 0.38 | 3259 | Train 51 | Test 59 | 0.79 | 0.28 | 0.73 | 0.41 |
| 1212 | Train 19 | Test 60 | 0.78 | 0.25 | 0.76 | 0.38 | 3260 | Train 51 | Test 60 | 0.79 | 0.28 | 0.73 | 0.41 |
| 1213 | Train 19 | Test 61 | 0.74 | 0.26 | 0.57 | 0.36 | 3261 | Train 51 | Test 61 | 0.73 | 0.24 | 0.68 | 0.35 |
| 1214 | Train 19 | Test 62 | 0.72 | 0.34 | 0.49 | 0.4 | 3262 | Train 51 | Test 62 | 0.71 | 0.32 | 0.57 | 0.41 |
| 1215 | Train 19 | Test 63 | 0.75 | 0.26 | 0.57 | 0.36 | 3263 | Train 51 | Test 63 | 0.74 | 0.24 | 0.69 | 0.36 |
| 1216 | Train 19 | Test 64 | 0.72 | 0.34 | 0.48 | 0.4 | 3264 | Train 51 | Test 64 | 0.71 | 0.32 | 0.57 | 0.41 |
| 1217 | Train 20 | Test 1 | 0.75 | 0.23 | 0.66 | 0.34 | 3265 | Train 52 | Test 1 | 0.73 | 0.23 | 0.63 | 0.33 |
| 1218 | Train 20 | Test 2 | 0.77 | 0.26 | 0.72 | 0.38 | 3266 | Train 52 | Test 2 | 0.72 | 0.25 | 0.59 | 0.35 |
| 1219 | Train 20 | Test 3 | 0.75 | 0.23 | 0.67 | 0.34 | 3267 | Train 52 | Test 3 | 0.73 | 0.23 | 0.63 | 0.33 |
| 1220 | Train 20 | Test 4 | 0.77 | 0.26 | 0.72 | 0.38 | 3268 | Train 52 | Test 4 | 0.72 | 0.25 | 0.59 | 0.35 |
| 1221 | Train 20 | Test 5 | 0.71 | 0.21 | 0.6 | 0.31 | 3269 | Train 52 | Test 5 | 0.7 | 0.21 | 0.58 | 0.31 |
| 1222 | Train 20 | Test 6 | 0.71 | 0.34 | 0.39 | 0.36 | 3270 | Train 52 | Test 6 | 0.69 | 0.29 | 0.47 | 0.36 |
| 1223 | Train 20 | Test 7 | 0.71 | 0.21 | 0.59 | 0.31 | 3271 | Train 52 | Test 7 | 0.7 | 0.21 | 0.58 | 0.31 |
| 1224 | Train 20 | Test 8 | 0.71 | 0.33 | 0.39 | 0.36 | 3272 | Train 52 | Test 8 | 0.69 | 0.29 | 0.47 | 0.36 |
| 1225 | Train 20 | Test 9 | 0.75 | 0.23 | 0.65 | 0.34 | 3273 | Train 52 | Test 9 | 0.73 | 0.23 | 0.6 | 0.33 |
| 1226 | Train 20 | Test 10 | 0.77 | 0.26 | 0.71 | 0.38 | 3274 | Train 52 | Test 10 | 0.72 | 0.26 | 0.56 | 0.35 |
| 1227 | Train 20 | Test 11 | 0.76 | 0.23 | 0.65 | 0.34 | 3275 | Train 52 | Test 11 | 0.73 | 0.23 | 0.6 | 0.33 |
| 1228 | Train 20 | Test 12 | 0.77 | 0.26 | 0.71 | 0.38 | 3276 | Train 52 | Test 12 | 0.72 | 0.26 | 0.56 | 0.35 |
| 1229 | Train 20 | Test 13 | 0.71 | 0.21 | 0.57 | 0.31 | 3277 | Train 52 | Test 13 | 0.7 | 0.21 | 0.55 | 0.3 |
| 1230 | Train 20 | Test 14 | 0.71 | 0.33 | 0.39 | 0.36 | 3278 | Train 52 | Test 14 | 0.69 | 0.29 | 0.47 | 0.36 |
| 1231 | Train 20 | Test 15 | 0.71 | 0.21 | 0.57 | 0.31 | 3279 | Train 52 | Test 15 | 0.7 | 0.21 | 0.54 | 0.3 |
| 1232 | Train 20 | Test 16 | 0.71 | 0.33 | 0.4 | 0.36 | 3280 | Train 52 | Test 16 | 0.69 | 0.29 | 0.47 | 0.36 |
| 1233 | Train 20 | Test 17 | 0.75 | 0.23 | 0.66 | 0.34 | 3281 | Train 52 | Test 17 | 0.73 | 0.23 | 0.63 | 0.33 |
| 1234 | Train 20 | Test 18 | 0.77 | 0.26 | 0.72 | 0.38 | 3282 | Train 52 | Test 18 | 0.72 | 0.25 | 0.59 | 0.35 |
| 1235 | Train 20 | Test 19 | 0.75 | 0.23 | 0.67 | 0.34 | 3283 | Train 52 | Test 19 | 0.73 | 0.23 | 0.63 | 0.33 |
| 1236 | Train 20 | Test 20 | 0.77 | 0.26 | 0.73 | 0.38 | 3284 | Train 52 | Test 20 | 0.72 | 0.25 | 0.59 | 0.35 |
| 1237 | Train 20 | Test 21 | 0.71 | 0.21 | 0.6 | 0.31 | 3285 | Train 52 | Test 21 | 0.7 | 0.21 | 0.58 | 0.31 |
| 1238 | Train 20 | Test 22 | 0.71 | 0.34 | 0.39 | 0.36 | 3286 | Train 52 | Test 22 | 0.69 | 0.29 | 0.47 | 0.36 |
| 1239 | Train 20 | Test 23 | 0.71 | 0.21 | 0.59 | 0.31 | 3287 | Train 52 | Test 23 | 0.7 | 0.21 | 0.58 | 0.31 |
| 1240 | Train 20 | Test 24 | 0.71 | 0.33 | 0.39 | 0.36 | 3288 | Train 52 | Test 24 | 0.69 | 0.29 | 0.47 | 0.36 |
| 1241 | Train 20 | Test 25 | 0.76 | 0.23 | 0.65 | 0.34 | 3289 | Train 52 | Test 25 | 0.73 | 0.23 | 0.6 | 0.33 |
| 1242 | Train 20 | Test 26 | 0.77 | 0.26 | 0.71 | 0.38 | 3290 | Train 52 | Test 26 | 0.72 | 0.26 | 0.56 | 0.35 |
| 1243 | Train 20 | Test 27 | 0.76 | 0.23 | 0.65 | 0.34 | 3291 | Train 52 | Test 27 | 0.73 | 0.23 | 0.6 | 0.33 |
| 1244 | Train 20 | Test 28 | 0.77 | 0.26 | 0.71 | 0.38 | 3292 | Train 52 | Test 28 | 0.72 | 0.26 | 0.56 | 0.35 |
| 1245 | Train 20 | Test 29 | 0.71 | 0.21 | 0.57 | 0.31 | 3293 | Train 52 | Test 29 | 0.7 | 0.21 | 0.55 | 0.3 |
| 1246 | Train 20 | Test 30 | 0.71 | 0.33 | 0.39 | 0.36 | 3294 | Train 52 | Test 30 | 0.69 | 0.29 | 0.47 | 0.36 |
| 1247 | Train 20 | Test 31 | 0.71 | 0.21 | 0.57 | 0.31 | 3295 | Train 52 | Test 31 | 0.7 | 0.21 | 0.54 | 0.3 |
| 1248 | Train 20 | Test 32 | 0.71 | 0.33 | 0.4 | 0.36 | 3296 | Train 52 | Test 32 | 0.69 | 0.29 | 0.47 | 0.36 |
| 1249 | Train 20 | Test 33 | 0.76 | 0.23 | 0.66 | 0.34 | 3297 | Train 52 | Test 33 | 0.73 | 0.23 | 0.63 | 0.33 |
| 1250 | Train 20 | Test 34 | 0.77 | 0.26 | 0.72 | 0.38 | 3298 | Train 52 | Test 34 | 0.72 | 0.25 | 0.59 | 0.35 |
| 1251 | Train 20 | Test 35 | 0.76 | 0.23 | 0.66 | 0.34 | 3299 | Train 52 | Test 35 | 0.74 | 0.23 | 0.63 | 0.33 |
| 1252 | Train 20 | Test 36 | 0.77 | 0.26 | 0.72 | 0.38 | 3300 | Train 52 | Test 36 | 0.72 | 0.25 | 0.59 | 0.35 |
| 1253 | Train 20 | Test 37 | 0.71 | 0.21 | 0.59 | 0.31 | 3301 | Train 52 | Test 37 | 0.7 | 0.21 | 0.58 | 0.31 |
| 1254 | Train 20 | Test 38 | 0.71 | 0.34 | 0.39 | 0.36 | 3302 | Train 52 | Test 38 | 0.69 | 0.29 | 0.47 | 0.36 |
| 1255 | Train 20 | Test 39 | 0.71 | 0.21 | 0.59 | 0.31 | 3303 | Train 52 | Test 39 | 0.7 | 0.21 | 0.57 | 0.31 |
| 1256 | Train 20 | Test 40 | 0.71 | 0.33 | 0.39 | 0.36 | 3304 | Train 52 | Test 40 | 0.69 | 0.29 | 0.47 | 0.36 |
| 1257 | Train 20 | Test 41 | 0.76 | 0.23 | 0.65 | 0.34 | 3305 | Train 52 | Test 41 | 0.74 | 0.23 | 0.6 | 0.33 |
| 1258 | Train 20 | Test 42 | 0.77 | 0.26 | 0.71 | 0.38 | 3306 | Train 52 | Test 42 | 0.72 | 0.25 | 0.56 | 0.35 |
| 1259 | Train 20 | Test 43 | 0.76 | 0.23 | 0.65 | 0.34 | 3307 | Train 52 | Test 43 | 0.74 | 0.23 | 0.6 | 0.33 |
| 1260 | Train 20 | Test 44 | 0.77 | 0.26 | 0.71 | 0.38 | 3308 | Train 52 | Test 44 | 0.72 | 0.25 | 0.56 | 0.35 |
| 1261 | Train 20 | Test 45 | 0.71 | 0.21 | 0.57 | 0.31 | 3309 | Train 52 | Test 45 | 0.7 | 0.21 | 0.54 | 0.3 |
| 1262 | Train 20 | Test 46 | 0.71 | 0.33 | 0.39 | 0.36 | 3310 | Train 52 | Test 46 | 0.69 | 0.29 | 0.47 | 0.36 |
| 1263 | Train 20 | Test 47 | 0.71 | 0.21 | 0.57 | 0.31 | 3311 | Train 52 | Test 47 | 0.7 | 0.21 | 0.54 | 0.3 |
| 1264 | Train 20 | Test 48 | 0.71 | 0.33 | 0.4 | 0.36 | 3312 | Train 52 | Test 48 | 0.69 | 0.29 | 0.47 | 0.36 |
| 1265 | Train 20 | Test 49 | 0.77 | 0.25 | 0.72 | 0.38 | 3313 | Train 52 | Test 49 | 0.78 | 0.27 | 0.75 | 0.4 |
| 1266 | Train 20 | Test 50 | 0.77 | 0.25 | 0.72 | 0.38 | 3314 | Train 52 | Test 50 | 0.78 | 0.27 | 0.74 | 0.4 |
| 1267 | Train 20 | Test 51 | 0.77 | 0.25 | 0.73 | 0.38 | 3315 | Train 52 | Test 51 | 0.78 | 0.27 | 0.75 | 0.4 |
| 1268 | Train 20 | Test 52 | 0.77 | 0.26 | 0.73 | 0.38 | 3316 | Train 52 | Test 52 | 0.78 | 0.27 | 0.74 | 0.4 |
| 1269 | Train 20 | Test 53 | 0.74 | 0.28 | 0.44 | 0.34 | 3317 | Train 52 | Test 53 | 0.73 | 0.24 | 0.64 | 0.35 |
| 1270 | Train 20 | Test 54 | 0.72 | 0.38 | 0.39 | 0.38 | 3318 | Train 52 | Test 54 | 0.71 | 0.34 | 0.52 | 0.41 |
| 1271 | Train 20 | Test 55 | 0.73 | 0.24 | 0.57 | 0.34 | 3319 | Train 52 | Test 55 | 0.73 | 0.24 | 0.64 | 0.35 |
| 1272 | Train 20 | Test 56 | 0.72 | 0.36 | 0.4 | 0.38 | 3320 | Train 52 | Test 56 | 0.71 | 0.33 | 0.52 | 0.4 |
| 1273 | Train 20 | Test 57 | 0.78 | 0.26 | 0.72 | 0.38 | 3321 | Train 52 | Test 57 | 0.79 | 0.28 | 0.73 | 0.4 |
| 1274 | Train 20 | Test 58 | 0.78 | 0.26 | 0.72 | 0.38 | 3322 | Train 52 | Test 58 | 0.79 | 0.28 | 0.72 | 0.4 |
| 1275 | Train 20 | Test 59 | 0.78 | 0.26 | 0.73 | 0.39 | 3323 | Train 52 | Test 59 | 0.79 | 0.28 | 0.73 | 0.41 |
| 1276 | Train 20 | Test 60 | 0.78 | 0.26 | 0.72 | 0.39 | 3324 | Train 52 | Test 60 | 0.79 | 0.28 | 0.73 | 0.41 |
| 1277 | Train 20 | Test 61 | 0.75 | 0.28 | 0.43 | 0.34 | 3325 | Train 52 | Test 61 | 0.73 | 0.24 | 0.62 | 0.35 |
| 1278 | Train 20 | Test 62 | 0.72 | 0.38 | 0.39 | 0.38 | 3326 | Train 52 | Test 62 | 0.71 | 0.33 | 0.53 | 0.41 |
| 1279 | Train 20 | Test 63 | 0.75 | 0.29 | 0.43 | 0.34 | 3327 | Train 52 | Test 63 | 0.74 | 0.24 | 0.62 | 0.35 |
| 1280 | Train 20 | Test 64 | 0.72 | 0.36 | 0.4 | 0.38 | 3328 | Train 52 | Test 64 | 0.71 | 0.33 | 0.53 | 0.4 |
| 1281 | Train 21 | Test 1 | 0.75 | 0.22 | 0.74 | 0.33 | 3329 | Train 53 | Test 1 | 0.73 | 0.18 | 0.81 | 0.3 |
| 1282 | Train 21 | Test 2 | 0.75 | 0.25 | 0.76 | 0.37 | 3330 | Train 53 | Test 2 | 0.73 | 0.21 | 0.82 | 0.34 |
| 1283 | Train 21 | Test 3 | 0.75 | 0.22 | 0.74 | 0.33 | 3331 | Train 53 | Test 3 | 0.73 | 0.18 | 0.81 | 0.3 |
| 1284 | Train 21 | Test 4 | 0.75 | 0.25 | 0.76 | 0.37 | 3332 | Train 53 | Test 4 | 0.73 | 0.21 | 0.82 | 0.34 |
| 1285 | Train 21 | Test 5 | 0.73 | 0.21 | 0.71 | 0.32 | 3333 | Train 53 | Test 5 | 0.71 | 0.18 | 0.77 | 0.29 |
| 1286 | Train 21 | Test 6 | 0.7 | 0.29 | 0.59 | 0.39 | 3334 | Train 53 | Test 6 | 0.7 | 0.27 | 0.67 | 0.38 |
| 1287 | Train 21 | Test 7 | 0.73 | 0.21 | 0.71 | 0.32 | 3335 | Train 53 | Test 7 | 0.71 | 0.18 | 0.77 | 0.29 |
| 1288 | Train 21 | Test 8 | 0.7 | 0.29 | 0.59 | 0.39 | 3336 | Train 53 | Test 8 | 0.7 | 0.26 | 0.67 | 0.38 |
| 1289 | Train 21 | Test 9 | 0.75 | 0.22 | 0.71 | 0.33 | 3337 | Train 53 | Test 9 | 0.73 | 0.19 | 0.8 | 0.3 |
| 1290 | Train 21 | Test 10 | 0.75 | 0.25 | 0.73 | 0.37 | 3338 | Train 53 | Test 10 | 0.73 | 0.22 | 0.8 | 0.34 |
| 1291 | Train 21 | Test 11 | 0.75 | 0.22 | 0.71 | 0.34 | 3339 | Train 53 | Test 11 | 0.73 | 0.19 | 0.8 | 0.3 |
| 1292 | Train 21 | Test 12 | 0.75 | 0.25 | 0.73 | 0.37 | 3340 | Train 53 | Test 12 | 0.73 | 0.22 | 0.8 | 0.34 |
| 1293 | Train 21 | Test 13 | 0.73 | 0.22 | 0.69 | 0.33 | 3341 | Train 53 | Test 13 | 0.71 | 0.19 | 0.75 | 0.3 |
| 1294 | Train 21 | Test 14 | 0.7 | 0.29 | 0.59 | 0.39 | 3342 | Train 53 | Test 14 | 0.7 | 0.27 | 0.67 | 0.38 |
| 1295 | Train 21 | Test 15 | 0.73 | 0.22 | 0.68 | 0.33 | 3343 | Train 53 | Test 15 | 0.71 | 0.19 | 0.75 | 0.3 |
| 1296 | Train 21 | Test 16 | 0.7 | 0.28 | 0.59 | 0.38 | 3344 | Train 53 | Test 16 | 0.71 | 0.26 | 0.67 | 0.38 |
| 1297 | Train 21 | Test 17 | 0.75 | 0.22 | 0.74 | 0.33 | 3345 | Train 53 | Test 17 | 0.73 | 0.18 | 0.81 | 0.3 |
| 1298 | Train 21 | Test 18 | 0.75 | 0.25 | 0.76 | 0.37 | 3346 | Train 53 | Test 18 | 0.73 | 0.21 | 0.82 | 0.34 |
| 1299 | Train 21 | Test 19 | 0.75 | 0.22 | 0.74 | 0.33 | 3347 | Train 53 | Test 19 | 0.73 | 0.18 | 0.81 | 0.3 |
| 1300 | Train 21 | Test 20 | 0.75 | 0.25 | 0.76 | 0.37 | 3348 | Train 53 | Test 20 | 0.73 | 0.21 | 0.82 | 0.34 |
| 1301 | Train 21 | Test 21 | 0.73 | 0.21 | 0.71 | 0.32 | 3349 | Train 53 | Test 21 | 0.71 | 0.18 | 0.77 | 0.3 |
| 1302 | Train 21 | Test 22 | 0.7 | 0.29 | 0.59 | 0.39 | 3350 | Train 53 | Test 22 | 0.7 | 0.27 | 0.67 | 0.38 |
| 1303 | Train 21 | Test 23 | 0.73 | 0.21 | 0.71 | 0.32 | 3351 | Train 53 | Test 23 | 0.71 | 0.18 | 0.77 | 0.29 |
| 1304 | Train 21 | Test 24 | 0.7 | 0.29 | 0.59 | 0.38 | 3352 | Train 53 | Test 24 | 0.7 | 0.26 | 0.67 | 0.38 |
| 1305 | Train 21 | Test 25 | 0.75 | 0.22 | 0.71 | 0.33 | 3353 | Train 53 | Test 25 | 0.73 | 0.19 | 0.8 | 0.3 |
| 1306 | Train 21 | Test 26 | 0.75 | 0.25 | 0.73 | 0.37 | 3354 | Train 53 | Test 26 | 0.73 | 0.22 | 0.8 | 0.34 |
| 1307 | Train 21 | Test 27 | 0.75 | 0.22 | 0.71 | 0.34 | 3355 | Train 53 | Test 27 | 0.73 | 0.19 | 0.8 | 0.3 |
| 1308 | Train 21 | Test 28 | 0.75 | 0.25 | 0.73 | 0.37 | 3356 | Train 53 | Test 28 | 0.73 | 0.22 | 0.8 | 0.34 |
| 1309 | Train 21 | Test 29 | 0.73 | 0.22 | 0.69 | 0.33 | 3357 | Train 53 | Test 29 | 0.72 | 0.19 | 0.75 | 0.3 |
| 1310 | Train 21 | Test 30 | 0.71 | 0.29 | 0.59 | 0.39 | 3358 | Train 53 | Test 30 | 0.71 | 0.27 | 0.67 | 0.38 |
| 1311 | Train 21 | Test 31 | 0.73 | 0.22 | 0.68 | 0.33 | 3359 | Train 53 | Test 31 | 0.71 | 0.19 | 0.75 | 0.3 |
| 1312 | Train 21 | Test 32 | 0.71 | 0.28 | 0.59 | 0.38 | 3360 | Train 53 | Test 32 | 0.71 | 0.26 | 0.67 | 0.38 |
| 1313 | Train 21 | Test 33 | 0.75 | 0.21 | 0.74 | 0.33 | 3361 | Train 53 | Test 33 | 0.73 | 0.18 | 0.81 | 0.3 |
| 1314 | Train 21 | Test 34 | 0.75 | 0.24 | 0.75 | 0.37 | 3362 | Train 53 | Test 34 | 0.74 | 0.21 | 0.82 | 0.34 |
| 1315 | Train 21 | Test 35 | 0.75 | 0.22 | 0.74 | 0.33 | 3363 | Train 53 | Test 35 | 0.73 | 0.18 | 0.81 | 0.3 |
| 1316 | Train 21 | Test 36 | 0.75 | 0.25 | 0.75 | 0.37 | 3364 | Train 53 | Test 36 | 0.74 | 0.21 | 0.82 | 0.34 |
| 1317 | Train 21 | Test 37 | 0.73 | 0.21 | 0.71 | 0.32 | 3365 | Train 53 | Test 37 | 0.72 | 0.18 | 0.77 | 0.29 |
| 1318 | Train 21 | Test 38 | 0.7 | 0.29 | 0.59 | 0.39 | 3366 | Train 53 | Test 38 | 0.7 | 0.27 | 0.67 | 0.38 |
| 1319 | Train 21 | Test 39 | 0.73 | 0.21 | 0.7 | 0.32 | 3367 | Train 53 | Test 39 | 0.71 | 0.18 | 0.76 | 0.29 |
| 1320 | Train 21 | Test 40 | 0.7 | 0.29 | 0.59 | 0.38 | 3368 | Train 53 | Test 40 | 0.7 | 0.26 | 0.67 | 0.38 |
| 1321 | Train 21 | Test 41 | 0.75 | 0.22 | 0.71 | 0.33 | 3369 | Train 53 | Test 41 | 0.73 | 0.19 | 0.8 | 0.3 |
| 1322 | Train 21 | Test 42 | 0.75 | 0.25 | 0.73 | 0.37 | 3370 | Train 53 | Test 42 | 0.74 | 0.22 | 0.8 | 0.34 |
| 1323 | Train 21 | Test 43 | 0.75 | 0.22 | 0.71 | 0.33 | 3371 | Train 53 | Test 43 | 0.73 | 0.19 | 0.8 | 0.3 |
| 1324 | Train 21 | Test 44 | 0.75 | 0.25 | 0.73 | 0.37 | 3372 | Train 53 | Test 44 | 0.74 | 0.22 | 0.8 | 0.34 |
| 1325 | Train 21 | Test 45 | 0.74 | 0.22 | 0.69 | 0.33 | 3373 | Train 53 | Test 45 | 0.72 | 0.19 | 0.75 | 0.3 |
| 1326 | Train 21 | Test 46 | 0.71 | 0.29 | 0.59 | 0.39 | 3374 | Train 53 | Test 46 | 0.71 | 0.27 | 0.67 | 0.38 |
| 1327 | Train 21 | Test 47 | 0.73 | 0.22 | 0.68 | 0.33 | 3375 | Train 53 | Test 47 | 0.72 | 0.19 | 0.75 | 0.3 |
| 1328 | Train 21 | Test 48 | 0.71 | 0.28 | 0.59 | 0.38 | 3376 | Train 53 | Test 48 | 0.71 | 0.26 | 0.67 | 0.38 |
| 1329 | Train 21 | Test 49 | 0.77 | 0.25 | 0.77 | 0.37 | 3377 | Train 53 | Test 49 | 0.77 | 0.25 | 0.79 | 0.38 |
| 1330 | Train 21 | Test 50 | 0.76 | 0.25 | 0.77 | 0.37 | 3378 | Train 53 | Test 50 | 0.77 | 0.25 | 0.78 | 0.38 |
| 1331 | Train 21 | Test 51 | 0.77 | 0.25 | 0.77 | 0.38 | 3379 | Train 53 | Test 51 | 0.77 | 0.25 | 0.79 | 0.38 |
| 1332 | Train 21 | Test 52 | 0.76 | 0.25 | 0.77 | 0.38 | 3380 | Train 53 | Test 52 | 0.77 | 0.25 | 0.79 | 0.38 |
| 1333 | Train 21 | Test 53 | 0.76 | 0.25 | 0.73 | 0.38 | 3381 | Train 53 | Test 53 | 0.77 | 0.25 | 0.72 | 0.37 |
| 1334 | Train 21 | Test 54 | 0.72 | 0.34 | 0.59 | 0.43 | 3382 | Train 53 | Test 54 | 0.72 | 0.33 | 0.65 | 0.43 |
| 1335 | Train 21 | Test 55 | 0.76 | 0.24 | 0.74 | 0.37 | 3383 | Train 53 | Test 55 | 0.77 | 0.24 | 0.75 | 0.36 |
| 1336 | Train 21 | Test 56 | 0.72 | 0.32 | 0.59 | 0.42 | 3384 | Train 53 | Test 56 | 0.72 | 0.31 | 0.65 | 0.42 |
| 1337 | Train 21 | Test 57 | 0.77 | 0.25 | 0.76 | 0.38 | 3385 | Train 53 | Test 57 | 0.78 | 0.25 | 0.77 | 0.38 |
| 1338 | Train 21 | Test 58 | 0.77 | 0.25 | 0.76 | 0.38 | 3386 | Train 53 | Test 58 | 0.77 | 0.25 | 0.77 | 0.38 |
| 1339 | Train 21 | Test 59 | 0.77 | 0.25 | 0.76 | 0.38 | 3387 | Train 53 | Test 59 | 0.78 | 0.25 | 0.77 | 0.38 |
| 1340 | Train 21 | Test 60 | 0.77 | 0.25 | 0.76 | 0.38 | 3388 | Train 53 | Test 60 | 0.77 | 0.25 | 0.77 | 0.38 |
| 1341 | Train 21 | Test 61 | 0.76 | 0.26 | 0.72 | 0.38 | 3389 | Train 53 | Test 61 | 0.77 | 0.25 | 0.72 | 0.37 |
| 1342 | Train 21 | Test 62 | 0.72 | 0.33 | 0.59 | 0.43 | 3390 | Train 53 | Test 62 | 0.72 | 0.32 | 0.65 | 0.43 |
| 1343 | Train 21 | Test 63 | 0.77 | 0.26 | 0.72 | 0.38 | 3391 | Train 53 | Test 63 | 0.77 | 0.25 | 0.72 | 0.37 |
| 1344 | Train 21 | Test 64 | 0.72 | 0.32 | 0.59 | 0.41 | 3392 | Train 53 | Test 64 | 0.72 | 0.31 | 0.65 | 0.42 |
| 1345 | Train 22 | Test 1 | 0.73 | 0.21 | 0.74 | 0.32 | 3393 | Train 54 | Test 1 | 0.72 | 0.18 | 0.81 | 0.29 |
| 1346 | Train 22 | Test 2 | 0.75 | 0.23 | 0.78 | 0.36 | 3394 | Train 54 | Test 2 | 0.73 | 0.21 | 0.83 | 0.33 |
| 1347 | Train 22 | Test 3 | 0.73 | 0.21 | 0.74 | 0.32 | 3395 | Train 54 | Test 3 | 0.72 | 0.18 | 0.81 | 0.29 |
| 1348 | Train 22 | Test 4 | 0.75 | 0.23 | 0.78 | 0.36 | 3396 | Train 54 | Test 4 | 0.73 | 0.21 | 0.83 | 0.33 |
| 1349 | Train 22 | Test 5 | 0.72 | 0.2 | 0.71 | 0.31 | 3397 | Train 54 | Test 5 | 0.71 | 0.18 | 0.77 | 0.29 |
| 1350 | Train 22 | Test 6 | 0.74 | 0.29 | 0.7 | 0.41 | 3398 | Train 54 | Test 6 | 0.73 | 0.27 | 0.74 | 0.4 |
| 1351 | Train 22 | Test 7 | 0.72 | 0.2 | 0.7 | 0.31 | 3399 | Train 54 | Test 7 | 0.7 | 0.18 | 0.77 | 0.29 |
| 1352 | Train 22 | Test 8 | 0.74 | 0.28 | 0.7 | 0.4 | 3400 | Train 54 | Test 8 | 0.73 | 0.26 | 0.74 | 0.39 |
| 1353 | Train 22 | Test 9 | 0.73 | 0.21 | 0.71 | 0.32 | 3401 | Train 54 | Test 9 | 0.72 | 0.18 | 0.8 | 0.3 |
| 1354 | Train 22 | Test 10 | 0.75 | 0.24 | 0.76 | 0.36 | 3402 | Train 54 | Test 10 | 0.73 | 0.21 | 0.83 | 0.34 |
| 1355 | Train 22 | Test 11 | 0.73 | 0.21 | 0.71 | 0.32 | 3403 | Train 54 | Test 11 | 0.72 | 0.18 | 0.8 | 0.3 |
| 1356 | Train 22 | Test 12 | 0.75 | 0.24 | 0.76 | 0.36 | 3404 | Train 54 | Test 12 | 0.73 | 0.21 | 0.83 | 0.34 |
| 1357 | Train 22 | Test 13 | 0.72 | 0.2 | 0.68 | 0.31 | 3405 | Train 54 | Test 13 | 0.71 | 0.18 | 0.76 | 0.3 |
| 1358 | Train 22 | Test 14 | 0.74 | 0.28 | 0.7 | 0.41 | 3406 | Train 54 | Test 14 | 0.73 | 0.27 | 0.74 | 0.39 |
| 1359 | Train 22 | Test 15 | 0.72 | 0.2 | 0.68 | 0.31 | 3407 | Train 54 | Test 15 | 0.7 | 0.18 | 0.76 | 0.3 |
| 1360 | Train 22 | Test 16 | 0.74 | 0.28 | 0.7 | 0.4 | 3408 | Train 54 | Test 16 | 0.73 | 0.26 | 0.74 | 0.39 |
| 1361 | Train 22 | Test 17 | 0.73 | 0.21 | 0.74 | 0.32 | 3409 | Train 54 | Test 17 | 0.72 | 0.18 | 0.81 | 0.29 |
| 1362 | Train 22 | Test 18 | 0.75 | 0.23 | 0.78 | 0.36 | 3410 | Train 54 | Test 18 | 0.73 | 0.21 | 0.83 | 0.33 |
| 1363 | Train 22 | Test 19 | 0.73 | 0.21 | 0.74 | 0.32 | 3411 | Train 54 | Test 19 | 0.72 | 0.18 | 0.81 | 0.29 |
| 1364 | Train 22 | Test 20 | 0.75 | 0.23 | 0.78 | 0.36 | 3412 | Train 54 | Test 20 | 0.73 | 0.21 | 0.83 | 0.33 |
| 1365 | Train 22 | Test 21 | 0.72 | 0.2 | 0.71 | 0.31 | 3413 | Train 54 | Test 21 | 0.71 | 0.18 | 0.78 | 0.29 |
| 1366 | Train 22 | Test 22 | 0.74 | 0.29 | 0.7 | 0.41 | 3414 | Train 54 | Test 22 | 0.73 | 0.27 | 0.74 | 0.4 |
| 1367 | Train 22 | Test 23 | 0.72 | 0.2 | 0.7 | 0.31 | 3415 | Train 54 | Test 23 | 0.7 | 0.18 | 0.77 | 0.29 |
| 1368 | Train 22 | Test 24 | 0.74 | 0.28 | 0.7 | 0.4 | 3416 | Train 54 | Test 24 | 0.73 | 0.26 | 0.74 | 0.39 |
| 1369 | Train 22 | Test 25 | 0.73 | 0.21 | 0.71 | 0.32 | 3417 | Train 54 | Test 25 | 0.72 | 0.18 | 0.8 | 0.3 |
| 1370 | Train 22 | Test 26 | 0.75 | 0.24 | 0.76 | 0.36 | 3418 | Train 54 | Test 26 | 0.73 | 0.21 | 0.83 | 0.34 |
| 1371 | Train 22 | Test 27 | 0.73 | 0.21 | 0.71 | 0.32 | 3419 | Train 54 | Test 27 | 0.72 | 0.18 | 0.8 | 0.3 |
| 1372 | Train 22 | Test 28 | 0.75 | 0.24 | 0.76 | 0.36 | 3420 | Train 54 | Test 28 | 0.73 | 0.21 | 0.83 | 0.34 |
| 1373 | Train 22 | Test 29 | 0.72 | 0.2 | 0.68 | 0.31 | 3421 | Train 54 | Test 29 | 0.71 | 0.18 | 0.76 | 0.3 |
| 1374 | Train 22 | Test 30 | 0.74 | 0.28 | 0.7 | 0.4 | 3422 | Train 54 | Test 30 | 0.73 | 0.27 | 0.74 | 0.39 |
| 1375 | Train 22 | Test 31 | 0.72 | 0.2 | 0.68 | 0.31 | 3423 | Train 54 | Test 31 | 0.7 | 0.18 | 0.76 | 0.3 |
| 1376 | Train 22 | Test 32 | 0.74 | 0.28 | 0.7 | 0.4 | 3424 | Train 54 | Test 32 | 0.73 | 0.26 | 0.74 | 0.39 |
| 1377 | Train 22 | Test 33 | 0.74 | 0.21 | 0.73 | 0.32 | 3425 | Train 54 | Test 33 | 0.72 | 0.18 | 0.81 | 0.29 |
| 1378 | Train 22 | Test 34 | 0.75 | 0.23 | 0.78 | 0.36 | 3426 | Train 54 | Test 34 | 0.73 | 0.21 | 0.83 | 0.33 |
| 1379 | Train 22 | Test 35 | 0.74 | 0.21 | 0.74 | 0.32 | 3427 | Train 54 | Test 35 | 0.72 | 0.18 | 0.81 | 0.29 |
| 1380 | Train 22 | Test 36 | 0.75 | 0.23 | 0.78 | 0.36 | 3428 | Train 54 | Test 36 | 0.73 | 0.21 | 0.83 | 0.33 |
| 1381 | Train 22 | Test 37 | 0.72 | 0.2 | 0.7 | 0.31 | 3429 | Train 54 | Test 37 | 0.71 | 0.18 | 0.77 | 0.29 |
| 1382 | Train 22 | Test 38 | 0.74 | 0.29 | 0.7 | 0.41 | 3430 | Train 54 | Test 38 | 0.73 | 0.27 | 0.74 | 0.4 |
| 1383 | Train 22 | Test 39 | 0.72 | 0.2 | 0.7 | 0.31 | 3431 | Train 54 | Test 39 | 0.71 | 0.18 | 0.77 | 0.29 |
| 1384 | Train 22 | Test 40 | 0.74 | 0.28 | 0.7 | 0.4 | 3432 | Train 54 | Test 40 | 0.73 | 0.26 | 0.74 | 0.39 |
| 1385 | Train 22 | Test 41 | 0.74 | 0.21 | 0.71 | 0.32 | 3433 | Train 54 | Test 41 | 0.72 | 0.18 | 0.8 | 0.3 |
| 1386 | Train 22 | Test 42 | 0.75 | 0.24 | 0.76 | 0.36 | 3434 | Train 54 | Test 42 | 0.74 | 0.21 | 0.83 | 0.34 |
| 1387 | Train 22 | Test 43 | 0.74 | 0.21 | 0.71 | 0.32 | 3435 | Train 54 | Test 43 | 0.72 | 0.18 | 0.8 | 0.3 |
| 1388 | Train 22 | Test 44 | 0.75 | 0.24 | 0.76 | 0.36 | 3436 | Train 54 | Test 44 | 0.74 | 0.21 | 0.83 | 0.34 |
| 1389 | Train 22 | Test 45 | 0.72 | 0.2 | 0.68 | 0.31 | 3437 | Train 54 | Test 45 | 0.71 | 0.18 | 0.76 | 0.3 |
| 1390 | Train 22 | Test 46 | 0.74 | 0.28 | 0.7 | 0.4 | 3438 | Train 54 | Test 46 | 0.73 | 0.27 | 0.74 | 0.39 |
| 1391 | Train 22 | Test 47 | 0.72 | 0.2 | 0.68 | 0.31 | 3439 | Train 54 | Test 47 | 0.71 | 0.18 | 0.76 | 0.3 |
| 1392 | Train 22 | Test 48 | 0.74 | 0.28 | 0.7 | 0.4 | 3440 | Train 54 | Test 48 | 0.73 | 0.26 | 0.74 | 0.39 |
| 1393 | Train 22 | Test 49 | 0.77 | 0.26 | 0.74 | 0.39 | 3441 | Train 54 | Test 49 | 0.77 | 0.26 | 0.75 | 0.38 |
| 1394 | Train 22 | Test 50 | 0.77 | 0.26 | 0.74 | 0.39 | 3442 | Train 54 | Test 50 | 0.77 | 0.26 | 0.75 | 0.38 |
| 1395 | Train 22 | Test 51 | 0.77 | 0.26 | 0.75 | 0.39 | 3443 | Train 54 | Test 51 | 0.77 | 0.26 | 0.75 | 0.38 |
| 1396 | Train 22 | Test 52 | 0.76 | 0.26 | 0.74 | 0.39 | 3444 | Train 54 | Test 52 | 0.77 | 0.26 | 0.75 | 0.39 |
| 1397 | Train 22 | Test 53 | 0.76 | 0.26 | 0.66 | 0.38 | 3445 | Train 54 | Test 53 | 0.76 | 0.27 | 0.67 | 0.38 |
| 1398 | Train 22 | Test 54 | 0.75 | 0.33 | 0.7 | 0.45 | 3446 | Train 54 | Test 54 | 0.75 | 0.33 | 0.72 | 0.45 |
| 1399 | Train 22 | Test 55 | 0.75 | 0.25 | 0.7 | 0.37 | 3447 | Train 54 | Test 55 | 0.76 | 0.25 | 0.73 | 0.37 |
| 1400 | Train 22 | Test 56 | 0.74 | 0.32 | 0.7 | 0.44 | 3448 | Train 54 | Test 56 | 0.75 | 0.32 | 0.72 | 0.44 |
| 1401 | Train 22 | Test 57 | 0.77 | 0.26 | 0.73 | 0.39 | 3449 | Train 54 | Test 57 | 0.78 | 0.26 | 0.75 | 0.39 |
| 1402 | Train 22 | Test 58 | 0.77 | 0.26 | 0.73 | 0.39 | 3450 | Train 54 | Test 58 | 0.78 | 0.27 | 0.74 | 0.39 |
| 1403 | Train 22 | Test 59 | 0.77 | 0.27 | 0.74 | 0.39 | 3451 | Train 54 | Test 59 | 0.78 | 0.27 | 0.75 | 0.39 |
| 1404 | Train 22 | Test 60 | 0.77 | 0.27 | 0.73 | 0.39 | 3452 | Train 54 | Test 60 | 0.78 | 0.27 | 0.75 | 0.39 |
| 1405 | Train 22 | Test 61 | 0.76 | 0.27 | 0.65 | 0.38 | 3453 | Train 54 | Test 61 | 0.77 | 0.27 | 0.66 | 0.39 |
| 1406 | Train 22 | Test 62 | 0.75 | 0.33 | 0.7 | 0.45 | 3454 | Train 54 | Test 62 | 0.75 | 0.33 | 0.72 | 0.45 |
| 1407 | Train 22 | Test 63 | 0.76 | 0.27 | 0.65 | 0.38 | 3455 | Train 54 | Test 63 | 0.77 | 0.27 | 0.66 | 0.39 |
| 1408 | Train 22 | Test 64 | 0.74 | 0.31 | 0.71 | 0.43 | 3456 | Train 54 | Test 64 | 0.75 | 0.31 | 0.72 | 0.44 |
| 1409 | Train 23 | Test 1 | 0.75 | 0.21 | 0.74 | 0.33 | 3457 | Train 55 | Test 1 | 0.73 | 0.19 | 0.79 | 0.3 |
| 1410 | Train 23 | Test 2 | 0.75 | 0.24 | 0.76 | 0.37 | 3458 | Train 55 | Test 2 | 0.73 | 0.22 | 0.79 | 0.34 |
| 1411 | Train 23 | Test 3 | 0.75 | 0.22 | 0.74 | 0.33 | 3459 | Train 55 | Test 3 | 0.73 | 0.19 | 0.79 | 0.3 |
| 1412 | Train 23 | Test 4 | 0.75 | 0.25 | 0.76 | 0.37 | 3460 | Train 55 | Test 4 | 0.73 | 0.22 | 0.79 | 0.34 |
| 1413 | Train 23 | Test 5 | 0.73 | 0.21 | 0.71 | 0.32 | 3461 | Train 55 | Test 5 | 0.71 | 0.19 | 0.74 | 0.3 |
| 1414 | Train 23 | Test 6 | 0.7 | 0.29 | 0.57 | 0.39 | 3462 | Train 55 | Test 6 | 0.7 | 0.25 | 0.73 | 0.37 |
| 1415 | Train 23 | Test 7 | 0.73 | 0.21 | 0.71 | 0.32 | 3463 | Train 55 | Test 7 | 0.71 | 0.19 | 0.73 | 0.3 |
| 1416 | Train 23 | Test 8 | 0.7 | 0.29 | 0.57 | 0.38 | 3464 | Train 55 | Test 8 | 0.7 | 0.25 | 0.73 | 0.37 |
| 1417 | Train 23 | Test 9 | 0.75 | 0.22 | 0.71 | 0.33 | 3465 | Train 55 | Test 9 | 0.73 | 0.19 | 0.78 | 0.31 |
| 1418 | Train 23 | Test 10 | 0.75 | 0.25 | 0.73 | 0.37 | 3466 | Train 55 | Test 10 | 0.73 | 0.22 | 0.78 | 0.35 |
| 1419 | Train 23 | Test 11 | 0.75 | 0.22 | 0.72 | 0.34 | 3467 | Train 55 | Test 11 | 0.73 | 0.19 | 0.78 | 0.31 |
| 1420 | Train 23 | Test 12 | 0.75 | 0.25 | 0.73 | 0.37 | 3468 | Train 55 | Test 12 | 0.73 | 0.22 | 0.78 | 0.35 |
| 1421 | Train 23 | Test 13 | 0.73 | 0.21 | 0.69 | 0.33 | 3469 | Train 55 | Test 13 | 0.71 | 0.19 | 0.72 | 0.3 |
| 1422 | Train 23 | Test 14 | 0.7 | 0.29 | 0.57 | 0.39 | 3470 | Train 55 | Test 14 | 0.7 | 0.25 | 0.73 | 0.37 |
| 1423 | Train 23 | Test 15 | 0.73 | 0.21 | 0.68 | 0.33 | 3471 | Train 55 | Test 15 | 0.71 | 0.19 | 0.72 | 0.3 |
| 1424 | Train 23 | Test 16 | 0.7 | 0.29 | 0.57 | 0.38 | 3472 | Train 55 | Test 16 | 0.7 | 0.25 | 0.73 | 0.37 |
| 1425 | Train 23 | Test 17 | 0.75 | 0.21 | 0.74 | 0.33 | 3473 | Train 55 | Test 17 | 0.73 | 0.19 | 0.79 | 0.3 |
| 1426 | Train 23 | Test 18 | 0.75 | 0.25 | 0.76 | 0.37 | 3474 | Train 55 | Test 18 | 0.73 | 0.22 | 0.79 | 0.34 |
| 1427 | Train 23 | Test 19 | 0.75 | 0.22 | 0.74 | 0.33 | 3475 | Train 55 | Test 19 | 0.73 | 0.19 | 0.79 | 0.3 |
| 1428 | Train 23 | Test 20 | 0.75 | 0.25 | 0.76 | 0.37 | 3476 | Train 55 | Test 20 | 0.73 | 0.22 | 0.79 | 0.34 |
| 1429 | Train 23 | Test 21 | 0.73 | 0.21 | 0.71 | 0.32 | 3477 | Train 55 | Test 21 | 0.71 | 0.19 | 0.74 | 0.3 |
| 1430 | Train 23 | Test 22 | 0.7 | 0.29 | 0.57 | 0.39 | 3478 | Train 55 | Test 22 | 0.7 | 0.25 | 0.73 | 0.37 |
| 1431 | Train 23 | Test 23 | 0.73 | 0.21 | 0.71 | 0.32 | 3479 | Train 55 | Test 23 | 0.71 | 0.19 | 0.73 | 0.3 |
| 1432 | Train 23 | Test 24 | 0.7 | 0.29 | 0.57 | 0.38 | 3480 | Train 55 | Test 24 | 0.7 | 0.25 | 0.73 | 0.37 |
| 1433 | Train 23 | Test 25 | 0.75 | 0.22 | 0.71 | 0.33 | 3481 | Train 55 | Test 25 | 0.73 | 0.19 | 0.78 | 0.31 |
| 1434 | Train 23 | Test 26 | 0.75 | 0.25 | 0.73 | 0.37 | 3482 | Train 55 | Test 26 | 0.73 | 0.22 | 0.78 | 0.35 |
| 1435 | Train 23 | Test 27 | 0.75 | 0.22 | 0.72 | 0.34 | 3483 | Train 55 | Test 27 | 0.73 | 0.19 | 0.78 | 0.31 |
| 1436 | Train 23 | Test 28 | 0.75 | 0.25 | 0.73 | 0.37 | 3484 | Train 55 | Test 28 | 0.73 | 0.22 | 0.78 | 0.35 |
| 1437 | Train 23 | Test 29 | 0.73 | 0.21 | 0.69 | 0.33 | 3485 | Train 55 | Test 29 | 0.71 | 0.19 | 0.72 | 0.3 |
| 1438 | Train 23 | Test 30 | 0.7 | 0.29 | 0.57 | 0.39 | 3486 | Train 55 | Test 30 | 0.7 | 0.25 | 0.73 | 0.37 |
| 1439 | Train 23 | Test 31 | 0.73 | 0.21 | 0.68 | 0.33 | 3487 | Train 55 | Test 31 | 0.71 | 0.19 | 0.72 | 0.3 |
| 1440 | Train 23 | Test 32 | 0.7 | 0.29 | 0.57 | 0.38 | 3488 | Train 55 | Test 32 | 0.7 | 0.25 | 0.73 | 0.37 |
| 1441 | Train 23 | Test 33 | 0.75 | 0.21 | 0.73 | 0.33 | 3489 | Train 55 | Test 33 | 0.73 | 0.19 | 0.79 | 0.3 |
| 1442 | Train 23 | Test 34 | 0.75 | 0.24 | 0.75 | 0.37 | 3490 | Train 55 | Test 34 | 0.73 | 0.22 | 0.79 | 0.34 |
| 1443 | Train 23 | Test 35 | 0.75 | 0.21 | 0.74 | 0.33 | 3491 | Train 55 | Test 35 | 0.73 | 0.19 | 0.79 | 0.3 |
| 1444 | Train 23 | Test 36 | 0.75 | 0.24 | 0.76 | 0.37 | 3492 | Train 55 | Test 36 | 0.73 | 0.22 | 0.79 | 0.34 |
| 1445 | Train 23 | Test 37 | 0.73 | 0.21 | 0.71 | 0.32 | 3493 | Train 55 | Test 37 | 0.72 | 0.19 | 0.74 | 0.3 |
| 1446 | Train 23 | Test 38 | 0.7 | 0.29 | 0.57 | 0.39 | 3494 | Train 55 | Test 38 | 0.7 | 0.25 | 0.73 | 0.37 |
| 1447 | Train 23 | Test 39 | 0.73 | 0.21 | 0.7 | 0.32 | 3495 | Train 55 | Test 39 | 0.71 | 0.19 | 0.73 | 0.3 |
| 1448 | Train 23 | Test 40 | 0.7 | 0.29 | 0.57 | 0.38 | 3496 | Train 55 | Test 40 | 0.7 | 0.25 | 0.73 | 0.37 |
| 1449 | Train 23 | Test 41 | 0.75 | 0.22 | 0.71 | 0.33 | 3497 | Train 55 | Test 41 | 0.73 | 0.19 | 0.77 | 0.31 |
| 1450 | Train 23 | Test 42 | 0.75 | 0.25 | 0.73 | 0.37 | 3498 | Train 55 | Test 42 | 0.73 | 0.22 | 0.78 | 0.35 |
| 1451 | Train 23 | Test 43 | 0.75 | 0.22 | 0.71 | 0.33 | 3499 | Train 55 | Test 43 | 0.73 | 0.19 | 0.77 | 0.31 |
| 1452 | Train 23 | Test 44 | 0.75 | 0.25 | 0.73 | 0.37 | 3500 | Train 55 | Test 44 | 0.73 | 0.22 | 0.78 | 0.35 |
| 1453 | Train 23 | Test 45 | 0.74 | 0.21 | 0.69 | 0.33 | 3501 | Train 55 | Test 45 | 0.72 | 0.19 | 0.72 | 0.3 |
| 1454 | Train 23 | Test 46 | 0.7 | 0.29 | 0.57 | 0.39 | 3502 | Train 55 | Test 46 | 0.7 | 0.25 | 0.73 | 0.37 |
| 1455 | Train 23 | Test 47 | 0.73 | 0.21 | 0.68 | 0.33 | 3503 | Train 55 | Test 47 | 0.71 | 0.19 | 0.71 | 0.3 |
| 1456 | Train 23 | Test 48 | 0.7 | 0.29 | 0.57 | 0.38 | 3504 | Train 55 | Test 48 | 0.7 | 0.25 | 0.73 | 0.37 |
| 1457 | Train 23 | Test 49 | 0.77 | 0.24 | 0.77 | 0.37 | 3505 | Train 55 | Test 49 | 0.77 | 0.25 | 0.76 | 0.38 |
| 1458 | Train 23 | Test 50 | 0.76 | 0.25 | 0.77 | 0.37 | 3506 | Train 55 | Test 50 | 0.77 | 0.25 | 0.76 | 0.38 |
| 1459 | Train 23 | Test 51 | 0.77 | 0.25 | 0.78 | 0.38 | 3507 | Train 55 | Test 51 | 0.77 | 0.25 | 0.77 | 0.38 |
| 1460 | Train 23 | Test 52 | 0.76 | 0.25 | 0.78 | 0.38 | 3508 | Train 55 | Test 52 | 0.77 | 0.25 | 0.76 | 0.38 |
| 1461 | Train 23 | Test 53 | 0.76 | 0.26 | 0.72 | 0.38 | 3509 | Train 55 | Test 53 | 0.77 | 0.23 | 0.8 | 0.36 |
| 1462 | Train 23 | Test 54 | 0.72 | 0.34 | 0.57 | 0.42 | 3510 | Train 55 | Test 54 | 0.71 | 0.31 | 0.71 | 0.43 |
| 1463 | Train 23 | Test 55 | 0.76 | 0.24 | 0.74 | 0.37 | 3511 | Train 55 | Test 55 | 0.77 | 0.25 | 0.73 | 0.37 |
| 1464 | Train 23 | Test 56 | 0.71 | 0.32 | 0.58 | 0.41 | 3512 | Train 55 | Test 56 | 0.71 | 0.29 | 0.71 | 0.41 |
| 1465 | Train 23 | Test 57 | 0.77 | 0.25 | 0.76 | 0.38 | 3513 | Train 55 | Test 57 | 0.78 | 0.26 | 0.76 | 0.39 |
| 1466 | Train 23 | Test 58 | 0.77 | 0.25 | 0.76 | 0.38 | 3514 | Train 55 | Test 58 | 0.78 | 0.26 | 0.76 | 0.39 |
| 1467 | Train 23 | Test 59 | 0.77 | 0.25 | 0.76 | 0.38 | 3515 | Train 55 | Test 59 | 0.78 | 0.26 | 0.76 | 0.39 |
| 1468 | Train 23 | Test 60 | 0.77 | 0.25 | 0.76 | 0.38 | 3516 | Train 55 | Test 60 | 0.78 | 0.26 | 0.76 | 0.39 |
| 1469 | Train 23 | Test 61 | 0.76 | 0.26 | 0.71 | 0.38 | 3517 | Train 55 | Test 61 | 0.77 | 0.24 | 0.79 | 0.36 |
| 1470 | Train 23 | Test 62 | 0.72 | 0.33 | 0.57 | 0.42 | 3518 | Train 55 | Test 62 | 0.72 | 0.3 | 0.71 | 0.43 |
| 1471 | Train 23 | Test 63 | 0.77 | 0.26 | 0.72 | 0.38 | 3519 | Train 55 | Test 63 | 0.77 | 0.24 | 0.79 | 0.36 |
| 1472 | Train 23 | Test 64 | 0.72 | 0.32 | 0.58 | 0.41 | 3520 | Train 55 | Test 64 | 0.71 | 0.29 | 0.72 | 0.41 |
| 1473 | Train 24 | Test 1 | 0.74 | 0.2 | 0.76 | 0.32 | 3521 | Train 56 | Test 1 | 0.72 | 0.17 | 0.84 | 0.29 |
| 1474 | Train 24 | Test 2 | 0.75 | 0.23 | 0.8 | 0.36 | 3522 | Train 56 | Test 2 | 0.73 | 0.2 | 0.85 | 0.33 |
| 1475 | Train 24 | Test 3 | 0.74 | 0.2 | 0.76 | 0.32 | 3523 | Train 56 | Test 3 | 0.72 | 0.17 | 0.84 | 0.29 |
| 1476 | Train 24 | Test 4 | 0.75 | 0.23 | 0.8 | 0.36 | 3524 | Train 56 | Test 4 | 0.73 | 0.2 | 0.85 | 0.33 |
| 1477 | Train 24 | Test 5 | 0.72 | 0.2 | 0.73 | 0.31 | 3525 | Train 56 | Test 5 | 0.7 | 0.17 | 0.81 | 0.28 |
| 1478 | Train 24 | Test 6 | 0.74 | 0.29 | 0.71 | 0.41 | 3526 | Train 56 | Test 6 | 0.72 | 0.27 | 0.73 | 0.39 |
| 1479 | Train 24 | Test 7 | 0.72 | 0.2 | 0.73 | 0.31 | 3527 | Train 56 | Test 7 | 0.7 | 0.17 | 0.81 | 0.28 |
| 1480 | Train 24 | Test 8 | 0.74 | 0.28 | 0.71 | 0.4 | 3528 | Train 56 | Test 8 | 0.72 | 0.26 | 0.73 | 0.39 |
| 1481 | Train 24 | Test 9 | 0.74 | 0.2 | 0.75 | 0.32 | 3529 | Train 56 | Test 9 | 0.72 | 0.17 | 0.83 | 0.29 |
| 1482 | Train 24 | Test 10 | 0.75 | 0.23 | 0.79 | 0.36 | 3530 | Train 56 | Test 10 | 0.73 | 0.2 | 0.84 | 0.33 |
| 1483 | Train 24 | Test 11 | 0.74 | 0.21 | 0.75 | 0.32 | 3531 | Train 56 | Test 11 | 0.72 | 0.17 | 0.83 | 0.29 |
| 1484 | Train 24 | Test 12 | 0.75 | 0.23 | 0.79 | 0.36 | 3532 | Train 56 | Test 12 | 0.73 | 0.2 | 0.84 | 0.33 |
| 1485 | Train 24 | Test 13 | 0.72 | 0.2 | 0.71 | 0.31 | 3533 | Train 56 | Test 13 | 0.7 | 0.18 | 0.79 | 0.29 |
| 1486 | Train 24 | Test 14 | 0.74 | 0.29 | 0.71 | 0.41 | 3534 | Train 56 | Test 14 | 0.72 | 0.27 | 0.73 | 0.39 |
| 1487 | Train 24 | Test 15 | 0.72 | 0.2 | 0.71 | 0.31 | 3535 | Train 56 | Test 15 | 0.7 | 0.18 | 0.79 | 0.29 |
| 1488 | Train 24 | Test 16 | 0.74 | 0.28 | 0.71 | 0.4 | 3536 | Train 56 | Test 16 | 0.72 | 0.26 | 0.73 | 0.39 |
| 1489 | Train 24 | Test 17 | 0.74 | 0.2 | 0.76 | 0.32 | 3537 | Train 56 | Test 17 | 0.72 | 0.17 | 0.84 | 0.29 |
| 1490 | Train 24 | Test 18 | 0.75 | 0.23 | 0.8 | 0.36 | 3538 | Train 56 | Test 18 | 0.73 | 0.2 | 0.85 | 0.33 |
| 1491 | Train 24 | Test 19 | 0.74 | 0.2 | 0.76 | 0.32 | 3539 | Train 56 | Test 19 | 0.72 | 0.17 | 0.84 | 0.29 |
| 1492 | Train 24 | Test 20 | 0.75 | 0.23 | 0.8 | 0.36 | 3540 | Train 56 | Test 20 | 0.73 | 0.2 | 0.85 | 0.33 |
| 1493 | Train 24 | Test 21 | 0.72 | 0.2 | 0.73 | 0.31 | 3541 | Train 56 | Test 21 | 0.7 | 0.17 | 0.81 | 0.28 |
| 1494 | Train 24 | Test 22 | 0.74 | 0.29 | 0.71 | 0.41 | 3542 | Train 56 | Test 22 | 0.72 | 0.27 | 0.73 | 0.39 |
| 1495 | Train 24 | Test 23 | 0.72 | 0.2 | 0.73 | 0.31 | 3543 | Train 56 | Test 23 | 0.7 | 0.17 | 0.81 | 0.28 |
| 1496 | Train 24 | Test 24 | 0.74 | 0.28 | 0.71 | 0.4 | 3544 | Train 56 | Test 24 | 0.72 | 0.26 | 0.73 | 0.39 |
| 1497 | Train 24 | Test 25 | 0.74 | 0.2 | 0.75 | 0.32 | 3545 | Train 56 | Test 25 | 0.72 | 0.17 | 0.83 | 0.29 |
| 1498 | Train 24 | Test 26 | 0.75 | 0.23 | 0.79 | 0.36 | 3546 | Train 56 | Test 26 | 0.73 | 0.2 | 0.84 | 0.33 |
| 1499 | Train 24 | Test 27 | 0.74 | 0.21 | 0.75 | 0.32 | 3547 | Train 56 | Test 27 | 0.72 | 0.17 | 0.83 | 0.29 |
| 1500 | Train 24 | Test 28 | 0.75 | 0.23 | 0.79 | 0.36 | 3548 | Train 56 | Test 28 | 0.73 | 0.2 | 0.84 | 0.33 |
| 1501 | Train 24 | Test 29 | 0.72 | 0.2 | 0.71 | 0.31 | 3549 | Train 56 | Test 29 | 0.7 | 0.18 | 0.79 | 0.29 |
| 1502 | Train 24 | Test 30 | 0.74 | 0.28 | 0.71 | 0.41 | 3550 | Train 56 | Test 30 | 0.72 | 0.27 | 0.73 | 0.39 |
| 1503 | Train 24 | Test 31 | 0.72 | 0.2 | 0.71 | 0.31 | 3551 | Train 56 | Test 31 | 0.7 | 0.18 | 0.79 | 0.29 |
| 1504 | Train 24 | Test 32 | 0.74 | 0.28 | 0.71 | 0.4 | 3552 | Train 56 | Test 32 | 0.72 | 0.26 | 0.73 | 0.39 |
| 1505 | Train 24 | Test 33 | 0.74 | 0.2 | 0.76 | 0.32 | 3553 | Train 56 | Test 33 | 0.72 | 0.17 | 0.84 | 0.29 |
| 1506 | Train 24 | Test 34 | 0.75 | 0.23 | 0.8 | 0.35 | 3554 | Train 56 | Test 34 | 0.73 | 0.2 | 0.85 | 0.33 |
| 1507 | Train 24 | Test 35 | 0.74 | 0.2 | 0.76 | 0.32 | 3555 | Train 56 | Test 35 | 0.72 | 0.17 | 0.84 | 0.29 |
| 1508 | Train 24 | Test 36 | 0.75 | 0.23 | 0.8 | 0.35 | 3556 | Train 56 | Test 36 | 0.73 | 0.2 | 0.85 | 0.33 |
| 1509 | Train 24 | Test 37 | 0.72 | 0.19 | 0.73 | 0.31 | 3557 | Train 56 | Test 37 | 0.7 | 0.17 | 0.81 | 0.28 |
| 1510 | Train 24 | Test 38 | 0.74 | 0.29 | 0.71 | 0.41 | 3558 | Train 56 | Test 38 | 0.72 | 0.27 | 0.73 | 0.39 |
| 1511 | Train 24 | Test 39 | 0.72 | 0.19 | 0.73 | 0.31 | 3559 | Train 56 | Test 39 | 0.7 | 0.17 | 0.8 | 0.28 |
| 1512 | Train 24 | Test 40 | 0.74 | 0.28 | 0.71 | 0.4 | 3560 | Train 56 | Test 40 | 0.72 | 0.26 | 0.73 | 0.39 |
| 1513 | Train 24 | Test 41 | 0.74 | 0.2 | 0.74 | 0.32 | 3561 | Train 56 | Test 41 | 0.72 | 0.17 | 0.83 | 0.29 |
| 1514 | Train 24 | Test 42 | 0.75 | 0.23 | 0.79 | 0.36 | 3562 | Train 56 | Test 42 | 0.73 | 0.2 | 0.84 | 0.33 |
| 1515 | Train 24 | Test 43 | 0.74 | 0.21 | 0.74 | 0.32 | 3563 | Train 56 | Test 43 | 0.72 | 0.17 | 0.83 | 0.29 |
| 1516 | Train 24 | Test 44 | 0.75 | 0.23 | 0.79 | 0.36 | 3564 | Train 56 | Test 44 | 0.73 | 0.2 | 0.84 | 0.33 |
| 1517 | Train 24 | Test 45 | 0.72 | 0.2 | 0.71 | 0.31 | 3565 | Train 56 | Test 45 | 0.7 | 0.18 | 0.79 | 0.29 |
| 1518 | Train 24 | Test 46 | 0.74 | 0.28 | 0.71 | 0.41 | 3566 | Train 56 | Test 46 | 0.72 | 0.27 | 0.73 | 0.39 |
| 1519 | Train 24 | Test 47 | 0.72 | 0.2 | 0.71 | 0.31 | 3567 | Train 56 | Test 47 | 0.7 | 0.18 | 0.79 | 0.29 |
| 1520 | Train 24 | Test 48 | 0.74 | 0.28 | 0.71 | 0.4 | 3568 | Train 56 | Test 48 | 0.72 | 0.26 | 0.73 | 0.39 |
| 1521 | Train 24 | Test 49 | 0.77 | 0.26 | 0.77 | 0.38 | 3569 | Train 56 | Test 49 | 0.77 | 0.25 | 0.78 | 0.38 |
| 1522 | Train 24 | Test 50 | 0.77 | 0.26 | 0.77 | 0.38 | 3570 | Train 56 | Test 50 | 0.77 | 0.25 | 0.78 | 0.38 |
| 1523 | Train 24 | Test 51 | 0.77 | 0.26 | 0.77 | 0.39 | 3571 | Train 56 | Test 51 | 0.77 | 0.25 | 0.78 | 0.38 |
| 1524 | Train 24 | Test 52 | 0.77 | 0.26 | 0.77 | 0.39 | 3572 | Train 56 | Test 52 | 0.77 | 0.25 | 0.78 | 0.38 |
| 1525 | Train 24 | Test 53 | 0.76 | 0.26 | 0.66 | 0.37 | 3573 | Train 56 | Test 53 | 0.76 | 0.27 | 0.68 | 0.38 |
| 1526 | Train 24 | Test 54 | 0.75 | 0.33 | 0.71 | 0.45 | 3574 | Train 56 | Test 54 | 0.75 | 0.33 | 0.72 | 0.46 |
| 1527 | Train 24 | Test 55 | 0.75 | 0.24 | 0.72 | 0.36 | 3575 | Train 56 | Test 55 | 0.76 | 0.24 | 0.76 | 0.36 |
| 1528 | Train 24 | Test 56 | 0.74 | 0.32 | 0.71 | 0.44 | 3576 | Train 56 | Test 56 | 0.75 | 0.32 | 0.72 | 0.44 |
| 1529 | Train 24 | Test 57 | 0.77 | 0.26 | 0.77 | 0.39 | 3577 | Train 56 | Test 57 | 0.78 | 0.26 | 0.78 | 0.39 |
| 1530 | Train 24 | Test 58 | 0.77 | 0.26 | 0.76 | 0.39 | 3578 | Train 56 | Test 58 | 0.78 | 0.26 | 0.78 | 0.39 |
| 1531 | Train 24 | Test 59 | 0.77 | 0.26 | 0.77 | 0.39 | 3579 | Train 56 | Test 59 | 0.78 | 0.26 | 0.78 | 0.39 |
| 1532 | Train 24 | Test 60 | 0.77 | 0.27 | 0.77 | 0.39 | 3580 | Train 56 | Test 60 | 0.78 | 0.26 | 0.78 | 0.39 |
| 1533 | Train 24 | Test 61 | 0.76 | 0.27 | 0.66 | 0.38 | 3581 | Train 56 | Test 61 | 0.77 | 0.27 | 0.67 | 0.39 |
| 1534 | Train 24 | Test 62 | 0.75 | 0.33 | 0.71 | 0.45 | 3582 | Train 56 | Test 62 | 0.75 | 0.33 | 0.72 | 0.45 |
| 1535 | Train 24 | Test 63 | 0.76 | 0.27 | 0.66 | 0.38 | 3583 | Train 56 | Test 63 | 0.77 | 0.27 | 0.67 | 0.39 |
| 1536 | Train 24 | Test 64 | 0.74 | 0.31 | 0.71 | 0.44 | 3584 | Train 56 | Test 64 | 0.75 | 0.32 | 0.72 | 0.44 |
| 1537 | Train 25 | Test 1 | 0.76 | 0.21 | 0.73 | 0.33 | 3585 | Train 57 | Test 1 | 0.74 | 0.21 | 0.72 | 0.32 |
| 1538 | Train 25 | Test 2 | 0.75 | 0.24 | 0.72 | 0.36 | 3586 | Train 57 | Test 2 | 0.72 | 0.23 | 0.68 | 0.35 |
| 1539 | Train 25 | Test 3 | 0.76 | 0.21 | 0.73 | 0.33 | 3587 | Train 57 | Test 3 | 0.74 | 0.21 | 0.72 | 0.33 |
| 1540 | Train 25 | Test 4 | 0.75 | 0.24 | 0.72 | 0.36 | 3588 | Train 57 | Test 4 | 0.72 | 0.24 | 0.68 | 0.35 |
| 1541 | Train 25 | Test 5 | 0.71 | 0.2 | 0.65 | 0.31 | 3589 | Train 57 | Test 5 | 0.7 | 0.19 | 0.68 | 0.3 |
| 1542 | Train 25 | Test 6 | 0.7 | 0.3 | 0.48 | 0.37 | 3590 | Train 57 | Test 6 | 0.69 | 0.28 | 0.58 | 0.37 |
| 1543 | Train 25 | Test 7 | 0.71 | 0.2 | 0.65 | 0.31 | 3591 | Train 57 | Test 7 | 0.7 | 0.19 | 0.68 | 0.3 |
| 1544 | Train 25 | Test 8 | 0.7 | 0.3 | 0.48 | 0.37 | 3592 | Train 57 | Test 8 | 0.69 | 0.27 | 0.58 | 0.37 |
| 1545 | Train 25 | Test 9 | 0.76 | 0.22 | 0.71 | 0.33 | 3593 | Train 57 | Test 9 | 0.74 | 0.21 | 0.69 | 0.33 |
| 1546 | Train 25 | Test 10 | 0.75 | 0.25 | 0.7 | 0.36 | 3594 | Train 57 | Test 10 | 0.72 | 0.24 | 0.65 | 0.35 |
| 1547 | Train 25 | Test 11 | 0.76 | 0.22 | 0.71 | 0.33 | 3595 | Train 57 | Test 11 | 0.74 | 0.21 | 0.7 | 0.33 |
| 1548 | Train 25 | Test 12 | 0.75 | 0.25 | 0.7 | 0.36 | 3596 | Train 57 | Test 12 | 0.72 | 0.24 | 0.66 | 0.35 |
| 1549 | Train 25 | Test 13 | 0.71 | 0.2 | 0.62 | 0.31 | 3597 | Train 57 | Test 13 | 0.7 | 0.2 | 0.66 | 0.3 |
| 1550 | Train 25 | Test 14 | 0.7 | 0.3 | 0.48 | 0.37 | 3598 | Train 57 | Test 14 | 0.69 | 0.27 | 0.58 | 0.37 |
| 1551 | Train 25 | Test 15 | 0.71 | 0.2 | 0.62 | 0.31 | 3599 | Train 57 | Test 15 | 0.7 | 0.2 | 0.65 | 0.3 |
| 1552 | Train 25 | Test 16 | 0.7 | 0.29 | 0.48 | 0.37 | 3600 | Train 57 | Test 16 | 0.69 | 0.27 | 0.58 | 0.37 |
| 1553 | Train 25 | Test 17 | 0.76 | 0.21 | 0.73 | 0.33 | 3601 | Train 57 | Test 17 | 0.74 | 0.21 | 0.72 | 0.32 |
| 1554 | Train 25 | Test 18 | 0.75 | 0.24 | 0.72 | 0.36 | 3602 | Train 57 | Test 18 | 0.72 | 0.23 | 0.68 | 0.35 |
| 1555 | Train 25 | Test 19 | 0.76 | 0.21 | 0.73 | 0.33 | 3603 | Train 57 | Test 19 | 0.74 | 0.21 | 0.72 | 0.33 |
| 1556 | Train 25 | Test 20 | 0.75 | 0.24 | 0.72 | 0.36 | 3604 | Train 57 | Test 20 | 0.72 | 0.24 | 0.68 | 0.35 |
| 1557 | Train 25 | Test 21 | 0.72 | 0.2 | 0.65 | 0.31 | 3605 | Train 57 | Test 21 | 0.7 | 0.19 | 0.68 | 0.3 |
| 1558 | Train 25 | Test 22 | 0.7 | 0.3 | 0.48 | 0.37 | 3606 | Train 57 | Test 22 | 0.69 | 0.28 | 0.58 | 0.37 |
| 1559 | Train 25 | Test 23 | 0.71 | 0.2 | 0.65 | 0.31 | 3607 | Train 57 | Test 23 | 0.7 | 0.19 | 0.68 | 0.3 |
| 1560 | Train 25 | Test 24 | 0.7 | 0.29 | 0.48 | 0.37 | 3608 | Train 57 | Test 24 | 0.69 | 0.27 | 0.58 | 0.37 |
| 1561 | Train 25 | Test 25 | 0.76 | 0.22 | 0.71 | 0.33 | 3609 | Train 57 | Test 25 | 0.74 | 0.21 | 0.69 | 0.33 |
| 1562 | Train 25 | Test 26 | 0.75 | 0.25 | 0.7 | 0.36 | 3610 | Train 57 | Test 26 | 0.72 | 0.24 | 0.65 | 0.35 |
| 1563 | Train 25 | Test 27 | 0.76 | 0.22 | 0.71 | 0.33 | 3611 | Train 57 | Test 27 | 0.74 | 0.21 | 0.7 | 0.33 |
| 1564 | Train 25 | Test 28 | 0.75 | 0.25 | 0.7 | 0.36 | 3612 | Train 57 | Test 28 | 0.72 | 0.24 | 0.66 | 0.35 |
| 1565 | Train 25 | Test 29 | 0.71 | 0.2 | 0.62 | 0.31 | 3613 | Train 57 | Test 29 | 0.7 | 0.2 | 0.66 | 0.3 |
| 1566 | Train 25 | Test 30 | 0.7 | 0.3 | 0.48 | 0.37 | 3614 | Train 57 | Test 30 | 0.69 | 0.27 | 0.58 | 0.37 |
| 1567 | Train 25 | Test 31 | 0.71 | 0.2 | 0.62 | 0.31 | 3615 | Train 57 | Test 31 | 0.7 | 0.2 | 0.65 | 0.3 |
| 1568 | Train 25 | Test 32 | 0.7 | 0.29 | 0.48 | 0.36 | 3616 | Train 57 | Test 32 | 0.69 | 0.27 | 0.58 | 0.37 |
| 1569 | Train 25 | Test 33 | 0.76 | 0.21 | 0.73 | 0.33 | 3617 | Train 57 | Test 33 | 0.74 | 0.21 | 0.72 | 0.32 |
| 1570 | Train 25 | Test 34 | 0.75 | 0.24 | 0.72 | 0.36 | 3618 | Train 57 | Test 34 | 0.72 | 0.23 | 0.68 | 0.35 |
| 1571 | Train 25 | Test 35 | 0.76 | 0.21 | 0.73 | 0.33 | 3619 | Train 57 | Test 35 | 0.74 | 0.21 | 0.72 | 0.32 |
| 1572 | Train 25 | Test 36 | 0.75 | 0.24 | 0.72 | 0.36 | 3620 | Train 57 | Test 36 | 0.73 | 0.23 | 0.68 | 0.35 |
| 1573 | Train 25 | Test 37 | 0.72 | 0.2 | 0.65 | 0.31 | 3621 | Train 57 | Test 37 | 0.71 | 0.19 | 0.68 | 0.3 |
| 1574 | Train 25 | Test 38 | 0.7 | 0.3 | 0.48 | 0.37 | 3622 | Train 57 | Test 38 | 0.69 | 0.28 | 0.58 | 0.37 |
| 1575 | Train 25 | Test 39 | 0.72 | 0.2 | 0.64 | 0.31 | 3623 | Train 57 | Test 39 | 0.71 | 0.19 | 0.68 | 0.3 |
| 1576 | Train 25 | Test 40 | 0.7 | 0.29 | 0.48 | 0.37 | 3624 | Train 57 | Test 40 | 0.69 | 0.27 | 0.58 | 0.37 |
| 1577 | Train 25 | Test 41 | 0.76 | 0.22 | 0.71 | 0.33 | 3625 | Train 57 | Test 41 | 0.74 | 0.21 | 0.69 | 0.32 |
| 1578 | Train 25 | Test 42 | 0.75 | 0.25 | 0.7 | 0.36 | 3626 | Train 57 | Test 42 | 0.73 | 0.24 | 0.65 | 0.35 |
| 1579 | Train 25 | Test 43 | 0.76 | 0.22 | 0.71 | 0.33 | 3627 | Train 57 | Test 43 | 0.74 | 0.21 | 0.69 | 0.33 |
| 1580 | Train 25 | Test 44 | 0.75 | 0.25 | 0.7 | 0.36 | 3628 | Train 57 | Test 44 | 0.73 | 0.24 | 0.65 | 0.35 |
| 1581 | Train 25 | Test 45 | 0.72 | 0.2 | 0.62 | 0.31 | 3629 | Train 57 | Test 45 | 0.71 | 0.2 | 0.66 | 0.3 |
| 1582 | Train 25 | Test 46 | 0.7 | 0.3 | 0.48 | 0.37 | 3630 | Train 57 | Test 46 | 0.69 | 0.27 | 0.58 | 0.37 |
| 1583 | Train 25 | Test 47 | 0.71 | 0.2 | 0.62 | 0.31 | 3631 | Train 57 | Test 47 | 0.7 | 0.2 | 0.65 | 0.3 |
| 1584 | Train 25 | Test 48 | 0.7 | 0.29 | 0.48 | 0.36 | 3632 | Train 57 | Test 48 | 0.7 | 0.27 | 0.58 | 0.37 |
| 1585 | Train 25 | Test 49 | 0.78 | 0.24 | 0.79 | 0.37 | 3633 | Train 57 | Test 49 | 0.78 | 0.27 | 0.76 | 0.39 |
| 1586 | Train 25 | Test 50 | 0.78 | 0.24 | 0.79 | 0.37 | 3634 | Train 57 | Test 50 | 0.78 | 0.27 | 0.76 | 0.4 |
| 1587 | Train 25 | Test 51 | 0.78 | 0.24 | 0.79 | 0.37 | 3635 | Train 57 | Test 51 | 0.78 | 0.27 | 0.76 | 0.4 |
| 1588 | Train 25 | Test 52 | 0.78 | 0.25 | 0.79 | 0.37 | 3636 | Train 57 | Test 52 | 0.78 | 0.27 | 0.76 | 0.4 |
| 1589 | Train 25 | Test 53 | 0.74 | 0.25 | 0.6 | 0.36 | 3637 | Train 57 | Test 53 | 0.73 | 0.23 | 0.71 | 0.34 |
| 1590 | Train 25 | Test 54 | 0.71 | 0.34 | 0.51 | 0.41 | 3638 | Train 57 | Test 54 | 0.71 | 0.32 | 0.61 | 0.42 |
| 1591 | Train 25 | Test 55 | 0.74 | 0.23 | 0.65 | 0.34 | 3639 | Train 57 | Test 55 | 0.73 | 0.23 | 0.71 | 0.34 |
| 1592 | Train 25 | Test 56 | 0.72 | 0.33 | 0.51 | 0.4 | 3640 | Train 57 | Test 56 | 0.71 | 0.31 | 0.61 | 0.41 |
| 1593 | Train 25 | Test 57 | 0.78 | 0.25 | 0.78 | 0.38 | 3641 | Train 57 | Test 57 | 0.79 | 0.27 | 0.74 | 0.4 |
| 1594 | Train 25 | Test 58 | 0.78 | 0.25 | 0.78 | 0.38 | 3642 | Train 57 | Test 58 | 0.79 | 0.27 | 0.74 | 0.4 |
| 1595 | Train 25 | Test 59 | 0.78 | 0.25 | 0.78 | 0.38 | 3643 | Train 57 | Test 59 | 0.79 | 0.27 | 0.75 | 0.4 |
| 1596 | Train 25 | Test 60 | 0.78 | 0.25 | 0.78 | 0.38 | 3644 | Train 57 | Test 60 | 0.79 | 0.27 | 0.74 | 0.4 |
| 1597 | Train 25 | Test 61 | 0.74 | 0.26 | 0.59 | 0.36 | 3645 | Train 57 | Test 61 | 0.73 | 0.23 | 0.7 | 0.35 |
| 1598 | Train 25 | Test 62 | 0.72 | 0.34 | 0.51 | 0.41 | 3646 | Train 57 | Test 62 | 0.71 | 0.32 | 0.61 | 0.42 |
| 1599 | Train 25 | Test 63 | 0.75 | 0.26 | 0.6 | 0.36 | 3647 | Train 57 | Test 63 | 0.73 | 0.23 | 0.7 | 0.35 |
| 1600 | Train 25 | Test 64 | 0.72 | 0.33 | 0.51 | 0.4 | 3648 | Train 57 | Test 64 | 0.72 | 0.31 | 0.61 | 0.41 |
| 1601 | Train 26 | Test 1 | 0.75 | 0.22 | 0.69 | 0.34 | 3649 | Train 58 | Test 1 | 0.74 | 0.22 | 0.68 | 0.33 |
| 1602 | Train 26 | Test 2 | 0.77 | 0.25 | 0.74 | 0.38 | 3650 | Train 58 | Test 2 | 0.72 | 0.24 | 0.65 | 0.35 |
| 1603 | Train 26 | Test 3 | 0.75 | 0.22 | 0.69 | 0.34 | 3651 | Train 58 | Test 3 | 0.74 | 0.22 | 0.68 | 0.33 |
| 1604 | Train 26 | Test 4 | 0.77 | 0.25 | 0.74 | 0.38 | 3652 | Train 58 | Test 4 | 0.72 | 0.24 | 0.65 | 0.36 |
| 1605 | Train 26 | Test 5 | 0.71 | 0.2 | 0.62 | 0.31 | 3653 | Train 58 | Test 5 | 0.7 | 0.2 | 0.63 | 0.31 |
| 1606 | Train 26 | Test 6 | 0.71 | 0.33 | 0.42 | 0.37 | 3654 | Train 58 | Test 6 | 0.69 | 0.29 | 0.52 | 0.37 |
| 1607 | Train 26 | Test 7 | 0.71 | 0.2 | 0.61 | 0.31 | 3655 | Train 58 | Test 7 | 0.7 | 0.2 | 0.63 | 0.31 |
| 1608 | Train 26 | Test 8 | 0.71 | 0.32 | 0.43 | 0.37 | 3656 | Train 58 | Test 8 | 0.69 | 0.28 | 0.52 | 0.37 |
| 1609 | Train 26 | Test 9 | 0.75 | 0.23 | 0.67 | 0.34 | 3657 | Train 58 | Test 9 | 0.74 | 0.22 | 0.65 | 0.33 |
| 1610 | Train 26 | Test 10 | 0.77 | 0.26 | 0.73 | 0.38 | 3658 | Train 58 | Test 10 | 0.72 | 0.25 | 0.62 | 0.36 |
| 1611 | Train 26 | Test 11 | 0.75 | 0.23 | 0.67 | 0.34 | 3659 | Train 58 | Test 11 | 0.74 | 0.22 | 0.65 | 0.33 |
| 1612 | Train 26 | Test 12 | 0.77 | 0.26 | 0.73 | 0.38 | 3660 | Train 58 | Test 12 | 0.72 | 0.25 | 0.62 | 0.36 |
| 1613 | Train 26 | Test 13 | 0.71 | 0.21 | 0.59 | 0.31 | 3661 | Train 58 | Test 13 | 0.7 | 0.21 | 0.6 | 0.31 |
| 1614 | Train 26 | Test 14 | 0.71 | 0.33 | 0.42 | 0.37 | 3662 | Train 58 | Test 14 | 0.69 | 0.29 | 0.52 | 0.37 |
| 1615 | Train 26 | Test 15 | 0.71 | 0.21 | 0.59 | 0.31 | 3663 | Train 58 | Test 15 | 0.7 | 0.21 | 0.6 | 0.31 |
| 1616 | Train 26 | Test 16 | 0.71 | 0.32 | 0.43 | 0.37 | 3664 | Train 58 | Test 16 | 0.69 | 0.28 | 0.52 | 0.36 |
| 1617 | Train 26 | Test 17 | 0.75 | 0.22 | 0.69 | 0.34 | 3665 | Train 58 | Test 17 | 0.74 | 0.22 | 0.68 | 0.33 |
| 1618 | Train 26 | Test 18 | 0.77 | 0.25 | 0.74 | 0.38 | 3666 | Train 58 | Test 18 | 0.72 | 0.24 | 0.65 | 0.35 |
| 1619 | Train 26 | Test 19 | 0.75 | 0.22 | 0.69 | 0.34 | 3667 | Train 58 | Test 19 | 0.74 | 0.22 | 0.68 | 0.33 |
| 1620 | Train 26 | Test 20 | 0.77 | 0.25 | 0.74 | 0.38 | 3668 | Train 58 | Test 20 | 0.72 | 0.24 | 0.65 | 0.36 |
| 1621 | Train 26 | Test 21 | 0.71 | 0.2 | 0.62 | 0.31 | 3669 | Train 58 | Test 21 | 0.7 | 0.2 | 0.63 | 0.31 |
| 1622 | Train 26 | Test 22 | 0.71 | 0.33 | 0.42 | 0.37 | 3670 | Train 58 | Test 22 | 0.69 | 0.29 | 0.52 | 0.37 |
| 1623 | Train 26 | Test 23 | 0.71 | 0.2 | 0.61 | 0.31 | 3671 | Train 58 | Test 23 | 0.7 | 0.2 | 0.63 | 0.31 |
| 1624 | Train 26 | Test 24 | 0.71 | 0.32 | 0.42 | 0.37 | 3672 | Train 58 | Test 24 | 0.69 | 0.28 | 0.52 | 0.37 |
| 1625 | Train 26 | Test 25 | 0.75 | 0.23 | 0.67 | 0.34 | 3673 | Train 58 | Test 25 | 0.74 | 0.22 | 0.65 | 0.33 |
| 1626 | Train 26 | Test 26 | 0.77 | 0.26 | 0.73 | 0.38 | 3674 | Train 58 | Test 26 | 0.72 | 0.25 | 0.62 | 0.36 |
| 1627 | Train 26 | Test 27 | 0.76 | 0.23 | 0.67 | 0.34 | 3675 | Train 58 | Test 27 | 0.74 | 0.22 | 0.65 | 0.33 |
| 1628 | Train 26 | Test 28 | 0.77 | 0.26 | 0.73 | 0.38 | 3676 | Train 58 | Test 28 | 0.72 | 0.25 | 0.62 | 0.36 |
| 1629 | Train 26 | Test 29 | 0.71 | 0.21 | 0.59 | 0.31 | 3677 | Train 58 | Test 29 | 0.7 | 0.21 | 0.6 | 0.31 |
| 1630 | Train 26 | Test 30 | 0.71 | 0.33 | 0.42 | 0.37 | 3678 | Train 58 | Test 30 | 0.69 | 0.28 | 0.52 | 0.37 |
| 1631 | Train 26 | Test 31 | 0.71 | 0.21 | 0.59 | 0.31 | 3679 | Train 58 | Test 31 | 0.7 | 0.21 | 0.6 | 0.31 |
| 1632 | Train 26 | Test 32 | 0.71 | 0.32 | 0.43 | 0.37 | 3680 | Train 58 | Test 32 | 0.69 | 0.28 | 0.52 | 0.36 |
| 1633 | Train 26 | Test 33 | 0.76 | 0.22 | 0.69 | 0.34 | 3681 | Train 58 | Test 33 | 0.74 | 0.22 | 0.67 | 0.33 |
| 1634 | Train 26 | Test 34 | 0.77 | 0.25 | 0.74 | 0.38 | 3682 | Train 58 | Test 34 | 0.73 | 0.24 | 0.64 | 0.35 |
| 1635 | Train 26 | Test 35 | 0.76 | 0.22 | 0.69 | 0.34 | 3683 | Train 58 | Test 35 | 0.74 | 0.22 | 0.68 | 0.33 |
| 1636 | Train 26 | Test 36 | 0.77 | 0.25 | 0.74 | 0.38 | 3684 | Train 58 | Test 36 | 0.73 | 0.24 | 0.65 | 0.35 |
| 1637 | Train 26 | Test 37 | 0.71 | 0.2 | 0.61 | 0.31 | 3685 | Train 58 | Test 37 | 0.7 | 0.2 | 0.63 | 0.31 |
| 1638 | Train 26 | Test 38 | 0.71 | 0.33 | 0.42 | 0.37 | 3686 | Train 58 | Test 38 | 0.69 | 0.29 | 0.52 | 0.37 |
| 1639 | Train 26 | Test 39 | 0.71 | 0.2 | 0.61 | 0.31 | 3687 | Train 58 | Test 39 | 0.7 | 0.2 | 0.63 | 0.31 |
| 1640 | Train 26 | Test 40 | 0.71 | 0.32 | 0.42 | 0.37 | 3688 | Train 58 | Test 40 | 0.69 | 0.28 | 0.52 | 0.37 |
| 1641 | Train 26 | Test 41 | 0.76 | 0.23 | 0.66 | 0.34 | 3689 | Train 58 | Test 41 | 0.74 | 0.22 | 0.65 | 0.33 |
| 1642 | Train 26 | Test 42 | 0.77 | 0.26 | 0.73 | 0.38 | 3690 | Train 58 | Test 42 | 0.73 | 0.25 | 0.62 | 0.36 |
| 1643 | Train 26 | Test 43 | 0.76 | 0.23 | 0.67 | 0.34 | 3691 | Train 58 | Test 43 | 0.74 | 0.22 | 0.65 | 0.33 |
| 1644 | Train 26 | Test 44 | 0.77 | 0.26 | 0.73 | 0.38 | 3692 | Train 58 | Test 44 | 0.73 | 0.25 | 0.62 | 0.36 |
| 1645 | Train 26 | Test 45 | 0.71 | 0.21 | 0.59 | 0.31 | 3693 | Train 58 | Test 45 | 0.7 | 0.21 | 0.6 | 0.31 |
| 1646 | Train 26 | Test 46 | 0.71 | 0.33 | 0.42 | 0.37 | 3694 | Train 58 | Test 46 | 0.69 | 0.28 | 0.52 | 0.37 |
| 1647 | Train 26 | Test 47 | 0.71 | 0.21 | 0.58 | 0.31 | 3695 | Train 58 | Test 47 | 0.7 | 0.21 | 0.6 | 0.31 |
| 1648 | Train 26 | Test 48 | 0.71 | 0.32 | 0.43 | 0.37 | 3696 | Train 58 | Test 48 | 0.7 | 0.28 | 0.52 | 0.36 |
| 1649 | Train 26 | Test 49 | 0.77 | 0.25 | 0.74 | 0.37 | 3697 | Train 58 | Test 49 | 0.78 | 0.27 | 0.76 | 0.39 |
| 1650 | Train 26 | Test 50 | 0.77 | 0.25 | 0.73 | 0.37 | 3698 | Train 58 | Test 50 | 0.78 | 0.27 | 0.76 | 0.39 |
| 1651 | Train 26 | Test 51 | 0.77 | 0.25 | 0.74 | 0.37 | 3699 | Train 58 | Test 51 | 0.78 | 0.27 | 0.76 | 0.39 |
| 1652 | Train 26 | Test 52 | 0.77 | 0.25 | 0.74 | 0.37 | 3700 | Train 58 | Test 52 | 0.78 | 0.27 | 0.76 | 0.39 |
| 1653 | Train 26 | Test 53 | 0.74 | 0.28 | 0.46 | 0.35 | 3701 | Train 58 | Test 53 | 0.73 | 0.23 | 0.67 | 0.35 |
| 1654 | Train 26 | Test 54 | 0.72 | 0.37 | 0.42 | 0.39 | 3702 | Train 58 | Test 54 | 0.71 | 0.33 | 0.56 | 0.42 |
| 1655 | Train 26 | Test 55 | 0.73 | 0.23 | 0.58 | 0.33 | 3703 | Train 58 | Test 55 | 0.73 | 0.23 | 0.67 | 0.35 |
| 1656 | Train 26 | Test 56 | 0.72 | 0.36 | 0.43 | 0.39 | 3704 | Train 58 | Test 56 | 0.71 | 0.32 | 0.56 | 0.41 |
| 1657 | Train 26 | Test 57 | 0.78 | 0.26 | 0.73 | 0.38 | 3705 | Train 58 | Test 57 | 0.79 | 0.27 | 0.75 | 0.4 |
| 1658 | Train 26 | Test 58 | 0.77 | 0.26 | 0.73 | 0.38 | 3706 | Train 58 | Test 58 | 0.79 | 0.27 | 0.74 | 0.4 |
| 1659 | Train 26 | Test 59 | 0.78 | 0.26 | 0.73 | 0.38 | 3707 | Train 58 | Test 59 | 0.79 | 0.27 | 0.75 | 0.4 |
| 1660 | Train 26 | Test 60 | 0.77 | 0.26 | 0.73 | 0.38 | 3708 | Train 58 | Test 60 | 0.79 | 0.27 | 0.74 | 0.4 |
| 1661 | Train 26 | Test 61 | 0.75 | 0.28 | 0.46 | 0.35 | 3709 | Train 58 | Test 61 | 0.73 | 0.24 | 0.66 | 0.35 |
| 1662 | Train 26 | Test 62 | 0.72 | 0.37 | 0.42 | 0.39 | 3710 | Train 58 | Test 62 | 0.71 | 0.33 | 0.56 | 0.41 |
| 1663 | Train 26 | Test 63 | 0.75 | 0.28 | 0.46 | 0.35 | 3711 | Train 58 | Test 63 | 0.73 | 0.24 | 0.66 | 0.35 |
| 1664 | Train 26 | Test 64 | 0.72 | 0.35 | 0.43 | 0.39 | 3712 | Train 58 | Test 64 | 0.71 | 0.32 | 0.56 | 0.41 |
| 1665 | Train 27 | Test 1 | 0.76 | 0.21 | 0.73 | 0.33 | 3713 | Train 59 | Test 1 | 0.74 | 0.21 | 0.71 | 0.33 |
| 1666 | Train 27 | Test 2 | 0.75 | 0.24 | 0.72 | 0.36 | 3714 | Train 59 | Test 2 | 0.72 | 0.24 | 0.67 | 0.35 |
| 1667 | Train 27 | Test 3 | 0.76 | 0.22 | 0.73 | 0.33 | 3715 | Train 59 | Test 3 | 0.74 | 0.21 | 0.71 | 0.33 |
| 1668 | Train 27 | Test 4 | 0.75 | 0.24 | 0.72 | 0.36 | 3716 | Train 59 | Test 4 | 0.72 | 0.24 | 0.67 | 0.35 |
| 1669 | Train 27 | Test 5 | 0.72 | 0.2 | 0.65 | 0.31 | 3717 | Train 59 | Test 5 | 0.7 | 0.2 | 0.68 | 0.31 |
| 1670 | Train 27 | Test 6 | 0.7 | 0.3 | 0.47 | 0.37 | 3718 | Train 59 | Test 6 | 0.69 | 0.28 | 0.56 | 0.37 |
| 1671 | Train 27 | Test 7 | 0.71 | 0.2 | 0.65 | 0.31 | 3719 | Train 59 | Test 7 | 0.7 | 0.2 | 0.67 | 0.31 |
| 1672 | Train 27 | Test 8 | 0.7 | 0.3 | 0.47 | 0.36 | 3720 | Train 59 | Test 8 | 0.69 | 0.27 | 0.56 | 0.37 |
| 1673 | Train 27 | Test 9 | 0.76 | 0.22 | 0.71 | 0.33 | 3721 | Train 59 | Test 9 | 0.74 | 0.21 | 0.68 | 0.32 |
| 1674 | Train 27 | Test 10 | 0.75 | 0.25 | 0.69 | 0.36 | 3722 | Train 59 | Test 10 | 0.72 | 0.24 | 0.63 | 0.35 |
| 1675 | Train 27 | Test 11 | 0.76 | 0.22 | 0.71 | 0.33 | 3723 | Train 59 | Test 11 | 0.74 | 0.21 | 0.68 | 0.33 |
| 1676 | Train 27 | Test 12 | 0.75 | 0.25 | 0.7 | 0.37 | 3724 | Train 59 | Test 12 | 0.72 | 0.24 | 0.64 | 0.35 |
| 1677 | Train 27 | Test 13 | 0.71 | 0.2 | 0.62 | 0.31 | 3725 | Train 59 | Test 13 | 0.7 | 0.2 | 0.65 | 0.3 |
| 1678 | Train 27 | Test 14 | 0.7 | 0.3 | 0.47 | 0.37 | 3726 | Train 59 | Test 14 | 0.69 | 0.28 | 0.56 | 0.37 |
| 1679 | Train 27 | Test 15 | 0.71 | 0.2 | 0.62 | 0.31 | 3727 | Train 59 | Test 15 | 0.7 | 0.2 | 0.64 | 0.3 |
| 1680 | Train 27 | Test 16 | 0.7 | 0.3 | 0.47 | 0.36 | 3728 | Train 59 | Test 16 | 0.69 | 0.27 | 0.56 | 0.37 |
| 1681 | Train 27 | Test 17 | 0.76 | 0.21 | 0.73 | 0.33 | 3729 | Train 59 | Test 17 | 0.74 | 0.21 | 0.71 | 0.33 |
| 1682 | Train 27 | Test 18 | 0.75 | 0.24 | 0.72 | 0.36 | 3730 | Train 59 | Test 18 | 0.72 | 0.24 | 0.67 | 0.35 |
| 1683 | Train 27 | Test 19 | 0.76 | 0.22 | 0.73 | 0.33 | 3731 | Train 59 | Test 19 | 0.74 | 0.21 | 0.71 | 0.33 |
| 1684 | Train 27 | Test 20 | 0.75 | 0.24 | 0.72 | 0.37 | 3732 | Train 59 | Test 20 | 0.72 | 0.24 | 0.67 | 0.35 |
| 1685 | Train 27 | Test 21 | 0.72 | 0.2 | 0.65 | 0.31 | 3733 | Train 59 | Test 21 | 0.7 | 0.2 | 0.68 | 0.31 |
| 1686 | Train 27 | Test 22 | 0.7 | 0.3 | 0.47 | 0.37 | 3734 | Train 59 | Test 22 | 0.69 | 0.28 | 0.56 | 0.37 |
| 1687 | Train 27 | Test 23 | 0.71 | 0.2 | 0.65 | 0.31 | 3735 | Train 59 | Test 23 | 0.7 | 0.2 | 0.67 | 0.31 |
| 1688 | Train 27 | Test 24 | 0.7 | 0.3 | 0.47 | 0.36 | 3736 | Train 59 | Test 24 | 0.69 | 0.27 | 0.56 | 0.37 |
| 1689 | Train 27 | Test 25 | 0.76 | 0.22 | 0.71 | 0.33 | 3737 | Train 59 | Test 25 | 0.74 | 0.21 | 0.68 | 0.32 |
| 1690 | Train 27 | Test 26 | 0.75 | 0.25 | 0.69 | 0.36 | 3738 | Train 59 | Test 26 | 0.72 | 0.24 | 0.63 | 0.35 |
| 1691 | Train 27 | Test 27 | 0.76 | 0.22 | 0.71 | 0.33 | 3739 | Train 59 | Test 27 | 0.74 | 0.21 | 0.68 | 0.33 |
| 1692 | Train 27 | Test 28 | 0.75 | 0.25 | 0.7 | 0.37 | 3740 | Train 59 | Test 28 | 0.72 | 0.24 | 0.64 | 0.35 |
| 1693 | Train 27 | Test 29 | 0.71 | 0.2 | 0.62 | 0.31 | 3741 | Train 59 | Test 29 | 0.7 | 0.2 | 0.65 | 0.3 |
| 1694 | Train 27 | Test 30 | 0.7 | 0.3 | 0.47 | 0.37 | 3742 | Train 59 | Test 30 | 0.69 | 0.27 | 0.56 | 0.37 |
| 1695 | Train 27 | Test 31 | 0.71 | 0.2 | 0.62 | 0.31 | 3743 | Train 59 | Test 31 | 0.7 | 0.2 | 0.64 | 0.3 |
| 1696 | Train 27 | Test 32 | 0.7 | 0.3 | 0.47 | 0.36 | 3744 | Train 59 | Test 32 | 0.69 | 0.27 | 0.56 | 0.37 |
| 1697 | Train 27 | Test 33 | 0.76 | 0.21 | 0.73 | 0.33 | 3745 | Train 59 | Test 33 | 0.74 | 0.21 | 0.71 | 0.32 |
| 1698 | Train 27 | Test 34 | 0.75 | 0.24 | 0.72 | 0.36 | 3746 | Train 59 | Test 34 | 0.72 | 0.23 | 0.67 | 0.35 |
| 1699 | Train 27 | Test 35 | 0.76 | 0.21 | 0.73 | 0.33 | 3747 | Train 59 | Test 35 | 0.74 | 0.21 | 0.71 | 0.32 |
| 1700 | Train 27 | Test 36 | 0.75 | 0.24 | 0.72 | 0.36 | 3748 | Train 59 | Test 36 | 0.72 | 0.24 | 0.67 | 0.35 |
| 1701 | Train 27 | Test 37 | 0.72 | 0.2 | 0.65 | 0.31 | 3749 | Train 59 | Test 37 | 0.71 | 0.2 | 0.68 | 0.31 |
| 1702 | Train 27 | Test 38 | 0.7 | 0.3 | 0.47 | 0.37 | 3750 | Train 59 | Test 38 | 0.69 | 0.28 | 0.56 | 0.37 |
| 1703 | Train 27 | Test 39 | 0.72 | 0.2 | 0.65 | 0.31 | 3751 | Train 59 | Test 39 | 0.71 | 0.2 | 0.67 | 0.31 |
| 1704 | Train 27 | Test 40 | 0.7 | 0.3 | 0.47 | 0.36 | 3752 | Train 59 | Test 40 | 0.69 | 0.27 | 0.56 | 0.37 |
| 1705 | Train 27 | Test 41 | 0.76 | 0.22 | 0.7 | 0.33 | 3753 | Train 59 | Test 41 | 0.74 | 0.21 | 0.68 | 0.32 |
| 1706 | Train 27 | Test 42 | 0.75 | 0.25 | 0.69 | 0.36 | 3754 | Train 59 | Test 42 | 0.72 | 0.24 | 0.63 | 0.35 |
| 1707 | Train 27 | Test 43 | 0.76 | 0.22 | 0.71 | 0.33 | 3755 | Train 59 | Test 43 | 0.74 | 0.21 | 0.68 | 0.32 |
| 1708 | Train 27 | Test 44 | 0.75 | 0.25 | 0.69 | 0.36 | 3756 | Train 59 | Test 44 | 0.72 | 0.24 | 0.63 | 0.35 |
| 1709 | Train 27 | Test 45 | 0.72 | 0.2 | 0.62 | 0.31 | 3757 | Train 59 | Test 45 | 0.71 | 0.2 | 0.65 | 0.3 |
| 1710 | Train 27 | Test 46 | 0.7 | 0.3 | 0.47 | 0.37 | 3758 | Train 59 | Test 46 | 0.69 | 0.27 | 0.56 | 0.37 |
| 1711 | Train 27 | Test 47 | 0.71 | 0.2 | 0.61 | 0.31 | 3759 | Train 59 | Test 47 | 0.7 | 0.2 | 0.64 | 0.3 |
| 1712 | Train 27 | Test 48 | 0.7 | 0.3 | 0.47 | 0.36 | 3760 | Train 59 | Test 48 | 0.69 | 0.27 | 0.56 | 0.37 |
| 1713 | Train 27 | Test 49 | 0.78 | 0.24 | 0.8 | 0.37 | 3761 | Train 59 | Test 49 | 0.78 | 0.27 | 0.75 | 0.39 |
| 1714 | Train 27 | Test 50 | 0.78 | 0.24 | 0.79 | 0.37 | 3762 | Train 59 | Test 50 | 0.78 | 0.27 | 0.75 | 0.39 |
| 1715 | Train 27 | Test 51 | 0.78 | 0.24 | 0.8 | 0.38 | 3763 | Train 59 | Test 51 | 0.78 | 0.27 | 0.76 | 0.4 |
| 1716 | Train 27 | Test 52 | 0.78 | 0.25 | 0.8 | 0.38 | 3764 | Train 59 | Test 52 | 0.78 | 0.27 | 0.75 | 0.4 |
| 1717 | Train 27 | Test 53 | 0.74 | 0.26 | 0.6 | 0.36 | 3765 | Train 59 | Test 53 | 0.73 | 0.23 | 0.71 | 0.35 |
| 1718 | Train 27 | Test 54 | 0.72 | 0.34 | 0.5 | 0.41 | 3766 | Train 59 | Test 54 | 0.71 | 0.32 | 0.59 | 0.42 |
| 1719 | Train 27 | Test 55 | 0.74 | 0.23 | 0.66 | 0.34 | 3767 | Train 59 | Test 55 | 0.73 | 0.23 | 0.72 | 0.35 |
| 1720 | Train 27 | Test 56 | 0.72 | 0.34 | 0.5 | 0.4 | 3768 | Train 59 | Test 56 | 0.71 | 0.31 | 0.59 | 0.41 |
| 1721 | Train 27 | Test 57 | 0.78 | 0.25 | 0.78 | 0.37 | 3769 | Train 59 | Test 57 | 0.79 | 0.27 | 0.74 | 0.4 |
| 1722 | Train 27 | Test 58 | 0.78 | 0.25 | 0.78 | 0.38 | 3770 | Train 59 | Test 58 | 0.79 | 0.27 | 0.74 | 0.4 |
| 1723 | Train 27 | Test 59 | 0.78 | 0.25 | 0.78 | 0.38 | 3771 | Train 59 | Test 59 | 0.79 | 0.27 | 0.74 | 0.4 |
| 1724 | Train 27 | Test 60 | 0.78 | 0.25 | 0.78 | 0.38 | 3772 | Train 59 | Test 60 | 0.79 | 0.27 | 0.74 | 0.4 |
| 1725 | Train 27 | Test 61 | 0.74 | 0.26 | 0.58 | 0.36 | 3773 | Train 59 | Test 61 | 0.73 | 0.23 | 0.7 | 0.35 |
| 1726 | Train 27 | Test 62 | 0.72 | 0.34 | 0.5 | 0.41 | 3774 | Train 59 | Test 62 | 0.71 | 0.32 | 0.59 | 0.41 |
| 1727 | Train 27 | Test 63 | 0.75 | 0.26 | 0.59 | 0.36 | 3775 | Train 59 | Test 63 | 0.73 | 0.23 | 0.71 | 0.35 |
| 1728 | Train 27 | Test 64 | 0.72 | 0.33 | 0.5 | 0.4 | 3776 | Train 59 | Test 64 | 0.71 | 0.31 | 0.59 | 0.41 |
| 1729 | Train 28 | Test 1 | 0.75 | 0.22 | 0.69 | 0.34 | 3777 | Train 60 | Test 1 | 0.74 | 0.22 | 0.67 | 0.33 |
| 1730 | Train 28 | Test 2 | 0.77 | 0.25 | 0.74 | 0.38 | 3778 | Train 60 | Test 2 | 0.72 | 0.24 | 0.63 | 0.35 |
| 1731 | Train 28 | Test 3 | 0.75 | 0.22 | 0.69 | 0.34 | 3779 | Train 60 | Test 3 | 0.74 | 0.22 | 0.67 | 0.33 |
| 1732 | Train 28 | Test 4 | 0.77 | 0.25 | 0.74 | 0.38 | 3780 | Train 60 | Test 4 | 0.72 | 0.25 | 0.63 | 0.35 |
| 1733 | Train 28 | Test 5 | 0.71 | 0.2 | 0.62 | 0.31 | 3781 | Train 60 | Test 5 | 0.7 | 0.2 | 0.62 | 0.31 |
| 1734 | Train 28 | Test 6 | 0.71 | 0.33 | 0.41 | 0.37 | 3782 | Train 60 | Test 6 | 0.69 | 0.29 | 0.5 | 0.37 |
| 1735 | Train 28 | Test 7 | 0.71 | 0.2 | 0.62 | 0.31 | 3783 | Train 60 | Test 7 | 0.7 | 0.2 | 0.61 | 0.31 |
| 1736 | Train 28 | Test 8 | 0.71 | 0.32 | 0.42 | 0.37 | 3784 | Train 60 | Test 8 | 0.69 | 0.28 | 0.5 | 0.36 |
| 1737 | Train 28 | Test 9 | 0.75 | 0.23 | 0.67 | 0.34 | 3785 | Train 60 | Test 9 | 0.74 | 0.22 | 0.64 | 0.33 |
| 1738 | Train 28 | Test 10 | 0.77 | 0.26 | 0.73 | 0.38 | 3786 | Train 60 | Test 10 | 0.72 | 0.25 | 0.6 | 0.35 |
| 1739 | Train 28 | Test 11 | 0.76 | 0.23 | 0.67 | 0.34 | 3787 | Train 60 | Test 11 | 0.74 | 0.23 | 0.64 | 0.33 |
| 1740 | Train 28 | Test 12 | 0.77 | 0.26 | 0.73 | 0.38 | 3788 | Train 60 | Test 12 | 0.72 | 0.25 | 0.6 | 0.35 |
| 1741 | Train 28 | Test 13 | 0.71 | 0.21 | 0.59 | 0.31 | 3789 | Train 60 | Test 13 | 0.7 | 0.21 | 0.59 | 0.31 |
| 1742 | Train 28 | Test 14 | 0.71 | 0.33 | 0.42 | 0.37 | 3790 | Train 60 | Test 14 | 0.69 | 0.29 | 0.5 | 0.37 |
| 1743 | Train 28 | Test 15 | 0.71 | 0.21 | 0.59 | 0.31 | 3791 | Train 60 | Test 15 | 0.7 | 0.21 | 0.58 | 0.31 |
| 1744 | Train 28 | Test 16 | 0.71 | 0.32 | 0.42 | 0.36 | 3792 | Train 60 | Test 16 | 0.69 | 0.28 | 0.5 | 0.36 |
| 1745 | Train 28 | Test 17 | 0.75 | 0.22 | 0.69 | 0.34 | 3793 | Train 60 | Test 17 | 0.74 | 0.22 | 0.67 | 0.33 |
| 1746 | Train 28 | Test 18 | 0.77 | 0.25 | 0.74 | 0.38 | 3794 | Train 60 | Test 18 | 0.72 | 0.24 | 0.63 | 0.35 |
| 1747 | Train 28 | Test 19 | 0.75 | 0.22 | 0.69 | 0.34 | 3795 | Train 60 | Test 19 | 0.74 | 0.22 | 0.67 | 0.33 |
| 1748 | Train 28 | Test 20 | 0.77 | 0.25 | 0.74 | 0.38 | 3796 | Train 60 | Test 20 | 0.72 | 0.25 | 0.63 | 0.35 |
| 1749 | Train 28 | Test 21 | 0.71 | 0.2 | 0.62 | 0.31 | 3797 | Train 60 | Test 21 | 0.7 | 0.2 | 0.62 | 0.31 |
| 1750 | Train 28 | Test 22 | 0.71 | 0.33 | 0.41 | 0.37 | 3798 | Train 60 | Test 22 | 0.69 | 0.29 | 0.5 | 0.37 |
| 1751 | Train 28 | Test 23 | 0.71 | 0.2 | 0.62 | 0.31 | 3799 | Train 60 | Test 23 | 0.7 | 0.2 | 0.61 | 0.31 |
| 1752 | Train 28 | Test 24 | 0.71 | 0.32 | 0.42 | 0.36 | 3800 | Train 60 | Test 24 | 0.69 | 0.28 | 0.5 | 0.36 |
| 1753 | Train 28 | Test 25 | 0.76 | 0.23 | 0.67 | 0.34 | 3801 | Train 60 | Test 25 | 0.74 | 0.22 | 0.64 | 0.33 |
| 1754 | Train 28 | Test 26 | 0.77 | 0.26 | 0.73 | 0.38 | 3802 | Train 60 | Test 26 | 0.72 | 0.25 | 0.6 | 0.35 |
| 1755 | Train 28 | Test 27 | 0.76 | 0.23 | 0.67 | 0.34 | 3803 | Train 60 | Test 27 | 0.74 | 0.23 | 0.64 | 0.33 |
| 1756 | Train 28 | Test 28 | 0.77 | 0.26 | 0.73 | 0.38 | 3804 | Train 60 | Test 28 | 0.72 | 0.25 | 0.6 | 0.35 |
| 1757 | Train 28 | Test 29 | 0.71 | 0.21 | 0.59 | 0.31 | 3805 | Train 60 | Test 29 | 0.7 | 0.21 | 0.59 | 0.31 |
| 1758 | Train 28 | Test 30 | 0.71 | 0.33 | 0.42 | 0.37 | 3806 | Train 60 | Test 30 | 0.69 | 0.29 | 0.5 | 0.36 |
| 1759 | Train 28 | Test 31 | 0.71 | 0.21 | 0.59 | 0.31 | 3807 | Train 60 | Test 31 | 0.7 | 0.21 | 0.58 | 0.31 |
| 1760 | Train 28 | Test 32 | 0.71 | 0.32 | 0.42 | 0.36 | 3808 | Train 60 | Test 32 | 0.69 | 0.28 | 0.5 | 0.36 |
| 1761 | Train 28 | Test 33 | 0.76 | 0.22 | 0.69 | 0.34 | 3809 | Train 60 | Test 33 | 0.74 | 0.22 | 0.66 | 0.33 |
| 1762 | Train 28 | Test 34 | 0.77 | 0.25 | 0.74 | 0.38 | 3810 | Train 60 | Test 34 | 0.72 | 0.24 | 0.63 | 0.35 |
| 1763 | Train 28 | Test 35 | 0.76 | 0.22 | 0.69 | 0.34 | 3811 | Train 60 | Test 35 | 0.74 | 0.22 | 0.66 | 0.33 |
| 1764 | Train 28 | Test 36 | 0.77 | 0.25 | 0.74 | 0.38 | 3812 | Train 60 | Test 36 | 0.72 | 0.24 | 0.63 | 0.35 |
| 1765 | Train 28 | Test 37 | 0.71 | 0.2 | 0.62 | 0.31 | 3813 | Train 60 | Test 37 | 0.71 | 0.2 | 0.62 | 0.31 |
| 1766 | Train 28 | Test 38 | 0.71 | 0.33 | 0.41 | 0.37 | 3814 | Train 60 | Test 38 | 0.69 | 0.29 | 0.5 | 0.37 |
| 1767 | Train 28 | Test 39 | 0.71 | 0.2 | 0.61 | 0.31 | 3815 | Train 60 | Test 39 | 0.7 | 0.2 | 0.61 | 0.31 |
| 1768 | Train 28 | Test 40 | 0.71 | 0.32 | 0.42 | 0.36 | 3816 | Train 60 | Test 40 | 0.69 | 0.28 | 0.5 | 0.36 |
| 1769 | Train 28 | Test 41 | 0.76 | 0.23 | 0.66 | 0.34 | 3817 | Train 60 | Test 41 | 0.74 | 0.22 | 0.63 | 0.33 |
| 1770 | Train 28 | Test 42 | 0.77 | 0.26 | 0.72 | 0.38 | 3818 | Train 60 | Test 42 | 0.72 | 0.25 | 0.6 | 0.35 |
| 1771 | Train 28 | Test 43 | 0.76 | 0.23 | 0.67 | 0.34 | 3819 | Train 60 | Test 43 | 0.74 | 0.22 | 0.64 | 0.33 |
| 1772 | Train 28 | Test 44 | 0.77 | 0.26 | 0.73 | 0.38 | 3820 | Train 60 | Test 44 | 0.72 | 0.25 | 0.6 | 0.35 |
| 1773 | Train 28 | Test 45 | 0.71 | 0.21 | 0.59 | 0.31 | 3821 | Train 60 | Test 45 | 0.7 | 0.21 | 0.59 | 0.31 |
| 1774 | Train 28 | Test 46 | 0.71 | 0.33 | 0.42 | 0.37 | 3822 | Train 60 | Test 46 | 0.69 | 0.29 | 0.5 | 0.36 |
| 1775 | Train 28 | Test 47 | 0.71 | 0.21 | 0.58 | 0.31 | 3823 | Train 60 | Test 47 | 0.7 | 0.21 | 0.58 | 0.31 |
| 1776 | Train 28 | Test 48 | 0.71 | 0.32 | 0.42 | 0.36 | 3824 | Train 60 | Test 48 | 0.69 | 0.28 | 0.5 | 0.36 |
| 1777 | Train 28 | Test 49 | 0.77 | 0.25 | 0.74 | 0.37 | 3825 | Train 60 | Test 49 | 0.78 | 0.27 | 0.76 | 0.39 |
| 1778 | Train 28 | Test 50 | 0.77 | 0.25 | 0.74 | 0.37 | 3826 | Train 60 | Test 50 | 0.78 | 0.27 | 0.76 | 0.4 |
| 1779 | Train 28 | Test 51 | 0.77 | 0.25 | 0.75 | 0.38 | 3827 | Train 60 | Test 51 | 0.78 | 0.27 | 0.76 | 0.4 |
| 1780 | Train 28 | Test 52 | 0.77 | 0.25 | 0.74 | 0.38 | 3828 | Train 60 | Test 52 | 0.78 | 0.27 | 0.76 | 0.4 |
| 1781 | Train 28 | Test 53 | 0.74 | 0.28 | 0.46 | 0.35 | 3829 | Train 60 | Test 53 | 0.73 | 0.24 | 0.67 | 0.35 |
| 1782 | Train 28 | Test 54 | 0.72 | 0.37 | 0.41 | 0.39 | 3830 | Train 60 | Test 54 | 0.71 | 0.33 | 0.55 | 0.41 |
| 1783 | Train 28 | Test 55 | 0.73 | 0.23 | 0.58 | 0.33 | 3831 | Train 60 | Test 55 | 0.73 | 0.24 | 0.67 | 0.35 |
| 1784 | Train 28 | Test 56 | 0.72 | 0.36 | 0.42 | 0.38 | 3832 | Train 60 | Test 56 | 0.71 | 0.32 | 0.55 | 0.41 |
| 1785 | Train 28 | Test 57 | 0.78 | 0.26 | 0.73 | 0.38 | 3833 | Train 60 | Test 57 | 0.79 | 0.27 | 0.75 | 0.4 |
| 1786 | Train 28 | Test 58 | 0.77 | 0.26 | 0.73 | 0.38 | 3834 | Train 60 | Test 58 | 0.79 | 0.27 | 0.74 | 0.4 |
| 1787 | Train 28 | Test 59 | 0.78 | 0.26 | 0.74 | 0.38 | 3835 | Train 60 | Test 59 | 0.79 | 0.27 | 0.75 | 0.4 |
| 1788 | Train 28 | Test 60 | 0.77 | 0.26 | 0.73 | 0.38 | 3836 | Train 60 | Test 60 | 0.79 | 0.27 | 0.74 | 0.4 |
| 1789 | Train 28 | Test 61 | 0.75 | 0.28 | 0.45 | 0.35 | 3837 | Train 60 | Test 61 | 0.73 | 0.24 | 0.66 | 0.35 |
| 1790 | Train 28 | Test 62 | 0.72 | 0.37 | 0.41 | 0.39 | 3838 | Train 60 | Test 62 | 0.71 | 0.33 | 0.55 | 0.41 |
| 1791 | Train 28 | Test 63 | 0.75 | 0.28 | 0.46 | 0.35 | 3839 | Train 60 | Test 63 | 0.74 | 0.24 | 0.66 | 0.35 |
| 1792 | Train 28 | Test 64 | 0.72 | 0.35 | 0.42 | 0.38 | 3840 | Train 60 | Test 64 | 0.71 | 0.32 | 0.55 | 0.41 |
| 1793 | Train 29 | Test 1 | 0.75 | 0.21 | 0.76 | 0.33 | 3841 | Train 61 | Test 1 | 0.73 | 0.18 | 0.82 | 0.3 |
| 1794 | Train 29 | Test 2 | 0.75 | 0.24 | 0.78 | 0.37 | 3842 | Train 61 | Test 2 | 0.73 | 0.21 | 0.83 | 0.34 |
| 1795 | Train 29 | Test 3 | 0.75 | 0.21 | 0.76 | 0.33 | 3843 | Train 61 | Test 3 | 0.73 | 0.18 | 0.82 | 0.3 |
| 1796 | Train 29 | Test 4 | 0.75 | 0.24 | 0.78 | 0.37 | 3844 | Train 61 | Test 4 | 0.73 | 0.21 | 0.83 | 0.34 |
| 1797 | Train 29 | Test 5 | 0.73 | 0.21 | 0.73 | 0.32 | 3845 | Train 61 | Test 5 | 0.72 | 0.18 | 0.79 | 0.29 |
| 1798 | Train 29 | Test 6 | 0.7 | 0.29 | 0.6 | 0.39 | 3846 | Train 61 | Test 6 | 0.7 | 0.27 | 0.68 | 0.38 |
| 1799 | Train 29 | Test 7 | 0.73 | 0.21 | 0.73 | 0.32 | 3847 | Train 61 | Test 7 | 0.71 | 0.18 | 0.78 | 0.29 |
| 1800 | Train 29 | Test 8 | 0.7 | 0.28 | 0.61 | 0.38 | 3848 | Train 61 | Test 8 | 0.7 | 0.26 | 0.68 | 0.38 |
| 1801 | Train 29 | Test 9 | 0.75 | 0.21 | 0.74 | 0.33 | 3849 | Train 61 | Test 9 | 0.73 | 0.18 | 0.81 | 0.3 |
| 1802 | Train 29 | Test 10 | 0.75 | 0.25 | 0.76 | 0.37 | 3850 | Train 61 | Test 10 | 0.73 | 0.21 | 0.81 | 0.34 |
| 1803 | Train 29 | Test 11 | 0.75 | 0.22 | 0.75 | 0.33 | 3851 | Train 61 | Test 11 | 0.73 | 0.19 | 0.81 | 0.3 |
| 1804 | Train 29 | Test 12 | 0.75 | 0.25 | 0.76 | 0.37 | 3852 | Train 61 | Test 12 | 0.73 | 0.21 | 0.81 | 0.34 |
| 1805 | Train 29 | Test 13 | 0.73 | 0.21 | 0.71 | 0.33 | 3853 | Train 61 | Test 13 | 0.72 | 0.18 | 0.77 | 0.3 |
| 1806 | Train 29 | Test 14 | 0.7 | 0.28 | 0.61 | 0.39 | 3854 | Train 61 | Test 14 | 0.7 | 0.26 | 0.69 | 0.38 |
| 1807 | Train 29 | Test 15 | 0.73 | 0.21 | 0.71 | 0.32 | 3855 | Train 61 | Test 15 | 0.71 | 0.18 | 0.76 | 0.3 |
| 1808 | Train 29 | Test 16 | 0.7 | 0.28 | 0.61 | 0.38 | 3856 | Train 61 | Test 16 | 0.71 | 0.26 | 0.69 | 0.38 |
| 1809 | Train 29 | Test 17 | 0.75 | 0.21 | 0.76 | 0.33 | 3857 | Train 61 | Test 17 | 0.73 | 0.18 | 0.82 | 0.3 |
| 1810 | Train 29 | Test 18 | 0.75 | 0.24 | 0.78 | 0.37 | 3858 | Train 61 | Test 18 | 0.73 | 0.21 | 0.83 | 0.34 |
| 1811 | Train 29 | Test 19 | 0.75 | 0.21 | 0.76 | 0.33 | 3859 | Train 61 | Test 19 | 0.73 | 0.18 | 0.83 | 0.3 |
| 1812 | Train 29 | Test 20 | 0.75 | 0.24 | 0.78 | 0.37 | 3860 | Train 61 | Test 20 | 0.73 | 0.21 | 0.83 | 0.34 |
| 1813 | Train 29 | Test 21 | 0.73 | 0.21 | 0.73 | 0.32 | 3861 | Train 61 | Test 21 | 0.72 | 0.18 | 0.79 | 0.29 |
| 1814 | Train 29 | Test 22 | 0.7 | 0.29 | 0.6 | 0.39 | 3862 | Train 61 | Test 22 | 0.7 | 0.26 | 0.68 | 0.38 |
| 1815 | Train 29 | Test 23 | 0.73 | 0.21 | 0.73 | 0.32 | 3863 | Train 61 | Test 23 | 0.71 | 0.18 | 0.78 | 0.29 |
| 1816 | Train 29 | Test 24 | 0.7 | 0.28 | 0.6 | 0.38 | 3864 | Train 61 | Test 24 | 0.7 | 0.26 | 0.68 | 0.38 |
| 1817 | Train 29 | Test 25 | 0.75 | 0.22 | 0.74 | 0.33 | 3865 | Train 61 | Test 25 | 0.73 | 0.18 | 0.81 | 0.3 |
| 1818 | Train 29 | Test 26 | 0.75 | 0.25 | 0.76 | 0.37 | 3866 | Train 61 | Test 26 | 0.73 | 0.21 | 0.81 | 0.34 |
| 1819 | Train 29 | Test 27 | 0.75 | 0.22 | 0.75 | 0.34 | 3867 | Train 61 | Test 27 | 0.73 | 0.19 | 0.81 | 0.3 |
| 1820 | Train 29 | Test 28 | 0.75 | 0.25 | 0.76 | 0.37 | 3868 | Train 61 | Test 28 | 0.73 | 0.21 | 0.81 | 0.34 |
| 1821 | Train 29 | Test 29 | 0.73 | 0.21 | 0.71 | 0.33 | 3869 | Train 61 | Test 29 | 0.72 | 0.18 | 0.77 | 0.3 |
| 1822 | Train 29 | Test 30 | 0.71 | 0.28 | 0.61 | 0.39 | 3870 | Train 61 | Test 30 | 0.71 | 0.26 | 0.69 | 0.38 |
| 1823 | Train 29 | Test 31 | 0.73 | 0.21 | 0.71 | 0.32 | 3871 | Train 61 | Test 31 | 0.71 | 0.18 | 0.76 | 0.3 |
| 1824 | Train 29 | Test 32 | 0.71 | 0.28 | 0.61 | 0.38 | 3872 | Train 61 | Test 32 | 0.71 | 0.26 | 0.69 | 0.38 |
| 1825 | Train 29 | Test 33 | 0.75 | 0.21 | 0.75 | 0.33 | 3873 | Train 61 | Test 33 | 0.73 | 0.18 | 0.82 | 0.3 |
| 1826 | Train 29 | Test 34 | 0.75 | 0.24 | 0.77 | 0.37 | 3874 | Train 61 | Test 34 | 0.74 | 0.21 | 0.83 | 0.33 |
| 1827 | Train 29 | Test 35 | 0.75 | 0.21 | 0.76 | 0.33 | 3875 | Train 61 | Test 35 | 0.73 | 0.18 | 0.82 | 0.3 |
| 1828 | Train 29 | Test 36 | 0.75 | 0.24 | 0.77 | 0.37 | 3876 | Train 61 | Test 36 | 0.74 | 0.21 | 0.83 | 0.34 |
| 1829 | Train 29 | Test 37 | 0.73 | 0.21 | 0.73 | 0.32 | 3877 | Train 61 | Test 37 | 0.72 | 0.18 | 0.78 | 0.29 |
| 1830 | Train 29 | Test 38 | 0.7 | 0.29 | 0.6 | 0.39 | 3878 | Train 61 | Test 38 | 0.7 | 0.26 | 0.68 | 0.38 |
| 1831 | Train 29 | Test 39 | 0.73 | 0.21 | 0.72 | 0.32 | 3879 | Train 61 | Test 39 | 0.72 | 0.18 | 0.78 | 0.29 |
| 1832 | Train 29 | Test 40 | 0.7 | 0.28 | 0.6 | 0.38 | 3880 | Train 61 | Test 40 | 0.7 | 0.26 | 0.68 | 0.38 |
| 1833 | Train 29 | Test 41 | 0.75 | 0.21 | 0.74 | 0.33 | 3881 | Train 61 | Test 41 | 0.73 | 0.18 | 0.81 | 0.3 |
| 1834 | Train 29 | Test 42 | 0.75 | 0.25 | 0.76 | 0.37 | 3882 | Train 61 | Test 42 | 0.74 | 0.21 | 0.81 | 0.34 |
| 1835 | Train 29 | Test 43 | 0.75 | 0.22 | 0.74 | 0.33 | 3883 | Train 61 | Test 43 | 0.73 | 0.18 | 0.81 | 0.3 |
| 1836 | Train 29 | Test 44 | 0.75 | 0.25 | 0.76 | 0.37 | 3884 | Train 61 | Test 44 | 0.74 | 0.21 | 0.81 | 0.34 |
| 1837 | Train 29 | Test 45 | 0.74 | 0.21 | 0.71 | 0.32 | 3885 | Train 61 | Test 45 | 0.72 | 0.18 | 0.77 | 0.3 |
| 1838 | Train 29 | Test 46 | 0.7 | 0.28 | 0.61 | 0.39 | 3886 | Train 61 | Test 46 | 0.71 | 0.26 | 0.69 | 0.38 |
| 1839 | Train 29 | Test 47 | 0.73 | 0.21 | 0.7 | 0.32 | 3887 | Train 61 | Test 47 | 0.72 | 0.18 | 0.76 | 0.3 |
| 1840 | Train 29 | Test 48 | 0.71 | 0.28 | 0.61 | 0.38 | 3888 | Train 61 | Test 48 | 0.71 | 0.26 | 0.69 | 0.38 |
| 1841 | Train 29 | Test 49 | 0.77 | 0.24 | 0.79 | 0.37 | 3889 | Train 61 | Test 49 | 0.77 | 0.24 | 0.79 | 0.37 |
| 1842 | Train 29 | Test 50 | 0.76 | 0.24 | 0.78 | 0.37 | 3890 | Train 61 | Test 50 | 0.77 | 0.25 | 0.79 | 0.37 |
| 1843 | Train 29 | Test 51 | 0.77 | 0.24 | 0.79 | 0.37 | 3891 | Train 61 | Test 51 | 0.77 | 0.25 | 0.8 | 0.38 |
| 1844 | Train 29 | Test 52 | 0.76 | 0.24 | 0.79 | 0.37 | 3892 | Train 61 | Test 52 | 0.77 | 0.25 | 0.79 | 0.38 |
| 1845 | Train 29 | Test 53 | 0.76 | 0.25 | 0.74 | 0.37 | 3893 | Train 61 | Test 53 | 0.77 | 0.25 | 0.74 | 0.37 |
| 1846 | Train 29 | Test 54 | 0.72 | 0.33 | 0.6 | 0.43 | 3894 | Train 61 | Test 54 | 0.72 | 0.32 | 0.66 | 0.43 |
| 1847 | Train 29 | Test 55 | 0.76 | 0.24 | 0.76 | 0.36 | 3895 | Train 61 | Test 55 | 0.77 | 0.24 | 0.77 | 0.36 |
| 1848 | Train 29 | Test 56 | 0.71 | 0.31 | 0.61 | 0.41 | 3896 | Train 61 | Test 56 | 0.72 | 0.31 | 0.66 | 0.42 |
| 1849 | Train 29 | Test 57 | 0.77 | 0.25 | 0.78 | 0.38 | 3897 | Train 61 | Test 57 | 0.78 | 0.25 | 0.78 | 0.38 |
| 1850 | Train 29 | Test 58 | 0.77 | 0.25 | 0.78 | 0.38 | 3898 | Train 61 | Test 58 | 0.78 | 0.25 | 0.78 | 0.38 |
| 1851 | Train 29 | Test 59 | 0.77 | 0.25 | 0.78 | 0.38 | 3899 | Train 61 | Test 59 | 0.78 | 0.25 | 0.78 | 0.38 |
| 1852 | Train 29 | Test 60 | 0.77 | 0.25 | 0.78 | 0.38 | 3900 | Train 61 | Test 60 | 0.77 | 0.25 | 0.78 | 0.38 |
| 1853 | Train 29 | Test 61 | 0.76 | 0.25 | 0.73 | 0.37 | 3901 | Train 61 | Test 61 | 0.77 | 0.25 | 0.73 | 0.37 |
| 1854 | Train 29 | Test 62 | 0.72 | 0.33 | 0.6 | 0.42 | 3902 | Train 61 | Test 62 | 0.72 | 0.32 | 0.66 | 0.43 |
| 1855 | Train 29 | Test 63 | 0.77 | 0.25 | 0.74 | 0.37 | 3903 | Train 61 | Test 63 | 0.77 | 0.25 | 0.73 | 0.37 |
| 1856 | Train 29 | Test 64 | 0.72 | 0.31 | 0.61 | 0.41 | 3904 | Train 61 | Test 64 | 0.72 | 0.31 | 0.66 | 0.42 |
| 1857 | Train 30 | Test 1 | 0.73 | 0.21 | 0.74 | 0.32 | 3905 | Train 62 | Test 1 | 0.72 | 0.18 | 0.81 | 0.29 |
| 1858 | Train 30 | Test 2 | 0.75 | 0.23 | 0.79 | 0.36 | 3906 | Train 62 | Test 2 | 0.73 | 0.21 | 0.83 | 0.33 |
| 1859 | Train 30 | Test 3 | 0.73 | 0.21 | 0.74 | 0.32 | 3907 | Train 62 | Test 3 | 0.72 | 0.18 | 0.81 | 0.29 |
| 1860 | Train 30 | Test 4 | 0.75 | 0.23 | 0.79 | 0.36 | 3908 | Train 62 | Test 4 | 0.73 | 0.21 | 0.83 | 0.33 |
| 1861 | Train 30 | Test 5 | 0.72 | 0.2 | 0.71 | 0.31 | 3909 | Train 62 | Test 5 | 0.71 | 0.18 | 0.78 | 0.29 |
| 1862 | Train 30 | Test 6 | 0.74 | 0.29 | 0.7 | 0.41 | 3910 | Train 62 | Test 6 | 0.73 | 0.27 | 0.74 | 0.4 |
| 1863 | Train 30 | Test 7 | 0.72 | 0.2 | 0.71 | 0.31 | 3911 | Train 62 | Test 7 | 0.7 | 0.18 | 0.78 | 0.29 |
| 1864 | Train 30 | Test 8 | 0.74 | 0.28 | 0.7 | 0.4 | 3912 | Train 62 | Test 8 | 0.73 | 0.26 | 0.74 | 0.39 |
| 1865 | Train 30 | Test 9 | 0.73 | 0.21 | 0.72 | 0.32 | 3913 | Train 62 | Test 9 | 0.72 | 0.18 | 0.8 | 0.3 |
| 1866 | Train 30 | Test 10 | 0.75 | 0.24 | 0.76 | 0.36 | 3914 | Train 62 | Test 10 | 0.73 | 0.21 | 0.83 | 0.34 |
| 1867 | Train 30 | Test 11 | 0.73 | 0.21 | 0.72 | 0.32 | 3915 | Train 62 | Test 11 | 0.72 | 0.18 | 0.8 | 0.3 |
| 1868 | Train 30 | Test 12 | 0.75 | 0.24 | 0.77 | 0.36 | 3916 | Train 62 | Test 12 | 0.73 | 0.21 | 0.83 | 0.34 |
| 1869 | Train 30 | Test 13 | 0.72 | 0.2 | 0.69 | 0.31 | 3917 | Train 62 | Test 13 | 0.71 | 0.18 | 0.76 | 0.3 |
| 1870 | Train 30 | Test 14 | 0.74 | 0.29 | 0.7 | 0.41 | 3918 | Train 62 | Test 14 | 0.73 | 0.27 | 0.74 | 0.39 |
| 1871 | Train 30 | Test 15 | 0.72 | 0.2 | 0.68 | 0.31 | 3919 | Train 62 | Test 15 | 0.7 | 0.18 | 0.76 | 0.3 |
| 1872 | Train 30 | Test 16 | 0.74 | 0.28 | 0.7 | 0.4 | 3920 | Train 62 | Test 16 | 0.73 | 0.26 | 0.74 | 0.39 |
| 1873 | Train 30 | Test 17 | 0.73 | 0.21 | 0.74 | 0.32 | 3921 | Train 62 | Test 17 | 0.72 | 0.18 | 0.81 | 0.29 |
| 1874 | Train 30 | Test 18 | 0.75 | 0.23 | 0.79 | 0.36 | 3922 | Train 62 | Test 18 | 0.73 | 0.21 | 0.83 | 0.33 |
| 1875 | Train 30 | Test 19 | 0.73 | 0.21 | 0.74 | 0.32 | 3923 | Train 62 | Test 19 | 0.72 | 0.18 | 0.81 | 0.29 |
| 1876 | Train 30 | Test 20 | 0.75 | 0.23 | 0.79 | 0.36 | 3924 | Train 62 | Test 20 | 0.73 | 0.21 | 0.83 | 0.33 |
| 1877 | Train 30 | Test 21 | 0.72 | 0.2 | 0.71 | 0.31 | 3925 | Train 62 | Test 21 | 0.71 | 0.18 | 0.78 | 0.29 |
| 1878 | Train 30 | Test 22 | 0.74 | 0.29 | 0.7 | 0.41 | 3926 | Train 62 | Test 22 | 0.73 | 0.27 | 0.74 | 0.4 |
| 1879 | Train 30 | Test 23 | 0.72 | 0.2 | 0.71 | 0.31 | 3927 | Train 62 | Test 23 | 0.7 | 0.18 | 0.78 | 0.29 |
| 1880 | Train 30 | Test 24 | 0.74 | 0.28 | 0.7 | 0.4 | 3928 | Train 62 | Test 24 | 0.73 | 0.26 | 0.74 | 0.39 |
| 1881 | Train 30 | Test 25 | 0.73 | 0.21 | 0.72 | 0.32 | 3929 | Train 62 | Test 25 | 0.72 | 0.18 | 0.8 | 0.3 |
| 1882 | Train 30 | Test 26 | 0.75 | 0.24 | 0.77 | 0.36 | 3930 | Train 62 | Test 26 | 0.74 | 0.21 | 0.83 | 0.34 |
| 1883 | Train 30 | Test 27 | 0.73 | 0.21 | 0.72 | 0.32 | 3931 | Train 62 | Test 27 | 0.72 | 0.18 | 0.8 | 0.3 |
| 1884 | Train 30 | Test 28 | 0.75 | 0.24 | 0.77 | 0.36 | 3932 | Train 62 | Test 28 | 0.73 | 0.21 | 0.83 | 0.34 |
| 1885 | Train 30 | Test 29 | 0.72 | 0.2 | 0.69 | 0.31 | 3933 | Train 62 | Test 29 | 0.71 | 0.18 | 0.76 | 0.3 |
| 1886 | Train 30 | Test 30 | 0.74 | 0.29 | 0.7 | 0.41 | 3934 | Train 62 | Test 30 | 0.73 | 0.27 | 0.74 | 0.39 |
| 1887 | Train 30 | Test 31 | 0.72 | 0.2 | 0.68 | 0.31 | 3935 | Train 62 | Test 31 | 0.7 | 0.18 | 0.76 | 0.3 |
| 1888 | Train 30 | Test 32 | 0.74 | 0.28 | 0.7 | 0.4 | 3936 | Train 62 | Test 32 | 0.73 | 0.26 | 0.74 | 0.39 |
| 1889 | Train 30 | Test 33 | 0.74 | 0.2 | 0.74 | 0.32 | 3937 | Train 62 | Test 33 | 0.72 | 0.18 | 0.81 | 0.29 |
| 1890 | Train 30 | Test 34 | 0.75 | 0.23 | 0.78 | 0.36 | 3938 | Train 62 | Test 34 | 0.74 | 0.21 | 0.83 | 0.33 |
| 1891 | Train 30 | Test 35 | 0.74 | 0.21 | 0.74 | 0.32 | 3939 | Train 62 | Test 35 | 0.72 | 0.18 | 0.81 | 0.29 |
| 1892 | Train 30 | Test 36 | 0.75 | 0.23 | 0.78 | 0.36 | 3940 | Train 62 | Test 36 | 0.74 | 0.21 | 0.83 | 0.33 |
| 1893 | Train 30 | Test 37 | 0.72 | 0.2 | 0.71 | 0.31 | 3941 | Train 62 | Test 37 | 0.71 | 0.18 | 0.78 | 0.29 |
| 1894 | Train 30 | Test 38 | 0.74 | 0.29 | 0.7 | 0.41 | 3942 | Train 62 | Test 38 | 0.73 | 0.27 | 0.74 | 0.4 |
| 1895 | Train 30 | Test 39 | 0.72 | 0.2 | 0.7 | 0.31 | 3943 | Train 62 | Test 39 | 0.71 | 0.18 | 0.77 | 0.29 |
| 1896 | Train 30 | Test 40 | 0.74 | 0.28 | 0.7 | 0.4 | 3944 | Train 62 | Test 40 | 0.73 | 0.26 | 0.74 | 0.39 |
| 1897 | Train 30 | Test 41 | 0.74 | 0.21 | 0.71 | 0.32 | 3945 | Train 62 | Test 41 | 0.72 | 0.18 | 0.8 | 0.3 |
| 1898 | Train 30 | Test 42 | 0.75 | 0.24 | 0.76 | 0.36 | 3946 | Train 62 | Test 42 | 0.74 | 0.21 | 0.83 | 0.34 |
| 1899 | Train 30 | Test 43 | 0.74 | 0.21 | 0.71 | 0.32 | 3947 | Train 62 | Test 43 | 0.72 | 0.18 | 0.8 | 0.3 |
| 1900 | Train 30 | Test 44 | 0.75 | 0.24 | 0.76 | 0.36 | 3948 | Train 62 | Test 44 | 0.74 | 0.21 | 0.83 | 0.34 |
| 1901 | Train 30 | Test 45 | 0.72 | 0.2 | 0.68 | 0.31 | 3949 | Train 62 | Test 45 | 0.71 | 0.18 | 0.76 | 0.3 |
| 1902 | Train 30 | Test 46 | 0.74 | 0.29 | 0.7 | 0.41 | 3950 | Train 62 | Test 46 | 0.73 | 0.27 | 0.74 | 0.39 |
| 1903 | Train 30 | Test 47 | 0.72 | 0.2 | 0.68 | 0.31 | 3951 | Train 62 | Test 47 | 0.71 | 0.18 | 0.76 | 0.3 |
| 1904 | Train 30 | Test 48 | 0.74 | 0.28 | 0.7 | 0.4 | 3952 | Train 62 | Test 48 | 0.73 | 0.26 | 0.74 | 0.39 |
| 1905 | Train 30 | Test 49 | 0.77 | 0.26 | 0.75 | 0.39 | 3953 | Train 62 | Test 49 | 0.77 | 0.26 | 0.75 | 0.38 |
| 1906 | Train 30 | Test 50 | 0.77 | 0.26 | 0.75 | 0.39 | 3954 | Train 62 | Test 50 | 0.77 | 0.26 | 0.75 | 0.38 |
| 1907 | Train 30 | Test 51 | 0.77 | 0.26 | 0.75 | 0.39 | 3955 | Train 62 | Test 51 | 0.77 | 0.26 | 0.75 | 0.38 |
| 1908 | Train 30 | Test 52 | 0.76 | 0.26 | 0.75 | 0.39 | 3956 | Train 62 | Test 52 | 0.77 | 0.26 | 0.75 | 0.38 |
| 1909 | Train 30 | Test 53 | 0.76 | 0.26 | 0.66 | 0.38 | 3957 | Train 62 | Test 53 | 0.76 | 0.27 | 0.66 | 0.38 |
| 1910 | Train 30 | Test 54 | 0.75 | 0.33 | 0.7 | 0.45 | 3958 | Train 62 | Test 54 | 0.75 | 0.33 | 0.71 | 0.45 |
| 1911 | Train 30 | Test 55 | 0.76 | 0.25 | 0.7 | 0.37 | 3959 | Train 62 | Test 55 | 0.76 | 0.25 | 0.72 | 0.37 |
| 1912 | Train 30 | Test 56 | 0.74 | 0.32 | 0.7 | 0.44 | 3960 | Train 62 | Test 56 | 0.75 | 0.32 | 0.72 | 0.44 |
| 1913 | Train 30 | Test 57 | 0.77 | 0.26 | 0.74 | 0.39 | 3961 | Train 62 | Test 57 | 0.78 | 0.26 | 0.75 | 0.39 |
| 1914 | Train 30 | Test 58 | 0.77 | 0.27 | 0.74 | 0.39 | 3962 | Train 62 | Test 58 | 0.78 | 0.26 | 0.75 | 0.39 |
| 1915 | Train 30 | Test 59 | 0.77 | 0.27 | 0.74 | 0.39 | 3963 | Train 62 | Test 59 | 0.78 | 0.27 | 0.75 | 0.39 |
| 1916 | Train 30 | Test 60 | 0.77 | 0.27 | 0.74 | 0.39 | 3964 | Train 62 | Test 60 | 0.77 | 0.27 | 0.75 | 0.39 |
| 1917 | Train 30 | Test 61 | 0.76 | 0.27 | 0.65 | 0.38 | 3965 | Train 62 | Test 61 | 0.77 | 0.27 | 0.66 | 0.39 |
| 1918 | Train 30 | Test 62 | 0.75 | 0.33 | 0.7 | 0.45 | 3966 | Train 62 | Test 62 | 0.75 | 0.33 | 0.72 | 0.45 |
| 1919 | Train 30 | Test 63 | 0.76 | 0.27 | 0.65 | 0.38 | 3967 | Train 62 | Test 63 | 0.77 | 0.27 | 0.66 | 0.39 |
| 1920 | Train 30 | Test 64 | 0.74 | 0.31 | 0.71 | 0.43 | 3968 | Train 62 | Test 64 | 0.75 | 0.31 | 0.72 | 0.44 |
| 1921 | Train 31 | Test 1 | 0.75 | 0.21 | 0.76 | 0.33 | 3969 | Train 63 | Test 1 | 0.73 | 0.18 | 0.83 | 0.3 |
| 1922 | Train 31 | Test 2 | 0.75 | 0.24 | 0.78 | 0.37 | 3970 | Train 63 | Test 2 | 0.73 | 0.21 | 0.83 | 0.34 |
| 1923 | Train 31 | Test 3 | 0.75 | 0.21 | 0.76 | 0.33 | 3971 | Train 63 | Test 3 | 0.73 | 0.18 | 0.83 | 0.3 |
| 1924 | Train 31 | Test 4 | 0.75 | 0.24 | 0.78 | 0.37 | 3972 | Train 63 | Test 4 | 0.73 | 0.21 | 0.83 | 0.34 |
| 1925 | Train 31 | Test 5 | 0.73 | 0.2 | 0.73 | 0.32 | 3973 | Train 63 | Test 5 | 0.72 | 0.18 | 0.78 | 0.29 |
| 1926 | Train 31 | Test 6 | 0.7 | 0.29 | 0.59 | 0.39 | 3974 | Train 63 | Test 6 | 0.7 | 0.27 | 0.67 | 0.38 |
| 1927 | Train 31 | Test 7 | 0.73 | 0.2 | 0.73 | 0.32 | 3975 | Train 63 | Test 7 | 0.72 | 0.18 | 0.78 | 0.29 |
| 1928 | Train 31 | Test 8 | 0.7 | 0.28 | 0.59 | 0.38 | 3976 | Train 63 | Test 8 | 0.7 | 0.26 | 0.66 | 0.38 |
| 1929 | Train 31 | Test 9 | 0.75 | 0.21 | 0.74 | 0.33 | 3977 | Train 63 | Test 9 | 0.73 | 0.19 | 0.81 | 0.3 |
| 1930 | Train 31 | Test 10 | 0.75 | 0.25 | 0.76 | 0.37 | 3978 | Train 63 | Test 10 | 0.73 | 0.22 | 0.82 | 0.34 |
| 1931 | Train 31 | Test 11 | 0.75 | 0.22 | 0.74 | 0.33 | 3979 | Train 63 | Test 11 | 0.73 | 0.19 | 0.82 | 0.3 |
| 1932 | Train 31 | Test 12 | 0.75 | 0.25 | 0.76 | 0.37 | 3980 | Train 63 | Test 12 | 0.73 | 0.22 | 0.82 | 0.34 |
| 1933 | Train 31 | Test 13 | 0.73 | 0.21 | 0.71 | 0.32 | 3981 | Train 63 | Test 13 | 0.72 | 0.18 | 0.77 | 0.3 |
| 1934 | Train 31 | Test 14 | 0.7 | 0.29 | 0.59 | 0.39 | 3982 | Train 63 | Test 14 | 0.7 | 0.27 | 0.67 | 0.38 |
| 1935 | Train 31 | Test 15 | 0.73 | 0.21 | 0.71 | 0.32 | 3983 | Train 63 | Test 15 | 0.72 | 0.18 | 0.76 | 0.3 |
| 1936 | Train 31 | Test 16 | 0.7 | 0.28 | 0.59 | 0.38 | 3984 | Train 63 | Test 16 | 0.7 | 0.26 | 0.67 | 0.38 |
| 1937 | Train 31 | Test 17 | 0.75 | 0.21 | 0.76 | 0.33 | 3985 | Train 63 | Test 17 | 0.73 | 0.18 | 0.83 | 0.3 |
| 1938 | Train 31 | Test 18 | 0.75 | 0.24 | 0.78 | 0.37 | 3986 | Train 63 | Test 18 | 0.73 | 0.21 | 0.83 | 0.34 |
| 1939 | Train 31 | Test 19 | 0.75 | 0.21 | 0.76 | 0.33 | 3987 | Train 63 | Test 19 | 0.73 | 0.18 | 0.83 | 0.3 |
| 1940 | Train 31 | Test 20 | 0.75 | 0.24 | 0.78 | 0.37 | 3988 | Train 63 | Test 20 | 0.73 | 0.21 | 0.83 | 0.34 |
| 1941 | Train 31 | Test 21 | 0.73 | 0.2 | 0.73 | 0.32 | 3989 | Train 63 | Test 21 | 0.72 | 0.18 | 0.78 | 0.29 |
| 1942 | Train 31 | Test 22 | 0.7 | 0.29 | 0.59 | 0.39 | 3990 | Train 63 | Test 22 | 0.7 | 0.27 | 0.66 | 0.38 |
| 1943 | Train 31 | Test 23 | 0.73 | 0.2 | 0.73 | 0.32 | 3991 | Train 63 | Test 23 | 0.72 | 0.18 | 0.78 | 0.29 |
| 1944 | Train 31 | Test 24 | 0.7 | 0.28 | 0.59 | 0.38 | 3992 | Train 63 | Test 24 | 0.7 | 0.26 | 0.66 | 0.38 |
| 1945 | Train 31 | Test 25 | 0.75 | 0.21 | 0.74 | 0.33 | 3993 | Train 63 | Test 25 | 0.73 | 0.19 | 0.81 | 0.3 |
| 1946 | Train 31 | Test 26 | 0.75 | 0.25 | 0.76 | 0.37 | 3994 | Train 63 | Test 26 | 0.73 | 0.22 | 0.82 | 0.34 |
| 1947 | Train 31 | Test 27 | 0.75 | 0.22 | 0.74 | 0.33 | 3995 | Train 63 | Test 27 | 0.73 | 0.19 | 0.82 | 0.3 |
| 1948 | Train 31 | Test 28 | 0.75 | 0.25 | 0.76 | 0.37 | 3996 | Train 63 | Test 28 | 0.73 | 0.22 | 0.82 | 0.34 |
| 1949 | Train 31 | Test 29 | 0.73 | 0.21 | 0.71 | 0.32 | 3997 | Train 63 | Test 29 | 0.72 | 0.18 | 0.77 | 0.3 |
| 1950 | Train 31 | Test 30 | 0.7 | 0.29 | 0.59 | 0.39 | 3998 | Train 63 | Test 30 | 0.7 | 0.27 | 0.67 | 0.38 |
| 1951 | Train 31 | Test 31 | 0.73 | 0.21 | 0.71 | 0.32 | 3999 | Train 63 | Test 31 | 0.72 | 0.19 | 0.76 | 0.3 |
| 1952 | Train 31 | Test 32 | 0.7 | 0.28 | 0.59 | 0.38 | 4000 | Train 63 | Test 32 | 0.7 | 0.26 | 0.67 | 0.38 |
| 1953 | Train 31 | Test 33 | 0.75 | 0.21 | 0.76 | 0.33 | 4001 | Train 63 | Test 33 | 0.73 | 0.18 | 0.82 | 0.3 |
| 1954 | Train 31 | Test 34 | 0.75 | 0.24 | 0.77 | 0.37 | 4002 | Train 63 | Test 34 | 0.74 | 0.21 | 0.83 | 0.34 |
| 1955 | Train 31 | Test 35 | 0.75 | 0.21 | 0.76 | 0.33 | 4003 | Train 63 | Test 35 | 0.73 | 0.18 | 0.82 | 0.3 |
| 1956 | Train 31 | Test 36 | 0.75 | 0.24 | 0.78 | 0.37 | 4004 | Train 63 | Test 36 | 0.74 | 0.21 | 0.83 | 0.34 |
| 1957 | Train 31 | Test 37 | 0.74 | 0.2 | 0.73 | 0.32 | 4005 | Train 63 | Test 37 | 0.72 | 0.18 | 0.78 | 0.29 |
| 1958 | Train 31 | Test 38 | 0.7 | 0.29 | 0.59 | 0.39 | 4006 | Train 63 | Test 38 | 0.7 | 0.27 | 0.66 | 0.38 |
| 1959 | Train 31 | Test 39 | 0.73 | 0.2 | 0.73 | 0.32 | 4007 | Train 63 | Test 39 | 0.72 | 0.18 | 0.77 | 0.29 |
| 1960 | Train 31 | Test 40 | 0.7 | 0.28 | 0.59 | 0.38 | 4008 | Train 63 | Test 40 | 0.7 | 0.26 | 0.66 | 0.38 |
| 1961 | Train 31 | Test 41 | 0.75 | 0.21 | 0.74 | 0.33 | 4009 | Train 63 | Test 41 | 0.74 | 0.19 | 0.81 | 0.3 |
| 1962 | Train 31 | Test 42 | 0.75 | 0.24 | 0.76 | 0.37 | 4010 | Train 63 | Test 42 | 0.74 | 0.22 | 0.82 | 0.34 |
| 1963 | Train 31 | Test 43 | 0.75 | 0.21 | 0.74 | 0.33 | 4011 | Train 63 | Test 43 | 0.74 | 0.19 | 0.81 | 0.3 |
| 1964 | Train 31 | Test 44 | 0.75 | 0.25 | 0.76 | 0.37 | 4012 | Train 63 | Test 44 | 0.74 | 0.22 | 0.82 | 0.34 |
| 1965 | Train 31 | Test 45 | 0.74 | 0.21 | 0.71 | 0.32 | 4013 | Train 63 | Test 45 | 0.72 | 0.18 | 0.76 | 0.3 |
| 1966 | Train 31 | Test 46 | 0.7 | 0.29 | 0.59 | 0.39 | 4014 | Train 63 | Test 46 | 0.7 | 0.27 | 0.67 | 0.38 |
| 1967 | Train 31 | Test 47 | 0.73 | 0.21 | 0.7 | 0.32 | 4015 | Train 63 | Test 47 | 0.72 | 0.19 | 0.76 | 0.3 |
| 1968 | Train 31 | Test 48 | 0.7 | 0.28 | 0.59 | 0.38 | 4016 | Train 63 | Test 48 | 0.71 | 0.26 | 0.67 | 0.38 |
| 1969 | Train 31 | Test 49 | 0.77 | 0.24 | 0.79 | 0.37 | 4017 | Train 63 | Test 49 | 0.77 | 0.24 | 0.8 | 0.37 |
| 1970 | Train 31 | Test 50 | 0.76 | 0.24 | 0.78 | 0.37 | 4018 | Train 63 | Test 50 | 0.77 | 0.24 | 0.8 | 0.37 |
| 1971 | Train 31 | Test 51 | 0.77 | 0.24 | 0.79 | 0.37 | 4019 | Train 63 | Test 51 | 0.77 | 0.24 | 0.8 | 0.37 |
| 1972 | Train 31 | Test 52 | 0.76 | 0.24 | 0.79 | 0.37 | 4020 | Train 63 | Test 52 | 0.77 | 0.24 | 0.8 | 0.37 |
| 1973 | Train 31 | Test 53 | 0.76 | 0.25 | 0.74 | 0.37 | 4021 | Train 63 | Test 53 | 0.77 | 0.25 | 0.74 | 0.37 |
| 1974 | Train 31 | Test 54 | 0.72 | 0.33 | 0.59 | 0.42 | 4022 | Train 63 | Test 54 | 0.72 | 0.32 | 0.64 | 0.43 |
| 1975 | Train 31 | Test 55 | 0.76 | 0.24 | 0.77 | 0.36 | 4023 | Train 63 | Test 55 | 0.77 | 0.24 | 0.77 | 0.36 |
| 1976 | Train 31 | Test 56 | 0.71 | 0.32 | 0.6 | 0.41 | 4024 | Train 63 | Test 56 | 0.72 | 0.31 | 0.65 | 0.42 |
| 1977 | Train 31 | Test 57 | 0.77 | 0.24 | 0.78 | 0.37 | 4025 | Train 63 | Test 57 | 0.78 | 0.25 | 0.79 | 0.38 |
| 1978 | Train 31 | Test 58 | 0.77 | 0.25 | 0.78 | 0.37 | 4026 | Train 63 | Test 58 | 0.78 | 0.25 | 0.79 | 0.38 |
| 1979 | Train 31 | Test 59 | 0.77 | 0.25 | 0.78 | 0.38 | 4027 | Train 63 | Test 59 | 0.78 | 0.25 | 0.79 | 0.38 |
| 1980 | Train 31 | Test 60 | 0.77 | 0.25 | 0.78 | 0.38 | 4028 | Train 63 | Test 60 | 0.78 | 0.25 | 0.79 | 0.38 |
| 1981 | Train 31 | Test 61 | 0.76 | 0.26 | 0.73 | 0.38 | 4029 | Train 63 | Test 61 | 0.77 | 0.25 | 0.73 | 0.38 |
| 1982 | Train 31 | Test 62 | 0.72 | 0.33 | 0.59 | 0.42 | 4030 | Train 63 | Test 62 | 0.72 | 0.32 | 0.65 | 0.43 |
| 1983 | Train 31 | Test 63 | 0.77 | 0.26 | 0.73 | 0.38 | 4031 | Train 63 | Test 63 | 0.77 | 0.25 | 0.74 | 0.38 |
| 1984 | Train 31 | Test 64 | 0.72 | 0.31 | 0.6 | 0.41 | 4032 | Train 63 | Test 64 | 0.72 | 0.31 | 0.65 | 0.42 |
| 1985 | Train 32 | Test 1 | 0.74 | 0.2 | 0.77 | 0.32 | 4033 | Train 64 | Test 1 | 0.72 | 0.17 | 0.84 | 0.28 |
| 1986 | Train 32 | Test 2 | 0.75 | 0.23 | 0.8 | 0.36 | 4034 | Train 64 | Test 2 | 0.73 | 0.2 | 0.85 | 0.32 |
| 1987 | Train 32 | Test 3 | 0.74 | 0.2 | 0.77 | 0.32 | 4035 | Train 64 | Test 3 | 0.72 | 0.17 | 0.84 | 0.29 |
| 1988 | Train 32 | Test 4 | 0.75 | 0.23 | 0.8 | 0.36 | 4036 | Train 64 | Test 4 | 0.73 | 0.2 | 0.85 | 0.33 |
| 1989 | Train 32 | Test 5 | 0.72 | 0.19 | 0.74 | 0.31 | 4037 | Train 64 | Test 5 | 0.7 | 0.17 | 0.81 | 0.28 |
| 1990 | Train 32 | Test 6 | 0.74 | 0.29 | 0.71 | 0.41 | 4038 | Train 64 | Test 6 | 0.72 | 0.27 | 0.73 | 0.39 |
| 1991 | Train 32 | Test 7 | 0.72 | 0.19 | 0.73 | 0.31 | 4039 | Train 64 | Test 7 | 0.7 | 0.17 | 0.81 | 0.28 |
| 1992 | Train 32 | Test 8 | 0.74 | 0.28 | 0.71 | 0.4 | 4040 | Train 64 | Test 8 | 0.72 | 0.26 | 0.73 | 0.39 |
| 1993 | Train 32 | Test 9 | 0.74 | 0.21 | 0.75 | 0.32 | 4041 | Train 64 | Test 9 | 0.72 | 0.17 | 0.84 | 0.29 |
| 1994 | Train 32 | Test 10 | 0.75 | 0.23 | 0.79 | 0.36 | 4042 | Train 64 | Test 10 | 0.73 | 0.2 | 0.85 | 0.33 |
| 1995 | Train 32 | Test 11 | 0.74 | 0.21 | 0.75 | 0.32 | 4043 | Train 64 | Test 11 | 0.72 | 0.17 | 0.84 | 0.29 |
| 1996 | Train 32 | Test 12 | 0.75 | 0.23 | 0.79 | 0.36 | 4044 | Train 64 | Test 12 | 0.73 | 0.2 | 0.85 | 0.33 |
| 1997 | Train 32 | Test 13 | 0.72 | 0.2 | 0.72 | 0.31 | 4045 | Train 64 | Test 13 | 0.7 | 0.18 | 0.8 | 0.29 |
| 1998 | Train 32 | Test 14 | 0.74 | 0.29 | 0.71 | 0.41 | 4046 | Train 64 | Test 14 | 0.72 | 0.27 | 0.73 | 0.39 |
| 1999 | Train 32 | Test 15 | 0.72 | 0.2 | 0.71 | 0.31 | 4047 | Train 64 | Test 15 | 0.7 | 0.18 | 0.79 | 0.29 |
| 2000 | Train 32 | Test 16 | 0.74 | 0.28 | 0.71 | 0.4 | 4048 | Train 64 | Test 16 | 0.72 | 0.26 | 0.73 | 0.39 |
| 2001 | Train 32 | Test 17 | 0.74 | 0.2 | 0.77 | 0.32 | 4049 | Train 64 | Test 17 | 0.72 | 0.17 | 0.84 | 0.28 |
| 2002 | Train 32 | Test 18 | 0.75 | 0.23 | 0.8 | 0.36 | 4050 | Train 64 | Test 18 | 0.73 | 0.2 | 0.85 | 0.32 |
| 2003 | Train 32 | Test 19 | 0.74 | 0.2 | 0.77 | 0.32 | 4051 | Train 64 | Test 19 | 0.72 | 0.17 | 0.84 | 0.29 |
| 2004 | Train 32 | Test 20 | 0.75 | 0.23 | 0.8 | 0.36 | 4052 | Train 64 | Test 20 | 0.73 | 0.2 | 0.85 | 0.33 |
| 2005 | Train 32 | Test 21 | 0.72 | 0.19 | 0.74 | 0.31 | 4053 | Train 64 | Test 21 | 0.7 | 0.17 | 0.81 | 0.28 |
| 2006 | Train 32 | Test 22 | 0.74 | 0.29 | 0.71 | 0.41 | 4054 | Train 64 | Test 22 | 0.72 | 0.27 | 0.73 | 0.39 |
| 2007 | Train 32 | Test 23 | 0.72 | 0.19 | 0.73 | 0.31 | 4055 | Train 64 | Test 23 | 0.7 | 0.17 | 0.81 | 0.28 |
| 2008 | Train 32 | Test 24 | 0.74 | 0.28 | 0.71 | 0.4 | 4056 | Train 64 | Test 24 | 0.72 | 0.26 | 0.73 | 0.39 |
| 2009 | Train 32 | Test 25 | 0.74 | 0.21 | 0.75 | 0.32 | 4057 | Train 64 | Test 25 | 0.72 | 0.17 | 0.84 | 0.29 |
| 2010 | Train 32 | Test 26 | 0.75 | 0.23 | 0.79 | 0.36 | 4058 | Train 64 | Test 26 | 0.73 | 0.2 | 0.85 | 0.33 |
| 2011 | Train 32 | Test 27 | 0.74 | 0.21 | 0.75 | 0.32 | 4059 | Train 64 | Test 27 | 0.72 | 0.18 | 0.84 | 0.29 |
| 2012 | Train 32 | Test 28 | 0.75 | 0.23 | 0.79 | 0.36 | 4060 | Train 64 | Test 28 | 0.73 | 0.2 | 0.85 | 0.33 |
| 2013 | Train 32 | Test 29 | 0.72 | 0.2 | 0.72 | 0.31 | 4061 | Train 64 | Test 29 | 0.7 | 0.18 | 0.8 | 0.29 |
| 2014 | Train 32 | Test 30 | 0.74 | 0.29 | 0.71 | 0.41 | 4062 | Train 64 | Test 30 | 0.72 | 0.27 | 0.73 | 0.39 |
| 2015 | Train 32 | Test 31 | 0.72 | 0.2 | 0.71 | 0.31 | 4063 | Train 64 | Test 31 | 0.7 | 0.18 | 0.79 | 0.29 |
| 2016 | Train 32 | Test 32 | 0.74 | 0.28 | 0.71 | 0.4 | 4064 | Train 64 | Test 32 | 0.72 | 0.26 | 0.73 | 0.39 |
| 2017 | Train 32 | Test 33 | 0.74 | 0.2 | 0.76 | 0.32 | 4065 | Train 64 | Test 33 | 0.72 | 0.17 | 0.84 | 0.28 |
| 2018 | Train 32 | Test 34 | 0.75 | 0.23 | 0.8 | 0.35 | 4066 | Train 64 | Test 34 | 0.73 | 0.2 | 0.85 | 0.32 |
| 2019 | Train 32 | Test 35 | 0.74 | 0.2 | 0.76 | 0.32 | 4067 | Train 64 | Test 35 | 0.72 | 0.17 | 0.84 | 0.29 |
| 2020 | Train 32 | Test 36 | 0.75 | 0.23 | 0.8 | 0.35 | 4068 | Train 64 | Test 36 | 0.73 | 0.2 | 0.85 | 0.33 |
| 2021 | Train 32 | Test 37 | 0.72 | 0.19 | 0.73 | 0.31 | 4069 | Train 64 | Test 37 | 0.7 | 0.17 | 0.81 | 0.28 |
| 2022 | Train 32 | Test 38 | 0.74 | 0.29 | 0.7 | 0.41 | 4070 | Train 64 | Test 38 | 0.72 | 0.27 | 0.73 | 0.39 |
| 2023 | Train 32 | Test 39 | 0.72 | 0.19 | 0.73 | 0.31 | 4071 | Train 64 | Test 39 | 0.7 | 0.17 | 0.81 | 0.28 |
| 2024 | Train 32 | Test 40 | 0.74 | 0.28 | 0.71 | 0.4 | 4072 | Train 64 | Test 40 | 0.72 | 0.26 | 0.73 | 0.39 |
| 2025 | Train 32 | Test 41 | 0.74 | 0.2 | 0.75 | 0.32 | 4073 | Train 64 | Test 41 | 0.72 | 0.17 | 0.84 | 0.29 |
| 2026 | Train 32 | Test 42 | 0.75 | 0.23 | 0.79 | 0.36 | 4074 | Train 64 | Test 42 | 0.73 | 0.2 | 0.85 | 0.33 |
| 2027 | Train 32 | Test 43 | 0.74 | 0.21 | 0.75 | 0.32 | 4075 | Train 64 | Test 43 | 0.72 | 0.17 | 0.84 | 0.29 |
| 2028 | Train 32 | Test 44 | 0.75 | 0.23 | 0.79 | 0.36 | 4076 | Train 64 | Test 44 | 0.73 | 0.2 | 0.85 | 0.33 |
| 2029 | Train 32 | Test 45 | 0.72 | 0.2 | 0.71 | 0.31 | 4077 | Train 64 | Test 45 | 0.7 | 0.18 | 0.79 | 0.29 |
| 2030 | Train 32 | Test 46 | 0.74 | 0.29 | 0.71 | 0.41 | 4078 | Train 64 | Test 46 | 0.72 | 0.27 | 0.73 | 0.39 |
| 2031 | Train 32 | Test 47 | 0.72 | 0.2 | 0.71 | 0.31 | 4079 | Train 64 | Test 47 | 0.7 | 0.18 | 0.79 | 0.29 |
| 2032 | Train 32 | Test 48 | 0.74 | 0.28 | 0.71 | 0.4 | 4080 | Train 64 | Test 48 | 0.72 | 0.26 | 0.73 | 0.39 |
| 2033 | Train 32 | Test 49 | 0.77 | 0.25 | 0.77 | 0.38 | 4081 | Train 64 | Test 49 | 0.77 | 0.25 | 0.78 | 0.38 |
| 2034 | Train 32 | Test 50 | 0.77 | 0.26 | 0.77 | 0.38 | 4082 | Train 64 | Test 50 | 0.77 | 0.25 | 0.78 | 0.38 |
| 2035 | Train 32 | Test 51 | 0.77 | 0.26 | 0.77 | 0.38 | 4083 | Train 64 | Test 51 | 0.77 | 0.25 | 0.78 | 0.38 |
| 2036 | Train 32 | Test 52 | 0.76 | 0.26 | 0.77 | 0.38 | 4084 | Train 64 | Test 52 | 0.77 | 0.25 | 0.78 | 0.38 |
| 2037 | Train 32 | Test 53 | 0.76 | 0.26 | 0.66 | 0.37 | 4085 | Train 64 | Test 53 | 0.76 | 0.27 | 0.68 | 0.38 |
| 2038 | Train 32 | Test 54 | 0.75 | 0.33 | 0.71 | 0.45 | 4086 | Train 64 | Test 54 | 0.75 | 0.33 | 0.72 | 0.46 |
| 2039 | Train 32 | Test 55 | 0.75 | 0.24 | 0.72 | 0.36 | 4087 | Train 64 | Test 55 | 0.76 | 0.24 | 0.76 | 0.36 |
| 2040 | Train 32 | Test 56 | 0.74 | 0.32 | 0.71 | 0.44 | 4088 | Train 64 | Test 56 | 0.75 | 0.32 | 0.72 | 0.44 |
| 2041 | Train 32 | Test 57 | 0.77 | 0.26 | 0.77 | 0.39 | 4089 | Train 64 | Test 57 | 0.78 | 0.26 | 0.78 | 0.39 |
| 2042 | Train 32 | Test 58 | 0.77 | 0.26 | 0.77 | 0.39 | 4090 | Train 64 | Test 58 | 0.78 | 0.26 | 0.78 | 0.39 |
| 2043 | Train 32 | Test 59 | 0.77 | 0.26 | 0.77 | 0.39 | 4091 | Train 64 | Test 59 | 0.78 | 0.26 | 0.78 | 0.39 |
| 2044 | Train 32 | Test 60 | 0.77 | 0.26 | 0.77 | 0.39 | 4092 | Train 64 | Test 60 | 0.78 | 0.26 | 0.78 | 0.39 |
| 2045 | Train 32 | Test 61 | 0.76 | 0.27 | 0.66 | 0.38 | 4093 | Train 64 | Test 61 | 0.77 | 0.27 | 0.67 | 0.39 |
| 2046 | Train 32 | Test 62 | 0.75 | 0.33 | 0.71 | 0.45 | 4094 | Train 64 | Test 62 | 0.75 | 0.33 | 0.72 | 0.45 |
| 2047 | Train 32 | Test 63 | 0.76 | 0.27 | 0.67 | 0.38 | 4095 | Train 64 | Test 63 | 0.77 | 0.27 | 0.67 | 0.39 |
| 2048 | Train 32 | Test 64 | 0.74 | 0.31 | 0.71 | 0.44 | 4096 | Train 64 | Test 64 | 0.75 | 0.32 | 0.72 | 0.44 |

Table 2. Random Forest (RF) Performance Metrics Across 64 Cohorts.

| Add Decisions | Train  Model | Test  Dataset | AUC (RF) | Precision (RF) | Recall (RF) | F1 (RF) | Add Decisions | Train  Model | Test  Dataset | AUC (RF) | Precision (RF) | Recall (RF) | F1 (RF) |
| --- | --- | --- | --- | --- | --- | --- | --- | --- | --- | --- | --- | --- | --- |
| 1 | Train 1 | Test 1 | 0.77 | 0.24 | 0.65 | 0.35 | 2049 | Train 33 | Test 1 | 0.77 | 0.24 | 0.65 | 0.35 |
| 2 | Train 1 | Test 2 | 0.74 | 0.26 | 0.58 | 0.36 | 2050 | Train 33 | Test 2 | 0.74 | 0.26 | 0.59 | 0.36 |
| 3 | Train 1 | Test 3 | 0.77 | 0.24 | 0.65 | 0.35 | 2051 | Train 33 | Test 3 | 0.77 | 0.24 | 0.65 | 0.35 |
| 4 | Train 1 | Test 4 | 0.74 | 0.26 | 0.58 | 0.36 | 2052 | Train 33 | Test 4 | 0.74 | 0.26 | 0.59 | 0.36 |
| 5 | Train 1 | Test 5 | 0.71 | 0.23 | 0.46 | 0.31 | 2053 | Train 33 | Test 5 | 0.71 | 0.23 | 0.47 | 0.31 |
| 6 | Train 1 | Test 6 | 0.69 | 0.32 | 0.27 | 0.29 | 2054 | Train 33 | Test 6 | 0.69 | 0.32 | 0.28 | 0.3 |
| 7 | Train 1 | Test 7 | 0.71 | 0.23 | 0.46 | 0.3 | 2055 | Train 33 | Test 7 | 0.71 | 0.23 | 0.46 | 0.3 |
| 8 | Train 1 | Test 8 | 0.69 | 0.31 | 0.27 | 0.29 | 2056 | Train 33 | Test 8 | 0.69 | 0.31 | 0.28 | 0.29 |
| 9 | Train 1 | Test 9 | 0.77 | 0.25 | 0.64 | 0.36 | 2057 | Train 33 | Test 9 | 0.77 | 0.24 | 0.64 | 0.35 |
| 10 | Train 1 | Test 10 | 0.75 | 0.27 | 0.57 | 0.37 | 2058 | Train 33 | Test 10 | 0.75 | 0.27 | 0.57 | 0.36 |
| 11 | Train 1 | Test 11 | 0.77 | 0.25 | 0.64 | 0.36 | 2059 | Train 33 | Test 11 | 0.77 | 0.24 | 0.64 | 0.35 |
| 12 | Train 1 | Test 12 | 0.75 | 0.27 | 0.57 | 0.37 | 2060 | Train 33 | Test 12 | 0.75 | 0.27 | 0.58 | 0.36 |
| 13 | Train 1 | Test 13 | 0.71 | 0.23 | 0.45 | 0.31 | 2061 | Train 33 | Test 13 | 0.71 | 0.23 | 0.45 | 0.31 |
| 14 | Train 1 | Test 14 | 0.69 | 0.32 | 0.27 | 0.29 | 2062 | Train 33 | Test 14 | 0.69 | 0.31 | 0.28 | 0.3 |
| 15 | Train 1 | Test 15 | 0.71 | 0.23 | 0.45 | 0.31 | 2063 | Train 33 | Test 15 | 0.71 | 0.23 | 0.45 | 0.3 |
| 16 | Train 1 | Test 16 | 0.69 | 0.31 | 0.27 | 0.29 | 2064 | Train 33 | Test 16 | 0.69 | 0.3 | 0.28 | 0.29 |
| 17 | Train 1 | Test 17 | 0.77 | 0.24 | 0.65 | 0.35 | 2065 | Train 33 | Test 17 | 0.77 | 0.24 | 0.65 | 0.35 |
| 18 | Train 1 | Test 18 | 0.74 | 0.26 | 0.58 | 0.36 | 2066 | Train 33 | Test 18 | 0.74 | 0.26 | 0.59 | 0.36 |
| 19 | Train 1 | Test 19 | 0.77 | 0.24 | 0.65 | 0.35 | 2067 | Train 33 | Test 19 | 0.77 | 0.24 | 0.65 | 0.35 |
| 20 | Train 1 | Test 20 | 0.74 | 0.26 | 0.58 | 0.36 | 2068 | Train 33 | Test 20 | 0.74 | 0.26 | 0.59 | 0.36 |
| 21 | Train 1 | Test 21 | 0.71 | 0.23 | 0.46 | 0.31 | 2069 | Train 33 | Test 21 | 0.71 | 0.23 | 0.47 | 0.31 |
| 22 | Train 1 | Test 22 | 0.69 | 0.32 | 0.27 | 0.29 | 2070 | Train 33 | Test 22 | 0.69 | 0.32 | 0.28 | 0.3 |
| 23 | Train 1 | Test 23 | 0.71 | 0.23 | 0.46 | 0.3 | 2071 | Train 33 | Test 23 | 0.71 | 0.23 | 0.46 | 0.3 |
| 24 | Train 1 | Test 24 | 0.69 | 0.31 | 0.27 | 0.29 | 2072 | Train 33 | Test 24 | 0.69 | 0.31 | 0.28 | 0.29 |
| 25 | Train 1 | Test 25 | 0.77 | 0.25 | 0.64 | 0.36 | 2073 | Train 33 | Test 25 | 0.77 | 0.24 | 0.64 | 0.35 |
| 26 | Train 1 | Test 26 | 0.75 | 0.27 | 0.57 | 0.37 | 2074 | Train 33 | Test 26 | 0.75 | 0.27 | 0.57 | 0.36 |
| 27 | Train 1 | Test 27 | 0.77 | 0.25 | 0.64 | 0.36 | 2075 | Train 33 | Test 27 | 0.77 | 0.24 | 0.64 | 0.35 |
| 28 | Train 1 | Test 28 | 0.75 | 0.27 | 0.57 | 0.37 | 2076 | Train 33 | Test 28 | 0.75 | 0.27 | 0.57 | 0.36 |
| 29 | Train 1 | Test 29 | 0.71 | 0.23 | 0.45 | 0.31 | 2077 | Train 33 | Test 29 | 0.71 | 0.23 | 0.45 | 0.31 |
| 30 | Train 1 | Test 30 | 0.69 | 0.32 | 0.27 | 0.29 | 2078 | Train 33 | Test 30 | 0.69 | 0.31 | 0.28 | 0.3 |
| 31 | Train 1 | Test 31 | 0.71 | 0.23 | 0.45 | 0.31 | 2079 | Train 33 | Test 31 | 0.71 | 0.23 | 0.45 | 0.3 |
| 32 | Train 1 | Test 32 | 0.69 | 0.31 | 0.27 | 0.29 | 2080 | Train 33 | Test 32 | 0.69 | 0.3 | 0.28 | 0.29 |
| 33 | Train 1 | Test 33 | 0.77 | 0.24 | 0.65 | 0.35 | 2081 | Train 33 | Test 33 | 0.77 | 0.24 | 0.65 | 0.35 |
| 34 | Train 1 | Test 34 | 0.75 | 0.26 | 0.58 | 0.36 | 2082 | Train 33 | Test 34 | 0.75 | 0.26 | 0.59 | 0.36 |
| 35 | Train 1 | Test 35 | 0.77 | 0.24 | 0.65 | 0.35 | 2083 | Train 33 | Test 35 | 0.77 | 0.24 | 0.65 | 0.35 |
| 36 | Train 1 | Test 36 | 0.75 | 0.26 | 0.58 | 0.36 | 2084 | Train 33 | Test 36 | 0.75 | 0.26 | 0.59 | 0.36 |
| 37 | Train 1 | Test 37 | 0.71 | 0.23 | 0.46 | 0.31 | 2085 | Train 33 | Test 37 | 0.71 | 0.23 | 0.47 | 0.31 |
| 38 | Train 1 | Test 38 | 0.69 | 0.32 | 0.27 | 0.29 | 2086 | Train 33 | Test 38 | 0.69 | 0.32 | 0.28 | 0.3 |
| 39 | Train 1 | Test 39 | 0.71 | 0.23 | 0.46 | 0.3 | 2087 | Train 33 | Test 39 | 0.71 | 0.23 | 0.46 | 0.3 |
| 40 | Train 1 | Test 40 | 0.69 | 0.31 | 0.27 | 0.29 | 2088 | Train 33 | Test 40 | 0.69 | 0.31 | 0.28 | 0.29 |
| 41 | Train 1 | Test 41 | 0.77 | 0.25 | 0.64 | 0.35 | 2089 | Train 33 | Test 41 | 0.77 | 0.24 | 0.64 | 0.35 |
| 42 | Train 1 | Test 42 | 0.75 | 0.27 | 0.57 | 0.36 | 2090 | Train 33 | Test 42 | 0.75 | 0.27 | 0.57 | 0.36 |
| 43 | Train 1 | Test 43 | 0.77 | 0.25 | 0.64 | 0.36 | 2091 | Train 33 | Test 43 | 0.77 | 0.24 | 0.64 | 0.35 |
| 44 | Train 1 | Test 44 | 0.75 | 0.27 | 0.57 | 0.37 | 2092 | Train 33 | Test 44 | 0.75 | 0.27 | 0.57 | 0.36 |
| 45 | Train 1 | Test 45 | 0.71 | 0.23 | 0.45 | 0.31 | 2093 | Train 33 | Test 45 | 0.71 | 0.23 | 0.45 | 0.31 |
| 46 | Train 1 | Test 46 | 0.69 | 0.32 | 0.27 | 0.29 | 2094 | Train 33 | Test 46 | 0.69 | 0.31 | 0.28 | 0.3 |
| 47 | Train 1 | Test 47 | 0.71 | 0.23 | 0.45 | 0.31 | 2095 | Train 33 | Test 47 | 0.71 | 0.23 | 0.45 | 0.3 |
| 48 | Train 1 | Test 48 | 0.69 | 0.31 | 0.27 | 0.29 | 2096 | Train 33 | Test 48 | 0.69 | 0.3 | 0.28 | 0.29 |
| 49 | Train 1 | Test 49 | 0.77 | 0.27 | 0.74 | 0.4 | 2097 | Train 33 | Test 49 | 0.77 | 0.27 | 0.74 | 0.4 |
| 50 | Train 1 | Test 50 | 0.77 | 0.27 | 0.74 | 0.4 | 2098 | Train 33 | Test 50 | 0.77 | 0.27 | 0.74 | 0.4 |
| 51 | Train 1 | Test 51 | 0.77 | 0.27 | 0.74 | 0.4 | 2099 | Train 33 | Test 51 | 0.77 | 0.27 | 0.74 | 0.4 |
| 52 | Train 1 | Test 52 | 0.77 | 0.27 | 0.74 | 0.4 | 2100 | Train 33 | Test 52 | 0.77 | 0.27 | 0.74 | 0.4 |
| 53 | Train 1 | Test 53 | 0.75 | 0.3 | 0.42 | 0.35 | 2101 | Train 33 | Test 53 | 0.75 | 0.29 | 0.44 | 0.35 |
| 54 | Train 1 | Test 54 | 0.71 | 0.37 | 0.27 | 0.31 | 2102 | Train 33 | Test 54 | 0.71 | 0.37 | 0.29 | 0.32 |
| 55 | Train 1 | Test 55 | 0.75 | 0.28 | 0.52 | 0.36 | 2103 | Train 33 | Test 55 | 0.75 | 0.27 | 0.51 | 0.36 |
| 56 | Train 1 | Test 56 | 0.7 | 0.34 | 0.28 | 0.31 | 2104 | Train 33 | Test 56 | 0.7 | 0.34 | 0.29 | 0.31 |
| 57 | Train 1 | Test 57 | 0.78 | 0.28 | 0.75 | 0.41 | 2105 | Train 33 | Test 57 | 0.78 | 0.28 | 0.74 | 0.4 |
| 58 | Train 1 | Test 58 | 0.78 | 0.28 | 0.74 | 0.41 | 2106 | Train 33 | Test 58 | 0.78 | 0.28 | 0.74 | 0.4 |
| 59 | Train 1 | Test 59 | 0.78 | 0.28 | 0.75 | 0.41 | 2107 | Train 33 | Test 59 | 0.78 | 0.28 | 0.74 | 0.41 |
| 60 | Train 1 | Test 60 | 0.78 | 0.28 | 0.74 | 0.41 | 2108 | Train 33 | Test 60 | 0.78 | 0.28 | 0.74 | 0.41 |
| 61 | Train 1 | Test 61 | 0.75 | 0.3 | 0.42 | 0.35 | 2109 | Train 33 | Test 61 | 0.75 | 0.3 | 0.44 | 0.35 |
| 62 | Train 1 | Test 62 | 0.71 | 0.37 | 0.27 | 0.31 | 2110 | Train 33 | Test 62 | 0.71 | 0.36 | 0.29 | 0.32 |
| 63 | Train 1 | Test 63 | 0.75 | 0.3 | 0.43 | 0.35 | 2111 | Train 33 | Test 63 | 0.75 | 0.3 | 0.44 | 0.35 |
| 64 | Train 1 | Test 64 | 0.7 | 0.34 | 0.28 | 0.31 | 2112 | Train 33 | Test 64 | 0.7 | 0.34 | 0.29 | 0.31 |
| 65 | Train 2 | Test 1 | 0.77 | 0.25 | 0.61 | 0.35 | 2113 | Train 34 | Test 1 | 0.77 | 0.25 | 0.62 | 0.35 |
| 66 | Train 2 | Test 2 | 0.78 | 0.28 | 0.67 | 0.39 | 2114 | Train 34 | Test 2 | 0.78 | 0.28 | 0.68 | 0.39 |
| 67 | Train 2 | Test 3 | 0.77 | 0.25 | 0.61 | 0.35 | 2115 | Train 34 | Test 3 | 0.77 | 0.25 | 0.62 | 0.36 |
| 68 | Train 2 | Test 4 | 0.78 | 0.28 | 0.67 | 0.39 | 2116 | Train 34 | Test 4 | 0.78 | 0.28 | 0.68 | 0.39 |
| 69 | Train 2 | Test 5 | 0.72 | 0.23 | 0.46 | 0.31 | 2117 | Train 34 | Test 5 | 0.72 | 0.23 | 0.46 | 0.31 |
| 70 | Train 2 | Test 6 | 0.71 | 0.35 | 0.33 | 0.34 | 2118 | Train 34 | Test 6 | 0.7 | 0.35 | 0.34 | 0.34 |
| 71 | Train 2 | Test 7 | 0.71 | 0.23 | 0.45 | 0.3 | 2119 | Train 34 | Test 7 | 0.71 | 0.23 | 0.46 | 0.3 |
| 72 | Train 2 | Test 8 | 0.7 | 0.34 | 0.33 | 0.33 | 2120 | Train 34 | Test 8 | 0.7 | 0.33 | 0.34 | 0.34 |
| 73 | Train 2 | Test 9 | 0.77 | 0.26 | 0.6 | 0.36 | 2121 | Train 34 | Test 9 | 0.77 | 0.25 | 0.6 | 0.36 |
| 74 | Train 2 | Test 10 | 0.78 | 0.29 | 0.66 | 0.4 | 2122 | Train 34 | Test 10 | 0.78 | 0.28 | 0.67 | 0.39 |
| 75 | Train 2 | Test 11 | 0.77 | 0.26 | 0.6 | 0.36 | 2123 | Train 34 | Test 11 | 0.77 | 0.25 | 0.61 | 0.36 |
| 76 | Train 2 | Test 12 | 0.78 | 0.29 | 0.66 | 0.4 | 2124 | Train 34 | Test 12 | 0.78 | 0.28 | 0.67 | 0.4 |
| 77 | Train 2 | Test 13 | 0.72 | 0.23 | 0.44 | 0.31 | 2125 | Train 34 | Test 13 | 0.72 | 0.23 | 0.45 | 0.31 |
| 78 | Train 2 | Test 14 | 0.71 | 0.35 | 0.33 | 0.34 | 2126 | Train 34 | Test 14 | 0.7 | 0.34 | 0.34 | 0.34 |
| 79 | Train 2 | Test 15 | 0.71 | 0.23 | 0.44 | 0.31 | 2127 | Train 34 | Test 15 | 0.71 | 0.23 | 0.45 | 0.3 |
| 80 | Train 2 | Test 16 | 0.71 | 0.34 | 0.33 | 0.33 | 2128 | Train 34 | Test 16 | 0.7 | 0.33 | 0.34 | 0.34 |
| 81 | Train 2 | Test 17 | 0.77 | 0.25 | 0.61 | 0.35 | 2129 | Train 34 | Test 17 | 0.77 | 0.25 | 0.62 | 0.35 |
| 82 | Train 2 | Test 18 | 0.78 | 0.28 | 0.67 | 0.39 | 2130 | Train 34 | Test 18 | 0.78 | 0.28 | 0.68 | 0.39 |
| 83 | Train 2 | Test 19 | 0.77 | 0.25 | 0.61 | 0.35 | 2131 | Train 34 | Test 19 | 0.77 | 0.25 | 0.62 | 0.36 |
| 84 | Train 2 | Test 20 | 0.78 | 0.28 | 0.67 | 0.39 | 2132 | Train 34 | Test 20 | 0.78 | 0.28 | 0.68 | 0.39 |
| 85 | Train 2 | Test 21 | 0.72 | 0.23 | 0.46 | 0.31 | 2133 | Train 34 | Test 21 | 0.72 | 0.23 | 0.46 | 0.31 |
| 86 | Train 2 | Test 22 | 0.71 | 0.35 | 0.33 | 0.34 | 2134 | Train 34 | Test 22 | 0.7 | 0.35 | 0.34 | 0.34 |
| 87 | Train 2 | Test 23 | 0.71 | 0.23 | 0.45 | 0.3 | 2135 | Train 34 | Test 23 | 0.71 | 0.23 | 0.46 | 0.3 |
| 88 | Train 2 | Test 24 | 0.71 | 0.34 | 0.33 | 0.33 | 2136 | Train 34 | Test 24 | 0.7 | 0.33 | 0.34 | 0.34 |
| 89 | Train 2 | Test 25 | 0.77 | 0.26 | 0.6 | 0.36 | 2137 | Train 34 | Test 25 | 0.77 | 0.25 | 0.61 | 0.36 |
| 90 | Train 2 | Test 26 | 0.78 | 0.29 | 0.66 | 0.4 | 2138 | Train 34 | Test 26 | 0.78 | 0.28 | 0.67 | 0.4 |
| 91 | Train 2 | Test 27 | 0.77 | 0.26 | 0.6 | 0.36 | 2139 | Train 34 | Test 27 | 0.77 | 0.25 | 0.61 | 0.36 |
| 92 | Train 2 | Test 28 | 0.78 | 0.29 | 0.66 | 0.4 | 2140 | Train 34 | Test 28 | 0.78 | 0.28 | 0.67 | 0.4 |
| 93 | Train 2 | Test 29 | 0.72 | 0.23 | 0.44 | 0.31 | 2141 | Train 34 | Test 29 | 0.72 | 0.23 | 0.45 | 0.31 |
| 94 | Train 2 | Test 30 | 0.71 | 0.35 | 0.33 | 0.34 | 2142 | Train 34 | Test 30 | 0.71 | 0.34 | 0.34 | 0.34 |
| 95 | Train 2 | Test 31 | 0.71 | 0.23 | 0.44 | 0.31 | 2143 | Train 34 | Test 31 | 0.71 | 0.23 | 0.45 | 0.3 |
| 96 | Train 2 | Test 32 | 0.71 | 0.34 | 0.33 | 0.33 | 2144 | Train 34 | Test 32 | 0.7 | 0.33 | 0.34 | 0.33 |
| 97 | Train 2 | Test 33 | 0.77 | 0.25 | 0.61 | 0.35 | 2145 | Train 34 | Test 33 | 0.77 | 0.25 | 0.62 | 0.35 |
| 98 | Train 2 | Test 34 | 0.78 | 0.28 | 0.67 | 0.39 | 2146 | Train 34 | Test 34 | 0.78 | 0.28 | 0.67 | 0.39 |
| 99 | Train 2 | Test 35 | 0.77 | 0.25 | 0.61 | 0.35 | 2147 | Train 34 | Test 35 | 0.77 | 0.25 | 0.62 | 0.35 |
| 100 | Train 2 | Test 36 | 0.78 | 0.28 | 0.67 | 0.39 | 2148 | Train 34 | Test 36 | 0.78 | 0.28 | 0.68 | 0.39 |
| 101 | Train 2 | Test 37 | 0.72 | 0.23 | 0.46 | 0.31 | 2149 | Train 34 | Test 37 | 0.72 | 0.23 | 0.46 | 0.31 |
| 102 | Train 2 | Test 38 | 0.71 | 0.35 | 0.33 | 0.34 | 2150 | Train 34 | Test 38 | 0.7 | 0.35 | 0.34 | 0.34 |
| 103 | Train 2 | Test 39 | 0.72 | 0.23 | 0.45 | 0.3 | 2151 | Train 34 | Test 39 | 0.72 | 0.23 | 0.46 | 0.3 |
| 104 | Train 2 | Test 40 | 0.71 | 0.34 | 0.33 | 0.33 | 2152 | Train 34 | Test 40 | 0.7 | 0.33 | 0.34 | 0.34 |
| 105 | Train 2 | Test 41 | 0.77 | 0.26 | 0.6 | 0.36 | 2153 | Train 34 | Test 41 | 0.77 | 0.25 | 0.6 | 0.36 |
| 106 | Train 2 | Test 42 | 0.78 | 0.29 | 0.66 | 0.4 | 2154 | Train 34 | Test 42 | 0.78 | 0.28 | 0.66 | 0.39 |
| 107 | Train 2 | Test 43 | 0.77 | 0.26 | 0.6 | 0.36 | 2155 | Train 34 | Test 43 | 0.77 | 0.25 | 0.61 | 0.36 |
| 108 | Train 2 | Test 44 | 0.78 | 0.29 | 0.66 | 0.4 | 2156 | Train 34 | Test 44 | 0.78 | 0.28 | 0.66 | 0.4 |
| 109 | Train 2 | Test 45 | 0.72 | 0.23 | 0.44 | 0.31 | 2157 | Train 34 | Test 45 | 0.72 | 0.23 | 0.45 | 0.31 |
| 110 | Train 2 | Test 46 | 0.71 | 0.35 | 0.33 | 0.34 | 2158 | Train 34 | Test 46 | 0.71 | 0.34 | 0.34 | 0.34 |
| 111 | Train 2 | Test 47 | 0.72 | 0.23 | 0.44 | 0.31 | 2159 | Train 34 | Test 47 | 0.72 | 0.23 | 0.45 | 0.3 |
| 112 | Train 2 | Test 48 | 0.71 | 0.34 | 0.33 | 0.33 | 2160 | Train 34 | Test 48 | 0.7 | 0.33 | 0.34 | 0.33 |
| 113 | Train 2 | Test 49 | 0.77 | 0.28 | 0.72 | 0.4 | 2161 | Train 34 | Test 49 | 0.77 | 0.27 | 0.73 | 0.4 |
| 114 | Train 2 | Test 50 | 0.77 | 0.28 | 0.72 | 0.4 | 2162 | Train 34 | Test 50 | 0.77 | 0.27 | 0.72 | 0.4 |
| 115 | Train 2 | Test 51 | 0.77 | 0.28 | 0.72 | 0.4 | 2163 | Train 34 | Test 51 | 0.77 | 0.28 | 0.73 | 0.4 |
| 116 | Train 2 | Test 52 | 0.77 | 0.28 | 0.72 | 0.4 | 2164 | Train 34 | Test 52 | 0.77 | 0.28 | 0.73 | 0.4 |
| 117 | Train 2 | Test 53 | 0.75 | 0.31 | 0.37 | 0.33 | 2165 | Train 34 | Test 53 | 0.75 | 0.31 | 0.38 | 0.34 |
| 118 | Train 2 | Test 54 | 0.72 | 0.4 | 0.31 | 0.35 | 2166 | Train 34 | Test 54 | 0.72 | 0.39 | 0.32 | 0.35 |
| 119 | Train 2 | Test 55 | 0.76 | 0.28 | 0.51 | 0.36 | 2167 | Train 34 | Test 55 | 0.75 | 0.28 | 0.51 | 0.36 |
| 120 | Train 2 | Test 56 | 0.71 | 0.37 | 0.32 | 0.34 | 2168 | Train 34 | Test 56 | 0.71 | 0.36 | 0.33 | 0.34 |
| 121 | Train 2 | Test 57 | 0.78 | 0.29 | 0.72 | 0.41 | 2169 | Train 34 | Test 57 | 0.78 | 0.28 | 0.72 | 0.4 |
| 122 | Train 2 | Test 58 | 0.78 | 0.29 | 0.72 | 0.41 | 2170 | Train 34 | Test 58 | 0.78 | 0.28 | 0.72 | 0.4 |
| 123 | Train 2 | Test 59 | 0.78 | 0.29 | 0.72 | 0.41 | 2171 | Train 34 | Test 59 | 0.78 | 0.28 | 0.73 | 0.41 |
| 124 | Train 2 | Test 60 | 0.78 | 0.29 | 0.72 | 0.41 | 2172 | Train 34 | Test 60 | 0.78 | 0.28 | 0.72 | 0.41 |
| 125 | Train 2 | Test 61 | 0.76 | 0.31 | 0.36 | 0.34 | 2173 | Train 34 | Test 61 | 0.76 | 0.31 | 0.38 | 0.34 |
| 126 | Train 2 | Test 62 | 0.72 | 0.39 | 0.31 | 0.35 | 2174 | Train 34 | Test 62 | 0.72 | 0.39 | 0.32 | 0.35 |
| 127 | Train 2 | Test 63 | 0.76 | 0.31 | 0.37 | 0.34 | 2175 | Train 34 | Test 63 | 0.76 | 0.31 | 0.39 | 0.34 |
| 128 | Train 2 | Test 64 | 0.72 | 0.36 | 0.32 | 0.34 | 2176 | Train 34 | Test 64 | 0.71 | 0.36 | 0.33 | 0.34 |
| 129 | Train 3 | Test 1 | 0.77 | 0.24 | 0.65 | 0.35 | 2177 | Train 35 | Test 1 | 0.77 | 0.24 | 0.65 | 0.35 |
| 130 | Train 3 | Test 2 | 0.75 | 0.27 | 0.58 | 0.36 | 2178 | Train 35 | Test 2 | 0.74 | 0.26 | 0.59 | 0.36 |
| 131 | Train 3 | Test 3 | 0.77 | 0.24 | 0.65 | 0.36 | 2179 | Train 35 | Test 3 | 0.77 | 0.24 | 0.65 | 0.35 |
| 132 | Train 3 | Test 4 | 0.75 | 0.27 | 0.59 | 0.37 | 2180 | Train 35 | Test 4 | 0.75 | 0.26 | 0.59 | 0.36 |
| 133 | Train 3 | Test 5 | 0.71 | 0.23 | 0.46 | 0.31 | 2181 | Train 35 | Test 5 | 0.71 | 0.23 | 0.46 | 0.3 |
| 134 | Train 3 | Test 6 | 0.69 | 0.31 | 0.25 | 0.28 | 2182 | Train 35 | Test 6 | 0.69 | 0.31 | 0.26 | 0.29 |
| 135 | Train 3 | Test 7 | 0.71 | 0.23 | 0.46 | 0.31 | 2183 | Train 35 | Test 7 | 0.71 | 0.23 | 0.46 | 0.3 |
| 136 | Train 3 | Test 8 | 0.69 | 0.3 | 0.25 | 0.28 | 2184 | Train 35 | Test 8 | 0.69 | 0.3 | 0.26 | 0.28 |
| 137 | Train 3 | Test 9 | 0.77 | 0.25 | 0.63 | 0.35 | 2185 | Train 35 | Test 9 | 0.77 | 0.24 | 0.64 | 0.35 |
| 138 | Train 3 | Test 10 | 0.75 | 0.27 | 0.56 | 0.36 | 2186 | Train 35 | Test 10 | 0.75 | 0.27 | 0.57 | 0.36 |
| 139 | Train 3 | Test 11 | 0.77 | 0.25 | 0.64 | 0.36 | 2187 | Train 35 | Test 11 | 0.77 | 0.24 | 0.64 | 0.35 |
| 140 | Train 3 | Test 12 | 0.75 | 0.27 | 0.56 | 0.36 | 2188 | Train 35 | Test 12 | 0.75 | 0.27 | 0.58 | 0.36 |
| 141 | Train 3 | Test 13 | 0.71 | 0.23 | 0.45 | 0.31 | 2189 | Train 35 | Test 13 | 0.71 | 0.23 | 0.45 | 0.3 |
| 142 | Train 3 | Test 14 | 0.69 | 0.31 | 0.25 | 0.28 | 2190 | Train 35 | Test 14 | 0.69 | 0.31 | 0.26 | 0.29 |
| 143 | Train 3 | Test 15 | 0.71 | 0.23 | 0.44 | 0.3 | 2191 | Train 35 | Test 15 | 0.71 | 0.23 | 0.45 | 0.3 |
| 144 | Train 3 | Test 16 | 0.69 | 0.3 | 0.25 | 0.28 | 2192 | Train 35 | Test 16 | 0.69 | 0.3 | 0.27 | 0.28 |
| 145 | Train 3 | Test 17 | 0.77 | 0.24 | 0.65 | 0.35 | 2193 | Train 35 | Test 17 | 0.77 | 0.24 | 0.65 | 0.35 |
| 146 | Train 3 | Test 18 | 0.75 | 0.26 | 0.58 | 0.36 | 2194 | Train 35 | Test 18 | 0.74 | 0.26 | 0.59 | 0.36 |
| 147 | Train 3 | Test 19 | 0.77 | 0.24 | 0.65 | 0.35 | 2195 | Train 35 | Test 19 | 0.77 | 0.24 | 0.65 | 0.35 |
| 148 | Train 3 | Test 20 | 0.75 | 0.27 | 0.59 | 0.37 | 2196 | Train 35 | Test 20 | 0.75 | 0.26 | 0.59 | 0.36 |
| 149 | Train 3 | Test 21 | 0.71 | 0.23 | 0.46 | 0.31 | 2197 | Train 35 | Test 21 | 0.71 | 0.23 | 0.46 | 0.3 |
| 150 | Train 3 | Test 22 | 0.69 | 0.31 | 0.25 | 0.28 | 2198 | Train 35 | Test 22 | 0.69 | 0.31 | 0.26 | 0.29 |
| 151 | Train 3 | Test 23 | 0.71 | 0.23 | 0.46 | 0.31 | 2199 | Train 35 | Test 23 | 0.71 | 0.23 | 0.46 | 0.3 |
| 152 | Train 3 | Test 24 | 0.69 | 0.3 | 0.25 | 0.28 | 2200 | Train 35 | Test 24 | 0.69 | 0.3 | 0.26 | 0.28 |
| 153 | Train 3 | Test 25 | 0.77 | 0.25 | 0.63 | 0.35 | 2201 | Train 35 | Test 25 | 0.77 | 0.24 | 0.64 | 0.35 |
| 154 | Train 3 | Test 26 | 0.75 | 0.27 | 0.56 | 0.36 | 2202 | Train 35 | Test 26 | 0.75 | 0.27 | 0.57 | 0.36 |
| 155 | Train 3 | Test 27 | 0.77 | 0.25 | 0.64 | 0.36 | 2203 | Train 35 | Test 27 | 0.77 | 0.24 | 0.64 | 0.35 |
| 156 | Train 3 | Test 28 | 0.75 | 0.27 | 0.56 | 0.36 | 2204 | Train 35 | Test 28 | 0.75 | 0.27 | 0.57 | 0.36 |
| 157 | Train 3 | Test 29 | 0.71 | 0.23 | 0.45 | 0.31 | 2205 | Train 35 | Test 29 | 0.71 | 0.23 | 0.45 | 0.3 |
| 158 | Train 3 | Test 30 | 0.69 | 0.31 | 0.25 | 0.28 | 2206 | Train 35 | Test 30 | 0.69 | 0.31 | 0.26 | 0.29 |
| 159 | Train 3 | Test 31 | 0.71 | 0.23 | 0.44 | 0.3 | 2207 | Train 35 | Test 31 | 0.71 | 0.23 | 0.45 | 0.3 |
| 160 | Train 3 | Test 32 | 0.69 | 0.3 | 0.25 | 0.28 | 2208 | Train 35 | Test 32 | 0.69 | 0.3 | 0.27 | 0.28 |
| 161 | Train 3 | Test 33 | 0.77 | 0.24 | 0.65 | 0.35 | 2209 | Train 35 | Test 33 | 0.77 | 0.24 | 0.65 | 0.35 |
| 162 | Train 3 | Test 34 | 0.75 | 0.26 | 0.58 | 0.36 | 2210 | Train 35 | Test 34 | 0.75 | 0.26 | 0.59 | 0.36 |
| 163 | Train 3 | Test 35 | 0.77 | 0.24 | 0.65 | 0.35 | 2211 | Train 35 | Test 35 | 0.77 | 0.24 | 0.65 | 0.35 |
| 164 | Train 3 | Test 36 | 0.75 | 0.26 | 0.58 | 0.36 | 2212 | Train 35 | Test 36 | 0.75 | 0.26 | 0.59 | 0.36 |
| 165 | Train 3 | Test 37 | 0.71 | 0.23 | 0.46 | 0.31 | 2213 | Train 35 | Test 37 | 0.71 | 0.23 | 0.46 | 0.3 |
| 166 | Train 3 | Test 38 | 0.69 | 0.31 | 0.25 | 0.28 | 2214 | Train 35 | Test 38 | 0.69 | 0.31 | 0.26 | 0.29 |
| 167 | Train 3 | Test 39 | 0.71 | 0.23 | 0.46 | 0.31 | 2215 | Train 35 | Test 39 | 0.71 | 0.23 | 0.46 | 0.3 |
| 168 | Train 3 | Test 40 | 0.69 | 0.3 | 0.25 | 0.28 | 2216 | Train 35 | Test 40 | 0.69 | 0.3 | 0.26 | 0.28 |
| 169 | Train 3 | Test 41 | 0.77 | 0.25 | 0.63 | 0.35 | 2217 | Train 35 | Test 41 | 0.77 | 0.24 | 0.64 | 0.35 |
| 170 | Train 3 | Test 42 | 0.75 | 0.27 | 0.56 | 0.36 | 2218 | Train 35 | Test 42 | 0.75 | 0.27 | 0.57 | 0.36 |
| 171 | Train 3 | Test 43 | 0.77 | 0.25 | 0.63 | 0.35 | 2219 | Train 35 | Test 43 | 0.77 | 0.24 | 0.64 | 0.35 |
| 172 | Train 3 | Test 44 | 0.75 | 0.27 | 0.56 | 0.36 | 2220 | Train 35 | Test 44 | 0.75 | 0.27 | 0.57 | 0.36 |
| 173 | Train 3 | Test 45 | 0.71 | 0.23 | 0.45 | 0.31 | 2221 | Train 35 | Test 45 | 0.71 | 0.23 | 0.45 | 0.3 |
| 174 | Train 3 | Test 46 | 0.69 | 0.31 | 0.25 | 0.28 | 2222 | Train 35 | Test 46 | 0.69 | 0.31 | 0.26 | 0.29 |
| 175 | Train 3 | Test 47 | 0.71 | 0.23 | 0.44 | 0.3 | 2223 | Train 35 | Test 47 | 0.71 | 0.23 | 0.45 | 0.3 |
| 176 | Train 3 | Test 48 | 0.69 | 0.3 | 0.25 | 0.28 | 2224 | Train 35 | Test 48 | 0.69 | 0.3 | 0.27 | 0.28 |
| 177 | Train 3 | Test 49 | 0.77 | 0.27 | 0.75 | 0.4 | 2225 | Train 35 | Test 49 | 0.77 | 0.27 | 0.75 | 0.4 |
| 178 | Train 3 | Test 50 | 0.77 | 0.27 | 0.75 | 0.4 | 2226 | Train 35 | Test 50 | 0.77 | 0.27 | 0.74 | 0.4 |
| 179 | Train 3 | Test 51 | 0.77 | 0.27 | 0.75 | 0.4 | 2227 | Train 35 | Test 51 | 0.77 | 0.27 | 0.75 | 0.4 |
| 180 | Train 3 | Test 52 | 0.77 | 0.27 | 0.75 | 0.4 | 2228 | Train 35 | Test 52 | 0.77 | 0.27 | 0.74 | 0.4 |
| 181 | Train 3 | Test 53 | 0.75 | 0.29 | 0.42 | 0.35 | 2229 | Train 35 | Test 53 | 0.75 | 0.29 | 0.44 | 0.35 |
| 182 | Train 3 | Test 54 | 0.7 | 0.36 | 0.26 | 0.3 | 2230 | Train 35 | Test 54 | 0.7 | 0.36 | 0.27 | 0.31 |
| 183 | Train 3 | Test 55 | 0.75 | 0.27 | 0.52 | 0.36 | 2231 | Train 35 | Test 55 | 0.75 | 0.27 | 0.52 | 0.36 |
| 184 | Train 3 | Test 56 | 0.7 | 0.34 | 0.26 | 0.3 | 2232 | Train 35 | Test 56 | 0.7 | 0.34 | 0.27 | 0.3 |
| 185 | Train 3 | Test 57 | 0.78 | 0.28 | 0.75 | 0.41 | 2233 | Train 35 | Test 57 | 0.78 | 0.27 | 0.75 | 0.4 |
| 186 | Train 3 | Test 58 | 0.78 | 0.28 | 0.75 | 0.41 | 2234 | Train 35 | Test 58 | 0.78 | 0.27 | 0.74 | 0.4 |
| 187 | Train 3 | Test 59 | 0.78 | 0.28 | 0.75 | 0.41 | 2235 | Train 35 | Test 59 | 0.78 | 0.28 | 0.75 | 0.4 |
| 188 | Train 3 | Test 60 | 0.78 | 0.28 | 0.75 | 0.41 | 2236 | Train 35 | Test 60 | 0.78 | 0.28 | 0.74 | 0.4 |
| 189 | Train 3 | Test 61 | 0.75 | 0.3 | 0.42 | 0.35 | 2237 | Train 35 | Test 61 | 0.75 | 0.3 | 0.43 | 0.35 |
| 190 | Train 3 | Test 62 | 0.71 | 0.36 | 0.26 | 0.3 | 2238 | Train 35 | Test 62 | 0.71 | 0.36 | 0.27 | 0.31 |
| 191 | Train 3 | Test 63 | 0.75 | 0.3 | 0.43 | 0.35 | 2239 | Train 35 | Test 63 | 0.75 | 0.3 | 0.44 | 0.35 |
| 192 | Train 3 | Test 64 | 0.7 | 0.34 | 0.27 | 0.3 | 2240 | Train 35 | Test 64 | 0.7 | 0.34 | 0.28 | 0.3 |
| 193 | Train 4 | Test 1 | 0.77 | 0.25 | 0.61 | 0.36 | 2241 | Train 36 | Test 1 | 0.77 | 0.25 | 0.61 | 0.35 |
| 194 | Train 4 | Test 2 | 0.78 | 0.28 | 0.67 | 0.39 | 2242 | Train 36 | Test 2 | 0.78 | 0.28 | 0.67 | 0.39 |
| 195 | Train 4 | Test 3 | 0.77 | 0.25 | 0.62 | 0.36 | 2243 | Train 36 | Test 3 | 0.77 | 0.25 | 0.61 | 0.35 |
| 196 | Train 4 | Test 4 | 0.78 | 0.28 | 0.67 | 0.4 | 2244 | Train 36 | Test 4 | 0.78 | 0.28 | 0.67 | 0.39 |
| 197 | Train 4 | Test 5 | 0.72 | 0.23 | 0.46 | 0.31 | 2245 | Train 36 | Test 5 | 0.72 | 0.23 | 0.46 | 0.31 |
| 198 | Train 4 | Test 6 | 0.7 | 0.35 | 0.32 | 0.33 | 2246 | Train 36 | Test 6 | 0.71 | 0.35 | 0.34 | 0.34 |
| 199 | Train 4 | Test 7 | 0.71 | 0.23 | 0.45 | 0.3 | 2247 | Train 36 | Test 7 | 0.71 | 0.23 | 0.46 | 0.3 |
| 200 | Train 4 | Test 8 | 0.7 | 0.34 | 0.32 | 0.33 | 2248 | Train 36 | Test 8 | 0.7 | 0.33 | 0.34 | 0.33 |
| 201 | Train 4 | Test 9 | 0.77 | 0.25 | 0.6 | 0.36 | 2249 | Train 36 | Test 9 | 0.77 | 0.25 | 0.6 | 0.35 |
| 202 | Train 4 | Test 10 | 0.78 | 0.28 | 0.66 | 0.4 | 2250 | Train 36 | Test 10 | 0.78 | 0.28 | 0.67 | 0.4 |
| 203 | Train 4 | Test 11 | 0.77 | 0.26 | 0.6 | 0.36 | 2251 | Train 36 | Test 11 | 0.77 | 0.25 | 0.61 | 0.36 |
| 204 | Train 4 | Test 12 | 0.78 | 0.28 | 0.66 | 0.4 | 2252 | Train 36 | Test 12 | 0.78 | 0.28 | 0.67 | 0.4 |
| 205 | Train 4 | Test 13 | 0.72 | 0.23 | 0.44 | 0.31 | 2253 | Train 36 | Test 13 | 0.72 | 0.23 | 0.45 | 0.31 |
| 206 | Train 4 | Test 14 | 0.71 | 0.35 | 0.32 | 0.33 | 2254 | Train 36 | Test 14 | 0.71 | 0.34 | 0.34 | 0.34 |
| 207 | Train 4 | Test 15 | 0.71 | 0.23 | 0.44 | 0.3 | 2255 | Train 36 | Test 15 | 0.71 | 0.23 | 0.45 | 0.3 |
| 208 | Train 4 | Test 16 | 0.7 | 0.33 | 0.32 | 0.33 | 2256 | Train 36 | Test 16 | 0.71 | 0.33 | 0.34 | 0.33 |
| 209 | Train 4 | Test 17 | 0.77 | 0.25 | 0.61 | 0.36 | 2257 | Train 36 | Test 17 | 0.77 | 0.25 | 0.61 | 0.35 |
| 210 | Train 4 | Test 18 | 0.78 | 0.28 | 0.67 | 0.39 | 2258 | Train 36 | Test 18 | 0.78 | 0.28 | 0.67 | 0.39 |
| 211 | Train 4 | Test 19 | 0.77 | 0.25 | 0.62 | 0.36 | 2259 | Train 36 | Test 19 | 0.77 | 0.25 | 0.61 | 0.35 |
| 212 | Train 4 | Test 20 | 0.78 | 0.28 | 0.67 | 0.4 | 2260 | Train 36 | Test 20 | 0.78 | 0.28 | 0.68 | 0.39 |
| 213 | Train 4 | Test 21 | 0.72 | 0.23 | 0.46 | 0.31 | 2261 | Train 36 | Test 21 | 0.72 | 0.23 | 0.46 | 0.31 |
| 214 | Train 4 | Test 22 | 0.71 | 0.35 | 0.32 | 0.33 | 2262 | Train 36 | Test 22 | 0.71 | 0.35 | 0.33 | 0.34 |
| 215 | Train 4 | Test 23 | 0.71 | 0.23 | 0.45 | 0.3 | 2263 | Train 36 | Test 23 | 0.71 | 0.23 | 0.46 | 0.3 |
| 216 | Train 4 | Test 24 | 0.7 | 0.34 | 0.32 | 0.33 | 2264 | Train 36 | Test 24 | 0.7 | 0.33 | 0.34 | 0.33 |
| 217 | Train 4 | Test 25 | 0.77 | 0.26 | 0.6 | 0.36 | 2265 | Train 36 | Test 25 | 0.77 | 0.25 | 0.6 | 0.36 |
| 218 | Train 4 | Test 26 | 0.78 | 0.28 | 0.66 | 0.4 | 2266 | Train 36 | Test 26 | 0.78 | 0.28 | 0.67 | 0.4 |
| 219 | Train 4 | Test 27 | 0.77 | 0.26 | 0.6 | 0.36 | 2267 | Train 36 | Test 27 | 0.77 | 0.25 | 0.61 | 0.36 |
| 220 | Train 4 | Test 28 | 0.78 | 0.28 | 0.66 | 0.4 | 2268 | Train 36 | Test 28 | 0.78 | 0.28 | 0.67 | 0.4 |
| 221 | Train 4 | Test 29 | 0.72 | 0.23 | 0.44 | 0.31 | 2269 | Train 36 | Test 29 | 0.72 | 0.23 | 0.45 | 0.31 |
| 222 | Train 4 | Test 30 | 0.71 | 0.35 | 0.32 | 0.33 | 2270 | Train 36 | Test 30 | 0.71 | 0.34 | 0.34 | 0.34 |
| 223 | Train 4 | Test 31 | 0.71 | 0.23 | 0.44 | 0.3 | 2271 | Train 36 | Test 31 | 0.72 | 0.23 | 0.45 | 0.3 |
| 224 | Train 4 | Test 32 | 0.71 | 0.33 | 0.32 | 0.33 | 2272 | Train 36 | Test 32 | 0.71 | 0.33 | 0.34 | 0.33 |
| 225 | Train 4 | Test 33 | 0.77 | 0.25 | 0.61 | 0.36 | 2273 | Train 36 | Test 33 | 0.77 | 0.25 | 0.61 | 0.35 |
| 226 | Train 4 | Test 34 | 0.78 | 0.28 | 0.67 | 0.39 | 2274 | Train 36 | Test 34 | 0.78 | 0.28 | 0.67 | 0.39 |
| 227 | Train 4 | Test 35 | 0.77 | 0.25 | 0.61 | 0.36 | 2275 | Train 36 | Test 35 | 0.77 | 0.25 | 0.61 | 0.35 |
| 228 | Train 4 | Test 36 | 0.78 | 0.28 | 0.67 | 0.39 | 2276 | Train 36 | Test 36 | 0.78 | 0.28 | 0.67 | 0.39 |
| 229 | Train 4 | Test 37 | 0.72 | 0.23 | 0.46 | 0.31 | 2277 | Train 36 | Test 37 | 0.72 | 0.23 | 0.46 | 0.3 |
| 230 | Train 4 | Test 38 | 0.71 | 0.35 | 0.32 | 0.33 | 2278 | Train 36 | Test 38 | 0.71 | 0.35 | 0.33 | 0.34 |
| 231 | Train 4 | Test 39 | 0.72 | 0.23 | 0.45 | 0.3 | 2279 | Train 36 | Test 39 | 0.72 | 0.23 | 0.46 | 0.3 |
| 232 | Train 4 | Test 40 | 0.7 | 0.34 | 0.32 | 0.33 | 2280 | Train 36 | Test 40 | 0.71 | 0.33 | 0.34 | 0.33 |
| 233 | Train 4 | Test 41 | 0.77 | 0.25 | 0.6 | 0.36 | 2281 | Train 36 | Test 41 | 0.77 | 0.25 | 0.6 | 0.35 |
| 234 | Train 4 | Test 42 | 0.78 | 0.28 | 0.66 | 0.4 | 2282 | Train 36 | Test 42 | 0.78 | 0.28 | 0.66 | 0.39 |
| 235 | Train 4 | Test 43 | 0.77 | 0.26 | 0.6 | 0.36 | 2283 | Train 36 | Test 43 | 0.77 | 0.25 | 0.6 | 0.36 |
| 236 | Train 4 | Test 44 | 0.78 | 0.28 | 0.66 | 0.4 | 2284 | Train 36 | Test 44 | 0.78 | 0.28 | 0.67 | 0.4 |
| 237 | Train 4 | Test 45 | 0.72 | 0.23 | 0.44 | 0.31 | 2285 | Train 36 | Test 45 | 0.72 | 0.23 | 0.45 | 0.31 |
| 238 | Train 4 | Test 46 | 0.71 | 0.35 | 0.32 | 0.33 | 2286 | Train 36 | Test 46 | 0.71 | 0.34 | 0.34 | 0.34 |
| 239 | Train 4 | Test 47 | 0.72 | 0.23 | 0.44 | 0.3 | 2287 | Train 36 | Test 47 | 0.72 | 0.23 | 0.45 | 0.3 |
| 240 | Train 4 | Test 48 | 0.71 | 0.33 | 0.32 | 0.33 | 2288 | Train 36 | Test 48 | 0.71 | 0.33 | 0.34 | 0.33 |
| 241 | Train 4 | Test 49 | 0.77 | 0.28 | 0.73 | 0.41 | 2289 | Train 36 | Test 49 | 0.77 | 0.27 | 0.72 | 0.4 |
| 242 | Train 4 | Test 50 | 0.77 | 0.28 | 0.73 | 0.4 | 2290 | Train 36 | Test 50 | 0.77 | 0.27 | 0.72 | 0.4 |
| 243 | Train 4 | Test 51 | 0.77 | 0.28 | 0.74 | 0.41 | 2291 | Train 36 | Test 51 | 0.77 | 0.27 | 0.73 | 0.4 |
| 244 | Train 4 | Test 52 | 0.77 | 0.28 | 0.73 | 0.41 | 2292 | Train 36 | Test 52 | 0.77 | 0.28 | 0.72 | 0.4 |
| 245 | Train 4 | Test 53 | 0.75 | 0.3 | 0.36 | 0.33 | 2293 | Train 36 | Test 53 | 0.75 | 0.3 | 0.38 | 0.34 |
| 246 | Train 4 | Test 54 | 0.72 | 0.39 | 0.3 | 0.34 | 2294 | Train 36 | Test 54 | 0.72 | 0.39 | 0.32 | 0.35 |
| 247 | Train 4 | Test 55 | 0.75 | 0.28 | 0.51 | 0.36 | 2295 | Train 36 | Test 55 | 0.76 | 0.28 | 0.51 | 0.36 |
| 248 | Train 4 | Test 56 | 0.71 | 0.36 | 0.31 | 0.33 | 2296 | Train 36 | Test 56 | 0.71 | 0.36 | 0.32 | 0.34 |
| 249 | Train 4 | Test 57 | 0.78 | 0.28 | 0.73 | 0.41 | 2297 | Train 36 | Test 57 | 0.78 | 0.28 | 0.72 | 0.4 |
| 250 | Train 4 | Test 58 | 0.78 | 0.28 | 0.72 | 0.41 | 2298 | Train 36 | Test 58 | 0.78 | 0.28 | 0.72 | 0.4 |
| 251 | Train 4 | Test 59 | 0.78 | 0.29 | 0.73 | 0.41 | 2299 | Train 36 | Test 59 | 0.78 | 0.28 | 0.72 | 0.41 |
| 252 | Train 4 | Test 60 | 0.78 | 0.29 | 0.72 | 0.41 | 2300 | Train 36 | Test 60 | 0.78 | 0.28 | 0.72 | 0.41 |
| 253 | Train 4 | Test 61 | 0.76 | 0.31 | 0.36 | 0.33 | 2301 | Train 36 | Test 61 | 0.76 | 0.3 | 0.38 | 0.34 |
| 254 | Train 4 | Test 62 | 0.72 | 0.39 | 0.3 | 0.34 | 2302 | Train 36 | Test 62 | 0.72 | 0.39 | 0.32 | 0.35 |
| 255 | Train 4 | Test 63 | 0.76 | 0.31 | 0.36 | 0.33 | 2303 | Train 36 | Test 63 | 0.76 | 0.3 | 0.38 | 0.34 |
| 256 | Train 4 | Test 64 | 0.72 | 0.36 | 0.31 | 0.33 | 2304 | Train 36 | Test 64 | 0.72 | 0.36 | 0.32 | 0.34 |
| 257 | Train 5 | Test 1 | 0.76 | 0.23 | 0.69 | 0.35 | 2305 | Train 37 | Test 1 | 0.76 | 0.23 | 0.69 | 0.34 |
| 258 | Train 5 | Test 2 | 0.76 | 0.26 | 0.69 | 0.38 | 2306 | Train 37 | Test 2 | 0.76 | 0.26 | 0.7 | 0.37 |
| 259 | Train 5 | Test 3 | 0.76 | 0.23 | 0.69 | 0.35 | 2307 | Train 37 | Test 3 | 0.76 | 0.23 | 0.69 | 0.34 |
| 260 | Train 5 | Test 4 | 0.76 | 0.26 | 0.7 | 0.38 | 2308 | Train 37 | Test 4 | 0.76 | 0.26 | 0.7 | 0.38 |
| 261 | Train 5 | Test 5 | 0.75 | 0.23 | 0.66 | 0.34 | 2309 | Train 37 | Test 5 | 0.75 | 0.23 | 0.66 | 0.34 |
| 262 | Train 5 | Test 6 | 0.7 | 0.3 | 0.49 | 0.37 | 2310 | Train 37 | Test 6 | 0.7 | 0.3 | 0.5 | 0.37 |
| 263 | Train 5 | Test 7 | 0.75 | 0.23 | 0.65 | 0.34 | 2311 | Train 37 | Test 7 | 0.75 | 0.22 | 0.65 | 0.33 |
| 264 | Train 5 | Test 8 | 0.7 | 0.29 | 0.49 | 0.37 | 2312 | Train 37 | Test 8 | 0.7 | 0.29 | 0.5 | 0.37 |
| 265 | Train 5 | Test 9 | 0.76 | 0.23 | 0.68 | 0.35 | 2313 | Train 37 | Test 9 | 0.76 | 0.23 | 0.68 | 0.34 |
| 266 | Train 5 | Test 10 | 0.76 | 0.26 | 0.69 | 0.38 | 2314 | Train 37 | Test 10 | 0.76 | 0.26 | 0.69 | 0.38 |
| 267 | Train 5 | Test 11 | 0.76 | 0.23 | 0.68 | 0.35 | 2315 | Train 37 | Test 11 | 0.76 | 0.23 | 0.68 | 0.35 |
| 268 | Train 5 | Test 12 | 0.76 | 0.26 | 0.69 | 0.38 | 2316 | Train 37 | Test 12 | 0.76 | 0.26 | 0.69 | 0.38 |
| 269 | Train 5 | Test 13 | 0.75 | 0.23 | 0.64 | 0.34 | 2317 | Train 37 | Test 13 | 0.75 | 0.23 | 0.64 | 0.34 |
| 270 | Train 5 | Test 14 | 0.7 | 0.3 | 0.49 | 0.37 | 2318 | Train 37 | Test 14 | 0.7 | 0.29 | 0.5 | 0.37 |
| 271 | Train 5 | Test 15 | 0.75 | 0.23 | 0.64 | 0.34 | 2319 | Train 37 | Test 15 | 0.75 | 0.23 | 0.64 | 0.34 |
| 272 | Train 5 | Test 16 | 0.7 | 0.29 | 0.49 | 0.37 | 2320 | Train 37 | Test 16 | 0.7 | 0.29 | 0.5 | 0.37 |
| 273 | Train 5 | Test 17 | 0.76 | 0.23 | 0.69 | 0.35 | 2321 | Train 37 | Test 17 | 0.76 | 0.23 | 0.69 | 0.34 |
| 274 | Train 5 | Test 18 | 0.76 | 0.26 | 0.69 | 0.38 | 2322 | Train 37 | Test 18 | 0.75 | 0.26 | 0.7 | 0.37 |
| 275 | Train 5 | Test 19 | 0.76 | 0.23 | 0.69 | 0.35 | 2323 | Train 37 | Test 19 | 0.76 | 0.23 | 0.69 | 0.34 |
| 276 | Train 5 | Test 20 | 0.76 | 0.26 | 0.7 | 0.38 | 2324 | Train 37 | Test 20 | 0.76 | 0.26 | 0.7 | 0.38 |
| 277 | Train 5 | Test 21 | 0.75 | 0.23 | 0.66 | 0.34 | 2325 | Train 37 | Test 21 | 0.75 | 0.23 | 0.66 | 0.34 |
| 278 | Train 5 | Test 22 | 0.7 | 0.3 | 0.49 | 0.37 | 2326 | Train 37 | Test 22 | 0.7 | 0.3 | 0.49 | 0.37 |
| 279 | Train 5 | Test 23 | 0.75 | 0.23 | 0.65 | 0.34 | 2327 | Train 37 | Test 23 | 0.75 | 0.22 | 0.65 | 0.33 |
| 280 | Train 5 | Test 24 | 0.7 | 0.29 | 0.49 | 0.37 | 2328 | Train 37 | Test 24 | 0.7 | 0.29 | 0.49 | 0.36 |
| 281 | Train 5 | Test 25 | 0.76 | 0.23 | 0.68 | 0.35 | 2329 | Train 37 | Test 25 | 0.76 | 0.23 | 0.68 | 0.34 |
| 282 | Train 5 | Test 26 | 0.76 | 0.26 | 0.69 | 0.38 | 2330 | Train 37 | Test 26 | 0.76 | 0.26 | 0.69 | 0.38 |
| 283 | Train 5 | Test 27 | 0.76 | 0.23 | 0.68 | 0.35 | 2331 | Train 37 | Test 27 | 0.76 | 0.23 | 0.68 | 0.35 |
| 284 | Train 5 | Test 28 | 0.76 | 0.26 | 0.69 | 0.38 | 2332 | Train 37 | Test 28 | 0.76 | 0.26 | 0.69 | 0.38 |
| 285 | Train 5 | Test 29 | 0.75 | 0.23 | 0.64 | 0.34 | 2333 | Train 37 | Test 29 | 0.75 | 0.23 | 0.64 | 0.34 |
| 286 | Train 5 | Test 30 | 0.7 | 0.3 | 0.49 | 0.37 | 2334 | Train 37 | Test 30 | 0.7 | 0.29 | 0.5 | 0.37 |
| 287 | Train 5 | Test 31 | 0.75 | 0.23 | 0.64 | 0.34 | 2335 | Train 37 | Test 31 | 0.75 | 0.23 | 0.64 | 0.34 |
| 288 | Train 5 | Test 32 | 0.7 | 0.29 | 0.49 | 0.37 | 2336 | Train 37 | Test 32 | 0.7 | 0.29 | 0.5 | 0.37 |
| 289 | Train 5 | Test 33 | 0.76 | 0.23 | 0.69 | 0.34 | 2337 | Train 37 | Test 33 | 0.76 | 0.23 | 0.69 | 0.34 |
| 290 | Train 5 | Test 34 | 0.76 | 0.26 | 0.69 | 0.38 | 2338 | Train 37 | Test 34 | 0.76 | 0.26 | 0.69 | 0.37 |
| 291 | Train 5 | Test 35 | 0.76 | 0.23 | 0.69 | 0.35 | 2339 | Train 37 | Test 35 | 0.76 | 0.23 | 0.69 | 0.34 |
| 292 | Train 5 | Test 36 | 0.76 | 0.26 | 0.69 | 0.38 | 2340 | Train 37 | Test 36 | 0.76 | 0.26 | 0.7 | 0.37 |
| 293 | Train 5 | Test 37 | 0.75 | 0.23 | 0.66 | 0.34 | 2341 | Train 37 | Test 37 | 0.75 | 0.23 | 0.66 | 0.34 |
| 294 | Train 5 | Test 38 | 0.7 | 0.3 | 0.49 | 0.37 | 2342 | Train 37 | Test 38 | 0.7 | 0.3 | 0.49 | 0.37 |
| 295 | Train 5 | Test 39 | 0.75 | 0.23 | 0.65 | 0.34 | 2343 | Train 37 | Test 39 | 0.75 | 0.22 | 0.65 | 0.33 |
| 296 | Train 5 | Test 40 | 0.7 | 0.29 | 0.49 | 0.37 | 2344 | Train 37 | Test 40 | 0.7 | 0.29 | 0.49 | 0.36 |
| 297 | Train 5 | Test 41 | 0.76 | 0.23 | 0.68 | 0.35 | 2345 | Train 37 | Test 41 | 0.76 | 0.23 | 0.68 | 0.34 |
| 298 | Train 5 | Test 42 | 0.76 | 0.26 | 0.68 | 0.38 | 2346 | Train 37 | Test 42 | 0.76 | 0.26 | 0.68 | 0.38 |
| 299 | Train 5 | Test 43 | 0.76 | 0.23 | 0.68 | 0.35 | 2347 | Train 37 | Test 43 | 0.76 | 0.23 | 0.68 | 0.34 |
| 300 | Train 5 | Test 44 | 0.76 | 0.26 | 0.68 | 0.38 | 2348 | Train 37 | Test 44 | 0.76 | 0.26 | 0.69 | 0.38 |
| 301 | Train 5 | Test 45 | 0.75 | 0.23 | 0.64 | 0.34 | 2349 | Train 37 | Test 45 | 0.75 | 0.23 | 0.64 | 0.34 |
| 302 | Train 5 | Test 46 | 0.7 | 0.3 | 0.49 | 0.37 | 2350 | Train 37 | Test 46 | 0.7 | 0.29 | 0.5 | 0.37 |
| 303 | Train 5 | Test 47 | 0.75 | 0.23 | 0.64 | 0.34 | 2351 | Train 37 | Test 47 | 0.75 | 0.23 | 0.63 | 0.34 |
| 304 | Train 5 | Test 48 | 0.7 | 0.29 | 0.49 | 0.37 | 2352 | Train 37 | Test 48 | 0.7 | 0.29 | 0.5 | 0.36 |
| 305 | Train 5 | Test 49 | 0.77 | 0.26 | 0.75 | 0.38 | 2353 | Train 37 | Test 49 | 0.77 | 0.26 | 0.75 | 0.38 |
| 306 | Train 5 | Test 50 | 0.77 | 0.26 | 0.75 | 0.38 | 2354 | Train 37 | Test 50 | 0.77 | 0.26 | 0.74 | 0.38 |
| 307 | Train 5 | Test 51 | 0.77 | 0.26 | 0.75 | 0.39 | 2355 | Train 37 | Test 51 | 0.77 | 0.26 | 0.75 | 0.38 |
| 308 | Train 5 | Test 52 | 0.77 | 0.26 | 0.75 | 0.39 | 2356 | Train 37 | Test 52 | 0.77 | 0.26 | 0.75 | 0.38 |
| 309 | Train 5 | Test 53 | 0.76 | 0.27 | 0.64 | 0.39 | 2357 | Train 37 | Test 53 | 0.76 | 0.27 | 0.64 | 0.38 |
| 310 | Train 5 | Test 54 | 0.71 | 0.36 | 0.49 | 0.41 | 2358 | Train 37 | Test 54 | 0.71 | 0.35 | 0.48 | 0.41 |
| 311 | Train 5 | Test 55 | 0.77 | 0.26 | 0.7 | 0.38 | 2359 | Train 37 | Test 55 | 0.77 | 0.26 | 0.7 | 0.38 |
| 312 | Train 5 | Test 56 | 0.71 | 0.34 | 0.49 | 0.4 | 2360 | Train 37 | Test 56 | 0.71 | 0.33 | 0.49 | 0.4 |
| 313 | Train 5 | Test 57 | 0.77 | 0.26 | 0.75 | 0.39 | 2361 | Train 37 | Test 57 | 0.77 | 0.26 | 0.75 | 0.39 |
| 314 | Train 5 | Test 58 | 0.77 | 0.26 | 0.75 | 0.39 | 2362 | Train 37 | Test 58 | 0.77 | 0.26 | 0.75 | 0.39 |
| 315 | Train 5 | Test 59 | 0.77 | 0.27 | 0.76 | 0.39 | 2363 | Train 37 | Test 59 | 0.77 | 0.26 | 0.76 | 0.39 |
| 316 | Train 5 | Test 60 | 0.77 | 0.27 | 0.75 | 0.39 | 2364 | Train 37 | Test 60 | 0.77 | 0.26 | 0.75 | 0.39 |
| 317 | Train 5 | Test 61 | 0.76 | 0.28 | 0.65 | 0.39 | 2365 | Train 37 | Test 61 | 0.76 | 0.27 | 0.65 | 0.39 |
| 318 | Train 5 | Test 62 | 0.71 | 0.35 | 0.49 | 0.41 | 2366 | Train 37 | Test 62 | 0.71 | 0.35 | 0.49 | 0.41 |
| 319 | Train 5 | Test 63 | 0.77 | 0.28 | 0.65 | 0.39 | 2367 | Train 37 | Test 63 | 0.77 | 0.27 | 0.65 | 0.39 |
| 320 | Train 5 | Test 64 | 0.71 | 0.34 | 0.5 | 0.4 | 2368 | Train 37 | Test 64 | 0.71 | 0.33 | 0.49 | 0.4 |
| 321 | Train 6 | Test 1 | 0.75 | 0.24 | 0.6 | 0.34 | 2369 | Train 38 | Test 1 | 0.75 | 0.24 | 0.6 | 0.34 |
| 322 | Train 6 | Test 2 | 0.76 | 0.27 | 0.67 | 0.38 | 2370 | Train 38 | Test 2 | 0.76 | 0.27 | 0.66 | 0.38 |
| 323 | Train 6 | Test 3 | 0.75 | 0.24 | 0.61 | 0.34 | 2371 | Train 38 | Test 3 | 0.75 | 0.24 | 0.6 | 0.34 |
| 324 | Train 6 | Test 4 | 0.76 | 0.27 | 0.67 | 0.38 | 2372 | Train 38 | Test 4 | 0.76 | 0.27 | 0.67 | 0.38 |
| 325 | Train 6 | Test 5 | 0.74 | 0.24 | 0.57 | 0.33 | 2373 | Train 38 | Test 5 | 0.74 | 0.23 | 0.57 | 0.33 |
| 326 | Train 6 | Test 6 | 0.76 | 0.31 | 0.7 | 0.43 | 2374 | Train 38 | Test 6 | 0.76 | 0.31 | 0.7 | 0.43 |
| 327 | Train 6 | Test 7 | 0.74 | 0.24 | 0.56 | 0.33 | 2375 | Train 38 | Test 7 | 0.74 | 0.23 | 0.56 | 0.33 |
| 328 | Train 6 | Test 8 | 0.75 | 0.3 | 0.7 | 0.42 | 2376 | Train 38 | Test 8 | 0.75 | 0.3 | 0.7 | 0.42 |
| 329 | Train 6 | Test 9 | 0.75 | 0.24 | 0.59 | 0.34 | 2377 | Train 38 | Test 9 | 0.75 | 0.24 | 0.58 | 0.34 |
| 330 | Train 6 | Test 10 | 0.76 | 0.27 | 0.66 | 0.38 | 2378 | Train 38 | Test 10 | 0.76 | 0.27 | 0.65 | 0.38 |
| 331 | Train 6 | Test 11 | 0.75 | 0.24 | 0.59 | 0.34 | 2379 | Train 38 | Test 11 | 0.75 | 0.24 | 0.58 | 0.34 |
| 332 | Train 6 | Test 12 | 0.76 | 0.27 | 0.66 | 0.38 | 2380 | Train 38 | Test 12 | 0.76 | 0.27 | 0.65 | 0.38 |
| 333 | Train 6 | Test 13 | 0.74 | 0.24 | 0.56 | 0.34 | 2381 | Train 38 | Test 13 | 0.74 | 0.24 | 0.55 | 0.33 |
| 334 | Train 6 | Test 14 | 0.76 | 0.31 | 0.7 | 0.43 | 2382 | Train 38 | Test 14 | 0.76 | 0.31 | 0.7 | 0.43 |
| 335 | Train 6 | Test 15 | 0.74 | 0.24 | 0.55 | 0.33 | 2383 | Train 38 | Test 15 | 0.74 | 0.24 | 0.55 | 0.33 |
| 336 | Train 6 | Test 16 | 0.75 | 0.3 | 0.7 | 0.42 | 2384 | Train 38 | Test 16 | 0.75 | 0.3 | 0.7 | 0.42 |
| 337 | Train 6 | Test 17 | 0.75 | 0.24 | 0.6 | 0.34 | 2385 | Train 38 | Test 17 | 0.75 | 0.24 | 0.6 | 0.34 |
| 338 | Train 6 | Test 18 | 0.76 | 0.27 | 0.67 | 0.38 | 2386 | Train 38 | Test 18 | 0.76 | 0.27 | 0.66 | 0.38 |
| 339 | Train 6 | Test 19 | 0.75 | 0.24 | 0.61 | 0.34 | 2387 | Train 38 | Test 19 | 0.75 | 0.24 | 0.6 | 0.34 |
| 340 | Train 6 | Test 20 | 0.76 | 0.27 | 0.67 | 0.38 | 2388 | Train 38 | Test 20 | 0.76 | 0.27 | 0.67 | 0.38 |
| 341 | Train 6 | Test 21 | 0.74 | 0.24 | 0.57 | 0.33 | 2389 | Train 38 | Test 21 | 0.74 | 0.23 | 0.57 | 0.33 |
| 342 | Train 6 | Test 22 | 0.76 | 0.31 | 0.7 | 0.43 | 2390 | Train 38 | Test 22 | 0.76 | 0.31 | 0.7 | 0.43 |
| 343 | Train 6 | Test 23 | 0.74 | 0.24 | 0.56 | 0.33 | 2391 | Train 38 | Test 23 | 0.74 | 0.23 | 0.56 | 0.33 |
| 344 | Train 6 | Test 24 | 0.75 | 0.3 | 0.7 | 0.42 | 2392 | Train 38 | Test 24 | 0.75 | 0.3 | 0.7 | 0.42 |
| 345 | Train 6 | Test 25 | 0.75 | 0.24 | 0.59 | 0.34 | 2393 | Train 38 | Test 25 | 0.75 | 0.24 | 0.58 | 0.34 |
| 346 | Train 6 | Test 26 | 0.76 | 0.27 | 0.66 | 0.38 | 2394 | Train 38 | Test 26 | 0.76 | 0.27 | 0.65 | 0.38 |
| 347 | Train 6 | Test 27 | 0.75 | 0.24 | 0.59 | 0.34 | 2395 | Train 38 | Test 27 | 0.75 | 0.24 | 0.59 | 0.34 |
| 348 | Train 6 | Test 28 | 0.76 | 0.27 | 0.66 | 0.38 | 2396 | Train 38 | Test 28 | 0.76 | 0.27 | 0.65 | 0.38 |
| 349 | Train 6 | Test 29 | 0.74 | 0.24 | 0.56 | 0.34 | 2397 | Train 38 | Test 29 | 0.74 | 0.24 | 0.55 | 0.33 |
| 350 | Train 6 | Test 30 | 0.76 | 0.31 | 0.7 | 0.42 | 2398 | Train 38 | Test 30 | 0.76 | 0.3 | 0.7 | 0.42 |
| 351 | Train 6 | Test 31 | 0.74 | 0.24 | 0.55 | 0.33 | 2399 | Train 38 | Test 31 | 0.74 | 0.24 | 0.55 | 0.33 |
| 352 | Train 6 | Test 32 | 0.75 | 0.3 | 0.7 | 0.42 | 2400 | Train 38 | Test 32 | 0.75 | 0.3 | 0.7 | 0.42 |
| 353 | Train 6 | Test 33 | 0.75 | 0.24 | 0.6 | 0.34 | 2401 | Train 38 | Test 33 | 0.75 | 0.24 | 0.59 | 0.34 |
| 354 | Train 6 | Test 34 | 0.76 | 0.27 | 0.67 | 0.38 | 2402 | Train 38 | Test 34 | 0.76 | 0.26 | 0.66 | 0.38 |
| 355 | Train 6 | Test 35 | 0.75 | 0.24 | 0.6 | 0.34 | 2403 | Train 38 | Test 35 | 0.75 | 0.24 | 0.6 | 0.34 |
| 356 | Train 6 | Test 36 | 0.76 | 0.27 | 0.67 | 0.38 | 2404 | Train 38 | Test 36 | 0.76 | 0.27 | 0.66 | 0.38 |
| 357 | Train 6 | Test 37 | 0.74 | 0.24 | 0.57 | 0.33 | 2405 | Train 38 | Test 37 | 0.74 | 0.23 | 0.56 | 0.33 |
| 358 | Train 6 | Test 38 | 0.76 | 0.31 | 0.7 | 0.43 | 2406 | Train 38 | Test 38 | 0.76 | 0.31 | 0.7 | 0.43 |
| 359 | Train 6 | Test 39 | 0.74 | 0.23 | 0.56 | 0.33 | 2407 | Train 38 | Test 39 | 0.74 | 0.23 | 0.56 | 0.33 |
| 360 | Train 6 | Test 40 | 0.75 | 0.3 | 0.7 | 0.42 | 2408 | Train 38 | Test 40 | 0.75 | 0.3 | 0.7 | 0.42 |
| 361 | Train 6 | Test 41 | 0.75 | 0.24 | 0.59 | 0.34 | 2409 | Train 38 | Test 41 | 0.75 | 0.24 | 0.58 | 0.34 |
| 362 | Train 6 | Test 42 | 0.76 | 0.27 | 0.66 | 0.38 | 2410 | Train 38 | Test 42 | 0.76 | 0.27 | 0.65 | 0.38 |
| 363 | Train 6 | Test 43 | 0.75 | 0.24 | 0.59 | 0.34 | 2411 | Train 38 | Test 43 | 0.75 | 0.24 | 0.58 | 0.34 |
| 364 | Train 6 | Test 44 | 0.76 | 0.27 | 0.66 | 0.38 | 2412 | Train 38 | Test 44 | 0.76 | 0.27 | 0.65 | 0.38 |
| 365 | Train 6 | Test 45 | 0.74 | 0.24 | 0.55 | 0.34 | 2413 | Train 38 | Test 45 | 0.74 | 0.24 | 0.55 | 0.33 |
| 366 | Train 6 | Test 46 | 0.76 | 0.31 | 0.7 | 0.43 | 2414 | Train 38 | Test 46 | 0.76 | 0.3 | 0.7 | 0.42 |
| 367 | Train 6 | Test 47 | 0.74 | 0.24 | 0.55 | 0.33 | 2415 | Train 38 | Test 47 | 0.74 | 0.24 | 0.54 | 0.33 |
| 368 | Train 6 | Test 48 | 0.75 | 0.3 | 0.7 | 0.42 | 2416 | Train 38 | Test 48 | 0.75 | 0.3 | 0.7 | 0.42 |
| 369 | Train 6 | Test 49 | 0.77 | 0.28 | 0.73 | 0.4 | 2417 | Train 38 | Test 49 | 0.77 | 0.28 | 0.73 | 0.4 |
| 370 | Train 6 | Test 50 | 0.77 | 0.28 | 0.73 | 0.4 | 2418 | Train 38 | Test 50 | 0.77 | 0.28 | 0.72 | 0.4 |
| 371 | Train 6 | Test 51 | 0.77 | 0.28 | 0.73 | 0.41 | 2419 | Train 38 | Test 51 | 0.77 | 0.28 | 0.73 | 0.4 |
| 372 | Train 6 | Test 52 | 0.77 | 0.28 | 0.73 | 0.41 | 2420 | Train 38 | Test 52 | 0.77 | 0.28 | 0.73 | 0.4 |
| 373 | Train 6 | Test 53 | 0.77 | 0.28 | 0.65 | 0.39 | 2421 | Train 38 | Test 53 | 0.77 | 0.28 | 0.65 | 0.39 |
| 374 | Train 6 | Test 54 | 0.76 | 0.34 | 0.73 | 0.46 | 2422 | Train 38 | Test 54 | 0.76 | 0.34 | 0.73 | 0.47 |
| 375 | Train 6 | Test 55 | 0.77 | 0.28 | 0.67 | 0.4 | 2423 | Train 38 | Test 55 | 0.77 | 0.28 | 0.67 | 0.39 |
| 376 | Train 6 | Test 56 | 0.75 | 0.32 | 0.73 | 0.45 | 2424 | Train 38 | Test 56 | 0.75 | 0.32 | 0.73 | 0.45 |
| 377 | Train 6 | Test 57 | 0.78 | 0.28 | 0.73 | 0.41 | 2425 | Train 38 | Test 57 | 0.78 | 0.28 | 0.73 | 0.41 |
| 378 | Train 6 | Test 58 | 0.78 | 0.28 | 0.72 | 0.41 | 2426 | Train 38 | Test 58 | 0.78 | 0.28 | 0.72 | 0.41 |
| 379 | Train 6 | Test 59 | 0.78 | 0.29 | 0.73 | 0.41 | 2427 | Train 38 | Test 59 | 0.78 | 0.28 | 0.73 | 0.41 |
| 380 | Train 6 | Test 60 | 0.78 | 0.29 | 0.72 | 0.41 | 2428 | Train 38 | Test 60 | 0.78 | 0.29 | 0.72 | 0.41 |
| 381 | Train 6 | Test 61 | 0.78 | 0.29 | 0.65 | 0.4 | 2429 | Train 38 | Test 61 | 0.78 | 0.29 | 0.65 | 0.4 |
| 382 | Train 6 | Test 62 | 0.76 | 0.34 | 0.73 | 0.46 | 2430 | Train 38 | Test 62 | 0.76 | 0.34 | 0.73 | 0.46 |
| 383 | Train 6 | Test 63 | 0.78 | 0.29 | 0.65 | 0.4 | 2431 | Train 38 | Test 63 | 0.78 | 0.29 | 0.65 | 0.4 |
| 384 | Train 6 | Test 64 | 0.75 | 0.32 | 0.73 | 0.45 | 2432 | Train 38 | Test 64 | 0.75 | 0.32 | 0.74 | 0.45 |
| 385 | Train 7 | Test 1 | 0.76 | 0.23 | 0.69 | 0.35 | 2433 | Train 39 | Test 1 | 0.76 | 0.23 | 0.7 | 0.35 |
| 386 | Train 7 | Test 2 | 0.76 | 0.26 | 0.69 | 0.38 | 2434 | Train 39 | Test 2 | 0.76 | 0.26 | 0.7 | 0.38 |
| 387 | Train 7 | Test 3 | 0.76 | 0.23 | 0.69 | 0.35 | 2435 | Train 39 | Test 3 | 0.76 | 0.23 | 0.7 | 0.35 |
| 388 | Train 7 | Test 4 | 0.76 | 0.26 | 0.7 | 0.38 | 2436 | Train 39 | Test 4 | 0.76 | 0.26 | 0.7 | 0.38 |
| 389 | Train 7 | Test 5 | 0.75 | 0.23 | 0.66 | 0.34 | 2437 | Train 39 | Test 5 | 0.75 | 0.23 | 0.66 | 0.34 |
| 390 | Train 7 | Test 6 | 0.7 | 0.3 | 0.48 | 0.37 | 2438 | Train 39 | Test 6 | 0.7 | 0.3 | 0.49 | 0.37 |
| 391 | Train 7 | Test 7 | 0.75 | 0.23 | 0.65 | 0.34 | 2439 | Train 39 | Test 7 | 0.75 | 0.23 | 0.66 | 0.34 |
| 392 | Train 7 | Test 8 | 0.7 | 0.29 | 0.48 | 0.36 | 2440 | Train 39 | Test 8 | 0.7 | 0.29 | 0.49 | 0.37 |
| 393 | Train 7 | Test 9 | 0.76 | 0.23 | 0.68 | 0.35 | 2441 | Train 39 | Test 9 | 0.76 | 0.23 | 0.68 | 0.35 |
| 394 | Train 7 | Test 10 | 0.76 | 0.26 | 0.69 | 0.38 | 2442 | Train 39 | Test 10 | 0.76 | 0.26 | 0.69 | 0.38 |
| 395 | Train 7 | Test 11 | 0.76 | 0.23 | 0.68 | 0.35 | 2443 | Train 39 | Test 11 | 0.76 | 0.23 | 0.68 | 0.35 |
| 396 | Train 7 | Test 12 | 0.76 | 0.26 | 0.69 | 0.38 | 2444 | Train 39 | Test 12 | 0.76 | 0.26 | 0.69 | 0.38 |
| 397 | Train 7 | Test 13 | 0.75 | 0.23 | 0.64 | 0.34 | 2445 | Train 39 | Test 13 | 0.75 | 0.23 | 0.65 | 0.34 |
| 398 | Train 7 | Test 14 | 0.7 | 0.3 | 0.48 | 0.37 | 2446 | Train 39 | Test 14 | 0.7 | 0.3 | 0.49 | 0.37 |
| 399 | Train 7 | Test 15 | 0.75 | 0.23 | 0.64 | 0.34 | 2447 | Train 39 | Test 15 | 0.75 | 0.23 | 0.64 | 0.34 |
| 400 | Train 7 | Test 16 | 0.7 | 0.29 | 0.48 | 0.36 | 2448 | Train 39 | Test 16 | 0.7 | 0.29 | 0.5 | 0.37 |
| 401 | Train 7 | Test 17 | 0.76 | 0.23 | 0.69 | 0.35 | 2449 | Train 39 | Test 17 | 0.76 | 0.23 | 0.7 | 0.35 |
| 402 | Train 7 | Test 18 | 0.76 | 0.26 | 0.69 | 0.38 | 2450 | Train 39 | Test 18 | 0.76 | 0.26 | 0.7 | 0.38 |
| 403 | Train 7 | Test 19 | 0.76 | 0.23 | 0.69 | 0.35 | 2451 | Train 39 | Test 19 | 0.76 | 0.23 | 0.7 | 0.35 |
| 404 | Train 7 | Test 20 | 0.76 | 0.26 | 0.7 | 0.38 | 2452 | Train 39 | Test 20 | 0.76 | 0.26 | 0.7 | 0.38 |
| 405 | Train 7 | Test 21 | 0.75 | 0.23 | 0.66 | 0.34 | 2453 | Train 39 | Test 21 | 0.75 | 0.23 | 0.66 | 0.34 |
| 406 | Train 7 | Test 22 | 0.7 | 0.3 | 0.48 | 0.37 | 2454 | Train 39 | Test 22 | 0.7 | 0.3 | 0.49 | 0.37 |
| 407 | Train 7 | Test 23 | 0.75 | 0.23 | 0.65 | 0.34 | 2455 | Train 39 | Test 23 | 0.75 | 0.23 | 0.66 | 0.34 |
| 408 | Train 7 | Test 24 | 0.7 | 0.29 | 0.48 | 0.36 | 2456 | Train 39 | Test 24 | 0.7 | 0.29 | 0.49 | 0.36 |
| 409 | Train 7 | Test 25 | 0.76 | 0.23 | 0.68 | 0.35 | 2457 | Train 39 | Test 25 | 0.76 | 0.23 | 0.68 | 0.35 |
| 410 | Train 7 | Test 26 | 0.76 | 0.26 | 0.68 | 0.38 | 2458 | Train 39 | Test 26 | 0.76 | 0.26 | 0.69 | 0.38 |
| 411 | Train 7 | Test 27 | 0.76 | 0.23 | 0.68 | 0.35 | 2459 | Train 39 | Test 27 | 0.76 | 0.23 | 0.69 | 0.35 |
| 412 | Train 7 | Test 28 | 0.76 | 0.26 | 0.69 | 0.38 | 2460 | Train 39 | Test 28 | 0.76 | 0.26 | 0.69 | 0.38 |
| 413 | Train 7 | Test 29 | 0.75 | 0.23 | 0.64 | 0.34 | 2461 | Train 39 | Test 29 | 0.75 | 0.23 | 0.65 | 0.34 |
| 414 | Train 7 | Test 30 | 0.7 | 0.3 | 0.48 | 0.37 | 2462 | Train 39 | Test 30 | 0.7 | 0.29 | 0.49 | 0.37 |
| 415 | Train 7 | Test 31 | 0.75 | 0.23 | 0.64 | 0.34 | 2463 | Train 39 | Test 31 | 0.75 | 0.23 | 0.64 | 0.34 |
| 416 | Train 7 | Test 32 | 0.7 | 0.29 | 0.48 | 0.36 | 2464 | Train 39 | Test 32 | 0.7 | 0.29 | 0.49 | 0.36 |
| 417 | Train 7 | Test 33 | 0.76 | 0.23 | 0.69 | 0.35 | 2465 | Train 39 | Test 33 | 0.76 | 0.23 | 0.69 | 0.34 |
| 418 | Train 7 | Test 34 | 0.76 | 0.26 | 0.69 | 0.38 | 2466 | Train 39 | Test 34 | 0.76 | 0.26 | 0.7 | 0.38 |
| 419 | Train 7 | Test 35 | 0.76 | 0.23 | 0.69 | 0.35 | 2467 | Train 39 | Test 35 | 0.76 | 0.23 | 0.69 | 0.34 |
| 420 | Train 7 | Test 36 | 0.76 | 0.26 | 0.69 | 0.38 | 2468 | Train 39 | Test 36 | 0.76 | 0.26 | 0.7 | 0.38 |
| 421 | Train 7 | Test 37 | 0.75 | 0.23 | 0.66 | 0.34 | 2469 | Train 39 | Test 37 | 0.75 | 0.23 | 0.66 | 0.34 |
| 422 | Train 7 | Test 38 | 0.7 | 0.3 | 0.48 | 0.37 | 2470 | Train 39 | Test 38 | 0.7 | 0.3 | 0.49 | 0.37 |
| 423 | Train 7 | Test 39 | 0.75 | 0.23 | 0.65 | 0.34 | 2471 | Train 39 | Test 39 | 0.75 | 0.23 | 0.65 | 0.33 |
| 424 | Train 7 | Test 40 | 0.7 | 0.29 | 0.48 | 0.36 | 2472 | Train 39 | Test 40 | 0.7 | 0.29 | 0.49 | 0.36 |
| 425 | Train 7 | Test 41 | 0.76 | 0.23 | 0.68 | 0.35 | 2473 | Train 39 | Test 41 | 0.76 | 0.23 | 0.68 | 0.35 |
| 426 | Train 7 | Test 42 | 0.76 | 0.26 | 0.68 | 0.38 | 2474 | Train 39 | Test 42 | 0.76 | 0.26 | 0.69 | 0.38 |
| 427 | Train 7 | Test 43 | 0.76 | 0.23 | 0.68 | 0.35 | 2475 | Train 39 | Test 43 | 0.76 | 0.23 | 0.68 | 0.35 |
| 428 | Train 7 | Test 44 | 0.76 | 0.26 | 0.68 | 0.38 | 2476 | Train 39 | Test 44 | 0.76 | 0.26 | 0.69 | 0.38 |
| 429 | Train 7 | Test 45 | 0.75 | 0.23 | 0.64 | 0.34 | 2477 | Train 39 | Test 45 | 0.75 | 0.23 | 0.65 | 0.34 |
| 430 | Train 7 | Test 46 | 0.7 | 0.3 | 0.48 | 0.37 | 2478 | Train 39 | Test 46 | 0.7 | 0.3 | 0.49 | 0.37 |
| 431 | Train 7 | Test 47 | 0.75 | 0.23 | 0.64 | 0.34 | 2479 | Train 39 | Test 47 | 0.75 | 0.23 | 0.64 | 0.34 |
| 432 | Train 7 | Test 48 | 0.7 | 0.29 | 0.48 | 0.36 | 2480 | Train 39 | Test 48 | 0.7 | 0.29 | 0.49 | 0.36 |
| 433 | Train 7 | Test 49 | 0.77 | 0.26 | 0.75 | 0.38 | 2481 | Train 39 | Test 49 | 0.77 | 0.26 | 0.75 | 0.38 |
| 434 | Train 7 | Test 50 | 0.77 | 0.26 | 0.75 | 0.38 | 2482 | Train 39 | Test 50 | 0.77 | 0.26 | 0.75 | 0.38 |
| 435 | Train 7 | Test 51 | 0.77 | 0.26 | 0.76 | 0.39 | 2483 | Train 39 | Test 51 | 0.77 | 0.26 | 0.76 | 0.39 |
| 436 | Train 7 | Test 52 | 0.77 | 0.26 | 0.75 | 0.39 | 2484 | Train 39 | Test 52 | 0.77 | 0.26 | 0.75 | 0.39 |
| 437 | Train 7 | Test 53 | 0.76 | 0.27 | 0.64 | 0.38 | 2485 | Train 39 | Test 53 | 0.76 | 0.27 | 0.64 | 0.38 |
| 438 | Train 7 | Test 54 | 0.71 | 0.36 | 0.48 | 0.41 | 2486 | Train 39 | Test 54 | 0.71 | 0.35 | 0.48 | 0.41 |
| 439 | Train 7 | Test 55 | 0.77 | 0.27 | 0.7 | 0.38 | 2487 | Train 39 | Test 55 | 0.77 | 0.26 | 0.7 | 0.38 |
| 440 | Train 7 | Test 56 | 0.7 | 0.34 | 0.48 | 0.4 | 2488 | Train 39 | Test 56 | 0.7 | 0.33 | 0.48 | 0.4 |
| 441 | Train 7 | Test 57 | 0.78 | 0.26 | 0.76 | 0.39 | 2489 | Train 39 | Test 57 | 0.77 | 0.26 | 0.75 | 0.39 |
| 442 | Train 7 | Test 58 | 0.77 | 0.26 | 0.75 | 0.39 | 2490 | Train 39 | Test 58 | 0.77 | 0.26 | 0.75 | 0.39 |
| 443 | Train 7 | Test 59 | 0.78 | 0.27 | 0.76 | 0.39 | 2491 | Train 39 | Test 59 | 0.78 | 0.27 | 0.76 | 0.39 |
| 444 | Train 7 | Test 60 | 0.77 | 0.27 | 0.76 | 0.39 | 2492 | Train 39 | Test 60 | 0.77 | 0.27 | 0.75 | 0.39 |
| 445 | Train 7 | Test 61 | 0.76 | 0.28 | 0.65 | 0.39 | 2493 | Train 39 | Test 61 | 0.76 | 0.28 | 0.65 | 0.39 |
| 446 | Train 7 | Test 62 | 0.71 | 0.36 | 0.48 | 0.41 | 2494 | Train 39 | Test 62 | 0.71 | 0.35 | 0.48 | 0.41 |
| 447 | Train 7 | Test 63 | 0.77 | 0.28 | 0.65 | 0.39 | 2495 | Train 39 | Test 63 | 0.77 | 0.28 | 0.65 | 0.39 |
| 448 | Train 7 | Test 64 | 0.71 | 0.34 | 0.49 | 0.4 | 2496 | Train 39 | Test 64 | 0.71 | 0.33 | 0.49 | 0.4 |
| 449 | Train 8 | Test 1 | 0.75 | 0.24 | 0.63 | 0.34 | 2497 | Train 40 | Test 1 | 0.75 | 0.24 | 0.63 | 0.34 |
| 450 | Train 8 | Test 2 | 0.76 | 0.26 | 0.69 | 0.38 | 2498 | Train 40 | Test 2 | 0.76 | 0.26 | 0.69 | 0.38 |
| 451 | Train 8 | Test 3 | 0.75 | 0.24 | 0.64 | 0.35 | 2499 | Train 40 | Test 3 | 0.75 | 0.24 | 0.63 | 0.35 |
| 452 | Train 8 | Test 4 | 0.76 | 0.26 | 0.69 | 0.38 | 2500 | Train 40 | Test 4 | 0.76 | 0.26 | 0.69 | 0.38 |
| 453 | Train 8 | Test 5 | 0.74 | 0.23 | 0.59 | 0.33 | 2501 | Train 40 | Test 5 | 0.74 | 0.23 | 0.59 | 0.33 |
| 454 | Train 8 | Test 6 | 0.76 | 0.31 | 0.7 | 0.43 | 2502 | Train 40 | Test 6 | 0.76 | 0.31 | 0.7 | 0.43 |
| 455 | Train 8 | Test 7 | 0.74 | 0.23 | 0.59 | 0.33 | 2503 | Train 40 | Test 7 | 0.74 | 0.23 | 0.59 | 0.33 |
| 456 | Train 8 | Test 8 | 0.76 | 0.3 | 0.7 | 0.42 | 2504 | Train 40 | Test 8 | 0.76 | 0.3 | 0.7 | 0.42 |
| 457 | Train 8 | Test 9 | 0.75 | 0.24 | 0.62 | 0.34 | 2505 | Train 40 | Test 9 | 0.75 | 0.24 | 0.62 | 0.34 |
| 458 | Train 8 | Test 10 | 0.76 | 0.27 | 0.67 | 0.38 | 2506 | Train 40 | Test 10 | 0.76 | 0.27 | 0.68 | 0.38 |
| 459 | Train 8 | Test 11 | 0.75 | 0.24 | 0.62 | 0.35 | 2507 | Train 40 | Test 11 | 0.75 | 0.24 | 0.62 | 0.35 |
| 460 | Train 8 | Test 12 | 0.76 | 0.27 | 0.67 | 0.38 | 2508 | Train 40 | Test 12 | 0.76 | 0.27 | 0.68 | 0.38 |
| 461 | Train 8 | Test 13 | 0.74 | 0.24 | 0.58 | 0.34 | 2509 | Train 40 | Test 13 | 0.74 | 0.24 | 0.58 | 0.34 |
| 462 | Train 8 | Test 14 | 0.76 | 0.3 | 0.7 | 0.42 | 2510 | Train 40 | Test 14 | 0.76 | 0.3 | 0.7 | 0.42 |
| 463 | Train 8 | Test 15 | 0.74 | 0.24 | 0.58 | 0.33 | 2511 | Train 40 | Test 15 | 0.74 | 0.24 | 0.57 | 0.33 |
| 464 | Train 8 | Test 16 | 0.76 | 0.3 | 0.7 | 0.42 | 2512 | Train 40 | Test 16 | 0.76 | 0.3 | 0.7 | 0.42 |
| 465 | Train 8 | Test 17 | 0.75 | 0.24 | 0.63 | 0.34 | 2513 | Train 40 | Test 17 | 0.75 | 0.24 | 0.63 | 0.34 |
| 466 | Train 8 | Test 18 | 0.76 | 0.26 | 0.69 | 0.38 | 2514 | Train 40 | Test 18 | 0.76 | 0.26 | 0.69 | 0.38 |
| 467 | Train 8 | Test 19 | 0.75 | 0.24 | 0.64 | 0.35 | 2515 | Train 40 | Test 19 | 0.75 | 0.24 | 0.63 | 0.35 |
| 468 | Train 8 | Test 20 | 0.76 | 0.26 | 0.69 | 0.38 | 2516 | Train 40 | Test 20 | 0.76 | 0.26 | 0.69 | 0.38 |
| 469 | Train 8 | Test 21 | 0.74 | 0.23 | 0.59 | 0.33 | 2517 | Train 40 | Test 21 | 0.74 | 0.23 | 0.59 | 0.33 |
| 470 | Train 8 | Test 22 | 0.76 | 0.3 | 0.7 | 0.42 | 2518 | Train 40 | Test 22 | 0.76 | 0.3 | 0.7 | 0.42 |
| 471 | Train 8 | Test 23 | 0.74 | 0.23 | 0.59 | 0.33 | 2519 | Train 40 | Test 23 | 0.74 | 0.23 | 0.59 | 0.33 |
| 472 | Train 8 | Test 24 | 0.76 | 0.3 | 0.7 | 0.42 | 2520 | Train 40 | Test 24 | 0.76 | 0.3 | 0.7 | 0.42 |
| 473 | Train 8 | Test 25 | 0.75 | 0.24 | 0.62 | 0.34 | 2521 | Train 40 | Test 25 | 0.75 | 0.24 | 0.62 | 0.34 |
| 474 | Train 8 | Test 26 | 0.76 | 0.27 | 0.67 | 0.38 | 2522 | Train 40 | Test 26 | 0.76 | 0.27 | 0.68 | 0.38 |
| 475 | Train 8 | Test 27 | 0.75 | 0.24 | 0.62 | 0.35 | 2523 | Train 40 | Test 27 | 0.75 | 0.24 | 0.62 | 0.35 |
| 476 | Train 8 | Test 28 | 0.76 | 0.27 | 0.67 | 0.38 | 2524 | Train 40 | Test 28 | 0.76 | 0.27 | 0.68 | 0.38 |
| 477 | Train 8 | Test 29 | 0.74 | 0.24 | 0.58 | 0.34 | 2525 | Train 40 | Test 29 | 0.74 | 0.24 | 0.58 | 0.34 |
| 478 | Train 8 | Test 30 | 0.76 | 0.3 | 0.7 | 0.42 | 2526 | Train 40 | Test 30 | 0.76 | 0.3 | 0.7 | 0.42 |
| 479 | Train 8 | Test 31 | 0.74 | 0.24 | 0.58 | 0.33 | 2527 | Train 40 | Test 31 | 0.74 | 0.24 | 0.57 | 0.33 |
| 480 | Train 8 | Test 32 | 0.76 | 0.3 | 0.7 | 0.42 | 2528 | Train 40 | Test 32 | 0.76 | 0.3 | 0.7 | 0.42 |
| 481 | Train 8 | Test 33 | 0.75 | 0.24 | 0.63 | 0.34 | 2529 | Train 40 | Test 33 | 0.75 | 0.24 | 0.63 | 0.34 |
| 482 | Train 8 | Test 34 | 0.76 | 0.26 | 0.69 | 0.38 | 2530 | Train 40 | Test 34 | 0.76 | 0.26 | 0.69 | 0.38 |
| 483 | Train 8 | Test 35 | 0.75 | 0.24 | 0.63 | 0.34 | 2531 | Train 40 | Test 35 | 0.75 | 0.24 | 0.63 | 0.35 |
| 484 | Train 8 | Test 36 | 0.76 | 0.26 | 0.69 | 0.38 | 2532 | Train 40 | Test 36 | 0.76 | 0.26 | 0.69 | 0.38 |
| 485 | Train 8 | Test 37 | 0.74 | 0.23 | 0.59 | 0.33 | 2533 | Train 40 | Test 37 | 0.74 | 0.23 | 0.59 | 0.33 |
| 486 | Train 8 | Test 38 | 0.76 | 0.31 | 0.7 | 0.43 | 2534 | Train 40 | Test 38 | 0.76 | 0.31 | 0.7 | 0.42 |
| 487 | Train 8 | Test 39 | 0.74 | 0.23 | 0.59 | 0.33 | 2535 | Train 40 | Test 39 | 0.74 | 0.23 | 0.59 | 0.33 |
| 488 | Train 8 | Test 40 | 0.76 | 0.3 | 0.7 | 0.42 | 2536 | Train 40 | Test 40 | 0.76 | 0.3 | 0.7 | 0.42 |
| 489 | Train 8 | Test 41 | 0.75 | 0.24 | 0.61 | 0.34 | 2537 | Train 40 | Test 41 | 0.75 | 0.24 | 0.61 | 0.34 |
| 490 | Train 8 | Test 42 | 0.76 | 0.27 | 0.67 | 0.38 | 2538 | Train 40 | Test 42 | 0.76 | 0.27 | 0.67 | 0.38 |
| 491 | Train 8 | Test 43 | 0.76 | 0.24 | 0.62 | 0.34 | 2539 | Train 40 | Test 43 | 0.76 | 0.24 | 0.61 | 0.34 |
| 492 | Train 8 | Test 44 | 0.76 | 0.27 | 0.67 | 0.38 | 2540 | Train 40 | Test 44 | 0.76 | 0.27 | 0.67 | 0.38 |
| 493 | Train 8 | Test 45 | 0.74 | 0.24 | 0.58 | 0.34 | 2541 | Train 40 | Test 45 | 0.74 | 0.24 | 0.58 | 0.34 |
| 494 | Train 8 | Test 46 | 0.76 | 0.3 | 0.7 | 0.42 | 2542 | Train 40 | Test 46 | 0.76 | 0.3 | 0.7 | 0.42 |
| 495 | Train 8 | Test 47 | 0.74 | 0.24 | 0.57 | 0.33 | 2543 | Train 40 | Test 47 | 0.74 | 0.24 | 0.57 | 0.33 |
| 496 | Train 8 | Test 48 | 0.76 | 0.3 | 0.7 | 0.42 | 2544 | Train 40 | Test 48 | 0.76 | 0.3 | 0.7 | 0.42 |
| 497 | Train 8 | Test 49 | 0.77 | 0.28 | 0.73 | 0.4 | 2545 | Train 40 | Test 49 | 0.77 | 0.27 | 0.74 | 0.4 |
| 498 | Train 8 | Test 50 | 0.77 | 0.28 | 0.73 | 0.4 | 2546 | Train 40 | Test 50 | 0.77 | 0.28 | 0.73 | 0.4 |
| 499 | Train 8 | Test 51 | 0.77 | 0.28 | 0.74 | 0.4 | 2547 | Train 40 | Test 51 | 0.77 | 0.28 | 0.74 | 0.4 |
| 500 | Train 8 | Test 52 | 0.77 | 0.28 | 0.73 | 0.4 | 2548 | Train 40 | Test 52 | 0.77 | 0.28 | 0.73 | 0.4 |
| 501 | Train 8 | Test 53 | 0.77 | 0.28 | 0.66 | 0.39 | 2549 | Train 40 | Test 53 | 0.77 | 0.28 | 0.66 | 0.39 |
| 502 | Train 8 | Test 54 | 0.76 | 0.34 | 0.73 | 0.47 | 2550 | Train 40 | Test 54 | 0.76 | 0.34 | 0.73 | 0.46 |
| 503 | Train 8 | Test 55 | 0.77 | 0.27 | 0.68 | 0.39 | 2551 | Train 40 | Test 55 | 0.77 | 0.27 | 0.68 | 0.39 |
| 504 | Train 8 | Test 56 | 0.76 | 0.33 | 0.73 | 0.45 | 2552 | Train 40 | Test 56 | 0.76 | 0.33 | 0.74 | 0.45 |
| 505 | Train 8 | Test 57 | 0.78 | 0.28 | 0.73 | 0.41 | 2553 | Train 40 | Test 57 | 0.78 | 0.28 | 0.74 | 0.41 |
| 506 | Train 8 | Test 58 | 0.78 | 0.28 | 0.73 | 0.4 | 2554 | Train 40 | Test 58 | 0.78 | 0.28 | 0.73 | 0.41 |
| 507 | Train 8 | Test 59 | 0.78 | 0.28 | 0.73 | 0.41 | 2555 | Train 40 | Test 59 | 0.78 | 0.28 | 0.74 | 0.41 |
| 508 | Train 8 | Test 60 | 0.78 | 0.28 | 0.73 | 0.41 | 2556 | Train 40 | Test 60 | 0.78 | 0.28 | 0.73 | 0.41 |
| 509 | Train 8 | Test 61 | 0.78 | 0.28 | 0.66 | 0.4 | 2557 | Train 40 | Test 61 | 0.78 | 0.28 | 0.66 | 0.4 |
| 510 | Train 8 | Test 62 | 0.76 | 0.34 | 0.73 | 0.46 | 2558 | Train 40 | Test 62 | 0.76 | 0.34 | 0.73 | 0.46 |
| 511 | Train 8 | Test 63 | 0.78 | 0.28 | 0.66 | 0.4 | 2559 | Train 40 | Test 63 | 0.78 | 0.28 | 0.66 | 0.4 |
| 512 | Train 8 | Test 64 | 0.76 | 0.32 | 0.74 | 0.45 | 2560 | Train 40 | Test 64 | 0.76 | 0.32 | 0.74 | 0.45 |
| 513 | Train 9 | Test 1 | 0.76 | 0.24 | 0.66 | 0.35 | 2561 | Train 41 | Test 1 | 0.76 | 0.24 | 0.66 | 0.35 |
| 514 | Train 9 | Test 2 | 0.74 | 0.26 | 0.6 | 0.36 | 2562 | Train 41 | Test 2 | 0.74 | 0.26 | 0.6 | 0.36 |
| 515 | Train 9 | Test 3 | 0.76 | 0.24 | 0.66 | 0.35 | 2563 | Train 41 | Test 3 | 0.76 | 0.24 | 0.67 | 0.35 |
| 516 | Train 9 | Test 4 | 0.74 | 0.26 | 0.6 | 0.36 | 2564 | Train 41 | Test 4 | 0.74 | 0.26 | 0.6 | 0.36 |
| 517 | Train 9 | Test 5 | 0.71 | 0.22 | 0.47 | 0.3 | 2565 | Train 41 | Test 5 | 0.71 | 0.22 | 0.48 | 0.3 |
| 518 | Train 9 | Test 6 | 0.69 | 0.31 | 0.28 | 0.3 | 2566 | Train 41 | Test 6 | 0.69 | 0.31 | 0.29 | 0.3 |
| 519 | Train 9 | Test 7 | 0.71 | 0.22 | 0.47 | 0.3 | 2567 | Train 41 | Test 7 | 0.71 | 0.22 | 0.47 | 0.3 |
| 520 | Train 9 | Test 8 | 0.69 | 0.3 | 0.28 | 0.29 | 2568 | Train 41 | Test 8 | 0.69 | 0.3 | 0.29 | 0.29 |
| 521 | Train 9 | Test 9 | 0.77 | 0.24 | 0.65 | 0.35 | 2569 | Train 41 | Test 9 | 0.77 | 0.24 | 0.65 | 0.35 |
| 522 | Train 9 | Test 10 | 0.75 | 0.27 | 0.59 | 0.37 | 2570 | Train 41 | Test 10 | 0.75 | 0.26 | 0.59 | 0.36 |
| 523 | Train 9 | Test 11 | 0.77 | 0.24 | 0.65 | 0.35 | 2571 | Train 41 | Test 11 | 0.77 | 0.24 | 0.65 | 0.35 |
| 524 | Train 9 | Test 12 | 0.75 | 0.27 | 0.59 | 0.37 | 2572 | Train 41 | Test 12 | 0.75 | 0.26 | 0.59 | 0.36 |
| 525 | Train 9 | Test 13 | 0.71 | 0.23 | 0.47 | 0.31 | 2573 | Train 41 | Test 13 | 0.71 | 0.23 | 0.47 | 0.31 |
| 526 | Train 9 | Test 14 | 0.69 | 0.31 | 0.28 | 0.3 | 2574 | Train 41 | Test 14 | 0.69 | 0.31 | 0.29 | 0.3 |
| 527 | Train 9 | Test 15 | 0.71 | 0.23 | 0.46 | 0.31 | 2575 | Train 41 | Test 15 | 0.71 | 0.23 | 0.46 | 0.3 |
| 528 | Train 9 | Test 16 | 0.69 | 0.3 | 0.28 | 0.29 | 2576 | Train 41 | Test 16 | 0.69 | 0.3 | 0.29 | 0.29 |
| 529 | Train 9 | Test 17 | 0.76 | 0.24 | 0.66 | 0.35 | 2577 | Train 41 | Test 17 | 0.76 | 0.24 | 0.66 | 0.35 |
| 530 | Train 9 | Test 18 | 0.74 | 0.26 | 0.6 | 0.36 | 2578 | Train 41 | Test 18 | 0.74 | 0.26 | 0.6 | 0.36 |
| 531 | Train 9 | Test 19 | 0.76 | 0.24 | 0.66 | 0.35 | 2579 | Train 41 | Test 19 | 0.76 | 0.24 | 0.67 | 0.35 |
| 532 | Train 9 | Test 20 | 0.74 | 0.26 | 0.6 | 0.36 | 2580 | Train 41 | Test 20 | 0.74 | 0.26 | 0.6 | 0.36 |
| 533 | Train 9 | Test 21 | 0.71 | 0.22 | 0.47 | 0.3 | 2581 | Train 41 | Test 21 | 0.71 | 0.22 | 0.48 | 0.3 |
| 534 | Train 9 | Test 22 | 0.69 | 0.31 | 0.28 | 0.3 | 2582 | Train 41 | Test 22 | 0.69 | 0.31 | 0.29 | 0.3 |
| 535 | Train 9 | Test 23 | 0.71 | 0.22 | 0.47 | 0.3 | 2583 | Train 41 | Test 23 | 0.71 | 0.22 | 0.47 | 0.3 |
| 536 | Train 9 | Test 24 | 0.69 | 0.3 | 0.28 | 0.29 | 2584 | Train 41 | Test 24 | 0.69 | 0.3 | 0.29 | 0.29 |
| 537 | Train 9 | Test 25 | 0.77 | 0.24 | 0.65 | 0.35 | 2585 | Train 41 | Test 25 | 0.77 | 0.24 | 0.65 | 0.35 |
| 538 | Train 9 | Test 26 | 0.75 | 0.27 | 0.59 | 0.37 | 2586 | Train 41 | Test 26 | 0.75 | 0.26 | 0.59 | 0.36 |
| 539 | Train 9 | Test 27 | 0.77 | 0.24 | 0.65 | 0.35 | 2587 | Train 41 | Test 27 | 0.77 | 0.24 | 0.65 | 0.35 |
| 540 | Train 9 | Test 28 | 0.75 | 0.27 | 0.59 | 0.37 | 2588 | Train 41 | Test 28 | 0.75 | 0.26 | 0.59 | 0.36 |
| 541 | Train 9 | Test 29 | 0.71 | 0.23 | 0.47 | 0.31 | 2589 | Train 41 | Test 29 | 0.71 | 0.23 | 0.47 | 0.31 |
| 542 | Train 9 | Test 30 | 0.69 | 0.31 | 0.28 | 0.3 | 2590 | Train 41 | Test 30 | 0.69 | 0.31 | 0.29 | 0.3 |
| 543 | Train 9 | Test 31 | 0.71 | 0.23 | 0.46 | 0.31 | 2591 | Train 41 | Test 31 | 0.71 | 0.23 | 0.46 | 0.3 |
| 544 | Train 9 | Test 32 | 0.69 | 0.3 | 0.28 | 0.29 | 2592 | Train 41 | Test 32 | 0.69 | 0.3 | 0.29 | 0.29 |
| 545 | Train 9 | Test 33 | 0.77 | 0.24 | 0.66 | 0.35 | 2593 | Train 41 | Test 33 | 0.77 | 0.24 | 0.66 | 0.35 |
| 546 | Train 9 | Test 34 | 0.75 | 0.26 | 0.6 | 0.36 | 2594 | Train 41 | Test 34 | 0.75 | 0.26 | 0.6 | 0.36 |
| 547 | Train 9 | Test 35 | 0.77 | 0.24 | 0.66 | 0.35 | 2595 | Train 41 | Test 35 | 0.77 | 0.24 | 0.66 | 0.35 |
| 548 | Train 9 | Test 36 | 0.75 | 0.26 | 0.6 | 0.36 | 2596 | Train 41 | Test 36 | 0.75 | 0.26 | 0.6 | 0.36 |
| 549 | Train 9 | Test 37 | 0.71 | 0.22 | 0.47 | 0.3 | 2597 | Train 41 | Test 37 | 0.71 | 0.22 | 0.48 | 0.3 |
| 550 | Train 9 | Test 38 | 0.69 | 0.31 | 0.28 | 0.3 | 2598 | Train 41 | Test 38 | 0.69 | 0.31 | 0.29 | 0.3 |
| 551 | Train 9 | Test 39 | 0.71 | 0.22 | 0.47 | 0.3 | 2599 | Train 41 | Test 39 | 0.71 | 0.22 | 0.47 | 0.3 |
| 552 | Train 9 | Test 40 | 0.69 | 0.3 | 0.28 | 0.29 | 2600 | Train 41 | Test 40 | 0.69 | 0.3 | 0.29 | 0.29 |
| 553 | Train 9 | Test 41 | 0.77 | 0.24 | 0.65 | 0.35 | 2601 | Train 41 | Test 41 | 0.77 | 0.24 | 0.65 | 0.35 |
| 554 | Train 9 | Test 42 | 0.75 | 0.27 | 0.58 | 0.36 | 2602 | Train 41 | Test 42 | 0.75 | 0.26 | 0.58 | 0.36 |
| 555 | Train 9 | Test 43 | 0.77 | 0.24 | 0.65 | 0.35 | 2603 | Train 41 | Test 43 | 0.77 | 0.24 | 0.65 | 0.35 |
| 556 | Train 9 | Test 44 | 0.75 | 0.27 | 0.59 | 0.37 | 2604 | Train 41 | Test 44 | 0.75 | 0.26 | 0.59 | 0.36 |
| 557 | Train 9 | Test 45 | 0.71 | 0.23 | 0.47 | 0.31 | 2605 | Train 41 | Test 45 | 0.71 | 0.23 | 0.47 | 0.31 |
| 558 | Train 9 | Test 46 | 0.69 | 0.31 | 0.28 | 0.3 | 2606 | Train 41 | Test 46 | 0.69 | 0.31 | 0.29 | 0.3 |
| 559 | Train 9 | Test 47 | 0.71 | 0.23 | 0.46 | 0.31 | 2607 | Train 41 | Test 47 | 0.71 | 0.23 | 0.46 | 0.3 |
| 560 | Train 9 | Test 48 | 0.69 | 0.3 | 0.28 | 0.29 | 2608 | Train 41 | Test 48 | 0.69 | 0.3 | 0.29 | 0.29 |
| 561 | Train 9 | Test 49 | 0.77 | 0.27 | 0.75 | 0.39 | 2609 | Train 41 | Test 49 | 0.77 | 0.27 | 0.75 | 0.4 |
| 562 | Train 9 | Test 50 | 0.77 | 0.27 | 0.74 | 0.39 | 2610 | Train 41 | Test 50 | 0.77 | 0.27 | 0.75 | 0.4 |
| 563 | Train 9 | Test 51 | 0.77 | 0.27 | 0.75 | 0.39 | 2611 | Train 41 | Test 51 | 0.77 | 0.27 | 0.76 | 0.4 |
| 564 | Train 9 | Test 52 | 0.77 | 0.27 | 0.74 | 0.39 | 2612 | Train 41 | Test 52 | 0.77 | 0.27 | 0.75 | 0.4 |
| 565 | Train 9 | Test 53 | 0.75 | 0.28 | 0.44 | 0.34 | 2613 | Train 41 | Test 53 | 0.75 | 0.28 | 0.44 | 0.34 |
| 566 | Train 9 | Test 54 | 0.71 | 0.36 | 0.29 | 0.32 | 2614 | Train 41 | Test 54 | 0.71 | 0.36 | 0.29 | 0.32 |
| 567 | Train 9 | Test 55 | 0.75 | 0.26 | 0.53 | 0.35 | 2615 | Train 41 | Test 55 | 0.75 | 0.27 | 0.53 | 0.36 |
| 568 | Train 9 | Test 56 | 0.7 | 0.34 | 0.29 | 0.31 | 2616 | Train 41 | Test 56 | 0.7 | 0.34 | 0.3 | 0.32 |
| 569 | Train 9 | Test 57 | 0.78 | 0.27 | 0.75 | 0.4 | 2617 | Train 41 | Test 57 | 0.78 | 0.27 | 0.75 | 0.4 |
| 570 | Train 9 | Test 58 | 0.78 | 0.27 | 0.74 | 0.4 | 2618 | Train 41 | Test 58 | 0.78 | 0.27 | 0.74 | 0.4 |
| 571 | Train 9 | Test 59 | 0.78 | 0.27 | 0.75 | 0.4 | 2619 | Train 41 | Test 59 | 0.78 | 0.27 | 0.75 | 0.4 |
| 572 | Train 9 | Test 60 | 0.78 | 0.27 | 0.74 | 0.4 | 2620 | Train 41 | Test 60 | 0.78 | 0.27 | 0.75 | 0.4 |
| 573 | Train 9 | Test 61 | 0.75 | 0.3 | 0.44 | 0.35 | 2621 | Train 41 | Test 61 | 0.75 | 0.29 | 0.44 | 0.35 |
| 574 | Train 9 | Test 62 | 0.71 | 0.36 | 0.29 | 0.32 | 2622 | Train 41 | Test 62 | 0.71 | 0.36 | 0.3 | 0.33 |
| 575 | Train 9 | Test 63 | 0.75 | 0.29 | 0.45 | 0.35 | 2623 | Train 41 | Test 63 | 0.75 | 0.29 | 0.44 | 0.35 |
| 576 | Train 9 | Test 64 | 0.7 | 0.34 | 0.3 | 0.32 | 2624 | Train 41 | Test 64 | 0.7 | 0.34 | 0.3 | 0.32 |
| 577 | Train 10 | Test 1 | 0.77 | 0.25 | 0.63 | 0.35 | 2625 | Train 42 | Test 1 | 0.76 | 0.24 | 0.63 | 0.35 |
| 578 | Train 10 | Test 2 | 0.78 | 0.28 | 0.69 | 0.39 | 2626 | Train 42 | Test 2 | 0.78 | 0.27 | 0.69 | 0.39 |
| 579 | Train 10 | Test 3 | 0.77 | 0.25 | 0.63 | 0.36 | 2627 | Train 42 | Test 3 | 0.76 | 0.24 | 0.63 | 0.35 |
| 580 | Train 10 | Test 4 | 0.78 | 0.28 | 0.69 | 0.39 | 2628 | Train 42 | Test 4 | 0.78 | 0.27 | 0.69 | 0.39 |
| 581 | Train 10 | Test 5 | 0.72 | 0.23 | 0.47 | 0.31 | 2629 | Train 42 | Test 5 | 0.72 | 0.23 | 0.48 | 0.31 |
| 582 | Train 10 | Test 6 | 0.71 | 0.34 | 0.34 | 0.34 | 2630 | Train 42 | Test 6 | 0.71 | 0.34 | 0.36 | 0.35 |
| 583 | Train 10 | Test 7 | 0.71 | 0.23 | 0.47 | 0.31 | 2631 | Train 42 | Test 7 | 0.71 | 0.23 | 0.48 | 0.31 |
| 584 | Train 10 | Test 8 | 0.71 | 0.33 | 0.34 | 0.34 | 2632 | Train 42 | Test 8 | 0.7 | 0.33 | 0.36 | 0.34 |
| 585 | Train 10 | Test 9 | 0.77 | 0.25 | 0.61 | 0.36 | 2633 | Train 42 | Test 9 | 0.77 | 0.25 | 0.61 | 0.35 |
| 586 | Train 10 | Test 10 | 0.78 | 0.28 | 0.68 | 0.4 | 2634 | Train 42 | Test 10 | 0.78 | 0.28 | 0.68 | 0.39 |
| 587 | Train 10 | Test 11 | 0.77 | 0.25 | 0.61 | 0.36 | 2635 | Train 42 | Test 11 | 0.77 | 0.25 | 0.62 | 0.35 |
| 588 | Train 10 | Test 12 | 0.78 | 0.28 | 0.68 | 0.4 | 2636 | Train 42 | Test 12 | 0.78 | 0.28 | 0.68 | 0.4 |
| 589 | Train 10 | Test 13 | 0.72 | 0.23 | 0.46 | 0.31 | 2637 | Train 42 | Test 13 | 0.72 | 0.23 | 0.47 | 0.31 |
| 590 | Train 10 | Test 14 | 0.71 | 0.34 | 0.34 | 0.34 | 2638 | Train 42 | Test 14 | 0.71 | 0.34 | 0.36 | 0.35 |
| 591 | Train 10 | Test 15 | 0.72 | 0.23 | 0.45 | 0.3 | 2639 | Train 42 | Test 15 | 0.72 | 0.23 | 0.46 | 0.31 |
| 592 | Train 10 | Test 16 | 0.71 | 0.33 | 0.34 | 0.34 | 2640 | Train 42 | Test 16 | 0.71 | 0.33 | 0.36 | 0.34 |
| 593 | Train 10 | Test 17 | 0.77 | 0.25 | 0.63 | 0.35 | 2641 | Train 42 | Test 17 | 0.76 | 0.24 | 0.63 | 0.35 |
| 594 | Train 10 | Test 18 | 0.78 | 0.28 | 0.69 | 0.39 | 2642 | Train 42 | Test 18 | 0.78 | 0.27 | 0.69 | 0.39 |
| 595 | Train 10 | Test 19 | 0.77 | 0.25 | 0.63 | 0.36 | 2643 | Train 42 | Test 19 | 0.76 | 0.25 | 0.63 | 0.35 |
| 596 | Train 10 | Test 20 | 0.78 | 0.28 | 0.69 | 0.39 | 2644 | Train 42 | Test 20 | 0.78 | 0.27 | 0.69 | 0.39 |
| 597 | Train 10 | Test 21 | 0.72 | 0.23 | 0.47 | 0.31 | 2645 | Train 42 | Test 21 | 0.72 | 0.23 | 0.49 | 0.31 |
| 598 | Train 10 | Test 22 | 0.71 | 0.34 | 0.34 | 0.34 | 2646 | Train 42 | Test 22 | 0.71 | 0.34 | 0.36 | 0.35 |
| 599 | Train 10 | Test 23 | 0.71 | 0.23 | 0.47 | 0.31 | 2647 | Train 42 | Test 23 | 0.71 | 0.23 | 0.48 | 0.31 |
| 600 | Train 10 | Test 24 | 0.71 | 0.33 | 0.34 | 0.34 | 2648 | Train 42 | Test 24 | 0.71 | 0.33 | 0.36 | 0.34 |
| 601 | Train 10 | Test 25 | 0.77 | 0.25 | 0.61 | 0.36 | 2649 | Train 42 | Test 25 | 0.77 | 0.25 | 0.61 | 0.35 |
| 602 | Train 10 | Test 26 | 0.78 | 0.28 | 0.68 | 0.4 | 2650 | Train 42 | Test 26 | 0.78 | 0.28 | 0.68 | 0.39 |
| 603 | Train 10 | Test 27 | 0.77 | 0.25 | 0.61 | 0.36 | 2651 | Train 42 | Test 27 | 0.77 | 0.25 | 0.62 | 0.36 |
| 604 | Train 10 | Test 28 | 0.78 | 0.28 | 0.68 | 0.4 | 2652 | Train 42 | Test 28 | 0.78 | 0.28 | 0.68 | 0.4 |
| 605 | Train 10 | Test 29 | 0.72 | 0.23 | 0.46 | 0.31 | 2653 | Train 42 | Test 29 | 0.72 | 0.23 | 0.47 | 0.31 |
| 606 | Train 10 | Test 30 | 0.71 | 0.34 | 0.34 | 0.34 | 2654 | Train 42 | Test 30 | 0.71 | 0.34 | 0.36 | 0.35 |
| 607 | Train 10 | Test 31 | 0.72 | 0.23 | 0.45 | 0.31 | 2655 | Train 42 | Test 31 | 0.72 | 0.23 | 0.46 | 0.31 |
| 608 | Train 10 | Test 32 | 0.71 | 0.33 | 0.34 | 0.33 | 2656 | Train 42 | Test 32 | 0.71 | 0.33 | 0.36 | 0.34 |
| 609 | Train 10 | Test 33 | 0.77 | 0.25 | 0.62 | 0.35 | 2657 | Train 42 | Test 33 | 0.77 | 0.24 | 0.63 | 0.35 |
| 610 | Train 10 | Test 34 | 0.78 | 0.28 | 0.68 | 0.39 | 2658 | Train 42 | Test 34 | 0.78 | 0.27 | 0.69 | 0.39 |
| 611 | Train 10 | Test 35 | 0.77 | 0.25 | 0.63 | 0.35 | 2659 | Train 42 | Test 35 | 0.77 | 0.24 | 0.63 | 0.35 |
| 612 | Train 10 | Test 36 | 0.78 | 0.28 | 0.69 | 0.39 | 2660 | Train 42 | Test 36 | 0.78 | 0.27 | 0.69 | 0.39 |
| 613 | Train 10 | Test 37 | 0.72 | 0.23 | 0.47 | 0.31 | 2661 | Train 42 | Test 37 | 0.72 | 0.23 | 0.48 | 0.31 |
| 614 | Train 10 | Test 38 | 0.71 | 0.34 | 0.34 | 0.34 | 2662 | Train 42 | Test 38 | 0.71 | 0.34 | 0.36 | 0.35 |
| 615 | Train 10 | Test 39 | 0.72 | 0.23 | 0.47 | 0.31 | 2663 | Train 42 | Test 39 | 0.72 | 0.23 | 0.48 | 0.31 |
| 616 | Train 10 | Test 40 | 0.71 | 0.33 | 0.34 | 0.34 | 2664 | Train 42 | Test 40 | 0.71 | 0.33 | 0.36 | 0.34 |
| 617 | Train 10 | Test 41 | 0.77 | 0.25 | 0.61 | 0.36 | 2665 | Train 42 | Test 41 | 0.77 | 0.25 | 0.61 | 0.35 |
| 618 | Train 10 | Test 42 | 0.78 | 0.28 | 0.67 | 0.4 | 2666 | Train 42 | Test 42 | 0.78 | 0.28 | 0.68 | 0.39 |
| 619 | Train 10 | Test 43 | 0.77 | 0.25 | 0.61 | 0.36 | 2667 | Train 42 | Test 43 | 0.77 | 0.25 | 0.61 | 0.35 |
| 620 | Train 10 | Test 44 | 0.78 | 0.28 | 0.67 | 0.4 | 2668 | Train 42 | Test 44 | 0.78 | 0.28 | 0.68 | 0.39 |
| 621 | Train 10 | Test 45 | 0.72 | 0.23 | 0.46 | 0.31 | 2669 | Train 42 | Test 45 | 0.72 | 0.23 | 0.47 | 0.31 |
| 622 | Train 10 | Test 46 | 0.71 | 0.34 | 0.34 | 0.34 | 2670 | Train 42 | Test 46 | 0.71 | 0.34 | 0.36 | 0.35 |
| 623 | Train 10 | Test 47 | 0.72 | 0.23 | 0.45 | 0.31 | 2671 | Train 42 | Test 47 | 0.72 | 0.23 | 0.46 | 0.31 |
| 624 | Train 10 | Test 48 | 0.71 | 0.33 | 0.34 | 0.34 | 2672 | Train 42 | Test 48 | 0.71 | 0.33 | 0.36 | 0.34 |
| 625 | Train 10 | Test 49 | 0.77 | 0.27 | 0.73 | 0.4 | 2673 | Train 42 | Test 49 | 0.77 | 0.27 | 0.74 | 0.4 |
| 626 | Train 10 | Test 50 | 0.77 | 0.27 | 0.73 | 0.4 | 2674 | Train 42 | Test 50 | 0.77 | 0.27 | 0.74 | 0.4 |
| 627 | Train 10 | Test 51 | 0.77 | 0.28 | 0.73 | 0.4 | 2675 | Train 42 | Test 51 | 0.77 | 0.28 | 0.74 | 0.4 |
| 628 | Train 10 | Test 52 | 0.77 | 0.28 | 0.73 | 0.4 | 2676 | Train 42 | Test 52 | 0.77 | 0.28 | 0.74 | 0.4 |
| 629 | Train 10 | Test 53 | 0.75 | 0.3 | 0.39 | 0.34 | 2677 | Train 42 | Test 53 | 0.75 | 0.31 | 0.42 | 0.35 |
| 630 | Train 10 | Test 54 | 0.72 | 0.39 | 0.32 | 0.35 | 2678 | Train 42 | Test 54 | 0.72 | 0.39 | 0.34 | 0.36 |
| 631 | Train 10 | Test 55 | 0.75 | 0.27 | 0.52 | 0.36 | 2679 | Train 42 | Test 55 | 0.76 | 0.28 | 0.55 | 0.37 |
| 632 | Train 10 | Test 56 | 0.71 | 0.36 | 0.33 | 0.34 | 2680 | Train 42 | Test 56 | 0.71 | 0.36 | 0.35 | 0.35 |
| 633 | Train 10 | Test 57 | 0.78 | 0.28 | 0.73 | 0.4 | 2681 | Train 42 | Test 57 | 0.78 | 0.28 | 0.73 | 0.4 |
| 634 | Train 10 | Test 58 | 0.78 | 0.28 | 0.73 | 0.4 | 2682 | Train 42 | Test 58 | 0.78 | 0.28 | 0.73 | 0.4 |
| 635 | Train 10 | Test 59 | 0.78 | 0.28 | 0.73 | 0.41 | 2683 | Train 42 | Test 59 | 0.78 | 0.28 | 0.74 | 0.41 |
| 636 | Train 10 | Test 60 | 0.78 | 0.28 | 0.73 | 0.41 | 2684 | Train 42 | Test 60 | 0.78 | 0.28 | 0.73 | 0.41 |
| 637 | Train 10 | Test 61 | 0.76 | 0.3 | 0.38 | 0.34 | 2685 | Train 42 | Test 61 | 0.76 | 0.31 | 0.41 | 0.36 |
| 638 | Train 10 | Test 62 | 0.72 | 0.38 | 0.32 | 0.35 | 2686 | Train 42 | Test 62 | 0.72 | 0.39 | 0.34 | 0.36 |
| 639 | Train 10 | Test 63 | 0.76 | 0.3 | 0.39 | 0.34 | 2687 | Train 42 | Test 63 | 0.76 | 0.31 | 0.42 | 0.36 |
| 640 | Train 10 | Test 64 | 0.72 | 0.35 | 0.33 | 0.34 | 2688 | Train 42 | Test 64 | 0.72 | 0.36 | 0.35 | 0.35 |
| 641 | Train 11 | Test 1 | 0.77 | 0.24 | 0.67 | 0.35 | 2689 | Train 43 | Test 1 | 0.76 | 0.24 | 0.67 | 0.35 |
| 642 | Train 11 | Test 2 | 0.75 | 0.26 | 0.61 | 0.37 | 2690 | Train 43 | Test 2 | 0.74 | 0.26 | 0.61 | 0.37 |
| 643 | Train 11 | Test 3 | 0.77 | 0.24 | 0.67 | 0.35 | 2691 | Train 43 | Test 3 | 0.76 | 0.24 | 0.67 | 0.35 |
| 644 | Train 11 | Test 4 | 0.75 | 0.26 | 0.62 | 0.37 | 2692 | Train 43 | Test 4 | 0.74 | 0.26 | 0.62 | 0.37 |
| 645 | Train 11 | Test 5 | 0.71 | 0.23 | 0.48 | 0.31 | 2693 | Train 43 | Test 5 | 0.71 | 0.22 | 0.48 | 0.31 |
| 646 | Train 11 | Test 6 | 0.69 | 0.32 | 0.27 | 0.29 | 2694 | Train 43 | Test 6 | 0.69 | 0.31 | 0.28 | 0.29 |
| 647 | Train 11 | Test 7 | 0.71 | 0.23 | 0.47 | 0.31 | 2695 | Train 43 | Test 7 | 0.71 | 0.22 | 0.48 | 0.3 |
| 648 | Train 11 | Test 8 | 0.69 | 0.31 | 0.28 | 0.29 | 2696 | Train 43 | Test 8 | 0.69 | 0.3 | 0.28 | 0.29 |
| 649 | Train 11 | Test 9 | 0.77 | 0.24 | 0.66 | 0.35 | 2697 | Train 43 | Test 9 | 0.77 | 0.24 | 0.65 | 0.35 |
| 650 | Train 11 | Test 10 | 0.75 | 0.27 | 0.6 | 0.37 | 2698 | Train 43 | Test 10 | 0.75 | 0.26 | 0.59 | 0.37 |
| 651 | Train 11 | Test 11 | 0.77 | 0.24 | 0.66 | 0.35 | 2699 | Train 43 | Test 11 | 0.77 | 0.24 | 0.66 | 0.35 |
| 652 | Train 11 | Test 12 | 0.75 | 0.27 | 0.6 | 0.37 | 2700 | Train 43 | Test 12 | 0.75 | 0.26 | 0.6 | 0.37 |
| 653 | Train 11 | Test 13 | 0.71 | 0.23 | 0.47 | 0.31 | 2701 | Train 43 | Test 13 | 0.71 | 0.23 | 0.47 | 0.31 |
| 654 | Train 11 | Test 14 | 0.69 | 0.32 | 0.28 | 0.29 | 2702 | Train 43 | Test 14 | 0.69 | 0.31 | 0.28 | 0.29 |
| 655 | Train 11 | Test 15 | 0.71 | 0.23 | 0.46 | 0.31 | 2703 | Train 43 | Test 15 | 0.71 | 0.23 | 0.46 | 0.3 |
| 656 | Train 11 | Test 16 | 0.69 | 0.31 | 0.28 | 0.29 | 2704 | Train 43 | Test 16 | 0.69 | 0.3 | 0.28 | 0.29 |
| 657 | Train 11 | Test 17 | 0.77 | 0.24 | 0.67 | 0.35 | 2705 | Train 43 | Test 17 | 0.76 | 0.24 | 0.67 | 0.35 |
| 658 | Train 11 | Test 18 | 0.75 | 0.26 | 0.61 | 0.37 | 2706 | Train 43 | Test 18 | 0.74 | 0.26 | 0.61 | 0.37 |
| 659 | Train 11 | Test 19 | 0.77 | 0.24 | 0.67 | 0.35 | 2707 | Train 43 | Test 19 | 0.76 | 0.24 | 0.67 | 0.35 |
| 660 | Train 11 | Test 20 | 0.75 | 0.26 | 0.62 | 0.37 | 2708 | Train 43 | Test 20 | 0.74 | 0.26 | 0.62 | 0.37 |
| 661 | Train 11 | Test 21 | 0.71 | 0.23 | 0.48 | 0.31 | 2709 | Train 43 | Test 21 | 0.71 | 0.22 | 0.48 | 0.31 |
| 662 | Train 11 | Test 22 | 0.69 | 0.32 | 0.27 | 0.29 | 2710 | Train 43 | Test 22 | 0.69 | 0.31 | 0.28 | 0.29 |
| 663 | Train 11 | Test 23 | 0.71 | 0.23 | 0.47 | 0.31 | 2711 | Train 43 | Test 23 | 0.71 | 0.22 | 0.48 | 0.3 |
| 664 | Train 11 | Test 24 | 0.69 | 0.31 | 0.28 | 0.29 | 2712 | Train 43 | Test 24 | 0.69 | 0.3 | 0.28 | 0.29 |
| 665 | Train 11 | Test 25 | 0.77 | 0.24 | 0.66 | 0.35 | 2713 | Train 43 | Test 25 | 0.77 | 0.24 | 0.65 | 0.35 |
| 666 | Train 11 | Test 26 | 0.75 | 0.27 | 0.6 | 0.37 | 2714 | Train 43 | Test 26 | 0.75 | 0.26 | 0.59 | 0.37 |
| 667 | Train 11 | Test 27 | 0.77 | 0.24 | 0.66 | 0.36 | 2715 | Train 43 | Test 27 | 0.77 | 0.24 | 0.66 | 0.35 |
| 668 | Train 11 | Test 28 | 0.75 | 0.27 | 0.6 | 0.37 | 2716 | Train 43 | Test 28 | 0.75 | 0.26 | 0.6 | 0.37 |
| 669 | Train 11 | Test 29 | 0.71 | 0.23 | 0.47 | 0.31 | 2717 | Train 43 | Test 29 | 0.71 | 0.23 | 0.47 | 0.31 |
| 670 | Train 11 | Test 30 | 0.69 | 0.32 | 0.28 | 0.29 | 2718 | Train 43 | Test 30 | 0.69 | 0.31 | 0.28 | 0.29 |
| 671 | Train 11 | Test 31 | 0.71 | 0.23 | 0.46 | 0.31 | 2719 | Train 43 | Test 31 | 0.71 | 0.23 | 0.46 | 0.3 |
| 672 | Train 11 | Test 32 | 0.69 | 0.31 | 0.28 | 0.29 | 2720 | Train 43 | Test 32 | 0.69 | 0.3 | 0.28 | 0.29 |
| 673 | Train 11 | Test 33 | 0.77 | 0.24 | 0.67 | 0.35 | 2721 | Train 43 | Test 33 | 0.77 | 0.24 | 0.67 | 0.35 |
| 674 | Train 11 | Test 34 | 0.75 | 0.26 | 0.61 | 0.37 | 2722 | Train 43 | Test 34 | 0.75 | 0.26 | 0.61 | 0.37 |
| 675 | Train 11 | Test 35 | 0.77 | 0.24 | 0.67 | 0.35 | 2723 | Train 43 | Test 35 | 0.77 | 0.24 | 0.67 | 0.35 |
| 676 | Train 11 | Test 36 | 0.75 | 0.26 | 0.61 | 0.37 | 2724 | Train 43 | Test 36 | 0.75 | 0.26 | 0.61 | 0.37 |
| 677 | Train 11 | Test 37 | 0.71 | 0.23 | 0.48 | 0.31 | 2725 | Train 43 | Test 37 | 0.71 | 0.22 | 0.48 | 0.31 |
| 678 | Train 11 | Test 38 | 0.69 | 0.32 | 0.27 | 0.29 | 2726 | Train 43 | Test 38 | 0.69 | 0.31 | 0.28 | 0.29 |
| 679 | Train 11 | Test 39 | 0.71 | 0.23 | 0.47 | 0.31 | 2727 | Train 43 | Test 39 | 0.71 | 0.22 | 0.48 | 0.3 |
| 680 | Train 11 | Test 40 | 0.69 | 0.31 | 0.28 | 0.29 | 2728 | Train 43 | Test 40 | 0.69 | 0.3 | 0.28 | 0.29 |
| 681 | Train 11 | Test 41 | 0.77 | 0.24 | 0.65 | 0.35 | 2729 | Train 43 | Test 41 | 0.77 | 0.24 | 0.65 | 0.35 |
| 682 | Train 11 | Test 42 | 0.75 | 0.27 | 0.6 | 0.37 | 2730 | Train 43 | Test 42 | 0.75 | 0.26 | 0.59 | 0.36 |
| 683 | Train 11 | Test 43 | 0.77 | 0.24 | 0.66 | 0.35 | 2731 | Train 43 | Test 43 | 0.77 | 0.24 | 0.65 | 0.35 |
| 684 | Train 11 | Test 44 | 0.75 | 0.27 | 0.6 | 0.37 | 2732 | Train 43 | Test 44 | 0.75 | 0.26 | 0.59 | 0.37 |
| 685 | Train 11 | Test 45 | 0.72 | 0.23 | 0.47 | 0.31 | 2733 | Train 43 | Test 45 | 0.72 | 0.23 | 0.47 | 0.31 |
| 686 | Train 11 | Test 46 | 0.69 | 0.32 | 0.28 | 0.29 | 2734 | Train 43 | Test 46 | 0.69 | 0.31 | 0.28 | 0.29 |
| 687 | Train 11 | Test 47 | 0.71 | 0.23 | 0.46 | 0.31 | 2735 | Train 43 | Test 47 | 0.71 | 0.23 | 0.46 | 0.3 |
| 688 | Train 11 | Test 48 | 0.69 | 0.31 | 0.28 | 0.29 | 2736 | Train 43 | Test 48 | 0.69 | 0.3 | 0.28 | 0.29 |
| 689 | Train 11 | Test 49 | 0.77 | 0.27 | 0.77 | 0.4 | 2737 | Train 43 | Test 49 | 0.77 | 0.27 | 0.76 | 0.39 |
| 690 | Train 11 | Test 50 | 0.77 | 0.27 | 0.76 | 0.4 | 2738 | Train 43 | Test 50 | 0.77 | 0.27 | 0.75 | 0.39 |
| 691 | Train 11 | Test 51 | 0.77 | 0.27 | 0.77 | 0.4 | 2739 | Train 43 | Test 51 | 0.77 | 0.27 | 0.76 | 0.39 |
| 692 | Train 11 | Test 52 | 0.77 | 0.27 | 0.76 | 0.4 | 2740 | Train 43 | Test 52 | 0.77 | 0.27 | 0.75 | 0.39 |
| 693 | Train 11 | Test 53 | 0.75 | 0.29 | 0.43 | 0.34 | 2741 | Train 43 | Test 53 | 0.75 | 0.28 | 0.44 | 0.34 |
| 694 | Train 11 | Test 54 | 0.71 | 0.37 | 0.28 | 0.32 | 2742 | Train 43 | Test 54 | 0.71 | 0.36 | 0.29 | 0.32 |
| 695 | Train 11 | Test 55 | 0.75 | 0.27 | 0.53 | 0.36 | 2743 | Train 43 | Test 55 | 0.75 | 0.27 | 0.53 | 0.36 |
| 696 | Train 11 | Test 56 | 0.7 | 0.34 | 0.29 | 0.31 | 2744 | Train 43 | Test 56 | 0.7 | 0.33 | 0.29 | 0.31 |
| 697 | Train 11 | Test 57 | 0.78 | 0.27 | 0.75 | 0.4 | 2745 | Train 43 | Test 57 | 0.78 | 0.27 | 0.75 | 0.4 |
| 698 | Train 11 | Test 58 | 0.78 | 0.27 | 0.75 | 0.4 | 2746 | Train 43 | Test 58 | 0.78 | 0.27 | 0.75 | 0.4 |
| 699 | Train 11 | Test 59 | 0.78 | 0.27 | 0.75 | 0.4 | 2747 | Train 43 | Test 59 | 0.78 | 0.27 | 0.75 | 0.4 |
| 700 | Train 11 | Test 60 | 0.78 | 0.27 | 0.75 | 0.4 | 2748 | Train 43 | Test 60 | 0.78 | 0.27 | 0.75 | 0.4 |
| 701 | Train 11 | Test 61 | 0.75 | 0.3 | 0.42 | 0.35 | 2749 | Train 43 | Test 61 | 0.75 | 0.29 | 0.44 | 0.35 |
| 702 | Train 11 | Test 62 | 0.71 | 0.37 | 0.29 | 0.32 | 2750 | Train 43 | Test 62 | 0.71 | 0.36 | 0.29 | 0.32 |
| 703 | Train 11 | Test 63 | 0.75 | 0.3 | 0.43 | 0.35 | 2751 | Train 43 | Test 63 | 0.75 | 0.29 | 0.45 | 0.35 |
| 704 | Train 11 | Test 64 | 0.7 | 0.34 | 0.29 | 0.31 | 2752 | Train 43 | Test 64 | 0.7 | 0.33 | 0.3 | 0.31 |
| 705 | Train 12 | Test 1 | 0.77 | 0.25 | 0.63 | 0.35 | 2753 | Train 44 | Test 1 | 0.76 | 0.24 | 0.63 | 0.35 |
| 706 | Train 12 | Test 2 | 0.78 | 0.27 | 0.69 | 0.39 | 2754 | Train 44 | Test 2 | 0.78 | 0.27 | 0.69 | 0.39 |
| 707 | Train 12 | Test 3 | 0.77 | 0.25 | 0.63 | 0.35 | 2755 | Train 44 | Test 3 | 0.76 | 0.25 | 0.63 | 0.35 |
| 708 | Train 12 | Test 4 | 0.78 | 0.28 | 0.69 | 0.39 | 2756 | Train 44 | Test 4 | 0.78 | 0.28 | 0.69 | 0.39 |
| 709 | Train 12 | Test 5 | 0.72 | 0.23 | 0.48 | 0.31 | 2757 | Train 44 | Test 5 | 0.72 | 0.23 | 0.48 | 0.31 |
| 710 | Train 12 | Test 6 | 0.71 | 0.34 | 0.34 | 0.34 | 2758 | Train 44 | Test 6 | 0.7 | 0.34 | 0.35 | 0.35 |
| 711 | Train 12 | Test 7 | 0.71 | 0.23 | 0.47 | 0.31 | 2759 | Train 44 | Test 7 | 0.71 | 0.23 | 0.48 | 0.31 |
| 712 | Train 12 | Test 8 | 0.7 | 0.33 | 0.34 | 0.34 | 2760 | Train 44 | Test 8 | 0.7 | 0.33 | 0.35 | 0.34 |
| 713 | Train 12 | Test 9 | 0.77 | 0.25 | 0.61 | 0.35 | 2761 | Train 44 | Test 9 | 0.77 | 0.25 | 0.62 | 0.35 |
| 714 | Train 12 | Test 10 | 0.78 | 0.28 | 0.67 | 0.39 | 2762 | Train 44 | Test 10 | 0.78 | 0.28 | 0.68 | 0.4 |
| 715 | Train 12 | Test 11 | 0.77 | 0.25 | 0.62 | 0.36 | 2763 | Train 44 | Test 11 | 0.77 | 0.25 | 0.62 | 0.36 |
| 716 | Train 12 | Test 12 | 0.78 | 0.28 | 0.68 | 0.4 | 2764 | Train 44 | Test 12 | 0.78 | 0.28 | 0.68 | 0.4 |
| 717 | Train 12 | Test 13 | 0.72 | 0.23 | 0.46 | 0.31 | 2765 | Train 44 | Test 13 | 0.72 | 0.23 | 0.47 | 0.31 |
| 718 | Train 12 | Test 14 | 0.71 | 0.34 | 0.34 | 0.34 | 2766 | Train 44 | Test 14 | 0.71 | 0.34 | 0.36 | 0.35 |
| 719 | Train 12 | Test 15 | 0.72 | 0.23 | 0.46 | 0.31 | 2767 | Train 44 | Test 15 | 0.72 | 0.23 | 0.47 | 0.31 |
| 720 | Train 12 | Test 16 | 0.71 | 0.33 | 0.34 | 0.34 | 2768 | Train 44 | Test 16 | 0.7 | 0.33 | 0.36 | 0.34 |
| 721 | Train 12 | Test 17 | 0.77 | 0.25 | 0.63 | 0.35 | 2769 | Train 44 | Test 17 | 0.76 | 0.24 | 0.63 | 0.35 |
| 722 | Train 12 | Test 18 | 0.78 | 0.27 | 0.69 | 0.39 | 2770 | Train 44 | Test 18 | 0.78 | 0.27 | 0.69 | 0.39 |
| 723 | Train 12 | Test 19 | 0.77 | 0.25 | 0.63 | 0.35 | 2771 | Train 44 | Test 19 | 0.76 | 0.25 | 0.63 | 0.35 |
| 724 | Train 12 | Test 20 | 0.78 | 0.28 | 0.69 | 0.39 | 2772 | Train 44 | Test 20 | 0.78 | 0.28 | 0.69 | 0.39 |
| 725 | Train 12 | Test 21 | 0.72 | 0.23 | 0.48 | 0.31 | 2773 | Train 44 | Test 21 | 0.72 | 0.23 | 0.48 | 0.31 |
| 726 | Train 12 | Test 22 | 0.71 | 0.34 | 0.34 | 0.34 | 2774 | Train 44 | Test 22 | 0.71 | 0.34 | 0.35 | 0.35 |
| 727 | Train 12 | Test 23 | 0.72 | 0.23 | 0.47 | 0.31 | 2775 | Train 44 | Test 23 | 0.71 | 0.23 | 0.48 | 0.31 |
| 728 | Train 12 | Test 24 | 0.71 | 0.33 | 0.34 | 0.34 | 2776 | Train 44 | Test 24 | 0.7 | 0.33 | 0.35 | 0.34 |
| 729 | Train 12 | Test 25 | 0.77 | 0.25 | 0.61 | 0.35 | 2777 | Train 44 | Test 25 | 0.77 | 0.25 | 0.62 | 0.35 |
| 730 | Train 12 | Test 26 | 0.78 | 0.28 | 0.67 | 0.39 | 2778 | Train 44 | Test 26 | 0.78 | 0.28 | 0.68 | 0.4 |
| 731 | Train 12 | Test 27 | 0.77 | 0.25 | 0.62 | 0.36 | 2779 | Train 44 | Test 27 | 0.77 | 0.25 | 0.62 | 0.36 |
| 732 | Train 12 | Test 28 | 0.78 | 0.28 | 0.68 | 0.4 | 2780 | Train 44 | Test 28 | 0.78 | 0.28 | 0.68 | 0.4 |
| 733 | Train 12 | Test 29 | 0.72 | 0.23 | 0.46 | 0.31 | 2781 | Train 44 | Test 29 | 0.72 | 0.23 | 0.47 | 0.31 |
| 734 | Train 12 | Test 30 | 0.71 | 0.34 | 0.34 | 0.34 | 2782 | Train 44 | Test 30 | 0.71 | 0.34 | 0.36 | 0.35 |
| 735 | Train 12 | Test 31 | 0.72 | 0.23 | 0.46 | 0.31 | 2783 | Train 44 | Test 31 | 0.72 | 0.23 | 0.47 | 0.31 |
| 736 | Train 12 | Test 32 | 0.71 | 0.33 | 0.34 | 0.33 | 2784 | Train 44 | Test 32 | 0.71 | 0.33 | 0.36 | 0.34 |
| 737 | Train 12 | Test 33 | 0.77 | 0.24 | 0.62 | 0.35 | 2785 | Train 44 | Test 33 | 0.77 | 0.24 | 0.63 | 0.35 |
| 738 | Train 12 | Test 34 | 0.78 | 0.27 | 0.68 | 0.39 | 2786 | Train 44 | Test 34 | 0.78 | 0.27 | 0.69 | 0.39 |
| 739 | Train 12 | Test 35 | 0.77 | 0.25 | 0.62 | 0.35 | 2787 | Train 44 | Test 35 | 0.77 | 0.24 | 0.63 | 0.35 |
| 740 | Train 12 | Test 36 | 0.78 | 0.27 | 0.68 | 0.39 | 2788 | Train 44 | Test 36 | 0.78 | 0.27 | 0.69 | 0.39 |
| 741 | Train 12 | Test 37 | 0.72 | 0.23 | 0.47 | 0.31 | 2789 | Train 44 | Test 37 | 0.72 | 0.23 | 0.48 | 0.31 |
| 742 | Train 12 | Test 38 | 0.71 | 0.34 | 0.34 | 0.34 | 2790 | Train 44 | Test 38 | 0.71 | 0.34 | 0.35 | 0.35 |
| 743 | Train 12 | Test 39 | 0.72 | 0.23 | 0.47 | 0.3 | 2791 | Train 44 | Test 39 | 0.72 | 0.23 | 0.48 | 0.31 |
| 744 | Train 12 | Test 40 | 0.71 | 0.33 | 0.34 | 0.34 | 2792 | Train 44 | Test 40 | 0.7 | 0.33 | 0.35 | 0.34 |
| 745 | Train 12 | Test 41 | 0.77 | 0.25 | 0.61 | 0.35 | 2793 | Train 44 | Test 41 | 0.77 | 0.25 | 0.61 | 0.35 |
| 746 | Train 12 | Test 42 | 0.78 | 0.28 | 0.67 | 0.39 | 2794 | Train 44 | Test 42 | 0.78 | 0.28 | 0.68 | 0.4 |
| 747 | Train 12 | Test 43 | 0.77 | 0.25 | 0.61 | 0.35 | 2795 | Train 44 | Test 43 | 0.77 | 0.25 | 0.62 | 0.35 |
| 748 | Train 12 | Test 44 | 0.78 | 0.28 | 0.67 | 0.39 | 2796 | Train 44 | Test 44 | 0.78 | 0.28 | 0.68 | 0.4 |
| 749 | Train 12 | Test 45 | 0.72 | 0.23 | 0.46 | 0.31 | 2797 | Train 44 | Test 45 | 0.72 | 0.23 | 0.47 | 0.31 |
| 750 | Train 12 | Test 46 | 0.71 | 0.34 | 0.34 | 0.34 | 2798 | Train 44 | Test 46 | 0.71 | 0.34 | 0.36 | 0.35 |
| 751 | Train 12 | Test 47 | 0.72 | 0.23 | 0.46 | 0.31 | 2799 | Train 44 | Test 47 | 0.72 | 0.23 | 0.46 | 0.31 |
| 752 | Train 12 | Test 48 | 0.71 | 0.33 | 0.34 | 0.34 | 2800 | Train 44 | Test 48 | 0.71 | 0.33 | 0.36 | 0.34 |
| 753 | Train 12 | Test 49 | 0.77 | 0.27 | 0.73 | 0.4 | 2801 | Train 44 | Test 49 | 0.77 | 0.27 | 0.74 | 0.4 |
| 754 | Train 12 | Test 50 | 0.77 | 0.27 | 0.73 | 0.4 | 2802 | Train 44 | Test 50 | 0.77 | 0.27 | 0.73 | 0.4 |
| 755 | Train 12 | Test 51 | 0.77 | 0.27 | 0.74 | 0.4 | 2803 | Train 44 | Test 51 | 0.77 | 0.27 | 0.74 | 0.4 |
| 756 | Train 12 | Test 52 | 0.77 | 0.27 | 0.73 | 0.4 | 2804 | Train 44 | Test 52 | 0.77 | 0.27 | 0.73 | 0.4 |
| 757 | Train 12 | Test 53 | 0.75 | 0.3 | 0.39 | 0.34 | 2805 | Train 44 | Test 53 | 0.75 | 0.3 | 0.41 | 0.35 |
| 758 | Train 12 | Test 54 | 0.72 | 0.39 | 0.32 | 0.35 | 2806 | Train 44 | Test 54 | 0.72 | 0.39 | 0.34 | 0.36 |
| 759 | Train 12 | Test 55 | 0.75 | 0.27 | 0.53 | 0.36 | 2807 | Train 44 | Test 55 | 0.75 | 0.27 | 0.54 | 0.36 |
| 760 | Train 12 | Test 56 | 0.71 | 0.36 | 0.33 | 0.34 | 2808 | Train 44 | Test 56 | 0.71 | 0.36 | 0.34 | 0.35 |
| 761 | Train 12 | Test 57 | 0.78 | 0.28 | 0.74 | 0.4 | 2809 | Train 44 | Test 57 | 0.78 | 0.28 | 0.74 | 0.4 |
| 762 | Train 12 | Test 58 | 0.78 | 0.28 | 0.73 | 0.4 | 2810 | Train 44 | Test 58 | 0.78 | 0.28 | 0.73 | 0.4 |
| 763 | Train 12 | Test 59 | 0.78 | 0.28 | 0.74 | 0.41 | 2811 | Train 44 | Test 59 | 0.78 | 0.28 | 0.74 | 0.41 |
| 764 | Train 12 | Test 60 | 0.78 | 0.28 | 0.73 | 0.41 | 2812 | Train 44 | Test 60 | 0.78 | 0.28 | 0.74 | 0.41 |
| 765 | Train 12 | Test 61 | 0.76 | 0.31 | 0.38 | 0.34 | 2813 | Train 44 | Test 61 | 0.76 | 0.31 | 0.4 | 0.35 |
| 766 | Train 12 | Test 62 | 0.72 | 0.39 | 0.33 | 0.35 | 2814 | Train 44 | Test 62 | 0.72 | 0.39 | 0.34 | 0.36 |
| 767 | Train 12 | Test 63 | 0.76 | 0.3 | 0.38 | 0.34 | 2815 | Train 44 | Test 63 | 0.76 | 0.31 | 0.41 | 0.35 |
| 768 | Train 12 | Test 64 | 0.72 | 0.36 | 0.33 | 0.34 | 2816 | Train 44 | Test 64 | 0.71 | 0.36 | 0.34 | 0.35 |
| 769 | Train 13 | Test 1 | 0.76 | 0.23 | 0.7 | 0.34 | 2817 | Train 45 | Test 1 | 0.76 | 0.23 | 0.71 | 0.34 |
| 770 | Train 13 | Test 2 | 0.76 | 0.25 | 0.71 | 0.37 | 2818 | Train 45 | Test 2 | 0.76 | 0.25 | 0.71 | 0.37 |
| 771 | Train 13 | Test 3 | 0.76 | 0.23 | 0.71 | 0.35 | 2819 | Train 45 | Test 3 | 0.76 | 0.23 | 0.71 | 0.34 |
| 772 | Train 13 | Test 4 | 0.76 | 0.26 | 0.71 | 0.38 | 2820 | Train 45 | Test 4 | 0.76 | 0.25 | 0.71 | 0.37 |
| 773 | Train 13 | Test 5 | 0.75 | 0.23 | 0.67 | 0.34 | 2821 | Train 45 | Test 5 | 0.75 | 0.22 | 0.68 | 0.33 |
| 774 | Train 13 | Test 6 | 0.7 | 0.29 | 0.52 | 0.37 | 2822 | Train 45 | Test 6 | 0.7 | 0.29 | 0.52 | 0.37 |
| 775 | Train 13 | Test 7 | 0.75 | 0.22 | 0.67 | 0.34 | 2823 | Train 45 | Test 7 | 0.75 | 0.22 | 0.67 | 0.33 |
| 776 | Train 13 | Test 8 | 0.7 | 0.29 | 0.51 | 0.37 | 2824 | Train 45 | Test 8 | 0.7 | 0.29 | 0.52 | 0.37 |
| 777 | Train 13 | Test 9 | 0.76 | 0.23 | 0.69 | 0.35 | 2825 | Train 45 | Test 9 | 0.76 | 0.23 | 0.69 | 0.34 |
| 778 | Train 13 | Test 10 | 0.76 | 0.26 | 0.7 | 0.38 | 2826 | Train 45 | Test 10 | 0.76 | 0.26 | 0.7 | 0.38 |
| 779 | Train 13 | Test 11 | 0.76 | 0.23 | 0.69 | 0.35 | 2827 | Train 45 | Test 11 | 0.76 | 0.23 | 0.7 | 0.34 |
| 780 | Train 13 | Test 12 | 0.76 | 0.26 | 0.7 | 0.38 | 2828 | Train 45 | Test 12 | 0.76 | 0.26 | 0.7 | 0.38 |
| 781 | Train 13 | Test 13 | 0.75 | 0.23 | 0.66 | 0.34 | 2829 | Train 45 | Test 13 | 0.75 | 0.23 | 0.66 | 0.34 |
| 782 | Train 13 | Test 14 | 0.7 | 0.29 | 0.52 | 0.37 | 2830 | Train 45 | Test 14 | 0.7 | 0.29 | 0.53 | 0.37 |
| 783 | Train 13 | Test 15 | 0.75 | 0.23 | 0.65 | 0.34 | 2831 | Train 45 | Test 15 | 0.75 | 0.23 | 0.65 | 0.34 |
| 784 | Train 13 | Test 16 | 0.7 | 0.29 | 0.52 | 0.37 | 2832 | Train 45 | Test 16 | 0.7 | 0.29 | 0.53 | 0.37 |
| 785 | Train 13 | Test 17 | 0.76 | 0.23 | 0.7 | 0.34 | 2833 | Train 45 | Test 17 | 0.76 | 0.23 | 0.71 | 0.34 |
| 786 | Train 13 | Test 18 | 0.76 | 0.25 | 0.71 | 0.37 | 2834 | Train 45 | Test 18 | 0.76 | 0.25 | 0.71 | 0.37 |
| 787 | Train 13 | Test 19 | 0.76 | 0.23 | 0.71 | 0.35 | 2835 | Train 45 | Test 19 | 0.76 | 0.23 | 0.71 | 0.34 |
| 788 | Train 13 | Test 20 | 0.76 | 0.26 | 0.71 | 0.38 | 2836 | Train 45 | Test 20 | 0.76 | 0.25 | 0.71 | 0.38 |
| 789 | Train 13 | Test 21 | 0.75 | 0.23 | 0.67 | 0.34 | 2837 | Train 45 | Test 21 | 0.75 | 0.22 | 0.68 | 0.34 |
| 790 | Train 13 | Test 22 | 0.7 | 0.29 | 0.52 | 0.37 | 2838 | Train 45 | Test 22 | 0.7 | 0.29 | 0.52 | 0.37 |
| 791 | Train 13 | Test 23 | 0.75 | 0.22 | 0.67 | 0.34 | 2839 | Train 45 | Test 23 | 0.75 | 0.22 | 0.67 | 0.33 |
| 792 | Train 13 | Test 24 | 0.7 | 0.29 | 0.51 | 0.37 | 2840 | Train 45 | Test 24 | 0.7 | 0.29 | 0.52 | 0.37 |
| 793 | Train 13 | Test 25 | 0.76 | 0.23 | 0.69 | 0.35 | 2841 | Train 45 | Test 25 | 0.76 | 0.23 | 0.69 | 0.34 |
| 794 | Train 13 | Test 26 | 0.76 | 0.26 | 0.7 | 0.38 | 2842 | Train 45 | Test 26 | 0.76 | 0.26 | 0.7 | 0.38 |
| 795 | Train 13 | Test 27 | 0.76 | 0.23 | 0.69 | 0.35 | 2843 | Train 45 | Test 27 | 0.76 | 0.23 | 0.7 | 0.34 |
| 796 | Train 13 | Test 28 | 0.76 | 0.26 | 0.7 | 0.38 | 2844 | Train 45 | Test 28 | 0.76 | 0.26 | 0.7 | 0.38 |
| 797 | Train 13 | Test 29 | 0.75 | 0.23 | 0.66 | 0.34 | 2845 | Train 45 | Test 29 | 0.75 | 0.23 | 0.66 | 0.34 |
| 798 | Train 13 | Test 30 | 0.7 | 0.29 | 0.52 | 0.37 | 2846 | Train 45 | Test 30 | 0.7 | 0.29 | 0.53 | 0.37 |
| 799 | Train 13 | Test 31 | 0.75 | 0.23 | 0.65 | 0.34 | 2847 | Train 45 | Test 31 | 0.75 | 0.23 | 0.65 | 0.34 |
| 800 | Train 13 | Test 32 | 0.7 | 0.29 | 0.52 | 0.37 | 2848 | Train 45 | Test 32 | 0.7 | 0.28 | 0.53 | 0.37 |
| 801 | Train 13 | Test 33 | 0.76 | 0.23 | 0.7 | 0.34 | 2849 | Train 45 | Test 33 | 0.76 | 0.23 | 0.7 | 0.34 |
| 802 | Train 13 | Test 34 | 0.76 | 0.25 | 0.71 | 0.37 | 2850 | Train 45 | Test 34 | 0.76 | 0.25 | 0.71 | 0.37 |
| 803 | Train 13 | Test 35 | 0.76 | 0.23 | 0.7 | 0.34 | 2851 | Train 45 | Test 35 | 0.76 | 0.23 | 0.71 | 0.34 |
| 804 | Train 13 | Test 36 | 0.76 | 0.25 | 0.71 | 0.37 | 2852 | Train 45 | Test 36 | 0.76 | 0.25 | 0.71 | 0.37 |
| 805 | Train 13 | Test 37 | 0.75 | 0.22 | 0.67 | 0.34 | 2853 | Train 45 | Test 37 | 0.75 | 0.22 | 0.67 | 0.33 |
| 806 | Train 13 | Test 38 | 0.7 | 0.29 | 0.51 | 0.37 | 2854 | Train 45 | Test 38 | 0.7 | 0.29 | 0.52 | 0.37 |
| 807 | Train 13 | Test 39 | 0.75 | 0.22 | 0.66 | 0.34 | 2855 | Train 45 | Test 39 | 0.75 | 0.22 | 0.67 | 0.33 |
| 808 | Train 13 | Test 40 | 0.7 | 0.29 | 0.51 | 0.37 | 2856 | Train 45 | Test 40 | 0.7 | 0.29 | 0.52 | 0.37 |
| 809 | Train 13 | Test 41 | 0.76 | 0.23 | 0.69 | 0.34 | 2857 | Train 45 | Test 41 | 0.76 | 0.23 | 0.69 | 0.34 |
| 810 | Train 13 | Test 42 | 0.76 | 0.26 | 0.69 | 0.38 | 2858 | Train 45 | Test 42 | 0.76 | 0.26 | 0.7 | 0.38 |
| 811 | Train 13 | Test 43 | 0.76 | 0.23 | 0.69 | 0.35 | 2859 | Train 45 | Test 43 | 0.76 | 0.23 | 0.69 | 0.34 |
| 812 | Train 13 | Test 44 | 0.76 | 0.26 | 0.7 | 0.38 | 2860 | Train 45 | Test 44 | 0.76 | 0.26 | 0.7 | 0.38 |
| 813 | Train 13 | Test 45 | 0.75 | 0.23 | 0.65 | 0.34 | 2861 | Train 45 | Test 45 | 0.75 | 0.23 | 0.66 | 0.34 |
| 814 | Train 13 | Test 46 | 0.7 | 0.29 | 0.52 | 0.37 | 2862 | Train 45 | Test 46 | 0.7 | 0.29 | 0.53 | 0.37 |
| 815 | Train 13 | Test 47 | 0.75 | 0.23 | 0.65 | 0.34 | 2863 | Train 45 | Test 47 | 0.75 | 0.23 | 0.65 | 0.33 |
| 816 | Train 13 | Test 48 | 0.7 | 0.29 | 0.52 | 0.37 | 2864 | Train 45 | Test 48 | 0.7 | 0.28 | 0.53 | 0.37 |
| 817 | Train 13 | Test 49 | 0.77 | 0.25 | 0.76 | 0.38 | 2865 | Train 45 | Test 49 | 0.77 | 0.26 | 0.76 | 0.38 |
| 818 | Train 13 | Test 50 | 0.77 | 0.26 | 0.75 | 0.38 | 2866 | Train 45 | Test 50 | 0.77 | 0.26 | 0.76 | 0.38 |
| 819 | Train 13 | Test 51 | 0.77 | 0.26 | 0.76 | 0.38 | 2867 | Train 45 | Test 51 | 0.77 | 0.26 | 0.76 | 0.38 |
| 820 | Train 13 | Test 52 | 0.77 | 0.26 | 0.76 | 0.38 | 2868 | Train 45 | Test 52 | 0.77 | 0.26 | 0.76 | 0.38 |
| 821 | Train 13 | Test 53 | 0.76 | 0.26 | 0.67 | 0.38 | 2869 | Train 45 | Test 53 | 0.76 | 0.26 | 0.67 | 0.38 |
| 822 | Train 13 | Test 54 | 0.71 | 0.35 | 0.51 | 0.42 | 2870 | Train 45 | Test 54 | 0.71 | 0.35 | 0.51 | 0.41 |
| 823 | Train 13 | Test 55 | 0.77 | 0.26 | 0.72 | 0.38 | 2871 | Train 45 | Test 55 | 0.77 | 0.26 | 0.72 | 0.38 |
| 824 | Train 13 | Test 56 | 0.71 | 0.33 | 0.51 | 0.4 | 2872 | Train 45 | Test 56 | 0.71 | 0.33 | 0.52 | 0.4 |
| 825 | Train 13 | Test 57 | 0.77 | 0.26 | 0.76 | 0.39 | 2873 | Train 45 | Test 57 | 0.78 | 0.26 | 0.76 | 0.39 |
| 826 | Train 13 | Test 58 | 0.77 | 0.26 | 0.75 | 0.39 | 2874 | Train 45 | Test 58 | 0.77 | 0.26 | 0.75 | 0.39 |
| 827 | Train 13 | Test 59 | 0.78 | 0.26 | 0.76 | 0.39 | 2875 | Train 45 | Test 59 | 0.78 | 0.26 | 0.76 | 0.39 |
| 828 | Train 13 | Test 60 | 0.77 | 0.26 | 0.76 | 0.39 | 2876 | Train 45 | Test 60 | 0.77 | 0.26 | 0.76 | 0.39 |
| 829 | Train 13 | Test 61 | 0.76 | 0.27 | 0.67 | 0.39 | 2877 | Train 45 | Test 61 | 0.76 | 0.27 | 0.67 | 0.39 |
| 830 | Train 13 | Test 62 | 0.71 | 0.35 | 0.51 | 0.42 | 2878 | Train 45 | Test 62 | 0.71 | 0.35 | 0.52 | 0.41 |
| 831 | Train 13 | Test 63 | 0.77 | 0.27 | 0.67 | 0.39 | 2879 | Train 45 | Test 63 | 0.77 | 0.27 | 0.67 | 0.39 |
| 832 | Train 13 | Test 64 | 0.71 | 0.33 | 0.52 | 0.41 | 2880 | Train 45 | Test 64 | 0.71 | 0.33 | 0.52 | 0.4 |
| 833 | Train 14 | Test 1 | 0.75 | 0.24 | 0.61 | 0.34 | 2881 | Train 46 | Test 1 | 0.75 | 0.24 | 0.6 | 0.34 |
| 834 | Train 14 | Test 2 | 0.76 | 0.27 | 0.67 | 0.38 | 2882 | Train 46 | Test 2 | 0.76 | 0.26 | 0.66 | 0.38 |
| 835 | Train 14 | Test 3 | 0.75 | 0.24 | 0.61 | 0.34 | 2883 | Train 46 | Test 3 | 0.75 | 0.24 | 0.6 | 0.34 |
| 836 | Train 14 | Test 4 | 0.76 | 0.27 | 0.68 | 0.38 | 2884 | Train 46 | Test 4 | 0.76 | 0.27 | 0.66 | 0.38 |
| 837 | Train 14 | Test 5 | 0.74 | 0.23 | 0.58 | 0.33 | 2885 | Train 46 | Test 5 | 0.74 | 0.23 | 0.57 | 0.33 |
| 838 | Train 14 | Test 6 | 0.76 | 0.31 | 0.69 | 0.43 | 2886 | Train 46 | Test 6 | 0.76 | 0.31 | 0.7 | 0.43 |
| 839 | Train 14 | Test 7 | 0.74 | 0.23 | 0.57 | 0.33 | 2887 | Train 46 | Test 7 | 0.74 | 0.23 | 0.56 | 0.33 |
| 840 | Train 14 | Test 8 | 0.75 | 0.3 | 0.69 | 0.42 | 2888 | Train 46 | Test 8 | 0.75 | 0.3 | 0.7 | 0.42 |
| 841 | Train 14 | Test 9 | 0.75 | 0.24 | 0.59 | 0.34 | 2889 | Train 46 | Test 9 | 0.75 | 0.24 | 0.58 | 0.34 |
| 842 | Train 14 | Test 10 | 0.76 | 0.27 | 0.66 | 0.38 | 2890 | Train 46 | Test 10 | 0.76 | 0.27 | 0.65 | 0.38 |
| 843 | Train 14 | Test 11 | 0.75 | 0.24 | 0.59 | 0.34 | 2891 | Train 46 | Test 11 | 0.75 | 0.24 | 0.59 | 0.34 |
| 844 | Train 14 | Test 12 | 0.76 | 0.27 | 0.66 | 0.38 | 2892 | Train 46 | Test 12 | 0.76 | 0.27 | 0.65 | 0.38 |
| 845 | Train 14 | Test 13 | 0.74 | 0.24 | 0.56 | 0.33 | 2893 | Train 46 | Test 13 | 0.74 | 0.24 | 0.55 | 0.33 |
| 846 | Train 14 | Test 14 | 0.76 | 0.31 | 0.7 | 0.43 | 2894 | Train 46 | Test 14 | 0.76 | 0.31 | 0.7 | 0.42 |
| 847 | Train 14 | Test 15 | 0.74 | 0.24 | 0.55 | 0.33 | 2895 | Train 46 | Test 15 | 0.74 | 0.24 | 0.55 | 0.33 |
| 848 | Train 14 | Test 16 | 0.75 | 0.3 | 0.7 | 0.42 | 2896 | Train 46 | Test 16 | 0.75 | 0.3 | 0.7 | 0.42 |
| 849 | Train 14 | Test 17 | 0.75 | 0.24 | 0.61 | 0.34 | 2897 | Train 46 | Test 17 | 0.75 | 0.24 | 0.6 | 0.34 |
| 850 | Train 14 | Test 18 | 0.76 | 0.27 | 0.68 | 0.38 | 2898 | Train 46 | Test 18 | 0.76 | 0.26 | 0.66 | 0.38 |
| 851 | Train 14 | Test 19 | 0.75 | 0.24 | 0.61 | 0.34 | 2899 | Train 46 | Test 19 | 0.75 | 0.24 | 0.6 | 0.34 |
| 852 | Train 14 | Test 20 | 0.76 | 0.27 | 0.68 | 0.38 | 2900 | Train 46 | Test 20 | 0.76 | 0.27 | 0.66 | 0.38 |
| 853 | Train 14 | Test 21 | 0.74 | 0.23 | 0.58 | 0.33 | 2901 | Train 46 | Test 21 | 0.74 | 0.23 | 0.57 | 0.33 |
| 854 | Train 14 | Test 22 | 0.76 | 0.31 | 0.69 | 0.43 | 2902 | Train 46 | Test 22 | 0.76 | 0.31 | 0.7 | 0.43 |
| 855 | Train 14 | Test 23 | 0.74 | 0.23 | 0.57 | 0.33 | 2903 | Train 46 | Test 23 | 0.74 | 0.23 | 0.56 | 0.33 |
| 856 | Train 14 | Test 24 | 0.75 | 0.3 | 0.69 | 0.42 | 2904 | Train 46 | Test 24 | 0.75 | 0.3 | 0.7 | 0.42 |
| 857 | Train 14 | Test 25 | 0.75 | 0.24 | 0.59 | 0.34 | 2905 | Train 46 | Test 25 | 0.75 | 0.24 | 0.58 | 0.34 |
| 858 | Train 14 | Test 26 | 0.76 | 0.27 | 0.66 | 0.38 | 2906 | Train 46 | Test 26 | 0.76 | 0.27 | 0.65 | 0.38 |
| 859 | Train 14 | Test 27 | 0.75 | 0.24 | 0.59 | 0.34 | 2907 | Train 46 | Test 27 | 0.75 | 0.24 | 0.59 | 0.34 |
| 860 | Train 14 | Test 28 | 0.76 | 0.27 | 0.66 | 0.38 | 2908 | Train 46 | Test 28 | 0.76 | 0.27 | 0.65 | 0.38 |
| 861 | Train 14 | Test 29 | 0.74 | 0.24 | 0.56 | 0.34 | 2909 | Train 46 | Test 29 | 0.74 | 0.24 | 0.55 | 0.33 |
| 862 | Train 14 | Test 30 | 0.76 | 0.31 | 0.7 | 0.42 | 2910 | Train 46 | Test 30 | 0.76 | 0.3 | 0.7 | 0.42 |
| 863 | Train 14 | Test 31 | 0.74 | 0.24 | 0.55 | 0.33 | 2911 | Train 46 | Test 31 | 0.74 | 0.24 | 0.55 | 0.33 |
| 864 | Train 14 | Test 32 | 0.75 | 0.3 | 0.7 | 0.42 | 2912 | Train 46 | Test 32 | 0.75 | 0.3 | 0.7 | 0.41 |
| 865 | Train 14 | Test 33 | 0.75 | 0.24 | 0.61 | 0.34 | 2913 | Train 46 | Test 33 | 0.75 | 0.24 | 0.59 | 0.34 |
| 866 | Train 14 | Test 34 | 0.76 | 0.27 | 0.67 | 0.38 | 2914 | Train 46 | Test 34 | 0.76 | 0.26 | 0.66 | 0.38 |
| 867 | Train 14 | Test 35 | 0.75 | 0.24 | 0.61 | 0.34 | 2915 | Train 46 | Test 35 | 0.75 | 0.24 | 0.6 | 0.34 |
| 868 | Train 14 | Test 36 | 0.76 | 0.27 | 0.67 | 0.38 | 2916 | Train 46 | Test 36 | 0.76 | 0.27 | 0.66 | 0.38 |
| 869 | Train 14 | Test 37 | 0.74 | 0.23 | 0.58 | 0.33 | 2917 | Train 46 | Test 37 | 0.74 | 0.23 | 0.56 | 0.33 |
| 870 | Train 14 | Test 38 | 0.76 | 0.31 | 0.69 | 0.43 | 2918 | Train 46 | Test 38 | 0.76 | 0.31 | 0.69 | 0.43 |
| 871 | Train 14 | Test 39 | 0.74 | 0.23 | 0.57 | 0.33 | 2919 | Train 46 | Test 39 | 0.74 | 0.23 | 0.56 | 0.33 |
| 872 | Train 14 | Test 40 | 0.75 | 0.3 | 0.69 | 0.42 | 2920 | Train 46 | Test 40 | 0.75 | 0.3 | 0.69 | 0.42 |
| 873 | Train 14 | Test 41 | 0.75 | 0.24 | 0.59 | 0.34 | 2921 | Train 46 | Test 41 | 0.75 | 0.24 | 0.58 | 0.34 |
| 874 | Train 14 | Test 42 | 0.76 | 0.27 | 0.66 | 0.38 | 2922 | Train 46 | Test 42 | 0.76 | 0.27 | 0.65 | 0.38 |
| 875 | Train 14 | Test 43 | 0.75 | 0.24 | 0.59 | 0.34 | 2923 | Train 46 | Test 43 | 0.75 | 0.24 | 0.58 | 0.34 |
| 876 | Train 14 | Test 44 | 0.76 | 0.27 | 0.66 | 0.38 | 2924 | Train 46 | Test 44 | 0.76 | 0.27 | 0.65 | 0.38 |
| 877 | Train 14 | Test 45 | 0.74 | 0.24 | 0.56 | 0.33 | 2925 | Train 46 | Test 45 | 0.74 | 0.24 | 0.55 | 0.33 |
| 878 | Train 14 | Test 46 | 0.76 | 0.31 | 0.7 | 0.42 | 2926 | Train 46 | Test 46 | 0.76 | 0.31 | 0.7 | 0.42 |
| 879 | Train 14 | Test 47 | 0.74 | 0.24 | 0.55 | 0.33 | 2927 | Train 46 | Test 47 | 0.74 | 0.24 | 0.55 | 0.33 |
| 880 | Train 14 | Test 48 | 0.75 | 0.3 | 0.69 | 0.42 | 2928 | Train 46 | Test 48 | 0.75 | 0.3 | 0.7 | 0.41 |
| 881 | Train 14 | Test 49 | 0.77 | 0.28 | 0.73 | 0.4 | 2929 | Train 46 | Test 49 | 0.77 | 0.28 | 0.73 | 0.4 |
| 882 | Train 14 | Test 50 | 0.77 | 0.28 | 0.73 | 0.4 | 2930 | Train 46 | Test 50 | 0.77 | 0.28 | 0.73 | 0.4 |
| 883 | Train 14 | Test 51 | 0.77 | 0.28 | 0.73 | 0.4 | 2931 | Train 46 | Test 51 | 0.77 | 0.28 | 0.73 | 0.4 |
| 884 | Train 14 | Test 52 | 0.77 | 0.28 | 0.73 | 0.4 | 2932 | Train 46 | Test 52 | 0.77 | 0.28 | 0.73 | 0.4 |
| 885 | Train 14 | Test 53 | 0.77 | 0.28 | 0.65 | 0.39 | 2933 | Train 46 | Test 53 | 0.77 | 0.28 | 0.66 | 0.39 |
| 886 | Train 14 | Test 54 | 0.76 | 0.34 | 0.73 | 0.47 | 2934 | Train 46 | Test 54 | 0.76 | 0.34 | 0.73 | 0.47 |
| 887 | Train 14 | Test 55 | 0.77 | 0.28 | 0.67 | 0.39 | 2935 | Train 46 | Test 55 | 0.77 | 0.28 | 0.66 | 0.39 |
| 888 | Train 14 | Test 56 | 0.75 | 0.33 | 0.73 | 0.45 | 2936 | Train 46 | Test 56 | 0.75 | 0.32 | 0.73 | 0.45 |
| 889 | Train 14 | Test 57 | 0.78 | 0.28 | 0.73 | 0.41 | 2937 | Train 46 | Test 57 | 0.78 | 0.28 | 0.72 | 0.41 |
| 890 | Train 14 | Test 58 | 0.78 | 0.28 | 0.72 | 0.41 | 2938 | Train 46 | Test 58 | 0.78 | 0.28 | 0.72 | 0.4 |
| 891 | Train 14 | Test 59 | 0.78 | 0.28 | 0.73 | 0.41 | 2939 | Train 46 | Test 59 | 0.78 | 0.28 | 0.72 | 0.41 |
| 892 | Train 14 | Test 60 | 0.78 | 0.28 | 0.72 | 0.41 | 2940 | Train 46 | Test 60 | 0.78 | 0.28 | 0.72 | 0.41 |
| 893 | Train 14 | Test 61 | 0.78 | 0.29 | 0.65 | 0.4 | 2941 | Train 46 | Test 61 | 0.78 | 0.29 | 0.65 | 0.4 |
| 894 | Train 14 | Test 62 | 0.76 | 0.34 | 0.73 | 0.46 | 2942 | Train 46 | Test 62 | 0.76 | 0.34 | 0.73 | 0.46 |
| 895 | Train 14 | Test 63 | 0.78 | 0.29 | 0.65 | 0.4 | 2943 | Train 46 | Test 63 | 0.78 | 0.28 | 0.65 | 0.4 |
| 896 | Train 14 | Test 64 | 0.76 | 0.32 | 0.73 | 0.45 | 2944 | Train 46 | Test 64 | 0.76 | 0.32 | 0.73 | 0.45 |
| 897 | Train 15 | Test 1 | 0.76 | 0.23 | 0.71 | 0.34 | 2945 | Train 47 | Test 1 | 0.76 | 0.23 | 0.71 | 0.34 |
| 898 | Train 15 | Test 2 | 0.76 | 0.26 | 0.71 | 0.38 | 2946 | Train 47 | Test 2 | 0.76 | 0.25 | 0.71 | 0.37 |
| 899 | Train 15 | Test 3 | 0.76 | 0.23 | 0.71 | 0.35 | 2947 | Train 47 | Test 3 | 0.76 | 0.23 | 0.71 | 0.34 |
| 900 | Train 15 | Test 4 | 0.76 | 0.26 | 0.71 | 0.38 | 2948 | Train 47 | Test 4 | 0.76 | 0.25 | 0.72 | 0.37 |
| 901 | Train 15 | Test 5 | 0.75 | 0.22 | 0.67 | 0.34 | 2949 | Train 47 | Test 5 | 0.75 | 0.22 | 0.68 | 0.33 |
| 902 | Train 15 | Test 6 | 0.7 | 0.29 | 0.51 | 0.37 | 2950 | Train 47 | Test 6 | 0.7 | 0.29 | 0.52 | 0.37 |
| 903 | Train 15 | Test 7 | 0.75 | 0.22 | 0.67 | 0.33 | 2951 | Train 47 | Test 7 | 0.75 | 0.22 | 0.67 | 0.33 |
| 904 | Train 15 | Test 8 | 0.7 | 0.29 | 0.51 | 0.37 | 2952 | Train 47 | Test 8 | 0.7 | 0.29 | 0.52 | 0.37 |
| 905 | Train 15 | Test 9 | 0.76 | 0.23 | 0.7 | 0.35 | 2953 | Train 47 | Test 9 | 0.76 | 0.23 | 0.7 | 0.34 |
| 906 | Train 15 | Test 10 | 0.76 | 0.26 | 0.7 | 0.38 | 2954 | Train 47 | Test 10 | 0.76 | 0.26 | 0.71 | 0.38 |
| 907 | Train 15 | Test 11 | 0.76 | 0.23 | 0.7 | 0.35 | 2955 | Train 47 | Test 11 | 0.76 | 0.23 | 0.7 | 0.35 |
| 908 | Train 15 | Test 12 | 0.76 | 0.26 | 0.7 | 0.38 | 2956 | Train 47 | Test 12 | 0.76 | 0.26 | 0.71 | 0.38 |
| 909 | Train 15 | Test 13 | 0.75 | 0.23 | 0.66 | 0.34 | 2957 | Train 47 | Test 13 | 0.75 | 0.23 | 0.67 | 0.34 |
| 910 | Train 15 | Test 14 | 0.7 | 0.29 | 0.52 | 0.37 | 2958 | Train 47 | Test 14 | 0.7 | 0.29 | 0.52 | 0.37 |
| 911 | Train 15 | Test 15 | 0.75 | 0.23 | 0.66 | 0.34 | 2959 | Train 47 | Test 15 | 0.75 | 0.23 | 0.66 | 0.34 |
| 912 | Train 15 | Test 16 | 0.7 | 0.29 | 0.51 | 0.37 | 2960 | Train 47 | Test 16 | 0.7 | 0.29 | 0.52 | 0.37 |
| 913 | Train 15 | Test 17 | 0.76 | 0.23 | 0.7 | 0.34 | 2961 | Train 47 | Test 17 | 0.76 | 0.23 | 0.71 | 0.34 |
| 914 | Train 15 | Test 18 | 0.76 | 0.26 | 0.71 | 0.38 | 2962 | Train 47 | Test 18 | 0.76 | 0.25 | 0.71 | 0.37 |
| 915 | Train 15 | Test 19 | 0.76 | 0.23 | 0.71 | 0.34 | 2963 | Train 47 | Test 19 | 0.76 | 0.23 | 0.71 | 0.34 |
| 916 | Train 15 | Test 20 | 0.76 | 0.26 | 0.71 | 0.38 | 2964 | Train 47 | Test 20 | 0.76 | 0.25 | 0.72 | 0.37 |
| 917 | Train 15 | Test 21 | 0.75 | 0.22 | 0.67 | 0.34 | 2965 | Train 47 | Test 21 | 0.75 | 0.22 | 0.68 | 0.33 |
| 918 | Train 15 | Test 22 | 0.7 | 0.29 | 0.51 | 0.37 | 2966 | Train 47 | Test 22 | 0.7 | 0.29 | 0.52 | 0.37 |
| 919 | Train 15 | Test 23 | 0.75 | 0.22 | 0.67 | 0.33 | 2967 | Train 47 | Test 23 | 0.75 | 0.22 | 0.67 | 0.33 |
| 920 | Train 15 | Test 24 | 0.7 | 0.29 | 0.51 | 0.37 | 2968 | Train 47 | Test 24 | 0.7 | 0.29 | 0.52 | 0.37 |
| 921 | Train 15 | Test 25 | 0.76 | 0.23 | 0.7 | 0.35 | 2969 | Train 47 | Test 25 | 0.76 | 0.23 | 0.7 | 0.34 |
| 922 | Train 15 | Test 26 | 0.76 | 0.26 | 0.7 | 0.38 | 2970 | Train 47 | Test 26 | 0.76 | 0.26 | 0.71 | 0.38 |
| 923 | Train 15 | Test 27 | 0.76 | 0.23 | 0.7 | 0.35 | 2971 | Train 47 | Test 27 | 0.76 | 0.23 | 0.7 | 0.35 |
| 924 | Train 15 | Test 28 | 0.76 | 0.26 | 0.7 | 0.38 | 2972 | Train 47 | Test 28 | 0.76 | 0.26 | 0.71 | 0.38 |
| 925 | Train 15 | Test 29 | 0.75 | 0.23 | 0.66 | 0.34 | 2973 | Train 47 | Test 29 | 0.75 | 0.23 | 0.67 | 0.34 |
| 926 | Train 15 | Test 30 | 0.7 | 0.29 | 0.52 | 0.37 | 2974 | Train 47 | Test 30 | 0.7 | 0.29 | 0.52 | 0.37 |
| 927 | Train 15 | Test 31 | 0.75 | 0.23 | 0.66 | 0.34 | 2975 | Train 47 | Test 31 | 0.75 | 0.23 | 0.66 | 0.34 |
| 928 | Train 15 | Test 32 | 0.7 | 0.29 | 0.51 | 0.37 | 2976 | Train 47 | Test 32 | 0.7 | 0.29 | 0.52 | 0.37 |
| 929 | Train 15 | Test 33 | 0.76 | 0.23 | 0.7 | 0.34 | 2977 | Train 47 | Test 33 | 0.76 | 0.22 | 0.7 | 0.34 |
| 930 | Train 15 | Test 34 | 0.76 | 0.25 | 0.71 | 0.37 | 2978 | Train 47 | Test 34 | 0.76 | 0.25 | 0.71 | 0.37 |
| 931 | Train 15 | Test 35 | 0.76 | 0.23 | 0.7 | 0.34 | 2979 | Train 47 | Test 35 | 0.76 | 0.23 | 0.71 | 0.34 |
| 932 | Train 15 | Test 36 | 0.76 | 0.25 | 0.71 | 0.37 | 2980 | Train 47 | Test 36 | 0.76 | 0.25 | 0.71 | 0.37 |
| 933 | Train 15 | Test 37 | 0.75 | 0.22 | 0.67 | 0.34 | 2981 | Train 47 | Test 37 | 0.75 | 0.22 | 0.68 | 0.33 |
| 934 | Train 15 | Test 38 | 0.7 | 0.29 | 0.51 | 0.37 | 2982 | Train 47 | Test 38 | 0.7 | 0.29 | 0.52 | 0.37 |
| 935 | Train 15 | Test 39 | 0.75 | 0.22 | 0.67 | 0.33 | 2983 | Train 47 | Test 39 | 0.75 | 0.22 | 0.67 | 0.33 |
| 936 | Train 15 | Test 40 | 0.7 | 0.29 | 0.51 | 0.37 | 2984 | Train 47 | Test 40 | 0.7 | 0.29 | 0.52 | 0.37 |
| 937 | Train 15 | Test 41 | 0.76 | 0.23 | 0.69 | 0.35 | 2985 | Train 47 | Test 41 | 0.76 | 0.23 | 0.69 | 0.34 |
| 938 | Train 15 | Test 42 | 0.76 | 0.26 | 0.7 | 0.38 | 2986 | Train 47 | Test 42 | 0.76 | 0.26 | 0.7 | 0.38 |
| 939 | Train 15 | Test 43 | 0.76 | 0.23 | 0.7 | 0.35 | 2987 | Train 47 | Test 43 | 0.76 | 0.23 | 0.7 | 0.34 |
| 940 | Train 15 | Test 44 | 0.76 | 0.26 | 0.7 | 0.38 | 2988 | Train 47 | Test 44 | 0.76 | 0.26 | 0.7 | 0.38 |
| 941 | Train 15 | Test 45 | 0.75 | 0.23 | 0.66 | 0.34 | 2989 | Train 47 | Test 45 | 0.75 | 0.23 | 0.66 | 0.34 |
| 942 | Train 15 | Test 46 | 0.7 | 0.29 | 0.51 | 0.37 | 2990 | Train 47 | Test 46 | 0.7 | 0.29 | 0.52 | 0.37 |
| 943 | Train 15 | Test 47 | 0.75 | 0.23 | 0.65 | 0.34 | 2991 | Train 47 | Test 47 | 0.75 | 0.23 | 0.66 | 0.34 |
| 944 | Train 15 | Test 48 | 0.7 | 0.29 | 0.51 | 0.37 | 2992 | Train 47 | Test 48 | 0.7 | 0.29 | 0.52 | 0.37 |
| 945 | Train 15 | Test 49 | 0.77 | 0.25 | 0.76 | 0.38 | 2993 | Train 47 | Test 49 | 0.77 | 0.26 | 0.76 | 0.38 |
| 946 | Train 15 | Test 50 | 0.77 | 0.25 | 0.76 | 0.38 | 2994 | Train 47 | Test 50 | 0.77 | 0.26 | 0.76 | 0.38 |
| 947 | Train 15 | Test 51 | 0.77 | 0.26 | 0.77 | 0.38 | 2995 | Train 47 | Test 51 | 0.77 | 0.26 | 0.76 | 0.38 |
| 948 | Train 15 | Test 52 | 0.77 | 0.26 | 0.76 | 0.38 | 2996 | Train 47 | Test 52 | 0.77 | 0.26 | 0.76 | 0.38 |
| 949 | Train 15 | Test 53 | 0.76 | 0.27 | 0.66 | 0.38 | 2997 | Train 47 | Test 53 | 0.76 | 0.26 | 0.67 | 0.38 |
| 950 | Train 15 | Test 54 | 0.71 | 0.35 | 0.51 | 0.42 | 2998 | Train 47 | Test 54 | 0.71 | 0.35 | 0.51 | 0.41 |
| 951 | Train 15 | Test 55 | 0.77 | 0.26 | 0.72 | 0.38 | 2999 | Train 47 | Test 55 | 0.77 | 0.26 | 0.72 | 0.38 |
| 952 | Train 15 | Test 56 | 0.71 | 0.34 | 0.51 | 0.4 | 3000 | Train 47 | Test 56 | 0.71 | 0.33 | 0.51 | 0.4 |
| 953 | Train 15 | Test 57 | 0.78 | 0.26 | 0.77 | 0.39 | 3001 | Train 47 | Test 57 | 0.78 | 0.26 | 0.76 | 0.39 |
| 954 | Train 15 | Test 58 | 0.77 | 0.26 | 0.76 | 0.39 | 3002 | Train 47 | Test 58 | 0.77 | 0.26 | 0.76 | 0.39 |
| 955 | Train 15 | Test 59 | 0.78 | 0.26 | 0.77 | 0.39 | 3003 | Train 47 | Test 59 | 0.78 | 0.26 | 0.76 | 0.39 |
| 956 | Train 15 | Test 60 | 0.77 | 0.26 | 0.77 | 0.39 | 3004 | Train 47 | Test 60 | 0.77 | 0.26 | 0.76 | 0.39 |
| 957 | Train 15 | Test 61 | 0.76 | 0.27 | 0.67 | 0.39 | 3005 | Train 47 | Test 61 | 0.76 | 0.27 | 0.67 | 0.39 |
| 958 | Train 15 | Test 62 | 0.71 | 0.35 | 0.51 | 0.42 | 3006 | Train 47 | Test 62 | 0.71 | 0.35 | 0.51 | 0.42 |
| 959 | Train 15 | Test 63 | 0.77 | 0.27 | 0.67 | 0.39 | 3007 | Train 47 | Test 63 | 0.77 | 0.27 | 0.67 | 0.39 |
| 960 | Train 15 | Test 64 | 0.71 | 0.33 | 0.51 | 0.4 | 3008 | Train 47 | Test 64 | 0.71 | 0.33 | 0.52 | 0.4 |
| 961 | Train 16 | Test 1 | 0.75 | 0.24 | 0.62 | 0.34 | 3009 | Train 48 | Test 1 | 0.75 | 0.24 | 0.62 | 0.34 |
| 962 | Train 16 | Test 2 | 0.76 | 0.26 | 0.68 | 0.38 | 3010 | Train 48 | Test 2 | 0.76 | 0.26 | 0.68 | 0.38 |
| 963 | Train 16 | Test 3 | 0.75 | 0.24 | 0.62 | 0.34 | 3011 | Train 48 | Test 3 | 0.75 | 0.24 | 0.62 | 0.34 |
| 964 | Train 16 | Test 4 | 0.76 | 0.26 | 0.68 | 0.38 | 3012 | Train 48 | Test 4 | 0.76 | 0.26 | 0.68 | 0.38 |
| 965 | Train 16 | Test 5 | 0.74 | 0.23 | 0.58 | 0.33 | 3013 | Train 48 | Test 5 | 0.74 | 0.23 | 0.58 | 0.33 |
| 966 | Train 16 | Test 6 | 0.76 | 0.31 | 0.7 | 0.43 | 3014 | Train 48 | Test 6 | 0.76 | 0.31 | 0.7 | 0.42 |
| 967 | Train 16 | Test 7 | 0.74 | 0.23 | 0.58 | 0.33 | 3015 | Train 48 | Test 7 | 0.74 | 0.23 | 0.58 | 0.33 |
| 968 | Train 16 | Test 8 | 0.76 | 0.3 | 0.7 | 0.42 | 3016 | Train 48 | Test 8 | 0.76 | 0.3 | 0.7 | 0.42 |
| 969 | Train 16 | Test 9 | 0.75 | 0.24 | 0.6 | 0.34 | 3017 | Train 48 | Test 9 | 0.75 | 0.24 | 0.6 | 0.34 |
| 970 | Train 16 | Test 10 | 0.76 | 0.27 | 0.67 | 0.38 | 3018 | Train 48 | Test 10 | 0.76 | 0.27 | 0.67 | 0.38 |
| 971 | Train 16 | Test 11 | 0.76 | 0.24 | 0.61 | 0.34 | 3019 | Train 48 | Test 11 | 0.76 | 0.24 | 0.6 | 0.34 |
| 972 | Train 16 | Test 12 | 0.76 | 0.27 | 0.67 | 0.38 | 3020 | Train 48 | Test 12 | 0.76 | 0.27 | 0.67 | 0.38 |
| 973 | Train 16 | Test 13 | 0.74 | 0.24 | 0.57 | 0.33 | 3021 | Train 48 | Test 13 | 0.74 | 0.24 | 0.57 | 0.33 |
| 974 | Train 16 | Test 14 | 0.76 | 0.3 | 0.7 | 0.42 | 3022 | Train 48 | Test 14 | 0.76 | 0.3 | 0.7 | 0.42 |
| 975 | Train 16 | Test 15 | 0.74 | 0.24 | 0.56 | 0.33 | 3023 | Train 48 | Test 15 | 0.74 | 0.24 | 0.56 | 0.33 |
| 976 | Train 16 | Test 16 | 0.76 | 0.3 | 0.7 | 0.42 | 3024 | Train 48 | Test 16 | 0.76 | 0.3 | 0.7 | 0.42 |
| 977 | Train 16 | Test 17 | 0.75 | 0.24 | 0.62 | 0.34 | 3025 | Train 48 | Test 17 | 0.75 | 0.24 | 0.62 | 0.34 |
| 978 | Train 16 | Test 18 | 0.76 | 0.26 | 0.68 | 0.38 | 3026 | Train 48 | Test 18 | 0.76 | 0.26 | 0.68 | 0.38 |
| 979 | Train 16 | Test 19 | 0.75 | 0.24 | 0.62 | 0.34 | 3027 | Train 48 | Test 19 | 0.75 | 0.24 | 0.62 | 0.34 |
| 980 | Train 16 | Test 20 | 0.76 | 0.26 | 0.68 | 0.38 | 3028 | Train 48 | Test 20 | 0.76 | 0.26 | 0.68 | 0.38 |
| 981 | Train 16 | Test 21 | 0.74 | 0.23 | 0.58 | 0.33 | 3029 | Train 48 | Test 21 | 0.74 | 0.23 | 0.58 | 0.33 |
| 982 | Train 16 | Test 22 | 0.76 | 0.31 | 0.7 | 0.43 | 3030 | Train 48 | Test 22 | 0.76 | 0.3 | 0.7 | 0.42 |
| 983 | Train 16 | Test 23 | 0.74 | 0.23 | 0.58 | 0.33 | 3031 | Train 48 | Test 23 | 0.74 | 0.23 | 0.58 | 0.33 |
| 984 | Train 16 | Test 24 | 0.76 | 0.3 | 0.7 | 0.42 | 3032 | Train 48 | Test 24 | 0.76 | 0.3 | 0.7 | 0.42 |
| 985 | Train 16 | Test 25 | 0.75 | 0.24 | 0.6 | 0.34 | 3033 | Train 48 | Test 25 | 0.75 | 0.24 | 0.6 | 0.34 |
| 986 | Train 16 | Test 26 | 0.76 | 0.27 | 0.67 | 0.38 | 3034 | Train 48 | Test 26 | 0.76 | 0.27 | 0.67 | 0.38 |
| 987 | Train 16 | Test 27 | 0.76 | 0.24 | 0.61 | 0.34 | 3035 | Train 48 | Test 27 | 0.76 | 0.24 | 0.6 | 0.34 |
| 988 | Train 16 | Test 28 | 0.76 | 0.27 | 0.67 | 0.38 | 3036 | Train 48 | Test 28 | 0.76 | 0.27 | 0.67 | 0.38 |
| 989 | Train 16 | Test 29 | 0.74 | 0.24 | 0.57 | 0.33 | 3037 | Train 48 | Test 29 | 0.74 | 0.24 | 0.57 | 0.33 |
| 990 | Train 16 | Test 30 | 0.76 | 0.3 | 0.7 | 0.42 | 3038 | Train 48 | Test 30 | 0.76 | 0.3 | 0.7 | 0.42 |
| 991 | Train 16 | Test 31 | 0.74 | 0.24 | 0.56 | 0.33 | 3039 | Train 48 | Test 31 | 0.74 | 0.24 | 0.56 | 0.33 |
| 992 | Train 16 | Test 32 | 0.76 | 0.3 | 0.7 | 0.42 | 3040 | Train 48 | Test 32 | 0.76 | 0.3 | 0.7 | 0.42 |
| 993 | Train 16 | Test 33 | 0.75 | 0.24 | 0.61 | 0.34 | 3041 | Train 48 | Test 33 | 0.75 | 0.23 | 0.61 | 0.34 |
| 994 | Train 16 | Test 34 | 0.76 | 0.26 | 0.67 | 0.38 | 3042 | Train 48 | Test 34 | 0.76 | 0.26 | 0.68 | 0.38 |
| 995 | Train 16 | Test 35 | 0.75 | 0.24 | 0.62 | 0.34 | 3043 | Train 48 | Test 35 | 0.75 | 0.24 | 0.62 | 0.34 |
| 996 | Train 16 | Test 36 | 0.76 | 0.26 | 0.68 | 0.38 | 3044 | Train 48 | Test 36 | 0.76 | 0.26 | 0.68 | 0.38 |
| 997 | Train 16 | Test 37 | 0.74 | 0.23 | 0.58 | 0.33 | 3045 | Train 48 | Test 37 | 0.74 | 0.23 | 0.58 | 0.33 |
| 998 | Train 16 | Test 38 | 0.76 | 0.31 | 0.7 | 0.43 | 3046 | Train 48 | Test 38 | 0.76 | 0.3 | 0.7 | 0.42 |
| 999 | Train 16 | Test 39 | 0.74 | 0.23 | 0.57 | 0.33 | 3047 | Train 48 | Test 39 | 0.74 | 0.23 | 0.58 | 0.33 |
| 1000 | Train 16 | Test 40 | 0.76 | 0.3 | 0.7 | 0.42 | 3048 | Train 48 | Test 40 | 0.76 | 0.3 | 0.7 | 0.42 |
| 1001 | Train 16 | Test 41 | 0.76 | 0.24 | 0.6 | 0.34 | 3049 | Train 48 | Test 41 | 0.76 | 0.24 | 0.6 | 0.34 |
| 1002 | Train 16 | Test 42 | 0.76 | 0.27 | 0.67 | 0.38 | 3050 | Train 48 | Test 42 | 0.76 | 0.27 | 0.66 | 0.38 |
| 1003 | Train 16 | Test 43 | 0.76 | 0.24 | 0.6 | 0.34 | 3051 | Train 48 | Test 43 | 0.76 | 0.24 | 0.6 | 0.34 |
| 1004 | Train 16 | Test 44 | 0.76 | 0.27 | 0.67 | 0.38 | 3052 | Train 48 | Test 44 | 0.76 | 0.27 | 0.67 | 0.38 |
| 1005 | Train 16 | Test 45 | 0.74 | 0.24 | 0.57 | 0.33 | 3053 | Train 48 | Test 45 | 0.74 | 0.24 | 0.57 | 0.33 |
| 1006 | Train 16 | Test 46 | 0.76 | 0.3 | 0.7 | 0.42 | 3054 | Train 48 | Test 46 | 0.76 | 0.3 | 0.7 | 0.42 |
| 1007 | Train 16 | Test 47 | 0.74 | 0.24 | 0.56 | 0.33 | 3055 | Train 48 | Test 47 | 0.74 | 0.24 | 0.56 | 0.33 |
| 1008 | Train 16 | Test 48 | 0.76 | 0.3 | 0.7 | 0.42 | 3056 | Train 48 | Test 48 | 0.76 | 0.3 | 0.7 | 0.42 |
| 1009 | Train 16 | Test 49 | 0.77 | 0.27 | 0.74 | 0.4 | 3057 | Train 48 | Test 49 | 0.77 | 0.27 | 0.74 | 0.4 |
| 1010 | Train 16 | Test 50 | 0.77 | 0.27 | 0.74 | 0.4 | 3058 | Train 48 | Test 50 | 0.77 | 0.27 | 0.74 | 0.4 |
| 1011 | Train 16 | Test 51 | 0.78 | 0.27 | 0.74 | 0.4 | 3059 | Train 48 | Test 51 | 0.77 | 0.27 | 0.74 | 0.4 |
| 1012 | Train 16 | Test 52 | 0.77 | 0.27 | 0.74 | 0.4 | 3060 | Train 48 | Test 52 | 0.77 | 0.27 | 0.74 | 0.4 |
| 1013 | Train 16 | Test 53 | 0.77 | 0.28 | 0.67 | 0.39 | 3061 | Train 48 | Test 53 | 0.77 | 0.28 | 0.66 | 0.39 |
| 1014 | Train 16 | Test 54 | 0.76 | 0.34 | 0.73 | 0.47 | 3062 | Train 48 | Test 54 | 0.76 | 0.34 | 0.73 | 0.46 |
| 1015 | Train 16 | Test 55 | 0.77 | 0.27 | 0.68 | 0.39 | 3063 | Train 48 | Test 55 | 0.77 | 0.27 | 0.68 | 0.39 |
| 1016 | Train 16 | Test 56 | 0.76 | 0.33 | 0.73 | 0.45 | 3064 | Train 48 | Test 56 | 0.76 | 0.33 | 0.73 | 0.45 |
| 1017 | Train 16 | Test 57 | 0.78 | 0.28 | 0.74 | 0.41 | 3065 | Train 48 | Test 57 | 0.78 | 0.28 | 0.74 | 0.4 |
| 1018 | Train 16 | Test 58 | 0.78 | 0.28 | 0.74 | 0.41 | 3066 | Train 48 | Test 58 | 0.78 | 0.28 | 0.73 | 0.4 |
| 1019 | Train 16 | Test 59 | 0.78 | 0.28 | 0.74 | 0.41 | 3067 | Train 48 | Test 59 | 0.78 | 0.28 | 0.74 | 0.41 |
| 1020 | Train 16 | Test 60 | 0.78 | 0.28 | 0.74 | 0.41 | 3068 | Train 48 | Test 60 | 0.78 | 0.28 | 0.73 | 0.41 |
| 1021 | Train 16 | Test 61 | 0.78 | 0.29 | 0.66 | 0.4 | 3069 | Train 48 | Test 61 | 0.78 | 0.28 | 0.65 | 0.4 |
| 1022 | Train 16 | Test 62 | 0.76 | 0.34 | 0.73 | 0.46 | 3070 | Train 48 | Test 62 | 0.76 | 0.34 | 0.73 | 0.46 |
| 1023 | Train 16 | Test 63 | 0.78 | 0.28 | 0.66 | 0.4 | 3071 | Train 48 | Test 63 | 0.78 | 0.28 | 0.66 | 0.39 |
| 1024 | Train 16 | Test 64 | 0.76 | 0.33 | 0.74 | 0.45 | 3072 | Train 48 | Test 64 | 0.76 | 0.32 | 0.73 | 0.45 |
| 1025 | Train 17 | Test 1 | 0.77 | 0.24 | 0.65 | 0.35 | 3073 | Train 49 | Test 1 | 0.72 | 0.27 | 0.34 | 0.3 |
| 1026 | Train 17 | Test 2 | 0.75 | 0.27 | 0.58 | 0.36 | 3074 | Train 49 | Test 2 | 0.71 | 0.27 | 0.26 | 0.27 |
| 1027 | Train 17 | Test 3 | 0.77 | 0.24 | 0.65 | 0.35 | 3075 | Train 49 | Test 3 | 0.72 | 0.28 | 0.35 | 0.31 |
| 1028 | Train 17 | Test 4 | 0.75 | 0.27 | 0.58 | 0.36 | 3076 | Train 49 | Test 4 | 0.71 | 0.27 | 0.27 | 0.27 |
| 1029 | Train 17 | Test 5 | 0.71 | 0.23 | 0.46 | 0.31 | 3077 | Train 49 | Test 5 | 0.69 | 0.26 | 0.21 | 0.23 |
| 1030 | Train 17 | Test 6 | 0.69 | 0.32 | 0.27 | 0.29 | 3078 | Train 49 | Test 6 | 0.68 | 0.32 | 0.11 | 0.17 |
| 1031 | Train 17 | Test 7 | 0.71 | 0.23 | 0.46 | 0.3 | 3079 | Train 49 | Test 7 | 0.69 | 0.26 | 0.21 | 0.23 |
| 1032 | Train 17 | Test 8 | 0.69 | 0.31 | 0.27 | 0.29 | 3080 | Train 49 | Test 8 | 0.68 | 0.31 | 0.12 | 0.17 |
| 1033 | Train 17 | Test 9 | 0.77 | 0.25 | 0.64 | 0.36 | 3081 | Train 49 | Test 9 | 0.72 | 0.28 | 0.34 | 0.31 |
| 1034 | Train 17 | Test 10 | 0.75 | 0.27 | 0.57 | 0.37 | 3082 | Train 49 | Test 10 | 0.71 | 0.28 | 0.26 | 0.27 |
| 1035 | Train 17 | Test 11 | 0.77 | 0.25 | 0.64 | 0.36 | 3083 | Train 49 | Test 11 | 0.72 | 0.28 | 0.34 | 0.31 |
| 1036 | Train 17 | Test 12 | 0.75 | 0.27 | 0.57 | 0.37 | 3084 | Train 49 | Test 12 | 0.71 | 0.28 | 0.26 | 0.27 |
| 1037 | Train 17 | Test 13 | 0.71 | 0.23 | 0.45 | 0.31 | 3085 | Train 49 | Test 13 | 0.69 | 0.27 | 0.21 | 0.23 |
| 1038 | Train 17 | Test 14 | 0.69 | 0.32 | 0.27 | 0.29 | 3086 | Train 49 | Test 14 | 0.68 | 0.32 | 0.12 | 0.17 |
| 1039 | Train 17 | Test 15 | 0.71 | 0.23 | 0.44 | 0.3 | 3087 | Train 49 | Test 15 | 0.69 | 0.27 | 0.21 | 0.23 |
| 1040 | Train 17 | Test 16 | 0.69 | 0.31 | 0.27 | 0.29 | 3088 | Train 49 | Test 16 | 0.68 | 0.31 | 0.12 | 0.17 |
| 1041 | Train 17 | Test 17 | 0.77 | 0.24 | 0.65 | 0.35 | 3089 | Train 49 | Test 17 | 0.72 | 0.27 | 0.34 | 0.3 |
| 1042 | Train 17 | Test 18 | 0.75 | 0.27 | 0.58 | 0.36 | 3090 | Train 49 | Test 18 | 0.71 | 0.27 | 0.26 | 0.27 |
| 1043 | Train 17 | Test 19 | 0.77 | 0.24 | 0.65 | 0.35 | 3091 | Train 49 | Test 19 | 0.72 | 0.27 | 0.35 | 0.31 |
| 1044 | Train 17 | Test 20 | 0.75 | 0.27 | 0.58 | 0.36 | 3092 | Train 49 | Test 20 | 0.71 | 0.27 | 0.27 | 0.27 |
| 1045 | Train 17 | Test 21 | 0.71 | 0.23 | 0.46 | 0.31 | 3093 | Train 49 | Test 21 | 0.69 | 0.26 | 0.21 | 0.23 |
| 1046 | Train 17 | Test 22 | 0.69 | 0.32 | 0.27 | 0.29 | 3094 | Train 49 | Test 22 | 0.68 | 0.32 | 0.11 | 0.17 |
| 1047 | Train 17 | Test 23 | 0.71 | 0.23 | 0.46 | 0.3 | 3095 | Train 49 | Test 23 | 0.69 | 0.26 | 0.21 | 0.23 |
| 1048 | Train 17 | Test 24 | 0.69 | 0.31 | 0.27 | 0.29 | 3096 | Train 49 | Test 24 | 0.68 | 0.31 | 0.12 | 0.17 |
| 1049 | Train 17 | Test 25 | 0.77 | 0.25 | 0.64 | 0.36 | 3097 | Train 49 | Test 25 | 0.72 | 0.28 | 0.34 | 0.31 |
| 1050 | Train 17 | Test 26 | 0.75 | 0.27 | 0.57 | 0.37 | 3098 | Train 49 | Test 26 | 0.71 | 0.28 | 0.26 | 0.27 |
| 1051 | Train 17 | Test 27 | 0.77 | 0.25 | 0.64 | 0.36 | 3099 | Train 49 | Test 27 | 0.72 | 0.28 | 0.34 | 0.31 |
| 1052 | Train 17 | Test 28 | 0.75 | 0.27 | 0.57 | 0.37 | 3100 | Train 49 | Test 28 | 0.71 | 0.28 | 0.26 | 0.27 |
| 1053 | Train 17 | Test 29 | 0.71 | 0.23 | 0.45 | 0.31 | 3101 | Train 49 | Test 29 | 0.69 | 0.27 | 0.21 | 0.23 |
| 1054 | Train 17 | Test 30 | 0.69 | 0.32 | 0.27 | 0.29 | 3102 | Train 49 | Test 30 | 0.68 | 0.32 | 0.12 | 0.17 |
| 1055 | Train 17 | Test 31 | 0.71 | 0.23 | 0.44 | 0.3 | 3103 | Train 49 | Test 31 | 0.69 | 0.27 | 0.21 | 0.23 |
| 1056 | Train 17 | Test 32 | 0.69 | 0.31 | 0.27 | 0.29 | 3104 | Train 49 | Test 32 | 0.68 | 0.31 | 0.12 | 0.17 |
| 1057 | Train 17 | Test 33 | 0.77 | 0.24 | 0.65 | 0.35 | 3105 | Train 49 | Test 33 | 0.72 | 0.27 | 0.34 | 0.3 |
| 1058 | Train 17 | Test 34 | 0.75 | 0.26 | 0.58 | 0.36 | 3106 | Train 49 | Test 34 | 0.71 | 0.27 | 0.26 | 0.27 |
| 1059 | Train 17 | Test 35 | 0.77 | 0.24 | 0.65 | 0.35 | 3107 | Train 49 | Test 35 | 0.72 | 0.27 | 0.34 | 0.31 |
| 1060 | Train 17 | Test 36 | 0.75 | 0.26 | 0.58 | 0.36 | 3108 | Train 49 | Test 36 | 0.71 | 0.27 | 0.27 | 0.27 |
| 1061 | Train 17 | Test 37 | 0.71 | 0.23 | 0.46 | 0.31 | 3109 | Train 49 | Test 37 | 0.69 | 0.26 | 0.21 | 0.23 |
| 1062 | Train 17 | Test 38 | 0.69 | 0.32 | 0.27 | 0.29 | 3110 | Train 49 | Test 38 | 0.68 | 0.32 | 0.11 | 0.17 |
| 1063 | Train 17 | Test 39 | 0.71 | 0.23 | 0.46 | 0.3 | 3111 | Train 49 | Test 39 | 0.69 | 0.26 | 0.21 | 0.23 |
| 1064 | Train 17 | Test 40 | 0.69 | 0.31 | 0.27 | 0.29 | 3112 | Train 49 | Test 40 | 0.68 | 0.31 | 0.12 | 0.17 |
| 1065 | Train 17 | Test 41 | 0.77 | 0.25 | 0.64 | 0.35 | 3113 | Train 49 | Test 41 | 0.72 | 0.28 | 0.34 | 0.3 |
| 1066 | Train 17 | Test 42 | 0.75 | 0.27 | 0.57 | 0.37 | 3114 | Train 49 | Test 42 | 0.71 | 0.28 | 0.26 | 0.27 |
| 1067 | Train 17 | Test 43 | 0.77 | 0.25 | 0.64 | 0.36 | 3115 | Train 49 | Test 43 | 0.72 | 0.28 | 0.34 | 0.31 |
| 1068 | Train 17 | Test 44 | 0.75 | 0.27 | 0.57 | 0.37 | 3116 | Train 49 | Test 44 | 0.71 | 0.28 | 0.26 | 0.27 |
| 1069 | Train 17 | Test 45 | 0.71 | 0.23 | 0.45 | 0.31 | 3117 | Train 49 | Test 45 | 0.69 | 0.27 | 0.21 | 0.23 |
| 1070 | Train 17 | Test 46 | 0.7 | 0.32 | 0.27 | 0.29 | 3118 | Train 49 | Test 46 | 0.68 | 0.32 | 0.12 | 0.17 |
| 1071 | Train 17 | Test 47 | 0.71 | 0.23 | 0.44 | 0.3 | 3119 | Train 49 | Test 47 | 0.69 | 0.27 | 0.21 | 0.23 |
| 1072 | Train 17 | Test 48 | 0.69 | 0.31 | 0.27 | 0.29 | 3120 | Train 49 | Test 48 | 0.68 | 0.31 | 0.12 | 0.17 |
| 1073 | Train 17 | Test 49 | 0.77 | 0.27 | 0.74 | 0.4 | 3121 | Train 49 | Test 49 | 0.77 | 0.29 | 0.65 | 0.4 |
| 1074 | Train 17 | Test 50 | 0.77 | 0.27 | 0.73 | 0.4 | 3122 | Train 49 | Test 50 | 0.77 | 0.29 | 0.65 | 0.4 |
| 1075 | Train 17 | Test 51 | 0.77 | 0.27 | 0.74 | 0.4 | 3123 | Train 49 | Test 51 | 0.77 | 0.29 | 0.65 | 0.4 |
| 1076 | Train 17 | Test 52 | 0.77 | 0.27 | 0.73 | 0.4 | 3124 | Train 49 | Test 52 | 0.77 | 0.29 | 0.65 | 0.4 |
| 1077 | Train 17 | Test 53 | 0.75 | 0.29 | 0.42 | 0.35 | 3125 | Train 49 | Test 53 | 0.73 | 0.28 | 0.38 | 0.32 |
| 1078 | Train 17 | Test 54 | 0.71 | 0.37 | 0.27 | 0.31 | 3126 | Train 49 | Test 54 | 0.69 | 0.33 | 0.16 | 0.22 |
| 1079 | Train 17 | Test 55 | 0.75 | 0.27 | 0.51 | 0.36 | 3127 | Train 49 | Test 55 | 0.74 | 0.29 | 0.39 | 0.33 |
| 1080 | Train 17 | Test 56 | 0.7 | 0.34 | 0.28 | 0.31 | 3128 | Train 49 | Test 56 | 0.69 | 0.32 | 0.16 | 0.22 |
| 1081 | Train 17 | Test 57 | 0.78 | 0.28 | 0.74 | 0.41 | 3129 | Train 49 | Test 57 | 0.78 | 0.29 | 0.64 | 0.4 |
| 1082 | Train 17 | Test 58 | 0.78 | 0.28 | 0.74 | 0.4 | 3130 | Train 49 | Test 58 | 0.77 | 0.29 | 0.64 | 0.4 |
| 1083 | Train 17 | Test 59 | 0.78 | 0.28 | 0.74 | 0.41 | 3131 | Train 49 | Test 59 | 0.78 | 0.3 | 0.65 | 0.41 |
| 1084 | Train 17 | Test 60 | 0.78 | 0.28 | 0.73 | 0.41 | 3132 | Train 49 | Test 60 | 0.78 | 0.3 | 0.64 | 0.41 |
| 1085 | Train 17 | Test 61 | 0.75 | 0.3 | 0.42 | 0.35 | 3133 | Train 49 | Test 61 | 0.74 | 0.28 | 0.38 | 0.33 |
| 1086 | Train 17 | Test 62 | 0.71 | 0.37 | 0.27 | 0.31 | 3134 | Train 49 | Test 62 | 0.69 | 0.33 | 0.16 | 0.22 |
| 1087 | Train 17 | Test 63 | 0.75 | 0.3 | 0.43 | 0.35 | 3135 | Train 49 | Test 63 | 0.74 | 0.29 | 0.39 | 0.33 |
| 1088 | Train 17 | Test 64 | 0.7 | 0.34 | 0.28 | 0.31 | 3136 | Train 49 | Test 64 | 0.69 | 0.32 | 0.17 | 0.22 |
| 1089 | Train 18 | Test 1 | 0.77 | 0.25 | 0.61 | 0.36 | 3137 | Train 50 | Test 1 | 0.7 | 0.27 | 0.34 | 0.3 |
| 1090 | Train 18 | Test 2 | 0.78 | 0.28 | 0.67 | 0.39 | 3138 | Train 50 | Test 2 | 0.7 | 0.27 | 0.26 | 0.27 |
| 1091 | Train 18 | Test 3 | 0.77 | 0.25 | 0.61 | 0.36 | 3139 | Train 50 | Test 3 | 0.7 | 0.28 | 0.35 | 0.31 |
| 1092 | Train 18 | Test 4 | 0.78 | 0.28 | 0.67 | 0.4 | 3140 | Train 50 | Test 4 | 0.7 | 0.27 | 0.26 | 0.27 |
| 1093 | Train 18 | Test 5 | 0.72 | 0.23 | 0.46 | 0.31 | 3141 | Train 50 | Test 5 | 0.68 | 0.26 | 0.2 | 0.23 |
| 1094 | Train 18 | Test 6 | 0.7 | 0.35 | 0.33 | 0.34 | 3142 | Train 50 | Test 6 | 0.67 | 0.31 | 0.11 | 0.16 |
| 1095 | Train 18 | Test 7 | 0.71 | 0.23 | 0.45 | 0.3 | 3143 | Train 50 | Test 7 | 0.67 | 0.26 | 0.2 | 0.23 |
| 1096 | Train 18 | Test 8 | 0.7 | 0.34 | 0.33 | 0.34 | 3144 | Train 50 | Test 8 | 0.67 | 0.3 | 0.11 | 0.16 |
| 1097 | Train 18 | Test 9 | 0.77 | 0.26 | 0.6 | 0.36 | 3145 | Train 50 | Test 9 | 0.7 | 0.28 | 0.34 | 0.31 |
| 1098 | Train 18 | Test 10 | 0.78 | 0.28 | 0.66 | 0.4 | 3146 | Train 50 | Test 10 | 0.7 | 0.28 | 0.26 | 0.27 |
| 1099 | Train 18 | Test 11 | 0.77 | 0.26 | 0.6 | 0.36 | 3147 | Train 50 | Test 11 | 0.7 | 0.28 | 0.35 | 0.31 |
| 1100 | Train 18 | Test 12 | 0.78 | 0.29 | 0.66 | 0.4 | 3148 | Train 50 | Test 12 | 0.7 | 0.28 | 0.26 | 0.27 |
| 1101 | Train 18 | Test 13 | 0.72 | 0.23 | 0.45 | 0.31 | 3149 | Train 50 | Test 13 | 0.68 | 0.26 | 0.2 | 0.23 |
| 1102 | Train 18 | Test 14 | 0.71 | 0.34 | 0.34 | 0.34 | 3150 | Train 50 | Test 14 | 0.67 | 0.31 | 0.11 | 0.16 |
| 1103 | Train 18 | Test 15 | 0.71 | 0.23 | 0.44 | 0.31 | 3151 | Train 50 | Test 15 | 0.68 | 0.27 | 0.2 | 0.23 |
| 1104 | Train 18 | Test 16 | 0.7 | 0.33 | 0.34 | 0.33 | 3152 | Train 50 | Test 16 | 0.67 | 0.3 | 0.11 | 0.16 |
| 1105 | Train 18 | Test 17 | 0.77 | 0.25 | 0.61 | 0.36 | 3153 | Train 50 | Test 17 | 0.7 | 0.27 | 0.34 | 0.3 |
| 1106 | Train 18 | Test 18 | 0.78 | 0.28 | 0.67 | 0.39 | 3154 | Train 50 | Test 18 | 0.7 | 0.27 | 0.26 | 0.27 |
| 1107 | Train 18 | Test 19 | 0.77 | 0.25 | 0.61 | 0.36 | 3155 | Train 50 | Test 19 | 0.7 | 0.28 | 0.35 | 0.31 |
| 1108 | Train 18 | Test 20 | 0.78 | 0.28 | 0.67 | 0.4 | 3156 | Train 50 | Test 20 | 0.7 | 0.27 | 0.26 | 0.27 |
| 1109 | Train 18 | Test 21 | 0.72 | 0.23 | 0.46 | 0.31 | 3157 | Train 50 | Test 21 | 0.68 | 0.26 | 0.2 | 0.23 |
| 1110 | Train 18 | Test 22 | 0.71 | 0.35 | 0.33 | 0.34 | 3158 | Train 50 | Test 22 | 0.67 | 0.31 | 0.11 | 0.16 |
| 1111 | Train 18 | Test 23 | 0.71 | 0.23 | 0.45 | 0.3 | 3159 | Train 50 | Test 23 | 0.68 | 0.26 | 0.2 | 0.23 |
| 1112 | Train 18 | Test 24 | 0.7 | 0.34 | 0.33 | 0.33 | 3160 | Train 50 | Test 24 | 0.67 | 0.3 | 0.11 | 0.16 |
| 1113 | Train 18 | Test 25 | 0.77 | 0.26 | 0.6 | 0.36 | 3161 | Train 50 | Test 25 | 0.7 | 0.28 | 0.34 | 0.31 |
| 1114 | Train 18 | Test 26 | 0.78 | 0.28 | 0.66 | 0.4 | 3162 | Train 50 | Test 26 | 0.7 | 0.28 | 0.26 | 0.27 |
| 1115 | Train 18 | Test 27 | 0.77 | 0.26 | 0.6 | 0.36 | 3163 | Train 50 | Test 27 | 0.7 | 0.28 | 0.35 | 0.31 |
| 1116 | Train 18 | Test 28 | 0.78 | 0.29 | 0.66 | 0.4 | 3164 | Train 50 | Test 28 | 0.7 | 0.28 | 0.26 | 0.27 |
| 1117 | Train 18 | Test 29 | 0.72 | 0.23 | 0.45 | 0.31 | 3165 | Train 50 | Test 29 | 0.68 | 0.26 | 0.2 | 0.23 |
| 1118 | Train 18 | Test 30 | 0.71 | 0.34 | 0.33 | 0.34 | 3166 | Train 50 | Test 30 | 0.67 | 0.31 | 0.11 | 0.16 |
| 1119 | Train 18 | Test 31 | 0.71 | 0.23 | 0.44 | 0.31 | 3167 | Train 50 | Test 31 | 0.68 | 0.27 | 0.2 | 0.23 |
| 1120 | Train 18 | Test 32 | 0.7 | 0.33 | 0.33 | 0.33 | 3168 | Train 50 | Test 32 | 0.67 | 0.3 | 0.11 | 0.16 |
| 1121 | Train 18 | Test 33 | 0.77 | 0.25 | 0.61 | 0.35 | 3169 | Train 50 | Test 33 | 0.7 | 0.27 | 0.34 | 0.3 |
| 1122 | Train 18 | Test 34 | 0.78 | 0.28 | 0.67 | 0.39 | 3170 | Train 50 | Test 34 | 0.7 | 0.27 | 0.26 | 0.26 |
| 1123 | Train 18 | Test 35 | 0.77 | 0.25 | 0.61 | 0.36 | 3171 | Train 50 | Test 35 | 0.7 | 0.28 | 0.34 | 0.31 |
| 1124 | Train 18 | Test 36 | 0.78 | 0.28 | 0.67 | 0.39 | 3172 | Train 50 | Test 36 | 0.7 | 0.27 | 0.26 | 0.27 |
| 1125 | Train 18 | Test 37 | 0.72 | 0.23 | 0.45 | 0.3 | 3173 | Train 50 | Test 37 | 0.68 | 0.26 | 0.2 | 0.23 |
| 1126 | Train 18 | Test 38 | 0.71 | 0.35 | 0.33 | 0.34 | 3174 | Train 50 | Test 38 | 0.67 | 0.31 | 0.11 | 0.16 |
| 1127 | Train 18 | Test 39 | 0.72 | 0.23 | 0.45 | 0.3 | 3175 | Train 50 | Test 39 | 0.68 | 0.26 | 0.2 | 0.23 |
| 1128 | Train 18 | Test 40 | 0.7 | 0.34 | 0.33 | 0.33 | 3176 | Train 50 | Test 40 | 0.67 | 0.3 | 0.11 | 0.16 |
| 1129 | Train 18 | Test 41 | 0.77 | 0.26 | 0.6 | 0.36 | 3177 | Train 50 | Test 41 | 0.7 | 0.28 | 0.34 | 0.31 |
| 1130 | Train 18 | Test 42 | 0.78 | 0.28 | 0.66 | 0.4 | 3178 | Train 50 | Test 42 | 0.7 | 0.28 | 0.26 | 0.27 |
| 1131 | Train 18 | Test 43 | 0.77 | 0.26 | 0.6 | 0.36 | 3179 | Train 50 | Test 43 | 0.71 | 0.28 | 0.35 | 0.31 |
| 1132 | Train 18 | Test 44 | 0.78 | 0.28 | 0.66 | 0.4 | 3180 | Train 50 | Test 44 | 0.7 | 0.28 | 0.26 | 0.27 |
| 1133 | Train 18 | Test 45 | 0.72 | 0.23 | 0.44 | 0.31 | 3181 | Train 50 | Test 45 | 0.68 | 0.26 | 0.2 | 0.23 |
| 1134 | Train 18 | Test 46 | 0.71 | 0.34 | 0.34 | 0.34 | 3182 | Train 50 | Test 46 | 0.67 | 0.31 | 0.11 | 0.16 |
| 1135 | Train 18 | Test 47 | 0.72 | 0.23 | 0.44 | 0.3 | 3183 | Train 50 | Test 47 | 0.68 | 0.27 | 0.2 | 0.23 |
| 1136 | Train 18 | Test 48 | 0.71 | 0.33 | 0.34 | 0.33 | 3184 | Train 50 | Test 48 | 0.67 | 0.3 | 0.11 | 0.16 |
| 1137 | Train 18 | Test 49 | 0.77 | 0.28 | 0.72 | 0.4 | 3185 | Train 50 | Test 49 | 0.77 | 0.29 | 0.66 | 0.4 |
| 1138 | Train 18 | Test 50 | 0.77 | 0.28 | 0.72 | 0.4 | 3186 | Train 50 | Test 50 | 0.77 | 0.29 | 0.65 | 0.4 |
| 1139 | Train 18 | Test 51 | 0.77 | 0.28 | 0.72 | 0.4 | 3187 | Train 50 | Test 51 | 0.77 | 0.29 | 0.66 | 0.4 |
| 1140 | Train 18 | Test 52 | 0.77 | 0.28 | 0.72 | 0.4 | 3188 | Train 50 | Test 52 | 0.77 | 0.29 | 0.65 | 0.4 |
| 1141 | Train 18 | Test 53 | 0.75 | 0.31 | 0.37 | 0.34 | 3189 | Train 50 | Test 53 | 0.71 | 0.28 | 0.38 | 0.32 |
| 1142 | Train 18 | Test 54 | 0.72 | 0.4 | 0.31 | 0.35 | 3190 | Train 50 | Test 54 | 0.68 | 0.33 | 0.15 | 0.21 |
| 1143 | Train 18 | Test 55 | 0.75 | 0.28 | 0.51 | 0.36 | 3191 | Train 50 | Test 55 | 0.72 | 0.29 | 0.38 | 0.33 |
| 1144 | Train 18 | Test 56 | 0.71 | 0.37 | 0.32 | 0.34 | 3192 | Train 50 | Test 56 | 0.68 | 0.32 | 0.16 | 0.21 |
| 1145 | Train 18 | Test 57 | 0.78 | 0.29 | 0.72 | 0.41 | 3193 | Train 50 | Test 57 | 0.78 | 0.3 | 0.66 | 0.41 |
| 1146 | Train 18 | Test 58 | 0.78 | 0.29 | 0.72 | 0.41 | 3194 | Train 50 | Test 58 | 0.77 | 0.3 | 0.65 | 0.41 |
| 1147 | Train 18 | Test 59 | 0.78 | 0.29 | 0.72 | 0.41 | 3195 | Train 50 | Test 59 | 0.78 | 0.3 | 0.66 | 0.41 |
| 1148 | Train 18 | Test 60 | 0.78 | 0.29 | 0.72 | 0.41 | 3196 | Train 50 | Test 60 | 0.77 | 0.3 | 0.65 | 0.41 |
| 1149 | Train 18 | Test 61 | 0.76 | 0.31 | 0.37 | 0.34 | 3197 | Train 50 | Test 61 | 0.72 | 0.28 | 0.38 | 0.32 |
| 1150 | Train 18 | Test 62 | 0.72 | 0.39 | 0.31 | 0.35 | 3198 | Train 50 | Test 62 | 0.68 | 0.33 | 0.16 | 0.21 |
| 1151 | Train 18 | Test 63 | 0.76 | 0.31 | 0.38 | 0.34 | 3199 | Train 50 | Test 63 | 0.72 | 0.29 | 0.38 | 0.33 |
| 1152 | Train 18 | Test 64 | 0.71 | 0.36 | 0.32 | 0.34 | 3200 | Train 50 | Test 64 | 0.68 | 0.32 | 0.16 | 0.21 |
| 1153 | Train 19 | Test 1 | 0.77 | 0.24 | 0.65 | 0.35 | 3201 | Train 51 | Test 1 | 0.72 | 0.26 | 0.35 | 0.3 |
| 1154 | Train 19 | Test 2 | 0.75 | 0.27 | 0.59 | 0.37 | 3202 | Train 51 | Test 2 | 0.7 | 0.26 | 0.27 | 0.26 |
| 1155 | Train 19 | Test 3 | 0.77 | 0.24 | 0.65 | 0.35 | 3203 | Train 51 | Test 3 | 0.72 | 0.27 | 0.35 | 0.3 |
| 1156 | Train 19 | Test 4 | 0.75 | 0.27 | 0.59 | 0.37 | 3204 | Train 51 | Test 4 | 0.71 | 0.26 | 0.27 | 0.27 |
| 1157 | Train 19 | Test 5 | 0.71 | 0.23 | 0.46 | 0.31 | 3205 | Train 51 | Test 5 | 0.69 | 0.25 | 0.21 | 0.23 |
| 1158 | Train 19 | Test 6 | 0.69 | 0.32 | 0.26 | 0.28 | 3206 | Train 51 | Test 6 | 0.68 | 0.31 | 0.12 | 0.17 |
| 1159 | Train 19 | Test 7 | 0.71 | 0.23 | 0.46 | 0.31 | 3207 | Train 51 | Test 7 | 0.69 | 0.25 | 0.21 | 0.23 |
| 1160 | Train 19 | Test 8 | 0.69 | 0.31 | 0.26 | 0.28 | 3208 | Train 51 | Test 8 | 0.68 | 0.3 | 0.12 | 0.17 |
| 1161 | Train 19 | Test 9 | 0.77 | 0.24 | 0.63 | 0.35 | 3209 | Train 51 | Test 9 | 0.72 | 0.27 | 0.34 | 0.3 |
| 1162 | Train 19 | Test 10 | 0.75 | 0.27 | 0.56 | 0.36 | 3210 | Train 51 | Test 10 | 0.71 | 0.27 | 0.26 | 0.27 |
| 1163 | Train 19 | Test 11 | 0.77 | 0.24 | 0.63 | 0.35 | 3211 | Train 51 | Test 11 | 0.72 | 0.27 | 0.35 | 0.3 |
| 1164 | Train 19 | Test 12 | 0.75 | 0.27 | 0.57 | 0.36 | 3212 | Train 51 | Test 12 | 0.71 | 0.27 | 0.27 | 0.27 |
| 1165 | Train 19 | Test 13 | 0.71 | 0.23 | 0.45 | 0.31 | 3213 | Train 51 | Test 13 | 0.69 | 0.26 | 0.21 | 0.23 |
| 1166 | Train 19 | Test 14 | 0.69 | 0.31 | 0.26 | 0.28 | 3214 | Train 51 | Test 14 | 0.68 | 0.31 | 0.12 | 0.17 |
| 1167 | Train 19 | Test 15 | 0.71 | 0.23 | 0.44 | 0.31 | 3215 | Train 51 | Test 15 | 0.69 | 0.26 | 0.21 | 0.23 |
| 1168 | Train 19 | Test 16 | 0.69 | 0.31 | 0.26 | 0.28 | 3216 | Train 51 | Test 16 | 0.68 | 0.3 | 0.12 | 0.17 |
| 1169 | Train 19 | Test 17 | 0.77 | 0.24 | 0.65 | 0.35 | 3217 | Train 51 | Test 17 | 0.72 | 0.26 | 0.35 | 0.3 |
| 1170 | Train 19 | Test 18 | 0.75 | 0.27 | 0.59 | 0.37 | 3218 | Train 51 | Test 18 | 0.7 | 0.26 | 0.27 | 0.26 |
| 1171 | Train 19 | Test 19 | 0.77 | 0.24 | 0.65 | 0.35 | 3219 | Train 51 | Test 19 | 0.72 | 0.27 | 0.35 | 0.3 |
| 1172 | Train 19 | Test 20 | 0.75 | 0.27 | 0.59 | 0.37 | 3220 | Train 51 | Test 20 | 0.71 | 0.26 | 0.27 | 0.27 |
| 1173 | Train 19 | Test 21 | 0.71 | 0.23 | 0.46 | 0.31 | 3221 | Train 51 | Test 21 | 0.69 | 0.25 | 0.21 | 0.23 |
| 1174 | Train 19 | Test 22 | 0.69 | 0.32 | 0.25 | 0.28 | 3222 | Train 51 | Test 22 | 0.68 | 0.31 | 0.12 | 0.17 |
| 1175 | Train 19 | Test 23 | 0.71 | 0.23 | 0.46 | 0.31 | 3223 | Train 51 | Test 23 | 0.69 | 0.25 | 0.21 | 0.23 |
| 1176 | Train 19 | Test 24 | 0.69 | 0.31 | 0.26 | 0.28 | 3224 | Train 51 | Test 24 | 0.68 | 0.3 | 0.12 | 0.17 |
| 1177 | Train 19 | Test 25 | 0.77 | 0.24 | 0.63 | 0.35 | 3225 | Train 51 | Test 25 | 0.72 | 0.27 | 0.34 | 0.3 |
| 1178 | Train 19 | Test 26 | 0.75 | 0.27 | 0.56 | 0.36 | 3226 | Train 51 | Test 26 | 0.71 | 0.27 | 0.26 | 0.27 |
| 1179 | Train 19 | Test 27 | 0.77 | 0.24 | 0.63 | 0.35 | 3227 | Train 51 | Test 27 | 0.72 | 0.27 | 0.35 | 0.3 |
| 1180 | Train 19 | Test 28 | 0.75 | 0.27 | 0.57 | 0.36 | 3228 | Train 51 | Test 28 | 0.71 | 0.27 | 0.26 | 0.27 |
| 1181 | Train 19 | Test 29 | 0.71 | 0.23 | 0.45 | 0.31 | 3229 | Train 51 | Test 29 | 0.69 | 0.26 | 0.21 | 0.23 |
| 1182 | Train 19 | Test 30 | 0.69 | 0.31 | 0.26 | 0.28 | 3230 | Train 51 | Test 30 | 0.68 | 0.31 | 0.12 | 0.17 |
| 1183 | Train 19 | Test 31 | 0.71 | 0.23 | 0.44 | 0.31 | 3231 | Train 51 | Test 31 | 0.69 | 0.26 | 0.21 | 0.23 |
| 1184 | Train 19 | Test 32 | 0.69 | 0.3 | 0.26 | 0.28 | 3232 | Train 51 | Test 32 | 0.68 | 0.3 | 0.12 | 0.17 |
| 1185 | Train 19 | Test 33 | 0.77 | 0.24 | 0.65 | 0.35 | 3233 | Train 51 | Test 33 | 0.72 | 0.26 | 0.34 | 0.3 |
| 1186 | Train 19 | Test 34 | 0.75 | 0.26 | 0.59 | 0.36 | 3234 | Train 51 | Test 34 | 0.71 | 0.26 | 0.26 | 0.26 |
| 1187 | Train 19 | Test 35 | 0.77 | 0.24 | 0.65 | 0.35 | 3235 | Train 51 | Test 35 | 0.72 | 0.26 | 0.35 | 0.3 |
| 1188 | Train 19 | Test 36 | 0.75 | 0.26 | 0.59 | 0.36 | 3236 | Train 51 | Test 36 | 0.71 | 0.26 | 0.27 | 0.27 |
| 1189 | Train 19 | Test 37 | 0.71 | 0.23 | 0.46 | 0.31 | 3237 | Train 51 | Test 37 | 0.69 | 0.25 | 0.21 | 0.23 |
| 1190 | Train 19 | Test 38 | 0.69 | 0.32 | 0.26 | 0.28 | 3238 | Train 51 | Test 38 | 0.68 | 0.31 | 0.12 | 0.17 |
| 1191 | Train 19 | Test 39 | 0.71 | 0.23 | 0.46 | 0.31 | 3239 | Train 51 | Test 39 | 0.69 | 0.25 | 0.21 | 0.23 |
| 1192 | Train 19 | Test 40 | 0.69 | 0.31 | 0.26 | 0.28 | 3240 | Train 51 | Test 40 | 0.68 | 0.3 | 0.12 | 0.17 |
| 1193 | Train 19 | Test 41 | 0.77 | 0.24 | 0.63 | 0.35 | 3241 | Train 51 | Test 41 | 0.72 | 0.27 | 0.34 | 0.3 |
| 1194 | Train 19 | Test 42 | 0.75 | 0.27 | 0.56 | 0.36 | 3242 | Train 51 | Test 42 | 0.71 | 0.27 | 0.26 | 0.26 |
| 1195 | Train 19 | Test 43 | 0.77 | 0.24 | 0.63 | 0.35 | 3243 | Train 51 | Test 43 | 0.72 | 0.27 | 0.35 | 0.3 |
| 1196 | Train 19 | Test 44 | 0.75 | 0.27 | 0.56 | 0.36 | 3244 | Train 51 | Test 44 | 0.71 | 0.27 | 0.26 | 0.27 |
| 1197 | Train 19 | Test 45 | 0.71 | 0.23 | 0.45 | 0.31 | 3245 | Train 51 | Test 45 | 0.69 | 0.26 | 0.21 | 0.23 |
| 1198 | Train 19 | Test 46 | 0.69 | 0.31 | 0.26 | 0.28 | 3246 | Train 51 | Test 46 | 0.68 | 0.31 | 0.12 | 0.17 |
| 1199 | Train 19 | Test 47 | 0.71 | 0.23 | 0.44 | 0.31 | 3247 | Train 51 | Test 47 | 0.69 | 0.26 | 0.21 | 0.23 |
| 1200 | Train 19 | Test 48 | 0.69 | 0.3 | 0.26 | 0.28 | 3248 | Train 51 | Test 48 | 0.68 | 0.3 | 0.12 | 0.17 |
| 1201 | Train 19 | Test 49 | 0.77 | 0.27 | 0.75 | 0.4 | 3249 | Train 51 | Test 49 | 0.77 | 0.28 | 0.66 | 0.4 |
| 1202 | Train 19 | Test 50 | 0.77 | 0.27 | 0.75 | 0.4 | 3250 | Train 51 | Test 50 | 0.77 | 0.28 | 0.65 | 0.39 |
| 1203 | Train 19 | Test 51 | 0.77 | 0.27 | 0.75 | 0.4 | 3251 | Train 51 | Test 51 | 0.77 | 0.29 | 0.66 | 0.4 |
| 1204 | Train 19 | Test 52 | 0.77 | 0.27 | 0.75 | 0.4 | 3252 | Train 51 | Test 52 | 0.77 | 0.29 | 0.65 | 0.4 |
| 1205 | Train 19 | Test 53 | 0.75 | 0.29 | 0.42 | 0.35 | 3253 | Train 51 | Test 53 | 0.73 | 0.28 | 0.38 | 0.32 |
| 1206 | Train 19 | Test 54 | 0.7 | 0.37 | 0.26 | 0.31 | 3254 | Train 51 | Test 54 | 0.69 | 0.33 | 0.17 | 0.22 |
| 1207 | Train 19 | Test 55 | 0.75 | 0.27 | 0.51 | 0.36 | 3255 | Train 51 | Test 55 | 0.74 | 0.28 | 0.39 | 0.32 |
| 1208 | Train 19 | Test 56 | 0.7 | 0.34 | 0.27 | 0.3 | 3256 | Train 51 | Test 56 | 0.69 | 0.32 | 0.17 | 0.22 |
| 1209 | Train 19 | Test 57 | 0.78 | 0.28 | 0.75 | 0.41 | 3257 | Train 51 | Test 57 | 0.78 | 0.29 | 0.65 | 0.4 |
| 1210 | Train 19 | Test 58 | 0.78 | 0.28 | 0.75 | 0.4 | 3258 | Train 51 | Test 58 | 0.77 | 0.29 | 0.65 | 0.4 |
| 1211 | Train 19 | Test 59 | 0.78 | 0.28 | 0.75 | 0.41 | 3259 | Train 51 | Test 59 | 0.78 | 0.29 | 0.65 | 0.4 |
| 1212 | Train 19 | Test 60 | 0.78 | 0.28 | 0.75 | 0.41 | 3260 | Train 51 | Test 60 | 0.77 | 0.29 | 0.65 | 0.4 |
| 1213 | Train 19 | Test 61 | 0.75 | 0.3 | 0.43 | 0.35 | 3261 | Train 51 | Test 61 | 0.74 | 0.28 | 0.38 | 0.32 |
| 1214 | Train 19 | Test 62 | 0.71 | 0.36 | 0.27 | 0.31 | 3262 | Train 51 | Test 62 | 0.7 | 0.33 | 0.17 | 0.22 |
| 1215 | Train 19 | Test 63 | 0.75 | 0.3 | 0.43 | 0.35 | 3263 | Train 51 | Test 63 | 0.74 | 0.28 | 0.39 | 0.33 |
| 1216 | Train 19 | Test 64 | 0.7 | 0.34 | 0.27 | 0.3 | 3264 | Train 51 | Test 64 | 0.69 | 0.32 | 0.17 | 0.22 |
| 1217 | Train 20 | Test 1 | 0.77 | 0.25 | 0.62 | 0.35 | 3265 | Train 52 | Test 1 | 0.69 | 0.26 | 0.35 | 0.3 |
| 1218 | Train 20 | Test 2 | 0.78 | 0.28 | 0.67 | 0.39 | 3266 | Train 52 | Test 2 | 0.69 | 0.26 | 0.27 | 0.26 |
| 1219 | Train 20 | Test 3 | 0.77 | 0.25 | 0.62 | 0.36 | 3267 | Train 52 | Test 3 | 0.7 | 0.27 | 0.35 | 0.3 |
| 1220 | Train 20 | Test 4 | 0.78 | 0.28 | 0.67 | 0.39 | 3268 | Train 52 | Test 4 | 0.69 | 0.27 | 0.27 | 0.27 |
| 1221 | Train 20 | Test 5 | 0.72 | 0.23 | 0.46 | 0.3 | 3269 | Train 52 | Test 5 | 0.67 | 0.25 | 0.2 | 0.23 |
| 1222 | Train 20 | Test 6 | 0.7 | 0.35 | 0.32 | 0.33 | 3270 | Train 52 | Test 6 | 0.67 | 0.31 | 0.11 | 0.16 |
| 1223 | Train 20 | Test 7 | 0.71 | 0.23 | 0.46 | 0.3 | 3271 | Train 52 | Test 7 | 0.67 | 0.25 | 0.2 | 0.23 |
| 1224 | Train 20 | Test 8 | 0.7 | 0.34 | 0.32 | 0.33 | 3272 | Train 52 | Test 8 | 0.67 | 0.3 | 0.11 | 0.16 |
| 1225 | Train 20 | Test 9 | 0.77 | 0.25 | 0.61 | 0.36 | 3273 | Train 52 | Test 9 | 0.7 | 0.27 | 0.34 | 0.3 |
| 1226 | Train 20 | Test 10 | 0.78 | 0.28 | 0.67 | 0.4 | 3274 | Train 52 | Test 10 | 0.69 | 0.27 | 0.26 | 0.27 |
| 1227 | Train 20 | Test 11 | 0.77 | 0.26 | 0.61 | 0.36 | 3275 | Train 52 | Test 11 | 0.7 | 0.27 | 0.35 | 0.31 |
| 1228 | Train 20 | Test 12 | 0.78 | 0.28 | 0.67 | 0.4 | 3276 | Train 52 | Test 12 | 0.69 | 0.27 | 0.26 | 0.27 |
| 1229 | Train 20 | Test 13 | 0.72 | 0.23 | 0.45 | 0.31 | 3277 | Train 52 | Test 13 | 0.68 | 0.26 | 0.2 | 0.23 |
| 1230 | Train 20 | Test 14 | 0.71 | 0.35 | 0.32 | 0.33 | 3278 | Train 52 | Test 14 | 0.67 | 0.31 | 0.11 | 0.16 |
| 1231 | Train 20 | Test 15 | 0.71 | 0.23 | 0.44 | 0.3 | 3279 | Train 52 | Test 15 | 0.68 | 0.26 | 0.2 | 0.23 |
| 1232 | Train 20 | Test 16 | 0.7 | 0.33 | 0.32 | 0.33 | 3280 | Train 52 | Test 16 | 0.67 | 0.3 | 0.11 | 0.16 |
| 1233 | Train 20 | Test 17 | 0.77 | 0.25 | 0.62 | 0.35 | 3281 | Train 52 | Test 17 | 0.69 | 0.26 | 0.35 | 0.3 |
| 1234 | Train 20 | Test 18 | 0.78 | 0.28 | 0.67 | 0.39 | 3282 | Train 52 | Test 18 | 0.69 | 0.26 | 0.27 | 0.26 |
| 1235 | Train 20 | Test 19 | 0.77 | 0.25 | 0.62 | 0.36 | 3283 | Train 52 | Test 19 | 0.7 | 0.27 | 0.35 | 0.3 |
| 1236 | Train 20 | Test 20 | 0.78 | 0.28 | 0.67 | 0.39 | 3284 | Train 52 | Test 20 | 0.69 | 0.27 | 0.27 | 0.27 |
| 1237 | Train 20 | Test 21 | 0.72 | 0.23 | 0.46 | 0.3 | 3285 | Train 52 | Test 21 | 0.67 | 0.25 | 0.2 | 0.22 |
| 1238 | Train 20 | Test 22 | 0.71 | 0.35 | 0.32 | 0.33 | 3286 | Train 52 | Test 22 | 0.67 | 0.31 | 0.11 | 0.16 |
| 1239 | Train 20 | Test 23 | 0.71 | 0.23 | 0.46 | 0.3 | 3287 | Train 52 | Test 23 | 0.67 | 0.25 | 0.2 | 0.23 |
| 1240 | Train 20 | Test 24 | 0.7 | 0.34 | 0.32 | 0.33 | 3288 | Train 52 | Test 24 | 0.67 | 0.3 | 0.11 | 0.16 |
| 1241 | Train 20 | Test 25 | 0.77 | 0.25 | 0.61 | 0.36 | 3289 | Train 52 | Test 25 | 0.7 | 0.27 | 0.34 | 0.3 |
| 1242 | Train 20 | Test 26 | 0.78 | 0.28 | 0.67 | 0.4 | 3290 | Train 52 | Test 26 | 0.69 | 0.27 | 0.26 | 0.27 |
| 1243 | Train 20 | Test 27 | 0.77 | 0.26 | 0.61 | 0.36 | 3291 | Train 52 | Test 27 | 0.7 | 0.27 | 0.35 | 0.3 |
| 1244 | Train 20 | Test 28 | 0.78 | 0.28 | 0.67 | 0.4 | 3292 | Train 52 | Test 28 | 0.69 | 0.27 | 0.26 | 0.27 |
| 1245 | Train 20 | Test 29 | 0.72 | 0.23 | 0.45 | 0.31 | 3293 | Train 52 | Test 29 | 0.68 | 0.26 | 0.2 | 0.23 |
| 1246 | Train 20 | Test 30 | 0.71 | 0.35 | 0.32 | 0.33 | 3294 | Train 52 | Test 30 | 0.67 | 0.31 | 0.11 | 0.16 |
| 1247 | Train 20 | Test 31 | 0.71 | 0.23 | 0.44 | 0.3 | 3295 | Train 52 | Test 31 | 0.68 | 0.26 | 0.2 | 0.23 |
| 1248 | Train 20 | Test 32 | 0.7 | 0.33 | 0.32 | 0.33 | 3296 | Train 52 | Test 32 | 0.67 | 0.3 | 0.11 | 0.16 |
| 1249 | Train 20 | Test 33 | 0.77 | 0.25 | 0.61 | 0.35 | 3297 | Train 52 | Test 33 | 0.7 | 0.26 | 0.34 | 0.3 |
| 1250 | Train 20 | Test 34 | 0.78 | 0.28 | 0.67 | 0.39 | 3298 | Train 52 | Test 34 | 0.69 | 0.26 | 0.27 | 0.26 |
| 1251 | Train 20 | Test 35 | 0.77 | 0.25 | 0.62 | 0.36 | 3299 | Train 52 | Test 35 | 0.7 | 0.27 | 0.35 | 0.3 |
| 1252 | Train 20 | Test 36 | 0.78 | 0.28 | 0.67 | 0.39 | 3300 | Train 52 | Test 36 | 0.7 | 0.26 | 0.27 | 0.27 |
| 1253 | Train 20 | Test 37 | 0.72 | 0.23 | 0.46 | 0.3 | 3301 | Train 52 | Test 37 | 0.68 | 0.25 | 0.2 | 0.22 |
| 1254 | Train 20 | Test 38 | 0.71 | 0.35 | 0.32 | 0.33 | 3302 | Train 52 | Test 38 | 0.67 | 0.31 | 0.11 | 0.16 |
| 1255 | Train 20 | Test 39 | 0.72 | 0.23 | 0.46 | 0.3 | 3303 | Train 52 | Test 39 | 0.68 | 0.25 | 0.2 | 0.23 |
| 1256 | Train 20 | Test 40 | 0.7 | 0.34 | 0.32 | 0.33 | 3304 | Train 52 | Test 40 | 0.67 | 0.3 | 0.11 | 0.16 |
| 1257 | Train 20 | Test 41 | 0.77 | 0.25 | 0.6 | 0.36 | 3305 | Train 52 | Test 41 | 0.7 | 0.27 | 0.34 | 0.3 |
| 1258 | Train 20 | Test 42 | 0.78 | 0.28 | 0.66 | 0.4 | 3306 | Train 52 | Test 42 | 0.7 | 0.27 | 0.26 | 0.27 |
| 1259 | Train 20 | Test 43 | 0.77 | 0.26 | 0.61 | 0.36 | 3307 | Train 52 | Test 43 | 0.7 | 0.27 | 0.34 | 0.3 |
| 1260 | Train 20 | Test 44 | 0.78 | 0.28 | 0.66 | 0.4 | 3308 | Train 52 | Test 44 | 0.7 | 0.27 | 0.26 | 0.27 |
| 1261 | Train 20 | Test 45 | 0.72 | 0.23 | 0.45 | 0.31 | 3309 | Train 52 | Test 45 | 0.68 | 0.26 | 0.2 | 0.23 |
| 1262 | Train 20 | Test 46 | 0.71 | 0.35 | 0.32 | 0.33 | 3310 | Train 52 | Test 46 | 0.67 | 0.31 | 0.11 | 0.16 |
| 1263 | Train 20 | Test 47 | 0.72 | 0.23 | 0.44 | 0.3 | 3311 | Train 52 | Test 47 | 0.68 | 0.26 | 0.2 | 0.23 |
| 1264 | Train 20 | Test 48 | 0.71 | 0.33 | 0.32 | 0.33 | 3312 | Train 52 | Test 48 | 0.67 | 0.3 | 0.11 | 0.16 |
| 1265 | Train 20 | Test 49 | 0.77 | 0.28 | 0.73 | 0.4 | 3313 | Train 52 | Test 49 | 0.77 | 0.28 | 0.66 | 0.39 |
| 1266 | Train 20 | Test 50 | 0.77 | 0.28 | 0.73 | 0.4 | 3314 | Train 52 | Test 50 | 0.77 | 0.28 | 0.65 | 0.39 |
| 1267 | Train 20 | Test 51 | 0.77 | 0.28 | 0.73 | 0.4 | 3315 | Train 52 | Test 51 | 0.77 | 0.28 | 0.66 | 0.4 |
| 1268 | Train 20 | Test 52 | 0.77 | 0.28 | 0.73 | 0.4 | 3316 | Train 52 | Test 52 | 0.77 | 0.28 | 0.65 | 0.4 |
| 1269 | Train 20 | Test 53 | 0.75 | 0.3 | 0.36 | 0.33 | 3317 | Train 52 | Test 53 | 0.71 | 0.28 | 0.37 | 0.32 |
| 1270 | Train 20 | Test 54 | 0.72 | 0.39 | 0.3 | 0.34 | 3318 | Train 52 | Test 54 | 0.68 | 0.33 | 0.16 | 0.22 |
| 1271 | Train 20 | Test 55 | 0.75 | 0.28 | 0.51 | 0.36 | 3319 | Train 52 | Test 55 | 0.71 | 0.28 | 0.38 | 0.32 |
| 1272 | Train 20 | Test 56 | 0.71 | 0.36 | 0.31 | 0.33 | 3320 | Train 52 | Test 56 | 0.68 | 0.32 | 0.16 | 0.21 |
| 1273 | Train 20 | Test 57 | 0.78 | 0.28 | 0.73 | 0.41 | 3321 | Train 52 | Test 57 | 0.78 | 0.29 | 0.65 | 0.4 |
| 1274 | Train 20 | Test 58 | 0.78 | 0.28 | 0.73 | 0.41 | 3322 | Train 52 | Test 58 | 0.77 | 0.29 | 0.65 | 0.4 |
| 1275 | Train 20 | Test 59 | 0.78 | 0.28 | 0.73 | 0.41 | 3323 | Train 52 | Test 59 | 0.78 | 0.29 | 0.65 | 0.4 |
| 1276 | Train 20 | Test 60 | 0.78 | 0.28 | 0.73 | 0.41 | 3324 | Train 52 | Test 60 | 0.77 | 0.29 | 0.65 | 0.4 |
| 1277 | Train 20 | Test 61 | 0.76 | 0.31 | 0.36 | 0.33 | 3325 | Train 52 | Test 61 | 0.71 | 0.28 | 0.38 | 0.32 |
| 1278 | Train 20 | Test 62 | 0.72 | 0.39 | 0.3 | 0.34 | 3326 | Train 52 | Test 62 | 0.68 | 0.33 | 0.16 | 0.22 |
| 1279 | Train 20 | Test 63 | 0.76 | 0.31 | 0.37 | 0.33 | 3327 | Train 52 | Test 63 | 0.72 | 0.28 | 0.38 | 0.32 |
| 1280 | Train 20 | Test 64 | 0.71 | 0.36 | 0.31 | 0.33 | 3328 | Train 52 | Test 64 | 0.68 | 0.32 | 0.16 | 0.22 |
| 1281 | Train 21 | Test 1 | 0.76 | 0.23 | 0.69 | 0.34 | 3329 | Train 53 | Test 1 | 0.73 | 0.23 | 0.57 | 0.33 |
| 1282 | Train 21 | Test 2 | 0.76 | 0.26 | 0.7 | 0.38 | 3330 | Train 53 | Test 2 | 0.72 | 0.25 | 0.52 | 0.34 |
| 1283 | Train 21 | Test 3 | 0.76 | 0.23 | 0.69 | 0.35 | 3331 | Train 53 | Test 3 | 0.73 | 0.23 | 0.57 | 0.33 |
| 1284 | Train 21 | Test 4 | 0.76 | 0.26 | 0.7 | 0.38 | 3332 | Train 53 | Test 4 | 0.72 | 0.25 | 0.52 | 0.34 |
| 1285 | Train 21 | Test 5 | 0.75 | 0.23 | 0.66 | 0.34 | 3333 | Train 53 | Test 5 | 0.71 | 0.23 | 0.5 | 0.31 |
| 1286 | Train 21 | Test 6 | 0.7 | 0.3 | 0.48 | 0.37 | 3334 | Train 53 | Test 6 | 0.68 | 0.29 | 0.36 | 0.32 |
| 1287 | Train 21 | Test 7 | 0.75 | 0.23 | 0.65 | 0.34 | 3335 | Train 53 | Test 7 | 0.71 | 0.23 | 0.49 | 0.31 |
| 1288 | Train 21 | Test 8 | 0.7 | 0.29 | 0.49 | 0.36 | 3336 | Train 53 | Test 8 | 0.68 | 0.28 | 0.37 | 0.32 |
| 1289 | Train 21 | Test 9 | 0.76 | 0.23 | 0.68 | 0.35 | 3337 | Train 53 | Test 9 | 0.73 | 0.24 | 0.54 | 0.33 |
| 1290 | Train 21 | Test 10 | 0.76 | 0.26 | 0.69 | 0.38 | 3338 | Train 53 | Test 10 | 0.72 | 0.26 | 0.5 | 0.34 |
| 1291 | Train 21 | Test 11 | 0.76 | 0.23 | 0.68 | 0.35 | 3339 | Train 53 | Test 11 | 0.73 | 0.24 | 0.54 | 0.33 |
| 1292 | Train 21 | Test 12 | 0.76 | 0.26 | 0.69 | 0.38 | 3340 | Train 53 | Test 12 | 0.72 | 0.26 | 0.5 | 0.34 |
| 1293 | Train 21 | Test 13 | 0.75 | 0.23 | 0.64 | 0.34 | 3341 | Train 53 | Test 13 | 0.71 | 0.24 | 0.48 | 0.32 |
| 1294 | Train 21 | Test 14 | 0.7 | 0.3 | 0.49 | 0.37 | 3342 | Train 53 | Test 14 | 0.68 | 0.29 | 0.37 | 0.32 |
| 1295 | Train 21 | Test 15 | 0.75 | 0.23 | 0.64 | 0.34 | 3343 | Train 53 | Test 15 | 0.71 | 0.24 | 0.47 | 0.32 |
| 1296 | Train 21 | Test 16 | 0.7 | 0.29 | 0.49 | 0.36 | 3344 | Train 53 | Test 16 | 0.68 | 0.28 | 0.37 | 0.32 |
| 1297 | Train 21 | Test 17 | 0.76 | 0.23 | 0.69 | 0.34 | 3345 | Train 53 | Test 17 | 0.73 | 0.23 | 0.57 | 0.33 |
| 1298 | Train 21 | Test 18 | 0.76 | 0.26 | 0.7 | 0.38 | 3346 | Train 53 | Test 18 | 0.72 | 0.25 | 0.52 | 0.34 |
| 1299 | Train 21 | Test 19 | 0.76 | 0.23 | 0.69 | 0.35 | 3347 | Train 53 | Test 19 | 0.73 | 0.23 | 0.57 | 0.33 |
| 1300 | Train 21 | Test 20 | 0.76 | 0.26 | 0.7 | 0.38 | 3348 | Train 53 | Test 20 | 0.72 | 0.25 | 0.52 | 0.34 |
| 1301 | Train 21 | Test 21 | 0.75 | 0.23 | 0.66 | 0.34 | 3349 | Train 53 | Test 21 | 0.71 | 0.23 | 0.5 | 0.31 |
| 1302 | Train 21 | Test 22 | 0.7 | 0.3 | 0.48 | 0.37 | 3350 | Train 53 | Test 22 | 0.68 | 0.29 | 0.36 | 0.32 |
| 1303 | Train 21 | Test 23 | 0.75 | 0.23 | 0.65 | 0.34 | 3351 | Train 53 | Test 23 | 0.71 | 0.23 | 0.49 | 0.31 |
| 1304 | Train 21 | Test 24 | 0.7 | 0.29 | 0.49 | 0.36 | 3352 | Train 53 | Test 24 | 0.68 | 0.28 | 0.36 | 0.32 |
| 1305 | Train 21 | Test 25 | 0.76 | 0.23 | 0.68 | 0.35 | 3353 | Train 53 | Test 25 | 0.73 | 0.24 | 0.54 | 0.33 |
| 1306 | Train 21 | Test 26 | 0.76 | 0.26 | 0.69 | 0.38 | 3354 | Train 53 | Test 26 | 0.72 | 0.26 | 0.5 | 0.34 |
| 1307 | Train 21 | Test 27 | 0.76 | 0.23 | 0.68 | 0.35 | 3355 | Train 53 | Test 27 | 0.73 | 0.24 | 0.54 | 0.33 |
| 1308 | Train 21 | Test 28 | 0.76 | 0.26 | 0.69 | 0.38 | 3356 | Train 53 | Test 28 | 0.72 | 0.26 | 0.5 | 0.34 |
| 1309 | Train 21 | Test 29 | 0.75 | 0.23 | 0.64 | 0.34 | 3357 | Train 53 | Test 29 | 0.71 | 0.24 | 0.48 | 0.32 |
| 1310 | Train 21 | Test 30 | 0.7 | 0.3 | 0.49 | 0.37 | 3358 | Train 53 | Test 30 | 0.69 | 0.29 | 0.37 | 0.32 |
| 1311 | Train 21 | Test 31 | 0.75 | 0.23 | 0.64 | 0.34 | 3359 | Train 53 | Test 31 | 0.71 | 0.24 | 0.47 | 0.32 |
| 1312 | Train 21 | Test 32 | 0.7 | 0.29 | 0.49 | 0.36 | 3360 | Train 53 | Test 32 | 0.68 | 0.28 | 0.37 | 0.32 |
| 1313 | Train 21 | Test 33 | 0.76 | 0.23 | 0.69 | 0.34 | 3361 | Train 53 | Test 33 | 0.73 | 0.23 | 0.56 | 0.33 |
| 1314 | Train 21 | Test 34 | 0.76 | 0.26 | 0.69 | 0.38 | 3362 | Train 53 | Test 34 | 0.72 | 0.25 | 0.52 | 0.34 |
| 1315 | Train 21 | Test 35 | 0.76 | 0.23 | 0.69 | 0.34 | 3363 | Train 53 | Test 35 | 0.73 | 0.23 | 0.57 | 0.33 |
| 1316 | Train 21 | Test 36 | 0.76 | 0.26 | 0.7 | 0.38 | 3364 | Train 53 | Test 36 | 0.72 | 0.25 | 0.52 | 0.34 |
| 1317 | Train 21 | Test 37 | 0.75 | 0.23 | 0.66 | 0.34 | 3365 | Train 53 | Test 37 | 0.71 | 0.23 | 0.5 | 0.31 |
| 1318 | Train 21 | Test 38 | 0.7 | 0.3 | 0.48 | 0.37 | 3366 | Train 53 | Test 38 | 0.68 | 0.29 | 0.36 | 0.32 |
| 1319 | Train 21 | Test 39 | 0.75 | 0.23 | 0.65 | 0.34 | 3367 | Train 53 | Test 39 | 0.71 | 0.23 | 0.49 | 0.31 |
| 1320 | Train 21 | Test 40 | 0.7 | 0.29 | 0.48 | 0.36 | 3368 | Train 53 | Test 40 | 0.68 | 0.28 | 0.37 | 0.32 |
| 1321 | Train 21 | Test 41 | 0.76 | 0.23 | 0.68 | 0.35 | 3369 | Train 53 | Test 41 | 0.73 | 0.24 | 0.54 | 0.33 |
| 1322 | Train 21 | Test 42 | 0.76 | 0.26 | 0.68 | 0.38 | 3370 | Train 53 | Test 42 | 0.72 | 0.26 | 0.49 | 0.34 |
| 1323 | Train 21 | Test 43 | 0.76 | 0.23 | 0.68 | 0.35 | 3371 | Train 53 | Test 43 | 0.73 | 0.24 | 0.54 | 0.33 |
| 1324 | Train 21 | Test 44 | 0.76 | 0.26 | 0.68 | 0.38 | 3372 | Train 53 | Test 44 | 0.72 | 0.26 | 0.5 | 0.34 |
| 1325 | Train 21 | Test 45 | 0.75 | 0.23 | 0.64 | 0.34 | 3373 | Train 53 | Test 45 | 0.71 | 0.24 | 0.48 | 0.32 |
| 1326 | Train 21 | Test 46 | 0.7 | 0.3 | 0.49 | 0.37 | 3374 | Train 53 | Test 46 | 0.69 | 0.29 | 0.37 | 0.32 |
| 1327 | Train 21 | Test 47 | 0.75 | 0.23 | 0.63 | 0.34 | 3375 | Train 53 | Test 47 | 0.71 | 0.24 | 0.47 | 0.32 |
| 1328 | Train 21 | Test 48 | 0.7 | 0.29 | 0.49 | 0.36 | 3376 | Train 53 | Test 48 | 0.69 | 0.28 | 0.37 | 0.32 |
| 1329 | Train 21 | Test 49 | 0.77 | 0.26 | 0.75 | 0.38 | 3377 | Train 53 | Test 49 | 0.77 | 0.27 | 0.66 | 0.39 |
| 1330 | Train 21 | Test 50 | 0.77 | 0.26 | 0.75 | 0.38 | 3378 | Train 53 | Test 50 | 0.76 | 0.27 | 0.65 | 0.38 |
| 1331 | Train 21 | Test 51 | 0.77 | 0.26 | 0.76 | 0.39 | 3379 | Train 53 | Test 51 | 0.77 | 0.27 | 0.66 | 0.39 |
| 1332 | Train 21 | Test 52 | 0.77 | 0.26 | 0.75 | 0.39 | 3380 | Train 53 | Test 52 | 0.76 | 0.28 | 0.66 | 0.39 |
| 1333 | Train 21 | Test 53 | 0.76 | 0.27 | 0.64 | 0.38 | 3381 | Train 53 | Test 53 | 0.77 | 0.27 | 0.65 | 0.39 |
| 1334 | Train 21 | Test 54 | 0.71 | 0.35 | 0.47 | 0.4 | 3382 | Train 53 | Test 54 | 0.7 | 0.35 | 0.38 | 0.36 |
| 1335 | Train 21 | Test 55 | 0.77 | 0.27 | 0.7 | 0.38 | 3383 | Train 53 | Test 55 | 0.77 | 0.28 | 0.63 | 0.38 |
| 1336 | Train 21 | Test 56 | 0.71 | 0.33 | 0.48 | 0.39 | 3384 | Train 53 | Test 56 | 0.7 | 0.33 | 0.38 | 0.35 |
| 1337 | Train 21 | Test 57 | 0.77 | 0.26 | 0.76 | 0.39 | 3385 | Train 53 | Test 57 | 0.77 | 0.28 | 0.65 | 0.4 |
| 1338 | Train 21 | Test 58 | 0.77 | 0.26 | 0.75 | 0.39 | 3386 | Train 53 | Test 58 | 0.77 | 0.29 | 0.65 | 0.4 |
| 1339 | Train 21 | Test 59 | 0.77 | 0.27 | 0.76 | 0.39 | 3387 | Train 53 | Test 59 | 0.77 | 0.29 | 0.66 | 0.4 |
| 1340 | Train 21 | Test 60 | 0.77 | 0.27 | 0.76 | 0.39 | 3388 | Train 53 | Test 60 | 0.77 | 0.29 | 0.65 | 0.4 |
| 1341 | Train 21 | Test 61 | 0.76 | 0.28 | 0.65 | 0.39 | 3389 | Train 53 | Test 61 | 0.77 | 0.28 | 0.65 | 0.39 |
| 1342 | Train 21 | Test 62 | 0.71 | 0.35 | 0.48 | 0.41 | 3390 | Train 53 | Test 62 | 0.7 | 0.34 | 0.38 | 0.36 |
| 1343 | Train 21 | Test 63 | 0.77 | 0.28 | 0.65 | 0.39 | 3391 | Train 53 | Test 63 | 0.77 | 0.28 | 0.65 | 0.39 |
| 1344 | Train 21 | Test 64 | 0.71 | 0.33 | 0.48 | 0.4 | 3392 | Train 53 | Test 64 | 0.7 | 0.32 | 0.39 | 0.35 |
| 1345 | Train 22 | Test 1 | 0.75 | 0.24 | 0.6 | 0.34 | 3393 | Train 54 | Test 1 | 0.73 | 0.21 | 0.68 | 0.32 |
| 1346 | Train 22 | Test 2 | 0.76 | 0.27 | 0.67 | 0.38 | 3394 | Train 54 | Test 2 | 0.74 | 0.24 | 0.72 | 0.35 |
| 1347 | Train 22 | Test 3 | 0.75 | 0.24 | 0.6 | 0.34 | 3395 | Train 54 | Test 3 | 0.73 | 0.21 | 0.68 | 0.32 |
| 1348 | Train 22 | Test 4 | 0.76 | 0.27 | 0.67 | 0.38 | 3396 | Train 54 | Test 4 | 0.74 | 0.24 | 0.72 | 0.36 |
| 1349 | Train 22 | Test 5 | 0.74 | 0.23 | 0.57 | 0.33 | 3397 | Train 54 | Test 5 | 0.71 | 0.21 | 0.61 | 0.31 |
| 1350 | Train 22 | Test 6 | 0.76 | 0.31 | 0.7 | 0.43 | 3398 | Train 54 | Test 6 | 0.73 | 0.29 | 0.66 | 0.4 |
| 1351 | Train 22 | Test 7 | 0.74 | 0.23 | 0.56 | 0.33 | 3399 | Train 54 | Test 7 | 0.71 | 0.21 | 0.6 | 0.31 |
| 1352 | Train 22 | Test 8 | 0.75 | 0.3 | 0.7 | 0.42 | 3400 | Train 54 | Test 8 | 0.73 | 0.28 | 0.66 | 0.39 |
| 1353 | Train 22 | Test 9 | 0.75 | 0.24 | 0.59 | 0.34 | 3401 | Train 54 | Test 9 | 0.73 | 0.21 | 0.67 | 0.32 |
| 1354 | Train 22 | Test 10 | 0.76 | 0.27 | 0.66 | 0.38 | 3402 | Train 54 | Test 10 | 0.74 | 0.24 | 0.71 | 0.36 |
| 1355 | Train 22 | Test 11 | 0.75 | 0.24 | 0.59 | 0.35 | 3403 | Train 54 | Test 11 | 0.73 | 0.21 | 0.67 | 0.32 |
| 1356 | Train 22 | Test 12 | 0.76 | 0.27 | 0.66 | 0.38 | 3404 | Train 54 | Test 12 | 0.74 | 0.24 | 0.71 | 0.36 |
| 1357 | Train 22 | Test 13 | 0.74 | 0.24 | 0.56 | 0.33 | 3405 | Train 54 | Test 13 | 0.72 | 0.22 | 0.59 | 0.32 |
| 1358 | Train 22 | Test 14 | 0.76 | 0.31 | 0.7 | 0.42 | 3406 | Train 54 | Test 14 | 0.73 | 0.29 | 0.66 | 0.4 |
| 1359 | Train 22 | Test 15 | 0.74 | 0.24 | 0.55 | 0.33 | 3407 | Train 54 | Test 15 | 0.71 | 0.22 | 0.59 | 0.31 |
| 1360 | Train 22 | Test 16 | 0.75 | 0.3 | 0.7 | 0.42 | 3408 | Train 54 | Test 16 | 0.73 | 0.28 | 0.66 | 0.39 |
| 1361 | Train 22 | Test 17 | 0.75 | 0.24 | 0.6 | 0.34 | 3409 | Train 54 | Test 17 | 0.73 | 0.21 | 0.68 | 0.32 |
| 1362 | Train 22 | Test 18 | 0.76 | 0.27 | 0.67 | 0.38 | 3410 | Train 54 | Test 18 | 0.74 | 0.24 | 0.72 | 0.35 |
| 1363 | Train 22 | Test 19 | 0.75 | 0.24 | 0.6 | 0.35 | 3411 | Train 54 | Test 19 | 0.73 | 0.21 | 0.68 | 0.32 |
| 1364 | Train 22 | Test 20 | 0.76 | 0.27 | 0.67 | 0.38 | 3412 | Train 54 | Test 20 | 0.74 | 0.24 | 0.72 | 0.36 |
| 1365 | Train 22 | Test 21 | 0.74 | 0.24 | 0.57 | 0.33 | 3413 | Train 54 | Test 21 | 0.71 | 0.21 | 0.61 | 0.31 |
| 1366 | Train 22 | Test 22 | 0.76 | 0.31 | 0.7 | 0.43 | 3414 | Train 54 | Test 22 | 0.73 | 0.29 | 0.66 | 0.4 |
| 1367 | Train 22 | Test 23 | 0.74 | 0.23 | 0.56 | 0.33 | 3415 | Train 54 | Test 23 | 0.71 | 0.21 | 0.6 | 0.31 |
| 1368 | Train 22 | Test 24 | 0.75 | 0.3 | 0.7 | 0.42 | 3416 | Train 54 | Test 24 | 0.73 | 0.28 | 0.66 | 0.39 |
| 1369 | Train 22 | Test 25 | 0.75 | 0.24 | 0.59 | 0.34 | 3417 | Train 54 | Test 25 | 0.73 | 0.21 | 0.67 | 0.32 |
| 1370 | Train 22 | Test 26 | 0.76 | 0.27 | 0.66 | 0.38 | 3418 | Train 54 | Test 26 | 0.74 | 0.24 | 0.71 | 0.36 |
| 1371 | Train 22 | Test 27 | 0.75 | 0.24 | 0.59 | 0.35 | 3419 | Train 54 | Test 27 | 0.73 | 0.21 | 0.67 | 0.32 |
| 1372 | Train 22 | Test 28 | 0.76 | 0.27 | 0.66 | 0.38 | 3420 | Train 54 | Test 28 | 0.74 | 0.24 | 0.71 | 0.36 |
| 1373 | Train 22 | Test 29 | 0.74 | 0.24 | 0.56 | 0.33 | 3421 | Train 54 | Test 29 | 0.72 | 0.22 | 0.59 | 0.32 |
| 1374 | Train 22 | Test 30 | 0.76 | 0.3 | 0.7 | 0.42 | 3422 | Train 54 | Test 30 | 0.73 | 0.29 | 0.66 | 0.4 |
| 1375 | Train 22 | Test 31 | 0.74 | 0.24 | 0.55 | 0.33 | 3423 | Train 54 | Test 31 | 0.71 | 0.22 | 0.59 | 0.31 |
| 1376 | Train 22 | Test 32 | 0.75 | 0.3 | 0.7 | 0.41 | 3424 | Train 54 | Test 32 | 0.73 | 0.28 | 0.66 | 0.39 |
| 1377 | Train 22 | Test 33 | 0.75 | 0.24 | 0.6 | 0.34 | 3425 | Train 54 | Test 33 | 0.73 | 0.21 | 0.68 | 0.32 |
| 1378 | Train 22 | Test 34 | 0.76 | 0.27 | 0.67 | 0.38 | 3426 | Train 54 | Test 34 | 0.74 | 0.24 | 0.72 | 0.35 |
| 1379 | Train 22 | Test 35 | 0.75 | 0.24 | 0.6 | 0.34 | 3427 | Train 54 | Test 35 | 0.73 | 0.21 | 0.68 | 0.32 |
| 1380 | Train 22 | Test 36 | 0.76 | 0.27 | 0.67 | 0.38 | 3428 | Train 54 | Test 36 | 0.74 | 0.24 | 0.72 | 0.36 |
| 1381 | Train 22 | Test 37 | 0.74 | 0.23 | 0.57 | 0.33 | 3429 | Train 54 | Test 37 | 0.72 | 0.21 | 0.61 | 0.31 |
| 1382 | Train 22 | Test 38 | 0.76 | 0.31 | 0.7 | 0.43 | 3430 | Train 54 | Test 38 | 0.73 | 0.29 | 0.66 | 0.4 |
| 1383 | Train 22 | Test 39 | 0.74 | 0.23 | 0.56 | 0.33 | 3431 | Train 54 | Test 39 | 0.71 | 0.21 | 0.6 | 0.31 |
| 1384 | Train 22 | Test 40 | 0.75 | 0.3 | 0.69 | 0.42 | 3432 | Train 54 | Test 40 | 0.73 | 0.28 | 0.65 | 0.39 |
| 1385 | Train 22 | Test 41 | 0.75 | 0.24 | 0.59 | 0.34 | 3433 | Train 54 | Test 41 | 0.73 | 0.21 | 0.67 | 0.32 |
| 1386 | Train 22 | Test 42 | 0.76 | 0.27 | 0.66 | 0.38 | 3434 | Train 54 | Test 42 | 0.74 | 0.24 | 0.71 | 0.36 |
| 1387 | Train 22 | Test 43 | 0.75 | 0.24 | 0.59 | 0.34 | 3435 | Train 54 | Test 43 | 0.73 | 0.21 | 0.67 | 0.32 |
| 1388 | Train 22 | Test 44 | 0.76 | 0.27 | 0.66 | 0.38 | 3436 | Train 54 | Test 44 | 0.74 | 0.24 | 0.71 | 0.36 |
| 1389 | Train 22 | Test 45 | 0.74 | 0.24 | 0.55 | 0.33 | 3437 | Train 54 | Test 45 | 0.72 | 0.22 | 0.59 | 0.32 |
| 1390 | Train 22 | Test 46 | 0.76 | 0.31 | 0.7 | 0.42 | 3438 | Train 54 | Test 46 | 0.73 | 0.29 | 0.66 | 0.4 |
| 1391 | Train 22 | Test 47 | 0.74 | 0.24 | 0.55 | 0.33 | 3439 | Train 54 | Test 47 | 0.72 | 0.22 | 0.58 | 0.31 |
| 1392 | Train 22 | Test 48 | 0.75 | 0.3 | 0.7 | 0.41 | 3440 | Train 54 | Test 48 | 0.73 | 0.28 | 0.66 | 0.39 |
| 1393 | Train 22 | Test 49 | 0.77 | 0.28 | 0.73 | 0.4 | 3441 | Train 54 | Test 49 | 0.77 | 0.28 | 0.7 | 0.4 |
| 1394 | Train 22 | Test 50 | 0.77 | 0.28 | 0.73 | 0.4 | 3442 | Train 54 | Test 50 | 0.77 | 0.28 | 0.69 | 0.4 |
| 1395 | Train 22 | Test 51 | 0.77 | 0.28 | 0.73 | 0.4 | 3443 | Train 54 | Test 51 | 0.77 | 0.28 | 0.7 | 0.4 |
| 1396 | Train 22 | Test 52 | 0.77 | 0.28 | 0.73 | 0.4 | 3444 | Train 54 | Test 52 | 0.77 | 0.28 | 0.7 | 0.4 |
| 1397 | Train 22 | Test 53 | 0.77 | 0.28 | 0.66 | 0.39 | 3445 | Train 54 | Test 53 | 0.77 | 0.28 | 0.62 | 0.39 |
| 1398 | Train 22 | Test 54 | 0.76 | 0.34 | 0.73 | 0.47 | 3446 | Train 54 | Test 54 | 0.76 | 0.35 | 0.71 | 0.47 |
| 1399 | Train 22 | Test 55 | 0.77 | 0.28 | 0.67 | 0.39 | 3447 | Train 54 | Test 55 | 0.77 | 0.28 | 0.64 | 0.39 |
| 1400 | Train 22 | Test 56 | 0.75 | 0.32 | 0.73 | 0.45 | 3448 | Train 54 | Test 56 | 0.76 | 0.33 | 0.71 | 0.45 |
| 1401 | Train 22 | Test 57 | 0.78 | 0.28 | 0.73 | 0.41 | 3449 | Train 54 | Test 57 | 0.78 | 0.29 | 0.7 | 0.41 |
| 1402 | Train 22 | Test 58 | 0.78 | 0.28 | 0.73 | 0.41 | 3450 | Train 54 | Test 58 | 0.77 | 0.29 | 0.69 | 0.41 |
| 1403 | Train 22 | Test 59 | 0.78 | 0.28 | 0.73 | 0.41 | 3451 | Train 54 | Test 59 | 0.78 | 0.29 | 0.7 | 0.41 |
| 1404 | Train 22 | Test 60 | 0.78 | 0.29 | 0.73 | 0.41 | 3452 | Train 54 | Test 60 | 0.77 | 0.29 | 0.69 | 0.41 |
| 1405 | Train 22 | Test 61 | 0.78 | 0.29 | 0.65 | 0.4 | 3453 | Train 54 | Test 61 | 0.78 | 0.29 | 0.62 | 0.39 |
| 1406 | Train 22 | Test 62 | 0.76 | 0.34 | 0.73 | 0.46 | 3454 | Train 54 | Test 62 | 0.76 | 0.35 | 0.71 | 0.47 |
| 1407 | Train 22 | Test 63 | 0.78 | 0.29 | 0.65 | 0.4 | 3455 | Train 54 | Test 63 | 0.77 | 0.29 | 0.62 | 0.39 |
| 1408 | Train 22 | Test 64 | 0.75 | 0.32 | 0.73 | 0.45 | 3456 | Train 54 | Test 64 | 0.76 | 0.33 | 0.71 | 0.45 |
| 1409 | Train 23 | Test 1 | 0.76 | 0.23 | 0.69 | 0.34 | 3457 | Train 55 | Test 1 | 0.73 | 0.23 | 0.59 | 0.33 |
| 1410 | Train 23 | Test 2 | 0.76 | 0.26 | 0.69 | 0.38 | 3458 | Train 55 | Test 2 | 0.72 | 0.26 | 0.56 | 0.35 |
| 1411 | Train 23 | Test 3 | 0.76 | 0.23 | 0.69 | 0.35 | 3459 | Train 55 | Test 3 | 0.73 | 0.23 | 0.6 | 0.33 |
| 1412 | Train 23 | Test 4 | 0.76 | 0.26 | 0.69 | 0.38 | 3460 | Train 55 | Test 4 | 0.72 | 0.26 | 0.56 | 0.35 |
| 1413 | Train 23 | Test 5 | 0.75 | 0.23 | 0.66 | 0.34 | 3461 | Train 55 | Test 5 | 0.71 | 0.23 | 0.52 | 0.32 |
| 1414 | Train 23 | Test 6 | 0.7 | 0.3 | 0.48 | 0.37 | 3462 | Train 55 | Test 6 | 0.68 | 0.3 | 0.4 | 0.34 |
| 1415 | Train 23 | Test 7 | 0.75 | 0.23 | 0.65 | 0.34 | 3463 | Train 55 | Test 7 | 0.71 | 0.23 | 0.51 | 0.31 |
| 1416 | Train 23 | Test 8 | 0.7 | 0.29 | 0.48 | 0.36 | 3464 | Train 55 | Test 8 | 0.68 | 0.29 | 0.4 | 0.34 |
| 1417 | Train 23 | Test 9 | 0.76 | 0.23 | 0.67 | 0.35 | 3465 | Train 55 | Test 9 | 0.73 | 0.23 | 0.56 | 0.33 |
| 1418 | Train 23 | Test 10 | 0.76 | 0.26 | 0.68 | 0.38 | 3466 | Train 55 | Test 10 | 0.73 | 0.26 | 0.54 | 0.35 |
| 1419 | Train 23 | Test 11 | 0.76 | 0.23 | 0.67 | 0.35 | 3467 | Train 55 | Test 11 | 0.73 | 0.24 | 0.57 | 0.33 |
| 1420 | Train 23 | Test 12 | 0.76 | 0.26 | 0.68 | 0.38 | 3468 | Train 55 | Test 12 | 0.73 | 0.26 | 0.54 | 0.35 |
| 1421 | Train 23 | Test 13 | 0.75 | 0.23 | 0.64 | 0.34 | 3469 | Train 55 | Test 13 | 0.71 | 0.23 | 0.49 | 0.32 |
| 1422 | Train 23 | Test 14 | 0.7 | 0.3 | 0.48 | 0.37 | 3470 | Train 55 | Test 14 | 0.69 | 0.3 | 0.4 | 0.34 |
| 1423 | Train 23 | Test 15 | 0.75 | 0.23 | 0.63 | 0.34 | 3471 | Train 55 | Test 15 | 0.71 | 0.23 | 0.48 | 0.31 |
| 1424 | Train 23 | Test 16 | 0.7 | 0.29 | 0.48 | 0.36 | 3472 | Train 55 | Test 16 | 0.69 | 0.29 | 0.4 | 0.34 |
| 1425 | Train 23 | Test 17 | 0.76 | 0.23 | 0.69 | 0.34 | 3473 | Train 55 | Test 17 | 0.73 | 0.23 | 0.59 | 0.33 |
| 1426 | Train 23 | Test 18 | 0.76 | 0.26 | 0.69 | 0.38 | 3474 | Train 55 | Test 18 | 0.72 | 0.26 | 0.56 | 0.35 |
| 1427 | Train 23 | Test 19 | 0.76 | 0.23 | 0.69 | 0.35 | 3475 | Train 55 | Test 19 | 0.73 | 0.23 | 0.6 | 0.33 |
| 1428 | Train 23 | Test 20 | 0.76 | 0.26 | 0.69 | 0.38 | 3476 | Train 55 | Test 20 | 0.72 | 0.26 | 0.56 | 0.35 |
| 1429 | Train 23 | Test 21 | 0.75 | 0.23 | 0.66 | 0.34 | 3477 | Train 55 | Test 21 | 0.71 | 0.23 | 0.52 | 0.32 |
| 1430 | Train 23 | Test 22 | 0.7 | 0.3 | 0.48 | 0.37 | 3478 | Train 55 | Test 22 | 0.69 | 0.3 | 0.4 | 0.34 |
| 1431 | Train 23 | Test 23 | 0.75 | 0.23 | 0.65 | 0.34 | 3479 | Train 55 | Test 23 | 0.71 | 0.23 | 0.51 | 0.31 |
| 1432 | Train 23 | Test 24 | 0.7 | 0.29 | 0.48 | 0.36 | 3480 | Train 55 | Test 24 | 0.69 | 0.29 | 0.4 | 0.34 |
| 1433 | Train 23 | Test 25 | 0.76 | 0.23 | 0.67 | 0.35 | 3481 | Train 55 | Test 25 | 0.73 | 0.23 | 0.56 | 0.33 |
| 1434 | Train 23 | Test 26 | 0.76 | 0.26 | 0.68 | 0.38 | 3482 | Train 55 | Test 26 | 0.73 | 0.26 | 0.54 | 0.35 |
| 1435 | Train 23 | Test 27 | 0.76 | 0.23 | 0.67 | 0.35 | 3483 | Train 55 | Test 27 | 0.73 | 0.24 | 0.57 | 0.33 |
| 1436 | Train 23 | Test 28 | 0.76 | 0.26 | 0.68 | 0.38 | 3484 | Train 55 | Test 28 | 0.73 | 0.26 | 0.54 | 0.35 |
| 1437 | Train 23 | Test 29 | 0.75 | 0.23 | 0.64 | 0.34 | 3485 | Train 55 | Test 29 | 0.71 | 0.23 | 0.49 | 0.32 |
| 1438 | Train 23 | Test 30 | 0.7 | 0.29 | 0.48 | 0.37 | 3486 | Train 55 | Test 30 | 0.69 | 0.3 | 0.4 | 0.34 |
| 1439 | Train 23 | Test 31 | 0.75 | 0.23 | 0.63 | 0.34 | 3487 | Train 55 | Test 31 | 0.71 | 0.23 | 0.48 | 0.31 |
| 1440 | Train 23 | Test 32 | 0.7 | 0.29 | 0.48 | 0.36 | 3488 | Train 55 | Test 32 | 0.69 | 0.29 | 0.4 | 0.34 |
| 1441 | Train 23 | Test 33 | 0.76 | 0.23 | 0.69 | 0.34 | 3489 | Train 55 | Test 33 | 0.73 | 0.23 | 0.59 | 0.33 |
| 1442 | Train 23 | Test 34 | 0.76 | 0.26 | 0.69 | 0.38 | 3490 | Train 55 | Test 34 | 0.73 | 0.25 | 0.56 | 0.35 |
| 1443 | Train 23 | Test 35 | 0.76 | 0.23 | 0.69 | 0.34 | 3491 | Train 55 | Test 35 | 0.74 | 0.23 | 0.59 | 0.33 |
| 1444 | Train 23 | Test 36 | 0.76 | 0.26 | 0.69 | 0.38 | 3492 | Train 55 | Test 36 | 0.73 | 0.26 | 0.56 | 0.35 |
| 1445 | Train 23 | Test 37 | 0.75 | 0.23 | 0.65 | 0.34 | 3493 | Train 55 | Test 37 | 0.71 | 0.23 | 0.52 | 0.32 |
| 1446 | Train 23 | Test 38 | 0.7 | 0.3 | 0.48 | 0.36 | 3494 | Train 55 | Test 38 | 0.69 | 0.3 | 0.4 | 0.34 |
| 1447 | Train 23 | Test 39 | 0.75 | 0.23 | 0.65 | 0.34 | 3495 | Train 55 | Test 39 | 0.71 | 0.23 | 0.51 | 0.31 |
| 1448 | Train 23 | Test 40 | 0.7 | 0.29 | 0.48 | 0.36 | 3496 | Train 55 | Test 40 | 0.69 | 0.29 | 0.4 | 0.34 |
| 1449 | Train 23 | Test 41 | 0.76 | 0.23 | 0.67 | 0.34 | 3497 | Train 55 | Test 41 | 0.74 | 0.23 | 0.56 | 0.33 |
| 1450 | Train 23 | Test 42 | 0.76 | 0.26 | 0.68 | 0.38 | 3498 | Train 55 | Test 42 | 0.73 | 0.26 | 0.53 | 0.35 |
| 1451 | Train 23 | Test 43 | 0.76 | 0.23 | 0.67 | 0.35 | 3499 | Train 55 | Test 43 | 0.74 | 0.23 | 0.56 | 0.33 |
| 1452 | Train 23 | Test 44 | 0.76 | 0.26 | 0.68 | 0.38 | 3500 | Train 55 | Test 44 | 0.73 | 0.26 | 0.54 | 0.35 |
| 1453 | Train 23 | Test 45 | 0.75 | 0.23 | 0.64 | 0.34 | 3501 | Train 55 | Test 45 | 0.72 | 0.23 | 0.49 | 0.32 |
| 1454 | Train 23 | Test 46 | 0.7 | 0.29 | 0.48 | 0.37 | 3502 | Train 55 | Test 46 | 0.69 | 0.29 | 0.4 | 0.34 |
| 1455 | Train 23 | Test 47 | 0.75 | 0.23 | 0.63 | 0.34 | 3503 | Train 55 | Test 47 | 0.71 | 0.23 | 0.48 | 0.31 |
| 1456 | Train 23 | Test 48 | 0.7 | 0.29 | 0.48 | 0.36 | 3504 | Train 55 | Test 48 | 0.69 | 0.29 | 0.4 | 0.34 |
| 1457 | Train 23 | Test 49 | 0.77 | 0.26 | 0.75 | 0.38 | 3505 | Train 55 | Test 49 | 0.77 | 0.27 | 0.72 | 0.39 |
| 1458 | Train 23 | Test 50 | 0.77 | 0.26 | 0.75 | 0.38 | 3506 | Train 55 | Test 50 | 0.77 | 0.27 | 0.72 | 0.39 |
| 1459 | Train 23 | Test 51 | 0.77 | 0.26 | 0.76 | 0.39 | 3507 | Train 55 | Test 51 | 0.77 | 0.27 | 0.73 | 0.39 |
| 1460 | Train 23 | Test 52 | 0.77 | 0.26 | 0.75 | 0.39 | 3508 | Train 55 | Test 52 | 0.77 | 0.27 | 0.72 | 0.39 |
| 1461 | Train 23 | Test 53 | 0.76 | 0.27 | 0.64 | 0.38 | 3509 | Train 55 | Test 53 | 0.77 | 0.28 | 0.65 | 0.39 |
| 1462 | Train 23 | Test 54 | 0.71 | 0.35 | 0.47 | 0.4 | 3510 | Train 55 | Test 54 | 0.7 | 0.35 | 0.41 | 0.38 |
| 1463 | Train 23 | Test 55 | 0.77 | 0.26 | 0.7 | 0.38 | 3511 | Train 55 | Test 55 | 0.77 | 0.27 | 0.66 | 0.39 |
| 1464 | Train 23 | Test 56 | 0.7 | 0.34 | 0.48 | 0.39 | 3512 | Train 55 | Test 56 | 0.7 | 0.33 | 0.42 | 0.37 |
| 1465 | Train 23 | Test 57 | 0.77 | 0.26 | 0.75 | 0.39 | 3513 | Train 55 | Test 57 | 0.78 | 0.28 | 0.72 | 0.4 |
| 1466 | Train 23 | Test 58 | 0.77 | 0.26 | 0.75 | 0.39 | 3514 | Train 55 | Test 58 | 0.77 | 0.28 | 0.72 | 0.4 |
| 1467 | Train 23 | Test 59 | 0.78 | 0.26 | 0.76 | 0.39 | 3515 | Train 55 | Test 59 | 0.78 | 0.28 | 0.72 | 0.4 |
| 1468 | Train 23 | Test 60 | 0.77 | 0.26 | 0.75 | 0.39 | 3516 | Train 55 | Test 60 | 0.78 | 0.28 | 0.72 | 0.4 |
| 1469 | Train 23 | Test 61 | 0.76 | 0.28 | 0.65 | 0.39 | 3517 | Train 55 | Test 61 | 0.77 | 0.28 | 0.65 | 0.4 |
| 1470 | Train 23 | Test 62 | 0.71 | 0.35 | 0.48 | 0.4 | 3518 | Train 55 | Test 62 | 0.71 | 0.35 | 0.42 | 0.38 |
| 1471 | Train 23 | Test 63 | 0.76 | 0.28 | 0.65 | 0.39 | 3519 | Train 55 | Test 63 | 0.77 | 0.28 | 0.65 | 0.4 |
| 1472 | Train 23 | Test 64 | 0.71 | 0.33 | 0.48 | 0.39 | 3520 | Train 55 | Test 64 | 0.7 | 0.33 | 0.42 | 0.37 |
| 1473 | Train 24 | Test 1 | 0.75 | 0.24 | 0.62 | 0.34 | 3521 | Train 56 | Test 1 | 0.73 | 0.21 | 0.69 | 0.32 |
| 1474 | Train 24 | Test 2 | 0.76 | 0.27 | 0.67 | 0.38 | 3522 | Train 56 | Test 2 | 0.74 | 0.24 | 0.72 | 0.36 |
| 1475 | Train 24 | Test 3 | 0.75 | 0.24 | 0.62 | 0.35 | 3523 | Train 56 | Test 3 | 0.73 | 0.21 | 0.69 | 0.32 |
| 1476 | Train 24 | Test 4 | 0.76 | 0.27 | 0.68 | 0.38 | 3524 | Train 56 | Test 4 | 0.74 | 0.24 | 0.73 | 0.36 |
| 1477 | Train 24 | Test 5 | 0.74 | 0.23 | 0.58 | 0.33 | 3525 | Train 56 | Test 5 | 0.72 | 0.21 | 0.62 | 0.31 |
| 1478 | Train 24 | Test 6 | 0.76 | 0.31 | 0.7 | 0.43 | 3526 | Train 56 | Test 6 | 0.73 | 0.28 | 0.66 | 0.4 |
| 1479 | Train 24 | Test 7 | 0.74 | 0.23 | 0.57 | 0.33 | 3527 | Train 56 | Test 7 | 0.71 | 0.21 | 0.61 | 0.31 |
| 1480 | Train 24 | Test 8 | 0.76 | 0.3 | 0.7 | 0.42 | 3528 | Train 56 | Test 8 | 0.73 | 0.28 | 0.65 | 0.39 |
| 1481 | Train 24 | Test 9 | 0.75 | 0.24 | 0.6 | 0.35 | 3529 | Train 56 | Test 9 | 0.73 | 0.21 | 0.68 | 0.32 |
| 1482 | Train 24 | Test 10 | 0.76 | 0.27 | 0.67 | 0.38 | 3530 | Train 56 | Test 10 | 0.74 | 0.24 | 0.71 | 0.36 |
| 1483 | Train 24 | Test 11 | 0.76 | 0.24 | 0.61 | 0.35 | 3531 | Train 56 | Test 11 | 0.73 | 0.21 | 0.68 | 0.32 |
| 1484 | Train 24 | Test 12 | 0.76 | 0.27 | 0.67 | 0.39 | 3532 | Train 56 | Test 12 | 0.74 | 0.24 | 0.71 | 0.36 |
| 1485 | Train 24 | Test 13 | 0.74 | 0.24 | 0.57 | 0.34 | 3533 | Train 56 | Test 13 | 0.72 | 0.21 | 0.6 | 0.32 |
| 1486 | Train 24 | Test 14 | 0.76 | 0.3 | 0.7 | 0.42 | 3534 | Train 56 | Test 14 | 0.73 | 0.28 | 0.66 | 0.4 |
| 1487 | Train 24 | Test 15 | 0.74 | 0.24 | 0.56 | 0.33 | 3535 | Train 56 | Test 15 | 0.72 | 0.21 | 0.6 | 0.32 |
| 1488 | Train 24 | Test 16 | 0.76 | 0.3 | 0.7 | 0.42 | 3536 | Train 56 | Test 16 | 0.73 | 0.28 | 0.65 | 0.39 |
| 1489 | Train 24 | Test 17 | 0.75 | 0.24 | 0.62 | 0.34 | 3537 | Train 56 | Test 17 | 0.73 | 0.21 | 0.69 | 0.32 |
| 1490 | Train 24 | Test 18 | 0.76 | 0.27 | 0.67 | 0.38 | 3538 | Train 56 | Test 18 | 0.74 | 0.24 | 0.72 | 0.36 |
| 1491 | Train 24 | Test 19 | 0.75 | 0.24 | 0.62 | 0.35 | 3539 | Train 56 | Test 19 | 0.73 | 0.21 | 0.69 | 0.32 |
| 1492 | Train 24 | Test 20 | 0.76 | 0.27 | 0.68 | 0.38 | 3540 | Train 56 | Test 20 | 0.74 | 0.24 | 0.73 | 0.36 |
| 1493 | Train 24 | Test 21 | 0.74 | 0.23 | 0.58 | 0.33 | 3541 | Train 56 | Test 21 | 0.72 | 0.21 | 0.62 | 0.31 |
| 1494 | Train 24 | Test 22 | 0.76 | 0.31 | 0.7 | 0.43 | 3542 | Train 56 | Test 22 | 0.73 | 0.28 | 0.66 | 0.4 |
| 1495 | Train 24 | Test 23 | 0.74 | 0.23 | 0.57 | 0.33 | 3543 | Train 56 | Test 23 | 0.72 | 0.21 | 0.61 | 0.31 |
| 1496 | Train 24 | Test 24 | 0.76 | 0.3 | 0.7 | 0.42 | 3544 | Train 56 | Test 24 | 0.73 | 0.28 | 0.65 | 0.39 |
| 1497 | Train 24 | Test 25 | 0.75 | 0.24 | 0.6 | 0.35 | 3545 | Train 56 | Test 25 | 0.73 | 0.21 | 0.68 | 0.32 |
| 1498 | Train 24 | Test 26 | 0.76 | 0.27 | 0.67 | 0.38 | 3546 | Train 56 | Test 26 | 0.74 | 0.24 | 0.71 | 0.36 |
| 1499 | Train 24 | Test 27 | 0.76 | 0.24 | 0.61 | 0.35 | 3547 | Train 56 | Test 27 | 0.73 | 0.21 | 0.68 | 0.32 |
| 1500 | Train 24 | Test 28 | 0.76 | 0.27 | 0.67 | 0.39 | 3548 | Train 56 | Test 28 | 0.74 | 0.24 | 0.71 | 0.36 |
| 1501 | Train 24 | Test 29 | 0.74 | 0.24 | 0.57 | 0.34 | 3549 | Train 56 | Test 29 | 0.72 | 0.21 | 0.6 | 0.32 |
| 1502 | Train 24 | Test 30 | 0.76 | 0.3 | 0.7 | 0.42 | 3550 | Train 56 | Test 30 | 0.73 | 0.28 | 0.66 | 0.4 |
| 1503 | Train 24 | Test 31 | 0.74 | 0.24 | 0.56 | 0.33 | 3551 | Train 56 | Test 31 | 0.72 | 0.21 | 0.6 | 0.32 |
| 1504 | Train 24 | Test 32 | 0.76 | 0.3 | 0.7 | 0.42 | 3552 | Train 56 | Test 32 | 0.73 | 0.28 | 0.66 | 0.39 |
| 1505 | Train 24 | Test 33 | 0.75 | 0.24 | 0.61 | 0.34 | 3553 | Train 56 | Test 33 | 0.73 | 0.21 | 0.69 | 0.32 |
| 1506 | Train 24 | Test 34 | 0.76 | 0.27 | 0.67 | 0.38 | 3554 | Train 56 | Test 34 | 0.74 | 0.23 | 0.72 | 0.35 |
| 1507 | Train 24 | Test 35 | 0.75 | 0.24 | 0.62 | 0.34 | 3555 | Train 56 | Test 35 | 0.73 | 0.21 | 0.69 | 0.32 |
| 1508 | Train 24 | Test 36 | 0.76 | 0.27 | 0.67 | 0.38 | 3556 | Train 56 | Test 36 | 0.74 | 0.24 | 0.72 | 0.36 |
| 1509 | Train 24 | Test 37 | 0.74 | 0.23 | 0.58 | 0.33 | 3557 | Train 56 | Test 37 | 0.72 | 0.21 | 0.62 | 0.31 |
| 1510 | Train 24 | Test 38 | 0.76 | 0.31 | 0.7 | 0.43 | 3558 | Train 56 | Test 38 | 0.73 | 0.28 | 0.66 | 0.4 |
| 1511 | Train 24 | Test 39 | 0.74 | 0.23 | 0.57 | 0.33 | 3559 | Train 56 | Test 39 | 0.72 | 0.21 | 0.61 | 0.31 |
| 1512 | Train 24 | Test 40 | 0.76 | 0.3 | 0.7 | 0.42 | 3560 | Train 56 | Test 40 | 0.73 | 0.28 | 0.65 | 0.39 |
| 1513 | Train 24 | Test 41 | 0.76 | 0.24 | 0.6 | 0.34 | 3561 | Train 56 | Test 41 | 0.73 | 0.21 | 0.67 | 0.32 |
| 1514 | Train 24 | Test 42 | 0.76 | 0.27 | 0.66 | 0.38 | 3562 | Train 56 | Test 42 | 0.74 | 0.24 | 0.71 | 0.36 |
| 1515 | Train 24 | Test 43 | 0.76 | 0.24 | 0.6 | 0.35 | 3563 | Train 56 | Test 43 | 0.73 | 0.21 | 0.67 | 0.32 |
| 1516 | Train 24 | Test 44 | 0.76 | 0.27 | 0.67 | 0.39 | 3564 | Train 56 | Test 44 | 0.74 | 0.24 | 0.71 | 0.36 |
| 1517 | Train 24 | Test 45 | 0.74 | 0.24 | 0.57 | 0.34 | 3565 | Train 56 | Test 45 | 0.72 | 0.21 | 0.6 | 0.32 |
| 1518 | Train 24 | Test 46 | 0.76 | 0.3 | 0.7 | 0.42 | 3566 | Train 56 | Test 46 | 0.73 | 0.28 | 0.66 | 0.4 |
| 1519 | Train 24 | Test 47 | 0.74 | 0.24 | 0.56 | 0.33 | 3567 | Train 56 | Test 47 | 0.72 | 0.21 | 0.59 | 0.32 |
| 1520 | Train 24 | Test 48 | 0.76 | 0.3 | 0.7 | 0.42 | 3568 | Train 56 | Test 48 | 0.73 | 0.28 | 0.65 | 0.39 |
| 1521 | Train 24 | Test 49 | 0.77 | 0.27 | 0.74 | 0.4 | 3569 | Train 56 | Test 49 | 0.77 | 0.28 | 0.71 | 0.4 |
| 1522 | Train 24 | Test 50 | 0.77 | 0.27 | 0.74 | 0.4 | 3570 | Train 56 | Test 50 | 0.77 | 0.28 | 0.71 | 0.4 |
| 1523 | Train 24 | Test 51 | 0.77 | 0.28 | 0.75 | 0.4 | 3571 | Train 56 | Test 51 | 0.77 | 0.28 | 0.72 | 0.4 |
| 1524 | Train 24 | Test 52 | 0.77 | 0.28 | 0.74 | 0.4 | 3572 | Train 56 | Test 52 | 0.77 | 0.28 | 0.71 | 0.4 |
| 1525 | Train 24 | Test 53 | 0.77 | 0.28 | 0.66 | 0.39 | 3573 | Train 56 | Test 53 | 0.77 | 0.28 | 0.64 | 0.39 |
| 1526 | Train 24 | Test 54 | 0.76 | 0.34 | 0.73 | 0.46 | 3574 | Train 56 | Test 54 | 0.76 | 0.35 | 0.71 | 0.46 |
| 1527 | Train 24 | Test 55 | 0.77 | 0.28 | 0.68 | 0.39 | 3575 | Train 56 | Test 55 | 0.77 | 0.27 | 0.64 | 0.39 |
| 1528 | Train 24 | Test 56 | 0.76 | 0.33 | 0.73 | 0.45 | 3576 | Train 56 | Test 56 | 0.76 | 0.33 | 0.71 | 0.45 |
| 1529 | Train 24 | Test 57 | 0.78 | 0.28 | 0.74 | 0.41 | 3577 | Train 56 | Test 57 | 0.78 | 0.28 | 0.7 | 0.4 |
| 1530 | Train 24 | Test 58 | 0.78 | 0.28 | 0.74 | 0.41 | 3578 | Train 56 | Test 58 | 0.77 | 0.28 | 0.7 | 0.4 |
| 1531 | Train 24 | Test 59 | 0.78 | 0.28 | 0.74 | 0.41 | 3579 | Train 56 | Test 59 | 0.78 | 0.28 | 0.71 | 0.4 |
| 1532 | Train 24 | Test 60 | 0.78 | 0.28 | 0.74 | 0.41 | 3580 | Train 56 | Test 60 | 0.78 | 0.28 | 0.7 | 0.4 |
| 1533 | Train 24 | Test 61 | 0.78 | 0.28 | 0.66 | 0.4 | 3581 | Train 56 | Test 61 | 0.78 | 0.29 | 0.64 | 0.4 |
| 1534 | Train 24 | Test 62 | 0.76 | 0.34 | 0.73 | 0.46 | 3582 | Train 56 | Test 62 | 0.76 | 0.34 | 0.71 | 0.46 |
| 1535 | Train 24 | Test 63 | 0.78 | 0.28 | 0.66 | 0.4 | 3583 | Train 56 | Test 63 | 0.78 | 0.29 | 0.64 | 0.39 |
| 1536 | Train 24 | Test 64 | 0.76 | 0.32 | 0.74 | 0.45 | 3584 | Train 56 | Test 64 | 0.77 | 0.33 | 0.71 | 0.45 |
| 1537 | Train 25 | Test 1 | 0.77 | 0.24 | 0.66 | 0.35 | 3585 | Train 57 | Test 1 | 0.71 | 0.27 | 0.36 | 0.31 |
| 1538 | Train 25 | Test 2 | 0.74 | 0.26 | 0.6 | 0.36 | 3586 | Train 57 | Test 2 | 0.7 | 0.27 | 0.28 | 0.28 |
| 1539 | Train 25 | Test 3 | 0.77 | 0.24 | 0.66 | 0.35 | 3587 | Train 57 | Test 3 | 0.71 | 0.27 | 0.36 | 0.31 |
| 1540 | Train 25 | Test 4 | 0.74 | 0.26 | 0.6 | 0.36 | 3588 | Train 57 | Test 4 | 0.7 | 0.27 | 0.29 | 0.28 |
| 1541 | Train 25 | Test 5 | 0.71 | 0.22 | 0.47 | 0.3 | 3589 | Train 57 | Test 5 | 0.69 | 0.25 | 0.22 | 0.24 |
| 1542 | Train 25 | Test 6 | 0.69 | 0.31 | 0.28 | 0.3 | 3590 | Train 57 | Test 6 | 0.67 | 0.32 | 0.13 | 0.18 |
| 1543 | Train 25 | Test 7 | 0.71 | 0.22 | 0.46 | 0.3 | 3591 | Train 57 | Test 7 | 0.69 | 0.26 | 0.22 | 0.24 |
| 1544 | Train 25 | Test 8 | 0.69 | 0.3 | 0.28 | 0.29 | 3592 | Train 57 | Test 8 | 0.67 | 0.31 | 0.13 | 0.18 |
| 1545 | Train 25 | Test 9 | 0.77 | 0.24 | 0.65 | 0.35 | 3593 | Train 57 | Test 9 | 0.71 | 0.27 | 0.35 | 0.3 |
| 1546 | Train 25 | Test 10 | 0.75 | 0.26 | 0.58 | 0.36 | 3594 | Train 57 | Test 10 | 0.7 | 0.27 | 0.27 | 0.27 |
| 1547 | Train 25 | Test 11 | 0.77 | 0.24 | 0.65 | 0.35 | 3595 | Train 57 | Test 11 | 0.72 | 0.27 | 0.35 | 0.31 |
| 1548 | Train 25 | Test 12 | 0.75 | 0.26 | 0.58 | 0.36 | 3596 | Train 57 | Test 12 | 0.71 | 0.27 | 0.28 | 0.28 |
| 1549 | Train 25 | Test 13 | 0.71 | 0.23 | 0.46 | 0.3 | 3597 | Train 57 | Test 13 | 0.69 | 0.26 | 0.21 | 0.23 |
| 1550 | Train 25 | Test 14 | 0.69 | 0.31 | 0.28 | 0.3 | 3598 | Train 57 | Test 14 | 0.68 | 0.32 | 0.13 | 0.18 |
| 1551 | Train 25 | Test 15 | 0.71 | 0.23 | 0.45 | 0.3 | 3599 | Train 57 | Test 15 | 0.69 | 0.26 | 0.21 | 0.24 |
| 1552 | Train 25 | Test 16 | 0.69 | 0.3 | 0.28 | 0.29 | 3600 | Train 57 | Test 16 | 0.68 | 0.31 | 0.13 | 0.18 |
| 1553 | Train 25 | Test 17 | 0.77 | 0.24 | 0.66 | 0.35 | 3601 | Train 57 | Test 17 | 0.71 | 0.27 | 0.36 | 0.31 |
| 1554 | Train 25 | Test 18 | 0.74 | 0.26 | 0.6 | 0.36 | 3602 | Train 57 | Test 18 | 0.7 | 0.27 | 0.28 | 0.28 |
| 1555 | Train 25 | Test 19 | 0.77 | 0.24 | 0.66 | 0.35 | 3603 | Train 57 | Test 19 | 0.71 | 0.27 | 0.36 | 0.31 |
| 1556 | Train 25 | Test 20 | 0.74 | 0.26 | 0.6 | 0.36 | 3604 | Train 57 | Test 20 | 0.7 | 0.27 | 0.29 | 0.28 |
| 1557 | Train 25 | Test 21 | 0.71 | 0.22 | 0.47 | 0.3 | 3605 | Train 57 | Test 21 | 0.69 | 0.25 | 0.22 | 0.24 |
| 1558 | Train 25 | Test 22 | 0.69 | 0.31 | 0.28 | 0.29 | 3606 | Train 57 | Test 22 | 0.67 | 0.32 | 0.13 | 0.18 |
| 1559 | Train 25 | Test 23 | 0.71 | 0.22 | 0.46 | 0.3 | 3607 | Train 57 | Test 23 | 0.69 | 0.26 | 0.22 | 0.24 |
| 1560 | Train 25 | Test 24 | 0.69 | 0.3 | 0.28 | 0.29 | 3608 | Train 57 | Test 24 | 0.67 | 0.31 | 0.13 | 0.18 |
| 1561 | Train 25 | Test 25 | 0.77 | 0.24 | 0.65 | 0.35 | 3609 | Train 57 | Test 25 | 0.71 | 0.27 | 0.35 | 0.3 |
| 1562 | Train 25 | Test 26 | 0.75 | 0.26 | 0.58 | 0.36 | 3610 | Train 57 | Test 26 | 0.7 | 0.27 | 0.27 | 0.27 |
| 1563 | Train 25 | Test 27 | 0.77 | 0.24 | 0.65 | 0.35 | 3611 | Train 57 | Test 27 | 0.72 | 0.27 | 0.35 | 0.31 |
| 1564 | Train 25 | Test 28 | 0.75 | 0.26 | 0.58 | 0.36 | 3612 | Train 57 | Test 28 | 0.71 | 0.27 | 0.28 | 0.27 |
| 1565 | Train 25 | Test 29 | 0.71 | 0.23 | 0.46 | 0.3 | 3613 | Train 57 | Test 29 | 0.69 | 0.26 | 0.21 | 0.23 |
| 1566 | Train 25 | Test 30 | 0.69 | 0.31 | 0.28 | 0.3 | 3614 | Train 57 | Test 30 | 0.68 | 0.32 | 0.13 | 0.18 |
| 1567 | Train 25 | Test 31 | 0.71 | 0.23 | 0.45 | 0.3 | 3615 | Train 57 | Test 31 | 0.69 | 0.26 | 0.21 | 0.24 |
| 1568 | Train 25 | Test 32 | 0.69 | 0.3 | 0.28 | 0.29 | 3616 | Train 57 | Test 32 | 0.68 | 0.31 | 0.13 | 0.18 |
| 1569 | Train 25 | Test 33 | 0.77 | 0.24 | 0.66 | 0.35 | 3617 | Train 57 | Test 33 | 0.71 | 0.27 | 0.36 | 0.3 |
| 1570 | Train 25 | Test 34 | 0.75 | 0.26 | 0.6 | 0.36 | 3618 | Train 57 | Test 34 | 0.7 | 0.27 | 0.28 | 0.28 |
| 1571 | Train 25 | Test 35 | 0.77 | 0.24 | 0.66 | 0.35 | 3619 | Train 57 | Test 35 | 0.72 | 0.27 | 0.36 | 0.31 |
| 1572 | Train 25 | Test 36 | 0.75 | 0.26 | 0.6 | 0.36 | 3620 | Train 57 | Test 36 | 0.71 | 0.27 | 0.28 | 0.28 |
| 1573 | Train 25 | Test 37 | 0.71 | 0.22 | 0.47 | 0.3 | 3621 | Train 57 | Test 37 | 0.69 | 0.25 | 0.22 | 0.24 |
| 1574 | Train 25 | Test 38 | 0.69 | 0.31 | 0.28 | 0.3 | 3622 | Train 57 | Test 38 | 0.68 | 0.32 | 0.13 | 0.18 |
| 1575 | Train 25 | Test 39 | 0.71 | 0.22 | 0.46 | 0.3 | 3623 | Train 57 | Test 39 | 0.69 | 0.26 | 0.22 | 0.24 |
| 1576 | Train 25 | Test 40 | 0.69 | 0.3 | 0.28 | 0.29 | 3624 | Train 57 | Test 40 | 0.68 | 0.3 | 0.13 | 0.18 |
| 1577 | Train 25 | Test 41 | 0.77 | 0.24 | 0.64 | 0.35 | 3625 | Train 57 | Test 41 | 0.72 | 0.27 | 0.35 | 0.3 |
| 1578 | Train 25 | Test 42 | 0.75 | 0.26 | 0.58 | 0.36 | 3626 | Train 57 | Test 42 | 0.71 | 0.27 | 0.27 | 0.27 |
| 1579 | Train 25 | Test 43 | 0.77 | 0.24 | 0.64 | 0.35 | 3627 | Train 57 | Test 43 | 0.72 | 0.27 | 0.35 | 0.31 |
| 1580 | Train 25 | Test 44 | 0.75 | 0.26 | 0.58 | 0.36 | 3628 | Train 57 | Test 44 | 0.71 | 0.27 | 0.27 | 0.27 |
| 1581 | Train 25 | Test 45 | 0.71 | 0.23 | 0.46 | 0.3 | 3629 | Train 57 | Test 45 | 0.69 | 0.26 | 0.21 | 0.23 |
| 1582 | Train 25 | Test 46 | 0.69 | 0.31 | 0.28 | 0.3 | 3630 | Train 57 | Test 46 | 0.68 | 0.32 | 0.13 | 0.18 |
| 1583 | Train 25 | Test 47 | 0.71 | 0.23 | 0.45 | 0.3 | 3631 | Train 57 | Test 47 | 0.69 | 0.26 | 0.21 | 0.23 |
| 1584 | Train 25 | Test 48 | 0.69 | 0.3 | 0.28 | 0.29 | 3632 | Train 57 | Test 48 | 0.68 | 0.31 | 0.13 | 0.18 |
| 1585 | Train 25 | Test 49 | 0.77 | 0.27 | 0.75 | 0.4 | 3633 | Train 57 | Test 49 | 0.77 | 0.28 | 0.65 | 0.39 |
| 1586 | Train 25 | Test 50 | 0.77 | 0.27 | 0.74 | 0.4 | 3634 | Train 57 | Test 50 | 0.77 | 0.28 | 0.65 | 0.39 |
| 1587 | Train 25 | Test 51 | 0.77 | 0.27 | 0.75 | 0.4 | 3635 | Train 57 | Test 51 | 0.77 | 0.28 | 0.66 | 0.4 |
| 1588 | Train 25 | Test 52 | 0.77 | 0.27 | 0.75 | 0.4 | 3636 | Train 57 | Test 52 | 0.77 | 0.28 | 0.65 | 0.4 |
| 1589 | Train 25 | Test 53 | 0.75 | 0.28 | 0.43 | 0.34 | 3637 | Train 57 | Test 53 | 0.73 | 0.27 | 0.39 | 0.32 |
| 1590 | Train 25 | Test 54 | 0.71 | 0.36 | 0.28 | 0.32 | 3638 | Train 57 | Test 54 | 0.69 | 0.33 | 0.17 | 0.23 |
| 1591 | Train 25 | Test 55 | 0.75 | 0.27 | 0.52 | 0.35 | 3639 | Train 57 | Test 55 | 0.73 | 0.28 | 0.39 | 0.32 |
| 1592 | Train 25 | Test 56 | 0.7 | 0.34 | 0.29 | 0.31 | 3640 | Train 57 | Test 56 | 0.69 | 0.32 | 0.18 | 0.23 |
| 1593 | Train 25 | Test 57 | 0.78 | 0.27 | 0.74 | 0.4 | 3641 | Train 57 | Test 57 | 0.78 | 0.29 | 0.65 | 0.4 |
| 1594 | Train 25 | Test 58 | 0.78 | 0.28 | 0.74 | 0.4 | 3642 | Train 57 | Test 58 | 0.77 | 0.29 | 0.64 | 0.4 |
| 1595 | Train 25 | Test 59 | 0.78 | 0.28 | 0.75 | 0.4 | 3643 | Train 57 | Test 59 | 0.78 | 0.29 | 0.65 | 0.4 |
| 1596 | Train 25 | Test 60 | 0.78 | 0.28 | 0.74 | 0.4 | 3644 | Train 57 | Test 60 | 0.77 | 0.29 | 0.65 | 0.4 |
| 1597 | Train 25 | Test 61 | 0.75 | 0.29 | 0.43 | 0.35 | 3645 | Train 57 | Test 61 | 0.73 | 0.28 | 0.39 | 0.32 |
| 1598 | Train 25 | Test 62 | 0.71 | 0.36 | 0.29 | 0.32 | 3646 | Train 57 | Test 62 | 0.69 | 0.33 | 0.18 | 0.23 |
| 1599 | Train 25 | Test 63 | 0.75 | 0.29 | 0.44 | 0.35 | 3647 | Train 57 | Test 63 | 0.74 | 0.28 | 0.39 | 0.33 |
| 1600 | Train 25 | Test 64 | 0.7 | 0.34 | 0.29 | 0.31 | 3648 | Train 57 | Test 64 | 0.69 | 0.32 | 0.18 | 0.23 |
| 1601 | Train 26 | Test 1 | 0.77 | 0.25 | 0.63 | 0.35 | 3649 | Train 58 | Test 1 | 0.69 | 0.26 | 0.35 | 0.3 |
| 1602 | Train 26 | Test 2 | 0.78 | 0.28 | 0.69 | 0.39 | 3650 | Train 58 | Test 2 | 0.69 | 0.27 | 0.28 | 0.27 |
| 1603 | Train 26 | Test 3 | 0.77 | 0.25 | 0.63 | 0.35 | 3651 | Train 58 | Test 3 | 0.69 | 0.27 | 0.36 | 0.3 |
| 1604 | Train 26 | Test 4 | 0.78 | 0.28 | 0.69 | 0.39 | 3652 | Train 58 | Test 4 | 0.69 | 0.27 | 0.28 | 0.27 |
| 1605 | Train 26 | Test 5 | 0.72 | 0.23 | 0.47 | 0.31 | 3653 | Train 58 | Test 5 | 0.67 | 0.25 | 0.21 | 0.23 |
| 1606 | Train 26 | Test 6 | 0.71 | 0.34 | 0.35 | 0.34 | 3654 | Train 58 | Test 6 | 0.67 | 0.31 | 0.13 | 0.18 |
| 1607 | Train 26 | Test 7 | 0.71 | 0.23 | 0.47 | 0.31 | 3655 | Train 58 | Test 7 | 0.67 | 0.25 | 0.21 | 0.23 |
| 1608 | Train 26 | Test 8 | 0.71 | 0.33 | 0.35 | 0.34 | 3656 | Train 58 | Test 8 | 0.66 | 0.3 | 0.13 | 0.18 |
| 1609 | Train 26 | Test 9 | 0.77 | 0.25 | 0.61 | 0.36 | 3657 | Train 58 | Test 9 | 0.69 | 0.27 | 0.35 | 0.3 |
| 1610 | Train 26 | Test 10 | 0.78 | 0.28 | 0.67 | 0.4 | 3658 | Train 58 | Test 10 | 0.69 | 0.27 | 0.27 | 0.27 |
| 1611 | Train 26 | Test 11 | 0.77 | 0.25 | 0.61 | 0.36 | 3659 | Train 58 | Test 11 | 0.69 | 0.27 | 0.36 | 0.31 |
| 1612 | Train 26 | Test 12 | 0.78 | 0.28 | 0.67 | 0.4 | 3660 | Train 58 | Test 12 | 0.69 | 0.27 | 0.28 | 0.27 |
| 1613 | Train 26 | Test 13 | 0.72 | 0.23 | 0.46 | 0.31 | 3661 | Train 58 | Test 13 | 0.67 | 0.26 | 0.21 | 0.23 |
| 1614 | Train 26 | Test 14 | 0.71 | 0.34 | 0.35 | 0.34 | 3662 | Train 58 | Test 14 | 0.67 | 0.31 | 0.13 | 0.18 |
| 1615 | Train 26 | Test 15 | 0.72 | 0.23 | 0.45 | 0.31 | 3663 | Train 58 | Test 15 | 0.67 | 0.26 | 0.21 | 0.23 |
| 1616 | Train 26 | Test 16 | 0.71 | 0.33 | 0.35 | 0.34 | 3664 | Train 58 | Test 16 | 0.67 | 0.3 | 0.13 | 0.18 |
| 1617 | Train 26 | Test 17 | 0.77 | 0.25 | 0.63 | 0.35 | 3665 | Train 58 | Test 17 | 0.69 | 0.26 | 0.35 | 0.3 |
| 1618 | Train 26 | Test 18 | 0.78 | 0.28 | 0.69 | 0.39 | 3666 | Train 58 | Test 18 | 0.69 | 0.26 | 0.28 | 0.27 |
| 1619 | Train 26 | Test 19 | 0.77 | 0.25 | 0.63 | 0.35 | 3667 | Train 58 | Test 19 | 0.69 | 0.27 | 0.36 | 0.3 |
| 1620 | Train 26 | Test 20 | 0.78 | 0.28 | 0.69 | 0.39 | 3668 | Train 58 | Test 20 | 0.69 | 0.27 | 0.28 | 0.27 |
| 1621 | Train 26 | Test 21 | 0.72 | 0.23 | 0.47 | 0.31 | 3669 | Train 58 | Test 21 | 0.67 | 0.25 | 0.21 | 0.23 |
| 1622 | Train 26 | Test 22 | 0.71 | 0.34 | 0.34 | 0.34 | 3670 | Train 58 | Test 22 | 0.67 | 0.31 | 0.12 | 0.18 |
| 1623 | Train 26 | Test 23 | 0.71 | 0.23 | 0.47 | 0.31 | 3671 | Train 58 | Test 23 | 0.67 | 0.25 | 0.21 | 0.23 |
| 1624 | Train 26 | Test 24 | 0.71 | 0.33 | 0.34 | 0.34 | 3672 | Train 58 | Test 24 | 0.67 | 0.3 | 0.13 | 0.18 |
| 1625 | Train 26 | Test 25 | 0.77 | 0.25 | 0.61 | 0.36 | 3673 | Train 58 | Test 25 | 0.69 | 0.27 | 0.35 | 0.3 |
| 1626 | Train 26 | Test 26 | 0.78 | 0.28 | 0.67 | 0.4 | 3674 | Train 58 | Test 26 | 0.69 | 0.27 | 0.27 | 0.27 |
| 1627 | Train 26 | Test 27 | 0.77 | 0.25 | 0.61 | 0.36 | 3675 | Train 58 | Test 27 | 0.69 | 0.27 | 0.36 | 0.31 |
| 1628 | Train 26 | Test 28 | 0.78 | 0.28 | 0.67 | 0.4 | 3676 | Train 58 | Test 28 | 0.69 | 0.27 | 0.28 | 0.27 |
| 1629 | Train 26 | Test 29 | 0.72 | 0.23 | 0.46 | 0.31 | 3677 | Train 58 | Test 29 | 0.67 | 0.26 | 0.21 | 0.23 |
| 1630 | Train 26 | Test 30 | 0.71 | 0.34 | 0.35 | 0.34 | 3678 | Train 58 | Test 30 | 0.67 | 0.31 | 0.12 | 0.18 |
| 1631 | Train 26 | Test 31 | 0.72 | 0.23 | 0.46 | 0.31 | 3679 | Train 58 | Test 31 | 0.67 | 0.26 | 0.21 | 0.23 |
| 1632 | Train 26 | Test 32 | 0.71 | 0.33 | 0.35 | 0.34 | 3680 | Train 58 | Test 32 | 0.67 | 0.3 | 0.13 | 0.18 |
| 1633 | Train 26 | Test 33 | 0.77 | 0.25 | 0.62 | 0.35 | 3681 | Train 58 | Test 33 | 0.69 | 0.26 | 0.35 | 0.3 |
| 1634 | Train 26 | Test 34 | 0.78 | 0.28 | 0.69 | 0.39 | 3682 | Train 58 | Test 34 | 0.69 | 0.26 | 0.28 | 0.27 |
| 1635 | Train 26 | Test 35 | 0.77 | 0.25 | 0.63 | 0.35 | 3683 | Train 58 | Test 35 | 0.7 | 0.26 | 0.36 | 0.3 |
| 1636 | Train 26 | Test 36 | 0.78 | 0.28 | 0.69 | 0.39 | 3684 | Train 58 | Test 36 | 0.69 | 0.27 | 0.28 | 0.27 |
| 1637 | Train 26 | Test 37 | 0.72 | 0.23 | 0.47 | 0.31 | 3685 | Train 58 | Test 37 | 0.67 | 0.25 | 0.21 | 0.23 |
| 1638 | Train 26 | Test 38 | 0.71 | 0.34 | 0.35 | 0.34 | 3686 | Train 58 | Test 38 | 0.67 | 0.31 | 0.13 | 0.18 |
| 1639 | Train 26 | Test 39 | 0.72 | 0.23 | 0.47 | 0.31 | 3687 | Train 58 | Test 39 | 0.67 | 0.25 | 0.21 | 0.23 |
| 1640 | Train 26 | Test 40 | 0.71 | 0.33 | 0.35 | 0.34 | 3688 | Train 58 | Test 40 | 0.67 | 0.3 | 0.13 | 0.18 |
| 1641 | Train 26 | Test 41 | 0.77 | 0.25 | 0.61 | 0.35 | 3689 | Train 58 | Test 41 | 0.7 | 0.27 | 0.35 | 0.3 |
| 1642 | Train 26 | Test 42 | 0.78 | 0.28 | 0.67 | 0.39 | 3690 | Train 58 | Test 42 | 0.7 | 0.27 | 0.27 | 0.27 |
| 1643 | Train 26 | Test 43 | 0.77 | 0.25 | 0.61 | 0.36 | 3691 | Train 58 | Test 43 | 0.7 | 0.27 | 0.36 | 0.31 |
| 1644 | Train 26 | Test 44 | 0.78 | 0.28 | 0.67 | 0.4 | 3692 | Train 58 | Test 44 | 0.7 | 0.27 | 0.28 | 0.27 |
| 1645 | Train 26 | Test 45 | 0.72 | 0.23 | 0.46 | 0.31 | 3693 | Train 58 | Test 45 | 0.67 | 0.26 | 0.21 | 0.23 |
| 1646 | Train 26 | Test 46 | 0.71 | 0.34 | 0.35 | 0.34 | 3694 | Train 58 | Test 46 | 0.67 | 0.31 | 0.13 | 0.18 |
| 1647 | Train 26 | Test 47 | 0.72 | 0.23 | 0.45 | 0.31 | 3695 | Train 58 | Test 47 | 0.67 | 0.26 | 0.21 | 0.23 |
| 1648 | Train 26 | Test 48 | 0.71 | 0.33 | 0.35 | 0.34 | 3696 | Train 58 | Test 48 | 0.67 | 0.3 | 0.13 | 0.18 |
| 1649 | Train 26 | Test 49 | 0.77 | 0.28 | 0.73 | 0.4 | 3697 | Train 58 | Test 49 | 0.77 | 0.28 | 0.66 | 0.39 |
| 1650 | Train 26 | Test 50 | 0.77 | 0.28 | 0.73 | 0.4 | 3698 | Train 58 | Test 50 | 0.77 | 0.28 | 0.65 | 0.39 |
| 1651 | Train 26 | Test 51 | 0.77 | 0.28 | 0.73 | 0.4 | 3699 | Train 58 | Test 51 | 0.77 | 0.28 | 0.66 | 0.39 |
| 1652 | Train 26 | Test 52 | 0.77 | 0.28 | 0.73 | 0.4 | 3700 | Train 58 | Test 52 | 0.77 | 0.28 | 0.65 | 0.39 |
| 1653 | Train 26 | Test 53 | 0.75 | 0.3 | 0.39 | 0.34 | 3701 | Train 58 | Test 53 | 0.7 | 0.27 | 0.38 | 0.32 |
| 1654 | Train 26 | Test 54 | 0.72 | 0.39 | 0.33 | 0.36 | 3702 | Train 58 | Test 54 | 0.68 | 0.33 | 0.17 | 0.23 |
| 1655 | Train 26 | Test 55 | 0.75 | 0.28 | 0.53 | 0.36 | 3703 | Train 58 | Test 55 | 0.71 | 0.28 | 0.39 | 0.32 |
| 1656 | Train 26 | Test 56 | 0.71 | 0.36 | 0.33 | 0.35 | 3704 | Train 58 | Test 56 | 0.67 | 0.31 | 0.18 | 0.23 |
| 1657 | Train 26 | Test 57 | 0.78 | 0.28 | 0.73 | 0.41 | 3705 | Train 58 | Test 57 | 0.78 | 0.29 | 0.66 | 0.4 |
| 1658 | Train 26 | Test 58 | 0.78 | 0.28 | 0.73 | 0.41 | 3706 | Train 58 | Test 58 | 0.77 | 0.29 | 0.66 | 0.4 |
| 1659 | Train 26 | Test 59 | 0.78 | 0.28 | 0.73 | 0.41 | 3707 | Train 58 | Test 59 | 0.78 | 0.29 | 0.66 | 0.4 |
| 1660 | Train 26 | Test 60 | 0.78 | 0.28 | 0.73 | 0.41 | 3708 | Train 58 | Test 60 | 0.77 | 0.29 | 0.66 | 0.4 |
| 1661 | Train 26 | Test 61 | 0.76 | 0.31 | 0.38 | 0.34 | 3709 | Train 58 | Test 61 | 0.71 | 0.28 | 0.38 | 0.32 |
| 1662 | Train 26 | Test 62 | 0.72 | 0.39 | 0.33 | 0.35 | 3710 | Train 58 | Test 62 | 0.68 | 0.33 | 0.17 | 0.23 |
| 1663 | Train 26 | Test 63 | 0.76 | 0.31 | 0.39 | 0.35 | 3711 | Train 58 | Test 63 | 0.71 | 0.28 | 0.39 | 0.33 |
| 1664 | Train 26 | Test 64 | 0.72 | 0.36 | 0.33 | 0.34 | 3712 | Train 58 | Test 64 | 0.68 | 0.31 | 0.18 | 0.23 |
| 1665 | Train 27 | Test 1 | 0.77 | 0.24 | 0.66 | 0.35 | 3713 | Train 59 | Test 1 | 0.72 | 0.26 | 0.36 | 0.3 |
| 1666 | Train 27 | Test 2 | 0.75 | 0.26 | 0.61 | 0.37 | 3714 | Train 59 | Test 2 | 0.7 | 0.27 | 0.29 | 0.28 |
| 1667 | Train 27 | Test 3 | 0.77 | 0.24 | 0.67 | 0.35 | 3715 | Train 59 | Test 3 | 0.72 | 0.26 | 0.36 | 0.3 |
| 1668 | Train 27 | Test 4 | 0.75 | 0.26 | 0.61 | 0.37 | 3716 | Train 59 | Test 4 | 0.71 | 0.27 | 0.29 | 0.28 |
| 1669 | Train 27 | Test 5 | 0.71 | 0.22 | 0.48 | 0.31 | 3717 | Train 59 | Test 5 | 0.69 | 0.25 | 0.22 | 0.24 |
| 1670 | Train 27 | Test 6 | 0.69 | 0.31 | 0.27 | 0.29 | 3718 | Train 59 | Test 6 | 0.67 | 0.31 | 0.13 | 0.19 |
| 1671 | Train 27 | Test 7 | 0.71 | 0.22 | 0.47 | 0.3 | 3719 | Train 59 | Test 7 | 0.69 | 0.25 | 0.22 | 0.24 |
| 1672 | Train 27 | Test 8 | 0.69 | 0.3 | 0.27 | 0.29 | 3720 | Train 59 | Test 8 | 0.68 | 0.3 | 0.14 | 0.19 |
| 1673 | Train 27 | Test 9 | 0.77 | 0.24 | 0.65 | 0.35 | 3721 | Train 59 | Test 9 | 0.72 | 0.27 | 0.35 | 0.3 |
| 1674 | Train 27 | Test 10 | 0.75 | 0.27 | 0.59 | 0.37 | 3722 | Train 59 | Test 10 | 0.71 | 0.27 | 0.28 | 0.28 |
| 1675 | Train 27 | Test 11 | 0.77 | 0.24 | 0.65 | 0.35 | 3723 | Train 59 | Test 11 | 0.72 | 0.27 | 0.36 | 0.31 |
| 1676 | Train 27 | Test 12 | 0.75 | 0.27 | 0.59 | 0.37 | 3724 | Train 59 | Test 12 | 0.71 | 0.27 | 0.28 | 0.28 |
| 1677 | Train 27 | Test 13 | 0.71 | 0.23 | 0.47 | 0.31 | 3725 | Train 59 | Test 13 | 0.69 | 0.26 | 0.22 | 0.24 |
| 1678 | Train 27 | Test 14 | 0.69 | 0.31 | 0.27 | 0.29 | 3726 | Train 59 | Test 14 | 0.68 | 0.31 | 0.13 | 0.19 |
| 1679 | Train 27 | Test 15 | 0.71 | 0.23 | 0.46 | 0.31 | 3727 | Train 59 | Test 15 | 0.69 | 0.26 | 0.22 | 0.24 |
| 1680 | Train 27 | Test 16 | 0.69 | 0.3 | 0.27 | 0.29 | 3728 | Train 59 | Test 16 | 0.68 | 0.3 | 0.14 | 0.19 |
| 1681 | Train 27 | Test 17 | 0.77 | 0.24 | 0.66 | 0.35 | 3729 | Train 59 | Test 17 | 0.72 | 0.26 | 0.36 | 0.3 |
| 1682 | Train 27 | Test 18 | 0.75 | 0.26 | 0.61 | 0.37 | 3730 | Train 59 | Test 18 | 0.7 | 0.27 | 0.29 | 0.27 |
| 1683 | Train 27 | Test 19 | 0.77 | 0.24 | 0.67 | 0.35 | 3731 | Train 59 | Test 19 | 0.72 | 0.26 | 0.36 | 0.3 |
| 1684 | Train 27 | Test 20 | 0.75 | 0.26 | 0.61 | 0.37 | 3732 | Train 59 | Test 20 | 0.71 | 0.27 | 0.29 | 0.28 |
| 1685 | Train 27 | Test 21 | 0.71 | 0.22 | 0.48 | 0.31 | 3733 | Train 59 | Test 21 | 0.69 | 0.25 | 0.22 | 0.24 |
| 1686 | Train 27 | Test 22 | 0.69 | 0.31 | 0.27 | 0.29 | 3734 | Train 59 | Test 22 | 0.68 | 0.31 | 0.13 | 0.19 |
| 1687 | Train 27 | Test 23 | 0.71 | 0.22 | 0.47 | 0.3 | 3735 | Train 59 | Test 23 | 0.69 | 0.25 | 0.22 | 0.24 |
| 1688 | Train 27 | Test 24 | 0.69 | 0.3 | 0.27 | 0.29 | 3736 | Train 59 | Test 24 | 0.68 | 0.3 | 0.13 | 0.19 |
| 1689 | Train 27 | Test 25 | 0.77 | 0.24 | 0.65 | 0.35 | 3737 | Train 59 | Test 25 | 0.72 | 0.27 | 0.35 | 0.3 |
| 1690 | Train 27 | Test 26 | 0.75 | 0.27 | 0.59 | 0.37 | 3738 | Train 59 | Test 26 | 0.71 | 0.27 | 0.28 | 0.28 |
| 1691 | Train 27 | Test 27 | 0.77 | 0.24 | 0.65 | 0.35 | 3739 | Train 59 | Test 27 | 0.72 | 0.27 | 0.36 | 0.31 |
| 1692 | Train 27 | Test 28 | 0.75 | 0.27 | 0.59 | 0.37 | 3740 | Train 59 | Test 28 | 0.71 | 0.27 | 0.28 | 0.28 |
| 1693 | Train 27 | Test 29 | 0.71 | 0.23 | 0.47 | 0.31 | 3741 | Train 59 | Test 29 | 0.69 | 0.26 | 0.22 | 0.24 |
| 1694 | Train 27 | Test 30 | 0.69 | 0.31 | 0.27 | 0.29 | 3742 | Train 59 | Test 30 | 0.68 | 0.31 | 0.13 | 0.19 |
| 1695 | Train 27 | Test 31 | 0.71 | 0.23 | 0.46 | 0.31 | 3743 | Train 59 | Test 31 | 0.69 | 0.26 | 0.22 | 0.24 |
| 1696 | Train 27 | Test 32 | 0.69 | 0.3 | 0.27 | 0.29 | 3744 | Train 59 | Test 32 | 0.68 | 0.3 | 0.14 | 0.19 |
| 1697 | Train 27 | Test 33 | 0.77 | 0.24 | 0.66 | 0.35 | 3745 | Train 59 | Test 33 | 0.72 | 0.26 | 0.35 | 0.3 |
| 1698 | Train 27 | Test 34 | 0.75 | 0.26 | 0.61 | 0.36 | 3746 | Train 59 | Test 34 | 0.71 | 0.26 | 0.28 | 0.27 |
| 1699 | Train 27 | Test 35 | 0.77 | 0.24 | 0.66 | 0.35 | 3747 | Train 59 | Test 35 | 0.72 | 0.26 | 0.36 | 0.3 |
| 1700 | Train 27 | Test 36 | 0.75 | 0.26 | 0.61 | 0.37 | 3748 | Train 59 | Test 36 | 0.71 | 0.27 | 0.29 | 0.28 |
| 1701 | Train 27 | Test 37 | 0.71 | 0.22 | 0.48 | 0.31 | 3749 | Train 59 | Test 37 | 0.69 | 0.25 | 0.22 | 0.23 |
| 1702 | Train 27 | Test 38 | 0.69 | 0.31 | 0.27 | 0.29 | 3750 | Train 59 | Test 38 | 0.68 | 0.31 | 0.13 | 0.19 |
| 1703 | Train 27 | Test 39 | 0.71 | 0.22 | 0.47 | 0.3 | 3751 | Train 59 | Test 39 | 0.69 | 0.25 | 0.22 | 0.24 |
| 1704 | Train 27 | Test 40 | 0.69 | 0.3 | 0.27 | 0.29 | 3752 | Train 59 | Test 40 | 0.68 | 0.3 | 0.13 | 0.19 |
| 1705 | Train 27 | Test 41 | 0.77 | 0.24 | 0.65 | 0.35 | 3753 | Train 59 | Test 41 | 0.72 | 0.26 | 0.35 | 0.3 |
| 1706 | Train 27 | Test 42 | 0.75 | 0.27 | 0.59 | 0.37 | 3754 | Train 59 | Test 42 | 0.71 | 0.27 | 0.28 | 0.27 |
| 1707 | Train 27 | Test 43 | 0.77 | 0.24 | 0.65 | 0.35 | 3755 | Train 59 | Test 43 | 0.72 | 0.27 | 0.36 | 0.3 |
| 1708 | Train 27 | Test 44 | 0.75 | 0.27 | 0.59 | 0.37 | 3756 | Train 59 | Test 44 | 0.71 | 0.27 | 0.28 | 0.28 |
| 1709 | Train 27 | Test 45 | 0.72 | 0.23 | 0.47 | 0.31 | 3757 | Train 59 | Test 45 | 0.69 | 0.26 | 0.22 | 0.24 |
| 1710 | Train 27 | Test 46 | 0.69 | 0.31 | 0.27 | 0.29 | 3758 | Train 59 | Test 46 | 0.68 | 0.31 | 0.13 | 0.19 |
| 1711 | Train 27 | Test 47 | 0.71 | 0.23 | 0.46 | 0.31 | 3759 | Train 59 | Test 47 | 0.69 | 0.26 | 0.22 | 0.24 |
| 1712 | Train 27 | Test 48 | 0.69 | 0.3 | 0.27 | 0.29 | 3760 | Train 59 | Test 48 | 0.68 | 0.3 | 0.14 | 0.19 |
| 1713 | Train 27 | Test 49 | 0.77 | 0.27 | 0.76 | 0.39 | 3761 | Train 59 | Test 49 | 0.77 | 0.28 | 0.66 | 0.39 |
| 1714 | Train 27 | Test 50 | 0.77 | 0.27 | 0.75 | 0.39 | 3762 | Train 59 | Test 50 | 0.77 | 0.28 | 0.65 | 0.39 |
| 1715 | Train 27 | Test 51 | 0.77 | 0.27 | 0.76 | 0.39 | 3763 | Train 59 | Test 51 | 0.77 | 0.28 | 0.66 | 0.39 |
| 1716 | Train 27 | Test 52 | 0.77 | 0.27 | 0.75 | 0.39 | 3764 | Train 59 | Test 52 | 0.77 | 0.28 | 0.66 | 0.39 |
| 1717 | Train 27 | Test 53 | 0.75 | 0.29 | 0.44 | 0.35 | 3765 | Train 59 | Test 53 | 0.73 | 0.27 | 0.39 | 0.32 |
| 1718 | Train 27 | Test 54 | 0.71 | 0.36 | 0.28 | 0.31 | 3766 | Train 59 | Test 54 | 0.69 | 0.33 | 0.18 | 0.23 |
| 1719 | Train 27 | Test 55 | 0.75 | 0.27 | 0.54 | 0.36 | 3767 | Train 59 | Test 55 | 0.73 | 0.27 | 0.4 | 0.32 |
| 1720 | Train 27 | Test 56 | 0.7 | 0.34 | 0.28 | 0.31 | 3768 | Train 59 | Test 56 | 0.69 | 0.31 | 0.18 | 0.23 |
| 1721 | Train 27 | Test 57 | 0.78 | 0.27 | 0.75 | 0.4 | 3769 | Train 59 | Test 57 | 0.78 | 0.28 | 0.65 | 0.39 |
| 1722 | Train 27 | Test 58 | 0.78 | 0.27 | 0.75 | 0.4 | 3770 | Train 59 | Test 58 | 0.77 | 0.28 | 0.65 | 0.39 |
| 1723 | Train 27 | Test 59 | 0.78 | 0.27 | 0.76 | 0.4 | 3771 | Train 59 | Test 59 | 0.78 | 0.29 | 0.66 | 0.4 |
| 1724 | Train 27 | Test 60 | 0.78 | 0.27 | 0.75 | 0.4 | 3772 | Train 59 | Test 60 | 0.78 | 0.29 | 0.65 | 0.4 |
| 1725 | Train 27 | Test 61 | 0.75 | 0.3 | 0.43 | 0.35 | 3773 | Train 59 | Test 61 | 0.74 | 0.28 | 0.39 | 0.32 |
| 1726 | Train 27 | Test 62 | 0.71 | 0.36 | 0.28 | 0.32 | 3774 | Train 59 | Test 62 | 0.69 | 0.33 | 0.18 | 0.23 |
| 1727 | Train 27 | Test 63 | 0.75 | 0.3 | 0.44 | 0.35 | 3775 | Train 59 | Test 63 | 0.74 | 0.28 | 0.39 | 0.33 |
| 1728 | Train 27 | Test 64 | 0.71 | 0.34 | 0.28 | 0.31 | 3776 | Train 59 | Test 64 | 0.69 | 0.31 | 0.18 | 0.23 |
| 1729 | Train 28 | Test 1 | 0.76 | 0.25 | 0.63 | 0.35 | 3777 | Train 60 | Test 1 | 0.69 | 0.26 | 0.36 | 0.3 |
| 1730 | Train 28 | Test 2 | 0.78 | 0.28 | 0.69 | 0.39 | 3778 | Train 60 | Test 2 | 0.69 | 0.27 | 0.29 | 0.28 |
| 1731 | Train 28 | Test 3 | 0.77 | 0.25 | 0.63 | 0.35 | 3779 | Train 60 | Test 3 | 0.69 | 0.26 | 0.36 | 0.31 |
| 1732 | Train 28 | Test 4 | 0.78 | 0.28 | 0.69 | 0.39 | 3780 | Train 60 | Test 4 | 0.69 | 0.27 | 0.29 | 0.28 |
| 1733 | Train 28 | Test 5 | 0.72 | 0.23 | 0.47 | 0.31 | 3781 | Train 60 | Test 5 | 0.67 | 0.25 | 0.22 | 0.23 |
| 1734 | Train 28 | Test 6 | 0.71 | 0.34 | 0.34 | 0.34 | 3782 | Train 60 | Test 6 | 0.67 | 0.31 | 0.13 | 0.18 |
| 1735 | Train 28 | Test 7 | 0.71 | 0.23 | 0.47 | 0.31 | 3783 | Train 60 | Test 7 | 0.67 | 0.25 | 0.22 | 0.24 |
| 1736 | Train 28 | Test 8 | 0.71 | 0.33 | 0.34 | 0.34 | 3784 | Train 60 | Test 8 | 0.67 | 0.3 | 0.13 | 0.19 |
| 1737 | Train 28 | Test 9 | 0.77 | 0.25 | 0.61 | 0.35 | 3785 | Train 60 | Test 9 | 0.69 | 0.27 | 0.36 | 0.31 |
| 1738 | Train 28 | Test 10 | 0.78 | 0.28 | 0.67 | 0.4 | 3786 | Train 60 | Test 10 | 0.69 | 0.27 | 0.28 | 0.28 |
| 1739 | Train 28 | Test 11 | 0.77 | 0.25 | 0.61 | 0.36 | 3787 | Train 60 | Test 11 | 0.69 | 0.27 | 0.36 | 0.31 |
| 1740 | Train 28 | Test 12 | 0.78 | 0.28 | 0.68 | 0.4 | 3788 | Train 60 | Test 12 | 0.69 | 0.27 | 0.29 | 0.28 |
| 1741 | Train 28 | Test 13 | 0.72 | 0.23 | 0.46 | 0.31 | 3789 | Train 60 | Test 13 | 0.67 | 0.26 | 0.22 | 0.23 |
| 1742 | Train 28 | Test 14 | 0.71 | 0.34 | 0.34 | 0.34 | 3790 | Train 60 | Test 14 | 0.67 | 0.31 | 0.13 | 0.18 |
| 1743 | Train 28 | Test 15 | 0.72 | 0.23 | 0.46 | 0.31 | 3791 | Train 60 | Test 15 | 0.67 | 0.26 | 0.22 | 0.24 |
| 1744 | Train 28 | Test 16 | 0.71 | 0.33 | 0.34 | 0.34 | 3792 | Train 60 | Test 16 | 0.67 | 0.3 | 0.13 | 0.19 |
| 1745 | Train 28 | Test 17 | 0.77 | 0.25 | 0.63 | 0.35 | 3793 | Train 60 | Test 17 | 0.69 | 0.26 | 0.36 | 0.3 |
| 1746 | Train 28 | Test 18 | 0.78 | 0.28 | 0.69 | 0.39 | 3794 | Train 60 | Test 18 | 0.69 | 0.27 | 0.29 | 0.28 |
| 1747 | Train 28 | Test 19 | 0.77 | 0.25 | 0.63 | 0.35 | 3795 | Train 60 | Test 19 | 0.69 | 0.26 | 0.36 | 0.31 |
| 1748 | Train 28 | Test 20 | 0.78 | 0.28 | 0.69 | 0.39 | 3796 | Train 60 | Test 20 | 0.69 | 0.27 | 0.29 | 0.28 |
| 1749 | Train 28 | Test 21 | 0.72 | 0.23 | 0.47 | 0.31 | 3797 | Train 60 | Test 21 | 0.67 | 0.25 | 0.22 | 0.23 |
| 1750 | Train 28 | Test 22 | 0.71 | 0.34 | 0.34 | 0.34 | 3798 | Train 60 | Test 22 | 0.67 | 0.31 | 0.13 | 0.18 |
| 1751 | Train 28 | Test 23 | 0.71 | 0.23 | 0.47 | 0.31 | 3799 | Train 60 | Test 23 | 0.67 | 0.25 | 0.22 | 0.23 |
| 1752 | Train 28 | Test 24 | 0.71 | 0.33 | 0.34 | 0.34 | 3800 | Train 60 | Test 24 | 0.67 | 0.3 | 0.13 | 0.19 |
| 1753 | Train 28 | Test 25 | 0.77 | 0.25 | 0.61 | 0.36 | 3801 | Train 60 | Test 25 | 0.69 | 0.27 | 0.36 | 0.3 |
| 1754 | Train 28 | Test 26 | 0.78 | 0.28 | 0.67 | 0.4 | 3802 | Train 60 | Test 26 | 0.69 | 0.27 | 0.28 | 0.28 |
| 1755 | Train 28 | Test 27 | 0.77 | 0.25 | 0.61 | 0.36 | 3803 | Train 60 | Test 27 | 0.69 | 0.27 | 0.36 | 0.31 |
| 1756 | Train 28 | Test 28 | 0.78 | 0.28 | 0.68 | 0.4 | 3804 | Train 60 | Test 28 | 0.69 | 0.27 | 0.29 | 0.28 |
| 1757 | Train 28 | Test 29 | 0.72 | 0.23 | 0.46 | 0.31 | 3805 | Train 60 | Test 29 | 0.67 | 0.26 | 0.22 | 0.23 |
| 1758 | Train 28 | Test 30 | 0.71 | 0.34 | 0.34 | 0.34 | 3806 | Train 60 | Test 30 | 0.67 | 0.31 | 0.13 | 0.18 |
| 1759 | Train 28 | Test 31 | 0.72 | 0.23 | 0.46 | 0.31 | 3807 | Train 60 | Test 31 | 0.67 | 0.26 | 0.22 | 0.24 |
| 1760 | Train 28 | Test 32 | 0.71 | 0.33 | 0.34 | 0.34 | 3808 | Train 60 | Test 32 | 0.67 | 0.3 | 0.13 | 0.19 |
| 1761 | Train 28 | Test 33 | 0.77 | 0.25 | 0.62 | 0.35 | 3809 | Train 60 | Test 33 | 0.69 | 0.26 | 0.36 | 0.3 |
| 1762 | Train 28 | Test 34 | 0.78 | 0.28 | 0.69 | 0.39 | 3810 | Train 60 | Test 34 | 0.69 | 0.27 | 0.29 | 0.27 |
| 1763 | Train 28 | Test 35 | 0.77 | 0.25 | 0.63 | 0.35 | 3811 | Train 60 | Test 35 | 0.69 | 0.26 | 0.36 | 0.3 |
| 1764 | Train 28 | Test 36 | 0.78 | 0.28 | 0.69 | 0.39 | 3812 | Train 60 | Test 36 | 0.69 | 0.27 | 0.29 | 0.28 |
| 1765 | Train 28 | Test 37 | 0.72 | 0.23 | 0.47 | 0.31 | 3813 | Train 60 | Test 37 | 0.67 | 0.25 | 0.22 | 0.23 |
| 1766 | Train 28 | Test 38 | 0.71 | 0.34 | 0.34 | 0.34 | 3814 | Train 60 | Test 38 | 0.67 | 0.31 | 0.13 | 0.18 |
| 1767 | Train 28 | Test 39 | 0.72 | 0.23 | 0.47 | 0.31 | 3815 | Train 60 | Test 39 | 0.67 | 0.25 | 0.22 | 0.23 |
| 1768 | Train 28 | Test 40 | 0.71 | 0.33 | 0.34 | 0.34 | 3816 | Train 60 | Test 40 | 0.67 | 0.3 | 0.13 | 0.19 |
| 1769 | Train 28 | Test 41 | 0.77 | 0.25 | 0.61 | 0.35 | 3817 | Train 60 | Test 41 | 0.69 | 0.26 | 0.36 | 0.3 |
| 1770 | Train 28 | Test 42 | 0.78 | 0.28 | 0.67 | 0.39 | 3818 | Train 60 | Test 42 | 0.69 | 0.27 | 0.28 | 0.28 |
| 1771 | Train 28 | Test 43 | 0.77 | 0.25 | 0.61 | 0.36 | 3819 | Train 60 | Test 43 | 0.7 | 0.27 | 0.36 | 0.31 |
| 1772 | Train 28 | Test 44 | 0.78 | 0.28 | 0.67 | 0.39 | 3820 | Train 60 | Test 44 | 0.7 | 0.27 | 0.29 | 0.28 |
| 1773 | Train 28 | Test 45 | 0.72 | 0.23 | 0.46 | 0.31 | 3821 | Train 60 | Test 45 | 0.68 | 0.26 | 0.22 | 0.23 |
| 1774 | Train 28 | Test 46 | 0.71 | 0.34 | 0.34 | 0.34 | 3822 | Train 60 | Test 46 | 0.67 | 0.31 | 0.13 | 0.18 |
| 1775 | Train 28 | Test 47 | 0.72 | 0.23 | 0.46 | 0.31 | 3823 | Train 60 | Test 47 | 0.67 | 0.26 | 0.22 | 0.23 |
| 1776 | Train 28 | Test 48 | 0.71 | 0.33 | 0.34 | 0.34 | 3824 | Train 60 | Test 48 | 0.67 | 0.3 | 0.13 | 0.19 |
| 1777 | Train 28 | Test 49 | 0.77 | 0.28 | 0.74 | 0.4 | 3825 | Train 60 | Test 49 | 0.77 | 0.28 | 0.67 | 0.39 |
| 1778 | Train 28 | Test 50 | 0.77 | 0.28 | 0.74 | 0.4 | 3826 | Train 60 | Test 50 | 0.77 | 0.28 | 0.66 | 0.39 |
| 1779 | Train 28 | Test 51 | 0.77 | 0.28 | 0.74 | 0.4 | 3827 | Train 60 | Test 51 | 0.77 | 0.28 | 0.67 | 0.39 |
| 1780 | Train 28 | Test 52 | 0.77 | 0.28 | 0.74 | 0.4 | 3828 | Train 60 | Test 52 | 0.77 | 0.28 | 0.66 | 0.39 |
| 1781 | Train 28 | Test 53 | 0.75 | 0.31 | 0.39 | 0.34 | 3829 | Train 60 | Test 53 | 0.71 | 0.27 | 0.39 | 0.32 |
| 1782 | Train 28 | Test 54 | 0.72 | 0.39 | 0.32 | 0.35 | 3830 | Train 60 | Test 54 | 0.68 | 0.33 | 0.18 | 0.23 |
| 1783 | Train 28 | Test 55 | 0.75 | 0.28 | 0.53 | 0.36 | 3831 | Train 60 | Test 55 | 0.71 | 0.27 | 0.39 | 0.32 |
| 1784 | Train 28 | Test 56 | 0.72 | 0.36 | 0.33 | 0.34 | 3832 | Train 60 | Test 56 | 0.68 | 0.31 | 0.18 | 0.23 |
| 1785 | Train 28 | Test 57 | 0.78 | 0.28 | 0.74 | 0.41 | 3833 | Train 60 | Test 57 | 0.78 | 0.28 | 0.67 | 0.4 |
| 1786 | Train 28 | Test 58 | 0.78 | 0.28 | 0.73 | 0.41 | 3834 | Train 60 | Test 58 | 0.77 | 0.28 | 0.66 | 0.4 |
| 1787 | Train 28 | Test 59 | 0.78 | 0.28 | 0.74 | 0.41 | 3835 | Train 60 | Test 59 | 0.78 | 0.29 | 0.67 | 0.4 |
| 1788 | Train 28 | Test 60 | 0.78 | 0.28 | 0.74 | 0.41 | 3836 | Train 60 | Test 60 | 0.78 | 0.29 | 0.67 | 0.4 |
| 1789 | Train 28 | Test 61 | 0.76 | 0.31 | 0.39 | 0.34 | 3837 | Train 60 | Test 61 | 0.71 | 0.27 | 0.38 | 0.32 |
| 1790 | Train 28 | Test 62 | 0.72 | 0.39 | 0.32 | 0.35 | 3838 | Train 60 | Test 62 | 0.68 | 0.33 | 0.18 | 0.23 |
| 1791 | Train 28 | Test 63 | 0.76 | 0.31 | 0.39 | 0.35 | 3839 | Train 60 | Test 63 | 0.71 | 0.28 | 0.39 | 0.33 |
| 1792 | Train 28 | Test 64 | 0.72 | 0.36 | 0.33 | 0.34 | 3840 | Train 60 | Test 64 | 0.68 | 0.31 | 0.19 | 0.23 |
| 1793 | Train 29 | Test 1 | 0.76 | 0.23 | 0.7 | 0.34 | 3841 | Train 61 | Test 1 | 0.72 | 0.23 | 0.58 | 0.33 |
| 1794 | Train 29 | Test 2 | 0.76 | 0.25 | 0.71 | 0.37 | 3842 | Train 61 | Test 2 | 0.71 | 0.25 | 0.54 | 0.34 |
| 1795 | Train 29 | Test 3 | 0.76 | 0.23 | 0.7 | 0.34 | 3843 | Train 61 | Test 3 | 0.72 | 0.23 | 0.58 | 0.33 |
| 1796 | Train 29 | Test 4 | 0.76 | 0.25 | 0.71 | 0.37 | 3844 | Train 61 | Test 4 | 0.71 | 0.25 | 0.54 | 0.35 |
| 1797 | Train 29 | Test 5 | 0.75 | 0.22 | 0.67 | 0.33 | 3845 | Train 61 | Test 5 | 0.71 | 0.23 | 0.51 | 0.32 |
| 1798 | Train 29 | Test 6 | 0.7 | 0.29 | 0.51 | 0.37 | 3846 | Train 61 | Test 6 | 0.68 | 0.29 | 0.4 | 0.34 |
| 1799 | Train 29 | Test 7 | 0.75 | 0.22 | 0.67 | 0.33 | 3847 | Train 61 | Test 7 | 0.71 | 0.23 | 0.51 | 0.31 |
| 1800 | Train 29 | Test 8 | 0.7 | 0.29 | 0.51 | 0.37 | 3848 | Train 61 | Test 8 | 0.68 | 0.28 | 0.4 | 0.33 |
| 1801 | Train 29 | Test 9 | 0.76 | 0.23 | 0.69 | 0.34 | 3849 | Train 61 | Test 9 | 0.73 | 0.23 | 0.57 | 0.33 |
| 1802 | Train 29 | Test 10 | 0.76 | 0.26 | 0.7 | 0.38 | 3850 | Train 61 | Test 10 | 0.72 | 0.26 | 0.53 | 0.35 |
| 1803 | Train 29 | Test 11 | 0.76 | 0.23 | 0.69 | 0.35 | 3851 | Train 61 | Test 11 | 0.73 | 0.23 | 0.57 | 0.33 |
| 1804 | Train 29 | Test 12 | 0.76 | 0.26 | 0.7 | 0.38 | 3852 | Train 61 | Test 12 | 0.72 | 0.26 | 0.53 | 0.35 |
| 1805 | Train 29 | Test 13 | 0.75 | 0.23 | 0.66 | 0.34 | 3853 | Train 61 | Test 13 | 0.71 | 0.23 | 0.5 | 0.32 |
| 1806 | Train 29 | Test 14 | 0.7 | 0.29 | 0.52 | 0.37 | 3854 | Train 61 | Test 14 | 0.68 | 0.29 | 0.41 | 0.34 |
| 1807 | Train 29 | Test 15 | 0.75 | 0.23 | 0.65 | 0.34 | 3855 | Train 61 | Test 15 | 0.71 | 0.23 | 0.5 | 0.32 |
| 1808 | Train 29 | Test 16 | 0.7 | 0.29 | 0.52 | 0.37 | 3856 | Train 61 | Test 16 | 0.68 | 0.28 | 0.41 | 0.33 |
| 1809 | Train 29 | Test 17 | 0.76 | 0.23 | 0.7 | 0.34 | 3857 | Train 61 | Test 17 | 0.72 | 0.23 | 0.58 | 0.33 |
| 1810 | Train 29 | Test 18 | 0.76 | 0.25 | 0.71 | 0.37 | 3858 | Train 61 | Test 18 | 0.71 | 0.25 | 0.54 | 0.34 |
| 1811 | Train 29 | Test 19 | 0.76 | 0.23 | 0.7 | 0.34 | 3859 | Train 61 | Test 19 | 0.72 | 0.23 | 0.58 | 0.33 |
| 1812 | Train 29 | Test 20 | 0.76 | 0.25 | 0.71 | 0.38 | 3860 | Train 61 | Test 20 | 0.71 | 0.25 | 0.54 | 0.34 |
| 1813 | Train 29 | Test 21 | 0.75 | 0.22 | 0.67 | 0.34 | 3861 | Train 61 | Test 21 | 0.71 | 0.23 | 0.51 | 0.32 |
| 1814 | Train 29 | Test 22 | 0.7 | 0.29 | 0.51 | 0.37 | 3862 | Train 61 | Test 22 | 0.68 | 0.29 | 0.4 | 0.34 |
| 1815 | Train 29 | Test 23 | 0.75 | 0.22 | 0.67 | 0.33 | 3863 | Train 61 | Test 23 | 0.71 | 0.23 | 0.51 | 0.31 |
| 1816 | Train 29 | Test 24 | 0.7 | 0.29 | 0.51 | 0.37 | 3864 | Train 61 | Test 24 | 0.68 | 0.28 | 0.4 | 0.33 |
| 1817 | Train 29 | Test 25 | 0.76 | 0.23 | 0.69 | 0.34 | 3865 | Train 61 | Test 25 | 0.73 | 0.23 | 0.57 | 0.33 |
| 1818 | Train 29 | Test 26 | 0.76 | 0.26 | 0.7 | 0.38 | 3866 | Train 61 | Test 26 | 0.72 | 0.26 | 0.53 | 0.35 |
| 1819 | Train 29 | Test 27 | 0.76 | 0.23 | 0.69 | 0.35 | 3867 | Train 61 | Test 27 | 0.73 | 0.23 | 0.57 | 0.33 |
| 1820 | Train 29 | Test 28 | 0.76 | 0.26 | 0.7 | 0.38 | 3868 | Train 61 | Test 28 | 0.72 | 0.26 | 0.53 | 0.35 |
| 1821 | Train 29 | Test 29 | 0.75 | 0.23 | 0.66 | 0.34 | 3869 | Train 61 | Test 29 | 0.71 | 0.23 | 0.5 | 0.32 |
| 1822 | Train 29 | Test 30 | 0.7 | 0.29 | 0.52 | 0.37 | 3870 | Train 61 | Test 30 | 0.68 | 0.29 | 0.4 | 0.34 |
| 1823 | Train 29 | Test 31 | 0.75 | 0.23 | 0.65 | 0.34 | 3871 | Train 61 | Test 31 | 0.71 | 0.23 | 0.5 | 0.32 |
| 1824 | Train 29 | Test 32 | 0.7 | 0.29 | 0.52 | 0.37 | 3872 | Train 61 | Test 32 | 0.68 | 0.28 | 0.41 | 0.33 |
| 1825 | Train 29 | Test 33 | 0.76 | 0.22 | 0.7 | 0.34 | 3873 | Train 61 | Test 33 | 0.73 | 0.23 | 0.57 | 0.33 |
| 1826 | Train 29 | Test 34 | 0.76 | 0.25 | 0.71 | 0.37 | 3874 | Train 61 | Test 34 | 0.72 | 0.25 | 0.53 | 0.34 |
| 1827 | Train 29 | Test 35 | 0.76 | 0.23 | 0.7 | 0.34 | 3875 | Train 61 | Test 35 | 0.73 | 0.23 | 0.58 | 0.33 |
| 1828 | Train 29 | Test 36 | 0.76 | 0.25 | 0.71 | 0.37 | 3876 | Train 61 | Test 36 | 0.72 | 0.25 | 0.54 | 0.34 |
| 1829 | Train 29 | Test 37 | 0.75 | 0.22 | 0.67 | 0.33 | 3877 | Train 61 | Test 37 | 0.71 | 0.23 | 0.51 | 0.32 |
| 1830 | Train 29 | Test 38 | 0.7 | 0.29 | 0.51 | 0.37 | 3878 | Train 61 | Test 38 | 0.68 | 0.29 | 0.4 | 0.34 |
| 1831 | Train 29 | Test 39 | 0.75 | 0.22 | 0.66 | 0.33 | 3879 | Train 61 | Test 39 | 0.71 | 0.23 | 0.51 | 0.31 |
| 1832 | Train 29 | Test 40 | 0.7 | 0.29 | 0.51 | 0.37 | 3880 | Train 61 | Test 40 | 0.68 | 0.28 | 0.4 | 0.33 |
| 1833 | Train 29 | Test 41 | 0.76 | 0.23 | 0.69 | 0.34 | 3881 | Train 61 | Test 41 | 0.73 | 0.23 | 0.56 | 0.33 |
| 1834 | Train 29 | Test 42 | 0.76 | 0.26 | 0.7 | 0.38 | 3882 | Train 61 | Test 42 | 0.72 | 0.26 | 0.52 | 0.35 |
| 1835 | Train 29 | Test 43 | 0.76 | 0.23 | 0.69 | 0.34 | 3883 | Train 61 | Test 43 | 0.73 | 0.23 | 0.57 | 0.33 |
| 1836 | Train 29 | Test 44 | 0.76 | 0.26 | 0.7 | 0.38 | 3884 | Train 61 | Test 44 | 0.72 | 0.26 | 0.53 | 0.35 |
| 1837 | Train 29 | Test 45 | 0.75 | 0.23 | 0.66 | 0.34 | 3885 | Train 61 | Test 45 | 0.71 | 0.23 | 0.5 | 0.32 |
| 1838 | Train 29 | Test 46 | 0.7 | 0.29 | 0.52 | 0.37 | 3886 | Train 61 | Test 46 | 0.68 | 0.29 | 0.4 | 0.34 |
| 1839 | Train 29 | Test 47 | 0.75 | 0.23 | 0.65 | 0.34 | 3887 | Train 61 | Test 47 | 0.71 | 0.23 | 0.5 | 0.32 |
| 1840 | Train 29 | Test 48 | 0.7 | 0.29 | 0.52 | 0.37 | 3888 | Train 61 | Test 48 | 0.68 | 0.28 | 0.41 | 0.33 |
| 1841 | Train 29 | Test 49 | 0.77 | 0.25 | 0.76 | 0.38 | 3889 | Train 61 | Test 49 | 0.77 | 0.27 | 0.67 | 0.39 |
| 1842 | Train 29 | Test 50 | 0.77 | 0.26 | 0.75 | 0.38 | 3890 | Train 61 | Test 50 | 0.76 | 0.27 | 0.67 | 0.39 |
| 1843 | Train 29 | Test 51 | 0.77 | 0.26 | 0.76 | 0.38 | 3891 | Train 61 | Test 51 | 0.77 | 0.28 | 0.67 | 0.39 |
| 1844 | Train 29 | Test 52 | 0.77 | 0.26 | 0.76 | 0.38 | 3892 | Train 61 | Test 52 | 0.77 | 0.28 | 0.67 | 0.39 |
| 1845 | Train 29 | Test 53 | 0.76 | 0.27 | 0.67 | 0.38 | 3893 | Train 61 | Test 53 | 0.77 | 0.27 | 0.67 | 0.39 |
| 1846 | Train 29 | Test 54 | 0.71 | 0.35 | 0.51 | 0.42 | 3894 | Train 61 | Test 54 | 0.7 | 0.34 | 0.42 | 0.38 |
| 1847 | Train 29 | Test 55 | 0.77 | 0.26 | 0.72 | 0.38 | 3895 | Train 61 | Test 55 | 0.76 | 0.27 | 0.65 | 0.38 |
| 1848 | Train 29 | Test 56 | 0.71 | 0.33 | 0.51 | 0.4 | 3896 | Train 61 | Test 56 | 0.69 | 0.32 | 0.42 | 0.37 |
| 1849 | Train 29 | Test 57 | 0.77 | 0.26 | 0.76 | 0.39 | 3897 | Train 61 | Test 57 | 0.77 | 0.28 | 0.67 | 0.4 |
| 1850 | Train 29 | Test 58 | 0.77 | 0.26 | 0.75 | 0.39 | 3898 | Train 61 | Test 58 | 0.77 | 0.28 | 0.67 | 0.4 |
| 1851 | Train 29 | Test 59 | 0.77 | 0.26 | 0.76 | 0.39 | 3899 | Train 61 | Test 59 | 0.77 | 0.28 | 0.68 | 0.4 |
| 1852 | Train 29 | Test 60 | 0.77 | 0.26 | 0.76 | 0.39 | 3900 | Train 61 | Test 60 | 0.77 | 0.28 | 0.67 | 0.4 |
| 1853 | Train 29 | Test 61 | 0.76 | 0.27 | 0.67 | 0.39 | 3901 | Train 61 | Test 61 | 0.77 | 0.28 | 0.66 | 0.39 |
| 1854 | Train 29 | Test 62 | 0.71 | 0.35 | 0.51 | 0.42 | 3902 | Train 61 | Test 62 | 0.7 | 0.34 | 0.42 | 0.38 |
| 1855 | Train 29 | Test 63 | 0.77 | 0.27 | 0.67 | 0.39 | 3903 | Train 61 | Test 63 | 0.77 | 0.28 | 0.66 | 0.39 |
| 1856 | Train 29 | Test 64 | 0.71 | 0.33 | 0.52 | 0.41 | 3904 | Train 61 | Test 64 | 0.7 | 0.32 | 0.43 | 0.37 |
| 1857 | Train 30 | Test 1 | 0.75 | 0.24 | 0.61 | 0.34 | 3905 | Train 62 | Test 1 | 0.73 | 0.21 | 0.69 | 0.32 |
| 1858 | Train 30 | Test 2 | 0.76 | 0.26 | 0.67 | 0.38 | 3906 | Train 62 | Test 2 | 0.74 | 0.24 | 0.72 | 0.36 |
| 1859 | Train 30 | Test 3 | 0.75 | 0.24 | 0.61 | 0.34 | 3907 | Train 62 | Test 3 | 0.73 | 0.21 | 0.69 | 0.32 |
| 1860 | Train 30 | Test 4 | 0.76 | 0.27 | 0.67 | 0.38 | 3908 | Train 62 | Test 4 | 0.74 | 0.24 | 0.72 | 0.36 |
| 1861 | Train 30 | Test 5 | 0.74 | 0.23 | 0.57 | 0.33 | 3909 | Train 62 | Test 5 | 0.72 | 0.21 | 0.61 | 0.32 |
| 1862 | Train 30 | Test 6 | 0.76 | 0.31 | 0.69 | 0.43 | 3910 | Train 62 | Test 6 | 0.73 | 0.29 | 0.66 | 0.4 |
| 1863 | Train 30 | Test 7 | 0.74 | 0.23 | 0.57 | 0.33 | 3911 | Train 62 | Test 7 | 0.71 | 0.21 | 0.61 | 0.32 |
| 1864 | Train 30 | Test 8 | 0.75 | 0.3 | 0.69 | 0.42 | 3912 | Train 62 | Test 8 | 0.73 | 0.28 | 0.66 | 0.4 |
| 1865 | Train 30 | Test 9 | 0.75 | 0.24 | 0.59 | 0.34 | 3913 | Train 62 | Test 9 | 0.73 | 0.21 | 0.67 | 0.32 |
| 1866 | Train 30 | Test 10 | 0.76 | 0.27 | 0.65 | 0.38 | 3914 | Train 62 | Test 10 | 0.74 | 0.24 | 0.71 | 0.36 |
| 1867 | Train 30 | Test 11 | 0.75 | 0.24 | 0.59 | 0.34 | 3915 | Train 62 | Test 11 | 0.73 | 0.21 | 0.67 | 0.32 |
| 1868 | Train 30 | Test 12 | 0.76 | 0.27 | 0.66 | 0.38 | 3916 | Train 62 | Test 12 | 0.74 | 0.24 | 0.71 | 0.36 |
| 1869 | Train 30 | Test 13 | 0.74 | 0.24 | 0.56 | 0.33 | 3917 | Train 62 | Test 13 | 0.72 | 0.22 | 0.6 | 0.32 |
| 1870 | Train 30 | Test 14 | 0.76 | 0.31 | 0.7 | 0.43 | 3918 | Train 62 | Test 14 | 0.73 | 0.29 | 0.66 | 0.4 |
| 1871 | Train 30 | Test 15 | 0.74 | 0.24 | 0.55 | 0.33 | 3919 | Train 62 | Test 15 | 0.71 | 0.22 | 0.59 | 0.32 |
| 1872 | Train 30 | Test 16 | 0.75 | 0.3 | 0.7 | 0.42 | 3920 | Train 62 | Test 16 | 0.73 | 0.28 | 0.66 | 0.39 |
| 1873 | Train 30 | Test 17 | 0.75 | 0.24 | 0.61 | 0.34 | 3921 | Train 62 | Test 17 | 0.73 | 0.21 | 0.69 | 0.32 |
| 1874 | Train 30 | Test 18 | 0.76 | 0.26 | 0.67 | 0.38 | 3922 | Train 62 | Test 18 | 0.74 | 0.24 | 0.72 | 0.36 |
| 1875 | Train 30 | Test 19 | 0.75 | 0.24 | 0.61 | 0.34 | 3923 | Train 62 | Test 19 | 0.73 | 0.21 | 0.69 | 0.32 |
| 1876 | Train 30 | Test 20 | 0.76 | 0.27 | 0.67 | 0.38 | 3924 | Train 62 | Test 20 | 0.74 | 0.24 | 0.72 | 0.36 |
| 1877 | Train 30 | Test 21 | 0.74 | 0.23 | 0.57 | 0.33 | 3925 | Train 62 | Test 21 | 0.72 | 0.21 | 0.61 | 0.32 |
| 1878 | Train 30 | Test 22 | 0.76 | 0.31 | 0.69 | 0.43 | 3926 | Train 62 | Test 22 | 0.73 | 0.29 | 0.66 | 0.4 |
| 1879 | Train 30 | Test 23 | 0.74 | 0.23 | 0.57 | 0.33 | 3927 | Train 62 | Test 23 | 0.71 | 0.21 | 0.61 | 0.32 |
| 1880 | Train 30 | Test 24 | 0.75 | 0.3 | 0.69 | 0.42 | 3928 | Train 62 | Test 24 | 0.73 | 0.28 | 0.66 | 0.39 |
| 1881 | Train 30 | Test 25 | 0.75 | 0.24 | 0.59 | 0.34 | 3929 | Train 62 | Test 25 | 0.73 | 0.21 | 0.67 | 0.32 |
| 1882 | Train 30 | Test 26 | 0.76 | 0.27 | 0.66 | 0.38 | 3930 | Train 62 | Test 26 | 0.74 | 0.24 | 0.71 | 0.36 |
| 1883 | Train 30 | Test 27 | 0.75 | 0.24 | 0.59 | 0.34 | 3931 | Train 62 | Test 27 | 0.73 | 0.21 | 0.67 | 0.32 |
| 1884 | Train 30 | Test 28 | 0.76 | 0.27 | 0.66 | 0.38 | 3932 | Train 62 | Test 28 | 0.74 | 0.24 | 0.71 | 0.36 |
| 1885 | Train 30 | Test 29 | 0.74 | 0.24 | 0.56 | 0.33 | 3933 | Train 62 | Test 29 | 0.72 | 0.22 | 0.6 | 0.32 |
| 1886 | Train 30 | Test 30 | 0.76 | 0.31 | 0.7 | 0.42 | 3934 | Train 62 | Test 30 | 0.73 | 0.29 | 0.66 | 0.4 |
| 1887 | Train 30 | Test 31 | 0.74 | 0.24 | 0.55 | 0.33 | 3935 | Train 62 | Test 31 | 0.71 | 0.22 | 0.59 | 0.32 |
| 1888 | Train 30 | Test 32 | 0.75 | 0.3 | 0.7 | 0.42 | 3936 | Train 62 | Test 32 | 0.73 | 0.28 | 0.66 | 0.39 |
| 1889 | Train 30 | Test 33 | 0.75 | 0.24 | 0.61 | 0.34 | 3937 | Train 62 | Test 33 | 0.73 | 0.21 | 0.68 | 0.32 |
| 1890 | Train 30 | Test 34 | 0.76 | 0.26 | 0.67 | 0.38 | 3938 | Train 62 | Test 34 | 0.74 | 0.24 | 0.72 | 0.36 |
| 1891 | Train 30 | Test 35 | 0.75 | 0.24 | 0.61 | 0.34 | 3939 | Train 62 | Test 35 | 0.73 | 0.21 | 0.69 | 0.32 |
| 1892 | Train 30 | Test 36 | 0.76 | 0.27 | 0.67 | 0.38 | 3940 | Train 62 | Test 36 | 0.74 | 0.24 | 0.72 | 0.36 |
| 1893 | Train 30 | Test 37 | 0.74 | 0.23 | 0.57 | 0.33 | 3941 | Train 62 | Test 37 | 0.72 | 0.21 | 0.61 | 0.32 |
| 1894 | Train 30 | Test 38 | 0.76 | 0.31 | 0.69 | 0.43 | 3942 | Train 62 | Test 38 | 0.73 | 0.29 | 0.66 | 0.4 |
| 1895 | Train 30 | Test 39 | 0.74 | 0.23 | 0.56 | 0.33 | 3943 | Train 62 | Test 39 | 0.71 | 0.21 | 0.6 | 0.32 |
| 1896 | Train 30 | Test 40 | 0.75 | 0.3 | 0.69 | 0.42 | 3944 | Train 62 | Test 40 | 0.73 | 0.28 | 0.66 | 0.39 |
| 1897 | Train 30 | Test 41 | 0.75 | 0.24 | 0.59 | 0.34 | 3945 | Train 62 | Test 41 | 0.73 | 0.21 | 0.67 | 0.32 |
| 1898 | Train 30 | Test 42 | 0.76 | 0.27 | 0.65 | 0.38 | 3946 | Train 62 | Test 42 | 0.74 | 0.24 | 0.71 | 0.36 |
| 1899 | Train 30 | Test 43 | 0.76 | 0.24 | 0.59 | 0.34 | 3947 | Train 62 | Test 43 | 0.73 | 0.21 | 0.67 | 0.32 |
| 1900 | Train 30 | Test 44 | 0.76 | 0.27 | 0.65 | 0.38 | 3948 | Train 62 | Test 44 | 0.74 | 0.24 | 0.71 | 0.36 |
| 1901 | Train 30 | Test 45 | 0.74 | 0.24 | 0.55 | 0.33 | 3949 | Train 62 | Test 45 | 0.72 | 0.22 | 0.59 | 0.32 |
| 1902 | Train 30 | Test 46 | 0.76 | 0.31 | 0.7 | 0.43 | 3950 | Train 62 | Test 46 | 0.73 | 0.29 | 0.66 | 0.4 |
| 1903 | Train 30 | Test 47 | 0.74 | 0.24 | 0.55 | 0.33 | 3951 | Train 62 | Test 47 | 0.72 | 0.22 | 0.59 | 0.32 |
| 1904 | Train 30 | Test 48 | 0.75 | 0.3 | 0.69 | 0.42 | 3952 | Train 62 | Test 48 | 0.73 | 0.28 | 0.66 | 0.39 |
| 1905 | Train 30 | Test 49 | 0.77 | 0.28 | 0.73 | 0.4 | 3953 | Train 62 | Test 49 | 0.77 | 0.28 | 0.71 | 0.4 |
| 1906 | Train 30 | Test 50 | 0.77 | 0.28 | 0.73 | 0.4 | 3954 | Train 62 | Test 50 | 0.77 | 0.28 | 0.71 | 0.4 |
| 1907 | Train 30 | Test 51 | 0.77 | 0.28 | 0.74 | 0.4 | 3955 | Train 62 | Test 51 | 0.77 | 0.28 | 0.71 | 0.41 |
| 1908 | Train 30 | Test 52 | 0.77 | 0.28 | 0.73 | 0.4 | 3956 | Train 62 | Test 52 | 0.77 | 0.28 | 0.71 | 0.41 |
| 1909 | Train 30 | Test 53 | 0.77 | 0.28 | 0.65 | 0.39 | 3957 | Train 62 | Test 53 | 0.77 | 0.28 | 0.63 | 0.39 |
| 1910 | Train 30 | Test 54 | 0.76 | 0.34 | 0.73 | 0.47 | 3958 | Train 62 | Test 54 | 0.76 | 0.35 | 0.71 | 0.47 |
| 1911 | Train 30 | Test 55 | 0.77 | 0.28 | 0.67 | 0.39 | 3959 | Train 62 | Test 55 | 0.77 | 0.28 | 0.64 | 0.39 |
| 1912 | Train 30 | Test 56 | 0.75 | 0.32 | 0.73 | 0.45 | 3960 | Train 62 | Test 56 | 0.76 | 0.33 | 0.71 | 0.45 |
| 1913 | Train 30 | Test 57 | 0.78 | 0.28 | 0.73 | 0.4 | 3961 | Train 62 | Test 57 | 0.78 | 0.29 | 0.71 | 0.41 |
| 1914 | Train 30 | Test 58 | 0.78 | 0.28 | 0.73 | 0.4 | 3962 | Train 62 | Test 58 | 0.77 | 0.29 | 0.7 | 0.41 |
| 1915 | Train 30 | Test 59 | 0.78 | 0.28 | 0.73 | 0.41 | 3963 | Train 62 | Test 59 | 0.78 | 0.29 | 0.71 | 0.41 |
| 1916 | Train 30 | Test 60 | 0.78 | 0.28 | 0.73 | 0.41 | 3964 | Train 62 | Test 60 | 0.77 | 0.29 | 0.7 | 0.41 |
| 1917 | Train 30 | Test 61 | 0.78 | 0.29 | 0.65 | 0.4 | 3965 | Train 62 | Test 61 | 0.78 | 0.29 | 0.63 | 0.4 |
| 1918 | Train 30 | Test 62 | 0.76 | 0.34 | 0.73 | 0.46 | 3966 | Train 62 | Test 62 | 0.76 | 0.35 | 0.71 | 0.47 |
| 1919 | Train 30 | Test 63 | 0.78 | 0.29 | 0.65 | 0.4 | 3967 | Train 62 | Test 63 | 0.78 | 0.29 | 0.63 | 0.4 |
| 1920 | Train 30 | Test 64 | 0.76 | 0.32 | 0.73 | 0.45 | 3968 | Train 62 | Test 64 | 0.76 | 0.33 | 0.71 | 0.45 |
| 1921 | Train 31 | Test 1 | 0.76 | 0.23 | 0.71 | 0.34 | 3969 | Train 63 | Test 1 | 0.72 | 0.23 | 0.57 | 0.33 |
| 1922 | Train 31 | Test 2 | 0.76 | 0.25 | 0.71 | 0.37 | 3970 | Train 63 | Test 2 | 0.72 | 0.26 | 0.54 | 0.35 |
| 1923 | Train 31 | Test 3 | 0.76 | 0.23 | 0.71 | 0.34 | 3971 | Train 63 | Test 3 | 0.72 | 0.23 | 0.58 | 0.33 |
| 1924 | Train 31 | Test 4 | 0.76 | 0.25 | 0.71 | 0.37 | 3972 | Train 63 | Test 4 | 0.72 | 0.26 | 0.55 | 0.35 |
| 1925 | Train 31 | Test 5 | 0.75 | 0.22 | 0.67 | 0.34 | 3973 | Train 63 | Test 5 | 0.71 | 0.23 | 0.51 | 0.32 |
| 1926 | Train 31 | Test 6 | 0.7 | 0.29 | 0.51 | 0.37 | 3974 | Train 63 | Test 6 | 0.68 | 0.29 | 0.4 | 0.33 |
| 1927 | Train 31 | Test 7 | 0.75 | 0.22 | 0.67 | 0.33 | 3975 | Train 63 | Test 7 | 0.71 | 0.23 | 0.51 | 0.31 |
| 1928 | Train 31 | Test 8 | 0.7 | 0.29 | 0.51 | 0.37 | 3976 | Train 63 | Test 8 | 0.68 | 0.28 | 0.4 | 0.33 |
| 1929 | Train 31 | Test 9 | 0.76 | 0.23 | 0.7 | 0.35 | 3977 | Train 63 | Test 9 | 0.72 | 0.23 | 0.56 | 0.33 |
| 1930 | Train 31 | Test 10 | 0.76 | 0.26 | 0.7 | 0.38 | 3978 | Train 63 | Test 10 | 0.72 | 0.26 | 0.53 | 0.35 |
| 1931 | Train 31 | Test 11 | 0.76 | 0.23 | 0.7 | 0.35 | 3979 | Train 63 | Test 11 | 0.73 | 0.23 | 0.56 | 0.33 |
| 1932 | Train 31 | Test 12 | 0.76 | 0.26 | 0.7 | 0.38 | 3980 | Train 63 | Test 12 | 0.72 | 0.26 | 0.53 | 0.35 |
| 1933 | Train 31 | Test 13 | 0.75 | 0.23 | 0.66 | 0.34 | 3981 | Train 63 | Test 13 | 0.71 | 0.23 | 0.5 | 0.32 |
| 1934 | Train 31 | Test 14 | 0.7 | 0.29 | 0.51 | 0.37 | 3982 | Train 63 | Test 14 | 0.68 | 0.29 | 0.4 | 0.33 |
| 1935 | Train 31 | Test 15 | 0.75 | 0.23 | 0.66 | 0.34 | 3983 | Train 63 | Test 15 | 0.71 | 0.23 | 0.49 | 0.32 |
| 1936 | Train 31 | Test 16 | 0.7 | 0.29 | 0.51 | 0.37 | 3984 | Train 63 | Test 16 | 0.68 | 0.28 | 0.4 | 0.33 |
| 1937 | Train 31 | Test 17 | 0.76 | 0.23 | 0.71 | 0.34 | 3985 | Train 63 | Test 17 | 0.72 | 0.23 | 0.57 | 0.33 |
| 1938 | Train 31 | Test 18 | 0.76 | 0.25 | 0.71 | 0.37 | 3986 | Train 63 | Test 18 | 0.72 | 0.26 | 0.54 | 0.35 |
| 1939 | Train 31 | Test 19 | 0.76 | 0.23 | 0.71 | 0.34 | 3987 | Train 63 | Test 19 | 0.72 | 0.23 | 0.58 | 0.33 |
| 1940 | Train 31 | Test 20 | 0.76 | 0.25 | 0.71 | 0.37 | 3988 | Train 63 | Test 20 | 0.72 | 0.26 | 0.55 | 0.35 |
| 1941 | Train 31 | Test 21 | 0.75 | 0.22 | 0.68 | 0.34 | 3989 | Train 63 | Test 21 | 0.71 | 0.23 | 0.51 | 0.32 |
| 1942 | Train 31 | Test 22 | 0.7 | 0.29 | 0.51 | 0.37 | 3990 | Train 63 | Test 22 | 0.68 | 0.29 | 0.4 | 0.33 |
| 1943 | Train 31 | Test 23 | 0.75 | 0.22 | 0.67 | 0.33 | 3991 | Train 63 | Test 23 | 0.71 | 0.23 | 0.51 | 0.31 |
| 1944 | Train 31 | Test 24 | 0.7 | 0.29 | 0.51 | 0.37 | 3992 | Train 63 | Test 24 | 0.68 | 0.28 | 0.4 | 0.33 |
| 1945 | Train 31 | Test 25 | 0.76 | 0.23 | 0.7 | 0.35 | 3993 | Train 63 | Test 25 | 0.72 | 0.23 | 0.56 | 0.33 |
| 1946 | Train 31 | Test 26 | 0.76 | 0.26 | 0.7 | 0.38 | 3994 | Train 63 | Test 26 | 0.72 | 0.26 | 0.53 | 0.35 |
| 1947 | Train 31 | Test 27 | 0.76 | 0.23 | 0.7 | 0.35 | 3995 | Train 63 | Test 27 | 0.73 | 0.23 | 0.56 | 0.33 |
| 1948 | Train 31 | Test 28 | 0.76 | 0.26 | 0.7 | 0.38 | 3996 | Train 63 | Test 28 | 0.72 | 0.26 | 0.53 | 0.35 |
| 1949 | Train 31 | Test 29 | 0.75 | 0.23 | 0.66 | 0.34 | 3997 | Train 63 | Test 29 | 0.71 | 0.23 | 0.5 | 0.32 |
| 1950 | Train 31 | Test 30 | 0.7 | 0.29 | 0.51 | 0.37 | 3998 | Train 63 | Test 30 | 0.68 | 0.29 | 0.4 | 0.33 |
| 1951 | Train 31 | Test 31 | 0.75 | 0.23 | 0.66 | 0.34 | 3999 | Train 63 | Test 31 | 0.71 | 0.23 | 0.49 | 0.32 |
| 1952 | Train 31 | Test 32 | 0.7 | 0.29 | 0.51 | 0.37 | 4000 | Train 63 | Test 32 | 0.68 | 0.28 | 0.4 | 0.33 |
| 1953 | Train 31 | Test 33 | 0.76 | 0.23 | 0.7 | 0.34 | 4001 | Train 63 | Test 33 | 0.72 | 0.23 | 0.57 | 0.33 |
| 1954 | Train 31 | Test 34 | 0.76 | 0.25 | 0.71 | 0.37 | 4002 | Train 63 | Test 34 | 0.72 | 0.26 | 0.54 | 0.35 |
| 1955 | Train 31 | Test 35 | 0.76 | 0.23 | 0.71 | 0.34 | 4003 | Train 63 | Test 35 | 0.73 | 0.23 | 0.57 | 0.33 |
| 1956 | Train 31 | Test 36 | 0.76 | 0.25 | 0.71 | 0.37 | 4004 | Train 63 | Test 36 | 0.72 | 0.26 | 0.55 | 0.35 |
| 1957 | Train 31 | Test 37 | 0.75 | 0.22 | 0.67 | 0.34 | 4005 | Train 63 | Test 37 | 0.71 | 0.23 | 0.51 | 0.32 |
| 1958 | Train 31 | Test 38 | 0.7 | 0.29 | 0.51 | 0.37 | 4006 | Train 63 | Test 38 | 0.68 | 0.29 | 0.4 | 0.33 |
| 1959 | Train 31 | Test 39 | 0.75 | 0.22 | 0.67 | 0.33 | 4007 | Train 63 | Test 39 | 0.71 | 0.23 | 0.51 | 0.31 |
| 1960 | Train 31 | Test 40 | 0.7 | 0.29 | 0.51 | 0.37 | 4008 | Train 63 | Test 40 | 0.68 | 0.28 | 0.4 | 0.33 |
| 1961 | Train 31 | Test 41 | 0.76 | 0.23 | 0.69 | 0.34 | 4009 | Train 63 | Test 41 | 0.73 | 0.23 | 0.56 | 0.33 |
| 1962 | Train 31 | Test 42 | 0.76 | 0.26 | 0.7 | 0.38 | 4010 | Train 63 | Test 42 | 0.72 | 0.26 | 0.53 | 0.35 |
| 1963 | Train 31 | Test 43 | 0.76 | 0.23 | 0.69 | 0.35 | 4011 | Train 63 | Test 43 | 0.73 | 0.23 | 0.56 | 0.33 |
| 1964 | Train 31 | Test 44 | 0.76 | 0.26 | 0.7 | 0.38 | 4012 | Train 63 | Test 44 | 0.72 | 0.26 | 0.53 | 0.35 |
| 1965 | Train 31 | Test 45 | 0.75 | 0.23 | 0.66 | 0.34 | 4013 | Train 63 | Test 45 | 0.71 | 0.23 | 0.5 | 0.32 |
| 1966 | Train 31 | Test 46 | 0.7 | 0.29 | 0.51 | 0.37 | 4014 | Train 63 | Test 46 | 0.68 | 0.29 | 0.4 | 0.33 |
| 1967 | Train 31 | Test 47 | 0.75 | 0.23 | 0.65 | 0.34 | 4015 | Train 63 | Test 47 | 0.71 | 0.23 | 0.49 | 0.32 |
| 1968 | Train 31 | Test 48 | 0.7 | 0.29 | 0.51 | 0.37 | 4016 | Train 63 | Test 48 | 0.68 | 0.28 | 0.4 | 0.33 |
| 1969 | Train 31 | Test 49 | 0.77 | 0.25 | 0.76 | 0.38 | 4017 | Train 63 | Test 49 | 0.76 | 0.27 | 0.68 | 0.39 |
| 1970 | Train 31 | Test 50 | 0.77 | 0.25 | 0.75 | 0.38 | 4018 | Train 63 | Test 50 | 0.76 | 0.27 | 0.68 | 0.39 |
| 1971 | Train 31 | Test 51 | 0.77 | 0.25 | 0.76 | 0.38 | 4019 | Train 63 | Test 51 | 0.77 | 0.27 | 0.69 | 0.39 |
| 1972 | Train 31 | Test 52 | 0.77 | 0.25 | 0.76 | 0.38 | 4020 | Train 63 | Test 52 | 0.76 | 0.27 | 0.68 | 0.39 |
| 1973 | Train 31 | Test 53 | 0.76 | 0.27 | 0.67 | 0.38 | 4021 | Train 63 | Test 53 | 0.77 | 0.27 | 0.67 | 0.39 |
| 1974 | Train 31 | Test 54 | 0.71 | 0.35 | 0.5 | 0.41 | 4022 | Train 63 | Test 54 | 0.7 | 0.34 | 0.41 | 0.37 |
| 1975 | Train 31 | Test 55 | 0.77 | 0.26 | 0.72 | 0.38 | 4023 | Train 63 | Test 55 | 0.76 | 0.27 | 0.64 | 0.38 |
| 1976 | Train 31 | Test 56 | 0.7 | 0.33 | 0.5 | 0.4 | 4024 | Train 63 | Test 56 | 0.69 | 0.32 | 0.42 | 0.36 |
| 1977 | Train 31 | Test 57 | 0.78 | 0.26 | 0.76 | 0.39 | 4025 | Train 63 | Test 57 | 0.77 | 0.28 | 0.68 | 0.39 |
| 1978 | Train 31 | Test 58 | 0.77 | 0.26 | 0.76 | 0.39 | 4026 | Train 63 | Test 58 | 0.77 | 0.28 | 0.68 | 0.39 |
| 1979 | Train 31 | Test 59 | 0.78 | 0.26 | 0.76 | 0.39 | 4027 | Train 63 | Test 59 | 0.77 | 0.28 | 0.68 | 0.4 |
| 1980 | Train 31 | Test 60 | 0.77 | 0.26 | 0.76 | 0.39 | 4028 | Train 63 | Test 60 | 0.77 | 0.28 | 0.68 | 0.4 |
| 1981 | Train 31 | Test 61 | 0.76 | 0.27 | 0.67 | 0.39 | 4029 | Train 63 | Test 61 | 0.77 | 0.28 | 0.67 | 0.39 |
| 1982 | Train 31 | Test 62 | 0.71 | 0.35 | 0.5 | 0.41 | 4030 | Train 63 | Test 62 | 0.7 | 0.34 | 0.42 | 0.37 |
| 1983 | Train 31 | Test 63 | 0.76 | 0.27 | 0.67 | 0.39 | 4031 | Train 63 | Test 63 | 0.77 | 0.28 | 0.67 | 0.39 |
| 1984 | Train 31 | Test 64 | 0.71 | 0.33 | 0.51 | 0.4 | 4032 | Train 63 | Test 64 | 0.7 | 0.32 | 0.42 | 0.36 |
| 1985 | Train 32 | Test 1 | 0.75 | 0.24 | 0.63 | 0.34 | 4033 | Train 64 | Test 1 | 0.73 | 0.21 | 0.7 | 0.32 |
| 1986 | Train 32 | Test 2 | 0.76 | 0.26 | 0.68 | 0.38 | 4034 | Train 64 | Test 2 | 0.74 | 0.23 | 0.73 | 0.35 |
| 1987 | Train 32 | Test 3 | 0.75 | 0.24 | 0.63 | 0.34 | 4035 | Train 64 | Test 3 | 0.73 | 0.21 | 0.7 | 0.32 |
| 1988 | Train 32 | Test 4 | 0.76 | 0.26 | 0.68 | 0.38 | 4036 | Train 64 | Test 4 | 0.74 | 0.24 | 0.73 | 0.36 |
| 1989 | Train 32 | Test 5 | 0.74 | 0.23 | 0.59 | 0.33 | 4037 | Train 64 | Test 5 | 0.72 | 0.21 | 0.63 | 0.31 |
| 1990 | Train 32 | Test 6 | 0.76 | 0.31 | 0.7 | 0.42 | 4038 | Train 64 | Test 6 | 0.73 | 0.29 | 0.66 | 0.4 |
| 1991 | Train 32 | Test 7 | 0.74 | 0.23 | 0.58 | 0.33 | 4039 | Train 64 | Test 7 | 0.72 | 0.21 | 0.62 | 0.31 |
| 1992 | Train 32 | Test 8 | 0.76 | 0.3 | 0.7 | 0.42 | 4040 | Train 64 | Test 8 | 0.73 | 0.28 | 0.65 | 0.39 |
| 1993 | Train 32 | Test 9 | 0.75 | 0.24 | 0.61 | 0.34 | 4041 | Train 64 | Test 9 | 0.73 | 0.21 | 0.68 | 0.32 |
| 1994 | Train 32 | Test 10 | 0.76 | 0.27 | 0.67 | 0.38 | 4042 | Train 64 | Test 10 | 0.74 | 0.24 | 0.72 | 0.36 |
| 1995 | Train 32 | Test 11 | 0.75 | 0.24 | 0.61 | 0.35 | 4043 | Train 64 | Test 11 | 0.73 | 0.21 | 0.69 | 0.32 |
| 1996 | Train 32 | Test 12 | 0.76 | 0.27 | 0.67 | 0.38 | 4044 | Train 64 | Test 12 | 0.74 | 0.24 | 0.72 | 0.36 |
| 1997 | Train 32 | Test 13 | 0.74 | 0.24 | 0.57 | 0.33 | 4045 | Train 64 | Test 13 | 0.72 | 0.21 | 0.61 | 0.32 |
| 1998 | Train 32 | Test 14 | 0.76 | 0.3 | 0.7 | 0.42 | 4046 | Train 64 | Test 14 | 0.73 | 0.28 | 0.66 | 0.4 |
| 1999 | Train 32 | Test 15 | 0.74 | 0.24 | 0.57 | 0.33 | 4047 | Train 64 | Test 15 | 0.72 | 0.21 | 0.61 | 0.32 |
| 2000 | Train 32 | Test 16 | 0.76 | 0.3 | 0.7 | 0.42 | 4048 | Train 64 | Test 16 | 0.73 | 0.28 | 0.66 | 0.39 |
| 2001 | Train 32 | Test 17 | 0.75 | 0.24 | 0.63 | 0.34 | 4049 | Train 64 | Test 17 | 0.73 | 0.21 | 0.7 | 0.32 |
| 2002 | Train 32 | Test 18 | 0.76 | 0.26 | 0.68 | 0.38 | 4050 | Train 64 | Test 18 | 0.74 | 0.23 | 0.73 | 0.35 |
| 2003 | Train 32 | Test 19 | 0.75 | 0.24 | 0.63 | 0.34 | 4051 | Train 64 | Test 19 | 0.73 | 0.21 | 0.7 | 0.32 |
| 2004 | Train 32 | Test 20 | 0.76 | 0.26 | 0.68 | 0.38 | 4052 | Train 64 | Test 20 | 0.74 | 0.24 | 0.73 | 0.36 |
| 2005 | Train 32 | Test 21 | 0.74 | 0.23 | 0.59 | 0.33 | 4053 | Train 64 | Test 21 | 0.72 | 0.21 | 0.63 | 0.31 |
| 2006 | Train 32 | Test 22 | 0.76 | 0.3 | 0.7 | 0.42 | 4054 | Train 64 | Test 22 | 0.73 | 0.28 | 0.66 | 0.4 |
| 2007 | Train 32 | Test 23 | 0.74 | 0.23 | 0.58 | 0.33 | 4055 | Train 64 | Test 23 | 0.72 | 0.21 | 0.62 | 0.31 |
| 2008 | Train 32 | Test 24 | 0.76 | 0.3 | 0.7 | 0.42 | 4056 | Train 64 | Test 24 | 0.73 | 0.28 | 0.65 | 0.39 |
| 2009 | Train 32 | Test 25 | 0.75 | 0.24 | 0.61 | 0.34 | 4057 | Train 64 | Test 25 | 0.73 | 0.21 | 0.69 | 0.32 |
| 2010 | Train 32 | Test 26 | 0.76 | 0.27 | 0.67 | 0.38 | 4058 | Train 64 | Test 26 | 0.74 | 0.24 | 0.72 | 0.36 |
| 2011 | Train 32 | Test 27 | 0.75 | 0.24 | 0.61 | 0.35 | 4059 | Train 64 | Test 27 | 0.73 | 0.21 | 0.69 | 0.32 |
| 2012 | Train 32 | Test 28 | 0.76 | 0.27 | 0.67 | 0.38 | 4060 | Train 64 | Test 28 | 0.74 | 0.24 | 0.72 | 0.36 |
| 2013 | Train 32 | Test 29 | 0.74 | 0.24 | 0.57 | 0.33 | 4061 | Train 64 | Test 29 | 0.72 | 0.21 | 0.61 | 0.32 |
| 2014 | Train 32 | Test 30 | 0.76 | 0.3 | 0.7 | 0.42 | 4062 | Train 64 | Test 30 | 0.73 | 0.28 | 0.66 | 0.4 |
| 2015 | Train 32 | Test 31 | 0.74 | 0.24 | 0.57 | 0.33 | 4063 | Train 64 | Test 31 | 0.72 | 0.21 | 0.61 | 0.32 |
| 2016 | Train 32 | Test 32 | 0.76 | 0.3 | 0.7 | 0.42 | 4064 | Train 64 | Test 32 | 0.73 | 0.28 | 0.66 | 0.39 |
| 2017 | Train 32 | Test 33 | 0.75 | 0.24 | 0.62 | 0.34 | 4065 | Train 64 | Test 33 | 0.73 | 0.2 | 0.69 | 0.32 |
| 2018 | Train 32 | Test 34 | 0.76 | 0.26 | 0.68 | 0.38 | 4066 | Train 64 | Test 34 | 0.74 | 0.23 | 0.73 | 0.35 |
| 2019 | Train 32 | Test 35 | 0.75 | 0.24 | 0.63 | 0.34 | 4067 | Train 64 | Test 35 | 0.73 | 0.21 | 0.7 | 0.32 |
| 2020 | Train 32 | Test 36 | 0.76 | 0.26 | 0.68 | 0.38 | 4068 | Train 64 | Test 36 | 0.74 | 0.23 | 0.73 | 0.36 |
| 2021 | Train 32 | Test 37 | 0.74 | 0.23 | 0.59 | 0.33 | 4069 | Train 64 | Test 37 | 0.72 | 0.21 | 0.63 | 0.31 |
| 2022 | Train 32 | Test 38 | 0.76 | 0.31 | 0.7 | 0.42 | 4070 | Train 64 | Test 38 | 0.73 | 0.28 | 0.66 | 0.4 |
| 2023 | Train 32 | Test 39 | 0.74 | 0.23 | 0.58 | 0.33 | 4071 | Train 64 | Test 39 | 0.72 | 0.21 | 0.62 | 0.31 |
| 2024 | Train 32 | Test 40 | 0.76 | 0.3 | 0.69 | 0.42 | 4072 | Train 64 | Test 40 | 0.73 | 0.28 | 0.65 | 0.39 |
| 2025 | Train 32 | Test 41 | 0.75 | 0.24 | 0.61 | 0.34 | 4073 | Train 64 | Test 41 | 0.73 | 0.21 | 0.68 | 0.32 |
| 2026 | Train 32 | Test 42 | 0.76 | 0.27 | 0.67 | 0.38 | 4074 | Train 64 | Test 42 | 0.74 | 0.24 | 0.72 | 0.36 |
| 2027 | Train 32 | Test 43 | 0.76 | 0.24 | 0.61 | 0.34 | 4075 | Train 64 | Test 43 | 0.73 | 0.21 | 0.68 | 0.32 |
| 2028 | Train 32 | Test 44 | 0.76 | 0.27 | 0.67 | 0.38 | 4076 | Train 64 | Test 44 | 0.74 | 0.24 | 0.72 | 0.36 |
| 2029 | Train 32 | Test 45 | 0.74 | 0.24 | 0.57 | 0.33 | 4077 | Train 64 | Test 45 | 0.72 | 0.21 | 0.61 | 0.32 |
| 2030 | Train 32 | Test 46 | 0.76 | 0.3 | 0.7 | 0.42 | 4078 | Train 64 | Test 46 | 0.73 | 0.28 | 0.66 | 0.4 |
| 2031 | Train 32 | Test 47 | 0.74 | 0.24 | 0.57 | 0.33 | 4079 | Train 64 | Test 47 | 0.72 | 0.21 | 0.61 | 0.32 |
| 2032 | Train 32 | Test 48 | 0.76 | 0.3 | 0.7 | 0.42 | 4080 | Train 64 | Test 48 | 0.73 | 0.28 | 0.65 | 0.39 |
| 2033 | Train 32 | Test 49 | 0.77 | 0.27 | 0.74 | 0.4 | 4081 | Train 64 | Test 49 | 0.77 | 0.27 | 0.71 | 0.39 |
| 2034 | Train 32 | Test 50 | 0.77 | 0.27 | 0.74 | 0.4 | 4082 | Train 64 | Test 50 | 0.77 | 0.27 | 0.71 | 0.39 |
| 2035 | Train 32 | Test 51 | 0.77 | 0.27 | 0.75 | 0.4 | 4083 | Train 64 | Test 51 | 0.77 | 0.28 | 0.72 | 0.4 |
| 2036 | Train 32 | Test 52 | 0.77 | 0.27 | 0.74 | 0.4 | 4084 | Train 64 | Test 52 | 0.77 | 0.28 | 0.72 | 0.4 |
| 2037 | Train 32 | Test 53 | 0.77 | 0.28 | 0.66 | 0.39 | 4085 | Train 64 | Test 53 | 0.77 | 0.28 | 0.64 | 0.39 |
| 2038 | Train 32 | Test 54 | 0.76 | 0.34 | 0.73 | 0.46 | 4086 | Train 64 | Test 54 | 0.76 | 0.35 | 0.71 | 0.47 |
| 2039 | Train 32 | Test 55 | 0.77 | 0.27 | 0.68 | 0.39 | 4087 | Train 64 | Test 55 | 0.77 | 0.27 | 0.65 | 0.38 |
| 2040 | Train 32 | Test 56 | 0.76 | 0.33 | 0.73 | 0.45 | 4088 | Train 64 | Test 56 | 0.76 | 0.33 | 0.71 | 0.45 |
| 2041 | Train 32 | Test 57 | 0.78 | 0.28 | 0.74 | 0.4 | 4089 | Train 64 | Test 57 | 0.78 | 0.28 | 0.71 | 0.4 |
| 2042 | Train 32 | Test 58 | 0.78 | 0.28 | 0.73 | 0.4 | 4090 | Train 64 | Test 58 | 0.77 | 0.28 | 0.71 | 0.4 |
| 2043 | Train 32 | Test 59 | 0.78 | 0.28 | 0.74 | 0.4 | 4091 | Train 64 | Test 59 | 0.78 | 0.28 | 0.71 | 0.4 |
| 2044 | Train 32 | Test 60 | 0.78 | 0.28 | 0.74 | 0.4 | 4092 | Train 64 | Test 60 | 0.78 | 0.28 | 0.71 | 0.4 |
| 2045 | Train 32 | Test 61 | 0.78 | 0.28 | 0.66 | 0.4 | 4093 | Train 64 | Test 61 | 0.78 | 0.29 | 0.64 | 0.4 |
| 2046 | Train 32 | Test 62 | 0.76 | 0.34 | 0.73 | 0.46 | 4094 | Train 64 | Test 62 | 0.76 | 0.34 | 0.71 | 0.46 |
| 2047 | Train 32 | Test 63 | 0.78 | 0.28 | 0.66 | 0.4 | 4095 | Train 64 | Test 63 | 0.78 | 0.29 | 0.64 | 0.39 |
| 2048 | Train 32 | Test 64 | 0.76 | 0.32 | 0.73 | 0.45 | 4096 | Train 64 | Test 64 | 0.77 | 0.33 | 0.71 | 0.45 |

Table 3. Gradient Boosting (GB) Performance Metrics Across 64 Cohorts.

| Add Decisions | Train  Model | Test  Dataset | AUC (GB) | Precision (GB) | Recall (GB) | F1 (GB) | Add Decisions | Train  Model | Test  Dataset | AUC (GB) | Precision (GB) | Recall (GB) | F1 (GB) |
| --- | --- | --- | --- | --- | --- | --- | --- | --- | --- | --- | --- | --- | --- |
| 1 | Train 1 | Test 1 | 0.76 | 0.39 | 0.11 | 0.18 | 2049 | Train 33 | Test 1 | 0.75 | 0.35 | 0.11 | 0.16 |
| 2 | Train 1 | Test 2 | 0.73 | 0.39 | 0.11 | 0.17 | 2050 | Train 33 | Test 2 | 0.72 | 0.34 | 0.09 | 0.15 |
| 3 | Train 1 | Test 3 | 0.75 | 0.39 | 0.12 | 0.18 | 2051 | Train 33 | Test 3 | 0.75 | 0.35 | 0.11 | 0.17 |
| 4 | Train 1 | Test 4 | 0.73 | 0.39 | 0.11 | 0.17 | 2052 | Train 33 | Test 4 | 0.73 | 0.34 | 0.1 | 0.15 |
| 5 | Train 1 | Test 5 | 0.69 | 0.26 | 0.12 | 0.17 | 2053 | Train 33 | Test 5 | 0.66 | 0.18 | 0.15 | 0.16 |
| 6 | Train 1 | Test 6 | 0.64 | 0.25 | 0.11 | 0.15 | 2054 | Train 33 | Test 6 | 0.63 | 0.21 | 0.12 | 0.15 |
| 7 | Train 1 | Test 7 | 0.69 | 0.26 | 0.12 | 0.17 | 2055 | Train 33 | Test 7 | 0.66 | 0.18 | 0.15 | 0.16 |
| 8 | Train 1 | Test 8 | 0.64 | 0.24 | 0.11 | 0.15 | 2056 | Train 33 | Test 8 | 0.63 | 0.21 | 0.12 | 0.15 |
| 9 | Train 1 | Test 9 | 0.76 | 0.41 | 0.1 | 0.17 | 2057 | Train 33 | Test 9 | 0.75 | 0.34 | 0.09 | 0.15 |
| 10 | Train 1 | Test 10 | 0.73 | 0.41 | 0.1 | 0.15 | 2058 | Train 33 | Test 10 | 0.73 | 0.33 | 0.08 | 0.13 |
| 11 | Train 1 | Test 11 | 0.76 | 0.42 | 0.11 | 0.17 | 2059 | Train 33 | Test 11 | 0.75 | 0.34 | 0.1 | 0.15 |
| 12 | Train 1 | Test 12 | 0.73 | 0.41 | 0.1 | 0.16 | 2060 | Train 33 | Test 12 | 0.73 | 0.34 | 0.08 | 0.14 |
| 13 | Train 1 | Test 13 | 0.69 | 0.25 | 0.11 | 0.15 | 2061 | Train 33 | Test 13 | 0.66 | 0.18 | 0.14 | 0.16 |
| 14 | Train 1 | Test 14 | 0.64 | 0.24 | 0.11 | 0.15 | 2062 | Train 33 | Test 14 | 0.63 | 0.21 | 0.12 | 0.15 |
| 15 | Train 1 | Test 15 | 0.69 | 0.25 | 0.11 | 0.15 | 2063 | Train 33 | Test 15 | 0.66 | 0.18 | 0.14 | 0.16 |
| 16 | Train 1 | Test 16 | 0.64 | 0.24 | 0.11 | 0.15 | 2064 | Train 33 | Test 16 | 0.63 | 0.21 | 0.12 | 0.15 |
| 17 | Train 1 | Test 17 | 0.76 | 0.39 | 0.11 | 0.18 | 2065 | Train 33 | Test 17 | 0.75 | 0.35 | 0.11 | 0.16 |
| 18 | Train 1 | Test 18 | 0.73 | 0.39 | 0.11 | 0.17 | 2066 | Train 33 | Test 18 | 0.72 | 0.34 | 0.09 | 0.15 |
| 19 | Train 1 | Test 19 | 0.76 | 0.39 | 0.12 | 0.18 | 2067 | Train 33 | Test 19 | 0.75 | 0.35 | 0.11 | 0.17 |
| 20 | Train 1 | Test 20 | 0.73 | 0.39 | 0.11 | 0.17 | 2068 | Train 33 | Test 20 | 0.73 | 0.34 | 0.1 | 0.15 |
| 21 | Train 1 | Test 21 | 0.69 | 0.26 | 0.12 | 0.17 | 2069 | Train 33 | Test 21 | 0.67 | 0.18 | 0.15 | 0.16 |
| 22 | Train 1 | Test 22 | 0.64 | 0.25 | 0.11 | 0.15 | 2070 | Train 33 | Test 22 | 0.63 | 0.21 | 0.12 | 0.15 |
| 23 | Train 1 | Test 23 | 0.69 | 0.26 | 0.12 | 0.17 | 2071 | Train 33 | Test 23 | 0.67 | 0.18 | 0.15 | 0.16 |
| 24 | Train 1 | Test 24 | 0.64 | 0.24 | 0.11 | 0.15 | 2072 | Train 33 | Test 24 | 0.63 | 0.21 | 0.12 | 0.15 |
| 25 | Train 1 | Test 25 | 0.76 | 0.41 | 0.1 | 0.17 | 2073 | Train 33 | Test 25 | 0.75 | 0.34 | 0.09 | 0.15 |
| 26 | Train 1 | Test 26 | 0.73 | 0.41 | 0.1 | 0.15 | 2074 | Train 33 | Test 26 | 0.73 | 0.33 | 0.08 | 0.13 |
| 27 | Train 1 | Test 27 | 0.76 | 0.42 | 0.11 | 0.17 | 2075 | Train 33 | Test 27 | 0.75 | 0.34 | 0.1 | 0.15 |
| 28 | Train 1 | Test 28 | 0.73 | 0.41 | 0.1 | 0.16 | 2076 | Train 33 | Test 28 | 0.73 | 0.34 | 0.08 | 0.14 |
| 29 | Train 1 | Test 29 | 0.69 | 0.25 | 0.11 | 0.15 | 2077 | Train 33 | Test 29 | 0.66 | 0.18 | 0.14 | 0.16 |
| 30 | Train 1 | Test 30 | 0.64 | 0.24 | 0.11 | 0.15 | 2078 | Train 33 | Test 30 | 0.63 | 0.21 | 0.12 | 0.15 |
| 31 | Train 1 | Test 31 | 0.69 | 0.25 | 0.11 | 0.15 | 2079 | Train 33 | Test 31 | 0.66 | 0.18 | 0.14 | 0.16 |
| 32 | Train 1 | Test 32 | 0.64 | 0.24 | 0.11 | 0.15 | 2080 | Train 33 | Test 32 | 0.63 | 0.21 | 0.12 | 0.15 |
| 33 | Train 1 | Test 33 | 0.76 | 0.39 | 0.11 | 0.18 | 2081 | Train 33 | Test 33 | 0.75 | 0.34 | 0.11 | 0.16 |
| 34 | Train 1 | Test 34 | 0.73 | 0.39 | 0.11 | 0.17 | 2082 | Train 33 | Test 34 | 0.73 | 0.33 | 0.09 | 0.15 |
| 35 | Train 1 | Test 35 | 0.76 | 0.39 | 0.12 | 0.18 | 2083 | Train 33 | Test 35 | 0.75 | 0.35 | 0.11 | 0.17 |
| 36 | Train 1 | Test 36 | 0.73 | 0.39 | 0.11 | 0.17 | 2084 | Train 33 | Test 36 | 0.73 | 0.34 | 0.09 | 0.15 |
| 37 | Train 1 | Test 37 | 0.69 | 0.26 | 0.12 | 0.17 | 2085 | Train 33 | Test 37 | 0.67 | 0.18 | 0.15 | 0.16 |
| 38 | Train 1 | Test 38 | 0.64 | 0.25 | 0.11 | 0.15 | 2086 | Train 33 | Test 38 | 0.63 | 0.21 | 0.12 | 0.15 |
| 39 | Train 1 | Test 39 | 0.69 | 0.26 | 0.12 | 0.17 | 2087 | Train 33 | Test 39 | 0.67 | 0.18 | 0.15 | 0.16 |
| 40 | Train 1 | Test 40 | 0.64 | 0.24 | 0.11 | 0.15 | 2088 | Train 33 | Test 40 | 0.63 | 0.21 | 0.12 | 0.15 |
| 41 | Train 1 | Test 41 | 0.76 | 0.41 | 0.1 | 0.16 | 2089 | Train 33 | Test 41 | 0.75 | 0.34 | 0.09 | 0.15 |
| 42 | Train 1 | Test 42 | 0.74 | 0.41 | 0.09 | 0.15 | 2090 | Train 33 | Test 42 | 0.73 | 0.33 | 0.08 | 0.13 |
| 43 | Train 1 | Test 43 | 0.76 | 0.41 | 0.11 | 0.17 | 2091 | Train 33 | Test 43 | 0.75 | 0.34 | 0.09 | 0.15 |
| 44 | Train 1 | Test 44 | 0.74 | 0.41 | 0.1 | 0.16 | 2092 | Train 33 | Test 44 | 0.73 | 0.33 | 0.08 | 0.13 |
| 45 | Train 1 | Test 45 | 0.69 | 0.25 | 0.11 | 0.15 | 2093 | Train 33 | Test 45 | 0.66 | 0.18 | 0.14 | 0.16 |
| 46 | Train 1 | Test 46 | 0.64 | 0.24 | 0.11 | 0.15 | 2094 | Train 33 | Test 46 | 0.63 | 0.21 | 0.12 | 0.15 |
| 47 | Train 1 | Test 47 | 0.69 | 0.25 | 0.11 | 0.15 | 2095 | Train 33 | Test 47 | 0.66 | 0.18 | 0.14 | 0.16 |
| 48 | Train 1 | Test 48 | 0.64 | 0.24 | 0.11 | 0.15 | 2096 | Train 33 | Test 48 | 0.63 | 0.21 | 0.12 | 0.15 |
| 49 | Train 1 | Test 49 | 0.77 | 0.44 | 0.16 | 0.24 | 2097 | Train 33 | Test 49 | 0.76 | 0.41 | 0.15 | 0.22 |
| 50 | Train 1 | Test 50 | 0.77 | 0.44 | 0.16 | 0.24 | 2098 | Train 33 | Test 50 | 0.76 | 0.41 | 0.15 | 0.22 |
| 51 | Train 1 | Test 51 | 0.77 | 0.44 | 0.17 | 0.24 | 2099 | Train 33 | Test 51 | 0.77 | 0.42 | 0.15 | 0.22 |
| 52 | Train 1 | Test 52 | 0.76 | 0.44 | 0.17 | 0.24 | 2100 | Train 33 | Test 52 | 0.76 | 0.42 | 0.15 | 0.22 |
| 53 | Train 1 | Test 53 | 0.64 | 0.2 | 0.13 | 0.16 | 2101 | Train 33 | Test 53 | 0.63 | 0.16 | 0.2 | 0.18 |
| 54 | Train 1 | Test 54 | 0.64 | 0.27 | 0.09 | 0.13 | 2102 | Train 33 | Test 54 | 0.63 | 0.23 | 0.13 | 0.16 |
| 55 | Train 1 | Test 55 | 0.7 | 0.27 | 0.14 | 0.18 | 2103 | Train 33 | Test 55 | 0.66 | 0.17 | 0.24 | 0.2 |
| 56 | Train 1 | Test 56 | 0.64 | 0.26 | 0.09 | 0.13 | 2104 | Train 33 | Test 56 | 0.63 | 0.23 | 0.13 | 0.16 |
| 57 | Train 1 | Test 57 | 0.77 | 0.46 | 0.16 | 0.24 | 2105 | Train 33 | Test 57 | 0.77 | 0.41 | 0.14 | 0.21 |
| 58 | Train 1 | Test 58 | 0.77 | 0.46 | 0.16 | 0.24 | 2106 | Train 33 | Test 58 | 0.77 | 0.4 | 0.14 | 0.2 |
| 59 | Train 1 | Test 59 | 0.77 | 0.47 | 0.16 | 0.24 | 2107 | Train 33 | Test 59 | 0.77 | 0.41 | 0.14 | 0.21 |
| 60 | Train 1 | Test 60 | 0.77 | 0.46 | 0.16 | 0.24 | 2108 | Train 33 | Test 60 | 0.77 | 0.41 | 0.14 | 0.21 |
| 61 | Train 1 | Test 61 | 0.64 | 0.19 | 0.13 | 0.16 | 2109 | Train 33 | Test 61 | 0.63 | 0.16 | 0.2 | 0.17 |
| 62 | Train 1 | Test 62 | 0.65 | 0.27 | 0.09 | 0.13 | 2110 | Train 33 | Test 62 | 0.63 | 0.23 | 0.13 | 0.17 |
| 63 | Train 1 | Test 63 | 0.64 | 0.2 | 0.13 | 0.16 | 2111 | Train 33 | Test 63 | 0.63 | 0.16 | 0.2 | 0.18 |
| 64 | Train 1 | Test 64 | 0.65 | 0.26 | 0.09 | 0.13 | 2112 | Train 33 | Test 64 | 0.63 | 0.23 | 0.13 | 0.16 |
| 65 | Train 2 | Test 1 | 0.75 | 0.38 | 0.11 | 0.16 | 2113 | Train 34 | Test 1 | 0.75 | 0.39 | 0.11 | 0.17 |
| 66 | Train 2 | Test 2 | 0.77 | 0.44 | 0.16 | 0.24 | 2114 | Train 34 | Test 2 | 0.76 | 0.45 | 0.16 | 0.24 |
| 67 | Train 2 | Test 3 | 0.75 | 0.38 | 0.11 | 0.17 | 2115 | Train 34 | Test 3 | 0.75 | 0.39 | 0.11 | 0.17 |
| 68 | Train 2 | Test 4 | 0.77 | 0.44 | 0.16 | 0.24 | 2116 | Train 34 | Test 4 | 0.76 | 0.45 | 0.16 | 0.24 |
| 69 | Train 2 | Test 5 | 0.7 | 0.28 | 0.11 | 0.16 | 2117 | Train 34 | Test 5 | 0.69 | 0.29 | 0.1 | 0.15 |
| 70 | Train 2 | Test 6 | 0.66 | 0.35 | 0.05 | 0.09 | 2118 | Train 34 | Test 6 | 0.66 | 0.32 | 0.06 | 0.1 |
| 71 | Train 2 | Test 7 | 0.7 | 0.28 | 0.11 | 0.16 | 2119 | Train 34 | Test 7 | 0.69 | 0.28 | 0.1 | 0.15 |
| 72 | Train 2 | Test 8 | 0.66 | 0.35 | 0.05 | 0.09 | 2120 | Train 34 | Test 8 | 0.66 | 0.32 | 0.06 | 0.1 |
| 73 | Train 2 | Test 9 | 0.76 | 0.4 | 0.1 | 0.16 | 2121 | Train 34 | Test 9 | 0.75 | 0.38 | 0.09 | 0.15 |
| 74 | Train 2 | Test 10 | 0.77 | 0.47 | 0.16 | 0.23 | 2122 | Train 34 | Test 10 | 0.76 | 0.46 | 0.15 | 0.23 |
| 75 | Train 2 | Test 11 | 0.76 | 0.4 | 0.1 | 0.16 | 2123 | Train 34 | Test 11 | 0.75 | 0.38 | 0.09 | 0.15 |
| 76 | Train 2 | Test 12 | 0.77 | 0.47 | 0.16 | 0.23 | 2124 | Train 34 | Test 12 | 0.76 | 0.46 | 0.15 | 0.23 |
| 77 | Train 2 | Test 13 | 0.7 | 0.3 | 0.11 | 0.16 | 2125 | Train 34 | Test 13 | 0.69 | 0.28 | 0.1 | 0.14 |
| 78 | Train 2 | Test 14 | 0.66 | 0.35 | 0.05 | 0.09 | 2126 | Train 34 | Test 14 | 0.66 | 0.31 | 0.06 | 0.1 |
| 79 | Train 2 | Test 15 | 0.7 | 0.29 | 0.1 | 0.15 | 2127 | Train 34 | Test 15 | 0.69 | 0.28 | 0.1 | 0.14 |
| 80 | Train 2 | Test 16 | 0.66 | 0.34 | 0.05 | 0.09 | 2128 | Train 34 | Test 16 | 0.66 | 0.31 | 0.06 | 0.1 |
| 81 | Train 2 | Test 17 | 0.75 | 0.38 | 0.1 | 0.16 | 2129 | Train 34 | Test 17 | 0.75 | 0.39 | 0.11 | 0.17 |
| 82 | Train 2 | Test 18 | 0.77 | 0.44 | 0.16 | 0.24 | 2130 | Train 34 | Test 18 | 0.76 | 0.45 | 0.16 | 0.24 |
| 83 | Train 2 | Test 19 | 0.75 | 0.38 | 0.11 | 0.17 | 2131 | Train 34 | Test 19 | 0.75 | 0.39 | 0.11 | 0.17 |
| 84 | Train 2 | Test 20 | 0.77 | 0.44 | 0.16 | 0.24 | 2132 | Train 34 | Test 20 | 0.76 | 0.45 | 0.16 | 0.24 |
| 85 | Train 2 | Test 21 | 0.7 | 0.28 | 0.11 | 0.16 | 2133 | Train 34 | Test 21 | 0.69 | 0.28 | 0.1 | 0.15 |
| 86 | Train 2 | Test 22 | 0.66 | 0.35 | 0.05 | 0.09 | 2134 | Train 34 | Test 22 | 0.66 | 0.32 | 0.06 | 0.1 |
| 87 | Train 2 | Test 23 | 0.7 | 0.28 | 0.11 | 0.16 | 2135 | Train 34 | Test 23 | 0.69 | 0.28 | 0.1 | 0.15 |
| 88 | Train 2 | Test 24 | 0.66 | 0.34 | 0.05 | 0.09 | 2136 | Train 34 | Test 24 | 0.66 | 0.32 | 0.06 | 0.1 |
| 89 | Train 2 | Test 25 | 0.76 | 0.4 | 0.1 | 0.16 | 2137 | Train 34 | Test 25 | 0.75 | 0.38 | 0.09 | 0.15 |
| 90 | Train 2 | Test 26 | 0.77 | 0.47 | 0.16 | 0.23 | 2138 | Train 34 | Test 26 | 0.76 | 0.46 | 0.15 | 0.23 |
| 91 | Train 2 | Test 27 | 0.76 | 0.4 | 0.1 | 0.16 | 2139 | Train 34 | Test 27 | 0.75 | 0.38 | 0.09 | 0.15 |
| 92 | Train 2 | Test 28 | 0.77 | 0.47 | 0.16 | 0.23 | 2140 | Train 34 | Test 28 | 0.76 | 0.46 | 0.15 | 0.22 |
| 93 | Train 2 | Test 29 | 0.7 | 0.3 | 0.1 | 0.16 | 2141 | Train 34 | Test 29 | 0.69 | 0.28 | 0.1 | 0.14 |
| 94 | Train 2 | Test 30 | 0.66 | 0.35 | 0.05 | 0.09 | 2142 | Train 34 | Test 30 | 0.66 | 0.31 | 0.06 | 0.1 |
| 95 | Train 2 | Test 31 | 0.7 | 0.29 | 0.1 | 0.15 | 2143 | Train 34 | Test 31 | 0.69 | 0.28 | 0.1 | 0.14 |
| 96 | Train 2 | Test 32 | 0.66 | 0.34 | 0.05 | 0.09 | 2144 | Train 34 | Test 32 | 0.66 | 0.31 | 0.06 | 0.1 |
| 97 | Train 2 | Test 33 | 0.76 | 0.38 | 0.1 | 0.16 | 2145 | Train 34 | Test 33 | 0.75 | 0.38 | 0.1 | 0.16 |
| 98 | Train 2 | Test 34 | 0.77 | 0.44 | 0.16 | 0.24 | 2146 | Train 34 | Test 34 | 0.77 | 0.44 | 0.16 | 0.24 |
| 99 | Train 2 | Test 35 | 0.76 | 0.38 | 0.11 | 0.16 | 2147 | Train 34 | Test 35 | 0.75 | 0.39 | 0.11 | 0.17 |
| 100 | Train 2 | Test 36 | 0.77 | 0.44 | 0.16 | 0.24 | 2148 | Train 34 | Test 36 | 0.77 | 0.44 | 0.16 | 0.24 |
| 101 | Train 2 | Test 37 | 0.7 | 0.28 | 0.11 | 0.16 | 2149 | Train 34 | Test 37 | 0.69 | 0.28 | 0.1 | 0.15 |
| 102 | Train 2 | Test 38 | 0.66 | 0.35 | 0.05 | 0.09 | 2150 | Train 34 | Test 38 | 0.66 | 0.32 | 0.06 | 0.1 |
| 103 | Train 2 | Test 39 | 0.7 | 0.27 | 0.11 | 0.15 | 2151 | Train 34 | Test 39 | 0.69 | 0.28 | 0.1 | 0.15 |
| 104 | Train 2 | Test 40 | 0.66 | 0.34 | 0.05 | 0.09 | 2152 | Train 34 | Test 40 | 0.66 | 0.32 | 0.06 | 0.1 |
| 105 | Train 2 | Test 41 | 0.76 | 0.39 | 0.1 | 0.16 | 2153 | Train 34 | Test 41 | 0.75 | 0.38 | 0.09 | 0.15 |
| 106 | Train 2 | Test 42 | 0.77 | 0.47 | 0.16 | 0.23 | 2154 | Train 34 | Test 42 | 0.76 | 0.45 | 0.15 | 0.22 |
| 107 | Train 2 | Test 43 | 0.76 | 0.4 | 0.1 | 0.16 | 2155 | Train 34 | Test 43 | 0.75 | 0.38 | 0.09 | 0.15 |
| 108 | Train 2 | Test 44 | 0.77 | 0.47 | 0.16 | 0.23 | 2156 | Train 34 | Test 44 | 0.76 | 0.45 | 0.15 | 0.22 |
| 109 | Train 2 | Test 45 | 0.7 | 0.3 | 0.1 | 0.15 | 2157 | Train 34 | Test 45 | 0.69 | 0.28 | 0.09 | 0.14 |
| 110 | Train 2 | Test 46 | 0.66 | 0.35 | 0.05 | 0.09 | 2158 | Train 34 | Test 46 | 0.66 | 0.31 | 0.06 | 0.1 |
| 111 | Train 2 | Test 47 | 0.7 | 0.29 | 0.1 | 0.15 | 2159 | Train 34 | Test 47 | 0.69 | 0.28 | 0.09 | 0.14 |
| 112 | Train 2 | Test 48 | 0.66 | 0.34 | 0.05 | 0.09 | 2160 | Train 34 | Test 48 | 0.66 | 0.31 | 0.06 | 0.1 |
| 113 | Train 2 | Test 49 | 0.76 | 0.47 | 0.17 | 0.25 | 2161 | Train 34 | Test 49 | 0.76 | 0.44 | 0.16 | 0.24 |
| 114 | Train 2 | Test 50 | 0.76 | 0.47 | 0.16 | 0.24 | 2162 | Train 34 | Test 50 | 0.76 | 0.44 | 0.16 | 0.24 |
| 115 | Train 2 | Test 51 | 0.76 | 0.47 | 0.17 | 0.25 | 2163 | Train 34 | Test 51 | 0.76 | 0.44 | 0.17 | 0.24 |
| 116 | Train 2 | Test 52 | 0.76 | 0.47 | 0.17 | 0.24 | 2164 | Train 34 | Test 52 | 0.76 | 0.44 | 0.17 | 0.24 |
| 117 | Train 2 | Test 53 | 0.69 | 0.27 | 0.08 | 0.12 | 2165 | Train 34 | Test 53 | 0.66 | 0.24 | 0.08 | 0.12 |
| 118 | Train 2 | Test 54 | 0.66 | 0.38 | 0.06 | 0.1 | 2166 | Train 34 | Test 54 | 0.66 | 0.34 | 0.05 | 0.09 |
| 119 | Train 2 | Test 55 | 0.73 | 0.36 | 0.12 | 0.18 | 2167 | Train 34 | Test 55 | 0.72 | 0.34 | 0.11 | 0.16 |
| 120 | Train 2 | Test 56 | 0.66 | 0.37 | 0.06 | 0.1 | 2168 | Train 34 | Test 56 | 0.65 | 0.34 | 0.05 | 0.09 |
| 121 | Train 2 | Test 57 | 0.77 | 0.49 | 0.15 | 0.23 | 2169 | Train 34 | Test 57 | 0.76 | 0.43 | 0.15 | 0.22 |
| 122 | Train 2 | Test 58 | 0.77 | 0.49 | 0.15 | 0.23 | 2170 | Train 34 | Test 58 | 0.76 | 0.43 | 0.14 | 0.22 |
| 123 | Train 2 | Test 59 | 0.77 | 0.49 | 0.15 | 0.23 | 2171 | Train 34 | Test 59 | 0.77 | 0.43 | 0.14 | 0.22 |
| 124 | Train 2 | Test 60 | 0.77 | 0.49 | 0.15 | 0.23 | 2172 | Train 34 | Test 60 | 0.76 | 0.43 | 0.14 | 0.21 |
| 125 | Train 2 | Test 61 | 0.69 | 0.29 | 0.08 | 0.12 | 2173 | Train 34 | Test 61 | 0.66 | 0.23 | 0.07 | 0.11 |
| 126 | Train 2 | Test 62 | 0.67 | 0.38 | 0.06 | 0.11 | 2174 | Train 34 | Test 62 | 0.66 | 0.34 | 0.05 | 0.09 |
| 127 | Train 2 | Test 63 | 0.69 | 0.29 | 0.08 | 0.12 | 2175 | Train 34 | Test 63 | 0.66 | 0.23 | 0.07 | 0.11 |
| 128 | Train 2 | Test 64 | 0.66 | 0.37 | 0.06 | 0.11 | 2176 | Train 34 | Test 64 | 0.66 | 0.34 | 0.05 | 0.09 |
| 129 | Train 3 | Test 1 | 0.76 | 0.39 | 0.11 | 0.18 | 2177 | Train 35 | Test 1 | 0.75 | 0.37 | 0.11 | 0.16 |
| 130 | Train 3 | Test 2 | 0.73 | 0.38 | 0.1 | 0.16 | 2178 | Train 35 | Test 2 | 0.73 | 0.36 | 0.1 | 0.16 |
| 131 | Train 3 | Test 3 | 0.76 | 0.39 | 0.11 | 0.18 | 2179 | Train 35 | Test 3 | 0.75 | 0.37 | 0.11 | 0.17 |
| 132 | Train 3 | Test 4 | 0.73 | 0.38 | 0.1 | 0.16 | 2180 | Train 35 | Test 4 | 0.73 | 0.37 | 0.1 | 0.16 |
| 133 | Train 3 | Test 5 | 0.68 | 0.2 | 0.15 | 0.17 | 2181 | Train 35 | Test 5 | 0.66 | 0.21 | 0.1 | 0.14 |
| 134 | Train 3 | Test 6 | 0.64 | 0.2 | 0.09 | 0.12 | 2182 | Train 35 | Test 6 | 0.66 | 0.25 | 0.13 | 0.17 |
| 135 | Train 3 | Test 7 | 0.68 | 0.21 | 0.15 | 0.17 | 2183 | Train 35 | Test 7 | 0.66 | 0.21 | 0.1 | 0.14 |
| 136 | Train 3 | Test 8 | 0.64 | 0.2 | 0.09 | 0.12 | 2184 | Train 35 | Test 8 | 0.66 | 0.24 | 0.13 | 0.17 |
| 137 | Train 3 | Test 9 | 0.76 | 0.36 | 0.09 | 0.14 | 2185 | Train 35 | Test 9 | 0.74 | 0.34 | 0.09 | 0.14 |
| 138 | Train 3 | Test 10 | 0.73 | 0.35 | 0.07 | 0.12 | 2186 | Train 35 | Test 10 | 0.73 | 0.35 | 0.09 | 0.14 |
| 139 | Train 3 | Test 11 | 0.76 | 0.36 | 0.09 | 0.14 | 2187 | Train 35 | Test 11 | 0.74 | 0.34 | 0.09 | 0.14 |
| 140 | Train 3 | Test 12 | 0.73 | 0.35 | 0.08 | 0.12 | 2188 | Train 35 | Test 12 | 0.73 | 0.35 | 0.09 | 0.14 |
| 141 | Train 3 | Test 13 | 0.68 | 0.19 | 0.14 | 0.16 | 2189 | Train 35 | Test 13 | 0.66 | 0.18 | 0.08 | 0.11 |
| 142 | Train 3 | Test 14 | 0.64 | 0.2 | 0.09 | 0.12 | 2190 | Train 35 | Test 14 | 0.66 | 0.24 | 0.13 | 0.17 |
| 143 | Train 3 | Test 15 | 0.68 | 0.19 | 0.14 | 0.16 | 2191 | Train 35 | Test 15 | 0.66 | 0.18 | 0.08 | 0.11 |
| 144 | Train 3 | Test 16 | 0.64 | 0.2 | 0.09 | 0.12 | 2192 | Train 35 | Test 16 | 0.66 | 0.24 | 0.13 | 0.16 |
| 145 | Train 3 | Test 17 | 0.76 | 0.39 | 0.11 | 0.18 | 2193 | Train 35 | Test 17 | 0.75 | 0.37 | 0.11 | 0.16 |
| 146 | Train 3 | Test 18 | 0.73 | 0.38 | 0.1 | 0.16 | 2194 | Train 35 | Test 18 | 0.73 | 0.36 | 0.1 | 0.16 |
| 147 | Train 3 | Test 19 | 0.76 | 0.39 | 0.11 | 0.18 | 2195 | Train 35 | Test 19 | 0.75 | 0.37 | 0.11 | 0.17 |
| 148 | Train 3 | Test 20 | 0.73 | 0.38 | 0.1 | 0.16 | 2196 | Train 35 | Test 20 | 0.73 | 0.37 | 0.1 | 0.16 |
| 149 | Train 3 | Test 21 | 0.68 | 0.2 | 0.15 | 0.17 | 2197 | Train 35 | Test 21 | 0.66 | 0.21 | 0.1 | 0.14 |
| 150 | Train 3 | Test 22 | 0.64 | 0.2 | 0.09 | 0.12 | 2198 | Train 35 | Test 22 | 0.66 | 0.25 | 0.13 | 0.17 |
| 151 | Train 3 | Test 23 | 0.68 | 0.21 | 0.15 | 0.17 | 2199 | Train 35 | Test 23 | 0.66 | 0.21 | 0.1 | 0.14 |
| 152 | Train 3 | Test 24 | 0.64 | 0.2 | 0.09 | 0.12 | 2200 | Train 35 | Test 24 | 0.66 | 0.24 | 0.12 | 0.17 |
| 153 | Train 3 | Test 25 | 0.76 | 0.36 | 0.09 | 0.14 | 2201 | Train 35 | Test 25 | 0.74 | 0.34 | 0.09 | 0.14 |
| 154 | Train 3 | Test 26 | 0.73 | 0.35 | 0.07 | 0.12 | 2202 | Train 35 | Test 26 | 0.73 | 0.35 | 0.09 | 0.14 |
| 155 | Train 3 | Test 27 | 0.76 | 0.36 | 0.09 | 0.14 | 2203 | Train 35 | Test 27 | 0.75 | 0.34 | 0.09 | 0.14 |
| 156 | Train 3 | Test 28 | 0.73 | 0.35 | 0.08 | 0.12 | 2204 | Train 35 | Test 28 | 0.73 | 0.35 | 0.09 | 0.14 |
| 157 | Train 3 | Test 29 | 0.68 | 0.19 | 0.14 | 0.16 | 2205 | Train 35 | Test 29 | 0.66 | 0.18 | 0.08 | 0.11 |
| 158 | Train 3 | Test 30 | 0.64 | 0.2 | 0.09 | 0.12 | 2206 | Train 35 | Test 30 | 0.66 | 0.24 | 0.13 | 0.17 |
| 159 | Train 3 | Test 31 | 0.68 | 0.2 | 0.14 | 0.16 | 2207 | Train 35 | Test 31 | 0.66 | 0.18 | 0.08 | 0.11 |
| 160 | Train 3 | Test 32 | 0.64 | 0.2 | 0.09 | 0.12 | 2208 | Train 35 | Test 32 | 0.66 | 0.24 | 0.12 | 0.16 |
| 161 | Train 3 | Test 33 | 0.76 | 0.38 | 0.11 | 0.17 | 2209 | Train 35 | Test 33 | 0.75 | 0.37 | 0.1 | 0.16 |
| 162 | Train 3 | Test 34 | 0.73 | 0.38 | 0.1 | 0.16 | 2210 | Train 35 | Test 34 | 0.73 | 0.36 | 0.1 | 0.16 |
| 163 | Train 3 | Test 35 | 0.76 | 0.38 | 0.11 | 0.17 | 2211 | Train 35 | Test 35 | 0.75 | 0.37 | 0.11 | 0.17 |
| 164 | Train 3 | Test 36 | 0.73 | 0.38 | 0.1 | 0.16 | 2212 | Train 35 | Test 36 | 0.73 | 0.37 | 0.1 | 0.16 |
| 165 | Train 3 | Test 37 | 0.68 | 0.2 | 0.15 | 0.17 | 2213 | Train 35 | Test 37 | 0.66 | 0.21 | 0.1 | 0.14 |
| 166 | Train 3 | Test 38 | 0.64 | 0.2 | 0.09 | 0.12 | 2214 | Train 35 | Test 38 | 0.66 | 0.25 | 0.13 | 0.17 |
| 167 | Train 3 | Test 39 | 0.68 | 0.21 | 0.15 | 0.17 | 2215 | Train 35 | Test 39 | 0.66 | 0.21 | 0.1 | 0.14 |
| 168 | Train 3 | Test 40 | 0.64 | 0.2 | 0.09 | 0.12 | 2216 | Train 35 | Test 40 | 0.66 | 0.24 | 0.13 | 0.17 |
| 169 | Train 3 | Test 41 | 0.76 | 0.36 | 0.09 | 0.14 | 2217 | Train 35 | Test 41 | 0.75 | 0.34 | 0.09 | 0.14 |
| 170 | Train 3 | Test 42 | 0.73 | 0.35 | 0.07 | 0.12 | 2218 | Train 35 | Test 42 | 0.73 | 0.35 | 0.09 | 0.14 |
| 171 | Train 3 | Test 43 | 0.76 | 0.36 | 0.09 | 0.14 | 2219 | Train 35 | Test 43 | 0.75 | 0.34 | 0.09 | 0.14 |
| 172 | Train 3 | Test 44 | 0.73 | 0.35 | 0.07 | 0.12 | 2220 | Train 35 | Test 44 | 0.73 | 0.35 | 0.09 | 0.14 |
| 173 | Train 3 | Test 45 | 0.68 | 0.19 | 0.14 | 0.16 | 2221 | Train 35 | Test 45 | 0.66 | 0.18 | 0.08 | 0.11 |
| 174 | Train 3 | Test 46 | 0.64 | 0.2 | 0.09 | 0.12 | 2222 | Train 35 | Test 46 | 0.66 | 0.24 | 0.13 | 0.17 |
| 175 | Train 3 | Test 47 | 0.68 | 0.19 | 0.14 | 0.16 | 2223 | Train 35 | Test 47 | 0.66 | 0.18 | 0.08 | 0.11 |
| 176 | Train 3 | Test 48 | 0.64 | 0.2 | 0.09 | 0.12 | 2224 | Train 35 | Test 48 | 0.66 | 0.24 | 0.12 | 0.16 |
| 177 | Train 3 | Test 49 | 0.77 | 0.44 | 0.18 | 0.25 | 2225 | Train 35 | Test 49 | 0.76 | 0.44 | 0.17 | 0.25 |
| 178 | Train 3 | Test 50 | 0.77 | 0.44 | 0.18 | 0.25 | 2226 | Train 35 | Test 50 | 0.76 | 0.44 | 0.17 | 0.25 |
| 179 | Train 3 | Test 51 | 0.77 | 0.44 | 0.18 | 0.25 | 2227 | Train 35 | Test 51 | 0.76 | 0.44 | 0.18 | 0.25 |
| 180 | Train 3 | Test 52 | 0.77 | 0.44 | 0.17 | 0.25 | 2228 | Train 35 | Test 52 | 0.76 | 0.44 | 0.17 | 0.25 |
| 181 | Train 3 | Test 53 | 0.66 | 0.16 | 0.19 | 0.17 | 2229 | Train 35 | Test 53 | 0.65 | 0.17 | 0.11 | 0.13 |
| 182 | Train 3 | Test 54 | 0.65 | 0.21 | 0.09 | 0.13 | 2230 | Train 35 | Test 54 | 0.65 | 0.26 | 0.15 | 0.19 |
| 183 | Train 3 | Test 55 | 0.68 | 0.18 | 0.23 | 0.21 | 2231 | Train 35 | Test 55 | 0.66 | 0.2 | 0.15 | 0.17 |
| 184 | Train 3 | Test 56 | 0.65 | 0.21 | 0.09 | 0.12 | 2232 | Train 35 | Test 56 | 0.65 | 0.26 | 0.14 | 0.18 |
| 185 | Train 3 | Test 57 | 0.77 | 0.4 | 0.14 | 0.21 | 2233 | Train 35 | Test 57 | 0.76 | 0.41 | 0.15 | 0.22 |
| 186 | Train 3 | Test 58 | 0.77 | 0.4 | 0.14 | 0.2 | 2234 | Train 35 | Test 58 | 0.76 | 0.41 | 0.14 | 0.21 |
| 187 | Train 3 | Test 59 | 0.77 | 0.4 | 0.14 | 0.21 | 2235 | Train 35 | Test 59 | 0.76 | 0.41 | 0.15 | 0.22 |
| 188 | Train 3 | Test 60 | 0.77 | 0.4 | 0.14 | 0.2 | 2236 | Train 35 | Test 60 | 0.76 | 0.41 | 0.15 | 0.22 |
| 189 | Train 3 | Test 61 | 0.66 | 0.16 | 0.19 | 0.17 | 2237 | Train 35 | Test 61 | 0.65 | 0.15 | 0.09 | 0.11 |
| 190 | Train 3 | Test 62 | 0.65 | 0.21 | 0.09 | 0.13 | 2238 | Train 35 | Test 62 | 0.65 | 0.26 | 0.15 | 0.19 |
| 191 | Train 3 | Test 63 | 0.66 | 0.16 | 0.19 | 0.17 | 2239 | Train 35 | Test 63 | 0.65 | 0.15 | 0.09 | 0.12 |
| 192 | Train 3 | Test 64 | 0.65 | 0.21 | 0.09 | 0.13 | 2240 | Train 35 | Test 64 | 0.65 | 0.26 | 0.14 | 0.18 |
| 193 | Train 4 | Test 1 | 0.75 | 0.39 | 0.11 | 0.17 | 2241 | Train 36 | Test 1 | 0.75 | 0.4 | 0.1 | 0.16 |
| 194 | Train 4 | Test 2 | 0.77 | 0.46 | 0.16 | 0.24 | 2242 | Train 36 | Test 2 | 0.77 | 0.47 | 0.16 | 0.24 |
| 195 | Train 4 | Test 3 | 0.75 | 0.39 | 0.1 | 0.17 | 2243 | Train 36 | Test 3 | 0.75 | 0.4 | 0.1 | 0.17 |
| 196 | Train 4 | Test 4 | 0.77 | 0.46 | 0.16 | 0.24 | 2244 | Train 36 | Test 4 | 0.77 | 0.47 | 0.16 | 0.24 |
| 197 | Train 4 | Test 5 | 0.69 | 0.28 | 0.11 | 0.16 | 2245 | Train 36 | Test 5 | 0.69 | 0.31 | 0.1 | 0.15 |
| 198 | Train 4 | Test 6 | 0.66 | 0.31 | 0.07 | 0.12 | 2246 | Train 36 | Test 6 | 0.66 | 0.34 | 0.07 | 0.11 |
| 199 | Train 4 | Test 7 | 0.69 | 0.28 | 0.11 | 0.15 | 2247 | Train 36 | Test 7 | 0.69 | 0.3 | 0.09 | 0.14 |
| 200 | Train 4 | Test 8 | 0.67 | 0.31 | 0.07 | 0.11 | 2248 | Train 36 | Test 8 | 0.66 | 0.33 | 0.06 | 0.11 |
| 201 | Train 4 | Test 9 | 0.75 | 0.39 | 0.09 | 0.15 | 2249 | Train 36 | Test 9 | 0.75 | 0.37 | 0.08 | 0.13 |
| 202 | Train 4 | Test 10 | 0.77 | 0.46 | 0.15 | 0.22 | 2250 | Train 36 | Test 10 | 0.77 | 0.46 | 0.14 | 0.22 |
| 203 | Train 4 | Test 11 | 0.75 | 0.38 | 0.09 | 0.15 | 2251 | Train 36 | Test 11 | 0.75 | 0.37 | 0.08 | 0.14 |
| 204 | Train 4 | Test 12 | 0.77 | 0.46 | 0.15 | 0.22 | 2252 | Train 36 | Test 12 | 0.77 | 0.46 | 0.14 | 0.22 |
| 205 | Train 4 | Test 13 | 0.69 | 0.29 | 0.1 | 0.15 | 2253 | Train 36 | Test 13 | 0.69 | 0.29 | 0.08 | 0.13 |
| 206 | Train 4 | Test 14 | 0.67 | 0.31 | 0.07 | 0.12 | 2254 | Train 36 | Test 14 | 0.66 | 0.33 | 0.07 | 0.11 |
| 207 | Train 4 | Test 15 | 0.69 | 0.29 | 0.1 | 0.15 | 2255 | Train 36 | Test 15 | 0.69 | 0.29 | 0.08 | 0.12 |
| 208 | Train 4 | Test 16 | 0.67 | 0.3 | 0.07 | 0.11 | 2256 | Train 36 | Test 16 | 0.66 | 0.32 | 0.06 | 0.11 |
| 209 | Train 4 | Test 17 | 0.75 | 0.39 | 0.1 | 0.17 | 2257 | Train 36 | Test 17 | 0.75 | 0.4 | 0.1 | 0.16 |
| 210 | Train 4 | Test 18 | 0.77 | 0.46 | 0.16 | 0.24 | 2258 | Train 36 | Test 18 | 0.77 | 0.47 | 0.16 | 0.24 |
| 211 | Train 4 | Test 19 | 0.75 | 0.39 | 0.1 | 0.17 | 2259 | Train 36 | Test 19 | 0.75 | 0.4 | 0.1 | 0.17 |
| 212 | Train 4 | Test 20 | 0.77 | 0.46 | 0.16 | 0.24 | 2260 | Train 36 | Test 20 | 0.77 | 0.47 | 0.16 | 0.24 |
| 213 | Train 4 | Test 21 | 0.69 | 0.28 | 0.11 | 0.16 | 2261 | Train 36 | Test 21 | 0.69 | 0.31 | 0.1 | 0.14 |
| 214 | Train 4 | Test 22 | 0.67 | 0.31 | 0.07 | 0.11 | 2262 | Train 36 | Test 22 | 0.66 | 0.34 | 0.07 | 0.11 |
| 215 | Train 4 | Test 23 | 0.69 | 0.28 | 0.11 | 0.15 | 2263 | Train 36 | Test 23 | 0.69 | 0.3 | 0.09 | 0.14 |
| 216 | Train 4 | Test 24 | 0.67 | 0.31 | 0.07 | 0.11 | 2264 | Train 36 | Test 24 | 0.67 | 0.33 | 0.06 | 0.11 |
| 217 | Train 4 | Test 25 | 0.75 | 0.39 | 0.09 | 0.15 | 2265 | Train 36 | Test 25 | 0.75 | 0.37 | 0.08 | 0.13 |
| 218 | Train 4 | Test 26 | 0.77 | 0.46 | 0.15 | 0.22 | 2266 | Train 36 | Test 26 | 0.77 | 0.47 | 0.14 | 0.22 |
| 219 | Train 4 | Test 27 | 0.75 | 0.38 | 0.09 | 0.15 | 2267 | Train 36 | Test 27 | 0.75 | 0.37 | 0.08 | 0.14 |
| 220 | Train 4 | Test 28 | 0.77 | 0.46 | 0.15 | 0.22 | 2268 | Train 36 | Test 28 | 0.77 | 0.46 | 0.14 | 0.22 |
| 221 | Train 4 | Test 29 | 0.69 | 0.29 | 0.1 | 0.15 | 2269 | Train 36 | Test 29 | 0.69 | 0.3 | 0.08 | 0.13 |
| 222 | Train 4 | Test 30 | 0.67 | 0.31 | 0.07 | 0.12 | 2270 | Train 36 | Test 30 | 0.67 | 0.33 | 0.07 | 0.11 |
| 223 | Train 4 | Test 31 | 0.69 | 0.29 | 0.1 | 0.15 | 2271 | Train 36 | Test 31 | 0.69 | 0.29 | 0.08 | 0.12 |
| 224 | Train 4 | Test 32 | 0.67 | 0.3 | 0.07 | 0.11 | 2272 | Train 36 | Test 32 | 0.67 | 0.32 | 0.06 | 0.11 |
| 225 | Train 4 | Test 33 | 0.76 | 0.39 | 0.1 | 0.16 | 2273 | Train 36 | Test 33 | 0.76 | 0.4 | 0.1 | 0.16 |
| 226 | Train 4 | Test 34 | 0.77 | 0.46 | 0.16 | 0.24 | 2274 | Train 36 | Test 34 | 0.77 | 0.47 | 0.16 | 0.24 |
| 227 | Train 4 | Test 35 | 0.76 | 0.39 | 0.1 | 0.16 | 2275 | Train 36 | Test 35 | 0.76 | 0.4 | 0.1 | 0.16 |
| 228 | Train 4 | Test 36 | 0.77 | 0.46 | 0.16 | 0.23 | 2276 | Train 36 | Test 36 | 0.77 | 0.47 | 0.16 | 0.24 |
| 229 | Train 4 | Test 37 | 0.69 | 0.28 | 0.11 | 0.16 | 2277 | Train 36 | Test 37 | 0.69 | 0.3 | 0.09 | 0.14 |
| 230 | Train 4 | Test 38 | 0.67 | 0.31 | 0.07 | 0.11 | 2278 | Train 36 | Test 38 | 0.66 | 0.33 | 0.07 | 0.11 |
| 231 | Train 4 | Test 39 | 0.69 | 0.28 | 0.11 | 0.15 | 2279 | Train 36 | Test 39 | 0.69 | 0.3 | 0.09 | 0.14 |
| 232 | Train 4 | Test 40 | 0.67 | 0.3 | 0.07 | 0.11 | 2280 | Train 36 | Test 40 | 0.66 | 0.33 | 0.06 | 0.11 |
| 233 | Train 4 | Test 41 | 0.75 | 0.38 | 0.09 | 0.15 | 2281 | Train 36 | Test 41 | 0.75 | 0.37 | 0.08 | 0.13 |
| 234 | Train 4 | Test 42 | 0.77 | 0.46 | 0.15 | 0.22 | 2282 | Train 36 | Test 42 | 0.77 | 0.46 | 0.14 | 0.21 |
| 235 | Train 4 | Test 43 | 0.75 | 0.38 | 0.09 | 0.15 | 2283 | Train 36 | Test 43 | 0.75 | 0.37 | 0.08 | 0.13 |
| 236 | Train 4 | Test 44 | 0.77 | 0.46 | 0.15 | 0.22 | 2284 | Train 36 | Test 44 | 0.77 | 0.46 | 0.14 | 0.22 |
| 237 | Train 4 | Test 45 | 0.69 | 0.29 | 0.1 | 0.15 | 2285 | Train 36 | Test 45 | 0.69 | 0.29 | 0.08 | 0.12 |
| 238 | Train 4 | Test 46 | 0.67 | 0.31 | 0.07 | 0.12 | 2286 | Train 36 | Test 46 | 0.67 | 0.33 | 0.07 | 0.11 |
| 239 | Train 4 | Test 47 | 0.69 | 0.29 | 0.1 | 0.15 | 2287 | Train 36 | Test 47 | 0.69 | 0.29 | 0.08 | 0.12 |
| 240 | Train 4 | Test 48 | 0.67 | 0.3 | 0.07 | 0.11 | 2288 | Train 36 | Test 48 | 0.67 | 0.32 | 0.06 | 0.11 |
| 241 | Train 4 | Test 49 | 0.77 | 0.45 | 0.16 | 0.24 | 2289 | Train 36 | Test 49 | 0.76 | 0.48 | 0.16 | 0.24 |
| 242 | Train 4 | Test 50 | 0.77 | 0.45 | 0.16 | 0.24 | 2290 | Train 36 | Test 50 | 0.76 | 0.48 | 0.16 | 0.24 |
| 243 | Train 4 | Test 51 | 0.77 | 0.45 | 0.16 | 0.24 | 2291 | Train 36 | Test 51 | 0.76 | 0.47 | 0.16 | 0.24 |
| 244 | Train 4 | Test 52 | 0.77 | 0.45 | 0.16 | 0.23 | 2292 | Train 36 | Test 52 | 0.76 | 0.47 | 0.16 | 0.24 |
| 245 | Train 4 | Test 53 | 0.67 | 0.26 | 0.06 | 0.1 | 2293 | Train 36 | Test 53 | 0.67 | 0.25 | 0.08 | 0.12 |
| 246 | Train 4 | Test 54 | 0.67 | 0.38 | 0.06 | 0.1 | 2294 | Train 36 | Test 54 | 0.67 | 0.33 | 0.06 | 0.1 |
| 247 | Train 4 | Test 55 | 0.71 | 0.36 | 0.11 | 0.17 | 2295 | Train 36 | Test 55 | 0.7 | 0.32 | 0.1 | 0.15 |
| 248 | Train 4 | Test 56 | 0.67 | 0.37 | 0.06 | 0.1 | 2296 | Train 36 | Test 56 | 0.66 | 0.32 | 0.06 | 0.1 |
| 249 | Train 4 | Test 57 | 0.77 | 0.45 | 0.14 | 0.21 | 2297 | Train 36 | Test 57 | 0.76 | 0.44 | 0.13 | 0.2 |
| 250 | Train 4 | Test 58 | 0.77 | 0.44 | 0.14 | 0.21 | 2298 | Train 36 | Test 58 | 0.76 | 0.44 | 0.13 | 0.2 |
| 251 | Train 4 | Test 59 | 0.77 | 0.44 | 0.14 | 0.21 | 2299 | Train 36 | Test 59 | 0.76 | 0.44 | 0.13 | 0.2 |
| 252 | Train 4 | Test 60 | 0.76 | 0.44 | 0.14 | 0.21 | 2300 | Train 36 | Test 60 | 0.76 | 0.44 | 0.13 | 0.2 |
| 253 | Train 4 | Test 61 | 0.67 | 0.27 | 0.06 | 0.1 | 2301 | Train 36 | Test 61 | 0.67 | 0.25 | 0.08 | 0.12 |
| 254 | Train 4 | Test 62 | 0.67 | 0.38 | 0.06 | 0.1 | 2302 | Train 36 | Test 62 | 0.67 | 0.33 | 0.06 | 0.1 |
| 255 | Train 4 | Test 63 | 0.67 | 0.27 | 0.06 | 0.1 | 2303 | Train 36 | Test 63 | 0.67 | 0.25 | 0.08 | 0.12 |
| 256 | Train 4 | Test 64 | 0.67 | 0.37 | 0.06 | 0.1 | 2304 | Train 36 | Test 64 | 0.67 | 0.32 | 0.06 | 0.1 |
| 257 | Train 5 | Test 1 | 0.76 | 0.4 | 0.09 | 0.14 | 2305 | Train 37 | Test 1 | 0.75 | 0.37 | 0.08 | 0.13 |
| 258 | Train 5 | Test 2 | 0.74 | 0.37 | 0.08 | 0.13 | 2306 | Train 37 | Test 2 | 0.74 | 0.38 | 0.08 | 0.13 |
| 259 | Train 5 | Test 3 | 0.76 | 0.4 | 0.09 | 0.14 | 2307 | Train 37 | Test 3 | 0.75 | 0.37 | 0.08 | 0.13 |
| 260 | Train 5 | Test 4 | 0.74 | 0.37 | 0.08 | 0.13 | 2308 | Train 37 | Test 4 | 0.74 | 0.38 | 0.08 | 0.13 |
| 261 | Train 5 | Test 5 | 0.74 | 0.39 | 0.09 | 0.15 | 2309 | Train 37 | Test 5 | 0.74 | 0.38 | 0.09 | 0.14 |
| 262 | Train 5 | Test 6 | 0.67 | 0.31 | 0.16 | 0.21 | 2310 | Train 37 | Test 6 | 0.68 | 0.34 | 0.1 | 0.15 |
| 263 | Train 5 | Test 7 | 0.74 | 0.39 | 0.09 | 0.15 | 2311 | Train 37 | Test 7 | 0.74 | 0.38 | 0.09 | 0.14 |
| 264 | Train 5 | Test 8 | 0.68 | 0.31 | 0.16 | 0.21 | 2312 | Train 37 | Test 8 | 0.68 | 0.34 | 0.09 | 0.15 |
| 265 | Train 5 | Test 9 | 0.75 | 0.41 | 0.08 | 0.13 | 2313 | Train 37 | Test 9 | 0.75 | 0.38 | 0.07 | 0.12 |
| 266 | Train 5 | Test 10 | 0.74 | 0.39 | 0.08 | 0.13 | 2314 | Train 37 | Test 10 | 0.74 | 0.39 | 0.07 | 0.12 |
| 267 | Train 5 | Test 11 | 0.76 | 0.41 | 0.08 | 0.13 | 2315 | Train 37 | Test 11 | 0.75 | 0.38 | 0.07 | 0.12 |
| 268 | Train 5 | Test 12 | 0.74 | 0.39 | 0.08 | 0.13 | 2316 | Train 37 | Test 12 | 0.74 | 0.39 | 0.07 | 0.12 |
| 269 | Train 5 | Test 13 | 0.74 | 0.41 | 0.08 | 0.14 | 2317 | Train 37 | Test 13 | 0.74 | 0.39 | 0.07 | 0.12 |
| 270 | Train 5 | Test 14 | 0.68 | 0.31 | 0.16 | 0.21 | 2318 | Train 37 | Test 14 | 0.68 | 0.33 | 0.1 | 0.15 |
| 271 | Train 5 | Test 15 | 0.74 | 0.41 | 0.08 | 0.14 | 2319 | Train 37 | Test 15 | 0.74 | 0.39 | 0.07 | 0.12 |
| 272 | Train 5 | Test 16 | 0.68 | 0.31 | 0.16 | 0.21 | 2320 | Train 37 | Test 16 | 0.68 | 0.33 | 0.09 | 0.15 |
| 273 | Train 5 | Test 17 | 0.76 | 0.4 | 0.09 | 0.14 | 2321 | Train 37 | Test 17 | 0.75 | 0.37 | 0.08 | 0.13 |
| 274 | Train 5 | Test 18 | 0.74 | 0.37 | 0.08 | 0.13 | 2322 | Train 37 | Test 18 | 0.74 | 0.38 | 0.08 | 0.13 |
| 275 | Train 5 | Test 19 | 0.76 | 0.4 | 0.09 | 0.14 | 2323 | Train 37 | Test 19 | 0.75 | 0.37 | 0.08 | 0.13 |
| 276 | Train 5 | Test 20 | 0.74 | 0.37 | 0.08 | 0.13 | 2324 | Train 37 | Test 20 | 0.74 | 0.38 | 0.08 | 0.13 |
| 277 | Train 5 | Test 21 | 0.74 | 0.39 | 0.09 | 0.15 | 2325 | Train 37 | Test 21 | 0.74 | 0.38 | 0.09 | 0.14 |
| 278 | Train 5 | Test 22 | 0.67 | 0.31 | 0.16 | 0.21 | 2326 | Train 37 | Test 22 | 0.68 | 0.33 | 0.1 | 0.15 |
| 279 | Train 5 | Test 23 | 0.74 | 0.39 | 0.09 | 0.15 | 2327 | Train 37 | Test 23 | 0.74 | 0.38 | 0.09 | 0.14 |
| 280 | Train 5 | Test 24 | 0.68 | 0.31 | 0.16 | 0.21 | 2328 | Train 37 | Test 24 | 0.68 | 0.33 | 0.1 | 0.15 |
| 281 | Train 5 | Test 25 | 0.75 | 0.41 | 0.08 | 0.13 | 2329 | Train 37 | Test 25 | 0.75 | 0.38 | 0.07 | 0.12 |
| 282 | Train 5 | Test 26 | 0.74 | 0.39 | 0.08 | 0.13 | 2330 | Train 37 | Test 26 | 0.74 | 0.39 | 0.07 | 0.12 |
| 283 | Train 5 | Test 27 | 0.76 | 0.41 | 0.08 | 0.13 | 2331 | Train 37 | Test 27 | 0.75 | 0.38 | 0.07 | 0.12 |
| 284 | Train 5 | Test 28 | 0.74 | 0.39 | 0.08 | 0.13 | 2332 | Train 37 | Test 28 | 0.74 | 0.39 | 0.07 | 0.12 |
| 285 | Train 5 | Test 29 | 0.75 | 0.41 | 0.08 | 0.14 | 2333 | Train 37 | Test 29 | 0.74 | 0.39 | 0.07 | 0.12 |
| 286 | Train 5 | Test 30 | 0.68 | 0.31 | 0.16 | 0.21 | 2334 | Train 37 | Test 30 | 0.68 | 0.33 | 0.1 | 0.15 |
| 287 | Train 5 | Test 31 | 0.74 | 0.41 | 0.08 | 0.14 | 2335 | Train 37 | Test 31 | 0.74 | 0.39 | 0.07 | 0.12 |
| 288 | Train 5 | Test 32 | 0.68 | 0.31 | 0.16 | 0.21 | 2336 | Train 37 | Test 32 | 0.68 | 0.33 | 0.1 | 0.15 |
| 289 | Train 5 | Test 33 | 0.76 | 0.4 | 0.09 | 0.14 | 2337 | Train 37 | Test 33 | 0.75 | 0.37 | 0.08 | 0.13 |
| 290 | Train 5 | Test 34 | 0.74 | 0.37 | 0.08 | 0.13 | 2338 | Train 37 | Test 34 | 0.74 | 0.38 | 0.08 | 0.13 |
| 291 | Train 5 | Test 35 | 0.76 | 0.4 | 0.09 | 0.14 | 2339 | Train 37 | Test 35 | 0.75 | 0.37 | 0.08 | 0.13 |
| 292 | Train 5 | Test 36 | 0.74 | 0.37 | 0.08 | 0.13 | 2340 | Train 37 | Test 36 | 0.74 | 0.39 | 0.08 | 0.13 |
| 293 | Train 5 | Test 37 | 0.75 | 0.39 | 0.09 | 0.15 | 2341 | Train 37 | Test 37 | 0.74 | 0.38 | 0.09 | 0.14 |
| 294 | Train 5 | Test 38 | 0.68 | 0.31 | 0.16 | 0.21 | 2342 | Train 37 | Test 38 | 0.68 | 0.33 | 0.1 | 0.15 |
| 295 | Train 5 | Test 39 | 0.74 | 0.39 | 0.09 | 0.15 | 2343 | Train 37 | Test 39 | 0.74 | 0.38 | 0.09 | 0.14 |
| 296 | Train 5 | Test 40 | 0.68 | 0.31 | 0.16 | 0.21 | 2344 | Train 37 | Test 40 | 0.68 | 0.33 | 0.09 | 0.15 |
| 297 | Train 5 | Test 41 | 0.76 | 0.41 | 0.08 | 0.13 | 2345 | Train 37 | Test 41 | 0.75 | 0.38 | 0.07 | 0.12 |
| 298 | Train 5 | Test 42 | 0.74 | 0.39 | 0.07 | 0.13 | 2346 | Train 37 | Test 42 | 0.74 | 0.39 | 0.07 | 0.12 |
| 299 | Train 5 | Test 43 | 0.76 | 0.41 | 0.08 | 0.13 | 2347 | Train 37 | Test 43 | 0.75 | 0.38 | 0.07 | 0.12 |
| 300 | Train 5 | Test 44 | 0.74 | 0.39 | 0.08 | 0.13 | 2348 | Train 37 | Test 44 | 0.74 | 0.39 | 0.07 | 0.12 |
| 301 | Train 5 | Test 45 | 0.75 | 0.41 | 0.08 | 0.14 | 2349 | Train 37 | Test 45 | 0.74 | 0.39 | 0.07 | 0.12 |
| 302 | Train 5 | Test 46 | 0.68 | 0.31 | 0.16 | 0.21 | 2350 | Train 37 | Test 46 | 0.68 | 0.33 | 0.1 | 0.15 |
| 303 | Train 5 | Test 47 | 0.74 | 0.4 | 0.08 | 0.14 | 2351 | Train 37 | Test 47 | 0.74 | 0.38 | 0.07 | 0.12 |
| 304 | Train 5 | Test 48 | 0.68 | 0.31 | 0.16 | 0.21 | 2352 | Train 37 | Test 48 | 0.68 | 0.33 | 0.09 | 0.15 |
| 305 | Train 5 | Test 49 | 0.77 | 0.45 | 0.14 | 0.22 | 2353 | Train 37 | Test 49 | 0.77 | 0.41 | 0.14 | 0.21 |
| 306 | Train 5 | Test 50 | 0.77 | 0.45 | 0.14 | 0.21 | 2354 | Train 37 | Test 50 | 0.76 | 0.4 | 0.14 | 0.21 |
| 307 | Train 5 | Test 51 | 0.77 | 0.45 | 0.14 | 0.22 | 2355 | Train 37 | Test 51 | 0.76 | 0.41 | 0.14 | 0.21 |
| 308 | Train 5 | Test 52 | 0.77 | 0.45 | 0.14 | 0.21 | 2356 | Train 37 | Test 52 | 0.76 | 0.41 | 0.14 | 0.21 |
| 309 | Train 5 | Test 53 | 0.75 | 0.39 | 0.18 | 0.25 | 2357 | Train 37 | Test 53 | 0.74 | 0.36 | 0.13 | 0.19 |
| 310 | Train 5 | Test 54 | 0.68 | 0.36 | 0.12 | 0.18 | 2358 | Train 37 | Test 54 | 0.69 | 0.38 | 0.09 | 0.14 |
| 311 | Train 5 | Test 55 | 0.77 | 0.44 | 0.13 | 0.2 | 2359 | Train 37 | Test 55 | 0.77 | 0.43 | 0.13 | 0.2 |
| 312 | Train 5 | Test 56 | 0.68 | 0.36 | 0.12 | 0.18 | 2360 | Train 37 | Test 56 | 0.69 | 0.38 | 0.08 | 0.14 |
| 313 | Train 5 | Test 57 | 0.77 | 0.48 | 0.14 | 0.21 | 2361 | Train 37 | Test 57 | 0.77 | 0.46 | 0.14 | 0.21 |
| 314 | Train 5 | Test 58 | 0.77 | 0.48 | 0.14 | 0.21 | 2362 | Train 37 | Test 58 | 0.77 | 0.46 | 0.14 | 0.21 |
| 315 | Train 5 | Test 59 | 0.77 | 0.48 | 0.14 | 0.21 | 2363 | Train 37 | Test 59 | 0.77 | 0.46 | 0.13 | 0.21 |
| 316 | Train 5 | Test 60 | 0.77 | 0.48 | 0.14 | 0.21 | 2364 | Train 37 | Test 60 | 0.77 | 0.45 | 0.13 | 0.2 |
| 317 | Train 5 | Test 61 | 0.75 | 0.39 | 0.16 | 0.23 | 2365 | Train 37 | Test 61 | 0.74 | 0.36 | 0.12 | 0.18 |
| 318 | Train 5 | Test 62 | 0.68 | 0.36 | 0.12 | 0.18 | 2366 | Train 37 | Test 62 | 0.69 | 0.37 | 0.09 | 0.14 |
| 319 | Train 5 | Test 63 | 0.75 | 0.39 | 0.16 | 0.23 | 2367 | Train 37 | Test 63 | 0.74 | 0.36 | 0.12 | 0.18 |
| 320 | Train 5 | Test 64 | 0.69 | 0.35 | 0.12 | 0.18 | 2368 | Train 37 | Test 64 | 0.69 | 0.37 | 0.08 | 0.14 |
| 321 | Train 6 | Test 1 | 0.73 | 0.34 | 0.13 | 0.19 | 2369 | Train 38 | Test 1 | 0.75 | 0.33 | 0.2 | 0.25 |
| 322 | Train 6 | Test 2 | 0.74 | 0.38 | 0.18 | 0.24 | 2370 | Train 38 | Test 2 | 0.75 | 0.37 | 0.23 | 0.28 |
| 323 | Train 6 | Test 3 | 0.73 | 0.34 | 0.13 | 0.19 | 2371 | Train 38 | Test 3 | 0.75 | 0.33 | 0.2 | 0.25 |
| 324 | Train 6 | Test 4 | 0.74 | 0.38 | 0.18 | 0.24 | 2372 | Train 38 | Test 4 | 0.75 | 0.37 | 0.23 | 0.28 |
| 325 | Train 6 | Test 5 | 0.73 | 0.35 | 0.13 | 0.19 | 2373 | Train 38 | Test 5 | 0.72 | 0.33 | 0.17 | 0.22 |
| 326 | Train 6 | Test 6 | 0.76 | 0.53 | 0.15 | 0.23 | 2374 | Train 38 | Test 6 | 0.76 | 0.53 | 0.15 | 0.24 |
| 327 | Train 6 | Test 7 | 0.73 | 0.34 | 0.13 | 0.19 | 2375 | Train 38 | Test 7 | 0.72 | 0.33 | 0.16 | 0.22 |
| 328 | Train 6 | Test 8 | 0.76 | 0.49 | 0.15 | 0.23 | 2376 | Train 38 | Test 8 | 0.76 | 0.49 | 0.15 | 0.23 |
| 329 | Train 6 | Test 9 | 0.73 | 0.35 | 0.12 | 0.18 | 2377 | Train 38 | Test 9 | 0.75 | 0.34 | 0.2 | 0.25 |
| 330 | Train 6 | Test 10 | 0.74 | 0.4 | 0.16 | 0.23 | 2378 | Train 38 | Test 10 | 0.76 | 0.38 | 0.22 | 0.28 |
| 331 | Train 6 | Test 11 | 0.74 | 0.35 | 0.11 | 0.17 | 2379 | Train 38 | Test 11 | 0.75 | 0.34 | 0.19 | 0.25 |
| 332 | Train 6 | Test 12 | 0.74 | 0.4 | 0.16 | 0.23 | 2380 | Train 38 | Test 12 | 0.76 | 0.38 | 0.21 | 0.27 |
| 333 | Train 6 | Test 13 | 0.73 | 0.35 | 0.12 | 0.18 | 2381 | Train 38 | Test 13 | 0.73 | 0.33 | 0.15 | 0.21 |
| 334 | Train 6 | Test 14 | 0.76 | 0.52 | 0.15 | 0.23 | 2382 | Train 38 | Test 14 | 0.76 | 0.53 | 0.15 | 0.23 |
| 335 | Train 6 | Test 15 | 0.73 | 0.35 | 0.12 | 0.18 | 2383 | Train 38 | Test 15 | 0.72 | 0.33 | 0.15 | 0.21 |
| 336 | Train 6 | Test 16 | 0.76 | 0.49 | 0.15 | 0.23 | 2384 | Train 38 | Test 16 | 0.76 | 0.49 | 0.15 | 0.23 |
| 337 | Train 6 | Test 17 | 0.73 | 0.34 | 0.13 | 0.19 | 2385 | Train 38 | Test 17 | 0.75 | 0.33 | 0.2 | 0.25 |
| 338 | Train 6 | Test 18 | 0.74 | 0.38 | 0.18 | 0.24 | 2386 | Train 38 | Test 18 | 0.75 | 0.37 | 0.23 | 0.28 |
| 339 | Train 6 | Test 19 | 0.73 | 0.34 | 0.13 | 0.19 | 2387 | Train 38 | Test 19 | 0.75 | 0.33 | 0.2 | 0.25 |
| 340 | Train 6 | Test 20 | 0.74 | 0.38 | 0.18 | 0.24 | 2388 | Train 38 | Test 20 | 0.75 | 0.37 | 0.23 | 0.28 |
| 341 | Train 6 | Test 21 | 0.73 | 0.35 | 0.13 | 0.19 | 2389 | Train 38 | Test 21 | 0.72 | 0.33 | 0.17 | 0.22 |
| 342 | Train 6 | Test 22 | 0.76 | 0.53 | 0.15 | 0.23 | 2390 | Train 38 | Test 22 | 0.76 | 0.53 | 0.15 | 0.24 |
| 343 | Train 6 | Test 23 | 0.73 | 0.34 | 0.13 | 0.19 | 2391 | Train 38 | Test 23 | 0.72 | 0.33 | 0.16 | 0.22 |
| 344 | Train 6 | Test 24 | 0.76 | 0.49 | 0.15 | 0.23 | 2392 | Train 38 | Test 24 | 0.76 | 0.49 | 0.15 | 0.23 |
| 345 | Train 6 | Test 25 | 0.73 | 0.35 | 0.12 | 0.18 | 2393 | Train 38 | Test 25 | 0.75 | 0.34 | 0.2 | 0.25 |
| 346 | Train 6 | Test 26 | 0.74 | 0.4 | 0.16 | 0.23 | 2394 | Train 38 | Test 26 | 0.76 | 0.38 | 0.22 | 0.28 |
| 347 | Train 6 | Test 27 | 0.73 | 0.35 | 0.12 | 0.17 | 2395 | Train 38 | Test 27 | 0.75 | 0.34 | 0.19 | 0.25 |
| 348 | Train 6 | Test 28 | 0.74 | 0.4 | 0.16 | 0.23 | 2396 | Train 38 | Test 28 | 0.76 | 0.38 | 0.22 | 0.27 |
| 349 | Train 6 | Test 29 | 0.73 | 0.35 | 0.12 | 0.18 | 2397 | Train 38 | Test 29 | 0.73 | 0.33 | 0.15 | 0.21 |
| 350 | Train 6 | Test 30 | 0.76 | 0.52 | 0.15 | 0.23 | 2398 | Train 38 | Test 30 | 0.76 | 0.53 | 0.15 | 0.23 |
| 351 | Train 6 | Test 31 | 0.73 | 0.35 | 0.12 | 0.18 | 2399 | Train 38 | Test 31 | 0.72 | 0.33 | 0.15 | 0.21 |
| 352 | Train 6 | Test 32 | 0.76 | 0.49 | 0.15 | 0.23 | 2400 | Train 38 | Test 32 | 0.76 | 0.49 | 0.15 | 0.23 |
| 353 | Train 6 | Test 33 | 0.73 | 0.34 | 0.13 | 0.18 | 2401 | Train 38 | Test 33 | 0.75 | 0.33 | 0.2 | 0.25 |
| 354 | Train 6 | Test 34 | 0.74 | 0.38 | 0.18 | 0.24 | 2402 | Train 38 | Test 34 | 0.75 | 0.37 | 0.23 | 0.28 |
| 355 | Train 6 | Test 35 | 0.73 | 0.34 | 0.13 | 0.18 | 2403 | Train 38 | Test 35 | 0.75 | 0.33 | 0.2 | 0.25 |
| 356 | Train 6 | Test 36 | 0.74 | 0.38 | 0.18 | 0.24 | 2404 | Train 38 | Test 36 | 0.75 | 0.37 | 0.23 | 0.28 |
| 357 | Train 6 | Test 37 | 0.73 | 0.34 | 0.13 | 0.19 | 2405 | Train 38 | Test 37 | 0.73 | 0.33 | 0.16 | 0.22 |
| 358 | Train 6 | Test 38 | 0.76 | 0.53 | 0.15 | 0.23 | 2406 | Train 38 | Test 38 | 0.76 | 0.53 | 0.15 | 0.23 |
| 359 | Train 6 | Test 39 | 0.73 | 0.34 | 0.13 | 0.19 | 2407 | Train 38 | Test 39 | 0.72 | 0.33 | 0.16 | 0.22 |
| 360 | Train 6 | Test 40 | 0.76 | 0.49 | 0.15 | 0.23 | 2408 | Train 38 | Test 40 | 0.76 | 0.49 | 0.15 | 0.23 |
| 361 | Train 6 | Test 41 | 0.74 | 0.35 | 0.12 | 0.17 | 2409 | Train 38 | Test 41 | 0.75 | 0.34 | 0.19 | 0.25 |
| 362 | Train 6 | Test 42 | 0.74 | 0.4 | 0.16 | 0.23 | 2410 | Train 38 | Test 42 | 0.76 | 0.38 | 0.21 | 0.27 |
| 363 | Train 6 | Test 43 | 0.74 | 0.35 | 0.11 | 0.17 | 2411 | Train 38 | Test 43 | 0.75 | 0.34 | 0.19 | 0.25 |
| 364 | Train 6 | Test 44 | 0.74 | 0.4 | 0.16 | 0.23 | 2412 | Train 38 | Test 44 | 0.76 | 0.38 | 0.21 | 0.27 |
| 365 | Train 6 | Test 45 | 0.73 | 0.35 | 0.12 | 0.18 | 2413 | Train 38 | Test 45 | 0.73 | 0.33 | 0.15 | 0.21 |
| 366 | Train 6 | Test 46 | 0.76 | 0.52 | 0.15 | 0.23 | 2414 | Train 38 | Test 46 | 0.76 | 0.53 | 0.15 | 0.23 |
| 367 | Train 6 | Test 47 | 0.73 | 0.35 | 0.12 | 0.18 | 2415 | Train 38 | Test 47 | 0.72 | 0.33 | 0.15 | 0.2 |
| 368 | Train 6 | Test 48 | 0.76 | 0.49 | 0.15 | 0.23 | 2416 | Train 38 | Test 48 | 0.76 | 0.49 | 0.15 | 0.23 |
| 369 | Train 6 | Test 49 | 0.75 | 0.35 | 0.21 | 0.26 | 2417 | Train 38 | Test 49 | 0.76 | 0.34 | 0.39 | 0.36 |
| 370 | Train 6 | Test 50 | 0.75 | 0.35 | 0.21 | 0.26 | 2418 | Train 38 | Test 50 | 0.76 | 0.34 | 0.39 | 0.36 |
| 371 | Train 6 | Test 51 | 0.75 | 0.35 | 0.21 | 0.26 | 2419 | Train 38 | Test 51 | 0.76 | 0.34 | 0.38 | 0.36 |
| 372 | Train 6 | Test 52 | 0.75 | 0.35 | 0.2 | 0.26 | 2420 | Train 38 | Test 52 | 0.76 | 0.34 | 0.38 | 0.36 |
| 373 | Train 6 | Test 53 | 0.77 | 0.45 | 0.14 | 0.21 | 2421 | Train 38 | Test 53 | 0.77 | 0.44 | 0.14 | 0.21 |
| 374 | Train 6 | Test 54 | 0.76 | 0.53 | 0.17 | 0.26 | 2422 | Train 38 | Test 54 | 0.76 | 0.56 | 0.18 | 0.27 |
| 375 | Train 6 | Test 55 | 0.75 | 0.38 | 0.17 | 0.24 | 2423 | Train 38 | Test 55 | 0.75 | 0.35 | 0.27 | 0.3 |
| 376 | Train 6 | Test 56 | 0.76 | 0.52 | 0.17 | 0.26 | 2424 | Train 38 | Test 56 | 0.76 | 0.53 | 0.18 | 0.27 |
| 377 | Train 6 | Test 57 | 0.75 | 0.37 | 0.2 | 0.26 | 2425 | Train 38 | Test 57 | 0.77 | 0.35 | 0.38 | 0.36 |
| 378 | Train 6 | Test 58 | 0.75 | 0.37 | 0.2 | 0.26 | 2426 | Train 38 | Test 58 | 0.77 | 0.35 | 0.38 | 0.36 |
| 379 | Train 6 | Test 59 | 0.75 | 0.37 | 0.19 | 0.25 | 2427 | Train 38 | Test 59 | 0.77 | 0.35 | 0.37 | 0.36 |
| 380 | Train 6 | Test 60 | 0.75 | 0.36 | 0.19 | 0.25 | 2428 | Train 38 | Test 60 | 0.77 | 0.35 | 0.37 | 0.36 |
| 381 | Train 6 | Test 61 | 0.77 | 0.44 | 0.12 | 0.19 | 2429 | Train 38 | Test 61 | 0.78 | 0.45 | 0.13 | 0.2 |
| 382 | Train 6 | Test 62 | 0.76 | 0.53 | 0.17 | 0.26 | 2430 | Train 38 | Test 62 | 0.76 | 0.55 | 0.18 | 0.27 |
| 383 | Train 6 | Test 63 | 0.77 | 0.44 | 0.12 | 0.19 | 2431 | Train 38 | Test 63 | 0.78 | 0.45 | 0.13 | 0.2 |
| 384 | Train 6 | Test 64 | 0.76 | 0.51 | 0.17 | 0.26 | 2432 | Train 38 | Test 64 | 0.76 | 0.53 | 0.18 | 0.26 |
| 385 | Train 7 | Test 1 | 0.76 | 0.38 | 0.08 | 0.13 | 2433 | Train 39 | Test 1 | 0.76 | 0.47 | 0.09 | 0.15 |
| 386 | Train 7 | Test 2 | 0.74 | 0.37 | 0.07 | 0.12 | 2434 | Train 39 | Test 2 | 0.75 | 0.44 | 0.08 | 0.14 |
| 387 | Train 7 | Test 3 | 0.76 | 0.38 | 0.08 | 0.13 | 2435 | Train 39 | Test 3 | 0.76 | 0.48 | 0.09 | 0.15 |
| 388 | Train 7 | Test 4 | 0.74 | 0.37 | 0.07 | 0.12 | 2436 | Train 39 | Test 4 | 0.75 | 0.44 | 0.08 | 0.14 |
| 389 | Train 7 | Test 5 | 0.75 | 0.4 | 0.09 | 0.15 | 2437 | Train 39 | Test 5 | 0.75 | 0.44 | 0.09 | 0.15 |
| 390 | Train 7 | Test 6 | 0.67 | 0.33 | 0.16 | 0.21 | 2438 | Train 39 | Test 6 | 0.68 | 0.35 | 0.1 | 0.16 |
| 391 | Train 7 | Test 7 | 0.75 | 0.4 | 0.09 | 0.15 | 2439 | Train 39 | Test 7 | 0.75 | 0.44 | 0.09 | 0.15 |
| 392 | Train 7 | Test 8 | 0.67 | 0.32 | 0.15 | 0.21 | 2440 | Train 39 | Test 8 | 0.68 | 0.35 | 0.1 | 0.15 |
| 393 | Train 7 | Test 9 | 0.76 | 0.43 | 0.08 | 0.13 | 2441 | Train 39 | Test 9 | 0.76 | 0.46 | 0.09 | 0.14 |
| 394 | Train 7 | Test 10 | 0.74 | 0.42 | 0.07 | 0.12 | 2442 | Train 39 | Test 10 | 0.75 | 0.44 | 0.08 | 0.13 |
| 395 | Train 7 | Test 11 | 0.76 | 0.43 | 0.08 | 0.13 | 2443 | Train 39 | Test 11 | 0.76 | 0.46 | 0.09 | 0.14 |
| 396 | Train 7 | Test 12 | 0.74 | 0.42 | 0.07 | 0.12 | 2444 | Train 39 | Test 12 | 0.75 | 0.44 | 0.08 | 0.13 |
| 397 | Train 7 | Test 13 | 0.75 | 0.42 | 0.08 | 0.13 | 2445 | Train 39 | Test 13 | 0.75 | 0.43 | 0.08 | 0.13 |
| 398 | Train 7 | Test 14 | 0.67 | 0.32 | 0.16 | 0.21 | 2446 | Train 39 | Test 14 | 0.68 | 0.34 | 0.1 | 0.16 |
| 399 | Train 7 | Test 15 | 0.75 | 0.42 | 0.08 | 0.13 | 2447 | Train 39 | Test 15 | 0.75 | 0.44 | 0.08 | 0.13 |
| 400 | Train 7 | Test 16 | 0.67 | 0.32 | 0.15 | 0.21 | 2448 | Train 39 | Test 16 | 0.68 | 0.35 | 0.1 | 0.15 |
| 401 | Train 7 | Test 17 | 0.76 | 0.37 | 0.08 | 0.13 | 2449 | Train 39 | Test 17 | 0.76 | 0.47 | 0.09 | 0.15 |
| 402 | Train 7 | Test 18 | 0.74 | 0.37 | 0.07 | 0.12 | 2450 | Train 39 | Test 18 | 0.75 | 0.44 | 0.08 | 0.14 |
| 403 | Train 7 | Test 19 | 0.76 | 0.38 | 0.08 | 0.13 | 2451 | Train 39 | Test 19 | 0.76 | 0.47 | 0.09 | 0.15 |
| 404 | Train 7 | Test 20 | 0.74 | 0.37 | 0.07 | 0.12 | 2452 | Train 39 | Test 20 | 0.75 | 0.44 | 0.08 | 0.14 |
| 405 | Train 7 | Test 21 | 0.75 | 0.4 | 0.09 | 0.15 | 2453 | Train 39 | Test 21 | 0.75 | 0.44 | 0.09 | 0.15 |
| 406 | Train 7 | Test 22 | 0.67 | 0.33 | 0.16 | 0.21 | 2454 | Train 39 | Test 22 | 0.68 | 0.34 | 0.1 | 0.16 |
| 407 | Train 7 | Test 23 | 0.75 | 0.4 | 0.09 | 0.15 | 2455 | Train 39 | Test 23 | 0.75 | 0.44 | 0.09 | 0.15 |
| 408 | Train 7 | Test 24 | 0.67 | 0.32 | 0.16 | 0.21 | 2456 | Train 39 | Test 24 | 0.68 | 0.35 | 0.1 | 0.15 |
| 409 | Train 7 | Test 25 | 0.76 | 0.43 | 0.08 | 0.13 | 2457 | Train 39 | Test 25 | 0.76 | 0.46 | 0.09 | 0.14 |
| 410 | Train 7 | Test 26 | 0.74 | 0.42 | 0.07 | 0.12 | 2458 | Train 39 | Test 26 | 0.75 | 0.44 | 0.08 | 0.13 |
| 411 | Train 7 | Test 27 | 0.76 | 0.43 | 0.07 | 0.13 | 2459 | Train 39 | Test 27 | 0.76 | 0.46 | 0.09 | 0.14 |
| 412 | Train 7 | Test 28 | 0.74 | 0.42 | 0.07 | 0.12 | 2460 | Train 39 | Test 28 | 0.75 | 0.44 | 0.08 | 0.13 |
| 413 | Train 7 | Test 29 | 0.75 | 0.41 | 0.08 | 0.13 | 2461 | Train 39 | Test 29 | 0.75 | 0.43 | 0.08 | 0.13 |
| 414 | Train 7 | Test 30 | 0.67 | 0.32 | 0.16 | 0.21 | 2462 | Train 39 | Test 30 | 0.68 | 0.34 | 0.1 | 0.16 |
| 415 | Train 7 | Test 31 | 0.75 | 0.42 | 0.08 | 0.13 | 2463 | Train 39 | Test 31 | 0.75 | 0.44 | 0.08 | 0.13 |
| 416 | Train 7 | Test 32 | 0.67 | 0.32 | 0.16 | 0.21 | 2464 | Train 39 | Test 32 | 0.68 | 0.34 | 0.1 | 0.15 |
| 417 | Train 7 | Test 33 | 0.76 | 0.38 | 0.08 | 0.13 | 2465 | Train 39 | Test 33 | 0.77 | 0.47 | 0.09 | 0.15 |
| 418 | Train 7 | Test 34 | 0.75 | 0.37 | 0.07 | 0.12 | 2466 | Train 39 | Test 34 | 0.75 | 0.44 | 0.08 | 0.14 |
| 419 | Train 7 | Test 35 | 0.76 | 0.38 | 0.08 | 0.13 | 2467 | Train 39 | Test 35 | 0.77 | 0.47 | 0.09 | 0.15 |
| 420 | Train 7 | Test 36 | 0.75 | 0.37 | 0.07 | 0.12 | 2468 | Train 39 | Test 36 | 0.75 | 0.44 | 0.08 | 0.14 |
| 421 | Train 7 | Test 37 | 0.75 | 0.4 | 0.09 | 0.15 | 2469 | Train 39 | Test 37 | 0.75 | 0.44 | 0.09 | 0.15 |
| 422 | Train 7 | Test 38 | 0.67 | 0.33 | 0.16 | 0.21 | 2470 | Train 39 | Test 38 | 0.68 | 0.35 | 0.1 | 0.16 |
| 423 | Train 7 | Test 39 | 0.75 | 0.4 | 0.09 | 0.15 | 2471 | Train 39 | Test 39 | 0.75 | 0.44 | 0.09 | 0.15 |
| 424 | Train 7 | Test 40 | 0.67 | 0.32 | 0.15 | 0.21 | 2472 | Train 39 | Test 40 | 0.68 | 0.35 | 0.1 | 0.15 |
| 425 | Train 7 | Test 41 | 0.76 | 0.42 | 0.08 | 0.13 | 2473 | Train 39 | Test 41 | 0.76 | 0.46 | 0.08 | 0.14 |
| 426 | Train 7 | Test 42 | 0.75 | 0.42 | 0.07 | 0.12 | 2474 | Train 39 | Test 42 | 0.75 | 0.44 | 0.08 | 0.13 |
| 427 | Train 7 | Test 43 | 0.76 | 0.42 | 0.07 | 0.13 | 2475 | Train 39 | Test 43 | 0.76 | 0.46 | 0.08 | 0.14 |
| 428 | Train 7 | Test 44 | 0.75 | 0.42 | 0.07 | 0.12 | 2476 | Train 39 | Test 44 | 0.75 | 0.44 | 0.08 | 0.13 |
| 429 | Train 7 | Test 45 | 0.75 | 0.41 | 0.08 | 0.13 | 2477 | Train 39 | Test 45 | 0.75 | 0.43 | 0.08 | 0.13 |
| 430 | Train 7 | Test 46 | 0.67 | 0.32 | 0.16 | 0.21 | 2478 | Train 39 | Test 46 | 0.68 | 0.34 | 0.1 | 0.16 |
| 431 | Train 7 | Test 47 | 0.75 | 0.42 | 0.07 | 0.13 | 2479 | Train 39 | Test 47 | 0.75 | 0.44 | 0.08 | 0.13 |
| 432 | Train 7 | Test 48 | 0.67 | 0.32 | 0.15 | 0.21 | 2480 | Train 39 | Test 48 | 0.68 | 0.34 | 0.1 | 0.15 |
| 433 | Train 7 | Test 49 | 0.77 | 0.42 | 0.13 | 0.2 | 2481 | Train 39 | Test 49 | 0.78 | 0.51 | 0.15 | 0.23 |
| 434 | Train 7 | Test 50 | 0.77 | 0.42 | 0.13 | 0.2 | 2482 | Train 39 | Test 50 | 0.78 | 0.51 | 0.15 | 0.23 |
| 435 | Train 7 | Test 51 | 0.77 | 0.42 | 0.13 | 0.2 | 2483 | Train 39 | Test 51 | 0.78 | 0.51 | 0.15 | 0.23 |
| 436 | Train 7 | Test 52 | 0.77 | 0.42 | 0.13 | 0.2 | 2484 | Train 39 | Test 52 | 0.78 | 0.51 | 0.14 | 0.23 |
| 437 | Train 7 | Test 53 | 0.73 | 0.37 | 0.14 | 0.21 | 2485 | Train 39 | Test 53 | 0.74 | 0.42 | 0.11 | 0.18 |
| 438 | Train 7 | Test 54 | 0.68 | 0.38 | 0.13 | 0.19 | 2486 | Train 39 | Test 54 | 0.69 | 0.39 | 0.08 | 0.14 |
| 439 | Train 7 | Test 55 | 0.77 | 0.44 | 0.12 | 0.19 | 2487 | Train 39 | Test 55 | 0.77 | 0.45 | 0.12 | 0.2 |
| 440 | Train 7 | Test 56 | 0.68 | 0.37 | 0.12 | 0.19 | 2488 | Train 39 | Test 56 | 0.69 | 0.39 | 0.08 | 0.13 |
| 441 | Train 7 | Test 57 | 0.77 | 0.5 | 0.14 | 0.22 | 2489 | Train 39 | Test 57 | 0.78 | 0.52 | 0.15 | 0.23 |
| 442 | Train 7 | Test 58 | 0.77 | 0.5 | 0.14 | 0.22 | 2490 | Train 39 | Test 58 | 0.78 | 0.51 | 0.15 | 0.23 |
| 443 | Train 7 | Test 59 | 0.77 | 0.5 | 0.14 | 0.21 | 2491 | Train 39 | Test 59 | 0.78 | 0.52 | 0.15 | 0.23 |
| 444 | Train 7 | Test 60 | 0.77 | 0.5 | 0.14 | 0.21 | 2492 | Train 39 | Test 60 | 0.78 | 0.52 | 0.15 | 0.23 |
| 445 | Train 7 | Test 61 | 0.74 | 0.38 | 0.13 | 0.2 | 2493 | Train 39 | Test 61 | 0.75 | 0.42 | 0.09 | 0.15 |
| 446 | Train 7 | Test 62 | 0.68 | 0.38 | 0.13 | 0.19 | 2494 | Train 39 | Test 62 | 0.69 | 0.38 | 0.08 | 0.14 |
| 447 | Train 7 | Test 63 | 0.74 | 0.37 | 0.13 | 0.19 | 2495 | Train 39 | Test 63 | 0.75 | 0.42 | 0.09 | 0.15 |
| 448 | Train 7 | Test 64 | 0.68 | 0.37 | 0.12 | 0.19 | 2496 | Train 39 | Test 64 | 0.69 | 0.39 | 0.08 | 0.14 |
| 449 | Train 8 | Test 1 | 0.74 | 0.27 | 0.14 | 0.18 | 2497 | Train 40 | Test 1 | 0.75 | 0.37 | 0.14 | 0.2 |
| 450 | Train 8 | Test 2 | 0.75 | 0.33 | 0.18 | 0.24 | 2498 | Train 40 | Test 2 | 0.76 | 0.41 | 0.18 | 0.25 |
| 451 | Train 8 | Test 3 | 0.74 | 0.27 | 0.14 | 0.19 | 2499 | Train 40 | Test 3 | 0.75 | 0.37 | 0.14 | 0.2 |
| 452 | Train 8 | Test 4 | 0.75 | 0.33 | 0.18 | 0.24 | 2500 | Train 40 | Test 4 | 0.76 | 0.4 | 0.18 | 0.25 |
| 453 | Train 8 | Test 5 | 0.73 | 0.29 | 0.13 | 0.18 | 2501 | Train 40 | Test 5 | 0.74 | 0.35 | 0.13 | 0.19 |
| 454 | Train 8 | Test 6 | 0.76 | 0.53 | 0.14 | 0.22 | 2502 | Train 40 | Test 6 | 0.76 | 0.53 | 0.14 | 0.22 |
| 455 | Train 8 | Test 7 | 0.73 | 0.3 | 0.13 | 0.18 | 2503 | Train 40 | Test 7 | 0.74 | 0.35 | 0.13 | 0.19 |
| 456 | Train 8 | Test 8 | 0.76 | 0.52 | 0.13 | 0.21 | 2504 | Train 40 | Test 8 | 0.76 | 0.53 | 0.14 | 0.22 |
| 457 | Train 8 | Test 9 | 0.74 | 0.27 | 0.12 | 0.17 | 2505 | Train 40 | Test 9 | 0.75 | 0.36 | 0.12 | 0.18 |
| 458 | Train 8 | Test 10 | 0.75 | 0.34 | 0.16 | 0.22 | 2506 | Train 40 | Test 10 | 0.76 | 0.41 | 0.16 | 0.23 |
| 459 | Train 8 | Test 11 | 0.74 | 0.27 | 0.12 | 0.17 | 2507 | Train 40 | Test 11 | 0.75 | 0.36 | 0.12 | 0.18 |
| 460 | Train 8 | Test 12 | 0.75 | 0.34 | 0.16 | 0.22 | 2508 | Train 40 | Test 12 | 0.76 | 0.41 | 0.16 | 0.23 |
| 461 | Train 8 | Test 13 | 0.73 | 0.29 | 0.11 | 0.16 | 2509 | Train 40 | Test 13 | 0.74 | 0.35 | 0.12 | 0.17 |
| 462 | Train 8 | Test 14 | 0.76 | 0.52 | 0.14 | 0.22 | 2510 | Train 40 | Test 14 | 0.76 | 0.53 | 0.14 | 0.22 |
| 463 | Train 8 | Test 15 | 0.73 | 0.29 | 0.11 | 0.16 | 2511 | Train 40 | Test 15 | 0.73 | 0.35 | 0.11 | 0.17 |
| 464 | Train 8 | Test 16 | 0.76 | 0.52 | 0.13 | 0.21 | 2512 | Train 40 | Test 16 | 0.76 | 0.52 | 0.14 | 0.22 |
| 465 | Train 8 | Test 17 | 0.74 | 0.27 | 0.14 | 0.18 | 2513 | Train 40 | Test 17 | 0.75 | 0.37 | 0.14 | 0.2 |
| 466 | Train 8 | Test 18 | 0.75 | 0.33 | 0.18 | 0.23 | 2514 | Train 40 | Test 18 | 0.76 | 0.41 | 0.18 | 0.25 |
| 467 | Train 8 | Test 19 | 0.74 | 0.27 | 0.14 | 0.19 | 2515 | Train 40 | Test 19 | 0.75 | 0.37 | 0.14 | 0.2 |
| 468 | Train 8 | Test 20 | 0.75 | 0.33 | 0.18 | 0.23 | 2516 | Train 40 | Test 20 | 0.76 | 0.4 | 0.18 | 0.25 |
| 469 | Train 8 | Test 21 | 0.73 | 0.29 | 0.13 | 0.18 | 2517 | Train 40 | Test 21 | 0.74 | 0.35 | 0.13 | 0.19 |
| 470 | Train 8 | Test 22 | 0.76 | 0.53 | 0.14 | 0.22 | 2518 | Train 40 | Test 22 | 0.76 | 0.53 | 0.14 | 0.22 |
| 471 | Train 8 | Test 23 | 0.73 | 0.29 | 0.13 | 0.18 | 2519 | Train 40 | Test 23 | 0.74 | 0.35 | 0.13 | 0.19 |
| 472 | Train 8 | Test 24 | 0.76 | 0.52 | 0.14 | 0.21 | 2520 | Train 40 | Test 24 | 0.76 | 0.53 | 0.14 | 0.22 |
| 473 | Train 8 | Test 25 | 0.74 | 0.27 | 0.12 | 0.17 | 2521 | Train 40 | Test 25 | 0.75 | 0.36 | 0.12 | 0.18 |
| 474 | Train 8 | Test 26 | 0.75 | 0.34 | 0.16 | 0.22 | 2522 | Train 40 | Test 26 | 0.76 | 0.41 | 0.16 | 0.23 |
| 475 | Train 8 | Test 27 | 0.74 | 0.27 | 0.12 | 0.17 | 2523 | Train 40 | Test 27 | 0.75 | 0.36 | 0.12 | 0.18 |
| 476 | Train 8 | Test 28 | 0.75 | 0.34 | 0.16 | 0.22 | 2524 | Train 40 | Test 28 | 0.76 | 0.41 | 0.16 | 0.23 |
| 477 | Train 8 | Test 29 | 0.73 | 0.29 | 0.11 | 0.16 | 2525 | Train 40 | Test 29 | 0.74 | 0.35 | 0.12 | 0.17 |
| 478 | Train 8 | Test 30 | 0.76 | 0.52 | 0.14 | 0.22 | 2526 | Train 40 | Test 30 | 0.76 | 0.53 | 0.14 | 0.22 |
| 479 | Train 8 | Test 31 | 0.73 | 0.29 | 0.11 | 0.16 | 2527 | Train 40 | Test 31 | 0.73 | 0.35 | 0.11 | 0.17 |
| 480 | Train 8 | Test 32 | 0.76 | 0.52 | 0.14 | 0.21 | 2528 | Train 40 | Test 32 | 0.76 | 0.52 | 0.14 | 0.22 |
| 481 | Train 8 | Test 33 | 0.74 | 0.27 | 0.14 | 0.18 | 2529 | Train 40 | Test 33 | 0.76 | 0.37 | 0.14 | 0.2 |
| 482 | Train 8 | Test 34 | 0.75 | 0.33 | 0.18 | 0.23 | 2530 | Train 40 | Test 34 | 0.76 | 0.4 | 0.18 | 0.25 |
| 483 | Train 8 | Test 35 | 0.74 | 0.27 | 0.14 | 0.18 | 2531 | Train 40 | Test 35 | 0.76 | 0.36 | 0.14 | 0.2 |
| 484 | Train 8 | Test 36 | 0.75 | 0.33 | 0.18 | 0.23 | 2532 | Train 40 | Test 36 | 0.76 | 0.4 | 0.18 | 0.25 |
| 485 | Train 8 | Test 37 | 0.73 | 0.29 | 0.13 | 0.18 | 2533 | Train 40 | Test 37 | 0.74 | 0.35 | 0.13 | 0.19 |
| 486 | Train 8 | Test 38 | 0.76 | 0.52 | 0.14 | 0.22 | 2534 | Train 40 | Test 38 | 0.76 | 0.53 | 0.14 | 0.22 |
| 487 | Train 8 | Test 39 | 0.73 | 0.3 | 0.13 | 0.18 | 2535 | Train 40 | Test 39 | 0.74 | 0.35 | 0.13 | 0.19 |
| 488 | Train 8 | Test 40 | 0.76 | 0.52 | 0.13 | 0.21 | 2536 | Train 40 | Test 40 | 0.76 | 0.53 | 0.14 | 0.22 |
| 489 | Train 8 | Test 41 | 0.74 | 0.26 | 0.12 | 0.17 | 2537 | Train 40 | Test 41 | 0.75 | 0.36 | 0.12 | 0.18 |
| 490 | Train 8 | Test 42 | 0.75 | 0.34 | 0.16 | 0.22 | 2538 | Train 40 | Test 42 | 0.76 | 0.41 | 0.16 | 0.23 |
| 491 | Train 8 | Test 43 | 0.74 | 0.27 | 0.12 | 0.17 | 2539 | Train 40 | Test 43 | 0.75 | 0.36 | 0.11 | 0.17 |
| 492 | Train 8 | Test 44 | 0.75 | 0.34 | 0.16 | 0.22 | 2540 | Train 40 | Test 44 | 0.76 | 0.41 | 0.16 | 0.23 |
| 493 | Train 8 | Test 45 | 0.73 | 0.29 | 0.11 | 0.16 | 2541 | Train 40 | Test 45 | 0.74 | 0.35 | 0.11 | 0.17 |
| 494 | Train 8 | Test 46 | 0.76 | 0.52 | 0.14 | 0.22 | 2542 | Train 40 | Test 46 | 0.76 | 0.53 | 0.14 | 0.22 |
| 495 | Train 8 | Test 47 | 0.73 | 0.29 | 0.11 | 0.16 | 2543 | Train 40 | Test 47 | 0.74 | 0.35 | 0.11 | 0.17 |
| 496 | Train 8 | Test 48 | 0.76 | 0.52 | 0.13 | 0.21 | 2544 | Train 40 | Test 48 | 0.76 | 0.52 | 0.14 | 0.22 |
| 497 | Train 8 | Test 49 | 0.73 | 0.26 | 0.24 | 0.25 | 2545 | Train 40 | Test 49 | 0.77 | 0.37 | 0.22 | 0.28 |
| 498 | Train 8 | Test 50 | 0.73 | 0.25 | 0.24 | 0.25 | 2546 | Train 40 | Test 50 | 0.77 | 0.37 | 0.22 | 0.27 |
| 499 | Train 8 | Test 51 | 0.73 | 0.26 | 0.24 | 0.25 | 2547 | Train 40 | Test 51 | 0.77 | 0.37 | 0.21 | 0.27 |
| 500 | Train 8 | Test 52 | 0.73 | 0.26 | 0.24 | 0.25 | 2548 | Train 40 | Test 52 | 0.77 | 0.37 | 0.21 | 0.27 |
| 501 | Train 8 | Test 53 | 0.77 | 0.44 | 0.13 | 0.2 | 2549 | Train 40 | Test 53 | 0.77 | 0.43 | 0.14 | 0.21 |
| 502 | Train 8 | Test 54 | 0.76 | 0.53 | 0.16 | 0.25 | 2550 | Train 40 | Test 54 | 0.76 | 0.54 | 0.17 | 0.25 |
| 503 | Train 8 | Test 55 | 0.75 | 0.3 | 0.19 | 0.23 | 2551 | Train 40 | Test 55 | 0.77 | 0.38 | 0.17 | 0.24 |
| 504 | Train 8 | Test 56 | 0.76 | 0.53 | 0.16 | 0.24 | 2552 | Train 40 | Test 56 | 0.76 | 0.54 | 0.16 | 0.25 |
| 505 | Train 8 | Test 57 | 0.74 | 0.26 | 0.21 | 0.23 | 2553 | Train 40 | Test 57 | 0.78 | 0.37 | 0.2 | 0.26 |
| 506 | Train 8 | Test 58 | 0.73 | 0.25 | 0.21 | 0.23 | 2554 | Train 40 | Test 58 | 0.78 | 0.37 | 0.19 | 0.26 |
| 507 | Train 8 | Test 59 | 0.74 | 0.26 | 0.21 | 0.23 | 2555 | Train 40 | Test 59 | 0.78 | 0.37 | 0.19 | 0.25 |
| 508 | Train 8 | Test 60 | 0.74 | 0.26 | 0.21 | 0.23 | 2556 | Train 40 | Test 60 | 0.77 | 0.37 | 0.19 | 0.25 |
| 509 | Train 8 | Test 61 | 0.78 | 0.43 | 0.12 | 0.19 | 2557 | Train 40 | Test 61 | 0.77 | 0.43 | 0.12 | 0.19 |
| 510 | Train 8 | Test 62 | 0.76 | 0.52 | 0.16 | 0.25 | 2558 | Train 40 | Test 62 | 0.76 | 0.53 | 0.16 | 0.25 |
| 511 | Train 8 | Test 63 | 0.78 | 0.43 | 0.12 | 0.19 | 2559 | Train 40 | Test 63 | 0.77 | 0.43 | 0.12 | 0.18 |
| 512 | Train 8 | Test 64 | 0.76 | 0.52 | 0.16 | 0.24 | 2560 | Train 40 | Test 64 | 0.76 | 0.53 | 0.16 | 0.25 |
| 513 | Train 9 | Test 1 | 0.74 | 0.31 | 0.13 | 0.18 | 2561 | Train 41 | Test 1 | 0.74 | 0.37 | 0.14 | 0.2 |
| 514 | Train 9 | Test 2 | 0.72 | 0.34 | 0.13 | 0.19 | 2562 | Train 41 | Test 2 | 0.72 | 0.38 | 0.12 | 0.19 |
| 515 | Train 9 | Test 3 | 0.74 | 0.31 | 0.13 | 0.18 | 2563 | Train 41 | Test 3 | 0.74 | 0.37 | 0.14 | 0.21 |
| 516 | Train 9 | Test 4 | 0.72 | 0.34 | 0.13 | 0.19 | 2564 | Train 41 | Test 4 | 0.72 | 0.38 | 0.12 | 0.19 |
| 517 | Train 9 | Test 5 | 0.66 | 0.18 | 0.16 | 0.17 | 2565 | Train 41 | Test 5 | 0.69 | 0.3 | 0.11 | 0.16 |
| 518 | Train 9 | Test 6 | 0.63 | 0.22 | 0.1 | 0.14 | 2566 | Train 41 | Test 6 | 0.64 | 0.29 | 0.1 | 0.14 |
| 519 | Train 9 | Test 7 | 0.66 | 0.18 | 0.16 | 0.17 | 2567 | Train 41 | Test 7 | 0.69 | 0.3 | 0.11 | 0.17 |
| 520 | Train 9 | Test 8 | 0.63 | 0.22 | 0.1 | 0.13 | 2568 | Train 41 | Test 8 | 0.64 | 0.28 | 0.1 | 0.14 |
| 521 | Train 9 | Test 9 | 0.75 | 0.36 | 0.12 | 0.18 | 2569 | Train 41 | Test 9 | 0.75 | 0.39 | 0.12 | 0.18 |
| 522 | Train 9 | Test 10 | 0.73 | 0.37 | 0.11 | 0.17 | 2570 | Train 41 | Test 10 | 0.72 | 0.4 | 0.1 | 0.17 |
| 523 | Train 9 | Test 11 | 0.75 | 0.36 | 0.12 | 0.18 | 2571 | Train 41 | Test 11 | 0.75 | 0.39 | 0.12 | 0.18 |
| 524 | Train 9 | Test 12 | 0.73 | 0.38 | 0.11 | 0.18 | 2572 | Train 41 | Test 12 | 0.72 | 0.4 | 0.11 | 0.17 |
| 525 | Train 9 | Test 13 | 0.67 | 0.18 | 0.15 | 0.16 | 2573 | Train 41 | Test 13 | 0.69 | 0.3 | 0.09 | 0.14 |
| 526 | Train 9 | Test 14 | 0.63 | 0.22 | 0.1 | 0.14 | 2574 | Train 41 | Test 14 | 0.64 | 0.28 | 0.1 | 0.15 |
| 527 | Train 9 | Test 15 | 0.67 | 0.19 | 0.15 | 0.17 | 2575 | Train 41 | Test 15 | 0.69 | 0.3 | 0.09 | 0.14 |
| 528 | Train 9 | Test 16 | 0.63 | 0.22 | 0.1 | 0.14 | 2576 | Train 41 | Test 16 | 0.64 | 0.28 | 0.1 | 0.14 |
| 529 | Train 9 | Test 17 | 0.74 | 0.31 | 0.13 | 0.18 | 2577 | Train 41 | Test 17 | 0.74 | 0.37 | 0.14 | 0.21 |
| 530 | Train 9 | Test 18 | 0.72 | 0.34 | 0.13 | 0.19 | 2578 | Train 41 | Test 18 | 0.72 | 0.38 | 0.12 | 0.19 |
| 531 | Train 9 | Test 19 | 0.74 | 0.31 | 0.13 | 0.18 | 2579 | Train 41 | Test 19 | 0.74 | 0.37 | 0.14 | 0.21 |
| 532 | Train 9 | Test 20 | 0.72 | 0.34 | 0.13 | 0.19 | 2580 | Train 41 | Test 20 | 0.72 | 0.38 | 0.12 | 0.19 |
| 533 | Train 9 | Test 21 | 0.66 | 0.18 | 0.16 | 0.17 | 2581 | Train 41 | Test 21 | 0.69 | 0.3 | 0.11 | 0.16 |
| 534 | Train 9 | Test 22 | 0.63 | 0.22 | 0.1 | 0.14 | 2582 | Train 41 | Test 22 | 0.64 | 0.28 | 0.1 | 0.14 |
| 535 | Train 9 | Test 23 | 0.66 | 0.18 | 0.16 | 0.17 | 2583 | Train 41 | Test 23 | 0.69 | 0.3 | 0.11 | 0.17 |
| 536 | Train 9 | Test 24 | 0.63 | 0.22 | 0.1 | 0.13 | 2584 | Train 41 | Test 24 | 0.64 | 0.28 | 0.1 | 0.14 |
| 537 | Train 9 | Test 25 | 0.75 | 0.36 | 0.12 | 0.18 | 2585 | Train 41 | Test 25 | 0.75 | 0.39 | 0.12 | 0.18 |
| 538 | Train 9 | Test 26 | 0.73 | 0.37 | 0.11 | 0.17 | 2586 | Train 41 | Test 26 | 0.72 | 0.4 | 0.11 | 0.17 |
| 539 | Train 9 | Test 27 | 0.75 | 0.36 | 0.12 | 0.18 | 2587 | Train 41 | Test 27 | 0.75 | 0.39 | 0.12 | 0.18 |
| 540 | Train 9 | Test 28 | 0.73 | 0.38 | 0.11 | 0.18 | 2588 | Train 41 | Test 28 | 0.72 | 0.4 | 0.11 | 0.17 |
| 541 | Train 9 | Test 29 | 0.67 | 0.18 | 0.15 | 0.16 | 2589 | Train 41 | Test 29 | 0.69 | 0.3 | 0.09 | 0.14 |
| 542 | Train 9 | Test 30 | 0.63 | 0.22 | 0.1 | 0.14 | 2590 | Train 41 | Test 30 | 0.64 | 0.28 | 0.1 | 0.15 |
| 543 | Train 9 | Test 31 | 0.67 | 0.18 | 0.15 | 0.16 | 2591 | Train 41 | Test 31 | 0.69 | 0.3 | 0.09 | 0.14 |
| 544 | Train 9 | Test 32 | 0.63 | 0.22 | 0.1 | 0.14 | 2592 | Train 41 | Test 32 | 0.64 | 0.28 | 0.1 | 0.14 |
| 545 | Train 9 | Test 33 | 0.74 | 0.31 | 0.13 | 0.18 | 2593 | Train 41 | Test 33 | 0.74 | 0.37 | 0.14 | 0.2 |
| 546 | Train 9 | Test 34 | 0.72 | 0.34 | 0.13 | 0.18 | 2594 | Train 41 | Test 34 | 0.72 | 0.38 | 0.12 | 0.19 |
| 547 | Train 9 | Test 35 | 0.74 | 0.31 | 0.13 | 0.18 | 2595 | Train 41 | Test 35 | 0.74 | 0.37 | 0.14 | 0.21 |
| 548 | Train 9 | Test 36 | 0.72 | 0.34 | 0.13 | 0.18 | 2596 | Train 41 | Test 36 | 0.72 | 0.38 | 0.12 | 0.19 |
| 549 | Train 9 | Test 37 | 0.67 | 0.18 | 0.16 | 0.17 | 2597 | Train 41 | Test 37 | 0.69 | 0.3 | 0.11 | 0.16 |
| 550 | Train 9 | Test 38 | 0.63 | 0.22 | 0.1 | 0.14 | 2598 | Train 41 | Test 38 | 0.64 | 0.28 | 0.1 | 0.14 |
| 551 | Train 9 | Test 39 | 0.67 | 0.18 | 0.16 | 0.17 | 2599 | Train 41 | Test 39 | 0.69 | 0.3 | 0.11 | 0.16 |
| 552 | Train 9 | Test 40 | 0.63 | 0.22 | 0.1 | 0.14 | 2600 | Train 41 | Test 40 | 0.64 | 0.28 | 0.1 | 0.14 |
| 553 | Train 9 | Test 41 | 0.75 | 0.36 | 0.12 | 0.18 | 2601 | Train 41 | Test 41 | 0.75 | 0.39 | 0.12 | 0.18 |
| 554 | Train 9 | Test 42 | 0.73 | 0.37 | 0.11 | 0.17 | 2602 | Train 41 | Test 42 | 0.72 | 0.4 | 0.1 | 0.16 |
| 555 | Train 9 | Test 43 | 0.75 | 0.36 | 0.12 | 0.18 | 2603 | Train 41 | Test 43 | 0.75 | 0.39 | 0.12 | 0.18 |
| 556 | Train 9 | Test 44 | 0.73 | 0.38 | 0.11 | 0.17 | 2604 | Train 41 | Test 44 | 0.73 | 0.4 | 0.1 | 0.17 |
| 557 | Train 9 | Test 45 | 0.67 | 0.18 | 0.15 | 0.16 | 2605 | Train 41 | Test 45 | 0.69 | 0.3 | 0.09 | 0.14 |
| 558 | Train 9 | Test 46 | 0.63 | 0.22 | 0.1 | 0.14 | 2606 | Train 41 | Test 46 | 0.64 | 0.28 | 0.1 | 0.14 |
| 559 | Train 9 | Test 47 | 0.67 | 0.19 | 0.15 | 0.17 | 2607 | Train 41 | Test 47 | 0.69 | 0.3 | 0.09 | 0.14 |
| 560 | Train 9 | Test 48 | 0.63 | 0.22 | 0.1 | 0.14 | 2608 | Train 41 | Test 48 | 0.64 | 0.28 | 0.1 | 0.14 |
| 561 | Train 9 | Test 49 | 0.75 | 0.37 | 0.19 | 0.26 | 2609 | Train 41 | Test 49 | 0.76 | 0.44 | 0.21 | 0.28 |
| 562 | Train 9 | Test 50 | 0.75 | 0.37 | 0.19 | 0.25 | 2610 | Train 41 | Test 50 | 0.75 | 0.44 | 0.21 | 0.28 |
| 563 | Train 9 | Test 51 | 0.75 | 0.37 | 0.19 | 0.25 | 2611 | Train 41 | Test 51 | 0.76 | 0.44 | 0.21 | 0.29 |
| 564 | Train 9 | Test 52 | 0.75 | 0.37 | 0.19 | 0.25 | 2612 | Train 41 | Test 52 | 0.75 | 0.44 | 0.21 | 0.28 |
| 565 | Train 9 | Test 53 | 0.61 | 0.14 | 0.16 | 0.15 | 2613 | Train 41 | Test 53 | 0.68 | 0.23 | 0.1 | 0.14 |
| 566 | Train 9 | Test 54 | 0.62 | 0.22 | 0.1 | 0.13 | 2614 | Train 41 | Test 54 | 0.66 | 0.33 | 0.08 | 0.12 |
| 567 | Train 9 | Test 55 | 0.64 | 0.16 | 0.21 | 0.19 | 2615 | Train 41 | Test 55 | 0.71 | 0.34 | 0.14 | 0.2 |
| 568 | Train 9 | Test 56 | 0.62 | 0.22 | 0.1 | 0.13 | 2616 | Train 41 | Test 56 | 0.66 | 0.32 | 0.08 | 0.12 |
| 569 | Train 9 | Test 57 | 0.76 | 0.45 | 0.19 | 0.27 | 2617 | Train 41 | Test 57 | 0.77 | 0.46 | 0.18 | 0.26 |
| 570 | Train 9 | Test 58 | 0.76 | 0.45 | 0.19 | 0.26 | 2618 | Train 41 | Test 58 | 0.77 | 0.45 | 0.18 | 0.26 |
| 571 | Train 9 | Test 59 | 0.76 | 0.45 | 0.19 | 0.27 | 2619 | Train 41 | Test 59 | 0.77 | 0.46 | 0.18 | 0.26 |
| 572 | Train 9 | Test 60 | 0.76 | 0.45 | 0.19 | 0.27 | 2620 | Train 41 | Test 60 | 0.77 | 0.46 | 0.18 | 0.26 |
| 573 | Train 9 | Test 61 | 0.61 | 0.14 | 0.17 | 0.15 | 2621 | Train 41 | Test 61 | 0.69 | 0.24 | 0.09 | 0.14 |
| 574 | Train 9 | Test 62 | 0.62 | 0.22 | 0.1 | 0.14 | 2622 | Train 41 | Test 62 | 0.66 | 0.33 | 0.08 | 0.12 |
| 575 | Train 9 | Test 63 | 0.61 | 0.14 | 0.17 | 0.15 | 2623 | Train 41 | Test 63 | 0.69 | 0.24 | 0.1 | 0.14 |
| 576 | Train 9 | Test 64 | 0.63 | 0.22 | 0.1 | 0.13 | 2624 | Train 41 | Test 64 | 0.66 | 0.32 | 0.08 | 0.12 |
| 577 | Train 10 | Test 1 | 0.75 | 0.36 | 0.12 | 0.18 | 2625 | Train 42 | Test 1 | 0.74 | 0.36 | 0.13 | 0.19 |
| 578 | Train 10 | Test 2 | 0.76 | 0.42 | 0.18 | 0.25 | 2626 | Train 42 | Test 2 | 0.76 | 0.42 | 0.18 | 0.26 |
| 579 | Train 10 | Test 3 | 0.75 | 0.36 | 0.13 | 0.19 | 2627 | Train 42 | Test 3 | 0.74 | 0.36 | 0.13 | 0.19 |
| 580 | Train 10 | Test 4 | 0.76 | 0.43 | 0.18 | 0.25 | 2628 | Train 42 | Test 4 | 0.76 | 0.42 | 0.18 | 0.26 |
| 581 | Train 10 | Test 5 | 0.69 | 0.29 | 0.13 | 0.18 | 2629 | Train 42 | Test 5 | 0.68 | 0.2 | 0.14 | 0.17 |
| 582 | Train 10 | Test 6 | 0.67 | 0.3 | 0.08 | 0.13 | 2630 | Train 42 | Test 6 | 0.66 | 0.27 | 0.08 | 0.12 |
| 583 | Train 10 | Test 7 | 0.69 | 0.29 | 0.13 | 0.18 | 2631 | Train 42 | Test 7 | 0.68 | 0.21 | 0.14 | 0.17 |
| 584 | Train 10 | Test 8 | 0.67 | 0.3 | 0.08 | 0.13 | 2632 | Train 42 | Test 8 | 0.66 | 0.27 | 0.08 | 0.12 |
| 585 | Train 10 | Test 9 | 0.75 | 0.36 | 0.11 | 0.16 | 2633 | Train 42 | Test 9 | 0.76 | 0.39 | 0.1 | 0.16 |
| 586 | Train 10 | Test 10 | 0.76 | 0.43 | 0.16 | 0.23 | 2634 | Train 42 | Test 10 | 0.77 | 0.45 | 0.16 | 0.23 |
| 587 | Train 10 | Test 11 | 0.75 | 0.36 | 0.1 | 0.16 | 2635 | Train 42 | Test 11 | 0.76 | 0.39 | 0.1 | 0.17 |
| 588 | Train 10 | Test 12 | 0.77 | 0.43 | 0.16 | 0.23 | 2636 | Train 42 | Test 12 | 0.77 | 0.45 | 0.16 | 0.23 |
| 589 | Train 10 | Test 13 | 0.7 | 0.28 | 0.11 | 0.16 | 2637 | Train 42 | Test 13 | 0.68 | 0.2 | 0.12 | 0.15 |
| 590 | Train 10 | Test 14 | 0.67 | 0.3 | 0.08 | 0.13 | 2638 | Train 42 | Test 14 | 0.66 | 0.26 | 0.08 | 0.12 |
| 591 | Train 10 | Test 15 | 0.69 | 0.28 | 0.11 | 0.16 | 2639 | Train 42 | Test 15 | 0.68 | 0.2 | 0.12 | 0.15 |
| 592 | Train 10 | Test 16 | 0.67 | 0.3 | 0.08 | 0.13 | 2640 | Train 42 | Test 16 | 0.66 | 0.26 | 0.08 | 0.12 |
| 593 | Train 10 | Test 17 | 0.75 | 0.36 | 0.12 | 0.18 | 2641 | Train 42 | Test 17 | 0.74 | 0.36 | 0.13 | 0.19 |
| 594 | Train 10 | Test 18 | 0.76 | 0.42 | 0.18 | 0.25 | 2642 | Train 42 | Test 18 | 0.76 | 0.43 | 0.18 | 0.26 |
| 595 | Train 10 | Test 19 | 0.75 | 0.36 | 0.13 | 0.19 | 2643 | Train 42 | Test 19 | 0.74 | 0.36 | 0.13 | 0.19 |
| 596 | Train 10 | Test 20 | 0.76 | 0.43 | 0.18 | 0.25 | 2644 | Train 42 | Test 20 | 0.76 | 0.43 | 0.18 | 0.26 |
| 597 | Train 10 | Test 21 | 0.69 | 0.29 | 0.13 | 0.18 | 2645 | Train 42 | Test 21 | 0.68 | 0.2 | 0.14 | 0.17 |
| 598 | Train 10 | Test 22 | 0.67 | 0.3 | 0.08 | 0.13 | 2646 | Train 42 | Test 22 | 0.66 | 0.27 | 0.08 | 0.12 |
| 599 | Train 10 | Test 23 | 0.69 | 0.29 | 0.13 | 0.18 | 2647 | Train 42 | Test 23 | 0.68 | 0.21 | 0.14 | 0.17 |
| 600 | Train 10 | Test 24 | 0.67 | 0.3 | 0.08 | 0.13 | 2648 | Train 42 | Test 24 | 0.66 | 0.27 | 0.08 | 0.12 |
| 601 | Train 10 | Test 25 | 0.75 | 0.36 | 0.11 | 0.16 | 2649 | Train 42 | Test 25 | 0.76 | 0.39 | 0.1 | 0.16 |
| 602 | Train 10 | Test 26 | 0.76 | 0.43 | 0.16 | 0.23 | 2650 | Train 42 | Test 26 | 0.77 | 0.45 | 0.16 | 0.23 |
| 603 | Train 10 | Test 27 | 0.75 | 0.36 | 0.1 | 0.16 | 2651 | Train 42 | Test 27 | 0.76 | 0.39 | 0.1 | 0.17 |
| 604 | Train 10 | Test 28 | 0.77 | 0.43 | 0.16 | 0.23 | 2652 | Train 42 | Test 28 | 0.77 | 0.45 | 0.16 | 0.23 |
| 605 | Train 10 | Test 29 | 0.7 | 0.28 | 0.11 | 0.16 | 2653 | Train 42 | Test 29 | 0.68 | 0.2 | 0.12 | 0.15 |
| 606 | Train 10 | Test 30 | 0.67 | 0.3 | 0.08 | 0.13 | 2654 | Train 42 | Test 30 | 0.66 | 0.26 | 0.08 | 0.12 |
| 607 | Train 10 | Test 31 | 0.69 | 0.28 | 0.11 | 0.16 | 2655 | Train 42 | Test 31 | 0.68 | 0.2 | 0.12 | 0.15 |
| 608 | Train 10 | Test 32 | 0.67 | 0.3 | 0.08 | 0.12 | 2656 | Train 42 | Test 32 | 0.66 | 0.26 | 0.08 | 0.12 |
| 609 | Train 10 | Test 33 | 0.75 | 0.36 | 0.12 | 0.18 | 2657 | Train 42 | Test 33 | 0.74 | 0.36 | 0.12 | 0.19 |
| 610 | Train 10 | Test 34 | 0.76 | 0.42 | 0.18 | 0.25 | 2658 | Train 42 | Test 34 | 0.76 | 0.42 | 0.18 | 0.25 |
| 611 | Train 10 | Test 35 | 0.75 | 0.36 | 0.12 | 0.19 | 2659 | Train 42 | Test 35 | 0.74 | 0.36 | 0.13 | 0.19 |
| 612 | Train 10 | Test 36 | 0.76 | 0.43 | 0.18 | 0.25 | 2660 | Train 42 | Test 36 | 0.76 | 0.42 | 0.18 | 0.25 |
| 613 | Train 10 | Test 37 | 0.69 | 0.29 | 0.13 | 0.18 | 2661 | Train 42 | Test 37 | 0.68 | 0.2 | 0.14 | 0.17 |
| 614 | Train 10 | Test 38 | 0.67 | 0.3 | 0.08 | 0.13 | 2662 | Train 42 | Test 38 | 0.66 | 0.27 | 0.08 | 0.12 |
| 615 | Train 10 | Test 39 | 0.69 | 0.29 | 0.13 | 0.18 | 2663 | Train 42 | Test 39 | 0.68 | 0.21 | 0.14 | 0.17 |
| 616 | Train 10 | Test 40 | 0.67 | 0.3 | 0.08 | 0.13 | 2664 | Train 42 | Test 40 | 0.66 | 0.27 | 0.08 | 0.12 |
| 617 | Train 10 | Test 41 | 0.75 | 0.36 | 0.1 | 0.16 | 2665 | Train 42 | Test 41 | 0.76 | 0.39 | 0.1 | 0.16 |
| 618 | Train 10 | Test 42 | 0.77 | 0.43 | 0.16 | 0.23 | 2666 | Train 42 | Test 42 | 0.77 | 0.45 | 0.15 | 0.23 |
| 619 | Train 10 | Test 43 | 0.75 | 0.36 | 0.1 | 0.16 | 2667 | Train 42 | Test 43 | 0.76 | 0.39 | 0.1 | 0.16 |
| 620 | Train 10 | Test 44 | 0.77 | 0.43 | 0.16 | 0.23 | 2668 | Train 42 | Test 44 | 0.77 | 0.45 | 0.16 | 0.23 |
| 621 | Train 10 | Test 45 | 0.7 | 0.28 | 0.11 | 0.16 | 2669 | Train 42 | Test 45 | 0.69 | 0.2 | 0.12 | 0.15 |
| 622 | Train 10 | Test 46 | 0.67 | 0.3 | 0.08 | 0.12 | 2670 | Train 42 | Test 46 | 0.66 | 0.26 | 0.08 | 0.12 |
| 623 | Train 10 | Test 47 | 0.7 | 0.28 | 0.11 | 0.16 | 2671 | Train 42 | Test 47 | 0.68 | 0.2 | 0.12 | 0.15 |
| 624 | Train 10 | Test 48 | 0.67 | 0.29 | 0.08 | 0.12 | 2672 | Train 42 | Test 48 | 0.66 | 0.26 | 0.08 | 0.12 |
| 625 | Train 10 | Test 49 | 0.76 | 0.46 | 0.19 | 0.27 | 2673 | Train 42 | Test 49 | 0.75 | 0.43 | 0.19 | 0.26 |
| 626 | Train 10 | Test 50 | 0.75 | 0.46 | 0.19 | 0.26 | 2674 | Train 42 | Test 50 | 0.75 | 0.43 | 0.19 | 0.26 |
| 627 | Train 10 | Test 51 | 0.76 | 0.46 | 0.19 | 0.27 | 2675 | Train 42 | Test 51 | 0.75 | 0.44 | 0.19 | 0.26 |
| 628 | Train 10 | Test 52 | 0.76 | 0.46 | 0.19 | 0.27 | 2676 | Train 42 | Test 52 | 0.75 | 0.43 | 0.19 | 0.26 |
| 629 | Train 10 | Test 53 | 0.66 | 0.22 | 0.11 | 0.15 | 2677 | Train 42 | Test 53 | 0.64 | 0.14 | 0.11 | 0.12 |
| 630 | Train 10 | Test 54 | 0.67 | 0.31 | 0.08 | 0.13 | 2678 | Train 42 | Test 54 | 0.66 | 0.24 | 0.07 | 0.11 |
| 631 | Train 10 | Test 55 | 0.71 | 0.35 | 0.14 | 0.2 | 2679 | Train 42 | Test 55 | 0.68 | 0.19 | 0.16 | 0.17 |
| 632 | Train 10 | Test 56 | 0.67 | 0.31 | 0.08 | 0.13 | 2680 | Train 42 | Test 56 | 0.66 | 0.24 | 0.07 | 0.11 |
| 633 | Train 10 | Test 57 | 0.76 | 0.45 | 0.16 | 0.24 | 2681 | Train 42 | Test 57 | 0.77 | 0.46 | 0.17 | 0.25 |
| 634 | Train 10 | Test 58 | 0.76 | 0.45 | 0.16 | 0.24 | 2682 | Train 42 | Test 58 | 0.77 | 0.45 | 0.17 | 0.25 |
| 635 | Train 10 | Test 59 | 0.77 | 0.45 | 0.16 | 0.24 | 2683 | Train 42 | Test 59 | 0.77 | 0.46 | 0.18 | 0.26 |
| 636 | Train 10 | Test 60 | 0.77 | 0.45 | 0.16 | 0.24 | 2684 | Train 42 | Test 60 | 0.77 | 0.46 | 0.18 | 0.25 |
| 637 | Train 10 | Test 61 | 0.67 | 0.23 | 0.11 | 0.15 | 2685 | Train 42 | Test 61 | 0.65 | 0.14 | 0.11 | 0.12 |
| 638 | Train 10 | Test 62 | 0.67 | 0.31 | 0.08 | 0.13 | 2686 | Train 42 | Test 62 | 0.66 | 0.23 | 0.07 | 0.11 |
| 639 | Train 10 | Test 63 | 0.67 | 0.23 | 0.11 | 0.15 | 2687 | Train 42 | Test 63 | 0.65 | 0.14 | 0.11 | 0.12 |
| 640 | Train 10 | Test 64 | 0.67 | 0.31 | 0.08 | 0.13 | 2688 | Train 42 | Test 64 | 0.66 | 0.23 | 0.07 | 0.11 |
| 641 | Train 11 | Test 1 | 0.74 | 0.35 | 0.13 | 0.19 | 2689 | Train 43 | Test 1 | 0.74 | 0.36 | 0.13 | 0.19 |
| 642 | Train 11 | Test 2 | 0.72 | 0.36 | 0.11 | 0.17 | 2690 | Train 43 | Test 2 | 0.72 | 0.36 | 0.12 | 0.18 |
| 643 | Train 11 | Test 3 | 0.74 | 0.35 | 0.13 | 0.19 | 2691 | Train 43 | Test 3 | 0.74 | 0.36 | 0.13 | 0.19 |
| 644 | Train 11 | Test 4 | 0.72 | 0.36 | 0.11 | 0.17 | 2692 | Train 43 | Test 4 | 0.72 | 0.36 | 0.12 | 0.18 |
| 645 | Train 11 | Test 5 | 0.68 | 0.2 | 0.13 | 0.16 | 2693 | Train 43 | Test 5 | 0.68 | 0.27 | 0.11 | 0.15 |
| 646 | Train 11 | Test 6 | 0.64 | 0.24 | 0.07 | 0.11 | 2694 | Train 43 | Test 6 | 0.64 | 0.26 | 0.1 | 0.15 |
| 647 | Train 11 | Test 7 | 0.68 | 0.21 | 0.13 | 0.16 | 2695 | Train 43 | Test 7 | 0.68 | 0.27 | 0.11 | 0.15 |
| 648 | Train 11 | Test 8 | 0.64 | 0.24 | 0.07 | 0.11 | 2696 | Train 43 | Test 8 | 0.64 | 0.25 | 0.1 | 0.14 |
| 649 | Train 11 | Test 9 | 0.75 | 0.37 | 0.12 | 0.18 | 2697 | Train 43 | Test 9 | 0.75 | 0.38 | 0.11 | 0.17 |
| 650 | Train 11 | Test 10 | 0.73 | 0.38 | 0.11 | 0.16 | 2698 | Train 43 | Test 10 | 0.72 | 0.37 | 0.11 | 0.16 |
| 651 | Train 11 | Test 11 | 0.75 | 0.37 | 0.12 | 0.18 | 2699 | Train 43 | Test 11 | 0.75 | 0.38 | 0.11 | 0.17 |
| 652 | Train 11 | Test 12 | 0.73 | 0.38 | 0.11 | 0.17 | 2700 | Train 43 | Test 12 | 0.72 | 0.38 | 0.11 | 0.17 |
| 653 | Train 11 | Test 13 | 0.68 | 0.2 | 0.13 | 0.16 | 2701 | Train 43 | Test 13 | 0.68 | 0.28 | 0.1 | 0.14 |
| 654 | Train 11 | Test 14 | 0.64 | 0.23 | 0.07 | 0.1 | 2702 | Train 43 | Test 14 | 0.64 | 0.25 | 0.1 | 0.15 |
| 655 | Train 11 | Test 15 | 0.68 | 0.21 | 0.13 | 0.16 | 2703 | Train 43 | Test 15 | 0.68 | 0.27 | 0.1 | 0.14 |
| 656 | Train 11 | Test 16 | 0.64 | 0.24 | 0.07 | 0.11 | 2704 | Train 43 | Test 16 | 0.64 | 0.25 | 0.1 | 0.14 |
| 657 | Train 11 | Test 17 | 0.74 | 0.35 | 0.13 | 0.19 | 2705 | Train 43 | Test 17 | 0.74 | 0.36 | 0.13 | 0.19 |
| 658 | Train 11 | Test 18 | 0.72 | 0.36 | 0.11 | 0.17 | 2706 | Train 43 | Test 18 | 0.72 | 0.36 | 0.12 | 0.18 |
| 659 | Train 11 | Test 19 | 0.74 | 0.35 | 0.13 | 0.19 | 2707 | Train 43 | Test 19 | 0.74 | 0.36 | 0.13 | 0.19 |
| 660 | Train 11 | Test 20 | 0.72 | 0.36 | 0.11 | 0.17 | 2708 | Train 43 | Test 20 | 0.72 | 0.36 | 0.12 | 0.18 |
| 661 | Train 11 | Test 21 | 0.68 | 0.2 | 0.13 | 0.16 | 2709 | Train 43 | Test 21 | 0.68 | 0.27 | 0.11 | 0.16 |
| 662 | Train 11 | Test 22 | 0.64 | 0.24 | 0.07 | 0.11 | 2710 | Train 43 | Test 22 | 0.64 | 0.25 | 0.1 | 0.15 |
| 663 | Train 11 | Test 23 | 0.68 | 0.2 | 0.13 | 0.16 | 2711 | Train 43 | Test 23 | 0.68 | 0.27 | 0.11 | 0.15 |
| 664 | Train 11 | Test 24 | 0.64 | 0.24 | 0.07 | 0.11 | 2712 | Train 43 | Test 24 | 0.65 | 0.25 | 0.1 | 0.14 |
| 665 | Train 11 | Test 25 | 0.75 | 0.37 | 0.12 | 0.18 | 2713 | Train 43 | Test 25 | 0.75 | 0.38 | 0.11 | 0.17 |
| 666 | Train 11 | Test 26 | 0.73 | 0.38 | 0.11 | 0.16 | 2714 | Train 43 | Test 26 | 0.72 | 0.37 | 0.11 | 0.16 |
| 667 | Train 11 | Test 27 | 0.75 | 0.37 | 0.12 | 0.18 | 2715 | Train 43 | Test 27 | 0.75 | 0.38 | 0.11 | 0.17 |
| 668 | Train 11 | Test 28 | 0.73 | 0.38 | 0.11 | 0.17 | 2716 | Train 43 | Test 28 | 0.72 | 0.38 | 0.11 | 0.17 |
| 669 | Train 11 | Test 29 | 0.68 | 0.2 | 0.13 | 0.16 | 2717 | Train 43 | Test 29 | 0.68 | 0.28 | 0.1 | 0.14 |
| 670 | Train 11 | Test 30 | 0.64 | 0.23 | 0.07 | 0.1 | 2718 | Train 43 | Test 30 | 0.64 | 0.25 | 0.1 | 0.15 |
| 671 | Train 11 | Test 31 | 0.68 | 0.21 | 0.13 | 0.16 | 2719 | Train 43 | Test 31 | 0.68 | 0.27 | 0.1 | 0.14 |
| 672 | Train 11 | Test 32 | 0.64 | 0.24 | 0.07 | 0.1 | 2720 | Train 43 | Test 32 | 0.65 | 0.25 | 0.1 | 0.14 |
| 673 | Train 11 | Test 33 | 0.74 | 0.35 | 0.13 | 0.19 | 2721 | Train 43 | Test 33 | 0.74 | 0.35 | 0.13 | 0.19 |
| 674 | Train 11 | Test 34 | 0.72 | 0.35 | 0.11 | 0.17 | 2722 | Train 43 | Test 34 | 0.72 | 0.35 | 0.12 | 0.18 |
| 675 | Train 11 | Test 35 | 0.74 | 0.35 | 0.13 | 0.19 | 2723 | Train 43 | Test 35 | 0.74 | 0.36 | 0.13 | 0.19 |
| 676 | Train 11 | Test 36 | 0.72 | 0.36 | 0.11 | 0.17 | 2724 | Train 43 | Test 36 | 0.72 | 0.36 | 0.12 | 0.18 |
| 677 | Train 11 | Test 37 | 0.68 | 0.2 | 0.13 | 0.16 | 2725 | Train 43 | Test 37 | 0.68 | 0.27 | 0.11 | 0.15 |
| 678 | Train 11 | Test 38 | 0.64 | 0.24 | 0.07 | 0.11 | 2726 | Train 43 | Test 38 | 0.64 | 0.25 | 0.1 | 0.15 |
| 679 | Train 11 | Test 39 | 0.68 | 0.21 | 0.13 | 0.16 | 2727 | Train 43 | Test 39 | 0.68 | 0.27 | 0.11 | 0.15 |
| 680 | Train 11 | Test 40 | 0.64 | 0.24 | 0.07 | 0.11 | 2728 | Train 43 | Test 40 | 0.65 | 0.25 | 0.1 | 0.14 |
| 681 | Train 11 | Test 41 | 0.75 | 0.37 | 0.12 | 0.18 | 2729 | Train 43 | Test 41 | 0.75 | 0.37 | 0.11 | 0.17 |
| 682 | Train 11 | Test 42 | 0.73 | 0.38 | 0.1 | 0.16 | 2730 | Train 43 | Test 42 | 0.73 | 0.37 | 0.1 | 0.16 |
| 683 | Train 11 | Test 43 | 0.75 | 0.37 | 0.12 | 0.18 | 2731 | Train 43 | Test 43 | 0.75 | 0.38 | 0.11 | 0.17 |
| 684 | Train 11 | Test 44 | 0.73 | 0.38 | 0.11 | 0.17 | 2732 | Train 43 | Test 44 | 0.73 | 0.37 | 0.11 | 0.16 |
| 685 | Train 11 | Test 45 | 0.68 | 0.2 | 0.13 | 0.16 | 2733 | Train 43 | Test 45 | 0.68 | 0.28 | 0.1 | 0.14 |
| 686 | Train 11 | Test 46 | 0.64 | 0.23 | 0.07 | 0.1 | 2734 | Train 43 | Test 46 | 0.64 | 0.25 | 0.1 | 0.15 |
| 687 | Train 11 | Test 47 | 0.68 | 0.21 | 0.13 | 0.16 | 2735 | Train 43 | Test 47 | 0.68 | 0.27 | 0.1 | 0.14 |
| 688 | Train 11 | Test 48 | 0.64 | 0.24 | 0.07 | 0.11 | 2736 | Train 43 | Test 48 | 0.64 | 0.25 | 0.1 | 0.14 |
| 689 | Train 11 | Test 49 | 0.75 | 0.41 | 0.19 | 0.26 | 2737 | Train 43 | Test 49 | 0.75 | 0.4 | 0.19 | 0.26 |
| 690 | Train 11 | Test 50 | 0.75 | 0.41 | 0.19 | 0.26 | 2738 | Train 43 | Test 50 | 0.75 | 0.4 | 0.19 | 0.25 |
| 691 | Train 11 | Test 51 | 0.75 | 0.42 | 0.19 | 0.26 | 2739 | Train 43 | Test 51 | 0.75 | 0.4 | 0.19 | 0.26 |
| 692 | Train 11 | Test 52 | 0.75 | 0.41 | 0.19 | 0.26 | 2740 | Train 43 | Test 52 | 0.75 | 0.4 | 0.19 | 0.26 |
| 693 | Train 11 | Test 53 | 0.65 | 0.15 | 0.12 | 0.14 | 2741 | Train 43 | Test 53 | 0.69 | 0.24 | 0.1 | 0.14 |
| 694 | Train 11 | Test 54 | 0.65 | 0.26 | 0.08 | 0.12 | 2742 | Train 43 | Test 54 | 0.66 | 0.32 | 0.09 | 0.15 |
| 695 | Train 11 | Test 55 | 0.68 | 0.19 | 0.16 | 0.18 | 2743 | Train 43 | Test 55 | 0.71 | 0.29 | 0.14 | 0.19 |
| 696 | Train 11 | Test 56 | 0.65 | 0.26 | 0.08 | 0.12 | 2744 | Train 43 | Test 56 | 0.66 | 0.31 | 0.09 | 0.14 |
| 697 | Train 11 | Test 57 | 0.77 | 0.45 | 0.19 | 0.26 | 2745 | Train 43 | Test 57 | 0.77 | 0.45 | 0.17 | 0.25 |
| 698 | Train 11 | Test 58 | 0.77 | 0.44 | 0.19 | 0.26 | 2746 | Train 43 | Test 58 | 0.77 | 0.45 | 0.17 | 0.25 |
| 699 | Train 11 | Test 59 | 0.77 | 0.44 | 0.19 | 0.26 | 2747 | Train 43 | Test 59 | 0.77 | 0.46 | 0.17 | 0.25 |
| 700 | Train 11 | Test 60 | 0.77 | 0.44 | 0.19 | 0.26 | 2748 | Train 43 | Test 60 | 0.77 | 0.45 | 0.17 | 0.25 |
| 701 | Train 11 | Test 61 | 0.65 | 0.15 | 0.12 | 0.13 | 2749 | Train 43 | Test 61 | 0.69 | 0.23 | 0.1 | 0.14 |
| 702 | Train 11 | Test 62 | 0.65 | 0.26 | 0.08 | 0.12 | 2750 | Train 43 | Test 62 | 0.66 | 0.32 | 0.09 | 0.15 |
| 703 | Train 11 | Test 63 | 0.66 | 0.16 | 0.12 | 0.14 | 2751 | Train 43 | Test 63 | 0.69 | 0.23 | 0.1 | 0.14 |
| 704 | Train 11 | Test 64 | 0.65 | 0.26 | 0.08 | 0.12 | 2752 | Train 43 | Test 64 | 0.66 | 0.31 | 0.09 | 0.14 |
| 705 | Train 12 | Test 1 | 0.75 | 0.36 | 0.13 | 0.19 | 2753 | Train 44 | Test 1 | 0.75 | 0.35 | 0.12 | 0.17 |
| 706 | Train 12 | Test 2 | 0.77 | 0.43 | 0.18 | 0.26 | 2754 | Train 44 | Test 2 | 0.76 | 0.42 | 0.18 | 0.25 |
| 707 | Train 12 | Test 3 | 0.75 | 0.36 | 0.13 | 0.19 | 2755 | Train 44 | Test 3 | 0.75 | 0.36 | 0.12 | 0.18 |
| 708 | Train 12 | Test 4 | 0.77 | 0.43 | 0.18 | 0.26 | 2756 | Train 44 | Test 4 | 0.76 | 0.42 | 0.18 | 0.25 |
| 709 | Train 12 | Test 5 | 0.68 | 0.3 | 0.11 | 0.16 | 2757 | Train 44 | Test 5 | 0.7 | 0.27 | 0.12 | 0.17 |
| 710 | Train 12 | Test 6 | 0.67 | 0.31 | 0.07 | 0.12 | 2758 | Train 44 | Test 6 | 0.67 | 0.3 | 0.08 | 0.13 |
| 711 | Train 12 | Test 7 | 0.68 | 0.3 | 0.11 | 0.16 | 2759 | Train 44 | Test 7 | 0.7 | 0.27 | 0.12 | 0.17 |
| 712 | Train 12 | Test 8 | 0.67 | 0.31 | 0.07 | 0.12 | 2760 | Train 44 | Test 8 | 0.67 | 0.3 | 0.08 | 0.13 |
| 713 | Train 12 | Test 9 | 0.76 | 0.37 | 0.11 | 0.17 | 2761 | Train 44 | Test 9 | 0.76 | 0.4 | 0.12 | 0.18 |
| 714 | Train 12 | Test 10 | 0.77 | 0.44 | 0.16 | 0.24 | 2762 | Train 44 | Test 10 | 0.77 | 0.46 | 0.17 | 0.25 |
| 715 | Train 12 | Test 11 | 0.76 | 0.37 | 0.11 | 0.17 | 2763 | Train 44 | Test 11 | 0.76 | 0.4 | 0.12 | 0.18 |
| 716 | Train 12 | Test 12 | 0.77 | 0.44 | 0.16 | 0.24 | 2764 | Train 44 | Test 12 | 0.77 | 0.46 | 0.17 | 0.25 |
| 717 | Train 12 | Test 13 | 0.68 | 0.3 | 0.09 | 0.14 | 2765 | Train 44 | Test 13 | 0.7 | 0.3 | 0.12 | 0.17 |
| 718 | Train 12 | Test 14 | 0.67 | 0.31 | 0.07 | 0.12 | 2766 | Train 44 | Test 14 | 0.67 | 0.3 | 0.08 | 0.13 |
| 719 | Train 12 | Test 15 | 0.68 | 0.3 | 0.09 | 0.14 | 2767 | Train 44 | Test 15 | 0.7 | 0.3 | 0.12 | 0.17 |
| 720 | Train 12 | Test 16 | 0.67 | 0.3 | 0.07 | 0.12 | 2768 | Train 44 | Test 16 | 0.67 | 0.29 | 0.08 | 0.13 |
| 721 | Train 12 | Test 17 | 0.75 | 0.36 | 0.13 | 0.19 | 2769 | Train 44 | Test 17 | 0.75 | 0.36 | 0.12 | 0.18 |
| 722 | Train 12 | Test 18 | 0.77 | 0.43 | 0.18 | 0.26 | 2770 | Train 44 | Test 18 | 0.76 | 0.42 | 0.18 | 0.25 |
| 723 | Train 12 | Test 19 | 0.75 | 0.36 | 0.13 | 0.19 | 2771 | Train 44 | Test 19 | 0.75 | 0.36 | 0.12 | 0.18 |
| 724 | Train 12 | Test 20 | 0.77 | 0.43 | 0.18 | 0.26 | 2772 | Train 44 | Test 20 | 0.76 | 0.42 | 0.18 | 0.25 |
| 725 | Train 12 | Test 21 | 0.68 | 0.3 | 0.11 | 0.16 | 2773 | Train 44 | Test 21 | 0.7 | 0.27 | 0.12 | 0.17 |
| 726 | Train 12 | Test 22 | 0.67 | 0.31 | 0.07 | 0.12 | 2774 | Train 44 | Test 22 | 0.67 | 0.3 | 0.08 | 0.13 |
| 727 | Train 12 | Test 23 | 0.68 | 0.3 | 0.11 | 0.16 | 2775 | Train 44 | Test 23 | 0.7 | 0.27 | 0.12 | 0.17 |
| 728 | Train 12 | Test 24 | 0.67 | 0.31 | 0.07 | 0.12 | 2776 | Train 44 | Test 24 | 0.67 | 0.3 | 0.08 | 0.13 |
| 729 | Train 12 | Test 25 | 0.76 | 0.37 | 0.11 | 0.17 | 2777 | Train 44 | Test 25 | 0.76 | 0.4 | 0.12 | 0.18 |
| 730 | Train 12 | Test 26 | 0.77 | 0.44 | 0.16 | 0.24 | 2778 | Train 44 | Test 26 | 0.77 | 0.46 | 0.17 | 0.25 |
| 731 | Train 12 | Test 27 | 0.76 | 0.37 | 0.11 | 0.17 | 2779 | Train 44 | Test 27 | 0.76 | 0.4 | 0.12 | 0.18 |
| 732 | Train 12 | Test 28 | 0.77 | 0.44 | 0.16 | 0.24 | 2780 | Train 44 | Test 28 | 0.77 | 0.46 | 0.17 | 0.25 |
| 733 | Train 12 | Test 29 | 0.68 | 0.29 | 0.09 | 0.14 | 2781 | Train 44 | Test 29 | 0.7 | 0.3 | 0.12 | 0.17 |
| 734 | Train 12 | Test 30 | 0.67 | 0.3 | 0.07 | 0.12 | 2782 | Train 44 | Test 30 | 0.67 | 0.29 | 0.08 | 0.13 |
| 735 | Train 12 | Test 31 | 0.68 | 0.29 | 0.09 | 0.14 | 2783 | Train 44 | Test 31 | 0.7 | 0.3 | 0.12 | 0.17 |
| 736 | Train 12 | Test 32 | 0.67 | 0.3 | 0.07 | 0.12 | 2784 | Train 44 | Test 32 | 0.67 | 0.29 | 0.08 | 0.13 |
| 737 | Train 12 | Test 33 | 0.75 | 0.36 | 0.12 | 0.19 | 2785 | Train 44 | Test 33 | 0.75 | 0.35 | 0.12 | 0.17 |
| 738 | Train 12 | Test 34 | 0.77 | 0.43 | 0.18 | 0.25 | 2786 | Train 44 | Test 34 | 0.77 | 0.42 | 0.18 | 0.25 |
| 739 | Train 12 | Test 35 | 0.75 | 0.36 | 0.12 | 0.19 | 2787 | Train 44 | Test 35 | 0.75 | 0.35 | 0.12 | 0.18 |
| 740 | Train 12 | Test 36 | 0.77 | 0.43 | 0.18 | 0.25 | 2788 | Train 44 | Test 36 | 0.77 | 0.42 | 0.18 | 0.25 |
| 741 | Train 12 | Test 37 | 0.68 | 0.3 | 0.11 | 0.16 | 2789 | Train 44 | Test 37 | 0.7 | 0.27 | 0.12 | 0.17 |
| 742 | Train 12 | Test 38 | 0.67 | 0.31 | 0.07 | 0.12 | 2790 | Train 44 | Test 38 | 0.67 | 0.3 | 0.08 | 0.13 |
| 743 | Train 12 | Test 39 | 0.68 | 0.3 | 0.11 | 0.16 | 2791 | Train 44 | Test 39 | 0.7 | 0.27 | 0.12 | 0.17 |
| 744 | Train 12 | Test 40 | 0.67 | 0.31 | 0.07 | 0.12 | 2792 | Train 44 | Test 40 | 0.67 | 0.3 | 0.08 | 0.13 |
| 745 | Train 12 | Test 41 | 0.76 | 0.37 | 0.11 | 0.16 | 2793 | Train 44 | Test 41 | 0.76 | 0.4 | 0.11 | 0.18 |
| 746 | Train 12 | Test 42 | 0.77 | 0.44 | 0.16 | 0.24 | 2794 | Train 44 | Test 42 | 0.77 | 0.46 | 0.17 | 0.24 |
| 747 | Train 12 | Test 43 | 0.76 | 0.37 | 0.11 | 0.16 | 2795 | Train 44 | Test 43 | 0.76 | 0.4 | 0.12 | 0.18 |
| 748 | Train 12 | Test 44 | 0.77 | 0.44 | 0.16 | 0.24 | 2796 | Train 44 | Test 44 | 0.77 | 0.45 | 0.17 | 0.25 |
| 749 | Train 12 | Test 45 | 0.68 | 0.29 | 0.09 | 0.14 | 2797 | Train 44 | Test 45 | 0.7 | 0.3 | 0.12 | 0.17 |
| 750 | Train 12 | Test 46 | 0.67 | 0.31 | 0.07 | 0.12 | 2798 | Train 44 | Test 46 | 0.67 | 0.29 | 0.08 | 0.13 |
| 751 | Train 12 | Test 47 | 0.68 | 0.3 | 0.09 | 0.14 | 2799 | Train 44 | Test 47 | 0.7 | 0.3 | 0.12 | 0.17 |
| 752 | Train 12 | Test 48 | 0.67 | 0.3 | 0.07 | 0.12 | 2800 | Train 44 | Test 48 | 0.67 | 0.29 | 0.08 | 0.13 |
| 753 | Train 12 | Test 49 | 0.76 | 0.44 | 0.18 | 0.26 | 2801 | Train 44 | Test 49 | 0.76 | 0.43 | 0.17 | 0.25 |
| 754 | Train 12 | Test 50 | 0.76 | 0.44 | 0.18 | 0.26 | 2802 | Train 44 | Test 50 | 0.76 | 0.43 | 0.17 | 0.25 |
| 755 | Train 12 | Test 51 | 0.76 | 0.45 | 0.18 | 0.26 | 2803 | Train 44 | Test 51 | 0.76 | 0.43 | 0.18 | 0.25 |
| 756 | Train 12 | Test 52 | 0.76 | 0.44 | 0.18 | 0.26 | 2804 | Train 44 | Test 52 | 0.76 | 0.43 | 0.17 | 0.25 |
| 757 | Train 12 | Test 53 | 0.67 | 0.3 | 0.1 | 0.15 | 2805 | Train 44 | Test 53 | 0.68 | 0.26 | 0.12 | 0.17 |
| 758 | Train 12 | Test 54 | 0.68 | 0.4 | 0.07 | 0.12 | 2806 | Train 44 | Test 54 | 0.67 | 0.34 | 0.08 | 0.13 |
| 759 | Train 12 | Test 55 | 0.71 | 0.35 | 0.14 | 0.2 | 2807 | Train 44 | Test 55 | 0.73 | 0.32 | 0.16 | 0.22 |
| 760 | Train 12 | Test 56 | 0.68 | 0.39 | 0.07 | 0.11 | 2808 | Train 44 | Test 56 | 0.67 | 0.33 | 0.08 | 0.13 |
| 761 | Train 12 | Test 57 | 0.77 | 0.43 | 0.17 | 0.24 | 2809 | Train 44 | Test 57 | 0.77 | 0.48 | 0.18 | 0.27 |
| 762 | Train 12 | Test 58 | 0.77 | 0.43 | 0.17 | 0.24 | 2810 | Train 44 | Test 58 | 0.77 | 0.48 | 0.18 | 0.26 |
| 763 | Train 12 | Test 59 | 0.77 | 0.43 | 0.17 | 0.24 | 2811 | Train 44 | Test 59 | 0.77 | 0.47 | 0.19 | 0.27 |
| 764 | Train 12 | Test 60 | 0.77 | 0.42 | 0.17 | 0.24 | 2812 | Train 44 | Test 60 | 0.77 | 0.47 | 0.19 | 0.27 |
| 765 | Train 12 | Test 61 | 0.68 | 0.31 | 0.1 | 0.15 | 2813 | Train 44 | Test 61 | 0.68 | 0.28 | 0.12 | 0.17 |
| 766 | Train 12 | Test 62 | 0.68 | 0.4 | 0.07 | 0.12 | 2814 | Train 44 | Test 62 | 0.67 | 0.33 | 0.08 | 0.13 |
| 767 | Train 12 | Test 63 | 0.68 | 0.32 | 0.1 | 0.15 | 2815 | Train 44 | Test 63 | 0.69 | 0.28 | 0.13 | 0.17 |
| 768 | Train 12 | Test 64 | 0.68 | 0.38 | 0.07 | 0.11 | 2816 | Train 44 | Test 64 | 0.67 | 0.33 | 0.08 | 0.13 |
| 769 | Train 13 | Test 1 | 0.74 | 0.4 | 0.1 | 0.16 | 2817 | Train 45 | Test 1 | 0.75 | 0.38 | 0.1 | 0.16 |
| 770 | Train 13 | Test 2 | 0.74 | 0.42 | 0.11 | 0.17 | 2818 | Train 45 | Test 2 | 0.74 | 0.38 | 0.09 | 0.15 |
| 771 | Train 13 | Test 3 | 0.75 | 0.4 | 0.1 | 0.17 | 2819 | Train 45 | Test 3 | 0.75 | 0.39 | 0.1 | 0.16 |
| 772 | Train 13 | Test 4 | 0.74 | 0.43 | 0.11 | 0.17 | 2820 | Train 45 | Test 4 | 0.74 | 0.39 | 0.09 | 0.15 |
| 773 | Train 13 | Test 5 | 0.74 | 0.38 | 0.1 | 0.16 | 2821 | Train 45 | Test 5 | 0.74 | 0.38 | 0.1 | 0.16 |
| 774 | Train 13 | Test 6 | 0.67 | 0.3 | 0.27 | 0.28 | 2822 | Train 45 | Test 6 | 0.66 | 0.32 | 0.17 | 0.22 |
| 775 | Train 13 | Test 7 | 0.74 | 0.39 | 0.1 | 0.16 | 2823 | Train 45 | Test 7 | 0.74 | 0.38 | 0.1 | 0.16 |
| 776 | Train 13 | Test 8 | 0.67 | 0.29 | 0.26 | 0.28 | 2824 | Train 45 | Test 8 | 0.67 | 0.31 | 0.17 | 0.22 |
| 777 | Train 13 | Test 9 | 0.75 | 0.42 | 0.09 | 0.15 | 2825 | Train 45 | Test 9 | 0.76 | 0.38 | 0.09 | 0.14 |
| 778 | Train 13 | Test 10 | 0.74 | 0.46 | 0.1 | 0.16 | 2826 | Train 45 | Test 10 | 0.74 | 0.38 | 0.08 | 0.13 |
| 779 | Train 13 | Test 11 | 0.75 | 0.43 | 0.09 | 0.15 | 2827 | Train 45 | Test 11 | 0.76 | 0.38 | 0.09 | 0.14 |
| 780 | Train 13 | Test 12 | 0.74 | 0.46 | 0.1 | 0.17 | 2828 | Train 45 | Test 12 | 0.74 | 0.38 | 0.08 | 0.13 |
| 781 | Train 13 | Test 13 | 0.74 | 0.41 | 0.1 | 0.15 | 2829 | Train 45 | Test 13 | 0.75 | 0.38 | 0.09 | 0.14 |
| 782 | Train 13 | Test 14 | 0.67 | 0.3 | 0.27 | 0.28 | 2830 | Train 45 | Test 14 | 0.67 | 0.31 | 0.17 | 0.22 |
| 783 | Train 13 | Test 15 | 0.74 | 0.41 | 0.09 | 0.15 | 2831 | Train 45 | Test 15 | 0.74 | 0.38 | 0.09 | 0.14 |
| 784 | Train 13 | Test 16 | 0.67 | 0.29 | 0.26 | 0.28 | 2832 | Train 45 | Test 16 | 0.67 | 0.31 | 0.17 | 0.22 |
| 785 | Train 13 | Test 17 | 0.74 | 0.4 | 0.1 | 0.17 | 2833 | Train 45 | Test 17 | 0.75 | 0.38 | 0.1 | 0.16 |
| 786 | Train 13 | Test 18 | 0.74 | 0.42 | 0.11 | 0.17 | 2834 | Train 45 | Test 18 | 0.74 | 0.38 | 0.09 | 0.15 |
| 787 | Train 13 | Test 19 | 0.75 | 0.4 | 0.11 | 0.17 | 2835 | Train 45 | Test 19 | 0.75 | 0.39 | 0.1 | 0.16 |
| 788 | Train 13 | Test 20 | 0.74 | 0.43 | 0.11 | 0.17 | 2836 | Train 45 | Test 20 | 0.74 | 0.39 | 0.09 | 0.15 |
| 789 | Train 13 | Test 21 | 0.74 | 0.38 | 0.1 | 0.16 | 2837 | Train 45 | Test 21 | 0.74 | 0.38 | 0.1 | 0.16 |
| 790 | Train 13 | Test 22 | 0.67 | 0.3 | 0.27 | 0.28 | 2838 | Train 45 | Test 22 | 0.67 | 0.32 | 0.17 | 0.22 |
| 791 | Train 13 | Test 23 | 0.74 | 0.39 | 0.1 | 0.16 | 2839 | Train 45 | Test 23 | 0.74 | 0.38 | 0.1 | 0.16 |
| 792 | Train 13 | Test 24 | 0.67 | 0.29 | 0.26 | 0.28 | 2840 | Train 45 | Test 24 | 0.67 | 0.31 | 0.17 | 0.22 |
| 793 | Train 13 | Test 25 | 0.75 | 0.42 | 0.09 | 0.15 | 2841 | Train 45 | Test 25 | 0.76 | 0.38 | 0.09 | 0.14 |
| 794 | Train 13 | Test 26 | 0.74 | 0.46 | 0.1 | 0.16 | 2842 | Train 45 | Test 26 | 0.74 | 0.38 | 0.08 | 0.13 |
| 795 | Train 13 | Test 27 | 0.75 | 0.42 | 0.09 | 0.15 | 2843 | Train 45 | Test 27 | 0.76 | 0.38 | 0.09 | 0.14 |
| 796 | Train 13 | Test 28 | 0.74 | 0.46 | 0.1 | 0.17 | 2844 | Train 45 | Test 28 | 0.74 | 0.38 | 0.08 | 0.13 |
| 797 | Train 13 | Test 29 | 0.74 | 0.41 | 0.1 | 0.15 | 2845 | Train 45 | Test 29 | 0.75 | 0.38 | 0.09 | 0.14 |
| 798 | Train 13 | Test 30 | 0.67 | 0.29 | 0.27 | 0.28 | 2846 | Train 45 | Test 30 | 0.67 | 0.31 | 0.17 | 0.22 |
| 799 | Train 13 | Test 31 | 0.74 | 0.41 | 0.09 | 0.15 | 2847 | Train 45 | Test 31 | 0.74 | 0.38 | 0.09 | 0.14 |
| 800 | Train 13 | Test 32 | 0.67 | 0.29 | 0.26 | 0.28 | 2848 | Train 45 | Test 32 | 0.67 | 0.31 | 0.17 | 0.22 |
| 801 | Train 13 | Test 33 | 0.75 | 0.39 | 0.1 | 0.16 | 2849 | Train 45 | Test 33 | 0.75 | 0.38 | 0.1 | 0.16 |
| 802 | Train 13 | Test 34 | 0.74 | 0.42 | 0.11 | 0.17 | 2850 | Train 45 | Test 34 | 0.74 | 0.38 | 0.09 | 0.15 |
| 803 | Train 13 | Test 35 | 0.75 | 0.4 | 0.1 | 0.16 | 2851 | Train 45 | Test 35 | 0.75 | 0.39 | 0.1 | 0.16 |
| 804 | Train 13 | Test 36 | 0.74 | 0.43 | 0.11 | 0.17 | 2852 | Train 45 | Test 36 | 0.74 | 0.38 | 0.09 | 0.15 |
| 805 | Train 13 | Test 37 | 0.74 | 0.38 | 0.1 | 0.16 | 2853 | Train 45 | Test 37 | 0.74 | 0.37 | 0.1 | 0.16 |
| 806 | Train 13 | Test 38 | 0.67 | 0.3 | 0.26 | 0.28 | 2854 | Train 45 | Test 38 | 0.67 | 0.32 | 0.17 | 0.22 |
| 807 | Train 13 | Test 39 | 0.74 | 0.38 | 0.1 | 0.16 | 2855 | Train 45 | Test 39 | 0.74 | 0.38 | 0.1 | 0.16 |
| 808 | Train 13 | Test 40 | 0.67 | 0.29 | 0.26 | 0.28 | 2856 | Train 45 | Test 40 | 0.67 | 0.31 | 0.17 | 0.22 |
| 809 | Train 13 | Test 41 | 0.75 | 0.42 | 0.09 | 0.15 | 2857 | Train 45 | Test 41 | 0.76 | 0.38 | 0.09 | 0.14 |
| 810 | Train 13 | Test 42 | 0.74 | 0.45 | 0.1 | 0.16 | 2858 | Train 45 | Test 42 | 0.74 | 0.37 | 0.08 | 0.13 |
| 811 | Train 13 | Test 43 | 0.75 | 0.42 | 0.09 | 0.15 | 2859 | Train 45 | Test 43 | 0.76 | 0.38 | 0.09 | 0.14 |
| 812 | Train 13 | Test 44 | 0.74 | 0.45 | 0.1 | 0.16 | 2860 | Train 45 | Test 44 | 0.74 | 0.38 | 0.08 | 0.13 |
| 813 | Train 13 | Test 45 | 0.74 | 0.41 | 0.09 | 0.15 | 2861 | Train 45 | Test 45 | 0.75 | 0.38 | 0.09 | 0.14 |
| 814 | Train 13 | Test 46 | 0.67 | 0.29 | 0.27 | 0.28 | 2862 | Train 45 | Test 46 | 0.67 | 0.31 | 0.17 | 0.22 |
| 815 | Train 13 | Test 47 | 0.74 | 0.41 | 0.09 | 0.15 | 2863 | Train 45 | Test 47 | 0.74 | 0.38 | 0.09 | 0.14 |
| 816 | Train 13 | Test 48 | 0.67 | 0.29 | 0.26 | 0.27 | 2864 | Train 45 | Test 48 | 0.67 | 0.31 | 0.17 | 0.22 |
| 817 | Train 13 | Test 49 | 0.75 | 0.45 | 0.17 | 0.25 | 2865 | Train 45 | Test 49 | 0.76 | 0.43 | 0.17 | 0.24 |
| 818 | Train 13 | Test 50 | 0.75 | 0.45 | 0.17 | 0.25 | 2866 | Train 45 | Test 50 | 0.76 | 0.43 | 0.16 | 0.24 |
| 819 | Train 13 | Test 51 | 0.76 | 0.46 | 0.17 | 0.25 | 2867 | Train 45 | Test 51 | 0.76 | 0.44 | 0.17 | 0.24 |
| 820 | Train 13 | Test 52 | 0.75 | 0.46 | 0.17 | 0.25 | 2868 | Train 45 | Test 52 | 0.76 | 0.43 | 0.17 | 0.24 |
| 821 | Train 13 | Test 53 | 0.73 | 0.33 | 0.17 | 0.22 | 2869 | Train 45 | Test 53 | 0.73 | 0.35 | 0.2 | 0.25 |
| 822 | Train 13 | Test 54 | 0.67 | 0.35 | 0.22 | 0.27 | 2870 | Train 45 | Test 54 | 0.68 | 0.37 | 0.15 | 0.21 |
| 823 | Train 13 | Test 55 | 0.76 | 0.43 | 0.16 | 0.23 | 2871 | Train 45 | Test 55 | 0.76 | 0.42 | 0.15 | 0.22 |
| 824 | Train 13 | Test 56 | 0.67 | 0.33 | 0.22 | 0.27 | 2872 | Train 45 | Test 56 | 0.68 | 0.35 | 0.15 | 0.21 |
| 825 | Train 13 | Test 57 | 0.77 | 0.49 | 0.16 | 0.24 | 2873 | Train 45 | Test 57 | 0.77 | 0.43 | 0.14 | 0.22 |
| 826 | Train 13 | Test 58 | 0.77 | 0.49 | 0.16 | 0.24 | 2874 | Train 45 | Test 58 | 0.77 | 0.43 | 0.14 | 0.21 |
| 827 | Train 13 | Test 59 | 0.77 | 0.49 | 0.16 | 0.24 | 2875 | Train 45 | Test 59 | 0.77 | 0.44 | 0.15 | 0.22 |
| 828 | Train 13 | Test 60 | 0.77 | 0.49 | 0.16 | 0.24 | 2876 | Train 45 | Test 60 | 0.77 | 0.43 | 0.14 | 0.22 |
| 829 | Train 13 | Test 61 | 0.73 | 0.34 | 0.16 | 0.22 | 2877 | Train 45 | Test 61 | 0.74 | 0.34 | 0.18 | 0.23 |
| 830 | Train 13 | Test 62 | 0.67 | 0.35 | 0.22 | 0.27 | 2878 | Train 45 | Test 62 | 0.68 | 0.36 | 0.15 | 0.21 |
| 831 | Train 13 | Test 63 | 0.73 | 0.32 | 0.16 | 0.21 | 2879 | Train 45 | Test 63 | 0.74 | 0.34 | 0.18 | 0.23 |
| 832 | Train 13 | Test 64 | 0.67 | 0.33 | 0.22 | 0.26 | 2880 | Train 45 | Test 64 | 0.68 | 0.35 | 0.15 | 0.21 |
| 833 | Train 14 | Test 1 | 0.74 | 0.32 | 0.12 | 0.17 | 2881 | Train 46 | Test 1 | 0.75 | 0.36 | 0.13 | 0.19 |
| 834 | Train 14 | Test 2 | 0.75 | 0.37 | 0.15 | 0.22 | 2882 | Train 46 | Test 2 | 0.76 | 0.4 | 0.17 | 0.24 |
| 835 | Train 14 | Test 3 | 0.74 | 0.32 | 0.12 | 0.17 | 2883 | Train 46 | Test 3 | 0.75 | 0.36 | 0.13 | 0.19 |
| 836 | Train 14 | Test 4 | 0.75 | 0.37 | 0.15 | 0.22 | 2884 | Train 46 | Test 4 | 0.76 | 0.4 | 0.17 | 0.24 |
| 837 | Train 14 | Test 5 | 0.73 | 0.34 | 0.11 | 0.17 | 2885 | Train 46 | Test 5 | 0.73 | 0.35 | 0.12 | 0.18 |
| 838 | Train 14 | Test 6 | 0.76 | 0.53 | 0.15 | 0.23 | 2886 | Train 46 | Test 6 | 0.76 | 0.54 | 0.15 | 0.24 |
| 839 | Train 14 | Test 7 | 0.73 | 0.33 | 0.11 | 0.17 | 2887 | Train 46 | Test 7 | 0.73 | 0.35 | 0.12 | 0.18 |
| 840 | Train 14 | Test 8 | 0.76 | 0.5 | 0.15 | 0.23 | 2888 | Train 46 | Test 8 | 0.76 | 0.5 | 0.15 | 0.23 |
| 841 | Train 14 | Test 9 | 0.74 | 0.34 | 0.12 | 0.18 | 2889 | Train 46 | Test 9 | 0.75 | 0.37 | 0.12 | 0.18 |
| 842 | Train 14 | Test 10 | 0.75 | 0.38 | 0.14 | 0.21 | 2890 | Train 46 | Test 10 | 0.76 | 0.4 | 0.16 | 0.22 |
| 843 | Train 14 | Test 11 | 0.74 | 0.34 | 0.12 | 0.18 | 2891 | Train 46 | Test 11 | 0.75 | 0.37 | 0.12 | 0.18 |
| 844 | Train 14 | Test 12 | 0.75 | 0.38 | 0.14 | 0.21 | 2892 | Train 46 | Test 12 | 0.76 | 0.41 | 0.16 | 0.22 |
| 845 | Train 14 | Test 13 | 0.73 | 0.36 | 0.11 | 0.17 | 2893 | Train 46 | Test 13 | 0.73 | 0.36 | 0.11 | 0.16 |
| 846 | Train 14 | Test 14 | 0.76 | 0.53 | 0.15 | 0.23 | 2894 | Train 46 | Test 14 | 0.76 | 0.53 | 0.15 | 0.23 |
| 847 | Train 14 | Test 15 | 0.73 | 0.36 | 0.11 | 0.17 | 2895 | Train 46 | Test 15 | 0.72 | 0.36 | 0.11 | 0.16 |
| 848 | Train 14 | Test 16 | 0.76 | 0.49 | 0.15 | 0.23 | 2896 | Train 46 | Test 16 | 0.76 | 0.5 | 0.15 | 0.23 |
| 849 | Train 14 | Test 17 | 0.74 | 0.32 | 0.12 | 0.17 | 2897 | Train 46 | Test 17 | 0.75 | 0.36 | 0.13 | 0.19 |
| 850 | Train 14 | Test 18 | 0.75 | 0.37 | 0.15 | 0.22 | 2898 | Train 46 | Test 18 | 0.75 | 0.4 | 0.17 | 0.24 |
| 851 | Train 14 | Test 19 | 0.74 | 0.32 | 0.12 | 0.17 | 2899 | Train 46 | Test 19 | 0.75 | 0.36 | 0.13 | 0.19 |
| 852 | Train 14 | Test 20 | 0.75 | 0.37 | 0.15 | 0.22 | 2900 | Train 46 | Test 20 | 0.75 | 0.4 | 0.17 | 0.24 |
| 853 | Train 14 | Test 21 | 0.73 | 0.34 | 0.11 | 0.17 | 2901 | Train 46 | Test 21 | 0.73 | 0.35 | 0.12 | 0.18 |
| 854 | Train 14 | Test 22 | 0.76 | 0.53 | 0.15 | 0.23 | 2902 | Train 46 | Test 22 | 0.76 | 0.53 | 0.15 | 0.24 |
| 855 | Train 14 | Test 23 | 0.73 | 0.33 | 0.11 | 0.17 | 2903 | Train 46 | Test 23 | 0.73 | 0.35 | 0.12 | 0.18 |
| 856 | Train 14 | Test 24 | 0.76 | 0.5 | 0.15 | 0.23 | 2904 | Train 46 | Test 24 | 0.76 | 0.5 | 0.15 | 0.23 |
| 857 | Train 14 | Test 25 | 0.74 | 0.34 | 0.12 | 0.18 | 2905 | Train 46 | Test 25 | 0.75 | 0.37 | 0.12 | 0.18 |
| 858 | Train 14 | Test 26 | 0.75 | 0.38 | 0.14 | 0.21 | 2906 | Train 46 | Test 26 | 0.75 | 0.4 | 0.16 | 0.22 |
| 859 | Train 14 | Test 27 | 0.74 | 0.34 | 0.12 | 0.18 | 2907 | Train 46 | Test 27 | 0.75 | 0.37 | 0.12 | 0.18 |
| 860 | Train 14 | Test 28 | 0.75 | 0.38 | 0.14 | 0.21 | 2908 | Train 46 | Test 28 | 0.75 | 0.4 | 0.16 | 0.22 |
| 861 | Train 14 | Test 29 | 0.73 | 0.36 | 0.11 | 0.17 | 2909 | Train 46 | Test 29 | 0.73 | 0.36 | 0.11 | 0.16 |
| 862 | Train 14 | Test 30 | 0.76 | 0.52 | 0.15 | 0.23 | 2910 | Train 46 | Test 30 | 0.76 | 0.53 | 0.15 | 0.23 |
| 863 | Train 14 | Test 31 | 0.73 | 0.36 | 0.11 | 0.17 | 2911 | Train 46 | Test 31 | 0.72 | 0.36 | 0.1 | 0.16 |
| 864 | Train 14 | Test 32 | 0.76 | 0.49 | 0.15 | 0.23 | 2912 | Train 46 | Test 32 | 0.76 | 0.5 | 0.15 | 0.23 |
| 865 | Train 14 | Test 33 | 0.74 | 0.31 | 0.12 | 0.17 | 2913 | Train 46 | Test 33 | 0.75 | 0.36 | 0.13 | 0.19 |
| 866 | Train 14 | Test 34 | 0.75 | 0.36 | 0.15 | 0.22 | 2914 | Train 46 | Test 34 | 0.76 | 0.39 | 0.17 | 0.23 |
| 867 | Train 14 | Test 35 | 0.74 | 0.32 | 0.12 | 0.17 | 2915 | Train 46 | Test 35 | 0.75 | 0.36 | 0.13 | 0.19 |
| 868 | Train 14 | Test 36 | 0.75 | 0.36 | 0.15 | 0.22 | 2916 | Train 46 | Test 36 | 0.76 | 0.39 | 0.17 | 0.23 |
| 869 | Train 14 | Test 37 | 0.73 | 0.33 | 0.11 | 0.17 | 2917 | Train 46 | Test 37 | 0.73 | 0.35 | 0.12 | 0.18 |
| 870 | Train 14 | Test 38 | 0.76 | 0.53 | 0.15 | 0.23 | 2918 | Train 46 | Test 38 | 0.76 | 0.54 | 0.15 | 0.24 |
| 871 | Train 14 | Test 39 | 0.73 | 0.33 | 0.11 | 0.17 | 2919 | Train 46 | Test 39 | 0.73 | 0.35 | 0.12 | 0.18 |
| 872 | Train 14 | Test 40 | 0.76 | 0.5 | 0.15 | 0.23 | 2920 | Train 46 | Test 40 | 0.76 | 0.5 | 0.15 | 0.23 |
| 873 | Train 14 | Test 41 | 0.74 | 0.34 | 0.12 | 0.17 | 2921 | Train 46 | Test 41 | 0.75 | 0.36 | 0.12 | 0.18 |
| 874 | Train 14 | Test 42 | 0.75 | 0.38 | 0.14 | 0.2 | 2922 | Train 46 | Test 42 | 0.76 | 0.4 | 0.15 | 0.22 |
| 875 | Train 14 | Test 43 | 0.74 | 0.34 | 0.12 | 0.17 | 2923 | Train 46 | Test 43 | 0.75 | 0.37 | 0.12 | 0.18 |
| 876 | Train 14 | Test 44 | 0.75 | 0.38 | 0.14 | 0.2 | 2924 | Train 46 | Test 44 | 0.76 | 0.4 | 0.15 | 0.22 |
| 877 | Train 14 | Test 45 | 0.73 | 0.36 | 0.11 | 0.17 | 2925 | Train 46 | Test 45 | 0.73 | 0.36 | 0.11 | 0.16 |
| 878 | Train 14 | Test 46 | 0.76 | 0.52 | 0.15 | 0.23 | 2926 | Train 46 | Test 46 | 0.76 | 0.53 | 0.15 | 0.23 |
| 879 | Train 14 | Test 47 | 0.73 | 0.36 | 0.11 | 0.17 | 2927 | Train 46 | Test 47 | 0.73 | 0.36 | 0.1 | 0.16 |
| 880 | Train 14 | Test 48 | 0.76 | 0.49 | 0.15 | 0.23 | 2928 | Train 46 | Test 48 | 0.76 | 0.5 | 0.15 | 0.23 |
| 881 | Train 14 | Test 49 | 0.76 | 0.35 | 0.21 | 0.26 | 2929 | Train 46 | Test 49 | 0.76 | 0.39 | 0.22 | 0.28 |
| 882 | Train 14 | Test 50 | 0.76 | 0.35 | 0.21 | 0.26 | 2930 | Train 46 | Test 50 | 0.76 | 0.39 | 0.22 | 0.28 |
| 883 | Train 14 | Test 51 | 0.77 | 0.36 | 0.21 | 0.27 | 2931 | Train 46 | Test 51 | 0.76 | 0.39 | 0.22 | 0.28 |
| 884 | Train 14 | Test 52 | 0.76 | 0.36 | 0.21 | 0.26 | 2932 | Train 46 | Test 52 | 0.76 | 0.39 | 0.22 | 0.28 |
| 885 | Train 14 | Test 53 | 0.77 | 0.44 | 0.14 | 0.22 | 2933 | Train 46 | Test 53 | 0.77 | 0.44 | 0.14 | 0.21 |
| 886 | Train 14 | Test 54 | 0.76 | 0.54 | 0.17 | 0.26 | 2934 | Train 46 | Test 54 | 0.76 | 0.54 | 0.18 | 0.27 |
| 887 | Train 14 | Test 55 | 0.76 | 0.37 | 0.17 | 0.23 | 2935 | Train 46 | Test 55 | 0.75 | 0.39 | 0.16 | 0.23 |
| 888 | Train 14 | Test 56 | 0.76 | 0.52 | 0.17 | 0.26 | 2936 | Train 46 | Test 56 | 0.76 | 0.52 | 0.18 | 0.26 |
| 889 | Train 14 | Test 57 | 0.78 | 0.37 | 0.2 | 0.26 | 2937 | Train 46 | Test 57 | 0.76 | 0.4 | 0.2 | 0.27 |
| 890 | Train 14 | Test 58 | 0.77 | 0.37 | 0.2 | 0.26 | 2938 | Train 46 | Test 58 | 0.76 | 0.39 | 0.2 | 0.27 |
| 891 | Train 14 | Test 59 | 0.78 | 0.37 | 0.2 | 0.26 | 2939 | Train 46 | Test 59 | 0.76 | 0.4 | 0.2 | 0.27 |
| 892 | Train 14 | Test 60 | 0.78 | 0.37 | 0.2 | 0.26 | 2940 | Train 46 | Test 60 | 0.76 | 0.4 | 0.2 | 0.27 |
| 893 | Train 14 | Test 61 | 0.78 | 0.46 | 0.14 | 0.21 | 2941 | Train 46 | Test 61 | 0.77 | 0.46 | 0.13 | 0.2 |
| 894 | Train 14 | Test 62 | 0.76 | 0.53 | 0.17 | 0.26 | 2942 | Train 46 | Test 62 | 0.76 | 0.54 | 0.18 | 0.27 |
| 895 | Train 14 | Test 63 | 0.78 | 0.46 | 0.14 | 0.21 | 2943 | Train 46 | Test 63 | 0.77 | 0.46 | 0.13 | 0.2 |
| 896 | Train 14 | Test 64 | 0.76 | 0.51 | 0.17 | 0.26 | 2944 | Train 46 | Test 64 | 0.76 | 0.51 | 0.18 | 0.26 |
| 897 | Train 15 | Test 1 | 0.75 | 0.4 | 0.1 | 0.17 | 2945 | Train 47 | Test 1 | 0.75 | 0.37 | 0.1 | 0.15 |
| 898 | Train 15 | Test 2 | 0.74 | 0.41 | 0.11 | 0.17 | 2946 | Train 47 | Test 2 | 0.74 | 0.37 | 0.09 | 0.15 |
| 899 | Train 15 | Test 3 | 0.75 | 0.4 | 0.1 | 0.16 | 2947 | Train 47 | Test 3 | 0.75 | 0.38 | 0.1 | 0.15 |
| 900 | Train 15 | Test 4 | 0.74 | 0.41 | 0.11 | 0.17 | 2948 | Train 47 | Test 4 | 0.74 | 0.38 | 0.09 | 0.15 |
| 901 | Train 15 | Test 5 | 0.74 | 0.39 | 0.1 | 0.16 | 2949 | Train 47 | Test 5 | 0.74 | 0.38 | 0.1 | 0.16 |
| 902 | Train 15 | Test 6 | 0.67 | 0.32 | 0.09 | 0.14 | 2950 | Train 47 | Test 6 | 0.66 | 0.3 | 0.27 | 0.28 |
| 903 | Train 15 | Test 7 | 0.74 | 0.39 | 0.1 | 0.16 | 2951 | Train 47 | Test 7 | 0.74 | 0.39 | 0.1 | 0.16 |
| 904 | Train 15 | Test 8 | 0.67 | 0.32 | 0.09 | 0.14 | 2952 | Train 47 | Test 8 | 0.66 | 0.29 | 0.26 | 0.28 |
| 905 | Train 15 | Test 9 | 0.76 | 0.39 | 0.08 | 0.14 | 2953 | Train 47 | Test 9 | 0.76 | 0.36 | 0.08 | 0.13 |
| 906 | Train 15 | Test 10 | 0.74 | 0.4 | 0.08 | 0.14 | 2954 | Train 47 | Test 10 | 0.74 | 0.37 | 0.08 | 0.13 |
| 907 | Train 15 | Test 11 | 0.76 | 0.39 | 0.08 | 0.14 | 2955 | Train 47 | Test 11 | 0.76 | 0.37 | 0.08 | 0.13 |
| 908 | Train 15 | Test 12 | 0.74 | 0.4 | 0.08 | 0.14 | 2956 | Train 47 | Test 12 | 0.74 | 0.37 | 0.08 | 0.14 |
| 909 | Train 15 | Test 13 | 0.75 | 0.38 | 0.08 | 0.14 | 2957 | Train 47 | Test 13 | 0.75 | 0.37 | 0.08 | 0.14 |
| 910 | Train 15 | Test 14 | 0.67 | 0.32 | 0.09 | 0.14 | 2958 | Train 47 | Test 14 | 0.66 | 0.29 | 0.27 | 0.28 |
| 911 | Train 15 | Test 15 | 0.75 | 0.39 | 0.09 | 0.14 | 2959 | Train 47 | Test 15 | 0.75 | 0.38 | 0.09 | 0.14 |
| 912 | Train 15 | Test 16 | 0.67 | 0.32 | 0.09 | 0.14 | 2960 | Train 47 | Test 16 | 0.66 | 0.29 | 0.26 | 0.28 |
| 913 | Train 15 | Test 17 | 0.75 | 0.4 | 0.1 | 0.17 | 2961 | Train 47 | Test 17 | 0.75 | 0.37 | 0.1 | 0.15 |
| 914 | Train 15 | Test 18 | 0.74 | 0.42 | 0.11 | 0.17 | 2962 | Train 47 | Test 18 | 0.74 | 0.37 | 0.09 | 0.15 |
| 915 | Train 15 | Test 19 | 0.75 | 0.4 | 0.1 | 0.17 | 2963 | Train 47 | Test 19 | 0.75 | 0.38 | 0.1 | 0.16 |
| 916 | Train 15 | Test 20 | 0.74 | 0.42 | 0.11 | 0.17 | 2964 | Train 47 | Test 20 | 0.74 | 0.38 | 0.09 | 0.15 |
| 917 | Train 15 | Test 21 | 0.74 | 0.39 | 0.1 | 0.16 | 2965 | Train 47 | Test 21 | 0.74 | 0.38 | 0.1 | 0.16 |
| 918 | Train 15 | Test 22 | 0.67 | 0.32 | 0.09 | 0.14 | 2966 | Train 47 | Test 22 | 0.66 | 0.29 | 0.27 | 0.28 |
| 919 | Train 15 | Test 23 | 0.74 | 0.39 | 0.1 | 0.16 | 2967 | Train 47 | Test 23 | 0.74 | 0.39 | 0.1 | 0.16 |
| 920 | Train 15 | Test 24 | 0.67 | 0.31 | 0.09 | 0.14 | 2968 | Train 47 | Test 24 | 0.66 | 0.29 | 0.26 | 0.28 |
| 921 | Train 15 | Test 25 | 0.76 | 0.39 | 0.08 | 0.14 | 2969 | Train 47 | Test 25 | 0.76 | 0.36 | 0.08 | 0.13 |
| 922 | Train 15 | Test 26 | 0.74 | 0.4 | 0.08 | 0.14 | 2970 | Train 47 | Test 26 | 0.74 | 0.37 | 0.08 | 0.13 |
| 923 | Train 15 | Test 27 | 0.76 | 0.39 | 0.08 | 0.14 | 2971 | Train 47 | Test 27 | 0.76 | 0.36 | 0.08 | 0.13 |
| 924 | Train 15 | Test 28 | 0.74 | 0.4 | 0.08 | 0.14 | 2972 | Train 47 | Test 28 | 0.74 | 0.37 | 0.08 | 0.14 |
| 925 | Train 15 | Test 29 | 0.75 | 0.38 | 0.08 | 0.14 | 2973 | Train 47 | Test 29 | 0.75 | 0.37 | 0.08 | 0.14 |
| 926 | Train 15 | Test 30 | 0.67 | 0.31 | 0.09 | 0.14 | 2974 | Train 47 | Test 30 | 0.66 | 0.29 | 0.27 | 0.28 |
| 927 | Train 15 | Test 31 | 0.75 | 0.38 | 0.09 | 0.14 | 2975 | Train 47 | Test 31 | 0.75 | 0.37 | 0.09 | 0.14 |
| 928 | Train 15 | Test 32 | 0.67 | 0.31 | 0.09 | 0.14 | 2976 | Train 47 | Test 32 | 0.66 | 0.29 | 0.26 | 0.27 |
| 929 | Train 15 | Test 33 | 0.75 | 0.4 | 0.1 | 0.16 | 2977 | Train 47 | Test 33 | 0.75 | 0.37 | 0.09 | 0.15 |
| 930 | Train 15 | Test 34 | 0.74 | 0.41 | 0.1 | 0.17 | 2978 | Train 47 | Test 34 | 0.74 | 0.37 | 0.09 | 0.15 |
| 931 | Train 15 | Test 35 | 0.75 | 0.4 | 0.1 | 0.16 | 2979 | Train 47 | Test 35 | 0.75 | 0.37 | 0.1 | 0.15 |
| 932 | Train 15 | Test 36 | 0.74 | 0.41 | 0.1 | 0.17 | 2980 | Train 47 | Test 36 | 0.74 | 0.37 | 0.09 | 0.15 |
| 933 | Train 15 | Test 37 | 0.74 | 0.39 | 0.1 | 0.16 | 2981 | Train 47 | Test 37 | 0.74 | 0.38 | 0.1 | 0.16 |
| 934 | Train 15 | Test 38 | 0.67 | 0.32 | 0.09 | 0.14 | 2982 | Train 47 | Test 38 | 0.66 | 0.29 | 0.27 | 0.28 |
| 935 | Train 15 | Test 39 | 0.74 | 0.39 | 0.1 | 0.16 | 2983 | Train 47 | Test 39 | 0.74 | 0.38 | 0.1 | 0.16 |
| 936 | Train 15 | Test 40 | 0.67 | 0.32 | 0.09 | 0.14 | 2984 | Train 47 | Test 40 | 0.66 | 0.29 | 0.26 | 0.28 |
| 937 | Train 15 | Test 41 | 0.76 | 0.39 | 0.08 | 0.14 | 2985 | Train 47 | Test 41 | 0.76 | 0.36 | 0.08 | 0.13 |
| 938 | Train 15 | Test 42 | 0.74 | 0.4 | 0.08 | 0.14 | 2986 | Train 47 | Test 42 | 0.74 | 0.37 | 0.08 | 0.13 |
| 939 | Train 15 | Test 43 | 0.76 | 0.39 | 0.08 | 0.13 | 2987 | Train 47 | Test 43 | 0.76 | 0.36 | 0.08 | 0.13 |
| 940 | Train 15 | Test 44 | 0.75 | 0.4 | 0.08 | 0.14 | 2988 | Train 47 | Test 44 | 0.75 | 0.37 | 0.08 | 0.14 |
| 941 | Train 15 | Test 45 | 0.75 | 0.38 | 0.08 | 0.14 | 2989 | Train 47 | Test 45 | 0.75 | 0.37 | 0.08 | 0.14 |
| 942 | Train 15 | Test 46 | 0.67 | 0.32 | 0.09 | 0.14 | 2990 | Train 47 | Test 46 | 0.66 | 0.29 | 0.27 | 0.28 |
| 943 | Train 15 | Test 47 | 0.75 | 0.38 | 0.08 | 0.14 | 2991 | Train 47 | Test 47 | 0.75 | 0.37 | 0.08 | 0.14 |
| 944 | Train 15 | Test 48 | 0.67 | 0.32 | 0.09 | 0.14 | 2992 | Train 47 | Test 48 | 0.66 | 0.28 | 0.26 | 0.27 |
| 945 | Train 15 | Test 49 | 0.77 | 0.43 | 0.16 | 0.24 | 2993 | Train 47 | Test 49 | 0.77 | 0.43 | 0.16 | 0.23 |
| 946 | Train 15 | Test 50 | 0.76 | 0.43 | 0.16 | 0.24 | 2994 | Train 47 | Test 50 | 0.76 | 0.42 | 0.16 | 0.23 |
| 947 | Train 15 | Test 51 | 0.77 | 0.43 | 0.16 | 0.23 | 2995 | Train 47 | Test 51 | 0.77 | 0.43 | 0.16 | 0.23 |
| 948 | Train 15 | Test 52 | 0.76 | 0.43 | 0.16 | 0.23 | 2996 | Train 47 | Test 52 | 0.77 | 0.43 | 0.16 | 0.23 |
| 949 | Train 15 | Test 53 | 0.73 | 0.29 | 0.16 | 0.2 | 2997 | Train 47 | Test 53 | 0.72 | 0.35 | 0.2 | 0.25 |
| 950 | Train 15 | Test 54 | 0.68 | 0.34 | 0.09 | 0.14 | 2998 | Train 47 | Test 54 | 0.66 | 0.36 | 0.2 | 0.26 |
| 951 | Train 15 | Test 55 | 0.77 | 0.41 | 0.14 | 0.21 | 2999 | Train 47 | Test 55 | 0.77 | 0.43 | 0.15 | 0.23 |
| 952 | Train 15 | Test 56 | 0.68 | 0.34 | 0.09 | 0.14 | 3000 | Train 47 | Test 56 | 0.66 | 0.34 | 0.21 | 0.26 |
| 953 | Train 15 | Test 57 | 0.78 | 0.45 | 0.14 | 0.21 | 3001 | Train 47 | Test 57 | 0.77 | 0.41 | 0.13 | 0.2 |
| 954 | Train 15 | Test 58 | 0.77 | 0.45 | 0.14 | 0.21 | 3002 | Train 47 | Test 58 | 0.77 | 0.41 | 0.13 | 0.2 |
| 955 | Train 15 | Test 59 | 0.78 | 0.45 | 0.14 | 0.21 | 3003 | Train 47 | Test 59 | 0.78 | 0.42 | 0.14 | 0.21 |
| 956 | Train 15 | Test 60 | 0.78 | 0.45 | 0.14 | 0.21 | 3004 | Train 47 | Test 60 | 0.77 | 0.42 | 0.14 | 0.2 |
| 957 | Train 15 | Test 61 | 0.74 | 0.29 | 0.14 | 0.19 | 3005 | Train 47 | Test 61 | 0.73 | 0.34 | 0.17 | 0.22 |
| 958 | Train 15 | Test 62 | 0.68 | 0.34 | 0.09 | 0.14 | 3006 | Train 47 | Test 62 | 0.66 | 0.36 | 0.2 | 0.26 |
| 959 | Train 15 | Test 63 | 0.74 | 0.29 | 0.14 | 0.19 | 3007 | Train 47 | Test 63 | 0.73 | 0.33 | 0.17 | 0.22 |
| 960 | Train 15 | Test 64 | 0.68 | 0.34 | 0.09 | 0.14 | 3008 | Train 47 | Test 64 | 0.66 | 0.33 | 0.2 | 0.25 |
| 961 | Train 16 | Test 1 | 0.75 | 0.36 | 0.12 | 0.18 | 3009 | Train 48 | Test 1 | 0.75 | 0.33 | 0.13 | 0.18 |
| 962 | Train 16 | Test 2 | 0.76 | 0.4 | 0.17 | 0.24 | 3010 | Train 48 | Test 2 | 0.76 | 0.37 | 0.16 | 0.23 |
| 963 | Train 16 | Test 3 | 0.75 | 0.37 | 0.13 | 0.19 | 3011 | Train 48 | Test 3 | 0.75 | 0.33 | 0.12 | 0.18 |
| 964 | Train 16 | Test 4 | 0.76 | 0.41 | 0.17 | 0.24 | 3012 | Train 48 | Test 4 | 0.76 | 0.37 | 0.16 | 0.23 |
| 965 | Train 16 | Test 5 | 0.73 | 0.36 | 0.12 | 0.18 | 3013 | Train 48 | Test 5 | 0.74 | 0.34 | 0.13 | 0.18 |
| 966 | Train 16 | Test 6 | 0.76 | 0.53 | 0.14 | 0.22 | 3014 | Train 48 | Test 6 | 0.76 | 0.52 | 0.14 | 0.22 |
| 967 | Train 16 | Test 7 | 0.73 | 0.36 | 0.12 | 0.18 | 3015 | Train 48 | Test 7 | 0.73 | 0.34 | 0.13 | 0.18 |
| 968 | Train 16 | Test 8 | 0.76 | 0.52 | 0.13 | 0.21 | 3016 | Train 48 | Test 8 | 0.76 | 0.52 | 0.14 | 0.22 |
| 969 | Train 16 | Test 9 | 0.75 | 0.36 | 0.11 | 0.16 | 3017 | Train 48 | Test 9 | 0.75 | 0.34 | 0.11 | 0.17 |
| 970 | Train 16 | Test 10 | 0.76 | 0.41 | 0.15 | 0.22 | 3018 | Train 48 | Test 10 | 0.76 | 0.39 | 0.15 | 0.22 |
| 971 | Train 16 | Test 11 | 0.75 | 0.36 | 0.11 | 0.17 | 3019 | Train 48 | Test 11 | 0.75 | 0.35 | 0.11 | 0.17 |
| 972 | Train 16 | Test 12 | 0.76 | 0.41 | 0.15 | 0.22 | 3020 | Train 48 | Test 12 | 0.76 | 0.39 | 0.15 | 0.22 |
| 973 | Train 16 | Test 13 | 0.73 | 0.36 | 0.1 | 0.16 | 3021 | Train 48 | Test 13 | 0.74 | 0.35 | 0.12 | 0.17 |
| 974 | Train 16 | Test 14 | 0.76 | 0.53 | 0.14 | 0.22 | 3022 | Train 48 | Test 14 | 0.76 | 0.52 | 0.14 | 0.22 |
| 975 | Train 16 | Test 15 | 0.73 | 0.36 | 0.1 | 0.16 | 3023 | Train 48 | Test 15 | 0.73 | 0.35 | 0.11 | 0.17 |
| 976 | Train 16 | Test 16 | 0.76 | 0.52 | 0.13 | 0.21 | 3024 | Train 48 | Test 16 | 0.76 | 0.52 | 0.14 | 0.22 |
| 977 | Train 16 | Test 17 | 0.75 | 0.36 | 0.12 | 0.18 | 3025 | Train 48 | Test 17 | 0.75 | 0.33 | 0.13 | 0.18 |
| 978 | Train 16 | Test 18 | 0.75 | 0.4 | 0.17 | 0.24 | 3026 | Train 48 | Test 18 | 0.76 | 0.37 | 0.16 | 0.23 |
| 979 | Train 16 | Test 19 | 0.75 | 0.37 | 0.13 | 0.19 | 3027 | Train 48 | Test 19 | 0.75 | 0.33 | 0.12 | 0.18 |
| 980 | Train 16 | Test 20 | 0.75 | 0.41 | 0.17 | 0.24 | 3028 | Train 48 | Test 20 | 0.76 | 0.37 | 0.16 | 0.23 |
| 981 | Train 16 | Test 21 | 0.73 | 0.36 | 0.12 | 0.18 | 3029 | Train 48 | Test 21 | 0.74 | 0.34 | 0.13 | 0.18 |
| 982 | Train 16 | Test 22 | 0.76 | 0.53 | 0.14 | 0.22 | 3030 | Train 48 | Test 22 | 0.76 | 0.52 | 0.14 | 0.22 |
| 983 | Train 16 | Test 23 | 0.73 | 0.36 | 0.12 | 0.18 | 3031 | Train 48 | Test 23 | 0.73 | 0.34 | 0.13 | 0.18 |
| 984 | Train 16 | Test 24 | 0.76 | 0.52 | 0.14 | 0.21 | 3032 | Train 48 | Test 24 | 0.76 | 0.52 | 0.14 | 0.22 |
| 985 | Train 16 | Test 25 | 0.75 | 0.36 | 0.11 | 0.16 | 3033 | Train 48 | Test 25 | 0.75 | 0.34 | 0.11 | 0.17 |
| 986 | Train 16 | Test 26 | 0.76 | 0.41 | 0.15 | 0.22 | 3034 | Train 48 | Test 26 | 0.76 | 0.39 | 0.15 | 0.22 |
| 987 | Train 16 | Test 27 | 0.75 | 0.36 | 0.11 | 0.17 | 3035 | Train 48 | Test 27 | 0.75 | 0.34 | 0.11 | 0.17 |
| 988 | Train 16 | Test 28 | 0.76 | 0.41 | 0.15 | 0.22 | 3036 | Train 48 | Test 28 | 0.76 | 0.39 | 0.15 | 0.22 |
| 989 | Train 16 | Test 29 | 0.73 | 0.36 | 0.1 | 0.16 | 3037 | Train 48 | Test 29 | 0.74 | 0.35 | 0.12 | 0.17 |
| 990 | Train 16 | Test 30 | 0.76 | 0.53 | 0.14 | 0.22 | 3038 | Train 48 | Test 30 | 0.76 | 0.52 | 0.14 | 0.22 |
| 991 | Train 16 | Test 31 | 0.73 | 0.36 | 0.1 | 0.16 | 3039 | Train 48 | Test 31 | 0.73 | 0.35 | 0.11 | 0.17 |
| 992 | Train 16 | Test 32 | 0.76 | 0.52 | 0.14 | 0.21 | 3040 | Train 48 | Test 32 | 0.76 | 0.52 | 0.14 | 0.22 |
| 993 | Train 16 | Test 33 | 0.75 | 0.36 | 0.12 | 0.18 | 3041 | Train 48 | Test 33 | 0.75 | 0.33 | 0.12 | 0.18 |
| 994 | Train 16 | Test 34 | 0.76 | 0.4 | 0.16 | 0.23 | 3042 | Train 48 | Test 34 | 0.76 | 0.37 | 0.16 | 0.22 |
| 995 | Train 16 | Test 35 | 0.75 | 0.36 | 0.12 | 0.19 | 3043 | Train 48 | Test 35 | 0.75 | 0.33 | 0.12 | 0.18 |
| 996 | Train 16 | Test 36 | 0.76 | 0.4 | 0.17 | 0.24 | 3044 | Train 48 | Test 36 | 0.76 | 0.37 | 0.16 | 0.22 |
| 997 | Train 16 | Test 37 | 0.73 | 0.36 | 0.12 | 0.18 | 3045 | Train 48 | Test 37 | 0.74 | 0.34 | 0.13 | 0.18 |
| 998 | Train 16 | Test 38 | 0.76 | 0.53 | 0.14 | 0.22 | 3046 | Train 48 | Test 38 | 0.76 | 0.52 | 0.14 | 0.22 |
| 999 | Train 16 | Test 39 | 0.73 | 0.36 | 0.12 | 0.18 | 3047 | Train 48 | Test 39 | 0.73 | 0.34 | 0.12 | 0.18 |
| 1000 | Train 16 | Test 40 | 0.76 | 0.52 | 0.13 | 0.21 | 3048 | Train 48 | Test 40 | 0.76 | 0.52 | 0.14 | 0.22 |
| 1001 | Train 16 | Test 41 | 0.75 | 0.35 | 0.11 | 0.16 | 3049 | Train 48 | Test 41 | 0.75 | 0.34 | 0.11 | 0.17 |
| 1002 | Train 16 | Test 42 | 0.76 | 0.4 | 0.15 | 0.22 | 3050 | Train 48 | Test 42 | 0.76 | 0.39 | 0.15 | 0.22 |
| 1003 | Train 16 | Test 43 | 0.75 | 0.36 | 0.11 | 0.16 | 3051 | Train 48 | Test 43 | 0.75 | 0.34 | 0.11 | 0.17 |
| 1004 | Train 16 | Test 44 | 0.76 | 0.41 | 0.15 | 0.22 | 3052 | Train 48 | Test 44 | 0.76 | 0.39 | 0.15 | 0.22 |
| 1005 | Train 16 | Test 45 | 0.73 | 0.36 | 0.1 | 0.16 | 3053 | Train 48 | Test 45 | 0.74 | 0.35 | 0.11 | 0.17 |
| 1006 | Train 16 | Test 46 | 0.76 | 0.52 | 0.14 | 0.22 | 3054 | Train 48 | Test 46 | 0.76 | 0.52 | 0.14 | 0.22 |
| 1007 | Train 16 | Test 47 | 0.73 | 0.36 | 0.1 | 0.16 | 3055 | Train 48 | Test 47 | 0.73 | 0.35 | 0.11 | 0.17 |
| 1008 | Train 16 | Test 48 | 0.76 | 0.52 | 0.13 | 0.21 | 3056 | Train 48 | Test 48 | 0.76 | 0.52 | 0.14 | 0.22 |
| 1009 | Train 16 | Test 49 | 0.76 | 0.39 | 0.23 | 0.29 | 3057 | Train 48 | Test 49 | 0.77 | 0.35 | 0.22 | 0.27 |
| 1010 | Train 16 | Test 50 | 0.76 | 0.39 | 0.23 | 0.29 | 3058 | Train 48 | Test 50 | 0.77 | 0.35 | 0.22 | 0.27 |
| 1011 | Train 16 | Test 51 | 0.76 | 0.4 | 0.23 | 0.29 | 3059 | Train 48 | Test 51 | 0.77 | 0.35 | 0.22 | 0.27 |
| 1012 | Train 16 | Test 52 | 0.76 | 0.39 | 0.23 | 0.29 | 3060 | Train 48 | Test 52 | 0.77 | 0.35 | 0.22 | 0.27 |
| 1013 | Train 16 | Test 53 | 0.77 | 0.47 | 0.15 | 0.23 | 3061 | Train 48 | Test 53 | 0.77 | 0.44 | 0.15 | 0.22 |
| 1014 | Train 16 | Test 54 | 0.76 | 0.54 | 0.16 | 0.25 | 3062 | Train 48 | Test 54 | 0.76 | 0.55 | 0.17 | 0.26 |
| 1015 | Train 16 | Test 55 | 0.76 | 0.42 | 0.19 | 0.26 | 3063 | Train 48 | Test 55 | 0.77 | 0.4 | 0.19 | 0.25 |
| 1016 | Train 16 | Test 56 | 0.76 | 0.53 | 0.16 | 0.25 | 3064 | Train 48 | Test 56 | 0.76 | 0.54 | 0.17 | 0.25 |
| 1017 | Train 16 | Test 57 | 0.77 | 0.39 | 0.2 | 0.26 | 3065 | Train 48 | Test 57 | 0.78 | 0.36 | 0.2 | 0.25 |
| 1018 | Train 16 | Test 58 | 0.77 | 0.39 | 0.2 | 0.26 | 3066 | Train 48 | Test 58 | 0.78 | 0.36 | 0.19 | 0.25 |
| 1019 | Train 16 | Test 59 | 0.77 | 0.39 | 0.2 | 0.26 | 3067 | Train 48 | Test 59 | 0.78 | 0.36 | 0.19 | 0.25 |
| 1020 | Train 16 | Test 60 | 0.77 | 0.39 | 0.2 | 0.26 | 3068 | Train 48 | Test 60 | 0.77 | 0.36 | 0.19 | 0.25 |
| 1021 | Train 16 | Test 61 | 0.77 | 0.45 | 0.13 | 0.2 | 3069 | Train 48 | Test 61 | 0.78 | 0.44 | 0.12 | 0.19 |
| 1022 | Train 16 | Test 62 | 0.76 | 0.54 | 0.16 | 0.25 | 3070 | Train 48 | Test 62 | 0.76 | 0.54 | 0.17 | 0.26 |
| 1023 | Train 16 | Test 63 | 0.77 | 0.45 | 0.13 | 0.2 | 3071 | Train 48 | Test 63 | 0.78 | 0.44 | 0.13 | 0.2 |
| 1024 | Train 16 | Test 64 | 0.76 | 0.53 | 0.16 | 0.25 | 3072 | Train 48 | Test 64 | 0.76 | 0.54 | 0.17 | 0.25 |
| 1025 | Train 17 | Test 1 | 0.75 | 0.37 | 0.11 | 0.17 | 3073 | Train 49 | Test 1 | 0.62 | 0.19 | 0.15 | 0.17 |
| 1026 | Train 17 | Test 2 | 0.72 | 0.37 | 0.1 | 0.15 | 3074 | Train 49 | Test 2 | 0.6 | 0.2 | 0.14 | 0.16 |
| 1027 | Train 17 | Test 3 | 0.75 | 0.37 | 0.11 | 0.17 | 3075 | Train 49 | Test 3 | 0.62 | 0.2 | 0.15 | 0.17 |
| 1028 | Train 17 | Test 4 | 0.72 | 0.37 | 0.1 | 0.15 | 3076 | Train 49 | Test 4 | 0.6 | 0.21 | 0.14 | 0.16 |
| 1029 | Train 17 | Test 5 | 0.69 | 0.28 | 0.1 | 0.15 | 3077 | Train 49 | Test 5 | 0.61 | 0.18 | 0.14 | 0.16 |
| 1030 | Train 17 | Test 6 | 0.65 | 0.29 | 0.06 | 0.1 | 3078 | Train 49 | Test 6 | 0.61 | 0.25 | 0.2 | 0.22 |
| 1031 | Train 17 | Test 7 | 0.69 | 0.28 | 0.1 | 0.15 | 3079 | Train 49 | Test 7 | 0.61 | 0.18 | 0.14 | 0.16 |
| 1032 | Train 17 | Test 8 | 0.65 | 0.29 | 0.06 | 0.1 | 3080 | Train 49 | Test 8 | 0.61 | 0.25 | 0.2 | 0.22 |
| 1033 | Train 17 | Test 9 | 0.75 | 0.37 | 0.09 | 0.15 | 3081 | Train 49 | Test 9 | 0.63 | 0.18 | 0.14 | 0.16 |
| 1034 | Train 17 | Test 10 | 0.73 | 0.36 | 0.08 | 0.13 | 3082 | Train 49 | Test 10 | 0.61 | 0.19 | 0.12 | 0.15 |
| 1035 | Train 17 | Test 11 | 0.75 | 0.37 | 0.09 | 0.15 | 3083 | Train 49 | Test 11 | 0.63 | 0.18 | 0.14 | 0.16 |
| 1036 | Train 17 | Test 12 | 0.73 | 0.36 | 0.08 | 0.13 | 3084 | Train 49 | Test 12 | 0.61 | 0.19 | 0.12 | 0.15 |
| 1037 | Train 17 | Test 13 | 0.68 | 0.28 | 0.09 | 0.14 | 3085 | Train 49 | Test 13 | 0.62 | 0.18 | 0.14 | 0.16 |
| 1038 | Train 17 | Test 14 | 0.65 | 0.29 | 0.06 | 0.1 | 3086 | Train 49 | Test 14 | 0.61 | 0.25 | 0.2 | 0.22 |
| 1039 | Train 17 | Test 15 | 0.68 | 0.28 | 0.09 | 0.14 | 3087 | Train 49 | Test 15 | 0.62 | 0.18 | 0.14 | 0.16 |
| 1040 | Train 17 | Test 16 | 0.65 | 0.29 | 0.06 | 0.1 | 3088 | Train 49 | Test 16 | 0.61 | 0.24 | 0.2 | 0.22 |
| 1041 | Train 17 | Test 17 | 0.75 | 0.37 | 0.11 | 0.17 | 3089 | Train 49 | Test 17 | 0.62 | 0.19 | 0.15 | 0.17 |
| 1042 | Train 17 | Test 18 | 0.72 | 0.37 | 0.1 | 0.15 | 3090 | Train 49 | Test 18 | 0.6 | 0.2 | 0.14 | 0.16 |
| 1043 | Train 17 | Test 19 | 0.75 | 0.37 | 0.11 | 0.17 | 3091 | Train 49 | Test 19 | 0.62 | 0.2 | 0.15 | 0.17 |
| 1044 | Train 17 | Test 20 | 0.72 | 0.37 | 0.1 | 0.15 | 3092 | Train 49 | Test 20 | 0.6 | 0.21 | 0.14 | 0.16 |
| 1045 | Train 17 | Test 21 | 0.69 | 0.28 | 0.1 | 0.15 | 3093 | Train 49 | Test 21 | 0.61 | 0.18 | 0.14 | 0.16 |
| 1046 | Train 17 | Test 22 | 0.65 | 0.29 | 0.06 | 0.1 | 3094 | Train 49 | Test 22 | 0.61 | 0.25 | 0.2 | 0.22 |
| 1047 | Train 17 | Test 23 | 0.69 | 0.28 | 0.1 | 0.15 | 3095 | Train 49 | Test 23 | 0.61 | 0.18 | 0.14 | 0.16 |
| 1048 | Train 17 | Test 24 | 0.65 | 0.29 | 0.06 | 0.1 | 3096 | Train 49 | Test 24 | 0.61 | 0.25 | 0.2 | 0.22 |
| 1049 | Train 17 | Test 25 | 0.75 | 0.37 | 0.09 | 0.15 | 3097 | Train 49 | Test 25 | 0.63 | 0.18 | 0.14 | 0.16 |
| 1050 | Train 17 | Test 26 | 0.73 | 0.36 | 0.08 | 0.13 | 3098 | Train 49 | Test 26 | 0.61 | 0.19 | 0.12 | 0.15 |
| 1051 | Train 17 | Test 27 | 0.75 | 0.37 | 0.09 | 0.15 | 3099 | Train 49 | Test 27 | 0.63 | 0.18 | 0.14 | 0.16 |
| 1052 | Train 17 | Test 28 | 0.73 | 0.36 | 0.08 | 0.13 | 3100 | Train 49 | Test 28 | 0.61 | 0.19 | 0.12 | 0.15 |
| 1053 | Train 17 | Test 29 | 0.69 | 0.28 | 0.09 | 0.14 | 3101 | Train 49 | Test 29 | 0.62 | 0.18 | 0.14 | 0.16 |
| 1054 | Train 17 | Test 30 | 0.65 | 0.29 | 0.06 | 0.1 | 3102 | Train 49 | Test 30 | 0.61 | 0.25 | 0.2 | 0.22 |
| 1055 | Train 17 | Test 31 | 0.68 | 0.28 | 0.09 | 0.14 | 3103 | Train 49 | Test 31 | 0.62 | 0.18 | 0.14 | 0.16 |
| 1056 | Train 17 | Test 32 | 0.65 | 0.29 | 0.06 | 0.1 | 3104 | Train 49 | Test 32 | 0.61 | 0.24 | 0.2 | 0.22 |
| 1057 | Train 17 | Test 33 | 0.75 | 0.37 | 0.11 | 0.17 | 3105 | Train 49 | Test 33 | 0.62 | 0.19 | 0.15 | 0.17 |
| 1058 | Train 17 | Test 34 | 0.72 | 0.37 | 0.1 | 0.15 | 3106 | Train 49 | Test 34 | 0.6 | 0.2 | 0.13 | 0.16 |
| 1059 | Train 17 | Test 35 | 0.75 | 0.37 | 0.11 | 0.17 | 3107 | Train 49 | Test 35 | 0.63 | 0.2 | 0.15 | 0.17 |
| 1060 | Train 17 | Test 36 | 0.73 | 0.37 | 0.09 | 0.15 | 3108 | Train 49 | Test 36 | 0.6 | 0.2 | 0.13 | 0.16 |
| 1061 | Train 17 | Test 37 | 0.69 | 0.28 | 0.1 | 0.15 | 3109 | Train 49 | Test 37 | 0.61 | 0.18 | 0.14 | 0.16 |
| 1062 | Train 17 | Test 38 | 0.65 | 0.29 | 0.06 | 0.1 | 3110 | Train 49 | Test 38 | 0.61 | 0.25 | 0.2 | 0.22 |
| 1063 | Train 17 | Test 39 | 0.69 | 0.28 | 0.1 | 0.15 | 3111 | Train 49 | Test 39 | 0.61 | 0.18 | 0.14 | 0.16 |
| 1064 | Train 17 | Test 40 | 0.65 | 0.29 | 0.06 | 0.1 | 3112 | Train 49 | Test 40 | 0.61 | 0.25 | 0.2 | 0.22 |
| 1065 | Train 17 | Test 41 | 0.75 | 0.36 | 0.09 | 0.15 | 3113 | Train 49 | Test 41 | 0.63 | 0.18 | 0.14 | 0.16 |
| 1066 | Train 17 | Test 42 | 0.73 | 0.36 | 0.08 | 0.13 | 3114 | Train 49 | Test 42 | 0.61 | 0.19 | 0.12 | 0.15 |
| 1067 | Train 17 | Test 43 | 0.75 | 0.36 | 0.09 | 0.15 | 3115 | Train 49 | Test 43 | 0.64 | 0.18 | 0.14 | 0.16 |
| 1068 | Train 17 | Test 44 | 0.73 | 0.36 | 0.08 | 0.13 | 3116 | Train 49 | Test 44 | 0.61 | 0.19 | 0.12 | 0.15 |
| 1069 | Train 17 | Test 45 | 0.69 | 0.28 | 0.09 | 0.14 | 3117 | Train 49 | Test 45 | 0.62 | 0.18 | 0.14 | 0.16 |
| 1070 | Train 17 | Test 46 | 0.65 | 0.29 | 0.06 | 0.1 | 3118 | Train 49 | Test 46 | 0.61 | 0.25 | 0.2 | 0.22 |
| 1071 | Train 17 | Test 47 | 0.69 | 0.28 | 0.09 | 0.14 | 3119 | Train 49 | Test 47 | 0.62 | 0.18 | 0.14 | 0.16 |
| 1072 | Train 17 | Test 48 | 0.65 | 0.28 | 0.06 | 0.1 | 3120 | Train 49 | Test 48 | 0.61 | 0.24 | 0.2 | 0.22 |
| 1073 | Train 17 | Test 49 | 0.76 | 0.47 | 0.19 | 0.27 | 3121 | Train 49 | Test 49 | 0.73 | 0.4 | 0.2 | 0.27 |
| 1074 | Train 17 | Test 50 | 0.76 | 0.46 | 0.19 | 0.27 | 3122 | Train 49 | Test 50 | 0.73 | 0.4 | 0.2 | 0.27 |
| 1075 | Train 17 | Test 51 | 0.76 | 0.46 | 0.19 | 0.26 | 3123 | Train 49 | Test 51 | 0.73 | 0.4 | 0.2 | 0.27 |
| 1076 | Train 17 | Test 52 | 0.76 | 0.46 | 0.18 | 0.26 | 3124 | Train 49 | Test 52 | 0.73 | 0.4 | 0.2 | 0.27 |
| 1077 | Train 17 | Test 53 | 0.68 | 0.25 | 0.07 | 0.11 | 3125 | Train 49 | Test 53 | 0.68 | 0.29 | 0.15 | 0.2 |
| 1078 | Train 17 | Test 54 | 0.67 | 0.36 | 0.07 | 0.12 | 3126 | Train 49 | Test 54 | 0.64 | 0.34 | 0.2 | 0.25 |
| 1079 | Train 17 | Test 55 | 0.7 | 0.35 | 0.11 | 0.17 | 3127 | Train 49 | Test 55 | 0.68 | 0.29 | 0.15 | 0.2 |
| 1080 | Train 17 | Test 56 | 0.66 | 0.36 | 0.07 | 0.12 | 3128 | Train 49 | Test 56 | 0.64 | 0.33 | 0.19 | 0.24 |
| 1081 | Train 17 | Test 57 | 0.76 | 0.44 | 0.15 | 0.22 | 3129 | Train 49 | Test 57 | 0.75 | 0.4 | 0.18 | 0.25 |
| 1082 | Train 17 | Test 58 | 0.76 | 0.43 | 0.15 | 0.22 | 3130 | Train 49 | Test 58 | 0.74 | 0.4 | 0.18 | 0.25 |
| 1083 | Train 17 | Test 59 | 0.76 | 0.44 | 0.15 | 0.22 | 3131 | Train 49 | Test 59 | 0.75 | 0.4 | 0.18 | 0.25 |
| 1084 | Train 17 | Test 60 | 0.76 | 0.43 | 0.14 | 0.22 | 3132 | Train 49 | Test 60 | 0.74 | 0.4 | 0.18 | 0.25 |
| 1085 | Train 17 | Test 61 | 0.68 | 0.26 | 0.07 | 0.11 | 3133 | Train 49 | Test 61 | 0.68 | 0.29 | 0.15 | 0.2 |
| 1086 | Train 17 | Test 62 | 0.67 | 0.35 | 0.07 | 0.12 | 3134 | Train 49 | Test 62 | 0.64 | 0.34 | 0.19 | 0.25 |
| 1087 | Train 17 | Test 63 | 0.68 | 0.26 | 0.07 | 0.11 | 3135 | Train 49 | Test 63 | 0.69 | 0.29 | 0.15 | 0.2 |
| 1088 | Train 17 | Test 64 | 0.67 | 0.35 | 0.07 | 0.12 | 3136 | Train 49 | Test 64 | 0.65 | 0.33 | 0.19 | 0.24 |
| 1089 | Train 18 | Test 1 | 0.75 | 0.36 | 0.1 | 0.16 | 3137 | Train 50 | Test 1 | 0.66 | 0.22 | 0.15 | 0.18 |
| 1090 | Train 18 | Test 2 | 0.76 | 0.44 | 0.17 | 0.24 | 3138 | Train 50 | Test 2 | 0.64 | 0.24 | 0.15 | 0.18 |
| 1091 | Train 18 | Test 3 | 0.75 | 0.36 | 0.1 | 0.16 | 3139 | Train 50 | Test 3 | 0.66 | 0.22 | 0.15 | 0.18 |
| 1092 | Train 18 | Test 4 | 0.76 | 0.44 | 0.17 | 0.24 | 3140 | Train 50 | Test 4 | 0.64 | 0.24 | 0.15 | 0.18 |
| 1093 | Train 18 | Test 5 | 0.69 | 0.28 | 0.1 | 0.15 | 3141 | Train 50 | Test 5 | 0.64 | 0.2 | 0.15 | 0.17 |
| 1094 | Train 18 | Test 6 | 0.66 | 0.33 | 0.07 | 0.11 | 3142 | Train 50 | Test 6 | 0.63 | 0.28 | 0.15 | 0.2 |
| 1095 | Train 18 | Test 7 | 0.69 | 0.28 | 0.1 | 0.14 | 3143 | Train 50 | Test 7 | 0.64 | 0.2 | 0.15 | 0.17 |
| 1096 | Train 18 | Test 8 | 0.66 | 0.33 | 0.07 | 0.11 | 3144 | Train 50 | Test 8 | 0.63 | 0.27 | 0.15 | 0.19 |
| 1097 | Train 18 | Test 9 | 0.75 | 0.36 | 0.08 | 0.14 | 3145 | Train 50 | Test 9 | 0.66 | 0.21 | 0.13 | 0.16 |
| 1098 | Train 18 | Test 10 | 0.76 | 0.46 | 0.14 | 0.22 | 3146 | Train 50 | Test 10 | 0.64 | 0.23 | 0.13 | 0.17 |
| 1099 | Train 18 | Test 11 | 0.75 | 0.36 | 0.08 | 0.14 | 3147 | Train 50 | Test 11 | 0.66 | 0.21 | 0.13 | 0.16 |
| 1100 | Train 18 | Test 12 | 0.76 | 0.46 | 0.14 | 0.22 | 3148 | Train 50 | Test 12 | 0.65 | 0.23 | 0.14 | 0.17 |
| 1101 | Train 18 | Test 13 | 0.69 | 0.28 | 0.09 | 0.13 | 3149 | Train 50 | Test 13 | 0.64 | 0.18 | 0.13 | 0.16 |
| 1102 | Train 18 | Test 14 | 0.66 | 0.33 | 0.07 | 0.11 | 3150 | Train 50 | Test 14 | 0.63 | 0.28 | 0.15 | 0.2 |
| 1103 | Train 18 | Test 15 | 0.68 | 0.28 | 0.08 | 0.13 | 3151 | Train 50 | Test 15 | 0.64 | 0.19 | 0.13 | 0.16 |
| 1104 | Train 18 | Test 16 | 0.66 | 0.32 | 0.06 | 0.11 | 3152 | Train 50 | Test 16 | 0.63 | 0.27 | 0.15 | 0.2 |
| 1105 | Train 18 | Test 17 | 0.75 | 0.36 | 0.1 | 0.16 | 3153 | Train 50 | Test 17 | 0.66 | 0.22 | 0.15 | 0.18 |
| 1106 | Train 18 | Test 18 | 0.76 | 0.44 | 0.17 | 0.24 | 3154 | Train 50 | Test 18 | 0.64 | 0.24 | 0.15 | 0.18 |
| 1107 | Train 18 | Test 19 | 0.75 | 0.36 | 0.1 | 0.16 | 3155 | Train 50 | Test 19 | 0.66 | 0.22 | 0.15 | 0.18 |
| 1108 | Train 18 | Test 20 | 0.76 | 0.44 | 0.17 | 0.24 | 3156 | Train 50 | Test 20 | 0.64 | 0.24 | 0.15 | 0.18 |
| 1109 | Train 18 | Test 21 | 0.69 | 0.28 | 0.1 | 0.15 | 3157 | Train 50 | Test 21 | 0.64 | 0.2 | 0.15 | 0.17 |
| 1110 | Train 18 | Test 22 | 0.66 | 0.33 | 0.07 | 0.11 | 3158 | Train 50 | Test 22 | 0.63 | 0.27 | 0.15 | 0.2 |
| 1111 | Train 18 | Test 23 | 0.69 | 0.28 | 0.1 | 0.14 | 3159 | Train 50 | Test 23 | 0.64 | 0.2 | 0.15 | 0.17 |
| 1112 | Train 18 | Test 24 | 0.66 | 0.33 | 0.07 | 0.11 | 3160 | Train 50 | Test 24 | 0.63 | 0.27 | 0.15 | 0.19 |
| 1113 | Train 18 | Test 25 | 0.75 | 0.37 | 0.09 | 0.14 | 3161 | Train 50 | Test 25 | 0.66 | 0.21 | 0.13 | 0.16 |
| 1114 | Train 18 | Test 26 | 0.76 | 0.46 | 0.15 | 0.22 | 3162 | Train 50 | Test 26 | 0.65 | 0.23 | 0.13 | 0.17 |
| 1115 | Train 18 | Test 27 | 0.75 | 0.37 | 0.09 | 0.14 | 3163 | Train 50 | Test 27 | 0.66 | 0.21 | 0.13 | 0.16 |
| 1116 | Train 18 | Test 28 | 0.76 | 0.46 | 0.14 | 0.22 | 3164 | Train 50 | Test 28 | 0.65 | 0.23 | 0.14 | 0.17 |
| 1117 | Train 18 | Test 29 | 0.69 | 0.28 | 0.09 | 0.13 | 3165 | Train 50 | Test 29 | 0.64 | 0.18 | 0.13 | 0.15 |
| 1118 | Train 18 | Test 30 | 0.66 | 0.33 | 0.07 | 0.11 | 3166 | Train 50 | Test 30 | 0.63 | 0.27 | 0.15 | 0.2 |
| 1119 | Train 18 | Test 31 | 0.68 | 0.28 | 0.08 | 0.13 | 3167 | Train 50 | Test 31 | 0.64 | 0.19 | 0.13 | 0.16 |
| 1120 | Train 18 | Test 32 | 0.66 | 0.32 | 0.07 | 0.11 | 3168 | Train 50 | Test 32 | 0.63 | 0.27 | 0.15 | 0.19 |
| 1121 | Train 18 | Test 33 | 0.75 | 0.36 | 0.1 | 0.16 | 3169 | Train 50 | Test 33 | 0.66 | 0.22 | 0.15 | 0.17 |
| 1122 | Train 18 | Test 34 | 0.77 | 0.44 | 0.16 | 0.24 | 3170 | Train 50 | Test 34 | 0.64 | 0.23 | 0.15 | 0.18 |
| 1123 | Train 18 | Test 35 | 0.75 | 0.36 | 0.1 | 0.16 | 3171 | Train 50 | Test 35 | 0.66 | 0.22 | 0.15 | 0.17 |
| 1124 | Train 18 | Test 36 | 0.77 | 0.44 | 0.16 | 0.24 | 3172 | Train 50 | Test 36 | 0.64 | 0.23 | 0.15 | 0.18 |
| 1125 | Train 18 | Test 37 | 0.69 | 0.28 | 0.1 | 0.14 | 3173 | Train 50 | Test 37 | 0.64 | 0.2 | 0.15 | 0.17 |
| 1126 | Train 18 | Test 38 | 0.66 | 0.33 | 0.07 | 0.11 | 3174 | Train 50 | Test 38 | 0.63 | 0.28 | 0.15 | 0.2 |
| 1127 | Train 18 | Test 39 | 0.69 | 0.28 | 0.1 | 0.14 | 3175 | Train 50 | Test 39 | 0.64 | 0.2 | 0.15 | 0.17 |
| 1128 | Train 18 | Test 40 | 0.66 | 0.33 | 0.07 | 0.11 | 3176 | Train 50 | Test 40 | 0.63 | 0.27 | 0.15 | 0.19 |
| 1129 | Train 18 | Test 41 | 0.75 | 0.36 | 0.08 | 0.14 | 3177 | Train 50 | Test 41 | 0.66 | 0.2 | 0.13 | 0.16 |
| 1130 | Train 18 | Test 42 | 0.76 | 0.46 | 0.14 | 0.22 | 3178 | Train 50 | Test 42 | 0.65 | 0.23 | 0.13 | 0.17 |
| 1131 | Train 18 | Test 43 | 0.75 | 0.36 | 0.08 | 0.14 | 3179 | Train 50 | Test 43 | 0.66 | 0.21 | 0.13 | 0.16 |
| 1132 | Train 18 | Test 44 | 0.76 | 0.45 | 0.14 | 0.22 | 3180 | Train 50 | Test 44 | 0.65 | 0.23 | 0.13 | 0.17 |
| 1133 | Train 18 | Test 45 | 0.69 | 0.28 | 0.08 | 0.13 | 3181 | Train 50 | Test 45 | 0.64 | 0.18 | 0.13 | 0.15 |
| 1134 | Train 18 | Test 46 | 0.66 | 0.32 | 0.07 | 0.11 | 3182 | Train 50 | Test 46 | 0.63 | 0.28 | 0.15 | 0.2 |
| 1135 | Train 18 | Test 47 | 0.68 | 0.28 | 0.08 | 0.13 | 3183 | Train 50 | Test 47 | 0.64 | 0.18 | 0.13 | 0.15 |
| 1136 | Train 18 | Test 48 | 0.66 | 0.32 | 0.06 | 0.11 | 3184 | Train 50 | Test 48 | 0.63 | 0.27 | 0.15 | 0.2 |
| 1137 | Train 18 | Test 49 | 0.76 | 0.42 | 0.16 | 0.23 | 3185 | Train 50 | Test 49 | 0.74 | 0.41 | 0.19 | 0.26 |
| 1138 | Train 18 | Test 50 | 0.76 | 0.42 | 0.15 | 0.23 | 3186 | Train 50 | Test 50 | 0.74 | 0.41 | 0.19 | 0.26 |
| 1139 | Train 18 | Test 51 | 0.76 | 0.42 | 0.16 | 0.23 | 3187 | Train 50 | Test 51 | 0.74 | 0.41 | 0.19 | 0.26 |
| 1140 | Train 18 | Test 52 | 0.76 | 0.42 | 0.15 | 0.23 | 3188 | Train 50 | Test 52 | 0.74 | 0.41 | 0.19 | 0.26 |
| 1141 | Train 18 | Test 53 | 0.68 | 0.27 | 0.08 | 0.12 | 3189 | Train 50 | Test 53 | 0.69 | 0.27 | 0.19 | 0.22 |
| 1142 | Train 18 | Test 54 | 0.66 | 0.38 | 0.06 | 0.11 | 3190 | Train 50 | Test 54 | 0.65 | 0.33 | 0.17 | 0.22 |
| 1143 | Train 18 | Test 55 | 0.71 | 0.35 | 0.11 | 0.17 | 3191 | Train 50 | Test 55 | 0.7 | 0.27 | 0.19 | 0.22 |
| 1144 | Train 18 | Test 56 | 0.66 | 0.38 | 0.06 | 0.11 | 3192 | Train 50 | Test 56 | 0.65 | 0.31 | 0.17 | 0.22 |
| 1145 | Train 18 | Test 57 | 0.76 | 0.44 | 0.13 | 0.2 | 3193 | Train 50 | Test 57 | 0.75 | 0.37 | 0.16 | 0.23 |
| 1146 | Train 18 | Test 58 | 0.75 | 0.44 | 0.13 | 0.2 | 3194 | Train 50 | Test 58 | 0.75 | 0.37 | 0.16 | 0.23 |
| 1147 | Train 18 | Test 59 | 0.76 | 0.44 | 0.13 | 0.2 | 3195 | Train 50 | Test 59 | 0.75 | 0.37 | 0.17 | 0.23 |
| 1148 | Train 18 | Test 60 | 0.75 | 0.44 | 0.13 | 0.2 | 3196 | Train 50 | Test 60 | 0.75 | 0.37 | 0.17 | 0.23 |
| 1149 | Train 18 | Test 61 | 0.68 | 0.29 | 0.08 | 0.12 | 3197 | Train 50 | Test 61 | 0.7 | 0.25 | 0.18 | 0.21 |
| 1150 | Train 18 | Test 62 | 0.67 | 0.38 | 0.06 | 0.11 | 3198 | Train 50 | Test 62 | 0.65 | 0.33 | 0.17 | 0.22 |
| 1151 | Train 18 | Test 63 | 0.68 | 0.28 | 0.08 | 0.12 | 3199 | Train 50 | Test 63 | 0.7 | 0.25 | 0.18 | 0.21 |
| 1152 | Train 18 | Test 64 | 0.66 | 0.37 | 0.06 | 0.11 | 3200 | Train 50 | Test 64 | 0.65 | 0.31 | 0.17 | 0.22 |
| 1153 | Train 19 | Test 1 | 0.75 | 0.4 | 0.12 | 0.18 | 3201 | Train 51 | Test 1 | 0.66 | 0.18 | 0.3 | 0.23 |
| 1154 | Train 19 | Test 2 | 0.73 | 0.39 | 0.11 | 0.17 | 3202 | Train 51 | Test 2 | 0.61 | 0.19 | 0.24 | 0.21 |
| 1155 | Train 19 | Test 3 | 0.75 | 0.4 | 0.12 | 0.19 | 3203 | Train 51 | Test 3 | 0.66 | 0.19 | 0.3 | 0.23 |
| 1156 | Train 19 | Test 4 | 0.73 | 0.4 | 0.11 | 0.17 | 3204 | Train 51 | Test 4 | 0.62 | 0.19 | 0.24 | 0.21 |
| 1157 | Train 19 | Test 5 | 0.67 | 0.18 | 0.18 | 0.18 | 3205 | Train 51 | Test 5 | 0.63 | 0.17 | 0.3 | 0.22 |
| 1158 | Train 19 | Test 6 | 0.63 | 0.21 | 0.12 | 0.15 | 3206 | Train 51 | Test 6 | 0.59 | 0.21 | 0.25 | 0.23 |
| 1159 | Train 19 | Test 7 | 0.67 | 0.18 | 0.18 | 0.18 | 3207 | Train 51 | Test 7 | 0.63 | 0.18 | 0.3 | 0.22 |
| 1160 | Train 19 | Test 8 | 0.63 | 0.21 | 0.12 | 0.15 | 3208 | Train 51 | Test 8 | 0.59 | 0.21 | 0.25 | 0.23 |
| 1161 | Train 19 | Test 9 | 0.75 | 0.38 | 0.1 | 0.15 | 3209 | Train 51 | Test 9 | 0.67 | 0.19 | 0.3 | 0.23 |
| 1162 | Train 19 | Test 10 | 0.73 | 0.37 | 0.09 | 0.14 | 3210 | Train 51 | Test 10 | 0.62 | 0.2 | 0.24 | 0.22 |
| 1163 | Train 19 | Test 11 | 0.75 | 0.38 | 0.1 | 0.16 | 3211 | Train 51 | Test 11 | 0.67 | 0.19 | 0.3 | 0.23 |
| 1164 | Train 19 | Test 12 | 0.73 | 0.37 | 0.09 | 0.14 | 3212 | Train 51 | Test 12 | 0.62 | 0.2 | 0.24 | 0.22 |
| 1165 | Train 19 | Test 13 | 0.67 | 0.18 | 0.17 | 0.18 | 3213 | Train 51 | Test 13 | 0.64 | 0.18 | 0.3 | 0.22 |
| 1166 | Train 19 | Test 14 | 0.63 | 0.21 | 0.12 | 0.15 | 3214 | Train 51 | Test 14 | 0.59 | 0.21 | 0.25 | 0.23 |
| 1167 | Train 19 | Test 15 | 0.67 | 0.18 | 0.17 | 0.17 | 3215 | Train 51 | Test 15 | 0.63 | 0.18 | 0.3 | 0.22 |
| 1168 | Train 19 | Test 16 | 0.63 | 0.21 | 0.12 | 0.15 | 3216 | Train 51 | Test 16 | 0.6 | 0.21 | 0.25 | 0.23 |
| 1169 | Train 19 | Test 17 | 0.75 | 0.4 | 0.12 | 0.18 | 3217 | Train 51 | Test 17 | 0.66 | 0.18 | 0.3 | 0.23 |
| 1170 | Train 19 | Test 18 | 0.73 | 0.39 | 0.11 | 0.17 | 3218 | Train 51 | Test 18 | 0.61 | 0.19 | 0.24 | 0.21 |
| 1171 | Train 19 | Test 19 | 0.75 | 0.4 | 0.12 | 0.19 | 3219 | Train 51 | Test 19 | 0.66 | 0.19 | 0.3 | 0.23 |
| 1172 | Train 19 | Test 20 | 0.73 | 0.4 | 0.11 | 0.17 | 3220 | Train 51 | Test 20 | 0.62 | 0.19 | 0.24 | 0.21 |
| 1173 | Train 19 | Test 21 | 0.67 | 0.18 | 0.18 | 0.18 | 3221 | Train 51 | Test 21 | 0.63 | 0.17 | 0.3 | 0.22 |
| 1174 | Train 19 | Test 22 | 0.63 | 0.21 | 0.12 | 0.15 | 3222 | Train 51 | Test 22 | 0.59 | 0.21 | 0.25 | 0.23 |
| 1175 | Train 19 | Test 23 | 0.67 | 0.19 | 0.18 | 0.18 | 3223 | Train 51 | Test 23 | 0.63 | 0.18 | 0.3 | 0.22 |
| 1176 | Train 19 | Test 24 | 0.64 | 0.21 | 0.12 | 0.15 | 3224 | Train 51 | Test 24 | 0.6 | 0.21 | 0.25 | 0.23 |
| 1177 | Train 19 | Test 25 | 0.75 | 0.38 | 0.1 | 0.15 | 3225 | Train 51 | Test 25 | 0.67 | 0.19 | 0.3 | 0.23 |
| 1178 | Train 19 | Test 26 | 0.73 | 0.37 | 0.09 | 0.14 | 3226 | Train 51 | Test 26 | 0.62 | 0.2 | 0.24 | 0.22 |
| 1179 | Train 19 | Test 27 | 0.75 | 0.38 | 0.1 | 0.16 | 3227 | Train 51 | Test 27 | 0.67 | 0.19 | 0.3 | 0.23 |
| 1180 | Train 19 | Test 28 | 0.73 | 0.37 | 0.09 | 0.14 | 3228 | Train 51 | Test 28 | 0.62 | 0.2 | 0.24 | 0.22 |
| 1181 | Train 19 | Test 29 | 0.67 | 0.18 | 0.17 | 0.18 | 3229 | Train 51 | Test 29 | 0.64 | 0.18 | 0.3 | 0.22 |
| 1182 | Train 19 | Test 30 | 0.63 | 0.21 | 0.12 | 0.15 | 3230 | Train 51 | Test 30 | 0.59 | 0.21 | 0.25 | 0.23 |
| 1183 | Train 19 | Test 31 | 0.67 | 0.18 | 0.17 | 0.18 | 3231 | Train 51 | Test 31 | 0.64 | 0.18 | 0.3 | 0.22 |
| 1184 | Train 19 | Test 32 | 0.63 | 0.21 | 0.12 | 0.15 | 3232 | Train 51 | Test 32 | 0.6 | 0.21 | 0.25 | 0.23 |
| 1185 | Train 19 | Test 33 | 0.75 | 0.4 | 0.12 | 0.18 | 3233 | Train 51 | Test 33 | 0.66 | 0.18 | 0.3 | 0.23 |
| 1186 | Train 19 | Test 34 | 0.73 | 0.39 | 0.11 | 0.17 | 3234 | Train 51 | Test 34 | 0.62 | 0.19 | 0.24 | 0.21 |
| 1187 | Train 19 | Test 35 | 0.75 | 0.4 | 0.12 | 0.19 | 3235 | Train 51 | Test 35 | 0.66 | 0.19 | 0.31 | 0.23 |
| 1188 | Train 19 | Test 36 | 0.73 | 0.39 | 0.11 | 0.17 | 3236 | Train 51 | Test 36 | 0.62 | 0.19 | 0.24 | 0.21 |
| 1189 | Train 19 | Test 37 | 0.67 | 0.18 | 0.18 | 0.18 | 3237 | Train 51 | Test 37 | 0.63 | 0.17 | 0.3 | 0.22 |
| 1190 | Train 19 | Test 38 | 0.63 | 0.21 | 0.12 | 0.15 | 3238 | Train 51 | Test 38 | 0.59 | 0.21 | 0.25 | 0.23 |
| 1191 | Train 19 | Test 39 | 0.67 | 0.18 | 0.18 | 0.18 | 3239 | Train 51 | Test 39 | 0.64 | 0.18 | 0.3 | 0.22 |
| 1192 | Train 19 | Test 40 | 0.64 | 0.21 | 0.12 | 0.15 | 3240 | Train 51 | Test 40 | 0.6 | 0.21 | 0.25 | 0.23 |
| 1193 | Train 19 | Test 41 | 0.75 | 0.38 | 0.1 | 0.15 | 3241 | Train 51 | Test 41 | 0.67 | 0.19 | 0.3 | 0.23 |
| 1194 | Train 19 | Test 42 | 0.73 | 0.36 | 0.09 | 0.14 | 3242 | Train 51 | Test 42 | 0.63 | 0.2 | 0.24 | 0.22 |
| 1195 | Train 19 | Test 43 | 0.75 | 0.38 | 0.1 | 0.15 | 3243 | Train 51 | Test 43 | 0.67 | 0.19 | 0.3 | 0.23 |
| 1196 | Train 19 | Test 44 | 0.73 | 0.37 | 0.09 | 0.14 | 3244 | Train 51 | Test 44 | 0.63 | 0.2 | 0.24 | 0.22 |
| 1197 | Train 19 | Test 45 | 0.67 | 0.18 | 0.17 | 0.17 | 3245 | Train 51 | Test 45 | 0.64 | 0.18 | 0.3 | 0.22 |
| 1198 | Train 19 | Test 46 | 0.63 | 0.21 | 0.12 | 0.15 | 3246 | Train 51 | Test 46 | 0.59 | 0.21 | 0.25 | 0.23 |
| 1199 | Train 19 | Test 47 | 0.67 | 0.18 | 0.17 | 0.17 | 3247 | Train 51 | Test 47 | 0.64 | 0.18 | 0.3 | 0.22 |
| 1200 | Train 19 | Test 48 | 0.63 | 0.21 | 0.12 | 0.15 | 3248 | Train 51 | Test 48 | 0.6 | 0.21 | 0.25 | 0.23 |
| 1201 | Train 19 | Test 49 | 0.76 | 0.46 | 0.18 | 0.26 | 3249 | Train 51 | Test 49 | 0.73 | 0.34 | 0.17 | 0.22 |
| 1202 | Train 19 | Test 50 | 0.76 | 0.46 | 0.18 | 0.26 | 3250 | Train 51 | Test 50 | 0.73 | 0.34 | 0.17 | 0.22 |
| 1203 | Train 19 | Test 51 | 0.76 | 0.46 | 0.19 | 0.27 | 3251 | Train 51 | Test 51 | 0.73 | 0.35 | 0.17 | 0.23 |
| 1204 | Train 19 | Test 52 | 0.76 | 0.46 | 0.19 | 0.26 | 3252 | Train 51 | Test 52 | 0.73 | 0.35 | 0.17 | 0.23 |
| 1205 | Train 19 | Test 53 | 0.62 | 0.15 | 0.27 | 0.2 | 3253 | Train 51 | Test 53 | 0.69 | 0.24 | 0.31 | 0.27 |
| 1206 | Train 19 | Test 54 | 0.63 | 0.21 | 0.13 | 0.16 | 3254 | Train 51 | Test 54 | 0.61 | 0.28 | 0.18 | 0.22 |
| 1207 | Train 19 | Test 55 | 0.65 | 0.17 | 0.31 | 0.22 | 3255 | Train 51 | Test 55 | 0.69 | 0.24 | 0.31 | 0.27 |
| 1208 | Train 19 | Test 56 | 0.63 | 0.21 | 0.13 | 0.16 | 3256 | Train 51 | Test 56 | 0.62 | 0.27 | 0.18 | 0.22 |
| 1209 | Train 19 | Test 57 | 0.76 | 0.42 | 0.15 | 0.22 | 3257 | Train 51 | Test 57 | 0.74 | 0.35 | 0.16 | 0.22 |
| 1210 | Train 19 | Test 58 | 0.76 | 0.41 | 0.15 | 0.22 | 3258 | Train 51 | Test 58 | 0.74 | 0.34 | 0.16 | 0.22 |
| 1211 | Train 19 | Test 59 | 0.76 | 0.42 | 0.15 | 0.22 | 3259 | Train 51 | Test 59 | 0.74 | 0.35 | 0.17 | 0.23 |
| 1212 | Train 19 | Test 60 | 0.76 | 0.42 | 0.15 | 0.22 | 3260 | Train 51 | Test 60 | 0.74 | 0.35 | 0.17 | 0.22 |
| 1213 | Train 19 | Test 61 | 0.62 | 0.15 | 0.27 | 0.2 | 3261 | Train 51 | Test 61 | 0.69 | 0.23 | 0.3 | 0.26 |
| 1214 | Train 19 | Test 62 | 0.63 | 0.21 | 0.13 | 0.16 | 3262 | Train 51 | Test 62 | 0.61 | 0.27 | 0.19 | 0.22 |
| 1215 | Train 19 | Test 63 | 0.63 | 0.16 | 0.27 | 0.2 | 3263 | Train 51 | Test 63 | 0.69 | 0.23 | 0.31 | 0.27 |
| 1216 | Train 19 | Test 64 | 0.63 | 0.21 | 0.13 | 0.16 | 3264 | Train 51 | Test 64 | 0.62 | 0.27 | 0.18 | 0.22 |
| 1217 | Train 20 | Test 1 | 0.75 | 0.4 | 0.12 | 0.18 | 3265 | Train 52 | Test 1 | 0.64 | 0.22 | 0.12 | 0.16 |
| 1218 | Train 20 | Test 2 | 0.77 | 0.45 | 0.17 | 0.24 | 3266 | Train 52 | Test 2 | 0.63 | 0.22 | 0.12 | 0.16 |
| 1219 | Train 20 | Test 3 | 0.75 | 0.4 | 0.12 | 0.18 | 3267 | Train 52 | Test 3 | 0.64 | 0.22 | 0.13 | 0.16 |
| 1220 | Train 20 | Test 4 | 0.77 | 0.45 | 0.17 | 0.24 | 3268 | Train 52 | Test 4 | 0.63 | 0.23 | 0.12 | 0.16 |
| 1221 | Train 20 | Test 5 | 0.68 | 0.26 | 0.1 | 0.14 | 3269 | Train 52 | Test 5 | 0.63 | 0.22 | 0.14 | 0.17 |
| 1222 | Train 20 | Test 6 | 0.65 | 0.29 | 0.07 | 0.11 | 3270 | Train 52 | Test 6 | 0.62 | 0.26 | 0.15 | 0.19 |
| 1223 | Train 20 | Test 7 | 0.68 | 0.26 | 0.1 | 0.14 | 3271 | Train 52 | Test 7 | 0.63 | 0.22 | 0.14 | 0.18 |
| 1224 | Train 20 | Test 8 | 0.65 | 0.29 | 0.07 | 0.11 | 3272 | Train 52 | Test 8 | 0.62 | 0.25 | 0.15 | 0.19 |
| 1225 | Train 20 | Test 9 | 0.76 | 0.43 | 0.11 | 0.18 | 3273 | Train 52 | Test 9 | 0.65 | 0.22 | 0.12 | 0.15 |
| 1226 | Train 20 | Test 10 | 0.77 | 0.47 | 0.16 | 0.24 | 3274 | Train 52 | Test 10 | 0.63 | 0.24 | 0.12 | 0.16 |
| 1227 | Train 20 | Test 11 | 0.76 | 0.43 | 0.11 | 0.17 | 3275 | Train 52 | Test 11 | 0.66 | 0.22 | 0.12 | 0.15 |
| 1228 | Train 20 | Test 12 | 0.77 | 0.47 | 0.16 | 0.24 | 3276 | Train 52 | Test 12 | 0.63 | 0.24 | 0.12 | 0.16 |
| 1229 | Train 20 | Test 13 | 0.69 | 0.27 | 0.09 | 0.13 | 3277 | Train 52 | Test 13 | 0.64 | 0.21 | 0.13 | 0.16 |
| 1230 | Train 20 | Test 14 | 0.65 | 0.29 | 0.07 | 0.11 | 3278 | Train 52 | Test 14 | 0.62 | 0.25 | 0.15 | 0.19 |
| 1231 | Train 20 | Test 15 | 0.69 | 0.27 | 0.09 | 0.13 | 3279 | Train 52 | Test 15 | 0.63 | 0.21 | 0.13 | 0.16 |
| 1232 | Train 20 | Test 16 | 0.65 | 0.29 | 0.07 | 0.11 | 3280 | Train 52 | Test 16 | 0.62 | 0.25 | 0.15 | 0.19 |
| 1233 | Train 20 | Test 17 | 0.75 | 0.4 | 0.12 | 0.18 | 3281 | Train 52 | Test 17 | 0.64 | 0.22 | 0.12 | 0.16 |
| 1234 | Train 20 | Test 18 | 0.77 | 0.45 | 0.17 | 0.24 | 3282 | Train 52 | Test 18 | 0.63 | 0.22 | 0.12 | 0.16 |
| 1235 | Train 20 | Test 19 | 0.75 | 0.4 | 0.12 | 0.18 | 3283 | Train 52 | Test 19 | 0.65 | 0.22 | 0.13 | 0.16 |
| 1236 | Train 20 | Test 20 | 0.77 | 0.45 | 0.17 | 0.24 | 3284 | Train 52 | Test 20 | 0.63 | 0.23 | 0.12 | 0.16 |
| 1237 | Train 20 | Test 21 | 0.69 | 0.26 | 0.1 | 0.14 | 3285 | Train 52 | Test 21 | 0.63 | 0.22 | 0.14 | 0.17 |
| 1238 | Train 20 | Test 22 | 0.65 | 0.29 | 0.07 | 0.11 | 3286 | Train 52 | Test 22 | 0.62 | 0.26 | 0.15 | 0.19 |
| 1239 | Train 20 | Test 23 | 0.68 | 0.26 | 0.1 | 0.14 | 3287 | Train 52 | Test 23 | 0.63 | 0.22 | 0.14 | 0.18 |
| 1240 | Train 20 | Test 24 | 0.65 | 0.29 | 0.07 | 0.11 | 3288 | Train 52 | Test 24 | 0.62 | 0.25 | 0.15 | 0.19 |
| 1241 | Train 20 | Test 25 | 0.76 | 0.43 | 0.11 | 0.18 | 3289 | Train 52 | Test 25 | 0.65 | 0.22 | 0.12 | 0.15 |
| 1242 | Train 20 | Test 26 | 0.77 | 0.48 | 0.16 | 0.24 | 3290 | Train 52 | Test 26 | 0.63 | 0.24 | 0.12 | 0.16 |
| 1243 | Train 20 | Test 27 | 0.76 | 0.43 | 0.11 | 0.18 | 3291 | Train 52 | Test 27 | 0.66 | 0.22 | 0.12 | 0.15 |
| 1244 | Train 20 | Test 28 | 0.77 | 0.48 | 0.16 | 0.24 | 3292 | Train 52 | Test 28 | 0.63 | 0.24 | 0.12 | 0.16 |
| 1245 | Train 20 | Test 29 | 0.69 | 0.27 | 0.09 | 0.14 | 3293 | Train 52 | Test 29 | 0.64 | 0.21 | 0.13 | 0.16 |
| 1246 | Train 20 | Test 30 | 0.66 | 0.29 | 0.07 | 0.11 | 3294 | Train 52 | Test 30 | 0.62 | 0.25 | 0.15 | 0.19 |
| 1247 | Train 20 | Test 31 | 0.69 | 0.27 | 0.09 | 0.13 | 3295 | Train 52 | Test 31 | 0.64 | 0.21 | 0.13 | 0.16 |
| 1248 | Train 20 | Test 32 | 0.66 | 0.28 | 0.07 | 0.11 | 3296 | Train 52 | Test 32 | 0.63 | 0.25 | 0.15 | 0.19 |
| 1249 | Train 20 | Test 33 | 0.76 | 0.4 | 0.12 | 0.18 | 3297 | Train 52 | Test 33 | 0.65 | 0.21 | 0.12 | 0.16 |
| 1250 | Train 20 | Test 34 | 0.77 | 0.45 | 0.17 | 0.24 | 3298 | Train 52 | Test 34 | 0.63 | 0.22 | 0.12 | 0.15 |
| 1251 | Train 20 | Test 35 | 0.76 | 0.4 | 0.12 | 0.18 | 3299 | Train 52 | Test 35 | 0.65 | 0.22 | 0.13 | 0.16 |
| 1252 | Train 20 | Test 36 | 0.77 | 0.45 | 0.17 | 0.24 | 3300 | Train 52 | Test 36 | 0.63 | 0.23 | 0.12 | 0.16 |
| 1253 | Train 20 | Test 37 | 0.69 | 0.26 | 0.1 | 0.14 | 3301 | Train 52 | Test 37 | 0.63 | 0.22 | 0.14 | 0.17 |
| 1254 | Train 20 | Test 38 | 0.65 | 0.29 | 0.07 | 0.11 | 3302 | Train 52 | Test 38 | 0.62 | 0.26 | 0.15 | 0.19 |
| 1255 | Train 20 | Test 39 | 0.69 | 0.26 | 0.1 | 0.14 | 3303 | Train 52 | Test 39 | 0.63 | 0.22 | 0.14 | 0.17 |
| 1256 | Train 20 | Test 40 | 0.65 | 0.29 | 0.07 | 0.11 | 3304 | Train 52 | Test 40 | 0.62 | 0.25 | 0.15 | 0.19 |
| 1257 | Train 20 | Test 41 | 0.76 | 0.43 | 0.11 | 0.17 | 3305 | Train 52 | Test 41 | 0.66 | 0.22 | 0.12 | 0.15 |
| 1258 | Train 20 | Test 42 | 0.77 | 0.47 | 0.16 | 0.24 | 3306 | Train 52 | Test 42 | 0.64 | 0.24 | 0.12 | 0.16 |
| 1259 | Train 20 | Test 43 | 0.76 | 0.43 | 0.11 | 0.17 | 3307 | Train 52 | Test 43 | 0.66 | 0.22 | 0.12 | 0.15 |
| 1260 | Train 20 | Test 44 | 0.77 | 0.47 | 0.16 | 0.23 | 3308 | Train 52 | Test 44 | 0.64 | 0.24 | 0.12 | 0.16 |
| 1261 | Train 20 | Test 45 | 0.69 | 0.27 | 0.09 | 0.13 | 3309 | Train 52 | Test 45 | 0.64 | 0.21 | 0.13 | 0.16 |
| 1262 | Train 20 | Test 46 | 0.66 | 0.29 | 0.07 | 0.12 | 3310 | Train 52 | Test 46 | 0.63 | 0.25 | 0.15 | 0.19 |
| 1263 | Train 20 | Test 47 | 0.69 | 0.27 | 0.09 | 0.13 | 3311 | Train 52 | Test 47 | 0.64 | 0.21 | 0.13 | 0.16 |
| 1264 | Train 20 | Test 48 | 0.65 | 0.29 | 0.07 | 0.11 | 3312 | Train 52 | Test 48 | 0.63 | 0.25 | 0.15 | 0.19 |
| 1265 | Train 20 | Test 49 | 0.77 | 0.49 | 0.18 | 0.27 | 3313 | Train 52 | Test 49 | 0.73 | 0.39 | 0.18 | 0.24 |
| 1266 | Train 20 | Test 50 | 0.77 | 0.49 | 0.18 | 0.26 | 3314 | Train 52 | Test 50 | 0.73 | 0.39 | 0.18 | 0.24 |
| 1267 | Train 20 | Test 51 | 0.77 | 0.49 | 0.18 | 0.26 | 3315 | Train 52 | Test 51 | 0.73 | 0.39 | 0.18 | 0.25 |
| 1268 | Train 20 | Test 52 | 0.76 | 0.49 | 0.18 | 0.26 | 3316 | Train 52 | Test 52 | 0.73 | 0.38 | 0.18 | 0.25 |
| 1269 | Train 20 | Test 53 | 0.66 | 0.21 | 0.09 | 0.12 | 3317 | Train 52 | Test 53 | 0.7 | 0.29 | 0.2 | 0.23 |
| 1270 | Train 20 | Test 54 | 0.65 | 0.32 | 0.06 | 0.1 | 3318 | Train 52 | Test 54 | 0.65 | 0.33 | 0.14 | 0.2 |
| 1271 | Train 20 | Test 55 | 0.72 | 0.32 | 0.12 | 0.18 | 3319 | Train 52 | Test 55 | 0.7 | 0.29 | 0.2 | 0.23 |
| 1272 | Train 20 | Test 56 | 0.65 | 0.31 | 0.06 | 0.1 | 3320 | Train 52 | Test 56 | 0.65 | 0.32 | 0.14 | 0.19 |
| 1273 | Train 20 | Test 57 | 0.77 | 0.5 | 0.16 | 0.25 | 3321 | Train 52 | Test 57 | 0.74 | 0.37 | 0.17 | 0.23 |
| 1274 | Train 20 | Test 58 | 0.77 | 0.5 | 0.16 | 0.25 | 3322 | Train 52 | Test 58 | 0.74 | 0.37 | 0.17 | 0.23 |
| 1275 | Train 20 | Test 59 | 0.77 | 0.5 | 0.16 | 0.24 | 3323 | Train 52 | Test 59 | 0.74 | 0.37 | 0.17 | 0.23 |
| 1276 | Train 20 | Test 60 | 0.77 | 0.5 | 0.16 | 0.24 | 3324 | Train 52 | Test 60 | 0.74 | 0.37 | 0.17 | 0.23 |
| 1277 | Train 20 | Test 61 | 0.66 | 0.22 | 0.09 | 0.12 | 3325 | Train 52 | Test 61 | 0.7 | 0.29 | 0.2 | 0.24 |
| 1278 | Train 20 | Test 62 | 0.65 | 0.32 | 0.06 | 0.1 | 3326 | Train 52 | Test 62 | 0.65 | 0.33 | 0.14 | 0.2 |
| 1279 | Train 20 | Test 63 | 0.66 | 0.22 | 0.09 | 0.12 | 3327 | Train 52 | Test 63 | 0.7 | 0.29 | 0.2 | 0.23 |
| 1280 | Train 20 | Test 64 | 0.65 | 0.31 | 0.06 | 0.1 | 3328 | Train 52 | Test 64 | 0.65 | 0.31 | 0.14 | 0.19 |
| 1281 | Train 21 | Test 1 | 0.76 | 0.37 | 0.08 | 0.14 | 3329 | Train 53 | Test 1 | 0.64 | 0.19 | 0.16 | 0.18 |
| 1282 | Train 21 | Test 2 | 0.74 | 0.39 | 0.08 | 0.13 | 3330 | Train 53 | Test 2 | 0.64 | 0.21 | 0.15 | 0.18 |
| 1283 | Train 21 | Test 3 | 0.76 | 0.38 | 0.09 | 0.14 | 3331 | Train 53 | Test 3 | 0.64 | 0.19 | 0.16 | 0.18 |
| 1284 | Train 21 | Test 4 | 0.74 | 0.39 | 0.08 | 0.14 | 3332 | Train 53 | Test 4 | 0.64 | 0.21 | 0.15 | 0.18 |
| 1285 | Train 21 | Test 5 | 0.75 | 0.4 | 0.1 | 0.15 | 3333 | Train 53 | Test 5 | 0.64 | 0.2 | 0.17 | 0.19 |
| 1286 | Train 21 | Test 6 | 0.66 | 0.34 | 0.08 | 0.13 | 3334 | Train 53 | Test 6 | 0.62 | 0.25 | 0.15 | 0.19 |
| 1287 | Train 21 | Test 7 | 0.75 | 0.39 | 0.1 | 0.15 | 3335 | Train 53 | Test 7 | 0.64 | 0.2 | 0.17 | 0.19 |
| 1288 | Train 21 | Test 8 | 0.67 | 0.34 | 0.08 | 0.13 | 3336 | Train 53 | Test 8 | 0.63 | 0.25 | 0.15 | 0.19 |
| 1289 | Train 21 | Test 9 | 0.76 | 0.36 | 0.07 | 0.12 | 3337 | Train 53 | Test 9 | 0.64 | 0.17 | 0.14 | 0.15 |
| 1290 | Train 21 | Test 10 | 0.74 | 0.38 | 0.07 | 0.12 | 3338 | Train 53 | Test 10 | 0.64 | 0.2 | 0.14 | 0.16 |
| 1291 | Train 21 | Test 11 | 0.76 | 0.36 | 0.07 | 0.12 | 3339 | Train 53 | Test 11 | 0.64 | 0.17 | 0.14 | 0.15 |
| 1292 | Train 21 | Test 12 | 0.74 | 0.38 | 0.07 | 0.12 | 3340 | Train 53 | Test 12 | 0.64 | 0.2 | 0.14 | 0.16 |
| 1293 | Train 21 | Test 13 | 0.75 | 0.37 | 0.07 | 0.12 | 3341 | Train 53 | Test 13 | 0.64 | 0.19 | 0.15 | 0.17 |
| 1294 | Train 21 | Test 14 | 0.67 | 0.34 | 0.08 | 0.13 | 3342 | Train 53 | Test 14 | 0.63 | 0.25 | 0.15 | 0.18 |
| 1295 | Train 21 | Test 15 | 0.75 | 0.37 | 0.08 | 0.13 | 3343 | Train 53 | Test 15 | 0.64 | 0.19 | 0.15 | 0.17 |
| 1296 | Train 21 | Test 16 | 0.67 | 0.34 | 0.08 | 0.13 | 3344 | Train 53 | Test 16 | 0.63 | 0.25 | 0.15 | 0.19 |
| 1297 | Train 21 | Test 17 | 0.76 | 0.37 | 0.08 | 0.14 | 3345 | Train 53 | Test 17 | 0.64 | 0.19 | 0.16 | 0.18 |
| 1298 | Train 21 | Test 18 | 0.74 | 0.39 | 0.08 | 0.13 | 3346 | Train 53 | Test 18 | 0.64 | 0.21 | 0.15 | 0.18 |
| 1299 | Train 21 | Test 19 | 0.76 | 0.37 | 0.09 | 0.14 | 3347 | Train 53 | Test 19 | 0.64 | 0.19 | 0.16 | 0.18 |
| 1300 | Train 21 | Test 20 | 0.74 | 0.39 | 0.08 | 0.14 | 3348 | Train 53 | Test 20 | 0.64 | 0.21 | 0.15 | 0.18 |
| 1301 | Train 21 | Test 21 | 0.75 | 0.4 | 0.1 | 0.15 | 3349 | Train 53 | Test 21 | 0.64 | 0.2 | 0.17 | 0.19 |
| 1302 | Train 21 | Test 22 | 0.66 | 0.34 | 0.08 | 0.13 | 3350 | Train 53 | Test 22 | 0.62 | 0.25 | 0.15 | 0.19 |
| 1303 | Train 21 | Test 23 | 0.75 | 0.39 | 0.1 | 0.15 | 3351 | Train 53 | Test 23 | 0.64 | 0.2 | 0.17 | 0.19 |
| 1304 | Train 21 | Test 24 | 0.67 | 0.34 | 0.08 | 0.13 | 3352 | Train 53 | Test 24 | 0.63 | 0.25 | 0.15 | 0.19 |
| 1305 | Train 21 | Test 25 | 0.76 | 0.36 | 0.07 | 0.12 | 3353 | Train 53 | Test 25 | 0.64 | 0.17 | 0.14 | 0.15 |
| 1306 | Train 21 | Test 26 | 0.74 | 0.38 | 0.07 | 0.12 | 3354 | Train 53 | Test 26 | 0.64 | 0.2 | 0.14 | 0.16 |
| 1307 | Train 21 | Test 27 | 0.76 | 0.36 | 0.07 | 0.12 | 3355 | Train 53 | Test 27 | 0.64 | 0.17 | 0.14 | 0.15 |
| 1308 | Train 21 | Test 28 | 0.74 | 0.38 | 0.07 | 0.12 | 3356 | Train 53 | Test 28 | 0.64 | 0.2 | 0.14 | 0.16 |
| 1309 | Train 21 | Test 29 | 0.75 | 0.37 | 0.07 | 0.12 | 3357 | Train 53 | Test 29 | 0.65 | 0.19 | 0.15 | 0.17 |
| 1310 | Train 21 | Test 30 | 0.67 | 0.34 | 0.08 | 0.13 | 3358 | Train 53 | Test 30 | 0.63 | 0.25 | 0.15 | 0.18 |
| 1311 | Train 21 | Test 31 | 0.75 | 0.37 | 0.07 | 0.12 | 3359 | Train 53 | Test 31 | 0.65 | 0.19 | 0.15 | 0.17 |
| 1312 | Train 21 | Test 32 | 0.67 | 0.34 | 0.08 | 0.13 | 3360 | Train 53 | Test 32 | 0.63 | 0.25 | 0.15 | 0.19 |
| 1313 | Train 21 | Test 33 | 0.76 | 0.37 | 0.08 | 0.14 | 3361 | Train 53 | Test 33 | 0.65 | 0.19 | 0.16 | 0.18 |
| 1314 | Train 21 | Test 34 | 0.74 | 0.39 | 0.08 | 0.13 | 3362 | Train 53 | Test 34 | 0.64 | 0.21 | 0.15 | 0.17 |
| 1315 | Train 21 | Test 35 | 0.76 | 0.37 | 0.08 | 0.14 | 3363 | Train 53 | Test 35 | 0.65 | 0.19 | 0.16 | 0.18 |
| 1316 | Train 21 | Test 36 | 0.74 | 0.39 | 0.08 | 0.13 | 3364 | Train 53 | Test 36 | 0.64 | 0.21 | 0.15 | 0.17 |
| 1317 | Train 21 | Test 37 | 0.75 | 0.39 | 0.09 | 0.15 | 3365 | Train 53 | Test 37 | 0.65 | 0.2 | 0.17 | 0.18 |
| 1318 | Train 21 | Test 38 | 0.67 | 0.34 | 0.08 | 0.13 | 3366 | Train 53 | Test 38 | 0.63 | 0.25 | 0.15 | 0.19 |
| 1319 | Train 21 | Test 39 | 0.75 | 0.39 | 0.1 | 0.15 | 3367 | Train 53 | Test 39 | 0.65 | 0.2 | 0.17 | 0.18 |
| 1320 | Train 21 | Test 40 | 0.67 | 0.34 | 0.08 | 0.13 | 3368 | Train 53 | Test 40 | 0.63 | 0.25 | 0.15 | 0.19 |
| 1321 | Train 21 | Test 41 | 0.76 | 0.36 | 0.07 | 0.12 | 3369 | Train 53 | Test 41 | 0.64 | 0.17 | 0.14 | 0.15 |
| 1322 | Train 21 | Test 42 | 0.74 | 0.38 | 0.07 | 0.11 | 3370 | Train 53 | Test 42 | 0.64 | 0.2 | 0.14 | 0.16 |
| 1323 | Train 21 | Test 43 | 0.76 | 0.36 | 0.07 | 0.12 | 3371 | Train 53 | Test 43 | 0.65 | 0.17 | 0.14 | 0.15 |
| 1324 | Train 21 | Test 44 | 0.74 | 0.38 | 0.07 | 0.11 | 3372 | Train 53 | Test 44 | 0.64 | 0.2 | 0.14 | 0.16 |
| 1325 | Train 21 | Test 45 | 0.75 | 0.37 | 0.07 | 0.12 | 3373 | Train 53 | Test 45 | 0.65 | 0.19 | 0.15 | 0.17 |
| 1326 | Train 21 | Test 46 | 0.67 | 0.34 | 0.08 | 0.13 | 3374 | Train 53 | Test 46 | 0.63 | 0.25 | 0.15 | 0.18 |
| 1327 | Train 21 | Test 47 | 0.75 | 0.37 | 0.07 | 0.12 | 3375 | Train 53 | Test 47 | 0.65 | 0.19 | 0.15 | 0.17 |
| 1328 | Train 21 | Test 48 | 0.67 | 0.34 | 0.08 | 0.13 | 3376 | Train 53 | Test 48 | 0.63 | 0.25 | 0.15 | 0.19 |
| 1329 | Train 21 | Test 49 | 0.77 | 0.43 | 0.13 | 0.2 | 3377 | Train 53 | Test 49 | 0.73 | 0.4 | 0.2 | 0.27 |
| 1330 | Train 21 | Test 50 | 0.77 | 0.42 | 0.13 | 0.2 | 3378 | Train 53 | Test 50 | 0.73 | 0.4 | 0.2 | 0.26 |
| 1331 | Train 21 | Test 51 | 0.77 | 0.43 | 0.13 | 0.2 | 3379 | Train 53 | Test 51 | 0.73 | 0.4 | 0.2 | 0.27 |
| 1332 | Train 21 | Test 52 | 0.77 | 0.42 | 0.13 | 0.2 | 3380 | Train 53 | Test 52 | 0.73 | 0.4 | 0.2 | 0.26 |
| 1333 | Train 21 | Test 53 | 0.75 | 0.41 | 0.12 | 0.18 | 3381 | Train 53 | Test 53 | 0.75 | 0.39 | 0.16 | 0.23 |
| 1334 | Train 21 | Test 54 | 0.69 | 0.42 | 0.07 | 0.12 | 3382 | Train 53 | Test 54 | 0.65 | 0.35 | 0.14 | 0.2 |
| 1335 | Train 21 | Test 55 | 0.77 | 0.43 | 0.12 | 0.19 | 3383 | Train 53 | Test 55 | 0.73 | 0.38 | 0.17 | 0.23 |
| 1336 | Train 21 | Test 56 | 0.69 | 0.42 | 0.07 | 0.12 | 3384 | Train 53 | Test 56 | 0.65 | 0.34 | 0.15 | 0.2 |
| 1337 | Train 21 | Test 57 | 0.78 | 0.41 | 0.12 | 0.18 | 3385 | Train 53 | Test 57 | 0.74 | 0.36 | 0.16 | 0.22 |
| 1338 | Train 21 | Test 58 | 0.78 | 0.41 | 0.12 | 0.18 | 3386 | Train 53 | Test 58 | 0.73 | 0.36 | 0.16 | 0.22 |
| 1339 | Train 21 | Test 59 | 0.78 | 0.41 | 0.12 | 0.18 | 3387 | Train 53 | Test 59 | 0.74 | 0.36 | 0.16 | 0.23 |
| 1340 | Train 21 | Test 60 | 0.78 | 0.41 | 0.12 | 0.18 | 3388 | Train 53 | Test 60 | 0.74 | 0.36 | 0.16 | 0.23 |
| 1341 | Train 21 | Test 61 | 0.75 | 0.42 | 0.1 | 0.16 | 3389 | Train 53 | Test 61 | 0.75 | 0.36 | 0.14 | 0.2 |
| 1342 | Train 21 | Test 62 | 0.69 | 0.43 | 0.07 | 0.12 | 3390 | Train 53 | Test 62 | 0.66 | 0.35 | 0.14 | 0.2 |
| 1343 | Train 21 | Test 63 | 0.75 | 0.42 | 0.1 | 0.17 | 3391 | Train 53 | Test 63 | 0.75 | 0.37 | 0.14 | 0.21 |
| 1344 | Train 21 | Test 64 | 0.7 | 0.42 | 0.07 | 0.12 | 3392 | Train 53 | Test 64 | 0.66 | 0.33 | 0.15 | 0.2 |
| 1345 | Train 22 | Test 1 | 0.74 | 0.34 | 0.12 | 0.18 | 3393 | Train 54 | Test 1 | 0.65 | 0.22 | 0.2 | 0.21 |
| 1346 | Train 22 | Test 2 | 0.75 | 0.38 | 0.15 | 0.22 | 3394 | Train 54 | Test 2 | 0.68 | 0.28 | 0.25 | 0.26 |
| 1347 | Train 22 | Test 3 | 0.74 | 0.34 | 0.12 | 0.18 | 3395 | Train 54 | Test 3 | 0.66 | 0.22 | 0.2 | 0.21 |
| 1348 | Train 22 | Test 4 | 0.75 | 0.38 | 0.15 | 0.22 | 3396 | Train 54 | Test 4 | 0.68 | 0.28 | 0.25 | 0.26 |
| 1349 | Train 22 | Test 5 | 0.73 | 0.34 | 0.12 | 0.18 | 3397 | Train 54 | Test 5 | 0.66 | 0.24 | 0.19 | 0.21 |
| 1350 | Train 22 | Test 6 | 0.76 | 0.53 | 0.15 | 0.23 | 3398 | Train 54 | Test 6 | 0.68 | 0.31 | 0.21 | 0.25 |
| 1351 | Train 22 | Test 7 | 0.73 | 0.34 | 0.12 | 0.18 | 3399 | Train 54 | Test 7 | 0.66 | 0.24 | 0.19 | 0.21 |
| 1352 | Train 22 | Test 8 | 0.76 | 0.49 | 0.15 | 0.23 | 3400 | Train 54 | Test 8 | 0.68 | 0.3 | 0.21 | 0.25 |
| 1353 | Train 22 | Test 9 | 0.74 | 0.34 | 0.1 | 0.16 | 3401 | Train 54 | Test 9 | 0.66 | 0.21 | 0.18 | 0.2 |
| 1354 | Train 22 | Test 10 | 0.75 | 0.41 | 0.14 | 0.21 | 3402 | Train 54 | Test 10 | 0.68 | 0.27 | 0.22 | 0.25 |
| 1355 | Train 22 | Test 11 | 0.74 | 0.34 | 0.1 | 0.16 | 3403 | Train 54 | Test 11 | 0.66 | 0.22 | 0.18 | 0.2 |
| 1356 | Train 22 | Test 12 | 0.75 | 0.41 | 0.14 | 0.21 | 3404 | Train 54 | Test 12 | 0.68 | 0.27 | 0.22 | 0.25 |
| 1357 | Train 22 | Test 13 | 0.73 | 0.36 | 0.1 | 0.16 | 3405 | Train 54 | Test 13 | 0.66 | 0.24 | 0.17 | 0.2 |
| 1358 | Train 22 | Test 14 | 0.76 | 0.52 | 0.15 | 0.23 | 3406 | Train 54 | Test 14 | 0.68 | 0.3 | 0.21 | 0.25 |
| 1359 | Train 22 | Test 15 | 0.73 | 0.35 | 0.1 | 0.16 | 3407 | Train 54 | Test 15 | 0.66 | 0.24 | 0.17 | 0.2 |
| 1360 | Train 22 | Test 16 | 0.76 | 0.49 | 0.15 | 0.23 | 3408 | Train 54 | Test 16 | 0.68 | 0.3 | 0.21 | 0.25 |
| 1361 | Train 22 | Test 17 | 0.74 | 0.33 | 0.12 | 0.18 | 3409 | Train 54 | Test 17 | 0.65 | 0.22 | 0.2 | 0.21 |
| 1362 | Train 22 | Test 18 | 0.75 | 0.38 | 0.15 | 0.22 | 3410 | Train 54 | Test 18 | 0.68 | 0.28 | 0.25 | 0.26 |
| 1363 | Train 22 | Test 19 | 0.74 | 0.34 | 0.12 | 0.18 | 3411 | Train 54 | Test 19 | 0.66 | 0.22 | 0.2 | 0.21 |
| 1364 | Train 22 | Test 20 | 0.75 | 0.38 | 0.15 | 0.22 | 3412 | Train 54 | Test 20 | 0.68 | 0.28 | 0.25 | 0.26 |
| 1365 | Train 22 | Test 21 | 0.73 | 0.34 | 0.12 | 0.18 | 3413 | Train 54 | Test 21 | 0.66 | 0.24 | 0.19 | 0.21 |
| 1366 | Train 22 | Test 22 | 0.76 | 0.53 | 0.15 | 0.23 | 3414 | Train 54 | Test 22 | 0.68 | 0.3 | 0.21 | 0.25 |
| 1367 | Train 22 | Test 23 | 0.73 | 0.34 | 0.12 | 0.18 | 3415 | Train 54 | Test 23 | 0.66 | 0.24 | 0.19 | 0.21 |
| 1368 | Train 22 | Test 24 | 0.76 | 0.49 | 0.15 | 0.23 | 3416 | Train 54 | Test 24 | 0.68 | 0.3 | 0.21 | 0.25 |
| 1369 | Train 22 | Test 25 | 0.74 | 0.34 | 0.1 | 0.16 | 3417 | Train 54 | Test 25 | 0.66 | 0.21 | 0.18 | 0.2 |
| 1370 | Train 22 | Test 26 | 0.75 | 0.41 | 0.14 | 0.21 | 3418 | Train 54 | Test 26 | 0.68 | 0.27 | 0.22 | 0.25 |
| 1371 | Train 22 | Test 27 | 0.74 | 0.34 | 0.1 | 0.16 | 3419 | Train 54 | Test 27 | 0.66 | 0.21 | 0.18 | 0.2 |
| 1372 | Train 22 | Test 28 | 0.75 | 0.41 | 0.14 | 0.21 | 3420 | Train 54 | Test 28 | 0.68 | 0.27 | 0.22 | 0.25 |
| 1373 | Train 22 | Test 29 | 0.73 | 0.36 | 0.1 | 0.16 | 3421 | Train 54 | Test 29 | 0.66 | 0.24 | 0.17 | 0.2 |
| 1374 | Train 22 | Test 30 | 0.76 | 0.52 | 0.15 | 0.23 | 3422 | Train 54 | Test 30 | 0.68 | 0.3 | 0.21 | 0.25 |
| 1375 | Train 22 | Test 31 | 0.73 | 0.35 | 0.1 | 0.16 | 3423 | Train 54 | Test 31 | 0.66 | 0.24 | 0.17 | 0.2 |
| 1376 | Train 22 | Test 32 | 0.76 | 0.49 | 0.15 | 0.23 | 3424 | Train 54 | Test 32 | 0.68 | 0.3 | 0.21 | 0.25 |
| 1377 | Train 22 | Test 33 | 0.74 | 0.33 | 0.12 | 0.18 | 3425 | Train 54 | Test 33 | 0.66 | 0.22 | 0.2 | 0.21 |
| 1378 | Train 22 | Test 34 | 0.75 | 0.37 | 0.15 | 0.21 | 3426 | Train 54 | Test 34 | 0.68 | 0.28 | 0.25 | 0.26 |
| 1379 | Train 22 | Test 35 | 0.74 | 0.33 | 0.12 | 0.18 | 3427 | Train 54 | Test 35 | 0.66 | 0.22 | 0.2 | 0.21 |
| 1380 | Train 22 | Test 36 | 0.75 | 0.37 | 0.15 | 0.21 | 3428 | Train 54 | Test 36 | 0.68 | 0.28 | 0.25 | 0.26 |
| 1381 | Train 22 | Test 37 | 0.73 | 0.34 | 0.12 | 0.17 | 3429 | Train 54 | Test 37 | 0.66 | 0.24 | 0.19 | 0.21 |
| 1382 | Train 22 | Test 38 | 0.76 | 0.53 | 0.15 | 0.23 | 3430 | Train 54 | Test 38 | 0.68 | 0.3 | 0.21 | 0.25 |
| 1383 | Train 22 | Test 39 | 0.73 | 0.34 | 0.12 | 0.17 | 3431 | Train 54 | Test 39 | 0.66 | 0.24 | 0.19 | 0.21 |
| 1384 | Train 22 | Test 40 | 0.76 | 0.49 | 0.15 | 0.23 | 3432 | Train 54 | Test 40 | 0.68 | 0.3 | 0.21 | 0.25 |
| 1385 | Train 22 | Test 41 | 0.74 | 0.34 | 0.1 | 0.16 | 3433 | Train 54 | Test 41 | 0.66 | 0.21 | 0.18 | 0.19 |
| 1386 | Train 22 | Test 42 | 0.76 | 0.4 | 0.14 | 0.21 | 3434 | Train 54 | Test 42 | 0.68 | 0.27 | 0.22 | 0.24 |
| 1387 | Train 22 | Test 43 | 0.74 | 0.34 | 0.1 | 0.16 | 3435 | Train 54 | Test 43 | 0.66 | 0.21 | 0.18 | 0.2 |
| 1388 | Train 22 | Test 44 | 0.76 | 0.4 | 0.14 | 0.21 | 3436 | Train 54 | Test 44 | 0.69 | 0.27 | 0.22 | 0.24 |
| 1389 | Train 22 | Test 45 | 0.73 | 0.35 | 0.1 | 0.16 | 3437 | Train 54 | Test 45 | 0.67 | 0.24 | 0.17 | 0.2 |
| 1390 | Train 22 | Test 46 | 0.76 | 0.52 | 0.15 | 0.23 | 3438 | Train 54 | Test 46 | 0.68 | 0.3 | 0.21 | 0.25 |
| 1391 | Train 22 | Test 47 | 0.73 | 0.35 | 0.1 | 0.16 | 3439 | Train 54 | Test 47 | 0.67 | 0.24 | 0.17 | 0.2 |
| 1392 | Train 22 | Test 48 | 0.76 | 0.49 | 0.15 | 0.23 | 3440 | Train 54 | Test 48 | 0.68 | 0.3 | 0.21 | 0.25 |
| 1393 | Train 22 | Test 49 | 0.76 | 0.35 | 0.2 | 0.25 | 3441 | Train 54 | Test 49 | 0.76 | 0.33 | 0.26 | 0.29 |
| 1394 | Train 22 | Test 50 | 0.76 | 0.35 | 0.2 | 0.25 | 3442 | Train 54 | Test 50 | 0.76 | 0.33 | 0.26 | 0.29 |
| 1395 | Train 22 | Test 51 | 0.76 | 0.35 | 0.2 | 0.26 | 3443 | Train 54 | Test 51 | 0.76 | 0.34 | 0.26 | 0.3 |
| 1396 | Train 22 | Test 52 | 0.76 | 0.35 | 0.2 | 0.25 | 3444 | Train 54 | Test 52 | 0.76 | 0.34 | 0.26 | 0.29 |
| 1397 | Train 22 | Test 53 | 0.77 | 0.43 | 0.14 | 0.21 | 3445 | Train 54 | Test 53 | 0.77 | 0.43 | 0.17 | 0.25 |
| 1398 | Train 22 | Test 54 | 0.76 | 0.54 | 0.17 | 0.26 | 3446 | Train 54 | Test 54 | 0.76 | 0.53 | 0.2 | 0.29 |
| 1399 | Train 22 | Test 55 | 0.75 | 0.39 | 0.16 | 0.23 | 3447 | Train 54 | Test 55 | 0.76 | 0.37 | 0.21 | 0.27 |
| 1400 | Train 22 | Test 56 | 0.76 | 0.51 | 0.17 | 0.26 | 3448 | Train 54 | Test 56 | 0.76 | 0.5 | 0.21 | 0.29 |
| 1401 | Train 22 | Test 57 | 0.77 | 0.37 | 0.19 | 0.25 | 3449 | Train 54 | Test 57 | 0.77 | 0.34 | 0.24 | 0.28 |
| 1402 | Train 22 | Test 58 | 0.77 | 0.37 | 0.19 | 0.25 | 3450 | Train 54 | Test 58 | 0.77 | 0.34 | 0.24 | 0.28 |
| 1403 | Train 22 | Test 59 | 0.77 | 0.38 | 0.19 | 0.25 | 3451 | Train 54 | Test 59 | 0.77 | 0.34 | 0.24 | 0.28 |
| 1404 | Train 22 | Test 60 | 0.77 | 0.38 | 0.19 | 0.25 | 3452 | Train 54 | Test 60 | 0.77 | 0.34 | 0.24 | 0.28 |
| 1405 | Train 22 | Test 61 | 0.77 | 0.45 | 0.13 | 0.2 | 3453 | Train 54 | Test 61 | 0.77 | 0.43 | 0.15 | 0.22 |
| 1406 | Train 22 | Test 62 | 0.76 | 0.53 | 0.17 | 0.26 | 3454 | Train 54 | Test 62 | 0.76 | 0.52 | 0.2 | 0.29 |
| 1407 | Train 22 | Test 63 | 0.77 | 0.46 | 0.13 | 0.2 | 3455 | Train 54 | Test 63 | 0.77 | 0.43 | 0.15 | 0.22 |
| 1408 | Train 22 | Test 64 | 0.76 | 0.51 | 0.17 | 0.26 | 3456 | Train 54 | Test 64 | 0.76 | 0.49 | 0.2 | 0.29 |
| 1409 | Train 23 | Test 1 | 0.76 | 0.4 | 0.09 | 0.14 | 3457 | Train 55 | Test 1 | 0.69 | 0.26 | 0.17 | 0.21 |
| 1410 | Train 23 | Test 2 | 0.74 | 0.41 | 0.08 | 0.13 | 3458 | Train 55 | Test 2 | 0.68 | 0.28 | 0.16 | 0.21 |
| 1411 | Train 23 | Test 3 | 0.76 | 0.41 | 0.09 | 0.14 | 3459 | Train 55 | Test 3 | 0.69 | 0.26 | 0.17 | 0.21 |
| 1412 | Train 23 | Test 4 | 0.74 | 0.41 | 0.08 | 0.13 | 3460 | Train 55 | Test 4 | 0.68 | 0.29 | 0.16 | 0.21 |
| 1413 | Train 23 | Test 5 | 0.75 | 0.39 | 0.09 | 0.14 | 3461 | Train 55 | Test 5 | 0.67 | 0.24 | 0.15 | 0.19 |
| 1414 | Train 23 | Test 6 | 0.66 | 0.29 | 0.25 | 0.27 | 3462 | Train 55 | Test 6 | 0.62 | 0.27 | 0.12 | 0.17 |
| 1415 | Train 23 | Test 7 | 0.75 | 0.4 | 0.09 | 0.14 | 3463 | Train 55 | Test 7 | 0.67 | 0.23 | 0.15 | 0.18 |
| 1416 | Train 23 | Test 8 | 0.66 | 0.29 | 0.25 | 0.26 | 3464 | Train 55 | Test 8 | 0.62 | 0.26 | 0.12 | 0.16 |
| 1417 | Train 23 | Test 9 | 0.76 | 0.42 | 0.08 | 0.13 | 3465 | Train 55 | Test 9 | 0.69 | 0.25 | 0.14 | 0.18 |
| 1418 | Train 23 | Test 10 | 0.74 | 0.4 | 0.07 | 0.12 | 3466 | Train 55 | Test 10 | 0.68 | 0.27 | 0.14 | 0.18 |
| 1419 | Train 23 | Test 11 | 0.76 | 0.42 | 0.08 | 0.13 | 3467 | Train 55 | Test 11 | 0.69 | 0.25 | 0.14 | 0.18 |
| 1420 | Train 23 | Test 12 | 0.74 | 0.4 | 0.07 | 0.12 | 3468 | Train 55 | Test 12 | 0.68 | 0.28 | 0.14 | 0.18 |
| 1421 | Train 23 | Test 13 | 0.75 | 0.39 | 0.07 | 0.12 | 3469 | Train 55 | Test 13 | 0.67 | 0.23 | 0.13 | 0.17 |
| 1422 | Train 23 | Test 14 | 0.66 | 0.29 | 0.25 | 0.27 | 3470 | Train 55 | Test 14 | 0.62 | 0.27 | 0.12 | 0.17 |
| 1423 | Train 23 | Test 15 | 0.75 | 0.4 | 0.07 | 0.12 | 3471 | Train 55 | Test 15 | 0.67 | 0.23 | 0.13 | 0.16 |
| 1424 | Train 23 | Test 16 | 0.66 | 0.28 | 0.25 | 0.26 | 3472 | Train 55 | Test 16 | 0.63 | 0.26 | 0.12 | 0.16 |
| 1425 | Train 23 | Test 17 | 0.76 | 0.4 | 0.09 | 0.14 | 3473 | Train 55 | Test 17 | 0.69 | 0.26 | 0.17 | 0.21 |
| 1426 | Train 23 | Test 18 | 0.74 | 0.41 | 0.08 | 0.13 | 3474 | Train 55 | Test 18 | 0.68 | 0.29 | 0.17 | 0.21 |
| 1427 | Train 23 | Test 19 | 0.76 | 0.41 | 0.09 | 0.14 | 3475 | Train 55 | Test 19 | 0.69 | 0.26 | 0.17 | 0.21 |
| 1428 | Train 23 | Test 20 | 0.74 | 0.41 | 0.08 | 0.13 | 3476 | Train 55 | Test 20 | 0.68 | 0.29 | 0.17 | 0.21 |
| 1429 | Train 23 | Test 21 | 0.75 | 0.39 | 0.09 | 0.14 | 3477 | Train 55 | Test 21 | 0.67 | 0.24 | 0.15 | 0.19 |
| 1430 | Train 23 | Test 22 | 0.66 | 0.29 | 0.25 | 0.27 | 3478 | Train 55 | Test 22 | 0.62 | 0.27 | 0.12 | 0.17 |
| 1431 | Train 23 | Test 23 | 0.75 | 0.4 | 0.09 | 0.14 | 3479 | Train 55 | Test 23 | 0.67 | 0.24 | 0.15 | 0.19 |
| 1432 | Train 23 | Test 24 | 0.66 | 0.28 | 0.25 | 0.26 | 3480 | Train 55 | Test 24 | 0.62 | 0.26 | 0.12 | 0.16 |
| 1433 | Train 23 | Test 25 | 0.76 | 0.42 | 0.08 | 0.13 | 3481 | Train 55 | Test 25 | 0.69 | 0.25 | 0.15 | 0.18 |
| 1434 | Train 23 | Test 26 | 0.74 | 0.4 | 0.07 | 0.12 | 3482 | Train 55 | Test 26 | 0.68 | 0.27 | 0.14 | 0.18 |
| 1435 | Train 23 | Test 27 | 0.76 | 0.42 | 0.08 | 0.13 | 3483 | Train 55 | Test 27 | 0.69 | 0.25 | 0.15 | 0.18 |
| 1436 | Train 23 | Test 28 | 0.74 | 0.4 | 0.07 | 0.12 | 3484 | Train 55 | Test 28 | 0.68 | 0.28 | 0.14 | 0.18 |
| 1437 | Train 23 | Test 29 | 0.75 | 0.39 | 0.07 | 0.12 | 3485 | Train 55 | Test 29 | 0.68 | 0.23 | 0.13 | 0.17 |
| 1438 | Train 23 | Test 30 | 0.66 | 0.29 | 0.25 | 0.27 | 3486 | Train 55 | Test 30 | 0.62 | 0.27 | 0.12 | 0.17 |
| 1439 | Train 23 | Test 31 | 0.75 | 0.4 | 0.07 | 0.12 | 3487 | Train 55 | Test 31 | 0.67 | 0.23 | 0.13 | 0.16 |
| 1440 | Train 23 | Test 32 | 0.66 | 0.28 | 0.25 | 0.26 | 3488 | Train 55 | Test 32 | 0.63 | 0.26 | 0.12 | 0.16 |
| 1441 | Train 23 | Test 33 | 0.76 | 0.4 | 0.08 | 0.14 | 3489 | Train 55 | Test 33 | 0.69 | 0.26 | 0.17 | 0.21 |
| 1442 | Train 23 | Test 34 | 0.74 | 0.41 | 0.08 | 0.13 | 3490 | Train 55 | Test 34 | 0.68 | 0.28 | 0.16 | 0.21 |
| 1443 | Train 23 | Test 35 | 0.76 | 0.41 | 0.09 | 0.14 | 3491 | Train 55 | Test 35 | 0.69 | 0.26 | 0.17 | 0.21 |
| 1444 | Train 23 | Test 36 | 0.74 | 0.41 | 0.08 | 0.13 | 3492 | Train 55 | Test 36 | 0.68 | 0.28 | 0.16 | 0.21 |
| 1445 | Train 23 | Test 37 | 0.75 | 0.39 | 0.08 | 0.14 | 3493 | Train 55 | Test 37 | 0.67 | 0.24 | 0.15 | 0.19 |
| 1446 | Train 23 | Test 38 | 0.66 | 0.29 | 0.25 | 0.27 | 3494 | Train 55 | Test 38 | 0.62 | 0.27 | 0.12 | 0.17 |
| 1447 | Train 23 | Test 39 | 0.75 | 0.4 | 0.08 | 0.14 | 3495 | Train 55 | Test 39 | 0.67 | 0.23 | 0.15 | 0.18 |
| 1448 | Train 23 | Test 40 | 0.66 | 0.28 | 0.25 | 0.26 | 3496 | Train 55 | Test 40 | 0.63 | 0.26 | 0.12 | 0.16 |
| 1449 | Train 23 | Test 41 | 0.76 | 0.41 | 0.08 | 0.13 | 3497 | Train 55 | Test 41 | 0.69 | 0.25 | 0.15 | 0.18 |
| 1450 | Train 23 | Test 42 | 0.74 | 0.4 | 0.07 | 0.12 | 3498 | Train 55 | Test 42 | 0.68 | 0.27 | 0.14 | 0.18 |
| 1451 | Train 23 | Test 43 | 0.76 | 0.41 | 0.08 | 0.13 | 3499 | Train 55 | Test 43 | 0.69 | 0.25 | 0.14 | 0.18 |
| 1452 | Train 23 | Test 44 | 0.74 | 0.4 | 0.07 | 0.12 | 3500 | Train 55 | Test 44 | 0.68 | 0.27 | 0.14 | 0.18 |
| 1453 | Train 23 | Test 45 | 0.75 | 0.39 | 0.07 | 0.12 | 3501 | Train 55 | Test 45 | 0.68 | 0.23 | 0.13 | 0.16 |
| 1454 | Train 23 | Test 46 | 0.66 | 0.29 | 0.25 | 0.27 | 3502 | Train 55 | Test 46 | 0.62 | 0.27 | 0.12 | 0.17 |
| 1455 | Train 23 | Test 47 | 0.75 | 0.4 | 0.07 | 0.12 | 3503 | Train 55 | Test 47 | 0.68 | 0.23 | 0.13 | 0.16 |
| 1456 | Train 23 | Test 48 | 0.66 | 0.28 | 0.25 | 0.26 | 3504 | Train 55 | Test 48 | 0.63 | 0.26 | 0.12 | 0.16 |
| 1457 | Train 23 | Test 49 | 0.77 | 0.44 | 0.14 | 0.21 | 3505 | Train 55 | Test 49 | 0.75 | 0.4 | 0.19 | 0.26 |
| 1458 | Train 23 | Test 50 | 0.77 | 0.43 | 0.14 | 0.21 | 3506 | Train 55 | Test 50 | 0.75 | 0.4 | 0.19 | 0.26 |
| 1459 | Train 23 | Test 51 | 0.77 | 0.44 | 0.14 | 0.22 | 3507 | Train 55 | Test 51 | 0.75 | 0.4 | 0.19 | 0.26 |
| 1460 | Train 23 | Test 52 | 0.77 | 0.44 | 0.14 | 0.22 | 3508 | Train 55 | Test 52 | 0.75 | 0.4 | 0.19 | 0.26 |
| 1461 | Train 23 | Test 53 | 0.74 | 0.32 | 0.14 | 0.2 | 3509 | Train 55 | Test 53 | 0.69 | 0.3 | 0.14 | 0.19 |
| 1462 | Train 23 | Test 54 | 0.67 | 0.35 | 0.19 | 0.24 | 3510 | Train 55 | Test 54 | 0.65 | 0.32 | 0.12 | 0.17 |
| 1463 | Train 23 | Test 55 | 0.77 | 0.42 | 0.12 | 0.18 | 3511 | Train 55 | Test 55 | 0.75 | 0.39 | 0.16 | 0.23 |
| 1464 | Train 23 | Test 56 | 0.66 | 0.33 | 0.19 | 0.24 | 3512 | Train 55 | Test 56 | 0.66 | 0.32 | 0.12 | 0.17 |
| 1465 | Train 23 | Test 57 | 0.77 | 0.45 | 0.13 | 0.2 | 3513 | Train 55 | Test 57 | 0.76 | 0.41 | 0.16 | 0.23 |
| 1466 | Train 23 | Test 58 | 0.77 | 0.45 | 0.13 | 0.2 | 3514 | Train 55 | Test 58 | 0.76 | 0.41 | 0.16 | 0.23 |
| 1467 | Train 23 | Test 59 | 0.78 | 0.45 | 0.13 | 0.2 | 3515 | Train 55 | Test 59 | 0.76 | 0.41 | 0.16 | 0.23 |
| 1468 | Train 23 | Test 60 | 0.77 | 0.45 | 0.13 | 0.2 | 3516 | Train 55 | Test 60 | 0.76 | 0.41 | 0.16 | 0.23 |
| 1469 | Train 23 | Test 61 | 0.74 | 0.33 | 0.14 | 0.19 | 3517 | Train 55 | Test 61 | 0.7 | 0.32 | 0.14 | 0.19 |
| 1470 | Train 23 | Test 62 | 0.67 | 0.35 | 0.19 | 0.24 | 3518 | Train 55 | Test 62 | 0.65 | 0.32 | 0.12 | 0.17 |
| 1471 | Train 23 | Test 63 | 0.74 | 0.31 | 0.14 | 0.19 | 3519 | Train 55 | Test 63 | 0.7 | 0.31 | 0.14 | 0.19 |
| 1472 | Train 23 | Test 64 | 0.66 | 0.32 | 0.19 | 0.24 | 3520 | Train 55 | Test 64 | 0.66 | 0.31 | 0.12 | 0.17 |
| 1473 | Train 24 | Test 1 | 0.75 | 0.34 | 0.14 | 0.19 | 3521 | Train 56 | Test 1 | 0.69 | 0.22 | 0.2 | 0.21 |
| 1474 | Train 24 | Test 2 | 0.75 | 0.39 | 0.18 | 0.24 | 3522 | Train 56 | Test 2 | 0.7 | 0.28 | 0.24 | 0.26 |
| 1475 | Train 24 | Test 3 | 0.75 | 0.35 | 0.13 | 0.19 | 3523 | Train 56 | Test 3 | 0.69 | 0.22 | 0.2 | 0.21 |
| 1476 | Train 24 | Test 4 | 0.75 | 0.39 | 0.18 | 0.24 | 3524 | Train 56 | Test 4 | 0.7 | 0.28 | 0.24 | 0.26 |
| 1477 | Train 24 | Test 5 | 0.73 | 0.34 | 0.13 | 0.18 | 3525 | Train 56 | Test 5 | 0.69 | 0.24 | 0.19 | 0.21 |
| 1478 | Train 24 | Test 6 | 0.76 | 0.53 | 0.14 | 0.22 | 3526 | Train 56 | Test 6 | 0.7 | 0.37 | 0.16 | 0.23 |
| 1479 | Train 24 | Test 7 | 0.73 | 0.35 | 0.12 | 0.18 | 3527 | Train 56 | Test 7 | 0.69 | 0.24 | 0.19 | 0.21 |
| 1480 | Train 24 | Test 8 | 0.76 | 0.53 | 0.14 | 0.22 | 3528 | Train 56 | Test 8 | 0.7 | 0.37 | 0.16 | 0.22 |
| 1481 | Train 24 | Test 9 | 0.75 | 0.35 | 0.12 | 0.18 | 3529 | Train 56 | Test 9 | 0.69 | 0.22 | 0.18 | 0.2 |
| 1482 | Train 24 | Test 10 | 0.76 | 0.39 | 0.16 | 0.23 | 3530 | Train 56 | Test 10 | 0.7 | 0.27 | 0.21 | 0.24 |
| 1483 | Train 24 | Test 11 | 0.75 | 0.35 | 0.12 | 0.18 | 3531 | Train 56 | Test 11 | 0.69 | 0.23 | 0.18 | 0.2 |
| 1484 | Train 24 | Test 12 | 0.76 | 0.39 | 0.16 | 0.23 | 3532 | Train 56 | Test 12 | 0.7 | 0.27 | 0.21 | 0.24 |
| 1485 | Train 24 | Test 13 | 0.73 | 0.36 | 0.11 | 0.17 | 3533 | Train 56 | Test 13 | 0.69 | 0.25 | 0.18 | 0.21 |
| 1486 | Train 24 | Test 14 | 0.76 | 0.53 | 0.14 | 0.22 | 3534 | Train 56 | Test 14 | 0.7 | 0.37 | 0.16 | 0.23 |
| 1487 | Train 24 | Test 15 | 0.73 | 0.36 | 0.11 | 0.17 | 3535 | Train 56 | Test 15 | 0.69 | 0.25 | 0.18 | 0.21 |
| 1488 | Train 24 | Test 16 | 0.76 | 0.52 | 0.14 | 0.22 | 3536 | Train 56 | Test 16 | 0.7 | 0.37 | 0.16 | 0.22 |
| 1489 | Train 24 | Test 17 | 0.75 | 0.34 | 0.14 | 0.19 | 3537 | Train 56 | Test 17 | 0.69 | 0.22 | 0.2 | 0.21 |
| 1490 | Train 24 | Test 18 | 0.75 | 0.39 | 0.18 | 0.24 | 3538 | Train 56 | Test 18 | 0.7 | 0.28 | 0.24 | 0.26 |
| 1491 | Train 24 | Test 19 | 0.75 | 0.34 | 0.13 | 0.19 | 3539 | Train 56 | Test 19 | 0.69 | 0.22 | 0.2 | 0.21 |
| 1492 | Train 24 | Test 20 | 0.75 | 0.39 | 0.18 | 0.24 | 3540 | Train 56 | Test 20 | 0.7 | 0.28 | 0.24 | 0.26 |
| 1493 | Train 24 | Test 21 | 0.73 | 0.34 | 0.12 | 0.18 | 3541 | Train 56 | Test 21 | 0.69 | 0.24 | 0.19 | 0.21 |
| 1494 | Train 24 | Test 22 | 0.76 | 0.53 | 0.14 | 0.22 | 3542 | Train 56 | Test 22 | 0.7 | 0.37 | 0.16 | 0.23 |
| 1495 | Train 24 | Test 23 | 0.73 | 0.35 | 0.12 | 0.18 | 3543 | Train 56 | Test 23 | 0.69 | 0.24 | 0.19 | 0.21 |
| 1496 | Train 24 | Test 24 | 0.76 | 0.53 | 0.14 | 0.22 | 3544 | Train 56 | Test 24 | 0.7 | 0.37 | 0.16 | 0.22 |
| 1497 | Train 24 | Test 25 | 0.75 | 0.35 | 0.12 | 0.18 | 3545 | Train 56 | Test 25 | 0.69 | 0.22 | 0.18 | 0.2 |
| 1498 | Train 24 | Test 26 | 0.76 | 0.39 | 0.16 | 0.23 | 3546 | Train 56 | Test 26 | 0.7 | 0.27 | 0.21 | 0.23 |
| 1499 | Train 24 | Test 27 | 0.75 | 0.35 | 0.12 | 0.18 | 3547 | Train 56 | Test 27 | 0.69 | 0.23 | 0.18 | 0.2 |
| 1500 | Train 24 | Test 28 | 0.76 | 0.39 | 0.16 | 0.23 | 3548 | Train 56 | Test 28 | 0.7 | 0.27 | 0.21 | 0.24 |
| 1501 | Train 24 | Test 29 | 0.73 | 0.36 | 0.11 | 0.17 | 3549 | Train 56 | Test 29 | 0.69 | 0.25 | 0.18 | 0.21 |
| 1502 | Train 24 | Test 30 | 0.76 | 0.53 | 0.14 | 0.22 | 3550 | Train 56 | Test 30 | 0.7 | 0.37 | 0.16 | 0.23 |
| 1503 | Train 24 | Test 31 | 0.73 | 0.35 | 0.11 | 0.17 | 3551 | Train 56 | Test 31 | 0.69 | 0.25 | 0.18 | 0.21 |
| 1504 | Train 24 | Test 32 | 0.76 | 0.52 | 0.14 | 0.22 | 3552 | Train 56 | Test 32 | 0.7 | 0.37 | 0.16 | 0.22 |
| 1505 | Train 24 | Test 33 | 0.75 | 0.34 | 0.13 | 0.19 | 3553 | Train 56 | Test 33 | 0.69 | 0.22 | 0.2 | 0.21 |
| 1506 | Train 24 | Test 34 | 0.76 | 0.39 | 0.18 | 0.24 | 3554 | Train 56 | Test 34 | 0.7 | 0.27 | 0.24 | 0.25 |
| 1507 | Train 24 | Test 35 | 0.75 | 0.34 | 0.13 | 0.19 | 3555 | Train 56 | Test 35 | 0.69 | 0.22 | 0.2 | 0.21 |
| 1508 | Train 24 | Test 36 | 0.76 | 0.39 | 0.17 | 0.24 | 3556 | Train 56 | Test 36 | 0.7 | 0.27 | 0.24 | 0.25 |
| 1509 | Train 24 | Test 37 | 0.73 | 0.34 | 0.12 | 0.18 | 3557 | Train 56 | Test 37 | 0.69 | 0.24 | 0.19 | 0.21 |
| 1510 | Train 24 | Test 38 | 0.76 | 0.53 | 0.14 | 0.22 | 3558 | Train 56 | Test 38 | 0.7 | 0.37 | 0.16 | 0.23 |
| 1511 | Train 24 | Test 39 | 0.73 | 0.35 | 0.12 | 0.18 | 3559 | Train 56 | Test 39 | 0.69 | 0.24 | 0.19 | 0.21 |
| 1512 | Train 24 | Test 40 | 0.76 | 0.52 | 0.14 | 0.22 | 3560 | Train 56 | Test 40 | 0.7 | 0.37 | 0.16 | 0.22 |
| 1513 | Train 24 | Test 41 | 0.75 | 0.34 | 0.12 | 0.18 | 3561 | Train 56 | Test 41 | 0.69 | 0.22 | 0.18 | 0.2 |
| 1514 | Train 24 | Test 42 | 0.76 | 0.39 | 0.16 | 0.23 | 3562 | Train 56 | Test 42 | 0.7 | 0.27 | 0.21 | 0.23 |
| 1515 | Train 24 | Test 43 | 0.75 | 0.34 | 0.12 | 0.18 | 3563 | Train 56 | Test 43 | 0.69 | 0.22 | 0.18 | 0.2 |
| 1516 | Train 24 | Test 44 | 0.76 | 0.39 | 0.16 | 0.22 | 3564 | Train 56 | Test 44 | 0.7 | 0.27 | 0.21 | 0.23 |
| 1517 | Train 24 | Test 45 | 0.73 | 0.36 | 0.11 | 0.17 | 3565 | Train 56 | Test 45 | 0.69 | 0.24 | 0.18 | 0.2 |
| 1518 | Train 24 | Test 46 | 0.76 | 0.53 | 0.14 | 0.22 | 3566 | Train 56 | Test 46 | 0.7 | 0.37 | 0.16 | 0.23 |
| 1519 | Train 24 | Test 47 | 0.73 | 0.36 | 0.11 | 0.17 | 3567 | Train 56 | Test 47 | 0.69 | 0.25 | 0.17 | 0.2 |
| 1520 | Train 24 | Test 48 | 0.76 | 0.52 | 0.14 | 0.22 | 3568 | Train 56 | Test 48 | 0.7 | 0.37 | 0.16 | 0.22 |
| 1521 | Train 24 | Test 49 | 0.77 | 0.37 | 0.23 | 0.29 | 3569 | Train 56 | Test 49 | 0.75 | 0.34 | 0.26 | 0.3 |
| 1522 | Train 24 | Test 50 | 0.77 | 0.37 | 0.23 | 0.28 | 3570 | Train 56 | Test 50 | 0.75 | 0.34 | 0.26 | 0.3 |
| 1523 | Train 24 | Test 51 | 0.77 | 0.37 | 0.23 | 0.28 | 3571 | Train 56 | Test 51 | 0.75 | 0.34 | 0.26 | 0.3 |
| 1524 | Train 24 | Test 52 | 0.77 | 0.37 | 0.23 | 0.28 | 3572 | Train 56 | Test 52 | 0.75 | 0.35 | 0.26 | 0.3 |
| 1525 | Train 24 | Test 53 | 0.77 | 0.45 | 0.15 | 0.22 | 3573 | Train 56 | Test 53 | 0.77 | 0.39 | 0.15 | 0.22 |
| 1526 | Train 24 | Test 54 | 0.76 | 0.54 | 0.17 | 0.26 | 3574 | Train 56 | Test 54 | 0.76 | 0.51 | 0.19 | 0.28 |
| 1527 | Train 24 | Test 55 | 0.76 | 0.4 | 0.19 | 0.26 | 3575 | Train 56 | Test 55 | 0.76 | 0.36 | 0.21 | 0.26 |
| 1528 | Train 24 | Test 56 | 0.76 | 0.54 | 0.16 | 0.25 | 3576 | Train 56 | Test 56 | 0.76 | 0.51 | 0.19 | 0.27 |
| 1529 | Train 24 | Test 57 | 0.77 | 0.36 | 0.21 | 0.26 | 3577 | Train 56 | Test 57 | 0.75 | 0.35 | 0.24 | 0.28 |
| 1530 | Train 24 | Test 58 | 0.77 | 0.35 | 0.21 | 0.26 | 3578 | Train 56 | Test 58 | 0.75 | 0.35 | 0.23 | 0.28 |
| 1531 | Train 24 | Test 59 | 0.77 | 0.36 | 0.2 | 0.26 | 3579 | Train 56 | Test 59 | 0.75 | 0.36 | 0.24 | 0.29 |
| 1532 | Train 24 | Test 60 | 0.77 | 0.35 | 0.2 | 0.26 | 3580 | Train 56 | Test 60 | 0.75 | 0.35 | 0.24 | 0.28 |
| 1533 | Train 24 | Test 61 | 0.78 | 0.45 | 0.13 | 0.2 | 3581 | Train 56 | Test 61 | 0.77 | 0.41 | 0.14 | 0.21 |
| 1534 | Train 24 | Test 62 | 0.76 | 0.54 | 0.17 | 0.26 | 3582 | Train 56 | Test 62 | 0.76 | 0.51 | 0.19 | 0.28 |
| 1535 | Train 24 | Test 63 | 0.78 | 0.45 | 0.13 | 0.2 | 3583 | Train 56 | Test 63 | 0.77 | 0.41 | 0.15 | 0.21 |
| 1536 | Train 24 | Test 64 | 0.76 | 0.53 | 0.16 | 0.25 | 3584 | Train 56 | Test 64 | 0.76 | 0.51 | 0.19 | 0.27 |
| 1537 | Train 25 | Test 1 | 0.74 | 0.36 | 0.14 | 0.2 | 3585 | Train 57 | Test 1 | 0.67 | 0.23 | 0.17 | 0.2 |
| 1538 | Train 25 | Test 2 | 0.72 | 0.36 | 0.12 | 0.19 | 3586 | Train 57 | Test 2 | 0.65 | 0.24 | 0.15 | 0.19 |
| 1539 | Train 25 | Test 3 | 0.74 | 0.36 | 0.14 | 0.2 | 3587 | Train 57 | Test 3 | 0.67 | 0.23 | 0.17 | 0.2 |
| 1540 | Train 25 | Test 4 | 0.72 | 0.37 | 0.13 | 0.19 | 3588 | Train 57 | Test 4 | 0.65 | 0.24 | 0.16 | 0.19 |
| 1541 | Train 25 | Test 5 | 0.69 | 0.26 | 0.13 | 0.18 | 3589 | Train 57 | Test 5 | 0.65 | 0.22 | 0.18 | 0.2 |
| 1542 | Train 25 | Test 6 | 0.65 | 0.3 | 0.07 | 0.11 | 3590 | Train 57 | Test 6 | 0.63 | 0.25 | 0.2 | 0.22 |
| 1543 | Train 25 | Test 7 | 0.69 | 0.27 | 0.13 | 0.18 | 3591 | Train 57 | Test 7 | 0.65 | 0.22 | 0.18 | 0.2 |
| 1544 | Train 25 | Test 8 | 0.65 | 0.3 | 0.07 | 0.11 | 3592 | Train 57 | Test 8 | 0.63 | 0.25 | 0.19 | 0.22 |
| 1545 | Train 25 | Test 9 | 0.75 | 0.37 | 0.11 | 0.18 | 3593 | Train 57 | Test 9 | 0.68 | 0.24 | 0.17 | 0.2 |
| 1546 | Train 25 | Test 10 | 0.73 | 0.38 | 0.11 | 0.17 | 3594 | Train 57 | Test 10 | 0.65 | 0.25 | 0.14 | 0.18 |
| 1547 | Train 25 | Test 11 | 0.75 | 0.37 | 0.12 | 0.18 | 3595 | Train 57 | Test 11 | 0.68 | 0.25 | 0.17 | 0.2 |
| 1548 | Train 25 | Test 12 | 0.73 | 0.38 | 0.11 | 0.17 | 3596 | Train 57 | Test 12 | 0.65 | 0.25 | 0.15 | 0.18 |
| 1549 | Train 25 | Test 13 | 0.69 | 0.27 | 0.12 | 0.16 | 3597 | Train 57 | Test 13 | 0.65 | 0.22 | 0.17 | 0.19 |
| 1550 | Train 25 | Test 14 | 0.65 | 0.3 | 0.07 | 0.11 | 3598 | Train 57 | Test 14 | 0.63 | 0.25 | 0.2 | 0.22 |
| 1551 | Train 25 | Test 15 | 0.69 | 0.27 | 0.12 | 0.16 | 3599 | Train 57 | Test 15 | 0.65 | 0.22 | 0.17 | 0.19 |
| 1552 | Train 25 | Test 16 | 0.65 | 0.29 | 0.07 | 0.11 | 3600 | Train 57 | Test 16 | 0.63 | 0.25 | 0.19 | 0.22 |
| 1553 | Train 25 | Test 17 | 0.74 | 0.36 | 0.14 | 0.2 | 3601 | Train 57 | Test 17 | 0.67 | 0.23 | 0.17 | 0.2 |
| 1554 | Train 25 | Test 18 | 0.72 | 0.36 | 0.13 | 0.19 | 3602 | Train 57 | Test 18 | 0.65 | 0.24 | 0.15 | 0.19 |
| 1555 | Train 25 | Test 19 | 0.74 | 0.36 | 0.14 | 0.2 | 3603 | Train 57 | Test 19 | 0.67 | 0.23 | 0.17 | 0.2 |
| 1556 | Train 25 | Test 20 | 0.72 | 0.37 | 0.13 | 0.19 | 3604 | Train 57 | Test 20 | 0.65 | 0.24 | 0.16 | 0.19 |
| 1557 | Train 25 | Test 21 | 0.69 | 0.27 | 0.13 | 0.18 | 3605 | Train 57 | Test 21 | 0.65 | 0.22 | 0.18 | 0.2 |
| 1558 | Train 25 | Test 22 | 0.65 | 0.3 | 0.07 | 0.11 | 3606 | Train 57 | Test 22 | 0.63 | 0.25 | 0.2 | 0.22 |
| 1559 | Train 25 | Test 23 | 0.69 | 0.27 | 0.13 | 0.18 | 3607 | Train 57 | Test 23 | 0.65 | 0.22 | 0.18 | 0.2 |
| 1560 | Train 25 | Test 24 | 0.66 | 0.3 | 0.07 | 0.11 | 3608 | Train 57 | Test 24 | 0.63 | 0.25 | 0.19 | 0.22 |
| 1561 | Train 25 | Test 25 | 0.75 | 0.37 | 0.11 | 0.18 | 3609 | Train 57 | Test 25 | 0.68 | 0.24 | 0.17 | 0.2 |
| 1562 | Train 25 | Test 26 | 0.73 | 0.38 | 0.11 | 0.17 | 3610 | Train 57 | Test 26 | 0.65 | 0.25 | 0.14 | 0.18 |
| 1563 | Train 25 | Test 27 | 0.75 | 0.37 | 0.12 | 0.18 | 3611 | Train 57 | Test 27 | 0.68 | 0.25 | 0.17 | 0.2 |
| 1564 | Train 25 | Test 28 | 0.73 | 0.38 | 0.11 | 0.17 | 3612 | Train 57 | Test 28 | 0.65 | 0.25 | 0.15 | 0.18 |
| 1565 | Train 25 | Test 29 | 0.69 | 0.27 | 0.12 | 0.16 | 3613 | Train 57 | Test 29 | 0.65 | 0.22 | 0.17 | 0.19 |
| 1566 | Train 25 | Test 30 | 0.65 | 0.29 | 0.07 | 0.11 | 3614 | Train 57 | Test 30 | 0.63 | 0.25 | 0.2 | 0.22 |
| 1567 | Train 25 | Test 31 | 0.69 | 0.27 | 0.12 | 0.16 | 3615 | Train 57 | Test 31 | 0.65 | 0.22 | 0.17 | 0.19 |
| 1568 | Train 25 | Test 32 | 0.66 | 0.29 | 0.07 | 0.11 | 3616 | Train 57 | Test 32 | 0.63 | 0.25 | 0.2 | 0.22 |
| 1569 | Train 25 | Test 33 | 0.74 | 0.36 | 0.13 | 0.2 | 3617 | Train 57 | Test 33 | 0.67 | 0.23 | 0.17 | 0.2 |
| 1570 | Train 25 | Test 34 | 0.73 | 0.36 | 0.12 | 0.18 | 3618 | Train 57 | Test 34 | 0.65 | 0.24 | 0.15 | 0.19 |
| 1571 | Train 25 | Test 35 | 0.74 | 0.36 | 0.14 | 0.2 | 3619 | Train 57 | Test 35 | 0.67 | 0.23 | 0.17 | 0.2 |
| 1572 | Train 25 | Test 36 | 0.73 | 0.36 | 0.13 | 0.19 | 3620 | Train 57 | Test 36 | 0.65 | 0.24 | 0.16 | 0.19 |
| 1573 | Train 25 | Test 37 | 0.69 | 0.26 | 0.13 | 0.17 | 3621 | Train 57 | Test 37 | 0.65 | 0.22 | 0.18 | 0.2 |
| 1574 | Train 25 | Test 38 | 0.65 | 0.3 | 0.07 | 0.11 | 3622 | Train 57 | Test 38 | 0.63 | 0.25 | 0.2 | 0.22 |
| 1575 | Train 25 | Test 39 | 0.69 | 0.26 | 0.13 | 0.17 | 3623 | Train 57 | Test 39 | 0.65 | 0.22 | 0.18 | 0.2 |
| 1576 | Train 25 | Test 40 | 0.66 | 0.3 | 0.07 | 0.11 | 3624 | Train 57 | Test 40 | 0.63 | 0.25 | 0.19 | 0.22 |
| 1577 | Train 25 | Test 41 | 0.75 | 0.37 | 0.11 | 0.17 | 3625 | Train 57 | Test 41 | 0.68 | 0.24 | 0.17 | 0.2 |
| 1578 | Train 25 | Test 42 | 0.73 | 0.38 | 0.11 | 0.17 | 3626 | Train 57 | Test 42 | 0.65 | 0.25 | 0.14 | 0.18 |
| 1579 | Train 25 | Test 43 | 0.75 | 0.37 | 0.11 | 0.18 | 3627 | Train 57 | Test 43 | 0.68 | 0.25 | 0.17 | 0.2 |
| 1580 | Train 25 | Test 44 | 0.73 | 0.38 | 0.11 | 0.17 | 3628 | Train 57 | Test 44 | 0.65 | 0.25 | 0.14 | 0.18 |
| 1581 | Train 25 | Test 45 | 0.69 | 0.26 | 0.12 | 0.16 | 3629 | Train 57 | Test 45 | 0.66 | 0.22 | 0.17 | 0.19 |
| 1582 | Train 25 | Test 46 | 0.65 | 0.29 | 0.07 | 0.11 | 3630 | Train 57 | Test 46 | 0.63 | 0.25 | 0.2 | 0.22 |
| 1583 | Train 25 | Test 47 | 0.69 | 0.26 | 0.12 | 0.16 | 3631 | Train 57 | Test 47 | 0.66 | 0.22 | 0.17 | 0.19 |
| 1584 | Train 25 | Test 48 | 0.66 | 0.29 | 0.07 | 0.11 | 3632 | Train 57 | Test 48 | 0.63 | 0.25 | 0.19 | 0.22 |
| 1585 | Train 25 | Test 49 | 0.75 | 0.41 | 0.2 | 0.27 | 3633 | Train 57 | Test 49 | 0.73 | 0.34 | 0.19 | 0.24 |
| 1586 | Train 25 | Test 50 | 0.75 | 0.41 | 0.2 | 0.27 | 3634 | Train 57 | Test 50 | 0.73 | 0.34 | 0.19 | 0.24 |
| 1587 | Train 25 | Test 51 | 0.75 | 0.41 | 0.2 | 0.27 | 3635 | Train 57 | Test 51 | 0.73 | 0.35 | 0.19 | 0.25 |
| 1588 | Train 25 | Test 52 | 0.75 | 0.41 | 0.2 | 0.27 | 3636 | Train 57 | Test 52 | 0.73 | 0.35 | 0.19 | 0.25 |
| 1589 | Train 25 | Test 53 | 0.68 | 0.25 | 0.12 | 0.16 | 3637 | Train 57 | Test 53 | 0.69 | 0.3 | 0.19 | 0.23 |
| 1590 | Train 25 | Test 54 | 0.67 | 0.34 | 0.08 | 0.13 | 3638 | Train 57 | Test 54 | 0.65 | 0.32 | 0.19 | 0.23 |
| 1591 | Train 25 | Test 55 | 0.71 | 0.29 | 0.17 | 0.21 | 3639 | Train 57 | Test 55 | 0.7 | 0.3 | 0.2 | 0.24 |
| 1592 | Train 25 | Test 56 | 0.67 | 0.34 | 0.08 | 0.13 | 3640 | Train 57 | Test 56 | 0.65 | 0.32 | 0.18 | 0.23 |
| 1593 | Train 25 | Test 57 | 0.76 | 0.46 | 0.18 | 0.26 | 3641 | Train 57 | Test 57 | 0.74 | 0.39 | 0.19 | 0.26 |
| 1594 | Train 25 | Test 58 | 0.76 | 0.45 | 0.18 | 0.26 | 3642 | Train 57 | Test 58 | 0.74 | 0.38 | 0.19 | 0.26 |
| 1595 | Train 25 | Test 59 | 0.76 | 0.46 | 0.18 | 0.26 | 3643 | Train 57 | Test 59 | 0.74 | 0.39 | 0.2 | 0.26 |
| 1596 | Train 25 | Test 60 | 0.76 | 0.45 | 0.18 | 0.26 | 3644 | Train 57 | Test 60 | 0.74 | 0.39 | 0.2 | 0.26 |
| 1597 | Train 25 | Test 61 | 0.68 | 0.25 | 0.11 | 0.16 | 3645 | Train 57 | Test 61 | 0.7 | 0.3 | 0.2 | 0.24 |
| 1598 | Train 25 | Test 62 | 0.67 | 0.34 | 0.08 | 0.13 | 3646 | Train 57 | Test 62 | 0.65 | 0.32 | 0.19 | 0.24 |
| 1599 | Train 25 | Test 63 | 0.69 | 0.25 | 0.11 | 0.16 | 3647 | Train 57 | Test 63 | 0.7 | 0.31 | 0.2 | 0.24 |
| 1600 | Train 25 | Test 64 | 0.67 | 0.33 | 0.08 | 0.13 | 3648 | Train 57 | Test 64 | 0.66 | 0.32 | 0.18 | 0.23 |
| 1601 | Train 26 | Test 1 | 0.75 | 0.36 | 0.12 | 0.18 | 3649 | Train 58 | Test 1 | 0.62 | 0.25 | 0.11 | 0.16 |
| 1602 | Train 26 | Test 2 | 0.76 | 0.43 | 0.18 | 0.25 | 3650 | Train 58 | Test 2 | 0.62 | 0.27 | 0.11 | 0.16 |
| 1603 | Train 26 | Test 3 | 0.75 | 0.36 | 0.12 | 0.18 | 3651 | Train 58 | Test 3 | 0.62 | 0.26 | 0.11 | 0.16 |
| 1604 | Train 26 | Test 4 | 0.76 | 0.43 | 0.18 | 0.25 | 3652 | Train 58 | Test 4 | 0.62 | 0.28 | 0.11 | 0.16 |
| 1605 | Train 26 | Test 5 | 0.68 | 0.22 | 0.14 | 0.17 | 3653 | Train 58 | Test 5 | 0.61 | 0.22 | 0.1 | 0.13 |
| 1606 | Train 26 | Test 6 | 0.67 | 0.28 | 0.1 | 0.15 | 3654 | Train 58 | Test 6 | 0.61 | 0.28 | 0.09 | 0.14 |
| 1607 | Train 26 | Test 7 | 0.68 | 0.22 | 0.14 | 0.17 | 3655 | Train 58 | Test 7 | 0.61 | 0.22 | 0.1 | 0.13 |
| 1608 | Train 26 | Test 8 | 0.67 | 0.28 | 0.1 | 0.15 | 3656 | Train 58 | Test 8 | 0.6 | 0.27 | 0.09 | 0.14 |
| 1609 | Train 26 | Test 9 | 0.76 | 0.38 | 0.1 | 0.16 | 3657 | Train 58 | Test 9 | 0.62 | 0.27 | 0.11 | 0.15 |
| 1610 | Train 26 | Test 10 | 0.77 | 0.44 | 0.16 | 0.24 | 3658 | Train 58 | Test 10 | 0.62 | 0.28 | 0.1 | 0.15 |
| 1611 | Train 26 | Test 11 | 0.76 | 0.38 | 0.1 | 0.16 | 3659 | Train 58 | Test 11 | 0.62 | 0.28 | 0.11 | 0.16 |
| 1612 | Train 26 | Test 12 | 0.77 | 0.45 | 0.16 | 0.24 | 3660 | Train 58 | Test 12 | 0.62 | 0.29 | 0.11 | 0.15 |
| 1613 | Train 26 | Test 13 | 0.69 | 0.21 | 0.13 | 0.16 | 3661 | Train 58 | Test 13 | 0.61 | 0.22 | 0.09 | 0.13 |
| 1614 | Train 26 | Test 14 | 0.67 | 0.28 | 0.1 | 0.15 | 3662 | Train 58 | Test 14 | 0.61 | 0.28 | 0.09 | 0.14 |
| 1615 | Train 26 | Test 15 | 0.69 | 0.21 | 0.13 | 0.16 | 3663 | Train 58 | Test 15 | 0.61 | 0.22 | 0.09 | 0.13 |
| 1616 | Train 26 | Test 16 | 0.67 | 0.27 | 0.1 | 0.15 | 3664 | Train 58 | Test 16 | 0.61 | 0.27 | 0.09 | 0.14 |
| 1617 | Train 26 | Test 17 | 0.75 | 0.36 | 0.12 | 0.18 | 3665 | Train 58 | Test 17 | 0.62 | 0.25 | 0.11 | 0.15 |
| 1618 | Train 26 | Test 18 | 0.76 | 0.43 | 0.18 | 0.25 | 3666 | Train 58 | Test 18 | 0.62 | 0.27 | 0.11 | 0.16 |
| 1619 | Train 26 | Test 19 | 0.75 | 0.37 | 0.12 | 0.18 | 3667 | Train 58 | Test 19 | 0.62 | 0.26 | 0.11 | 0.16 |
| 1620 | Train 26 | Test 20 | 0.76 | 0.43 | 0.18 | 0.25 | 3668 | Train 58 | Test 20 | 0.62 | 0.28 | 0.11 | 0.16 |
| 1621 | Train 26 | Test 21 | 0.68 | 0.22 | 0.14 | 0.17 | 3669 | Train 58 | Test 21 | 0.61 | 0.22 | 0.1 | 0.13 |
| 1622 | Train 26 | Test 22 | 0.67 | 0.28 | 0.1 | 0.15 | 3670 | Train 58 | Test 22 | 0.61 | 0.28 | 0.09 | 0.14 |
| 1623 | Train 26 | Test 23 | 0.68 | 0.22 | 0.14 | 0.17 | 3671 | Train 58 | Test 23 | 0.61 | 0.22 | 0.1 | 0.13 |
| 1624 | Train 26 | Test 24 | 0.67 | 0.28 | 0.1 | 0.15 | 3672 | Train 58 | Test 24 | 0.6 | 0.27 | 0.09 | 0.14 |
| 1625 | Train 26 | Test 25 | 0.76 | 0.38 | 0.1 | 0.16 | 3673 | Train 58 | Test 25 | 0.62 | 0.27 | 0.11 | 0.15 |
| 1626 | Train 26 | Test 26 | 0.77 | 0.44 | 0.16 | 0.24 | 3674 | Train 58 | Test 26 | 0.62 | 0.28 | 0.1 | 0.15 |
| 1627 | Train 26 | Test 27 | 0.76 | 0.38 | 0.1 | 0.16 | 3675 | Train 58 | Test 27 | 0.62 | 0.28 | 0.11 | 0.16 |
| 1628 | Train 26 | Test 28 | 0.77 | 0.45 | 0.16 | 0.24 | 3676 | Train 58 | Test 28 | 0.62 | 0.29 | 0.11 | 0.15 |
| 1629 | Train 26 | Test 29 | 0.69 | 0.21 | 0.13 | 0.16 | 3677 | Train 58 | Test 29 | 0.61 | 0.22 | 0.09 | 0.13 |
| 1630 | Train 26 | Test 30 | 0.67 | 0.28 | 0.1 | 0.15 | 3678 | Train 58 | Test 30 | 0.61 | 0.28 | 0.09 | 0.14 |
| 1631 | Train 26 | Test 31 | 0.69 | 0.21 | 0.13 | 0.16 | 3679 | Train 58 | Test 31 | 0.61 | 0.22 | 0.09 | 0.13 |
| 1632 | Train 26 | Test 32 | 0.67 | 0.27 | 0.1 | 0.15 | 3680 | Train 58 | Test 32 | 0.61 | 0.27 | 0.09 | 0.14 |
| 1633 | Train 26 | Test 33 | 0.75 | 0.36 | 0.12 | 0.18 | 3681 | Train 58 | Test 33 | 0.62 | 0.25 | 0.11 | 0.15 |
| 1634 | Train 26 | Test 34 | 0.76 | 0.43 | 0.18 | 0.25 | 3682 | Train 58 | Test 34 | 0.62 | 0.27 | 0.11 | 0.16 |
| 1635 | Train 26 | Test 35 | 0.75 | 0.36 | 0.12 | 0.18 | 3683 | Train 58 | Test 35 | 0.62 | 0.26 | 0.11 | 0.16 |
| 1636 | Train 26 | Test 36 | 0.76 | 0.43 | 0.18 | 0.25 | 3684 | Train 58 | Test 36 | 0.62 | 0.28 | 0.11 | 0.16 |
| 1637 | Train 26 | Test 37 | 0.68 | 0.22 | 0.14 | 0.17 | 3685 | Train 58 | Test 37 | 0.61 | 0.22 | 0.1 | 0.13 |
| 1638 | Train 26 | Test 38 | 0.67 | 0.28 | 0.1 | 0.15 | 3686 | Train 58 | Test 38 | 0.61 | 0.28 | 0.09 | 0.14 |
| 1639 | Train 26 | Test 39 | 0.68 | 0.22 | 0.14 | 0.17 | 3687 | Train 58 | Test 39 | 0.61 | 0.22 | 0.1 | 0.13 |
| 1640 | Train 26 | Test 40 | 0.67 | 0.28 | 0.1 | 0.15 | 3688 | Train 58 | Test 40 | 0.6 | 0.27 | 0.09 | 0.14 |
| 1641 | Train 26 | Test 41 | 0.76 | 0.38 | 0.1 | 0.16 | 3689 | Train 58 | Test 41 | 0.62 | 0.27 | 0.11 | 0.15 |
| 1642 | Train 26 | Test 42 | 0.77 | 0.44 | 0.16 | 0.23 | 3690 | Train 58 | Test 42 | 0.62 | 0.28 | 0.1 | 0.15 |
| 1643 | Train 26 | Test 43 | 0.76 | 0.38 | 0.1 | 0.16 | 3691 | Train 58 | Test 43 | 0.62 | 0.28 | 0.11 | 0.16 |
| 1644 | Train 26 | Test 44 | 0.77 | 0.44 | 0.16 | 0.23 | 3692 | Train 58 | Test 44 | 0.62 | 0.29 | 0.11 | 0.15 |
| 1645 | Train 26 | Test 45 | 0.69 | 0.21 | 0.13 | 0.16 | 3693 | Train 58 | Test 45 | 0.61 | 0.22 | 0.09 | 0.13 |
| 1646 | Train 26 | Test 46 | 0.67 | 0.28 | 0.1 | 0.15 | 3694 | Train 58 | Test 46 | 0.61 | 0.28 | 0.09 | 0.14 |
| 1647 | Train 26 | Test 47 | 0.69 | 0.21 | 0.13 | 0.16 | 3695 | Train 58 | Test 47 | 0.61 | 0.22 | 0.09 | 0.13 |
| 1648 | Train 26 | Test 48 | 0.67 | 0.27 | 0.1 | 0.15 | 3696 | Train 58 | Test 48 | 0.61 | 0.27 | 0.09 | 0.14 |
| 1649 | Train 26 | Test 49 | 0.76 | 0.44 | 0.18 | 0.26 | 3697 | Train 58 | Test 49 | 0.74 | 0.38 | 0.21 | 0.27 |
| 1650 | Train 26 | Test 50 | 0.76 | 0.43 | 0.18 | 0.25 | 3698 | Train 58 | Test 50 | 0.74 | 0.38 | 0.21 | 0.27 |
| 1651 | Train 26 | Test 51 | 0.76 | 0.44 | 0.18 | 0.25 | 3699 | Train 58 | Test 51 | 0.74 | 0.38 | 0.21 | 0.27 |
| 1652 | Train 26 | Test 52 | 0.76 | 0.43 | 0.18 | 0.25 | 3700 | Train 58 | Test 52 | 0.74 | 0.38 | 0.21 | 0.27 |
| 1653 | Train 26 | Test 53 | 0.66 | 0.18 | 0.12 | 0.15 | 3701 | Train 58 | Test 53 | 0.66 | 0.27 | 0.13 | 0.18 |
| 1654 | Train 26 | Test 54 | 0.67 | 0.28 | 0.09 | 0.13 | 3702 | Train 58 | Test 54 | 0.62 | 0.32 | 0.11 | 0.16 |
| 1655 | Train 26 | Test 55 | 0.69 | 0.23 | 0.17 | 0.2 | 3703 | Train 58 | Test 55 | 0.66 | 0.28 | 0.14 | 0.18 |
| 1656 | Train 26 | Test 56 | 0.67 | 0.27 | 0.09 | 0.13 | 3704 | Train 58 | Test 56 | 0.62 | 0.31 | 0.11 | 0.16 |
| 1657 | Train 26 | Test 57 | 0.77 | 0.47 | 0.17 | 0.25 | 3705 | Train 58 | Test 57 | 0.75 | 0.43 | 0.2 | 0.27 |
| 1658 | Train 26 | Test 58 | 0.77 | 0.47 | 0.17 | 0.25 | 3706 | Train 58 | Test 58 | 0.75 | 0.42 | 0.2 | 0.27 |
| 1659 | Train 26 | Test 59 | 0.77 | 0.47 | 0.17 | 0.25 | 3707 | Train 58 | Test 59 | 0.75 | 0.42 | 0.2 | 0.27 |
| 1660 | Train 26 | Test 60 | 0.77 | 0.47 | 0.17 | 0.25 | 3708 | Train 58 | Test 60 | 0.75 | 0.42 | 0.2 | 0.27 |
| 1661 | Train 26 | Test 61 | 0.66 | 0.18 | 0.11 | 0.14 | 3709 | Train 58 | Test 61 | 0.66 | 0.29 | 0.13 | 0.18 |
| 1662 | Train 26 | Test 62 | 0.67 | 0.28 | 0.09 | 0.13 | 3710 | Train 58 | Test 62 | 0.62 | 0.32 | 0.11 | 0.16 |
| 1663 | Train 26 | Test 63 | 0.66 | 0.18 | 0.11 | 0.14 | 3711 | Train 58 | Test 63 | 0.66 | 0.3 | 0.14 | 0.19 |
| 1664 | Train 26 | Test 64 | 0.67 | 0.27 | 0.09 | 0.13 | 3712 | Train 58 | Test 64 | 0.62 | 0.31 | 0.11 | 0.16 |
| 1665 | Train 27 | Test 1 | 0.75 | 0.35 | 0.13 | 0.19 | 3713 | Train 59 | Test 1 | 0.65 | 0.21 | 0.16 | 0.18 |
| 1666 | Train 27 | Test 2 | 0.72 | 0.36 | 0.12 | 0.18 | 3714 | Train 59 | Test 2 | 0.63 | 0.22 | 0.14 | 0.17 |
| 1667 | Train 27 | Test 3 | 0.75 | 0.35 | 0.13 | 0.19 | 3715 | Train 59 | Test 3 | 0.65 | 0.21 | 0.16 | 0.18 |
| 1668 | Train 27 | Test 4 | 0.72 | 0.36 | 0.12 | 0.18 | 3716 | Train 59 | Test 4 | 0.63 | 0.22 | 0.14 | 0.17 |
| 1669 | Train 27 | Test 5 | 0.68 | 0.29 | 0.11 | 0.16 | 3717 | Train 59 | Test 5 | 0.63 | 0.19 | 0.15 | 0.17 |
| 1670 | Train 27 | Test 6 | 0.63 | 0.25 | 0.09 | 0.13 | 3718 | Train 59 | Test 6 | 0.6 | 0.22 | 0.15 | 0.18 |
| 1671 | Train 27 | Test 7 | 0.68 | 0.29 | 0.11 | 0.16 | 3719 | Train 59 | Test 7 | 0.63 | 0.2 | 0.16 | 0.17 |
| 1672 | Train 27 | Test 8 | 0.63 | 0.24 | 0.09 | 0.13 | 3720 | Train 59 | Test 8 | 0.6 | 0.22 | 0.15 | 0.18 |
| 1673 | Train 27 | Test 9 | 0.76 | 0.34 | 0.11 | 0.17 | 3721 | Train 59 | Test 9 | 0.66 | 0.21 | 0.14 | 0.17 |
| 1674 | Train 27 | Test 10 | 0.73 | 0.34 | 0.09 | 0.15 | 3722 | Train 59 | Test 10 | 0.64 | 0.22 | 0.13 | 0.16 |
| 1675 | Train 27 | Test 11 | 0.76 | 0.34 | 0.11 | 0.17 | 3723 | Train 59 | Test 11 | 0.66 | 0.22 | 0.14 | 0.17 |
| 1676 | Train 27 | Test 12 | 0.73 | 0.34 | 0.09 | 0.15 | 3724 | Train 59 | Test 12 | 0.64 | 0.23 | 0.13 | 0.17 |
| 1677 | Train 27 | Test 13 | 0.68 | 0.28 | 0.09 | 0.14 | 3725 | Train 59 | Test 13 | 0.64 | 0.19 | 0.14 | 0.16 |
| 1678 | Train 27 | Test 14 | 0.63 | 0.24 | 0.09 | 0.13 | 3726 | Train 59 | Test 14 | 0.6 | 0.22 | 0.15 | 0.18 |
| 1679 | Train 27 | Test 15 | 0.68 | 0.28 | 0.09 | 0.14 | 3727 | Train 59 | Test 15 | 0.64 | 0.19 | 0.14 | 0.16 |
| 1680 | Train 27 | Test 16 | 0.63 | 0.24 | 0.09 | 0.13 | 3728 | Train 59 | Test 16 | 0.6 | 0.22 | 0.15 | 0.18 |
| 1681 | Train 27 | Test 17 | 0.75 | 0.35 | 0.13 | 0.19 | 3729 | Train 59 | Test 17 | 0.65 | 0.21 | 0.16 | 0.18 |
| 1682 | Train 27 | Test 18 | 0.72 | 0.36 | 0.12 | 0.18 | 3730 | Train 59 | Test 18 | 0.63 | 0.22 | 0.14 | 0.17 |
| 1683 | Train 27 | Test 19 | 0.75 | 0.35 | 0.13 | 0.19 | 3731 | Train 59 | Test 19 | 0.65 | 0.22 | 0.16 | 0.18 |
| 1684 | Train 27 | Test 20 | 0.72 | 0.36 | 0.12 | 0.18 | 3732 | Train 59 | Test 20 | 0.63 | 0.22 | 0.14 | 0.17 |
| 1685 | Train 27 | Test 21 | 0.68 | 0.29 | 0.11 | 0.16 | 3733 | Train 59 | Test 21 | 0.63 | 0.19 | 0.15 | 0.17 |
| 1686 | Train 27 | Test 22 | 0.63 | 0.24 | 0.09 | 0.13 | 3734 | Train 59 | Test 22 | 0.6 | 0.22 | 0.15 | 0.18 |
| 1687 | Train 27 | Test 23 | 0.68 | 0.29 | 0.11 | 0.16 | 3735 | Train 59 | Test 23 | 0.63 | 0.2 | 0.16 | 0.17 |
| 1688 | Train 27 | Test 24 | 0.63 | 0.24 | 0.09 | 0.13 | 3736 | Train 59 | Test 24 | 0.6 | 0.22 | 0.15 | 0.18 |
| 1689 | Train 27 | Test 25 | 0.76 | 0.34 | 0.11 | 0.17 | 3737 | Train 59 | Test 25 | 0.66 | 0.21 | 0.14 | 0.17 |
| 1690 | Train 27 | Test 26 | 0.73 | 0.34 | 0.09 | 0.15 | 3738 | Train 59 | Test 26 | 0.64 | 0.22 | 0.13 | 0.16 |
| 1691 | Train 27 | Test 27 | 0.76 | 0.34 | 0.11 | 0.17 | 3739 | Train 59 | Test 27 | 0.66 | 0.22 | 0.15 | 0.17 |
| 1692 | Train 27 | Test 28 | 0.73 | 0.34 | 0.09 | 0.15 | 3740 | Train 59 | Test 28 | 0.64 | 0.23 | 0.13 | 0.17 |
| 1693 | Train 27 | Test 29 | 0.68 | 0.28 | 0.09 | 0.14 | 3741 | Train 59 | Test 29 | 0.64 | 0.19 | 0.14 | 0.16 |
| 1694 | Train 27 | Test 30 | 0.63 | 0.24 | 0.09 | 0.13 | 3742 | Train 59 | Test 30 | 0.6 | 0.22 | 0.15 | 0.18 |
| 1695 | Train 27 | Test 31 | 0.68 | 0.28 | 0.09 | 0.14 | 3743 | Train 59 | Test 31 | 0.64 | 0.19 | 0.14 | 0.16 |
| 1696 | Train 27 | Test 32 | 0.63 | 0.24 | 0.09 | 0.13 | 3744 | Train 59 | Test 32 | 0.6 | 0.22 | 0.15 | 0.18 |
| 1697 | Train 27 | Test 33 | 0.75 | 0.35 | 0.13 | 0.19 | 3745 | Train 59 | Test 33 | 0.65 | 0.21 | 0.15 | 0.18 |
| 1698 | Train 27 | Test 34 | 0.73 | 0.36 | 0.12 | 0.17 | 3746 | Train 59 | Test 34 | 0.63 | 0.22 | 0.14 | 0.17 |
| 1699 | Train 27 | Test 35 | 0.75 | 0.35 | 0.13 | 0.19 | 3747 | Train 59 | Test 35 | 0.65 | 0.21 | 0.16 | 0.18 |
| 1700 | Train 27 | Test 36 | 0.73 | 0.36 | 0.12 | 0.18 | 3748 | Train 59 | Test 36 | 0.63 | 0.22 | 0.14 | 0.17 |
| 1701 | Train 27 | Test 37 | 0.68 | 0.29 | 0.11 | 0.16 | 3749 | Train 59 | Test 37 | 0.63 | 0.19 | 0.15 | 0.17 |
| 1702 | Train 27 | Test 38 | 0.63 | 0.24 | 0.09 | 0.13 | 3750 | Train 59 | Test 38 | 0.6 | 0.22 | 0.15 | 0.18 |
| 1703 | Train 27 | Test 39 | 0.68 | 0.29 | 0.11 | 0.16 | 3751 | Train 59 | Test 39 | 0.63 | 0.19 | 0.15 | 0.17 |
| 1704 | Train 27 | Test 40 | 0.63 | 0.24 | 0.09 | 0.13 | 3752 | Train 59 | Test 40 | 0.6 | 0.22 | 0.15 | 0.18 |
| 1705 | Train 27 | Test 41 | 0.76 | 0.34 | 0.11 | 0.17 | 3753 | Train 59 | Test 41 | 0.66 | 0.21 | 0.14 | 0.17 |
| 1706 | Train 27 | Test 42 | 0.73 | 0.34 | 0.09 | 0.15 | 3754 | Train 59 | Test 42 | 0.64 | 0.22 | 0.13 | 0.16 |
| 1707 | Train 27 | Test 43 | 0.76 | 0.34 | 0.11 | 0.16 | 3755 | Train 59 | Test 43 | 0.66 | 0.22 | 0.14 | 0.17 |
| 1708 | Train 27 | Test 44 | 0.73 | 0.34 | 0.09 | 0.15 | 3756 | Train 59 | Test 44 | 0.64 | 0.23 | 0.13 | 0.17 |
| 1709 | Train 27 | Test 45 | 0.68 | 0.28 | 0.09 | 0.14 | 3757 | Train 59 | Test 45 | 0.64 | 0.19 | 0.14 | 0.16 |
| 1710 | Train 27 | Test 46 | 0.63 | 0.24 | 0.09 | 0.13 | 3758 | Train 59 | Test 46 | 0.6 | 0.22 | 0.15 | 0.18 |
| 1711 | Train 27 | Test 47 | 0.68 | 0.28 | 0.09 | 0.14 | 3759 | Train 59 | Test 47 | 0.64 | 0.19 | 0.14 | 0.16 |
| 1712 | Train 27 | Test 48 | 0.63 | 0.24 | 0.09 | 0.13 | 3760 | Train 59 | Test 48 | 0.6 | 0.22 | 0.15 | 0.18 |
| 1713 | Train 27 | Test 49 | 0.76 | 0.41 | 0.19 | 0.26 | 3761 | Train 59 | Test 49 | 0.74 | 0.37 | 0.21 | 0.27 |
| 1714 | Train 27 | Test 50 | 0.76 | 0.41 | 0.19 | 0.25 | 3762 | Train 59 | Test 50 | 0.74 | 0.37 | 0.21 | 0.27 |
| 1715 | Train 27 | Test 51 | 0.76 | 0.41 | 0.19 | 0.26 | 3763 | Train 59 | Test 51 | 0.74 | 0.37 | 0.21 | 0.27 |
| 1716 | Train 27 | Test 52 | 0.76 | 0.41 | 0.18 | 0.25 | 3764 | Train 59 | Test 52 | 0.74 | 0.37 | 0.21 | 0.27 |
| 1717 | Train 27 | Test 53 | 0.67 | 0.28 | 0.09 | 0.14 | 3765 | Train 59 | Test 53 | 0.7 | 0.26 | 0.2 | 0.23 |
| 1718 | Train 27 | Test 54 | 0.64 | 0.3 | 0.07 | 0.12 | 3766 | Train 59 | Test 54 | 0.62 | 0.29 | 0.12 | 0.17 |
| 1719 | Train 27 | Test 55 | 0.7 | 0.35 | 0.14 | 0.2 | 3767 | Train 59 | Test 55 | 0.7 | 0.27 | 0.2 | 0.23 |
| 1720 | Train 27 | Test 56 | 0.64 | 0.29 | 0.07 | 0.12 | 3768 | Train 59 | Test 56 | 0.62 | 0.28 | 0.12 | 0.17 |
| 1721 | Train 27 | Test 57 | 0.77 | 0.42 | 0.16 | 0.24 | 3769 | Train 59 | Test 57 | 0.75 | 0.38 | 0.19 | 0.25 |
| 1722 | Train 27 | Test 58 | 0.77 | 0.41 | 0.16 | 0.23 | 3770 | Train 59 | Test 58 | 0.75 | 0.39 | 0.19 | 0.25 |
| 1723 | Train 27 | Test 59 | 0.77 | 0.41 | 0.16 | 0.23 | 3771 | Train 59 | Test 59 | 0.75 | 0.39 | 0.19 | 0.26 |
| 1724 | Train 27 | Test 60 | 0.77 | 0.41 | 0.16 | 0.23 | 3772 | Train 59 | Test 60 | 0.75 | 0.39 | 0.19 | 0.26 |
| 1725 | Train 27 | Test 61 | 0.67 | 0.3 | 0.09 | 0.14 | 3773 | Train 59 | Test 61 | 0.69 | 0.26 | 0.19 | 0.22 |
| 1726 | Train 27 | Test 62 | 0.65 | 0.3 | 0.07 | 0.12 | 3774 | Train 59 | Test 62 | 0.62 | 0.28 | 0.12 | 0.17 |
| 1727 | Train 27 | Test 63 | 0.68 | 0.3 | 0.09 | 0.14 | 3775 | Train 59 | Test 63 | 0.7 | 0.26 | 0.19 | 0.22 |
| 1728 | Train 27 | Test 64 | 0.64 | 0.29 | 0.07 | 0.12 | 3776 | Train 59 | Test 64 | 0.62 | 0.28 | 0.12 | 0.17 |
| 1729 | Train 28 | Test 1 | 0.75 | 0.38 | 0.13 | 0.2 | 3777 | Train 60 | Test 1 | 0.69 | 0.23 | 0.22 | 0.22 |
| 1730 | Train 28 | Test 2 | 0.76 | 0.42 | 0.18 | 0.25 | 3778 | Train 60 | Test 2 | 0.65 | 0.25 | 0.22 | 0.24 |
| 1731 | Train 28 | Test 3 | 0.75 | 0.38 | 0.14 | 0.2 | 3779 | Train 60 | Test 3 | 0.69 | 0.23 | 0.23 | 0.23 |
| 1732 | Train 28 | Test 4 | 0.76 | 0.43 | 0.18 | 0.25 | 3780 | Train 60 | Test 4 | 0.65 | 0.25 | 0.22 | 0.24 |
| 1733 | Train 28 | Test 5 | 0.7 | 0.28 | 0.13 | 0.18 | 3781 | Train 60 | Test 5 | 0.65 | 0.21 | 0.23 | 0.22 |
| 1734 | Train 28 | Test 6 | 0.68 | 0.33 | 0.07 | 0.12 | 3782 | Train 60 | Test 6 | 0.61 | 0.25 | 0.2 | 0.22 |
| 1735 | Train 28 | Test 7 | 0.7 | 0.28 | 0.13 | 0.18 | 3783 | Train 60 | Test 7 | 0.65 | 0.21 | 0.23 | 0.22 |
| 1736 | Train 28 | Test 8 | 0.67 | 0.32 | 0.07 | 0.12 | 3784 | Train 60 | Test 8 | 0.62 | 0.25 | 0.19 | 0.22 |
| 1737 | Train 28 | Test 9 | 0.76 | 0.42 | 0.12 | 0.18 | 3785 | Train 60 | Test 9 | 0.69 | 0.24 | 0.23 | 0.23 |
| 1738 | Train 28 | Test 10 | 0.77 | 0.46 | 0.17 | 0.24 | 3786 | Train 60 | Test 10 | 0.65 | 0.25 | 0.22 | 0.23 |
| 1739 | Train 28 | Test 11 | 0.76 | 0.42 | 0.12 | 0.19 | 3787 | Train 60 | Test 11 | 0.69 | 0.24 | 0.23 | 0.23 |
| 1740 | Train 28 | Test 12 | 0.77 | 0.46 | 0.17 | 0.25 | 3788 | Train 60 | Test 12 | 0.65 | 0.25 | 0.22 | 0.23 |
| 1741 | Train 28 | Test 13 | 0.7 | 0.3 | 0.12 | 0.17 | 3789 | Train 60 | Test 13 | 0.65 | 0.22 | 0.22 | 0.22 |
| 1742 | Train 28 | Test 14 | 0.68 | 0.32 | 0.07 | 0.11 | 3790 | Train 60 | Test 14 | 0.61 | 0.25 | 0.2 | 0.22 |
| 1743 | Train 28 | Test 15 | 0.7 | 0.3 | 0.12 | 0.17 | 3791 | Train 60 | Test 15 | 0.65 | 0.22 | 0.22 | 0.22 |
| 1744 | Train 28 | Test 16 | 0.67 | 0.32 | 0.07 | 0.11 | 3792 | Train 60 | Test 16 | 0.62 | 0.25 | 0.19 | 0.22 |
| 1745 | Train 28 | Test 17 | 0.75 | 0.38 | 0.13 | 0.2 | 3793 | Train 60 | Test 17 | 0.69 | 0.23 | 0.22 | 0.23 |
| 1746 | Train 28 | Test 18 | 0.76 | 0.42 | 0.18 | 0.25 | 3794 | Train 60 | Test 18 | 0.65 | 0.25 | 0.22 | 0.24 |
| 1747 | Train 28 | Test 19 | 0.75 | 0.38 | 0.14 | 0.2 | 3795 | Train 60 | Test 19 | 0.69 | 0.23 | 0.23 | 0.23 |
| 1748 | Train 28 | Test 20 | 0.76 | 0.43 | 0.18 | 0.25 | 3796 | Train 60 | Test 20 | 0.65 | 0.25 | 0.22 | 0.24 |
| 1749 | Train 28 | Test 21 | 0.7 | 0.28 | 0.13 | 0.18 | 3797 | Train 60 | Test 21 | 0.65 | 0.21 | 0.23 | 0.22 |
| 1750 | Train 28 | Test 22 | 0.68 | 0.33 | 0.07 | 0.12 | 3798 | Train 60 | Test 22 | 0.61 | 0.25 | 0.2 | 0.22 |
| 1751 | Train 28 | Test 23 | 0.7 | 0.28 | 0.13 | 0.18 | 3799 | Train 60 | Test 23 | 0.65 | 0.21 | 0.23 | 0.22 |
| 1752 | Train 28 | Test 24 | 0.68 | 0.32 | 0.07 | 0.12 | 3800 | Train 60 | Test 24 | 0.62 | 0.25 | 0.19 | 0.22 |
| 1753 | Train 28 | Test 25 | 0.76 | 0.42 | 0.12 | 0.18 | 3801 | Train 60 | Test 25 | 0.69 | 0.24 | 0.23 | 0.23 |
| 1754 | Train 28 | Test 26 | 0.77 | 0.46 | 0.17 | 0.24 | 3802 | Train 60 | Test 26 | 0.65 | 0.25 | 0.22 | 0.23 |
| 1755 | Train 28 | Test 27 | 0.76 | 0.42 | 0.12 | 0.19 | 3803 | Train 60 | Test 27 | 0.69 | 0.24 | 0.23 | 0.23 |
| 1756 | Train 28 | Test 28 | 0.77 | 0.46 | 0.17 | 0.25 | 3804 | Train 60 | Test 28 | 0.65 | 0.25 | 0.22 | 0.23 |
| 1757 | Train 28 | Test 29 | 0.7 | 0.29 | 0.12 | 0.17 | 3805 | Train 60 | Test 29 | 0.66 | 0.22 | 0.22 | 0.22 |
| 1758 | Train 28 | Test 30 | 0.68 | 0.32 | 0.07 | 0.11 | 3806 | Train 60 | Test 30 | 0.61 | 0.25 | 0.2 | 0.22 |
| 1759 | Train 28 | Test 31 | 0.7 | 0.3 | 0.12 | 0.17 | 3807 | Train 60 | Test 31 | 0.65 | 0.22 | 0.22 | 0.22 |
| 1760 | Train 28 | Test 32 | 0.68 | 0.32 | 0.07 | 0.11 | 3808 | Train 60 | Test 32 | 0.62 | 0.25 | 0.19 | 0.22 |
| 1761 | Train 28 | Test 33 | 0.75 | 0.38 | 0.13 | 0.2 | 3809 | Train 60 | Test 33 | 0.69 | 0.23 | 0.22 | 0.22 |
| 1762 | Train 28 | Test 34 | 0.77 | 0.42 | 0.18 | 0.25 | 3810 | Train 60 | Test 34 | 0.65 | 0.25 | 0.22 | 0.24 |
| 1763 | Train 28 | Test 35 | 0.75 | 0.38 | 0.13 | 0.2 | 3811 | Train 60 | Test 35 | 0.69 | 0.23 | 0.22 | 0.23 |
| 1764 | Train 28 | Test 36 | 0.77 | 0.43 | 0.18 | 0.25 | 3812 | Train 60 | Test 36 | 0.65 | 0.25 | 0.22 | 0.24 |
| 1765 | Train 28 | Test 37 | 0.7 | 0.28 | 0.13 | 0.18 | 3813 | Train 60 | Test 37 | 0.66 | 0.21 | 0.23 | 0.22 |
| 1766 | Train 28 | Test 38 | 0.68 | 0.33 | 0.07 | 0.12 | 3814 | Train 60 | Test 38 | 0.61 | 0.25 | 0.19 | 0.22 |
| 1767 | Train 28 | Test 39 | 0.7 | 0.28 | 0.13 | 0.18 | 3815 | Train 60 | Test 39 | 0.66 | 0.21 | 0.23 | 0.22 |
| 1768 | Train 28 | Test 40 | 0.68 | 0.32 | 0.07 | 0.12 | 3816 | Train 60 | Test 40 | 0.62 | 0.25 | 0.19 | 0.22 |
| 1769 | Train 28 | Test 41 | 0.76 | 0.41 | 0.12 | 0.18 | 3817 | Train 60 | Test 41 | 0.69 | 0.24 | 0.23 | 0.23 |
| 1770 | Train 28 | Test 42 | 0.77 | 0.46 | 0.17 | 0.24 | 3818 | Train 60 | Test 42 | 0.65 | 0.25 | 0.22 | 0.23 |
| 1771 | Train 28 | Test 43 | 0.76 | 0.42 | 0.12 | 0.19 | 3819 | Train 60 | Test 43 | 0.69 | 0.24 | 0.23 | 0.23 |
| 1772 | Train 28 | Test 44 | 0.77 | 0.46 | 0.17 | 0.25 | 3820 | Train 60 | Test 44 | 0.65 | 0.25 | 0.22 | 0.23 |
| 1773 | Train 28 | Test 45 | 0.7 | 0.29 | 0.12 | 0.17 | 3821 | Train 60 | Test 45 | 0.66 | 0.22 | 0.22 | 0.22 |
| 1774 | Train 28 | Test 46 | 0.68 | 0.32 | 0.07 | 0.11 | 3822 | Train 60 | Test 46 | 0.62 | 0.25 | 0.2 | 0.22 |
| 1775 | Train 28 | Test 47 | 0.7 | 0.3 | 0.12 | 0.17 | 3823 | Train 60 | Test 47 | 0.66 | 0.22 | 0.22 | 0.22 |
| 1776 | Train 28 | Test 48 | 0.68 | 0.32 | 0.07 | 0.11 | 3824 | Train 60 | Test 48 | 0.62 | 0.25 | 0.19 | 0.22 |
| 1777 | Train 28 | Test 49 | 0.77 | 0.46 | 0.2 | 0.28 | 3825 | Train 60 | Test 49 | 0.73 | 0.38 | 0.21 | 0.27 |
| 1778 | Train 28 | Test 50 | 0.76 | 0.45 | 0.2 | 0.28 | 3826 | Train 60 | Test 50 | 0.73 | 0.38 | 0.21 | 0.27 |
| 1779 | Train 28 | Test 51 | 0.77 | 0.46 | 0.21 | 0.28 | 3827 | Train 60 | Test 51 | 0.73 | 0.39 | 0.21 | 0.28 |
| 1780 | Train 28 | Test 52 | 0.76 | 0.46 | 0.2 | 0.28 | 3828 | Train 60 | Test 52 | 0.73 | 0.39 | 0.21 | 0.28 |
| 1781 | Train 28 | Test 53 | 0.7 | 0.25 | 0.09 | 0.14 | 3829 | Train 60 | Test 53 | 0.71 | 0.32 | 0.26 | 0.29 |
| 1782 | Train 28 | Test 54 | 0.69 | 0.34 | 0.07 | 0.12 | 3830 | Train 60 | Test 54 | 0.63 | 0.33 | 0.15 | 0.2 |
| 1783 | Train 28 | Test 55 | 0.73 | 0.31 | 0.13 | 0.18 | 3831 | Train 60 | Test 55 | 0.72 | 0.32 | 0.26 | 0.29 |
| 1784 | Train 28 | Test 56 | 0.68 | 0.34 | 0.07 | 0.12 | 3832 | Train 60 | Test 56 | 0.63 | 0.32 | 0.15 | 0.2 |
| 1785 | Train 28 | Test 57 | 0.77 | 0.49 | 0.19 | 0.28 | 3833 | Train 60 | Test 57 | 0.74 | 0.42 | 0.2 | 0.27 |
| 1786 | Train 28 | Test 58 | 0.77 | 0.49 | 0.19 | 0.27 | 3834 | Train 60 | Test 58 | 0.74 | 0.42 | 0.2 | 0.27 |
| 1787 | Train 28 | Test 59 | 0.77 | 0.5 | 0.2 | 0.28 | 3835 | Train 60 | Test 59 | 0.75 | 0.42 | 0.21 | 0.28 |
| 1788 | Train 28 | Test 60 | 0.77 | 0.5 | 0.2 | 0.28 | 3836 | Train 60 | Test 60 | 0.75 | 0.42 | 0.21 | 0.28 |
| 1789 | Train 28 | Test 61 | 0.7 | 0.26 | 0.1 | 0.15 | 3837 | Train 60 | Test 61 | 0.72 | 0.32 | 0.25 | 0.28 |
| 1790 | Train 28 | Test 62 | 0.69 | 0.34 | 0.07 | 0.12 | 3838 | Train 60 | Test 62 | 0.63 | 0.33 | 0.15 | 0.21 |
| 1791 | Train 28 | Test 63 | 0.71 | 0.27 | 0.11 | 0.15 | 3839 | Train 60 | Test 63 | 0.72 | 0.33 | 0.25 | 0.28 |
| 1792 | Train 28 | Test 64 | 0.68 | 0.33 | 0.07 | 0.12 | 3840 | Train 60 | Test 64 | 0.64 | 0.32 | 0.15 | 0.2 |
| 1793 | Train 29 | Test 1 | 0.75 | 0.39 | 0.1 | 0.15 | 3841 | Train 61 | Test 1 | 0.66 | 0.23 | 0.15 | 0.18 |
| 1794 | Train 29 | Test 2 | 0.74 | 0.41 | 0.1 | 0.16 | 3842 | Train 61 | Test 2 | 0.65 | 0.25 | 0.15 | 0.19 |
| 1795 | Train 29 | Test 3 | 0.75 | 0.39 | 0.1 | 0.16 | 3843 | Train 61 | Test 3 | 0.66 | 0.23 | 0.15 | 0.18 |
| 1796 | Train 29 | Test 4 | 0.74 | 0.42 | 0.1 | 0.16 | 3844 | Train 61 | Test 4 | 0.65 | 0.26 | 0.15 | 0.19 |
| 1797 | Train 29 | Test 5 | 0.74 | 0.39 | 0.1 | 0.16 | 3845 | Train 61 | Test 5 | 0.66 | 0.23 | 0.15 | 0.18 |
| 1798 | Train 29 | Test 6 | 0.69 | 0.35 | 0.13 | 0.19 | 3846 | Train 61 | Test 6 | 0.62 | 0.29 | 0.1 | 0.15 |
| 1799 | Train 29 | Test 7 | 0.74 | 0.39 | 0.1 | 0.16 | 3847 | Train 61 | Test 7 | 0.66 | 0.23 | 0.15 | 0.18 |
| 1800 | Train 29 | Test 8 | 0.69 | 0.34 | 0.13 | 0.19 | 3848 | Train 61 | Test 8 | 0.62 | 0.28 | 0.1 | 0.15 |
| 1801 | Train 29 | Test 9 | 0.75 | 0.38 | 0.08 | 0.13 | 3849 | Train 61 | Test 9 | 0.67 | 0.22 | 0.13 | 0.17 |
| 1802 | Train 29 | Test 10 | 0.74 | 0.4 | 0.08 | 0.14 | 3850 | Train 61 | Test 10 | 0.66 | 0.26 | 0.15 | 0.19 |
| 1803 | Train 29 | Test 11 | 0.75 | 0.38 | 0.08 | 0.13 | 3851 | Train 61 | Test 11 | 0.67 | 0.22 | 0.14 | 0.17 |
| 1804 | Train 29 | Test 12 | 0.74 | 0.41 | 0.08 | 0.14 | 3852 | Train 61 | Test 12 | 0.66 | 0.26 | 0.15 | 0.19 |
| 1805 | Train 29 | Test 13 | 0.75 | 0.37 | 0.09 | 0.14 | 3853 | Train 61 | Test 13 | 0.66 | 0.22 | 0.13 | 0.17 |
| 1806 | Train 29 | Test 14 | 0.69 | 0.34 | 0.14 | 0.19 | 3854 | Train 61 | Test 14 | 0.62 | 0.29 | 0.1 | 0.15 |
| 1807 | Train 29 | Test 15 | 0.74 | 0.37 | 0.09 | 0.14 | 3855 | Train 61 | Test 15 | 0.66 | 0.22 | 0.13 | 0.17 |
| 1808 | Train 29 | Test 16 | 0.69 | 0.34 | 0.13 | 0.19 | 3856 | Train 61 | Test 16 | 0.62 | 0.28 | 0.1 | 0.15 |
| 1809 | Train 29 | Test 17 | 0.75 | 0.39 | 0.1 | 0.15 | 3857 | Train 61 | Test 17 | 0.66 | 0.23 | 0.15 | 0.18 |
| 1810 | Train 29 | Test 18 | 0.74 | 0.42 | 0.1 | 0.16 | 3858 | Train 61 | Test 18 | 0.65 | 0.25 | 0.15 | 0.19 |
| 1811 | Train 29 | Test 19 | 0.75 | 0.39 | 0.1 | 0.16 | 3859 | Train 61 | Test 19 | 0.66 | 0.23 | 0.15 | 0.18 |
| 1812 | Train 29 | Test 20 | 0.74 | 0.42 | 0.1 | 0.16 | 3860 | Train 61 | Test 20 | 0.65 | 0.26 | 0.15 | 0.19 |
| 1813 | Train 29 | Test 21 | 0.74 | 0.39 | 0.1 | 0.16 | 3861 | Train 61 | Test 21 | 0.66 | 0.23 | 0.15 | 0.18 |
| 1814 | Train 29 | Test 22 | 0.69 | 0.34 | 0.13 | 0.19 | 3862 | Train 61 | Test 22 | 0.62 | 0.29 | 0.1 | 0.15 |
| 1815 | Train 29 | Test 23 | 0.74 | 0.39 | 0.1 | 0.16 | 3863 | Train 61 | Test 23 | 0.66 | 0.23 | 0.15 | 0.18 |
| 1816 | Train 29 | Test 24 | 0.69 | 0.34 | 0.13 | 0.19 | 3864 | Train 61 | Test 24 | 0.62 | 0.28 | 0.1 | 0.15 |
| 1817 | Train 29 | Test 25 | 0.75 | 0.37 | 0.08 | 0.13 | 3865 | Train 61 | Test 25 | 0.67 | 0.22 | 0.13 | 0.17 |
| 1818 | Train 29 | Test 26 | 0.74 | 0.4 | 0.08 | 0.14 | 3866 | Train 61 | Test 26 | 0.66 | 0.26 | 0.15 | 0.19 |
| 1819 | Train 29 | Test 27 | 0.75 | 0.38 | 0.08 | 0.13 | 3867 | Train 61 | Test 27 | 0.67 | 0.22 | 0.14 | 0.17 |
| 1820 | Train 29 | Test 28 | 0.74 | 0.41 | 0.08 | 0.14 | 3868 | Train 61 | Test 28 | 0.66 | 0.26 | 0.15 | 0.19 |
| 1821 | Train 29 | Test 29 | 0.75 | 0.37 | 0.09 | 0.14 | 3869 | Train 61 | Test 29 | 0.66 | 0.22 | 0.13 | 0.17 |
| 1822 | Train 29 | Test 30 | 0.69 | 0.34 | 0.14 | 0.19 | 3870 | Train 61 | Test 30 | 0.62 | 0.29 | 0.1 | 0.15 |
| 1823 | Train 29 | Test 31 | 0.74 | 0.37 | 0.09 | 0.14 | 3871 | Train 61 | Test 31 | 0.66 | 0.23 | 0.14 | 0.17 |
| 1824 | Train 29 | Test 32 | 0.69 | 0.34 | 0.14 | 0.19 | 3872 | Train 61 | Test 32 | 0.62 | 0.28 | 0.1 | 0.15 |
| 1825 | Train 29 | Test 33 | 0.75 | 0.39 | 0.1 | 0.15 | 3873 | Train 61 | Test 33 | 0.67 | 0.23 | 0.15 | 0.18 |
| 1826 | Train 29 | Test 34 | 0.74 | 0.41 | 0.1 | 0.16 | 3874 | Train 61 | Test 34 | 0.66 | 0.25 | 0.15 | 0.19 |
| 1827 | Train 29 | Test 35 | 0.75 | 0.39 | 0.1 | 0.16 | 3875 | Train 61 | Test 35 | 0.67 | 0.23 | 0.15 | 0.18 |
| 1828 | Train 29 | Test 36 | 0.74 | 0.42 | 0.1 | 0.16 | 3876 | Train 61 | Test 36 | 0.66 | 0.26 | 0.15 | 0.19 |
| 1829 | Train 29 | Test 37 | 0.74 | 0.39 | 0.1 | 0.16 | 3877 | Train 61 | Test 37 | 0.66 | 0.23 | 0.15 | 0.18 |
| 1830 | Train 29 | Test 38 | 0.69 | 0.35 | 0.13 | 0.19 | 3878 | Train 61 | Test 38 | 0.62 | 0.29 | 0.1 | 0.15 |
| 1831 | Train 29 | Test 39 | 0.74 | 0.39 | 0.1 | 0.16 | 3879 | Train 61 | Test 39 | 0.66 | 0.23 | 0.15 | 0.18 |
| 1832 | Train 29 | Test 40 | 0.69 | 0.34 | 0.13 | 0.19 | 3880 | Train 61 | Test 40 | 0.62 | 0.28 | 0.1 | 0.15 |
| 1833 | Train 29 | Test 41 | 0.76 | 0.37 | 0.08 | 0.13 | 3881 | Train 61 | Test 41 | 0.67 | 0.22 | 0.13 | 0.17 |
| 1834 | Train 29 | Test 42 | 0.74 | 0.4 | 0.08 | 0.13 | 3882 | Train 61 | Test 42 | 0.66 | 0.25 | 0.15 | 0.19 |
| 1835 | Train 29 | Test 43 | 0.76 | 0.38 | 0.08 | 0.13 | 3883 | Train 61 | Test 43 | 0.67 | 0.22 | 0.14 | 0.17 |
| 1836 | Train 29 | Test 44 | 0.74 | 0.41 | 0.08 | 0.14 | 3884 | Train 61 | Test 44 | 0.66 | 0.26 | 0.15 | 0.19 |
| 1837 | Train 29 | Test 45 | 0.75 | 0.37 | 0.09 | 0.14 | 3885 | Train 61 | Test 45 | 0.66 | 0.22 | 0.13 | 0.17 |
| 1838 | Train 29 | Test 46 | 0.69 | 0.34 | 0.14 | 0.19 | 3886 | Train 61 | Test 46 | 0.62 | 0.29 | 0.1 | 0.15 |
| 1839 | Train 29 | Test 47 | 0.74 | 0.37 | 0.09 | 0.14 | 3887 | Train 61 | Test 47 | 0.66 | 0.22 | 0.13 | 0.17 |
| 1840 | Train 29 | Test 48 | 0.69 | 0.34 | 0.13 | 0.19 | 3888 | Train 61 | Test 48 | 0.62 | 0.28 | 0.1 | 0.15 |
| 1841 | Train 29 | Test 49 | 0.76 | 0.45 | 0.16 | 0.23 | 3889 | Train 61 | Test 49 | 0.73 | 0.34 | 0.19 | 0.25 |
| 1842 | Train 29 | Test 50 | 0.76 | 0.44 | 0.15 | 0.23 | 3890 | Train 61 | Test 50 | 0.73 | 0.34 | 0.19 | 0.24 |
| 1843 | Train 29 | Test 51 | 0.76 | 0.45 | 0.16 | 0.23 | 3891 | Train 61 | Test 51 | 0.73 | 0.35 | 0.19 | 0.25 |
| 1844 | Train 29 | Test 52 | 0.76 | 0.45 | 0.16 | 0.23 | 3892 | Train 61 | Test 52 | 0.73 | 0.35 | 0.19 | 0.25 |
| 1845 | Train 29 | Test 53 | 0.73 | 0.37 | 0.17 | 0.23 | 3893 | Train 61 | Test 53 | 0.75 | 0.39 | 0.18 | 0.24 |
| 1846 | Train 29 | Test 54 | 0.69 | 0.4 | 0.1 | 0.17 | 3894 | Train 61 | Test 54 | 0.64 | 0.34 | 0.1 | 0.15 |
| 1847 | Train 29 | Test 55 | 0.77 | 0.43 | 0.14 | 0.21 | 3895 | Train 61 | Test 55 | 0.74 | 0.37 | 0.19 | 0.25 |
| 1848 | Train 29 | Test 56 | 0.69 | 0.4 | 0.1 | 0.17 | 3896 | Train 61 | Test 56 | 0.64 | 0.33 | 0.1 | 0.15 |
| 1849 | Train 29 | Test 57 | 0.77 | 0.44 | 0.14 | 0.21 | 3897 | Train 61 | Test 57 | 0.75 | 0.38 | 0.18 | 0.25 |
| 1850 | Train 29 | Test 58 | 0.77 | 0.44 | 0.14 | 0.21 | 3898 | Train 61 | Test 58 | 0.74 | 0.38 | 0.18 | 0.25 |
| 1851 | Train 29 | Test 59 | 0.77 | 0.45 | 0.15 | 0.22 | 3899 | Train 61 | Test 59 | 0.75 | 0.39 | 0.19 | 0.25 |
| 1852 | Train 29 | Test 60 | 0.77 | 0.45 | 0.14 | 0.22 | 3900 | Train 61 | Test 60 | 0.75 | 0.39 | 0.19 | 0.25 |
| 1853 | Train 29 | Test 61 | 0.74 | 0.35 | 0.15 | 0.21 | 3901 | Train 61 | Test 61 | 0.76 | 0.4 | 0.16 | 0.23 |
| 1854 | Train 29 | Test 62 | 0.69 | 0.4 | 0.1 | 0.16 | 3902 | Train 61 | Test 62 | 0.64 | 0.34 | 0.1 | 0.15 |
| 1855 | Train 29 | Test 63 | 0.74 | 0.35 | 0.16 | 0.22 | 3903 | Train 61 | Test 63 | 0.76 | 0.41 | 0.16 | 0.23 |
| 1856 | Train 29 | Test 64 | 0.69 | 0.39 | 0.1 | 0.16 | 3904 | Train 61 | Test 64 | 0.65 | 0.33 | 0.1 | 0.15 |
| 1857 | Train 30 | Test 1 | 0.73 | 0.32 | 0.2 | 0.24 | 3905 | Train 62 | Test 1 | 0.69 | 0.21 | 0.3 | 0.25 |
| 1858 | Train 30 | Test 2 | 0.74 | 0.35 | 0.22 | 0.27 | 3906 | Train 62 | Test 2 | 0.7 | 0.25 | 0.32 | 0.28 |
| 1859 | Train 30 | Test 3 | 0.73 | 0.32 | 0.2 | 0.24 | 3907 | Train 62 | Test 3 | 0.69 | 0.21 | 0.3 | 0.25 |
| 1860 | Train 30 | Test 4 | 0.74 | 0.35 | 0.22 | 0.27 | 3908 | Train 62 | Test 4 | 0.7 | 0.26 | 0.32 | 0.29 |
| 1861 | Train 30 | Test 5 | 0.73 | 0.32 | 0.17 | 0.23 | 3909 | Train 62 | Test 5 | 0.68 | 0.22 | 0.27 | 0.24 |
| 1862 | Train 30 | Test 6 | 0.76 | 0.52 | 0.15 | 0.23 | 3910 | Train 62 | Test 6 | 0.7 | 0.33 | 0.23 | 0.27 |
| 1863 | Train 30 | Test 7 | 0.72 | 0.32 | 0.17 | 0.22 | 3911 | Train 62 | Test 7 | 0.68 | 0.22 | 0.26 | 0.24 |
| 1864 | Train 30 | Test 8 | 0.76 | 0.49 | 0.15 | 0.23 | 3912 | Train 62 | Test 8 | 0.7 | 0.33 | 0.23 | 0.27 |
| 1865 | Train 30 | Test 9 | 0.74 | 0.34 | 0.2 | 0.25 | 3913 | Train 62 | Test 9 | 0.69 | 0.21 | 0.28 | 0.24 |
| 1866 | Train 30 | Test 10 | 0.74 | 0.38 | 0.22 | 0.28 | 3914 | Train 62 | Test 10 | 0.7 | 0.26 | 0.3 | 0.28 |
| 1867 | Train 30 | Test 11 | 0.74 | 0.34 | 0.2 | 0.25 | 3915 | Train 62 | Test 11 | 0.69 | 0.21 | 0.28 | 0.24 |
| 1868 | Train 30 | Test 12 | 0.74 | 0.38 | 0.22 | 0.28 | 3916 | Train 62 | Test 12 | 0.7 | 0.26 | 0.3 | 0.28 |
| 1869 | Train 30 | Test 13 | 0.73 | 0.33 | 0.15 | 0.21 | 3917 | Train 62 | Test 13 | 0.68 | 0.22 | 0.24 | 0.23 |
| 1870 | Train 30 | Test 14 | 0.76 | 0.52 | 0.15 | 0.23 | 3918 | Train 62 | Test 14 | 0.7 | 0.33 | 0.22 | 0.27 |
| 1871 | Train 30 | Test 15 | 0.72 | 0.33 | 0.15 | 0.21 | 3919 | Train 62 | Test 15 | 0.68 | 0.22 | 0.24 | 0.23 |
| 1872 | Train 30 | Test 16 | 0.76 | 0.49 | 0.15 | 0.23 | 3920 | Train 62 | Test 16 | 0.7 | 0.32 | 0.22 | 0.26 |
| 1873 | Train 30 | Test 17 | 0.73 | 0.32 | 0.2 | 0.24 | 3921 | Train 62 | Test 17 | 0.69 | 0.21 | 0.3 | 0.25 |
| 1874 | Train 30 | Test 18 | 0.74 | 0.35 | 0.22 | 0.27 | 3922 | Train 62 | Test 18 | 0.7 | 0.25 | 0.32 | 0.29 |
| 1875 | Train 30 | Test 19 | 0.73 | 0.32 | 0.2 | 0.24 | 3923 | Train 62 | Test 19 | 0.69 | 0.21 | 0.3 | 0.25 |
| 1876 | Train 30 | Test 20 | 0.74 | 0.35 | 0.22 | 0.27 | 3924 | Train 62 | Test 20 | 0.7 | 0.26 | 0.32 | 0.29 |
| 1877 | Train 30 | Test 21 | 0.73 | 0.32 | 0.17 | 0.23 | 3925 | Train 62 | Test 21 | 0.68 | 0.22 | 0.27 | 0.24 |
| 1878 | Train 30 | Test 22 | 0.76 | 0.52 | 0.15 | 0.23 | 3926 | Train 62 | Test 22 | 0.7 | 0.33 | 0.23 | 0.27 |
| 1879 | Train 30 | Test 23 | 0.72 | 0.32 | 0.17 | 0.22 | 3927 | Train 62 | Test 23 | 0.68 | 0.22 | 0.26 | 0.24 |
| 1880 | Train 30 | Test 24 | 0.76 | 0.49 | 0.15 | 0.23 | 3928 | Train 62 | Test 24 | 0.7 | 0.33 | 0.22 | 0.27 |
| 1881 | Train 30 | Test 25 | 0.74 | 0.34 | 0.2 | 0.25 | 3929 | Train 62 | Test 25 | 0.69 | 0.21 | 0.28 | 0.24 |
| 1882 | Train 30 | Test 26 | 0.74 | 0.38 | 0.22 | 0.28 | 3930 | Train 62 | Test 26 | 0.7 | 0.26 | 0.3 | 0.28 |
| 1883 | Train 30 | Test 27 | 0.74 | 0.34 | 0.2 | 0.25 | 3931 | Train 62 | Test 27 | 0.69 | 0.21 | 0.28 | 0.24 |
| 1884 | Train 30 | Test 28 | 0.74 | 0.38 | 0.22 | 0.28 | 3932 | Train 62 | Test 28 | 0.7 | 0.26 | 0.3 | 0.28 |
| 1885 | Train 30 | Test 29 | 0.73 | 0.33 | 0.15 | 0.21 | 3933 | Train 62 | Test 29 | 0.68 | 0.22 | 0.24 | 0.23 |
| 1886 | Train 30 | Test 30 | 0.76 | 0.52 | 0.15 | 0.23 | 3934 | Train 62 | Test 30 | 0.7 | 0.33 | 0.22 | 0.26 |
| 1887 | Train 30 | Test 31 | 0.72 | 0.33 | 0.15 | 0.21 | 3935 | Train 62 | Test 31 | 0.68 | 0.22 | 0.24 | 0.23 |
| 1888 | Train 30 | Test 32 | 0.76 | 0.49 | 0.15 | 0.23 | 3936 | Train 62 | Test 32 | 0.7 | 0.32 | 0.22 | 0.26 |
| 1889 | Train 30 | Test 33 | 0.73 | 0.31 | 0.2 | 0.24 | 3937 | Train 62 | Test 33 | 0.69 | 0.21 | 0.3 | 0.25 |
| 1890 | Train 30 | Test 34 | 0.74 | 0.35 | 0.22 | 0.27 | 3938 | Train 62 | Test 34 | 0.7 | 0.25 | 0.32 | 0.28 |
| 1891 | Train 30 | Test 35 | 0.73 | 0.31 | 0.2 | 0.24 | 3939 | Train 62 | Test 35 | 0.69 | 0.21 | 0.3 | 0.25 |
| 1892 | Train 30 | Test 36 | 0.74 | 0.35 | 0.22 | 0.27 | 3940 | Train 62 | Test 36 | 0.7 | 0.25 | 0.32 | 0.28 |
| 1893 | Train 30 | Test 37 | 0.73 | 0.32 | 0.17 | 0.22 | 3941 | Train 62 | Test 37 | 0.68 | 0.22 | 0.26 | 0.24 |
| 1894 | Train 30 | Test 38 | 0.76 | 0.52 | 0.15 | 0.23 | 3942 | Train 62 | Test 38 | 0.7 | 0.33 | 0.22 | 0.27 |
| 1895 | Train 30 | Test 39 | 0.72 | 0.32 | 0.17 | 0.22 | 3943 | Train 62 | Test 39 | 0.68 | 0.22 | 0.26 | 0.24 |
| 1896 | Train 30 | Test 40 | 0.76 | 0.49 | 0.15 | 0.23 | 3944 | Train 62 | Test 40 | 0.7 | 0.32 | 0.22 | 0.27 |
| 1897 | Train 30 | Test 41 | 0.74 | 0.34 | 0.2 | 0.25 | 3945 | Train 62 | Test 41 | 0.69 | 0.21 | 0.28 | 0.24 |
| 1898 | Train 30 | Test 42 | 0.74 | 0.38 | 0.22 | 0.28 | 3946 | Train 62 | Test 42 | 0.7 | 0.25 | 0.3 | 0.28 |
| 1899 | Train 30 | Test 43 | 0.74 | 0.34 | 0.19 | 0.25 | 3947 | Train 62 | Test 43 | 0.69 | 0.21 | 0.28 | 0.24 |
| 1900 | Train 30 | Test 44 | 0.74 | 0.38 | 0.22 | 0.28 | 3948 | Train 62 | Test 44 | 0.7 | 0.25 | 0.3 | 0.28 |
| 1901 | Train 30 | Test 45 | 0.73 | 0.33 | 0.15 | 0.21 | 3949 | Train 62 | Test 45 | 0.68 | 0.22 | 0.24 | 0.23 |
| 1902 | Train 30 | Test 46 | 0.76 | 0.52 | 0.15 | 0.23 | 3950 | Train 62 | Test 46 | 0.7 | 0.33 | 0.22 | 0.26 |
| 1903 | Train 30 | Test 47 | 0.72 | 0.33 | 0.15 | 0.2 | 3951 | Train 62 | Test 47 | 0.68 | 0.22 | 0.23 | 0.23 |
| 1904 | Train 30 | Test 48 | 0.76 | 0.49 | 0.15 | 0.23 | 3952 | Train 62 | Test 48 | 0.7 | 0.32 | 0.22 | 0.26 |
| 1905 | Train 30 | Test 49 | 0.76 | 0.31 | 0.37 | 0.34 | 3953 | Train 62 | Test 49 | 0.75 | 0.32 | 0.26 | 0.29 |
| 1906 | Train 30 | Test 50 | 0.76 | 0.31 | 0.37 | 0.34 | 3954 | Train 62 | Test 50 | 0.75 | 0.32 | 0.26 | 0.28 |
| 1907 | Train 30 | Test 51 | 0.76 | 0.32 | 0.36 | 0.34 | 3955 | Train 62 | Test 51 | 0.75 | 0.33 | 0.26 | 0.29 |
| 1908 | Train 30 | Test 52 | 0.76 | 0.31 | 0.36 | 0.34 | 3956 | Train 62 | Test 52 | 0.75 | 0.32 | 0.26 | 0.29 |
| 1909 | Train 30 | Test 53 | 0.77 | 0.39 | 0.13 | 0.19 | 3957 | Train 62 | Test 53 | 0.77 | 0.39 | 0.16 | 0.23 |
| 1910 | Train 30 | Test 54 | 0.76 | 0.53 | 0.17 | 0.26 | 3958 | Train 62 | Test 54 | 0.76 | 0.51 | 0.21 | 0.29 |
| 1911 | Train 30 | Test 55 | 0.75 | 0.32 | 0.26 | 0.29 | 3959 | Train 62 | Test 55 | 0.75 | 0.35 | 0.2 | 0.25 |
| 1912 | Train 30 | Test 56 | 0.76 | 0.51 | 0.17 | 0.26 | 3960 | Train 62 | Test 56 | 0.76 | 0.49 | 0.21 | 0.29 |
| 1913 | Train 30 | Test 57 | 0.77 | 0.34 | 0.38 | 0.36 | 3961 | Train 62 | Test 57 | 0.77 | 0.32 | 0.23 | 0.26 |
| 1914 | Train 30 | Test 58 | 0.77 | 0.34 | 0.37 | 0.36 | 3962 | Train 62 | Test 58 | 0.76 | 0.32 | 0.22 | 0.26 |
| 1915 | Train 30 | Test 59 | 0.77 | 0.34 | 0.37 | 0.35 | 3963 | Train 62 | Test 59 | 0.76 | 0.32 | 0.22 | 0.26 |
| 1916 | Train 30 | Test 60 | 0.77 | 0.34 | 0.36 | 0.35 | 3964 | Train 62 | Test 60 | 0.76 | 0.32 | 0.22 | 0.26 |
| 1917 | Train 30 | Test 61 | 0.77 | 0.41 | 0.11 | 0.18 | 3965 | Train 62 | Test 61 | 0.77 | 0.39 | 0.14 | 0.21 |
| 1918 | Train 30 | Test 62 | 0.76 | 0.53 | 0.17 | 0.26 | 3966 | Train 62 | Test 62 | 0.76 | 0.51 | 0.2 | 0.29 |
| 1919 | Train 30 | Test 63 | 0.78 | 0.41 | 0.11 | 0.18 | 3967 | Train 62 | Test 63 | 0.77 | 0.39 | 0.14 | 0.21 |
| 1920 | Train 30 | Test 64 | 0.76 | 0.51 | 0.17 | 0.26 | 3968 | Train 62 | Test 64 | 0.76 | 0.48 | 0.21 | 0.29 |
| 1921 | Train 31 | Test 1 | 0.75 | 0.37 | 0.1 | 0.15 | 3969 | Train 63 | Test 1 | 0.68 | 0.23 | 0.15 | 0.18 |
| 1922 | Train 31 | Test 2 | 0.74 | 0.38 | 0.09 | 0.15 | 3970 | Train 63 | Test 2 | 0.67 | 0.26 | 0.15 | 0.19 |
| 1923 | Train 31 | Test 3 | 0.75 | 0.38 | 0.1 | 0.16 | 3971 | Train 63 | Test 3 | 0.68 | 0.24 | 0.15 | 0.18 |
| 1924 | Train 31 | Test 4 | 0.75 | 0.38 | 0.1 | 0.15 | 3972 | Train 63 | Test 4 | 0.67 | 0.26 | 0.15 | 0.19 |
| 1925 | Train 31 | Test 5 | 0.75 | 0.37 | 0.1 | 0.16 | 3973 | Train 63 | Test 5 | 0.67 | 0.24 | 0.16 | 0.19 |
| 1926 | Train 31 | Test 6 | 0.66 | 0.29 | 0.16 | 0.21 | 3974 | Train 63 | Test 6 | 0.63 | 0.31 | 0.12 | 0.17 |
| 1927 | Train 31 | Test 7 | 0.74 | 0.38 | 0.1 | 0.16 | 3975 | Train 63 | Test 7 | 0.67 | 0.24 | 0.16 | 0.19 |
| 1928 | Train 31 | Test 8 | 0.67 | 0.29 | 0.16 | 0.21 | 3976 | Train 63 | Test 8 | 0.63 | 0.3 | 0.12 | 0.17 |
| 1929 | Train 31 | Test 9 | 0.76 | 0.4 | 0.08 | 0.13 | 3977 | Train 63 | Test 9 | 0.68 | 0.25 | 0.14 | 0.18 |
| 1930 | Train 31 | Test 10 | 0.75 | 0.41 | 0.08 | 0.13 | 3978 | Train 63 | Test 10 | 0.67 | 0.27 | 0.15 | 0.19 |
| 1931 | Train 31 | Test 11 | 0.76 | 0.4 | 0.08 | 0.13 | 3979 | Train 63 | Test 11 | 0.68 | 0.25 | 0.15 | 0.19 |
| 1932 | Train 31 | Test 12 | 0.75 | 0.41 | 0.08 | 0.13 | 3980 | Train 63 | Test 12 | 0.67 | 0.28 | 0.15 | 0.2 |
| 1933 | Train 31 | Test 13 | 0.75 | 0.4 | 0.09 | 0.15 | 3981 | Train 63 | Test 13 | 0.67 | 0.24 | 0.14 | 0.18 |
| 1934 | Train 31 | Test 14 | 0.66 | 0.28 | 0.16 | 0.21 | 3982 | Train 63 | Test 14 | 0.64 | 0.3 | 0.12 | 0.17 |
| 1935 | Train 31 | Test 15 | 0.75 | 0.41 | 0.09 | 0.15 | 3983 | Train 63 | Test 15 | 0.67 | 0.24 | 0.15 | 0.18 |
| 1936 | Train 31 | Test 16 | 0.67 | 0.28 | 0.16 | 0.21 | 3984 | Train 63 | Test 16 | 0.64 | 0.3 | 0.12 | 0.17 |
| 1937 | Train 31 | Test 17 | 0.75 | 0.37 | 0.1 | 0.15 | 3985 | Train 63 | Test 17 | 0.68 | 0.23 | 0.15 | 0.18 |
| 1938 | Train 31 | Test 18 | 0.74 | 0.38 | 0.09 | 0.15 | 3986 | Train 63 | Test 18 | 0.67 | 0.26 | 0.15 | 0.19 |
| 1939 | Train 31 | Test 19 | 0.75 | 0.37 | 0.1 | 0.16 | 3987 | Train 63 | Test 19 | 0.68 | 0.24 | 0.15 | 0.18 |
| 1940 | Train 31 | Test 20 | 0.75 | 0.38 | 0.1 | 0.15 | 3988 | Train 63 | Test 20 | 0.67 | 0.26 | 0.15 | 0.19 |
| 1941 | Train 31 | Test 21 | 0.75 | 0.37 | 0.1 | 0.16 | 3989 | Train 63 | Test 21 | 0.67 | 0.24 | 0.16 | 0.19 |
| 1942 | Train 31 | Test 22 | 0.66 | 0.29 | 0.16 | 0.21 | 3990 | Train 63 | Test 22 | 0.63 | 0.31 | 0.12 | 0.17 |
| 1943 | Train 31 | Test 23 | 0.74 | 0.37 | 0.1 | 0.16 | 3991 | Train 63 | Test 23 | 0.67 | 0.24 | 0.16 | 0.19 |
| 1944 | Train 31 | Test 24 | 0.67 | 0.29 | 0.16 | 0.21 | 3992 | Train 63 | Test 24 | 0.64 | 0.3 | 0.12 | 0.17 |
| 1945 | Train 31 | Test 25 | 0.76 | 0.4 | 0.08 | 0.13 | 3993 | Train 63 | Test 25 | 0.68 | 0.25 | 0.14 | 0.18 |
| 1946 | Train 31 | Test 26 | 0.75 | 0.4 | 0.08 | 0.13 | 3994 | Train 63 | Test 26 | 0.67 | 0.27 | 0.15 | 0.19 |
| 1947 | Train 31 | Test 27 | 0.76 | 0.4 | 0.08 | 0.13 | 3995 | Train 63 | Test 27 | 0.68 | 0.25 | 0.15 | 0.19 |
| 1948 | Train 31 | Test 28 | 0.75 | 0.41 | 0.08 | 0.13 | 3996 | Train 63 | Test 28 | 0.67 | 0.28 | 0.15 | 0.2 |
| 1949 | Train 31 | Test 29 | 0.75 | 0.4 | 0.09 | 0.15 | 3997 | Train 63 | Test 29 | 0.67 | 0.24 | 0.14 | 0.18 |
| 1950 | Train 31 | Test 30 | 0.66 | 0.28 | 0.16 | 0.21 | 3998 | Train 63 | Test 30 | 0.64 | 0.3 | 0.12 | 0.17 |
| 1951 | Train 31 | Test 31 | 0.75 | 0.41 | 0.09 | 0.15 | 3999 | Train 63 | Test 31 | 0.67 | 0.24 | 0.15 | 0.18 |
| 1952 | Train 31 | Test 32 | 0.67 | 0.28 | 0.16 | 0.21 | 4000 | Train 63 | Test 32 | 0.64 | 0.3 | 0.12 | 0.17 |
| 1953 | Train 31 | Test 33 | 0.76 | 0.37 | 0.1 | 0.15 | 4001 | Train 63 | Test 33 | 0.68 | 0.23 | 0.15 | 0.18 |
| 1954 | Train 31 | Test 34 | 0.75 | 0.38 | 0.09 | 0.15 | 4002 | Train 63 | Test 34 | 0.67 | 0.26 | 0.15 | 0.19 |
| 1955 | Train 31 | Test 35 | 0.76 | 0.37 | 0.1 | 0.16 | 4003 | Train 63 | Test 35 | 0.68 | 0.24 | 0.15 | 0.18 |
| 1956 | Train 31 | Test 36 | 0.75 | 0.38 | 0.09 | 0.15 | 4004 | Train 63 | Test 36 | 0.67 | 0.26 | 0.15 | 0.19 |
| 1957 | Train 31 | Test 37 | 0.75 | 0.37 | 0.1 | 0.16 | 4005 | Train 63 | Test 37 | 0.67 | 0.24 | 0.16 | 0.19 |
| 1958 | Train 31 | Test 38 | 0.67 | 0.29 | 0.16 | 0.21 | 4006 | Train 63 | Test 38 | 0.63 | 0.31 | 0.12 | 0.17 |
| 1959 | Train 31 | Test 39 | 0.75 | 0.37 | 0.1 | 0.16 | 4007 | Train 63 | Test 39 | 0.67 | 0.24 | 0.16 | 0.19 |
| 1960 | Train 31 | Test 40 | 0.67 | 0.29 | 0.16 | 0.21 | 4008 | Train 63 | Test 40 | 0.64 | 0.3 | 0.12 | 0.17 |
| 1961 | Train 31 | Test 41 | 0.76 | 0.39 | 0.08 | 0.13 | 4009 | Train 63 | Test 41 | 0.68 | 0.25 | 0.14 | 0.18 |
| 1962 | Train 31 | Test 42 | 0.75 | 0.4 | 0.08 | 0.13 | 4010 | Train 63 | Test 42 | 0.67 | 0.27 | 0.15 | 0.19 |
| 1963 | Train 31 | Test 43 | 0.76 | 0.4 | 0.08 | 0.13 | 4011 | Train 63 | Test 43 | 0.68 | 0.25 | 0.15 | 0.19 |
| 1964 | Train 31 | Test 44 | 0.75 | 0.41 | 0.08 | 0.13 | 4012 | Train 63 | Test 44 | 0.67 | 0.28 | 0.15 | 0.2 |
| 1965 | Train 31 | Test 45 | 0.75 | 0.4 | 0.09 | 0.14 | 4013 | Train 63 | Test 45 | 0.68 | 0.23 | 0.14 | 0.18 |
| 1966 | Train 31 | Test 46 | 0.67 | 0.28 | 0.16 | 0.21 | 4014 | Train 63 | Test 46 | 0.64 | 0.3 | 0.12 | 0.17 |
| 1967 | Train 31 | Test 47 | 0.75 | 0.4 | 0.09 | 0.14 | 4015 | Train 63 | Test 47 | 0.68 | 0.24 | 0.15 | 0.18 |
| 1968 | Train 31 | Test 48 | 0.67 | 0.28 | 0.16 | 0.21 | 4016 | Train 63 | Test 48 | 0.64 | 0.3 | 0.12 | 0.17 |
| 1969 | Train 31 | Test 49 | 0.77 | 0.41 | 0.15 | 0.22 | 4017 | Train 63 | Test 49 | 0.73 | 0.36 | 0.16 | 0.22 |
| 1970 | Train 31 | Test 50 | 0.76 | 0.41 | 0.15 | 0.21 | 4018 | Train 63 | Test 50 | 0.73 | 0.36 | 0.16 | 0.22 |
| 1971 | Train 31 | Test 51 | 0.77 | 0.42 | 0.15 | 0.22 | 4019 | Train 63 | Test 51 | 0.74 | 0.37 | 0.16 | 0.23 |
| 1972 | Train 31 | Test 52 | 0.77 | 0.42 | 0.15 | 0.22 | 4020 | Train 63 | Test 52 | 0.73 | 0.37 | 0.16 | 0.23 |
| 1973 | Train 31 | Test 53 | 0.69 | 0.25 | 0.22 | 0.24 | 4021 | Train 63 | Test 53 | 0.75 | 0.38 | 0.16 | 0.23 |
| 1974 | Train 31 | Test 54 | 0.66 | 0.3 | 0.15 | 0.2 | 4022 | Train 63 | Test 54 | 0.65 | 0.37 | 0.11 | 0.17 |
| 1975 | Train 31 | Test 55 | 0.77 | 0.43 | 0.15 | 0.22 | 4023 | Train 63 | Test 55 | 0.75 | 0.37 | 0.17 | 0.23 |
| 1976 | Train 31 | Test 56 | 0.66 | 0.3 | 0.15 | 0.2 | 4024 | Train 63 | Test 56 | 0.65 | 0.36 | 0.11 | 0.17 |
| 1977 | Train 31 | Test 57 | 0.78 | 0.44 | 0.13 | 0.2 | 4025 | Train 63 | Test 57 | 0.75 | 0.41 | 0.16 | 0.23 |
| 1978 | Train 31 | Test 58 | 0.77 | 0.43 | 0.13 | 0.2 | 4026 | Train 63 | Test 58 | 0.75 | 0.41 | 0.16 | 0.23 |
| 1979 | Train 31 | Test 59 | 0.78 | 0.45 | 0.13 | 0.2 | 4027 | Train 63 | Test 59 | 0.75 | 0.41 | 0.17 | 0.24 |
| 1980 | Train 31 | Test 60 | 0.78 | 0.44 | 0.13 | 0.2 | 4028 | Train 63 | Test 60 | 0.75 | 0.41 | 0.17 | 0.24 |
| 1981 | Train 31 | Test 61 | 0.69 | 0.24 | 0.2 | 0.22 | 4029 | Train 63 | Test 61 | 0.75 | 0.41 | 0.15 | 0.22 |
| 1982 | Train 31 | Test 62 | 0.66 | 0.3 | 0.15 | 0.2 | 4030 | Train 63 | Test 62 | 0.65 | 0.36 | 0.11 | 0.17 |
| 1983 | Train 31 | Test 63 | 0.69 | 0.25 | 0.2 | 0.22 | 4031 | Train 63 | Test 63 | 0.76 | 0.41 | 0.16 | 0.23 |
| 1984 | Train 31 | Test 64 | 0.66 | 0.3 | 0.15 | 0.2 | 4032 | Train 63 | Test 64 | 0.66 | 0.36 | 0.11 | 0.17 |
| 1985 | Train 32 | Test 1 | 0.75 | 0.32 | 0.11 | 0.16 | 4033 | Train 64 | Test 1 | 0.68 | 0.24 | 0.23 | 0.23 |
| 1986 | Train 32 | Test 2 | 0.76 | 0.38 | 0.16 | 0.23 | 4034 | Train 64 | Test 2 | 0.69 | 0.28 | 0.24 | 0.26 |
| 1987 | Train 32 | Test 3 | 0.75 | 0.32 | 0.11 | 0.16 | 4035 | Train 64 | Test 3 | 0.68 | 0.24 | 0.23 | 0.23 |
| 1988 | Train 32 | Test 4 | 0.76 | 0.38 | 0.16 | 0.22 | 4036 | Train 64 | Test 4 | 0.69 | 0.28 | 0.24 | 0.26 |
| 1989 | Train 32 | Test 5 | 0.72 | 0.27 | 0.13 | 0.18 | 4037 | Train 64 | Test 5 | 0.69 | 0.24 | 0.22 | 0.23 |
| 1990 | Train 32 | Test 6 | 0.76 | 0.52 | 0.14 | 0.22 | 4038 | Train 64 | Test 6 | 0.69 | 0.34 | 0.18 | 0.24 |
| 1991 | Train 32 | Test 7 | 0.72 | 0.27 | 0.13 | 0.18 | 4039 | Train 64 | Test 7 | 0.69 | 0.24 | 0.22 | 0.23 |
| 1992 | Train 32 | Test 8 | 0.76 | 0.51 | 0.13 | 0.21 | 4040 | Train 64 | Test 8 | 0.7 | 0.34 | 0.18 | 0.24 |
| 1993 | Train 32 | Test 9 | 0.75 | 0.31 | 0.09 | 0.14 | 4041 | Train 64 | Test 9 | 0.69 | 0.24 | 0.22 | 0.23 |
| 1994 | Train 32 | Test 10 | 0.76 | 0.38 | 0.14 | 0.2 | 4042 | Train 64 | Test 10 | 0.7 | 0.27 | 0.22 | 0.24 |
| 1995 | Train 32 | Test 11 | 0.75 | 0.3 | 0.09 | 0.14 | 4043 | Train 64 | Test 11 | 0.69 | 0.24 | 0.21 | 0.23 |
| 1996 | Train 32 | Test 12 | 0.76 | 0.37 | 0.14 | 0.2 | 4044 | Train 64 | Test 12 | 0.7 | 0.27 | 0.22 | 0.24 |
| 1997 | Train 32 | Test 13 | 0.72 | 0.26 | 0.11 | 0.16 | 4045 | Train 64 | Test 13 | 0.69 | 0.24 | 0.21 | 0.22 |
| 1998 | Train 32 | Test 14 | 0.76 | 0.51 | 0.14 | 0.22 | 4046 | Train 64 | Test 14 | 0.69 | 0.34 | 0.18 | 0.24 |
| 1999 | Train 32 | Test 15 | 0.72 | 0.26 | 0.11 | 0.16 | 4047 | Train 64 | Test 15 | 0.69 | 0.24 | 0.2 | 0.22 |
| 2000 | Train 32 | Test 16 | 0.76 | 0.51 | 0.13 | 0.21 | 4048 | Train 64 | Test 16 | 0.7 | 0.34 | 0.18 | 0.23 |
| 2001 | Train 32 | Test 17 | 0.75 | 0.32 | 0.11 | 0.16 | 4049 | Train 64 | Test 17 | 0.68 | 0.24 | 0.23 | 0.23 |
| 2002 | Train 32 | Test 18 | 0.76 | 0.38 | 0.16 | 0.23 | 4050 | Train 64 | Test 18 | 0.69 | 0.28 | 0.24 | 0.26 |
| 2003 | Train 32 | Test 19 | 0.75 | 0.32 | 0.11 | 0.16 | 4051 | Train 64 | Test 19 | 0.68 | 0.24 | 0.23 | 0.23 |
| 2004 | Train 32 | Test 20 | 0.76 | 0.38 | 0.16 | 0.22 | 4052 | Train 64 | Test 20 | 0.69 | 0.28 | 0.24 | 0.26 |
| 2005 | Train 32 | Test 21 | 0.72 | 0.27 | 0.13 | 0.18 | 4053 | Train 64 | Test 21 | 0.69 | 0.24 | 0.22 | 0.23 |
| 2006 | Train 32 | Test 22 | 0.76 | 0.52 | 0.14 | 0.22 | 4054 | Train 64 | Test 22 | 0.69 | 0.34 | 0.18 | 0.24 |
| 2007 | Train 32 | Test 23 | 0.72 | 0.27 | 0.13 | 0.18 | 4055 | Train 64 | Test 23 | 0.69 | 0.24 | 0.22 | 0.23 |
| 2008 | Train 32 | Test 24 | 0.76 | 0.51 | 0.13 | 0.21 | 4056 | Train 64 | Test 24 | 0.7 | 0.34 | 0.18 | 0.23 |
| 2009 | Train 32 | Test 25 | 0.75 | 0.31 | 0.09 | 0.14 | 4057 | Train 64 | Test 25 | 0.69 | 0.24 | 0.22 | 0.23 |
| 2010 | Train 32 | Test 26 | 0.76 | 0.38 | 0.14 | 0.2 | 4058 | Train 64 | Test 26 | 0.7 | 0.27 | 0.22 | 0.24 |
| 2011 | Train 32 | Test 27 | 0.75 | 0.3 | 0.09 | 0.14 | 4059 | Train 64 | Test 27 | 0.69 | 0.24 | 0.21 | 0.23 |
| 2012 | Train 32 | Test 28 | 0.76 | 0.37 | 0.14 | 0.2 | 4060 | Train 64 | Test 28 | 0.7 | 0.27 | 0.22 | 0.24 |
| 2013 | Train 32 | Test 29 | 0.72 | 0.26 | 0.12 | 0.16 | 4061 | Train 64 | Test 29 | 0.69 | 0.24 | 0.21 | 0.22 |
| 2014 | Train 32 | Test 30 | 0.76 | 0.51 | 0.14 | 0.22 | 4062 | Train 64 | Test 30 | 0.69 | 0.34 | 0.18 | 0.24 |
| 2015 | Train 32 | Test 31 | 0.72 | 0.26 | 0.11 | 0.16 | 4063 | Train 64 | Test 31 | 0.69 | 0.24 | 0.2 | 0.22 |
| 2016 | Train 32 | Test 32 | 0.76 | 0.51 | 0.13 | 0.21 | 4064 | Train 64 | Test 32 | 0.7 | 0.34 | 0.18 | 0.23 |
| 2017 | Train 32 | Test 33 | 0.75 | 0.31 | 0.11 | 0.16 | 4065 | Train 64 | Test 33 | 0.69 | 0.24 | 0.23 | 0.23 |
| 2018 | Train 32 | Test 34 | 0.76 | 0.38 | 0.16 | 0.22 | 4066 | Train 64 | Test 34 | 0.69 | 0.27 | 0.24 | 0.26 |
| 2019 | Train 32 | Test 35 | 0.75 | 0.31 | 0.11 | 0.16 | 4067 | Train 64 | Test 35 | 0.69 | 0.24 | 0.23 | 0.23 |
| 2020 | Train 32 | Test 36 | 0.76 | 0.38 | 0.16 | 0.22 | 4068 | Train 64 | Test 36 | 0.69 | 0.27 | 0.24 | 0.26 |
| 2021 | Train 32 | Test 37 | 0.72 | 0.27 | 0.13 | 0.18 | 4069 | Train 64 | Test 37 | 0.69 | 0.24 | 0.22 | 0.23 |
| 2022 | Train 32 | Test 38 | 0.76 | 0.52 | 0.14 | 0.22 | 4070 | Train 64 | Test 38 | 0.69 | 0.34 | 0.18 | 0.24 |
| 2023 | Train 32 | Test 39 | 0.72 | 0.27 | 0.13 | 0.18 | 4071 | Train 64 | Test 39 | 0.69 | 0.24 | 0.22 | 0.23 |
| 2024 | Train 32 | Test 40 | 0.76 | 0.51 | 0.13 | 0.21 | 4072 | Train 64 | Test 40 | 0.7 | 0.34 | 0.18 | 0.24 |
| 2025 | Train 32 | Test 41 | 0.75 | 0.3 | 0.09 | 0.14 | 4073 | Train 64 | Test 41 | 0.7 | 0.24 | 0.22 | 0.23 |
| 2026 | Train 32 | Test 42 | 0.76 | 0.37 | 0.14 | 0.2 | 4074 | Train 64 | Test 42 | 0.7 | 0.27 | 0.22 | 0.24 |
| 2027 | Train 32 | Test 43 | 0.75 | 0.3 | 0.09 | 0.14 | 4075 | Train 64 | Test 43 | 0.7 | 0.24 | 0.21 | 0.23 |
| 2028 | Train 32 | Test 44 | 0.76 | 0.37 | 0.14 | 0.2 | 4076 | Train 64 | Test 44 | 0.7 | 0.27 | 0.22 | 0.24 |
| 2029 | Train 32 | Test 45 | 0.73 | 0.26 | 0.11 | 0.16 | 4077 | Train 64 | Test 45 | 0.69 | 0.24 | 0.21 | 0.22 |
| 2030 | Train 32 | Test 46 | 0.76 | 0.51 | 0.14 | 0.21 | 4078 | Train 64 | Test 46 | 0.69 | 0.34 | 0.18 | 0.24 |
| 2031 | Train 32 | Test 47 | 0.72 | 0.26 | 0.11 | 0.16 | 4079 | Train 64 | Test 47 | 0.69 | 0.24 | 0.2 | 0.22 |
| 2032 | Train 32 | Test 48 | 0.76 | 0.51 | 0.13 | 0.21 | 4080 | Train 64 | Test 48 | 0.7 | 0.34 | 0.18 | 0.23 |
| 2033 | Train 32 | Test 49 | 0.77 | 0.34 | 0.19 | 0.24 | 4081 | Train 64 | Test 49 | 0.75 | 0.34 | 0.27 | 0.3 |
| 2034 | Train 32 | Test 50 | 0.77 | 0.34 | 0.19 | 0.24 | 4082 | Train 64 | Test 50 | 0.75 | 0.34 | 0.27 | 0.3 |
| 2035 | Train 32 | Test 51 | 0.77 | 0.34 | 0.18 | 0.24 | 4083 | Train 64 | Test 51 | 0.75 | 0.34 | 0.26 | 0.3 |
| 2036 | Train 32 | Test 52 | 0.77 | 0.34 | 0.18 | 0.24 | 4084 | Train 64 | Test 52 | 0.75 | 0.34 | 0.26 | 0.3 |
| 2037 | Train 32 | Test 53 | 0.77 | 0.46 | 0.13 | 0.21 | 4085 | Train 64 | Test 53 | 0.77 | 0.39 | 0.15 | 0.22 |
| 2038 | Train 32 | Test 54 | 0.76 | 0.54 | 0.16 | 0.25 | 4086 | Train 64 | Test 54 | 0.76 | 0.51 | 0.19 | 0.28 |
| 2039 | Train 32 | Test 55 | 0.74 | 0.27 | 0.16 | 0.2 | 4087 | Train 64 | Test 55 | 0.76 | 0.36 | 0.21 | 0.26 |
| 2040 | Train 32 | Test 56 | 0.76 | 0.53 | 0.16 | 0.24 | 4088 | Train 64 | Test 56 | 0.76 | 0.51 | 0.19 | 0.27 |
| 2041 | Train 32 | Test 57 | 0.78 | 0.35 | 0.18 | 0.23 | 4089 | Train 64 | Test 57 | 0.76 | 0.36 | 0.25 | 0.3 |
| 2042 | Train 32 | Test 58 | 0.77 | 0.35 | 0.17 | 0.23 | 4090 | Train 64 | Test 58 | 0.76 | 0.36 | 0.25 | 0.29 |
| 2043 | Train 32 | Test 59 | 0.78 | 0.35 | 0.17 | 0.23 | 4091 | Train 64 | Test 59 | 0.76 | 0.36 | 0.24 | 0.29 |
| 2044 | Train 32 | Test 60 | 0.77 | 0.35 | 0.17 | 0.23 | 4092 | Train 64 | Test 60 | 0.76 | 0.36 | 0.24 | 0.29 |
| 2045 | Train 32 | Test 61 | 0.78 | 0.44 | 0.11 | 0.18 | 4093 | Train 64 | Test 61 | 0.77 | 0.41 | 0.14 | 0.21 |
| 2046 | Train 32 | Test 62 | 0.76 | 0.53 | 0.16 | 0.25 | 4094 | Train 64 | Test 62 | 0.76 | 0.51 | 0.19 | 0.28 |
| 2047 | Train 32 | Test 63 | 0.78 | 0.43 | 0.11 | 0.17 | 4095 | Train 64 | Test 63 | 0.77 | 0.42 | 0.14 | 0.21 |
| 2048 | Train 32 | Test 64 | 0.76 | 0.52 | 0.16 | 0.24 | 4096 | Train 64 | Test 64 | 0.76 | 0.51 | 0.19 | 0.27 |
