# Supplementary material for: A Systematic Evaluation of Cohort Selection Criteria and Their Impact on Machine Learning Model Performance and Demographic Disparities in COVID-19 Outcomes: Cohort Study
Source: JMIR Form Res. 2026 Aug 20;10:e88406. doi: 10.2196/88406 (PMC13492188; doi:10.2196/88406)
Supplement: Supplementary file 4 [file formative-v10-e88406-s004.docx]

Appendix 4

Appendix 4 presents AUC values stratified by demographic groups—Male, Female, Black, White, Asian, Hispanic, and Non-Hispanic—across all 64 cohorts, providing insights into model performance differences across key demographic subgroups.

Table 1. AUC Performance of the ML Models— LR, RF, and GB —for Male Patients Trained on Cohorts 1–32 and Tested Across 64 Cohorts.

| Male | Train  Model | Test  Dataset | AUC (LR) | AUC (RF) | AUC (GB) | Male | Train  Model | Test  Dataset | AUC (LR) | AUC (RF) | AUC (GB) |
| --- | --- | --- | --- | --- | --- | --- | --- | --- | --- | --- | --- |
| 1 | Train 1 | Test Male 1 | 0.76 | 0.84 | 0.86 | 1025 | Train 17 | Test Male 1 | 0.76 | 0.84 | 0.86 |
| 2 | Train 1 | Test Male 2 | 0.74 | 0.79 | 0.81 | 1026 | Train 17 | Test Male 2 | 0.74 | 0.8 | 0.8 |
| 3 | Train 1 | Test Male 3 | 0.76 | 0.84 | 0.85 | 1027 | Train 17 | Test Male 3 | 0.76 | 0.84 | 0.85 |
| 4 | Train 1 | Test Male 4 | 0.74 | 0.79 | 0.81 | 1028 | Train 17 | Test Male 4 | 0.74 | 0.8 | 0.8 |
| 5 | Train 1 | Test Male 5 | 0.72 | 0.75 | 0.74 | 1029 | Train 17 | Test Male 5 | 0.72 | 0.75 | 0.74 |
| 6 | Train 1 | Test Male 6 | 0.69 | 0.7 | 0.64 | 1030 | Train 17 | Test Male 6 | 0.69 | 0.7 | 0.65 |
| 7 | Train 1 | Test Male 7 | 0.72 | 0.75 | 0.73 | 1031 | Train 17 | Test Male 7 | 0.72 | 0.75 | 0.73 |
| 8 | Train 1 | Test Male 8 | 0.69 | 0.7 | 0.64 | 1032 | Train 17 | Test Male 8 | 0.69 | 0.7 | 0.65 |
| 9 | Train 1 | Test Male 9 | 0.76 | 0.83 | 0.82 | 1033 | Train 17 | Test Male 9 | 0.76 | 0.83 | 0.82 |
| 10 | Train 1 | Test Male 10 | 0.74 | 0.79 | 0.78 | 1034 | Train 17 | Test Male 10 | 0.74 | 0.79 | 0.78 |
| 11 | Train 1 | Test Male 11 | 0.76 | 0.83 | 0.82 | 1035 | Train 17 | Test Male 11 | 0.76 | 0.83 | 0.82 |
| 12 | Train 1 | Test Male 12 | 0.74 | 0.79 | 0.78 | 1036 | Train 17 | Test Male 12 | 0.74 | 0.79 | 0.78 |
| 13 | Train 1 | Test Male 13 | 0.72 | 0.74 | 0.72 | 1037 | Train 17 | Test Male 13 | 0.72 | 0.74 | 0.72 |
| 14 | Train 1 | Test Male 14 | 0.69 | 0.7 | 0.64 | 1038 | Train 17 | Test Male 14 | 0.69 | 0.7 | 0.65 |
| 15 | Train 1 | Test Male 15 | 0.71 | 0.74 | 0.71 | 1039 | Train 17 | Test Male 15 | 0.71 | 0.74 | 0.72 |
| 16 | Train 1 | Test Male 16 | 0.69 | 0.7 | 0.64 | 1040 | Train 17 | Test Male 16 | 0.69 | 0.7 | 0.65 |
| 17 | Train 1 | Test Male 17 | 0.76 | 0.84 | 0.86 | 1041 | Train 17 | Test Male 17 | 0.76 | 0.84 | 0.86 |
| 18 | Train 1 | Test Male 18 | 0.74 | 0.79 | 0.81 | 1042 | Train 17 | Test Male 18 | 0.74 | 0.8 | 0.8 |
| 19 | Train 1 | Test Male 19 | 0.76 | 0.84 | 0.85 | 1043 | Train 17 | Test Male 19 | 0.76 | 0.84 | 0.85 |
| 20 | Train 1 | Test Male 20 | 0.74 | 0.79 | 0.81 | 1044 | Train 17 | Test Male 20 | 0.74 | 0.8 | 0.8 |
| 21 | Train 1 | Test Male 21 | 0.72 | 0.75 | 0.74 | 1045 | Train 17 | Test Male 21 | 0.72 | 0.75 | 0.74 |
| 22 | Train 1 | Test Male 22 | 0.69 | 0.7 | 0.64 | 1046 | Train 17 | Test Male 22 | 0.69 | 0.7 | 0.65 |
| 23 | Train 1 | Test Male 23 | 0.72 | 0.75 | 0.73 | 1047 | Train 17 | Test Male 23 | 0.72 | 0.75 | 0.73 |
| 24 | Train 1 | Test Male 24 | 0.69 | 0.7 | 0.64 | 1048 | Train 17 | Test Male 24 | 0.69 | 0.7 | 0.65 |
| 25 | Train 1 | Test Male 25 | 0.76 | 0.83 | 0.82 | 1049 | Train 17 | Test Male 25 | 0.76 | 0.83 | 0.82 |
| 26 | Train 1 | Test Male 26 | 0.74 | 0.79 | 0.78 | 1050 | Train 17 | Test Male 26 | 0.74 | 0.79 | 0.78 |
| 27 | Train 1 | Test Male 27 | 0.76 | 0.83 | 0.82 | 1051 | Train 17 | Test Male 27 | 0.76 | 0.83 | 0.82 |
| 28 | Train 1 | Test Male 28 | 0.74 | 0.78 | 0.78 | 1052 | Train 17 | Test Male 28 | 0.74 | 0.79 | 0.78 |
| 29 | Train 1 | Test Male 29 | 0.72 | 0.74 | 0.72 | 1053 | Train 17 | Test Male 29 | 0.72 | 0.74 | 0.72 |
| 30 | Train 1 | Test Male 30 | 0.69 | 0.7 | 0.64 | 1054 | Train 17 | Test Male 30 | 0.69 | 0.7 | 0.65 |
| 31 | Train 1 | Test Male 31 | 0.71 | 0.74 | 0.71 | 1055 | Train 17 | Test Male 31 | 0.71 | 0.74 | 0.72 |
| 32 | Train 1 | Test Male 32 | 0.69 | 0.7 | 0.64 | 1056 | Train 17 | Test Male 32 | 0.69 | 0.7 | 0.65 |
| 33 | Train 1 | Test Male 33 | 0.76 | 0.84 | 0.85 | 1057 | Train 17 | Test Male 33 | 0.76 | 0.84 | 0.85 |
| 34 | Train 1 | Test Male 34 | 0.74 | 0.79 | 0.81 | 1058 | Train 17 | Test Male 34 | 0.74 | 0.8 | 0.8 |
| 35 | Train 1 | Test Male 35 | 0.76 | 0.84 | 0.85 | 1059 | Train 17 | Test Male 35 | 0.76 | 0.84 | 0.85 |
| 36 | Train 1 | Test Male 36 | 0.74 | 0.79 | 0.8 | 1060 | Train 17 | Test Male 36 | 0.74 | 0.79 | 0.8 |
| 37 | Train 1 | Test Male 37 | 0.72 | 0.75 | 0.74 | 1061 | Train 17 | Test Male 37 | 0.72 | 0.75 | 0.73 |
| 38 | Train 1 | Test Male 38 | 0.69 | 0.7 | 0.64 | 1062 | Train 17 | Test Male 38 | 0.69 | 0.7 | 0.65 |
| 39 | Train 1 | Test Male 39 | 0.71 | 0.74 | 0.73 | 1063 | Train 17 | Test Male 39 | 0.71 | 0.74 | 0.73 |
| 40 | Train 1 | Test Male 40 | 0.69 | 0.7 | 0.64 | 1064 | Train 17 | Test Male 40 | 0.69 | 0.7 | 0.65 |
| 41 | Train 1 | Test Male 41 | 0.76 | 0.83 | 0.82 | 1065 | Train 17 | Test Male 41 | 0.76 | 0.83 | 0.82 |
| 42 | Train 1 | Test Male 42 | 0.74 | 0.78 | 0.78 | 1066 | Train 17 | Test Male 42 | 0.74 | 0.79 | 0.78 |
| 43 | Train 1 | Test Male 43 | 0.76 | 0.82 | 0.82 | 1067 | Train 17 | Test Male 43 | 0.76 | 0.82 | 0.82 |
| 44 | Train 1 | Test Male 44 | 0.74 | 0.78 | 0.78 | 1068 | Train 17 | Test Male 44 | 0.74 | 0.79 | 0.78 |
| 45 | Train 1 | Test Male 45 | 0.71 | 0.74 | 0.72 | 1069 | Train 17 | Test Male 45 | 0.71 | 0.74 | 0.72 |
| 46 | Train 1 | Test Male 46 | 0.69 | 0.7 | 0.64 | 1070 | Train 17 | Test Male 46 | 0.69 | 0.7 | 0.65 |
| 47 | Train 1 | Test Male 47 | 0.71 | 0.74 | 0.71 | 1071 | Train 17 | Test Male 47 | 0.71 | 0.74 | 0.72 |
| 48 | Train 1 | Test Male 48 | 0.69 | 0.7 | 0.64 | 1072 | Train 17 | Test Male 48 | 0.69 | 0.7 | 0.65 |
| 49 | Train 1 | Test Male 49 | 0.77 | 0.83 | 0.86 | 1073 | Train 17 | Test Male 49 | 0.77 | 0.83 | 0.86 |
| 50 | Train 1 | Test Male 50 | 0.77 | 0.83 | 0.86 | 1074 | Train 17 | Test Male 50 | 0.77 | 0.83 | 0.86 |
| 51 | Train 1 | Test Male 51 | 0.77 | 0.83 | 0.85 | 1075 | Train 17 | Test Male 51 | 0.77 | 0.83 | 0.85 |
| 52 | Train 1 | Test Male 52 | 0.77 | 0.83 | 0.85 | 1076 | Train 17 | Test Male 52 | 0.77 | 0.83 | 0.85 |
| 53 | Train 1 | Test Male 53 | 0.73 | 0.77 | 0.65 | 1077 | Train 17 | Test Male 53 | 0.73 | 0.77 | 0.71 |
| 54 | Train 1 | Test Male 54 | 0.71 | 0.71 | 0.64 | 1078 | Train 17 | Test Male 54 | 0.71 | 0.71 | 0.66 |
| 55 | Train 1 | Test Male 55 | 0.73 | 0.77 | 0.65 | 1079 | Train 17 | Test Male 55 | 0.73 | 0.77 | 0.71 |
| 56 | Train 1 | Test Male 56 | 0.71 | 0.71 | 0.64 | 1080 | Train 17 | Test Male 56 | 0.71 | 0.71 | 0.66 |
| 57 | Train 1 | Test Male 57 | 0.77 | 0.82 | 0.82 | 1081 | Train 17 | Test Male 57 | 0.77 | 0.82 | 0.82 |
| 58 | Train 1 | Test Male 58 | 0.77 | 0.82 | 0.82 | 1082 | Train 17 | Test Male 58 | 0.77 | 0.82 | 0.82 |
| 59 | Train 1 | Test Male 59 | 0.77 | 0.82 | 0.82 | 1083 | Train 17 | Test Male 59 | 0.77 | 0.82 | 0.82 |
| 60 | Train 1 | Test Male 60 | 0.77 | 0.82 | 0.82 | 1084 | Train 17 | Test Male 60 | 0.77 | 0.82 | 0.82 |
| 61 | Train 1 | Test Male 61 | 0.73 | 0.77 | 0.64 | 1085 | Train 17 | Test Male 61 | 0.73 | 0.77 | 0.69 |
| 62 | Train 1 | Test Male 62 | 0.71 | 0.71 | 0.64 | 1086 | Train 17 | Test Male 62 | 0.71 | 0.71 | 0.66 |
| 63 | Train 1 | Test Male 63 | 0.73 | 0.77 | 0.65 | 1087 | Train 17 | Test Male 63 | 0.73 | 0.77 | 0.7 |
| 64 | Train 1 | Test Male 64 | 0.71 | 0.71 | 0.64 | 1088 | Train 17 | Test Male 64 | 0.71 | 0.71 | 0.66 |
| 65 | Train 2 | Test Male 1 | 0.75 | 0.83 | 0.84 | 1089 | Train 18 | Test Male 1 | 0.75 | 0.83 | 0.84 |
| 66 | Train 2 | Test Male 2 | 0.76 | 0.83 | 0.85 | 1090 | Train 18 | Test Male 2 | 0.76 | 0.83 | 0.85 |
| 67 | Train 2 | Test Male 3 | 0.75 | 0.83 | 0.84 | 1091 | Train 18 | Test Male 3 | 0.75 | 0.83 | 0.84 |
| 68 | Train 2 | Test Male 4 | 0.76 | 0.83 | 0.85 | 1092 | Train 18 | Test Male 4 | 0.76 | 0.83 | 0.85 |
| 69 | Train 2 | Test Male 5 | 0.72 | 0.75 | 0.74 | 1093 | Train 18 | Test Male 5 | 0.72 | 0.75 | 0.74 |
| 70 | Train 2 | Test Male 6 | 0.7 | 0.71 | 0.66 | 1094 | Train 18 | Test Male 6 | 0.7 | 0.71 | 0.67 |
| 71 | Train 2 | Test Male 7 | 0.72 | 0.75 | 0.74 | 1095 | Train 18 | Test Male 7 | 0.72 | 0.75 | 0.73 |
| 72 | Train 2 | Test Male 8 | 0.7 | 0.71 | 0.66 | 1096 | Train 18 | Test Male 8 | 0.7 | 0.71 | 0.67 |
| 73 | Train 2 | Test Male 9 | 0.75 | 0.82 | 0.81 | 1097 | Train 18 | Test Male 9 | 0.75 | 0.82 | 0.81 |
| 74 | Train 2 | Test Male 10 | 0.76 | 0.82 | 0.82 | 1098 | Train 18 | Test Male 10 | 0.76 | 0.82 | 0.82 |
| 75 | Train 2 | Test Male 11 | 0.75 | 0.82 | 0.81 | 1099 | Train 18 | Test Male 11 | 0.75 | 0.82 | 0.81 |
| 76 | Train 2 | Test Male 12 | 0.76 | 0.82 | 0.82 | 1100 | Train 18 | Test Male 12 | 0.76 | 0.82 | 0.82 |
| 77 | Train 2 | Test Male 13 | 0.72 | 0.74 | 0.73 | 1101 | Train 18 | Test Male 13 | 0.72 | 0.74 | 0.72 |
| 78 | Train 2 | Test Male 14 | 0.71 | 0.71 | 0.66 | 1102 | Train 18 | Test Male 14 | 0.71 | 0.71 | 0.67 |
| 79 | Train 2 | Test Male 15 | 0.71 | 0.74 | 0.72 | 1103 | Train 18 | Test Male 15 | 0.71 | 0.74 | 0.71 |
| 80 | Train 2 | Test Male 16 | 0.7 | 0.71 | 0.66 | 1104 | Train 18 | Test Male 16 | 0.7 | 0.71 | 0.67 |
| 81 | Train 2 | Test Male 17 | 0.75 | 0.83 | 0.84 | 1105 | Train 18 | Test Male 17 | 0.75 | 0.83 | 0.84 |
| 82 | Train 2 | Test Male 18 | 0.76 | 0.83 | 0.85 | 1106 | Train 18 | Test Male 18 | 0.76 | 0.83 | 0.85 |
| 83 | Train 2 | Test Male 19 | 0.75 | 0.83 | 0.84 | 1107 | Train 18 | Test Male 19 | 0.75 | 0.83 | 0.84 |
| 84 | Train 2 | Test Male 20 | 0.76 | 0.83 | 0.85 | 1108 | Train 18 | Test Male 20 | 0.76 | 0.83 | 0.85 |
| 85 | Train 2 | Test Male 21 | 0.72 | 0.75 | 0.74 | 1109 | Train 18 | Test Male 21 | 0.72 | 0.75 | 0.74 |
| 86 | Train 2 | Test Male 22 | 0.7 | 0.71 | 0.66 | 1110 | Train 18 | Test Male 22 | 0.7 | 0.71 | 0.67 |
| 87 | Train 2 | Test Male 23 | 0.72 | 0.75 | 0.74 | 1111 | Train 18 | Test Male 23 | 0.72 | 0.75 | 0.73 |
| 88 | Train 2 | Test Male 24 | 0.7 | 0.71 | 0.66 | 1112 | Train 18 | Test Male 24 | 0.7 | 0.71 | 0.67 |
| 89 | Train 2 | Test Male 25 | 0.75 | 0.82 | 0.81 | 1113 | Train 18 | Test Male 25 | 0.75 | 0.82 | 0.81 |
| 90 | Train 2 | Test Male 26 | 0.76 | 0.82 | 0.82 | 1114 | Train 18 | Test Male 26 | 0.76 | 0.82 | 0.82 |
| 91 | Train 2 | Test Male 27 | 0.75 | 0.82 | 0.81 | 1115 | Train 18 | Test Male 27 | 0.75 | 0.82 | 0.81 |
| 92 | Train 2 | Test Male 28 | 0.76 | 0.82 | 0.82 | 1116 | Train 18 | Test Male 28 | 0.76 | 0.82 | 0.82 |
| 93 | Train 2 | Test Male 29 | 0.72 | 0.74 | 0.73 | 1117 | Train 18 | Test Male 29 | 0.72 | 0.74 | 0.72 |
| 94 | Train 2 | Test Male 30 | 0.71 | 0.71 | 0.66 | 1118 | Train 18 | Test Male 30 | 0.71 | 0.71 | 0.67 |
| 95 | Train 2 | Test Male 31 | 0.71 | 0.74 | 0.72 | 1119 | Train 18 | Test Male 31 | 0.71 | 0.74 | 0.71 |
| 96 | Train 2 | Test Male 32 | 0.71 | 0.71 | 0.66 | 1120 | Train 18 | Test Male 32 | 0.71 | 0.71 | 0.67 |
| 97 | Train 2 | Test Male 33 | 0.75 | 0.83 | 0.84 | 1121 | Train 18 | Test Male 33 | 0.75 | 0.83 | 0.84 |
| 98 | Train 2 | Test Male 34 | 0.76 | 0.83 | 0.85 | 1122 | Train 18 | Test Male 34 | 0.76 | 0.83 | 0.85 |
| 99 | Train 2 | Test Male 35 | 0.75 | 0.83 | 0.84 | 1123 | Train 18 | Test Male 35 | 0.75 | 0.83 | 0.84 |
| 100 | Train 2 | Test Male 36 | 0.76 | 0.83 | 0.85 | 1124 | Train 18 | Test Male 36 | 0.76 | 0.83 | 0.85 |
| 101 | Train 2 | Test Male 37 | 0.72 | 0.75 | 0.74 | 1125 | Train 18 | Test Male 37 | 0.72 | 0.75 | 0.73 |
| 102 | Train 2 | Test Male 38 | 0.7 | 0.71 | 0.66 | 1126 | Train 18 | Test Male 38 | 0.7 | 0.71 | 0.67 |
| 103 | Train 2 | Test Male 39 | 0.72 | 0.75 | 0.74 | 1127 | Train 18 | Test Male 39 | 0.72 | 0.75 | 0.73 |
| 104 | Train 2 | Test Male 40 | 0.7 | 0.71 | 0.66 | 1128 | Train 18 | Test Male 40 | 0.7 | 0.71 | 0.67 |
| 105 | Train 2 | Test Male 41 | 0.75 | 0.82 | 0.81 | 1129 | Train 18 | Test Male 41 | 0.75 | 0.82 | 0.81 |
| 106 | Train 2 | Test Male 42 | 0.76 | 0.82 | 0.82 | 1130 | Train 18 | Test Male 42 | 0.76 | 0.82 | 0.82 |
| 107 | Train 2 | Test Male 43 | 0.75 | 0.82 | 0.81 | 1131 | Train 18 | Test Male 43 | 0.75 | 0.82 | 0.81 |
| 108 | Train 2 | Test Male 44 | 0.76 | 0.82 | 0.82 | 1132 | Train 18 | Test Male 44 | 0.76 | 0.82 | 0.82 |
| 109 | Train 2 | Test Male 45 | 0.72 | 0.74 | 0.72 | 1133 | Train 18 | Test Male 45 | 0.72 | 0.74 | 0.72 |
| 110 | Train 2 | Test Male 46 | 0.71 | 0.71 | 0.66 | 1134 | Train 18 | Test Male 46 | 0.71 | 0.71 | 0.67 |
| 111 | Train 2 | Test Male 47 | 0.71 | 0.74 | 0.72 | 1135 | Train 18 | Test Male 47 | 0.71 | 0.74 | 0.71 |
| 112 | Train 2 | Test Male 48 | 0.7 | 0.71 | 0.66 | 1136 | Train 18 | Test Male 48 | 0.7 | 0.71 | 0.67 |
| 113 | Train 2 | Test Male 49 | 0.77 | 0.82 | 0.85 | 1137 | Train 18 | Test Male 49 | 0.77 | 0.83 | 0.85 |
| 114 | Train 2 | Test Male 50 | 0.77 | 0.82 | 0.85 | 1138 | Train 18 | Test Male 50 | 0.77 | 0.83 | 0.85 |
| 115 | Train 2 | Test Male 51 | 0.77 | 0.82 | 0.84 | 1139 | Train 18 | Test Male 51 | 0.77 | 0.82 | 0.84 |
| 116 | Train 2 | Test Male 52 | 0.76 | 0.82 | 0.84 | 1140 | Train 18 | Test Male 52 | 0.76 | 0.82 | 0.84 |
| 117 | Train 2 | Test Male 53 | 0.74 | 0.78 | 0.71 | 1141 | Train 18 | Test Male 53 | 0.74 | 0.78 | 0.72 |
| 118 | Train 2 | Test Male 54 | 0.71 | 0.72 | 0.66 | 1142 | Train 18 | Test Male 54 | 0.71 | 0.72 | 0.67 |
| 119 | Train 2 | Test Male 55 | 0.74 | 0.78 | 0.71 | 1143 | Train 18 | Test Male 55 | 0.74 | 0.78 | 0.72 |
| 120 | Train 2 | Test Male 56 | 0.71 | 0.71 | 0.66 | 1144 | Train 18 | Test Male 56 | 0.71 | 0.71 | 0.66 |
| 121 | Train 2 | Test Male 57 | 0.77 | 0.82 | 0.82 | 1145 | Train 18 | Test Male 57 | 0.77 | 0.82 | 0.82 |
| 122 | Train 2 | Test Male 58 | 0.77 | 0.82 | 0.82 | 1146 | Train 18 | Test Male 58 | 0.77 | 0.82 | 0.82 |
| 123 | Train 2 | Test Male 59 | 0.77 | 0.81 | 0.81 | 1147 | Train 18 | Test Male 59 | 0.76 | 0.81 | 0.81 |
| 124 | Train 2 | Test Male 60 | 0.76 | 0.81 | 0.81 | 1148 | Train 18 | Test Male 60 | 0.76 | 0.81 | 0.81 |
| 125 | Train 2 | Test Male 61 | 0.73 | 0.78 | 0.67 | 1149 | Train 18 | Test Male 61 | 0.73 | 0.77 | 0.66 |
| 126 | Train 2 | Test Male 62 | 0.71 | 0.72 | 0.66 | 1150 | Train 18 | Test Male 62 | 0.71 | 0.72 | 0.67 |
| 127 | Train 2 | Test Male 63 | 0.73 | 0.78 | 0.7 | 1151 | Train 18 | Test Male 63 | 0.73 | 0.77 | 0.71 |
| 128 | Train 2 | Test Male 64 | 0.71 | 0.71 | 0.66 | 1152 | Train 18 | Test Male 64 | 0.71 | 0.71 | 0.66 |
| 129 | Train 3 | Test Male 1 | 0.76 | 0.84 | 0.86 | 1153 | Train 19 | Test Male 1 | 0.76 | 0.84 | 0.85 |
| 130 | Train 3 | Test Male 2 | 0.74 | 0.79 | 0.8 | 1154 | Train 19 | Test Male 2 | 0.74 | 0.79 | 0.8 |
| 131 | Train 3 | Test Male 3 | 0.76 | 0.84 | 0.86 | 1155 | Train 19 | Test Male 3 | 0.76 | 0.84 | 0.85 |
| 132 | Train 3 | Test Male 4 | 0.74 | 0.79 | 0.8 | 1156 | Train 19 | Test Male 4 | 0.74 | 0.8 | 0.8 |
| 133 | Train 3 | Test Male 5 | 0.72 | 0.75 | 0.72 | 1157 | Train 19 | Test Male 5 | 0.72 | 0.75 | 0.71 |
| 134 | Train 3 | Test Male 6 | 0.69 | 0.7 | 0.64 | 1158 | Train 19 | Test Male 6 | 0.69 | 0.7 | 0.63 |
| 135 | Train 3 | Test Male 7 | 0.72 | 0.75 | 0.72 | 1159 | Train 19 | Test Male 7 | 0.71 | 0.75 | 0.71 |
| 136 | Train 3 | Test Male 8 | 0.69 | 0.7 | 0.64 | 1160 | Train 19 | Test Male 8 | 0.69 | 0.7 | 0.63 |
| 137 | Train 3 | Test Male 9 | 0.76 | 0.83 | 0.82 | 1161 | Train 19 | Test Male 9 | 0.76 | 0.83 | 0.82 |
| 138 | Train 3 | Test Male 10 | 0.74 | 0.78 | 0.77 | 1162 | Train 19 | Test Male 10 | 0.74 | 0.79 | 0.77 |
| 139 | Train 3 | Test Male 11 | 0.76 | 0.83 | 0.82 | 1163 | Train 19 | Test Male 11 | 0.76 | 0.83 | 0.82 |
| 140 | Train 3 | Test Male 12 | 0.74 | 0.79 | 0.77 | 1164 | Train 19 | Test Male 12 | 0.74 | 0.79 | 0.77 |
| 141 | Train 3 | Test Male 13 | 0.71 | 0.74 | 0.7 | 1165 | Train 19 | Test Male 13 | 0.71 | 0.74 | 0.69 |
| 142 | Train 3 | Test Male 14 | 0.69 | 0.7 | 0.64 | 1166 | Train 19 | Test Male 14 | 0.69 | 0.7 | 0.63 |
| 143 | Train 3 | Test Male 15 | 0.71 | 0.74 | 0.7 | 1167 | Train 19 | Test Male 15 | 0.71 | 0.74 | 0.69 |
| 144 | Train 3 | Test Male 16 | 0.69 | 0.7 | 0.64 | 1168 | Train 19 | Test Male 16 | 0.69 | 0.7 | 0.63 |
| 145 | Train 3 | Test Male 17 | 0.76 | 0.84 | 0.86 | 1169 | Train 19 | Test Male 17 | 0.76 | 0.84 | 0.85 |
| 146 | Train 3 | Test Male 18 | 0.74 | 0.79 | 0.8 | 1170 | Train 19 | Test Male 18 | 0.74 | 0.79 | 0.8 |
| 147 | Train 3 | Test Male 19 | 0.76 | 0.84 | 0.86 | 1171 | Train 19 | Test Male 19 | 0.76 | 0.84 | 0.85 |
| 148 | Train 3 | Test Male 20 | 0.74 | 0.79 | 0.8 | 1172 | Train 19 | Test Male 20 | 0.74 | 0.8 | 0.8 |
| 149 | Train 3 | Test Male 21 | 0.72 | 0.75 | 0.72 | 1173 | Train 19 | Test Male 21 | 0.72 | 0.75 | 0.71 |
| 150 | Train 3 | Test Male 22 | 0.69 | 0.7 | 0.64 | 1174 | Train 19 | Test Male 22 | 0.69 | 0.7 | 0.63 |
| 151 | Train 3 | Test Male 23 | 0.72 | 0.75 | 0.72 | 1175 | Train 19 | Test Male 23 | 0.71 | 0.75 | 0.71 |
| 152 | Train 3 | Test Male 24 | 0.69 | 0.7 | 0.64 | 1176 | Train 19 | Test Male 24 | 0.69 | 0.7 | 0.63 |
| 153 | Train 3 | Test Male 25 | 0.76 | 0.83 | 0.82 | 1177 | Train 19 | Test Male 25 | 0.76 | 0.83 | 0.82 |
| 154 | Train 3 | Test Male 26 | 0.74 | 0.78 | 0.77 | 1178 | Train 19 | Test Male 26 | 0.74 | 0.79 | 0.77 |
| 155 | Train 3 | Test Male 27 | 0.76 | 0.83 | 0.82 | 1179 | Train 19 | Test Male 27 | 0.76 | 0.83 | 0.82 |
| 156 | Train 3 | Test Male 28 | 0.74 | 0.79 | 0.77 | 1180 | Train 19 | Test Male 28 | 0.74 | 0.79 | 0.77 |
| 157 | Train 3 | Test Male 29 | 0.72 | 0.74 | 0.7 | 1181 | Train 19 | Test Male 29 | 0.71 | 0.74 | 0.69 |
| 158 | Train 3 | Test Male 30 | 0.69 | 0.7 | 0.64 | 1182 | Train 19 | Test Male 30 | 0.69 | 0.7 | 0.63 |
| 159 | Train 3 | Test Male 31 | 0.71 | 0.74 | 0.7 | 1183 | Train 19 | Test Male 31 | 0.71 | 0.74 | 0.69 |
| 160 | Train 3 | Test Male 32 | 0.69 | 0.7 | 0.64 | 1184 | Train 19 | Test Male 32 | 0.69 | 0.7 | 0.63 |
| 161 | Train 3 | Test Male 33 | 0.76 | 0.84 | 0.85 | 1185 | Train 19 | Test Male 33 | 0.76 | 0.84 | 0.85 |
| 162 | Train 3 | Test Male 34 | 0.74 | 0.79 | 0.8 | 1186 | Train 19 | Test Male 34 | 0.74 | 0.79 | 0.8 |
| 163 | Train 3 | Test Male 35 | 0.76 | 0.84 | 0.85 | 1187 | Train 19 | Test Male 35 | 0.76 | 0.84 | 0.85 |
| 164 | Train 3 | Test Male 36 | 0.74 | 0.79 | 0.8 | 1188 | Train 19 | Test Male 36 | 0.74 | 0.79 | 0.8 |
| 165 | Train 3 | Test Male 37 | 0.72 | 0.75 | 0.72 | 1189 | Train 19 | Test Male 37 | 0.72 | 0.75 | 0.71 |
| 166 | Train 3 | Test Male 38 | 0.69 | 0.7 | 0.64 | 1190 | Train 19 | Test Male 38 | 0.69 | 0.7 | 0.63 |
| 167 | Train 3 | Test Male 39 | 0.71 | 0.74 | 0.72 | 1191 | Train 19 | Test Male 39 | 0.71 | 0.75 | 0.71 |
| 168 | Train 3 | Test Male 40 | 0.69 | 0.7 | 0.64 | 1192 | Train 19 | Test Male 40 | 0.69 | 0.7 | 0.63 |
| 169 | Train 3 | Test Male 41 | 0.76 | 0.83 | 0.82 | 1193 | Train 19 | Test Male 41 | 0.76 | 0.83 | 0.82 |
| 170 | Train 3 | Test Male 42 | 0.74 | 0.78 | 0.77 | 1194 | Train 19 | Test Male 42 | 0.74 | 0.78 | 0.77 |
| 171 | Train 3 | Test Male 43 | 0.76 | 0.83 | 0.82 | 1195 | Train 19 | Test Male 43 | 0.76 | 0.83 | 0.82 |
| 172 | Train 3 | Test Male 44 | 0.74 | 0.78 | 0.77 | 1196 | Train 19 | Test Male 44 | 0.74 | 0.79 | 0.77 |
| 173 | Train 3 | Test Male 45 | 0.71 | 0.74 | 0.7 | 1197 | Train 19 | Test Male 45 | 0.71 | 0.74 | 0.69 |
| 174 | Train 3 | Test Male 46 | 0.69 | 0.7 | 0.63 | 1198 | Train 19 | Test Male 46 | 0.69 | 0.7 | 0.63 |
| 175 | Train 3 | Test Male 47 | 0.71 | 0.74 | 0.7 | 1199 | Train 19 | Test Male 47 | 0.71 | 0.74 | 0.69 |
| 176 | Train 3 | Test Male 48 | 0.69 | 0.7 | 0.64 | 1200 | Train 19 | Test Male 48 | 0.69 | 0.7 | 0.63 |
| 177 | Train 3 | Test Male 49 | 0.77 | 0.83 | 0.86 | 1201 | Train 19 | Test Male 49 | 0.77 | 0.83 | 0.85 |
| 178 | Train 3 | Test Male 50 | 0.77 | 0.83 | 0.86 | 1202 | Train 19 | Test Male 50 | 0.77 | 0.83 | 0.85 |
| 179 | Train 3 | Test Male 51 | 0.77 | 0.83 | 0.86 | 1203 | Train 19 | Test Male 51 | 0.77 | 0.83 | 0.85 |
| 180 | Train 3 | Test Male 52 | 0.77 | 0.83 | 0.86 | 1204 | Train 19 | Test Male 52 | 0.77 | 0.83 | 0.85 |
| 181 | Train 3 | Test Male 53 | 0.73 | 0.77 | 0.67 | 1205 | Train 19 | Test Male 53 | 0.73 | 0.77 | 0.64 |
| 182 | Train 3 | Test Male 54 | 0.71 | 0.71 | 0.65 | 1206 | Train 19 | Test Male 54 | 0.71 | 0.71 | 0.62 |
| 183 | Train 3 | Test Male 55 | 0.73 | 0.78 | 0.67 | 1207 | Train 19 | Test Male 55 | 0.73 | 0.77 | 0.64 |
| 184 | Train 3 | Test Male 56 | 0.71 | 0.7 | 0.65 | 1208 | Train 19 | Test Male 56 | 0.71 | 0.7 | 0.63 |
| 185 | Train 3 | Test Male 57 | 0.77 | 0.82 | 0.82 | 1209 | Train 19 | Test Male 57 | 0.77 | 0.82 | 0.82 |
| 186 | Train 3 | Test Male 58 | 0.77 | 0.82 | 0.82 | 1210 | Train 19 | Test Male 58 | 0.77 | 0.82 | 0.82 |
| 187 | Train 3 | Test Male 59 | 0.77 | 0.82 | 0.82 | 1211 | Train 19 | Test Male 59 | 0.77 | 0.82 | 0.82 |
| 188 | Train 3 | Test Male 60 | 0.77 | 0.82 | 0.82 | 1212 | Train 19 | Test Male 60 | 0.77 | 0.82 | 0.82 |
| 189 | Train 3 | Test Male 61 | 0.73 | 0.77 | 0.65 | 1213 | Train 19 | Test Male 61 | 0.73 | 0.77 | 0.62 |
| 190 | Train 3 | Test Male 62 | 0.71 | 0.71 | 0.65 | 1214 | Train 19 | Test Male 62 | 0.71 | 0.71 | 0.62 |
| 191 | Train 3 | Test Male 63 | 0.73 | 0.77 | 0.66 | 1215 | Train 19 | Test Male 63 | 0.73 | 0.77 | 0.63 |
| 192 | Train 3 | Test Male 64 | 0.71 | 0.7 | 0.65 | 1216 | Train 19 | Test Male 64 | 0.71 | 0.7 | 0.63 |
| 193 | Train 4 | Test Male 1 | 0.75 | 0.83 | 0.84 | 1217 | Train 20 | Test Male 1 | 0.75 | 0.83 | 0.84 |
| 194 | Train 4 | Test Male 2 | 0.76 | 0.83 | 0.85 | 1218 | Train 20 | Test Male 2 | 0.76 | 0.83 | 0.85 |
| 195 | Train 4 | Test Male 3 | 0.75 | 0.83 | 0.84 | 1219 | Train 20 | Test Male 3 | 0.75 | 0.83 | 0.84 |
| 196 | Train 4 | Test Male 4 | 0.76 | 0.83 | 0.85 | 1220 | Train 20 | Test Male 4 | 0.76 | 0.83 | 0.85 |
| 197 | Train 4 | Test Male 5 | 0.72 | 0.75 | 0.73 | 1221 | Train 20 | Test Male 5 | 0.72 | 0.75 | 0.73 |
| 198 | Train 4 | Test Male 6 | 0.7 | 0.71 | 0.66 | 1222 | Train 20 | Test Male 6 | 0.7 | 0.71 | 0.66 |
| 199 | Train 4 | Test Male 7 | 0.72 | 0.75 | 0.73 | 1223 | Train 20 | Test Male 7 | 0.72 | 0.75 | 0.73 |
| 200 | Train 4 | Test Male 8 | 0.7 | 0.71 | 0.66 | 1224 | Train 20 | Test Male 8 | 0.7 | 0.71 | 0.66 |
| 201 | Train 4 | Test Male 9 | 0.75 | 0.82 | 0.81 | 1225 | Train 20 | Test Male 9 | 0.75 | 0.82 | 0.82 |
| 202 | Train 4 | Test Male 10 | 0.76 | 0.82 | 0.82 | 1226 | Train 20 | Test Male 10 | 0.76 | 0.82 | 0.82 |
| 203 | Train 4 | Test Male 11 | 0.75 | 0.82 | 0.81 | 1227 | Train 20 | Test Male 11 | 0.75 | 0.82 | 0.82 |
| 204 | Train 4 | Test Male 12 | 0.76 | 0.82 | 0.82 | 1228 | Train 20 | Test Male 12 | 0.76 | 0.82 | 0.82 |
| 205 | Train 4 | Test Male 13 | 0.72 | 0.74 | 0.72 | 1229 | Train 20 | Test Male 13 | 0.72 | 0.74 | 0.72 |
| 206 | Train 4 | Test Male 14 | 0.7 | 0.71 | 0.66 | 1230 | Train 20 | Test Male 14 | 0.7 | 0.71 | 0.66 |
| 207 | Train 4 | Test Male 15 | 0.71 | 0.74 | 0.72 | 1231 | Train 20 | Test Male 15 | 0.71 | 0.74 | 0.71 |
| 208 | Train 4 | Test Male 16 | 0.7 | 0.71 | 0.66 | 1232 | Train 20 | Test Male 16 | 0.7 | 0.71 | 0.66 |
| 209 | Train 4 | Test Male 17 | 0.75 | 0.83 | 0.84 | 1233 | Train 20 | Test Male 17 | 0.75 | 0.83 | 0.84 |
| 210 | Train 4 | Test Male 18 | 0.76 | 0.83 | 0.85 | 1234 | Train 20 | Test Male 18 | 0.76 | 0.83 | 0.85 |
| 211 | Train 4 | Test Male 19 | 0.75 | 0.83 | 0.84 | 1235 | Train 20 | Test Male 19 | 0.75 | 0.83 | 0.84 |
| 212 | Train 4 | Test Male 20 | 0.76 | 0.83 | 0.85 | 1236 | Train 20 | Test Male 20 | 0.76 | 0.83 | 0.85 |
| 213 | Train 4 | Test Male 21 | 0.72 | 0.75 | 0.73 | 1237 | Train 20 | Test Male 21 | 0.72 | 0.75 | 0.73 |
| 214 | Train 4 | Test Male 22 | 0.7 | 0.71 | 0.66 | 1238 | Train 20 | Test Male 22 | 0.7 | 0.71 | 0.66 |
| 215 | Train 4 | Test Male 23 | 0.72 | 0.75 | 0.73 | 1239 | Train 20 | Test Male 23 | 0.72 | 0.75 | 0.73 |
| 216 | Train 4 | Test Male 24 | 0.7 | 0.71 | 0.66 | 1240 | Train 20 | Test Male 24 | 0.7 | 0.71 | 0.66 |
| 217 | Train 4 | Test Male 25 | 0.75 | 0.82 | 0.81 | 1241 | Train 20 | Test Male 25 | 0.75 | 0.82 | 0.82 |
| 218 | Train 4 | Test Male 26 | 0.76 | 0.82 | 0.82 | 1242 | Train 20 | Test Male 26 | 0.76 | 0.82 | 0.82 |
| 219 | Train 4 | Test Male 27 | 0.75 | 0.82 | 0.81 | 1243 | Train 20 | Test Male 27 | 0.75 | 0.82 | 0.82 |
| 220 | Train 4 | Test Male 28 | 0.76 | 0.82 | 0.82 | 1244 | Train 20 | Test Male 28 | 0.76 | 0.82 | 0.82 |
| 221 | Train 4 | Test Male 29 | 0.72 | 0.74 | 0.72 | 1245 | Train 20 | Test Male 29 | 0.72 | 0.74 | 0.72 |
| 222 | Train 4 | Test Male 30 | 0.7 | 0.71 | 0.66 | 1246 | Train 20 | Test Male 30 | 0.71 | 0.71 | 0.66 |
| 223 | Train 4 | Test Male 31 | 0.71 | 0.74 | 0.72 | 1247 | Train 20 | Test Male 31 | 0.71 | 0.74 | 0.71 |
| 224 | Train 4 | Test Male 32 | 0.7 | 0.71 | 0.66 | 1248 | Train 20 | Test Male 32 | 0.7 | 0.71 | 0.66 |
| 225 | Train 4 | Test Male 33 | 0.75 | 0.83 | 0.84 | 1249 | Train 20 | Test Male 33 | 0.75 | 0.83 | 0.84 |
| 226 | Train 4 | Test Male 34 | 0.76 | 0.83 | 0.85 | 1250 | Train 20 | Test Male 34 | 0.76 | 0.83 | 0.85 |
| 227 | Train 4 | Test Male 35 | 0.75 | 0.83 | 0.84 | 1251 | Train 20 | Test Male 35 | 0.75 | 0.83 | 0.84 |
| 228 | Train 4 | Test Male 36 | 0.76 | 0.83 | 0.85 | 1252 | Train 20 | Test Male 36 | 0.76 | 0.83 | 0.85 |
| 229 | Train 4 | Test Male 37 | 0.72 | 0.75 | 0.73 | 1253 | Train 20 | Test Male 37 | 0.72 | 0.75 | 0.73 |
| 230 | Train 4 | Test Male 38 | 0.7 | 0.71 | 0.66 | 1254 | Train 20 | Test Male 38 | 0.7 | 0.71 | 0.66 |
| 231 | Train 4 | Test Male 39 | 0.72 | 0.75 | 0.73 | 1255 | Train 20 | Test Male 39 | 0.72 | 0.75 | 0.73 |
| 232 | Train 4 | Test Male 40 | 0.7 | 0.71 | 0.66 | 1256 | Train 20 | Test Male 40 | 0.7 | 0.71 | 0.66 |
| 233 | Train 4 | Test Male 41 | 0.75 | 0.82 | 0.81 | 1257 | Train 20 | Test Male 41 | 0.75 | 0.82 | 0.82 |
| 234 | Train 4 | Test Male 42 | 0.76 | 0.82 | 0.82 | 1258 | Train 20 | Test Male 42 | 0.76 | 0.82 | 0.82 |
| 235 | Train 4 | Test Male 43 | 0.75 | 0.82 | 0.81 | 1259 | Train 20 | Test Male 43 | 0.75 | 0.82 | 0.82 |
| 236 | Train 4 | Test Male 44 | 0.76 | 0.82 | 0.82 | 1260 | Train 20 | Test Male 44 | 0.76 | 0.82 | 0.82 |
| 237 | Train 4 | Test Male 45 | 0.71 | 0.74 | 0.72 | 1261 | Train 20 | Test Male 45 | 0.72 | 0.74 | 0.71 |
| 238 | Train 4 | Test Male 46 | 0.7 | 0.71 | 0.66 | 1262 | Train 20 | Test Male 46 | 0.7 | 0.71 | 0.66 |
| 239 | Train 4 | Test Male 47 | 0.71 | 0.74 | 0.72 | 1263 | Train 20 | Test Male 47 | 0.71 | 0.74 | 0.71 |
| 240 | Train 4 | Test Male 48 | 0.7 | 0.71 | 0.66 | 1264 | Train 20 | Test Male 48 | 0.7 | 0.71 | 0.66 |
| 241 | Train 4 | Test Male 49 | 0.77 | 0.83 | 0.85 | 1265 | Train 20 | Test Male 49 | 0.77 | 0.82 | 0.85 |
| 242 | Train 4 | Test Male 50 | 0.77 | 0.82 | 0.85 | 1266 | Train 20 | Test Male 50 | 0.77 | 0.82 | 0.85 |
| 243 | Train 4 | Test Male 51 | 0.77 | 0.82 | 0.85 | 1267 | Train 20 | Test Male 51 | 0.77 | 0.82 | 0.85 |
| 244 | Train 4 | Test Male 52 | 0.76 | 0.82 | 0.85 | 1268 | Train 20 | Test Male 52 | 0.76 | 0.82 | 0.85 |
| 245 | Train 4 | Test Male 53 | 0.74 | 0.78 | 0.7 | 1269 | Train 20 | Test Male 53 | 0.74 | 0.78 | 0.69 |
| 246 | Train 4 | Test Male 54 | 0.71 | 0.72 | 0.66 | 1270 | Train 20 | Test Male 54 | 0.71 | 0.72 | 0.66 |
| 247 | Train 4 | Test Male 55 | 0.73 | 0.78 | 0.7 | 1271 | Train 20 | Test Male 55 | 0.74 | 0.78 | 0.69 |
| 248 | Train 4 | Test Male 56 | 0.71 | 0.71 | 0.66 | 1272 | Train 20 | Test Male 56 | 0.71 | 0.71 | 0.65 |
| 249 | Train 4 | Test Male 57 | 0.77 | 0.82 | 0.82 | 1273 | Train 20 | Test Male 57 | 0.77 | 0.82 | 0.82 |
| 250 | Train 4 | Test Male 58 | 0.77 | 0.82 | 0.82 | 1274 | Train 20 | Test Male 58 | 0.77 | 0.82 | 0.82 |
| 251 | Train 4 | Test Male 59 | 0.77 | 0.82 | 0.82 | 1275 | Train 20 | Test Male 59 | 0.77 | 0.81 | 0.82 |
| 252 | Train 4 | Test Male 60 | 0.76 | 0.82 | 0.82 | 1276 | Train 20 | Test Male 60 | 0.76 | 0.81 | 0.82 |
| 253 | Train 4 | Test Male 61 | 0.73 | 0.77 | 0.68 | 1277 | Train 20 | Test Male 61 | 0.73 | 0.77 | 0.67 |
| 254 | Train 4 | Test Male 62 | 0.71 | 0.72 | 0.66 | 1278 | Train 20 | Test Male 62 | 0.71 | 0.72 | 0.66 |
| 255 | Train 4 | Test Male 63 | 0.73 | 0.77 | 0.69 | 1279 | Train 20 | Test Male 63 | 0.73 | 0.77 | 0.69 |
| 256 | Train 4 | Test Male 64 | 0.71 | 0.71 | 0.66 | 1280 | Train 20 | Test Male 64 | 0.71 | 0.71 | 0.65 |
| 257 | Train 5 | Test Male 1 | 0.75 | 0.81 | 0.82 | 1281 | Train 21 | Test Male 1 | 0.75 | 0.81 | 0.82 |
| 258 | Train 5 | Test Male 2 | 0.75 | 0.79 | 0.79 | 1282 | Train 21 | Test Male 2 | 0.75 | 0.79 | 0.78 |
| 259 | Train 5 | Test Male 3 | 0.75 | 0.81 | 0.82 | 1283 | Train 21 | Test Male 3 | 0.75 | 0.81 | 0.82 |
| 260 | Train 5 | Test Male 4 | 0.75 | 0.79 | 0.78 | 1284 | Train 21 | Test Male 4 | 0.75 | 0.79 | 0.78 |
| 261 | Train 5 | Test Male 5 | 0.74 | 0.81 | 0.82 | 1285 | Train 21 | Test Male 5 | 0.74 | 0.81 | 0.82 |
| 262 | Train 5 | Test Male 6 | 0.7 | 0.72 | 0.69 | 1286 | Train 21 | Test Male 6 | 0.7 | 0.71 | 0.68 |
| 263 | Train 5 | Test Male 7 | 0.74 | 0.81 | 0.82 | 1287 | Train 21 | Test Male 7 | 0.74 | 0.81 | 0.82 |
| 264 | Train 5 | Test Male 8 | 0.7 | 0.71 | 0.69 | 1288 | Train 21 | Test Male 8 | 0.7 | 0.71 | 0.68 |
| 265 | Train 5 | Test Male 9 | 0.75 | 0.8 | 0.8 | 1289 | Train 21 | Test Male 9 | 0.75 | 0.8 | 0.8 |
| 266 | Train 5 | Test Male 10 | 0.75 | 0.78 | 0.77 | 1290 | Train 21 | Test Male 10 | 0.75 | 0.78 | 0.77 |
| 267 | Train 5 | Test Male 11 | 0.75 | 0.8 | 0.8 | 1291 | Train 21 | Test Male 11 | 0.75 | 0.8 | 0.8 |
| 268 | Train 5 | Test Male 12 | 0.75 | 0.78 | 0.77 | 1292 | Train 21 | Test Male 12 | 0.75 | 0.78 | 0.77 |
| 269 | Train 5 | Test Male 13 | 0.74 | 0.8 | 0.8 | 1293 | Train 21 | Test Male 13 | 0.74 | 0.8 | 0.8 |
| 270 | Train 5 | Test Male 14 | 0.7 | 0.72 | 0.69 | 1294 | Train 21 | Test Male 14 | 0.7 | 0.71 | 0.68 |
| 271 | Train 5 | Test Male 15 | 0.74 | 0.8 | 0.79 | 1295 | Train 21 | Test Male 15 | 0.74 | 0.8 | 0.79 |
| 272 | Train 5 | Test Male 16 | 0.7 | 0.72 | 0.69 | 1296 | Train 21 | Test Male 16 | 0.7 | 0.71 | 0.68 |
| 273 | Train 5 | Test Male 17 | 0.75 | 0.81 | 0.82 | 1297 | Train 21 | Test Male 17 | 0.75 | 0.81 | 0.82 |
| 274 | Train 5 | Test Male 18 | 0.75 | 0.79 | 0.79 | 1298 | Train 21 | Test Male 18 | 0.75 | 0.79 | 0.78 |
| 275 | Train 5 | Test Male 19 | 0.75 | 0.81 | 0.82 | 1299 | Train 21 | Test Male 19 | 0.75 | 0.81 | 0.82 |
| 276 | Train 5 | Test Male 20 | 0.75 | 0.79 | 0.78 | 1300 | Train 21 | Test Male 20 | 0.75 | 0.79 | 0.78 |
| 277 | Train 5 | Test Male 21 | 0.74 | 0.81 | 0.82 | 1301 | Train 21 | Test Male 21 | 0.74 | 0.81 | 0.82 |
| 278 | Train 5 | Test Male 22 | 0.7 | 0.72 | 0.69 | 1302 | Train 21 | Test Male 22 | 0.7 | 0.71 | 0.68 |
| 279 | Train 5 | Test Male 23 | 0.74 | 0.81 | 0.82 | 1303 | Train 21 | Test Male 23 | 0.74 | 0.81 | 0.82 |
| 280 | Train 5 | Test Male 24 | 0.7 | 0.72 | 0.69 | 1304 | Train 21 | Test Male 24 | 0.7 | 0.71 | 0.68 |
| 281 | Train 5 | Test Male 25 | 0.75 | 0.8 | 0.8 | 1305 | Train 21 | Test Male 25 | 0.75 | 0.8 | 0.8 |
| 282 | Train 5 | Test Male 26 | 0.75 | 0.78 | 0.77 | 1306 | Train 21 | Test Male 26 | 0.75 | 0.78 | 0.77 |
| 283 | Train 5 | Test Male 27 | 0.75 | 0.8 | 0.8 | 1307 | Train 21 | Test Male 27 | 0.75 | 0.8 | 0.8 |
| 284 | Train 5 | Test Male 28 | 0.75 | 0.78 | 0.77 | 1308 | Train 21 | Test Male 28 | 0.75 | 0.78 | 0.77 |
| 285 | Train 5 | Test Male 29 | 0.74 | 0.8 | 0.8 | 1309 | Train 21 | Test Male 29 | 0.74 | 0.8 | 0.8 |
| 286 | Train 5 | Test Male 30 | 0.7 | 0.72 | 0.69 | 1310 | Train 21 | Test Male 30 | 0.7 | 0.71 | 0.68 |
| 287 | Train 5 | Test Male 31 | 0.74 | 0.8 | 0.79 | 1311 | Train 21 | Test Male 31 | 0.74 | 0.8 | 0.79 |
| 288 | Train 5 | Test Male 32 | 0.7 | 0.72 | 0.69 | 1312 | Train 21 | Test Male 32 | 0.7 | 0.71 | 0.68 |
| 289 | Train 5 | Test Male 33 | 0.75 | 0.81 | 0.82 | 1313 | Train 21 | Test Male 33 | 0.75 | 0.81 | 0.82 |
| 290 | Train 5 | Test Male 34 | 0.75 | 0.79 | 0.78 | 1314 | Train 21 | Test Male 34 | 0.75 | 0.79 | 0.78 |
| 291 | Train 5 | Test Male 35 | 0.75 | 0.81 | 0.82 | 1315 | Train 21 | Test Male 35 | 0.75 | 0.81 | 0.82 |
| 292 | Train 5 | Test Male 36 | 0.75 | 0.79 | 0.78 | 1316 | Train 21 | Test Male 36 | 0.75 | 0.79 | 0.78 |
| 293 | Train 5 | Test Male 37 | 0.74 | 0.81 | 0.82 | 1317 | Train 21 | Test Male 37 | 0.74 | 0.81 | 0.82 |
| 294 | Train 5 | Test Male 38 | 0.7 | 0.72 | 0.69 | 1318 | Train 21 | Test Male 38 | 0.7 | 0.71 | 0.68 |
| 295 | Train 5 | Test Male 39 | 0.74 | 0.81 | 0.81 | 1319 | Train 21 | Test Male 39 | 0.74 | 0.81 | 0.82 |
| 296 | Train 5 | Test Male 40 | 0.7 | 0.71 | 0.69 | 1320 | Train 21 | Test Male 40 | 0.7 | 0.71 | 0.68 |
| 297 | Train 5 | Test Male 41 | 0.75 | 0.8 | 0.8 | 1321 | Train 21 | Test Male 41 | 0.75 | 0.8 | 0.8 |
| 298 | Train 5 | Test Male 42 | 0.75 | 0.78 | 0.77 | 1322 | Train 21 | Test Male 42 | 0.75 | 0.78 | 0.77 |
| 299 | Train 5 | Test Male 43 | 0.75 | 0.8 | 0.8 | 1323 | Train 21 | Test Male 43 | 0.75 | 0.8 | 0.8 |
| 300 | Train 5 | Test Male 44 | 0.75 | 0.78 | 0.77 | 1324 | Train 21 | Test Male 44 | 0.75 | 0.78 | 0.77 |
| 301 | Train 5 | Test Male 45 | 0.74 | 0.8 | 0.8 | 1325 | Train 21 | Test Male 45 | 0.74 | 0.8 | 0.8 |
| 302 | Train 5 | Test Male 46 | 0.7 | 0.72 | 0.69 | 1326 | Train 21 | Test Male 46 | 0.7 | 0.71 | 0.68 |
| 303 | Train 5 | Test Male 47 | 0.74 | 0.8 | 0.79 | 1327 | Train 21 | Test Male 47 | 0.74 | 0.8 | 0.79 |
| 304 | Train 5 | Test Male 48 | 0.7 | 0.72 | 0.69 | 1328 | Train 21 | Test Male 48 | 0.7 | 0.71 | 0.68 |
| 305 | Train 5 | Test Male 49 | 0.76 | 0.81 | 0.83 | 1329 | Train 21 | Test Male 49 | 0.76 | 0.81 | 0.83 |
| 306 | Train 5 | Test Male 50 | 0.76 | 0.81 | 0.83 | 1330 | Train 21 | Test Male 50 | 0.76 | 0.81 | 0.83 |
| 307 | Train 5 | Test Male 51 | 0.76 | 0.81 | 0.82 | 1331 | Train 21 | Test Male 51 | 0.76 | 0.81 | 0.83 |
| 308 | Train 5 | Test Male 52 | 0.76 | 0.81 | 0.82 | 1332 | Train 21 | Test Male 52 | 0.76 | 0.81 | 0.82 |
| 309 | Train 5 | Test Male 53 | 0.76 | 0.81 | 0.79 | 1333 | Train 21 | Test Male 53 | 0.76 | 0.81 | 0.79 |
| 310 | Train 5 | Test Male 54 | 0.71 | 0.72 | 0.69 | 1334 | Train 21 | Test Male 54 | 0.71 | 0.71 | 0.7 |
| 311 | Train 5 | Test Male 55 | 0.76 | 0.81 | 0.78 | 1335 | Train 21 | Test Male 55 | 0.76 | 0.8 | 0.79 |
| 312 | Train 5 | Test Male 56 | 0.71 | 0.71 | 0.7 | 1336 | Train 21 | Test Male 56 | 0.71 | 0.71 | 0.7 |
| 313 | Train 5 | Test Male 57 | 0.76 | 0.81 | 0.81 | 1337 | Train 21 | Test Male 57 | 0.76 | 0.81 | 0.81 |
| 314 | Train 5 | Test Male 58 | 0.76 | 0.81 | 0.8 | 1338 | Train 21 | Test Male 58 | 0.76 | 0.81 | 0.81 |
| 315 | Train 5 | Test Male 59 | 0.76 | 0.8 | 0.8 | 1339 | Train 21 | Test Male 59 | 0.76 | 0.8 | 0.8 |
| 316 | Train 5 | Test Male 60 | 0.76 | 0.8 | 0.8 | 1340 | Train 21 | Test Male 60 | 0.76 | 0.8 | 0.8 |
| 317 | Train 5 | Test Male 61 | 0.76 | 0.8 | 0.74 | 1341 | Train 21 | Test Male 61 | 0.76 | 0.8 | 0.77 |
| 318 | Train 5 | Test Male 62 | 0.71 | 0.72 | 0.69 | 1342 | Train 21 | Test Male 62 | 0.71 | 0.71 | 0.7 |
| 319 | Train 5 | Test Male 63 | 0.75 | 0.8 | 0.78 | 1343 | Train 21 | Test Male 63 | 0.75 | 0.8 | 0.78 |
| 320 | Train 5 | Test Male 64 | 0.71 | 0.71 | 0.69 | 1344 | Train 21 | Test Male 64 | 0.71 | 0.71 | 0.7 |
| 321 | Train 6 | Test Male 1 | 0.73 | 0.78 | 0.76 | 1345 | Train 22 | Test Male 1 | 0.73 | 0.78 | 0.77 |
| 322 | Train 6 | Test Male 2 | 0.74 | 0.78 | 0.76 | 1346 | Train 22 | Test Male 2 | 0.74 | 0.78 | 0.77 |
| 323 | Train 6 | Test Male 3 | 0.73 | 0.78 | 0.76 | 1347 | Train 22 | Test Male 3 | 0.73 | 0.78 | 0.77 |
| 324 | Train 6 | Test Male 4 | 0.74 | 0.78 | 0.76 | 1348 | Train 22 | Test Male 4 | 0.74 | 0.78 | 0.77 |
| 325 | Train 6 | Test Male 5 | 0.72 | 0.77 | 0.76 | 1349 | Train 22 | Test Male 5 | 0.72 | 0.77 | 0.76 |
| 326 | Train 6 | Test Male 6 | 0.73 | 0.79 | 0.79 | 1350 | Train 22 | Test Male 6 | 0.73 | 0.79 | 0.79 |
| 327 | Train 6 | Test Male 7 | 0.72 | 0.77 | 0.76 | 1351 | Train 22 | Test Male 7 | 0.72 | 0.77 | 0.76 |
| 328 | Train 6 | Test Male 8 | 0.73 | 0.78 | 0.78 | 1352 | Train 22 | Test Male 8 | 0.73 | 0.78 | 0.78 |
| 329 | Train 6 | Test Male 9 | 0.73 | 0.78 | 0.76 | 1353 | Train 22 | Test Male 9 | 0.73 | 0.78 | 0.77 |
| 330 | Train 6 | Test Male 10 | 0.74 | 0.79 | 0.77 | 1354 | Train 22 | Test Male 10 | 0.74 | 0.78 | 0.78 |
| 331 | Train 6 | Test Male 11 | 0.73 | 0.78 | 0.76 | 1355 | Train 22 | Test Male 11 | 0.73 | 0.78 | 0.77 |
| 332 | Train 6 | Test Male 12 | 0.74 | 0.78 | 0.77 | 1356 | Train 22 | Test Male 12 | 0.74 | 0.78 | 0.78 |
| 333 | Train 6 | Test Male 13 | 0.72 | 0.78 | 0.76 | 1357 | Train 22 | Test Male 13 | 0.72 | 0.78 | 0.76 |
| 334 | Train 6 | Test Male 14 | 0.74 | 0.78 | 0.79 | 1358 | Train 22 | Test Male 14 | 0.74 | 0.78 | 0.79 |
| 335 | Train 6 | Test Male 15 | 0.72 | 0.77 | 0.76 | 1359 | Train 22 | Test Male 15 | 0.72 | 0.77 | 0.76 |
| 336 | Train 6 | Test Male 16 | 0.73 | 0.78 | 0.78 | 1360 | Train 22 | Test Male 16 | 0.73 | 0.78 | 0.78 |
| 337 | Train 6 | Test Male 17 | 0.73 | 0.78 | 0.76 | 1361 | Train 22 | Test Male 17 | 0.73 | 0.78 | 0.77 |
| 338 | Train 6 | Test Male 18 | 0.74 | 0.78 | 0.76 | 1362 | Train 22 | Test Male 18 | 0.74 | 0.78 | 0.77 |
| 339 | Train 6 | Test Male 19 | 0.73 | 0.78 | 0.76 | 1363 | Train 22 | Test Male 19 | 0.73 | 0.78 | 0.77 |
| 340 | Train 6 | Test Male 20 | 0.74 | 0.78 | 0.76 | 1364 | Train 22 | Test Male 20 | 0.74 | 0.78 | 0.77 |
| 341 | Train 6 | Test Male 21 | 0.72 | 0.77 | 0.76 | 1365 | Train 22 | Test Male 21 | 0.72 | 0.77 | 0.76 |
| 342 | Train 6 | Test Male 22 | 0.73 | 0.79 | 0.79 | 1366 | Train 22 | Test Male 22 | 0.73 | 0.79 | 0.79 |
| 343 | Train 6 | Test Male 23 | 0.72 | 0.77 | 0.76 | 1367 | Train 22 | Test Male 23 | 0.72 | 0.77 | 0.76 |
| 344 | Train 6 | Test Male 24 | 0.73 | 0.78 | 0.78 | 1368 | Train 22 | Test Male 24 | 0.73 | 0.78 | 0.78 |
| 345 | Train 6 | Test Male 25 | 0.73 | 0.78 | 0.76 | 1369 | Train 22 | Test Male 25 | 0.73 | 0.78 | 0.77 |
| 346 | Train 6 | Test Male 26 | 0.74 | 0.79 | 0.77 | 1370 | Train 22 | Test Male 26 | 0.74 | 0.78 | 0.78 |
| 347 | Train 6 | Test Male 27 | 0.73 | 0.78 | 0.76 | 1371 | Train 22 | Test Male 27 | 0.73 | 0.78 | 0.77 |
| 348 | Train 6 | Test Male 28 | 0.74 | 0.78 | 0.77 | 1372 | Train 22 | Test Male 28 | 0.74 | 0.78 | 0.78 |
| 349 | Train 6 | Test Male 29 | 0.72 | 0.78 | 0.76 | 1373 | Train 22 | Test Male 29 | 0.72 | 0.78 | 0.76 |
| 350 | Train 6 | Test Male 30 | 0.74 | 0.78 | 0.79 | 1374 | Train 22 | Test Male 30 | 0.74 | 0.78 | 0.79 |
| 351 | Train 6 | Test Male 31 | 0.72 | 0.77 | 0.76 | 1375 | Train 22 | Test Male 31 | 0.72 | 0.77 | 0.76 |
| 352 | Train 6 | Test Male 32 | 0.73 | 0.78 | 0.78 | 1376 | Train 22 | Test Male 32 | 0.73 | 0.78 | 0.78 |
| 353 | Train 6 | Test Male 33 | 0.73 | 0.78 | 0.76 | 1377 | Train 22 | Test Male 33 | 0.73 | 0.78 | 0.77 |
| 354 | Train 6 | Test Male 34 | 0.74 | 0.78 | 0.76 | 1378 | Train 22 | Test Male 34 | 0.74 | 0.78 | 0.77 |
| 355 | Train 6 | Test Male 35 | 0.73 | 0.78 | 0.76 | 1379 | Train 22 | Test Male 35 | 0.73 | 0.78 | 0.77 |
| 356 | Train 6 | Test Male 36 | 0.74 | 0.78 | 0.76 | 1380 | Train 22 | Test Male 36 | 0.74 | 0.78 | 0.77 |
| 357 | Train 6 | Test Male 37 | 0.72 | 0.77 | 0.76 | 1381 | Train 22 | Test Male 37 | 0.72 | 0.77 | 0.76 |
| 358 | Train 6 | Test Male 38 | 0.73 | 0.79 | 0.79 | 1382 | Train 22 | Test Male 38 | 0.73 | 0.79 | 0.79 |
| 359 | Train 6 | Test Male 39 | 0.72 | 0.77 | 0.76 | 1383 | Train 22 | Test Male 39 | 0.72 | 0.77 | 0.76 |
| 360 | Train 6 | Test Male 40 | 0.73 | 0.78 | 0.78 | 1384 | Train 22 | Test Male 40 | 0.73 | 0.78 | 0.78 |
| 361 | Train 6 | Test Male 41 | 0.73 | 0.78 | 0.76 | 1385 | Train 22 | Test Male 41 | 0.73 | 0.78 | 0.77 |
| 362 | Train 6 | Test Male 42 | 0.74 | 0.78 | 0.77 | 1386 | Train 22 | Test Male 42 | 0.74 | 0.78 | 0.78 |
| 363 | Train 6 | Test Male 43 | 0.73 | 0.78 | 0.76 | 1387 | Train 22 | Test Male 43 | 0.73 | 0.78 | 0.77 |
| 364 | Train 6 | Test Male 44 | 0.74 | 0.78 | 0.77 | 1388 | Train 22 | Test Male 44 | 0.74 | 0.78 | 0.78 |
| 365 | Train 6 | Test Male 45 | 0.72 | 0.77 | 0.76 | 1389 | Train 22 | Test Male 45 | 0.72 | 0.77 | 0.76 |
| 366 | Train 6 | Test Male 46 | 0.74 | 0.78 | 0.79 | 1390 | Train 22 | Test Male 46 | 0.74 | 0.78 | 0.79 |
| 367 | Train 6 | Test Male 47 | 0.72 | 0.77 | 0.76 | 1391 | Train 22 | Test Male 47 | 0.72 | 0.77 | 0.76 |
| 368 | Train 6 | Test Male 48 | 0.73 | 0.78 | 0.78 | 1392 | Train 22 | Test Male 48 | 0.73 | 0.78 | 0.78 |
| 369 | Train 6 | Test Male 49 | 0.76 | 0.8 | 0.77 | 1393 | Train 22 | Test Male 49 | 0.76 | 0.8 | 0.78 |
| 370 | Train 6 | Test Male 50 | 0.76 | 0.8 | 0.77 | 1394 | Train 22 | Test Male 50 | 0.76 | 0.8 | 0.78 |
| 371 | Train 6 | Test Male 51 | 0.76 | 0.8 | 0.77 | 1395 | Train 22 | Test Male 51 | 0.76 | 0.79 | 0.78 |
| 372 | Train 6 | Test Male 52 | 0.76 | 0.79 | 0.77 | 1396 | Train 22 | Test Male 52 | 0.76 | 0.79 | 0.78 |
| 373 | Train 6 | Test Male 53 | 0.75 | 0.8 | 0.8 | 1397 | Train 22 | Test Male 53 | 0.75 | 0.8 | 0.8 |
| 374 | Train 6 | Test Male 54 | 0.74 | 0.78 | 0.79 | 1398 | Train 22 | Test Male 54 | 0.74 | 0.78 | 0.79 |
| 375 | Train 6 | Test Male 55 | 0.75 | 0.8 | 0.8 | 1399 | Train 22 | Test Male 55 | 0.75 | 0.8 | 0.8 |
| 376 | Train 6 | Test Male 56 | 0.74 | 0.78 | 0.78 | 1400 | Train 22 | Test Male 56 | 0.74 | 0.78 | 0.78 |
| 377 | Train 6 | Test Male 57 | 0.76 | 0.8 | 0.78 | 1401 | Train 22 | Test Male 57 | 0.76 | 0.8 | 0.79 |
| 378 | Train 6 | Test Male 58 | 0.76 | 0.8 | 0.78 | 1402 | Train 22 | Test Male 58 | 0.76 | 0.8 | 0.79 |
| 379 | Train 6 | Test Male 59 | 0.76 | 0.8 | 0.78 | 1403 | Train 22 | Test Male 59 | 0.76 | 0.8 | 0.79 |
| 380 | Train 6 | Test Male 60 | 0.76 | 0.8 | 0.78 | 1404 | Train 22 | Test Male 60 | 0.76 | 0.8 | 0.79 |
| 381 | Train 6 | Test Male 61 | 0.75 | 0.81 | 0.77 | 1405 | Train 22 | Test Male 61 | 0.75 | 0.81 | 0.78 |
| 382 | Train 6 | Test Male 62 | 0.74 | 0.78 | 0.79 | 1406 | Train 22 | Test Male 62 | 0.74 | 0.78 | 0.79 |
| 383 | Train 6 | Test Male 63 | 0.75 | 0.8 | 0.8 | 1407 | Train 22 | Test Male 63 | 0.75 | 0.8 | 0.81 |
| 384 | Train 6 | Test Male 64 | 0.74 | 0.78 | 0.78 | 1408 | Train 22 | Test Male 64 | 0.74 | 0.77 | 0.78 |
| 385 | Train 7 | Test Male 1 | 0.75 | 0.81 | 0.82 | 1409 | Train 23 | Test Male 1 | 0.75 | 0.81 | 0.82 |
| 386 | Train 7 | Test Male 2 | 0.75 | 0.79 | 0.79 | 1410 | Train 23 | Test Male 2 | 0.75 | 0.79 | 0.78 |
| 387 | Train 7 | Test Male 3 | 0.75 | 0.81 | 0.82 | 1411 | Train 23 | Test Male 3 | 0.75 | 0.81 | 0.82 |
| 388 | Train 7 | Test Male 4 | 0.75 | 0.79 | 0.79 | 1412 | Train 23 | Test Male 4 | 0.75 | 0.79 | 0.78 |
| 389 | Train 7 | Test Male 5 | 0.74 | 0.81 | 0.82 | 1413 | Train 23 | Test Male 5 | 0.74 | 0.81 | 0.82 |
| 390 | Train 7 | Test Male 6 | 0.7 | 0.71 | 0.68 | 1414 | Train 23 | Test Male 6 | 0.7 | 0.71 | 0.67 |
| 391 | Train 7 | Test Male 7 | 0.74 | 0.81 | 0.82 | 1415 | Train 23 | Test Male 7 | 0.74 | 0.81 | 0.82 |
| 392 | Train 7 | Test Male 8 | 0.7 | 0.71 | 0.68 | 1416 | Train 23 | Test Male 8 | 0.7 | 0.71 | 0.67 |
| 393 | Train 7 | Test Male 9 | 0.75 | 0.8 | 0.8 | 1417 | Train 23 | Test Male 9 | 0.75 | 0.8 | 0.8 |
| 394 | Train 7 | Test Male 10 | 0.75 | 0.78 | 0.77 | 1418 | Train 23 | Test Male 10 | 0.75 | 0.78 | 0.77 |
| 395 | Train 7 | Test Male 11 | 0.75 | 0.8 | 0.8 | 1419 | Train 23 | Test Male 11 | 0.75 | 0.8 | 0.8 |
| 396 | Train 7 | Test Male 12 | 0.75 | 0.78 | 0.77 | 1420 | Train 23 | Test Male 12 | 0.75 | 0.78 | 0.77 |
| 397 | Train 7 | Test Male 13 | 0.74 | 0.8 | 0.8 | 1421 | Train 23 | Test Male 13 | 0.74 | 0.8 | 0.8 |
| 398 | Train 7 | Test Male 14 | 0.7 | 0.71 | 0.68 | 1422 | Train 23 | Test Male 14 | 0.7 | 0.71 | 0.67 |
| 399 | Train 7 | Test Male 15 | 0.74 | 0.8 | 0.79 | 1423 | Train 23 | Test Male 15 | 0.74 | 0.8 | 0.8 |
| 400 | Train 7 | Test Male 16 | 0.7 | 0.71 | 0.68 | 1424 | Train 23 | Test Male 16 | 0.7 | 0.71 | 0.67 |
| 401 | Train 7 | Test Male 17 | 0.75 | 0.81 | 0.82 | 1425 | Train 23 | Test Male 17 | 0.75 | 0.81 | 0.82 |
| 402 | Train 7 | Test Male 18 | 0.75 | 0.79 | 0.79 | 1426 | Train 23 | Test Male 18 | 0.75 | 0.79 | 0.78 |
| 403 | Train 7 | Test Male 19 | 0.75 | 0.81 | 0.82 | 1427 | Train 23 | Test Male 19 | 0.75 | 0.81 | 0.82 |
| 404 | Train 7 | Test Male 20 | 0.75 | 0.79 | 0.79 | 1428 | Train 23 | Test Male 20 | 0.75 | 0.79 | 0.78 |
| 405 | Train 7 | Test Male 21 | 0.74 | 0.81 | 0.82 | 1429 | Train 23 | Test Male 21 | 0.74 | 0.81 | 0.82 |
| 406 | Train 7 | Test Male 22 | 0.7 | 0.71 | 0.68 | 1430 | Train 23 | Test Male 22 | 0.7 | 0.71 | 0.67 |
| 407 | Train 7 | Test Male 23 | 0.74 | 0.81 | 0.82 | 1431 | Train 23 | Test Male 23 | 0.74 | 0.81 | 0.82 |
| 408 | Train 7 | Test Male 24 | 0.7 | 0.71 | 0.68 | 1432 | Train 23 | Test Male 24 | 0.7 | 0.71 | 0.67 |
| 409 | Train 7 | Test Male 25 | 0.75 | 0.8 | 0.8 | 1433 | Train 23 | Test Male 25 | 0.75 | 0.8 | 0.8 |
| 410 | Train 7 | Test Male 26 | 0.75 | 0.78 | 0.77 | 1434 | Train 23 | Test Male 26 | 0.75 | 0.78 | 0.77 |
| 411 | Train 7 | Test Male 27 | 0.75 | 0.8 | 0.8 | 1435 | Train 23 | Test Male 27 | 0.75 | 0.8 | 0.8 |
| 412 | Train 7 | Test Male 28 | 0.75 | 0.78 | 0.77 | 1436 | Train 23 | Test Male 28 | 0.75 | 0.78 | 0.77 |
| 413 | Train 7 | Test Male 29 | 0.74 | 0.8 | 0.8 | 1437 | Train 23 | Test Male 29 | 0.74 | 0.8 | 0.8 |
| 414 | Train 7 | Test Male 30 | 0.7 | 0.71 | 0.68 | 1438 | Train 23 | Test Male 30 | 0.7 | 0.71 | 0.67 |
| 415 | Train 7 | Test Male 31 | 0.74 | 0.8 | 0.79 | 1439 | Train 23 | Test Male 31 | 0.74 | 0.8 | 0.8 |
| 416 | Train 7 | Test Male 32 | 0.7 | 0.71 | 0.68 | 1440 | Train 23 | Test Male 32 | 0.7 | 0.71 | 0.67 |
| 417 | Train 7 | Test Male 33 | 0.75 | 0.81 | 0.82 | 1441 | Train 23 | Test Male 33 | 0.75 | 0.81 | 0.82 |
| 418 | Train 7 | Test Male 34 | 0.75 | 0.79 | 0.79 | 1442 | Train 23 | Test Male 34 | 0.75 | 0.79 | 0.78 |
| 419 | Train 7 | Test Male 35 | 0.75 | 0.81 | 0.82 | 1443 | Train 23 | Test Male 35 | 0.75 | 0.81 | 0.82 |
| 420 | Train 7 | Test Male 36 | 0.75 | 0.79 | 0.79 | 1444 | Train 23 | Test Male 36 | 0.75 | 0.79 | 0.78 |
| 421 | Train 7 | Test Male 37 | 0.74 | 0.81 | 0.82 | 1445 | Train 23 | Test Male 37 | 0.74 | 0.81 | 0.82 |
| 422 | Train 7 | Test Male 38 | 0.7 | 0.71 | 0.68 | 1446 | Train 23 | Test Male 38 | 0.7 | 0.71 | 0.67 |
| 423 | Train 7 | Test Male 39 | 0.74 | 0.81 | 0.82 | 1447 | Train 23 | Test Male 39 | 0.74 | 0.81 | 0.82 |
| 424 | Train 7 | Test Male 40 | 0.7 | 0.71 | 0.68 | 1448 | Train 23 | Test Male 40 | 0.7 | 0.71 | 0.67 |
| 425 | Train 7 | Test Male 41 | 0.75 | 0.8 | 0.8 | 1449 | Train 23 | Test Male 41 | 0.75 | 0.8 | 0.8 |
| 426 | Train 7 | Test Male 42 | 0.75 | 0.78 | 0.77 | 1450 | Train 23 | Test Male 42 | 0.75 | 0.78 | 0.77 |
| 427 | Train 7 | Test Male 43 | 0.75 | 0.8 | 0.8 | 1451 | Train 23 | Test Male 43 | 0.75 | 0.8 | 0.8 |
| 428 | Train 7 | Test Male 44 | 0.75 | 0.78 | 0.77 | 1452 | Train 23 | Test Male 44 | 0.75 | 0.78 | 0.77 |
| 429 | Train 7 | Test Male 45 | 0.74 | 0.8 | 0.79 | 1453 | Train 23 | Test Male 45 | 0.74 | 0.8 | 0.8 |
| 430 | Train 7 | Test Male 46 | 0.7 | 0.71 | 0.68 | 1454 | Train 23 | Test Male 46 | 0.7 | 0.71 | 0.67 |
| 431 | Train 7 | Test Male 47 | 0.74 | 0.8 | 0.79 | 1455 | Train 23 | Test Male 47 | 0.74 | 0.8 | 0.8 |
| 432 | Train 7 | Test Male 48 | 0.7 | 0.71 | 0.68 | 1456 | Train 23 | Test Male 48 | 0.7 | 0.71 | 0.67 |
| 433 | Train 7 | Test Male 49 | 0.76 | 0.81 | 0.83 | 1457 | Train 23 | Test Male 49 | 0.76 | 0.81 | 0.83 |
| 434 | Train 7 | Test Male 50 | 0.76 | 0.81 | 0.83 | 1458 | Train 23 | Test Male 50 | 0.76 | 0.81 | 0.83 |
| 435 | Train 7 | Test Male 51 | 0.76 | 0.81 | 0.83 | 1459 | Train 23 | Test Male 51 | 0.76 | 0.81 | 0.83 |
| 436 | Train 7 | Test Male 52 | 0.76 | 0.81 | 0.82 | 1460 | Train 23 | Test Male 52 | 0.76 | 0.81 | 0.83 |
| 437 | Train 7 | Test Male 53 | 0.76 | 0.8 | 0.77 | 1461 | Train 23 | Test Male 53 | 0.76 | 0.8 | 0.78 |
| 438 | Train 7 | Test Male 54 | 0.71 | 0.71 | 0.69 | 1462 | Train 23 | Test Male 54 | 0.71 | 0.71 | 0.67 |
| 439 | Train 7 | Test Male 55 | 0.76 | 0.8 | 0.76 | 1463 | Train 23 | Test Male 55 | 0.76 | 0.8 | 0.78 |
| 440 | Train 7 | Test Male 56 | 0.71 | 0.71 | 0.68 | 1464 | Train 23 | Test Male 56 | 0.71 | 0.71 | 0.66 |
| 441 | Train 7 | Test Male 57 | 0.76 | 0.81 | 0.81 | 1465 | Train 23 | Test Male 57 | 0.76 | 0.81 | 0.81 |
| 442 | Train 7 | Test Male 58 | 0.76 | 0.81 | 0.81 | 1466 | Train 23 | Test Male 58 | 0.76 | 0.81 | 0.81 |
| 443 | Train 7 | Test Male 59 | 0.76 | 0.8 | 0.81 | 1467 | Train 23 | Test Male 59 | 0.76 | 0.81 | 0.81 |
| 444 | Train 7 | Test Male 60 | 0.76 | 0.8 | 0.8 | 1468 | Train 23 | Test Male 60 | 0.76 | 0.8 | 0.81 |
| 445 | Train 7 | Test Male 61 | 0.76 | 0.8 | 0.67 | 1469 | Train 23 | Test Male 61 | 0.76 | 0.79 | 0.76 |
| 446 | Train 7 | Test Male 62 | 0.71 | 0.71 | 0.68 | 1470 | Train 23 | Test Male 62 | 0.71 | 0.71 | 0.67 |
| 447 | Train 7 | Test Male 63 | 0.76 | 0.8 | 0.76 | 1471 | Train 23 | Test Male 63 | 0.76 | 0.8 | 0.77 |
| 448 | Train 7 | Test Male 64 | 0.71 | 0.71 | 0.68 | 1472 | Train 23 | Test Male 64 | 0.71 | 0.71 | 0.66 |
| 449 | Train 8 | Test Male 1 | 0.74 | 0.78 | 0.76 | 1473 | Train 24 | Test Male 1 | 0.74 | 0.78 | 0.77 |
| 450 | Train 8 | Test Male 2 | 0.74 | 0.78 | 0.77 | 1474 | Train 24 | Test Male 2 | 0.75 | 0.78 | 0.77 |
| 451 | Train 8 | Test Male 3 | 0.73 | 0.78 | 0.76 | 1475 | Train 24 | Test Male 3 | 0.74 | 0.78 | 0.77 |
| 452 | Train 8 | Test Male 4 | 0.74 | 0.78 | 0.77 | 1476 | Train 24 | Test Male 4 | 0.74 | 0.78 | 0.77 |
| 453 | Train 8 | Test Male 5 | 0.72 | 0.77 | 0.75 | 1477 | Train 24 | Test Male 5 | 0.72 | 0.77 | 0.76 |
| 454 | Train 8 | Test Male 6 | 0.73 | 0.79 | 0.79 | 1478 | Train 24 | Test Male 6 | 0.73 | 0.79 | 0.79 |
| 455 | Train 8 | Test Male 7 | 0.72 | 0.77 | 0.75 | 1479 | Train 24 | Test Male 7 | 0.72 | 0.77 | 0.76 |
| 456 | Train 8 | Test Male 8 | 0.73 | 0.78 | 0.79 | 1480 | Train 24 | Test Male 8 | 0.73 | 0.78 | 0.79 |
| 457 | Train 8 | Test Male 9 | 0.73 | 0.78 | 0.77 | 1481 | Train 24 | Test Male 9 | 0.73 | 0.78 | 0.77 |
| 458 | Train 8 | Test Male 10 | 0.75 | 0.78 | 0.77 | 1482 | Train 24 | Test Male 10 | 0.75 | 0.78 | 0.78 |
| 459 | Train 8 | Test Male 11 | 0.73 | 0.78 | 0.77 | 1483 | Train 24 | Test Male 11 | 0.73 | 0.78 | 0.77 |
| 460 | Train 8 | Test Male 12 | 0.74 | 0.78 | 0.77 | 1484 | Train 24 | Test Male 12 | 0.74 | 0.78 | 0.78 |
| 461 | Train 8 | Test Male 13 | 0.72 | 0.77 | 0.76 | 1485 | Train 24 | Test Male 13 | 0.72 | 0.77 | 0.76 |
| 462 | Train 8 | Test Male 14 | 0.73 | 0.78 | 0.79 | 1486 | Train 24 | Test Male 14 | 0.73 | 0.78 | 0.79 |
| 463 | Train 8 | Test Male 15 | 0.72 | 0.77 | 0.76 | 1487 | Train 24 | Test Male 15 | 0.72 | 0.77 | 0.76 |
| 464 | Train 8 | Test Male 16 | 0.73 | 0.78 | 0.79 | 1488 | Train 24 | Test Male 16 | 0.73 | 0.78 | 0.79 |
| 465 | Train 8 | Test Male 17 | 0.74 | 0.78 | 0.76 | 1489 | Train 24 | Test Male 17 | 0.74 | 0.78 | 0.77 |
| 466 | Train 8 | Test Male 18 | 0.74 | 0.78 | 0.76 | 1490 | Train 24 | Test Male 18 | 0.75 | 0.78 | 0.77 |
| 467 | Train 8 | Test Male 19 | 0.73 | 0.78 | 0.76 | 1491 | Train 24 | Test Male 19 | 0.74 | 0.78 | 0.77 |
| 468 | Train 8 | Test Male 20 | 0.74 | 0.78 | 0.76 | 1492 | Train 24 | Test Male 20 | 0.74 | 0.78 | 0.77 |
| 469 | Train 8 | Test Male 21 | 0.72 | 0.77 | 0.75 | 1493 | Train 24 | Test Male 21 | 0.72 | 0.77 | 0.76 |
| 470 | Train 8 | Test Male 22 | 0.73 | 0.78 | 0.79 | 1494 | Train 24 | Test Male 22 | 0.73 | 0.79 | 0.79 |
| 471 | Train 8 | Test Male 23 | 0.72 | 0.77 | 0.75 | 1495 | Train 24 | Test Male 23 | 0.72 | 0.77 | 0.76 |
| 472 | Train 8 | Test Male 24 | 0.73 | 0.78 | 0.79 | 1496 | Train 24 | Test Male 24 | 0.73 | 0.78 | 0.79 |
| 473 | Train 8 | Test Male 25 | 0.73 | 0.78 | 0.77 | 1497 | Train 24 | Test Male 25 | 0.73 | 0.78 | 0.77 |
| 474 | Train 8 | Test Male 26 | 0.75 | 0.78 | 0.77 | 1498 | Train 24 | Test Male 26 | 0.75 | 0.78 | 0.78 |
| 475 | Train 8 | Test Male 27 | 0.73 | 0.78 | 0.77 | 1499 | Train 24 | Test Male 27 | 0.73 | 0.78 | 0.77 |
| 476 | Train 8 | Test Male 28 | 0.74 | 0.78 | 0.77 | 1500 | Train 24 | Test Male 28 | 0.74 | 0.78 | 0.78 |
| 477 | Train 8 | Test Male 29 | 0.72 | 0.77 | 0.76 | 1501 | Train 24 | Test Male 29 | 0.72 | 0.77 | 0.76 |
| 478 | Train 8 | Test Male 30 | 0.73 | 0.78 | 0.79 | 1502 | Train 24 | Test Male 30 | 0.73 | 0.78 | 0.79 |
| 479 | Train 8 | Test Male 31 | 0.72 | 0.77 | 0.76 | 1503 | Train 24 | Test Male 31 | 0.72 | 0.77 | 0.76 |
| 480 | Train 8 | Test Male 32 | 0.73 | 0.78 | 0.79 | 1504 | Train 24 | Test Male 32 | 0.73 | 0.78 | 0.79 |
| 481 | Train 8 | Test Male 33 | 0.73 | 0.78 | 0.76 | 1505 | Train 24 | Test Male 33 | 0.74 | 0.78 | 0.77 |
| 482 | Train 8 | Test Male 34 | 0.74 | 0.78 | 0.76 | 1506 | Train 24 | Test Male 34 | 0.74 | 0.78 | 0.77 |
| 483 | Train 8 | Test Male 35 | 0.73 | 0.78 | 0.76 | 1507 | Train 24 | Test Male 35 | 0.73 | 0.78 | 0.77 |
| 484 | Train 8 | Test Male 36 | 0.74 | 0.78 | 0.76 | 1508 | Train 24 | Test Male 36 | 0.74 | 0.78 | 0.77 |
| 485 | Train 8 | Test Male 37 | 0.72 | 0.77 | 0.75 | 1509 | Train 24 | Test Male 37 | 0.72 | 0.77 | 0.76 |
| 486 | Train 8 | Test Male 38 | 0.73 | 0.78 | 0.79 | 1510 | Train 24 | Test Male 38 | 0.73 | 0.78 | 0.79 |
| 487 | Train 8 | Test Male 39 | 0.72 | 0.77 | 0.75 | 1511 | Train 24 | Test Male 39 | 0.72 | 0.77 | 0.76 |
| 488 | Train 8 | Test Male 40 | 0.73 | 0.78 | 0.79 | 1512 | Train 24 | Test Male 40 | 0.73 | 0.78 | 0.79 |
| 489 | Train 8 | Test Male 41 | 0.73 | 0.78 | 0.77 | 1513 | Train 24 | Test Male 41 | 0.73 | 0.78 | 0.77 |
| 490 | Train 8 | Test Male 42 | 0.74 | 0.78 | 0.77 | 1514 | Train 24 | Test Male 42 | 0.75 | 0.78 | 0.78 |
| 491 | Train 8 | Test Male 43 | 0.73 | 0.78 | 0.77 | 1515 | Train 24 | Test Male 43 | 0.73 | 0.78 | 0.77 |
| 492 | Train 8 | Test Male 44 | 0.74 | 0.78 | 0.77 | 1516 | Train 24 | Test Male 44 | 0.74 | 0.78 | 0.78 |
| 493 | Train 8 | Test Male 45 | 0.72 | 0.77 | 0.76 | 1517 | Train 24 | Test Male 45 | 0.72 | 0.77 | 0.76 |
| 494 | Train 8 | Test Male 46 | 0.73 | 0.78 | 0.79 | 1518 | Train 24 | Test Male 46 | 0.73 | 0.78 | 0.79 |
| 495 | Train 8 | Test Male 47 | 0.72 | 0.77 | 0.76 | 1519 | Train 24 | Test Male 47 | 0.72 | 0.77 | 0.76 |
| 496 | Train 8 | Test Male 48 | 0.73 | 0.78 | 0.79 | 1520 | Train 24 | Test Male 48 | 0.73 | 0.78 | 0.79 |
| 497 | Train 8 | Test Male 49 | 0.76 | 0.8 | 0.74 | 1521 | Train 24 | Test Male 49 | 0.76 | 0.8 | 0.79 |
| 498 | Train 8 | Test Male 50 | 0.76 | 0.8 | 0.74 | 1522 | Train 24 | Test Male 50 | 0.76 | 0.8 | 0.79 |
| 499 | Train 8 | Test Male 51 | 0.76 | 0.8 | 0.74 | 1523 | Train 24 | Test Male 51 | 0.76 | 0.8 | 0.79 |
| 500 | Train 8 | Test Male 52 | 0.76 | 0.79 | 0.74 | 1524 | Train 24 | Test Male 52 | 0.76 | 0.8 | 0.79 |
| 501 | Train 8 | Test Male 53 | 0.75 | 0.8 | 0.8 | 1525 | Train 24 | Test Male 53 | 0.75 | 0.8 | 0.8 |
| 502 | Train 8 | Test Male 54 | 0.74 | 0.78 | 0.79 | 1526 | Train 24 | Test Male 54 | 0.74 | 0.78 | 0.79 |
| 503 | Train 8 | Test Male 55 | 0.75 | 0.8 | 0.8 | 1527 | Train 24 | Test Male 55 | 0.75 | 0.8 | 0.8 |
| 504 | Train 8 | Test Male 56 | 0.74 | 0.78 | 0.79 | 1528 | Train 24 | Test Male 56 | 0.74 | 0.78 | 0.79 |
| 505 | Train 8 | Test Male 57 | 0.76 | 0.8 | 0.75 | 1529 | Train 24 | Test Male 57 | 0.76 | 0.8 | 0.79 |
| 506 | Train 8 | Test Male 58 | 0.76 | 0.8 | 0.75 | 1530 | Train 24 | Test Male 58 | 0.76 | 0.8 | 0.79 |
| 507 | Train 8 | Test Male 59 | 0.76 | 0.8 | 0.75 | 1531 | Train 24 | Test Male 59 | 0.76 | 0.8 | 0.79 |
| 508 | Train 8 | Test Male 60 | 0.76 | 0.8 | 0.75 | 1532 | Train 24 | Test Male 60 | 0.76 | 0.8 | 0.79 |
| 509 | Train 8 | Test Male 61 | 0.75 | 0.81 | 0.75 | 1533 | Train 24 | Test Male 61 | 0.75 | 0.81 | 0.78 |
| 510 | Train 8 | Test Male 62 | 0.74 | 0.78 | 0.79 | 1534 | Train 24 | Test Male 62 | 0.74 | 0.78 | 0.79 |
| 511 | Train 8 | Test Male 63 | 0.75 | 0.8 | 0.81 | 1535 | Train 24 | Test Male 63 | 0.75 | 0.8 | 0.81 |
| 512 | Train 8 | Test Male 64 | 0.74 | 0.78 | 0.79 | 1536 | Train 24 | Test Male 64 | 0.74 | 0.78 | 0.79 |
| 513 | Train 9 | Test Male 1 | 0.76 | 0.83 | 0.82 | 1537 | Train 25 | Test Male 1 | 0.76 | 0.83 | 0.82 |
| 514 | Train 9 | Test Male 2 | 0.75 | 0.79 | 0.77 | 1538 | Train 25 | Test Male 2 | 0.75 | 0.79 | 0.78 |
| 515 | Train 9 | Test Male 3 | 0.76 | 0.83 | 0.82 | 1539 | Train 25 | Test Male 3 | 0.76 | 0.83 | 0.82 |
| 516 | Train 9 | Test Male 4 | 0.75 | 0.79 | 0.77 | 1540 | Train 25 | Test Male 4 | 0.74 | 0.79 | 0.78 |
| 517 | Train 9 | Test Male 5 | 0.72 | 0.75 | 0.68 | 1541 | Train 25 | Test Male 5 | 0.72 | 0.74 | 0.72 |
| 518 | Train 9 | Test Male 6 | 0.69 | 0.7 | 0.63 | 1542 | Train 25 | Test Male 6 | 0.69 | 0.7 | 0.66 |
| 519 | Train 9 | Test Male 7 | 0.72 | 0.74 | 0.68 | 1543 | Train 25 | Test Male 7 | 0.72 | 0.74 | 0.72 |
| 520 | Train 9 | Test Male 8 | 0.69 | 0.7 | 0.63 | 1544 | Train 25 | Test Male 8 | 0.69 | 0.7 | 0.66 |
| 521 | Train 9 | Test Male 9 | 0.76 | 0.84 | 0.85 | 1545 | Train 25 | Test Male 9 | 0.76 | 0.84 | 0.85 |
| 522 | Train 9 | Test Male 10 | 0.74 | 0.79 | 0.8 | 1546 | Train 25 | Test Male 10 | 0.74 | 0.79 | 0.81 |
| 523 | Train 9 | Test Male 11 | 0.76 | 0.84 | 0.85 | 1547 | Train 25 | Test Male 11 | 0.76 | 0.84 | 0.85 |
| 524 | Train 9 | Test Male 12 | 0.74 | 0.79 | 0.8 | 1548 | Train 25 | Test Male 12 | 0.74 | 0.79 | 0.8 |
| 525 | Train 9 | Test Male 13 | 0.72 | 0.75 | 0.7 | 1549 | Train 25 | Test Male 13 | 0.72 | 0.75 | 0.74 |
| 526 | Train 9 | Test Male 14 | 0.69 | 0.7 | 0.63 | 1550 | Train 25 | Test Male 14 | 0.69 | 0.7 | 0.66 |
| 527 | Train 9 | Test Male 15 | 0.71 | 0.75 | 0.7 | 1551 | Train 25 | Test Male 15 | 0.71 | 0.74 | 0.74 |
| 528 | Train 9 | Test Male 16 | 0.69 | 0.7 | 0.63 | 1552 | Train 25 | Test Male 16 | 0.69 | 0.7 | 0.66 |
| 529 | Train 9 | Test Male 17 | 0.76 | 0.83 | 0.82 | 1553 | Train 25 | Test Male 17 | 0.76 | 0.83 | 0.82 |
| 530 | Train 9 | Test Male 18 | 0.75 | 0.79 | 0.77 | 1554 | Train 25 | Test Male 18 | 0.75 | 0.79 | 0.78 |
| 531 | Train 9 | Test Male 19 | 0.76 | 0.83 | 0.82 | 1555 | Train 25 | Test Male 19 | 0.76 | 0.83 | 0.82 |
| 532 | Train 9 | Test Male 20 | 0.75 | 0.79 | 0.77 | 1556 | Train 25 | Test Male 20 | 0.74 | 0.79 | 0.78 |
| 533 | Train 9 | Test Male 21 | 0.72 | 0.75 | 0.68 | 1557 | Train 25 | Test Male 21 | 0.72 | 0.74 | 0.72 |
| 534 | Train 9 | Test Male 22 | 0.69 | 0.7 | 0.63 | 1558 | Train 25 | Test Male 22 | 0.69 | 0.7 | 0.66 |
| 535 | Train 9 | Test Male 23 | 0.72 | 0.74 | 0.68 | 1559 | Train 25 | Test Male 23 | 0.72 | 0.74 | 0.72 |
| 536 | Train 9 | Test Male 24 | 0.69 | 0.7 | 0.63 | 1560 | Train 25 | Test Male 24 | 0.69 | 0.7 | 0.66 |
| 537 | Train 9 | Test Male 25 | 0.76 | 0.84 | 0.85 | 1561 | Train 25 | Test Male 25 | 0.76 | 0.84 | 0.85 |
| 538 | Train 9 | Test Male 26 | 0.74 | 0.79 | 0.8 | 1562 | Train 25 | Test Male 26 | 0.74 | 0.79 | 0.81 |
| 539 | Train 9 | Test Male 27 | 0.76 | 0.84 | 0.85 | 1563 | Train 25 | Test Male 27 | 0.76 | 0.84 | 0.85 |
| 540 | Train 9 | Test Male 28 | 0.74 | 0.79 | 0.8 | 1564 | Train 25 | Test Male 28 | 0.74 | 0.79 | 0.8 |
| 541 | Train 9 | Test Male 29 | 0.72 | 0.75 | 0.7 | 1565 | Train 25 | Test Male 29 | 0.72 | 0.75 | 0.74 |
| 542 | Train 9 | Test Male 30 | 0.69 | 0.7 | 0.63 | 1566 | Train 25 | Test Male 30 | 0.69 | 0.7 | 0.66 |
| 543 | Train 9 | Test Male 31 | 0.71 | 0.75 | 0.7 | 1567 | Train 25 | Test Male 31 | 0.71 | 0.74 | 0.74 |
| 544 | Train 9 | Test Male 32 | 0.69 | 0.7 | 0.63 | 1568 | Train 25 | Test Male 32 | 0.69 | 0.7 | 0.66 |
| 545 | Train 9 | Test Male 33 | 0.76 | 0.83 | 0.82 | 1569 | Train 25 | Test Male 33 | 0.76 | 0.83 | 0.82 |
| 546 | Train 9 | Test Male 34 | 0.74 | 0.79 | 0.77 | 1570 | Train 25 | Test Male 34 | 0.74 | 0.79 | 0.78 |
| 547 | Train 9 | Test Male 35 | 0.76 | 0.83 | 0.82 | 1571 | Train 25 | Test Male 35 | 0.76 | 0.83 | 0.82 |
| 548 | Train 9 | Test Male 36 | 0.74 | 0.79 | 0.77 | 1572 | Train 25 | Test Male 36 | 0.74 | 0.79 | 0.78 |
| 549 | Train 9 | Test Male 37 | 0.72 | 0.74 | 0.68 | 1573 | Train 25 | Test Male 37 | 0.72 | 0.74 | 0.72 |
| 550 | Train 9 | Test Male 38 | 0.69 | 0.7 | 0.63 | 1574 | Train 25 | Test Male 38 | 0.69 | 0.7 | 0.66 |
| 551 | Train 9 | Test Male 39 | 0.72 | 0.74 | 0.68 | 1575 | Train 25 | Test Male 39 | 0.71 | 0.74 | 0.72 |
| 552 | Train 9 | Test Male 40 | 0.69 | 0.7 | 0.63 | 1576 | Train 25 | Test Male 40 | 0.69 | 0.7 | 0.66 |
| 553 | Train 9 | Test Male 41 | 0.76 | 0.84 | 0.85 | 1577 | Train 25 | Test Male 41 | 0.76 | 0.84 | 0.85 |
| 554 | Train 9 | Test Male 42 | 0.74 | 0.79 | 0.8 | 1578 | Train 25 | Test Male 42 | 0.74 | 0.79 | 0.8 |
| 555 | Train 9 | Test Male 43 | 0.76 | 0.84 | 0.85 | 1579 | Train 25 | Test Male 43 | 0.76 | 0.84 | 0.85 |
| 556 | Train 9 | Test Male 44 | 0.74 | 0.79 | 0.8 | 1580 | Train 25 | Test Male 44 | 0.74 | 0.79 | 0.8 |
| 557 | Train 9 | Test Male 45 | 0.71 | 0.75 | 0.7 | 1581 | Train 25 | Test Male 45 | 0.71 | 0.75 | 0.74 |
| 558 | Train 9 | Test Male 46 | 0.69 | 0.7 | 0.63 | 1582 | Train 25 | Test Male 46 | 0.69 | 0.7 | 0.66 |
| 559 | Train 9 | Test Male 47 | 0.71 | 0.74 | 0.7 | 1583 | Train 25 | Test Male 47 | 0.71 | 0.74 | 0.73 |
| 560 | Train 9 | Test Male 48 | 0.69 | 0.7 | 0.63 | 1584 | Train 25 | Test Male 48 | 0.69 | 0.7 | 0.66 |
| 561 | Train 9 | Test Male 49 | 0.77 | 0.82 | 0.82 | 1585 | Train 25 | Test Male 49 | 0.77 | 0.82 | 0.82 |
| 562 | Train 9 | Test Male 50 | 0.77 | 0.82 | 0.82 | 1586 | Train 25 | Test Male 50 | 0.77 | 0.82 | 0.82 |
| 563 | Train 9 | Test Male 51 | 0.77 | 0.82 | 0.82 | 1587 | Train 25 | Test Male 51 | 0.77 | 0.82 | 0.82 |
| 564 | Train 9 | Test Male 52 | 0.77 | 0.82 | 0.82 | 1588 | Train 25 | Test Male 52 | 0.77 | 0.82 | 0.81 |
| 565 | Train 9 | Test Male 53 | 0.73 | 0.77 | 0.61 | 1589 | Train 25 | Test Male 53 | 0.73 | 0.77 | 0.71 |
| 566 | Train 9 | Test Male 54 | 0.71 | 0.71 | 0.62 | 1590 | Train 25 | Test Male 54 | 0.71 | 0.71 | 0.67 |
| 567 | Train 9 | Test Male 55 | 0.73 | 0.77 | 0.61 | 1591 | Train 25 | Test Male 55 | 0.73 | 0.77 | 0.71 |
| 568 | Train 9 | Test Male 56 | 0.71 | 0.71 | 0.62 | 1592 | Train 25 | Test Male 56 | 0.71 | 0.71 | 0.67 |
| 569 | Train 9 | Test Male 57 | 0.77 | 0.83 | 0.86 | 1593 | Train 25 | Test Male 57 | 0.77 | 0.83 | 0.86 |
| 570 | Train 9 | Test Male 58 | 0.77 | 0.83 | 0.85 | 1594 | Train 25 | Test Male 58 | 0.77 | 0.83 | 0.85 |
| 571 | Train 9 | Test Male 59 | 0.77 | 0.83 | 0.85 | 1595 | Train 25 | Test Male 59 | 0.77 | 0.83 | 0.85 |
| 572 | Train 9 | Test Male 60 | 0.77 | 0.83 | 0.85 | 1596 | Train 25 | Test Male 60 | 0.77 | 0.83 | 0.85 |
| 573 | Train 9 | Test Male 61 | 0.73 | 0.78 | 0.6 | 1597 | Train 25 | Test Male 61 | 0.73 | 0.78 | 0.69 |
| 574 | Train 9 | Test Male 62 | 0.71 | 0.71 | 0.62 | 1598 | Train 25 | Test Male 62 | 0.71 | 0.71 | 0.67 |
| 575 | Train 9 | Test Male 63 | 0.73 | 0.78 | 0.62 | 1599 | Train 25 | Test Male 63 | 0.73 | 0.77 | 0.71 |
| 576 | Train 9 | Test Male 64 | 0.71 | 0.71 | 0.62 | 1600 | Train 25 | Test Male 64 | 0.71 | 0.71 | 0.67 |
| 577 | Train 10 | Test Male 1 | 0.75 | 0.82 | 0.82 | 1601 | Train 26 | Test Male 1 | 0.75 | 0.82 | 0.81 |
| 578 | Train 10 | Test Male 2 | 0.76 | 0.83 | 0.82 | 1602 | Train 26 | Test Male 2 | 0.76 | 0.83 | 0.82 |
| 579 | Train 10 | Test Male 3 | 0.75 | 0.82 | 0.81 | 1603 | Train 26 | Test Male 3 | 0.75 | 0.82 | 0.81 |
| 580 | Train 10 | Test Male 4 | 0.76 | 0.83 | 0.82 | 1604 | Train 26 | Test Male 4 | 0.76 | 0.83 | 0.82 |
| 581 | Train 10 | Test Male 5 | 0.72 | 0.75 | 0.73 | 1605 | Train 26 | Test Male 5 | 0.72 | 0.75 | 0.72 |
| 582 | Train 10 | Test Male 6 | 0.7 | 0.72 | 0.67 | 1606 | Train 26 | Test Male 6 | 0.7 | 0.72 | 0.67 |
| 583 | Train 10 | Test Male 7 | 0.72 | 0.74 | 0.72 | 1607 | Train 26 | Test Male 7 | 0.72 | 0.74 | 0.71 |
| 584 | Train 10 | Test Male 8 | 0.7 | 0.71 | 0.67 | 1608 | Train 26 | Test Male 8 | 0.7 | 0.71 | 0.67 |
| 585 | Train 10 | Test Male 9 | 0.75 | 0.83 | 0.84 | 1609 | Train 26 | Test Male 9 | 0.75 | 0.83 | 0.84 |
| 586 | Train 10 | Test Male 10 | 0.76 | 0.83 | 0.85 | 1610 | Train 26 | Test Male 10 | 0.76 | 0.83 | 0.84 |
| 587 | Train 10 | Test Male 11 | 0.75 | 0.83 | 0.84 | 1611 | Train 26 | Test Male 11 | 0.75 | 0.83 | 0.84 |
| 588 | Train 10 | Test Male 12 | 0.76 | 0.83 | 0.84 | 1612 | Train 26 | Test Male 12 | 0.76 | 0.83 | 0.84 |
| 589 | Train 10 | Test Male 13 | 0.72 | 0.75 | 0.74 | 1613 | Train 26 | Test Male 13 | 0.72 | 0.75 | 0.72 |
| 590 | Train 10 | Test Male 14 | 0.7 | 0.72 | 0.67 | 1614 | Train 26 | Test Male 14 | 0.71 | 0.72 | 0.67 |
| 591 | Train 10 | Test Male 15 | 0.72 | 0.75 | 0.73 | 1615 | Train 26 | Test Male 15 | 0.72 | 0.75 | 0.72 |
| 592 | Train 10 | Test Male 16 | 0.7 | 0.72 | 0.67 | 1616 | Train 26 | Test Male 16 | 0.71 | 0.72 | 0.67 |
| 593 | Train 10 | Test Male 17 | 0.75 | 0.82 | 0.81 | 1617 | Train 26 | Test Male 17 | 0.75 | 0.82 | 0.81 |
| 594 | Train 10 | Test Male 18 | 0.76 | 0.83 | 0.82 | 1618 | Train 26 | Test Male 18 | 0.76 | 0.83 | 0.82 |
| 595 | Train 10 | Test Male 19 | 0.75 | 0.82 | 0.81 | 1619 | Train 26 | Test Male 19 | 0.75 | 0.82 | 0.81 |
| 596 | Train 10 | Test Male 20 | 0.76 | 0.83 | 0.82 | 1620 | Train 26 | Test Male 20 | 0.76 | 0.83 | 0.82 |
| 597 | Train 10 | Test Male 21 | 0.72 | 0.75 | 0.73 | 1621 | Train 26 | Test Male 21 | 0.72 | 0.75 | 0.72 |
| 598 | Train 10 | Test Male 22 | 0.7 | 0.72 | 0.67 | 1622 | Train 26 | Test Male 22 | 0.71 | 0.72 | 0.67 |
| 599 | Train 10 | Test Male 23 | 0.72 | 0.75 | 0.72 | 1623 | Train 26 | Test Male 23 | 0.72 | 0.75 | 0.71 |
| 600 | Train 10 | Test Male 24 | 0.7 | 0.71 | 0.67 | 1624 | Train 26 | Test Male 24 | 0.7 | 0.71 | 0.67 |
| 601 | Train 10 | Test Male 25 | 0.75 | 0.83 | 0.84 | 1625 | Train 26 | Test Male 25 | 0.75 | 0.83 | 0.84 |
| 602 | Train 10 | Test Male 26 | 0.76 | 0.83 | 0.85 | 1626 | Train 26 | Test Male 26 | 0.76 | 0.83 | 0.84 |
| 603 | Train 10 | Test Male 27 | 0.75 | 0.83 | 0.84 | 1627 | Train 26 | Test Male 27 | 0.75 | 0.83 | 0.84 |
| 604 | Train 10 | Test Male 28 | 0.76 | 0.83 | 0.84 | 1628 | Train 26 | Test Male 28 | 0.76 | 0.83 | 0.84 |
| 605 | Train 10 | Test Male 29 | 0.72 | 0.75 | 0.74 | 1629 | Train 26 | Test Male 29 | 0.72 | 0.75 | 0.72 |
| 606 | Train 10 | Test Male 30 | 0.71 | 0.72 | 0.67 | 1630 | Train 26 | Test Male 30 | 0.71 | 0.72 | 0.67 |
| 607 | Train 10 | Test Male 31 | 0.72 | 0.75 | 0.73 | 1631 | Train 26 | Test Male 31 | 0.72 | 0.75 | 0.72 |
| 608 | Train 10 | Test Male 32 | 0.7 | 0.72 | 0.67 | 1632 | Train 26 | Test Male 32 | 0.71 | 0.72 | 0.67 |
| 609 | Train 10 | Test Male 33 | 0.75 | 0.82 | 0.81 | 1633 | Train 26 | Test Male 33 | 0.75 | 0.82 | 0.81 |
| 610 | Train 10 | Test Male 34 | 0.76 | 0.83 | 0.82 | 1634 | Train 26 | Test Male 34 | 0.76 | 0.83 | 0.82 |
| 611 | Train 10 | Test Male 35 | 0.75 | 0.82 | 0.81 | 1635 | Train 26 | Test Male 35 | 0.75 | 0.82 | 0.81 |
| 612 | Train 10 | Test Male 36 | 0.76 | 0.83 | 0.82 | 1636 | Train 26 | Test Male 36 | 0.76 | 0.83 | 0.82 |
| 613 | Train 10 | Test Male 37 | 0.72 | 0.75 | 0.73 | 1637 | Train 26 | Test Male 37 | 0.72 | 0.75 | 0.71 |
| 614 | Train 10 | Test Male 38 | 0.7 | 0.72 | 0.67 | 1638 | Train 26 | Test Male 38 | 0.7 | 0.72 | 0.67 |
| 615 | Train 10 | Test Male 39 | 0.72 | 0.74 | 0.72 | 1639 | Train 26 | Test Male 39 | 0.72 | 0.74 | 0.71 |
| 616 | Train 10 | Test Male 40 | 0.7 | 0.71 | 0.67 | 1640 | Train 26 | Test Male 40 | 0.7 | 0.71 | 0.67 |
| 617 | Train 10 | Test Male 41 | 0.75 | 0.83 | 0.84 | 1641 | Train 26 | Test Male 41 | 0.75 | 0.83 | 0.84 |
| 618 | Train 10 | Test Male 42 | 0.76 | 0.83 | 0.84 | 1642 | Train 26 | Test Male 42 | 0.76 | 0.83 | 0.84 |
| 619 | Train 10 | Test Male 43 | 0.75 | 0.83 | 0.84 | 1643 | Train 26 | Test Male 43 | 0.75 | 0.83 | 0.84 |
| 620 | Train 10 | Test Male 44 | 0.76 | 0.83 | 0.84 | 1644 | Train 26 | Test Male 44 | 0.76 | 0.83 | 0.84 |
| 621 | Train 10 | Test Male 45 | 0.72 | 0.75 | 0.74 | 1645 | Train 26 | Test Male 45 | 0.72 | 0.75 | 0.72 |
| 622 | Train 10 | Test Male 46 | 0.7 | 0.72 | 0.67 | 1646 | Train 26 | Test Male 46 | 0.71 | 0.72 | 0.67 |
| 623 | Train 10 | Test Male 47 | 0.71 | 0.75 | 0.73 | 1647 | Train 26 | Test Male 47 | 0.71 | 0.75 | 0.72 |
| 624 | Train 10 | Test Male 48 | 0.7 | 0.71 | 0.67 | 1648 | Train 26 | Test Male 48 | 0.71 | 0.72 | 0.67 |
| 625 | Train 10 | Test Male 49 | 0.77 | 0.82 | 0.82 | 1649 | Train 26 | Test Male 49 | 0.77 | 0.82 | 0.81 |
| 626 | Train 10 | Test Male 50 | 0.77 | 0.82 | 0.82 | 1650 | Train 26 | Test Male 50 | 0.77 | 0.82 | 0.81 |
| 627 | Train 10 | Test Male 51 | 0.77 | 0.82 | 0.81 | 1651 | Train 26 | Test Male 51 | 0.77 | 0.82 | 0.81 |
| 628 | Train 10 | Test Male 52 | 0.76 | 0.81 | 0.81 | 1652 | Train 26 | Test Male 52 | 0.76 | 0.82 | 0.81 |
| 629 | Train 10 | Test Male 53 | 0.74 | 0.78 | 0.69 | 1653 | Train 26 | Test Male 53 | 0.74 | 0.78 | 0.67 |
| 630 | Train 10 | Test Male 54 | 0.71 | 0.72 | 0.67 | 1654 | Train 26 | Test Male 54 | 0.71 | 0.72 | 0.66 |
| 631 | Train 10 | Test Male 55 | 0.74 | 0.78 | 0.69 | 1655 | Train 26 | Test Male 55 | 0.74 | 0.78 | 0.67 |
| 632 | Train 10 | Test Male 56 | 0.71 | 0.71 | 0.67 | 1656 | Train 26 | Test Male 56 | 0.71 | 0.71 | 0.66 |
| 633 | Train 10 | Test Male 57 | 0.77 | 0.82 | 0.85 | 1657 | Train 26 | Test Male 57 | 0.77 | 0.83 | 0.84 |
| 634 | Train 10 | Test Male 58 | 0.77 | 0.82 | 0.85 | 1658 | Train 26 | Test Male 58 | 0.77 | 0.83 | 0.84 |
| 635 | Train 10 | Test Male 59 | 0.77 | 0.82 | 0.84 | 1659 | Train 26 | Test Male 59 | 0.76 | 0.82 | 0.84 |
| 636 | Train 10 | Test Male 60 | 0.76 | 0.82 | 0.84 | 1660 | Train 26 | Test Male 60 | 0.76 | 0.82 | 0.84 |
| 637 | Train 10 | Test Male 61 | 0.74 | 0.78 | 0.68 | 1661 | Train 26 | Test Male 61 | 0.74 | 0.78 | 0.66 |
| 638 | Train 10 | Test Male 62 | 0.71 | 0.72 | 0.67 | 1662 | Train 26 | Test Male 62 | 0.71 | 0.72 | 0.66 |
| 639 | Train 10 | Test Male 63 | 0.73 | 0.78 | 0.7 | 1663 | Train 26 | Test Male 63 | 0.74 | 0.78 | 0.67 |
| 640 | Train 10 | Test Male 64 | 0.71 | 0.71 | 0.67 | 1664 | Train 26 | Test Male 64 | 0.71 | 0.71 | 0.66 |
| 641 | Train 11 | Test Male 1 | 0.76 | 0.83 | 0.82 | 1665 | Train 27 | Test Male 1 | 0.76 | 0.83 | 0.82 |
| 642 | Train 11 | Test Male 2 | 0.75 | 0.79 | 0.78 | 1666 | Train 27 | Test Male 2 | 0.75 | 0.79 | 0.77 |
| 643 | Train 11 | Test Male 3 | 0.76 | 0.83 | 0.82 | 1667 | Train 27 | Test Male 3 | 0.76 | 0.83 | 0.82 |
| 644 | Train 11 | Test Male 4 | 0.75 | 0.79 | 0.78 | 1668 | Train 27 | Test Male 4 | 0.75 | 0.79 | 0.77 |
| 645 | Train 11 | Test Male 5 | 0.72 | 0.75 | 0.71 | 1669 | Train 27 | Test Male 5 | 0.72 | 0.75 | 0.71 |
| 646 | Train 11 | Test Male 6 | 0.69 | 0.7 | 0.65 | 1670 | Train 27 | Test Male 6 | 0.69 | 0.7 | 0.63 |
| 647 | Train 11 | Test Male 7 | 0.72 | 0.74 | 0.71 | 1671 | Train 27 | Test Male 7 | 0.72 | 0.74 | 0.71 |
| 648 | Train 11 | Test Male 8 | 0.69 | 0.7 | 0.65 | 1672 | Train 27 | Test Male 8 | 0.69 | 0.7 | 0.63 |
| 649 | Train 11 | Test Male 9 | 0.76 | 0.84 | 0.85 | 1673 | Train 27 | Test Male 9 | 0.76 | 0.84 | 0.85 |
| 650 | Train 11 | Test Male 10 | 0.74 | 0.79 | 0.8 | 1674 | Train 27 | Test Male 10 | 0.74 | 0.79 | 0.8 |
| 651 | Train 11 | Test Male 11 | 0.76 | 0.84 | 0.85 | 1675 | Train 27 | Test Male 11 | 0.76 | 0.84 | 0.85 |
| 652 | Train 11 | Test Male 12 | 0.74 | 0.79 | 0.8 | 1676 | Train 27 | Test Male 12 | 0.74 | 0.79 | 0.8 |
| 653 | Train 11 | Test Male 13 | 0.71 | 0.75 | 0.72 | 1677 | Train 27 | Test Male 13 | 0.72 | 0.75 | 0.72 |
| 654 | Train 11 | Test Male 14 | 0.69 | 0.7 | 0.65 | 1678 | Train 27 | Test Male 14 | 0.69 | 0.7 | 0.63 |
| 655 | Train 11 | Test Male 15 | 0.71 | 0.75 | 0.72 | 1679 | Train 27 | Test Male 15 | 0.71 | 0.75 | 0.72 |
| 656 | Train 11 | Test Male 16 | 0.69 | 0.7 | 0.65 | 1680 | Train 27 | Test Male 16 | 0.69 | 0.7 | 0.63 |
| 657 | Train 11 | Test Male 17 | 0.76 | 0.83 | 0.82 | 1681 | Train 27 | Test Male 17 | 0.76 | 0.83 | 0.82 |
| 658 | Train 11 | Test Male 18 | 0.75 | 0.79 | 0.78 | 1682 | Train 27 | Test Male 18 | 0.75 | 0.79 | 0.77 |
| 659 | Train 11 | Test Male 19 | 0.76 | 0.83 | 0.82 | 1683 | Train 27 | Test Male 19 | 0.76 | 0.83 | 0.82 |
| 660 | Train 11 | Test Male 20 | 0.75 | 0.79 | 0.78 | 1684 | Train 27 | Test Male 20 | 0.75 | 0.79 | 0.77 |
| 661 | Train 11 | Test Male 21 | 0.72 | 0.75 | 0.71 | 1685 | Train 27 | Test Male 21 | 0.72 | 0.75 | 0.71 |
| 662 | Train 11 | Test Male 22 | 0.69 | 0.7 | 0.65 | 1686 | Train 27 | Test Male 22 | 0.69 | 0.7 | 0.63 |
| 663 | Train 11 | Test Male 23 | 0.72 | 0.74 | 0.71 | 1687 | Train 27 | Test Male 23 | 0.72 | 0.74 | 0.71 |
| 664 | Train 11 | Test Male 24 | 0.69 | 0.7 | 0.65 | 1688 | Train 27 | Test Male 24 | 0.69 | 0.7 | 0.63 |
| 665 | Train 11 | Test Male 25 | 0.76 | 0.84 | 0.85 | 1689 | Train 27 | Test Male 25 | 0.76 | 0.84 | 0.85 |
| 666 | Train 11 | Test Male 26 | 0.74 | 0.79 | 0.8 | 1690 | Train 27 | Test Male 26 | 0.74 | 0.79 | 0.8 |
| 667 | Train 11 | Test Male 27 | 0.76 | 0.84 | 0.85 | 1691 | Train 27 | Test Male 27 | 0.76 | 0.84 | 0.85 |
| 668 | Train 11 | Test Male 28 | 0.74 | 0.79 | 0.8 | 1692 | Train 27 | Test Male 28 | 0.74 | 0.79 | 0.8 |
| 669 | Train 11 | Test Male 29 | 0.72 | 0.75 | 0.72 | 1693 | Train 27 | Test Male 29 | 0.72 | 0.75 | 0.72 |
| 670 | Train 11 | Test Male 30 | 0.69 | 0.7 | 0.65 | 1694 | Train 27 | Test Male 30 | 0.69 | 0.7 | 0.63 |
| 671 | Train 11 | Test Male 31 | 0.71 | 0.75 | 0.72 | 1695 | Train 27 | Test Male 31 | 0.71 | 0.75 | 0.72 |
| 672 | Train 11 | Test Male 32 | 0.69 | 0.7 | 0.65 | 1696 | Train 27 | Test Male 32 | 0.69 | 0.7 | 0.63 |
| 673 | Train 11 | Test Male 33 | 0.76 | 0.83 | 0.82 | 1697 | Train 27 | Test Male 33 | 0.76 | 0.83 | 0.82 |
| 674 | Train 11 | Test Male 34 | 0.74 | 0.79 | 0.78 | 1698 | Train 27 | Test Male 34 | 0.74 | 0.79 | 0.77 |
| 675 | Train 11 | Test Male 35 | 0.76 | 0.83 | 0.82 | 1699 | Train 27 | Test Male 35 | 0.76 | 0.83 | 0.82 |
| 676 | Train 11 | Test Male 36 | 0.74 | 0.79 | 0.78 | 1700 | Train 27 | Test Male 36 | 0.74 | 0.79 | 0.77 |
| 677 | Train 11 | Test Male 37 | 0.72 | 0.74 | 0.71 | 1701 | Train 27 | Test Male 37 | 0.72 | 0.74 | 0.7 |
| 678 | Train 11 | Test Male 38 | 0.69 | 0.7 | 0.65 | 1702 | Train 27 | Test Male 38 | 0.69 | 0.7 | 0.63 |
| 679 | Train 11 | Test Male 39 | 0.71 | 0.74 | 0.71 | 1703 | Train 27 | Test Male 39 | 0.71 | 0.74 | 0.7 |
| 680 | Train 11 | Test Male 40 | 0.69 | 0.7 | 0.65 | 1704 | Train 27 | Test Male 40 | 0.69 | 0.7 | 0.63 |
| 681 | Train 11 | Test Male 41 | 0.76 | 0.84 | 0.85 | 1705 | Train 27 | Test Male 41 | 0.76 | 0.84 | 0.85 |
| 682 | Train 11 | Test Male 42 | 0.74 | 0.79 | 0.8 | 1706 | Train 27 | Test Male 42 | 0.74 | 0.79 | 0.8 |
| 683 | Train 11 | Test Male 43 | 0.76 | 0.84 | 0.85 | 1707 | Train 27 | Test Male 43 | 0.76 | 0.84 | 0.85 |
| 684 | Train 11 | Test Male 44 | 0.74 | 0.79 | 0.8 | 1708 | Train 27 | Test Male 44 | 0.74 | 0.79 | 0.8 |
| 685 | Train 11 | Test Male 45 | 0.71 | 0.75 | 0.72 | 1709 | Train 27 | Test Male 45 | 0.71 | 0.75 | 0.72 |
| 686 | Train 11 | Test Male 46 | 0.69 | 0.7 | 0.65 | 1710 | Train 27 | Test Male 46 | 0.69 | 0.7 | 0.63 |
| 687 | Train 11 | Test Male 47 | 0.71 | 0.74 | 0.72 | 1711 | Train 27 | Test Male 47 | 0.71 | 0.75 | 0.72 |
| 688 | Train 11 | Test Male 48 | 0.69 | 0.7 | 0.65 | 1712 | Train 27 | Test Male 48 | 0.69 | 0.7 | 0.63 |
| 689 | Train 11 | Test Male 49 | 0.77 | 0.82 | 0.81 | 1713 | Train 27 | Test Male 49 | 0.77 | 0.82 | 0.82 |
| 690 | Train 11 | Test Male 50 | 0.77 | 0.82 | 0.81 | 1714 | Train 27 | Test Male 50 | 0.77 | 0.82 | 0.82 |
| 691 | Train 11 | Test Male 51 | 0.77 | 0.82 | 0.81 | 1715 | Train 27 | Test Male 51 | 0.77 | 0.82 | 0.82 |
| 692 | Train 11 | Test Male 52 | 0.77 | 0.82 | 0.81 | 1716 | Train 27 | Test Male 52 | 0.77 | 0.82 | 0.82 |
| 693 | Train 11 | Test Male 53 | 0.73 | 0.77 | 0.66 | 1717 | Train 27 | Test Male 53 | 0.73 | 0.77 | 0.68 |
| 694 | Train 11 | Test Male 54 | 0.71 | 0.71 | 0.65 | 1718 | Train 27 | Test Male 54 | 0.71 | 0.71 | 0.64 |
| 695 | Train 11 | Test Male 55 | 0.73 | 0.77 | 0.67 | 1719 | Train 27 | Test Male 55 | 0.73 | 0.77 | 0.68 |
| 696 | Train 11 | Test Male 56 | 0.71 | 0.71 | 0.65 | 1720 | Train 27 | Test Male 56 | 0.71 | 0.71 | 0.63 |
| 697 | Train 11 | Test Male 57 | 0.77 | 0.83 | 0.85 | 1721 | Train 27 | Test Male 57 | 0.77 | 0.83 | 0.85 |
| 698 | Train 11 | Test Male 58 | 0.77 | 0.83 | 0.85 | 1722 | Train 27 | Test Male 58 | 0.77 | 0.83 | 0.85 |
| 699 | Train 11 | Test Male 59 | 0.77 | 0.83 | 0.85 | 1723 | Train 27 | Test Male 59 | 0.77 | 0.83 | 0.85 |
| 700 | Train 11 | Test Male 60 | 0.77 | 0.83 | 0.85 | 1724 | Train 27 | Test Male 60 | 0.77 | 0.83 | 0.85 |
| 701 | Train 11 | Test Male 61 | 0.73 | 0.78 | 0.65 | 1725 | Train 27 | Test Male 61 | 0.73 | 0.78 | 0.66 |
| 702 | Train 11 | Test Male 62 | 0.71 | 0.71 | 0.65 | 1726 | Train 27 | Test Male 62 | 0.71 | 0.71 | 0.64 |
| 703 | Train 11 | Test Male 63 | 0.73 | 0.78 | 0.67 | 1727 | Train 27 | Test Male 63 | 0.73 | 0.78 | 0.69 |
| 704 | Train 11 | Test Male 64 | 0.71 | 0.71 | 0.65 | 1728 | Train 27 | Test Male 64 | 0.71 | 0.71 | 0.63 |
| 705 | Train 12 | Test Male 1 | 0.75 | 0.82 | 0.81 | 1729 | Train 28 | Test Male 1 | 0.75 | 0.82 | 0.81 |
| 706 | Train 12 | Test Male 2 | 0.76 | 0.83 | 0.82 | 1730 | Train 28 | Test Male 2 | 0.76 | 0.83 | 0.82 |
| 707 | Train 12 | Test Male 3 | 0.75 | 0.82 | 0.81 | 1731 | Train 28 | Test Male 3 | 0.75 | 0.82 | 0.81 |
| 708 | Train 12 | Test Male 4 | 0.76 | 0.83 | 0.82 | 1732 | Train 28 | Test Male 4 | 0.76 | 0.83 | 0.82 |
| 709 | Train 12 | Test Male 5 | 0.72 | 0.75 | 0.72 | 1733 | Train 28 | Test Male 5 | 0.72 | 0.75 | 0.72 |
| 710 | Train 12 | Test Male 6 | 0.7 | 0.72 | 0.68 | 1734 | Train 28 | Test Male 6 | 0.7 | 0.72 | 0.68 |
| 711 | Train 12 | Test Male 7 | 0.72 | 0.75 | 0.71 | 1735 | Train 28 | Test Male 7 | 0.72 | 0.75 | 0.72 |
| 712 | Train 12 | Test Male 8 | 0.7 | 0.71 | 0.68 | 1736 | Train 28 | Test Male 8 | 0.7 | 0.71 | 0.68 |
| 713 | Train 12 | Test Male 9 | 0.75 | 0.83 | 0.84 | 1737 | Train 28 | Test Male 9 | 0.75 | 0.83 | 0.84 |
| 714 | Train 12 | Test Male 10 | 0.76 | 0.83 | 0.85 | 1738 | Train 28 | Test Male 10 | 0.76 | 0.83 | 0.84 |
| 715 | Train 12 | Test Male 11 | 0.75 | 0.83 | 0.84 | 1739 | Train 28 | Test Male 11 | 0.75 | 0.83 | 0.84 |
| 716 | Train 12 | Test Male 12 | 0.76 | 0.83 | 0.85 | 1740 | Train 28 | Test Male 12 | 0.76 | 0.83 | 0.84 |
| 717 | Train 12 | Test Male 13 | 0.72 | 0.75 | 0.72 | 1741 | Train 28 | Test Male 13 | 0.72 | 0.75 | 0.73 |
| 718 | Train 12 | Test Male 14 | 0.71 | 0.72 | 0.68 | 1742 | Train 28 | Test Male 14 | 0.7 | 0.72 | 0.68 |
| 719 | Train 12 | Test Male 15 | 0.72 | 0.75 | 0.72 | 1743 | Train 28 | Test Male 15 | 0.72 | 0.75 | 0.73 |
| 720 | Train 12 | Test Male 16 | 0.7 | 0.71 | 0.68 | 1744 | Train 28 | Test Male 16 | 0.7 | 0.72 | 0.68 |
| 721 | Train 12 | Test Male 17 | 0.75 | 0.82 | 0.81 | 1745 | Train 28 | Test Male 17 | 0.75 | 0.82 | 0.81 |
| 722 | Train 12 | Test Male 18 | 0.76 | 0.83 | 0.82 | 1746 | Train 28 | Test Male 18 | 0.76 | 0.83 | 0.82 |
| 723 | Train 12 | Test Male 19 | 0.75 | 0.82 | 0.81 | 1747 | Train 28 | Test Male 19 | 0.75 | 0.82 | 0.81 |
| 724 | Train 12 | Test Male 20 | 0.76 | 0.83 | 0.82 | 1748 | Train 28 | Test Male 20 | 0.76 | 0.83 | 0.82 |
| 725 | Train 12 | Test Male 21 | 0.72 | 0.75 | 0.72 | 1749 | Train 28 | Test Male 21 | 0.72 | 0.75 | 0.72 |
| 726 | Train 12 | Test Male 22 | 0.7 | 0.72 | 0.68 | 1750 | Train 28 | Test Male 22 | 0.7 | 0.72 | 0.68 |
| 727 | Train 12 | Test Male 23 | 0.72 | 0.75 | 0.71 | 1751 | Train 28 | Test Male 23 | 0.72 | 0.75 | 0.72 |
| 728 | Train 12 | Test Male 24 | 0.7 | 0.71 | 0.68 | 1752 | Train 28 | Test Male 24 | 0.7 | 0.71 | 0.68 |
| 729 | Train 12 | Test Male 25 | 0.75 | 0.83 | 0.84 | 1753 | Train 28 | Test Male 25 | 0.75 | 0.83 | 0.84 |
| 730 | Train 12 | Test Male 26 | 0.76 | 0.83 | 0.85 | 1754 | Train 28 | Test Male 26 | 0.76 | 0.83 | 0.84 |
| 731 | Train 12 | Test Male 27 | 0.75 | 0.83 | 0.84 | 1755 | Train 28 | Test Male 27 | 0.75 | 0.83 | 0.84 |
| 732 | Train 12 | Test Male 28 | 0.76 | 0.83 | 0.85 | 1756 | Train 28 | Test Male 28 | 0.76 | 0.83 | 0.84 |
| 733 | Train 12 | Test Male 29 | 0.72 | 0.75 | 0.72 | 1757 | Train 28 | Test Male 29 | 0.72 | 0.75 | 0.73 |
| 734 | Train 12 | Test Male 30 | 0.71 | 0.72 | 0.68 | 1758 | Train 28 | Test Male 30 | 0.71 | 0.72 | 0.68 |
| 735 | Train 12 | Test Male 31 | 0.72 | 0.75 | 0.72 | 1759 | Train 28 | Test Male 31 | 0.72 | 0.75 | 0.73 |
| 736 | Train 12 | Test Male 32 | 0.71 | 0.71 | 0.68 | 1760 | Train 28 | Test Male 32 | 0.7 | 0.72 | 0.68 |
| 737 | Train 12 | Test Male 33 | 0.75 | 0.82 | 0.81 | 1761 | Train 28 | Test Male 33 | 0.75 | 0.82 | 0.81 |
| 738 | Train 12 | Test Male 34 | 0.76 | 0.83 | 0.82 | 1762 | Train 28 | Test Male 34 | 0.76 | 0.83 | 0.82 |
| 739 | Train 12 | Test Male 35 | 0.75 | 0.82 | 0.81 | 1763 | Train 28 | Test Male 35 | 0.75 | 0.82 | 0.81 |
| 740 | Train 12 | Test Male 36 | 0.76 | 0.83 | 0.82 | 1764 | Train 28 | Test Male 36 | 0.76 | 0.83 | 0.82 |
| 741 | Train 12 | Test Male 37 | 0.72 | 0.75 | 0.71 | 1765 | Train 28 | Test Male 37 | 0.72 | 0.75 | 0.72 |
| 742 | Train 12 | Test Male 38 | 0.7 | 0.71 | 0.68 | 1766 | Train 28 | Test Male 38 | 0.7 | 0.72 | 0.68 |
| 743 | Train 12 | Test Male 39 | 0.72 | 0.74 | 0.71 | 1767 | Train 28 | Test Male 39 | 0.72 | 0.74 | 0.72 |
| 744 | Train 12 | Test Male 40 | 0.7 | 0.71 | 0.68 | 1768 | Train 28 | Test Male 40 | 0.7 | 0.71 | 0.68 |
| 745 | Train 12 | Test Male 41 | 0.75 | 0.83 | 0.84 | 1769 | Train 28 | Test Male 41 | 0.75 | 0.83 | 0.84 |
| 746 | Train 12 | Test Male 42 | 0.76 | 0.83 | 0.84 | 1770 | Train 28 | Test Male 42 | 0.76 | 0.83 | 0.84 |
| 747 | Train 12 | Test Male 43 | 0.75 | 0.83 | 0.84 | 1771 | Train 28 | Test Male 43 | 0.75 | 0.83 | 0.84 |
| 748 | Train 12 | Test Male 44 | 0.76 | 0.83 | 0.84 | 1772 | Train 28 | Test Male 44 | 0.76 | 0.83 | 0.84 |
| 749 | Train 12 | Test Male 45 | 0.72 | 0.75 | 0.72 | 1773 | Train 28 | Test Male 45 | 0.72 | 0.75 | 0.73 |
| 750 | Train 12 | Test Male 46 | 0.7 | 0.72 | 0.68 | 1774 | Train 28 | Test Male 46 | 0.7 | 0.72 | 0.68 |
| 751 | Train 12 | Test Male 47 | 0.71 | 0.75 | 0.72 | 1775 | Train 28 | Test Male 47 | 0.71 | 0.75 | 0.73 |
| 752 | Train 12 | Test Male 48 | 0.7 | 0.71 | 0.68 | 1776 | Train 28 | Test Male 48 | 0.7 | 0.71 | 0.68 |
| 753 | Train 12 | Test Male 49 | 0.77 | 0.82 | 0.81 | 1777 | Train 28 | Test Male 49 | 0.77 | 0.82 | 0.82 |
| 754 | Train 12 | Test Male 50 | 0.77 | 0.82 | 0.81 | 1778 | Train 28 | Test Male 50 | 0.77 | 0.82 | 0.81 |
| 755 | Train 12 | Test Male 51 | 0.77 | 0.82 | 0.81 | 1779 | Train 28 | Test Male 51 | 0.77 | 0.82 | 0.81 |
| 756 | Train 12 | Test Male 52 | 0.76 | 0.82 | 0.81 | 1780 | Train 28 | Test Male 52 | 0.76 | 0.82 | 0.81 |
| 757 | Train 12 | Test Male 53 | 0.74 | 0.78 | 0.71 | 1781 | Train 28 | Test Male 53 | 0.74 | 0.78 | 0.72 |
| 758 | Train 12 | Test Male 54 | 0.71 | 0.72 | 0.69 | 1782 | Train 28 | Test Male 54 | 0.71 | 0.72 | 0.68 |
| 759 | Train 12 | Test Male 55 | 0.74 | 0.78 | 0.71 | 1783 | Train 28 | Test Male 55 | 0.74 | 0.78 | 0.72 |
| 760 | Train 12 | Test Male 56 | 0.71 | 0.71 | 0.68 | 1784 | Train 28 | Test Male 56 | 0.71 | 0.71 | 0.67 |
| 761 | Train 12 | Test Male 57 | 0.77 | 0.82 | 0.84 | 1785 | Train 28 | Test Male 57 | 0.77 | 0.82 | 0.84 |
| 762 | Train 12 | Test Male 58 | 0.77 | 0.82 | 0.84 | 1786 | Train 28 | Test Male 58 | 0.77 | 0.82 | 0.84 |
| 763 | Train 12 | Test Male 59 | 0.77 | 0.82 | 0.84 | 1787 | Train 28 | Test Male 59 | 0.77 | 0.82 | 0.84 |
| 764 | Train 12 | Test Male 60 | 0.76 | 0.82 | 0.84 | 1788 | Train 28 | Test Male 60 | 0.76 | 0.82 | 0.84 |
| 765 | Train 12 | Test Male 61 | 0.74 | 0.78 | 0.69 | 1789 | Train 28 | Test Male 61 | 0.74 | 0.78 | 0.69 |
| 766 | Train 12 | Test Male 62 | 0.71 | 0.72 | 0.69 | 1790 | Train 28 | Test Male 62 | 0.71 | 0.72 | 0.68 |
| 767 | Train 12 | Test Male 63 | 0.74 | 0.78 | 0.72 | 1791 | Train 28 | Test Male 63 | 0.74 | 0.78 | 0.72 |
| 768 | Train 12 | Test Male 64 | 0.71 | 0.71 | 0.69 | 1792 | Train 28 | Test Male 64 | 0.71 | 0.71 | 0.67 |
| 769 | Train 13 | Test Male 1 | 0.75 | 0.8 | 0.8 | 1793 | Train 29 | Test Male 1 | 0.75 | 0.8 | 0.8 |
| 770 | Train 13 | Test Male 2 | 0.75 | 0.79 | 0.78 | 1794 | Train 29 | Test Male 2 | 0.75 | 0.79 | 0.77 |
| 771 | Train 13 | Test Male 3 | 0.75 | 0.8 | 0.8 | 1795 | Train 29 | Test Male 3 | 0.75 | 0.8 | 0.8 |
| 772 | Train 13 | Test Male 4 | 0.75 | 0.79 | 0.77 | 1796 | Train 29 | Test Male 4 | 0.75 | 0.79 | 0.77 |
| 773 | Train 13 | Test Male 5 | 0.74 | 0.8 | 0.8 | 1797 | Train 29 | Test Male 5 | 0.74 | 0.8 | 0.8 |
| 774 | Train 13 | Test Male 6 | 0.7 | 0.72 | 0.68 | 1798 | Train 29 | Test Male 6 | 0.7 | 0.72 | 0.7 |
| 775 | Train 13 | Test Male 7 | 0.74 | 0.8 | 0.8 | 1799 | Train 29 | Test Male 7 | 0.74 | 0.8 | 0.79 |
| 776 | Train 13 | Test Male 8 | 0.7 | 0.72 | 0.68 | 1800 | Train 29 | Test Male 8 | 0.7 | 0.72 | 0.7 |
| 777 | Train 13 | Test Male 9 | 0.75 | 0.81 | 0.82 | 1801 | Train 29 | Test Male 9 | 0.75 | 0.81 | 0.82 |
| 778 | Train 13 | Test Male 10 | 0.75 | 0.79 | 0.79 | 1802 | Train 29 | Test Male 10 | 0.75 | 0.79 | 0.79 |
| 779 | Train 13 | Test Male 11 | 0.75 | 0.81 | 0.82 | 1803 | Train 29 | Test Male 11 | 0.75 | 0.81 | 0.82 |
| 780 | Train 13 | Test Male 12 | 0.75 | 0.79 | 0.79 | 1804 | Train 29 | Test Male 12 | 0.75 | 0.79 | 0.79 |
| 781 | Train 13 | Test Male 13 | 0.74 | 0.81 | 0.82 | 1805 | Train 29 | Test Male 13 | 0.74 | 0.81 | 0.82 |
| 782 | Train 13 | Test Male 14 | 0.7 | 0.72 | 0.68 | 1806 | Train 29 | Test Male 14 | 0.7 | 0.72 | 0.7 |
| 783 | Train 13 | Test Male 15 | 0.74 | 0.81 | 0.81 | 1807 | Train 29 | Test Male 15 | 0.74 | 0.81 | 0.81 |
| 784 | Train 13 | Test Male 16 | 0.7 | 0.72 | 0.68 | 1808 | Train 29 | Test Male 16 | 0.7 | 0.72 | 0.7 |
| 785 | Train 13 | Test Male 17 | 0.75 | 0.8 | 0.8 | 1809 | Train 29 | Test Male 17 | 0.75 | 0.8 | 0.8 |
| 786 | Train 13 | Test Male 18 | 0.75 | 0.79 | 0.78 | 1810 | Train 29 | Test Male 18 | 0.75 | 0.79 | 0.77 |
| 787 | Train 13 | Test Male 19 | 0.75 | 0.8 | 0.8 | 1811 | Train 29 | Test Male 19 | 0.75 | 0.8 | 0.8 |
| 788 | Train 13 | Test Male 20 | 0.75 | 0.79 | 0.77 | 1812 | Train 29 | Test Male 20 | 0.75 | 0.79 | 0.77 |
| 789 | Train 13 | Test Male 21 | 0.74 | 0.8 | 0.8 | 1813 | Train 29 | Test Male 21 | 0.74 | 0.8 | 0.8 |
| 790 | Train 13 | Test Male 22 | 0.7 | 0.72 | 0.68 | 1814 | Train 29 | Test Male 22 | 0.7 | 0.72 | 0.7 |
| 791 | Train 13 | Test Male 23 | 0.74 | 0.8 | 0.8 | 1815 | Train 29 | Test Male 23 | 0.74 | 0.8 | 0.79 |
| 792 | Train 13 | Test Male 24 | 0.7 | 0.72 | 0.68 | 1816 | Train 29 | Test Male 24 | 0.7 | 0.72 | 0.7 |
| 793 | Train 13 | Test Male 25 | 0.75 | 0.81 | 0.82 | 1817 | Train 29 | Test Male 25 | 0.75 | 0.81 | 0.82 |
| 794 | Train 13 | Test Male 26 | 0.75 | 0.79 | 0.79 | 1818 | Train 29 | Test Male 26 | 0.75 | 0.79 | 0.79 |
| 795 | Train 13 | Test Male 27 | 0.75 | 0.81 | 0.82 | 1819 | Train 29 | Test Male 27 | 0.75 | 0.81 | 0.82 |
| 796 | Train 13 | Test Male 28 | 0.75 | 0.79 | 0.79 | 1820 | Train 29 | Test Male 28 | 0.75 | 0.79 | 0.79 |
| 797 | Train 13 | Test Male 29 | 0.74 | 0.81 | 0.82 | 1821 | Train 29 | Test Male 29 | 0.74 | 0.81 | 0.82 |
| 798 | Train 13 | Test Male 30 | 0.7 | 0.72 | 0.68 | 1822 | Train 29 | Test Male 30 | 0.7 | 0.72 | 0.7 |
| 799 | Train 13 | Test Male 31 | 0.74 | 0.81 | 0.81 | 1823 | Train 29 | Test Male 31 | 0.74 | 0.81 | 0.81 |
| 800 | Train 13 | Test Male 32 | 0.7 | 0.72 | 0.68 | 1824 | Train 29 | Test Male 32 | 0.7 | 0.72 | 0.7 |
| 801 | Train 13 | Test Male 33 | 0.75 | 0.8 | 0.8 | 1825 | Train 29 | Test Male 33 | 0.75 | 0.8 | 0.8 |
| 802 | Train 13 | Test Male 34 | 0.75 | 0.79 | 0.77 | 1826 | Train 29 | Test Male 34 | 0.75 | 0.79 | 0.77 |
| 803 | Train 13 | Test Male 35 | 0.75 | 0.8 | 0.8 | 1827 | Train 29 | Test Male 35 | 0.75 | 0.8 | 0.8 |
| 804 | Train 13 | Test Male 36 | 0.75 | 0.78 | 0.77 | 1828 | Train 29 | Test Male 36 | 0.75 | 0.78 | 0.77 |
| 805 | Train 13 | Test Male 37 | 0.74 | 0.8 | 0.8 | 1829 | Train 29 | Test Male 37 | 0.74 | 0.8 | 0.8 |
| 806 | Train 13 | Test Male 38 | 0.7 | 0.72 | 0.68 | 1830 | Train 29 | Test Male 38 | 0.7 | 0.72 | 0.7 |
| 807 | Train 13 | Test Male 39 | 0.74 | 0.8 | 0.8 | 1831 | Train 29 | Test Male 39 | 0.74 | 0.8 | 0.79 |
| 808 | Train 13 | Test Male 40 | 0.7 | 0.72 | 0.68 | 1832 | Train 29 | Test Male 40 | 0.7 | 0.72 | 0.7 |
| 809 | Train 13 | Test Male 41 | 0.75 | 0.81 | 0.82 | 1833 | Train 29 | Test Male 41 | 0.75 | 0.81 | 0.82 |
| 810 | Train 13 | Test Male 42 | 0.75 | 0.79 | 0.79 | 1834 | Train 29 | Test Male 42 | 0.75 | 0.79 | 0.78 |
| 811 | Train 13 | Test Male 43 | 0.75 | 0.81 | 0.82 | 1835 | Train 29 | Test Male 43 | 0.75 | 0.81 | 0.81 |
| 812 | Train 13 | Test Male 44 | 0.75 | 0.79 | 0.79 | 1836 | Train 29 | Test Male 44 | 0.75 | 0.79 | 0.78 |
| 813 | Train 13 | Test Male 45 | 0.74 | 0.81 | 0.82 | 1837 | Train 29 | Test Male 45 | 0.74 | 0.81 | 0.82 |
| 814 | Train 13 | Test Male 46 | 0.7 | 0.72 | 0.68 | 1838 | Train 29 | Test Male 46 | 0.7 | 0.72 | 0.7 |
| 815 | Train 13 | Test Male 47 | 0.74 | 0.8 | 0.81 | 1839 | Train 29 | Test Male 47 | 0.74 | 0.8 | 0.81 |
| 816 | Train 13 | Test Male 48 | 0.7 | 0.72 | 0.68 | 1840 | Train 29 | Test Male 48 | 0.7 | 0.72 | 0.7 |
| 817 | Train 13 | Test Male 49 | 0.76 | 0.81 | 0.81 | 1841 | Train 29 | Test Male 49 | 0.76 | 0.81 | 0.8 |
| 818 | Train 13 | Test Male 50 | 0.76 | 0.81 | 0.8 | 1842 | Train 29 | Test Male 50 | 0.76 | 0.81 | 0.8 |
| 819 | Train 13 | Test Male 51 | 0.76 | 0.81 | 0.8 | 1843 | Train 29 | Test Male 51 | 0.76 | 0.81 | 0.8 |
| 820 | Train 13 | Test Male 52 | 0.76 | 0.8 | 0.8 | 1844 | Train 29 | Test Male 52 | 0.76 | 0.8 | 0.8 |
| 821 | Train 13 | Test Male 53 | 0.76 | 0.81 | 0.78 | 1845 | Train 29 | Test Male 53 | 0.76 | 0.8 | 0.78 |
| 822 | Train 13 | Test Male 54 | 0.71 | 0.72 | 0.68 | 1846 | Train 29 | Test Male 54 | 0.71 | 0.72 | 0.71 |
| 823 | Train 13 | Test Male 55 | 0.76 | 0.8 | 0.77 | 1847 | Train 29 | Test Male 55 | 0.76 | 0.8 | 0.77 |
| 824 | Train 13 | Test Male 56 | 0.71 | 0.71 | 0.67 | 1848 | Train 29 | Test Male 56 | 0.71 | 0.71 | 0.71 |
| 825 | Train 13 | Test Male 57 | 0.76 | 0.81 | 0.83 | 1849 | Train 29 | Test Male 57 | 0.76 | 0.81 | 0.83 |
| 826 | Train 13 | Test Male 58 | 0.76 | 0.81 | 0.83 | 1850 | Train 29 | Test Male 58 | 0.76 | 0.81 | 0.83 |
| 827 | Train 13 | Test Male 59 | 0.76 | 0.81 | 0.82 | 1851 | Train 29 | Test Male 59 | 0.76 | 0.81 | 0.82 |
| 828 | Train 13 | Test Male 60 | 0.76 | 0.81 | 0.82 | 1852 | Train 29 | Test Male 60 | 0.76 | 0.81 | 0.82 |
| 829 | Train 13 | Test Male 61 | 0.76 | 0.8 | 0.74 | 1853 | Train 29 | Test Male 61 | 0.76 | 0.8 | 0.76 |
| 830 | Train 13 | Test Male 62 | 0.71 | 0.72 | 0.68 | 1854 | Train 29 | Test Male 62 | 0.71 | 0.72 | 0.71 |
| 831 | Train 13 | Test Male 63 | 0.76 | 0.81 | 0.78 | 1855 | Train 29 | Test Male 63 | 0.76 | 0.81 | 0.79 |
| 832 | Train 13 | Test Male 64 | 0.71 | 0.72 | 0.67 | 1856 | Train 29 | Test Male 64 | 0.71 | 0.71 | 0.71 |
| 833 | Train 14 | Test Male 1 | 0.73 | 0.78 | 0.76 | 1857 | Train 30 | Test Male 1 | 0.73 | 0.78 | 0.76 |
| 834 | Train 14 | Test Male 2 | 0.74 | 0.78 | 0.77 | 1858 | Train 30 | Test Male 2 | 0.74 | 0.78 | 0.76 |
| 835 | Train 14 | Test Male 3 | 0.73 | 0.78 | 0.76 | 1859 | Train 30 | Test Male 3 | 0.73 | 0.78 | 0.76 |
| 836 | Train 14 | Test Male 4 | 0.74 | 0.78 | 0.77 | 1860 | Train 30 | Test Male 4 | 0.74 | 0.78 | 0.76 |
| 837 | Train 14 | Test Male 5 | 0.72 | 0.77 | 0.76 | 1861 | Train 30 | Test Male 5 | 0.72 | 0.77 | 0.76 |
| 838 | Train 14 | Test Male 6 | 0.73 | 0.79 | 0.79 | 1862 | Train 30 | Test Male 6 | 0.73 | 0.79 | 0.79 |
| 839 | Train 14 | Test Male 7 | 0.72 | 0.77 | 0.75 | 1863 | Train 30 | Test Male 7 | 0.72 | 0.77 | 0.75 |
| 840 | Train 14 | Test Male 8 | 0.73 | 0.78 | 0.78 | 1864 | Train 30 | Test Male 8 | 0.73 | 0.78 | 0.78 |
| 841 | Train 14 | Test Male 9 | 0.73 | 0.78 | 0.77 | 1865 | Train 30 | Test Male 9 | 0.73 | 0.78 | 0.76 |
| 842 | Train 14 | Test Male 10 | 0.74 | 0.79 | 0.77 | 1866 | Train 30 | Test Male 10 | 0.74 | 0.79 | 0.77 |
| 843 | Train 14 | Test Male 11 | 0.73 | 0.78 | 0.77 | 1867 | Train 30 | Test Male 11 | 0.73 | 0.78 | 0.76 |
| 844 | Train 14 | Test Male 12 | 0.74 | 0.79 | 0.77 | 1868 | Train 30 | Test Male 12 | 0.74 | 0.79 | 0.77 |
| 845 | Train 14 | Test Male 13 | 0.72 | 0.78 | 0.76 | 1869 | Train 30 | Test Male 13 | 0.72 | 0.78 | 0.76 |
| 846 | Train 14 | Test Male 14 | 0.74 | 0.79 | 0.79 | 1870 | Train 30 | Test Male 14 | 0.74 | 0.79 | 0.79 |
| 847 | Train 14 | Test Male 15 | 0.72 | 0.77 | 0.76 | 1871 | Train 30 | Test Male 15 | 0.72 | 0.77 | 0.76 |
| 848 | Train 14 | Test Male 16 | 0.73 | 0.78 | 0.78 | 1872 | Train 30 | Test Male 16 | 0.73 | 0.78 | 0.78 |
| 849 | Train 14 | Test Male 17 | 0.73 | 0.78 | 0.76 | 1873 | Train 30 | Test Male 17 | 0.73 | 0.78 | 0.76 |
| 850 | Train 14 | Test Male 18 | 0.74 | 0.78 | 0.77 | 1874 | Train 30 | Test Male 18 | 0.74 | 0.78 | 0.76 |
| 851 | Train 14 | Test Male 19 | 0.73 | 0.78 | 0.76 | 1875 | Train 30 | Test Male 19 | 0.73 | 0.78 | 0.76 |
| 852 | Train 14 | Test Male 20 | 0.74 | 0.78 | 0.77 | 1876 | Train 30 | Test Male 20 | 0.74 | 0.78 | 0.76 |
| 853 | Train 14 | Test Male 21 | 0.72 | 0.77 | 0.76 | 1877 | Train 30 | Test Male 21 | 0.72 | 0.77 | 0.76 |
| 854 | Train 14 | Test Male 22 | 0.73 | 0.79 | 0.79 | 1878 | Train 30 | Test Male 22 | 0.73 | 0.79 | 0.79 |
| 855 | Train 14 | Test Male 23 | 0.72 | 0.77 | 0.75 | 1879 | Train 30 | Test Male 23 | 0.72 | 0.77 | 0.75 |
| 856 | Train 14 | Test Male 24 | 0.73 | 0.78 | 0.78 | 1880 | Train 30 | Test Male 24 | 0.73 | 0.78 | 0.78 |
| 857 | Train 14 | Test Male 25 | 0.73 | 0.78 | 0.77 | 1881 | Train 30 | Test Male 25 | 0.73 | 0.78 | 0.76 |
| 858 | Train 14 | Test Male 26 | 0.74 | 0.79 | 0.77 | 1882 | Train 30 | Test Male 26 | 0.74 | 0.79 | 0.77 |
| 859 | Train 14 | Test Male 27 | 0.73 | 0.78 | 0.77 | 1883 | Train 30 | Test Male 27 | 0.73 | 0.78 | 0.76 |
| 860 | Train 14 | Test Male 28 | 0.74 | 0.79 | 0.77 | 1884 | Train 30 | Test Male 28 | 0.74 | 0.79 | 0.77 |
| 861 | Train 14 | Test Male 29 | 0.72 | 0.78 | 0.76 | 1885 | Train 30 | Test Male 29 | 0.72 | 0.78 | 0.76 |
| 862 | Train 14 | Test Male 30 | 0.74 | 0.79 | 0.79 | 1886 | Train 30 | Test Male 30 | 0.74 | 0.79 | 0.79 |
| 863 | Train 14 | Test Male 31 | 0.72 | 0.77 | 0.76 | 1887 | Train 30 | Test Male 31 | 0.72 | 0.77 | 0.76 |
| 864 | Train 14 | Test Male 32 | 0.73 | 0.78 | 0.78 | 1888 | Train 30 | Test Male 32 | 0.73 | 0.78 | 0.78 |
| 865 | Train 14 | Test Male 33 | 0.73 | 0.78 | 0.76 | 1889 | Train 30 | Test Male 33 | 0.73 | 0.78 | 0.76 |
| 866 | Train 14 | Test Male 34 | 0.74 | 0.78 | 0.77 | 1890 | Train 30 | Test Male 34 | 0.74 | 0.78 | 0.76 |
| 867 | Train 14 | Test Male 35 | 0.73 | 0.78 | 0.76 | 1891 | Train 30 | Test Male 35 | 0.73 | 0.78 | 0.76 |
| 868 | Train 14 | Test Male 36 | 0.74 | 0.78 | 0.76 | 1892 | Train 30 | Test Male 36 | 0.74 | 0.78 | 0.76 |
| 869 | Train 14 | Test Male 37 | 0.72 | 0.77 | 0.75 | 1893 | Train 30 | Test Male 37 | 0.72 | 0.77 | 0.75 |
| 870 | Train 14 | Test Male 38 | 0.73 | 0.79 | 0.79 | 1894 | Train 30 | Test Male 38 | 0.73 | 0.79 | 0.79 |
| 871 | Train 14 | Test Male 39 | 0.72 | 0.77 | 0.75 | 1895 | Train 30 | Test Male 39 | 0.72 | 0.77 | 0.75 |
| 872 | Train 14 | Test Male 40 | 0.73 | 0.78 | 0.78 | 1896 | Train 30 | Test Male 40 | 0.73 | 0.78 | 0.78 |
| 873 | Train 14 | Test Male 41 | 0.73 | 0.78 | 0.77 | 1897 | Train 30 | Test Male 41 | 0.73 | 0.78 | 0.76 |
| 874 | Train 14 | Test Male 42 | 0.74 | 0.79 | 0.77 | 1898 | Train 30 | Test Male 42 | 0.74 | 0.79 | 0.77 |
| 875 | Train 14 | Test Male 43 | 0.73 | 0.78 | 0.77 | 1899 | Train 30 | Test Male 43 | 0.73 | 0.78 | 0.76 |
| 876 | Train 14 | Test Male 44 | 0.74 | 0.78 | 0.77 | 1900 | Train 30 | Test Male 44 | 0.74 | 0.78 | 0.77 |
| 877 | Train 14 | Test Male 45 | 0.72 | 0.77 | 0.76 | 1901 | Train 30 | Test Male 45 | 0.72 | 0.77 | 0.76 |
| 878 | Train 14 | Test Male 46 | 0.74 | 0.79 | 0.79 | 1902 | Train 30 | Test Male 46 | 0.74 | 0.79 | 0.79 |
| 879 | Train 14 | Test Male 47 | 0.72 | 0.77 | 0.76 | 1903 | Train 30 | Test Male 47 | 0.72 | 0.77 | 0.76 |
| 880 | Train 14 | Test Male 48 | 0.73 | 0.78 | 0.78 | 1904 | Train 30 | Test Male 48 | 0.73 | 0.78 | 0.78 |
| 881 | Train 14 | Test Male 49 | 0.76 | 0.8 | 0.79 | 1905 | Train 30 | Test Male 49 | 0.76 | 0.8 | 0.78 |
| 882 | Train 14 | Test Male 50 | 0.76 | 0.8 | 0.79 | 1906 | Train 30 | Test Male 50 | 0.76 | 0.8 | 0.78 |
| 883 | Train 14 | Test Male 51 | 0.76 | 0.79 | 0.79 | 1907 | Train 30 | Test Male 51 | 0.76 | 0.79 | 0.78 |
| 884 | Train 14 | Test Male 52 | 0.76 | 0.79 | 0.78 | 1908 | Train 30 | Test Male 52 | 0.76 | 0.79 | 0.78 |
| 885 | Train 14 | Test Male 53 | 0.75 | 0.8 | 0.8 | 1909 | Train 30 | Test Male 53 | 0.75 | 0.8 | 0.8 |
| 886 | Train 14 | Test Male 54 | 0.74 | 0.78 | 0.79 | 1910 | Train 30 | Test Male 54 | 0.74 | 0.78 | 0.79 |
| 887 | Train 14 | Test Male 55 | 0.75 | 0.8 | 0.8 | 1911 | Train 30 | Test Male 55 | 0.75 | 0.8 | 0.8 |
| 888 | Train 14 | Test Male 56 | 0.74 | 0.78 | 0.78 | 1912 | Train 30 | Test Male 56 | 0.74 | 0.78 | 0.78 |
| 889 | Train 14 | Test Male 57 | 0.76 | 0.8 | 0.8 | 1913 | Train 30 | Test Male 57 | 0.76 | 0.8 | 0.8 |
| 890 | Train 14 | Test Male 58 | 0.76 | 0.8 | 0.8 | 1914 | Train 30 | Test Male 58 | 0.76 | 0.8 | 0.8 |
| 891 | Train 14 | Test Male 59 | 0.76 | 0.8 | 0.8 | 1915 | Train 30 | Test Male 59 | 0.76 | 0.8 | 0.79 |
| 892 | Train 14 | Test Male 60 | 0.76 | 0.8 | 0.8 | 1916 | Train 30 | Test Male 60 | 0.76 | 0.8 | 0.79 |
| 893 | Train 14 | Test Male 61 | 0.75 | 0.81 | 0.76 | 1917 | Train 30 | Test Male 61 | 0.75 | 0.81 | 0.77 |
| 894 | Train 14 | Test Male 62 | 0.74 | 0.78 | 0.79 | 1918 | Train 30 | Test Male 62 | 0.74 | 0.78 | 0.79 |
| 895 | Train 14 | Test Male 63 | 0.75 | 0.81 | 0.81 | 1919 | Train 30 | Test Male 63 | 0.75 | 0.81 | 0.81 |
| 896 | Train 14 | Test Male 64 | 0.74 | 0.78 | 0.78 | 1920 | Train 30 | Test Male 64 | 0.74 | 0.78 | 0.78 |
| 897 | Train 15 | Test Male 1 | 0.75 | 0.8 | 0.8 | 1921 | Train 31 | Test Male 1 | 0.75 | 0.8 | 0.8 |
| 898 | Train 15 | Test Male 2 | 0.75 | 0.79 | 0.77 | 1922 | Train 31 | Test Male 2 | 0.75 | 0.79 | 0.78 |
| 899 | Train 15 | Test Male 3 | 0.75 | 0.8 | 0.8 | 1923 | Train 31 | Test Male 3 | 0.75 | 0.8 | 0.8 |
| 900 | Train 15 | Test Male 4 | 0.75 | 0.79 | 0.77 | 1924 | Train 31 | Test Male 4 | 0.75 | 0.79 | 0.78 |
| 901 | Train 15 | Test Male 5 | 0.74 | 0.8 | 0.8 | 1925 | Train 31 | Test Male 5 | 0.74 | 0.8 | 0.8 |
| 902 | Train 15 | Test Male 6 | 0.7 | 0.72 | 0.69 | 1926 | Train 31 | Test Male 6 | 0.7 | 0.71 | 0.68 |
| 903 | Train 15 | Test Male 7 | 0.74 | 0.8 | 0.8 | 1927 | Train 31 | Test Male 7 | 0.74 | 0.8 | 0.8 |
| 904 | Train 15 | Test Male 8 | 0.7 | 0.72 | 0.69 | 1928 | Train 31 | Test Male 8 | 0.7 | 0.71 | 0.68 |
| 905 | Train 15 | Test Male 9 | 0.75 | 0.81 | 0.82 | 1929 | Train 31 | Test Male 9 | 0.75 | 0.81 | 0.82 |
| 906 | Train 15 | Test Male 10 | 0.75 | 0.79 | 0.78 | 1930 | Train 31 | Test Male 10 | 0.75 | 0.79 | 0.79 |
| 907 | Train 15 | Test Male 11 | 0.75 | 0.81 | 0.82 | 1931 | Train 31 | Test Male 11 | 0.75 | 0.81 | 0.82 |
| 908 | Train 15 | Test Male 12 | 0.75 | 0.79 | 0.78 | 1932 | Train 31 | Test Male 12 | 0.75 | 0.79 | 0.79 |
| 909 | Train 15 | Test Male 13 | 0.74 | 0.81 | 0.81 | 1933 | Train 31 | Test Male 13 | 0.74 | 0.81 | 0.82 |
| 910 | Train 15 | Test Male 14 | 0.7 | 0.72 | 0.69 | 1934 | Train 31 | Test Male 14 | 0.7 | 0.71 | 0.68 |
| 911 | Train 15 | Test Male 15 | 0.74 | 0.81 | 0.81 | 1935 | Train 31 | Test Male 15 | 0.74 | 0.81 | 0.82 |
| 912 | Train 15 | Test Male 16 | 0.7 | 0.72 | 0.69 | 1936 | Train 31 | Test Male 16 | 0.7 | 0.72 | 0.68 |
| 913 | Train 15 | Test Male 17 | 0.75 | 0.8 | 0.8 | 1937 | Train 31 | Test Male 17 | 0.75 | 0.8 | 0.8 |
| 914 | Train 15 | Test Male 18 | 0.75 | 0.79 | 0.77 | 1938 | Train 31 | Test Male 18 | 0.75 | 0.79 | 0.78 |
| 915 | Train 15 | Test Male 19 | 0.75 | 0.8 | 0.8 | 1939 | Train 31 | Test Male 19 | 0.75 | 0.8 | 0.8 |
| 916 | Train 15 | Test Male 20 | 0.75 | 0.79 | 0.77 | 1940 | Train 31 | Test Male 20 | 0.75 | 0.79 | 0.78 |
| 917 | Train 15 | Test Male 21 | 0.74 | 0.8 | 0.8 | 1941 | Train 31 | Test Male 21 | 0.74 | 0.8 | 0.8 |
| 918 | Train 15 | Test Male 22 | 0.7 | 0.72 | 0.69 | 1942 | Train 31 | Test Male 22 | 0.7 | 0.71 | 0.68 |
| 919 | Train 15 | Test Male 23 | 0.74 | 0.8 | 0.8 | 1943 | Train 31 | Test Male 23 | 0.74 | 0.8 | 0.8 |
| 920 | Train 15 | Test Male 24 | 0.7 | 0.72 | 0.69 | 1944 | Train 31 | Test Male 24 | 0.7 | 0.71 | 0.68 |
| 921 | Train 15 | Test Male 25 | 0.75 | 0.81 | 0.82 | 1945 | Train 31 | Test Male 25 | 0.75 | 0.81 | 0.82 |
| 922 | Train 15 | Test Male 26 | 0.75 | 0.79 | 0.78 | 1946 | Train 31 | Test Male 26 | 0.75 | 0.79 | 0.79 |
| 923 | Train 15 | Test Male 27 | 0.75 | 0.81 | 0.82 | 1947 | Train 31 | Test Male 27 | 0.75 | 0.81 | 0.82 |
| 924 | Train 15 | Test Male 28 | 0.75 | 0.79 | 0.78 | 1948 | Train 31 | Test Male 28 | 0.75 | 0.79 | 0.79 |
| 925 | Train 15 | Test Male 29 | 0.74 | 0.81 | 0.81 | 1949 | Train 31 | Test Male 29 | 0.74 | 0.81 | 0.82 |
| 926 | Train 15 | Test Male 30 | 0.7 | 0.72 | 0.69 | 1950 | Train 31 | Test Male 30 | 0.7 | 0.71 | 0.68 |
| 927 | Train 15 | Test Male 31 | 0.74 | 0.81 | 0.81 | 1951 | Train 31 | Test Male 31 | 0.74 | 0.81 | 0.82 |
| 928 | Train 15 | Test Male 32 | 0.7 | 0.72 | 0.69 | 1952 | Train 31 | Test Male 32 | 0.7 | 0.72 | 0.68 |
| 929 | Train 15 | Test Male 33 | 0.75 | 0.8 | 0.8 | 1953 | Train 31 | Test Male 33 | 0.75 | 0.8 | 0.8 |
| 930 | Train 15 | Test Male 34 | 0.75 | 0.79 | 0.77 | 1954 | Train 31 | Test Male 34 | 0.75 | 0.79 | 0.78 |
| 931 | Train 15 | Test Male 35 | 0.75 | 0.8 | 0.8 | 1955 | Train 31 | Test Male 35 | 0.75 | 0.8 | 0.8 |
| 932 | Train 15 | Test Male 36 | 0.75 | 0.79 | 0.77 | 1956 | Train 31 | Test Male 36 | 0.75 | 0.79 | 0.78 |
| 933 | Train 15 | Test Male 37 | 0.74 | 0.8 | 0.8 | 1957 | Train 31 | Test Male 37 | 0.74 | 0.8 | 0.8 |
| 934 | Train 15 | Test Male 38 | 0.7 | 0.72 | 0.69 | 1958 | Train 31 | Test Male 38 | 0.7 | 0.71 | 0.68 |
| 935 | Train 15 | Test Male 39 | 0.74 | 0.8 | 0.8 | 1959 | Train 31 | Test Male 39 | 0.74 | 0.8 | 0.8 |
| 936 | Train 15 | Test Male 40 | 0.7 | 0.72 | 0.69 | 1960 | Train 31 | Test Male 40 | 0.7 | 0.71 | 0.68 |
| 937 | Train 15 | Test Male 41 | 0.75 | 0.81 | 0.81 | 1961 | Train 31 | Test Male 41 | 0.75 | 0.81 | 0.82 |
| 938 | Train 15 | Test Male 42 | 0.75 | 0.79 | 0.78 | 1962 | Train 31 | Test Male 42 | 0.75 | 0.79 | 0.79 |
| 939 | Train 15 | Test Male 43 | 0.75 | 0.81 | 0.81 | 1963 | Train 31 | Test Male 43 | 0.75 | 0.81 | 0.82 |
| 940 | Train 15 | Test Male 44 | 0.75 | 0.79 | 0.78 | 1964 | Train 31 | Test Male 44 | 0.75 | 0.79 | 0.79 |
| 941 | Train 15 | Test Male 45 | 0.74 | 0.81 | 0.81 | 1965 | Train 31 | Test Male 45 | 0.74 | 0.81 | 0.82 |
| 942 | Train 15 | Test Male 46 | 0.7 | 0.72 | 0.69 | 1966 | Train 31 | Test Male 46 | 0.7 | 0.71 | 0.68 |
| 943 | Train 15 | Test Male 47 | 0.74 | 0.81 | 0.81 | 1967 | Train 31 | Test Male 47 | 0.74 | 0.81 | 0.82 |
| 944 | Train 15 | Test Male 48 | 0.7 | 0.72 | 0.69 | 1968 | Train 31 | Test Male 48 | 0.7 | 0.72 | 0.68 |
| 945 | Train 15 | Test Male 49 | 0.76 | 0.81 | 0.8 | 1969 | Train 31 | Test Male 49 | 0.76 | 0.81 | 0.81 |
| 946 | Train 15 | Test Male 50 | 0.76 | 0.81 | 0.8 | 1970 | Train 31 | Test Male 50 | 0.76 | 0.81 | 0.81 |
| 947 | Train 15 | Test Male 51 | 0.76 | 0.81 | 0.8 | 1971 | Train 31 | Test Male 51 | 0.76 | 0.81 | 0.81 |
| 948 | Train 15 | Test Male 52 | 0.76 | 0.81 | 0.8 | 1972 | Train 31 | Test Male 52 | 0.76 | 0.81 | 0.81 |
| 949 | Train 15 | Test Male 53 | 0.76 | 0.8 | 0.78 | 1973 | Train 31 | Test Male 53 | 0.76 | 0.8 | 0.72 |
| 950 | Train 15 | Test Male 54 | 0.71 | 0.71 | 0.69 | 1974 | Train 31 | Test Male 54 | 0.71 | 0.71 | 0.67 |
| 951 | Train 15 | Test Male 55 | 0.76 | 0.8 | 0.77 | 1975 | Train 31 | Test Male 55 | 0.76 | 0.8 | 0.72 |
| 952 | Train 15 | Test Male 56 | 0.71 | 0.71 | 0.69 | 1976 | Train 31 | Test Male 56 | 0.71 | 0.71 | 0.68 |
| 953 | Train 15 | Test Male 57 | 0.76 | 0.81 | 0.83 | 1977 | Train 31 | Test Male 57 | 0.76 | 0.81 | 0.83 |
| 954 | Train 15 | Test Male 58 | 0.76 | 0.81 | 0.82 | 1978 | Train 31 | Test Male 58 | 0.76 | 0.81 | 0.83 |
| 955 | Train 15 | Test Male 59 | 0.76 | 0.81 | 0.82 | 1979 | Train 31 | Test Male 59 | 0.76 | 0.81 | 0.83 |
| 956 | Train 15 | Test Male 60 | 0.76 | 0.81 | 0.82 | 1980 | Train 31 | Test Male 60 | 0.76 | 0.81 | 0.83 |
| 957 | Train 15 | Test Male 61 | 0.76 | 0.8 | 0.77 | 1981 | Train 31 | Test Male 61 | 0.76 | 0.8 | 0.71 |
| 958 | Train 15 | Test Male 62 | 0.71 | 0.72 | 0.69 | 1982 | Train 31 | Test Male 62 | 0.71 | 0.71 | 0.67 |
| 959 | Train 15 | Test Male 63 | 0.76 | 0.81 | 0.79 | 1983 | Train 31 | Test Male 63 | 0.76 | 0.81 | 0.73 |
| 960 | Train 15 | Test Male 64 | 0.71 | 0.71 | 0.69 | 1984 | Train 31 | Test Male 64 | 0.71 | 0.71 | 0.68 |
| 961 | Train 16 | Test Male 1 | 0.74 | 0.78 | 0.77 | 1985 | Train 32 | Test Male 1 | 0.74 | 0.78 | 0.77 |
| 962 | Train 16 | Test Male 2 | 0.75 | 0.78 | 0.77 | 1986 | Train 32 | Test Male 2 | 0.75 | 0.78 | 0.77 |
| 963 | Train 16 | Test Male 3 | 0.74 | 0.78 | 0.77 | 1987 | Train 32 | Test Male 3 | 0.73 | 0.78 | 0.77 |
| 964 | Train 16 | Test Male 4 | 0.74 | 0.78 | 0.77 | 1988 | Train 32 | Test Male 4 | 0.74 | 0.78 | 0.77 |
| 965 | Train 16 | Test Male 5 | 0.72 | 0.77 | 0.76 | 1989 | Train 32 | Test Male 5 | 0.72 | 0.77 | 0.75 |
| 966 | Train 16 | Test Male 6 | 0.73 | 0.78 | 0.79 | 1990 | Train 32 | Test Male 6 | 0.73 | 0.78 | 0.79 |
| 967 | Train 16 | Test Male 7 | 0.72 | 0.77 | 0.76 | 1991 | Train 32 | Test Male 7 | 0.72 | 0.77 | 0.74 |
| 968 | Train 16 | Test Male 8 | 0.73 | 0.78 | 0.79 | 1992 | Train 32 | Test Male 8 | 0.73 | 0.78 | 0.79 |
| 969 | Train 16 | Test Male 9 | 0.74 | 0.78 | 0.77 | 1993 | Train 32 | Test Male 9 | 0.73 | 0.78 | 0.77 |
| 970 | Train 16 | Test Male 10 | 0.75 | 0.79 | 0.78 | 1994 | Train 32 | Test Male 10 | 0.75 | 0.78 | 0.78 |
| 971 | Train 16 | Test Male 11 | 0.73 | 0.78 | 0.77 | 1995 | Train 32 | Test Male 11 | 0.73 | 0.78 | 0.77 |
| 972 | Train 16 | Test Male 12 | 0.75 | 0.78 | 0.78 | 1996 | Train 32 | Test Male 12 | 0.74 | 0.78 | 0.78 |
| 973 | Train 16 | Test Male 13 | 0.72 | 0.77 | 0.76 | 1997 | Train 32 | Test Male 13 | 0.72 | 0.77 | 0.75 |
| 974 | Train 16 | Test Male 14 | 0.73 | 0.78 | 0.79 | 1998 | Train 32 | Test Male 14 | 0.73 | 0.78 | 0.79 |
| 975 | Train 16 | Test Male 15 | 0.72 | 0.77 | 0.76 | 1999 | Train 32 | Test Male 15 | 0.72 | 0.77 | 0.75 |
| 976 | Train 16 | Test Male 16 | 0.73 | 0.78 | 0.79 | 2000 | Train 32 | Test Male 16 | 0.73 | 0.78 | 0.79 |
| 977 | Train 16 | Test Male 17 | 0.74 | 0.78 | 0.77 | 2001 | Train 32 | Test Male 17 | 0.74 | 0.78 | 0.77 |
| 978 | Train 16 | Test Male 18 | 0.75 | 0.78 | 0.77 | 2002 | Train 32 | Test Male 18 | 0.75 | 0.78 | 0.77 |
| 979 | Train 16 | Test Male 19 | 0.74 | 0.78 | 0.77 | 2003 | Train 32 | Test Male 19 | 0.73 | 0.78 | 0.77 |
| 980 | Train 16 | Test Male 20 | 0.74 | 0.78 | 0.77 | 2004 | Train 32 | Test Male 20 | 0.74 | 0.78 | 0.77 |
| 981 | Train 16 | Test Male 21 | 0.72 | 0.77 | 0.76 | 2005 | Train 32 | Test Male 21 | 0.72 | 0.77 | 0.75 |
| 982 | Train 16 | Test Male 22 | 0.73 | 0.78 | 0.79 | 2006 | Train 32 | Test Male 22 | 0.73 | 0.78 | 0.79 |
| 983 | Train 16 | Test Male 23 | 0.72 | 0.77 | 0.76 | 2007 | Train 32 | Test Male 23 | 0.72 | 0.77 | 0.74 |
| 984 | Train 16 | Test Male 24 | 0.73 | 0.78 | 0.79 | 2008 | Train 32 | Test Male 24 | 0.73 | 0.78 | 0.79 |
| 985 | Train 16 | Test Male 25 | 0.74 | 0.78 | 0.77 | 2009 | Train 32 | Test Male 25 | 0.73 | 0.78 | 0.77 |
| 986 | Train 16 | Test Male 26 | 0.75 | 0.79 | 0.78 | 2010 | Train 32 | Test Male 26 | 0.75 | 0.78 | 0.78 |
| 987 | Train 16 | Test Male 27 | 0.73 | 0.78 | 0.77 | 2011 | Train 32 | Test Male 27 | 0.73 | 0.78 | 0.77 |
| 988 | Train 16 | Test Male 28 | 0.75 | 0.78 | 0.78 | 2012 | Train 32 | Test Male 28 | 0.74 | 0.78 | 0.78 |
| 989 | Train 16 | Test Male 29 | 0.72 | 0.77 | 0.76 | 2013 | Train 32 | Test Male 29 | 0.72 | 0.77 | 0.75 |
| 990 | Train 16 | Test Male 30 | 0.73 | 0.78 | 0.79 | 2014 | Train 32 | Test Male 30 | 0.73 | 0.78 | 0.79 |
| 991 | Train 16 | Test Male 31 | 0.72 | 0.77 | 0.76 | 2015 | Train 32 | Test Male 31 | 0.72 | 0.77 | 0.75 |
| 992 | Train 16 | Test Male 32 | 0.73 | 0.78 | 0.79 | 2016 | Train 32 | Test Male 32 | 0.73 | 0.78 | 0.79 |
| 993 | Train 16 | Test Male 33 | 0.74 | 0.78 | 0.77 | 2017 | Train 32 | Test Male 33 | 0.74 | 0.78 | 0.76 |
| 994 | Train 16 | Test Male 34 | 0.75 | 0.78 | 0.77 | 2018 | Train 32 | Test Male 34 | 0.74 | 0.78 | 0.77 |
| 995 | Train 16 | Test Male 35 | 0.73 | 0.78 | 0.77 | 2019 | Train 32 | Test Male 35 | 0.73 | 0.78 | 0.76 |
| 996 | Train 16 | Test Male 36 | 0.74 | 0.78 | 0.77 | 2020 | Train 32 | Test Male 36 | 0.74 | 0.78 | 0.77 |
| 997 | Train 16 | Test Male 37 | 0.72 | 0.77 | 0.76 | 2021 | Train 32 | Test Male 37 | 0.72 | 0.77 | 0.74 |
| 998 | Train 16 | Test Male 38 | 0.73 | 0.78 | 0.79 | 2022 | Train 32 | Test Male 38 | 0.73 | 0.78 | 0.79 |
| 999 | Train 16 | Test Male 39 | 0.72 | 0.77 | 0.76 | 2023 | Train 32 | Test Male 39 | 0.72 | 0.77 | 0.74 |
| 1000 | Train 16 | Test Male 40 | 0.73 | 0.78 | 0.79 | 2024 | Train 32 | Test Male 40 | 0.73 | 0.78 | 0.79 |
| 1001 | Train 16 | Test Male 41 | 0.73 | 0.78 | 0.77 | 2025 | Train 32 | Test Male 41 | 0.73 | 0.78 | 0.77 |
| 1002 | Train 16 | Test Male 42 | 0.75 | 0.78 | 0.78 | 2026 | Train 32 | Test Male 42 | 0.75 | 0.78 | 0.77 |
| 1003 | Train 16 | Test Male 43 | 0.73 | 0.78 | 0.77 | 2027 | Train 32 | Test Male 43 | 0.73 | 0.78 | 0.77 |
| 1004 | Train 16 | Test Male 44 | 0.74 | 0.78 | 0.78 | 2028 | Train 32 | Test Male 44 | 0.74 | 0.78 | 0.77 |
| 1005 | Train 16 | Test Male 45 | 0.72 | 0.77 | 0.76 | 2029 | Train 32 | Test Male 45 | 0.72 | 0.77 | 0.75 |
| 1006 | Train 16 | Test Male 46 | 0.73 | 0.78 | 0.79 | 2030 | Train 32 | Test Male 46 | 0.73 | 0.78 | 0.79 |
| 1007 | Train 16 | Test Male 47 | 0.72 | 0.77 | 0.76 | 2031 | Train 32 | Test Male 47 | 0.72 | 0.77 | 0.75 |
| 1008 | Train 16 | Test Male 48 | 0.73 | 0.78 | 0.79 | 2032 | Train 32 | Test Male 48 | 0.73 | 0.78 | 0.79 |
| 1009 | Train 16 | Test Male 49 | 0.76 | 0.8 | 0.79 | 2033 | Train 32 | Test Male 49 | 0.76 | 0.8 | 0.79 |
| 1010 | Train 16 | Test Male 50 | 0.76 | 0.8 | 0.78 | 2034 | Train 32 | Test Male 50 | 0.76 | 0.8 | 0.79 |
| 1011 | Train 16 | Test Male 51 | 0.76 | 0.8 | 0.79 | 2035 | Train 32 | Test Male 51 | 0.76 | 0.8 | 0.79 |
| 1012 | Train 16 | Test Male 52 | 0.76 | 0.8 | 0.78 | 2036 | Train 32 | Test Male 52 | 0.76 | 0.79 | 0.79 |
| 1013 | Train 16 | Test Male 53 | 0.75 | 0.8 | 0.8 | 2037 | Train 32 | Test Male 53 | 0.75 | 0.8 | 0.8 |
| 1014 | Train 16 | Test Male 54 | 0.74 | 0.78 | 0.79 | 2038 | Train 32 | Test Male 54 | 0.74 | 0.78 | 0.79 |
| 1015 | Train 16 | Test Male 55 | 0.75 | 0.8 | 0.8 | 2039 | Train 32 | Test Male 55 | 0.75 | 0.8 | 0.8 |
| 1016 | Train 16 | Test Male 56 | 0.74 | 0.78 | 0.79 | 2040 | Train 32 | Test Male 56 | 0.74 | 0.78 | 0.79 |
| 1017 | Train 16 | Test Male 57 | 0.76 | 0.8 | 0.8 | 2041 | Train 32 | Test Male 57 | 0.76 | 0.8 | 0.79 |
| 1018 | Train 16 | Test Male 58 | 0.76 | 0.8 | 0.79 | 2042 | Train 32 | Test Male 58 | 0.76 | 0.8 | 0.79 |
| 1019 | Train 16 | Test Male 59 | 0.76 | 0.8 | 0.79 | 2043 | Train 32 | Test Male 59 | 0.76 | 0.8 | 0.79 |
| 1020 | Train 16 | Test Male 60 | 0.76 | 0.8 | 0.79 | 2044 | Train 32 | Test Male 60 | 0.76 | 0.8 | 0.79 |
| 1021 | Train 16 | Test Male 61 | 0.75 | 0.81 | 0.76 | 2045 | Train 32 | Test Male 61 | 0.75 | 0.81 | 0.78 |
| 1022 | Train 16 | Test Male 62 | 0.74 | 0.78 | 0.79 | 2046 | Train 32 | Test Male 62 | 0.74 | 0.78 | 0.79 |
| 1023 | Train 16 | Test Male 63 | 0.75 | 0.81 | 0.81 | 2047 | Train 32 | Test Male 63 | 0.75 | 0.81 | 0.81 |
| 1024 | Train 16 | Test Male 64 | 0.74 | 0.78 | 0.79 | 2048 | Train 32 | Test Male 64 | 0.74 | 0.78 | 0.79 |

Table 2. AUC Performance of the ML Models— LR, RF, and GB —for Male Patients Trained on Cohorts 33–64 and Tested Across 64 Cohorts.

| Male | Train  Model | Test  Dataset | AUC (LR) | AUC (RF) | AUC (GB) | Male | Train  Model | Test  Dataset | AUC (LR) | AUC (RF) | AUC (GB) |
| --- | --- | --- | --- | --- | --- | --- | --- | --- | --- | --- | --- |
| 2049 | Train 33 | Test Male 1 | 0.76 | 0.84 | 0.85 | 3073 | Train 49 | Test Male 1 | 0.74 | 0.76 | 0.68 |
| 2050 | Train 33 | Test Male 2 | 0.74 | 0.79 | 0.8 | 3074 | Train 49 | Test Male 2 | 0.72 | 0.73 | 0.63 |
| 2051 | Train 33 | Test Male 3 | 0.76 | 0.84 | 0.85 | 3075 | Train 49 | Test Male 3 | 0.74 | 0.76 | 0.68 |
| 2052 | Train 33 | Test Male 4 | 0.74 | 0.79 | 0.8 | 3076 | Train 49 | Test Male 4 | 0.72 | 0.73 | 0.63 |
| 2053 | Train 33 | Test Male 5 | 0.72 | 0.75 | 0.71 | 3077 | Train 49 | Test Male 5 | 0.7 | 0.72 | 0.64 |
| 2054 | Train 33 | Test Male 6 | 0.69 | 0.7 | 0.63 | 3078 | Train 49 | Test Male 6 | 0.68 | 0.68 | 0.61 |
| 2055 | Train 33 | Test Male 7 | 0.72 | 0.74 | 0.7 | 3079 | Train 49 | Test Male 7 | 0.7 | 0.72 | 0.64 |
| 2056 | Train 33 | Test Male 8 | 0.69 | 0.7 | 0.63 | 3080 | Train 49 | Test Male 8 | 0.68 | 0.68 | 0.61 |
| 2057 | Train 33 | Test Male 9 | 0.76 | 0.83 | 0.82 | 3081 | Train 49 | Test Male 9 | 0.74 | 0.75 | 0.66 |
| 2058 | Train 33 | Test Male 10 | 0.74 | 0.79 | 0.78 | 3082 | Train 49 | Test Male 10 | 0.72 | 0.73 | 0.62 |
| 2059 | Train 33 | Test Male 11 | 0.76 | 0.82 | 0.81 | 3083 | Train 49 | Test Male 11 | 0.74 | 0.75 | 0.66 |
| 2060 | Train 33 | Test Male 12 | 0.74 | 0.78 | 0.78 | 3084 | Train 49 | Test Male 12 | 0.72 | 0.73 | 0.62 |
| 2061 | Train 33 | Test Male 13 | 0.72 | 0.74 | 0.69 | 3085 | Train 49 | Test Male 13 | 0.7 | 0.72 | 0.63 |
| 2062 | Train 33 | Test Male 14 | 0.69 | 0.7 | 0.63 | 3086 | Train 49 | Test Male 14 | 0.68 | 0.68 | 0.61 |
| 2063 | Train 33 | Test Male 15 | 0.71 | 0.74 | 0.69 | 3087 | Train 49 | Test Male 15 | 0.7 | 0.71 | 0.63 |
| 2064 | Train 33 | Test Male 16 | 0.69 | 0.7 | 0.63 | 3088 | Train 49 | Test Male 16 | 0.68 | 0.68 | 0.61 |
| 2065 | Train 33 | Test Male 17 | 0.76 | 0.84 | 0.85 | 3089 | Train 49 | Test Male 17 | 0.74 | 0.76 | 0.68 |
| 2066 | Train 33 | Test Male 18 | 0.74 | 0.79 | 0.8 | 3090 | Train 49 | Test Male 18 | 0.72 | 0.73 | 0.63 |
| 2067 | Train 33 | Test Male 19 | 0.76 | 0.84 | 0.85 | 3091 | Train 49 | Test Male 19 | 0.74 | 0.76 | 0.68 |
| 2068 | Train 33 | Test Male 20 | 0.74 | 0.79 | 0.8 | 3092 | Train 49 | Test Male 20 | 0.72 | 0.73 | 0.63 |
| 2069 | Train 33 | Test Male 21 | 0.72 | 0.75 | 0.71 | 3093 | Train 49 | Test Male 21 | 0.7 | 0.72 | 0.64 |
| 2070 | Train 33 | Test Male 22 | 0.69 | 0.7 | 0.63 | 3094 | Train 49 | Test Male 22 | 0.68 | 0.68 | 0.61 |
| 2071 | Train 33 | Test Male 23 | 0.72 | 0.74 | 0.7 | 3095 | Train 49 | Test Male 23 | 0.7 | 0.72 | 0.64 |
| 2072 | Train 33 | Test Male 24 | 0.69 | 0.7 | 0.63 | 3096 | Train 49 | Test Male 24 | 0.68 | 0.68 | 0.61 |
| 2073 | Train 33 | Test Male 25 | 0.76 | 0.83 | 0.82 | 3097 | Train 49 | Test Male 25 | 0.74 | 0.75 | 0.66 |
| 2074 | Train 33 | Test Male 26 | 0.74 | 0.79 | 0.78 | 3098 | Train 49 | Test Male 26 | 0.72 | 0.73 | 0.62 |
| 2075 | Train 33 | Test Male 27 | 0.76 | 0.82 | 0.81 | 3099 | Train 49 | Test Male 27 | 0.74 | 0.75 | 0.66 |
| 2076 | Train 33 | Test Male 28 | 0.74 | 0.78 | 0.78 | 3100 | Train 49 | Test Male 28 | 0.72 | 0.73 | 0.63 |
| 2077 | Train 33 | Test Male 29 | 0.72 | 0.74 | 0.69 | 3101 | Train 49 | Test Male 29 | 0.7 | 0.72 | 0.63 |
| 2078 | Train 33 | Test Male 30 | 0.69 | 0.7 | 0.63 | 3102 | Train 49 | Test Male 30 | 0.68 | 0.68 | 0.61 |
| 2079 | Train 33 | Test Male 31 | 0.71 | 0.74 | 0.69 | 3103 | Train 49 | Test Male 31 | 0.7 | 0.71 | 0.63 |
| 2080 | Train 33 | Test Male 32 | 0.69 | 0.7 | 0.63 | 3104 | Train 49 | Test Male 32 | 0.68 | 0.68 | 0.61 |
| 2081 | Train 33 | Test Male 33 | 0.76 | 0.84 | 0.85 | 3105 | Train 49 | Test Male 33 | 0.74 | 0.75 | 0.68 |
| 2082 | Train 33 | Test Male 34 | 0.74 | 0.79 | 0.8 | 3106 | Train 49 | Test Male 34 | 0.72 | 0.73 | 0.63 |
| 2083 | Train 33 | Test Male 35 | 0.76 | 0.84 | 0.85 | 3107 | Train 49 | Test Male 35 | 0.74 | 0.75 | 0.68 |
| 2084 | Train 33 | Test Male 36 | 0.74 | 0.79 | 0.8 | 3108 | Train 49 | Test Male 36 | 0.72 | 0.73 | 0.63 |
| 2085 | Train 33 | Test Male 37 | 0.72 | 0.75 | 0.71 | 3109 | Train 49 | Test Male 37 | 0.7 | 0.72 | 0.64 |
| 2086 | Train 33 | Test Male 38 | 0.69 | 0.7 | 0.63 | 3110 | Train 49 | Test Male 38 | 0.68 | 0.68 | 0.61 |
| 2087 | Train 33 | Test Male 39 | 0.71 | 0.74 | 0.7 | 3111 | Train 49 | Test Male 39 | 0.7 | 0.72 | 0.64 |
| 2088 | Train 33 | Test Male 40 | 0.69 | 0.7 | 0.63 | 3112 | Train 49 | Test Male 40 | 0.68 | 0.68 | 0.61 |
| 2089 | Train 33 | Test Male 41 | 0.76 | 0.83 | 0.82 | 3113 | Train 49 | Test Male 41 | 0.74 | 0.75 | 0.66 |
| 2090 | Train 33 | Test Male 42 | 0.74 | 0.79 | 0.78 | 3114 | Train 49 | Test Male 42 | 0.72 | 0.73 | 0.62 |
| 2091 | Train 33 | Test Male 43 | 0.76 | 0.82 | 0.81 | 3115 | Train 49 | Test Male 43 | 0.74 | 0.75 | 0.66 |
| 2092 | Train 33 | Test Male 44 | 0.74 | 0.78 | 0.78 | 3116 | Train 49 | Test Male 44 | 0.72 | 0.73 | 0.62 |
| 2093 | Train 33 | Test Male 45 | 0.71 | 0.74 | 0.69 | 3117 | Train 49 | Test Male 45 | 0.7 | 0.72 | 0.63 |
| 2094 | Train 33 | Test Male 46 | 0.69 | 0.7 | 0.63 | 3118 | Train 49 | Test Male 46 | 0.68 | 0.68 | 0.61 |
| 2095 | Train 33 | Test Male 47 | 0.71 | 0.74 | 0.69 | 3119 | Train 49 | Test Male 47 | 0.7 | 0.71 | 0.63 |
| 2096 | Train 33 | Test Male 48 | 0.69 | 0.7 | 0.63 | 3120 | Train 49 | Test Male 48 | 0.68 | 0.68 | 0.61 |
| 2097 | Train 33 | Test Male 49 | 0.77 | 0.83 | 0.85 | 3121 | Train 49 | Test Male 49 | 0.78 | 0.85 | 0.89 |
| 2098 | Train 33 | Test Male 50 | 0.77 | 0.83 | 0.85 | 3122 | Train 49 | Test Male 50 | 0.78 | 0.85 | 0.89 |
| 2099 | Train 33 | Test Male 51 | 0.77 | 0.83 | 0.85 | 3123 | Train 49 | Test Male 51 | 0.78 | 0.85 | 0.89 |
| 2100 | Train 33 | Test Male 52 | 0.77 | 0.83 | 0.84 | 3124 | Train 49 | Test Male 52 | 0.78 | 0.85 | 0.89 |
| 2101 | Train 33 | Test Male 53 | 0.73 | 0.78 | 0.64 | 3125 | Train 49 | Test Male 53 | 0.72 | 0.78 | 0.73 |
| 2102 | Train 33 | Test Male 54 | 0.71 | 0.71 | 0.62 | 3126 | Train 49 | Test Male 54 | 0.69 | 0.7 | 0.65 |
| 2103 | Train 33 | Test Male 55 | 0.73 | 0.77 | 0.64 | 3127 | Train 49 | Test Male 55 | 0.72 | 0.78 | 0.73 |
| 2104 | Train 33 | Test Male 56 | 0.71 | 0.71 | 0.63 | 3128 | Train 49 | Test Male 56 | 0.7 | 0.7 | 0.65 |
| 2105 | Train 33 | Test Male 57 | 0.77 | 0.82 | 0.82 | 3129 | Train 49 | Test Male 57 | 0.78 | 0.84 | 0.83 |
| 2106 | Train 33 | Test Male 58 | 0.77 | 0.82 | 0.82 | 3130 | Train 49 | Test Male 58 | 0.78 | 0.84 | 0.83 |
| 2107 | Train 33 | Test Male 59 | 0.77 | 0.82 | 0.82 | 3131 | Train 49 | Test Male 59 | 0.78 | 0.83 | 0.82 |
| 2108 | Train 33 | Test Male 60 | 0.77 | 0.82 | 0.81 | 3132 | Train 49 | Test Male 60 | 0.78 | 0.83 | 0.82 |
| 2109 | Train 33 | Test Male 61 | 0.73 | 0.77 | 0.6 | 3133 | Train 49 | Test Male 61 | 0.72 | 0.77 | 0.72 |
| 2110 | Train 33 | Test Male 62 | 0.71 | 0.71 | 0.62 | 3134 | Train 49 | Test Male 62 | 0.69 | 0.7 | 0.65 |
| 2111 | Train 33 | Test Male 63 | 0.73 | 0.77 | 0.63 | 3135 | Train 49 | Test Male 63 | 0.72 | 0.77 | 0.72 |
| 2112 | Train 33 | Test Male 64 | 0.71 | 0.71 | 0.63 | 3136 | Train 49 | Test Male 64 | 0.7 | 0.7 | 0.65 |
| 2113 | Train 34 | Test Male 1 | 0.75 | 0.83 | 0.84 | 3137 | Train 50 | Test Male 1 | 0.74 | 0.73 | 0.71 |
| 2114 | Train 34 | Test Male 2 | 0.76 | 0.83 | 0.84 | 3138 | Train 50 | Test Male 2 | 0.72 | 0.72 | 0.68 |
| 2115 | Train 34 | Test Male 3 | 0.75 | 0.83 | 0.84 | 3139 | Train 50 | Test Male 3 | 0.74 | 0.73 | 0.71 |
| 2116 | Train 34 | Test Male 4 | 0.76 | 0.83 | 0.84 | 3140 | Train 50 | Test Male 4 | 0.72 | 0.72 | 0.68 |
| 2117 | Train 34 | Test Male 5 | 0.72 | 0.75 | 0.73 | 3141 | Train 50 | Test Male 5 | 0.7 | 0.7 | 0.67 |
| 2118 | Train 34 | Test Male 6 | 0.7 | 0.71 | 0.66 | 3142 | Train 50 | Test Male 6 | 0.68 | 0.67 | 0.63 |
| 2119 | Train 34 | Test Male 7 | 0.72 | 0.75 | 0.73 | 3143 | Train 50 | Test Male 7 | 0.7 | 0.7 | 0.67 |
| 2120 | Train 34 | Test Male 8 | 0.7 | 0.71 | 0.66 | 3144 | Train 50 | Test Male 8 | 0.68 | 0.67 | 0.63 |
| 2121 | Train 34 | Test Male 9 | 0.75 | 0.82 | 0.81 | 3145 | Train 50 | Test Male 9 | 0.73 | 0.73 | 0.7 |
| 2122 | Train 34 | Test Male 10 | 0.76 | 0.82 | 0.82 | 3146 | Train 50 | Test Male 10 | 0.72 | 0.71 | 0.66 |
| 2123 | Train 34 | Test Male 11 | 0.75 | 0.82 | 0.81 | 3147 | Train 50 | Test Male 11 | 0.73 | 0.73 | 0.69 |
| 2124 | Train 34 | Test Male 12 | 0.76 | 0.82 | 0.82 | 3148 | Train 50 | Test Male 12 | 0.72 | 0.71 | 0.66 |
| 2125 | Train 34 | Test Male 13 | 0.72 | 0.74 | 0.72 | 3149 | Train 50 | Test Male 13 | 0.7 | 0.7 | 0.66 |
| 2126 | Train 34 | Test Male 14 | 0.71 | 0.71 | 0.66 | 3150 | Train 50 | Test Male 14 | 0.68 | 0.67 | 0.63 |
| 2127 | Train 34 | Test Male 15 | 0.71 | 0.74 | 0.72 | 3151 | Train 50 | Test Male 15 | 0.7 | 0.7 | 0.66 |
| 2128 | Train 34 | Test Male 16 | 0.71 | 0.71 | 0.66 | 3152 | Train 50 | Test Male 16 | 0.68 | 0.67 | 0.63 |
| 2129 | Train 34 | Test Male 17 | 0.75 | 0.83 | 0.84 | 3153 | Train 50 | Test Male 17 | 0.74 | 0.73 | 0.71 |
| 2130 | Train 34 | Test Male 18 | 0.76 | 0.83 | 0.84 | 3154 | Train 50 | Test Male 18 | 0.72 | 0.72 | 0.68 |
| 2131 | Train 34 | Test Male 19 | 0.75 | 0.83 | 0.84 | 3155 | Train 50 | Test Male 19 | 0.74 | 0.73 | 0.71 |
| 2132 | Train 34 | Test Male 20 | 0.76 | 0.83 | 0.84 | 3156 | Train 50 | Test Male 20 | 0.72 | 0.72 | 0.68 |
| 2133 | Train 34 | Test Male 21 | 0.72 | 0.75 | 0.73 | 3157 | Train 50 | Test Male 21 | 0.7 | 0.7 | 0.67 |
| 2134 | Train 34 | Test Male 22 | 0.7 | 0.71 | 0.66 | 3158 | Train 50 | Test Male 22 | 0.68 | 0.67 | 0.63 |
| 2135 | Train 34 | Test Male 23 | 0.72 | 0.75 | 0.73 | 3159 | Train 50 | Test Male 23 | 0.7 | 0.7 | 0.67 |
| 2136 | Train 34 | Test Male 24 | 0.7 | 0.71 | 0.66 | 3160 | Train 50 | Test Male 24 | 0.68 | 0.67 | 0.63 |
| 2137 | Train 34 | Test Male 25 | 0.75 | 0.82 | 0.81 | 3161 | Train 50 | Test Male 25 | 0.73 | 0.73 | 0.7 |
| 2138 | Train 34 | Test Male 26 | 0.76 | 0.82 | 0.82 | 3162 | Train 50 | Test Male 26 | 0.72 | 0.71 | 0.66 |
| 2139 | Train 34 | Test Male 27 | 0.75 | 0.82 | 0.81 | 3163 | Train 50 | Test Male 27 | 0.73 | 0.73 | 0.69 |
| 2140 | Train 34 | Test Male 28 | 0.76 | 0.82 | 0.82 | 3164 | Train 50 | Test Male 28 | 0.72 | 0.71 | 0.66 |
| 2141 | Train 34 | Test Male 29 | 0.72 | 0.74 | 0.72 | 3165 | Train 50 | Test Male 29 | 0.7 | 0.7 | 0.66 |
| 2142 | Train 34 | Test Male 30 | 0.71 | 0.71 | 0.66 | 3166 | Train 50 | Test Male 30 | 0.68 | 0.68 | 0.63 |
| 2143 | Train 34 | Test Male 31 | 0.71 | 0.74 | 0.72 | 3167 | Train 50 | Test Male 31 | 0.7 | 0.7 | 0.66 |
| 2144 | Train 34 | Test Male 32 | 0.71 | 0.71 | 0.66 | 3168 | Train 50 | Test Male 32 | 0.68 | 0.67 | 0.64 |
| 2145 | Train 34 | Test Male 33 | 0.75 | 0.83 | 0.84 | 3169 | Train 50 | Test Male 33 | 0.73 | 0.73 | 0.71 |
| 2146 | Train 34 | Test Male 34 | 0.76 | 0.83 | 0.84 | 3170 | Train 50 | Test Male 34 | 0.72 | 0.72 | 0.68 |
| 2147 | Train 34 | Test Male 35 | 0.75 | 0.83 | 0.84 | 3171 | Train 50 | Test Male 35 | 0.73 | 0.73 | 0.71 |
| 2148 | Train 34 | Test Male 36 | 0.76 | 0.83 | 0.84 | 3172 | Train 50 | Test Male 36 | 0.72 | 0.72 | 0.68 |
| 2149 | Train 34 | Test Male 37 | 0.72 | 0.75 | 0.73 | 3173 | Train 50 | Test Male 37 | 0.7 | 0.7 | 0.67 |
| 2150 | Train 34 | Test Male 38 | 0.7 | 0.71 | 0.66 | 3174 | Train 50 | Test Male 38 | 0.68 | 0.67 | 0.63 |
| 2151 | Train 34 | Test Male 39 | 0.72 | 0.75 | 0.73 | 3175 | Train 50 | Test Male 39 | 0.7 | 0.7 | 0.67 |
| 2152 | Train 34 | Test Male 40 | 0.7 | 0.71 | 0.66 | 3176 | Train 50 | Test Male 40 | 0.68 | 0.67 | 0.63 |
| 2153 | Train 34 | Test Male 41 | 0.75 | 0.82 | 0.81 | 3177 | Train 50 | Test Male 41 | 0.73 | 0.73 | 0.69 |
| 2154 | Train 34 | Test Male 42 | 0.76 | 0.82 | 0.82 | 3178 | Train 50 | Test Male 42 | 0.72 | 0.71 | 0.66 |
| 2155 | Train 34 | Test Male 43 | 0.75 | 0.82 | 0.81 | 3179 | Train 50 | Test Male 43 | 0.73 | 0.73 | 0.69 |
| 2156 | Train 34 | Test Male 44 | 0.76 | 0.82 | 0.82 | 3180 | Train 50 | Test Male 44 | 0.72 | 0.71 | 0.66 |
| 2157 | Train 34 | Test Male 45 | 0.72 | 0.74 | 0.72 | 3181 | Train 50 | Test Male 45 | 0.7 | 0.7 | 0.66 |
| 2158 | Train 34 | Test Male 46 | 0.71 | 0.71 | 0.66 | 3182 | Train 50 | Test Male 46 | 0.68 | 0.67 | 0.63 |
| 2159 | Train 34 | Test Male 47 | 0.71 | 0.74 | 0.72 | 3183 | Train 50 | Test Male 47 | 0.7 | 0.7 | 0.66 |
| 2160 | Train 34 | Test Male 48 | 0.7 | 0.71 | 0.66 | 3184 | Train 50 | Test Male 48 | 0.68 | 0.67 | 0.63 |
| 2161 | Train 34 | Test Male 49 | 0.77 | 0.82 | 0.85 | 3185 | Train 50 | Test Male 49 | 0.78 | 0.85 | 0.9 |
| 2162 | Train 34 | Test Male 50 | 0.77 | 0.82 | 0.85 | 3186 | Train 50 | Test Male 50 | 0.78 | 0.85 | 0.9 |
| 2163 | Train 34 | Test Male 51 | 0.76 | 0.82 | 0.84 | 3187 | Train 50 | Test Male 51 | 0.78 | 0.85 | 0.89 |
| 2164 | Train 34 | Test Male 52 | 0.76 | 0.82 | 0.84 | 3188 | Train 50 | Test Male 52 | 0.78 | 0.85 | 0.89 |
| 2165 | Train 34 | Test Male 53 | 0.74 | 0.78 | 0.68 | 3189 | Train 50 | Test Male 53 | 0.72 | 0.75 | 0.74 |
| 2166 | Train 34 | Test Male 54 | 0.71 | 0.71 | 0.66 | 3190 | Train 50 | Test Male 54 | 0.69 | 0.69 | 0.65 |
| 2167 | Train 34 | Test Male 55 | 0.74 | 0.78 | 0.68 | 3191 | Train 50 | Test Male 55 | 0.72 | 0.75 | 0.74 |
| 2168 | Train 34 | Test Male 56 | 0.71 | 0.71 | 0.65 | 3192 | Train 50 | Test Male 56 | 0.7 | 0.68 | 0.65 |
| 2169 | Train 34 | Test Male 57 | 0.77 | 0.82 | 0.82 | 3193 | Train 50 | Test Male 57 | 0.78 | 0.84 | 0.83 |
| 2170 | Train 34 | Test Male 58 | 0.77 | 0.82 | 0.82 | 3194 | Train 50 | Test Male 58 | 0.78 | 0.84 | 0.83 |
| 2171 | Train 34 | Test Male 59 | 0.76 | 0.81 | 0.82 | 3195 | Train 50 | Test Male 59 | 0.78 | 0.83 | 0.83 |
| 2172 | Train 34 | Test Male 60 | 0.76 | 0.81 | 0.82 | 3196 | Train 50 | Test Male 60 | 0.78 | 0.83 | 0.83 |
| 2173 | Train 34 | Test Male 61 | 0.74 | 0.77 | 0.67 | 3197 | Train 50 | Test Male 61 | 0.72 | 0.75 | 0.73 |
| 2174 | Train 34 | Test Male 62 | 0.71 | 0.71 | 0.66 | 3198 | Train 50 | Test Male 62 | 0.69 | 0.69 | 0.65 |
| 2175 | Train 34 | Test Male 63 | 0.73 | 0.77 | 0.68 | 3199 | Train 50 | Test Male 63 | 0.72 | 0.75 | 0.73 |
| 2176 | Train 34 | Test Male 64 | 0.71 | 0.71 | 0.65 | 3200 | Train 50 | Test Male 64 | 0.7 | 0.68 | 0.65 |
| 2177 | Train 35 | Test Male 1 | 0.76 | 0.84 | 0.85 | 3201 | Train 51 | Test Male 1 | 0.74 | 0.75 | 0.71 |
| 2178 | Train 35 | Test Male 2 | 0.74 | 0.79 | 0.8 | 3202 | Train 51 | Test Male 2 | 0.72 | 0.73 | 0.64 |
| 2179 | Train 35 | Test Male 3 | 0.76 | 0.84 | 0.85 | 3203 | Train 51 | Test Male 3 | 0.74 | 0.76 | 0.71 |
| 2180 | Train 35 | Test Male 4 | 0.74 | 0.79 | 0.8 | 3204 | Train 51 | Test Male 4 | 0.72 | 0.73 | 0.64 |
| 2181 | Train 35 | Test Male 5 | 0.72 | 0.75 | 0.7 | 3205 | Train 51 | Test Male 5 | 0.7 | 0.72 | 0.65 |
| 2182 | Train 35 | Test Male 6 | 0.69 | 0.7 | 0.65 | 3206 | Train 51 | Test Male 6 | 0.68 | 0.68 | 0.59 |
| 2183 | Train 35 | Test Male 7 | 0.72 | 0.75 | 0.7 | 3207 | Train 51 | Test Male 7 | 0.7 | 0.71 | 0.65 |
| 2184 | Train 35 | Test Male 8 | 0.69 | 0.7 | 0.65 | 3208 | Train 51 | Test Male 8 | 0.68 | 0.68 | 0.59 |
| 2185 | Train 35 | Test Male 9 | 0.76 | 0.83 | 0.81 | 3209 | Train 51 | Test Male 9 | 0.74 | 0.75 | 0.69 |
| 2186 | Train 35 | Test Male 10 | 0.74 | 0.78 | 0.77 | 3210 | Train 51 | Test Male 10 | 0.71 | 0.72 | 0.63 |
| 2187 | Train 35 | Test Male 11 | 0.76 | 0.83 | 0.81 | 3211 | Train 51 | Test Male 11 | 0.74 | 0.75 | 0.69 |
| 2188 | Train 35 | Test Male 12 | 0.74 | 0.79 | 0.77 | 3212 | Train 51 | Test Male 12 | 0.71 | 0.72 | 0.63 |
| 2189 | Train 35 | Test Male 13 | 0.72 | 0.74 | 0.68 | 3213 | Train 51 | Test Male 13 | 0.7 | 0.71 | 0.64 |
| 2190 | Train 35 | Test Male 14 | 0.69 | 0.7 | 0.65 | 3214 | Train 51 | Test Male 14 | 0.68 | 0.68 | 0.59 |
| 2191 | Train 35 | Test Male 15 | 0.71 | 0.74 | 0.68 | 3215 | Train 51 | Test Male 15 | 0.7 | 0.71 | 0.64 |
| 2192 | Train 35 | Test Male 16 | 0.69 | 0.7 | 0.65 | 3216 | Train 51 | Test Male 16 | 0.68 | 0.68 | 0.59 |
| 2193 | Train 35 | Test Male 17 | 0.76 | 0.84 | 0.85 | 3217 | Train 51 | Test Male 17 | 0.74 | 0.75 | 0.71 |
| 2194 | Train 35 | Test Male 18 | 0.74 | 0.79 | 0.8 | 3218 | Train 51 | Test Male 18 | 0.72 | 0.73 | 0.64 |
| 2195 | Train 35 | Test Male 19 | 0.76 | 0.84 | 0.85 | 3219 | Train 51 | Test Male 19 | 0.74 | 0.76 | 0.71 |
| 2196 | Train 35 | Test Male 20 | 0.74 | 0.79 | 0.8 | 3220 | Train 51 | Test Male 20 | 0.72 | 0.73 | 0.64 |
| 2197 | Train 35 | Test Male 21 | 0.72 | 0.75 | 0.7 | 3221 | Train 51 | Test Male 21 | 0.7 | 0.72 | 0.65 |
| 2198 | Train 35 | Test Male 22 | 0.69 | 0.7 | 0.65 | 3222 | Train 51 | Test Male 22 | 0.68 | 0.68 | 0.59 |
| 2199 | Train 35 | Test Male 23 | 0.72 | 0.75 | 0.7 | 3223 | Train 51 | Test Male 23 | 0.7 | 0.71 | 0.65 |
| 2200 | Train 35 | Test Male 24 | 0.69 | 0.7 | 0.66 | 3224 | Train 51 | Test Male 24 | 0.68 | 0.68 | 0.59 |
| 2201 | Train 35 | Test Male 25 | 0.76 | 0.83 | 0.81 | 3225 | Train 51 | Test Male 25 | 0.74 | 0.75 | 0.69 |
| 2202 | Train 35 | Test Male 26 | 0.74 | 0.78 | 0.77 | 3226 | Train 51 | Test Male 26 | 0.71 | 0.72 | 0.63 |
| 2203 | Train 35 | Test Male 27 | 0.76 | 0.83 | 0.81 | 3227 | Train 51 | Test Male 27 | 0.74 | 0.75 | 0.69 |
| 2204 | Train 35 | Test Male 28 | 0.74 | 0.79 | 0.77 | 3228 | Train 51 | Test Male 28 | 0.71 | 0.72 | 0.63 |
| 2205 | Train 35 | Test Male 29 | 0.72 | 0.74 | 0.68 | 3229 | Train 51 | Test Male 29 | 0.7 | 0.71 | 0.64 |
| 2206 | Train 35 | Test Male 30 | 0.69 | 0.7 | 0.65 | 3230 | Train 51 | Test Male 30 | 0.68 | 0.68 | 0.59 |
| 2207 | Train 35 | Test Male 31 | 0.71 | 0.74 | 0.68 | 3231 | Train 51 | Test Male 31 | 0.7 | 0.71 | 0.64 |
| 2208 | Train 35 | Test Male 32 | 0.69 | 0.7 | 0.65 | 3232 | Train 51 | Test Male 32 | 0.68 | 0.68 | 0.59 |
| 2209 | Train 35 | Test Male 33 | 0.76 | 0.84 | 0.85 | 3233 | Train 51 | Test Male 33 | 0.74 | 0.75 | 0.71 |
| 2210 | Train 35 | Test Male 34 | 0.74 | 0.79 | 0.8 | 3234 | Train 51 | Test Male 34 | 0.72 | 0.73 | 0.64 |
| 2211 | Train 35 | Test Male 35 | 0.76 | 0.84 | 0.85 | 3235 | Train 51 | Test Male 35 | 0.74 | 0.76 | 0.71 |
| 2212 | Train 35 | Test Male 36 | 0.74 | 0.79 | 0.8 | 3236 | Train 51 | Test Male 36 | 0.72 | 0.73 | 0.64 |
| 2213 | Train 35 | Test Male 37 | 0.72 | 0.75 | 0.7 | 3237 | Train 51 | Test Male 37 | 0.7 | 0.71 | 0.65 |
| 2214 | Train 35 | Test Male 38 | 0.69 | 0.7 | 0.65 | 3238 | Train 51 | Test Male 38 | 0.68 | 0.68 | 0.59 |
| 2215 | Train 35 | Test Male 39 | 0.71 | 0.74 | 0.7 | 3239 | Train 51 | Test Male 39 | 0.7 | 0.71 | 0.65 |
| 2216 | Train 35 | Test Male 40 | 0.69 | 0.7 | 0.65 | 3240 | Train 51 | Test Male 40 | 0.68 | 0.68 | 0.59 |
| 2217 | Train 35 | Test Male 41 | 0.76 | 0.83 | 0.81 | 3241 | Train 51 | Test Male 41 | 0.74 | 0.75 | 0.69 |
| 2218 | Train 35 | Test Male 42 | 0.74 | 0.78 | 0.77 | 3242 | Train 51 | Test Male 42 | 0.71 | 0.72 | 0.63 |
| 2219 | Train 35 | Test Male 43 | 0.76 | 0.83 | 0.81 | 3243 | Train 51 | Test Male 43 | 0.74 | 0.75 | 0.69 |
| 2220 | Train 35 | Test Male 44 | 0.74 | 0.79 | 0.77 | 3244 | Train 51 | Test Male 44 | 0.71 | 0.72 | 0.63 |
| 2221 | Train 35 | Test Male 45 | 0.71 | 0.74 | 0.68 | 3245 | Train 51 | Test Male 45 | 0.7 | 0.71 | 0.64 |
| 2222 | Train 35 | Test Male 46 | 0.69 | 0.7 | 0.65 | 3246 | Train 51 | Test Male 46 | 0.68 | 0.68 | 0.59 |
| 2223 | Train 35 | Test Male 47 | 0.71 | 0.74 | 0.68 | 3247 | Train 51 | Test Male 47 | 0.7 | 0.71 | 0.64 |
| 2224 | Train 35 | Test Male 48 | 0.69 | 0.7 | 0.65 | 3248 | Train 51 | Test Male 48 | 0.68 | 0.68 | 0.59 |
| 2225 | Train 35 | Test Male 49 | 0.77 | 0.83 | 0.85 | 3249 | Train 51 | Test Male 49 | 0.78 | 0.85 | 0.89 |
| 2226 | Train 35 | Test Male 50 | 0.77 | 0.83 | 0.85 | 3250 | Train 51 | Test Male 50 | 0.78 | 0.85 | 0.89 |
| 2227 | Train 35 | Test Male 51 | 0.77 | 0.83 | 0.85 | 3251 | Train 51 | Test Male 51 | 0.78 | 0.85 | 0.89 |
| 2228 | Train 35 | Test Male 52 | 0.77 | 0.83 | 0.85 | 3252 | Train 51 | Test Male 52 | 0.78 | 0.85 | 0.89 |
| 2229 | Train 35 | Test Male 53 | 0.73 | 0.77 | 0.65 | 3253 | Train 51 | Test Male 53 | 0.72 | 0.78 | 0.73 |
| 2230 | Train 35 | Test Male 54 | 0.71 | 0.71 | 0.64 | 3254 | Train 51 | Test Male 54 | 0.69 | 0.7 | 0.62 |
| 2231 | Train 35 | Test Male 55 | 0.73 | 0.77 | 0.66 | 3255 | Train 51 | Test Male 55 | 0.72 | 0.78 | 0.73 |
| 2232 | Train 35 | Test Male 56 | 0.71 | 0.7 | 0.65 | 3256 | Train 51 | Test Male 56 | 0.7 | 0.7 | 0.62 |
| 2233 | Train 35 | Test Male 57 | 0.77 | 0.82 | 0.82 | 3257 | Train 51 | Test Male 57 | 0.78 | 0.84 | 0.83 |
| 2234 | Train 35 | Test Male 58 | 0.77 | 0.82 | 0.82 | 3258 | Train 51 | Test Male 58 | 0.78 | 0.84 | 0.83 |
| 2235 | Train 35 | Test Male 59 | 0.77 | 0.82 | 0.82 | 3259 | Train 51 | Test Male 59 | 0.78 | 0.84 | 0.83 |
| 2236 | Train 35 | Test Male 60 | 0.77 | 0.82 | 0.82 | 3260 | Train 51 | Test Male 60 | 0.78 | 0.84 | 0.83 |
| 2237 | Train 35 | Test Male 61 | 0.73 | 0.77 | 0.62 | 3261 | Train 51 | Test Male 61 | 0.72 | 0.77 | 0.71 |
| 2238 | Train 35 | Test Male 62 | 0.71 | 0.71 | 0.64 | 3262 | Train 51 | Test Male 62 | 0.69 | 0.7 | 0.62 |
| 2239 | Train 35 | Test Male 63 | 0.73 | 0.77 | 0.65 | 3263 | Train 51 | Test Male 63 | 0.72 | 0.77 | 0.72 |
| 2240 | Train 35 | Test Male 64 | 0.71 | 0.7 | 0.65 | 3264 | Train 51 | Test Male 64 | 0.7 | 0.7 | 0.62 |
| 2241 | Train 36 | Test Male 1 | 0.75 | 0.83 | 0.84 | 3265 | Train 52 | Test Male 1 | 0.73 | 0.72 | 0.69 |
| 2242 | Train 36 | Test Male 2 | 0.76 | 0.83 | 0.85 | 3266 | Train 52 | Test Male 2 | 0.72 | 0.71 | 0.66 |
| 2243 | Train 36 | Test Male 3 | 0.75 | 0.83 | 0.84 | 3267 | Train 52 | Test Male 3 | 0.74 | 0.72 | 0.7 |
| 2244 | Train 36 | Test Male 4 | 0.76 | 0.83 | 0.85 | 3268 | Train 52 | Test Male 4 | 0.72 | 0.71 | 0.66 |
| 2245 | Train 36 | Test Male 5 | 0.72 | 0.75 | 0.73 | 3269 | Train 52 | Test Male 5 | 0.7 | 0.7 | 0.66 |
| 2246 | Train 36 | Test Male 6 | 0.7 | 0.71 | 0.66 | 3270 | Train 52 | Test Male 6 | 0.68 | 0.67 | 0.62 |
| 2247 | Train 36 | Test Male 7 | 0.72 | 0.75 | 0.73 | 3271 | Train 52 | Test Male 7 | 0.7 | 0.7 | 0.66 |
| 2248 | Train 36 | Test Male 8 | 0.7 | 0.71 | 0.66 | 3272 | Train 52 | Test Male 8 | 0.68 | 0.67 | 0.63 |
| 2249 | Train 36 | Test Male 9 | 0.75 | 0.82 | 0.81 | 3273 | Train 52 | Test Male 9 | 0.73 | 0.72 | 0.68 |
| 2250 | Train 36 | Test Male 10 | 0.76 | 0.82 | 0.82 | 3274 | Train 52 | Test Male 10 | 0.72 | 0.71 | 0.65 |
| 2251 | Train 36 | Test Male 11 | 0.75 | 0.82 | 0.81 | 3275 | Train 52 | Test Male 11 | 0.73 | 0.72 | 0.68 |
| 2252 | Train 36 | Test Male 12 | 0.76 | 0.82 | 0.82 | 3276 | Train 52 | Test Male 12 | 0.72 | 0.71 | 0.65 |
| 2253 | Train 36 | Test Male 13 | 0.72 | 0.75 | 0.72 | 3277 | Train 52 | Test Male 13 | 0.7 | 0.7 | 0.65 |
| 2254 | Train 36 | Test Male 14 | 0.71 | 0.71 | 0.66 | 3278 | Train 52 | Test Male 14 | 0.68 | 0.67 | 0.63 |
| 2255 | Train 36 | Test Male 15 | 0.71 | 0.74 | 0.72 | 3279 | Train 52 | Test Male 15 | 0.7 | 0.69 | 0.65 |
| 2256 | Train 36 | Test Male 16 | 0.7 | 0.71 | 0.66 | 3280 | Train 52 | Test Male 16 | 0.68 | 0.67 | 0.63 |
| 2257 | Train 36 | Test Male 17 | 0.75 | 0.83 | 0.84 | 3281 | Train 52 | Test Male 17 | 0.73 | 0.72 | 0.69 |
| 2258 | Train 36 | Test Male 18 | 0.76 | 0.83 | 0.85 | 3282 | Train 52 | Test Male 18 | 0.72 | 0.71 | 0.66 |
| 2259 | Train 36 | Test Male 19 | 0.75 | 0.83 | 0.84 | 3283 | Train 52 | Test Male 19 | 0.74 | 0.72 | 0.7 |
| 2260 | Train 36 | Test Male 20 | 0.76 | 0.83 | 0.85 | 3284 | Train 52 | Test Male 20 | 0.72 | 0.71 | 0.66 |
| 2261 | Train 36 | Test Male 21 | 0.72 | 0.75 | 0.73 | 3285 | Train 52 | Test Male 21 | 0.7 | 0.7 | 0.66 |
| 2262 | Train 36 | Test Male 22 | 0.7 | 0.71 | 0.66 | 3286 | Train 52 | Test Male 22 | 0.68 | 0.67 | 0.62 |
| 2263 | Train 36 | Test Male 23 | 0.72 | 0.75 | 0.73 | 3287 | Train 52 | Test Male 23 | 0.7 | 0.7 | 0.66 |
| 2264 | Train 36 | Test Male 24 | 0.7 | 0.71 | 0.66 | 3288 | Train 52 | Test Male 24 | 0.68 | 0.67 | 0.63 |
| 2265 | Train 36 | Test Male 25 | 0.75 | 0.82 | 0.81 | 3289 | Train 52 | Test Male 25 | 0.73 | 0.72 | 0.68 |
| 2266 | Train 36 | Test Male 26 | 0.76 | 0.82 | 0.82 | 3290 | Train 52 | Test Male 26 | 0.72 | 0.71 | 0.65 |
| 2267 | Train 36 | Test Male 27 | 0.75 | 0.82 | 0.81 | 3291 | Train 52 | Test Male 27 | 0.73 | 0.72 | 0.68 |
| 2268 | Train 36 | Test Male 28 | 0.76 | 0.82 | 0.82 | 3292 | Train 52 | Test Male 28 | 0.72 | 0.71 | 0.65 |
| 2269 | Train 36 | Test Male 29 | 0.72 | 0.75 | 0.72 | 3293 | Train 52 | Test Male 29 | 0.7 | 0.7 | 0.65 |
| 2270 | Train 36 | Test Male 30 | 0.71 | 0.71 | 0.66 | 3294 | Train 52 | Test Male 30 | 0.68 | 0.67 | 0.63 |
| 2271 | Train 36 | Test Male 31 | 0.71 | 0.74 | 0.72 | 3295 | Train 52 | Test Male 31 | 0.7 | 0.69 | 0.65 |
| 2272 | Train 36 | Test Male 32 | 0.7 | 0.71 | 0.66 | 3296 | Train 52 | Test Male 32 | 0.68 | 0.67 | 0.63 |
| 2273 | Train 36 | Test Male 33 | 0.75 | 0.83 | 0.84 | 3297 | Train 52 | Test Male 33 | 0.73 | 0.72 | 0.69 |
| 2274 | Train 36 | Test Male 34 | 0.76 | 0.83 | 0.85 | 3298 | Train 52 | Test Male 34 | 0.72 | 0.71 | 0.66 |
| 2275 | Train 36 | Test Male 35 | 0.75 | 0.83 | 0.84 | 3299 | Train 52 | Test Male 35 | 0.73 | 0.72 | 0.69 |
| 2276 | Train 36 | Test Male 36 | 0.76 | 0.83 | 0.85 | 3300 | Train 52 | Test Male 36 | 0.72 | 0.71 | 0.66 |
| 2277 | Train 36 | Test Male 37 | 0.72 | 0.75 | 0.73 | 3301 | Train 52 | Test Male 37 | 0.7 | 0.7 | 0.66 |
| 2278 | Train 36 | Test Male 38 | 0.7 | 0.71 | 0.66 | 3302 | Train 52 | Test Male 38 | 0.68 | 0.67 | 0.62 |
| 2279 | Train 36 | Test Male 39 | 0.72 | 0.75 | 0.73 | 3303 | Train 52 | Test Male 39 | 0.7 | 0.69 | 0.66 |
| 2280 | Train 36 | Test Male 40 | 0.7 | 0.71 | 0.66 | 3304 | Train 52 | Test Male 40 | 0.68 | 0.67 | 0.63 |
| 2281 | Train 36 | Test Male 41 | 0.75 | 0.82 | 0.81 | 3305 | Train 52 | Test Male 41 | 0.73 | 0.72 | 0.68 |
| 2282 | Train 36 | Test Male 42 | 0.76 | 0.82 | 0.82 | 3306 | Train 52 | Test Male 42 | 0.72 | 0.71 | 0.65 |
| 2283 | Train 36 | Test Male 43 | 0.75 | 0.82 | 0.81 | 3307 | Train 52 | Test Male 43 | 0.73 | 0.72 | 0.68 |
| 2284 | Train 36 | Test Male 44 | 0.76 | 0.82 | 0.82 | 3308 | Train 52 | Test Male 44 | 0.72 | 0.71 | 0.65 |
| 2285 | Train 36 | Test Male 45 | 0.72 | 0.74 | 0.72 | 3309 | Train 52 | Test Male 45 | 0.7 | 0.7 | 0.65 |
| 2286 | Train 36 | Test Male 46 | 0.7 | 0.71 | 0.66 | 3310 | Train 52 | Test Male 46 | 0.68 | 0.67 | 0.63 |
| 2287 | Train 36 | Test Male 47 | 0.71 | 0.74 | 0.71 | 3311 | Train 52 | Test Male 47 | 0.7 | 0.69 | 0.65 |
| 2288 | Train 36 | Test Male 48 | 0.7 | 0.71 | 0.66 | 3312 | Train 52 | Test Male 48 | 0.68 | 0.67 | 0.63 |
| 2289 | Train 36 | Test Male 49 | 0.77 | 0.82 | 0.84 | 3313 | Train 52 | Test Male 49 | 0.78 | 0.85 | 0.89 |
| 2290 | Train 36 | Test Male 50 | 0.77 | 0.82 | 0.84 | 3314 | Train 52 | Test Male 50 | 0.78 | 0.85 | 0.89 |
| 2291 | Train 36 | Test Male 51 | 0.77 | 0.82 | 0.84 | 3315 | Train 52 | Test Male 51 | 0.78 | 0.85 | 0.89 |
| 2292 | Train 36 | Test Male 52 | 0.76 | 0.82 | 0.84 | 3316 | Train 52 | Test Male 52 | 0.78 | 0.85 | 0.89 |
| 2293 | Train 36 | Test Male 53 | 0.74 | 0.78 | 0.69 | 3317 | Train 52 | Test Male 53 | 0.72 | 0.74 | 0.75 |
| 2294 | Train 36 | Test Male 54 | 0.71 | 0.72 | 0.66 | 3318 | Train 52 | Test Male 54 | 0.69 | 0.69 | 0.66 |
| 2295 | Train 36 | Test Male 55 | 0.74 | 0.78 | 0.69 | 3319 | Train 52 | Test Male 55 | 0.72 | 0.75 | 0.75 |
| 2296 | Train 36 | Test Male 56 | 0.71 | 0.71 | 0.66 | 3320 | Train 52 | Test Male 56 | 0.7 | 0.68 | 0.66 |
| 2297 | Train 36 | Test Male 57 | 0.77 | 0.82 | 0.82 | 3321 | Train 52 | Test Male 57 | 0.78 | 0.84 | 0.83 |
| 2298 | Train 36 | Test Male 58 | 0.77 | 0.82 | 0.82 | 3322 | Train 52 | Test Male 58 | 0.78 | 0.84 | 0.83 |
| 2299 | Train 36 | Test Male 59 | 0.77 | 0.81 | 0.82 | 3323 | Train 52 | Test Male 59 | 0.78 | 0.84 | 0.83 |
| 2300 | Train 36 | Test Male 60 | 0.76 | 0.81 | 0.82 | 3324 | Train 52 | Test Male 60 | 0.78 | 0.84 | 0.83 |
| 2301 | Train 36 | Test Male 61 | 0.73 | 0.77 | 0.66 | 3325 | Train 52 | Test Male 61 | 0.72 | 0.74 | 0.74 |
| 2302 | Train 36 | Test Male 62 | 0.71 | 0.72 | 0.66 | 3326 | Train 52 | Test Male 62 | 0.69 | 0.69 | 0.66 |
| 2303 | Train 36 | Test Male 63 | 0.73 | 0.77 | 0.69 | 3327 | Train 52 | Test Male 63 | 0.72 | 0.74 | 0.74 |
| 2304 | Train 36 | Test Male 64 | 0.71 | 0.71 | 0.66 | 3328 | Train 52 | Test Male 64 | 0.69 | 0.68 | 0.66 |
| 2305 | Train 37 | Test Male 1 | 0.75 | 0.81 | 0.82 | 3329 | Train 53 | Test Male 1 | 0.73 | 0.75 | 0.67 |
| 2306 | Train 37 | Test Male 2 | 0.75 | 0.79 | 0.79 | 3330 | Train 53 | Test Male 2 | 0.73 | 0.73 | 0.65 |
| 2307 | Train 37 | Test Male 3 | 0.75 | 0.81 | 0.82 | 3331 | Train 53 | Test Male 3 | 0.73 | 0.75 | 0.67 |
| 2308 | Train 37 | Test Male 4 | 0.75 | 0.79 | 0.79 | 3332 | Train 53 | Test Male 4 | 0.73 | 0.73 | 0.65 |
| 2309 | Train 37 | Test Male 5 | 0.74 | 0.81 | 0.82 | 3333 | Train 53 | Test Male 5 | 0.72 | 0.75 | 0.68 |
| 2310 | Train 37 | Test Male 6 | 0.7 | 0.71 | 0.69 | 3334 | Train 53 | Test Male 6 | 0.7 | 0.69 | 0.63 |
| 2311 | Train 37 | Test Male 7 | 0.74 | 0.81 | 0.82 | 3335 | Train 53 | Test Male 7 | 0.72 | 0.74 | 0.68 |
| 2312 | Train 37 | Test Male 8 | 0.7 | 0.71 | 0.69 | 3336 | Train 53 | Test Male 8 | 0.7 | 0.69 | 0.64 |
| 2313 | Train 37 | Test Male 9 | 0.75 | 0.8 | 0.8 | 3337 | Train 53 | Test Male 9 | 0.73 | 0.76 | 0.67 |
| 2314 | Train 37 | Test Male 10 | 0.75 | 0.78 | 0.77 | 3338 | Train 53 | Test Male 10 | 0.73 | 0.73 | 0.66 |
| 2315 | Train 37 | Test Male 11 | 0.75 | 0.8 | 0.8 | 3339 | Train 53 | Test Male 11 | 0.73 | 0.75 | 0.67 |
| 2316 | Train 37 | Test Male 12 | 0.75 | 0.78 | 0.77 | 3340 | Train 53 | Test Male 12 | 0.73 | 0.73 | 0.66 |
| 2317 | Train 37 | Test Male 13 | 0.74 | 0.8 | 0.8 | 3341 | Train 53 | Test Male 13 | 0.72 | 0.75 | 0.68 |
| 2318 | Train 37 | Test Male 14 | 0.7 | 0.71 | 0.69 | 3342 | Train 53 | Test Male 14 | 0.7 | 0.69 | 0.64 |
| 2319 | Train 37 | Test Male 15 | 0.74 | 0.8 | 0.79 | 3343 | Train 53 | Test Male 15 | 0.72 | 0.74 | 0.68 |
| 2320 | Train 37 | Test Male 16 | 0.7 | 0.71 | 0.69 | 3344 | Train 53 | Test Male 16 | 0.7 | 0.69 | 0.64 |
| 2321 | Train 37 | Test Male 17 | 0.75 | 0.81 | 0.82 | 3345 | Train 53 | Test Male 17 | 0.73 | 0.76 | 0.67 |
| 2322 | Train 37 | Test Male 18 | 0.75 | 0.79 | 0.79 | 3346 | Train 53 | Test Male 18 | 0.73 | 0.73 | 0.65 |
| 2323 | Train 37 | Test Male 19 | 0.75 | 0.81 | 0.82 | 3347 | Train 53 | Test Male 19 | 0.73 | 0.75 | 0.67 |
| 2324 | Train 37 | Test Male 20 | 0.75 | 0.79 | 0.79 | 3348 | Train 53 | Test Male 20 | 0.73 | 0.73 | 0.65 |
| 2325 | Train 37 | Test Male 21 | 0.74 | 0.81 | 0.82 | 3349 | Train 53 | Test Male 21 | 0.72 | 0.75 | 0.68 |
| 2326 | Train 37 | Test Male 22 | 0.7 | 0.71 | 0.69 | 3350 | Train 53 | Test Male 22 | 0.7 | 0.69 | 0.63 |
| 2327 | Train 37 | Test Male 23 | 0.74 | 0.81 | 0.82 | 3351 | Train 53 | Test Male 23 | 0.72 | 0.74 | 0.68 |
| 2328 | Train 37 | Test Male 24 | 0.7 | 0.71 | 0.69 | 3352 | Train 53 | Test Male 24 | 0.7 | 0.69 | 0.64 |
| 2329 | Train 37 | Test Male 25 | 0.75 | 0.8 | 0.8 | 3353 | Train 53 | Test Male 25 | 0.73 | 0.76 | 0.67 |
| 2330 | Train 37 | Test Male 26 | 0.75 | 0.78 | 0.77 | 3354 | Train 53 | Test Male 26 | 0.73 | 0.73 | 0.66 |
| 2331 | Train 37 | Test Male 27 | 0.75 | 0.8 | 0.8 | 3355 | Train 53 | Test Male 27 | 0.73 | 0.75 | 0.67 |
| 2332 | Train 37 | Test Male 28 | 0.75 | 0.78 | 0.77 | 3356 | Train 53 | Test Male 28 | 0.73 | 0.73 | 0.66 |
| 2333 | Train 37 | Test Male 29 | 0.74 | 0.8 | 0.8 | 3357 | Train 53 | Test Male 29 | 0.72 | 0.75 | 0.68 |
| 2334 | Train 37 | Test Male 30 | 0.7 | 0.71 | 0.69 | 3358 | Train 53 | Test Male 30 | 0.7 | 0.69 | 0.64 |
| 2335 | Train 37 | Test Male 31 | 0.74 | 0.8 | 0.79 | 3359 | Train 53 | Test Male 31 | 0.72 | 0.75 | 0.68 |
| 2336 | Train 37 | Test Male 32 | 0.7 | 0.71 | 0.69 | 3360 | Train 53 | Test Male 32 | 0.7 | 0.69 | 0.64 |
| 2337 | Train 37 | Test Male 33 | 0.75 | 0.81 | 0.82 | 3361 | Train 53 | Test Male 33 | 0.73 | 0.75 | 0.67 |
| 2338 | Train 37 | Test Male 34 | 0.75 | 0.79 | 0.79 | 3362 | Train 53 | Test Male 34 | 0.73 | 0.73 | 0.65 |
| 2339 | Train 37 | Test Male 35 | 0.75 | 0.81 | 0.82 | 3363 | Train 53 | Test Male 35 | 0.73 | 0.75 | 0.67 |
| 2340 | Train 37 | Test Male 36 | 0.75 | 0.79 | 0.79 | 3364 | Train 53 | Test Male 36 | 0.73 | 0.73 | 0.65 |
| 2341 | Train 37 | Test Male 37 | 0.74 | 0.81 | 0.82 | 3365 | Train 53 | Test Male 37 | 0.72 | 0.75 | 0.68 |
| 2342 | Train 37 | Test Male 38 | 0.7 | 0.71 | 0.69 | 3366 | Train 53 | Test Male 38 | 0.7 | 0.69 | 0.63 |
| 2343 | Train 37 | Test Male 39 | 0.74 | 0.81 | 0.82 | 3367 | Train 53 | Test Male 39 | 0.72 | 0.74 | 0.68 |
| 2344 | Train 37 | Test Male 40 | 0.7 | 0.71 | 0.69 | 3368 | Train 53 | Test Male 40 | 0.7 | 0.69 | 0.64 |
| 2345 | Train 37 | Test Male 41 | 0.75 | 0.8 | 0.8 | 3369 | Train 53 | Test Male 41 | 0.73 | 0.75 | 0.66 |
| 2346 | Train 37 | Test Male 42 | 0.75 | 0.78 | 0.77 | 3370 | Train 53 | Test Male 42 | 0.73 | 0.73 | 0.66 |
| 2347 | Train 37 | Test Male 43 | 0.75 | 0.8 | 0.8 | 3371 | Train 53 | Test Male 43 | 0.73 | 0.75 | 0.66 |
| 2348 | Train 37 | Test Male 44 | 0.75 | 0.78 | 0.77 | 3372 | Train 53 | Test Male 44 | 0.73 | 0.73 | 0.66 |
| 2349 | Train 37 | Test Male 45 | 0.74 | 0.8 | 0.8 | 3373 | Train 53 | Test Male 45 | 0.72 | 0.75 | 0.68 |
| 2350 | Train 37 | Test Male 46 | 0.7 | 0.71 | 0.69 | 3374 | Train 53 | Test Male 46 | 0.7 | 0.69 | 0.64 |
| 2351 | Train 37 | Test Male 47 | 0.73 | 0.8 | 0.79 | 3375 | Train 53 | Test Male 47 | 0.72 | 0.74 | 0.68 |
| 2352 | Train 37 | Test Male 48 | 0.7 | 0.71 | 0.69 | 3376 | Train 53 | Test Male 48 | 0.7 | 0.69 | 0.64 |
| 2353 | Train 37 | Test Male 49 | 0.76 | 0.81 | 0.83 | 3377 | Train 53 | Test Male 49 | 0.77 | 0.82 | 0.8 |
| 2354 | Train 37 | Test Male 50 | 0.76 | 0.81 | 0.83 | 3378 | Train 53 | Test Male 50 | 0.77 | 0.82 | 0.8 |
| 2355 | Train 37 | Test Male 51 | 0.76 | 0.81 | 0.82 | 3379 | Train 53 | Test Male 51 | 0.77 | 0.82 | 0.8 |
| 2356 | Train 37 | Test Male 52 | 0.76 | 0.81 | 0.82 | 3380 | Train 53 | Test Male 52 | 0.77 | 0.82 | 0.8 |
| 2357 | Train 37 | Test Male 53 | 0.76 | 0.81 | 0.79 | 3381 | Train 53 | Test Male 53 | 0.77 | 0.84 | 0.87 |
| 2358 | Train 37 | Test Male 54 | 0.71 | 0.71 | 0.69 | 3382 | Train 53 | Test Male 54 | 0.71 | 0.72 | 0.67 |
| 2359 | Train 37 | Test Male 55 | 0.76 | 0.81 | 0.78 | 3383 | Train 53 | Test Male 55 | 0.76 | 0.84 | 0.86 |
| 2360 | Train 37 | Test Male 56 | 0.71 | 0.71 | 0.7 | 3384 | Train 53 | Test Male 56 | 0.71 | 0.71 | 0.67 |
| 2361 | Train 37 | Test Male 57 | 0.76 | 0.81 | 0.81 | 3385 | Train 53 | Test Male 57 | 0.77 | 0.82 | 0.8 |
| 2362 | Train 37 | Test Male 58 | 0.76 | 0.81 | 0.81 | 3386 | Train 53 | Test Male 58 | 0.77 | 0.82 | 0.8 |
| 2363 | Train 37 | Test Male 59 | 0.76 | 0.8 | 0.8 | 3387 | Train 53 | Test Male 59 | 0.77 | 0.82 | 0.8 |
| 2364 | Train 37 | Test Male 60 | 0.76 | 0.8 | 0.8 | 3388 | Train 53 | Test Male 60 | 0.77 | 0.81 | 0.8 |
| 2365 | Train 37 | Test Male 61 | 0.75 | 0.8 | 0.73 | 3389 | Train 53 | Test Male 61 | 0.76 | 0.83 | 0.75 |
| 2366 | Train 37 | Test Male 62 | 0.71 | 0.71 | 0.69 | 3390 | Train 53 | Test Male 62 | 0.71 | 0.72 | 0.67 |
| 2367 | Train 37 | Test Male 63 | 0.75 | 0.8 | 0.78 | 3391 | Train 53 | Test Male 63 | 0.76 | 0.83 | 0.83 |
| 2368 | Train 37 | Test Male 64 | 0.71 | 0.71 | 0.7 | 3392 | Train 53 | Test Male 64 | 0.71 | 0.71 | 0.67 |
| 2369 | Train 38 | Test Male 1 | 0.73 | 0.78 | 0.76 | 3393 | Train 54 | Test Male 1 | 0.72 | 0.74 | 0.67 |
| 2370 | Train 38 | Test Male 2 | 0.74 | 0.78 | 0.77 | 3394 | Train 54 | Test Male 2 | 0.73 | 0.75 | 0.68 |
| 2371 | Train 38 | Test Male 3 | 0.73 | 0.78 | 0.76 | 3395 | Train 54 | Test Male 3 | 0.71 | 0.74 | 0.67 |
| 2372 | Train 38 | Test Male 4 | 0.74 | 0.78 | 0.77 | 3396 | Train 54 | Test Male 4 | 0.72 | 0.75 | 0.68 |
| 2373 | Train 38 | Test Male 5 | 0.72 | 0.77 | 0.76 | 3397 | Train 54 | Test Male 5 | 0.71 | 0.74 | 0.68 |
| 2374 | Train 38 | Test Male 6 | 0.73 | 0.79 | 0.79 | 3398 | Train 54 | Test Male 6 | 0.72 | 0.75 | 0.7 |
| 2375 | Train 38 | Test Male 7 | 0.72 | 0.77 | 0.75 | 3399 | Train 54 | Test Male 7 | 0.71 | 0.74 | 0.68 |
| 2376 | Train 38 | Test Male 8 | 0.73 | 0.78 | 0.78 | 3400 | Train 54 | Test Male 8 | 0.72 | 0.75 | 0.7 |
| 2377 | Train 38 | Test Male 9 | 0.73 | 0.78 | 0.77 | 3401 | Train 54 | Test Male 9 | 0.71 | 0.75 | 0.68 |
| 2378 | Train 38 | Test Male 10 | 0.74 | 0.78 | 0.77 | 3402 | Train 54 | Test Male 10 | 0.73 | 0.75 | 0.69 |
| 2379 | Train 38 | Test Male 11 | 0.73 | 0.78 | 0.77 | 3403 | Train 54 | Test Male 11 | 0.71 | 0.75 | 0.68 |
| 2380 | Train 38 | Test Male 12 | 0.74 | 0.78 | 0.77 | 3404 | Train 54 | Test Male 12 | 0.72 | 0.75 | 0.68 |
| 2381 | Train 38 | Test Male 13 | 0.72 | 0.77 | 0.76 | 3405 | Train 54 | Test Male 13 | 0.71 | 0.74 | 0.69 |
| 2382 | Train 38 | Test Male 14 | 0.74 | 0.78 | 0.79 | 3406 | Train 54 | Test Male 14 | 0.72 | 0.75 | 0.7 |
| 2383 | Train 38 | Test Male 15 | 0.72 | 0.77 | 0.76 | 3407 | Train 54 | Test Male 15 | 0.71 | 0.74 | 0.69 |
| 2384 | Train 38 | Test Male 16 | 0.73 | 0.78 | 0.78 | 3408 | Train 54 | Test Male 16 | 0.72 | 0.75 | 0.7 |
| 2385 | Train 38 | Test Male 17 | 0.73 | 0.78 | 0.76 | 3409 | Train 54 | Test Male 17 | 0.72 | 0.74 | 0.67 |
| 2386 | Train 38 | Test Male 18 | 0.74 | 0.78 | 0.77 | 3410 | Train 54 | Test Male 18 | 0.73 | 0.75 | 0.68 |
| 2387 | Train 38 | Test Male 19 | 0.73 | 0.78 | 0.76 | 3411 | Train 54 | Test Male 19 | 0.71 | 0.74 | 0.67 |
| 2388 | Train 38 | Test Male 20 | 0.74 | 0.78 | 0.77 | 3412 | Train 54 | Test Male 20 | 0.72 | 0.75 | 0.68 |
| 2389 | Train 38 | Test Male 21 | 0.72 | 0.77 | 0.76 | 3413 | Train 54 | Test Male 21 | 0.71 | 0.74 | 0.68 |
| 2390 | Train 38 | Test Male 22 | 0.73 | 0.79 | 0.79 | 3414 | Train 54 | Test Male 22 | 0.72 | 0.75 | 0.7 |
| 2391 | Train 38 | Test Male 23 | 0.72 | 0.77 | 0.75 | 3415 | Train 54 | Test Male 23 | 0.71 | 0.74 | 0.68 |
| 2392 | Train 38 | Test Male 24 | 0.73 | 0.78 | 0.78 | 3416 | Train 54 | Test Male 24 | 0.72 | 0.75 | 0.7 |
| 2393 | Train 38 | Test Male 25 | 0.73 | 0.78 | 0.77 | 3417 | Train 54 | Test Male 25 | 0.71 | 0.75 | 0.68 |
| 2394 | Train 38 | Test Male 26 | 0.74 | 0.78 | 0.77 | 3418 | Train 54 | Test Male 26 | 0.73 | 0.75 | 0.69 |
| 2395 | Train 38 | Test Male 27 | 0.73 | 0.78 | 0.77 | 3419 | Train 54 | Test Male 27 | 0.71 | 0.75 | 0.68 |
| 2396 | Train 38 | Test Male 28 | 0.74 | 0.78 | 0.77 | 3420 | Train 54 | Test Male 28 | 0.72 | 0.75 | 0.68 |
| 2397 | Train 38 | Test Male 29 | 0.72 | 0.77 | 0.76 | 3421 | Train 54 | Test Male 29 | 0.71 | 0.74 | 0.69 |
| 2398 | Train 38 | Test Male 30 | 0.74 | 0.78 | 0.79 | 3422 | Train 54 | Test Male 30 | 0.72 | 0.75 | 0.7 |
| 2399 | Train 38 | Test Male 31 | 0.72 | 0.77 | 0.76 | 3423 | Train 54 | Test Male 31 | 0.71 | 0.74 | 0.69 |
| 2400 | Train 38 | Test Male 32 | 0.73 | 0.78 | 0.78 | 3424 | Train 54 | Test Male 32 | 0.72 | 0.75 | 0.7 |
| 2401 | Train 38 | Test Male 33 | 0.73 | 0.78 | 0.76 | 3425 | Train 54 | Test Male 33 | 0.72 | 0.74 | 0.67 |
| 2402 | Train 38 | Test Male 34 | 0.74 | 0.78 | 0.77 | 3426 | Train 54 | Test Male 34 | 0.72 | 0.75 | 0.68 |
| 2403 | Train 38 | Test Male 35 | 0.73 | 0.78 | 0.76 | 3427 | Train 54 | Test Male 35 | 0.71 | 0.74 | 0.67 |
| 2404 | Train 38 | Test Male 36 | 0.74 | 0.78 | 0.77 | 3428 | Train 54 | Test Male 36 | 0.72 | 0.75 | 0.68 |
| 2405 | Train 38 | Test Male 37 | 0.72 | 0.77 | 0.76 | 3429 | Train 54 | Test Male 37 | 0.71 | 0.74 | 0.68 |
| 2406 | Train 38 | Test Male 38 | 0.73 | 0.79 | 0.79 | 3430 | Train 54 | Test Male 38 | 0.72 | 0.75 | 0.7 |
| 2407 | Train 38 | Test Male 39 | 0.72 | 0.77 | 0.75 | 3431 | Train 54 | Test Male 39 | 0.71 | 0.74 | 0.68 |
| 2408 | Train 38 | Test Male 40 | 0.73 | 0.78 | 0.78 | 3432 | Train 54 | Test Male 40 | 0.72 | 0.75 | 0.7 |
| 2409 | Train 38 | Test Male 41 | 0.73 | 0.78 | 0.77 | 3433 | Train 54 | Test Male 41 | 0.71 | 0.75 | 0.68 |
| 2410 | Train 38 | Test Male 42 | 0.74 | 0.78 | 0.77 | 3434 | Train 54 | Test Male 42 | 0.73 | 0.75 | 0.69 |
| 2411 | Train 38 | Test Male 43 | 0.73 | 0.78 | 0.77 | 3435 | Train 54 | Test Male 43 | 0.71 | 0.75 | 0.68 |
| 2412 | Train 38 | Test Male 44 | 0.74 | 0.78 | 0.77 | 3436 | Train 54 | Test Male 44 | 0.72 | 0.75 | 0.68 |
| 2413 | Train 38 | Test Male 45 | 0.72 | 0.77 | 0.76 | 3437 | Train 54 | Test Male 45 | 0.71 | 0.74 | 0.69 |
| 2414 | Train 38 | Test Male 46 | 0.74 | 0.78 | 0.79 | 3438 | Train 54 | Test Male 46 | 0.72 | 0.75 | 0.7 |
| 2415 | Train 38 | Test Male 47 | 0.72 | 0.77 | 0.76 | 3439 | Train 54 | Test Male 47 | 0.71 | 0.74 | 0.69 |
| 2416 | Train 38 | Test Male 48 | 0.73 | 0.78 | 0.78 | 3440 | Train 54 | Test Male 48 | 0.72 | 0.75 | 0.7 |
| 2417 | Train 38 | Test Male 49 | 0.76 | 0.8 | 0.78 | 3441 | Train 54 | Test Male 49 | 0.77 | 0.8 | 0.79 |
| 2418 | Train 38 | Test Male 50 | 0.76 | 0.8 | 0.78 | 3442 | Train 54 | Test Male 50 | 0.77 | 0.8 | 0.79 |
| 2419 | Train 38 | Test Male 51 | 0.76 | 0.79 | 0.78 | 3443 | Train 54 | Test Male 51 | 0.77 | 0.8 | 0.79 |
| 2420 | Train 38 | Test Male 52 | 0.76 | 0.79 | 0.78 | 3444 | Train 54 | Test Male 52 | 0.77 | 0.8 | 0.79 |
| 2421 | Train 38 | Test Male 53 | 0.75 | 0.81 | 0.8 | 3445 | Train 54 | Test Male 53 | 0.76 | 0.82 | 0.81 |
| 2422 | Train 38 | Test Male 54 | 0.74 | 0.78 | 0.79 | 3446 | Train 54 | Test Male 54 | 0.75 | 0.8 | 0.81 |
| 2423 | Train 38 | Test Male 55 | 0.75 | 0.8 | 0.8 | 3447 | Train 54 | Test Male 55 | 0.76 | 0.82 | 0.81 |
| 2424 | Train 38 | Test Male 56 | 0.74 | 0.78 | 0.78 | 3448 | Train 54 | Test Male 56 | 0.74 | 0.8 | 0.8 |
| 2425 | Train 38 | Test Male 57 | 0.76 | 0.8 | 0.79 | 3449 | Train 54 | Test Male 57 | 0.77 | 0.81 | 0.8 |
| 2426 | Train 38 | Test Male 58 | 0.76 | 0.8 | 0.79 | 3450 | Train 54 | Test Male 58 | 0.77 | 0.81 | 0.8 |
| 2427 | Train 38 | Test Male 59 | 0.76 | 0.8 | 0.79 | 3451 | Train 54 | Test Male 59 | 0.77 | 0.8 | 0.8 |
| 2428 | Train 38 | Test Male 60 | 0.76 | 0.8 | 0.79 | 3452 | Train 54 | Test Male 60 | 0.77 | 0.8 | 0.8 |
| 2429 | Train 38 | Test Male 61 | 0.75 | 0.81 | 0.76 | 3453 | Train 54 | Test Male 61 | 0.76 | 0.82 | 0.76 |
| 2430 | Train 38 | Test Male 62 | 0.74 | 0.78 | 0.79 | 3454 | Train 54 | Test Male 62 | 0.75 | 0.8 | 0.81 |
| 2431 | Train 38 | Test Male 63 | 0.75 | 0.8 | 0.81 | 3455 | Train 54 | Test Male 63 | 0.76 | 0.82 | 0.82 |
| 2432 | Train 38 | Test Male 64 | 0.74 | 0.78 | 0.78 | 3456 | Train 54 | Test Male 64 | 0.74 | 0.79 | 0.8 |
| 2433 | Train 39 | Test Male 1 | 0.75 | 0.81 | 0.82 | 3457 | Train 55 | Test Male 1 | 0.73 | 0.76 | 0.72 |
| 2434 | Train 39 | Test Male 2 | 0.75 | 0.79 | 0.79 | 3458 | Train 55 | Test Male 2 | 0.73 | 0.74 | 0.7 |
| 2435 | Train 39 | Test Male 3 | 0.75 | 0.81 | 0.82 | 3459 | Train 55 | Test Male 3 | 0.73 | 0.76 | 0.72 |
| 2436 | Train 39 | Test Male 4 | 0.75 | 0.79 | 0.79 | 3460 | Train 55 | Test Male 4 | 0.73 | 0.74 | 0.7 |
| 2437 | Train 39 | Test Male 5 | 0.74 | 0.81 | 0.82 | 3461 | Train 55 | Test Male 5 | 0.72 | 0.75 | 0.72 |
| 2438 | Train 39 | Test Male 6 | 0.7 | 0.71 | 0.69 | 3462 | Train 55 | Test Male 6 | 0.69 | 0.7 | 0.63 |
| 2439 | Train 39 | Test Male 7 | 0.74 | 0.81 | 0.82 | 3463 | Train 55 | Test Male 7 | 0.72 | 0.75 | 0.72 |
| 2440 | Train 39 | Test Male 8 | 0.7 | 0.71 | 0.69 | 3464 | Train 55 | Test Male 8 | 0.69 | 0.69 | 0.63 |
| 2441 | Train 39 | Test Male 9 | 0.75 | 0.8 | 0.8 | 3465 | Train 55 | Test Male 9 | 0.73 | 0.76 | 0.71 |
| 2442 | Train 39 | Test Male 10 | 0.75 | 0.78 | 0.77 | 3466 | Train 55 | Test Male 10 | 0.73 | 0.74 | 0.69 |
| 2443 | Train 39 | Test Male 11 | 0.75 | 0.8 | 0.8 | 3467 | Train 55 | Test Male 11 | 0.73 | 0.76 | 0.71 |
| 2444 | Train 39 | Test Male 12 | 0.75 | 0.78 | 0.77 | 3468 | Train 55 | Test Male 12 | 0.73 | 0.74 | 0.69 |
| 2445 | Train 39 | Test Male 13 | 0.74 | 0.8 | 0.8 | 3469 | Train 55 | Test Male 13 | 0.72 | 0.75 | 0.71 |
| 2446 | Train 39 | Test Male 14 | 0.7 | 0.71 | 0.69 | 3470 | Train 55 | Test Male 14 | 0.69 | 0.7 | 0.63 |
| 2447 | Train 39 | Test Male 15 | 0.74 | 0.8 | 0.8 | 3471 | Train 55 | Test Male 15 | 0.72 | 0.75 | 0.71 |
| 2448 | Train 39 | Test Male 16 | 0.7 | 0.71 | 0.69 | 3472 | Train 55 | Test Male 16 | 0.69 | 0.7 | 0.63 |
| 2449 | Train 39 | Test Male 17 | 0.75 | 0.81 | 0.82 | 3473 | Train 55 | Test Male 17 | 0.73 | 0.76 | 0.72 |
| 2450 | Train 39 | Test Male 18 | 0.75 | 0.79 | 0.79 | 3474 | Train 55 | Test Male 18 | 0.73 | 0.74 | 0.7 |
| 2451 | Train 39 | Test Male 19 | 0.75 | 0.81 | 0.82 | 3475 | Train 55 | Test Male 19 | 0.73 | 0.76 | 0.72 |
| 2452 | Train 39 | Test Male 20 | 0.75 | 0.79 | 0.79 | 3476 | Train 55 | Test Male 20 | 0.73 | 0.74 | 0.7 |
| 2453 | Train 39 | Test Male 21 | 0.74 | 0.81 | 0.82 | 3477 | Train 55 | Test Male 21 | 0.72 | 0.75 | 0.72 |
| 2454 | Train 39 | Test Male 22 | 0.7 | 0.71 | 0.69 | 3478 | Train 55 | Test Male 22 | 0.69 | 0.7 | 0.63 |
| 2455 | Train 39 | Test Male 23 | 0.74 | 0.81 | 0.82 | 3479 | Train 55 | Test Male 23 | 0.72 | 0.75 | 0.72 |
| 2456 | Train 39 | Test Male 24 | 0.7 | 0.71 | 0.69 | 3480 | Train 55 | Test Male 24 | 0.69 | 0.7 | 0.63 |
| 2457 | Train 39 | Test Male 25 | 0.75 | 0.8 | 0.8 | 3481 | Train 55 | Test Male 25 | 0.73 | 0.76 | 0.71 |
| 2458 | Train 39 | Test Male 26 | 0.75 | 0.78 | 0.77 | 3482 | Train 55 | Test Male 26 | 0.73 | 0.74 | 0.69 |
| 2459 | Train 39 | Test Male 27 | 0.75 | 0.8 | 0.8 | 3483 | Train 55 | Test Male 27 | 0.73 | 0.76 | 0.71 |
| 2460 | Train 39 | Test Male 28 | 0.75 | 0.78 | 0.77 | 3484 | Train 55 | Test Male 28 | 0.73 | 0.74 | 0.69 |
| 2461 | Train 39 | Test Male 29 | 0.74 | 0.8 | 0.8 | 3485 | Train 55 | Test Male 29 | 0.72 | 0.75 | 0.71 |
| 2462 | Train 39 | Test Male 30 | 0.7 | 0.71 | 0.69 | 3486 | Train 55 | Test Male 30 | 0.69 | 0.7 | 0.63 |
| 2463 | Train 39 | Test Male 31 | 0.74 | 0.8 | 0.8 | 3487 | Train 55 | Test Male 31 | 0.72 | 0.75 | 0.71 |
| 2464 | Train 39 | Test Male 32 | 0.7 | 0.71 | 0.69 | 3488 | Train 55 | Test Male 32 | 0.69 | 0.7 | 0.63 |
| 2465 | Train 39 | Test Male 33 | 0.75 | 0.81 | 0.82 | 3489 | Train 55 | Test Male 33 | 0.73 | 0.76 | 0.72 |
| 2466 | Train 39 | Test Male 34 | 0.75 | 0.79 | 0.79 | 3490 | Train 55 | Test Male 34 | 0.73 | 0.74 | 0.69 |
| 2467 | Train 39 | Test Male 35 | 0.75 | 0.81 | 0.82 | 3491 | Train 55 | Test Male 35 | 0.73 | 0.76 | 0.72 |
| 2468 | Train 39 | Test Male 36 | 0.75 | 0.79 | 0.79 | 3492 | Train 55 | Test Male 36 | 0.73 | 0.74 | 0.7 |
| 2469 | Train 39 | Test Male 37 | 0.74 | 0.81 | 0.82 | 3493 | Train 55 | Test Male 37 | 0.72 | 0.75 | 0.72 |
| 2470 | Train 39 | Test Male 38 | 0.7 | 0.71 | 0.69 | 3494 | Train 55 | Test Male 38 | 0.69 | 0.7 | 0.63 |
| 2471 | Train 39 | Test Male 39 | 0.74 | 0.81 | 0.82 | 3495 | Train 55 | Test Male 39 | 0.72 | 0.75 | 0.72 |
| 2472 | Train 39 | Test Male 40 | 0.7 | 0.71 | 0.69 | 3496 | Train 55 | Test Male 40 | 0.69 | 0.7 | 0.63 |
| 2473 | Train 39 | Test Male 41 | 0.75 | 0.8 | 0.8 | 3497 | Train 55 | Test Male 41 | 0.73 | 0.76 | 0.71 |
| 2474 | Train 39 | Test Male 42 | 0.75 | 0.78 | 0.77 | 3498 | Train 55 | Test Male 42 | 0.73 | 0.74 | 0.69 |
| 2475 | Train 39 | Test Male 43 | 0.75 | 0.8 | 0.8 | 3499 | Train 55 | Test Male 43 | 0.73 | 0.76 | 0.71 |
| 2476 | Train 39 | Test Male 44 | 0.75 | 0.78 | 0.77 | 3500 | Train 55 | Test Male 44 | 0.73 | 0.74 | 0.69 |
| 2477 | Train 39 | Test Male 45 | 0.74 | 0.8 | 0.8 | 3501 | Train 55 | Test Male 45 | 0.72 | 0.75 | 0.71 |
| 2478 | Train 39 | Test Male 46 | 0.7 | 0.71 | 0.69 | 3502 | Train 55 | Test Male 46 | 0.69 | 0.7 | 0.63 |
| 2479 | Train 39 | Test Male 47 | 0.74 | 0.8 | 0.79 | 3503 | Train 55 | Test Male 47 | 0.72 | 0.75 | 0.71 |
| 2480 | Train 39 | Test Male 48 | 0.7 | 0.71 | 0.69 | 3504 | Train 55 | Test Male 48 | 0.69 | 0.7 | 0.63 |
| 2481 | Train 39 | Test Male 49 | 0.76 | 0.81 | 0.83 | 3505 | Train 55 | Test Male 49 | 0.77 | 0.83 | 0.83 |
| 2482 | Train 39 | Test Male 50 | 0.76 | 0.81 | 0.83 | 3506 | Train 55 | Test Male 50 | 0.77 | 0.83 | 0.83 |
| 2483 | Train 39 | Test Male 51 | 0.76 | 0.81 | 0.83 | 3507 | Train 55 | Test Male 51 | 0.77 | 0.83 | 0.83 |
| 2484 | Train 39 | Test Male 52 | 0.76 | 0.81 | 0.83 | 3508 | Train 55 | Test Male 52 | 0.77 | 0.83 | 0.83 |
| 2485 | Train 39 | Test Male 53 | 0.76 | 0.81 | 0.78 | 3509 | Train 55 | Test Male 53 | 0.77 | 0.84 | 0.77 |
| 2486 | Train 39 | Test Male 54 | 0.71 | 0.71 | 0.7 | 3510 | Train 55 | Test Male 54 | 0.71 | 0.72 | 0.66 |
| 2487 | Train 39 | Test Male 55 | 0.76 | 0.8 | 0.78 | 3511 | Train 55 | Test Male 55 | 0.76 | 0.84 | 0.76 |
| 2488 | Train 39 | Test Male 56 | 0.71 | 0.71 | 0.7 | 3512 | Train 55 | Test Male 56 | 0.7 | 0.71 | 0.66 |
| 2489 | Train 39 | Test Male 57 | 0.76 | 0.81 | 0.81 | 3513 | Train 55 | Test Male 57 | 0.77 | 0.83 | 0.82 |
| 2490 | Train 39 | Test Male 58 | 0.76 | 0.81 | 0.81 | 3514 | Train 55 | Test Male 58 | 0.77 | 0.83 | 0.82 |
| 2491 | Train 39 | Test Male 59 | 0.76 | 0.8 | 0.81 | 3515 | Train 55 | Test Male 59 | 0.77 | 0.83 | 0.82 |
| 2492 | Train 39 | Test Male 60 | 0.76 | 0.8 | 0.81 | 3516 | Train 55 | Test Male 60 | 0.77 | 0.83 | 0.82 |
| 2493 | Train 39 | Test Male 61 | 0.76 | 0.8 | 0.74 | 3517 | Train 55 | Test Male 61 | 0.76 | 0.83 | 0.71 |
| 2494 | Train 39 | Test Male 62 | 0.71 | 0.71 | 0.7 | 3518 | Train 55 | Test Male 62 | 0.71 | 0.72 | 0.65 |
| 2495 | Train 39 | Test Male 63 | 0.75 | 0.8 | 0.77 | 3519 | Train 55 | Test Male 63 | 0.76 | 0.83 | 0.74 |
| 2496 | Train 39 | Test Male 64 | 0.71 | 0.71 | 0.7 | 3520 | Train 55 | Test Male 64 | 0.7 | 0.71 | 0.66 |
| 2497 | Train 40 | Test Male 1 | 0.74 | 0.78 | 0.78 | 3521 | Train 56 | Test Male 1 | 0.72 | 0.74 | 0.71 |
| 2498 | Train 40 | Test Male 2 | 0.75 | 0.78 | 0.78 | 3522 | Train 56 | Test Male 2 | 0.72 | 0.75 | 0.71 |
| 2499 | Train 40 | Test Male 3 | 0.73 | 0.78 | 0.78 | 3523 | Train 56 | Test Male 3 | 0.72 | 0.75 | 0.71 |
| 2500 | Train 40 | Test Male 4 | 0.74 | 0.78 | 0.78 | 3524 | Train 56 | Test Male 4 | 0.72 | 0.75 | 0.71 |
| 2501 | Train 40 | Test Male 5 | 0.72 | 0.77 | 0.77 | 3525 | Train 56 | Test Male 5 | 0.71 | 0.74 | 0.71 |
| 2502 | Train 40 | Test Male 6 | 0.73 | 0.78 | 0.79 | 3526 | Train 56 | Test Male 6 | 0.72 | 0.75 | 0.72 |
| 2503 | Train 40 | Test Male 7 | 0.72 | 0.77 | 0.77 | 3527 | Train 56 | Test Male 7 | 0.71 | 0.74 | 0.71 |
| 2504 | Train 40 | Test Male 8 | 0.73 | 0.78 | 0.79 | 3528 | Train 56 | Test Male 8 | 0.72 | 0.75 | 0.72 |
| 2505 | Train 40 | Test Male 9 | 0.73 | 0.78 | 0.78 | 3529 | Train 56 | Test Male 9 | 0.71 | 0.75 | 0.72 |
| 2506 | Train 40 | Test Male 10 | 0.75 | 0.78 | 0.78 | 3530 | Train 56 | Test Male 10 | 0.72 | 0.75 | 0.71 |
| 2507 | Train 40 | Test Male 11 | 0.73 | 0.78 | 0.78 | 3531 | Train 56 | Test Male 11 | 0.71 | 0.75 | 0.72 |
| 2508 | Train 40 | Test Male 12 | 0.74 | 0.78 | 0.78 | 3532 | Train 56 | Test Male 12 | 0.72 | 0.75 | 0.71 |
| 2509 | Train 40 | Test Male 13 | 0.72 | 0.77 | 0.77 | 3533 | Train 56 | Test Male 13 | 0.71 | 0.74 | 0.72 |
| 2510 | Train 40 | Test Male 14 | 0.73 | 0.78 | 0.79 | 3534 | Train 56 | Test Male 14 | 0.72 | 0.75 | 0.72 |
| 2511 | Train 40 | Test Male 15 | 0.72 | 0.77 | 0.77 | 3535 | Train 56 | Test Male 15 | 0.7 | 0.74 | 0.72 |
| 2512 | Train 40 | Test Male 16 | 0.73 | 0.78 | 0.79 | 3536 | Train 56 | Test Male 16 | 0.72 | 0.75 | 0.72 |
| 2513 | Train 40 | Test Male 17 | 0.74 | 0.78 | 0.78 | 3537 | Train 56 | Test Male 17 | 0.72 | 0.74 | 0.71 |
| 2514 | Train 40 | Test Male 18 | 0.75 | 0.78 | 0.78 | 3538 | Train 56 | Test Male 18 | 0.72 | 0.75 | 0.71 |
| 2515 | Train 40 | Test Male 19 | 0.73 | 0.78 | 0.78 | 3539 | Train 56 | Test Male 19 | 0.72 | 0.75 | 0.71 |
| 2516 | Train 40 | Test Male 20 | 0.74 | 0.78 | 0.78 | 3540 | Train 56 | Test Male 20 | 0.72 | 0.75 | 0.71 |
| 2517 | Train 40 | Test Male 21 | 0.72 | 0.77 | 0.77 | 3541 | Train 56 | Test Male 21 | 0.71 | 0.74 | 0.71 |
| 2518 | Train 40 | Test Male 22 | 0.73 | 0.78 | 0.79 | 3542 | Train 56 | Test Male 22 | 0.72 | 0.75 | 0.72 |
| 2519 | Train 40 | Test Male 23 | 0.72 | 0.77 | 0.77 | 3543 | Train 56 | Test Male 23 | 0.71 | 0.74 | 0.71 |
| 2520 | Train 40 | Test Male 24 | 0.73 | 0.78 | 0.79 | 3544 | Train 56 | Test Male 24 | 0.72 | 0.75 | 0.72 |
| 2521 | Train 40 | Test Male 25 | 0.73 | 0.78 | 0.78 | 3545 | Train 56 | Test Male 25 | 0.71 | 0.75 | 0.72 |
| 2522 | Train 40 | Test Male 26 | 0.75 | 0.78 | 0.78 | 3546 | Train 56 | Test Male 26 | 0.72 | 0.75 | 0.71 |
| 2523 | Train 40 | Test Male 27 | 0.73 | 0.78 | 0.78 | 3547 | Train 56 | Test Male 27 | 0.71 | 0.75 | 0.72 |
| 2524 | Train 40 | Test Male 28 | 0.74 | 0.78 | 0.78 | 3548 | Train 56 | Test Male 28 | 0.72 | 0.75 | 0.71 |
| 2525 | Train 40 | Test Male 29 | 0.72 | 0.77 | 0.77 | 3549 | Train 56 | Test Male 29 | 0.71 | 0.74 | 0.72 |
| 2526 | Train 40 | Test Male 30 | 0.73 | 0.78 | 0.79 | 3550 | Train 56 | Test Male 30 | 0.72 | 0.75 | 0.72 |
| 2527 | Train 40 | Test Male 31 | 0.72 | 0.77 | 0.77 | 3551 | Train 56 | Test Male 31 | 0.7 | 0.74 | 0.72 |
| 2528 | Train 40 | Test Male 32 | 0.73 | 0.78 | 0.79 | 3552 | Train 56 | Test Male 32 | 0.72 | 0.75 | 0.72 |
| 2529 | Train 40 | Test Male 33 | 0.73 | 0.78 | 0.78 | 3553 | Train 56 | Test Male 33 | 0.72 | 0.74 | 0.71 |
| 2530 | Train 40 | Test Male 34 | 0.74 | 0.78 | 0.78 | 3554 | Train 56 | Test Male 34 | 0.72 | 0.75 | 0.71 |
| 2531 | Train 40 | Test Male 35 | 0.73 | 0.78 | 0.78 | 3555 | Train 56 | Test Male 35 | 0.71 | 0.74 | 0.71 |
| 2532 | Train 40 | Test Male 36 | 0.74 | 0.78 | 0.78 | 3556 | Train 56 | Test Male 36 | 0.72 | 0.75 | 0.71 |
| 2533 | Train 40 | Test Male 37 | 0.72 | 0.77 | 0.77 | 3557 | Train 56 | Test Male 37 | 0.71 | 0.74 | 0.71 |
| 2534 | Train 40 | Test Male 38 | 0.73 | 0.78 | 0.79 | 3558 | Train 56 | Test Male 38 | 0.72 | 0.75 | 0.72 |
| 2535 | Train 40 | Test Male 39 | 0.72 | 0.77 | 0.77 | 3559 | Train 56 | Test Male 39 | 0.71 | 0.74 | 0.71 |
| 2536 | Train 40 | Test Male 40 | 0.73 | 0.78 | 0.79 | 3560 | Train 56 | Test Male 40 | 0.72 | 0.75 | 0.72 |
| 2537 | Train 40 | Test Male 41 | 0.73 | 0.78 | 0.78 | 3561 | Train 56 | Test Male 41 | 0.71 | 0.75 | 0.71 |
| 2538 | Train 40 | Test Male 42 | 0.74 | 0.78 | 0.78 | 3562 | Train 56 | Test Male 42 | 0.72 | 0.75 | 0.71 |
| 2539 | Train 40 | Test Male 43 | 0.73 | 0.78 | 0.78 | 3563 | Train 56 | Test Male 43 | 0.71 | 0.75 | 0.72 |
| 2540 | Train 40 | Test Male 44 | 0.74 | 0.78 | 0.78 | 3564 | Train 56 | Test Male 44 | 0.72 | 0.75 | 0.71 |
| 2541 | Train 40 | Test Male 45 | 0.72 | 0.77 | 0.77 | 3565 | Train 56 | Test Male 45 | 0.71 | 0.74 | 0.72 |
| 2542 | Train 40 | Test Male 46 | 0.73 | 0.78 | 0.79 | 3566 | Train 56 | Test Male 46 | 0.72 | 0.75 | 0.72 |
| 2543 | Train 40 | Test Male 47 | 0.72 | 0.77 | 0.77 | 3567 | Train 56 | Test Male 47 | 0.7 | 0.74 | 0.72 |
| 2544 | Train 40 | Test Male 48 | 0.73 | 0.78 | 0.79 | 3568 | Train 56 | Test Male 48 | 0.72 | 0.75 | 0.72 |
| 2545 | Train 40 | Test Male 49 | 0.76 | 0.8 | 0.79 | 3569 | Train 56 | Test Male 49 | 0.77 | 0.8 | 0.78 |
| 2546 | Train 40 | Test Male 50 | 0.76 | 0.8 | 0.79 | 3570 | Train 56 | Test Male 50 | 0.77 | 0.8 | 0.78 |
| 2547 | Train 40 | Test Male 51 | 0.76 | 0.8 | 0.79 | 3571 | Train 56 | Test Male 51 | 0.77 | 0.8 | 0.78 |
| 2548 | Train 40 | Test Male 52 | 0.76 | 0.8 | 0.79 | 3572 | Train 56 | Test Male 52 | 0.77 | 0.8 | 0.78 |
| 2549 | Train 40 | Test Male 53 | 0.75 | 0.8 | 0.8 | 3573 | Train 56 | Test Male 53 | 0.76 | 0.82 | 0.81 |
| 2550 | Train 40 | Test Male 54 | 0.74 | 0.78 | 0.79 | 3574 | Train 56 | Test Male 54 | 0.74 | 0.8 | 0.8 |
| 2551 | Train 40 | Test Male 55 | 0.75 | 0.8 | 0.8 | 3575 | Train 56 | Test Male 55 | 0.76 | 0.82 | 0.81 |
| 2552 | Train 40 | Test Male 56 | 0.74 | 0.78 | 0.79 | 3576 | Train 56 | Test Male 56 | 0.74 | 0.8 | 0.81 |
| 2553 | Train 40 | Test Male 57 | 0.76 | 0.8 | 0.8 | 3577 | Train 56 | Test Male 57 | 0.77 | 0.8 | 0.8 |
| 2554 | Train 40 | Test Male 58 | 0.76 | 0.8 | 0.8 | 3578 | Train 56 | Test Male 58 | 0.77 | 0.8 | 0.8 |
| 2555 | Train 40 | Test Male 59 | 0.76 | 0.8 | 0.8 | 3579 | Train 56 | Test Male 59 | 0.77 | 0.8 | 0.8 |
| 2556 | Train 40 | Test Male 60 | 0.76 | 0.8 | 0.8 | 3580 | Train 56 | Test Male 60 | 0.77 | 0.8 | 0.8 |
| 2557 | Train 40 | Test Male 61 | 0.75 | 0.81 | 0.76 | 3581 | Train 56 | Test Male 61 | 0.76 | 0.82 | 0.77 |
| 2558 | Train 40 | Test Male 62 | 0.74 | 0.78 | 0.79 | 3582 | Train 56 | Test Male 62 | 0.74 | 0.8 | 0.8 |
| 2559 | Train 40 | Test Male 63 | 0.75 | 0.81 | 0.81 | 3583 | Train 56 | Test Male 63 | 0.76 | 0.82 | 0.82 |
| 2560 | Train 40 | Test Male 64 | 0.74 | 0.78 | 0.79 | 3584 | Train 56 | Test Male 64 | 0.74 | 0.8 | 0.8 |
| 2561 | Train 41 | Test Male 1 | 0.76 | 0.83 | 0.82 | 3585 | Train 57 | Test Male 1 | 0.74 | 0.75 | 0.72 |
| 2562 | Train 41 | Test Male 2 | 0.75 | 0.79 | 0.77 | 3586 | Train 57 | Test Male 2 | 0.72 | 0.72 | 0.68 |
| 2563 | Train 41 | Test Male 3 | 0.76 | 0.83 | 0.82 | 3587 | Train 57 | Test Male 3 | 0.74 | 0.75 | 0.72 |
| 2564 | Train 41 | Test Male 4 | 0.74 | 0.79 | 0.77 | 3588 | Train 57 | Test Male 4 | 0.72 | 0.72 | 0.68 |
| 2565 | Train 41 | Test Male 5 | 0.72 | 0.74 | 0.72 | 3589 | Train 57 | Test Male 5 | 0.7 | 0.71 | 0.68 |
| 2566 | Train 41 | Test Male 6 | 0.69 | 0.7 | 0.64 | 3590 | Train 57 | Test Male 6 | 0.68 | 0.68 | 0.64 |
| 2567 | Train 41 | Test Male 7 | 0.72 | 0.74 | 0.72 | 3591 | Train 57 | Test Male 7 | 0.7 | 0.71 | 0.68 |
| 2568 | Train 41 | Test Male 8 | 0.69 | 0.7 | 0.64 | 3592 | Train 57 | Test Male 8 | 0.68 | 0.68 | 0.64 |
| 2569 | Train 41 | Test Male 9 | 0.76 | 0.84 | 0.85 | 3593 | Train 57 | Test Male 9 | 0.74 | 0.75 | 0.74 |
| 2570 | Train 41 | Test Male 10 | 0.74 | 0.79 | 0.79 | 3594 | Train 57 | Test Male 10 | 0.72 | 0.73 | 0.69 |
| 2571 | Train 41 | Test Male 11 | 0.76 | 0.84 | 0.85 | 3595 | Train 57 | Test Male 11 | 0.74 | 0.75 | 0.74 |
| 2572 | Train 41 | Test Male 12 | 0.74 | 0.79 | 0.79 | 3596 | Train 57 | Test Male 12 | 0.72 | 0.73 | 0.69 |
| 2573 | Train 41 | Test Male 13 | 0.72 | 0.75 | 0.74 | 3597 | Train 57 | Test Male 13 | 0.7 | 0.71 | 0.69 |
| 2574 | Train 41 | Test Male 14 | 0.69 | 0.7 | 0.64 | 3598 | Train 57 | Test Male 14 | 0.68 | 0.68 | 0.64 |
| 2575 | Train 41 | Test Male 15 | 0.71 | 0.74 | 0.73 | 3599 | Train 57 | Test Male 15 | 0.7 | 0.71 | 0.69 |
| 2576 | Train 41 | Test Male 16 | 0.69 | 0.7 | 0.64 | 3600 | Train 57 | Test Male 16 | 0.68 | 0.68 | 0.64 |
| 2577 | Train 41 | Test Male 17 | 0.76 | 0.83 | 0.82 | 3601 | Train 57 | Test Male 17 | 0.74 | 0.75 | 0.72 |
| 2578 | Train 41 | Test Male 18 | 0.75 | 0.79 | 0.77 | 3602 | Train 57 | Test Male 18 | 0.72 | 0.72 | 0.68 |
| 2579 | Train 41 | Test Male 19 | 0.76 | 0.83 | 0.82 | 3603 | Train 57 | Test Male 19 | 0.74 | 0.75 | 0.72 |
| 2580 | Train 41 | Test Male 20 | 0.74 | 0.79 | 0.77 | 3604 | Train 57 | Test Male 20 | 0.72 | 0.72 | 0.68 |
| 2581 | Train 41 | Test Male 21 | 0.72 | 0.74 | 0.72 | 3605 | Train 57 | Test Male 21 | 0.7 | 0.71 | 0.68 |
| 2582 | Train 41 | Test Male 22 | 0.69 | 0.7 | 0.64 | 3606 | Train 57 | Test Male 22 | 0.68 | 0.68 | 0.64 |
| 2583 | Train 41 | Test Male 23 | 0.72 | 0.74 | 0.72 | 3607 | Train 57 | Test Male 23 | 0.7 | 0.71 | 0.68 |
| 2584 | Train 41 | Test Male 24 | 0.69 | 0.7 | 0.64 | 3608 | Train 57 | Test Male 24 | 0.68 | 0.68 | 0.64 |
| 2585 | Train 41 | Test Male 25 | 0.76 | 0.84 | 0.85 | 3609 | Train 57 | Test Male 25 | 0.74 | 0.75 | 0.74 |
| 2586 | Train 41 | Test Male 26 | 0.74 | 0.79 | 0.79 | 3610 | Train 57 | Test Male 26 | 0.72 | 0.73 | 0.69 |
| 2587 | Train 41 | Test Male 27 | 0.76 | 0.84 | 0.85 | 3611 | Train 57 | Test Male 27 | 0.74 | 0.75 | 0.74 |
| 2588 | Train 41 | Test Male 28 | 0.74 | 0.79 | 0.79 | 3612 | Train 57 | Test Male 28 | 0.72 | 0.73 | 0.69 |
| 2589 | Train 41 | Test Male 29 | 0.72 | 0.75 | 0.74 | 3613 | Train 57 | Test Male 29 | 0.7 | 0.71 | 0.69 |
| 2590 | Train 41 | Test Male 30 | 0.69 | 0.7 | 0.64 | 3614 | Train 57 | Test Male 30 | 0.68 | 0.68 | 0.64 |
| 2591 | Train 41 | Test Male 31 | 0.71 | 0.74 | 0.73 | 3615 | Train 57 | Test Male 31 | 0.7 | 0.71 | 0.69 |
| 2592 | Train 41 | Test Male 32 | 0.69 | 0.7 | 0.64 | 3616 | Train 57 | Test Male 32 | 0.68 | 0.68 | 0.64 |
| 2593 | Train 41 | Test Male 33 | 0.76 | 0.83 | 0.82 | 3617 | Train 57 | Test Male 33 | 0.74 | 0.74 | 0.71 |
| 2594 | Train 41 | Test Male 34 | 0.74 | 0.79 | 0.77 | 3618 | Train 57 | Test Male 34 | 0.72 | 0.72 | 0.67 |
| 2595 | Train 41 | Test Male 35 | 0.76 | 0.83 | 0.82 | 3619 | Train 57 | Test Male 35 | 0.74 | 0.74 | 0.71 |
| 2596 | Train 41 | Test Male 36 | 0.74 | 0.79 | 0.77 | 3620 | Train 57 | Test Male 36 | 0.72 | 0.72 | 0.68 |
| 2597 | Train 41 | Test Male 37 | 0.72 | 0.74 | 0.72 | 3621 | Train 57 | Test Male 37 | 0.7 | 0.71 | 0.68 |
| 2598 | Train 41 | Test Male 38 | 0.69 | 0.7 | 0.64 | 3622 | Train 57 | Test Male 38 | 0.68 | 0.68 | 0.64 |
| 2599 | Train 41 | Test Male 39 | 0.72 | 0.74 | 0.72 | 3623 | Train 57 | Test Male 39 | 0.7 | 0.71 | 0.68 |
| 2600 | Train 41 | Test Male 40 | 0.69 | 0.7 | 0.64 | 3624 | Train 57 | Test Male 40 | 0.68 | 0.68 | 0.64 |
| 2601 | Train 41 | Test Male 41 | 0.76 | 0.84 | 0.85 | 3625 | Train 57 | Test Male 41 | 0.74 | 0.75 | 0.74 |
| 2602 | Train 41 | Test Male 42 | 0.74 | 0.79 | 0.79 | 3626 | Train 57 | Test Male 42 | 0.72 | 0.73 | 0.69 |
| 2603 | Train 41 | Test Male 43 | 0.76 | 0.84 | 0.85 | 3627 | Train 57 | Test Male 43 | 0.74 | 0.75 | 0.74 |
| 2604 | Train 41 | Test Male 44 | 0.74 | 0.79 | 0.79 | 3628 | Train 57 | Test Male 44 | 0.72 | 0.73 | 0.69 |
| 2605 | Train 41 | Test Male 45 | 0.71 | 0.75 | 0.74 | 3629 | Train 57 | Test Male 45 | 0.7 | 0.71 | 0.69 |
| 2606 | Train 41 | Test Male 46 | 0.69 | 0.7 | 0.64 | 3630 | Train 57 | Test Male 46 | 0.68 | 0.68 | 0.64 |
| 2607 | Train 41 | Test Male 47 | 0.71 | 0.74 | 0.73 | 3631 | Train 57 | Test Male 47 | 0.7 | 0.71 | 0.69 |
| 2608 | Train 41 | Test Male 48 | 0.69 | 0.7 | 0.64 | 3632 | Train 57 | Test Male 48 | 0.68 | 0.68 | 0.64 |
| 2609 | Train 41 | Test Male 49 | 0.77 | 0.82 | 0.82 | 3633 | Train 57 | Test Male 49 | 0.78 | 0.84 | 0.84 |
| 2610 | Train 41 | Test Male 50 | 0.77 | 0.82 | 0.82 | 3634 | Train 57 | Test Male 50 | 0.78 | 0.84 | 0.83 |
| 2611 | Train 41 | Test Male 51 | 0.77 | 0.82 | 0.81 | 3635 | Train 57 | Test Male 51 | 0.78 | 0.84 | 0.83 |
| 2612 | Train 41 | Test Male 52 | 0.77 | 0.82 | 0.81 | 3636 | Train 57 | Test Male 52 | 0.78 | 0.84 | 0.83 |
| 2613 | Train 41 | Test Male 53 | 0.73 | 0.77 | 0.71 | 3637 | Train 57 | Test Male 53 | 0.72 | 0.77 | 0.76 |
| 2614 | Train 41 | Test Male 54 | 0.71 | 0.71 | 0.66 | 3638 | Train 57 | Test Male 54 | 0.69 | 0.69 | 0.67 |
| 2615 | Train 41 | Test Male 55 | 0.73 | 0.77 | 0.71 | 3639 | Train 57 | Test Male 55 | 0.72 | 0.77 | 0.76 |
| 2616 | Train 41 | Test Male 56 | 0.71 | 0.71 | 0.65 | 3640 | Train 57 | Test Male 56 | 0.7 | 0.69 | 0.67 |
| 2617 | Train 41 | Test Male 57 | 0.77 | 0.83 | 0.86 | 3641 | Train 57 | Test Male 57 | 0.78 | 0.85 | 0.9 |
| 2618 | Train 41 | Test Male 58 | 0.77 | 0.83 | 0.85 | 3642 | Train 57 | Test Male 58 | 0.78 | 0.85 | 0.9 |
| 2619 | Train 41 | Test Male 59 | 0.77 | 0.83 | 0.85 | 3643 | Train 57 | Test Male 59 | 0.78 | 0.85 | 0.89 |
| 2620 | Train 41 | Test Male 60 | 0.77 | 0.83 | 0.85 | 3644 | Train 57 | Test Male 60 | 0.78 | 0.85 | 0.89 |
| 2621 | Train 41 | Test Male 61 | 0.73 | 0.78 | 0.69 | 3645 | Train 57 | Test Male 61 | 0.71 | 0.77 | 0.77 |
| 2622 | Train 41 | Test Male 62 | 0.71 | 0.71 | 0.66 | 3646 | Train 57 | Test Male 62 | 0.69 | 0.69 | 0.67 |
| 2623 | Train 41 | Test Male 63 | 0.73 | 0.78 | 0.71 | 3647 | Train 57 | Test Male 63 | 0.72 | 0.77 | 0.77 |
| 2624 | Train 41 | Test Male 64 | 0.71 | 0.71 | 0.66 | 3648 | Train 57 | Test Male 64 | 0.7 | 0.69 | 0.67 |
| 2625 | Train 42 | Test Male 1 | 0.75 | 0.82 | 0.81 | 3649 | Train 58 | Test Male 1 | 0.74 | 0.72 | 0.65 |
| 2626 | Train 42 | Test Male 2 | 0.76 | 0.83 | 0.82 | 3650 | Train 58 | Test Male 2 | 0.72 | 0.71 | 0.64 |
| 2627 | Train 42 | Test Male 3 | 0.75 | 0.82 | 0.81 | 3651 | Train 58 | Test Male 3 | 0.74 | 0.72 | 0.65 |
| 2628 | Train 42 | Test Male 4 | 0.76 | 0.82 | 0.82 | 3652 | Train 58 | Test Male 4 | 0.72 | 0.71 | 0.64 |
| 2629 | Train 42 | Test Male 5 | 0.72 | 0.75 | 0.71 | 3653 | Train 58 | Test Male 5 | 0.7 | 0.69 | 0.62 |
| 2630 | Train 42 | Test Male 6 | 0.7 | 0.72 | 0.66 | 3654 | Train 58 | Test Male 6 | 0.68 | 0.67 | 0.6 |
| 2631 | Train 42 | Test Male 7 | 0.72 | 0.75 | 0.7 | 3655 | Train 58 | Test Male 7 | 0.7 | 0.69 | 0.62 |
| 2632 | Train 42 | Test Male 8 | 0.7 | 0.71 | 0.66 | 3656 | Train 58 | Test Male 8 | 0.68 | 0.67 | 0.6 |
| 2633 | Train 42 | Test Male 9 | 0.75 | 0.83 | 0.84 | 3657 | Train 58 | Test Male 9 | 0.74 | 0.72 | 0.67 |
| 2634 | Train 42 | Test Male 10 | 0.76 | 0.83 | 0.84 | 3658 | Train 58 | Test Male 10 | 0.72 | 0.71 | 0.65 |
| 2635 | Train 42 | Test Male 11 | 0.75 | 0.83 | 0.84 | 3659 | Train 58 | Test Male 11 | 0.74 | 0.72 | 0.67 |
| 2636 | Train 42 | Test Male 12 | 0.76 | 0.83 | 0.84 | 3660 | Train 58 | Test Male 12 | 0.72 | 0.71 | 0.65 |
| 2637 | Train 42 | Test Male 13 | 0.72 | 0.75 | 0.72 | 3661 | Train 58 | Test Male 13 | 0.7 | 0.7 | 0.63 |
| 2638 | Train 42 | Test Male 14 | 0.71 | 0.72 | 0.66 | 3662 | Train 58 | Test Male 14 | 0.68 | 0.67 | 0.61 |
| 2639 | Train 42 | Test Male 15 | 0.72 | 0.75 | 0.71 | 3663 | Train 58 | Test Male 15 | 0.7 | 0.69 | 0.63 |
| 2640 | Train 42 | Test Male 16 | 0.71 | 0.71 | 0.66 | 3664 | Train 58 | Test Male 16 | 0.68 | 0.67 | 0.61 |
| 2641 | Train 42 | Test Male 17 | 0.75 | 0.82 | 0.81 | 3665 | Train 58 | Test Male 17 | 0.74 | 0.72 | 0.65 |
| 2642 | Train 42 | Test Male 18 | 0.76 | 0.83 | 0.82 | 3666 | Train 58 | Test Male 18 | 0.72 | 0.71 | 0.64 |
| 2643 | Train 42 | Test Male 19 | 0.75 | 0.82 | 0.81 | 3667 | Train 58 | Test Male 19 | 0.74 | 0.72 | 0.65 |
| 2644 | Train 42 | Test Male 20 | 0.76 | 0.82 | 0.82 | 3668 | Train 58 | Test Male 20 | 0.72 | 0.71 | 0.64 |
| 2645 | Train 42 | Test Male 21 | 0.72 | 0.75 | 0.71 | 3669 | Train 58 | Test Male 21 | 0.7 | 0.69 | 0.62 |
| 2646 | Train 42 | Test Male 22 | 0.71 | 0.72 | 0.66 | 3670 | Train 58 | Test Male 22 | 0.68 | 0.67 | 0.6 |
| 2647 | Train 42 | Test Male 23 | 0.72 | 0.75 | 0.7 | 3671 | Train 58 | Test Male 23 | 0.7 | 0.69 | 0.62 |
| 2648 | Train 42 | Test Male 24 | 0.7 | 0.71 | 0.66 | 3672 | Train 58 | Test Male 24 | 0.68 | 0.67 | 0.6 |
| 2649 | Train 42 | Test Male 25 | 0.75 | 0.83 | 0.84 | 3673 | Train 58 | Test Male 25 | 0.74 | 0.72 | 0.67 |
| 2650 | Train 42 | Test Male 26 | 0.76 | 0.83 | 0.84 | 3674 | Train 58 | Test Male 26 | 0.72 | 0.71 | 0.65 |
| 2651 | Train 42 | Test Male 27 | 0.75 | 0.83 | 0.84 | 3675 | Train 58 | Test Male 27 | 0.74 | 0.72 | 0.67 |
| 2652 | Train 42 | Test Male 28 | 0.76 | 0.83 | 0.84 | 3676 | Train 58 | Test Male 28 | 0.72 | 0.71 | 0.65 |
| 2653 | Train 42 | Test Male 29 | 0.72 | 0.75 | 0.72 | 3677 | Train 58 | Test Male 29 | 0.7 | 0.7 | 0.63 |
| 2654 | Train 42 | Test Male 30 | 0.71 | 0.72 | 0.66 | 3678 | Train 58 | Test Male 30 | 0.68 | 0.67 | 0.61 |
| 2655 | Train 42 | Test Male 31 | 0.72 | 0.75 | 0.71 | 3679 | Train 58 | Test Male 31 | 0.7 | 0.69 | 0.63 |
| 2656 | Train 42 | Test Male 32 | 0.71 | 0.71 | 0.66 | 3680 | Train 58 | Test Male 32 | 0.68 | 0.67 | 0.61 |
| 2657 | Train 42 | Test Male 33 | 0.75 | 0.82 | 0.81 | 3681 | Train 58 | Test Male 33 | 0.74 | 0.72 | 0.65 |
| 2658 | Train 42 | Test Male 34 | 0.76 | 0.83 | 0.82 | 3682 | Train 58 | Test Male 34 | 0.72 | 0.71 | 0.63 |
| 2659 | Train 42 | Test Male 35 | 0.75 | 0.82 | 0.81 | 3683 | Train 58 | Test Male 35 | 0.74 | 0.72 | 0.65 |
| 2660 | Train 42 | Test Male 36 | 0.76 | 0.82 | 0.82 | 3684 | Train 58 | Test Male 36 | 0.72 | 0.71 | 0.64 |
| 2661 | Train 42 | Test Male 37 | 0.72 | 0.75 | 0.71 | 3685 | Train 58 | Test Male 37 | 0.7 | 0.69 | 0.62 |
| 2662 | Train 42 | Test Male 38 | 0.7 | 0.72 | 0.66 | 3686 | Train 58 | Test Male 38 | 0.68 | 0.67 | 0.6 |
| 2663 | Train 42 | Test Male 39 | 0.72 | 0.74 | 0.7 | 3687 | Train 58 | Test Male 39 | 0.7 | 0.69 | 0.62 |
| 2664 | Train 42 | Test Male 40 | 0.7 | 0.71 | 0.66 | 3688 | Train 58 | Test Male 40 | 0.68 | 0.67 | 0.6 |
| 2665 | Train 42 | Test Male 41 | 0.75 | 0.83 | 0.84 | 3689 | Train 58 | Test Male 41 | 0.74 | 0.72 | 0.67 |
| 2666 | Train 42 | Test Male 42 | 0.76 | 0.83 | 0.84 | 3690 | Train 58 | Test Male 42 | 0.72 | 0.71 | 0.65 |
| 2667 | Train 42 | Test Male 43 | 0.75 | 0.83 | 0.84 | 3691 | Train 58 | Test Male 43 | 0.74 | 0.72 | 0.67 |
| 2668 | Train 42 | Test Male 44 | 0.76 | 0.83 | 0.84 | 3692 | Train 58 | Test Male 44 | 0.72 | 0.71 | 0.65 |
| 2669 | Train 42 | Test Male 45 | 0.72 | 0.75 | 0.72 | 3693 | Train 58 | Test Male 45 | 0.7 | 0.69 | 0.63 |
| 2670 | Train 42 | Test Male 46 | 0.71 | 0.72 | 0.66 | 3694 | Train 58 | Test Male 46 | 0.68 | 0.67 | 0.61 |
| 2671 | Train 42 | Test Male 47 | 0.71 | 0.75 | 0.71 | 3695 | Train 58 | Test Male 47 | 0.7 | 0.69 | 0.63 |
| 2672 | Train 42 | Test Male 48 | 0.71 | 0.71 | 0.66 | 3696 | Train 58 | Test Male 48 | 0.68 | 0.67 | 0.61 |
| 2673 | Train 42 | Test Male 49 | 0.77 | 0.82 | 0.81 | 3697 | Train 58 | Test Male 49 | 0.78 | 0.84 | 0.84 |
| 2674 | Train 42 | Test Male 50 | 0.77 | 0.82 | 0.81 | 3698 | Train 58 | Test Male 50 | 0.78 | 0.84 | 0.84 |
| 2675 | Train 42 | Test Male 51 | 0.77 | 0.82 | 0.81 | 3699 | Train 58 | Test Male 51 | 0.78 | 0.84 | 0.83 |
| 2676 | Train 42 | Test Male 52 | 0.76 | 0.81 | 0.81 | 3700 | Train 58 | Test Male 52 | 0.78 | 0.84 | 0.83 |
| 2677 | Train 42 | Test Male 53 | 0.74 | 0.78 | 0.66 | 3701 | Train 58 | Test Male 53 | 0.72 | 0.74 | 0.7 |
| 2678 | Train 42 | Test Male 54 | 0.71 | 0.72 | 0.65 | 3702 | Train 58 | Test Male 54 | 0.69 | 0.68 | 0.62 |
| 2679 | Train 42 | Test Male 55 | 0.74 | 0.78 | 0.66 | 3703 | Train 58 | Test Male 55 | 0.72 | 0.74 | 0.7 |
| 2680 | Train 42 | Test Male 56 | 0.71 | 0.71 | 0.65 | 3704 | Train 58 | Test Male 56 | 0.7 | 0.68 | 0.62 |
| 2681 | Train 42 | Test Male 57 | 0.77 | 0.82 | 0.85 | 3705 | Train 58 | Test Male 57 | 0.78 | 0.85 | 0.9 |
| 2682 | Train 42 | Test Male 58 | 0.77 | 0.82 | 0.85 | 3706 | Train 58 | Test Male 58 | 0.78 | 0.85 | 0.9 |
| 2683 | Train 42 | Test Male 59 | 0.76 | 0.82 | 0.84 | 3707 | Train 58 | Test Male 59 | 0.78 | 0.85 | 0.89 |
| 2684 | Train 42 | Test Male 60 | 0.76 | 0.82 | 0.84 | 3708 | Train 58 | Test Male 60 | 0.78 | 0.85 | 0.89 |
| 2685 | Train 42 | Test Male 61 | 0.74 | 0.78 | 0.65 | 3709 | Train 58 | Test Male 61 | 0.72 | 0.75 | 0.71 |
| 2686 | Train 42 | Test Male 62 | 0.71 | 0.72 | 0.65 | 3710 | Train 58 | Test Male 62 | 0.69 | 0.68 | 0.62 |
| 2687 | Train 42 | Test Male 63 | 0.74 | 0.78 | 0.66 | 3711 | Train 58 | Test Male 63 | 0.72 | 0.75 | 0.71 |
| 2688 | Train 42 | Test Male 64 | 0.71 | 0.71 | 0.65 | 3712 | Train 58 | Test Male 64 | 0.7 | 0.68 | 0.62 |
| 2689 | Train 43 | Test Male 1 | 0.76 | 0.83 | 0.82 | 3713 | Train 59 | Test Male 1 | 0.74 | 0.75 | 0.68 |
| 2690 | Train 43 | Test Male 2 | 0.74 | 0.79 | 0.78 | 3714 | Train 59 | Test Male 2 | 0.72 | 0.72 | 0.64 |
| 2691 | Train 43 | Test Male 3 | 0.76 | 0.83 | 0.82 | 3715 | Train 59 | Test Male 3 | 0.74 | 0.75 | 0.68 |
| 2692 | Train 43 | Test Male 4 | 0.74 | 0.79 | 0.78 | 3716 | Train 59 | Test Male 4 | 0.72 | 0.73 | 0.65 |
| 2693 | Train 43 | Test Male 5 | 0.72 | 0.74 | 0.72 | 3717 | Train 59 | Test Male 5 | 0.7 | 0.71 | 0.65 |
| 2694 | Train 43 | Test Male 6 | 0.69 | 0.7 | 0.65 | 3718 | Train 59 | Test Male 6 | 0.68 | 0.68 | 0.6 |
| 2695 | Train 43 | Test Male 7 | 0.72 | 0.74 | 0.71 | 3719 | Train 59 | Test Male 7 | 0.7 | 0.71 | 0.65 |
| 2696 | Train 43 | Test Male 8 | 0.69 | 0.7 | 0.65 | 3720 | Train 59 | Test Male 8 | 0.68 | 0.68 | 0.61 |
| 2697 | Train 43 | Test Male 9 | 0.76 | 0.84 | 0.85 | 3721 | Train 59 | Test Male 9 | 0.74 | 0.75 | 0.7 |
| 2698 | Train 43 | Test Male 10 | 0.74 | 0.79 | 0.8 | 3722 | Train 59 | Test Male 10 | 0.72 | 0.73 | 0.66 |
| 2699 | Train 43 | Test Male 11 | 0.76 | 0.84 | 0.85 | 3723 | Train 59 | Test Male 11 | 0.74 | 0.75 | 0.71 |
| 2700 | Train 43 | Test Male 12 | 0.74 | 0.79 | 0.8 | 3724 | Train 59 | Test Male 12 | 0.72 | 0.73 | 0.67 |
| 2701 | Train 43 | Test Male 13 | 0.72 | 0.75 | 0.73 | 3725 | Train 59 | Test Male 13 | 0.7 | 0.72 | 0.66 |
| 2702 | Train 43 | Test Male 14 | 0.69 | 0.7 | 0.65 | 3726 | Train 59 | Test Male 14 | 0.68 | 0.68 | 0.6 |
| 2703 | Train 43 | Test Male 15 | 0.71 | 0.75 | 0.73 | 3727 | Train 59 | Test Male 15 | 0.7 | 0.71 | 0.66 |
| 2704 | Train 43 | Test Male 16 | 0.69 | 0.7 | 0.65 | 3728 | Train 59 | Test Male 16 | 0.68 | 0.68 | 0.61 |
| 2705 | Train 43 | Test Male 17 | 0.76 | 0.83 | 0.82 | 3729 | Train 59 | Test Male 17 | 0.74 | 0.75 | 0.68 |
| 2706 | Train 43 | Test Male 18 | 0.74 | 0.79 | 0.78 | 3730 | Train 59 | Test Male 18 | 0.72 | 0.72 | 0.64 |
| 2707 | Train 43 | Test Male 19 | 0.76 | 0.83 | 0.82 | 3731 | Train 59 | Test Male 19 | 0.74 | 0.75 | 0.68 |
| 2708 | Train 43 | Test Male 20 | 0.74 | 0.79 | 0.78 | 3732 | Train 59 | Test Male 20 | 0.72 | 0.73 | 0.65 |
| 2709 | Train 43 | Test Male 21 | 0.72 | 0.74 | 0.72 | 3733 | Train 59 | Test Male 21 | 0.7 | 0.71 | 0.65 |
| 2710 | Train 43 | Test Male 22 | 0.69 | 0.7 | 0.65 | 3734 | Train 59 | Test Male 22 | 0.68 | 0.68 | 0.6 |
| 2711 | Train 43 | Test Male 23 | 0.72 | 0.74 | 0.71 | 3735 | Train 59 | Test Male 23 | 0.7 | 0.71 | 0.65 |
| 2712 | Train 43 | Test Male 24 | 0.69 | 0.7 | 0.65 | 3736 | Train 59 | Test Male 24 | 0.68 | 0.68 | 0.61 |
| 2713 | Train 43 | Test Male 25 | 0.76 | 0.84 | 0.85 | 3737 | Train 59 | Test Male 25 | 0.74 | 0.75 | 0.7 |
| 2714 | Train 43 | Test Male 26 | 0.74 | 0.79 | 0.8 | 3738 | Train 59 | Test Male 26 | 0.72 | 0.73 | 0.66 |
| 2715 | Train 43 | Test Male 27 | 0.76 | 0.84 | 0.85 | 3739 | Train 59 | Test Male 27 | 0.74 | 0.76 | 0.71 |
| 2716 | Train 43 | Test Male 28 | 0.74 | 0.79 | 0.8 | 3740 | Train 59 | Test Male 28 | 0.72 | 0.73 | 0.67 |
| 2717 | Train 43 | Test Male 29 | 0.72 | 0.75 | 0.73 | 3741 | Train 59 | Test Male 29 | 0.7 | 0.72 | 0.66 |
| 2718 | Train 43 | Test Male 30 | 0.69 | 0.7 | 0.65 | 3742 | Train 59 | Test Male 30 | 0.68 | 0.68 | 0.6 |
| 2719 | Train 43 | Test Male 31 | 0.71 | 0.75 | 0.73 | 3743 | Train 59 | Test Male 31 | 0.7 | 0.71 | 0.66 |
| 2720 | Train 43 | Test Male 32 | 0.69 | 0.7 | 0.65 | 3744 | Train 59 | Test Male 32 | 0.68 | 0.68 | 0.61 |
| 2721 | Train 43 | Test Male 33 | 0.76 | 0.83 | 0.82 | 3745 | Train 59 | Test Male 33 | 0.74 | 0.75 | 0.68 |
| 2722 | Train 43 | Test Male 34 | 0.74 | 0.79 | 0.78 | 3746 | Train 59 | Test Male 34 | 0.72 | 0.72 | 0.64 |
| 2723 | Train 43 | Test Male 35 | 0.76 | 0.83 | 0.82 | 3747 | Train 59 | Test Male 35 | 0.74 | 0.75 | 0.68 |
| 2724 | Train 43 | Test Male 36 | 0.74 | 0.79 | 0.78 | 3748 | Train 59 | Test Male 36 | 0.72 | 0.73 | 0.65 |
| 2725 | Train 43 | Test Male 37 | 0.72 | 0.74 | 0.72 | 3749 | Train 59 | Test Male 37 | 0.7 | 0.71 | 0.65 |
| 2726 | Train 43 | Test Male 38 | 0.69 | 0.7 | 0.65 | 3750 | Train 59 | Test Male 38 | 0.68 | 0.68 | 0.6 |
| 2727 | Train 43 | Test Male 39 | 0.72 | 0.74 | 0.71 | 3751 | Train 59 | Test Male 39 | 0.7 | 0.71 | 0.65 |
| 2728 | Train 43 | Test Male 40 | 0.69 | 0.7 | 0.65 | 3752 | Train 59 | Test Male 40 | 0.68 | 0.68 | 0.61 |
| 2729 | Train 43 | Test Male 41 | 0.76 | 0.84 | 0.85 | 3753 | Train 59 | Test Male 41 | 0.74 | 0.75 | 0.7 |
| 2730 | Train 43 | Test Male 42 | 0.74 | 0.79 | 0.8 | 3754 | Train 59 | Test Male 42 | 0.72 | 0.73 | 0.66 |
| 2731 | Train 43 | Test Male 43 | 0.76 | 0.84 | 0.85 | 3755 | Train 59 | Test Male 43 | 0.74 | 0.75 | 0.71 |
| 2732 | Train 43 | Test Male 44 | 0.74 | 0.79 | 0.8 | 3756 | Train 59 | Test Male 44 | 0.72 | 0.73 | 0.67 |
| 2733 | Train 43 | Test Male 45 | 0.71 | 0.75 | 0.73 | 3757 | Train 59 | Test Male 45 | 0.7 | 0.71 | 0.66 |
| 2734 | Train 43 | Test Male 46 | 0.69 | 0.7 | 0.65 | 3758 | Train 59 | Test Male 46 | 0.68 | 0.68 | 0.6 |
| 2735 | Train 43 | Test Male 47 | 0.71 | 0.75 | 0.73 | 3759 | Train 59 | Test Male 47 | 0.7 | 0.71 | 0.66 |
| 2736 | Train 43 | Test Male 48 | 0.69 | 0.7 | 0.65 | 3760 | Train 59 | Test Male 48 | 0.68 | 0.68 | 0.61 |
| 2737 | Train 43 | Test Male 49 | 0.77 | 0.82 | 0.82 | 3761 | Train 59 | Test Male 49 | 0.78 | 0.84 | 0.83 |
| 2738 | Train 43 | Test Male 50 | 0.77 | 0.82 | 0.82 | 3762 | Train 59 | Test Male 50 | 0.78 | 0.84 | 0.83 |
| 2739 | Train 43 | Test Male 51 | 0.77 | 0.82 | 0.82 | 3763 | Train 59 | Test Male 51 | 0.78 | 0.84 | 0.83 |
| 2740 | Train 43 | Test Male 52 | 0.77 | 0.82 | 0.82 | 3764 | Train 59 | Test Male 52 | 0.78 | 0.84 | 0.83 |
| 2741 | Train 43 | Test Male 53 | 0.73 | 0.77 | 0.71 | 3765 | Train 59 | Test Male 53 | 0.72 | 0.77 | 0.73 |
| 2742 | Train 43 | Test Male 54 | 0.71 | 0.71 | 0.67 | 3766 | Train 59 | Test Male 54 | 0.69 | 0.7 | 0.63 |
| 2743 | Train 43 | Test Male 55 | 0.73 | 0.77 | 0.71 | 3767 | Train 59 | Test Male 55 | 0.72 | 0.78 | 0.74 |
| 2744 | Train 43 | Test Male 56 | 0.71 | 0.71 | 0.66 | 3768 | Train 59 | Test Male 56 | 0.69 | 0.69 | 0.63 |
| 2745 | Train 43 | Test Male 57 | 0.77 | 0.83 | 0.85 | 3769 | Train 59 | Test Male 57 | 0.78 | 0.85 | 0.89 |
| 2746 | Train 43 | Test Male 58 | 0.77 | 0.83 | 0.85 | 3770 | Train 59 | Test Male 58 | 0.78 | 0.85 | 0.89 |
| 2747 | Train 43 | Test Male 59 | 0.77 | 0.83 | 0.85 | 3771 | Train 59 | Test Male 59 | 0.78 | 0.85 | 0.89 |
| 2748 | Train 43 | Test Male 60 | 0.77 | 0.83 | 0.85 | 3772 | Train 59 | Test Male 60 | 0.78 | 0.85 | 0.89 |
| 2749 | Train 43 | Test Male 61 | 0.73 | 0.78 | 0.68 | 3773 | Train 59 | Test Male 61 | 0.72 | 0.78 | 0.75 |
| 2750 | Train 43 | Test Male 62 | 0.71 | 0.71 | 0.67 | 3774 | Train 59 | Test Male 62 | 0.69 | 0.7 | 0.63 |
| 2751 | Train 43 | Test Male 63 | 0.73 | 0.78 | 0.71 | 3775 | Train 59 | Test Male 63 | 0.72 | 0.78 | 0.75 |
| 2752 | Train 43 | Test Male 64 | 0.71 | 0.71 | 0.66 | 3776 | Train 59 | Test Male 64 | 0.69 | 0.69 | 0.63 |
| 2753 | Train 44 | Test Male 1 | 0.75 | 0.82 | 0.81 | 3777 | Train 60 | Test Male 1 | 0.74 | 0.72 | 0.7 |
| 2754 | Train 44 | Test Male 2 | 0.76 | 0.83 | 0.82 | 3778 | Train 60 | Test Male 2 | 0.72 | 0.7 | 0.65 |
| 2755 | Train 44 | Test Male 3 | 0.75 | 0.82 | 0.81 | 3779 | Train 60 | Test Male 3 | 0.74 | 0.72 | 0.71 |
| 2756 | Train 44 | Test Male 4 | 0.76 | 0.83 | 0.82 | 3780 | Train 60 | Test Male 4 | 0.72 | 0.71 | 0.65 |
| 2757 | Train 44 | Test Male 5 | 0.72 | 0.75 | 0.73 | 3781 | Train 60 | Test Male 5 | 0.7 | 0.69 | 0.66 |
| 2758 | Train 44 | Test Male 6 | 0.7 | 0.71 | 0.68 | 3782 | Train 60 | Test Male 6 | 0.68 | 0.67 | 0.61 |
| 2759 | Train 44 | Test Male 7 | 0.72 | 0.74 | 0.73 | 3783 | Train 60 | Test Male 7 | 0.7 | 0.69 | 0.66 |
| 2760 | Train 44 | Test Male 8 | 0.7 | 0.71 | 0.68 | 3784 | Train 60 | Test Male 8 | 0.68 | 0.67 | 0.61 |
| 2761 | Train 44 | Test Male 9 | 0.75 | 0.83 | 0.84 | 3785 | Train 60 | Test Male 9 | 0.74 | 0.72 | 0.73 |
| 2762 | Train 44 | Test Male 10 | 0.76 | 0.83 | 0.84 | 3786 | Train 60 | Test Male 10 | 0.72 | 0.71 | 0.67 |
| 2763 | Train 44 | Test Male 11 | 0.75 | 0.83 | 0.84 | 3787 | Train 60 | Test Male 11 | 0.74 | 0.72 | 0.73 |
| 2764 | Train 44 | Test Male 12 | 0.76 | 0.83 | 0.84 | 3788 | Train 60 | Test Male 12 | 0.72 | 0.71 | 0.67 |
| 2765 | Train 44 | Test Male 13 | 0.72 | 0.75 | 0.74 | 3789 | Train 60 | Test Male 13 | 0.7 | 0.7 | 0.67 |
| 2766 | Train 44 | Test Male 14 | 0.71 | 0.71 | 0.68 | 3790 | Train 60 | Test Male 14 | 0.68 | 0.67 | 0.61 |
| 2767 | Train 44 | Test Male 15 | 0.72 | 0.75 | 0.74 | 3791 | Train 60 | Test Male 15 | 0.7 | 0.69 | 0.67 |
| 2768 | Train 44 | Test Male 16 | 0.7 | 0.71 | 0.68 | 3792 | Train 60 | Test Male 16 | 0.68 | 0.67 | 0.61 |
| 2769 | Train 44 | Test Male 17 | 0.75 | 0.82 | 0.81 | 3793 | Train 60 | Test Male 17 | 0.74 | 0.72 | 0.7 |
| 2770 | Train 44 | Test Male 18 | 0.76 | 0.83 | 0.82 | 3794 | Train 60 | Test Male 18 | 0.72 | 0.7 | 0.65 |
| 2771 | Train 44 | Test Male 19 | 0.75 | 0.82 | 0.81 | 3795 | Train 60 | Test Male 19 | 0.74 | 0.72 | 0.71 |
| 2772 | Train 44 | Test Male 20 | 0.76 | 0.83 | 0.82 | 3796 | Train 60 | Test Male 20 | 0.72 | 0.71 | 0.65 |
| 2773 | Train 44 | Test Male 21 | 0.72 | 0.75 | 0.73 | 3797 | Train 60 | Test Male 21 | 0.7 | 0.69 | 0.66 |
| 2774 | Train 44 | Test Male 22 | 0.7 | 0.71 | 0.68 | 3798 | Train 60 | Test Male 22 | 0.68 | 0.67 | 0.61 |
| 2775 | Train 44 | Test Male 23 | 0.72 | 0.74 | 0.73 | 3799 | Train 60 | Test Male 23 | 0.7 | 0.69 | 0.66 |
| 2776 | Train 44 | Test Male 24 | 0.7 | 0.71 | 0.68 | 3800 | Train 60 | Test Male 24 | 0.68 | 0.67 | 0.61 |
| 2777 | Train 44 | Test Male 25 | 0.75 | 0.83 | 0.84 | 3801 | Train 60 | Test Male 25 | 0.74 | 0.72 | 0.73 |
| 2778 | Train 44 | Test Male 26 | 0.76 | 0.83 | 0.84 | 3802 | Train 60 | Test Male 26 | 0.72 | 0.71 | 0.67 |
| 2779 | Train 44 | Test Male 27 | 0.75 | 0.83 | 0.84 | 3803 | Train 60 | Test Male 27 | 0.74 | 0.72 | 0.73 |
| 2780 | Train 44 | Test Male 28 | 0.76 | 0.83 | 0.84 | 3804 | Train 60 | Test Male 28 | 0.72 | 0.71 | 0.67 |
| 2781 | Train 44 | Test Male 29 | 0.72 | 0.75 | 0.74 | 3805 | Train 60 | Test Male 29 | 0.7 | 0.7 | 0.67 |
| 2782 | Train 44 | Test Male 30 | 0.71 | 0.71 | 0.68 | 3806 | Train 60 | Test Male 30 | 0.68 | 0.67 | 0.61 |
| 2783 | Train 44 | Test Male 31 | 0.72 | 0.75 | 0.74 | 3807 | Train 60 | Test Male 31 | 0.7 | 0.69 | 0.67 |
| 2784 | Train 44 | Test Male 32 | 0.7 | 0.71 | 0.68 | 3808 | Train 60 | Test Male 32 | 0.68 | 0.67 | 0.61 |
| 2785 | Train 44 | Test Male 33 | 0.75 | 0.82 | 0.81 | 3809 | Train 60 | Test Male 33 | 0.74 | 0.71 | 0.7 |
| 2786 | Train 44 | Test Male 34 | 0.76 | 0.83 | 0.82 | 3810 | Train 60 | Test Male 34 | 0.72 | 0.7 | 0.65 |
| 2787 | Train 44 | Test Male 35 | 0.75 | 0.82 | 0.81 | 3811 | Train 60 | Test Male 35 | 0.74 | 0.72 | 0.71 |
| 2788 | Train 44 | Test Male 36 | 0.76 | 0.83 | 0.82 | 3812 | Train 60 | Test Male 36 | 0.72 | 0.71 | 0.65 |
| 2789 | Train 44 | Test Male 37 | 0.72 | 0.75 | 0.73 | 3813 | Train 60 | Test Male 37 | 0.7 | 0.69 | 0.66 |
| 2790 | Train 44 | Test Male 38 | 0.7 | 0.71 | 0.68 | 3814 | Train 60 | Test Male 38 | 0.68 | 0.67 | 0.61 |
| 2791 | Train 44 | Test Male 39 | 0.72 | 0.74 | 0.72 | 3815 | Train 60 | Test Male 39 | 0.7 | 0.69 | 0.66 |
| 2792 | Train 44 | Test Male 40 | 0.7 | 0.71 | 0.68 | 3816 | Train 60 | Test Male 40 | 0.68 | 0.67 | 0.61 |
| 2793 | Train 44 | Test Male 41 | 0.75 | 0.83 | 0.84 | 3817 | Train 60 | Test Male 41 | 0.74 | 0.72 | 0.73 |
| 2794 | Train 44 | Test Male 42 | 0.76 | 0.83 | 0.84 | 3818 | Train 60 | Test Male 42 | 0.72 | 0.71 | 0.67 |
| 2795 | Train 44 | Test Male 43 | 0.75 | 0.83 | 0.84 | 3819 | Train 60 | Test Male 43 | 0.74 | 0.72 | 0.73 |
| 2796 | Train 44 | Test Male 44 | 0.76 | 0.83 | 0.84 | 3820 | Train 60 | Test Male 44 | 0.72 | 0.71 | 0.67 |
| 2797 | Train 44 | Test Male 45 | 0.72 | 0.75 | 0.74 | 3821 | Train 60 | Test Male 45 | 0.7 | 0.69 | 0.67 |
| 2798 | Train 44 | Test Male 46 | 0.7 | 0.71 | 0.68 | 3822 | Train 60 | Test Male 46 | 0.68 | 0.67 | 0.61 |
| 2799 | Train 44 | Test Male 47 | 0.71 | 0.75 | 0.74 | 3823 | Train 60 | Test Male 47 | 0.7 | 0.69 | 0.67 |
| 2800 | Train 44 | Test Male 48 | 0.7 | 0.71 | 0.68 | 3824 | Train 60 | Test Male 48 | 0.68 | 0.67 | 0.61 |
| 2801 | Train 44 | Test Male 49 | 0.77 | 0.82 | 0.81 | 3825 | Train 60 | Test Male 49 | 0.78 | 0.84 | 0.83 |
| 2802 | Train 44 | Test Male 50 | 0.77 | 0.82 | 0.81 | 3826 | Train 60 | Test Male 50 | 0.78 | 0.84 | 0.83 |
| 2803 | Train 44 | Test Male 51 | 0.77 | 0.82 | 0.81 | 3827 | Train 60 | Test Male 51 | 0.78 | 0.84 | 0.83 |
| 2804 | Train 44 | Test Male 52 | 0.76 | 0.82 | 0.81 | 3828 | Train 60 | Test Male 52 | 0.78 | 0.84 | 0.83 |
| 2805 | Train 44 | Test Male 53 | 0.74 | 0.78 | 0.71 | 3829 | Train 60 | Test Male 53 | 0.72 | 0.74 | 0.76 |
| 2806 | Train 44 | Test Male 54 | 0.71 | 0.72 | 0.67 | 3830 | Train 60 | Test Male 54 | 0.69 | 0.69 | 0.64 |
| 2807 | Train 44 | Test Male 55 | 0.74 | 0.78 | 0.71 | 3831 | Train 60 | Test Male 55 | 0.72 | 0.75 | 0.76 |
| 2808 | Train 44 | Test Male 56 | 0.71 | 0.71 | 0.67 | 3832 | Train 60 | Test Male 56 | 0.7 | 0.68 | 0.64 |
| 2809 | Train 44 | Test Male 57 | 0.77 | 0.82 | 0.84 | 3833 | Train 60 | Test Male 57 | 0.78 | 0.85 | 0.89 |
| 2810 | Train 44 | Test Male 58 | 0.77 | 0.82 | 0.84 | 3834 | Train 60 | Test Male 58 | 0.78 | 0.85 | 0.89 |
| 2811 | Train 44 | Test Male 59 | 0.77 | 0.82 | 0.85 | 3835 | Train 60 | Test Male 59 | 0.78 | 0.85 | 0.89 |
| 2812 | Train 44 | Test Male 60 | 0.76 | 0.82 | 0.84 | 3836 | Train 60 | Test Male 60 | 0.78 | 0.85 | 0.89 |
| 2813 | Train 44 | Test Male 61 | 0.74 | 0.78 | 0.65 | 3837 | Train 60 | Test Male 61 | 0.72 | 0.74 | 0.77 |
| 2814 | Train 44 | Test Male 62 | 0.71 | 0.72 | 0.67 | 3838 | Train 60 | Test Male 62 | 0.69 | 0.69 | 0.64 |
| 2815 | Train 44 | Test Male 63 | 0.74 | 0.78 | 0.72 | 3839 | Train 60 | Test Male 63 | 0.72 | 0.75 | 0.77 |
| 2816 | Train 44 | Test Male 64 | 0.71 | 0.71 | 0.67 | 3840 | Train 60 | Test Male 64 | 0.7 | 0.68 | 0.64 |
| 2817 | Train 45 | Test Male 1 | 0.75 | 0.8 | 0.8 | 3841 | Train 61 | Test Male 1 | 0.73 | 0.75 | 0.69 |
| 2818 | Train 45 | Test Male 2 | 0.75 | 0.79 | 0.77 | 3842 | Train 61 | Test Male 2 | 0.73 | 0.73 | 0.67 |
| 2819 | Train 45 | Test Male 3 | 0.75 | 0.8 | 0.8 | 3843 | Train 61 | Test Male 3 | 0.73 | 0.75 | 0.69 |
| 2820 | Train 45 | Test Male 4 | 0.75 | 0.79 | 0.77 | 3844 | Train 61 | Test Male 4 | 0.73 | 0.73 | 0.67 |
| 2821 | Train 45 | Test Male 5 | 0.74 | 0.8 | 0.8 | 3845 | Train 61 | Test Male 5 | 0.72 | 0.74 | 0.7 |
| 2822 | Train 45 | Test Male 6 | 0.7 | 0.72 | 0.68 | 3846 | Train 61 | Test Male 6 | 0.69 | 0.69 | 0.63 |
| 2823 | Train 45 | Test Male 7 | 0.74 | 0.8 | 0.79 | 3847 | Train 61 | Test Male 7 | 0.72 | 0.74 | 0.7 |
| 2824 | Train 45 | Test Male 8 | 0.7 | 0.72 | 0.68 | 3848 | Train 61 | Test Male 8 | 0.69 | 0.69 | 0.63 |
| 2825 | Train 45 | Test Male 9 | 0.75 | 0.81 | 0.82 | 3849 | Train 61 | Test Male 9 | 0.73 | 0.75 | 0.71 |
| 2826 | Train 45 | Test Male 10 | 0.75 | 0.79 | 0.79 | 3850 | Train 61 | Test Male 10 | 0.73 | 0.73 | 0.69 |
| 2827 | Train 45 | Test Male 11 | 0.75 | 0.81 | 0.82 | 3851 | Train 61 | Test Male 11 | 0.73 | 0.75 | 0.71 |
| 2828 | Train 45 | Test Male 12 | 0.75 | 0.79 | 0.79 | 3852 | Train 61 | Test Male 12 | 0.73 | 0.73 | 0.69 |
| 2829 | Train 45 | Test Male 13 | 0.74 | 0.81 | 0.82 | 3853 | Train 61 | Test Male 13 | 0.72 | 0.75 | 0.71 |
| 2830 | Train 45 | Test Male 14 | 0.7 | 0.72 | 0.68 | 3854 | Train 61 | Test Male 14 | 0.7 | 0.69 | 0.63 |
| 2831 | Train 45 | Test Male 15 | 0.74 | 0.8 | 0.81 | 3855 | Train 61 | Test Male 15 | 0.72 | 0.75 | 0.71 |
| 2832 | Train 45 | Test Male 16 | 0.7 | 0.72 | 0.68 | 3856 | Train 61 | Test Male 16 | 0.7 | 0.69 | 0.63 |
| 2833 | Train 45 | Test Male 17 | 0.75 | 0.8 | 0.8 | 3857 | Train 61 | Test Male 17 | 0.73 | 0.75 | 0.69 |
| 2834 | Train 45 | Test Male 18 | 0.75 | 0.79 | 0.77 | 3858 | Train 61 | Test Male 18 | 0.73 | 0.73 | 0.67 |
| 2835 | Train 45 | Test Male 19 | 0.75 | 0.8 | 0.8 | 3859 | Train 61 | Test Male 19 | 0.73 | 0.75 | 0.69 |
| 2836 | Train 45 | Test Male 20 | 0.75 | 0.79 | 0.77 | 3860 | Train 61 | Test Male 20 | 0.73 | 0.73 | 0.67 |
| 2837 | Train 45 | Test Male 21 | 0.74 | 0.8 | 0.8 | 3861 | Train 61 | Test Male 21 | 0.72 | 0.74 | 0.7 |
| 2838 | Train 45 | Test Male 22 | 0.7 | 0.72 | 0.68 | 3862 | Train 61 | Test Male 22 | 0.69 | 0.69 | 0.63 |
| 2839 | Train 45 | Test Male 23 | 0.74 | 0.8 | 0.79 | 3863 | Train 61 | Test Male 23 | 0.72 | 0.74 | 0.7 |
| 2840 | Train 45 | Test Male 24 | 0.7 | 0.72 | 0.68 | 3864 | Train 61 | Test Male 24 | 0.69 | 0.69 | 0.63 |
| 2841 | Train 45 | Test Male 25 | 0.75 | 0.81 | 0.82 | 3865 | Train 61 | Test Male 25 | 0.73 | 0.75 | 0.71 |
| 2842 | Train 45 | Test Male 26 | 0.75 | 0.79 | 0.79 | 3866 | Train 61 | Test Male 26 | 0.73 | 0.73 | 0.69 |
| 2843 | Train 45 | Test Male 27 | 0.75 | 0.81 | 0.82 | 3867 | Train 61 | Test Male 27 | 0.73 | 0.75 | 0.71 |
| 2844 | Train 45 | Test Male 28 | 0.75 | 0.79 | 0.79 | 3868 | Train 61 | Test Male 28 | 0.73 | 0.73 | 0.69 |
| 2845 | Train 45 | Test Male 29 | 0.74 | 0.81 | 0.82 | 3869 | Train 61 | Test Male 29 | 0.72 | 0.75 | 0.71 |
| 2846 | Train 45 | Test Male 30 | 0.7 | 0.72 | 0.68 | 3870 | Train 61 | Test Male 30 | 0.7 | 0.69 | 0.63 |
| 2847 | Train 45 | Test Male 31 | 0.74 | 0.81 | 0.81 | 3871 | Train 61 | Test Male 31 | 0.72 | 0.75 | 0.71 |
| 2848 | Train 45 | Test Male 32 | 0.7 | 0.72 | 0.68 | 3872 | Train 61 | Test Male 32 | 0.7 | 0.69 | 0.63 |
| 2849 | Train 45 | Test Male 33 | 0.75 | 0.8 | 0.8 | 3873 | Train 61 | Test Male 33 | 0.73 | 0.75 | 0.69 |
| 2850 | Train 45 | Test Male 34 | 0.75 | 0.79 | 0.77 | 3874 | Train 61 | Test Male 34 | 0.73 | 0.73 | 0.67 |
| 2851 | Train 45 | Test Male 35 | 0.75 | 0.8 | 0.8 | 3875 | Train 61 | Test Male 35 | 0.73 | 0.75 | 0.69 |
| 2852 | Train 45 | Test Male 36 | 0.75 | 0.79 | 0.77 | 3876 | Train 61 | Test Male 36 | 0.73 | 0.73 | 0.67 |
| 2853 | Train 45 | Test Male 37 | 0.74 | 0.8 | 0.8 | 3877 | Train 61 | Test Male 37 | 0.72 | 0.74 | 0.7 |
| 2854 | Train 45 | Test Male 38 | 0.7 | 0.72 | 0.68 | 3878 | Train 61 | Test Male 38 | 0.69 | 0.69 | 0.63 |
| 2855 | Train 45 | Test Male 39 | 0.74 | 0.8 | 0.79 | 3879 | Train 61 | Test Male 39 | 0.72 | 0.74 | 0.7 |
| 2856 | Train 45 | Test Male 40 | 0.7 | 0.72 | 0.68 | 3880 | Train 61 | Test Male 40 | 0.69 | 0.69 | 0.63 |
| 2857 | Train 45 | Test Male 41 | 0.75 | 0.81 | 0.82 | 3881 | Train 61 | Test Male 41 | 0.73 | 0.75 | 0.71 |
| 2858 | Train 45 | Test Male 42 | 0.75 | 0.79 | 0.79 | 3882 | Train 61 | Test Male 42 | 0.73 | 0.73 | 0.69 |
| 2859 | Train 45 | Test Male 43 | 0.75 | 0.81 | 0.82 | 3883 | Train 61 | Test Male 43 | 0.73 | 0.75 | 0.71 |
| 2860 | Train 45 | Test Male 44 | 0.75 | 0.79 | 0.79 | 3884 | Train 61 | Test Male 44 | 0.73 | 0.73 | 0.69 |
| 2861 | Train 45 | Test Male 45 | 0.74 | 0.81 | 0.81 | 3885 | Train 61 | Test Male 45 | 0.72 | 0.75 | 0.71 |
| 2862 | Train 45 | Test Male 46 | 0.7 | 0.72 | 0.68 | 3886 | Train 61 | Test Male 46 | 0.7 | 0.69 | 0.63 |
| 2863 | Train 45 | Test Male 47 | 0.74 | 0.8 | 0.81 | 3887 | Train 61 | Test Male 47 | 0.72 | 0.75 | 0.71 |
| 2864 | Train 45 | Test Male 48 | 0.7 | 0.72 | 0.68 | 3888 | Train 61 | Test Male 48 | 0.7 | 0.69 | 0.63 |
| 2865 | Train 45 | Test Male 49 | 0.76 | 0.81 | 0.8 | 3889 | Train 61 | Test Male 49 | 0.77 | 0.82 | 0.79 |
| 2866 | Train 45 | Test Male 50 | 0.76 | 0.81 | 0.8 | 3890 | Train 61 | Test Male 50 | 0.77 | 0.82 | 0.79 |
| 2867 | Train 45 | Test Male 51 | 0.76 | 0.81 | 0.8 | 3891 | Train 61 | Test Male 51 | 0.77 | 0.81 | 0.79 |
| 2868 | Train 45 | Test Male 52 | 0.76 | 0.8 | 0.8 | 3892 | Train 61 | Test Male 52 | 0.77 | 0.81 | 0.79 |
| 2869 | Train 45 | Test Male 53 | 0.76 | 0.81 | 0.77 | 3893 | Train 61 | Test Male 53 | 0.77 | 0.84 | 0.84 |
| 2870 | Train 45 | Test Male 54 | 0.71 | 0.72 | 0.69 | 3894 | Train 61 | Test Male 54 | 0.71 | 0.71 | 0.66 |
| 2871 | Train 45 | Test Male 55 | 0.76 | 0.81 | 0.77 | 3895 | Train 61 | Test Male 55 | 0.77 | 0.83 | 0.83 |
| 2872 | Train 45 | Test Male 56 | 0.71 | 0.71 | 0.69 | 3896 | Train 61 | Test Male 56 | 0.71 | 0.71 | 0.66 |
| 2873 | Train 45 | Test Male 57 | 0.76 | 0.81 | 0.83 | 3897 | Train 61 | Test Male 57 | 0.77 | 0.82 | 0.83 |
| 2874 | Train 45 | Test Male 58 | 0.76 | 0.81 | 0.83 | 3898 | Train 61 | Test Male 58 | 0.77 | 0.82 | 0.82 |
| 2875 | Train 45 | Test Male 59 | 0.76 | 0.81 | 0.82 | 3899 | Train 61 | Test Male 59 | 0.77 | 0.82 | 0.82 |
| 2876 | Train 45 | Test Male 60 | 0.76 | 0.81 | 0.82 | 3900 | Train 61 | Test Male 60 | 0.77 | 0.82 | 0.82 |
| 2877 | Train 45 | Test Male 61 | 0.76 | 0.81 | 0.77 | 3901 | Train 61 | Test Male 61 | 0.77 | 0.84 | 0.82 |
| 2878 | Train 45 | Test Male 62 | 0.71 | 0.72 | 0.69 | 3902 | Train 61 | Test Male 62 | 0.71 | 0.72 | 0.66 |
| 2879 | Train 45 | Test Male 63 | 0.75 | 0.81 | 0.78 | 3903 | Train 61 | Test Male 63 | 0.76 | 0.84 | 0.86 |
| 2880 | Train 45 | Test Male 64 | 0.71 | 0.72 | 0.69 | 3904 | Train 61 | Test Male 64 | 0.71 | 0.71 | 0.66 |
| 2881 | Train 46 | Test Male 1 | 0.73 | 0.78 | 0.77 | 3905 | Train 62 | Test Male 1 | 0.72 | 0.74 | 0.7 |
| 2882 | Train 46 | Test Male 2 | 0.74 | 0.78 | 0.77 | 3906 | Train 62 | Test Male 2 | 0.73 | 0.75 | 0.7 |
| 2883 | Train 46 | Test Male 3 | 0.73 | 0.78 | 0.77 | 3907 | Train 62 | Test Male 3 | 0.72 | 0.74 | 0.7 |
| 2884 | Train 46 | Test Male 4 | 0.74 | 0.78 | 0.77 | 3908 | Train 62 | Test Male 4 | 0.72 | 0.75 | 0.7 |
| 2885 | Train 46 | Test Male 5 | 0.72 | 0.77 | 0.76 | 3909 | Train 62 | Test Male 5 | 0.71 | 0.74 | 0.71 |
| 2886 | Train 46 | Test Male 6 | 0.73 | 0.79 | 0.79 | 3910 | Train 62 | Test Male 6 | 0.72 | 0.75 | 0.72 |
| 2887 | Train 46 | Test Male 7 | 0.72 | 0.77 | 0.76 | 3911 | Train 62 | Test Male 7 | 0.71 | 0.74 | 0.7 |
| 2888 | Train 46 | Test Male 8 | 0.73 | 0.78 | 0.78 | 3912 | Train 62 | Test Male 8 | 0.72 | 0.75 | 0.72 |
| 2889 | Train 46 | Test Male 9 | 0.73 | 0.78 | 0.77 | 3913 | Train 62 | Test Male 9 | 0.72 | 0.75 | 0.71 |
| 2890 | Train 46 | Test Male 10 | 0.74 | 0.79 | 0.78 | 3914 | Train 62 | Test Male 10 | 0.73 | 0.75 | 0.7 |
| 2891 | Train 46 | Test Male 11 | 0.73 | 0.78 | 0.77 | 3915 | Train 62 | Test Male 11 | 0.71 | 0.75 | 0.71 |
| 2892 | Train 46 | Test Male 12 | 0.74 | 0.78 | 0.78 | 3916 | Train 62 | Test Male 12 | 0.73 | 0.75 | 0.7 |
| 2893 | Train 46 | Test Male 13 | 0.72 | 0.78 | 0.76 | 3917 | Train 62 | Test Male 13 | 0.71 | 0.74 | 0.71 |
| 2894 | Train 46 | Test Male 14 | 0.74 | 0.79 | 0.79 | 3918 | Train 62 | Test Male 14 | 0.72 | 0.75 | 0.72 |
| 2895 | Train 46 | Test Male 15 | 0.72 | 0.77 | 0.76 | 3919 | Train 62 | Test Male 15 | 0.71 | 0.74 | 0.71 |
| 2896 | Train 46 | Test Male 16 | 0.73 | 0.78 | 0.78 | 3920 | Train 62 | Test Male 16 | 0.72 | 0.75 | 0.72 |
| 2897 | Train 46 | Test Male 17 | 0.73 | 0.78 | 0.77 | 3921 | Train 62 | Test Male 17 | 0.72 | 0.74 | 0.7 |
| 2898 | Train 46 | Test Male 18 | 0.74 | 0.78 | 0.77 | 3922 | Train 62 | Test Male 18 | 0.73 | 0.75 | 0.7 |
| 2899 | Train 46 | Test Male 19 | 0.73 | 0.78 | 0.77 | 3923 | Train 62 | Test Male 19 | 0.72 | 0.74 | 0.7 |
| 2900 | Train 46 | Test Male 20 | 0.74 | 0.78 | 0.77 | 3924 | Train 62 | Test Male 20 | 0.72 | 0.75 | 0.7 |
| 2901 | Train 46 | Test Male 21 | 0.72 | 0.77 | 0.76 | 3925 | Train 62 | Test Male 21 | 0.71 | 0.74 | 0.71 |
| 2902 | Train 46 | Test Male 22 | 0.73 | 0.79 | 0.79 | 3926 | Train 62 | Test Male 22 | 0.72 | 0.75 | 0.72 |
| 2903 | Train 46 | Test Male 23 | 0.72 | 0.77 | 0.76 | 3927 | Train 62 | Test Male 23 | 0.71 | 0.74 | 0.7 |
| 2904 | Train 46 | Test Male 24 | 0.73 | 0.78 | 0.78 | 3928 | Train 62 | Test Male 24 | 0.72 | 0.75 | 0.72 |
| 2905 | Train 46 | Test Male 25 | 0.73 | 0.78 | 0.77 | 3929 | Train 62 | Test Male 25 | 0.72 | 0.75 | 0.71 |
| 2906 | Train 46 | Test Male 26 | 0.74 | 0.79 | 0.78 | 3930 | Train 62 | Test Male 26 | 0.73 | 0.75 | 0.7 |
| 2907 | Train 46 | Test Male 27 | 0.73 | 0.78 | 0.77 | 3931 | Train 62 | Test Male 27 | 0.71 | 0.75 | 0.71 |
| 2908 | Train 46 | Test Male 28 | 0.74 | 0.78 | 0.78 | 3932 | Train 62 | Test Male 28 | 0.73 | 0.75 | 0.7 |
| 2909 | Train 46 | Test Male 29 | 0.72 | 0.78 | 0.76 | 3933 | Train 62 | Test Male 29 | 0.71 | 0.74 | 0.71 |
| 2910 | Train 46 | Test Male 30 | 0.74 | 0.79 | 0.79 | 3934 | Train 62 | Test Male 30 | 0.72 | 0.75 | 0.72 |
| 2911 | Train 46 | Test Male 31 | 0.72 | 0.77 | 0.76 | 3935 | Train 62 | Test Male 31 | 0.71 | 0.74 | 0.71 |
| 2912 | Train 46 | Test Male 32 | 0.73 | 0.78 | 0.78 | 3936 | Train 62 | Test Male 32 | 0.72 | 0.75 | 0.72 |
| 2913 | Train 46 | Test Male 33 | 0.73 | 0.78 | 0.77 | 3937 | Train 62 | Test Male 33 | 0.72 | 0.74 | 0.7 |
| 2914 | Train 46 | Test Male 34 | 0.74 | 0.78 | 0.77 | 3938 | Train 62 | Test Male 34 | 0.73 | 0.75 | 0.7 |
| 2915 | Train 46 | Test Male 35 | 0.73 | 0.78 | 0.77 | 3939 | Train 62 | Test Male 35 | 0.71 | 0.74 | 0.7 |
| 2916 | Train 46 | Test Male 36 | 0.74 | 0.78 | 0.77 | 3940 | Train 62 | Test Male 36 | 0.72 | 0.75 | 0.7 |
| 2917 | Train 46 | Test Male 37 | 0.72 | 0.77 | 0.76 | 3941 | Train 62 | Test Male 37 | 0.71 | 0.74 | 0.71 |
| 2918 | Train 46 | Test Male 38 | 0.73 | 0.79 | 0.79 | 3942 | Train 62 | Test Male 38 | 0.72 | 0.75 | 0.72 |
| 2919 | Train 46 | Test Male 39 | 0.72 | 0.77 | 0.76 | 3943 | Train 62 | Test Male 39 | 0.71 | 0.74 | 0.7 |
| 2920 | Train 46 | Test Male 40 | 0.73 | 0.78 | 0.78 | 3944 | Train 62 | Test Male 40 | 0.72 | 0.75 | 0.72 |
| 2921 | Train 46 | Test Male 41 | 0.73 | 0.78 | 0.77 | 3945 | Train 62 | Test Male 41 | 0.71 | 0.75 | 0.71 |
| 2922 | Train 46 | Test Male 42 | 0.74 | 0.78 | 0.78 | 3946 | Train 62 | Test Male 42 | 0.73 | 0.75 | 0.7 |
| 2923 | Train 46 | Test Male 43 | 0.73 | 0.78 | 0.77 | 3947 | Train 62 | Test Male 43 | 0.71 | 0.75 | 0.71 |
| 2924 | Train 46 | Test Male 44 | 0.74 | 0.78 | 0.78 | 3948 | Train 62 | Test Male 44 | 0.73 | 0.75 | 0.7 |
| 2925 | Train 46 | Test Male 45 | 0.72 | 0.77 | 0.76 | 3949 | Train 62 | Test Male 45 | 0.71 | 0.74 | 0.71 |
| 2926 | Train 46 | Test Male 46 | 0.74 | 0.79 | 0.79 | 3950 | Train 62 | Test Male 46 | 0.72 | 0.75 | 0.72 |
| 2927 | Train 46 | Test Male 47 | 0.72 | 0.77 | 0.76 | 3951 | Train 62 | Test Male 47 | 0.71 | 0.74 | 0.71 |
| 2928 | Train 46 | Test Male 48 | 0.73 | 0.78 | 0.78 | 3952 | Train 62 | Test Male 48 | 0.72 | 0.75 | 0.72 |
| 2929 | Train 46 | Test Male 49 | 0.76 | 0.8 | 0.79 | 3953 | Train 62 | Test Male 49 | 0.77 | 0.8 | 0.79 |
| 2930 | Train 46 | Test Male 50 | 0.76 | 0.8 | 0.79 | 3954 | Train 62 | Test Male 50 | 0.77 | 0.8 | 0.79 |
| 2931 | Train 46 | Test Male 51 | 0.76 | 0.79 | 0.78 | 3955 | Train 62 | Test Male 51 | 0.77 | 0.8 | 0.79 |
| 2932 | Train 46 | Test Male 52 | 0.76 | 0.79 | 0.78 | 3956 | Train 62 | Test Male 52 | 0.77 | 0.8 | 0.79 |
| 2933 | Train 46 | Test Male 53 | 0.75 | 0.8 | 0.8 | 3957 | Train 62 | Test Male 53 | 0.76 | 0.82 | 0.81 |
| 2934 | Train 46 | Test Male 54 | 0.74 | 0.78 | 0.79 | 3958 | Train 62 | Test Male 54 | 0.75 | 0.8 | 0.81 |
| 2935 | Train 46 | Test Male 55 | 0.75 | 0.8 | 0.8 | 3959 | Train 62 | Test Male 55 | 0.76 | 0.82 | 0.81 |
| 2936 | Train 46 | Test Male 56 | 0.74 | 0.78 | 0.78 | 3960 | Train 62 | Test Male 56 | 0.74 | 0.8 | 0.8 |
| 2937 | Train 46 | Test Male 57 | 0.76 | 0.8 | 0.8 | 3961 | Train 62 | Test Male 57 | 0.77 | 0.81 | 0.81 |
| 2938 | Train 46 | Test Male 58 | 0.76 | 0.8 | 0.8 | 3962 | Train 62 | Test Male 58 | 0.77 | 0.81 | 0.81 |
| 2939 | Train 46 | Test Male 59 | 0.76 | 0.8 | 0.8 | 3963 | Train 62 | Test Male 59 | 0.77 | 0.8 | 0.81 |
| 2940 | Train 46 | Test Male 60 | 0.76 | 0.8 | 0.8 | 3964 | Train 62 | Test Male 60 | 0.77 | 0.8 | 0.81 |
| 2941 | Train 46 | Test Male 61 | 0.75 | 0.81 | 0.78 | 3965 | Train 62 | Test Male 61 | 0.76 | 0.82 | 0.76 |
| 2942 | Train 46 | Test Male 62 | 0.74 | 0.78 | 0.79 | 3966 | Train 62 | Test Male 62 | 0.75 | 0.8 | 0.81 |
| 2943 | Train 46 | Test Male 63 | 0.75 | 0.81 | 0.81 | 3967 | Train 62 | Test Male 63 | 0.76 | 0.82 | 0.83 |
| 2944 | Train 46 | Test Male 64 | 0.74 | 0.78 | 0.78 | 3968 | Train 62 | Test Male 64 | 0.74 | 0.8 | 0.8 |
| 2945 | Train 47 | Test Male 1 | 0.75 | 0.8 | 0.8 | 3969 | Train 63 | Test Male 1 | 0.73 | 0.75 | 0.7 |
| 2946 | Train 47 | Test Male 2 | 0.75 | 0.79 | 0.77 | 3970 | Train 63 | Test Male 2 | 0.73 | 0.73 | 0.68 |
| 2947 | Train 47 | Test Male 3 | 0.75 | 0.8 | 0.8 | 3971 | Train 63 | Test Male 3 | 0.73 | 0.75 | 0.7 |
| 2948 | Train 47 | Test Male 4 | 0.75 | 0.79 | 0.77 | 3972 | Train 63 | Test Male 4 | 0.73 | 0.73 | 0.68 |
| 2949 | Train 47 | Test Male 5 | 0.74 | 0.8 | 0.8 | 3973 | Train 63 | Test Male 5 | 0.72 | 0.74 | 0.7 |
| 2950 | Train 47 | Test Male 6 | 0.7 | 0.71 | 0.67 | 3974 | Train 63 | Test Male 6 | 0.69 | 0.69 | 0.65 |
| 2951 | Train 47 | Test Male 7 | 0.74 | 0.8 | 0.8 | 3975 | Train 63 | Test Male 7 | 0.72 | 0.74 | 0.7 |
| 2952 | Train 47 | Test Male 8 | 0.7 | 0.72 | 0.67 | 3976 | Train 63 | Test Male 8 | 0.69 | 0.69 | 0.65 |
| 2953 | Train 47 | Test Male 9 | 0.75 | 0.81 | 0.82 | 3977 | Train 63 | Test Male 9 | 0.73 | 0.75 | 0.72 |
| 2954 | Train 47 | Test Male 10 | 0.75 | 0.79 | 0.78 | 3978 | Train 63 | Test Male 10 | 0.73 | 0.73 | 0.69 |
| 2955 | Train 47 | Test Male 11 | 0.75 | 0.81 | 0.82 | 3979 | Train 63 | Test Male 11 | 0.73 | 0.75 | 0.72 |
| 2956 | Train 47 | Test Male 12 | 0.75 | 0.79 | 0.79 | 3980 | Train 63 | Test Male 12 | 0.73 | 0.73 | 0.69 |
| 2957 | Train 47 | Test Male 13 | 0.74 | 0.81 | 0.82 | 3981 | Train 63 | Test Male 13 | 0.72 | 0.75 | 0.72 |
| 2958 | Train 47 | Test Male 14 | 0.7 | 0.72 | 0.67 | 3982 | Train 63 | Test Male 14 | 0.7 | 0.69 | 0.65 |
| 2959 | Train 47 | Test Male 15 | 0.74 | 0.81 | 0.81 | 3983 | Train 63 | Test Male 15 | 0.72 | 0.75 | 0.72 |
| 2960 | Train 47 | Test Male 16 | 0.7 | 0.72 | 0.67 | 3984 | Train 63 | Test Male 16 | 0.7 | 0.69 | 0.65 |
| 2961 | Train 47 | Test Male 17 | 0.75 | 0.8 | 0.8 | 3985 | Train 63 | Test Male 17 | 0.73 | 0.75 | 0.7 |
| 2962 | Train 47 | Test Male 18 | 0.75 | 0.79 | 0.77 | 3986 | Train 63 | Test Male 18 | 0.73 | 0.73 | 0.68 |
| 2963 | Train 47 | Test Male 19 | 0.75 | 0.8 | 0.8 | 3987 | Train 63 | Test Male 19 | 0.73 | 0.75 | 0.7 |
| 2964 | Train 47 | Test Male 20 | 0.75 | 0.79 | 0.77 | 3988 | Train 63 | Test Male 20 | 0.73 | 0.73 | 0.68 |
| 2965 | Train 47 | Test Male 21 | 0.74 | 0.8 | 0.8 | 3989 | Train 63 | Test Male 21 | 0.72 | 0.74 | 0.7 |
| 2966 | Train 47 | Test Male 22 | 0.7 | 0.71 | 0.67 | 3990 | Train 63 | Test Male 22 | 0.69 | 0.69 | 0.65 |
| 2967 | Train 47 | Test Male 23 | 0.74 | 0.8 | 0.8 | 3991 | Train 63 | Test Male 23 | 0.72 | 0.74 | 0.7 |
| 2968 | Train 47 | Test Male 24 | 0.7 | 0.72 | 0.67 | 3992 | Train 63 | Test Male 24 | 0.69 | 0.69 | 0.65 |
| 2969 | Train 47 | Test Male 25 | 0.75 | 0.81 | 0.82 | 3993 | Train 63 | Test Male 25 | 0.73 | 0.75 | 0.72 |
| 2970 | Train 47 | Test Male 26 | 0.75 | 0.79 | 0.78 | 3994 | Train 63 | Test Male 26 | 0.73 | 0.73 | 0.69 |
| 2971 | Train 47 | Test Male 27 | 0.75 | 0.81 | 0.82 | 3995 | Train 63 | Test Male 27 | 0.73 | 0.75 | 0.72 |
| 2972 | Train 47 | Test Male 28 | 0.75 | 0.79 | 0.79 | 3996 | Train 63 | Test Male 28 | 0.73 | 0.73 | 0.69 |
| 2973 | Train 47 | Test Male 29 | 0.74 | 0.81 | 0.82 | 3997 | Train 63 | Test Male 29 | 0.72 | 0.75 | 0.72 |
| 2974 | Train 47 | Test Male 30 | 0.7 | 0.72 | 0.67 | 3998 | Train 63 | Test Male 30 | 0.7 | 0.69 | 0.65 |
| 2975 | Train 47 | Test Male 31 | 0.74 | 0.81 | 0.81 | 3999 | Train 63 | Test Male 31 | 0.72 | 0.75 | 0.72 |
| 2976 | Train 47 | Test Male 32 | 0.7 | 0.72 | 0.67 | 4000 | Train 63 | Test Male 32 | 0.7 | 0.69 | 0.65 |
| 2977 | Train 47 | Test Male 33 | 0.75 | 0.8 | 0.8 | 4001 | Train 63 | Test Male 33 | 0.73 | 0.75 | 0.7 |
| 2978 | Train 47 | Test Male 34 | 0.75 | 0.79 | 0.77 | 4002 | Train 63 | Test Male 34 | 0.73 | 0.73 | 0.68 |
| 2979 | Train 47 | Test Male 35 | 0.75 | 0.8 | 0.8 | 4003 | Train 63 | Test Male 35 | 0.73 | 0.75 | 0.7 |
| 2980 | Train 47 | Test Male 36 | 0.75 | 0.79 | 0.77 | 4004 | Train 63 | Test Male 36 | 0.73 | 0.73 | 0.68 |
| 2981 | Train 47 | Test Male 37 | 0.74 | 0.8 | 0.8 | 4005 | Train 63 | Test Male 37 | 0.72 | 0.74 | 0.7 |
| 2982 | Train 47 | Test Male 38 | 0.7 | 0.71 | 0.67 | 4006 | Train 63 | Test Male 38 | 0.69 | 0.69 | 0.65 |
| 2983 | Train 47 | Test Male 39 | 0.74 | 0.8 | 0.8 | 4007 | Train 63 | Test Male 39 | 0.72 | 0.74 | 0.7 |
| 2984 | Train 47 | Test Male 40 | 0.7 | 0.72 | 0.67 | 4008 | Train 63 | Test Male 40 | 0.69 | 0.69 | 0.65 |
| 2985 | Train 47 | Test Male 41 | 0.75 | 0.81 | 0.82 | 4009 | Train 63 | Test Male 41 | 0.73 | 0.75 | 0.72 |
| 2986 | Train 47 | Test Male 42 | 0.75 | 0.79 | 0.78 | 4010 | Train 63 | Test Male 42 | 0.73 | 0.73 | 0.69 |
| 2987 | Train 47 | Test Male 43 | 0.75 | 0.81 | 0.82 | 4011 | Train 63 | Test Male 43 | 0.73 | 0.75 | 0.72 |
| 2988 | Train 47 | Test Male 44 | 0.75 | 0.79 | 0.78 | 4012 | Train 63 | Test Male 44 | 0.73 | 0.73 | 0.69 |
| 2989 | Train 47 | Test Male 45 | 0.74 | 0.81 | 0.81 | 4013 | Train 63 | Test Male 45 | 0.72 | 0.75 | 0.72 |
| 2990 | Train 47 | Test Male 46 | 0.7 | 0.72 | 0.67 | 4014 | Train 63 | Test Male 46 | 0.7 | 0.69 | 0.65 |
| 2991 | Train 47 | Test Male 47 | 0.74 | 0.81 | 0.81 | 4015 | Train 63 | Test Male 47 | 0.72 | 0.75 | 0.72 |
| 2992 | Train 47 | Test Male 48 | 0.7 | 0.72 | 0.67 | 4016 | Train 63 | Test Male 48 | 0.7 | 0.69 | 0.65 |
| 2993 | Train 47 | Test Male 49 | 0.76 | 0.81 | 0.8 | 4017 | Train 63 | Test Male 49 | 0.77 | 0.81 | 0.8 |
| 2994 | Train 47 | Test Male 50 | 0.76 | 0.81 | 0.8 | 4018 | Train 63 | Test Male 50 | 0.77 | 0.81 | 0.8 |
| 2995 | Train 47 | Test Male 51 | 0.76 | 0.81 | 0.8 | 4019 | Train 63 | Test Male 51 | 0.77 | 0.81 | 0.8 |
| 2996 | Train 47 | Test Male 52 | 0.76 | 0.81 | 0.8 | 4020 | Train 63 | Test Male 52 | 0.77 | 0.81 | 0.8 |
| 2997 | Train 47 | Test Male 53 | 0.76 | 0.8 | 0.76 | 4021 | Train 63 | Test Male 53 | 0.77 | 0.84 | 0.84 |
| 2998 | Train 47 | Test Male 54 | 0.71 | 0.71 | 0.67 | 4022 | Train 63 | Test Male 54 | 0.71 | 0.71 | 0.68 |
| 2999 | Train 47 | Test Male 55 | 0.76 | 0.8 | 0.76 | 4023 | Train 63 | Test Male 55 | 0.77 | 0.84 | 0.84 |
| 3000 | Train 47 | Test Male 56 | 0.71 | 0.71 | 0.67 | 4024 | Train 63 | Test Male 56 | 0.71 | 0.71 | 0.68 |
| 3001 | Train 47 | Test Male 57 | 0.76 | 0.81 | 0.83 | 4025 | Train 63 | Test Male 57 | 0.77 | 0.82 | 0.83 |
| 3002 | Train 47 | Test Male 58 | 0.76 | 0.81 | 0.82 | 4026 | Train 63 | Test Male 58 | 0.77 | 0.82 | 0.83 |
| 3003 | Train 47 | Test Male 59 | 0.76 | 0.81 | 0.83 | 4027 | Train 63 | Test Male 59 | 0.77 | 0.82 | 0.83 |
| 3004 | Train 47 | Test Male 60 | 0.76 | 0.81 | 0.83 | 4028 | Train 63 | Test Male 60 | 0.77 | 0.82 | 0.83 |
| 3005 | Train 47 | Test Male 61 | 0.76 | 0.8 | 0.68 | 4029 | Train 63 | Test Male 61 | 0.77 | 0.84 | 0.81 |
| 3006 | Train 47 | Test Male 62 | 0.71 | 0.71 | 0.67 | 4030 | Train 63 | Test Male 62 | 0.71 | 0.71 | 0.68 |
| 3007 | Train 47 | Test Male 63 | 0.76 | 0.81 | 0.77 | 4031 | Train 63 | Test Male 63 | 0.76 | 0.84 | 0.86 |
| 3008 | Train 47 | Test Male 64 | 0.71 | 0.71 | 0.67 | 4032 | Train 63 | Test Male 64 | 0.71 | 0.71 | 0.68 |
| 3009 | Train 48 | Test Male 1 | 0.74 | 0.78 | 0.77 | 4033 | Train 64 | Test Male 1 | 0.72 | 0.75 | 0.71 |
| 3010 | Train 48 | Test Male 2 | 0.75 | 0.78 | 0.77 | 4034 | Train 64 | Test Male 2 | 0.72 | 0.75 | 0.71 |
| 3011 | Train 48 | Test Male 3 | 0.73 | 0.78 | 0.77 | 4035 | Train 64 | Test Male 3 | 0.72 | 0.75 | 0.71 |
| 3012 | Train 48 | Test Male 4 | 0.74 | 0.78 | 0.77 | 4036 | Train 64 | Test Male 4 | 0.72 | 0.75 | 0.71 |
| 3013 | Train 48 | Test Male 5 | 0.72 | 0.77 | 0.76 | 4037 | Train 64 | Test Male 5 | 0.71 | 0.74 | 0.72 |
| 3014 | Train 48 | Test Male 6 | 0.73 | 0.78 | 0.79 | 4038 | Train 64 | Test Male 6 | 0.72 | 0.75 | 0.72 |
| 3015 | Train 48 | Test Male 7 | 0.72 | 0.77 | 0.76 | 4039 | Train 64 | Test Male 7 | 0.71 | 0.74 | 0.72 |
| 3016 | Train 48 | Test Male 8 | 0.73 | 0.78 | 0.79 | 4040 | Train 64 | Test Male 8 | 0.72 | 0.75 | 0.72 |
| 3017 | Train 48 | Test Male 9 | 0.73 | 0.78 | 0.78 | 4041 | Train 64 | Test Male 9 | 0.72 | 0.75 | 0.72 |
| 3018 | Train 48 | Test Male 10 | 0.75 | 0.78 | 0.78 | 4042 | Train 64 | Test Male 10 | 0.72 | 0.75 | 0.71 |
| 3019 | Train 48 | Test Male 11 | 0.73 | 0.78 | 0.78 | 4043 | Train 64 | Test Male 11 | 0.71 | 0.75 | 0.72 |
| 3020 | Train 48 | Test Male 12 | 0.74 | 0.78 | 0.78 | 4044 | Train 64 | Test Male 12 | 0.72 | 0.75 | 0.71 |
| 3021 | Train 48 | Test Male 13 | 0.72 | 0.77 | 0.77 | 4045 | Train 64 | Test Male 13 | 0.71 | 0.74 | 0.72 |
| 3022 | Train 48 | Test Male 14 | 0.73 | 0.78 | 0.79 | 4046 | Train 64 | Test Male 14 | 0.72 | 0.75 | 0.72 |
| 3023 | Train 48 | Test Male 15 | 0.72 | 0.77 | 0.77 | 4047 | Train 64 | Test Male 15 | 0.7 | 0.74 | 0.72 |
| 3024 | Train 48 | Test Male 16 | 0.73 | 0.78 | 0.79 | 4048 | Train 64 | Test Male 16 | 0.72 | 0.75 | 0.72 |
| 3025 | Train 48 | Test Male 17 | 0.74 | 0.78 | 0.77 | 4049 | Train 64 | Test Male 17 | 0.72 | 0.75 | 0.71 |
| 3026 | Train 48 | Test Male 18 | 0.75 | 0.78 | 0.77 | 4050 | Train 64 | Test Male 18 | 0.72 | 0.75 | 0.71 |
| 3027 | Train 48 | Test Male 19 | 0.73 | 0.78 | 0.77 | 4051 | Train 64 | Test Male 19 | 0.72 | 0.75 | 0.71 |
| 3028 | Train 48 | Test Male 20 | 0.74 | 0.78 | 0.77 | 4052 | Train 64 | Test Male 20 | 0.72 | 0.75 | 0.71 |
| 3029 | Train 48 | Test Male 21 | 0.72 | 0.77 | 0.76 | 4053 | Train 64 | Test Male 21 | 0.71 | 0.74 | 0.72 |
| 3030 | Train 48 | Test Male 22 | 0.73 | 0.78 | 0.79 | 4054 | Train 64 | Test Male 22 | 0.72 | 0.75 | 0.72 |
| 3031 | Train 48 | Test Male 23 | 0.72 | 0.77 | 0.76 | 4055 | Train 64 | Test Male 23 | 0.71 | 0.74 | 0.72 |
| 3032 | Train 48 | Test Male 24 | 0.73 | 0.78 | 0.79 | 4056 | Train 64 | Test Male 24 | 0.72 | 0.75 | 0.72 |
| 3033 | Train 48 | Test Male 25 | 0.73 | 0.78 | 0.78 | 4057 | Train 64 | Test Male 25 | 0.72 | 0.75 | 0.72 |
| 3034 | Train 48 | Test Male 26 | 0.75 | 0.78 | 0.78 | 4058 | Train 64 | Test Male 26 | 0.72 | 0.75 | 0.71 |
| 3035 | Train 48 | Test Male 27 | 0.73 | 0.78 | 0.78 | 4059 | Train 64 | Test Male 27 | 0.71 | 0.75 | 0.72 |
| 3036 | Train 48 | Test Male 28 | 0.74 | 0.78 | 0.78 | 4060 | Train 64 | Test Male 28 | 0.72 | 0.75 | 0.71 |
| 3037 | Train 48 | Test Male 29 | 0.72 | 0.77 | 0.77 | 4061 | Train 64 | Test Male 29 | 0.71 | 0.74 | 0.72 |
| 3038 | Train 48 | Test Male 30 | 0.73 | 0.78 | 0.79 | 4062 | Train 64 | Test Male 30 | 0.72 | 0.75 | 0.72 |
| 3039 | Train 48 | Test Male 31 | 0.72 | 0.77 | 0.77 | 4063 | Train 64 | Test Male 31 | 0.7 | 0.74 | 0.72 |
| 3040 | Train 48 | Test Male 32 | 0.73 | 0.78 | 0.79 | 4064 | Train 64 | Test Male 32 | 0.72 | 0.75 | 0.72 |
| 3041 | Train 48 | Test Male 33 | 0.74 | 0.78 | 0.77 | 4065 | Train 64 | Test Male 33 | 0.72 | 0.75 | 0.71 |
| 3042 | Train 48 | Test Male 34 | 0.74 | 0.78 | 0.77 | 4066 | Train 64 | Test Male 34 | 0.72 | 0.75 | 0.7 |
| 3043 | Train 48 | Test Male 35 | 0.73 | 0.78 | 0.77 | 4067 | Train 64 | Test Male 35 | 0.72 | 0.75 | 0.71 |
| 3044 | Train 48 | Test Male 36 | 0.74 | 0.78 | 0.77 | 4068 | Train 64 | Test Male 36 | 0.72 | 0.75 | 0.7 |
| 3045 | Train 48 | Test Male 37 | 0.72 | 0.77 | 0.76 | 4069 | Train 64 | Test Male 37 | 0.71 | 0.74 | 0.72 |
| 3046 | Train 48 | Test Male 38 | 0.73 | 0.78 | 0.79 | 4070 | Train 64 | Test Male 38 | 0.72 | 0.75 | 0.72 |
| 3047 | Train 48 | Test Male 39 | 0.72 | 0.77 | 0.76 | 4071 | Train 64 | Test Male 39 | 0.71 | 0.74 | 0.72 |
| 3048 | Train 48 | Test Male 40 | 0.73 | 0.78 | 0.79 | 4072 | Train 64 | Test Male 40 | 0.72 | 0.75 | 0.72 |
| 3049 | Train 48 | Test Male 41 | 0.73 | 0.78 | 0.78 | 4073 | Train 64 | Test Male 41 | 0.71 | 0.75 | 0.72 |
| 3050 | Train 48 | Test Male 42 | 0.75 | 0.78 | 0.78 | 4074 | Train 64 | Test Male 42 | 0.72 | 0.75 | 0.71 |
| 3051 | Train 48 | Test Male 43 | 0.73 | 0.78 | 0.78 | 4075 | Train 64 | Test Male 43 | 0.71 | 0.75 | 0.72 |
| 3052 | Train 48 | Test Male 44 | 0.74 | 0.78 | 0.78 | 4076 | Train 64 | Test Male 44 | 0.72 | 0.75 | 0.71 |
| 3053 | Train 48 | Test Male 45 | 0.72 | 0.77 | 0.77 | 4077 | Train 64 | Test Male 45 | 0.71 | 0.74 | 0.72 |
| 3054 | Train 48 | Test Male 46 | 0.73 | 0.78 | 0.79 | 4078 | Train 64 | Test Male 46 | 0.72 | 0.75 | 0.72 |
| 3055 | Train 48 | Test Male 47 | 0.72 | 0.77 | 0.76 | 4079 | Train 64 | Test Male 47 | 0.7 | 0.74 | 0.72 |
| 3056 | Train 48 | Test Male 48 | 0.73 | 0.78 | 0.79 | 4080 | Train 64 | Test Male 48 | 0.72 | 0.75 | 0.72 |
| 3057 | Train 48 | Test Male 49 | 0.76 | 0.8 | 0.79 | 4081 | Train 64 | Test Male 49 | 0.77 | 0.8 | 0.79 |
| 3058 | Train 48 | Test Male 50 | 0.76 | 0.8 | 0.79 | 4082 | Train 64 | Test Male 50 | 0.77 | 0.8 | 0.79 |
| 3059 | Train 48 | Test Male 51 | 0.76 | 0.8 | 0.79 | 4083 | Train 64 | Test Male 51 | 0.77 | 0.8 | 0.79 |
| 3060 | Train 48 | Test Male 52 | 0.76 | 0.79 | 0.79 | 4084 | Train 64 | Test Male 52 | 0.77 | 0.8 | 0.79 |
| 3061 | Train 48 | Test Male 53 | 0.75 | 0.8 | 0.8 | 4085 | Train 64 | Test Male 53 | 0.76 | 0.82 | 0.82 |
| 3062 | Train 48 | Test Male 54 | 0.74 | 0.78 | 0.79 | 4086 | Train 64 | Test Male 54 | 0.74 | 0.8 | 0.81 |
| 3063 | Train 48 | Test Male 55 | 0.75 | 0.8 | 0.8 | 4087 | Train 64 | Test Male 55 | 0.76 | 0.82 | 0.82 |
| 3064 | Train 48 | Test Male 56 | 0.74 | 0.78 | 0.79 | 4088 | Train 64 | Test Male 56 | 0.74 | 0.8 | 0.81 |
| 3065 | Train 48 | Test Male 57 | 0.76 | 0.8 | 0.8 | 4089 | Train 64 | Test Male 57 | 0.77 | 0.81 | 0.81 |
| 3066 | Train 48 | Test Male 58 | 0.76 | 0.8 | 0.8 | 4090 | Train 64 | Test Male 58 | 0.77 | 0.81 | 0.81 |
| 3067 | Train 48 | Test Male 59 | 0.76 | 0.8 | 0.8 | 4091 | Train 64 | Test Male 59 | 0.77 | 0.8 | 0.81 |
| 3068 | Train 48 | Test Male 60 | 0.76 | 0.8 | 0.8 | 4092 | Train 64 | Test Male 60 | 0.77 | 0.8 | 0.81 |
| 3069 | Train 48 | Test Male 61 | 0.75 | 0.81 | 0.77 | 4093 | Train 64 | Test Male 61 | 0.76 | 0.82 | 0.71 |
| 3070 | Train 48 | Test Male 62 | 0.74 | 0.78 | 0.79 | 4094 | Train 64 | Test Male 62 | 0.74 | 0.8 | 0.81 |
| 3071 | Train 48 | Test Male 63 | 0.75 | 0.81 | 0.81 | 4095 | Train 64 | Test Male 63 | 0.76 | 0.82 | 0.83 |
| 3072 | Train 48 | Test Male 64 | 0.74 | 0.78 | 0.79 | 4096 | Train 64 | Test Male 64 | 0.74 | 0.8 | 0.81 |

Table 3. AUC Performance of the ML Models— LR, RF, and GB —for Female Patients Trained on Cohorts 1–32 and Tested Across 64 Cohorts.

| Female | Train  Model | Test  Dataset | AUC (LR) | AUC (RF) | AUC (GB) | Female | Train  Model | Test  Dataset | AUC (LR) | AUC (RF) | AUC (GB) |
| --- | --- | --- | --- | --- | --- | --- | --- | --- | --- | --- | --- |
| 1 | Train 1 | Test Female 1 | 0.76 | 0.84 | 0.86 | 1025 | Train 17 | Test Female 1 | 0.76 | 0.84 | 0.86 |
| 2 | Train 1 | Test Female 2 | 0.76 | 0.81 | 0.81 | 1026 | Train 17 | Test Female 2 | 0.76 | 0.81 | 0.8 |
| 3 | Train 1 | Test Female 3 | 0.77 | 0.84 | 0.86 | 1027 | Train 17 | Test Female 3 | 0.77 | 0.84 | 0.86 |
| 4 | Train 1 | Test Female 4 | 0.76 | 0.81 | 0.81 | 1028 | Train 17 | Test Female 4 | 0.76 | 0.81 | 0.8 |
| 5 | Train 1 | Test Female 5 | 0.73 | 0.76 | 0.75 | 1029 | Train 17 | Test Female 5 | 0.73 | 0.76 | 0.75 |
| 6 | Train 1 | Test Female 6 | 0.71 | 0.71 | 0.66 | 1030 | Train 17 | Test Female 6 | 0.71 | 0.72 | 0.68 |
| 7 | Train 1 | Test Female 7 | 0.73 | 0.76 | 0.75 | 1031 | Train 17 | Test Female 7 | 0.73 | 0.76 | 0.74 |
| 8 | Train 1 | Test Female 8 | 0.71 | 0.71 | 0.66 | 1032 | Train 17 | Test Female 8 | 0.71 | 0.72 | 0.67 |
| 9 | Train 1 | Test Female 9 | 0.77 | 0.83 | 0.82 | 1033 | Train 17 | Test Female 9 | 0.77 | 0.83 | 0.83 |
| 10 | Train 1 | Test Female 10 | 0.76 | 0.8 | 0.79 | 1034 | Train 17 | Test Female 10 | 0.76 | 0.8 | 0.78 |
| 11 | Train 1 | Test Female 11 | 0.77 | 0.83 | 0.82 | 1035 | Train 17 | Test Female 11 | 0.77 | 0.83 | 0.82 |
| 12 | Train 1 | Test Female 12 | 0.76 | 0.8 | 0.79 | 1036 | Train 17 | Test Female 12 | 0.76 | 0.8 | 0.78 |
| 13 | Train 1 | Test Female 13 | 0.73 | 0.75 | 0.69 | 1037 | Train 17 | Test Female 13 | 0.73 | 0.75 | 0.71 |
| 14 | Train 1 | Test Female 14 | 0.71 | 0.72 | 0.66 | 1038 | Train 17 | Test Female 14 | 0.71 | 0.72 | 0.68 |
| 15 | Train 1 | Test Female 15 | 0.73 | 0.75 | 0.73 | 1039 | Train 17 | Test Female 15 | 0.73 | 0.75 | 0.73 |
| 16 | Train 1 | Test Female 16 | 0.72 | 0.72 | 0.66 | 1040 | Train 17 | Test Female 16 | 0.72 | 0.72 | 0.67 |
| 17 | Train 1 | Test Female 17 | 0.76 | 0.84 | 0.86 | 1041 | Train 17 | Test Female 17 | 0.76 | 0.84 | 0.86 |
| 18 | Train 1 | Test Female 18 | 0.76 | 0.81 | 0.81 | 1042 | Train 17 | Test Female 18 | 0.76 | 0.81 | 0.81 |
| 19 | Train 1 | Test Female 19 | 0.77 | 0.84 | 0.86 | 1043 | Train 17 | Test Female 19 | 0.77 | 0.84 | 0.86 |
| 20 | Train 1 | Test Female 20 | 0.76 | 0.81 | 0.81 | 1044 | Train 17 | Test Female 20 | 0.76 | 0.81 | 0.8 |
| 21 | Train 1 | Test Female 21 | 0.73 | 0.76 | 0.75 | 1045 | Train 17 | Test Female 21 | 0.73 | 0.76 | 0.75 |
| 22 | Train 1 | Test Female 22 | 0.71 | 0.72 | 0.67 | 1046 | Train 17 | Test Female 22 | 0.71 | 0.72 | 0.68 |
| 23 | Train 1 | Test Female 23 | 0.73 | 0.76 | 0.75 | 1047 | Train 17 | Test Female 23 | 0.73 | 0.76 | 0.74 |
| 24 | Train 1 | Test Female 24 | 0.71 | 0.71 | 0.67 | 1048 | Train 17 | Test Female 24 | 0.71 | 0.72 | 0.67 |
| 25 | Train 1 | Test Female 25 | 0.77 | 0.83 | 0.82 | 1049 | Train 17 | Test Female 25 | 0.77 | 0.83 | 0.83 |
| 26 | Train 1 | Test Female 26 | 0.76 | 0.8 | 0.79 | 1050 | Train 17 | Test Female 26 | 0.76 | 0.8 | 0.78 |
| 27 | Train 1 | Test Female 27 | 0.77 | 0.83 | 0.82 | 1051 | Train 17 | Test Female 27 | 0.77 | 0.83 | 0.82 |
| 28 | Train 1 | Test Female 28 | 0.76 | 0.8 | 0.79 | 1052 | Train 17 | Test Female 28 | 0.76 | 0.8 | 0.78 |
| 29 | Train 1 | Test Female 29 | 0.73 | 0.75 | 0.69 | 1053 | Train 17 | Test Female 29 | 0.73 | 0.75 | 0.71 |
| 30 | Train 1 | Test Female 30 | 0.72 | 0.72 | 0.67 | 1054 | Train 17 | Test Female 30 | 0.71 | 0.72 | 0.68 |
| 31 | Train 1 | Test Female 31 | 0.73 | 0.75 | 0.73 | 1055 | Train 17 | Test Female 31 | 0.73 | 0.75 | 0.73 |
| 32 | Train 1 | Test Female 32 | 0.72 | 0.72 | 0.67 | 1056 | Train 17 | Test Female 32 | 0.72 | 0.72 | 0.68 |
| 33 | Train 1 | Test Female 33 | 0.77 | 0.85 | 0.86 | 1057 | Train 17 | Test Female 33 | 0.77 | 0.85 | 0.86 |
| 34 | Train 1 | Test Female 34 | 0.76 | 0.81 | 0.82 | 1058 | Train 17 | Test Female 34 | 0.76 | 0.81 | 0.81 |
| 35 | Train 1 | Test Female 35 | 0.77 | 0.85 | 0.86 | 1059 | Train 17 | Test Female 35 | 0.77 | 0.85 | 0.86 |
| 36 | Train 1 | Test Female 36 | 0.76 | 0.81 | 0.81 | 1060 | Train 17 | Test Female 36 | 0.76 | 0.81 | 0.81 |
| 37 | Train 1 | Test Female 37 | 0.73 | 0.76 | 0.75 | 1061 | Train 17 | Test Female 37 | 0.73 | 0.76 | 0.75 |
| 38 | Train 1 | Test Female 38 | 0.71 | 0.72 | 0.67 | 1062 | Train 17 | Test Female 38 | 0.71 | 0.72 | 0.68 |
| 39 | Train 1 | Test Female 39 | 0.73 | 0.76 | 0.75 | 1063 | Train 17 | Test Female 39 | 0.73 | 0.76 | 0.74 |
| 40 | Train 1 | Test Female 40 | 0.72 | 0.72 | 0.67 | 1064 | Train 17 | Test Female 40 | 0.71 | 0.72 | 0.68 |
| 41 | Train 1 | Test Female 41 | 0.77 | 0.84 | 0.83 | 1065 | Train 17 | Test Female 41 | 0.77 | 0.84 | 0.83 |
| 42 | Train 1 | Test Female 42 | 0.76 | 0.8 | 0.79 | 1066 | Train 17 | Test Female 42 | 0.76 | 0.8 | 0.78 |
| 43 | Train 1 | Test Female 43 | 0.77 | 0.84 | 0.83 | 1067 | Train 17 | Test Female 43 | 0.77 | 0.84 | 0.83 |
| 44 | Train 1 | Test Female 44 | 0.76 | 0.8 | 0.79 | 1068 | Train 17 | Test Female 44 | 0.76 | 0.8 | 0.78 |
| 45 | Train 1 | Test Female 45 | 0.73 | 0.75 | 0.69 | 1069 | Train 17 | Test Female 45 | 0.73 | 0.75 | 0.72 |
| 46 | Train 1 | Test Female 46 | 0.72 | 0.72 | 0.67 | 1070 | Train 17 | Test Female 46 | 0.72 | 0.72 | 0.68 |
| 47 | Train 1 | Test Female 47 | 0.73 | 0.75 | 0.73 | 1071 | Train 17 | Test Female 47 | 0.73 | 0.76 | 0.73 |
| 48 | Train 1 | Test Female 48 | 0.72 | 0.72 | 0.67 | 1072 | Train 17 | Test Female 48 | 0.72 | 0.72 | 0.68 |
| 49 | Train 1 | Test Female 49 | 0.79 | 0.84 | 0.87 | 1073 | Train 17 | Test Female 49 | 0.79 | 0.84 | 0.87 |
| 50 | Train 1 | Test Female 50 | 0.79 | 0.84 | 0.87 | 1074 | Train 17 | Test Female 50 | 0.79 | 0.84 | 0.87 |
| 51 | Train 1 | Test Female 51 | 0.79 | 0.84 | 0.86 | 1075 | Train 17 | Test Female 51 | 0.79 | 0.84 | 0.86 |
| 52 | Train 1 | Test Female 52 | 0.79 | 0.84 | 0.86 | 1076 | Train 17 | Test Female 52 | 0.79 | 0.84 | 0.86 |
| 53 | Train 1 | Test Female 53 | 0.74 | 0.77 | 0.73 | 1077 | Train 17 | Test Female 53 | 0.74 | 0.77 | 0.72 |
| 54 | Train 1 | Test Female 54 | 0.73 | 0.73 | 0.67 | 1078 | Train 17 | Test Female 54 | 0.73 | 0.73 | 0.69 |
| 55 | Train 1 | Test Female 55 | 0.74 | 0.77 | 0.73 | 1079 | Train 17 | Test Female 55 | 0.74 | 0.78 | 0.72 |
| 56 | Train 1 | Test Female 56 | 0.73 | 0.72 | 0.67 | 1080 | Train 17 | Test Female 56 | 0.73 | 0.72 | 0.68 |
| 57 | Train 1 | Test Female 57 | 0.79 | 0.84 | 0.83 | 1081 | Train 17 | Test Female 57 | 0.79 | 0.83 | 0.83 |
| 58 | Train 1 | Test Female 58 | 0.79 | 0.83 | 0.83 | 1082 | Train 17 | Test Female 58 | 0.79 | 0.83 | 0.83 |
| 59 | Train 1 | Test Female 59 | 0.79 | 0.83 | 0.83 | 1083 | Train 17 | Test Female 59 | 0.79 | 0.83 | 0.83 |
| 60 | Train 1 | Test Female 60 | 0.79 | 0.83 | 0.83 | 1084 | Train 17 | Test Female 60 | 0.79 | 0.83 | 0.83 |
| 61 | Train 1 | Test Female 61 | 0.74 | 0.77 | 0.73 | 1085 | Train 17 | Test Female 61 | 0.74 | 0.77 | 0.72 |
| 62 | Train 1 | Test Female 62 | 0.73 | 0.73 | 0.67 | 1086 | Train 17 | Test Female 62 | 0.73 | 0.73 | 0.69 |
| 63 | Train 1 | Test Female 63 | 0.75 | 0.77 | 0.73 | 1087 | Train 17 | Test Female 63 | 0.75 | 0.77 | 0.72 |
| 64 | Train 1 | Test Female 64 | 0.73 | 0.72 | 0.67 | 1088 | Train 17 | Test Female 64 | 0.73 | 0.72 | 0.68 |
| 65 | Train 2 | Test Female 1 | 0.76 | 0.83 | 0.85 | 1089 | Train 18 | Test Female 1 | 0.76 | 0.83 | 0.85 |
| 66 | Train 2 | Test Female 2 | 0.77 | 0.84 | 0.86 | 1090 | Train 18 | Test Female 2 | 0.77 | 0.84 | 0.86 |
| 67 | Train 2 | Test Female 3 | 0.76 | 0.83 | 0.85 | 1091 | Train 18 | Test Female 3 | 0.76 | 0.83 | 0.85 |
| 68 | Train 2 | Test Female 4 | 0.77 | 0.84 | 0.86 | 1092 | Train 18 | Test Female 4 | 0.77 | 0.84 | 0.86 |
| 69 | Train 2 | Test Female 5 | 0.73 | 0.76 | 0.76 | 1093 | Train 18 | Test Female 5 | 0.73 | 0.76 | 0.76 |
| 70 | Train 2 | Test Female 6 | 0.73 | 0.73 | 0.68 | 1094 | Train 18 | Test Female 6 | 0.73 | 0.73 | 0.69 |
| 71 | Train 2 | Test Female 7 | 0.73 | 0.76 | 0.75 | 1095 | Train 18 | Test Female 7 | 0.73 | 0.76 | 0.75 |
| 72 | Train 2 | Test Female 8 | 0.72 | 0.73 | 0.68 | 1096 | Train 18 | Test Female 8 | 0.72 | 0.73 | 0.69 |
| 73 | Train 2 | Test Female 9 | 0.76 | 0.82 | 0.82 | 1097 | Train 18 | Test Female 9 | 0.76 | 0.82 | 0.82 |
| 74 | Train 2 | Test Female 10 | 0.77 | 0.83 | 0.83 | 1098 | Train 18 | Test Female 10 | 0.77 | 0.83 | 0.83 |
| 75 | Train 2 | Test Female 11 | 0.76 | 0.82 | 0.82 | 1099 | Train 18 | Test Female 11 | 0.76 | 0.82 | 0.82 |
| 76 | Train 2 | Test Female 12 | 0.78 | 0.83 | 0.83 | 1100 | Train 18 | Test Female 12 | 0.78 | 0.83 | 0.83 |
| 77 | Train 2 | Test Female 13 | 0.73 | 0.75 | 0.69 | 1101 | Train 18 | Test Female 13 | 0.73 | 0.75 | 0.71 |
| 78 | Train 2 | Test Female 14 | 0.73 | 0.73 | 0.68 | 1102 | Train 18 | Test Female 14 | 0.73 | 0.73 | 0.69 |
| 79 | Train 2 | Test Female 15 | 0.73 | 0.76 | 0.74 | 1103 | Train 18 | Test Female 15 | 0.73 | 0.76 | 0.73 |
| 80 | Train 2 | Test Female 16 | 0.73 | 0.73 | 0.68 | 1104 | Train 18 | Test Female 16 | 0.73 | 0.73 | 0.69 |
| 81 | Train 2 | Test Female 17 | 0.76 | 0.83 | 0.85 | 1105 | Train 18 | Test Female 17 | 0.76 | 0.83 | 0.85 |
| 82 | Train 2 | Test Female 18 | 0.77 | 0.84 | 0.86 | 1106 | Train 18 | Test Female 18 | 0.77 | 0.84 | 0.86 |
| 83 | Train 2 | Test Female 19 | 0.76 | 0.83 | 0.85 | 1107 | Train 18 | Test Female 19 | 0.76 | 0.83 | 0.85 |
| 84 | Train 2 | Test Female 20 | 0.77 | 0.84 | 0.86 | 1108 | Train 18 | Test Female 20 | 0.77 | 0.84 | 0.86 |
| 85 | Train 2 | Test Female 21 | 0.73 | 0.76 | 0.76 | 1109 | Train 18 | Test Female 21 | 0.73 | 0.76 | 0.76 |
| 86 | Train 2 | Test Female 22 | 0.73 | 0.73 | 0.68 | 1110 | Train 18 | Test Female 22 | 0.73 | 0.73 | 0.69 |
| 87 | Train 2 | Test Female 23 | 0.73 | 0.76 | 0.75 | 1111 | Train 18 | Test Female 23 | 0.73 | 0.76 | 0.75 |
| 88 | Train 2 | Test Female 24 | 0.73 | 0.73 | 0.68 | 1112 | Train 18 | Test Female 24 | 0.73 | 0.73 | 0.69 |
| 89 | Train 2 | Test Female 25 | 0.76 | 0.82 | 0.82 | 1113 | Train 18 | Test Female 25 | 0.76 | 0.82 | 0.82 |
| 90 | Train 2 | Test Female 26 | 0.77 | 0.83 | 0.83 | 1114 | Train 18 | Test Female 26 | 0.77 | 0.83 | 0.83 |
| 91 | Train 2 | Test Female 27 | 0.76 | 0.82 | 0.82 | 1115 | Train 18 | Test Female 27 | 0.76 | 0.82 | 0.82 |
| 92 | Train 2 | Test Female 28 | 0.78 | 0.83 | 0.83 | 1116 | Train 18 | Test Female 28 | 0.78 | 0.83 | 0.83 |
| 93 | Train 2 | Test Female 29 | 0.73 | 0.75 | 0.69 | 1117 | Train 18 | Test Female 29 | 0.73 | 0.75 | 0.71 |
| 94 | Train 2 | Test Female 30 | 0.73 | 0.73 | 0.68 | 1118 | Train 18 | Test Female 30 | 0.73 | 0.73 | 0.69 |
| 95 | Train 2 | Test Female 31 | 0.73 | 0.76 | 0.74 | 1119 | Train 18 | Test Female 31 | 0.73 | 0.76 | 0.73 |
| 96 | Train 2 | Test Female 32 | 0.73 | 0.73 | 0.68 | 1120 | Train 18 | Test Female 32 | 0.73 | 0.73 | 0.69 |
| 97 | Train 2 | Test Female 33 | 0.76 | 0.84 | 0.85 | 1121 | Train 18 | Test Female 33 | 0.76 | 0.84 | 0.85 |
| 98 | Train 2 | Test Female 34 | 0.78 | 0.85 | 0.86 | 1122 | Train 18 | Test Female 34 | 0.78 | 0.85 | 0.86 |
| 99 | Train 2 | Test Female 35 | 0.76 | 0.84 | 0.85 | 1123 | Train 18 | Test Female 35 | 0.76 | 0.84 | 0.85 |
| 100 | Train 2 | Test Female 36 | 0.78 | 0.85 | 0.86 | 1124 | Train 18 | Test Female 36 | 0.78 | 0.85 | 0.86 |
| 101 | Train 2 | Test Female 37 | 0.73 | 0.77 | 0.76 | 1125 | Train 18 | Test Female 37 | 0.73 | 0.77 | 0.76 |
| 102 | Train 2 | Test Female 38 | 0.73 | 0.73 | 0.68 | 1126 | Train 18 | Test Female 38 | 0.73 | 0.73 | 0.69 |
| 103 | Train 2 | Test Female 39 | 0.73 | 0.76 | 0.76 | 1127 | Train 18 | Test Female 39 | 0.73 | 0.76 | 0.75 |
| 104 | Train 2 | Test Female 40 | 0.73 | 0.73 | 0.68 | 1128 | Train 18 | Test Female 40 | 0.73 | 0.73 | 0.69 |
| 105 | Train 2 | Test Female 41 | 0.76 | 0.83 | 0.82 | 1129 | Train 18 | Test Female 41 | 0.76 | 0.83 | 0.82 |
| 106 | Train 2 | Test Female 42 | 0.78 | 0.84 | 0.83 | 1130 | Train 18 | Test Female 42 | 0.78 | 0.84 | 0.83 |
| 107 | Train 2 | Test Female 43 | 0.77 | 0.83 | 0.82 | 1131 | Train 18 | Test Female 43 | 0.77 | 0.83 | 0.82 |
| 108 | Train 2 | Test Female 44 | 0.78 | 0.84 | 0.83 | 1132 | Train 18 | Test Female 44 | 0.78 | 0.84 | 0.83 |
| 109 | Train 2 | Test Female 45 | 0.73 | 0.75 | 0.7 | 1133 | Train 18 | Test Female 45 | 0.73 | 0.75 | 0.71 |
| 110 | Train 2 | Test Female 46 | 0.73 | 0.73 | 0.68 | 1134 | Train 18 | Test Female 46 | 0.73 | 0.73 | 0.69 |
| 111 | Train 2 | Test Female 47 | 0.73 | 0.76 | 0.74 | 1135 | Train 18 | Test Female 47 | 0.73 | 0.76 | 0.73 |
| 112 | Train 2 | Test Female 48 | 0.73 | 0.73 | 0.68 | 1136 | Train 18 | Test Female 48 | 0.73 | 0.73 | 0.69 |
| 113 | Train 2 | Test Female 49 | 0.78 | 0.84 | 0.86 | 1137 | Train 18 | Test Female 49 | 0.78 | 0.84 | 0.86 |
| 114 | Train 2 | Test Female 50 | 0.78 | 0.84 | 0.86 | 1138 | Train 18 | Test Female 50 | 0.78 | 0.84 | 0.86 |
| 115 | Train 2 | Test Female 51 | 0.78 | 0.83 | 0.85 | 1139 | Train 18 | Test Female 51 | 0.78 | 0.83 | 0.85 |
| 116 | Train 2 | Test Female 52 | 0.78 | 0.83 | 0.85 | 1140 | Train 18 | Test Female 52 | 0.78 | 0.83 | 0.85 |
| 117 | Train 2 | Test Female 53 | 0.74 | 0.78 | 0.75 | 1141 | Train 18 | Test Female 53 | 0.74 | 0.78 | 0.75 |
| 118 | Train 2 | Test Female 54 | 0.73 | 0.74 | 0.69 | 1142 | Train 18 | Test Female 54 | 0.73 | 0.74 | 0.69 |
| 119 | Train 2 | Test Female 55 | 0.75 | 0.78 | 0.75 | 1143 | Train 18 | Test Female 55 | 0.75 | 0.78 | 0.75 |
| 120 | Train 2 | Test Female 56 | 0.73 | 0.73 | 0.68 | 1144 | Train 18 | Test Female 56 | 0.73 | 0.73 | 0.68 |
| 121 | Train 2 | Test Female 57 | 0.78 | 0.83 | 0.83 | 1145 | Train 18 | Test Female 57 | 0.78 | 0.83 | 0.82 |
| 122 | Train 2 | Test Female 58 | 0.78 | 0.83 | 0.83 | 1146 | Train 18 | Test Female 58 | 0.78 | 0.83 | 0.82 |
| 123 | Train 2 | Test Female 59 | 0.79 | 0.83 | 0.82 | 1147 | Train 18 | Test Female 59 | 0.79 | 0.83 | 0.82 |
| 124 | Train 2 | Test Female 60 | 0.79 | 0.83 | 0.82 | 1148 | Train 18 | Test Female 60 | 0.79 | 0.83 | 0.82 |
| 125 | Train 2 | Test Female 61 | 0.75 | 0.78 | 0.75 | 1149 | Train 18 | Test Female 61 | 0.75 | 0.78 | 0.74 |
| 126 | Train 2 | Test Female 62 | 0.74 | 0.74 | 0.69 | 1150 | Train 18 | Test Female 62 | 0.74 | 0.74 | 0.69 |
| 127 | Train 2 | Test Female 63 | 0.75 | 0.78 | 0.75 | 1151 | Train 18 | Test Female 63 | 0.75 | 0.78 | 0.74 |
| 128 | Train 2 | Test Female 64 | 0.73 | 0.73 | 0.69 | 1152 | Train 18 | Test Female 64 | 0.73 | 0.73 | 0.69 |
| 129 | Train 3 | Test Female 1 | 0.76 | 0.84 | 0.86 | 1153 | Train 19 | Test Female 1 | 0.76 | 0.84 | 0.86 |
| 130 | Train 3 | Test Female 2 | 0.76 | 0.81 | 0.81 | 1154 | Train 19 | Test Female 2 | 0.76 | 0.81 | 0.81 |
| 131 | Train 3 | Test Female 3 | 0.77 | 0.84 | 0.86 | 1155 | Train 19 | Test Female 3 | 0.77 | 0.84 | 0.86 |
| 132 | Train 3 | Test Female 4 | 0.76 | 0.81 | 0.81 | 1156 | Train 19 | Test Female 4 | 0.76 | 0.81 | 0.81 |
| 133 | Train 3 | Test Female 5 | 0.73 | 0.76 | 0.75 | 1157 | Train 19 | Test Female 5 | 0.73 | 0.76 | 0.74 |
| 134 | Train 3 | Test Female 6 | 0.71 | 0.71 | 0.66 | 1158 | Train 19 | Test Female 6 | 0.71 | 0.71 | 0.66 |
| 135 | Train 3 | Test Female 7 | 0.73 | 0.76 | 0.75 | 1159 | Train 19 | Test Female 7 | 0.73 | 0.76 | 0.74 |
| 136 | Train 3 | Test Female 8 | 0.71 | 0.71 | 0.66 | 1160 | Train 19 | Test Female 8 | 0.71 | 0.71 | 0.66 |
| 137 | Train 3 | Test Female 9 | 0.77 | 0.83 | 0.83 | 1161 | Train 19 | Test Female 9 | 0.77 | 0.83 | 0.83 |
| 138 | Train 3 | Test Female 10 | 0.76 | 0.8 | 0.78 | 1162 | Train 19 | Test Female 10 | 0.76 | 0.8 | 0.79 |
| 139 | Train 3 | Test Female 11 | 0.77 | 0.83 | 0.83 | 1163 | Train 19 | Test Female 11 | 0.77 | 0.83 | 0.83 |
| 140 | Train 3 | Test Female 12 | 0.76 | 0.8 | 0.78 | 1164 | Train 19 | Test Female 12 | 0.76 | 0.8 | 0.79 |
| 141 | Train 3 | Test Female 13 | 0.73 | 0.75 | 0.71 | 1165 | Train 19 | Test Female 13 | 0.73 | 0.75 | 0.69 |
| 142 | Train 3 | Test Female 14 | 0.71 | 0.72 | 0.66 | 1166 | Train 19 | Test Female 14 | 0.71 | 0.72 | 0.66 |
| 143 | Train 3 | Test Female 15 | 0.73 | 0.75 | 0.73 | 1167 | Train 19 | Test Female 15 | 0.73 | 0.75 | 0.72 |
| 144 | Train 3 | Test Female 16 | 0.71 | 0.71 | 0.66 | 1168 | Train 19 | Test Female 16 | 0.71 | 0.72 | 0.66 |
| 145 | Train 3 | Test Female 17 | 0.76 | 0.84 | 0.86 | 1169 | Train 19 | Test Female 17 | 0.76 | 0.84 | 0.86 |
| 146 | Train 3 | Test Female 18 | 0.76 | 0.81 | 0.81 | 1170 | Train 19 | Test Female 18 | 0.76 | 0.81 | 0.81 |
| 147 | Train 3 | Test Female 19 | 0.77 | 0.84 | 0.86 | 1171 | Train 19 | Test Female 19 | 0.77 | 0.84 | 0.86 |
| 148 | Train 3 | Test Female 20 | 0.76 | 0.81 | 0.81 | 1172 | Train 19 | Test Female 20 | 0.76 | 0.81 | 0.81 |
| 149 | Train 3 | Test Female 21 | 0.73 | 0.76 | 0.75 | 1173 | Train 19 | Test Female 21 | 0.73 | 0.76 | 0.74 |
| 150 | Train 3 | Test Female 22 | 0.71 | 0.71 | 0.66 | 1174 | Train 19 | Test Female 22 | 0.71 | 0.72 | 0.66 |
| 151 | Train 3 | Test Female 23 | 0.73 | 0.76 | 0.75 | 1175 | Train 19 | Test Female 23 | 0.73 | 0.76 | 0.74 |
| 152 | Train 3 | Test Female 24 | 0.71 | 0.71 | 0.66 | 1176 | Train 19 | Test Female 24 | 0.71 | 0.71 | 0.66 |
| 153 | Train 3 | Test Female 25 | 0.77 | 0.83 | 0.82 | 1177 | Train 19 | Test Female 25 | 0.77 | 0.83 | 0.83 |
| 154 | Train 3 | Test Female 26 | 0.76 | 0.8 | 0.78 | 1178 | Train 19 | Test Female 26 | 0.76 | 0.8 | 0.79 |
| 155 | Train 3 | Test Female 27 | 0.77 | 0.83 | 0.83 | 1179 | Train 19 | Test Female 27 | 0.77 | 0.83 | 0.83 |
| 156 | Train 3 | Test Female 28 | 0.76 | 0.8 | 0.78 | 1180 | Train 19 | Test Female 28 | 0.76 | 0.8 | 0.79 |
| 157 | Train 3 | Test Female 29 | 0.73 | 0.75 | 0.71 | 1181 | Train 19 | Test Female 29 | 0.73 | 0.75 | 0.69 |
| 158 | Train 3 | Test Female 30 | 0.71 | 0.72 | 0.66 | 1182 | Train 19 | Test Female 30 | 0.71 | 0.72 | 0.66 |
| 159 | Train 3 | Test Female 31 | 0.73 | 0.75 | 0.73 | 1183 | Train 19 | Test Female 31 | 0.73 | 0.75 | 0.72 |
| 160 | Train 3 | Test Female 32 | 0.72 | 0.72 | 0.66 | 1184 | Train 19 | Test Female 32 | 0.72 | 0.72 | 0.66 |
| 161 | Train 3 | Test Female 33 | 0.77 | 0.85 | 0.86 | 1185 | Train 19 | Test Female 33 | 0.77 | 0.85 | 0.86 |
| 162 | Train 3 | Test Female 34 | 0.76 | 0.81 | 0.81 | 1186 | Train 19 | Test Female 34 | 0.76 | 0.81 | 0.81 |
| 163 | Train 3 | Test Female 35 | 0.77 | 0.85 | 0.86 | 1187 | Train 19 | Test Female 35 | 0.77 | 0.85 | 0.86 |
| 164 | Train 3 | Test Female 36 | 0.76 | 0.81 | 0.81 | 1188 | Train 19 | Test Female 36 | 0.76 | 0.81 | 0.81 |
| 165 | Train 3 | Test Female 37 | 0.73 | 0.76 | 0.75 | 1189 | Train 19 | Test Female 37 | 0.73 | 0.76 | 0.74 |
| 166 | Train 3 | Test Female 38 | 0.71 | 0.72 | 0.66 | 1190 | Train 19 | Test Female 38 | 0.71 | 0.72 | 0.66 |
| 167 | Train 3 | Test Female 39 | 0.73 | 0.76 | 0.75 | 1191 | Train 19 | Test Female 39 | 0.73 | 0.76 | 0.74 |
| 168 | Train 3 | Test Female 40 | 0.71 | 0.72 | 0.67 | 1192 | Train 19 | Test Female 40 | 0.71 | 0.72 | 0.66 |
| 169 | Train 3 | Test Female 41 | 0.77 | 0.84 | 0.83 | 1193 | Train 19 | Test Female 41 | 0.77 | 0.84 | 0.83 |
| 170 | Train 3 | Test Female 42 | 0.76 | 0.8 | 0.78 | 1194 | Train 19 | Test Female 42 | 0.76 | 0.8 | 0.79 |
| 171 | Train 3 | Test Female 43 | 0.77 | 0.84 | 0.83 | 1195 | Train 19 | Test Female 43 | 0.77 | 0.84 | 0.83 |
| 172 | Train 3 | Test Female 44 | 0.76 | 0.8 | 0.79 | 1196 | Train 19 | Test Female 44 | 0.76 | 0.8 | 0.79 |
| 173 | Train 3 | Test Female 45 | 0.73 | 0.75 | 0.71 | 1197 | Train 19 | Test Female 45 | 0.73 | 0.75 | 0.7 |
| 174 | Train 3 | Test Female 46 | 0.71 | 0.72 | 0.66 | 1198 | Train 19 | Test Female 46 | 0.71 | 0.72 | 0.66 |
| 175 | Train 3 | Test Female 47 | 0.73 | 0.76 | 0.74 | 1199 | Train 19 | Test Female 47 | 0.73 | 0.76 | 0.73 |
| 176 | Train 3 | Test Female 48 | 0.72 | 0.72 | 0.66 | 1200 | Train 19 | Test Female 48 | 0.72 | 0.72 | 0.66 |
| 177 | Train 3 | Test Female 49 | 0.79 | 0.84 | 0.87 | 1201 | Train 19 | Test Female 49 | 0.79 | 0.84 | 0.87 |
| 178 | Train 3 | Test Female 50 | 0.79 | 0.84 | 0.87 | 1202 | Train 19 | Test Female 50 | 0.79 | 0.84 | 0.86 |
| 179 | Train 3 | Test Female 51 | 0.79 | 0.84 | 0.87 | 1203 | Train 19 | Test Female 51 | 0.79 | 0.84 | 0.87 |
| 180 | Train 3 | Test Female 52 | 0.79 | 0.84 | 0.87 | 1204 | Train 19 | Test Female 52 | 0.79 | 0.84 | 0.87 |
| 181 | Train 3 | Test Female 53 | 0.74 | 0.77 | 0.73 | 1205 | Train 19 | Test Female 53 | 0.74 | 0.77 | 0.71 |
| 182 | Train 3 | Test Female 54 | 0.73 | 0.72 | 0.67 | 1206 | Train 19 | Test Female 54 | 0.73 | 0.73 | 0.66 |
| 183 | Train 3 | Test Female 55 | 0.74 | 0.77 | 0.74 | 1207 | Train 19 | Test Female 55 | 0.74 | 0.78 | 0.71 |
| 184 | Train 3 | Test Female 56 | 0.73 | 0.72 | 0.67 | 1208 | Train 19 | Test Female 56 | 0.73 | 0.72 | 0.66 |
| 185 | Train 3 | Test Female 57 | 0.79 | 0.83 | 0.83 | 1209 | Train 19 | Test Female 57 | 0.79 | 0.83 | 0.84 |
| 186 | Train 3 | Test Female 58 | 0.79 | 0.83 | 0.83 | 1210 | Train 19 | Test Female 58 | 0.79 | 0.83 | 0.84 |
| 187 | Train 3 | Test Female 59 | 0.79 | 0.84 | 0.83 | 1211 | Train 19 | Test Female 59 | 0.79 | 0.84 | 0.84 |
| 188 | Train 3 | Test Female 60 | 0.79 | 0.84 | 0.83 | 1212 | Train 19 | Test Female 60 | 0.79 | 0.84 | 0.84 |
| 189 | Train 3 | Test Female 61 | 0.74 | 0.77 | 0.73 | 1213 | Train 19 | Test Female 61 | 0.74 | 0.77 | 0.71 |
| 190 | Train 3 | Test Female 62 | 0.73 | 0.73 | 0.68 | 1214 | Train 19 | Test Female 62 | 0.73 | 0.73 | 0.66 |
| 191 | Train 3 | Test Female 63 | 0.74 | 0.77 | 0.73 | 1215 | Train 19 | Test Female 63 | 0.74 | 0.77 | 0.71 |
| 192 | Train 3 | Test Female 64 | 0.73 | 0.72 | 0.68 | 1216 | Train 19 | Test Female 64 | 0.73 | 0.72 | 0.66 |
| 193 | Train 4 | Test Female 1 | 0.76 | 0.83 | 0.85 | 1217 | Train 20 | Test Female 1 | 0.76 | 0.83 | 0.85 |
| 194 | Train 4 | Test Female 2 | 0.77 | 0.84 | 0.86 | 1218 | Train 20 | Test Female 2 | 0.77 | 0.84 | 0.86 |
| 195 | Train 4 | Test Female 3 | 0.76 | 0.83 | 0.85 | 1219 | Train 20 | Test Female 3 | 0.76 | 0.83 | 0.85 |
| 196 | Train 4 | Test Female 4 | 0.77 | 0.84 | 0.86 | 1220 | Train 20 | Test Female 4 | 0.77 | 0.84 | 0.86 |
| 197 | Train 4 | Test Female 5 | 0.73 | 0.76 | 0.75 | 1221 | Train 20 | Test Female 5 | 0.73 | 0.76 | 0.75 |
| 198 | Train 4 | Test Female 6 | 0.72 | 0.73 | 0.69 | 1222 | Train 20 | Test Female 6 | 0.73 | 0.73 | 0.68 |
| 199 | Train 4 | Test Female 7 | 0.73 | 0.76 | 0.75 | 1223 | Train 20 | Test Female 7 | 0.73 | 0.76 | 0.74 |
| 200 | Train 4 | Test Female 8 | 0.72 | 0.73 | 0.69 | 1224 | Train 20 | Test Female 8 | 0.72 | 0.73 | 0.68 |
| 201 | Train 4 | Test Female 9 | 0.76 | 0.82 | 0.82 | 1225 | Train 20 | Test Female 9 | 0.76 | 0.82 | 0.82 |
| 202 | Train 4 | Test Female 10 | 0.77 | 0.83 | 0.83 | 1226 | Train 20 | Test Female 10 | 0.77 | 0.83 | 0.83 |
| 203 | Train 4 | Test Female 11 | 0.76 | 0.82 | 0.82 | 1227 | Train 20 | Test Female 11 | 0.76 | 0.82 | 0.82 |
| 204 | Train 4 | Test Female 12 | 0.77 | 0.83 | 0.83 | 1228 | Train 20 | Test Female 12 | 0.77 | 0.83 | 0.83 |
| 205 | Train 4 | Test Female 13 | 0.73 | 0.75 | 0.7 | 1229 | Train 20 | Test Female 13 | 0.73 | 0.75 | 0.69 |
| 206 | Train 4 | Test Female 14 | 0.73 | 0.73 | 0.69 | 1230 | Train 20 | Test Female 14 | 0.73 | 0.73 | 0.68 |
| 207 | Train 4 | Test Female 15 | 0.73 | 0.76 | 0.73 | 1231 | Train 20 | Test Female 15 | 0.73 | 0.76 | 0.73 |
| 208 | Train 4 | Test Female 16 | 0.73 | 0.73 | 0.69 | 1232 | Train 20 | Test Female 16 | 0.73 | 0.73 | 0.68 |
| 209 | Train 4 | Test Female 17 | 0.76 | 0.83 | 0.85 | 1233 | Train 20 | Test Female 17 | 0.76 | 0.83 | 0.85 |
| 210 | Train 4 | Test Female 18 | 0.77 | 0.84 | 0.86 | 1234 | Train 20 | Test Female 18 | 0.77 | 0.84 | 0.86 |
| 211 | Train 4 | Test Female 19 | 0.76 | 0.83 | 0.85 | 1235 | Train 20 | Test Female 19 | 0.76 | 0.83 | 0.85 |
| 212 | Train 4 | Test Female 20 | 0.77 | 0.84 | 0.86 | 1236 | Train 20 | Test Female 20 | 0.77 | 0.84 | 0.86 |
| 213 | Train 4 | Test Female 21 | 0.73 | 0.76 | 0.75 | 1237 | Train 20 | Test Female 21 | 0.73 | 0.76 | 0.75 |
| 214 | Train 4 | Test Female 22 | 0.73 | 0.73 | 0.69 | 1238 | Train 20 | Test Female 22 | 0.73 | 0.73 | 0.68 |
| 215 | Train 4 | Test Female 23 | 0.73 | 0.76 | 0.75 | 1239 | Train 20 | Test Female 23 | 0.73 | 0.76 | 0.75 |
| 216 | Train 4 | Test Female 24 | 0.72 | 0.73 | 0.69 | 1240 | Train 20 | Test Female 24 | 0.73 | 0.73 | 0.68 |
| 217 | Train 4 | Test Female 25 | 0.76 | 0.82 | 0.82 | 1241 | Train 20 | Test Female 25 | 0.76 | 0.82 | 0.82 |
| 218 | Train 4 | Test Female 26 | 0.77 | 0.83 | 0.83 | 1242 | Train 20 | Test Female 26 | 0.77 | 0.83 | 0.83 |
| 219 | Train 4 | Test Female 27 | 0.76 | 0.82 | 0.82 | 1243 | Train 20 | Test Female 27 | 0.76 | 0.82 | 0.82 |
| 220 | Train 4 | Test Female 28 | 0.78 | 0.83 | 0.83 | 1244 | Train 20 | Test Female 28 | 0.78 | 0.83 | 0.83 |
| 221 | Train 4 | Test Female 29 | 0.73 | 0.75 | 0.7 | 1245 | Train 20 | Test Female 29 | 0.73 | 0.75 | 0.69 |
| 222 | Train 4 | Test Female 30 | 0.73 | 0.73 | 0.69 | 1246 | Train 20 | Test Female 30 | 0.73 | 0.73 | 0.68 |
| 223 | Train 4 | Test Female 31 | 0.73 | 0.76 | 0.73 | 1247 | Train 20 | Test Female 31 | 0.73 | 0.76 | 0.73 |
| 224 | Train 4 | Test Female 32 | 0.73 | 0.73 | 0.69 | 1248 | Train 20 | Test Female 32 | 0.73 | 0.73 | 0.68 |
| 225 | Train 4 | Test Female 33 | 0.76 | 0.84 | 0.85 | 1249 | Train 20 | Test Female 33 | 0.76 | 0.84 | 0.85 |
| 226 | Train 4 | Test Female 34 | 0.78 | 0.85 | 0.86 | 1250 | Train 20 | Test Female 34 | 0.78 | 0.85 | 0.86 |
| 227 | Train 4 | Test Female 35 | 0.76 | 0.84 | 0.85 | 1251 | Train 20 | Test Female 35 | 0.76 | 0.84 | 0.85 |
| 228 | Train 4 | Test Female 36 | 0.78 | 0.85 | 0.86 | 1252 | Train 20 | Test Female 36 | 0.78 | 0.85 | 0.86 |
| 229 | Train 4 | Test Female 37 | 0.73 | 0.77 | 0.75 | 1253 | Train 20 | Test Female 37 | 0.73 | 0.77 | 0.75 |
| 230 | Train 4 | Test Female 38 | 0.73 | 0.73 | 0.69 | 1254 | Train 20 | Test Female 38 | 0.73 | 0.73 | 0.68 |
| 231 | Train 4 | Test Female 39 | 0.73 | 0.76 | 0.75 | 1255 | Train 20 | Test Female 39 | 0.73 | 0.76 | 0.75 |
| 232 | Train 4 | Test Female 40 | 0.73 | 0.73 | 0.69 | 1256 | Train 20 | Test Female 40 | 0.73 | 0.73 | 0.68 |
| 233 | Train 4 | Test Female 41 | 0.76 | 0.83 | 0.82 | 1257 | Train 20 | Test Female 41 | 0.76 | 0.83 | 0.82 |
| 234 | Train 4 | Test Female 42 | 0.78 | 0.84 | 0.83 | 1258 | Train 20 | Test Female 42 | 0.78 | 0.84 | 0.83 |
| 235 | Train 4 | Test Female 43 | 0.77 | 0.83 | 0.82 | 1259 | Train 20 | Test Female 43 | 0.77 | 0.83 | 0.82 |
| 236 | Train 4 | Test Female 44 | 0.78 | 0.84 | 0.83 | 1260 | Train 20 | Test Female 44 | 0.78 | 0.84 | 0.83 |
| 237 | Train 4 | Test Female 45 | 0.73 | 0.75 | 0.7 | 1261 | Train 20 | Test Female 45 | 0.73 | 0.75 | 0.69 |
| 238 | Train 4 | Test Female 46 | 0.73 | 0.73 | 0.69 | 1262 | Train 20 | Test Female 46 | 0.73 | 0.73 | 0.68 |
| 239 | Train 4 | Test Female 47 | 0.73 | 0.76 | 0.73 | 1263 | Train 20 | Test Female 47 | 0.73 | 0.76 | 0.73 |
| 240 | Train 4 | Test Female 48 | 0.73 | 0.73 | 0.69 | 1264 | Train 20 | Test Female 48 | 0.73 | 0.73 | 0.68 |
| 241 | Train 4 | Test Female 49 | 0.78 | 0.83 | 0.86 | 1265 | Train 20 | Test Female 49 | 0.78 | 0.83 | 0.86 |
| 242 | Train 4 | Test Female 50 | 0.78 | 0.83 | 0.86 | 1266 | Train 20 | Test Female 50 | 0.78 | 0.83 | 0.86 |
| 243 | Train 4 | Test Female 51 | 0.78 | 0.84 | 0.86 | 1267 | Train 20 | Test Female 51 | 0.78 | 0.84 | 0.86 |
| 244 | Train 4 | Test Female 52 | 0.78 | 0.84 | 0.86 | 1268 | Train 20 | Test Female 52 | 0.78 | 0.84 | 0.86 |
| 245 | Train 4 | Test Female 53 | 0.74 | 0.78 | 0.74 | 1269 | Train 20 | Test Female 53 | 0.74 | 0.78 | 0.74 |
| 246 | Train 4 | Test Female 54 | 0.73 | 0.74 | 0.69 | 1270 | Train 20 | Test Female 54 | 0.73 | 0.74 | 0.67 |
| 247 | Train 4 | Test Female 55 | 0.75 | 0.78 | 0.74 | 1271 | Train 20 | Test Female 55 | 0.75 | 0.78 | 0.75 |
| 248 | Train 4 | Test Female 56 | 0.73 | 0.73 | 0.69 | 1272 | Train 20 | Test Female 56 | 0.73 | 0.73 | 0.67 |
| 249 | Train 4 | Test Female 57 | 0.78 | 0.83 | 0.83 | 1273 | Train 20 | Test Female 57 | 0.78 | 0.83 | 0.83 |
| 250 | Train 4 | Test Female 58 | 0.78 | 0.83 | 0.83 | 1274 | Train 20 | Test Female 58 | 0.78 | 0.83 | 0.83 |
| 251 | Train 4 | Test Female 59 | 0.79 | 0.83 | 0.83 | 1275 | Train 20 | Test Female 59 | 0.79 | 0.83 | 0.83 |
| 252 | Train 4 | Test Female 60 | 0.79 | 0.83 | 0.83 | 1276 | Train 20 | Test Female 60 | 0.79 | 0.83 | 0.83 |
| 253 | Train 4 | Test Female 61 | 0.74 | 0.78 | 0.74 | 1277 | Train 20 | Test Female 61 | 0.75 | 0.78 | 0.74 |
| 254 | Train 4 | Test Female 62 | 0.74 | 0.74 | 0.69 | 1278 | Train 20 | Test Female 62 | 0.74 | 0.74 | 0.68 |
| 255 | Train 4 | Test Female 63 | 0.75 | 0.78 | 0.74 | 1279 | Train 20 | Test Female 63 | 0.75 | 0.78 | 0.74 |
| 256 | Train 4 | Test Female 64 | 0.73 | 0.73 | 0.69 | 1280 | Train 20 | Test Female 64 | 0.73 | 0.73 | 0.67 |
| 257 | Train 5 | Test Female 1 | 0.75 | 0.82 | 0.83 | 1281 | Train 21 | Test Female 1 | 0.75 | 0.82 | 0.83 |
| 258 | Train 5 | Test Female 2 | 0.76 | 0.8 | 0.8 | 1282 | Train 21 | Test Female 2 | 0.76 | 0.8 | 0.8 |
| 259 | Train 5 | Test Female 3 | 0.76 | 0.82 | 0.83 | 1283 | Train 21 | Test Female 3 | 0.76 | 0.82 | 0.83 |
| 260 | Train 5 | Test Female 4 | 0.76 | 0.8 | 0.8 | 1284 | Train 21 | Test Female 4 | 0.76 | 0.8 | 0.8 |
| 261 | Train 5 | Test Female 5 | 0.75 | 0.82 | 0.83 | 1285 | Train 21 | Test Female 5 | 0.75 | 0.82 | 0.83 |
| 262 | Train 5 | Test Female 6 | 0.72 | 0.73 | 0.7 | 1286 | Train 21 | Test Female 6 | 0.72 | 0.73 | 0.69 |
| 263 | Train 5 | Test Female 7 | 0.75 | 0.81 | 0.83 | 1287 | Train 21 | Test Female 7 | 0.75 | 0.81 | 0.83 |
| 264 | Train 5 | Test Female 8 | 0.72 | 0.73 | 0.7 | 1288 | Train 21 | Test Female 8 | 0.72 | 0.73 | 0.7 |
| 265 | Train 5 | Test Female 9 | 0.76 | 0.81 | 0.81 | 1289 | Train 21 | Test Female 9 | 0.76 | 0.81 | 0.81 |
| 266 | Train 5 | Test Female 10 | 0.76 | 0.8 | 0.79 | 1290 | Train 21 | Test Female 10 | 0.76 | 0.8 | 0.78 |
| 267 | Train 5 | Test Female 11 | 0.76 | 0.81 | 0.81 | 1291 | Train 21 | Test Female 11 | 0.76 | 0.81 | 0.81 |
| 268 | Train 5 | Test Female 12 | 0.76 | 0.8 | 0.78 | 1292 | Train 21 | Test Female 12 | 0.76 | 0.8 | 0.78 |
| 269 | Train 5 | Test Female 13 | 0.75 | 0.79 | 0.77 | 1293 | Train 21 | Test Female 13 | 0.75 | 0.79 | 0.77 |
| 270 | Train 5 | Test Female 14 | 0.72 | 0.73 | 0.7 | 1294 | Train 21 | Test Female 14 | 0.72 | 0.73 | 0.69 |
| 271 | Train 5 | Test Female 15 | 0.75 | 0.8 | 0.8 | 1295 | Train 21 | Test Female 15 | 0.75 | 0.8 | 0.81 |
| 272 | Train 5 | Test Female 16 | 0.72 | 0.73 | 0.7 | 1296 | Train 21 | Test Female 16 | 0.72 | 0.73 | 0.7 |
| 273 | Train 5 | Test Female 17 | 0.76 | 0.82 | 0.83 | 1297 | Train 21 | Test Female 17 | 0.76 | 0.82 | 0.83 |
| 274 | Train 5 | Test Female 18 | 0.76 | 0.8 | 0.8 | 1298 | Train 21 | Test Female 18 | 0.76 | 0.8 | 0.8 |
| 275 | Train 5 | Test Female 19 | 0.76 | 0.82 | 0.83 | 1299 | Train 21 | Test Female 19 | 0.76 | 0.82 | 0.83 |
| 276 | Train 5 | Test Female 20 | 0.76 | 0.8 | 0.8 | 1300 | Train 21 | Test Female 20 | 0.76 | 0.8 | 0.8 |
| 277 | Train 5 | Test Female 21 | 0.75 | 0.82 | 0.83 | 1301 | Train 21 | Test Female 21 | 0.75 | 0.82 | 0.83 |
| 278 | Train 5 | Test Female 22 | 0.72 | 0.73 | 0.7 | 1302 | Train 21 | Test Female 22 | 0.72 | 0.73 | 0.69 |
| 279 | Train 5 | Test Female 23 | 0.75 | 0.81 | 0.83 | 1303 | Train 21 | Test Female 23 | 0.75 | 0.81 | 0.83 |
| 280 | Train 5 | Test Female 24 | 0.72 | 0.73 | 0.7 | 1304 | Train 21 | Test Female 24 | 0.72 | 0.73 | 0.7 |
| 281 | Train 5 | Test Female 25 | 0.76 | 0.81 | 0.81 | 1305 | Train 21 | Test Female 25 | 0.76 | 0.81 | 0.81 |
| 282 | Train 5 | Test Female 26 | 0.76 | 0.8 | 0.79 | 1306 | Train 21 | Test Female 26 | 0.76 | 0.8 | 0.78 |
| 283 | Train 5 | Test Female 27 | 0.76 | 0.81 | 0.81 | 1307 | Train 21 | Test Female 27 | 0.76 | 0.81 | 0.81 |
| 284 | Train 5 | Test Female 28 | 0.76 | 0.8 | 0.78 | 1308 | Train 21 | Test Female 28 | 0.76 | 0.8 | 0.78 |
| 285 | Train 5 | Test Female 29 | 0.75 | 0.79 | 0.77 | 1309 | Train 21 | Test Female 29 | 0.75 | 0.79 | 0.77 |
| 286 | Train 5 | Test Female 30 | 0.72 | 0.73 | 0.7 | 1310 | Train 21 | Test Female 30 | 0.72 | 0.73 | 0.7 |
| 287 | Train 5 | Test Female 31 | 0.75 | 0.8 | 0.8 | 1311 | Train 21 | Test Female 31 | 0.75 | 0.81 | 0.81 |
| 288 | Train 5 | Test Female 32 | 0.72 | 0.73 | 0.71 | 1312 | Train 21 | Test Female 32 | 0.72 | 0.73 | 0.7 |
| 289 | Train 5 | Test Female 33 | 0.76 | 0.82 | 0.83 | 1313 | Train 21 | Test Female 33 | 0.76 | 0.82 | 0.84 |
| 290 | Train 5 | Test Female 34 | 0.77 | 0.81 | 0.8 | 1314 | Train 21 | Test Female 34 | 0.77 | 0.81 | 0.8 |
| 291 | Train 5 | Test Female 35 | 0.76 | 0.82 | 0.83 | 1315 | Train 21 | Test Female 35 | 0.76 | 0.82 | 0.83 |
| 292 | Train 5 | Test Female 36 | 0.77 | 0.81 | 0.8 | 1316 | Train 21 | Test Female 36 | 0.77 | 0.81 | 0.8 |
| 293 | Train 5 | Test Female 37 | 0.75 | 0.82 | 0.83 | 1317 | Train 21 | Test Female 37 | 0.75 | 0.82 | 0.83 |
| 294 | Train 5 | Test Female 38 | 0.72 | 0.73 | 0.71 | 1318 | Train 21 | Test Female 38 | 0.72 | 0.73 | 0.7 |
| 295 | Train 5 | Test Female 39 | 0.75 | 0.82 | 0.83 | 1319 | Train 21 | Test Female 39 | 0.75 | 0.82 | 0.83 |
| 296 | Train 5 | Test Female 40 | 0.72 | 0.73 | 0.71 | 1320 | Train 21 | Test Female 40 | 0.72 | 0.73 | 0.7 |
| 297 | Train 5 | Test Female 41 | 0.76 | 0.81 | 0.82 | 1321 | Train 21 | Test Female 41 | 0.76 | 0.81 | 0.81 |
| 298 | Train 5 | Test Female 42 | 0.77 | 0.8 | 0.79 | 1322 | Train 21 | Test Female 42 | 0.77 | 0.8 | 0.79 |
| 299 | Train 5 | Test Female 43 | 0.76 | 0.81 | 0.81 | 1323 | Train 21 | Test Female 43 | 0.76 | 0.81 | 0.81 |
| 300 | Train 5 | Test Female 44 | 0.77 | 0.8 | 0.79 | 1324 | Train 21 | Test Female 44 | 0.77 | 0.8 | 0.79 |
| 301 | Train 5 | Test Female 45 | 0.75 | 0.79 | 0.77 | 1325 | Train 21 | Test Female 45 | 0.75 | 0.79 | 0.78 |
| 302 | Train 5 | Test Female 46 | 0.72 | 0.73 | 0.71 | 1326 | Train 21 | Test Female 46 | 0.72 | 0.73 | 0.7 |
| 303 | Train 5 | Test Female 47 | 0.75 | 0.81 | 0.81 | 1327 | Train 21 | Test Female 47 | 0.75 | 0.81 | 0.81 |
| 304 | Train 5 | Test Female 48 | 0.72 | 0.73 | 0.71 | 1328 | Train 21 | Test Female 48 | 0.72 | 0.73 | 0.7 |
| 305 | Train 5 | Test Female 49 | 0.78 | 0.83 | 0.85 | 1329 | Train 21 | Test Female 49 | 0.78 | 0.83 | 0.85 |
| 306 | Train 5 | Test Female 50 | 0.78 | 0.83 | 0.85 | 1330 | Train 21 | Test Female 50 | 0.78 | 0.83 | 0.85 |
| 307 | Train 5 | Test Female 51 | 0.78 | 0.83 | 0.84 | 1331 | Train 21 | Test Female 51 | 0.78 | 0.83 | 0.84 |
| 308 | Train 5 | Test Female 52 | 0.78 | 0.83 | 0.84 | 1332 | Train 21 | Test Female 52 | 0.78 | 0.83 | 0.84 |
| 309 | Train 5 | Test Female 53 | 0.77 | 0.82 | 0.83 | 1333 | Train 21 | Test Female 53 | 0.77 | 0.82 | 0.83 |
| 310 | Train 5 | Test Female 54 | 0.74 | 0.74 | 0.71 | 1334 | Train 21 | Test Female 54 | 0.74 | 0.74 | 0.72 |
| 311 | Train 5 | Test Female 55 | 0.77 | 0.82 | 0.83 | 1335 | Train 21 | Test Female 55 | 0.77 | 0.82 | 0.83 |
| 312 | Train 5 | Test Female 56 | 0.73 | 0.74 | 0.71 | 1336 | Train 21 | Test Female 56 | 0.73 | 0.74 | 0.72 |
| 313 | Train 5 | Test Female 57 | 0.78 | 0.82 | 0.82 | 1337 | Train 21 | Test Female 57 | 0.78 | 0.82 | 0.83 |
| 314 | Train 5 | Test Female 58 | 0.78 | 0.82 | 0.82 | 1338 | Train 21 | Test Female 58 | 0.78 | 0.82 | 0.83 |
| 315 | Train 5 | Test Female 59 | 0.78 | 0.82 | 0.82 | 1339 | Train 21 | Test Female 59 | 0.78 | 0.82 | 0.83 |
| 316 | Train 5 | Test Female 60 | 0.78 | 0.82 | 0.82 | 1340 | Train 21 | Test Female 60 | 0.78 | 0.82 | 0.82 |
| 317 | Train 5 | Test Female 61 | 0.77 | 0.82 | 0.82 | 1341 | Train 21 | Test Female 61 | 0.77 | 0.82 | 0.82 |
| 318 | Train 5 | Test Female 62 | 0.74 | 0.74 | 0.71 | 1342 | Train 21 | Test Female 62 | 0.74 | 0.74 | 0.72 |
| 319 | Train 5 | Test Female 63 | 0.77 | 0.82 | 0.82 | 1343 | Train 21 | Test Female 63 | 0.77 | 0.82 | 0.82 |
| 320 | Train 5 | Test Female 64 | 0.73 | 0.74 | 0.71 | 1344 | Train 21 | Test Female 64 | 0.73 | 0.74 | 0.72 |
| 321 | Train 6 | Test Female 1 | 0.74 | 0.78 | 0.75 | 1345 | Train 22 | Test Female 1 | 0.74 | 0.78 | 0.76 |
| 322 | Train 6 | Test Female 2 | 0.76 | 0.79 | 0.77 | 1346 | Train 22 | Test Female 2 | 0.76 | 0.79 | 0.78 |
| 323 | Train 6 | Test Female 3 | 0.74 | 0.78 | 0.75 | 1347 | Train 22 | Test Female 3 | 0.74 | 0.78 | 0.76 |
| 324 | Train 6 | Test Female 4 | 0.76 | 0.79 | 0.77 | 1348 | Train 22 | Test Female 4 | 0.76 | 0.79 | 0.78 |
| 325 | Train 6 | Test Female 5 | 0.73 | 0.78 | 0.76 | 1349 | Train 22 | Test Female 5 | 0.73 | 0.78 | 0.76 |
| 326 | Train 6 | Test Female 6 | 0.75 | 0.8 | 0.8 | 1350 | Train 22 | Test Female 6 | 0.75 | 0.8 | 0.81 |
| 327 | Train 6 | Test Female 7 | 0.73 | 0.78 | 0.76 | 1351 | Train 22 | Test Female 7 | 0.73 | 0.78 | 0.76 |
| 328 | Train 6 | Test Female 8 | 0.75 | 0.79 | 0.8 | 1352 | Train 22 | Test Female 8 | 0.75 | 0.79 | 0.8 |
| 329 | Train 6 | Test Female 9 | 0.74 | 0.79 | 0.76 | 1353 | Train 22 | Test Female 9 | 0.74 | 0.79 | 0.77 |
| 330 | Train 6 | Test Female 10 | 0.76 | 0.79 | 0.78 | 1354 | Train 22 | Test Female 10 | 0.76 | 0.79 | 0.78 |
| 331 | Train 6 | Test Female 11 | 0.74 | 0.79 | 0.76 | 1355 | Train 22 | Test Female 11 | 0.74 | 0.79 | 0.77 |
| 332 | Train 6 | Test Female 12 | 0.76 | 0.79 | 0.77 | 1356 | Train 22 | Test Female 12 | 0.76 | 0.79 | 0.78 |
| 333 | Train 6 | Test Female 13 | 0.73 | 0.79 | 0.8 | 1357 | Train 22 | Test Female 13 | 0.73 | 0.79 | 0.8 |
| 334 | Train 6 | Test Female 14 | 0.75 | 0.8 | 0.81 | 1358 | Train 22 | Test Female 14 | 0.75 | 0.8 | 0.81 |
| 335 | Train 6 | Test Female 15 | 0.73 | 0.78 | 0.77 | 1359 | Train 22 | Test Female 15 | 0.73 | 0.78 | 0.77 |
| 336 | Train 6 | Test Female 16 | 0.75 | 0.79 | 0.8 | 1360 | Train 22 | Test Female 16 | 0.75 | 0.79 | 0.8 |
| 337 | Train 6 | Test Female 17 | 0.74 | 0.78 | 0.75 | 1361 | Train 22 | Test Female 17 | 0.74 | 0.78 | 0.76 |
| 338 | Train 6 | Test Female 18 | 0.76 | 0.79 | 0.77 | 1362 | Train 22 | Test Female 18 | 0.76 | 0.79 | 0.78 |
| 339 | Train 6 | Test Female 19 | 0.74 | 0.78 | 0.75 | 1363 | Train 22 | Test Female 19 | 0.74 | 0.78 | 0.76 |
| 340 | Train 6 | Test Female 20 | 0.76 | 0.79 | 0.77 | 1364 | Train 22 | Test Female 20 | 0.76 | 0.79 | 0.78 |
| 341 | Train 6 | Test Female 21 | 0.73 | 0.78 | 0.76 | 1365 | Train 22 | Test Female 21 | 0.73 | 0.78 | 0.76 |
| 342 | Train 6 | Test Female 22 | 0.75 | 0.8 | 0.81 | 1366 | Train 22 | Test Female 22 | 0.75 | 0.8 | 0.81 |
| 343 | Train 6 | Test Female 23 | 0.73 | 0.78 | 0.76 | 1367 | Train 22 | Test Female 23 | 0.73 | 0.78 | 0.76 |
| 344 | Train 6 | Test Female 24 | 0.75 | 0.79 | 0.8 | 1368 | Train 22 | Test Female 24 | 0.75 | 0.79 | 0.8 |
| 345 | Train 6 | Test Female 25 | 0.74 | 0.79 | 0.76 | 1369 | Train 22 | Test Female 25 | 0.74 | 0.79 | 0.77 |
| 346 | Train 6 | Test Female 26 | 0.76 | 0.79 | 0.78 | 1370 | Train 22 | Test Female 26 | 0.76 | 0.8 | 0.78 |
| 347 | Train 6 | Test Female 27 | 0.74 | 0.79 | 0.76 | 1371 | Train 22 | Test Female 27 | 0.74 | 0.79 | 0.77 |
| 348 | Train 6 | Test Female 28 | 0.76 | 0.79 | 0.77 | 1372 | Train 22 | Test Female 28 | 0.76 | 0.79 | 0.78 |
| 349 | Train 6 | Test Female 29 | 0.73 | 0.79 | 0.8 | 1373 | Train 22 | Test Female 29 | 0.73 | 0.79 | 0.8 |
| 350 | Train 6 | Test Female 30 | 0.75 | 0.8 | 0.81 | 1374 | Train 22 | Test Female 30 | 0.75 | 0.8 | 0.81 |
| 351 | Train 6 | Test Female 31 | 0.73 | 0.78 | 0.77 | 1375 | Train 22 | Test Female 31 | 0.73 | 0.78 | 0.77 |
| 352 | Train 6 | Test Female 32 | 0.75 | 0.79 | 0.8 | 1376 | Train 22 | Test Female 32 | 0.75 | 0.79 | 0.8 |
| 353 | Train 6 | Test Female 33 | 0.75 | 0.79 | 0.76 | 1377 | Train 22 | Test Female 33 | 0.75 | 0.79 | 0.77 |
| 354 | Train 6 | Test Female 34 | 0.77 | 0.8 | 0.77 | 1378 | Train 22 | Test Female 34 | 0.77 | 0.8 | 0.78 |
| 355 | Train 6 | Test Female 35 | 0.75 | 0.79 | 0.76 | 1379 | Train 22 | Test Female 35 | 0.75 | 0.79 | 0.77 |
| 356 | Train 6 | Test Female 36 | 0.77 | 0.8 | 0.77 | 1380 | Train 22 | Test Female 36 | 0.77 | 0.8 | 0.78 |
| 357 | Train 6 | Test Female 37 | 0.74 | 0.78 | 0.77 | 1381 | Train 22 | Test Female 37 | 0.74 | 0.78 | 0.77 |
| 358 | Train 6 | Test Female 38 | 0.75 | 0.8 | 0.81 | 1382 | Train 22 | Test Female 38 | 0.75 | 0.8 | 0.81 |
| 359 | Train 6 | Test Female 39 | 0.74 | 0.78 | 0.76 | 1383 | Train 22 | Test Female 39 | 0.74 | 0.78 | 0.77 |
| 360 | Train 6 | Test Female 40 | 0.75 | 0.79 | 0.8 | 1384 | Train 22 | Test Female 40 | 0.75 | 0.79 | 0.8 |
| 361 | Train 6 | Test Female 41 | 0.75 | 0.79 | 0.77 | 1385 | Train 22 | Test Female 41 | 0.75 | 0.79 | 0.77 |
| 362 | Train 6 | Test Female 42 | 0.77 | 0.8 | 0.78 | 1386 | Train 22 | Test Female 42 | 0.77 | 0.8 | 0.79 |
| 363 | Train 6 | Test Female 43 | 0.75 | 0.79 | 0.77 | 1387 | Train 22 | Test Female 43 | 0.75 | 0.79 | 0.77 |
| 364 | Train 6 | Test Female 44 | 0.77 | 0.8 | 0.78 | 1388 | Train 22 | Test Female 44 | 0.77 | 0.8 | 0.79 |
| 365 | Train 6 | Test Female 45 | 0.74 | 0.79 | 0.8 | 1389 | Train 22 | Test Female 45 | 0.74 | 0.79 | 0.8 |
| 366 | Train 6 | Test Female 46 | 0.76 | 0.8 | 0.81 | 1390 | Train 22 | Test Female 46 | 0.76 | 0.8 | 0.81 |
| 367 | Train 6 | Test Female 47 | 0.74 | 0.78 | 0.77 | 1391 | Train 22 | Test Female 47 | 0.74 | 0.78 | 0.77 |
| 368 | Train 6 | Test Female 48 | 0.75 | 0.79 | 0.8 | 1392 | Train 22 | Test Female 48 | 0.75 | 0.79 | 0.8 |
| 369 | Train 6 | Test Female 49 | 0.78 | 0.81 | 0.79 | 1393 | Train 22 | Test Female 49 | 0.78 | 0.81 | 0.8 |
| 370 | Train 6 | Test Female 50 | 0.78 | 0.81 | 0.79 | 1394 | Train 22 | Test Female 50 | 0.78 | 0.81 | 0.8 |
| 371 | Train 6 | Test Female 51 | 0.78 | 0.81 | 0.79 | 1395 | Train 22 | Test Female 51 | 0.78 | 0.81 | 0.8 |
| 372 | Train 6 | Test Female 52 | 0.78 | 0.81 | 0.79 | 1396 | Train 22 | Test Female 52 | 0.78 | 0.81 | 0.8 |
| 373 | Train 6 | Test Female 53 | 0.76 | 0.81 | 0.79 | 1397 | Train 22 | Test Female 53 | 0.76 | 0.81 | 0.8 |
| 374 | Train 6 | Test Female 54 | 0.76 | 0.8 | 0.8 | 1398 | Train 22 | Test Female 54 | 0.76 | 0.8 | 0.8 |
| 375 | Train 6 | Test Female 55 | 0.76 | 0.81 | 0.79 | 1399 | Train 22 | Test Female 55 | 0.76 | 0.81 | 0.8 |
| 376 | Train 6 | Test Female 56 | 0.76 | 0.79 | 0.8 | 1400 | Train 22 | Test Female 56 | 0.76 | 0.79 | 0.8 |
| 377 | Train 6 | Test Female 57 | 0.78 | 0.81 | 0.81 | 1401 | Train 22 | Test Female 57 | 0.78 | 0.81 | 0.81 |
| 378 | Train 6 | Test Female 58 | 0.78 | 0.81 | 0.81 | 1402 | Train 22 | Test Female 58 | 0.78 | 0.81 | 0.81 |
| 379 | Train 6 | Test Female 59 | 0.78 | 0.81 | 0.81 | 1403 | Train 22 | Test Female 59 | 0.78 | 0.81 | 0.81 |
| 380 | Train 6 | Test Female 60 | 0.78 | 0.81 | 0.8 | 1404 | Train 22 | Test Female 60 | 0.78 | 0.81 | 0.81 |
| 381 | Train 6 | Test Female 61 | 0.77 | 0.81 | 0.8 | 1405 | Train 22 | Test Female 61 | 0.77 | 0.81 | 0.8 |
| 382 | Train 6 | Test Female 62 | 0.76 | 0.8 | 0.8 | 1406 | Train 22 | Test Female 62 | 0.76 | 0.8 | 0.8 |
| 383 | Train 6 | Test Female 63 | 0.77 | 0.81 | 0.8 | 1407 | Train 22 | Test Female 63 | 0.77 | 0.81 | 0.8 |
| 384 | Train 6 | Test Female 64 | 0.76 | 0.79 | 0.8 | 1408 | Train 22 | Test Female 64 | 0.76 | 0.79 | 0.8 |
| 385 | Train 7 | Test Female 1 | 0.75 | 0.82 | 0.83 | 1409 | Train 23 | Test Female 1 | 0.75 | 0.82 | 0.83 |
| 386 | Train 7 | Test Female 2 | 0.76 | 0.8 | 0.81 | 1410 | Train 23 | Test Female 2 | 0.76 | 0.8 | 0.8 |
| 387 | Train 7 | Test Female 3 | 0.76 | 0.82 | 0.83 | 1411 | Train 23 | Test Female 3 | 0.76 | 0.82 | 0.83 |
| 388 | Train 7 | Test Female 4 | 0.76 | 0.8 | 0.81 | 1412 | Train 23 | Test Female 4 | 0.76 | 0.8 | 0.8 |
| 389 | Train 7 | Test Female 5 | 0.75 | 0.82 | 0.83 | 1413 | Train 23 | Test Female 5 | 0.75 | 0.82 | 0.83 |
| 390 | Train 7 | Test Female 6 | 0.72 | 0.73 | 0.7 | 1414 | Train 23 | Test Female 6 | 0.72 | 0.73 | 0.69 |
| 391 | Train 7 | Test Female 7 | 0.75 | 0.82 | 0.83 | 1415 | Train 23 | Test Female 7 | 0.75 | 0.82 | 0.83 |
| 392 | Train 7 | Test Female 8 | 0.72 | 0.73 | 0.7 | 1416 | Train 23 | Test Female 8 | 0.72 | 0.73 | 0.69 |
| 393 | Train 7 | Test Female 9 | 0.76 | 0.81 | 0.81 | 1417 | Train 23 | Test Female 9 | 0.76 | 0.81 | 0.81 |
| 394 | Train 7 | Test Female 10 | 0.76 | 0.8 | 0.79 | 1418 | Train 23 | Test Female 10 | 0.76 | 0.8 | 0.78 |
| 395 | Train 7 | Test Female 11 | 0.76 | 0.81 | 0.81 | 1419 | Train 23 | Test Female 11 | 0.76 | 0.81 | 0.81 |
| 396 | Train 7 | Test Female 12 | 0.76 | 0.8 | 0.79 | 1420 | Train 23 | Test Female 12 | 0.76 | 0.8 | 0.78 |
| 397 | Train 7 | Test Female 13 | 0.75 | 0.79 | 0.75 | 1421 | Train 23 | Test Female 13 | 0.75 | 0.79 | 0.74 |
| 398 | Train 7 | Test Female 14 | 0.72 | 0.73 | 0.7 | 1422 | Train 23 | Test Female 14 | 0.72 | 0.73 | 0.69 |
| 399 | Train 7 | Test Female 15 | 0.75 | 0.81 | 0.81 | 1423 | Train 23 | Test Female 15 | 0.75 | 0.81 | 0.81 |
| 400 | Train 7 | Test Female 16 | 0.72 | 0.73 | 0.7 | 1424 | Train 23 | Test Female 16 | 0.72 | 0.73 | 0.69 |
| 401 | Train 7 | Test Female 17 | 0.76 | 0.82 | 0.83 | 1425 | Train 23 | Test Female 17 | 0.76 | 0.82 | 0.83 |
| 402 | Train 7 | Test Female 18 | 0.76 | 0.8 | 0.81 | 1426 | Train 23 | Test Female 18 | 0.76 | 0.8 | 0.8 |
| 403 | Train 7 | Test Female 19 | 0.76 | 0.82 | 0.83 | 1427 | Train 23 | Test Female 19 | 0.76 | 0.82 | 0.83 |
| 404 | Train 7 | Test Female 20 | 0.76 | 0.8 | 0.81 | 1428 | Train 23 | Test Female 20 | 0.76 | 0.8 | 0.8 |
| 405 | Train 7 | Test Female 21 | 0.75 | 0.82 | 0.83 | 1429 | Train 23 | Test Female 21 | 0.75 | 0.82 | 0.83 |
| 406 | Train 7 | Test Female 22 | 0.72 | 0.73 | 0.7 | 1430 | Train 23 | Test Female 22 | 0.72 | 0.73 | 0.69 |
| 407 | Train 7 | Test Female 23 | 0.75 | 0.82 | 0.83 | 1431 | Train 23 | Test Female 23 | 0.75 | 0.82 | 0.83 |
| 408 | Train 7 | Test Female 24 | 0.72 | 0.73 | 0.7 | 1432 | Train 23 | Test Female 24 | 0.72 | 0.73 | 0.69 |
| 409 | Train 7 | Test Female 25 | 0.76 | 0.81 | 0.81 | 1433 | Train 23 | Test Female 25 | 0.76 | 0.81 | 0.81 |
| 410 | Train 7 | Test Female 26 | 0.76 | 0.8 | 0.79 | 1434 | Train 23 | Test Female 26 | 0.76 | 0.8 | 0.78 |
| 411 | Train 7 | Test Female 27 | 0.76 | 0.81 | 0.81 | 1435 | Train 23 | Test Female 27 | 0.76 | 0.81 | 0.81 |
| 412 | Train 7 | Test Female 28 | 0.76 | 0.8 | 0.79 | 1436 | Train 23 | Test Female 28 | 0.76 | 0.8 | 0.78 |
| 413 | Train 7 | Test Female 29 | 0.75 | 0.79 | 0.75 | 1437 | Train 23 | Test Female 29 | 0.75 | 0.79 | 0.74 |
| 414 | Train 7 | Test Female 30 | 0.72 | 0.73 | 0.7 | 1438 | Train 23 | Test Female 30 | 0.72 | 0.73 | 0.69 |
| 415 | Train 7 | Test Female 31 | 0.75 | 0.81 | 0.81 | 1439 | Train 23 | Test Female 31 | 0.75 | 0.81 | 0.81 |
| 416 | Train 7 | Test Female 32 | 0.72 | 0.73 | 0.7 | 1440 | Train 23 | Test Female 32 | 0.72 | 0.73 | 0.69 |
| 417 | Train 7 | Test Female 33 | 0.76 | 0.82 | 0.84 | 1441 | Train 23 | Test Female 33 | 0.76 | 0.82 | 0.83 |
| 418 | Train 7 | Test Female 34 | 0.77 | 0.81 | 0.81 | 1442 | Train 23 | Test Female 34 | 0.77 | 0.81 | 0.8 |
| 419 | Train 7 | Test Female 35 | 0.76 | 0.82 | 0.84 | 1443 | Train 23 | Test Female 35 | 0.76 | 0.82 | 0.83 |
| 420 | Train 7 | Test Female 36 | 0.77 | 0.81 | 0.81 | 1444 | Train 23 | Test Female 36 | 0.77 | 0.81 | 0.8 |
| 421 | Train 7 | Test Female 37 | 0.75 | 0.82 | 0.83 | 1445 | Train 23 | Test Female 37 | 0.75 | 0.82 | 0.83 |
| 422 | Train 7 | Test Female 38 | 0.72 | 0.73 | 0.7 | 1446 | Train 23 | Test Female 38 | 0.72 | 0.73 | 0.69 |
| 423 | Train 7 | Test Female 39 | 0.75 | 0.82 | 0.83 | 1447 | Train 23 | Test Female 39 | 0.75 | 0.82 | 0.83 |
| 424 | Train 7 | Test Female 40 | 0.72 | 0.73 | 0.7 | 1448 | Train 23 | Test Female 40 | 0.72 | 0.73 | 0.69 |
| 425 | Train 7 | Test Female 41 | 0.76 | 0.81 | 0.81 | 1449 | Train 23 | Test Female 41 | 0.76 | 0.81 | 0.81 |
| 426 | Train 7 | Test Female 42 | 0.77 | 0.8 | 0.79 | 1450 | Train 23 | Test Female 42 | 0.77 | 0.8 | 0.79 |
| 427 | Train 7 | Test Female 43 | 0.76 | 0.81 | 0.81 | 1451 | Train 23 | Test Female 43 | 0.76 | 0.81 | 0.81 |
| 428 | Train 7 | Test Female 44 | 0.77 | 0.8 | 0.79 | 1452 | Train 23 | Test Female 44 | 0.77 | 0.8 | 0.79 |
| 429 | Train 7 | Test Female 45 | 0.75 | 0.79 | 0.76 | 1453 | Train 23 | Test Female 45 | 0.75 | 0.79 | 0.75 |
| 430 | Train 7 | Test Female 46 | 0.72 | 0.73 | 0.7 | 1454 | Train 23 | Test Female 46 | 0.72 | 0.73 | 0.69 |
| 431 | Train 7 | Test Female 47 | 0.75 | 0.81 | 0.81 | 1455 | Train 23 | Test Female 47 | 0.75 | 0.81 | 0.81 |
| 432 | Train 7 | Test Female 48 | 0.72 | 0.73 | 0.7 | 1456 | Train 23 | Test Female 48 | 0.72 | 0.73 | 0.69 |
| 433 | Train 7 | Test Female 49 | 0.78 | 0.83 | 0.85 | 1457 | Train 23 | Test Female 49 | 0.78 | 0.83 | 0.84 |
| 434 | Train 7 | Test Female 50 | 0.78 | 0.83 | 0.85 | 1458 | Train 23 | Test Female 50 | 0.78 | 0.83 | 0.84 |
| 435 | Train 7 | Test Female 51 | 0.78 | 0.83 | 0.85 | 1459 | Train 23 | Test Female 51 | 0.78 | 0.83 | 0.85 |
| 436 | Train 7 | Test Female 52 | 0.78 | 0.83 | 0.85 | 1460 | Train 23 | Test Female 52 | 0.78 | 0.83 | 0.85 |
| 437 | Train 7 | Test Female 53 | 0.77 | 0.82 | 0.83 | 1461 | Train 23 | Test Female 53 | 0.77 | 0.82 | 0.82 |
| 438 | Train 7 | Test Female 54 | 0.73 | 0.74 | 0.71 | 1462 | Train 23 | Test Female 54 | 0.73 | 0.74 | 0.7 |
| 439 | Train 7 | Test Female 55 | 0.77 | 0.82 | 0.83 | 1463 | Train 23 | Test Female 55 | 0.77 | 0.82 | 0.83 |
| 440 | Train 7 | Test Female 56 | 0.73 | 0.74 | 0.71 | 1464 | Train 23 | Test Female 56 | 0.73 | 0.73 | 0.69 |
| 441 | Train 7 | Test Female 57 | 0.78 | 0.82 | 0.82 | 1465 | Train 23 | Test Female 57 | 0.78 | 0.82 | 0.82 |
| 442 | Train 7 | Test Female 58 | 0.78 | 0.82 | 0.82 | 1466 | Train 23 | Test Female 58 | 0.78 | 0.82 | 0.82 |
| 443 | Train 7 | Test Female 59 | 0.78 | 0.82 | 0.82 | 1467 | Train 23 | Test Female 59 | 0.78 | 0.82 | 0.82 |
| 444 | Train 7 | Test Female 60 | 0.78 | 0.82 | 0.82 | 1468 | Train 23 | Test Female 60 | 0.78 | 0.82 | 0.82 |
| 445 | Train 7 | Test Female 61 | 0.77 | 0.82 | 0.82 | 1469 | Train 23 | Test Female 61 | 0.77 | 0.82 | 0.82 |
| 446 | Train 7 | Test Female 62 | 0.74 | 0.74 | 0.71 | 1470 | Train 23 | Test Female 62 | 0.74 | 0.74 | 0.7 |
| 447 | Train 7 | Test Female 63 | 0.77 | 0.82 | 0.82 | 1471 | Train 23 | Test Female 63 | 0.77 | 0.82 | 0.82 |
| 448 | Train 7 | Test Female 64 | 0.73 | 0.74 | 0.71 | 1472 | Train 23 | Test Female 64 | 0.73 | 0.74 | 0.69 |
| 449 | Train 8 | Test Female 1 | 0.74 | 0.78 | 0.77 | 1473 | Train 24 | Test Female 1 | 0.74 | 0.78 | 0.77 |
| 450 | Train 8 | Test Female 2 | 0.76 | 0.79 | 0.78 | 1474 | Train 24 | Test Female 2 | 0.76 | 0.79 | 0.78 |
| 451 | Train 8 | Test Female 3 | 0.74 | 0.78 | 0.77 | 1475 | Train 24 | Test Female 3 | 0.75 | 0.78 | 0.77 |
| 452 | Train 8 | Test Female 4 | 0.76 | 0.79 | 0.78 | 1476 | Train 24 | Test Female 4 | 0.76 | 0.79 | 0.78 |
| 453 | Train 8 | Test Female 5 | 0.73 | 0.78 | 0.77 | 1477 | Train 24 | Test Female 5 | 0.73 | 0.78 | 0.77 |
| 454 | Train 8 | Test Female 6 | 0.75 | 0.8 | 0.8 | 1478 | Train 24 | Test Female 6 | 0.75 | 0.8 | 0.8 |
| 455 | Train 8 | Test Female 7 | 0.73 | 0.78 | 0.77 | 1479 | Train 24 | Test Female 7 | 0.73 | 0.78 | 0.77 |
| 456 | Train 8 | Test Female 8 | 0.75 | 0.8 | 0.8 | 1480 | Train 24 | Test Female 8 | 0.75 | 0.8 | 0.8 |
| 457 | Train 8 | Test Female 9 | 0.74 | 0.79 | 0.78 | 1481 | Train 24 | Test Female 9 | 0.75 | 0.79 | 0.78 |
| 458 | Train 8 | Test Female 10 | 0.76 | 0.79 | 0.79 | 1482 | Train 24 | Test Female 10 | 0.76 | 0.79 | 0.79 |
| 459 | Train 8 | Test Female 11 | 0.75 | 0.79 | 0.78 | 1483 | Train 24 | Test Female 11 | 0.75 | 0.79 | 0.78 |
| 460 | Train 8 | Test Female 12 | 0.76 | 0.79 | 0.79 | 1484 | Train 24 | Test Female 12 | 0.76 | 0.79 | 0.79 |
| 461 | Train 8 | Test Female 13 | 0.73 | 0.79 | 0.8 | 1485 | Train 24 | Test Female 13 | 0.73 | 0.79 | 0.8 |
| 462 | Train 8 | Test Female 14 | 0.75 | 0.8 | 0.8 | 1486 | Train 24 | Test Female 14 | 0.75 | 0.8 | 0.8 |
| 463 | Train 8 | Test Female 15 | 0.73 | 0.78 | 0.77 | 1487 | Train 24 | Test Female 15 | 0.73 | 0.78 | 0.78 |
| 464 | Train 8 | Test Female 16 | 0.75 | 0.8 | 0.8 | 1488 | Train 24 | Test Female 16 | 0.75 | 0.8 | 0.8 |
| 465 | Train 8 | Test Female 17 | 0.74 | 0.78 | 0.77 | 1489 | Train 24 | Test Female 17 | 0.74 | 0.78 | 0.77 |
| 466 | Train 8 | Test Female 18 | 0.76 | 0.79 | 0.78 | 1490 | Train 24 | Test Female 18 | 0.76 | 0.79 | 0.78 |
| 467 | Train 8 | Test Female 19 | 0.74 | 0.78 | 0.77 | 1491 | Train 24 | Test Female 19 | 0.75 | 0.78 | 0.77 |
| 468 | Train 8 | Test Female 20 | 0.76 | 0.79 | 0.78 | 1492 | Train 24 | Test Female 20 | 0.76 | 0.79 | 0.78 |
| 469 | Train 8 | Test Female 21 | 0.73 | 0.78 | 0.77 | 1493 | Train 24 | Test Female 21 | 0.73 | 0.78 | 0.77 |
| 470 | Train 8 | Test Female 22 | 0.75 | 0.8 | 0.8 | 1494 | Train 24 | Test Female 22 | 0.75 | 0.8 | 0.8 |
| 471 | Train 8 | Test Female 23 | 0.73 | 0.78 | 0.77 | 1495 | Train 24 | Test Female 23 | 0.73 | 0.78 | 0.77 |
| 472 | Train 8 | Test Female 24 | 0.75 | 0.8 | 0.8 | 1496 | Train 24 | Test Female 24 | 0.75 | 0.8 | 0.8 |
| 473 | Train 8 | Test Female 25 | 0.74 | 0.79 | 0.78 | 1497 | Train 24 | Test Female 25 | 0.75 | 0.79 | 0.78 |
| 474 | Train 8 | Test Female 26 | 0.76 | 0.79 | 0.79 | 1498 | Train 24 | Test Female 26 | 0.76 | 0.79 | 0.79 |
| 475 | Train 8 | Test Female 27 | 0.75 | 0.79 | 0.78 | 1499 | Train 24 | Test Female 27 | 0.75 | 0.79 | 0.78 |
| 476 | Train 8 | Test Female 28 | 0.76 | 0.8 | 0.79 | 1500 | Train 24 | Test Female 28 | 0.76 | 0.79 | 0.79 |
| 477 | Train 8 | Test Female 29 | 0.73 | 0.79 | 0.8 | 1501 | Train 24 | Test Female 29 | 0.73 | 0.79 | 0.8 |
| 478 | Train 8 | Test Female 30 | 0.75 | 0.8 | 0.8 | 1502 | Train 24 | Test Female 30 | 0.75 | 0.8 | 0.8 |
| 479 | Train 8 | Test Female 31 | 0.73 | 0.78 | 0.77 | 1503 | Train 24 | Test Female 31 | 0.73 | 0.78 | 0.78 |
| 480 | Train 8 | Test Female 32 | 0.75 | 0.8 | 0.8 | 1504 | Train 24 | Test Female 32 | 0.75 | 0.8 | 0.8 |
| 481 | Train 8 | Test Female 33 | 0.75 | 0.79 | 0.77 | 1505 | Train 24 | Test Female 33 | 0.75 | 0.79 | 0.78 |
| 482 | Train 8 | Test Female 34 | 0.77 | 0.8 | 0.79 | 1506 | Train 24 | Test Female 34 | 0.77 | 0.8 | 0.79 |
| 483 | Train 8 | Test Female 35 | 0.75 | 0.79 | 0.78 | 1507 | Train 24 | Test Female 35 | 0.75 | 0.79 | 0.78 |
| 484 | Train 8 | Test Female 36 | 0.77 | 0.8 | 0.79 | 1508 | Train 24 | Test Female 36 | 0.77 | 0.8 | 0.79 |
| 485 | Train 8 | Test Female 37 | 0.74 | 0.78 | 0.77 | 1509 | Train 24 | Test Female 37 | 0.74 | 0.78 | 0.77 |
| 486 | Train 8 | Test Female 38 | 0.75 | 0.8 | 0.8 | 1510 | Train 24 | Test Female 38 | 0.75 | 0.8 | 0.8 |
| 487 | Train 8 | Test Female 39 | 0.74 | 0.78 | 0.77 | 1511 | Train 24 | Test Female 39 | 0.74 | 0.78 | 0.77 |
| 488 | Train 8 | Test Female 40 | 0.75 | 0.8 | 0.8 | 1512 | Train 24 | Test Female 40 | 0.75 | 0.8 | 0.8 |
| 489 | Train 8 | Test Female 41 | 0.75 | 0.79 | 0.78 | 1513 | Train 24 | Test Female 41 | 0.75 | 0.79 | 0.79 |
| 490 | Train 8 | Test Female 42 | 0.77 | 0.8 | 0.79 | 1514 | Train 24 | Test Female 42 | 0.77 | 0.8 | 0.79 |
| 491 | Train 8 | Test Female 43 | 0.75 | 0.79 | 0.78 | 1515 | Train 24 | Test Female 43 | 0.75 | 0.79 | 0.79 |
| 492 | Train 8 | Test Female 44 | 0.77 | 0.8 | 0.79 | 1516 | Train 24 | Test Female 44 | 0.77 | 0.8 | 0.79 |
| 493 | Train 8 | Test Female 45 | 0.74 | 0.79 | 0.8 | 1517 | Train 24 | Test Female 45 | 0.74 | 0.79 | 0.8 |
| 494 | Train 8 | Test Female 46 | 0.75 | 0.8 | 0.8 | 1518 | Train 24 | Test Female 46 | 0.75 | 0.8 | 0.81 |
| 495 | Train 8 | Test Female 47 | 0.74 | 0.78 | 0.78 | 1519 | Train 24 | Test Female 47 | 0.74 | 0.78 | 0.78 |
| 496 | Train 8 | Test Female 48 | 0.75 | 0.8 | 0.8 | 1520 | Train 24 | Test Female 48 | 0.75 | 0.8 | 0.8 |
| 497 | Train 8 | Test Female 49 | 0.78 | 0.81 | 0.77 | 1521 | Train 24 | Test Female 49 | 0.78 | 0.81 | 0.8 |
| 498 | Train 8 | Test Female 50 | 0.78 | 0.81 | 0.77 | 1522 | Train 24 | Test Female 50 | 0.78 | 0.81 | 0.8 |
| 499 | Train 8 | Test Female 51 | 0.78 | 0.81 | 0.78 | 1523 | Train 24 | Test Female 51 | 0.78 | 0.81 | 0.8 |
| 500 | Train 8 | Test Female 52 | 0.78 | 0.81 | 0.77 | 1524 | Train 24 | Test Female 52 | 0.78 | 0.81 | 0.8 |
| 501 | Train 8 | Test Female 53 | 0.76 | 0.8 | 0.79 | 1525 | Train 24 | Test Female 53 | 0.76 | 0.81 | 0.8 |
| 502 | Train 8 | Test Female 54 | 0.76 | 0.8 | 0.8 | 1526 | Train 24 | Test Female 54 | 0.76 | 0.8 | 0.8 |
| 503 | Train 8 | Test Female 55 | 0.76 | 0.81 | 0.79 | 1527 | Train 24 | Test Female 55 | 0.76 | 0.81 | 0.8 |
| 504 | Train 8 | Test Female 56 | 0.76 | 0.8 | 0.8 | 1528 | Train 24 | Test Female 56 | 0.76 | 0.8 | 0.8 |
| 505 | Train 8 | Test Female 57 | 0.78 | 0.81 | 0.79 | 1529 | Train 24 | Test Female 57 | 0.78 | 0.81 | 0.81 |
| 506 | Train 8 | Test Female 58 | 0.78 | 0.81 | 0.79 | 1530 | Train 24 | Test Female 58 | 0.78 | 0.81 | 0.81 |
| 507 | Train 8 | Test Female 59 | 0.78 | 0.81 | 0.79 | 1531 | Train 24 | Test Female 59 | 0.78 | 0.81 | 0.81 |
| 508 | Train 8 | Test Female 60 | 0.78 | 0.81 | 0.79 | 1532 | Train 24 | Test Female 60 | 0.78 | 0.81 | 0.81 |
| 509 | Train 8 | Test Female 61 | 0.76 | 0.81 | 0.79 | 1533 | Train 24 | Test Female 61 | 0.76 | 0.81 | 0.81 |
| 510 | Train 8 | Test Female 62 | 0.76 | 0.8 | 0.8 | 1534 | Train 24 | Test Female 62 | 0.76 | 0.8 | 0.8 |
| 511 | Train 8 | Test Female 63 | 0.76 | 0.81 | 0.8 | 1535 | Train 24 | Test Female 63 | 0.76 | 0.81 | 0.81 |
| 512 | Train 8 | Test Female 64 | 0.76 | 0.8 | 0.8 | 1536 | Train 24 | Test Female 64 | 0.76 | 0.8 | 0.81 |
| 513 | Train 9 | Test Female 1 | 0.76 | 0.84 | 0.82 | 1537 | Train 25 | Test Female 1 | 0.76 | 0.84 | 0.82 |
| 514 | Train 9 | Test Female 2 | 0.76 | 0.8 | 0.78 | 1538 | Train 25 | Test Female 2 | 0.76 | 0.8 | 0.78 |
| 515 | Train 9 | Test Female 3 | 0.76 | 0.83 | 0.82 | 1539 | Train 25 | Test Female 3 | 0.76 | 0.84 | 0.82 |
| 516 | Train 9 | Test Female 4 | 0.76 | 0.8 | 0.78 | 1540 | Train 25 | Test Female 4 | 0.76 | 0.8 | 0.78 |
| 517 | Train 9 | Test Female 5 | 0.73 | 0.76 | 0.72 | 1541 | Train 25 | Test Female 5 | 0.73 | 0.76 | 0.74 |
| 518 | Train 9 | Test Female 6 | 0.71 | 0.72 | 0.66 | 1542 | Train 25 | Test Female 6 | 0.71 | 0.72 | 0.68 |
| 519 | Train 9 | Test Female 7 | 0.73 | 0.75 | 0.72 | 1543 | Train 25 | Test Female 7 | 0.73 | 0.75 | 0.73 |
| 520 | Train 9 | Test Female 8 | 0.71 | 0.72 | 0.66 | 1544 | Train 25 | Test Female 8 | 0.71 | 0.72 | 0.68 |
| 521 | Train 9 | Test Female 9 | 0.76 | 0.84 | 0.86 | 1545 | Train 25 | Test Female 9 | 0.76 | 0.84 | 0.86 |
| 522 | Train 9 | Test Female 10 | 0.76 | 0.81 | 0.81 | 1546 | Train 25 | Test Female 10 | 0.76 | 0.81 | 0.81 |
| 523 | Train 9 | Test Female 11 | 0.77 | 0.84 | 0.86 | 1547 | Train 25 | Test Female 11 | 0.77 | 0.84 | 0.86 |
| 524 | Train 9 | Test Female 12 | 0.76 | 0.81 | 0.81 | 1548 | Train 25 | Test Female 12 | 0.76 | 0.81 | 0.81 |
| 525 | Train 9 | Test Female 13 | 0.73 | 0.76 | 0.71 | 1549 | Train 25 | Test Female 13 | 0.73 | 0.76 | 0.73 |
| 526 | Train 9 | Test Female 14 | 0.71 | 0.72 | 0.66 | 1550 | Train 25 | Test Female 14 | 0.71 | 0.72 | 0.68 |
| 527 | Train 9 | Test Female 15 | 0.73 | 0.76 | 0.74 | 1551 | Train 25 | Test Female 15 | 0.73 | 0.76 | 0.75 |
| 528 | Train 9 | Test Female 16 | 0.72 | 0.72 | 0.66 | 1552 | Train 25 | Test Female 16 | 0.71 | 0.72 | 0.68 |
| 529 | Train 9 | Test Female 17 | 0.76 | 0.84 | 0.82 | 1553 | Train 25 | Test Female 17 | 0.76 | 0.84 | 0.82 |
| 530 | Train 9 | Test Female 18 | 0.76 | 0.8 | 0.78 | 1554 | Train 25 | Test Female 18 | 0.76 | 0.8 | 0.78 |
| 531 | Train 9 | Test Female 19 | 0.77 | 0.83 | 0.82 | 1555 | Train 25 | Test Female 19 | 0.77 | 0.84 | 0.82 |
| 532 | Train 9 | Test Female 20 | 0.76 | 0.8 | 0.78 | 1556 | Train 25 | Test Female 20 | 0.76 | 0.8 | 0.78 |
| 533 | Train 9 | Test Female 21 | 0.73 | 0.76 | 0.72 | 1557 | Train 25 | Test Female 21 | 0.73 | 0.76 | 0.74 |
| 534 | Train 9 | Test Female 22 | 0.71 | 0.72 | 0.66 | 1558 | Train 25 | Test Female 22 | 0.71 | 0.72 | 0.68 |
| 535 | Train 9 | Test Female 23 | 0.73 | 0.75 | 0.72 | 1559 | Train 25 | Test Female 23 | 0.73 | 0.75 | 0.73 |
| 536 | Train 9 | Test Female 24 | 0.71 | 0.72 | 0.66 | 1560 | Train 25 | Test Female 24 | 0.71 | 0.72 | 0.68 |
| 537 | Train 9 | Test Female 25 | 0.77 | 0.84 | 0.86 | 1561 | Train 25 | Test Female 25 | 0.77 | 0.84 | 0.86 |
| 538 | Train 9 | Test Female 26 | 0.76 | 0.81 | 0.81 | 1562 | Train 25 | Test Female 26 | 0.76 | 0.81 | 0.81 |
| 539 | Train 9 | Test Female 27 | 0.77 | 0.84 | 0.86 | 1563 | Train 25 | Test Female 27 | 0.77 | 0.84 | 0.86 |
| 540 | Train 9 | Test Female 28 | 0.76 | 0.81 | 0.81 | 1564 | Train 25 | Test Female 28 | 0.76 | 0.81 | 0.81 |
| 541 | Train 9 | Test Female 29 | 0.73 | 0.76 | 0.71 | 1565 | Train 25 | Test Female 29 | 0.73 | 0.76 | 0.73 |
| 542 | Train 9 | Test Female 30 | 0.71 | 0.72 | 0.66 | 1566 | Train 25 | Test Female 30 | 0.71 | 0.72 | 0.68 |
| 543 | Train 9 | Test Female 31 | 0.73 | 0.76 | 0.74 | 1567 | Train 25 | Test Female 31 | 0.73 | 0.76 | 0.75 |
| 544 | Train 9 | Test Female 32 | 0.72 | 0.72 | 0.66 | 1568 | Train 25 | Test Female 32 | 0.71 | 0.72 | 0.68 |
| 545 | Train 9 | Test Female 33 | 0.77 | 0.84 | 0.83 | 1569 | Train 25 | Test Female 33 | 0.77 | 0.84 | 0.82 |
| 546 | Train 9 | Test Female 34 | 0.76 | 0.8 | 0.79 | 1570 | Train 25 | Test Female 34 | 0.76 | 0.8 | 0.79 |
| 547 | Train 9 | Test Female 35 | 0.77 | 0.84 | 0.83 | 1571 | Train 25 | Test Female 35 | 0.77 | 0.84 | 0.82 |
| 548 | Train 9 | Test Female 36 | 0.76 | 0.8 | 0.78 | 1572 | Train 25 | Test Female 36 | 0.76 | 0.8 | 0.79 |
| 549 | Train 9 | Test Female 37 | 0.73 | 0.76 | 0.73 | 1573 | Train 25 | Test Female 37 | 0.73 | 0.76 | 0.74 |
| 550 | Train 9 | Test Female 38 | 0.71 | 0.72 | 0.66 | 1574 | Train 25 | Test Female 38 | 0.71 | 0.72 | 0.68 |
| 551 | Train 9 | Test Female 39 | 0.73 | 0.76 | 0.73 | 1575 | Train 25 | Test Female 39 | 0.73 | 0.76 | 0.74 |
| 552 | Train 9 | Test Female 40 | 0.72 | 0.72 | 0.66 | 1576 | Train 25 | Test Female 40 | 0.71 | 0.72 | 0.68 |
| 553 | Train 9 | Test Female 41 | 0.77 | 0.85 | 0.87 | 1577 | Train 25 | Test Female 41 | 0.77 | 0.85 | 0.86 |
| 554 | Train 9 | Test Female 42 | 0.76 | 0.81 | 0.81 | 1578 | Train 25 | Test Female 42 | 0.76 | 0.81 | 0.82 |
| 555 | Train 9 | Test Female 43 | 0.77 | 0.85 | 0.86 | 1579 | Train 25 | Test Female 43 | 0.77 | 0.85 | 0.86 |
| 556 | Train 9 | Test Female 44 | 0.76 | 0.81 | 0.81 | 1580 | Train 25 | Test Female 44 | 0.76 | 0.81 | 0.81 |
| 557 | Train 9 | Test Female 45 | 0.73 | 0.76 | 0.71 | 1581 | Train 25 | Test Female 45 | 0.73 | 0.76 | 0.73 |
| 558 | Train 9 | Test Female 46 | 0.72 | 0.72 | 0.66 | 1582 | Train 25 | Test Female 46 | 0.71 | 0.72 | 0.68 |
| 559 | Train 9 | Test Female 47 | 0.73 | 0.76 | 0.74 | 1583 | Train 25 | Test Female 47 | 0.73 | 0.76 | 0.76 |
| 560 | Train 9 | Test Female 48 | 0.72 | 0.72 | 0.66 | 1584 | Train 25 | Test Female 48 | 0.72 | 0.72 | 0.68 |
| 561 | Train 9 | Test Female 49 | 0.79 | 0.84 | 0.83 | 1585 | Train 25 | Test Female 49 | 0.79 | 0.84 | 0.83 |
| 562 | Train 9 | Test Female 50 | 0.79 | 0.84 | 0.83 | 1586 | Train 25 | Test Female 50 | 0.79 | 0.84 | 0.83 |
| 563 | Train 9 | Test Female 51 | 0.79 | 0.83 | 0.82 | 1587 | Train 25 | Test Female 51 | 0.79 | 0.84 | 0.82 |
| 564 | Train 9 | Test Female 52 | 0.79 | 0.83 | 0.82 | 1588 | Train 25 | Test Female 52 | 0.79 | 0.83 | 0.82 |
| 565 | Train 9 | Test Female 53 | 0.74 | 0.77 | 0.72 | 1589 | Train 25 | Test Female 53 | 0.74 | 0.77 | 0.75 |
| 566 | Train 9 | Test Female 54 | 0.73 | 0.73 | 0.66 | 1590 | Train 25 | Test Female 54 | 0.73 | 0.73 | 0.69 |
| 567 | Train 9 | Test Female 55 | 0.74 | 0.78 | 0.72 | 1591 | Train 25 | Test Female 55 | 0.74 | 0.78 | 0.75 |
| 568 | Train 9 | Test Female 56 | 0.73 | 0.72 | 0.66 | 1592 | Train 25 | Test Female 56 | 0.73 | 0.72 | 0.69 |
| 569 | Train 9 | Test Female 57 | 0.79 | 0.85 | 0.87 | 1593 | Train 25 | Test Female 57 | 0.79 | 0.85 | 0.87 |
| 570 | Train 9 | Test Female 58 | 0.79 | 0.84 | 0.87 | 1594 | Train 25 | Test Female 58 | 0.79 | 0.85 | 0.87 |
| 571 | Train 9 | Test Female 59 | 0.79 | 0.84 | 0.86 | 1595 | Train 25 | Test Female 59 | 0.79 | 0.84 | 0.86 |
| 572 | Train 9 | Test Female 60 | 0.79 | 0.84 | 0.86 | 1596 | Train 25 | Test Female 60 | 0.79 | 0.84 | 0.86 |
| 573 | Train 9 | Test Female 61 | 0.74 | 0.78 | 0.73 | 1597 | Train 25 | Test Female 61 | 0.74 | 0.78 | 0.76 |
| 574 | Train 9 | Test Female 62 | 0.73 | 0.73 | 0.67 | 1598 | Train 25 | Test Female 62 | 0.73 | 0.73 | 0.7 |
| 575 | Train 9 | Test Female 63 | 0.75 | 0.78 | 0.73 | 1599 | Train 25 | Test Female 63 | 0.74 | 0.78 | 0.76 |
| 576 | Train 9 | Test Female 64 | 0.73 | 0.73 | 0.67 | 1600 | Train 25 | Test Female 64 | 0.73 | 0.73 | 0.69 |
| 577 | Train 10 | Test Female 1 | 0.76 | 0.83 | 0.82 | 1601 | Train 26 | Test Female 1 | 0.76 | 0.83 | 0.82 |
| 578 | Train 10 | Test Female 2 | 0.77 | 0.84 | 0.83 | 1602 | Train 26 | Test Female 2 | 0.77 | 0.84 | 0.83 |
| 579 | Train 10 | Test Female 3 | 0.76 | 0.83 | 0.81 | 1603 | Train 26 | Test Female 3 | 0.76 | 0.83 | 0.82 |
| 580 | Train 10 | Test Female 4 | 0.77 | 0.84 | 0.83 | 1604 | Train 26 | Test Female 4 | 0.77 | 0.84 | 0.83 |
| 581 | Train 10 | Test Female 5 | 0.73 | 0.76 | 0.74 | 1605 | Train 26 | Test Female 5 | 0.73 | 0.76 | 0.74 |
| 582 | Train 10 | Test Female 6 | 0.72 | 0.73 | 0.69 | 1606 | Train 26 | Test Female 6 | 0.73 | 0.73 | 0.7 |
| 583 | Train 10 | Test Female 7 | 0.73 | 0.76 | 0.73 | 1607 | Train 26 | Test Female 7 | 0.73 | 0.76 | 0.74 |
| 584 | Train 10 | Test Female 8 | 0.72 | 0.73 | 0.69 | 1608 | Train 26 | Test Female 8 | 0.73 | 0.73 | 0.7 |
| 585 | Train 10 | Test Female 9 | 0.76 | 0.83 | 0.85 | 1609 | Train 26 | Test Female 9 | 0.76 | 0.83 | 0.85 |
| 586 | Train 10 | Test Female 10 | 0.77 | 0.84 | 0.86 | 1610 | Train 26 | Test Female 10 | 0.77 | 0.84 | 0.86 |
| 587 | Train 10 | Test Female 11 | 0.76 | 0.83 | 0.84 | 1611 | Train 26 | Test Female 11 | 0.76 | 0.83 | 0.84 |
| 588 | Train 10 | Test Female 12 | 0.77 | 0.84 | 0.85 | 1612 | Train 26 | Test Female 12 | 0.77 | 0.84 | 0.86 |
| 589 | Train 10 | Test Female 13 | 0.73 | 0.76 | 0.72 | 1613 | Train 26 | Test Female 13 | 0.73 | 0.76 | 0.72 |
| 590 | Train 10 | Test Female 14 | 0.73 | 0.74 | 0.7 | 1614 | Train 26 | Test Female 14 | 0.73 | 0.74 | 0.7 |
| 591 | Train 10 | Test Female 15 | 0.73 | 0.76 | 0.75 | 1615 | Train 26 | Test Female 15 | 0.73 | 0.76 | 0.75 |
| 592 | Train 10 | Test Female 16 | 0.73 | 0.73 | 0.7 | 1616 | Train 26 | Test Female 16 | 0.73 | 0.73 | 0.7 |
| 593 | Train 10 | Test Female 17 | 0.76 | 0.83 | 0.82 | 1617 | Train 26 | Test Female 17 | 0.76 | 0.83 | 0.82 |
| 594 | Train 10 | Test Female 18 | 0.77 | 0.84 | 0.83 | 1618 | Train 26 | Test Female 18 | 0.77 | 0.84 | 0.83 |
| 595 | Train 10 | Test Female 19 | 0.76 | 0.83 | 0.81 | 1619 | Train 26 | Test Female 19 | 0.76 | 0.83 | 0.82 |
| 596 | Train 10 | Test Female 20 | 0.77 | 0.84 | 0.83 | 1620 | Train 26 | Test Female 20 | 0.77 | 0.84 | 0.83 |
| 597 | Train 10 | Test Female 21 | 0.73 | 0.76 | 0.74 | 1621 | Train 26 | Test Female 21 | 0.73 | 0.76 | 0.74 |
| 598 | Train 10 | Test Female 22 | 0.73 | 0.73 | 0.7 | 1622 | Train 26 | Test Female 22 | 0.73 | 0.73 | 0.7 |
| 599 | Train 10 | Test Female 23 | 0.73 | 0.76 | 0.73 | 1623 | Train 26 | Test Female 23 | 0.73 | 0.76 | 0.74 |
| 600 | Train 10 | Test Female 24 | 0.73 | 0.73 | 0.7 | 1624 | Train 26 | Test Female 24 | 0.73 | 0.73 | 0.7 |
| 601 | Train 10 | Test Female 25 | 0.76 | 0.83 | 0.85 | 1625 | Train 26 | Test Female 25 | 0.76 | 0.83 | 0.85 |
| 602 | Train 10 | Test Female 26 | 0.77 | 0.84 | 0.86 | 1626 | Train 26 | Test Female 26 | 0.77 | 0.84 | 0.86 |
| 603 | Train 10 | Test Female 27 | 0.76 | 0.83 | 0.84 | 1627 | Train 26 | Test Female 27 | 0.76 | 0.83 | 0.84 |
| 604 | Train 10 | Test Female 28 | 0.78 | 0.84 | 0.85 | 1628 | Train 26 | Test Female 28 | 0.78 | 0.84 | 0.86 |
| 605 | Train 10 | Test Female 29 | 0.73 | 0.76 | 0.72 | 1629 | Train 26 | Test Female 29 | 0.73 | 0.76 | 0.72 |
| 606 | Train 10 | Test Female 30 | 0.73 | 0.74 | 0.7 | 1630 | Train 26 | Test Female 30 | 0.73 | 0.74 | 0.7 |
| 607 | Train 10 | Test Female 31 | 0.73 | 0.76 | 0.75 | 1631 | Train 26 | Test Female 31 | 0.73 | 0.76 | 0.75 |
| 608 | Train 10 | Test Female 32 | 0.73 | 0.73 | 0.7 | 1632 | Train 26 | Test Female 32 | 0.73 | 0.73 | 0.7 |
| 609 | Train 10 | Test Female 33 | 0.76 | 0.83 | 0.82 | 1633 | Train 26 | Test Female 33 | 0.76 | 0.83 | 0.82 |
| 610 | Train 10 | Test Female 34 | 0.78 | 0.84 | 0.83 | 1634 | Train 26 | Test Female 34 | 0.78 | 0.84 | 0.83 |
| 611 | Train 10 | Test Female 35 | 0.76 | 0.83 | 0.82 | 1635 | Train 26 | Test Female 35 | 0.76 | 0.83 | 0.82 |
| 612 | Train 10 | Test Female 36 | 0.78 | 0.84 | 0.83 | 1636 | Train 26 | Test Female 36 | 0.78 | 0.84 | 0.83 |
| 613 | Train 10 | Test Female 37 | 0.73 | 0.76 | 0.74 | 1637 | Train 26 | Test Female 37 | 0.73 | 0.76 | 0.74 |
| 614 | Train 10 | Test Female 38 | 0.73 | 0.74 | 0.7 | 1638 | Train 26 | Test Female 38 | 0.73 | 0.74 | 0.7 |
| 615 | Train 10 | Test Female 39 | 0.73 | 0.76 | 0.74 | 1639 | Train 26 | Test Female 39 | 0.73 | 0.76 | 0.74 |
| 616 | Train 10 | Test Female 40 | 0.73 | 0.73 | 0.7 | 1640 | Train 26 | Test Female 40 | 0.73 | 0.73 | 0.7 |
| 617 | Train 10 | Test Female 41 | 0.76 | 0.84 | 0.85 | 1641 | Train 26 | Test Female 41 | 0.76 | 0.84 | 0.85 |
| 618 | Train 10 | Test Female 42 | 0.78 | 0.85 | 0.86 | 1642 | Train 26 | Test Female 42 | 0.78 | 0.85 | 0.86 |
| 619 | Train 10 | Test Female 43 | 0.77 | 0.84 | 0.85 | 1643 | Train 26 | Test Female 43 | 0.77 | 0.84 | 0.85 |
| 620 | Train 10 | Test Female 44 | 0.78 | 0.84 | 0.86 | 1644 | Train 26 | Test Female 44 | 0.78 | 0.85 | 0.86 |
| 621 | Train 10 | Test Female 45 | 0.73 | 0.76 | 0.72 | 1645 | Train 26 | Test Female 45 | 0.73 | 0.76 | 0.72 |
| 622 | Train 10 | Test Female 46 | 0.73 | 0.74 | 0.7 | 1646 | Train 26 | Test Female 46 | 0.73 | 0.74 | 0.7 |
| 623 | Train 10 | Test Female 47 | 0.73 | 0.77 | 0.75 | 1647 | Train 26 | Test Female 47 | 0.73 | 0.76 | 0.75 |
| 624 | Train 10 | Test Female 48 | 0.73 | 0.74 | 0.7 | 1648 | Train 26 | Test Female 48 | 0.73 | 0.74 | 0.7 |
| 625 | Train 10 | Test Female 49 | 0.78 | 0.83 | 0.82 | 1649 | Train 26 | Test Female 49 | 0.78 | 0.83 | 0.83 |
| 626 | Train 10 | Test Female 50 | 0.78 | 0.83 | 0.82 | 1650 | Train 26 | Test Female 50 | 0.78 | 0.83 | 0.83 |
| 627 | Train 10 | Test Female 51 | 0.78 | 0.83 | 0.82 | 1651 | Train 26 | Test Female 51 | 0.78 | 0.83 | 0.83 |
| 628 | Train 10 | Test Female 52 | 0.78 | 0.83 | 0.82 | 1652 | Train 26 | Test Female 52 | 0.78 | 0.83 | 0.83 |
| 629 | Train 10 | Test Female 53 | 0.74 | 0.78 | 0.75 | 1653 | Train 26 | Test Female 53 | 0.75 | 0.78 | 0.74 |
| 630 | Train 10 | Test Female 54 | 0.73 | 0.74 | 0.7 | 1654 | Train 26 | Test Female 54 | 0.74 | 0.74 | 0.7 |
| 631 | Train 10 | Test Female 55 | 0.75 | 0.78 | 0.75 | 1655 | Train 26 | Test Female 55 | 0.75 | 0.78 | 0.74 |
| 632 | Train 10 | Test Female 56 | 0.73 | 0.73 | 0.7 | 1656 | Train 26 | Test Female 56 | 0.73 | 0.73 | 0.69 |
| 633 | Train 10 | Test Female 57 | 0.78 | 0.84 | 0.86 | 1657 | Train 26 | Test Female 57 | 0.78 | 0.84 | 0.86 |
| 634 | Train 10 | Test Female 58 | 0.78 | 0.84 | 0.86 | 1658 | Train 26 | Test Female 58 | 0.78 | 0.84 | 0.86 |
| 635 | Train 10 | Test Female 59 | 0.79 | 0.84 | 0.85 | 1659 | Train 26 | Test Female 59 | 0.79 | 0.84 | 0.85 |
| 636 | Train 10 | Test Female 60 | 0.79 | 0.84 | 0.85 | 1660 | Train 26 | Test Female 60 | 0.79 | 0.84 | 0.85 |
| 637 | Train 10 | Test Female 61 | 0.75 | 0.78 | 0.76 | 1661 | Train 26 | Test Female 61 | 0.75 | 0.78 | 0.75 |
| 638 | Train 10 | Test Female 62 | 0.74 | 0.74 | 0.7 | 1662 | Train 26 | Test Female 62 | 0.74 | 0.74 | 0.7 |
| 639 | Train 10 | Test Female 63 | 0.75 | 0.78 | 0.76 | 1663 | Train 26 | Test Female 63 | 0.75 | 0.78 | 0.75 |
| 640 | Train 10 | Test Female 64 | 0.73 | 0.73 | 0.7 | 1664 | Train 26 | Test Female 64 | 0.73 | 0.73 | 0.7 |
| 641 | Train 11 | Test Female 1 | 0.76 | 0.83 | 0.82 | 1665 | Train 27 | Test Female 1 | 0.76 | 0.83 | 0.82 |
| 642 | Train 11 | Test Female 2 | 0.76 | 0.8 | 0.78 | 1666 | Train 27 | Test Female 2 | 0.76 | 0.8 | 0.78 |
| 643 | Train 11 | Test Female 3 | 0.76 | 0.84 | 0.82 | 1667 | Train 27 | Test Female 3 | 0.76 | 0.84 | 0.82 |
| 644 | Train 11 | Test Female 4 | 0.76 | 0.8 | 0.78 | 1668 | Train 27 | Test Female 4 | 0.76 | 0.8 | 0.78 |
| 645 | Train 11 | Test Female 5 | 0.73 | 0.76 | 0.73 | 1669 | Train 27 | Test Female 5 | 0.73 | 0.76 | 0.73 |
| 646 | Train 11 | Test Female 6 | 0.71 | 0.72 | 0.67 | 1670 | Train 27 | Test Female 6 | 0.71 | 0.72 | 0.66 |
| 647 | Train 11 | Test Female 7 | 0.73 | 0.75 | 0.73 | 1671 | Train 27 | Test Female 7 | 0.73 | 0.75 | 0.72 |
| 648 | Train 11 | Test Female 8 | 0.71 | 0.72 | 0.67 | 1672 | Train 27 | Test Female 8 | 0.71 | 0.72 | 0.66 |
| 649 | Train 11 | Test Female 9 | 0.76 | 0.84 | 0.86 | 1673 | Train 27 | Test Female 9 | 0.76 | 0.84 | 0.86 |
| 650 | Train 11 | Test Female 10 | 0.76 | 0.81 | 0.8 | 1674 | Train 27 | Test Female 10 | 0.76 | 0.81 | 0.81 |
| 651 | Train 11 | Test Female 11 | 0.77 | 0.84 | 0.86 | 1675 | Train 27 | Test Female 11 | 0.77 | 0.84 | 0.86 |
| 652 | Train 11 | Test Female 12 | 0.76 | 0.81 | 0.81 | 1676 | Train 27 | Test Female 12 | 0.76 | 0.81 | 0.81 |
| 653 | Train 11 | Test Female 13 | 0.73 | 0.76 | 0.72 | 1677 | Train 27 | Test Female 13 | 0.73 | 0.76 | 0.72 |
| 654 | Train 11 | Test Female 14 | 0.71 | 0.72 | 0.67 | 1678 | Train 27 | Test Female 14 | 0.71 | 0.72 | 0.66 |
| 655 | Train 11 | Test Female 15 | 0.73 | 0.76 | 0.74 | 1679 | Train 27 | Test Female 15 | 0.73 | 0.76 | 0.74 |
| 656 | Train 11 | Test Female 16 | 0.71 | 0.72 | 0.67 | 1680 | Train 27 | Test Female 16 | 0.71 | 0.72 | 0.66 |
| 657 | Train 11 | Test Female 17 | 0.76 | 0.83 | 0.82 | 1681 | Train 27 | Test Female 17 | 0.76 | 0.83 | 0.82 |
| 658 | Train 11 | Test Female 18 | 0.76 | 0.8 | 0.78 | 1682 | Train 27 | Test Female 18 | 0.76 | 0.8 | 0.78 |
| 659 | Train 11 | Test Female 19 | 0.77 | 0.84 | 0.82 | 1683 | Train 27 | Test Female 19 | 0.77 | 0.84 | 0.82 |
| 660 | Train 11 | Test Female 20 | 0.76 | 0.8 | 0.78 | 1684 | Train 27 | Test Female 20 | 0.76 | 0.8 | 0.78 |
| 661 | Train 11 | Test Female 21 | 0.73 | 0.76 | 0.73 | 1685 | Train 27 | Test Female 21 | 0.73 | 0.76 | 0.73 |
| 662 | Train 11 | Test Female 22 | 0.71 | 0.72 | 0.67 | 1686 | Train 27 | Test Female 22 | 0.71 | 0.72 | 0.66 |
| 663 | Train 11 | Test Female 23 | 0.73 | 0.75 | 0.73 | 1687 | Train 27 | Test Female 23 | 0.73 | 0.76 | 0.73 |
| 664 | Train 11 | Test Female 24 | 0.71 | 0.72 | 0.67 | 1688 | Train 27 | Test Female 24 | 0.71 | 0.72 | 0.66 |
| 665 | Train 11 | Test Female 25 | 0.77 | 0.84 | 0.86 | 1689 | Train 27 | Test Female 25 | 0.77 | 0.84 | 0.86 |
| 666 | Train 11 | Test Female 26 | 0.76 | 0.81 | 0.8 | 1690 | Train 27 | Test Female 26 | 0.76 | 0.81 | 0.81 |
| 667 | Train 11 | Test Female 27 | 0.77 | 0.84 | 0.86 | 1691 | Train 27 | Test Female 27 | 0.77 | 0.84 | 0.86 |
| 668 | Train 11 | Test Female 28 | 0.76 | 0.81 | 0.81 | 1692 | Train 27 | Test Female 28 | 0.76 | 0.81 | 0.81 |
| 669 | Train 11 | Test Female 29 | 0.73 | 0.76 | 0.72 | 1693 | Train 27 | Test Female 29 | 0.73 | 0.76 | 0.72 |
| 670 | Train 11 | Test Female 30 | 0.71 | 0.72 | 0.67 | 1694 | Train 27 | Test Female 30 | 0.71 | 0.72 | 0.66 |
| 671 | Train 11 | Test Female 31 | 0.73 | 0.76 | 0.74 | 1695 | Train 27 | Test Female 31 | 0.73 | 0.76 | 0.74 |
| 672 | Train 11 | Test Female 32 | 0.72 | 0.72 | 0.67 | 1696 | Train 27 | Test Female 32 | 0.72 | 0.72 | 0.66 |
| 673 | Train 11 | Test Female 33 | 0.77 | 0.84 | 0.82 | 1697 | Train 27 | Test Female 33 | 0.77 | 0.84 | 0.83 |
| 674 | Train 11 | Test Female 34 | 0.76 | 0.81 | 0.78 | 1698 | Train 27 | Test Female 34 | 0.76 | 0.81 | 0.78 |
| 675 | Train 11 | Test Female 35 | 0.77 | 0.84 | 0.82 | 1699 | Train 27 | Test Female 35 | 0.77 | 0.84 | 0.83 |
| 676 | Train 11 | Test Female 36 | 0.76 | 0.81 | 0.78 | 1700 | Train 27 | Test Female 36 | 0.76 | 0.81 | 0.78 |
| 677 | Train 11 | Test Female 37 | 0.73 | 0.76 | 0.73 | 1701 | Train 27 | Test Female 37 | 0.73 | 0.76 | 0.73 |
| 678 | Train 11 | Test Female 38 | 0.71 | 0.72 | 0.67 | 1702 | Train 27 | Test Female 38 | 0.71 | 0.72 | 0.66 |
| 679 | Train 11 | Test Female 39 | 0.73 | 0.76 | 0.73 | 1703 | Train 27 | Test Female 39 | 0.73 | 0.76 | 0.73 |
| 680 | Train 11 | Test Female 40 | 0.71 | 0.72 | 0.67 | 1704 | Train 27 | Test Female 40 | 0.71 | 0.72 | 0.66 |
| 681 | Train 11 | Test Female 41 | 0.77 | 0.85 | 0.86 | 1705 | Train 27 | Test Female 41 | 0.77 | 0.85 | 0.86 |
| 682 | Train 11 | Test Female 42 | 0.76 | 0.81 | 0.81 | 1706 | Train 27 | Test Female 42 | 0.76 | 0.81 | 0.81 |
| 683 | Train 11 | Test Female 43 | 0.77 | 0.85 | 0.86 | 1707 | Train 27 | Test Female 43 | 0.77 | 0.85 | 0.86 |
| 684 | Train 11 | Test Female 44 | 0.76 | 0.81 | 0.81 | 1708 | Train 27 | Test Female 44 | 0.76 | 0.81 | 0.81 |
| 685 | Train 11 | Test Female 45 | 0.73 | 0.76 | 0.73 | 1709 | Train 27 | Test Female 45 | 0.73 | 0.76 | 0.72 |
| 686 | Train 11 | Test Female 46 | 0.71 | 0.72 | 0.68 | 1710 | Train 27 | Test Female 46 | 0.71 | 0.72 | 0.66 |
| 687 | Train 11 | Test Female 47 | 0.73 | 0.76 | 0.75 | 1711 | Train 27 | Test Female 47 | 0.73 | 0.76 | 0.74 |
| 688 | Train 11 | Test Female 48 | 0.72 | 0.72 | 0.68 | 1712 | Train 27 | Test Female 48 | 0.72 | 0.72 | 0.66 |
| 689 | Train 11 | Test Female 49 | 0.79 | 0.84 | 0.83 | 1713 | Train 27 | Test Female 49 | 0.79 | 0.84 | 0.83 |
| 690 | Train 11 | Test Female 50 | 0.79 | 0.84 | 0.83 | 1714 | Train 27 | Test Female 50 | 0.79 | 0.83 | 0.83 |
| 691 | Train 11 | Test Female 51 | 0.79 | 0.84 | 0.83 | 1715 | Train 27 | Test Female 51 | 0.79 | 0.84 | 0.83 |
| 692 | Train 11 | Test Female 52 | 0.79 | 0.84 | 0.83 | 1716 | Train 27 | Test Female 52 | 0.79 | 0.84 | 0.83 |
| 693 | Train 11 | Test Female 53 | 0.74 | 0.77 | 0.72 | 1717 | Train 27 | Test Female 53 | 0.74 | 0.77 | 0.74 |
| 694 | Train 11 | Test Female 54 | 0.73 | 0.73 | 0.68 | 1718 | Train 27 | Test Female 54 | 0.73 | 0.73 | 0.68 |
| 695 | Train 11 | Test Female 55 | 0.74 | 0.78 | 0.72 | 1719 | Train 27 | Test Female 55 | 0.74 | 0.78 | 0.74 |
| 696 | Train 11 | Test Female 56 | 0.73 | 0.72 | 0.68 | 1720 | Train 27 | Test Female 56 | 0.73 | 0.72 | 0.68 |
| 697 | Train 11 | Test Female 57 | 0.79 | 0.85 | 0.86 | 1721 | Train 27 | Test Female 57 | 0.79 | 0.84 | 0.87 |
| 698 | Train 11 | Test Female 58 | 0.79 | 0.84 | 0.86 | 1722 | Train 27 | Test Female 58 | 0.79 | 0.84 | 0.87 |
| 699 | Train 11 | Test Female 59 | 0.79 | 0.85 | 0.87 | 1723 | Train 27 | Test Female 59 | 0.79 | 0.85 | 0.87 |
| 700 | Train 11 | Test Female 60 | 0.79 | 0.85 | 0.87 | 1724 | Train 27 | Test Female 60 | 0.79 | 0.84 | 0.87 |
| 701 | Train 11 | Test Female 61 | 0.74 | 0.78 | 0.73 | 1725 | Train 27 | Test Female 61 | 0.74 | 0.78 | 0.75 |
| 702 | Train 11 | Test Female 62 | 0.73 | 0.73 | 0.68 | 1726 | Train 27 | Test Female 62 | 0.73 | 0.73 | 0.68 |
| 703 | Train 11 | Test Female 63 | 0.74 | 0.78 | 0.74 | 1727 | Train 27 | Test Female 63 | 0.74 | 0.78 | 0.75 |
| 704 | Train 11 | Test Female 64 | 0.73 | 0.73 | 0.68 | 1728 | Train 27 | Test Female 64 | 0.73 | 0.73 | 0.68 |
| 705 | Train 12 | Test Female 1 | 0.76 | 0.83 | 0.82 | 1729 | Train 28 | Test Female 1 | 0.76 | 0.83 | 0.82 |
| 706 | Train 12 | Test Female 2 | 0.77 | 0.84 | 0.83 | 1730 | Train 28 | Test Female 2 | 0.77 | 0.84 | 0.83 |
| 707 | Train 12 | Test Female 3 | 0.76 | 0.83 | 0.82 | 1731 | Train 28 | Test Female 3 | 0.76 | 0.83 | 0.82 |
| 708 | Train 12 | Test Female 4 | 0.77 | 0.84 | 0.83 | 1732 | Train 28 | Test Female 4 | 0.77 | 0.84 | 0.83 |
| 709 | Train 12 | Test Female 5 | 0.73 | 0.76 | 0.73 | 1733 | Train 28 | Test Female 5 | 0.73 | 0.76 | 0.74 |
| 710 | Train 12 | Test Female 6 | 0.73 | 0.73 | 0.69 | 1734 | Train 28 | Test Female 6 | 0.72 | 0.73 | 0.7 |
| 711 | Train 12 | Test Female 7 | 0.73 | 0.76 | 0.72 | 1735 | Train 28 | Test Female 7 | 0.73 | 0.76 | 0.74 |
| 712 | Train 12 | Test Female 8 | 0.72 | 0.73 | 0.69 | 1736 | Train 28 | Test Female 8 | 0.72 | 0.73 | 0.7 |
| 713 | Train 12 | Test Female 9 | 0.76 | 0.83 | 0.85 | 1737 | Train 28 | Test Female 9 | 0.76 | 0.83 | 0.85 |
| 714 | Train 12 | Test Female 10 | 0.77 | 0.84 | 0.86 | 1738 | Train 28 | Test Female 10 | 0.77 | 0.84 | 0.86 |
| 715 | Train 12 | Test Female 11 | 0.76 | 0.83 | 0.85 | 1739 | Train 28 | Test Female 11 | 0.76 | 0.83 | 0.85 |
| 716 | Train 12 | Test Female 12 | 0.77 | 0.84 | 0.86 | 1740 | Train 28 | Test Female 12 | 0.77 | 0.84 | 0.86 |
| 717 | Train 12 | Test Female 13 | 0.73 | 0.76 | 0.7 | 1741 | Train 28 | Test Female 13 | 0.73 | 0.76 | 0.73 |
| 718 | Train 12 | Test Female 14 | 0.73 | 0.73 | 0.69 | 1742 | Train 28 | Test Female 14 | 0.73 | 0.74 | 0.7 |
| 719 | Train 12 | Test Female 15 | 0.73 | 0.76 | 0.74 | 1743 | Train 28 | Test Female 15 | 0.73 | 0.76 | 0.75 |
| 720 | Train 12 | Test Female 16 | 0.73 | 0.73 | 0.69 | 1744 | Train 28 | Test Female 16 | 0.73 | 0.73 | 0.7 |
| 721 | Train 12 | Test Female 17 | 0.76 | 0.83 | 0.82 | 1745 | Train 28 | Test Female 17 | 0.76 | 0.83 | 0.82 |
| 722 | Train 12 | Test Female 18 | 0.77 | 0.84 | 0.83 | 1746 | Train 28 | Test Female 18 | 0.77 | 0.84 | 0.83 |
| 723 | Train 12 | Test Female 19 | 0.76 | 0.83 | 0.82 | 1747 | Train 28 | Test Female 19 | 0.76 | 0.83 | 0.82 |
| 724 | Train 12 | Test Female 20 | 0.77 | 0.84 | 0.83 | 1748 | Train 28 | Test Female 20 | 0.77 | 0.84 | 0.83 |
| 725 | Train 12 | Test Female 21 | 0.73 | 0.76 | 0.73 | 1749 | Train 28 | Test Female 21 | 0.73 | 0.76 | 0.74 |
| 726 | Train 12 | Test Female 22 | 0.73 | 0.73 | 0.69 | 1750 | Train 28 | Test Female 22 | 0.73 | 0.73 | 0.7 |
| 727 | Train 12 | Test Female 23 | 0.73 | 0.76 | 0.73 | 1751 | Train 28 | Test Female 23 | 0.73 | 0.76 | 0.74 |
| 728 | Train 12 | Test Female 24 | 0.73 | 0.73 | 0.69 | 1752 | Train 28 | Test Female 24 | 0.73 | 0.73 | 0.7 |
| 729 | Train 12 | Test Female 25 | 0.76 | 0.83 | 0.85 | 1753 | Train 28 | Test Female 25 | 0.76 | 0.83 | 0.85 |
| 730 | Train 12 | Test Female 26 | 0.77 | 0.84 | 0.86 | 1754 | Train 28 | Test Female 26 | 0.77 | 0.84 | 0.86 |
| 731 | Train 12 | Test Female 27 | 0.76 | 0.83 | 0.85 | 1755 | Train 28 | Test Female 27 | 0.76 | 0.83 | 0.85 |
| 732 | Train 12 | Test Female 28 | 0.77 | 0.84 | 0.86 | 1756 | Train 28 | Test Female 28 | 0.78 | 0.84 | 0.86 |
| 733 | Train 12 | Test Female 29 | 0.73 | 0.76 | 0.7 | 1757 | Train 28 | Test Female 29 | 0.73 | 0.76 | 0.73 |
| 734 | Train 12 | Test Female 30 | 0.73 | 0.73 | 0.69 | 1758 | Train 28 | Test Female 30 | 0.73 | 0.74 | 0.71 |
| 735 | Train 12 | Test Female 31 | 0.73 | 0.76 | 0.74 | 1759 | Train 28 | Test Female 31 | 0.73 | 0.76 | 0.76 |
| 736 | Train 12 | Test Female 32 | 0.73 | 0.73 | 0.69 | 1760 | Train 28 | Test Female 32 | 0.73 | 0.73 | 0.7 |
| 737 | Train 12 | Test Female 33 | 0.76 | 0.83 | 0.82 | 1761 | Train 28 | Test Female 33 | 0.76 | 0.83 | 0.82 |
| 738 | Train 12 | Test Female 34 | 0.78 | 0.84 | 0.83 | 1762 | Train 28 | Test Female 34 | 0.78 | 0.84 | 0.83 |
| 739 | Train 12 | Test Female 35 | 0.76 | 0.83 | 0.82 | 1763 | Train 28 | Test Female 35 | 0.76 | 0.83 | 0.82 |
| 740 | Train 12 | Test Female 36 | 0.78 | 0.84 | 0.83 | 1764 | Train 28 | Test Female 36 | 0.78 | 0.84 | 0.84 |
| 741 | Train 12 | Test Female 37 | 0.73 | 0.76 | 0.73 | 1765 | Train 28 | Test Female 37 | 0.73 | 0.76 | 0.75 |
| 742 | Train 12 | Test Female 38 | 0.73 | 0.73 | 0.69 | 1766 | Train 28 | Test Female 38 | 0.73 | 0.73 | 0.7 |
| 743 | Train 12 | Test Female 39 | 0.73 | 0.76 | 0.73 | 1767 | Train 28 | Test Female 39 | 0.73 | 0.76 | 0.74 |
| 744 | Train 12 | Test Female 40 | 0.73 | 0.73 | 0.69 | 1768 | Train 28 | Test Female 40 | 0.73 | 0.73 | 0.7 |
| 745 | Train 12 | Test Female 41 | 0.76 | 0.84 | 0.85 | 1769 | Train 28 | Test Female 41 | 0.76 | 0.84 | 0.85 |
| 746 | Train 12 | Test Female 42 | 0.78 | 0.84 | 0.86 | 1770 | Train 28 | Test Female 42 | 0.78 | 0.84 | 0.86 |
| 747 | Train 12 | Test Female 43 | 0.77 | 0.84 | 0.85 | 1771 | Train 28 | Test Female 43 | 0.77 | 0.84 | 0.85 |
| 748 | Train 12 | Test Female 44 | 0.78 | 0.85 | 0.86 | 1772 | Train 28 | Test Female 44 | 0.78 | 0.84 | 0.86 |
| 749 | Train 12 | Test Female 45 | 0.73 | 0.76 | 0.7 | 1773 | Train 28 | Test Female 45 | 0.73 | 0.76 | 0.73 |
| 750 | Train 12 | Test Female 46 | 0.73 | 0.74 | 0.69 | 1774 | Train 28 | Test Female 46 | 0.73 | 0.74 | 0.71 |
| 751 | Train 12 | Test Female 47 | 0.73 | 0.77 | 0.74 | 1775 | Train 28 | Test Female 47 | 0.73 | 0.76 | 0.76 |
| 752 | Train 12 | Test Female 48 | 0.73 | 0.73 | 0.69 | 1776 | Train 28 | Test Female 48 | 0.73 | 0.74 | 0.7 |
| 753 | Train 12 | Test Female 49 | 0.78 | 0.83 | 0.83 | 1777 | Train 28 | Test Female 49 | 0.78 | 0.83 | 0.83 |
| 754 | Train 12 | Test Female 50 | 0.78 | 0.83 | 0.83 | 1778 | Train 28 | Test Female 50 | 0.78 | 0.83 | 0.83 |
| 755 | Train 12 | Test Female 51 | 0.78 | 0.83 | 0.83 | 1779 | Train 28 | Test Female 51 | 0.78 | 0.83 | 0.83 |
| 756 | Train 12 | Test Female 52 | 0.78 | 0.83 | 0.83 | 1780 | Train 28 | Test Female 52 | 0.78 | 0.83 | 0.83 |
| 757 | Train 12 | Test Female 53 | 0.74 | 0.78 | 0.74 | 1781 | Train 28 | Test Female 53 | 0.74 | 0.78 | 0.75 |
| 758 | Train 12 | Test Female 54 | 0.73 | 0.74 | 0.69 | 1782 | Train 28 | Test Female 54 | 0.73 | 0.74 | 0.71 |
| 759 | Train 12 | Test Female 55 | 0.75 | 0.78 | 0.75 | 1783 | Train 28 | Test Female 55 | 0.75 | 0.78 | 0.76 |
| 760 | Train 12 | Test Female 56 | 0.73 | 0.73 | 0.69 | 1784 | Train 28 | Test Female 56 | 0.73 | 0.73 | 0.71 |
| 761 | Train 12 | Test Female 57 | 0.78 | 0.84 | 0.86 | 1785 | Train 28 | Test Female 57 | 0.78 | 0.84 | 0.86 |
| 762 | Train 12 | Test Female 58 | 0.78 | 0.84 | 0.86 | 1786 | Train 28 | Test Female 58 | 0.78 | 0.84 | 0.86 |
| 763 | Train 12 | Test Female 59 | 0.79 | 0.84 | 0.86 | 1787 | Train 28 | Test Female 59 | 0.79 | 0.84 | 0.86 |
| 764 | Train 12 | Test Female 60 | 0.79 | 0.84 | 0.86 | 1788 | Train 28 | Test Female 60 | 0.79 | 0.84 | 0.86 |
| 765 | Train 12 | Test Female 61 | 0.75 | 0.78 | 0.75 | 1789 | Train 28 | Test Female 61 | 0.75 | 0.78 | 0.76 |
| 766 | Train 12 | Test Female 62 | 0.74 | 0.74 | 0.69 | 1790 | Train 28 | Test Female 62 | 0.74 | 0.74 | 0.71 |
| 767 | Train 12 | Test Female 63 | 0.75 | 0.78 | 0.75 | 1791 | Train 28 | Test Female 63 | 0.75 | 0.78 | 0.77 |
| 768 | Train 12 | Test Female 64 | 0.73 | 0.73 | 0.69 | 1792 | Train 28 | Test Female 64 | 0.73 | 0.73 | 0.71 |
| 769 | Train 13 | Test Female 1 | 0.75 | 0.81 | 0.8 | 1793 | Train 29 | Test Female 1 | 0.75 | 0.81 | 0.81 |
| 770 | Train 13 | Test Female 2 | 0.76 | 0.8 | 0.79 | 1794 | Train 29 | Test Female 2 | 0.76 | 0.8 | 0.78 |
| 771 | Train 13 | Test Female 3 | 0.76 | 0.81 | 0.8 | 1795 | Train 29 | Test Female 3 | 0.76 | 0.81 | 0.81 |
| 772 | Train 13 | Test Female 4 | 0.76 | 0.8 | 0.78 | 1796 | Train 29 | Test Female 4 | 0.76 | 0.8 | 0.78 |
| 773 | Train 13 | Test Female 5 | 0.75 | 0.81 | 0.81 | 1797 | Train 29 | Test Female 5 | 0.75 | 0.81 | 0.81 |
| 774 | Train 13 | Test Female 6 | 0.72 | 0.73 | 0.7 | 1798 | Train 29 | Test Female 6 | 0.72 | 0.73 | 0.72 |
| 775 | Train 13 | Test Female 7 | 0.75 | 0.81 | 0.8 | 1799 | Train 29 | Test Female 7 | 0.75 | 0.81 | 0.8 |
| 776 | Train 13 | Test Female 8 | 0.72 | 0.73 | 0.7 | 1800 | Train 29 | Test Female 8 | 0.72 | 0.73 | 0.72 |
| 777 | Train 13 | Test Female 9 | 0.76 | 0.82 | 0.83 | 1801 | Train 29 | Test Female 9 | 0.76 | 0.82 | 0.83 |
| 778 | Train 13 | Test Female 10 | 0.76 | 0.8 | 0.8 | 1802 | Train 29 | Test Female 10 | 0.76 | 0.8 | 0.8 |
| 779 | Train 13 | Test Female 11 | 0.76 | 0.82 | 0.82 | 1803 | Train 29 | Test Female 11 | 0.76 | 0.82 | 0.83 |
| 780 | Train 13 | Test Female 12 | 0.76 | 0.8 | 0.8 | 1804 | Train 29 | Test Female 12 | 0.76 | 0.8 | 0.8 |
| 781 | Train 13 | Test Female 13 | 0.75 | 0.8 | 0.75 | 1805 | Train 29 | Test Female 13 | 0.75 | 0.8 | 0.79 |
| 782 | Train 13 | Test Female 14 | 0.72 | 0.73 | 0.7 | 1806 | Train 29 | Test Female 14 | 0.72 | 0.73 | 0.72 |
| 783 | Train 13 | Test Female 15 | 0.75 | 0.81 | 0.82 | 1807 | Train 29 | Test Female 15 | 0.75 | 0.81 | 0.83 |
| 784 | Train 13 | Test Female 16 | 0.72 | 0.73 | 0.7 | 1808 | Train 29 | Test Female 16 | 0.72 | 0.73 | 0.72 |
| 785 | Train 13 | Test Female 17 | 0.75 | 0.81 | 0.8 | 1809 | Train 29 | Test Female 17 | 0.75 | 0.81 | 0.81 |
| 786 | Train 13 | Test Female 18 | 0.76 | 0.8 | 0.79 | 1810 | Train 29 | Test Female 18 | 0.76 | 0.8 | 0.78 |
| 787 | Train 13 | Test Female 19 | 0.76 | 0.81 | 0.8 | 1811 | Train 29 | Test Female 19 | 0.76 | 0.81 | 0.81 |
| 788 | Train 13 | Test Female 20 | 0.76 | 0.8 | 0.78 | 1812 | Train 29 | Test Female 20 | 0.76 | 0.8 | 0.78 |
| 789 | Train 13 | Test Female 21 | 0.75 | 0.81 | 0.81 | 1813 | Train 29 | Test Female 21 | 0.75 | 0.81 | 0.81 |
| 790 | Train 13 | Test Female 22 | 0.72 | 0.73 | 0.7 | 1814 | Train 29 | Test Female 22 | 0.72 | 0.73 | 0.72 |
| 791 | Train 13 | Test Female 23 | 0.75 | 0.81 | 0.8 | 1815 | Train 29 | Test Female 23 | 0.75 | 0.81 | 0.8 |
| 792 | Train 13 | Test Female 24 | 0.72 | 0.73 | 0.7 | 1816 | Train 29 | Test Female 24 | 0.72 | 0.73 | 0.72 |
| 793 | Train 13 | Test Female 25 | 0.76 | 0.82 | 0.83 | 1817 | Train 29 | Test Female 25 | 0.76 | 0.82 | 0.83 |
| 794 | Train 13 | Test Female 26 | 0.76 | 0.8 | 0.8 | 1818 | Train 29 | Test Female 26 | 0.76 | 0.8 | 0.8 |
| 795 | Train 13 | Test Female 27 | 0.76 | 0.82 | 0.82 | 1819 | Train 29 | Test Female 27 | 0.76 | 0.82 | 0.83 |
| 796 | Train 13 | Test Female 28 | 0.76 | 0.8 | 0.8 | 1820 | Train 29 | Test Female 28 | 0.76 | 0.8 | 0.8 |
| 797 | Train 13 | Test Female 29 | 0.75 | 0.8 | 0.75 | 1821 | Train 29 | Test Female 29 | 0.75 | 0.8 | 0.79 |
| 798 | Train 13 | Test Female 30 | 0.72 | 0.74 | 0.7 | 1822 | Train 29 | Test Female 30 | 0.72 | 0.73 | 0.72 |
| 799 | Train 13 | Test Female 31 | 0.75 | 0.81 | 0.82 | 1823 | Train 29 | Test Female 31 | 0.75 | 0.81 | 0.83 |
| 800 | Train 13 | Test Female 32 | 0.72 | 0.74 | 0.7 | 1824 | Train 29 | Test Female 32 | 0.72 | 0.73 | 0.72 |
| 801 | Train 13 | Test Female 33 | 0.76 | 0.81 | 0.81 | 1825 | Train 29 | Test Female 33 | 0.76 | 0.81 | 0.81 |
| 802 | Train 13 | Test Female 34 | 0.77 | 0.8 | 0.79 | 1826 | Train 29 | Test Female 34 | 0.77 | 0.8 | 0.79 |
| 803 | Train 13 | Test Female 35 | 0.76 | 0.82 | 0.81 | 1827 | Train 29 | Test Female 35 | 0.76 | 0.81 | 0.81 |
| 804 | Train 13 | Test Female 36 | 0.77 | 0.8 | 0.79 | 1828 | Train 29 | Test Female 36 | 0.77 | 0.8 | 0.79 |
| 805 | Train 13 | Test Female 37 | 0.75 | 0.81 | 0.81 | 1829 | Train 29 | Test Female 37 | 0.75 | 0.81 | 0.81 |
| 806 | Train 13 | Test Female 38 | 0.72 | 0.74 | 0.7 | 1830 | Train 29 | Test Female 38 | 0.72 | 0.73 | 0.72 |
| 807 | Train 13 | Test Female 39 | 0.75 | 0.81 | 0.8 | 1831 | Train 29 | Test Female 39 | 0.75 | 0.81 | 0.81 |
| 808 | Train 13 | Test Female 40 | 0.72 | 0.74 | 0.7 | 1832 | Train 29 | Test Female 40 | 0.72 | 0.73 | 0.72 |
| 809 | Train 13 | Test Female 41 | 0.76 | 0.82 | 0.83 | 1833 | Train 29 | Test Female 41 | 0.76 | 0.82 | 0.83 |
| 810 | Train 13 | Test Female 42 | 0.77 | 0.81 | 0.8 | 1834 | Train 29 | Test Female 42 | 0.77 | 0.81 | 0.81 |
| 811 | Train 13 | Test Female 43 | 0.76 | 0.82 | 0.83 | 1835 | Train 29 | Test Female 43 | 0.76 | 0.82 | 0.83 |
| 812 | Train 13 | Test Female 44 | 0.77 | 0.81 | 0.8 | 1836 | Train 29 | Test Female 44 | 0.77 | 0.81 | 0.81 |
| 813 | Train 13 | Test Female 45 | 0.75 | 0.8 | 0.76 | 1837 | Train 29 | Test Female 45 | 0.75 | 0.8 | 0.79 |
| 814 | Train 13 | Test Female 46 | 0.72 | 0.74 | 0.7 | 1838 | Train 29 | Test Female 46 | 0.72 | 0.74 | 0.72 |
| 815 | Train 13 | Test Female 47 | 0.75 | 0.82 | 0.82 | 1839 | Train 29 | Test Female 47 | 0.75 | 0.81 | 0.83 |
| 816 | Train 13 | Test Female 48 | 0.72 | 0.74 | 0.7 | 1840 | Train 29 | Test Female 48 | 0.72 | 0.74 | 0.72 |
| 817 | Train 13 | Test Female 49 | 0.78 | 0.82 | 0.82 | 1841 | Train 29 | Test Female 49 | 0.78 | 0.82 | 0.82 |
| 818 | Train 13 | Test Female 50 | 0.78 | 0.82 | 0.82 | 1842 | Train 29 | Test Female 50 | 0.78 | 0.82 | 0.82 |
| 819 | Train 13 | Test Female 51 | 0.78 | 0.82 | 0.82 | 1843 | Train 29 | Test Female 51 | 0.78 | 0.82 | 0.82 |
| 820 | Train 13 | Test Female 52 | 0.78 | 0.82 | 0.82 | 1844 | Train 29 | Test Female 52 | 0.78 | 0.82 | 0.82 |
| 821 | Train 13 | Test Female 53 | 0.77 | 0.82 | 0.82 | 1845 | Train 29 | Test Female 53 | 0.77 | 0.82 | 0.83 |
| 822 | Train 13 | Test Female 54 | 0.74 | 0.74 | 0.71 | 1846 | Train 29 | Test Female 54 | 0.73 | 0.74 | 0.73 |
| 823 | Train 13 | Test Female 55 | 0.77 | 0.82 | 0.82 | 1847 | Train 29 | Test Female 55 | 0.77 | 0.82 | 0.83 |
| 824 | Train 13 | Test Female 56 | 0.73 | 0.74 | 0.7 | 1848 | Train 29 | Test Female 56 | 0.73 | 0.74 | 0.73 |
| 825 | Train 13 | Test Female 57 | 0.78 | 0.83 | 0.84 | 1849 | Train 29 | Test Female 57 | 0.78 | 0.83 | 0.85 |
| 826 | Train 13 | Test Female 58 | 0.78 | 0.83 | 0.84 | 1850 | Train 29 | Test Female 58 | 0.78 | 0.83 | 0.85 |
| 827 | Train 13 | Test Female 59 | 0.78 | 0.83 | 0.84 | 1851 | Train 29 | Test Female 59 | 0.78 | 0.83 | 0.85 |
| 828 | Train 13 | Test Female 60 | 0.78 | 0.83 | 0.84 | 1852 | Train 29 | Test Female 60 | 0.78 | 0.83 | 0.84 |
| 829 | Train 13 | Test Female 61 | 0.77 | 0.83 | 0.84 | 1853 | Train 29 | Test Female 61 | 0.77 | 0.83 | 0.84 |
| 830 | Train 13 | Test Female 62 | 0.74 | 0.75 | 0.71 | 1854 | Train 29 | Test Female 62 | 0.74 | 0.74 | 0.73 |
| 831 | Train 13 | Test Female 63 | 0.77 | 0.83 | 0.84 | 1855 | Train 29 | Test Female 63 | 0.77 | 0.83 | 0.84 |
| 832 | Train 13 | Test Female 64 | 0.73 | 0.74 | 0.7 | 1856 | Train 29 | Test Female 64 | 0.73 | 0.74 | 0.73 |
| 833 | Train 14 | Test Female 1 | 0.74 | 0.78 | 0.76 | 1857 | Train 30 | Test Female 1 | 0.74 | 0.78 | 0.75 |
| 834 | Train 14 | Test Female 2 | 0.76 | 0.79 | 0.77 | 1858 | Train 30 | Test Female 2 | 0.76 | 0.79 | 0.76 |
| 835 | Train 14 | Test Female 3 | 0.74 | 0.78 | 0.76 | 1859 | Train 30 | Test Female 3 | 0.74 | 0.78 | 0.75 |
| 836 | Train 14 | Test Female 4 | 0.76 | 0.79 | 0.77 | 1860 | Train 30 | Test Female 4 | 0.76 | 0.79 | 0.76 |
| 837 | Train 14 | Test Female 5 | 0.73 | 0.78 | 0.76 | 1861 | Train 30 | Test Female 5 | 0.73 | 0.78 | 0.76 |
| 838 | Train 14 | Test Female 6 | 0.75 | 0.8 | 0.8 | 1862 | Train 30 | Test Female 6 | 0.75 | 0.8 | 0.8 |
| 839 | Train 14 | Test Female 7 | 0.73 | 0.78 | 0.76 | 1863 | Train 30 | Test Female 7 | 0.73 | 0.78 | 0.75 |
| 840 | Train 14 | Test Female 8 | 0.75 | 0.79 | 0.8 | 1864 | Train 30 | Test Female 8 | 0.75 | 0.79 | 0.8 |
| 841 | Train 14 | Test Female 9 | 0.74 | 0.79 | 0.77 | 1865 | Train 30 | Test Female 9 | 0.74 | 0.79 | 0.76 |
| 842 | Train 14 | Test Female 10 | 0.76 | 0.8 | 0.78 | 1866 | Train 30 | Test Female 10 | 0.76 | 0.8 | 0.77 |
| 843 | Train 14 | Test Female 11 | 0.74 | 0.79 | 0.77 | 1867 | Train 30 | Test Female 11 | 0.74 | 0.79 | 0.76 |
| 844 | Train 14 | Test Female 12 | 0.76 | 0.8 | 0.78 | 1868 | Train 30 | Test Female 12 | 0.76 | 0.8 | 0.77 |
| 845 | Train 14 | Test Female 13 | 0.73 | 0.79 | 0.8 | 1869 | Train 30 | Test Female 13 | 0.73 | 0.79 | 0.8 |
| 846 | Train 14 | Test Female 14 | 0.75 | 0.8 | 0.81 | 1870 | Train 30 | Test Female 14 | 0.75 | 0.8 | 0.81 |
| 847 | Train 14 | Test Female 15 | 0.73 | 0.78 | 0.77 | 1871 | Train 30 | Test Female 15 | 0.73 | 0.78 | 0.77 |
| 848 | Train 14 | Test Female 16 | 0.75 | 0.79 | 0.8 | 1872 | Train 30 | Test Female 16 | 0.75 | 0.79 | 0.8 |
| 849 | Train 14 | Test Female 17 | 0.74 | 0.78 | 0.76 | 1873 | Train 30 | Test Female 17 | 0.74 | 0.78 | 0.75 |
| 850 | Train 14 | Test Female 18 | 0.76 | 0.79 | 0.77 | 1874 | Train 30 | Test Female 18 | 0.76 | 0.79 | 0.76 |
| 851 | Train 14 | Test Female 19 | 0.74 | 0.78 | 0.76 | 1875 | Train 30 | Test Female 19 | 0.74 | 0.78 | 0.75 |
| 852 | Train 14 | Test Female 20 | 0.76 | 0.79 | 0.77 | 1876 | Train 30 | Test Female 20 | 0.76 | 0.79 | 0.76 |
| 853 | Train 14 | Test Female 21 | 0.73 | 0.78 | 0.76 | 1877 | Train 30 | Test Female 21 | 0.73 | 0.78 | 0.76 |
| 854 | Train 14 | Test Female 22 | 0.75 | 0.8 | 0.8 | 1878 | Train 30 | Test Female 22 | 0.75 | 0.8 | 0.8 |
| 855 | Train 14 | Test Female 23 | 0.73 | 0.78 | 0.76 | 1879 | Train 30 | Test Female 23 | 0.73 | 0.78 | 0.76 |
| 856 | Train 14 | Test Female 24 | 0.75 | 0.79 | 0.8 | 1880 | Train 30 | Test Female 24 | 0.75 | 0.79 | 0.8 |
| 857 | Train 14 | Test Female 25 | 0.74 | 0.79 | 0.77 | 1881 | Train 30 | Test Female 25 | 0.74 | 0.79 | 0.76 |
| 858 | Train 14 | Test Female 26 | 0.76 | 0.8 | 0.78 | 1882 | Train 30 | Test Female 26 | 0.76 | 0.8 | 0.77 |
| 859 | Train 14 | Test Female 27 | 0.74 | 0.79 | 0.77 | 1883 | Train 30 | Test Female 27 | 0.74 | 0.79 | 0.76 |
| 860 | Train 14 | Test Female 28 | 0.76 | 0.8 | 0.78 | 1884 | Train 30 | Test Female 28 | 0.76 | 0.8 | 0.77 |
| 861 | Train 14 | Test Female 29 | 0.73 | 0.79 | 0.8 | 1885 | Train 30 | Test Female 29 | 0.73 | 0.79 | 0.8 |
| 862 | Train 14 | Test Female 30 | 0.75 | 0.8 | 0.81 | 1886 | Train 30 | Test Female 30 | 0.75 | 0.8 | 0.81 |
| 863 | Train 14 | Test Female 31 | 0.73 | 0.78 | 0.77 | 1887 | Train 30 | Test Female 31 | 0.73 | 0.78 | 0.77 |
| 864 | Train 14 | Test Female 32 | 0.75 | 0.79 | 0.8 | 1888 | Train 30 | Test Female 32 | 0.75 | 0.79 | 0.8 |
| 865 | Train 14 | Test Female 33 | 0.75 | 0.79 | 0.76 | 1889 | Train 30 | Test Female 33 | 0.75 | 0.79 | 0.76 |
| 866 | Train 14 | Test Female 34 | 0.77 | 0.8 | 0.77 | 1890 | Train 30 | Test Female 34 | 0.77 | 0.8 | 0.77 |
| 867 | Train 14 | Test Female 35 | 0.75 | 0.79 | 0.76 | 1891 | Train 30 | Test Female 35 | 0.75 | 0.79 | 0.76 |
| 868 | Train 14 | Test Female 36 | 0.77 | 0.8 | 0.77 | 1892 | Train 30 | Test Female 36 | 0.77 | 0.8 | 0.77 |
| 869 | Train 14 | Test Female 37 | 0.74 | 0.78 | 0.77 | 1893 | Train 30 | Test Female 37 | 0.74 | 0.78 | 0.76 |
| 870 | Train 14 | Test Female 38 | 0.75 | 0.8 | 0.81 | 1894 | Train 30 | Test Female 38 | 0.75 | 0.8 | 0.8 |
| 871 | Train 14 | Test Female 39 | 0.74 | 0.78 | 0.76 | 1895 | Train 30 | Test Female 39 | 0.74 | 0.78 | 0.76 |
| 872 | Train 14 | Test Female 40 | 0.75 | 0.79 | 0.8 | 1896 | Train 30 | Test Female 40 | 0.75 | 0.79 | 0.8 |
| 873 | Train 14 | Test Female 41 | 0.75 | 0.79 | 0.77 | 1897 | Train 30 | Test Female 41 | 0.75 | 0.79 | 0.77 |
| 874 | Train 14 | Test Female 42 | 0.77 | 0.8 | 0.78 | 1898 | Train 30 | Test Female 42 | 0.77 | 0.8 | 0.78 |
| 875 | Train 14 | Test Female 43 | 0.75 | 0.79 | 0.77 | 1899 | Train 30 | Test Female 43 | 0.75 | 0.79 | 0.77 |
| 876 | Train 14 | Test Female 44 | 0.77 | 0.8 | 0.78 | 1900 | Train 30 | Test Female 44 | 0.77 | 0.8 | 0.78 |
| 877 | Train 14 | Test Female 45 | 0.74 | 0.79 | 0.8 | 1901 | Train 30 | Test Female 45 | 0.74 | 0.79 | 0.8 |
| 878 | Train 14 | Test Female 46 | 0.76 | 0.8 | 0.81 | 1902 | Train 30 | Test Female 46 | 0.76 | 0.8 | 0.81 |
| 879 | Train 14 | Test Female 47 | 0.74 | 0.78 | 0.77 | 1903 | Train 30 | Test Female 47 | 0.74 | 0.78 | 0.77 |
| 880 | Train 14 | Test Female 48 | 0.75 | 0.8 | 0.8 | 1904 | Train 30 | Test Female 48 | 0.75 | 0.8 | 0.8 |
| 881 | Train 14 | Test Female 49 | 0.78 | 0.81 | 0.8 | 1905 | Train 30 | Test Female 49 | 0.78 | 0.81 | 0.8 |
| 882 | Train 14 | Test Female 50 | 0.78 | 0.81 | 0.8 | 1906 | Train 30 | Test Female 50 | 0.78 | 0.81 | 0.79 |
| 883 | Train 14 | Test Female 51 | 0.78 | 0.81 | 0.8 | 1907 | Train 30 | Test Female 51 | 0.78 | 0.81 | 0.79 |
| 884 | Train 14 | Test Female 52 | 0.78 | 0.81 | 0.8 | 1908 | Train 30 | Test Female 52 | 0.78 | 0.81 | 0.79 |
| 885 | Train 14 | Test Female 53 | 0.76 | 0.81 | 0.8 | 1909 | Train 30 | Test Female 53 | 0.76 | 0.81 | 0.79 |
| 886 | Train 14 | Test Female 54 | 0.76 | 0.8 | 0.8 | 1910 | Train 30 | Test Female 54 | 0.76 | 0.8 | 0.8 |
| 887 | Train 14 | Test Female 55 | 0.76 | 0.81 | 0.8 | 1911 | Train 30 | Test Female 55 | 0.76 | 0.81 | 0.79 |
| 888 | Train 14 | Test Female 56 | 0.76 | 0.79 | 0.8 | 1912 | Train 30 | Test Female 56 | 0.76 | 0.79 | 0.8 |
| 889 | Train 14 | Test Female 57 | 0.78 | 0.82 | 0.81 | 1913 | Train 30 | Test Female 57 | 0.78 | 0.82 | 0.82 |
| 890 | Train 14 | Test Female 58 | 0.78 | 0.81 | 0.81 | 1914 | Train 30 | Test Female 58 | 0.78 | 0.81 | 0.81 |
| 891 | Train 14 | Test Female 59 | 0.78 | 0.81 | 0.81 | 1915 | Train 30 | Test Female 59 | 0.78 | 0.81 | 0.81 |
| 892 | Train 14 | Test Female 60 | 0.78 | 0.81 | 0.81 | 1916 | Train 30 | Test Female 60 | 0.78 | 0.81 | 0.81 |
| 893 | Train 14 | Test Female 61 | 0.77 | 0.81 | 0.81 | 1917 | Train 30 | Test Female 61 | 0.77 | 0.81 | 0.8 |
| 894 | Train 14 | Test Female 62 | 0.76 | 0.8 | 0.8 | 1918 | Train 30 | Test Female 62 | 0.76 | 0.8 | 0.81 |
| 895 | Train 14 | Test Female 63 | 0.77 | 0.81 | 0.81 | 1919 | Train 30 | Test Female 63 | 0.77 | 0.81 | 0.8 |
| 896 | Train 14 | Test Female 64 | 0.76 | 0.79 | 0.8 | 1920 | Train 30 | Test Female 64 | 0.76 | 0.79 | 0.8 |
| 897 | Train 15 | Test Female 1 | 0.75 | 0.81 | 0.81 | 1921 | Train 31 | Test Female 1 | 0.75 | 0.81 | 0.81 |
| 898 | Train 15 | Test Female 2 | 0.76 | 0.8 | 0.78 | 1922 | Train 31 | Test Female 2 | 0.76 | 0.8 | 0.79 |
| 899 | Train 15 | Test Female 3 | 0.76 | 0.81 | 0.81 | 1923 | Train 31 | Test Female 3 | 0.76 | 0.81 | 0.81 |
| 900 | Train 15 | Test Female 4 | 0.76 | 0.8 | 0.78 | 1924 | Train 31 | Test Female 4 | 0.76 | 0.8 | 0.79 |
| 901 | Train 15 | Test Female 5 | 0.75 | 0.81 | 0.81 | 1925 | Train 31 | Test Female 5 | 0.75 | 0.81 | 0.81 |
| 902 | Train 15 | Test Female 6 | 0.72 | 0.73 | 0.7 | 1926 | Train 31 | Test Female 6 | 0.72 | 0.73 | 0.7 |
| 903 | Train 15 | Test Female 7 | 0.75 | 0.81 | 0.8 | 1927 | Train 31 | Test Female 7 | 0.75 | 0.81 | 0.81 |
| 904 | Train 15 | Test Female 8 | 0.72 | 0.73 | 0.7 | 1928 | Train 31 | Test Female 8 | 0.72 | 0.73 | 0.7 |
| 905 | Train 15 | Test Female 9 | 0.76 | 0.82 | 0.83 | 1929 | Train 31 | Test Female 9 | 0.76 | 0.81 | 0.83 |
| 906 | Train 15 | Test Female 10 | 0.76 | 0.8 | 0.8 | 1930 | Train 31 | Test Female 10 | 0.76 | 0.8 | 0.81 |
| 907 | Train 15 | Test Female 11 | 0.76 | 0.82 | 0.83 | 1931 | Train 31 | Test Female 11 | 0.76 | 0.82 | 0.83 |
| 908 | Train 15 | Test Female 12 | 0.76 | 0.8 | 0.8 | 1932 | Train 31 | Test Female 12 | 0.76 | 0.8 | 0.81 |
| 909 | Train 15 | Test Female 13 | 0.75 | 0.8 | 0.78 | 1933 | Train 31 | Test Female 13 | 0.75 | 0.8 | 0.75 |
| 910 | Train 15 | Test Female 14 | 0.72 | 0.73 | 0.7 | 1934 | Train 31 | Test Female 14 | 0.72 | 0.73 | 0.7 |
| 911 | Train 15 | Test Female 15 | 0.75 | 0.82 | 0.83 | 1935 | Train 31 | Test Female 15 | 0.75 | 0.82 | 0.83 |
| 912 | Train 15 | Test Female 16 | 0.72 | 0.73 | 0.71 | 1936 | Train 31 | Test Female 16 | 0.72 | 0.73 | 0.7 |
| 913 | Train 15 | Test Female 17 | 0.75 | 0.81 | 0.81 | 1937 | Train 31 | Test Female 17 | 0.75 | 0.81 | 0.81 |
| 914 | Train 15 | Test Female 18 | 0.76 | 0.8 | 0.78 | 1938 | Train 31 | Test Female 18 | 0.76 | 0.8 | 0.79 |
| 915 | Train 15 | Test Female 19 | 0.76 | 0.81 | 0.81 | 1939 | Train 31 | Test Female 19 | 0.76 | 0.81 | 0.81 |
| 916 | Train 15 | Test Female 20 | 0.76 | 0.8 | 0.78 | 1940 | Train 31 | Test Female 20 | 0.76 | 0.8 | 0.79 |
| 917 | Train 15 | Test Female 21 | 0.75 | 0.81 | 0.81 | 1941 | Train 31 | Test Female 21 | 0.75 | 0.81 | 0.81 |
| 918 | Train 15 | Test Female 22 | 0.72 | 0.73 | 0.7 | 1942 | Train 31 | Test Female 22 | 0.72 | 0.73 | 0.7 |
| 919 | Train 15 | Test Female 23 | 0.75 | 0.81 | 0.81 | 1943 | Train 31 | Test Female 23 | 0.75 | 0.81 | 0.81 |
| 920 | Train 15 | Test Female 24 | 0.72 | 0.73 | 0.71 | 1944 | Train 31 | Test Female 24 | 0.72 | 0.73 | 0.7 |
| 921 | Train 15 | Test Female 25 | 0.76 | 0.82 | 0.83 | 1945 | Train 31 | Test Female 25 | 0.76 | 0.82 | 0.83 |
| 922 | Train 15 | Test Female 26 | 0.76 | 0.8 | 0.8 | 1946 | Train 31 | Test Female 26 | 0.76 | 0.8 | 0.81 |
| 923 | Train 15 | Test Female 27 | 0.76 | 0.82 | 0.83 | 1947 | Train 31 | Test Female 27 | 0.76 | 0.82 | 0.83 |
| 924 | Train 15 | Test Female 28 | 0.76 | 0.8 | 0.8 | 1948 | Train 31 | Test Female 28 | 0.76 | 0.8 | 0.81 |
| 925 | Train 15 | Test Female 29 | 0.75 | 0.8 | 0.78 | 1949 | Train 31 | Test Female 29 | 0.75 | 0.8 | 0.75 |
| 926 | Train 15 | Test Female 30 | 0.72 | 0.73 | 0.7 | 1950 | Train 31 | Test Female 30 | 0.72 | 0.73 | 0.7 |
| 927 | Train 15 | Test Female 31 | 0.75 | 0.82 | 0.83 | 1951 | Train 31 | Test Female 31 | 0.75 | 0.82 | 0.83 |
| 928 | Train 15 | Test Female 32 | 0.72 | 0.73 | 0.71 | 1952 | Train 31 | Test Female 32 | 0.72 | 0.73 | 0.7 |
| 929 | Train 15 | Test Female 33 | 0.76 | 0.81 | 0.81 | 1953 | Train 31 | Test Female 33 | 0.76 | 0.81 | 0.81 |
| 930 | Train 15 | Test Female 34 | 0.77 | 0.8 | 0.79 | 1954 | Train 31 | Test Female 34 | 0.77 | 0.8 | 0.8 |
| 931 | Train 15 | Test Female 35 | 0.76 | 0.82 | 0.81 | 1955 | Train 31 | Test Female 35 | 0.76 | 0.82 | 0.81 |
| 932 | Train 15 | Test Female 36 | 0.77 | 0.8 | 0.79 | 1956 | Train 31 | Test Female 36 | 0.77 | 0.8 | 0.8 |
| 933 | Train 15 | Test Female 37 | 0.75 | 0.81 | 0.81 | 1957 | Train 31 | Test Female 37 | 0.75 | 0.81 | 0.81 |
| 934 | Train 15 | Test Female 38 | 0.72 | 0.73 | 0.7 | 1958 | Train 31 | Test Female 38 | 0.72 | 0.73 | 0.7 |
| 935 | Train 15 | Test Female 39 | 0.75 | 0.81 | 0.81 | 1959 | Train 31 | Test Female 39 | 0.75 | 0.81 | 0.81 |
| 936 | Train 15 | Test Female 40 | 0.72 | 0.73 | 0.71 | 1960 | Train 31 | Test Female 40 | 0.72 | 0.73 | 0.7 |
| 937 | Train 15 | Test Female 41 | 0.76 | 0.82 | 0.83 | 1961 | Train 31 | Test Female 41 | 0.76 | 0.82 | 0.83 |
| 938 | Train 15 | Test Female 42 | 0.77 | 0.81 | 0.81 | 1962 | Train 31 | Test Female 42 | 0.77 | 0.81 | 0.81 |
| 939 | Train 15 | Test Female 43 | 0.76 | 0.82 | 0.83 | 1963 | Train 31 | Test Female 43 | 0.76 | 0.82 | 0.83 |
| 940 | Train 15 | Test Female 44 | 0.77 | 0.81 | 0.81 | 1964 | Train 31 | Test Female 44 | 0.77 | 0.81 | 0.81 |
| 941 | Train 15 | Test Female 45 | 0.75 | 0.8 | 0.78 | 1965 | Train 31 | Test Female 45 | 0.75 | 0.8 | 0.76 |
| 942 | Train 15 | Test Female 46 | 0.72 | 0.74 | 0.7 | 1966 | Train 31 | Test Female 46 | 0.72 | 0.73 | 0.7 |
| 943 | Train 15 | Test Female 47 | 0.75 | 0.82 | 0.83 | 1967 | Train 31 | Test Female 47 | 0.75 | 0.82 | 0.83 |
| 944 | Train 15 | Test Female 48 | 0.72 | 0.74 | 0.71 | 1968 | Train 31 | Test Female 48 | 0.72 | 0.74 | 0.7 |
| 945 | Train 15 | Test Female 49 | 0.78 | 0.82 | 0.82 | 1969 | Train 31 | Test Female 49 | 0.78 | 0.82 | 0.82 |
| 946 | Train 15 | Test Female 50 | 0.78 | 0.82 | 0.82 | 1970 | Train 31 | Test Female 50 | 0.78 | 0.82 | 0.82 |
| 947 | Train 15 | Test Female 51 | 0.78 | 0.82 | 0.83 | 1971 | Train 31 | Test Female 51 | 0.78 | 0.82 | 0.82 |
| 948 | Train 15 | Test Female 52 | 0.78 | 0.82 | 0.83 | 1972 | Train 31 | Test Female 52 | 0.78 | 0.82 | 0.82 |
| 949 | Train 15 | Test Female 53 | 0.77 | 0.82 | 0.83 | 1973 | Train 31 | Test Female 53 | 0.77 | 0.82 | 0.83 |
| 950 | Train 15 | Test Female 54 | 0.73 | 0.74 | 0.71 | 1974 | Train 31 | Test Female 54 | 0.73 | 0.74 | 0.7 |
| 951 | Train 15 | Test Female 55 | 0.77 | 0.82 | 0.83 | 1975 | Train 31 | Test Female 55 | 0.77 | 0.82 | 0.83 |
| 952 | Train 15 | Test Female 56 | 0.73 | 0.74 | 0.71 | 1976 | Train 31 | Test Female 56 | 0.73 | 0.74 | 0.7 |
| 953 | Train 15 | Test Female 57 | 0.78 | 0.83 | 0.85 | 1977 | Train 31 | Test Female 57 | 0.78 | 0.83 | 0.84 |
| 954 | Train 15 | Test Female 58 | 0.78 | 0.83 | 0.85 | 1978 | Train 31 | Test Female 58 | 0.78 | 0.83 | 0.84 |
| 955 | Train 15 | Test Female 59 | 0.78 | 0.83 | 0.85 | 1979 | Train 31 | Test Female 59 | 0.78 | 0.83 | 0.85 |
| 956 | Train 15 | Test Female 60 | 0.78 | 0.83 | 0.85 | 1980 | Train 31 | Test Female 60 | 0.78 | 0.83 | 0.85 |
| 957 | Train 15 | Test Female 61 | 0.77 | 0.83 | 0.84 | 1981 | Train 31 | Test Female 61 | 0.77 | 0.83 | 0.84 |
| 958 | Train 15 | Test Female 62 | 0.74 | 0.74 | 0.71 | 1982 | Train 31 | Test Female 62 | 0.74 | 0.74 | 0.7 |
| 959 | Train 15 | Test Female 63 | 0.77 | 0.83 | 0.84 | 1983 | Train 31 | Test Female 63 | 0.77 | 0.83 | 0.84 |
| 960 | Train 15 | Test Female 64 | 0.73 | 0.74 | 0.72 | 1984 | Train 31 | Test Female 64 | 0.73 | 0.74 | 0.7 |
| 961 | Train 16 | Test Female 1 | 0.74 | 0.78 | 0.77 | 1985 | Train 32 | Test Female 1 | 0.74 | 0.78 | 0.77 |
| 962 | Train 16 | Test Female 2 | 0.76 | 0.79 | 0.78 | 1986 | Train 32 | Test Female 2 | 0.76 | 0.79 | 0.78 |
| 963 | Train 16 | Test Female 3 | 0.75 | 0.78 | 0.77 | 1987 | Train 32 | Test Female 3 | 0.75 | 0.78 | 0.77 |
| 964 | Train 16 | Test Female 4 | 0.76 | 0.79 | 0.78 | 1988 | Train 32 | Test Female 4 | 0.76 | 0.79 | 0.78 |
| 965 | Train 16 | Test Female 5 | 0.73 | 0.78 | 0.76 | 1989 | Train 32 | Test Female 5 | 0.73 | 0.78 | 0.76 |
| 966 | Train 16 | Test Female 6 | 0.75 | 0.8 | 0.8 | 1990 | Train 32 | Test Female 6 | 0.75 | 0.8 | 0.8 |
| 967 | Train 16 | Test Female 7 | 0.73 | 0.78 | 0.76 | 1991 | Train 32 | Test Female 7 | 0.73 | 0.78 | 0.76 |
| 968 | Train 16 | Test Female 8 | 0.75 | 0.8 | 0.8 | 1992 | Train 32 | Test Female 8 | 0.75 | 0.8 | 0.8 |
| 969 | Train 16 | Test Female 9 | 0.75 | 0.78 | 0.78 | 1993 | Train 32 | Test Female 9 | 0.75 | 0.79 | 0.78 |
| 970 | Train 16 | Test Female 10 | 0.76 | 0.79 | 0.79 | 1994 | Train 32 | Test Female 10 | 0.76 | 0.79 | 0.79 |
| 971 | Train 16 | Test Female 11 | 0.75 | 0.79 | 0.78 | 1995 | Train 32 | Test Female 11 | 0.75 | 0.79 | 0.78 |
| 972 | Train 16 | Test Female 12 | 0.76 | 0.79 | 0.79 | 1996 | Train 32 | Test Female 12 | 0.76 | 0.79 | 0.79 |
| 973 | Train 16 | Test Female 13 | 0.73 | 0.79 | 0.8 | 1997 | Train 32 | Test Female 13 | 0.73 | 0.79 | 0.8 |
| 974 | Train 16 | Test Female 14 | 0.75 | 0.8 | 0.8 | 1998 | Train 32 | Test Female 14 | 0.75 | 0.8 | 0.8 |
| 975 | Train 16 | Test Female 15 | 0.73 | 0.78 | 0.77 | 1999 | Train 32 | Test Female 15 | 0.73 | 0.78 | 0.76 |
| 976 | Train 16 | Test Female 16 | 0.75 | 0.8 | 0.8 | 2000 | Train 32 | Test Female 16 | 0.75 | 0.8 | 0.8 |
| 977 | Train 16 | Test Female 17 | 0.74 | 0.78 | 0.77 | 2001 | Train 32 | Test Female 17 | 0.74 | 0.78 | 0.77 |
| 978 | Train 16 | Test Female 18 | 0.76 | 0.79 | 0.78 | 2002 | Train 32 | Test Female 18 | 0.76 | 0.79 | 0.78 |
| 979 | Train 16 | Test Female 19 | 0.75 | 0.78 | 0.77 | 2003 | Train 32 | Test Female 19 | 0.75 | 0.78 | 0.77 |
| 980 | Train 16 | Test Female 20 | 0.76 | 0.79 | 0.78 | 2004 | Train 32 | Test Female 20 | 0.76 | 0.79 | 0.78 |
| 981 | Train 16 | Test Female 21 | 0.73 | 0.78 | 0.76 | 2005 | Train 32 | Test Female 21 | 0.73 | 0.78 | 0.76 |
| 982 | Train 16 | Test Female 22 | 0.75 | 0.8 | 0.8 | 2006 | Train 32 | Test Female 22 | 0.75 | 0.8 | 0.8 |
| 983 | Train 16 | Test Female 23 | 0.73 | 0.78 | 0.76 | 2007 | Train 32 | Test Female 23 | 0.73 | 0.78 | 0.76 |
| 984 | Train 16 | Test Female 24 | 0.75 | 0.8 | 0.8 | 2008 | Train 32 | Test Female 24 | 0.75 | 0.8 | 0.8 |
| 985 | Train 16 | Test Female 25 | 0.75 | 0.78 | 0.78 | 2009 | Train 32 | Test Female 25 | 0.75 | 0.79 | 0.78 |
| 986 | Train 16 | Test Female 26 | 0.76 | 0.79 | 0.79 | 2010 | Train 32 | Test Female 26 | 0.76 | 0.79 | 0.79 |
| 987 | Train 16 | Test Female 27 | 0.75 | 0.79 | 0.78 | 2011 | Train 32 | Test Female 27 | 0.75 | 0.79 | 0.78 |
| 988 | Train 16 | Test Female 28 | 0.76 | 0.79 | 0.79 | 2012 | Train 32 | Test Female 28 | 0.76 | 0.79 | 0.79 |
| 989 | Train 16 | Test Female 29 | 0.73 | 0.79 | 0.8 | 2013 | Train 32 | Test Female 29 | 0.73 | 0.79 | 0.8 |
| 990 | Train 16 | Test Female 30 | 0.75 | 0.8 | 0.8 | 2014 | Train 32 | Test Female 30 | 0.75 | 0.8 | 0.8 |
| 991 | Train 16 | Test Female 31 | 0.73 | 0.78 | 0.77 | 2015 | Train 32 | Test Female 31 | 0.73 | 0.78 | 0.76 |
| 992 | Train 16 | Test Female 32 | 0.75 | 0.8 | 0.8 | 2016 | Train 32 | Test Female 32 | 0.75 | 0.8 | 0.8 |
| 993 | Train 16 | Test Female 33 | 0.75 | 0.79 | 0.77 | 2017 | Train 32 | Test Female 33 | 0.75 | 0.79 | 0.77 |
| 994 | Train 16 | Test Female 34 | 0.77 | 0.8 | 0.78 | 2018 | Train 32 | Test Female 34 | 0.77 | 0.79 | 0.78 |
| 995 | Train 16 | Test Female 35 | 0.75 | 0.79 | 0.77 | 2019 | Train 32 | Test Female 35 | 0.75 | 0.79 | 0.77 |
| 996 | Train 16 | Test Female 36 | 0.77 | 0.8 | 0.79 | 2020 | Train 32 | Test Female 36 | 0.77 | 0.8 | 0.78 |
| 997 | Train 16 | Test Female 37 | 0.74 | 0.78 | 0.77 | 2021 | Train 32 | Test Female 37 | 0.74 | 0.78 | 0.76 |
| 998 | Train 16 | Test Female 38 | 0.75 | 0.8 | 0.8 | 2022 | Train 32 | Test Female 38 | 0.75 | 0.8 | 0.8 |
| 999 | Train 16 | Test Female 39 | 0.74 | 0.78 | 0.77 | 2023 | Train 32 | Test Female 39 | 0.74 | 0.78 | 0.76 |
| 1000 | Train 16 | Test Female 40 | 0.75 | 0.8 | 0.8 | 2024 | Train 32 | Test Female 40 | 0.75 | 0.8 | 0.8 |
| 1001 | Train 16 | Test Female 41 | 0.75 | 0.79 | 0.78 | 2025 | Train 32 | Test Female 41 | 0.75 | 0.79 | 0.78 |
| 1002 | Train 16 | Test Female 42 | 0.77 | 0.8 | 0.79 | 2026 | Train 32 | Test Female 42 | 0.77 | 0.8 | 0.79 |
| 1003 | Train 16 | Test Female 43 | 0.75 | 0.79 | 0.78 | 2027 | Train 32 | Test Female 43 | 0.75 | 0.79 | 0.78 |
| 1004 | Train 16 | Test Female 44 | 0.77 | 0.8 | 0.79 | 2028 | Train 32 | Test Female 44 | 0.77 | 0.8 | 0.79 |
| 1005 | Train 16 | Test Female 45 | 0.74 | 0.79 | 0.8 | 2029 | Train 32 | Test Female 45 | 0.74 | 0.79 | 0.8 |
| 1006 | Train 16 | Test Female 46 | 0.75 | 0.8 | 0.81 | 2030 | Train 32 | Test Female 46 | 0.75 | 0.8 | 0.8 |
| 1007 | Train 16 | Test Female 47 | 0.74 | 0.78 | 0.78 | 2031 | Train 32 | Test Female 47 | 0.74 | 0.78 | 0.77 |
| 1008 | Train 16 | Test Female 48 | 0.75 | 0.8 | 0.81 | 2032 | Train 32 | Test Female 48 | 0.75 | 0.8 | 0.8 |
| 1009 | Train 16 | Test Female 49 | 0.78 | 0.81 | 0.8 | 2033 | Train 32 | Test Female 49 | 0.78 | 0.81 | 0.8 |
| 1010 | Train 16 | Test Female 50 | 0.78 | 0.81 | 0.8 | 2034 | Train 32 | Test Female 50 | 0.78 | 0.81 | 0.8 |
| 1011 | Train 16 | Test Female 51 | 0.78 | 0.81 | 0.8 | 2035 | Train 32 | Test Female 51 | 0.78 | 0.81 | 0.8 |
| 1012 | Train 16 | Test Female 52 | 0.78 | 0.81 | 0.8 | 2036 | Train 32 | Test Female 52 | 0.78 | 0.81 | 0.8 |
| 1013 | Train 16 | Test Female 53 | 0.76 | 0.8 | 0.8 | 2037 | Train 32 | Test Female 53 | 0.76 | 0.8 | 0.78 |
| 1014 | Train 16 | Test Female 54 | 0.76 | 0.8 | 0.8 | 2038 | Train 32 | Test Female 54 | 0.76 | 0.8 | 0.8 |
| 1015 | Train 16 | Test Female 55 | 0.76 | 0.81 | 0.8 | 2039 | Train 32 | Test Female 55 | 0.76 | 0.81 | 0.78 |
| 1016 | Train 16 | Test Female 56 | 0.76 | 0.8 | 0.8 | 2040 | Train 32 | Test Female 56 | 0.76 | 0.8 | 0.8 |
| 1017 | Train 16 | Test Female 57 | 0.78 | 0.81 | 0.81 | 2041 | Train 32 | Test Female 57 | 0.78 | 0.81 | 0.81 |
| 1018 | Train 16 | Test Female 58 | 0.78 | 0.81 | 0.81 | 2042 | Train 32 | Test Female 58 | 0.78 | 0.81 | 0.81 |
| 1019 | Train 16 | Test Female 59 | 0.78 | 0.81 | 0.81 | 2043 | Train 32 | Test Female 59 | 0.78 | 0.81 | 0.81 |
| 1020 | Train 16 | Test Female 60 | 0.78 | 0.81 | 0.81 | 2044 | Train 32 | Test Female 60 | 0.78 | 0.81 | 0.81 |
| 1021 | Train 16 | Test Female 61 | 0.76 | 0.81 | 0.81 | 2045 | Train 32 | Test Female 61 | 0.76 | 0.81 | 0.79 |
| 1022 | Train 16 | Test Female 62 | 0.76 | 0.8 | 0.8 | 2046 | Train 32 | Test Female 62 | 0.76 | 0.8 | 0.8 |
| 1023 | Train 16 | Test Female 63 | 0.76 | 0.81 | 0.81 | 2047 | Train 32 | Test Female 63 | 0.76 | 0.81 | 0.79 |
| 1024 | Train 16 | Test Female 64 | 0.76 | 0.8 | 0.81 | 2048 | Train 32 | Test Female 64 | 0.76 | 0.8 | 0.8 |

Table 4. AUC Performance of the ML Models— LR, RF, and GB —for Female Patients Trained on Cohorts 33–64 and Tested Across 64 Cohorts.

| Female | Train  Model | Test  Dataset | AUC (LR) | AUC (RF) | AUC (GB) | Female | Train  Model | Test  Dataset | AUC (LR) | AUC (RF) | AUC (GB) |
| --- | --- | --- | --- | --- | --- | --- | --- | --- | --- | --- | --- |
| 2049 | Train 33 | Test Female 1 | 0.76 | 0.84 | 0.86 | 3073 | Train 49 | Test Female 1 | 0.74 | 0.75 | 0.68 |
| 2050 | Train 33 | Test Female 2 | 0.76 | 0.8 | 0.81 | 3074 | Train 49 | Test Female 2 | 0.73 | 0.73 | 0.64 |
| 2051 | Train 33 | Test Female 3 | 0.77 | 0.84 | 0.86 | 3075 | Train 49 | Test Female 3 | 0.74 | 0.75 | 0.68 |
| 2052 | Train 33 | Test Female 4 | 0.76 | 0.8 | 0.81 | 3076 | Train 49 | Test Female 4 | 0.73 | 0.73 | 0.64 |
| 2053 | Train 33 | Test Female 5 | 0.73 | 0.76 | 0.73 | 3077 | Train 49 | Test Female 5 | 0.72 | 0.72 | 0.64 |
| 2054 | Train 33 | Test Female 6 | 0.71 | 0.71 | 0.65 | 3078 | Train 49 | Test Female 6 | 0.71 | 0.7 | 0.62 |
| 2055 | Train 33 | Test Female 7 | 0.73 | 0.76 | 0.73 | 3079 | Train 49 | Test Female 7 | 0.72 | 0.72 | 0.64 |
| 2056 | Train 33 | Test Female 8 | 0.71 | 0.71 | 0.66 | 3080 | Train 49 | Test Female 8 | 0.71 | 0.7 | 0.63 |
| 2057 | Train 33 | Test Female 9 | 0.77 | 0.83 | 0.82 | 3081 | Train 49 | Test Female 9 | 0.74 | 0.75 | 0.66 |
| 2058 | Train 33 | Test Female 10 | 0.76 | 0.8 | 0.78 | 3082 | Train 49 | Test Female 10 | 0.73 | 0.73 | 0.63 |
| 2059 | Train 33 | Test Female 11 | 0.77 | 0.83 | 0.82 | 3083 | Train 49 | Test Female 11 | 0.74 | 0.75 | 0.66 |
| 2060 | Train 33 | Test Female 12 | 0.76 | 0.8 | 0.78 | 3084 | Train 49 | Test Female 12 | 0.73 | 0.73 | 0.63 |
| 2061 | Train 33 | Test Female 13 | 0.73 | 0.75 | 0.69 | 3085 | Train 49 | Test Female 13 | 0.72 | 0.72 | 0.64 |
| 2062 | Train 33 | Test Female 14 | 0.71 | 0.72 | 0.65 | 3086 | Train 49 | Test Female 14 | 0.71 | 0.7 | 0.63 |
| 2063 | Train 33 | Test Female 15 | 0.73 | 0.75 | 0.71 | 3087 | Train 49 | Test Female 15 | 0.72 | 0.72 | 0.64 |
| 2064 | Train 33 | Test Female 16 | 0.72 | 0.72 | 0.66 | 3088 | Train 49 | Test Female 16 | 0.71 | 0.7 | 0.63 |
| 2065 | Train 33 | Test Female 17 | 0.76 | 0.84 | 0.86 | 3089 | Train 49 | Test Female 17 | 0.74 | 0.75 | 0.68 |
| 2066 | Train 33 | Test Female 18 | 0.76 | 0.81 | 0.81 | 3090 | Train 49 | Test Female 18 | 0.73 | 0.73 | 0.64 |
| 2067 | Train 33 | Test Female 19 | 0.77 | 0.84 | 0.86 | 3091 | Train 49 | Test Female 19 | 0.74 | 0.75 | 0.68 |
| 2068 | Train 33 | Test Female 20 | 0.76 | 0.8 | 0.81 | 3092 | Train 49 | Test Female 20 | 0.73 | 0.73 | 0.64 |
| 2069 | Train 33 | Test Female 21 | 0.73 | 0.76 | 0.73 | 3093 | Train 49 | Test Female 21 | 0.72 | 0.72 | 0.64 |
| 2070 | Train 33 | Test Female 22 | 0.71 | 0.72 | 0.65 | 3094 | Train 49 | Test Female 22 | 0.71 | 0.7 | 0.63 |
| 2071 | Train 33 | Test Female 23 | 0.73 | 0.76 | 0.73 | 3095 | Train 49 | Test Female 23 | 0.72 | 0.72 | 0.64 |
| 2072 | Train 33 | Test Female 24 | 0.71 | 0.71 | 0.66 | 3096 | Train 49 | Test Female 24 | 0.71 | 0.7 | 0.63 |
| 2073 | Train 33 | Test Female 25 | 0.77 | 0.83 | 0.82 | 3097 | Train 49 | Test Female 25 | 0.74 | 0.75 | 0.66 |
| 2074 | Train 33 | Test Female 26 | 0.76 | 0.8 | 0.78 | 3098 | Train 49 | Test Female 26 | 0.73 | 0.73 | 0.63 |
| 2075 | Train 33 | Test Female 27 | 0.77 | 0.83 | 0.82 | 3099 | Train 49 | Test Female 27 | 0.74 | 0.75 | 0.66 |
| 2076 | Train 33 | Test Female 28 | 0.76 | 0.8 | 0.78 | 3100 | Train 49 | Test Female 28 | 0.73 | 0.73 | 0.63 |
| 2077 | Train 33 | Test Female 29 | 0.73 | 0.75 | 0.69 | 3101 | Train 49 | Test Female 29 | 0.72 | 0.72 | 0.64 |
| 2078 | Train 33 | Test Female 30 | 0.71 | 0.72 | 0.65 | 3102 | Train 49 | Test Female 30 | 0.71 | 0.7 | 0.63 |
| 2079 | Train 33 | Test Female 31 | 0.73 | 0.75 | 0.71 | 3103 | Train 49 | Test Female 31 | 0.72 | 0.72 | 0.64 |
| 2080 | Train 33 | Test Female 32 | 0.72 | 0.72 | 0.66 | 3104 | Train 49 | Test Female 32 | 0.71 | 0.7 | 0.63 |
| 2081 | Train 33 | Test Female 33 | 0.77 | 0.85 | 0.86 | 3105 | Train 49 | Test Female 33 | 0.75 | 0.76 | 0.69 |
| 2082 | Train 33 | Test Female 34 | 0.76 | 0.81 | 0.81 | 3106 | Train 49 | Test Female 34 | 0.74 | 0.74 | 0.65 |
| 2083 | Train 33 | Test Female 35 | 0.77 | 0.85 | 0.86 | 3107 | Train 49 | Test Female 35 | 0.75 | 0.76 | 0.69 |
| 2084 | Train 33 | Test Female 36 | 0.76 | 0.81 | 0.81 | 3108 | Train 49 | Test Female 36 | 0.74 | 0.74 | 0.65 |
| 2085 | Train 33 | Test Female 37 | 0.73 | 0.76 | 0.74 | 3109 | Train 49 | Test Female 37 | 0.72 | 0.72 | 0.65 |
| 2086 | Train 33 | Test Female 38 | 0.71 | 0.72 | 0.66 | 3110 | Train 49 | Test Female 38 | 0.71 | 0.7 | 0.63 |
| 2087 | Train 33 | Test Female 39 | 0.73 | 0.76 | 0.73 | 3111 | Train 49 | Test Female 39 | 0.72 | 0.72 | 0.65 |
| 2088 | Train 33 | Test Female 40 | 0.72 | 0.72 | 0.66 | 3112 | Train 49 | Test Female 40 | 0.71 | 0.7 | 0.63 |
| 2089 | Train 33 | Test Female 41 | 0.77 | 0.84 | 0.83 | 3113 | Train 49 | Test Female 41 | 0.75 | 0.76 | 0.67 |
| 2090 | Train 33 | Test Female 42 | 0.76 | 0.8 | 0.79 | 3114 | Train 49 | Test Female 42 | 0.74 | 0.74 | 0.63 |
| 2091 | Train 33 | Test Female 43 | 0.77 | 0.83 | 0.83 | 3115 | Train 49 | Test Female 43 | 0.75 | 0.76 | 0.67 |
| 2092 | Train 33 | Test Female 44 | 0.76 | 0.8 | 0.79 | 3116 | Train 49 | Test Female 44 | 0.74 | 0.74 | 0.64 |
| 2093 | Train 33 | Test Female 45 | 0.73 | 0.75 | 0.69 | 3117 | Train 49 | Test Female 45 | 0.72 | 0.72 | 0.64 |
| 2094 | Train 33 | Test Female 46 | 0.72 | 0.72 | 0.66 | 3118 | Train 49 | Test Female 46 | 0.71 | 0.7 | 0.63 |
| 2095 | Train 33 | Test Female 47 | 0.73 | 0.76 | 0.72 | 3119 | Train 49 | Test Female 47 | 0.72 | 0.72 | 0.64 |
| 2096 | Train 33 | Test Female 48 | 0.72 | 0.72 | 0.66 | 3120 | Train 49 | Test Female 48 | 0.71 | 0.7 | 0.63 |
| 2097 | Train 33 | Test Female 49 | 0.79 | 0.84 | 0.87 | 3121 | Train 49 | Test Female 49 | 0.8 | 0.87 | 0.91 |
| 2098 | Train 33 | Test Female 50 | 0.79 | 0.84 | 0.87 | 3122 | Train 49 | Test Female 50 | 0.8 | 0.87 | 0.91 |
| 2099 | Train 33 | Test Female 51 | 0.79 | 0.84 | 0.87 | 3123 | Train 49 | Test Female 51 | 0.8 | 0.86 | 0.9 |
| 2100 | Train 33 | Test Female 52 | 0.79 | 0.84 | 0.87 | 3124 | Train 49 | Test Female 52 | 0.8 | 0.86 | 0.9 |
| 2101 | Train 33 | Test Female 53 | 0.74 | 0.78 | 0.69 | 3125 | Train 49 | Test Female 53 | 0.74 | 0.78 | 0.72 |
| 2102 | Train 33 | Test Female 54 | 0.73 | 0.73 | 0.65 | 3126 | Train 49 | Test Female 54 | 0.73 | 0.72 | 0.67 |
| 2103 | Train 33 | Test Female 55 | 0.74 | 0.78 | 0.7 | 3127 | Train 49 | Test Female 55 | 0.74 | 0.78 | 0.72 |
| 2104 | Train 33 | Test Female 56 | 0.73 | 0.72 | 0.65 | 3128 | Train 49 | Test Female 56 | 0.73 | 0.72 | 0.67 |
| 2105 | Train 33 | Test Female 57 | 0.79 | 0.84 | 0.84 | 3129 | Train 49 | Test Female 57 | 0.8 | 0.85 | 0.85 |
| 2106 | Train 33 | Test Female 58 | 0.79 | 0.83 | 0.84 | 3130 | Train 49 | Test Female 58 | 0.8 | 0.85 | 0.85 |
| 2107 | Train 33 | Test Female 59 | 0.79 | 0.83 | 0.83 | 3131 | Train 49 | Test Female 59 | 0.8 | 0.85 | 0.85 |
| 2108 | Train 33 | Test Female 60 | 0.79 | 0.83 | 0.83 | 3132 | Train 49 | Test Female 60 | 0.8 | 0.85 | 0.85 |
| 2109 | Train 33 | Test Female 61 | 0.74 | 0.78 | 0.69 | 3133 | Train 49 | Test Female 61 | 0.74 | 0.78 | 0.72 |
| 2110 | Train 33 | Test Female 62 | 0.73 | 0.73 | 0.65 | 3134 | Train 49 | Test Female 62 | 0.73 | 0.72 | 0.67 |
| 2111 | Train 33 | Test Female 63 | 0.75 | 0.78 | 0.69 | 3135 | Train 49 | Test Female 63 | 0.75 | 0.78 | 0.72 |
| 2112 | Train 33 | Test Female 64 | 0.73 | 0.73 | 0.66 | 3136 | Train 49 | Test Female 64 | 0.73 | 0.72 | 0.67 |
| 2113 | Train 34 | Test Female 1 | 0.76 | 0.83 | 0.85 | 3137 | Train 50 | Test Female 1 | 0.74 | 0.73 | 0.7 |
| 2114 | Train 34 | Test Female 2 | 0.77 | 0.84 | 0.86 | 3138 | Train 50 | Test Female 2 | 0.73 | 0.72 | 0.67 |
| 2115 | Train 34 | Test Female 3 | 0.76 | 0.83 | 0.84 | 3139 | Train 50 | Test Female 3 | 0.74 | 0.73 | 0.7 |
| 2116 | Train 34 | Test Female 4 | 0.77 | 0.84 | 0.86 | 3140 | Train 50 | Test Female 4 | 0.73 | 0.72 | 0.67 |
| 2117 | Train 34 | Test Female 5 | 0.73 | 0.76 | 0.75 | 3141 | Train 50 | Test Female 5 | 0.71 | 0.7 | 0.67 |
| 2118 | Train 34 | Test Female 6 | 0.73 | 0.73 | 0.68 | 3142 | Train 50 | Test Female 6 | 0.71 | 0.69 | 0.65 |
| 2119 | Train 34 | Test Female 7 | 0.73 | 0.76 | 0.75 | 3143 | Train 50 | Test Female 7 | 0.71 | 0.7 | 0.67 |
| 2120 | Train 34 | Test Female 8 | 0.73 | 0.73 | 0.68 | 3144 | Train 50 | Test Female 8 | 0.71 | 0.69 | 0.65 |
| 2121 | Train 34 | Test Female 9 | 0.76 | 0.82 | 0.82 | 3145 | Train 50 | Test Female 9 | 0.74 | 0.73 | 0.68 |
| 2122 | Train 34 | Test Female 10 | 0.77 | 0.83 | 0.83 | 3146 | Train 50 | Test Female 10 | 0.73 | 0.72 | 0.66 |
| 2123 | Train 34 | Test Female 11 | 0.76 | 0.82 | 0.82 | 3147 | Train 50 | Test Female 11 | 0.74 | 0.73 | 0.68 |
| 2124 | Train 34 | Test Female 12 | 0.78 | 0.83 | 0.83 | 3148 | Train 50 | Test Female 12 | 0.73 | 0.72 | 0.66 |
| 2125 | Train 34 | Test Female 13 | 0.73 | 0.75 | 0.69 | 3149 | Train 50 | Test Female 13 | 0.71 | 0.7 | 0.66 |
| 2126 | Train 34 | Test Female 14 | 0.73 | 0.73 | 0.68 | 3150 | Train 50 | Test Female 14 | 0.71 | 0.69 | 0.65 |
| 2127 | Train 34 | Test Female 15 | 0.73 | 0.76 | 0.73 | 3151 | Train 50 | Test Female 15 | 0.71 | 0.7 | 0.66 |
| 2128 | Train 34 | Test Female 16 | 0.73 | 0.73 | 0.68 | 3152 | Train 50 | Test Female 16 | 0.71 | 0.69 | 0.65 |
| 2129 | Train 34 | Test Female 17 | 0.76 | 0.83 | 0.85 | 3153 | Train 50 | Test Female 17 | 0.74 | 0.73 | 0.7 |
| 2130 | Train 34 | Test Female 18 | 0.77 | 0.84 | 0.86 | 3154 | Train 50 | Test Female 18 | 0.73 | 0.72 | 0.67 |
| 2131 | Train 34 | Test Female 19 | 0.76 | 0.83 | 0.84 | 3155 | Train 50 | Test Female 19 | 0.74 | 0.73 | 0.7 |
| 2132 | Train 34 | Test Female 20 | 0.77 | 0.84 | 0.86 | 3156 | Train 50 | Test Female 20 | 0.73 | 0.72 | 0.67 |
| 2133 | Train 34 | Test Female 21 | 0.73 | 0.76 | 0.75 | 3157 | Train 50 | Test Female 21 | 0.71 | 0.7 | 0.67 |
| 2134 | Train 34 | Test Female 22 | 0.73 | 0.73 | 0.68 | 3158 | Train 50 | Test Female 22 | 0.71 | 0.69 | 0.65 |
| 2135 | Train 34 | Test Female 23 | 0.73 | 0.76 | 0.75 | 3159 | Train 50 | Test Female 23 | 0.71 | 0.7 | 0.67 |
| 2136 | Train 34 | Test Female 24 | 0.73 | 0.73 | 0.68 | 3160 | Train 50 | Test Female 24 | 0.71 | 0.69 | 0.65 |
| 2137 | Train 34 | Test Female 25 | 0.76 | 0.82 | 0.82 | 3161 | Train 50 | Test Female 25 | 0.74 | 0.73 | 0.68 |
| 2138 | Train 34 | Test Female 26 | 0.78 | 0.83 | 0.83 | 3162 | Train 50 | Test Female 26 | 0.73 | 0.72 | 0.66 |
| 2139 | Train 34 | Test Female 27 | 0.76 | 0.82 | 0.82 | 3163 | Train 50 | Test Female 27 | 0.74 | 0.73 | 0.68 |
| 2140 | Train 34 | Test Female 28 | 0.78 | 0.83 | 0.83 | 3164 | Train 50 | Test Female 28 | 0.73 | 0.72 | 0.66 |
| 2141 | Train 34 | Test Female 29 | 0.73 | 0.75 | 0.69 | 3165 | Train 50 | Test Female 29 | 0.71 | 0.7 | 0.66 |
| 2142 | Train 34 | Test Female 30 | 0.73 | 0.73 | 0.68 | 3166 | Train 50 | Test Female 30 | 0.71 | 0.7 | 0.65 |
| 2143 | Train 34 | Test Female 31 | 0.73 | 0.76 | 0.73 | 3167 | Train 50 | Test Female 31 | 0.71 | 0.7 | 0.66 |
| 2144 | Train 34 | Test Female 32 | 0.73 | 0.73 | 0.68 | 3168 | Train 50 | Test Female 32 | 0.71 | 0.69 | 0.65 |
| 2145 | Train 34 | Test Female 33 | 0.76 | 0.84 | 0.85 | 3169 | Train 50 | Test Female 33 | 0.75 | 0.74 | 0.7 |
| 2146 | Train 34 | Test Female 34 | 0.78 | 0.85 | 0.86 | 3170 | Train 50 | Test Female 34 | 0.74 | 0.73 | 0.68 |
| 2147 | Train 34 | Test Female 35 | 0.77 | 0.84 | 0.85 | 3171 | Train 50 | Test Female 35 | 0.75 | 0.74 | 0.7 |
| 2148 | Train 34 | Test Female 36 | 0.78 | 0.85 | 0.86 | 3172 | Train 50 | Test Female 36 | 0.74 | 0.73 | 0.68 |
| 2149 | Train 34 | Test Female 37 | 0.73 | 0.77 | 0.75 | 3173 | Train 50 | Test Female 37 | 0.72 | 0.71 | 0.67 |
| 2150 | Train 34 | Test Female 38 | 0.73 | 0.73 | 0.68 | 3174 | Train 50 | Test Female 38 | 0.71 | 0.69 | 0.65 |
| 2151 | Train 34 | Test Female 39 | 0.73 | 0.76 | 0.75 | 3175 | Train 50 | Test Female 39 | 0.72 | 0.71 | 0.67 |
| 2152 | Train 34 | Test Female 40 | 0.73 | 0.73 | 0.68 | 3176 | Train 50 | Test Female 40 | 0.71 | 0.69 | 0.65 |
| 2153 | Train 34 | Test Female 41 | 0.76 | 0.83 | 0.82 | 3177 | Train 50 | Test Female 41 | 0.75 | 0.74 | 0.69 |
| 2154 | Train 34 | Test Female 42 | 0.78 | 0.84 | 0.83 | 3178 | Train 50 | Test Female 42 | 0.74 | 0.72 | 0.67 |
| 2155 | Train 34 | Test Female 43 | 0.77 | 0.83 | 0.82 | 3179 | Train 50 | Test Female 43 | 0.75 | 0.74 | 0.69 |
| 2156 | Train 34 | Test Female 44 | 0.78 | 0.84 | 0.83 | 3180 | Train 50 | Test Female 44 | 0.74 | 0.72 | 0.67 |
| 2157 | Train 34 | Test Female 45 | 0.73 | 0.75 | 0.7 | 3181 | Train 50 | Test Female 45 | 0.72 | 0.71 | 0.67 |
| 2158 | Train 34 | Test Female 46 | 0.73 | 0.73 | 0.68 | 3182 | Train 50 | Test Female 46 | 0.71 | 0.7 | 0.65 |
| 2159 | Train 34 | Test Female 47 | 0.73 | 0.76 | 0.74 | 3183 | Train 50 | Test Female 47 | 0.72 | 0.71 | 0.67 |
| 2160 | Train 34 | Test Female 48 | 0.73 | 0.73 | 0.68 | 3184 | Train 50 | Test Female 48 | 0.71 | 0.7 | 0.65 |
| 2161 | Train 34 | Test Female 49 | 0.78 | 0.84 | 0.86 | 3185 | Train 50 | Test Female 49 | 0.8 | 0.87 | 0.91 |
| 2162 | Train 34 | Test Female 50 | 0.78 | 0.84 | 0.86 | 3186 | Train 50 | Test Female 50 | 0.8 | 0.87 | 0.91 |
| 2163 | Train 34 | Test Female 51 | 0.78 | 0.83 | 0.86 | 3187 | Train 50 | Test Female 51 | 0.8 | 0.86 | 0.9 |
| 2164 | Train 34 | Test Female 52 | 0.78 | 0.83 | 0.86 | 3188 | Train 50 | Test Female 52 | 0.8 | 0.86 | 0.9 |
| 2165 | Train 34 | Test Female 53 | 0.74 | 0.78 | 0.75 | 3189 | Train 50 | Test Female 53 | 0.74 | 0.76 | 0.75 |
| 2166 | Train 34 | Test Female 54 | 0.74 | 0.74 | 0.68 | 3190 | Train 50 | Test Female 54 | 0.73 | 0.71 | 0.67 |
| 2167 | Train 34 | Test Female 55 | 0.75 | 0.78 | 0.75 | 3191 | Train 50 | Test Female 55 | 0.74 | 0.76 | 0.75 |
| 2168 | Train 34 | Test Female 56 | 0.73 | 0.73 | 0.68 | 3192 | Train 50 | Test Female 56 | 0.73 | 0.71 | 0.67 |
| 2169 | Train 34 | Test Female 57 | 0.78 | 0.83 | 0.83 | 3193 | Train 50 | Test Female 57 | 0.8 | 0.85 | 0.85 |
| 2170 | Train 34 | Test Female 58 | 0.78 | 0.83 | 0.83 | 3194 | Train 50 | Test Female 58 | 0.8 | 0.85 | 0.85 |
| 2171 | Train 34 | Test Female 59 | 0.79 | 0.83 | 0.83 | 3195 | Train 50 | Test Female 59 | 0.8 | 0.85 | 0.85 |
| 2172 | Train 34 | Test Female 60 | 0.79 | 0.83 | 0.83 | 3196 | Train 50 | Test Female 60 | 0.8 | 0.85 | 0.85 |
| 2173 | Train 34 | Test Female 61 | 0.75 | 0.78 | 0.74 | 3197 | Train 50 | Test Female 61 | 0.74 | 0.76 | 0.74 |
| 2174 | Train 34 | Test Female 62 | 0.74 | 0.74 | 0.68 | 3198 | Train 50 | Test Female 62 | 0.73 | 0.71 | 0.67 |
| 2175 | Train 34 | Test Female 63 | 0.75 | 0.78 | 0.74 | 3199 | Train 50 | Test Female 63 | 0.74 | 0.76 | 0.74 |
| 2176 | Train 34 | Test Female 64 | 0.73 | 0.73 | 0.68 | 3200 | Train 50 | Test Female 64 | 0.73 | 0.71 | 0.67 |
| 2177 | Train 35 | Test Female 1 | 0.76 | 0.84 | 0.85 | 3201 | Train 51 | Test Female 1 | 0.74 | 0.75 | 0.72 |
| 2178 | Train 35 | Test Female 2 | 0.76 | 0.81 | 0.81 | 3202 | Train 51 | Test Female 2 | 0.73 | 0.73 | 0.67 |
| 2179 | Train 35 | Test Female 3 | 0.77 | 0.84 | 0.85 | 3203 | Train 51 | Test Female 3 | 0.74 | 0.76 | 0.72 |
| 2180 | Train 35 | Test Female 4 | 0.76 | 0.81 | 0.81 | 3204 | Train 51 | Test Female 4 | 0.73 | 0.74 | 0.67 |
| 2181 | Train 35 | Test Female 5 | 0.73 | 0.76 | 0.73 | 3205 | Train 51 | Test Female 5 | 0.72 | 0.72 | 0.66 |
| 2182 | Train 35 | Test Female 6 | 0.71 | 0.71 | 0.67 | 3206 | Train 51 | Test Female 6 | 0.7 | 0.7 | 0.6 |
| 2183 | Train 35 | Test Female 7 | 0.73 | 0.76 | 0.73 | 3207 | Train 51 | Test Female 7 | 0.72 | 0.72 | 0.66 |
| 2184 | Train 35 | Test Female 8 | 0.71 | 0.71 | 0.68 | 3208 | Train 51 | Test Female 8 | 0.71 | 0.7 | 0.6 |
| 2185 | Train 35 | Test Female 9 | 0.77 | 0.83 | 0.82 | 3209 | Train 51 | Test Female 9 | 0.74 | 0.75 | 0.7 |
| 2186 | Train 35 | Test Female 10 | 0.76 | 0.8 | 0.78 | 3210 | Train 51 | Test Female 10 | 0.73 | 0.73 | 0.65 |
| 2187 | Train 35 | Test Female 11 | 0.77 | 0.83 | 0.82 | 3211 | Train 51 | Test Female 11 | 0.74 | 0.75 | 0.7 |
| 2188 | Train 35 | Test Female 12 | 0.76 | 0.8 | 0.78 | 3212 | Train 51 | Test Female 12 | 0.73 | 0.73 | 0.65 |
| 2189 | Train 35 | Test Female 13 | 0.73 | 0.75 | 0.69 | 3213 | Train 51 | Test Female 13 | 0.72 | 0.72 | 0.65 |
| 2190 | Train 35 | Test Female 14 | 0.71 | 0.72 | 0.67 | 3214 | Train 51 | Test Female 14 | 0.71 | 0.7 | 0.6 |
| 2191 | Train 35 | Test Female 15 | 0.73 | 0.75 | 0.71 | 3215 | Train 51 | Test Female 15 | 0.72 | 0.72 | 0.65 |
| 2192 | Train 35 | Test Female 16 | 0.72 | 0.72 | 0.68 | 3216 | Train 51 | Test Female 16 | 0.71 | 0.7 | 0.61 |
| 2193 | Train 35 | Test Female 17 | 0.76 | 0.84 | 0.85 | 3217 | Train 51 | Test Female 17 | 0.74 | 0.75 | 0.72 |
| 2194 | Train 35 | Test Female 18 | 0.76 | 0.81 | 0.81 | 3218 | Train 51 | Test Female 18 | 0.73 | 0.73 | 0.67 |
| 2195 | Train 35 | Test Female 19 | 0.77 | 0.84 | 0.86 | 3219 | Train 51 | Test Female 19 | 0.74 | 0.76 | 0.72 |
| 2196 | Train 35 | Test Female 20 | 0.76 | 0.81 | 0.81 | 3220 | Train 51 | Test Female 20 | 0.73 | 0.74 | 0.67 |
| 2197 | Train 35 | Test Female 21 | 0.73 | 0.76 | 0.73 | 3221 | Train 51 | Test Female 21 | 0.72 | 0.72 | 0.66 |
| 2198 | Train 35 | Test Female 22 | 0.71 | 0.72 | 0.68 | 3222 | Train 51 | Test Female 22 | 0.7 | 0.7 | 0.6 |
| 2199 | Train 35 | Test Female 23 | 0.73 | 0.76 | 0.73 | 3223 | Train 51 | Test Female 23 | 0.72 | 0.72 | 0.66 |
| 2200 | Train 35 | Test Female 24 | 0.71 | 0.72 | 0.68 | 3224 | Train 51 | Test Female 24 | 0.71 | 0.7 | 0.61 |
| 2201 | Train 35 | Test Female 25 | 0.77 | 0.83 | 0.82 | 3225 | Train 51 | Test Female 25 | 0.74 | 0.75 | 0.7 |
| 2202 | Train 35 | Test Female 26 | 0.76 | 0.8 | 0.78 | 3226 | Train 51 | Test Female 26 | 0.73 | 0.73 | 0.65 |
| 2203 | Train 35 | Test Female 27 | 0.77 | 0.83 | 0.82 | 3227 | Train 51 | Test Female 27 | 0.74 | 0.75 | 0.7 |
| 2204 | Train 35 | Test Female 28 | 0.76 | 0.8 | 0.78 | 3228 | Train 51 | Test Female 28 | 0.73 | 0.73 | 0.65 |
| 2205 | Train 35 | Test Female 29 | 0.73 | 0.75 | 0.69 | 3229 | Train 51 | Test Female 29 | 0.72 | 0.72 | 0.65 |
| 2206 | Train 35 | Test Female 30 | 0.71 | 0.72 | 0.68 | 3230 | Train 51 | Test Female 30 | 0.71 | 0.7 | 0.6 |
| 2207 | Train 35 | Test Female 31 | 0.73 | 0.75 | 0.71 | 3231 | Train 51 | Test Female 31 | 0.72 | 0.72 | 0.65 |
| 2208 | Train 35 | Test Female 32 | 0.72 | 0.72 | 0.68 | 3232 | Train 51 | Test Female 32 | 0.71 | 0.7 | 0.61 |
| 2209 | Train 35 | Test Female 33 | 0.77 | 0.85 | 0.86 | 3233 | Train 51 | Test Female 33 | 0.75 | 0.76 | 0.73 |
| 2210 | Train 35 | Test Female 34 | 0.76 | 0.81 | 0.81 | 3234 | Train 51 | Test Female 34 | 0.74 | 0.74 | 0.67 |
| 2211 | Train 35 | Test Female 35 | 0.77 | 0.85 | 0.86 | 3235 | Train 51 | Test Female 35 | 0.75 | 0.76 | 0.73 |
| 2212 | Train 35 | Test Female 36 | 0.76 | 0.81 | 0.81 | 3236 | Train 51 | Test Female 36 | 0.74 | 0.74 | 0.68 |
| 2213 | Train 35 | Test Female 37 | 0.73 | 0.76 | 0.73 | 3237 | Train 51 | Test Female 37 | 0.72 | 0.72 | 0.67 |
| 2214 | Train 35 | Test Female 38 | 0.71 | 0.72 | 0.68 | 3238 | Train 51 | Test Female 38 | 0.71 | 0.7 | 0.6 |
| 2215 | Train 35 | Test Female 39 | 0.73 | 0.76 | 0.73 | 3239 | Train 51 | Test Female 39 | 0.72 | 0.72 | 0.67 |
| 2216 | Train 35 | Test Female 40 | 0.72 | 0.72 | 0.68 | 3240 | Train 51 | Test Female 40 | 0.71 | 0.7 | 0.61 |
| 2217 | Train 35 | Test Female 41 | 0.77 | 0.84 | 0.83 | 3241 | Train 51 | Test Female 41 | 0.75 | 0.76 | 0.71 |
| 2218 | Train 35 | Test Female 42 | 0.76 | 0.8 | 0.79 | 3242 | Train 51 | Test Female 42 | 0.74 | 0.74 | 0.66 |
| 2219 | Train 35 | Test Female 43 | 0.77 | 0.84 | 0.83 | 3243 | Train 51 | Test Female 43 | 0.75 | 0.76 | 0.71 |
| 2220 | Train 35 | Test Female 44 | 0.76 | 0.8 | 0.79 | 3244 | Train 51 | Test Female 44 | 0.74 | 0.74 | 0.66 |
| 2221 | Train 35 | Test Female 45 | 0.73 | 0.75 | 0.69 | 3245 | Train 51 | Test Female 45 | 0.72 | 0.72 | 0.66 |
| 2222 | Train 35 | Test Female 46 | 0.72 | 0.72 | 0.68 | 3246 | Train 51 | Test Female 46 | 0.71 | 0.7 | 0.6 |
| 2223 | Train 35 | Test Female 47 | 0.73 | 0.76 | 0.71 | 3247 | Train 51 | Test Female 47 | 0.72 | 0.72 | 0.66 |
| 2224 | Train 35 | Test Female 48 | 0.72 | 0.72 | 0.68 | 3248 | Train 51 | Test Female 48 | 0.71 | 0.7 | 0.61 |
| 2225 | Train 35 | Test Female 49 | 0.79 | 0.84 | 0.86 | 3249 | Train 51 | Test Female 49 | 0.8 | 0.87 | 0.91 |
| 2226 | Train 35 | Test Female 50 | 0.79 | 0.84 | 0.86 | 3250 | Train 51 | Test Female 50 | 0.8 | 0.87 | 0.91 |
| 2227 | Train 35 | Test Female 51 | 0.79 | 0.84 | 0.87 | 3251 | Train 51 | Test Female 51 | 0.8 | 0.87 | 0.91 |
| 2228 | Train 35 | Test Female 52 | 0.79 | 0.84 | 0.87 | 3252 | Train 51 | Test Female 52 | 0.8 | 0.87 | 0.91 |
| 2229 | Train 35 | Test Female 53 | 0.74 | 0.77 | 0.7 | 3253 | Train 51 | Test Female 53 | 0.74 | 0.78 | 0.74 |
| 2230 | Train 35 | Test Female 54 | 0.73 | 0.73 | 0.68 | 3254 | Train 51 | Test Female 54 | 0.73 | 0.72 | 0.64 |
| 2231 | Train 35 | Test Female 55 | 0.74 | 0.78 | 0.71 | 3255 | Train 51 | Test Female 55 | 0.75 | 0.78 | 0.74 |
| 2232 | Train 35 | Test Female 56 | 0.73 | 0.72 | 0.68 | 3256 | Train 51 | Test Female 56 | 0.73 | 0.72 | 0.64 |
| 2233 | Train 35 | Test Female 57 | 0.79 | 0.83 | 0.83 | 3257 | Train 51 | Test Female 57 | 0.8 | 0.85 | 0.85 |
| 2234 | Train 35 | Test Female 58 | 0.79 | 0.83 | 0.83 | 3258 | Train 51 | Test Female 58 | 0.8 | 0.85 | 0.85 |
| 2235 | Train 35 | Test Female 59 | 0.79 | 0.84 | 0.83 | 3259 | Train 51 | Test Female 59 | 0.8 | 0.86 | 0.85 |
| 2236 | Train 35 | Test Female 60 | 0.79 | 0.84 | 0.83 | 3260 | Train 51 | Test Female 60 | 0.8 | 0.86 | 0.85 |
| 2237 | Train 35 | Test Female 61 | 0.74 | 0.77 | 0.7 | 3261 | Train 51 | Test Female 61 | 0.74 | 0.78 | 0.73 |
| 2238 | Train 35 | Test Female 62 | 0.73 | 0.73 | 0.68 | 3262 | Train 51 | Test Female 62 | 0.73 | 0.72 | 0.64 |
| 2239 | Train 35 | Test Female 63 | 0.75 | 0.78 | 0.7 | 3263 | Train 51 | Test Female 63 | 0.75 | 0.78 | 0.73 |
| 2240 | Train 35 | Test Female 64 | 0.73 | 0.72 | 0.68 | 3264 | Train 51 | Test Female 64 | 0.73 | 0.72 | 0.64 |
| 2241 | Train 36 | Test Female 1 | 0.76 | 0.83 | 0.85 | 3265 | Train 52 | Test Female 1 | 0.74 | 0.73 | 0.71 |
| 2242 | Train 36 | Test Female 2 | 0.77 | 0.84 | 0.86 | 3266 | Train 52 | Test Female 2 | 0.73 | 0.72 | 0.69 |
| 2243 | Train 36 | Test Female 3 | 0.76 | 0.83 | 0.85 | 3267 | Train 52 | Test Female 3 | 0.74 | 0.73 | 0.72 |
| 2244 | Train 36 | Test Female 4 | 0.77 | 0.84 | 0.86 | 3268 | Train 52 | Test Female 4 | 0.73 | 0.72 | 0.69 |
| 2245 | Train 36 | Test Female 5 | 0.73 | 0.76 | 0.74 | 3269 | Train 52 | Test Female 5 | 0.71 | 0.7 | 0.68 |
| 2246 | Train 36 | Test Female 6 | 0.73 | 0.73 | 0.69 | 3270 | Train 52 | Test Female 6 | 0.7 | 0.69 | 0.65 |
| 2247 | Train 36 | Test Female 7 | 0.73 | 0.76 | 0.74 | 3271 | Train 52 | Test Female 7 | 0.71 | 0.7 | 0.68 |
| 2248 | Train 36 | Test Female 8 | 0.72 | 0.73 | 0.69 | 3272 | Train 52 | Test Female 8 | 0.71 | 0.69 | 0.64 |
| 2249 | Train 36 | Test Female 9 | 0.76 | 0.82 | 0.82 | 3273 | Train 52 | Test Female 9 | 0.74 | 0.73 | 0.69 |
| 2250 | Train 36 | Test Female 10 | 0.77 | 0.83 | 0.83 | 3274 | Train 52 | Test Female 10 | 0.73 | 0.72 | 0.67 |
| 2251 | Train 36 | Test Female 11 | 0.76 | 0.82 | 0.82 | 3275 | Train 52 | Test Female 11 | 0.74 | 0.73 | 0.7 |
| 2252 | Train 36 | Test Female 12 | 0.78 | 0.83 | 0.83 | 3276 | Train 52 | Test Female 12 | 0.73 | 0.72 | 0.67 |
| 2253 | Train 36 | Test Female 13 | 0.73 | 0.75 | 0.69 | 3277 | Train 52 | Test Female 13 | 0.71 | 0.7 | 0.67 |
| 2254 | Train 36 | Test Female 14 | 0.73 | 0.73 | 0.69 | 3278 | Train 52 | Test Female 14 | 0.71 | 0.69 | 0.65 |
| 2255 | Train 36 | Test Female 15 | 0.73 | 0.76 | 0.73 | 3279 | Train 52 | Test Female 15 | 0.71 | 0.7 | 0.67 |
| 2256 | Train 36 | Test Female 16 | 0.73 | 0.73 | 0.69 | 3280 | Train 52 | Test Female 16 | 0.71 | 0.69 | 0.65 |
| 2257 | Train 36 | Test Female 17 | 0.76 | 0.83 | 0.85 | 3281 | Train 52 | Test Female 17 | 0.74 | 0.73 | 0.71 |
| 2258 | Train 36 | Test Female 18 | 0.77 | 0.84 | 0.86 | 3282 | Train 52 | Test Female 18 | 0.73 | 0.72 | 0.69 |
| 2259 | Train 36 | Test Female 19 | 0.76 | 0.83 | 0.85 | 3283 | Train 52 | Test Female 19 | 0.74 | 0.73 | 0.72 |
| 2260 | Train 36 | Test Female 20 | 0.77 | 0.84 | 0.86 | 3284 | Train 52 | Test Female 20 | 0.73 | 0.72 | 0.69 |
| 2261 | Train 36 | Test Female 21 | 0.73 | 0.76 | 0.75 | 3285 | Train 52 | Test Female 21 | 0.71 | 0.7 | 0.68 |
| 2262 | Train 36 | Test Female 22 | 0.73 | 0.73 | 0.69 | 3286 | Train 52 | Test Female 22 | 0.7 | 0.69 | 0.65 |
| 2263 | Train 36 | Test Female 23 | 0.73 | 0.76 | 0.74 | 3287 | Train 52 | Test Female 23 | 0.71 | 0.7 | 0.68 |
| 2264 | Train 36 | Test Female 24 | 0.73 | 0.73 | 0.69 | 3288 | Train 52 | Test Female 24 | 0.71 | 0.69 | 0.65 |
| 2265 | Train 36 | Test Female 25 | 0.76 | 0.82 | 0.82 | 3289 | Train 52 | Test Female 25 | 0.74 | 0.73 | 0.69 |
| 2266 | Train 36 | Test Female 26 | 0.77 | 0.83 | 0.83 | 3290 | Train 52 | Test Female 26 | 0.73 | 0.72 | 0.67 |
| 2267 | Train 36 | Test Female 27 | 0.76 | 0.82 | 0.82 | 3291 | Train 52 | Test Female 27 | 0.74 | 0.73 | 0.7 |
| 2268 | Train 36 | Test Female 28 | 0.78 | 0.83 | 0.83 | 3292 | Train 52 | Test Female 28 | 0.73 | 0.72 | 0.67 |
| 2269 | Train 36 | Test Female 29 | 0.73 | 0.75 | 0.69 | 3293 | Train 52 | Test Female 29 | 0.71 | 0.7 | 0.67 |
| 2270 | Train 36 | Test Female 30 | 0.73 | 0.73 | 0.69 | 3294 | Train 52 | Test Female 30 | 0.71 | 0.7 | 0.65 |
| 2271 | Train 36 | Test Female 31 | 0.73 | 0.76 | 0.73 | 3295 | Train 52 | Test Female 31 | 0.71 | 0.7 | 0.67 |
| 2272 | Train 36 | Test Female 32 | 0.73 | 0.73 | 0.69 | 3296 | Train 52 | Test Female 32 | 0.71 | 0.69 | 0.65 |
| 2273 | Train 36 | Test Female 33 | 0.76 | 0.84 | 0.85 | 3297 | Train 52 | Test Female 33 | 0.75 | 0.74 | 0.72 |
| 2274 | Train 36 | Test Female 34 | 0.78 | 0.85 | 0.86 | 3298 | Train 52 | Test Female 34 | 0.74 | 0.72 | 0.69 |
| 2275 | Train 36 | Test Female 35 | 0.77 | 0.84 | 0.85 | 3299 | Train 52 | Test Female 35 | 0.75 | 0.74 | 0.72 |
| 2276 | Train 36 | Test Female 36 | 0.78 | 0.85 | 0.86 | 3300 | Train 52 | Test Female 36 | 0.74 | 0.73 | 0.69 |
| 2277 | Train 36 | Test Female 37 | 0.73 | 0.77 | 0.75 | 3301 | Train 52 | Test Female 37 | 0.72 | 0.71 | 0.68 |
| 2278 | Train 36 | Test Female 38 | 0.73 | 0.73 | 0.69 | 3302 | Train 52 | Test Female 38 | 0.71 | 0.69 | 0.65 |
| 2279 | Train 36 | Test Female 39 | 0.73 | 0.76 | 0.75 | 3303 | Train 52 | Test Female 39 | 0.72 | 0.71 | 0.68 |
| 2280 | Train 36 | Test Female 40 | 0.73 | 0.73 | 0.69 | 3304 | Train 52 | Test Female 40 | 0.71 | 0.69 | 0.65 |
| 2281 | Train 36 | Test Female 41 | 0.76 | 0.83 | 0.82 | 3305 | Train 52 | Test Female 41 | 0.75 | 0.73 | 0.7 |
| 2282 | Train 36 | Test Female 42 | 0.78 | 0.84 | 0.84 | 3306 | Train 52 | Test Female 42 | 0.74 | 0.72 | 0.68 |
| 2283 | Train 36 | Test Female 43 | 0.77 | 0.83 | 0.82 | 3307 | Train 52 | Test Female 43 | 0.75 | 0.74 | 0.7 |
| 2284 | Train 36 | Test Female 44 | 0.78 | 0.84 | 0.84 | 3308 | Train 52 | Test Female 44 | 0.74 | 0.72 | 0.68 |
| 2285 | Train 36 | Test Female 45 | 0.73 | 0.75 | 0.7 | 3309 | Train 52 | Test Female 45 | 0.72 | 0.71 | 0.67 |
| 2286 | Train 36 | Test Female 46 | 0.73 | 0.73 | 0.69 | 3310 | Train 52 | Test Female 46 | 0.71 | 0.7 | 0.65 |
| 2287 | Train 36 | Test Female 47 | 0.73 | 0.76 | 0.73 | 3311 | Train 52 | Test Female 47 | 0.72 | 0.71 | 0.67 |
| 2288 | Train 36 | Test Female 48 | 0.73 | 0.73 | 0.69 | 3312 | Train 52 | Test Female 48 | 0.71 | 0.7 | 0.65 |
| 2289 | Train 36 | Test Female 49 | 0.78 | 0.84 | 0.86 | 3313 | Train 52 | Test Female 49 | 0.8 | 0.87 | 0.91 |
| 2290 | Train 36 | Test Female 50 | 0.78 | 0.84 | 0.86 | 3314 | Train 52 | Test Female 50 | 0.8 | 0.87 | 0.91 |
| 2291 | Train 36 | Test Female 51 | 0.78 | 0.84 | 0.86 | 3315 | Train 52 | Test Female 51 | 0.8 | 0.87 | 0.91 |
| 2292 | Train 36 | Test Female 52 | 0.78 | 0.84 | 0.86 | 3316 | Train 52 | Test Female 52 | 0.8 | 0.87 | 0.91 |
| 2293 | Train 36 | Test Female 53 | 0.74 | 0.78 | 0.73 | 3317 | Train 52 | Test Female 53 | 0.74 | 0.75 | 0.76 |
| 2294 | Train 36 | Test Female 54 | 0.73 | 0.74 | 0.69 | 3318 | Train 52 | Test Female 54 | 0.72 | 0.71 | 0.68 |
| 2295 | Train 36 | Test Female 55 | 0.75 | 0.78 | 0.73 | 3319 | Train 52 | Test Female 55 | 0.74 | 0.76 | 0.76 |
| 2296 | Train 36 | Test Female 56 | 0.73 | 0.73 | 0.69 | 3320 | Train 52 | Test Female 56 | 0.73 | 0.71 | 0.68 |
| 2297 | Train 36 | Test Female 57 | 0.78 | 0.83 | 0.83 | 3321 | Train 52 | Test Female 57 | 0.8 | 0.85 | 0.85 |
| 2298 | Train 36 | Test Female 58 | 0.78 | 0.83 | 0.83 | 3322 | Train 52 | Test Female 58 | 0.8 | 0.85 | 0.85 |
| 2299 | Train 36 | Test Female 59 | 0.79 | 0.83 | 0.83 | 3323 | Train 52 | Test Female 59 | 0.8 | 0.86 | 0.85 |
| 2300 | Train 36 | Test Female 60 | 0.79 | 0.83 | 0.83 | 3324 | Train 52 | Test Female 60 | 0.8 | 0.86 | 0.85 |
| 2301 | Train 36 | Test Female 61 | 0.75 | 0.78 | 0.73 | 3325 | Train 52 | Test Female 61 | 0.74 | 0.75 | 0.75 |
| 2302 | Train 36 | Test Female 62 | 0.74 | 0.74 | 0.69 | 3326 | Train 52 | Test Female 62 | 0.73 | 0.71 | 0.68 |
| 2303 | Train 36 | Test Female 63 | 0.75 | 0.78 | 0.73 | 3327 | Train 52 | Test Female 63 | 0.74 | 0.76 | 0.76 |
| 2304 | Train 36 | Test Female 64 | 0.73 | 0.73 | 0.69 | 3328 | Train 52 | Test Female 64 | 0.73 | 0.71 | 0.68 |
| 2305 | Train 37 | Test Female 1 | 0.75 | 0.81 | 0.83 | 3329 | Train 53 | Test Female 1 | 0.74 | 0.76 | 0.69 |
| 2306 | Train 37 | Test Female 2 | 0.76 | 0.8 | 0.8 | 3330 | Train 53 | Test Female 2 | 0.74 | 0.74 | 0.67 |
| 2307 | Train 37 | Test Female 3 | 0.76 | 0.81 | 0.83 | 3331 | Train 53 | Test Female 3 | 0.74 | 0.76 | 0.69 |
| 2308 | Train 37 | Test Female 4 | 0.76 | 0.8 | 0.8 | 3332 | Train 53 | Test Female 4 | 0.74 | 0.74 | 0.67 |
| 2309 | Train 37 | Test Female 5 | 0.75 | 0.82 | 0.83 | 3333 | Train 53 | Test Female 5 | 0.73 | 0.75 | 0.7 |
| 2310 | Train 37 | Test Female 6 | 0.72 | 0.73 | 0.71 | 3334 | Train 53 | Test Female 6 | 0.72 | 0.71 | 0.65 |
| 2311 | Train 37 | Test Female 7 | 0.75 | 0.81 | 0.83 | 3335 | Train 53 | Test Female 7 | 0.73 | 0.75 | 0.7 |
| 2312 | Train 37 | Test Female 8 | 0.72 | 0.73 | 0.71 | 3336 | Train 53 | Test Female 8 | 0.72 | 0.71 | 0.65 |
| 2313 | Train 37 | Test Female 9 | 0.76 | 0.81 | 0.81 | 3337 | Train 53 | Test Female 9 | 0.74 | 0.76 | 0.69 |
| 2314 | Train 37 | Test Female 10 | 0.76 | 0.8 | 0.79 | 3338 | Train 53 | Test Female 10 | 0.75 | 0.75 | 0.67 |
| 2315 | Train 37 | Test Female 11 | 0.76 | 0.81 | 0.81 | 3339 | Train 53 | Test Female 11 | 0.74 | 0.76 | 0.69 |
| 2316 | Train 37 | Test Female 12 | 0.76 | 0.8 | 0.79 | 3340 | Train 53 | Test Female 12 | 0.75 | 0.75 | 0.67 |
| 2317 | Train 37 | Test Female 13 | 0.75 | 0.79 | 0.77 | 3341 | Train 53 | Test Female 13 | 0.73 | 0.76 | 0.71 |
| 2318 | Train 37 | Test Female 14 | 0.72 | 0.73 | 0.71 | 3342 | Train 53 | Test Female 14 | 0.72 | 0.71 | 0.65 |
| 2319 | Train 37 | Test Female 15 | 0.75 | 0.8 | 0.8 | 3343 | Train 53 | Test Female 15 | 0.73 | 0.75 | 0.7 |
| 2320 | Train 37 | Test Female 16 | 0.72 | 0.73 | 0.71 | 3344 | Train 53 | Test Female 16 | 0.72 | 0.71 | 0.65 |
| 2321 | Train 37 | Test Female 17 | 0.76 | 0.82 | 0.83 | 3345 | Train 53 | Test Female 17 | 0.74 | 0.76 | 0.69 |
| 2322 | Train 37 | Test Female 18 | 0.76 | 0.8 | 0.8 | 3346 | Train 53 | Test Female 18 | 0.75 | 0.74 | 0.67 |
| 2323 | Train 37 | Test Female 19 | 0.76 | 0.81 | 0.83 | 3347 | Train 53 | Test Female 19 | 0.74 | 0.76 | 0.69 |
| 2324 | Train 37 | Test Female 20 | 0.76 | 0.8 | 0.8 | 3348 | Train 53 | Test Female 20 | 0.74 | 0.74 | 0.67 |
| 2325 | Train 37 | Test Female 21 | 0.75 | 0.82 | 0.83 | 3349 | Train 53 | Test Female 21 | 0.73 | 0.76 | 0.7 |
| 2326 | Train 37 | Test Female 22 | 0.72 | 0.73 | 0.71 | 3350 | Train 53 | Test Female 22 | 0.72 | 0.71 | 0.65 |
| 2327 | Train 37 | Test Female 23 | 0.75 | 0.81 | 0.83 | 3351 | Train 53 | Test Female 23 | 0.73 | 0.75 | 0.7 |
| 2328 | Train 37 | Test Female 24 | 0.72 | 0.73 | 0.71 | 3352 | Train 53 | Test Female 24 | 0.72 | 0.71 | 0.65 |
| 2329 | Train 37 | Test Female 25 | 0.76 | 0.81 | 0.81 | 3353 | Train 53 | Test Female 25 | 0.74 | 0.76 | 0.69 |
| 2330 | Train 37 | Test Female 26 | 0.76 | 0.8 | 0.79 | 3354 | Train 53 | Test Female 26 | 0.75 | 0.75 | 0.67 |
| 2331 | Train 37 | Test Female 27 | 0.76 | 0.81 | 0.81 | 3355 | Train 53 | Test Female 27 | 0.74 | 0.76 | 0.69 |
| 2332 | Train 37 | Test Female 28 | 0.76 | 0.8 | 0.79 | 3356 | Train 53 | Test Female 28 | 0.75 | 0.75 | 0.67 |
| 2333 | Train 37 | Test Female 29 | 0.75 | 0.79 | 0.77 | 3357 | Train 53 | Test Female 29 | 0.73 | 0.76 | 0.71 |
| 2334 | Train 37 | Test Female 30 | 0.72 | 0.73 | 0.71 | 3358 | Train 53 | Test Female 30 | 0.72 | 0.71 | 0.65 |
| 2335 | Train 37 | Test Female 31 | 0.75 | 0.8 | 0.8 | 3359 | Train 53 | Test Female 31 | 0.73 | 0.75 | 0.7 |
| 2336 | Train 37 | Test Female 32 | 0.72 | 0.73 | 0.71 | 3360 | Train 53 | Test Female 32 | 0.72 | 0.71 | 0.65 |
| 2337 | Train 37 | Test Female 33 | 0.76 | 0.82 | 0.83 | 3361 | Train 53 | Test Female 33 | 0.75 | 0.77 | 0.7 |
| 2338 | Train 37 | Test Female 34 | 0.77 | 0.81 | 0.81 | 3362 | Train 53 | Test Female 34 | 0.75 | 0.75 | 0.67 |
| 2339 | Train 37 | Test Female 35 | 0.76 | 0.82 | 0.83 | 3363 | Train 53 | Test Female 35 | 0.75 | 0.77 | 0.7 |
| 2340 | Train 37 | Test Female 36 | 0.77 | 0.81 | 0.81 | 3364 | Train 53 | Test Female 36 | 0.75 | 0.75 | 0.67 |
| 2341 | Train 37 | Test Female 37 | 0.75 | 0.82 | 0.83 | 3365 | Train 53 | Test Female 37 | 0.73 | 0.76 | 0.7 |
| 2342 | Train 37 | Test Female 38 | 0.72 | 0.73 | 0.71 | 3366 | Train 53 | Test Female 38 | 0.72 | 0.71 | 0.65 |
| 2343 | Train 37 | Test Female 39 | 0.75 | 0.82 | 0.83 | 3367 | Train 53 | Test Female 39 | 0.73 | 0.76 | 0.7 |
| 2344 | Train 37 | Test Female 40 | 0.72 | 0.73 | 0.71 | 3368 | Train 53 | Test Female 40 | 0.72 | 0.71 | 0.65 |
| 2345 | Train 37 | Test Female 41 | 0.76 | 0.81 | 0.81 | 3369 | Train 53 | Test Female 41 | 0.75 | 0.77 | 0.7 |
| 2346 | Train 37 | Test Female 42 | 0.77 | 0.8 | 0.79 | 3370 | Train 53 | Test Female 42 | 0.75 | 0.75 | 0.68 |
| 2347 | Train 37 | Test Female 43 | 0.76 | 0.81 | 0.81 | 3371 | Train 53 | Test Female 43 | 0.75 | 0.77 | 0.7 |
| 2348 | Train 37 | Test Female 44 | 0.77 | 0.8 | 0.79 | 3372 | Train 53 | Test Female 44 | 0.75 | 0.75 | 0.68 |
| 2349 | Train 37 | Test Female 45 | 0.75 | 0.79 | 0.77 | 3373 | Train 53 | Test Female 45 | 0.73 | 0.76 | 0.71 |
| 2350 | Train 37 | Test Female 46 | 0.72 | 0.73 | 0.71 | 3374 | Train 53 | Test Female 46 | 0.72 | 0.71 | 0.65 |
| 2351 | Train 37 | Test Female 47 | 0.75 | 0.81 | 0.81 | 3375 | Train 53 | Test Female 47 | 0.73 | 0.76 | 0.7 |
| 2352 | Train 37 | Test Female 48 | 0.72 | 0.73 | 0.71 | 3376 | Train 53 | Test Female 48 | 0.72 | 0.71 | 0.65 |
| 2353 | Train 37 | Test Female 49 | 0.78 | 0.83 | 0.84 | 3377 | Train 53 | Test Female 49 | 0.79 | 0.84 | 0.83 |
| 2354 | Train 37 | Test Female 50 | 0.78 | 0.83 | 0.84 | 3378 | Train 53 | Test Female 50 | 0.79 | 0.84 | 0.83 |
| 2355 | Train 37 | Test Female 51 | 0.78 | 0.83 | 0.84 | 3379 | Train 53 | Test Female 51 | 0.79 | 0.84 | 0.82 |
| 2356 | Train 37 | Test Female 52 | 0.78 | 0.83 | 0.84 | 3380 | Train 53 | Test Female 52 | 0.79 | 0.83 | 0.82 |
| 2357 | Train 37 | Test Female 53 | 0.77 | 0.82 | 0.83 | 3381 | Train 53 | Test Female 53 | 0.78 | 0.85 | 0.86 |
| 2358 | Train 37 | Test Female 54 | 0.74 | 0.74 | 0.71 | 3382 | Train 53 | Test Female 54 | 0.74 | 0.74 | 0.69 |
| 2359 | Train 37 | Test Female 55 | 0.77 | 0.82 | 0.83 | 3383 | Train 53 | Test Female 55 | 0.78 | 0.84 | 0.85 |
| 2360 | Train 37 | Test Female 56 | 0.73 | 0.74 | 0.72 | 3384 | Train 53 | Test Female 56 | 0.74 | 0.74 | 0.68 |
| 2361 | Train 37 | Test Female 57 | 0.78 | 0.82 | 0.82 | 3385 | Train 53 | Test Female 57 | 0.79 | 0.84 | 0.83 |
| 2362 | Train 37 | Test Female 58 | 0.78 | 0.82 | 0.82 | 3386 | Train 53 | Test Female 58 | 0.79 | 0.84 | 0.83 |
| 2363 | Train 37 | Test Female 59 | 0.78 | 0.82 | 0.82 | 3387 | Train 53 | Test Female 59 | 0.79 | 0.83 | 0.82 |
| 2364 | Train 37 | Test Female 60 | 0.78 | 0.82 | 0.82 | 3388 | Train 53 | Test Female 60 | 0.79 | 0.83 | 0.82 |
| 2365 | Train 37 | Test Female 61 | 0.77 | 0.82 | 0.82 | 3389 | Train 53 | Test Female 61 | 0.78 | 0.84 | 0.83 |
| 2366 | Train 37 | Test Female 62 | 0.74 | 0.74 | 0.72 | 3390 | Train 53 | Test Female 62 | 0.74 | 0.74 | 0.69 |
| 2367 | Train 37 | Test Female 63 | 0.77 | 0.82 | 0.82 | 3391 | Train 53 | Test Female 63 | 0.78 | 0.84 | 0.83 |
| 2368 | Train 37 | Test Female 64 | 0.73 | 0.74 | 0.72 | 3392 | Train 53 | Test Female 64 | 0.74 | 0.74 | 0.69 |
| 2369 | Train 38 | Test Female 1 | 0.74 | 0.78 | 0.77 | 3393 | Train 54 | Test Female 1 | 0.73 | 0.74 | 0.67 |
| 2370 | Train 38 | Test Female 2 | 0.76 | 0.79 | 0.78 | 3394 | Train 54 | Test Female 2 | 0.74 | 0.75 | 0.69 |
| 2371 | Train 38 | Test Female 3 | 0.74 | 0.78 | 0.77 | 3395 | Train 54 | Test Female 3 | 0.73 | 0.74 | 0.67 |
| 2372 | Train 38 | Test Female 4 | 0.76 | 0.79 | 0.78 | 3396 | Train 54 | Test Female 4 | 0.74 | 0.75 | 0.69 |
| 2373 | Train 38 | Test Female 5 | 0.73 | 0.78 | 0.76 | 3397 | Train 54 | Test Female 5 | 0.72 | 0.74 | 0.69 |
| 2374 | Train 38 | Test Female 6 | 0.75 | 0.8 | 0.8 | 3398 | Train 54 | Test Female 6 | 0.74 | 0.77 | 0.72 |
| 2375 | Train 38 | Test Female 7 | 0.73 | 0.78 | 0.76 | 3399 | Train 54 | Test Female 7 | 0.72 | 0.74 | 0.69 |
| 2376 | Train 38 | Test Female 8 | 0.75 | 0.79 | 0.8 | 3400 | Train 54 | Test Female 8 | 0.74 | 0.76 | 0.72 |
| 2377 | Train 38 | Test Female 9 | 0.74 | 0.79 | 0.78 | 3401 | Train 54 | Test Female 9 | 0.73 | 0.75 | 0.68 |
| 2378 | Train 38 | Test Female 10 | 0.76 | 0.79 | 0.79 | 3402 | Train 54 | Test Female 10 | 0.74 | 0.76 | 0.7 |
| 2379 | Train 38 | Test Female 11 | 0.74 | 0.79 | 0.78 | 3403 | Train 54 | Test Female 11 | 0.73 | 0.75 | 0.68 |
| 2380 | Train 38 | Test Female 12 | 0.76 | 0.79 | 0.79 | 3404 | Train 54 | Test Female 12 | 0.74 | 0.76 | 0.7 |
| 2381 | Train 38 | Test Female 13 | 0.73 | 0.79 | 0.8 | 3405 | Train 54 | Test Female 13 | 0.72 | 0.75 | 0.69 |
| 2382 | Train 38 | Test Female 14 | 0.75 | 0.8 | 0.8 | 3406 | Train 54 | Test Female 14 | 0.74 | 0.77 | 0.72 |
| 2383 | Train 38 | Test Female 15 | 0.73 | 0.78 | 0.77 | 3407 | Train 54 | Test Female 15 | 0.72 | 0.74 | 0.7 |
| 2384 | Train 38 | Test Female 16 | 0.75 | 0.79 | 0.8 | 3408 | Train 54 | Test Female 16 | 0.74 | 0.76 | 0.72 |
| 2385 | Train 38 | Test Female 17 | 0.74 | 0.78 | 0.77 | 3409 | Train 54 | Test Female 17 | 0.73 | 0.74 | 0.67 |
| 2386 | Train 38 | Test Female 18 | 0.76 | 0.79 | 0.78 | 3410 | Train 54 | Test Female 18 | 0.74 | 0.75 | 0.69 |
| 2387 | Train 38 | Test Female 19 | 0.74 | 0.78 | 0.77 | 3411 | Train 54 | Test Female 19 | 0.73 | 0.74 | 0.67 |
| 2388 | Train 38 | Test Female 20 | 0.76 | 0.79 | 0.78 | 3412 | Train 54 | Test Female 20 | 0.74 | 0.75 | 0.69 |
| 2389 | Train 38 | Test Female 21 | 0.73 | 0.78 | 0.76 | 3413 | Train 54 | Test Female 21 | 0.72 | 0.75 | 0.69 |
| 2390 | Train 38 | Test Female 22 | 0.75 | 0.8 | 0.8 | 3414 | Train 54 | Test Female 22 | 0.74 | 0.77 | 0.72 |
| 2391 | Train 38 | Test Female 23 | 0.73 | 0.78 | 0.76 | 3415 | Train 54 | Test Female 23 | 0.72 | 0.74 | 0.69 |
| 2392 | Train 38 | Test Female 24 | 0.75 | 0.79 | 0.8 | 3416 | Train 54 | Test Female 24 | 0.74 | 0.76 | 0.72 |
| 2393 | Train 38 | Test Female 25 | 0.74 | 0.79 | 0.78 | 3417 | Train 54 | Test Female 25 | 0.73 | 0.75 | 0.68 |
| 2394 | Train 38 | Test Female 26 | 0.76 | 0.79 | 0.79 | 3418 | Train 54 | Test Female 26 | 0.74 | 0.76 | 0.7 |
| 2395 | Train 38 | Test Female 27 | 0.74 | 0.79 | 0.78 | 3419 | Train 54 | Test Female 27 | 0.73 | 0.75 | 0.68 |
| 2396 | Train 38 | Test Female 28 | 0.76 | 0.79 | 0.79 | 3420 | Train 54 | Test Female 28 | 0.74 | 0.76 | 0.7 |
| 2397 | Train 38 | Test Female 29 | 0.73 | 0.79 | 0.8 | 3421 | Train 54 | Test Female 29 | 0.72 | 0.75 | 0.69 |
| 2398 | Train 38 | Test Female 30 | 0.75 | 0.8 | 0.8 | 3422 | Train 54 | Test Female 30 | 0.74 | 0.77 | 0.72 |
| 2399 | Train 38 | Test Female 31 | 0.73 | 0.78 | 0.77 | 3423 | Train 54 | Test Female 31 | 0.72 | 0.75 | 0.7 |
| 2400 | Train 38 | Test Female 32 | 0.75 | 0.79 | 0.8 | 3424 | Train 54 | Test Female 32 | 0.74 | 0.76 | 0.72 |
| 2401 | Train 38 | Test Female 33 | 0.75 | 0.79 | 0.78 | 3425 | Train 54 | Test Female 33 | 0.73 | 0.75 | 0.67 |
| 2402 | Train 38 | Test Female 34 | 0.77 | 0.8 | 0.79 | 3426 | Train 54 | Test Female 34 | 0.75 | 0.76 | 0.69 |
| 2403 | Train 38 | Test Female 35 | 0.75 | 0.79 | 0.78 | 3427 | Train 54 | Test Female 35 | 0.73 | 0.75 | 0.68 |
| 2404 | Train 38 | Test Female 36 | 0.77 | 0.8 | 0.79 | 3428 | Train 54 | Test Female 36 | 0.75 | 0.76 | 0.69 |
| 2405 | Train 38 | Test Female 37 | 0.74 | 0.78 | 0.77 | 3429 | Train 54 | Test Female 37 | 0.72 | 0.75 | 0.7 |
| 2406 | Train 38 | Test Female 38 | 0.75 | 0.8 | 0.81 | 3430 | Train 54 | Test Female 38 | 0.74 | 0.77 | 0.72 |
| 2407 | Train 38 | Test Female 39 | 0.74 | 0.78 | 0.77 | 3431 | Train 54 | Test Female 39 | 0.72 | 0.75 | 0.69 |
| 2408 | Train 38 | Test Female 40 | 0.75 | 0.79 | 0.8 | 3432 | Train 54 | Test Female 40 | 0.74 | 0.77 | 0.73 |
| 2409 | Train 38 | Test Female 41 | 0.75 | 0.79 | 0.79 | 3433 | Train 54 | Test Female 41 | 0.73 | 0.75 | 0.69 |
| 2410 | Train 38 | Test Female 42 | 0.77 | 0.8 | 0.8 | 3434 | Train 54 | Test Female 42 | 0.75 | 0.76 | 0.7 |
| 2411 | Train 38 | Test Female 43 | 0.75 | 0.79 | 0.79 | 3435 | Train 54 | Test Female 43 | 0.74 | 0.75 | 0.69 |
| 2412 | Train 38 | Test Female 44 | 0.77 | 0.8 | 0.79 | 3436 | Train 54 | Test Female 44 | 0.75 | 0.76 | 0.7 |
| 2413 | Train 38 | Test Female 45 | 0.74 | 0.79 | 0.8 | 3437 | Train 54 | Test Female 45 | 0.72 | 0.75 | 0.69 |
| 2414 | Train 38 | Test Female 46 | 0.76 | 0.8 | 0.81 | 3438 | Train 54 | Test Female 46 | 0.74 | 0.77 | 0.73 |
| 2415 | Train 38 | Test Female 47 | 0.74 | 0.78 | 0.77 | 3439 | Train 54 | Test Female 47 | 0.72 | 0.75 | 0.7 |
| 2416 | Train 38 | Test Female 48 | 0.75 | 0.8 | 0.8 | 3440 | Train 54 | Test Female 48 | 0.74 | 0.77 | 0.73 |
| 2417 | Train 38 | Test Female 49 | 0.78 | 0.81 | 0.8 | 3441 | Train 54 | Test Female 49 | 0.78 | 0.81 | 0.8 |
| 2418 | Train 38 | Test Female 50 | 0.78 | 0.81 | 0.8 | 3442 | Train 54 | Test Female 50 | 0.78 | 0.81 | 0.8 |
| 2419 | Train 38 | Test Female 51 | 0.78 | 0.81 | 0.8 | 3443 | Train 54 | Test Female 51 | 0.78 | 0.81 | 0.8 |
| 2420 | Train 38 | Test Female 52 | 0.78 | 0.81 | 0.8 | 3444 | Train 54 | Test Female 52 | 0.78 | 0.81 | 0.8 |
| 2421 | Train 38 | Test Female 53 | 0.76 | 0.81 | 0.79 | 3445 | Train 54 | Test Female 53 | 0.77 | 0.82 | 0.81 |
| 2422 | Train 38 | Test Female 54 | 0.76 | 0.8 | 0.8 | 3446 | Train 54 | Test Female 54 | 0.77 | 0.82 | 0.82 |
| 2423 | Train 38 | Test Female 55 | 0.77 | 0.81 | 0.79 | 3447 | Train 54 | Test Female 55 | 0.77 | 0.82 | 0.81 |
| 2424 | Train 38 | Test Female 56 | 0.76 | 0.79 | 0.8 | 3448 | Train 54 | Test Female 56 | 0.76 | 0.81 | 0.82 |
| 2425 | Train 38 | Test Female 57 | 0.78 | 0.81 | 0.81 | 3449 | Train 54 | Test Female 57 | 0.79 | 0.82 | 0.82 |
| 2426 | Train 38 | Test Female 58 | 0.78 | 0.81 | 0.81 | 3450 | Train 54 | Test Female 58 | 0.78 | 0.82 | 0.82 |
| 2427 | Train 38 | Test Female 59 | 0.78 | 0.81 | 0.81 | 3451 | Train 54 | Test Female 59 | 0.79 | 0.82 | 0.82 |
| 2428 | Train 38 | Test Female 60 | 0.78 | 0.81 | 0.81 | 3452 | Train 54 | Test Female 60 | 0.79 | 0.82 | 0.82 |
| 2429 | Train 38 | Test Female 61 | 0.77 | 0.81 | 0.8 | 3453 | Train 54 | Test Female 61 | 0.77 | 0.82 | 0.82 |
| 2430 | Train 38 | Test Female 62 | 0.76 | 0.8 | 0.8 | 3454 | Train 54 | Test Female 62 | 0.77 | 0.82 | 0.82 |
| 2431 | Train 38 | Test Female 63 | 0.77 | 0.81 | 0.8 | 3455 | Train 54 | Test Female 63 | 0.77 | 0.82 | 0.81 |
| 2432 | Train 38 | Test Female 64 | 0.76 | 0.79 | 0.8 | 3456 | Train 54 | Test Female 64 | 0.76 | 0.81 | 0.82 |
| 2433 | Train 39 | Test Female 1 | 0.75 | 0.81 | 0.83 | 3457 | Train 55 | Test Female 1 | 0.74 | 0.77 | 0.73 |
| 2434 | Train 39 | Test Female 2 | 0.76 | 0.8 | 0.8 | 3458 | Train 55 | Test Female 2 | 0.75 | 0.75 | 0.71 |
| 2435 | Train 39 | Test Female 3 | 0.76 | 0.81 | 0.83 | 3459 | Train 55 | Test Female 3 | 0.74 | 0.77 | 0.73 |
| 2436 | Train 39 | Test Female 4 | 0.76 | 0.8 | 0.8 | 3460 | Train 55 | Test Female 4 | 0.74 | 0.75 | 0.71 |
| 2437 | Train 39 | Test Female 5 | 0.75 | 0.82 | 0.83 | 3461 | Train 55 | Test Female 5 | 0.73 | 0.76 | 0.72 |
| 2438 | Train 39 | Test Female 6 | 0.72 | 0.73 | 0.7 | 3462 | Train 55 | Test Female 6 | 0.71 | 0.71 | 0.64 |
| 2439 | Train 39 | Test Female 7 | 0.75 | 0.81 | 0.83 | 3463 | Train 55 | Test Female 7 | 0.73 | 0.76 | 0.72 |
| 2440 | Train 39 | Test Female 8 | 0.72 | 0.73 | 0.71 | 3464 | Train 55 | Test Female 8 | 0.71 | 0.71 | 0.65 |
| 2441 | Train 39 | Test Female 9 | 0.76 | 0.81 | 0.81 | 3465 | Train 55 | Test Female 9 | 0.74 | 0.77 | 0.73 |
| 2442 | Train 39 | Test Female 10 | 0.76 | 0.8 | 0.79 | 3466 | Train 55 | Test Female 10 | 0.75 | 0.75 | 0.71 |
| 2443 | Train 39 | Test Female 11 | 0.76 | 0.81 | 0.81 | 3467 | Train 55 | Test Female 11 | 0.74 | 0.77 | 0.73 |
| 2444 | Train 39 | Test Female 12 | 0.76 | 0.8 | 0.79 | 3468 | Train 55 | Test Female 12 | 0.75 | 0.76 | 0.71 |
| 2445 | Train 39 | Test Female 13 | 0.75 | 0.79 | 0.76 | 3469 | Train 55 | Test Female 13 | 0.73 | 0.75 | 0.66 |
| 2446 | Train 39 | Test Female 14 | 0.72 | 0.73 | 0.7 | 3470 | Train 55 | Test Female 14 | 0.72 | 0.71 | 0.64 |
| 2447 | Train 39 | Test Female 15 | 0.75 | 0.81 | 0.81 | 3471 | Train 55 | Test Female 15 | 0.73 | 0.76 | 0.72 |
| 2448 | Train 39 | Test Female 16 | 0.72 | 0.73 | 0.71 | 3472 | Train 55 | Test Female 16 | 0.72 | 0.71 | 0.65 |
| 2449 | Train 39 | Test Female 17 | 0.76 | 0.81 | 0.83 | 3473 | Train 55 | Test Female 17 | 0.74 | 0.77 | 0.73 |
| 2450 | Train 39 | Test Female 18 | 0.76 | 0.8 | 0.8 | 3474 | Train 55 | Test Female 18 | 0.75 | 0.75 | 0.71 |
| 2451 | Train 39 | Test Female 19 | 0.76 | 0.81 | 0.83 | 3475 | Train 55 | Test Female 19 | 0.74 | 0.77 | 0.73 |
| 2452 | Train 39 | Test Female 20 | 0.76 | 0.8 | 0.8 | 3476 | Train 55 | Test Female 20 | 0.75 | 0.75 | 0.71 |
| 2453 | Train 39 | Test Female 21 | 0.75 | 0.82 | 0.83 | 3477 | Train 55 | Test Female 21 | 0.73 | 0.76 | 0.72 |
| 2454 | Train 39 | Test Female 22 | 0.72 | 0.73 | 0.7 | 3478 | Train 55 | Test Female 22 | 0.71 | 0.71 | 0.64 |
| 2455 | Train 39 | Test Female 23 | 0.75 | 0.81 | 0.83 | 3479 | Train 55 | Test Female 23 | 0.73 | 0.76 | 0.72 |
| 2456 | Train 39 | Test Female 24 | 0.72 | 0.73 | 0.71 | 3480 | Train 55 | Test Female 24 | 0.71 | 0.71 | 0.65 |
| 2457 | Train 39 | Test Female 25 | 0.76 | 0.81 | 0.81 | 3481 | Train 55 | Test Female 25 | 0.74 | 0.77 | 0.73 |
| 2458 | Train 39 | Test Female 26 | 0.76 | 0.8 | 0.79 | 3482 | Train 55 | Test Female 26 | 0.75 | 0.75 | 0.71 |
| 2459 | Train 39 | Test Female 27 | 0.76 | 0.81 | 0.81 | 3483 | Train 55 | Test Female 27 | 0.74 | 0.77 | 0.73 |
| 2460 | Train 39 | Test Female 28 | 0.76 | 0.8 | 0.79 | 3484 | Train 55 | Test Female 28 | 0.75 | 0.76 | 0.71 |
| 2461 | Train 39 | Test Female 29 | 0.75 | 0.79 | 0.76 | 3485 | Train 55 | Test Female 29 | 0.73 | 0.75 | 0.66 |
| 2462 | Train 39 | Test Female 30 | 0.72 | 0.73 | 0.71 | 3486 | Train 55 | Test Female 30 | 0.72 | 0.71 | 0.64 |
| 2463 | Train 39 | Test Female 31 | 0.75 | 0.81 | 0.81 | 3487 | Train 55 | Test Female 31 | 0.73 | 0.76 | 0.72 |
| 2464 | Train 39 | Test Female 32 | 0.72 | 0.73 | 0.71 | 3488 | Train 55 | Test Female 32 | 0.72 | 0.71 | 0.65 |
| 2465 | Train 39 | Test Female 33 | 0.76 | 0.82 | 0.83 | 3489 | Train 55 | Test Female 33 | 0.75 | 0.78 | 0.74 |
| 2466 | Train 39 | Test Female 34 | 0.77 | 0.81 | 0.81 | 3490 | Train 55 | Test Female 34 | 0.75 | 0.76 | 0.71 |
| 2467 | Train 39 | Test Female 35 | 0.76 | 0.82 | 0.83 | 3491 | Train 55 | Test Female 35 | 0.75 | 0.78 | 0.74 |
| 2468 | Train 39 | Test Female 36 | 0.77 | 0.81 | 0.81 | 3492 | Train 55 | Test Female 36 | 0.75 | 0.76 | 0.72 |
| 2469 | Train 39 | Test Female 37 | 0.75 | 0.82 | 0.83 | 3493 | Train 55 | Test Female 37 | 0.73 | 0.76 | 0.73 |
| 2470 | Train 39 | Test Female 38 | 0.72 | 0.73 | 0.71 | 3494 | Train 55 | Test Female 38 | 0.72 | 0.71 | 0.65 |
| 2471 | Train 39 | Test Female 39 | 0.75 | 0.82 | 0.83 | 3495 | Train 55 | Test Female 39 | 0.73 | 0.76 | 0.72 |
| 2472 | Train 39 | Test Female 40 | 0.72 | 0.73 | 0.71 | 3496 | Train 55 | Test Female 40 | 0.72 | 0.71 | 0.65 |
| 2473 | Train 39 | Test Female 41 | 0.76 | 0.81 | 0.81 | 3497 | Train 55 | Test Female 41 | 0.75 | 0.78 | 0.74 |
| 2474 | Train 39 | Test Female 42 | 0.77 | 0.8 | 0.79 | 3498 | Train 55 | Test Female 42 | 0.75 | 0.76 | 0.71 |
| 2475 | Train 39 | Test Female 43 | 0.76 | 0.81 | 0.81 | 3499 | Train 55 | Test Female 43 | 0.75 | 0.78 | 0.74 |
| 2476 | Train 39 | Test Female 44 | 0.77 | 0.8 | 0.79 | 3500 | Train 55 | Test Female 44 | 0.75 | 0.76 | 0.72 |
| 2477 | Train 39 | Test Female 45 | 0.75 | 0.79 | 0.77 | 3501 | Train 55 | Test Female 45 | 0.73 | 0.76 | 0.67 |
| 2478 | Train 39 | Test Female 46 | 0.72 | 0.73 | 0.71 | 3502 | Train 55 | Test Female 46 | 0.72 | 0.71 | 0.65 |
| 2479 | Train 39 | Test Female 47 | 0.75 | 0.81 | 0.81 | 3503 | Train 55 | Test Female 47 | 0.73 | 0.76 | 0.73 |
| 2480 | Train 39 | Test Female 48 | 0.72 | 0.73 | 0.71 | 3504 | Train 55 | Test Female 48 | 0.72 | 0.71 | 0.65 |
| 2481 | Train 39 | Test Female 49 | 0.78 | 0.83 | 0.84 | 3505 | Train 55 | Test Female 49 | 0.79 | 0.84 | 0.85 |
| 2482 | Train 39 | Test Female 50 | 0.78 | 0.83 | 0.84 | 3506 | Train 55 | Test Female 50 | 0.79 | 0.84 | 0.85 |
| 2483 | Train 39 | Test Female 51 | 0.78 | 0.83 | 0.85 | 3507 | Train 55 | Test Female 51 | 0.79 | 0.85 | 0.85 |
| 2484 | Train 39 | Test Female 52 | 0.78 | 0.83 | 0.85 | 3508 | Train 55 | Test Female 52 | 0.79 | 0.85 | 0.85 |
| 2485 | Train 39 | Test Female 53 | 0.77 | 0.82 | 0.83 | 3509 | Train 55 | Test Female 53 | 0.78 | 0.85 | 0.87 |
| 2486 | Train 39 | Test Female 54 | 0.74 | 0.74 | 0.72 | 3510 | Train 55 | Test Female 54 | 0.73 | 0.74 | 0.68 |
| 2487 | Train 39 | Test Female 55 | 0.77 | 0.82 | 0.83 | 3511 | Train 55 | Test Female 55 | 0.78 | 0.85 | 0.88 |
| 2488 | Train 39 | Test Female 56 | 0.73 | 0.74 | 0.72 | 3512 | Train 55 | Test Female 56 | 0.73 | 0.74 | 0.69 |
| 2489 | Train 39 | Test Female 57 | 0.78 | 0.82 | 0.82 | 3513 | Train 55 | Test Female 57 | 0.79 | 0.84 | 0.85 |
| 2490 | Train 39 | Test Female 58 | 0.78 | 0.82 | 0.82 | 3514 | Train 55 | Test Female 58 | 0.79 | 0.84 | 0.85 |
| 2491 | Train 39 | Test Female 59 | 0.78 | 0.82 | 0.82 | 3515 | Train 55 | Test Female 59 | 0.79 | 0.84 | 0.85 |
| 2492 | Train 39 | Test Female 60 | 0.78 | 0.82 | 0.82 | 3516 | Train 55 | Test Female 60 | 0.79 | 0.84 | 0.85 |
| 2493 | Train 39 | Test Female 61 | 0.77 | 0.82 | 0.82 | 3517 | Train 55 | Test Female 61 | 0.78 | 0.84 | 0.85 |
| 2494 | Train 39 | Test Female 62 | 0.74 | 0.74 | 0.72 | 3518 | Train 55 | Test Female 62 | 0.74 | 0.74 | 0.69 |
| 2495 | Train 39 | Test Female 63 | 0.77 | 0.82 | 0.82 | 3519 | Train 55 | Test Female 63 | 0.78 | 0.84 | 0.85 |
| 2496 | Train 39 | Test Female 64 | 0.73 | 0.74 | 0.72 | 3520 | Train 55 | Test Female 64 | 0.73 | 0.74 | 0.69 |
| 2497 | Train 40 | Test Female 1 | 0.74 | 0.78 | 0.78 | 3521 | Train 56 | Test Female 1 | 0.73 | 0.74 | 0.72 |
| 2498 | Train 40 | Test Female 2 | 0.76 | 0.79 | 0.79 | 3522 | Train 56 | Test Female 2 | 0.74 | 0.75 | 0.72 |
| 2499 | Train 40 | Test Female 3 | 0.74 | 0.78 | 0.78 | 3523 | Train 56 | Test Female 3 | 0.73 | 0.75 | 0.72 |
| 2500 | Train 40 | Test Female 4 | 0.76 | 0.79 | 0.79 | 3524 | Train 56 | Test Female 4 | 0.74 | 0.75 | 0.72 |
| 2501 | Train 40 | Test Female 5 | 0.73 | 0.78 | 0.78 | 3525 | Train 56 | Test Female 5 | 0.72 | 0.75 | 0.72 |
| 2502 | Train 40 | Test Female 6 | 0.75 | 0.8 | 0.8 | 3526 | Train 56 | Test Female 6 | 0.73 | 0.76 | 0.74 |
| 2503 | Train 40 | Test Female 7 | 0.73 | 0.78 | 0.77 | 3527 | Train 56 | Test Female 7 | 0.72 | 0.74 | 0.72 |
| 2504 | Train 40 | Test Female 8 | 0.75 | 0.8 | 0.8 | 3528 | Train 56 | Test Female 8 | 0.73 | 0.76 | 0.74 |
| 2505 | Train 40 | Test Female 9 | 0.74 | 0.78 | 0.79 | 3529 | Train 56 | Test Female 9 | 0.73 | 0.75 | 0.73 |
| 2506 | Train 40 | Test Female 10 | 0.76 | 0.79 | 0.8 | 3530 | Train 56 | Test Female 10 | 0.74 | 0.76 | 0.73 |
| 2507 | Train 40 | Test Female 11 | 0.75 | 0.79 | 0.79 | 3531 | Train 56 | Test Female 11 | 0.73 | 0.75 | 0.73 |
| 2508 | Train 40 | Test Female 12 | 0.76 | 0.79 | 0.8 | 3532 | Train 56 | Test Female 12 | 0.74 | 0.76 | 0.73 |
| 2509 | Train 40 | Test Female 13 | 0.73 | 0.79 | 0.8 | 3533 | Train 56 | Test Female 13 | 0.72 | 0.75 | 0.73 |
| 2510 | Train 40 | Test Female 14 | 0.75 | 0.8 | 0.8 | 3534 | Train 56 | Test Female 14 | 0.73 | 0.76 | 0.74 |
| 2511 | Train 40 | Test Female 15 | 0.73 | 0.78 | 0.78 | 3535 | Train 56 | Test Female 15 | 0.72 | 0.75 | 0.73 |
| 2512 | Train 40 | Test Female 16 | 0.75 | 0.8 | 0.8 | 3536 | Train 56 | Test Female 16 | 0.74 | 0.77 | 0.74 |
| 2513 | Train 40 | Test Female 17 | 0.74 | 0.78 | 0.78 | 3537 | Train 56 | Test Female 17 | 0.73 | 0.74 | 0.72 |
| 2514 | Train 40 | Test Female 18 | 0.76 | 0.79 | 0.79 | 3538 | Train 56 | Test Female 18 | 0.74 | 0.75 | 0.72 |
| 2515 | Train 40 | Test Female 19 | 0.75 | 0.78 | 0.78 | 3539 | Train 56 | Test Female 19 | 0.73 | 0.75 | 0.72 |
| 2516 | Train 40 | Test Female 20 | 0.76 | 0.79 | 0.79 | 3540 | Train 56 | Test Female 20 | 0.74 | 0.75 | 0.72 |
| 2517 | Train 40 | Test Female 21 | 0.73 | 0.78 | 0.78 | 3541 | Train 56 | Test Female 21 | 0.72 | 0.75 | 0.72 |
| 2518 | Train 40 | Test Female 22 | 0.75 | 0.8 | 0.8 | 3542 | Train 56 | Test Female 22 | 0.73 | 0.76 | 0.74 |
| 2519 | Train 40 | Test Female 23 | 0.73 | 0.78 | 0.77 | 3543 | Train 56 | Test Female 23 | 0.72 | 0.74 | 0.72 |
| 2520 | Train 40 | Test Female 24 | 0.75 | 0.8 | 0.8 | 3544 | Train 56 | Test Female 24 | 0.73 | 0.76 | 0.74 |
| 2521 | Train 40 | Test Female 25 | 0.75 | 0.79 | 0.79 | 3545 | Train 56 | Test Female 25 | 0.73 | 0.75 | 0.73 |
| 2522 | Train 40 | Test Female 26 | 0.76 | 0.79 | 0.8 | 3546 | Train 56 | Test Female 26 | 0.74 | 0.76 | 0.73 |
| 2523 | Train 40 | Test Female 27 | 0.75 | 0.79 | 0.79 | 3547 | Train 56 | Test Female 27 | 0.73 | 0.75 | 0.73 |
| 2524 | Train 40 | Test Female 28 | 0.76 | 0.79 | 0.8 | 3548 | Train 56 | Test Female 28 | 0.74 | 0.76 | 0.73 |
| 2525 | Train 40 | Test Female 29 | 0.73 | 0.79 | 0.8 | 3549 | Train 56 | Test Female 29 | 0.72 | 0.75 | 0.73 |
| 2526 | Train 40 | Test Female 30 | 0.75 | 0.8 | 0.8 | 3550 | Train 56 | Test Female 30 | 0.74 | 0.76 | 0.74 |
| 2527 | Train 40 | Test Female 31 | 0.73 | 0.78 | 0.78 | 3551 | Train 56 | Test Female 31 | 0.72 | 0.75 | 0.73 |
| 2528 | Train 40 | Test Female 32 | 0.75 | 0.8 | 0.8 | 3552 | Train 56 | Test Female 32 | 0.74 | 0.77 | 0.74 |
| 2529 | Train 40 | Test Female 33 | 0.75 | 0.79 | 0.78 | 3553 | Train 56 | Test Female 33 | 0.74 | 0.75 | 0.72 |
| 2530 | Train 40 | Test Female 34 | 0.77 | 0.8 | 0.79 | 3554 | Train 56 | Test Female 34 | 0.74 | 0.76 | 0.73 |
| 2531 | Train 40 | Test Female 35 | 0.75 | 0.79 | 0.78 | 3555 | Train 56 | Test Female 35 | 0.74 | 0.75 | 0.73 |
| 2532 | Train 40 | Test Female 36 | 0.77 | 0.8 | 0.79 | 3556 | Train 56 | Test Female 36 | 0.74 | 0.76 | 0.73 |
| 2533 | Train 40 | Test Female 37 | 0.74 | 0.78 | 0.78 | 3557 | Train 56 | Test Female 37 | 0.72 | 0.75 | 0.73 |
| 2534 | Train 40 | Test Female 38 | 0.75 | 0.8 | 0.8 | 3558 | Train 56 | Test Female 38 | 0.73 | 0.77 | 0.74 |
| 2535 | Train 40 | Test Female 39 | 0.74 | 0.78 | 0.78 | 3559 | Train 56 | Test Female 39 | 0.72 | 0.75 | 0.73 |
| 2536 | Train 40 | Test Female 40 | 0.75 | 0.8 | 0.8 | 3560 | Train 56 | Test Female 40 | 0.73 | 0.77 | 0.74 |
| 2537 | Train 40 | Test Female 41 | 0.75 | 0.79 | 0.79 | 3561 | Train 56 | Test Female 41 | 0.74 | 0.76 | 0.73 |
| 2538 | Train 40 | Test Female 42 | 0.77 | 0.8 | 0.8 | 3562 | Train 56 | Test Female 42 | 0.74 | 0.76 | 0.74 |
| 2539 | Train 40 | Test Female 43 | 0.75 | 0.79 | 0.79 | 3563 | Train 56 | Test Female 43 | 0.74 | 0.76 | 0.74 |
| 2540 | Train 40 | Test Female 44 | 0.77 | 0.8 | 0.8 | 3564 | Train 56 | Test Female 44 | 0.74 | 0.76 | 0.74 |
| 2541 | Train 40 | Test Female 45 | 0.74 | 0.79 | 0.8 | 3565 | Train 56 | Test Female 45 | 0.72 | 0.75 | 0.74 |
| 2542 | Train 40 | Test Female 46 | 0.75 | 0.8 | 0.8 | 3566 | Train 56 | Test Female 46 | 0.74 | 0.77 | 0.74 |
| 2543 | Train 40 | Test Female 47 | 0.74 | 0.78 | 0.79 | 3567 | Train 56 | Test Female 47 | 0.72 | 0.75 | 0.74 |
| 2544 | Train 40 | Test Female 48 | 0.75 | 0.8 | 0.8 | 3568 | Train 56 | Test Female 48 | 0.74 | 0.77 | 0.74 |
| 2545 | Train 40 | Test Female 49 | 0.78 | 0.81 | 0.8 | 3569 | Train 56 | Test Female 49 | 0.79 | 0.81 | 0.8 |
| 2546 | Train 40 | Test Female 50 | 0.78 | 0.81 | 0.8 | 3570 | Train 56 | Test Female 50 | 0.79 | 0.81 | 0.8 |
| 2547 | Train 40 | Test Female 51 | 0.78 | 0.81 | 0.8 | 3571 | Train 56 | Test Female 51 | 0.79 | 0.81 | 0.8 |
| 2548 | Train 40 | Test Female 52 | 0.78 | 0.81 | 0.8 | 3572 | Train 56 | Test Female 52 | 0.79 | 0.81 | 0.8 |
| 2549 | Train 40 | Test Female 53 | 0.76 | 0.81 | 0.8 | 3573 | Train 56 | Test Female 53 | 0.77 | 0.81 | 0.81 |
| 2550 | Train 40 | Test Female 54 | 0.76 | 0.8 | 0.8 | 3574 | Train 56 | Test Female 54 | 0.76 | 0.82 | 0.82 |
| 2551 | Train 40 | Test Female 55 | 0.76 | 0.81 | 0.81 | 3575 | Train 56 | Test Female 55 | 0.77 | 0.82 | 0.81 |
| 2552 | Train 40 | Test Female 56 | 0.76 | 0.8 | 0.8 | 3576 | Train 56 | Test Female 56 | 0.76 | 0.82 | 0.82 |
| 2553 | Train 40 | Test Female 57 | 0.78 | 0.81 | 0.82 | 3577 | Train 56 | Test Female 57 | 0.79 | 0.82 | 0.82 |
| 2554 | Train 40 | Test Female 58 | 0.78 | 0.81 | 0.82 | 3578 | Train 56 | Test Female 58 | 0.79 | 0.82 | 0.82 |
| 2555 | Train 40 | Test Female 59 | 0.78 | 0.81 | 0.82 | 3579 | Train 56 | Test Female 59 | 0.79 | 0.82 | 0.82 |
| 2556 | Train 40 | Test Female 60 | 0.78 | 0.81 | 0.82 | 3580 | Train 56 | Test Female 60 | 0.79 | 0.82 | 0.82 |
| 2557 | Train 40 | Test Female 61 | 0.76 | 0.81 | 0.81 | 3581 | Train 56 | Test Female 61 | 0.77 | 0.82 | 0.82 |
| 2558 | Train 40 | Test Female 62 | 0.76 | 0.8 | 0.8 | 3582 | Train 56 | Test Female 62 | 0.77 | 0.82 | 0.82 |
| 2559 | Train 40 | Test Female 63 | 0.76 | 0.81 | 0.81 | 3583 | Train 56 | Test Female 63 | 0.77 | 0.82 | 0.82 |
| 2560 | Train 40 | Test Female 64 | 0.76 | 0.8 | 0.8 | 3584 | Train 56 | Test Female 64 | 0.76 | 0.82 | 0.82 |
| 2561 | Train 41 | Test Female 1 | 0.76 | 0.83 | 0.82 | 3585 | Train 57 | Test Female 1 | 0.74 | 0.75 | 0.71 |
| 2562 | Train 41 | Test Female 2 | 0.76 | 0.8 | 0.78 | 3586 | Train 57 | Test Female 2 | 0.73 | 0.73 | 0.67 |
| 2563 | Train 41 | Test Female 3 | 0.76 | 0.83 | 0.82 | 3587 | Train 57 | Test Female 3 | 0.74 | 0.75 | 0.71 |
| 2564 | Train 41 | Test Female 4 | 0.76 | 0.8 | 0.78 | 3588 | Train 57 | Test Female 4 | 0.73 | 0.73 | 0.67 |
| 2565 | Train 41 | Test Female 5 | 0.73 | 0.76 | 0.74 | 3589 | Train 57 | Test Female 5 | 0.72 | 0.71 | 0.67 |
| 2566 | Train 41 | Test Female 6 | 0.71 | 0.72 | 0.67 | 3590 | Train 57 | Test Female 6 | 0.71 | 0.7 | 0.63 |
| 2567 | Train 41 | Test Female 7 | 0.73 | 0.75 | 0.73 | 3591 | Train 57 | Test Female 7 | 0.72 | 0.71 | 0.67 |
| 2568 | Train 41 | Test Female 8 | 0.71 | 0.72 | 0.67 | 3592 | Train 57 | Test Female 8 | 0.71 | 0.7 | 0.64 |
| 2569 | Train 41 | Test Female 9 | 0.77 | 0.84 | 0.86 | 3593 | Train 57 | Test Female 9 | 0.74 | 0.75 | 0.73 |
| 2570 | Train 41 | Test Female 10 | 0.76 | 0.81 | 0.81 | 3594 | Train 57 | Test Female 10 | 0.73 | 0.73 | 0.68 |
| 2571 | Train 41 | Test Female 11 | 0.77 | 0.84 | 0.85 | 3595 | Train 57 | Test Female 11 | 0.74 | 0.75 | 0.73 |
| 2572 | Train 41 | Test Female 12 | 0.76 | 0.81 | 0.8 | 3596 | Train 57 | Test Female 12 | 0.74 | 0.73 | 0.68 |
| 2573 | Train 41 | Test Female 13 | 0.73 | 0.75 | 0.73 | 3597 | Train 57 | Test Female 13 | 0.72 | 0.72 | 0.68 |
| 2574 | Train 41 | Test Female 14 | 0.71 | 0.72 | 0.67 | 3598 | Train 57 | Test Female 14 | 0.71 | 0.7 | 0.63 |
| 2575 | Train 41 | Test Female 15 | 0.73 | 0.76 | 0.75 | 3599 | Train 57 | Test Female 15 | 0.71 | 0.72 | 0.68 |
| 2576 | Train 41 | Test Female 16 | 0.72 | 0.72 | 0.67 | 3600 | Train 57 | Test Female 16 | 0.71 | 0.7 | 0.64 |
| 2577 | Train 41 | Test Female 17 | 0.76 | 0.83 | 0.82 | 3601 | Train 57 | Test Female 17 | 0.74 | 0.75 | 0.71 |
| 2578 | Train 41 | Test Female 18 | 0.76 | 0.8 | 0.78 | 3602 | Train 57 | Test Female 18 | 0.73 | 0.73 | 0.67 |
| 2579 | Train 41 | Test Female 19 | 0.77 | 0.83 | 0.82 | 3603 | Train 57 | Test Female 19 | 0.74 | 0.75 | 0.71 |
| 2580 | Train 41 | Test Female 20 | 0.76 | 0.8 | 0.78 | 3604 | Train 57 | Test Female 20 | 0.74 | 0.73 | 0.67 |
| 2581 | Train 41 | Test Female 21 | 0.73 | 0.76 | 0.74 | 3605 | Train 57 | Test Female 21 | 0.72 | 0.71 | 0.67 |
| 2582 | Train 41 | Test Female 22 | 0.71 | 0.72 | 0.67 | 3606 | Train 57 | Test Female 22 | 0.71 | 0.7 | 0.63 |
| 2583 | Train 41 | Test Female 23 | 0.73 | 0.75 | 0.73 | 3607 | Train 57 | Test Female 23 | 0.72 | 0.71 | 0.67 |
| 2584 | Train 41 | Test Female 24 | 0.71 | 0.72 | 0.67 | 3608 | Train 57 | Test Female 24 | 0.71 | 0.7 | 0.64 |
| 2585 | Train 41 | Test Female 25 | 0.77 | 0.84 | 0.86 | 3609 | Train 57 | Test Female 25 | 0.74 | 0.75 | 0.73 |
| 2586 | Train 41 | Test Female 26 | 0.76 | 0.81 | 0.81 | 3610 | Train 57 | Test Female 26 | 0.73 | 0.73 | 0.68 |
| 2587 | Train 41 | Test Female 27 | 0.77 | 0.84 | 0.85 | 3611 | Train 57 | Test Female 27 | 0.74 | 0.75 | 0.73 |
| 2588 | Train 41 | Test Female 28 | 0.76 | 0.81 | 0.8 | 3612 | Train 57 | Test Female 28 | 0.74 | 0.73 | 0.68 |
| 2589 | Train 41 | Test Female 29 | 0.73 | 0.76 | 0.73 | 3613 | Train 57 | Test Female 29 | 0.72 | 0.72 | 0.68 |
| 2590 | Train 41 | Test Female 30 | 0.72 | 0.72 | 0.67 | 3614 | Train 57 | Test Female 30 | 0.71 | 0.7 | 0.63 |
| 2591 | Train 41 | Test Female 31 | 0.73 | 0.76 | 0.75 | 3615 | Train 57 | Test Female 31 | 0.72 | 0.72 | 0.68 |
| 2592 | Train 41 | Test Female 32 | 0.72 | 0.72 | 0.67 | 3616 | Train 57 | Test Female 32 | 0.71 | 0.7 | 0.64 |
| 2593 | Train 41 | Test Female 33 | 0.77 | 0.84 | 0.83 | 3617 | Train 57 | Test Female 33 | 0.75 | 0.75 | 0.71 |
| 2594 | Train 41 | Test Female 34 | 0.76 | 0.8 | 0.79 | 3618 | Train 57 | Test Female 34 | 0.74 | 0.73 | 0.68 |
| 2595 | Train 41 | Test Female 35 | 0.77 | 0.84 | 0.82 | 3619 | Train 57 | Test Female 35 | 0.75 | 0.75 | 0.71 |
| 2596 | Train 41 | Test Female 36 | 0.76 | 0.81 | 0.79 | 3620 | Train 57 | Test Female 36 | 0.74 | 0.73 | 0.68 |
| 2597 | Train 41 | Test Female 37 | 0.73 | 0.76 | 0.74 | 3621 | Train 57 | Test Female 37 | 0.72 | 0.72 | 0.67 |
| 2598 | Train 41 | Test Female 38 | 0.71 | 0.72 | 0.67 | 3622 | Train 57 | Test Female 38 | 0.71 | 0.7 | 0.64 |
| 2599 | Train 41 | Test Female 39 | 0.73 | 0.76 | 0.74 | 3623 | Train 57 | Test Female 39 | 0.72 | 0.72 | 0.67 |
| 2600 | Train 41 | Test Female 40 | 0.72 | 0.72 | 0.67 | 3624 | Train 57 | Test Female 40 | 0.71 | 0.7 | 0.64 |
| 2601 | Train 41 | Test Female 41 | 0.77 | 0.85 | 0.86 | 3625 | Train 57 | Test Female 41 | 0.75 | 0.76 | 0.73 |
| 2602 | Train 41 | Test Female 42 | 0.76 | 0.81 | 0.81 | 3626 | Train 57 | Test Female 42 | 0.74 | 0.74 | 0.69 |
| 2603 | Train 41 | Test Female 43 | 0.77 | 0.85 | 0.86 | 3627 | Train 57 | Test Female 43 | 0.75 | 0.76 | 0.73 |
| 2604 | Train 41 | Test Female 44 | 0.76 | 0.81 | 0.81 | 3628 | Train 57 | Test Female 44 | 0.74 | 0.74 | 0.69 |
| 2605 | Train 41 | Test Female 45 | 0.73 | 0.76 | 0.73 | 3629 | Train 57 | Test Female 45 | 0.72 | 0.72 | 0.68 |
| 2606 | Train 41 | Test Female 46 | 0.72 | 0.72 | 0.67 | 3630 | Train 57 | Test Female 46 | 0.71 | 0.7 | 0.64 |
| 2607 | Train 41 | Test Female 47 | 0.73 | 0.76 | 0.75 | 3631 | Train 57 | Test Female 47 | 0.72 | 0.72 | 0.68 |
| 2608 | Train 41 | Test Female 48 | 0.72 | 0.72 | 0.68 | 3632 | Train 57 | Test Female 48 | 0.71 | 0.7 | 0.64 |
| 2609 | Train 41 | Test Female 49 | 0.79 | 0.84 | 0.83 | 3633 | Train 57 | Test Female 49 | 0.8 | 0.86 | 0.85 |
| 2610 | Train 41 | Test Female 50 | 0.79 | 0.84 | 0.83 | 3634 | Train 57 | Test Female 50 | 0.8 | 0.86 | 0.85 |
| 2611 | Train 41 | Test Female 51 | 0.79 | 0.84 | 0.82 | 3635 | Train 57 | Test Female 51 | 0.8 | 0.86 | 0.85 |
| 2612 | Train 41 | Test Female 52 | 0.79 | 0.84 | 0.82 | 3636 | Train 57 | Test Female 52 | 0.8 | 0.86 | 0.85 |
| 2613 | Train 41 | Test Female 53 | 0.74 | 0.78 | 0.75 | 3637 | Train 57 | Test Female 53 | 0.74 | 0.78 | 0.75 |
| 2614 | Train 41 | Test Female 54 | 0.73 | 0.73 | 0.69 | 3638 | Train 57 | Test Female 54 | 0.73 | 0.72 | 0.68 |
| 2615 | Train 41 | Test Female 55 | 0.75 | 0.78 | 0.75 | 3639 | Train 57 | Test Female 55 | 0.74 | 0.78 | 0.75 |
| 2616 | Train 41 | Test Female 56 | 0.73 | 0.73 | 0.69 | 3640 | Train 57 | Test Female 56 | 0.73 | 0.72 | 0.68 |
| 2617 | Train 41 | Test Female 57 | 0.79 | 0.85 | 0.87 | 3641 | Train 57 | Test Female 57 | 0.8 | 0.87 | 0.91 |
| 2618 | Train 41 | Test Female 58 | 0.79 | 0.85 | 0.86 | 3642 | Train 57 | Test Female 58 | 0.8 | 0.87 | 0.91 |
| 2619 | Train 41 | Test Female 59 | 0.79 | 0.84 | 0.86 | 3643 | Train 57 | Test Female 59 | 0.8 | 0.87 | 0.9 |
| 2620 | Train 41 | Test Female 60 | 0.79 | 0.84 | 0.86 | 3644 | Train 57 | Test Female 60 | 0.8 | 0.87 | 0.9 |
| 2621 | Train 41 | Test Female 61 | 0.74 | 0.78 | 0.76 | 3645 | Train 57 | Test Female 61 | 0.74 | 0.78 | 0.77 |
| 2622 | Train 41 | Test Female 62 | 0.73 | 0.73 | 0.69 | 3646 | Train 57 | Test Female 62 | 0.73 | 0.72 | 0.68 |
| 2623 | Train 41 | Test Female 63 | 0.75 | 0.78 | 0.76 | 3647 | Train 57 | Test Female 63 | 0.74 | 0.78 | 0.77 |
| 2624 | Train 41 | Test Female 64 | 0.73 | 0.73 | 0.69 | 3648 | Train 57 | Test Female 64 | 0.74 | 0.72 | 0.68 |
| 2625 | Train 42 | Test Female 1 | 0.76 | 0.83 | 0.81 | 3649 | Train 58 | Test Female 1 | 0.74 | 0.72 | 0.64 |
| 2626 | Train 42 | Test Female 2 | 0.77 | 0.84 | 0.83 | 3650 | Train 58 | Test Female 2 | 0.74 | 0.71 | 0.64 |
| 2627 | Train 42 | Test Female 3 | 0.76 | 0.82 | 0.81 | 3651 | Train 58 | Test Female 3 | 0.74 | 0.72 | 0.65 |
| 2628 | Train 42 | Test Female 4 | 0.77 | 0.83 | 0.82 | 3652 | Train 58 | Test Female 4 | 0.74 | 0.71 | 0.64 |
| 2629 | Train 42 | Test Female 5 | 0.73 | 0.76 | 0.72 | 3653 | Train 58 | Test Female 5 | 0.71 | 0.7 | 0.63 |
| 2630 | Train 42 | Test Female 6 | 0.73 | 0.73 | 0.69 | 3654 | Train 58 | Test Female 6 | 0.71 | 0.69 | 0.62 |
| 2631 | Train 42 | Test Female 7 | 0.73 | 0.76 | 0.72 | 3655 | Train 58 | Test Female 7 | 0.71 | 0.7 | 0.63 |
| 2632 | Train 42 | Test Female 8 | 0.73 | 0.73 | 0.69 | 3656 | Train 58 | Test Female 8 | 0.71 | 0.69 | 0.62 |
| 2633 | Train 42 | Test Female 9 | 0.76 | 0.83 | 0.85 | 3657 | Train 58 | Test Female 9 | 0.74 | 0.72 | 0.66 |
| 2634 | Train 42 | Test Female 10 | 0.77 | 0.84 | 0.86 | 3658 | Train 58 | Test Female 10 | 0.74 | 0.72 | 0.65 |
| 2635 | Train 42 | Test Female 11 | 0.76 | 0.83 | 0.84 | 3659 | Train 58 | Test Female 11 | 0.74 | 0.73 | 0.66 |
| 2636 | Train 42 | Test Female 12 | 0.78 | 0.84 | 0.85 | 3660 | Train 58 | Test Female 12 | 0.74 | 0.72 | 0.65 |
| 2637 | Train 42 | Test Female 13 | 0.73 | 0.76 | 0.7 | 3661 | Train 58 | Test Female 13 | 0.71 | 0.7 | 0.64 |
| 2638 | Train 42 | Test Female 14 | 0.73 | 0.73 | 0.69 | 3662 | Train 58 | Test Female 14 | 0.71 | 0.69 | 0.63 |
| 2639 | Train 42 | Test Female 15 | 0.73 | 0.76 | 0.73 | 3663 | Train 58 | Test Female 15 | 0.71 | 0.7 | 0.64 |
| 2640 | Train 42 | Test Female 16 | 0.73 | 0.73 | 0.69 | 3664 | Train 58 | Test Female 16 | 0.71 | 0.69 | 0.63 |
| 2641 | Train 42 | Test Female 17 | 0.76 | 0.83 | 0.81 | 3665 | Train 58 | Test Female 17 | 0.74 | 0.72 | 0.64 |
| 2642 | Train 42 | Test Female 18 | 0.77 | 0.84 | 0.83 | 3666 | Train 58 | Test Female 18 | 0.74 | 0.71 | 0.64 |
| 2643 | Train 42 | Test Female 19 | 0.76 | 0.82 | 0.81 | 3667 | Train 58 | Test Female 19 | 0.74 | 0.72 | 0.65 |
| 2644 | Train 42 | Test Female 20 | 0.77 | 0.84 | 0.82 | 3668 | Train 58 | Test Female 20 | 0.74 | 0.71 | 0.64 |
| 2645 | Train 42 | Test Female 21 | 0.73 | 0.76 | 0.72 | 3669 | Train 58 | Test Female 21 | 0.72 | 0.7 | 0.63 |
| 2646 | Train 42 | Test Female 22 | 0.73 | 0.73 | 0.69 | 3670 | Train 58 | Test Female 22 | 0.71 | 0.69 | 0.63 |
| 2647 | Train 42 | Test Female 23 | 0.73 | 0.76 | 0.72 | 3671 | Train 58 | Test Female 23 | 0.71 | 0.7 | 0.63 |
| 2648 | Train 42 | Test Female 24 | 0.73 | 0.73 | 0.69 | 3672 | Train 58 | Test Female 24 | 0.71 | 0.69 | 0.62 |
| 2649 | Train 42 | Test Female 25 | 0.76 | 0.83 | 0.85 | 3673 | Train 58 | Test Female 25 | 0.74 | 0.72 | 0.66 |
| 2650 | Train 42 | Test Female 26 | 0.77 | 0.84 | 0.86 | 3674 | Train 58 | Test Female 26 | 0.74 | 0.72 | 0.65 |
| 2651 | Train 42 | Test Female 27 | 0.76 | 0.83 | 0.84 | 3675 | Train 58 | Test Female 27 | 0.74 | 0.73 | 0.66 |
| 2652 | Train 42 | Test Female 28 | 0.78 | 0.84 | 0.85 | 3676 | Train 58 | Test Female 28 | 0.74 | 0.72 | 0.65 |
| 2653 | Train 42 | Test Female 29 | 0.73 | 0.76 | 0.7 | 3677 | Train 58 | Test Female 29 | 0.71 | 0.7 | 0.64 |
| 2654 | Train 42 | Test Female 30 | 0.73 | 0.74 | 0.69 | 3678 | Train 58 | Test Female 30 | 0.71 | 0.69 | 0.63 |
| 2655 | Train 42 | Test Female 31 | 0.73 | 0.76 | 0.73 | 3679 | Train 58 | Test Female 31 | 0.71 | 0.7 | 0.64 |
| 2656 | Train 42 | Test Female 32 | 0.73 | 0.73 | 0.69 | 3680 | Train 58 | Test Female 32 | 0.71 | 0.69 | 0.63 |
| 2657 | Train 42 | Test Female 33 | 0.76 | 0.83 | 0.82 | 3681 | Train 58 | Test Female 33 | 0.75 | 0.73 | 0.65 |
| 2658 | Train 42 | Test Female 34 | 0.78 | 0.84 | 0.83 | 3682 | Train 58 | Test Female 34 | 0.74 | 0.72 | 0.64 |
| 2659 | Train 42 | Test Female 35 | 0.77 | 0.83 | 0.82 | 3683 | Train 58 | Test Female 35 | 0.75 | 0.73 | 0.65 |
| 2660 | Train 42 | Test Female 36 | 0.78 | 0.84 | 0.83 | 3684 | Train 58 | Test Female 36 | 0.74 | 0.72 | 0.64 |
| 2661 | Train 42 | Test Female 37 | 0.73 | 0.76 | 0.73 | 3685 | Train 58 | Test Female 37 | 0.72 | 0.7 | 0.63 |
| 2662 | Train 42 | Test Female 38 | 0.73 | 0.73 | 0.69 | 3686 | Train 58 | Test Female 38 | 0.71 | 0.69 | 0.63 |
| 2663 | Train 42 | Test Female 39 | 0.73 | 0.76 | 0.73 | 3687 | Train 58 | Test Female 39 | 0.72 | 0.7 | 0.63 |
| 2664 | Train 42 | Test Female 40 | 0.73 | 0.73 | 0.69 | 3688 | Train 58 | Test Female 40 | 0.71 | 0.69 | 0.63 |
| 2665 | Train 42 | Test Female 41 | 0.76 | 0.84 | 0.85 | 3689 | Train 58 | Test Female 41 | 0.75 | 0.73 | 0.67 |
| 2666 | Train 42 | Test Female 42 | 0.78 | 0.85 | 0.86 | 3690 | Train 58 | Test Female 42 | 0.74 | 0.72 | 0.65 |
| 2667 | Train 42 | Test Female 43 | 0.77 | 0.84 | 0.85 | 3691 | Train 58 | Test Female 43 | 0.75 | 0.73 | 0.67 |
| 2668 | Train 42 | Test Female 44 | 0.78 | 0.85 | 0.86 | 3692 | Train 58 | Test Female 44 | 0.74 | 0.72 | 0.65 |
| 2669 | Train 42 | Test Female 45 | 0.73 | 0.76 | 0.71 | 3693 | Train 58 | Test Female 45 | 0.72 | 0.71 | 0.64 |
| 2670 | Train 42 | Test Female 46 | 0.73 | 0.74 | 0.69 | 3694 | Train 58 | Test Female 46 | 0.71 | 0.69 | 0.63 |
| 2671 | Train 42 | Test Female 47 | 0.73 | 0.77 | 0.74 | 3695 | Train 58 | Test Female 47 | 0.72 | 0.7 | 0.64 |
| 2672 | Train 42 | Test Female 48 | 0.73 | 0.74 | 0.69 | 3696 | Train 58 | Test Female 48 | 0.71 | 0.69 | 0.63 |
| 2673 | Train 42 | Test Female 49 | 0.78 | 0.83 | 0.83 | 3697 | Train 58 | Test Female 49 | 0.8 | 0.86 | 0.85 |
| 2674 | Train 42 | Test Female 50 | 0.78 | 0.83 | 0.83 | 3698 | Train 58 | Test Female 50 | 0.8 | 0.86 | 0.85 |
| 2675 | Train 42 | Test Female 51 | 0.78 | 0.83 | 0.82 | 3699 | Train 58 | Test Female 51 | 0.8 | 0.85 | 0.85 |
| 2676 | Train 42 | Test Female 52 | 0.78 | 0.83 | 0.82 | 3700 | Train 58 | Test Female 52 | 0.8 | 0.85 | 0.85 |
| 2677 | Train 42 | Test Female 53 | 0.75 | 0.78 | 0.72 | 3701 | Train 58 | Test Female 53 | 0.74 | 0.75 | 0.71 |
| 2678 | Train 42 | Test Female 54 | 0.74 | 0.74 | 0.69 | 3702 | Train 58 | Test Female 54 | 0.73 | 0.71 | 0.65 |
| 2679 | Train 42 | Test Female 55 | 0.75 | 0.78 | 0.72 | 3703 | Train 58 | Test Female 55 | 0.74 | 0.75 | 0.71 |
| 2680 | Train 42 | Test Female 56 | 0.73 | 0.73 | 0.68 | 3704 | Train 58 | Test Female 56 | 0.73 | 0.7 | 0.65 |
| 2681 | Train 42 | Test Female 57 | 0.78 | 0.84 | 0.86 | 3705 | Train 58 | Test Female 57 | 0.8 | 0.87 | 0.91 |
| 2682 | Train 42 | Test Female 58 | 0.78 | 0.84 | 0.86 | 3706 | Train 58 | Test Female 58 | 0.8 | 0.87 | 0.91 |
| 2683 | Train 42 | Test Female 59 | 0.79 | 0.84 | 0.86 | 3707 | Train 58 | Test Female 59 | 0.8 | 0.87 | 0.9 |
| 2684 | Train 42 | Test Female 60 | 0.79 | 0.84 | 0.86 | 3708 | Train 58 | Test Female 60 | 0.8 | 0.86 | 0.9 |
| 2685 | Train 42 | Test Female 61 | 0.75 | 0.78 | 0.72 | 3709 | Train 58 | Test Female 61 | 0.74 | 0.75 | 0.72 |
| 2686 | Train 42 | Test Female 62 | 0.74 | 0.74 | 0.69 | 3710 | Train 58 | Test Female 62 | 0.73 | 0.71 | 0.65 |
| 2687 | Train 42 | Test Female 63 | 0.75 | 0.78 | 0.72 | 3711 | Train 58 | Test Female 63 | 0.74 | 0.76 | 0.72 |
| 2688 | Train 42 | Test Female 64 | 0.73 | 0.73 | 0.69 | 3712 | Train 58 | Test Female 64 | 0.73 | 0.71 | 0.65 |
| 2689 | Train 43 | Test Female 1 | 0.76 | 0.83 | 0.82 | 3713 | Train 59 | Test Female 1 | 0.74 | 0.75 | 0.68 |
| 2690 | Train 43 | Test Female 2 | 0.76 | 0.8 | 0.78 | 3714 | Train 59 | Test Female 2 | 0.73 | 0.73 | 0.66 |
| 2691 | Train 43 | Test Female 3 | 0.76 | 0.83 | 0.82 | 3715 | Train 59 | Test Female 3 | 0.74 | 0.75 | 0.69 |
| 2692 | Train 43 | Test Female 4 | 0.76 | 0.8 | 0.78 | 3716 | Train 59 | Test Female 4 | 0.73 | 0.73 | 0.66 |
| 2693 | Train 43 | Test Female 5 | 0.73 | 0.76 | 0.73 | 3717 | Train 59 | Test Female 5 | 0.72 | 0.72 | 0.65 |
| 2694 | Train 43 | Test Female 6 | 0.71 | 0.72 | 0.67 | 3718 | Train 59 | Test Female 6 | 0.7 | 0.7 | 0.61 |
| 2695 | Train 43 | Test Female 7 | 0.73 | 0.76 | 0.73 | 3719 | Train 59 | Test Female 7 | 0.72 | 0.72 | 0.65 |
| 2696 | Train 43 | Test Female 8 | 0.71 | 0.72 | 0.67 | 3720 | Train 59 | Test Female 8 | 0.71 | 0.7 | 0.61 |
| 2697 | Train 43 | Test Female 9 | 0.76 | 0.84 | 0.86 | 3721 | Train 59 | Test Female 9 | 0.74 | 0.76 | 0.7 |
| 2698 | Train 43 | Test Female 10 | 0.76 | 0.8 | 0.81 | 3722 | Train 59 | Test Female 10 | 0.73 | 0.74 | 0.67 |
| 2699 | Train 43 | Test Female 11 | 0.77 | 0.84 | 0.86 | 3723 | Train 59 | Test Female 11 | 0.75 | 0.76 | 0.7 |
| 2700 | Train 43 | Test Female 12 | 0.76 | 0.81 | 0.81 | 3724 | Train 59 | Test Female 12 | 0.73 | 0.74 | 0.67 |
| 2701 | Train 43 | Test Female 13 | 0.73 | 0.75 | 0.72 | 3725 | Train 59 | Test Female 13 | 0.72 | 0.72 | 0.66 |
| 2702 | Train 43 | Test Female 14 | 0.71 | 0.72 | 0.67 | 3726 | Train 59 | Test Female 14 | 0.71 | 0.7 | 0.61 |
| 2703 | Train 43 | Test Female 15 | 0.73 | 0.76 | 0.75 | 3727 | Train 59 | Test Female 15 | 0.72 | 0.72 | 0.66 |
| 2704 | Train 43 | Test Female 16 | 0.72 | 0.72 | 0.67 | 3728 | Train 59 | Test Female 16 | 0.71 | 0.7 | 0.61 |
| 2705 | Train 43 | Test Female 17 | 0.76 | 0.83 | 0.82 | 3729 | Train 59 | Test Female 17 | 0.74 | 0.75 | 0.68 |
| 2706 | Train 43 | Test Female 18 | 0.76 | 0.8 | 0.78 | 3730 | Train 59 | Test Female 18 | 0.73 | 0.73 | 0.66 |
| 2707 | Train 43 | Test Female 19 | 0.77 | 0.83 | 0.82 | 3731 | Train 59 | Test Female 19 | 0.74 | 0.75 | 0.69 |
| 2708 | Train 43 | Test Female 20 | 0.76 | 0.8 | 0.78 | 3732 | Train 59 | Test Female 20 | 0.73 | 0.73 | 0.66 |
| 2709 | Train 43 | Test Female 21 | 0.73 | 0.76 | 0.73 | 3733 | Train 59 | Test Female 21 | 0.72 | 0.72 | 0.65 |
| 2710 | Train 43 | Test Female 22 | 0.71 | 0.72 | 0.67 | 3734 | Train 59 | Test Female 22 | 0.7 | 0.7 | 0.61 |
| 2711 | Train 43 | Test Female 23 | 0.73 | 0.76 | 0.73 | 3735 | Train 59 | Test Female 23 | 0.72 | 0.72 | 0.65 |
| 2712 | Train 43 | Test Female 24 | 0.71 | 0.72 | 0.67 | 3736 | Train 59 | Test Female 24 | 0.71 | 0.7 | 0.61 |
| 2713 | Train 43 | Test Female 25 | 0.77 | 0.84 | 0.86 | 3737 | Train 59 | Test Female 25 | 0.74 | 0.76 | 0.7 |
| 2714 | Train 43 | Test Female 26 | 0.76 | 0.8 | 0.81 | 3738 | Train 59 | Test Female 26 | 0.73 | 0.74 | 0.67 |
| 2715 | Train 43 | Test Female 27 | 0.77 | 0.84 | 0.86 | 3739 | Train 59 | Test Female 27 | 0.75 | 0.76 | 0.7 |
| 2716 | Train 43 | Test Female 28 | 0.76 | 0.81 | 0.81 | 3740 | Train 59 | Test Female 28 | 0.73 | 0.74 | 0.67 |
| 2717 | Train 43 | Test Female 29 | 0.73 | 0.76 | 0.72 | 3741 | Train 59 | Test Female 29 | 0.72 | 0.72 | 0.66 |
| 2718 | Train 43 | Test Female 30 | 0.71 | 0.72 | 0.67 | 3742 | Train 59 | Test Female 30 | 0.71 | 0.7 | 0.61 |
| 2719 | Train 43 | Test Female 31 | 0.73 | 0.76 | 0.75 | 3743 | Train 59 | Test Female 31 | 0.72 | 0.72 | 0.66 |
| 2720 | Train 43 | Test Female 32 | 0.72 | 0.72 | 0.67 | 3744 | Train 59 | Test Female 32 | 0.71 | 0.7 | 0.61 |
| 2721 | Train 43 | Test Female 33 | 0.77 | 0.84 | 0.83 | 3745 | Train 59 | Test Female 33 | 0.75 | 0.76 | 0.69 |
| 2722 | Train 43 | Test Female 34 | 0.76 | 0.8 | 0.79 | 3746 | Train 59 | Test Female 34 | 0.74 | 0.74 | 0.66 |
| 2723 | Train 43 | Test Female 35 | 0.77 | 0.84 | 0.83 | 3747 | Train 59 | Test Female 35 | 0.75 | 0.76 | 0.69 |
| 2724 | Train 43 | Test Female 36 | 0.76 | 0.81 | 0.79 | 3748 | Train 59 | Test Female 36 | 0.74 | 0.74 | 0.67 |
| 2725 | Train 43 | Test Female 37 | 0.73 | 0.76 | 0.74 | 3749 | Train 59 | Test Female 37 | 0.72 | 0.72 | 0.65 |
| 2726 | Train 43 | Test Female 38 | 0.71 | 0.72 | 0.67 | 3750 | Train 59 | Test Female 38 | 0.71 | 0.7 | 0.61 |
| 2727 | Train 43 | Test Female 39 | 0.73 | 0.76 | 0.74 | 3751 | Train 59 | Test Female 39 | 0.72 | 0.72 | 0.66 |
| 2728 | Train 43 | Test Female 40 | 0.72 | 0.72 | 0.67 | 3752 | Train 59 | Test Female 40 | 0.71 | 0.7 | 0.61 |
| 2729 | Train 43 | Test Female 41 | 0.77 | 0.85 | 0.86 | 3753 | Train 59 | Test Female 41 | 0.75 | 0.76 | 0.7 |
| 2730 | Train 43 | Test Female 42 | 0.76 | 0.81 | 0.81 | 3754 | Train 59 | Test Female 42 | 0.74 | 0.74 | 0.67 |
| 2731 | Train 43 | Test Female 43 | 0.77 | 0.85 | 0.86 | 3755 | Train 59 | Test Female 43 | 0.75 | 0.77 | 0.71 |
| 2732 | Train 43 | Test Female 44 | 0.76 | 0.81 | 0.81 | 3756 | Train 59 | Test Female 44 | 0.74 | 0.74 | 0.68 |
| 2733 | Train 43 | Test Female 45 | 0.73 | 0.76 | 0.72 | 3757 | Train 59 | Test Female 45 | 0.72 | 0.73 | 0.66 |
| 2734 | Train 43 | Test Female 46 | 0.72 | 0.72 | 0.67 | 3758 | Train 59 | Test Female 46 | 0.71 | 0.71 | 0.61 |
| 2735 | Train 43 | Test Female 47 | 0.73 | 0.76 | 0.75 | 3759 | Train 59 | Test Female 47 | 0.72 | 0.73 | 0.66 |
| 2736 | Train 43 | Test Female 48 | 0.72 | 0.72 | 0.67 | 3760 | Train 59 | Test Female 48 | 0.71 | 0.7 | 0.62 |
| 2737 | Train 43 | Test Female 49 | 0.79 | 0.84 | 0.83 | 3761 | Train 59 | Test Female 49 | 0.8 | 0.86 | 0.86 |
| 2738 | Train 43 | Test Female 50 | 0.79 | 0.84 | 0.83 | 3762 | Train 59 | Test Female 50 | 0.8 | 0.86 | 0.85 |
| 2739 | Train 43 | Test Female 51 | 0.79 | 0.84 | 0.83 | 3763 | Train 59 | Test Female 51 | 0.8 | 0.86 | 0.86 |
| 2740 | Train 43 | Test Female 52 | 0.79 | 0.84 | 0.83 | 3764 | Train 59 | Test Female 52 | 0.8 | 0.86 | 0.86 |
| 2741 | Train 43 | Test Female 53 | 0.74 | 0.78 | 0.76 | 3765 | Train 59 | Test Female 53 | 0.74 | 0.78 | 0.74 |
| 2742 | Train 43 | Test Female 54 | 0.73 | 0.73 | 0.69 | 3766 | Train 59 | Test Female 54 | 0.73 | 0.72 | 0.64 |
| 2743 | Train 43 | Test Female 55 | 0.74 | 0.78 | 0.76 | 3767 | Train 59 | Test Female 55 | 0.75 | 0.78 | 0.74 |
| 2744 | Train 43 | Test Female 56 | 0.73 | 0.72 | 0.69 | 3768 | Train 59 | Test Female 56 | 0.73 | 0.72 | 0.65 |
| 2745 | Train 43 | Test Female 57 | 0.79 | 0.85 | 0.87 | 3769 | Train 59 | Test Female 57 | 0.8 | 0.87 | 0.91 |
| 2746 | Train 43 | Test Female 58 | 0.79 | 0.85 | 0.86 | 3770 | Train 59 | Test Female 58 | 0.8 | 0.87 | 0.91 |
| 2747 | Train 43 | Test Female 59 | 0.79 | 0.85 | 0.87 | 3771 | Train 59 | Test Female 59 | 0.8 | 0.87 | 0.91 |
| 2748 | Train 43 | Test Female 60 | 0.79 | 0.85 | 0.87 | 3772 | Train 59 | Test Female 60 | 0.8 | 0.87 | 0.91 |
| 2749 | Train 43 | Test Female 61 | 0.74 | 0.78 | 0.77 | 3773 | Train 59 | Test Female 61 | 0.74 | 0.78 | 0.76 |
| 2750 | Train 43 | Test Female 62 | 0.73 | 0.73 | 0.69 | 3774 | Train 59 | Test Female 62 | 0.73 | 0.73 | 0.65 |
| 2751 | Train 43 | Test Female 63 | 0.75 | 0.78 | 0.77 | 3775 | Train 59 | Test Female 63 | 0.75 | 0.79 | 0.76 |
| 2752 | Train 43 | Test Female 64 | 0.73 | 0.73 | 0.69 | 3776 | Train 59 | Test Female 64 | 0.73 | 0.72 | 0.65 |
| 2753 | Train 44 | Test Female 1 | 0.76 | 0.82 | 0.81 | 3777 | Train 60 | Test Female 1 | 0.74 | 0.72 | 0.72 |
| 2754 | Train 44 | Test Female 2 | 0.77 | 0.84 | 0.83 | 3778 | Train 60 | Test Female 2 | 0.73 | 0.71 | 0.68 |
| 2755 | Train 44 | Test Female 3 | 0.76 | 0.83 | 0.81 | 3779 | Train 60 | Test Female 3 | 0.74 | 0.72 | 0.72 |
| 2756 | Train 44 | Test Female 4 | 0.77 | 0.84 | 0.83 | 3780 | Train 60 | Test Female 4 | 0.73 | 0.71 | 0.68 |
| 2757 | Train 44 | Test Female 5 | 0.73 | 0.76 | 0.74 | 3781 | Train 60 | Test Female 5 | 0.72 | 0.7 | 0.68 |
| 2758 | Train 44 | Test Female 6 | 0.73 | 0.73 | 0.69 | 3782 | Train 60 | Test Female 6 | 0.7 | 0.69 | 0.63 |
| 2759 | Train 44 | Test Female 7 | 0.73 | 0.76 | 0.74 | 3783 | Train 60 | Test Female 7 | 0.71 | 0.7 | 0.68 |
| 2760 | Train 44 | Test Female 8 | 0.72 | 0.73 | 0.69 | 3784 | Train 60 | Test Female 8 | 0.71 | 0.69 | 0.63 |
| 2761 | Train 44 | Test Female 9 | 0.76 | 0.83 | 0.84 | 3785 | Train 60 | Test Female 9 | 0.74 | 0.72 | 0.74 |
| 2762 | Train 44 | Test Female 10 | 0.77 | 0.84 | 0.86 | 3786 | Train 60 | Test Female 10 | 0.73 | 0.71 | 0.69 |
| 2763 | Train 44 | Test Female 11 | 0.76 | 0.83 | 0.84 | 3787 | Train 60 | Test Female 11 | 0.74 | 0.73 | 0.74 |
| 2764 | Train 44 | Test Female 12 | 0.78 | 0.84 | 0.86 | 3788 | Train 60 | Test Female 12 | 0.73 | 0.72 | 0.69 |
| 2765 | Train 44 | Test Female 13 | 0.73 | 0.75 | 0.71 | 3789 | Train 60 | Test Female 13 | 0.71 | 0.7 | 0.69 |
| 2766 | Train 44 | Test Female 14 | 0.73 | 0.73 | 0.69 | 3790 | Train 60 | Test Female 14 | 0.71 | 0.69 | 0.63 |
| 2767 | Train 44 | Test Female 15 | 0.73 | 0.76 | 0.76 | 3791 | Train 60 | Test Female 15 | 0.71 | 0.7 | 0.69 |
| 2768 | Train 44 | Test Female 16 | 0.73 | 0.73 | 0.69 | 3792 | Train 60 | Test Female 16 | 0.71 | 0.69 | 0.64 |
| 2769 | Train 44 | Test Female 17 | 0.76 | 0.82 | 0.81 | 3793 | Train 60 | Test Female 17 | 0.74 | 0.72 | 0.72 |
| 2770 | Train 44 | Test Female 18 | 0.77 | 0.84 | 0.83 | 3794 | Train 60 | Test Female 18 | 0.73 | 0.71 | 0.68 |
| 2771 | Train 44 | Test Female 19 | 0.76 | 0.83 | 0.81 | 3795 | Train 60 | Test Female 19 | 0.74 | 0.72 | 0.72 |
| 2772 | Train 44 | Test Female 20 | 0.77 | 0.84 | 0.83 | 3796 | Train 60 | Test Female 20 | 0.73 | 0.71 | 0.68 |
| 2773 | Train 44 | Test Female 21 | 0.73 | 0.76 | 0.74 | 3797 | Train 60 | Test Female 21 | 0.72 | 0.7 | 0.68 |
| 2774 | Train 44 | Test Female 22 | 0.73 | 0.73 | 0.69 | 3798 | Train 60 | Test Female 22 | 0.71 | 0.69 | 0.63 |
| 2775 | Train 44 | Test Female 23 | 0.73 | 0.76 | 0.74 | 3799 | Train 60 | Test Female 23 | 0.71 | 0.7 | 0.68 |
| 2776 | Train 44 | Test Female 24 | 0.73 | 0.73 | 0.69 | 3800 | Train 60 | Test Female 24 | 0.71 | 0.69 | 0.64 |
| 2777 | Train 44 | Test Female 25 | 0.76 | 0.83 | 0.84 | 3801 | Train 60 | Test Female 25 | 0.74 | 0.72 | 0.74 |
| 2778 | Train 44 | Test Female 26 | 0.77 | 0.84 | 0.86 | 3802 | Train 60 | Test Female 26 | 0.73 | 0.71 | 0.69 |
| 2779 | Train 44 | Test Female 27 | 0.76 | 0.83 | 0.84 | 3803 | Train 60 | Test Female 27 | 0.74 | 0.73 | 0.74 |
| 2780 | Train 44 | Test Female 28 | 0.78 | 0.84 | 0.86 | 3804 | Train 60 | Test Female 28 | 0.73 | 0.72 | 0.69 |
| 2781 | Train 44 | Test Female 29 | 0.73 | 0.75 | 0.71 | 3805 | Train 60 | Test Female 29 | 0.72 | 0.7 | 0.69 |
| 2782 | Train 44 | Test Female 30 | 0.73 | 0.73 | 0.7 | 3806 | Train 60 | Test Female 30 | 0.71 | 0.69 | 0.64 |
| 2783 | Train 44 | Test Female 31 | 0.73 | 0.76 | 0.76 | 3807 | Train 60 | Test Female 31 | 0.71 | 0.7 | 0.69 |
| 2784 | Train 44 | Test Female 32 | 0.73 | 0.73 | 0.7 | 3808 | Train 60 | Test Female 32 | 0.71 | 0.69 | 0.64 |
| 2785 | Train 44 | Test Female 33 | 0.76 | 0.83 | 0.82 | 3809 | Train 60 | Test Female 33 | 0.75 | 0.73 | 0.73 |
| 2786 | Train 44 | Test Female 34 | 0.78 | 0.84 | 0.83 | 3810 | Train 60 | Test Female 34 | 0.74 | 0.72 | 0.68 |
| 2787 | Train 44 | Test Female 35 | 0.77 | 0.83 | 0.82 | 3811 | Train 60 | Test Female 35 | 0.75 | 0.73 | 0.73 |
| 2788 | Train 44 | Test Female 36 | 0.78 | 0.84 | 0.83 | 3812 | Train 60 | Test Female 36 | 0.74 | 0.72 | 0.68 |
| 2789 | Train 44 | Test Female 37 | 0.73 | 0.76 | 0.75 | 3813 | Train 60 | Test Female 37 | 0.72 | 0.7 | 0.69 |
| 2790 | Train 44 | Test Female 38 | 0.73 | 0.73 | 0.69 | 3814 | Train 60 | Test Female 38 | 0.71 | 0.69 | 0.63 |
| 2791 | Train 44 | Test Female 39 | 0.73 | 0.76 | 0.74 | 3815 | Train 60 | Test Female 39 | 0.72 | 0.7 | 0.69 |
| 2792 | Train 44 | Test Female 40 | 0.73 | 0.73 | 0.69 | 3816 | Train 60 | Test Female 40 | 0.71 | 0.69 | 0.64 |
| 2793 | Train 44 | Test Female 41 | 0.76 | 0.84 | 0.85 | 3817 | Train 60 | Test Female 41 | 0.75 | 0.73 | 0.75 |
| 2794 | Train 44 | Test Female 42 | 0.78 | 0.84 | 0.86 | 3818 | Train 60 | Test Female 42 | 0.74 | 0.72 | 0.7 |
| 2795 | Train 44 | Test Female 43 | 0.77 | 0.84 | 0.85 | 3819 | Train 60 | Test Female 43 | 0.75 | 0.73 | 0.75 |
| 2796 | Train 44 | Test Female 44 | 0.78 | 0.85 | 0.86 | 3820 | Train 60 | Test Female 44 | 0.74 | 0.72 | 0.7 |
| 2797 | Train 44 | Test Female 45 | 0.73 | 0.76 | 0.72 | 3821 | Train 60 | Test Female 45 | 0.72 | 0.7 | 0.7 |
| 2798 | Train 44 | Test Female 46 | 0.73 | 0.73 | 0.7 | 3822 | Train 60 | Test Female 46 | 0.71 | 0.69 | 0.64 |
| 2799 | Train 44 | Test Female 47 | 0.73 | 0.77 | 0.76 | 3823 | Train 60 | Test Female 47 | 0.72 | 0.7 | 0.7 |
| 2800 | Train 44 | Test Female 48 | 0.73 | 0.73 | 0.7 | 3824 | Train 60 | Test Female 48 | 0.71 | 0.69 | 0.64 |
| 2801 | Train 44 | Test Female 49 | 0.78 | 0.83 | 0.82 | 3825 | Train 60 | Test Female 49 | 0.8 | 0.86 | 0.85 |
| 2802 | Train 44 | Test Female 50 | 0.78 | 0.83 | 0.82 | 3826 | Train 60 | Test Female 50 | 0.79 | 0.86 | 0.85 |
| 2803 | Train 44 | Test Female 51 | 0.78 | 0.83 | 0.82 | 3827 | Train 60 | Test Female 51 | 0.8 | 0.86 | 0.85 |
| 2804 | Train 44 | Test Female 52 | 0.78 | 0.83 | 0.82 | 3828 | Train 60 | Test Female 52 | 0.8 | 0.86 | 0.85 |
| 2805 | Train 44 | Test Female 53 | 0.75 | 0.78 | 0.77 | 3829 | Train 60 | Test Female 53 | 0.74 | 0.75 | 0.77 |
| 2806 | Train 44 | Test Female 54 | 0.73 | 0.74 | 0.69 | 3830 | Train 60 | Test Female 54 | 0.73 | 0.71 | 0.66 |
| 2807 | Train 44 | Test Female 55 | 0.75 | 0.78 | 0.77 | 3831 | Train 60 | Test Female 55 | 0.74 | 0.75 | 0.78 |
| 2808 | Train 44 | Test Female 56 | 0.73 | 0.73 | 0.69 | 3832 | Train 60 | Test Female 56 | 0.73 | 0.7 | 0.67 |
| 2809 | Train 44 | Test Female 57 | 0.78 | 0.84 | 0.86 | 3833 | Train 60 | Test Female 57 | 0.8 | 0.87 | 0.91 |
| 2810 | Train 44 | Test Female 58 | 0.78 | 0.84 | 0.86 | 3834 | Train 60 | Test Female 58 | 0.8 | 0.87 | 0.91 |
| 2811 | Train 44 | Test Female 59 | 0.79 | 0.84 | 0.86 | 3835 | Train 60 | Test Female 59 | 0.8 | 0.87 | 0.91 |
| 2812 | Train 44 | Test Female 60 | 0.79 | 0.84 | 0.86 | 3836 | Train 60 | Test Female 60 | 0.8 | 0.87 | 0.91 |
| 2813 | Train 44 | Test Female 61 | 0.75 | 0.78 | 0.77 | 3837 | Train 60 | Test Female 61 | 0.74 | 0.75 | 0.79 |
| 2814 | Train 44 | Test Female 62 | 0.74 | 0.74 | 0.69 | 3838 | Train 60 | Test Female 62 | 0.73 | 0.71 | 0.67 |
| 2815 | Train 44 | Test Female 63 | 0.75 | 0.78 | 0.77 | 3839 | Train 60 | Test Female 63 | 0.75 | 0.76 | 0.79 |
| 2816 | Train 44 | Test Female 64 | 0.73 | 0.73 | 0.69 | 3840 | Train 60 | Test Female 64 | 0.73 | 0.71 | 0.67 |
| 2817 | Train 45 | Test Female 1 | 0.75 | 0.81 | 0.81 | 3841 | Train 61 | Test Female 1 | 0.74 | 0.76 | 0.71 |
| 2818 | Train 45 | Test Female 2 | 0.76 | 0.8 | 0.78 | 3842 | Train 61 | Test Female 2 | 0.74 | 0.74 | 0.69 |
| 2819 | Train 45 | Test Female 3 | 0.76 | 0.81 | 0.8 | 3843 | Train 61 | Test Female 3 | 0.74 | 0.76 | 0.71 |
| 2820 | Train 45 | Test Female 4 | 0.76 | 0.8 | 0.78 | 3844 | Train 61 | Test Female 4 | 0.74 | 0.74 | 0.69 |
| 2821 | Train 45 | Test Female 5 | 0.75 | 0.81 | 0.81 | 3845 | Train 61 | Test Female 5 | 0.73 | 0.75 | 0.7 |
| 2822 | Train 45 | Test Female 6 | 0.72 | 0.73 | 0.7 | 3846 | Train 61 | Test Female 6 | 0.71 | 0.71 | 0.64 |
| 2823 | Train 45 | Test Female 7 | 0.75 | 0.81 | 0.8 | 3847 | Train 61 | Test Female 7 | 0.73 | 0.75 | 0.7 |
| 2824 | Train 45 | Test Female 8 | 0.72 | 0.73 | 0.7 | 3848 | Train 61 | Test Female 8 | 0.71 | 0.71 | 0.65 |
| 2825 | Train 45 | Test Female 9 | 0.76 | 0.81 | 0.83 | 3849 | Train 61 | Test Female 9 | 0.74 | 0.76 | 0.72 |
| 2826 | Train 45 | Test Female 10 | 0.76 | 0.8 | 0.8 | 3850 | Train 61 | Test Female 10 | 0.75 | 0.75 | 0.7 |
| 2827 | Train 45 | Test Female 11 | 0.76 | 0.81 | 0.83 | 3851 | Train 61 | Test Female 11 | 0.74 | 0.76 | 0.72 |
| 2828 | Train 45 | Test Female 12 | 0.76 | 0.8 | 0.8 | 3852 | Train 61 | Test Female 12 | 0.75 | 0.75 | 0.7 |
| 2829 | Train 45 | Test Female 13 | 0.75 | 0.8 | 0.76 | 3853 | Train 61 | Test Female 13 | 0.73 | 0.76 | 0.73 |
| 2830 | Train 45 | Test Female 14 | 0.72 | 0.73 | 0.7 | 3854 | Train 61 | Test Female 14 | 0.72 | 0.71 | 0.65 |
| 2831 | Train 45 | Test Female 15 | 0.75 | 0.81 | 0.82 | 3855 | Train 61 | Test Female 15 | 0.73 | 0.75 | 0.72 |
| 2832 | Train 45 | Test Female 16 | 0.72 | 0.73 | 0.7 | 3856 | Train 61 | Test Female 16 | 0.72 | 0.71 | 0.65 |
| 2833 | Train 45 | Test Female 17 | 0.75 | 0.81 | 0.81 | 3857 | Train 61 | Test Female 17 | 0.74 | 0.76 | 0.71 |
| 2834 | Train 45 | Test Female 18 | 0.76 | 0.8 | 0.78 | 3858 | Train 61 | Test Female 18 | 0.74 | 0.74 | 0.69 |
| 2835 | Train 45 | Test Female 19 | 0.76 | 0.81 | 0.8 | 3859 | Train 61 | Test Female 19 | 0.74 | 0.76 | 0.71 |
| 2836 | Train 45 | Test Female 20 | 0.76 | 0.8 | 0.78 | 3860 | Train 61 | Test Female 20 | 0.74 | 0.74 | 0.69 |
| 2837 | Train 45 | Test Female 21 | 0.75 | 0.81 | 0.81 | 3861 | Train 61 | Test Female 21 | 0.73 | 0.75 | 0.7 |
| 2838 | Train 45 | Test Female 22 | 0.72 | 0.73 | 0.7 | 3862 | Train 61 | Test Female 22 | 0.72 | 0.71 | 0.65 |
| 2839 | Train 45 | Test Female 23 | 0.75 | 0.81 | 0.8 | 3863 | Train 61 | Test Female 23 | 0.73 | 0.75 | 0.7 |
| 2840 | Train 45 | Test Female 24 | 0.72 | 0.73 | 0.7 | 3864 | Train 61 | Test Female 24 | 0.72 | 0.71 | 0.65 |
| 2841 | Train 45 | Test Female 25 | 0.76 | 0.81 | 0.83 | 3865 | Train 61 | Test Female 25 | 0.74 | 0.76 | 0.72 |
| 2842 | Train 45 | Test Female 26 | 0.76 | 0.8 | 0.8 | 3866 | Train 61 | Test Female 26 | 0.75 | 0.75 | 0.7 |
| 2843 | Train 45 | Test Female 27 | 0.76 | 0.81 | 0.83 | 3867 | Train 61 | Test Female 27 | 0.74 | 0.76 | 0.72 |
| 2844 | Train 45 | Test Female 28 | 0.76 | 0.8 | 0.8 | 3868 | Train 61 | Test Female 28 | 0.75 | 0.75 | 0.7 |
| 2845 | Train 45 | Test Female 29 | 0.75 | 0.8 | 0.76 | 3869 | Train 61 | Test Female 29 | 0.73 | 0.76 | 0.73 |
| 2846 | Train 45 | Test Female 30 | 0.72 | 0.74 | 0.7 | 3870 | Train 61 | Test Female 30 | 0.72 | 0.71 | 0.65 |
| 2847 | Train 45 | Test Female 31 | 0.75 | 0.81 | 0.82 | 3871 | Train 61 | Test Female 31 | 0.73 | 0.75 | 0.72 |
| 2848 | Train 45 | Test Female 32 | 0.72 | 0.74 | 0.7 | 3872 | Train 61 | Test Female 32 | 0.72 | 0.71 | 0.65 |
| 2849 | Train 45 | Test Female 33 | 0.76 | 0.81 | 0.81 | 3873 | Train 61 | Test Female 33 | 0.75 | 0.77 | 0.72 |
| 2850 | Train 45 | Test Female 34 | 0.77 | 0.8 | 0.78 | 3874 | Train 61 | Test Female 34 | 0.75 | 0.75 | 0.69 |
| 2851 | Train 45 | Test Female 35 | 0.76 | 0.81 | 0.81 | 3875 | Train 61 | Test Female 35 | 0.75 | 0.77 | 0.72 |
| 2852 | Train 45 | Test Female 36 | 0.77 | 0.8 | 0.78 | 3876 | Train 61 | Test Female 36 | 0.75 | 0.75 | 0.69 |
| 2853 | Train 45 | Test Female 37 | 0.75 | 0.81 | 0.81 | 3877 | Train 61 | Test Female 37 | 0.73 | 0.76 | 0.71 |
| 2854 | Train 45 | Test Female 38 | 0.72 | 0.74 | 0.7 | 3878 | Train 61 | Test Female 38 | 0.72 | 0.71 | 0.65 |
| 2855 | Train 45 | Test Female 39 | 0.75 | 0.81 | 0.81 | 3879 | Train 61 | Test Female 39 | 0.73 | 0.76 | 0.71 |
| 2856 | Train 45 | Test Female 40 | 0.72 | 0.74 | 0.7 | 3880 | Train 61 | Test Female 40 | 0.72 | 0.71 | 0.65 |
| 2857 | Train 45 | Test Female 41 | 0.76 | 0.82 | 0.83 | 3881 | Train 61 | Test Female 41 | 0.75 | 0.77 | 0.73 |
| 2858 | Train 45 | Test Female 42 | 0.77 | 0.81 | 0.8 | 3882 | Train 61 | Test Female 42 | 0.75 | 0.75 | 0.7 |
| 2859 | Train 45 | Test Female 43 | 0.76 | 0.82 | 0.83 | 3883 | Train 61 | Test Female 43 | 0.75 | 0.77 | 0.73 |
| 2860 | Train 45 | Test Female 44 | 0.77 | 0.81 | 0.8 | 3884 | Train 61 | Test Female 44 | 0.75 | 0.75 | 0.7 |
| 2861 | Train 45 | Test Female 45 | 0.75 | 0.8 | 0.77 | 3885 | Train 61 | Test Female 45 | 0.73 | 0.76 | 0.74 |
| 2862 | Train 45 | Test Female 46 | 0.72 | 0.74 | 0.7 | 3886 | Train 61 | Test Female 46 | 0.72 | 0.71 | 0.65 |
| 2863 | Train 45 | Test Female 47 | 0.75 | 0.82 | 0.83 | 3887 | Train 61 | Test Female 47 | 0.73 | 0.76 | 0.72 |
| 2864 | Train 45 | Test Female 48 | 0.72 | 0.74 | 0.7 | 3888 | Train 61 | Test Female 48 | 0.72 | 0.71 | 0.65 |
| 2865 | Train 45 | Test Female 49 | 0.78 | 0.82 | 0.82 | 3889 | Train 61 | Test Female 49 | 0.79 | 0.84 | 0.81 |
| 2866 | Train 45 | Test Female 50 | 0.78 | 0.82 | 0.82 | 3890 | Train 61 | Test Female 50 | 0.79 | 0.83 | 0.81 |
| 2867 | Train 45 | Test Female 51 | 0.78 | 0.82 | 0.82 | 3891 | Train 61 | Test Female 51 | 0.79 | 0.83 | 0.81 |
| 2868 | Train 45 | Test Female 52 | 0.78 | 0.82 | 0.82 | 3892 | Train 61 | Test Female 52 | 0.79 | 0.83 | 0.81 |
| 2869 | Train 45 | Test Female 53 | 0.77 | 0.82 | 0.83 | 3893 | Train 61 | Test Female 53 | 0.78 | 0.84 | 0.83 |
| 2870 | Train 45 | Test Female 54 | 0.74 | 0.74 | 0.71 | 3894 | Train 61 | Test Female 54 | 0.74 | 0.74 | 0.68 |
| 2871 | Train 45 | Test Female 55 | 0.77 | 0.82 | 0.83 | 3895 | Train 61 | Test Female 55 | 0.78 | 0.84 | 0.83 |
| 2872 | Train 45 | Test Female 56 | 0.73 | 0.74 | 0.71 | 3896 | Train 61 | Test Female 56 | 0.74 | 0.74 | 0.68 |
| 2873 | Train 45 | Test Female 57 | 0.78 | 0.83 | 0.84 | 3897 | Train 61 | Test Female 57 | 0.79 | 0.84 | 0.85 |
| 2874 | Train 45 | Test Female 58 | 0.78 | 0.83 | 0.84 | 3898 | Train 61 | Test Female 58 | 0.79 | 0.84 | 0.85 |
| 2875 | Train 45 | Test Female 59 | 0.78 | 0.83 | 0.84 | 3899 | Train 61 | Test Female 59 | 0.79 | 0.84 | 0.85 |
| 2876 | Train 45 | Test Female 60 | 0.78 | 0.83 | 0.84 | 3900 | Train 61 | Test Female 60 | 0.79 | 0.84 | 0.84 |
| 2877 | Train 45 | Test Female 61 | 0.77 | 0.83 | 0.84 | 3901 | Train 61 | Test Female 61 | 0.78 | 0.85 | 0.85 |
| 2878 | Train 45 | Test Female 62 | 0.74 | 0.75 | 0.71 | 3902 | Train 61 | Test Female 62 | 0.74 | 0.74 | 0.68 |
| 2879 | Train 45 | Test Female 63 | 0.77 | 0.83 | 0.84 | 3903 | Train 61 | Test Female 63 | 0.78 | 0.85 | 0.85 |
| 2880 | Train 45 | Test Female 64 | 0.73 | 0.74 | 0.71 | 3904 | Train 61 | Test Female 64 | 0.74 | 0.74 | 0.68 |
| 2881 | Train 46 | Test Female 1 | 0.74 | 0.78 | 0.77 | 3905 | Train 62 | Test Female 1 | 0.73 | 0.74 | 0.71 |
| 2882 | Train 46 | Test Female 2 | 0.76 | 0.79 | 0.78 | 3906 | Train 62 | Test Female 2 | 0.74 | 0.75 | 0.71 |
| 2883 | Train 46 | Test Female 3 | 0.74 | 0.78 | 0.77 | 3907 | Train 62 | Test Female 3 | 0.73 | 0.74 | 0.71 |
| 2884 | Train 46 | Test Female 4 | 0.76 | 0.79 | 0.78 | 3908 | Train 62 | Test Female 4 | 0.74 | 0.75 | 0.71 |
| 2885 | Train 46 | Test Female 5 | 0.73 | 0.78 | 0.77 | 3909 | Train 62 | Test Female 5 | 0.72 | 0.74 | 0.71 |
| 2886 | Train 46 | Test Female 6 | 0.75 | 0.8 | 0.8 | 3910 | Train 62 | Test Female 6 | 0.74 | 0.77 | 0.74 |
| 2887 | Train 46 | Test Female 7 | 0.73 | 0.78 | 0.76 | 3911 | Train 62 | Test Female 7 | 0.72 | 0.74 | 0.71 |
| 2888 | Train 46 | Test Female 8 | 0.75 | 0.79 | 0.8 | 3912 | Train 62 | Test Female 8 | 0.74 | 0.76 | 0.74 |
| 2889 | Train 46 | Test Female 9 | 0.74 | 0.78 | 0.78 | 3913 | Train 62 | Test Female 9 | 0.73 | 0.75 | 0.72 |
| 2890 | Train 46 | Test Female 10 | 0.76 | 0.79 | 0.79 | 3914 | Train 62 | Test Female 10 | 0.74 | 0.76 | 0.72 |
| 2891 | Train 46 | Test Female 11 | 0.74 | 0.79 | 0.78 | 3915 | Train 62 | Test Female 11 | 0.73 | 0.75 | 0.72 |
| 2892 | Train 46 | Test Female 12 | 0.76 | 0.79 | 0.79 | 3916 | Train 62 | Test Female 12 | 0.74 | 0.76 | 0.72 |
| 2893 | Train 46 | Test Female 13 | 0.73 | 0.79 | 0.8 | 3917 | Train 62 | Test Female 13 | 0.72 | 0.75 | 0.72 |
| 2894 | Train 46 | Test Female 14 | 0.75 | 0.8 | 0.81 | 3918 | Train 62 | Test Female 14 | 0.74 | 0.77 | 0.74 |
| 2895 | Train 46 | Test Female 15 | 0.73 | 0.78 | 0.77 | 3919 | Train 62 | Test Female 15 | 0.72 | 0.75 | 0.72 |
| 2896 | Train 46 | Test Female 16 | 0.75 | 0.79 | 0.8 | 3920 | Train 62 | Test Female 16 | 0.74 | 0.76 | 0.74 |
| 2897 | Train 46 | Test Female 17 | 0.74 | 0.78 | 0.77 | 3921 | Train 62 | Test Female 17 | 0.73 | 0.74 | 0.71 |
| 2898 | Train 46 | Test Female 18 | 0.76 | 0.79 | 0.78 | 3922 | Train 62 | Test Female 18 | 0.74 | 0.75 | 0.71 |
| 2899 | Train 46 | Test Female 19 | 0.74 | 0.78 | 0.77 | 3923 | Train 62 | Test Female 19 | 0.73 | 0.74 | 0.71 |
| 2900 | Train 46 | Test Female 20 | 0.76 | 0.79 | 0.78 | 3924 | Train 62 | Test Female 20 | 0.74 | 0.75 | 0.71 |
| 2901 | Train 46 | Test Female 21 | 0.73 | 0.78 | 0.77 | 3925 | Train 62 | Test Female 21 | 0.72 | 0.74 | 0.71 |
| 2902 | Train 46 | Test Female 22 | 0.75 | 0.8 | 0.8 | 3926 | Train 62 | Test Female 22 | 0.74 | 0.77 | 0.74 |
| 2903 | Train 46 | Test Female 23 | 0.73 | 0.78 | 0.76 | 3927 | Train 62 | Test Female 23 | 0.72 | 0.74 | 0.71 |
| 2904 | Train 46 | Test Female 24 | 0.75 | 0.79 | 0.8 | 3928 | Train 62 | Test Female 24 | 0.74 | 0.76 | 0.74 |
| 2905 | Train 46 | Test Female 25 | 0.74 | 0.79 | 0.78 | 3929 | Train 62 | Test Female 25 | 0.73 | 0.75 | 0.72 |
| 2906 | Train 46 | Test Female 26 | 0.76 | 0.79 | 0.79 | 3930 | Train 62 | Test Female 26 | 0.74 | 0.76 | 0.72 |
| 2907 | Train 46 | Test Female 27 | 0.74 | 0.79 | 0.78 | 3931 | Train 62 | Test Female 27 | 0.73 | 0.75 | 0.72 |
| 2908 | Train 46 | Test Female 28 | 0.76 | 0.79 | 0.79 | 3932 | Train 62 | Test Female 28 | 0.74 | 0.76 | 0.72 |
| 2909 | Train 46 | Test Female 29 | 0.73 | 0.79 | 0.8 | 3933 | Train 62 | Test Female 29 | 0.72 | 0.75 | 0.72 |
| 2910 | Train 46 | Test Female 30 | 0.75 | 0.8 | 0.81 | 3934 | Train 62 | Test Female 30 | 0.74 | 0.77 | 0.74 |
| 2911 | Train 46 | Test Female 31 | 0.73 | 0.78 | 0.77 | 3935 | Train 62 | Test Female 31 | 0.72 | 0.75 | 0.72 |
| 2912 | Train 46 | Test Female 32 | 0.75 | 0.79 | 0.8 | 3936 | Train 62 | Test Female 32 | 0.74 | 0.76 | 0.74 |
| 2913 | Train 46 | Test Female 33 | 0.75 | 0.79 | 0.77 | 3937 | Train 62 | Test Female 33 | 0.73 | 0.75 | 0.72 |
| 2914 | Train 46 | Test Female 34 | 0.77 | 0.8 | 0.78 | 3938 | Train 62 | Test Female 34 | 0.75 | 0.76 | 0.72 |
| 2915 | Train 46 | Test Female 35 | 0.75 | 0.79 | 0.77 | 3939 | Train 62 | Test Female 35 | 0.74 | 0.75 | 0.72 |
| 2916 | Train 46 | Test Female 36 | 0.77 | 0.8 | 0.78 | 3940 | Train 62 | Test Female 36 | 0.75 | 0.76 | 0.72 |
| 2917 | Train 46 | Test Female 37 | 0.74 | 0.78 | 0.77 | 3941 | Train 62 | Test Female 37 | 0.73 | 0.75 | 0.72 |
| 2918 | Train 46 | Test Female 38 | 0.75 | 0.8 | 0.81 | 3942 | Train 62 | Test Female 38 | 0.74 | 0.77 | 0.74 |
| 2919 | Train 46 | Test Female 39 | 0.74 | 0.78 | 0.77 | 3943 | Train 62 | Test Female 39 | 0.72 | 0.75 | 0.72 |
| 2920 | Train 46 | Test Female 40 | 0.75 | 0.79 | 0.8 | 3944 | Train 62 | Test Female 40 | 0.74 | 0.76 | 0.74 |
| 2921 | Train 46 | Test Female 41 | 0.75 | 0.79 | 0.78 | 3945 | Train 62 | Test Female 41 | 0.74 | 0.76 | 0.73 |
| 2922 | Train 46 | Test Female 42 | 0.77 | 0.8 | 0.79 | 3946 | Train 62 | Test Female 42 | 0.75 | 0.76 | 0.73 |
| 2923 | Train 46 | Test Female 43 | 0.75 | 0.79 | 0.78 | 3947 | Train 62 | Test Female 43 | 0.74 | 0.76 | 0.73 |
| 2924 | Train 46 | Test Female 44 | 0.77 | 0.8 | 0.79 | 3948 | Train 62 | Test Female 44 | 0.75 | 0.76 | 0.73 |
| 2925 | Train 46 | Test Female 45 | 0.74 | 0.8 | 0.8 | 3949 | Train 62 | Test Female 45 | 0.73 | 0.75 | 0.72 |
| 2926 | Train 46 | Test Female 46 | 0.76 | 0.8 | 0.81 | 3950 | Train 62 | Test Female 46 | 0.74 | 0.77 | 0.74 |
| 2927 | Train 46 | Test Female 47 | 0.74 | 0.78 | 0.78 | 3951 | Train 62 | Test Female 47 | 0.72 | 0.75 | 0.73 |
| 2928 | Train 46 | Test Female 48 | 0.75 | 0.8 | 0.8 | 3952 | Train 62 | Test Female 48 | 0.74 | 0.77 | 0.74 |
| 2929 | Train 46 | Test Female 49 | 0.78 | 0.81 | 0.8 | 3953 | Train 62 | Test Female 49 | 0.78 | 0.81 | 0.81 |
| 2930 | Train 46 | Test Female 50 | 0.78 | 0.81 | 0.8 | 3954 | Train 62 | Test Female 50 | 0.78 | 0.81 | 0.81 |
| 2931 | Train 46 | Test Female 51 | 0.78 | 0.81 | 0.8 | 3955 | Train 62 | Test Female 51 | 0.78 | 0.81 | 0.81 |
| 2932 | Train 46 | Test Female 52 | 0.78 | 0.81 | 0.8 | 3956 | Train 62 | Test Female 52 | 0.78 | 0.81 | 0.81 |
| 2933 | Train 46 | Test Female 53 | 0.76 | 0.81 | 0.79 | 3957 | Train 62 | Test Female 53 | 0.77 | 0.82 | 0.81 |
| 2934 | Train 46 | Test Female 54 | 0.76 | 0.8 | 0.8 | 3958 | Train 62 | Test Female 54 | 0.77 | 0.82 | 0.82 |
| 2935 | Train 46 | Test Female 55 | 0.77 | 0.81 | 0.79 | 3959 | Train 62 | Test Female 55 | 0.77 | 0.82 | 0.81 |
| 2936 | Train 46 | Test Female 56 | 0.76 | 0.79 | 0.8 | 3960 | Train 62 | Test Female 56 | 0.76 | 0.81 | 0.82 |
| 2937 | Train 46 | Test Female 57 | 0.78 | 0.81 | 0.82 | 3961 | Train 62 | Test Female 57 | 0.79 | 0.82 | 0.83 |
| 2938 | Train 46 | Test Female 58 | 0.78 | 0.81 | 0.82 | 3962 | Train 62 | Test Female 58 | 0.78 | 0.82 | 0.83 |
| 2939 | Train 46 | Test Female 59 | 0.78 | 0.81 | 0.81 | 3963 | Train 62 | Test Female 59 | 0.79 | 0.82 | 0.83 |
| 2940 | Train 46 | Test Female 60 | 0.78 | 0.81 | 0.81 | 3964 | Train 62 | Test Female 60 | 0.79 | 0.82 | 0.83 |
| 2941 | Train 46 | Test Female 61 | 0.77 | 0.81 | 0.8 | 3965 | Train 62 | Test Female 61 | 0.77 | 0.82 | 0.83 |
| 2942 | Train 46 | Test Female 62 | 0.76 | 0.8 | 0.8 | 3966 | Train 62 | Test Female 62 | 0.77 | 0.82 | 0.83 |
| 2943 | Train 46 | Test Female 63 | 0.77 | 0.81 | 0.8 | 3967 | Train 62 | Test Female 63 | 0.77 | 0.82 | 0.83 |
| 2944 | Train 46 | Test Female 64 | 0.76 | 0.79 | 0.8 | 3968 | Train 62 | Test Female 64 | 0.76 | 0.81 | 0.82 |
| 2945 | Train 47 | Test Female 1 | 0.75 | 0.81 | 0.81 | 3969 | Train 63 | Test Female 1 | 0.74 | 0.76 | 0.72 |
| 2946 | Train 47 | Test Female 2 | 0.76 | 0.8 | 0.79 | 3970 | Train 63 | Test Female 2 | 0.75 | 0.74 | 0.69 |
| 2947 | Train 47 | Test Female 3 | 0.76 | 0.81 | 0.81 | 3971 | Train 63 | Test Female 3 | 0.74 | 0.76 | 0.72 |
| 2948 | Train 47 | Test Female 4 | 0.76 | 0.8 | 0.79 | 3972 | Train 63 | Test Female 4 | 0.75 | 0.75 | 0.69 |
| 2949 | Train 47 | Test Female 5 | 0.75 | 0.81 | 0.81 | 3973 | Train 63 | Test Female 5 | 0.73 | 0.75 | 0.71 |
| 2950 | Train 47 | Test Female 6 | 0.72 | 0.73 | 0.68 | 3974 | Train 63 | Test Female 6 | 0.71 | 0.71 | 0.66 |
| 2951 | Train 47 | Test Female 7 | 0.75 | 0.81 | 0.81 | 3975 | Train 63 | Test Female 7 | 0.73 | 0.75 | 0.71 |
| 2952 | Train 47 | Test Female 8 | 0.72 | 0.73 | 0.68 | 3976 | Train 63 | Test Female 8 | 0.71 | 0.71 | 0.66 |
| 2953 | Train 47 | Test Female 9 | 0.76 | 0.81 | 0.83 | 3977 | Train 63 | Test Female 9 | 0.74 | 0.76 | 0.73 |
| 2954 | Train 47 | Test Female 10 | 0.76 | 0.8 | 0.8 | 3978 | Train 63 | Test Female 10 | 0.75 | 0.75 | 0.7 |
| 2955 | Train 47 | Test Female 11 | 0.76 | 0.81 | 0.83 | 3979 | Train 63 | Test Female 11 | 0.74 | 0.76 | 0.74 |
| 2956 | Train 47 | Test Female 12 | 0.76 | 0.8 | 0.8 | 3980 | Train 63 | Test Female 12 | 0.75 | 0.75 | 0.71 |
| 2957 | Train 47 | Test Female 13 | 0.75 | 0.8 | 0.75 | 3981 | Train 63 | Test Female 13 | 0.73 | 0.76 | 0.74 |
| 2958 | Train 47 | Test Female 14 | 0.72 | 0.73 | 0.68 | 3982 | Train 63 | Test Female 14 | 0.72 | 0.71 | 0.66 |
| 2959 | Train 47 | Test Female 15 | 0.75 | 0.81 | 0.83 | 3983 | Train 63 | Test Female 15 | 0.73 | 0.75 | 0.73 |
| 2960 | Train 47 | Test Female 16 | 0.72 | 0.73 | 0.68 | 3984 | Train 63 | Test Female 16 | 0.72 | 0.71 | 0.66 |
| 2961 | Train 47 | Test Female 17 | 0.75 | 0.81 | 0.81 | 3985 | Train 63 | Test Female 17 | 0.74 | 0.76 | 0.72 |
| 2962 | Train 47 | Test Female 18 | 0.76 | 0.8 | 0.79 | 3986 | Train 63 | Test Female 18 | 0.75 | 0.74 | 0.69 |
| 2963 | Train 47 | Test Female 19 | 0.76 | 0.81 | 0.81 | 3987 | Train 63 | Test Female 19 | 0.74 | 0.76 | 0.72 |
| 2964 | Train 47 | Test Female 20 | 0.76 | 0.8 | 0.79 | 3988 | Train 63 | Test Female 20 | 0.75 | 0.75 | 0.69 |
| 2965 | Train 47 | Test Female 21 | 0.75 | 0.81 | 0.81 | 3989 | Train 63 | Test Female 21 | 0.73 | 0.75 | 0.71 |
| 2966 | Train 47 | Test Female 22 | 0.72 | 0.73 | 0.68 | 3990 | Train 63 | Test Female 22 | 0.72 | 0.71 | 0.66 |
| 2967 | Train 47 | Test Female 23 | 0.75 | 0.81 | 0.81 | 3991 | Train 63 | Test Female 23 | 0.73 | 0.75 | 0.71 |
| 2968 | Train 47 | Test Female 24 | 0.72 | 0.73 | 0.68 | 3992 | Train 63 | Test Female 24 | 0.71 | 0.71 | 0.66 |
| 2969 | Train 47 | Test Female 25 | 0.76 | 0.81 | 0.83 | 3993 | Train 63 | Test Female 25 | 0.74 | 0.76 | 0.73 |
| 2970 | Train 47 | Test Female 26 | 0.76 | 0.8 | 0.8 | 3994 | Train 63 | Test Female 26 | 0.75 | 0.75 | 0.7 |
| 2971 | Train 47 | Test Female 27 | 0.76 | 0.81 | 0.83 | 3995 | Train 63 | Test Female 27 | 0.74 | 0.76 | 0.74 |
| 2972 | Train 47 | Test Female 28 | 0.76 | 0.8 | 0.8 | 3996 | Train 63 | Test Female 28 | 0.75 | 0.75 | 0.71 |
| 2973 | Train 47 | Test Female 29 | 0.75 | 0.8 | 0.76 | 3997 | Train 63 | Test Female 29 | 0.73 | 0.76 | 0.74 |
| 2974 | Train 47 | Test Female 30 | 0.72 | 0.73 | 0.69 | 3998 | Train 63 | Test Female 30 | 0.72 | 0.71 | 0.66 |
| 2975 | Train 47 | Test Female 31 | 0.75 | 0.81 | 0.83 | 3999 | Train 63 | Test Female 31 | 0.73 | 0.75 | 0.73 |
| 2976 | Train 47 | Test Female 32 | 0.72 | 0.74 | 0.69 | 4000 | Train 63 | Test Female 32 | 0.72 | 0.71 | 0.66 |
| 2977 | Train 47 | Test Female 33 | 0.76 | 0.81 | 0.81 | 4001 | Train 63 | Test Female 33 | 0.75 | 0.76 | 0.73 |
| 2978 | Train 47 | Test Female 34 | 0.77 | 0.8 | 0.79 | 4002 | Train 63 | Test Female 34 | 0.75 | 0.75 | 0.7 |
| 2979 | Train 47 | Test Female 35 | 0.76 | 0.82 | 0.81 | 4003 | Train 63 | Test Female 35 | 0.75 | 0.77 | 0.73 |
| 2980 | Train 47 | Test Female 36 | 0.77 | 0.8 | 0.79 | 4004 | Train 63 | Test Female 36 | 0.75 | 0.75 | 0.7 |
| 2981 | Train 47 | Test Female 37 | 0.75 | 0.81 | 0.81 | 4005 | Train 63 | Test Female 37 | 0.73 | 0.76 | 0.72 |
| 2982 | Train 47 | Test Female 38 | 0.72 | 0.73 | 0.69 | 4006 | Train 63 | Test Female 38 | 0.72 | 0.71 | 0.66 |
| 2983 | Train 47 | Test Female 39 | 0.75 | 0.81 | 0.81 | 4007 | Train 63 | Test Female 39 | 0.73 | 0.76 | 0.72 |
| 2984 | Train 47 | Test Female 40 | 0.72 | 0.74 | 0.69 | 4008 | Train 63 | Test Female 40 | 0.72 | 0.71 | 0.66 |
| 2985 | Train 47 | Test Female 41 | 0.76 | 0.82 | 0.83 | 4009 | Train 63 | Test Female 41 | 0.75 | 0.77 | 0.74 |
| 2986 | Train 47 | Test Female 42 | 0.77 | 0.81 | 0.81 | 4010 | Train 63 | Test Female 42 | 0.75 | 0.75 | 0.71 |
| 2987 | Train 47 | Test Female 43 | 0.76 | 0.82 | 0.83 | 4011 | Train 63 | Test Female 43 | 0.75 | 0.77 | 0.74 |
| 2988 | Train 47 | Test Female 44 | 0.77 | 0.81 | 0.81 | 4012 | Train 63 | Test Female 44 | 0.75 | 0.75 | 0.71 |
| 2989 | Train 47 | Test Female 45 | 0.75 | 0.8 | 0.76 | 4013 | Train 63 | Test Female 45 | 0.73 | 0.76 | 0.74 |
| 2990 | Train 47 | Test Female 46 | 0.72 | 0.74 | 0.69 | 4014 | Train 63 | Test Female 46 | 0.72 | 0.71 | 0.66 |
| 2991 | Train 47 | Test Female 47 | 0.75 | 0.82 | 0.83 | 4015 | Train 63 | Test Female 47 | 0.73 | 0.76 | 0.74 |
| 2992 | Train 47 | Test Female 48 | 0.72 | 0.74 | 0.69 | 4016 | Train 63 | Test Female 48 | 0.72 | 0.71 | 0.66 |
| 2993 | Train 47 | Test Female 49 | 0.78 | 0.82 | 0.82 | 4017 | Train 63 | Test Female 49 | 0.79 | 0.83 | 0.82 |
| 2994 | Train 47 | Test Female 50 | 0.78 | 0.82 | 0.82 | 4018 | Train 63 | Test Female 50 | 0.79 | 0.83 | 0.82 |
| 2995 | Train 47 | Test Female 51 | 0.78 | 0.82 | 0.82 | 4019 | Train 63 | Test Female 51 | 0.79 | 0.83 | 0.82 |
| 2996 | Train 47 | Test Female 52 | 0.78 | 0.82 | 0.82 | 4020 | Train 63 | Test Female 52 | 0.79 | 0.83 | 0.82 |
| 2997 | Train 47 | Test Female 53 | 0.77 | 0.82 | 0.83 | 4021 | Train 63 | Test Female 53 | 0.78 | 0.84 | 0.83 |
| 2998 | Train 47 | Test Female 54 | 0.74 | 0.74 | 0.69 | 4022 | Train 63 | Test Female 54 | 0.74 | 0.74 | 0.7 |
| 2999 | Train 47 | Test Female 55 | 0.77 | 0.83 | 0.83 | 4023 | Train 63 | Test Female 55 | 0.78 | 0.84 | 0.84 |
| 3000 | Train 47 | Test Female 56 | 0.73 | 0.74 | 0.69 | 4024 | Train 63 | Test Female 56 | 0.73 | 0.74 | 0.7 |
| 3001 | Train 47 | Test Female 57 | 0.78 | 0.83 | 0.85 | 4025 | Train 63 | Test Female 57 | 0.79 | 0.84 | 0.86 |
| 3002 | Train 47 | Test Female 58 | 0.78 | 0.83 | 0.85 | 4026 | Train 63 | Test Female 58 | 0.79 | 0.84 | 0.86 |
| 3003 | Train 47 | Test Female 59 | 0.78 | 0.83 | 0.85 | 4027 | Train 63 | Test Female 59 | 0.79 | 0.84 | 0.86 |
| 3004 | Train 47 | Test Female 60 | 0.78 | 0.83 | 0.85 | 4028 | Train 63 | Test Female 60 | 0.79 | 0.84 | 0.86 |
| 3005 | Train 47 | Test Female 61 | 0.77 | 0.83 | 0.84 | 4029 | Train 63 | Test Female 61 | 0.78 | 0.84 | 0.86 |
| 3006 | Train 47 | Test Female 62 | 0.74 | 0.74 | 0.69 | 4030 | Train 63 | Test Female 62 | 0.74 | 0.74 | 0.7 |
| 3007 | Train 47 | Test Female 63 | 0.77 | 0.83 | 0.84 | 4031 | Train 63 | Test Female 63 | 0.78 | 0.84 | 0.86 |
| 3008 | Train 47 | Test Female 64 | 0.73 | 0.74 | 0.69 | 4032 | Train 63 | Test Female 64 | 0.74 | 0.74 | 0.7 |
| 3009 | Train 48 | Test Female 1 | 0.74 | 0.78 | 0.77 | 4033 | Train 64 | Test Female 1 | 0.73 | 0.75 | 0.71 |
| 3010 | Train 48 | Test Female 2 | 0.76 | 0.79 | 0.78 | 4034 | Train 64 | Test Female 2 | 0.74 | 0.75 | 0.71 |
| 3011 | Train 48 | Test Female 3 | 0.74 | 0.78 | 0.77 | 4035 | Train 64 | Test Female 3 | 0.73 | 0.75 | 0.71 |
| 3012 | Train 48 | Test Female 4 | 0.76 | 0.79 | 0.78 | 4036 | Train 64 | Test Female 4 | 0.74 | 0.75 | 0.71 |
| 3013 | Train 48 | Test Female 5 | 0.73 | 0.78 | 0.77 | 4037 | Train 64 | Test Female 5 | 0.72 | 0.75 | 0.71 |
| 3014 | Train 48 | Test Female 6 | 0.75 | 0.8 | 0.8 | 4038 | Train 64 | Test Female 6 | 0.73 | 0.76 | 0.73 |
| 3015 | Train 48 | Test Female 7 | 0.73 | 0.78 | 0.77 | 4039 | Train 64 | Test Female 7 | 0.72 | 0.74 | 0.71 |
| 3016 | Train 48 | Test Female 8 | 0.75 | 0.8 | 0.8 | 4040 | Train 64 | Test Female 8 | 0.73 | 0.76 | 0.73 |
| 3017 | Train 48 | Test Female 9 | 0.74 | 0.78 | 0.78 | 4041 | Train 64 | Test Female 9 | 0.73 | 0.75 | 0.72 |
| 3018 | Train 48 | Test Female 10 | 0.76 | 0.79 | 0.79 | 4042 | Train 64 | Test Female 10 | 0.74 | 0.76 | 0.72 |
| 3019 | Train 48 | Test Female 11 | 0.75 | 0.79 | 0.78 | 4043 | Train 64 | Test Female 11 | 0.73 | 0.75 | 0.72 |
| 3020 | Train 48 | Test Female 12 | 0.76 | 0.79 | 0.79 | 4044 | Train 64 | Test Female 12 | 0.74 | 0.76 | 0.72 |
| 3021 | Train 48 | Test Female 13 | 0.73 | 0.79 | 0.8 | 4045 | Train 64 | Test Female 13 | 0.72 | 0.75 | 0.72 |
| 3022 | Train 48 | Test Female 14 | 0.75 | 0.8 | 0.8 | 4046 | Train 64 | Test Female 14 | 0.73 | 0.77 | 0.73 |
| 3023 | Train 48 | Test Female 15 | 0.73 | 0.78 | 0.78 | 4047 | Train 64 | Test Female 15 | 0.72 | 0.75 | 0.72 |
| 3024 | Train 48 | Test Female 16 | 0.75 | 0.8 | 0.8 | 4048 | Train 64 | Test Female 16 | 0.73 | 0.77 | 0.74 |
| 3025 | Train 48 | Test Female 17 | 0.74 | 0.78 | 0.77 | 4049 | Train 64 | Test Female 17 | 0.73 | 0.75 | 0.71 |
| 3026 | Train 48 | Test Female 18 | 0.76 | 0.79 | 0.78 | 4050 | Train 64 | Test Female 18 | 0.74 | 0.75 | 0.71 |
| 3027 | Train 48 | Test Female 19 | 0.75 | 0.78 | 0.77 | 4051 | Train 64 | Test Female 19 | 0.73 | 0.75 | 0.71 |
| 3028 | Train 48 | Test Female 20 | 0.76 | 0.79 | 0.78 | 4052 | Train 64 | Test Female 20 | 0.74 | 0.76 | 0.71 |
| 3029 | Train 48 | Test Female 21 | 0.73 | 0.78 | 0.77 | 4053 | Train 64 | Test Female 21 | 0.72 | 0.75 | 0.71 |
| 3030 | Train 48 | Test Female 22 | 0.75 | 0.8 | 0.8 | 4054 | Train 64 | Test Female 22 | 0.73 | 0.76 | 0.73 |
| 3031 | Train 48 | Test Female 23 | 0.73 | 0.78 | 0.77 | 4055 | Train 64 | Test Female 23 | 0.72 | 0.74 | 0.71 |
| 3032 | Train 48 | Test Female 24 | 0.75 | 0.8 | 0.8 | 4056 | Train 64 | Test Female 24 | 0.73 | 0.76 | 0.74 |
| 3033 | Train 48 | Test Female 25 | 0.75 | 0.78 | 0.78 | 4057 | Train 64 | Test Female 25 | 0.73 | 0.75 | 0.72 |
| 3034 | Train 48 | Test Female 26 | 0.76 | 0.79 | 0.79 | 4058 | Train 64 | Test Female 26 | 0.74 | 0.76 | 0.72 |
| 3035 | Train 48 | Test Female 27 | 0.75 | 0.79 | 0.78 | 4059 | Train 64 | Test Female 27 | 0.73 | 0.75 | 0.72 |
| 3036 | Train 48 | Test Female 28 | 0.76 | 0.79 | 0.79 | 4060 | Train 64 | Test Female 28 | 0.74 | 0.76 | 0.72 |
| 3037 | Train 48 | Test Female 29 | 0.73 | 0.79 | 0.8 | 4061 | Train 64 | Test Female 29 | 0.72 | 0.75 | 0.72 |
| 3038 | Train 48 | Test Female 30 | 0.75 | 0.8 | 0.8 | 4062 | Train 64 | Test Female 30 | 0.74 | 0.77 | 0.73 |
| 3039 | Train 48 | Test Female 31 | 0.73 | 0.78 | 0.78 | 4063 | Train 64 | Test Female 31 | 0.72 | 0.75 | 0.72 |
| 3040 | Train 48 | Test Female 32 | 0.75 | 0.8 | 0.8 | 4064 | Train 64 | Test Female 32 | 0.74 | 0.77 | 0.74 |
| 3041 | Train 48 | Test Female 33 | 0.75 | 0.79 | 0.78 | 4065 | Train 64 | Test Female 33 | 0.74 | 0.75 | 0.71 |
| 3042 | Train 48 | Test Female 34 | 0.77 | 0.8 | 0.79 | 4066 | Train 64 | Test Female 34 | 0.74 | 0.76 | 0.72 |
| 3043 | Train 48 | Test Female 35 | 0.75 | 0.79 | 0.78 | 4067 | Train 64 | Test Female 35 | 0.74 | 0.75 | 0.72 |
| 3044 | Train 48 | Test Female 36 | 0.77 | 0.8 | 0.79 | 4068 | Train 64 | Test Female 36 | 0.74 | 0.76 | 0.72 |
| 3045 | Train 48 | Test Female 37 | 0.74 | 0.78 | 0.77 | 4069 | Train 64 | Test Female 37 | 0.72 | 0.75 | 0.72 |
| 3046 | Train 48 | Test Female 38 | 0.75 | 0.8 | 0.8 | 4070 | Train 64 | Test Female 38 | 0.73 | 0.77 | 0.73 |
| 3047 | Train 48 | Test Female 39 | 0.74 | 0.78 | 0.77 | 4071 | Train 64 | Test Female 39 | 0.72 | 0.75 | 0.72 |
| 3048 | Train 48 | Test Female 40 | 0.75 | 0.8 | 0.8 | 4072 | Train 64 | Test Female 40 | 0.73 | 0.77 | 0.74 |
| 3049 | Train 48 | Test Female 41 | 0.75 | 0.79 | 0.79 | 4073 | Train 64 | Test Female 41 | 0.74 | 0.76 | 0.73 |
| 3050 | Train 48 | Test Female 42 | 0.77 | 0.8 | 0.79 | 4074 | Train 64 | Test Female 42 | 0.74 | 0.76 | 0.72 |
| 3051 | Train 48 | Test Female 43 | 0.75 | 0.79 | 0.79 | 4075 | Train 64 | Test Female 43 | 0.74 | 0.76 | 0.73 |
| 3052 | Train 48 | Test Female 44 | 0.77 | 0.8 | 0.8 | 4076 | Train 64 | Test Female 44 | 0.75 | 0.76 | 0.72 |
| 3053 | Train 48 | Test Female 45 | 0.74 | 0.79 | 0.8 | 4077 | Train 64 | Test Female 45 | 0.72 | 0.75 | 0.73 |
| 3054 | Train 48 | Test Female 46 | 0.75 | 0.8 | 0.8 | 4078 | Train 64 | Test Female 46 | 0.74 | 0.77 | 0.73 |
| 3055 | Train 48 | Test Female 47 | 0.74 | 0.78 | 0.78 | 4079 | Train 64 | Test Female 47 | 0.72 | 0.75 | 0.73 |
| 3056 | Train 48 | Test Female 48 | 0.75 | 0.8 | 0.8 | 4080 | Train 64 | Test Female 48 | 0.74 | 0.77 | 0.74 |
| 3057 | Train 48 | Test Female 49 | 0.78 | 0.81 | 0.8 | 4081 | Train 64 | Test Female 49 | 0.79 | 0.81 | 0.81 |
| 3058 | Train 48 | Test Female 50 | 0.78 | 0.81 | 0.8 | 4082 | Train 64 | Test Female 50 | 0.79 | 0.81 | 0.81 |
| 3059 | Train 48 | Test Female 51 | 0.78 | 0.81 | 0.8 | 4083 | Train 64 | Test Female 51 | 0.79 | 0.81 | 0.81 |
| 3060 | Train 48 | Test Female 52 | 0.78 | 0.81 | 0.8 | 4084 | Train 64 | Test Female 52 | 0.79 | 0.81 | 0.81 |
| 3061 | Train 48 | Test Female 53 | 0.76 | 0.8 | 0.8 | 4085 | Train 64 | Test Female 53 | 0.77 | 0.81 | 0.81 |
| 3062 | Train 48 | Test Female 54 | 0.76 | 0.8 | 0.8 | 4086 | Train 64 | Test Female 54 | 0.76 | 0.82 | 0.82 |
| 3063 | Train 48 | Test Female 55 | 0.76 | 0.81 | 0.8 | 4087 | Train 64 | Test Female 55 | 0.77 | 0.82 | 0.81 |
| 3064 | Train 48 | Test Female 56 | 0.76 | 0.8 | 0.8 | 4088 | Train 64 | Test Female 56 | 0.76 | 0.82 | 0.82 |
| 3065 | Train 48 | Test Female 57 | 0.78 | 0.81 | 0.81 | 4089 | Train 64 | Test Female 57 | 0.79 | 0.82 | 0.83 |
| 3066 | Train 48 | Test Female 58 | 0.78 | 0.81 | 0.81 | 4090 | Train 64 | Test Female 58 | 0.79 | 0.82 | 0.83 |
| 3067 | Train 48 | Test Female 59 | 0.78 | 0.81 | 0.81 | 4091 | Train 64 | Test Female 59 | 0.79 | 0.82 | 0.83 |
| 3068 | Train 48 | Test Female 60 | 0.78 | 0.81 | 0.81 | 4092 | Train 64 | Test Female 60 | 0.79 | 0.82 | 0.83 |
| 3069 | Train 48 | Test Female 61 | 0.76 | 0.81 | 0.81 | 4093 | Train 64 | Test Female 61 | 0.77 | 0.82 | 0.82 |
| 3070 | Train 48 | Test Female 62 | 0.76 | 0.8 | 0.8 | 4094 | Train 64 | Test Female 62 | 0.77 | 0.82 | 0.82 |
| 3071 | Train 48 | Test Female 63 | 0.77 | 0.81 | 0.81 | 4095 | Train 64 | Test Female 63 | 0.77 | 0.82 | 0.83 |
| 3072 | Train 48 | Test Female 64 | 0.76 | 0.8 | 0.8 | 4096 | Train 64 | Test Female 64 | 0.76 | 0.82 | 0.82 |

Table 5. AUC Performance of the ML Models— LR, RF, and GB —for Black Patients Trained on Cohorts 1–32 and Tested Across 64 Cohorts.

| Black | Train  Model | Test  Dataset | AUC (LR) | AUC (RF) | AUC (GB) | Black | Train  Model | Test  Dataset | AUC (LR) | AUC (RF) | AUC (GB) |
| --- | --- | --- | --- | --- | --- | --- | --- | --- | --- | --- | --- |
| 1 | Train 1 | Test Black 1 | 0.76 | 0.86 | 0.86 | 1025 | Train 17 | Test Black 1 | 0.76 | 0.86 | 0.86 |
| 2 | Train 1 | Test Black 2 | 0.76 | 0.84 | 0.84 | 1026 | Train 17 | Test Black 2 | 0.76 | 0.84 | 0.84 |
| 3 | Train 1 | Test Black 3 | 0.76 | 0.86 | 0.86 | 1027 | Train 17 | Test Black 3 | 0.76 | 0.86 | 0.86 |
| 4 | Train 1 | Test Black 4 | 0.76 | 0.84 | 0.84 | 1028 | Train 17 | Test Black 4 | 0.76 | 0.84 | 0.84 |
| 5 | Train 1 | Test Black 5 | 0.73 | 0.76 | 0.78 | 1029 | Train 17 | Test Black 5 | 0.73 | 0.77 | 0.79 |
| 6 | Train 1 | Test Black 6 | 0.71 | 0.71 | 0.68 | 1030 | Train 17 | Test Black 6 | 0.71 | 0.71 | 0.69 |
| 7 | Train 1 | Test Black 7 | 0.73 | 0.76 | 0.77 | 1031 | Train 17 | Test Black 7 | 0.73 | 0.76 | 0.78 |
| 8 | Train 1 | Test Black 8 | 0.71 | 0.71 | 0.68 | 1032 | Train 17 | Test Black 8 | 0.71 | 0.71 | 0.69 |
| 9 | Train 1 | Test Black 9 | 0.76 | 0.85 | 0.83 | 1033 | Train 17 | Test Black 9 | 0.76 | 0.85 | 0.83 |
| 10 | Train 1 | Test Black 10 | 0.76 | 0.83 | 0.81 | 1034 | Train 17 | Test Black 10 | 0.76 | 0.83 | 0.82 |
| 11 | Train 1 | Test Black 11 | 0.76 | 0.85 | 0.83 | 1035 | Train 17 | Test Black 11 | 0.76 | 0.85 | 0.83 |
| 12 | Train 1 | Test Black 12 | 0.76 | 0.83 | 0.81 | 1036 | Train 17 | Test Black 12 | 0.76 | 0.83 | 0.81 |
| 13 | Train 1 | Test Black 13 | 0.73 | 0.76 | 0.76 | 1037 | Train 17 | Test Black 13 | 0.73 | 0.76 | 0.77 |
| 14 | Train 1 | Test Black 14 | 0.71 | 0.71 | 0.68 | 1038 | Train 17 | Test Black 14 | 0.71 | 0.71 | 0.69 |
| 15 | Train 1 | Test Black 15 | 0.73 | 0.75 | 0.75 | 1039 | Train 17 | Test Black 15 | 0.73 | 0.75 | 0.76 |
| 16 | Train 1 | Test Black 16 | 0.71 | 0.71 | 0.68 | 1040 | Train 17 | Test Black 16 | 0.71 | 0.71 | 0.69 |
| 17 | Train 1 | Test Black 17 | 0.76 | 0.86 | 0.86 | 1041 | Train 17 | Test Black 17 | 0.76 | 0.86 | 0.86 |
| 18 | Train 1 | Test Black 18 | 0.76 | 0.84 | 0.84 | 1042 | Train 17 | Test Black 18 | 0.76 | 0.84 | 0.84 |
| 19 | Train 1 | Test Black 19 | 0.76 | 0.86 | 0.86 | 1043 | Train 17 | Test Black 19 | 0.76 | 0.86 | 0.86 |
| 20 | Train 1 | Test Black 20 | 0.76 | 0.84 | 0.84 | 1044 | Train 17 | Test Black 20 | 0.76 | 0.84 | 0.84 |
| 21 | Train 1 | Test Black 21 | 0.73 | 0.76 | 0.78 | 1045 | Train 17 | Test Black 21 | 0.73 | 0.77 | 0.79 |
| 22 | Train 1 | Test Black 22 | 0.71 | 0.71 | 0.68 | 1046 | Train 17 | Test Black 22 | 0.71 | 0.71 | 0.69 |
| 23 | Train 1 | Test Black 23 | 0.73 | 0.76 | 0.77 | 1047 | Train 17 | Test Black 23 | 0.73 | 0.76 | 0.78 |
| 24 | Train 1 | Test Black 24 | 0.71 | 0.71 | 0.68 | 1048 | Train 17 | Test Black 24 | 0.71 | 0.71 | 0.69 |
| 25 | Train 1 | Test Black 25 | 0.76 | 0.85 | 0.83 | 1049 | Train 17 | Test Black 25 | 0.76 | 0.85 | 0.83 |
| 26 | Train 1 | Test Black 26 | 0.76 | 0.83 | 0.81 | 1050 | Train 17 | Test Black 26 | 0.76 | 0.83 | 0.82 |
| 27 | Train 1 | Test Black 27 | 0.76 | 0.85 | 0.83 | 1051 | Train 17 | Test Black 27 | 0.76 | 0.85 | 0.83 |
| 28 | Train 1 | Test Black 28 | 0.76 | 0.83 | 0.81 | 1052 | Train 17 | Test Black 28 | 0.76 | 0.83 | 0.81 |
| 29 | Train 1 | Test Black 29 | 0.73 | 0.76 | 0.76 | 1053 | Train 17 | Test Black 29 | 0.73 | 0.76 | 0.77 |
| 30 | Train 1 | Test Black 30 | 0.71 | 0.71 | 0.68 | 1054 | Train 17 | Test Black 30 | 0.71 | 0.71 | 0.69 |
| 31 | Train 1 | Test Black 31 | 0.73 | 0.75 | 0.75 | 1055 | Train 17 | Test Black 31 | 0.73 | 0.75 | 0.76 |
| 32 | Train 1 | Test Black 32 | 0.71 | 0.71 | 0.68 | 1056 | Train 17 | Test Black 32 | 0.71 | 0.71 | 0.69 |
| 33 | Train 1 | Test Black 33 | 0.76 | 0.86 | 0.86 | 1057 | Train 17 | Test Black 33 | 0.76 | 0.86 | 0.86 |
| 34 | Train 1 | Test Black 34 | 0.76 | 0.84 | 0.84 | 1058 | Train 17 | Test Black 34 | 0.76 | 0.84 | 0.84 |
| 35 | Train 1 | Test Black 35 | 0.76 | 0.86 | 0.86 | 1059 | Train 17 | Test Black 35 | 0.76 | 0.86 | 0.86 |
| 36 | Train 1 | Test Black 36 | 0.76 | 0.84 | 0.84 | 1060 | Train 17 | Test Black 36 | 0.76 | 0.84 | 0.84 |
| 37 | Train 1 | Test Black 37 | 0.73 | 0.76 | 0.78 | 1061 | Train 17 | Test Black 37 | 0.73 | 0.77 | 0.79 |
| 38 | Train 1 | Test Black 38 | 0.71 | 0.71 | 0.68 | 1062 | Train 17 | Test Black 38 | 0.71 | 0.71 | 0.69 |
| 39 | Train 1 | Test Black 39 | 0.73 | 0.76 | 0.77 | 1063 | Train 17 | Test Black 39 | 0.73 | 0.76 | 0.78 |
| 40 | Train 1 | Test Black 40 | 0.71 | 0.71 | 0.68 | 1064 | Train 17 | Test Black 40 | 0.71 | 0.71 | 0.69 |
| 41 | Train 1 | Test Black 41 | 0.76 | 0.85 | 0.83 | 1065 | Train 17 | Test Black 41 | 0.76 | 0.85 | 0.83 |
| 42 | Train 1 | Test Black 42 | 0.76 | 0.83 | 0.81 | 1066 | Train 17 | Test Black 42 | 0.76 | 0.83 | 0.81 |
| 43 | Train 1 | Test Black 43 | 0.76 | 0.85 | 0.83 | 1067 | Train 17 | Test Black 43 | 0.76 | 0.85 | 0.83 |
| 44 | Train 1 | Test Black 44 | 0.76 | 0.83 | 0.81 | 1068 | Train 17 | Test Black 44 | 0.76 | 0.83 | 0.81 |
| 45 | Train 1 | Test Black 45 | 0.73 | 0.75 | 0.76 | 1069 | Train 17 | Test Black 45 | 0.73 | 0.76 | 0.77 |
| 46 | Train 1 | Test Black 46 | 0.71 | 0.71 | 0.68 | 1070 | Train 17 | Test Black 46 | 0.71 | 0.71 | 0.69 |
| 47 | Train 1 | Test Black 47 | 0.73 | 0.75 | 0.75 | 1071 | Train 17 | Test Black 47 | 0.73 | 0.75 | 0.76 |
| 48 | Train 1 | Test Black 48 | 0.71 | 0.71 | 0.68 | 1072 | Train 17 | Test Black 48 | 0.71 | 0.71 | 0.69 |
| 49 | Train 1 | Test Black 49 | 0.77 | 0.82 | 0.84 | 1073 | Train 17 | Test Black 49 | 0.77 | 0.82 | 0.83 |
| 50 | Train 1 | Test Black 50 | 0.77 | 0.82 | 0.84 | 1074 | Train 17 | Test Black 50 | 0.77 | 0.82 | 0.83 |
| 51 | Train 1 | Test Black 51 | 0.77 | 0.82 | 0.84 | 1075 | Train 17 | Test Black 51 | 0.77 | 0.82 | 0.82 |
| 52 | Train 1 | Test Black 52 | 0.77 | 0.82 | 0.84 | 1076 | Train 17 | Test Black 52 | 0.77 | 0.82 | 0.82 |
| 53 | Train 1 | Test Black 53 | 0.76 | 0.78 | 0.77 | 1077 | Train 17 | Test Black 53 | 0.76 | 0.79 | 0.77 |
| 54 | Train 1 | Test Black 54 | 0.71 | 0.71 | 0.66 | 1078 | Train 17 | Test Black 54 | 0.71 | 0.71 | 0.67 |
| 55 | Train 1 | Test Black 55 | 0.76 | 0.78 | 0.77 | 1079 | Train 17 | Test Black 55 | 0.76 | 0.79 | 0.77 |
| 56 | Train 1 | Test Black 56 | 0.71 | 0.71 | 0.66 | 1080 | Train 17 | Test Black 56 | 0.71 | 0.71 | 0.67 |
| 57 | Train 1 | Test Black 57 | 0.77 | 0.81 | 0.8 | 1081 | Train 17 | Test Black 57 | 0.77 | 0.81 | 0.8 |
| 58 | Train 1 | Test Black 58 | 0.77 | 0.81 | 0.8 | 1082 | Train 17 | Test Black 58 | 0.77 | 0.81 | 0.8 |
| 59 | Train 1 | Test Black 59 | 0.77 | 0.81 | 0.8 | 1083 | Train 17 | Test Black 59 | 0.77 | 0.81 | 0.79 |
| 60 | Train 1 | Test Black 60 | 0.77 | 0.81 | 0.8 | 1084 | Train 17 | Test Black 60 | 0.77 | 0.81 | 0.79 |
| 61 | Train 1 | Test Black 61 | 0.76 | 0.78 | 0.75 | 1085 | Train 17 | Test Black 61 | 0.75 | 0.78 | 0.76 |
| 62 | Train 1 | Test Black 62 | 0.71 | 0.71 | 0.66 | 1086 | Train 17 | Test Black 62 | 0.71 | 0.71 | 0.67 |
| 63 | Train 1 | Test Black 63 | 0.76 | 0.78 | 0.75 | 1087 | Train 17 | Test Black 63 | 0.76 | 0.78 | 0.76 |
| 64 | Train 1 | Test Black 64 | 0.71 | 0.71 | 0.66 | 1088 | Train 17 | Test Black 64 | 0.71 | 0.71 | 0.67 |
| 65 | Train 2 | Test Black 1 | 0.75 | 0.85 | 0.85 | 1089 | Train 18 | Test Black 1 | 0.75 | 0.85 | 0.85 |
| 66 | Train 2 | Test Black 2 | 0.76 | 0.86 | 0.86 | 1090 | Train 18 | Test Black 2 | 0.76 | 0.86 | 0.86 |
| 67 | Train 2 | Test Black 3 | 0.75 | 0.85 | 0.85 | 1091 | Train 18 | Test Black 3 | 0.75 | 0.85 | 0.85 |
| 68 | Train 2 | Test Black 4 | 0.76 | 0.86 | 0.85 | 1092 | Train 18 | Test Black 4 | 0.76 | 0.86 | 0.85 |
| 69 | Train 2 | Test Black 5 | 0.73 | 0.77 | 0.78 | 1093 | Train 18 | Test Black 5 | 0.73 | 0.77 | 0.78 |
| 70 | Train 2 | Test Black 6 | 0.71 | 0.72 | 0.69 | 1094 | Train 18 | Test Black 6 | 0.71 | 0.72 | 0.68 |
| 71 | Train 2 | Test Black 7 | 0.73 | 0.76 | 0.77 | 1095 | Train 18 | Test Black 7 | 0.73 | 0.77 | 0.78 |
| 72 | Train 2 | Test Black 8 | 0.71 | 0.72 | 0.68 | 1096 | Train 18 | Test Black 8 | 0.71 | 0.72 | 0.68 |
| 73 | Train 2 | Test Black 9 | 0.75 | 0.84 | 0.82 | 1097 | Train 18 | Test Black 9 | 0.75 | 0.84 | 0.82 |
| 74 | Train 2 | Test Black 10 | 0.76 | 0.84 | 0.83 | 1098 | Train 18 | Test Black 10 | 0.76 | 0.84 | 0.82 |
| 75 | Train 2 | Test Black 11 | 0.75 | 0.84 | 0.82 | 1099 | Train 18 | Test Black 11 | 0.75 | 0.84 | 0.82 |
| 76 | Train 2 | Test Black 12 | 0.76 | 0.84 | 0.82 | 1100 | Train 18 | Test Black 12 | 0.76 | 0.84 | 0.82 |
| 77 | Train 2 | Test Black 13 | 0.73 | 0.76 | 0.76 | 1101 | Train 18 | Test Black 13 | 0.73 | 0.76 | 0.76 |
| 78 | Train 2 | Test Black 14 | 0.71 | 0.72 | 0.69 | 1102 | Train 18 | Test Black 14 | 0.71 | 0.72 | 0.68 |
| 79 | Train 2 | Test Black 15 | 0.73 | 0.76 | 0.75 | 1103 | Train 18 | Test Black 15 | 0.73 | 0.76 | 0.76 |
| 80 | Train 2 | Test Black 16 | 0.71 | 0.72 | 0.68 | 1104 | Train 18 | Test Black 16 | 0.71 | 0.72 | 0.68 |
| 81 | Train 2 | Test Black 17 | 0.75 | 0.85 | 0.85 | 1105 | Train 18 | Test Black 17 | 0.75 | 0.85 | 0.85 |
| 82 | Train 2 | Test Black 18 | 0.76 | 0.86 | 0.86 | 1106 | Train 18 | Test Black 18 | 0.76 | 0.86 | 0.86 |
| 83 | Train 2 | Test Black 19 | 0.75 | 0.85 | 0.85 | 1107 | Train 18 | Test Black 19 | 0.75 | 0.85 | 0.85 |
| 84 | Train 2 | Test Black 20 | 0.76 | 0.86 | 0.85 | 1108 | Train 18 | Test Black 20 | 0.76 | 0.86 | 0.85 |
| 85 | Train 2 | Test Black 21 | 0.73 | 0.77 | 0.78 | 1109 | Train 18 | Test Black 21 | 0.73 | 0.77 | 0.78 |
| 86 | Train 2 | Test Black 22 | 0.71 | 0.72 | 0.69 | 1110 | Train 18 | Test Black 22 | 0.71 | 0.72 | 0.68 |
| 87 | Train 2 | Test Black 23 | 0.73 | 0.77 | 0.77 | 1111 | Train 18 | Test Black 23 | 0.73 | 0.77 | 0.78 |
| 88 | Train 2 | Test Black 24 | 0.71 | 0.72 | 0.69 | 1112 | Train 18 | Test Black 24 | 0.71 | 0.72 | 0.68 |
| 89 | Train 2 | Test Black 25 | 0.75 | 0.84 | 0.82 | 1113 | Train 18 | Test Black 25 | 0.75 | 0.84 | 0.82 |
| 90 | Train 2 | Test Black 26 | 0.76 | 0.84 | 0.83 | 1114 | Train 18 | Test Black 26 | 0.76 | 0.84 | 0.82 |
| 91 | Train 2 | Test Black 27 | 0.75 | 0.84 | 0.82 | 1115 | Train 18 | Test Black 27 | 0.75 | 0.84 | 0.82 |
| 92 | Train 2 | Test Black 28 | 0.76 | 0.84 | 0.82 | 1116 | Train 18 | Test Black 28 | 0.76 | 0.84 | 0.82 |
| 93 | Train 2 | Test Black 29 | 0.73 | 0.76 | 0.76 | 1117 | Train 18 | Test Black 29 | 0.73 | 0.76 | 0.76 |
| 94 | Train 2 | Test Black 30 | 0.71 | 0.72 | 0.69 | 1118 | Train 18 | Test Black 30 | 0.71 | 0.72 | 0.68 |
| 95 | Train 2 | Test Black 31 | 0.73 | 0.76 | 0.75 | 1119 | Train 18 | Test Black 31 | 0.73 | 0.76 | 0.76 |
| 96 | Train 2 | Test Black 32 | 0.71 | 0.72 | 0.68 | 1120 | Train 18 | Test Black 32 | 0.71 | 0.72 | 0.68 |
| 97 | Train 2 | Test Black 33 | 0.75 | 0.85 | 0.85 | 1121 | Train 18 | Test Black 33 | 0.75 | 0.85 | 0.85 |
| 98 | Train 2 | Test Black 34 | 0.75 | 0.85 | 0.85 | 1122 | Train 18 | Test Black 34 | 0.75 | 0.85 | 0.85 |
| 99 | Train 2 | Test Black 35 | 0.75 | 0.85 | 0.85 | 1123 | Train 18 | Test Black 35 | 0.75 | 0.85 | 0.85 |
| 100 | Train 2 | Test Black 36 | 0.75 | 0.85 | 0.85 | 1124 | Train 18 | Test Black 36 | 0.75 | 0.85 | 0.85 |
| 101 | Train 2 | Test Black 37 | 0.73 | 0.77 | 0.78 | 1125 | Train 18 | Test Black 37 | 0.73 | 0.77 | 0.78 |
| 102 | Train 2 | Test Black 38 | 0.71 | 0.72 | 0.69 | 1126 | Train 18 | Test Black 38 | 0.71 | 0.72 | 0.68 |
| 103 | Train 2 | Test Black 39 | 0.73 | 0.77 | 0.77 | 1127 | Train 18 | Test Black 39 | 0.73 | 0.77 | 0.78 |
| 104 | Train 2 | Test Black 40 | 0.71 | 0.72 | 0.68 | 1128 | Train 18 | Test Black 40 | 0.71 | 0.72 | 0.68 |
| 105 | Train 2 | Test Black 41 | 0.75 | 0.84 | 0.82 | 1129 | Train 18 | Test Black 41 | 0.75 | 0.84 | 0.82 |
| 106 | Train 2 | Test Black 42 | 0.75 | 0.84 | 0.83 | 1130 | Train 18 | Test Black 42 | 0.75 | 0.84 | 0.82 |
| 107 | Train 2 | Test Black 43 | 0.75 | 0.84 | 0.82 | 1131 | Train 18 | Test Black 43 | 0.75 | 0.84 | 0.82 |
| 108 | Train 2 | Test Black 44 | 0.75 | 0.84 | 0.82 | 1132 | Train 18 | Test Black 44 | 0.75 | 0.84 | 0.82 |
| 109 | Train 2 | Test Black 45 | 0.73 | 0.76 | 0.76 | 1133 | Train 18 | Test Black 45 | 0.73 | 0.76 | 0.76 |
| 110 | Train 2 | Test Black 46 | 0.71 | 0.72 | 0.69 | 1134 | Train 18 | Test Black 46 | 0.71 | 0.72 | 0.68 |
| 111 | Train 2 | Test Black 47 | 0.73 | 0.76 | 0.75 | 1135 | Train 18 | Test Black 47 | 0.73 | 0.76 | 0.76 |
| 112 | Train 2 | Test Black 48 | 0.71 | 0.72 | 0.68 | 1136 | Train 18 | Test Black 48 | 0.71 | 0.72 | 0.68 |
| 113 | Train 2 | Test Black 49 | 0.77 | 0.82 | 0.81 | 1137 | Train 18 | Test Black 49 | 0.77 | 0.82 | 0.82 |
| 114 | Train 2 | Test Black 50 | 0.77 | 0.82 | 0.81 | 1138 | Train 18 | Test Black 50 | 0.77 | 0.82 | 0.82 |
| 115 | Train 2 | Test Black 51 | 0.77 | 0.82 | 0.81 | 1139 | Train 18 | Test Black 51 | 0.77 | 0.82 | 0.81 |
| 116 | Train 2 | Test Black 52 | 0.77 | 0.82 | 0.81 | 1140 | Train 18 | Test Black 52 | 0.77 | 0.82 | 0.81 |
| 117 | Train 2 | Test Black 53 | 0.75 | 0.79 | 0.76 | 1141 | Train 18 | Test Black 53 | 0.75 | 0.79 | 0.77 |
| 118 | Train 2 | Test Black 54 | 0.71 | 0.72 | 0.66 | 1142 | Train 18 | Test Black 54 | 0.71 | 0.72 | 0.66 |
| 119 | Train 2 | Test Black 55 | 0.75 | 0.79 | 0.76 | 1143 | Train 18 | Test Black 55 | 0.75 | 0.79 | 0.77 |
| 120 | Train 2 | Test Black 56 | 0.71 | 0.71 | 0.66 | 1144 | Train 18 | Test Black 56 | 0.71 | 0.71 | 0.66 |
| 121 | Train 2 | Test Black 57 | 0.77 | 0.81 | 0.78 | 1145 | Train 18 | Test Black 57 | 0.77 | 0.81 | 0.78 |
| 122 | Train 2 | Test Black 58 | 0.77 | 0.81 | 0.78 | 1146 | Train 18 | Test Black 58 | 0.77 | 0.81 | 0.78 |
| 123 | Train 2 | Test Black 59 | 0.77 | 0.81 | 0.78 | 1147 | Train 18 | Test Black 59 | 0.77 | 0.81 | 0.77 |
| 124 | Train 2 | Test Black 60 | 0.77 | 0.81 | 0.78 | 1148 | Train 18 | Test Black 60 | 0.77 | 0.81 | 0.77 |
| 125 | Train 2 | Test Black 61 | 0.75 | 0.79 | 0.75 | 1149 | Train 18 | Test Black 61 | 0.75 | 0.79 | 0.76 |
| 126 | Train 2 | Test Black 62 | 0.71 | 0.72 | 0.66 | 1150 | Train 18 | Test Black 62 | 0.71 | 0.72 | 0.67 |
| 127 | Train 2 | Test Black 63 | 0.75 | 0.79 | 0.75 | 1151 | Train 18 | Test Black 63 | 0.75 | 0.79 | 0.76 |
| 128 | Train 2 | Test Black 64 | 0.71 | 0.71 | 0.66 | 1152 | Train 18 | Test Black 64 | 0.71 | 0.71 | 0.66 |
| 129 | Train 3 | Test Black 1 | 0.76 | 0.86 | 0.87 | 1153 | Train 19 | Test Black 1 | 0.76 | 0.86 | 0.86 |
| 130 | Train 3 | Test Black 2 | 0.76 | 0.84 | 0.84 | 1154 | Train 19 | Test Black 2 | 0.76 | 0.84 | 0.84 |
| 131 | Train 3 | Test Black 3 | 0.76 | 0.86 | 0.87 | 1155 | Train 19 | Test Black 3 | 0.76 | 0.86 | 0.86 |
| 132 | Train 3 | Test Black 4 | 0.76 | 0.84 | 0.84 | 1156 | Train 19 | Test Black 4 | 0.76 | 0.84 | 0.84 |
| 133 | Train 3 | Test Black 5 | 0.73 | 0.77 | 0.78 | 1157 | Train 19 | Test Black 5 | 0.73 | 0.77 | 0.77 |
| 134 | Train 3 | Test Black 6 | 0.71 | 0.71 | 0.69 | 1158 | Train 19 | Test Black 6 | 0.71 | 0.71 | 0.68 |
| 135 | Train 3 | Test Black 7 | 0.73 | 0.76 | 0.77 | 1159 | Train 19 | Test Black 7 | 0.73 | 0.76 | 0.77 |
| 136 | Train 3 | Test Black 8 | 0.71 | 0.71 | 0.69 | 1160 | Train 19 | Test Black 8 | 0.71 | 0.71 | 0.68 |
| 137 | Train 3 | Test Black 9 | 0.76 | 0.85 | 0.83 | 1161 | Train 19 | Test Black 9 | 0.76 | 0.85 | 0.83 |
| 138 | Train 3 | Test Black 10 | 0.76 | 0.83 | 0.81 | 1162 | Train 19 | Test Black 10 | 0.76 | 0.83 | 0.81 |
| 139 | Train 3 | Test Black 11 | 0.76 | 0.85 | 0.83 | 1163 | Train 19 | Test Black 11 | 0.76 | 0.85 | 0.83 |
| 140 | Train 3 | Test Black 12 | 0.76 | 0.83 | 0.81 | 1164 | Train 19 | Test Black 12 | 0.76 | 0.83 | 0.81 |
| 141 | Train 3 | Test Black 13 | 0.73 | 0.76 | 0.76 | 1165 | Train 19 | Test Black 13 | 0.73 | 0.76 | 0.76 |
| 142 | Train 3 | Test Black 14 | 0.71 | 0.71 | 0.69 | 1166 | Train 19 | Test Black 14 | 0.71 | 0.71 | 0.68 |
| 143 | Train 3 | Test Black 15 | 0.73 | 0.75 | 0.75 | 1167 | Train 19 | Test Black 15 | 0.73 | 0.75 | 0.75 |
| 144 | Train 3 | Test Black 16 | 0.71 | 0.71 | 0.69 | 1168 | Train 19 | Test Black 16 | 0.71 | 0.71 | 0.68 |
| 145 | Train 3 | Test Black 17 | 0.76 | 0.86 | 0.87 | 1169 | Train 19 | Test Black 17 | 0.76 | 0.86 | 0.86 |
| 146 | Train 3 | Test Black 18 | 0.76 | 0.84 | 0.84 | 1170 | Train 19 | Test Black 18 | 0.76 | 0.84 | 0.84 |
| 147 | Train 3 | Test Black 19 | 0.76 | 0.86 | 0.87 | 1171 | Train 19 | Test Black 19 | 0.76 | 0.86 | 0.86 |
| 148 | Train 3 | Test Black 20 | 0.76 | 0.84 | 0.84 | 1172 | Train 19 | Test Black 20 | 0.76 | 0.84 | 0.84 |
| 149 | Train 3 | Test Black 21 | 0.73 | 0.77 | 0.78 | 1173 | Train 19 | Test Black 21 | 0.73 | 0.77 | 0.77 |
| 150 | Train 3 | Test Black 22 | 0.71 | 0.71 | 0.69 | 1174 | Train 19 | Test Black 22 | 0.71 | 0.71 | 0.68 |
| 151 | Train 3 | Test Black 23 | 0.73 | 0.76 | 0.77 | 1175 | Train 19 | Test Black 23 | 0.73 | 0.76 | 0.77 |
| 152 | Train 3 | Test Black 24 | 0.71 | 0.71 | 0.69 | 1176 | Train 19 | Test Black 24 | 0.71 | 0.71 | 0.68 |
| 153 | Train 3 | Test Black 25 | 0.76 | 0.85 | 0.83 | 1177 | Train 19 | Test Black 25 | 0.76 | 0.85 | 0.83 |
| 154 | Train 3 | Test Black 26 | 0.76 | 0.83 | 0.81 | 1178 | Train 19 | Test Black 26 | 0.76 | 0.83 | 0.81 |
| 155 | Train 3 | Test Black 27 | 0.76 | 0.85 | 0.83 | 1179 | Train 19 | Test Black 27 | 0.76 | 0.85 | 0.83 |
| 156 | Train 3 | Test Black 28 | 0.76 | 0.83 | 0.81 | 1180 | Train 19 | Test Black 28 | 0.76 | 0.83 | 0.81 |
| 157 | Train 3 | Test Black 29 | 0.73 | 0.76 | 0.76 | 1181 | Train 19 | Test Black 29 | 0.73 | 0.76 | 0.76 |
| 158 | Train 3 | Test Black 30 | 0.71 | 0.71 | 0.69 | 1182 | Train 19 | Test Black 30 | 0.71 | 0.71 | 0.68 |
| 159 | Train 3 | Test Black 31 | 0.73 | 0.75 | 0.75 | 1183 | Train 19 | Test Black 31 | 0.73 | 0.75 | 0.75 |
| 160 | Train 3 | Test Black 32 | 0.71 | 0.71 | 0.69 | 1184 | Train 19 | Test Black 32 | 0.71 | 0.71 | 0.68 |
| 161 | Train 3 | Test Black 33 | 0.76 | 0.86 | 0.86 | 1185 | Train 19 | Test Black 33 | 0.76 | 0.86 | 0.86 |
| 162 | Train 3 | Test Black 34 | 0.76 | 0.84 | 0.84 | 1186 | Train 19 | Test Black 34 | 0.76 | 0.84 | 0.83 |
| 163 | Train 3 | Test Black 35 | 0.76 | 0.86 | 0.86 | 1187 | Train 19 | Test Black 35 | 0.76 | 0.86 | 0.86 |
| 164 | Train 3 | Test Black 36 | 0.76 | 0.84 | 0.84 | 1188 | Train 19 | Test Black 36 | 0.76 | 0.84 | 0.83 |
| 165 | Train 3 | Test Black 37 | 0.73 | 0.76 | 0.78 | 1189 | Train 19 | Test Black 37 | 0.73 | 0.77 | 0.77 |
| 166 | Train 3 | Test Black 38 | 0.71 | 0.71 | 0.69 | 1190 | Train 19 | Test Black 38 | 0.71 | 0.71 | 0.68 |
| 167 | Train 3 | Test Black 39 | 0.73 | 0.76 | 0.77 | 1191 | Train 19 | Test Black 39 | 0.73 | 0.76 | 0.77 |
| 168 | Train 3 | Test Black 40 | 0.71 | 0.71 | 0.69 | 1192 | Train 19 | Test Black 40 | 0.71 | 0.71 | 0.68 |
| 169 | Train 3 | Test Black 41 | 0.76 | 0.85 | 0.83 | 1193 | Train 19 | Test Black 41 | 0.76 | 0.85 | 0.83 |
| 170 | Train 3 | Test Black 42 | 0.76 | 0.83 | 0.81 | 1194 | Train 19 | Test Black 42 | 0.76 | 0.83 | 0.81 |
| 171 | Train 3 | Test Black 43 | 0.76 | 0.85 | 0.83 | 1195 | Train 19 | Test Black 43 | 0.76 | 0.85 | 0.83 |
| 172 | Train 3 | Test Black 44 | 0.76 | 0.83 | 0.81 | 1196 | Train 19 | Test Black 44 | 0.76 | 0.83 | 0.81 |
| 173 | Train 3 | Test Black 45 | 0.73 | 0.76 | 0.76 | 1197 | Train 19 | Test Black 45 | 0.73 | 0.76 | 0.76 |
| 174 | Train 3 | Test Black 46 | 0.71 | 0.71 | 0.69 | 1198 | Train 19 | Test Black 46 | 0.71 | 0.71 | 0.68 |
| 175 | Train 3 | Test Black 47 | 0.73 | 0.75 | 0.75 | 1199 | Train 19 | Test Black 47 | 0.73 | 0.75 | 0.75 |
| 176 | Train 3 | Test Black 48 | 0.71 | 0.71 | 0.69 | 1200 | Train 19 | Test Black 48 | 0.71 | 0.71 | 0.68 |
| 177 | Train 3 | Test Black 49 | 0.77 | 0.82 | 0.83 | 1201 | Train 19 | Test Black 49 | 0.77 | 0.82 | 0.82 |
| 178 | Train 3 | Test Black 50 | 0.77 | 0.82 | 0.83 | 1202 | Train 19 | Test Black 50 | 0.77 | 0.82 | 0.82 |
| 179 | Train 3 | Test Black 51 | 0.77 | 0.82 | 0.83 | 1203 | Train 19 | Test Black 51 | 0.77 | 0.82 | 0.83 |
| 180 | Train 3 | Test Black 52 | 0.77 | 0.82 | 0.83 | 1204 | Train 19 | Test Black 52 | 0.77 | 0.82 | 0.83 |
| 181 | Train 3 | Test Black 53 | 0.75 | 0.78 | 0.77 | 1205 | Train 19 | Test Black 53 | 0.75 | 0.79 | 0.76 |
| 182 | Train 3 | Test Black 54 | 0.71 | 0.71 | 0.67 | 1206 | Train 19 | Test Black 54 | 0.71 | 0.71 | 0.66 |
| 183 | Train 3 | Test Black 55 | 0.76 | 0.79 | 0.77 | 1207 | Train 19 | Test Black 55 | 0.76 | 0.79 | 0.76 |
| 184 | Train 3 | Test Black 56 | 0.71 | 0.7 | 0.67 | 1208 | Train 19 | Test Black 56 | 0.71 | 0.7 | 0.66 |
| 185 | Train 3 | Test Black 57 | 0.77 | 0.81 | 0.79 | 1209 | Train 19 | Test Black 57 | 0.77 | 0.81 | 0.8 |
| 186 | Train 3 | Test Black 58 | 0.77 | 0.81 | 0.79 | 1210 | Train 19 | Test Black 58 | 0.77 | 0.81 | 0.8 |
| 187 | Train 3 | Test Black 59 | 0.77 | 0.81 | 0.79 | 1211 | Train 19 | Test Black 59 | 0.77 | 0.81 | 0.8 |
| 188 | Train 3 | Test Black 60 | 0.77 | 0.81 | 0.8 | 1212 | Train 19 | Test Black 60 | 0.77 | 0.81 | 0.8 |
| 189 | Train 3 | Test Black 61 | 0.75 | 0.78 | 0.76 | 1213 | Train 19 | Test Black 61 | 0.75 | 0.78 | 0.76 |
| 190 | Train 3 | Test Black 62 | 0.71 | 0.71 | 0.67 | 1214 | Train 19 | Test Black 62 | 0.71 | 0.71 | 0.66 |
| 191 | Train 3 | Test Black 63 | 0.75 | 0.78 | 0.76 | 1215 | Train 19 | Test Black 63 | 0.75 | 0.78 | 0.76 |
| 192 | Train 3 | Test Black 64 | 0.71 | 0.7 | 0.67 | 1216 | Train 19 | Test Black 64 | 0.71 | 0.71 | 0.66 |
| 193 | Train 4 | Test Black 1 | 0.75 | 0.85 | 0.86 | 1217 | Train 20 | Test Black 1 | 0.75 | 0.85 | 0.85 |
| 194 | Train 4 | Test Black 2 | 0.76 | 0.86 | 0.86 | 1218 | Train 20 | Test Black 2 | 0.76 | 0.86 | 0.85 |
| 195 | Train 4 | Test Black 3 | 0.75 | 0.85 | 0.86 | 1219 | Train 20 | Test Black 3 | 0.75 | 0.85 | 0.85 |
| 196 | Train 4 | Test Black 4 | 0.76 | 0.86 | 0.86 | 1220 | Train 20 | Test Black 4 | 0.76 | 0.86 | 0.85 |
| 197 | Train 4 | Test Black 5 | 0.73 | 0.77 | 0.78 | 1221 | Train 20 | Test Black 5 | 0.73 | 0.77 | 0.75 |
| 198 | Train 4 | Test Black 6 | 0.71 | 0.72 | 0.69 | 1222 | Train 20 | Test Black 6 | 0.71 | 0.72 | 0.68 |
| 199 | Train 4 | Test Black 7 | 0.73 | 0.76 | 0.77 | 1223 | Train 20 | Test Black 7 | 0.73 | 0.77 | 0.75 |
| 200 | Train 4 | Test Black 8 | 0.71 | 0.72 | 0.69 | 1224 | Train 20 | Test Black 8 | 0.71 | 0.72 | 0.68 |
| 201 | Train 4 | Test Black 9 | 0.75 | 0.84 | 0.83 | 1225 | Train 20 | Test Black 9 | 0.75 | 0.84 | 0.83 |
| 202 | Train 4 | Test Black 10 | 0.76 | 0.84 | 0.83 | 1226 | Train 20 | Test Black 10 | 0.76 | 0.84 | 0.83 |
| 203 | Train 4 | Test Black 11 | 0.75 | 0.84 | 0.83 | 1227 | Train 20 | Test Black 11 | 0.75 | 0.84 | 0.83 |
| 204 | Train 4 | Test Black 12 | 0.76 | 0.84 | 0.83 | 1228 | Train 20 | Test Black 12 | 0.76 | 0.84 | 0.83 |
| 205 | Train 4 | Test Black 13 | 0.73 | 0.76 | 0.76 | 1229 | Train 20 | Test Black 13 | 0.73 | 0.76 | 0.73 |
| 206 | Train 4 | Test Black 14 | 0.71 | 0.72 | 0.69 | 1230 | Train 20 | Test Black 14 | 0.71 | 0.72 | 0.68 |
| 207 | Train 4 | Test Black 15 | 0.73 | 0.76 | 0.75 | 1231 | Train 20 | Test Black 15 | 0.73 | 0.76 | 0.73 |
| 208 | Train 4 | Test Black 16 | 0.71 | 0.72 | 0.69 | 1232 | Train 20 | Test Black 16 | 0.71 | 0.72 | 0.68 |
| 209 | Train 4 | Test Black 17 | 0.75 | 0.85 | 0.86 | 1233 | Train 20 | Test Black 17 | 0.75 | 0.85 | 0.85 |
| 210 | Train 4 | Test Black 18 | 0.76 | 0.86 | 0.86 | 1234 | Train 20 | Test Black 18 | 0.76 | 0.86 | 0.85 |
| 211 | Train 4 | Test Black 19 | 0.75 | 0.85 | 0.86 | 1235 | Train 20 | Test Black 19 | 0.75 | 0.85 | 0.85 |
| 212 | Train 4 | Test Black 20 | 0.76 | 0.86 | 0.86 | 1236 | Train 20 | Test Black 20 | 0.76 | 0.86 | 0.85 |
| 213 | Train 4 | Test Black 21 | 0.73 | 0.77 | 0.78 | 1237 | Train 20 | Test Black 21 | 0.73 | 0.77 | 0.75 |
| 214 | Train 4 | Test Black 22 | 0.71 | 0.72 | 0.69 | 1238 | Train 20 | Test Black 22 | 0.71 | 0.72 | 0.68 |
| 215 | Train 4 | Test Black 23 | 0.73 | 0.76 | 0.77 | 1239 | Train 20 | Test Black 23 | 0.73 | 0.77 | 0.75 |
| 216 | Train 4 | Test Black 24 | 0.71 | 0.72 | 0.69 | 1240 | Train 20 | Test Black 24 | 0.71 | 0.72 | 0.68 |
| 217 | Train 4 | Test Black 25 | 0.75 | 0.84 | 0.83 | 1241 | Train 20 | Test Black 25 | 0.75 | 0.84 | 0.83 |
| 218 | Train 4 | Test Black 26 | 0.76 | 0.84 | 0.83 | 1242 | Train 20 | Test Black 26 | 0.76 | 0.84 | 0.83 |
| 219 | Train 4 | Test Black 27 | 0.75 | 0.84 | 0.83 | 1243 | Train 20 | Test Black 27 | 0.75 | 0.84 | 0.82 |
| 220 | Train 4 | Test Black 28 | 0.76 | 0.84 | 0.83 | 1244 | Train 20 | Test Black 28 | 0.76 | 0.84 | 0.83 |
| 221 | Train 4 | Test Black 29 | 0.73 | 0.76 | 0.76 | 1245 | Train 20 | Test Black 29 | 0.73 | 0.76 | 0.73 |
| 222 | Train 4 | Test Black 30 | 0.71 | 0.72 | 0.69 | 1246 | Train 20 | Test Black 30 | 0.71 | 0.72 | 0.68 |
| 223 | Train 4 | Test Black 31 | 0.73 | 0.76 | 0.76 | 1247 | Train 20 | Test Black 31 | 0.73 | 0.76 | 0.73 |
| 224 | Train 4 | Test Black 32 | 0.71 | 0.72 | 0.69 | 1248 | Train 20 | Test Black 32 | 0.71 | 0.72 | 0.68 |
| 225 | Train 4 | Test Black 33 | 0.75 | 0.85 | 0.85 | 1249 | Train 20 | Test Black 33 | 0.75 | 0.85 | 0.85 |
| 226 | Train 4 | Test Black 34 | 0.75 | 0.85 | 0.85 | 1250 | Train 20 | Test Black 34 | 0.75 | 0.85 | 0.85 |
| 227 | Train 4 | Test Black 35 | 0.75 | 0.85 | 0.85 | 1251 | Train 20 | Test Black 35 | 0.75 | 0.85 | 0.85 |
| 228 | Train 4 | Test Black 36 | 0.75 | 0.85 | 0.85 | 1252 | Train 20 | Test Black 36 | 0.75 | 0.85 | 0.85 |
| 229 | Train 4 | Test Black 37 | 0.73 | 0.77 | 0.78 | 1253 | Train 20 | Test Black 37 | 0.73 | 0.77 | 0.75 |
| 230 | Train 4 | Test Black 38 | 0.71 | 0.72 | 0.69 | 1254 | Train 20 | Test Black 38 | 0.71 | 0.72 | 0.68 |
| 231 | Train 4 | Test Black 39 | 0.73 | 0.76 | 0.77 | 1255 | Train 20 | Test Black 39 | 0.73 | 0.77 | 0.75 |
| 232 | Train 4 | Test Black 40 | 0.71 | 0.72 | 0.69 | 1256 | Train 20 | Test Black 40 | 0.71 | 0.72 | 0.68 |
| 233 | Train 4 | Test Black 41 | 0.75 | 0.84 | 0.83 | 1257 | Train 20 | Test Black 41 | 0.75 | 0.84 | 0.82 |
| 234 | Train 4 | Test Black 42 | 0.75 | 0.84 | 0.83 | 1258 | Train 20 | Test Black 42 | 0.75 | 0.84 | 0.83 |
| 235 | Train 4 | Test Black 43 | 0.75 | 0.84 | 0.82 | 1259 | Train 20 | Test Black 43 | 0.75 | 0.84 | 0.82 |
| 236 | Train 4 | Test Black 44 | 0.75 | 0.84 | 0.83 | 1260 | Train 20 | Test Black 44 | 0.75 | 0.84 | 0.82 |
| 237 | Train 4 | Test Black 45 | 0.73 | 0.76 | 0.76 | 1261 | Train 20 | Test Black 45 | 0.73 | 0.76 | 0.73 |
| 238 | Train 4 | Test Black 46 | 0.71 | 0.72 | 0.69 | 1262 | Train 20 | Test Black 46 | 0.71 | 0.72 | 0.68 |
| 239 | Train 4 | Test Black 47 | 0.73 | 0.76 | 0.75 | 1263 | Train 20 | Test Black 47 | 0.73 | 0.76 | 0.73 |
| 240 | Train 4 | Test Black 48 | 0.71 | 0.72 | 0.69 | 1264 | Train 20 | Test Black 48 | 0.71 | 0.72 | 0.68 |
| 241 | Train 4 | Test Black 49 | 0.77 | 0.82 | 0.82 | 1265 | Train 20 | Test Black 49 | 0.77 | 0.82 | 0.83 |
| 242 | Train 4 | Test Black 50 | 0.77 | 0.82 | 0.82 | 1266 | Train 20 | Test Black 50 | 0.77 | 0.82 | 0.83 |
| 243 | Train 4 | Test Black 51 | 0.77 | 0.82 | 0.82 | 1267 | Train 20 | Test Black 51 | 0.77 | 0.82 | 0.83 |
| 244 | Train 4 | Test Black 52 | 0.77 | 0.82 | 0.82 | 1268 | Train 20 | Test Black 52 | 0.77 | 0.82 | 0.83 |
| 245 | Train 4 | Test Black 53 | 0.75 | 0.79 | 0.76 | 1269 | Train 20 | Test Black 53 | 0.75 | 0.79 | 0.77 |
| 246 | Train 4 | Test Black 54 | 0.71 | 0.72 | 0.67 | 1270 | Train 20 | Test Black 54 | 0.71 | 0.72 | 0.67 |
| 247 | Train 4 | Test Black 55 | 0.75 | 0.79 | 0.76 | 1271 | Train 20 | Test Black 55 | 0.75 | 0.79 | 0.77 |
| 248 | Train 4 | Test Black 56 | 0.71 | 0.71 | 0.67 | 1272 | Train 20 | Test Black 56 | 0.71 | 0.71 | 0.67 |
| 249 | Train 4 | Test Black 57 | 0.77 | 0.81 | 0.79 | 1273 | Train 20 | Test Black 57 | 0.77 | 0.81 | 0.79 |
| 250 | Train 4 | Test Black 58 | 0.77 | 0.81 | 0.79 | 1274 | Train 20 | Test Black 58 | 0.77 | 0.81 | 0.8 |
| 251 | Train 4 | Test Black 59 | 0.77 | 0.81 | 0.79 | 1275 | Train 20 | Test Black 59 | 0.77 | 0.81 | 0.79 |
| 252 | Train 4 | Test Black 60 | 0.77 | 0.81 | 0.79 | 1276 | Train 20 | Test Black 60 | 0.77 | 0.81 | 0.79 |
| 253 | Train 4 | Test Black 61 | 0.75 | 0.79 | 0.75 | 1277 | Train 20 | Test Black 61 | 0.75 | 0.79 | 0.77 |
| 254 | Train 4 | Test Black 62 | 0.71 | 0.72 | 0.67 | 1278 | Train 20 | Test Black 62 | 0.71 | 0.72 | 0.67 |
| 255 | Train 4 | Test Black 63 | 0.75 | 0.79 | 0.76 | 1279 | Train 20 | Test Black 63 | 0.75 | 0.79 | 0.77 |
| 256 | Train 4 | Test Black 64 | 0.71 | 0.72 | 0.67 | 1280 | Train 20 | Test Black 64 | 0.71 | 0.71 | 0.67 |
| 257 | Train 5 | Test Black 1 | 0.75 | 0.83 | 0.83 | 1281 | Train 21 | Test Black 1 | 0.75 | 0.83 | 0.83 |
| 258 | Train 5 | Test Black 2 | 0.75 | 0.82 | 0.82 | 1282 | Train 21 | Test Black 2 | 0.75 | 0.82 | 0.82 |
| 259 | Train 5 | Test Black 3 | 0.75 | 0.83 | 0.83 | 1283 | Train 21 | Test Black 3 | 0.75 | 0.83 | 0.83 |
| 260 | Train 5 | Test Black 4 | 0.75 | 0.82 | 0.82 | 1284 | Train 21 | Test Black 4 | 0.75 | 0.82 | 0.82 |
| 261 | Train 5 | Test Black 5 | 0.74 | 0.82 | 0.82 | 1285 | Train 21 | Test Black 5 | 0.74 | 0.82 | 0.82 |
| 262 | Train 5 | Test Black 6 | 0.7 | 0.73 | 0.7 | 1286 | Train 21 | Test Black 6 | 0.7 | 0.73 | 0.7 |
| 263 | Train 5 | Test Black 7 | 0.73 | 0.81 | 0.81 | 1287 | Train 21 | Test Black 7 | 0.73 | 0.81 | 0.81 |
| 264 | Train 5 | Test Black 8 | 0.7 | 0.73 | 0.7 | 1288 | Train 21 | Test Black 8 | 0.7 | 0.73 | 0.7 |
| 265 | Train 5 | Test Black 9 | 0.75 | 0.82 | 0.81 | 1289 | Train 21 | Test Black 9 | 0.75 | 0.82 | 0.81 |
| 266 | Train 5 | Test Black 10 | 0.75 | 0.81 | 0.8 | 1290 | Train 21 | Test Black 10 | 0.75 | 0.82 | 0.8 |
| 267 | Train 5 | Test Black 11 | 0.75 | 0.82 | 0.81 | 1291 | Train 21 | Test Black 11 | 0.75 | 0.82 | 0.81 |
| 268 | Train 5 | Test Black 12 | 0.75 | 0.81 | 0.8 | 1292 | Train 21 | Test Black 12 | 0.75 | 0.81 | 0.8 |
| 269 | Train 5 | Test Black 13 | 0.73 | 0.81 | 0.8 | 1293 | Train 21 | Test Black 13 | 0.74 | 0.81 | 0.8 |
| 270 | Train 5 | Test Black 14 | 0.7 | 0.73 | 0.7 | 1294 | Train 21 | Test Black 14 | 0.7 | 0.73 | 0.7 |
| 271 | Train 5 | Test Black 15 | 0.73 | 0.8 | 0.79 | 1295 | Train 21 | Test Black 15 | 0.73 | 0.8 | 0.79 |
| 272 | Train 5 | Test Black 16 | 0.7 | 0.73 | 0.7 | 1296 | Train 21 | Test Black 16 | 0.71 | 0.73 | 0.7 |
| 273 | Train 5 | Test Black 17 | 0.75 | 0.83 | 0.83 | 1297 | Train 21 | Test Black 17 | 0.75 | 0.83 | 0.83 |
| 274 | Train 5 | Test Black 18 | 0.75 | 0.82 | 0.82 | 1298 | Train 21 | Test Black 18 | 0.75 | 0.82 | 0.82 |
| 275 | Train 5 | Test Black 19 | 0.75 | 0.83 | 0.83 | 1299 | Train 21 | Test Black 19 | 0.75 | 0.83 | 0.83 |
| 276 | Train 5 | Test Black 20 | 0.75 | 0.82 | 0.82 | 1300 | Train 21 | Test Black 20 | 0.75 | 0.82 | 0.82 |
| 277 | Train 5 | Test Black 21 | 0.74 | 0.82 | 0.82 | 1301 | Train 21 | Test Black 21 | 0.74 | 0.82 | 0.82 |
| 278 | Train 5 | Test Black 22 | 0.7 | 0.73 | 0.7 | 1302 | Train 21 | Test Black 22 | 0.7 | 0.73 | 0.7 |
| 279 | Train 5 | Test Black 23 | 0.73 | 0.81 | 0.81 | 1303 | Train 21 | Test Black 23 | 0.73 | 0.81 | 0.81 |
| 280 | Train 5 | Test Black 24 | 0.7 | 0.73 | 0.7 | 1304 | Train 21 | Test Black 24 | 0.7 | 0.73 | 0.7 |
| 281 | Train 5 | Test Black 25 | 0.75 | 0.82 | 0.81 | 1305 | Train 21 | Test Black 25 | 0.75 | 0.82 | 0.81 |
| 282 | Train 5 | Test Black 26 | 0.75 | 0.81 | 0.8 | 1306 | Train 21 | Test Black 26 | 0.75 | 0.82 | 0.8 |
| 283 | Train 5 | Test Black 27 | 0.75 | 0.82 | 0.81 | 1307 | Train 21 | Test Black 27 | 0.75 | 0.82 | 0.81 |
| 284 | Train 5 | Test Black 28 | 0.75 | 0.81 | 0.8 | 1308 | Train 21 | Test Black 28 | 0.75 | 0.81 | 0.8 |
| 285 | Train 5 | Test Black 29 | 0.74 | 0.81 | 0.8 | 1309 | Train 21 | Test Black 29 | 0.74 | 0.81 | 0.8 |
| 286 | Train 5 | Test Black 30 | 0.7 | 0.73 | 0.7 | 1310 | Train 21 | Test Black 30 | 0.7 | 0.73 | 0.7 |
| 287 | Train 5 | Test Black 31 | 0.73 | 0.8 | 0.79 | 1311 | Train 21 | Test Black 31 | 0.73 | 0.8 | 0.79 |
| 288 | Train 5 | Test Black 32 | 0.71 | 0.73 | 0.7 | 1312 | Train 21 | Test Black 32 | 0.71 | 0.73 | 0.7 |
| 289 | Train 5 | Test Black 33 | 0.75 | 0.83 | 0.83 | 1313 | Train 21 | Test Black 33 | 0.75 | 0.83 | 0.83 |
| 290 | Train 5 | Test Black 34 | 0.75 | 0.82 | 0.82 | 1314 | Train 21 | Test Black 34 | 0.75 | 0.82 | 0.82 |
| 291 | Train 5 | Test Black 35 | 0.75 | 0.83 | 0.83 | 1315 | Train 21 | Test Black 35 | 0.75 | 0.83 | 0.83 |
| 292 | Train 5 | Test Black 36 | 0.75 | 0.82 | 0.82 | 1316 | Train 21 | Test Black 36 | 0.75 | 0.82 | 0.81 |
| 293 | Train 5 | Test Black 37 | 0.74 | 0.82 | 0.82 | 1317 | Train 21 | Test Black 37 | 0.74 | 0.82 | 0.82 |
| 294 | Train 5 | Test Black 38 | 0.7 | 0.73 | 0.7 | 1318 | Train 21 | Test Black 38 | 0.7 | 0.73 | 0.7 |
| 295 | Train 5 | Test Black 39 | 0.73 | 0.81 | 0.81 | 1319 | Train 21 | Test Black 39 | 0.73 | 0.81 | 0.81 |
| 296 | Train 5 | Test Black 40 | 0.7 | 0.73 | 0.7 | 1320 | Train 21 | Test Black 40 | 0.7 | 0.73 | 0.7 |
| 297 | Train 5 | Test Black 41 | 0.75 | 0.82 | 0.81 | 1321 | Train 21 | Test Black 41 | 0.75 | 0.82 | 0.81 |
| 298 | Train 5 | Test Black 42 | 0.75 | 0.81 | 0.8 | 1322 | Train 21 | Test Black 42 | 0.75 | 0.81 | 0.8 |
| 299 | Train 5 | Test Black 43 | 0.75 | 0.82 | 0.81 | 1323 | Train 21 | Test Black 43 | 0.75 | 0.82 | 0.81 |
| 300 | Train 5 | Test Black 44 | 0.75 | 0.81 | 0.8 | 1324 | Train 21 | Test Black 44 | 0.75 | 0.81 | 0.79 |
| 301 | Train 5 | Test Black 45 | 0.74 | 0.81 | 0.8 | 1325 | Train 21 | Test Black 45 | 0.74 | 0.81 | 0.8 |
| 302 | Train 5 | Test Black 46 | 0.7 | 0.73 | 0.7 | 1326 | Train 21 | Test Black 46 | 0.7 | 0.73 | 0.7 |
| 303 | Train 5 | Test Black 47 | 0.73 | 0.8 | 0.79 | 1327 | Train 21 | Test Black 47 | 0.73 | 0.8 | 0.79 |
| 304 | Train 5 | Test Black 48 | 0.7 | 0.73 | 0.7 | 1328 | Train 21 | Test Black 48 | 0.71 | 0.73 | 0.7 |
| 305 | Train 5 | Test Black 49 | 0.76 | 0.8 | 0.8 | 1329 | Train 21 | Test Black 49 | 0.76 | 0.8 | 0.8 |
| 306 | Train 5 | Test Black 50 | 0.76 | 0.8 | 0.8 | 1330 | Train 21 | Test Black 50 | 0.76 | 0.8 | 0.8 |
| 307 | Train 5 | Test Black 51 | 0.76 | 0.8 | 0.79 | 1331 | Train 21 | Test Black 51 | 0.76 | 0.8 | 0.8 |
| 308 | Train 5 | Test Black 52 | 0.76 | 0.8 | 0.79 | 1332 | Train 21 | Test Black 52 | 0.76 | 0.8 | 0.8 |
| 309 | Train 5 | Test Black 53 | 0.76 | 0.8 | 0.79 | 1333 | Train 21 | Test Black 53 | 0.76 | 0.8 | 0.79 |
| 310 | Train 5 | Test Black 54 | 0.71 | 0.73 | 0.68 | 1334 | Train 21 | Test Black 54 | 0.71 | 0.72 | 0.68 |
| 311 | Train 5 | Test Black 55 | 0.76 | 0.8 | 0.78 | 1335 | Train 21 | Test Black 55 | 0.76 | 0.8 | 0.79 |
| 312 | Train 5 | Test Black 56 | 0.71 | 0.72 | 0.68 | 1336 | Train 21 | Test Black 56 | 0.71 | 0.72 | 0.68 |
| 313 | Train 5 | Test Black 57 | 0.76 | 0.79 | 0.77 | 1337 | Train 21 | Test Black 57 | 0.76 | 0.79 | 0.78 |
| 314 | Train 5 | Test Black 58 | 0.76 | 0.79 | 0.77 | 1338 | Train 21 | Test Black 58 | 0.76 | 0.79 | 0.78 |
| 315 | Train 5 | Test Black 59 | 0.76 | 0.79 | 0.77 | 1339 | Train 21 | Test Black 59 | 0.76 | 0.79 | 0.78 |
| 316 | Train 5 | Test Black 60 | 0.76 | 0.79 | 0.77 | 1340 | Train 21 | Test Black 60 | 0.76 | 0.79 | 0.78 |
| 317 | Train 5 | Test Black 61 | 0.76 | 0.8 | 0.78 | 1341 | Train 21 | Test Black 61 | 0.76 | 0.8 | 0.78 |
| 318 | Train 5 | Test Black 62 | 0.71 | 0.73 | 0.68 | 1342 | Train 21 | Test Black 62 | 0.71 | 0.73 | 0.68 |
| 319 | Train 5 | Test Black 63 | 0.76 | 0.8 | 0.78 | 1343 | Train 21 | Test Black 63 | 0.76 | 0.8 | 0.78 |
| 320 | Train 5 | Test Black 64 | 0.71 | 0.72 | 0.68 | 1344 | Train 21 | Test Black 64 | 0.71 | 0.72 | 0.68 |
| 321 | Train 6 | Test Black 1 | 0.73 | 0.79 | 0.75 | 1345 | Train 22 | Test Black 1 | 0.73 | 0.79 | 0.76 |
| 322 | Train 6 | Test Black 2 | 0.74 | 0.8 | 0.75 | 1346 | Train 22 | Test Black 2 | 0.74 | 0.8 | 0.77 |
| 323 | Train 6 | Test Black 3 | 0.73 | 0.79 | 0.75 | 1347 | Train 22 | Test Black 3 | 0.73 | 0.79 | 0.76 |
| 324 | Train 6 | Test Black 4 | 0.74 | 0.8 | 0.75 | 1348 | Train 22 | Test Black 4 | 0.74 | 0.8 | 0.77 |
| 325 | Train 6 | Test Black 5 | 0.72 | 0.78 | 0.74 | 1349 | Train 22 | Test Black 5 | 0.72 | 0.78 | 0.75 |
| 326 | Train 6 | Test Black 6 | 0.73 | 0.78 | 0.76 | 1350 | Train 22 | Test Black 6 | 0.73 | 0.78 | 0.77 |
| 327 | Train 6 | Test Black 7 | 0.72 | 0.77 | 0.74 | 1351 | Train 22 | Test Black 7 | 0.72 | 0.78 | 0.75 |
| 328 | Train 6 | Test Black 8 | 0.73 | 0.78 | 0.75 | 1352 | Train 22 | Test Black 8 | 0.73 | 0.78 | 0.76 |
| 329 | Train 6 | Test Black 9 | 0.73 | 0.8 | 0.76 | 1353 | Train 22 | Test Black 9 | 0.73 | 0.8 | 0.77 |
| 330 | Train 6 | Test Black 10 | 0.74 | 0.8 | 0.76 | 1354 | Train 22 | Test Black 10 | 0.74 | 0.8 | 0.77 |
| 331 | Train 6 | Test Black 11 | 0.73 | 0.8 | 0.76 | 1355 | Train 22 | Test Black 11 | 0.73 | 0.8 | 0.77 |
| 332 | Train 6 | Test Black 12 | 0.74 | 0.8 | 0.76 | 1356 | Train 22 | Test Black 12 | 0.74 | 0.8 | 0.77 |
| 333 | Train 6 | Test Black 13 | 0.72 | 0.78 | 0.75 | 1357 | Train 22 | Test Black 13 | 0.72 | 0.78 | 0.76 |
| 334 | Train 6 | Test Black 14 | 0.73 | 0.78 | 0.76 | 1358 | Train 22 | Test Black 14 | 0.73 | 0.78 | 0.77 |
| 335 | Train 6 | Test Black 15 | 0.72 | 0.78 | 0.75 | 1359 | Train 22 | Test Black 15 | 0.72 | 0.78 | 0.76 |
| 336 | Train 6 | Test Black 16 | 0.73 | 0.78 | 0.76 | 1360 | Train 22 | Test Black 16 | 0.73 | 0.78 | 0.76 |
| 337 | Train 6 | Test Black 17 | 0.73 | 0.79 | 0.75 | 1361 | Train 22 | Test Black 17 | 0.73 | 0.79 | 0.76 |
| 338 | Train 6 | Test Black 18 | 0.74 | 0.8 | 0.75 | 1362 | Train 22 | Test Black 18 | 0.74 | 0.8 | 0.77 |
| 339 | Train 6 | Test Black 19 | 0.73 | 0.79 | 0.75 | 1363 | Train 22 | Test Black 19 | 0.73 | 0.79 | 0.76 |
| 340 | Train 6 | Test Black 20 | 0.74 | 0.8 | 0.75 | 1364 | Train 22 | Test Black 20 | 0.74 | 0.8 | 0.77 |
| 341 | Train 6 | Test Black 21 | 0.72 | 0.78 | 0.74 | 1365 | Train 22 | Test Black 21 | 0.72 | 0.78 | 0.75 |
| 342 | Train 6 | Test Black 22 | 0.73 | 0.78 | 0.76 | 1366 | Train 22 | Test Black 22 | 0.73 | 0.78 | 0.77 |
| 343 | Train 6 | Test Black 23 | 0.72 | 0.77 | 0.74 | 1367 | Train 22 | Test Black 23 | 0.72 | 0.78 | 0.75 |
| 344 | Train 6 | Test Black 24 | 0.73 | 0.78 | 0.76 | 1368 | Train 22 | Test Black 24 | 0.73 | 0.78 | 0.76 |
| 345 | Train 6 | Test Black 25 | 0.73 | 0.8 | 0.76 | 1369 | Train 22 | Test Black 25 | 0.73 | 0.8 | 0.77 |
| 346 | Train 6 | Test Black 26 | 0.74 | 0.8 | 0.76 | 1370 | Train 22 | Test Black 26 | 0.74 | 0.8 | 0.77 |
| 347 | Train 6 | Test Black 27 | 0.73 | 0.8 | 0.76 | 1371 | Train 22 | Test Black 27 | 0.73 | 0.8 | 0.77 |
| 348 | Train 6 | Test Black 28 | 0.74 | 0.8 | 0.76 | 1372 | Train 22 | Test Black 28 | 0.74 | 0.8 | 0.77 |
| 349 | Train 6 | Test Black 29 | 0.72 | 0.78 | 0.75 | 1373 | Train 22 | Test Black 29 | 0.72 | 0.78 | 0.76 |
| 350 | Train 6 | Test Black 30 | 0.73 | 0.78 | 0.76 | 1374 | Train 22 | Test Black 30 | 0.73 | 0.79 | 0.77 |
| 351 | Train 6 | Test Black 31 | 0.72 | 0.78 | 0.75 | 1375 | Train 22 | Test Black 31 | 0.72 | 0.78 | 0.76 |
| 352 | Train 6 | Test Black 32 | 0.73 | 0.78 | 0.76 | 1376 | Train 22 | Test Black 32 | 0.73 | 0.78 | 0.76 |
| 353 | Train 6 | Test Black 33 | 0.73 | 0.79 | 0.75 | 1377 | Train 22 | Test Black 33 | 0.73 | 0.79 | 0.76 |
| 354 | Train 6 | Test Black 34 | 0.74 | 0.8 | 0.75 | 1378 | Train 22 | Test Black 34 | 0.74 | 0.8 | 0.77 |
| 355 | Train 6 | Test Black 35 | 0.73 | 0.79 | 0.75 | 1379 | Train 22 | Test Black 35 | 0.73 | 0.79 | 0.76 |
| 356 | Train 6 | Test Black 36 | 0.74 | 0.8 | 0.75 | 1380 | Train 22 | Test Black 36 | 0.74 | 0.8 | 0.77 |
| 357 | Train 6 | Test Black 37 | 0.72 | 0.78 | 0.74 | 1381 | Train 22 | Test Black 37 | 0.72 | 0.78 | 0.75 |
| 358 | Train 6 | Test Black 38 | 0.73 | 0.78 | 0.76 | 1382 | Train 22 | Test Black 38 | 0.73 | 0.78 | 0.77 |
| 359 | Train 6 | Test Black 39 | 0.72 | 0.77 | 0.74 | 1383 | Train 22 | Test Black 39 | 0.72 | 0.78 | 0.75 |
| 360 | Train 6 | Test Black 40 | 0.73 | 0.78 | 0.75 | 1384 | Train 22 | Test Black 40 | 0.73 | 0.78 | 0.76 |
| 361 | Train 6 | Test Black 41 | 0.73 | 0.8 | 0.76 | 1385 | Train 22 | Test Black 41 | 0.73 | 0.8 | 0.77 |
| 362 | Train 6 | Test Black 42 | 0.74 | 0.8 | 0.76 | 1386 | Train 22 | Test Black 42 | 0.74 | 0.8 | 0.77 |
| 363 | Train 6 | Test Black 43 | 0.73 | 0.8 | 0.75 | 1387 | Train 22 | Test Black 43 | 0.73 | 0.8 | 0.77 |
| 364 | Train 6 | Test Black 44 | 0.74 | 0.8 | 0.76 | 1388 | Train 22 | Test Black 44 | 0.74 | 0.8 | 0.77 |
| 365 | Train 6 | Test Black 45 | 0.72 | 0.78 | 0.75 | 1389 | Train 22 | Test Black 45 | 0.72 | 0.78 | 0.76 |
| 366 | Train 6 | Test Black 46 | 0.73 | 0.78 | 0.76 | 1390 | Train 22 | Test Black 46 | 0.73 | 0.78 | 0.77 |
| 367 | Train 6 | Test Black 47 | 0.72 | 0.78 | 0.75 | 1391 | Train 22 | Test Black 47 | 0.72 | 0.78 | 0.76 |
| 368 | Train 6 | Test Black 48 | 0.73 | 0.78 | 0.76 | 1392 | Train 22 | Test Black 48 | 0.73 | 0.78 | 0.76 |
| 369 | Train 6 | Test Black 49 | 0.76 | 0.8 | 0.75 | 1393 | Train 22 | Test Black 49 | 0.76 | 0.8 | 0.78 |
| 370 | Train 6 | Test Black 50 | 0.76 | 0.8 | 0.75 | 1394 | Train 22 | Test Black 50 | 0.76 | 0.8 | 0.77 |
| 371 | Train 6 | Test Black 51 | 0.76 | 0.8 | 0.75 | 1395 | Train 22 | Test Black 51 | 0.76 | 0.8 | 0.77 |
| 372 | Train 6 | Test Black 52 | 0.76 | 0.8 | 0.75 | 1396 | Train 22 | Test Black 52 | 0.76 | 0.8 | 0.77 |
| 373 | Train 6 | Test Black 53 | 0.76 | 0.8 | 0.75 | 1397 | Train 22 | Test Black 53 | 0.76 | 0.8 | 0.78 |
| 374 | Train 6 | Test Black 54 | 0.74 | 0.78 | 0.78 | 1398 | Train 22 | Test Black 54 | 0.74 | 0.78 | 0.78 |
| 375 | Train 6 | Test Black 55 | 0.76 | 0.8 | 0.75 | 1399 | Train 22 | Test Black 55 | 0.76 | 0.8 | 0.78 |
| 376 | Train 6 | Test Black 56 | 0.74 | 0.78 | 0.78 | 1400 | Train 22 | Test Black 56 | 0.74 | 0.78 | 0.78 |
| 377 | Train 6 | Test Black 57 | 0.76 | 0.8 | 0.76 | 1401 | Train 22 | Test Black 57 | 0.76 | 0.8 | 0.79 |
| 378 | Train 6 | Test Black 58 | 0.76 | 0.8 | 0.76 | 1402 | Train 22 | Test Black 58 | 0.76 | 0.8 | 0.79 |
| 379 | Train 6 | Test Black 59 | 0.76 | 0.8 | 0.76 | 1403 | Train 22 | Test Black 59 | 0.76 | 0.8 | 0.79 |
| 380 | Train 6 | Test Black 60 | 0.76 | 0.8 | 0.76 | 1404 | Train 22 | Test Black 60 | 0.76 | 0.8 | 0.79 |
| 381 | Train 6 | Test Black 61 | 0.76 | 0.8 | 0.76 | 1405 | Train 22 | Test Black 61 | 0.76 | 0.8 | 0.79 |
| 382 | Train 6 | Test Black 62 | 0.74 | 0.78 | 0.78 | 1406 | Train 22 | Test Black 62 | 0.74 | 0.78 | 0.78 |
| 383 | Train 6 | Test Black 63 | 0.75 | 0.8 | 0.76 | 1407 | Train 22 | Test Black 63 | 0.75 | 0.8 | 0.79 |
| 384 | Train 6 | Test Black 64 | 0.74 | 0.78 | 0.78 | 1408 | Train 22 | Test Black 64 | 0.74 | 0.78 | 0.78 |
| 385 | Train 7 | Test Black 1 | 0.75 | 0.83 | 0.83 | 1409 | Train 23 | Test Black 1 | 0.75 | 0.83 | 0.83 |
| 386 | Train 7 | Test Black 2 | 0.75 | 0.82 | 0.81 | 1410 | Train 23 | Test Black 2 | 0.75 | 0.82 | 0.82 |
| 387 | Train 7 | Test Black 3 | 0.75 | 0.83 | 0.83 | 1411 | Train 23 | Test Black 3 | 0.75 | 0.83 | 0.83 |
| 388 | Train 7 | Test Black 4 | 0.75 | 0.82 | 0.81 | 1412 | Train 23 | Test Black 4 | 0.75 | 0.82 | 0.82 |
| 389 | Train 7 | Test Black 5 | 0.74 | 0.82 | 0.82 | 1413 | Train 23 | Test Black 5 | 0.74 | 0.82 | 0.82 |
| 390 | Train 7 | Test Black 6 | 0.7 | 0.73 | 0.71 | 1414 | Train 23 | Test Black 6 | 0.7 | 0.73 | 0.71 |
| 391 | Train 7 | Test Black 7 | 0.73 | 0.82 | 0.82 | 1415 | Train 23 | Test Black 7 | 0.73 | 0.82 | 0.82 |
| 392 | Train 7 | Test Black 8 | 0.7 | 0.73 | 0.71 | 1416 | Train 23 | Test Black 8 | 0.7 | 0.73 | 0.71 |
| 393 | Train 7 | Test Black 9 | 0.75 | 0.82 | 0.81 | 1417 | Train 23 | Test Black 9 | 0.75 | 0.82 | 0.81 |
| 394 | Train 7 | Test Black 10 | 0.75 | 0.82 | 0.8 | 1418 | Train 23 | Test Black 10 | 0.75 | 0.81 | 0.8 |
| 395 | Train 7 | Test Black 11 | 0.75 | 0.82 | 0.81 | 1419 | Train 23 | Test Black 11 | 0.75 | 0.82 | 0.81 |
| 396 | Train 7 | Test Black 12 | 0.75 | 0.81 | 0.8 | 1420 | Train 23 | Test Black 12 | 0.75 | 0.81 | 0.8 |
| 397 | Train 7 | Test Black 13 | 0.73 | 0.81 | 0.8 | 1421 | Train 23 | Test Black 13 | 0.73 | 0.81 | 0.8 |
| 398 | Train 7 | Test Black 14 | 0.7 | 0.73 | 0.71 | 1422 | Train 23 | Test Black 14 | 0.7 | 0.73 | 0.71 |
| 399 | Train 7 | Test Black 15 | 0.73 | 0.81 | 0.79 | 1423 | Train 23 | Test Black 15 | 0.73 | 0.81 | 0.8 |
| 400 | Train 7 | Test Black 16 | 0.7 | 0.73 | 0.71 | 1424 | Train 23 | Test Black 16 | 0.7 | 0.73 | 0.71 |
| 401 | Train 7 | Test Black 17 | 0.75 | 0.83 | 0.83 | 1425 | Train 23 | Test Black 17 | 0.75 | 0.83 | 0.83 |
| 402 | Train 7 | Test Black 18 | 0.75 | 0.82 | 0.81 | 1426 | Train 23 | Test Black 18 | 0.75 | 0.82 | 0.82 |
| 403 | Train 7 | Test Black 19 | 0.75 | 0.83 | 0.83 | 1427 | Train 23 | Test Black 19 | 0.75 | 0.83 | 0.83 |
| 404 | Train 7 | Test Black 20 | 0.75 | 0.82 | 0.81 | 1428 | Train 23 | Test Black 20 | 0.75 | 0.82 | 0.82 |
| 405 | Train 7 | Test Black 21 | 0.74 | 0.82 | 0.82 | 1429 | Train 23 | Test Black 21 | 0.74 | 0.82 | 0.82 |
| 406 | Train 7 | Test Black 22 | 0.7 | 0.73 | 0.71 | 1430 | Train 23 | Test Black 22 | 0.7 | 0.73 | 0.71 |
| 407 | Train 7 | Test Black 23 | 0.73 | 0.82 | 0.82 | 1431 | Train 23 | Test Black 23 | 0.73 | 0.82 | 0.82 |
| 408 | Train 7 | Test Black 24 | 0.7 | 0.73 | 0.71 | 1432 | Train 23 | Test Black 24 | 0.7 | 0.73 | 0.71 |
| 409 | Train 7 | Test Black 25 | 0.75 | 0.82 | 0.81 | 1433 | Train 23 | Test Black 25 | 0.75 | 0.82 | 0.81 |
| 410 | Train 7 | Test Black 26 | 0.75 | 0.82 | 0.8 | 1434 | Train 23 | Test Black 26 | 0.75 | 0.81 | 0.8 |
| 411 | Train 7 | Test Black 27 | 0.75 | 0.82 | 0.81 | 1435 | Train 23 | Test Black 27 | 0.75 | 0.82 | 0.81 |
| 412 | Train 7 | Test Black 28 | 0.75 | 0.81 | 0.8 | 1436 | Train 23 | Test Black 28 | 0.75 | 0.81 | 0.8 |
| 413 | Train 7 | Test Black 29 | 0.74 | 0.81 | 0.8 | 1437 | Train 23 | Test Black 29 | 0.74 | 0.81 | 0.8 |
| 414 | Train 7 | Test Black 30 | 0.7 | 0.73 | 0.71 | 1438 | Train 23 | Test Black 30 | 0.7 | 0.73 | 0.71 |
| 415 | Train 7 | Test Black 31 | 0.73 | 0.81 | 0.79 | 1439 | Train 23 | Test Black 31 | 0.73 | 0.81 | 0.8 |
| 416 | Train 7 | Test Black 32 | 0.7 | 0.73 | 0.71 | 1440 | Train 23 | Test Black 32 | 0.7 | 0.73 | 0.71 |
| 417 | Train 7 | Test Black 33 | 0.75 | 0.83 | 0.83 | 1441 | Train 23 | Test Black 33 | 0.75 | 0.83 | 0.83 |
| 418 | Train 7 | Test Black 34 | 0.75 | 0.82 | 0.81 | 1442 | Train 23 | Test Black 34 | 0.75 | 0.82 | 0.82 |
| 419 | Train 7 | Test Black 35 | 0.75 | 0.83 | 0.83 | 1443 | Train 23 | Test Black 35 | 0.75 | 0.83 | 0.83 |
| 420 | Train 7 | Test Black 36 | 0.75 | 0.82 | 0.81 | 1444 | Train 23 | Test Black 36 | 0.75 | 0.82 | 0.82 |
| 421 | Train 7 | Test Black 37 | 0.74 | 0.82 | 0.82 | 1445 | Train 23 | Test Black 37 | 0.74 | 0.82 | 0.82 |
| 422 | Train 7 | Test Black 38 | 0.7 | 0.73 | 0.71 | 1446 | Train 23 | Test Black 38 | 0.7 | 0.73 | 0.71 |
| 423 | Train 7 | Test Black 39 | 0.73 | 0.82 | 0.82 | 1447 | Train 23 | Test Black 39 | 0.73 | 0.82 | 0.82 |
| 424 | Train 7 | Test Black 40 | 0.7 | 0.73 | 0.71 | 1448 | Train 23 | Test Black 40 | 0.7 | 0.73 | 0.71 |
| 425 | Train 7 | Test Black 41 | 0.75 | 0.82 | 0.81 | 1449 | Train 23 | Test Black 41 | 0.75 | 0.82 | 0.81 |
| 426 | Train 7 | Test Black 42 | 0.75 | 0.81 | 0.79 | 1450 | Train 23 | Test Black 42 | 0.75 | 0.81 | 0.8 |
| 427 | Train 7 | Test Black 43 | 0.75 | 0.82 | 0.81 | 1451 | Train 23 | Test Black 43 | 0.75 | 0.82 | 0.81 |
| 428 | Train 7 | Test Black 44 | 0.75 | 0.81 | 0.8 | 1452 | Train 23 | Test Black 44 | 0.75 | 0.81 | 0.8 |
| 429 | Train 7 | Test Black 45 | 0.74 | 0.81 | 0.8 | 1453 | Train 23 | Test Black 45 | 0.74 | 0.81 | 0.8 |
| 430 | Train 7 | Test Black 46 | 0.7 | 0.73 | 0.71 | 1454 | Train 23 | Test Black 46 | 0.7 | 0.73 | 0.71 |
| 431 | Train 7 | Test Black 47 | 0.73 | 0.81 | 0.79 | 1455 | Train 23 | Test Black 47 | 0.73 | 0.81 | 0.8 |
| 432 | Train 7 | Test Black 48 | 0.7 | 0.73 | 0.71 | 1456 | Train 23 | Test Black 48 | 0.7 | 0.73 | 0.71 |
| 433 | Train 7 | Test Black 49 | 0.76 | 0.8 | 0.81 | 1457 | Train 23 | Test Black 49 | 0.76 | 0.8 | 0.82 |
| 434 | Train 7 | Test Black 50 | 0.76 | 0.8 | 0.81 | 1458 | Train 23 | Test Black 50 | 0.76 | 0.8 | 0.82 |
| 435 | Train 7 | Test Black 51 | 0.76 | 0.8 | 0.81 | 1459 | Train 23 | Test Black 51 | 0.76 | 0.8 | 0.82 |
| 436 | Train 7 | Test Black 52 | 0.76 | 0.8 | 0.81 | 1460 | Train 23 | Test Black 52 | 0.76 | 0.8 | 0.82 |
| 437 | Train 7 | Test Black 53 | 0.76 | 0.8 | 0.79 | 1461 | Train 23 | Test Black 53 | 0.76 | 0.8 | 0.8 |
| 438 | Train 7 | Test Black 54 | 0.71 | 0.72 | 0.67 | 1462 | Train 23 | Test Black 54 | 0.71 | 0.72 | 0.66 |
| 439 | Train 7 | Test Black 55 | 0.76 | 0.8 | 0.79 | 1463 | Train 23 | Test Black 55 | 0.76 | 0.8 | 0.8 |
| 440 | Train 7 | Test Black 56 | 0.71 | 0.72 | 0.66 | 1464 | Train 23 | Test Black 56 | 0.71 | 0.72 | 0.66 |
| 441 | Train 7 | Test Black 57 | 0.76 | 0.79 | 0.79 | 1465 | Train 23 | Test Black 57 | 0.76 | 0.79 | 0.79 |
| 442 | Train 7 | Test Black 58 | 0.76 | 0.79 | 0.79 | 1466 | Train 23 | Test Black 58 | 0.76 | 0.79 | 0.79 |
| 443 | Train 7 | Test Black 59 | 0.76 | 0.79 | 0.79 | 1467 | Train 23 | Test Black 59 | 0.76 | 0.79 | 0.79 |
| 444 | Train 7 | Test Black 60 | 0.76 | 0.79 | 0.79 | 1468 | Train 23 | Test Black 60 | 0.76 | 0.79 | 0.79 |
| 445 | Train 7 | Test Black 61 | 0.76 | 0.8 | 0.79 | 1469 | Train 23 | Test Black 61 | 0.76 | 0.8 | 0.8 |
| 446 | Train 7 | Test Black 62 | 0.71 | 0.72 | 0.67 | 1470 | Train 23 | Test Black 62 | 0.71 | 0.72 | 0.66 |
| 447 | Train 7 | Test Black 63 | 0.76 | 0.8 | 0.79 | 1471 | Train 23 | Test Black 63 | 0.76 | 0.8 | 0.8 |
| 448 | Train 7 | Test Black 64 | 0.71 | 0.72 | 0.67 | 1472 | Train 23 | Test Black 64 | 0.71 | 0.72 | 0.66 |
| 449 | Train 8 | Test Black 1 | 0.73 | 0.79 | 0.77 | 1473 | Train 24 | Test Black 1 | 0.73 | 0.79 | 0.77 |
| 450 | Train 8 | Test Black 2 | 0.74 | 0.8 | 0.77 | 1474 | Train 24 | Test Black 2 | 0.74 | 0.79 | 0.77 |
| 451 | Train 8 | Test Black 3 | 0.73 | 0.79 | 0.77 | 1475 | Train 24 | Test Black 3 | 0.73 | 0.79 | 0.77 |
| 452 | Train 8 | Test Black 4 | 0.74 | 0.8 | 0.77 | 1476 | Train 24 | Test Black 4 | 0.74 | 0.79 | 0.77 |
| 453 | Train 8 | Test Black 5 | 0.72 | 0.78 | 0.76 | 1477 | Train 24 | Test Black 5 | 0.72 | 0.78 | 0.76 |
| 454 | Train 8 | Test Black 6 | 0.73 | 0.78 | 0.77 | 1478 | Train 24 | Test Black 6 | 0.73 | 0.78 | 0.77 |
| 455 | Train 8 | Test Black 7 | 0.72 | 0.77 | 0.75 | 1479 | Train 24 | Test Black 7 | 0.72 | 0.77 | 0.76 |
| 456 | Train 8 | Test Black 8 | 0.73 | 0.78 | 0.77 | 1480 | Train 24 | Test Black 8 | 0.73 | 0.78 | 0.77 |
| 457 | Train 8 | Test Black 9 | 0.73 | 0.8 | 0.78 | 1481 | Train 24 | Test Black 9 | 0.73 | 0.8 | 0.78 |
| 458 | Train 8 | Test Black 10 | 0.74 | 0.8 | 0.78 | 1482 | Train 24 | Test Black 10 | 0.74 | 0.8 | 0.78 |
| 459 | Train 8 | Test Black 11 | 0.73 | 0.8 | 0.78 | 1483 | Train 24 | Test Black 11 | 0.73 | 0.8 | 0.78 |
| 460 | Train 8 | Test Black 12 | 0.74 | 0.8 | 0.78 | 1484 | Train 24 | Test Black 12 | 0.74 | 0.8 | 0.78 |
| 461 | Train 8 | Test Black 13 | 0.72 | 0.78 | 0.76 | 1485 | Train 24 | Test Black 13 | 0.72 | 0.78 | 0.77 |
| 462 | Train 8 | Test Black 14 | 0.73 | 0.78 | 0.77 | 1486 | Train 24 | Test Black 14 | 0.73 | 0.78 | 0.77 |
| 463 | Train 8 | Test Black 15 | 0.72 | 0.78 | 0.76 | 1487 | Train 24 | Test Black 15 | 0.72 | 0.78 | 0.76 |
| 464 | Train 8 | Test Black 16 | 0.73 | 0.78 | 0.77 | 1488 | Train 24 | Test Black 16 | 0.73 | 0.78 | 0.77 |
| 465 | Train 8 | Test Black 17 | 0.73 | 0.79 | 0.77 | 1489 | Train 24 | Test Black 17 | 0.73 | 0.79 | 0.77 |
| 466 | Train 8 | Test Black 18 | 0.74 | 0.8 | 0.77 | 1490 | Train 24 | Test Black 18 | 0.74 | 0.79 | 0.77 |
| 467 | Train 8 | Test Black 19 | 0.73 | 0.79 | 0.77 | 1491 | Train 24 | Test Black 19 | 0.73 | 0.79 | 0.77 |
| 468 | Train 8 | Test Black 20 | 0.74 | 0.8 | 0.77 | 1492 | Train 24 | Test Black 20 | 0.74 | 0.79 | 0.77 |
| 469 | Train 8 | Test Black 21 | 0.72 | 0.78 | 0.76 | 1493 | Train 24 | Test Black 21 | 0.72 | 0.78 | 0.76 |
| 470 | Train 8 | Test Black 22 | 0.73 | 0.78 | 0.77 | 1494 | Train 24 | Test Black 22 | 0.73 | 0.78 | 0.77 |
| 471 | Train 8 | Test Black 23 | 0.72 | 0.77 | 0.75 | 1495 | Train 24 | Test Black 23 | 0.72 | 0.77 | 0.76 |
| 472 | Train 8 | Test Black 24 | 0.73 | 0.78 | 0.77 | 1496 | Train 24 | Test Black 24 | 0.73 | 0.78 | 0.77 |
| 473 | Train 8 | Test Black 25 | 0.73 | 0.8 | 0.78 | 1497 | Train 24 | Test Black 25 | 0.73 | 0.8 | 0.78 |
| 474 | Train 8 | Test Black 26 | 0.74 | 0.8 | 0.78 | 1498 | Train 24 | Test Black 26 | 0.74 | 0.8 | 0.78 |
| 475 | Train 8 | Test Black 27 | 0.73 | 0.8 | 0.78 | 1499 | Train 24 | Test Black 27 | 0.73 | 0.8 | 0.78 |
| 476 | Train 8 | Test Black 28 | 0.74 | 0.8 | 0.78 | 1500 | Train 24 | Test Black 28 | 0.74 | 0.8 | 0.78 |
| 477 | Train 8 | Test Black 29 | 0.72 | 0.78 | 0.76 | 1501 | Train 24 | Test Black 29 | 0.72 | 0.78 | 0.77 |
| 478 | Train 8 | Test Black 30 | 0.73 | 0.78 | 0.77 | 1502 | Train 24 | Test Black 30 | 0.73 | 0.78 | 0.77 |
| 479 | Train 8 | Test Black 31 | 0.72 | 0.78 | 0.76 | 1503 | Train 24 | Test Black 31 | 0.72 | 0.78 | 0.76 |
| 480 | Train 8 | Test Black 32 | 0.73 | 0.78 | 0.77 | 1504 | Train 24 | Test Black 32 | 0.73 | 0.78 | 0.77 |
| 481 | Train 8 | Test Black 33 | 0.73 | 0.79 | 0.77 | 1505 | Train 24 | Test Black 33 | 0.73 | 0.79 | 0.77 |
| 482 | Train 8 | Test Black 34 | 0.74 | 0.79 | 0.77 | 1506 | Train 24 | Test Black 34 | 0.74 | 0.79 | 0.77 |
| 483 | Train 8 | Test Black 35 | 0.73 | 0.79 | 0.77 | 1507 | Train 24 | Test Black 35 | 0.73 | 0.79 | 0.77 |
| 484 | Train 8 | Test Black 36 | 0.74 | 0.79 | 0.77 | 1508 | Train 24 | Test Black 36 | 0.74 | 0.79 | 0.77 |
| 485 | Train 8 | Test Black 37 | 0.72 | 0.78 | 0.76 | 1509 | Train 24 | Test Black 37 | 0.72 | 0.78 | 0.76 |
| 486 | Train 8 | Test Black 38 | 0.73 | 0.78 | 0.77 | 1510 | Train 24 | Test Black 38 | 0.73 | 0.78 | 0.77 |
| 487 | Train 8 | Test Black 39 | 0.72 | 0.77 | 0.75 | 1511 | Train 24 | Test Black 39 | 0.72 | 0.77 | 0.76 |
| 488 | Train 8 | Test Black 40 | 0.73 | 0.78 | 0.77 | 1512 | Train 24 | Test Black 40 | 0.73 | 0.78 | 0.77 |
| 489 | Train 8 | Test Black 41 | 0.73 | 0.8 | 0.78 | 1513 | Train 24 | Test Black 41 | 0.73 | 0.79 | 0.78 |
| 490 | Train 8 | Test Black 42 | 0.74 | 0.8 | 0.78 | 1514 | Train 24 | Test Black 42 | 0.74 | 0.8 | 0.78 |
| 491 | Train 8 | Test Black 43 | 0.73 | 0.8 | 0.78 | 1515 | Train 24 | Test Black 43 | 0.73 | 0.79 | 0.78 |
| 492 | Train 8 | Test Black 44 | 0.74 | 0.8 | 0.78 | 1516 | Train 24 | Test Black 44 | 0.74 | 0.8 | 0.78 |
| 493 | Train 8 | Test Black 45 | 0.72 | 0.78 | 0.76 | 1517 | Train 24 | Test Black 45 | 0.72 | 0.78 | 0.77 |
| 494 | Train 8 | Test Black 46 | 0.73 | 0.78 | 0.77 | 1518 | Train 24 | Test Black 46 | 0.73 | 0.78 | 0.77 |
| 495 | Train 8 | Test Black 47 | 0.72 | 0.78 | 0.76 | 1519 | Train 24 | Test Black 47 | 0.72 | 0.78 | 0.76 |
| 496 | Train 8 | Test Black 48 | 0.73 | 0.78 | 0.77 | 1520 | Train 24 | Test Black 48 | 0.73 | 0.78 | 0.77 |
| 497 | Train 8 | Test Black 49 | 0.76 | 0.8 | 0.74 | 1521 | Train 24 | Test Black 49 | 0.76 | 0.8 | 0.78 |
| 498 | Train 8 | Test Black 50 | 0.76 | 0.8 | 0.74 | 1522 | Train 24 | Test Black 50 | 0.76 | 0.8 | 0.78 |
| 499 | Train 8 | Test Black 51 | 0.76 | 0.8 | 0.74 | 1523 | Train 24 | Test Black 51 | 0.76 | 0.8 | 0.78 |
| 500 | Train 8 | Test Black 52 | 0.76 | 0.8 | 0.74 | 1524 | Train 24 | Test Black 52 | 0.76 | 0.8 | 0.78 |
| 501 | Train 8 | Test Black 53 | 0.75 | 0.8 | 0.75 | 1525 | Train 24 | Test Black 53 | 0.75 | 0.8 | 0.79 |
| 502 | Train 8 | Test Black 54 | 0.74 | 0.78 | 0.78 | 1526 | Train 24 | Test Black 54 | 0.74 | 0.78 | 0.78 |
| 503 | Train 8 | Test Black 55 | 0.75 | 0.8 | 0.75 | 1527 | Train 24 | Test Black 55 | 0.75 | 0.8 | 0.79 |
| 504 | Train 8 | Test Black 56 | 0.74 | 0.78 | 0.78 | 1528 | Train 24 | Test Black 56 | 0.74 | 0.78 | 0.78 |
| 505 | Train 8 | Test Black 57 | 0.76 | 0.8 | 0.75 | 1529 | Train 24 | Test Black 57 | 0.76 | 0.8 | 0.79 |
| 506 | Train 8 | Test Black 58 | 0.76 | 0.8 | 0.75 | 1530 | Train 24 | Test Black 58 | 0.76 | 0.8 | 0.79 |
| 507 | Train 8 | Test Black 59 | 0.76 | 0.8 | 0.75 | 1531 | Train 24 | Test Black 59 | 0.76 | 0.8 | 0.79 |
| 508 | Train 8 | Test Black 60 | 0.76 | 0.8 | 0.75 | 1532 | Train 24 | Test Black 60 | 0.76 | 0.8 | 0.79 |
| 509 | Train 8 | Test Black 61 | 0.75 | 0.8 | 0.76 | 1533 | Train 24 | Test Black 61 | 0.75 | 0.8 | 0.79 |
| 510 | Train 8 | Test Black 62 | 0.74 | 0.78 | 0.78 | 1534 | Train 24 | Test Black 62 | 0.74 | 0.78 | 0.78 |
| 511 | Train 8 | Test Black 63 | 0.75 | 0.8 | 0.76 | 1535 | Train 24 | Test Black 63 | 0.75 | 0.8 | 0.79 |
| 512 | Train 8 | Test Black 64 | 0.74 | 0.78 | 0.78 | 1536 | Train 24 | Test Black 64 | 0.74 | 0.78 | 0.78 |
| 513 | Train 9 | Test Black 1 | 0.76 | 0.85 | 0.83 | 1537 | Train 25 | Test Black 1 | 0.76 | 0.85 | 0.83 |
| 514 | Train 9 | Test Black 2 | 0.76 | 0.83 | 0.81 | 1538 | Train 25 | Test Black 2 | 0.76 | 0.83 | 0.81 |
| 515 | Train 9 | Test Black 3 | 0.76 | 0.85 | 0.83 | 1539 | Train 25 | Test Black 3 | 0.76 | 0.85 | 0.83 |
| 516 | Train 9 | Test Black 4 | 0.76 | 0.83 | 0.81 | 1540 | Train 25 | Test Black 4 | 0.76 | 0.83 | 0.81 |
| 517 | Train 9 | Test Black 5 | 0.73 | 0.76 | 0.75 | 1541 | Train 25 | Test Black 5 | 0.73 | 0.76 | 0.77 |
| 518 | Train 9 | Test Black 6 | 0.71 | 0.71 | 0.69 | 1542 | Train 25 | Test Black 6 | 0.71 | 0.71 | 0.71 |
| 519 | Train 9 | Test Black 7 | 0.73 | 0.76 | 0.74 | 1543 | Train 25 | Test Black 7 | 0.73 | 0.76 | 0.76 |
| 520 | Train 9 | Test Black 8 | 0.71 | 0.71 | 0.69 | 1544 | Train 25 | Test Black 8 | 0.71 | 0.71 | 0.71 |
| 521 | Train 9 | Test Black 9 | 0.76 | 0.86 | 0.86 | 1545 | Train 25 | Test Black 9 | 0.76 | 0.86 | 0.86 |
| 522 | Train 9 | Test Black 10 | 0.76 | 0.84 | 0.84 | 1546 | Train 25 | Test Black 10 | 0.76 | 0.84 | 0.84 |
| 523 | Train 9 | Test Black 11 | 0.76 | 0.86 | 0.86 | 1547 | Train 25 | Test Black 11 | 0.76 | 0.86 | 0.86 |
| 524 | Train 9 | Test Black 12 | 0.76 | 0.84 | 0.84 | 1548 | Train 25 | Test Black 12 | 0.76 | 0.84 | 0.84 |
| 525 | Train 9 | Test Black 13 | 0.73 | 0.77 | 0.77 | 1549 | Train 25 | Test Black 13 | 0.73 | 0.77 | 0.78 |
| 526 | Train 9 | Test Black 14 | 0.71 | 0.72 | 0.69 | 1550 | Train 25 | Test Black 14 | 0.71 | 0.72 | 0.71 |
| 527 | Train 9 | Test Black 15 | 0.73 | 0.76 | 0.76 | 1551 | Train 25 | Test Black 15 | 0.73 | 0.76 | 0.78 |
| 528 | Train 9 | Test Black 16 | 0.71 | 0.71 | 0.69 | 1552 | Train 25 | Test Black 16 | 0.71 | 0.72 | 0.71 |
| 529 | Train 9 | Test Black 17 | 0.76 | 0.85 | 0.83 | 1553 | Train 25 | Test Black 17 | 0.76 | 0.85 | 0.83 |
| 530 | Train 9 | Test Black 18 | 0.76 | 0.83 | 0.81 | 1554 | Train 25 | Test Black 18 | 0.76 | 0.83 | 0.81 |
| 531 | Train 9 | Test Black 19 | 0.76 | 0.85 | 0.83 | 1555 | Train 25 | Test Black 19 | 0.76 | 0.85 | 0.83 |
| 532 | Train 9 | Test Black 20 | 0.76 | 0.83 | 0.81 | 1556 | Train 25 | Test Black 20 | 0.76 | 0.83 | 0.81 |
| 533 | Train 9 | Test Black 21 | 0.73 | 0.76 | 0.75 | 1557 | Train 25 | Test Black 21 | 0.73 | 0.76 | 0.77 |
| 534 | Train 9 | Test Black 22 | 0.71 | 0.71 | 0.69 | 1558 | Train 25 | Test Black 22 | 0.71 | 0.71 | 0.71 |
| 535 | Train 9 | Test Black 23 | 0.73 | 0.76 | 0.74 | 1559 | Train 25 | Test Black 23 | 0.73 | 0.76 | 0.76 |
| 536 | Train 9 | Test Black 24 | 0.71 | 0.71 | 0.69 | 1560 | Train 25 | Test Black 24 | 0.71 | 0.71 | 0.71 |
| 537 | Train 9 | Test Black 25 | 0.76 | 0.86 | 0.86 | 1561 | Train 25 | Test Black 25 | 0.76 | 0.86 | 0.86 |
| 538 | Train 9 | Test Black 26 | 0.76 | 0.84 | 0.84 | 1562 | Train 25 | Test Black 26 | 0.76 | 0.84 | 0.84 |
| 539 | Train 9 | Test Black 27 | 0.76 | 0.86 | 0.86 | 1563 | Train 25 | Test Black 27 | 0.76 | 0.86 | 0.86 |
| 540 | Train 9 | Test Black 28 | 0.76 | 0.84 | 0.84 | 1564 | Train 25 | Test Black 28 | 0.76 | 0.84 | 0.84 |
| 541 | Train 9 | Test Black 29 | 0.73 | 0.77 | 0.77 | 1565 | Train 25 | Test Black 29 | 0.73 | 0.77 | 0.78 |
| 542 | Train 9 | Test Black 30 | 0.71 | 0.72 | 0.69 | 1566 | Train 25 | Test Black 30 | 0.71 | 0.72 | 0.71 |
| 543 | Train 9 | Test Black 31 | 0.73 | 0.76 | 0.76 | 1567 | Train 25 | Test Black 31 | 0.73 | 0.76 | 0.78 |
| 544 | Train 9 | Test Black 32 | 0.71 | 0.72 | 0.69 | 1568 | Train 25 | Test Black 32 | 0.71 | 0.72 | 0.71 |
| 545 | Train 9 | Test Black 33 | 0.76 | 0.85 | 0.83 | 1569 | Train 25 | Test Black 33 | 0.76 | 0.85 | 0.83 |
| 546 | Train 9 | Test Black 34 | 0.76 | 0.83 | 0.81 | 1570 | Train 25 | Test Black 34 | 0.76 | 0.83 | 0.81 |
| 547 | Train 9 | Test Black 35 | 0.76 | 0.85 | 0.83 | 1571 | Train 25 | Test Black 35 | 0.76 | 0.85 | 0.83 |
| 548 | Train 9 | Test Black 36 | 0.76 | 0.83 | 0.81 | 1572 | Train 25 | Test Black 36 | 0.76 | 0.83 | 0.81 |
| 549 | Train 9 | Test Black 37 | 0.73 | 0.76 | 0.75 | 1573 | Train 25 | Test Black 37 | 0.73 | 0.76 | 0.77 |
| 550 | Train 9 | Test Black 38 | 0.71 | 0.71 | 0.69 | 1574 | Train 25 | Test Black 38 | 0.71 | 0.71 | 0.71 |
| 551 | Train 9 | Test Black 39 | 0.73 | 0.76 | 0.74 | 1575 | Train 25 | Test Black 39 | 0.73 | 0.76 | 0.76 |
| 552 | Train 9 | Test Black 40 | 0.71 | 0.71 | 0.69 | 1576 | Train 25 | Test Black 40 | 0.71 | 0.71 | 0.71 |
| 553 | Train 9 | Test Black 41 | 0.76 | 0.86 | 0.86 | 1577 | Train 25 | Test Black 41 | 0.76 | 0.86 | 0.86 |
| 554 | Train 9 | Test Black 42 | 0.76 | 0.84 | 0.84 | 1578 | Train 25 | Test Black 42 | 0.76 | 0.84 | 0.84 |
| 555 | Train 9 | Test Black 43 | 0.76 | 0.86 | 0.86 | 1579 | Train 25 | Test Black 43 | 0.76 | 0.86 | 0.86 |
| 556 | Train 9 | Test Black 44 | 0.76 | 0.84 | 0.84 | 1580 | Train 25 | Test Black 44 | 0.76 | 0.84 | 0.84 |
| 557 | Train 9 | Test Black 45 | 0.73 | 0.77 | 0.77 | 1581 | Train 25 | Test Black 45 | 0.73 | 0.77 | 0.78 |
| 558 | Train 9 | Test Black 46 | 0.71 | 0.71 | 0.69 | 1582 | Train 25 | Test Black 46 | 0.71 | 0.72 | 0.71 |
| 559 | Train 9 | Test Black 47 | 0.73 | 0.76 | 0.76 | 1583 | Train 25 | Test Black 47 | 0.73 | 0.76 | 0.78 |
| 560 | Train 9 | Test Black 48 | 0.71 | 0.71 | 0.69 | 1584 | Train 25 | Test Black 48 | 0.71 | 0.72 | 0.71 |
| 561 | Train 9 | Test Black 49 | 0.77 | 0.82 | 0.8 | 1585 | Train 25 | Test Black 49 | 0.77 | 0.82 | 0.8 |
| 562 | Train 9 | Test Black 50 | 0.77 | 0.82 | 0.8 | 1586 | Train 25 | Test Black 50 | 0.77 | 0.82 | 0.8 |
| 563 | Train 9 | Test Black 51 | 0.77 | 0.82 | 0.8 | 1587 | Train 25 | Test Black 51 | 0.77 | 0.81 | 0.8 |
| 564 | Train 9 | Test Black 52 | 0.77 | 0.82 | 0.8 | 1588 | Train 25 | Test Black 52 | 0.77 | 0.81 | 0.8 |
| 565 | Train 9 | Test Black 53 | 0.76 | 0.79 | 0.73 | 1589 | Train 25 | Test Black 53 | 0.75 | 0.79 | 0.77 |
| 566 | Train 9 | Test Black 54 | 0.71 | 0.71 | 0.64 | 1590 | Train 25 | Test Black 54 | 0.71 | 0.71 | 0.68 |
| 567 | Train 9 | Test Black 55 | 0.76 | 0.79 | 0.73 | 1591 | Train 25 | Test Black 55 | 0.76 | 0.79 | 0.77 |
| 568 | Train 9 | Test Black 56 | 0.71 | 0.71 | 0.64 | 1592 | Train 25 | Test Black 56 | 0.71 | 0.71 | 0.68 |
| 569 | Train 9 | Test Black 57 | 0.77 | 0.83 | 0.83 | 1593 | Train 25 | Test Black 57 | 0.77 | 0.83 | 0.84 |
| 570 | Train 9 | Test Black 58 | 0.77 | 0.83 | 0.83 | 1594 | Train 25 | Test Black 58 | 0.77 | 0.83 | 0.84 |
| 571 | Train 9 | Test Black 59 | 0.77 | 0.82 | 0.83 | 1595 | Train 25 | Test Black 59 | 0.77 | 0.82 | 0.83 |
| 572 | Train 9 | Test Black 60 | 0.77 | 0.82 | 0.83 | 1596 | Train 25 | Test Black 60 | 0.77 | 0.82 | 0.83 |
| 573 | Train 9 | Test Black 61 | 0.75 | 0.79 | 0.75 | 1597 | Train 25 | Test Black 61 | 0.75 | 0.79 | 0.78 |
| 574 | Train 9 | Test Black 62 | 0.71 | 0.71 | 0.65 | 1598 | Train 25 | Test Black 62 | 0.71 | 0.71 | 0.68 |
| 575 | Train 9 | Test Black 63 | 0.76 | 0.79 | 0.74 | 1599 | Train 25 | Test Black 63 | 0.75 | 0.79 | 0.78 |
| 576 | Train 9 | Test Black 64 | 0.71 | 0.71 | 0.65 | 1600 | Train 25 | Test Black 64 | 0.71 | 0.71 | 0.68 |
| 577 | Train 10 | Test Black 1 | 0.75 | 0.84 | 0.82 | 1601 | Train 26 | Test Black 1 | 0.75 | 0.84 | 0.83 |
| 578 | Train 10 | Test Black 2 | 0.76 | 0.85 | 0.82 | 1602 | Train 26 | Test Black 2 | 0.76 | 0.85 | 0.83 |
| 579 | Train 10 | Test Black 3 | 0.75 | 0.84 | 0.82 | 1603 | Train 26 | Test Black 3 | 0.75 | 0.84 | 0.82 |
| 580 | Train 10 | Test Black 4 | 0.76 | 0.85 | 0.82 | 1604 | Train 26 | Test Black 4 | 0.76 | 0.85 | 0.83 |
| 581 | Train 10 | Test Black 5 | 0.73 | 0.77 | 0.76 | 1605 | Train 26 | Test Black 5 | 0.73 | 0.76 | 0.76 |
| 582 | Train 10 | Test Black 6 | 0.71 | 0.73 | 0.71 | 1606 | Train 26 | Test Black 6 | 0.71 | 0.73 | 0.7 |
| 583 | Train 10 | Test Black 7 | 0.73 | 0.76 | 0.76 | 1607 | Train 26 | Test Black 7 | 0.73 | 0.76 | 0.75 |
| 584 | Train 10 | Test Black 8 | 0.71 | 0.72 | 0.71 | 1608 | Train 26 | Test Black 8 | 0.71 | 0.72 | 0.7 |
| 585 | Train 10 | Test Black 9 | 0.75 | 0.85 | 0.85 | 1609 | Train 26 | Test Black 9 | 0.75 | 0.85 | 0.85 |
| 586 | Train 10 | Test Black 10 | 0.76 | 0.86 | 0.85 | 1610 | Train 26 | Test Black 10 | 0.76 | 0.86 | 0.86 |
| 587 | Train 10 | Test Black 11 | 0.75 | 0.85 | 0.85 | 1611 | Train 26 | Test Black 11 | 0.75 | 0.85 | 0.85 |
| 588 | Train 10 | Test Black 12 | 0.76 | 0.86 | 0.85 | 1612 | Train 26 | Test Black 12 | 0.76 | 0.86 | 0.85 |
| 589 | Train 10 | Test Black 13 | 0.73 | 0.77 | 0.78 | 1613 | Train 26 | Test Black 13 | 0.73 | 0.77 | 0.77 |
| 590 | Train 10 | Test Black 14 | 0.71 | 0.73 | 0.71 | 1614 | Train 26 | Test Black 14 | 0.71 | 0.73 | 0.7 |
| 591 | Train 10 | Test Black 15 | 0.73 | 0.76 | 0.77 | 1615 | Train 26 | Test Black 15 | 0.73 | 0.76 | 0.77 |
| 592 | Train 10 | Test Black 16 | 0.71 | 0.73 | 0.71 | 1616 | Train 26 | Test Black 16 | 0.71 | 0.73 | 0.7 |
| 593 | Train 10 | Test Black 17 | 0.75 | 0.84 | 0.82 | 1617 | Train 26 | Test Black 17 | 0.75 | 0.84 | 0.83 |
| 594 | Train 10 | Test Black 18 | 0.76 | 0.85 | 0.82 | 1618 | Train 26 | Test Black 18 | 0.76 | 0.85 | 0.83 |
| 595 | Train 10 | Test Black 19 | 0.75 | 0.84 | 0.82 | 1619 | Train 26 | Test Black 19 | 0.75 | 0.84 | 0.82 |
| 596 | Train 10 | Test Black 20 | 0.76 | 0.85 | 0.82 | 1620 | Train 26 | Test Black 20 | 0.76 | 0.85 | 0.83 |
| 597 | Train 10 | Test Black 21 | 0.73 | 0.77 | 0.76 | 1621 | Train 26 | Test Black 21 | 0.73 | 0.77 | 0.76 |
| 598 | Train 10 | Test Black 22 | 0.71 | 0.73 | 0.71 | 1622 | Train 26 | Test Black 22 | 0.71 | 0.73 | 0.7 |
| 599 | Train 10 | Test Black 23 | 0.73 | 0.76 | 0.76 | 1623 | Train 26 | Test Black 23 | 0.73 | 0.76 | 0.75 |
| 600 | Train 10 | Test Black 24 | 0.71 | 0.72 | 0.71 | 1624 | Train 26 | Test Black 24 | 0.71 | 0.72 | 0.7 |
| 601 | Train 10 | Test Black 25 | 0.75 | 0.85 | 0.85 | 1625 | Train 26 | Test Black 25 | 0.75 | 0.85 | 0.85 |
| 602 | Train 10 | Test Black 26 | 0.76 | 0.86 | 0.85 | 1626 | Train 26 | Test Black 26 | 0.76 | 0.86 | 0.86 |
| 603 | Train 10 | Test Black 27 | 0.75 | 0.85 | 0.85 | 1627 | Train 26 | Test Black 27 | 0.75 | 0.85 | 0.85 |
| 604 | Train 10 | Test Black 28 | 0.76 | 0.86 | 0.85 | 1628 | Train 26 | Test Black 28 | 0.76 | 0.86 | 0.85 |
| 605 | Train 10 | Test Black 29 | 0.73 | 0.77 | 0.78 | 1629 | Train 26 | Test Black 29 | 0.73 | 0.77 | 0.77 |
| 606 | Train 10 | Test Black 30 | 0.71 | 0.73 | 0.71 | 1630 | Train 26 | Test Black 30 | 0.71 | 0.73 | 0.7 |
| 607 | Train 10 | Test Black 31 | 0.73 | 0.76 | 0.77 | 1631 | Train 26 | Test Black 31 | 0.73 | 0.76 | 0.77 |
| 608 | Train 10 | Test Black 32 | 0.71 | 0.73 | 0.71 | 1632 | Train 26 | Test Black 32 | 0.71 | 0.73 | 0.7 |
| 609 | Train 10 | Test Black 33 | 0.75 | 0.84 | 0.82 | 1633 | Train 26 | Test Black 33 | 0.75 | 0.84 | 0.82 |
| 610 | Train 10 | Test Black 34 | 0.75 | 0.85 | 0.82 | 1634 | Train 26 | Test Black 34 | 0.75 | 0.85 | 0.83 |
| 611 | Train 10 | Test Black 35 | 0.75 | 0.84 | 0.82 | 1635 | Train 26 | Test Black 35 | 0.75 | 0.84 | 0.82 |
| 612 | Train 10 | Test Black 36 | 0.75 | 0.85 | 0.82 | 1636 | Train 26 | Test Black 36 | 0.75 | 0.85 | 0.83 |
| 613 | Train 10 | Test Black 37 | 0.73 | 0.77 | 0.76 | 1637 | Train 26 | Test Black 37 | 0.73 | 0.76 | 0.76 |
| 614 | Train 10 | Test Black 38 | 0.71 | 0.73 | 0.71 | 1638 | Train 26 | Test Black 38 | 0.71 | 0.73 | 0.7 |
| 615 | Train 10 | Test Black 39 | 0.73 | 0.76 | 0.76 | 1639 | Train 26 | Test Black 39 | 0.73 | 0.76 | 0.75 |
| 616 | Train 10 | Test Black 40 | 0.71 | 0.72 | 0.71 | 1640 | Train 26 | Test Black 40 | 0.71 | 0.72 | 0.7 |
| 617 | Train 10 | Test Black 41 | 0.75 | 0.85 | 0.85 | 1641 | Train 26 | Test Black 41 | 0.75 | 0.85 | 0.85 |
| 618 | Train 10 | Test Black 42 | 0.75 | 0.85 | 0.85 | 1642 | Train 26 | Test Black 42 | 0.75 | 0.85 | 0.85 |
| 619 | Train 10 | Test Black 43 | 0.75 | 0.85 | 0.85 | 1643 | Train 26 | Test Black 43 | 0.75 | 0.85 | 0.85 |
| 620 | Train 10 | Test Black 44 | 0.75 | 0.85 | 0.85 | 1644 | Train 26 | Test Black 44 | 0.75 | 0.85 | 0.85 |
| 621 | Train 10 | Test Black 45 | 0.73 | 0.77 | 0.78 | 1645 | Train 26 | Test Black 45 | 0.73 | 0.77 | 0.77 |
| 622 | Train 10 | Test Black 46 | 0.71 | 0.73 | 0.71 | 1646 | Train 26 | Test Black 46 | 0.71 | 0.73 | 0.7 |
| 623 | Train 10 | Test Black 47 | 0.73 | 0.76 | 0.77 | 1647 | Train 26 | Test Black 47 | 0.73 | 0.76 | 0.77 |
| 624 | Train 10 | Test Black 48 | 0.71 | 0.73 | 0.71 | 1648 | Train 26 | Test Black 48 | 0.71 | 0.73 | 0.7 |
| 625 | Train 10 | Test Black 49 | 0.77 | 0.81 | 0.76 | 1649 | Train 26 | Test Black 49 | 0.77 | 0.81 | 0.78 |
| 626 | Train 10 | Test Black 50 | 0.77 | 0.81 | 0.76 | 1650 | Train 26 | Test Black 50 | 0.77 | 0.82 | 0.78 |
| 627 | Train 10 | Test Black 51 | 0.77 | 0.81 | 0.76 | 1651 | Train 26 | Test Black 51 | 0.77 | 0.81 | 0.78 |
| 628 | Train 10 | Test Black 52 | 0.77 | 0.81 | 0.76 | 1652 | Train 26 | Test Black 52 | 0.77 | 0.81 | 0.78 |
| 629 | Train 10 | Test Black 53 | 0.75 | 0.79 | 0.75 | 1653 | Train 26 | Test Black 53 | 0.75 | 0.79 | 0.74 |
| 630 | Train 10 | Test Black 54 | 0.71 | 0.72 | 0.68 | 1654 | Train 26 | Test Black 54 | 0.71 | 0.72 | 0.68 |
| 631 | Train 10 | Test Black 55 | 0.75 | 0.79 | 0.75 | 1655 | Train 26 | Test Black 55 | 0.75 | 0.79 | 0.74 |
| 632 | Train 10 | Test Black 56 | 0.71 | 0.72 | 0.68 | 1656 | Train 26 | Test Black 56 | 0.71 | 0.72 | 0.68 |
| 633 | Train 10 | Test Black 57 | 0.77 | 0.82 | 0.8 | 1657 | Train 26 | Test Black 57 | 0.77 | 0.82 | 0.82 |
| 634 | Train 10 | Test Black 58 | 0.77 | 0.82 | 0.8 | 1658 | Train 26 | Test Black 58 | 0.77 | 0.82 | 0.82 |
| 635 | Train 10 | Test Black 59 | 0.77 | 0.82 | 0.79 | 1659 | Train 26 | Test Black 59 | 0.77 | 0.82 | 0.81 |
| 636 | Train 10 | Test Black 60 | 0.77 | 0.82 | 0.79 | 1660 | Train 26 | Test Black 60 | 0.77 | 0.82 | 0.81 |
| 637 | Train 10 | Test Black 61 | 0.75 | 0.79 | 0.76 | 1661 | Train 26 | Test Black 61 | 0.75 | 0.8 | 0.76 |
| 638 | Train 10 | Test Black 62 | 0.71 | 0.72 | 0.68 | 1662 | Train 26 | Test Black 62 | 0.71 | 0.72 | 0.68 |
| 639 | Train 10 | Test Black 63 | 0.75 | 0.79 | 0.76 | 1663 | Train 26 | Test Black 63 | 0.75 | 0.79 | 0.76 |
| 640 | Train 10 | Test Black 64 | 0.71 | 0.72 | 0.68 | 1664 | Train 26 | Test Black 64 | 0.71 | 0.72 | 0.68 |
| 641 | Train 11 | Test Black 1 | 0.76 | 0.85 | 0.83 | 1665 | Train 27 | Test Black 1 | 0.76 | 0.85 | 0.83 |
| 642 | Train 11 | Test Black 2 | 0.76 | 0.83 | 0.81 | 1666 | Train 27 | Test Black 2 | 0.76 | 0.83 | 0.81 |
| 643 | Train 11 | Test Black 3 | 0.76 | 0.85 | 0.83 | 1667 | Train 27 | Test Black 3 | 0.76 | 0.85 | 0.83 |
| 644 | Train 11 | Test Black 4 | 0.76 | 0.83 | 0.81 | 1668 | Train 27 | Test Black 4 | 0.76 | 0.83 | 0.81 |
| 645 | Train 11 | Test Black 5 | 0.73 | 0.76 | 0.75 | 1669 | Train 27 | Test Black 5 | 0.73 | 0.76 | 0.72 |
| 646 | Train 11 | Test Black 6 | 0.71 | 0.71 | 0.69 | 1670 | Train 27 | Test Black 6 | 0.71 | 0.71 | 0.66 |
| 647 | Train 11 | Test Black 7 | 0.73 | 0.76 | 0.75 | 1671 | Train 27 | Test Black 7 | 0.73 | 0.76 | 0.72 |
| 648 | Train 11 | Test Black 8 | 0.71 | 0.71 | 0.69 | 1672 | Train 27 | Test Black 8 | 0.71 | 0.71 | 0.66 |
| 649 | Train 11 | Test Black 9 | 0.76 | 0.86 | 0.86 | 1673 | Train 27 | Test Black 9 | 0.76 | 0.86 | 0.86 |
| 650 | Train 11 | Test Black 10 | 0.76 | 0.84 | 0.83 | 1674 | Train 27 | Test Black 10 | 0.76 | 0.84 | 0.84 |
| 651 | Train 11 | Test Black 11 | 0.76 | 0.86 | 0.86 | 1675 | Train 27 | Test Black 11 | 0.76 | 0.86 | 0.86 |
| 652 | Train 11 | Test Black 12 | 0.76 | 0.84 | 0.84 | 1676 | Train 27 | Test Black 12 | 0.76 | 0.84 | 0.84 |
| 653 | Train 11 | Test Black 13 | 0.73 | 0.76 | 0.77 | 1677 | Train 27 | Test Black 13 | 0.73 | 0.77 | 0.74 |
| 654 | Train 11 | Test Black 14 | 0.71 | 0.71 | 0.69 | 1678 | Train 27 | Test Black 14 | 0.71 | 0.72 | 0.66 |
| 655 | Train 11 | Test Black 15 | 0.73 | 0.76 | 0.77 | 1679 | Train 27 | Test Black 15 | 0.73 | 0.76 | 0.74 |
| 656 | Train 11 | Test Black 16 | 0.71 | 0.71 | 0.69 | 1680 | Train 27 | Test Black 16 | 0.71 | 0.72 | 0.66 |
| 657 | Train 11 | Test Black 17 | 0.76 | 0.85 | 0.83 | 1681 | Train 27 | Test Black 17 | 0.76 | 0.85 | 0.83 |
| 658 | Train 11 | Test Black 18 | 0.76 | 0.83 | 0.81 | 1682 | Train 27 | Test Black 18 | 0.76 | 0.83 | 0.81 |
| 659 | Train 11 | Test Black 19 | 0.76 | 0.85 | 0.83 | 1683 | Train 27 | Test Black 19 | 0.76 | 0.85 | 0.83 |
| 660 | Train 11 | Test Black 20 | 0.76 | 0.83 | 0.81 | 1684 | Train 27 | Test Black 20 | 0.76 | 0.83 | 0.81 |
| 661 | Train 11 | Test Black 21 | 0.73 | 0.76 | 0.75 | 1685 | Train 27 | Test Black 21 | 0.73 | 0.76 | 0.72 |
| 662 | Train 11 | Test Black 22 | 0.71 | 0.71 | 0.69 | 1686 | Train 27 | Test Black 22 | 0.71 | 0.71 | 0.66 |
| 663 | Train 11 | Test Black 23 | 0.73 | 0.76 | 0.75 | 1687 | Train 27 | Test Black 23 | 0.73 | 0.76 | 0.72 |
| 664 | Train 11 | Test Black 24 | 0.71 | 0.71 | 0.69 | 1688 | Train 27 | Test Black 24 | 0.71 | 0.71 | 0.66 |
| 665 | Train 11 | Test Black 25 | 0.76 | 0.86 | 0.86 | 1689 | Train 27 | Test Black 25 | 0.76 | 0.86 | 0.86 |
| 666 | Train 11 | Test Black 26 | 0.76 | 0.84 | 0.83 | 1690 | Train 27 | Test Black 26 | 0.76 | 0.84 | 0.84 |
| 667 | Train 11 | Test Black 27 | 0.76 | 0.86 | 0.86 | 1691 | Train 27 | Test Black 27 | 0.76 | 0.86 | 0.86 |
| 668 | Train 11 | Test Black 28 | 0.76 | 0.84 | 0.84 | 1692 | Train 27 | Test Black 28 | 0.76 | 0.84 | 0.84 |
| 669 | Train 11 | Test Black 29 | 0.73 | 0.76 | 0.77 | 1693 | Train 27 | Test Black 29 | 0.73 | 0.77 | 0.74 |
| 670 | Train 11 | Test Black 30 | 0.71 | 0.71 | 0.69 | 1694 | Train 27 | Test Black 30 | 0.71 | 0.72 | 0.66 |
| 671 | Train 11 | Test Black 31 | 0.73 | 0.76 | 0.77 | 1695 | Train 27 | Test Black 31 | 0.73 | 0.76 | 0.74 |
| 672 | Train 11 | Test Black 32 | 0.71 | 0.71 | 0.69 | 1696 | Train 27 | Test Black 32 | 0.71 | 0.72 | 0.66 |
| 673 | Train 11 | Test Black 33 | 0.76 | 0.85 | 0.82 | 1697 | Train 27 | Test Black 33 | 0.76 | 0.85 | 0.83 |
| 674 | Train 11 | Test Black 34 | 0.76 | 0.83 | 0.8 | 1698 | Train 27 | Test Black 34 | 0.76 | 0.83 | 0.81 |
| 675 | Train 11 | Test Black 35 | 0.76 | 0.85 | 0.83 | 1699 | Train 27 | Test Black 35 | 0.76 | 0.85 | 0.83 |
| 676 | Train 11 | Test Black 36 | 0.76 | 0.83 | 0.8 | 1700 | Train 27 | Test Black 36 | 0.76 | 0.83 | 0.81 |
| 677 | Train 11 | Test Black 37 | 0.73 | 0.76 | 0.75 | 1701 | Train 27 | Test Black 37 | 0.73 | 0.76 | 0.72 |
| 678 | Train 11 | Test Black 38 | 0.71 | 0.71 | 0.69 | 1702 | Train 27 | Test Black 38 | 0.71 | 0.71 | 0.66 |
| 679 | Train 11 | Test Black 39 | 0.73 | 0.76 | 0.75 | 1703 | Train 27 | Test Black 39 | 0.73 | 0.76 | 0.72 |
| 680 | Train 11 | Test Black 40 | 0.71 | 0.71 | 0.69 | 1704 | Train 27 | Test Black 40 | 0.71 | 0.71 | 0.66 |
| 681 | Train 11 | Test Black 41 | 0.76 | 0.86 | 0.86 | 1705 | Train 27 | Test Black 41 | 0.76 | 0.86 | 0.86 |
| 682 | Train 11 | Test Black 42 | 0.76 | 0.84 | 0.83 | 1706 | Train 27 | Test Black 42 | 0.76 | 0.84 | 0.84 |
| 683 | Train 11 | Test Black 43 | 0.76 | 0.86 | 0.86 | 1707 | Train 27 | Test Black 43 | 0.76 | 0.86 | 0.86 |
| 684 | Train 11 | Test Black 44 | 0.76 | 0.84 | 0.83 | 1708 | Train 27 | Test Black 44 | 0.76 | 0.84 | 0.84 |
| 685 | Train 11 | Test Black 45 | 0.73 | 0.76 | 0.77 | 1709 | Train 27 | Test Black 45 | 0.73 | 0.77 | 0.74 |
| 686 | Train 11 | Test Black 46 | 0.71 | 0.71 | 0.69 | 1710 | Train 27 | Test Black 46 | 0.71 | 0.72 | 0.66 |
| 687 | Train 11 | Test Black 47 | 0.73 | 0.76 | 0.76 | 1711 | Train 27 | Test Black 47 | 0.73 | 0.76 | 0.74 |
| 688 | Train 11 | Test Black 48 | 0.71 | 0.71 | 0.69 | 1712 | Train 27 | Test Black 48 | 0.71 | 0.72 | 0.66 |
| 689 | Train 11 | Test Black 49 | 0.77 | 0.82 | 0.77 | 1713 | Train 27 | Test Black 49 | 0.77 | 0.81 | 0.8 |
| 690 | Train 11 | Test Black 50 | 0.77 | 0.82 | 0.77 | 1714 | Train 27 | Test Black 50 | 0.77 | 0.82 | 0.8 |
| 691 | Train 11 | Test Black 51 | 0.77 | 0.82 | 0.78 | 1715 | Train 27 | Test Black 51 | 0.77 | 0.82 | 0.8 |
| 692 | Train 11 | Test Black 52 | 0.77 | 0.82 | 0.78 | 1716 | Train 27 | Test Black 52 | 0.77 | 0.82 | 0.8 |
| 693 | Train 11 | Test Black 53 | 0.75 | 0.79 | 0.72 | 1717 | Train 27 | Test Black 53 | 0.75 | 0.79 | 0.77 |
| 694 | Train 11 | Test Black 54 | 0.71 | 0.71 | 0.67 | 1718 | Train 27 | Test Black 54 | 0.71 | 0.71 | 0.64 |
| 695 | Train 11 | Test Black 55 | 0.76 | 0.79 | 0.72 | 1719 | Train 27 | Test Black 55 | 0.76 | 0.79 | 0.77 |
| 696 | Train 11 | Test Black 56 | 0.71 | 0.71 | 0.67 | 1720 | Train 27 | Test Black 56 | 0.71 | 0.71 | 0.64 |
| 697 | Train 11 | Test Black 57 | 0.77 | 0.83 | 0.81 | 1721 | Train 27 | Test Black 57 | 0.77 | 0.82 | 0.84 |
| 698 | Train 11 | Test Black 58 | 0.77 | 0.83 | 0.81 | 1722 | Train 27 | Test Black 58 | 0.77 | 0.82 | 0.83 |
| 699 | Train 11 | Test Black 59 | 0.77 | 0.83 | 0.81 | 1723 | Train 27 | Test Black 59 | 0.77 | 0.82 | 0.83 |
| 700 | Train 11 | Test Black 60 | 0.77 | 0.83 | 0.81 | 1724 | Train 27 | Test Black 60 | 0.77 | 0.82 | 0.83 |
| 701 | Train 11 | Test Black 61 | 0.75 | 0.79 | 0.74 | 1725 | Train 27 | Test Black 61 | 0.75 | 0.79 | 0.78 |
| 702 | Train 11 | Test Black 62 | 0.71 | 0.71 | 0.67 | 1726 | Train 27 | Test Black 62 | 0.71 | 0.71 | 0.65 |
| 703 | Train 11 | Test Black 63 | 0.75 | 0.79 | 0.74 | 1727 | Train 27 | Test Black 63 | 0.75 | 0.79 | 0.78 |
| 704 | Train 11 | Test Black 64 | 0.71 | 0.71 | 0.67 | 1728 | Train 27 | Test Black 64 | 0.71 | 0.71 | 0.65 |
| 705 | Train 12 | Test Black 1 | 0.75 | 0.84 | 0.82 | 1729 | Train 28 | Test Black 1 | 0.75 | 0.84 | 0.82 |
| 706 | Train 12 | Test Black 2 | 0.76 | 0.85 | 0.83 | 1730 | Train 28 | Test Black 2 | 0.76 | 0.85 | 0.83 |
| 707 | Train 12 | Test Black 3 | 0.75 | 0.84 | 0.82 | 1731 | Train 28 | Test Black 3 | 0.75 | 0.84 | 0.82 |
| 708 | Train 12 | Test Black 4 | 0.76 | 0.85 | 0.83 | 1732 | Train 28 | Test Black 4 | 0.76 | 0.85 | 0.83 |
| 709 | Train 12 | Test Black 5 | 0.73 | 0.76 | 0.71 | 1733 | Train 28 | Test Black 5 | 0.73 | 0.77 | 0.76 |
| 710 | Train 12 | Test Black 6 | 0.71 | 0.73 | 0.69 | 1734 | Train 28 | Test Black 6 | 0.71 | 0.73 | 0.71 |
| 711 | Train 12 | Test Black 7 | 0.73 | 0.76 | 0.71 | 1735 | Train 28 | Test Black 7 | 0.73 | 0.76 | 0.75 |
| 712 | Train 12 | Test Black 8 | 0.71 | 0.72 | 0.69 | 1736 | Train 28 | Test Black 8 | 0.71 | 0.72 | 0.71 |
| 713 | Train 12 | Test Black 9 | 0.75 | 0.85 | 0.85 | 1737 | Train 28 | Test Black 9 | 0.75 | 0.85 | 0.85 |
| 714 | Train 12 | Test Black 10 | 0.76 | 0.85 | 0.85 | 1738 | Train 28 | Test Black 10 | 0.76 | 0.85 | 0.85 |
| 715 | Train 12 | Test Black 11 | 0.75 | 0.85 | 0.85 | 1739 | Train 28 | Test Black 11 | 0.75 | 0.85 | 0.85 |
| 716 | Train 12 | Test Black 12 | 0.76 | 0.85 | 0.85 | 1740 | Train 28 | Test Black 12 | 0.76 | 0.85 | 0.85 |
| 717 | Train 12 | Test Black 13 | 0.73 | 0.77 | 0.73 | 1741 | Train 28 | Test Black 13 | 0.73 | 0.77 | 0.78 |
| 718 | Train 12 | Test Black 14 | 0.71 | 0.73 | 0.69 | 1742 | Train 28 | Test Black 14 | 0.71 | 0.73 | 0.71 |
| 719 | Train 12 | Test Black 15 | 0.73 | 0.76 | 0.73 | 1743 | Train 28 | Test Black 15 | 0.73 | 0.76 | 0.77 |
| 720 | Train 12 | Test Black 16 | 0.71 | 0.73 | 0.69 | 1744 | Train 28 | Test Black 16 | 0.71 | 0.73 | 0.71 |
| 721 | Train 12 | Test Black 17 | 0.75 | 0.84 | 0.82 | 1745 | Train 28 | Test Black 17 | 0.75 | 0.84 | 0.82 |
| 722 | Train 12 | Test Black 18 | 0.76 | 0.85 | 0.83 | 1746 | Train 28 | Test Black 18 | 0.76 | 0.85 | 0.83 |
| 723 | Train 12 | Test Black 19 | 0.75 | 0.84 | 0.82 | 1747 | Train 28 | Test Black 19 | 0.75 | 0.84 | 0.82 |
| 724 | Train 12 | Test Black 20 | 0.76 | 0.85 | 0.83 | 1748 | Train 28 | Test Black 20 | 0.76 | 0.85 | 0.83 |
| 725 | Train 12 | Test Black 21 | 0.73 | 0.76 | 0.71 | 1749 | Train 28 | Test Black 21 | 0.73 | 0.77 | 0.76 |
| 726 | Train 12 | Test Black 22 | 0.71 | 0.73 | 0.69 | 1750 | Train 28 | Test Black 22 | 0.71 | 0.73 | 0.71 |
| 727 | Train 12 | Test Black 23 | 0.73 | 0.76 | 0.71 | 1751 | Train 28 | Test Black 23 | 0.73 | 0.76 | 0.75 |
| 728 | Train 12 | Test Black 24 | 0.71 | 0.73 | 0.69 | 1752 | Train 28 | Test Black 24 | 0.71 | 0.72 | 0.71 |
| 729 | Train 12 | Test Black 25 | 0.75 | 0.85 | 0.85 | 1753 | Train 28 | Test Black 25 | 0.75 | 0.85 | 0.85 |
| 730 | Train 12 | Test Black 26 | 0.76 | 0.85 | 0.85 | 1754 | Train 28 | Test Black 26 | 0.76 | 0.85 | 0.85 |
| 731 | Train 12 | Test Black 27 | 0.75 | 0.85 | 0.85 | 1755 | Train 28 | Test Black 27 | 0.75 | 0.85 | 0.85 |
| 732 | Train 12 | Test Black 28 | 0.76 | 0.85 | 0.85 | 1756 | Train 28 | Test Black 28 | 0.76 | 0.85 | 0.85 |
| 733 | Train 12 | Test Black 29 | 0.73 | 0.77 | 0.73 | 1757 | Train 28 | Test Black 29 | 0.73 | 0.77 | 0.78 |
| 734 | Train 12 | Test Black 30 | 0.71 | 0.73 | 0.69 | 1758 | Train 28 | Test Black 30 | 0.71 | 0.73 | 0.71 |
| 735 | Train 12 | Test Black 31 | 0.73 | 0.76 | 0.73 | 1759 | Train 28 | Test Black 31 | 0.73 | 0.76 | 0.77 |
| 736 | Train 12 | Test Black 32 | 0.71 | 0.73 | 0.69 | 1760 | Train 28 | Test Black 32 | 0.71 | 0.73 | 0.71 |
| 737 | Train 12 | Test Black 33 | 0.75 | 0.84 | 0.82 | 1761 | Train 28 | Test Black 33 | 0.75 | 0.84 | 0.82 |
| 738 | Train 12 | Test Black 34 | 0.75 | 0.85 | 0.83 | 1762 | Train 28 | Test Black 34 | 0.75 | 0.85 | 0.83 |
| 739 | Train 12 | Test Black 35 | 0.75 | 0.84 | 0.82 | 1763 | Train 28 | Test Black 35 | 0.75 | 0.84 | 0.82 |
| 740 | Train 12 | Test Black 36 | 0.75 | 0.85 | 0.83 | 1764 | Train 28 | Test Black 36 | 0.75 | 0.85 | 0.83 |
| 741 | Train 12 | Test Black 37 | 0.73 | 0.76 | 0.71 | 1765 | Train 28 | Test Black 37 | 0.73 | 0.76 | 0.76 |
| 742 | Train 12 | Test Black 38 | 0.71 | 0.73 | 0.69 | 1766 | Train 28 | Test Black 38 | 0.71 | 0.73 | 0.71 |
| 743 | Train 12 | Test Black 39 | 0.73 | 0.76 | 0.71 | 1767 | Train 28 | Test Black 39 | 0.73 | 0.76 | 0.76 |
| 744 | Train 12 | Test Black 40 | 0.71 | 0.72 | 0.69 | 1768 | Train 28 | Test Black 40 | 0.71 | 0.72 | 0.71 |
| 745 | Train 12 | Test Black 41 | 0.75 | 0.85 | 0.85 | 1769 | Train 28 | Test Black 41 | 0.75 | 0.85 | 0.85 |
| 746 | Train 12 | Test Black 42 | 0.75 | 0.85 | 0.85 | 1770 | Train 28 | Test Black 42 | 0.75 | 0.85 | 0.85 |
| 747 | Train 12 | Test Black 43 | 0.75 | 0.85 | 0.85 | 1771 | Train 28 | Test Black 43 | 0.75 | 0.85 | 0.85 |
| 748 | Train 12 | Test Black 44 | 0.75 | 0.85 | 0.85 | 1772 | Train 28 | Test Black 44 | 0.75 | 0.85 | 0.85 |
| 749 | Train 12 | Test Black 45 | 0.73 | 0.77 | 0.73 | 1773 | Train 28 | Test Black 45 | 0.73 | 0.77 | 0.78 |
| 750 | Train 12 | Test Black 46 | 0.71 | 0.73 | 0.69 | 1774 | Train 28 | Test Black 46 | 0.71 | 0.73 | 0.71 |
| 751 | Train 12 | Test Black 47 | 0.73 | 0.76 | 0.73 | 1775 | Train 28 | Test Black 47 | 0.73 | 0.76 | 0.77 |
| 752 | Train 12 | Test Black 48 | 0.71 | 0.73 | 0.69 | 1776 | Train 28 | Test Black 48 | 0.71 | 0.73 | 0.71 |
| 753 | Train 12 | Test Black 49 | 0.77 | 0.81 | 0.8 | 1777 | Train 28 | Test Black 49 | 0.77 | 0.81 | 0.8 |
| 754 | Train 12 | Test Black 50 | 0.77 | 0.81 | 0.8 | 1778 | Train 28 | Test Black 50 | 0.77 | 0.81 | 0.8 |
| 755 | Train 12 | Test Black 51 | 0.77 | 0.81 | 0.8 | 1779 | Train 28 | Test Black 51 | 0.77 | 0.81 | 0.8 |
| 756 | Train 12 | Test Black 52 | 0.77 | 0.81 | 0.8 | 1780 | Train 28 | Test Black 52 | 0.77 | 0.81 | 0.8 |
| 757 | Train 12 | Test Black 53 | 0.75 | 0.79 | 0.77 | 1781 | Train 28 | Test Black 53 | 0.75 | 0.79 | 0.77 |
| 758 | Train 12 | Test Black 54 | 0.71 | 0.72 | 0.68 | 1782 | Train 28 | Test Black 54 | 0.71 | 0.72 | 0.69 |
| 759 | Train 12 | Test Black 55 | 0.75 | 0.79 | 0.77 | 1783 | Train 28 | Test Black 55 | 0.75 | 0.79 | 0.77 |
| 760 | Train 12 | Test Black 56 | 0.71 | 0.72 | 0.68 | 1784 | Train 28 | Test Black 56 | 0.71 | 0.72 | 0.69 |
| 761 | Train 12 | Test Black 57 | 0.77 | 0.82 | 0.83 | 1785 | Train 28 | Test Black 57 | 0.77 | 0.82 | 0.82 |
| 762 | Train 12 | Test Black 58 | 0.77 | 0.82 | 0.83 | 1786 | Train 28 | Test Black 58 | 0.77 | 0.82 | 0.83 |
| 763 | Train 12 | Test Black 59 | 0.77 | 0.82 | 0.83 | 1787 | Train 28 | Test Black 59 | 0.77 | 0.82 | 0.83 |
| 764 | Train 12 | Test Black 60 | 0.77 | 0.82 | 0.83 | 1788 | Train 28 | Test Black 60 | 0.77 | 0.82 | 0.83 |
| 765 | Train 12 | Test Black 61 | 0.75 | 0.79 | 0.78 | 1789 | Train 28 | Test Black 61 | 0.75 | 0.79 | 0.78 |
| 766 | Train 12 | Test Black 62 | 0.71 | 0.72 | 0.68 | 1790 | Train 28 | Test Black 62 | 0.71 | 0.72 | 0.69 |
| 767 | Train 12 | Test Black 63 | 0.75 | 0.79 | 0.78 | 1791 | Train 28 | Test Black 63 | 0.75 | 0.79 | 0.78 |
| 768 | Train 12 | Test Black 64 | 0.71 | 0.72 | 0.68 | 1792 | Train 28 | Test Black 64 | 0.71 | 0.72 | 0.69 |
| 769 | Train 13 | Test Black 1 | 0.75 | 0.82 | 0.81 | 1793 | Train 29 | Test Black 1 | 0.75 | 0.82 | 0.81 |
| 770 | Train 13 | Test Black 2 | 0.75 | 0.82 | 0.8 | 1794 | Train 29 | Test Black 2 | 0.75 | 0.82 | 0.8 |
| 771 | Train 13 | Test Black 3 | 0.75 | 0.82 | 0.81 | 1795 | Train 29 | Test Black 3 | 0.75 | 0.82 | 0.81 |
| 772 | Train 13 | Test Black 4 | 0.75 | 0.82 | 0.8 | 1796 | Train 29 | Test Black 4 | 0.75 | 0.82 | 0.8 |
| 773 | Train 13 | Test Black 5 | 0.74 | 0.81 | 0.8 | 1797 | Train 29 | Test Black 5 | 0.74 | 0.81 | 0.8 |
| 774 | Train 13 | Test Black 6 | 0.7 | 0.74 | 0.72 | 1798 | Train 29 | Test Black 6 | 0.7 | 0.73 | 0.73 |
| 775 | Train 13 | Test Black 7 | 0.73 | 0.81 | 0.8 | 1799 | Train 29 | Test Black 7 | 0.73 | 0.81 | 0.79 |
| 776 | Train 13 | Test Black 8 | 0.7 | 0.73 | 0.72 | 1800 | Train 29 | Test Black 8 | 0.7 | 0.73 | 0.72 |
| 777 | Train 13 | Test Black 9 | 0.75 | 0.83 | 0.83 | 1801 | Train 29 | Test Black 9 | 0.75 | 0.83 | 0.83 |
| 778 | Train 13 | Test Black 10 | 0.75 | 0.82 | 0.82 | 1802 | Train 29 | Test Black 10 | 0.75 | 0.82 | 0.82 |
| 779 | Train 13 | Test Black 11 | 0.75 | 0.83 | 0.83 | 1803 | Train 29 | Test Black 11 | 0.75 | 0.83 | 0.83 |
| 780 | Train 13 | Test Black 12 | 0.75 | 0.82 | 0.82 | 1804 | Train 29 | Test Black 12 | 0.75 | 0.82 | 0.82 |
| 781 | Train 13 | Test Black 13 | 0.73 | 0.82 | 0.82 | 1805 | Train 29 | Test Black 13 | 0.73 | 0.82 | 0.82 |
| 782 | Train 13 | Test Black 14 | 0.7 | 0.74 | 0.72 | 1806 | Train 29 | Test Black 14 | 0.7 | 0.74 | 0.73 |
| 783 | Train 13 | Test Black 15 | 0.73 | 0.81 | 0.81 | 1807 | Train 29 | Test Black 15 | 0.73 | 0.81 | 0.81 |
| 784 | Train 13 | Test Black 16 | 0.71 | 0.74 | 0.72 | 1808 | Train 29 | Test Black 16 | 0.7 | 0.74 | 0.73 |
| 785 | Train 13 | Test Black 17 | 0.75 | 0.82 | 0.81 | 1809 | Train 29 | Test Black 17 | 0.75 | 0.82 | 0.81 |
| 786 | Train 13 | Test Black 18 | 0.75 | 0.82 | 0.8 | 1810 | Train 29 | Test Black 18 | 0.75 | 0.82 | 0.8 |
| 787 | Train 13 | Test Black 19 | 0.75 | 0.82 | 0.81 | 1811 | Train 29 | Test Black 19 | 0.75 | 0.82 | 0.81 |
| 788 | Train 13 | Test Black 20 | 0.75 | 0.82 | 0.8 | 1812 | Train 29 | Test Black 20 | 0.75 | 0.82 | 0.8 |
| 789 | Train 13 | Test Black 21 | 0.74 | 0.81 | 0.8 | 1813 | Train 29 | Test Black 21 | 0.74 | 0.81 | 0.8 |
| 790 | Train 13 | Test Black 22 | 0.7 | 0.74 | 0.72 | 1814 | Train 29 | Test Black 22 | 0.7 | 0.74 | 0.73 |
| 791 | Train 13 | Test Black 23 | 0.73 | 0.81 | 0.8 | 1815 | Train 29 | Test Black 23 | 0.73 | 0.81 | 0.79 |
| 792 | Train 13 | Test Black 24 | 0.7 | 0.73 | 0.72 | 1816 | Train 29 | Test Black 24 | 0.7 | 0.73 | 0.72 |
| 793 | Train 13 | Test Black 25 | 0.75 | 0.83 | 0.83 | 1817 | Train 29 | Test Black 25 | 0.75 | 0.83 | 0.83 |
| 794 | Train 13 | Test Black 26 | 0.75 | 0.82 | 0.82 | 1818 | Train 29 | Test Black 26 | 0.75 | 0.82 | 0.82 |
| 795 | Train 13 | Test Black 27 | 0.75 | 0.83 | 0.83 | 1819 | Train 29 | Test Black 27 | 0.75 | 0.83 | 0.83 |
| 796 | Train 13 | Test Black 28 | 0.75 | 0.82 | 0.82 | 1820 | Train 29 | Test Black 28 | 0.75 | 0.82 | 0.82 |
| 797 | Train 13 | Test Black 29 | 0.73 | 0.82 | 0.82 | 1821 | Train 29 | Test Black 29 | 0.73 | 0.82 | 0.82 |
| 798 | Train 13 | Test Black 30 | 0.7 | 0.74 | 0.72 | 1822 | Train 29 | Test Black 30 | 0.7 | 0.74 | 0.73 |
| 799 | Train 13 | Test Black 31 | 0.73 | 0.81 | 0.81 | 1823 | Train 29 | Test Black 31 | 0.73 | 0.81 | 0.81 |
| 800 | Train 13 | Test Black 32 | 0.71 | 0.74 | 0.72 | 1824 | Train 29 | Test Black 32 | 0.71 | 0.74 | 0.73 |
| 801 | Train 13 | Test Black 33 | 0.75 | 0.82 | 0.81 | 1825 | Train 29 | Test Black 33 | 0.75 | 0.82 | 0.81 |
| 802 | Train 13 | Test Black 34 | 0.75 | 0.82 | 0.8 | 1826 | Train 29 | Test Black 34 | 0.75 | 0.82 | 0.8 |
| 803 | Train 13 | Test Black 35 | 0.75 | 0.82 | 0.81 | 1827 | Train 29 | Test Black 35 | 0.75 | 0.82 | 0.81 |
| 804 | Train 13 | Test Black 36 | 0.75 | 0.82 | 0.8 | 1828 | Train 29 | Test Black 36 | 0.75 | 0.82 | 0.8 |
| 805 | Train 13 | Test Black 37 | 0.74 | 0.81 | 0.8 | 1829 | Train 29 | Test Black 37 | 0.74 | 0.81 | 0.8 |
| 806 | Train 13 | Test Black 38 | 0.7 | 0.74 | 0.72 | 1830 | Train 29 | Test Black 38 | 0.7 | 0.73 | 0.73 |
| 807 | Train 13 | Test Black 39 | 0.73 | 0.81 | 0.79 | 1831 | Train 29 | Test Black 39 | 0.73 | 0.81 | 0.79 |
| 808 | Train 13 | Test Black 40 | 0.7 | 0.73 | 0.72 | 1832 | Train 29 | Test Black 40 | 0.7 | 0.73 | 0.72 |
| 809 | Train 13 | Test Black 41 | 0.75 | 0.83 | 0.83 | 1833 | Train 29 | Test Black 41 | 0.75 | 0.83 | 0.83 |
| 810 | Train 13 | Test Black 42 | 0.75 | 0.82 | 0.82 | 1834 | Train 29 | Test Black 42 | 0.75 | 0.82 | 0.82 |
| 811 | Train 13 | Test Black 43 | 0.75 | 0.83 | 0.83 | 1835 | Train 29 | Test Black 43 | 0.75 | 0.83 | 0.82 |
| 812 | Train 13 | Test Black 44 | 0.75 | 0.82 | 0.82 | 1836 | Train 29 | Test Black 44 | 0.75 | 0.82 | 0.82 |
| 813 | Train 13 | Test Black 45 | 0.74 | 0.82 | 0.82 | 1837 | Train 29 | Test Black 45 | 0.74 | 0.82 | 0.82 |
| 814 | Train 13 | Test Black 46 | 0.7 | 0.74 | 0.72 | 1838 | Train 29 | Test Black 46 | 0.7 | 0.74 | 0.73 |
| 815 | Train 13 | Test Black 47 | 0.73 | 0.81 | 0.81 | 1839 | Train 29 | Test Black 47 | 0.73 | 0.81 | 0.81 |
| 816 | Train 13 | Test Black 48 | 0.71 | 0.74 | 0.72 | 1840 | Train 29 | Test Black 48 | 0.7 | 0.74 | 0.73 |
| 817 | Train 13 | Test Black 49 | 0.77 | 0.79 | 0.77 | 1841 | Train 29 | Test Black 49 | 0.77 | 0.79 | 0.77 |
| 818 | Train 13 | Test Black 50 | 0.77 | 0.79 | 0.77 | 1842 | Train 29 | Test Black 50 | 0.76 | 0.79 | 0.76 |
| 819 | Train 13 | Test Black 51 | 0.77 | 0.79 | 0.76 | 1843 | Train 29 | Test Black 51 | 0.77 | 0.79 | 0.76 |
| 820 | Train 13 | Test Black 52 | 0.77 | 0.79 | 0.76 | 1844 | Train 29 | Test Black 52 | 0.77 | 0.79 | 0.76 |
| 821 | Train 13 | Test Black 53 | 0.76 | 0.8 | 0.79 | 1845 | Train 29 | Test Black 53 | 0.76 | 0.8 | 0.78 |
| 822 | Train 13 | Test Black 54 | 0.71 | 0.73 | 0.67 | 1846 | Train 29 | Test Black 54 | 0.71 | 0.73 | 0.7 |
| 823 | Train 13 | Test Black 55 | 0.76 | 0.8 | 0.78 | 1847 | Train 29 | Test Black 55 | 0.76 | 0.8 | 0.78 |
| 824 | Train 13 | Test Black 56 | 0.71 | 0.72 | 0.67 | 1848 | Train 29 | Test Black 56 | 0.71 | 0.72 | 0.7 |
| 825 | Train 13 | Test Black 57 | 0.76 | 0.8 | 0.79 | 1849 | Train 29 | Test Black 57 | 0.76 | 0.8 | 0.79 |
| 826 | Train 13 | Test Black 58 | 0.76 | 0.8 | 0.79 | 1850 | Train 29 | Test Black 58 | 0.76 | 0.8 | 0.79 |
| 827 | Train 13 | Test Black 59 | 0.76 | 0.8 | 0.79 | 1851 | Train 29 | Test Black 59 | 0.76 | 0.8 | 0.79 |
| 828 | Train 13 | Test Black 60 | 0.76 | 0.8 | 0.79 | 1852 | Train 29 | Test Black 60 | 0.76 | 0.8 | 0.79 |
| 829 | Train 13 | Test Black 61 | 0.76 | 0.81 | 0.8 | 1853 | Train 29 | Test Black 61 | 0.76 | 0.81 | 0.8 |
| 830 | Train 13 | Test Black 62 | 0.71 | 0.73 | 0.67 | 1854 | Train 29 | Test Black 62 | 0.71 | 0.73 | 0.7 |
| 831 | Train 13 | Test Black 63 | 0.76 | 0.81 | 0.8 | 1855 | Train 29 | Test Black 63 | 0.76 | 0.81 | 0.79 |
| 832 | Train 13 | Test Black 64 | 0.71 | 0.72 | 0.67 | 1856 | Train 29 | Test Black 64 | 0.71 | 0.72 | 0.7 |
| 833 | Train 14 | Test Black 1 | 0.73 | 0.79 | 0.76 | 1857 | Train 30 | Test Black 1 | 0.73 | 0.79 | 0.76 |
| 834 | Train 14 | Test Black 2 | 0.74 | 0.8 | 0.76 | 1858 | Train 30 | Test Black 2 | 0.74 | 0.8 | 0.76 |
| 835 | Train 14 | Test Black 3 | 0.73 | 0.79 | 0.76 | 1859 | Train 30 | Test Black 3 | 0.73 | 0.79 | 0.76 |
| 836 | Train 14 | Test Black 4 | 0.74 | 0.79 | 0.76 | 1860 | Train 30 | Test Black 4 | 0.74 | 0.79 | 0.76 |
| 837 | Train 14 | Test Black 5 | 0.72 | 0.78 | 0.75 | 1861 | Train 30 | Test Black 5 | 0.72 | 0.78 | 0.75 |
| 838 | Train 14 | Test Black 6 | 0.73 | 0.78 | 0.77 | 1862 | Train 30 | Test Black 6 | 0.73 | 0.78 | 0.76 |
| 839 | Train 14 | Test Black 7 | 0.72 | 0.77 | 0.75 | 1863 | Train 30 | Test Black 7 | 0.72 | 0.77 | 0.75 |
| 840 | Train 14 | Test Black 8 | 0.73 | 0.78 | 0.76 | 1864 | Train 30 | Test Black 8 | 0.73 | 0.77 | 0.75 |
| 841 | Train 14 | Test Black 9 | 0.73 | 0.8 | 0.77 | 1865 | Train 30 | Test Black 9 | 0.73 | 0.8 | 0.77 |
| 842 | Train 14 | Test Black 10 | 0.74 | 0.8 | 0.77 | 1866 | Train 30 | Test Black 10 | 0.74 | 0.8 | 0.77 |
| 843 | Train 14 | Test Black 11 | 0.73 | 0.8 | 0.77 | 1867 | Train 30 | Test Black 11 | 0.73 | 0.8 | 0.77 |
| 844 | Train 14 | Test Black 12 | 0.74 | 0.8 | 0.77 | 1868 | Train 30 | Test Black 12 | 0.74 | 0.8 | 0.77 |
| 845 | Train 14 | Test Black 13 | 0.72 | 0.78 | 0.76 | 1869 | Train 30 | Test Black 13 | 0.72 | 0.78 | 0.76 |
| 846 | Train 14 | Test Black 14 | 0.73 | 0.79 | 0.77 | 1870 | Train 30 | Test Black 14 | 0.73 | 0.78 | 0.76 |
| 847 | Train 14 | Test Black 15 | 0.72 | 0.78 | 0.75 | 1871 | Train 30 | Test Black 15 | 0.72 | 0.78 | 0.75 |
| 848 | Train 14 | Test Black 16 | 0.73 | 0.78 | 0.76 | 1872 | Train 30 | Test Black 16 | 0.73 | 0.78 | 0.75 |
| 849 | Train 14 | Test Black 17 | 0.73 | 0.79 | 0.76 | 1873 | Train 30 | Test Black 17 | 0.73 | 0.79 | 0.76 |
| 850 | Train 14 | Test Black 18 | 0.74 | 0.8 | 0.76 | 1874 | Train 30 | Test Black 18 | 0.74 | 0.8 | 0.76 |
| 851 | Train 14 | Test Black 19 | 0.73 | 0.79 | 0.76 | 1875 | Train 30 | Test Black 19 | 0.73 | 0.79 | 0.76 |
| 852 | Train 14 | Test Black 20 | 0.74 | 0.79 | 0.76 | 1876 | Train 30 | Test Black 20 | 0.74 | 0.79 | 0.76 |
| 853 | Train 14 | Test Black 21 | 0.72 | 0.78 | 0.75 | 1877 | Train 30 | Test Black 21 | 0.72 | 0.78 | 0.75 |
| 854 | Train 14 | Test Black 22 | 0.73 | 0.78 | 0.77 | 1878 | Train 30 | Test Black 22 | 0.73 | 0.78 | 0.76 |
| 855 | Train 14 | Test Black 23 | 0.72 | 0.77 | 0.75 | 1879 | Train 30 | Test Black 23 | 0.72 | 0.77 | 0.75 |
| 856 | Train 14 | Test Black 24 | 0.73 | 0.78 | 0.76 | 1880 | Train 30 | Test Black 24 | 0.73 | 0.78 | 0.75 |
| 857 | Train 14 | Test Black 25 | 0.73 | 0.8 | 0.77 | 1881 | Train 30 | Test Black 25 | 0.73 | 0.8 | 0.77 |
| 858 | Train 14 | Test Black 26 | 0.74 | 0.8 | 0.77 | 1882 | Train 30 | Test Black 26 | 0.74 | 0.8 | 0.77 |
| 859 | Train 14 | Test Black 27 | 0.73 | 0.8 | 0.77 | 1883 | Train 30 | Test Black 27 | 0.73 | 0.8 | 0.77 |
| 860 | Train 14 | Test Black 28 | 0.74 | 0.8 | 0.77 | 1884 | Train 30 | Test Black 28 | 0.74 | 0.8 | 0.77 |
| 861 | Train 14 | Test Black 29 | 0.72 | 0.78 | 0.76 | 1885 | Train 30 | Test Black 29 | 0.72 | 0.78 | 0.76 |
| 862 | Train 14 | Test Black 30 | 0.73 | 0.79 | 0.77 | 1886 | Train 30 | Test Black 30 | 0.73 | 0.78 | 0.76 |
| 863 | Train 14 | Test Black 31 | 0.72 | 0.78 | 0.75 | 1887 | Train 30 | Test Black 31 | 0.72 | 0.78 | 0.75 |
| 864 | Train 14 | Test Black 32 | 0.73 | 0.78 | 0.76 | 1888 | Train 30 | Test Black 32 | 0.73 | 0.78 | 0.75 |
| 865 | Train 14 | Test Black 33 | 0.73 | 0.79 | 0.76 | 1889 | Train 30 | Test Black 33 | 0.73 | 0.79 | 0.76 |
| 866 | Train 14 | Test Black 34 | 0.74 | 0.79 | 0.76 | 1890 | Train 30 | Test Black 34 | 0.74 | 0.79 | 0.76 |
| 867 | Train 14 | Test Black 35 | 0.73 | 0.79 | 0.76 | 1891 | Train 30 | Test Black 35 | 0.73 | 0.79 | 0.76 |
| 868 | Train 14 | Test Black 36 | 0.74 | 0.79 | 0.76 | 1892 | Train 30 | Test Black 36 | 0.74 | 0.79 | 0.76 |
| 869 | Train 14 | Test Black 37 | 0.72 | 0.78 | 0.75 | 1893 | Train 30 | Test Black 37 | 0.72 | 0.78 | 0.75 |
| 870 | Train 14 | Test Black 38 | 0.73 | 0.78 | 0.77 | 1894 | Train 30 | Test Black 38 | 0.73 | 0.78 | 0.76 |
| 871 | Train 14 | Test Black 39 | 0.72 | 0.77 | 0.75 | 1895 | Train 30 | Test Black 39 | 0.72 | 0.77 | 0.75 |
| 872 | Train 14 | Test Black 40 | 0.73 | 0.78 | 0.76 | 1896 | Train 30 | Test Black 40 | 0.73 | 0.78 | 0.75 |
| 873 | Train 14 | Test Black 41 | 0.73 | 0.8 | 0.77 | 1897 | Train 30 | Test Black 41 | 0.73 | 0.8 | 0.77 |
| 874 | Train 14 | Test Black 42 | 0.74 | 0.8 | 0.77 | 1898 | Train 30 | Test Black 42 | 0.74 | 0.8 | 0.77 |
| 875 | Train 14 | Test Black 43 | 0.73 | 0.8 | 0.77 | 1899 | Train 30 | Test Black 43 | 0.73 | 0.8 | 0.77 |
| 876 | Train 14 | Test Black 44 | 0.74 | 0.8 | 0.77 | 1900 | Train 30 | Test Black 44 | 0.74 | 0.8 | 0.77 |
| 877 | Train 14 | Test Black 45 | 0.72 | 0.78 | 0.76 | 1901 | Train 30 | Test Black 45 | 0.72 | 0.78 | 0.76 |
| 878 | Train 14 | Test Black 46 | 0.73 | 0.79 | 0.77 | 1902 | Train 30 | Test Black 46 | 0.73 | 0.78 | 0.76 |
| 879 | Train 14 | Test Black 47 | 0.72 | 0.78 | 0.75 | 1903 | Train 30 | Test Black 47 | 0.72 | 0.78 | 0.75 |
| 880 | Train 14 | Test Black 48 | 0.73 | 0.78 | 0.76 | 1904 | Train 30 | Test Black 48 | 0.73 | 0.78 | 0.75 |
| 881 | Train 14 | Test Black 49 | 0.76 | 0.8 | 0.78 | 1905 | Train 30 | Test Black 49 | 0.76 | 0.8 | 0.78 |
| 882 | Train 14 | Test Black 50 | 0.76 | 0.8 | 0.79 | 1906 | Train 30 | Test Black 50 | 0.76 | 0.8 | 0.78 |
| 883 | Train 14 | Test Black 51 | 0.76 | 0.8 | 0.78 | 1907 | Train 30 | Test Black 51 | 0.76 | 0.8 | 0.78 |
| 884 | Train 14 | Test Black 52 | 0.76 | 0.8 | 0.78 | 1908 | Train 30 | Test Black 52 | 0.76 | 0.8 | 0.78 |
| 885 | Train 14 | Test Black 53 | 0.76 | 0.8 | 0.78 | 1909 | Train 30 | Test Black 53 | 0.76 | 0.8 | 0.78 |
| 886 | Train 14 | Test Black 54 | 0.74 | 0.78 | 0.78 | 1910 | Train 30 | Test Black 54 | 0.74 | 0.78 | 0.78 |
| 887 | Train 14 | Test Black 55 | 0.75 | 0.8 | 0.78 | 1911 | Train 30 | Test Black 55 | 0.76 | 0.8 | 0.78 |
| 888 | Train 14 | Test Black 56 | 0.74 | 0.78 | 0.78 | 1912 | Train 30 | Test Black 56 | 0.74 | 0.78 | 0.78 |
| 889 | Train 14 | Test Black 57 | 0.76 | 0.8 | 0.79 | 1913 | Train 30 | Test Black 57 | 0.76 | 0.8 | 0.79 |
| 890 | Train 14 | Test Black 58 | 0.76 | 0.8 | 0.8 | 1914 | Train 30 | Test Black 58 | 0.76 | 0.8 | 0.79 |
| 891 | Train 14 | Test Black 59 | 0.76 | 0.8 | 0.79 | 1915 | Train 30 | Test Black 59 | 0.76 | 0.8 | 0.79 |
| 892 | Train 14 | Test Black 60 | 0.76 | 0.8 | 0.79 | 1916 | Train 30 | Test Black 60 | 0.76 | 0.8 | 0.79 |
| 893 | Train 14 | Test Black 61 | 0.75 | 0.8 | 0.79 | 1917 | Train 30 | Test Black 61 | 0.76 | 0.8 | 0.79 |
| 894 | Train 14 | Test Black 62 | 0.74 | 0.78 | 0.78 | 1918 | Train 30 | Test Black 62 | 0.74 | 0.78 | 0.78 |
| 895 | Train 14 | Test Black 63 | 0.75 | 0.8 | 0.79 | 1919 | Train 30 | Test Black 63 | 0.75 | 0.8 | 0.79 |
| 896 | Train 14 | Test Black 64 | 0.74 | 0.78 | 0.78 | 1920 | Train 30 | Test Black 64 | 0.74 | 0.78 | 0.78 |
| 897 | Train 15 | Test Black 1 | 0.75 | 0.82 | 0.81 | 1921 | Train 31 | Test Black 1 | 0.75 | 0.82 | 0.81 |
| 898 | Train 15 | Test Black 2 | 0.75 | 0.82 | 0.8 | 1922 | Train 31 | Test Black 2 | 0.75 | 0.82 | 0.8 |
| 899 | Train 15 | Test Black 3 | 0.75 | 0.82 | 0.81 | 1923 | Train 31 | Test Black 3 | 0.75 | 0.82 | 0.81 |
| 900 | Train 15 | Test Black 4 | 0.75 | 0.82 | 0.8 | 1924 | Train 31 | Test Black 4 | 0.75 | 0.82 | 0.8 |
| 901 | Train 15 | Test Black 5 | 0.74 | 0.81 | 0.8 | 1925 | Train 31 | Test Black 5 | 0.74 | 0.81 | 0.8 |
| 902 | Train 15 | Test Black 6 | 0.7 | 0.74 | 0.7 | 1926 | Train 31 | Test Black 6 | 0.7 | 0.74 | 0.7 |
| 903 | Train 15 | Test Black 7 | 0.73 | 0.81 | 0.79 | 1927 | Train 31 | Test Black 7 | 0.73 | 0.81 | 0.8 |
| 904 | Train 15 | Test Black 8 | 0.7 | 0.74 | 0.7 | 1928 | Train 31 | Test Black 8 | 0.7 | 0.74 | 0.7 |
| 905 | Train 15 | Test Black 9 | 0.75 | 0.83 | 0.83 | 1929 | Train 31 | Test Black 9 | 0.75 | 0.83 | 0.83 |
| 906 | Train 15 | Test Black 10 | 0.75 | 0.82 | 0.81 | 1930 | Train 31 | Test Black 10 | 0.75 | 0.82 | 0.82 |
| 907 | Train 15 | Test Black 11 | 0.75 | 0.83 | 0.83 | 1931 | Train 31 | Test Black 11 | 0.75 | 0.83 | 0.83 |
| 908 | Train 15 | Test Black 12 | 0.75 | 0.82 | 0.81 | 1932 | Train 31 | Test Black 12 | 0.75 | 0.82 | 0.82 |
| 909 | Train 15 | Test Black 13 | 0.73 | 0.82 | 0.82 | 1933 | Train 31 | Test Black 13 | 0.73 | 0.82 | 0.82 |
| 910 | Train 15 | Test Black 14 | 0.7 | 0.74 | 0.7 | 1934 | Train 31 | Test Black 14 | 0.7 | 0.74 | 0.7 |
| 911 | Train 15 | Test Black 15 | 0.73 | 0.82 | 0.82 | 1935 | Train 31 | Test Black 15 | 0.73 | 0.82 | 0.82 |
| 912 | Train 15 | Test Black 16 | 0.7 | 0.74 | 0.71 | 1936 | Train 31 | Test Black 16 | 0.7 | 0.74 | 0.7 |
| 913 | Train 15 | Test Black 17 | 0.75 | 0.82 | 0.81 | 1937 | Train 31 | Test Black 17 | 0.75 | 0.82 | 0.81 |
| 914 | Train 15 | Test Black 18 | 0.75 | 0.82 | 0.8 | 1938 | Train 31 | Test Black 18 | 0.75 | 0.82 | 0.8 |
| 915 | Train 15 | Test Black 19 | 0.75 | 0.82 | 0.81 | 1939 | Train 31 | Test Black 19 | 0.75 | 0.82 | 0.81 |
| 916 | Train 15 | Test Black 20 | 0.75 | 0.82 | 0.8 | 1940 | Train 31 | Test Black 20 | 0.75 | 0.82 | 0.8 |
| 917 | Train 15 | Test Black 21 | 0.74 | 0.81 | 0.8 | 1941 | Train 31 | Test Black 21 | 0.74 | 0.81 | 0.8 |
| 918 | Train 15 | Test Black 22 | 0.7 | 0.74 | 0.7 | 1942 | Train 31 | Test Black 22 | 0.7 | 0.74 | 0.7 |
| 919 | Train 15 | Test Black 23 | 0.73 | 0.81 | 0.79 | 1943 | Train 31 | Test Black 23 | 0.73 | 0.81 | 0.8 |
| 920 | Train 15 | Test Black 24 | 0.7 | 0.74 | 0.7 | 1944 | Train 31 | Test Black 24 | 0.7 | 0.74 | 0.7 |
| 921 | Train 15 | Test Black 25 | 0.75 | 0.83 | 0.83 | 1945 | Train 31 | Test Black 25 | 0.75 | 0.83 | 0.83 |
| 922 | Train 15 | Test Black 26 | 0.75 | 0.82 | 0.81 | 1946 | Train 31 | Test Black 26 | 0.75 | 0.82 | 0.82 |
| 923 | Train 15 | Test Black 27 | 0.75 | 0.83 | 0.83 | 1947 | Train 31 | Test Black 27 | 0.75 | 0.83 | 0.83 |
| 924 | Train 15 | Test Black 28 | 0.75 | 0.82 | 0.81 | 1948 | Train 31 | Test Black 28 | 0.75 | 0.82 | 0.82 |
| 925 | Train 15 | Test Black 29 | 0.73 | 0.82 | 0.82 | 1949 | Train 31 | Test Black 29 | 0.73 | 0.82 | 0.82 |
| 926 | Train 15 | Test Black 30 | 0.7 | 0.74 | 0.7 | 1950 | Train 31 | Test Black 30 | 0.7 | 0.74 | 0.7 |
| 927 | Train 15 | Test Black 31 | 0.73 | 0.82 | 0.82 | 1951 | Train 31 | Test Black 31 | 0.73 | 0.82 | 0.82 |
| 928 | Train 15 | Test Black 32 | 0.7 | 0.74 | 0.71 | 1952 | Train 31 | Test Black 32 | 0.7 | 0.74 | 0.7 |
| 929 | Train 15 | Test Black 33 | 0.75 | 0.82 | 0.81 | 1953 | Train 31 | Test Black 33 | 0.75 | 0.82 | 0.81 |
| 930 | Train 15 | Test Black 34 | 0.75 | 0.82 | 0.79 | 1954 | Train 31 | Test Black 34 | 0.75 | 0.82 | 0.8 |
| 931 | Train 15 | Test Black 35 | 0.75 | 0.82 | 0.81 | 1955 | Train 31 | Test Black 35 | 0.75 | 0.82 | 0.81 |
| 932 | Train 15 | Test Black 36 | 0.75 | 0.82 | 0.79 | 1956 | Train 31 | Test Black 36 | 0.75 | 0.82 | 0.8 |
| 933 | Train 15 | Test Black 37 | 0.74 | 0.81 | 0.8 | 1957 | Train 31 | Test Black 37 | 0.74 | 0.81 | 0.8 |
| 934 | Train 15 | Test Black 38 | 0.7 | 0.74 | 0.7 | 1958 | Train 31 | Test Black 38 | 0.7 | 0.74 | 0.7 |
| 935 | Train 15 | Test Black 39 | 0.73 | 0.81 | 0.79 | 1959 | Train 31 | Test Black 39 | 0.73 | 0.81 | 0.8 |
| 936 | Train 15 | Test Black 40 | 0.7 | 0.74 | 0.7 | 1960 | Train 31 | Test Black 40 | 0.7 | 0.74 | 0.7 |
| 937 | Train 15 | Test Black 41 | 0.75 | 0.83 | 0.83 | 1961 | Train 31 | Test Black 41 | 0.75 | 0.83 | 0.83 |
| 938 | Train 15 | Test Black 42 | 0.75 | 0.82 | 0.81 | 1962 | Train 31 | Test Black 42 | 0.75 | 0.82 | 0.82 |
| 939 | Train 15 | Test Black 43 | 0.75 | 0.83 | 0.83 | 1963 | Train 31 | Test Black 43 | 0.75 | 0.83 | 0.83 |
| 940 | Train 15 | Test Black 44 | 0.75 | 0.82 | 0.81 | 1964 | Train 31 | Test Black 44 | 0.75 | 0.82 | 0.82 |
| 941 | Train 15 | Test Black 45 | 0.73 | 0.82 | 0.82 | 1965 | Train 31 | Test Black 45 | 0.73 | 0.82 | 0.82 |
| 942 | Train 15 | Test Black 46 | 0.7 | 0.74 | 0.7 | 1966 | Train 31 | Test Black 46 | 0.7 | 0.74 | 0.7 |
| 943 | Train 15 | Test Black 47 | 0.73 | 0.82 | 0.82 | 1967 | Train 31 | Test Black 47 | 0.73 | 0.81 | 0.82 |
| 944 | Train 15 | Test Black 48 | 0.7 | 0.74 | 0.7 | 1968 | Train 31 | Test Black 48 | 0.7 | 0.74 | 0.7 |
| 945 | Train 15 | Test Black 49 | 0.76 | 0.79 | 0.78 | 1969 | Train 31 | Test Black 49 | 0.76 | 0.79 | 0.8 |
| 946 | Train 15 | Test Black 50 | 0.76 | 0.79 | 0.78 | 1970 | Train 31 | Test Black 50 | 0.76 | 0.8 | 0.8 |
| 947 | Train 15 | Test Black 51 | 0.77 | 0.79 | 0.78 | 1971 | Train 31 | Test Black 51 | 0.77 | 0.79 | 0.8 |
| 948 | Train 15 | Test Black 52 | 0.77 | 0.79 | 0.78 | 1972 | Train 31 | Test Black 52 | 0.77 | 0.79 | 0.8 |
| 949 | Train 15 | Test Black 53 | 0.76 | 0.8 | 0.8 | 1973 | Train 31 | Test Black 53 | 0.76 | 0.8 | 0.8 |
| 950 | Train 15 | Test Black 54 | 0.71 | 0.72 | 0.68 | 1974 | Train 31 | Test Black 54 | 0.71 | 0.72 | 0.67 |
| 951 | Train 15 | Test Black 55 | 0.76 | 0.8 | 0.8 | 1975 | Train 31 | Test Black 55 | 0.76 | 0.8 | 0.8 |
| 952 | Train 15 | Test Black 56 | 0.71 | 0.72 | 0.68 | 1976 | Train 31 | Test Black 56 | 0.71 | 0.72 | 0.67 |
| 953 | Train 15 | Test Black 57 | 0.76 | 0.8 | 0.8 | 1977 | Train 31 | Test Black 57 | 0.76 | 0.8 | 0.82 |
| 954 | Train 15 | Test Black 58 | 0.76 | 0.8 | 0.8 | 1978 | Train 31 | Test Black 58 | 0.76 | 0.8 | 0.82 |
| 955 | Train 15 | Test Black 59 | 0.76 | 0.8 | 0.8 | 1979 | Train 31 | Test Black 59 | 0.76 | 0.8 | 0.82 |
| 956 | Train 15 | Test Black 60 | 0.76 | 0.8 | 0.8 | 1980 | Train 31 | Test Black 60 | 0.76 | 0.8 | 0.82 |
| 957 | Train 15 | Test Black 61 | 0.76 | 0.81 | 0.81 | 1981 | Train 31 | Test Black 61 | 0.76 | 0.81 | 0.82 |
| 958 | Train 15 | Test Black 62 | 0.71 | 0.72 | 0.68 | 1982 | Train 31 | Test Black 62 | 0.71 | 0.72 | 0.67 |
| 959 | Train 15 | Test Black 63 | 0.76 | 0.81 | 0.81 | 1983 | Train 31 | Test Black 63 | 0.76 | 0.81 | 0.82 |
| 960 | Train 15 | Test Black 64 | 0.71 | 0.72 | 0.68 | 1984 | Train 31 | Test Black 64 | 0.71 | 0.72 | 0.67 |
| 961 | Train 16 | Test Black 1 | 0.73 | 0.79 | 0.77 | 1985 | Train 32 | Test Black 1 | 0.73 | 0.79 | 0.77 |
| 962 | Train 16 | Test Black 2 | 0.74 | 0.79 | 0.77 | 1986 | Train 32 | Test Black 2 | 0.74 | 0.79 | 0.77 |
| 963 | Train 16 | Test Black 3 | 0.73 | 0.79 | 0.77 | 1987 | Train 32 | Test Black 3 | 0.73 | 0.79 | 0.77 |
| 964 | Train 16 | Test Black 4 | 0.74 | 0.79 | 0.77 | 1988 | Train 32 | Test Black 4 | 0.74 | 0.79 | 0.77 |
| 965 | Train 16 | Test Black 5 | 0.72 | 0.78 | 0.76 | 1989 | Train 32 | Test Black 5 | 0.72 | 0.77 | 0.75 |
| 966 | Train 16 | Test Black 6 | 0.73 | 0.78 | 0.77 | 1990 | Train 32 | Test Black 6 | 0.73 | 0.78 | 0.75 |
| 967 | Train 16 | Test Black 7 | 0.72 | 0.77 | 0.75 | 1991 | Train 32 | Test Black 7 | 0.72 | 0.77 | 0.75 |
| 968 | Train 16 | Test Black 8 | 0.73 | 0.78 | 0.77 | 1992 | Train 32 | Test Black 8 | 0.73 | 0.78 | 0.75 |
| 969 | Train 16 | Test Black 9 | 0.73 | 0.79 | 0.78 | 1993 | Train 32 | Test Black 9 | 0.73 | 0.79 | 0.78 |
| 970 | Train 16 | Test Black 10 | 0.74 | 0.8 | 0.78 | 1994 | Train 32 | Test Black 10 | 0.74 | 0.8 | 0.78 |
| 971 | Train 16 | Test Black 11 | 0.73 | 0.79 | 0.78 | 1995 | Train 32 | Test Black 11 | 0.73 | 0.79 | 0.78 |
| 972 | Train 16 | Test Black 12 | 0.74 | 0.8 | 0.78 | 1996 | Train 32 | Test Black 12 | 0.74 | 0.8 | 0.78 |
| 973 | Train 16 | Test Black 13 | 0.72 | 0.78 | 0.76 | 1997 | Train 32 | Test Black 13 | 0.72 | 0.78 | 0.75 |
| 974 | Train 16 | Test Black 14 | 0.73 | 0.78 | 0.77 | 1998 | Train 32 | Test Black 14 | 0.73 | 0.78 | 0.76 |
| 975 | Train 16 | Test Black 15 | 0.72 | 0.78 | 0.76 | 1999 | Train 32 | Test Black 15 | 0.72 | 0.78 | 0.75 |
| 976 | Train 16 | Test Black 16 | 0.73 | 0.78 | 0.77 | 2000 | Train 32 | Test Black 16 | 0.73 | 0.78 | 0.76 |
| 977 | Train 16 | Test Black 17 | 0.73 | 0.79 | 0.77 | 2001 | Train 32 | Test Black 17 | 0.73 | 0.79 | 0.77 |
| 978 | Train 16 | Test Black 18 | 0.74 | 0.79 | 0.77 | 2002 | Train 32 | Test Black 18 | 0.74 | 0.79 | 0.77 |
| 979 | Train 16 | Test Black 19 | 0.73 | 0.79 | 0.77 | 2003 | Train 32 | Test Black 19 | 0.73 | 0.79 | 0.77 |
| 980 | Train 16 | Test Black 20 | 0.74 | 0.79 | 0.77 | 2004 | Train 32 | Test Black 20 | 0.74 | 0.79 | 0.77 |
| 981 | Train 16 | Test Black 21 | 0.72 | 0.78 | 0.76 | 2005 | Train 32 | Test Black 21 | 0.72 | 0.77 | 0.75 |
| 982 | Train 16 | Test Black 22 | 0.73 | 0.78 | 0.77 | 2006 | Train 32 | Test Black 22 | 0.73 | 0.78 | 0.75 |
| 983 | Train 16 | Test Black 23 | 0.72 | 0.77 | 0.75 | 2007 | Train 32 | Test Black 23 | 0.72 | 0.77 | 0.75 |
| 984 | Train 16 | Test Black 24 | 0.73 | 0.78 | 0.77 | 2008 | Train 32 | Test Black 24 | 0.73 | 0.78 | 0.75 |
| 985 | Train 16 | Test Black 25 | 0.73 | 0.79 | 0.78 | 2009 | Train 32 | Test Black 25 | 0.73 | 0.79 | 0.78 |
| 986 | Train 16 | Test Black 26 | 0.74 | 0.8 | 0.78 | 2010 | Train 32 | Test Black 26 | 0.74 | 0.8 | 0.78 |
| 987 | Train 16 | Test Black 27 | 0.73 | 0.79 | 0.78 | 2011 | Train 32 | Test Black 27 | 0.73 | 0.79 | 0.78 |
| 988 | Train 16 | Test Black 28 | 0.74 | 0.8 | 0.78 | 2012 | Train 32 | Test Black 28 | 0.74 | 0.8 | 0.78 |
| 989 | Train 16 | Test Black 29 | 0.72 | 0.78 | 0.76 | 2013 | Train 32 | Test Black 29 | 0.72 | 0.78 | 0.75 |
| 990 | Train 16 | Test Black 30 | 0.73 | 0.78 | 0.77 | 2014 | Train 32 | Test Black 30 | 0.73 | 0.78 | 0.76 |
| 991 | Train 16 | Test Black 31 | 0.72 | 0.78 | 0.76 | 2015 | Train 32 | Test Black 31 | 0.72 | 0.78 | 0.75 |
| 992 | Train 16 | Test Black 32 | 0.73 | 0.78 | 0.77 | 2016 | Train 32 | Test Black 32 | 0.73 | 0.78 | 0.76 |
| 993 | Train 16 | Test Black 33 | 0.73 | 0.79 | 0.77 | 2017 | Train 32 | Test Black 33 | 0.73 | 0.79 | 0.77 |
| 994 | Train 16 | Test Black 34 | 0.74 | 0.79 | 0.77 | 2018 | Train 32 | Test Black 34 | 0.74 | 0.79 | 0.77 |
| 995 | Train 16 | Test Black 35 | 0.73 | 0.79 | 0.77 | 2019 | Train 32 | Test Black 35 | 0.73 | 0.79 | 0.77 |
| 996 | Train 16 | Test Black 36 | 0.74 | 0.79 | 0.77 | 2020 | Train 32 | Test Black 36 | 0.74 | 0.79 | 0.77 |
| 997 | Train 16 | Test Black 37 | 0.72 | 0.77 | 0.76 | 2021 | Train 32 | Test Black 37 | 0.72 | 0.77 | 0.75 |
| 998 | Train 16 | Test Black 38 | 0.73 | 0.78 | 0.77 | 2022 | Train 32 | Test Black 38 | 0.73 | 0.78 | 0.75 |
| 999 | Train 16 | Test Black 39 | 0.72 | 0.77 | 0.75 | 2023 | Train 32 | Test Black 39 | 0.72 | 0.77 | 0.75 |
| 1000 | Train 16 | Test Black 40 | 0.73 | 0.78 | 0.77 | 2024 | Train 32 | Test Black 40 | 0.73 | 0.78 | 0.75 |
| 1001 | Train 16 | Test Black 41 | 0.73 | 0.79 | 0.77 | 2025 | Train 32 | Test Black 41 | 0.73 | 0.79 | 0.77 |
| 1002 | Train 16 | Test Black 42 | 0.74 | 0.8 | 0.78 | 2026 | Train 32 | Test Black 42 | 0.74 | 0.8 | 0.78 |
| 1003 | Train 16 | Test Black 43 | 0.73 | 0.79 | 0.77 | 2027 | Train 32 | Test Black 43 | 0.73 | 0.79 | 0.78 |
| 1004 | Train 16 | Test Black 44 | 0.74 | 0.8 | 0.78 | 2028 | Train 32 | Test Black 44 | 0.74 | 0.8 | 0.78 |
| 1005 | Train 16 | Test Black 45 | 0.72 | 0.78 | 0.76 | 2029 | Train 32 | Test Black 45 | 0.72 | 0.78 | 0.75 |
| 1006 | Train 16 | Test Black 46 | 0.73 | 0.78 | 0.77 | 2030 | Train 32 | Test Black 46 | 0.73 | 0.78 | 0.76 |
| 1007 | Train 16 | Test Black 47 | 0.72 | 0.78 | 0.76 | 2031 | Train 32 | Test Black 47 | 0.72 | 0.77 | 0.75 |
| 1008 | Train 16 | Test Black 48 | 0.73 | 0.78 | 0.77 | 2032 | Train 32 | Test Black 48 | 0.73 | 0.78 | 0.76 |
| 1009 | Train 16 | Test Black 49 | 0.76 | 0.8 | 0.78 | 2033 | Train 32 | Test Black 49 | 0.76 | 0.8 | 0.78 |
| 1010 | Train 16 | Test Black 50 | 0.76 | 0.8 | 0.78 | 2034 | Train 32 | Test Black 50 | 0.76 | 0.8 | 0.78 |
| 1011 | Train 16 | Test Black 51 | 0.76 | 0.8 | 0.78 | 2035 | Train 32 | Test Black 51 | 0.76 | 0.8 | 0.78 |
| 1012 | Train 16 | Test Black 52 | 0.76 | 0.8 | 0.78 | 2036 | Train 32 | Test Black 52 | 0.76 | 0.8 | 0.78 |
| 1013 | Train 16 | Test Black 53 | 0.75 | 0.8 | 0.78 | 2037 | Train 32 | Test Black 53 | 0.75 | 0.8 | 0.77 |
| 1014 | Train 16 | Test Black 54 | 0.74 | 0.78 | 0.78 | 2038 | Train 32 | Test Black 54 | 0.74 | 0.78 | 0.78 |
| 1015 | Train 16 | Test Black 55 | 0.75 | 0.8 | 0.78 | 2039 | Train 32 | Test Black 55 | 0.75 | 0.8 | 0.77 |
| 1016 | Train 16 | Test Black 56 | 0.74 | 0.78 | 0.78 | 2040 | Train 32 | Test Black 56 | 0.74 | 0.78 | 0.78 |
| 1017 | Train 16 | Test Black 57 | 0.76 | 0.8 | 0.79 | 2041 | Train 32 | Test Black 57 | 0.76 | 0.8 | 0.79 |
| 1018 | Train 16 | Test Black 58 | 0.76 | 0.8 | 0.79 | 2042 | Train 32 | Test Black 58 | 0.76 | 0.8 | 0.79 |
| 1019 | Train 16 | Test Black 59 | 0.76 | 0.8 | 0.79 | 2043 | Train 32 | Test Black 59 | 0.76 | 0.8 | 0.79 |
| 1020 | Train 16 | Test Black 60 | 0.76 | 0.8 | 0.79 | 2044 | Train 32 | Test Black 60 | 0.76 | 0.8 | 0.79 |
| 1021 | Train 16 | Test Black 61 | 0.75 | 0.8 | 0.78 | 2045 | Train 32 | Test Black 61 | 0.75 | 0.8 | 0.77 |
| 1022 | Train 16 | Test Black 62 | 0.74 | 0.78 | 0.78 | 2046 | Train 32 | Test Black 62 | 0.74 | 0.78 | 0.78 |
| 1023 | Train 16 | Test Black 63 | 0.75 | 0.8 | 0.78 | 2047 | Train 32 | Test Black 63 | 0.75 | 0.8 | 0.77 |
| 1024 | Train 16 | Test Black 64 | 0.74 | 0.78 | 0.78 | 2048 | Train 32 | Test Black 64 | 0.74 | 0.78 | 0.78 |

Table 6. AUC Performance of the ML Models— LR, RF, and GB —for Black Patients Trained on Cohorts 33–64 and Tested Across 64 Cohorts.

| Black | Train  Model | Test  Dataset | AUC (LR) | AUC (RF) | AUC (GB) | Black | Train  Model | Test  Dataset | AUC (LR) | AUC (RF) | AUC (GB) |
| --- | --- | --- | --- | --- | --- | --- | --- | --- | --- | --- | --- |
| 2049 | Train 33 | Test Black 1 | 0.76 | 0.86 | 0.86 | 3073 | Train 49 | Test Black 1 | 0.73 | 0.76 | 0.68 |
| 2050 | Train 33 | Test Black 2 | 0.76 | 0.84 | 0.84 | 3074 | Train 49 | Test Black 2 | 0.72 | 0.75 | 0.67 |
| 2051 | Train 33 | Test Black 3 | 0.76 | 0.86 | 0.86 | 3075 | Train 49 | Test Black 3 | 0.73 | 0.76 | 0.68 |
| 2052 | Train 33 | Test Black 4 | 0.76 | 0.84 | 0.84 | 3076 | Train 49 | Test Black 4 | 0.72 | 0.75 | 0.67 |
| 2053 | Train 33 | Test Black 5 | 0.73 | 0.76 | 0.78 | 3077 | Train 49 | Test Black 5 | 0.71 | 0.71 | 0.66 |
| 2054 | Train 33 | Test Black 6 | 0.71 | 0.71 | 0.69 | 3078 | Train 49 | Test Black 6 | 0.7 | 0.69 | 0.64 |
| 2055 | Train 33 | Test Black 7 | 0.73 | 0.76 | 0.77 | 3079 | Train 49 | Test Black 7 | 0.71 | 0.7 | 0.65 |
| 2056 | Train 33 | Test Black 8 | 0.71 | 0.71 | 0.69 | 3080 | Train 49 | Test Black 8 | 0.7 | 0.69 | 0.64 |
| 2057 | Train 33 | Test Black 9 | 0.76 | 0.85 | 0.83 | 3081 | Train 49 | Test Black 9 | 0.73 | 0.76 | 0.66 |
| 2058 | Train 33 | Test Black 10 | 0.76 | 0.83 | 0.81 | 3082 | Train 49 | Test Black 10 | 0.72 | 0.75 | 0.66 |
| 2059 | Train 33 | Test Black 11 | 0.76 | 0.84 | 0.83 | 3083 | Train 49 | Test Black 11 | 0.73 | 0.76 | 0.66 |
| 2060 | Train 33 | Test Black 12 | 0.76 | 0.83 | 0.81 | 3084 | Train 49 | Test Black 12 | 0.72 | 0.75 | 0.66 |
| 2061 | Train 33 | Test Black 13 | 0.73 | 0.76 | 0.76 | 3085 | Train 49 | Test Black 13 | 0.71 | 0.71 | 0.65 |
| 2062 | Train 33 | Test Black 14 | 0.71 | 0.71 | 0.69 | 3086 | Train 49 | Test Black 14 | 0.7 | 0.69 | 0.64 |
| 2063 | Train 33 | Test Black 15 | 0.73 | 0.75 | 0.75 | 3087 | Train 49 | Test Black 15 | 0.7 | 0.7 | 0.65 |
| 2064 | Train 33 | Test Black 16 | 0.71 | 0.71 | 0.69 | 3088 | Train 49 | Test Black 16 | 0.7 | 0.69 | 0.64 |
| 2065 | Train 33 | Test Black 17 | 0.76 | 0.86 | 0.86 | 3089 | Train 49 | Test Black 17 | 0.73 | 0.76 | 0.68 |
| 2066 | Train 33 | Test Black 18 | 0.76 | 0.84 | 0.84 | 3090 | Train 49 | Test Black 18 | 0.72 | 0.75 | 0.67 |
| 2067 | Train 33 | Test Black 19 | 0.76 | 0.86 | 0.86 | 3091 | Train 49 | Test Black 19 | 0.73 | 0.76 | 0.68 |
| 2068 | Train 33 | Test Black 20 | 0.76 | 0.84 | 0.84 | 3092 | Train 49 | Test Black 20 | 0.72 | 0.75 | 0.67 |
| 2069 | Train 33 | Test Black 21 | 0.73 | 0.76 | 0.78 | 3093 | Train 49 | Test Black 21 | 0.71 | 0.71 | 0.66 |
| 2070 | Train 33 | Test Black 22 | 0.71 | 0.71 | 0.69 | 3094 | Train 49 | Test Black 22 | 0.7 | 0.69 | 0.64 |
| 2071 | Train 33 | Test Black 23 | 0.73 | 0.76 | 0.77 | 3095 | Train 49 | Test Black 23 | 0.71 | 0.7 | 0.65 |
| 2072 | Train 33 | Test Black 24 | 0.71 | 0.71 | 0.69 | 3096 | Train 49 | Test Black 24 | 0.7 | 0.69 | 0.64 |
| 2073 | Train 33 | Test Black 25 | 0.76 | 0.85 | 0.83 | 3097 | Train 49 | Test Black 25 | 0.73 | 0.76 | 0.66 |
| 2074 | Train 33 | Test Black 26 | 0.76 | 0.83 | 0.81 | 3098 | Train 49 | Test Black 26 | 0.72 | 0.75 | 0.66 |
| 2075 | Train 33 | Test Black 27 | 0.76 | 0.84 | 0.83 | 3099 | Train 49 | Test Black 27 | 0.73 | 0.76 | 0.66 |
| 2076 | Train 33 | Test Black 28 | 0.76 | 0.83 | 0.81 | 3100 | Train 49 | Test Black 28 | 0.72 | 0.75 | 0.66 |
| 2077 | Train 33 | Test Black 29 | 0.73 | 0.76 | 0.76 | 3101 | Train 49 | Test Black 29 | 0.71 | 0.71 | 0.65 |
| 2078 | Train 33 | Test Black 30 | 0.71 | 0.71 | 0.69 | 3102 | Train 49 | Test Black 30 | 0.7 | 0.69 | 0.64 |
| 2079 | Train 33 | Test Black 31 | 0.73 | 0.75 | 0.75 | 3103 | Train 49 | Test Black 31 | 0.7 | 0.7 | 0.65 |
| 2080 | Train 33 | Test Black 32 | 0.71 | 0.71 | 0.69 | 3104 | Train 49 | Test Black 32 | 0.7 | 0.69 | 0.64 |
| 2081 | Train 33 | Test Black 33 | 0.76 | 0.86 | 0.86 | 3105 | Train 49 | Test Black 33 | 0.73 | 0.76 | 0.68 |
| 2082 | Train 33 | Test Black 34 | 0.76 | 0.84 | 0.84 | 3106 | Train 49 | Test Black 34 | 0.72 | 0.76 | 0.67 |
| 2083 | Train 33 | Test Black 35 | 0.76 | 0.86 | 0.86 | 3107 | Train 49 | Test Black 35 | 0.73 | 0.76 | 0.68 |
| 2084 | Train 33 | Test Black 36 | 0.76 | 0.84 | 0.84 | 3108 | Train 49 | Test Black 36 | 0.72 | 0.76 | 0.67 |
| 2085 | Train 33 | Test Black 37 | 0.73 | 0.76 | 0.78 | 3109 | Train 49 | Test Black 37 | 0.71 | 0.71 | 0.66 |
| 2086 | Train 33 | Test Black 38 | 0.71 | 0.71 | 0.69 | 3110 | Train 49 | Test Black 38 | 0.7 | 0.69 | 0.64 |
| 2087 | Train 33 | Test Black 39 | 0.73 | 0.76 | 0.77 | 3111 | Train 49 | Test Black 39 | 0.71 | 0.71 | 0.65 |
| 2088 | Train 33 | Test Black 40 | 0.71 | 0.71 | 0.69 | 3112 | Train 49 | Test Black 40 | 0.7 | 0.69 | 0.64 |
| 2089 | Train 33 | Test Black 41 | 0.76 | 0.85 | 0.83 | 3113 | Train 49 | Test Black 41 | 0.73 | 0.76 | 0.66 |
| 2090 | Train 33 | Test Black 42 | 0.76 | 0.83 | 0.81 | 3114 | Train 49 | Test Black 42 | 0.72 | 0.75 | 0.66 |
| 2091 | Train 33 | Test Black 43 | 0.76 | 0.85 | 0.83 | 3115 | Train 49 | Test Black 43 | 0.73 | 0.76 | 0.66 |
| 2092 | Train 33 | Test Black 44 | 0.76 | 0.83 | 0.81 | 3116 | Train 49 | Test Black 44 | 0.72 | 0.75 | 0.66 |
| 2093 | Train 33 | Test Black 45 | 0.73 | 0.76 | 0.76 | 3117 | Train 49 | Test Black 45 | 0.71 | 0.71 | 0.65 |
| 2094 | Train 33 | Test Black 46 | 0.71 | 0.71 | 0.69 | 3118 | Train 49 | Test Black 46 | 0.7 | 0.69 | 0.64 |
| 2095 | Train 33 | Test Black 47 | 0.73 | 0.75 | 0.76 | 3119 | Train 49 | Test Black 47 | 0.71 | 0.7 | 0.65 |
| 2096 | Train 33 | Test Black 48 | 0.71 | 0.71 | 0.69 | 3120 | Train 49 | Test Black 48 | 0.7 | 0.69 | 0.64 |
| 2097 | Train 33 | Test Black 49 | 0.77 | 0.82 | 0.83 | 3121 | Train 49 | Test Black 49 | 0.78 | 0.85 | 0.88 |
| 2098 | Train 33 | Test Black 50 | 0.77 | 0.82 | 0.83 | 3122 | Train 49 | Test Black 50 | 0.78 | 0.85 | 0.89 |
| 2099 | Train 33 | Test Black 51 | 0.77 | 0.82 | 0.83 | 3123 | Train 49 | Test Black 51 | 0.78 | 0.85 | 0.88 |
| 2100 | Train 33 | Test Black 52 | 0.77 | 0.82 | 0.83 | 3124 | Train 49 | Test Black 52 | 0.78 | 0.85 | 0.88 |
| 2101 | Train 33 | Test Black 53 | 0.76 | 0.79 | 0.78 | 3125 | Train 49 | Test Black 53 | 0.75 | 0.8 | 0.78 |
| 2102 | Train 33 | Test Black 54 | 0.71 | 0.71 | 0.66 | 3126 | Train 49 | Test Black 54 | 0.71 | 0.7 | 0.67 |
| 2103 | Train 33 | Test Black 55 | 0.76 | 0.79 | 0.78 | 3127 | Train 49 | Test Black 55 | 0.75 | 0.8 | 0.78 |
| 2104 | Train 33 | Test Black 56 | 0.71 | 0.71 | 0.66 | 3128 | Train 49 | Test Black 56 | 0.71 | 0.7 | 0.67 |
| 2105 | Train 33 | Test Black 57 | 0.77 | 0.81 | 0.81 | 3129 | Train 49 | Test Black 57 | 0.78 | 0.84 | 0.82 |
| 2106 | Train 33 | Test Black 58 | 0.77 | 0.81 | 0.81 | 3130 | Train 49 | Test Black 58 | 0.78 | 0.84 | 0.82 |
| 2107 | Train 33 | Test Black 59 | 0.77 | 0.81 | 0.8 | 3131 | Train 49 | Test Black 59 | 0.78 | 0.84 | 0.81 |
| 2108 | Train 33 | Test Black 60 | 0.77 | 0.81 | 0.8 | 3132 | Train 49 | Test Black 60 | 0.78 | 0.84 | 0.81 |
| 2109 | Train 33 | Test Black 61 | 0.75 | 0.78 | 0.77 | 3133 | Train 49 | Test Black 61 | 0.74 | 0.79 | 0.76 |
| 2110 | Train 33 | Test Black 62 | 0.71 | 0.71 | 0.66 | 3134 | Train 49 | Test Black 62 | 0.71 | 0.7 | 0.67 |
| 2111 | Train 33 | Test Black 63 | 0.76 | 0.78 | 0.77 | 3135 | Train 49 | Test Black 63 | 0.74 | 0.79 | 0.76 |
| 2112 | Train 33 | Test Black 64 | 0.71 | 0.71 | 0.66 | 3136 | Train 49 | Test Black 64 | 0.72 | 0.7 | 0.67 |
| 2113 | Train 34 | Test Black 1 | 0.75 | 0.85 | 0.85 | 3137 | Train 50 | Test Black 1 | 0.72 | 0.74 | 0.71 |
| 2114 | Train 34 | Test Black 2 | 0.76 | 0.85 | 0.85 | 3138 | Train 50 | Test Black 2 | 0.72 | 0.74 | 0.7 |
| 2115 | Train 34 | Test Black 3 | 0.75 | 0.85 | 0.85 | 3139 | Train 50 | Test Black 3 | 0.72 | 0.74 | 0.71 |
| 2116 | Train 34 | Test Black 4 | 0.76 | 0.85 | 0.85 | 3140 | Train 50 | Test Black 4 | 0.72 | 0.74 | 0.7 |
| 2117 | Train 34 | Test Black 5 | 0.73 | 0.77 | 0.78 | 3141 | Train 50 | Test Black 5 | 0.71 | 0.7 | 0.67 |
| 2118 | Train 34 | Test Black 6 | 0.71 | 0.72 | 0.68 | 3142 | Train 50 | Test Black 6 | 0.7 | 0.68 | 0.64 |
| 2119 | Train 34 | Test Black 7 | 0.73 | 0.76 | 0.78 | 3143 | Train 50 | Test Black 7 | 0.7 | 0.69 | 0.67 |
| 2120 | Train 34 | Test Black 8 | 0.71 | 0.72 | 0.68 | 3144 | Train 50 | Test Black 8 | 0.7 | 0.68 | 0.65 |
| 2121 | Train 34 | Test Black 9 | 0.75 | 0.84 | 0.82 | 3145 | Train 50 | Test Black 9 | 0.72 | 0.74 | 0.69 |
| 2122 | Train 34 | Test Black 10 | 0.76 | 0.84 | 0.82 | 3146 | Train 50 | Test Black 10 | 0.72 | 0.74 | 0.69 |
| 2123 | Train 34 | Test Black 11 | 0.75 | 0.84 | 0.82 | 3147 | Train 50 | Test Black 11 | 0.72 | 0.74 | 0.69 |
| 2124 | Train 34 | Test Black 12 | 0.76 | 0.84 | 0.82 | 3148 | Train 50 | Test Black 12 | 0.72 | 0.73 | 0.69 |
| 2125 | Train 34 | Test Black 13 | 0.73 | 0.76 | 0.76 | 3149 | Train 50 | Test Black 13 | 0.7 | 0.7 | 0.66 |
| 2126 | Train 34 | Test Black 14 | 0.71 | 0.72 | 0.68 | 3150 | Train 50 | Test Black 14 | 0.7 | 0.68 | 0.65 |
| 2127 | Train 34 | Test Black 15 | 0.73 | 0.76 | 0.76 | 3151 | Train 50 | Test Black 15 | 0.7 | 0.69 | 0.66 |
| 2128 | Train 34 | Test Black 16 | 0.71 | 0.72 | 0.68 | 3152 | Train 50 | Test Black 16 | 0.7 | 0.68 | 0.65 |
| 2129 | Train 34 | Test Black 17 | 0.75 | 0.85 | 0.85 | 3153 | Train 50 | Test Black 17 | 0.72 | 0.74 | 0.71 |
| 2130 | Train 34 | Test Black 18 | 0.76 | 0.85 | 0.85 | 3154 | Train 50 | Test Black 18 | 0.72 | 0.74 | 0.7 |
| 2131 | Train 34 | Test Black 19 | 0.75 | 0.85 | 0.85 | 3155 | Train 50 | Test Black 19 | 0.72 | 0.74 | 0.71 |
| 2132 | Train 34 | Test Black 20 | 0.76 | 0.85 | 0.85 | 3156 | Train 50 | Test Black 20 | 0.72 | 0.74 | 0.7 |
| 2133 | Train 34 | Test Black 21 | 0.73 | 0.77 | 0.78 | 3157 | Train 50 | Test Black 21 | 0.71 | 0.7 | 0.67 |
| 2134 | Train 34 | Test Black 22 | 0.71 | 0.72 | 0.68 | 3158 | Train 50 | Test Black 22 | 0.7 | 0.68 | 0.64 |
| 2135 | Train 34 | Test Black 23 | 0.73 | 0.76 | 0.78 | 3159 | Train 50 | Test Black 23 | 0.7 | 0.69 | 0.67 |
| 2136 | Train 34 | Test Black 24 | 0.71 | 0.72 | 0.68 | 3160 | Train 50 | Test Black 24 | 0.7 | 0.68 | 0.65 |
| 2137 | Train 34 | Test Black 25 | 0.75 | 0.84 | 0.82 | 3161 | Train 50 | Test Black 25 | 0.72 | 0.74 | 0.69 |
| 2138 | Train 34 | Test Black 26 | 0.76 | 0.84 | 0.82 | 3162 | Train 50 | Test Black 26 | 0.72 | 0.74 | 0.69 |
| 2139 | Train 34 | Test Black 27 | 0.75 | 0.84 | 0.82 | 3163 | Train 50 | Test Black 27 | 0.72 | 0.74 | 0.69 |
| 2140 | Train 34 | Test Black 28 | 0.76 | 0.84 | 0.82 | 3164 | Train 50 | Test Black 28 | 0.72 | 0.73 | 0.69 |
| 2141 | Train 34 | Test Black 29 | 0.73 | 0.76 | 0.76 | 3165 | Train 50 | Test Black 29 | 0.7 | 0.7 | 0.66 |
| 2142 | Train 34 | Test Black 30 | 0.71 | 0.72 | 0.68 | 3166 | Train 50 | Test Black 30 | 0.7 | 0.68 | 0.65 |
| 2143 | Train 34 | Test Black 31 | 0.73 | 0.76 | 0.76 | 3167 | Train 50 | Test Black 31 | 0.7 | 0.69 | 0.66 |
| 2144 | Train 34 | Test Black 32 | 0.71 | 0.72 | 0.68 | 3168 | Train 50 | Test Black 32 | 0.7 | 0.68 | 0.65 |
| 2145 | Train 34 | Test Black 33 | 0.75 | 0.85 | 0.85 | 3169 | Train 50 | Test Black 33 | 0.72 | 0.74 | 0.7 |
| 2146 | Train 34 | Test Black 34 | 0.75 | 0.85 | 0.85 | 3170 | Train 50 | Test Black 34 | 0.72 | 0.74 | 0.7 |
| 2147 | Train 34 | Test Black 35 | 0.75 | 0.85 | 0.85 | 3171 | Train 50 | Test Black 35 | 0.72 | 0.74 | 0.7 |
| 2148 | Train 34 | Test Black 36 | 0.76 | 0.85 | 0.85 | 3172 | Train 50 | Test Black 36 | 0.72 | 0.74 | 0.7 |
| 2149 | Train 34 | Test Black 37 | 0.73 | 0.77 | 0.78 | 3173 | Train 50 | Test Black 37 | 0.71 | 0.7 | 0.67 |
| 2150 | Train 34 | Test Black 38 | 0.71 | 0.72 | 0.68 | 3174 | Train 50 | Test Black 38 | 0.7 | 0.68 | 0.64 |
| 2151 | Train 34 | Test Black 39 | 0.73 | 0.77 | 0.78 | 3175 | Train 50 | Test Black 39 | 0.7 | 0.7 | 0.67 |
| 2152 | Train 34 | Test Black 40 | 0.71 | 0.72 | 0.68 | 3176 | Train 50 | Test Black 40 | 0.7 | 0.68 | 0.65 |
| 2153 | Train 34 | Test Black 41 | 0.75 | 0.84 | 0.82 | 3177 | Train 50 | Test Black 41 | 0.72 | 0.74 | 0.69 |
| 2154 | Train 34 | Test Black 42 | 0.75 | 0.84 | 0.82 | 3178 | Train 50 | Test Black 42 | 0.72 | 0.74 | 0.69 |
| 2155 | Train 34 | Test Black 43 | 0.75 | 0.84 | 0.82 | 3179 | Train 50 | Test Black 43 | 0.72 | 0.74 | 0.69 |
| 2156 | Train 34 | Test Black 44 | 0.76 | 0.84 | 0.82 | 3180 | Train 50 | Test Black 44 | 0.72 | 0.74 | 0.69 |
| 2157 | Train 34 | Test Black 45 | 0.73 | 0.76 | 0.76 | 3181 | Train 50 | Test Black 45 | 0.71 | 0.7 | 0.66 |
| 2158 | Train 34 | Test Black 46 | 0.71 | 0.72 | 0.68 | 3182 | Train 50 | Test Black 46 | 0.7 | 0.68 | 0.65 |
| 2159 | Train 34 | Test Black 47 | 0.73 | 0.76 | 0.76 | 3183 | Train 50 | Test Black 47 | 0.7 | 0.69 | 0.66 |
| 2160 | Train 34 | Test Black 48 | 0.71 | 0.72 | 0.68 | 3184 | Train 50 | Test Black 48 | 0.7 | 0.68 | 0.65 |
| 2161 | Train 34 | Test Black 49 | 0.77 | 0.82 | 0.83 | 3185 | Train 50 | Test Black 49 | 0.78 | 0.85 | 0.89 |
| 2162 | Train 34 | Test Black 50 | 0.77 | 0.82 | 0.83 | 3186 | Train 50 | Test Black 50 | 0.78 | 0.85 | 0.9 |
| 2163 | Train 34 | Test Black 51 | 0.77 | 0.82 | 0.82 | 3187 | Train 50 | Test Black 51 | 0.78 | 0.85 | 0.89 |
| 2164 | Train 34 | Test Black 52 | 0.77 | 0.82 | 0.82 | 3188 | Train 50 | Test Black 52 | 0.78 | 0.85 | 0.89 |
| 2165 | Train 34 | Test Black 53 | 0.75 | 0.79 | 0.77 | 3189 | Train 50 | Test Black 53 | 0.75 | 0.79 | 0.77 |
| 2166 | Train 34 | Test Black 54 | 0.71 | 0.72 | 0.66 | 3190 | Train 50 | Test Black 54 | 0.71 | 0.69 | 0.65 |
| 2167 | Train 34 | Test Black 55 | 0.75 | 0.79 | 0.77 | 3191 | Train 50 | Test Black 55 | 0.75 | 0.79 | 0.77 |
| 2168 | Train 34 | Test Black 56 | 0.71 | 0.71 | 0.66 | 3192 | Train 50 | Test Black 56 | 0.71 | 0.69 | 0.65 |
| 2169 | Train 34 | Test Black 57 | 0.77 | 0.81 | 0.79 | 3193 | Train 50 | Test Black 57 | 0.78 | 0.84 | 0.83 |
| 2170 | Train 34 | Test Black 58 | 0.77 | 0.81 | 0.79 | 3194 | Train 50 | Test Black 58 | 0.78 | 0.84 | 0.83 |
| 2171 | Train 34 | Test Black 59 | 0.77 | 0.81 | 0.79 | 3195 | Train 50 | Test Black 59 | 0.78 | 0.84 | 0.82 |
| 2172 | Train 34 | Test Black 60 | 0.77 | 0.81 | 0.79 | 3196 | Train 50 | Test Black 60 | 0.78 | 0.84 | 0.82 |
| 2173 | Train 34 | Test Black 61 | 0.75 | 0.79 | 0.76 | 3197 | Train 50 | Test Black 61 | 0.75 | 0.79 | 0.76 |
| 2174 | Train 34 | Test Black 62 | 0.71 | 0.72 | 0.67 | 3198 | Train 50 | Test Black 62 | 0.71 | 0.69 | 0.66 |
| 2175 | Train 34 | Test Black 63 | 0.75 | 0.79 | 0.76 | 3199 | Train 50 | Test Black 63 | 0.75 | 0.79 | 0.75 |
| 2176 | Train 34 | Test Black 64 | 0.71 | 0.71 | 0.66 | 3200 | Train 50 | Test Black 64 | 0.71 | 0.69 | 0.66 |
| 2177 | Train 35 | Test Black 1 | 0.76 | 0.86 | 0.86 | 3201 | Train 51 | Test Black 1 | 0.73 | 0.76 | 0.72 |
| 2178 | Train 35 | Test Black 2 | 0.76 | 0.84 | 0.84 | 3202 | Train 51 | Test Black 2 | 0.72 | 0.75 | 0.7 |
| 2179 | Train 35 | Test Black 3 | 0.76 | 0.86 | 0.86 | 3203 | Train 51 | Test Black 3 | 0.73 | 0.76 | 0.72 |
| 2180 | Train 35 | Test Black 4 | 0.76 | 0.84 | 0.84 | 3204 | Train 51 | Test Black 4 | 0.72 | 0.76 | 0.7 |
| 2181 | Train 35 | Test Black 5 | 0.73 | 0.76 | 0.73 | 3205 | Train 51 | Test Black 5 | 0.71 | 0.71 | 0.67 |
| 2182 | Train 35 | Test Black 6 | 0.71 | 0.71 | 0.67 | 3206 | Train 51 | Test Black 6 | 0.7 | 0.69 | 0.61 |
| 2183 | Train 35 | Test Black 7 | 0.73 | 0.76 | 0.73 | 3207 | Train 51 | Test Black 7 | 0.71 | 0.71 | 0.67 |
| 2184 | Train 35 | Test Black 8 | 0.71 | 0.71 | 0.67 | 3208 | Train 51 | Test Black 8 | 0.7 | 0.69 | 0.61 |
| 2185 | Train 35 | Test Black 9 | 0.76 | 0.85 | 0.83 | 3209 | Train 51 | Test Black 9 | 0.73 | 0.76 | 0.71 |
| 2186 | Train 35 | Test Black 10 | 0.76 | 0.83 | 0.81 | 3210 | Train 51 | Test Black 10 | 0.72 | 0.75 | 0.69 |
| 2187 | Train 35 | Test Black 11 | 0.76 | 0.85 | 0.83 | 3211 | Train 51 | Test Black 11 | 0.73 | 0.76 | 0.71 |
| 2188 | Train 35 | Test Black 12 | 0.76 | 0.83 | 0.81 | 3212 | Train 51 | Test Black 12 | 0.72 | 0.75 | 0.69 |
| 2189 | Train 35 | Test Black 13 | 0.73 | 0.76 | 0.71 | 3213 | Train 51 | Test Black 13 | 0.71 | 0.71 | 0.66 |
| 2190 | Train 35 | Test Black 14 | 0.71 | 0.71 | 0.67 | 3214 | Train 51 | Test Black 14 | 0.7 | 0.69 | 0.61 |
| 2191 | Train 35 | Test Black 15 | 0.73 | 0.75 | 0.71 | 3215 | Train 51 | Test Black 15 | 0.7 | 0.7 | 0.66 |
| 2192 | Train 35 | Test Black 16 | 0.71 | 0.71 | 0.67 | 3216 | Train 51 | Test Black 16 | 0.7 | 0.69 | 0.61 |
| 2193 | Train 35 | Test Black 17 | 0.76 | 0.86 | 0.86 | 3217 | Train 51 | Test Black 17 | 0.73 | 0.76 | 0.72 |
| 2194 | Train 35 | Test Black 18 | 0.76 | 0.84 | 0.84 | 3218 | Train 51 | Test Black 18 | 0.72 | 0.75 | 0.7 |
| 2195 | Train 35 | Test Black 19 | 0.76 | 0.86 | 0.86 | 3219 | Train 51 | Test Black 19 | 0.73 | 0.76 | 0.72 |
| 2196 | Train 35 | Test Black 20 | 0.76 | 0.84 | 0.84 | 3220 | Train 51 | Test Black 20 | 0.72 | 0.76 | 0.7 |
| 2197 | Train 35 | Test Black 21 | 0.73 | 0.76 | 0.73 | 3221 | Train 51 | Test Black 21 | 0.71 | 0.71 | 0.67 |
| 2198 | Train 35 | Test Black 22 | 0.71 | 0.71 | 0.67 | 3222 | Train 51 | Test Black 22 | 0.7 | 0.69 | 0.61 |
| 2199 | Train 35 | Test Black 23 | 0.73 | 0.76 | 0.73 | 3223 | Train 51 | Test Black 23 | 0.71 | 0.71 | 0.67 |
| 2200 | Train 35 | Test Black 24 | 0.71 | 0.71 | 0.67 | 3224 | Train 51 | Test Black 24 | 0.7 | 0.69 | 0.61 |
| 2201 | Train 35 | Test Black 25 | 0.76 | 0.85 | 0.83 | 3225 | Train 51 | Test Black 25 | 0.73 | 0.76 | 0.71 |
| 2202 | Train 35 | Test Black 26 | 0.76 | 0.83 | 0.81 | 3226 | Train 51 | Test Black 26 | 0.72 | 0.75 | 0.69 |
| 2203 | Train 35 | Test Black 27 | 0.76 | 0.85 | 0.83 | 3227 | Train 51 | Test Black 27 | 0.73 | 0.76 | 0.71 |
| 2204 | Train 35 | Test Black 28 | 0.76 | 0.83 | 0.81 | 3228 | Train 51 | Test Black 28 | 0.72 | 0.75 | 0.69 |
| 2205 | Train 35 | Test Black 29 | 0.73 | 0.76 | 0.71 | 3229 | Train 51 | Test Black 29 | 0.71 | 0.71 | 0.66 |
| 2206 | Train 35 | Test Black 30 | 0.71 | 0.71 | 0.67 | 3230 | Train 51 | Test Black 30 | 0.7 | 0.69 | 0.61 |
| 2207 | Train 35 | Test Black 31 | 0.73 | 0.75 | 0.71 | 3231 | Train 51 | Test Black 31 | 0.7 | 0.7 | 0.66 |
| 2208 | Train 35 | Test Black 32 | 0.71 | 0.71 | 0.67 | 3232 | Train 51 | Test Black 32 | 0.7 | 0.69 | 0.61 |
| 2209 | Train 35 | Test Black 33 | 0.76 | 0.86 | 0.86 | 3233 | Train 51 | Test Black 33 | 0.73 | 0.76 | 0.72 |
| 2210 | Train 35 | Test Black 34 | 0.76 | 0.84 | 0.84 | 3234 | Train 51 | Test Black 34 | 0.72 | 0.76 | 0.7 |
| 2211 | Train 35 | Test Black 35 | 0.76 | 0.86 | 0.86 | 3235 | Train 51 | Test Black 35 | 0.73 | 0.76 | 0.72 |
| 2212 | Train 35 | Test Black 36 | 0.76 | 0.84 | 0.84 | 3236 | Train 51 | Test Black 36 | 0.72 | 0.76 | 0.7 |
| 2213 | Train 35 | Test Black 37 | 0.73 | 0.77 | 0.74 | 3237 | Train 51 | Test Black 37 | 0.71 | 0.71 | 0.67 |
| 2214 | Train 35 | Test Black 38 | 0.71 | 0.71 | 0.67 | 3238 | Train 51 | Test Black 38 | 0.7 | 0.69 | 0.61 |
| 2215 | Train 35 | Test Black 39 | 0.73 | 0.76 | 0.73 | 3239 | Train 51 | Test Black 39 | 0.71 | 0.71 | 0.67 |
| 2216 | Train 35 | Test Black 40 | 0.71 | 0.71 | 0.67 | 3240 | Train 51 | Test Black 40 | 0.7 | 0.69 | 0.61 |
| 2217 | Train 35 | Test Black 41 | 0.76 | 0.85 | 0.83 | 3241 | Train 51 | Test Black 41 | 0.73 | 0.76 | 0.71 |
| 2218 | Train 35 | Test Black 42 | 0.76 | 0.83 | 0.81 | 3242 | Train 51 | Test Black 42 | 0.72 | 0.75 | 0.69 |
| 2219 | Train 35 | Test Black 43 | 0.76 | 0.85 | 0.83 | 3243 | Train 51 | Test Black 43 | 0.73 | 0.76 | 0.71 |
| 2220 | Train 35 | Test Black 44 | 0.76 | 0.83 | 0.81 | 3244 | Train 51 | Test Black 44 | 0.72 | 0.75 | 0.69 |
| 2221 | Train 35 | Test Black 45 | 0.73 | 0.76 | 0.71 | 3245 | Train 51 | Test Black 45 | 0.71 | 0.71 | 0.66 |
| 2222 | Train 35 | Test Black 46 | 0.71 | 0.71 | 0.67 | 3246 | Train 51 | Test Black 46 | 0.7 | 0.69 | 0.61 |
| 2223 | Train 35 | Test Black 47 | 0.73 | 0.75 | 0.71 | 3247 | Train 51 | Test Black 47 | 0.71 | 0.7 | 0.66 |
| 2224 | Train 35 | Test Black 48 | 0.71 | 0.71 | 0.67 | 3248 | Train 51 | Test Black 48 | 0.7 | 0.69 | 0.61 |
| 2225 | Train 35 | Test Black 49 | 0.77 | 0.82 | 0.84 | 3249 | Train 51 | Test Black 49 | 0.78 | 0.85 | 0.88 |
| 2226 | Train 35 | Test Black 50 | 0.77 | 0.82 | 0.84 | 3250 | Train 51 | Test Black 50 | 0.78 | 0.85 | 0.88 |
| 2227 | Train 35 | Test Black 51 | 0.77 | 0.82 | 0.84 | 3251 | Train 51 | Test Black 51 | 0.78 | 0.85 | 0.88 |
| 2228 | Train 35 | Test Black 52 | 0.77 | 0.82 | 0.84 | 3252 | Train 51 | Test Black 52 | 0.78 | 0.85 | 0.88 |
| 2229 | Train 35 | Test Black 53 | 0.76 | 0.79 | 0.75 | 3253 | Train 51 | Test Black 53 | 0.75 | 0.8 | 0.76 |
| 2230 | Train 35 | Test Black 54 | 0.71 | 0.71 | 0.67 | 3254 | Train 51 | Test Black 54 | 0.71 | 0.7 | 0.63 |
| 2231 | Train 35 | Test Black 55 | 0.76 | 0.79 | 0.76 | 3255 | Train 51 | Test Black 55 | 0.75 | 0.8 | 0.76 |
| 2232 | Train 35 | Test Black 56 | 0.71 | 0.71 | 0.67 | 3256 | Train 51 | Test Black 56 | 0.71 | 0.7 | 0.63 |
| 2233 | Train 35 | Test Black 57 | 0.77 | 0.81 | 0.81 | 3257 | Train 51 | Test Black 57 | 0.78 | 0.84 | 0.82 |
| 2234 | Train 35 | Test Black 58 | 0.77 | 0.81 | 0.81 | 3258 | Train 51 | Test Black 58 | 0.78 | 0.84 | 0.82 |
| 2235 | Train 35 | Test Black 59 | 0.77 | 0.81 | 0.81 | 3259 | Train 51 | Test Black 59 | 0.78 | 0.84 | 0.82 |
| 2236 | Train 35 | Test Black 60 | 0.77 | 0.81 | 0.81 | 3260 | Train 51 | Test Black 60 | 0.78 | 0.84 | 0.82 |
| 2237 | Train 35 | Test Black 61 | 0.75 | 0.78 | 0.75 | 3261 | Train 51 | Test Black 61 | 0.74 | 0.79 | 0.74 |
| 2238 | Train 35 | Test Black 62 | 0.71 | 0.71 | 0.67 | 3262 | Train 51 | Test Black 62 | 0.71 | 0.7 | 0.63 |
| 2239 | Train 35 | Test Black 63 | 0.75 | 0.78 | 0.75 | 3263 | Train 51 | Test Black 63 | 0.74 | 0.8 | 0.74 |
| 2240 | Train 35 | Test Black 64 | 0.71 | 0.71 | 0.67 | 3264 | Train 51 | Test Black 64 | 0.71 | 0.7 | 0.64 |
| 2241 | Train 36 | Test Black 1 | 0.75 | 0.85 | 0.85 | 3265 | Train 52 | Test Black 1 | 0.72 | 0.73 | 0.72 |
| 2242 | Train 36 | Test Black 2 | 0.76 | 0.85 | 0.86 | 3266 | Train 52 | Test Black 2 | 0.72 | 0.73 | 0.71 |
| 2243 | Train 36 | Test Black 3 | 0.75 | 0.85 | 0.85 | 3267 | Train 52 | Test Black 3 | 0.72 | 0.74 | 0.72 |
| 2244 | Train 36 | Test Black 4 | 0.76 | 0.85 | 0.86 | 3268 | Train 52 | Test Black 4 | 0.72 | 0.73 | 0.71 |
| 2245 | Train 36 | Test Black 5 | 0.73 | 0.77 | 0.78 | 3269 | Train 52 | Test Black 5 | 0.71 | 0.7 | 0.66 |
| 2246 | Train 36 | Test Black 6 | 0.71 | 0.72 | 0.69 | 3270 | Train 52 | Test Black 6 | 0.7 | 0.68 | 0.64 |
| 2247 | Train 36 | Test Black 7 | 0.73 | 0.76 | 0.77 | 3271 | Train 52 | Test Black 7 | 0.7 | 0.69 | 0.66 |
| 2248 | Train 36 | Test Black 8 | 0.71 | 0.72 | 0.69 | 3272 | Train 52 | Test Black 8 | 0.7 | 0.68 | 0.64 |
| 2249 | Train 36 | Test Black 9 | 0.75 | 0.84 | 0.83 | 3273 | Train 52 | Test Black 9 | 0.72 | 0.73 | 0.7 |
| 2250 | Train 36 | Test Black 10 | 0.76 | 0.84 | 0.83 | 3274 | Train 52 | Test Black 10 | 0.72 | 0.73 | 0.69 |
| 2251 | Train 36 | Test Black 11 | 0.75 | 0.84 | 0.83 | 3275 | Train 52 | Test Black 11 | 0.72 | 0.73 | 0.7 |
| 2252 | Train 36 | Test Black 12 | 0.76 | 0.84 | 0.83 | 3276 | Train 52 | Test Black 12 | 0.72 | 0.73 | 0.69 |
| 2253 | Train 36 | Test Black 13 | 0.73 | 0.76 | 0.76 | 3277 | Train 52 | Test Black 13 | 0.7 | 0.7 | 0.65 |
| 2254 | Train 36 | Test Black 14 | 0.71 | 0.72 | 0.69 | 3278 | Train 52 | Test Black 14 | 0.7 | 0.68 | 0.64 |
| 2255 | Train 36 | Test Black 15 | 0.73 | 0.76 | 0.76 | 3279 | Train 52 | Test Black 15 | 0.7 | 0.69 | 0.65 |
| 2256 | Train 36 | Test Black 16 | 0.71 | 0.72 | 0.69 | 3280 | Train 52 | Test Black 16 | 0.7 | 0.68 | 0.64 |
| 2257 | Train 36 | Test Black 17 | 0.75 | 0.85 | 0.85 | 3281 | Train 52 | Test Black 17 | 0.72 | 0.74 | 0.72 |
| 2258 | Train 36 | Test Black 18 | 0.76 | 0.85 | 0.86 | 3282 | Train 52 | Test Black 18 | 0.72 | 0.73 | 0.71 |
| 2259 | Train 36 | Test Black 19 | 0.75 | 0.85 | 0.85 | 3283 | Train 52 | Test Black 19 | 0.72 | 0.74 | 0.72 |
| 2260 | Train 36 | Test Black 20 | 0.76 | 0.85 | 0.86 | 3284 | Train 52 | Test Black 20 | 0.72 | 0.73 | 0.71 |
| 2261 | Train 36 | Test Black 21 | 0.73 | 0.77 | 0.78 | 3285 | Train 52 | Test Black 21 | 0.71 | 0.7 | 0.66 |
| 2262 | Train 36 | Test Black 22 | 0.71 | 0.72 | 0.69 | 3286 | Train 52 | Test Black 22 | 0.7 | 0.68 | 0.64 |
| 2263 | Train 36 | Test Black 23 | 0.73 | 0.76 | 0.77 | 3287 | Train 52 | Test Black 23 | 0.7 | 0.69 | 0.66 |
| 2264 | Train 36 | Test Black 24 | 0.71 | 0.72 | 0.69 | 3288 | Train 52 | Test Black 24 | 0.7 | 0.68 | 0.64 |
| 2265 | Train 36 | Test Black 25 | 0.75 | 0.84 | 0.83 | 3289 | Train 52 | Test Black 25 | 0.72 | 0.73 | 0.7 |
| 2266 | Train 36 | Test Black 26 | 0.76 | 0.84 | 0.83 | 3290 | Train 52 | Test Black 26 | 0.72 | 0.73 | 0.69 |
| 2267 | Train 36 | Test Black 27 | 0.75 | 0.84 | 0.83 | 3291 | Train 52 | Test Black 27 | 0.72 | 0.73 | 0.7 |
| 2268 | Train 36 | Test Black 28 | 0.76 | 0.84 | 0.83 | 3292 | Train 52 | Test Black 28 | 0.72 | 0.73 | 0.69 |
| 2269 | Train 36 | Test Black 29 | 0.73 | 0.76 | 0.76 | 3293 | Train 52 | Test Black 29 | 0.7 | 0.7 | 0.65 |
| 2270 | Train 36 | Test Black 30 | 0.71 | 0.72 | 0.69 | 3294 | Train 52 | Test Black 30 | 0.7 | 0.68 | 0.64 |
| 2271 | Train 36 | Test Black 31 | 0.73 | 0.76 | 0.76 | 3295 | Train 52 | Test Black 31 | 0.7 | 0.69 | 0.65 |
| 2272 | Train 36 | Test Black 32 | 0.71 | 0.72 | 0.69 | 3296 | Train 52 | Test Black 32 | 0.7 | 0.68 | 0.64 |
| 2273 | Train 36 | Test Black 33 | 0.75 | 0.85 | 0.85 | 3297 | Train 52 | Test Black 33 | 0.72 | 0.74 | 0.72 |
| 2274 | Train 36 | Test Black 34 | 0.75 | 0.85 | 0.86 | 3298 | Train 52 | Test Black 34 | 0.72 | 0.73 | 0.71 |
| 2275 | Train 36 | Test Black 35 | 0.75 | 0.85 | 0.85 | 3299 | Train 52 | Test Black 35 | 0.73 | 0.74 | 0.72 |
| 2276 | Train 36 | Test Black 36 | 0.76 | 0.85 | 0.86 | 3300 | Train 52 | Test Black 36 | 0.72 | 0.74 | 0.71 |
| 2277 | Train 36 | Test Black 37 | 0.73 | 0.77 | 0.78 | 3301 | Train 52 | Test Black 37 | 0.71 | 0.7 | 0.66 |
| 2278 | Train 36 | Test Black 38 | 0.71 | 0.72 | 0.69 | 3302 | Train 52 | Test Black 38 | 0.7 | 0.68 | 0.64 |
| 2279 | Train 36 | Test Black 39 | 0.73 | 0.77 | 0.77 | 3303 | Train 52 | Test Black 39 | 0.7 | 0.69 | 0.66 |
| 2280 | Train 36 | Test Black 40 | 0.71 | 0.72 | 0.69 | 3304 | Train 52 | Test Black 40 | 0.7 | 0.68 | 0.64 |
| 2281 | Train 36 | Test Black 41 | 0.75 | 0.84 | 0.83 | 3305 | Train 52 | Test Black 41 | 0.72 | 0.73 | 0.7 |
| 2282 | Train 36 | Test Black 42 | 0.75 | 0.84 | 0.83 | 3306 | Train 52 | Test Black 42 | 0.72 | 0.73 | 0.69 |
| 2283 | Train 36 | Test Black 43 | 0.75 | 0.84 | 0.83 | 3307 | Train 52 | Test Black 43 | 0.72 | 0.74 | 0.7 |
| 2284 | Train 36 | Test Black 44 | 0.76 | 0.84 | 0.83 | 3308 | Train 52 | Test Black 44 | 0.72 | 0.73 | 0.69 |
| 2285 | Train 36 | Test Black 45 | 0.73 | 0.76 | 0.76 | 3309 | Train 52 | Test Black 45 | 0.71 | 0.7 | 0.65 |
| 2286 | Train 36 | Test Black 46 | 0.71 | 0.72 | 0.69 | 3310 | Train 52 | Test Black 46 | 0.7 | 0.68 | 0.64 |
| 2287 | Train 36 | Test Black 47 | 0.73 | 0.76 | 0.76 | 3311 | Train 52 | Test Black 47 | 0.7 | 0.69 | 0.65 |
| 2288 | Train 36 | Test Black 48 | 0.71 | 0.72 | 0.69 | 3312 | Train 52 | Test Black 48 | 0.7 | 0.68 | 0.64 |
| 2289 | Train 36 | Test Black 49 | 0.77 | 0.82 | 0.81 | 3313 | Train 52 | Test Black 49 | 0.78 | 0.85 | 0.89 |
| 2290 | Train 36 | Test Black 50 | 0.77 | 0.82 | 0.81 | 3314 | Train 52 | Test Black 50 | 0.78 | 0.85 | 0.89 |
| 2291 | Train 36 | Test Black 51 | 0.77 | 0.82 | 0.81 | 3315 | Train 52 | Test Black 51 | 0.78 | 0.85 | 0.89 |
| 2292 | Train 36 | Test Black 52 | 0.77 | 0.82 | 0.81 | 3316 | Train 52 | Test Black 52 | 0.78 | 0.85 | 0.89 |
| 2293 | Train 36 | Test Black 53 | 0.75 | 0.79 | 0.77 | 3317 | Train 52 | Test Black 53 | 0.75 | 0.79 | 0.79 |
| 2294 | Train 36 | Test Black 54 | 0.71 | 0.72 | 0.68 | 3318 | Train 52 | Test Black 54 | 0.71 | 0.68 | 0.66 |
| 2295 | Train 36 | Test Black 55 | 0.75 | 0.79 | 0.77 | 3319 | Train 52 | Test Black 55 | 0.75 | 0.79 | 0.79 |
| 2296 | Train 36 | Test Black 56 | 0.71 | 0.71 | 0.67 | 3320 | Train 52 | Test Black 56 | 0.71 | 0.68 | 0.66 |
| 2297 | Train 36 | Test Black 57 | 0.77 | 0.81 | 0.78 | 3321 | Train 52 | Test Black 57 | 0.78 | 0.84 | 0.83 |
| 2298 | Train 36 | Test Black 58 | 0.77 | 0.81 | 0.78 | 3322 | Train 52 | Test Black 58 | 0.78 | 0.84 | 0.83 |
| 2299 | Train 36 | Test Black 59 | 0.77 | 0.81 | 0.78 | 3323 | Train 52 | Test Black 59 | 0.78 | 0.84 | 0.83 |
| 2300 | Train 36 | Test Black 60 | 0.77 | 0.81 | 0.78 | 3324 | Train 52 | Test Black 60 | 0.78 | 0.84 | 0.83 |
| 2301 | Train 36 | Test Black 61 | 0.75 | 0.79 | 0.76 | 3325 | Train 52 | Test Black 61 | 0.75 | 0.78 | 0.77 |
| 2302 | Train 36 | Test Black 62 | 0.71 | 0.72 | 0.68 | 3326 | Train 52 | Test Black 62 | 0.71 | 0.68 | 0.66 |
| 2303 | Train 36 | Test Black 63 | 0.75 | 0.79 | 0.76 | 3327 | Train 52 | Test Black 63 | 0.75 | 0.79 | 0.77 |
| 2304 | Train 36 | Test Black 64 | 0.71 | 0.71 | 0.68 | 3328 | Train 52 | Test Black 64 | 0.71 | 0.68 | 0.66 |
| 2305 | Train 37 | Test Black 1 | 0.75 | 0.83 | 0.83 | 3329 | Train 53 | Test Black 1 | 0.72 | 0.76 | 0.67 |
| 2306 | Train 37 | Test Black 2 | 0.75 | 0.82 | 0.82 | 3330 | Train 53 | Test Black 2 | 0.73 | 0.76 | 0.67 |
| 2307 | Train 37 | Test Black 3 | 0.75 | 0.83 | 0.83 | 3331 | Train 53 | Test Black 3 | 0.72 | 0.76 | 0.67 |
| 2308 | Train 37 | Test Black 4 | 0.75 | 0.82 | 0.82 | 3332 | Train 53 | Test Black 4 | 0.73 | 0.76 | 0.67 |
| 2309 | Train 37 | Test Black 5 | 0.74 | 0.82 | 0.82 | 3333 | Train 53 | Test Black 5 | 0.71 | 0.73 | 0.67 |
| 2310 | Train 37 | Test Black 6 | 0.7 | 0.73 | 0.72 | 3334 | Train 53 | Test Black 6 | 0.69 | 0.7 | 0.64 |
| 2311 | Train 37 | Test Black 7 | 0.73 | 0.81 | 0.82 | 3335 | Train 53 | Test Black 7 | 0.71 | 0.73 | 0.67 |
| 2312 | Train 37 | Test Black 8 | 0.7 | 0.73 | 0.72 | 3336 | Train 53 | Test Black 8 | 0.69 | 0.7 | 0.64 |
| 2313 | Train 37 | Test Black 9 | 0.75 | 0.82 | 0.81 | 3337 | Train 53 | Test Black 9 | 0.72 | 0.76 | 0.68 |
| 2314 | Train 37 | Test Black 10 | 0.75 | 0.81 | 0.8 | 3338 | Train 53 | Test Black 10 | 0.73 | 0.76 | 0.68 |
| 2315 | Train 37 | Test Black 11 | 0.75 | 0.82 | 0.81 | 3339 | Train 53 | Test Black 11 | 0.72 | 0.76 | 0.68 |
| 2316 | Train 37 | Test Black 12 | 0.75 | 0.81 | 0.8 | 3340 | Train 53 | Test Black 12 | 0.73 | 0.76 | 0.68 |
| 2317 | Train 37 | Test Black 13 | 0.73 | 0.81 | 0.8 | 3341 | Train 53 | Test Black 13 | 0.71 | 0.73 | 0.68 |
| 2318 | Train 37 | Test Black 14 | 0.7 | 0.73 | 0.72 | 3342 | Train 53 | Test Black 14 | 0.7 | 0.7 | 0.64 |
| 2319 | Train 37 | Test Black 15 | 0.73 | 0.8 | 0.79 | 3343 | Train 53 | Test Black 15 | 0.71 | 0.73 | 0.68 |
| 2320 | Train 37 | Test Black 16 | 0.71 | 0.73 | 0.72 | 3344 | Train 53 | Test Black 16 | 0.7 | 0.7 | 0.64 |
| 2321 | Train 37 | Test Black 17 | 0.75 | 0.83 | 0.83 | 3345 | Train 53 | Test Black 17 | 0.72 | 0.76 | 0.67 |
| 2322 | Train 37 | Test Black 18 | 0.75 | 0.82 | 0.82 | 3346 | Train 53 | Test Black 18 | 0.73 | 0.76 | 0.67 |
| 2323 | Train 37 | Test Black 19 | 0.75 | 0.83 | 0.83 | 3347 | Train 53 | Test Black 19 | 0.72 | 0.76 | 0.67 |
| 2324 | Train 37 | Test Black 20 | 0.75 | 0.82 | 0.82 | 3348 | Train 53 | Test Black 20 | 0.73 | 0.76 | 0.67 |
| 2325 | Train 37 | Test Black 21 | 0.74 | 0.82 | 0.82 | 3349 | Train 53 | Test Black 21 | 0.71 | 0.73 | 0.67 |
| 2326 | Train 37 | Test Black 22 | 0.7 | 0.73 | 0.72 | 3350 | Train 53 | Test Black 22 | 0.69 | 0.7 | 0.64 |
| 2327 | Train 37 | Test Black 23 | 0.73 | 0.81 | 0.82 | 3351 | Train 53 | Test Black 23 | 0.71 | 0.73 | 0.67 |
| 2328 | Train 37 | Test Black 24 | 0.7 | 0.73 | 0.72 | 3352 | Train 53 | Test Black 24 | 0.69 | 0.7 | 0.64 |
| 2329 | Train 37 | Test Black 25 | 0.75 | 0.82 | 0.81 | 3353 | Train 53 | Test Black 25 | 0.72 | 0.76 | 0.68 |
| 2330 | Train 37 | Test Black 26 | 0.75 | 0.81 | 0.8 | 3354 | Train 53 | Test Black 26 | 0.73 | 0.76 | 0.68 |
| 2331 | Train 37 | Test Black 27 | 0.75 | 0.82 | 0.81 | 3355 | Train 53 | Test Black 27 | 0.72 | 0.76 | 0.68 |
| 2332 | Train 37 | Test Black 28 | 0.75 | 0.81 | 0.8 | 3356 | Train 53 | Test Black 28 | 0.73 | 0.76 | 0.68 |
| 2333 | Train 37 | Test Black 29 | 0.74 | 0.81 | 0.8 | 3357 | Train 53 | Test Black 29 | 0.71 | 0.73 | 0.68 |
| 2334 | Train 37 | Test Black 30 | 0.7 | 0.73 | 0.72 | 3358 | Train 53 | Test Black 30 | 0.7 | 0.7 | 0.64 |
| 2335 | Train 37 | Test Black 31 | 0.73 | 0.8 | 0.79 | 3359 | Train 53 | Test Black 31 | 0.71 | 0.73 | 0.68 |
| 2336 | Train 37 | Test Black 32 | 0.71 | 0.73 | 0.72 | 3360 | Train 53 | Test Black 32 | 0.7 | 0.7 | 0.64 |
| 2337 | Train 37 | Test Black 33 | 0.75 | 0.83 | 0.83 | 3361 | Train 53 | Test Black 33 | 0.72 | 0.76 | 0.67 |
| 2338 | Train 37 | Test Black 34 | 0.75 | 0.82 | 0.83 | 3362 | Train 53 | Test Black 34 | 0.73 | 0.76 | 0.67 |
| 2339 | Train 37 | Test Black 35 | 0.75 | 0.83 | 0.83 | 3363 | Train 53 | Test Black 35 | 0.72 | 0.76 | 0.67 |
| 2340 | Train 37 | Test Black 36 | 0.75 | 0.82 | 0.82 | 3364 | Train 53 | Test Black 36 | 0.73 | 0.76 | 0.67 |
| 2341 | Train 37 | Test Black 37 | 0.74 | 0.82 | 0.83 | 3365 | Train 53 | Test Black 37 | 0.71 | 0.73 | 0.68 |
| 2342 | Train 37 | Test Black 38 | 0.7 | 0.73 | 0.72 | 3366 | Train 53 | Test Black 38 | 0.69 | 0.7 | 0.64 |
| 2343 | Train 37 | Test Black 39 | 0.73 | 0.81 | 0.82 | 3367 | Train 53 | Test Black 39 | 0.71 | 0.73 | 0.67 |
| 2344 | Train 37 | Test Black 40 | 0.7 | 0.73 | 0.72 | 3368 | Train 53 | Test Black 40 | 0.69 | 0.7 | 0.64 |
| 2345 | Train 37 | Test Black 41 | 0.75 | 0.82 | 0.81 | 3369 | Train 53 | Test Black 41 | 0.72 | 0.76 | 0.68 |
| 2346 | Train 37 | Test Black 42 | 0.75 | 0.81 | 0.8 | 3370 | Train 53 | Test Black 42 | 0.73 | 0.76 | 0.68 |
| 2347 | Train 37 | Test Black 43 | 0.75 | 0.82 | 0.81 | 3371 | Train 53 | Test Black 43 | 0.72 | 0.76 | 0.68 |
| 2348 | Train 37 | Test Black 44 | 0.75 | 0.81 | 0.8 | 3372 | Train 53 | Test Black 44 | 0.73 | 0.76 | 0.68 |
| 2349 | Train 37 | Test Black 45 | 0.74 | 0.81 | 0.8 | 3373 | Train 53 | Test Black 45 | 0.71 | 0.73 | 0.68 |
| 2350 | Train 37 | Test Black 46 | 0.7 | 0.73 | 0.72 | 3374 | Train 53 | Test Black 46 | 0.7 | 0.7 | 0.64 |
| 2351 | Train 37 | Test Black 47 | 0.73 | 0.8 | 0.79 | 3375 | Train 53 | Test Black 47 | 0.71 | 0.73 | 0.68 |
| 2352 | Train 37 | Test Black 48 | 0.71 | 0.73 | 0.72 | 3376 | Train 53 | Test Black 48 | 0.7 | 0.7 | 0.64 |
| 2353 | Train 37 | Test Black 49 | 0.76 | 0.8 | 0.81 | 3377 | Train 53 | Test Black 49 | 0.77 | 0.81 | 0.79 |
| 2354 | Train 37 | Test Black 50 | 0.76 | 0.8 | 0.81 | 3378 | Train 53 | Test Black 50 | 0.77 | 0.81 | 0.79 |
| 2355 | Train 37 | Test Black 51 | 0.76 | 0.8 | 0.8 | 3379 | Train 53 | Test Black 51 | 0.77 | 0.81 | 0.79 |
| 2356 | Train 37 | Test Black 52 | 0.76 | 0.8 | 0.8 | 3380 | Train 53 | Test Black 52 | 0.77 | 0.81 | 0.79 |
| 2357 | Train 37 | Test Black 53 | 0.76 | 0.8 | 0.8 | 3381 | Train 53 | Test Black 53 | 0.77 | 0.83 | 0.83 |
| 2358 | Train 37 | Test Black 54 | 0.71 | 0.73 | 0.69 | 3382 | Train 53 | Test Black 54 | 0.71 | 0.72 | 0.67 |
| 2359 | Train 37 | Test Black 55 | 0.76 | 0.8 | 0.79 | 3383 | Train 53 | Test Black 55 | 0.77 | 0.82 | 0.82 |
| 2360 | Train 37 | Test Black 56 | 0.71 | 0.72 | 0.69 | 3384 | Train 53 | Test Black 56 | 0.71 | 0.72 | 0.66 |
| 2361 | Train 37 | Test Black 57 | 0.76 | 0.79 | 0.79 | 3385 | Train 53 | Test Black 57 | 0.77 | 0.81 | 0.79 |
| 2362 | Train 37 | Test Black 58 | 0.76 | 0.79 | 0.79 | 3386 | Train 53 | Test Black 58 | 0.77 | 0.81 | 0.79 |
| 2363 | Train 37 | Test Black 59 | 0.76 | 0.79 | 0.78 | 3387 | Train 53 | Test Black 59 | 0.77 | 0.81 | 0.79 |
| 2364 | Train 37 | Test Black 60 | 0.76 | 0.79 | 0.79 | 3388 | Train 53 | Test Black 60 | 0.77 | 0.81 | 0.79 |
| 2365 | Train 37 | Test Black 61 | 0.76 | 0.8 | 0.79 | 3389 | Train 53 | Test Black 61 | 0.77 | 0.82 | 0.8 |
| 2366 | Train 37 | Test Black 62 | 0.71 | 0.73 | 0.7 | 3390 | Train 53 | Test Black 62 | 0.71 | 0.72 | 0.67 |
| 2367 | Train 37 | Test Black 63 | 0.76 | 0.8 | 0.79 | 3391 | Train 53 | Test Black 63 | 0.76 | 0.82 | 0.8 |
| 2368 | Train 37 | Test Black 64 | 0.71 | 0.72 | 0.7 | 3392 | Train 53 | Test Black 64 | 0.71 | 0.72 | 0.67 |
| 2369 | Train 38 | Test Black 1 | 0.73 | 0.79 | 0.77 | 3393 | Train 54 | Test Black 1 | 0.71 | 0.74 | 0.65 |
| 2370 | Train 38 | Test Black 2 | 0.74 | 0.8 | 0.77 | 3394 | Train 54 | Test Black 2 | 0.72 | 0.74 | 0.66 |
| 2371 | Train 38 | Test Black 3 | 0.73 | 0.79 | 0.77 | 3395 | Train 54 | Test Black 3 | 0.71 | 0.74 | 0.65 |
| 2372 | Train 38 | Test Black 4 | 0.74 | 0.8 | 0.77 | 3396 | Train 54 | Test Black 4 | 0.72 | 0.74 | 0.66 |
| 2373 | Train 38 | Test Black 5 | 0.72 | 0.78 | 0.75 | 3397 | Train 54 | Test Black 5 | 0.7 | 0.72 | 0.64 |
| 2374 | Train 38 | Test Black 6 | 0.73 | 0.78 | 0.77 | 3398 | Train 54 | Test Black 6 | 0.72 | 0.75 | 0.7 |
| 2375 | Train 38 | Test Black 7 | 0.72 | 0.77 | 0.75 | 3399 | Train 54 | Test Black 7 | 0.7 | 0.72 | 0.64 |
| 2376 | Train 38 | Test Black 8 | 0.73 | 0.78 | 0.76 | 3400 | Train 54 | Test Black 8 | 0.72 | 0.75 | 0.69 |
| 2377 | Train 38 | Test Black 9 | 0.73 | 0.8 | 0.78 | 3401 | Train 54 | Test Black 9 | 0.71 | 0.74 | 0.66 |
| 2378 | Train 38 | Test Black 10 | 0.74 | 0.8 | 0.78 | 3402 | Train 54 | Test Black 10 | 0.72 | 0.75 | 0.67 |
| 2379 | Train 38 | Test Black 11 | 0.73 | 0.8 | 0.78 | 3403 | Train 54 | Test Black 11 | 0.71 | 0.74 | 0.66 |
| 2380 | Train 38 | Test Black 12 | 0.74 | 0.8 | 0.78 | 3404 | Train 54 | Test Black 12 | 0.72 | 0.75 | 0.67 |
| 2381 | Train 38 | Test Black 13 | 0.72 | 0.78 | 0.76 | 3405 | Train 54 | Test Black 13 | 0.7 | 0.72 | 0.64 |
| 2382 | Train 38 | Test Black 14 | 0.73 | 0.78 | 0.77 | 3406 | Train 54 | Test Black 14 | 0.72 | 0.76 | 0.7 |
| 2383 | Train 38 | Test Black 15 | 0.72 | 0.78 | 0.75 | 3407 | Train 54 | Test Black 15 | 0.7 | 0.72 | 0.64 |
| 2384 | Train 38 | Test Black 16 | 0.73 | 0.78 | 0.76 | 3408 | Train 54 | Test Black 16 | 0.72 | 0.75 | 0.7 |
| 2385 | Train 38 | Test Black 17 | 0.73 | 0.79 | 0.77 | 3409 | Train 54 | Test Black 17 | 0.71 | 0.74 | 0.65 |
| 2386 | Train 38 | Test Black 18 | 0.74 | 0.8 | 0.77 | 3410 | Train 54 | Test Black 18 | 0.72 | 0.74 | 0.66 |
| 2387 | Train 38 | Test Black 19 | 0.73 | 0.79 | 0.77 | 3411 | Train 54 | Test Black 19 | 0.71 | 0.74 | 0.65 |
| 2388 | Train 38 | Test Black 20 | 0.74 | 0.8 | 0.77 | 3412 | Train 54 | Test Black 20 | 0.72 | 0.74 | 0.66 |
| 2389 | Train 38 | Test Black 21 | 0.72 | 0.78 | 0.75 | 3413 | Train 54 | Test Black 21 | 0.7 | 0.72 | 0.64 |
| 2390 | Train 38 | Test Black 22 | 0.73 | 0.78 | 0.77 | 3414 | Train 54 | Test Black 22 | 0.72 | 0.75 | 0.7 |
| 2391 | Train 38 | Test Black 23 | 0.72 | 0.77 | 0.75 | 3415 | Train 54 | Test Black 23 | 0.7 | 0.72 | 0.64 |
| 2392 | Train 38 | Test Black 24 | 0.73 | 0.78 | 0.76 | 3416 | Train 54 | Test Black 24 | 0.72 | 0.75 | 0.69 |
| 2393 | Train 38 | Test Black 25 | 0.73 | 0.8 | 0.78 | 3417 | Train 54 | Test Black 25 | 0.71 | 0.74 | 0.66 |
| 2394 | Train 38 | Test Black 26 | 0.74 | 0.8 | 0.78 | 3418 | Train 54 | Test Black 26 | 0.72 | 0.75 | 0.67 |
| 2395 | Train 38 | Test Black 27 | 0.73 | 0.8 | 0.78 | 3419 | Train 54 | Test Black 27 | 0.71 | 0.74 | 0.66 |
| 2396 | Train 38 | Test Black 28 | 0.74 | 0.8 | 0.78 | 3420 | Train 54 | Test Black 28 | 0.72 | 0.75 | 0.67 |
| 2397 | Train 38 | Test Black 29 | 0.72 | 0.78 | 0.76 | 3421 | Train 54 | Test Black 29 | 0.7 | 0.72 | 0.64 |
| 2398 | Train 38 | Test Black 30 | 0.73 | 0.78 | 0.77 | 3422 | Train 54 | Test Black 30 | 0.72 | 0.76 | 0.7 |
| 2399 | Train 38 | Test Black 31 | 0.72 | 0.78 | 0.75 | 3423 | Train 54 | Test Black 31 | 0.7 | 0.72 | 0.64 |
| 2400 | Train 38 | Test Black 32 | 0.73 | 0.78 | 0.76 | 3424 | Train 54 | Test Black 32 | 0.72 | 0.75 | 0.7 |
| 2401 | Train 38 | Test Black 33 | 0.73 | 0.79 | 0.77 | 3425 | Train 54 | Test Black 33 | 0.71 | 0.74 | 0.65 |
| 2402 | Train 38 | Test Black 34 | 0.74 | 0.8 | 0.77 | 3426 | Train 54 | Test Black 34 | 0.72 | 0.75 | 0.67 |
| 2403 | Train 38 | Test Black 35 | 0.73 | 0.79 | 0.77 | 3427 | Train 54 | Test Black 35 | 0.71 | 0.74 | 0.65 |
| 2404 | Train 38 | Test Black 36 | 0.74 | 0.8 | 0.77 | 3428 | Train 54 | Test Black 36 | 0.72 | 0.74 | 0.66 |
| 2405 | Train 38 | Test Black 37 | 0.72 | 0.78 | 0.75 | 3429 | Train 54 | Test Black 37 | 0.7 | 0.72 | 0.64 |
| 2406 | Train 38 | Test Black 38 | 0.73 | 0.78 | 0.77 | 3430 | Train 54 | Test Black 38 | 0.72 | 0.75 | 0.7 |
| 2407 | Train 38 | Test Black 39 | 0.72 | 0.78 | 0.75 | 3431 | Train 54 | Test Black 39 | 0.7 | 0.72 | 0.64 |
| 2408 | Train 38 | Test Black 40 | 0.73 | 0.78 | 0.76 | 3432 | Train 54 | Test Black 40 | 0.72 | 0.75 | 0.7 |
| 2409 | Train 38 | Test Black 41 | 0.73 | 0.8 | 0.78 | 3433 | Train 54 | Test Black 41 | 0.71 | 0.74 | 0.66 |
| 2410 | Train 38 | Test Black 42 | 0.74 | 0.8 | 0.78 | 3434 | Train 54 | Test Black 42 | 0.72 | 0.75 | 0.67 |
| 2411 | Train 38 | Test Black 43 | 0.73 | 0.8 | 0.78 | 3435 | Train 54 | Test Black 43 | 0.71 | 0.74 | 0.66 |
| 2412 | Train 38 | Test Black 44 | 0.74 | 0.8 | 0.78 | 3436 | Train 54 | Test Black 44 | 0.72 | 0.75 | 0.67 |
| 2413 | Train 38 | Test Black 45 | 0.72 | 0.78 | 0.76 | 3437 | Train 54 | Test Black 45 | 0.7 | 0.73 | 0.65 |
| 2414 | Train 38 | Test Black 46 | 0.73 | 0.79 | 0.77 | 3438 | Train 54 | Test Black 46 | 0.72 | 0.76 | 0.7 |
| 2415 | Train 38 | Test Black 47 | 0.72 | 0.78 | 0.75 | 3439 | Train 54 | Test Black 47 | 0.7 | 0.72 | 0.65 |
| 2416 | Train 38 | Test Black 48 | 0.73 | 0.78 | 0.76 | 3440 | Train 54 | Test Black 48 | 0.72 | 0.75 | 0.7 |
| 2417 | Train 38 | Test Black 49 | 0.76 | 0.8 | 0.78 | 3441 | Train 54 | Test Black 49 | 0.77 | 0.8 | 0.77 |
| 2418 | Train 38 | Test Black 50 | 0.76 | 0.8 | 0.78 | 3442 | Train 54 | Test Black 50 | 0.77 | 0.8 | 0.77 |
| 2419 | Train 38 | Test Black 51 | 0.76 | 0.8 | 0.78 | 3443 | Train 54 | Test Black 51 | 0.77 | 0.8 | 0.77 |
| 2420 | Train 38 | Test Black 52 | 0.76 | 0.8 | 0.77 | 3444 | Train 54 | Test Black 52 | 0.77 | 0.8 | 0.77 |
| 2421 | Train 38 | Test Black 53 | 0.76 | 0.8 | 0.77 | 3445 | Train 54 | Test Black 53 | 0.76 | 0.81 | 0.78 |
| 2422 | Train 38 | Test Black 54 | 0.74 | 0.78 | 0.78 | 3446 | Train 54 | Test Black 54 | 0.75 | 0.8 | 0.81 |
| 2423 | Train 38 | Test Black 55 | 0.75 | 0.8 | 0.77 | 3447 | Train 54 | Test Black 55 | 0.76 | 0.81 | 0.78 |
| 2424 | Train 38 | Test Black 56 | 0.74 | 0.78 | 0.78 | 3448 | Train 54 | Test Black 56 | 0.74 | 0.8 | 0.8 |
| 2425 | Train 38 | Test Black 57 | 0.76 | 0.8 | 0.79 | 3449 | Train 54 | Test Black 57 | 0.77 | 0.81 | 0.78 |
| 2426 | Train 38 | Test Black 58 | 0.76 | 0.8 | 0.79 | 3450 | Train 54 | Test Black 58 | 0.77 | 0.81 | 0.78 |
| 2427 | Train 38 | Test Black 59 | 0.76 | 0.8 | 0.79 | 3451 | Train 54 | Test Black 59 | 0.77 | 0.81 | 0.78 |
| 2428 | Train 38 | Test Black 60 | 0.76 | 0.8 | 0.79 | 3452 | Train 54 | Test Black 60 | 0.77 | 0.81 | 0.78 |
| 2429 | Train 38 | Test Black 61 | 0.75 | 0.8 | 0.78 | 3453 | Train 54 | Test Black 61 | 0.76 | 0.81 | 0.79 |
| 2430 | Train 38 | Test Black 62 | 0.74 | 0.78 | 0.78 | 3454 | Train 54 | Test Black 62 | 0.75 | 0.8 | 0.8 |
| 2431 | Train 38 | Test Black 63 | 0.75 | 0.8 | 0.78 | 3455 | Train 54 | Test Black 63 | 0.76 | 0.81 | 0.78 |
| 2432 | Train 38 | Test Black 64 | 0.74 | 0.78 | 0.78 | 3456 | Train 54 | Test Black 64 | 0.75 | 0.8 | 0.8 |
| 2433 | Train 39 | Test Black 1 | 0.75 | 0.83 | 0.83 | 3457 | Train 55 | Test Black 1 | 0.73 | 0.77 | 0.72 |
| 2434 | Train 39 | Test Black 2 | 0.75 | 0.82 | 0.82 | 3458 | Train 55 | Test Black 2 | 0.73 | 0.76 | 0.72 |
| 2435 | Train 39 | Test Black 3 | 0.75 | 0.83 | 0.83 | 3459 | Train 55 | Test Black 3 | 0.72 | 0.77 | 0.72 |
| 2436 | Train 39 | Test Black 4 | 0.75 | 0.82 | 0.82 | 3460 | Train 55 | Test Black 4 | 0.73 | 0.76 | 0.72 |
| 2437 | Train 39 | Test Black 5 | 0.74 | 0.82 | 0.82 | 3461 | Train 55 | Test Black 5 | 0.71 | 0.73 | 0.7 |
| 2438 | Train 39 | Test Black 6 | 0.7 | 0.73 | 0.7 | 3462 | Train 55 | Test Black 6 | 0.69 | 0.7 | 0.64 |
| 2439 | Train 39 | Test Black 7 | 0.73 | 0.81 | 0.82 | 3463 | Train 55 | Test Black 7 | 0.71 | 0.73 | 0.7 |
| 2440 | Train 39 | Test Black 8 | 0.7 | 0.73 | 0.7 | 3464 | Train 55 | Test Black 8 | 0.69 | 0.7 | 0.64 |
| 2441 | Train 39 | Test Black 9 | 0.75 | 0.82 | 0.8 | 3465 | Train 55 | Test Black 9 | 0.72 | 0.77 | 0.73 |
| 2442 | Train 39 | Test Black 10 | 0.75 | 0.81 | 0.8 | 3466 | Train 55 | Test Black 10 | 0.73 | 0.77 | 0.72 |
| 2443 | Train 39 | Test Black 11 | 0.75 | 0.82 | 0.8 | 3467 | Train 55 | Test Black 11 | 0.72 | 0.77 | 0.73 |
| 2444 | Train 39 | Test Black 12 | 0.75 | 0.81 | 0.8 | 3468 | Train 55 | Test Black 12 | 0.73 | 0.77 | 0.72 |
| 2445 | Train 39 | Test Black 13 | 0.73 | 0.81 | 0.8 | 3469 | Train 55 | Test Black 13 | 0.71 | 0.73 | 0.71 |
| 2446 | Train 39 | Test Black 14 | 0.7 | 0.73 | 0.7 | 3470 | Train 55 | Test Black 14 | 0.7 | 0.71 | 0.65 |
| 2447 | Train 39 | Test Black 15 | 0.73 | 0.8 | 0.79 | 3471 | Train 55 | Test Black 15 | 0.71 | 0.73 | 0.7 |
| 2448 | Train 39 | Test Black 16 | 0.71 | 0.73 | 0.7 | 3472 | Train 55 | Test Black 16 | 0.69 | 0.71 | 0.64 |
| 2449 | Train 39 | Test Black 17 | 0.75 | 0.83 | 0.83 | 3473 | Train 55 | Test Black 17 | 0.73 | 0.77 | 0.72 |
| 2450 | Train 39 | Test Black 18 | 0.75 | 0.82 | 0.82 | 3474 | Train 55 | Test Black 18 | 0.73 | 0.76 | 0.72 |
| 2451 | Train 39 | Test Black 19 | 0.75 | 0.83 | 0.83 | 3475 | Train 55 | Test Black 19 | 0.72 | 0.77 | 0.72 |
| 2452 | Train 39 | Test Black 20 | 0.75 | 0.82 | 0.82 | 3476 | Train 55 | Test Black 20 | 0.73 | 0.76 | 0.72 |
| 2453 | Train 39 | Test Black 21 | 0.74 | 0.82 | 0.82 | 3477 | Train 55 | Test Black 21 | 0.71 | 0.73 | 0.7 |
| 2454 | Train 39 | Test Black 22 | 0.7 | 0.73 | 0.7 | 3478 | Train 55 | Test Black 22 | 0.69 | 0.7 | 0.64 |
| 2455 | Train 39 | Test Black 23 | 0.73 | 0.81 | 0.82 | 3479 | Train 55 | Test Black 23 | 0.71 | 0.73 | 0.7 |
| 2456 | Train 39 | Test Black 24 | 0.7 | 0.73 | 0.7 | 3480 | Train 55 | Test Black 24 | 0.69 | 0.7 | 0.64 |
| 2457 | Train 39 | Test Black 25 | 0.75 | 0.82 | 0.8 | 3481 | Train 55 | Test Black 25 | 0.72 | 0.77 | 0.73 |
| 2458 | Train 39 | Test Black 26 | 0.75 | 0.81 | 0.8 | 3482 | Train 55 | Test Black 26 | 0.73 | 0.77 | 0.72 |
| 2459 | Train 39 | Test Black 27 | 0.75 | 0.82 | 0.8 | 3483 | Train 55 | Test Black 27 | 0.72 | 0.77 | 0.73 |
| 2460 | Train 39 | Test Black 28 | 0.75 | 0.81 | 0.8 | 3484 | Train 55 | Test Black 28 | 0.73 | 0.77 | 0.72 |
| 2461 | Train 39 | Test Black 29 | 0.73 | 0.81 | 0.8 | 3485 | Train 55 | Test Black 29 | 0.71 | 0.73 | 0.71 |
| 2462 | Train 39 | Test Black 30 | 0.7 | 0.73 | 0.7 | 3486 | Train 55 | Test Black 30 | 0.7 | 0.71 | 0.65 |
| 2463 | Train 39 | Test Black 31 | 0.73 | 0.8 | 0.79 | 3487 | Train 55 | Test Black 31 | 0.71 | 0.73 | 0.7 |
| 2464 | Train 39 | Test Black 32 | 0.71 | 0.73 | 0.7 | 3488 | Train 55 | Test Black 32 | 0.7 | 0.71 | 0.64 |
| 2465 | Train 39 | Test Black 33 | 0.75 | 0.83 | 0.83 | 3489 | Train 55 | Test Black 33 | 0.73 | 0.77 | 0.72 |
| 2466 | Train 39 | Test Black 34 | 0.75 | 0.82 | 0.82 | 3490 | Train 55 | Test Black 34 | 0.73 | 0.76 | 0.72 |
| 2467 | Train 39 | Test Black 35 | 0.75 | 0.83 | 0.83 | 3491 | Train 55 | Test Black 35 | 0.73 | 0.77 | 0.73 |
| 2468 | Train 39 | Test Black 36 | 0.75 | 0.82 | 0.82 | 3492 | Train 55 | Test Black 36 | 0.73 | 0.76 | 0.72 |
| 2469 | Train 39 | Test Black 37 | 0.74 | 0.82 | 0.82 | 3493 | Train 55 | Test Black 37 | 0.71 | 0.73 | 0.71 |
| 2470 | Train 39 | Test Black 38 | 0.7 | 0.73 | 0.7 | 3494 | Train 55 | Test Black 38 | 0.69 | 0.7 | 0.64 |
| 2471 | Train 39 | Test Black 39 | 0.73 | 0.82 | 0.82 | 3495 | Train 55 | Test Black 39 | 0.71 | 0.73 | 0.7 |
| 2472 | Train 39 | Test Black 40 | 0.7 | 0.73 | 0.7 | 3496 | Train 55 | Test Black 40 | 0.69 | 0.7 | 0.64 |
| 2473 | Train 39 | Test Black 41 | 0.75 | 0.82 | 0.81 | 3497 | Train 55 | Test Black 41 | 0.73 | 0.77 | 0.73 |
| 2474 | Train 39 | Test Black 42 | 0.75 | 0.81 | 0.8 | 3498 | Train 55 | Test Black 42 | 0.73 | 0.77 | 0.72 |
| 2475 | Train 39 | Test Black 43 | 0.75 | 0.82 | 0.81 | 3499 | Train 55 | Test Black 43 | 0.73 | 0.77 | 0.73 |
| 2476 | Train 39 | Test Black 44 | 0.75 | 0.81 | 0.8 | 3500 | Train 55 | Test Black 44 | 0.73 | 0.77 | 0.72 |
| 2477 | Train 39 | Test Black 45 | 0.74 | 0.81 | 0.8 | 3501 | Train 55 | Test Black 45 | 0.71 | 0.73 | 0.71 |
| 2478 | Train 39 | Test Black 46 | 0.7 | 0.73 | 0.7 | 3502 | Train 55 | Test Black 46 | 0.7 | 0.71 | 0.65 |
| 2479 | Train 39 | Test Black 47 | 0.73 | 0.8 | 0.79 | 3503 | Train 55 | Test Black 47 | 0.71 | 0.73 | 0.7 |
| 2480 | Train 39 | Test Black 48 | 0.71 | 0.73 | 0.7 | 3504 | Train 55 | Test Black 48 | 0.7 | 0.71 | 0.64 |
| 2481 | Train 39 | Test Black 49 | 0.76 | 0.8 | 0.81 | 3505 | Train 55 | Test Black 49 | 0.77 | 0.82 | 0.82 |
| 2482 | Train 39 | Test Black 50 | 0.76 | 0.8 | 0.81 | 3506 | Train 55 | Test Black 50 | 0.77 | 0.82 | 0.82 |
| 2483 | Train 39 | Test Black 51 | 0.76 | 0.8 | 0.81 | 3507 | Train 55 | Test Black 51 | 0.77 | 0.82 | 0.82 |
| 2484 | Train 39 | Test Black 52 | 0.76 | 0.8 | 0.81 | 3508 | Train 55 | Test Black 52 | 0.77 | 0.82 | 0.82 |
| 2485 | Train 39 | Test Black 53 | 0.76 | 0.8 | 0.8 | 3509 | Train 55 | Test Black 53 | 0.77 | 0.84 | 0.85 |
| 2486 | Train 39 | Test Black 54 | 0.71 | 0.72 | 0.69 | 3510 | Train 55 | Test Black 54 | 0.71 | 0.72 | 0.67 |
| 2487 | Train 39 | Test Black 55 | 0.76 | 0.8 | 0.8 | 3511 | Train 55 | Test Black 55 | 0.77 | 0.84 | 0.85 |
| 2488 | Train 39 | Test Black 56 | 0.71 | 0.72 | 0.68 | 3512 | Train 55 | Test Black 56 | 0.71 | 0.72 | 0.67 |
| 2489 | Train 39 | Test Black 57 | 0.76 | 0.79 | 0.79 | 3513 | Train 55 | Test Black 57 | 0.77 | 0.82 | 0.82 |
| 2490 | Train 39 | Test Black 58 | 0.76 | 0.79 | 0.79 | 3514 | Train 55 | Test Black 58 | 0.77 | 0.82 | 0.82 |
| 2491 | Train 39 | Test Black 59 | 0.76 | 0.79 | 0.79 | 3515 | Train 55 | Test Black 59 | 0.77 | 0.82 | 0.82 |
| 2492 | Train 39 | Test Black 60 | 0.76 | 0.79 | 0.79 | 3516 | Train 55 | Test Black 60 | 0.77 | 0.82 | 0.82 |
| 2493 | Train 39 | Test Black 61 | 0.76 | 0.8 | 0.79 | 3517 | Train 55 | Test Black 61 | 0.77 | 0.83 | 0.83 |
| 2494 | Train 39 | Test Black 62 | 0.71 | 0.72 | 0.69 | 3518 | Train 55 | Test Black 62 | 0.71 | 0.72 | 0.67 |
| 2495 | Train 39 | Test Black 63 | 0.76 | 0.8 | 0.79 | 3519 | Train 55 | Test Black 63 | 0.77 | 0.83 | 0.82 |
| 2496 | Train 39 | Test Black 64 | 0.71 | 0.72 | 0.68 | 3520 | Train 55 | Test Black 64 | 0.71 | 0.72 | 0.67 |
| 2497 | Train 40 | Test Black 1 | 0.73 | 0.79 | 0.78 | 3521 | Train 56 | Test Black 1 | 0.71 | 0.74 | 0.69 |
| 2498 | Train 40 | Test Black 2 | 0.74 | 0.8 | 0.78 | 3522 | Train 56 | Test Black 2 | 0.72 | 0.74 | 0.7 |
| 2499 | Train 40 | Test Black 3 | 0.73 | 0.79 | 0.78 | 3523 | Train 56 | Test Black 3 | 0.71 | 0.74 | 0.69 |
| 2500 | Train 40 | Test Black 4 | 0.74 | 0.8 | 0.78 | 3524 | Train 56 | Test Black 4 | 0.72 | 0.74 | 0.7 |
| 2501 | Train 40 | Test Black 5 | 0.72 | 0.78 | 0.76 | 3525 | Train 56 | Test Black 5 | 0.7 | 0.72 | 0.69 |
| 2502 | Train 40 | Test Black 6 | 0.73 | 0.78 | 0.77 | 3526 | Train 56 | Test Black 6 | 0.72 | 0.75 | 0.73 |
| 2503 | Train 40 | Test Black 7 | 0.72 | 0.77 | 0.76 | 3527 | Train 56 | Test Black 7 | 0.7 | 0.72 | 0.68 |
| 2504 | Train 40 | Test Black 8 | 0.73 | 0.78 | 0.77 | 3528 | Train 56 | Test Black 8 | 0.71 | 0.75 | 0.73 |
| 2505 | Train 40 | Test Black 9 | 0.73 | 0.8 | 0.78 | 3529 | Train 56 | Test Black 9 | 0.71 | 0.74 | 0.7 |
| 2506 | Train 40 | Test Black 10 | 0.74 | 0.8 | 0.78 | 3530 | Train 56 | Test Black 10 | 0.72 | 0.75 | 0.71 |
| 2507 | Train 40 | Test Black 11 | 0.73 | 0.8 | 0.78 | 3531 | Train 56 | Test Black 11 | 0.71 | 0.74 | 0.7 |
| 2508 | Train 40 | Test Black 12 | 0.74 | 0.8 | 0.78 | 3532 | Train 56 | Test Black 12 | 0.72 | 0.75 | 0.71 |
| 2509 | Train 40 | Test Black 13 | 0.72 | 0.78 | 0.77 | 3533 | Train 56 | Test Black 13 | 0.7 | 0.73 | 0.69 |
| 2510 | Train 40 | Test Black 14 | 0.73 | 0.78 | 0.77 | 3534 | Train 56 | Test Black 14 | 0.72 | 0.75 | 0.73 |
| 2511 | Train 40 | Test Black 15 | 0.72 | 0.78 | 0.76 | 3535 | Train 56 | Test Black 15 | 0.69 | 0.72 | 0.69 |
| 2512 | Train 40 | Test Black 16 | 0.73 | 0.78 | 0.77 | 3536 | Train 56 | Test Black 16 | 0.72 | 0.75 | 0.73 |
| 2513 | Train 40 | Test Black 17 | 0.73 | 0.79 | 0.78 | 3537 | Train 56 | Test Black 17 | 0.71 | 0.74 | 0.69 |
| 2514 | Train 40 | Test Black 18 | 0.74 | 0.8 | 0.78 | 3538 | Train 56 | Test Black 18 | 0.72 | 0.74 | 0.7 |
| 2515 | Train 40 | Test Black 19 | 0.73 | 0.79 | 0.78 | 3539 | Train 56 | Test Black 19 | 0.71 | 0.74 | 0.69 |
| 2516 | Train 40 | Test Black 20 | 0.74 | 0.8 | 0.78 | 3540 | Train 56 | Test Black 20 | 0.72 | 0.74 | 0.7 |
| 2517 | Train 40 | Test Black 21 | 0.72 | 0.78 | 0.76 | 3541 | Train 56 | Test Black 21 | 0.7 | 0.72 | 0.69 |
| 2518 | Train 40 | Test Black 22 | 0.73 | 0.78 | 0.77 | 3542 | Train 56 | Test Black 22 | 0.72 | 0.75 | 0.73 |
| 2519 | Train 40 | Test Black 23 | 0.72 | 0.77 | 0.76 | 3543 | Train 56 | Test Black 23 | 0.7 | 0.72 | 0.68 |
| 2520 | Train 40 | Test Black 24 | 0.73 | 0.78 | 0.77 | 3544 | Train 56 | Test Black 24 | 0.72 | 0.75 | 0.73 |
| 2521 | Train 40 | Test Black 25 | 0.73 | 0.8 | 0.78 | 3545 | Train 56 | Test Black 25 | 0.71 | 0.74 | 0.7 |
| 2522 | Train 40 | Test Black 26 | 0.74 | 0.8 | 0.78 | 3546 | Train 56 | Test Black 26 | 0.72 | 0.75 | 0.71 |
| 2523 | Train 40 | Test Black 27 | 0.73 | 0.8 | 0.78 | 3547 | Train 56 | Test Black 27 | 0.71 | 0.74 | 0.7 |
| 2524 | Train 40 | Test Black 28 | 0.74 | 0.8 | 0.78 | 3548 | Train 56 | Test Black 28 | 0.72 | 0.75 | 0.71 |
| 2525 | Train 40 | Test Black 29 | 0.72 | 0.78 | 0.77 | 3549 | Train 56 | Test Black 29 | 0.7 | 0.73 | 0.69 |
| 2526 | Train 40 | Test Black 30 | 0.73 | 0.78 | 0.77 | 3550 | Train 56 | Test Black 30 | 0.72 | 0.75 | 0.73 |
| 2527 | Train 40 | Test Black 31 | 0.72 | 0.78 | 0.76 | 3551 | Train 56 | Test Black 31 | 0.69 | 0.72 | 0.69 |
| 2528 | Train 40 | Test Black 32 | 0.73 | 0.78 | 0.77 | 3552 | Train 56 | Test Black 32 | 0.72 | 0.75 | 0.73 |
| 2529 | Train 40 | Test Black 33 | 0.73 | 0.79 | 0.78 | 3553 | Train 56 | Test Black 33 | 0.71 | 0.74 | 0.69 |
| 2530 | Train 40 | Test Black 34 | 0.74 | 0.8 | 0.78 | 3554 | Train 56 | Test Black 34 | 0.72 | 0.75 | 0.7 |
| 2531 | Train 40 | Test Black 35 | 0.73 | 0.79 | 0.78 | 3555 | Train 56 | Test Black 35 | 0.71 | 0.74 | 0.69 |
| 2532 | Train 40 | Test Black 36 | 0.74 | 0.8 | 0.78 | 3556 | Train 56 | Test Black 36 | 0.72 | 0.75 | 0.7 |
| 2533 | Train 40 | Test Black 37 | 0.72 | 0.78 | 0.76 | 3557 | Train 56 | Test Black 37 | 0.7 | 0.73 | 0.69 |
| 2534 | Train 40 | Test Black 38 | 0.73 | 0.78 | 0.77 | 3558 | Train 56 | Test Black 38 | 0.72 | 0.75 | 0.73 |
| 2535 | Train 40 | Test Black 39 | 0.72 | 0.78 | 0.76 | 3559 | Train 56 | Test Black 39 | 0.7 | 0.72 | 0.68 |
| 2536 | Train 40 | Test Black 40 | 0.73 | 0.78 | 0.77 | 3560 | Train 56 | Test Black 40 | 0.71 | 0.75 | 0.73 |
| 2537 | Train 40 | Test Black 41 | 0.73 | 0.8 | 0.78 | 3561 | Train 56 | Test Black 41 | 0.71 | 0.74 | 0.7 |
| 2538 | Train 40 | Test Black 42 | 0.74 | 0.8 | 0.79 | 3562 | Train 56 | Test Black 42 | 0.72 | 0.75 | 0.71 |
| 2539 | Train 40 | Test Black 43 | 0.73 | 0.8 | 0.78 | 3563 | Train 56 | Test Black 43 | 0.71 | 0.74 | 0.7 |
| 2540 | Train 40 | Test Black 44 | 0.74 | 0.8 | 0.79 | 3564 | Train 56 | Test Black 44 | 0.72 | 0.75 | 0.71 |
| 2541 | Train 40 | Test Black 45 | 0.72 | 0.78 | 0.77 | 3565 | Train 56 | Test Black 45 | 0.7 | 0.73 | 0.7 |
| 2542 | Train 40 | Test Black 46 | 0.73 | 0.78 | 0.77 | 3566 | Train 56 | Test Black 46 | 0.72 | 0.75 | 0.73 |
| 2543 | Train 40 | Test Black 47 | 0.72 | 0.78 | 0.76 | 3567 | Train 56 | Test Black 47 | 0.7 | 0.72 | 0.69 |
| 2544 | Train 40 | Test Black 48 | 0.73 | 0.78 | 0.77 | 3568 | Train 56 | Test Black 48 | 0.72 | 0.75 | 0.73 |
| 2545 | Train 40 | Test Black 49 | 0.76 | 0.8 | 0.78 | 3569 | Train 56 | Test Black 49 | 0.77 | 0.8 | 0.77 |
| 2546 | Train 40 | Test Black 50 | 0.76 | 0.8 | 0.78 | 3570 | Train 56 | Test Black 50 | 0.77 | 0.8 | 0.77 |
| 2547 | Train 40 | Test Black 51 | 0.76 | 0.8 | 0.78 | 3571 | Train 56 | Test Black 51 | 0.77 | 0.8 | 0.77 |
| 2548 | Train 40 | Test Black 52 | 0.76 | 0.8 | 0.77 | 3572 | Train 56 | Test Black 52 | 0.77 | 0.8 | 0.77 |
| 2549 | Train 40 | Test Black 53 | 0.75 | 0.8 | 0.77 | 3573 | Train 56 | Test Black 53 | 0.76 | 0.8 | 0.78 |
| 2550 | Train 40 | Test Black 54 | 0.74 | 0.78 | 0.78 | 3574 | Train 56 | Test Black 54 | 0.74 | 0.8 | 0.8 |
| 2551 | Train 40 | Test Black 55 | 0.75 | 0.8 | 0.77 | 3575 | Train 56 | Test Black 55 | 0.76 | 0.81 | 0.78 |
| 2552 | Train 40 | Test Black 56 | 0.74 | 0.78 | 0.78 | 3576 | Train 56 | Test Black 56 | 0.74 | 0.8 | 0.8 |
| 2553 | Train 40 | Test Black 57 | 0.76 | 0.8 | 0.79 | 3577 | Train 56 | Test Black 57 | 0.77 | 0.81 | 0.79 |
| 2554 | Train 40 | Test Black 58 | 0.76 | 0.8 | 0.79 | 3578 | Train 56 | Test Black 58 | 0.77 | 0.81 | 0.79 |
| 2555 | Train 40 | Test Black 59 | 0.76 | 0.8 | 0.79 | 3579 | Train 56 | Test Black 59 | 0.77 | 0.81 | 0.79 |
| 2556 | Train 40 | Test Black 60 | 0.76 | 0.8 | 0.79 | 3580 | Train 56 | Test Black 60 | 0.77 | 0.81 | 0.79 |
| 2557 | Train 40 | Test Black 61 | 0.75 | 0.8 | 0.78 | 3581 | Train 56 | Test Black 61 | 0.76 | 0.81 | 0.79 |
| 2558 | Train 40 | Test Black 62 | 0.74 | 0.78 | 0.78 | 3582 | Train 56 | Test Black 62 | 0.75 | 0.8 | 0.8 |
| 2559 | Train 40 | Test Black 63 | 0.75 | 0.8 | 0.78 | 3583 | Train 56 | Test Black 63 | 0.76 | 0.81 | 0.79 |
| 2560 | Train 40 | Test Black 64 | 0.74 | 0.78 | 0.78 | 3584 | Train 56 | Test Black 64 | 0.74 | 0.8 | 0.8 |
| 2561 | Train 41 | Test Black 1 | 0.76 | 0.85 | 0.83 | 3585 | Train 57 | Test Black 1 | 0.73 | 0.75 | 0.7 |
| 2562 | Train 41 | Test Black 2 | 0.76 | 0.83 | 0.81 | 3586 | Train 57 | Test Black 2 | 0.72 | 0.75 | 0.7 |
| 2563 | Train 41 | Test Black 3 | 0.76 | 0.85 | 0.82 | 3587 | Train 57 | Test Black 3 | 0.73 | 0.75 | 0.7 |
| 2564 | Train 41 | Test Black 4 | 0.76 | 0.83 | 0.81 | 3588 | Train 57 | Test Black 4 | 0.72 | 0.74 | 0.69 |
| 2565 | Train 41 | Test Black 5 | 0.73 | 0.76 | 0.75 | 3589 | Train 57 | Test Black 5 | 0.71 | 0.7 | 0.69 |
| 2566 | Train 41 | Test Black 6 | 0.71 | 0.71 | 0.67 | 3590 | Train 57 | Test Black 6 | 0.7 | 0.69 | 0.65 |
| 2567 | Train 41 | Test Black 7 | 0.73 | 0.76 | 0.74 | 3591 | Train 57 | Test Black 7 | 0.71 | 0.7 | 0.68 |
| 2568 | Train 41 | Test Black 8 | 0.71 | 0.71 | 0.67 | 3592 | Train 57 | Test Black 8 | 0.7 | 0.69 | 0.65 |
| 2569 | Train 41 | Test Black 9 | 0.76 | 0.86 | 0.86 | 3593 | Train 57 | Test Black 9 | 0.73 | 0.76 | 0.72 |
| 2570 | Train 41 | Test Black 10 | 0.76 | 0.84 | 0.84 | 3594 | Train 57 | Test Black 10 | 0.72 | 0.75 | 0.71 |
| 2571 | Train 41 | Test Black 11 | 0.76 | 0.86 | 0.86 | 3595 | Train 57 | Test Black 11 | 0.73 | 0.76 | 0.72 |
| 2572 | Train 41 | Test Black 12 | 0.76 | 0.84 | 0.84 | 3596 | Train 57 | Test Black 12 | 0.72 | 0.75 | 0.71 |
| 2573 | Train 41 | Test Black 13 | 0.73 | 0.77 | 0.77 | 3597 | Train 57 | Test Black 13 | 0.71 | 0.71 | 0.69 |
| 2574 | Train 41 | Test Black 14 | 0.71 | 0.72 | 0.67 | 3598 | Train 57 | Test Black 14 | 0.7 | 0.69 | 0.65 |
| 2575 | Train 41 | Test Black 15 | 0.73 | 0.76 | 0.76 | 3599 | Train 57 | Test Black 15 | 0.7 | 0.7 | 0.69 |
| 2576 | Train 41 | Test Black 16 | 0.71 | 0.72 | 0.67 | 3600 | Train 57 | Test Black 16 | 0.7 | 0.69 | 0.65 |
| 2577 | Train 41 | Test Black 17 | 0.76 | 0.85 | 0.83 | 3601 | Train 57 | Test Black 17 | 0.73 | 0.75 | 0.7 |
| 2578 | Train 41 | Test Black 18 | 0.76 | 0.83 | 0.81 | 3602 | Train 57 | Test Black 18 | 0.72 | 0.75 | 0.7 |
| 2579 | Train 41 | Test Black 19 | 0.76 | 0.85 | 0.82 | 3603 | Train 57 | Test Black 19 | 0.73 | 0.75 | 0.7 |
| 2580 | Train 41 | Test Black 20 | 0.76 | 0.83 | 0.81 | 3604 | Train 57 | Test Black 20 | 0.72 | 0.74 | 0.69 |
| 2581 | Train 41 | Test Black 21 | 0.73 | 0.76 | 0.75 | 3605 | Train 57 | Test Black 21 | 0.71 | 0.7 | 0.69 |
| 2582 | Train 41 | Test Black 22 | 0.71 | 0.71 | 0.67 | 3606 | Train 57 | Test Black 22 | 0.7 | 0.69 | 0.65 |
| 2583 | Train 41 | Test Black 23 | 0.73 | 0.76 | 0.74 | 3607 | Train 57 | Test Black 23 | 0.71 | 0.7 | 0.68 |
| 2584 | Train 41 | Test Black 24 | 0.71 | 0.71 | 0.67 | 3608 | Train 57 | Test Black 24 | 0.7 | 0.69 | 0.65 |
| 2585 | Train 41 | Test Black 25 | 0.76 | 0.86 | 0.86 | 3609 | Train 57 | Test Black 25 | 0.73 | 0.76 | 0.72 |
| 2586 | Train 41 | Test Black 26 | 0.76 | 0.84 | 0.84 | 3610 | Train 57 | Test Black 26 | 0.72 | 0.75 | 0.71 |
| 2587 | Train 41 | Test Black 27 | 0.76 | 0.86 | 0.86 | 3611 | Train 57 | Test Black 27 | 0.73 | 0.76 | 0.72 |
| 2588 | Train 41 | Test Black 28 | 0.76 | 0.84 | 0.83 | 3612 | Train 57 | Test Black 28 | 0.72 | 0.75 | 0.71 |
| 2589 | Train 41 | Test Black 29 | 0.73 | 0.77 | 0.77 | 3613 | Train 57 | Test Black 29 | 0.71 | 0.71 | 0.69 |
| 2590 | Train 41 | Test Black 30 | 0.71 | 0.72 | 0.67 | 3614 | Train 57 | Test Black 30 | 0.7 | 0.69 | 0.65 |
| 2591 | Train 41 | Test Black 31 | 0.73 | 0.76 | 0.76 | 3615 | Train 57 | Test Black 31 | 0.7 | 0.7 | 0.69 |
| 2592 | Train 41 | Test Black 32 | 0.71 | 0.72 | 0.67 | 3616 | Train 57 | Test Black 32 | 0.7 | 0.69 | 0.65 |
| 2593 | Train 41 | Test Black 33 | 0.76 | 0.85 | 0.82 | 3617 | Train 57 | Test Black 33 | 0.73 | 0.75 | 0.7 |
| 2594 | Train 41 | Test Black 34 | 0.76 | 0.83 | 0.81 | 3618 | Train 57 | Test Black 34 | 0.72 | 0.75 | 0.69 |
| 2595 | Train 41 | Test Black 35 | 0.76 | 0.85 | 0.82 | 3619 | Train 57 | Test Black 35 | 0.73 | 0.75 | 0.7 |
| 2596 | Train 41 | Test Black 36 | 0.76 | 0.83 | 0.81 | 3620 | Train 57 | Test Black 36 | 0.72 | 0.75 | 0.69 |
| 2597 | Train 41 | Test Black 37 | 0.73 | 0.76 | 0.75 | 3621 | Train 57 | Test Black 37 | 0.71 | 0.71 | 0.68 |
| 2598 | Train 41 | Test Black 38 | 0.71 | 0.71 | 0.67 | 3622 | Train 57 | Test Black 38 | 0.7 | 0.69 | 0.65 |
| 2599 | Train 41 | Test Black 39 | 0.73 | 0.76 | 0.74 | 3623 | Train 57 | Test Black 39 | 0.71 | 0.7 | 0.68 |
| 2600 | Train 41 | Test Black 40 | 0.71 | 0.71 | 0.67 | 3624 | Train 57 | Test Black 40 | 0.7 | 0.69 | 0.65 |
| 2601 | Train 41 | Test Black 41 | 0.76 | 0.86 | 0.86 | 3625 | Train 57 | Test Black 41 | 0.73 | 0.76 | 0.72 |
| 2602 | Train 41 | Test Black 42 | 0.76 | 0.84 | 0.84 | 3626 | Train 57 | Test Black 42 | 0.72 | 0.75 | 0.71 |
| 2603 | Train 41 | Test Black 43 | 0.76 | 0.86 | 0.86 | 3627 | Train 57 | Test Black 43 | 0.73 | 0.76 | 0.72 |
| 2604 | Train 41 | Test Black 44 | 0.76 | 0.84 | 0.84 | 3628 | Train 57 | Test Black 44 | 0.72 | 0.75 | 0.71 |
| 2605 | Train 41 | Test Black 45 | 0.73 | 0.77 | 0.77 | 3629 | Train 57 | Test Black 45 | 0.71 | 0.71 | 0.69 |
| 2606 | Train 41 | Test Black 46 | 0.71 | 0.72 | 0.67 | 3630 | Train 57 | Test Black 46 | 0.7 | 0.69 | 0.65 |
| 2607 | Train 41 | Test Black 47 | 0.73 | 0.76 | 0.76 | 3631 | Train 57 | Test Black 47 | 0.71 | 0.71 | 0.69 |
| 2608 | Train 41 | Test Black 48 | 0.71 | 0.72 | 0.67 | 3632 | Train 57 | Test Black 48 | 0.7 | 0.69 | 0.65 |
| 2609 | Train 41 | Test Black 49 | 0.77 | 0.82 | 0.8 | 3633 | Train 57 | Test Black 49 | 0.78 | 0.84 | 0.83 |
| 2610 | Train 41 | Test Black 50 | 0.77 | 0.82 | 0.8 | 3634 | Train 57 | Test Black 50 | 0.78 | 0.84 | 0.83 |
| 2611 | Train 41 | Test Black 51 | 0.77 | 0.82 | 0.8 | 3635 | Train 57 | Test Black 51 | 0.78 | 0.84 | 0.82 |
| 2612 | Train 41 | Test Black 52 | 0.77 | 0.82 | 0.8 | 3636 | Train 57 | Test Black 52 | 0.78 | 0.84 | 0.82 |
| 2613 | Train 41 | Test Black 53 | 0.76 | 0.79 | 0.78 | 3637 | Train 57 | Test Black 53 | 0.74 | 0.8 | 0.78 |
| 2614 | Train 41 | Test Black 54 | 0.71 | 0.71 | 0.66 | 3638 | Train 57 | Test Black 54 | 0.71 | 0.7 | 0.66 |
| 2615 | Train 41 | Test Black 55 | 0.76 | 0.79 | 0.78 | 3639 | Train 57 | Test Black 55 | 0.74 | 0.8 | 0.78 |
| 2616 | Train 41 | Test Black 56 | 0.71 | 0.71 | 0.66 | 3640 | Train 57 | Test Black 56 | 0.71 | 0.7 | 0.66 |
| 2617 | Train 41 | Test Black 57 | 0.77 | 0.83 | 0.84 | 3641 | Train 57 | Test Black 57 | 0.78 | 0.86 | 0.88 |
| 2618 | Train 41 | Test Black 58 | 0.77 | 0.83 | 0.84 | 3642 | Train 57 | Test Black 58 | 0.78 | 0.86 | 0.88 |
| 2619 | Train 41 | Test Black 59 | 0.77 | 0.82 | 0.83 | 3643 | Train 57 | Test Black 59 | 0.78 | 0.85 | 0.87 |
| 2620 | Train 41 | Test Black 60 | 0.77 | 0.83 | 0.83 | 3644 | Train 57 | Test Black 60 | 0.78 | 0.85 | 0.87 |
| 2621 | Train 41 | Test Black 61 | 0.76 | 0.79 | 0.79 | 3645 | Train 57 | Test Black 61 | 0.74 | 0.81 | 0.8 |
| 2622 | Train 41 | Test Black 62 | 0.71 | 0.71 | 0.66 | 3646 | Train 57 | Test Black 62 | 0.71 | 0.7 | 0.66 |
| 2623 | Train 41 | Test Black 63 | 0.76 | 0.79 | 0.79 | 3647 | Train 57 | Test Black 63 | 0.74 | 0.8 | 0.8 |
| 2624 | Train 41 | Test Black 64 | 0.71 | 0.71 | 0.66 | 3648 | Train 57 | Test Black 64 | 0.72 | 0.7 | 0.66 |
| 2625 | Train 42 | Test Black 1 | 0.75 | 0.84 | 0.82 | 3649 | Train 58 | Test Black 1 | 0.72 | 0.73 | 0.65 |
| 2626 | Train 42 | Test Black 2 | 0.76 | 0.85 | 0.82 | 3650 | Train 58 | Test Black 2 | 0.72 | 0.73 | 0.65 |
| 2627 | Train 42 | Test Black 3 | 0.75 | 0.84 | 0.82 | 3651 | Train 58 | Test Black 3 | 0.72 | 0.73 | 0.66 |
| 2628 | Train 42 | Test Black 4 | 0.76 | 0.85 | 0.82 | 3652 | Train 58 | Test Black 4 | 0.72 | 0.73 | 0.65 |
| 2629 | Train 42 | Test Black 5 | 0.73 | 0.76 | 0.75 | 3653 | Train 58 | Test Black 5 | 0.71 | 0.7 | 0.64 |
| 2630 | Train 42 | Test Black 6 | 0.71 | 0.73 | 0.7 | 3654 | Train 58 | Test Black 6 | 0.7 | 0.68 | 0.62 |
| 2631 | Train 42 | Test Black 7 | 0.73 | 0.76 | 0.74 | 3655 | Train 58 | Test Black 7 | 0.7 | 0.69 | 0.63 |
| 2632 | Train 42 | Test Black 8 | 0.71 | 0.73 | 0.7 | 3656 | Train 58 | Test Black 8 | 0.7 | 0.68 | 0.62 |
| 2633 | Train 42 | Test Black 9 | 0.75 | 0.85 | 0.85 | 3657 | Train 58 | Test Black 9 | 0.72 | 0.74 | 0.67 |
| 2634 | Train 42 | Test Black 10 | 0.76 | 0.85 | 0.85 | 3658 | Train 58 | Test Black 10 | 0.72 | 0.73 | 0.67 |
| 2635 | Train 42 | Test Black 11 | 0.75 | 0.85 | 0.85 | 3659 | Train 58 | Test Black 11 | 0.72 | 0.74 | 0.67 |
| 2636 | Train 42 | Test Black 12 | 0.76 | 0.85 | 0.85 | 3660 | Train 58 | Test Black 12 | 0.72 | 0.73 | 0.67 |
| 2637 | Train 42 | Test Black 13 | 0.73 | 0.77 | 0.76 | 3661 | Train 58 | Test Black 13 | 0.7 | 0.7 | 0.65 |
| 2638 | Train 42 | Test Black 14 | 0.71 | 0.73 | 0.7 | 3662 | Train 58 | Test Black 14 | 0.7 | 0.68 | 0.62 |
| 2639 | Train 42 | Test Black 15 | 0.73 | 0.76 | 0.76 | 3663 | Train 58 | Test Black 15 | 0.7 | 0.69 | 0.64 |
| 2640 | Train 42 | Test Black 16 | 0.71 | 0.73 | 0.69 | 3664 | Train 58 | Test Black 16 | 0.7 | 0.68 | 0.62 |
| 2641 | Train 42 | Test Black 17 | 0.75 | 0.84 | 0.82 | 3665 | Train 58 | Test Black 17 | 0.72 | 0.73 | 0.66 |
| 2642 | Train 42 | Test Black 18 | 0.76 | 0.85 | 0.82 | 3666 | Train 58 | Test Black 18 | 0.72 | 0.73 | 0.65 |
| 2643 | Train 42 | Test Black 19 | 0.75 | 0.84 | 0.82 | 3667 | Train 58 | Test Black 19 | 0.72 | 0.73 | 0.66 |
| 2644 | Train 42 | Test Black 20 | 0.76 | 0.85 | 0.82 | 3668 | Train 58 | Test Black 20 | 0.72 | 0.73 | 0.65 |
| 2645 | Train 42 | Test Black 21 | 0.73 | 0.76 | 0.75 | 3669 | Train 58 | Test Black 21 | 0.71 | 0.7 | 0.64 |
| 2646 | Train 42 | Test Black 22 | 0.71 | 0.73 | 0.7 | 3670 | Train 58 | Test Black 22 | 0.7 | 0.68 | 0.62 |
| 2647 | Train 42 | Test Black 23 | 0.73 | 0.76 | 0.74 | 3671 | Train 58 | Test Black 23 | 0.7 | 0.69 | 0.63 |
| 2648 | Train 42 | Test Black 24 | 0.71 | 0.73 | 0.7 | 3672 | Train 58 | Test Black 24 | 0.7 | 0.68 | 0.62 |
| 2649 | Train 42 | Test Black 25 | 0.75 | 0.85 | 0.85 | 3673 | Train 58 | Test Black 25 | 0.72 | 0.74 | 0.67 |
| 2650 | Train 42 | Test Black 26 | 0.76 | 0.85 | 0.85 | 3674 | Train 58 | Test Black 26 | 0.72 | 0.73 | 0.67 |
| 2651 | Train 42 | Test Black 27 | 0.75 | 0.85 | 0.85 | 3675 | Train 58 | Test Black 27 | 0.72 | 0.74 | 0.67 |
| 2652 | Train 42 | Test Black 28 | 0.76 | 0.85 | 0.85 | 3676 | Train 58 | Test Black 28 | 0.72 | 0.73 | 0.67 |
| 2653 | Train 42 | Test Black 29 | 0.73 | 0.77 | 0.76 | 3677 | Train 58 | Test Black 29 | 0.71 | 0.7 | 0.65 |
| 2654 | Train 42 | Test Black 30 | 0.71 | 0.73 | 0.7 | 3678 | Train 58 | Test Black 30 | 0.7 | 0.68 | 0.62 |
| 2655 | Train 42 | Test Black 31 | 0.73 | 0.76 | 0.76 | 3679 | Train 58 | Test Black 31 | 0.7 | 0.69 | 0.64 |
| 2656 | Train 42 | Test Black 32 | 0.71 | 0.73 | 0.7 | 3680 | Train 58 | Test Black 32 | 0.7 | 0.68 | 0.62 |
| 2657 | Train 42 | Test Black 33 | 0.75 | 0.84 | 0.82 | 3681 | Train 58 | Test Black 33 | 0.73 | 0.73 | 0.65 |
| 2658 | Train 42 | Test Black 34 | 0.75 | 0.85 | 0.82 | 3682 | Train 58 | Test Black 34 | 0.72 | 0.73 | 0.65 |
| 2659 | Train 42 | Test Black 35 | 0.75 | 0.84 | 0.82 | 3683 | Train 58 | Test Black 35 | 0.73 | 0.73 | 0.65 |
| 2660 | Train 42 | Test Black 36 | 0.76 | 0.85 | 0.82 | 3684 | Train 58 | Test Black 36 | 0.72 | 0.73 | 0.65 |
| 2661 | Train 42 | Test Black 37 | 0.73 | 0.76 | 0.75 | 3685 | Train 58 | Test Black 37 | 0.71 | 0.7 | 0.64 |
| 2662 | Train 42 | Test Black 38 | 0.71 | 0.73 | 0.7 | 3686 | Train 58 | Test Black 38 | 0.7 | 0.68 | 0.62 |
| 2663 | Train 42 | Test Black 39 | 0.73 | 0.76 | 0.74 | 3687 | Train 58 | Test Black 39 | 0.7 | 0.69 | 0.63 |
| 2664 | Train 42 | Test Black 40 | 0.71 | 0.73 | 0.7 | 3688 | Train 58 | Test Black 40 | 0.7 | 0.68 | 0.62 |
| 2665 | Train 42 | Test Black 41 | 0.75 | 0.85 | 0.85 | 3689 | Train 58 | Test Black 41 | 0.73 | 0.74 | 0.67 |
| 2666 | Train 42 | Test Black 42 | 0.75 | 0.86 | 0.85 | 3690 | Train 58 | Test Black 42 | 0.72 | 0.74 | 0.66 |
| 2667 | Train 42 | Test Black 43 | 0.75 | 0.85 | 0.85 | 3691 | Train 58 | Test Black 43 | 0.73 | 0.74 | 0.67 |
| 2668 | Train 42 | Test Black 44 | 0.76 | 0.85 | 0.85 | 3692 | Train 58 | Test Black 44 | 0.72 | 0.74 | 0.67 |
| 2669 | Train 42 | Test Black 45 | 0.73 | 0.77 | 0.76 | 3693 | Train 58 | Test Black 45 | 0.71 | 0.7 | 0.65 |
| 2670 | Train 42 | Test Black 46 | 0.71 | 0.73 | 0.7 | 3694 | Train 58 | Test Black 46 | 0.7 | 0.68 | 0.62 |
| 2671 | Train 42 | Test Black 47 | 0.73 | 0.76 | 0.76 | 3695 | Train 58 | Test Black 47 | 0.7 | 0.69 | 0.64 |
| 2672 | Train 42 | Test Black 48 | 0.71 | 0.73 | 0.7 | 3696 | Train 58 | Test Black 48 | 0.7 | 0.68 | 0.62 |
| 2673 | Train 42 | Test Black 49 | 0.77 | 0.81 | 0.77 | 3697 | Train 58 | Test Black 49 | 0.78 | 0.84 | 0.84 |
| 2674 | Train 42 | Test Black 50 | 0.77 | 0.81 | 0.77 | 3698 | Train 58 | Test Black 50 | 0.78 | 0.84 | 0.84 |
| 2675 | Train 42 | Test Black 51 | 0.77 | 0.81 | 0.77 | 3699 | Train 58 | Test Black 51 | 0.78 | 0.84 | 0.83 |
| 2676 | Train 42 | Test Black 52 | 0.77 | 0.81 | 0.77 | 3700 | Train 58 | Test Black 52 | 0.78 | 0.84 | 0.83 |
| 2677 | Train 42 | Test Black 53 | 0.75 | 0.79 | 0.72 | 3701 | Train 58 | Test Black 53 | 0.75 | 0.79 | 0.77 |
| 2678 | Train 42 | Test Black 54 | 0.71 | 0.72 | 0.68 | 3702 | Train 58 | Test Black 54 | 0.71 | 0.68 | 0.63 |
| 2679 | Train 42 | Test Black 55 | 0.75 | 0.79 | 0.72 | 3703 | Train 58 | Test Black 55 | 0.75 | 0.79 | 0.77 |
| 2680 | Train 42 | Test Black 56 | 0.71 | 0.72 | 0.68 | 3704 | Train 58 | Test Black 56 | 0.71 | 0.68 | 0.63 |
| 2681 | Train 42 | Test Black 57 | 0.77 | 0.82 | 0.81 | 3705 | Train 58 | Test Black 57 | 0.78 | 0.85 | 0.89 |
| 2682 | Train 42 | Test Black 58 | 0.77 | 0.82 | 0.81 | 3706 | Train 58 | Test Black 58 | 0.78 | 0.85 | 0.89 |
| 2683 | Train 42 | Test Black 59 | 0.77 | 0.82 | 0.8 | 3707 | Train 58 | Test Black 59 | 0.78 | 0.85 | 0.88 |
| 2684 | Train 42 | Test Black 60 | 0.77 | 0.82 | 0.8 | 3708 | Train 58 | Test Black 60 | 0.78 | 0.85 | 0.88 |
| 2685 | Train 42 | Test Black 61 | 0.75 | 0.79 | 0.74 | 3709 | Train 58 | Test Black 61 | 0.75 | 0.79 | 0.79 |
| 2686 | Train 42 | Test Black 62 | 0.71 | 0.72 | 0.68 | 3710 | Train 58 | Test Black 62 | 0.71 | 0.69 | 0.63 |
| 2687 | Train 42 | Test Black 63 | 0.75 | 0.79 | 0.74 | 3711 | Train 58 | Test Black 63 | 0.75 | 0.79 | 0.79 |
| 2688 | Train 42 | Test Black 64 | 0.71 | 0.72 | 0.68 | 3712 | Train 58 | Test Black 64 | 0.71 | 0.68 | 0.63 |
| 2689 | Train 43 | Test Black 1 | 0.76 | 0.85 | 0.83 | 3713 | Train 59 | Test Black 1 | 0.73 | 0.76 | 0.69 |
| 2690 | Train 43 | Test Black 2 | 0.76 | 0.83 | 0.81 | 3714 | Train 59 | Test Black 2 | 0.72 | 0.75 | 0.68 |
| 2691 | Train 43 | Test Black 3 | 0.76 | 0.85 | 0.83 | 3715 | Train 59 | Test Black 3 | 0.73 | 0.76 | 0.69 |
| 2692 | Train 43 | Test Black 4 | 0.76 | 0.83 | 0.81 | 3716 | Train 59 | Test Black 4 | 0.72 | 0.75 | 0.68 |
| 2693 | Train 43 | Test Black 5 | 0.73 | 0.76 | 0.73 | 3717 | Train 59 | Test Black 5 | 0.71 | 0.71 | 0.66 |
| 2694 | Train 43 | Test Black 6 | 0.71 | 0.71 | 0.69 | 3718 | Train 59 | Test Black 6 | 0.7 | 0.69 | 0.62 |
| 2695 | Train 43 | Test Black 7 | 0.73 | 0.76 | 0.73 | 3719 | Train 59 | Test Black 7 | 0.71 | 0.7 | 0.66 |
| 2696 | Train 43 | Test Black 8 | 0.71 | 0.71 | 0.69 | 3720 | Train 59 | Test Black 8 | 0.7 | 0.69 | 0.62 |
| 2697 | Train 43 | Test Black 9 | 0.76 | 0.86 | 0.86 | 3721 | Train 59 | Test Black 9 | 0.73 | 0.77 | 0.71 |
| 2698 | Train 43 | Test Black 10 | 0.76 | 0.84 | 0.84 | 3722 | Train 59 | Test Black 10 | 0.72 | 0.76 | 0.69 |
| 2699 | Train 43 | Test Black 11 | 0.76 | 0.86 | 0.86 | 3723 | Train 59 | Test Black 11 | 0.73 | 0.77 | 0.71 |
| 2700 | Train 43 | Test Black 12 | 0.76 | 0.84 | 0.84 | 3724 | Train 59 | Test Black 12 | 0.72 | 0.76 | 0.69 |
| 2701 | Train 43 | Test Black 13 | 0.73 | 0.77 | 0.75 | 3725 | Train 59 | Test Black 13 | 0.71 | 0.71 | 0.67 |
| 2702 | Train 43 | Test Black 14 | 0.71 | 0.72 | 0.69 | 3726 | Train 59 | Test Black 14 | 0.7 | 0.69 | 0.62 |
| 2703 | Train 43 | Test Black 15 | 0.73 | 0.76 | 0.75 | 3727 | Train 59 | Test Black 15 | 0.7 | 0.71 | 0.67 |
| 2704 | Train 43 | Test Black 16 | 0.71 | 0.72 | 0.69 | 3728 | Train 59 | Test Black 16 | 0.7 | 0.69 | 0.62 |
| 2705 | Train 43 | Test Black 17 | 0.76 | 0.85 | 0.83 | 3729 | Train 59 | Test Black 17 | 0.73 | 0.76 | 0.69 |
| 2706 | Train 43 | Test Black 18 | 0.76 | 0.83 | 0.81 | 3730 | Train 59 | Test Black 18 | 0.72 | 0.75 | 0.68 |
| 2707 | Train 43 | Test Black 19 | 0.76 | 0.85 | 0.83 | 3731 | Train 59 | Test Black 19 | 0.73 | 0.76 | 0.69 |
| 2708 | Train 43 | Test Black 20 | 0.76 | 0.83 | 0.81 | 3732 | Train 59 | Test Black 20 | 0.72 | 0.75 | 0.68 |
| 2709 | Train 43 | Test Black 21 | 0.73 | 0.76 | 0.73 | 3733 | Train 59 | Test Black 21 | 0.71 | 0.71 | 0.66 |
| 2710 | Train 43 | Test Black 22 | 0.71 | 0.71 | 0.69 | 3734 | Train 59 | Test Black 22 | 0.7 | 0.69 | 0.62 |
| 2711 | Train 43 | Test Black 23 | 0.73 | 0.76 | 0.73 | 3735 | Train 59 | Test Black 23 | 0.71 | 0.7 | 0.66 |
| 2712 | Train 43 | Test Black 24 | 0.71 | 0.71 | 0.69 | 3736 | Train 59 | Test Black 24 | 0.7 | 0.69 | 0.62 |
| 2713 | Train 43 | Test Black 25 | 0.76 | 0.86 | 0.86 | 3737 | Train 59 | Test Black 25 | 0.73 | 0.77 | 0.71 |
| 2714 | Train 43 | Test Black 26 | 0.76 | 0.84 | 0.84 | 3738 | Train 59 | Test Black 26 | 0.72 | 0.76 | 0.69 |
| 2715 | Train 43 | Test Black 27 | 0.76 | 0.86 | 0.86 | 3739 | Train 59 | Test Black 27 | 0.73 | 0.77 | 0.71 |
| 2716 | Train 43 | Test Black 28 | 0.76 | 0.84 | 0.84 | 3740 | Train 59 | Test Black 28 | 0.72 | 0.76 | 0.69 |
| 2717 | Train 43 | Test Black 29 | 0.73 | 0.77 | 0.75 | 3741 | Train 59 | Test Black 29 | 0.71 | 0.71 | 0.67 |
| 2718 | Train 43 | Test Black 30 | 0.71 | 0.72 | 0.69 | 3742 | Train 59 | Test Black 30 | 0.7 | 0.69 | 0.62 |
| 2719 | Train 43 | Test Black 31 | 0.73 | 0.76 | 0.75 | 3743 | Train 59 | Test Black 31 | 0.7 | 0.71 | 0.67 |
| 2720 | Train 43 | Test Black 32 | 0.71 | 0.72 | 0.69 | 3744 | Train 59 | Test Black 32 | 0.7 | 0.69 | 0.62 |
| 2721 | Train 43 | Test Black 33 | 0.76 | 0.85 | 0.83 | 3745 | Train 59 | Test Black 33 | 0.73 | 0.76 | 0.69 |
| 2722 | Train 43 | Test Black 34 | 0.76 | 0.83 | 0.81 | 3746 | Train 59 | Test Black 34 | 0.72 | 0.75 | 0.68 |
| 2723 | Train 43 | Test Black 35 | 0.76 | 0.85 | 0.83 | 3747 | Train 59 | Test Black 35 | 0.73 | 0.76 | 0.69 |
| 2724 | Train 43 | Test Black 36 | 0.76 | 0.83 | 0.81 | 3748 | Train 59 | Test Black 36 | 0.72 | 0.75 | 0.68 |
| 2725 | Train 43 | Test Black 37 | 0.73 | 0.76 | 0.73 | 3749 | Train 59 | Test Black 37 | 0.71 | 0.71 | 0.66 |
| 2726 | Train 43 | Test Black 38 | 0.71 | 0.71 | 0.69 | 3750 | Train 59 | Test Black 38 | 0.7 | 0.69 | 0.62 |
| 2727 | Train 43 | Test Black 39 | 0.73 | 0.76 | 0.73 | 3751 | Train 59 | Test Black 39 | 0.71 | 0.71 | 0.66 |
| 2728 | Train 43 | Test Black 40 | 0.71 | 0.71 | 0.69 | 3752 | Train 59 | Test Black 40 | 0.7 | 0.69 | 0.62 |
| 2729 | Train 43 | Test Black 41 | 0.76 | 0.86 | 0.86 | 3753 | Train 59 | Test Black 41 | 0.73 | 0.77 | 0.7 |
| 2730 | Train 43 | Test Black 42 | 0.76 | 0.84 | 0.84 | 3754 | Train 59 | Test Black 42 | 0.72 | 0.76 | 0.69 |
| 2731 | Train 43 | Test Black 43 | 0.76 | 0.86 | 0.86 | 3755 | Train 59 | Test Black 43 | 0.73 | 0.77 | 0.71 |
| 2732 | Train 43 | Test Black 44 | 0.76 | 0.84 | 0.84 | 3756 | Train 59 | Test Black 44 | 0.72 | 0.76 | 0.69 |
| 2733 | Train 43 | Test Black 45 | 0.73 | 0.77 | 0.76 | 3757 | Train 59 | Test Black 45 | 0.71 | 0.71 | 0.67 |
| 2734 | Train 43 | Test Black 46 | 0.71 | 0.72 | 0.69 | 3758 | Train 59 | Test Black 46 | 0.7 | 0.69 | 0.62 |
| 2735 | Train 43 | Test Black 47 | 0.73 | 0.76 | 0.75 | 3759 | Train 59 | Test Black 47 | 0.71 | 0.71 | 0.67 |
| 2736 | Train 43 | Test Black 48 | 0.71 | 0.72 | 0.69 | 3760 | Train 59 | Test Black 48 | 0.7 | 0.69 | 0.62 |
| 2737 | Train 43 | Test Black 49 | 0.77 | 0.82 | 0.79 | 3761 | Train 59 | Test Black 49 | 0.78 | 0.84 | 0.81 |
| 2738 | Train 43 | Test Black 50 | 0.77 | 0.82 | 0.79 | 3762 | Train 59 | Test Black 50 | 0.78 | 0.84 | 0.81 |
| 2739 | Train 43 | Test Black 51 | 0.77 | 0.82 | 0.79 | 3763 | Train 59 | Test Black 51 | 0.78 | 0.84 | 0.81 |
| 2740 | Train 43 | Test Black 52 | 0.77 | 0.82 | 0.79 | 3764 | Train 59 | Test Black 52 | 0.78 | 0.85 | 0.82 |
| 2741 | Train 43 | Test Black 53 | 0.76 | 0.79 | 0.77 | 3765 | Train 59 | Test Black 53 | 0.74 | 0.8 | 0.77 |
| 2742 | Train 43 | Test Black 54 | 0.71 | 0.71 | 0.67 | 3766 | Train 59 | Test Black 54 | 0.71 | 0.7 | 0.63 |
| 2743 | Train 43 | Test Black 55 | 0.76 | 0.79 | 0.77 | 3767 | Train 59 | Test Black 55 | 0.74 | 0.8 | 0.77 |
| 2744 | Train 43 | Test Black 56 | 0.71 | 0.71 | 0.67 | 3768 | Train 59 | Test Black 56 | 0.71 | 0.7 | 0.63 |
| 2745 | Train 43 | Test Black 57 | 0.77 | 0.83 | 0.83 | 3769 | Train 59 | Test Black 57 | 0.78 | 0.85 | 0.88 |
| 2746 | Train 43 | Test Black 58 | 0.77 | 0.83 | 0.83 | 3770 | Train 59 | Test Black 58 | 0.78 | 0.85 | 0.88 |
| 2747 | Train 43 | Test Black 59 | 0.77 | 0.83 | 0.83 | 3771 | Train 59 | Test Black 59 | 0.78 | 0.86 | 0.88 |
| 2748 | Train 43 | Test Black 60 | 0.77 | 0.83 | 0.83 | 3772 | Train 59 | Test Black 60 | 0.78 | 0.86 | 0.88 |
| 2749 | Train 43 | Test Black 61 | 0.75 | 0.79 | 0.78 | 3773 | Train 59 | Test Black 61 | 0.74 | 0.81 | 0.79 |
| 2750 | Train 43 | Test Black 62 | 0.71 | 0.71 | 0.68 | 3774 | Train 59 | Test Black 62 | 0.71 | 0.7 | 0.63 |
| 2751 | Train 43 | Test Black 63 | 0.76 | 0.79 | 0.78 | 3775 | Train 59 | Test Black 63 | 0.74 | 0.81 | 0.79 |
| 2752 | Train 43 | Test Black 64 | 0.71 | 0.71 | 0.67 | 3776 | Train 59 | Test Black 64 | 0.71 | 0.7 | 0.63 |
| 2753 | Train 44 | Test Black 1 | 0.75 | 0.84 | 0.82 | 3777 | Train 60 | Test Black 1 | 0.72 | 0.73 | 0.71 |
| 2754 | Train 44 | Test Black 2 | 0.76 | 0.85 | 0.82 | 3778 | Train 60 | Test Black 2 | 0.72 | 0.73 | 0.7 |
| 2755 | Train 44 | Test Black 3 | 0.75 | 0.84 | 0.82 | 3779 | Train 60 | Test Black 3 | 0.73 | 0.73 | 0.71 |
| 2756 | Train 44 | Test Black 4 | 0.76 | 0.85 | 0.82 | 3780 | Train 60 | Test Black 4 | 0.72 | 0.73 | 0.7 |
| 2757 | Train 44 | Test Black 5 | 0.73 | 0.76 | 0.75 | 3781 | Train 60 | Test Black 5 | 0.7 | 0.69 | 0.68 |
| 2758 | Train 44 | Test Black 6 | 0.71 | 0.73 | 0.69 | 3782 | Train 60 | Test Black 6 | 0.7 | 0.68 | 0.63 |
| 2759 | Train 44 | Test Black 7 | 0.73 | 0.76 | 0.74 | 3783 | Train 60 | Test Black 7 | 0.7 | 0.69 | 0.68 |
| 2760 | Train 44 | Test Black 8 | 0.71 | 0.72 | 0.69 | 3784 | Train 60 | Test Black 8 | 0.7 | 0.68 | 0.63 |
| 2761 | Train 44 | Test Black 9 | 0.75 | 0.85 | 0.85 | 3785 | Train 60 | Test Black 9 | 0.72 | 0.73 | 0.73 |
| 2762 | Train 44 | Test Black 10 | 0.76 | 0.85 | 0.85 | 3786 | Train 60 | Test Black 10 | 0.72 | 0.73 | 0.71 |
| 2763 | Train 44 | Test Black 11 | 0.75 | 0.85 | 0.84 | 3787 | Train 60 | Test Black 11 | 0.72 | 0.73 | 0.73 |
| 2764 | Train 44 | Test Black 12 | 0.76 | 0.85 | 0.85 | 3788 | Train 60 | Test Black 12 | 0.72 | 0.73 | 0.71 |
| 2765 | Train 44 | Test Black 13 | 0.73 | 0.77 | 0.77 | 3789 | Train 60 | Test Black 13 | 0.7 | 0.7 | 0.69 |
| 2766 | Train 44 | Test Black 14 | 0.71 | 0.73 | 0.7 | 3790 | Train 60 | Test Black 14 | 0.7 | 0.68 | 0.63 |
| 2767 | Train 44 | Test Black 15 | 0.73 | 0.76 | 0.76 | 3791 | Train 60 | Test Black 15 | 0.7 | 0.69 | 0.69 |
| 2768 | Train 44 | Test Black 16 | 0.71 | 0.73 | 0.7 | 3792 | Train 60 | Test Black 16 | 0.7 | 0.68 | 0.63 |
| 2769 | Train 44 | Test Black 17 | 0.75 | 0.84 | 0.82 | 3793 | Train 60 | Test Black 17 | 0.72 | 0.73 | 0.71 |
| 2770 | Train 44 | Test Black 18 | 0.76 | 0.85 | 0.82 | 3794 | Train 60 | Test Black 18 | 0.72 | 0.73 | 0.7 |
| 2771 | Train 44 | Test Black 19 | 0.75 | 0.84 | 0.82 | 3795 | Train 60 | Test Black 19 | 0.73 | 0.73 | 0.71 |
| 2772 | Train 44 | Test Black 20 | 0.76 | 0.85 | 0.82 | 3796 | Train 60 | Test Black 20 | 0.72 | 0.73 | 0.7 |
| 2773 | Train 44 | Test Black 21 | 0.73 | 0.76 | 0.75 | 3797 | Train 60 | Test Black 21 | 0.7 | 0.69 | 0.68 |
| 2774 | Train 44 | Test Black 22 | 0.71 | 0.73 | 0.69 | 3798 | Train 60 | Test Black 22 | 0.7 | 0.68 | 0.62 |
| 2775 | Train 44 | Test Black 23 | 0.73 | 0.76 | 0.74 | 3799 | Train 60 | Test Black 23 | 0.7 | 0.69 | 0.68 |
| 2776 | Train 44 | Test Black 24 | 0.71 | 0.72 | 0.69 | 3800 | Train 60 | Test Black 24 | 0.7 | 0.68 | 0.63 |
| 2777 | Train 44 | Test Black 25 | 0.75 | 0.85 | 0.85 | 3801 | Train 60 | Test Black 25 | 0.72 | 0.73 | 0.73 |
| 2778 | Train 44 | Test Black 26 | 0.76 | 0.85 | 0.85 | 3802 | Train 60 | Test Black 26 | 0.72 | 0.73 | 0.71 |
| 2779 | Train 44 | Test Black 27 | 0.75 | 0.85 | 0.84 | 3803 | Train 60 | Test Black 27 | 0.72 | 0.73 | 0.73 |
| 2780 | Train 44 | Test Black 28 | 0.76 | 0.85 | 0.85 | 3804 | Train 60 | Test Black 28 | 0.72 | 0.73 | 0.71 |
| 2781 | Train 44 | Test Black 29 | 0.73 | 0.77 | 0.77 | 3805 | Train 60 | Test Black 29 | 0.7 | 0.7 | 0.69 |
| 2782 | Train 44 | Test Black 30 | 0.71 | 0.73 | 0.7 | 3806 | Train 60 | Test Black 30 | 0.7 | 0.68 | 0.63 |
| 2783 | Train 44 | Test Black 31 | 0.73 | 0.76 | 0.76 | 3807 | Train 60 | Test Black 31 | 0.7 | 0.69 | 0.69 |
| 2784 | Train 44 | Test Black 32 | 0.71 | 0.73 | 0.7 | 3808 | Train 60 | Test Black 32 | 0.7 | 0.68 | 0.63 |
| 2785 | Train 44 | Test Black 33 | 0.75 | 0.84 | 0.82 | 3809 | Train 60 | Test Black 33 | 0.73 | 0.73 | 0.71 |
| 2786 | Train 44 | Test Black 34 | 0.75 | 0.85 | 0.82 | 3810 | Train 60 | Test Black 34 | 0.72 | 0.73 | 0.7 |
| 2787 | Train 44 | Test Black 35 | 0.75 | 0.84 | 0.82 | 3811 | Train 60 | Test Black 35 | 0.73 | 0.73 | 0.71 |
| 2788 | Train 44 | Test Black 36 | 0.76 | 0.85 | 0.82 | 3812 | Train 60 | Test Black 36 | 0.72 | 0.73 | 0.7 |
| 2789 | Train 44 | Test Black 37 | 0.73 | 0.76 | 0.75 | 3813 | Train 60 | Test Black 37 | 0.71 | 0.7 | 0.68 |
| 2790 | Train 44 | Test Black 38 | 0.71 | 0.73 | 0.69 | 3814 | Train 60 | Test Black 38 | 0.7 | 0.68 | 0.63 |
| 2791 | Train 44 | Test Black 39 | 0.73 | 0.76 | 0.75 | 3815 | Train 60 | Test Black 39 | 0.7 | 0.69 | 0.68 |
| 2792 | Train 44 | Test Black 40 | 0.71 | 0.72 | 0.69 | 3816 | Train 60 | Test Black 40 | 0.7 | 0.68 | 0.63 |
| 2793 | Train 44 | Test Black 41 | 0.75 | 0.85 | 0.85 | 3817 | Train 60 | Test Black 41 | 0.73 | 0.73 | 0.73 |
| 2794 | Train 44 | Test Black 42 | 0.75 | 0.86 | 0.85 | 3818 | Train 60 | Test Black 42 | 0.72 | 0.73 | 0.71 |
| 2795 | Train 44 | Test Black 43 | 0.75 | 0.85 | 0.85 | 3819 | Train 60 | Test Black 43 | 0.73 | 0.73 | 0.73 |
| 2796 | Train 44 | Test Black 44 | 0.76 | 0.86 | 0.85 | 3820 | Train 60 | Test Black 44 | 0.72 | 0.73 | 0.71 |
| 2797 | Train 44 | Test Black 45 | 0.73 | 0.77 | 0.77 | 3821 | Train 60 | Test Black 45 | 0.71 | 0.7 | 0.69 |
| 2798 | Train 44 | Test Black 46 | 0.71 | 0.73 | 0.7 | 3822 | Train 60 | Test Black 46 | 0.7 | 0.68 | 0.63 |
| 2799 | Train 44 | Test Black 47 | 0.73 | 0.76 | 0.76 | 3823 | Train 60 | Test Black 47 | 0.7 | 0.69 | 0.69 |
| 2800 | Train 44 | Test Black 48 | 0.71 | 0.73 | 0.7 | 3824 | Train 60 | Test Black 48 | 0.7 | 0.68 | 0.63 |
| 2801 | Train 44 | Test Black 49 | 0.77 | 0.81 | 0.8 | 3825 | Train 60 | Test Black 49 | 0.78 | 0.84 | 0.82 |
| 2802 | Train 44 | Test Black 50 | 0.77 | 0.81 | 0.8 | 3826 | Train 60 | Test Black 50 | 0.78 | 0.84 | 0.82 |
| 2803 | Train 44 | Test Black 51 | 0.77 | 0.81 | 0.8 | 3827 | Train 60 | Test Black 51 | 0.78 | 0.84 | 0.82 |
| 2804 | Train 44 | Test Black 52 | 0.77 | 0.81 | 0.8 | 3828 | Train 60 | Test Black 52 | 0.78 | 0.84 | 0.82 |
| 2805 | Train 44 | Test Black 53 | 0.75 | 0.79 | 0.78 | 3829 | Train 60 | Test Black 53 | 0.75 | 0.79 | 0.78 |
| 2806 | Train 44 | Test Black 54 | 0.71 | 0.72 | 0.67 | 3830 | Train 60 | Test Black 54 | 0.71 | 0.68 | 0.64 |
| 2807 | Train 44 | Test Black 55 | 0.75 | 0.79 | 0.78 | 3831 | Train 60 | Test Black 55 | 0.75 | 0.79 | 0.78 |
| 2808 | Train 44 | Test Black 56 | 0.71 | 0.72 | 0.67 | 3832 | Train 60 | Test Black 56 | 0.71 | 0.68 | 0.64 |
| 2809 | Train 44 | Test Black 57 | 0.77 | 0.82 | 0.83 | 3833 | Train 60 | Test Black 57 | 0.78 | 0.85 | 0.88 |
| 2810 | Train 44 | Test Black 58 | 0.77 | 0.82 | 0.83 | 3834 | Train 60 | Test Black 58 | 0.78 | 0.85 | 0.88 |
| 2811 | Train 44 | Test Black 59 | 0.77 | 0.82 | 0.82 | 3835 | Train 60 | Test Black 59 | 0.78 | 0.85 | 0.88 |
| 2812 | Train 44 | Test Black 60 | 0.77 | 0.82 | 0.82 | 3836 | Train 60 | Test Black 60 | 0.78 | 0.86 | 0.88 |
| 2813 | Train 44 | Test Black 61 | 0.75 | 0.79 | 0.78 | 3837 | Train 60 | Test Black 61 | 0.75 | 0.79 | 0.8 |
| 2814 | Train 44 | Test Black 62 | 0.71 | 0.72 | 0.68 | 3838 | Train 60 | Test Black 62 | 0.71 | 0.69 | 0.64 |
| 2815 | Train 44 | Test Black 63 | 0.75 | 0.8 | 0.78 | 3839 | Train 60 | Test Black 63 | 0.75 | 0.8 | 0.8 |
| 2816 | Train 44 | Test Black 64 | 0.71 | 0.72 | 0.68 | 3840 | Train 60 | Test Black 64 | 0.71 | 0.69 | 0.64 |
| 2817 | Train 45 | Test Black 1 | 0.75 | 0.82 | 0.81 | 3841 | Train 61 | Test Black 1 | 0.72 | 0.75 | 0.7 |
| 2818 | Train 45 | Test Black 2 | 0.75 | 0.82 | 0.8 | 3842 | Train 61 | Test Black 2 | 0.73 | 0.75 | 0.69 |
| 2819 | Train 45 | Test Black 3 | 0.75 | 0.82 | 0.81 | 3843 | Train 61 | Test Black 3 | 0.72 | 0.75 | 0.7 |
| 2820 | Train 45 | Test Black 4 | 0.75 | 0.82 | 0.8 | 3844 | Train 61 | Test Black 4 | 0.73 | 0.75 | 0.69 |
| 2821 | Train 45 | Test Black 5 | 0.74 | 0.81 | 0.8 | 3845 | Train 61 | Test Black 5 | 0.71 | 0.72 | 0.69 |
| 2822 | Train 45 | Test Black 6 | 0.7 | 0.74 | 0.71 | 3846 | Train 61 | Test Black 6 | 0.69 | 0.7 | 0.64 |
| 2823 | Train 45 | Test Black 7 | 0.73 | 0.81 | 0.79 | 3847 | Train 61 | Test Black 7 | 0.71 | 0.72 | 0.69 |
| 2824 | Train 45 | Test Black 8 | 0.7 | 0.73 | 0.71 | 3848 | Train 61 | Test Black 8 | 0.69 | 0.7 | 0.64 |
| 2825 | Train 45 | Test Black 9 | 0.75 | 0.83 | 0.83 | 3849 | Train 61 | Test Black 9 | 0.72 | 0.76 | 0.71 |
| 2826 | Train 45 | Test Black 10 | 0.75 | 0.82 | 0.82 | 3850 | Train 61 | Test Black 10 | 0.73 | 0.76 | 0.71 |
| 2827 | Train 45 | Test Black 11 | 0.75 | 0.83 | 0.83 | 3851 | Train 61 | Test Black 11 | 0.72 | 0.76 | 0.71 |
| 2828 | Train 45 | Test Black 12 | 0.75 | 0.82 | 0.82 | 3852 | Train 61 | Test Black 12 | 0.73 | 0.76 | 0.7 |
| 2829 | Train 45 | Test Black 13 | 0.73 | 0.82 | 0.82 | 3853 | Train 61 | Test Black 13 | 0.71 | 0.73 | 0.7 |
| 2830 | Train 45 | Test Black 14 | 0.7 | 0.74 | 0.71 | 3854 | Train 61 | Test Black 14 | 0.7 | 0.7 | 0.64 |
| 2831 | Train 45 | Test Black 15 | 0.73 | 0.81 | 0.81 | 3855 | Train 61 | Test Black 15 | 0.71 | 0.73 | 0.7 |
| 2832 | Train 45 | Test Black 16 | 0.71 | 0.74 | 0.71 | 3856 | Train 61 | Test Black 16 | 0.69 | 0.7 | 0.64 |
| 2833 | Train 45 | Test Black 17 | 0.75 | 0.82 | 0.81 | 3857 | Train 61 | Test Black 17 | 0.72 | 0.75 | 0.7 |
| 2834 | Train 45 | Test Black 18 | 0.75 | 0.82 | 0.8 | 3858 | Train 61 | Test Black 18 | 0.73 | 0.75 | 0.69 |
| 2835 | Train 45 | Test Black 19 | 0.75 | 0.82 | 0.81 | 3859 | Train 61 | Test Black 19 | 0.72 | 0.75 | 0.7 |
| 2836 | Train 45 | Test Black 20 | 0.75 | 0.82 | 0.8 | 3860 | Train 61 | Test Black 20 | 0.73 | 0.75 | 0.69 |
| 2837 | Train 45 | Test Black 21 | 0.74 | 0.81 | 0.8 | 3861 | Train 61 | Test Black 21 | 0.71 | 0.72 | 0.69 |
| 2838 | Train 45 | Test Black 22 | 0.7 | 0.74 | 0.71 | 3862 | Train 61 | Test Black 22 | 0.69 | 0.7 | 0.64 |
| 2839 | Train 45 | Test Black 23 | 0.73 | 0.81 | 0.79 | 3863 | Train 61 | Test Black 23 | 0.71 | 0.72 | 0.69 |
| 2840 | Train 45 | Test Black 24 | 0.7 | 0.74 | 0.71 | 3864 | Train 61 | Test Black 24 | 0.69 | 0.7 | 0.64 |
| 2841 | Train 45 | Test Black 25 | 0.75 | 0.83 | 0.83 | 3865 | Train 61 | Test Black 25 | 0.72 | 0.76 | 0.71 |
| 2842 | Train 45 | Test Black 26 | 0.75 | 0.82 | 0.82 | 3866 | Train 61 | Test Black 26 | 0.73 | 0.76 | 0.71 |
| 2843 | Train 45 | Test Black 27 | 0.75 | 0.83 | 0.83 | 3867 | Train 61 | Test Black 27 | 0.72 | 0.76 | 0.71 |
| 2844 | Train 45 | Test Black 28 | 0.75 | 0.82 | 0.82 | 3868 | Train 61 | Test Black 28 | 0.73 | 0.76 | 0.71 |
| 2845 | Train 45 | Test Black 29 | 0.73 | 0.82 | 0.82 | 3869 | Train 61 | Test Black 29 | 0.71 | 0.73 | 0.7 |
| 2846 | Train 45 | Test Black 30 | 0.71 | 0.74 | 0.71 | 3870 | Train 61 | Test Black 30 | 0.7 | 0.7 | 0.64 |
| 2847 | Train 45 | Test Black 31 | 0.73 | 0.81 | 0.81 | 3871 | Train 61 | Test Black 31 | 0.71 | 0.73 | 0.7 |
| 2848 | Train 45 | Test Black 32 | 0.71 | 0.74 | 0.71 | 3872 | Train 61 | Test Black 32 | 0.7 | 0.7 | 0.64 |
| 2849 | Train 45 | Test Black 33 | 0.75 | 0.82 | 0.81 | 3873 | Train 61 | Test Black 33 | 0.72 | 0.75 | 0.7 |
| 2850 | Train 45 | Test Black 34 | 0.75 | 0.82 | 0.8 | 3874 | Train 61 | Test Black 34 | 0.73 | 0.75 | 0.69 |
| 2851 | Train 45 | Test Black 35 | 0.75 | 0.82 | 0.81 | 3875 | Train 61 | Test Black 35 | 0.72 | 0.75 | 0.7 |
| 2852 | Train 45 | Test Black 36 | 0.75 | 0.82 | 0.8 | 3876 | Train 61 | Test Black 36 | 0.73 | 0.75 | 0.69 |
| 2853 | Train 45 | Test Black 37 | 0.74 | 0.81 | 0.8 | 3877 | Train 61 | Test Black 37 | 0.71 | 0.72 | 0.69 |
| 2854 | Train 45 | Test Black 38 | 0.7 | 0.74 | 0.71 | 3878 | Train 61 | Test Black 38 | 0.69 | 0.7 | 0.64 |
| 2855 | Train 45 | Test Black 39 | 0.73 | 0.81 | 0.79 | 3879 | Train 61 | Test Black 39 | 0.71 | 0.72 | 0.69 |
| 2856 | Train 45 | Test Black 40 | 0.7 | 0.74 | 0.71 | 3880 | Train 61 | Test Black 40 | 0.69 | 0.7 | 0.64 |
| 2857 | Train 45 | Test Black 41 | 0.75 | 0.83 | 0.83 | 3881 | Train 61 | Test Black 41 | 0.72 | 0.76 | 0.71 |
| 2858 | Train 45 | Test Black 42 | 0.75 | 0.82 | 0.82 | 3882 | Train 61 | Test Black 42 | 0.73 | 0.76 | 0.71 |
| 2859 | Train 45 | Test Black 43 | 0.75 | 0.83 | 0.83 | 3883 | Train 61 | Test Black 43 | 0.72 | 0.76 | 0.71 |
| 2860 | Train 45 | Test Black 44 | 0.75 | 0.82 | 0.82 | 3884 | Train 61 | Test Black 44 | 0.73 | 0.76 | 0.71 |
| 2861 | Train 45 | Test Black 45 | 0.74 | 0.82 | 0.82 | 3885 | Train 61 | Test Black 45 | 0.71 | 0.73 | 0.71 |
| 2862 | Train 45 | Test Black 46 | 0.7 | 0.74 | 0.71 | 3886 | Train 61 | Test Black 46 | 0.7 | 0.7 | 0.64 |
| 2863 | Train 45 | Test Black 47 | 0.73 | 0.81 | 0.81 | 3887 | Train 61 | Test Black 47 | 0.71 | 0.73 | 0.7 |
| 2864 | Train 45 | Test Black 48 | 0.71 | 0.74 | 0.71 | 3888 | Train 61 | Test Black 48 | 0.7 | 0.7 | 0.64 |
| 2865 | Train 45 | Test Black 49 | 0.77 | 0.8 | 0.78 | 3889 | Train 61 | Test Black 49 | 0.77 | 0.81 | 0.79 |
| 2866 | Train 45 | Test Black 50 | 0.76 | 0.8 | 0.78 | 3890 | Train 61 | Test Black 50 | 0.77 | 0.81 | 0.79 |
| 2867 | Train 45 | Test Black 51 | 0.77 | 0.8 | 0.78 | 3891 | Train 61 | Test Black 51 | 0.77 | 0.81 | 0.79 |
| 2868 | Train 45 | Test Black 52 | 0.77 | 0.8 | 0.77 | 3892 | Train 61 | Test Black 52 | 0.77 | 0.81 | 0.79 |
| 2869 | Train 45 | Test Black 53 | 0.76 | 0.81 | 0.79 | 3893 | Train 61 | Test Black 53 | 0.77 | 0.82 | 0.81 |
| 2870 | Train 45 | Test Black 54 | 0.71 | 0.73 | 0.69 | 3894 | Train 61 | Test Black 54 | 0.71 | 0.72 | 0.66 |
| 2871 | Train 45 | Test Black 55 | 0.76 | 0.8 | 0.79 | 3895 | Train 61 | Test Black 55 | 0.77 | 0.82 | 0.8 |
| 2872 | Train 45 | Test Black 56 | 0.71 | 0.72 | 0.69 | 3896 | Train 61 | Test Black 56 | 0.71 | 0.71 | 0.66 |
| 2873 | Train 45 | Test Black 57 | 0.76 | 0.8 | 0.81 | 3897 | Train 61 | Test Black 57 | 0.77 | 0.82 | 0.82 |
| 2874 | Train 45 | Test Black 58 | 0.76 | 0.8 | 0.81 | 3898 | Train 61 | Test Black 58 | 0.77 | 0.82 | 0.82 |
| 2875 | Train 45 | Test Black 59 | 0.76 | 0.8 | 0.81 | 3899 | Train 61 | Test Black 59 | 0.77 | 0.82 | 0.82 |
| 2876 | Train 45 | Test Black 60 | 0.76 | 0.8 | 0.81 | 3900 | Train 61 | Test Black 60 | 0.77 | 0.82 | 0.82 |
| 2877 | Train 45 | Test Black 61 | 0.76 | 0.81 | 0.81 | 3901 | Train 61 | Test Black 61 | 0.77 | 0.83 | 0.83 |
| 2878 | Train 45 | Test Black 62 | 0.71 | 0.73 | 0.69 | 3902 | Train 61 | Test Black 62 | 0.71 | 0.72 | 0.66 |
| 2879 | Train 45 | Test Black 63 | 0.76 | 0.81 | 0.81 | 3903 | Train 61 | Test Black 63 | 0.77 | 0.83 | 0.83 |
| 2880 | Train 45 | Test Black 64 | 0.71 | 0.73 | 0.69 | 3904 | Train 61 | Test Black 64 | 0.71 | 0.72 | 0.66 |
| 2881 | Train 46 | Test Black 1 | 0.73 | 0.79 | 0.77 | 3905 | Train 62 | Test Black 1 | 0.71 | 0.74 | 0.7 |
| 2882 | Train 46 | Test Black 2 | 0.74 | 0.79 | 0.77 | 3906 | Train 62 | Test Black 2 | 0.72 | 0.74 | 0.7 |
| 2883 | Train 46 | Test Black 3 | 0.73 | 0.79 | 0.77 | 3907 | Train 62 | Test Black 3 | 0.71 | 0.74 | 0.7 |
| 2884 | Train 46 | Test Black 4 | 0.74 | 0.79 | 0.77 | 3908 | Train 62 | Test Black 4 | 0.72 | 0.74 | 0.7 |
| 2885 | Train 46 | Test Black 5 | 0.72 | 0.78 | 0.76 | 3909 | Train 62 | Test Black 5 | 0.7 | 0.72 | 0.7 |
| 2886 | Train 46 | Test Black 6 | 0.73 | 0.78 | 0.76 | 3910 | Train 62 | Test Black 6 | 0.72 | 0.75 | 0.72 |
| 2887 | Train 46 | Test Black 7 | 0.72 | 0.77 | 0.75 | 3911 | Train 62 | Test Black 7 | 0.7 | 0.71 | 0.69 |
| 2888 | Train 46 | Test Black 8 | 0.73 | 0.77 | 0.75 | 3912 | Train 62 | Test Black 8 | 0.72 | 0.75 | 0.72 |
| 2889 | Train 46 | Test Black 9 | 0.73 | 0.8 | 0.78 | 3913 | Train 62 | Test Black 9 | 0.71 | 0.74 | 0.71 |
| 2890 | Train 46 | Test Black 10 | 0.74 | 0.8 | 0.78 | 3914 | Train 62 | Test Black 10 | 0.72 | 0.75 | 0.71 |
| 2891 | Train 46 | Test Black 11 | 0.73 | 0.8 | 0.78 | 3915 | Train 62 | Test Black 11 | 0.71 | 0.74 | 0.71 |
| 2892 | Train 46 | Test Black 12 | 0.74 | 0.8 | 0.78 | 3916 | Train 62 | Test Black 12 | 0.72 | 0.75 | 0.71 |
| 2893 | Train 46 | Test Black 13 | 0.72 | 0.78 | 0.76 | 3917 | Train 62 | Test Black 13 | 0.7 | 0.72 | 0.71 |
| 2894 | Train 46 | Test Black 14 | 0.73 | 0.78 | 0.77 | 3918 | Train 62 | Test Black 14 | 0.72 | 0.75 | 0.73 |
| 2895 | Train 46 | Test Black 15 | 0.72 | 0.78 | 0.76 | 3919 | Train 62 | Test Black 15 | 0.7 | 0.72 | 0.7 |
| 2896 | Train 46 | Test Black 16 | 0.73 | 0.78 | 0.76 | 3920 | Train 62 | Test Black 16 | 0.72 | 0.75 | 0.73 |
| 2897 | Train 46 | Test Black 17 | 0.73 | 0.79 | 0.77 | 3921 | Train 62 | Test Black 17 | 0.71 | 0.74 | 0.7 |
| 2898 | Train 46 | Test Black 18 | 0.74 | 0.79 | 0.77 | 3922 | Train 62 | Test Black 18 | 0.72 | 0.74 | 0.7 |
| 2899 | Train 46 | Test Black 19 | 0.73 | 0.79 | 0.77 | 3923 | Train 62 | Test Black 19 | 0.71 | 0.74 | 0.7 |
| 2900 | Train 46 | Test Black 20 | 0.74 | 0.79 | 0.77 | 3924 | Train 62 | Test Black 20 | 0.72 | 0.74 | 0.7 |
| 2901 | Train 46 | Test Black 21 | 0.72 | 0.78 | 0.76 | 3925 | Train 62 | Test Black 21 | 0.7 | 0.72 | 0.7 |
| 2902 | Train 46 | Test Black 22 | 0.73 | 0.78 | 0.76 | 3926 | Train 62 | Test Black 22 | 0.72 | 0.75 | 0.72 |
| 2903 | Train 46 | Test Black 23 | 0.72 | 0.77 | 0.75 | 3927 | Train 62 | Test Black 23 | 0.7 | 0.71 | 0.69 |
| 2904 | Train 46 | Test Black 24 | 0.73 | 0.78 | 0.75 | 3928 | Train 62 | Test Black 24 | 0.72 | 0.75 | 0.72 |
| 2905 | Train 46 | Test Black 25 | 0.73 | 0.8 | 0.78 | 3929 | Train 62 | Test Black 25 | 0.71 | 0.74 | 0.71 |
| 2906 | Train 46 | Test Black 26 | 0.74 | 0.8 | 0.78 | 3930 | Train 62 | Test Black 26 | 0.72 | 0.75 | 0.71 |
| 2907 | Train 46 | Test Black 27 | 0.73 | 0.8 | 0.78 | 3931 | Train 62 | Test Black 27 | 0.71 | 0.74 | 0.71 |
| 2908 | Train 46 | Test Black 28 | 0.74 | 0.8 | 0.78 | 3932 | Train 62 | Test Black 28 | 0.72 | 0.75 | 0.71 |
| 2909 | Train 46 | Test Black 29 | 0.72 | 0.78 | 0.76 | 3933 | Train 62 | Test Black 29 | 0.7 | 0.72 | 0.71 |
| 2910 | Train 46 | Test Black 30 | 0.73 | 0.78 | 0.77 | 3934 | Train 62 | Test Black 30 | 0.72 | 0.75 | 0.73 |
| 2911 | Train 46 | Test Black 31 | 0.72 | 0.78 | 0.76 | 3935 | Train 62 | Test Black 31 | 0.7 | 0.72 | 0.7 |
| 2912 | Train 46 | Test Black 32 | 0.73 | 0.78 | 0.76 | 3936 | Train 62 | Test Black 32 | 0.72 | 0.75 | 0.73 |
| 2913 | Train 46 | Test Black 33 | 0.73 | 0.79 | 0.77 | 3937 | Train 62 | Test Black 33 | 0.71 | 0.74 | 0.7 |
| 2914 | Train 46 | Test Black 34 | 0.74 | 0.79 | 0.77 | 3938 | Train 62 | Test Black 34 | 0.72 | 0.74 | 0.7 |
| 2915 | Train 46 | Test Black 35 | 0.73 | 0.79 | 0.77 | 3939 | Train 62 | Test Black 35 | 0.71 | 0.74 | 0.7 |
| 2916 | Train 46 | Test Black 36 | 0.74 | 0.79 | 0.77 | 3940 | Train 62 | Test Black 36 | 0.72 | 0.74 | 0.7 |
| 2917 | Train 46 | Test Black 37 | 0.72 | 0.78 | 0.76 | 3941 | Train 62 | Test Black 37 | 0.7 | 0.72 | 0.7 |
| 2918 | Train 46 | Test Black 38 | 0.73 | 0.78 | 0.76 | 3942 | Train 62 | Test Black 38 | 0.72 | 0.75 | 0.72 |
| 2919 | Train 46 | Test Black 39 | 0.72 | 0.77 | 0.75 | 3943 | Train 62 | Test Black 39 | 0.7 | 0.72 | 0.7 |
| 2920 | Train 46 | Test Black 40 | 0.73 | 0.78 | 0.75 | 3944 | Train 62 | Test Black 40 | 0.72 | 0.75 | 0.72 |
| 2921 | Train 46 | Test Black 41 | 0.73 | 0.8 | 0.78 | 3945 | Train 62 | Test Black 41 | 0.71 | 0.74 | 0.71 |
| 2922 | Train 46 | Test Black 42 | 0.74 | 0.8 | 0.78 | 3946 | Train 62 | Test Black 42 | 0.72 | 0.75 | 0.71 |
| 2923 | Train 46 | Test Black 43 | 0.73 | 0.8 | 0.78 | 3947 | Train 62 | Test Black 43 | 0.71 | 0.74 | 0.71 |
| 2924 | Train 46 | Test Black 44 | 0.74 | 0.8 | 0.78 | 3948 | Train 62 | Test Black 44 | 0.72 | 0.75 | 0.71 |
| 2925 | Train 46 | Test Black 45 | 0.72 | 0.78 | 0.76 | 3949 | Train 62 | Test Black 45 | 0.7 | 0.72 | 0.71 |
| 2926 | Train 46 | Test Black 46 | 0.73 | 0.78 | 0.77 | 3950 | Train 62 | Test Black 46 | 0.72 | 0.75 | 0.73 |
| 2927 | Train 46 | Test Black 47 | 0.72 | 0.78 | 0.76 | 3951 | Train 62 | Test Black 47 | 0.7 | 0.72 | 0.7 |
| 2928 | Train 46 | Test Black 48 | 0.73 | 0.78 | 0.76 | 3952 | Train 62 | Test Black 48 | 0.72 | 0.75 | 0.73 |
| 2929 | Train 46 | Test Black 49 | 0.76 | 0.8 | 0.78 | 3953 | Train 62 | Test Black 49 | 0.77 | 0.8 | 0.76 |
| 2930 | Train 46 | Test Black 50 | 0.76 | 0.8 | 0.78 | 3954 | Train 62 | Test Black 50 | 0.77 | 0.8 | 0.76 |
| 2931 | Train 46 | Test Black 51 | 0.76 | 0.8 | 0.78 | 3955 | Train 62 | Test Black 51 | 0.77 | 0.8 | 0.76 |
| 2932 | Train 46 | Test Black 52 | 0.76 | 0.8 | 0.78 | 3956 | Train 62 | Test Black 52 | 0.77 | 0.8 | 0.76 |
| 2933 | Train 46 | Test Black 53 | 0.76 | 0.8 | 0.77 | 3957 | Train 62 | Test Black 53 | 0.76 | 0.81 | 0.78 |
| 2934 | Train 46 | Test Black 54 | 0.74 | 0.78 | 0.78 | 3958 | Train 62 | Test Black 54 | 0.75 | 0.8 | 0.81 |
| 2935 | Train 46 | Test Black 55 | 0.75 | 0.8 | 0.77 | 3959 | Train 62 | Test Black 55 | 0.76 | 0.81 | 0.78 |
| 2936 | Train 46 | Test Black 56 | 0.74 | 0.78 | 0.78 | 3960 | Train 62 | Test Black 56 | 0.74 | 0.8 | 0.8 |
| 2937 | Train 46 | Test Black 57 | 0.76 | 0.81 | 0.79 | 3961 | Train 62 | Test Black 57 | 0.77 | 0.81 | 0.78 |
| 2938 | Train 46 | Test Black 58 | 0.76 | 0.81 | 0.79 | 3962 | Train 62 | Test Black 58 | 0.77 | 0.81 | 0.78 |
| 2939 | Train 46 | Test Black 59 | 0.76 | 0.8 | 0.79 | 3963 | Train 62 | Test Black 59 | 0.77 | 0.81 | 0.78 |
| 2940 | Train 46 | Test Black 60 | 0.76 | 0.8 | 0.79 | 3964 | Train 62 | Test Black 60 | 0.77 | 0.81 | 0.78 |
| 2941 | Train 46 | Test Black 61 | 0.75 | 0.8 | 0.78 | 3965 | Train 62 | Test Black 61 | 0.76 | 0.81 | 0.8 |
| 2942 | Train 46 | Test Black 62 | 0.74 | 0.78 | 0.78 | 3966 | Train 62 | Test Black 62 | 0.75 | 0.8 | 0.81 |
| 2943 | Train 46 | Test Black 63 | 0.75 | 0.8 | 0.78 | 3967 | Train 62 | Test Black 63 | 0.76 | 0.81 | 0.79 |
| 2944 | Train 46 | Test Black 64 | 0.74 | 0.78 | 0.78 | 3968 | Train 62 | Test Black 64 | 0.75 | 0.8 | 0.8 |
| 2945 | Train 47 | Test Black 1 | 0.75 | 0.82 | 0.81 | 3969 | Train 63 | Test Black 1 | 0.73 | 0.75 | 0.7 |
| 2946 | Train 47 | Test Black 2 | 0.75 | 0.82 | 0.8 | 3970 | Train 63 | Test Black 2 | 0.73 | 0.75 | 0.69 |
| 2947 | Train 47 | Test Black 3 | 0.75 | 0.82 | 0.81 | 3971 | Train 63 | Test Black 3 | 0.73 | 0.75 | 0.7 |
| 2948 | Train 47 | Test Black 4 | 0.75 | 0.82 | 0.8 | 3972 | Train 63 | Test Black 4 | 0.73 | 0.75 | 0.69 |
| 2949 | Train 47 | Test Black 5 | 0.74 | 0.81 | 0.8 | 3973 | Train 63 | Test Black 5 | 0.71 | 0.72 | 0.69 |
| 2950 | Train 47 | Test Black 6 | 0.7 | 0.74 | 0.7 | 3974 | Train 63 | Test Black 6 | 0.69 | 0.69 | 0.64 |
| 2951 | Train 47 | Test Black 7 | 0.73 | 0.81 | 0.8 | 3975 | Train 63 | Test Black 7 | 0.71 | 0.72 | 0.69 |
| 2952 | Train 47 | Test Black 8 | 0.7 | 0.74 | 0.7 | 3976 | Train 63 | Test Black 8 | 0.69 | 0.69 | 0.64 |
| 2953 | Train 47 | Test Black 9 | 0.75 | 0.83 | 0.83 | 3977 | Train 63 | Test Black 9 | 0.72 | 0.76 | 0.71 |
| 2954 | Train 47 | Test Black 10 | 0.75 | 0.82 | 0.82 | 3978 | Train 63 | Test Black 10 | 0.73 | 0.75 | 0.71 |
| 2955 | Train 47 | Test Black 11 | 0.75 | 0.83 | 0.83 | 3979 | Train 63 | Test Black 11 | 0.72 | 0.76 | 0.71 |
| 2956 | Train 47 | Test Black 12 | 0.75 | 0.82 | 0.82 | 3980 | Train 63 | Test Black 12 | 0.73 | 0.76 | 0.71 |
| 2957 | Train 47 | Test Black 13 | 0.73 | 0.82 | 0.82 | 3981 | Train 63 | Test Black 13 | 0.71 | 0.73 | 0.71 |
| 2958 | Train 47 | Test Black 14 | 0.7 | 0.74 | 0.7 | 3982 | Train 63 | Test Black 14 | 0.7 | 0.7 | 0.64 |
| 2959 | Train 47 | Test Black 15 | 0.73 | 0.81 | 0.82 | 3983 | Train 63 | Test Black 15 | 0.71 | 0.72 | 0.71 |
| 2960 | Train 47 | Test Black 16 | 0.7 | 0.74 | 0.7 | 3984 | Train 63 | Test Black 16 | 0.69 | 0.7 | 0.64 |
| 2961 | Train 47 | Test Black 17 | 0.75 | 0.82 | 0.81 | 3985 | Train 63 | Test Black 17 | 0.73 | 0.75 | 0.7 |
| 2962 | Train 47 | Test Black 18 | 0.75 | 0.82 | 0.8 | 3986 | Train 63 | Test Black 18 | 0.73 | 0.75 | 0.69 |
| 2963 | Train 47 | Test Black 19 | 0.75 | 0.82 | 0.81 | 3987 | Train 63 | Test Black 19 | 0.73 | 0.75 | 0.7 |
| 2964 | Train 47 | Test Black 20 | 0.75 | 0.82 | 0.8 | 3988 | Train 63 | Test Black 20 | 0.73 | 0.75 | 0.69 |
| 2965 | Train 47 | Test Black 21 | 0.74 | 0.81 | 0.8 | 3989 | Train 63 | Test Black 21 | 0.71 | 0.72 | 0.69 |
| 2966 | Train 47 | Test Black 22 | 0.7 | 0.74 | 0.7 | 3990 | Train 63 | Test Black 22 | 0.69 | 0.7 | 0.64 |
| 2967 | Train 47 | Test Black 23 | 0.73 | 0.81 | 0.8 | 3991 | Train 63 | Test Black 23 | 0.71 | 0.72 | 0.69 |
| 2968 | Train 47 | Test Black 24 | 0.7 | 0.74 | 0.7 | 3992 | Train 63 | Test Black 24 | 0.69 | 0.69 | 0.64 |
| 2969 | Train 47 | Test Black 25 | 0.75 | 0.83 | 0.83 | 3993 | Train 63 | Test Black 25 | 0.72 | 0.76 | 0.71 |
| 2970 | Train 47 | Test Black 26 | 0.75 | 0.82 | 0.82 | 3994 | Train 63 | Test Black 26 | 0.73 | 0.75 | 0.71 |
| 2971 | Train 47 | Test Black 27 | 0.75 | 0.83 | 0.83 | 3995 | Train 63 | Test Black 27 | 0.72 | 0.76 | 0.71 |
| 2972 | Train 47 | Test Black 28 | 0.75 | 0.82 | 0.82 | 3996 | Train 63 | Test Black 28 | 0.73 | 0.76 | 0.71 |
| 2973 | Train 47 | Test Black 29 | 0.73 | 0.82 | 0.82 | 3997 | Train 63 | Test Black 29 | 0.71 | 0.73 | 0.71 |
| 2974 | Train 47 | Test Black 30 | 0.7 | 0.74 | 0.7 | 3998 | Train 63 | Test Black 30 | 0.7 | 0.7 | 0.64 |
| 2975 | Train 47 | Test Black 31 | 0.73 | 0.81 | 0.82 | 3999 | Train 63 | Test Black 31 | 0.71 | 0.72 | 0.71 |
| 2976 | Train 47 | Test Black 32 | 0.71 | 0.74 | 0.7 | 4000 | Train 63 | Test Black 32 | 0.69 | 0.7 | 0.64 |
| 2977 | Train 47 | Test Black 33 | 0.75 | 0.82 | 0.81 | 4001 | Train 63 | Test Black 33 | 0.73 | 0.75 | 0.7 |
| 2978 | Train 47 | Test Black 34 | 0.75 | 0.82 | 0.8 | 4002 | Train 63 | Test Black 34 | 0.73 | 0.75 | 0.69 |
| 2979 | Train 47 | Test Black 35 | 0.75 | 0.82 | 0.81 | 4003 | Train 63 | Test Black 35 | 0.73 | 0.75 | 0.7 |
| 2980 | Train 47 | Test Black 36 | 0.75 | 0.82 | 0.8 | 4004 | Train 63 | Test Black 36 | 0.73 | 0.75 | 0.69 |
| 2981 | Train 47 | Test Black 37 | 0.74 | 0.81 | 0.8 | 4005 | Train 63 | Test Black 37 | 0.71 | 0.72 | 0.69 |
| 2982 | Train 47 | Test Black 38 | 0.7 | 0.74 | 0.7 | 4006 | Train 63 | Test Black 38 | 0.69 | 0.7 | 0.64 |
| 2983 | Train 47 | Test Black 39 | 0.73 | 0.81 | 0.8 | 4007 | Train 63 | Test Black 39 | 0.71 | 0.72 | 0.69 |
| 2984 | Train 47 | Test Black 40 | 0.7 | 0.74 | 0.7 | 4008 | Train 63 | Test Black 40 | 0.69 | 0.69 | 0.64 |
| 2985 | Train 47 | Test Black 41 | 0.75 | 0.83 | 0.83 | 4009 | Train 63 | Test Black 41 | 0.73 | 0.76 | 0.71 |
| 2986 | Train 47 | Test Black 42 | 0.75 | 0.82 | 0.82 | 4010 | Train 63 | Test Black 42 | 0.73 | 0.75 | 0.71 |
| 2987 | Train 47 | Test Black 43 | 0.75 | 0.83 | 0.83 | 4011 | Train 63 | Test Black 43 | 0.73 | 0.76 | 0.72 |
| 2988 | Train 47 | Test Black 44 | 0.75 | 0.82 | 0.82 | 4012 | Train 63 | Test Black 44 | 0.73 | 0.76 | 0.71 |
| 2989 | Train 47 | Test Black 45 | 0.73 | 0.82 | 0.82 | 4013 | Train 63 | Test Black 45 | 0.71 | 0.73 | 0.71 |
| 2990 | Train 47 | Test Black 46 | 0.7 | 0.74 | 0.7 | 4014 | Train 63 | Test Black 46 | 0.7 | 0.7 | 0.64 |
| 2991 | Train 47 | Test Black 47 | 0.73 | 0.81 | 0.82 | 4015 | Train 63 | Test Black 47 | 0.71 | 0.72 | 0.71 |
| 2992 | Train 47 | Test Black 48 | 0.7 | 0.74 | 0.7 | 4016 | Train 63 | Test Black 48 | 0.69 | 0.7 | 0.64 |
| 2993 | Train 47 | Test Black 49 | 0.76 | 0.8 | 0.79 | 4017 | Train 63 | Test Black 49 | 0.77 | 0.8 | 0.79 |
| 2994 | Train 47 | Test Black 50 | 0.76 | 0.8 | 0.79 | 4018 | Train 63 | Test Black 50 | 0.77 | 0.81 | 0.79 |
| 2995 | Train 47 | Test Black 51 | 0.76 | 0.8 | 0.79 | 4019 | Train 63 | Test Black 51 | 0.77 | 0.81 | 0.79 |
| 2996 | Train 47 | Test Black 52 | 0.76 | 0.8 | 0.79 | 4020 | Train 63 | Test Black 52 | 0.77 | 0.81 | 0.79 |
| 2997 | Train 47 | Test Black 53 | 0.76 | 0.8 | 0.8 | 4021 | Train 63 | Test Black 53 | 0.77 | 0.82 | 0.81 |
| 2998 | Train 47 | Test Black 54 | 0.71 | 0.72 | 0.65 | 4022 | Train 63 | Test Black 54 | 0.71 | 0.71 | 0.67 |
| 2999 | Train 47 | Test Black 55 | 0.76 | 0.8 | 0.8 | 4023 | Train 63 | Test Black 55 | 0.77 | 0.82 | 0.81 |
| 3000 | Train 47 | Test Black 56 | 0.71 | 0.72 | 0.64 | 4024 | Train 63 | Test Black 56 | 0.71 | 0.71 | 0.67 |
| 3001 | Train 47 | Test Black 57 | 0.76 | 0.8 | 0.81 | 4025 | Train 63 | Test Black 57 | 0.77 | 0.81 | 0.83 |
| 3002 | Train 47 | Test Black 58 | 0.76 | 0.8 | 0.81 | 4026 | Train 63 | Test Black 58 | 0.77 | 0.81 | 0.83 |
| 3003 | Train 47 | Test Black 59 | 0.76 | 0.8 | 0.81 | 4027 | Train 63 | Test Black 59 | 0.77 | 0.81 | 0.83 |
| 3004 | Train 47 | Test Black 60 | 0.76 | 0.8 | 0.81 | 4028 | Train 63 | Test Black 60 | 0.77 | 0.81 | 0.83 |
| 3005 | Train 47 | Test Black 61 | 0.76 | 0.81 | 0.81 | 4029 | Train 63 | Test Black 61 | 0.77 | 0.82 | 0.83 |
| 3006 | Train 47 | Test Black 62 | 0.71 | 0.72 | 0.65 | 4030 | Train 63 | Test Black 62 | 0.71 | 0.71 | 0.67 |
| 3007 | Train 47 | Test Black 63 | 0.76 | 0.81 | 0.81 | 4031 | Train 63 | Test Black 63 | 0.77 | 0.83 | 0.83 |
| 3008 | Train 47 | Test Black 64 | 0.71 | 0.72 | 0.64 | 4032 | Train 63 | Test Black 64 | 0.71 | 0.71 | 0.67 |
| 3009 | Train 48 | Test Black 1 | 0.73 | 0.79 | 0.77 | 4033 | Train 64 | Test Black 1 | 0.71 | 0.74 | 0.7 |
| 3010 | Train 48 | Test Black 2 | 0.74 | 0.79 | 0.77 | 4034 | Train 64 | Test Black 2 | 0.72 | 0.75 | 0.7 |
| 3011 | Train 48 | Test Black 3 | 0.73 | 0.79 | 0.77 | 4035 | Train 64 | Test Black 3 | 0.71 | 0.74 | 0.7 |
| 3012 | Train 48 | Test Black 4 | 0.74 | 0.79 | 0.77 | 4036 | Train 64 | Test Black 4 | 0.72 | 0.75 | 0.7 |
| 3013 | Train 48 | Test Black 5 | 0.72 | 0.78 | 0.76 | 4037 | Train 64 | Test Black 5 | 0.7 | 0.72 | 0.7 |
| 3014 | Train 48 | Test Black 6 | 0.73 | 0.78 | 0.77 | 4038 | Train 64 | Test Black 6 | 0.72 | 0.75 | 0.73 |
| 3015 | Train 48 | Test Black 7 | 0.72 | 0.77 | 0.76 | 4039 | Train 64 | Test Black 7 | 0.7 | 0.72 | 0.7 |
| 3016 | Train 48 | Test Black 8 | 0.73 | 0.78 | 0.77 | 4040 | Train 64 | Test Black 8 | 0.71 | 0.75 | 0.73 |
| 3017 | Train 48 | Test Black 9 | 0.73 | 0.8 | 0.78 | 4041 | Train 64 | Test Black 9 | 0.71 | 0.75 | 0.71 |
| 3018 | Train 48 | Test Black 10 | 0.74 | 0.8 | 0.78 | 4042 | Train 64 | Test Black 10 | 0.72 | 0.75 | 0.71 |
| 3019 | Train 48 | Test Black 11 | 0.73 | 0.8 | 0.78 | 4043 | Train 64 | Test Black 11 | 0.71 | 0.75 | 0.71 |
| 3020 | Train 48 | Test Black 12 | 0.74 | 0.8 | 0.78 | 4044 | Train 64 | Test Black 12 | 0.72 | 0.75 | 0.71 |
| 3021 | Train 48 | Test Black 13 | 0.72 | 0.78 | 0.76 | 4045 | Train 64 | Test Black 13 | 0.7 | 0.73 | 0.71 |
| 3022 | Train 48 | Test Black 14 | 0.73 | 0.78 | 0.77 | 4046 | Train 64 | Test Black 14 | 0.72 | 0.75 | 0.73 |
| 3023 | Train 48 | Test Black 15 | 0.72 | 0.78 | 0.76 | 4047 | Train 64 | Test Black 15 | 0.69 | 0.72 | 0.71 |
| 3024 | Train 48 | Test Black 16 | 0.73 | 0.78 | 0.77 | 4048 | Train 64 | Test Black 16 | 0.72 | 0.75 | 0.73 |
| 3025 | Train 48 | Test Black 17 | 0.73 | 0.79 | 0.77 | 4049 | Train 64 | Test Black 17 | 0.71 | 0.74 | 0.7 |
| 3026 | Train 48 | Test Black 18 | 0.74 | 0.79 | 0.77 | 4050 | Train 64 | Test Black 18 | 0.72 | 0.75 | 0.7 |
| 3027 | Train 48 | Test Black 19 | 0.73 | 0.79 | 0.77 | 4051 | Train 64 | Test Black 19 | 0.71 | 0.74 | 0.7 |
| 3028 | Train 48 | Test Black 20 | 0.74 | 0.79 | 0.77 | 4052 | Train 64 | Test Black 20 | 0.72 | 0.75 | 0.7 |
| 3029 | Train 48 | Test Black 21 | 0.72 | 0.78 | 0.76 | 4053 | Train 64 | Test Black 21 | 0.7 | 0.72 | 0.7 |
| 3030 | Train 48 | Test Black 22 | 0.73 | 0.78 | 0.77 | 4054 | Train 64 | Test Black 22 | 0.72 | 0.75 | 0.73 |
| 3031 | Train 48 | Test Black 23 | 0.72 | 0.77 | 0.76 | 4055 | Train 64 | Test Black 23 | 0.7 | 0.72 | 0.7 |
| 3032 | Train 48 | Test Black 24 | 0.73 | 0.78 | 0.77 | 4056 | Train 64 | Test Black 24 | 0.72 | 0.75 | 0.73 |
| 3033 | Train 48 | Test Black 25 | 0.73 | 0.79 | 0.78 | 4057 | Train 64 | Test Black 25 | 0.71 | 0.74 | 0.71 |
| 3034 | Train 48 | Test Black 26 | 0.74 | 0.8 | 0.78 | 4058 | Train 64 | Test Black 26 | 0.72 | 0.75 | 0.71 |
| 3035 | Train 48 | Test Black 27 | 0.73 | 0.8 | 0.78 | 4059 | Train 64 | Test Black 27 | 0.71 | 0.75 | 0.71 |
| 3036 | Train 48 | Test Black 28 | 0.74 | 0.8 | 0.78 | 4060 | Train 64 | Test Black 28 | 0.72 | 0.75 | 0.71 |
| 3037 | Train 48 | Test Black 29 | 0.72 | 0.78 | 0.76 | 4061 | Train 64 | Test Black 29 | 0.7 | 0.73 | 0.71 |
| 3038 | Train 48 | Test Black 30 | 0.73 | 0.78 | 0.77 | 4062 | Train 64 | Test Black 30 | 0.72 | 0.75 | 0.73 |
| 3039 | Train 48 | Test Black 31 | 0.72 | 0.78 | 0.76 | 4063 | Train 64 | Test Black 31 | 0.69 | 0.72 | 0.71 |
| 3040 | Train 48 | Test Black 32 | 0.73 | 0.78 | 0.77 | 4064 | Train 64 | Test Black 32 | 0.72 | 0.75 | 0.73 |
| 3041 | Train 48 | Test Black 33 | 0.73 | 0.79 | 0.77 | 4065 | Train 64 | Test Black 33 | 0.71 | 0.74 | 0.7 |
| 3042 | Train 48 | Test Black 34 | 0.74 | 0.79 | 0.77 | 4066 | Train 64 | Test Black 34 | 0.72 | 0.75 | 0.7 |
| 3043 | Train 48 | Test Black 35 | 0.73 | 0.79 | 0.77 | 4067 | Train 64 | Test Black 35 | 0.71 | 0.74 | 0.7 |
| 3044 | Train 48 | Test Black 36 | 0.74 | 0.79 | 0.77 | 4068 | Train 64 | Test Black 36 | 0.72 | 0.75 | 0.7 |
| 3045 | Train 48 | Test Black 37 | 0.72 | 0.78 | 0.76 | 4069 | Train 64 | Test Black 37 | 0.7 | 0.73 | 0.7 |
| 3046 | Train 48 | Test Black 38 | 0.73 | 0.78 | 0.77 | 4070 | Train 64 | Test Black 38 | 0.72 | 0.75 | 0.73 |
| 3047 | Train 48 | Test Black 39 | 0.72 | 0.77 | 0.76 | 4071 | Train 64 | Test Black 39 | 0.7 | 0.72 | 0.7 |
| 3048 | Train 48 | Test Black 40 | 0.73 | 0.78 | 0.77 | 4072 | Train 64 | Test Black 40 | 0.71 | 0.75 | 0.73 |
| 3049 | Train 48 | Test Black 41 | 0.73 | 0.79 | 0.78 | 4073 | Train 64 | Test Black 41 | 0.71 | 0.75 | 0.71 |
| 3050 | Train 48 | Test Black 42 | 0.74 | 0.8 | 0.78 | 4074 | Train 64 | Test Black 42 | 0.72 | 0.75 | 0.71 |
| 3051 | Train 48 | Test Black 43 | 0.73 | 0.79 | 0.78 | 4075 | Train 64 | Test Black 43 | 0.71 | 0.75 | 0.71 |
| 3052 | Train 48 | Test Black 44 | 0.74 | 0.8 | 0.78 | 4076 | Train 64 | Test Black 44 | 0.72 | 0.75 | 0.71 |
| 3053 | Train 48 | Test Black 45 | 0.72 | 0.78 | 0.76 | 4077 | Train 64 | Test Black 45 | 0.7 | 0.73 | 0.71 |
| 3054 | Train 48 | Test Black 46 | 0.73 | 0.78 | 0.77 | 4078 | Train 64 | Test Black 46 | 0.72 | 0.75 | 0.73 |
| 3055 | Train 48 | Test Black 47 | 0.72 | 0.78 | 0.76 | 4079 | Train 64 | Test Black 47 | 0.7 | 0.73 | 0.71 |
| 3056 | Train 48 | Test Black 48 | 0.73 | 0.78 | 0.77 | 4080 | Train 64 | Test Black 48 | 0.72 | 0.75 | 0.73 |
| 3057 | Train 48 | Test Black 49 | 0.76 | 0.8 | 0.78 | 4081 | Train 64 | Test Black 49 | 0.77 | 0.8 | 0.78 |
| 3058 | Train 48 | Test Black 50 | 0.76 | 0.8 | 0.78 | 4082 | Train 64 | Test Black 50 | 0.77 | 0.8 | 0.78 |
| 3059 | Train 48 | Test Black 51 | 0.76 | 0.8 | 0.78 | 4083 | Train 64 | Test Black 51 | 0.77 | 0.8 | 0.78 |
| 3060 | Train 48 | Test Black 52 | 0.76 | 0.8 | 0.78 | 4084 | Train 64 | Test Black 52 | 0.77 | 0.8 | 0.78 |
| 3061 | Train 48 | Test Black 53 | 0.75 | 0.8 | 0.78 | 4085 | Train 64 | Test Black 53 | 0.76 | 0.81 | 0.79 |
| 3062 | Train 48 | Test Black 54 | 0.74 | 0.78 | 0.78 | 4086 | Train 64 | Test Black 54 | 0.74 | 0.8 | 0.8 |
| 3063 | Train 48 | Test Black 55 | 0.75 | 0.8 | 0.78 | 4087 | Train 64 | Test Black 55 | 0.76 | 0.81 | 0.78 |
| 3064 | Train 48 | Test Black 56 | 0.74 | 0.78 | 0.78 | 4088 | Train 64 | Test Black 56 | 0.74 | 0.8 | 0.8 |
| 3065 | Train 48 | Test Black 57 | 0.76 | 0.8 | 0.79 | 4089 | Train 64 | Test Black 57 | 0.77 | 0.81 | 0.8 |
| 3066 | Train 48 | Test Black 58 | 0.76 | 0.8 | 0.79 | 4090 | Train 64 | Test Black 58 | 0.77 | 0.81 | 0.8 |
| 3067 | Train 48 | Test Black 59 | 0.76 | 0.8 | 0.79 | 4091 | Train 64 | Test Black 59 | 0.77 | 0.81 | 0.8 |
| 3068 | Train 48 | Test Black 60 | 0.76 | 0.8 | 0.79 | 4092 | Train 64 | Test Black 60 | 0.77 | 0.81 | 0.8 |
| 3069 | Train 48 | Test Black 61 | 0.75 | 0.8 | 0.78 | 4093 | Train 64 | Test Black 61 | 0.76 | 0.81 | 0.8 |
| 3070 | Train 48 | Test Black 62 | 0.74 | 0.78 | 0.78 | 4094 | Train 64 | Test Black 62 | 0.75 | 0.8 | 0.8 |
| 3071 | Train 48 | Test Black 63 | 0.75 | 0.8 | 0.78 | 4095 | Train 64 | Test Black 63 | 0.76 | 0.81 | 0.8 |
| 3072 | Train 48 | Test Black 64 | 0.74 | 0.78 | 0.78 | 4096 | Train 64 | Test Black 64 | 0.74 | 0.8 | 0.8 |

Table 7. AUC Performance of the ML Models— LR, RF, and GB —for White Patients Trained on Cohorts 1–32 and Tested Across 64 Cohorts.

| White | Train  Model | Test  Dataset | AUC (LR) | AUC (RF) | AUC (GB) | White | Train  Model | Test  Dataset | AUC (LR) | AUC (RF) | AUC (GB) |
| --- | --- | --- | --- | --- | --- | --- | --- | --- | --- | --- | --- |
| 1 | Train 1 | Test White 1 | 0.74 | 0.82 | 0.84 | 1025 | Train 17 | Test White 1 | 0.74 | 0.82 | 0.84 |
| 2 | Train 1 | Test White 2 | 0.73 | 0.76 | 0.78 | 1026 | Train 17 | Test White 2 | 0.73 | 0.76 | 0.78 |
| 3 | Train 1 | Test White 3 | 0.74 | 0.82 | 0.84 | 1027 | Train 17 | Test White 3 | 0.74 | 0.81 | 0.84 |
| 4 | Train 1 | Test White 4 | 0.73 | 0.76 | 0.78 | 1028 | Train 17 | Test White 4 | 0.73 | 0.76 | 0.78 |
| 5 | Train 1 | Test White 5 | 0.7 | 0.73 | 0.69 | 1029 | Train 17 | Test White 5 | 0.7 | 0.74 | 0.71 |
| 6 | Train 1 | Test White 6 | 0.69 | 0.7 | 0.64 | 1030 | Train 17 | Test White 6 | 0.69 | 0.7 | 0.66 |
| 7 | Train 1 | Test White 7 | 0.7 | 0.73 | 0.69 | 1031 | Train 17 | Test White 7 | 0.7 | 0.73 | 0.71 |
| 8 | Train 1 | Test White 8 | 0.69 | 0.69 | 0.64 | 1032 | Train 17 | Test White 8 | 0.69 | 0.7 | 0.66 |
| 9 | Train 1 | Test White 9 | 0.74 | 0.8 | 0.8 | 1033 | Train 17 | Test White 9 | 0.74 | 0.8 | 0.8 |
| 10 | Train 1 | Test White 10 | 0.73 | 0.75 | 0.76 | 1034 | Train 17 | Test White 10 | 0.73 | 0.76 | 0.76 |
| 11 | Train 1 | Test White 11 | 0.75 | 0.8 | 0.8 | 1035 | Train 17 | Test White 11 | 0.75 | 0.8 | 0.8 |
| 12 | Train 1 | Test White 12 | 0.73 | 0.75 | 0.76 | 1036 | Train 17 | Test White 12 | 0.73 | 0.76 | 0.76 |
| 13 | Train 1 | Test White 13 | 0.7 | 0.73 | 0.67 | 1037 | Train 17 | Test White 13 | 0.7 | 0.73 | 0.69 |
| 14 | Train 1 | Test White 14 | 0.69 | 0.7 | 0.64 | 1038 | Train 17 | Test White 14 | 0.69 | 0.7 | 0.66 |
| 15 | Train 1 | Test White 15 | 0.7 | 0.73 | 0.67 | 1039 | Train 17 | Test White 15 | 0.7 | 0.73 | 0.69 |
| 16 | Train 1 | Test White 16 | 0.69 | 0.7 | 0.64 | 1040 | Train 17 | Test White 16 | 0.69 | 0.7 | 0.66 |
| 17 | Train 1 | Test White 17 | 0.74 | 0.82 | 0.84 | 1041 | Train 17 | Test White 17 | 0.74 | 0.82 | 0.84 |
| 18 | Train 1 | Test White 18 | 0.73 | 0.76 | 0.78 | 1042 | Train 17 | Test White 18 | 0.73 | 0.76 | 0.78 |
| 19 | Train 1 | Test White 19 | 0.74 | 0.82 | 0.84 | 1043 | Train 17 | Test White 19 | 0.74 | 0.81 | 0.84 |
| 20 | Train 1 | Test White 20 | 0.73 | 0.76 | 0.78 | 1044 | Train 17 | Test White 20 | 0.73 | 0.76 | 0.78 |
| 21 | Train 1 | Test White 21 | 0.71 | 0.73 | 0.69 | 1045 | Train 17 | Test White 21 | 0.71 | 0.74 | 0.71 |
| 22 | Train 1 | Test White 22 | 0.69 | 0.7 | 0.64 | 1046 | Train 17 | Test White 22 | 0.69 | 0.7 | 0.66 |
| 23 | Train 1 | Test White 23 | 0.71 | 0.73 | 0.69 | 1047 | Train 17 | Test White 23 | 0.71 | 0.73 | 0.71 |
| 24 | Train 1 | Test White 24 | 0.69 | 0.7 | 0.64 | 1048 | Train 17 | Test White 24 | 0.69 | 0.7 | 0.66 |
| 25 | Train 1 | Test White 25 | 0.74 | 0.8 | 0.8 | 1049 | Train 17 | Test White 25 | 0.74 | 0.8 | 0.8 |
| 26 | Train 1 | Test White 26 | 0.73 | 0.75 | 0.76 | 1050 | Train 17 | Test White 26 | 0.73 | 0.76 | 0.76 |
| 27 | Train 1 | Test White 27 | 0.75 | 0.8 | 0.8 | 1051 | Train 17 | Test White 27 | 0.75 | 0.8 | 0.8 |
| 28 | Train 1 | Test White 28 | 0.73 | 0.75 | 0.76 | 1052 | Train 17 | Test White 28 | 0.73 | 0.76 | 0.76 |
| 29 | Train 1 | Test White 29 | 0.7 | 0.73 | 0.67 | 1053 | Train 17 | Test White 29 | 0.7 | 0.73 | 0.7 |
| 30 | Train 1 | Test White 30 | 0.69 | 0.7 | 0.64 | 1054 | Train 17 | Test White 30 | 0.69 | 0.7 | 0.66 |
| 31 | Train 1 | Test White 31 | 0.7 | 0.73 | 0.67 | 1055 | Train 17 | Test White 31 | 0.7 | 0.73 | 0.7 |
| 32 | Train 1 | Test White 32 | 0.7 | 0.7 | 0.64 | 1056 | Train 17 | Test White 32 | 0.69 | 0.7 | 0.66 |
| 33 | Train 1 | Test White 33 | 0.75 | 0.82 | 0.84 | 1057 | Train 17 | Test White 33 | 0.75 | 0.82 | 0.84 |
| 34 | Train 1 | Test White 34 | 0.73 | 0.76 | 0.78 | 1058 | Train 17 | Test White 34 | 0.73 | 0.76 | 0.78 |
| 35 | Train 1 | Test White 35 | 0.75 | 0.82 | 0.84 | 1059 | Train 17 | Test White 35 | 0.75 | 0.82 | 0.84 |
| 36 | Train 1 | Test White 36 | 0.73 | 0.76 | 0.78 | 1060 | Train 17 | Test White 36 | 0.73 | 0.76 | 0.78 |
| 37 | Train 1 | Test White 37 | 0.71 | 0.73 | 0.69 | 1061 | Train 17 | Test White 37 | 0.71 | 0.74 | 0.71 |
| 38 | Train 1 | Test White 38 | 0.69 | 0.7 | 0.64 | 1062 | Train 17 | Test White 38 | 0.69 | 0.7 | 0.66 |
| 39 | Train 1 | Test White 39 | 0.71 | 0.73 | 0.69 | 1063 | Train 17 | Test White 39 | 0.71 | 0.74 | 0.71 |
| 40 | Train 1 | Test White 40 | 0.69 | 0.7 | 0.64 | 1064 | Train 17 | Test White 40 | 0.69 | 0.7 | 0.66 |
| 41 | Train 1 | Test White 41 | 0.75 | 0.81 | 0.8 | 1065 | Train 17 | Test White 41 | 0.75 | 0.8 | 0.8 |
| 42 | Train 1 | Test White 42 | 0.73 | 0.76 | 0.76 | 1066 | Train 17 | Test White 42 | 0.73 | 0.76 | 0.76 |
| 43 | Train 1 | Test White 43 | 0.75 | 0.8 | 0.8 | 1067 | Train 17 | Test White 43 | 0.75 | 0.8 | 0.8 |
| 44 | Train 1 | Test White 44 | 0.73 | 0.76 | 0.76 | 1068 | Train 17 | Test White 44 | 0.73 | 0.76 | 0.76 |
| 45 | Train 1 | Test White 45 | 0.71 | 0.73 | 0.67 | 1069 | Train 17 | Test White 45 | 0.71 | 0.73 | 0.7 |
| 46 | Train 1 | Test White 46 | 0.69 | 0.7 | 0.64 | 1070 | Train 17 | Test White 46 | 0.69 | 0.7 | 0.66 |
| 47 | Train 1 | Test White 47 | 0.71 | 0.73 | 0.67 | 1071 | Train 17 | Test White 47 | 0.71 | 0.73 | 0.7 |
| 48 | Train 1 | Test White 48 | 0.7 | 0.7 | 0.64 | 1072 | Train 17 | Test White 48 | 0.69 | 0.7 | 0.66 |
| 49 | Train 1 | Test White 49 | 0.73 | 0.76 | 0.81 | 1073 | Train 17 | Test White 49 | 0.73 | 0.77 | 0.8 |
| 50 | Train 1 | Test White 50 | 0.73 | 0.76 | 0.81 | 1074 | Train 17 | Test White 50 | 0.73 | 0.77 | 0.8 |
| 51 | Train 1 | Test White 51 | 0.73 | 0.76 | 0.8 | 1075 | Train 17 | Test White 51 | 0.73 | 0.77 | 0.8 |
| 52 | Train 1 | Test White 52 | 0.73 | 0.76 | 0.8 | 1076 | Train 17 | Test White 52 | 0.73 | 0.76 | 0.8 |
| 53 | Train 1 | Test White 53 | 0.7 | 0.73 | 0.63 | 1077 | Train 17 | Test White 53 | 0.7 | 0.73 | 0.66 |
| 54 | Train 1 | Test White 54 | 0.7 | 0.7 | 0.64 | 1078 | Train 17 | Test White 54 | 0.7 | 0.7 | 0.67 |
| 55 | Train 1 | Test White 55 | 0.7 | 0.73 | 0.63 | 1079 | Train 17 | Test White 55 | 0.7 | 0.74 | 0.66 |
| 56 | Train 1 | Test White 56 | 0.7 | 0.69 | 0.64 | 1080 | Train 17 | Test White 56 | 0.7 | 0.7 | 0.66 |
| 57 | Train 1 | Test White 57 | 0.73 | 0.76 | 0.77 | 1081 | Train 17 | Test White 57 | 0.73 | 0.76 | 0.77 |
| 58 | Train 1 | Test White 58 | 0.73 | 0.76 | 0.77 | 1082 | Train 17 | Test White 58 | 0.73 | 0.76 | 0.77 |
| 59 | Train 1 | Test White 59 | 0.73 | 0.76 | 0.77 | 1083 | Train 17 | Test White 59 | 0.73 | 0.76 | 0.76 |
| 60 | Train 1 | Test White 60 | 0.73 | 0.76 | 0.77 | 1084 | Train 17 | Test White 60 | 0.73 | 0.76 | 0.76 |
| 61 | Train 1 | Test White 61 | 0.7 | 0.73 | 0.63 | 1085 | Train 17 | Test White 61 | 0.7 | 0.73 | 0.66 |
| 62 | Train 1 | Test White 62 | 0.7 | 0.7 | 0.64 | 1086 | Train 17 | Test White 62 | 0.7 | 0.7 | 0.67 |
| 63 | Train 1 | Test White 63 | 0.7 | 0.73 | 0.63 | 1087 | Train 17 | Test White 63 | 0.7 | 0.73 | 0.66 |
| 64 | Train 1 | Test White 64 | 0.7 | 0.7 | 0.64 | 1088 | Train 17 | Test White 64 | 0.7 | 0.7 | 0.66 |
| 65 | Train 2 | Test White 1 | 0.74 | 0.81 | 0.82 | 1089 | Train 18 | Test White 1 | 0.74 | 0.81 | 0.82 |
| 66 | Train 2 | Test White 2 | 0.75 | 0.81 | 0.83 | 1090 | Train 18 | Test White 2 | 0.75 | 0.81 | 0.83 |
| 67 | Train 2 | Test White 3 | 0.74 | 0.81 | 0.82 | 1091 | Train 18 | Test White 3 | 0.74 | 0.8 | 0.82 |
| 68 | Train 2 | Test White 4 | 0.75 | 0.81 | 0.83 | 1092 | Train 18 | Test White 4 | 0.75 | 0.81 | 0.83 |
| 69 | Train 2 | Test White 5 | 0.71 | 0.73 | 0.7 | 1093 | Train 18 | Test White 5 | 0.71 | 0.73 | 0.7 |
| 70 | Train 2 | Test White 6 | 0.7 | 0.71 | 0.67 | 1094 | Train 18 | Test White 6 | 0.7 | 0.71 | 0.67 |
| 71 | Train 2 | Test White 7 | 0.71 | 0.73 | 0.7 | 1095 | Train 18 | Test White 7 | 0.71 | 0.73 | 0.7 |
| 72 | Train 2 | Test White 8 | 0.7 | 0.71 | 0.66 | 1096 | Train 18 | Test White 8 | 0.7 | 0.71 | 0.67 |
| 73 | Train 2 | Test White 9 | 0.74 | 0.8 | 0.79 | 1097 | Train 18 | Test White 9 | 0.74 | 0.8 | 0.79 |
| 74 | Train 2 | Test White 10 | 0.75 | 0.8 | 0.8 | 1098 | Train 18 | Test White 10 | 0.75 | 0.8 | 0.8 |
| 75 | Train 2 | Test White 11 | 0.74 | 0.8 | 0.79 | 1099 | Train 18 | Test White 11 | 0.74 | 0.8 | 0.79 |
| 76 | Train 2 | Test White 12 | 0.75 | 0.8 | 0.8 | 1100 | Train 18 | Test White 12 | 0.75 | 0.8 | 0.8 |
| 77 | Train 2 | Test White 13 | 0.7 | 0.73 | 0.68 | 1101 | Train 18 | Test White 13 | 0.7 | 0.73 | 0.69 |
| 78 | Train 2 | Test White 14 | 0.7 | 0.71 | 0.67 | 1102 | Train 18 | Test White 14 | 0.7 | 0.71 | 0.67 |
| 79 | Train 2 | Test White 15 | 0.7 | 0.73 | 0.68 | 1103 | Train 18 | Test White 15 | 0.71 | 0.73 | 0.69 |
| 80 | Train 2 | Test White 16 | 0.7 | 0.71 | 0.66 | 1104 | Train 18 | Test White 16 | 0.7 | 0.71 | 0.67 |
| 81 | Train 2 | Test White 17 | 0.74 | 0.81 | 0.82 | 1105 | Train 18 | Test White 17 | 0.74 | 0.81 | 0.82 |
| 82 | Train 2 | Test White 18 | 0.75 | 0.81 | 0.83 | 1106 | Train 18 | Test White 18 | 0.75 | 0.81 | 0.83 |
| 83 | Train 2 | Test White 19 | 0.74 | 0.81 | 0.82 | 1107 | Train 18 | Test White 19 | 0.74 | 0.8 | 0.82 |
| 84 | Train 2 | Test White 20 | 0.75 | 0.81 | 0.83 | 1108 | Train 18 | Test White 20 | 0.75 | 0.81 | 0.83 |
| 85 | Train 2 | Test White 21 | 0.71 | 0.73 | 0.7 | 1109 | Train 18 | Test White 21 | 0.71 | 0.73 | 0.7 |
| 86 | Train 2 | Test White 22 | 0.7 | 0.71 | 0.67 | 1110 | Train 18 | Test White 22 | 0.7 | 0.71 | 0.67 |
| 87 | Train 2 | Test White 23 | 0.71 | 0.73 | 0.7 | 1111 | Train 18 | Test White 23 | 0.71 | 0.73 | 0.7 |
| 88 | Train 2 | Test White 24 | 0.7 | 0.71 | 0.66 | 1112 | Train 18 | Test White 24 | 0.7 | 0.71 | 0.67 |
| 89 | Train 2 | Test White 25 | 0.74 | 0.8 | 0.79 | 1113 | Train 18 | Test White 25 | 0.74 | 0.8 | 0.79 |
| 90 | Train 2 | Test White 26 | 0.75 | 0.8 | 0.8 | 1114 | Train 18 | Test White 26 | 0.75 | 0.8 | 0.8 |
| 91 | Train 2 | Test White 27 | 0.74 | 0.8 | 0.79 | 1115 | Train 18 | Test White 27 | 0.74 | 0.8 | 0.79 |
| 92 | Train 2 | Test White 28 | 0.75 | 0.8 | 0.8 | 1116 | Train 18 | Test White 28 | 0.75 | 0.8 | 0.8 |
| 93 | Train 2 | Test White 29 | 0.71 | 0.73 | 0.68 | 1117 | Train 18 | Test White 29 | 0.71 | 0.73 | 0.69 |
| 94 | Train 2 | Test White 30 | 0.71 | 0.71 | 0.67 | 1118 | Train 18 | Test White 30 | 0.71 | 0.71 | 0.67 |
| 95 | Train 2 | Test White 31 | 0.71 | 0.73 | 0.68 | 1119 | Train 18 | Test White 31 | 0.71 | 0.73 | 0.69 |
| 96 | Train 2 | Test White 32 | 0.7 | 0.71 | 0.66 | 1120 | Train 18 | Test White 32 | 0.7 | 0.71 | 0.67 |
| 97 | Train 2 | Test White 33 | 0.74 | 0.81 | 0.83 | 1121 | Train 18 | Test White 33 | 0.74 | 0.81 | 0.82 |
| 98 | Train 2 | Test White 34 | 0.75 | 0.81 | 0.83 | 1122 | Train 18 | Test White 34 | 0.75 | 0.81 | 0.83 |
| 99 | Train 2 | Test White 35 | 0.74 | 0.81 | 0.82 | 1123 | Train 18 | Test White 35 | 0.74 | 0.81 | 0.82 |
| 100 | Train 2 | Test White 36 | 0.75 | 0.81 | 0.83 | 1124 | Train 18 | Test White 36 | 0.75 | 0.81 | 0.83 |
| 101 | Train 2 | Test White 37 | 0.71 | 0.73 | 0.7 | 1125 | Train 18 | Test White 37 | 0.71 | 0.73 | 0.7 |
| 102 | Train 2 | Test White 38 | 0.7 | 0.71 | 0.67 | 1126 | Train 18 | Test White 38 | 0.7 | 0.71 | 0.67 |
| 103 | Train 2 | Test White 39 | 0.71 | 0.73 | 0.7 | 1127 | Train 18 | Test White 39 | 0.71 | 0.73 | 0.7 |
| 104 | Train 2 | Test White 40 | 0.7 | 0.71 | 0.66 | 1128 | Train 18 | Test White 40 | 0.7 | 0.71 | 0.67 |
| 105 | Train 2 | Test White 41 | 0.74 | 0.8 | 0.79 | 1129 | Train 18 | Test White 41 | 0.74 | 0.8 | 0.79 |
| 106 | Train 2 | Test White 42 | 0.75 | 0.81 | 0.81 | 1130 | Train 18 | Test White 42 | 0.75 | 0.81 | 0.81 |
| 107 | Train 2 | Test White 43 | 0.74 | 0.8 | 0.79 | 1131 | Train 18 | Test White 43 | 0.74 | 0.8 | 0.79 |
| 108 | Train 2 | Test White 44 | 0.75 | 0.81 | 0.8 | 1132 | Train 18 | Test White 44 | 0.75 | 0.8 | 0.8 |
| 109 | Train 2 | Test White 45 | 0.71 | 0.73 | 0.68 | 1133 | Train 18 | Test White 45 | 0.71 | 0.73 | 0.69 |
| 110 | Train 2 | Test White 46 | 0.71 | 0.71 | 0.67 | 1134 | Train 18 | Test White 46 | 0.71 | 0.71 | 0.67 |
| 111 | Train 2 | Test White 47 | 0.71 | 0.73 | 0.68 | 1135 | Train 18 | Test White 47 | 0.71 | 0.73 | 0.69 |
| 112 | Train 2 | Test White 48 | 0.7 | 0.71 | 0.66 | 1136 | Train 18 | Test White 48 | 0.7 | 0.71 | 0.67 |
| 113 | Train 2 | Test White 49 | 0.73 | 0.76 | 0.8 | 1137 | Train 18 | Test White 49 | 0.73 | 0.76 | 0.79 |
| 114 | Train 2 | Test White 50 | 0.73 | 0.76 | 0.8 | 1138 | Train 18 | Test White 50 | 0.73 | 0.76 | 0.79 |
| 115 | Train 2 | Test White 51 | 0.73 | 0.76 | 0.79 | 1139 | Train 18 | Test White 51 | 0.73 | 0.76 | 0.79 |
| 116 | Train 2 | Test White 52 | 0.73 | 0.76 | 0.79 | 1140 | Train 18 | Test White 52 | 0.73 | 0.76 | 0.79 |
| 117 | Train 2 | Test White 53 | 0.7 | 0.74 | 0.64 | 1141 | Train 18 | Test White 53 | 0.7 | 0.74 | 0.62 |
| 118 | Train 2 | Test White 54 | 0.71 | 0.71 | 0.67 | 1142 | Train 18 | Test White 54 | 0.71 | 0.71 | 0.67 |
| 119 | Train 2 | Test White 55 | 0.7 | 0.74 | 0.64 | 1143 | Train 18 | Test White 55 | 0.7 | 0.74 | 0.62 |
| 120 | Train 2 | Test White 56 | 0.7 | 0.7 | 0.66 | 1144 | Train 18 | Test White 56 | 0.7 | 0.7 | 0.66 |
| 121 | Train 2 | Test White 57 | 0.73 | 0.75 | 0.77 | 1145 | Train 18 | Test White 57 | 0.73 | 0.75 | 0.76 |
| 122 | Train 2 | Test White 58 | 0.73 | 0.75 | 0.77 | 1146 | Train 18 | Test White 58 | 0.73 | 0.75 | 0.76 |
| 123 | Train 2 | Test White 59 | 0.73 | 0.75 | 0.77 | 1147 | Train 18 | Test White 59 | 0.73 | 0.75 | 0.76 |
| 124 | Train 2 | Test White 60 | 0.73 | 0.75 | 0.76 | 1148 | Train 18 | Test White 60 | 0.73 | 0.75 | 0.76 |
| 125 | Train 2 | Test White 61 | 0.7 | 0.74 | 0.64 | 1149 | Train 18 | Test White 61 | 0.7 | 0.74 | 0.62 |
| 126 | Train 2 | Test White 62 | 0.71 | 0.71 | 0.67 | 1150 | Train 18 | Test White 62 | 0.71 | 0.71 | 0.67 |
| 127 | Train 2 | Test White 63 | 0.7 | 0.74 | 0.64 | 1151 | Train 18 | Test White 63 | 0.7 | 0.74 | 0.62 |
| 128 | Train 2 | Test White 64 | 0.7 | 0.7 | 0.66 | 1152 | Train 18 | Test White 64 | 0.7 | 0.7 | 0.66 |
| 129 | Train 3 | Test White 1 | 0.74 | 0.82 | 0.84 | 1153 | Train 19 | Test White 1 | 0.74 | 0.82 | 0.84 |
| 130 | Train 3 | Test White 2 | 0.73 | 0.76 | 0.78 | 1154 | Train 19 | Test White 2 | 0.73 | 0.76 | 0.77 |
| 131 | Train 3 | Test White 3 | 0.74 | 0.82 | 0.84 | 1155 | Train 19 | Test White 3 | 0.74 | 0.82 | 0.84 |
| 132 | Train 3 | Test White 4 | 0.73 | 0.76 | 0.78 | 1156 | Train 19 | Test White 4 | 0.73 | 0.76 | 0.77 |
| 133 | Train 3 | Test White 5 | 0.7 | 0.73 | 0.69 | 1157 | Train 19 | Test White 5 | 0.7 | 0.73 | 0.67 |
| 134 | Train 3 | Test White 6 | 0.69 | 0.69 | 0.64 | 1158 | Train 19 | Test White 6 | 0.69 | 0.69 | 0.62 |
| 135 | Train 3 | Test White 7 | 0.7 | 0.73 | 0.69 | 1159 | Train 19 | Test White 7 | 0.7 | 0.73 | 0.67 |
| 136 | Train 3 | Test White 8 | 0.69 | 0.69 | 0.64 | 1160 | Train 19 | Test White 8 | 0.69 | 0.69 | 0.62 |
| 137 | Train 3 | Test White 9 | 0.74 | 0.8 | 0.8 | 1161 | Train 19 | Test White 9 | 0.74 | 0.8 | 0.8 |
| 138 | Train 3 | Test White 10 | 0.73 | 0.76 | 0.76 | 1162 | Train 19 | Test White 10 | 0.73 | 0.76 | 0.75 |
| 139 | Train 3 | Test White 11 | 0.75 | 0.8 | 0.8 | 1163 | Train 19 | Test White 11 | 0.75 | 0.8 | 0.8 |
| 140 | Train 3 | Test White 12 | 0.73 | 0.76 | 0.76 | 1164 | Train 19 | Test White 12 | 0.73 | 0.76 | 0.75 |
| 141 | Train 3 | Test White 13 | 0.7 | 0.73 | 0.67 | 1165 | Train 19 | Test White 13 | 0.7 | 0.73 | 0.66 |
| 142 | Train 3 | Test White 14 | 0.69 | 0.7 | 0.64 | 1166 | Train 19 | Test White 14 | 0.69 | 0.7 | 0.62 |
| 143 | Train 3 | Test White 15 | 0.7 | 0.73 | 0.67 | 1167 | Train 19 | Test White 15 | 0.7 | 0.73 | 0.66 |
| 144 | Train 3 | Test White 16 | 0.69 | 0.7 | 0.64 | 1168 | Train 19 | Test White 16 | 0.69 | 0.7 | 0.62 |
| 145 | Train 3 | Test White 17 | 0.74 | 0.82 | 0.84 | 1169 | Train 19 | Test White 17 | 0.74 | 0.82 | 0.84 |
| 146 | Train 3 | Test White 18 | 0.73 | 0.76 | 0.78 | 1170 | Train 19 | Test White 18 | 0.73 | 0.76 | 0.77 |
| 147 | Train 3 | Test White 19 | 0.74 | 0.82 | 0.84 | 1171 | Train 19 | Test White 19 | 0.74 | 0.82 | 0.84 |
| 148 | Train 3 | Test White 20 | 0.73 | 0.76 | 0.78 | 1172 | Train 19 | Test White 20 | 0.73 | 0.76 | 0.78 |
| 149 | Train 3 | Test White 21 | 0.7 | 0.73 | 0.69 | 1173 | Train 19 | Test White 21 | 0.7 | 0.73 | 0.67 |
| 150 | Train 3 | Test White 22 | 0.69 | 0.69 | 0.64 | 1174 | Train 19 | Test White 22 | 0.69 | 0.7 | 0.62 |
| 151 | Train 3 | Test White 23 | 0.7 | 0.73 | 0.69 | 1175 | Train 19 | Test White 23 | 0.7 | 0.73 | 0.67 |
| 152 | Train 3 | Test White 24 | 0.69 | 0.69 | 0.64 | 1176 | Train 19 | Test White 24 | 0.69 | 0.69 | 0.63 |
| 153 | Train 3 | Test White 25 | 0.74 | 0.8 | 0.8 | 1177 | Train 19 | Test White 25 | 0.74 | 0.8 | 0.8 |
| 154 | Train 3 | Test White 26 | 0.73 | 0.76 | 0.76 | 1178 | Train 19 | Test White 26 | 0.73 | 0.76 | 0.75 |
| 155 | Train 3 | Test White 27 | 0.75 | 0.8 | 0.8 | 1179 | Train 19 | Test White 27 | 0.75 | 0.8 | 0.8 |
| 156 | Train 3 | Test White 28 | 0.73 | 0.76 | 0.76 | 1180 | Train 19 | Test White 28 | 0.73 | 0.76 | 0.75 |
| 157 | Train 3 | Test White 29 | 0.7 | 0.73 | 0.67 | 1181 | Train 19 | Test White 29 | 0.7 | 0.73 | 0.66 |
| 158 | Train 3 | Test White 30 | 0.69 | 0.7 | 0.64 | 1182 | Train 19 | Test White 30 | 0.69 | 0.7 | 0.62 |
| 159 | Train 3 | Test White 31 | 0.7 | 0.73 | 0.67 | 1183 | Train 19 | Test White 31 | 0.7 | 0.73 | 0.66 |
| 160 | Train 3 | Test White 32 | 0.69 | 0.7 | 0.64 | 1184 | Train 19 | Test White 32 | 0.69 | 0.7 | 0.63 |
| 161 | Train 3 | Test White 33 | 0.75 | 0.82 | 0.84 | 1185 | Train 19 | Test White 33 | 0.75 | 0.82 | 0.84 |
| 162 | Train 3 | Test White 34 | 0.73 | 0.76 | 0.78 | 1186 | Train 19 | Test White 34 | 0.73 | 0.76 | 0.78 |
| 163 | Train 3 | Test White 35 | 0.75 | 0.82 | 0.84 | 1187 | Train 19 | Test White 35 | 0.75 | 0.82 | 0.84 |
| 164 | Train 3 | Test White 36 | 0.73 | 0.76 | 0.78 | 1188 | Train 19 | Test White 36 | 0.73 | 0.76 | 0.78 |
| 165 | Train 3 | Test White 37 | 0.71 | 0.73 | 0.69 | 1189 | Train 19 | Test White 37 | 0.71 | 0.73 | 0.67 |
| 166 | Train 3 | Test White 38 | 0.69 | 0.69 | 0.64 | 1190 | Train 19 | Test White 38 | 0.69 | 0.7 | 0.62 |
| 167 | Train 3 | Test White 39 | 0.71 | 0.73 | 0.69 | 1191 | Train 19 | Test White 39 | 0.71 | 0.73 | 0.67 |
| 168 | Train 3 | Test White 40 | 0.69 | 0.69 | 0.64 | 1192 | Train 19 | Test White 40 | 0.69 | 0.69 | 0.63 |
| 169 | Train 3 | Test White 41 | 0.75 | 0.81 | 0.8 | 1193 | Train 19 | Test White 41 | 0.75 | 0.81 | 0.8 |
| 170 | Train 3 | Test White 42 | 0.73 | 0.76 | 0.76 | 1194 | Train 19 | Test White 42 | 0.73 | 0.76 | 0.75 |
| 171 | Train 3 | Test White 43 | 0.75 | 0.81 | 0.8 | 1195 | Train 19 | Test White 43 | 0.75 | 0.81 | 0.8 |
| 172 | Train 3 | Test White 44 | 0.73 | 0.76 | 0.76 | 1196 | Train 19 | Test White 44 | 0.73 | 0.76 | 0.75 |
| 173 | Train 3 | Test White 45 | 0.71 | 0.73 | 0.67 | 1197 | Train 19 | Test White 45 | 0.71 | 0.73 | 0.66 |
| 174 | Train 3 | Test White 46 | 0.69 | 0.7 | 0.64 | 1198 | Train 19 | Test White 46 | 0.69 | 0.7 | 0.62 |
| 175 | Train 3 | Test White 47 | 0.71 | 0.73 | 0.67 | 1199 | Train 19 | Test White 47 | 0.71 | 0.73 | 0.66 |
| 176 | Train 3 | Test White 48 | 0.69 | 0.7 | 0.64 | 1200 | Train 19 | Test White 48 | 0.69 | 0.7 | 0.63 |
| 177 | Train 3 | Test White 49 | 0.73 | 0.77 | 0.81 | 1201 | Train 19 | Test White 49 | 0.73 | 0.77 | 0.8 |
| 178 | Train 3 | Test White 50 | 0.73 | 0.77 | 0.81 | 1202 | Train 19 | Test White 50 | 0.73 | 0.77 | 0.8 |
| 179 | Train 3 | Test White 51 | 0.73 | 0.77 | 0.81 | 1203 | Train 19 | Test White 51 | 0.73 | 0.77 | 0.8 |
| 180 | Train 3 | Test White 52 | 0.73 | 0.77 | 0.81 | 1204 | Train 19 | Test White 52 | 0.73 | 0.77 | 0.8 |
| 181 | Train 3 | Test White 53 | 0.7 | 0.73 | 0.61 | 1205 | Train 19 | Test White 53 | 0.7 | 0.73 | 0.58 |
| 182 | Train 3 | Test White 54 | 0.7 | 0.7 | 0.63 | 1206 | Train 19 | Test White 54 | 0.7 | 0.7 | 0.61 |
| 183 | Train 3 | Test White 55 | 0.7 | 0.74 | 0.61 | 1207 | Train 19 | Test White 55 | 0.7 | 0.73 | 0.58 |
| 184 | Train 3 | Test White 56 | 0.7 | 0.69 | 0.63 | 1208 | Train 19 | Test White 56 | 0.7 | 0.69 | 0.61 |
| 185 | Train 3 | Test White 57 | 0.73 | 0.76 | 0.77 | 1209 | Train 19 | Test White 57 | 0.73 | 0.76 | 0.77 |
| 186 | Train 3 | Test White 58 | 0.73 | 0.76 | 0.77 | 1210 | Train 19 | Test White 58 | 0.73 | 0.76 | 0.77 |
| 187 | Train 3 | Test White 59 | 0.74 | 0.76 | 0.77 | 1211 | Train 19 | Test White 59 | 0.74 | 0.76 | 0.77 |
| 188 | Train 3 | Test White 60 | 0.74 | 0.76 | 0.77 | 1212 | Train 19 | Test White 60 | 0.74 | 0.76 | 0.77 |
| 189 | Train 3 | Test White 61 | 0.7 | 0.73 | 0.61 | 1213 | Train 19 | Test White 61 | 0.7 | 0.73 | 0.57 |
| 190 | Train 3 | Test White 62 | 0.7 | 0.7 | 0.63 | 1214 | Train 19 | Test White 62 | 0.7 | 0.7 | 0.61 |
| 191 | Train 3 | Test White 63 | 0.7 | 0.73 | 0.61 | 1215 | Train 19 | Test White 63 | 0.7 | 0.73 | 0.58 |
| 192 | Train 3 | Test White 64 | 0.7 | 0.69 | 0.63 | 1216 | Train 19 | Test White 64 | 0.7 | 0.7 | 0.61 |
| 193 | Train 4 | Test White 1 | 0.74 | 0.8 | 0.82 | 1217 | Train 20 | Test White 1 | 0.74 | 0.8 | 0.83 |
| 194 | Train 4 | Test White 2 | 0.75 | 0.81 | 0.83 | 1218 | Train 20 | Test White 2 | 0.75 | 0.81 | 0.83 |
| 195 | Train 4 | Test White 3 | 0.74 | 0.81 | 0.82 | 1219 | Train 20 | Test White 3 | 0.74 | 0.81 | 0.83 |
| 196 | Train 4 | Test White 4 | 0.75 | 0.81 | 0.83 | 1220 | Train 20 | Test White 4 | 0.75 | 0.81 | 0.83 |
| 197 | Train 4 | Test White 5 | 0.71 | 0.73 | 0.69 | 1221 | Train 20 | Test White 5 | 0.71 | 0.73 | 0.69 |
| 198 | Train 4 | Test White 6 | 0.7 | 0.71 | 0.67 | 1222 | Train 20 | Test White 6 | 0.7 | 0.71 | 0.66 |
| 199 | Train 4 | Test White 7 | 0.71 | 0.73 | 0.69 | 1223 | Train 20 | Test White 7 | 0.71 | 0.73 | 0.69 |
| 200 | Train 4 | Test White 8 | 0.7 | 0.71 | 0.67 | 1224 | Train 20 | Test White 8 | 0.7 | 0.71 | 0.66 |
| 201 | Train 4 | Test White 9 | 0.74 | 0.8 | 0.79 | 1225 | Train 20 | Test White 9 | 0.74 | 0.8 | 0.8 |
| 202 | Train 4 | Test White 10 | 0.75 | 0.8 | 0.8 | 1226 | Train 20 | Test White 10 | 0.75 | 0.8 | 0.81 |
| 203 | Train 4 | Test White 11 | 0.74 | 0.8 | 0.79 | 1227 | Train 20 | Test White 11 | 0.74 | 0.8 | 0.8 |
| 204 | Train 4 | Test White 12 | 0.75 | 0.8 | 0.8 | 1228 | Train 20 | Test White 12 | 0.75 | 0.8 | 0.81 |
| 205 | Train 4 | Test White 13 | 0.7 | 0.73 | 0.68 | 1229 | Train 20 | Test White 13 | 0.7 | 0.73 | 0.68 |
| 206 | Train 4 | Test White 14 | 0.7 | 0.71 | 0.67 | 1230 | Train 20 | Test White 14 | 0.7 | 0.71 | 0.66 |
| 207 | Train 4 | Test White 15 | 0.7 | 0.73 | 0.68 | 1231 | Train 20 | Test White 15 | 0.71 | 0.73 | 0.68 |
| 208 | Train 4 | Test White 16 | 0.7 | 0.71 | 0.67 | 1232 | Train 20 | Test White 16 | 0.7 | 0.71 | 0.66 |
| 209 | Train 4 | Test White 17 | 0.74 | 0.8 | 0.82 | 1233 | Train 20 | Test White 17 | 0.74 | 0.81 | 0.83 |
| 210 | Train 4 | Test White 18 | 0.75 | 0.81 | 0.83 | 1234 | Train 20 | Test White 18 | 0.75 | 0.81 | 0.83 |
| 211 | Train 4 | Test White 19 | 0.74 | 0.81 | 0.82 | 1235 | Train 20 | Test White 19 | 0.74 | 0.81 | 0.83 |
| 212 | Train 4 | Test White 20 | 0.75 | 0.81 | 0.83 | 1236 | Train 20 | Test White 20 | 0.75 | 0.81 | 0.83 |
| 213 | Train 4 | Test White 21 | 0.71 | 0.73 | 0.69 | 1237 | Train 20 | Test White 21 | 0.71 | 0.73 | 0.69 |
| 214 | Train 4 | Test White 22 | 0.7 | 0.71 | 0.67 | 1238 | Train 20 | Test White 22 | 0.7 | 0.71 | 0.66 |
| 215 | Train 4 | Test White 23 | 0.71 | 0.73 | 0.69 | 1239 | Train 20 | Test White 23 | 0.71 | 0.73 | 0.69 |
| 216 | Train 4 | Test White 24 | 0.7 | 0.71 | 0.67 | 1240 | Train 20 | Test White 24 | 0.7 | 0.71 | 0.66 |
| 217 | Train 4 | Test White 25 | 0.74 | 0.8 | 0.79 | 1241 | Train 20 | Test White 25 | 0.74 | 0.8 | 0.8 |
| 218 | Train 4 | Test White 26 | 0.75 | 0.8 | 0.8 | 1242 | Train 20 | Test White 26 | 0.75 | 0.8 | 0.81 |
| 219 | Train 4 | Test White 27 | 0.74 | 0.8 | 0.79 | 1243 | Train 20 | Test White 27 | 0.74 | 0.8 | 0.8 |
| 220 | Train 4 | Test White 28 | 0.75 | 0.8 | 0.8 | 1244 | Train 20 | Test White 28 | 0.75 | 0.8 | 0.81 |
| 221 | Train 4 | Test White 29 | 0.71 | 0.73 | 0.68 | 1245 | Train 20 | Test White 29 | 0.71 | 0.73 | 0.68 |
| 222 | Train 4 | Test White 30 | 0.7 | 0.71 | 0.67 | 1246 | Train 20 | Test White 30 | 0.71 | 0.71 | 0.66 |
| 223 | Train 4 | Test White 31 | 0.71 | 0.73 | 0.68 | 1247 | Train 20 | Test White 31 | 0.71 | 0.73 | 0.68 |
| 224 | Train 4 | Test White 32 | 0.7 | 0.71 | 0.67 | 1248 | Train 20 | Test White 32 | 0.7 | 0.71 | 0.66 |
| 225 | Train 4 | Test White 33 | 0.74 | 0.81 | 0.82 | 1249 | Train 20 | Test White 33 | 0.74 | 0.81 | 0.83 |
| 226 | Train 4 | Test White 34 | 0.75 | 0.81 | 0.83 | 1250 | Train 20 | Test White 34 | 0.75 | 0.81 | 0.83 |
| 227 | Train 4 | Test White 35 | 0.74 | 0.81 | 0.82 | 1251 | Train 20 | Test White 35 | 0.74 | 0.81 | 0.83 |
| 228 | Train 4 | Test White 36 | 0.75 | 0.81 | 0.83 | 1252 | Train 20 | Test White 36 | 0.75 | 0.81 | 0.83 |
| 229 | Train 4 | Test White 37 | 0.71 | 0.73 | 0.69 | 1253 | Train 20 | Test White 37 | 0.71 | 0.73 | 0.69 |
| 230 | Train 4 | Test White 38 | 0.7 | 0.71 | 0.67 | 1254 | Train 20 | Test White 38 | 0.7 | 0.71 | 0.66 |
| 231 | Train 4 | Test White 39 | 0.71 | 0.73 | 0.69 | 1255 | Train 20 | Test White 39 | 0.71 | 0.73 | 0.69 |
| 232 | Train 4 | Test White 40 | 0.7 | 0.71 | 0.67 | 1256 | Train 20 | Test White 40 | 0.7 | 0.71 | 0.66 |
| 233 | Train 4 | Test White 41 | 0.74 | 0.8 | 0.79 | 1257 | Train 20 | Test White 41 | 0.74 | 0.8 | 0.8 |
| 234 | Train 4 | Test White 42 | 0.75 | 0.81 | 0.8 | 1258 | Train 20 | Test White 42 | 0.75 | 0.81 | 0.81 |
| 235 | Train 4 | Test White 43 | 0.74 | 0.8 | 0.79 | 1259 | Train 20 | Test White 43 | 0.74 | 0.8 | 0.8 |
| 236 | Train 4 | Test White 44 | 0.75 | 0.81 | 0.8 | 1260 | Train 20 | Test White 44 | 0.75 | 0.81 | 0.81 |
| 237 | Train 4 | Test White 45 | 0.71 | 0.73 | 0.68 | 1261 | Train 20 | Test White 45 | 0.71 | 0.73 | 0.68 |
| 238 | Train 4 | Test White 46 | 0.7 | 0.71 | 0.67 | 1262 | Train 20 | Test White 46 | 0.71 | 0.71 | 0.66 |
| 239 | Train 4 | Test White 47 | 0.71 | 0.73 | 0.68 | 1263 | Train 20 | Test White 47 | 0.71 | 0.73 | 0.68 |
| 240 | Train 4 | Test White 48 | 0.7 | 0.71 | 0.67 | 1264 | Train 20 | Test White 48 | 0.7 | 0.71 | 0.66 |
| 241 | Train 4 | Test White 49 | 0.73 | 0.76 | 0.79 | 1265 | Train 20 | Test White 49 | 0.73 | 0.76 | 0.8 |
| 242 | Train 4 | Test White 50 | 0.73 | 0.76 | 0.79 | 1266 | Train 20 | Test White 50 | 0.73 | 0.76 | 0.8 |
| 243 | Train 4 | Test White 51 | 0.73 | 0.76 | 0.79 | 1267 | Train 20 | Test White 51 | 0.73 | 0.76 | 0.8 |
| 244 | Train 4 | Test White 52 | 0.73 | 0.76 | 0.79 | 1268 | Train 20 | Test White 52 | 0.73 | 0.76 | 0.8 |
| 245 | Train 4 | Test White 53 | 0.7 | 0.74 | 0.65 | 1269 | Train 20 | Test White 53 | 0.7 | 0.74 | 0.63 |
| 246 | Train 4 | Test White 54 | 0.71 | 0.71 | 0.67 | 1270 | Train 20 | Test White 54 | 0.71 | 0.71 | 0.66 |
| 247 | Train 4 | Test White 55 | 0.7 | 0.74 | 0.65 | 1271 | Train 20 | Test White 55 | 0.7 | 0.74 | 0.63 |
| 248 | Train 4 | Test White 56 | 0.7 | 0.7 | 0.67 | 1272 | Train 20 | Test White 56 | 0.7 | 0.7 | 0.65 |
| 249 | Train 4 | Test White 57 | 0.73 | 0.75 | 0.76 | 1273 | Train 20 | Test White 57 | 0.73 | 0.75 | 0.77 |
| 250 | Train 4 | Test White 58 | 0.73 | 0.75 | 0.76 | 1274 | Train 20 | Test White 58 | 0.73 | 0.75 | 0.77 |
| 251 | Train 4 | Test White 59 | 0.73 | 0.75 | 0.76 | 1275 | Train 20 | Test White 59 | 0.73 | 0.75 | 0.77 |
| 252 | Train 4 | Test White 60 | 0.73 | 0.75 | 0.76 | 1276 | Train 20 | Test White 60 | 0.73 | 0.75 | 0.77 |
| 253 | Train 4 | Test White 61 | 0.7 | 0.74 | 0.65 | 1277 | Train 20 | Test White 61 | 0.7 | 0.74 | 0.62 |
| 254 | Train 4 | Test White 62 | 0.71 | 0.71 | 0.67 | 1278 | Train 20 | Test White 62 | 0.71 | 0.71 | 0.66 |
| 255 | Train 4 | Test White 63 | 0.7 | 0.74 | 0.65 | 1279 | Train 20 | Test White 63 | 0.7 | 0.74 | 0.62 |
| 256 | Train 4 | Test White 64 | 0.7 | 0.7 | 0.67 | 1280 | Train 20 | Test White 64 | 0.7 | 0.7 | 0.65 |
| 257 | Train 5 | Test White 1 | 0.74 | 0.79 | 0.81 | 1281 | Train 21 | Test White 1 | 0.74 | 0.79 | 0.81 |
| 258 | Train 5 | Test White 2 | 0.74 | 0.77 | 0.77 | 1282 | Train 21 | Test White 2 | 0.74 | 0.77 | 0.77 |
| 259 | Train 5 | Test White 3 | 0.74 | 0.79 | 0.81 | 1283 | Train 21 | Test White 3 | 0.74 | 0.79 | 0.81 |
| 260 | Train 5 | Test White 4 | 0.74 | 0.77 | 0.77 | 1284 | Train 21 | Test White 4 | 0.74 | 0.77 | 0.77 |
| 261 | Train 5 | Test White 5 | 0.73 | 0.78 | 0.77 | 1285 | Train 21 | Test White 5 | 0.73 | 0.78 | 0.78 |
| 262 | Train 5 | Test White 6 | 0.7 | 0.71 | 0.69 | 1286 | Train 21 | Test White 6 | 0.7 | 0.71 | 0.68 |
| 263 | Train 5 | Test White 7 | 0.73 | 0.78 | 0.77 | 1287 | Train 21 | Test White 7 | 0.73 | 0.78 | 0.78 |
| 264 | Train 5 | Test White 8 | 0.7 | 0.71 | 0.7 | 1288 | Train 21 | Test White 8 | 0.7 | 0.71 | 0.68 |
| 265 | Train 5 | Test White 9 | 0.74 | 0.78 | 0.78 | 1289 | Train 21 | Test White 9 | 0.74 | 0.78 | 0.78 |
| 266 | Train 5 | Test White 10 | 0.74 | 0.76 | 0.76 | 1290 | Train 21 | Test White 10 | 0.74 | 0.76 | 0.76 |
| 267 | Train 5 | Test White 11 | 0.74 | 0.78 | 0.78 | 1291 | Train 21 | Test White 11 | 0.74 | 0.78 | 0.78 |
| 268 | Train 5 | Test White 12 | 0.74 | 0.76 | 0.76 | 1292 | Train 21 | Test White 12 | 0.74 | 0.76 | 0.76 |
| 269 | Train 5 | Test White 13 | 0.73 | 0.77 | 0.75 | 1293 | Train 21 | Test White 13 | 0.73 | 0.77 | 0.76 |
| 270 | Train 5 | Test White 14 | 0.7 | 0.71 | 0.7 | 1294 | Train 21 | Test White 14 | 0.7 | 0.71 | 0.68 |
| 271 | Train 5 | Test White 15 | 0.73 | 0.77 | 0.75 | 1295 | Train 21 | Test White 15 | 0.73 | 0.77 | 0.76 |
| 272 | Train 5 | Test White 16 | 0.7 | 0.71 | 0.7 | 1296 | Train 21 | Test White 16 | 0.7 | 0.71 | 0.69 |
| 273 | Train 5 | Test White 17 | 0.74 | 0.79 | 0.81 | 1297 | Train 21 | Test White 17 | 0.74 | 0.79 | 0.81 |
| 274 | Train 5 | Test White 18 | 0.74 | 0.77 | 0.77 | 1298 | Train 21 | Test White 18 | 0.74 | 0.77 | 0.77 |
| 275 | Train 5 | Test White 19 | 0.74 | 0.79 | 0.81 | 1299 | Train 21 | Test White 19 | 0.74 | 0.79 | 0.81 |
| 276 | Train 5 | Test White 20 | 0.74 | 0.77 | 0.77 | 1300 | Train 21 | Test White 20 | 0.74 | 0.77 | 0.77 |
| 277 | Train 5 | Test White 21 | 0.73 | 0.78 | 0.77 | 1301 | Train 21 | Test White 21 | 0.73 | 0.78 | 0.78 |
| 278 | Train 5 | Test White 22 | 0.7 | 0.71 | 0.69 | 1302 | Train 21 | Test White 22 | 0.7 | 0.71 | 0.68 |
| 279 | Train 5 | Test White 23 | 0.73 | 0.78 | 0.77 | 1303 | Train 21 | Test White 23 | 0.73 | 0.78 | 0.78 |
| 280 | Train 5 | Test White 24 | 0.7 | 0.71 | 0.7 | 1304 | Train 21 | Test White 24 | 0.7 | 0.71 | 0.68 |
| 281 | Train 5 | Test White 25 | 0.74 | 0.78 | 0.78 | 1305 | Train 21 | Test White 25 | 0.74 | 0.78 | 0.78 |
| 282 | Train 5 | Test White 26 | 0.74 | 0.76 | 0.76 | 1306 | Train 21 | Test White 26 | 0.74 | 0.76 | 0.76 |
| 283 | Train 5 | Test White 27 | 0.74 | 0.78 | 0.78 | 1307 | Train 21 | Test White 27 | 0.74 | 0.78 | 0.78 |
| 284 | Train 5 | Test White 28 | 0.74 | 0.76 | 0.76 | 1308 | Train 21 | Test White 28 | 0.74 | 0.76 | 0.76 |
| 285 | Train 5 | Test White 29 | 0.73 | 0.77 | 0.75 | 1309 | Train 21 | Test White 29 | 0.73 | 0.77 | 0.76 |
| 286 | Train 5 | Test White 30 | 0.7 | 0.71 | 0.7 | 1310 | Train 21 | Test White 30 | 0.7 | 0.71 | 0.68 |
| 287 | Train 5 | Test White 31 | 0.73 | 0.77 | 0.75 | 1311 | Train 21 | Test White 31 | 0.73 | 0.77 | 0.76 |
| 288 | Train 5 | Test White 32 | 0.7 | 0.71 | 0.7 | 1312 | Train 21 | Test White 32 | 0.7 | 0.71 | 0.69 |
| 289 | Train 5 | Test White 33 | 0.74 | 0.79 | 0.81 | 1313 | Train 21 | Test White 33 | 0.74 | 0.79 | 0.81 |
| 290 | Train 5 | Test White 34 | 0.74 | 0.77 | 0.77 | 1314 | Train 21 | Test White 34 | 0.74 | 0.77 | 0.77 |
| 291 | Train 5 | Test White 35 | 0.74 | 0.79 | 0.81 | 1315 | Train 21 | Test White 35 | 0.74 | 0.79 | 0.81 |
| 292 | Train 5 | Test White 36 | 0.74 | 0.77 | 0.77 | 1316 | Train 21 | Test White 36 | 0.74 | 0.77 | 0.77 |
| 293 | Train 5 | Test White 37 | 0.73 | 0.78 | 0.77 | 1317 | Train 21 | Test White 37 | 0.73 | 0.78 | 0.78 |
| 294 | Train 5 | Test White 38 | 0.7 | 0.71 | 0.69 | 1318 | Train 21 | Test White 38 | 0.7 | 0.71 | 0.68 |
| 295 | Train 5 | Test White 39 | 0.73 | 0.78 | 0.77 | 1319 | Train 21 | Test White 39 | 0.73 | 0.78 | 0.78 |
| 296 | Train 5 | Test White 40 | 0.7 | 0.71 | 0.7 | 1320 | Train 21 | Test White 40 | 0.7 | 0.71 | 0.68 |
| 297 | Train 5 | Test White 41 | 0.74 | 0.78 | 0.79 | 1321 | Train 21 | Test White 41 | 0.74 | 0.78 | 0.79 |
| 298 | Train 5 | Test White 42 | 0.74 | 0.76 | 0.76 | 1322 | Train 21 | Test White 42 | 0.74 | 0.76 | 0.76 |
| 299 | Train 5 | Test White 43 | 0.74 | 0.78 | 0.78 | 1323 | Train 21 | Test White 43 | 0.74 | 0.78 | 0.79 |
| 300 | Train 5 | Test White 44 | 0.74 | 0.76 | 0.76 | 1324 | Train 21 | Test White 44 | 0.74 | 0.76 | 0.76 |
| 301 | Train 5 | Test White 45 | 0.73 | 0.77 | 0.75 | 1325 | Train 21 | Test White 45 | 0.73 | 0.77 | 0.76 |
| 302 | Train 5 | Test White 46 | 0.7 | 0.71 | 0.7 | 1326 | Train 21 | Test White 46 | 0.7 | 0.71 | 0.68 |
| 303 | Train 5 | Test White 47 | 0.73 | 0.77 | 0.75 | 1327 | Train 21 | Test White 47 | 0.73 | 0.77 | 0.76 |
| 304 | Train 5 | Test White 48 | 0.7 | 0.71 | 0.7 | 1328 | Train 21 | Test White 48 | 0.7 | 0.71 | 0.69 |
| 305 | Train 5 | Test White 49 | 0.73 | 0.74 | 0.77 | 1329 | Train 21 | Test White 49 | 0.73 | 0.74 | 0.78 |
| 306 | Train 5 | Test White 50 | 0.73 | 0.74 | 0.77 | 1330 | Train 21 | Test White 50 | 0.73 | 0.74 | 0.78 |
| 307 | Train 5 | Test White 51 | 0.73 | 0.74 | 0.76 | 1331 | Train 21 | Test White 51 | 0.73 | 0.74 | 0.78 |
| 308 | Train 5 | Test White 52 | 0.73 | 0.74 | 0.76 | 1332 | Train 21 | Test White 52 | 0.73 | 0.74 | 0.78 |
| 309 | Train 5 | Test White 53 | 0.73 | 0.77 | 0.74 | 1333 | Train 21 | Test White 53 | 0.73 | 0.77 | 0.76 |
| 310 | Train 5 | Test White 54 | 0.71 | 0.71 | 0.69 | 1334 | Train 21 | Test White 54 | 0.71 | 0.71 | 0.7 |
| 311 | Train 5 | Test White 55 | 0.73 | 0.77 | 0.73 | 1335 | Train 21 | Test White 55 | 0.73 | 0.77 | 0.75 |
| 312 | Train 5 | Test White 56 | 0.7 | 0.71 | 0.69 | 1336 | Train 21 | Test White 56 | 0.7 | 0.71 | 0.7 |
| 313 | Train 5 | Test White 57 | 0.73 | 0.74 | 0.74 | 1337 | Train 21 | Test White 57 | 0.73 | 0.74 | 0.76 |
| 314 | Train 5 | Test White 58 | 0.73 | 0.74 | 0.74 | 1338 | Train 21 | Test White 58 | 0.73 | 0.74 | 0.76 |
| 315 | Train 5 | Test White 59 | 0.73 | 0.74 | 0.74 | 1339 | Train 21 | Test White 59 | 0.73 | 0.74 | 0.76 |
| 316 | Train 5 | Test White 60 | 0.73 | 0.74 | 0.74 | 1340 | Train 21 | Test White 60 | 0.73 | 0.74 | 0.76 |
| 317 | Train 5 | Test White 61 | 0.73 | 0.77 | 0.73 | 1341 | Train 21 | Test White 61 | 0.73 | 0.77 | 0.75 |
| 318 | Train 5 | Test White 62 | 0.71 | 0.71 | 0.69 | 1342 | Train 21 | Test White 62 | 0.71 | 0.71 | 0.7 |
| 319 | Train 5 | Test White 63 | 0.73 | 0.77 | 0.73 | 1343 | Train 21 | Test White 63 | 0.73 | 0.77 | 0.75 |
| 320 | Train 5 | Test White 64 | 0.71 | 0.71 | 0.7 | 1344 | Train 21 | Test White 64 | 0.71 | 0.71 | 0.7 |
| 321 | Train 6 | Test White 1 | 0.72 | 0.75 | 0.74 | 1345 | Train 22 | Test White 1 | 0.72 | 0.75 | 0.75 |
| 322 | Train 6 | Test White 2 | 0.74 | 0.76 | 0.75 | 1346 | Train 22 | Test White 2 | 0.74 | 0.76 | 0.75 |
| 323 | Train 6 | Test White 3 | 0.72 | 0.75 | 0.74 | 1347 | Train 22 | Test White 3 | 0.72 | 0.75 | 0.75 |
| 324 | Train 6 | Test White 4 | 0.74 | 0.76 | 0.75 | 1348 | Train 22 | Test White 4 | 0.74 | 0.76 | 0.75 |
| 325 | Train 6 | Test White 5 | 0.72 | 0.77 | 0.77 | 1349 | Train 22 | Test White 5 | 0.72 | 0.77 | 0.77 |
| 326 | Train 6 | Test White 6 | 0.73 | 0.77 | 0.78 | 1350 | Train 22 | Test White 6 | 0.73 | 0.77 | 0.78 |
| 327 | Train 6 | Test White 7 | 0.72 | 0.77 | 0.77 | 1351 | Train 22 | Test White 7 | 0.72 | 0.77 | 0.77 |
| 328 | Train 6 | Test White 8 | 0.73 | 0.77 | 0.78 | 1352 | Train 22 | Test White 8 | 0.73 | 0.77 | 0.78 |
| 329 | Train 6 | Test White 9 | 0.72 | 0.75 | 0.75 | 1353 | Train 22 | Test White 9 | 0.72 | 0.75 | 0.75 |
| 330 | Train 6 | Test White 10 | 0.74 | 0.76 | 0.75 | 1354 | Train 22 | Test White 10 | 0.74 | 0.76 | 0.76 |
| 331 | Train 6 | Test White 11 | 0.72 | 0.75 | 0.75 | 1355 | Train 22 | Test White 11 | 0.72 | 0.75 | 0.75 |
| 332 | Train 6 | Test White 12 | 0.74 | 0.76 | 0.75 | 1356 | Train 22 | Test White 12 | 0.74 | 0.76 | 0.76 |
| 333 | Train 6 | Test White 13 | 0.72 | 0.77 | 0.78 | 1357 | Train 22 | Test White 13 | 0.72 | 0.77 | 0.78 |
| 334 | Train 6 | Test White 14 | 0.73 | 0.77 | 0.78 | 1358 | Train 22 | Test White 14 | 0.73 | 0.77 | 0.78 |
| 335 | Train 6 | Test White 15 | 0.72 | 0.77 | 0.78 | 1359 | Train 22 | Test White 15 | 0.72 | 0.77 | 0.78 |
| 336 | Train 6 | Test White 16 | 0.73 | 0.77 | 0.78 | 1360 | Train 22 | Test White 16 | 0.73 | 0.77 | 0.78 |
| 337 | Train 6 | Test White 17 | 0.72 | 0.75 | 0.74 | 1361 | Train 22 | Test White 17 | 0.72 | 0.75 | 0.75 |
| 338 | Train 6 | Test White 18 | 0.74 | 0.76 | 0.75 | 1362 | Train 22 | Test White 18 | 0.74 | 0.76 | 0.75 |
| 339 | Train 6 | Test White 19 | 0.72 | 0.75 | 0.74 | 1363 | Train 22 | Test White 19 | 0.72 | 0.75 | 0.75 |
| 340 | Train 6 | Test White 20 | 0.74 | 0.76 | 0.75 | 1364 | Train 22 | Test White 20 | 0.74 | 0.76 | 0.75 |
| 341 | Train 6 | Test White 21 | 0.72 | 0.77 | 0.77 | 1365 | Train 22 | Test White 21 | 0.72 | 0.77 | 0.77 |
| 342 | Train 6 | Test White 22 | 0.73 | 0.77 | 0.78 | 1366 | Train 22 | Test White 22 | 0.73 | 0.77 | 0.78 |
| 343 | Train 6 | Test White 23 | 0.72 | 0.77 | 0.77 | 1367 | Train 22 | Test White 23 | 0.72 | 0.77 | 0.77 |
| 344 | Train 6 | Test White 24 | 0.73 | 0.77 | 0.78 | 1368 | Train 22 | Test White 24 | 0.73 | 0.77 | 0.78 |
| 345 | Train 6 | Test White 25 | 0.72 | 0.75 | 0.75 | 1369 | Train 22 | Test White 25 | 0.72 | 0.75 | 0.75 |
| 346 | Train 6 | Test White 26 | 0.74 | 0.76 | 0.75 | 1370 | Train 22 | Test White 26 | 0.74 | 0.76 | 0.76 |
| 347 | Train 6 | Test White 27 | 0.72 | 0.75 | 0.75 | 1371 | Train 22 | Test White 27 | 0.72 | 0.75 | 0.75 |
| 348 | Train 6 | Test White 28 | 0.74 | 0.76 | 0.75 | 1372 | Train 22 | Test White 28 | 0.74 | 0.76 | 0.76 |
| 349 | Train 6 | Test White 29 | 0.72 | 0.77 | 0.78 | 1373 | Train 22 | Test White 29 | 0.72 | 0.77 | 0.78 |
| 350 | Train 6 | Test White 30 | 0.73 | 0.77 | 0.78 | 1374 | Train 22 | Test White 30 | 0.73 | 0.77 | 0.78 |
| 351 | Train 6 | Test White 31 | 0.72 | 0.77 | 0.78 | 1375 | Train 22 | Test White 31 | 0.72 | 0.77 | 0.78 |
| 352 | Train 6 | Test White 32 | 0.73 | 0.77 | 0.78 | 1376 | Train 22 | Test White 32 | 0.73 | 0.77 | 0.78 |
| 353 | Train 6 | Test White 33 | 0.73 | 0.75 | 0.74 | 1377 | Train 22 | Test White 33 | 0.73 | 0.75 | 0.75 |
| 354 | Train 6 | Test White 34 | 0.74 | 0.76 | 0.75 | 1378 | Train 22 | Test White 34 | 0.74 | 0.76 | 0.76 |
| 355 | Train 6 | Test White 35 | 0.73 | 0.75 | 0.74 | 1379 | Train 22 | Test White 35 | 0.73 | 0.75 | 0.75 |
| 356 | Train 6 | Test White 36 | 0.74 | 0.76 | 0.75 | 1380 | Train 22 | Test White 36 | 0.74 | 0.76 | 0.75 |
| 357 | Train 6 | Test White 37 | 0.72 | 0.77 | 0.77 | 1381 | Train 22 | Test White 37 | 0.72 | 0.77 | 0.77 |
| 358 | Train 6 | Test White 38 | 0.73 | 0.77 | 0.78 | 1382 | Train 22 | Test White 38 | 0.73 | 0.77 | 0.78 |
| 359 | Train 6 | Test White 39 | 0.72 | 0.77 | 0.77 | 1383 | Train 22 | Test White 39 | 0.72 | 0.77 | 0.77 |
| 360 | Train 6 | Test White 40 | 0.73 | 0.77 | 0.78 | 1384 | Train 22 | Test White 40 | 0.73 | 0.77 | 0.78 |
| 361 | Train 6 | Test White 41 | 0.73 | 0.76 | 0.75 | 1385 | Train 22 | Test White 41 | 0.73 | 0.76 | 0.75 |
| 362 | Train 6 | Test White 42 | 0.74 | 0.76 | 0.76 | 1386 | Train 22 | Test White 42 | 0.74 | 0.76 | 0.76 |
| 363 | Train 6 | Test White 43 | 0.73 | 0.76 | 0.75 | 1387 | Train 22 | Test White 43 | 0.73 | 0.76 | 0.75 |
| 364 | Train 6 | Test White 44 | 0.74 | 0.76 | 0.76 | 1388 | Train 22 | Test White 44 | 0.74 | 0.76 | 0.76 |
| 365 | Train 6 | Test White 45 | 0.72 | 0.77 | 0.78 | 1389 | Train 22 | Test White 45 | 0.72 | 0.77 | 0.78 |
| 366 | Train 6 | Test White 46 | 0.73 | 0.77 | 0.78 | 1390 | Train 22 | Test White 46 | 0.73 | 0.77 | 0.78 |
| 367 | Train 6 | Test White 47 | 0.72 | 0.77 | 0.78 | 1391 | Train 22 | Test White 47 | 0.72 | 0.77 | 0.78 |
| 368 | Train 6 | Test White 48 | 0.73 | 0.77 | 0.78 | 1392 | Train 22 | Test White 48 | 0.73 | 0.77 | 0.78 |
| 369 | Train 6 | Test White 49 | 0.72 | 0.76 | 0.75 | 1393 | Train 22 | Test White 49 | 0.72 | 0.76 | 0.75 |
| 370 | Train 6 | Test White 50 | 0.72 | 0.76 | 0.75 | 1394 | Train 22 | Test White 50 | 0.73 | 0.76 | 0.75 |
| 371 | Train 6 | Test White 51 | 0.72 | 0.76 | 0.74 | 1395 | Train 22 | Test White 51 | 0.72 | 0.75 | 0.75 |
| 372 | Train 6 | Test White 52 | 0.72 | 0.76 | 0.74 | 1396 | Train 22 | Test White 52 | 0.73 | 0.75 | 0.75 |
| 373 | Train 6 | Test White 53 | 0.73 | 0.78 | 0.75 | 1397 | Train 22 | Test White 53 | 0.73 | 0.78 | 0.76 |
| 374 | Train 6 | Test White 54 | 0.74 | 0.77 | 0.78 | 1398 | Train 22 | Test White 54 | 0.74 | 0.77 | 0.78 |
| 375 | Train 6 | Test White 55 | 0.73 | 0.78 | 0.75 | 1399 | Train 22 | Test White 55 | 0.73 | 0.78 | 0.76 |
| 376 | Train 6 | Test White 56 | 0.73 | 0.76 | 0.78 | 1400 | Train 22 | Test White 56 | 0.73 | 0.76 | 0.78 |
| 377 | Train 6 | Test White 57 | 0.73 | 0.76 | 0.76 | 1401 | Train 22 | Test White 57 | 0.73 | 0.76 | 0.76 |
| 378 | Train 6 | Test White 58 | 0.73 | 0.76 | 0.76 | 1402 | Train 22 | Test White 58 | 0.73 | 0.76 | 0.76 |
| 379 | Train 6 | Test White 59 | 0.73 | 0.76 | 0.76 | 1403 | Train 22 | Test White 59 | 0.73 | 0.76 | 0.76 |
| 380 | Train 6 | Test White 60 | 0.73 | 0.76 | 0.76 | 1404 | Train 22 | Test White 60 | 0.73 | 0.76 | 0.76 |
| 381 | Train 6 | Test White 61 | 0.73 | 0.78 | 0.76 | 1405 | Train 22 | Test White 61 | 0.73 | 0.78 | 0.77 |
| 382 | Train 6 | Test White 62 | 0.74 | 0.77 | 0.78 | 1406 | Train 22 | Test White 62 | 0.74 | 0.77 | 0.78 |
| 383 | Train 6 | Test White 63 | 0.73 | 0.78 | 0.75 | 1407 | Train 22 | Test White 63 | 0.73 | 0.78 | 0.77 |
| 384 | Train 6 | Test White 64 | 0.73 | 0.76 | 0.78 | 1408 | Train 22 | Test White 64 | 0.73 | 0.76 | 0.77 |
| 385 | Train 7 | Test White 1 | 0.74 | 0.79 | 0.81 | 1409 | Train 23 | Test White 1 | 0.74 | 0.79 | 0.8 |
| 386 | Train 7 | Test White 2 | 0.74 | 0.77 | 0.77 | 1410 | Train 23 | Test White 2 | 0.74 | 0.77 | 0.77 |
| 387 | Train 7 | Test White 3 | 0.74 | 0.79 | 0.81 | 1411 | Train 23 | Test White 3 | 0.74 | 0.79 | 0.81 |
| 388 | Train 7 | Test White 4 | 0.74 | 0.77 | 0.77 | 1412 | Train 23 | Test White 4 | 0.74 | 0.77 | 0.77 |
| 389 | Train 7 | Test White 5 | 0.73 | 0.78 | 0.75 | 1413 | Train 23 | Test White 5 | 0.73 | 0.78 | 0.73 |
| 390 | Train 7 | Test White 6 | 0.7 | 0.71 | 0.69 | 1414 | Train 23 | Test White 6 | 0.7 | 0.7 | 0.67 |
| 391 | Train 7 | Test White 7 | 0.73 | 0.78 | 0.75 | 1415 | Train 23 | Test White 7 | 0.73 | 0.78 | 0.73 |
| 392 | Train 7 | Test White 8 | 0.7 | 0.71 | 0.69 | 1416 | Train 23 | Test White 8 | 0.7 | 0.71 | 0.67 |
| 393 | Train 7 | Test White 9 | 0.74 | 0.78 | 0.78 | 1417 | Train 23 | Test White 9 | 0.74 | 0.78 | 0.78 |
| 394 | Train 7 | Test White 10 | 0.74 | 0.76 | 0.76 | 1418 | Train 23 | Test White 10 | 0.74 | 0.76 | 0.76 |
| 395 | Train 7 | Test White 11 | 0.74 | 0.78 | 0.78 | 1419 | Train 23 | Test White 11 | 0.74 | 0.78 | 0.78 |
| 396 | Train 7 | Test White 12 | 0.74 | 0.76 | 0.76 | 1420 | Train 23 | Test White 12 | 0.74 | 0.76 | 0.76 |
| 397 | Train 7 | Test White 13 | 0.73 | 0.77 | 0.73 | 1421 | Train 23 | Test White 13 | 0.73 | 0.77 | 0.72 |
| 398 | Train 7 | Test White 14 | 0.7 | 0.71 | 0.69 | 1422 | Train 23 | Test White 14 | 0.7 | 0.71 | 0.67 |
| 399 | Train 7 | Test White 15 | 0.73 | 0.77 | 0.73 | 1423 | Train 23 | Test White 15 | 0.73 | 0.77 | 0.72 |
| 400 | Train 7 | Test White 16 | 0.7 | 0.71 | 0.69 | 1424 | Train 23 | Test White 16 | 0.7 | 0.71 | 0.67 |
| 401 | Train 7 | Test White 17 | 0.74 | 0.79 | 0.81 | 1425 | Train 23 | Test White 17 | 0.74 | 0.79 | 0.81 |
| 402 | Train 7 | Test White 18 | 0.74 | 0.77 | 0.77 | 1426 | Train 23 | Test White 18 | 0.74 | 0.77 | 0.77 |
| 403 | Train 7 | Test White 19 | 0.74 | 0.79 | 0.81 | 1427 | Train 23 | Test White 19 | 0.74 | 0.79 | 0.81 |
| 404 | Train 7 | Test White 20 | 0.74 | 0.77 | 0.77 | 1428 | Train 23 | Test White 20 | 0.74 | 0.77 | 0.77 |
| 405 | Train 7 | Test White 21 | 0.73 | 0.78 | 0.75 | 1429 | Train 23 | Test White 21 | 0.73 | 0.78 | 0.74 |
| 406 | Train 7 | Test White 22 | 0.7 | 0.71 | 0.69 | 1430 | Train 23 | Test White 22 | 0.7 | 0.71 | 0.67 |
| 407 | Train 7 | Test White 23 | 0.73 | 0.78 | 0.75 | 1431 | Train 23 | Test White 23 | 0.73 | 0.78 | 0.73 |
| 408 | Train 7 | Test White 24 | 0.7 | 0.71 | 0.69 | 1432 | Train 23 | Test White 24 | 0.7 | 0.71 | 0.67 |
| 409 | Train 7 | Test White 25 | 0.74 | 0.78 | 0.78 | 1433 | Train 23 | Test White 25 | 0.74 | 0.78 | 0.78 |
| 410 | Train 7 | Test White 26 | 0.74 | 0.76 | 0.76 | 1434 | Train 23 | Test White 26 | 0.74 | 0.76 | 0.76 |
| 411 | Train 7 | Test White 27 | 0.74 | 0.78 | 0.78 | 1435 | Train 23 | Test White 27 | 0.74 | 0.78 | 0.78 |
| 412 | Train 7 | Test White 28 | 0.74 | 0.76 | 0.76 | 1436 | Train 23 | Test White 28 | 0.74 | 0.76 | 0.76 |
| 413 | Train 7 | Test White 29 | 0.73 | 0.77 | 0.73 | 1437 | Train 23 | Test White 29 | 0.73 | 0.77 | 0.72 |
| 414 | Train 7 | Test White 30 | 0.7 | 0.71 | 0.69 | 1438 | Train 23 | Test White 30 | 0.7 | 0.71 | 0.67 |
| 415 | Train 7 | Test White 31 | 0.73 | 0.77 | 0.73 | 1439 | Train 23 | Test White 31 | 0.73 | 0.77 | 0.72 |
| 416 | Train 7 | Test White 32 | 0.7 | 0.71 | 0.69 | 1440 | Train 23 | Test White 32 | 0.7 | 0.71 | 0.67 |
| 417 | Train 7 | Test White 33 | 0.74 | 0.79 | 0.81 | 1441 | Train 23 | Test White 33 | 0.74 | 0.79 | 0.81 |
| 418 | Train 7 | Test White 34 | 0.74 | 0.77 | 0.77 | 1442 | Train 23 | Test White 34 | 0.74 | 0.77 | 0.77 |
| 419 | Train 7 | Test White 35 | 0.74 | 0.79 | 0.81 | 1443 | Train 23 | Test White 35 | 0.74 | 0.79 | 0.81 |
| 420 | Train 7 | Test White 36 | 0.74 | 0.77 | 0.77 | 1444 | Train 23 | Test White 36 | 0.74 | 0.77 | 0.77 |
| 421 | Train 7 | Test White 37 | 0.73 | 0.78 | 0.75 | 1445 | Train 23 | Test White 37 | 0.73 | 0.78 | 0.74 |
| 422 | Train 7 | Test White 38 | 0.7 | 0.71 | 0.69 | 1446 | Train 23 | Test White 38 | 0.7 | 0.71 | 0.67 |
| 423 | Train 7 | Test White 39 | 0.73 | 0.78 | 0.75 | 1447 | Train 23 | Test White 39 | 0.73 | 0.78 | 0.73 |
| 424 | Train 7 | Test White 40 | 0.7 | 0.71 | 0.69 | 1448 | Train 23 | Test White 40 | 0.7 | 0.71 | 0.67 |
| 425 | Train 7 | Test White 41 | 0.74 | 0.78 | 0.78 | 1449 | Train 23 | Test White 41 | 0.74 | 0.78 | 0.78 |
| 426 | Train 7 | Test White 42 | 0.74 | 0.77 | 0.76 | 1450 | Train 23 | Test White 42 | 0.74 | 0.77 | 0.76 |
| 427 | Train 7 | Test White 43 | 0.74 | 0.78 | 0.78 | 1451 | Train 23 | Test White 43 | 0.74 | 0.78 | 0.78 |
| 428 | Train 7 | Test White 44 | 0.74 | 0.77 | 0.76 | 1452 | Train 23 | Test White 44 | 0.74 | 0.77 | 0.76 |
| 429 | Train 7 | Test White 45 | 0.73 | 0.77 | 0.73 | 1453 | Train 23 | Test White 45 | 0.73 | 0.77 | 0.72 |
| 430 | Train 7 | Test White 46 | 0.7 | 0.71 | 0.69 | 1454 | Train 23 | Test White 46 | 0.7 | 0.71 | 0.67 |
| 431 | Train 7 | Test White 47 | 0.73 | 0.77 | 0.73 | 1455 | Train 23 | Test White 47 | 0.73 | 0.77 | 0.72 |
| 432 | Train 7 | Test White 48 | 0.7 | 0.71 | 0.69 | 1456 | Train 23 | Test White 48 | 0.7 | 0.71 | 0.67 |
| 433 | Train 7 | Test White 49 | 0.73 | 0.74 | 0.78 | 1457 | Train 23 | Test White 49 | 0.72 | 0.74 | 0.78 |
| 434 | Train 7 | Test White 50 | 0.73 | 0.74 | 0.78 | 1458 | Train 23 | Test White 50 | 0.72 | 0.74 | 0.78 |
| 435 | Train 7 | Test White 51 | 0.73 | 0.74 | 0.78 | 1459 | Train 23 | Test White 51 | 0.72 | 0.74 | 0.78 |
| 436 | Train 7 | Test White 52 | 0.73 | 0.74 | 0.78 | 1460 | Train 23 | Test White 52 | 0.72 | 0.74 | 0.78 |
| 437 | Train 7 | Test White 53 | 0.73 | 0.77 | 0.7 | 1461 | Train 23 | Test White 53 | 0.73 | 0.77 | 0.75 |
| 438 | Train 7 | Test White 54 | 0.71 | 0.7 | 0.69 | 1462 | Train 23 | Test White 54 | 0.71 | 0.7 | 0.67 |
| 439 | Train 7 | Test White 55 | 0.73 | 0.77 | 0.7 | 1463 | Train 23 | Test White 55 | 0.73 | 0.77 | 0.74 |
| 440 | Train 7 | Test White 56 | 0.7 | 0.7 | 0.69 | 1464 | Train 23 | Test White 56 | 0.7 | 0.7 | 0.66 |
| 441 | Train 7 | Test White 57 | 0.73 | 0.74 | 0.76 | 1465 | Train 23 | Test White 57 | 0.73 | 0.74 | 0.76 |
| 442 | Train 7 | Test White 58 | 0.73 | 0.74 | 0.76 | 1466 | Train 23 | Test White 58 | 0.73 | 0.74 | 0.76 |
| 443 | Train 7 | Test White 59 | 0.73 | 0.74 | 0.76 | 1467 | Train 23 | Test White 59 | 0.73 | 0.74 | 0.76 |
| 444 | Train 7 | Test White 60 | 0.73 | 0.74 | 0.76 | 1468 | Train 23 | Test White 60 | 0.73 | 0.74 | 0.76 |
| 445 | Train 7 | Test White 61 | 0.74 | 0.77 | 0.7 | 1469 | Train 23 | Test White 61 | 0.74 | 0.77 | 0.74 |
| 446 | Train 7 | Test White 62 | 0.71 | 0.71 | 0.69 | 1470 | Train 23 | Test White 62 | 0.71 | 0.7 | 0.67 |
| 447 | Train 7 | Test White 63 | 0.74 | 0.77 | 0.69 | 1471 | Train 23 | Test White 63 | 0.74 | 0.77 | 0.74 |
| 448 | Train 7 | Test White 64 | 0.71 | 0.71 | 0.69 | 1472 | Train 23 | Test White 64 | 0.71 | 0.7 | 0.66 |
| 449 | Train 8 | Test White 1 | 0.73 | 0.75 | 0.75 | 1473 | Train 24 | Test White 1 | 0.73 | 0.75 | 0.75 |
| 450 | Train 8 | Test White 2 | 0.74 | 0.76 | 0.76 | 1474 | Train 24 | Test White 2 | 0.74 | 0.76 | 0.76 |
| 451 | Train 8 | Test White 3 | 0.73 | 0.75 | 0.75 | 1475 | Train 24 | Test White 3 | 0.73 | 0.75 | 0.75 |
| 452 | Train 8 | Test White 4 | 0.74 | 0.76 | 0.76 | 1476 | Train 24 | Test White 4 | 0.74 | 0.76 | 0.76 |
| 453 | Train 8 | Test White 5 | 0.72 | 0.77 | 0.77 | 1477 | Train 24 | Test White 5 | 0.72 | 0.77 | 0.77 |
| 454 | Train 8 | Test White 6 | 0.73 | 0.77 | 0.78 | 1478 | Train 24 | Test White 6 | 0.73 | 0.77 | 0.78 |
| 455 | Train 8 | Test White 7 | 0.72 | 0.77 | 0.77 | 1479 | Train 24 | Test White 7 | 0.72 | 0.77 | 0.77 |
| 456 | Train 8 | Test White 8 | 0.73 | 0.77 | 0.78 | 1480 | Train 24 | Test White 8 | 0.73 | 0.77 | 0.78 |
| 457 | Train 8 | Test White 9 | 0.73 | 0.75 | 0.76 | 1481 | Train 24 | Test White 9 | 0.73 | 0.75 | 0.76 |
| 458 | Train 8 | Test White 10 | 0.74 | 0.76 | 0.77 | 1482 | Train 24 | Test White 10 | 0.74 | 0.76 | 0.76 |
| 459 | Train 8 | Test White 11 | 0.73 | 0.76 | 0.76 | 1483 | Train 24 | Test White 11 | 0.73 | 0.76 | 0.76 |
| 460 | Train 8 | Test White 12 | 0.74 | 0.76 | 0.77 | 1484 | Train 24 | Test White 12 | 0.74 | 0.76 | 0.76 |
| 461 | Train 8 | Test White 13 | 0.72 | 0.77 | 0.78 | 1485 | Train 24 | Test White 13 | 0.72 | 0.77 | 0.78 |
| 462 | Train 8 | Test White 14 | 0.73 | 0.77 | 0.78 | 1486 | Train 24 | Test White 14 | 0.73 | 0.77 | 0.78 |
| 463 | Train 8 | Test White 15 | 0.72 | 0.77 | 0.78 | 1487 | Train 24 | Test White 15 | 0.72 | 0.77 | 0.78 |
| 464 | Train 8 | Test White 16 | 0.73 | 0.77 | 0.78 | 1488 | Train 24 | Test White 16 | 0.73 | 0.77 | 0.78 |
| 465 | Train 8 | Test White 17 | 0.73 | 0.75 | 0.75 | 1489 | Train 24 | Test White 17 | 0.73 | 0.75 | 0.75 |
| 466 | Train 8 | Test White 18 | 0.74 | 0.76 | 0.76 | 1490 | Train 24 | Test White 18 | 0.74 | 0.76 | 0.75 |
| 467 | Train 8 | Test White 19 | 0.73 | 0.75 | 0.75 | 1491 | Train 24 | Test White 19 | 0.73 | 0.75 | 0.75 |
| 468 | Train 8 | Test White 20 | 0.74 | 0.76 | 0.76 | 1492 | Train 24 | Test White 20 | 0.74 | 0.76 | 0.75 |
| 469 | Train 8 | Test White 21 | 0.72 | 0.77 | 0.77 | 1493 | Train 24 | Test White 21 | 0.72 | 0.77 | 0.77 |
| 470 | Train 8 | Test White 22 | 0.73 | 0.77 | 0.78 | 1494 | Train 24 | Test White 22 | 0.73 | 0.77 | 0.78 |
| 471 | Train 8 | Test White 23 | 0.72 | 0.77 | 0.77 | 1495 | Train 24 | Test White 23 | 0.72 | 0.77 | 0.77 |
| 472 | Train 8 | Test White 24 | 0.73 | 0.77 | 0.78 | 1496 | Train 24 | Test White 24 | 0.73 | 0.77 | 0.78 |
| 473 | Train 8 | Test White 25 | 0.73 | 0.75 | 0.76 | 1497 | Train 24 | Test White 25 | 0.73 | 0.75 | 0.76 |
| 474 | Train 8 | Test White 26 | 0.74 | 0.76 | 0.77 | 1498 | Train 24 | Test White 26 | 0.74 | 0.76 | 0.76 |
| 475 | Train 8 | Test White 27 | 0.73 | 0.76 | 0.76 | 1499 | Train 24 | Test White 27 | 0.73 | 0.76 | 0.76 |
| 476 | Train 8 | Test White 28 | 0.74 | 0.76 | 0.77 | 1500 | Train 24 | Test White 28 | 0.74 | 0.76 | 0.76 |
| 477 | Train 8 | Test White 29 | 0.72 | 0.77 | 0.78 | 1501 | Train 24 | Test White 29 | 0.72 | 0.77 | 0.78 |
| 478 | Train 8 | Test White 30 | 0.73 | 0.77 | 0.78 | 1502 | Train 24 | Test White 30 | 0.73 | 0.77 | 0.78 |
| 479 | Train 8 | Test White 31 | 0.72 | 0.77 | 0.78 | 1503 | Train 24 | Test White 31 | 0.72 | 0.77 | 0.78 |
| 480 | Train 8 | Test White 32 | 0.73 | 0.77 | 0.78 | 1504 | Train 24 | Test White 32 | 0.73 | 0.77 | 0.78 |
| 481 | Train 8 | Test White 33 | 0.73 | 0.75 | 0.75 | 1505 | Train 24 | Test White 33 | 0.73 | 0.75 | 0.75 |
| 482 | Train 8 | Test White 34 | 0.74 | 0.76 | 0.76 | 1506 | Train 24 | Test White 34 | 0.74 | 0.76 | 0.76 |
| 483 | Train 8 | Test White 35 | 0.73 | 0.75 | 0.75 | 1507 | Train 24 | Test White 35 | 0.73 | 0.75 | 0.75 |
| 484 | Train 8 | Test White 36 | 0.74 | 0.76 | 0.76 | 1508 | Train 24 | Test White 36 | 0.74 | 0.76 | 0.76 |
| 485 | Train 8 | Test White 37 | 0.72 | 0.77 | 0.77 | 1509 | Train 24 | Test White 37 | 0.72 | 0.77 | 0.77 |
| 486 | Train 8 | Test White 38 | 0.73 | 0.77 | 0.78 | 1510 | Train 24 | Test White 38 | 0.73 | 0.77 | 0.78 |
| 487 | Train 8 | Test White 39 | 0.72 | 0.77 | 0.77 | 1511 | Train 24 | Test White 39 | 0.72 | 0.77 | 0.77 |
| 488 | Train 8 | Test White 40 | 0.73 | 0.77 | 0.78 | 1512 | Train 24 | Test White 40 | 0.73 | 0.77 | 0.78 |
| 489 | Train 8 | Test White 41 | 0.73 | 0.76 | 0.76 | 1513 | Train 24 | Test White 41 | 0.73 | 0.76 | 0.76 |
| 490 | Train 8 | Test White 42 | 0.74 | 0.76 | 0.77 | 1514 | Train 24 | Test White 42 | 0.74 | 0.76 | 0.76 |
| 491 | Train 8 | Test White 43 | 0.73 | 0.76 | 0.76 | 1515 | Train 24 | Test White 43 | 0.73 | 0.76 | 0.76 |
| 492 | Train 8 | Test White 44 | 0.74 | 0.76 | 0.77 | 1516 | Train 24 | Test White 44 | 0.74 | 0.76 | 0.76 |
| 493 | Train 8 | Test White 45 | 0.72 | 0.77 | 0.78 | 1517 | Train 24 | Test White 45 | 0.72 | 0.77 | 0.78 |
| 494 | Train 8 | Test White 46 | 0.73 | 0.77 | 0.78 | 1518 | Train 24 | Test White 46 | 0.73 | 0.77 | 0.78 |
| 495 | Train 8 | Test White 47 | 0.72 | 0.77 | 0.78 | 1519 | Train 24 | Test White 47 | 0.72 | 0.77 | 0.78 |
| 496 | Train 8 | Test White 48 | 0.73 | 0.77 | 0.78 | 1520 | Train 24 | Test White 48 | 0.73 | 0.77 | 0.78 |
| 497 | Train 8 | Test White 49 | 0.73 | 0.76 | 0.73 | 1521 | Train 24 | Test White 49 | 0.73 | 0.76 | 0.74 |
| 498 | Train 8 | Test White 50 | 0.73 | 0.76 | 0.73 | 1522 | Train 24 | Test White 50 | 0.73 | 0.76 | 0.74 |
| 499 | Train 8 | Test White 51 | 0.73 | 0.76 | 0.73 | 1523 | Train 24 | Test White 51 | 0.73 | 0.76 | 0.74 |
| 500 | Train 8 | Test White 52 | 0.73 | 0.76 | 0.73 | 1524 | Train 24 | Test White 52 | 0.73 | 0.76 | 0.74 |
| 501 | Train 8 | Test White 53 | 0.73 | 0.78 | 0.73 | 1525 | Train 24 | Test White 53 | 0.73 | 0.78 | 0.76 |
| 502 | Train 8 | Test White 54 | 0.73 | 0.77 | 0.78 | 1526 | Train 24 | Test White 54 | 0.73 | 0.77 | 0.78 |
| 503 | Train 8 | Test White 55 | 0.73 | 0.78 | 0.73 | 1527 | Train 24 | Test White 55 | 0.73 | 0.78 | 0.76 |
| 504 | Train 8 | Test White 56 | 0.73 | 0.77 | 0.78 | 1528 | Train 24 | Test White 56 | 0.73 | 0.77 | 0.78 |
| 505 | Train 8 | Test White 57 | 0.73 | 0.76 | 0.74 | 1529 | Train 24 | Test White 57 | 0.73 | 0.76 | 0.76 |
| 506 | Train 8 | Test White 58 | 0.73 | 0.76 | 0.74 | 1530 | Train 24 | Test White 58 | 0.73 | 0.76 | 0.76 |
| 507 | Train 8 | Test White 59 | 0.73 | 0.76 | 0.75 | 1531 | Train 24 | Test White 59 | 0.73 | 0.76 | 0.76 |
| 508 | Train 8 | Test White 60 | 0.73 | 0.76 | 0.75 | 1532 | Train 24 | Test White 60 | 0.73 | 0.76 | 0.76 |
| 509 | Train 8 | Test White 61 | 0.73 | 0.78 | 0.73 | 1533 | Train 24 | Test White 61 | 0.73 | 0.78 | 0.77 |
| 510 | Train 8 | Test White 62 | 0.74 | 0.77 | 0.78 | 1534 | Train 24 | Test White 62 | 0.74 | 0.77 | 0.78 |
| 511 | Train 8 | Test White 63 | 0.73 | 0.78 | 0.73 | 1535 | Train 24 | Test White 63 | 0.73 | 0.78 | 0.77 |
| 512 | Train 8 | Test White 64 | 0.73 | 0.77 | 0.78 | 1536 | Train 24 | Test White 64 | 0.73 | 0.77 | 0.78 |
| 513 | Train 9 | Test White 1 | 0.74 | 0.81 | 0.8 | 1537 | Train 25 | Test White 1 | 0.74 | 0.81 | 0.8 |
| 514 | Train 9 | Test White 2 | 0.73 | 0.76 | 0.75 | 1538 | Train 25 | Test White 2 | 0.73 | 0.76 | 0.76 |
| 515 | Train 9 | Test White 3 | 0.74 | 0.81 | 0.8 | 1539 | Train 25 | Test White 3 | 0.74 | 0.81 | 0.8 |
| 516 | Train 9 | Test White 4 | 0.73 | 0.76 | 0.75 | 1540 | Train 25 | Test White 4 | 0.73 | 0.76 | 0.76 |
| 517 | Train 9 | Test White 5 | 0.71 | 0.73 | 0.66 | 1541 | Train 25 | Test White 5 | 0.7 | 0.73 | 0.7 |
| 518 | Train 9 | Test White 6 | 0.69 | 0.7 | 0.64 | 1542 | Train 25 | Test White 6 | 0.69 | 0.7 | 0.67 |
| 519 | Train 9 | Test White 7 | 0.71 | 0.73 | 0.66 | 1543 | Train 25 | Test White 7 | 0.7 | 0.73 | 0.7 |
| 520 | Train 9 | Test White 8 | 0.69 | 0.7 | 0.64 | 1544 | Train 25 | Test White 8 | 0.69 | 0.7 | 0.67 |
| 521 | Train 9 | Test White 9 | 0.75 | 0.81 | 0.84 | 1545 | Train 25 | Test White 9 | 0.74 | 0.82 | 0.84 |
| 522 | Train 9 | Test White 10 | 0.73 | 0.76 | 0.78 | 1546 | Train 25 | Test White 10 | 0.73 | 0.76 | 0.79 |
| 523 | Train 9 | Test White 11 | 0.75 | 0.81 | 0.84 | 1547 | Train 25 | Test White 11 | 0.75 | 0.81 | 0.84 |
| 524 | Train 9 | Test White 12 | 0.73 | 0.76 | 0.78 | 1548 | Train 25 | Test White 12 | 0.73 | 0.76 | 0.79 |
| 525 | Train 9 | Test White 13 | 0.7 | 0.73 | 0.67 | 1549 | Train 25 | Test White 13 | 0.7 | 0.73 | 0.71 |
| 526 | Train 9 | Test White 14 | 0.69 | 0.7 | 0.64 | 1550 | Train 25 | Test White 14 | 0.69 | 0.7 | 0.67 |
| 527 | Train 9 | Test White 15 | 0.71 | 0.73 | 0.67 | 1551 | Train 25 | Test White 15 | 0.7 | 0.73 | 0.71 |
| 528 | Train 9 | Test White 16 | 0.7 | 0.7 | 0.64 | 1552 | Train 25 | Test White 16 | 0.69 | 0.7 | 0.67 |
| 529 | Train 9 | Test White 17 | 0.74 | 0.81 | 0.8 | 1553 | Train 25 | Test White 17 | 0.74 | 0.81 | 0.8 |
| 530 | Train 9 | Test White 18 | 0.73 | 0.76 | 0.75 | 1554 | Train 25 | Test White 18 | 0.73 | 0.76 | 0.76 |
| 531 | Train 9 | Test White 19 | 0.75 | 0.81 | 0.8 | 1555 | Train 25 | Test White 19 | 0.75 | 0.81 | 0.8 |
| 532 | Train 9 | Test White 20 | 0.73 | 0.76 | 0.75 | 1556 | Train 25 | Test White 20 | 0.73 | 0.76 | 0.76 |
| 533 | Train 9 | Test White 21 | 0.71 | 0.73 | 0.66 | 1557 | Train 25 | Test White 21 | 0.71 | 0.73 | 0.7 |
| 534 | Train 9 | Test White 22 | 0.69 | 0.7 | 0.64 | 1558 | Train 25 | Test White 22 | 0.69 | 0.7 | 0.67 |
| 535 | Train 9 | Test White 23 | 0.71 | 0.73 | 0.66 | 1559 | Train 25 | Test White 23 | 0.71 | 0.73 | 0.7 |
| 536 | Train 9 | Test White 24 | 0.69 | 0.7 | 0.64 | 1560 | Train 25 | Test White 24 | 0.69 | 0.7 | 0.67 |
| 537 | Train 9 | Test White 25 | 0.75 | 0.81 | 0.84 | 1561 | Train 25 | Test White 25 | 0.75 | 0.82 | 0.84 |
| 538 | Train 9 | Test White 26 | 0.73 | 0.76 | 0.78 | 1562 | Train 25 | Test White 26 | 0.73 | 0.76 | 0.79 |
| 539 | Train 9 | Test White 27 | 0.75 | 0.81 | 0.84 | 1563 | Train 25 | Test White 27 | 0.75 | 0.81 | 0.84 |
| 540 | Train 9 | Test White 28 | 0.73 | 0.76 | 0.78 | 1564 | Train 25 | Test White 28 | 0.73 | 0.76 | 0.79 |
| 541 | Train 9 | Test White 29 | 0.71 | 0.73 | 0.67 | 1565 | Train 25 | Test White 29 | 0.7 | 0.73 | 0.71 |
| 542 | Train 9 | Test White 30 | 0.69 | 0.7 | 0.64 | 1566 | Train 25 | Test White 30 | 0.69 | 0.7 | 0.67 |
| 543 | Train 9 | Test White 31 | 0.71 | 0.73 | 0.67 | 1567 | Train 25 | Test White 31 | 0.7 | 0.73 | 0.71 |
| 544 | Train 9 | Test White 32 | 0.7 | 0.7 | 0.64 | 1568 | Train 25 | Test White 32 | 0.69 | 0.7 | 0.67 |
| 545 | Train 9 | Test White 33 | 0.75 | 0.81 | 0.8 | 1569 | Train 25 | Test White 33 | 0.75 | 0.81 | 0.8 |
| 546 | Train 9 | Test White 34 | 0.73 | 0.76 | 0.75 | 1570 | Train 25 | Test White 34 | 0.73 | 0.76 | 0.76 |
| 547 | Train 9 | Test White 35 | 0.75 | 0.81 | 0.8 | 1571 | Train 25 | Test White 35 | 0.75 | 0.81 | 0.8 |
| 548 | Train 9 | Test White 36 | 0.73 | 0.76 | 0.75 | 1572 | Train 25 | Test White 36 | 0.73 | 0.76 | 0.76 |
| 549 | Train 9 | Test White 37 | 0.71 | 0.73 | 0.66 | 1573 | Train 25 | Test White 37 | 0.71 | 0.73 | 0.7 |
| 550 | Train 9 | Test White 38 | 0.69 | 0.7 | 0.64 | 1574 | Train 25 | Test White 38 | 0.69 | 0.7 | 0.67 |
| 551 | Train 9 | Test White 39 | 0.71 | 0.73 | 0.66 | 1575 | Train 25 | Test White 39 | 0.71 | 0.73 | 0.7 |
| 552 | Train 9 | Test White 40 | 0.69 | 0.7 | 0.64 | 1576 | Train 25 | Test White 40 | 0.69 | 0.7 | 0.67 |
| 553 | Train 9 | Test White 41 | 0.75 | 0.82 | 0.84 | 1577 | Train 25 | Test White 41 | 0.75 | 0.82 | 0.84 |
| 554 | Train 9 | Test White 42 | 0.73 | 0.76 | 0.78 | 1578 | Train 25 | Test White 42 | 0.73 | 0.76 | 0.79 |
| 555 | Train 9 | Test White 43 | 0.75 | 0.82 | 0.84 | 1579 | Train 25 | Test White 43 | 0.75 | 0.82 | 0.84 |
| 556 | Train 9 | Test White 44 | 0.73 | 0.76 | 0.78 | 1580 | Train 25 | Test White 44 | 0.73 | 0.76 | 0.79 |
| 557 | Train 9 | Test White 45 | 0.71 | 0.73 | 0.68 | 1581 | Train 25 | Test White 45 | 0.71 | 0.73 | 0.71 |
| 558 | Train 9 | Test White 46 | 0.69 | 0.7 | 0.64 | 1582 | Train 25 | Test White 46 | 0.69 | 0.7 | 0.67 |
| 559 | Train 9 | Test White 47 | 0.71 | 0.73 | 0.68 | 1583 | Train 25 | Test White 47 | 0.71 | 0.73 | 0.71 |
| 560 | Train 9 | Test White 48 | 0.7 | 0.7 | 0.64 | 1584 | Train 25 | Test White 48 | 0.69 | 0.7 | 0.67 |
| 561 | Train 9 | Test White 49 | 0.73 | 0.76 | 0.76 | 1585 | Train 25 | Test White 49 | 0.73 | 0.76 | 0.76 |
| 562 | Train 9 | Test White 50 | 0.73 | 0.76 | 0.76 | 1586 | Train 25 | Test White 50 | 0.73 | 0.76 | 0.76 |
| 563 | Train 9 | Test White 51 | 0.73 | 0.76 | 0.76 | 1587 | Train 25 | Test White 51 | 0.73 | 0.76 | 0.76 |
| 564 | Train 9 | Test White 52 | 0.73 | 0.76 | 0.76 | 1588 | Train 25 | Test White 52 | 0.73 | 0.76 | 0.76 |
| 565 | Train 9 | Test White 53 | 0.7 | 0.73 | 0.58 | 1589 | Train 25 | Test White 53 | 0.7 | 0.73 | 0.66 |
| 566 | Train 9 | Test White 54 | 0.7 | 0.7 | 0.62 | 1590 | Train 25 | Test White 54 | 0.7 | 0.7 | 0.67 |
| 567 | Train 9 | Test White 55 | 0.7 | 0.73 | 0.58 | 1591 | Train 25 | Test White 55 | 0.7 | 0.73 | 0.66 |
| 568 | Train 9 | Test White 56 | 0.7 | 0.7 | 0.63 | 1592 | Train 25 | Test White 56 | 0.7 | 0.69 | 0.67 |
| 569 | Train 9 | Test White 57 | 0.73 | 0.77 | 0.81 | 1593 | Train 25 | Test White 57 | 0.73 | 0.77 | 0.81 |
| 570 | Train 9 | Test White 58 | 0.73 | 0.77 | 0.8 | 1594 | Train 25 | Test White 58 | 0.73 | 0.77 | 0.8 |
| 571 | Train 9 | Test White 59 | 0.73 | 0.77 | 0.8 | 1595 | Train 25 | Test White 59 | 0.73 | 0.77 | 0.8 |
| 572 | Train 9 | Test White 60 | 0.73 | 0.77 | 0.8 | 1596 | Train 25 | Test White 60 | 0.73 | 0.77 | 0.8 |
| 573 | Train 9 | Test White 61 | 0.7 | 0.74 | 0.58 | 1597 | Train 25 | Test White 61 | 0.7 | 0.74 | 0.67 |
| 574 | Train 9 | Test White 62 | 0.7 | 0.7 | 0.62 | 1598 | Train 25 | Test White 62 | 0.7 | 0.7 | 0.67 |
| 575 | Train 9 | Test White 63 | 0.7 | 0.74 | 0.59 | 1599 | Train 25 | Test White 63 | 0.7 | 0.74 | 0.67 |
| 576 | Train 9 | Test White 64 | 0.7 | 0.7 | 0.63 | 1600 | Train 25 | Test White 64 | 0.7 | 0.7 | 0.67 |
| 577 | Train 10 | Test White 1 | 0.74 | 0.8 | 0.79 | 1601 | Train 26 | Test White 1 | 0.74 | 0.8 | 0.79 |
| 578 | Train 10 | Test White 2 | 0.75 | 0.81 | 0.8 | 1602 | Train 26 | Test White 2 | 0.75 | 0.81 | 0.8 |
| 579 | Train 10 | Test White 3 | 0.74 | 0.8 | 0.79 | 1603 | Train 26 | Test White 3 | 0.74 | 0.8 | 0.79 |
| 580 | Train 10 | Test White 4 | 0.75 | 0.81 | 0.8 | 1604 | Train 26 | Test White 4 | 0.75 | 0.81 | 0.8 |
| 581 | Train 10 | Test White 5 | 0.71 | 0.73 | 0.68 | 1605 | Train 26 | Test White 5 | 0.71 | 0.73 | 0.68 |
| 582 | Train 10 | Test White 6 | 0.7 | 0.71 | 0.67 | 1606 | Train 26 | Test White 6 | 0.7 | 0.71 | 0.67 |
| 583 | Train 10 | Test White 7 | 0.71 | 0.73 | 0.68 | 1607 | Train 26 | Test White 7 | 0.71 | 0.73 | 0.68 |
| 584 | Train 10 | Test White 8 | 0.7 | 0.71 | 0.67 | 1608 | Train 26 | Test White 8 | 0.7 | 0.71 | 0.67 |
| 585 | Train 10 | Test White 9 | 0.74 | 0.8 | 0.82 | 1609 | Train 26 | Test White 9 | 0.74 | 0.8 | 0.82 |
| 586 | Train 10 | Test White 10 | 0.75 | 0.81 | 0.83 | 1610 | Train 26 | Test White 10 | 0.75 | 0.81 | 0.83 |
| 587 | Train 10 | Test White 11 | 0.74 | 0.8 | 0.82 | 1611 | Train 26 | Test White 11 | 0.74 | 0.8 | 0.82 |
| 588 | Train 10 | Test White 12 | 0.75 | 0.81 | 0.83 | 1612 | Train 26 | Test White 12 | 0.75 | 0.81 | 0.83 |
| 589 | Train 10 | Test White 13 | 0.71 | 0.73 | 0.69 | 1613 | Train 26 | Test White 13 | 0.71 | 0.73 | 0.69 |
| 590 | Train 10 | Test White 14 | 0.7 | 0.71 | 0.67 | 1614 | Train 26 | Test White 14 | 0.71 | 0.71 | 0.67 |
| 591 | Train 10 | Test White 15 | 0.71 | 0.73 | 0.69 | 1615 | Train 26 | Test White 15 | 0.71 | 0.73 | 0.69 |
| 592 | Train 10 | Test White 16 | 0.7 | 0.71 | 0.67 | 1616 | Train 26 | Test White 16 | 0.7 | 0.71 | 0.67 |
| 593 | Train 10 | Test White 17 | 0.74 | 0.8 | 0.79 | 1617 | Train 26 | Test White 17 | 0.74 | 0.8 | 0.79 |
| 594 | Train 10 | Test White 18 | 0.75 | 0.81 | 0.8 | 1618 | Train 26 | Test White 18 | 0.75 | 0.81 | 0.8 |
| 595 | Train 10 | Test White 19 | 0.74 | 0.8 | 0.79 | 1619 | Train 26 | Test White 19 | 0.74 | 0.8 | 0.79 |
| 596 | Train 10 | Test White 20 | 0.75 | 0.81 | 0.8 | 1620 | Train 26 | Test White 20 | 0.75 | 0.81 | 0.8 |
| 597 | Train 10 | Test White 21 | 0.71 | 0.73 | 0.68 | 1621 | Train 26 | Test White 21 | 0.71 | 0.73 | 0.68 |
| 598 | Train 10 | Test White 22 | 0.7 | 0.71 | 0.67 | 1622 | Train 26 | Test White 22 | 0.7 | 0.71 | 0.67 |
| 599 | Train 10 | Test White 23 | 0.71 | 0.73 | 0.68 | 1623 | Train 26 | Test White 23 | 0.71 | 0.73 | 0.68 |
| 600 | Train 10 | Test White 24 | 0.7 | 0.71 | 0.67 | 1624 | Train 26 | Test White 24 | 0.7 | 0.71 | 0.67 |
| 601 | Train 10 | Test White 25 | 0.74 | 0.8 | 0.82 | 1625 | Train 26 | Test White 25 | 0.74 | 0.8 | 0.82 |
| 602 | Train 10 | Test White 26 | 0.75 | 0.81 | 0.83 | 1626 | Train 26 | Test White 26 | 0.75 | 0.81 | 0.83 |
| 603 | Train 10 | Test White 27 | 0.74 | 0.8 | 0.82 | 1627 | Train 26 | Test White 27 | 0.74 | 0.8 | 0.82 |
| 604 | Train 10 | Test White 28 | 0.75 | 0.81 | 0.83 | 1628 | Train 26 | Test White 28 | 0.75 | 0.81 | 0.83 |
| 605 | Train 10 | Test White 29 | 0.71 | 0.73 | 0.69 | 1629 | Train 26 | Test White 29 | 0.71 | 0.73 | 0.69 |
| 606 | Train 10 | Test White 30 | 0.71 | 0.71 | 0.67 | 1630 | Train 26 | Test White 30 | 0.71 | 0.71 | 0.67 |
| 607 | Train 10 | Test White 31 | 0.71 | 0.73 | 0.69 | 1631 | Train 26 | Test White 31 | 0.71 | 0.73 | 0.69 |
| 608 | Train 10 | Test White 32 | 0.7 | 0.71 | 0.67 | 1632 | Train 26 | Test White 32 | 0.71 | 0.71 | 0.67 |
| 609 | Train 10 | Test White 33 | 0.74 | 0.8 | 0.8 | 1633 | Train 26 | Test White 33 | 0.74 | 0.8 | 0.79 |
| 610 | Train 10 | Test White 34 | 0.75 | 0.81 | 0.8 | 1634 | Train 26 | Test White 34 | 0.75 | 0.81 | 0.8 |
| 611 | Train 10 | Test White 35 | 0.74 | 0.8 | 0.79 | 1635 | Train 26 | Test White 35 | 0.74 | 0.8 | 0.79 |
| 612 | Train 10 | Test White 36 | 0.75 | 0.81 | 0.8 | 1636 | Train 26 | Test White 36 | 0.75 | 0.81 | 0.8 |
| 613 | Train 10 | Test White 37 | 0.71 | 0.73 | 0.68 | 1637 | Train 26 | Test White 37 | 0.71 | 0.73 | 0.68 |
| 614 | Train 10 | Test White 38 | 0.7 | 0.71 | 0.67 | 1638 | Train 26 | Test White 38 | 0.7 | 0.71 | 0.67 |
| 615 | Train 10 | Test White 39 | 0.71 | 0.73 | 0.68 | 1639 | Train 26 | Test White 39 | 0.71 | 0.73 | 0.68 |
| 616 | Train 10 | Test White 40 | 0.7 | 0.71 | 0.67 | 1640 | Train 26 | Test White 40 | 0.7 | 0.71 | 0.67 |
| 617 | Train 10 | Test White 41 | 0.74 | 0.81 | 0.82 | 1641 | Train 26 | Test White 41 | 0.74 | 0.81 | 0.82 |
| 618 | Train 10 | Test White 42 | 0.75 | 0.81 | 0.83 | 1642 | Train 26 | Test White 42 | 0.75 | 0.81 | 0.83 |
| 619 | Train 10 | Test White 43 | 0.74 | 0.81 | 0.82 | 1643 | Train 26 | Test White 43 | 0.74 | 0.81 | 0.82 |
| 620 | Train 10 | Test White 44 | 0.75 | 0.81 | 0.83 | 1644 | Train 26 | Test White 44 | 0.75 | 0.81 | 0.83 |
| 621 | Train 10 | Test White 45 | 0.71 | 0.73 | 0.69 | 1645 | Train 26 | Test White 45 | 0.71 | 0.73 | 0.69 |
| 622 | Train 10 | Test White 46 | 0.71 | 0.71 | 0.67 | 1646 | Train 26 | Test White 46 | 0.71 | 0.71 | 0.67 |
| 623 | Train 10 | Test White 47 | 0.71 | 0.73 | 0.7 | 1647 | Train 26 | Test White 47 | 0.71 | 0.73 | 0.69 |
| 624 | Train 10 | Test White 48 | 0.7 | 0.71 | 0.67 | 1648 | Train 26 | Test White 48 | 0.71 | 0.71 | 0.67 |
| 625 | Train 10 | Test White 49 | 0.73 | 0.76 | 0.75 | 1649 | Train 26 | Test White 49 | 0.73 | 0.76 | 0.76 |
| 626 | Train 10 | Test White 50 | 0.73 | 0.76 | 0.75 | 1650 | Train 26 | Test White 50 | 0.73 | 0.76 | 0.76 |
| 627 | Train 10 | Test White 51 | 0.73 | 0.76 | 0.75 | 1651 | Train 26 | Test White 51 | 0.73 | 0.76 | 0.76 |
| 628 | Train 10 | Test White 52 | 0.73 | 0.76 | 0.75 | 1652 | Train 26 | Test White 52 | 0.73 | 0.76 | 0.76 |
| 629 | Train 10 | Test White 53 | 0.7 | 0.74 | 0.66 | 1653 | Train 26 | Test White 53 | 0.7 | 0.74 | 0.62 |
| 630 | Train 10 | Test White 54 | 0.71 | 0.71 | 0.67 | 1654 | Train 26 | Test White 54 | 0.71 | 0.71 | 0.66 |
| 631 | Train 10 | Test White 55 | 0.7 | 0.74 | 0.66 | 1655 | Train 26 | Test White 55 | 0.71 | 0.74 | 0.62 |
| 632 | Train 10 | Test White 56 | 0.7 | 0.7 | 0.67 | 1656 | Train 26 | Test White 56 | 0.7 | 0.7 | 0.66 |
| 633 | Train 10 | Test White 57 | 0.73 | 0.76 | 0.79 | 1657 | Train 26 | Test White 57 | 0.73 | 0.76 | 0.79 |
| 634 | Train 10 | Test White 58 | 0.73 | 0.76 | 0.79 | 1658 | Train 26 | Test White 58 | 0.73 | 0.76 | 0.79 |
| 635 | Train 10 | Test White 59 | 0.73 | 0.76 | 0.79 | 1659 | Train 26 | Test White 59 | 0.73 | 0.76 | 0.79 |
| 636 | Train 10 | Test White 60 | 0.73 | 0.76 | 0.79 | 1660 | Train 26 | Test White 60 | 0.73 | 0.76 | 0.79 |
| 637 | Train 10 | Test White 61 | 0.7 | 0.74 | 0.66 | 1661 | Train 26 | Test White 61 | 0.71 | 0.74 | 0.62 |
| 638 | Train 10 | Test White 62 | 0.71 | 0.71 | 0.67 | 1662 | Train 26 | Test White 62 | 0.71 | 0.71 | 0.66 |
| 639 | Train 10 | Test White 63 | 0.71 | 0.74 | 0.66 | 1663 | Train 26 | Test White 63 | 0.71 | 0.74 | 0.62 |
| 640 | Train 10 | Test White 64 | 0.7 | 0.71 | 0.67 | 1664 | Train 26 | Test White 64 | 0.7 | 0.71 | 0.66 |
| 641 | Train 11 | Test White 1 | 0.74 | 0.81 | 0.8 | 1665 | Train 27 | Test White 1 | 0.74 | 0.81 | 0.8 |
| 642 | Train 11 | Test White 2 | 0.73 | 0.76 | 0.75 | 1666 | Train 27 | Test White 2 | 0.73 | 0.76 | 0.75 |
| 643 | Train 11 | Test White 3 | 0.74 | 0.81 | 0.8 | 1667 | Train 27 | Test White 3 | 0.74 | 0.81 | 0.8 |
| 644 | Train 11 | Test White 4 | 0.73 | 0.76 | 0.75 | 1668 | Train 27 | Test White 4 | 0.73 | 0.76 | 0.75 |
| 645 | Train 11 | Test White 5 | 0.7 | 0.73 | 0.68 | 1669 | Train 27 | Test White 5 | 0.71 | 0.73 | 0.68 |
| 646 | Train 11 | Test White 6 | 0.69 | 0.7 | 0.65 | 1670 | Train 27 | Test White 6 | 0.69 | 0.7 | 0.64 |
| 647 | Train 11 | Test White 7 | 0.7 | 0.73 | 0.68 | 1671 | Train 27 | Test White 7 | 0.71 | 0.73 | 0.68 |
| 648 | Train 11 | Test White 8 | 0.69 | 0.7 | 0.65 | 1672 | Train 27 | Test White 8 | 0.69 | 0.7 | 0.64 |
| 649 | Train 11 | Test White 9 | 0.74 | 0.81 | 0.83 | 1673 | Train 27 | Test White 9 | 0.74 | 0.81 | 0.84 |
| 650 | Train 11 | Test White 10 | 0.73 | 0.76 | 0.78 | 1674 | Train 27 | Test White 10 | 0.73 | 0.76 | 0.78 |
| 651 | Train 11 | Test White 11 | 0.75 | 0.82 | 0.83 | 1675 | Train 27 | Test White 11 | 0.75 | 0.82 | 0.84 |
| 652 | Train 11 | Test White 12 | 0.73 | 0.76 | 0.78 | 1676 | Train 27 | Test White 12 | 0.73 | 0.76 | 0.78 |
| 653 | Train 11 | Test White 13 | 0.7 | 0.73 | 0.69 | 1677 | Train 27 | Test White 13 | 0.7 | 0.73 | 0.69 |
| 654 | Train 11 | Test White 14 | 0.69 | 0.7 | 0.65 | 1678 | Train 27 | Test White 14 | 0.69 | 0.7 | 0.64 |
| 655 | Train 11 | Test White 15 | 0.7 | 0.73 | 0.69 | 1679 | Train 27 | Test White 15 | 0.7 | 0.73 | 0.69 |
| 656 | Train 11 | Test White 16 | 0.69 | 0.7 | 0.65 | 1680 | Train 27 | Test White 16 | 0.69 | 0.7 | 0.64 |
| 657 | Train 11 | Test White 17 | 0.74 | 0.81 | 0.8 | 1681 | Train 27 | Test White 17 | 0.74 | 0.81 | 0.8 |
| 658 | Train 11 | Test White 18 | 0.73 | 0.76 | 0.75 | 1682 | Train 27 | Test White 18 | 0.73 | 0.76 | 0.75 |
| 659 | Train 11 | Test White 19 | 0.75 | 0.81 | 0.8 | 1683 | Train 27 | Test White 19 | 0.75 | 0.81 | 0.8 |
| 660 | Train 11 | Test White 20 | 0.73 | 0.76 | 0.75 | 1684 | Train 27 | Test White 20 | 0.73 | 0.76 | 0.75 |
| 661 | Train 11 | Test White 21 | 0.7 | 0.73 | 0.68 | 1685 | Train 27 | Test White 21 | 0.71 | 0.73 | 0.68 |
| 662 | Train 11 | Test White 22 | 0.69 | 0.7 | 0.65 | 1686 | Train 27 | Test White 22 | 0.69 | 0.7 | 0.64 |
| 663 | Train 11 | Test White 23 | 0.7 | 0.73 | 0.68 | 1687 | Train 27 | Test White 23 | 0.71 | 0.73 | 0.68 |
| 664 | Train 11 | Test White 24 | 0.69 | 0.7 | 0.65 | 1688 | Train 27 | Test White 24 | 0.69 | 0.7 | 0.64 |
| 665 | Train 11 | Test White 25 | 0.75 | 0.81 | 0.83 | 1689 | Train 27 | Test White 25 | 0.75 | 0.81 | 0.84 |
| 666 | Train 11 | Test White 26 | 0.73 | 0.76 | 0.78 | 1690 | Train 27 | Test White 26 | 0.73 | 0.76 | 0.78 |
| 667 | Train 11 | Test White 27 | 0.75 | 0.82 | 0.83 | 1691 | Train 27 | Test White 27 | 0.75 | 0.82 | 0.84 |
| 668 | Train 11 | Test White 28 | 0.73 | 0.76 | 0.78 | 1692 | Train 27 | Test White 28 | 0.73 | 0.76 | 0.78 |
| 669 | Train 11 | Test White 29 | 0.7 | 0.73 | 0.69 | 1693 | Train 27 | Test White 29 | 0.7 | 0.74 | 0.69 |
| 670 | Train 11 | Test White 30 | 0.69 | 0.7 | 0.65 | 1694 | Train 27 | Test White 30 | 0.69 | 0.7 | 0.64 |
| 671 | Train 11 | Test White 31 | 0.7 | 0.73 | 0.69 | 1695 | Train 27 | Test White 31 | 0.71 | 0.74 | 0.69 |
| 672 | Train 11 | Test White 32 | 0.69 | 0.7 | 0.65 | 1696 | Train 27 | Test White 32 | 0.69 | 0.7 | 0.64 |
| 673 | Train 11 | Test White 33 | 0.75 | 0.81 | 0.8 | 1697 | Train 27 | Test White 33 | 0.75 | 0.81 | 0.8 |
| 674 | Train 11 | Test White 34 | 0.73 | 0.76 | 0.75 | 1698 | Train 27 | Test White 34 | 0.73 | 0.76 | 0.75 |
| 675 | Train 11 | Test White 35 | 0.75 | 0.81 | 0.8 | 1699 | Train 27 | Test White 35 | 0.75 | 0.81 | 0.8 |
| 676 | Train 11 | Test White 36 | 0.73 | 0.76 | 0.75 | 1700 | Train 27 | Test White 36 | 0.73 | 0.76 | 0.75 |
| 677 | Train 11 | Test White 37 | 0.71 | 0.73 | 0.68 | 1701 | Train 27 | Test White 37 | 0.71 | 0.73 | 0.68 |
| 678 | Train 11 | Test White 38 | 0.69 | 0.7 | 0.65 | 1702 | Train 27 | Test White 38 | 0.69 | 0.7 | 0.64 |
| 679 | Train 11 | Test White 39 | 0.71 | 0.73 | 0.68 | 1703 | Train 27 | Test White 39 | 0.71 | 0.73 | 0.68 |
| 680 | Train 11 | Test White 40 | 0.69 | 0.7 | 0.65 | 1704 | Train 27 | Test White 40 | 0.69 | 0.7 | 0.64 |
| 681 | Train 11 | Test White 41 | 0.75 | 0.82 | 0.83 | 1705 | Train 27 | Test White 41 | 0.75 | 0.82 | 0.84 |
| 682 | Train 11 | Test White 42 | 0.73 | 0.77 | 0.78 | 1706 | Train 27 | Test White 42 | 0.73 | 0.77 | 0.78 |
| 683 | Train 11 | Test White 43 | 0.75 | 0.82 | 0.83 | 1707 | Train 27 | Test White 43 | 0.75 | 0.82 | 0.84 |
| 684 | Train 11 | Test White 44 | 0.73 | 0.77 | 0.78 | 1708 | Train 27 | Test White 44 | 0.73 | 0.77 | 0.78 |
| 685 | Train 11 | Test White 45 | 0.71 | 0.73 | 0.69 | 1709 | Train 27 | Test White 45 | 0.71 | 0.74 | 0.69 |
| 686 | Train 11 | Test White 46 | 0.69 | 0.7 | 0.65 | 1710 | Train 27 | Test White 46 | 0.69 | 0.7 | 0.64 |
| 687 | Train 11 | Test White 47 | 0.71 | 0.73 | 0.69 | 1711 | Train 27 | Test White 47 | 0.71 | 0.74 | 0.69 |
| 688 | Train 11 | Test White 48 | 0.69 | 0.7 | 0.65 | 1712 | Train 27 | Test White 48 | 0.7 | 0.7 | 0.64 |
| 689 | Train 11 | Test White 49 | 0.73 | 0.76 | 0.76 | 1713 | Train 27 | Test White 49 | 0.73 | 0.76 | 0.77 |
| 690 | Train 11 | Test White 50 | 0.73 | 0.76 | 0.76 | 1714 | Train 27 | Test White 50 | 0.73 | 0.76 | 0.77 |
| 691 | Train 11 | Test White 51 | 0.73 | 0.76 | 0.76 | 1715 | Train 27 | Test White 51 | 0.73 | 0.76 | 0.77 |
| 692 | Train 11 | Test White 52 | 0.73 | 0.76 | 0.76 | 1716 | Train 27 | Test White 52 | 0.73 | 0.76 | 0.77 |
| 693 | Train 11 | Test White 53 | 0.7 | 0.73 | 0.62 | 1717 | Train 27 | Test White 53 | 0.7 | 0.73 | 0.64 |
| 694 | Train 11 | Test White 54 | 0.7 | 0.7 | 0.64 | 1718 | Train 27 | Test White 54 | 0.7 | 0.7 | 0.65 |
| 695 | Train 11 | Test White 55 | 0.7 | 0.73 | 0.62 | 1719 | Train 27 | Test White 55 | 0.7 | 0.74 | 0.64 |
| 696 | Train 11 | Test White 56 | 0.7 | 0.7 | 0.65 | 1720 | Train 27 | Test White 56 | 0.7 | 0.7 | 0.64 |
| 697 | Train 11 | Test White 57 | 0.73 | 0.77 | 0.79 | 1721 | Train 27 | Test White 57 | 0.73 | 0.77 | 0.81 |
| 698 | Train 11 | Test White 58 | 0.73 | 0.77 | 0.79 | 1722 | Train 27 | Test White 58 | 0.73 | 0.77 | 0.81 |
| 699 | Train 11 | Test White 59 | 0.74 | 0.77 | 0.79 | 1723 | Train 27 | Test White 59 | 0.74 | 0.77 | 0.81 |
| 700 | Train 11 | Test White 60 | 0.74 | 0.77 | 0.79 | 1724 | Train 27 | Test White 60 | 0.74 | 0.77 | 0.81 |
| 701 | Train 11 | Test White 61 | 0.7 | 0.74 | 0.62 | 1725 | Train 27 | Test White 61 | 0.7 | 0.74 | 0.65 |
| 702 | Train 11 | Test White 62 | 0.7 | 0.7 | 0.65 | 1726 | Train 27 | Test White 62 | 0.7 | 0.7 | 0.65 |
| 703 | Train 11 | Test White 63 | 0.7 | 0.74 | 0.62 | 1727 | Train 27 | Test White 63 | 0.7 | 0.74 | 0.65 |
| 704 | Train 11 | Test White 64 | 0.7 | 0.7 | 0.65 | 1728 | Train 27 | Test White 64 | 0.7 | 0.7 | 0.64 |
| 705 | Train 12 | Test White 1 | 0.74 | 0.8 | 0.79 | 1729 | Train 28 | Test White 1 | 0.74 | 0.8 | 0.79 |
| 706 | Train 12 | Test White 2 | 0.75 | 0.81 | 0.8 | 1730 | Train 28 | Test White 2 | 0.75 | 0.81 | 0.81 |
| 707 | Train 12 | Test White 3 | 0.74 | 0.8 | 0.79 | 1731 | Train 28 | Test White 3 | 0.74 | 0.8 | 0.79 |
| 708 | Train 12 | Test White 4 | 0.75 | 0.81 | 0.8 | 1732 | Train 28 | Test White 4 | 0.75 | 0.81 | 0.81 |
| 709 | Train 12 | Test White 5 | 0.71 | 0.73 | 0.69 | 1733 | Train 28 | Test White 5 | 0.71 | 0.73 | 0.7 |
| 710 | Train 12 | Test White 6 | 0.7 | 0.71 | 0.68 | 1734 | Train 28 | Test White 6 | 0.7 | 0.71 | 0.68 |
| 711 | Train 12 | Test White 7 | 0.71 | 0.73 | 0.69 | 1735 | Train 28 | Test White 7 | 0.71 | 0.73 | 0.7 |
| 712 | Train 12 | Test White 8 | 0.7 | 0.71 | 0.68 | 1736 | Train 28 | Test White 8 | 0.7 | 0.71 | 0.68 |
| 713 | Train 12 | Test White 9 | 0.74 | 0.8 | 0.82 | 1737 | Train 28 | Test White 9 | 0.74 | 0.8 | 0.82 |
| 714 | Train 12 | Test White 10 | 0.75 | 0.81 | 0.83 | 1738 | Train 28 | Test White 10 | 0.75 | 0.81 | 0.83 |
| 715 | Train 12 | Test White 11 | 0.74 | 0.8 | 0.82 | 1739 | Train 28 | Test White 11 | 0.74 | 0.8 | 0.82 |
| 716 | Train 12 | Test White 12 | 0.75 | 0.81 | 0.83 | 1740 | Train 28 | Test White 12 | 0.75 | 0.81 | 0.83 |
| 717 | Train 12 | Test White 13 | 0.71 | 0.73 | 0.7 | 1741 | Train 28 | Test White 13 | 0.71 | 0.73 | 0.71 |
| 718 | Train 12 | Test White 14 | 0.71 | 0.71 | 0.68 | 1742 | Train 28 | Test White 14 | 0.7 | 0.71 | 0.68 |
| 719 | Train 12 | Test White 15 | 0.71 | 0.73 | 0.7 | 1743 | Train 28 | Test White 15 | 0.71 | 0.73 | 0.71 |
| 720 | Train 12 | Test White 16 | 0.7 | 0.71 | 0.68 | 1744 | Train 28 | Test White 16 | 0.7 | 0.71 | 0.68 |
| 721 | Train 12 | Test White 17 | 0.74 | 0.8 | 0.79 | 1745 | Train 28 | Test White 17 | 0.74 | 0.8 | 0.79 |
| 722 | Train 12 | Test White 18 | 0.75 | 0.81 | 0.8 | 1746 | Train 28 | Test White 18 | 0.75 | 0.81 | 0.81 |
| 723 | Train 12 | Test White 19 | 0.74 | 0.8 | 0.79 | 1747 | Train 28 | Test White 19 | 0.74 | 0.8 | 0.79 |
| 724 | Train 12 | Test White 20 | 0.75 | 0.81 | 0.8 | 1748 | Train 28 | Test White 20 | 0.75 | 0.81 | 0.81 |
| 725 | Train 12 | Test White 21 | 0.71 | 0.73 | 0.69 | 1749 | Train 28 | Test White 21 | 0.71 | 0.73 | 0.7 |
| 726 | Train 12 | Test White 22 | 0.7 | 0.71 | 0.68 | 1750 | Train 28 | Test White 22 | 0.7 | 0.71 | 0.68 |
| 727 | Train 12 | Test White 23 | 0.71 | 0.73 | 0.69 | 1751 | Train 28 | Test White 23 | 0.71 | 0.73 | 0.7 |
| 728 | Train 12 | Test White 24 | 0.7 | 0.71 | 0.68 | 1752 | Train 28 | Test White 24 | 0.7 | 0.71 | 0.68 |
| 729 | Train 12 | Test White 25 | 0.74 | 0.8 | 0.82 | 1753 | Train 28 | Test White 25 | 0.74 | 0.8 | 0.82 |
| 730 | Train 12 | Test White 26 | 0.75 | 0.81 | 0.83 | 1754 | Train 28 | Test White 26 | 0.75 | 0.81 | 0.83 |
| 731 | Train 12 | Test White 27 | 0.74 | 0.8 | 0.82 | 1755 | Train 28 | Test White 27 | 0.74 | 0.8 | 0.82 |
| 732 | Train 12 | Test White 28 | 0.75 | 0.81 | 0.83 | 1756 | Train 28 | Test White 28 | 0.75 | 0.81 | 0.83 |
| 733 | Train 12 | Test White 29 | 0.71 | 0.73 | 0.7 | 1757 | Train 28 | Test White 29 | 0.71 | 0.73 | 0.71 |
| 734 | Train 12 | Test White 30 | 0.71 | 0.71 | 0.68 | 1758 | Train 28 | Test White 30 | 0.71 | 0.71 | 0.68 |
| 735 | Train 12 | Test White 31 | 0.71 | 0.73 | 0.7 | 1759 | Train 28 | Test White 31 | 0.71 | 0.73 | 0.71 |
| 736 | Train 12 | Test White 32 | 0.7 | 0.71 | 0.68 | 1760 | Train 28 | Test White 32 | 0.7 | 0.71 | 0.68 |
| 737 | Train 12 | Test White 33 | 0.74 | 0.8 | 0.79 | 1761 | Train 28 | Test White 33 | 0.74 | 0.8 | 0.8 |
| 738 | Train 12 | Test White 34 | 0.75 | 0.81 | 0.81 | 1762 | Train 28 | Test White 34 | 0.75 | 0.81 | 0.81 |
| 739 | Train 12 | Test White 35 | 0.74 | 0.8 | 0.79 | 1763 | Train 28 | Test White 35 | 0.74 | 0.8 | 0.8 |
| 740 | Train 12 | Test White 36 | 0.75 | 0.81 | 0.81 | 1764 | Train 28 | Test White 36 | 0.75 | 0.81 | 0.81 |
| 741 | Train 12 | Test White 37 | 0.71 | 0.73 | 0.69 | 1765 | Train 28 | Test White 37 | 0.71 | 0.73 | 0.7 |
| 742 | Train 12 | Test White 38 | 0.7 | 0.71 | 0.68 | 1766 | Train 28 | Test White 38 | 0.7 | 0.71 | 0.68 |
| 743 | Train 12 | Test White 39 | 0.71 | 0.73 | 0.69 | 1767 | Train 28 | Test White 39 | 0.71 | 0.73 | 0.7 |
| 744 | Train 12 | Test White 40 | 0.7 | 0.71 | 0.68 | 1768 | Train 28 | Test White 40 | 0.7 | 0.71 | 0.68 |
| 745 | Train 12 | Test White 41 | 0.74 | 0.81 | 0.82 | 1769 | Train 28 | Test White 41 | 0.74 | 0.81 | 0.82 |
| 746 | Train 12 | Test White 42 | 0.75 | 0.81 | 0.83 | 1770 | Train 28 | Test White 42 | 0.75 | 0.81 | 0.83 |
| 747 | Train 12 | Test White 43 | 0.74 | 0.81 | 0.82 | 1771 | Train 28 | Test White 43 | 0.74 | 0.81 | 0.82 |
| 748 | Train 12 | Test White 44 | 0.75 | 0.81 | 0.83 | 1772 | Train 28 | Test White 44 | 0.75 | 0.81 | 0.83 |
| 749 | Train 12 | Test White 45 | 0.71 | 0.73 | 0.7 | 1773 | Train 28 | Test White 45 | 0.71 | 0.73 | 0.71 |
| 750 | Train 12 | Test White 46 | 0.71 | 0.71 | 0.68 | 1774 | Train 28 | Test White 46 | 0.71 | 0.71 | 0.68 |
| 751 | Train 12 | Test White 47 | 0.71 | 0.73 | 0.7 | 1775 | Train 28 | Test White 47 | 0.71 | 0.73 | 0.71 |
| 752 | Train 12 | Test White 48 | 0.7 | 0.71 | 0.68 | 1776 | Train 28 | Test White 48 | 0.7 | 0.71 | 0.68 |
| 753 | Train 12 | Test White 49 | 0.73 | 0.76 | 0.76 | 1777 | Train 28 | Test White 49 | 0.73 | 0.76 | 0.76 |
| 754 | Train 12 | Test White 50 | 0.73 | 0.76 | 0.76 | 1778 | Train 28 | Test White 50 | 0.73 | 0.75 | 0.76 |
| 755 | Train 12 | Test White 51 | 0.73 | 0.76 | 0.76 | 1779 | Train 28 | Test White 51 | 0.73 | 0.76 | 0.76 |
| 756 | Train 12 | Test White 52 | 0.73 | 0.76 | 0.76 | 1780 | Train 28 | Test White 52 | 0.73 | 0.76 | 0.76 |
| 757 | Train 12 | Test White 53 | 0.7 | 0.74 | 0.65 | 1781 | Train 28 | Test White 53 | 0.7 | 0.74 | 0.68 |
| 758 | Train 12 | Test White 54 | 0.71 | 0.71 | 0.68 | 1782 | Train 28 | Test White 54 | 0.71 | 0.71 | 0.69 |
| 759 | Train 12 | Test White 55 | 0.71 | 0.74 | 0.65 | 1783 | Train 28 | Test White 55 | 0.7 | 0.74 | 0.68 |
| 760 | Train 12 | Test White 56 | 0.7 | 0.7 | 0.68 | 1784 | Train 28 | Test White 56 | 0.7 | 0.7 | 0.68 |
| 761 | Train 12 | Test White 57 | 0.73 | 0.77 | 0.79 | 1785 | Train 28 | Test White 57 | 0.73 | 0.76 | 0.8 |
| 762 | Train 12 | Test White 58 | 0.73 | 0.76 | 0.79 | 1786 | Train 28 | Test White 58 | 0.73 | 0.76 | 0.8 |
| 763 | Train 12 | Test White 59 | 0.73 | 0.77 | 0.8 | 1787 | Train 28 | Test White 59 | 0.73 | 0.76 | 0.8 |
| 764 | Train 12 | Test White 60 | 0.73 | 0.77 | 0.79 | 1788 | Train 28 | Test White 60 | 0.73 | 0.76 | 0.8 |
| 765 | Train 12 | Test White 61 | 0.71 | 0.74 | 0.66 | 1789 | Train 28 | Test White 61 | 0.71 | 0.74 | 0.68 |
| 766 | Train 12 | Test White 62 | 0.71 | 0.71 | 0.68 | 1790 | Train 28 | Test White 62 | 0.71 | 0.71 | 0.69 |
| 767 | Train 12 | Test White 63 | 0.71 | 0.74 | 0.66 | 1791 | Train 28 | Test White 63 | 0.71 | 0.74 | 0.68 |
| 768 | Train 12 | Test White 64 | 0.7 | 0.7 | 0.68 | 1792 | Train 28 | Test White 64 | 0.7 | 0.71 | 0.68 |
| 769 | Train 13 | Test White 1 | 0.74 | 0.78 | 0.78 | 1793 | Train 29 | Test White 1 | 0.74 | 0.78 | 0.78 |
| 770 | Train 13 | Test White 2 | 0.74 | 0.76 | 0.76 | 1794 | Train 29 | Test White 2 | 0.74 | 0.76 | 0.76 |
| 771 | Train 13 | Test White 3 | 0.74 | 0.78 | 0.78 | 1795 | Train 29 | Test White 3 | 0.74 | 0.78 | 0.78 |
| 772 | Train 13 | Test White 4 | 0.74 | 0.76 | 0.76 | 1796 | Train 29 | Test White 4 | 0.74 | 0.76 | 0.76 |
| 773 | Train 13 | Test White 5 | 0.73 | 0.77 | 0.72 | 1797 | Train 29 | Test White 5 | 0.73 | 0.77 | 0.75 |
| 774 | Train 13 | Test White 6 | 0.7 | 0.71 | 0.68 | 1798 | Train 29 | Test White 6 | 0.7 | 0.71 | 0.7 |
| 775 | Train 13 | Test White 7 | 0.73 | 0.77 | 0.72 | 1799 | Train 29 | Test White 7 | 0.73 | 0.77 | 0.75 |
| 776 | Train 13 | Test White 8 | 0.7 | 0.71 | 0.68 | 1800 | Train 29 | Test White 8 | 0.7 | 0.71 | 0.7 |
| 777 | Train 13 | Test White 9 | 0.74 | 0.79 | 0.8 | 1801 | Train 29 | Test White 9 | 0.74 | 0.79 | 0.8 |
| 778 | Train 13 | Test White 10 | 0.74 | 0.77 | 0.78 | 1802 | Train 29 | Test White 10 | 0.74 | 0.77 | 0.78 |
| 779 | Train 13 | Test White 11 | 0.74 | 0.79 | 0.8 | 1803 | Train 29 | Test White 11 | 0.74 | 0.79 | 0.8 |
| 780 | Train 13 | Test White 12 | 0.74 | 0.77 | 0.77 | 1804 | Train 29 | Test White 12 | 0.74 | 0.77 | 0.78 |
| 781 | Train 13 | Test White 13 | 0.73 | 0.78 | 0.73 | 1805 | Train 29 | Test White 13 | 0.73 | 0.78 | 0.77 |
| 782 | Train 13 | Test White 14 | 0.7 | 0.71 | 0.68 | 1806 | Train 29 | Test White 14 | 0.7 | 0.71 | 0.7 |
| 783 | Train 13 | Test White 15 | 0.73 | 0.78 | 0.73 | 1807 | Train 29 | Test White 15 | 0.73 | 0.78 | 0.77 |
| 784 | Train 13 | Test White 16 | 0.7 | 0.71 | 0.68 | 1808 | Train 29 | Test White 16 | 0.7 | 0.71 | 0.7 |
| 785 | Train 13 | Test White 17 | 0.74 | 0.78 | 0.78 | 1809 | Train 29 | Test White 17 | 0.74 | 0.78 | 0.78 |
| 786 | Train 13 | Test White 18 | 0.74 | 0.76 | 0.76 | 1810 | Train 29 | Test White 18 | 0.74 | 0.76 | 0.76 |
| 787 | Train 13 | Test White 19 | 0.74 | 0.78 | 0.78 | 1811 | Train 29 | Test White 19 | 0.74 | 0.78 | 0.78 |
| 788 | Train 13 | Test White 20 | 0.74 | 0.76 | 0.76 | 1812 | Train 29 | Test White 20 | 0.74 | 0.76 | 0.76 |
| 789 | Train 13 | Test White 21 | 0.73 | 0.77 | 0.72 | 1813 | Train 29 | Test White 21 | 0.73 | 0.77 | 0.75 |
| 790 | Train 13 | Test White 22 | 0.7 | 0.71 | 0.68 | 1814 | Train 29 | Test White 22 | 0.7 | 0.71 | 0.7 |
| 791 | Train 13 | Test White 23 | 0.73 | 0.77 | 0.72 | 1815 | Train 29 | Test White 23 | 0.73 | 0.77 | 0.75 |
| 792 | Train 13 | Test White 24 | 0.7 | 0.71 | 0.68 | 1816 | Train 29 | Test White 24 | 0.7 | 0.71 | 0.7 |
| 793 | Train 13 | Test White 25 | 0.74 | 0.79 | 0.8 | 1817 | Train 29 | Test White 25 | 0.74 | 0.79 | 0.8 |
| 794 | Train 13 | Test White 26 | 0.74 | 0.77 | 0.78 | 1818 | Train 29 | Test White 26 | 0.74 | 0.77 | 0.78 |
| 795 | Train 13 | Test White 27 | 0.74 | 0.79 | 0.8 | 1819 | Train 29 | Test White 27 | 0.74 | 0.79 | 0.8 |
| 796 | Train 13 | Test White 28 | 0.74 | 0.77 | 0.77 | 1820 | Train 29 | Test White 28 | 0.74 | 0.77 | 0.78 |
| 797 | Train 13 | Test White 29 | 0.73 | 0.78 | 0.73 | 1821 | Train 29 | Test White 29 | 0.73 | 0.78 | 0.77 |
| 798 | Train 13 | Test White 30 | 0.7 | 0.71 | 0.68 | 1822 | Train 29 | Test White 30 | 0.7 | 0.71 | 0.7 |
| 799 | Train 13 | Test White 31 | 0.73 | 0.78 | 0.73 | 1823 | Train 29 | Test White 31 | 0.73 | 0.78 | 0.77 |
| 800 | Train 13 | Test White 32 | 0.7 | 0.71 | 0.68 | 1824 | Train 29 | Test White 32 | 0.7 | 0.71 | 0.7 |
| 801 | Train 13 | Test White 33 | 0.74 | 0.78 | 0.78 | 1825 | Train 29 | Test White 33 | 0.74 | 0.78 | 0.78 |
| 802 | Train 13 | Test White 34 | 0.74 | 0.77 | 0.76 | 1826 | Train 29 | Test White 34 | 0.74 | 0.77 | 0.76 |
| 803 | Train 13 | Test White 35 | 0.74 | 0.78 | 0.78 | 1827 | Train 29 | Test White 35 | 0.74 | 0.78 | 0.78 |
| 804 | Train 13 | Test White 36 | 0.74 | 0.77 | 0.76 | 1828 | Train 29 | Test White 36 | 0.74 | 0.77 | 0.76 |
| 805 | Train 13 | Test White 37 | 0.73 | 0.77 | 0.72 | 1829 | Train 29 | Test White 37 | 0.73 | 0.77 | 0.75 |
| 806 | Train 13 | Test White 38 | 0.7 | 0.71 | 0.68 | 1830 | Train 29 | Test White 38 | 0.7 | 0.71 | 0.7 |
| 807 | Train 13 | Test White 39 | 0.73 | 0.77 | 0.72 | 1831 | Train 29 | Test White 39 | 0.73 | 0.77 | 0.75 |
| 808 | Train 13 | Test White 40 | 0.7 | 0.71 | 0.68 | 1832 | Train 29 | Test White 40 | 0.7 | 0.71 | 0.7 |
| 809 | Train 13 | Test White 41 | 0.74 | 0.79 | 0.8 | 1833 | Train 29 | Test White 41 | 0.74 | 0.79 | 0.8 |
| 810 | Train 13 | Test White 42 | 0.74 | 0.77 | 0.78 | 1834 | Train 29 | Test White 42 | 0.74 | 0.77 | 0.78 |
| 811 | Train 13 | Test White 43 | 0.74 | 0.79 | 0.8 | 1835 | Train 29 | Test White 43 | 0.74 | 0.79 | 0.8 |
| 812 | Train 13 | Test White 44 | 0.74 | 0.77 | 0.78 | 1836 | Train 29 | Test White 44 | 0.74 | 0.77 | 0.78 |
| 813 | Train 13 | Test White 45 | 0.73 | 0.78 | 0.73 | 1837 | Train 29 | Test White 45 | 0.73 | 0.78 | 0.77 |
| 814 | Train 13 | Test White 46 | 0.7 | 0.71 | 0.68 | 1838 | Train 29 | Test White 46 | 0.7 | 0.71 | 0.7 |
| 815 | Train 13 | Test White 47 | 0.73 | 0.78 | 0.73 | 1839 | Train 29 | Test White 47 | 0.73 | 0.78 | 0.77 |
| 816 | Train 13 | Test White 48 | 0.7 | 0.71 | 0.68 | 1840 | Train 29 | Test White 48 | 0.7 | 0.71 | 0.71 |
| 817 | Train 13 | Test White 49 | 0.73 | 0.74 | 0.74 | 1841 | Train 29 | Test White 49 | 0.73 | 0.74 | 0.74 |
| 818 | Train 13 | Test White 50 | 0.73 | 0.74 | 0.74 | 1842 | Train 29 | Test White 50 | 0.73 | 0.74 | 0.74 |
| 819 | Train 13 | Test White 51 | 0.73 | 0.74 | 0.74 | 1843 | Train 29 | Test White 51 | 0.73 | 0.74 | 0.74 |
| 820 | Train 13 | Test White 52 | 0.73 | 0.74 | 0.74 | 1844 | Train 29 | Test White 52 | 0.73 | 0.74 | 0.74 |
| 821 | Train 13 | Test White 53 | 0.73 | 0.77 | 0.71 | 1845 | Train 29 | Test White 53 | 0.73 | 0.77 | 0.73 |
| 822 | Train 13 | Test White 54 | 0.71 | 0.71 | 0.67 | 1846 | Train 29 | Test White 54 | 0.71 | 0.71 | 0.7 |
| 823 | Train 13 | Test White 55 | 0.73 | 0.77 | 0.71 | 1847 | Train 29 | Test White 55 | 0.73 | 0.77 | 0.73 |
| 824 | Train 13 | Test White 56 | 0.7 | 0.71 | 0.67 | 1848 | Train 29 | Test White 56 | 0.7 | 0.71 | 0.71 |
| 825 | Train 13 | Test White 57 | 0.73 | 0.74 | 0.77 | 1849 | Train 29 | Test White 57 | 0.73 | 0.74 | 0.76 |
| 826 | Train 13 | Test White 58 | 0.73 | 0.74 | 0.77 | 1850 | Train 29 | Test White 58 | 0.73 | 0.74 | 0.76 |
| 827 | Train 13 | Test White 59 | 0.73 | 0.74 | 0.77 | 1851 | Train 29 | Test White 59 | 0.73 | 0.74 | 0.76 |
| 828 | Train 13 | Test White 60 | 0.73 | 0.74 | 0.77 | 1852 | Train 29 | Test White 60 | 0.73 | 0.74 | 0.76 |
| 829 | Train 13 | Test White 61 | 0.74 | 0.78 | 0.73 | 1853 | Train 29 | Test White 61 | 0.74 | 0.78 | 0.75 |
| 830 | Train 13 | Test White 62 | 0.71 | 0.71 | 0.67 | 1854 | Train 29 | Test White 62 | 0.71 | 0.71 | 0.71 |
| 831 | Train 13 | Test White 63 | 0.74 | 0.78 | 0.72 | 1855 | Train 29 | Test White 63 | 0.74 | 0.78 | 0.74 |
| 832 | Train 13 | Test White 64 | 0.71 | 0.71 | 0.67 | 1856 | Train 29 | Test White 64 | 0.71 | 0.71 | 0.71 |
| 833 | Train 14 | Test White 1 | 0.72 | 0.75 | 0.74 | 1857 | Train 30 | Test White 1 | 0.72 | 0.75 | 0.73 |
| 834 | Train 14 | Test White 2 | 0.74 | 0.76 | 0.75 | 1858 | Train 30 | Test White 2 | 0.74 | 0.76 | 0.74 |
| 835 | Train 14 | Test White 3 | 0.72 | 0.75 | 0.74 | 1859 | Train 30 | Test White 3 | 0.72 | 0.75 | 0.73 |
| 836 | Train 14 | Test White 4 | 0.74 | 0.76 | 0.75 | 1860 | Train 30 | Test White 4 | 0.74 | 0.76 | 0.74 |
| 837 | Train 14 | Test White 5 | 0.72 | 0.77 | 0.77 | 1861 | Train 30 | Test White 5 | 0.72 | 0.77 | 0.77 |
| 838 | Train 14 | Test White 6 | 0.73 | 0.77 | 0.78 | 1862 | Train 30 | Test White 6 | 0.73 | 0.77 | 0.78 |
| 839 | Train 14 | Test White 7 | 0.72 | 0.77 | 0.77 | 1863 | Train 30 | Test White 7 | 0.72 | 0.77 | 0.77 |
| 840 | Train 14 | Test White 8 | 0.73 | 0.77 | 0.78 | 1864 | Train 30 | Test White 8 | 0.73 | 0.77 | 0.78 |
| 841 | Train 14 | Test White 9 | 0.72 | 0.76 | 0.75 | 1865 | Train 30 | Test White 9 | 0.72 | 0.76 | 0.74 |
| 842 | Train 14 | Test White 10 | 0.74 | 0.76 | 0.75 | 1866 | Train 30 | Test White 10 | 0.74 | 0.76 | 0.75 |
| 843 | Train 14 | Test White 11 | 0.72 | 0.76 | 0.75 | 1867 | Train 30 | Test White 11 | 0.72 | 0.76 | 0.74 |
| 844 | Train 14 | Test White 12 | 0.74 | 0.76 | 0.75 | 1868 | Train 30 | Test White 12 | 0.74 | 0.76 | 0.75 |
| 845 | Train 14 | Test White 13 | 0.72 | 0.77 | 0.78 | 1869 | Train 30 | Test White 13 | 0.72 | 0.77 | 0.78 |
| 846 | Train 14 | Test White 14 | 0.73 | 0.77 | 0.78 | 1870 | Train 30 | Test White 14 | 0.73 | 0.78 | 0.78 |
| 847 | Train 14 | Test White 15 | 0.72 | 0.77 | 0.78 | 1871 | Train 30 | Test White 15 | 0.72 | 0.77 | 0.78 |
| 848 | Train 14 | Test White 16 | 0.73 | 0.77 | 0.78 | 1872 | Train 30 | Test White 16 | 0.73 | 0.77 | 0.78 |
| 849 | Train 14 | Test White 17 | 0.72 | 0.75 | 0.74 | 1873 | Train 30 | Test White 17 | 0.72 | 0.75 | 0.73 |
| 850 | Train 14 | Test White 18 | 0.74 | 0.76 | 0.74 | 1874 | Train 30 | Test White 18 | 0.74 | 0.76 | 0.74 |
| 851 | Train 14 | Test White 19 | 0.72 | 0.75 | 0.74 | 1875 | Train 30 | Test White 19 | 0.72 | 0.75 | 0.73 |
| 852 | Train 14 | Test White 20 | 0.74 | 0.76 | 0.74 | 1876 | Train 30 | Test White 20 | 0.74 | 0.76 | 0.74 |
| 853 | Train 14 | Test White 21 | 0.72 | 0.77 | 0.77 | 1877 | Train 30 | Test White 21 | 0.72 | 0.77 | 0.77 |
| 854 | Train 14 | Test White 22 | 0.73 | 0.77 | 0.78 | 1878 | Train 30 | Test White 22 | 0.73 | 0.77 | 0.78 |
| 855 | Train 14 | Test White 23 | 0.72 | 0.77 | 0.77 | 1879 | Train 30 | Test White 23 | 0.72 | 0.77 | 0.77 |
| 856 | Train 14 | Test White 24 | 0.73 | 0.77 | 0.78 | 1880 | Train 30 | Test White 24 | 0.73 | 0.77 | 0.78 |
| 857 | Train 14 | Test White 25 | 0.72 | 0.76 | 0.75 | 1881 | Train 30 | Test White 25 | 0.72 | 0.76 | 0.74 |
| 858 | Train 14 | Test White 26 | 0.74 | 0.76 | 0.75 | 1882 | Train 30 | Test White 26 | 0.74 | 0.76 | 0.75 |
| 859 | Train 14 | Test White 27 | 0.72 | 0.76 | 0.75 | 1883 | Train 30 | Test White 27 | 0.72 | 0.76 | 0.74 |
| 860 | Train 14 | Test White 28 | 0.74 | 0.76 | 0.75 | 1884 | Train 30 | Test White 28 | 0.74 | 0.76 | 0.75 |
| 861 | Train 14 | Test White 29 | 0.72 | 0.77 | 0.78 | 1885 | Train 30 | Test White 29 | 0.72 | 0.77 | 0.78 |
| 862 | Train 14 | Test White 30 | 0.73 | 0.78 | 0.78 | 1886 | Train 30 | Test White 30 | 0.73 | 0.78 | 0.78 |
| 863 | Train 14 | Test White 31 | 0.72 | 0.77 | 0.78 | 1887 | Train 30 | Test White 31 | 0.72 | 0.77 | 0.78 |
| 864 | Train 14 | Test White 32 | 0.73 | 0.77 | 0.78 | 1888 | Train 30 | Test White 32 | 0.73 | 0.77 | 0.78 |
| 865 | Train 14 | Test White 33 | 0.73 | 0.75 | 0.74 | 1889 | Train 30 | Test White 33 | 0.73 | 0.75 | 0.74 |
| 866 | Train 14 | Test White 34 | 0.74 | 0.76 | 0.75 | 1890 | Train 30 | Test White 34 | 0.74 | 0.76 | 0.74 |
| 867 | Train 14 | Test White 35 | 0.73 | 0.75 | 0.74 | 1891 | Train 30 | Test White 35 | 0.73 | 0.75 | 0.74 |
| 868 | Train 14 | Test White 36 | 0.74 | 0.76 | 0.75 | 1892 | Train 30 | Test White 36 | 0.74 | 0.76 | 0.74 |
| 869 | Train 14 | Test White 37 | 0.72 | 0.77 | 0.77 | 1893 | Train 30 | Test White 37 | 0.72 | 0.77 | 0.77 |
| 870 | Train 14 | Test White 38 | 0.73 | 0.77 | 0.78 | 1894 | Train 30 | Test White 38 | 0.73 | 0.77 | 0.78 |
| 871 | Train 14 | Test White 39 | 0.72 | 0.77 | 0.77 | 1895 | Train 30 | Test White 39 | 0.72 | 0.77 | 0.77 |
| 872 | Train 14 | Test White 40 | 0.73 | 0.77 | 0.78 | 1896 | Train 30 | Test White 40 | 0.73 | 0.77 | 0.78 |
| 873 | Train 14 | Test White 41 | 0.73 | 0.76 | 0.75 | 1897 | Train 30 | Test White 41 | 0.73 | 0.76 | 0.74 |
| 874 | Train 14 | Test White 42 | 0.74 | 0.77 | 0.75 | 1898 | Train 30 | Test White 42 | 0.74 | 0.77 | 0.75 |
| 875 | Train 14 | Test White 43 | 0.73 | 0.76 | 0.75 | 1899 | Train 30 | Test White 43 | 0.73 | 0.76 | 0.74 |
| 876 | Train 14 | Test White 44 | 0.74 | 0.77 | 0.75 | 1900 | Train 30 | Test White 44 | 0.74 | 0.77 | 0.75 |
| 877 | Train 14 | Test White 45 | 0.72 | 0.77 | 0.78 | 1901 | Train 30 | Test White 45 | 0.72 | 0.77 | 0.78 |
| 878 | Train 14 | Test White 46 | 0.73 | 0.78 | 0.78 | 1902 | Train 30 | Test White 46 | 0.73 | 0.78 | 0.78 |
| 879 | Train 14 | Test White 47 | 0.72 | 0.77 | 0.78 | 1903 | Train 30 | Test White 47 | 0.72 | 0.77 | 0.78 |
| 880 | Train 14 | Test White 48 | 0.73 | 0.77 | 0.78 | 1904 | Train 30 | Test White 48 | 0.73 | 0.77 | 0.78 |
| 881 | Train 14 | Test White 49 | 0.72 | 0.76 | 0.75 | 1905 | Train 30 | Test White 49 | 0.72 | 0.76 | 0.75 |
| 882 | Train 14 | Test White 50 | 0.72 | 0.76 | 0.75 | 1906 | Train 30 | Test White 50 | 0.72 | 0.76 | 0.75 |
| 883 | Train 14 | Test White 51 | 0.72 | 0.76 | 0.75 | 1907 | Train 30 | Test White 51 | 0.72 | 0.76 | 0.75 |
| 884 | Train 14 | Test White 52 | 0.72 | 0.76 | 0.75 | 1908 | Train 30 | Test White 52 | 0.72 | 0.76 | 0.75 |
| 885 | Train 14 | Test White 53 | 0.73 | 0.78 | 0.74 | 1909 | Train 30 | Test White 53 | 0.73 | 0.78 | 0.74 |
| 886 | Train 14 | Test White 54 | 0.74 | 0.77 | 0.78 | 1910 | Train 30 | Test White 54 | 0.74 | 0.77 | 0.78 |
| 887 | Train 14 | Test White 55 | 0.73 | 0.78 | 0.74 | 1911 | Train 30 | Test White 55 | 0.73 | 0.78 | 0.74 |
| 888 | Train 14 | Test White 56 | 0.73 | 0.76 | 0.78 | 1912 | Train 30 | Test White 56 | 0.73 | 0.76 | 0.78 |
| 889 | Train 14 | Test White 57 | 0.73 | 0.76 | 0.77 | 1913 | Train 30 | Test White 57 | 0.73 | 0.76 | 0.77 |
| 890 | Train 14 | Test White 58 | 0.73 | 0.76 | 0.77 | 1914 | Train 30 | Test White 58 | 0.73 | 0.76 | 0.77 |
| 891 | Train 14 | Test White 59 | 0.73 | 0.76 | 0.77 | 1915 | Train 30 | Test White 59 | 0.73 | 0.76 | 0.76 |
| 892 | Train 14 | Test White 60 | 0.73 | 0.76 | 0.77 | 1916 | Train 30 | Test White 60 | 0.73 | 0.76 | 0.76 |
| 893 | Train 14 | Test White 61 | 0.73 | 0.78 | 0.75 | 1917 | Train 30 | Test White 61 | 0.73 | 0.78 | 0.76 |
| 894 | Train 14 | Test White 62 | 0.74 | 0.77 | 0.78 | 1918 | Train 30 | Test White 62 | 0.74 | 0.77 | 0.78 |
| 895 | Train 14 | Test White 63 | 0.73 | 0.78 | 0.75 | 1919 | Train 30 | Test White 63 | 0.73 | 0.78 | 0.75 |
| 896 | Train 14 | Test White 64 | 0.73 | 0.76 | 0.78 | 1920 | Train 30 | Test White 64 | 0.73 | 0.76 | 0.78 |
| 897 | Train 15 | Test White 1 | 0.74 | 0.78 | 0.78 | 1921 | Train 31 | Test White 1 | 0.74 | 0.78 | 0.79 |
| 898 | Train 15 | Test White 2 | 0.74 | 0.76 | 0.76 | 1922 | Train 31 | Test White 2 | 0.74 | 0.77 | 0.76 |
| 899 | Train 15 | Test White 3 | 0.74 | 0.78 | 0.78 | 1923 | Train 31 | Test White 3 | 0.74 | 0.78 | 0.79 |
| 900 | Train 15 | Test White 4 | 0.74 | 0.77 | 0.76 | 1924 | Train 31 | Test White 4 | 0.74 | 0.77 | 0.76 |
| 901 | Train 15 | Test White 5 | 0.73 | 0.77 | 0.75 | 1925 | Train 31 | Test White 5 | 0.73 | 0.77 | 0.71 |
| 902 | Train 15 | Test White 6 | 0.7 | 0.71 | 0.69 | 1926 | Train 31 | Test White 6 | 0.7 | 0.71 | 0.68 |
| 903 | Train 15 | Test White 7 | 0.73 | 0.77 | 0.75 | 1927 | Train 31 | Test White 7 | 0.73 | 0.77 | 0.71 |
| 904 | Train 15 | Test White 8 | 0.7 | 0.71 | 0.69 | 1928 | Train 31 | Test White 8 | 0.7 | 0.71 | 0.68 |
| 905 | Train 15 | Test White 9 | 0.74 | 0.79 | 0.8 | 1929 | Train 31 | Test White 9 | 0.74 | 0.79 | 0.8 |
| 906 | Train 15 | Test White 10 | 0.74 | 0.77 | 0.77 | 1930 | Train 31 | Test White 10 | 0.74 | 0.77 | 0.78 |
| 907 | Train 15 | Test White 11 | 0.74 | 0.79 | 0.8 | 1931 | Train 31 | Test White 11 | 0.74 | 0.79 | 0.81 |
| 908 | Train 15 | Test White 12 | 0.74 | 0.77 | 0.77 | 1932 | Train 31 | Test White 12 | 0.74 | 0.77 | 0.78 |
| 909 | Train 15 | Test White 13 | 0.73 | 0.78 | 0.76 | 1933 | Train 31 | Test White 13 | 0.73 | 0.78 | 0.73 |
| 910 | Train 15 | Test White 14 | 0.7 | 0.71 | 0.69 | 1934 | Train 31 | Test White 14 | 0.7 | 0.71 | 0.68 |
| 911 | Train 15 | Test White 15 | 0.73 | 0.78 | 0.76 | 1935 | Train 31 | Test White 15 | 0.73 | 0.78 | 0.73 |
| 912 | Train 15 | Test White 16 | 0.7 | 0.71 | 0.69 | 1936 | Train 31 | Test White 16 | 0.7 | 0.71 | 0.68 |
| 913 | Train 15 | Test White 17 | 0.74 | 0.78 | 0.78 | 1937 | Train 31 | Test White 17 | 0.74 | 0.78 | 0.79 |
| 914 | Train 15 | Test White 18 | 0.74 | 0.77 | 0.76 | 1938 | Train 31 | Test White 18 | 0.74 | 0.77 | 0.76 |
| 915 | Train 15 | Test White 19 | 0.74 | 0.78 | 0.78 | 1939 | Train 31 | Test White 19 | 0.74 | 0.78 | 0.79 |
| 916 | Train 15 | Test White 20 | 0.74 | 0.77 | 0.76 | 1940 | Train 31 | Test White 20 | 0.74 | 0.77 | 0.76 |
| 917 | Train 15 | Test White 21 | 0.73 | 0.77 | 0.75 | 1941 | Train 31 | Test White 21 | 0.73 | 0.77 | 0.71 |
| 918 | Train 15 | Test White 22 | 0.7 | 0.71 | 0.69 | 1942 | Train 31 | Test White 22 | 0.7 | 0.71 | 0.68 |
| 919 | Train 15 | Test White 23 | 0.73 | 0.77 | 0.75 | 1943 | Train 31 | Test White 23 | 0.73 | 0.77 | 0.71 |
| 920 | Train 15 | Test White 24 | 0.7 | 0.71 | 0.69 | 1944 | Train 31 | Test White 24 | 0.7 | 0.71 | 0.68 |
| 921 | Train 15 | Test White 25 | 0.74 | 0.79 | 0.8 | 1945 | Train 31 | Test White 25 | 0.74 | 0.79 | 0.8 |
| 922 | Train 15 | Test White 26 | 0.74 | 0.77 | 0.77 | 1946 | Train 31 | Test White 26 | 0.74 | 0.77 | 0.78 |
| 923 | Train 15 | Test White 27 | 0.74 | 0.79 | 0.8 | 1947 | Train 31 | Test White 27 | 0.74 | 0.79 | 0.81 |
| 924 | Train 15 | Test White 28 | 0.74 | 0.77 | 0.77 | 1948 | Train 31 | Test White 28 | 0.74 | 0.77 | 0.78 |
| 925 | Train 15 | Test White 29 | 0.73 | 0.78 | 0.76 | 1949 | Train 31 | Test White 29 | 0.73 | 0.78 | 0.73 |
| 926 | Train 15 | Test White 30 | 0.7 | 0.71 | 0.69 | 1950 | Train 31 | Test White 30 | 0.7 | 0.71 | 0.68 |
| 927 | Train 15 | Test White 31 | 0.73 | 0.78 | 0.76 | 1951 | Train 31 | Test White 31 | 0.73 | 0.78 | 0.73 |
| 928 | Train 15 | Test White 32 | 0.7 | 0.71 | 0.69 | 1952 | Train 31 | Test White 32 | 0.7 | 0.71 | 0.68 |
| 929 | Train 15 | Test White 33 | 0.74 | 0.78 | 0.78 | 1953 | Train 31 | Test White 33 | 0.74 | 0.78 | 0.79 |
| 930 | Train 15 | Test White 34 | 0.74 | 0.77 | 0.76 | 1954 | Train 31 | Test White 34 | 0.74 | 0.77 | 0.76 |
| 931 | Train 15 | Test White 35 | 0.74 | 0.78 | 0.78 | 1955 | Train 31 | Test White 35 | 0.74 | 0.78 | 0.79 |
| 932 | Train 15 | Test White 36 | 0.74 | 0.77 | 0.76 | 1956 | Train 31 | Test White 36 | 0.74 | 0.77 | 0.76 |
| 933 | Train 15 | Test White 37 | 0.73 | 0.77 | 0.75 | 1957 | Train 31 | Test White 37 | 0.73 | 0.77 | 0.71 |
| 934 | Train 15 | Test White 38 | 0.7 | 0.71 | 0.69 | 1958 | Train 31 | Test White 38 | 0.7 | 0.71 | 0.68 |
| 935 | Train 15 | Test White 39 | 0.73 | 0.77 | 0.75 | 1959 | Train 31 | Test White 39 | 0.73 | 0.77 | 0.71 |
| 936 | Train 15 | Test White 40 | 0.7 | 0.71 | 0.69 | 1960 | Train 31 | Test White 40 | 0.7 | 0.71 | 0.68 |
| 937 | Train 15 | Test White 41 | 0.74 | 0.79 | 0.8 | 1961 | Train 31 | Test White 41 | 0.74 | 0.79 | 0.81 |
| 938 | Train 15 | Test White 42 | 0.74 | 0.77 | 0.77 | 1962 | Train 31 | Test White 42 | 0.74 | 0.77 | 0.78 |
| 939 | Train 15 | Test White 43 | 0.74 | 0.79 | 0.8 | 1963 | Train 31 | Test White 43 | 0.74 | 0.79 | 0.81 |
| 940 | Train 15 | Test White 44 | 0.74 | 0.77 | 0.77 | 1964 | Train 31 | Test White 44 | 0.74 | 0.77 | 0.78 |
| 941 | Train 15 | Test White 45 | 0.73 | 0.78 | 0.76 | 1965 | Train 31 | Test White 45 | 0.73 | 0.78 | 0.73 |
| 942 | Train 15 | Test White 46 | 0.7 | 0.71 | 0.69 | 1966 | Train 31 | Test White 46 | 0.7 | 0.71 | 0.68 |
| 943 | Train 15 | Test White 47 | 0.73 | 0.78 | 0.76 | 1967 | Train 31 | Test White 47 | 0.73 | 0.78 | 0.73 |
| 944 | Train 15 | Test White 48 | 0.7 | 0.71 | 0.69 | 1968 | Train 31 | Test White 48 | 0.7 | 0.71 | 0.68 |
| 945 | Train 15 | Test White 49 | 0.73 | 0.74 | 0.76 | 1969 | Train 31 | Test White 49 | 0.73 | 0.74 | 0.75 |
| 946 | Train 15 | Test White 50 | 0.73 | 0.74 | 0.76 | 1970 | Train 31 | Test White 50 | 0.73 | 0.74 | 0.75 |
| 947 | Train 15 | Test White 51 | 0.73 | 0.74 | 0.76 | 1971 | Train 31 | Test White 51 | 0.73 | 0.74 | 0.75 |
| 948 | Train 15 | Test White 52 | 0.73 | 0.74 | 0.76 | 1972 | Train 31 | Test White 52 | 0.73 | 0.74 | 0.75 |
| 949 | Train 15 | Test White 53 | 0.73 | 0.77 | 0.75 | 1973 | Train 31 | Test White 53 | 0.73 | 0.77 | 0.68 |
| 950 | Train 15 | Test White 54 | 0.71 | 0.71 | 0.69 | 1974 | Train 31 | Test White 54 | 0.71 | 0.7 | 0.67 |
| 951 | Train 15 | Test White 55 | 0.73 | 0.77 | 0.75 | 1975 | Train 31 | Test White 55 | 0.73 | 0.77 | 0.68 |
| 952 | Train 15 | Test White 56 | 0.7 | 0.71 | 0.69 | 1976 | Train 31 | Test White 56 | 0.7 | 0.7 | 0.68 |
| 953 | Train 15 | Test White 57 | 0.73 | 0.75 | 0.78 | 1977 | Train 31 | Test White 57 | 0.73 | 0.75 | 0.78 |
| 954 | Train 15 | Test White 58 | 0.73 | 0.74 | 0.78 | 1978 | Train 31 | Test White 58 | 0.73 | 0.75 | 0.78 |
| 955 | Train 15 | Test White 59 | 0.73 | 0.75 | 0.78 | 1979 | Train 31 | Test White 59 | 0.73 | 0.75 | 0.78 |
| 956 | Train 15 | Test White 60 | 0.73 | 0.75 | 0.78 | 1980 | Train 31 | Test White 60 | 0.73 | 0.75 | 0.78 |
| 957 | Train 15 | Test White 61 | 0.74 | 0.78 | 0.76 | 1981 | Train 31 | Test White 61 | 0.74 | 0.78 | 0.69 |
| 958 | Train 15 | Test White 62 | 0.71 | 0.71 | 0.69 | 1982 | Train 31 | Test White 62 | 0.71 | 0.71 | 0.67 |
| 959 | Train 15 | Test White 63 | 0.74 | 0.78 | 0.76 | 1983 | Train 31 | Test White 63 | 0.74 | 0.78 | 0.69 |
| 960 | Train 15 | Test White 64 | 0.71 | 0.71 | 0.69 | 1984 | Train 31 | Test White 64 | 0.71 | 0.71 | 0.68 |
| 961 | Train 16 | Test White 1 | 0.73 | 0.75 | 0.75 | 1985 | Train 32 | Test White 1 | 0.73 | 0.75 | 0.74 |
| 962 | Train 16 | Test White 2 | 0.74 | 0.76 | 0.75 | 1986 | Train 32 | Test White 2 | 0.74 | 0.76 | 0.75 |
| 963 | Train 16 | Test White 3 | 0.73 | 0.75 | 0.75 | 1987 | Train 32 | Test White 3 | 0.73 | 0.75 | 0.74 |
| 964 | Train 16 | Test White 4 | 0.74 | 0.76 | 0.75 | 1988 | Train 32 | Test White 4 | 0.74 | 0.76 | 0.75 |
| 965 | Train 16 | Test White 5 | 0.72 | 0.77 | 0.77 | 1989 | Train 32 | Test White 5 | 0.72 | 0.77 | 0.77 |
| 966 | Train 16 | Test White 6 | 0.73 | 0.77 | 0.78 | 1990 | Train 32 | Test White 6 | 0.73 | 0.77 | 0.78 |
| 967 | Train 16 | Test White 7 | 0.72 | 0.77 | 0.77 | 1991 | Train 32 | Test White 7 | 0.72 | 0.77 | 0.77 |
| 968 | Train 16 | Test White 8 | 0.73 | 0.77 | 0.78 | 1992 | Train 32 | Test White 8 | 0.73 | 0.77 | 0.78 |
| 969 | Train 16 | Test White 9 | 0.73 | 0.76 | 0.76 | 1993 | Train 32 | Test White 9 | 0.73 | 0.76 | 0.75 |
| 970 | Train 16 | Test White 10 | 0.74 | 0.76 | 0.76 | 1994 | Train 32 | Test White 10 | 0.74 | 0.76 | 0.76 |
| 971 | Train 16 | Test White 11 | 0.73 | 0.76 | 0.76 | 1995 | Train 32 | Test White 11 | 0.73 | 0.76 | 0.75 |
| 972 | Train 16 | Test White 12 | 0.74 | 0.76 | 0.76 | 1996 | Train 32 | Test White 12 | 0.74 | 0.76 | 0.76 |
| 973 | Train 16 | Test White 13 | 0.72 | 0.77 | 0.78 | 1997 | Train 32 | Test White 13 | 0.72 | 0.77 | 0.78 |
| 974 | Train 16 | Test White 14 | 0.73 | 0.77 | 0.78 | 1998 | Train 32 | Test White 14 | 0.73 | 0.77 | 0.78 |
| 975 | Train 16 | Test White 15 | 0.72 | 0.77 | 0.78 | 1999 | Train 32 | Test White 15 | 0.72 | 0.77 | 0.78 |
| 976 | Train 16 | Test White 16 | 0.73 | 0.77 | 0.78 | 2000 | Train 32 | Test White 16 | 0.73 | 0.77 | 0.78 |
| 977 | Train 16 | Test White 17 | 0.73 | 0.75 | 0.75 | 2001 | Train 32 | Test White 17 | 0.73 | 0.75 | 0.74 |
| 978 | Train 16 | Test White 18 | 0.74 | 0.76 | 0.75 | 2002 | Train 32 | Test White 18 | 0.74 | 0.76 | 0.75 |
| 979 | Train 16 | Test White 19 | 0.73 | 0.75 | 0.75 | 2003 | Train 32 | Test White 19 | 0.73 | 0.75 | 0.74 |
| 980 | Train 16 | Test White 20 | 0.74 | 0.76 | 0.75 | 2004 | Train 32 | Test White 20 | 0.74 | 0.76 | 0.75 |
| 981 | Train 16 | Test White 21 | 0.72 | 0.77 | 0.77 | 2005 | Train 32 | Test White 21 | 0.72 | 0.77 | 0.77 |
| 982 | Train 16 | Test White 22 | 0.73 | 0.77 | 0.78 | 2006 | Train 32 | Test White 22 | 0.73 | 0.77 | 0.78 |
| 983 | Train 16 | Test White 23 | 0.72 | 0.77 | 0.77 | 2007 | Train 32 | Test White 23 | 0.72 | 0.77 | 0.77 |
| 984 | Train 16 | Test White 24 | 0.73 | 0.77 | 0.78 | 2008 | Train 32 | Test White 24 | 0.73 | 0.77 | 0.78 |
| 985 | Train 16 | Test White 25 | 0.73 | 0.76 | 0.76 | 2009 | Train 32 | Test White 25 | 0.73 | 0.75 | 0.75 |
| 986 | Train 16 | Test White 26 | 0.74 | 0.76 | 0.76 | 2010 | Train 32 | Test White 26 | 0.74 | 0.76 | 0.76 |
| 987 | Train 16 | Test White 27 | 0.73 | 0.76 | 0.76 | 2011 | Train 32 | Test White 27 | 0.73 | 0.76 | 0.75 |
| 988 | Train 16 | Test White 28 | 0.74 | 0.76 | 0.76 | 2012 | Train 32 | Test White 28 | 0.74 | 0.76 | 0.76 |
| 989 | Train 16 | Test White 29 | 0.72 | 0.77 | 0.78 | 2013 | Train 32 | Test White 29 | 0.72 | 0.77 | 0.78 |
| 990 | Train 16 | Test White 30 | 0.73 | 0.77 | 0.78 | 2014 | Train 32 | Test White 30 | 0.73 | 0.77 | 0.78 |
| 991 | Train 16 | Test White 31 | 0.72 | 0.77 | 0.78 | 2015 | Train 32 | Test White 31 | 0.72 | 0.77 | 0.78 |
| 992 | Train 16 | Test White 32 | 0.73 | 0.77 | 0.78 | 2016 | Train 32 | Test White 32 | 0.73 | 0.77 | 0.78 |
| 993 | Train 16 | Test White 33 | 0.73 | 0.75 | 0.75 | 2017 | Train 32 | Test White 33 | 0.73 | 0.75 | 0.74 |
| 994 | Train 16 | Test White 34 | 0.74 | 0.76 | 0.75 | 2018 | Train 32 | Test White 34 | 0.74 | 0.76 | 0.75 |
| 995 | Train 16 | Test White 35 | 0.73 | 0.75 | 0.75 | 2019 | Train 32 | Test White 35 | 0.73 | 0.75 | 0.74 |
| 996 | Train 16 | Test White 36 | 0.74 | 0.76 | 0.75 | 2020 | Train 32 | Test White 36 | 0.74 | 0.76 | 0.75 |
| 997 | Train 16 | Test White 37 | 0.72 | 0.77 | 0.77 | 2021 | Train 32 | Test White 37 | 0.72 | 0.77 | 0.77 |
| 998 | Train 16 | Test White 38 | 0.73 | 0.77 | 0.78 | 2022 | Train 32 | Test White 38 | 0.73 | 0.77 | 0.78 |
| 999 | Train 16 | Test White 39 | 0.72 | 0.77 | 0.77 | 2023 | Train 32 | Test White 39 | 0.72 | 0.77 | 0.77 |
| 1000 | Train 16 | Test White 40 | 0.73 | 0.77 | 0.78 | 2024 | Train 32 | Test White 40 | 0.73 | 0.77 | 0.78 |
| 1001 | Train 16 | Test White 41 | 0.73 | 0.76 | 0.76 | 2025 | Train 32 | Test White 41 | 0.73 | 0.76 | 0.75 |
| 1002 | Train 16 | Test White 42 | 0.74 | 0.76 | 0.76 | 2026 | Train 32 | Test White 42 | 0.74 | 0.76 | 0.76 |
| 1003 | Train 16 | Test White 43 | 0.73 | 0.76 | 0.76 | 2027 | Train 32 | Test White 43 | 0.73 | 0.76 | 0.75 |
| 1004 | Train 16 | Test White 44 | 0.74 | 0.76 | 0.76 | 2028 | Train 32 | Test White 44 | 0.74 | 0.76 | 0.76 |
| 1005 | Train 16 | Test White 45 | 0.72 | 0.77 | 0.78 | 2029 | Train 32 | Test White 45 | 0.72 | 0.77 | 0.78 |
| 1006 | Train 16 | Test White 46 | 0.73 | 0.77 | 0.78 | 2030 | Train 32 | Test White 46 | 0.73 | 0.77 | 0.78 |
| 1007 | Train 16 | Test White 47 | 0.72 | 0.77 | 0.78 | 2031 | Train 32 | Test White 47 | 0.72 | 0.77 | 0.78 |
| 1008 | Train 16 | Test White 48 | 0.73 | 0.77 | 0.78 | 2032 | Train 32 | Test White 48 | 0.73 | 0.77 | 0.78 |
| 1009 | Train 16 | Test White 49 | 0.73 | 0.76 | 0.75 | 2033 | Train 32 | Test White 49 | 0.73 | 0.76 | 0.75 |
| 1010 | Train 16 | Test White 50 | 0.73 | 0.76 | 0.75 | 2034 | Train 32 | Test White 50 | 0.73 | 0.76 | 0.75 |
| 1011 | Train 16 | Test White 51 | 0.73 | 0.76 | 0.75 | 2035 | Train 32 | Test White 51 | 0.73 | 0.76 | 0.75 |
| 1012 | Train 16 | Test White 52 | 0.73 | 0.76 | 0.75 | 2036 | Train 32 | Test White 52 | 0.73 | 0.76 | 0.75 |
| 1013 | Train 16 | Test White 53 | 0.73 | 0.78 | 0.75 | 2037 | Train 32 | Test White 53 | 0.73 | 0.78 | 0.76 |
| 1014 | Train 16 | Test White 54 | 0.73 | 0.77 | 0.78 | 2038 | Train 32 | Test White 54 | 0.73 | 0.77 | 0.78 |
| 1015 | Train 16 | Test White 55 | 0.73 | 0.78 | 0.75 | 2039 | Train 32 | Test White 55 | 0.73 | 0.78 | 0.76 |
| 1016 | Train 16 | Test White 56 | 0.73 | 0.77 | 0.78 | 2040 | Train 32 | Test White 56 | 0.73 | 0.77 | 0.78 |
| 1017 | Train 16 | Test White 57 | 0.73 | 0.76 | 0.76 | 2041 | Train 32 | Test White 57 | 0.73 | 0.76 | 0.77 |
| 1018 | Train 16 | Test White 58 | 0.73 | 0.76 | 0.76 | 2042 | Train 32 | Test White 58 | 0.73 | 0.76 | 0.77 |
| 1019 | Train 16 | Test White 59 | 0.73 | 0.76 | 0.76 | 2043 | Train 32 | Test White 59 | 0.73 | 0.76 | 0.76 |
| 1020 | Train 16 | Test White 60 | 0.73 | 0.76 | 0.76 | 2044 | Train 32 | Test White 60 | 0.73 | 0.76 | 0.76 |
| 1021 | Train 16 | Test White 61 | 0.73 | 0.78 | 0.76 | 2045 | Train 32 | Test White 61 | 0.73 | 0.78 | 0.77 |
| 1022 | Train 16 | Test White 62 | 0.74 | 0.77 | 0.78 | 2046 | Train 32 | Test White 62 | 0.74 | 0.77 | 0.78 |
| 1023 | Train 16 | Test White 63 | 0.73 | 0.78 | 0.76 | 2047 | Train 32 | Test White 63 | 0.73 | 0.78 | 0.77 |
| 1024 | Train 16 | Test White 64 | 0.73 | 0.77 | 0.78 | 2048 | Train 32 | Test White 64 | 0.73 | 0.77 | 0.78 |

Table 8. AUC Performance of the ML Models— LR, RF, and GB —for White Patients Trained on Cohorts 33–64 and Tested Across 64 Cohorts.

| White | Train  Model | Test  Dataset | AUC (LR) | AUC (RF) | AUC (GB) | White | Train  Model | Test  Dataset | AUC (LR) | AUC (RF) | AUC (GB) |
| --- | --- | --- | --- | --- | --- | --- | --- | --- | --- | --- | --- |
| 2049 | Train 33 | Test White 1 | 0.74 | 0.81 | 0.84 | 3073 | Train 49 | Test White 1 | 0.73 | 0.72 | 0.66 |
| 2050 | Train 33 | Test White 2 | 0.73 | 0.76 | 0.78 | 3074 | Train 49 | Test White 2 | 0.71 | 0.69 | 0.61 |
| 2051 | Train 33 | Test White 3 | 0.74 | 0.81 | 0.84 | 3075 | Train 49 | Test White 3 | 0.73 | 0.72 | 0.66 |
| 2052 | Train 33 | Test White 4 | 0.73 | 0.76 | 0.78 | 3076 | Train 49 | Test White 4 | 0.71 | 0.69 | 0.61 |
| 2053 | Train 33 | Test White 5 | 0.7 | 0.73 | 0.67 | 3077 | Train 49 | Test White 5 | 0.7 | 0.7 | 0.63 |
| 2054 | Train 33 | Test White 6 | 0.69 | 0.69 | 0.63 | 3078 | Train 49 | Test White 6 | 0.68 | 0.68 | 0.6 |
| 2055 | Train 33 | Test White 7 | 0.71 | 0.73 | 0.67 | 3079 | Train 49 | Test White 7 | 0.7 | 0.7 | 0.63 |
| 2056 | Train 33 | Test White 8 | 0.69 | 0.69 | 0.64 | 3080 | Train 49 | Test White 8 | 0.68 | 0.68 | 0.61 |
| 2057 | Train 33 | Test White 9 | 0.74 | 0.8 | 0.8 | 3081 | Train 49 | Test White 9 | 0.73 | 0.71 | 0.64 |
| 2058 | Train 33 | Test White 10 | 0.73 | 0.75 | 0.76 | 3082 | Train 49 | Test White 10 | 0.71 | 0.69 | 0.6 |
| 2059 | Train 33 | Test White 11 | 0.74 | 0.8 | 0.8 | 3083 | Train 49 | Test White 11 | 0.73 | 0.71 | 0.64 |
| 2060 | Train 33 | Test White 12 | 0.73 | 0.75 | 0.76 | 3084 | Train 49 | Test White 12 | 0.71 | 0.69 | 0.6 |
| 2061 | Train 33 | Test White 13 | 0.7 | 0.73 | 0.66 | 3085 | Train 49 | Test White 13 | 0.7 | 0.7 | 0.63 |
| 2062 | Train 33 | Test White 14 | 0.69 | 0.7 | 0.63 | 3086 | Train 49 | Test White 14 | 0.68 | 0.68 | 0.6 |
| 2063 | Train 33 | Test White 15 | 0.7 | 0.73 | 0.66 | 3087 | Train 49 | Test White 15 | 0.7 | 0.7 | 0.63 |
| 2064 | Train 33 | Test White 16 | 0.69 | 0.69 | 0.64 | 3088 | Train 49 | Test White 16 | 0.69 | 0.68 | 0.61 |
| 2065 | Train 33 | Test White 17 | 0.74 | 0.81 | 0.84 | 3089 | Train 49 | Test White 17 | 0.73 | 0.72 | 0.66 |
| 2066 | Train 33 | Test White 18 | 0.73 | 0.76 | 0.78 | 3090 | Train 49 | Test White 18 | 0.71 | 0.69 | 0.61 |
| 2067 | Train 33 | Test White 19 | 0.74 | 0.81 | 0.83 | 3091 | Train 49 | Test White 19 | 0.73 | 0.72 | 0.66 |
| 2068 | Train 33 | Test White 20 | 0.73 | 0.76 | 0.78 | 3092 | Train 49 | Test White 20 | 0.71 | 0.69 | 0.61 |
| 2069 | Train 33 | Test White 21 | 0.71 | 0.73 | 0.67 | 3093 | Train 49 | Test White 21 | 0.7 | 0.7 | 0.63 |
| 2070 | Train 33 | Test White 22 | 0.69 | 0.69 | 0.63 | 3094 | Train 49 | Test White 22 | 0.68 | 0.68 | 0.6 |
| 2071 | Train 33 | Test White 23 | 0.71 | 0.73 | 0.67 | 3095 | Train 49 | Test White 23 | 0.7 | 0.7 | 0.63 |
| 2072 | Train 33 | Test White 24 | 0.69 | 0.69 | 0.64 | 3096 | Train 49 | Test White 24 | 0.68 | 0.68 | 0.61 |
| 2073 | Train 33 | Test White 25 | 0.74 | 0.8 | 0.8 | 3097 | Train 49 | Test White 25 | 0.73 | 0.71 | 0.64 |
| 2074 | Train 33 | Test White 26 | 0.73 | 0.75 | 0.76 | 3098 | Train 49 | Test White 26 | 0.71 | 0.69 | 0.6 |
| 2075 | Train 33 | Test White 27 | 0.74 | 0.8 | 0.8 | 3099 | Train 49 | Test White 27 | 0.73 | 0.71 | 0.64 |
| 2076 | Train 33 | Test White 28 | 0.73 | 0.75 | 0.76 | 3100 | Train 49 | Test White 28 | 0.71 | 0.69 | 0.6 |
| 2077 | Train 33 | Test White 29 | 0.7 | 0.73 | 0.66 | 3101 | Train 49 | Test White 29 | 0.7 | 0.7 | 0.63 |
| 2078 | Train 33 | Test White 30 | 0.69 | 0.7 | 0.63 | 3102 | Train 49 | Test White 30 | 0.68 | 0.68 | 0.61 |
| 2079 | Train 33 | Test White 31 | 0.7 | 0.73 | 0.66 | 3103 | Train 49 | Test White 31 | 0.7 | 0.7 | 0.63 |
| 2080 | Train 33 | Test White 32 | 0.69 | 0.7 | 0.64 | 3104 | Train 49 | Test White 32 | 0.69 | 0.68 | 0.61 |
| 2081 | Train 33 | Test White 33 | 0.75 | 0.82 | 0.84 | 3105 | Train 49 | Test White 33 | 0.73 | 0.72 | 0.66 |
| 2082 | Train 33 | Test White 34 | 0.73 | 0.76 | 0.78 | 3106 | Train 49 | Test White 34 | 0.71 | 0.69 | 0.61 |
| 2083 | Train 33 | Test White 35 | 0.75 | 0.82 | 0.84 | 3107 | Train 49 | Test White 35 | 0.73 | 0.72 | 0.66 |
| 2084 | Train 33 | Test White 36 | 0.73 | 0.76 | 0.78 | 3108 | Train 49 | Test White 36 | 0.71 | 0.69 | 0.61 |
| 2085 | Train 33 | Test White 37 | 0.71 | 0.73 | 0.67 | 3109 | Train 49 | Test White 37 | 0.7 | 0.7 | 0.63 |
| 2086 | Train 33 | Test White 38 | 0.69 | 0.69 | 0.63 | 3110 | Train 49 | Test White 38 | 0.68 | 0.68 | 0.6 |
| 2087 | Train 33 | Test White 39 | 0.71 | 0.73 | 0.67 | 3111 | Train 49 | Test White 39 | 0.7 | 0.7 | 0.63 |
| 2088 | Train 33 | Test White 40 | 0.69 | 0.69 | 0.64 | 3112 | Train 49 | Test White 40 | 0.68 | 0.68 | 0.61 |
| 2089 | Train 33 | Test White 41 | 0.75 | 0.8 | 0.8 | 3113 | Train 49 | Test White 41 | 0.73 | 0.72 | 0.65 |
| 2090 | Train 33 | Test White 42 | 0.73 | 0.76 | 0.76 | 3114 | Train 49 | Test White 42 | 0.71 | 0.69 | 0.61 |
| 2091 | Train 33 | Test White 43 | 0.75 | 0.8 | 0.8 | 3115 | Train 49 | Test White 43 | 0.73 | 0.72 | 0.65 |
| 2092 | Train 33 | Test White 44 | 0.73 | 0.76 | 0.76 | 3116 | Train 49 | Test White 44 | 0.71 | 0.69 | 0.61 |
| 2093 | Train 33 | Test White 45 | 0.71 | 0.73 | 0.66 | 3117 | Train 49 | Test White 45 | 0.7 | 0.7 | 0.63 |
| 2094 | Train 33 | Test White 46 | 0.69 | 0.7 | 0.63 | 3118 | Train 49 | Test White 46 | 0.68 | 0.68 | 0.61 |
| 2095 | Train 33 | Test White 47 | 0.71 | 0.73 | 0.66 | 3119 | Train 49 | Test White 47 | 0.7 | 0.7 | 0.63 |
| 2096 | Train 33 | Test White 48 | 0.69 | 0.7 | 0.64 | 3120 | Train 49 | Test White 48 | 0.69 | 0.68 | 0.61 |
| 2097 | Train 33 | Test White 49 | 0.73 | 0.77 | 0.81 | 3121 | Train 49 | Test White 49 | 0.75 | 0.79 | 0.85 |
| 2098 | Train 33 | Test White 50 | 0.73 | 0.77 | 0.8 | 3122 | Train 49 | Test White 50 | 0.75 | 0.79 | 0.85 |
| 2099 | Train 33 | Test White 51 | 0.73 | 0.76 | 0.8 | 3123 | Train 49 | Test White 51 | 0.75 | 0.79 | 0.85 |
| 2100 | Train 33 | Test White 52 | 0.73 | 0.76 | 0.8 | 3124 | Train 49 | Test White 52 | 0.75 | 0.79 | 0.85 |
| 2101 | Train 33 | Test White 53 | 0.7 | 0.73 | 0.58 | 3125 | Train 49 | Test White 53 | 0.69 | 0.74 | 0.69 |
| 2102 | Train 33 | Test White 54 | 0.7 | 0.7 | 0.62 | 3126 | Train 49 | Test White 54 | 0.69 | 0.69 | 0.64 |
| 2103 | Train 33 | Test White 55 | 0.7 | 0.74 | 0.58 | 3127 | Train 49 | Test White 55 | 0.69 | 0.74 | 0.7 |
| 2104 | Train 33 | Test White 56 | 0.7 | 0.69 | 0.62 | 3128 | Train 49 | Test White 56 | 0.69 | 0.68 | 0.64 |
| 2105 | Train 33 | Test White 57 | 0.73 | 0.76 | 0.77 | 3129 | Train 49 | Test White 57 | 0.75 | 0.78 | 0.78 |
| 2106 | Train 33 | Test White 58 | 0.73 | 0.76 | 0.77 | 3130 | Train 49 | Test White 58 | 0.75 | 0.78 | 0.78 |
| 2107 | Train 33 | Test White 59 | 0.73 | 0.76 | 0.77 | 3131 | Train 49 | Test White 59 | 0.75 | 0.78 | 0.78 |
| 2108 | Train 33 | Test White 60 | 0.73 | 0.76 | 0.77 | 3132 | Train 49 | Test White 60 | 0.75 | 0.78 | 0.78 |
| 2109 | Train 33 | Test White 61 | 0.7 | 0.73 | 0.58 | 3133 | Train 49 | Test White 61 | 0.69 | 0.73 | 0.69 |
| 2110 | Train 33 | Test White 62 | 0.7 | 0.7 | 0.62 | 3134 | Train 49 | Test White 62 | 0.69 | 0.69 | 0.64 |
| 2111 | Train 33 | Test White 63 | 0.7 | 0.73 | 0.58 | 3135 | Train 49 | Test White 63 | 0.69 | 0.74 | 0.69 |
| 2112 | Train 33 | Test White 64 | 0.7 | 0.7 | 0.62 | 3136 | Train 49 | Test White 64 | 0.7 | 0.69 | 0.64 |
| 2113 | Train 34 | Test White 1 | 0.74 | 0.8 | 0.82 | 3137 | Train 50 | Test White 1 | 0.73 | 0.69 | 0.68 |
| 2114 | Train 34 | Test White 2 | 0.75 | 0.81 | 0.83 | 3138 | Train 50 | Test White 2 | 0.71 | 0.68 | 0.65 |
| 2115 | Train 34 | Test White 3 | 0.74 | 0.8 | 0.82 | 3139 | Train 50 | Test White 3 | 0.73 | 0.69 | 0.68 |
| 2116 | Train 34 | Test White 4 | 0.75 | 0.81 | 0.83 | 3140 | Train 50 | Test White 4 | 0.71 | 0.68 | 0.65 |
| 2117 | Train 34 | Test White 5 | 0.71 | 0.73 | 0.68 | 3141 | Train 50 | Test White 5 | 0.7 | 0.69 | 0.66 |
| 2118 | Train 34 | Test White 6 | 0.7 | 0.7 | 0.66 | 3142 | Train 50 | Test White 6 | 0.68 | 0.67 | 0.63 |
| 2119 | Train 34 | Test White 7 | 0.71 | 0.73 | 0.68 | 3143 | Train 50 | Test White 7 | 0.7 | 0.69 | 0.66 |
| 2120 | Train 34 | Test White 8 | 0.7 | 0.7 | 0.66 | 3144 | Train 50 | Test White 8 | 0.69 | 0.67 | 0.63 |
| 2121 | Train 34 | Test White 9 | 0.74 | 0.79 | 0.79 | 3145 | Train 50 | Test White 9 | 0.73 | 0.69 | 0.67 |
| 2122 | Train 34 | Test White 10 | 0.75 | 0.8 | 0.8 | 3146 | Train 50 | Test White 10 | 0.71 | 0.68 | 0.64 |
| 2123 | Train 34 | Test White 11 | 0.74 | 0.79 | 0.79 | 3147 | Train 50 | Test White 11 | 0.73 | 0.69 | 0.67 |
| 2124 | Train 34 | Test White 12 | 0.75 | 0.8 | 0.8 | 3148 | Train 50 | Test White 12 | 0.71 | 0.68 | 0.64 |
| 2125 | Train 34 | Test White 13 | 0.71 | 0.73 | 0.67 | 3149 | Train 50 | Test White 13 | 0.7 | 0.69 | 0.66 |
| 2126 | Train 34 | Test White 14 | 0.71 | 0.71 | 0.66 | 3150 | Train 50 | Test White 14 | 0.69 | 0.68 | 0.64 |
| 2127 | Train 34 | Test White 15 | 0.71 | 0.73 | 0.67 | 3151 | Train 50 | Test White 15 | 0.7 | 0.69 | 0.66 |
| 2128 | Train 34 | Test White 16 | 0.7 | 0.7 | 0.66 | 3152 | Train 50 | Test White 16 | 0.69 | 0.67 | 0.64 |
| 2129 | Train 34 | Test White 17 | 0.74 | 0.8 | 0.82 | 3153 | Train 50 | Test White 17 | 0.73 | 0.69 | 0.68 |
| 2130 | Train 34 | Test White 18 | 0.75 | 0.81 | 0.83 | 3154 | Train 50 | Test White 18 | 0.71 | 0.68 | 0.65 |
| 2131 | Train 34 | Test White 19 | 0.74 | 0.8 | 0.82 | 3155 | Train 50 | Test White 19 | 0.73 | 0.69 | 0.68 |
| 2132 | Train 34 | Test White 20 | 0.75 | 0.81 | 0.83 | 3156 | Train 50 | Test White 20 | 0.71 | 0.68 | 0.65 |
| 2133 | Train 34 | Test White 21 | 0.71 | 0.73 | 0.68 | 3157 | Train 50 | Test White 21 | 0.7 | 0.69 | 0.66 |
| 2134 | Train 34 | Test White 22 | 0.7 | 0.71 | 0.66 | 3158 | Train 50 | Test White 22 | 0.68 | 0.67 | 0.64 |
| 2135 | Train 34 | Test White 23 | 0.71 | 0.73 | 0.68 | 3159 | Train 50 | Test White 23 | 0.7 | 0.69 | 0.66 |
| 2136 | Train 34 | Test White 24 | 0.7 | 0.7 | 0.66 | 3160 | Train 50 | Test White 24 | 0.69 | 0.67 | 0.64 |
| 2137 | Train 34 | Test White 25 | 0.74 | 0.79 | 0.79 | 3161 | Train 50 | Test White 25 | 0.73 | 0.69 | 0.67 |
| 2138 | Train 34 | Test White 26 | 0.75 | 0.8 | 0.8 | 3162 | Train 50 | Test White 26 | 0.71 | 0.68 | 0.64 |
| 2139 | Train 34 | Test White 27 | 0.74 | 0.79 | 0.79 | 3163 | Train 50 | Test White 27 | 0.73 | 0.69 | 0.67 |
| 2140 | Train 34 | Test White 28 | 0.75 | 0.8 | 0.8 | 3164 | Train 50 | Test White 28 | 0.71 | 0.68 | 0.64 |
| 2141 | Train 34 | Test White 29 | 0.71 | 0.73 | 0.67 | 3165 | Train 50 | Test White 29 | 0.7 | 0.69 | 0.66 |
| 2142 | Train 34 | Test White 30 | 0.71 | 0.71 | 0.66 | 3166 | Train 50 | Test White 30 | 0.69 | 0.68 | 0.64 |
| 2143 | Train 34 | Test White 31 | 0.71 | 0.73 | 0.67 | 3167 | Train 50 | Test White 31 | 0.7 | 0.69 | 0.66 |
| 2144 | Train 34 | Test White 32 | 0.71 | 0.7 | 0.66 | 3168 | Train 50 | Test White 32 | 0.69 | 0.67 | 0.64 |
| 2145 | Train 34 | Test White 33 | 0.74 | 0.81 | 0.82 | 3169 | Train 50 | Test White 33 | 0.73 | 0.7 | 0.68 |
| 2146 | Train 34 | Test White 34 | 0.75 | 0.81 | 0.83 | 3170 | Train 50 | Test White 34 | 0.71 | 0.68 | 0.65 |
| 2147 | Train 34 | Test White 35 | 0.74 | 0.81 | 0.82 | 3171 | Train 50 | Test White 35 | 0.73 | 0.7 | 0.68 |
| 2148 | Train 34 | Test White 36 | 0.75 | 0.81 | 0.83 | 3172 | Train 50 | Test White 36 | 0.71 | 0.68 | 0.65 |
| 2149 | Train 34 | Test White 37 | 0.71 | 0.73 | 0.68 | 3173 | Train 50 | Test White 37 | 0.7 | 0.69 | 0.66 |
| 2150 | Train 34 | Test White 38 | 0.7 | 0.71 | 0.66 | 3174 | Train 50 | Test White 38 | 0.68 | 0.67 | 0.63 |
| 2151 | Train 34 | Test White 39 | 0.71 | 0.73 | 0.68 | 3175 | Train 50 | Test White 39 | 0.7 | 0.69 | 0.66 |
| 2152 | Train 34 | Test White 40 | 0.7 | 0.7 | 0.66 | 3176 | Train 50 | Test White 40 | 0.69 | 0.67 | 0.64 |
| 2153 | Train 34 | Test White 41 | 0.74 | 0.8 | 0.79 | 3177 | Train 50 | Test White 41 | 0.73 | 0.7 | 0.67 |
| 2154 | Train 34 | Test White 42 | 0.75 | 0.8 | 0.8 | 3178 | Train 50 | Test White 42 | 0.71 | 0.68 | 0.64 |
| 2155 | Train 34 | Test White 43 | 0.74 | 0.8 | 0.79 | 3179 | Train 50 | Test White 43 | 0.73 | 0.7 | 0.67 |
| 2156 | Train 34 | Test White 44 | 0.75 | 0.8 | 0.8 | 3180 | Train 50 | Test White 44 | 0.71 | 0.68 | 0.64 |
| 2157 | Train 34 | Test White 45 | 0.71 | 0.73 | 0.67 | 3181 | Train 50 | Test White 45 | 0.7 | 0.69 | 0.66 |
| 2158 | Train 34 | Test White 46 | 0.71 | 0.71 | 0.66 | 3182 | Train 50 | Test White 46 | 0.69 | 0.68 | 0.64 |
| 2159 | Train 34 | Test White 47 | 0.71 | 0.73 | 0.67 | 3183 | Train 50 | Test White 47 | 0.7 | 0.69 | 0.66 |
| 2160 | Train 34 | Test White 48 | 0.7 | 0.7 | 0.66 | 3184 | Train 50 | Test White 48 | 0.69 | 0.67 | 0.64 |
| 2161 | Train 34 | Test White 49 | 0.73 | 0.76 | 0.8 | 3185 | Train 50 | Test White 49 | 0.75 | 0.8 | 0.86 |
| 2162 | Train 34 | Test White 50 | 0.73 | 0.76 | 0.8 | 3186 | Train 50 | Test White 50 | 0.75 | 0.8 | 0.86 |
| 2163 | Train 34 | Test White 51 | 0.73 | 0.76 | 0.8 | 3187 | Train 50 | Test White 51 | 0.75 | 0.79 | 0.85 |
| 2164 | Train 34 | Test White 52 | 0.73 | 0.76 | 0.8 | 3188 | Train 50 | Test White 52 | 0.75 | 0.79 | 0.85 |
| 2165 | Train 34 | Test White 53 | 0.7 | 0.74 | 0.67 | 3189 | Train 50 | Test White 53 | 0.69 | 0.72 | 0.72 |
| 2166 | Train 34 | Test White 54 | 0.71 | 0.7 | 0.66 | 3190 | Train 50 | Test White 54 | 0.69 | 0.68 | 0.65 |
| 2167 | Train 34 | Test White 55 | 0.7 | 0.74 | 0.67 | 3191 | Train 50 | Test White 55 | 0.69 | 0.72 | 0.72 |
| 2168 | Train 34 | Test White 56 | 0.7 | 0.7 | 0.65 | 3192 | Train 50 | Test White 56 | 0.69 | 0.67 | 0.65 |
| 2169 | Train 34 | Test White 57 | 0.73 | 0.75 | 0.77 | 3193 | Train 50 | Test White 57 | 0.75 | 0.78 | 0.78 |
| 2170 | Train 34 | Test White 58 | 0.73 | 0.75 | 0.77 | 3194 | Train 50 | Test White 58 | 0.75 | 0.78 | 0.78 |
| 2171 | Train 34 | Test White 59 | 0.73 | 0.75 | 0.77 | 3195 | Train 50 | Test White 59 | 0.75 | 0.78 | 0.78 |
| 2172 | Train 34 | Test White 60 | 0.73 | 0.75 | 0.77 | 3196 | Train 50 | Test White 60 | 0.75 | 0.78 | 0.78 |
| 2173 | Train 34 | Test White 61 | 0.7 | 0.74 | 0.67 | 3197 | Train 50 | Test White 61 | 0.69 | 0.72 | 0.71 |
| 2174 | Train 34 | Test White 62 | 0.71 | 0.71 | 0.66 | 3198 | Train 50 | Test White 62 | 0.69 | 0.68 | 0.65 |
| 2175 | Train 34 | Test White 63 | 0.71 | 0.74 | 0.67 | 3199 | Train 50 | Test White 63 | 0.69 | 0.72 | 0.71 |
| 2176 | Train 34 | Test White 64 | 0.7 | 0.7 | 0.66 | 3200 | Train 50 | Test White 64 | 0.69 | 0.68 | 0.65 |
| 2177 | Train 35 | Test White 1 | 0.74 | 0.81 | 0.83 | 3201 | Train 51 | Test White 1 | 0.73 | 0.72 | 0.67 |
| 2178 | Train 35 | Test White 2 | 0.73 | 0.76 | 0.78 | 3202 | Train 51 | Test White 2 | 0.71 | 0.69 | 0.61 |
| 2179 | Train 35 | Test White 3 | 0.74 | 0.81 | 0.83 | 3203 | Train 51 | Test White 3 | 0.73 | 0.72 | 0.67 |
| 2180 | Train 35 | Test White 4 | 0.73 | 0.76 | 0.78 | 3204 | Train 51 | Test White 4 | 0.71 | 0.69 | 0.61 |
| 2181 | Train 35 | Test White 5 | 0.7 | 0.73 | 0.68 | 3205 | Train 51 | Test White 5 | 0.7 | 0.7 | 0.63 |
| 2182 | Train 35 | Test White 6 | 0.69 | 0.69 | 0.65 | 3206 | Train 51 | Test White 6 | 0.68 | 0.68 | 0.58 |
| 2183 | Train 35 | Test White 7 | 0.71 | 0.73 | 0.68 | 3207 | Train 51 | Test White 7 | 0.7 | 0.7 | 0.64 |
| 2184 | Train 35 | Test White 8 | 0.69 | 0.69 | 0.65 | 3208 | Train 51 | Test White 8 | 0.68 | 0.68 | 0.58 |
| 2185 | Train 35 | Test White 9 | 0.74 | 0.8 | 0.8 | 3209 | Train 51 | Test White 9 | 0.73 | 0.71 | 0.66 |
| 2186 | Train 35 | Test White 10 | 0.73 | 0.75 | 0.75 | 3210 | Train 51 | Test White 10 | 0.71 | 0.69 | 0.6 |
| 2187 | Train 35 | Test White 11 | 0.74 | 0.8 | 0.8 | 3211 | Train 51 | Test White 11 | 0.73 | 0.71 | 0.66 |
| 2188 | Train 35 | Test White 12 | 0.73 | 0.76 | 0.75 | 3212 | Train 51 | Test White 12 | 0.71 | 0.69 | 0.6 |
| 2189 | Train 35 | Test White 13 | 0.7 | 0.73 | 0.66 | 3213 | Train 51 | Test White 13 | 0.7 | 0.7 | 0.63 |
| 2190 | Train 35 | Test White 14 | 0.69 | 0.7 | 0.65 | 3214 | Train 51 | Test White 14 | 0.68 | 0.68 | 0.58 |
| 2191 | Train 35 | Test White 15 | 0.7 | 0.73 | 0.66 | 3215 | Train 51 | Test White 15 | 0.7 | 0.7 | 0.63 |
| 2192 | Train 35 | Test White 16 | 0.69 | 0.69 | 0.65 | 3216 | Train 51 | Test White 16 | 0.68 | 0.68 | 0.58 |
| 2193 | Train 35 | Test White 17 | 0.74 | 0.81 | 0.83 | 3217 | Train 51 | Test White 17 | 0.73 | 0.72 | 0.67 |
| 2194 | Train 35 | Test White 18 | 0.73 | 0.76 | 0.78 | 3218 | Train 51 | Test White 18 | 0.71 | 0.69 | 0.61 |
| 2195 | Train 35 | Test White 19 | 0.74 | 0.81 | 0.83 | 3219 | Train 51 | Test White 19 | 0.73 | 0.72 | 0.67 |
| 2196 | Train 35 | Test White 20 | 0.73 | 0.76 | 0.78 | 3220 | Train 51 | Test White 20 | 0.71 | 0.69 | 0.61 |
| 2197 | Train 35 | Test White 21 | 0.71 | 0.73 | 0.68 | 3221 | Train 51 | Test White 21 | 0.7 | 0.7 | 0.64 |
| 2198 | Train 35 | Test White 22 | 0.69 | 0.69 | 0.65 | 3222 | Train 51 | Test White 22 | 0.68 | 0.68 | 0.58 |
| 2199 | Train 35 | Test White 23 | 0.71 | 0.73 | 0.68 | 3223 | Train 51 | Test White 23 | 0.7 | 0.7 | 0.64 |
| 2200 | Train 35 | Test White 24 | 0.69 | 0.69 | 0.66 | 3224 | Train 51 | Test White 24 | 0.68 | 0.68 | 0.58 |
| 2201 | Train 35 | Test White 25 | 0.74 | 0.8 | 0.8 | 3225 | Train 51 | Test White 25 | 0.73 | 0.71 | 0.66 |
| 2202 | Train 35 | Test White 26 | 0.73 | 0.75 | 0.75 | 3226 | Train 51 | Test White 26 | 0.71 | 0.69 | 0.6 |
| 2203 | Train 35 | Test White 27 | 0.75 | 0.8 | 0.8 | 3227 | Train 51 | Test White 27 | 0.73 | 0.71 | 0.66 |
| 2204 | Train 35 | Test White 28 | 0.73 | 0.76 | 0.75 | 3228 | Train 51 | Test White 28 | 0.71 | 0.69 | 0.6 |
| 2205 | Train 35 | Test White 29 | 0.7 | 0.73 | 0.66 | 3229 | Train 51 | Test White 29 | 0.7 | 0.7 | 0.63 |
| 2206 | Train 35 | Test White 30 | 0.69 | 0.7 | 0.66 | 3230 | Train 51 | Test White 30 | 0.68 | 0.68 | 0.58 |
| 2207 | Train 35 | Test White 31 | 0.7 | 0.73 | 0.66 | 3231 | Train 51 | Test White 31 | 0.7 | 0.7 | 0.63 |
| 2208 | Train 35 | Test White 32 | 0.69 | 0.7 | 0.66 | 3232 | Train 51 | Test White 32 | 0.68 | 0.68 | 0.58 |
| 2209 | Train 35 | Test White 33 | 0.75 | 0.82 | 0.83 | 3233 | Train 51 | Test White 33 | 0.73 | 0.72 | 0.67 |
| 2210 | Train 35 | Test White 34 | 0.73 | 0.76 | 0.78 | 3234 | Train 51 | Test White 34 | 0.71 | 0.69 | 0.61 |
| 2211 | Train 35 | Test White 35 | 0.75 | 0.82 | 0.83 | 3235 | Train 51 | Test White 35 | 0.73 | 0.72 | 0.68 |
| 2212 | Train 35 | Test White 36 | 0.73 | 0.76 | 0.78 | 3236 | Train 51 | Test White 36 | 0.71 | 0.69 | 0.62 |
| 2213 | Train 35 | Test White 37 | 0.71 | 0.73 | 0.68 | 3237 | Train 51 | Test White 37 | 0.7 | 0.7 | 0.64 |
| 2214 | Train 35 | Test White 38 | 0.69 | 0.69 | 0.65 | 3238 | Train 51 | Test White 38 | 0.68 | 0.68 | 0.58 |
| 2215 | Train 35 | Test White 39 | 0.71 | 0.73 | 0.68 | 3239 | Train 51 | Test White 39 | 0.7 | 0.7 | 0.64 |
| 2216 | Train 35 | Test White 40 | 0.69 | 0.69 | 0.66 | 3240 | Train 51 | Test White 40 | 0.68 | 0.68 | 0.58 |
| 2217 | Train 35 | Test White 41 | 0.75 | 0.8 | 0.8 | 3241 | Train 51 | Test White 41 | 0.73 | 0.72 | 0.66 |
| 2218 | Train 35 | Test White 42 | 0.73 | 0.76 | 0.76 | 3242 | Train 51 | Test White 42 | 0.71 | 0.69 | 0.6 |
| 2219 | Train 35 | Test White 43 | 0.75 | 0.81 | 0.8 | 3243 | Train 51 | Test White 43 | 0.73 | 0.72 | 0.66 |
| 2220 | Train 35 | Test White 44 | 0.73 | 0.76 | 0.76 | 3244 | Train 51 | Test White 44 | 0.71 | 0.69 | 0.6 |
| 2221 | Train 35 | Test White 45 | 0.71 | 0.73 | 0.66 | 3245 | Train 51 | Test White 45 | 0.7 | 0.7 | 0.63 |
| 2222 | Train 35 | Test White 46 | 0.69 | 0.7 | 0.65 | 3246 | Train 51 | Test White 46 | 0.68 | 0.68 | 0.58 |
| 2223 | Train 35 | Test White 47 | 0.71 | 0.73 | 0.66 | 3247 | Train 51 | Test White 47 | 0.7 | 0.7 | 0.63 |
| 2224 | Train 35 | Test White 48 | 0.69 | 0.7 | 0.66 | 3248 | Train 51 | Test White 48 | 0.68 | 0.68 | 0.58 |
| 2225 | Train 35 | Test White 49 | 0.73 | 0.77 | 0.81 | 3249 | Train 51 | Test White 49 | 0.75 | 0.8 | 0.84 |
| 2226 | Train 35 | Test White 50 | 0.73 | 0.76 | 0.81 | 3250 | Train 51 | Test White 50 | 0.75 | 0.8 | 0.84 |
| 2227 | Train 35 | Test White 51 | 0.73 | 0.77 | 0.81 | 3251 | Train 51 | Test White 51 | 0.75 | 0.8 | 0.84 |
| 2228 | Train 35 | Test White 52 | 0.73 | 0.77 | 0.81 | 3252 | Train 51 | Test White 52 | 0.75 | 0.8 | 0.84 |
| 2229 | Train 35 | Test White 53 | 0.7 | 0.73 | 0.59 | 3253 | Train 51 | Test White 53 | 0.69 | 0.73 | 0.69 |
| 2230 | Train 35 | Test White 54 | 0.7 | 0.7 | 0.64 | 3254 | Train 51 | Test White 54 | 0.68 | 0.68 | 0.6 |
| 2231 | Train 35 | Test White 55 | 0.7 | 0.73 | 0.59 | 3255 | Train 51 | Test White 55 | 0.69 | 0.74 | 0.69 |
| 2232 | Train 35 | Test White 56 | 0.7 | 0.69 | 0.64 | 3256 | Train 51 | Test White 56 | 0.69 | 0.68 | 0.61 |
| 2233 | Train 35 | Test White 57 | 0.73 | 0.76 | 0.78 | 3257 | Train 51 | Test White 57 | 0.75 | 0.78 | 0.77 |
| 2234 | Train 35 | Test White 58 | 0.73 | 0.76 | 0.78 | 3258 | Train 51 | Test White 58 | 0.75 | 0.78 | 0.77 |
| 2235 | Train 35 | Test White 59 | 0.73 | 0.76 | 0.78 | 3259 | Train 51 | Test White 59 | 0.75 | 0.78 | 0.77 |
| 2236 | Train 35 | Test White 60 | 0.73 | 0.76 | 0.78 | 3260 | Train 51 | Test White 60 | 0.75 | 0.78 | 0.77 |
| 2237 | Train 35 | Test White 61 | 0.7 | 0.73 | 0.59 | 3261 | Train 51 | Test White 61 | 0.69 | 0.73 | 0.68 |
| 2238 | Train 35 | Test White 62 | 0.7 | 0.7 | 0.64 | 3262 | Train 51 | Test White 62 | 0.68 | 0.69 | 0.6 |
| 2239 | Train 35 | Test White 63 | 0.7 | 0.73 | 0.59 | 3263 | Train 51 | Test White 63 | 0.69 | 0.74 | 0.68 |
| 2240 | Train 35 | Test White 64 | 0.7 | 0.69 | 0.64 | 3264 | Train 51 | Test White 64 | 0.69 | 0.69 | 0.61 |
| 2241 | Train 36 | Test White 1 | 0.74 | 0.8 | 0.82 | 3265 | Train 52 | Test White 1 | 0.72 | 0.69 | 0.67 |
| 2242 | Train 36 | Test White 2 | 0.75 | 0.81 | 0.83 | 3266 | Train 52 | Test White 2 | 0.71 | 0.67 | 0.64 |
| 2243 | Train 36 | Test White 3 | 0.74 | 0.8 | 0.82 | 3267 | Train 52 | Test White 3 | 0.73 | 0.69 | 0.67 |
| 2244 | Train 36 | Test White 4 | 0.75 | 0.81 | 0.83 | 3268 | Train 52 | Test White 4 | 0.71 | 0.67 | 0.64 |
| 2245 | Train 36 | Test White 5 | 0.71 | 0.73 | 0.68 | 3269 | Train 52 | Test White 5 | 0.7 | 0.68 | 0.66 |
| 2246 | Train 36 | Test White 6 | 0.7 | 0.71 | 0.66 | 3270 | Train 52 | Test White 6 | 0.68 | 0.67 | 0.62 |
| 2247 | Train 36 | Test White 7 | 0.71 | 0.73 | 0.68 | 3271 | Train 52 | Test White 7 | 0.7 | 0.68 | 0.66 |
| 2248 | Train 36 | Test White 8 | 0.7 | 0.71 | 0.66 | 3272 | Train 52 | Test White 8 | 0.68 | 0.67 | 0.62 |
| 2249 | Train 36 | Test White 9 | 0.74 | 0.8 | 0.79 | 3273 | Train 52 | Test White 9 | 0.72 | 0.69 | 0.66 |
| 2250 | Train 36 | Test White 10 | 0.75 | 0.8 | 0.8 | 3274 | Train 52 | Test White 10 | 0.71 | 0.67 | 0.63 |
| 2251 | Train 36 | Test White 11 | 0.74 | 0.8 | 0.79 | 3275 | Train 52 | Test White 11 | 0.73 | 0.69 | 0.66 |
| 2252 | Train 36 | Test White 12 | 0.75 | 0.8 | 0.8 | 3276 | Train 52 | Test White 12 | 0.71 | 0.68 | 0.63 |
| 2253 | Train 36 | Test White 13 | 0.71 | 0.73 | 0.67 | 3277 | Train 52 | Test White 13 | 0.7 | 0.68 | 0.66 |
| 2254 | Train 36 | Test White 14 | 0.7 | 0.71 | 0.66 | 3278 | Train 52 | Test White 14 | 0.68 | 0.67 | 0.63 |
| 2255 | Train 36 | Test White 15 | 0.71 | 0.73 | 0.67 | 3279 | Train 52 | Test White 15 | 0.7 | 0.69 | 0.66 |
| 2256 | Train 36 | Test White 16 | 0.7 | 0.71 | 0.66 | 3280 | Train 52 | Test White 16 | 0.68 | 0.67 | 0.63 |
| 2257 | Train 36 | Test White 17 | 0.74 | 0.8 | 0.82 | 3281 | Train 52 | Test White 17 | 0.72 | 0.69 | 0.67 |
| 2258 | Train 36 | Test White 18 | 0.75 | 0.81 | 0.83 | 3282 | Train 52 | Test White 18 | 0.71 | 0.67 | 0.64 |
| 2259 | Train 36 | Test White 19 | 0.74 | 0.8 | 0.82 | 3283 | Train 52 | Test White 19 | 0.73 | 0.69 | 0.67 |
| 2260 | Train 36 | Test White 20 | 0.75 | 0.81 | 0.83 | 3284 | Train 52 | Test White 20 | 0.71 | 0.67 | 0.64 |
| 2261 | Train 36 | Test White 21 | 0.71 | 0.73 | 0.68 | 3285 | Train 52 | Test White 21 | 0.7 | 0.68 | 0.66 |
| 2262 | Train 36 | Test White 22 | 0.7 | 0.71 | 0.66 | 3286 | Train 52 | Test White 22 | 0.68 | 0.67 | 0.62 |
| 2263 | Train 36 | Test White 23 | 0.71 | 0.73 | 0.68 | 3287 | Train 52 | Test White 23 | 0.7 | 0.68 | 0.66 |
| 2264 | Train 36 | Test White 24 | 0.7 | 0.71 | 0.66 | 3288 | Train 52 | Test White 24 | 0.68 | 0.67 | 0.63 |
| 2265 | Train 36 | Test White 25 | 0.74 | 0.8 | 0.79 | 3289 | Train 52 | Test White 25 | 0.73 | 0.69 | 0.66 |
| 2266 | Train 36 | Test White 26 | 0.75 | 0.8 | 0.8 | 3290 | Train 52 | Test White 26 | 0.71 | 0.67 | 0.63 |
| 2267 | Train 36 | Test White 27 | 0.74 | 0.8 | 0.79 | 3291 | Train 52 | Test White 27 | 0.73 | 0.69 | 0.66 |
| 2268 | Train 36 | Test White 28 | 0.75 | 0.8 | 0.8 | 3292 | Train 52 | Test White 28 | 0.71 | 0.68 | 0.63 |
| 2269 | Train 36 | Test White 29 | 0.71 | 0.73 | 0.67 | 3293 | Train 52 | Test White 29 | 0.7 | 0.68 | 0.66 |
| 2270 | Train 36 | Test White 30 | 0.71 | 0.71 | 0.67 | 3294 | Train 52 | Test White 30 | 0.68 | 0.68 | 0.63 |
| 2271 | Train 36 | Test White 31 | 0.71 | 0.73 | 0.67 | 3295 | Train 52 | Test White 31 | 0.7 | 0.69 | 0.66 |
| 2272 | Train 36 | Test White 32 | 0.7 | 0.71 | 0.67 | 3296 | Train 52 | Test White 32 | 0.69 | 0.67 | 0.63 |
| 2273 | Train 36 | Test White 33 | 0.74 | 0.81 | 0.82 | 3297 | Train 52 | Test White 33 | 0.73 | 0.69 | 0.67 |
| 2274 | Train 36 | Test White 34 | 0.75 | 0.81 | 0.83 | 3298 | Train 52 | Test White 34 | 0.71 | 0.68 | 0.64 |
| 2275 | Train 36 | Test White 35 | 0.74 | 0.81 | 0.82 | 3299 | Train 52 | Test White 35 | 0.73 | 0.69 | 0.67 |
| 2276 | Train 36 | Test White 36 | 0.75 | 0.81 | 0.83 | 3300 | Train 52 | Test White 36 | 0.71 | 0.68 | 0.64 |
| 2277 | Train 36 | Test White 37 | 0.71 | 0.73 | 0.68 | 3301 | Train 52 | Test White 37 | 0.7 | 0.68 | 0.66 |
| 2278 | Train 36 | Test White 38 | 0.7 | 0.71 | 0.66 | 3302 | Train 52 | Test White 38 | 0.68 | 0.67 | 0.62 |
| 2279 | Train 36 | Test White 39 | 0.71 | 0.73 | 0.68 | 3303 | Train 52 | Test White 39 | 0.7 | 0.69 | 0.66 |
| 2280 | Train 36 | Test White 40 | 0.7 | 0.71 | 0.66 | 3304 | Train 52 | Test White 40 | 0.68 | 0.67 | 0.62 |
| 2281 | Train 36 | Test White 41 | 0.74 | 0.8 | 0.79 | 3305 | Train 52 | Test White 41 | 0.73 | 0.69 | 0.66 |
| 2282 | Train 36 | Test White 42 | 0.75 | 0.81 | 0.81 | 3306 | Train 52 | Test White 42 | 0.71 | 0.68 | 0.63 |
| 2283 | Train 36 | Test White 43 | 0.74 | 0.8 | 0.79 | 3307 | Train 52 | Test White 43 | 0.73 | 0.69 | 0.66 |
| 2284 | Train 36 | Test White 44 | 0.75 | 0.81 | 0.81 | 3308 | Train 52 | Test White 44 | 0.71 | 0.68 | 0.63 |
| 2285 | Train 36 | Test White 45 | 0.71 | 0.73 | 0.67 | 3309 | Train 52 | Test White 45 | 0.7 | 0.69 | 0.66 |
| 2286 | Train 36 | Test White 46 | 0.71 | 0.71 | 0.67 | 3310 | Train 52 | Test White 46 | 0.68 | 0.67 | 0.63 |
| 2287 | Train 36 | Test White 47 | 0.71 | 0.73 | 0.67 | 3311 | Train 52 | Test White 47 | 0.7 | 0.69 | 0.66 |
| 2288 | Train 36 | Test White 48 | 0.7 | 0.71 | 0.67 | 3312 | Train 52 | Test White 48 | 0.69 | 0.67 | 0.63 |
| 2289 | Train 36 | Test White 49 | 0.73 | 0.76 | 0.8 | 3313 | Train 52 | Test White 49 | 0.75 | 0.8 | 0.84 |
| 2290 | Train 36 | Test White 50 | 0.73 | 0.76 | 0.8 | 3314 | Train 52 | Test White 50 | 0.75 | 0.79 | 0.84 |
| 2291 | Train 36 | Test White 51 | 0.73 | 0.76 | 0.8 | 3315 | Train 52 | Test White 51 | 0.75 | 0.8 | 0.84 |
| 2292 | Train 36 | Test White 52 | 0.73 | 0.76 | 0.8 | 3316 | Train 52 | Test White 52 | 0.75 | 0.8 | 0.84 |
| 2293 | Train 36 | Test White 53 | 0.7 | 0.74 | 0.63 | 3317 | Train 52 | Test White 53 | 0.69 | 0.72 | 0.73 |
| 2294 | Train 36 | Test White 54 | 0.71 | 0.71 | 0.66 | 3318 | Train 52 | Test White 54 | 0.68 | 0.68 | 0.65 |
| 2295 | Train 36 | Test White 55 | 0.7 | 0.74 | 0.63 | 3319 | Train 52 | Test White 55 | 0.69 | 0.72 | 0.73 |
| 2296 | Train 36 | Test White 56 | 0.7 | 0.7 | 0.66 | 3320 | Train 52 | Test White 56 | 0.69 | 0.67 | 0.65 |
| 2297 | Train 36 | Test White 57 | 0.73 | 0.75 | 0.77 | 3321 | Train 52 | Test White 57 | 0.75 | 0.78 | 0.77 |
| 2298 | Train 36 | Test White 58 | 0.73 | 0.75 | 0.76 | 3322 | Train 52 | Test White 58 | 0.75 | 0.78 | 0.77 |
| 2299 | Train 36 | Test White 59 | 0.73 | 0.76 | 0.77 | 3323 | Train 52 | Test White 59 | 0.75 | 0.78 | 0.78 |
| 2300 | Train 36 | Test White 60 | 0.73 | 0.76 | 0.77 | 3324 | Train 52 | Test White 60 | 0.75 | 0.78 | 0.78 |
| 2301 | Train 36 | Test White 61 | 0.7 | 0.74 | 0.63 | 3325 | Train 52 | Test White 61 | 0.69 | 0.71 | 0.72 |
| 2302 | Train 36 | Test White 62 | 0.71 | 0.71 | 0.67 | 3326 | Train 52 | Test White 62 | 0.69 | 0.68 | 0.65 |
| 2303 | Train 36 | Test White 63 | 0.7 | 0.74 | 0.63 | 3327 | Train 52 | Test White 63 | 0.69 | 0.72 | 0.73 |
| 2304 | Train 36 | Test White 64 | 0.7 | 0.7 | 0.66 | 3328 | Train 52 | Test White 64 | 0.69 | 0.67 | 0.65 |
| 2305 | Train 37 | Test White 1 | 0.74 | 0.79 | 0.81 | 3329 | Train 53 | Test White 1 | 0.72 | 0.73 | 0.65 |
| 2306 | Train 37 | Test White 2 | 0.74 | 0.77 | 0.77 | 3330 | Train 53 | Test White 2 | 0.72 | 0.7 | 0.63 |
| 2307 | Train 37 | Test White 3 | 0.74 | 0.79 | 0.8 | 3331 | Train 53 | Test White 3 | 0.72 | 0.73 | 0.65 |
| 2308 | Train 37 | Test White 4 | 0.74 | 0.77 | 0.77 | 3332 | Train 53 | Test White 4 | 0.72 | 0.7 | 0.63 |
| 2309 | Train 37 | Test White 5 | 0.73 | 0.78 | 0.77 | 3333 | Train 53 | Test White 5 | 0.72 | 0.74 | 0.69 |
| 2310 | Train 37 | Test White 6 | 0.7 | 0.71 | 0.69 | 3334 | Train 53 | Test White 6 | 0.7 | 0.69 | 0.63 |
| 2311 | Train 37 | Test White 7 | 0.73 | 0.78 | 0.77 | 3335 | Train 53 | Test White 7 | 0.72 | 0.74 | 0.68 |
| 2312 | Train 37 | Test White 8 | 0.7 | 0.71 | 0.69 | 3336 | Train 53 | Test White 8 | 0.7 | 0.69 | 0.63 |
| 2313 | Train 37 | Test White 9 | 0.74 | 0.78 | 0.78 | 3337 | Train 53 | Test White 9 | 0.72 | 0.73 | 0.65 |
| 2314 | Train 37 | Test White 10 | 0.74 | 0.76 | 0.76 | 3338 | Train 53 | Test White 10 | 0.72 | 0.7 | 0.64 |
| 2315 | Train 37 | Test White 11 | 0.74 | 0.78 | 0.78 | 3339 | Train 53 | Test White 11 | 0.72 | 0.73 | 0.65 |
| 2316 | Train 37 | Test White 12 | 0.74 | 0.76 | 0.76 | 3340 | Train 53 | Test White 12 | 0.72 | 0.7 | 0.64 |
| 2317 | Train 37 | Test White 13 | 0.73 | 0.77 | 0.75 | 3341 | Train 53 | Test White 13 | 0.72 | 0.75 | 0.68 |
| 2318 | Train 37 | Test White 14 | 0.7 | 0.71 | 0.69 | 3342 | Train 53 | Test White 14 | 0.7 | 0.69 | 0.63 |
| 2319 | Train 37 | Test White 15 | 0.73 | 0.77 | 0.75 | 3343 | Train 53 | Test White 15 | 0.71 | 0.74 | 0.68 |
| 2320 | Train 37 | Test White 16 | 0.7 | 0.71 | 0.69 | 3344 | Train 53 | Test White 16 | 0.7 | 0.69 | 0.63 |
| 2321 | Train 37 | Test White 17 | 0.74 | 0.79 | 0.81 | 3345 | Train 53 | Test White 17 | 0.72 | 0.73 | 0.65 |
| 2322 | Train 37 | Test White 18 | 0.74 | 0.77 | 0.77 | 3346 | Train 53 | Test White 18 | 0.72 | 0.7 | 0.63 |
| 2323 | Train 37 | Test White 19 | 0.74 | 0.79 | 0.8 | 3347 | Train 53 | Test White 19 | 0.72 | 0.73 | 0.65 |
| 2324 | Train 37 | Test White 20 | 0.74 | 0.77 | 0.77 | 3348 | Train 53 | Test White 20 | 0.72 | 0.7 | 0.63 |
| 2325 | Train 37 | Test White 21 | 0.73 | 0.78 | 0.77 | 3349 | Train 53 | Test White 21 | 0.72 | 0.74 | 0.69 |
| 2326 | Train 37 | Test White 22 | 0.7 | 0.71 | 0.69 | 3350 | Train 53 | Test White 22 | 0.7 | 0.69 | 0.63 |
| 2327 | Train 37 | Test White 23 | 0.73 | 0.78 | 0.77 | 3351 | Train 53 | Test White 23 | 0.72 | 0.74 | 0.68 |
| 2328 | Train 37 | Test White 24 | 0.7 | 0.71 | 0.69 | 3352 | Train 53 | Test White 24 | 0.7 | 0.69 | 0.63 |
| 2329 | Train 37 | Test White 25 | 0.74 | 0.78 | 0.78 | 3353 | Train 53 | Test White 25 | 0.72 | 0.73 | 0.65 |
| 2330 | Train 37 | Test White 26 | 0.74 | 0.76 | 0.76 | 3354 | Train 53 | Test White 26 | 0.72 | 0.7 | 0.64 |
| 2331 | Train 37 | Test White 27 | 0.74 | 0.78 | 0.78 | 3355 | Train 53 | Test White 27 | 0.72 | 0.73 | 0.65 |
| 2332 | Train 37 | Test White 28 | 0.74 | 0.76 | 0.76 | 3356 | Train 53 | Test White 28 | 0.72 | 0.7 | 0.64 |
| 2333 | Train 37 | Test White 29 | 0.73 | 0.77 | 0.75 | 3357 | Train 53 | Test White 29 | 0.72 | 0.75 | 0.68 |
| 2334 | Train 37 | Test White 30 | 0.7 | 0.71 | 0.69 | 3358 | Train 53 | Test White 30 | 0.7 | 0.69 | 0.63 |
| 2335 | Train 37 | Test White 31 | 0.73 | 0.77 | 0.75 | 3359 | Train 53 | Test White 31 | 0.72 | 0.74 | 0.68 |
| 2336 | Train 37 | Test White 32 | 0.7 | 0.71 | 0.69 | 3360 | Train 53 | Test White 32 | 0.7 | 0.69 | 0.63 |
| 2337 | Train 37 | Test White 33 | 0.74 | 0.79 | 0.81 | 3361 | Train 53 | Test White 33 | 0.73 | 0.73 | 0.65 |
| 2338 | Train 37 | Test White 34 | 0.74 | 0.77 | 0.78 | 3362 | Train 53 | Test White 34 | 0.72 | 0.7 | 0.63 |
| 2339 | Train 37 | Test White 35 | 0.74 | 0.79 | 0.81 | 3363 | Train 53 | Test White 35 | 0.73 | 0.73 | 0.65 |
| 2340 | Train 37 | Test White 36 | 0.74 | 0.77 | 0.78 | 3364 | Train 53 | Test White 36 | 0.72 | 0.7 | 0.63 |
| 2341 | Train 37 | Test White 37 | 0.73 | 0.78 | 0.77 | 3365 | Train 53 | Test White 37 | 0.72 | 0.75 | 0.69 |
| 2342 | Train 37 | Test White 38 | 0.7 | 0.71 | 0.69 | 3366 | Train 53 | Test White 38 | 0.7 | 0.69 | 0.63 |
| 2343 | Train 37 | Test White 39 | 0.73 | 0.78 | 0.77 | 3367 | Train 53 | Test White 39 | 0.72 | 0.74 | 0.69 |
| 2344 | Train 37 | Test White 40 | 0.7 | 0.71 | 0.69 | 3368 | Train 53 | Test White 40 | 0.7 | 0.69 | 0.63 |
| 2345 | Train 37 | Test White 41 | 0.74 | 0.78 | 0.79 | 3369 | Train 53 | Test White 41 | 0.73 | 0.73 | 0.65 |
| 2346 | Train 37 | Test White 42 | 0.74 | 0.76 | 0.76 | 3370 | Train 53 | Test White 42 | 0.73 | 0.7 | 0.64 |
| 2347 | Train 37 | Test White 43 | 0.74 | 0.78 | 0.78 | 3371 | Train 53 | Test White 43 | 0.73 | 0.73 | 0.65 |
| 2348 | Train 37 | Test White 44 | 0.74 | 0.76 | 0.76 | 3372 | Train 53 | Test White 44 | 0.73 | 0.7 | 0.64 |
| 2349 | Train 37 | Test White 45 | 0.73 | 0.77 | 0.75 | 3373 | Train 53 | Test White 45 | 0.72 | 0.75 | 0.68 |
| 2350 | Train 37 | Test White 46 | 0.7 | 0.71 | 0.69 | 3374 | Train 53 | Test White 46 | 0.7 | 0.69 | 0.63 |
| 2351 | Train 37 | Test White 47 | 0.73 | 0.77 | 0.75 | 3375 | Train 53 | Test White 47 | 0.72 | 0.75 | 0.68 |
| 2352 | Train 37 | Test White 48 | 0.7 | 0.71 | 0.69 | 3376 | Train 53 | Test White 48 | 0.7 | 0.69 | 0.63 |
| 2353 | Train 37 | Test White 49 | 0.72 | 0.74 | 0.77 | 3377 | Train 53 | Test White 49 | 0.73 | 0.76 | 0.76 |
| 2354 | Train 37 | Test White 50 | 0.72 | 0.74 | 0.77 | 3378 | Train 53 | Test White 50 | 0.73 | 0.75 | 0.76 |
| 2355 | Train 37 | Test White 51 | 0.72 | 0.74 | 0.77 | 3379 | Train 53 | Test White 51 | 0.73 | 0.75 | 0.76 |
| 2356 | Train 37 | Test White 52 | 0.72 | 0.74 | 0.77 | 3380 | Train 53 | Test White 52 | 0.73 | 0.75 | 0.76 |
| 2357 | Train 37 | Test White 53 | 0.73 | 0.77 | 0.74 | 3381 | Train 53 | Test White 53 | 0.74 | 0.82 | 0.78 |
| 2358 | Train 37 | Test White 54 | 0.71 | 0.71 | 0.69 | 3382 | Train 53 | Test White 54 | 0.71 | 0.7 | 0.65 |
| 2359 | Train 37 | Test White 55 | 0.73 | 0.77 | 0.74 | 3383 | Train 53 | Test White 55 | 0.74 | 0.82 | 0.78 |
| 2360 | Train 37 | Test White 56 | 0.7 | 0.71 | 0.69 | 3384 | Train 53 | Test White 56 | 0.71 | 0.7 | 0.65 |
| 2361 | Train 37 | Test White 57 | 0.73 | 0.74 | 0.75 | 3385 | Train 53 | Test White 57 | 0.73 | 0.75 | 0.76 |
| 2362 | Train 37 | Test White 58 | 0.73 | 0.74 | 0.75 | 3386 | Train 53 | Test White 58 | 0.73 | 0.75 | 0.76 |
| 2363 | Train 37 | Test White 59 | 0.73 | 0.74 | 0.75 | 3387 | Train 53 | Test White 59 | 0.73 | 0.75 | 0.76 |
| 2364 | Train 37 | Test White 60 | 0.73 | 0.74 | 0.75 | 3388 | Train 53 | Test White 60 | 0.73 | 0.75 | 0.76 |
| 2365 | Train 37 | Test White 61 | 0.73 | 0.77 | 0.74 | 3389 | Train 53 | Test White 61 | 0.75 | 0.81 | 0.75 |
| 2366 | Train 37 | Test White 62 | 0.71 | 0.71 | 0.69 | 3390 | Train 53 | Test White 62 | 0.71 | 0.7 | 0.66 |
| 2367 | Train 37 | Test White 63 | 0.73 | 0.77 | 0.74 | 3391 | Train 53 | Test White 63 | 0.75 | 0.81 | 0.75 |
| 2368 | Train 37 | Test White 64 | 0.71 | 0.71 | 0.69 | 3392 | Train 53 | Test White 64 | 0.71 | 0.7 | 0.66 |
| 2369 | Train 38 | Test White 1 | 0.72 | 0.75 | 0.75 | 3393 | Train 54 | Test White 1 | 0.71 | 0.72 | 0.64 |
| 2370 | Train 38 | Test White 2 | 0.74 | 0.76 | 0.75 | 3394 | Train 54 | Test White 2 | 0.72 | 0.73 | 0.66 |
| 2371 | Train 38 | Test White 3 | 0.72 | 0.75 | 0.75 | 3395 | Train 54 | Test White 3 | 0.71 | 0.72 | 0.64 |
| 2372 | Train 38 | Test White 4 | 0.74 | 0.76 | 0.75 | 3396 | Train 54 | Test White 4 | 0.72 | 0.73 | 0.66 |
| 2373 | Train 38 | Test White 5 | 0.72 | 0.77 | 0.77 | 3397 | Train 54 | Test White 5 | 0.71 | 0.73 | 0.66 |
| 2374 | Train 38 | Test White 6 | 0.73 | 0.77 | 0.78 | 3398 | Train 54 | Test White 6 | 0.72 | 0.75 | 0.7 |
| 2375 | Train 38 | Test White 7 | 0.72 | 0.77 | 0.77 | 3399 | Train 54 | Test White 7 | 0.71 | 0.73 | 0.66 |
| 2376 | Train 38 | Test White 8 | 0.73 | 0.77 | 0.78 | 3400 | Train 54 | Test White 8 | 0.72 | 0.74 | 0.7 |
| 2377 | Train 38 | Test White 9 | 0.72 | 0.75 | 0.76 | 3401 | Train 54 | Test White 9 | 0.71 | 0.72 | 0.65 |
| 2378 | Train 38 | Test White 10 | 0.74 | 0.76 | 0.76 | 3402 | Train 54 | Test White 10 | 0.73 | 0.73 | 0.67 |
| 2379 | Train 38 | Test White 11 | 0.72 | 0.75 | 0.76 | 3403 | Train 54 | Test White 11 | 0.71 | 0.72 | 0.65 |
| 2380 | Train 38 | Test White 12 | 0.74 | 0.76 | 0.76 | 3404 | Train 54 | Test White 12 | 0.73 | 0.73 | 0.67 |
| 2381 | Train 38 | Test White 13 | 0.72 | 0.77 | 0.78 | 3405 | Train 54 | Test White 13 | 0.71 | 0.74 | 0.67 |
| 2382 | Train 38 | Test White 14 | 0.73 | 0.77 | 0.78 | 3406 | Train 54 | Test White 14 | 0.72 | 0.75 | 0.7 |
| 2383 | Train 38 | Test White 15 | 0.72 | 0.77 | 0.78 | 3407 | Train 54 | Test White 15 | 0.71 | 0.74 | 0.67 |
| 2384 | Train 38 | Test White 16 | 0.73 | 0.77 | 0.78 | 3408 | Train 54 | Test White 16 | 0.72 | 0.74 | 0.7 |
| 2385 | Train 38 | Test White 17 | 0.72 | 0.75 | 0.75 | 3409 | Train 54 | Test White 17 | 0.71 | 0.72 | 0.64 |
| 2386 | Train 38 | Test White 18 | 0.74 | 0.76 | 0.75 | 3410 | Train 54 | Test White 18 | 0.72 | 0.73 | 0.66 |
| 2387 | Train 38 | Test White 19 | 0.72 | 0.75 | 0.75 | 3411 | Train 54 | Test White 19 | 0.71 | 0.72 | 0.64 |
| 2388 | Train 38 | Test White 20 | 0.74 | 0.76 | 0.75 | 3412 | Train 54 | Test White 20 | 0.72 | 0.73 | 0.66 |
| 2389 | Train 38 | Test White 21 | 0.72 | 0.77 | 0.77 | 3413 | Train 54 | Test White 21 | 0.71 | 0.74 | 0.66 |
| 2390 | Train 38 | Test White 22 | 0.73 | 0.77 | 0.78 | 3414 | Train 54 | Test White 22 | 0.72 | 0.75 | 0.7 |
| 2391 | Train 38 | Test White 23 | 0.72 | 0.77 | 0.77 | 3415 | Train 54 | Test White 23 | 0.71 | 0.73 | 0.66 |
| 2392 | Train 38 | Test White 24 | 0.73 | 0.77 | 0.78 | 3416 | Train 54 | Test White 24 | 0.72 | 0.74 | 0.7 |
| 2393 | Train 38 | Test White 25 | 0.72 | 0.75 | 0.76 | 3417 | Train 54 | Test White 25 | 0.71 | 0.72 | 0.65 |
| 2394 | Train 38 | Test White 26 | 0.74 | 0.76 | 0.76 | 3418 | Train 54 | Test White 26 | 0.73 | 0.73 | 0.67 |
| 2395 | Train 38 | Test White 27 | 0.72 | 0.75 | 0.76 | 3419 | Train 54 | Test White 27 | 0.71 | 0.72 | 0.65 |
| 2396 | Train 38 | Test White 28 | 0.74 | 0.76 | 0.76 | 3420 | Train 54 | Test White 28 | 0.73 | 0.73 | 0.67 |
| 2397 | Train 38 | Test White 29 | 0.72 | 0.77 | 0.78 | 3421 | Train 54 | Test White 29 | 0.71 | 0.74 | 0.67 |
| 2398 | Train 38 | Test White 30 | 0.73 | 0.77 | 0.78 | 3422 | Train 54 | Test White 30 | 0.72 | 0.75 | 0.7 |
| 2399 | Train 38 | Test White 31 | 0.72 | 0.77 | 0.78 | 3423 | Train 54 | Test White 31 | 0.71 | 0.74 | 0.67 |
| 2400 | Train 38 | Test White 32 | 0.73 | 0.77 | 0.78 | 3424 | Train 54 | Test White 32 | 0.72 | 0.74 | 0.7 |
| 2401 | Train 38 | Test White 33 | 0.73 | 0.75 | 0.75 | 3425 | Train 54 | Test White 33 | 0.71 | 0.72 | 0.64 |
| 2402 | Train 38 | Test White 34 | 0.74 | 0.76 | 0.75 | 3426 | Train 54 | Test White 34 | 0.73 | 0.73 | 0.66 |
| 2403 | Train 38 | Test White 35 | 0.73 | 0.75 | 0.75 | 3427 | Train 54 | Test White 35 | 0.71 | 0.72 | 0.64 |
| 2404 | Train 38 | Test White 36 | 0.74 | 0.76 | 0.75 | 3428 | Train 54 | Test White 36 | 0.73 | 0.73 | 0.66 |
| 2405 | Train 38 | Test White 37 | 0.72 | 0.77 | 0.77 | 3429 | Train 54 | Test White 37 | 0.71 | 0.74 | 0.66 |
| 2406 | Train 38 | Test White 38 | 0.73 | 0.77 | 0.78 | 3430 | Train 54 | Test White 38 | 0.72 | 0.75 | 0.7 |
| 2407 | Train 38 | Test White 39 | 0.72 | 0.77 | 0.77 | 3431 | Train 54 | Test White 39 | 0.71 | 0.74 | 0.66 |
| 2408 | Train 38 | Test White 40 | 0.73 | 0.77 | 0.78 | 3432 | Train 54 | Test White 40 | 0.72 | 0.74 | 0.7 |
| 2409 | Train 38 | Test White 41 | 0.73 | 0.75 | 0.76 | 3433 | Train 54 | Test White 41 | 0.71 | 0.72 | 0.66 |
| 2410 | Train 38 | Test White 42 | 0.74 | 0.76 | 0.76 | 3434 | Train 54 | Test White 42 | 0.73 | 0.73 | 0.67 |
| 2411 | Train 38 | Test White 43 | 0.73 | 0.75 | 0.76 | 3435 | Train 54 | Test White 43 | 0.71 | 0.72 | 0.66 |
| 2412 | Train 38 | Test White 44 | 0.74 | 0.76 | 0.76 | 3436 | Train 54 | Test White 44 | 0.73 | 0.73 | 0.67 |
| 2413 | Train 38 | Test White 45 | 0.72 | 0.77 | 0.78 | 3437 | Train 54 | Test White 45 | 0.71 | 0.74 | 0.67 |
| 2414 | Train 38 | Test White 46 | 0.73 | 0.77 | 0.78 | 3438 | Train 54 | Test White 46 | 0.72 | 0.75 | 0.7 |
| 2415 | Train 38 | Test White 47 | 0.72 | 0.77 | 0.78 | 3439 | Train 54 | Test White 47 | 0.71 | 0.74 | 0.67 |
| 2416 | Train 38 | Test White 48 | 0.73 | 0.77 | 0.78 | 3440 | Train 54 | Test White 48 | 0.72 | 0.74 | 0.7 |
| 2417 | Train 38 | Test White 49 | 0.73 | 0.76 | 0.75 | 3441 | Train 54 | Test White 49 | 0.73 | 0.76 | 0.74 |
| 2418 | Train 38 | Test White 50 | 0.73 | 0.76 | 0.75 | 3442 | Train 54 | Test White 50 | 0.74 | 0.76 | 0.74 |
| 2419 | Train 38 | Test White 51 | 0.73 | 0.76 | 0.75 | 3443 | Train 54 | Test White 51 | 0.73 | 0.76 | 0.74 |
| 2420 | Train 38 | Test White 52 | 0.73 | 0.76 | 0.75 | 3444 | Train 54 | Test White 52 | 0.74 | 0.76 | 0.74 |
| 2421 | Train 38 | Test White 53 | 0.73 | 0.78 | 0.74 | 3445 | Train 54 | Test White 53 | 0.74 | 0.8 | 0.76 |
| 2422 | Train 38 | Test White 54 | 0.74 | 0.77 | 0.78 | 3446 | Train 54 | Test White 54 | 0.74 | 0.79 | 0.8 |
| 2423 | Train 38 | Test White 55 | 0.73 | 0.78 | 0.74 | 3447 | Train 54 | Test White 55 | 0.74 | 0.8 | 0.76 |
| 2424 | Train 38 | Test White 56 | 0.73 | 0.76 | 0.78 | 3448 | Train 54 | Test White 56 | 0.74 | 0.78 | 0.79 |
| 2425 | Train 38 | Test White 57 | 0.73 | 0.76 | 0.76 | 3449 | Train 54 | Test White 57 | 0.74 | 0.76 | 0.76 |
| 2426 | Train 38 | Test White 58 | 0.73 | 0.76 | 0.76 | 3450 | Train 54 | Test White 58 | 0.74 | 0.77 | 0.76 |
| 2427 | Train 38 | Test White 59 | 0.73 | 0.76 | 0.76 | 3451 | Train 54 | Test White 59 | 0.74 | 0.76 | 0.76 |
| 2428 | Train 38 | Test White 60 | 0.73 | 0.76 | 0.76 | 3452 | Train 54 | Test White 60 | 0.74 | 0.76 | 0.76 |
| 2429 | Train 38 | Test White 61 | 0.73 | 0.79 | 0.75 | 3453 | Train 54 | Test White 61 | 0.74 | 0.8 | 0.77 |
| 2430 | Train 38 | Test White 62 | 0.74 | 0.77 | 0.78 | 3454 | Train 54 | Test White 62 | 0.74 | 0.79 | 0.8 |
| 2431 | Train 38 | Test White 63 | 0.73 | 0.78 | 0.75 | 3455 | Train 54 | Test White 63 | 0.74 | 0.8 | 0.77 |
| 2432 | Train 38 | Test White 64 | 0.73 | 0.76 | 0.78 | 3456 | Train 54 | Test White 64 | 0.74 | 0.78 | 0.79 |
| 2433 | Train 39 | Test White 1 | 0.74 | 0.79 | 0.81 | 3457 | Train 55 | Test White 1 | 0.72 | 0.74 | 0.7 |
| 2434 | Train 39 | Test White 2 | 0.74 | 0.77 | 0.78 | 3458 | Train 55 | Test White 2 | 0.72 | 0.71 | 0.67 |
| 2435 | Train 39 | Test White 3 | 0.74 | 0.79 | 0.81 | 3459 | Train 55 | Test White 3 | 0.72 | 0.74 | 0.7 |
| 2436 | Train 39 | Test White 4 | 0.74 | 0.77 | 0.78 | 3460 | Train 55 | Test White 4 | 0.72 | 0.71 | 0.68 |
| 2437 | Train 39 | Test White 5 | 0.73 | 0.78 | 0.77 | 3461 | Train 55 | Test White 5 | 0.72 | 0.74 | 0.66 |
| 2438 | Train 39 | Test White 6 | 0.7 | 0.71 | 0.69 | 3462 | Train 55 | Test White 6 | 0.69 | 0.69 | 0.62 |
| 2439 | Train 39 | Test White 7 | 0.73 | 0.78 | 0.77 | 3463 | Train 55 | Test White 7 | 0.72 | 0.74 | 0.66 |
| 2440 | Train 39 | Test White 8 | 0.7 | 0.71 | 0.69 | 3464 | Train 55 | Test White 8 | 0.69 | 0.69 | 0.63 |
| 2441 | Train 39 | Test White 9 | 0.74 | 0.78 | 0.78 | 3465 | Train 55 | Test White 9 | 0.72 | 0.74 | 0.7 |
| 2442 | Train 39 | Test White 10 | 0.74 | 0.76 | 0.76 | 3466 | Train 55 | Test White 10 | 0.73 | 0.71 | 0.67 |
| 2443 | Train 39 | Test White 11 | 0.74 | 0.78 | 0.78 | 3467 | Train 55 | Test White 11 | 0.72 | 0.74 | 0.7 |
| 2444 | Train 39 | Test White 12 | 0.74 | 0.76 | 0.76 | 3468 | Train 55 | Test White 12 | 0.73 | 0.72 | 0.68 |
| 2445 | Train 39 | Test White 13 | 0.73 | 0.77 | 0.75 | 3469 | Train 55 | Test White 13 | 0.72 | 0.74 | 0.65 |
| 2446 | Train 39 | Test White 14 | 0.7 | 0.71 | 0.69 | 3470 | Train 55 | Test White 14 | 0.7 | 0.69 | 0.63 |
| 2447 | Train 39 | Test White 15 | 0.73 | 0.77 | 0.75 | 3471 | Train 55 | Test White 15 | 0.72 | 0.74 | 0.65 |
| 2448 | Train 39 | Test White 16 | 0.7 | 0.71 | 0.69 | 3472 | Train 55 | Test White 16 | 0.7 | 0.69 | 0.63 |
| 2449 | Train 39 | Test White 17 | 0.74 | 0.79 | 0.81 | 3473 | Train 55 | Test White 17 | 0.72 | 0.74 | 0.7 |
| 2450 | Train 39 | Test White 18 | 0.74 | 0.77 | 0.78 | 3474 | Train 55 | Test White 18 | 0.72 | 0.71 | 0.67 |
| 2451 | Train 39 | Test White 19 | 0.74 | 0.79 | 0.81 | 3475 | Train 55 | Test White 19 | 0.72 | 0.74 | 0.7 |
| 2452 | Train 39 | Test White 20 | 0.74 | 0.77 | 0.78 | 3476 | Train 55 | Test White 20 | 0.72 | 0.71 | 0.68 |
| 2453 | Train 39 | Test White 21 | 0.73 | 0.78 | 0.77 | 3477 | Train 55 | Test White 21 | 0.72 | 0.74 | 0.66 |
| 2454 | Train 39 | Test White 22 | 0.7 | 0.71 | 0.69 | 3478 | Train 55 | Test White 22 | 0.7 | 0.69 | 0.62 |
| 2455 | Train 39 | Test White 23 | 0.73 | 0.78 | 0.77 | 3479 | Train 55 | Test White 23 | 0.72 | 0.74 | 0.66 |
| 2456 | Train 39 | Test White 24 | 0.7 | 0.71 | 0.69 | 3480 | Train 55 | Test White 24 | 0.7 | 0.69 | 0.63 |
| 2457 | Train 39 | Test White 25 | 0.74 | 0.78 | 0.78 | 3481 | Train 55 | Test White 25 | 0.72 | 0.74 | 0.7 |
| 2458 | Train 39 | Test White 26 | 0.74 | 0.76 | 0.76 | 3482 | Train 55 | Test White 26 | 0.73 | 0.71 | 0.67 |
| 2459 | Train 39 | Test White 27 | 0.74 | 0.78 | 0.78 | 3483 | Train 55 | Test White 27 | 0.72 | 0.74 | 0.7 |
| 2460 | Train 39 | Test White 28 | 0.74 | 0.76 | 0.76 | 3484 | Train 55 | Test White 28 | 0.73 | 0.72 | 0.68 |
| 2461 | Train 39 | Test White 29 | 0.73 | 0.77 | 0.75 | 3485 | Train 55 | Test White 29 | 0.72 | 0.74 | 0.65 |
| 2462 | Train 39 | Test White 30 | 0.7 | 0.71 | 0.69 | 3486 | Train 55 | Test White 30 | 0.7 | 0.69 | 0.63 |
| 2463 | Train 39 | Test White 31 | 0.73 | 0.77 | 0.75 | 3487 | Train 55 | Test White 31 | 0.72 | 0.74 | 0.65 |
| 2464 | Train 39 | Test White 32 | 0.7 | 0.71 | 0.69 | 3488 | Train 55 | Test White 32 | 0.7 | 0.69 | 0.63 |
| 2465 | Train 39 | Test White 33 | 0.74 | 0.79 | 0.81 | 3489 | Train 55 | Test White 33 | 0.73 | 0.74 | 0.7 |
| 2466 | Train 39 | Test White 34 | 0.74 | 0.77 | 0.78 | 3490 | Train 55 | Test White 34 | 0.73 | 0.71 | 0.68 |
| 2467 | Train 39 | Test White 35 | 0.74 | 0.79 | 0.81 | 3491 | Train 55 | Test White 35 | 0.73 | 0.74 | 0.7 |
| 2468 | Train 39 | Test White 36 | 0.74 | 0.77 | 0.78 | 3492 | Train 55 | Test White 36 | 0.73 | 0.71 | 0.68 |
| 2469 | Train 39 | Test White 37 | 0.73 | 0.78 | 0.77 | 3493 | Train 55 | Test White 37 | 0.72 | 0.74 | 0.66 |
| 2470 | Train 39 | Test White 38 | 0.7 | 0.71 | 0.69 | 3494 | Train 55 | Test White 38 | 0.7 | 0.69 | 0.63 |
| 2471 | Train 39 | Test White 39 | 0.73 | 0.78 | 0.77 | 3495 | Train 55 | Test White 39 | 0.72 | 0.74 | 0.66 |
| 2472 | Train 39 | Test White 40 | 0.7 | 0.71 | 0.69 | 3496 | Train 55 | Test White 40 | 0.7 | 0.69 | 0.63 |
| 2473 | Train 39 | Test White 41 | 0.74 | 0.78 | 0.79 | 3497 | Train 55 | Test White 41 | 0.73 | 0.74 | 0.7 |
| 2474 | Train 39 | Test White 42 | 0.74 | 0.77 | 0.76 | 3498 | Train 55 | Test White 42 | 0.73 | 0.72 | 0.68 |
| 2475 | Train 39 | Test White 43 | 0.74 | 0.78 | 0.79 | 3499 | Train 55 | Test White 43 | 0.73 | 0.74 | 0.7 |
| 2476 | Train 39 | Test White 44 | 0.74 | 0.77 | 0.76 | 3500 | Train 55 | Test White 44 | 0.73 | 0.72 | 0.68 |
| 2477 | Train 39 | Test White 45 | 0.73 | 0.77 | 0.75 | 3501 | Train 55 | Test White 45 | 0.72 | 0.74 | 0.65 |
| 2478 | Train 39 | Test White 46 | 0.7 | 0.71 | 0.69 | 3502 | Train 55 | Test White 46 | 0.7 | 0.69 | 0.63 |
| 2479 | Train 39 | Test White 47 | 0.73 | 0.77 | 0.75 | 3503 | Train 55 | Test White 47 | 0.72 | 0.74 | 0.65 |
| 2480 | Train 39 | Test White 48 | 0.7 | 0.71 | 0.69 | 3504 | Train 55 | Test White 48 | 0.7 | 0.69 | 0.63 |
| 2481 | Train 39 | Test White 49 | 0.72 | 0.74 | 0.79 | 3505 | Train 55 | Test White 49 | 0.73 | 0.77 | 0.79 |
| 2482 | Train 39 | Test White 50 | 0.72 | 0.74 | 0.79 | 3506 | Train 55 | Test White 50 | 0.73 | 0.77 | 0.79 |
| 2483 | Train 39 | Test White 51 | 0.72 | 0.74 | 0.79 | 3507 | Train 55 | Test White 51 | 0.74 | 0.77 | 0.79 |
| 2484 | Train 39 | Test White 52 | 0.72 | 0.74 | 0.79 | 3508 | Train 55 | Test White 52 | 0.74 | 0.77 | 0.79 |
| 2485 | Train 39 | Test White 53 | 0.73 | 0.77 | 0.72 | 3509 | Train 55 | Test White 53 | 0.74 | 0.81 | 0.73 |
| 2486 | Train 39 | Test White 54 | 0.71 | 0.7 | 0.7 | 3510 | Train 55 | Test White 54 | 0.7 | 0.71 | 0.65 |
| 2487 | Train 39 | Test White 55 | 0.73 | 0.77 | 0.72 | 3511 | Train 55 | Test White 55 | 0.74 | 0.81 | 0.73 |
| 2488 | Train 39 | Test White 56 | 0.7 | 0.7 | 0.7 | 3512 | Train 55 | Test White 56 | 0.7 | 0.71 | 0.66 |
| 2489 | Train 39 | Test White 57 | 0.73 | 0.74 | 0.77 | 3513 | Train 55 | Test White 57 | 0.74 | 0.77 | 0.78 |
| 2490 | Train 39 | Test White 58 | 0.73 | 0.74 | 0.77 | 3514 | Train 55 | Test White 58 | 0.74 | 0.77 | 0.78 |
| 2491 | Train 39 | Test White 59 | 0.73 | 0.74 | 0.77 | 3515 | Train 55 | Test White 59 | 0.74 | 0.77 | 0.78 |
| 2492 | Train 39 | Test White 60 | 0.73 | 0.74 | 0.77 | 3516 | Train 55 | Test White 60 | 0.74 | 0.77 | 0.78 |
| 2493 | Train 39 | Test White 61 | 0.73 | 0.77 | 0.71 | 3517 | Train 55 | Test White 61 | 0.74 | 0.81 | 0.7 |
| 2494 | Train 39 | Test White 62 | 0.71 | 0.71 | 0.7 | 3518 | Train 55 | Test White 62 | 0.7 | 0.71 | 0.65 |
| 2495 | Train 39 | Test White 63 | 0.73 | 0.77 | 0.71 | 3519 | Train 55 | Test White 63 | 0.74 | 0.81 | 0.7 |
| 2496 | Train 39 | Test White 64 | 0.71 | 0.71 | 0.7 | 3520 | Train 55 | Test White 64 | 0.7 | 0.71 | 0.66 |
| 2497 | Train 40 | Test White 1 | 0.73 | 0.75 | 0.76 | 3521 | Train 56 | Test White 1 | 0.71 | 0.72 | 0.69 |
| 2498 | Train 40 | Test White 2 | 0.74 | 0.76 | 0.76 | 3522 | Train 56 | Test White 2 | 0.72 | 0.73 | 0.7 |
| 2499 | Train 40 | Test White 3 | 0.73 | 0.75 | 0.76 | 3523 | Train 56 | Test White 3 | 0.71 | 0.72 | 0.7 |
| 2500 | Train 40 | Test White 4 | 0.74 | 0.76 | 0.76 | 3524 | Train 56 | Test White 4 | 0.72 | 0.73 | 0.7 |
| 2501 | Train 40 | Test White 5 | 0.72 | 0.77 | 0.77 | 3525 | Train 56 | Test White 5 | 0.7 | 0.74 | 0.71 |
| 2502 | Train 40 | Test White 6 | 0.73 | 0.77 | 0.78 | 3526 | Train 56 | Test White 6 | 0.71 | 0.74 | 0.71 |
| 2503 | Train 40 | Test White 7 | 0.72 | 0.77 | 0.77 | 3527 | Train 56 | Test White 7 | 0.7 | 0.74 | 0.71 |
| 2504 | Train 40 | Test White 8 | 0.73 | 0.77 | 0.78 | 3528 | Train 56 | Test White 8 | 0.71 | 0.74 | 0.72 |
| 2505 | Train 40 | Test White 9 | 0.73 | 0.76 | 0.76 | 3529 | Train 56 | Test White 9 | 0.71 | 0.72 | 0.7 |
| 2506 | Train 40 | Test White 10 | 0.74 | 0.76 | 0.77 | 3530 | Train 56 | Test White 10 | 0.72 | 0.73 | 0.7 |
| 2507 | Train 40 | Test White 11 | 0.73 | 0.76 | 0.76 | 3531 | Train 56 | Test White 11 | 0.71 | 0.72 | 0.7 |
| 2508 | Train 40 | Test White 12 | 0.74 | 0.76 | 0.77 | 3532 | Train 56 | Test White 12 | 0.72 | 0.73 | 0.7 |
| 2509 | Train 40 | Test White 13 | 0.72 | 0.77 | 0.78 | 3533 | Train 56 | Test White 13 | 0.7 | 0.74 | 0.72 |
| 2510 | Train 40 | Test White 14 | 0.73 | 0.77 | 0.78 | 3534 | Train 56 | Test White 14 | 0.71 | 0.74 | 0.71 |
| 2511 | Train 40 | Test White 15 | 0.72 | 0.77 | 0.78 | 3535 | Train 56 | Test White 15 | 0.7 | 0.74 | 0.72 |
| 2512 | Train 40 | Test White 16 | 0.73 | 0.77 | 0.78 | 3536 | Train 56 | Test White 16 | 0.72 | 0.75 | 0.72 |
| 2513 | Train 40 | Test White 17 | 0.73 | 0.75 | 0.76 | 3537 | Train 56 | Test White 17 | 0.71 | 0.72 | 0.69 |
| 2514 | Train 40 | Test White 18 | 0.74 | 0.76 | 0.76 | 3538 | Train 56 | Test White 18 | 0.72 | 0.73 | 0.7 |
| 2515 | Train 40 | Test White 19 | 0.73 | 0.75 | 0.76 | 3539 | Train 56 | Test White 19 | 0.71 | 0.72 | 0.69 |
| 2516 | Train 40 | Test White 20 | 0.74 | 0.76 | 0.76 | 3540 | Train 56 | Test White 20 | 0.72 | 0.73 | 0.7 |
| 2517 | Train 40 | Test White 21 | 0.72 | 0.77 | 0.77 | 3541 | Train 56 | Test White 21 | 0.7 | 0.74 | 0.71 |
| 2518 | Train 40 | Test White 22 | 0.73 | 0.77 | 0.78 | 3542 | Train 56 | Test White 22 | 0.71 | 0.74 | 0.71 |
| 2519 | Train 40 | Test White 23 | 0.72 | 0.77 | 0.77 | 3543 | Train 56 | Test White 23 | 0.7 | 0.74 | 0.71 |
| 2520 | Train 40 | Test White 24 | 0.73 | 0.77 | 0.78 | 3544 | Train 56 | Test White 24 | 0.71 | 0.74 | 0.72 |
| 2521 | Train 40 | Test White 25 | 0.73 | 0.76 | 0.76 | 3545 | Train 56 | Test White 25 | 0.71 | 0.72 | 0.7 |
| 2522 | Train 40 | Test White 26 | 0.74 | 0.76 | 0.77 | 3546 | Train 56 | Test White 26 | 0.72 | 0.73 | 0.7 |
| 2523 | Train 40 | Test White 27 | 0.73 | 0.76 | 0.76 | 3547 | Train 56 | Test White 27 | 0.71 | 0.72 | 0.7 |
| 2524 | Train 40 | Test White 28 | 0.74 | 0.76 | 0.77 | 3548 | Train 56 | Test White 28 | 0.72 | 0.73 | 0.7 |
| 2525 | Train 40 | Test White 29 | 0.72 | 0.77 | 0.78 | 3549 | Train 56 | Test White 29 | 0.7 | 0.74 | 0.72 |
| 2526 | Train 40 | Test White 30 | 0.73 | 0.77 | 0.78 | 3550 | Train 56 | Test White 30 | 0.71 | 0.74 | 0.71 |
| 2527 | Train 40 | Test White 31 | 0.72 | 0.77 | 0.78 | 3551 | Train 56 | Test White 31 | 0.7 | 0.74 | 0.72 |
| 2528 | Train 40 | Test White 32 | 0.73 | 0.77 | 0.78 | 3552 | Train 56 | Test White 32 | 0.72 | 0.75 | 0.72 |
| 2529 | Train 40 | Test White 33 | 0.73 | 0.75 | 0.76 | 3553 | Train 56 | Test White 33 | 0.71 | 0.72 | 0.7 |
| 2530 | Train 40 | Test White 34 | 0.74 | 0.76 | 0.76 | 3554 | Train 56 | Test White 34 | 0.72 | 0.73 | 0.7 |
| 2531 | Train 40 | Test White 35 | 0.73 | 0.75 | 0.76 | 3555 | Train 56 | Test White 35 | 0.71 | 0.72 | 0.7 |
| 2532 | Train 40 | Test White 36 | 0.74 | 0.76 | 0.76 | 3556 | Train 56 | Test White 36 | 0.72 | 0.73 | 0.7 |
| 2533 | Train 40 | Test White 37 | 0.72 | 0.77 | 0.77 | 3557 | Train 56 | Test White 37 | 0.7 | 0.74 | 0.71 |
| 2534 | Train 40 | Test White 38 | 0.73 | 0.77 | 0.78 | 3558 | Train 56 | Test White 38 | 0.71 | 0.74 | 0.71 |
| 2535 | Train 40 | Test White 39 | 0.72 | 0.77 | 0.77 | 3559 | Train 56 | Test White 39 | 0.7 | 0.74 | 0.71 |
| 2536 | Train 40 | Test White 40 | 0.73 | 0.77 | 0.78 | 3560 | Train 56 | Test White 40 | 0.71 | 0.75 | 0.72 |
| 2537 | Train 40 | Test White 41 | 0.73 | 0.76 | 0.76 | 3561 | Train 56 | Test White 41 | 0.71 | 0.72 | 0.7 |
| 2538 | Train 40 | Test White 42 | 0.74 | 0.76 | 0.77 | 3562 | Train 56 | Test White 42 | 0.72 | 0.73 | 0.71 |
| 2539 | Train 40 | Test White 43 | 0.73 | 0.76 | 0.76 | 3563 | Train 56 | Test White 43 | 0.71 | 0.73 | 0.7 |
| 2540 | Train 40 | Test White 44 | 0.74 | 0.76 | 0.77 | 3564 | Train 56 | Test White 44 | 0.72 | 0.73 | 0.71 |
| 2541 | Train 40 | Test White 45 | 0.72 | 0.77 | 0.78 | 3565 | Train 56 | Test White 45 | 0.7 | 0.74 | 0.72 |
| 2542 | Train 40 | Test White 46 | 0.73 | 0.77 | 0.78 | 3566 | Train 56 | Test White 46 | 0.71 | 0.74 | 0.71 |
| 2543 | Train 40 | Test White 47 | 0.72 | 0.77 | 0.78 | 3567 | Train 56 | Test White 47 | 0.7 | 0.74 | 0.72 |
| 2544 | Train 40 | Test White 48 | 0.73 | 0.77 | 0.78 | 3568 | Train 56 | Test White 48 | 0.72 | 0.75 | 0.72 |
| 2545 | Train 40 | Test White 49 | 0.73 | 0.76 | 0.75 | 3569 | Train 56 | Test White 49 | 0.74 | 0.76 | 0.74 |
| 2546 | Train 40 | Test White 50 | 0.73 | 0.76 | 0.75 | 3570 | Train 56 | Test White 50 | 0.74 | 0.76 | 0.74 |
| 2547 | Train 40 | Test White 51 | 0.73 | 0.76 | 0.75 | 3571 | Train 56 | Test White 51 | 0.74 | 0.76 | 0.74 |
| 2548 | Train 40 | Test White 52 | 0.73 | 0.76 | 0.75 | 3572 | Train 56 | Test White 52 | 0.74 | 0.76 | 0.74 |
| 2549 | Train 40 | Test White 53 | 0.73 | 0.78 | 0.75 | 3573 | Train 56 | Test White 53 | 0.74 | 0.8 | 0.74 |
| 2550 | Train 40 | Test White 54 | 0.73 | 0.77 | 0.78 | 3574 | Train 56 | Test White 54 | 0.74 | 0.79 | 0.8 |
| 2551 | Train 40 | Test White 55 | 0.73 | 0.78 | 0.75 | 3575 | Train 56 | Test White 55 | 0.74 | 0.8 | 0.74 |
| 2552 | Train 40 | Test White 56 | 0.73 | 0.77 | 0.78 | 3576 | Train 56 | Test White 56 | 0.74 | 0.79 | 0.8 |
| 2553 | Train 40 | Test White 57 | 0.73 | 0.76 | 0.76 | 3577 | Train 56 | Test White 57 | 0.74 | 0.76 | 0.76 |
| 2554 | Train 40 | Test White 58 | 0.73 | 0.76 | 0.76 | 3578 | Train 56 | Test White 58 | 0.74 | 0.76 | 0.76 |
| 2555 | Train 40 | Test White 59 | 0.73 | 0.76 | 0.76 | 3579 | Train 56 | Test White 59 | 0.74 | 0.77 | 0.76 |
| 2556 | Train 40 | Test White 60 | 0.73 | 0.76 | 0.76 | 3580 | Train 56 | Test White 60 | 0.74 | 0.77 | 0.76 |
| 2557 | Train 40 | Test White 61 | 0.73 | 0.78 | 0.76 | 3581 | Train 56 | Test White 61 | 0.74 | 0.8 | 0.76 |
| 2558 | Train 40 | Test White 62 | 0.73 | 0.77 | 0.78 | 3582 | Train 56 | Test White 62 | 0.74 | 0.79 | 0.8 |
| 2559 | Train 40 | Test White 63 | 0.73 | 0.78 | 0.76 | 3583 | Train 56 | Test White 63 | 0.74 | 0.8 | 0.76 |
| 2560 | Train 40 | Test White 64 | 0.73 | 0.77 | 0.78 | 3584 | Train 56 | Test White 64 | 0.74 | 0.79 | 0.8 |
| 2561 | Train 41 | Test White 1 | 0.74 | 0.81 | 0.8 | 3585 | Train 57 | Test White 1 | 0.73 | 0.71 | 0.69 |
| 2562 | Train 41 | Test White 2 | 0.73 | 0.76 | 0.75 | 3586 | Train 57 | Test White 2 | 0.71 | 0.68 | 0.64 |
| 2563 | Train 41 | Test White 3 | 0.74 | 0.81 | 0.8 | 3587 | Train 57 | Test White 3 | 0.73 | 0.71 | 0.69 |
| 2564 | Train 41 | Test White 4 | 0.73 | 0.76 | 0.75 | 3588 | Train 57 | Test White 4 | 0.71 | 0.68 | 0.64 |
| 2565 | Train 41 | Test White 5 | 0.71 | 0.73 | 0.69 | 3589 | Train 57 | Test White 5 | 0.7 | 0.7 | 0.66 |
| 2566 | Train 41 | Test White 6 | 0.69 | 0.7 | 0.65 | 3590 | Train 57 | Test White 6 | 0.68 | 0.68 | 0.62 |
| 2567 | Train 41 | Test White 7 | 0.71 | 0.73 | 0.69 | 3591 | Train 57 | Test White 7 | 0.7 | 0.7 | 0.66 |
| 2568 | Train 41 | Test White 8 | 0.69 | 0.69 | 0.65 | 3592 | Train 57 | Test White 8 | 0.68 | 0.68 | 0.63 |
| 2569 | Train 41 | Test White 9 | 0.74 | 0.81 | 0.83 | 3593 | Train 57 | Test White 9 | 0.73 | 0.71 | 0.71 |
| 2570 | Train 41 | Test White 10 | 0.73 | 0.76 | 0.78 | 3594 | Train 57 | Test White 10 | 0.71 | 0.69 | 0.65 |
| 2571 | Train 41 | Test White 11 | 0.75 | 0.81 | 0.83 | 3595 | Train 57 | Test White 11 | 0.73 | 0.71 | 0.71 |
| 2572 | Train 41 | Test White 12 | 0.73 | 0.76 | 0.78 | 3596 | Train 57 | Test White 12 | 0.71 | 0.69 | 0.65 |
| 2573 | Train 41 | Test White 13 | 0.71 | 0.73 | 0.7 | 3597 | Train 57 | Test White 13 | 0.7 | 0.7 | 0.67 |
| 2574 | Train 41 | Test White 14 | 0.69 | 0.7 | 0.65 | 3598 | Train 57 | Test White 14 | 0.68 | 0.68 | 0.62 |
| 2575 | Train 41 | Test White 15 | 0.71 | 0.73 | 0.7 | 3599 | Train 57 | Test White 15 | 0.7 | 0.7 | 0.67 |
| 2576 | Train 41 | Test White 16 | 0.7 | 0.7 | 0.65 | 3600 | Train 57 | Test White 16 | 0.69 | 0.68 | 0.63 |
| 2577 | Train 41 | Test White 17 | 0.74 | 0.81 | 0.8 | 3601 | Train 57 | Test White 17 | 0.73 | 0.71 | 0.69 |
| 2578 | Train 41 | Test White 18 | 0.73 | 0.76 | 0.75 | 3602 | Train 57 | Test White 18 | 0.71 | 0.68 | 0.64 |
| 2579 | Train 41 | Test White 19 | 0.74 | 0.81 | 0.8 | 3603 | Train 57 | Test White 19 | 0.73 | 0.71 | 0.69 |
| 2580 | Train 41 | Test White 20 | 0.73 | 0.76 | 0.75 | 3604 | Train 57 | Test White 20 | 0.71 | 0.68 | 0.64 |
| 2581 | Train 41 | Test White 21 | 0.71 | 0.73 | 0.69 | 3605 | Train 57 | Test White 21 | 0.7 | 0.7 | 0.66 |
| 2582 | Train 41 | Test White 22 | 0.69 | 0.7 | 0.65 | 3606 | Train 57 | Test White 22 | 0.68 | 0.68 | 0.62 |
| 2583 | Train 41 | Test White 23 | 0.71 | 0.73 | 0.69 | 3607 | Train 57 | Test White 23 | 0.7 | 0.7 | 0.66 |
| 2584 | Train 41 | Test White 24 | 0.69 | 0.7 | 0.65 | 3608 | Train 57 | Test White 24 | 0.68 | 0.68 | 0.63 |
| 2585 | Train 41 | Test White 25 | 0.75 | 0.81 | 0.83 | 3609 | Train 57 | Test White 25 | 0.73 | 0.71 | 0.71 |
| 2586 | Train 41 | Test White 26 | 0.73 | 0.76 | 0.78 | 3610 | Train 57 | Test White 26 | 0.71 | 0.69 | 0.65 |
| 2587 | Train 41 | Test White 27 | 0.75 | 0.81 | 0.83 | 3611 | Train 57 | Test White 27 | 0.73 | 0.71 | 0.71 |
| 2588 | Train 41 | Test White 28 | 0.73 | 0.76 | 0.78 | 3612 | Train 57 | Test White 28 | 0.71 | 0.69 | 0.65 |
| 2589 | Train 41 | Test White 29 | 0.71 | 0.73 | 0.7 | 3613 | Train 57 | Test White 29 | 0.7 | 0.7 | 0.67 |
| 2590 | Train 41 | Test White 30 | 0.69 | 0.7 | 0.65 | 3614 | Train 57 | Test White 30 | 0.68 | 0.68 | 0.62 |
| 2591 | Train 41 | Test White 31 | 0.71 | 0.73 | 0.7 | 3615 | Train 57 | Test White 31 | 0.7 | 0.7 | 0.67 |
| 2592 | Train 41 | Test White 32 | 0.7 | 0.7 | 0.65 | 3616 | Train 57 | Test White 32 | 0.69 | 0.68 | 0.63 |
| 2593 | Train 41 | Test White 33 | 0.75 | 0.81 | 0.8 | 3617 | Train 57 | Test White 33 | 0.73 | 0.71 | 0.69 |
| 2594 | Train 41 | Test White 34 | 0.73 | 0.76 | 0.75 | 3618 | Train 57 | Test White 34 | 0.71 | 0.69 | 0.64 |
| 2595 | Train 41 | Test White 35 | 0.75 | 0.81 | 0.8 | 3619 | Train 57 | Test White 35 | 0.73 | 0.71 | 0.69 |
| 2596 | Train 41 | Test White 36 | 0.73 | 0.76 | 0.75 | 3620 | Train 57 | Test White 36 | 0.71 | 0.69 | 0.64 |
| 2597 | Train 41 | Test White 37 | 0.71 | 0.73 | 0.69 | 3621 | Train 57 | Test White 37 | 0.7 | 0.7 | 0.66 |
| 2598 | Train 41 | Test White 38 | 0.69 | 0.7 | 0.65 | 3622 | Train 57 | Test White 38 | 0.68 | 0.68 | 0.62 |
| 2599 | Train 41 | Test White 39 | 0.71 | 0.73 | 0.69 | 3623 | Train 57 | Test White 39 | 0.7 | 0.7 | 0.66 |
| 2600 | Train 41 | Test White 40 | 0.69 | 0.7 | 0.65 | 3624 | Train 57 | Test White 40 | 0.68 | 0.68 | 0.63 |
| 2601 | Train 41 | Test White 41 | 0.75 | 0.82 | 0.84 | 3625 | Train 57 | Test White 41 | 0.73 | 0.71 | 0.71 |
| 2602 | Train 41 | Test White 42 | 0.73 | 0.76 | 0.78 | 3626 | Train 57 | Test White 42 | 0.71 | 0.69 | 0.66 |
| 2603 | Train 41 | Test White 43 | 0.75 | 0.82 | 0.83 | 3627 | Train 57 | Test White 43 | 0.73 | 0.72 | 0.71 |
| 2604 | Train 41 | Test White 44 | 0.73 | 0.76 | 0.78 | 3628 | Train 57 | Test White 44 | 0.71 | 0.69 | 0.66 |
| 2605 | Train 41 | Test White 45 | 0.71 | 0.73 | 0.71 | 3629 | Train 57 | Test White 45 | 0.7 | 0.7 | 0.67 |
| 2606 | Train 41 | Test White 46 | 0.69 | 0.7 | 0.65 | 3630 | Train 57 | Test White 46 | 0.68 | 0.68 | 0.62 |
| 2607 | Train 41 | Test White 47 | 0.71 | 0.73 | 0.7 | 3631 | Train 57 | Test White 47 | 0.7 | 0.7 | 0.67 |
| 2608 | Train 41 | Test White 48 | 0.7 | 0.7 | 0.65 | 3632 | Train 57 | Test White 48 | 0.69 | 0.68 | 0.63 |
| 2609 | Train 41 | Test White 49 | 0.73 | 0.76 | 0.77 | 3633 | Train 57 | Test White 49 | 0.75 | 0.79 | 0.79 |
| 2610 | Train 41 | Test White 50 | 0.73 | 0.76 | 0.77 | 3634 | Train 57 | Test White 50 | 0.75 | 0.79 | 0.79 |
| 2611 | Train 41 | Test White 51 | 0.73 | 0.76 | 0.76 | 3635 | Train 57 | Test White 51 | 0.75 | 0.79 | 0.79 |
| 2612 | Train 41 | Test White 52 | 0.73 | 0.76 | 0.76 | 3636 | Train 57 | Test White 52 | 0.75 | 0.79 | 0.79 |
| 2613 | Train 41 | Test White 53 | 0.7 | 0.73 | 0.67 | 3637 | Train 57 | Test White 53 | 0.69 | 0.73 | 0.72 |
| 2614 | Train 41 | Test White 54 | 0.7 | 0.7 | 0.66 | 3638 | Train 57 | Test White 54 | 0.68 | 0.68 | 0.66 |
| 2615 | Train 41 | Test White 55 | 0.7 | 0.73 | 0.67 | 3639 | Train 57 | Test White 55 | 0.69 | 0.73 | 0.72 |
| 2616 | Train 41 | Test White 56 | 0.7 | 0.69 | 0.66 | 3640 | Train 57 | Test White 56 | 0.69 | 0.68 | 0.66 |
| 2617 | Train 41 | Test White 57 | 0.73 | 0.77 | 0.81 | 3641 | Train 57 | Test White 57 | 0.75 | 0.8 | 0.85 |
| 2618 | Train 41 | Test White 58 | 0.73 | 0.77 | 0.81 | 3642 | Train 57 | Test White 58 | 0.75 | 0.8 | 0.85 |
| 2619 | Train 41 | Test White 59 | 0.73 | 0.77 | 0.81 | 3643 | Train 57 | Test White 59 | 0.75 | 0.8 | 0.84 |
| 2620 | Train 41 | Test White 60 | 0.73 | 0.77 | 0.81 | 3644 | Train 57 | Test White 60 | 0.75 | 0.8 | 0.84 |
| 2621 | Train 41 | Test White 61 | 0.7 | 0.74 | 0.67 | 3645 | Train 57 | Test White 61 | 0.69 | 0.73 | 0.73 |
| 2622 | Train 41 | Test White 62 | 0.7 | 0.7 | 0.66 | 3646 | Train 57 | Test White 62 | 0.69 | 0.68 | 0.66 |
| 2623 | Train 41 | Test White 63 | 0.7 | 0.74 | 0.67 | 3647 | Train 57 | Test White 63 | 0.69 | 0.73 | 0.73 |
| 2624 | Train 41 | Test White 64 | 0.71 | 0.7 | 0.66 | 3648 | Train 57 | Test White 64 | 0.69 | 0.68 | 0.66 |
| 2625 | Train 42 | Test White 1 | 0.74 | 0.8 | 0.79 | 3649 | Train 58 | Test White 1 | 0.73 | 0.68 | 0.64 |
| 2626 | Train 42 | Test White 2 | 0.75 | 0.81 | 0.8 | 3650 | Train 58 | Test White 2 | 0.71 | 0.67 | 0.62 |
| 2627 | Train 42 | Test White 3 | 0.74 | 0.8 | 0.79 | 3651 | Train 58 | Test White 3 | 0.73 | 0.68 | 0.64 |
| 2628 | Train 42 | Test White 4 | 0.75 | 0.81 | 0.8 | 3652 | Train 58 | Test White 4 | 0.71 | 0.67 | 0.62 |
| 2629 | Train 42 | Test White 5 | 0.71 | 0.73 | 0.67 | 3653 | Train 58 | Test White 5 | 0.7 | 0.68 | 0.62 |
| 2630 | Train 42 | Test White 6 | 0.7 | 0.71 | 0.66 | 3654 | Train 58 | Test White 6 | 0.68 | 0.67 | 0.62 |
| 2631 | Train 42 | Test White 7 | 0.71 | 0.73 | 0.67 | 3655 | Train 58 | Test White 7 | 0.7 | 0.68 | 0.63 |
| 2632 | Train 42 | Test White 8 | 0.7 | 0.71 | 0.66 | 3656 | Train 58 | Test White 8 | 0.69 | 0.66 | 0.61 |
| 2633 | Train 42 | Test White 9 | 0.74 | 0.8 | 0.82 | 3657 | Train 58 | Test White 9 | 0.73 | 0.69 | 0.65 |
| 2634 | Train 42 | Test White 10 | 0.75 | 0.81 | 0.83 | 3658 | Train 58 | Test White 10 | 0.71 | 0.68 | 0.64 |
| 2635 | Train 42 | Test White 11 | 0.74 | 0.8 | 0.82 | 3659 | Train 58 | Test White 11 | 0.73 | 0.69 | 0.65 |
| 2636 | Train 42 | Test White 12 | 0.75 | 0.81 | 0.83 | 3660 | Train 58 | Test White 12 | 0.71 | 0.68 | 0.64 |
| 2637 | Train 42 | Test White 13 | 0.71 | 0.73 | 0.68 | 3661 | Train 58 | Test White 13 | 0.7 | 0.68 | 0.63 |
| 2638 | Train 42 | Test White 14 | 0.71 | 0.71 | 0.66 | 3662 | Train 58 | Test White 14 | 0.69 | 0.67 | 0.62 |
| 2639 | Train 42 | Test White 15 | 0.71 | 0.73 | 0.68 | 3663 | Train 58 | Test White 15 | 0.7 | 0.68 | 0.63 |
| 2640 | Train 42 | Test White 16 | 0.7 | 0.71 | 0.66 | 3664 | Train 58 | Test White 16 | 0.69 | 0.67 | 0.62 |
| 2641 | Train 42 | Test White 17 | 0.74 | 0.8 | 0.79 | 3665 | Train 58 | Test White 17 | 0.73 | 0.68 | 0.64 |
| 2642 | Train 42 | Test White 18 | 0.75 | 0.81 | 0.8 | 3666 | Train 58 | Test White 18 | 0.71 | 0.67 | 0.62 |
| 2643 | Train 42 | Test White 19 | 0.74 | 0.8 | 0.79 | 3667 | Train 58 | Test White 19 | 0.73 | 0.68 | 0.64 |
| 2644 | Train 42 | Test White 20 | 0.75 | 0.81 | 0.8 | 3668 | Train 58 | Test White 20 | 0.71 | 0.67 | 0.62 |
| 2645 | Train 42 | Test White 21 | 0.71 | 0.73 | 0.67 | 3669 | Train 58 | Test White 21 | 0.7 | 0.68 | 0.62 |
| 2646 | Train 42 | Test White 22 | 0.7 | 0.71 | 0.66 | 3670 | Train 58 | Test White 22 | 0.68 | 0.67 | 0.62 |
| 2647 | Train 42 | Test White 23 | 0.71 | 0.73 | 0.67 | 3671 | Train 58 | Test White 23 | 0.7 | 0.68 | 0.63 |
| 2648 | Train 42 | Test White 24 | 0.7 | 0.71 | 0.66 | 3672 | Train 58 | Test White 24 | 0.69 | 0.67 | 0.61 |
| 2649 | Train 42 | Test White 25 | 0.74 | 0.8 | 0.82 | 3673 | Train 58 | Test White 25 | 0.73 | 0.69 | 0.65 |
| 2650 | Train 42 | Test White 26 | 0.75 | 0.81 | 0.83 | 3674 | Train 58 | Test White 26 | 0.71 | 0.68 | 0.64 |
| 2651 | Train 42 | Test White 27 | 0.74 | 0.8 | 0.82 | 3675 | Train 58 | Test White 27 | 0.73 | 0.69 | 0.65 |
| 2652 | Train 42 | Test White 28 | 0.75 | 0.81 | 0.83 | 3676 | Train 58 | Test White 28 | 0.71 | 0.68 | 0.64 |
| 2653 | Train 42 | Test White 29 | 0.71 | 0.73 | 0.68 | 3677 | Train 58 | Test White 29 | 0.7 | 0.68 | 0.63 |
| 2654 | Train 42 | Test White 30 | 0.71 | 0.71 | 0.66 | 3678 | Train 58 | Test White 30 | 0.69 | 0.67 | 0.62 |
| 2655 | Train 42 | Test White 31 | 0.71 | 0.73 | 0.68 | 3679 | Train 58 | Test White 31 | 0.7 | 0.68 | 0.63 |
| 2656 | Train 42 | Test White 32 | 0.71 | 0.71 | 0.66 | 3680 | Train 58 | Test White 32 | 0.69 | 0.67 | 0.62 |
| 2657 | Train 42 | Test White 33 | 0.74 | 0.8 | 0.79 | 3681 | Train 58 | Test White 33 | 0.73 | 0.68 | 0.64 |
| 2658 | Train 42 | Test White 34 | 0.75 | 0.81 | 0.8 | 3682 | Train 58 | Test White 34 | 0.72 | 0.67 | 0.62 |
| 2659 | Train 42 | Test White 35 | 0.74 | 0.8 | 0.79 | 3683 | Train 58 | Test White 35 | 0.73 | 0.68 | 0.64 |
| 2660 | Train 42 | Test White 36 | 0.75 | 0.81 | 0.8 | 3684 | Train 58 | Test White 36 | 0.72 | 0.67 | 0.63 |
| 2661 | Train 42 | Test White 37 | 0.71 | 0.73 | 0.67 | 3685 | Train 58 | Test White 37 | 0.7 | 0.68 | 0.63 |
| 2662 | Train 42 | Test White 38 | 0.7 | 0.71 | 0.66 | 3686 | Train 58 | Test White 38 | 0.68 | 0.67 | 0.62 |
| 2663 | Train 42 | Test White 39 | 0.71 | 0.73 | 0.67 | 3687 | Train 58 | Test White 39 | 0.7 | 0.68 | 0.63 |
| 2664 | Train 42 | Test White 40 | 0.7 | 0.71 | 0.66 | 3688 | Train 58 | Test White 40 | 0.69 | 0.67 | 0.61 |
| 2665 | Train 42 | Test White 41 | 0.74 | 0.81 | 0.82 | 3689 | Train 58 | Test White 41 | 0.73 | 0.69 | 0.65 |
| 2666 | Train 42 | Test White 42 | 0.75 | 0.81 | 0.83 | 3690 | Train 58 | Test White 42 | 0.72 | 0.68 | 0.64 |
| 2667 | Train 42 | Test White 43 | 0.74 | 0.8 | 0.82 | 3691 | Train 58 | Test White 43 | 0.73 | 0.69 | 0.65 |
| 2668 | Train 42 | Test White 44 | 0.75 | 0.81 | 0.83 | 3692 | Train 58 | Test White 44 | 0.72 | 0.68 | 0.64 |
| 2669 | Train 42 | Test White 45 | 0.71 | 0.73 | 0.68 | 3693 | Train 58 | Test White 45 | 0.7 | 0.68 | 0.63 |
| 2670 | Train 42 | Test White 46 | 0.71 | 0.71 | 0.66 | 3694 | Train 58 | Test White 46 | 0.69 | 0.67 | 0.62 |
| 2671 | Train 42 | Test White 47 | 0.71 | 0.73 | 0.68 | 3695 | Train 58 | Test White 47 | 0.7 | 0.68 | 0.63 |
| 2672 | Train 42 | Test White 48 | 0.71 | 0.71 | 0.66 | 3696 | Train 58 | Test White 48 | 0.69 | 0.67 | 0.62 |
| 2673 | Train 42 | Test White 49 | 0.73 | 0.76 | 0.75 | 3697 | Train 58 | Test White 49 | 0.75 | 0.78 | 0.79 |
| 2674 | Train 42 | Test White 50 | 0.73 | 0.76 | 0.75 | 3698 | Train 58 | Test White 50 | 0.75 | 0.78 | 0.79 |
| 2675 | Train 42 | Test White 51 | 0.73 | 0.76 | 0.75 | 3699 | Train 58 | Test White 51 | 0.75 | 0.78 | 0.78 |
| 2676 | Train 42 | Test White 52 | 0.73 | 0.76 | 0.75 | 3700 | Train 58 | Test White 52 | 0.75 | 0.78 | 0.78 |
| 2677 | Train 42 | Test White 53 | 0.7 | 0.74 | 0.61 | 3701 | Train 58 | Test White 53 | 0.69 | 0.71 | 0.68 |
| 2678 | Train 42 | Test White 54 | 0.71 | 0.71 | 0.65 | 3702 | Train 58 | Test White 54 | 0.69 | 0.67 | 0.63 |
| 2679 | Train 42 | Test White 55 | 0.71 | 0.74 | 0.62 | 3703 | Train 58 | Test White 55 | 0.69 | 0.71 | 0.68 |
| 2680 | Train 42 | Test White 56 | 0.7 | 0.7 | 0.65 | 3704 | Train 58 | Test White 56 | 0.69 | 0.67 | 0.63 |
| 2681 | Train 42 | Test White 57 | 0.73 | 0.76 | 0.79 | 3705 | Train 58 | Test White 57 | 0.75 | 0.79 | 0.85 |
| 2682 | Train 42 | Test White 58 | 0.73 | 0.76 | 0.79 | 3706 | Train 58 | Test White 58 | 0.75 | 0.79 | 0.85 |
| 2683 | Train 42 | Test White 59 | 0.73 | 0.76 | 0.79 | 3707 | Train 58 | Test White 59 | 0.75 | 0.79 | 0.85 |
| 2684 | Train 42 | Test White 60 | 0.73 | 0.76 | 0.79 | 3708 | Train 58 | Test White 60 | 0.75 | 0.79 | 0.85 |
| 2685 | Train 42 | Test White 61 | 0.71 | 0.74 | 0.62 | 3709 | Train 58 | Test White 61 | 0.69 | 0.71 | 0.69 |
| 2686 | Train 42 | Test White 62 | 0.71 | 0.71 | 0.65 | 3710 | Train 58 | Test White 62 | 0.69 | 0.68 | 0.63 |
| 2687 | Train 42 | Test White 63 | 0.71 | 0.74 | 0.62 | 3711 | Train 58 | Test White 63 | 0.69 | 0.71 | 0.69 |
| 2688 | Train 42 | Test White 64 | 0.7 | 0.71 | 0.65 | 3712 | Train 58 | Test White 64 | 0.69 | 0.67 | 0.63 |
| 2689 | Train 43 | Test White 1 | 0.74 | 0.81 | 0.8 | 3713 | Train 59 | Test White 1 | 0.73 | 0.71 | 0.65 |
| 2690 | Train 43 | Test White 2 | 0.73 | 0.76 | 0.75 | 3714 | Train 59 | Test White 2 | 0.71 | 0.69 | 0.62 |
| 2691 | Train 43 | Test White 3 | 0.74 | 0.81 | 0.8 | 3715 | Train 59 | Test White 3 | 0.73 | 0.71 | 0.65 |
| 2692 | Train 43 | Test White 4 | 0.73 | 0.76 | 0.75 | 3716 | Train 59 | Test White 4 | 0.71 | 0.69 | 0.62 |
| 2693 | Train 43 | Test White 5 | 0.71 | 0.73 | 0.7 | 3717 | Train 59 | Test White 5 | 0.7 | 0.7 | 0.64 |
| 2694 | Train 43 | Test White 6 | 0.69 | 0.7 | 0.65 | 3718 | Train 59 | Test White 6 | 0.68 | 0.68 | 0.6 |
| 2695 | Train 43 | Test White 7 | 0.71 | 0.73 | 0.7 | 3719 | Train 59 | Test White 7 | 0.7 | 0.7 | 0.64 |
| 2696 | Train 43 | Test White 8 | 0.69 | 0.7 | 0.65 | 3720 | Train 59 | Test White 8 | 0.68 | 0.68 | 0.6 |
| 2697 | Train 43 | Test White 9 | 0.74 | 0.81 | 0.83 | 3721 | Train 59 | Test White 9 | 0.73 | 0.72 | 0.67 |
| 2698 | Train 43 | Test White 10 | 0.73 | 0.76 | 0.77 | 3722 | Train 59 | Test White 10 | 0.71 | 0.69 | 0.64 |
| 2699 | Train 43 | Test White 11 | 0.75 | 0.81 | 0.83 | 3723 | Train 59 | Test White 11 | 0.73 | 0.72 | 0.67 |
| 2700 | Train 43 | Test White 12 | 0.73 | 0.76 | 0.77 | 3724 | Train 59 | Test White 12 | 0.71 | 0.69 | 0.64 |
| 2701 | Train 43 | Test White 13 | 0.71 | 0.73 | 0.71 | 3725 | Train 59 | Test White 13 | 0.7 | 0.7 | 0.64 |
| 2702 | Train 43 | Test White 14 | 0.69 | 0.7 | 0.65 | 3726 | Train 59 | Test White 14 | 0.68 | 0.68 | 0.6 |
| 2703 | Train 43 | Test White 15 | 0.71 | 0.73 | 0.71 | 3727 | Train 59 | Test White 15 | 0.7 | 0.7 | 0.64 |
| 2704 | Train 43 | Test White 16 | 0.69 | 0.7 | 0.65 | 3728 | Train 59 | Test White 16 | 0.68 | 0.68 | 0.6 |
| 2705 | Train 43 | Test White 17 | 0.74 | 0.81 | 0.8 | 3729 | Train 59 | Test White 17 | 0.73 | 0.71 | 0.65 |
| 2706 | Train 43 | Test White 18 | 0.73 | 0.76 | 0.75 | 3730 | Train 59 | Test White 18 | 0.71 | 0.69 | 0.62 |
| 2707 | Train 43 | Test White 19 | 0.74 | 0.81 | 0.8 | 3731 | Train 59 | Test White 19 | 0.73 | 0.71 | 0.65 |
| 2708 | Train 43 | Test White 20 | 0.73 | 0.76 | 0.75 | 3732 | Train 59 | Test White 20 | 0.71 | 0.69 | 0.62 |
| 2709 | Train 43 | Test White 21 | 0.71 | 0.73 | 0.7 | 3733 | Train 59 | Test White 21 | 0.7 | 0.7 | 0.64 |
| 2710 | Train 43 | Test White 22 | 0.69 | 0.7 | 0.65 | 3734 | Train 59 | Test White 22 | 0.68 | 0.68 | 0.6 |
| 2711 | Train 43 | Test White 23 | 0.71 | 0.73 | 0.7 | 3735 | Train 59 | Test White 23 | 0.7 | 0.7 | 0.64 |
| 2712 | Train 43 | Test White 24 | 0.69 | 0.7 | 0.65 | 3736 | Train 59 | Test White 24 | 0.68 | 0.68 | 0.6 |
| 2713 | Train 43 | Test White 25 | 0.74 | 0.81 | 0.83 | 3737 | Train 59 | Test White 25 | 0.73 | 0.72 | 0.67 |
| 2714 | Train 43 | Test White 26 | 0.73 | 0.76 | 0.77 | 3738 | Train 59 | Test White 26 | 0.71 | 0.69 | 0.64 |
| 2715 | Train 43 | Test White 27 | 0.75 | 0.81 | 0.83 | 3739 | Train 59 | Test White 27 | 0.73 | 0.72 | 0.67 |
| 2716 | Train 43 | Test White 28 | 0.73 | 0.76 | 0.77 | 3740 | Train 59 | Test White 28 | 0.71 | 0.69 | 0.64 |
| 2717 | Train 43 | Test White 29 | 0.71 | 0.73 | 0.71 | 3741 | Train 59 | Test White 29 | 0.7 | 0.7 | 0.64 |
| 2718 | Train 43 | Test White 30 | 0.69 | 0.7 | 0.65 | 3742 | Train 59 | Test White 30 | 0.68 | 0.68 | 0.6 |
| 2719 | Train 43 | Test White 31 | 0.71 | 0.73 | 0.71 | 3743 | Train 59 | Test White 31 | 0.7 | 0.7 | 0.64 |
| 2720 | Train 43 | Test White 32 | 0.7 | 0.7 | 0.65 | 3744 | Train 59 | Test White 32 | 0.68 | 0.68 | 0.6 |
| 2721 | Train 43 | Test White 33 | 0.75 | 0.81 | 0.8 | 3745 | Train 59 | Test White 33 | 0.73 | 0.71 | 0.65 |
| 2722 | Train 43 | Test White 34 | 0.73 | 0.76 | 0.75 | 3746 | Train 59 | Test White 34 | 0.71 | 0.69 | 0.62 |
| 2723 | Train 43 | Test White 35 | 0.75 | 0.81 | 0.8 | 3747 | Train 59 | Test White 35 | 0.73 | 0.71 | 0.66 |
| 2724 | Train 43 | Test White 36 | 0.73 | 0.76 | 0.75 | 3748 | Train 59 | Test White 36 | 0.71 | 0.69 | 0.62 |
| 2725 | Train 43 | Test White 37 | 0.71 | 0.73 | 0.7 | 3749 | Train 59 | Test White 37 | 0.7 | 0.7 | 0.64 |
| 2726 | Train 43 | Test White 38 | 0.69 | 0.7 | 0.65 | 3750 | Train 59 | Test White 38 | 0.68 | 0.68 | 0.6 |
| 2727 | Train 43 | Test White 39 | 0.71 | 0.73 | 0.7 | 3751 | Train 59 | Test White 39 | 0.7 | 0.7 | 0.64 |
| 2728 | Train 43 | Test White 40 | 0.69 | 0.7 | 0.65 | 3752 | Train 59 | Test White 40 | 0.68 | 0.68 | 0.6 |
| 2729 | Train 43 | Test White 41 | 0.75 | 0.82 | 0.84 | 3753 | Train 59 | Test White 41 | 0.73 | 0.72 | 0.67 |
| 2730 | Train 43 | Test White 42 | 0.73 | 0.76 | 0.78 | 3754 | Train 59 | Test White 42 | 0.71 | 0.69 | 0.64 |
| 2731 | Train 43 | Test White 43 | 0.75 | 0.82 | 0.84 | 3755 | Train 59 | Test White 43 | 0.73 | 0.72 | 0.67 |
| 2732 | Train 43 | Test White 44 | 0.73 | 0.76 | 0.78 | 3756 | Train 59 | Test White 44 | 0.71 | 0.69 | 0.64 |
| 2733 | Train 43 | Test White 45 | 0.71 | 0.73 | 0.71 | 3757 | Train 59 | Test White 45 | 0.7 | 0.7 | 0.64 |
| 2734 | Train 43 | Test White 46 | 0.69 | 0.7 | 0.65 | 3758 | Train 59 | Test White 46 | 0.68 | 0.68 | 0.6 |
| 2735 | Train 43 | Test White 47 | 0.71 | 0.73 | 0.71 | 3759 | Train 59 | Test White 47 | 0.7 | 0.7 | 0.64 |
| 2736 | Train 43 | Test White 48 | 0.7 | 0.7 | 0.65 | 3760 | Train 59 | Test White 48 | 0.68 | 0.68 | 0.6 |
| 2737 | Train 43 | Test White 49 | 0.73 | 0.76 | 0.76 | 3761 | Train 59 | Test White 49 | 0.75 | 0.79 | 0.78 |
| 2738 | Train 43 | Test White 50 | 0.73 | 0.76 | 0.76 | 3762 | Train 59 | Test White 50 | 0.75 | 0.79 | 0.78 |
| 2739 | Train 43 | Test White 51 | 0.73 | 0.76 | 0.76 | 3763 | Train 59 | Test White 51 | 0.75 | 0.79 | 0.78 |
| 2740 | Train 43 | Test White 52 | 0.73 | 0.76 | 0.76 | 3764 | Train 59 | Test White 52 | 0.75 | 0.79 | 0.78 |
| 2741 | Train 43 | Test White 53 | 0.7 | 0.73 | 0.66 | 3765 | Train 59 | Test White 53 | 0.69 | 0.73 | 0.7 |
| 2742 | Train 43 | Test White 54 | 0.7 | 0.7 | 0.67 | 3766 | Train 59 | Test White 54 | 0.68 | 0.69 | 0.62 |
| 2743 | Train 43 | Test White 55 | 0.7 | 0.73 | 0.66 | 3767 | Train 59 | Test White 55 | 0.69 | 0.73 | 0.7 |
| 2744 | Train 43 | Test White 56 | 0.7 | 0.69 | 0.67 | 3768 | Train 59 | Test White 56 | 0.69 | 0.68 | 0.62 |
| 2745 | Train 43 | Test White 57 | 0.73 | 0.77 | 0.8 | 3769 | Train 59 | Test White 57 | 0.75 | 0.8 | 0.84 |
| 2746 | Train 43 | Test White 58 | 0.73 | 0.77 | 0.8 | 3770 | Train 59 | Test White 58 | 0.75 | 0.8 | 0.84 |
| 2747 | Train 43 | Test White 59 | 0.73 | 0.77 | 0.8 | 3771 | Train 59 | Test White 59 | 0.75 | 0.8 | 0.84 |
| 2748 | Train 43 | Test White 60 | 0.73 | 0.77 | 0.8 | 3772 | Train 59 | Test White 60 | 0.75 | 0.8 | 0.84 |
| 2749 | Train 43 | Test White 61 | 0.7 | 0.74 | 0.66 | 3773 | Train 59 | Test White 61 | 0.69 | 0.74 | 0.72 |
| 2750 | Train 43 | Test White 62 | 0.7 | 0.7 | 0.67 | 3774 | Train 59 | Test White 62 | 0.68 | 0.69 | 0.62 |
| 2751 | Train 43 | Test White 63 | 0.7 | 0.74 | 0.66 | 3775 | Train 59 | Test White 63 | 0.69 | 0.74 | 0.72 |
| 2752 | Train 43 | Test White 64 | 0.71 | 0.7 | 0.67 | 3776 | Train 59 | Test White 64 | 0.69 | 0.69 | 0.63 |
| 2753 | Train 44 | Test White 1 | 0.74 | 0.8 | 0.79 | 3777 | Train 60 | Test White 1 | 0.73 | 0.68 | 0.69 |
| 2754 | Train 44 | Test White 2 | 0.75 | 0.81 | 0.8 | 3778 | Train 60 | Test White 2 | 0.71 | 0.67 | 0.64 |
| 2755 | Train 44 | Test White 3 | 0.74 | 0.8 | 0.79 | 3779 | Train 60 | Test White 3 | 0.73 | 0.68 | 0.69 |
| 2756 | Train 44 | Test White 4 | 0.75 | 0.81 | 0.8 | 3780 | Train 60 | Test White 4 | 0.71 | 0.67 | 0.64 |
| 2757 | Train 44 | Test White 5 | 0.71 | 0.73 | 0.69 | 3781 | Train 60 | Test White 5 | 0.7 | 0.68 | 0.67 |
| 2758 | Train 44 | Test White 6 | 0.7 | 0.71 | 0.68 | 3782 | Train 60 | Test White 6 | 0.68 | 0.67 | 0.61 |
| 2759 | Train 44 | Test White 7 | 0.71 | 0.73 | 0.69 | 3783 | Train 60 | Test White 7 | 0.7 | 0.68 | 0.67 |
| 2760 | Train 44 | Test White 8 | 0.7 | 0.71 | 0.68 | 3784 | Train 60 | Test White 8 | 0.68 | 0.67 | 0.61 |
| 2761 | Train 44 | Test White 9 | 0.74 | 0.8 | 0.82 | 3785 | Train 60 | Test White 9 | 0.73 | 0.69 | 0.71 |
| 2762 | Train 44 | Test White 10 | 0.75 | 0.81 | 0.83 | 3786 | Train 60 | Test White 10 | 0.71 | 0.68 | 0.65 |
| 2763 | Train 44 | Test White 11 | 0.74 | 0.8 | 0.82 | 3787 | Train 60 | Test White 11 | 0.73 | 0.69 | 0.71 |
| 2764 | Train 44 | Test White 12 | 0.75 | 0.81 | 0.83 | 3788 | Train 60 | Test White 12 | 0.71 | 0.68 | 0.65 |
| 2765 | Train 44 | Test White 13 | 0.71 | 0.73 | 0.7 | 3789 | Train 60 | Test White 13 | 0.7 | 0.68 | 0.68 |
| 2766 | Train 44 | Test White 14 | 0.7 | 0.71 | 0.68 | 3790 | Train 60 | Test White 14 | 0.68 | 0.67 | 0.61 |
| 2767 | Train 44 | Test White 15 | 0.71 | 0.73 | 0.7 | 3791 | Train 60 | Test White 15 | 0.7 | 0.68 | 0.68 |
| 2768 | Train 44 | Test White 16 | 0.7 | 0.71 | 0.68 | 3792 | Train 60 | Test White 16 | 0.69 | 0.67 | 0.61 |
| 2769 | Train 44 | Test White 17 | 0.74 | 0.8 | 0.79 | 3793 | Train 60 | Test White 17 | 0.73 | 0.68 | 0.69 |
| 2770 | Train 44 | Test White 18 | 0.75 | 0.81 | 0.8 | 3794 | Train 60 | Test White 18 | 0.71 | 0.67 | 0.64 |
| 2771 | Train 44 | Test White 19 | 0.74 | 0.8 | 0.79 | 3795 | Train 60 | Test White 19 | 0.73 | 0.68 | 0.69 |
| 2772 | Train 44 | Test White 20 | 0.75 | 0.81 | 0.8 | 3796 | Train 60 | Test White 20 | 0.71 | 0.67 | 0.64 |
| 2773 | Train 44 | Test White 21 | 0.71 | 0.73 | 0.69 | 3797 | Train 60 | Test White 21 | 0.7 | 0.68 | 0.67 |
| 2774 | Train 44 | Test White 22 | 0.7 | 0.71 | 0.68 | 3798 | Train 60 | Test White 22 | 0.68 | 0.67 | 0.61 |
| 2775 | Train 44 | Test White 23 | 0.71 | 0.73 | 0.69 | 3799 | Train 60 | Test White 23 | 0.7 | 0.68 | 0.67 |
| 2776 | Train 44 | Test White 24 | 0.7 | 0.71 | 0.68 | 3800 | Train 60 | Test White 24 | 0.68 | 0.67 | 0.61 |
| 2777 | Train 44 | Test White 25 | 0.74 | 0.8 | 0.82 | 3801 | Train 60 | Test White 25 | 0.73 | 0.69 | 0.71 |
| 2778 | Train 44 | Test White 26 | 0.75 | 0.81 | 0.83 | 3802 | Train 60 | Test White 26 | 0.71 | 0.68 | 0.65 |
| 2779 | Train 44 | Test White 27 | 0.74 | 0.8 | 0.82 | 3803 | Train 60 | Test White 27 | 0.73 | 0.69 | 0.71 |
| 2780 | Train 44 | Test White 28 | 0.75 | 0.81 | 0.83 | 3804 | Train 60 | Test White 28 | 0.71 | 0.68 | 0.65 |
| 2781 | Train 44 | Test White 29 | 0.71 | 0.73 | 0.7 | 3805 | Train 60 | Test White 29 | 0.7 | 0.68 | 0.68 |
| 2782 | Train 44 | Test White 30 | 0.71 | 0.71 | 0.68 | 3806 | Train 60 | Test White 30 | 0.68 | 0.67 | 0.61 |
| 2783 | Train 44 | Test White 31 | 0.71 | 0.73 | 0.7 | 3807 | Train 60 | Test White 31 | 0.7 | 0.68 | 0.68 |
| 2784 | Train 44 | Test White 32 | 0.7 | 0.71 | 0.68 | 3808 | Train 60 | Test White 32 | 0.69 | 0.67 | 0.61 |
| 2785 | Train 44 | Test White 33 | 0.74 | 0.8 | 0.79 | 3809 | Train 60 | Test White 33 | 0.73 | 0.68 | 0.69 |
| 2786 | Train 44 | Test White 34 | 0.75 | 0.81 | 0.8 | 3810 | Train 60 | Test White 34 | 0.71 | 0.67 | 0.64 |
| 2787 | Train 44 | Test White 35 | 0.74 | 0.8 | 0.79 | 3811 | Train 60 | Test White 35 | 0.73 | 0.69 | 0.69 |
| 2788 | Train 44 | Test White 36 | 0.75 | 0.81 | 0.8 | 3812 | Train 60 | Test White 36 | 0.71 | 0.67 | 0.64 |
| 2789 | Train 44 | Test White 37 | 0.71 | 0.73 | 0.69 | 3813 | Train 60 | Test White 37 | 0.7 | 0.68 | 0.67 |
| 2790 | Train 44 | Test White 38 | 0.7 | 0.71 | 0.68 | 3814 | Train 60 | Test White 38 | 0.68 | 0.67 | 0.61 |
| 2791 | Train 44 | Test White 39 | 0.71 | 0.73 | 0.69 | 3815 | Train 60 | Test White 39 | 0.7 | 0.68 | 0.67 |
| 2792 | Train 44 | Test White 40 | 0.7 | 0.71 | 0.68 | 3816 | Train 60 | Test White 40 | 0.68 | 0.67 | 0.61 |
| 2793 | Train 44 | Test White 41 | 0.74 | 0.8 | 0.82 | 3817 | Train 60 | Test White 41 | 0.73 | 0.69 | 0.71 |
| 2794 | Train 44 | Test White 42 | 0.75 | 0.81 | 0.83 | 3818 | Train 60 | Test White 42 | 0.71 | 0.68 | 0.65 |
| 2795 | Train 44 | Test White 43 | 0.74 | 0.81 | 0.82 | 3819 | Train 60 | Test White 43 | 0.73 | 0.69 | 0.71 |
| 2796 | Train 44 | Test White 44 | 0.75 | 0.81 | 0.83 | 3820 | Train 60 | Test White 44 | 0.71 | 0.68 | 0.65 |
| 2797 | Train 44 | Test White 45 | 0.71 | 0.73 | 0.7 | 3821 | Train 60 | Test White 45 | 0.7 | 0.68 | 0.68 |
| 2798 | Train 44 | Test White 46 | 0.71 | 0.71 | 0.68 | 3822 | Train 60 | Test White 46 | 0.68 | 0.67 | 0.61 |
| 2799 | Train 44 | Test White 47 | 0.71 | 0.73 | 0.7 | 3823 | Train 60 | Test White 47 | 0.7 | 0.68 | 0.68 |
| 2800 | Train 44 | Test White 48 | 0.7 | 0.71 | 0.68 | 3824 | Train 60 | Test White 48 | 0.69 | 0.67 | 0.61 |
| 2801 | Train 44 | Test White 49 | 0.73 | 0.76 | 0.76 | 3825 | Train 60 | Test White 49 | 0.75 | 0.79 | 0.77 |
| 2802 | Train 44 | Test White 50 | 0.73 | 0.76 | 0.76 | 3826 | Train 60 | Test White 50 | 0.75 | 0.79 | 0.77 |
| 2803 | Train 44 | Test White 51 | 0.73 | 0.76 | 0.76 | 3827 | Train 60 | Test White 51 | 0.75 | 0.79 | 0.77 |
| 2804 | Train 44 | Test White 52 | 0.73 | 0.76 | 0.76 | 3828 | Train 60 | Test White 52 | 0.75 | 0.79 | 0.77 |
| 2805 | Train 44 | Test White 53 | 0.7 | 0.74 | 0.66 | 3829 | Train 60 | Test White 53 | 0.69 | 0.71 | 0.74 |
| 2806 | Train 44 | Test White 54 | 0.71 | 0.71 | 0.67 | 3830 | Train 60 | Test White 54 | 0.68 | 0.68 | 0.64 |
| 2807 | Train 44 | Test White 55 | 0.7 | 0.74 | 0.66 | 3831 | Train 60 | Test White 55 | 0.69 | 0.71 | 0.74 |
| 2808 | Train 44 | Test White 56 | 0.7 | 0.7 | 0.67 | 3832 | Train 60 | Test White 56 | 0.69 | 0.67 | 0.64 |
| 2809 | Train 44 | Test White 57 | 0.73 | 0.76 | 0.8 | 3833 | Train 60 | Test White 57 | 0.75 | 0.8 | 0.84 |
| 2810 | Train 44 | Test White 58 | 0.73 | 0.76 | 0.8 | 3834 | Train 60 | Test White 58 | 0.75 | 0.8 | 0.84 |
| 2811 | Train 44 | Test White 59 | 0.73 | 0.76 | 0.8 | 3835 | Train 60 | Test White 59 | 0.75 | 0.8 | 0.84 |
| 2812 | Train 44 | Test White 60 | 0.73 | 0.76 | 0.8 | 3836 | Train 60 | Test White 60 | 0.75 | 0.8 | 0.84 |
| 2813 | Train 44 | Test White 61 | 0.71 | 0.74 | 0.66 | 3837 | Train 60 | Test White 61 | 0.69 | 0.71 | 0.75 |
| 2814 | Train 44 | Test White 62 | 0.71 | 0.71 | 0.67 | 3838 | Train 60 | Test White 62 | 0.69 | 0.68 | 0.64 |
| 2815 | Train 44 | Test White 63 | 0.71 | 0.74 | 0.66 | 3839 | Train 60 | Test White 63 | 0.69 | 0.72 | 0.76 |
| 2816 | Train 44 | Test White 64 | 0.7 | 0.7 | 0.67 | 3840 | Train 60 | Test White 64 | 0.69 | 0.67 | 0.64 |
| 2817 | Train 45 | Test White 1 | 0.74 | 0.78 | 0.78 | 3841 | Train 61 | Test White 1 | 0.72 | 0.73 | 0.67 |
| 2818 | Train 45 | Test White 2 | 0.74 | 0.76 | 0.76 | 3842 | Train 61 | Test White 2 | 0.72 | 0.7 | 0.65 |
| 2819 | Train 45 | Test White 3 | 0.74 | 0.78 | 0.78 | 3843 | Train 61 | Test White 3 | 0.72 | 0.73 | 0.67 |
| 2820 | Train 45 | Test White 4 | 0.74 | 0.76 | 0.76 | 3844 | Train 61 | Test White 4 | 0.72 | 0.7 | 0.65 |
| 2821 | Train 45 | Test White 5 | 0.73 | 0.77 | 0.73 | 3845 | Train 61 | Test White 5 | 0.72 | 0.74 | 0.7 |
| 2822 | Train 45 | Test White 6 | 0.7 | 0.71 | 0.68 | 3846 | Train 61 | Test White 6 | 0.7 | 0.69 | 0.63 |
| 2823 | Train 45 | Test White 7 | 0.73 | 0.77 | 0.73 | 3847 | Train 61 | Test White 7 | 0.72 | 0.74 | 0.7 |
| 2824 | Train 45 | Test White 8 | 0.7 | 0.71 | 0.68 | 3848 | Train 61 | Test White 8 | 0.7 | 0.69 | 0.63 |
| 2825 | Train 45 | Test White 9 | 0.74 | 0.79 | 0.8 | 3849 | Train 61 | Test White 9 | 0.72 | 0.73 | 0.69 |
| 2826 | Train 45 | Test White 10 | 0.74 | 0.77 | 0.77 | 3850 | Train 61 | Test White 10 | 0.72 | 0.7 | 0.66 |
| 2827 | Train 45 | Test White 11 | 0.74 | 0.79 | 0.8 | 3851 | Train 61 | Test White 11 | 0.72 | 0.73 | 0.69 |
| 2828 | Train 45 | Test White 12 | 0.74 | 0.77 | 0.77 | 3852 | Train 61 | Test White 12 | 0.72 | 0.7 | 0.66 |
| 2829 | Train 45 | Test White 13 | 0.73 | 0.78 | 0.74 | 3853 | Train 61 | Test White 13 | 0.72 | 0.75 | 0.72 |
| 2830 | Train 45 | Test White 14 | 0.7 | 0.71 | 0.68 | 3854 | Train 61 | Test White 14 | 0.7 | 0.69 | 0.63 |
| 2831 | Train 45 | Test White 15 | 0.73 | 0.78 | 0.74 | 3855 | Train 61 | Test White 15 | 0.72 | 0.75 | 0.72 |
| 2832 | Train 45 | Test White 16 | 0.7 | 0.71 | 0.68 | 3856 | Train 61 | Test White 16 | 0.7 | 0.69 | 0.63 |
| 2833 | Train 45 | Test White 17 | 0.74 | 0.78 | 0.78 | 3857 | Train 61 | Test White 17 | 0.72 | 0.73 | 0.67 |
| 2834 | Train 45 | Test White 18 | 0.74 | 0.76 | 0.76 | 3858 | Train 61 | Test White 18 | 0.72 | 0.7 | 0.65 |
| 2835 | Train 45 | Test White 19 | 0.74 | 0.78 | 0.78 | 3859 | Train 61 | Test White 19 | 0.72 | 0.73 | 0.67 |
| 2836 | Train 45 | Test White 20 | 0.74 | 0.76 | 0.76 | 3860 | Train 61 | Test White 20 | 0.72 | 0.7 | 0.65 |
| 2837 | Train 45 | Test White 21 | 0.73 | 0.77 | 0.73 | 3861 | Train 61 | Test White 21 | 0.72 | 0.74 | 0.7 |
| 2838 | Train 45 | Test White 22 | 0.7 | 0.71 | 0.68 | 3862 | Train 61 | Test White 22 | 0.7 | 0.69 | 0.63 |
| 2839 | Train 45 | Test White 23 | 0.73 | 0.77 | 0.73 | 3863 | Train 61 | Test White 23 | 0.72 | 0.74 | 0.7 |
| 2840 | Train 45 | Test White 24 | 0.7 | 0.71 | 0.68 | 3864 | Train 61 | Test White 24 | 0.7 | 0.69 | 0.63 |
| 2841 | Train 45 | Test White 25 | 0.74 | 0.79 | 0.8 | 3865 | Train 61 | Test White 25 | 0.72 | 0.73 | 0.69 |
| 2842 | Train 45 | Test White 26 | 0.74 | 0.77 | 0.77 | 3866 | Train 61 | Test White 26 | 0.72 | 0.7 | 0.67 |
| 2843 | Train 45 | Test White 27 | 0.74 | 0.79 | 0.8 | 3867 | Train 61 | Test White 27 | 0.72 | 0.73 | 0.69 |
| 2844 | Train 45 | Test White 28 | 0.74 | 0.77 | 0.77 | 3868 | Train 61 | Test White 28 | 0.72 | 0.7 | 0.67 |
| 2845 | Train 45 | Test White 29 | 0.73 | 0.78 | 0.74 | 3869 | Train 61 | Test White 29 | 0.72 | 0.75 | 0.72 |
| 2846 | Train 45 | Test White 30 | 0.7 | 0.71 | 0.68 | 3870 | Train 61 | Test White 30 | 0.7 | 0.69 | 0.63 |
| 2847 | Train 45 | Test White 31 | 0.73 | 0.78 | 0.74 | 3871 | Train 61 | Test White 31 | 0.72 | 0.75 | 0.72 |
| 2848 | Train 45 | Test White 32 | 0.7 | 0.71 | 0.68 | 3872 | Train 61 | Test White 32 | 0.7 | 0.69 | 0.63 |
| 2849 | Train 45 | Test White 33 | 0.74 | 0.78 | 0.79 | 3873 | Train 61 | Test White 33 | 0.73 | 0.73 | 0.68 |
| 2850 | Train 45 | Test White 34 | 0.74 | 0.77 | 0.76 | 3874 | Train 61 | Test White 34 | 0.72 | 0.7 | 0.66 |
| 2851 | Train 45 | Test White 35 | 0.74 | 0.78 | 0.79 | 3875 | Train 61 | Test White 35 | 0.73 | 0.73 | 0.68 |
| 2852 | Train 45 | Test White 36 | 0.74 | 0.77 | 0.76 | 3876 | Train 61 | Test White 36 | 0.72 | 0.7 | 0.66 |
| 2853 | Train 45 | Test White 37 | 0.73 | 0.77 | 0.73 | 3877 | Train 61 | Test White 37 | 0.72 | 0.74 | 0.7 |
| 2854 | Train 45 | Test White 38 | 0.7 | 0.71 | 0.68 | 3878 | Train 61 | Test White 38 | 0.7 | 0.69 | 0.63 |
| 2855 | Train 45 | Test White 39 | 0.73 | 0.77 | 0.73 | 3879 | Train 61 | Test White 39 | 0.72 | 0.74 | 0.7 |
| 2856 | Train 45 | Test White 40 | 0.7 | 0.71 | 0.68 | 3880 | Train 61 | Test White 40 | 0.7 | 0.69 | 0.63 |
| 2857 | Train 45 | Test White 41 | 0.74 | 0.79 | 0.81 | 3881 | Train 61 | Test White 41 | 0.73 | 0.73 | 0.69 |
| 2858 | Train 45 | Test White 42 | 0.74 | 0.77 | 0.78 | 3882 | Train 61 | Test White 42 | 0.73 | 0.7 | 0.67 |
| 2859 | Train 45 | Test White 43 | 0.74 | 0.79 | 0.8 | 3883 | Train 61 | Test White 43 | 0.73 | 0.73 | 0.69 |
| 2860 | Train 45 | Test White 44 | 0.74 | 0.77 | 0.77 | 3884 | Train 61 | Test White 44 | 0.73 | 0.7 | 0.67 |
| 2861 | Train 45 | Test White 45 | 0.73 | 0.78 | 0.74 | 3885 | Train 61 | Test White 45 | 0.72 | 0.75 | 0.72 |
| 2862 | Train 45 | Test White 46 | 0.7 | 0.71 | 0.68 | 3886 | Train 61 | Test White 46 | 0.7 | 0.69 | 0.63 |
| 2863 | Train 45 | Test White 47 | 0.73 | 0.78 | 0.74 | 3887 | Train 61 | Test White 47 | 0.72 | 0.75 | 0.72 |
| 2864 | Train 45 | Test White 48 | 0.7 | 0.71 | 0.68 | 3888 | Train 61 | Test White 48 | 0.7 | 0.69 | 0.63 |
| 2865 | Train 45 | Test White 49 | 0.73 | 0.74 | 0.75 | 3889 | Train 61 | Test White 49 | 0.73 | 0.75 | 0.76 |
| 2866 | Train 45 | Test White 50 | 0.73 | 0.74 | 0.75 | 3890 | Train 61 | Test White 50 | 0.73 | 0.75 | 0.75 |
| 2867 | Train 45 | Test White 51 | 0.73 | 0.74 | 0.75 | 3891 | Train 61 | Test White 51 | 0.73 | 0.75 | 0.75 |
| 2868 | Train 45 | Test White 52 | 0.73 | 0.74 | 0.75 | 3892 | Train 61 | Test White 52 | 0.73 | 0.75 | 0.75 |
| 2869 | Train 45 | Test White 53 | 0.73 | 0.77 | 0.75 | 3893 | Train 61 | Test White 53 | 0.75 | 0.81 | 0.78 |
| 2870 | Train 45 | Test White 54 | 0.71 | 0.71 | 0.68 | 3894 | Train 61 | Test White 54 | 0.71 | 0.7 | 0.65 |
| 2871 | Train 45 | Test White 55 | 0.73 | 0.77 | 0.74 | 3895 | Train 61 | Test White 55 | 0.75 | 0.81 | 0.78 |
| 2872 | Train 45 | Test White 56 | 0.7 | 0.71 | 0.68 | 3896 | Train 61 | Test White 56 | 0.7 | 0.7 | 0.66 |
| 2873 | Train 45 | Test White 57 | 0.73 | 0.74 | 0.78 | 3897 | Train 61 | Test White 57 | 0.74 | 0.76 | 0.79 |
| 2874 | Train 45 | Test White 58 | 0.73 | 0.74 | 0.78 | 3898 | Train 61 | Test White 58 | 0.74 | 0.76 | 0.79 |
| 2875 | Train 45 | Test White 59 | 0.73 | 0.74 | 0.78 | 3899 | Train 61 | Test White 59 | 0.74 | 0.76 | 0.79 |
| 2876 | Train 45 | Test White 60 | 0.73 | 0.74 | 0.78 | 3900 | Train 61 | Test White 60 | 0.74 | 0.76 | 0.79 |
| 2877 | Train 45 | Test White 61 | 0.73 | 0.78 | 0.76 | 3901 | Train 61 | Test White 61 | 0.75 | 0.82 | 0.81 |
| 2878 | Train 45 | Test White 62 | 0.71 | 0.71 | 0.68 | 3902 | Train 61 | Test White 62 | 0.71 | 0.7 | 0.65 |
| 2879 | Train 45 | Test White 63 | 0.73 | 0.78 | 0.76 | 3903 | Train 61 | Test White 63 | 0.75 | 0.82 | 0.81 |
| 2880 | Train 45 | Test White 64 | 0.71 | 0.71 | 0.69 | 3904 | Train 61 | Test White 64 | 0.71 | 0.7 | 0.66 |
| 2881 | Train 46 | Test White 1 | 0.72 | 0.75 | 0.75 | 3905 | Train 62 | Test White 1 | 0.71 | 0.72 | 0.68 |
| 2882 | Train 46 | Test White 2 | 0.74 | 0.76 | 0.76 | 3906 | Train 62 | Test White 2 | 0.73 | 0.73 | 0.68 |
| 2883 | Train 46 | Test White 3 | 0.72 | 0.75 | 0.75 | 3907 | Train 62 | Test White 3 | 0.71 | 0.72 | 0.68 |
| 2884 | Train 46 | Test White 4 | 0.74 | 0.76 | 0.76 | 3908 | Train 62 | Test White 4 | 0.73 | 0.73 | 0.68 |
| 2885 | Train 46 | Test White 5 | 0.72 | 0.77 | 0.77 | 3909 | Train 62 | Test White 5 | 0.71 | 0.74 | 0.69 |
| 2886 | Train 46 | Test White 6 | 0.73 | 0.77 | 0.78 | 3910 | Train 62 | Test White 6 | 0.72 | 0.75 | 0.71 |
| 2887 | Train 46 | Test White 7 | 0.72 | 0.77 | 0.77 | 3911 | Train 62 | Test White 7 | 0.71 | 0.73 | 0.69 |
| 2888 | Train 46 | Test White 8 | 0.73 | 0.77 | 0.78 | 3912 | Train 62 | Test White 8 | 0.72 | 0.74 | 0.71 |
| 2889 | Train 46 | Test White 9 | 0.72 | 0.75 | 0.76 | 3913 | Train 62 | Test White 9 | 0.71 | 0.72 | 0.69 |
| 2890 | Train 46 | Test White 10 | 0.74 | 0.76 | 0.76 | 3914 | Train 62 | Test White 10 | 0.73 | 0.73 | 0.69 |
| 2891 | Train 46 | Test White 11 | 0.72 | 0.75 | 0.76 | 3915 | Train 62 | Test White 11 | 0.71 | 0.72 | 0.69 |
| 2892 | Train 46 | Test White 12 | 0.74 | 0.76 | 0.76 | 3916 | Train 62 | Test White 12 | 0.73 | 0.73 | 0.69 |
| 2893 | Train 46 | Test White 13 | 0.72 | 0.77 | 0.78 | 3917 | Train 62 | Test White 13 | 0.71 | 0.74 | 0.7 |
| 2894 | Train 46 | Test White 14 | 0.73 | 0.77 | 0.78 | 3918 | Train 62 | Test White 14 | 0.72 | 0.75 | 0.72 |
| 2895 | Train 46 | Test White 15 | 0.72 | 0.77 | 0.78 | 3919 | Train 62 | Test White 15 | 0.71 | 0.74 | 0.7 |
| 2896 | Train 46 | Test White 16 | 0.73 | 0.77 | 0.78 | 3920 | Train 62 | Test White 16 | 0.72 | 0.74 | 0.72 |
| 2897 | Train 46 | Test White 17 | 0.72 | 0.75 | 0.75 | 3921 | Train 62 | Test White 17 | 0.71 | 0.72 | 0.68 |
| 2898 | Train 46 | Test White 18 | 0.74 | 0.76 | 0.75 | 3922 | Train 62 | Test White 18 | 0.73 | 0.73 | 0.68 |
| 2899 | Train 46 | Test White 19 | 0.72 | 0.75 | 0.75 | 3923 | Train 62 | Test White 19 | 0.71 | 0.72 | 0.68 |
| 2900 | Train 46 | Test White 20 | 0.74 | 0.76 | 0.75 | 3924 | Train 62 | Test White 20 | 0.73 | 0.73 | 0.68 |
| 2901 | Train 46 | Test White 21 | 0.72 | 0.77 | 0.77 | 3925 | Train 62 | Test White 21 | 0.71 | 0.74 | 0.69 |
| 2902 | Train 46 | Test White 22 | 0.73 | 0.77 | 0.78 | 3926 | Train 62 | Test White 22 | 0.72 | 0.75 | 0.71 |
| 2903 | Train 46 | Test White 23 | 0.72 | 0.77 | 0.77 | 3927 | Train 62 | Test White 23 | 0.71 | 0.73 | 0.69 |
| 2904 | Train 46 | Test White 24 | 0.73 | 0.77 | 0.78 | 3928 | Train 62 | Test White 24 | 0.72 | 0.74 | 0.71 |
| 2905 | Train 46 | Test White 25 | 0.72 | 0.75 | 0.76 | 3929 | Train 62 | Test White 25 | 0.71 | 0.72 | 0.69 |
| 2906 | Train 46 | Test White 26 | 0.74 | 0.76 | 0.76 | 3930 | Train 62 | Test White 26 | 0.73 | 0.73 | 0.69 |
| 2907 | Train 46 | Test White 27 | 0.72 | 0.75 | 0.76 | 3931 | Train 62 | Test White 27 | 0.71 | 0.72 | 0.69 |
| 2908 | Train 46 | Test White 28 | 0.74 | 0.76 | 0.76 | 3932 | Train 62 | Test White 28 | 0.73 | 0.73 | 0.69 |
| 2909 | Train 46 | Test White 29 | 0.72 | 0.77 | 0.78 | 3933 | Train 62 | Test White 29 | 0.71 | 0.74 | 0.7 |
| 2910 | Train 46 | Test White 30 | 0.73 | 0.77 | 0.78 | 3934 | Train 62 | Test White 30 | 0.73 | 0.75 | 0.72 |
| 2911 | Train 46 | Test White 31 | 0.72 | 0.77 | 0.78 | 3935 | Train 62 | Test White 31 | 0.71 | 0.74 | 0.7 |
| 2912 | Train 46 | Test White 32 | 0.73 | 0.77 | 0.78 | 3936 | Train 62 | Test White 32 | 0.72 | 0.74 | 0.72 |
| 2913 | Train 46 | Test White 33 | 0.73 | 0.75 | 0.75 | 3937 | Train 62 | Test White 33 | 0.71 | 0.72 | 0.68 |
| 2914 | Train 46 | Test White 34 | 0.74 | 0.76 | 0.76 | 3938 | Train 62 | Test White 34 | 0.73 | 0.73 | 0.68 |
| 2915 | Train 46 | Test White 35 | 0.73 | 0.75 | 0.75 | 3939 | Train 62 | Test White 35 | 0.71 | 0.72 | 0.68 |
| 2916 | Train 46 | Test White 36 | 0.74 | 0.76 | 0.76 | 3940 | Train 62 | Test White 36 | 0.73 | 0.73 | 0.68 |
| 2917 | Train 46 | Test White 37 | 0.72 | 0.77 | 0.77 | 3941 | Train 62 | Test White 37 | 0.71 | 0.74 | 0.69 |
| 2918 | Train 46 | Test White 38 | 0.73 | 0.77 | 0.78 | 3942 | Train 62 | Test White 38 | 0.72 | 0.75 | 0.71 |
| 2919 | Train 46 | Test White 39 | 0.72 | 0.77 | 0.77 | 3943 | Train 62 | Test White 39 | 0.71 | 0.74 | 0.69 |
| 2920 | Train 46 | Test White 40 | 0.73 | 0.77 | 0.78 | 3944 | Train 62 | Test White 40 | 0.72 | 0.74 | 0.71 |
| 2921 | Train 46 | Test White 41 | 0.73 | 0.76 | 0.76 | 3945 | Train 62 | Test White 41 | 0.71 | 0.73 | 0.69 |
| 2922 | Train 46 | Test White 42 | 0.74 | 0.76 | 0.76 | 3946 | Train 62 | Test White 42 | 0.73 | 0.73 | 0.69 |
| 2923 | Train 46 | Test White 43 | 0.73 | 0.76 | 0.76 | 3947 | Train 62 | Test White 43 | 0.71 | 0.73 | 0.69 |
| 2924 | Train 46 | Test White 44 | 0.74 | 0.76 | 0.76 | 3948 | Train 62 | Test White 44 | 0.73 | 0.73 | 0.69 |
| 2925 | Train 46 | Test White 45 | 0.72 | 0.77 | 0.78 | 3949 | Train 62 | Test White 45 | 0.71 | 0.74 | 0.7 |
| 2926 | Train 46 | Test White 46 | 0.73 | 0.78 | 0.78 | 3950 | Train 62 | Test White 46 | 0.72 | 0.75 | 0.72 |
| 2927 | Train 46 | Test White 47 | 0.72 | 0.77 | 0.78 | 3951 | Train 62 | Test White 47 | 0.71 | 0.74 | 0.7 |
| 2928 | Train 46 | Test White 48 | 0.73 | 0.77 | 0.78 | 3952 | Train 62 | Test White 48 | 0.72 | 0.74 | 0.72 |
| 2929 | Train 46 | Test White 49 | 0.72 | 0.76 | 0.75 | 3953 | Train 62 | Test White 49 | 0.73 | 0.76 | 0.75 |
| 2930 | Train 46 | Test White 50 | 0.72 | 0.76 | 0.75 | 3954 | Train 62 | Test White 50 | 0.73 | 0.76 | 0.75 |
| 2931 | Train 46 | Test White 51 | 0.72 | 0.76 | 0.75 | 3955 | Train 62 | Test White 51 | 0.73 | 0.76 | 0.75 |
| 2932 | Train 46 | Test White 52 | 0.73 | 0.76 | 0.75 | 3956 | Train 62 | Test White 52 | 0.73 | 0.76 | 0.75 |
| 2933 | Train 46 | Test White 53 | 0.73 | 0.78 | 0.76 | 3957 | Train 62 | Test White 53 | 0.74 | 0.8 | 0.73 |
| 2934 | Train 46 | Test White 54 | 0.74 | 0.77 | 0.78 | 3958 | Train 62 | Test White 54 | 0.74 | 0.79 | 0.8 |
| 2935 | Train 46 | Test White 55 | 0.73 | 0.78 | 0.76 | 3959 | Train 62 | Test White 55 | 0.74 | 0.79 | 0.73 |
| 2936 | Train 46 | Test White 56 | 0.73 | 0.76 | 0.77 | 3960 | Train 62 | Test White 56 | 0.74 | 0.78 | 0.79 |
| 2937 | Train 46 | Test White 57 | 0.73 | 0.76 | 0.77 | 3961 | Train 62 | Test White 57 | 0.74 | 0.77 | 0.77 |
| 2938 | Train 46 | Test White 58 | 0.73 | 0.76 | 0.77 | 3962 | Train 62 | Test White 58 | 0.74 | 0.77 | 0.77 |
| 2939 | Train 46 | Test White 59 | 0.73 | 0.76 | 0.77 | 3963 | Train 62 | Test White 59 | 0.74 | 0.76 | 0.77 |
| 2940 | Train 46 | Test White 60 | 0.73 | 0.76 | 0.77 | 3964 | Train 62 | Test White 60 | 0.74 | 0.76 | 0.77 |
| 2941 | Train 46 | Test White 61 | 0.73 | 0.78 | 0.77 | 3965 | Train 62 | Test White 61 | 0.74 | 0.8 | 0.74 |
| 2942 | Train 46 | Test White 62 | 0.74 | 0.77 | 0.78 | 3966 | Train 62 | Test White 62 | 0.74 | 0.79 | 0.8 |
| 2943 | Train 46 | Test White 63 | 0.73 | 0.78 | 0.77 | 3967 | Train 62 | Test White 63 | 0.74 | 0.8 | 0.74 |
| 2944 | Train 46 | Test White 64 | 0.73 | 0.76 | 0.77 | 3968 | Train 62 | Test White 64 | 0.74 | 0.78 | 0.79 |
| 2945 | Train 47 | Test White 1 | 0.74 | 0.78 | 0.78 | 3969 | Train 63 | Test White 1 | 0.73 | 0.72 | 0.69 |
| 2946 | Train 47 | Test White 2 | 0.74 | 0.76 | 0.76 | 3970 | Train 63 | Test White 2 | 0.72 | 0.7 | 0.66 |
| 2947 | Train 47 | Test White 3 | 0.74 | 0.78 | 0.78 | 3971 | Train 63 | Test White 3 | 0.73 | 0.73 | 0.69 |
| 2948 | Train 47 | Test White 4 | 0.74 | 0.77 | 0.76 | 3972 | Train 63 | Test White 4 | 0.72 | 0.7 | 0.66 |
| 2949 | Train 47 | Test White 5 | 0.73 | 0.77 | 0.72 | 3973 | Train 63 | Test White 5 | 0.72 | 0.74 | 0.7 |
| 2950 | Train 47 | Test White 6 | 0.7 | 0.71 | 0.68 | 3974 | Train 63 | Test White 6 | 0.7 | 0.69 | 0.64 |
| 2951 | Train 47 | Test White 7 | 0.73 | 0.77 | 0.71 | 3975 | Train 63 | Test White 7 | 0.72 | 0.74 | 0.7 |
| 2952 | Train 47 | Test White 8 | 0.7 | 0.71 | 0.68 | 3976 | Train 63 | Test White 8 | 0.7 | 0.69 | 0.64 |
| 2953 | Train 47 | Test White 9 | 0.74 | 0.79 | 0.8 | 3977 | Train 63 | Test White 9 | 0.73 | 0.73 | 0.7 |
| 2954 | Train 47 | Test White 10 | 0.74 | 0.77 | 0.78 | 3978 | Train 63 | Test White 10 | 0.72 | 0.71 | 0.67 |
| 2955 | Train 47 | Test White 11 | 0.74 | 0.79 | 0.8 | 3979 | Train 63 | Test White 11 | 0.73 | 0.73 | 0.7 |
| 2956 | Train 47 | Test White 12 | 0.74 | 0.77 | 0.78 | 3980 | Train 63 | Test White 12 | 0.72 | 0.71 | 0.67 |
| 2957 | Train 47 | Test White 13 | 0.73 | 0.78 | 0.73 | 3981 | Train 63 | Test White 13 | 0.72 | 0.75 | 0.72 |
| 2958 | Train 47 | Test White 14 | 0.7 | 0.71 | 0.68 | 3982 | Train 63 | Test White 14 | 0.7 | 0.69 | 0.64 |
| 2959 | Train 47 | Test White 15 | 0.73 | 0.78 | 0.73 | 3983 | Train 63 | Test White 15 | 0.72 | 0.75 | 0.72 |
| 2960 | Train 47 | Test White 16 | 0.7 | 0.71 | 0.68 | 3984 | Train 63 | Test White 16 | 0.7 | 0.69 | 0.65 |
| 2961 | Train 47 | Test White 17 | 0.74 | 0.78 | 0.78 | 3985 | Train 63 | Test White 17 | 0.73 | 0.72 | 0.69 |
| 2962 | Train 47 | Test White 18 | 0.74 | 0.76 | 0.76 | 3986 | Train 63 | Test White 18 | 0.72 | 0.7 | 0.66 |
| 2963 | Train 47 | Test White 19 | 0.74 | 0.78 | 0.78 | 3987 | Train 63 | Test White 19 | 0.73 | 0.73 | 0.69 |
| 2964 | Train 47 | Test White 20 | 0.74 | 0.77 | 0.76 | 3988 | Train 63 | Test White 20 | 0.72 | 0.7 | 0.66 |
| 2965 | Train 47 | Test White 21 | 0.73 | 0.77 | 0.72 | 3989 | Train 63 | Test White 21 | 0.72 | 0.74 | 0.7 |
| 2966 | Train 47 | Test White 22 | 0.7 | 0.71 | 0.68 | 3990 | Train 63 | Test White 22 | 0.7 | 0.69 | 0.64 |
| 2967 | Train 47 | Test White 23 | 0.73 | 0.77 | 0.72 | 3991 | Train 63 | Test White 23 | 0.72 | 0.74 | 0.7 |
| 2968 | Train 47 | Test White 24 | 0.7 | 0.71 | 0.68 | 3992 | Train 63 | Test White 24 | 0.7 | 0.69 | 0.64 |
| 2969 | Train 47 | Test White 25 | 0.74 | 0.79 | 0.8 | 3993 | Train 63 | Test White 25 | 0.73 | 0.73 | 0.7 |
| 2970 | Train 47 | Test White 26 | 0.74 | 0.77 | 0.78 | 3994 | Train 63 | Test White 26 | 0.72 | 0.71 | 0.67 |
| 2971 | Train 47 | Test White 27 | 0.74 | 0.79 | 0.8 | 3995 | Train 63 | Test White 27 | 0.73 | 0.73 | 0.7 |
| 2972 | Train 47 | Test White 28 | 0.74 | 0.77 | 0.78 | 3996 | Train 63 | Test White 28 | 0.72 | 0.71 | 0.67 |
| 2973 | Train 47 | Test White 29 | 0.73 | 0.78 | 0.73 | 3997 | Train 63 | Test White 29 | 0.72 | 0.75 | 0.72 |
| 2974 | Train 47 | Test White 30 | 0.7 | 0.71 | 0.68 | 3998 | Train 63 | Test White 30 | 0.7 | 0.69 | 0.64 |
| 2975 | Train 47 | Test White 31 | 0.73 | 0.78 | 0.73 | 3999 | Train 63 | Test White 31 | 0.72 | 0.75 | 0.72 |
| 2976 | Train 47 | Test White 32 | 0.7 | 0.71 | 0.68 | 4000 | Train 63 | Test White 32 | 0.7 | 0.69 | 0.65 |
| 2977 | Train 47 | Test White 33 | 0.74 | 0.78 | 0.78 | 4001 | Train 63 | Test White 33 | 0.73 | 0.73 | 0.69 |
| 2978 | Train 47 | Test White 34 | 0.74 | 0.77 | 0.76 | 4002 | Train 63 | Test White 34 | 0.72 | 0.7 | 0.66 |
| 2979 | Train 47 | Test White 35 | 0.74 | 0.78 | 0.78 | 4003 | Train 63 | Test White 35 | 0.73 | 0.73 | 0.69 |
| 2980 | Train 47 | Test White 36 | 0.74 | 0.77 | 0.76 | 4004 | Train 63 | Test White 36 | 0.72 | 0.7 | 0.66 |
| 2981 | Train 47 | Test White 37 | 0.73 | 0.77 | 0.72 | 4005 | Train 63 | Test White 37 | 0.72 | 0.74 | 0.7 |
| 2982 | Train 47 | Test White 38 | 0.7 | 0.71 | 0.68 | 4006 | Train 63 | Test White 38 | 0.7 | 0.69 | 0.64 |
| 2983 | Train 47 | Test White 39 | 0.73 | 0.77 | 0.72 | 4007 | Train 63 | Test White 39 | 0.72 | 0.74 | 0.7 |
| 2984 | Train 47 | Test White 40 | 0.7 | 0.71 | 0.68 | 4008 | Train 63 | Test White 40 | 0.7 | 0.69 | 0.65 |
| 2985 | Train 47 | Test White 41 | 0.74 | 0.79 | 0.8 | 4009 | Train 63 | Test White 41 | 0.73 | 0.73 | 0.7 |
| 2986 | Train 47 | Test White 42 | 0.74 | 0.77 | 0.78 | 4010 | Train 63 | Test White 42 | 0.73 | 0.71 | 0.67 |
| 2987 | Train 47 | Test White 43 | 0.74 | 0.79 | 0.8 | 4011 | Train 63 | Test White 43 | 0.73 | 0.73 | 0.7 |
| 2988 | Train 47 | Test White 44 | 0.74 | 0.77 | 0.78 | 4012 | Train 63 | Test White 44 | 0.73 | 0.71 | 0.67 |
| 2989 | Train 47 | Test White 45 | 0.73 | 0.78 | 0.73 | 4013 | Train 63 | Test White 45 | 0.72 | 0.75 | 0.72 |
| 2990 | Train 47 | Test White 46 | 0.7 | 0.71 | 0.68 | 4014 | Train 63 | Test White 46 | 0.7 | 0.69 | 0.64 |
| 2991 | Train 47 | Test White 47 | 0.73 | 0.78 | 0.73 | 4015 | Train 63 | Test White 47 | 0.72 | 0.75 | 0.72 |
| 2992 | Train 47 | Test White 48 | 0.7 | 0.71 | 0.68 | 4016 | Train 63 | Test White 48 | 0.7 | 0.69 | 0.65 |
| 2993 | Train 47 | Test White 49 | 0.73 | 0.74 | 0.75 | 4017 | Train 63 | Test White 49 | 0.73 | 0.75 | 0.75 |
| 2994 | Train 47 | Test White 50 | 0.72 | 0.74 | 0.75 | 4018 | Train 63 | Test White 50 | 0.73 | 0.75 | 0.75 |
| 2995 | Train 47 | Test White 51 | 0.73 | 0.74 | 0.75 | 4019 | Train 63 | Test White 51 | 0.73 | 0.75 | 0.75 |
| 2996 | Train 47 | Test White 52 | 0.73 | 0.74 | 0.75 | 4020 | Train 63 | Test White 52 | 0.73 | 0.75 | 0.75 |
| 2997 | Train 47 | Test White 53 | 0.73 | 0.77 | 0.69 | 4021 | Train 63 | Test White 53 | 0.75 | 0.81 | 0.78 |
| 2998 | Train 47 | Test White 54 | 0.71 | 0.71 | 0.67 | 4022 | Train 63 | Test White 54 | 0.7 | 0.7 | 0.66 |
| 2999 | Train 47 | Test White 55 | 0.73 | 0.77 | 0.68 | 4023 | Train 63 | Test White 55 | 0.75 | 0.81 | 0.78 |
| 3000 | Train 47 | Test White 56 | 0.7 | 0.71 | 0.67 | 4024 | Train 63 | Test White 56 | 0.7 | 0.7 | 0.67 |
| 3001 | Train 47 | Test White 57 | 0.73 | 0.74 | 0.78 | 4025 | Train 63 | Test White 57 | 0.74 | 0.76 | 0.79 |
| 3002 | Train 47 | Test White 58 | 0.73 | 0.74 | 0.78 | 4026 | Train 63 | Test White 58 | 0.74 | 0.76 | 0.79 |
| 3003 | Train 47 | Test White 59 | 0.73 | 0.75 | 0.78 | 4027 | Train 63 | Test White 59 | 0.74 | 0.76 | 0.79 |
| 3004 | Train 47 | Test White 60 | 0.73 | 0.75 | 0.78 | 4028 | Train 63 | Test White 60 | 0.74 | 0.76 | 0.79 |
| 3005 | Train 47 | Test White 61 | 0.74 | 0.78 | 0.7 | 4029 | Train 63 | Test White 61 | 0.75 | 0.82 | 0.81 |
| 3006 | Train 47 | Test White 62 | 0.71 | 0.71 | 0.67 | 4030 | Train 63 | Test White 62 | 0.71 | 0.7 | 0.67 |
| 3007 | Train 47 | Test White 63 | 0.74 | 0.78 | 0.7 | 4031 | Train 63 | Test White 63 | 0.75 | 0.82 | 0.81 |
| 3008 | Train 47 | Test White 64 | 0.71 | 0.71 | 0.67 | 4032 | Train 63 | Test White 64 | 0.7 | 0.7 | 0.67 |
| 3009 | Train 48 | Test White 1 | 0.73 | 0.75 | 0.75 | 4033 | Train 64 | Test White 1 | 0.71 | 0.72 | 0.69 |
| 3010 | Train 48 | Test White 2 | 0.74 | 0.76 | 0.76 | 4034 | Train 64 | Test White 2 | 0.72 | 0.73 | 0.69 |
| 3011 | Train 48 | Test White 3 | 0.73 | 0.75 | 0.75 | 4035 | Train 64 | Test White 3 | 0.71 | 0.72 | 0.69 |
| 3012 | Train 48 | Test White 4 | 0.74 | 0.76 | 0.76 | 4036 | Train 64 | Test White 4 | 0.72 | 0.73 | 0.69 |
| 3013 | Train 48 | Test White 5 | 0.72 | 0.77 | 0.77 | 4037 | Train 64 | Test White 5 | 0.7 | 0.74 | 0.7 |
| 3014 | Train 48 | Test White 6 | 0.73 | 0.77 | 0.78 | 4038 | Train 64 | Test White 6 | 0.71 | 0.74 | 0.71 |
| 3015 | Train 48 | Test White 7 | 0.72 | 0.77 | 0.77 | 4039 | Train 64 | Test White 7 | 0.7 | 0.74 | 0.7 |
| 3016 | Train 48 | Test White 8 | 0.73 | 0.77 | 0.78 | 4040 | Train 64 | Test White 8 | 0.71 | 0.74 | 0.71 |
| 3017 | Train 48 | Test White 9 | 0.73 | 0.75 | 0.76 | 4041 | Train 64 | Test White 9 | 0.71 | 0.73 | 0.7 |
| 3018 | Train 48 | Test White 10 | 0.74 | 0.76 | 0.76 | 4042 | Train 64 | Test White 10 | 0.72 | 0.73 | 0.69 |
| 3019 | Train 48 | Test White 11 | 0.73 | 0.75 | 0.76 | 4043 | Train 64 | Test White 11 | 0.71 | 0.73 | 0.7 |
| 3020 | Train 48 | Test White 12 | 0.74 | 0.76 | 0.76 | 4044 | Train 64 | Test White 12 | 0.72 | 0.73 | 0.69 |
| 3021 | Train 48 | Test White 13 | 0.72 | 0.77 | 0.78 | 4045 | Train 64 | Test White 13 | 0.7 | 0.74 | 0.71 |
| 3022 | Train 48 | Test White 14 | 0.73 | 0.77 | 0.78 | 4046 | Train 64 | Test White 14 | 0.71 | 0.74 | 0.71 |
| 3023 | Train 48 | Test White 15 | 0.72 | 0.77 | 0.78 | 4047 | Train 64 | Test White 15 | 0.7 | 0.74 | 0.71 |
| 3024 | Train 48 | Test White 16 | 0.73 | 0.77 | 0.78 | 4048 | Train 64 | Test White 16 | 0.72 | 0.75 | 0.71 |
| 3025 | Train 48 | Test White 17 | 0.73 | 0.75 | 0.75 | 4049 | Train 64 | Test White 17 | 0.71 | 0.72 | 0.69 |
| 3026 | Train 48 | Test White 18 | 0.74 | 0.76 | 0.76 | 4050 | Train 64 | Test White 18 | 0.72 | 0.73 | 0.69 |
| 3027 | Train 48 | Test White 19 | 0.73 | 0.75 | 0.75 | 4051 | Train 64 | Test White 19 | 0.71 | 0.72 | 0.69 |
| 3028 | Train 48 | Test White 20 | 0.74 | 0.76 | 0.75 | 4052 | Train 64 | Test White 20 | 0.72 | 0.73 | 0.69 |
| 3029 | Train 48 | Test White 21 | 0.72 | 0.77 | 0.77 | 4053 | Train 64 | Test White 21 | 0.7 | 0.74 | 0.7 |
| 3030 | Train 48 | Test White 22 | 0.73 | 0.77 | 0.78 | 4054 | Train 64 | Test White 22 | 0.71 | 0.74 | 0.71 |
| 3031 | Train 48 | Test White 23 | 0.72 | 0.77 | 0.77 | 4055 | Train 64 | Test White 23 | 0.7 | 0.74 | 0.7 |
| 3032 | Train 48 | Test White 24 | 0.73 | 0.77 | 0.78 | 4056 | Train 64 | Test White 24 | 0.71 | 0.74 | 0.71 |
| 3033 | Train 48 | Test White 25 | 0.73 | 0.75 | 0.76 | 4057 | Train 64 | Test White 25 | 0.71 | 0.73 | 0.7 |
| 3034 | Train 48 | Test White 26 | 0.74 | 0.76 | 0.76 | 4058 | Train 64 | Test White 26 | 0.72 | 0.73 | 0.69 |
| 3035 | Train 48 | Test White 27 | 0.73 | 0.75 | 0.76 | 4059 | Train 64 | Test White 27 | 0.71 | 0.73 | 0.7 |
| 3036 | Train 48 | Test White 28 | 0.74 | 0.76 | 0.76 | 4060 | Train 64 | Test White 28 | 0.72 | 0.73 | 0.69 |
| 3037 | Train 48 | Test White 29 | 0.72 | 0.77 | 0.78 | 4061 | Train 64 | Test White 29 | 0.7 | 0.74 | 0.71 |
| 3038 | Train 48 | Test White 30 | 0.73 | 0.77 | 0.78 | 4062 | Train 64 | Test White 30 | 0.71 | 0.74 | 0.71 |
| 3039 | Train 48 | Test White 31 | 0.72 | 0.77 | 0.78 | 4063 | Train 64 | Test White 31 | 0.7 | 0.74 | 0.71 |
| 3040 | Train 48 | Test White 32 | 0.73 | 0.77 | 0.78 | 4064 | Train 64 | Test White 32 | 0.72 | 0.75 | 0.71 |
| 3041 | Train 48 | Test White 33 | 0.73 | 0.75 | 0.75 | 4065 | Train 64 | Test White 33 | 0.71 | 0.72 | 0.69 |
| 3042 | Train 48 | Test White 34 | 0.74 | 0.76 | 0.76 | 4066 | Train 64 | Test White 34 | 0.72 | 0.73 | 0.69 |
| 3043 | Train 48 | Test White 35 | 0.73 | 0.75 | 0.75 | 4067 | Train 64 | Test White 35 | 0.71 | 0.72 | 0.69 |
| 3044 | Train 48 | Test White 36 | 0.74 | 0.76 | 0.76 | 4068 | Train 64 | Test White 36 | 0.72 | 0.73 | 0.69 |
| 3045 | Train 48 | Test White 37 | 0.72 | 0.77 | 0.77 | 4069 | Train 64 | Test White 37 | 0.7 | 0.74 | 0.7 |
| 3046 | Train 48 | Test White 38 | 0.73 | 0.77 | 0.78 | 4070 | Train 64 | Test White 38 | 0.71 | 0.74 | 0.71 |
| 3047 | Train 48 | Test White 39 | 0.72 | 0.77 | 0.77 | 4071 | Train 64 | Test White 39 | 0.7 | 0.74 | 0.7 |
| 3048 | Train 48 | Test White 40 | 0.73 | 0.77 | 0.78 | 4072 | Train 64 | Test White 40 | 0.71 | 0.75 | 0.71 |
| 3049 | Train 48 | Test White 41 | 0.73 | 0.76 | 0.76 | 4073 | Train 64 | Test White 41 | 0.71 | 0.73 | 0.7 |
| 3050 | Train 48 | Test White 42 | 0.74 | 0.76 | 0.76 | 4074 | Train 64 | Test White 42 | 0.72 | 0.73 | 0.69 |
| 3051 | Train 48 | Test White 43 | 0.73 | 0.76 | 0.76 | 4075 | Train 64 | Test White 43 | 0.71 | 0.73 | 0.7 |
| 3052 | Train 48 | Test White 44 | 0.74 | 0.76 | 0.76 | 4076 | Train 64 | Test White 44 | 0.72 | 0.73 | 0.69 |
| 3053 | Train 48 | Test White 45 | 0.72 | 0.77 | 0.78 | 4077 | Train 64 | Test White 45 | 0.7 | 0.74 | 0.71 |
| 3054 | Train 48 | Test White 46 | 0.73 | 0.77 | 0.78 | 4078 | Train 64 | Test White 46 | 0.71 | 0.74 | 0.71 |
| 3055 | Train 48 | Test White 47 | 0.72 | 0.77 | 0.78 | 4079 | Train 64 | Test White 47 | 0.7 | 0.74 | 0.71 |
| 3056 | Train 48 | Test White 48 | 0.73 | 0.77 | 0.78 | 4080 | Train 64 | Test White 48 | 0.72 | 0.75 | 0.71 |
| 3057 | Train 48 | Test White 49 | 0.73 | 0.76 | 0.75 | 4081 | Train 64 | Test White 49 | 0.74 | 0.76 | 0.74 |
| 3058 | Train 48 | Test White 50 | 0.73 | 0.76 | 0.75 | 4082 | Train 64 | Test White 50 | 0.74 | 0.76 | 0.74 |
| 3059 | Train 48 | Test White 51 | 0.73 | 0.76 | 0.75 | 4083 | Train 64 | Test White 51 | 0.74 | 0.76 | 0.74 |
| 3060 | Train 48 | Test White 52 | 0.73 | 0.76 | 0.75 | 4084 | Train 64 | Test White 52 | 0.74 | 0.76 | 0.74 |
| 3061 | Train 48 | Test White 53 | 0.73 | 0.78 | 0.76 | 4085 | Train 64 | Test White 53 | 0.74 | 0.8 | 0.74 |
| 3062 | Train 48 | Test White 54 | 0.73 | 0.77 | 0.78 | 4086 | Train 64 | Test White 54 | 0.74 | 0.79 | 0.8 |
| 3063 | Train 48 | Test White 55 | 0.73 | 0.78 | 0.76 | 4087 | Train 64 | Test White 55 | 0.74 | 0.8 | 0.74 |
| 3064 | Train 48 | Test White 56 | 0.73 | 0.77 | 0.78 | 4088 | Train 64 | Test White 56 | 0.74 | 0.79 | 0.8 |
| 3065 | Train 48 | Test White 57 | 0.73 | 0.76 | 0.76 | 4089 | Train 64 | Test White 57 | 0.74 | 0.77 | 0.76 |
| 3066 | Train 48 | Test White 58 | 0.73 | 0.76 | 0.76 | 4090 | Train 64 | Test White 58 | 0.74 | 0.77 | 0.76 |
| 3067 | Train 48 | Test White 59 | 0.73 | 0.76 | 0.76 | 4091 | Train 64 | Test White 59 | 0.74 | 0.77 | 0.76 |
| 3068 | Train 48 | Test White 60 | 0.73 | 0.76 | 0.77 | 4092 | Train 64 | Test White 60 | 0.74 | 0.77 | 0.76 |
| 3069 | Train 48 | Test White 61 | 0.73 | 0.78 | 0.76 | 4093 | Train 64 | Test White 61 | 0.74 | 0.8 | 0.75 |
| 3070 | Train 48 | Test White 62 | 0.74 | 0.77 | 0.78 | 4094 | Train 64 | Test White 62 | 0.74 | 0.79 | 0.8 |
| 3071 | Train 48 | Test White 63 | 0.73 | 0.78 | 0.76 | 4095 | Train 64 | Test White 63 | 0.74 | 0.8 | 0.75 |
| 3072 | Train 48 | Test White 64 | 0.73 | 0.77 | 0.78 | 4096 | Train 64 | Test White 64 | 0.74 | 0.79 | 0.8 |

Table 9. AUC Performance of the ML Models— LR, RF, and GB —for Asian Patients Trained on Cohorts 1–32 and Tested Across 64 Cohorts.

| Asian | Train  Model | Test  Dataset | AUC (LR) | AUC (RF) | AUC (GB) | Asian | Train  Model | Test  Dataset | AUC (LR) | AUC (RF) | AUC (GB) |
| --- | --- | --- | --- | --- | --- | --- | --- | --- | --- | --- | --- |
| 1 | Train 1 | Test Asian 1 | 0.8 | 0.93 | 0.93 | 1025 | Train 17 | Test Asian 1 | 0.8 | 0.92 | 0.92 |
| 2 | Train 1 | Test Asian 2 | 0.8 | 0.9 | 0.9 | 1026 | Train 17 | Test Asian 2 | 0.8 | 0.9 | 0.88 |
| 3 | Train 1 | Test Asian 3 | 0.8 | 0.93 | 0.93 | 1027 | Train 17 | Test Asian 3 | 0.8 | 0.92 | 0.92 |
| 4 | Train 1 | Test Asian 4 | 0.8 | 0.9 | 0.9 | 1028 | Train 17 | Test Asian 4 | 0.8 | 0.9 | 0.88 |
| 5 | Train 1 | Test Asian 5 | 0.75 | 0.76 | 0.7 | 1029 | Train 17 | Test Asian 5 | 0.75 | 0.76 | 0.69 |
| 6 | Train 1 | Test Asian 6 | 0.75 | 0.75 | 0.69 | 1030 | Train 17 | Test Asian 6 | 0.75 | 0.75 | 0.68 |
| 7 | Train 1 | Test Asian 7 | 0.74 | 0.75 | 0.7 | 1031 | Train 17 | Test Asian 7 | 0.74 | 0.75 | 0.69 |
| 8 | Train 1 | Test Asian 8 | 0.75 | 0.74 | 0.7 | 1032 | Train 17 | Test Asian 8 | 0.75 | 0.75 | 0.68 |
| 9 | Train 1 | Test Asian 9 | 0.79 | 0.9 | 0.87 | 1033 | Train 17 | Test Asian 9 | 0.79 | 0.9 | 0.87 |
| 10 | Train 1 | Test Asian 10 | 0.79 | 0.88 | 0.85 | 1034 | Train 17 | Test Asian 10 | 0.79 | 0.87 | 0.83 |
| 11 | Train 1 | Test Asian 11 | 0.79 | 0.9 | 0.87 | 1035 | Train 17 | Test Asian 11 | 0.79 | 0.9 | 0.87 |
| 12 | Train 1 | Test Asian 12 | 0.79 | 0.88 | 0.85 | 1036 | Train 17 | Test Asian 12 | 0.79 | 0.87 | 0.83 |
| 13 | Train 1 | Test Asian 13 | 0.74 | 0.75 | 0.7 | 1037 | Train 17 | Test Asian 13 | 0.74 | 0.75 | 0.68 |
| 14 | Train 1 | Test Asian 14 | 0.75 | 0.73 | 0.59 | 1038 | Train 17 | Test Asian 14 | 0.75 | 0.73 | 0.6 |
| 15 | Train 1 | Test Asian 15 | 0.74 | 0.75 | 0.69 | 1039 | Train 17 | Test Asian 15 | 0.74 | 0.75 | 0.68 |
| 16 | Train 1 | Test Asian 16 | 0.75 | 0.74 | 0.69 | 1040 | Train 17 | Test Asian 16 | 0.75 | 0.75 | 0.68 |
| 17 | Train 1 | Test Asian 17 | 0.8 | 0.93 | 0.93 | 1041 | Train 17 | Test Asian 17 | 0.8 | 0.92 | 0.92 |
| 18 | Train 1 | Test Asian 18 | 0.8 | 0.9 | 0.9 | 1042 | Train 17 | Test Asian 18 | 0.8 | 0.9 | 0.88 |
| 19 | Train 1 | Test Asian 19 | 0.8 | 0.93 | 0.93 | 1043 | Train 17 | Test Asian 19 | 0.8 | 0.92 | 0.92 |
| 20 | Train 1 | Test Asian 20 | 0.8 | 0.9 | 0.9 | 1044 | Train 17 | Test Asian 20 | 0.8 | 0.9 | 0.88 |
| 21 | Train 1 | Test Asian 21 | 0.75 | 0.76 | 0.7 | 1045 | Train 17 | Test Asian 21 | 0.75 | 0.76 | 0.69 |
| 22 | Train 1 | Test Asian 22 | 0.75 | 0.75 | 0.69 | 1046 | Train 17 | Test Asian 22 | 0.75 | 0.75 | 0.68 |
| 23 | Train 1 | Test Asian 23 | 0.74 | 0.76 | 0.7 | 1047 | Train 17 | Test Asian 23 | 0.74 | 0.75 | 0.69 |
| 24 | Train 1 | Test Asian 24 | 0.75 | 0.75 | 0.7 | 1048 | Train 17 | Test Asian 24 | 0.75 | 0.75 | 0.68 |
| 25 | Train 1 | Test Asian 25 | 0.79 | 0.9 | 0.87 | 1049 | Train 17 | Test Asian 25 | 0.79 | 0.9 | 0.87 |
| 26 | Train 1 | Test Asian 26 | 0.79 | 0.88 | 0.85 | 1050 | Train 17 | Test Asian 26 | 0.79 | 0.87 | 0.83 |
| 27 | Train 1 | Test Asian 27 | 0.79 | 0.9 | 0.87 | 1051 | Train 17 | Test Asian 27 | 0.79 | 0.9 | 0.87 |
| 28 | Train 1 | Test Asian 28 | 0.79 | 0.88 | 0.85 | 1052 | Train 17 | Test Asian 28 | 0.79 | 0.87 | 0.83 |
| 29 | Train 1 | Test Asian 29 | 0.74 | 0.75 | 0.7 | 1053 | Train 17 | Test Asian 29 | 0.74 | 0.75 | 0.68 |
| 30 | Train 1 | Test Asian 30 | 0.75 | 0.73 | 0.59 | 1054 | Train 17 | Test Asian 30 | 0.75 | 0.73 | 0.6 |
| 31 | Train 1 | Test Asian 31 | 0.74 | 0.75 | 0.69 | 1055 | Train 17 | Test Asian 31 | 0.74 | 0.75 | 0.68 |
| 32 | Train 1 | Test Asian 32 | 0.75 | 0.75 | 0.69 | 1056 | Train 17 | Test Asian 32 | 0.75 | 0.75 | 0.68 |
| 33 | Train 1 | Test Asian 33 | 0.8 | 0.92 | 0.93 | 1057 | Train 17 | Test Asian 33 | 0.8 | 0.92 | 0.92 |
| 34 | Train 1 | Test Asian 34 | 0.8 | 0.9 | 0.9 | 1058 | Train 17 | Test Asian 34 | 0.8 | 0.9 | 0.88 |
| 35 | Train 1 | Test Asian 35 | 0.8 | 0.92 | 0.93 | 1059 | Train 17 | Test Asian 35 | 0.8 | 0.92 | 0.92 |
| 36 | Train 1 | Test Asian 36 | 0.8 | 0.9 | 0.9 | 1060 | Train 17 | Test Asian 36 | 0.8 | 0.9 | 0.88 |
| 37 | Train 1 | Test Asian 37 | 0.75 | 0.75 | 0.7 | 1061 | Train 17 | Test Asian 37 | 0.75 | 0.75 | 0.69 |
| 38 | Train 1 | Test Asian 38 | 0.75 | 0.74 | 0.69 | 1062 | Train 17 | Test Asian 38 | 0.75 | 0.75 | 0.68 |
| 39 | Train 1 | Test Asian 39 | 0.74 | 0.75 | 0.7 | 1063 | Train 17 | Test Asian 39 | 0.74 | 0.75 | 0.69 |
| 40 | Train 1 | Test Asian 40 | 0.75 | 0.74 | 0.7 | 1064 | Train 17 | Test Asian 40 | 0.75 | 0.74 | 0.68 |
| 41 | Train 1 | Test Asian 41 | 0.79 | 0.9 | 0.87 | 1065 | Train 17 | Test Asian 41 | 0.79 | 0.9 | 0.87 |
| 42 | Train 1 | Test Asian 42 | 0.79 | 0.88 | 0.85 | 1066 | Train 17 | Test Asian 42 | 0.79 | 0.87 | 0.83 |
| 43 | Train 1 | Test Asian 43 | 0.79 | 0.9 | 0.87 | 1067 | Train 17 | Test Asian 43 | 0.79 | 0.9 | 0.87 |
| 44 | Train 1 | Test Asian 44 | 0.79 | 0.88 | 0.85 | 1068 | Train 17 | Test Asian 44 | 0.79 | 0.87 | 0.83 |
| 45 | Train 1 | Test Asian 45 | 0.74 | 0.75 | 0.69 | 1069 | Train 17 | Test Asian 45 | 0.74 | 0.75 | 0.68 |
| 46 | Train 1 | Test Asian 46 | 0.75 | 0.74 | 0.69 | 1070 | Train 17 | Test Asian 46 | 0.75 | 0.75 | 0.68 |
| 47 | Train 1 | Test Asian 47 | 0.74 | 0.74 | 0.69 | 1071 | Train 17 | Test Asian 47 | 0.74 | 0.75 | 0.68 |
| 48 | Train 1 | Test Asian 48 | 0.75 | 0.74 | 0.69 | 1072 | Train 17 | Test Asian 48 | 0.75 | 0.74 | 0.68 |
| 49 | Train 1 | Test Asian 49 | 0.77 | 0.88 | 0.91 | 1073 | Train 17 | Test Asian 49 | 0.77 | 0.88 | 0.87 |
| 50 | Train 1 | Test Asian 50 | 0.77 | 0.88 | 0.91 | 1074 | Train 17 | Test Asian 50 | 0.77 | 0.88 | 0.87 |
| 51 | Train 1 | Test Asian 51 | 0.77 | 0.88 | 0.91 | 1075 | Train 17 | Test Asian 51 | 0.77 | 0.88 | 0.87 |
| 52 | Train 1 | Test Asian 52 | 0.77 | 0.88 | 0.91 | 1076 | Train 17 | Test Asian 52 | 0.77 | 0.88 | 0.87 |
| 53 | Train 1 | Test Asian 53 | 0.79 | 0.8 | 0.69 | 1077 | Train 17 | Test Asian 53 | 0.79 | 0.8 | 0.62 |
| 54 | Train 1 | Test Asian 54 | 0.78 | 0.75 | 0.61 | 1078 | Train 17 | Test Asian 54 | 0.78 | 0.75 | 0.6 |
| 55 | Train 1 | Test Asian 55 | 0.79 | 0.8 | 0.68 | 1079 | Train 17 | Test Asian 55 | 0.79 | 0.8 | 0.62 |
| 56 | Train 1 | Test Asian 56 | 0.78 | 0.74 | 0.61 | 1080 | Train 17 | Test Asian 56 | 0.78 | 0.75 | 0.6 |
| 57 | Train 1 | Test Asian 57 | 0.76 | 0.85 | 0.82 | 1081 | Train 17 | Test Asian 57 | 0.76 | 0.84 | 0.82 |
| 58 | Train 1 | Test Asian 58 | 0.76 | 0.85 | 0.83 | 1082 | Train 17 | Test Asian 58 | 0.76 | 0.84 | 0.82 |
| 59 | Train 1 | Test Asian 59 | 0.76 | 0.85 | 0.82 | 1083 | Train 17 | Test Asian 59 | 0.76 | 0.84 | 0.82 |
| 60 | Train 1 | Test Asian 60 | 0.76 | 0.85 | 0.83 | 1084 | Train 17 | Test Asian 60 | 0.76 | 0.84 | 0.82 |
| 61 | Train 1 | Test Asian 61 | 0.79 | 0.79 | 0.68 | 1085 | Train 17 | Test Asian 61 | 0.79 | 0.79 | 0.59 |
| 62 | Train 1 | Test Asian 62 | 0.78 | 0.75 | 0.61 | 1086 | Train 17 | Test Asian 62 | 0.78 | 0.75 | 0.6 |
| 63 | Train 1 | Test Asian 63 | 0.78 | 0.79 | 0.67 | 1087 | Train 17 | Test Asian 63 | 0.78 | 0.79 | 0.59 |
| 64 | Train 1 | Test Asian 64 | 0.78 | 0.74 | 0.61 | 1088 | Train 17 | Test Asian 64 | 0.78 | 0.74 | 0.6 |
| 65 | Train 2 | Test Asian 1 | 0.79 | 0.91 | 0.92 | 1089 | Train 18 | Test Asian 1 | 0.79 | 0.91 | 0.91 |
| 66 | Train 2 | Test Asian 2 | 0.81 | 0.92 | 0.93 | 1090 | Train 18 | Test Asian 2 | 0.81 | 0.92 | 0.92 |
| 67 | Train 2 | Test Asian 3 | 0.79 | 0.91 | 0.92 | 1091 | Train 18 | Test Asian 3 | 0.79 | 0.91 | 0.91 |
| 68 | Train 2 | Test Asian 4 | 0.81 | 0.92 | 0.93 | 1092 | Train 18 | Test Asian 4 | 0.81 | 0.92 | 0.92 |
| 69 | Train 2 | Test Asian 5 | 0.75 | 0.76 | 0.75 | 1093 | Train 18 | Test Asian 5 | 0.75 | 0.76 | 0.76 |
| 70 | Train 2 | Test Asian 6 | 0.75 | 0.75 | 0.72 | 1094 | Train 18 | Test Asian 6 | 0.75 | 0.75 | 0.7 |
| 71 | Train 2 | Test Asian 7 | 0.75 | 0.76 | 0.74 | 1095 | Train 18 | Test Asian 7 | 0.75 | 0.76 | 0.75 |
| 72 | Train 2 | Test Asian 8 | 0.75 | 0.75 | 0.71 | 1096 | Train 18 | Test Asian 8 | 0.75 | 0.75 | 0.71 |
| 73 | Train 2 | Test Asian 9 | 0.78 | 0.89 | 0.88 | 1097 | Train 18 | Test Asian 9 | 0.78 | 0.89 | 0.87 |
| 74 | Train 2 | Test Asian 10 | 0.8 | 0.9 | 0.89 | 1098 | Train 18 | Test Asian 10 | 0.8 | 0.9 | 0.88 |
| 75 | Train 2 | Test Asian 11 | 0.78 | 0.89 | 0.88 | 1099 | Train 18 | Test Asian 11 | 0.78 | 0.89 | 0.87 |
| 76 | Train 2 | Test Asian 12 | 0.8 | 0.9 | 0.89 | 1100 | Train 18 | Test Asian 12 | 0.8 | 0.9 | 0.88 |
| 77 | Train 2 | Test Asian 13 | 0.75 | 0.76 | 0.73 | 1101 | Train 18 | Test Asian 13 | 0.75 | 0.76 | 0.74 |
| 78 | Train 2 | Test Asian 14 | 0.75 | 0.74 | 0.57 | 1102 | Train 18 | Test Asian 14 | 0.75 | 0.73 | 0.56 |
| 79 | Train 2 | Test Asian 15 | 0.75 | 0.75 | 0.73 | 1103 | Train 18 | Test Asian 15 | 0.75 | 0.75 | 0.73 |
| 80 | Train 2 | Test Asian 16 | 0.75 | 0.75 | 0.71 | 1104 | Train 18 | Test Asian 16 | 0.75 | 0.75 | 0.71 |
| 81 | Train 2 | Test Asian 17 | 0.79 | 0.91 | 0.92 | 1105 | Train 18 | Test Asian 17 | 0.79 | 0.91 | 0.91 |
| 82 | Train 2 | Test Asian 18 | 0.81 | 0.92 | 0.93 | 1106 | Train 18 | Test Asian 18 | 0.81 | 0.92 | 0.92 |
| 83 | Train 2 | Test Asian 19 | 0.79 | 0.91 | 0.92 | 1107 | Train 18 | Test Asian 19 | 0.79 | 0.91 | 0.91 |
| 84 | Train 2 | Test Asian 20 | 0.81 | 0.92 | 0.93 | 1108 | Train 18 | Test Asian 20 | 0.81 | 0.92 | 0.92 |
| 85 | Train 2 | Test Asian 21 | 0.75 | 0.76 | 0.75 | 1109 | Train 18 | Test Asian 21 | 0.75 | 0.76 | 0.76 |
| 86 | Train 2 | Test Asian 22 | 0.75 | 0.75 | 0.72 | 1110 | Train 18 | Test Asian 22 | 0.75 | 0.75 | 0.71 |
| 87 | Train 2 | Test Asian 23 | 0.75 | 0.76 | 0.74 | 1111 | Train 18 | Test Asian 23 | 0.75 | 0.76 | 0.75 |
| 88 | Train 2 | Test Asian 24 | 0.75 | 0.75 | 0.72 | 1112 | Train 18 | Test Asian 24 | 0.75 | 0.75 | 0.71 |
| 89 | Train 2 | Test Asian 25 | 0.78 | 0.89 | 0.88 | 1113 | Train 18 | Test Asian 25 | 0.78 | 0.89 | 0.87 |
| 90 | Train 2 | Test Asian 26 | 0.8 | 0.9 | 0.89 | 1114 | Train 18 | Test Asian 26 | 0.8 | 0.9 | 0.88 |
| 91 | Train 2 | Test Asian 27 | 0.78 | 0.89 | 0.88 | 1115 | Train 18 | Test Asian 27 | 0.78 | 0.89 | 0.87 |
| 92 | Train 2 | Test Asian 28 | 0.8 | 0.9 | 0.89 | 1116 | Train 18 | Test Asian 28 | 0.8 | 0.9 | 0.88 |
| 93 | Train 2 | Test Asian 29 | 0.75 | 0.76 | 0.73 | 1117 | Train 18 | Test Asian 29 | 0.75 | 0.76 | 0.74 |
| 94 | Train 2 | Test Asian 30 | 0.75 | 0.74 | 0.57 | 1118 | Train 18 | Test Asian 30 | 0.75 | 0.73 | 0.56 |
| 95 | Train 2 | Test Asian 31 | 0.75 | 0.75 | 0.73 | 1119 | Train 18 | Test Asian 31 | 0.75 | 0.75 | 0.73 |
| 96 | Train 2 | Test Asian 32 | 0.75 | 0.75 | 0.72 | 1120 | Train 18 | Test Asian 32 | 0.75 | 0.75 | 0.71 |
| 97 | Train 2 | Test Asian 33 | 0.79 | 0.91 | 0.92 | 1121 | Train 18 | Test Asian 33 | 0.79 | 0.91 | 0.91 |
| 98 | Train 2 | Test Asian 34 | 0.81 | 0.92 | 0.93 | 1122 | Train 18 | Test Asian 34 | 0.81 | 0.92 | 0.92 |
| 99 | Train 2 | Test Asian 35 | 0.79 | 0.91 | 0.92 | 1123 | Train 18 | Test Asian 35 | 0.79 | 0.91 | 0.91 |
| 100 | Train 2 | Test Asian 36 | 0.81 | 0.92 | 0.93 | 1124 | Train 18 | Test Asian 36 | 0.81 | 0.92 | 0.92 |
| 101 | Train 2 | Test Asian 37 | 0.75 | 0.76 | 0.75 | 1125 | Train 18 | Test Asian 37 | 0.75 | 0.76 | 0.76 |
| 102 | Train 2 | Test Asian 38 | 0.74 | 0.75 | 0.71 | 1126 | Train 18 | Test Asian 38 | 0.74 | 0.75 | 0.7 |
| 103 | Train 2 | Test Asian 39 | 0.75 | 0.76 | 0.74 | 1127 | Train 18 | Test Asian 39 | 0.75 | 0.76 | 0.75 |
| 104 | Train 2 | Test Asian 40 | 0.74 | 0.75 | 0.71 | 1128 | Train 18 | Test Asian 40 | 0.74 | 0.74 | 0.71 |
| 105 | Train 2 | Test Asian 41 | 0.78 | 0.89 | 0.88 | 1129 | Train 18 | Test Asian 41 | 0.78 | 0.89 | 0.87 |
| 106 | Train 2 | Test Asian 42 | 0.8 | 0.9 | 0.88 | 1130 | Train 18 | Test Asian 42 | 0.8 | 0.9 | 0.88 |
| 107 | Train 2 | Test Asian 43 | 0.78 | 0.89 | 0.88 | 1131 | Train 18 | Test Asian 43 | 0.78 | 0.89 | 0.87 |
| 108 | Train 2 | Test Asian 44 | 0.8 | 0.9 | 0.88 | 1132 | Train 18 | Test Asian 44 | 0.8 | 0.9 | 0.88 |
| 109 | Train 2 | Test Asian 45 | 0.75 | 0.76 | 0.73 | 1133 | Train 18 | Test Asian 45 | 0.75 | 0.76 | 0.74 |
| 110 | Train 2 | Test Asian 46 | 0.74 | 0.75 | 0.72 | 1134 | Train 18 | Test Asian 46 | 0.74 | 0.75 | 0.71 |
| 111 | Train 2 | Test Asian 47 | 0.75 | 0.75 | 0.73 | 1135 | Train 18 | Test Asian 47 | 0.75 | 0.75 | 0.73 |
| 112 | Train 2 | Test Asian 48 | 0.75 | 0.75 | 0.71 | 1136 | Train 18 | Test Asian 48 | 0.75 | 0.74 | 0.71 |
| 113 | Train 2 | Test Asian 49 | 0.77 | 0.86 | 0.85 | 1137 | Train 18 | Test Asian 49 | 0.77 | 0.86 | 0.87 |
| 114 | Train 2 | Test Asian 50 | 0.77 | 0.86 | 0.85 | 1138 | Train 18 | Test Asian 50 | 0.77 | 0.86 | 0.87 |
| 115 | Train 2 | Test Asian 51 | 0.77 | 0.86 | 0.85 | 1139 | Train 18 | Test Asian 51 | 0.77 | 0.86 | 0.87 |
| 116 | Train 2 | Test Asian 52 | 0.77 | 0.86 | 0.85 | 1140 | Train 18 | Test Asian 52 | 0.77 | 0.86 | 0.87 |
| 117 | Train 2 | Test Asian 53 | 0.79 | 0.81 | 0.68 | 1141 | Train 18 | Test Asian 53 | 0.79 | 0.81 | 0.62 |
| 118 | Train 2 | Test Asian 54 | 0.77 | 0.75 | 0.59 | 1142 | Train 18 | Test Asian 54 | 0.77 | 0.74 | 0.55 |
| 119 | Train 2 | Test Asian 55 | 0.79 | 0.81 | 0.68 | 1143 | Train 18 | Test Asian 55 | 0.79 | 0.81 | 0.61 |
| 120 | Train 2 | Test Asian 56 | 0.76 | 0.75 | 0.59 | 1144 | Train 18 | Test Asian 56 | 0.76 | 0.73 | 0.56 |
| 121 | Train 2 | Test Asian 57 | 0.76 | 0.83 | 0.82 | 1145 | Train 18 | Test Asian 57 | 0.76 | 0.83 | 0.81 |
| 122 | Train 2 | Test Asian 58 | 0.76 | 0.84 | 0.82 | 1146 | Train 18 | Test Asian 58 | 0.76 | 0.83 | 0.81 |
| 123 | Train 2 | Test Asian 59 | 0.76 | 0.83 | 0.82 | 1147 | Train 18 | Test Asian 59 | 0.76 | 0.83 | 0.81 |
| 124 | Train 2 | Test Asian 60 | 0.76 | 0.84 | 0.82 | 1148 | Train 18 | Test Asian 60 | 0.76 | 0.83 | 0.81 |
| 125 | Train 2 | Test Asian 61 | 0.79 | 0.8 | 0.68 | 1149 | Train 18 | Test Asian 61 | 0.79 | 0.8 | 0.61 |
| 126 | Train 2 | Test Asian 62 | 0.77 | 0.75 | 0.59 | 1150 | Train 18 | Test Asian 62 | 0.77 | 0.74 | 0.56 |
| 127 | Train 2 | Test Asian 63 | 0.78 | 0.8 | 0.67 | 1151 | Train 18 | Test Asian 63 | 0.78 | 0.8 | 0.61 |
| 128 | Train 2 | Test Asian 64 | 0.76 | 0.74 | 0.59 | 1152 | Train 18 | Test Asian 64 | 0.76 | 0.73 | 0.57 |
| 129 | Train 3 | Test Asian 1 | 0.8 | 0.92 | 0.92 | 1153 | Train 19 | Test Asian 1 | 0.8 | 0.92 | 0.93 |
| 130 | Train 3 | Test Asian 2 | 0.8 | 0.9 | 0.89 | 1154 | Train 19 | Test Asian 2 | 0.8 | 0.9 | 0.87 |
| 131 | Train 3 | Test Asian 3 | 0.8 | 0.92 | 0.92 | 1155 | Train 19 | Test Asian 3 | 0.8 | 0.92 | 0.93 |
| 132 | Train 3 | Test Asian 4 | 0.8 | 0.9 | 0.89 | 1156 | Train 19 | Test Asian 4 | 0.81 | 0.9 | 0.87 |
| 133 | Train 3 | Test Asian 5 | 0.75 | 0.76 | 0.72 | 1157 | Train 19 | Test Asian 5 | 0.75 | 0.76 | 0.71 |
| 134 | Train 3 | Test Asian 6 | 0.75 | 0.75 | 0.71 | 1158 | Train 19 | Test Asian 6 | 0.75 | 0.75 | 0.68 |
| 135 | Train 3 | Test Asian 7 | 0.74 | 0.75 | 0.72 | 1159 | Train 19 | Test Asian 7 | 0.74 | 0.75 | 0.71 |
| 136 | Train 3 | Test Asian 8 | 0.75 | 0.75 | 0.71 | 1160 | Train 19 | Test Asian 8 | 0.75 | 0.74 | 0.68 |
| 137 | Train 3 | Test Asian 9 | 0.79 | 0.9 | 0.88 | 1161 | Train 19 | Test Asian 9 | 0.79 | 0.9 | 0.89 |
| 138 | Train 3 | Test Asian 10 | 0.79 | 0.87 | 0.85 | 1162 | Train 19 | Test Asian 10 | 0.79 | 0.87 | 0.84 |
| 139 | Train 3 | Test Asian 11 | 0.79 | 0.9 | 0.88 | 1163 | Train 19 | Test Asian 11 | 0.79 | 0.9 | 0.89 |
| 140 | Train 3 | Test Asian 12 | 0.79 | 0.87 | 0.85 | 1164 | Train 19 | Test Asian 12 | 0.79 | 0.87 | 0.84 |
| 141 | Train 3 | Test Asian 13 | 0.74 | 0.75 | 0.72 | 1165 | Train 19 | Test Asian 13 | 0.74 | 0.75 | 0.71 |
| 142 | Train 3 | Test Asian 14 | 0.75 | 0.73 | 0.64 | 1166 | Train 19 | Test Asian 14 | 0.75 | 0.72 | 0.61 |
| 143 | Train 3 | Test Asian 15 | 0.74 | 0.75 | 0.71 | 1167 | Train 19 | Test Asian 15 | 0.74 | 0.75 | 0.7 |
| 144 | Train 3 | Test Asian 16 | 0.75 | 0.74 | 0.71 | 1168 | Train 19 | Test Asian 16 | 0.75 | 0.74 | 0.68 |
| 145 | Train 3 | Test Asian 17 | 0.8 | 0.92 | 0.92 | 1169 | Train 19 | Test Asian 17 | 0.8 | 0.92 | 0.93 |
| 146 | Train 3 | Test Asian 18 | 0.8 | 0.9 | 0.89 | 1170 | Train 19 | Test Asian 18 | 0.81 | 0.9 | 0.87 |
| 147 | Train 3 | Test Asian 19 | 0.8 | 0.92 | 0.92 | 1171 | Train 19 | Test Asian 19 | 0.8 | 0.92 | 0.93 |
| 148 | Train 3 | Test Asian 20 | 0.8 | 0.9 | 0.89 | 1172 | Train 19 | Test Asian 20 | 0.81 | 0.9 | 0.88 |
| 149 | Train 3 | Test Asian 21 | 0.75 | 0.76 | 0.73 | 1173 | Train 19 | Test Asian 21 | 0.75 | 0.76 | 0.71 |
| 150 | Train 3 | Test Asian 22 | 0.75 | 0.75 | 0.72 | 1174 | Train 19 | Test Asian 22 | 0.75 | 0.75 | 0.68 |
| 151 | Train 3 | Test Asian 23 | 0.75 | 0.75 | 0.72 | 1175 | Train 19 | Test Asian 23 | 0.74 | 0.75 | 0.71 |
| 152 | Train 3 | Test Asian 24 | 0.75 | 0.75 | 0.72 | 1176 | Train 19 | Test Asian 24 | 0.75 | 0.75 | 0.68 |
| 153 | Train 3 | Test Asian 25 | 0.79 | 0.9 | 0.88 | 1177 | Train 19 | Test Asian 25 | 0.79 | 0.9 | 0.89 |
| 154 | Train 3 | Test Asian 26 | 0.79 | 0.88 | 0.85 | 1178 | Train 19 | Test Asian 26 | 0.79 | 0.87 | 0.84 |
| 155 | Train 3 | Test Asian 27 | 0.79 | 0.9 | 0.88 | 1179 | Train 19 | Test Asian 27 | 0.79 | 0.9 | 0.89 |
| 156 | Train 3 | Test Asian 28 | 0.79 | 0.88 | 0.85 | 1180 | Train 19 | Test Asian 28 | 0.79 | 0.88 | 0.85 |
| 157 | Train 3 | Test Asian 29 | 0.74 | 0.75 | 0.72 | 1181 | Train 19 | Test Asian 29 | 0.74 | 0.75 | 0.71 |
| 158 | Train 3 | Test Asian 30 | 0.75 | 0.73 | 0.65 | 1182 | Train 19 | Test Asian 30 | 0.75 | 0.72 | 0.61 |
| 159 | Train 3 | Test Asian 31 | 0.74 | 0.75 | 0.71 | 1183 | Train 19 | Test Asian 31 | 0.74 | 0.75 | 0.7 |
| 160 | Train 3 | Test Asian 32 | 0.75 | 0.75 | 0.72 | 1184 | Train 19 | Test Asian 32 | 0.75 | 0.74 | 0.69 |
| 161 | Train 3 | Test Asian 33 | 0.8 | 0.92 | 0.92 | 1185 | Train 19 | Test Asian 33 | 0.8 | 0.92 | 0.93 |
| 162 | Train 3 | Test Asian 34 | 0.8 | 0.9 | 0.89 | 1186 | Train 19 | Test Asian 34 | 0.8 | 0.9 | 0.87 |
| 163 | Train 3 | Test Asian 35 | 0.8 | 0.92 | 0.92 | 1187 | Train 19 | Test Asian 35 | 0.8 | 0.92 | 0.93 |
| 164 | Train 3 | Test Asian 36 | 0.8 | 0.9 | 0.89 | 1188 | Train 19 | Test Asian 36 | 0.8 | 0.9 | 0.87 |
| 165 | Train 3 | Test Asian 37 | 0.75 | 0.75 | 0.72 | 1189 | Train 19 | Test Asian 37 | 0.75 | 0.75 | 0.71 |
| 166 | Train 3 | Test Asian 38 | 0.75 | 0.74 | 0.71 | 1190 | Train 19 | Test Asian 38 | 0.75 | 0.74 | 0.68 |
| 167 | Train 3 | Test Asian 39 | 0.74 | 0.75 | 0.72 | 1191 | Train 19 | Test Asian 39 | 0.74 | 0.75 | 0.71 |
| 168 | Train 3 | Test Asian 40 | 0.75 | 0.74 | 0.71 | 1192 | Train 19 | Test Asian 40 | 0.75 | 0.74 | 0.68 |
| 169 | Train 3 | Test Asian 41 | 0.79 | 0.9 | 0.88 | 1193 | Train 19 | Test Asian 41 | 0.79 | 0.9 | 0.89 |
| 170 | Train 3 | Test Asian 42 | 0.79 | 0.87 | 0.85 | 1194 | Train 19 | Test Asian 42 | 0.79 | 0.87 | 0.84 |
| 171 | Train 3 | Test Asian 43 | 0.79 | 0.9 | 0.88 | 1195 | Train 19 | Test Asian 43 | 0.79 | 0.9 | 0.89 |
| 172 | Train 3 | Test Asian 44 | 0.79 | 0.87 | 0.85 | 1196 | Train 19 | Test Asian 44 | 0.79 | 0.87 | 0.84 |
| 173 | Train 3 | Test Asian 45 | 0.74 | 0.75 | 0.71 | 1197 | Train 19 | Test Asian 45 | 0.74 | 0.75 | 0.7 |
| 174 | Train 3 | Test Asian 46 | 0.75 | 0.74 | 0.72 | 1198 | Train 19 | Test Asian 46 | 0.75 | 0.74 | 0.69 |
| 175 | Train 3 | Test Asian 47 | 0.74 | 0.75 | 0.71 | 1199 | Train 19 | Test Asian 47 | 0.74 | 0.74 | 0.7 |
| 176 | Train 3 | Test Asian 48 | 0.75 | 0.74 | 0.71 | 1200 | Train 19 | Test Asian 48 | 0.75 | 0.74 | 0.69 |
| 177 | Train 3 | Test Asian 49 | 0.77 | 0.88 | 0.88 | 1201 | Train 19 | Test Asian 49 | 0.77 | 0.88 | 0.9 |
| 178 | Train 3 | Test Asian 50 | 0.77 | 0.88 | 0.88 | 1202 | Train 19 | Test Asian 50 | 0.77 | 0.88 | 0.9 |
| 179 | Train 3 | Test Asian 51 | 0.77 | 0.88 | 0.88 | 1203 | Train 19 | Test Asian 51 | 0.77 | 0.88 | 0.9 |
| 180 | Train 3 | Test Asian 52 | 0.77 | 0.88 | 0.88 | 1204 | Train 19 | Test Asian 52 | 0.77 | 0.88 | 0.9 |
| 181 | Train 3 | Test Asian 53 | 0.79 | 0.8 | 0.72 | 1205 | Train 19 | Test Asian 53 | 0.79 | 0.81 | 0.63 |
| 182 | Train 3 | Test Asian 54 | 0.78 | 0.74 | 0.68 | 1206 | Train 19 | Test Asian 54 | 0.78 | 0.74 | 0.59 |
| 183 | Train 3 | Test Asian 55 | 0.79 | 0.8 | 0.71 | 1207 | Train 19 | Test Asian 55 | 0.79 | 0.8 | 0.62 |
| 184 | Train 3 | Test Asian 56 | 0.78 | 0.74 | 0.68 | 1208 | Train 19 | Test Asian 56 | 0.78 | 0.73 | 0.6 |
| 185 | Train 3 | Test Asian 57 | 0.76 | 0.85 | 0.81 | 1209 | Train 19 | Test Asian 57 | 0.76 | 0.85 | 0.9 |
| 186 | Train 3 | Test Asian 58 | 0.76 | 0.85 | 0.81 | 1210 | Train 19 | Test Asian 58 | 0.76 | 0.85 | 0.89 |
| 187 | Train 3 | Test Asian 59 | 0.76 | 0.85 | 0.81 | 1211 | Train 19 | Test Asian 59 | 0.76 | 0.85 | 0.9 |
| 188 | Train 3 | Test Asian 60 | 0.76 | 0.85 | 0.81 | 1212 | Train 19 | Test Asian 60 | 0.76 | 0.85 | 0.89 |
| 189 | Train 3 | Test Asian 61 | 0.79 | 0.79 | 0.71 | 1213 | Train 19 | Test Asian 61 | 0.78 | 0.8 | 0.62 |
| 190 | Train 3 | Test Asian 62 | 0.78 | 0.74 | 0.68 | 1214 | Train 19 | Test Asian 62 | 0.78 | 0.74 | 0.6 |
| 191 | Train 3 | Test Asian 63 | 0.78 | 0.79 | 0.7 | 1215 | Train 19 | Test Asian 63 | 0.78 | 0.79 | 0.62 |
| 192 | Train 3 | Test Asian 64 | 0.78 | 0.74 | 0.68 | 1216 | Train 19 | Test Asian 64 | 0.78 | 0.73 | 0.6 |
| 193 | Train 4 | Test Asian 1 | 0.79 | 0.91 | 0.92 | 1217 | Train 20 | Test Asian 1 | 0.79 | 0.91 | 0.92 |
| 194 | Train 4 | Test Asian 2 | 0.81 | 0.92 | 0.92 | 1218 | Train 20 | Test Asian 2 | 0.81 | 0.92 | 0.93 |
| 195 | Train 4 | Test Asian 3 | 0.79 | 0.91 | 0.92 | 1219 | Train 20 | Test Asian 3 | 0.79 | 0.91 | 0.92 |
| 196 | Train 4 | Test Asian 4 | 0.81 | 0.92 | 0.92 | 1220 | Train 20 | Test Asian 4 | 0.81 | 0.92 | 0.93 |
| 197 | Train 4 | Test Asian 5 | 0.75 | 0.76 | 0.74 | 1221 | Train 20 | Test Asian 5 | 0.75 | 0.76 | 0.73 |
| 198 | Train 4 | Test Asian 6 | 0.74 | 0.75 | 0.71 | 1222 | Train 20 | Test Asian 6 | 0.74 | 0.75 | 0.7 |
| 199 | Train 4 | Test Asian 7 | 0.75 | 0.76 | 0.73 | 1223 | Train 20 | Test Asian 7 | 0.75 | 0.76 | 0.72 |
| 200 | Train 4 | Test Asian 8 | 0.75 | 0.75 | 0.71 | 1224 | Train 20 | Test Asian 8 | 0.75 | 0.74 | 0.7 |
| 201 | Train 4 | Test Asian 9 | 0.78 | 0.89 | 0.89 | 1225 | Train 20 | Test Asian 9 | 0.78 | 0.89 | 0.89 |
| 202 | Train 4 | Test Asian 10 | 0.8 | 0.9 | 0.89 | 1226 | Train 20 | Test Asian 10 | 0.8 | 0.9 | 0.88 |
| 203 | Train 4 | Test Asian 11 | 0.78 | 0.89 | 0.89 | 1227 | Train 20 | Test Asian 11 | 0.78 | 0.89 | 0.89 |
| 204 | Train 4 | Test Asian 12 | 0.8 | 0.9 | 0.89 | 1228 | Train 20 | Test Asian 12 | 0.8 | 0.9 | 0.88 |
| 205 | Train 4 | Test Asian 13 | 0.75 | 0.76 | 0.73 | 1229 | Train 20 | Test Asian 13 | 0.75 | 0.76 | 0.72 |
| 206 | Train 4 | Test Asian 14 | 0.75 | 0.74 | 0.65 | 1230 | Train 20 | Test Asian 14 | 0.75 | 0.73 | 0.52 |
| 207 | Train 4 | Test Asian 15 | 0.75 | 0.75 | 0.73 | 1231 | Train 20 | Test Asian 15 | 0.75 | 0.75 | 0.72 |
| 208 | Train 4 | Test Asian 16 | 0.75 | 0.75 | 0.71 | 1232 | Train 20 | Test Asian 16 | 0.75 | 0.74 | 0.7 |
| 209 | Train 4 | Test Asian 17 | 0.79 | 0.91 | 0.92 | 1233 | Train 20 | Test Asian 17 | 0.79 | 0.91 | 0.92 |
| 210 | Train 4 | Test Asian 18 | 0.81 | 0.92 | 0.92 | 1234 | Train 20 | Test Asian 18 | 0.81 | 0.92 | 0.93 |
| 211 | Train 4 | Test Asian 19 | 0.79 | 0.91 | 0.92 | 1235 | Train 20 | Test Asian 19 | 0.79 | 0.91 | 0.92 |
| 212 | Train 4 | Test Asian 20 | 0.81 | 0.92 | 0.92 | 1236 | Train 20 | Test Asian 20 | 0.81 | 0.92 | 0.93 |
| 213 | Train 4 | Test Asian 21 | 0.75 | 0.76 | 0.74 | 1237 | Train 20 | Test Asian 21 | 0.75 | 0.76 | 0.73 |
| 214 | Train 4 | Test Asian 22 | 0.75 | 0.75 | 0.71 | 1238 | Train 20 | Test Asian 22 | 0.75 | 0.75 | 0.7 |
| 215 | Train 4 | Test Asian 23 | 0.75 | 0.76 | 0.73 | 1239 | Train 20 | Test Asian 23 | 0.75 | 0.76 | 0.72 |
| 216 | Train 4 | Test Asian 24 | 0.75 | 0.75 | 0.71 | 1240 | Train 20 | Test Asian 24 | 0.75 | 0.74 | 0.7 |
| 217 | Train 4 | Test Asian 25 | 0.78 | 0.89 | 0.89 | 1241 | Train 20 | Test Asian 25 | 0.78 | 0.89 | 0.89 |
| 218 | Train 4 | Test Asian 26 | 0.8 | 0.9 | 0.89 | 1242 | Train 20 | Test Asian 26 | 0.8 | 0.9 | 0.88 |
| 219 | Train 4 | Test Asian 27 | 0.78 | 0.89 | 0.89 | 1243 | Train 20 | Test Asian 27 | 0.78 | 0.89 | 0.89 |
| 220 | Train 4 | Test Asian 28 | 0.8 | 0.9 | 0.89 | 1244 | Train 20 | Test Asian 28 | 0.8 | 0.9 | 0.88 |
| 221 | Train 4 | Test Asian 29 | 0.75 | 0.76 | 0.74 | 1245 | Train 20 | Test Asian 29 | 0.75 | 0.76 | 0.72 |
| 222 | Train 4 | Test Asian 30 | 0.75 | 0.74 | 0.65 | 1246 | Train 20 | Test Asian 30 | 0.75 | 0.74 | 0.52 |
| 223 | Train 4 | Test Asian 31 | 0.75 | 0.75 | 0.73 | 1247 | Train 20 | Test Asian 31 | 0.75 | 0.75 | 0.72 |
| 224 | Train 4 | Test Asian 32 | 0.75 | 0.75 | 0.71 | 1248 | Train 20 | Test Asian 32 | 0.75 | 0.74 | 0.7 |
| 225 | Train 4 | Test Asian 33 | 0.79 | 0.91 | 0.92 | 1249 | Train 20 | Test Asian 33 | 0.79 | 0.91 | 0.92 |
| 226 | Train 4 | Test Asian 34 | 0.81 | 0.92 | 0.92 | 1250 | Train 20 | Test Asian 34 | 0.81 | 0.92 | 0.93 |
| 227 | Train 4 | Test Asian 35 | 0.79 | 0.91 | 0.92 | 1251 | Train 20 | Test Asian 35 | 0.79 | 0.91 | 0.92 |
| 228 | Train 4 | Test Asian 36 | 0.81 | 0.92 | 0.92 | 1252 | Train 20 | Test Asian 36 | 0.81 | 0.92 | 0.93 |
| 229 | Train 4 | Test Asian 37 | 0.75 | 0.76 | 0.74 | 1253 | Train 20 | Test Asian 37 | 0.75 | 0.76 | 0.73 |
| 230 | Train 4 | Test Asian 38 | 0.74 | 0.75 | 0.71 | 1254 | Train 20 | Test Asian 38 | 0.74 | 0.75 | 0.7 |
| 231 | Train 4 | Test Asian 39 | 0.75 | 0.76 | 0.73 | 1255 | Train 20 | Test Asian 39 | 0.75 | 0.76 | 0.72 |
| 232 | Train 4 | Test Asian 40 | 0.74 | 0.74 | 0.71 | 1256 | Train 20 | Test Asian 40 | 0.74 | 0.74 | 0.7 |
| 233 | Train 4 | Test Asian 41 | 0.78 | 0.89 | 0.89 | 1257 | Train 20 | Test Asian 41 | 0.78 | 0.89 | 0.89 |
| 234 | Train 4 | Test Asian 42 | 0.8 | 0.9 | 0.89 | 1258 | Train 20 | Test Asian 42 | 0.8 | 0.89 | 0.88 |
| 235 | Train 4 | Test Asian 43 | 0.78 | 0.89 | 0.89 | 1259 | Train 20 | Test Asian 43 | 0.78 | 0.89 | 0.89 |
| 236 | Train 4 | Test Asian 44 | 0.8 | 0.9 | 0.89 | 1260 | Train 20 | Test Asian 44 | 0.8 | 0.89 | 0.88 |
| 237 | Train 4 | Test Asian 45 | 0.75 | 0.76 | 0.73 | 1261 | Train 20 | Test Asian 45 | 0.75 | 0.75 | 0.72 |
| 238 | Train 4 | Test Asian 46 | 0.74 | 0.75 | 0.71 | 1262 | Train 20 | Test Asian 46 | 0.74 | 0.75 | 0.7 |
| 239 | Train 4 | Test Asian 47 | 0.75 | 0.75 | 0.73 | 1263 | Train 20 | Test Asian 47 | 0.75 | 0.75 | 0.72 |
| 240 | Train 4 | Test Asian 48 | 0.75 | 0.74 | 0.71 | 1264 | Train 20 | Test Asian 48 | 0.75 | 0.74 | 0.7 |
| 241 | Train 4 | Test Asian 49 | 0.77 | 0.86 | 0.88 | 1265 | Train 20 | Test Asian 49 | 0.77 | 0.86 | 0.87 |
| 242 | Train 4 | Test Asian 50 | 0.77 | 0.86 | 0.88 | 1266 | Train 20 | Test Asian 50 | 0.77 | 0.86 | 0.87 |
| 243 | Train 4 | Test Asian 51 | 0.77 | 0.86 | 0.88 | 1267 | Train 20 | Test Asian 51 | 0.77 | 0.86 | 0.87 |
| 244 | Train 4 | Test Asian 52 | 0.77 | 0.86 | 0.88 | 1268 | Train 20 | Test Asian 52 | 0.77 | 0.86 | 0.87 |
| 245 | Train 4 | Test Asian 53 | 0.79 | 0.81 | 0.7 | 1269 | Train 20 | Test Asian 53 | 0.79 | 0.81 | 0.56 |
| 246 | Train 4 | Test Asian 54 | 0.77 | 0.75 | 0.64 | 1270 | Train 20 | Test Asian 54 | 0.77 | 0.74 | 0.5 |
| 247 | Train 4 | Test Asian 55 | 0.79 | 0.81 | 0.7 | 1271 | Train 20 | Test Asian 55 | 0.79 | 0.81 | 0.55 |
| 248 | Train 4 | Test Asian 56 | 0.76 | 0.74 | 0.64 | 1272 | Train 20 | Test Asian 56 | 0.76 | 0.74 | 0.5 |
| 249 | Train 4 | Test Asian 57 | 0.76 | 0.83 | 0.82 | 1273 | Train 20 | Test Asian 57 | 0.76 | 0.83 | 0.83 |
| 250 | Train 4 | Test Asian 58 | 0.76 | 0.83 | 0.82 | 1274 | Train 20 | Test Asian 58 | 0.76 | 0.83 | 0.83 |
| 251 | Train 4 | Test Asian 59 | 0.76 | 0.83 | 0.82 | 1275 | Train 20 | Test Asian 59 | 0.76 | 0.83 | 0.83 |
| 252 | Train 4 | Test Asian 60 | 0.76 | 0.83 | 0.82 | 1276 | Train 20 | Test Asian 60 | 0.76 | 0.83 | 0.83 |
| 253 | Train 4 | Test Asian 61 | 0.79 | 0.8 | 0.69 | 1277 | Train 20 | Test Asian 61 | 0.79 | 0.8 | 0.55 |
| 254 | Train 4 | Test Asian 62 | 0.77 | 0.75 | 0.64 | 1278 | Train 20 | Test Asian 62 | 0.77 | 0.74 | 0.5 |
| 255 | Train 4 | Test Asian 63 | 0.78 | 0.8 | 0.69 | 1279 | Train 20 | Test Asian 63 | 0.78 | 0.8 | 0.55 |
| 256 | Train 4 | Test Asian 64 | 0.76 | 0.74 | 0.64 | 1280 | Train 20 | Test Asian 64 | 0.76 | 0.74 | 0.51 |
| 257 | Train 5 | Test Asian 1 | 0.78 | 0.87 | 0.86 | 1281 | Train 21 | Test Asian 1 | 0.78 | 0.87 | 0.87 |
| 258 | Train 5 | Test Asian 2 | 0.8 | 0.87 | 0.85 | 1282 | Train 21 | Test Asian 2 | 0.8 | 0.87 | 0.85 |
| 259 | Train 5 | Test Asian 3 | 0.78 | 0.87 | 0.86 | 1283 | Train 21 | Test Asian 3 | 0.78 | 0.87 | 0.87 |
| 260 | Train 5 | Test Asian 4 | 0.8 | 0.87 | 0.85 | 1284 | Train 21 | Test Asian 4 | 0.8 | 0.87 | 0.85 |
| 261 | Train 5 | Test Asian 5 | 0.76 | 0.85 | 0.86 | 1285 | Train 21 | Test Asian 5 | 0.76 | 0.85 | 0.86 |
| 262 | Train 5 | Test Asian 6 | 0.76 | 0.78 | 0.75 | 1286 | Train 21 | Test Asian 6 | 0.76 | 0.78 | 0.76 |
| 263 | Train 5 | Test Asian 7 | 0.76 | 0.85 | 0.85 | 1287 | Train 21 | Test Asian 7 | 0.76 | 0.85 | 0.85 |
| 264 | Train 5 | Test Asian 8 | 0.76 | 0.78 | 0.75 | 1288 | Train 21 | Test Asian 8 | 0.76 | 0.78 | 0.76 |
| 265 | Train 5 | Test Asian 9 | 0.77 | 0.85 | 0.83 | 1289 | Train 21 | Test Asian 9 | 0.77 | 0.85 | 0.83 |
| 266 | Train 5 | Test Asian 10 | 0.79 | 0.85 | 0.82 | 1290 | Train 21 | Test Asian 10 | 0.79 | 0.85 | 0.82 |
| 267 | Train 5 | Test Asian 11 | 0.77 | 0.85 | 0.83 | 1291 | Train 21 | Test Asian 11 | 0.77 | 0.85 | 0.83 |
| 268 | Train 5 | Test Asian 12 | 0.79 | 0.85 | 0.82 | 1292 | Train 21 | Test Asian 12 | 0.79 | 0.85 | 0.82 |
| 269 | Train 5 | Test Asian 13 | 0.76 | 0.84 | 0.83 | 1293 | Train 21 | Test Asian 13 | 0.76 | 0.84 | 0.83 |
| 270 | Train 5 | Test Asian 14 | 0.76 | 0.76 | 0.7 | 1294 | Train 21 | Test Asian 14 | 0.76 | 0.76 | 0.74 |
| 271 | Train 5 | Test Asian 15 | 0.76 | 0.83 | 0.82 | 1295 | Train 21 | Test Asian 15 | 0.76 | 0.83 | 0.82 |
| 272 | Train 5 | Test Asian 16 | 0.76 | 0.78 | 0.74 | 1296 | Train 21 | Test Asian 16 | 0.76 | 0.78 | 0.76 |
| 273 | Train 5 | Test Asian 17 | 0.78 | 0.87 | 0.86 | 1297 | Train 21 | Test Asian 17 | 0.78 | 0.87 | 0.87 |
| 274 | Train 5 | Test Asian 18 | 0.8 | 0.87 | 0.85 | 1298 | Train 21 | Test Asian 18 | 0.8 | 0.87 | 0.85 |
| 275 | Train 5 | Test Asian 19 | 0.78 | 0.87 | 0.86 | 1299 | Train 21 | Test Asian 19 | 0.78 | 0.87 | 0.87 |
| 276 | Train 5 | Test Asian 20 | 0.8 | 0.87 | 0.85 | 1300 | Train 21 | Test Asian 20 | 0.8 | 0.87 | 0.85 |
| 277 | Train 5 | Test Asian 21 | 0.77 | 0.85 | 0.86 | 1301 | Train 21 | Test Asian 21 | 0.77 | 0.85 | 0.86 |
| 278 | Train 5 | Test Asian 22 | 0.76 | 0.78 | 0.75 | 1302 | Train 21 | Test Asian 22 | 0.76 | 0.78 | 0.76 |
| 279 | Train 5 | Test Asian 23 | 0.76 | 0.85 | 0.85 | 1303 | Train 21 | Test Asian 23 | 0.76 | 0.85 | 0.85 |
| 280 | Train 5 | Test Asian 24 | 0.76 | 0.78 | 0.75 | 1304 | Train 21 | Test Asian 24 | 0.76 | 0.78 | 0.76 |
| 281 | Train 5 | Test Asian 25 | 0.77 | 0.85 | 0.83 | 1305 | Train 21 | Test Asian 25 | 0.77 | 0.85 | 0.83 |
| 282 | Train 5 | Test Asian 26 | 0.79 | 0.85 | 0.82 | 1306 | Train 21 | Test Asian 26 | 0.79 | 0.85 | 0.82 |
| 283 | Train 5 | Test Asian 27 | 0.77 | 0.85 | 0.83 | 1307 | Train 21 | Test Asian 27 | 0.77 | 0.85 | 0.83 |
| 284 | Train 5 | Test Asian 28 | 0.79 | 0.85 | 0.82 | 1308 | Train 21 | Test Asian 28 | 0.79 | 0.85 | 0.82 |
| 285 | Train 5 | Test Asian 29 | 0.76 | 0.84 | 0.83 | 1309 | Train 21 | Test Asian 29 | 0.76 | 0.84 | 0.83 |
| 286 | Train 5 | Test Asian 30 | 0.76 | 0.76 | 0.7 | 1310 | Train 21 | Test Asian 30 | 0.76 | 0.76 | 0.74 |
| 287 | Train 5 | Test Asian 31 | 0.76 | 0.83 | 0.82 | 1311 | Train 21 | Test Asian 31 | 0.76 | 0.83 | 0.82 |
| 288 | Train 5 | Test Asian 32 | 0.76 | 0.78 | 0.74 | 1312 | Train 21 | Test Asian 32 | 0.76 | 0.78 | 0.76 |
| 289 | Train 5 | Test Asian 33 | 0.78 | 0.87 | 0.86 | 1313 | Train 21 | Test Asian 33 | 0.78 | 0.87 | 0.87 |
| 290 | Train 5 | Test Asian 34 | 0.8 | 0.87 | 0.85 | 1314 | Train 21 | Test Asian 34 | 0.8 | 0.87 | 0.86 |
| 291 | Train 5 | Test Asian 35 | 0.78 | 0.87 | 0.86 | 1315 | Train 21 | Test Asian 35 | 0.78 | 0.87 | 0.87 |
| 292 | Train 5 | Test Asian 36 | 0.8 | 0.87 | 0.85 | 1316 | Train 21 | Test Asian 36 | 0.8 | 0.87 | 0.86 |
| 293 | Train 5 | Test Asian 37 | 0.76 | 0.85 | 0.86 | 1317 | Train 21 | Test Asian 37 | 0.76 | 0.85 | 0.86 |
| 294 | Train 5 | Test Asian 38 | 0.75 | 0.78 | 0.75 | 1318 | Train 21 | Test Asian 38 | 0.75 | 0.78 | 0.76 |
| 295 | Train 5 | Test Asian 39 | 0.76 | 0.84 | 0.85 | 1319 | Train 21 | Test Asian 39 | 0.76 | 0.84 | 0.85 |
| 296 | Train 5 | Test Asian 40 | 0.76 | 0.78 | 0.75 | 1320 | Train 21 | Test Asian 40 | 0.76 | 0.78 | 0.76 |
| 297 | Train 5 | Test Asian 41 | 0.77 | 0.85 | 0.83 | 1321 | Train 21 | Test Asian 41 | 0.77 | 0.85 | 0.84 |
| 298 | Train 5 | Test Asian 42 | 0.8 | 0.85 | 0.82 | 1322 | Train 21 | Test Asian 42 | 0.8 | 0.85 | 0.82 |
| 299 | Train 5 | Test Asian 43 | 0.77 | 0.85 | 0.83 | 1323 | Train 21 | Test Asian 43 | 0.77 | 0.85 | 0.84 |
| 300 | Train 5 | Test Asian 44 | 0.8 | 0.85 | 0.82 | 1324 | Train 21 | Test Asian 44 | 0.8 | 0.85 | 0.82 |
| 301 | Train 5 | Test Asian 45 | 0.76 | 0.83 | 0.83 | 1325 | Train 21 | Test Asian 45 | 0.76 | 0.83 | 0.83 |
| 302 | Train 5 | Test Asian 46 | 0.76 | 0.78 | 0.74 | 1326 | Train 21 | Test Asian 46 | 0.75 | 0.78 | 0.76 |
| 303 | Train 5 | Test Asian 47 | 0.76 | 0.83 | 0.82 | 1327 | Train 21 | Test Asian 47 | 0.76 | 0.83 | 0.82 |
| 304 | Train 5 | Test Asian 48 | 0.76 | 0.78 | 0.74 | 1328 | Train 21 | Test Asian 48 | 0.76 | 0.78 | 0.76 |
| 305 | Train 5 | Test Asian 49 | 0.76 | 0.84 | 0.81 | 1329 | Train 21 | Test Asian 49 | 0.76 | 0.85 | 0.87 |
| 306 | Train 5 | Test Asian 50 | 0.76 | 0.84 | 0.81 | 1330 | Train 21 | Test Asian 50 | 0.76 | 0.85 | 0.87 |
| 307 | Train 5 | Test Asian 51 | 0.76 | 0.84 | 0.81 | 1331 | Train 21 | Test Asian 51 | 0.76 | 0.85 | 0.87 |
| 308 | Train 5 | Test Asian 52 | 0.76 | 0.84 | 0.81 | 1332 | Train 21 | Test Asian 52 | 0.76 | 0.85 | 0.87 |
| 309 | Train 5 | Test Asian 53 | 0.8 | 0.86 | 0.79 | 1333 | Train 21 | Test Asian 53 | 0.8 | 0.86 | 0.79 |
| 310 | Train 5 | Test Asian 54 | 0.77 | 0.77 | 0.69 | 1334 | Train 21 | Test Asian 54 | 0.77 | 0.76 | 0.72 |
| 311 | Train 5 | Test Asian 55 | 0.8 | 0.85 | 0.78 | 1335 | Train 21 | Test Asian 55 | 0.8 | 0.85 | 0.78 |
| 312 | Train 5 | Test Asian 56 | 0.77 | 0.76 | 0.69 | 1336 | Train 21 | Test Asian 56 | 0.77 | 0.76 | 0.73 |
| 313 | Train 5 | Test Asian 57 | 0.74 | 0.82 | 0.78 | 1337 | Train 21 | Test Asian 57 | 0.75 | 0.82 | 0.8 |
| 314 | Train 5 | Test Asian 58 | 0.74 | 0.82 | 0.78 | 1338 | Train 21 | Test Asian 58 | 0.74 | 0.82 | 0.8 |
| 315 | Train 5 | Test Asian 59 | 0.74 | 0.82 | 0.78 | 1339 | Train 21 | Test Asian 59 | 0.75 | 0.82 | 0.8 |
| 316 | Train 5 | Test Asian 60 | 0.74 | 0.82 | 0.78 | 1340 | Train 21 | Test Asian 60 | 0.74 | 0.82 | 0.8 |
| 317 | Train 5 | Test Asian 61 | 0.79 | 0.85 | 0.78 | 1341 | Train 21 | Test Asian 61 | 0.79 | 0.85 | 0.78 |
| 318 | Train 5 | Test Asian 62 | 0.77 | 0.76 | 0.69 | 1342 | Train 21 | Test Asian 62 | 0.77 | 0.76 | 0.73 |
| 319 | Train 5 | Test Asian 63 | 0.79 | 0.84 | 0.77 | 1343 | Train 21 | Test Asian 63 | 0.79 | 0.84 | 0.77 |
| 320 | Train 5 | Test Asian 64 | 0.77 | 0.76 | 0.69 | 1344 | Train 21 | Test Asian 64 | 0.77 | 0.76 | 0.73 |
| 321 | Train 6 | Test Asian 1 | 0.76 | 0.84 | 0.82 | 1345 | Train 22 | Test Asian 1 | 0.76 | 0.84 | 0.75 |
| 322 | Train 6 | Test Asian 2 | 0.79 | 0.86 | 0.84 | 1346 | Train 22 | Test Asian 2 | 0.79 | 0.86 | 0.78 |
| 323 | Train 6 | Test Asian 3 | 0.76 | 0.84 | 0.82 | 1347 | Train 22 | Test Asian 3 | 0.76 | 0.84 | 0.75 |
| 324 | Train 6 | Test Asian 4 | 0.79 | 0.86 | 0.84 | 1348 | Train 22 | Test Asian 4 | 0.79 | 0.86 | 0.78 |
| 325 | Train 6 | Test Asian 5 | 0.76 | 0.81 | 0.8 | 1349 | Train 22 | Test Asian 5 | 0.76 | 0.81 | 0.73 |
| 326 | Train 6 | Test Asian 6 | 0.76 | 0.82 | 0.8 | 1350 | Train 22 | Test Asian 6 | 0.76 | 0.82 | 0.76 |
| 327 | Train 6 | Test Asian 7 | 0.75 | 0.8 | 0.8 | 1351 | Train 22 | Test Asian 7 | 0.75 | 0.8 | 0.73 |
| 328 | Train 6 | Test Asian 8 | 0.76 | 0.82 | 0.8 | 1352 | Train 22 | Test Asian 8 | 0.76 | 0.81 | 0.75 |
| 329 | Train 6 | Test Asian 9 | 0.75 | 0.84 | 0.82 | 1353 | Train 22 | Test Asian 9 | 0.75 | 0.84 | 0.75 |
| 330 | Train 6 | Test Asian 10 | 0.78 | 0.86 | 0.84 | 1354 | Train 22 | Test Asian 10 | 0.78 | 0.86 | 0.79 |
| 331 | Train 6 | Test Asian 11 | 0.75 | 0.84 | 0.82 | 1355 | Train 22 | Test Asian 11 | 0.75 | 0.84 | 0.75 |
| 332 | Train 6 | Test Asian 12 | 0.78 | 0.86 | 0.84 | 1356 | Train 22 | Test Asian 12 | 0.78 | 0.86 | 0.79 |
| 333 | Train 6 | Test Asian 13 | 0.75 | 0.81 | 0.81 | 1357 | Train 22 | Test Asian 13 | 0.75 | 0.81 | 0.74 |
| 334 | Train 6 | Test Asian 14 | 0.76 | 0.83 | 0.84 | 1358 | Train 22 | Test Asian 14 | 0.76 | 0.83 | 0.84 |
| 335 | Train 6 | Test Asian 15 | 0.75 | 0.8 | 0.8 | 1359 | Train 22 | Test Asian 15 | 0.75 | 0.8 | 0.74 |
| 336 | Train 6 | Test Asian 16 | 0.76 | 0.81 | 0.79 | 1360 | Train 22 | Test Asian 16 | 0.76 | 0.81 | 0.75 |
| 337 | Train 6 | Test Asian 17 | 0.76 | 0.84 | 0.82 | 1361 | Train 22 | Test Asian 17 | 0.76 | 0.84 | 0.75 |
| 338 | Train 6 | Test Asian 18 | 0.79 | 0.86 | 0.84 | 1362 | Train 22 | Test Asian 18 | 0.79 | 0.86 | 0.78 |
| 339 | Train 6 | Test Asian 19 | 0.76 | 0.84 | 0.82 | 1363 | Train 22 | Test Asian 19 | 0.76 | 0.84 | 0.75 |
| 340 | Train 6 | Test Asian 20 | 0.79 | 0.86 | 0.84 | 1364 | Train 22 | Test Asian 20 | 0.79 | 0.86 | 0.78 |
| 341 | Train 6 | Test Asian 21 | 0.76 | 0.81 | 0.8 | 1365 | Train 22 | Test Asian 21 | 0.76 | 0.81 | 0.73 |
| 342 | Train 6 | Test Asian 22 | 0.77 | 0.82 | 0.8 | 1366 | Train 22 | Test Asian 22 | 0.77 | 0.82 | 0.76 |
| 343 | Train 6 | Test Asian 23 | 0.75 | 0.8 | 0.8 | 1367 | Train 22 | Test Asian 23 | 0.75 | 0.8 | 0.73 |
| 344 | Train 6 | Test Asian 24 | 0.77 | 0.82 | 0.8 | 1368 | Train 22 | Test Asian 24 | 0.77 | 0.81 | 0.76 |
| 345 | Train 6 | Test Asian 25 | 0.75 | 0.84 | 0.82 | 1369 | Train 22 | Test Asian 25 | 0.75 | 0.84 | 0.75 |
| 346 | Train 6 | Test Asian 26 | 0.78 | 0.86 | 0.84 | 1370 | Train 22 | Test Asian 26 | 0.78 | 0.86 | 0.79 |
| 347 | Train 6 | Test Asian 27 | 0.75 | 0.84 | 0.82 | 1371 | Train 22 | Test Asian 27 | 0.75 | 0.84 | 0.75 |
| 348 | Train 6 | Test Asian 28 | 0.78 | 0.86 | 0.84 | 1372 | Train 22 | Test Asian 28 | 0.78 | 0.86 | 0.79 |
| 349 | Train 6 | Test Asian 29 | 0.75 | 0.81 | 0.81 | 1373 | Train 22 | Test Asian 29 | 0.75 | 0.81 | 0.74 |
| 350 | Train 6 | Test Asian 30 | 0.76 | 0.83 | 0.84 | 1374 | Train 22 | Test Asian 30 | 0.76 | 0.83 | 0.84 |
| 351 | Train 6 | Test Asian 31 | 0.75 | 0.8 | 0.8 | 1375 | Train 22 | Test Asian 31 | 0.75 | 0.8 | 0.74 |
| 352 | Train 6 | Test Asian 32 | 0.76 | 0.81 | 0.79 | 1376 | Train 22 | Test Asian 32 | 0.76 | 0.81 | 0.75 |
| 353 | Train 6 | Test Asian 33 | 0.76 | 0.84 | 0.82 | 1377 | Train 22 | Test Asian 33 | 0.76 | 0.84 | 0.75 |
| 354 | Train 6 | Test Asian 34 | 0.78 | 0.86 | 0.84 | 1378 | Train 22 | Test Asian 34 | 0.78 | 0.86 | 0.78 |
| 355 | Train 6 | Test Asian 35 | 0.76 | 0.84 | 0.82 | 1379 | Train 22 | Test Asian 35 | 0.76 | 0.84 | 0.75 |
| 356 | Train 6 | Test Asian 36 | 0.78 | 0.86 | 0.84 | 1380 | Train 22 | Test Asian 36 | 0.78 | 0.86 | 0.78 |
| 357 | Train 6 | Test Asian 37 | 0.75 | 0.81 | 0.8 | 1381 | Train 22 | Test Asian 37 | 0.75 | 0.8 | 0.73 |
| 358 | Train 6 | Test Asian 38 | 0.76 | 0.82 | 0.8 | 1382 | Train 22 | Test Asian 38 | 0.76 | 0.82 | 0.76 |
| 359 | Train 6 | Test Asian 39 | 0.75 | 0.8 | 0.79 | 1383 | Train 22 | Test Asian 39 | 0.75 | 0.8 | 0.73 |
| 360 | Train 6 | Test Asian 40 | 0.76 | 0.81 | 0.79 | 1384 | Train 22 | Test Asian 40 | 0.76 | 0.81 | 0.75 |
| 361 | Train 6 | Test Asian 41 | 0.75 | 0.84 | 0.82 | 1385 | Train 22 | Test Asian 41 | 0.75 | 0.84 | 0.75 |
| 362 | Train 6 | Test Asian 42 | 0.78 | 0.86 | 0.84 | 1386 | Train 22 | Test Asian 42 | 0.78 | 0.86 | 0.79 |
| 363 | Train 6 | Test Asian 43 | 0.75 | 0.84 | 0.82 | 1387 | Train 22 | Test Asian 43 | 0.75 | 0.84 | 0.75 |
| 364 | Train 6 | Test Asian 44 | 0.78 | 0.86 | 0.84 | 1388 | Train 22 | Test Asian 44 | 0.78 | 0.86 | 0.79 |
| 365 | Train 6 | Test Asian 45 | 0.75 | 0.81 | 0.8 | 1389 | Train 22 | Test Asian 45 | 0.75 | 0.8 | 0.74 |
| 366 | Train 6 | Test Asian 46 | 0.76 | 0.82 | 0.79 | 1390 | Train 22 | Test Asian 46 | 0.76 | 0.81 | 0.76 |
| 367 | Train 6 | Test Asian 47 | 0.75 | 0.8 | 0.8 | 1391 | Train 22 | Test Asian 47 | 0.75 | 0.8 | 0.73 |
| 368 | Train 6 | Test Asian 48 | 0.76 | 0.81 | 0.79 | 1392 | Train 22 | Test Asian 48 | 0.76 | 0.81 | 0.75 |
| 369 | Train 6 | Test Asian 49 | 0.77 | 0.84 | 0.78 | 1393 | Train 22 | Test Asian 49 | 0.77 | 0.83 | 0.8 |
| 370 | Train 6 | Test Asian 50 | 0.77 | 0.84 | 0.79 | 1394 | Train 22 | Test Asian 50 | 0.77 | 0.84 | 0.81 |
| 371 | Train 6 | Test Asian 51 | 0.77 | 0.84 | 0.78 | 1395 | Train 22 | Test Asian 51 | 0.77 | 0.83 | 0.8 |
| 372 | Train 6 | Test Asian 52 | 0.77 | 0.84 | 0.79 | 1396 | Train 22 | Test Asian 52 | 0.77 | 0.84 | 0.81 |
| 373 | Train 6 | Test Asian 53 | 0.8 | 0.85 | 0.79 | 1397 | Train 22 | Test Asian 53 | 0.8 | 0.84 | 0.8 |
| 374 | Train 6 | Test Asian 54 | 0.79 | 0.83 | 0.84 | 1398 | Train 22 | Test Asian 54 | 0.79 | 0.83 | 0.84 |
| 375 | Train 6 | Test Asian 55 | 0.79 | 0.84 | 0.78 | 1399 | Train 22 | Test Asian 55 | 0.79 | 0.84 | 0.79 |
| 376 | Train 6 | Test Asian 56 | 0.78 | 0.83 | 0.83 | 1400 | Train 22 | Test Asian 56 | 0.78 | 0.83 | 0.83 |
| 377 | Train 6 | Test Asian 57 | 0.76 | 0.84 | 0.82 | 1401 | Train 22 | Test Asian 57 | 0.76 | 0.83 | 0.83 |
| 378 | Train 6 | Test Asian 58 | 0.76 | 0.84 | 0.82 | 1402 | Train 22 | Test Asian 58 | 0.76 | 0.83 | 0.83 |
| 379 | Train 6 | Test Asian 59 | 0.76 | 0.84 | 0.82 | 1403 | Train 22 | Test Asian 59 | 0.76 | 0.83 | 0.83 |
| 380 | Train 6 | Test Asian 60 | 0.76 | 0.84 | 0.82 | 1404 | Train 22 | Test Asian 60 | 0.76 | 0.83 | 0.83 |
| 381 | Train 6 | Test Asian 61 | 0.79 | 0.84 | 0.8 | 1405 | Train 22 | Test Asian 61 | 0.79 | 0.84 | 0.8 |
| 382 | Train 6 | Test Asian 62 | 0.79 | 0.83 | 0.84 | 1406 | Train 22 | Test Asian 62 | 0.79 | 0.83 | 0.84 |
| 383 | Train 6 | Test Asian 63 | 0.78 | 0.84 | 0.79 | 1407 | Train 22 | Test Asian 63 | 0.78 | 0.84 | 0.79 |
| 384 | Train 6 | Test Asian 64 | 0.78 | 0.83 | 0.83 | 1408 | Train 22 | Test Asian 64 | 0.78 | 0.83 | 0.83 |
| 385 | Train 7 | Test Asian 1 | 0.78 | 0.87 | 0.87 | 1409 | Train 23 | Test Asian 1 | 0.78 | 0.87 | 0.85 |
| 386 | Train 7 | Test Asian 2 | 0.8 | 0.87 | 0.85 | 1410 | Train 23 | Test Asian 2 | 0.8 | 0.87 | 0.85 |
| 387 | Train 7 | Test Asian 3 | 0.78 | 0.87 | 0.87 | 1411 | Train 23 | Test Asian 3 | 0.78 | 0.87 | 0.85 |
| 388 | Train 7 | Test Asian 4 | 0.8 | 0.87 | 0.85 | 1412 | Train 23 | Test Asian 4 | 0.8 | 0.87 | 0.85 |
| 389 | Train 7 | Test Asian 5 | 0.76 | 0.85 | 0.87 | 1413 | Train 23 | Test Asian 5 | 0.76 | 0.85 | 0.85 |
| 390 | Train 7 | Test Asian 6 | 0.75 | 0.78 | 0.74 | 1414 | Train 23 | Test Asian 6 | 0.75 | 0.78 | 0.76 |
| 391 | Train 7 | Test Asian 7 | 0.76 | 0.85 | 0.87 | 1415 | Train 23 | Test Asian 7 | 0.76 | 0.85 | 0.85 |
| 392 | Train 7 | Test Asian 8 | 0.75 | 0.78 | 0.75 | 1416 | Train 23 | Test Asian 8 | 0.75 | 0.78 | 0.76 |
| 393 | Train 7 | Test Asian 9 | 0.77 | 0.85 | 0.84 | 1417 | Train 23 | Test Asian 9 | 0.77 | 0.85 | 0.84 |
| 394 | Train 7 | Test Asian 10 | 0.79 | 0.85 | 0.81 | 1418 | Train 23 | Test Asian 10 | 0.79 | 0.85 | 0.83 |
| 395 | Train 7 | Test Asian 11 | 0.77 | 0.85 | 0.84 | 1419 | Train 23 | Test Asian 11 | 0.77 | 0.85 | 0.84 |
| 396 | Train 7 | Test Asian 12 | 0.79 | 0.85 | 0.81 | 1420 | Train 23 | Test Asian 12 | 0.79 | 0.85 | 0.83 |
| 397 | Train 7 | Test Asian 13 | 0.76 | 0.83 | 0.84 | 1421 | Train 23 | Test Asian 13 | 0.76 | 0.83 | 0.83 |
| 398 | Train 7 | Test Asian 14 | 0.75 | 0.76 | 0.72 | 1422 | Train 23 | Test Asian 14 | 0.75 | 0.75 | 0.74 |
| 399 | Train 7 | Test Asian 15 | 0.76 | 0.83 | 0.83 | 1423 | Train 23 | Test Asian 15 | 0.76 | 0.83 | 0.83 |
| 400 | Train 7 | Test Asian 16 | 0.75 | 0.78 | 0.74 | 1424 | Train 23 | Test Asian 16 | 0.75 | 0.78 | 0.76 |
| 401 | Train 7 | Test Asian 17 | 0.78 | 0.87 | 0.87 | 1425 | Train 23 | Test Asian 17 | 0.78 | 0.87 | 0.85 |
| 402 | Train 7 | Test Asian 18 | 0.8 | 0.87 | 0.85 | 1426 | Train 23 | Test Asian 18 | 0.8 | 0.87 | 0.85 |
| 403 | Train 7 | Test Asian 19 | 0.78 | 0.87 | 0.87 | 1427 | Train 23 | Test Asian 19 | 0.78 | 0.87 | 0.85 |
| 404 | Train 7 | Test Asian 20 | 0.8 | 0.87 | 0.85 | 1428 | Train 23 | Test Asian 20 | 0.8 | 0.87 | 0.85 |
| 405 | Train 7 | Test Asian 21 | 0.77 | 0.85 | 0.87 | 1429 | Train 23 | Test Asian 21 | 0.77 | 0.85 | 0.85 |
| 406 | Train 7 | Test Asian 22 | 0.75 | 0.78 | 0.74 | 1430 | Train 23 | Test Asian 22 | 0.75 | 0.78 | 0.76 |
| 407 | Train 7 | Test Asian 23 | 0.76 | 0.85 | 0.87 | 1431 | Train 23 | Test Asian 23 | 0.76 | 0.85 | 0.85 |
| 408 | Train 7 | Test Asian 24 | 0.76 | 0.78 | 0.75 | 1432 | Train 23 | Test Asian 24 | 0.76 | 0.78 | 0.76 |
| 409 | Train 7 | Test Asian 25 | 0.77 | 0.85 | 0.84 | 1433 | Train 23 | Test Asian 25 | 0.77 | 0.85 | 0.84 |
| 410 | Train 7 | Test Asian 26 | 0.79 | 0.85 | 0.81 | 1434 | Train 23 | Test Asian 26 | 0.79 | 0.85 | 0.83 |
| 411 | Train 7 | Test Asian 27 | 0.77 | 0.85 | 0.84 | 1435 | Train 23 | Test Asian 27 | 0.77 | 0.85 | 0.84 |
| 412 | Train 7 | Test Asian 28 | 0.79 | 0.85 | 0.81 | 1436 | Train 23 | Test Asian 28 | 0.79 | 0.85 | 0.83 |
| 413 | Train 7 | Test Asian 29 | 0.76 | 0.83 | 0.84 | 1437 | Train 23 | Test Asian 29 | 0.76 | 0.83 | 0.83 |
| 414 | Train 7 | Test Asian 30 | 0.75 | 0.76 | 0.72 | 1438 | Train 23 | Test Asian 30 | 0.75 | 0.75 | 0.74 |
| 415 | Train 7 | Test Asian 31 | 0.76 | 0.83 | 0.83 | 1439 | Train 23 | Test Asian 31 | 0.76 | 0.83 | 0.83 |
| 416 | Train 7 | Test Asian 32 | 0.76 | 0.78 | 0.74 | 1440 | Train 23 | Test Asian 32 | 0.76 | 0.78 | 0.76 |
| 417 | Train 7 | Test Asian 33 | 0.78 | 0.87 | 0.87 | 1441 | Train 23 | Test Asian 33 | 0.78 | 0.87 | 0.85 |
| 418 | Train 7 | Test Asian 34 | 0.8 | 0.87 | 0.85 | 1442 | Train 23 | Test Asian 34 | 0.8 | 0.87 | 0.85 |
| 419 | Train 7 | Test Asian 35 | 0.78 | 0.87 | 0.87 | 1443 | Train 23 | Test Asian 35 | 0.78 | 0.87 | 0.85 |
| 420 | Train 7 | Test Asian 36 | 0.8 | 0.87 | 0.85 | 1444 | Train 23 | Test Asian 36 | 0.8 | 0.87 | 0.85 |
| 421 | Train 7 | Test Asian 37 | 0.76 | 0.85 | 0.87 | 1445 | Train 23 | Test Asian 37 | 0.76 | 0.85 | 0.85 |
| 422 | Train 7 | Test Asian 38 | 0.75 | 0.78 | 0.74 | 1446 | Train 23 | Test Asian 38 | 0.75 | 0.78 | 0.76 |
| 423 | Train 7 | Test Asian 39 | 0.76 | 0.84 | 0.86 | 1447 | Train 23 | Test Asian 39 | 0.76 | 0.84 | 0.85 |
| 424 | Train 7 | Test Asian 40 | 0.75 | 0.78 | 0.75 | 1448 | Train 23 | Test Asian 40 | 0.75 | 0.78 | 0.76 |
| 425 | Train 7 | Test Asian 41 | 0.77 | 0.85 | 0.84 | 1449 | Train 23 | Test Asian 41 | 0.77 | 0.85 | 0.84 |
| 426 | Train 7 | Test Asian 42 | 0.8 | 0.85 | 0.8 | 1450 | Train 23 | Test Asian 42 | 0.8 | 0.85 | 0.83 |
| 427 | Train 7 | Test Asian 43 | 0.77 | 0.85 | 0.84 | 1451 | Train 23 | Test Asian 43 | 0.77 | 0.85 | 0.84 |
| 428 | Train 7 | Test Asian 44 | 0.8 | 0.85 | 0.8 | 1452 | Train 23 | Test Asian 44 | 0.8 | 0.85 | 0.83 |
| 429 | Train 7 | Test Asian 45 | 0.76 | 0.83 | 0.83 | 1453 | Train 23 | Test Asian 45 | 0.76 | 0.83 | 0.83 |
| 430 | Train 7 | Test Asian 46 | 0.75 | 0.78 | 0.74 | 1454 | Train 23 | Test Asian 46 | 0.75 | 0.78 | 0.76 |
| 431 | Train 7 | Test Asian 47 | 0.76 | 0.83 | 0.83 | 1455 | Train 23 | Test Asian 47 | 0.76 | 0.83 | 0.83 |
| 432 | Train 7 | Test Asian 48 | 0.75 | 0.78 | 0.74 | 1456 | Train 23 | Test Asian 48 | 0.75 | 0.77 | 0.76 |
| 433 | Train 7 | Test Asian 49 | 0.76 | 0.84 | 0.88 | 1457 | Train 23 | Test Asian 49 | 0.76 | 0.84 | 0.8 |
| 434 | Train 7 | Test Asian 50 | 0.76 | 0.84 | 0.88 | 1458 | Train 23 | Test Asian 50 | 0.76 | 0.84 | 0.8 |
| 435 | Train 7 | Test Asian 51 | 0.76 | 0.84 | 0.88 | 1459 | Train 23 | Test Asian 51 | 0.76 | 0.84 | 0.8 |
| 436 | Train 7 | Test Asian 52 | 0.76 | 0.84 | 0.88 | 1460 | Train 23 | Test Asian 52 | 0.76 | 0.84 | 0.8 |
| 437 | Train 7 | Test Asian 53 | 0.8 | 0.85 | 0.78 | 1461 | Train 23 | Test Asian 53 | 0.8 | 0.85 | 0.82 |
| 438 | Train 7 | Test Asian 54 | 0.77 | 0.75 | 0.68 | 1462 | Train 23 | Test Asian 54 | 0.77 | 0.75 | 0.72 |
| 439 | Train 7 | Test Asian 55 | 0.8 | 0.85 | 0.77 | 1463 | Train 23 | Test Asian 55 | 0.8 | 0.85 | 0.81 |
| 440 | Train 7 | Test Asian 56 | 0.77 | 0.75 | 0.68 | 1464 | Train 23 | Test Asian 56 | 0.77 | 0.74 | 0.72 |
| 441 | Train 7 | Test Asian 57 | 0.74 | 0.82 | 0.85 | 1465 | Train 23 | Test Asian 57 | 0.74 | 0.82 | 0.8 |
| 442 | Train 7 | Test Asian 58 | 0.74 | 0.82 | 0.84 | 1466 | Train 23 | Test Asian 58 | 0.74 | 0.82 | 0.8 |
| 443 | Train 7 | Test Asian 59 | 0.74 | 0.82 | 0.85 | 1467 | Train 23 | Test Asian 59 | 0.74 | 0.82 | 0.8 |
| 444 | Train 7 | Test Asian 60 | 0.74 | 0.82 | 0.84 | 1468 | Train 23 | Test Asian 60 | 0.74 | 0.82 | 0.8 |
| 445 | Train 7 | Test Asian 61 | 0.79 | 0.85 | 0.77 | 1469 | Train 23 | Test Asian 61 | 0.79 | 0.84 | 0.82 |
| 446 | Train 7 | Test Asian 62 | 0.77 | 0.75 | 0.68 | 1470 | Train 23 | Test Asian 62 | 0.77 | 0.75 | 0.72 |
| 447 | Train 7 | Test Asian 63 | 0.79 | 0.84 | 0.76 | 1471 | Train 23 | Test Asian 63 | 0.79 | 0.84 | 0.81 |
| 448 | Train 7 | Test Asian 64 | 0.77 | 0.75 | 0.68 | 1472 | Train 23 | Test Asian 64 | 0.77 | 0.74 | 0.72 |
| 449 | Train 8 | Test Asian 1 | 0.76 | 0.84 | 0.82 | 1473 | Train 24 | Test Asian 1 | 0.76 | 0.85 | 0.78 |
| 450 | Train 8 | Test Asian 2 | 0.79 | 0.86 | 0.84 | 1474 | Train 24 | Test Asian 2 | 0.79 | 0.86 | 0.8 |
| 451 | Train 8 | Test Asian 3 | 0.76 | 0.84 | 0.82 | 1475 | Train 24 | Test Asian 3 | 0.76 | 0.85 | 0.78 |
| 452 | Train 8 | Test Asian 4 | 0.79 | 0.86 | 0.84 | 1476 | Train 24 | Test Asian 4 | 0.79 | 0.86 | 0.8 |
| 453 | Train 8 | Test Asian 5 | 0.75 | 0.8 | 0.79 | 1477 | Train 24 | Test Asian 5 | 0.75 | 0.8 | 0.75 |
| 454 | Train 8 | Test Asian 6 | 0.76 | 0.82 | 0.79 | 1478 | Train 24 | Test Asian 6 | 0.76 | 0.82 | 0.77 |
| 455 | Train 8 | Test Asian 7 | 0.75 | 0.8 | 0.78 | 1479 | Train 24 | Test Asian 7 | 0.75 | 0.8 | 0.74 |
| 456 | Train 8 | Test Asian 8 | 0.76 | 0.82 | 0.79 | 1480 | Train 24 | Test Asian 8 | 0.76 | 0.82 | 0.77 |
| 457 | Train 8 | Test Asian 9 | 0.76 | 0.84 | 0.83 | 1481 | Train 24 | Test Asian 9 | 0.76 | 0.84 | 0.77 |
| 458 | Train 8 | Test Asian 10 | 0.78 | 0.86 | 0.84 | 1482 | Train 24 | Test Asian 10 | 0.78 | 0.86 | 0.8 |
| 459 | Train 8 | Test Asian 11 | 0.76 | 0.84 | 0.83 | 1483 | Train 24 | Test Asian 11 | 0.76 | 0.84 | 0.77 |
| 460 | Train 8 | Test Asian 12 | 0.78 | 0.86 | 0.84 | 1484 | Train 24 | Test Asian 12 | 0.78 | 0.86 | 0.8 |
| 461 | Train 8 | Test Asian 13 | 0.75 | 0.8 | 0.79 | 1485 | Train 24 | Test Asian 13 | 0.75 | 0.8 | 0.76 |
| 462 | Train 8 | Test Asian 14 | 0.76 | 0.83 | 0.83 | 1486 | Train 24 | Test Asian 14 | 0.76 | 0.83 | 0.83 |
| 463 | Train 8 | Test Asian 15 | 0.75 | 0.8 | 0.79 | 1487 | Train 24 | Test Asian 15 | 0.75 | 0.8 | 0.75 |
| 464 | Train 8 | Test Asian 16 | 0.76 | 0.81 | 0.79 | 1488 | Train 24 | Test Asian 16 | 0.76 | 0.81 | 0.77 |
| 465 | Train 8 | Test Asian 17 | 0.76 | 0.84 | 0.82 | 1489 | Train 24 | Test Asian 17 | 0.76 | 0.85 | 0.77 |
| 466 | Train 8 | Test Asian 18 | 0.79 | 0.86 | 0.84 | 1490 | Train 24 | Test Asian 18 | 0.79 | 0.86 | 0.8 |
| 467 | Train 8 | Test Asian 19 | 0.76 | 0.84 | 0.82 | 1491 | Train 24 | Test Asian 19 | 0.76 | 0.85 | 0.77 |
| 468 | Train 8 | Test Asian 20 | 0.79 | 0.86 | 0.84 | 1492 | Train 24 | Test Asian 20 | 0.79 | 0.86 | 0.8 |
| 469 | Train 8 | Test Asian 21 | 0.76 | 0.8 | 0.79 | 1493 | Train 24 | Test Asian 21 | 0.76 | 0.8 | 0.75 |
| 470 | Train 8 | Test Asian 22 | 0.76 | 0.82 | 0.79 | 1494 | Train 24 | Test Asian 22 | 0.76 | 0.82 | 0.77 |
| 471 | Train 8 | Test Asian 23 | 0.75 | 0.8 | 0.78 | 1495 | Train 24 | Test Asian 23 | 0.75 | 0.8 | 0.75 |
| 472 | Train 8 | Test Asian 24 | 0.77 | 0.82 | 0.79 | 1496 | Train 24 | Test Asian 24 | 0.77 | 0.82 | 0.77 |
| 473 | Train 8 | Test Asian 25 | 0.76 | 0.84 | 0.83 | 1497 | Train 24 | Test Asian 25 | 0.76 | 0.84 | 0.77 |
| 474 | Train 8 | Test Asian 26 | 0.78 | 0.86 | 0.84 | 1498 | Train 24 | Test Asian 26 | 0.78 | 0.86 | 0.8 |
| 475 | Train 8 | Test Asian 27 | 0.76 | 0.84 | 0.83 | 1499 | Train 24 | Test Asian 27 | 0.76 | 0.84 | 0.77 |
| 476 | Train 8 | Test Asian 28 | 0.78 | 0.86 | 0.84 | 1500 | Train 24 | Test Asian 28 | 0.78 | 0.86 | 0.8 |
| 477 | Train 8 | Test Asian 29 | 0.75 | 0.8 | 0.79 | 1501 | Train 24 | Test Asian 29 | 0.75 | 0.8 | 0.76 |
| 478 | Train 8 | Test Asian 30 | 0.76 | 0.83 | 0.83 | 1502 | Train 24 | Test Asian 30 | 0.76 | 0.83 | 0.83 |
| 479 | Train 8 | Test Asian 31 | 0.75 | 0.8 | 0.79 | 1503 | Train 24 | Test Asian 31 | 0.75 | 0.8 | 0.75 |
| 480 | Train 8 | Test Asian 32 | 0.76 | 0.81 | 0.79 | 1504 | Train 24 | Test Asian 32 | 0.76 | 0.81 | 0.77 |
| 481 | Train 8 | Test Asian 33 | 0.76 | 0.84 | 0.82 | 1505 | Train 24 | Test Asian 33 | 0.76 | 0.84 | 0.77 |
| 482 | Train 8 | Test Asian 34 | 0.78 | 0.86 | 0.84 | 1506 | Train 24 | Test Asian 34 | 0.78 | 0.86 | 0.79 |
| 483 | Train 8 | Test Asian 35 | 0.76 | 0.84 | 0.82 | 1507 | Train 24 | Test Asian 35 | 0.76 | 0.84 | 0.77 |
| 484 | Train 8 | Test Asian 36 | 0.78 | 0.86 | 0.84 | 1508 | Train 24 | Test Asian 36 | 0.78 | 0.86 | 0.79 |
| 485 | Train 8 | Test Asian 37 | 0.75 | 0.8 | 0.78 | 1509 | Train 24 | Test Asian 37 | 0.75 | 0.8 | 0.75 |
| 486 | Train 8 | Test Asian 38 | 0.76 | 0.81 | 0.79 | 1510 | Train 24 | Test Asian 38 | 0.76 | 0.81 | 0.77 |
| 487 | Train 8 | Test Asian 39 | 0.75 | 0.8 | 0.78 | 1511 | Train 24 | Test Asian 39 | 0.75 | 0.8 | 0.74 |
| 488 | Train 8 | Test Asian 40 | 0.76 | 0.81 | 0.79 | 1512 | Train 24 | Test Asian 40 | 0.76 | 0.81 | 0.77 |
| 489 | Train 8 | Test Asian 41 | 0.76 | 0.84 | 0.83 | 1513 | Train 24 | Test Asian 41 | 0.76 | 0.84 | 0.77 |
| 490 | Train 8 | Test Asian 42 | 0.78 | 0.85 | 0.84 | 1514 | Train 24 | Test Asian 42 | 0.78 | 0.86 | 0.79 |
| 491 | Train 8 | Test Asian 43 | 0.76 | 0.84 | 0.83 | 1515 | Train 24 | Test Asian 43 | 0.76 | 0.84 | 0.77 |
| 492 | Train 8 | Test Asian 44 | 0.78 | 0.85 | 0.84 | 1516 | Train 24 | Test Asian 44 | 0.78 | 0.86 | 0.79 |
| 493 | Train 8 | Test Asian 45 | 0.75 | 0.8 | 0.79 | 1517 | Train 24 | Test Asian 45 | 0.75 | 0.8 | 0.76 |
| 494 | Train 8 | Test Asian 46 | 0.76 | 0.81 | 0.79 | 1518 | Train 24 | Test Asian 46 | 0.76 | 0.81 | 0.77 |
| 495 | Train 8 | Test Asian 47 | 0.75 | 0.8 | 0.79 | 1519 | Train 24 | Test Asian 47 | 0.75 | 0.8 | 0.75 |
| 496 | Train 8 | Test Asian 48 | 0.76 | 0.81 | 0.79 | 1520 | Train 24 | Test Asian 48 | 0.76 | 0.81 | 0.77 |
| 497 | Train 8 | Test Asian 49 | 0.77 | 0.83 | 0.73 | 1521 | Train 24 | Test Asian 49 | 0.77 | 0.84 | 0.78 |
| 498 | Train 8 | Test Asian 50 | 0.77 | 0.83 | 0.73 | 1522 | Train 24 | Test Asian 50 | 0.77 | 0.84 | 0.79 |
| 499 | Train 8 | Test Asian 51 | 0.77 | 0.83 | 0.73 | 1523 | Train 24 | Test Asian 51 | 0.77 | 0.84 | 0.78 |
| 500 | Train 8 | Test Asian 52 | 0.77 | 0.83 | 0.73 | 1524 | Train 24 | Test Asian 52 | 0.77 | 0.84 | 0.79 |
| 501 | Train 8 | Test Asian 53 | 0.8 | 0.84 | 0.77 | 1525 | Train 24 | Test Asian 53 | 0.8 | 0.84 | 0.79 |
| 502 | Train 8 | Test Asian 54 | 0.79 | 0.83 | 0.83 | 1526 | Train 24 | Test Asian 54 | 0.79 | 0.83 | 0.84 |
| 503 | Train 8 | Test Asian 55 | 0.79 | 0.83 | 0.76 | 1527 | Train 24 | Test Asian 55 | 0.79 | 0.84 | 0.78 |
| 504 | Train 8 | Test Asian 56 | 0.79 | 0.83 | 0.83 | 1528 | Train 24 | Test Asian 56 | 0.79 | 0.83 | 0.84 |
| 505 | Train 8 | Test Asian 57 | 0.77 | 0.83 | 0.75 | 1529 | Train 24 | Test Asian 57 | 0.76 | 0.84 | 0.8 |
| 506 | Train 8 | Test Asian 58 | 0.76 | 0.83 | 0.75 | 1530 | Train 24 | Test Asian 58 | 0.76 | 0.84 | 0.8 |
| 507 | Train 8 | Test Asian 59 | 0.77 | 0.83 | 0.75 | 1531 | Train 24 | Test Asian 59 | 0.76 | 0.84 | 0.8 |
| 508 | Train 8 | Test Asian 60 | 0.76 | 0.83 | 0.75 | 1532 | Train 24 | Test Asian 60 | 0.76 | 0.84 | 0.8 |
| 509 | Train 8 | Test Asian 61 | 0.79 | 0.84 | 0.77 | 1533 | Train 24 | Test Asian 61 | 0.79 | 0.84 | 0.78 |
| 510 | Train 8 | Test Asian 62 | 0.79 | 0.83 | 0.83 | 1534 | Train 24 | Test Asian 62 | 0.79 | 0.83 | 0.84 |
| 511 | Train 8 | Test Asian 63 | 0.78 | 0.83 | 0.76 | 1535 | Train 24 | Test Asian 63 | 0.78 | 0.83 | 0.77 |
| 512 | Train 8 | Test Asian 64 | 0.79 | 0.83 | 0.83 | 1536 | Train 24 | Test Asian 64 | 0.79 | 0.83 | 0.84 |
| 513 | Train 9 | Test Asian 1 | 0.8 | 0.92 | 0.89 | 1537 | Train 25 | Test Asian 1 | 0.8 | 0.91 | 0.87 |
| 514 | Train 9 | Test Asian 2 | 0.81 | 0.89 | 0.84 | 1538 | Train 25 | Test Asian 2 | 0.81 | 0.89 | 0.84 |
| 515 | Train 9 | Test Asian 3 | 0.8 | 0.92 | 0.89 | 1539 | Train 25 | Test Asian 3 | 0.8 | 0.91 | 0.87 |
| 516 | Train 9 | Test Asian 4 | 0.81 | 0.89 | 0.84 | 1540 | Train 25 | Test Asian 4 | 0.81 | 0.89 | 0.84 |
| 517 | Train 9 | Test Asian 5 | 0.75 | 0.75 | 0.67 | 1541 | Train 25 | Test Asian 5 | 0.75 | 0.75 | 0.72 |
| 518 | Train 9 | Test Asian 6 | 0.75 | 0.75 | 0.67 | 1542 | Train 25 | Test Asian 6 | 0.75 | 0.75 | 0.69 |
| 519 | Train 9 | Test Asian 7 | 0.75 | 0.75 | 0.67 | 1543 | Train 25 | Test Asian 7 | 0.74 | 0.75 | 0.71 |
| 520 | Train 9 | Test Asian 8 | 0.75 | 0.75 | 0.68 | 1544 | Train 25 | Test Asian 8 | 0.75 | 0.75 | 0.69 |
| 521 | Train 9 | Test Asian 9 | 0.79 | 0.92 | 0.93 | 1545 | Train 25 | Test Asian 9 | 0.79 | 0.92 | 0.92 |
| 522 | Train 9 | Test Asian 10 | 0.79 | 0.89 | 0.88 | 1546 | Train 25 | Test Asian 10 | 0.8 | 0.89 | 0.87 |
| 523 | Train 9 | Test Asian 11 | 0.79 | 0.92 | 0.93 | 1547 | Train 25 | Test Asian 11 | 0.79 | 0.92 | 0.92 |
| 524 | Train 9 | Test Asian 12 | 0.8 | 0.89 | 0.88 | 1548 | Train 25 | Test Asian 12 | 0.8 | 0.89 | 0.87 |
| 525 | Train 9 | Test Asian 13 | 0.74 | 0.76 | 0.68 | 1549 | Train 25 | Test Asian 13 | 0.74 | 0.75 | 0.72 |
| 526 | Train 9 | Test Asian 14 | 0.75 | 0.73 | 0.65 | 1550 | Train 25 | Test Asian 14 | 0.75 | 0.73 | 0.67 |
| 527 | Train 9 | Test Asian 15 | 0.74 | 0.75 | 0.68 | 1551 | Train 25 | Test Asian 15 | 0.74 | 0.75 | 0.72 |
| 528 | Train 9 | Test Asian 16 | 0.75 | 0.75 | 0.68 | 1552 | Train 25 | Test Asian 16 | 0.75 | 0.75 | 0.69 |
| 529 | Train 9 | Test Asian 17 | 0.8 | 0.92 | 0.89 | 1553 | Train 25 | Test Asian 17 | 0.8 | 0.91 | 0.87 |
| 530 | Train 9 | Test Asian 18 | 0.81 | 0.89 | 0.84 | 1554 | Train 25 | Test Asian 18 | 0.81 | 0.89 | 0.84 |
| 531 | Train 9 | Test Asian 19 | 0.8 | 0.92 | 0.89 | 1555 | Train 25 | Test Asian 19 | 0.8 | 0.91 | 0.87 |
| 532 | Train 9 | Test Asian 20 | 0.81 | 0.89 | 0.84 | 1556 | Train 25 | Test Asian 20 | 0.81 | 0.89 | 0.84 |
| 533 | Train 9 | Test Asian 21 | 0.75 | 0.75 | 0.67 | 1557 | Train 25 | Test Asian 21 | 0.75 | 0.75 | 0.72 |
| 534 | Train 9 | Test Asian 22 | 0.75 | 0.75 | 0.68 | 1558 | Train 25 | Test Asian 22 | 0.75 | 0.75 | 0.69 |
| 535 | Train 9 | Test Asian 23 | 0.75 | 0.75 | 0.67 | 1559 | Train 25 | Test Asian 23 | 0.75 | 0.75 | 0.71 |
| 536 | Train 9 | Test Asian 24 | 0.75 | 0.75 | 0.68 | 1560 | Train 25 | Test Asian 24 | 0.75 | 0.75 | 0.69 |
| 537 | Train 9 | Test Asian 25 | 0.79 | 0.92 | 0.93 | 1561 | Train 25 | Test Asian 25 | 0.79 | 0.92 | 0.92 |
| 538 | Train 9 | Test Asian 26 | 0.79 | 0.89 | 0.88 | 1562 | Train 25 | Test Asian 26 | 0.8 | 0.89 | 0.87 |
| 539 | Train 9 | Test Asian 27 | 0.79 | 0.92 | 0.93 | 1563 | Train 25 | Test Asian 27 | 0.79 | 0.92 | 0.92 |
| 540 | Train 9 | Test Asian 28 | 0.79 | 0.89 | 0.88 | 1564 | Train 25 | Test Asian 28 | 0.8 | 0.89 | 0.87 |
| 541 | Train 9 | Test Asian 29 | 0.75 | 0.76 | 0.68 | 1565 | Train 25 | Test Asian 29 | 0.75 | 0.75 | 0.72 |
| 542 | Train 9 | Test Asian 30 | 0.75 | 0.73 | 0.65 | 1566 | Train 25 | Test Asian 30 | 0.75 | 0.73 | 0.67 |
| 543 | Train 9 | Test Asian 31 | 0.74 | 0.75 | 0.68 | 1567 | Train 25 | Test Asian 31 | 0.74 | 0.75 | 0.72 |
| 544 | Train 9 | Test Asian 32 | 0.75 | 0.75 | 0.68 | 1568 | Train 25 | Test Asian 32 | 0.75 | 0.75 | 0.69 |
| 545 | Train 9 | Test Asian 33 | 0.8 | 0.92 | 0.89 | 1569 | Train 25 | Test Asian 33 | 0.8 | 0.91 | 0.87 |
| 546 | Train 9 | Test Asian 34 | 0.8 | 0.89 | 0.84 | 1570 | Train 25 | Test Asian 34 | 0.8 | 0.89 | 0.84 |
| 547 | Train 9 | Test Asian 35 | 0.8 | 0.92 | 0.89 | 1571 | Train 25 | Test Asian 35 | 0.8 | 0.91 | 0.87 |
| 548 | Train 9 | Test Asian 36 | 0.8 | 0.89 | 0.84 | 1572 | Train 25 | Test Asian 36 | 0.8 | 0.89 | 0.84 |
| 549 | Train 9 | Test Asian 37 | 0.75 | 0.75 | 0.67 | 1573 | Train 25 | Test Asian 37 | 0.75 | 0.75 | 0.71 |
| 550 | Train 9 | Test Asian 38 | 0.75 | 0.75 | 0.68 | 1574 | Train 25 | Test Asian 38 | 0.75 | 0.74 | 0.69 |
| 551 | Train 9 | Test Asian 39 | 0.74 | 0.75 | 0.67 | 1575 | Train 25 | Test Asian 39 | 0.74 | 0.74 | 0.71 |
| 552 | Train 9 | Test Asian 40 | 0.75 | 0.74 | 0.68 | 1576 | Train 25 | Test Asian 40 | 0.75 | 0.74 | 0.69 |
| 553 | Train 9 | Test Asian 41 | 0.79 | 0.92 | 0.93 | 1577 | Train 25 | Test Asian 41 | 0.79 | 0.92 | 0.92 |
| 554 | Train 9 | Test Asian 42 | 0.79 | 0.89 | 0.88 | 1578 | Train 25 | Test Asian 42 | 0.79 | 0.89 | 0.87 |
| 555 | Train 9 | Test Asian 43 | 0.79 | 0.92 | 0.93 | 1579 | Train 25 | Test Asian 43 | 0.79 | 0.92 | 0.92 |
| 556 | Train 9 | Test Asian 44 | 0.79 | 0.89 | 0.88 | 1580 | Train 25 | Test Asian 44 | 0.79 | 0.89 | 0.87 |
| 557 | Train 9 | Test Asian 45 | 0.74 | 0.75 | 0.68 | 1581 | Train 25 | Test Asian 45 | 0.74 | 0.75 | 0.72 |
| 558 | Train 9 | Test Asian 46 | 0.75 | 0.75 | 0.67 | 1582 | Train 25 | Test Asian 46 | 0.75 | 0.75 | 0.69 |
| 559 | Train 9 | Test Asian 47 | 0.74 | 0.75 | 0.68 | 1583 | Train 25 | Test Asian 47 | 0.74 | 0.75 | 0.72 |
| 560 | Train 9 | Test Asian 48 | 0.75 | 0.75 | 0.68 | 1584 | Train 25 | Test Asian 48 | 0.75 | 0.74 | 0.69 |
| 561 | Train 9 | Test Asian 49 | 0.77 | 0.87 | 0.85 | 1585 | Train 25 | Test Asian 49 | 0.77 | 0.86 | 0.84 |
| 562 | Train 9 | Test Asian 50 | 0.77 | 0.87 | 0.85 | 1586 | Train 25 | Test Asian 50 | 0.77 | 0.87 | 0.84 |
| 563 | Train 9 | Test Asian 51 | 0.77 | 0.87 | 0.85 | 1587 | Train 25 | Test Asian 51 | 0.77 | 0.86 | 0.84 |
| 564 | Train 9 | Test Asian 52 | 0.77 | 0.87 | 0.85 | 1588 | Train 25 | Test Asian 52 | 0.77 | 0.87 | 0.84 |
| 565 | Train 9 | Test Asian 53 | 0.79 | 0.81 | 0.67 | 1589 | Train 25 | Test Asian 53 | 0.79 | 0.8 | 0.7 |
| 566 | Train 9 | Test Asian 54 | 0.78 | 0.75 | 0.65 | 1590 | Train 25 | Test Asian 54 | 0.78 | 0.75 | 0.65 |
| 567 | Train 9 | Test Asian 55 | 0.79 | 0.8 | 0.67 | 1591 | Train 25 | Test Asian 55 | 0.79 | 0.8 | 0.7 |
| 568 | Train 9 | Test Asian 56 | 0.78 | 0.74 | 0.66 | 1592 | Train 25 | Test Asian 56 | 0.78 | 0.74 | 0.65 |
| 569 | Train 9 | Test Asian 57 | 0.76 | 0.88 | 0.91 | 1593 | Train 25 | Test Asian 57 | 0.76 | 0.88 | 0.89 |
| 570 | Train 9 | Test Asian 58 | 0.76 | 0.88 | 0.91 | 1594 | Train 25 | Test Asian 58 | 0.76 | 0.88 | 0.89 |
| 571 | Train 9 | Test Asian 59 | 0.76 | 0.88 | 0.91 | 1595 | Train 25 | Test Asian 59 | 0.76 | 0.88 | 0.89 |
| 572 | Train 9 | Test Asian 60 | 0.76 | 0.88 | 0.91 | 1596 | Train 25 | Test Asian 60 | 0.76 | 0.88 | 0.89 |
| 573 | Train 9 | Test Asian 61 | 0.79 | 0.81 | 0.67 | 1597 | Train 25 | Test Asian 61 | 0.79 | 0.8 | 0.7 |
| 574 | Train 9 | Test Asian 62 | 0.78 | 0.75 | 0.66 | 1598 | Train 25 | Test Asian 62 | 0.78 | 0.75 | 0.65 |
| 575 | Train 9 | Test Asian 63 | 0.78 | 0.8 | 0.67 | 1599 | Train 25 | Test Asian 63 | 0.78 | 0.8 | 0.7 |
| 576 | Train 9 | Test Asian 64 | 0.78 | 0.74 | 0.66 | 1600 | Train 25 | Test Asian 64 | 0.78 | 0.74 | 0.65 |
| 577 | Train 10 | Test Asian 1 | 0.79 | 0.9 | 0.89 | 1601 | Train 26 | Test Asian 1 | 0.79 | 0.9 | 0.88 |
| 578 | Train 10 | Test Asian 2 | 0.81 | 0.91 | 0.9 | 1602 | Train 26 | Test Asian 2 | 0.81 | 0.91 | 0.89 |
| 579 | Train 10 | Test Asian 3 | 0.79 | 0.9 | 0.89 | 1603 | Train 26 | Test Asian 3 | 0.79 | 0.9 | 0.88 |
| 580 | Train 10 | Test Asian 4 | 0.81 | 0.91 | 0.9 | 1604 | Train 26 | Test Asian 4 | 0.81 | 0.91 | 0.89 |
| 581 | Train 10 | Test Asian 5 | 0.75 | 0.76 | 0.71 | 1605 | Train 26 | Test Asian 5 | 0.75 | 0.76 | 0.72 |
| 582 | Train 10 | Test Asian 6 | 0.75 | 0.75 | 0.7 | 1606 | Train 26 | Test Asian 6 | 0.75 | 0.75 | 0.7 |
| 583 | Train 10 | Test Asian 7 | 0.75 | 0.76 | 0.71 | 1607 | Train 26 | Test Asian 7 | 0.75 | 0.76 | 0.71 |
| 584 | Train 10 | Test Asian 8 | 0.75 | 0.75 | 0.69 | 1608 | Train 26 | Test Asian 8 | 0.75 | 0.75 | 0.7 |
| 585 | Train 10 | Test Asian 9 | 0.78 | 0.91 | 0.91 | 1609 | Train 26 | Test Asian 9 | 0.78 | 0.91 | 0.9 |
| 586 | Train 10 | Test Asian 10 | 0.8 | 0.91 | 0.92 | 1610 | Train 26 | Test Asian 10 | 0.8 | 0.91 | 0.91 |
| 587 | Train 10 | Test Asian 11 | 0.78 | 0.91 | 0.91 | 1611 | Train 26 | Test Asian 11 | 0.78 | 0.91 | 0.9 |
| 588 | Train 10 | Test Asian 12 | 0.8 | 0.91 | 0.92 | 1612 | Train 26 | Test Asian 12 | 0.8 | 0.91 | 0.91 |
| 589 | Train 10 | Test Asian 13 | 0.75 | 0.76 | 0.71 | 1613 | Train 26 | Test Asian 13 | 0.75 | 0.76 | 0.72 |
| 590 | Train 10 | Test Asian 14 | 0.75 | 0.74 | 0.68 | 1614 | Train 26 | Test Asian 14 | 0.75 | 0.74 | 0.69 |
| 591 | Train 10 | Test Asian 15 | 0.75 | 0.76 | 0.71 | 1615 | Train 26 | Test Asian 15 | 0.75 | 0.76 | 0.71 |
| 592 | Train 10 | Test Asian 16 | 0.75 | 0.75 | 0.69 | 1616 | Train 26 | Test Asian 16 | 0.75 | 0.75 | 0.7 |
| 593 | Train 10 | Test Asian 17 | 0.79 | 0.9 | 0.89 | 1617 | Train 26 | Test Asian 17 | 0.79 | 0.9 | 0.88 |
| 594 | Train 10 | Test Asian 18 | 0.81 | 0.91 | 0.9 | 1618 | Train 26 | Test Asian 18 | 0.81 | 0.91 | 0.89 |
| 595 | Train 10 | Test Asian 19 | 0.79 | 0.9 | 0.89 | 1619 | Train 26 | Test Asian 19 | 0.79 | 0.9 | 0.88 |
| 596 | Train 10 | Test Asian 20 | 0.81 | 0.91 | 0.9 | 1620 | Train 26 | Test Asian 20 | 0.81 | 0.91 | 0.89 |
| 597 | Train 10 | Test Asian 21 | 0.75 | 0.76 | 0.72 | 1621 | Train 26 | Test Asian 21 | 0.75 | 0.76 | 0.72 |
| 598 | Train 10 | Test Asian 22 | 0.75 | 0.75 | 0.7 | 1622 | Train 26 | Test Asian 22 | 0.75 | 0.75 | 0.71 |
| 599 | Train 10 | Test Asian 23 | 0.75 | 0.76 | 0.71 | 1623 | Train 26 | Test Asian 23 | 0.75 | 0.76 | 0.71 |
| 600 | Train 10 | Test Asian 24 | 0.75 | 0.75 | 0.69 | 1624 | Train 26 | Test Asian 24 | 0.75 | 0.75 | 0.7 |
| 601 | Train 10 | Test Asian 25 | 0.78 | 0.91 | 0.91 | 1625 | Train 26 | Test Asian 25 | 0.78 | 0.91 | 0.9 |
| 602 | Train 10 | Test Asian 26 | 0.8 | 0.91 | 0.92 | 1626 | Train 26 | Test Asian 26 | 0.8 | 0.91 | 0.91 |
| 603 | Train 10 | Test Asian 27 | 0.78 | 0.91 | 0.91 | 1627 | Train 26 | Test Asian 27 | 0.78 | 0.91 | 0.9 |
| 604 | Train 10 | Test Asian 28 | 0.8 | 0.91 | 0.92 | 1628 | Train 26 | Test Asian 28 | 0.8 | 0.91 | 0.91 |
| 605 | Train 10 | Test Asian 29 | 0.75 | 0.76 | 0.71 | 1629 | Train 26 | Test Asian 29 | 0.75 | 0.76 | 0.72 |
| 606 | Train 10 | Test Asian 30 | 0.75 | 0.74 | 0.68 | 1630 | Train 26 | Test Asian 30 | 0.75 | 0.74 | 0.69 |
| 607 | Train 10 | Test Asian 31 | 0.75 | 0.76 | 0.71 | 1631 | Train 26 | Test Asian 31 | 0.75 | 0.76 | 0.72 |
| 608 | Train 10 | Test Asian 32 | 0.75 | 0.75 | 0.7 | 1632 | Train 26 | Test Asian 32 | 0.75 | 0.75 | 0.7 |
| 609 | Train 10 | Test Asian 33 | 0.79 | 0.9 | 0.89 | 1633 | Train 26 | Test Asian 33 | 0.79 | 0.9 | 0.88 |
| 610 | Train 10 | Test Asian 34 | 0.81 | 0.91 | 0.9 | 1634 | Train 26 | Test Asian 34 | 0.81 | 0.91 | 0.89 |
| 611 | Train 10 | Test Asian 35 | 0.79 | 0.9 | 0.89 | 1635 | Train 26 | Test Asian 35 | 0.79 | 0.9 | 0.88 |
| 612 | Train 10 | Test Asian 36 | 0.81 | 0.91 | 0.9 | 1636 | Train 26 | Test Asian 36 | 0.81 | 0.91 | 0.89 |
| 613 | Train 10 | Test Asian 37 | 0.75 | 0.76 | 0.71 | 1637 | Train 26 | Test Asian 37 | 0.75 | 0.76 | 0.72 |
| 614 | Train 10 | Test Asian 38 | 0.74 | 0.75 | 0.7 | 1638 | Train 26 | Test Asian 38 | 0.74 | 0.75 | 0.71 |
| 615 | Train 10 | Test Asian 39 | 0.75 | 0.75 | 0.71 | 1639 | Train 26 | Test Asian 39 | 0.75 | 0.76 | 0.71 |
| 616 | Train 10 | Test Asian 40 | 0.75 | 0.75 | 0.69 | 1640 | Train 26 | Test Asian 40 | 0.75 | 0.75 | 0.7 |
| 617 | Train 10 | Test Asian 41 | 0.78 | 0.9 | 0.91 | 1641 | Train 26 | Test Asian 41 | 0.78 | 0.91 | 0.9 |
| 618 | Train 10 | Test Asian 42 | 0.8 | 0.91 | 0.92 | 1642 | Train 26 | Test Asian 42 | 0.8 | 0.91 | 0.91 |
| 619 | Train 10 | Test Asian 43 | 0.78 | 0.9 | 0.91 | 1643 | Train 26 | Test Asian 43 | 0.78 | 0.91 | 0.9 |
| 620 | Train 10 | Test Asian 44 | 0.8 | 0.91 | 0.92 | 1644 | Train 26 | Test Asian 44 | 0.8 | 0.91 | 0.91 |
| 621 | Train 10 | Test Asian 45 | 0.75 | 0.76 | 0.71 | 1645 | Train 26 | Test Asian 45 | 0.75 | 0.76 | 0.72 |
| 622 | Train 10 | Test Asian 46 | 0.75 | 0.75 | 0.7 | 1646 | Train 26 | Test Asian 46 | 0.75 | 0.75 | 0.71 |
| 623 | Train 10 | Test Asian 47 | 0.75 | 0.76 | 0.71 | 1647 | Train 26 | Test Asian 47 | 0.75 | 0.76 | 0.72 |
| 624 | Train 10 | Test Asian 48 | 0.75 | 0.75 | 0.7 | 1648 | Train 26 | Test Asian 48 | 0.75 | 0.75 | 0.71 |
| 625 | Train 10 | Test Asian 49 | 0.77 | 0.85 | 0.82 | 1649 | Train 26 | Test Asian 49 | 0.77 | 0.85 | 0.82 |
| 626 | Train 10 | Test Asian 50 | 0.77 | 0.85 | 0.82 | 1650 | Train 26 | Test Asian 50 | 0.77 | 0.86 | 0.82 |
| 627 | Train 10 | Test Asian 51 | 0.77 | 0.85 | 0.82 | 1651 | Train 26 | Test Asian 51 | 0.77 | 0.85 | 0.82 |
| 628 | Train 10 | Test Asian 52 | 0.77 | 0.85 | 0.82 | 1652 | Train 26 | Test Asian 52 | 0.77 | 0.86 | 0.82 |
| 629 | Train 10 | Test Asian 53 | 0.8 | 0.81 | 0.66 | 1653 | Train 26 | Test Asian 53 | 0.8 | 0.81 | 0.7 |
| 630 | Train 10 | Test Asian 54 | 0.77 | 0.74 | 0.67 | 1654 | Train 26 | Test Asian 54 | 0.77 | 0.75 | 0.68 |
| 631 | Train 10 | Test Asian 55 | 0.79 | 0.8 | 0.65 | 1655 | Train 26 | Test Asian 55 | 0.79 | 0.81 | 0.7 |
| 632 | Train 10 | Test Asian 56 | 0.76 | 0.74 | 0.67 | 1656 | Train 26 | Test Asian 56 | 0.76 | 0.74 | 0.69 |
| 633 | Train 10 | Test Asian 57 | 0.76 | 0.86 | 0.86 | 1657 | Train 26 | Test Asian 57 | 0.76 | 0.86 | 0.85 |
| 634 | Train 10 | Test Asian 58 | 0.76 | 0.86 | 0.86 | 1658 | Train 26 | Test Asian 58 | 0.76 | 0.87 | 0.85 |
| 635 | Train 10 | Test Asian 59 | 0.76 | 0.86 | 0.86 | 1659 | Train 26 | Test Asian 59 | 0.76 | 0.86 | 0.85 |
| 636 | Train 10 | Test Asian 60 | 0.76 | 0.86 | 0.86 | 1660 | Train 26 | Test Asian 60 | 0.76 | 0.87 | 0.85 |
| 637 | Train 10 | Test Asian 61 | 0.79 | 0.81 | 0.66 | 1661 | Train 26 | Test Asian 61 | 0.79 | 0.81 | 0.7 |
| 638 | Train 10 | Test Asian 62 | 0.77 | 0.75 | 0.68 | 1662 | Train 26 | Test Asian 62 | 0.77 | 0.75 | 0.68 |
| 639 | Train 10 | Test Asian 63 | 0.78 | 0.81 | 0.66 | 1663 | Train 26 | Test Asian 63 | 0.78 | 0.81 | 0.69 |
| 640 | Train 10 | Test Asian 64 | 0.76 | 0.74 | 0.67 | 1664 | Train 26 | Test Asian 64 | 0.76 | 0.74 | 0.69 |
| 641 | Train 11 | Test Asian 1 | 0.8 | 0.91 | 0.89 | 1665 | Train 27 | Test Asian 1 | 0.8 | 0.91 | 0.89 |
| 642 | Train 11 | Test Asian 2 | 0.81 | 0.89 | 0.84 | 1666 | Train 27 | Test Asian 2 | 0.81 | 0.89 | 0.85 |
| 643 | Train 11 | Test Asian 3 | 0.8 | 0.91 | 0.89 | 1667 | Train 27 | Test Asian 3 | 0.8 | 0.91 | 0.89 |
| 644 | Train 11 | Test Asian 4 | 0.81 | 0.89 | 0.84 | 1668 | Train 27 | Test Asian 4 | 0.81 | 0.89 | 0.85 |
| 645 | Train 11 | Test Asian 5 | 0.75 | 0.76 | 0.69 | 1669 | Train 27 | Test Asian 5 | 0.75 | 0.75 | 0.68 |
| 646 | Train 11 | Test Asian 6 | 0.75 | 0.75 | 0.67 | 1670 | Train 27 | Test Asian 6 | 0.75 | 0.75 | 0.67 |
| 647 | Train 11 | Test Asian 7 | 0.75 | 0.75 | 0.69 | 1671 | Train 27 | Test Asian 7 | 0.75 | 0.75 | 0.67 |
| 648 | Train 11 | Test Asian 8 | 0.75 | 0.75 | 0.67 | 1672 | Train 27 | Test Asian 8 | 0.75 | 0.75 | 0.67 |
| 649 | Train 11 | Test Asian 9 | 0.79 | 0.92 | 0.92 | 1673 | Train 27 | Test Asian 9 | 0.79 | 0.92 | 0.94 |
| 650 | Train 11 | Test Asian 10 | 0.8 | 0.89 | 0.86 | 1674 | Train 27 | Test Asian 10 | 0.8 | 0.89 | 0.88 |
| 651 | Train 11 | Test Asian 11 | 0.79 | 0.92 | 0.92 | 1675 | Train 27 | Test Asian 11 | 0.79 | 0.92 | 0.94 |
| 652 | Train 11 | Test Asian 12 | 0.8 | 0.89 | 0.86 | 1676 | Train 27 | Test Asian 12 | 0.8 | 0.89 | 0.88 |
| 653 | Train 11 | Test Asian 13 | 0.74 | 0.76 | 0.7 | 1677 | Train 27 | Test Asian 13 | 0.74 | 0.76 | 0.69 |
| 654 | Train 11 | Test Asian 14 | 0.75 | 0.73 | 0.65 | 1678 | Train 27 | Test Asian 14 | 0.75 | 0.73 | 0.66 |
| 655 | Train 11 | Test Asian 15 | 0.74 | 0.76 | 0.7 | 1679 | Train 27 | Test Asian 15 | 0.74 | 0.75 | 0.68 |
| 656 | Train 11 | Test Asian 16 | 0.75 | 0.75 | 0.67 | 1680 | Train 27 | Test Asian 16 | 0.75 | 0.75 | 0.67 |
| 657 | Train 11 | Test Asian 17 | 0.8 | 0.91 | 0.89 | 1681 | Train 27 | Test Asian 17 | 0.8 | 0.91 | 0.89 |
| 658 | Train 11 | Test Asian 18 | 0.81 | 0.89 | 0.84 | 1682 | Train 27 | Test Asian 18 | 0.81 | 0.89 | 0.85 |
| 659 | Train 11 | Test Asian 19 | 0.8 | 0.91 | 0.89 | 1683 | Train 27 | Test Asian 19 | 0.8 | 0.91 | 0.89 |
| 660 | Train 11 | Test Asian 20 | 0.81 | 0.89 | 0.84 | 1684 | Train 27 | Test Asian 20 | 0.81 | 0.89 | 0.85 |
| 661 | Train 11 | Test Asian 21 | 0.75 | 0.76 | 0.69 | 1685 | Train 27 | Test Asian 21 | 0.75 | 0.75 | 0.68 |
| 662 | Train 11 | Test Asian 22 | 0.75 | 0.75 | 0.67 | 1686 | Train 27 | Test Asian 22 | 0.75 | 0.75 | 0.67 |
| 663 | Train 11 | Test Asian 23 | 0.75 | 0.75 | 0.69 | 1687 | Train 27 | Test Asian 23 | 0.75 | 0.75 | 0.67 |
| 664 | Train 11 | Test Asian 24 | 0.75 | 0.75 | 0.67 | 1688 | Train 27 | Test Asian 24 | 0.75 | 0.75 | 0.67 |
| 665 | Train 11 | Test Asian 25 | 0.79 | 0.92 | 0.92 | 1689 | Train 27 | Test Asian 25 | 0.79 | 0.92 | 0.94 |
| 666 | Train 11 | Test Asian 26 | 0.8 | 0.89 | 0.85 | 1690 | Train 27 | Test Asian 26 | 0.79 | 0.89 | 0.88 |
| 667 | Train 11 | Test Asian 27 | 0.79 | 0.92 | 0.92 | 1691 | Train 27 | Test Asian 27 | 0.79 | 0.92 | 0.94 |
| 668 | Train 11 | Test Asian 28 | 0.8 | 0.89 | 0.85 | 1692 | Train 27 | Test Asian 28 | 0.79 | 0.89 | 0.88 |
| 669 | Train 11 | Test Asian 29 | 0.75 | 0.76 | 0.7 | 1693 | Train 27 | Test Asian 29 | 0.75 | 0.76 | 0.69 |
| 670 | Train 11 | Test Asian 30 | 0.75 | 0.73 | 0.65 | 1694 | Train 27 | Test Asian 30 | 0.75 | 0.73 | 0.66 |
| 671 | Train 11 | Test Asian 31 | 0.74 | 0.76 | 0.7 | 1695 | Train 27 | Test Asian 31 | 0.74 | 0.75 | 0.68 |
| 672 | Train 11 | Test Asian 32 | 0.75 | 0.75 | 0.67 | 1696 | Train 27 | Test Asian 32 | 0.75 | 0.75 | 0.67 |
| 673 | Train 11 | Test Asian 33 | 0.8 | 0.91 | 0.89 | 1697 | Train 27 | Test Asian 33 | 0.8 | 0.91 | 0.89 |
| 674 | Train 11 | Test Asian 34 | 0.8 | 0.89 | 0.84 | 1698 | Train 27 | Test Asian 34 | 0.8 | 0.89 | 0.85 |
| 675 | Train 11 | Test Asian 35 | 0.8 | 0.91 | 0.89 | 1699 | Train 27 | Test Asian 35 | 0.8 | 0.91 | 0.89 |
| 676 | Train 11 | Test Asian 36 | 0.8 | 0.89 | 0.84 | 1700 | Train 27 | Test Asian 36 | 0.8 | 0.89 | 0.85 |
| 677 | Train 11 | Test Asian 37 | 0.75 | 0.75 | 0.69 | 1701 | Train 27 | Test Asian 37 | 0.75 | 0.75 | 0.68 |
| 678 | Train 11 | Test Asian 38 | 0.75 | 0.75 | 0.67 | 1702 | Train 27 | Test Asian 38 | 0.75 | 0.75 | 0.67 |
| 679 | Train 11 | Test Asian 39 | 0.74 | 0.75 | 0.69 | 1703 | Train 27 | Test Asian 39 | 0.74 | 0.75 | 0.67 |
| 680 | Train 11 | Test Asian 40 | 0.75 | 0.75 | 0.67 | 1704 | Train 27 | Test Asian 40 | 0.75 | 0.74 | 0.67 |
| 681 | Train 11 | Test Asian 41 | 0.79 | 0.92 | 0.92 | 1705 | Train 27 | Test Asian 41 | 0.79 | 0.92 | 0.93 |
| 682 | Train 11 | Test Asian 42 | 0.79 | 0.89 | 0.85 | 1706 | Train 27 | Test Asian 42 | 0.79 | 0.89 | 0.87 |
| 683 | Train 11 | Test Asian 43 | 0.79 | 0.92 | 0.92 | 1707 | Train 27 | Test Asian 43 | 0.79 | 0.92 | 0.93 |
| 684 | Train 11 | Test Asian 44 | 0.79 | 0.89 | 0.85 | 1708 | Train 27 | Test Asian 44 | 0.79 | 0.89 | 0.87 |
| 685 | Train 11 | Test Asian 45 | 0.74 | 0.76 | 0.7 | 1709 | Train 27 | Test Asian 45 | 0.74 | 0.75 | 0.69 |
| 686 | Train 11 | Test Asian 46 | 0.75 | 0.75 | 0.67 | 1710 | Train 27 | Test Asian 46 | 0.75 | 0.75 | 0.67 |
| 687 | Train 11 | Test Asian 47 | 0.74 | 0.75 | 0.7 | 1711 | Train 27 | Test Asian 47 | 0.74 | 0.75 | 0.68 |
| 688 | Train 11 | Test Asian 48 | 0.75 | 0.75 | 0.67 | 1712 | Train 27 | Test Asian 48 | 0.75 | 0.75 | 0.67 |
| 689 | Train 11 | Test Asian 49 | 0.77 | 0.87 | 0.8 | 1713 | Train 27 | Test Asian 49 | 0.77 | 0.86 | 0.83 |
| 690 | Train 11 | Test Asian 50 | 0.77 | 0.87 | 0.79 | 1714 | Train 27 | Test Asian 50 | 0.77 | 0.86 | 0.82 |
| 691 | Train 11 | Test Asian 51 | 0.77 | 0.87 | 0.8 | 1715 | Train 27 | Test Asian 51 | 0.77 | 0.86 | 0.83 |
| 692 | Train 11 | Test Asian 52 | 0.77 | 0.87 | 0.79 | 1716 | Train 27 | Test Asian 52 | 0.77 | 0.86 | 0.82 |
| 693 | Train 11 | Test Asian 53 | 0.79 | 0.81 | 0.68 | 1717 | Train 27 | Test Asian 53 | 0.79 | 0.81 | 0.67 |
| 694 | Train 11 | Test Asian 54 | 0.78 | 0.75 | 0.63 | 1718 | Train 27 | Test Asian 54 | 0.78 | 0.75 | 0.66 |
| 695 | Train 11 | Test Asian 55 | 0.79 | 0.8 | 0.68 | 1719 | Train 27 | Test Asian 55 | 0.79 | 0.8 | 0.66 |
| 696 | Train 11 | Test Asian 56 | 0.78 | 0.74 | 0.63 | 1720 | Train 27 | Test Asian 56 | 0.78 | 0.75 | 0.66 |
| 697 | Train 11 | Test Asian 57 | 0.76 | 0.88 | 0.88 | 1721 | Train 27 | Test Asian 57 | 0.76 | 0.88 | 0.89 |
| 698 | Train 11 | Test Asian 58 | 0.76 | 0.88 | 0.87 | 1722 | Train 27 | Test Asian 58 | 0.76 | 0.88 | 0.89 |
| 699 | Train 11 | Test Asian 59 | 0.76 | 0.88 | 0.88 | 1723 | Train 27 | Test Asian 59 | 0.76 | 0.88 | 0.89 |
| 700 | Train 11 | Test Asian 60 | 0.76 | 0.88 | 0.87 | 1724 | Train 27 | Test Asian 60 | 0.76 | 0.88 | 0.89 |
| 701 | Train 11 | Test Asian 61 | 0.78 | 0.81 | 0.69 | 1725 | Train 27 | Test Asian 61 | 0.79 | 0.8 | 0.68 |
| 702 | Train 11 | Test Asian 62 | 0.78 | 0.75 | 0.63 | 1726 | Train 27 | Test Asian 62 | 0.78 | 0.75 | 0.66 |
| 703 | Train 11 | Test Asian 63 | 0.78 | 0.8 | 0.69 | 1727 | Train 27 | Test Asian 63 | 0.78 | 0.8 | 0.67 |
| 704 | Train 11 | Test Asian 64 | 0.78 | 0.74 | 0.63 | 1728 | Train 27 | Test Asian 64 | 0.78 | 0.75 | 0.66 |
| 705 | Train 12 | Test Asian 1 | 0.79 | 0.9 | 0.88 | 1729 | Train 28 | Test Asian 1 | 0.79 | 0.9 | 0.88 |
| 706 | Train 12 | Test Asian 2 | 0.81 | 0.91 | 0.89 | 1730 | Train 28 | Test Asian 2 | 0.81 | 0.91 | 0.88 |
| 707 | Train 12 | Test Asian 3 | 0.79 | 0.9 | 0.88 | 1731 | Train 28 | Test Asian 3 | 0.79 | 0.9 | 0.88 |
| 708 | Train 12 | Test Asian 4 | 0.81 | 0.91 | 0.89 | 1732 | Train 28 | Test Asian 4 | 0.81 | 0.91 | 0.88 |
| 709 | Train 12 | Test Asian 5 | 0.75 | 0.76 | 0.72 | 1733 | Train 28 | Test Asian 5 | 0.75 | 0.76 | 0.72 |
| 710 | Train 12 | Test Asian 6 | 0.75 | 0.75 | 0.71 | 1734 | Train 28 | Test Asian 6 | 0.75 | 0.75 | 0.71 |
| 711 | Train 12 | Test Asian 7 | 0.75 | 0.76 | 0.71 | 1735 | Train 28 | Test Asian 7 | 0.75 | 0.76 | 0.71 |
| 712 | Train 12 | Test Asian 8 | 0.75 | 0.75 | 0.7 | 1736 | Train 28 | Test Asian 8 | 0.75 | 0.75 | 0.71 |
| 713 | Train 12 | Test Asian 9 | 0.78 | 0.91 | 0.91 | 1737 | Train 28 | Test Asian 9 | 0.78 | 0.91 | 0.9 |
| 714 | Train 12 | Test Asian 10 | 0.8 | 0.91 | 0.91 | 1738 | Train 28 | Test Asian 10 | 0.8 | 0.91 | 0.91 |
| 715 | Train 12 | Test Asian 11 | 0.78 | 0.91 | 0.91 | 1739 | Train 28 | Test Asian 11 | 0.78 | 0.91 | 0.9 |
| 716 | Train 12 | Test Asian 12 | 0.8 | 0.91 | 0.91 | 1740 | Train 28 | Test Asian 12 | 0.8 | 0.91 | 0.91 |
| 717 | Train 12 | Test Asian 13 | 0.75 | 0.76 | 0.72 | 1741 | Train 28 | Test Asian 13 | 0.75 | 0.76 | 0.73 |
| 718 | Train 12 | Test Asian 14 | 0.75 | 0.74 | 0.66 | 1742 | Train 28 | Test Asian 14 | 0.75 | 0.74 | 0.66 |
| 719 | Train 12 | Test Asian 15 | 0.75 | 0.76 | 0.71 | 1743 | Train 28 | Test Asian 15 | 0.75 | 0.76 | 0.73 |
| 720 | Train 12 | Test Asian 16 | 0.75 | 0.75 | 0.7 | 1744 | Train 28 | Test Asian 16 | 0.75 | 0.75 | 0.71 |
| 721 | Train 12 | Test Asian 17 | 0.79 | 0.9 | 0.88 | 1745 | Train 28 | Test Asian 17 | 0.79 | 0.9 | 0.88 |
| 722 | Train 12 | Test Asian 18 | 0.81 | 0.91 | 0.89 | 1746 | Train 28 | Test Asian 18 | 0.81 | 0.91 | 0.88 |
| 723 | Train 12 | Test Asian 19 | 0.79 | 0.9 | 0.88 | 1747 | Train 28 | Test Asian 19 | 0.79 | 0.9 | 0.88 |
| 724 | Train 12 | Test Asian 20 | 0.81 | 0.91 | 0.89 | 1748 | Train 28 | Test Asian 20 | 0.81 | 0.91 | 0.88 |
| 725 | Train 12 | Test Asian 21 | 0.75 | 0.76 | 0.72 | 1749 | Train 28 | Test Asian 21 | 0.75 | 0.76 | 0.72 |
| 726 | Train 12 | Test Asian 22 | 0.75 | 0.75 | 0.71 | 1750 | Train 28 | Test Asian 22 | 0.75 | 0.75 | 0.71 |
| 727 | Train 12 | Test Asian 23 | 0.75 | 0.76 | 0.71 | 1751 | Train 28 | Test Asian 23 | 0.75 | 0.76 | 0.71 |
| 728 | Train 12 | Test Asian 24 | 0.75 | 0.75 | 0.7 | 1752 | Train 28 | Test Asian 24 | 0.75 | 0.75 | 0.71 |
| 729 | Train 12 | Test Asian 25 | 0.78 | 0.91 | 0.91 | 1753 | Train 28 | Test Asian 25 | 0.78 | 0.91 | 0.9 |
| 730 | Train 12 | Test Asian 26 | 0.8 | 0.91 | 0.91 | 1754 | Train 28 | Test Asian 26 | 0.8 | 0.91 | 0.91 |
| 731 | Train 12 | Test Asian 27 | 0.78 | 0.91 | 0.91 | 1755 | Train 28 | Test Asian 27 | 0.78 | 0.91 | 0.9 |
| 732 | Train 12 | Test Asian 28 | 0.8 | 0.91 | 0.91 | 1756 | Train 28 | Test Asian 28 | 0.8 | 0.91 | 0.91 |
| 733 | Train 12 | Test Asian 29 | 0.75 | 0.76 | 0.72 | 1757 | Train 28 | Test Asian 29 | 0.75 | 0.76 | 0.73 |
| 734 | Train 12 | Test Asian 30 | 0.75 | 0.74 | 0.66 | 1758 | Train 28 | Test Asian 30 | 0.75 | 0.74 | 0.66 |
| 735 | Train 12 | Test Asian 31 | 0.75 | 0.76 | 0.71 | 1759 | Train 28 | Test Asian 31 | 0.75 | 0.76 | 0.73 |
| 736 | Train 12 | Test Asian 32 | 0.75 | 0.75 | 0.7 | 1760 | Train 28 | Test Asian 32 | 0.75 | 0.75 | 0.71 |
| 737 | Train 12 | Test Asian 33 | 0.79 | 0.9 | 0.88 | 1761 | Train 28 | Test Asian 33 | 0.79 | 0.9 | 0.88 |
| 738 | Train 12 | Test Asian 34 | 0.81 | 0.91 | 0.89 | 1762 | Train 28 | Test Asian 34 | 0.81 | 0.91 | 0.88 |
| 739 | Train 12 | Test Asian 35 | 0.79 | 0.9 | 0.88 | 1763 | Train 28 | Test Asian 35 | 0.79 | 0.9 | 0.88 |
| 740 | Train 12 | Test Asian 36 | 0.81 | 0.91 | 0.89 | 1764 | Train 28 | Test Asian 36 | 0.81 | 0.91 | 0.88 |
| 741 | Train 12 | Test Asian 37 | 0.75 | 0.76 | 0.72 | 1765 | Train 28 | Test Asian 37 | 0.75 | 0.76 | 0.72 |
| 742 | Train 12 | Test Asian 38 | 0.74 | 0.75 | 0.71 | 1766 | Train 28 | Test Asian 38 | 0.74 | 0.75 | 0.71 |
| 743 | Train 12 | Test Asian 39 | 0.75 | 0.76 | 0.71 | 1767 | Train 28 | Test Asian 39 | 0.75 | 0.76 | 0.71 |
| 744 | Train 12 | Test Asian 40 | 0.75 | 0.75 | 0.7 | 1768 | Train 28 | Test Asian 40 | 0.74 | 0.74 | 0.71 |
| 745 | Train 12 | Test Asian 41 | 0.78 | 0.91 | 0.91 | 1769 | Train 28 | Test Asian 41 | 0.78 | 0.9 | 0.9 |
| 746 | Train 12 | Test Asian 42 | 0.8 | 0.91 | 0.9 | 1770 | Train 28 | Test Asian 42 | 0.8 | 0.91 | 0.91 |
| 747 | Train 12 | Test Asian 43 | 0.78 | 0.91 | 0.91 | 1771 | Train 28 | Test Asian 43 | 0.78 | 0.9 | 0.9 |
| 748 | Train 12 | Test Asian 44 | 0.8 | 0.91 | 0.9 | 1772 | Train 28 | Test Asian 44 | 0.8 | 0.91 | 0.91 |
| 749 | Train 12 | Test Asian 45 | 0.75 | 0.76 | 0.72 | 1773 | Train 28 | Test Asian 45 | 0.75 | 0.76 | 0.73 |
| 750 | Train 12 | Test Asian 46 | 0.75 | 0.75 | 0.71 | 1774 | Train 28 | Test Asian 46 | 0.75 | 0.75 | 0.71 |
| 751 | Train 12 | Test Asian 47 | 0.75 | 0.76 | 0.71 | 1775 | Train 28 | Test Asian 47 | 0.75 | 0.76 | 0.73 |
| 752 | Train 12 | Test Asian 48 | 0.75 | 0.75 | 0.7 | 1776 | Train 28 | Test Asian 48 | 0.75 | 0.75 | 0.71 |
| 753 | Train 12 | Test Asian 49 | 0.77 | 0.85 | 0.83 | 1777 | Train 28 | Test Asian 49 | 0.77 | 0.85 | 0.83 |
| 754 | Train 12 | Test Asian 50 | 0.77 | 0.85 | 0.83 | 1778 | Train 28 | Test Asian 50 | 0.77 | 0.85 | 0.83 |
| 755 | Train 12 | Test Asian 51 | 0.77 | 0.85 | 0.83 | 1779 | Train 28 | Test Asian 51 | 0.77 | 0.85 | 0.83 |
| 756 | Train 12 | Test Asian 52 | 0.77 | 0.85 | 0.83 | 1780 | Train 28 | Test Asian 52 | 0.77 | 0.85 | 0.83 |
| 757 | Train 12 | Test Asian 53 | 0.8 | 0.81 | 0.7 | 1781 | Train 28 | Test Asian 53 | 0.79 | 0.81 | 0.62 |
| 758 | Train 12 | Test Asian 54 | 0.77 | 0.74 | 0.67 | 1782 | Train 28 | Test Asian 54 | 0.77 | 0.75 | 0.62 |
| 759 | Train 12 | Test Asian 55 | 0.79 | 0.81 | 0.7 | 1783 | Train 28 | Test Asian 55 | 0.79 | 0.81 | 0.62 |
| 760 | Train 12 | Test Asian 56 | 0.76 | 0.74 | 0.66 | 1784 | Train 28 | Test Asian 56 | 0.76 | 0.74 | 0.63 |
| 761 | Train 12 | Test Asian 57 | 0.76 | 0.86 | 0.86 | 1785 | Train 28 | Test Asian 57 | 0.76 | 0.86 | 0.89 |
| 762 | Train 12 | Test Asian 58 | 0.76 | 0.87 | 0.86 | 1786 | Train 28 | Test Asian 58 | 0.76 | 0.86 | 0.89 |
| 763 | Train 12 | Test Asian 59 | 0.76 | 0.86 | 0.86 | 1787 | Train 28 | Test Asian 59 | 0.76 | 0.86 | 0.89 |
| 764 | Train 12 | Test Asian 60 | 0.76 | 0.87 | 0.86 | 1788 | Train 28 | Test Asian 60 | 0.76 | 0.86 | 0.89 |
| 765 | Train 12 | Test Asian 61 | 0.79 | 0.81 | 0.7 | 1789 | Train 28 | Test Asian 61 | 0.79 | 0.81 | 0.62 |
| 766 | Train 12 | Test Asian 62 | 0.77 | 0.74 | 0.67 | 1790 | Train 28 | Test Asian 62 | 0.77 | 0.75 | 0.62 |
| 767 | Train 12 | Test Asian 63 | 0.78 | 0.81 | 0.7 | 1791 | Train 28 | Test Asian 63 | 0.78 | 0.81 | 0.61 |
| 768 | Train 12 | Test Asian 64 | 0.76 | 0.74 | 0.67 | 1792 | Train 28 | Test Asian 64 | 0.76 | 0.74 | 0.63 |
| 769 | Train 13 | Test Asian 1 | 0.78 | 0.86 | 0.86 | 1793 | Train 29 | Test Asian 1 | 0.78 | 0.86 | 0.86 |
| 770 | Train 13 | Test Asian 2 | 0.8 | 0.86 | 0.85 | 1794 | Train 29 | Test Asian 2 | 0.8 | 0.86 | 0.85 |
| 771 | Train 13 | Test Asian 3 | 0.78 | 0.86 | 0.86 | 1795 | Train 29 | Test Asian 3 | 0.78 | 0.86 | 0.86 |
| 772 | Train 13 | Test Asian 4 | 0.8 | 0.86 | 0.85 | 1796 | Train 29 | Test Asian 4 | 0.8 | 0.86 | 0.85 |
| 773 | Train 13 | Test Asian 5 | 0.76 | 0.85 | 0.84 | 1797 | Train 29 | Test Asian 5 | 0.76 | 0.84 | 0.83 |
| 774 | Train 13 | Test Asian 6 | 0.76 | 0.79 | 0.78 | 1798 | Train 29 | Test Asian 6 | 0.75 | 0.79 | 0.79 |
| 775 | Train 13 | Test Asian 7 | 0.76 | 0.84 | 0.83 | 1799 | Train 29 | Test Asian 7 | 0.76 | 0.84 | 0.82 |
| 776 | Train 13 | Test Asian 8 | 0.76 | 0.78 | 0.77 | 1800 | Train 29 | Test Asian 8 | 0.76 | 0.78 | 0.79 |
| 777 | Train 13 | Test Asian 9 | 0.77 | 0.87 | 0.87 | 1801 | Train 29 | Test Asian 9 | 0.77 | 0.87 | 0.88 |
| 778 | Train 13 | Test Asian 10 | 0.79 | 0.86 | 0.86 | 1802 | Train 29 | Test Asian 10 | 0.79 | 0.86 | 0.87 |
| 779 | Train 13 | Test Asian 11 | 0.77 | 0.87 | 0.87 | 1803 | Train 29 | Test Asian 11 | 0.77 | 0.87 | 0.88 |
| 780 | Train 13 | Test Asian 12 | 0.79 | 0.86 | 0.86 | 1804 | Train 29 | Test Asian 12 | 0.79 | 0.86 | 0.87 |
| 781 | Train 13 | Test Asian 13 | 0.76 | 0.85 | 0.86 | 1805 | Train 29 | Test Asian 13 | 0.76 | 0.85 | 0.85 |
| 782 | Train 13 | Test Asian 14 | 0.76 | 0.77 | 0.72 | 1806 | Train 29 | Test Asian 14 | 0.75 | 0.77 | 0.75 |
| 783 | Train 13 | Test Asian 15 | 0.76 | 0.84 | 0.86 | 1807 | Train 29 | Test Asian 15 | 0.76 | 0.84 | 0.85 |
| 784 | Train 13 | Test Asian 16 | 0.76 | 0.78 | 0.77 | 1808 | Train 29 | Test Asian 16 | 0.76 | 0.78 | 0.78 |
| 785 | Train 13 | Test Asian 17 | 0.78 | 0.86 | 0.86 | 1809 | Train 29 | Test Asian 17 | 0.78 | 0.86 | 0.86 |
| 786 | Train 13 | Test Asian 18 | 0.8 | 0.86 | 0.85 | 1810 | Train 29 | Test Asian 18 | 0.8 | 0.86 | 0.85 |
| 787 | Train 13 | Test Asian 19 | 0.78 | 0.86 | 0.86 | 1811 | Train 29 | Test Asian 19 | 0.78 | 0.86 | 0.86 |
| 788 | Train 13 | Test Asian 20 | 0.8 | 0.86 | 0.85 | 1812 | Train 29 | Test Asian 20 | 0.8 | 0.86 | 0.85 |
| 789 | Train 13 | Test Asian 21 | 0.76 | 0.85 | 0.84 | 1813 | Train 29 | Test Asian 21 | 0.77 | 0.85 | 0.83 |
| 790 | Train 13 | Test Asian 22 | 0.76 | 0.79 | 0.78 | 1814 | Train 29 | Test Asian 22 | 0.76 | 0.79 | 0.79 |
| 791 | Train 13 | Test Asian 23 | 0.76 | 0.84 | 0.83 | 1815 | Train 29 | Test Asian 23 | 0.76 | 0.84 | 0.82 |
| 792 | Train 13 | Test Asian 24 | 0.76 | 0.79 | 0.77 | 1816 | Train 29 | Test Asian 24 | 0.76 | 0.78 | 0.79 |
| 793 | Train 13 | Test Asian 25 | 0.77 | 0.87 | 0.88 | 1817 | Train 29 | Test Asian 25 | 0.77 | 0.87 | 0.88 |
| 794 | Train 13 | Test Asian 26 | 0.79 | 0.86 | 0.86 | 1818 | Train 29 | Test Asian 26 | 0.79 | 0.86 | 0.86 |
| 795 | Train 13 | Test Asian 27 | 0.77 | 0.87 | 0.88 | 1819 | Train 29 | Test Asian 27 | 0.77 | 0.87 | 0.88 |
| 796 | Train 13 | Test Asian 28 | 0.79 | 0.86 | 0.86 | 1820 | Train 29 | Test Asian 28 | 0.79 | 0.86 | 0.86 |
| 797 | Train 13 | Test Asian 29 | 0.76 | 0.85 | 0.86 | 1821 | Train 29 | Test Asian 29 | 0.76 | 0.85 | 0.85 |
| 798 | Train 13 | Test Asian 30 | 0.76 | 0.77 | 0.72 | 1822 | Train 29 | Test Asian 30 | 0.76 | 0.77 | 0.76 |
| 799 | Train 13 | Test Asian 31 | 0.76 | 0.84 | 0.86 | 1823 | Train 29 | Test Asian 31 | 0.76 | 0.84 | 0.85 |
| 800 | Train 13 | Test Asian 32 | 0.76 | 0.78 | 0.77 | 1824 | Train 29 | Test Asian 32 | 0.76 | 0.78 | 0.78 |
| 801 | Train 13 | Test Asian 33 | 0.78 | 0.86 | 0.86 | 1825 | Train 29 | Test Asian 33 | 0.78 | 0.86 | 0.86 |
| 802 | Train 13 | Test Asian 34 | 0.8 | 0.86 | 0.85 | 1826 | Train 29 | Test Asian 34 | 0.8 | 0.86 | 0.86 |
| 803 | Train 13 | Test Asian 35 | 0.78 | 0.86 | 0.86 | 1827 | Train 29 | Test Asian 35 | 0.78 | 0.86 | 0.86 |
| 804 | Train 13 | Test Asian 36 | 0.8 | 0.86 | 0.85 | 1828 | Train 29 | Test Asian 36 | 0.8 | 0.86 | 0.86 |
| 805 | Train 13 | Test Asian 37 | 0.76 | 0.84 | 0.84 | 1829 | Train 29 | Test Asian 37 | 0.76 | 0.84 | 0.83 |
| 806 | Train 13 | Test Asian 38 | 0.75 | 0.79 | 0.77 | 1830 | Train 29 | Test Asian 38 | 0.75 | 0.78 | 0.79 |
| 807 | Train 13 | Test Asian 39 | 0.76 | 0.84 | 0.83 | 1831 | Train 29 | Test Asian 39 | 0.76 | 0.84 | 0.82 |
| 808 | Train 13 | Test Asian 40 | 0.76 | 0.78 | 0.77 | 1832 | Train 29 | Test Asian 40 | 0.75 | 0.78 | 0.78 |
| 809 | Train 13 | Test Asian 41 | 0.77 | 0.87 | 0.87 | 1833 | Train 29 | Test Asian 41 | 0.77 | 0.87 | 0.88 |
| 810 | Train 13 | Test Asian 42 | 0.8 | 0.86 | 0.85 | 1834 | Train 29 | Test Asian 42 | 0.8 | 0.86 | 0.86 |
| 811 | Train 13 | Test Asian 43 | 0.77 | 0.87 | 0.87 | 1835 | Train 29 | Test Asian 43 | 0.77 | 0.87 | 0.88 |
| 812 | Train 13 | Test Asian 44 | 0.8 | 0.86 | 0.85 | 1836 | Train 29 | Test Asian 44 | 0.8 | 0.86 | 0.86 |
| 813 | Train 13 | Test Asian 45 | 0.76 | 0.84 | 0.86 | 1837 | Train 29 | Test Asian 45 | 0.76 | 0.84 | 0.85 |
| 814 | Train 13 | Test Asian 46 | 0.75 | 0.78 | 0.77 | 1838 | Train 29 | Test Asian 46 | 0.75 | 0.78 | 0.78 |
| 815 | Train 13 | Test Asian 47 | 0.76 | 0.84 | 0.85 | 1839 | Train 29 | Test Asian 47 | 0.76 | 0.84 | 0.85 |
| 816 | Train 13 | Test Asian 48 | 0.76 | 0.78 | 0.77 | 1840 | Train 29 | Test Asian 48 | 0.75 | 0.78 | 0.78 |
| 817 | Train 13 | Test Asian 49 | 0.76 | 0.83 | 0.8 | 1841 | Train 29 | Test Asian 49 | 0.76 | 0.84 | 0.8 |
| 818 | Train 13 | Test Asian 50 | 0.76 | 0.83 | 0.8 | 1842 | Train 29 | Test Asian 50 | 0.76 | 0.84 | 0.8 |
| 819 | Train 13 | Test Asian 51 | 0.76 | 0.83 | 0.8 | 1843 | Train 29 | Test Asian 51 | 0.76 | 0.84 | 0.8 |
| 820 | Train 13 | Test Asian 52 | 0.76 | 0.83 | 0.8 | 1844 | Train 29 | Test Asian 52 | 0.76 | 0.84 | 0.8 |
| 821 | Train 13 | Test Asian 53 | 0.8 | 0.86 | 0.79 | 1845 | Train 29 | Test Asian 53 | 0.8 | 0.86 | 0.79 |
| 822 | Train 13 | Test Asian 54 | 0.77 | 0.77 | 0.69 | 1846 | Train 29 | Test Asian 54 | 0.77 | 0.77 | 0.75 |
| 823 | Train 13 | Test Asian 55 | 0.8 | 0.85 | 0.78 | 1847 | Train 29 | Test Asian 55 | 0.8 | 0.85 | 0.79 |
| 824 | Train 13 | Test Asian 56 | 0.77 | 0.77 | 0.69 | 1848 | Train 29 | Test Asian 56 | 0.77 | 0.76 | 0.75 |
| 825 | Train 13 | Test Asian 57 | 0.74 | 0.84 | 0.83 | 1849 | Train 29 | Test Asian 57 | 0.74 | 0.85 | 0.84 |
| 826 | Train 13 | Test Asian 58 | 0.74 | 0.84 | 0.83 | 1850 | Train 29 | Test Asian 58 | 0.74 | 0.85 | 0.84 |
| 827 | Train 13 | Test Asian 59 | 0.74 | 0.84 | 0.83 | 1851 | Train 29 | Test Asian 59 | 0.74 | 0.85 | 0.84 |
| 828 | Train 13 | Test Asian 60 | 0.74 | 0.84 | 0.83 | 1852 | Train 29 | Test Asian 60 | 0.74 | 0.85 | 0.84 |
| 829 | Train 13 | Test Asian 61 | 0.79 | 0.86 | 0.79 | 1853 | Train 29 | Test Asian 61 | 0.79 | 0.86 | 0.79 |
| 830 | Train 13 | Test Asian 62 | 0.77 | 0.77 | 0.7 | 1854 | Train 29 | Test Asian 62 | 0.77 | 0.77 | 0.75 |
| 831 | Train 13 | Test Asian 63 | 0.79 | 0.86 | 0.78 | 1855 | Train 29 | Test Asian 63 | 0.79 | 0.85 | 0.78 |
| 832 | Train 13 | Test Asian 64 | 0.77 | 0.76 | 0.69 | 1856 | Train 29 | Test Asian 64 | 0.77 | 0.76 | 0.75 |
| 833 | Train 14 | Test Asian 1 | 0.76 | 0.84 | 0.79 | 1857 | Train 30 | Test Asian 1 | 0.76 | 0.84 | 0.8 |
| 834 | Train 14 | Test Asian 2 | 0.79 | 0.86 | 0.81 | 1858 | Train 30 | Test Asian 2 | 0.79 | 0.86 | 0.82 |
| 835 | Train 14 | Test Asian 3 | 0.76 | 0.84 | 0.79 | 1859 | Train 30 | Test Asian 3 | 0.76 | 0.84 | 0.8 |
| 836 | Train 14 | Test Asian 4 | 0.79 | 0.86 | 0.81 | 1860 | Train 30 | Test Asian 4 | 0.79 | 0.86 | 0.82 |
| 837 | Train 14 | Test Asian 5 | 0.76 | 0.81 | 0.77 | 1861 | Train 30 | Test Asian 5 | 0.76 | 0.8 | 0.77 |
| 838 | Train 14 | Test Asian 6 | 0.76 | 0.82 | 0.79 | 1862 | Train 30 | Test Asian 6 | 0.76 | 0.82 | 0.79 |
| 839 | Train 14 | Test Asian 7 | 0.75 | 0.8 | 0.77 | 1863 | Train 30 | Test Asian 7 | 0.75 | 0.8 | 0.77 |
| 840 | Train 14 | Test Asian 8 | 0.76 | 0.81 | 0.78 | 1864 | Train 30 | Test Asian 8 | 0.76 | 0.81 | 0.78 |
| 841 | Train 14 | Test Asian 9 | 0.75 | 0.84 | 0.81 | 1865 | Train 30 | Test Asian 9 | 0.75 | 0.85 | 0.82 |
| 842 | Train 14 | Test Asian 10 | 0.78 | 0.86 | 0.83 | 1866 | Train 30 | Test Asian 10 | 0.78 | 0.86 | 0.83 |
| 843 | Train 14 | Test Asian 11 | 0.75 | 0.84 | 0.81 | 1867 | Train 30 | Test Asian 11 | 0.75 | 0.85 | 0.82 |
| 844 | Train 14 | Test Asian 12 | 0.78 | 0.86 | 0.83 | 1868 | Train 30 | Test Asian 12 | 0.78 | 0.86 | 0.83 |
| 845 | Train 14 | Test Asian 13 | 0.75 | 0.81 | 0.79 | 1869 | Train 30 | Test Asian 13 | 0.75 | 0.81 | 0.79 |
| 846 | Train 14 | Test Asian 14 | 0.76 | 0.83 | 0.84 | 1870 | Train 30 | Test Asian 14 | 0.76 | 0.83 | 0.83 |
| 847 | Train 14 | Test Asian 15 | 0.75 | 0.8 | 0.78 | 1871 | Train 30 | Test Asian 15 | 0.75 | 0.8 | 0.78 |
| 848 | Train 14 | Test Asian 16 | 0.76 | 0.81 | 0.78 | 1872 | Train 30 | Test Asian 16 | 0.76 | 0.81 | 0.78 |
| 849 | Train 14 | Test Asian 17 | 0.76 | 0.84 | 0.79 | 1873 | Train 30 | Test Asian 17 | 0.76 | 0.84 | 0.8 |
| 850 | Train 14 | Test Asian 18 | 0.79 | 0.86 | 0.81 | 1874 | Train 30 | Test Asian 18 | 0.79 | 0.86 | 0.82 |
| 851 | Train 14 | Test Asian 19 | 0.76 | 0.84 | 0.79 | 1875 | Train 30 | Test Asian 19 | 0.76 | 0.84 | 0.8 |
| 852 | Train 14 | Test Asian 20 | 0.79 | 0.86 | 0.81 | 1876 | Train 30 | Test Asian 20 | 0.79 | 0.86 | 0.82 |
| 853 | Train 14 | Test Asian 21 | 0.76 | 0.81 | 0.77 | 1877 | Train 30 | Test Asian 21 | 0.76 | 0.81 | 0.77 |
| 854 | Train 14 | Test Asian 22 | 0.77 | 0.82 | 0.79 | 1878 | Train 30 | Test Asian 22 | 0.77 | 0.82 | 0.79 |
| 855 | Train 14 | Test Asian 23 | 0.75 | 0.8 | 0.77 | 1879 | Train 30 | Test Asian 23 | 0.75 | 0.8 | 0.77 |
| 856 | Train 14 | Test Asian 24 | 0.77 | 0.81 | 0.78 | 1880 | Train 30 | Test Asian 24 | 0.77 | 0.81 | 0.78 |
| 857 | Train 14 | Test Asian 25 | 0.75 | 0.84 | 0.81 | 1881 | Train 30 | Test Asian 25 | 0.75 | 0.85 | 0.82 |
| 858 | Train 14 | Test Asian 26 | 0.78 | 0.86 | 0.83 | 1882 | Train 30 | Test Asian 26 | 0.78 | 0.86 | 0.83 |
| 859 | Train 14 | Test Asian 27 | 0.75 | 0.84 | 0.81 | 1883 | Train 30 | Test Asian 27 | 0.75 | 0.85 | 0.82 |
| 860 | Train 14 | Test Asian 28 | 0.78 | 0.86 | 0.83 | 1884 | Train 30 | Test Asian 28 | 0.78 | 0.86 | 0.83 |
| 861 | Train 14 | Test Asian 29 | 0.75 | 0.81 | 0.79 | 1885 | Train 30 | Test Asian 29 | 0.75 | 0.81 | 0.79 |
| 862 | Train 14 | Test Asian 30 | 0.76 | 0.83 | 0.84 | 1886 | Train 30 | Test Asian 30 | 0.76 | 0.83 | 0.83 |
| 863 | Train 14 | Test Asian 31 | 0.75 | 0.8 | 0.78 | 1887 | Train 30 | Test Asian 31 | 0.75 | 0.8 | 0.78 |
| 864 | Train 14 | Test Asian 32 | 0.76 | 0.81 | 0.78 | 1888 | Train 30 | Test Asian 32 | 0.76 | 0.81 | 0.78 |
| 865 | Train 14 | Test Asian 33 | 0.76 | 0.84 | 0.79 | 1889 | Train 30 | Test Asian 33 | 0.76 | 0.84 | 0.79 |
| 866 | Train 14 | Test Asian 34 | 0.78 | 0.86 | 0.81 | 1890 | Train 30 | Test Asian 34 | 0.78 | 0.86 | 0.81 |
| 867 | Train 14 | Test Asian 35 | 0.76 | 0.84 | 0.79 | 1891 | Train 30 | Test Asian 35 | 0.76 | 0.84 | 0.79 |
| 868 | Train 14 | Test Asian 36 | 0.78 | 0.86 | 0.81 | 1892 | Train 30 | Test Asian 36 | 0.78 | 0.86 | 0.81 |
| 869 | Train 14 | Test Asian 37 | 0.75 | 0.8 | 0.77 | 1893 | Train 30 | Test Asian 37 | 0.75 | 0.8 | 0.77 |
| 870 | Train 14 | Test Asian 38 | 0.76 | 0.82 | 0.79 | 1894 | Train 30 | Test Asian 38 | 0.76 | 0.82 | 0.79 |
| 871 | Train 14 | Test Asian 39 | 0.75 | 0.8 | 0.77 | 1895 | Train 30 | Test Asian 39 | 0.75 | 0.8 | 0.76 |
| 872 | Train 14 | Test Asian 40 | 0.76 | 0.81 | 0.78 | 1896 | Train 30 | Test Asian 40 | 0.76 | 0.81 | 0.78 |
| 873 | Train 14 | Test Asian 41 | 0.75 | 0.84 | 0.81 | 1897 | Train 30 | Test Asian 41 | 0.75 | 0.85 | 0.81 |
| 874 | Train 14 | Test Asian 42 | 0.78 | 0.86 | 0.83 | 1898 | Train 30 | Test Asian 42 | 0.78 | 0.86 | 0.83 |
| 875 | Train 14 | Test Asian 43 | 0.75 | 0.84 | 0.81 | 1899 | Train 30 | Test Asian 43 | 0.75 | 0.85 | 0.81 |
| 876 | Train 14 | Test Asian 44 | 0.78 | 0.86 | 0.83 | 1900 | Train 30 | Test Asian 44 | 0.78 | 0.86 | 0.83 |
| 877 | Train 14 | Test Asian 45 | 0.75 | 0.8 | 0.78 | 1901 | Train 30 | Test Asian 45 | 0.75 | 0.81 | 0.78 |
| 878 | Train 14 | Test Asian 46 | 0.76 | 0.82 | 0.79 | 1902 | Train 30 | Test Asian 46 | 0.76 | 0.82 | 0.78 |
| 879 | Train 14 | Test Asian 47 | 0.75 | 0.8 | 0.78 | 1903 | Train 30 | Test Asian 47 | 0.75 | 0.8 | 0.78 |
| 880 | Train 14 | Test Asian 48 | 0.76 | 0.81 | 0.78 | 1904 | Train 30 | Test Asian 48 | 0.76 | 0.81 | 0.78 |
| 881 | Train 14 | Test Asian 49 | 0.77 | 0.83 | 0.82 | 1905 | Train 30 | Test Asian 49 | 0.77 | 0.84 | 0.77 |
| 882 | Train 14 | Test Asian 50 | 0.77 | 0.84 | 0.82 | 1906 | Train 30 | Test Asian 50 | 0.77 | 0.84 | 0.78 |
| 883 | Train 14 | Test Asian 51 | 0.77 | 0.83 | 0.82 | 1907 | Train 30 | Test Asian 51 | 0.77 | 0.84 | 0.77 |
| 884 | Train 14 | Test Asian 52 | 0.77 | 0.84 | 0.82 | 1908 | Train 30 | Test Asian 52 | 0.77 | 0.84 | 0.78 |
| 885 | Train 14 | Test Asian 53 | 0.8 | 0.84 | 0.77 | 1909 | Train 30 | Test Asian 53 | 0.8 | 0.84 | 0.77 |
| 886 | Train 14 | Test Asian 54 | 0.79 | 0.83 | 0.84 | 1910 | Train 30 | Test Asian 54 | 0.79 | 0.83 | 0.83 |
| 887 | Train 14 | Test Asian 55 | 0.79 | 0.84 | 0.75 | 1911 | Train 30 | Test Asian 55 | 0.79 | 0.84 | 0.77 |
| 888 | Train 14 | Test Asian 56 | 0.78 | 0.82 | 0.83 | 1912 | Train 30 | Test Asian 56 | 0.78 | 0.83 | 0.83 |
| 889 | Train 14 | Test Asian 57 | 0.76 | 0.84 | 0.82 | 1913 | Train 30 | Test Asian 57 | 0.76 | 0.85 | 0.83 |
| 890 | Train 14 | Test Asian 58 | 0.76 | 0.84 | 0.82 | 1914 | Train 30 | Test Asian 58 | 0.76 | 0.85 | 0.83 |
| 891 | Train 14 | Test Asian 59 | 0.76 | 0.84 | 0.82 | 1915 | Train 30 | Test Asian 59 | 0.76 | 0.85 | 0.83 |
| 892 | Train 14 | Test Asian 60 | 0.76 | 0.84 | 0.82 | 1916 | Train 30 | Test Asian 60 | 0.76 | 0.85 | 0.83 |
| 893 | Train 14 | Test Asian 61 | 0.79 | 0.84 | 0.77 | 1917 | Train 30 | Test Asian 61 | 0.79 | 0.84 | 0.78 |
| 894 | Train 14 | Test Asian 62 | 0.79 | 0.83 | 0.84 | 1918 | Train 30 | Test Asian 62 | 0.79 | 0.83 | 0.83 |
| 895 | Train 14 | Test Asian 63 | 0.78 | 0.84 | 0.76 | 1919 | Train 30 | Test Asian 63 | 0.78 | 0.84 | 0.77 |
| 896 | Train 14 | Test Asian 64 | 0.78 | 0.83 | 0.83 | 1920 | Train 30 | Test Asian 64 | 0.78 | 0.83 | 0.83 |
| 897 | Train 15 | Test Asian 1 | 0.78 | 0.86 | 0.85 | 1921 | Train 31 | Test Asian 1 | 0.78 | 0.86 | 0.86 |
| 898 | Train 15 | Test Asian 2 | 0.8 | 0.86 | 0.84 | 1922 | Train 31 | Test Asian 2 | 0.8 | 0.86 | 0.86 |
| 899 | Train 15 | Test Asian 3 | 0.78 | 0.86 | 0.85 | 1923 | Train 31 | Test Asian 3 | 0.78 | 0.86 | 0.86 |
| 900 | Train 15 | Test Asian 4 | 0.8 | 0.86 | 0.84 | 1924 | Train 31 | Test Asian 4 | 0.8 | 0.86 | 0.86 |
| 901 | Train 15 | Test Asian 5 | 0.76 | 0.85 | 0.84 | 1925 | Train 31 | Test Asian 5 | 0.76 | 0.84 | 0.84 |
| 902 | Train 15 | Test Asian 6 | 0.75 | 0.78 | 0.78 | 1926 | Train 31 | Test Asian 6 | 0.75 | 0.78 | 0.79 |
| 903 | Train 15 | Test Asian 7 | 0.76 | 0.84 | 0.84 | 1927 | Train 31 | Test Asian 7 | 0.76 | 0.84 | 0.84 |
| 904 | Train 15 | Test Asian 8 | 0.75 | 0.78 | 0.77 | 1928 | Train 31 | Test Asian 8 | 0.75 | 0.78 | 0.78 |
| 905 | Train 15 | Test Asian 9 | 0.77 | 0.87 | 0.87 | 1929 | Train 31 | Test Asian 9 | 0.77 | 0.87 | 0.87 |
| 906 | Train 15 | Test Asian 10 | 0.79 | 0.86 | 0.85 | 1930 | Train 31 | Test Asian 10 | 0.79 | 0.86 | 0.86 |
| 907 | Train 15 | Test Asian 11 | 0.77 | 0.87 | 0.87 | 1931 | Train 31 | Test Asian 11 | 0.77 | 0.87 | 0.87 |
| 908 | Train 15 | Test Asian 12 | 0.79 | 0.86 | 0.85 | 1932 | Train 31 | Test Asian 12 | 0.79 | 0.86 | 0.86 |
| 909 | Train 15 | Test Asian 13 | 0.76 | 0.85 | 0.86 | 1933 | Train 31 | Test Asian 13 | 0.76 | 0.85 | 0.85 |
| 910 | Train 15 | Test Asian 14 | 0.75 | 0.76 | 0.76 | 1934 | Train 31 | Test Asian 14 | 0.75 | 0.76 | 0.74 |
| 911 | Train 15 | Test Asian 15 | 0.76 | 0.84 | 0.86 | 1935 | Train 31 | Test Asian 15 | 0.76 | 0.84 | 0.85 |
| 912 | Train 15 | Test Asian 16 | 0.75 | 0.78 | 0.77 | 1936 | Train 31 | Test Asian 16 | 0.75 | 0.78 | 0.78 |
| 913 | Train 15 | Test Asian 17 | 0.78 | 0.86 | 0.85 | 1937 | Train 31 | Test Asian 17 | 0.78 | 0.86 | 0.86 |
| 914 | Train 15 | Test Asian 18 | 0.8 | 0.86 | 0.84 | 1938 | Train 31 | Test Asian 18 | 0.8 | 0.86 | 0.86 |
| 915 | Train 15 | Test Asian 19 | 0.78 | 0.86 | 0.85 | 1939 | Train 31 | Test Asian 19 | 0.78 | 0.86 | 0.86 |
| 916 | Train 15 | Test Asian 20 | 0.8 | 0.86 | 0.84 | 1940 | Train 31 | Test Asian 20 | 0.8 | 0.86 | 0.86 |
| 917 | Train 15 | Test Asian 21 | 0.77 | 0.85 | 0.84 | 1941 | Train 31 | Test Asian 21 | 0.77 | 0.85 | 0.84 |
| 918 | Train 15 | Test Asian 22 | 0.75 | 0.78 | 0.78 | 1942 | Train 31 | Test Asian 22 | 0.75 | 0.79 | 0.79 |
| 919 | Train 15 | Test Asian 23 | 0.76 | 0.84 | 0.84 | 1943 | Train 31 | Test Asian 23 | 0.76 | 0.84 | 0.84 |
| 920 | Train 15 | Test Asian 24 | 0.76 | 0.78 | 0.78 | 1944 | Train 31 | Test Asian 24 | 0.75 | 0.78 | 0.78 |
| 921 | Train 15 | Test Asian 25 | 0.77 | 0.87 | 0.87 | 1945 | Train 31 | Test Asian 25 | 0.77 | 0.87 | 0.87 |
| 922 | Train 15 | Test Asian 26 | 0.79 | 0.86 | 0.85 | 1946 | Train 31 | Test Asian 26 | 0.79 | 0.86 | 0.86 |
| 923 | Train 15 | Test Asian 27 | 0.77 | 0.87 | 0.87 | 1947 | Train 31 | Test Asian 27 | 0.77 | 0.87 | 0.87 |
| 924 | Train 15 | Test Asian 28 | 0.79 | 0.86 | 0.85 | 1948 | Train 31 | Test Asian 28 | 0.79 | 0.86 | 0.86 |
| 925 | Train 15 | Test Asian 29 | 0.76 | 0.85 | 0.86 | 1949 | Train 31 | Test Asian 29 | 0.76 | 0.85 | 0.85 |
| 926 | Train 15 | Test Asian 30 | 0.75 | 0.76 | 0.76 | 1950 | Train 31 | Test Asian 30 | 0.75 | 0.76 | 0.74 |
| 927 | Train 15 | Test Asian 31 | 0.76 | 0.84 | 0.86 | 1951 | Train 31 | Test Asian 31 | 0.76 | 0.84 | 0.85 |
| 928 | Train 15 | Test Asian 32 | 0.76 | 0.78 | 0.77 | 1952 | Train 31 | Test Asian 32 | 0.76 | 0.78 | 0.78 |
| 929 | Train 15 | Test Asian 33 | 0.78 | 0.86 | 0.85 | 1953 | Train 31 | Test Asian 33 | 0.78 | 0.86 | 0.86 |
| 930 | Train 15 | Test Asian 34 | 0.8 | 0.86 | 0.84 | 1954 | Train 31 | Test Asian 34 | 0.8 | 0.86 | 0.86 |
| 931 | Train 15 | Test Asian 35 | 0.78 | 0.86 | 0.85 | 1955 | Train 31 | Test Asian 35 | 0.78 | 0.86 | 0.86 |
| 932 | Train 15 | Test Asian 36 | 0.8 | 0.86 | 0.84 | 1956 | Train 31 | Test Asian 36 | 0.8 | 0.86 | 0.86 |
| 933 | Train 15 | Test Asian 37 | 0.76 | 0.84 | 0.84 | 1957 | Train 31 | Test Asian 37 | 0.76 | 0.84 | 0.84 |
| 934 | Train 15 | Test Asian 38 | 0.75 | 0.78 | 0.77 | 1958 | Train 31 | Test Asian 38 | 0.75 | 0.78 | 0.78 |
| 935 | Train 15 | Test Asian 39 | 0.76 | 0.84 | 0.83 | 1959 | Train 31 | Test Asian 39 | 0.76 | 0.84 | 0.83 |
| 936 | Train 15 | Test Asian 40 | 0.75 | 0.78 | 0.77 | 1960 | Train 31 | Test Asian 40 | 0.75 | 0.78 | 0.78 |
| 937 | Train 15 | Test Asian 41 | 0.77 | 0.87 | 0.87 | 1961 | Train 31 | Test Asian 41 | 0.77 | 0.87 | 0.87 |
| 938 | Train 15 | Test Asian 42 | 0.8 | 0.86 | 0.85 | 1962 | Train 31 | Test Asian 42 | 0.8 | 0.86 | 0.86 |
| 939 | Train 15 | Test Asian 43 | 0.77 | 0.87 | 0.87 | 1963 | Train 31 | Test Asian 43 | 0.77 | 0.87 | 0.87 |
| 940 | Train 15 | Test Asian 44 | 0.8 | 0.86 | 0.85 | 1964 | Train 31 | Test Asian 44 | 0.8 | 0.86 | 0.86 |
| 941 | Train 15 | Test Asian 45 | 0.76 | 0.84 | 0.86 | 1965 | Train 31 | Test Asian 45 | 0.76 | 0.84 | 0.85 |
| 942 | Train 15 | Test Asian 46 | 0.75 | 0.78 | 0.77 | 1966 | Train 31 | Test Asian 46 | 0.75 | 0.78 | 0.78 |
| 943 | Train 15 | Test Asian 47 | 0.76 | 0.84 | 0.85 | 1967 | Train 31 | Test Asian 47 | 0.76 | 0.84 | 0.84 |
| 944 | Train 15 | Test Asian 48 | 0.75 | 0.78 | 0.77 | 1968 | Train 31 | Test Asian 48 | 0.75 | 0.78 | 0.78 |
| 945 | Train 15 | Test Asian 49 | 0.76 | 0.83 | 0.81 | 1969 | Train 31 | Test Asian 49 | 0.76 | 0.83 | 0.85 |
| 946 | Train 15 | Test Asian 50 | 0.76 | 0.83 | 0.81 | 1970 | Train 31 | Test Asian 50 | 0.76 | 0.83 | 0.84 |
| 947 | Train 15 | Test Asian 51 | 0.76 | 0.83 | 0.81 | 1971 | Train 31 | Test Asian 51 | 0.76 | 0.83 | 0.85 |
| 948 | Train 15 | Test Asian 52 | 0.76 | 0.83 | 0.81 | 1972 | Train 31 | Test Asian 52 | 0.76 | 0.83 | 0.84 |
| 949 | Train 15 | Test Asian 53 | 0.8 | 0.86 | 0.8 | 1973 | Train 31 | Test Asian 53 | 0.8 | 0.85 | 0.77 |
| 950 | Train 15 | Test Asian 54 | 0.77 | 0.76 | 0.72 | 1974 | Train 31 | Test Asian 54 | 0.77 | 0.75 | 0.69 |
| 951 | Train 15 | Test Asian 55 | 0.8 | 0.85 | 0.79 | 1975 | Train 31 | Test Asian 55 | 0.8 | 0.85 | 0.76 |
| 952 | Train 15 | Test Asian 56 | 0.77 | 0.75 | 0.73 | 1976 | Train 31 | Test Asian 56 | 0.77 | 0.75 | 0.69 |
| 953 | Train 15 | Test Asian 57 | 0.75 | 0.84 | 0.83 | 1977 | Train 31 | Test Asian 57 | 0.75 | 0.85 | 0.87 |
| 954 | Train 15 | Test Asian 58 | 0.75 | 0.84 | 0.83 | 1978 | Train 31 | Test Asian 58 | 0.74 | 0.85 | 0.87 |
| 955 | Train 15 | Test Asian 59 | 0.75 | 0.84 | 0.83 | 1979 | Train 31 | Test Asian 59 | 0.75 | 0.85 | 0.87 |
| 956 | Train 15 | Test Asian 60 | 0.75 | 0.84 | 0.83 | 1980 | Train 31 | Test Asian 60 | 0.74 | 0.85 | 0.87 |
| 957 | Train 15 | Test Asian 61 | 0.79 | 0.86 | 0.8 | 1981 | Train 31 | Test Asian 61 | 0.79 | 0.86 | 0.77 |
| 958 | Train 15 | Test Asian 62 | 0.77 | 0.76 | 0.72 | 1982 | Train 31 | Test Asian 62 | 0.77 | 0.76 | 0.7 |
| 959 | Train 15 | Test Asian 63 | 0.79 | 0.85 | 0.8 | 1983 | Train 31 | Test Asian 63 | 0.79 | 0.85 | 0.77 |
| 960 | Train 15 | Test Asian 64 | 0.77 | 0.75 | 0.73 | 1984 | Train 31 | Test Asian 64 | 0.77 | 0.75 | 0.7 |
| 961 | Train 16 | Test Asian 1 | 0.76 | 0.84 | 0.83 | 1985 | Train 32 | Test Asian 1 | 0.76 | 0.84 | 0.8 |
| 962 | Train 16 | Test Asian 2 | 0.79 | 0.86 | 0.85 | 1986 | Train 32 | Test Asian 2 | 0.79 | 0.86 | 0.82 |
| 963 | Train 16 | Test Asian 3 | 0.76 | 0.84 | 0.83 | 1987 | Train 32 | Test Asian 3 | 0.76 | 0.84 | 0.8 |
| 964 | Train 16 | Test Asian 4 | 0.79 | 0.86 | 0.85 | 1988 | Train 32 | Test Asian 4 | 0.79 | 0.86 | 0.82 |
| 965 | Train 16 | Test Asian 5 | 0.76 | 0.8 | 0.79 | 1989 | Train 32 | Test Asian 5 | 0.75 | 0.8 | 0.76 |
| 966 | Train 16 | Test Asian 6 | 0.76 | 0.82 | 0.8 | 1990 | Train 32 | Test Asian 6 | 0.76 | 0.82 | 0.77 |
| 967 | Train 16 | Test Asian 7 | 0.75 | 0.8 | 0.78 | 1991 | Train 32 | Test Asian 7 | 0.75 | 0.8 | 0.76 |
| 968 | Train 16 | Test Asian 8 | 0.76 | 0.81 | 0.8 | 1992 | Train 32 | Test Asian 8 | 0.76 | 0.81 | 0.77 |
| 969 | Train 16 | Test Asian 9 | 0.76 | 0.85 | 0.83 | 1993 | Train 32 | Test Asian 9 | 0.76 | 0.84 | 0.8 |
| 970 | Train 16 | Test Asian 10 | 0.78 | 0.86 | 0.84 | 1994 | Train 32 | Test Asian 10 | 0.78 | 0.86 | 0.82 |
| 971 | Train 16 | Test Asian 11 | 0.76 | 0.85 | 0.83 | 1995 | Train 32 | Test Asian 11 | 0.76 | 0.84 | 0.8 |
| 972 | Train 16 | Test Asian 12 | 0.78 | 0.86 | 0.84 | 1996 | Train 32 | Test Asian 12 | 0.78 | 0.86 | 0.82 |
| 973 | Train 16 | Test Asian 13 | 0.75 | 0.81 | 0.79 | 1997 | Train 32 | Test Asian 13 | 0.75 | 0.8 | 0.77 |
| 974 | Train 16 | Test Asian 14 | 0.76 | 0.83 | 0.83 | 1998 | Train 32 | Test Asian 14 | 0.76 | 0.83 | 0.83 |
| 975 | Train 16 | Test Asian 15 | 0.75 | 0.8 | 0.79 | 1999 | Train 32 | Test Asian 15 | 0.75 | 0.8 | 0.76 |
| 976 | Train 16 | Test Asian 16 | 0.76 | 0.81 | 0.8 | 2000 | Train 32 | Test Asian 16 | 0.76 | 0.81 | 0.77 |
| 977 | Train 16 | Test Asian 17 | 0.76 | 0.84 | 0.83 | 2001 | Train 32 | Test Asian 17 | 0.76 | 0.84 | 0.8 |
| 978 | Train 16 | Test Asian 18 | 0.79 | 0.86 | 0.85 | 2002 | Train 32 | Test Asian 18 | 0.79 | 0.86 | 0.82 |
| 979 | Train 16 | Test Asian 19 | 0.76 | 0.84 | 0.83 | 2003 | Train 32 | Test Asian 19 | 0.76 | 0.84 | 0.8 |
| 980 | Train 16 | Test Asian 20 | 0.79 | 0.86 | 0.85 | 2004 | Train 32 | Test Asian 20 | 0.79 | 0.86 | 0.82 |
| 981 | Train 16 | Test Asian 21 | 0.76 | 0.8 | 0.79 | 2005 | Train 32 | Test Asian 21 | 0.76 | 0.8 | 0.76 |
| 982 | Train 16 | Test Asian 22 | 0.76 | 0.82 | 0.8 | 2006 | Train 32 | Test Asian 22 | 0.76 | 0.82 | 0.77 |
| 983 | Train 16 | Test Asian 23 | 0.75 | 0.8 | 0.78 | 2007 | Train 32 | Test Asian 23 | 0.75 | 0.8 | 0.76 |
| 984 | Train 16 | Test Asian 24 | 0.77 | 0.82 | 0.8 | 2008 | Train 32 | Test Asian 24 | 0.77 | 0.82 | 0.77 |
| 985 | Train 16 | Test Asian 25 | 0.76 | 0.85 | 0.83 | 2009 | Train 32 | Test Asian 25 | 0.76 | 0.84 | 0.8 |
| 986 | Train 16 | Test Asian 26 | 0.78 | 0.86 | 0.84 | 2010 | Train 32 | Test Asian 26 | 0.78 | 0.86 | 0.82 |
| 987 | Train 16 | Test Asian 27 | 0.76 | 0.85 | 0.83 | 2011 | Train 32 | Test Asian 27 | 0.76 | 0.84 | 0.8 |
| 988 | Train 16 | Test Asian 28 | 0.78 | 0.86 | 0.84 | 2012 | Train 32 | Test Asian 28 | 0.78 | 0.86 | 0.82 |
| 989 | Train 16 | Test Asian 29 | 0.75 | 0.81 | 0.79 | 2013 | Train 32 | Test Asian 29 | 0.75 | 0.8 | 0.77 |
| 990 | Train 16 | Test Asian 30 | 0.76 | 0.83 | 0.83 | 2014 | Train 32 | Test Asian 30 | 0.76 | 0.83 | 0.83 |
| 991 | Train 16 | Test Asian 31 | 0.75 | 0.8 | 0.79 | 2015 | Train 32 | Test Asian 31 | 0.75 | 0.8 | 0.77 |
| 992 | Train 16 | Test Asian 32 | 0.76 | 0.81 | 0.8 | 2016 | Train 32 | Test Asian 32 | 0.76 | 0.81 | 0.77 |
| 993 | Train 16 | Test Asian 33 | 0.76 | 0.84 | 0.83 | 2017 | Train 32 | Test Asian 33 | 0.76 | 0.84 | 0.8 |
| 994 | Train 16 | Test Asian 34 | 0.79 | 0.86 | 0.85 | 2018 | Train 32 | Test Asian 34 | 0.78 | 0.86 | 0.82 |
| 995 | Train 16 | Test Asian 35 | 0.76 | 0.84 | 0.83 | 2019 | Train 32 | Test Asian 35 | 0.76 | 0.84 | 0.8 |
| 996 | Train 16 | Test Asian 36 | 0.79 | 0.86 | 0.85 | 2020 | Train 32 | Test Asian 36 | 0.78 | 0.86 | 0.82 |
| 997 | Train 16 | Test Asian 37 | 0.75 | 0.8 | 0.78 | 2021 | Train 32 | Test Asian 37 | 0.75 | 0.8 | 0.76 |
| 998 | Train 16 | Test Asian 38 | 0.76 | 0.81 | 0.8 | 2022 | Train 32 | Test Asian 38 | 0.76 | 0.81 | 0.77 |
| 999 | Train 16 | Test Asian 39 | 0.75 | 0.8 | 0.78 | 2023 | Train 32 | Test Asian 39 | 0.75 | 0.8 | 0.76 |
| 1000 | Train 16 | Test Asian 40 | 0.76 | 0.81 | 0.8 | 2024 | Train 32 | Test Asian 40 | 0.76 | 0.81 | 0.77 |
| 1001 | Train 16 | Test Asian 41 | 0.76 | 0.84 | 0.83 | 2025 | Train 32 | Test Asian 41 | 0.76 | 0.84 | 0.8 |
| 1002 | Train 16 | Test Asian 42 | 0.78 | 0.86 | 0.84 | 2026 | Train 32 | Test Asian 42 | 0.78 | 0.86 | 0.82 |
| 1003 | Train 16 | Test Asian 43 | 0.76 | 0.84 | 0.83 | 2027 | Train 32 | Test Asian 43 | 0.76 | 0.84 | 0.8 |
| 1004 | Train 16 | Test Asian 44 | 0.78 | 0.86 | 0.84 | 2028 | Train 32 | Test Asian 44 | 0.78 | 0.86 | 0.82 |
| 1005 | Train 16 | Test Asian 45 | 0.75 | 0.8 | 0.79 | 2029 | Train 32 | Test Asian 45 | 0.75 | 0.8 | 0.77 |
| 1006 | Train 16 | Test Asian 46 | 0.76 | 0.81 | 0.79 | 2030 | Train 32 | Test Asian 46 | 0.76 | 0.81 | 0.77 |
| 1007 | Train 16 | Test Asian 47 | 0.75 | 0.8 | 0.79 | 2031 | Train 32 | Test Asian 47 | 0.75 | 0.8 | 0.76 |
| 1008 | Train 16 | Test Asian 48 | 0.76 | 0.81 | 0.79 | 2032 | Train 32 | Test Asian 48 | 0.76 | 0.81 | 0.77 |
| 1009 | Train 16 | Test Asian 49 | 0.77 | 0.84 | 0.8 | 2033 | Train 32 | Test Asian 49 | 0.77 | 0.83 | 0.77 |
| 1010 | Train 16 | Test Asian 50 | 0.77 | 0.84 | 0.8 | 2034 | Train 32 | Test Asian 50 | 0.77 | 0.84 | 0.77 |
| 1011 | Train 16 | Test Asian 51 | 0.77 | 0.84 | 0.8 | 2035 | Train 32 | Test Asian 51 | 0.77 | 0.83 | 0.77 |
| 1012 | Train 16 | Test Asian 52 | 0.77 | 0.84 | 0.8 | 2036 | Train 32 | Test Asian 52 | 0.77 | 0.84 | 0.77 |
| 1013 | Train 16 | Test Asian 53 | 0.8 | 0.84 | 0.79 | 2037 | Train 32 | Test Asian 53 | 0.8 | 0.84 | 0.81 |
| 1014 | Train 16 | Test Asian 54 | 0.79 | 0.83 | 0.82 | 2038 | Train 32 | Test Asian 54 | 0.79 | 0.83 | 0.83 |
| 1015 | Train 16 | Test Asian 55 | 0.79 | 0.84 | 0.78 | 2039 | Train 32 | Test Asian 55 | 0.79 | 0.84 | 0.8 |
| 1016 | Train 16 | Test Asian 56 | 0.79 | 0.83 | 0.83 | 2040 | Train 32 | Test Asian 56 | 0.79 | 0.83 | 0.84 |
| 1017 | Train 16 | Test Asian 57 | 0.76 | 0.85 | 0.78 | 2041 | Train 32 | Test Asian 57 | 0.76 | 0.84 | 0.78 |
| 1018 | Train 16 | Test Asian 58 | 0.76 | 0.85 | 0.78 | 2042 | Train 32 | Test Asian 58 | 0.76 | 0.84 | 0.79 |
| 1019 | Train 16 | Test Asian 59 | 0.76 | 0.85 | 0.78 | 2043 | Train 32 | Test Asian 59 | 0.76 | 0.84 | 0.78 |
| 1020 | Train 16 | Test Asian 60 | 0.76 | 0.85 | 0.78 | 2044 | Train 32 | Test Asian 60 | 0.76 | 0.84 | 0.79 |
| 1021 | Train 16 | Test Asian 61 | 0.79 | 0.84 | 0.78 | 2045 | Train 32 | Test Asian 61 | 0.79 | 0.84 | 0.82 |
| 1022 | Train 16 | Test Asian 62 | 0.79 | 0.83 | 0.83 | 2046 | Train 32 | Test Asian 62 | 0.79 | 0.83 | 0.83 |
| 1023 | Train 16 | Test Asian 63 | 0.78 | 0.84 | 0.77 | 2047 | Train 32 | Test Asian 63 | 0.78 | 0.84 | 0.81 |
| 1024 | Train 16 | Test Asian 64 | 0.79 | 0.83 | 0.83 | 2048 | Train 32 | Test Asian 64 | 0.79 | 0.83 | 0.84 |

Table 10. AUC Performance of the ML Models— LR, RF, and GB —for Asian Patients Trained on Cohorts 33–64 and Tested Across 64 Cohorts.

| Asian | Train  Model | Test  Dataset | AUC (LR) | AUC (RF) | AUC (GB) | Asian | Train  Model | Test  Dataset | AUC (LR) | AUC (RF) | AUC (GB) |
| --- | --- | --- | --- | --- | --- | --- | --- | --- | --- | --- | --- |
| 2049 | Train 33 | Test Asian 1 | 0.8 | 0.92 | 0.95 | 3073 | Train 49 | Test Asian 1 | 0.75 | 0.81 | 0.69 |
| 2050 | Train 33 | Test Asian 2 | 0.8 | 0.89 | 0.92 | 3074 | Train 49 | Test Asian 2 | 0.75 | 0.82 | 0.66 |
| 2051 | Train 33 | Test Asian 3 | 0.8 | 0.92 | 0.95 | 3075 | Train 49 | Test Asian 3 | 0.75 | 0.81 | 0.69 |
| 2052 | Train 33 | Test Asian 4 | 0.8 | 0.89 | 0.92 | 3076 | Train 49 | Test Asian 4 | 0.75 | 0.82 | 0.66 |
| 2053 | Train 33 | Test Asian 5 | 0.75 | 0.76 | 0.7 | 3077 | Train 49 | Test Asian 5 | 0.73 | 0.72 | 0.56 |
| 2054 | Train 33 | Test Asian 6 | 0.75 | 0.75 | 0.67 | 3078 | Train 49 | Test Asian 6 | 0.73 | 0.71 | 0.59 |
| 2055 | Train 33 | Test Asian 7 | 0.74 | 0.76 | 0.7 | 3079 | Train 49 | Test Asian 7 | 0.73 | 0.72 | 0.56 |
| 2056 | Train 33 | Test Asian 8 | 0.75 | 0.75 | 0.67 | 3080 | Train 49 | Test Asian 8 | 0.73 | 0.71 | 0.59 |
| 2057 | Train 33 | Test Asian 9 | 0.79 | 0.9 | 0.88 | 3081 | Train 49 | Test Asian 9 | 0.74 | 0.8 | 0.66 |
| 2058 | Train 33 | Test Asian 10 | 0.79 | 0.87 | 0.85 | 3082 | Train 49 | Test Asian 10 | 0.74 | 0.81 | 0.64 |
| 2059 | Train 33 | Test Asian 11 | 0.79 | 0.9 | 0.88 | 3083 | Train 49 | Test Asian 11 | 0.74 | 0.8 | 0.66 |
| 2060 | Train 33 | Test Asian 12 | 0.79 | 0.87 | 0.85 | 3084 | Train 49 | Test Asian 12 | 0.74 | 0.81 | 0.64 |
| 2061 | Train 33 | Test Asian 13 | 0.74 | 0.75 | 0.68 | 3085 | Train 49 | Test Asian 13 | 0.72 | 0.71 | 0.56 |
| 2062 | Train 33 | Test Asian 14 | 0.75 | 0.73 | 0.59 | 3086 | Train 49 | Test Asian 14 | 0.73 | 0.71 | 0.58 |
| 2063 | Train 33 | Test Asian 15 | 0.74 | 0.75 | 0.67 | 3087 | Train 49 | Test Asian 15 | 0.72 | 0.71 | 0.56 |
| 2064 | Train 33 | Test Asian 16 | 0.75 | 0.75 | 0.67 | 3088 | Train 49 | Test Asian 16 | 0.73 | 0.71 | 0.59 |
| 2065 | Train 33 | Test Asian 17 | 0.8 | 0.92 | 0.95 | 3089 | Train 49 | Test Asian 17 | 0.75 | 0.81 | 0.69 |
| 2066 | Train 33 | Test Asian 18 | 0.8 | 0.89 | 0.92 | 3090 | Train 49 | Test Asian 18 | 0.75 | 0.82 | 0.66 |
| 2067 | Train 33 | Test Asian 19 | 0.8 | 0.92 | 0.95 | 3091 | Train 49 | Test Asian 19 | 0.75 | 0.81 | 0.69 |
| 2068 | Train 33 | Test Asian 20 | 0.8 | 0.9 | 0.92 | 3092 | Train 49 | Test Asian 20 | 0.75 | 0.82 | 0.66 |
| 2069 | Train 33 | Test Asian 21 | 0.75 | 0.76 | 0.7 | 3093 | Train 49 | Test Asian 21 | 0.73 | 0.72 | 0.56 |
| 2070 | Train 33 | Test Asian 22 | 0.75 | 0.75 | 0.67 | 3094 | Train 49 | Test Asian 22 | 0.73 | 0.71 | 0.59 |
| 2071 | Train 33 | Test Asian 23 | 0.74 | 0.76 | 0.7 | 3095 | Train 49 | Test Asian 23 | 0.73 | 0.72 | 0.56 |
| 2072 | Train 33 | Test Asian 24 | 0.75 | 0.75 | 0.67 | 3096 | Train 49 | Test Asian 24 | 0.73 | 0.71 | 0.59 |
| 2073 | Train 33 | Test Asian 25 | 0.79 | 0.9 | 0.88 | 3097 | Train 49 | Test Asian 25 | 0.74 | 0.8 | 0.66 |
| 2074 | Train 33 | Test Asian 26 | 0.79 | 0.87 | 0.85 | 3098 | Train 49 | Test Asian 26 | 0.74 | 0.81 | 0.64 |
| 2075 | Train 33 | Test Asian 27 | 0.79 | 0.9 | 0.88 | 3099 | Train 49 | Test Asian 27 | 0.74 | 0.8 | 0.66 |
| 2076 | Train 33 | Test Asian 28 | 0.79 | 0.87 | 0.85 | 3100 | Train 49 | Test Asian 28 | 0.74 | 0.81 | 0.64 |
| 2077 | Train 33 | Test Asian 29 | 0.74 | 0.75 | 0.68 | 3101 | Train 49 | Test Asian 29 | 0.73 | 0.71 | 0.56 |
| 2078 | Train 33 | Test Asian 30 | 0.75 | 0.73 | 0.59 | 3102 | Train 49 | Test Asian 30 | 0.73 | 0.72 | 0.59 |
| 2079 | Train 33 | Test Asian 31 | 0.74 | 0.75 | 0.67 | 3103 | Train 49 | Test Asian 31 | 0.72 | 0.71 | 0.56 |
| 2080 | Train 33 | Test Asian 32 | 0.75 | 0.75 | 0.67 | 3104 | Train 49 | Test Asian 32 | 0.73 | 0.71 | 0.59 |
| 2081 | Train 33 | Test Asian 33 | 0.8 | 0.92 | 0.95 | 3105 | Train 49 | Test Asian 33 | 0.75 | 0.81 | 0.69 |
| 2082 | Train 33 | Test Asian 34 | 0.8 | 0.89 | 0.92 | 3106 | Train 49 | Test Asian 34 | 0.75 | 0.82 | 0.66 |
| 2083 | Train 33 | Test Asian 35 | 0.8 | 0.92 | 0.95 | 3107 | Train 49 | Test Asian 35 | 0.75 | 0.81 | 0.69 |
| 2084 | Train 33 | Test Asian 36 | 0.8 | 0.89 | 0.92 | 3108 | Train 49 | Test Asian 36 | 0.75 | 0.82 | 0.66 |
| 2085 | Train 33 | Test Asian 37 | 0.75 | 0.76 | 0.7 | 3109 | Train 49 | Test Asian 37 | 0.73 | 0.72 | 0.56 |
| 2086 | Train 33 | Test Asian 38 | 0.75 | 0.75 | 0.67 | 3110 | Train 49 | Test Asian 38 | 0.73 | 0.71 | 0.59 |
| 2087 | Train 33 | Test Asian 39 | 0.74 | 0.75 | 0.69 | 3111 | Train 49 | Test Asian 39 | 0.73 | 0.72 | 0.56 |
| 2088 | Train 33 | Test Asian 40 | 0.75 | 0.75 | 0.67 | 3112 | Train 49 | Test Asian 40 | 0.73 | 0.71 | 0.59 |
| 2089 | Train 33 | Test Asian 41 | 0.79 | 0.9 | 0.88 | 3113 | Train 49 | Test Asian 41 | 0.74 | 0.8 | 0.66 |
| 2090 | Train 33 | Test Asian 42 | 0.79 | 0.87 | 0.85 | 3114 | Train 49 | Test Asian 42 | 0.74 | 0.81 | 0.64 |
| 2091 | Train 33 | Test Asian 43 | 0.79 | 0.9 | 0.88 | 3115 | Train 49 | Test Asian 43 | 0.74 | 0.8 | 0.66 |
| 2092 | Train 33 | Test Asian 44 | 0.79 | 0.87 | 0.85 | 3116 | Train 49 | Test Asian 44 | 0.74 | 0.81 | 0.64 |
| 2093 | Train 33 | Test Asian 45 | 0.74 | 0.75 | 0.67 | 3117 | Train 49 | Test Asian 45 | 0.73 | 0.71 | 0.56 |
| 2094 | Train 33 | Test Asian 46 | 0.75 | 0.75 | 0.67 | 3118 | Train 49 | Test Asian 46 | 0.73 | 0.71 | 0.58 |
| 2095 | Train 33 | Test Asian 47 | 0.74 | 0.75 | 0.67 | 3119 | Train 49 | Test Asian 47 | 0.72 | 0.71 | 0.56 |
| 2096 | Train 33 | Test Asian 48 | 0.75 | 0.75 | 0.67 | 3120 | Train 49 | Test Asian 48 | 0.73 | 0.71 | 0.59 |
| 2097 | Train 33 | Test Asian 49 | 0.77 | 0.88 | 0.95 | 3121 | Train 49 | Test Asian 49 | 0.77 | 0.93 | 0.97 |
| 2098 | Train 33 | Test Asian 50 | 0.77 | 0.88 | 0.95 | 3122 | Train 49 | Test Asian 50 | 0.77 | 0.93 | 0.97 |
| 2099 | Train 33 | Test Asian 51 | 0.77 | 0.88 | 0.95 | 3123 | Train 49 | Test Asian 51 | 0.77 | 0.93 | 0.97 |
| 2100 | Train 33 | Test Asian 52 | 0.77 | 0.88 | 0.95 | 3124 | Train 49 | Test Asian 52 | 0.77 | 0.93 | 0.97 |
| 2101 | Train 33 | Test Asian 53 | 0.79 | 0.81 | 0.6 | 3125 | Train 49 | Test Asian 53 | 0.77 | 0.75 | 0.6 |
| 2102 | Train 33 | Test Asian 54 | 0.78 | 0.76 | 0.56 | 3126 | Train 49 | Test Asian 54 | 0.74 | 0.74 | 0.63 |
| 2103 | Train 33 | Test Asian 55 | 0.79 | 0.8 | 0.61 | 3127 | Train 49 | Test Asian 55 | 0.77 | 0.75 | 0.61 |
| 2104 | Train 33 | Test Asian 56 | 0.78 | 0.75 | 0.57 | 3128 | Train 49 | Test Asian 56 | 0.75 | 0.73 | 0.63 |
| 2105 | Train 33 | Test Asian 57 | 0.76 | 0.84 | 0.89 | 3129 | Train 49 | Test Asian 57 | 0.76 | 0.89 | 0.91 |
| 2106 | Train 33 | Test Asian 58 | 0.76 | 0.85 | 0.88 | 3130 | Train 49 | Test Asian 58 | 0.76 | 0.9 | 0.91 |
| 2107 | Train 33 | Test Asian 59 | 0.76 | 0.84 | 0.89 | 3131 | Train 49 | Test Asian 59 | 0.76 | 0.89 | 0.91 |
| 2108 | Train 33 | Test Asian 60 | 0.76 | 0.85 | 0.88 | 3132 | Train 49 | Test Asian 60 | 0.76 | 0.9 | 0.91 |
| 2109 | Train 33 | Test Asian 61 | 0.79 | 0.8 | 0.58 | 3133 | Train 49 | Test Asian 61 | 0.76 | 0.74 | 0.61 |
| 2110 | Train 33 | Test Asian 62 | 0.78 | 0.76 | 0.56 | 3134 | Train 49 | Test Asian 62 | 0.75 | 0.74 | 0.64 |
| 2111 | Train 33 | Test Asian 63 | 0.78 | 0.79 | 0.58 | 3135 | Train 49 | Test Asian 63 | 0.76 | 0.74 | 0.61 |
| 2112 | Train 33 | Test Asian 64 | 0.78 | 0.75 | 0.57 | 3136 | Train 49 | Test Asian 64 | 0.75 | 0.73 | 0.64 |
| 2113 | Train 34 | Test Asian 1 | 0.79 | 0.91 | 0.91 | 3137 | Train 50 | Test Asian 1 | 0.75 | 0.76 | 0.71 |
| 2114 | Train 34 | Test Asian 2 | 0.81 | 0.92 | 0.92 | 3138 | Train 50 | Test Asian 2 | 0.76 | 0.78 | 0.71 |
| 2115 | Train 34 | Test Asian 3 | 0.79 | 0.91 | 0.91 | 3139 | Train 50 | Test Asian 3 | 0.75 | 0.76 | 0.71 |
| 2116 | Train 34 | Test Asian 4 | 0.81 | 0.92 | 0.92 | 3140 | Train 50 | Test Asian 4 | 0.76 | 0.78 | 0.71 |
| 2117 | Train 34 | Test Asian 5 | 0.75 | 0.76 | 0.72 | 3141 | Train 50 | Test Asian 5 | 0.73 | 0.68 | 0.59 |
| 2118 | Train 34 | Test Asian 6 | 0.74 | 0.75 | 0.7 | 3142 | Train 50 | Test Asian 6 | 0.73 | 0.7 | 0.62 |
| 2119 | Train 34 | Test Asian 7 | 0.75 | 0.76 | 0.72 | 3143 | Train 50 | Test Asian 7 | 0.73 | 0.69 | 0.59 |
| 2120 | Train 34 | Test Asian 8 | 0.75 | 0.75 | 0.7 | 3144 | Train 50 | Test Asian 8 | 0.73 | 0.69 | 0.62 |
| 2121 | Train 34 | Test Asian 9 | 0.78 | 0.89 | 0.87 | 3145 | Train 50 | Test Asian 9 | 0.74 | 0.76 | 0.66 |
| 2122 | Train 34 | Test Asian 10 | 0.8 | 0.9 | 0.88 | 3146 | Train 50 | Test Asian 10 | 0.75 | 0.78 | 0.68 |
| 2123 | Train 34 | Test Asian 11 | 0.78 | 0.89 | 0.87 | 3147 | Train 50 | Test Asian 11 | 0.74 | 0.76 | 0.66 |
| 2124 | Train 34 | Test Asian 12 | 0.8 | 0.9 | 0.88 | 3148 | Train 50 | Test Asian 12 | 0.75 | 0.78 | 0.68 |
| 2125 | Train 34 | Test Asian 13 | 0.75 | 0.76 | 0.71 | 3149 | Train 50 | Test Asian 13 | 0.72 | 0.68 | 0.59 |
| 2126 | Train 34 | Test Asian 14 | 0.75 | 0.73 | 0.67 | 3150 | Train 50 | Test Asian 14 | 0.73 | 0.7 | 0.62 |
| 2127 | Train 34 | Test Asian 15 | 0.75 | 0.75 | 0.71 | 3151 | Train 50 | Test Asian 15 | 0.72 | 0.68 | 0.59 |
| 2128 | Train 34 | Test Asian 16 | 0.75 | 0.75 | 0.7 | 3152 | Train 50 | Test Asian 16 | 0.73 | 0.69 | 0.63 |
| 2129 | Train 34 | Test Asian 17 | 0.79 | 0.91 | 0.91 | 3153 | Train 50 | Test Asian 17 | 0.75 | 0.76 | 0.71 |
| 2130 | Train 34 | Test Asian 18 | 0.81 | 0.92 | 0.92 | 3154 | Train 50 | Test Asian 18 | 0.76 | 0.78 | 0.71 |
| 2131 | Train 34 | Test Asian 19 | 0.79 | 0.91 | 0.91 | 3155 | Train 50 | Test Asian 19 | 0.75 | 0.76 | 0.71 |
| 2132 | Train 34 | Test Asian 20 | 0.81 | 0.92 | 0.92 | 3156 | Train 50 | Test Asian 20 | 0.76 | 0.78 | 0.71 |
| 2133 | Train 34 | Test Asian 21 | 0.75 | 0.76 | 0.72 | 3157 | Train 50 | Test Asian 21 | 0.73 | 0.68 | 0.59 |
| 2134 | Train 34 | Test Asian 22 | 0.75 | 0.75 | 0.7 | 3158 | Train 50 | Test Asian 22 | 0.73 | 0.7 | 0.62 |
| 2135 | Train 34 | Test Asian 23 | 0.75 | 0.76 | 0.72 | 3159 | Train 50 | Test Asian 23 | 0.73 | 0.69 | 0.59 |
| 2136 | Train 34 | Test Asian 24 | 0.75 | 0.75 | 0.7 | 3160 | Train 50 | Test Asian 24 | 0.73 | 0.69 | 0.63 |
| 2137 | Train 34 | Test Asian 25 | 0.78 | 0.89 | 0.87 | 3161 | Train 50 | Test Asian 25 | 0.74 | 0.76 | 0.66 |
| 2138 | Train 34 | Test Asian 26 | 0.8 | 0.9 | 0.88 | 3162 | Train 50 | Test Asian 26 | 0.75 | 0.78 | 0.68 |
| 2139 | Train 34 | Test Asian 27 | 0.78 | 0.89 | 0.87 | 3163 | Train 50 | Test Asian 27 | 0.74 | 0.76 | 0.66 |
| 2140 | Train 34 | Test Asian 28 | 0.8 | 0.9 | 0.88 | 3164 | Train 50 | Test Asian 28 | 0.75 | 0.78 | 0.68 |
| 2141 | Train 34 | Test Asian 29 | 0.75 | 0.76 | 0.71 | 3165 | Train 50 | Test Asian 29 | 0.72 | 0.68 | 0.59 |
| 2142 | Train 34 | Test Asian 30 | 0.75 | 0.73 | 0.67 | 3166 | Train 50 | Test Asian 30 | 0.73 | 0.7 | 0.62 |
| 2143 | Train 34 | Test Asian 31 | 0.75 | 0.75 | 0.71 | 3167 | Train 50 | Test Asian 31 | 0.72 | 0.68 | 0.59 |
| 2144 | Train 34 | Test Asian 32 | 0.75 | 0.75 | 0.7 | 3168 | Train 50 | Test Asian 32 | 0.73 | 0.7 | 0.63 |
| 2145 | Train 34 | Test Asian 33 | 0.79 | 0.91 | 0.91 | 3169 | Train 50 | Test Asian 33 | 0.75 | 0.76 | 0.71 |
| 2146 | Train 34 | Test Asian 34 | 0.81 | 0.92 | 0.92 | 3170 | Train 50 | Test Asian 34 | 0.75 | 0.78 | 0.72 |
| 2147 | Train 34 | Test Asian 35 | 0.79 | 0.91 | 0.91 | 3171 | Train 50 | Test Asian 35 | 0.75 | 0.76 | 0.71 |
| 2148 | Train 34 | Test Asian 36 | 0.81 | 0.92 | 0.92 | 3172 | Train 50 | Test Asian 36 | 0.75 | 0.78 | 0.72 |
| 2149 | Train 34 | Test Asian 37 | 0.75 | 0.76 | 0.72 | 3173 | Train 50 | Test Asian 37 | 0.73 | 0.68 | 0.58 |
| 2150 | Train 34 | Test Asian 38 | 0.74 | 0.75 | 0.7 | 3174 | Train 50 | Test Asian 38 | 0.73 | 0.69 | 0.62 |
| 2151 | Train 34 | Test Asian 39 | 0.75 | 0.76 | 0.72 | 3175 | Train 50 | Test Asian 39 | 0.73 | 0.68 | 0.59 |
| 2152 | Train 34 | Test Asian 40 | 0.74 | 0.75 | 0.7 | 3176 | Train 50 | Test Asian 40 | 0.73 | 0.69 | 0.62 |
| 2153 | Train 34 | Test Asian 41 | 0.78 | 0.89 | 0.87 | 3177 | Train 50 | Test Asian 41 | 0.74 | 0.76 | 0.66 |
| 2154 | Train 34 | Test Asian 42 | 0.8 | 0.89 | 0.88 | 3178 | Train 50 | Test Asian 42 | 0.74 | 0.77 | 0.68 |
| 2155 | Train 34 | Test Asian 43 | 0.78 | 0.89 | 0.87 | 3179 | Train 50 | Test Asian 43 | 0.74 | 0.76 | 0.66 |
| 2156 | Train 34 | Test Asian 44 | 0.8 | 0.89 | 0.88 | 3180 | Train 50 | Test Asian 44 | 0.74 | 0.77 | 0.68 |
| 2157 | Train 34 | Test Asian 45 | 0.75 | 0.76 | 0.71 | 3181 | Train 50 | Test Asian 45 | 0.72 | 0.68 | 0.58 |
| 2158 | Train 34 | Test Asian 46 | 0.74 | 0.75 | 0.7 | 3182 | Train 50 | Test Asian 46 | 0.73 | 0.7 | 0.62 |
| 2159 | Train 34 | Test Asian 47 | 0.75 | 0.75 | 0.71 | 3183 | Train 50 | Test Asian 47 | 0.72 | 0.68 | 0.59 |
| 2160 | Train 34 | Test Asian 48 | 0.75 | 0.75 | 0.7 | 3184 | Train 50 | Test Asian 48 | 0.73 | 0.69 | 0.63 |
| 2161 | Train 34 | Test Asian 49 | 0.77 | 0.86 | 0.85 | 3185 | Train 50 | Test Asian 49 | 0.77 | 0.92 | 0.96 |
| 2162 | Train 34 | Test Asian 50 | 0.77 | 0.86 | 0.85 | 3186 | Train 50 | Test Asian 50 | 0.77 | 0.92 | 0.96 |
| 2163 | Train 34 | Test Asian 51 | 0.77 | 0.86 | 0.85 | 3187 | Train 50 | Test Asian 51 | 0.77 | 0.92 | 0.96 |
| 2164 | Train 34 | Test Asian 52 | 0.77 | 0.86 | 0.85 | 3188 | Train 50 | Test Asian 52 | 0.77 | 0.92 | 0.96 |
| 2165 | Train 34 | Test Asian 53 | 0.79 | 0.81 | 0.68 | 3189 | Train 50 | Test Asian 53 | 0.77 | 0.69 | 0.61 |
| 2166 | Train 34 | Test Asian 54 | 0.77 | 0.74 | 0.66 | 3190 | Train 50 | Test Asian 54 | 0.75 | 0.71 | 0.64 |
| 2167 | Train 34 | Test Asian 55 | 0.79 | 0.81 | 0.68 | 3191 | Train 50 | Test Asian 55 | 0.76 | 0.7 | 0.61 |
| 2168 | Train 34 | Test Asian 56 | 0.76 | 0.73 | 0.65 | 3192 | Train 50 | Test Asian 56 | 0.75 | 0.69 | 0.64 |
| 2169 | Train 34 | Test Asian 57 | 0.76 | 0.83 | 0.82 | 3193 | Train 50 | Test Asian 57 | 0.76 | 0.89 | 0.86 |
| 2170 | Train 34 | Test Asian 58 | 0.76 | 0.83 | 0.81 | 3194 | Train 50 | Test Asian 58 | 0.76 | 0.89 | 0.87 |
| 2171 | Train 34 | Test Asian 59 | 0.76 | 0.83 | 0.82 | 3195 | Train 50 | Test Asian 59 | 0.76 | 0.89 | 0.86 |
| 2172 | Train 34 | Test Asian 60 | 0.76 | 0.83 | 0.81 | 3196 | Train 50 | Test Asian 60 | 0.76 | 0.89 | 0.87 |
| 2173 | Train 34 | Test Asian 61 | 0.79 | 0.8 | 0.67 | 3197 | Train 50 | Test Asian 61 | 0.76 | 0.69 | 0.6 |
| 2174 | Train 34 | Test Asian 62 | 0.77 | 0.74 | 0.66 | 3198 | Train 50 | Test Asian 62 | 0.75 | 0.71 | 0.64 |
| 2175 | Train 34 | Test Asian 63 | 0.78 | 0.8 | 0.67 | 3199 | Train 50 | Test Asian 63 | 0.76 | 0.69 | 0.6 |
| 2176 | Train 34 | Test Asian 64 | 0.76 | 0.73 | 0.66 | 3200 | Train 50 | Test Asian 64 | 0.75 | 0.69 | 0.65 |
| 2177 | Train 35 | Test Asian 1 | 0.8 | 0.92 | 0.89 | 3201 | Train 51 | Test Asian 1 | 0.75 | 0.81 | 0.75 |
| 2178 | Train 35 | Test Asian 2 | 0.8 | 0.9 | 0.86 | 3202 | Train 51 | Test Asian 2 | 0.75 | 0.83 | 0.69 |
| 2179 | Train 35 | Test Asian 3 | 0.8 | 0.92 | 0.89 | 3203 | Train 51 | Test Asian 3 | 0.75 | 0.81 | 0.75 |
| 2180 | Train 35 | Test Asian 4 | 0.8 | 0.9 | 0.86 | 3204 | Train 51 | Test Asian 4 | 0.75 | 0.83 | 0.69 |
| 2181 | Train 35 | Test Asian 5 | 0.75 | 0.76 | 0.71 | 3205 | Train 51 | Test Asian 5 | 0.73 | 0.71 | 0.62 |
| 2182 | Train 35 | Test Asian 6 | 0.75 | 0.75 | 0.7 | 3206 | Train 51 | Test Asian 6 | 0.73 | 0.72 | 0.59 |
| 2183 | Train 35 | Test Asian 7 | 0.74 | 0.76 | 0.71 | 3207 | Train 51 | Test Asian 7 | 0.73 | 0.71 | 0.62 |
| 2184 | Train 35 | Test Asian 8 | 0.75 | 0.75 | 0.7 | 3208 | Train 51 | Test Asian 8 | 0.73 | 0.72 | 0.59 |
| 2185 | Train 35 | Test Asian 9 | 0.79 | 0.9 | 0.86 | 3209 | Train 51 | Test Asian 9 | 0.74 | 0.8 | 0.71 |
| 2186 | Train 35 | Test Asian 10 | 0.79 | 0.87 | 0.83 | 3210 | Train 51 | Test Asian 10 | 0.74 | 0.81 | 0.66 |
| 2187 | Train 35 | Test Asian 11 | 0.79 | 0.9 | 0.86 | 3211 | Train 51 | Test Asian 11 | 0.74 | 0.8 | 0.71 |
| 2188 | Train 35 | Test Asian 12 | 0.79 | 0.87 | 0.83 | 3212 | Train 51 | Test Asian 12 | 0.74 | 0.81 | 0.66 |
| 2189 | Train 35 | Test Asian 13 | 0.74 | 0.75 | 0.7 | 3213 | Train 51 | Test Asian 13 | 0.73 | 0.71 | 0.62 |
| 2190 | Train 35 | Test Asian 14 | 0.75 | 0.73 | 0.65 | 3214 | Train 51 | Test Asian 14 | 0.73 | 0.72 | 0.59 |
| 2191 | Train 35 | Test Asian 15 | 0.74 | 0.75 | 0.69 | 3215 | Train 51 | Test Asian 15 | 0.72 | 0.71 | 0.61 |
| 2192 | Train 35 | Test Asian 16 | 0.75 | 0.75 | 0.7 | 3216 | Train 51 | Test Asian 16 | 0.73 | 0.72 | 0.59 |
| 2193 | Train 35 | Test Asian 17 | 0.8 | 0.92 | 0.89 | 3217 | Train 51 | Test Asian 17 | 0.75 | 0.81 | 0.75 |
| 2194 | Train 35 | Test Asian 18 | 0.8 | 0.9 | 0.86 | 3218 | Train 51 | Test Asian 18 | 0.75 | 0.83 | 0.69 |
| 2195 | Train 35 | Test Asian 19 | 0.8 | 0.92 | 0.89 | 3219 | Train 51 | Test Asian 19 | 0.75 | 0.81 | 0.75 |
| 2196 | Train 35 | Test Asian 20 | 0.8 | 0.9 | 0.86 | 3220 | Train 51 | Test Asian 20 | 0.75 | 0.83 | 0.69 |
| 2197 | Train 35 | Test Asian 21 | 0.75 | 0.76 | 0.71 | 3221 | Train 51 | Test Asian 21 | 0.73 | 0.72 | 0.62 |
| 2198 | Train 35 | Test Asian 22 | 0.75 | 0.75 | 0.7 | 3222 | Train 51 | Test Asian 22 | 0.73 | 0.72 | 0.59 |
| 2199 | Train 35 | Test Asian 23 | 0.75 | 0.76 | 0.71 | 3223 | Train 51 | Test Asian 23 | 0.73 | 0.72 | 0.62 |
| 2200 | Train 35 | Test Asian 24 | 0.75 | 0.75 | 0.7 | 3224 | Train 51 | Test Asian 24 | 0.73 | 0.72 | 0.59 |
| 2201 | Train 35 | Test Asian 25 | 0.79 | 0.9 | 0.86 | 3225 | Train 51 | Test Asian 25 | 0.74 | 0.8 | 0.71 |
| 2202 | Train 35 | Test Asian 26 | 0.79 | 0.87 | 0.83 | 3226 | Train 51 | Test Asian 26 | 0.74 | 0.81 | 0.66 |
| 2203 | Train 35 | Test Asian 27 | 0.79 | 0.9 | 0.86 | 3227 | Train 51 | Test Asian 27 | 0.74 | 0.8 | 0.71 |
| 2204 | Train 35 | Test Asian 28 | 0.79 | 0.87 | 0.83 | 3228 | Train 51 | Test Asian 28 | 0.74 | 0.81 | 0.66 |
| 2205 | Train 35 | Test Asian 29 | 0.74 | 0.75 | 0.7 | 3229 | Train 51 | Test Asian 29 | 0.73 | 0.71 | 0.62 |
| 2206 | Train 35 | Test Asian 30 | 0.75 | 0.73 | 0.65 | 3230 | Train 51 | Test Asian 30 | 0.73 | 0.72 | 0.59 |
| 2207 | Train 35 | Test Asian 31 | 0.74 | 0.75 | 0.69 | 3231 | Train 51 | Test Asian 31 | 0.72 | 0.71 | 0.61 |
| 2208 | Train 35 | Test Asian 32 | 0.75 | 0.75 | 0.7 | 3232 | Train 51 | Test Asian 32 | 0.73 | 0.72 | 0.59 |
| 2209 | Train 35 | Test Asian 33 | 0.8 | 0.92 | 0.89 | 3233 | Train 51 | Test Asian 33 | 0.75 | 0.81 | 0.75 |
| 2210 | Train 35 | Test Asian 34 | 0.8 | 0.9 | 0.86 | 3234 | Train 51 | Test Asian 34 | 0.75 | 0.82 | 0.69 |
| 2211 | Train 35 | Test Asian 35 | 0.8 | 0.92 | 0.89 | 3235 | Train 51 | Test Asian 35 | 0.75 | 0.81 | 0.75 |
| 2212 | Train 35 | Test Asian 36 | 0.8 | 0.9 | 0.86 | 3236 | Train 51 | Test Asian 36 | 0.75 | 0.82 | 0.69 |
| 2213 | Train 35 | Test Asian 37 | 0.75 | 0.76 | 0.71 | 3237 | Train 51 | Test Asian 37 | 0.73 | 0.71 | 0.62 |
| 2214 | Train 35 | Test Asian 38 | 0.75 | 0.75 | 0.7 | 3238 | Train 51 | Test Asian 38 | 0.73 | 0.72 | 0.59 |
| 2215 | Train 35 | Test Asian 39 | 0.74 | 0.75 | 0.7 | 3239 | Train 51 | Test Asian 39 | 0.73 | 0.71 | 0.62 |
| 2216 | Train 35 | Test Asian 40 | 0.75 | 0.75 | 0.7 | 3240 | Train 51 | Test Asian 40 | 0.73 | 0.72 | 0.6 |
| 2217 | Train 35 | Test Asian 41 | 0.79 | 0.9 | 0.86 | 3241 | Train 51 | Test Asian 41 | 0.74 | 0.8 | 0.71 |
| 2218 | Train 35 | Test Asian 42 | 0.79 | 0.87 | 0.82 | 3242 | Train 51 | Test Asian 42 | 0.74 | 0.81 | 0.66 |
| 2219 | Train 35 | Test Asian 43 | 0.79 | 0.9 | 0.86 | 3243 | Train 51 | Test Asian 43 | 0.74 | 0.8 | 0.71 |
| 2220 | Train 35 | Test Asian 44 | 0.79 | 0.87 | 0.82 | 3244 | Train 51 | Test Asian 44 | 0.74 | 0.81 | 0.66 |
| 2221 | Train 35 | Test Asian 45 | 0.74 | 0.75 | 0.7 | 3245 | Train 51 | Test Asian 45 | 0.73 | 0.71 | 0.62 |
| 2222 | Train 35 | Test Asian 46 | 0.75 | 0.75 | 0.69 | 3246 | Train 51 | Test Asian 46 | 0.73 | 0.72 | 0.59 |
| 2223 | Train 35 | Test Asian 47 | 0.74 | 0.75 | 0.69 | 3247 | Train 51 | Test Asian 47 | 0.72 | 0.71 | 0.61 |
| 2224 | Train 35 | Test Asian 48 | 0.75 | 0.74 | 0.69 | 3248 | Train 51 | Test Asian 48 | 0.73 | 0.72 | 0.59 |
| 2225 | Train 35 | Test Asian 49 | 0.77 | 0.88 | 0.84 | 3249 | Train 51 | Test Asian 49 | 0.77 | 0.92 | 0.95 |
| 2226 | Train 35 | Test Asian 50 | 0.77 | 0.88 | 0.83 | 3250 | Train 51 | Test Asian 50 | 0.77 | 0.93 | 0.95 |
| 2227 | Train 35 | Test Asian 51 | 0.77 | 0.88 | 0.84 | 3251 | Train 51 | Test Asian 51 | 0.77 | 0.92 | 0.95 |
| 2228 | Train 35 | Test Asian 52 | 0.77 | 0.88 | 0.83 | 3252 | Train 51 | Test Asian 52 | 0.77 | 0.93 | 0.95 |
| 2229 | Train 35 | Test Asian 53 | 0.79 | 0.81 | 0.62 | 3253 | Train 51 | Test Asian 53 | 0.77 | 0.74 | 0.74 |
| 2230 | Train 35 | Test Asian 54 | 0.78 | 0.75 | 0.68 | 3254 | Train 51 | Test Asian 54 | 0.74 | 0.74 | 0.66 |
| 2231 | Train 35 | Test Asian 55 | 0.79 | 0.8 | 0.62 | 3255 | Train 51 | Test Asian 55 | 0.76 | 0.74 | 0.73 |
| 2232 | Train 35 | Test Asian 56 | 0.78 | 0.75 | 0.69 | 3256 | Train 51 | Test Asian 56 | 0.75 | 0.73 | 0.66 |
| 2233 | Train 35 | Test Asian 57 | 0.76 | 0.85 | 0.81 | 3257 | Train 51 | Test Asian 57 | 0.76 | 0.89 | 0.89 |
| 2234 | Train 35 | Test Asian 58 | 0.76 | 0.85 | 0.8 | 3258 | Train 51 | Test Asian 58 | 0.76 | 0.89 | 0.89 |
| 2235 | Train 35 | Test Asian 59 | 0.76 | 0.85 | 0.81 | 3259 | Train 51 | Test Asian 59 | 0.76 | 0.89 | 0.89 |
| 2236 | Train 35 | Test Asian 60 | 0.76 | 0.85 | 0.8 | 3260 | Train 51 | Test Asian 60 | 0.76 | 0.89 | 0.89 |
| 2237 | Train 35 | Test Asian 61 | 0.79 | 0.8 | 0.62 | 3261 | Train 51 | Test Asian 61 | 0.76 | 0.74 | 0.74 |
| 2238 | Train 35 | Test Asian 62 | 0.78 | 0.75 | 0.69 | 3262 | Train 51 | Test Asian 62 | 0.75 | 0.74 | 0.66 |
| 2239 | Train 35 | Test Asian 63 | 0.78 | 0.8 | 0.62 | 3263 | Train 51 | Test Asian 63 | 0.76 | 0.74 | 0.74 |
| 2240 | Train 35 | Test Asian 64 | 0.78 | 0.75 | 0.69 | 3264 | Train 51 | Test Asian 64 | 0.76 | 0.73 | 0.66 |
| 2241 | Train 36 | Test Asian 1 | 0.79 | 0.91 | 0.91 | 3265 | Train 52 | Test Asian 1 | 0.75 | 0.76 | 0.73 |
| 2242 | Train 36 | Test Asian 2 | 0.81 | 0.92 | 0.92 | 3266 | Train 52 | Test Asian 2 | 0.76 | 0.78 | 0.73 |
| 2243 | Train 36 | Test Asian 3 | 0.79 | 0.91 | 0.91 | 3267 | Train 52 | Test Asian 3 | 0.75 | 0.76 | 0.73 |
| 2244 | Train 36 | Test Asian 4 | 0.81 | 0.92 | 0.92 | 3268 | Train 52 | Test Asian 4 | 0.76 | 0.78 | 0.73 |
| 2245 | Train 36 | Test Asian 5 | 0.75 | 0.76 | 0.73 | 3269 | Train 52 | Test Asian 5 | 0.73 | 0.68 | 0.62 |
| 2246 | Train 36 | Test Asian 6 | 0.74 | 0.75 | 0.71 | 3270 | Train 52 | Test Asian 6 | 0.73 | 0.69 | 0.63 |
| 2247 | Train 36 | Test Asian 7 | 0.75 | 0.76 | 0.73 | 3271 | Train 52 | Test Asian 7 | 0.73 | 0.68 | 0.62 |
| 2248 | Train 36 | Test Asian 8 | 0.75 | 0.75 | 0.71 | 3272 | Train 52 | Test Asian 8 | 0.73 | 0.69 | 0.63 |
| 2249 | Train 36 | Test Asian 9 | 0.78 | 0.89 | 0.88 | 3273 | Train 52 | Test Asian 9 | 0.74 | 0.75 | 0.71 |
| 2250 | Train 36 | Test Asian 10 | 0.8 | 0.9 | 0.89 | 3274 | Train 52 | Test Asian 10 | 0.75 | 0.78 | 0.71 |
| 2251 | Train 36 | Test Asian 11 | 0.78 | 0.89 | 0.88 | 3275 | Train 52 | Test Asian 11 | 0.74 | 0.75 | 0.71 |
| 2252 | Train 36 | Test Asian 12 | 0.8 | 0.9 | 0.89 | 3276 | Train 52 | Test Asian 12 | 0.75 | 0.78 | 0.71 |
| 2253 | Train 36 | Test Asian 13 | 0.75 | 0.76 | 0.73 | 3277 | Train 52 | Test Asian 13 | 0.72 | 0.67 | 0.63 |
| 2254 | Train 36 | Test Asian 14 | 0.75 | 0.74 | 0.66 | 3278 | Train 52 | Test Asian 14 | 0.73 | 0.69 | 0.63 |
| 2255 | Train 36 | Test Asian 15 | 0.75 | 0.75 | 0.72 | 3279 | Train 52 | Test Asian 15 | 0.72 | 0.68 | 0.63 |
| 2256 | Train 36 | Test Asian 16 | 0.75 | 0.75 | 0.71 | 3280 | Train 52 | Test Asian 16 | 0.73 | 0.69 | 0.63 |
| 2257 | Train 36 | Test Asian 17 | 0.79 | 0.91 | 0.91 | 3281 | Train 52 | Test Asian 17 | 0.75 | 0.76 | 0.73 |
| 2258 | Train 36 | Test Asian 18 | 0.81 | 0.92 | 0.92 | 3282 | Train 52 | Test Asian 18 | 0.76 | 0.78 | 0.73 |
| 2259 | Train 36 | Test Asian 19 | 0.79 | 0.91 | 0.91 | 3283 | Train 52 | Test Asian 19 | 0.75 | 0.76 | 0.73 |
| 2260 | Train 36 | Test Asian 20 | 0.81 | 0.92 | 0.92 | 3284 | Train 52 | Test Asian 20 | 0.76 | 0.78 | 0.73 |
| 2261 | Train 36 | Test Asian 21 | 0.75 | 0.77 | 0.73 | 3285 | Train 52 | Test Asian 21 | 0.73 | 0.68 | 0.62 |
| 2262 | Train 36 | Test Asian 22 | 0.75 | 0.75 | 0.71 | 3286 | Train 52 | Test Asian 22 | 0.73 | 0.69 | 0.63 |
| 2263 | Train 36 | Test Asian 23 | 0.75 | 0.76 | 0.73 | 3287 | Train 52 | Test Asian 23 | 0.73 | 0.68 | 0.62 |
| 2264 | Train 36 | Test Asian 24 | 0.75 | 0.75 | 0.71 | 3288 | Train 52 | Test Asian 24 | 0.73 | 0.69 | 0.63 |
| 2265 | Train 36 | Test Asian 25 | 0.78 | 0.89 | 0.88 | 3289 | Train 52 | Test Asian 25 | 0.74 | 0.75 | 0.71 |
| 2266 | Train 36 | Test Asian 26 | 0.8 | 0.9 | 0.89 | 3290 | Train 52 | Test Asian 26 | 0.75 | 0.78 | 0.71 |
| 2267 | Train 36 | Test Asian 27 | 0.78 | 0.89 | 0.88 | 3291 | Train 52 | Test Asian 27 | 0.74 | 0.75 | 0.71 |
| 2268 | Train 36 | Test Asian 28 | 0.8 | 0.9 | 0.89 | 3292 | Train 52 | Test Asian 28 | 0.75 | 0.78 | 0.71 |
| 2269 | Train 36 | Test Asian 29 | 0.75 | 0.76 | 0.73 | 3293 | Train 52 | Test Asian 29 | 0.72 | 0.68 | 0.63 |
| 2270 | Train 36 | Test Asian 30 | 0.75 | 0.74 | 0.66 | 3294 | Train 52 | Test Asian 30 | 0.73 | 0.69 | 0.63 |
| 2271 | Train 36 | Test Asian 31 | 0.75 | 0.75 | 0.72 | 3295 | Train 52 | Test Asian 31 | 0.72 | 0.68 | 0.63 |
| 2272 | Train 36 | Test Asian 32 | 0.75 | 0.75 | 0.71 | 3296 | Train 52 | Test Asian 32 | 0.73 | 0.69 | 0.63 |
| 2273 | Train 36 | Test Asian 33 | 0.79 | 0.91 | 0.91 | 3297 | Train 52 | Test Asian 33 | 0.75 | 0.76 | 0.73 |
| 2274 | Train 36 | Test Asian 34 | 0.81 | 0.92 | 0.92 | 3298 | Train 52 | Test Asian 34 | 0.75 | 0.78 | 0.73 |
| 2275 | Train 36 | Test Asian 35 | 0.79 | 0.91 | 0.91 | 3299 | Train 52 | Test Asian 35 | 0.75 | 0.76 | 0.73 |
| 2276 | Train 36 | Test Asian 36 | 0.81 | 0.92 | 0.92 | 3300 | Train 52 | Test Asian 36 | 0.75 | 0.78 | 0.73 |
| 2277 | Train 36 | Test Asian 37 | 0.75 | 0.76 | 0.73 | 3301 | Train 52 | Test Asian 37 | 0.73 | 0.68 | 0.62 |
| 2278 | Train 36 | Test Asian 38 | 0.74 | 0.75 | 0.71 | 3302 | Train 52 | Test Asian 38 | 0.73 | 0.69 | 0.63 |
| 2279 | Train 36 | Test Asian 39 | 0.75 | 0.76 | 0.73 | 3303 | Train 52 | Test Asian 39 | 0.73 | 0.68 | 0.62 |
| 2280 | Train 36 | Test Asian 40 | 0.74 | 0.75 | 0.71 | 3304 | Train 52 | Test Asian 40 | 0.73 | 0.69 | 0.63 |
| 2281 | Train 36 | Test Asian 41 | 0.78 | 0.89 | 0.88 | 3305 | Train 52 | Test Asian 41 | 0.74 | 0.75 | 0.71 |
| 2282 | Train 36 | Test Asian 42 | 0.8 | 0.9 | 0.89 | 3306 | Train 52 | Test Asian 42 | 0.75 | 0.77 | 0.71 |
| 2283 | Train 36 | Test Asian 43 | 0.78 | 0.89 | 0.88 | 3307 | Train 52 | Test Asian 43 | 0.74 | 0.75 | 0.71 |
| 2284 | Train 36 | Test Asian 44 | 0.8 | 0.9 | 0.89 | 3308 | Train 52 | Test Asian 44 | 0.75 | 0.77 | 0.71 |
| 2285 | Train 36 | Test Asian 45 | 0.75 | 0.76 | 0.73 | 3309 | Train 52 | Test Asian 45 | 0.72 | 0.67 | 0.63 |
| 2286 | Train 36 | Test Asian 46 | 0.74 | 0.75 | 0.71 | 3310 | Train 52 | Test Asian 46 | 0.73 | 0.69 | 0.63 |
| 2287 | Train 36 | Test Asian 47 | 0.75 | 0.75 | 0.72 | 3311 | Train 52 | Test Asian 47 | 0.72 | 0.67 | 0.63 |
| 2288 | Train 36 | Test Asian 48 | 0.75 | 0.75 | 0.71 | 3312 | Train 52 | Test Asian 48 | 0.73 | 0.69 | 0.63 |
| 2289 | Train 36 | Test Asian 49 | 0.77 | 0.86 | 0.88 | 3313 | Train 52 | Test Asian 49 | 0.77 | 0.92 | 0.94 |
| 2290 | Train 36 | Test Asian 50 | 0.77 | 0.86 | 0.88 | 3314 | Train 52 | Test Asian 50 | 0.77 | 0.92 | 0.94 |
| 2291 | Train 36 | Test Asian 51 | 0.77 | 0.86 | 0.88 | 3315 | Train 52 | Test Asian 51 | 0.77 | 0.92 | 0.94 |
| 2292 | Train 36 | Test Asian 52 | 0.77 | 0.86 | 0.88 | 3316 | Train 52 | Test Asian 52 | 0.77 | 0.92 | 0.94 |
| 2293 | Train 36 | Test Asian 53 | 0.79 | 0.81 | 0.74 | 3317 | Train 52 | Test Asian 53 | 0.76 | 0.67 | 0.66 |
| 2294 | Train 36 | Test Asian 54 | 0.77 | 0.74 | 0.67 | 3318 | Train 52 | Test Asian 54 | 0.74 | 0.69 | 0.66 |
| 2295 | Train 36 | Test Asian 55 | 0.79 | 0.81 | 0.73 | 3319 | Train 52 | Test Asian 55 | 0.76 | 0.68 | 0.66 |
| 2296 | Train 36 | Test Asian 56 | 0.76 | 0.74 | 0.68 | 3320 | Train 52 | Test Asian 56 | 0.75 | 0.68 | 0.65 |
| 2297 | Train 36 | Test Asian 57 | 0.76 | 0.83 | 0.83 | 3321 | Train 52 | Test Asian 57 | 0.76 | 0.89 | 0.9 |
| 2298 | Train 36 | Test Asian 58 | 0.76 | 0.83 | 0.84 | 3322 | Train 52 | Test Asian 58 | 0.76 | 0.89 | 0.9 |
| 2299 | Train 36 | Test Asian 59 | 0.76 | 0.83 | 0.83 | 3323 | Train 52 | Test Asian 59 | 0.76 | 0.89 | 0.9 |
| 2300 | Train 36 | Test Asian 60 | 0.76 | 0.83 | 0.84 | 3324 | Train 52 | Test Asian 60 | 0.76 | 0.89 | 0.9 |
| 2301 | Train 36 | Test Asian 61 | 0.79 | 0.8 | 0.73 | 3325 | Train 52 | Test Asian 61 | 0.76 | 0.67 | 0.66 |
| 2302 | Train 36 | Test Asian 62 | 0.77 | 0.74 | 0.67 | 3326 | Train 52 | Test Asian 62 | 0.75 | 0.69 | 0.66 |
| 2303 | Train 36 | Test Asian 63 | 0.78 | 0.8 | 0.73 | 3327 | Train 52 | Test Asian 63 | 0.75 | 0.67 | 0.66 |
| 2304 | Train 36 | Test Asian 64 | 0.76 | 0.74 | 0.68 | 3328 | Train 52 | Test Asian 64 | 0.75 | 0.68 | 0.65 |
| 2305 | Train 37 | Test Asian 1 | 0.78 | 0.87 | 0.86 | 3329 | Train 53 | Test Asian 1 | 0.73 | 0.8 | 0.67 |
| 2306 | Train 37 | Test Asian 2 | 0.8 | 0.86 | 0.85 | 3330 | Train 53 | Test Asian 2 | 0.75 | 0.8 | 0.67 |
| 2307 | Train 37 | Test Asian 3 | 0.78 | 0.87 | 0.86 | 3331 | Train 53 | Test Asian 3 | 0.73 | 0.8 | 0.67 |
| 2308 | Train 37 | Test Asian 4 | 0.8 | 0.86 | 0.85 | 3332 | Train 53 | Test Asian 4 | 0.75 | 0.8 | 0.67 |
| 2309 | Train 37 | Test Asian 5 | 0.77 | 0.85 | 0.86 | 3333 | Train 53 | Test Asian 5 | 0.73 | 0.78 | 0.68 |
| 2310 | Train 37 | Test Asian 6 | 0.76 | 0.78 | 0.78 | 3334 | Train 53 | Test Asian 6 | 0.72 | 0.72 | 0.66 |
| 2311 | Train 37 | Test Asian 7 | 0.76 | 0.85 | 0.85 | 3335 | Train 53 | Test Asian 7 | 0.73 | 0.78 | 0.68 |
| 2312 | Train 37 | Test Asian 8 | 0.76 | 0.78 | 0.78 | 3336 | Train 53 | Test Asian 8 | 0.72 | 0.72 | 0.66 |
| 2313 | Train 37 | Test Asian 9 | 0.77 | 0.85 | 0.84 | 3337 | Train 53 | Test Asian 9 | 0.73 | 0.79 | 0.61 |
| 2314 | Train 37 | Test Asian 10 | 0.79 | 0.84 | 0.83 | 3338 | Train 53 | Test Asian 10 | 0.75 | 0.79 | 0.63 |
| 2315 | Train 37 | Test Asian 11 | 0.77 | 0.85 | 0.84 | 3339 | Train 53 | Test Asian 11 | 0.73 | 0.79 | 0.61 |
| 2316 | Train 37 | Test Asian 12 | 0.79 | 0.84 | 0.83 | 3340 | Train 53 | Test Asian 12 | 0.75 | 0.79 | 0.63 |
| 2317 | Train 37 | Test Asian 13 | 0.76 | 0.83 | 0.83 | 3341 | Train 53 | Test Asian 13 | 0.73 | 0.78 | 0.67 |
| 2318 | Train 37 | Test Asian 14 | 0.76 | 0.76 | 0.74 | 3342 | Train 53 | Test Asian 14 | 0.72 | 0.72 | 0.68 |
| 2319 | Train 37 | Test Asian 15 | 0.76 | 0.83 | 0.83 | 3343 | Train 53 | Test Asian 15 | 0.72 | 0.77 | 0.67 |
| 2320 | Train 37 | Test Asian 16 | 0.76 | 0.78 | 0.78 | 3344 | Train 53 | Test Asian 16 | 0.73 | 0.72 | 0.66 |
| 2321 | Train 37 | Test Asian 17 | 0.78 | 0.87 | 0.86 | 3345 | Train 53 | Test Asian 17 | 0.74 | 0.8 | 0.67 |
| 2322 | Train 37 | Test Asian 18 | 0.8 | 0.86 | 0.85 | 3346 | Train 53 | Test Asian 18 | 0.75 | 0.8 | 0.67 |
| 2323 | Train 37 | Test Asian 19 | 0.78 | 0.87 | 0.86 | 3347 | Train 53 | Test Asian 19 | 0.74 | 0.8 | 0.67 |
| 2324 | Train 37 | Test Asian 20 | 0.8 | 0.86 | 0.85 | 3348 | Train 53 | Test Asian 20 | 0.75 | 0.8 | 0.67 |
| 2325 | Train 37 | Test Asian 21 | 0.77 | 0.85 | 0.86 | 3349 | Train 53 | Test Asian 21 | 0.73 | 0.78 | 0.68 |
| 2326 | Train 37 | Test Asian 22 | 0.76 | 0.78 | 0.78 | 3350 | Train 53 | Test Asian 22 | 0.72 | 0.72 | 0.66 |
| 2327 | Train 37 | Test Asian 23 | 0.76 | 0.85 | 0.85 | 3351 | Train 53 | Test Asian 23 | 0.73 | 0.78 | 0.68 |
| 2328 | Train 37 | Test Asian 24 | 0.76 | 0.78 | 0.78 | 3352 | Train 53 | Test Asian 24 | 0.73 | 0.72 | 0.66 |
| 2329 | Train 37 | Test Asian 25 | 0.77 | 0.85 | 0.84 | 3353 | Train 53 | Test Asian 25 | 0.73 | 0.79 | 0.61 |
| 2330 | Train 37 | Test Asian 26 | 0.79 | 0.84 | 0.83 | 3354 | Train 53 | Test Asian 26 | 0.75 | 0.79 | 0.63 |
| 2331 | Train 37 | Test Asian 27 | 0.77 | 0.85 | 0.84 | 3355 | Train 53 | Test Asian 27 | 0.73 | 0.79 | 0.61 |
| 2332 | Train 37 | Test Asian 28 | 0.79 | 0.84 | 0.83 | 3356 | Train 53 | Test Asian 28 | 0.75 | 0.79 | 0.63 |
| 2333 | Train 37 | Test Asian 29 | 0.76 | 0.83 | 0.83 | 3357 | Train 53 | Test Asian 29 | 0.73 | 0.78 | 0.67 |
| 2334 | Train 37 | Test Asian 30 | 0.76 | 0.76 | 0.74 | 3358 | Train 53 | Test Asian 30 | 0.72 | 0.73 | 0.68 |
| 2335 | Train 37 | Test Asian 31 | 0.76 | 0.83 | 0.83 | 3359 | Train 53 | Test Asian 31 | 0.73 | 0.77 | 0.67 |
| 2336 | Train 37 | Test Asian 32 | 0.76 | 0.78 | 0.78 | 3360 | Train 53 | Test Asian 32 | 0.73 | 0.72 | 0.66 |
| 2337 | Train 37 | Test Asian 33 | 0.78 | 0.87 | 0.86 | 3361 | Train 53 | Test Asian 33 | 0.74 | 0.8 | 0.67 |
| 2338 | Train 37 | Test Asian 34 | 0.8 | 0.87 | 0.85 | 3362 | Train 53 | Test Asian 34 | 0.76 | 0.8 | 0.68 |
| 2339 | Train 37 | Test Asian 35 | 0.78 | 0.87 | 0.86 | 3363 | Train 53 | Test Asian 35 | 0.74 | 0.8 | 0.67 |
| 2340 | Train 37 | Test Asian 36 | 0.8 | 0.87 | 0.85 | 3364 | Train 53 | Test Asian 36 | 0.76 | 0.8 | 0.68 |
| 2341 | Train 37 | Test Asian 37 | 0.76 | 0.85 | 0.86 | 3365 | Train 53 | Test Asian 37 | 0.73 | 0.78 | 0.68 |
| 2342 | Train 37 | Test Asian 38 | 0.75 | 0.78 | 0.78 | 3366 | Train 53 | Test Asian 38 | 0.72 | 0.72 | 0.66 |
| 2343 | Train 37 | Test Asian 39 | 0.76 | 0.84 | 0.85 | 3367 | Train 53 | Test Asian 39 | 0.73 | 0.78 | 0.68 |
| 2344 | Train 37 | Test Asian 40 | 0.76 | 0.78 | 0.78 | 3368 | Train 53 | Test Asian 40 | 0.72 | 0.72 | 0.66 |
| 2345 | Train 37 | Test Asian 41 | 0.77 | 0.85 | 0.84 | 3369 | Train 53 | Test Asian 41 | 0.73 | 0.8 | 0.61 |
| 2346 | Train 37 | Test Asian 42 | 0.8 | 0.84 | 0.83 | 3370 | Train 53 | Test Asian 42 | 0.75 | 0.79 | 0.64 |
| 2347 | Train 37 | Test Asian 43 | 0.77 | 0.85 | 0.84 | 3371 | Train 53 | Test Asian 43 | 0.73 | 0.8 | 0.61 |
| 2348 | Train 37 | Test Asian 44 | 0.8 | 0.84 | 0.83 | 3372 | Train 53 | Test Asian 44 | 0.75 | 0.79 | 0.64 |
| 2349 | Train 37 | Test Asian 45 | 0.76 | 0.83 | 0.83 | 3373 | Train 53 | Test Asian 45 | 0.73 | 0.78 | 0.67 |
| 2350 | Train 37 | Test Asian 46 | 0.76 | 0.78 | 0.78 | 3374 | Train 53 | Test Asian 46 | 0.72 | 0.72 | 0.66 |
| 2351 | Train 37 | Test Asian 47 | 0.76 | 0.83 | 0.82 | 3375 | Train 53 | Test Asian 47 | 0.72 | 0.77 | 0.67 |
| 2352 | Train 37 | Test Asian 48 | 0.76 | 0.78 | 0.77 | 3376 | Train 53 | Test Asian 48 | 0.73 | 0.72 | 0.66 |
| 2353 | Train 37 | Test Asian 49 | 0.76 | 0.84 | 0.82 | 3377 | Train 53 | Test Asian 49 | 0.75 | 0.87 | 0.88 |
| 2354 | Train 37 | Test Asian 50 | 0.76 | 0.85 | 0.82 | 3378 | Train 53 | Test Asian 50 | 0.75 | 0.87 | 0.88 |
| 2355 | Train 37 | Test Asian 51 | 0.76 | 0.84 | 0.82 | 3379 | Train 53 | Test Asian 51 | 0.75 | 0.87 | 0.88 |
| 2356 | Train 37 | Test Asian 52 | 0.76 | 0.85 | 0.82 | 3380 | Train 53 | Test Asian 52 | 0.75 | 0.87 | 0.88 |
| 2357 | Train 37 | Test Asian 53 | 0.8 | 0.86 | 0.79 | 3381 | Train 53 | Test Asian 53 | 0.8 | 0.9 | 0.83 |
| 2358 | Train 37 | Test Asian 54 | 0.77 | 0.76 | 0.72 | 3382 | Train 53 | Test Asian 54 | 0.77 | 0.78 | 0.76 |
| 2359 | Train 37 | Test Asian 55 | 0.8 | 0.86 | 0.78 | 3383 | Train 53 | Test Asian 55 | 0.8 | 0.9 | 0.83 |
| 2360 | Train 37 | Test Asian 56 | 0.77 | 0.76 | 0.72 | 3384 | Train 53 | Test Asian 56 | 0.77 | 0.77 | 0.76 |
| 2361 | Train 37 | Test Asian 57 | 0.75 | 0.82 | 0.8 | 3385 | Train 53 | Test Asian 57 | 0.74 | 0.86 | 0.86 |
| 2362 | Train 37 | Test Asian 58 | 0.75 | 0.82 | 0.8 | 3386 | Train 53 | Test Asian 58 | 0.74 | 0.86 | 0.86 |
| 2363 | Train 37 | Test Asian 59 | 0.75 | 0.82 | 0.8 | 3387 | Train 53 | Test Asian 59 | 0.74 | 0.86 | 0.86 |
| 2364 | Train 37 | Test Asian 60 | 0.75 | 0.82 | 0.8 | 3388 | Train 53 | Test Asian 60 | 0.74 | 0.86 | 0.86 |
| 2365 | Train 37 | Test Asian 61 | 0.79 | 0.85 | 0.78 | 3389 | Train 53 | Test Asian 61 | 0.8 | 0.89 | 0.78 |
| 2366 | Train 37 | Test Asian 62 | 0.77 | 0.76 | 0.72 | 3390 | Train 53 | Test Asian 62 | 0.77 | 0.78 | 0.76 |
| 2367 | Train 37 | Test Asian 63 | 0.79 | 0.85 | 0.78 | 3391 | Train 53 | Test Asian 63 | 0.79 | 0.88 | 0.78 |
| 2368 | Train 37 | Test Asian 64 | 0.77 | 0.75 | 0.72 | 3392 | Train 53 | Test Asian 64 | 0.77 | 0.77 | 0.75 |
| 2369 | Train 38 | Test Asian 1 | 0.76 | 0.84 | 0.81 | 3393 | Train 54 | Test Asian 1 | 0.72 | 0.78 | 0.76 |
| 2370 | Train 38 | Test Asian 2 | 0.79 | 0.86 | 0.83 | 3394 | Train 54 | Test Asian 2 | 0.75 | 0.8 | 0.78 |
| 2371 | Train 38 | Test Asian 3 | 0.76 | 0.84 | 0.81 | 3395 | Train 54 | Test Asian 3 | 0.72 | 0.78 | 0.76 |
| 2372 | Train 38 | Test Asian 4 | 0.79 | 0.86 | 0.83 | 3396 | Train 54 | Test Asian 4 | 0.75 | 0.8 | 0.78 |
| 2373 | Train 38 | Test Asian 5 | 0.75 | 0.81 | 0.77 | 3397 | Train 54 | Test Asian 5 | 0.74 | 0.75 | 0.74 |
| 2374 | Train 38 | Test Asian 6 | 0.76 | 0.82 | 0.8 | 3398 | Train 54 | Test Asian 6 | 0.75 | 0.78 | 0.75 |
| 2375 | Train 38 | Test Asian 7 | 0.75 | 0.8 | 0.77 | 3399 | Train 54 | Test Asian 7 | 0.74 | 0.75 | 0.73 |
| 2376 | Train 38 | Test Asian 8 | 0.76 | 0.81 | 0.79 | 3400 | Train 54 | Test Asian 8 | 0.75 | 0.78 | 0.75 |
| 2377 | Train 38 | Test Asian 9 | 0.75 | 0.84 | 0.81 | 3401 | Train 54 | Test Asian 9 | 0.71 | 0.77 | 0.76 |
| 2378 | Train 38 | Test Asian 10 | 0.78 | 0.86 | 0.83 | 3402 | Train 54 | Test Asian 10 | 0.74 | 0.8 | 0.77 |
| 2379 | Train 38 | Test Asian 11 | 0.75 | 0.84 | 0.81 | 3403 | Train 54 | Test Asian 11 | 0.71 | 0.77 | 0.76 |
| 2380 | Train 38 | Test Asian 12 | 0.78 | 0.86 | 0.83 | 3404 | Train 54 | Test Asian 12 | 0.74 | 0.8 | 0.77 |
| 2381 | Train 38 | Test Asian 13 | 0.75 | 0.81 | 0.79 | 3405 | Train 54 | Test Asian 13 | 0.74 | 0.75 | 0.74 |
| 2382 | Train 38 | Test Asian 14 | 0.76 | 0.83 | 0.83 | 3406 | Train 54 | Test Asian 14 | 0.75 | 0.79 | 0.76 |
| 2383 | Train 38 | Test Asian 15 | 0.75 | 0.8 | 0.78 | 3407 | Train 54 | Test Asian 15 | 0.73 | 0.75 | 0.73 |
| 2384 | Train 38 | Test Asian 16 | 0.76 | 0.81 | 0.79 | 3408 | Train 54 | Test Asian 16 | 0.75 | 0.78 | 0.75 |
| 2385 | Train 38 | Test Asian 17 | 0.76 | 0.84 | 0.81 | 3409 | Train 54 | Test Asian 17 | 0.72 | 0.78 | 0.76 |
| 2386 | Train 38 | Test Asian 18 | 0.79 | 0.86 | 0.83 | 3410 | Train 54 | Test Asian 18 | 0.75 | 0.8 | 0.78 |
| 2387 | Train 38 | Test Asian 19 | 0.76 | 0.84 | 0.81 | 3411 | Train 54 | Test Asian 19 | 0.72 | 0.78 | 0.76 |
| 2388 | Train 38 | Test Asian 20 | 0.79 | 0.86 | 0.83 | 3412 | Train 54 | Test Asian 20 | 0.75 | 0.8 | 0.78 |
| 2389 | Train 38 | Test Asian 21 | 0.76 | 0.81 | 0.77 | 3413 | Train 54 | Test Asian 21 | 0.74 | 0.75 | 0.74 |
| 2390 | Train 38 | Test Asian 22 | 0.77 | 0.82 | 0.8 | 3414 | Train 54 | Test Asian 22 | 0.75 | 0.78 | 0.76 |
| 2391 | Train 38 | Test Asian 23 | 0.75 | 0.8 | 0.77 | 3415 | Train 54 | Test Asian 23 | 0.74 | 0.75 | 0.73 |
| 2392 | Train 38 | Test Asian 24 | 0.77 | 0.81 | 0.79 | 3416 | Train 54 | Test Asian 24 | 0.75 | 0.78 | 0.75 |
| 2393 | Train 38 | Test Asian 25 | 0.75 | 0.84 | 0.81 | 3417 | Train 54 | Test Asian 25 | 0.71 | 0.77 | 0.76 |
| 2394 | Train 38 | Test Asian 26 | 0.78 | 0.86 | 0.83 | 3418 | Train 54 | Test Asian 26 | 0.74 | 0.8 | 0.77 |
| 2395 | Train 38 | Test Asian 27 | 0.75 | 0.84 | 0.81 | 3419 | Train 54 | Test Asian 27 | 0.71 | 0.77 | 0.76 |
| 2396 | Train 38 | Test Asian 28 | 0.78 | 0.86 | 0.83 | 3420 | Train 54 | Test Asian 28 | 0.74 | 0.8 | 0.77 |
| 2397 | Train 38 | Test Asian 29 | 0.75 | 0.81 | 0.79 | 3421 | Train 54 | Test Asian 29 | 0.74 | 0.75 | 0.74 |
| 2398 | Train 38 | Test Asian 30 | 0.76 | 0.83 | 0.83 | 3422 | Train 54 | Test Asian 30 | 0.75 | 0.79 | 0.76 |
| 2399 | Train 38 | Test Asian 31 | 0.75 | 0.8 | 0.78 | 3423 | Train 54 | Test Asian 31 | 0.73 | 0.75 | 0.73 |
| 2400 | Train 38 | Test Asian 32 | 0.76 | 0.81 | 0.79 | 3424 | Train 54 | Test Asian 32 | 0.75 | 0.78 | 0.75 |
| 2401 | Train 38 | Test Asian 33 | 0.76 | 0.84 | 0.81 | 3425 | Train 54 | Test Asian 33 | 0.72 | 0.78 | 0.76 |
| 2402 | Train 38 | Test Asian 34 | 0.78 | 0.86 | 0.83 | 3426 | Train 54 | Test Asian 34 | 0.75 | 0.8 | 0.78 |
| 2403 | Train 38 | Test Asian 35 | 0.76 | 0.84 | 0.81 | 3427 | Train 54 | Test Asian 35 | 0.72 | 0.78 | 0.76 |
| 2404 | Train 38 | Test Asian 36 | 0.78 | 0.86 | 0.83 | 3428 | Train 54 | Test Asian 36 | 0.75 | 0.8 | 0.78 |
| 2405 | Train 38 | Test Asian 37 | 0.75 | 0.8 | 0.77 | 3429 | Train 54 | Test Asian 37 | 0.74 | 0.75 | 0.73 |
| 2406 | Train 38 | Test Asian 38 | 0.76 | 0.82 | 0.8 | 3430 | Train 54 | Test Asian 38 | 0.75 | 0.78 | 0.75 |
| 2407 | Train 38 | Test Asian 39 | 0.75 | 0.8 | 0.77 | 3431 | Train 54 | Test Asian 39 | 0.74 | 0.75 | 0.73 |
| 2408 | Train 38 | Test Asian 40 | 0.76 | 0.81 | 0.79 | 3432 | Train 54 | Test Asian 40 | 0.75 | 0.78 | 0.75 |
| 2409 | Train 38 | Test Asian 41 | 0.75 | 0.84 | 0.81 | 3433 | Train 54 | Test Asian 41 | 0.71 | 0.77 | 0.75 |
| 2410 | Train 38 | Test Asian 42 | 0.78 | 0.86 | 0.83 | 3434 | Train 54 | Test Asian 42 | 0.74 | 0.8 | 0.77 |
| 2411 | Train 38 | Test Asian 43 | 0.75 | 0.84 | 0.81 | 3435 | Train 54 | Test Asian 43 | 0.71 | 0.77 | 0.75 |
| 2412 | Train 38 | Test Asian 44 | 0.78 | 0.86 | 0.83 | 3436 | Train 54 | Test Asian 44 | 0.74 | 0.8 | 0.77 |
| 2413 | Train 38 | Test Asian 45 | 0.75 | 0.8 | 0.79 | 3437 | Train 54 | Test Asian 45 | 0.74 | 0.75 | 0.73 |
| 2414 | Train 38 | Test Asian 46 | 0.76 | 0.82 | 0.79 | 3438 | Train 54 | Test Asian 46 | 0.75 | 0.78 | 0.75 |
| 2415 | Train 38 | Test Asian 47 | 0.75 | 0.8 | 0.78 | 3439 | Train 54 | Test Asian 47 | 0.73 | 0.75 | 0.73 |
| 2416 | Train 38 | Test Asian 48 | 0.76 | 0.81 | 0.79 | 3440 | Train 54 | Test Asian 48 | 0.75 | 0.78 | 0.75 |
| 2417 | Train 38 | Test Asian 49 | 0.77 | 0.84 | 0.74 | 3441 | Train 54 | Test Asian 49 | 0.77 | 0.86 | 0.84 |
| 2418 | Train 38 | Test Asian 50 | 0.77 | 0.84 | 0.75 | 3442 | Train 54 | Test Asian 50 | 0.77 | 0.86 | 0.84 |
| 2419 | Train 38 | Test Asian 51 | 0.77 | 0.84 | 0.74 | 3443 | Train 54 | Test Asian 51 | 0.77 | 0.86 | 0.84 |
| 2420 | Train 38 | Test Asian 52 | 0.77 | 0.84 | 0.75 | 3444 | Train 54 | Test Asian 52 | 0.77 | 0.86 | 0.84 |
| 2421 | Train 38 | Test Asian 53 | 0.8 | 0.85 | 0.73 | 3445 | Train 54 | Test Asian 53 | 0.8 | 0.87 | 0.81 |
| 2422 | Train 38 | Test Asian 54 | 0.79 | 0.83 | 0.83 | 3446 | Train 54 | Test Asian 54 | 0.79 | 0.86 | 0.87 |
| 2423 | Train 38 | Test Asian 55 | 0.79 | 0.84 | 0.72 | 3447 | Train 54 | Test Asian 55 | 0.79 | 0.86 | 0.81 |
| 2424 | Train 38 | Test Asian 56 | 0.78 | 0.83 | 0.82 | 3448 | Train 54 | Test Asian 56 | 0.78 | 0.85 | 0.86 |
| 2425 | Train 38 | Test Asian 57 | 0.76 | 0.84 | 0.78 | 3449 | Train 54 | Test Asian 57 | 0.76 | 0.86 | 0.85 |
| 2426 | Train 38 | Test Asian 58 | 0.76 | 0.84 | 0.78 | 3450 | Train 54 | Test Asian 58 | 0.76 | 0.86 | 0.85 |
| 2427 | Train 38 | Test Asian 59 | 0.76 | 0.84 | 0.78 | 3451 | Train 54 | Test Asian 59 | 0.76 | 0.86 | 0.85 |
| 2428 | Train 38 | Test Asian 60 | 0.76 | 0.84 | 0.78 | 3452 | Train 54 | Test Asian 60 | 0.76 | 0.86 | 0.85 |
| 2429 | Train 38 | Test Asian 61 | 0.79 | 0.85 | 0.74 | 3453 | Train 54 | Test Asian 61 | 0.79 | 0.86 | 0.82 |
| 2430 | Train 38 | Test Asian 62 | 0.79 | 0.83 | 0.83 | 3454 | Train 54 | Test Asian 62 | 0.79 | 0.85 | 0.87 |
| 2431 | Train 38 | Test Asian 63 | 0.78 | 0.84 | 0.73 | 3455 | Train 54 | Test Asian 63 | 0.78 | 0.86 | 0.81 |
| 2432 | Train 38 | Test Asian 64 | 0.78 | 0.83 | 0.82 | 3456 | Train 54 | Test Asian 64 | 0.78 | 0.85 | 0.86 |
| 2433 | Train 39 | Test Asian 1 | 0.78 | 0.87 | 0.86 | 3457 | Train 55 | Test Asian 1 | 0.74 | 0.8 | 0.76 |
| 2434 | Train 39 | Test Asian 2 | 0.8 | 0.86 | 0.85 | 3458 | Train 55 | Test Asian 2 | 0.76 | 0.8 | 0.76 |
| 2435 | Train 39 | Test Asian 3 | 0.78 | 0.87 | 0.86 | 3459 | Train 55 | Test Asian 3 | 0.74 | 0.8 | 0.76 |
| 2436 | Train 39 | Test Asian 4 | 0.8 | 0.86 | 0.85 | 3460 | Train 55 | Test Asian 4 | 0.76 | 0.8 | 0.76 |
| 2437 | Train 39 | Test Asian 5 | 0.76 | 0.85 | 0.85 | 3461 | Train 55 | Test Asian 5 | 0.73 | 0.78 | 0.74 |
| 2438 | Train 39 | Test Asian 6 | 0.75 | 0.78 | 0.77 | 3462 | Train 55 | Test Asian 6 | 0.72 | 0.73 | 0.68 |
| 2439 | Train 39 | Test Asian 7 | 0.76 | 0.85 | 0.85 | 3463 | Train 55 | Test Asian 7 | 0.73 | 0.78 | 0.73 |
| 2440 | Train 39 | Test Asian 8 | 0.75 | 0.78 | 0.77 | 3464 | Train 55 | Test Asian 8 | 0.73 | 0.73 | 0.68 |
| 2441 | Train 39 | Test Asian 9 | 0.77 | 0.85 | 0.84 | 3465 | Train 55 | Test Asian 9 | 0.73 | 0.8 | 0.72 |
| 2442 | Train 39 | Test Asian 10 | 0.79 | 0.84 | 0.83 | 3466 | Train 55 | Test Asian 10 | 0.75 | 0.79 | 0.72 |
| 2443 | Train 39 | Test Asian 11 | 0.77 | 0.85 | 0.84 | 3467 | Train 55 | Test Asian 11 | 0.73 | 0.8 | 0.72 |
| 2444 | Train 39 | Test Asian 12 | 0.79 | 0.84 | 0.83 | 3468 | Train 55 | Test Asian 12 | 0.75 | 0.79 | 0.72 |
| 2445 | Train 39 | Test Asian 13 | 0.76 | 0.83 | 0.83 | 3469 | Train 55 | Test Asian 13 | 0.73 | 0.78 | 0.73 |
| 2446 | Train 39 | Test Asian 14 | 0.75 | 0.75 | 0.75 | 3470 | Train 55 | Test Asian 14 | 0.72 | 0.73 | 0.66 |
| 2447 | Train 39 | Test Asian 15 | 0.76 | 0.83 | 0.83 | 3471 | Train 55 | Test Asian 15 | 0.73 | 0.77 | 0.73 |
| 2448 | Train 39 | Test Asian 16 | 0.75 | 0.78 | 0.76 | 3472 | Train 55 | Test Asian 16 | 0.73 | 0.73 | 0.68 |
| 2449 | Train 39 | Test Asian 17 | 0.78 | 0.87 | 0.86 | 3473 | Train 55 | Test Asian 17 | 0.74 | 0.8 | 0.76 |
| 2450 | Train 39 | Test Asian 18 | 0.8 | 0.86 | 0.85 | 3474 | Train 55 | Test Asian 18 | 0.76 | 0.8 | 0.76 |
| 2451 | Train 39 | Test Asian 19 | 0.78 | 0.87 | 0.86 | 3475 | Train 55 | Test Asian 19 | 0.74 | 0.8 | 0.76 |
| 2452 | Train 39 | Test Asian 20 | 0.8 | 0.86 | 0.85 | 3476 | Train 55 | Test Asian 20 | 0.76 | 0.8 | 0.76 |
| 2453 | Train 39 | Test Asian 21 | 0.77 | 0.85 | 0.86 | 3477 | Train 55 | Test Asian 21 | 0.73 | 0.78 | 0.74 |
| 2454 | Train 39 | Test Asian 22 | 0.75 | 0.78 | 0.77 | 3478 | Train 55 | Test Asian 22 | 0.72 | 0.73 | 0.68 |
| 2455 | Train 39 | Test Asian 23 | 0.76 | 0.85 | 0.85 | 3479 | Train 55 | Test Asian 23 | 0.73 | 0.78 | 0.73 |
| 2456 | Train 39 | Test Asian 24 | 0.76 | 0.78 | 0.77 | 3480 | Train 55 | Test Asian 24 | 0.73 | 0.73 | 0.68 |
| 2457 | Train 39 | Test Asian 25 | 0.77 | 0.85 | 0.84 | 3481 | Train 55 | Test Asian 25 | 0.73 | 0.8 | 0.72 |
| 2458 | Train 39 | Test Asian 26 | 0.79 | 0.84 | 0.83 | 3482 | Train 55 | Test Asian 26 | 0.75 | 0.79 | 0.72 |
| 2459 | Train 39 | Test Asian 27 | 0.77 | 0.85 | 0.84 | 3483 | Train 55 | Test Asian 27 | 0.73 | 0.8 | 0.72 |
| 2460 | Train 39 | Test Asian 28 | 0.79 | 0.84 | 0.83 | 3484 | Train 55 | Test Asian 28 | 0.75 | 0.79 | 0.72 |
| 2461 | Train 39 | Test Asian 29 | 0.76 | 0.83 | 0.83 | 3485 | Train 55 | Test Asian 29 | 0.73 | 0.78 | 0.73 |
| 2462 | Train 39 | Test Asian 30 | 0.76 | 0.75 | 0.75 | 3486 | Train 55 | Test Asian 30 | 0.73 | 0.73 | 0.66 |
| 2463 | Train 39 | Test Asian 31 | 0.76 | 0.83 | 0.83 | 3487 | Train 55 | Test Asian 31 | 0.73 | 0.77 | 0.73 |
| 2464 | Train 39 | Test Asian 32 | 0.76 | 0.78 | 0.77 | 3488 | Train 55 | Test Asian 32 | 0.73 | 0.73 | 0.68 |
| 2465 | Train 39 | Test Asian 33 | 0.78 | 0.87 | 0.86 | 3489 | Train 55 | Test Asian 33 | 0.74 | 0.81 | 0.76 |
| 2466 | Train 39 | Test Asian 34 | 0.8 | 0.87 | 0.85 | 3490 | Train 55 | Test Asian 34 | 0.76 | 0.81 | 0.76 |
| 2467 | Train 39 | Test Asian 35 | 0.78 | 0.87 | 0.86 | 3491 | Train 55 | Test Asian 35 | 0.74 | 0.81 | 0.76 |
| 2468 | Train 39 | Test Asian 36 | 0.8 | 0.87 | 0.85 | 3492 | Train 55 | Test Asian 36 | 0.76 | 0.81 | 0.76 |
| 2469 | Train 39 | Test Asian 37 | 0.76 | 0.85 | 0.85 | 3493 | Train 55 | Test Asian 37 | 0.73 | 0.78 | 0.73 |
| 2470 | Train 39 | Test Asian 38 | 0.75 | 0.78 | 0.77 | 3494 | Train 55 | Test Asian 38 | 0.72 | 0.73 | 0.68 |
| 2471 | Train 39 | Test Asian 39 | 0.76 | 0.84 | 0.85 | 3495 | Train 55 | Test Asian 39 | 0.73 | 0.78 | 0.73 |
| 2472 | Train 39 | Test Asian 40 | 0.75 | 0.78 | 0.76 | 3496 | Train 55 | Test Asian 40 | 0.73 | 0.73 | 0.68 |
| 2473 | Train 39 | Test Asian 41 | 0.77 | 0.85 | 0.85 | 3497 | Train 55 | Test Asian 41 | 0.74 | 0.8 | 0.72 |
| 2474 | Train 39 | Test Asian 42 | 0.8 | 0.84 | 0.83 | 3498 | Train 55 | Test Asian 42 | 0.75 | 0.8 | 0.72 |
| 2475 | Train 39 | Test Asian 43 | 0.77 | 0.85 | 0.85 | 3499 | Train 55 | Test Asian 43 | 0.74 | 0.8 | 0.72 |
| 2476 | Train 39 | Test Asian 44 | 0.8 | 0.84 | 0.83 | 3500 | Train 55 | Test Asian 44 | 0.75 | 0.8 | 0.72 |
| 2477 | Train 39 | Test Asian 45 | 0.76 | 0.83 | 0.83 | 3501 | Train 55 | Test Asian 45 | 0.73 | 0.78 | 0.73 |
| 2478 | Train 39 | Test Asian 46 | 0.75 | 0.78 | 0.77 | 3502 | Train 55 | Test Asian 46 | 0.73 | 0.73 | 0.67 |
| 2479 | Train 39 | Test Asian 47 | 0.76 | 0.83 | 0.83 | 3503 | Train 55 | Test Asian 47 | 0.73 | 0.77 | 0.73 |
| 2480 | Train 39 | Test Asian 48 | 0.75 | 0.78 | 0.76 | 3504 | Train 55 | Test Asian 48 | 0.73 | 0.73 | 0.68 |
| 2481 | Train 39 | Test Asian 49 | 0.76 | 0.84 | 0.85 | 3505 | Train 55 | Test Asian 49 | 0.76 | 0.89 | 0.9 |
| 2482 | Train 39 | Test Asian 50 | 0.76 | 0.84 | 0.85 | 3506 | Train 55 | Test Asian 50 | 0.76 | 0.89 | 0.9 |
| 2483 | Train 39 | Test Asian 51 | 0.76 | 0.84 | 0.85 | 3507 | Train 55 | Test Asian 51 | 0.76 | 0.89 | 0.9 |
| 2484 | Train 39 | Test Asian 52 | 0.76 | 0.84 | 0.85 | 3508 | Train 55 | Test Asian 52 | 0.76 | 0.89 | 0.9 |
| 2485 | Train 39 | Test Asian 53 | 0.8 | 0.86 | 0.79 | 3509 | Train 55 | Test Asian 53 | 0.8 | 0.9 | 0.84 |
| 2486 | Train 39 | Test Asian 54 | 0.77 | 0.75 | 0.72 | 3510 | Train 55 | Test Asian 54 | 0.77 | 0.79 | 0.7 |
| 2487 | Train 39 | Test Asian 55 | 0.8 | 0.85 | 0.78 | 3511 | Train 55 | Test Asian 55 | 0.8 | 0.89 | 0.84 |
| 2488 | Train 39 | Test Asian 56 | 0.77 | 0.75 | 0.72 | 3512 | Train 55 | Test Asian 56 | 0.76 | 0.78 | 0.71 |
| 2489 | Train 39 | Test Asian 57 | 0.75 | 0.81 | 0.84 | 3513 | Train 55 | Test Asian 57 | 0.74 | 0.87 | 0.87 |
| 2490 | Train 39 | Test Asian 58 | 0.75 | 0.82 | 0.84 | 3514 | Train 55 | Test Asian 58 | 0.75 | 0.88 | 0.87 |
| 2491 | Train 39 | Test Asian 59 | 0.75 | 0.81 | 0.84 | 3515 | Train 55 | Test Asian 59 | 0.74 | 0.87 | 0.87 |
| 2492 | Train 39 | Test Asian 60 | 0.75 | 0.82 | 0.84 | 3516 | Train 55 | Test Asian 60 | 0.75 | 0.88 | 0.87 |
| 2493 | Train 39 | Test Asian 61 | 0.79 | 0.85 | 0.78 | 3517 | Train 55 | Test Asian 61 | 0.8 | 0.88 | 0.82 |
| 2494 | Train 39 | Test Asian 62 | 0.77 | 0.75 | 0.72 | 3518 | Train 55 | Test Asian 62 | 0.77 | 0.79 | 0.7 |
| 2495 | Train 39 | Test Asian 63 | 0.79 | 0.84 | 0.77 | 3519 | Train 55 | Test Asian 63 | 0.79 | 0.88 | 0.81 |
| 2496 | Train 39 | Test Asian 64 | 0.77 | 0.74 | 0.72 | 3520 | Train 55 | Test Asian 64 | 0.76 | 0.77 | 0.71 |
| 2497 | Train 40 | Test Asian 1 | 0.76 | 0.84 | 0.82 | 3521 | Train 56 | Test Asian 1 | 0.73 | 0.78 | 0.74 |
| 2498 | Train 40 | Test Asian 2 | 0.79 | 0.86 | 0.84 | 3522 | Train 56 | Test Asian 2 | 0.75 | 0.8 | 0.76 |
| 2499 | Train 40 | Test Asian 3 | 0.76 | 0.84 | 0.82 | 3523 | Train 56 | Test Asian 3 | 0.73 | 0.78 | 0.74 |
| 2500 | Train 40 | Test Asian 4 | 0.79 | 0.86 | 0.84 | 3524 | Train 56 | Test Asian 4 | 0.75 | 0.8 | 0.76 |
| 2501 | Train 40 | Test Asian 5 | 0.76 | 0.8 | 0.8 | 3525 | Train 56 | Test Asian 5 | 0.74 | 0.75 | 0.74 |
| 2502 | Train 40 | Test Asian 6 | 0.76 | 0.82 | 0.81 | 3526 | Train 56 | Test Asian 6 | 0.74 | 0.78 | 0.77 |
| 2503 | Train 40 | Test Asian 7 | 0.75 | 0.8 | 0.8 | 3527 | Train 56 | Test Asian 7 | 0.73 | 0.75 | 0.74 |
| 2504 | Train 40 | Test Asian 8 | 0.77 | 0.82 | 0.81 | 3528 | Train 56 | Test Asian 8 | 0.75 | 0.78 | 0.77 |
| 2505 | Train 40 | Test Asian 9 | 0.76 | 0.84 | 0.83 | 3529 | Train 56 | Test Asian 9 | 0.72 | 0.77 | 0.74 |
| 2506 | Train 40 | Test Asian 10 | 0.78 | 0.86 | 0.85 | 3530 | Train 56 | Test Asian 10 | 0.74 | 0.8 | 0.76 |
| 2507 | Train 40 | Test Asian 11 | 0.76 | 0.84 | 0.83 | 3531 | Train 56 | Test Asian 11 | 0.72 | 0.77 | 0.74 |
| 2508 | Train 40 | Test Asian 12 | 0.78 | 0.86 | 0.85 | 3532 | Train 56 | Test Asian 12 | 0.74 | 0.8 | 0.76 |
| 2509 | Train 40 | Test Asian 13 | 0.75 | 0.8 | 0.81 | 3533 | Train 56 | Test Asian 13 | 0.73 | 0.75 | 0.75 |
| 2510 | Train 40 | Test Asian 14 | 0.76 | 0.83 | 0.83 | 3534 | Train 56 | Test Asian 14 | 0.74 | 0.79 | 0.77 |
| 2511 | Train 40 | Test Asian 15 | 0.75 | 0.8 | 0.81 | 3535 | Train 56 | Test Asian 15 | 0.73 | 0.75 | 0.75 |
| 2512 | Train 40 | Test Asian 16 | 0.76 | 0.81 | 0.8 | 3536 | Train 56 | Test Asian 16 | 0.75 | 0.78 | 0.77 |
| 2513 | Train 40 | Test Asian 17 | 0.76 | 0.84 | 0.82 | 3537 | Train 56 | Test Asian 17 | 0.73 | 0.78 | 0.74 |
| 2514 | Train 40 | Test Asian 18 | 0.79 | 0.86 | 0.84 | 3538 | Train 56 | Test Asian 18 | 0.75 | 0.8 | 0.76 |
| 2515 | Train 40 | Test Asian 19 | 0.76 | 0.84 | 0.82 | 3539 | Train 56 | Test Asian 19 | 0.73 | 0.78 | 0.74 |
| 2516 | Train 40 | Test Asian 20 | 0.79 | 0.86 | 0.84 | 3540 | Train 56 | Test Asian 20 | 0.75 | 0.8 | 0.76 |
| 2517 | Train 40 | Test Asian 21 | 0.76 | 0.8 | 0.8 | 3541 | Train 56 | Test Asian 21 | 0.74 | 0.75 | 0.74 |
| 2518 | Train 40 | Test Asian 22 | 0.76 | 0.82 | 0.81 | 3542 | Train 56 | Test Asian 22 | 0.74 | 0.78 | 0.77 |
| 2519 | Train 40 | Test Asian 23 | 0.75 | 0.8 | 0.8 | 3543 | Train 56 | Test Asian 23 | 0.74 | 0.75 | 0.74 |
| 2520 | Train 40 | Test Asian 24 | 0.77 | 0.82 | 0.81 | 3544 | Train 56 | Test Asian 24 | 0.75 | 0.78 | 0.77 |
| 2521 | Train 40 | Test Asian 25 | 0.76 | 0.84 | 0.83 | 3545 | Train 56 | Test Asian 25 | 0.72 | 0.77 | 0.74 |
| 2522 | Train 40 | Test Asian 26 | 0.78 | 0.86 | 0.84 | 3546 | Train 56 | Test Asian 26 | 0.74 | 0.8 | 0.76 |
| 2523 | Train 40 | Test Asian 27 | 0.76 | 0.84 | 0.83 | 3547 | Train 56 | Test Asian 27 | 0.72 | 0.77 | 0.74 |
| 2524 | Train 40 | Test Asian 28 | 0.78 | 0.86 | 0.84 | 3548 | Train 56 | Test Asian 28 | 0.74 | 0.8 | 0.76 |
| 2525 | Train 40 | Test Asian 29 | 0.75 | 0.8 | 0.81 | 3549 | Train 56 | Test Asian 29 | 0.73 | 0.75 | 0.75 |
| 2526 | Train 40 | Test Asian 30 | 0.76 | 0.83 | 0.83 | 3550 | Train 56 | Test Asian 30 | 0.74 | 0.79 | 0.78 |
| 2527 | Train 40 | Test Asian 31 | 0.75 | 0.8 | 0.81 | 3551 | Train 56 | Test Asian 31 | 0.73 | 0.75 | 0.75 |
| 2528 | Train 40 | Test Asian 32 | 0.77 | 0.81 | 0.8 | 3552 | Train 56 | Test Asian 32 | 0.75 | 0.78 | 0.77 |
| 2529 | Train 40 | Test Asian 33 | 0.76 | 0.84 | 0.82 | 3553 | Train 56 | Test Asian 33 | 0.73 | 0.78 | 0.74 |
| 2530 | Train 40 | Test Asian 34 | 0.79 | 0.86 | 0.84 | 3554 | Train 56 | Test Asian 34 | 0.75 | 0.8 | 0.76 |
| 2531 | Train 40 | Test Asian 35 | 0.76 | 0.84 | 0.82 | 3555 | Train 56 | Test Asian 35 | 0.73 | 0.78 | 0.74 |
| 2532 | Train 40 | Test Asian 36 | 0.79 | 0.86 | 0.84 | 3556 | Train 56 | Test Asian 36 | 0.75 | 0.8 | 0.76 |
| 2533 | Train 40 | Test Asian 37 | 0.75 | 0.8 | 0.8 | 3557 | Train 56 | Test Asian 37 | 0.74 | 0.75 | 0.74 |
| 2534 | Train 40 | Test Asian 38 | 0.76 | 0.82 | 0.81 | 3558 | Train 56 | Test Asian 38 | 0.74 | 0.78 | 0.77 |
| 2535 | Train 40 | Test Asian 39 | 0.75 | 0.8 | 0.79 | 3559 | Train 56 | Test Asian 39 | 0.73 | 0.75 | 0.74 |
| 2536 | Train 40 | Test Asian 40 | 0.76 | 0.82 | 0.8 | 3560 | Train 56 | Test Asian 40 | 0.75 | 0.78 | 0.77 |
| 2537 | Train 40 | Test Asian 41 | 0.76 | 0.84 | 0.83 | 3561 | Train 56 | Test Asian 41 | 0.72 | 0.77 | 0.74 |
| 2538 | Train 40 | Test Asian 42 | 0.78 | 0.86 | 0.84 | 3562 | Train 56 | Test Asian 42 | 0.74 | 0.8 | 0.76 |
| 2539 | Train 40 | Test Asian 43 | 0.76 | 0.84 | 0.83 | 3563 | Train 56 | Test Asian 43 | 0.72 | 0.77 | 0.74 |
| 2540 | Train 40 | Test Asian 44 | 0.78 | 0.86 | 0.84 | 3564 | Train 56 | Test Asian 44 | 0.74 | 0.8 | 0.76 |
| 2541 | Train 40 | Test Asian 45 | 0.75 | 0.8 | 0.81 | 3565 | Train 56 | Test Asian 45 | 0.73 | 0.75 | 0.75 |
| 2542 | Train 40 | Test Asian 46 | 0.76 | 0.81 | 0.8 | 3566 | Train 56 | Test Asian 46 | 0.74 | 0.78 | 0.77 |
| 2543 | Train 40 | Test Asian 47 | 0.75 | 0.8 | 0.8 | 3567 | Train 56 | Test Asian 47 | 0.73 | 0.75 | 0.74 |
| 2544 | Train 40 | Test Asian 48 | 0.76 | 0.81 | 0.8 | 3568 | Train 56 | Test Asian 48 | 0.75 | 0.78 | 0.77 |
| 2545 | Train 40 | Test Asian 49 | 0.77 | 0.84 | 0.8 | 3569 | Train 56 | Test Asian 49 | 0.77 | 0.86 | 0.77 |
| 2546 | Train 40 | Test Asian 50 | 0.77 | 0.84 | 0.8 | 3570 | Train 56 | Test Asian 50 | 0.77 | 0.86 | 0.77 |
| 2547 | Train 40 | Test Asian 51 | 0.77 | 0.84 | 0.8 | 3571 | Train 56 | Test Asian 51 | 0.77 | 0.86 | 0.77 |
| 2548 | Train 40 | Test Asian 52 | 0.77 | 0.84 | 0.8 | 3572 | Train 56 | Test Asian 52 | 0.77 | 0.86 | 0.77 |
| 2549 | Train 40 | Test Asian 53 | 0.8 | 0.85 | 0.75 | 3573 | Train 56 | Test Asian 53 | 0.8 | 0.87 | 0.8 |
| 2550 | Train 40 | Test Asian 54 | 0.79 | 0.83 | 0.83 | 3574 | Train 56 | Test Asian 54 | 0.78 | 0.86 | 0.86 |
| 2551 | Train 40 | Test Asian 55 | 0.79 | 0.84 | 0.74 | 3575 | Train 56 | Test Asian 55 | 0.79 | 0.86 | 0.8 |
| 2552 | Train 40 | Test Asian 56 | 0.79 | 0.83 | 0.83 | 3576 | Train 56 | Test Asian 56 | 0.78 | 0.86 | 0.86 |
| 2553 | Train 40 | Test Asian 57 | 0.77 | 0.84 | 0.81 | 3577 | Train 56 | Test Asian 57 | 0.76 | 0.85 | 0.8 |
| 2554 | Train 40 | Test Asian 58 | 0.77 | 0.84 | 0.81 | 3578 | Train 56 | Test Asian 58 | 0.76 | 0.85 | 0.8 |
| 2555 | Train 40 | Test Asian 59 | 0.77 | 0.84 | 0.81 | 3579 | Train 56 | Test Asian 59 | 0.76 | 0.85 | 0.8 |
| 2556 | Train 40 | Test Asian 60 | 0.77 | 0.84 | 0.81 | 3580 | Train 56 | Test Asian 60 | 0.76 | 0.85 | 0.8 |
| 2557 | Train 40 | Test Asian 61 | 0.79 | 0.84 | 0.76 | 3581 | Train 56 | Test Asian 61 | 0.79 | 0.87 | 0.81 |
| 2558 | Train 40 | Test Asian 62 | 0.79 | 0.83 | 0.83 | 3582 | Train 56 | Test Asian 62 | 0.78 | 0.86 | 0.86 |
| 2559 | Train 40 | Test Asian 63 | 0.78 | 0.84 | 0.75 | 3583 | Train 56 | Test Asian 63 | 0.78 | 0.86 | 0.8 |
| 2560 | Train 40 | Test Asian 64 | 0.79 | 0.83 | 0.83 | 3584 | Train 56 | Test Asian 64 | 0.78 | 0.86 | 0.86 |
| 2561 | Train 41 | Test Asian 1 | 0.8 | 0.91 | 0.9 | 3585 | Train 57 | Test Asian 1 | 0.75 | 0.8 | 0.71 |
| 2562 | Train 41 | Test Asian 2 | 0.8 | 0.89 | 0.86 | 3586 | Train 57 | Test Asian 2 | 0.75 | 0.81 | 0.71 |
| 2563 | Train 41 | Test Asian 3 | 0.8 | 0.91 | 0.9 | 3587 | Train 57 | Test Asian 3 | 0.75 | 0.8 | 0.71 |
| 2564 | Train 41 | Test Asian 4 | 0.8 | 0.89 | 0.86 | 3588 | Train 57 | Test Asian 4 | 0.75 | 0.81 | 0.71 |
| 2565 | Train 41 | Test Asian 5 | 0.75 | 0.75 | 0.71 | 3589 | Train 57 | Test Asian 5 | 0.73 | 0.71 | 0.62 |
| 2566 | Train 41 | Test Asian 6 | 0.75 | 0.75 | 0.7 | 3590 | Train 57 | Test Asian 6 | 0.73 | 0.71 | 0.62 |
| 2567 | Train 41 | Test Asian 7 | 0.75 | 0.75 | 0.7 | 3591 | Train 57 | Test Asian 7 | 0.73 | 0.71 | 0.61 |
| 2568 | Train 41 | Test Asian 8 | 0.75 | 0.75 | 0.7 | 3592 | Train 57 | Test Asian 8 | 0.73 | 0.71 | 0.62 |
| 2569 | Train 41 | Test Asian 9 | 0.79 | 0.92 | 0.93 | 3593 | Train 57 | Test Asian 9 | 0.74 | 0.8 | 0.73 |
| 2570 | Train 41 | Test Asian 10 | 0.79 | 0.89 | 0.87 | 3594 | Train 57 | Test Asian 10 | 0.74 | 0.81 | 0.72 |
| 2571 | Train 41 | Test Asian 11 | 0.79 | 0.92 | 0.93 | 3595 | Train 57 | Test Asian 11 | 0.74 | 0.8 | 0.73 |
| 2572 | Train 41 | Test Asian 12 | 0.79 | 0.89 | 0.87 | 3596 | Train 57 | Test Asian 12 | 0.74 | 0.81 | 0.72 |
| 2573 | Train 41 | Test Asian 13 | 0.75 | 0.76 | 0.72 | 3597 | Train 57 | Test Asian 13 | 0.72 | 0.71 | 0.61 |
| 2574 | Train 41 | Test Asian 14 | 0.75 | 0.73 | 0.67 | 3598 | Train 57 | Test Asian 14 | 0.72 | 0.71 | 0.62 |
| 2575 | Train 41 | Test Asian 15 | 0.74 | 0.75 | 0.72 | 3599 | Train 57 | Test Asian 15 | 0.72 | 0.71 | 0.61 |
| 2576 | Train 41 | Test Asian 16 | 0.75 | 0.75 | 0.7 | 3600 | Train 57 | Test Asian 16 | 0.73 | 0.71 | 0.62 |
| 2577 | Train 41 | Test Asian 17 | 0.8 | 0.91 | 0.9 | 3601 | Train 57 | Test Asian 17 | 0.75 | 0.8 | 0.71 |
| 2578 | Train 41 | Test Asian 18 | 0.8 | 0.89 | 0.86 | 3602 | Train 57 | Test Asian 18 | 0.75 | 0.81 | 0.71 |
| 2579 | Train 41 | Test Asian 19 | 0.8 | 0.91 | 0.9 | 3603 | Train 57 | Test Asian 19 | 0.75 | 0.8 | 0.71 |
| 2580 | Train 41 | Test Asian 20 | 0.8 | 0.89 | 0.86 | 3604 | Train 57 | Test Asian 20 | 0.75 | 0.81 | 0.71 |
| 2581 | Train 41 | Test Asian 21 | 0.75 | 0.75 | 0.71 | 3605 | Train 57 | Test Asian 21 | 0.73 | 0.71 | 0.62 |
| 2582 | Train 41 | Test Asian 22 | 0.75 | 0.75 | 0.7 | 3606 | Train 57 | Test Asian 22 | 0.73 | 0.71 | 0.62 |
| 2583 | Train 41 | Test Asian 23 | 0.75 | 0.75 | 0.7 | 3607 | Train 57 | Test Asian 23 | 0.73 | 0.71 | 0.62 |
| 2584 | Train 41 | Test Asian 24 | 0.75 | 0.75 | 0.7 | 3608 | Train 57 | Test Asian 24 | 0.73 | 0.71 | 0.62 |
| 2585 | Train 41 | Test Asian 25 | 0.79 | 0.92 | 0.93 | 3609 | Train 57 | Test Asian 25 | 0.74 | 0.8 | 0.73 |
| 2586 | Train 41 | Test Asian 26 | 0.79 | 0.89 | 0.87 | 3610 | Train 57 | Test Asian 26 | 0.74 | 0.82 | 0.72 |
| 2587 | Train 41 | Test Asian 27 | 0.79 | 0.92 | 0.93 | 3611 | Train 57 | Test Asian 27 | 0.74 | 0.8 | 0.73 |
| 2588 | Train 41 | Test Asian 28 | 0.79 | 0.89 | 0.87 | 3612 | Train 57 | Test Asian 28 | 0.74 | 0.82 | 0.72 |
| 2589 | Train 41 | Test Asian 29 | 0.75 | 0.76 | 0.72 | 3613 | Train 57 | Test Asian 29 | 0.73 | 0.71 | 0.61 |
| 2590 | Train 41 | Test Asian 30 | 0.75 | 0.73 | 0.67 | 3614 | Train 57 | Test Asian 30 | 0.73 | 0.72 | 0.62 |
| 2591 | Train 41 | Test Asian 31 | 0.74 | 0.75 | 0.72 | 3615 | Train 57 | Test Asian 31 | 0.72 | 0.71 | 0.61 |
| 2592 | Train 41 | Test Asian 32 | 0.76 | 0.75 | 0.7 | 3616 | Train 57 | Test Asian 32 | 0.73 | 0.71 | 0.62 |
| 2593 | Train 41 | Test Asian 33 | 0.8 | 0.91 | 0.9 | 3617 | Train 57 | Test Asian 33 | 0.75 | 0.8 | 0.71 |
| 2594 | Train 41 | Test Asian 34 | 0.8 | 0.89 | 0.87 | 3618 | Train 57 | Test Asian 34 | 0.75 | 0.81 | 0.71 |
| 2595 | Train 41 | Test Asian 35 | 0.8 | 0.91 | 0.9 | 3619 | Train 57 | Test Asian 35 | 0.75 | 0.8 | 0.71 |
| 2596 | Train 41 | Test Asian 36 | 0.8 | 0.89 | 0.87 | 3620 | Train 57 | Test Asian 36 | 0.75 | 0.81 | 0.71 |
| 2597 | Train 41 | Test Asian 37 | 0.75 | 0.75 | 0.71 | 3621 | Train 57 | Test Asian 37 | 0.73 | 0.7 | 0.62 |
| 2598 | Train 41 | Test Asian 38 | 0.75 | 0.75 | 0.7 | 3622 | Train 57 | Test Asian 38 | 0.72 | 0.71 | 0.62 |
| 2599 | Train 41 | Test Asian 39 | 0.74 | 0.75 | 0.7 | 3623 | Train 57 | Test Asian 39 | 0.73 | 0.7 | 0.61 |
| 2600 | Train 41 | Test Asian 40 | 0.75 | 0.74 | 0.7 | 3624 | Train 57 | Test Asian 40 | 0.73 | 0.71 | 0.62 |
| 2601 | Train 41 | Test Asian 41 | 0.79 | 0.92 | 0.92 | 3625 | Train 57 | Test Asian 41 | 0.74 | 0.8 | 0.73 |
| 2602 | Train 41 | Test Asian 42 | 0.79 | 0.89 | 0.87 | 3626 | Train 57 | Test Asian 42 | 0.74 | 0.81 | 0.72 |
| 2603 | Train 41 | Test Asian 43 | 0.79 | 0.92 | 0.92 | 3627 | Train 57 | Test Asian 43 | 0.74 | 0.8 | 0.73 |
| 2604 | Train 41 | Test Asian 44 | 0.79 | 0.89 | 0.87 | 3628 | Train 57 | Test Asian 44 | 0.74 | 0.81 | 0.72 |
| 2605 | Train 41 | Test Asian 45 | 0.74 | 0.75 | 0.72 | 3629 | Train 57 | Test Asian 45 | 0.73 | 0.71 | 0.61 |
| 2606 | Train 41 | Test Asian 46 | 0.75 | 0.75 | 0.7 | 3630 | Train 57 | Test Asian 46 | 0.72 | 0.71 | 0.62 |
| 2607 | Train 41 | Test Asian 47 | 0.74 | 0.75 | 0.71 | 3631 | Train 57 | Test Asian 47 | 0.72 | 0.71 | 0.61 |
| 2608 | Train 41 | Test Asian 48 | 0.75 | 0.75 | 0.7 | 3632 | Train 57 | Test Asian 48 | 0.73 | 0.71 | 0.62 |
| 2609 | Train 41 | Test Asian 49 | 0.77 | 0.86 | 0.82 | 3633 | Train 57 | Test Asian 49 | 0.77 | 0.91 | 0.87 |
| 2610 | Train 41 | Test Asian 50 | 0.77 | 0.86 | 0.82 | 3634 | Train 57 | Test Asian 50 | 0.76 | 0.91 | 0.86 |
| 2611 | Train 41 | Test Asian 51 | 0.77 | 0.86 | 0.82 | 3635 | Train 57 | Test Asian 51 | 0.77 | 0.91 | 0.87 |
| 2612 | Train 41 | Test Asian 52 | 0.77 | 0.86 | 0.82 | 3636 | Train 57 | Test Asian 52 | 0.76 | 0.91 | 0.86 |
| 2613 | Train 41 | Test Asian 53 | 0.79 | 0.8 | 0.72 | 3637 | Train 57 | Test Asian 53 | 0.77 | 0.72 | 0.66 |
| 2614 | Train 41 | Test Asian 54 | 0.78 | 0.75 | 0.67 | 3638 | Train 57 | Test Asian 54 | 0.74 | 0.73 | 0.66 |
| 2615 | Train 41 | Test Asian 55 | 0.79 | 0.8 | 0.72 | 3639 | Train 57 | Test Asian 55 | 0.77 | 0.72 | 0.66 |
| 2616 | Train 41 | Test Asian 56 | 0.78 | 0.74 | 0.67 | 3640 | Train 57 | Test Asian 56 | 0.75 | 0.72 | 0.66 |
| 2617 | Train 41 | Test Asian 57 | 0.76 | 0.88 | 0.86 | 3641 | Train 57 | Test Asian 57 | 0.76 | 0.93 | 0.97 |
| 2618 | Train 41 | Test Asian 58 | 0.76 | 0.88 | 0.85 | 3642 | Train 57 | Test Asian 58 | 0.76 | 0.93 | 0.97 |
| 2619 | Train 41 | Test Asian 59 | 0.76 | 0.88 | 0.86 | 3643 | Train 57 | Test Asian 59 | 0.76 | 0.93 | 0.97 |
| 2620 | Train 41 | Test Asian 60 | 0.76 | 0.88 | 0.85 | 3644 | Train 57 | Test Asian 60 | 0.76 | 0.93 | 0.97 |
| 2621 | Train 41 | Test Asian 61 | 0.79 | 0.8 | 0.72 | 3645 | Train 57 | Test Asian 61 | 0.76 | 0.73 | 0.66 |
| 2622 | Train 41 | Test Asian 62 | 0.78 | 0.75 | 0.67 | 3646 | Train 57 | Test Asian 62 | 0.74 | 0.74 | 0.67 |
| 2623 | Train 41 | Test Asian 63 | 0.78 | 0.8 | 0.72 | 3647 | Train 57 | Test Asian 63 | 0.76 | 0.73 | 0.66 |
| 2624 | Train 41 | Test Asian 64 | 0.78 | 0.74 | 0.67 | 3648 | Train 57 | Test Asian 64 | 0.75 | 0.72 | 0.67 |
| 2625 | Train 42 | Test Asian 1 | 0.79 | 0.9 | 0.88 | 3649 | Train 58 | Test Asian 1 | 0.75 | 0.75 | 0.62 |
| 2626 | Train 42 | Test Asian 2 | 0.81 | 0.91 | 0.89 | 3650 | Train 58 | Test Asian 2 | 0.76 | 0.77 | 0.64 |
| 2627 | Train 42 | Test Asian 3 | 0.79 | 0.9 | 0.88 | 3651 | Train 58 | Test Asian 3 | 0.75 | 0.75 | 0.62 |
| 2628 | Train 42 | Test Asian 4 | 0.81 | 0.91 | 0.89 | 3652 | Train 58 | Test Asian 4 | 0.76 | 0.77 | 0.64 |
| 2629 | Train 42 | Test Asian 5 | 0.75 | 0.76 | 0.71 | 3653 | Train 58 | Test Asian 5 | 0.73 | 0.67 | 0.57 |
| 2630 | Train 42 | Test Asian 6 | 0.75 | 0.75 | 0.7 | 3654 | Train 58 | Test Asian 6 | 0.73 | 0.69 | 0.6 |
| 2631 | Train 42 | Test Asian 7 | 0.75 | 0.76 | 0.7 | 3655 | Train 58 | Test Asian 7 | 0.73 | 0.67 | 0.57 |
| 2632 | Train 42 | Test Asian 8 | 0.75 | 0.75 | 0.69 | 3656 | Train 58 | Test Asian 8 | 0.73 | 0.68 | 0.6 |
| 2633 | Train 42 | Test Asian 9 | 0.78 | 0.91 | 0.91 | 3657 | Train 58 | Test Asian 9 | 0.74 | 0.75 | 0.62 |
| 2634 | Train 42 | Test Asian 10 | 0.8 | 0.91 | 0.92 | 3658 | Train 58 | Test Asian 10 | 0.75 | 0.77 | 0.65 |
| 2635 | Train 42 | Test Asian 11 | 0.78 | 0.91 | 0.91 | 3659 | Train 58 | Test Asian 11 | 0.74 | 0.75 | 0.62 |
| 2636 | Train 42 | Test Asian 12 | 0.8 | 0.91 | 0.92 | 3660 | Train 58 | Test Asian 12 | 0.75 | 0.77 | 0.65 |
| 2637 | Train 42 | Test Asian 13 | 0.75 | 0.76 | 0.73 | 3661 | Train 58 | Test Asian 13 | 0.72 | 0.67 | 0.58 |
| 2638 | Train 42 | Test Asian 14 | 0.75 | 0.74 | 0.67 | 3662 | Train 58 | Test Asian 14 | 0.73 | 0.69 | 0.6 |
| 2639 | Train 42 | Test Asian 15 | 0.75 | 0.76 | 0.72 | 3663 | Train 58 | Test Asian 15 | 0.72 | 0.67 | 0.58 |
| 2640 | Train 42 | Test Asian 16 | 0.75 | 0.75 | 0.69 | 3664 | Train 58 | Test Asian 16 | 0.73 | 0.68 | 0.6 |
| 2641 | Train 42 | Test Asian 17 | 0.79 | 0.9 | 0.88 | 3665 | Train 58 | Test Asian 17 | 0.75 | 0.75 | 0.62 |
| 2642 | Train 42 | Test Asian 18 | 0.81 | 0.91 | 0.89 | 3666 | Train 58 | Test Asian 18 | 0.76 | 0.77 | 0.64 |
| 2643 | Train 42 | Test Asian 19 | 0.79 | 0.9 | 0.88 | 3667 | Train 58 | Test Asian 19 | 0.75 | 0.75 | 0.62 |
| 2644 | Train 42 | Test Asian 20 | 0.81 | 0.91 | 0.89 | 3668 | Train 58 | Test Asian 20 | 0.76 | 0.77 | 0.64 |
| 2645 | Train 42 | Test Asian 21 | 0.75 | 0.76 | 0.71 | 3669 | Train 58 | Test Asian 21 | 0.73 | 0.67 | 0.57 |
| 2646 | Train 42 | Test Asian 22 | 0.75 | 0.75 | 0.7 | 3670 | Train 58 | Test Asian 22 | 0.73 | 0.69 | 0.6 |
| 2647 | Train 42 | Test Asian 23 | 0.75 | 0.76 | 0.7 | 3671 | Train 58 | Test Asian 23 | 0.73 | 0.67 | 0.57 |
| 2648 | Train 42 | Test Asian 24 | 0.75 | 0.75 | 0.69 | 3672 | Train 58 | Test Asian 24 | 0.73 | 0.68 | 0.6 |
| 2649 | Train 42 | Test Asian 25 | 0.78 | 0.91 | 0.91 | 3673 | Train 58 | Test Asian 25 | 0.74 | 0.75 | 0.62 |
| 2650 | Train 42 | Test Asian 26 | 0.8 | 0.91 | 0.92 | 3674 | Train 58 | Test Asian 26 | 0.75 | 0.77 | 0.65 |
| 2651 | Train 42 | Test Asian 27 | 0.78 | 0.91 | 0.91 | 3675 | Train 58 | Test Asian 27 | 0.74 | 0.75 | 0.62 |
| 2652 | Train 42 | Test Asian 28 | 0.8 | 0.91 | 0.92 | 3676 | Train 58 | Test Asian 28 | 0.75 | 0.77 | 0.65 |
| 2653 | Train 42 | Test Asian 29 | 0.75 | 0.76 | 0.73 | 3677 | Train 58 | Test Asian 29 | 0.72 | 0.67 | 0.58 |
| 2654 | Train 42 | Test Asian 30 | 0.75 | 0.74 | 0.67 | 3678 | Train 58 | Test Asian 30 | 0.73 | 0.69 | 0.6 |
| 2655 | Train 42 | Test Asian 31 | 0.75 | 0.76 | 0.72 | 3679 | Train 58 | Test Asian 31 | 0.72 | 0.67 | 0.58 |
| 2656 | Train 42 | Test Asian 32 | 0.75 | 0.75 | 0.7 | 3680 | Train 58 | Test Asian 32 | 0.73 | 0.69 | 0.6 |
| 2657 | Train 42 | Test Asian 33 | 0.79 | 0.9 | 0.88 | 3681 | Train 58 | Test Asian 33 | 0.75 | 0.75 | 0.62 |
| 2658 | Train 42 | Test Asian 34 | 0.81 | 0.91 | 0.89 | 3682 | Train 58 | Test Asian 34 | 0.75 | 0.77 | 0.64 |
| 2659 | Train 42 | Test Asian 35 | 0.79 | 0.9 | 0.88 | 3683 | Train 58 | Test Asian 35 | 0.75 | 0.75 | 0.62 |
| 2660 | Train 42 | Test Asian 36 | 0.81 | 0.91 | 0.89 | 3684 | Train 58 | Test Asian 36 | 0.75 | 0.77 | 0.64 |
| 2661 | Train 42 | Test Asian 37 | 0.75 | 0.76 | 0.71 | 3685 | Train 58 | Test Asian 37 | 0.73 | 0.67 | 0.57 |
| 2662 | Train 42 | Test Asian 38 | 0.74 | 0.75 | 0.7 | 3686 | Train 58 | Test Asian 38 | 0.73 | 0.68 | 0.6 |
| 2663 | Train 42 | Test Asian 39 | 0.75 | 0.76 | 0.7 | 3687 | Train 58 | Test Asian 39 | 0.73 | 0.67 | 0.57 |
| 2664 | Train 42 | Test Asian 40 | 0.75 | 0.75 | 0.69 | 3688 | Train 58 | Test Asian 40 | 0.73 | 0.68 | 0.6 |
| 2665 | Train 42 | Test Asian 41 | 0.78 | 0.91 | 0.91 | 3689 | Train 58 | Test Asian 41 | 0.74 | 0.75 | 0.62 |
| 2666 | Train 42 | Test Asian 42 | 0.8 | 0.91 | 0.92 | 3690 | Train 58 | Test Asian 42 | 0.75 | 0.77 | 0.65 |
| 2667 | Train 42 | Test Asian 43 | 0.78 | 0.91 | 0.91 | 3691 | Train 58 | Test Asian 43 | 0.74 | 0.75 | 0.62 |
| 2668 | Train 42 | Test Asian 44 | 0.8 | 0.91 | 0.92 | 3692 | Train 58 | Test Asian 44 | 0.75 | 0.77 | 0.65 |
| 2669 | Train 42 | Test Asian 45 | 0.75 | 0.76 | 0.73 | 3693 | Train 58 | Test Asian 45 | 0.72 | 0.67 | 0.58 |
| 2670 | Train 42 | Test Asian 46 | 0.75 | 0.75 | 0.7 | 3694 | Train 58 | Test Asian 46 | 0.73 | 0.69 | 0.6 |
| 2671 | Train 42 | Test Asian 47 | 0.75 | 0.76 | 0.72 | 3695 | Train 58 | Test Asian 47 | 0.72 | 0.67 | 0.58 |
| 2672 | Train 42 | Test Asian 48 | 0.75 | 0.75 | 0.69 | 3696 | Train 58 | Test Asian 48 | 0.73 | 0.68 | 0.6 |
| 2673 | Train 42 | Test Asian 49 | 0.77 | 0.85 | 0.8 | 3697 | Train 58 | Test Asian 49 | 0.77 | 0.91 | 0.9 |
| 2674 | Train 42 | Test Asian 50 | 0.77 | 0.85 | 0.81 | 3698 | Train 58 | Test Asian 50 | 0.77 | 0.91 | 0.9 |
| 2675 | Train 42 | Test Asian 51 | 0.77 | 0.85 | 0.8 | 3699 | Train 58 | Test Asian 51 | 0.77 | 0.91 | 0.9 |
| 2676 | Train 42 | Test Asian 52 | 0.77 | 0.85 | 0.81 | 3700 | Train 58 | Test Asian 52 | 0.77 | 0.91 | 0.9 |
| 2677 | Train 42 | Test Asian 53 | 0.8 | 0.81 | 0.69 | 3701 | Train 58 | Test Asian 53 | 0.77 | 0.66 | 0.58 |
| 2678 | Train 42 | Test Asian 54 | 0.77 | 0.74 | 0.66 | 3702 | Train 58 | Test Asian 54 | 0.74 | 0.68 | 0.62 |
| 2679 | Train 42 | Test Asian 55 | 0.79 | 0.81 | 0.69 | 3703 | Train 58 | Test Asian 55 | 0.76 | 0.67 | 0.59 |
| 2680 | Train 42 | Test Asian 56 | 0.76 | 0.73 | 0.66 | 3704 | Train 58 | Test Asian 56 | 0.75 | 0.67 | 0.61 |
| 2681 | Train 42 | Test Asian 57 | 0.76 | 0.86 | 0.87 | 3705 | Train 58 | Test Asian 57 | 0.76 | 0.92 | 0.94 |
| 2682 | Train 42 | Test Asian 58 | 0.76 | 0.86 | 0.87 | 3706 | Train 58 | Test Asian 58 | 0.76 | 0.93 | 0.94 |
| 2683 | Train 42 | Test Asian 59 | 0.76 | 0.86 | 0.87 | 3707 | Train 58 | Test Asian 59 | 0.76 | 0.92 | 0.94 |
| 2684 | Train 42 | Test Asian 60 | 0.76 | 0.86 | 0.87 | 3708 | Train 58 | Test Asian 60 | 0.76 | 0.93 | 0.94 |
| 2685 | Train 42 | Test Asian 61 | 0.79 | 0.81 | 0.69 | 3709 | Train 58 | Test Asian 61 | 0.76 | 0.67 | 0.58 |
| 2686 | Train 42 | Test Asian 62 | 0.77 | 0.74 | 0.66 | 3710 | Train 58 | Test Asian 62 | 0.75 | 0.69 | 0.62 |
| 2687 | Train 42 | Test Asian 63 | 0.78 | 0.81 | 0.69 | 3711 | Train 58 | Test Asian 63 | 0.76 | 0.67 | 0.58 |
| 2688 | Train 42 | Test Asian 64 | 0.76 | 0.73 | 0.66 | 3712 | Train 58 | Test Asian 64 | 0.75 | 0.67 | 0.61 |
| 2689 | Train 43 | Test Asian 1 | 0.8 | 0.91 | 0.9 | 3713 | Train 59 | Test Asian 1 | 0.75 | 0.81 | 0.7 |
| 2690 | Train 43 | Test Asian 2 | 0.8 | 0.89 | 0.86 | 3714 | Train 59 | Test Asian 2 | 0.75 | 0.82 | 0.69 |
| 2691 | Train 43 | Test Asian 3 | 0.8 | 0.91 | 0.9 | 3715 | Train 59 | Test Asian 3 | 0.75 | 0.81 | 0.7 |
| 2692 | Train 43 | Test Asian 4 | 0.8 | 0.89 | 0.86 | 3716 | Train 59 | Test Asian 4 | 0.75 | 0.82 | 0.69 |
| 2693 | Train 43 | Test Asian 5 | 0.75 | 0.75 | 0.65 | 3717 | Train 59 | Test Asian 5 | 0.73 | 0.71 | 0.59 |
| 2694 | Train 43 | Test Asian 6 | 0.75 | 0.75 | 0.66 | 3718 | Train 59 | Test Asian 6 | 0.73 | 0.71 | 0.58 |
| 2695 | Train 43 | Test Asian 7 | 0.75 | 0.75 | 0.65 | 3719 | Train 59 | Test Asian 7 | 0.73 | 0.71 | 0.6 |
| 2696 | Train 43 | Test Asian 8 | 0.75 | 0.75 | 0.66 | 3720 | Train 59 | Test Asian 8 | 0.73 | 0.71 | 0.59 |
| 2697 | Train 43 | Test Asian 9 | 0.79 | 0.92 | 0.92 | 3721 | Train 59 | Test Asian 9 | 0.74 | 0.81 | 0.72 |
| 2698 | Train 43 | Test Asian 10 | 0.79 | 0.89 | 0.87 | 3722 | Train 59 | Test Asian 10 | 0.74 | 0.82 | 0.7 |
| 2699 | Train 43 | Test Asian 11 | 0.79 | 0.92 | 0.92 | 3723 | Train 59 | Test Asian 11 | 0.74 | 0.81 | 0.72 |
| 2700 | Train 43 | Test Asian 12 | 0.79 | 0.89 | 0.87 | 3724 | Train 59 | Test Asian 12 | 0.74 | 0.82 | 0.7 |
| 2701 | Train 43 | Test Asian 13 | 0.75 | 0.76 | 0.66 | 3725 | Train 59 | Test Asian 13 | 0.72 | 0.71 | 0.61 |
| 2702 | Train 43 | Test Asian 14 | 0.75 | 0.73 | 0.61 | 3726 | Train 59 | Test Asian 14 | 0.72 | 0.71 | 0.59 |
| 2703 | Train 43 | Test Asian 15 | 0.74 | 0.75 | 0.66 | 3727 | Train 59 | Test Asian 15 | 0.72 | 0.71 | 0.61 |
| 2704 | Train 43 | Test Asian 16 | 0.75 | 0.75 | 0.66 | 3728 | Train 59 | Test Asian 16 | 0.73 | 0.71 | 0.59 |
| 2705 | Train 43 | Test Asian 17 | 0.8 | 0.91 | 0.9 | 3729 | Train 59 | Test Asian 17 | 0.75 | 0.81 | 0.7 |
| 2706 | Train 43 | Test Asian 18 | 0.8 | 0.89 | 0.86 | 3730 | Train 59 | Test Asian 18 | 0.75 | 0.82 | 0.69 |
| 2707 | Train 43 | Test Asian 19 | 0.8 | 0.91 | 0.9 | 3731 | Train 59 | Test Asian 19 | 0.75 | 0.81 | 0.7 |
| 2708 | Train 43 | Test Asian 20 | 0.8 | 0.89 | 0.86 | 3732 | Train 59 | Test Asian 20 | 0.75 | 0.82 | 0.69 |
| 2709 | Train 43 | Test Asian 21 | 0.75 | 0.75 | 0.65 | 3733 | Train 59 | Test Asian 21 | 0.73 | 0.71 | 0.6 |
| 2710 | Train 43 | Test Asian 22 | 0.75 | 0.75 | 0.66 | 3734 | Train 59 | Test Asian 22 | 0.73 | 0.71 | 0.59 |
| 2711 | Train 43 | Test Asian 23 | 0.75 | 0.75 | 0.65 | 3735 | Train 59 | Test Asian 23 | 0.73 | 0.71 | 0.6 |
| 2712 | Train 43 | Test Asian 24 | 0.75 | 0.75 | 0.66 | 3736 | Train 59 | Test Asian 24 | 0.73 | 0.71 | 0.59 |
| 2713 | Train 43 | Test Asian 25 | 0.79 | 0.92 | 0.92 | 3737 | Train 59 | Test Asian 25 | 0.74 | 0.81 | 0.72 |
| 2714 | Train 43 | Test Asian 26 | 0.79 | 0.89 | 0.87 | 3738 | Train 59 | Test Asian 26 | 0.74 | 0.82 | 0.7 |
| 2715 | Train 43 | Test Asian 27 | 0.79 | 0.92 | 0.92 | 3739 | Train 59 | Test Asian 27 | 0.74 | 0.81 | 0.72 |
| 2716 | Train 43 | Test Asian 28 | 0.79 | 0.89 | 0.87 | 3740 | Train 59 | Test Asian 28 | 0.74 | 0.82 | 0.7 |
| 2717 | Train 43 | Test Asian 29 | 0.75 | 0.76 | 0.66 | 3741 | Train 59 | Test Asian 29 | 0.73 | 0.71 | 0.61 |
| 2718 | Train 43 | Test Asian 30 | 0.75 | 0.73 | 0.61 | 3742 | Train 59 | Test Asian 30 | 0.73 | 0.71 | 0.59 |
| 2719 | Train 43 | Test Asian 31 | 0.74 | 0.75 | 0.66 | 3743 | Train 59 | Test Asian 31 | 0.72 | 0.71 | 0.61 |
| 2720 | Train 43 | Test Asian 32 | 0.75 | 0.75 | 0.66 | 3744 | Train 59 | Test Asian 32 | 0.73 | 0.71 | 0.59 |
| 2721 | Train 43 | Test Asian 33 | 0.8 | 0.91 | 0.9 | 3745 | Train 59 | Test Asian 33 | 0.75 | 0.81 | 0.7 |
| 2722 | Train 43 | Test Asian 34 | 0.8 | 0.89 | 0.86 | 3746 | Train 59 | Test Asian 34 | 0.75 | 0.82 | 0.69 |
| 2723 | Train 43 | Test Asian 35 | 0.8 | 0.91 | 0.9 | 3747 | Train 59 | Test Asian 35 | 0.75 | 0.81 | 0.7 |
| 2724 | Train 43 | Test Asian 36 | 0.8 | 0.89 | 0.86 | 3748 | Train 59 | Test Asian 36 | 0.75 | 0.82 | 0.69 |
| 2725 | Train 43 | Test Asian 37 | 0.75 | 0.75 | 0.65 | 3749 | Train 59 | Test Asian 37 | 0.73 | 0.71 | 0.59 |
| 2726 | Train 43 | Test Asian 38 | 0.75 | 0.75 | 0.66 | 3750 | Train 59 | Test Asian 38 | 0.72 | 0.71 | 0.59 |
| 2727 | Train 43 | Test Asian 39 | 0.74 | 0.75 | 0.65 | 3751 | Train 59 | Test Asian 39 | 0.73 | 0.71 | 0.59 |
| 2728 | Train 43 | Test Asian 40 | 0.75 | 0.74 | 0.66 | 3752 | Train 59 | Test Asian 40 | 0.73 | 0.71 | 0.59 |
| 2729 | Train 43 | Test Asian 41 | 0.79 | 0.92 | 0.92 | 3753 | Train 59 | Test Asian 41 | 0.74 | 0.81 | 0.72 |
| 2730 | Train 43 | Test Asian 42 | 0.79 | 0.89 | 0.87 | 3754 | Train 59 | Test Asian 42 | 0.74 | 0.82 | 0.7 |
| 2731 | Train 43 | Test Asian 43 | 0.79 | 0.92 | 0.92 | 3755 | Train 59 | Test Asian 43 | 0.74 | 0.81 | 0.72 |
| 2732 | Train 43 | Test Asian 44 | 0.79 | 0.89 | 0.87 | 3756 | Train 59 | Test Asian 44 | 0.74 | 0.82 | 0.7 |
| 2733 | Train 43 | Test Asian 45 | 0.74 | 0.75 | 0.66 | 3757 | Train 59 | Test Asian 45 | 0.73 | 0.71 | 0.61 |
| 2734 | Train 43 | Test Asian 46 | 0.75 | 0.75 | 0.66 | 3758 | Train 59 | Test Asian 46 | 0.72 | 0.71 | 0.59 |
| 2735 | Train 43 | Test Asian 47 | 0.74 | 0.75 | 0.66 | 3759 | Train 59 | Test Asian 47 | 0.72 | 0.71 | 0.61 |
| 2736 | Train 43 | Test Asian 48 | 0.75 | 0.74 | 0.66 | 3760 | Train 59 | Test Asian 48 | 0.73 | 0.71 | 0.59 |
| 2737 | Train 43 | Test Asian 49 | 0.77 | 0.86 | 0.85 | 3761 | Train 59 | Test Asian 49 | 0.77 | 0.91 | 0.87 |
| 2738 | Train 43 | Test Asian 50 | 0.77 | 0.86 | 0.84 | 3762 | Train 59 | Test Asian 50 | 0.77 | 0.91 | 0.87 |
| 2739 | Train 43 | Test Asian 51 | 0.77 | 0.86 | 0.85 | 3763 | Train 59 | Test Asian 51 | 0.77 | 0.91 | 0.87 |
| 2740 | Train 43 | Test Asian 52 | 0.77 | 0.86 | 0.84 | 3764 | Train 59 | Test Asian 52 | 0.77 | 0.91 | 0.87 |
| 2741 | Train 43 | Test Asian 53 | 0.79 | 0.81 | 0.61 | 3765 | Train 59 | Test Asian 53 | 0.77 | 0.74 | 0.63 |
| 2742 | Train 43 | Test Asian 54 | 0.78 | 0.74 | 0.6 | 3766 | Train 59 | Test Asian 54 | 0.74 | 0.74 | 0.62 |
| 2743 | Train 43 | Test Asian 55 | 0.79 | 0.8 | 0.61 | 3767 | Train 59 | Test Asian 55 | 0.76 | 0.74 | 0.63 |
| 2744 | Train 43 | Test Asian 56 | 0.78 | 0.74 | 0.61 | 3768 | Train 59 | Test Asian 56 | 0.75 | 0.73 | 0.62 |
| 2745 | Train 43 | Test Asian 57 | 0.76 | 0.87 | 0.89 | 3769 | Train 59 | Test Asian 57 | 0.76 | 0.93 | 0.96 |
| 2746 | Train 43 | Test Asian 58 | 0.76 | 0.88 | 0.89 | 3770 | Train 59 | Test Asian 58 | 0.76 | 0.93 | 0.96 |
| 2747 | Train 43 | Test Asian 59 | 0.76 | 0.87 | 0.89 | 3771 | Train 59 | Test Asian 59 | 0.76 | 0.93 | 0.96 |
| 2748 | Train 43 | Test Asian 60 | 0.76 | 0.88 | 0.89 | 3772 | Train 59 | Test Asian 60 | 0.76 | 0.93 | 0.96 |
| 2749 | Train 43 | Test Asian 61 | 0.79 | 0.8 | 0.63 | 3773 | Train 59 | Test Asian 61 | 0.76 | 0.75 | 0.67 |
| 2750 | Train 43 | Test Asian 62 | 0.78 | 0.75 | 0.61 | 3774 | Train 59 | Test Asian 62 | 0.74 | 0.74 | 0.62 |
| 2751 | Train 43 | Test Asian 63 | 0.78 | 0.8 | 0.62 | 3775 | Train 59 | Test Asian 63 | 0.76 | 0.75 | 0.67 |
| 2752 | Train 43 | Test Asian 64 | 0.78 | 0.74 | 0.61 | 3776 | Train 59 | Test Asian 64 | 0.75 | 0.73 | 0.62 |
| 2753 | Train 44 | Test Asian 1 | 0.79 | 0.9 | 0.88 | 3777 | Train 60 | Test Asian 1 | 0.75 | 0.75 | 0.77 |
| 2754 | Train 44 | Test Asian 2 | 0.81 | 0.91 | 0.89 | 3778 | Train 60 | Test Asian 2 | 0.76 | 0.78 | 0.72 |
| 2755 | Train 44 | Test Asian 3 | 0.79 | 0.9 | 0.88 | 3779 | Train 60 | Test Asian 3 | 0.75 | 0.75 | 0.77 |
| 2756 | Train 44 | Test Asian 4 | 0.81 | 0.91 | 0.89 | 3780 | Train 60 | Test Asian 4 | 0.76 | 0.78 | 0.72 |
| 2757 | Train 44 | Test Asian 5 | 0.75 | 0.76 | 0.71 | 3781 | Train 60 | Test Asian 5 | 0.73 | 0.68 | 0.6 |
| 2758 | Train 44 | Test Asian 6 | 0.75 | 0.75 | 0.72 | 3782 | Train 60 | Test Asian 6 | 0.73 | 0.69 | 0.61 |
| 2759 | Train 44 | Test Asian 7 | 0.75 | 0.76 | 0.71 | 3783 | Train 60 | Test Asian 7 | 0.73 | 0.68 | 0.6 |
| 2760 | Train 44 | Test Asian 8 | 0.75 | 0.75 | 0.72 | 3784 | Train 60 | Test Asian 8 | 0.73 | 0.69 | 0.61 |
| 2761 | Train 44 | Test Asian 9 | 0.78 | 0.91 | 0.92 | 3785 | Train 60 | Test Asian 9 | 0.74 | 0.76 | 0.78 |
| 2762 | Train 44 | Test Asian 10 | 0.8 | 0.91 | 0.93 | 3786 | Train 60 | Test Asian 10 | 0.75 | 0.78 | 0.72 |
| 2763 | Train 44 | Test Asian 11 | 0.78 | 0.91 | 0.92 | 3787 | Train 60 | Test Asian 11 | 0.74 | 0.76 | 0.78 |
| 2764 | Train 44 | Test Asian 12 | 0.8 | 0.91 | 0.93 | 3788 | Train 60 | Test Asian 12 | 0.75 | 0.78 | 0.72 |
| 2765 | Train 44 | Test Asian 13 | 0.75 | 0.76 | 0.73 | 3789 | Train 60 | Test Asian 13 | 0.72 | 0.68 | 0.61 |
| 2766 | Train 44 | Test Asian 14 | 0.75 | 0.74 | 0.7 | 3790 | Train 60 | Test Asian 14 | 0.73 | 0.69 | 0.6 |
| 2767 | Train 44 | Test Asian 15 | 0.75 | 0.76 | 0.73 | 3791 | Train 60 | Test Asian 15 | 0.72 | 0.68 | 0.61 |
| 2768 | Train 44 | Test Asian 16 | 0.75 | 0.75 | 0.72 | 3792 | Train 60 | Test Asian 16 | 0.73 | 0.69 | 0.6 |
| 2769 | Train 44 | Test Asian 17 | 0.79 | 0.9 | 0.88 | 3793 | Train 60 | Test Asian 17 | 0.75 | 0.75 | 0.77 |
| 2770 | Train 44 | Test Asian 18 | 0.81 | 0.91 | 0.89 | 3794 | Train 60 | Test Asian 18 | 0.76 | 0.78 | 0.72 |
| 2771 | Train 44 | Test Asian 19 | 0.79 | 0.9 | 0.88 | 3795 | Train 60 | Test Asian 19 | 0.75 | 0.75 | 0.77 |
| 2772 | Train 44 | Test Asian 20 | 0.81 | 0.91 | 0.89 | 3796 | Train 60 | Test Asian 20 | 0.76 | 0.78 | 0.72 |
| 2773 | Train 44 | Test Asian 21 | 0.76 | 0.76 | 0.71 | 3797 | Train 60 | Test Asian 21 | 0.73 | 0.68 | 0.6 |
| 2774 | Train 44 | Test Asian 22 | 0.75 | 0.75 | 0.72 | 3798 | Train 60 | Test Asian 22 | 0.73 | 0.69 | 0.61 |
| 2775 | Train 44 | Test Asian 23 | 0.75 | 0.76 | 0.71 | 3799 | Train 60 | Test Asian 23 | 0.73 | 0.68 | 0.6 |
| 2776 | Train 44 | Test Asian 24 | 0.75 | 0.75 | 0.72 | 3800 | Train 60 | Test Asian 24 | 0.73 | 0.69 | 0.61 |
| 2777 | Train 44 | Test Asian 25 | 0.78 | 0.91 | 0.92 | 3801 | Train 60 | Test Asian 25 | 0.74 | 0.76 | 0.78 |
| 2778 | Train 44 | Test Asian 26 | 0.8 | 0.91 | 0.93 | 3802 | Train 60 | Test Asian 26 | 0.75 | 0.78 | 0.72 |
| 2779 | Train 44 | Test Asian 27 | 0.78 | 0.91 | 0.92 | 3803 | Train 60 | Test Asian 27 | 0.74 | 0.76 | 0.78 |
| 2780 | Train 44 | Test Asian 28 | 0.8 | 0.91 | 0.93 | 3804 | Train 60 | Test Asian 28 | 0.75 | 0.78 | 0.72 |
| 2781 | Train 44 | Test Asian 29 | 0.75 | 0.76 | 0.73 | 3805 | Train 60 | Test Asian 29 | 0.72 | 0.68 | 0.61 |
| 2782 | Train 44 | Test Asian 30 | 0.75 | 0.74 | 0.7 | 3806 | Train 60 | Test Asian 30 | 0.73 | 0.69 | 0.61 |
| 2783 | Train 44 | Test Asian 31 | 0.75 | 0.76 | 0.73 | 3807 | Train 60 | Test Asian 31 | 0.72 | 0.68 | 0.61 |
| 2784 | Train 44 | Test Asian 32 | 0.75 | 0.75 | 0.72 | 3808 | Train 60 | Test Asian 32 | 0.73 | 0.69 | 0.6 |
| 2785 | Train 44 | Test Asian 33 | 0.79 | 0.9 | 0.88 | 3809 | Train 60 | Test Asian 33 | 0.75 | 0.75 | 0.77 |
| 2786 | Train 44 | Test Asian 34 | 0.81 | 0.91 | 0.89 | 3810 | Train 60 | Test Asian 34 | 0.75 | 0.77 | 0.72 |
| 2787 | Train 44 | Test Asian 35 | 0.79 | 0.9 | 0.88 | 3811 | Train 60 | Test Asian 35 | 0.75 | 0.75 | 0.77 |
| 2788 | Train 44 | Test Asian 36 | 0.81 | 0.91 | 0.89 | 3812 | Train 60 | Test Asian 36 | 0.75 | 0.77 | 0.72 |
| 2789 | Train 44 | Test Asian 37 | 0.75 | 0.76 | 0.71 | 3813 | Train 60 | Test Asian 37 | 0.73 | 0.68 | 0.6 |
| 2790 | Train 44 | Test Asian 38 | 0.74 | 0.75 | 0.72 | 3814 | Train 60 | Test Asian 38 | 0.73 | 0.69 | 0.61 |
| 2791 | Train 44 | Test Asian 39 | 0.75 | 0.76 | 0.7 | 3815 | Train 60 | Test Asian 39 | 0.73 | 0.68 | 0.6 |
| 2792 | Train 44 | Test Asian 40 | 0.74 | 0.75 | 0.72 | 3816 | Train 60 | Test Asian 40 | 0.73 | 0.69 | 0.61 |
| 2793 | Train 44 | Test Asian 41 | 0.78 | 0.91 | 0.92 | 3817 | Train 60 | Test Asian 41 | 0.74 | 0.76 | 0.78 |
| 2794 | Train 44 | Test Asian 42 | 0.8 | 0.91 | 0.92 | 3818 | Train 60 | Test Asian 42 | 0.75 | 0.78 | 0.72 |
| 2795 | Train 44 | Test Asian 43 | 0.78 | 0.91 | 0.92 | 3819 | Train 60 | Test Asian 43 | 0.74 | 0.76 | 0.78 |
| 2796 | Train 44 | Test Asian 44 | 0.8 | 0.91 | 0.92 | 3820 | Train 60 | Test Asian 44 | 0.75 | 0.78 | 0.72 |
| 2797 | Train 44 | Test Asian 45 | 0.75 | 0.76 | 0.73 | 3821 | Train 60 | Test Asian 45 | 0.72 | 0.68 | 0.61 |
| 2798 | Train 44 | Test Asian 46 | 0.74 | 0.75 | 0.72 | 3822 | Train 60 | Test Asian 46 | 0.73 | 0.69 | 0.61 |
| 2799 | Train 44 | Test Asian 47 | 0.75 | 0.76 | 0.73 | 3823 | Train 60 | Test Asian 47 | 0.72 | 0.68 | 0.61 |
| 2800 | Train 44 | Test Asian 48 | 0.75 | 0.75 | 0.72 | 3824 | Train 60 | Test Asian 48 | 0.73 | 0.69 | 0.6 |
| 2801 | Train 44 | Test Asian 49 | 0.77 | 0.85 | 0.8 | 3825 | Train 60 | Test Asian 49 | 0.77 | 0.91 | 0.87 |
| 2802 | Train 44 | Test Asian 50 | 0.77 | 0.85 | 0.8 | 3826 | Train 60 | Test Asian 50 | 0.77 | 0.91 | 0.88 |
| 2803 | Train 44 | Test Asian 51 | 0.77 | 0.85 | 0.8 | 3827 | Train 60 | Test Asian 51 | 0.77 | 0.91 | 0.87 |
| 2804 | Train 44 | Test Asian 52 | 0.77 | 0.85 | 0.8 | 3828 | Train 60 | Test Asian 52 | 0.77 | 0.91 | 0.88 |
| 2805 | Train 44 | Test Asian 53 | 0.8 | 0.81 | 0.61 | 3829 | Train 60 | Test Asian 53 | 0.76 | 0.68 | 0.73 |
| 2806 | Train 44 | Test Asian 54 | 0.77 | 0.74 | 0.68 | 3830 | Train 60 | Test Asian 54 | 0.74 | 0.69 | 0.7 |
| 2807 | Train 44 | Test Asian 55 | 0.79 | 0.8 | 0.62 | 3831 | Train 60 | Test Asian 55 | 0.76 | 0.68 | 0.73 |
| 2808 | Train 44 | Test Asian 56 | 0.76 | 0.73 | 0.68 | 3832 | Train 60 | Test Asian 56 | 0.75 | 0.68 | 0.69 |
| 2809 | Train 44 | Test Asian 57 | 0.76 | 0.86 | 0.91 | 3833 | Train 60 | Test Asian 57 | 0.76 | 0.93 | 0.97 |
| 2810 | Train 44 | Test Asian 58 | 0.76 | 0.86 | 0.91 | 3834 | Train 60 | Test Asian 58 | 0.76 | 0.93 | 0.97 |
| 2811 | Train 44 | Test Asian 59 | 0.76 | 0.86 | 0.91 | 3835 | Train 60 | Test Asian 59 | 0.76 | 0.93 | 0.97 |
| 2812 | Train 44 | Test Asian 60 | 0.76 | 0.86 | 0.91 | 3836 | Train 60 | Test Asian 60 | 0.76 | 0.93 | 0.97 |
| 2813 | Train 44 | Test Asian 61 | 0.79 | 0.81 | 0.62 | 3837 | Train 60 | Test Asian 61 | 0.76 | 0.68 | 0.75 |
| 2814 | Train 44 | Test Asian 62 | 0.77 | 0.74 | 0.69 | 3838 | Train 60 | Test Asian 62 | 0.74 | 0.7 | 0.7 |
| 2815 | Train 44 | Test Asian 63 | 0.78 | 0.8 | 0.63 | 3839 | Train 60 | Test Asian 63 | 0.76 | 0.68 | 0.74 |
| 2816 | Train 44 | Test Asian 64 | 0.76 | 0.73 | 0.69 | 3840 | Train 60 | Test Asian 64 | 0.75 | 0.68 | 0.69 |
| 2817 | Train 45 | Test Asian 1 | 0.78 | 0.86 | 0.86 | 3841 | Train 61 | Test Asian 1 | 0.74 | 0.8 | 0.65 |
| 2818 | Train 45 | Test Asian 2 | 0.8 | 0.86 | 0.85 | 3842 | Train 61 | Test Asian 2 | 0.75 | 0.8 | 0.66 |
| 2819 | Train 45 | Test Asian 3 | 0.78 | 0.86 | 0.86 | 3843 | Train 61 | Test Asian 3 | 0.74 | 0.8 | 0.65 |
| 2820 | Train 45 | Test Asian 4 | 0.8 | 0.86 | 0.85 | 3844 | Train 61 | Test Asian 4 | 0.75 | 0.8 | 0.66 |
| 2821 | Train 45 | Test Asian 5 | 0.76 | 0.84 | 0.84 | 3845 | Train 61 | Test Asian 5 | 0.73 | 0.78 | 0.65 |
| 2822 | Train 45 | Test Asian 6 | 0.75 | 0.79 | 0.77 | 3846 | Train 61 | Test Asian 6 | 0.72 | 0.72 | 0.62 |
| 2823 | Train 45 | Test Asian 7 | 0.76 | 0.84 | 0.83 | 3847 | Train 61 | Test Asian 7 | 0.73 | 0.78 | 0.65 |
| 2824 | Train 45 | Test Asian 8 | 0.76 | 0.78 | 0.77 | 3848 | Train 61 | Test Asian 8 | 0.72 | 0.72 | 0.62 |
| 2825 | Train 45 | Test Asian 9 | 0.77 | 0.87 | 0.87 | 3849 | Train 61 | Test Asian 9 | 0.73 | 0.81 | 0.63 |
| 2826 | Train 45 | Test Asian 10 | 0.79 | 0.86 | 0.86 | 3850 | Train 61 | Test Asian 10 | 0.75 | 0.79 | 0.63 |
| 2827 | Train 45 | Test Asian 11 | 0.77 | 0.87 | 0.87 | 3851 | Train 61 | Test Asian 11 | 0.73 | 0.81 | 0.63 |
| 2828 | Train 45 | Test Asian 12 | 0.79 | 0.86 | 0.86 | 3852 | Train 61 | Test Asian 12 | 0.75 | 0.79 | 0.63 |
| 2829 | Train 45 | Test Asian 13 | 0.76 | 0.85 | 0.85 | 3853 | Train 61 | Test Asian 13 | 0.73 | 0.78 | 0.66 |
| 2830 | Train 45 | Test Asian 14 | 0.76 | 0.77 | 0.72 | 3854 | Train 61 | Test Asian 14 | 0.72 | 0.72 | 0.66 |
| 2831 | Train 45 | Test Asian 15 | 0.76 | 0.84 | 0.84 | 3855 | Train 61 | Test Asian 15 | 0.73 | 0.78 | 0.66 |
| 2832 | Train 45 | Test Asian 16 | 0.76 | 0.78 | 0.77 | 3856 | Train 61 | Test Asian 16 | 0.72 | 0.72 | 0.61 |
| 2833 | Train 45 | Test Asian 17 | 0.78 | 0.86 | 0.86 | 3857 | Train 61 | Test Asian 17 | 0.74 | 0.8 | 0.65 |
| 2834 | Train 45 | Test Asian 18 | 0.8 | 0.86 | 0.85 | 3858 | Train 61 | Test Asian 18 | 0.75 | 0.8 | 0.66 |
| 2835 | Train 45 | Test Asian 19 | 0.78 | 0.86 | 0.86 | 3859 | Train 61 | Test Asian 19 | 0.74 | 0.8 | 0.65 |
| 2836 | Train 45 | Test Asian 20 | 0.8 | 0.86 | 0.85 | 3860 | Train 61 | Test Asian 20 | 0.75 | 0.8 | 0.66 |
| 2837 | Train 45 | Test Asian 21 | 0.77 | 0.84 | 0.84 | 3861 | Train 61 | Test Asian 21 | 0.73 | 0.78 | 0.65 |
| 2838 | Train 45 | Test Asian 22 | 0.76 | 0.79 | 0.77 | 3862 | Train 61 | Test Asian 22 | 0.72 | 0.72 | 0.62 |
| 2839 | Train 45 | Test Asian 23 | 0.76 | 0.84 | 0.83 | 3863 | Train 61 | Test Asian 23 | 0.73 | 0.78 | 0.65 |
| 2840 | Train 45 | Test Asian 24 | 0.76 | 0.79 | 0.77 | 3864 | Train 61 | Test Asian 24 | 0.72 | 0.72 | 0.62 |
| 2841 | Train 45 | Test Asian 25 | 0.77 | 0.87 | 0.87 | 3865 | Train 61 | Test Asian 25 | 0.73 | 0.81 | 0.63 |
| 2842 | Train 45 | Test Asian 26 | 0.79 | 0.86 | 0.86 | 3866 | Train 61 | Test Asian 26 | 0.75 | 0.8 | 0.63 |
| 2843 | Train 45 | Test Asian 27 | 0.77 | 0.87 | 0.87 | 3867 | Train 61 | Test Asian 27 | 0.73 | 0.81 | 0.63 |
| 2844 | Train 45 | Test Asian 28 | 0.79 | 0.86 | 0.86 | 3868 | Train 61 | Test Asian 28 | 0.75 | 0.8 | 0.63 |
| 2845 | Train 45 | Test Asian 29 | 0.76 | 0.85 | 0.85 | 3869 | Train 61 | Test Asian 29 | 0.73 | 0.78 | 0.66 |
| 2846 | Train 45 | Test Asian 30 | 0.76 | 0.77 | 0.72 | 3870 | Train 61 | Test Asian 30 | 0.72 | 0.72 | 0.67 |
| 2847 | Train 45 | Test Asian 31 | 0.76 | 0.84 | 0.84 | 3871 | Train 61 | Test Asian 31 | 0.73 | 0.78 | 0.66 |
| 2848 | Train 45 | Test Asian 32 | 0.76 | 0.78 | 0.77 | 3872 | Train 61 | Test Asian 32 | 0.72 | 0.72 | 0.61 |
| 2849 | Train 45 | Test Asian 33 | 0.78 | 0.86 | 0.86 | 3873 | Train 61 | Test Asian 33 | 0.74 | 0.8 | 0.65 |
| 2850 | Train 45 | Test Asian 34 | 0.8 | 0.86 | 0.85 | 3874 | Train 61 | Test Asian 34 | 0.75 | 0.8 | 0.66 |
| 2851 | Train 45 | Test Asian 35 | 0.78 | 0.86 | 0.86 | 3875 | Train 61 | Test Asian 35 | 0.74 | 0.8 | 0.65 |
| 2852 | Train 45 | Test Asian 36 | 0.8 | 0.86 | 0.85 | 3876 | Train 61 | Test Asian 36 | 0.75 | 0.8 | 0.66 |
| 2853 | Train 45 | Test Asian 37 | 0.76 | 0.84 | 0.83 | 3877 | Train 61 | Test Asian 37 | 0.73 | 0.78 | 0.65 |
| 2854 | Train 45 | Test Asian 38 | 0.75 | 0.79 | 0.77 | 3878 | Train 61 | Test Asian 38 | 0.72 | 0.72 | 0.61 |
| 2855 | Train 45 | Test Asian 39 | 0.76 | 0.84 | 0.83 | 3879 | Train 61 | Test Asian 39 | 0.73 | 0.77 | 0.65 |
| 2856 | Train 45 | Test Asian 40 | 0.75 | 0.78 | 0.77 | 3880 | Train 61 | Test Asian 40 | 0.72 | 0.72 | 0.61 |
| 2857 | Train 45 | Test Asian 41 | 0.77 | 0.87 | 0.87 | 3881 | Train 61 | Test Asian 41 | 0.73 | 0.81 | 0.63 |
| 2858 | Train 45 | Test Asian 42 | 0.8 | 0.86 | 0.86 | 3882 | Train 61 | Test Asian 42 | 0.75 | 0.8 | 0.63 |
| 2859 | Train 45 | Test Asian 43 | 0.77 | 0.87 | 0.87 | 3883 | Train 61 | Test Asian 43 | 0.73 | 0.81 | 0.63 |
| 2860 | Train 45 | Test Asian 44 | 0.8 | 0.86 | 0.86 | 3884 | Train 61 | Test Asian 44 | 0.75 | 0.8 | 0.63 |
| 2861 | Train 45 | Test Asian 45 | 0.76 | 0.85 | 0.85 | 3885 | Train 61 | Test Asian 45 | 0.73 | 0.78 | 0.66 |
| 2862 | Train 45 | Test Asian 46 | 0.75 | 0.79 | 0.77 | 3886 | Train 61 | Test Asian 46 | 0.72 | 0.72 | 0.61 |
| 2863 | Train 45 | Test Asian 47 | 0.76 | 0.84 | 0.84 | 3887 | Train 61 | Test Asian 47 | 0.72 | 0.78 | 0.66 |
| 2864 | Train 45 | Test Asian 48 | 0.76 | 0.78 | 0.77 | 3888 | Train 61 | Test Asian 48 | 0.72 | 0.72 | 0.61 |
| 2865 | Train 45 | Test Asian 49 | 0.76 | 0.83 | 0.85 | 3889 | Train 61 | Test Asian 49 | 0.76 | 0.87 | 0.75 |
| 2866 | Train 45 | Test Asian 50 | 0.76 | 0.83 | 0.85 | 3890 | Train 61 | Test Asian 50 | 0.76 | 0.87 | 0.75 |
| 2867 | Train 45 | Test Asian 51 | 0.76 | 0.83 | 0.85 | 3891 | Train 61 | Test Asian 51 | 0.76 | 0.87 | 0.75 |
| 2868 | Train 45 | Test Asian 52 | 0.76 | 0.83 | 0.85 | 3892 | Train 61 | Test Asian 52 | 0.76 | 0.87 | 0.75 |
| 2869 | Train 45 | Test Asian 53 | 0.8 | 0.86 | 0.8 | 3893 | Train 61 | Test Asian 53 | 0.8 | 0.9 | 0.85 |
| 2870 | Train 45 | Test Asian 54 | 0.77 | 0.76 | 0.71 | 3894 | Train 61 | Test Asian 54 | 0.77 | 0.77 | 0.7 |
| 2871 | Train 45 | Test Asian 55 | 0.8 | 0.86 | 0.79 | 3895 | Train 61 | Test Asian 55 | 0.8 | 0.89 | 0.84 |
| 2872 | Train 45 | Test Asian 56 | 0.77 | 0.76 | 0.7 | 3896 | Train 61 | Test Asian 56 | 0.77 | 0.76 | 0.7 |
| 2873 | Train 45 | Test Asian 57 | 0.75 | 0.84 | 0.86 | 3897 | Train 61 | Test Asian 57 | 0.74 | 0.89 | 0.83 |
| 2874 | Train 45 | Test Asian 58 | 0.75 | 0.84 | 0.86 | 3898 | Train 61 | Test Asian 58 | 0.74 | 0.89 | 0.82 |
| 2875 | Train 45 | Test Asian 59 | 0.75 | 0.84 | 0.86 | 3899 | Train 61 | Test Asian 59 | 0.74 | 0.89 | 0.83 |
| 2876 | Train 45 | Test Asian 60 | 0.75 | 0.84 | 0.86 | 3900 | Train 61 | Test Asian 60 | 0.74 | 0.89 | 0.82 |
| 2877 | Train 45 | Test Asian 61 | 0.79 | 0.86 | 0.8 | 3901 | Train 61 | Test Asian 61 | 0.8 | 0.9 | 0.87 |
| 2878 | Train 45 | Test Asian 62 | 0.77 | 0.76 | 0.71 | 3902 | Train 61 | Test Asian 62 | 0.77 | 0.77 | 0.7 |
| 2879 | Train 45 | Test Asian 63 | 0.79 | 0.86 | 0.79 | 3903 | Train 61 | Test Asian 63 | 0.79 | 0.89 | 0.87 |
| 2880 | Train 45 | Test Asian 64 | 0.77 | 0.76 | 0.7 | 3904 | Train 61 | Test Asian 64 | 0.77 | 0.76 | 0.7 |
| 2881 | Train 46 | Test Asian 1 | 0.76 | 0.84 | 0.81 | 3905 | Train 62 | Test Asian 1 | 0.72 | 0.78 | 0.75 |
| 2882 | Train 46 | Test Asian 2 | 0.79 | 0.86 | 0.83 | 3906 | Train 62 | Test Asian 2 | 0.75 | 0.8 | 0.76 |
| 2883 | Train 46 | Test Asian 3 | 0.76 | 0.84 | 0.81 | 3907 | Train 62 | Test Asian 3 | 0.72 | 0.78 | 0.75 |
| 2884 | Train 46 | Test Asian 4 | 0.79 | 0.86 | 0.83 | 3908 | Train 62 | Test Asian 4 | 0.75 | 0.8 | 0.76 |
| 2885 | Train 46 | Test Asian 5 | 0.76 | 0.8 | 0.79 | 3909 | Train 62 | Test Asian 5 | 0.74 | 0.75 | 0.71 |
| 2886 | Train 46 | Test Asian 6 | 0.76 | 0.82 | 0.79 | 3910 | Train 62 | Test Asian 6 | 0.75 | 0.78 | 0.75 |
| 2887 | Train 46 | Test Asian 7 | 0.75 | 0.8 | 0.78 | 3911 | Train 62 | Test Asian 7 | 0.74 | 0.75 | 0.71 |
| 2888 | Train 46 | Test Asian 8 | 0.76 | 0.81 | 0.79 | 3912 | Train 62 | Test Asian 8 | 0.75 | 0.78 | 0.75 |
| 2889 | Train 46 | Test Asian 9 | 0.75 | 0.84 | 0.81 | 3913 | Train 62 | Test Asian 9 | 0.71 | 0.78 | 0.75 |
| 2890 | Train 46 | Test Asian 10 | 0.78 | 0.86 | 0.83 | 3914 | Train 62 | Test Asian 10 | 0.74 | 0.8 | 0.76 |
| 2891 | Train 46 | Test Asian 11 | 0.75 | 0.84 | 0.81 | 3915 | Train 62 | Test Asian 11 | 0.71 | 0.78 | 0.75 |
| 2892 | Train 46 | Test Asian 12 | 0.78 | 0.86 | 0.83 | 3916 | Train 62 | Test Asian 12 | 0.74 | 0.8 | 0.76 |
| 2893 | Train 46 | Test Asian 13 | 0.75 | 0.81 | 0.79 | 3917 | Train 62 | Test Asian 13 | 0.74 | 0.75 | 0.72 |
| 2894 | Train 46 | Test Asian 14 | 0.76 | 0.83 | 0.83 | 3918 | Train 62 | Test Asian 14 | 0.75 | 0.79 | 0.76 |
| 2895 | Train 46 | Test Asian 15 | 0.75 | 0.8 | 0.78 | 3919 | Train 62 | Test Asian 15 | 0.74 | 0.75 | 0.72 |
| 2896 | Train 46 | Test Asian 16 | 0.76 | 0.81 | 0.79 | 3920 | Train 62 | Test Asian 16 | 0.75 | 0.77 | 0.75 |
| 2897 | Train 46 | Test Asian 17 | 0.76 | 0.84 | 0.81 | 3921 | Train 62 | Test Asian 17 | 0.72 | 0.78 | 0.75 |
| 2898 | Train 46 | Test Asian 18 | 0.79 | 0.86 | 0.83 | 3922 | Train 62 | Test Asian 18 | 0.75 | 0.8 | 0.76 |
| 2899 | Train 46 | Test Asian 19 | 0.76 | 0.84 | 0.81 | 3923 | Train 62 | Test Asian 19 | 0.72 | 0.78 | 0.75 |
| 2900 | Train 46 | Test Asian 20 | 0.79 | 0.86 | 0.83 | 3924 | Train 62 | Test Asian 20 | 0.75 | 0.8 | 0.76 |
| 2901 | Train 46 | Test Asian 21 | 0.76 | 0.8 | 0.79 | 3925 | Train 62 | Test Asian 21 | 0.74 | 0.75 | 0.71 |
| 2902 | Train 46 | Test Asian 22 | 0.77 | 0.82 | 0.79 | 3926 | Train 62 | Test Asian 22 | 0.75 | 0.78 | 0.75 |
| 2903 | Train 46 | Test Asian 23 | 0.75 | 0.8 | 0.78 | 3927 | Train 62 | Test Asian 23 | 0.74 | 0.75 | 0.71 |
| 2904 | Train 46 | Test Asian 24 | 0.77 | 0.81 | 0.79 | 3928 | Train 62 | Test Asian 24 | 0.75 | 0.78 | 0.75 |
| 2905 | Train 46 | Test Asian 25 | 0.75 | 0.84 | 0.81 | 3929 | Train 62 | Test Asian 25 | 0.71 | 0.78 | 0.75 |
| 2906 | Train 46 | Test Asian 26 | 0.78 | 0.86 | 0.83 | 3930 | Train 62 | Test Asian 26 | 0.74 | 0.8 | 0.76 |
| 2907 | Train 46 | Test Asian 27 | 0.75 | 0.84 | 0.81 | 3931 | Train 62 | Test Asian 27 | 0.71 | 0.78 | 0.75 |
| 2908 | Train 46 | Test Asian 28 | 0.78 | 0.86 | 0.83 | 3932 | Train 62 | Test Asian 28 | 0.75 | 0.8 | 0.76 |
| 2909 | Train 46 | Test Asian 29 | 0.75 | 0.81 | 0.79 | 3933 | Train 62 | Test Asian 29 | 0.74 | 0.75 | 0.72 |
| 2910 | Train 46 | Test Asian 30 | 0.76 | 0.83 | 0.83 | 3934 | Train 62 | Test Asian 30 | 0.75 | 0.79 | 0.76 |
| 2911 | Train 46 | Test Asian 31 | 0.75 | 0.8 | 0.78 | 3935 | Train 62 | Test Asian 31 | 0.74 | 0.75 | 0.72 |
| 2912 | Train 46 | Test Asian 32 | 0.76 | 0.81 | 0.79 | 3936 | Train 62 | Test Asian 32 | 0.75 | 0.78 | 0.75 |
| 2913 | Train 46 | Test Asian 33 | 0.76 | 0.84 | 0.81 | 3937 | Train 62 | Test Asian 33 | 0.72 | 0.78 | 0.75 |
| 2914 | Train 46 | Test Asian 34 | 0.78 | 0.86 | 0.83 | 3938 | Train 62 | Test Asian 34 | 0.75 | 0.8 | 0.76 |
| 2915 | Train 46 | Test Asian 35 | 0.76 | 0.84 | 0.81 | 3939 | Train 62 | Test Asian 35 | 0.72 | 0.78 | 0.75 |
| 2916 | Train 46 | Test Asian 36 | 0.78 | 0.86 | 0.83 | 3940 | Train 62 | Test Asian 36 | 0.75 | 0.8 | 0.76 |
| 2917 | Train 46 | Test Asian 37 | 0.75 | 0.8 | 0.78 | 3941 | Train 62 | Test Asian 37 | 0.74 | 0.75 | 0.71 |
| 2918 | Train 46 | Test Asian 38 | 0.76 | 0.82 | 0.79 | 3942 | Train 62 | Test Asian 38 | 0.75 | 0.78 | 0.75 |
| 2919 | Train 46 | Test Asian 39 | 0.75 | 0.8 | 0.78 | 3943 | Train 62 | Test Asian 39 | 0.74 | 0.75 | 0.71 |
| 2920 | Train 46 | Test Asian 40 | 0.76 | 0.81 | 0.79 | 3944 | Train 62 | Test Asian 40 | 0.75 | 0.77 | 0.75 |
| 2921 | Train 46 | Test Asian 41 | 0.75 | 0.84 | 0.81 | 3945 | Train 62 | Test Asian 41 | 0.71 | 0.78 | 0.75 |
| 2922 | Train 46 | Test Asian 42 | 0.78 | 0.86 | 0.83 | 3946 | Train 62 | Test Asian 42 | 0.75 | 0.8 | 0.75 |
| 2923 | Train 46 | Test Asian 43 | 0.75 | 0.84 | 0.81 | 3947 | Train 62 | Test Asian 43 | 0.71 | 0.78 | 0.75 |
| 2924 | Train 46 | Test Asian 44 | 0.78 | 0.86 | 0.83 | 3948 | Train 62 | Test Asian 44 | 0.75 | 0.8 | 0.75 |
| 2925 | Train 46 | Test Asian 45 | 0.75 | 0.8 | 0.79 | 3949 | Train 62 | Test Asian 45 | 0.74 | 0.75 | 0.72 |
| 2926 | Train 46 | Test Asian 46 | 0.76 | 0.82 | 0.79 | 3950 | Train 62 | Test Asian 46 | 0.75 | 0.78 | 0.75 |
| 2927 | Train 46 | Test Asian 47 | 0.75 | 0.8 | 0.78 | 3951 | Train 62 | Test Asian 47 | 0.73 | 0.75 | 0.72 |
| 2928 | Train 46 | Test Asian 48 | 0.76 | 0.81 | 0.78 | 3952 | Train 62 | Test Asian 48 | 0.75 | 0.77 | 0.75 |
| 2929 | Train 46 | Test Asian 49 | 0.77 | 0.84 | 0.78 | 3953 | Train 62 | Test Asian 49 | 0.77 | 0.85 | 0.82 |
| 2930 | Train 46 | Test Asian 50 | 0.77 | 0.84 | 0.78 | 3954 | Train 62 | Test Asian 50 | 0.77 | 0.86 | 0.82 |
| 2931 | Train 46 | Test Asian 51 | 0.77 | 0.84 | 0.78 | 3955 | Train 62 | Test Asian 51 | 0.77 | 0.85 | 0.82 |
| 2932 | Train 46 | Test Asian 52 | 0.77 | 0.84 | 0.78 | 3956 | Train 62 | Test Asian 52 | 0.77 | 0.86 | 0.82 |
| 2933 | Train 46 | Test Asian 53 | 0.8 | 0.85 | 0.76 | 3957 | Train 62 | Test Asian 53 | 0.8 | 0.87 | 0.77 |
| 2934 | Train 46 | Test Asian 54 | 0.79 | 0.83 | 0.83 | 3958 | Train 62 | Test Asian 54 | 0.79 | 0.86 | 0.87 |
| 2935 | Train 46 | Test Asian 55 | 0.79 | 0.84 | 0.75 | 3959 | Train 62 | Test Asian 55 | 0.79 | 0.86 | 0.77 |
| 2936 | Train 46 | Test Asian 56 | 0.78 | 0.83 | 0.83 | 3960 | Train 62 | Test Asian 56 | 0.78 | 0.85 | 0.86 |
| 2937 | Train 46 | Test Asian 57 | 0.76 | 0.85 | 0.81 | 3961 | Train 62 | Test Asian 57 | 0.76 | 0.86 | 0.88 |
| 2938 | Train 46 | Test Asian 58 | 0.76 | 0.85 | 0.81 | 3962 | Train 62 | Test Asian 58 | 0.76 | 0.86 | 0.88 |
| 2939 | Train 46 | Test Asian 59 | 0.76 | 0.85 | 0.81 | 3963 | Train 62 | Test Asian 59 | 0.76 | 0.86 | 0.88 |
| 2940 | Train 46 | Test Asian 60 | 0.76 | 0.85 | 0.81 | 3964 | Train 62 | Test Asian 60 | 0.76 | 0.86 | 0.88 |
| 2941 | Train 46 | Test Asian 61 | 0.79 | 0.85 | 0.78 | 3965 | Train 62 | Test Asian 61 | 0.79 | 0.87 | 0.78 |
| 2942 | Train 46 | Test Asian 62 | 0.79 | 0.83 | 0.84 | 3966 | Train 62 | Test Asian 62 | 0.79 | 0.86 | 0.87 |
| 2943 | Train 46 | Test Asian 63 | 0.78 | 0.84 | 0.77 | 3967 | Train 62 | Test Asian 63 | 0.78 | 0.86 | 0.78 |
| 2944 | Train 46 | Test Asian 64 | 0.78 | 0.83 | 0.83 | 3968 | Train 62 | Test Asian 64 | 0.78 | 0.85 | 0.86 |
| 2945 | Train 47 | Test Asian 1 | 0.78 | 0.86 | 0.85 | 3969 | Train 63 | Test Asian 1 | 0.74 | 0.8 | 0.76 |
| 2946 | Train 47 | Test Asian 2 | 0.8 | 0.86 | 0.84 | 3970 | Train 63 | Test Asian 2 | 0.76 | 0.8 | 0.75 |
| 2947 | Train 47 | Test Asian 3 | 0.78 | 0.86 | 0.85 | 3971 | Train 63 | Test Asian 3 | 0.74 | 0.8 | 0.76 |
| 2948 | Train 47 | Test Asian 4 | 0.8 | 0.86 | 0.84 | 3972 | Train 63 | Test Asian 4 | 0.76 | 0.8 | 0.75 |
| 2949 | Train 47 | Test Asian 5 | 0.76 | 0.84 | 0.84 | 3973 | Train 63 | Test Asian 5 | 0.73 | 0.78 | 0.72 |
| 2950 | Train 47 | Test Asian 6 | 0.75 | 0.78 | 0.77 | 3974 | Train 63 | Test Asian 6 | 0.72 | 0.72 | 0.69 |
| 2951 | Train 47 | Test Asian 7 | 0.76 | 0.84 | 0.83 | 3975 | Train 63 | Test Asian 7 | 0.73 | 0.78 | 0.72 |
| 2952 | Train 47 | Test Asian 8 | 0.75 | 0.78 | 0.77 | 3976 | Train 63 | Test Asian 8 | 0.72 | 0.72 | 0.69 |
| 2953 | Train 47 | Test Asian 9 | 0.77 | 0.87 | 0.86 | 3977 | Train 63 | Test Asian 9 | 0.73 | 0.8 | 0.73 |
| 2954 | Train 47 | Test Asian 10 | 0.79 | 0.86 | 0.85 | 3978 | Train 63 | Test Asian 10 | 0.75 | 0.8 | 0.72 |
| 2955 | Train 47 | Test Asian 11 | 0.77 | 0.87 | 0.86 | 3979 | Train 63 | Test Asian 11 | 0.73 | 0.8 | 0.73 |
| 2956 | Train 47 | Test Asian 12 | 0.79 | 0.86 | 0.85 | 3980 | Train 63 | Test Asian 12 | 0.75 | 0.8 | 0.72 |
| 2957 | Train 47 | Test Asian 13 | 0.76 | 0.85 | 0.85 | 3981 | Train 63 | Test Asian 13 | 0.73 | 0.78 | 0.71 |
| 2958 | Train 47 | Test Asian 14 | 0.75 | 0.76 | 0.71 | 3982 | Train 63 | Test Asian 14 | 0.72 | 0.73 | 0.68 |
| 2959 | Train 47 | Test Asian 15 | 0.76 | 0.84 | 0.85 | 3983 | Train 63 | Test Asian 15 | 0.73 | 0.78 | 0.71 |
| 2960 | Train 47 | Test Asian 16 | 0.75 | 0.78 | 0.77 | 3984 | Train 63 | Test Asian 16 | 0.73 | 0.72 | 0.69 |
| 2961 | Train 47 | Test Asian 17 | 0.78 | 0.86 | 0.85 | 3985 | Train 63 | Test Asian 17 | 0.74 | 0.8 | 0.76 |
| 2962 | Train 47 | Test Asian 18 | 0.8 | 0.86 | 0.84 | 3986 | Train 63 | Test Asian 18 | 0.76 | 0.8 | 0.75 |
| 2963 | Train 47 | Test Asian 19 | 0.78 | 0.86 | 0.85 | 3987 | Train 63 | Test Asian 19 | 0.74 | 0.8 | 0.76 |
| 2964 | Train 47 | Test Asian 20 | 0.8 | 0.86 | 0.84 | 3988 | Train 63 | Test Asian 20 | 0.76 | 0.8 | 0.75 |
| 2965 | Train 47 | Test Asian 21 | 0.77 | 0.84 | 0.84 | 3989 | Train 63 | Test Asian 21 | 0.73 | 0.78 | 0.72 |
| 2966 | Train 47 | Test Asian 22 | 0.75 | 0.78 | 0.77 | 3990 | Train 63 | Test Asian 22 | 0.72 | 0.72 | 0.69 |
| 2967 | Train 47 | Test Asian 23 | 0.76 | 0.84 | 0.83 | 3991 | Train 63 | Test Asian 23 | 0.73 | 0.78 | 0.72 |
| 2968 | Train 47 | Test Asian 24 | 0.76 | 0.78 | 0.77 | 3992 | Train 63 | Test Asian 24 | 0.73 | 0.72 | 0.69 |
| 2969 | Train 47 | Test Asian 25 | 0.77 | 0.87 | 0.86 | 3993 | Train 63 | Test Asian 25 | 0.73 | 0.8 | 0.73 |
| 2970 | Train 47 | Test Asian 26 | 0.79 | 0.86 | 0.85 | 3994 | Train 63 | Test Asian 26 | 0.75 | 0.8 | 0.72 |
| 2971 | Train 47 | Test Asian 27 | 0.77 | 0.87 | 0.86 | 3995 | Train 63 | Test Asian 27 | 0.73 | 0.8 | 0.73 |
| 2972 | Train 47 | Test Asian 28 | 0.79 | 0.86 | 0.85 | 3996 | Train 63 | Test Asian 28 | 0.75 | 0.8 | 0.72 |
| 2973 | Train 47 | Test Asian 29 | 0.76 | 0.85 | 0.85 | 3997 | Train 63 | Test Asian 29 | 0.73 | 0.78 | 0.71 |
| 2974 | Train 47 | Test Asian 30 | 0.75 | 0.76 | 0.71 | 3998 | Train 63 | Test Asian 30 | 0.72 | 0.73 | 0.68 |
| 2975 | Train 47 | Test Asian 31 | 0.76 | 0.84 | 0.85 | 3999 | Train 63 | Test Asian 31 | 0.73 | 0.78 | 0.72 |
| 2976 | Train 47 | Test Asian 32 | 0.76 | 0.78 | 0.77 | 4000 | Train 63 | Test Asian 32 | 0.73 | 0.72 | 0.69 |
| 2977 | Train 47 | Test Asian 33 | 0.78 | 0.86 | 0.85 | 4001 | Train 63 | Test Asian 33 | 0.74 | 0.8 | 0.76 |
| 2978 | Train 47 | Test Asian 34 | 0.8 | 0.86 | 0.84 | 4002 | Train 63 | Test Asian 34 | 0.76 | 0.8 | 0.75 |
| 2979 | Train 47 | Test Asian 35 | 0.78 | 0.86 | 0.85 | 4003 | Train 63 | Test Asian 35 | 0.74 | 0.8 | 0.77 |
| 2980 | Train 47 | Test Asian 36 | 0.8 | 0.86 | 0.84 | 4004 | Train 63 | Test Asian 36 | 0.76 | 0.8 | 0.75 |
| 2981 | Train 47 | Test Asian 37 | 0.76 | 0.84 | 0.84 | 4005 | Train 63 | Test Asian 37 | 0.73 | 0.78 | 0.72 |
| 2982 | Train 47 | Test Asian 38 | 0.75 | 0.78 | 0.77 | 4006 | Train 63 | Test Asian 38 | 0.72 | 0.72 | 0.69 |
| 2983 | Train 47 | Test Asian 39 | 0.76 | 0.84 | 0.83 | 4007 | Train 63 | Test Asian 39 | 0.73 | 0.78 | 0.72 |
| 2984 | Train 47 | Test Asian 40 | 0.75 | 0.78 | 0.77 | 4008 | Train 63 | Test Asian 40 | 0.72 | 0.72 | 0.69 |
| 2985 | Train 47 | Test Asian 41 | 0.77 | 0.87 | 0.86 | 4009 | Train 63 | Test Asian 41 | 0.74 | 0.8 | 0.73 |
| 2986 | Train 47 | Test Asian 42 | 0.8 | 0.86 | 0.85 | 4010 | Train 63 | Test Asian 42 | 0.75 | 0.8 | 0.73 |
| 2987 | Train 47 | Test Asian 43 | 0.77 | 0.87 | 0.86 | 4011 | Train 63 | Test Asian 43 | 0.74 | 0.8 | 0.73 |
| 2988 | Train 47 | Test Asian 44 | 0.8 | 0.86 | 0.85 | 4012 | Train 63 | Test Asian 44 | 0.75 | 0.8 | 0.73 |
| 2989 | Train 47 | Test Asian 45 | 0.76 | 0.84 | 0.85 | 4013 | Train 63 | Test Asian 45 | 0.73 | 0.78 | 0.71 |
| 2990 | Train 47 | Test Asian 46 | 0.75 | 0.78 | 0.77 | 4014 | Train 63 | Test Asian 46 | 0.72 | 0.72 | 0.69 |
| 2991 | Train 47 | Test Asian 47 | 0.76 | 0.84 | 0.85 | 4015 | Train 63 | Test Asian 47 | 0.73 | 0.78 | 0.72 |
| 2992 | Train 47 | Test Asian 48 | 0.75 | 0.78 | 0.77 | 4016 | Train 63 | Test Asian 48 | 0.73 | 0.72 | 0.69 |
| 2993 | Train 47 | Test Asian 49 | 0.76 | 0.83 | 0.84 | 4017 | Train 63 | Test Asian 49 | 0.76 | 0.87 | 0.83 |
| 2994 | Train 47 | Test Asian 50 | 0.76 | 0.83 | 0.84 | 4018 | Train 63 | Test Asian 50 | 0.76 | 0.87 | 0.83 |
| 2995 | Train 47 | Test Asian 51 | 0.76 | 0.83 | 0.84 | 4019 | Train 63 | Test Asian 51 | 0.76 | 0.87 | 0.83 |
| 2996 | Train 47 | Test Asian 52 | 0.76 | 0.83 | 0.84 | 4020 | Train 63 | Test Asian 52 | 0.76 | 0.87 | 0.83 |
| 2997 | Train 47 | Test Asian 53 | 0.8 | 0.86 | 0.78 | 4021 | Train 63 | Test Asian 53 | 0.8 | 0.89 | 0.86 |
| 2998 | Train 47 | Test Asian 54 | 0.77 | 0.75 | 0.69 | 4022 | Train 63 | Test Asian 54 | 0.77 | 0.78 | 0.75 |
| 2999 | Train 47 | Test Asian 55 | 0.8 | 0.85 | 0.77 | 4023 | Train 63 | Test Asian 55 | 0.8 | 0.89 | 0.85 |
| 3000 | Train 47 | Test Asian 56 | 0.77 | 0.75 | 0.68 | 4024 | Train 63 | Test Asian 56 | 0.77 | 0.77 | 0.74 |
| 3001 | Train 47 | Test Asian 57 | 0.75 | 0.84 | 0.88 | 4025 | Train 63 | Test Asian 57 | 0.74 | 0.88 | 0.89 |
| 3002 | Train 47 | Test Asian 58 | 0.75 | 0.84 | 0.87 | 4026 | Train 63 | Test Asian 58 | 0.74 | 0.88 | 0.89 |
| 3003 | Train 47 | Test Asian 59 | 0.75 | 0.84 | 0.88 | 4027 | Train 63 | Test Asian 59 | 0.74 | 0.88 | 0.89 |
| 3004 | Train 47 | Test Asian 60 | 0.75 | 0.84 | 0.87 | 4028 | Train 63 | Test Asian 60 | 0.74 | 0.88 | 0.89 |
| 3005 | Train 47 | Test Asian 61 | 0.79 | 0.86 | 0.79 | 4029 | Train 63 | Test Asian 61 | 0.8 | 0.9 | 0.86 |
| 3006 | Train 47 | Test Asian 62 | 0.77 | 0.75 | 0.69 | 4030 | Train 63 | Test Asian 62 | 0.77 | 0.78 | 0.75 |
| 3007 | Train 47 | Test Asian 63 | 0.79 | 0.85 | 0.78 | 4031 | Train 63 | Test Asian 63 | 0.79 | 0.89 | 0.85 |
| 3008 | Train 47 | Test Asian 64 | 0.77 | 0.75 | 0.68 | 4032 | Train 63 | Test Asian 64 | 0.77 | 0.77 | 0.74 |
| 3009 | Train 48 | Test Asian 1 | 0.76 | 0.84 | 0.85 | 4033 | Train 64 | Test Asian 1 | 0.73 | 0.78 | 0.77 |
| 3010 | Train 48 | Test Asian 2 | 0.79 | 0.86 | 0.86 | 4034 | Train 64 | Test Asian 2 | 0.75 | 0.81 | 0.79 |
| 3011 | Train 48 | Test Asian 3 | 0.76 | 0.84 | 0.85 | 4035 | Train 64 | Test Asian 3 | 0.73 | 0.78 | 0.77 |
| 3012 | Train 48 | Test Asian 4 | 0.79 | 0.86 | 0.86 | 4036 | Train 64 | Test Asian 4 | 0.75 | 0.81 | 0.79 |
| 3013 | Train 48 | Test Asian 5 | 0.75 | 0.8 | 0.79 | 4037 | Train 64 | Test Asian 5 | 0.74 | 0.75 | 0.75 |
| 3014 | Train 48 | Test Asian 6 | 0.76 | 0.81 | 0.81 | 4038 | Train 64 | Test Asian 6 | 0.74 | 0.78 | 0.77 |
| 3015 | Train 48 | Test Asian 7 | 0.75 | 0.8 | 0.79 | 4039 | Train 64 | Test Asian 7 | 0.73 | 0.75 | 0.75 |
| 3016 | Train 48 | Test Asian 8 | 0.76 | 0.81 | 0.81 | 4040 | Train 64 | Test Asian 8 | 0.75 | 0.78 | 0.77 |
| 3017 | Train 48 | Test Asian 9 | 0.76 | 0.84 | 0.85 | 4041 | Train 64 | Test Asian 9 | 0.72 | 0.78 | 0.78 |
| 3018 | Train 48 | Test Asian 10 | 0.78 | 0.86 | 0.86 | 4042 | Train 64 | Test Asian 10 | 0.74 | 0.8 | 0.79 |
| 3019 | Train 48 | Test Asian 11 | 0.76 | 0.84 | 0.85 | 4043 | Train 64 | Test Asian 11 | 0.72 | 0.78 | 0.78 |
| 3020 | Train 48 | Test Asian 12 | 0.78 | 0.86 | 0.86 | 4044 | Train 64 | Test Asian 12 | 0.74 | 0.8 | 0.79 |
| 3021 | Train 48 | Test Asian 13 | 0.75 | 0.8 | 0.8 | 4045 | Train 64 | Test Asian 13 | 0.73 | 0.75 | 0.75 |
| 3022 | Train 48 | Test Asian 14 | 0.76 | 0.83 | 0.83 | 4046 | Train 64 | Test Asian 14 | 0.74 | 0.79 | 0.77 |
| 3023 | Train 48 | Test Asian 15 | 0.75 | 0.8 | 0.8 | 4047 | Train 64 | Test Asian 15 | 0.73 | 0.75 | 0.75 |
| 3024 | Train 48 | Test Asian 16 | 0.76 | 0.81 | 0.81 | 4048 | Train 64 | Test Asian 16 | 0.75 | 0.78 | 0.77 |
| 3025 | Train 48 | Test Asian 17 | 0.76 | 0.84 | 0.85 | 4049 | Train 64 | Test Asian 17 | 0.73 | 0.78 | 0.77 |
| 3026 | Train 48 | Test Asian 18 | 0.79 | 0.86 | 0.86 | 4050 | Train 64 | Test Asian 18 | 0.75 | 0.81 | 0.79 |
| 3027 | Train 48 | Test Asian 19 | 0.76 | 0.84 | 0.85 | 4051 | Train 64 | Test Asian 19 | 0.73 | 0.78 | 0.77 |
| 3028 | Train 48 | Test Asian 20 | 0.79 | 0.86 | 0.86 | 4052 | Train 64 | Test Asian 20 | 0.75 | 0.81 | 0.79 |
| 3029 | Train 48 | Test Asian 21 | 0.76 | 0.8 | 0.79 | 4053 | Train 64 | Test Asian 21 | 0.74 | 0.75 | 0.75 |
| 3030 | Train 48 | Test Asian 22 | 0.76 | 0.81 | 0.81 | 4054 | Train 64 | Test Asian 22 | 0.74 | 0.78 | 0.77 |
| 3031 | Train 48 | Test Asian 23 | 0.75 | 0.8 | 0.79 | 4055 | Train 64 | Test Asian 23 | 0.74 | 0.75 | 0.75 |
| 3032 | Train 48 | Test Asian 24 | 0.77 | 0.81 | 0.81 | 4056 | Train 64 | Test Asian 24 | 0.75 | 0.78 | 0.77 |
| 3033 | Train 48 | Test Asian 25 | 0.76 | 0.85 | 0.85 | 4057 | Train 64 | Test Asian 25 | 0.72 | 0.78 | 0.78 |
| 3034 | Train 48 | Test Asian 26 | 0.78 | 0.86 | 0.86 | 4058 | Train 64 | Test Asian 26 | 0.74 | 0.8 | 0.79 |
| 3035 | Train 48 | Test Asian 27 | 0.76 | 0.85 | 0.85 | 4059 | Train 64 | Test Asian 27 | 0.72 | 0.78 | 0.78 |
| 3036 | Train 48 | Test Asian 28 | 0.78 | 0.86 | 0.86 | 4060 | Train 64 | Test Asian 28 | 0.74 | 0.8 | 0.79 |
| 3037 | Train 48 | Test Asian 29 | 0.75 | 0.8 | 0.8 | 4061 | Train 64 | Test Asian 29 | 0.73 | 0.75 | 0.75 |
| 3038 | Train 48 | Test Asian 30 | 0.76 | 0.83 | 0.83 | 4062 | Train 64 | Test Asian 30 | 0.74 | 0.79 | 0.77 |
| 3039 | Train 48 | Test Asian 31 | 0.75 | 0.8 | 0.8 | 4063 | Train 64 | Test Asian 31 | 0.73 | 0.75 | 0.75 |
| 3040 | Train 48 | Test Asian 32 | 0.76 | 0.81 | 0.81 | 4064 | Train 64 | Test Asian 32 | 0.75 | 0.78 | 0.77 |
| 3041 | Train 48 | Test Asian 33 | 0.76 | 0.84 | 0.85 | 4065 | Train 64 | Test Asian 33 | 0.73 | 0.78 | 0.77 |
| 3042 | Train 48 | Test Asian 34 | 0.78 | 0.86 | 0.86 | 4066 | Train 64 | Test Asian 34 | 0.75 | 0.81 | 0.79 |
| 3043 | Train 48 | Test Asian 35 | 0.76 | 0.84 | 0.85 | 4067 | Train 64 | Test Asian 35 | 0.73 | 0.78 | 0.77 |
| 3044 | Train 48 | Test Asian 36 | 0.78 | 0.86 | 0.86 | 4068 | Train 64 | Test Asian 36 | 0.75 | 0.81 | 0.79 |
| 3045 | Train 48 | Test Asian 37 | 0.75 | 0.8 | 0.79 | 4069 | Train 64 | Test Asian 37 | 0.74 | 0.75 | 0.75 |
| 3046 | Train 48 | Test Asian 38 | 0.76 | 0.81 | 0.81 | 4070 | Train 64 | Test Asian 38 | 0.74 | 0.78 | 0.77 |
| 3047 | Train 48 | Test Asian 39 | 0.75 | 0.8 | 0.79 | 4071 | Train 64 | Test Asian 39 | 0.73 | 0.75 | 0.75 |
| 3048 | Train 48 | Test Asian 40 | 0.76 | 0.81 | 0.81 | 4072 | Train 64 | Test Asian 40 | 0.75 | 0.78 | 0.77 |
| 3049 | Train 48 | Test Asian 41 | 0.76 | 0.84 | 0.85 | 4073 | Train 64 | Test Asian 41 | 0.72 | 0.78 | 0.78 |
| 3050 | Train 48 | Test Asian 42 | 0.78 | 0.86 | 0.86 | 4074 | Train 64 | Test Asian 42 | 0.74 | 0.8 | 0.79 |
| 3051 | Train 48 | Test Asian 43 | 0.76 | 0.84 | 0.85 | 4075 | Train 64 | Test Asian 43 | 0.72 | 0.78 | 0.78 |
| 3052 | Train 48 | Test Asian 44 | 0.78 | 0.86 | 0.86 | 4076 | Train 64 | Test Asian 44 | 0.74 | 0.8 | 0.79 |
| 3053 | Train 48 | Test Asian 45 | 0.75 | 0.8 | 0.8 | 4077 | Train 64 | Test Asian 45 | 0.73 | 0.75 | 0.75 |
| 3054 | Train 48 | Test Asian 46 | 0.76 | 0.81 | 0.8 | 4078 | Train 64 | Test Asian 46 | 0.74 | 0.78 | 0.77 |
| 3055 | Train 48 | Test Asian 47 | 0.75 | 0.8 | 0.8 | 4079 | Train 64 | Test Asian 47 | 0.73 | 0.75 | 0.75 |
| 3056 | Train 48 | Test Asian 48 | 0.76 | 0.81 | 0.81 | 4080 | Train 64 | Test Asian 48 | 0.75 | 0.78 | 0.77 |
| 3057 | Train 48 | Test Asian 49 | 0.77 | 0.84 | 0.79 | 4081 | Train 64 | Test Asian 49 | 0.77 | 0.86 | 0.76 |
| 3058 | Train 48 | Test Asian 50 | 0.77 | 0.84 | 0.79 | 4082 | Train 64 | Test Asian 50 | 0.77 | 0.86 | 0.76 |
| 3059 | Train 48 | Test Asian 51 | 0.77 | 0.84 | 0.79 | 4083 | Train 64 | Test Asian 51 | 0.77 | 0.86 | 0.76 |
| 3060 | Train 48 | Test Asian 52 | 0.77 | 0.84 | 0.79 | 4084 | Train 64 | Test Asian 52 | 0.77 | 0.86 | 0.76 |
| 3061 | Train 48 | Test Asian 53 | 0.8 | 0.84 | 0.78 | 4085 | Train 64 | Test Asian 53 | 0.8 | 0.87 | 0.77 |
| 3062 | Train 48 | Test Asian 54 | 0.79 | 0.83 | 0.83 | 4086 | Train 64 | Test Asian 54 | 0.78 | 0.86 | 0.85 |
| 3063 | Train 48 | Test Asian 55 | 0.79 | 0.84 | 0.77 | 4087 | Train 64 | Test Asian 55 | 0.79 | 0.86 | 0.77 |
| 3064 | Train 48 | Test Asian 56 | 0.79 | 0.83 | 0.84 | 4088 | Train 64 | Test Asian 56 | 0.79 | 0.86 | 0.85 |
| 3065 | Train 48 | Test Asian 57 | 0.76 | 0.84 | 0.8 | 4089 | Train 64 | Test Asian 57 | 0.76 | 0.87 | 0.8 |
| 3066 | Train 48 | Test Asian 58 | 0.76 | 0.84 | 0.79 | 4090 | Train 64 | Test Asian 58 | 0.76 | 0.87 | 0.8 |
| 3067 | Train 48 | Test Asian 59 | 0.76 | 0.84 | 0.8 | 4091 | Train 64 | Test Asian 59 | 0.76 | 0.87 | 0.8 |
| 3068 | Train 48 | Test Asian 60 | 0.76 | 0.84 | 0.79 | 4092 | Train 64 | Test Asian 60 | 0.76 | 0.87 | 0.8 |
| 3069 | Train 48 | Test Asian 61 | 0.79 | 0.84 | 0.78 | 4093 | Train 64 | Test Asian 61 | 0.79 | 0.87 | 0.78 |
| 3070 | Train 48 | Test Asian 62 | 0.79 | 0.83 | 0.83 | 4094 | Train 64 | Test Asian 62 | 0.78 | 0.86 | 0.85 |
| 3071 | Train 48 | Test Asian 63 | 0.78 | 0.84 | 0.77 | 4095 | Train 64 | Test Asian 63 | 0.79 | 0.86 | 0.77 |
| 3072 | Train 48 | Test Asian 64 | 0.79 | 0.83 | 0.84 | 4096 | Train 64 | Test Asian 64 | 0.78 | 0.86 | 0.85 |

Table 11. AUC Performance of the ML Models— LR, RF, and GB —for Hispanic Patients Trained on Cohorts 1–32 and Tested Across 64 Cohorts.

| Hispanic | Train  Model | Test  Dataset | AUC (LR) | AUC (RF) | AUC (GB) | Hispanic | Train  Model | Test  Dataset | AUC (LR) | AUC (RF) | AUC (GB) |
| --- | --- | --- | --- | --- | --- | --- | --- | --- | --- | --- | --- |
| 1 | Train 1 | Test Hispanic 1 | 0.79 | 0.89 | 0.88 | 1025 | Train 17 | Test Hispanic 1 | 0.79 | 0.89 | 0.88 |
| 2 | Train 1 | Test Hispanic 2 | 0.78 | 0.87 | 0.86 | 1026 | Train 17 | Test Hispanic 2 | 0.78 | 0.87 | 0.85 |
| 3 | Train 1 | Test Hispanic 3 | 0.79 | 0.89 | 0.88 | 1027 | Train 17 | Test Hispanic 3 | 0.79 | 0.89 | 0.87 |
| 4 | Train 1 | Test Hispanic 4 | 0.78 | 0.87 | 0.86 | 1028 | Train 17 | Test Hispanic 4 | 0.78 | 0.87 | 0.85 |
| 5 | Train 1 | Test Hispanic 5 | 0.74 | 0.79 | 0.76 | 1029 | Train 17 | Test Hispanic 5 | 0.74 | 0.79 | 0.76 |
| 6 | Train 1 | Test Hispanic 6 | 0.72 | 0.73 | 0.7 | 1030 | Train 17 | Test Hispanic 6 | 0.72 | 0.73 | 0.7 |
| 7 | Train 1 | Test Hispanic 7 | 0.74 | 0.78 | 0.75 | 1031 | Train 17 | Test Hispanic 7 | 0.74 | 0.78 | 0.75 |
| 8 | Train 1 | Test Hispanic 8 | 0.72 | 0.73 | 0.7 | 1032 | Train 17 | Test Hispanic 8 | 0.72 | 0.73 | 0.7 |
| 9 | Train 1 | Test Hispanic 9 | 0.79 | 0.88 | 0.85 | 1033 | Train 17 | Test Hispanic 9 | 0.79 | 0.88 | 0.85 |
| 10 | Train 1 | Test Hispanic 10 | 0.78 | 0.86 | 0.83 | 1034 | Train 17 | Test Hispanic 10 | 0.78 | 0.86 | 0.83 |
| 11 | Train 1 | Test Hispanic 11 | 0.79 | 0.87 | 0.85 | 1035 | Train 17 | Test Hispanic 11 | 0.79 | 0.88 | 0.84 |
| 12 | Train 1 | Test Hispanic 12 | 0.78 | 0.86 | 0.83 | 1036 | Train 17 | Test Hispanic 12 | 0.78 | 0.86 | 0.82 |
| 13 | Train 1 | Test Hispanic 13 | 0.74 | 0.78 | 0.75 | 1037 | Train 17 | Test Hispanic 13 | 0.74 | 0.78 | 0.74 |
| 14 | Train 1 | Test Hispanic 14 | 0.72 | 0.73 | 0.7 | 1038 | Train 17 | Test Hispanic 14 | 0.72 | 0.73 | 0.7 |
| 15 | Train 1 | Test Hispanic 15 | 0.74 | 0.77 | 0.74 | 1039 | Train 17 | Test Hispanic 15 | 0.74 | 0.77 | 0.73 |
| 16 | Train 1 | Test Hispanic 16 | 0.72 | 0.73 | 0.7 | 1040 | Train 17 | Test Hispanic 16 | 0.72 | 0.73 | 0.7 |
| 17 | Train 1 | Test Hispanic 17 | 0.79 | 0.89 | 0.88 | 1041 | Train 17 | Test Hispanic 17 | 0.79 | 0.89 | 0.88 |
| 18 | Train 1 | Test Hispanic 18 | 0.78 | 0.87 | 0.86 | 1042 | Train 17 | Test Hispanic 18 | 0.78 | 0.87 | 0.85 |
| 19 | Train 1 | Test Hispanic 19 | 0.79 | 0.89 | 0.88 | 1043 | Train 17 | Test Hispanic 19 | 0.79 | 0.89 | 0.87 |
| 20 | Train 1 | Test Hispanic 20 | 0.78 | 0.87 | 0.86 | 1044 | Train 17 | Test Hispanic 20 | 0.78 | 0.87 | 0.85 |
| 21 | Train 1 | Test Hispanic 21 | 0.74 | 0.79 | 0.76 | 1045 | Train 17 | Test Hispanic 21 | 0.74 | 0.79 | 0.76 |
| 22 | Train 1 | Test Hispanic 22 | 0.72 | 0.73 | 0.7 | 1046 | Train 17 | Test Hispanic 22 | 0.72 | 0.73 | 0.7 |
| 23 | Train 1 | Test Hispanic 23 | 0.74 | 0.78 | 0.75 | 1047 | Train 17 | Test Hispanic 23 | 0.74 | 0.78 | 0.75 |
| 24 | Train 1 | Test Hispanic 24 | 0.72 | 0.73 | 0.7 | 1048 | Train 17 | Test Hispanic 24 | 0.72 | 0.73 | 0.7 |
| 25 | Train 1 | Test Hispanic 25 | 0.79 | 0.88 | 0.85 | 1049 | Train 17 | Test Hispanic 25 | 0.79 | 0.88 | 0.85 |
| 26 | Train 1 | Test Hispanic 26 | 0.78 | 0.86 | 0.83 | 1050 | Train 17 | Test Hispanic 26 | 0.78 | 0.86 | 0.83 |
| 27 | Train 1 | Test Hispanic 27 | 0.79 | 0.87 | 0.85 | 1051 | Train 17 | Test Hispanic 27 | 0.79 | 0.88 | 0.84 |
| 28 | Train 1 | Test Hispanic 28 | 0.78 | 0.86 | 0.83 | 1052 | Train 17 | Test Hispanic 28 | 0.78 | 0.86 | 0.82 |
| 29 | Train 1 | Test Hispanic 29 | 0.74 | 0.78 | 0.75 | 1053 | Train 17 | Test Hispanic 29 | 0.74 | 0.78 | 0.74 |
| 30 | Train 1 | Test Hispanic 30 | 0.72 | 0.73 | 0.7 | 1054 | Train 17 | Test Hispanic 30 | 0.72 | 0.73 | 0.7 |
| 31 | Train 1 | Test Hispanic 31 | 0.74 | 0.77 | 0.74 | 1055 | Train 17 | Test Hispanic 31 | 0.74 | 0.77 | 0.73 |
| 32 | Train 1 | Test Hispanic 32 | 0.72 | 0.73 | 0.7 | 1056 | Train 17 | Test Hispanic 32 | 0.72 | 0.73 | 0.7 |
| 33 | Train 1 | Test Hispanic 33 | 0.8 | 0.89 | 0.89 | 1057 | Train 17 | Test Hispanic 33 | 0.8 | 0.89 | 0.88 |
| 34 | Train 1 | Test Hispanic 34 | 0.79 | 0.87 | 0.87 | 1058 | Train 17 | Test Hispanic 34 | 0.79 | 0.87 | 0.85 |
| 35 | Train 1 | Test Hispanic 35 | 0.8 | 0.89 | 0.88 | 1059 | Train 17 | Test Hispanic 35 | 0.8 | 0.89 | 0.88 |
| 36 | Train 1 | Test Hispanic 36 | 0.79 | 0.87 | 0.86 | 1060 | Train 17 | Test Hispanic 36 | 0.79 | 0.87 | 0.85 |
| 37 | Train 1 | Test Hispanic 37 | 0.74 | 0.79 | 0.77 | 1061 | Train 17 | Test Hispanic 37 | 0.74 | 0.79 | 0.76 |
| 38 | Train 1 | Test Hispanic 38 | 0.72 | 0.73 | 0.71 | 1062 | Train 17 | Test Hispanic 38 | 0.72 | 0.73 | 0.7 |
| 39 | Train 1 | Test Hispanic 39 | 0.74 | 0.78 | 0.76 | 1063 | Train 17 | Test Hispanic 39 | 0.74 | 0.78 | 0.75 |
| 40 | Train 1 | Test Hispanic 40 | 0.73 | 0.73 | 0.7 | 1064 | Train 17 | Test Hispanic 40 | 0.72 | 0.73 | 0.7 |
| 41 | Train 1 | Test Hispanic 41 | 0.8 | 0.88 | 0.85 | 1065 | Train 17 | Test Hispanic 41 | 0.8 | 0.88 | 0.85 |
| 42 | Train 1 | Test Hispanic 42 | 0.79 | 0.86 | 0.84 | 1066 | Train 17 | Test Hispanic 42 | 0.79 | 0.86 | 0.83 |
| 43 | Train 1 | Test Hispanic 43 | 0.8 | 0.88 | 0.85 | 1067 | Train 17 | Test Hispanic 43 | 0.8 | 0.88 | 0.85 |
| 44 | Train 1 | Test Hispanic 44 | 0.79 | 0.86 | 0.84 | 1068 | Train 17 | Test Hispanic 44 | 0.79 | 0.86 | 0.83 |
| 45 | Train 1 | Test Hispanic 45 | 0.74 | 0.78 | 0.75 | 1069 | Train 17 | Test Hispanic 45 | 0.74 | 0.78 | 0.74 |
| 46 | Train 1 | Test Hispanic 46 | 0.72 | 0.73 | 0.71 | 1070 | Train 17 | Test Hispanic 46 | 0.72 | 0.73 | 0.71 |
| 47 | Train 1 | Test Hispanic 47 | 0.74 | 0.78 | 0.74 | 1071 | Train 17 | Test Hispanic 47 | 0.74 | 0.78 | 0.73 |
| 48 | Train 1 | Test Hispanic 48 | 0.73 | 0.73 | 0.7 | 1072 | Train 17 | Test Hispanic 48 | 0.72 | 0.73 | 0.71 |
| 49 | Train 1 | Test Hispanic 49 | 0.8 | 0.88 | 0.88 | 1073 | Train 17 | Test Hispanic 49 | 0.8 | 0.88 | 0.88 |
| 50 | Train 1 | Test Hispanic 50 | 0.8 | 0.87 | 0.88 | 1074 | Train 17 | Test Hispanic 50 | 0.8 | 0.88 | 0.87 |
| 51 | Train 1 | Test Hispanic 51 | 0.81 | 0.88 | 0.88 | 1075 | Train 17 | Test Hispanic 51 | 0.81 | 0.88 | 0.87 |
| 52 | Train 1 | Test Hispanic 52 | 0.8 | 0.87 | 0.87 | 1076 | Train 17 | Test Hispanic 52 | 0.8 | 0.87 | 0.86 |
| 53 | Train 1 | Test Hispanic 53 | 0.78 | 0.83 | 0.78 | 1077 | Train 17 | Test Hispanic 53 | 0.78 | 0.83 | 0.79 |
| 54 | Train 1 | Test Hispanic 54 | 0.75 | 0.74 | 0.71 | 1078 | Train 17 | Test Hispanic 54 | 0.75 | 0.74 | 0.73 |
| 55 | Train 1 | Test Hispanic 55 | 0.78 | 0.83 | 0.78 | 1079 | Train 17 | Test Hispanic 55 | 0.78 | 0.83 | 0.79 |
| 56 | Train 1 | Test Hispanic 56 | 0.76 | 0.74 | 0.71 | 1080 | Train 17 | Test Hispanic 56 | 0.76 | 0.74 | 0.73 |
| 57 | Train 1 | Test Hispanic 57 | 0.81 | 0.87 | 0.85 | 1081 | Train 17 | Test Hispanic 57 | 0.81 | 0.87 | 0.84 |
| 58 | Train 1 | Test Hispanic 58 | 0.8 | 0.86 | 0.85 | 1082 | Train 17 | Test Hispanic 58 | 0.8 | 0.86 | 0.84 |
| 59 | Train 1 | Test Hispanic 59 | 0.81 | 0.87 | 0.85 | 1083 | Train 17 | Test Hispanic 59 | 0.81 | 0.87 | 0.84 |
| 60 | Train 1 | Test Hispanic 60 | 0.81 | 0.86 | 0.84 | 1084 | Train 17 | Test Hispanic 60 | 0.81 | 0.86 | 0.83 |
| 61 | Train 1 | Test Hispanic 61 | 0.78 | 0.82 | 0.78 | 1085 | Train 17 | Test Hispanic 61 | 0.78 | 0.83 | 0.78 |
| 62 | Train 1 | Test Hispanic 62 | 0.75 | 0.74 | 0.71 | 1086 | Train 17 | Test Hispanic 62 | 0.75 | 0.74 | 0.73 |
| 63 | Train 1 | Test Hispanic 63 | 0.79 | 0.83 | 0.78 | 1087 | Train 17 | Test Hispanic 63 | 0.79 | 0.83 | 0.78 |
| 64 | Train 1 | Test Hispanic 64 | 0.76 | 0.74 | 0.71 | 1088 | Train 17 | Test Hispanic 64 | 0.76 | 0.74 | 0.73 |
| 65 | Train 2 | Test Hispanic 1 | 0.79 | 0.89 | 0.87 | 1089 | Train 18 | Test Hispanic 1 | 0.79 | 0.89 | 0.87 |
| 66 | Train 2 | Test Hispanic 2 | 0.78 | 0.89 | 0.87 | 1090 | Train 18 | Test Hispanic 2 | 0.78 | 0.89 | 0.87 |
| 67 | Train 2 | Test Hispanic 3 | 0.79 | 0.89 | 0.87 | 1091 | Train 18 | Test Hispanic 3 | 0.79 | 0.89 | 0.87 |
| 68 | Train 2 | Test Hispanic 4 | 0.79 | 0.89 | 0.87 | 1092 | Train 18 | Test Hispanic 4 | 0.79 | 0.89 | 0.87 |
| 69 | Train 2 | Test Hispanic 5 | 0.74 | 0.8 | 0.75 | 1093 | Train 18 | Test Hispanic 5 | 0.74 | 0.8 | 0.76 |
| 70 | Train 2 | Test Hispanic 6 | 0.73 | 0.74 | 0.7 | 1094 | Train 18 | Test Hispanic 6 | 0.73 | 0.74 | 0.7 |
| 71 | Train 2 | Test Hispanic 7 | 0.74 | 0.79 | 0.75 | 1095 | Train 18 | Test Hispanic 7 | 0.74 | 0.79 | 0.75 |
| 72 | Train 2 | Test Hispanic 8 | 0.73 | 0.74 | 0.7 | 1096 | Train 18 | Test Hispanic 8 | 0.73 | 0.74 | 0.7 |
| 73 | Train 2 | Test Hispanic 9 | 0.79 | 0.87 | 0.84 | 1097 | Train 18 | Test Hispanic 9 | 0.79 | 0.87 | 0.84 |
| 74 | Train 2 | Test Hispanic 10 | 0.78 | 0.88 | 0.85 | 1098 | Train 18 | Test Hispanic 10 | 0.78 | 0.88 | 0.84 |
| 75 | Train 2 | Test Hispanic 11 | 0.79 | 0.87 | 0.83 | 1099 | Train 18 | Test Hispanic 11 | 0.79 | 0.87 | 0.84 |
| 76 | Train 2 | Test Hispanic 12 | 0.79 | 0.88 | 0.84 | 1100 | Train 18 | Test Hispanic 12 | 0.79 | 0.88 | 0.84 |
| 77 | Train 2 | Test Hispanic 13 | 0.74 | 0.79 | 0.74 | 1101 | Train 18 | Test Hispanic 13 | 0.74 | 0.79 | 0.74 |
| 78 | Train 2 | Test Hispanic 14 | 0.73 | 0.75 | 0.71 | 1102 | Train 18 | Test Hispanic 14 | 0.73 | 0.74 | 0.7 |
| 79 | Train 2 | Test Hispanic 15 | 0.74 | 0.78 | 0.73 | 1103 | Train 18 | Test Hispanic 15 | 0.74 | 0.78 | 0.74 |
| 80 | Train 2 | Test Hispanic 16 | 0.73 | 0.74 | 0.7 | 1104 | Train 18 | Test Hispanic 16 | 0.73 | 0.74 | 0.7 |
| 81 | Train 2 | Test Hispanic 17 | 0.79 | 0.89 | 0.87 | 1105 | Train 18 | Test Hispanic 17 | 0.79 | 0.89 | 0.87 |
| 82 | Train 2 | Test Hispanic 18 | 0.78 | 0.89 | 0.87 | 1106 | Train 18 | Test Hispanic 18 | 0.78 | 0.89 | 0.87 |
| 83 | Train 2 | Test Hispanic 19 | 0.79 | 0.89 | 0.87 | 1107 | Train 18 | Test Hispanic 19 | 0.79 | 0.89 | 0.87 |
| 84 | Train 2 | Test Hispanic 20 | 0.79 | 0.89 | 0.87 | 1108 | Train 18 | Test Hispanic 20 | 0.79 | 0.89 | 0.87 |
| 85 | Train 2 | Test Hispanic 21 | 0.74 | 0.8 | 0.76 | 1109 | Train 18 | Test Hispanic 21 | 0.74 | 0.8 | 0.76 |
| 86 | Train 2 | Test Hispanic 22 | 0.73 | 0.75 | 0.7 | 1110 | Train 18 | Test Hispanic 22 | 0.73 | 0.75 | 0.7 |
| 87 | Train 2 | Test Hispanic 23 | 0.74 | 0.79 | 0.75 | 1111 | Train 18 | Test Hispanic 23 | 0.74 | 0.79 | 0.75 |
| 88 | Train 2 | Test Hispanic 24 | 0.73 | 0.74 | 0.7 | 1112 | Train 18 | Test Hispanic 24 | 0.73 | 0.74 | 0.7 |
| 89 | Train 2 | Test Hispanic 25 | 0.79 | 0.87 | 0.84 | 1113 | Train 18 | Test Hispanic 25 | 0.79 | 0.87 | 0.84 |
| 90 | Train 2 | Test Hispanic 26 | 0.78 | 0.88 | 0.85 | 1114 | Train 18 | Test Hispanic 26 | 0.78 | 0.88 | 0.84 |
| 91 | Train 2 | Test Hispanic 27 | 0.79 | 0.87 | 0.83 | 1115 | Train 18 | Test Hispanic 27 | 0.79 | 0.87 | 0.84 |
| 92 | Train 2 | Test Hispanic 28 | 0.79 | 0.88 | 0.84 | 1116 | Train 18 | Test Hispanic 28 | 0.79 | 0.88 | 0.84 |
| 93 | Train 2 | Test Hispanic 29 | 0.74 | 0.79 | 0.74 | 1117 | Train 18 | Test Hispanic 29 | 0.74 | 0.79 | 0.74 |
| 94 | Train 2 | Test Hispanic 30 | 0.73 | 0.75 | 0.71 | 1118 | Train 18 | Test Hispanic 30 | 0.73 | 0.75 | 0.71 |
| 95 | Train 2 | Test Hispanic 31 | 0.74 | 0.78 | 0.73 | 1119 | Train 18 | Test Hispanic 31 | 0.74 | 0.78 | 0.74 |
| 96 | Train 2 | Test Hispanic 32 | 0.73 | 0.74 | 0.71 | 1120 | Train 18 | Test Hispanic 32 | 0.73 | 0.74 | 0.7 |
| 97 | Train 2 | Test Hispanic 33 | 0.79 | 0.89 | 0.87 | 1121 | Train 18 | Test Hispanic 33 | 0.79 | 0.89 | 0.87 |
| 98 | Train 2 | Test Hispanic 34 | 0.79 | 0.89 | 0.88 | 1122 | Train 18 | Test Hispanic 34 | 0.79 | 0.89 | 0.87 |
| 99 | Train 2 | Test Hispanic 35 | 0.8 | 0.89 | 0.87 | 1123 | Train 18 | Test Hispanic 35 | 0.8 | 0.89 | 0.87 |
| 100 | Train 2 | Test Hispanic 36 | 0.79 | 0.89 | 0.87 | 1124 | Train 18 | Test Hispanic 36 | 0.79 | 0.89 | 0.87 |
| 101 | Train 2 | Test Hispanic 37 | 0.75 | 0.8 | 0.76 | 1125 | Train 18 | Test Hispanic 37 | 0.75 | 0.8 | 0.76 |
| 102 | Train 2 | Test Hispanic 38 | 0.73 | 0.75 | 0.71 | 1126 | Train 18 | Test Hispanic 38 | 0.73 | 0.75 | 0.7 |
| 103 | Train 2 | Test Hispanic 39 | 0.75 | 0.79 | 0.75 | 1127 | Train 18 | Test Hispanic 39 | 0.75 | 0.79 | 0.76 |
| 104 | Train 2 | Test Hispanic 40 | 0.73 | 0.74 | 0.71 | 1128 | Train 18 | Test Hispanic 40 | 0.73 | 0.74 | 0.7 |
| 105 | Train 2 | Test Hispanic 41 | 0.79 | 0.88 | 0.84 | 1129 | Train 18 | Test Hispanic 41 | 0.79 | 0.88 | 0.84 |
| 106 | Train 2 | Test Hispanic 42 | 0.79 | 0.88 | 0.85 | 1130 | Train 18 | Test Hispanic 42 | 0.79 | 0.88 | 0.85 |
| 107 | Train 2 | Test Hispanic 43 | 0.8 | 0.88 | 0.84 | 1131 | Train 18 | Test Hispanic 43 | 0.8 | 0.88 | 0.84 |
| 108 | Train 2 | Test Hispanic 44 | 0.8 | 0.88 | 0.84 | 1132 | Train 18 | Test Hispanic 44 | 0.8 | 0.88 | 0.84 |
| 109 | Train 2 | Test Hispanic 45 | 0.75 | 0.79 | 0.74 | 1133 | Train 18 | Test Hispanic 45 | 0.75 | 0.79 | 0.75 |
| 110 | Train 2 | Test Hispanic 46 | 0.73 | 0.75 | 0.71 | 1134 | Train 18 | Test Hispanic 46 | 0.73 | 0.75 | 0.71 |
| 111 | Train 2 | Test Hispanic 47 | 0.75 | 0.79 | 0.74 | 1135 | Train 18 | Test Hispanic 47 | 0.75 | 0.79 | 0.74 |
| 112 | Train 2 | Test Hispanic 48 | 0.73 | 0.75 | 0.71 | 1136 | Train 18 | Test Hispanic 48 | 0.73 | 0.74 | 0.71 |
| 113 | Train 2 | Test Hispanic 49 | 0.8 | 0.87 | 0.87 | 1137 | Train 18 | Test Hispanic 49 | 0.8 | 0.87 | 0.87 |
| 114 | Train 2 | Test Hispanic 50 | 0.79 | 0.87 | 0.87 | 1138 | Train 18 | Test Hispanic 50 | 0.79 | 0.87 | 0.87 |
| 115 | Train 2 | Test Hispanic 51 | 0.8 | 0.87 | 0.86 | 1139 | Train 18 | Test Hispanic 51 | 0.8 | 0.87 | 0.87 |
| 116 | Train 2 | Test Hispanic 52 | 0.8 | 0.87 | 0.86 | 1140 | Train 18 | Test Hispanic 52 | 0.8 | 0.87 | 0.87 |
| 117 | Train 2 | Test Hispanic 53 | 0.78 | 0.83 | 0.79 | 1141 | Train 18 | Test Hispanic 53 | 0.78 | 0.83 | 0.79 |
| 118 | Train 2 | Test Hispanic 54 | 0.75 | 0.76 | 0.73 | 1142 | Train 18 | Test Hispanic 54 | 0.75 | 0.76 | 0.73 |
| 119 | Train 2 | Test Hispanic 55 | 0.78 | 0.83 | 0.79 | 1143 | Train 18 | Test Hispanic 55 | 0.78 | 0.83 | 0.79 |
| 120 | Train 2 | Test Hispanic 56 | 0.75 | 0.76 | 0.73 | 1144 | Train 18 | Test Hispanic 56 | 0.75 | 0.76 | 0.73 |
| 121 | Train 2 | Test Hispanic 57 | 0.8 | 0.86 | 0.84 | 1145 | Train 18 | Test Hispanic 57 | 0.8 | 0.86 | 0.83 |
| 122 | Train 2 | Test Hispanic 58 | 0.79 | 0.86 | 0.83 | 1146 | Train 18 | Test Hispanic 58 | 0.79 | 0.86 | 0.83 |
| 123 | Train 2 | Test Hispanic 59 | 0.81 | 0.86 | 0.83 | 1147 | Train 18 | Test Hispanic 59 | 0.81 | 0.86 | 0.83 |
| 124 | Train 2 | Test Hispanic 60 | 0.8 | 0.86 | 0.83 | 1148 | Train 18 | Test Hispanic 60 | 0.8 | 0.86 | 0.83 |
| 125 | Train 2 | Test Hispanic 61 | 0.78 | 0.83 | 0.79 | 1149 | Train 18 | Test Hispanic 61 | 0.78 | 0.83 | 0.79 |
| 126 | Train 2 | Test Hispanic 62 | 0.75 | 0.76 | 0.73 | 1150 | Train 18 | Test Hispanic 62 | 0.75 | 0.76 | 0.73 |
| 127 | Train 2 | Test Hispanic 63 | 0.79 | 0.83 | 0.79 | 1151 | Train 18 | Test Hispanic 63 | 0.79 | 0.83 | 0.79 |
| 128 | Train 2 | Test Hispanic 64 | 0.75 | 0.76 | 0.73 | 1152 | Train 18 | Test Hispanic 64 | 0.75 | 0.76 | 0.73 |
| 129 | Train 3 | Test Hispanic 1 | 0.79 | 0.89 | 0.88 | 1153 | Train 19 | Test Hispanic 1 | 0.79 | 0.89 | 0.87 |
| 130 | Train 3 | Test Hispanic 2 | 0.78 | 0.87 | 0.85 | 1154 | Train 19 | Test Hispanic 2 | 0.78 | 0.87 | 0.85 |
| 131 | Train 3 | Test Hispanic 3 | 0.79 | 0.89 | 0.88 | 1155 | Train 19 | Test Hispanic 3 | 0.79 | 0.89 | 0.88 |
| 132 | Train 3 | Test Hispanic 4 | 0.78 | 0.87 | 0.85 | 1156 | Train 19 | Test Hispanic 4 | 0.78 | 0.87 | 0.85 |
| 133 | Train 3 | Test Hispanic 5 | 0.74 | 0.79 | 0.76 | 1157 | Train 19 | Test Hispanic 5 | 0.74 | 0.79 | 0.74 |
| 134 | Train 3 | Test Hispanic 6 | 0.72 | 0.73 | 0.7 | 1158 | Train 19 | Test Hispanic 6 | 0.72 | 0.73 | 0.69 |
| 135 | Train 3 | Test Hispanic 7 | 0.74 | 0.78 | 0.76 | 1159 | Train 19 | Test Hispanic 7 | 0.74 | 0.78 | 0.74 |
| 136 | Train 3 | Test Hispanic 8 | 0.72 | 0.73 | 0.7 | 1160 | Train 19 | Test Hispanic 8 | 0.72 | 0.73 | 0.69 |
| 137 | Train 3 | Test Hispanic 9 | 0.79 | 0.87 | 0.84 | 1161 | Train 19 | Test Hispanic 9 | 0.79 | 0.87 | 0.84 |
| 138 | Train 3 | Test Hispanic 10 | 0.78 | 0.86 | 0.82 | 1162 | Train 19 | Test Hispanic 10 | 0.78 | 0.86 | 0.82 |
| 139 | Train 3 | Test Hispanic 11 | 0.79 | 0.87 | 0.85 | 1163 | Train 19 | Test Hispanic 11 | 0.79 | 0.87 | 0.84 |
| 140 | Train 3 | Test Hispanic 12 | 0.78 | 0.86 | 0.83 | 1164 | Train 19 | Test Hispanic 12 | 0.78 | 0.86 | 0.82 |
| 141 | Train 3 | Test Hispanic 13 | 0.74 | 0.78 | 0.74 | 1165 | Train 19 | Test Hispanic 13 | 0.74 | 0.78 | 0.73 |
| 142 | Train 3 | Test Hispanic 14 | 0.72 | 0.73 | 0.7 | 1166 | Train 19 | Test Hispanic 14 | 0.72 | 0.73 | 0.69 |
| 143 | Train 3 | Test Hispanic 15 | 0.74 | 0.78 | 0.74 | 1167 | Train 19 | Test Hispanic 15 | 0.74 | 0.77 | 0.72 |
| 144 | Train 3 | Test Hispanic 16 | 0.72 | 0.73 | 0.7 | 1168 | Train 19 | Test Hispanic 16 | 0.72 | 0.73 | 0.69 |
| 145 | Train 3 | Test Hispanic 17 | 0.79 | 0.89 | 0.88 | 1169 | Train 19 | Test Hispanic 17 | 0.79 | 0.89 | 0.87 |
| 146 | Train 3 | Test Hispanic 18 | 0.78 | 0.87 | 0.85 | 1170 | Train 19 | Test Hispanic 18 | 0.78 | 0.87 | 0.85 |
| 147 | Train 3 | Test Hispanic 19 | 0.79 | 0.89 | 0.88 | 1171 | Train 19 | Test Hispanic 19 | 0.79 | 0.89 | 0.88 |
| 148 | Train 3 | Test Hispanic 20 | 0.78 | 0.87 | 0.85 | 1172 | Train 19 | Test Hispanic 20 | 0.78 | 0.87 | 0.85 |
| 149 | Train 3 | Test Hispanic 21 | 0.74 | 0.79 | 0.76 | 1173 | Train 19 | Test Hispanic 21 | 0.74 | 0.79 | 0.74 |
| 150 | Train 3 | Test Hispanic 22 | 0.72 | 0.73 | 0.7 | 1174 | Train 19 | Test Hispanic 22 | 0.72 | 0.73 | 0.69 |
| 151 | Train 3 | Test Hispanic 23 | 0.74 | 0.78 | 0.76 | 1175 | Train 19 | Test Hispanic 23 | 0.74 | 0.78 | 0.74 |
| 152 | Train 3 | Test Hispanic 24 | 0.72 | 0.73 | 0.7 | 1176 | Train 19 | Test Hispanic 24 | 0.72 | 0.73 | 0.69 |
| 153 | Train 3 | Test Hispanic 25 | 0.79 | 0.87 | 0.84 | 1177 | Train 19 | Test Hispanic 25 | 0.79 | 0.87 | 0.84 |
| 154 | Train 3 | Test Hispanic 26 | 0.78 | 0.86 | 0.82 | 1178 | Train 19 | Test Hispanic 26 | 0.78 | 0.86 | 0.82 |
| 155 | Train 3 | Test Hispanic 27 | 0.79 | 0.87 | 0.85 | 1179 | Train 19 | Test Hispanic 27 | 0.79 | 0.87 | 0.84 |
| 156 | Train 3 | Test Hispanic 28 | 0.78 | 0.86 | 0.83 | 1180 | Train 19 | Test Hispanic 28 | 0.78 | 0.86 | 0.82 |
| 157 | Train 3 | Test Hispanic 29 | 0.74 | 0.78 | 0.74 | 1181 | Train 19 | Test Hispanic 29 | 0.74 | 0.78 | 0.73 |
| 158 | Train 3 | Test Hispanic 30 | 0.72 | 0.73 | 0.7 | 1182 | Train 19 | Test Hispanic 30 | 0.72 | 0.73 | 0.69 |
| 159 | Train 3 | Test Hispanic 31 | 0.74 | 0.78 | 0.74 | 1183 | Train 19 | Test Hispanic 31 | 0.74 | 0.77 | 0.72 |
| 160 | Train 3 | Test Hispanic 32 | 0.72 | 0.73 | 0.7 | 1184 | Train 19 | Test Hispanic 32 | 0.72 | 0.73 | 0.69 |
| 161 | Train 3 | Test Hispanic 33 | 0.8 | 0.89 | 0.88 | 1185 | Train 19 | Test Hispanic 33 | 0.8 | 0.89 | 0.88 |
| 162 | Train 3 | Test Hispanic 34 | 0.78 | 0.87 | 0.85 | 1186 | Train 19 | Test Hispanic 34 | 0.78 | 0.87 | 0.85 |
| 163 | Train 3 | Test Hispanic 35 | 0.8 | 0.89 | 0.88 | 1187 | Train 19 | Test Hispanic 35 | 0.8 | 0.89 | 0.88 |
| 164 | Train 3 | Test Hispanic 36 | 0.79 | 0.87 | 0.85 | 1188 | Train 19 | Test Hispanic 36 | 0.79 | 0.87 | 0.85 |
| 165 | Train 3 | Test Hispanic 37 | 0.74 | 0.79 | 0.77 | 1189 | Train 19 | Test Hispanic 37 | 0.74 | 0.79 | 0.75 |
| 166 | Train 3 | Test Hispanic 38 | 0.72 | 0.73 | 0.7 | 1190 | Train 19 | Test Hispanic 38 | 0.72 | 0.73 | 0.69 |
| 167 | Train 3 | Test Hispanic 39 | 0.74 | 0.78 | 0.76 | 1191 | Train 19 | Test Hispanic 39 | 0.74 | 0.78 | 0.75 |
| 168 | Train 3 | Test Hispanic 40 | 0.72 | 0.73 | 0.71 | 1192 | Train 19 | Test Hispanic 40 | 0.72 | 0.73 | 0.69 |
| 169 | Train 3 | Test Hispanic 41 | 0.8 | 0.88 | 0.85 | 1193 | Train 19 | Test Hispanic 41 | 0.8 | 0.88 | 0.85 |
| 170 | Train 3 | Test Hispanic 42 | 0.79 | 0.86 | 0.83 | 1194 | Train 19 | Test Hispanic 42 | 0.79 | 0.86 | 0.83 |
| 171 | Train 3 | Test Hispanic 43 | 0.8 | 0.88 | 0.85 | 1195 | Train 19 | Test Hispanic 43 | 0.8 | 0.88 | 0.85 |
| 172 | Train 3 | Test Hispanic 44 | 0.79 | 0.86 | 0.83 | 1196 | Train 19 | Test Hispanic 44 | 0.79 | 0.86 | 0.83 |
| 173 | Train 3 | Test Hispanic 45 | 0.74 | 0.78 | 0.75 | 1197 | Train 19 | Test Hispanic 45 | 0.74 | 0.78 | 0.73 |
| 174 | Train 3 | Test Hispanic 46 | 0.72 | 0.73 | 0.7 | 1198 | Train 19 | Test Hispanic 46 | 0.72 | 0.73 | 0.69 |
| 175 | Train 3 | Test Hispanic 47 | 0.74 | 0.78 | 0.75 | 1199 | Train 19 | Test Hispanic 47 | 0.74 | 0.78 | 0.73 |
| 176 | Train 3 | Test Hispanic 48 | 0.72 | 0.73 | 0.71 | 1200 | Train 19 | Test Hispanic 48 | 0.72 | 0.73 | 0.69 |
| 177 | Train 3 | Test Hispanic 49 | 0.8 | 0.87 | 0.88 | 1201 | Train 19 | Test Hispanic 49 | 0.8 | 0.87 | 0.88 |
| 178 | Train 3 | Test Hispanic 50 | 0.8 | 0.87 | 0.88 | 1202 | Train 19 | Test Hispanic 50 | 0.8 | 0.87 | 0.87 |
| 179 | Train 3 | Test Hispanic 51 | 0.81 | 0.88 | 0.88 | 1203 | Train 19 | Test Hispanic 51 | 0.81 | 0.88 | 0.88 |
| 180 | Train 3 | Test Hispanic 52 | 0.8 | 0.87 | 0.88 | 1204 | Train 19 | Test Hispanic 52 | 0.8 | 0.87 | 0.87 |
| 181 | Train 3 | Test Hispanic 53 | 0.78 | 0.82 | 0.8 | 1205 | Train 19 | Test Hispanic 53 | 0.78 | 0.82 | 0.78 |
| 182 | Train 3 | Test Hispanic 54 | 0.75 | 0.74 | 0.72 | 1206 | Train 19 | Test Hispanic 54 | 0.75 | 0.74 | 0.7 |
| 183 | Train 3 | Test Hispanic 55 | 0.78 | 0.83 | 0.8 | 1207 | Train 19 | Test Hispanic 55 | 0.78 | 0.83 | 0.79 |
| 184 | Train 3 | Test Hispanic 56 | 0.75 | 0.74 | 0.73 | 1208 | Train 19 | Test Hispanic 56 | 0.75 | 0.74 | 0.71 |
| 185 | Train 3 | Test Hispanic 57 | 0.81 | 0.86 | 0.85 | 1209 | Train 19 | Test Hispanic 57 | 0.81 | 0.86 | 0.84 |
| 186 | Train 3 | Test Hispanic 58 | 0.8 | 0.86 | 0.84 | 1210 | Train 19 | Test Hispanic 58 | 0.8 | 0.86 | 0.84 |
| 187 | Train 3 | Test Hispanic 59 | 0.81 | 0.86 | 0.85 | 1211 | Train 19 | Test Hispanic 59 | 0.81 | 0.86 | 0.84 |
| 188 | Train 3 | Test Hispanic 60 | 0.81 | 0.86 | 0.85 | 1212 | Train 19 | Test Hispanic 60 | 0.81 | 0.86 | 0.84 |
| 189 | Train 3 | Test Hispanic 61 | 0.78 | 0.82 | 0.79 | 1213 | Train 19 | Test Hispanic 61 | 0.78 | 0.82 | 0.77 |
| 190 | Train 3 | Test Hispanic 62 | 0.75 | 0.74 | 0.72 | 1214 | Train 19 | Test Hispanic 62 | 0.75 | 0.74 | 0.7 |
| 191 | Train 3 | Test Hispanic 63 | 0.79 | 0.83 | 0.8 | 1215 | Train 19 | Test Hispanic 63 | 0.79 | 0.83 | 0.78 |
| 192 | Train 3 | Test Hispanic 64 | 0.76 | 0.74 | 0.73 | 1216 | Train 19 | Test Hispanic 64 | 0.76 | 0.74 | 0.71 |
| 193 | Train 4 | Test Hispanic 1 | 0.79 | 0.88 | 0.87 | 1217 | Train 20 | Test Hispanic 1 | 0.79 | 0.88 | 0.87 |
| 194 | Train 4 | Test Hispanic 2 | 0.78 | 0.89 | 0.87 | 1218 | Train 20 | Test Hispanic 2 | 0.78 | 0.88 | 0.87 |
| 195 | Train 4 | Test Hispanic 3 | 0.79 | 0.88 | 0.87 | 1219 | Train 20 | Test Hispanic 3 | 0.79 | 0.88 | 0.87 |
| 196 | Train 4 | Test Hispanic 4 | 0.78 | 0.89 | 0.87 | 1220 | Train 20 | Test Hispanic 4 | 0.78 | 0.88 | 0.88 |
| 197 | Train 4 | Test Hispanic 5 | 0.74 | 0.79 | 0.77 | 1221 | Train 20 | Test Hispanic 5 | 0.74 | 0.8 | 0.76 |
| 198 | Train 4 | Test Hispanic 6 | 0.72 | 0.74 | 0.71 | 1222 | Train 20 | Test Hispanic 6 | 0.73 | 0.74 | 0.7 |
| 199 | Train 4 | Test Hispanic 7 | 0.74 | 0.79 | 0.76 | 1223 | Train 20 | Test Hispanic 7 | 0.74 | 0.79 | 0.76 |
| 200 | Train 4 | Test Hispanic 8 | 0.73 | 0.74 | 0.71 | 1224 | Train 20 | Test Hispanic 8 | 0.73 | 0.74 | 0.7 |
| 201 | Train 4 | Test Hispanic 9 | 0.79 | 0.87 | 0.84 | 1225 | Train 20 | Test Hispanic 9 | 0.79 | 0.87 | 0.85 |
| 202 | Train 4 | Test Hispanic 10 | 0.78 | 0.87 | 0.84 | 1226 | Train 20 | Test Hispanic 10 | 0.78 | 0.87 | 0.85 |
| 203 | Train 4 | Test Hispanic 11 | 0.79 | 0.87 | 0.84 | 1227 | Train 20 | Test Hispanic 11 | 0.79 | 0.87 | 0.85 |
| 204 | Train 4 | Test Hispanic 12 | 0.79 | 0.87 | 0.84 | 1228 | Train 20 | Test Hispanic 12 | 0.79 | 0.87 | 0.85 |
| 205 | Train 4 | Test Hispanic 13 | 0.74 | 0.79 | 0.75 | 1229 | Train 20 | Test Hispanic 13 | 0.74 | 0.79 | 0.75 |
| 206 | Train 4 | Test Hispanic 14 | 0.73 | 0.74 | 0.71 | 1230 | Train 20 | Test Hispanic 14 | 0.73 | 0.74 | 0.7 |
| 207 | Train 4 | Test Hispanic 15 | 0.74 | 0.78 | 0.75 | 1231 | Train 20 | Test Hispanic 15 | 0.74 | 0.78 | 0.75 |
| 208 | Train 4 | Test Hispanic 16 | 0.73 | 0.74 | 0.71 | 1232 | Train 20 | Test Hispanic 16 | 0.73 | 0.74 | 0.71 |
| 209 | Train 4 | Test Hispanic 17 | 0.79 | 0.88 | 0.87 | 1233 | Train 20 | Test Hispanic 17 | 0.79 | 0.88 | 0.87 |
| 210 | Train 4 | Test Hispanic 18 | 0.78 | 0.89 | 0.87 | 1234 | Train 20 | Test Hispanic 18 | 0.78 | 0.88 | 0.87 |
| 211 | Train 4 | Test Hispanic 19 | 0.79 | 0.88 | 0.87 | 1235 | Train 20 | Test Hispanic 19 | 0.79 | 0.88 | 0.87 |
| 212 | Train 4 | Test Hispanic 20 | 0.78 | 0.89 | 0.87 | 1236 | Train 20 | Test Hispanic 20 | 0.78 | 0.88 | 0.88 |
| 213 | Train 4 | Test Hispanic 21 | 0.74 | 0.79 | 0.77 | 1237 | Train 20 | Test Hispanic 21 | 0.74 | 0.8 | 0.76 |
| 214 | Train 4 | Test Hispanic 22 | 0.73 | 0.74 | 0.71 | 1238 | Train 20 | Test Hispanic 22 | 0.73 | 0.74 | 0.7 |
| 215 | Train 4 | Test Hispanic 23 | 0.74 | 0.79 | 0.76 | 1239 | Train 20 | Test Hispanic 23 | 0.74 | 0.79 | 0.76 |
| 216 | Train 4 | Test Hispanic 24 | 0.73 | 0.74 | 0.71 | 1240 | Train 20 | Test Hispanic 24 | 0.73 | 0.74 | 0.7 |
| 217 | Train 4 | Test Hispanic 25 | 0.79 | 0.87 | 0.84 | 1241 | Train 20 | Test Hispanic 25 | 0.79 | 0.87 | 0.85 |
| 218 | Train 4 | Test Hispanic 26 | 0.78 | 0.87 | 0.84 | 1242 | Train 20 | Test Hispanic 26 | 0.78 | 0.87 | 0.85 |
| 219 | Train 4 | Test Hispanic 27 | 0.79 | 0.87 | 0.84 | 1243 | Train 20 | Test Hispanic 27 | 0.79 | 0.87 | 0.85 |
| 220 | Train 4 | Test Hispanic 28 | 0.79 | 0.87 | 0.84 | 1244 | Train 20 | Test Hispanic 28 | 0.79 | 0.87 | 0.85 |
| 221 | Train 4 | Test Hispanic 29 | 0.74 | 0.79 | 0.75 | 1245 | Train 20 | Test Hispanic 29 | 0.74 | 0.79 | 0.75 |
| 222 | Train 4 | Test Hispanic 30 | 0.73 | 0.75 | 0.71 | 1246 | Train 20 | Test Hispanic 30 | 0.73 | 0.74 | 0.7 |
| 223 | Train 4 | Test Hispanic 31 | 0.74 | 0.78 | 0.75 | 1247 | Train 20 | Test Hispanic 31 | 0.74 | 0.78 | 0.75 |
| 224 | Train 4 | Test Hispanic 32 | 0.73 | 0.74 | 0.71 | 1248 | Train 20 | Test Hispanic 32 | 0.73 | 0.74 | 0.71 |
| 225 | Train 4 | Test Hispanic 33 | 0.79 | 0.88 | 0.87 | 1249 | Train 20 | Test Hispanic 33 | 0.79 | 0.88 | 0.87 |
| 226 | Train 4 | Test Hispanic 34 | 0.79 | 0.89 | 0.87 | 1250 | Train 20 | Test Hispanic 34 | 0.79 | 0.89 | 0.88 |
| 227 | Train 4 | Test Hispanic 35 | 0.8 | 0.89 | 0.88 | 1251 | Train 20 | Test Hispanic 35 | 0.8 | 0.88 | 0.88 |
| 228 | Train 4 | Test Hispanic 36 | 0.79 | 0.89 | 0.87 | 1252 | Train 20 | Test Hispanic 36 | 0.79 | 0.89 | 0.88 |
| 229 | Train 4 | Test Hispanic 37 | 0.75 | 0.8 | 0.77 | 1253 | Train 20 | Test Hispanic 37 | 0.75 | 0.8 | 0.76 |
| 230 | Train 4 | Test Hispanic 38 | 0.73 | 0.74 | 0.71 | 1254 | Train 20 | Test Hispanic 38 | 0.73 | 0.74 | 0.7 |
| 231 | Train 4 | Test Hispanic 39 | 0.75 | 0.79 | 0.77 | 1255 | Train 20 | Test Hispanic 39 | 0.75 | 0.79 | 0.76 |
| 232 | Train 4 | Test Hispanic 40 | 0.73 | 0.74 | 0.72 | 1256 | Train 20 | Test Hispanic 40 | 0.73 | 0.74 | 0.71 |
| 233 | Train 4 | Test Hispanic 41 | 0.79 | 0.87 | 0.85 | 1257 | Train 20 | Test Hispanic 41 | 0.79 | 0.87 | 0.85 |
| 234 | Train 4 | Test Hispanic 42 | 0.79 | 0.87 | 0.85 | 1258 | Train 20 | Test Hispanic 42 | 0.79 | 0.87 | 0.86 |
| 235 | Train 4 | Test Hispanic 43 | 0.8 | 0.87 | 0.85 | 1259 | Train 20 | Test Hispanic 43 | 0.8 | 0.87 | 0.85 |
| 236 | Train 4 | Test Hispanic 44 | 0.79 | 0.88 | 0.85 | 1260 | Train 20 | Test Hispanic 44 | 0.79 | 0.87 | 0.85 |
| 237 | Train 4 | Test Hispanic 45 | 0.75 | 0.79 | 0.76 | 1261 | Train 20 | Test Hispanic 45 | 0.75 | 0.79 | 0.75 |
| 238 | Train 4 | Test Hispanic 46 | 0.73 | 0.75 | 0.72 | 1262 | Train 20 | Test Hispanic 46 | 0.73 | 0.75 | 0.7 |
| 239 | Train 4 | Test Hispanic 47 | 0.75 | 0.79 | 0.75 | 1263 | Train 20 | Test Hispanic 47 | 0.75 | 0.79 | 0.75 |
| 240 | Train 4 | Test Hispanic 48 | 0.73 | 0.74 | 0.72 | 1264 | Train 20 | Test Hispanic 48 | 0.73 | 0.74 | 0.71 |
| 241 | Train 4 | Test Hispanic 49 | 0.8 | 0.87 | 0.88 | 1265 | Train 20 | Test Hispanic 49 | 0.8 | 0.87 | 0.87 |
| 242 | Train 4 | Test Hispanic 50 | 0.79 | 0.87 | 0.87 | 1266 | Train 20 | Test Hispanic 50 | 0.79 | 0.87 | 0.87 |
| 243 | Train 4 | Test Hispanic 51 | 0.8 | 0.87 | 0.88 | 1267 | Train 20 | Test Hispanic 51 | 0.8 | 0.87 | 0.87 |
| 244 | Train 4 | Test Hispanic 52 | 0.8 | 0.87 | 0.88 | 1268 | Train 20 | Test Hispanic 52 | 0.8 | 0.87 | 0.87 |
| 245 | Train 4 | Test Hispanic 53 | 0.78 | 0.82 | 0.8 | 1269 | Train 20 | Test Hispanic 53 | 0.78 | 0.82 | 0.79 |
| 246 | Train 4 | Test Hispanic 54 | 0.75 | 0.76 | 0.74 | 1270 | Train 20 | Test Hispanic 54 | 0.75 | 0.76 | 0.71 |
| 247 | Train 4 | Test Hispanic 55 | 0.78 | 0.83 | 0.81 | 1271 | Train 20 | Test Hispanic 55 | 0.78 | 0.83 | 0.8 |
| 248 | Train 4 | Test Hispanic 56 | 0.75 | 0.76 | 0.74 | 1272 | Train 20 | Test Hispanic 56 | 0.75 | 0.75 | 0.72 |
| 249 | Train 4 | Test Hispanic 57 | 0.8 | 0.86 | 0.84 | 1273 | Train 20 | Test Hispanic 57 | 0.8 | 0.86 | 0.84 |
| 250 | Train 4 | Test Hispanic 58 | 0.79 | 0.86 | 0.84 | 1274 | Train 20 | Test Hispanic 58 | 0.79 | 0.85 | 0.84 |
| 251 | Train 4 | Test Hispanic 59 | 0.81 | 0.86 | 0.85 | 1275 | Train 20 | Test Hispanic 59 | 0.81 | 0.86 | 0.84 |
| 252 | Train 4 | Test Hispanic 60 | 0.8 | 0.86 | 0.84 | 1276 | Train 20 | Test Hispanic 60 | 0.8 | 0.86 | 0.84 |
| 253 | Train 4 | Test Hispanic 61 | 0.78 | 0.82 | 0.8 | 1277 | Train 20 | Test Hispanic 61 | 0.78 | 0.82 | 0.79 |
| 254 | Train 4 | Test Hispanic 62 | 0.75 | 0.76 | 0.74 | 1278 | Train 20 | Test Hispanic 62 | 0.75 | 0.76 | 0.72 |
| 255 | Train 4 | Test Hispanic 63 | 0.79 | 0.83 | 0.8 | 1279 | Train 20 | Test Hispanic 63 | 0.79 | 0.83 | 0.8 |
| 256 | Train 4 | Test Hispanic 64 | 0.75 | 0.76 | 0.74 | 1280 | Train 20 | Test Hispanic 64 | 0.75 | 0.76 | 0.72 |
| 257 | Train 5 | Test Hispanic 1 | 0.77 | 0.86 | 0.85 | 1281 | Train 21 | Test Hispanic 1 | 0.77 | 0.86 | 0.85 |
| 258 | Train 5 | Test Hispanic 2 | 0.77 | 0.85 | 0.83 | 1282 | Train 21 | Test Hispanic 2 | 0.77 | 0.85 | 0.84 |
| 259 | Train 5 | Test Hispanic 3 | 0.78 | 0.86 | 0.85 | 1283 | Train 21 | Test Hispanic 3 | 0.78 | 0.86 | 0.85 |
| 260 | Train 5 | Test Hispanic 4 | 0.77 | 0.85 | 0.83 | 1284 | Train 21 | Test Hispanic 4 | 0.77 | 0.85 | 0.83 |
| 261 | Train 5 | Test Hispanic 5 | 0.75 | 0.86 | 0.84 | 1285 | Train 21 | Test Hispanic 5 | 0.75 | 0.86 | 0.84 |
| 262 | Train 5 | Test Hispanic 6 | 0.72 | 0.77 | 0.76 | 1286 | Train 21 | Test Hispanic 6 | 0.72 | 0.77 | 0.74 |
| 263 | Train 5 | Test Hispanic 7 | 0.75 | 0.85 | 0.83 | 1287 | Train 21 | Test Hispanic 7 | 0.75 | 0.85 | 0.84 |
| 264 | Train 5 | Test Hispanic 8 | 0.72 | 0.76 | 0.76 | 1288 | Train 21 | Test Hispanic 8 | 0.72 | 0.76 | 0.74 |
| 265 | Train 5 | Test Hispanic 9 | 0.77 | 0.85 | 0.83 | 1289 | Train 21 | Test Hispanic 9 | 0.77 | 0.85 | 0.82 |
| 266 | Train 5 | Test Hispanic 10 | 0.77 | 0.84 | 0.81 | 1290 | Train 21 | Test Hispanic 10 | 0.77 | 0.84 | 0.82 |
| 267 | Train 5 | Test Hispanic 11 | 0.78 | 0.85 | 0.83 | 1291 | Train 21 | Test Hispanic 11 | 0.78 | 0.85 | 0.83 |
| 268 | Train 5 | Test Hispanic 12 | 0.77 | 0.84 | 0.81 | 1292 | Train 21 | Test Hispanic 12 | 0.77 | 0.84 | 0.82 |
| 269 | Train 5 | Test Hispanic 13 | 0.75 | 0.85 | 0.82 | 1293 | Train 21 | Test Hispanic 13 | 0.75 | 0.85 | 0.82 |
| 270 | Train 5 | Test Hispanic 14 | 0.72 | 0.77 | 0.76 | 1294 | Train 21 | Test Hispanic 14 | 0.72 | 0.77 | 0.74 |
| 271 | Train 5 | Test Hispanic 15 | 0.75 | 0.84 | 0.82 | 1295 | Train 21 | Test Hispanic 15 | 0.75 | 0.84 | 0.82 |
| 272 | Train 5 | Test Hispanic 16 | 0.72 | 0.76 | 0.76 | 1296 | Train 21 | Test Hispanic 16 | 0.72 | 0.76 | 0.74 |
| 273 | Train 5 | Test Hispanic 17 | 0.77 | 0.86 | 0.85 | 1297 | Train 21 | Test Hispanic 17 | 0.77 | 0.86 | 0.85 |
| 274 | Train 5 | Test Hispanic 18 | 0.77 | 0.85 | 0.83 | 1298 | Train 21 | Test Hispanic 18 | 0.77 | 0.85 | 0.83 |
| 275 | Train 5 | Test Hispanic 19 | 0.78 | 0.86 | 0.85 | 1299 | Train 21 | Test Hispanic 19 | 0.78 | 0.86 | 0.85 |
| 276 | Train 5 | Test Hispanic 20 | 0.77 | 0.85 | 0.83 | 1300 | Train 21 | Test Hispanic 20 | 0.77 | 0.85 | 0.83 |
| 277 | Train 5 | Test Hispanic 21 | 0.75 | 0.86 | 0.84 | 1301 | Train 21 | Test Hispanic 21 | 0.75 | 0.86 | 0.84 |
| 278 | Train 5 | Test Hispanic 22 | 0.72 | 0.77 | 0.76 | 1302 | Train 21 | Test Hispanic 22 | 0.72 | 0.77 | 0.74 |
| 279 | Train 5 | Test Hispanic 23 | 0.75 | 0.85 | 0.83 | 1303 | Train 21 | Test Hispanic 23 | 0.75 | 0.85 | 0.84 |
| 280 | Train 5 | Test Hispanic 24 | 0.72 | 0.77 | 0.76 | 1304 | Train 21 | Test Hispanic 24 | 0.72 | 0.76 | 0.74 |
| 281 | Train 5 | Test Hispanic 25 | 0.77 | 0.85 | 0.83 | 1305 | Train 21 | Test Hispanic 25 | 0.77 | 0.85 | 0.82 |
| 282 | Train 5 | Test Hispanic 26 | 0.77 | 0.84 | 0.81 | 1306 | Train 21 | Test Hispanic 26 | 0.77 | 0.84 | 0.82 |
| 283 | Train 5 | Test Hispanic 27 | 0.78 | 0.85 | 0.83 | 1307 | Train 21 | Test Hispanic 27 | 0.78 | 0.85 | 0.83 |
| 284 | Train 5 | Test Hispanic 28 | 0.77 | 0.84 | 0.81 | 1308 | Train 21 | Test Hispanic 28 | 0.77 | 0.84 | 0.82 |
| 285 | Train 5 | Test Hispanic 29 | 0.75 | 0.85 | 0.82 | 1309 | Train 21 | Test Hispanic 29 | 0.75 | 0.85 | 0.82 |
| 286 | Train 5 | Test Hispanic 30 | 0.72 | 0.77 | 0.76 | 1310 | Train 21 | Test Hispanic 30 | 0.72 | 0.77 | 0.74 |
| 287 | Train 5 | Test Hispanic 31 | 0.75 | 0.84 | 0.82 | 1311 | Train 21 | Test Hispanic 31 | 0.75 | 0.84 | 0.82 |
| 288 | Train 5 | Test Hispanic 32 | 0.72 | 0.77 | 0.76 | 1312 | Train 21 | Test Hispanic 32 | 0.72 | 0.76 | 0.74 |
| 289 | Train 5 | Test Hispanic 33 | 0.78 | 0.86 | 0.85 | 1313 | Train 21 | Test Hispanic 33 | 0.78 | 0.86 | 0.85 |
| 290 | Train 5 | Test Hispanic 34 | 0.78 | 0.85 | 0.83 | 1314 | Train 21 | Test Hispanic 34 | 0.78 | 0.85 | 0.84 |
| 291 | Train 5 | Test Hispanic 35 | 0.79 | 0.86 | 0.85 | 1315 | Train 21 | Test Hispanic 35 | 0.79 | 0.86 | 0.85 |
| 292 | Train 5 | Test Hispanic 36 | 0.78 | 0.85 | 0.83 | 1316 | Train 21 | Test Hispanic 36 | 0.78 | 0.85 | 0.84 |
| 293 | Train 5 | Test Hispanic 37 | 0.75 | 0.86 | 0.84 | 1317 | Train 21 | Test Hispanic 37 | 0.75 | 0.86 | 0.84 |
| 294 | Train 5 | Test Hispanic 38 | 0.72 | 0.77 | 0.76 | 1318 | Train 21 | Test Hispanic 38 | 0.72 | 0.77 | 0.74 |
| 295 | Train 5 | Test Hispanic 39 | 0.75 | 0.85 | 0.84 | 1319 | Train 21 | Test Hispanic 39 | 0.75 | 0.85 | 0.84 |
| 296 | Train 5 | Test Hispanic 40 | 0.73 | 0.77 | 0.76 | 1320 | Train 21 | Test Hispanic 40 | 0.73 | 0.76 | 0.75 |
| 297 | Train 5 | Test Hispanic 41 | 0.78 | 0.85 | 0.83 | 1321 | Train 21 | Test Hispanic 41 | 0.78 | 0.85 | 0.83 |
| 298 | Train 5 | Test Hispanic 42 | 0.78 | 0.84 | 0.82 | 1322 | Train 21 | Test Hispanic 42 | 0.78 | 0.84 | 0.82 |
| 299 | Train 5 | Test Hispanic 43 | 0.79 | 0.85 | 0.83 | 1323 | Train 21 | Test Hispanic 43 | 0.79 | 0.85 | 0.83 |
| 300 | Train 5 | Test Hispanic 44 | 0.78 | 0.84 | 0.82 | 1324 | Train 21 | Test Hispanic 44 | 0.78 | 0.84 | 0.82 |
| 301 | Train 5 | Test Hispanic 45 | 0.75 | 0.85 | 0.83 | 1325 | Train 21 | Test Hispanic 45 | 0.75 | 0.85 | 0.82 |
| 302 | Train 5 | Test Hispanic 46 | 0.72 | 0.77 | 0.76 | 1326 | Train 21 | Test Hispanic 46 | 0.72 | 0.77 | 0.75 |
| 303 | Train 5 | Test Hispanic 47 | 0.75 | 0.84 | 0.82 | 1327 | Train 21 | Test Hispanic 47 | 0.75 | 0.84 | 0.82 |
| 304 | Train 5 | Test Hispanic 48 | 0.73 | 0.77 | 0.76 | 1328 | Train 21 | Test Hispanic 48 | 0.73 | 0.76 | 0.75 |
| 305 | Train 5 | Test Hispanic 49 | 0.79 | 0.86 | 0.86 | 1329 | Train 21 | Test Hispanic 49 | 0.79 | 0.86 | 0.85 |
| 306 | Train 5 | Test Hispanic 50 | 0.79 | 0.86 | 0.85 | 1330 | Train 21 | Test Hispanic 50 | 0.79 | 0.86 | 0.85 |
| 307 | Train 5 | Test Hispanic 51 | 0.8 | 0.86 | 0.86 | 1331 | Train 21 | Test Hispanic 51 | 0.8 | 0.86 | 0.85 |
| 308 | Train 5 | Test Hispanic 52 | 0.79 | 0.86 | 0.85 | 1332 | Train 21 | Test Hispanic 52 | 0.79 | 0.86 | 0.85 |
| 309 | Train 5 | Test Hispanic 53 | 0.78 | 0.86 | 0.84 | 1333 | Train 21 | Test Hispanic 53 | 0.78 | 0.86 | 0.84 |
| 310 | Train 5 | Test Hispanic 54 | 0.74 | 0.77 | 0.75 | 1334 | Train 21 | Test Hispanic 54 | 0.74 | 0.76 | 0.73 |
| 311 | Train 5 | Test Hispanic 55 | 0.78 | 0.86 | 0.84 | 1335 | Train 21 | Test Hispanic 55 | 0.78 | 0.86 | 0.84 |
| 312 | Train 5 | Test Hispanic 56 | 0.74 | 0.76 | 0.75 | 1336 | Train 21 | Test Hispanic 56 | 0.74 | 0.75 | 0.74 |
| 313 | Train 5 | Test Hispanic 57 | 0.79 | 0.85 | 0.84 | 1337 | Train 21 | Test Hispanic 57 | 0.79 | 0.85 | 0.83 |
| 314 | Train 5 | Test Hispanic 58 | 0.79 | 0.85 | 0.83 | 1338 | Train 21 | Test Hispanic 58 | 0.79 | 0.85 | 0.83 |
| 315 | Train 5 | Test Hispanic 59 | 0.8 | 0.85 | 0.84 | 1339 | Train 21 | Test Hispanic 59 | 0.8 | 0.85 | 0.84 |
| 316 | Train 5 | Test Hispanic 60 | 0.8 | 0.85 | 0.83 | 1340 | Train 21 | Test Hispanic 60 | 0.8 | 0.85 | 0.83 |
| 317 | Train 5 | Test Hispanic 61 | 0.78 | 0.85 | 0.83 | 1341 | Train 21 | Test Hispanic 61 | 0.78 | 0.86 | 0.83 |
| 318 | Train 5 | Test Hispanic 62 | 0.74 | 0.77 | 0.75 | 1342 | Train 21 | Test Hispanic 62 | 0.74 | 0.76 | 0.74 |
| 319 | Train 5 | Test Hispanic 63 | 0.78 | 0.86 | 0.83 | 1343 | Train 21 | Test Hispanic 63 | 0.78 | 0.86 | 0.83 |
| 320 | Train 5 | Test Hispanic 64 | 0.74 | 0.76 | 0.75 | 1344 | Train 21 | Test Hispanic 64 | 0.74 | 0.75 | 0.74 |
| 321 | Train 6 | Test Hispanic 1 | 0.77 | 0.84 | 0.77 | 1345 | Train 22 | Test Hispanic 1 | 0.77 | 0.84 | 0.8 |
| 322 | Train 6 | Test Hispanic 2 | 0.77 | 0.84 | 0.78 | 1346 | Train 22 | Test Hispanic 2 | 0.77 | 0.84 | 0.8 |
| 323 | Train 6 | Test Hispanic 3 | 0.77 | 0.84 | 0.77 | 1347 | Train 22 | Test Hispanic 3 | 0.77 | 0.84 | 0.8 |
| 324 | Train 6 | Test Hispanic 4 | 0.77 | 0.84 | 0.78 | 1348 | Train 22 | Test Hispanic 4 | 0.77 | 0.84 | 0.8 |
| 325 | Train 6 | Test Hispanic 5 | 0.74 | 0.83 | 0.78 | 1349 | Train 22 | Test Hispanic 5 | 0.74 | 0.83 | 0.79 |
| 326 | Train 6 | Test Hispanic 6 | 0.75 | 0.84 | 0.79 | 1350 | Train 22 | Test Hispanic 6 | 0.75 | 0.84 | 0.79 |
| 327 | Train 6 | Test Hispanic 7 | 0.74 | 0.82 | 0.77 | 1351 | Train 22 | Test Hispanic 7 | 0.74 | 0.82 | 0.78 |
| 328 | Train 6 | Test Hispanic 8 | 0.75 | 0.83 | 0.78 | 1352 | Train 22 | Test Hispanic 8 | 0.75 | 0.83 | 0.79 |
| 329 | Train 6 | Test Hispanic 9 | 0.77 | 0.84 | 0.78 | 1353 | Train 22 | Test Hispanic 9 | 0.77 | 0.85 | 0.8 |
| 330 | Train 6 | Test Hispanic 10 | 0.77 | 0.85 | 0.78 | 1354 | Train 22 | Test Hispanic 10 | 0.77 | 0.85 | 0.8 |
| 331 | Train 6 | Test Hispanic 11 | 0.77 | 0.85 | 0.78 | 1355 | Train 22 | Test Hispanic 11 | 0.77 | 0.85 | 0.8 |
| 332 | Train 6 | Test Hispanic 12 | 0.77 | 0.85 | 0.79 | 1356 | Train 22 | Test Hispanic 12 | 0.77 | 0.85 | 0.8 |
| 333 | Train 6 | Test Hispanic 13 | 0.74 | 0.84 | 0.79 | 1357 | Train 22 | Test Hispanic 13 | 0.74 | 0.84 | 0.79 |
| 334 | Train 6 | Test Hispanic 14 | 0.75 | 0.84 | 0.79 | 1358 | Train 22 | Test Hispanic 14 | 0.75 | 0.84 | 0.79 |
| 335 | Train 6 | Test Hispanic 15 | 0.74 | 0.83 | 0.78 | 1359 | Train 22 | Test Hispanic 15 | 0.74 | 0.83 | 0.79 |
| 336 | Train 6 | Test Hispanic 16 | 0.75 | 0.83 | 0.78 | 1360 | Train 22 | Test Hispanic 16 | 0.75 | 0.83 | 0.79 |
| 337 | Train 6 | Test Hispanic 17 | 0.77 | 0.84 | 0.77 | 1361 | Train 22 | Test Hispanic 17 | 0.77 | 0.84 | 0.79 |
| 338 | Train 6 | Test Hispanic 18 | 0.77 | 0.84 | 0.78 | 1362 | Train 22 | Test Hispanic 18 | 0.77 | 0.84 | 0.8 |
| 339 | Train 6 | Test Hispanic 19 | 0.77 | 0.84 | 0.77 | 1363 | Train 22 | Test Hispanic 19 | 0.77 | 0.84 | 0.8 |
| 340 | Train 6 | Test Hispanic 20 | 0.77 | 0.84 | 0.78 | 1364 | Train 22 | Test Hispanic 20 | 0.77 | 0.84 | 0.8 |
| 341 | Train 6 | Test Hispanic 21 | 0.74 | 0.83 | 0.78 | 1365 | Train 22 | Test Hispanic 21 | 0.74 | 0.83 | 0.79 |
| 342 | Train 6 | Test Hispanic 22 | 0.75 | 0.84 | 0.79 | 1366 | Train 22 | Test Hispanic 22 | 0.75 | 0.84 | 0.79 |
| 343 | Train 6 | Test Hispanic 23 | 0.74 | 0.82 | 0.77 | 1367 | Train 22 | Test Hispanic 23 | 0.74 | 0.82 | 0.78 |
| 344 | Train 6 | Test Hispanic 24 | 0.75 | 0.83 | 0.78 | 1368 | Train 22 | Test Hispanic 24 | 0.75 | 0.83 | 0.79 |
| 345 | Train 6 | Test Hispanic 25 | 0.77 | 0.84 | 0.78 | 1369 | Train 22 | Test Hispanic 25 | 0.77 | 0.85 | 0.8 |
| 346 | Train 6 | Test Hispanic 26 | 0.77 | 0.85 | 0.78 | 1370 | Train 22 | Test Hispanic 26 | 0.77 | 0.85 | 0.8 |
| 347 | Train 6 | Test Hispanic 27 | 0.77 | 0.85 | 0.78 | 1371 | Train 22 | Test Hispanic 27 | 0.77 | 0.85 | 0.8 |
| 348 | Train 6 | Test Hispanic 28 | 0.77 | 0.85 | 0.79 | 1372 | Train 22 | Test Hispanic 28 | 0.77 | 0.85 | 0.8 |
| 349 | Train 6 | Test Hispanic 29 | 0.74 | 0.84 | 0.79 | 1373 | Train 22 | Test Hispanic 29 | 0.74 | 0.84 | 0.79 |
| 350 | Train 6 | Test Hispanic 30 | 0.75 | 0.84 | 0.79 | 1374 | Train 22 | Test Hispanic 30 | 0.75 | 0.84 | 0.79 |
| 351 | Train 6 | Test Hispanic 31 | 0.74 | 0.83 | 0.78 | 1375 | Train 22 | Test Hispanic 31 | 0.74 | 0.83 | 0.79 |
| 352 | Train 6 | Test Hispanic 32 | 0.75 | 0.83 | 0.78 | 1376 | Train 22 | Test Hispanic 32 | 0.75 | 0.83 | 0.79 |
| 353 | Train 6 | Test Hispanic 33 | 0.78 | 0.85 | 0.78 | 1377 | Train 22 | Test Hispanic 33 | 0.78 | 0.85 | 0.8 |
| 354 | Train 6 | Test Hispanic 34 | 0.78 | 0.84 | 0.78 | 1378 | Train 22 | Test Hispanic 34 | 0.78 | 0.85 | 0.8 |
| 355 | Train 6 | Test Hispanic 35 | 0.78 | 0.85 | 0.78 | 1379 | Train 22 | Test Hispanic 35 | 0.78 | 0.85 | 0.8 |
| 356 | Train 6 | Test Hispanic 36 | 0.78 | 0.85 | 0.78 | 1380 | Train 22 | Test Hispanic 36 | 0.78 | 0.85 | 0.8 |
| 357 | Train 6 | Test Hispanic 37 | 0.75 | 0.83 | 0.78 | 1381 | Train 22 | Test Hispanic 37 | 0.75 | 0.83 | 0.79 |
| 358 | Train 6 | Test Hispanic 38 | 0.75 | 0.84 | 0.79 | 1382 | Train 22 | Test Hispanic 38 | 0.75 | 0.84 | 0.8 |
| 359 | Train 6 | Test Hispanic 39 | 0.75 | 0.83 | 0.78 | 1383 | Train 22 | Test Hispanic 39 | 0.75 | 0.83 | 0.79 |
| 360 | Train 6 | Test Hispanic 40 | 0.75 | 0.83 | 0.79 | 1384 | Train 22 | Test Hispanic 40 | 0.75 | 0.83 | 0.79 |
| 361 | Train 6 | Test Hispanic 41 | 0.78 | 0.85 | 0.79 | 1385 | Train 22 | Test Hispanic 41 | 0.78 | 0.85 | 0.81 |
| 362 | Train 6 | Test Hispanic 42 | 0.78 | 0.85 | 0.79 | 1386 | Train 22 | Test Hispanic 42 | 0.78 | 0.85 | 0.81 |
| 363 | Train 6 | Test Hispanic 43 | 0.78 | 0.85 | 0.79 | 1387 | Train 22 | Test Hispanic 43 | 0.78 | 0.85 | 0.81 |
| 364 | Train 6 | Test Hispanic 44 | 0.78 | 0.85 | 0.79 | 1388 | Train 22 | Test Hispanic 44 | 0.78 | 0.85 | 0.81 |
| 365 | Train 6 | Test Hispanic 45 | 0.75 | 0.84 | 0.79 | 1389 | Train 22 | Test Hispanic 45 | 0.75 | 0.84 | 0.79 |
| 366 | Train 6 | Test Hispanic 46 | 0.75 | 0.84 | 0.79 | 1390 | Train 22 | Test Hispanic 46 | 0.75 | 0.84 | 0.79 |
| 367 | Train 6 | Test Hispanic 47 | 0.75 | 0.83 | 0.78 | 1391 | Train 22 | Test Hispanic 47 | 0.75 | 0.83 | 0.79 |
| 368 | Train 6 | Test Hispanic 48 | 0.75 | 0.83 | 0.78 | 1392 | Train 22 | Test Hispanic 48 | 0.75 | 0.83 | 0.79 |
| 369 | Train 6 | Test Hispanic 49 | 0.79 | 0.85 | 0.81 | 1393 | Train 22 | Test Hispanic 49 | 0.79 | 0.85 | 0.81 |
| 370 | Train 6 | Test Hispanic 50 | 0.79 | 0.85 | 0.8 | 1394 | Train 22 | Test Hispanic 50 | 0.79 | 0.85 | 0.8 |
| 371 | Train 6 | Test Hispanic 51 | 0.8 | 0.85 | 0.81 | 1395 | Train 22 | Test Hispanic 51 | 0.8 | 0.85 | 0.81 |
| 372 | Train 6 | Test Hispanic 52 | 0.79 | 0.85 | 0.81 | 1396 | Train 22 | Test Hispanic 52 | 0.79 | 0.85 | 0.81 |
| 373 | Train 6 | Test Hispanic 53 | 0.77 | 0.86 | 0.79 | 1397 | Train 22 | Test Hispanic 53 | 0.77 | 0.86 | 0.8 |
| 374 | Train 6 | Test Hispanic 54 | 0.78 | 0.85 | 0.79 | 1398 | Train 22 | Test Hispanic 54 | 0.78 | 0.85 | 0.8 |
| 375 | Train 6 | Test Hispanic 55 | 0.78 | 0.86 | 0.8 | 1399 | Train 22 | Test Hispanic 55 | 0.78 | 0.86 | 0.81 |
| 376 | Train 6 | Test Hispanic 56 | 0.77 | 0.84 | 0.79 | 1400 | Train 22 | Test Hispanic 56 | 0.77 | 0.84 | 0.79 |
| 377 | Train 6 | Test Hispanic 57 | 0.79 | 0.86 | 0.82 | 1401 | Train 22 | Test Hispanic 57 | 0.79 | 0.86 | 0.82 |
| 378 | Train 6 | Test Hispanic 58 | 0.79 | 0.86 | 0.82 | 1402 | Train 22 | Test Hispanic 58 | 0.79 | 0.86 | 0.82 |
| 379 | Train 6 | Test Hispanic 59 | 0.8 | 0.86 | 0.82 | 1403 | Train 22 | Test Hispanic 59 | 0.8 | 0.86 | 0.82 |
| 380 | Train 6 | Test Hispanic 60 | 0.79 | 0.86 | 0.82 | 1404 | Train 22 | Test Hispanic 60 | 0.79 | 0.86 | 0.82 |
| 381 | Train 6 | Test Hispanic 61 | 0.77 | 0.86 | 0.8 | 1405 | Train 22 | Test Hispanic 61 | 0.77 | 0.86 | 0.81 |
| 382 | Train 6 | Test Hispanic 62 | 0.78 | 0.85 | 0.79 | 1406 | Train 22 | Test Hispanic 62 | 0.78 | 0.85 | 0.8 |
| 383 | Train 6 | Test Hispanic 63 | 0.78 | 0.86 | 0.81 | 1407 | Train 22 | Test Hispanic 63 | 0.78 | 0.86 | 0.81 |
| 384 | Train 6 | Test Hispanic 64 | 0.77 | 0.84 | 0.79 | 1408 | Train 22 | Test Hispanic 64 | 0.77 | 0.84 | 0.79 |
| 385 | Train 7 | Test Hispanic 1 | 0.77 | 0.86 | 0.85 | 1409 | Train 23 | Test Hispanic 1 | 0.77 | 0.86 | 0.85 |
| 386 | Train 7 | Test Hispanic 2 | 0.77 | 0.85 | 0.83 | 1410 | Train 23 | Test Hispanic 2 | 0.77 | 0.84 | 0.83 |
| 387 | Train 7 | Test Hispanic 3 | 0.78 | 0.86 | 0.85 | 1411 | Train 23 | Test Hispanic 3 | 0.78 | 0.86 | 0.85 |
| 388 | Train 7 | Test Hispanic 4 | 0.77 | 0.85 | 0.84 | 1412 | Train 23 | Test Hispanic 4 | 0.77 | 0.85 | 0.83 |
| 389 | Train 7 | Test Hispanic 5 | 0.75 | 0.86 | 0.84 | 1413 | Train 23 | Test Hispanic 5 | 0.75 | 0.86 | 0.84 |
| 390 | Train 7 | Test Hispanic 6 | 0.72 | 0.77 | 0.75 | 1414 | Train 23 | Test Hispanic 6 | 0.72 | 0.77 | 0.76 |
| 391 | Train 7 | Test Hispanic 7 | 0.75 | 0.86 | 0.84 | 1415 | Train 23 | Test Hispanic 7 | 0.75 | 0.86 | 0.84 |
| 392 | Train 7 | Test Hispanic 8 | 0.72 | 0.77 | 0.75 | 1416 | Train 23 | Test Hispanic 8 | 0.72 | 0.77 | 0.76 |
| 393 | Train 7 | Test Hispanic 9 | 0.77 | 0.84 | 0.82 | 1417 | Train 23 | Test Hispanic 9 | 0.77 | 0.84 | 0.83 |
| 394 | Train 7 | Test Hispanic 10 | 0.77 | 0.84 | 0.81 | 1418 | Train 23 | Test Hispanic 10 | 0.77 | 0.84 | 0.82 |
| 395 | Train 7 | Test Hispanic 11 | 0.78 | 0.85 | 0.83 | 1419 | Train 23 | Test Hispanic 11 | 0.78 | 0.84 | 0.83 |
| 396 | Train 7 | Test Hispanic 12 | 0.77 | 0.84 | 0.82 | 1420 | Train 23 | Test Hispanic 12 | 0.77 | 0.84 | 0.82 |
| 397 | Train 7 | Test Hispanic 13 | 0.75 | 0.85 | 0.82 | 1421 | Train 23 | Test Hispanic 13 | 0.75 | 0.85 | 0.82 |
| 398 | Train 7 | Test Hispanic 14 | 0.72 | 0.77 | 0.75 | 1422 | Train 23 | Test Hispanic 14 | 0.72 | 0.77 | 0.76 |
| 399 | Train 7 | Test Hispanic 15 | 0.75 | 0.84 | 0.82 | 1423 | Train 23 | Test Hispanic 15 | 0.75 | 0.84 | 0.82 |
| 400 | Train 7 | Test Hispanic 16 | 0.72 | 0.77 | 0.75 | 1424 | Train 23 | Test Hispanic 16 | 0.72 | 0.77 | 0.76 |
| 401 | Train 7 | Test Hispanic 17 | 0.77 | 0.86 | 0.85 | 1425 | Train 23 | Test Hispanic 17 | 0.77 | 0.86 | 0.85 |
| 402 | Train 7 | Test Hispanic 18 | 0.77 | 0.85 | 0.83 | 1426 | Train 23 | Test Hispanic 18 | 0.77 | 0.84 | 0.83 |
| 403 | Train 7 | Test Hispanic 19 | 0.78 | 0.86 | 0.85 | 1427 | Train 23 | Test Hispanic 19 | 0.78 | 0.86 | 0.85 |
| 404 | Train 7 | Test Hispanic 20 | 0.77 | 0.85 | 0.84 | 1428 | Train 23 | Test Hispanic 20 | 0.77 | 0.85 | 0.83 |
| 405 | Train 7 | Test Hispanic 21 | 0.75 | 0.86 | 0.84 | 1429 | Train 23 | Test Hispanic 21 | 0.75 | 0.86 | 0.84 |
| 406 | Train 7 | Test Hispanic 22 | 0.72 | 0.77 | 0.75 | 1430 | Train 23 | Test Hispanic 22 | 0.72 | 0.77 | 0.76 |
| 407 | Train 7 | Test Hispanic 23 | 0.75 | 0.86 | 0.84 | 1431 | Train 23 | Test Hispanic 23 | 0.75 | 0.86 | 0.84 |
| 408 | Train 7 | Test Hispanic 24 | 0.72 | 0.77 | 0.75 | 1432 | Train 23 | Test Hispanic 24 | 0.72 | 0.77 | 0.76 |
| 409 | Train 7 | Test Hispanic 25 | 0.77 | 0.84 | 0.82 | 1433 | Train 23 | Test Hispanic 25 | 0.77 | 0.84 | 0.83 |
| 410 | Train 7 | Test Hispanic 26 | 0.77 | 0.84 | 0.81 | 1434 | Train 23 | Test Hispanic 26 | 0.77 | 0.84 | 0.82 |
| 411 | Train 7 | Test Hispanic 27 | 0.78 | 0.85 | 0.83 | 1435 | Train 23 | Test Hispanic 27 | 0.78 | 0.84 | 0.83 |
| 412 | Train 7 | Test Hispanic 28 | 0.77 | 0.84 | 0.82 | 1436 | Train 23 | Test Hispanic 28 | 0.77 | 0.84 | 0.82 |
| 413 | Train 7 | Test Hispanic 29 | 0.75 | 0.85 | 0.82 | 1437 | Train 23 | Test Hispanic 29 | 0.75 | 0.85 | 0.82 |
| 414 | Train 7 | Test Hispanic 30 | 0.72 | 0.77 | 0.75 | 1438 | Train 23 | Test Hispanic 30 | 0.72 | 0.77 | 0.76 |
| 415 | Train 7 | Test Hispanic 31 | 0.75 | 0.84 | 0.82 | 1439 | Train 23 | Test Hispanic 31 | 0.75 | 0.84 | 0.82 |
| 416 | Train 7 | Test Hispanic 32 | 0.72 | 0.77 | 0.75 | 1440 | Train 23 | Test Hispanic 32 | 0.72 | 0.77 | 0.76 |
| 417 | Train 7 | Test Hispanic 33 | 0.78 | 0.86 | 0.85 | 1441 | Train 23 | Test Hispanic 33 | 0.78 | 0.86 | 0.85 |
| 418 | Train 7 | Test Hispanic 34 | 0.78 | 0.85 | 0.84 | 1442 | Train 23 | Test Hispanic 34 | 0.78 | 0.85 | 0.83 |
| 419 | Train 7 | Test Hispanic 35 | 0.79 | 0.86 | 0.85 | 1443 | Train 23 | Test Hispanic 35 | 0.79 | 0.86 | 0.85 |
| 420 | Train 7 | Test Hispanic 36 | 0.78 | 0.85 | 0.84 | 1444 | Train 23 | Test Hispanic 36 | 0.78 | 0.85 | 0.84 |
| 421 | Train 7 | Test Hispanic 37 | 0.75 | 0.86 | 0.84 | 1445 | Train 23 | Test Hispanic 37 | 0.75 | 0.86 | 0.84 |
| 422 | Train 7 | Test Hispanic 38 | 0.72 | 0.77 | 0.75 | 1446 | Train 23 | Test Hispanic 38 | 0.72 | 0.77 | 0.76 |
| 423 | Train 7 | Test Hispanic 39 | 0.75 | 0.86 | 0.84 | 1447 | Train 23 | Test Hispanic 39 | 0.75 | 0.86 | 0.84 |
| 424 | Train 7 | Test Hispanic 40 | 0.72 | 0.77 | 0.75 | 1448 | Train 23 | Test Hispanic 40 | 0.72 | 0.77 | 0.76 |
| 425 | Train 7 | Test Hispanic 41 | 0.78 | 0.85 | 0.83 | 1449 | Train 23 | Test Hispanic 41 | 0.78 | 0.85 | 0.84 |
| 426 | Train 7 | Test Hispanic 42 | 0.78 | 0.84 | 0.82 | 1450 | Train 23 | Test Hispanic 42 | 0.78 | 0.84 | 0.82 |
| 427 | Train 7 | Test Hispanic 43 | 0.79 | 0.85 | 0.83 | 1451 | Train 23 | Test Hispanic 43 | 0.79 | 0.85 | 0.84 |
| 428 | Train 7 | Test Hispanic 44 | 0.78 | 0.84 | 0.82 | 1452 | Train 23 | Test Hispanic 44 | 0.78 | 0.84 | 0.82 |
| 429 | Train 7 | Test Hispanic 45 | 0.75 | 0.85 | 0.82 | 1453 | Train 23 | Test Hispanic 45 | 0.75 | 0.85 | 0.83 |
| 430 | Train 7 | Test Hispanic 46 | 0.72 | 0.77 | 0.75 | 1454 | Train 23 | Test Hispanic 46 | 0.72 | 0.77 | 0.76 |
| 431 | Train 7 | Test Hispanic 47 | 0.75 | 0.85 | 0.82 | 1455 | Train 23 | Test Hispanic 47 | 0.75 | 0.85 | 0.83 |
| 432 | Train 7 | Test Hispanic 48 | 0.73 | 0.77 | 0.75 | 1456 | Train 23 | Test Hispanic 48 | 0.73 | 0.77 | 0.76 |
| 433 | Train 7 | Test Hispanic 49 | 0.79 | 0.86 | 0.86 | 1457 | Train 23 | Test Hispanic 49 | 0.79 | 0.86 | 0.86 |
| 434 | Train 7 | Test Hispanic 50 | 0.79 | 0.86 | 0.85 | 1458 | Train 23 | Test Hispanic 50 | 0.79 | 0.86 | 0.85 |
| 435 | Train 7 | Test Hispanic 51 | 0.8 | 0.86 | 0.86 | 1459 | Train 23 | Test Hispanic 51 | 0.8 | 0.86 | 0.86 |
| 436 | Train 7 | Test Hispanic 52 | 0.79 | 0.86 | 0.86 | 1460 | Train 23 | Test Hispanic 52 | 0.79 | 0.86 | 0.86 |
| 437 | Train 7 | Test Hispanic 53 | 0.78 | 0.86 | 0.83 | 1461 | Train 23 | Test Hispanic 53 | 0.78 | 0.86 | 0.84 |
| 438 | Train 7 | Test Hispanic 54 | 0.74 | 0.76 | 0.74 | 1462 | Train 23 | Test Hispanic 54 | 0.74 | 0.76 | 0.75 |
| 439 | Train 7 | Test Hispanic 55 | 0.78 | 0.86 | 0.84 | 1463 | Train 23 | Test Hispanic 55 | 0.78 | 0.86 | 0.84 |
| 440 | Train 7 | Test Hispanic 56 | 0.74 | 0.76 | 0.74 | 1464 | Train 23 | Test Hispanic 56 | 0.74 | 0.75 | 0.75 |
| 441 | Train 7 | Test Hispanic 57 | 0.79 | 0.85 | 0.84 | 1465 | Train 23 | Test Hispanic 57 | 0.79 | 0.85 | 0.84 |
| 442 | Train 7 | Test Hispanic 58 | 0.79 | 0.85 | 0.83 | 1466 | Train 23 | Test Hispanic 58 | 0.79 | 0.85 | 0.83 |
| 443 | Train 7 | Test Hispanic 59 | 0.8 | 0.85 | 0.84 | 1467 | Train 23 | Test Hispanic 59 | 0.8 | 0.85 | 0.84 |
| 444 | Train 7 | Test Hispanic 60 | 0.79 | 0.85 | 0.84 | 1468 | Train 23 | Test Hispanic 60 | 0.79 | 0.85 | 0.84 |
| 445 | Train 7 | Test Hispanic 61 | 0.78 | 0.85 | 0.83 | 1469 | Train 23 | Test Hispanic 61 | 0.78 | 0.85 | 0.83 |
| 446 | Train 7 | Test Hispanic 62 | 0.74 | 0.77 | 0.74 | 1470 | Train 23 | Test Hispanic 62 | 0.74 | 0.76 | 0.76 |
| 447 | Train 7 | Test Hispanic 63 | 0.78 | 0.86 | 0.83 | 1471 | Train 23 | Test Hispanic 63 | 0.78 | 0.85 | 0.84 |
| 448 | Train 7 | Test Hispanic 64 | 0.74 | 0.76 | 0.74 | 1472 | Train 23 | Test Hispanic 64 | 0.74 | 0.75 | 0.75 |
| 449 | Train 8 | Test Hispanic 1 | 0.77 | 0.84 | 0.77 | 1473 | Train 24 | Test Hispanic 1 | 0.77 | 0.84 | 0.8 |
| 450 | Train 8 | Test Hispanic 2 | 0.77 | 0.84 | 0.78 | 1474 | Train 24 | Test Hispanic 2 | 0.77 | 0.84 | 0.8 |
| 451 | Train 8 | Test Hispanic 3 | 0.78 | 0.84 | 0.77 | 1475 | Train 24 | Test Hispanic 3 | 0.78 | 0.84 | 0.8 |
| 452 | Train 8 | Test Hispanic 4 | 0.78 | 0.84 | 0.78 | 1476 | Train 24 | Test Hispanic 4 | 0.77 | 0.84 | 0.8 |
| 453 | Train 8 | Test Hispanic 5 | 0.74 | 0.83 | 0.77 | 1477 | Train 24 | Test Hispanic 5 | 0.74 | 0.83 | 0.78 |
| 454 | Train 8 | Test Hispanic 6 | 0.75 | 0.84 | 0.79 | 1478 | Train 24 | Test Hispanic 6 | 0.75 | 0.84 | 0.79 |
| 455 | Train 8 | Test Hispanic 7 | 0.74 | 0.83 | 0.77 | 1479 | Train 24 | Test Hispanic 7 | 0.74 | 0.83 | 0.78 |
| 456 | Train 8 | Test Hispanic 8 | 0.75 | 0.83 | 0.79 | 1480 | Train 24 | Test Hispanic 8 | 0.75 | 0.83 | 0.79 |
| 457 | Train 8 | Test Hispanic 9 | 0.77 | 0.84 | 0.78 | 1481 | Train 24 | Test Hispanic 9 | 0.77 | 0.84 | 0.81 |
| 458 | Train 8 | Test Hispanic 10 | 0.77 | 0.84 | 0.79 | 1482 | Train 24 | Test Hispanic 10 | 0.77 | 0.84 | 0.81 |
| 459 | Train 8 | Test Hispanic 11 | 0.78 | 0.84 | 0.78 | 1483 | Train 24 | Test Hispanic 11 | 0.78 | 0.84 | 0.81 |
| 460 | Train 8 | Test Hispanic 12 | 0.78 | 0.84 | 0.79 | 1484 | Train 24 | Test Hispanic 12 | 0.78 | 0.84 | 0.81 |
| 461 | Train 8 | Test Hispanic 13 | 0.74 | 0.83 | 0.78 | 1485 | Train 24 | Test Hispanic 13 | 0.74 | 0.83 | 0.79 |
| 462 | Train 8 | Test Hispanic 14 | 0.75 | 0.84 | 0.79 | 1486 | Train 24 | Test Hispanic 14 | 0.75 | 0.84 | 0.79 |
| 463 | Train 8 | Test Hispanic 15 | 0.74 | 0.83 | 0.78 | 1487 | Train 24 | Test Hispanic 15 | 0.74 | 0.83 | 0.79 |
| 464 | Train 8 | Test Hispanic 16 | 0.75 | 0.83 | 0.79 | 1488 | Train 24 | Test Hispanic 16 | 0.75 | 0.83 | 0.79 |
| 465 | Train 8 | Test Hispanic 17 | 0.77 | 0.84 | 0.77 | 1489 | Train 24 | Test Hispanic 17 | 0.77 | 0.84 | 0.8 |
| 466 | Train 8 | Test Hispanic 18 | 0.77 | 0.84 | 0.78 | 1490 | Train 24 | Test Hispanic 18 | 0.77 | 0.84 | 0.8 |
| 467 | Train 8 | Test Hispanic 19 | 0.78 | 0.84 | 0.77 | 1491 | Train 24 | Test Hispanic 19 | 0.78 | 0.84 | 0.8 |
| 468 | Train 8 | Test Hispanic 20 | 0.78 | 0.84 | 0.78 | 1492 | Train 24 | Test Hispanic 20 | 0.77 | 0.84 | 0.8 |
| 469 | Train 8 | Test Hispanic 21 | 0.74 | 0.83 | 0.77 | 1493 | Train 24 | Test Hispanic 21 | 0.74 | 0.83 | 0.78 |
| 470 | Train 8 | Test Hispanic 22 | 0.75 | 0.84 | 0.79 | 1494 | Train 24 | Test Hispanic 22 | 0.75 | 0.84 | 0.79 |
| 471 | Train 8 | Test Hispanic 23 | 0.74 | 0.83 | 0.77 | 1495 | Train 24 | Test Hispanic 23 | 0.74 | 0.83 | 0.78 |
| 472 | Train 8 | Test Hispanic 24 | 0.75 | 0.84 | 0.79 | 1496 | Train 24 | Test Hispanic 24 | 0.75 | 0.83 | 0.79 |
| 473 | Train 8 | Test Hispanic 25 | 0.77 | 0.84 | 0.78 | 1497 | Train 24 | Test Hispanic 25 | 0.77 | 0.84 | 0.81 |
| 474 | Train 8 | Test Hispanic 26 | 0.77 | 0.84 | 0.79 | 1498 | Train 24 | Test Hispanic 26 | 0.77 | 0.84 | 0.81 |
| 475 | Train 8 | Test Hispanic 27 | 0.78 | 0.84 | 0.78 | 1499 | Train 24 | Test Hispanic 27 | 0.78 | 0.84 | 0.81 |
| 476 | Train 8 | Test Hispanic 28 | 0.78 | 0.84 | 0.79 | 1500 | Train 24 | Test Hispanic 28 | 0.78 | 0.84 | 0.81 |
| 477 | Train 8 | Test Hispanic 29 | 0.74 | 0.83 | 0.78 | 1501 | Train 24 | Test Hispanic 29 | 0.74 | 0.83 | 0.79 |
| 478 | Train 8 | Test Hispanic 30 | 0.75 | 0.84 | 0.79 | 1502 | Train 24 | Test Hispanic 30 | 0.75 | 0.84 | 0.79 |
| 479 | Train 8 | Test Hispanic 31 | 0.74 | 0.83 | 0.78 | 1503 | Train 24 | Test Hispanic 31 | 0.74 | 0.83 | 0.79 |
| 480 | Train 8 | Test Hispanic 32 | 0.75 | 0.83 | 0.79 | 1504 | Train 24 | Test Hispanic 32 | 0.75 | 0.83 | 0.79 |
| 481 | Train 8 | Test Hispanic 33 | 0.78 | 0.84 | 0.78 | 1505 | Train 24 | Test Hispanic 33 | 0.78 | 0.84 | 0.81 |
| 482 | Train 8 | Test Hispanic 34 | 0.78 | 0.84 | 0.78 | 1506 | Train 24 | Test Hispanic 34 | 0.78 | 0.84 | 0.8 |
| 483 | Train 8 | Test Hispanic 35 | 0.79 | 0.84 | 0.78 | 1507 | Train 24 | Test Hispanic 35 | 0.79 | 0.84 | 0.81 |
| 484 | Train 8 | Test Hispanic 36 | 0.78 | 0.84 | 0.79 | 1508 | Train 24 | Test Hispanic 36 | 0.78 | 0.84 | 0.81 |
| 485 | Train 8 | Test Hispanic 37 | 0.75 | 0.83 | 0.78 | 1509 | Train 24 | Test Hispanic 37 | 0.75 | 0.83 | 0.78 |
| 486 | Train 8 | Test Hispanic 38 | 0.75 | 0.84 | 0.79 | 1510 | Train 24 | Test Hispanic 38 | 0.75 | 0.84 | 0.79 |
| 487 | Train 8 | Test Hispanic 39 | 0.75 | 0.83 | 0.78 | 1511 | Train 24 | Test Hispanic 39 | 0.75 | 0.83 | 0.78 |
| 488 | Train 8 | Test Hispanic 40 | 0.76 | 0.84 | 0.79 | 1512 | Train 24 | Test Hispanic 40 | 0.76 | 0.84 | 0.79 |
| 489 | Train 8 | Test Hispanic 41 | 0.78 | 0.85 | 0.79 | 1513 | Train 24 | Test Hispanic 41 | 0.78 | 0.85 | 0.81 |
| 490 | Train 8 | Test Hispanic 42 | 0.78 | 0.85 | 0.79 | 1514 | Train 24 | Test Hispanic 42 | 0.78 | 0.85 | 0.81 |
| 491 | Train 8 | Test Hispanic 43 | 0.79 | 0.85 | 0.79 | 1515 | Train 24 | Test Hispanic 43 | 0.79 | 0.85 | 0.81 |
| 492 | Train 8 | Test Hispanic 44 | 0.79 | 0.85 | 0.8 | 1516 | Train 24 | Test Hispanic 44 | 0.78 | 0.85 | 0.81 |
| 493 | Train 8 | Test Hispanic 45 | 0.75 | 0.83 | 0.79 | 1517 | Train 24 | Test Hispanic 45 | 0.75 | 0.83 | 0.79 |
| 494 | Train 8 | Test Hispanic 46 | 0.75 | 0.84 | 0.79 | 1518 | Train 24 | Test Hispanic 46 | 0.75 | 0.84 | 0.79 |
| 495 | Train 8 | Test Hispanic 47 | 0.75 | 0.83 | 0.79 | 1519 | Train 24 | Test Hispanic 47 | 0.75 | 0.83 | 0.79 |
| 496 | Train 8 | Test Hispanic 48 | 0.76 | 0.83 | 0.79 | 1520 | Train 24 | Test Hispanic 48 | 0.75 | 0.83 | 0.79 |
| 497 | Train 8 | Test Hispanic 49 | 0.79 | 0.85 | 0.77 | 1521 | Train 24 | Test Hispanic 49 | 0.8 | 0.85 | 0.81 |
| 498 | Train 8 | Test Hispanic 50 | 0.79 | 0.85 | 0.76 | 1522 | Train 24 | Test Hispanic 50 | 0.79 | 0.85 | 0.81 |
| 499 | Train 8 | Test Hispanic 51 | 0.8 | 0.85 | 0.77 | 1523 | Train 24 | Test Hispanic 51 | 0.8 | 0.85 | 0.82 |
| 500 | Train 8 | Test Hispanic 52 | 0.8 | 0.85 | 0.77 | 1524 | Train 24 | Test Hispanic 52 | 0.8 | 0.85 | 0.81 |
| 501 | Train 8 | Test Hispanic 53 | 0.77 | 0.85 | 0.78 | 1525 | Train 24 | Test Hispanic 53 | 0.77 | 0.85 | 0.8 |
| 502 | Train 8 | Test Hispanic 54 | 0.78 | 0.85 | 0.78 | 1526 | Train 24 | Test Hispanic 54 | 0.78 | 0.85 | 0.8 |
| 503 | Train 8 | Test Hispanic 55 | 0.78 | 0.85 | 0.78 | 1527 | Train 24 | Test Hispanic 55 | 0.78 | 0.85 | 0.81 |
| 504 | Train 8 | Test Hispanic 56 | 0.78 | 0.84 | 0.78 | 1528 | Train 24 | Test Hispanic 56 | 0.78 | 0.84 | 0.8 |
| 505 | Train 8 | Test Hispanic 57 | 0.8 | 0.86 | 0.78 | 1529 | Train 24 | Test Hispanic 57 | 0.8 | 0.86 | 0.83 |
| 506 | Train 8 | Test Hispanic 58 | 0.79 | 0.85 | 0.78 | 1530 | Train 24 | Test Hispanic 58 | 0.79 | 0.85 | 0.82 |
| 507 | Train 8 | Test Hispanic 59 | 0.8 | 0.86 | 0.79 | 1531 | Train 24 | Test Hispanic 59 | 0.8 | 0.86 | 0.83 |
| 508 | Train 8 | Test Hispanic 60 | 0.8 | 0.86 | 0.78 | 1532 | Train 24 | Test Hispanic 60 | 0.8 | 0.86 | 0.83 |
| 509 | Train 8 | Test Hispanic 61 | 0.78 | 0.86 | 0.78 | 1533 | Train 24 | Test Hispanic 61 | 0.78 | 0.86 | 0.81 |
| 510 | Train 8 | Test Hispanic 62 | 0.78 | 0.85 | 0.78 | 1534 | Train 24 | Test Hispanic 62 | 0.78 | 0.85 | 0.8 |
| 511 | Train 8 | Test Hispanic 63 | 0.78 | 0.86 | 0.79 | 1535 | Train 24 | Test Hispanic 63 | 0.78 | 0.86 | 0.82 |
| 512 | Train 8 | Test Hispanic 64 | 0.78 | 0.84 | 0.78 | 1536 | Train 24 | Test Hispanic 64 | 0.78 | 0.84 | 0.8 |
| 513 | Train 9 | Test Hispanic 1 | 0.79 | 0.88 | 0.85 | 1537 | Train 25 | Test Hispanic 1 | 0.79 | 0.88 | 0.85 |
| 514 | Train 9 | Test Hispanic 2 | 0.78 | 0.86 | 0.83 | 1538 | Train 25 | Test Hispanic 2 | 0.78 | 0.86 | 0.83 |
| 515 | Train 9 | Test Hispanic 3 | 0.79 | 0.88 | 0.85 | 1539 | Train 25 | Test Hispanic 3 | 0.79 | 0.88 | 0.84 |
| 516 | Train 9 | Test Hispanic 4 | 0.78 | 0.86 | 0.83 | 1540 | Train 25 | Test Hispanic 4 | 0.78 | 0.86 | 0.83 |
| 517 | Train 9 | Test Hispanic 5 | 0.74 | 0.78 | 0.73 | 1541 | Train 25 | Test Hispanic 5 | 0.74 | 0.78 | 0.74 |
| 518 | Train 9 | Test Hispanic 6 | 0.72 | 0.73 | 0.69 | 1542 | Train 25 | Test Hispanic 6 | 0.72 | 0.73 | 0.7 |
| 519 | Train 9 | Test Hispanic 7 | 0.74 | 0.78 | 0.72 | 1543 | Train 25 | Test Hispanic 7 | 0.74 | 0.78 | 0.74 |
| 520 | Train 9 | Test Hispanic 8 | 0.72 | 0.73 | 0.69 | 1544 | Train 25 | Test Hispanic 8 | 0.72 | 0.73 | 0.7 |
| 521 | Train 9 | Test Hispanic 9 | 0.79 | 0.89 | 0.88 | 1545 | Train 25 | Test Hispanic 9 | 0.79 | 0.89 | 0.88 |
| 522 | Train 9 | Test Hispanic 10 | 0.78 | 0.87 | 0.85 | 1546 | Train 25 | Test Hispanic 10 | 0.78 | 0.87 | 0.86 |
| 523 | Train 9 | Test Hispanic 11 | 0.79 | 0.89 | 0.87 | 1547 | Train 25 | Test Hispanic 11 | 0.79 | 0.89 | 0.87 |
| 524 | Train 9 | Test Hispanic 12 | 0.78 | 0.87 | 0.85 | 1548 | Train 25 | Test Hispanic 12 | 0.78 | 0.87 | 0.85 |
| 525 | Train 9 | Test Hispanic 13 | 0.74 | 0.79 | 0.74 | 1549 | Train 25 | Test Hispanic 13 | 0.74 | 0.79 | 0.76 |
| 526 | Train 9 | Test Hispanic 14 | 0.72 | 0.73 | 0.69 | 1550 | Train 25 | Test Hispanic 14 | 0.72 | 0.73 | 0.7 |
| 527 | Train 9 | Test Hispanic 15 | 0.74 | 0.78 | 0.73 | 1551 | Train 25 | Test Hispanic 15 | 0.74 | 0.78 | 0.75 |
| 528 | Train 9 | Test Hispanic 16 | 0.72 | 0.73 | 0.69 | 1552 | Train 25 | Test Hispanic 16 | 0.72 | 0.73 | 0.7 |
| 529 | Train 9 | Test Hispanic 17 | 0.79 | 0.88 | 0.85 | 1553 | Train 25 | Test Hispanic 17 | 0.79 | 0.88 | 0.85 |
| 530 | Train 9 | Test Hispanic 18 | 0.78 | 0.86 | 0.83 | 1554 | Train 25 | Test Hispanic 18 | 0.78 | 0.86 | 0.83 |
| 531 | Train 9 | Test Hispanic 19 | 0.79 | 0.88 | 0.85 | 1555 | Train 25 | Test Hispanic 19 | 0.79 | 0.88 | 0.84 |
| 532 | Train 9 | Test Hispanic 20 | 0.78 | 0.86 | 0.83 | 1556 | Train 25 | Test Hispanic 20 | 0.78 | 0.86 | 0.83 |
| 533 | Train 9 | Test Hispanic 21 | 0.74 | 0.78 | 0.73 | 1557 | Train 25 | Test Hispanic 21 | 0.74 | 0.78 | 0.74 |
| 534 | Train 9 | Test Hispanic 22 | 0.72 | 0.73 | 0.69 | 1558 | Train 25 | Test Hispanic 22 | 0.72 | 0.73 | 0.7 |
| 535 | Train 9 | Test Hispanic 23 | 0.74 | 0.78 | 0.72 | 1559 | Train 25 | Test Hispanic 23 | 0.74 | 0.78 | 0.74 |
| 536 | Train 9 | Test Hispanic 24 | 0.72 | 0.73 | 0.69 | 1560 | Train 25 | Test Hispanic 24 | 0.72 | 0.73 | 0.7 |
| 537 | Train 9 | Test Hispanic 25 | 0.79 | 0.89 | 0.88 | 1561 | Train 25 | Test Hispanic 25 | 0.79 | 0.89 | 0.88 |
| 538 | Train 9 | Test Hispanic 26 | 0.78 | 0.87 | 0.85 | 1562 | Train 25 | Test Hispanic 26 | 0.78 | 0.87 | 0.86 |
| 539 | Train 9 | Test Hispanic 27 | 0.79 | 0.89 | 0.87 | 1563 | Train 25 | Test Hispanic 27 | 0.79 | 0.89 | 0.87 |
| 540 | Train 9 | Test Hispanic 28 | 0.78 | 0.87 | 0.85 | 1564 | Train 25 | Test Hispanic 28 | 0.78 | 0.87 | 0.85 |
| 541 | Train 9 | Test Hispanic 29 | 0.74 | 0.79 | 0.74 | 1565 | Train 25 | Test Hispanic 29 | 0.74 | 0.79 | 0.76 |
| 542 | Train 9 | Test Hispanic 30 | 0.72 | 0.73 | 0.69 | 1566 | Train 25 | Test Hispanic 30 | 0.72 | 0.73 | 0.7 |
| 543 | Train 9 | Test Hispanic 31 | 0.74 | 0.78 | 0.73 | 1567 | Train 25 | Test Hispanic 31 | 0.74 | 0.78 | 0.75 |
| 544 | Train 9 | Test Hispanic 32 | 0.72 | 0.73 | 0.69 | 1568 | Train 25 | Test Hispanic 32 | 0.72 | 0.73 | 0.7 |
| 545 | Train 9 | Test Hispanic 33 | 0.8 | 0.88 | 0.85 | 1569 | Train 25 | Test Hispanic 33 | 0.8 | 0.88 | 0.85 |
| 546 | Train 9 | Test Hispanic 34 | 0.79 | 0.87 | 0.83 | 1570 | Train 25 | Test Hispanic 34 | 0.78 | 0.87 | 0.83 |
| 547 | Train 9 | Test Hispanic 35 | 0.8 | 0.88 | 0.85 | 1571 | Train 25 | Test Hispanic 35 | 0.8 | 0.88 | 0.85 |
| 548 | Train 9 | Test Hispanic 36 | 0.79 | 0.87 | 0.83 | 1572 | Train 25 | Test Hispanic 36 | 0.79 | 0.87 | 0.83 |
| 549 | Train 9 | Test Hispanic 37 | 0.74 | 0.78 | 0.73 | 1573 | Train 25 | Test Hispanic 37 | 0.74 | 0.78 | 0.74 |
| 550 | Train 9 | Test Hispanic 38 | 0.72 | 0.73 | 0.69 | 1574 | Train 25 | Test Hispanic 38 | 0.72 | 0.73 | 0.7 |
| 551 | Train 9 | Test Hispanic 39 | 0.74 | 0.78 | 0.72 | 1575 | Train 25 | Test Hispanic 39 | 0.74 | 0.78 | 0.74 |
| 552 | Train 9 | Test Hispanic 40 | 0.73 | 0.73 | 0.69 | 1576 | Train 25 | Test Hispanic 40 | 0.72 | 0.73 | 0.7 |
| 553 | Train 9 | Test Hispanic 41 | 0.8 | 0.89 | 0.88 | 1577 | Train 25 | Test Hispanic 41 | 0.8 | 0.89 | 0.88 |
| 554 | Train 9 | Test Hispanic 42 | 0.79 | 0.87 | 0.86 | 1578 | Train 25 | Test Hispanic 42 | 0.79 | 0.87 | 0.86 |
| 555 | Train 9 | Test Hispanic 43 | 0.8 | 0.89 | 0.88 | 1579 | Train 25 | Test Hispanic 43 | 0.8 | 0.89 | 0.88 |
| 556 | Train 9 | Test Hispanic 44 | 0.79 | 0.87 | 0.86 | 1580 | Train 25 | Test Hispanic 44 | 0.79 | 0.87 | 0.86 |
| 557 | Train 9 | Test Hispanic 45 | 0.74 | 0.79 | 0.74 | 1581 | Train 25 | Test Hispanic 45 | 0.74 | 0.79 | 0.76 |
| 558 | Train 9 | Test Hispanic 46 | 0.72 | 0.73 | 0.69 | 1582 | Train 25 | Test Hispanic 46 | 0.72 | 0.73 | 0.7 |
| 559 | Train 9 | Test Hispanic 47 | 0.74 | 0.78 | 0.74 | 1583 | Train 25 | Test Hispanic 47 | 0.74 | 0.78 | 0.75 |
| 560 | Train 9 | Test Hispanic 48 | 0.73 | 0.73 | 0.69 | 1584 | Train 25 | Test Hispanic 48 | 0.72 | 0.73 | 0.7 |
| 561 | Train 9 | Test Hispanic 49 | 0.8 | 0.87 | 0.84 | 1585 | Train 25 | Test Hispanic 49 | 0.8 | 0.87 | 0.84 |
| 562 | Train 9 | Test Hispanic 50 | 0.8 | 0.86 | 0.83 | 1586 | Train 25 | Test Hispanic 50 | 0.8 | 0.86 | 0.84 |
| 563 | Train 9 | Test Hispanic 51 | 0.81 | 0.87 | 0.84 | 1587 | Train 25 | Test Hispanic 51 | 0.81 | 0.87 | 0.84 |
| 564 | Train 9 | Test Hispanic 52 | 0.8 | 0.86 | 0.83 | 1588 | Train 25 | Test Hispanic 52 | 0.8 | 0.86 | 0.84 |
| 565 | Train 9 | Test Hispanic 53 | 0.78 | 0.83 | 0.77 | 1589 | Train 25 | Test Hispanic 53 | 0.78 | 0.83 | 0.8 |
| 566 | Train 9 | Test Hispanic 54 | 0.75 | 0.75 | 0.7 | 1590 | Train 25 | Test Hispanic 54 | 0.75 | 0.75 | 0.73 |
| 567 | Train 9 | Test Hispanic 55 | 0.78 | 0.83 | 0.77 | 1591 | Train 25 | Test Hispanic 55 | 0.79 | 0.83 | 0.8 |
| 568 | Train 9 | Test Hispanic 56 | 0.75 | 0.75 | 0.71 | 1592 | Train 25 | Test Hispanic 56 | 0.75 | 0.75 | 0.73 |
| 569 | Train 9 | Test Hispanic 57 | 0.81 | 0.88 | 0.87 | 1593 | Train 25 | Test Hispanic 57 | 0.81 | 0.88 | 0.88 |
| 570 | Train 9 | Test Hispanic 58 | 0.8 | 0.88 | 0.87 | 1594 | Train 25 | Test Hispanic 58 | 0.8 | 0.88 | 0.88 |
| 571 | Train 9 | Test Hispanic 59 | 0.81 | 0.88 | 0.87 | 1595 | Train 25 | Test Hispanic 59 | 0.81 | 0.88 | 0.88 |
| 572 | Train 9 | Test Hispanic 60 | 0.81 | 0.88 | 0.86 | 1596 | Train 25 | Test Hispanic 60 | 0.81 | 0.88 | 0.87 |
| 573 | Train 9 | Test Hispanic 61 | 0.78 | 0.84 | 0.78 | 1597 | Train 25 | Test Hispanic 61 | 0.78 | 0.84 | 0.81 |
| 574 | Train 9 | Test Hispanic 62 | 0.75 | 0.75 | 0.7 | 1598 | Train 25 | Test Hispanic 62 | 0.75 | 0.75 | 0.73 |
| 575 | Train 9 | Test Hispanic 63 | 0.79 | 0.84 | 0.78 | 1599 | Train 25 | Test Hispanic 63 | 0.79 | 0.84 | 0.81 |
| 576 | Train 9 | Test Hispanic 64 | 0.76 | 0.75 | 0.71 | 1600 | Train 25 | Test Hispanic 64 | 0.76 | 0.75 | 0.73 |
| 577 | Train 10 | Test Hispanic 1 | 0.79 | 0.88 | 0.84 | 1601 | Train 26 | Test Hispanic 1 | 0.79 | 0.88 | 0.84 |
| 578 | Train 10 | Test Hispanic 2 | 0.78 | 0.88 | 0.85 | 1602 | Train 26 | Test Hispanic 2 | 0.78 | 0.88 | 0.85 |
| 579 | Train 10 | Test Hispanic 3 | 0.79 | 0.88 | 0.84 | 1603 | Train 26 | Test Hispanic 3 | 0.79 | 0.88 | 0.84 |
| 580 | Train 10 | Test Hispanic 4 | 0.78 | 0.88 | 0.84 | 1604 | Train 26 | Test Hispanic 4 | 0.79 | 0.88 | 0.85 |
| 581 | Train 10 | Test Hispanic 5 | 0.74 | 0.79 | 0.75 | 1605 | Train 26 | Test Hispanic 5 | 0.74 | 0.79 | 0.76 |
| 582 | Train 10 | Test Hispanic 6 | 0.72 | 0.75 | 0.72 | 1606 | Train 26 | Test Hispanic 6 | 0.73 | 0.75 | 0.72 |
| 583 | Train 10 | Test Hispanic 7 | 0.74 | 0.79 | 0.75 | 1607 | Train 26 | Test Hispanic 7 | 0.74 | 0.78 | 0.75 |
| 584 | Train 10 | Test Hispanic 8 | 0.73 | 0.75 | 0.72 | 1608 | Train 26 | Test Hispanic 8 | 0.73 | 0.75 | 0.72 |
| 585 | Train 10 | Test Hispanic 9 | 0.79 | 0.89 | 0.87 | 1609 | Train 26 | Test Hispanic 9 | 0.79 | 0.88 | 0.87 |
| 586 | Train 10 | Test Hispanic 10 | 0.78 | 0.89 | 0.87 | 1610 | Train 26 | Test Hispanic 10 | 0.78 | 0.89 | 0.87 |
| 587 | Train 10 | Test Hispanic 11 | 0.79 | 0.88 | 0.87 | 1611 | Train 26 | Test Hispanic 11 | 0.79 | 0.88 | 0.86 |
| 588 | Train 10 | Test Hispanic 12 | 0.79 | 0.89 | 0.87 | 1612 | Train 26 | Test Hispanic 12 | 0.79 | 0.89 | 0.87 |
| 589 | Train 10 | Test Hispanic 13 | 0.74 | 0.8 | 0.77 | 1613 | Train 26 | Test Hispanic 13 | 0.74 | 0.8 | 0.77 |
| 590 | Train 10 | Test Hispanic 14 | 0.73 | 0.75 | 0.73 | 1614 | Train 26 | Test Hispanic 14 | 0.73 | 0.75 | 0.72 |
| 591 | Train 10 | Test Hispanic 15 | 0.74 | 0.79 | 0.76 | 1615 | Train 26 | Test Hispanic 15 | 0.74 | 0.79 | 0.76 |
| 592 | Train 10 | Test Hispanic 16 | 0.73 | 0.75 | 0.73 | 1616 | Train 26 | Test Hispanic 16 | 0.73 | 0.75 | 0.72 |
| 593 | Train 10 | Test Hispanic 17 | 0.79 | 0.88 | 0.84 | 1617 | Train 26 | Test Hispanic 17 | 0.79 | 0.88 | 0.84 |
| 594 | Train 10 | Test Hispanic 18 | 0.78 | 0.88 | 0.85 | 1618 | Train 26 | Test Hispanic 18 | 0.78 | 0.88 | 0.85 |
| 595 | Train 10 | Test Hispanic 19 | 0.79 | 0.88 | 0.84 | 1619 | Train 26 | Test Hispanic 19 | 0.79 | 0.88 | 0.84 |
| 596 | Train 10 | Test Hispanic 20 | 0.78 | 0.88 | 0.84 | 1620 | Train 26 | Test Hispanic 20 | 0.79 | 0.88 | 0.85 |
| 597 | Train 10 | Test Hispanic 21 | 0.74 | 0.79 | 0.75 | 1621 | Train 26 | Test Hispanic 21 | 0.74 | 0.79 | 0.76 |
| 598 | Train 10 | Test Hispanic 22 | 0.73 | 0.75 | 0.72 | 1622 | Train 26 | Test Hispanic 22 | 0.73 | 0.75 | 0.72 |
| 599 | Train 10 | Test Hispanic 23 | 0.74 | 0.79 | 0.75 | 1623 | Train 26 | Test Hispanic 23 | 0.74 | 0.79 | 0.75 |
| 600 | Train 10 | Test Hispanic 24 | 0.73 | 0.75 | 0.72 | 1624 | Train 26 | Test Hispanic 24 | 0.73 | 0.75 | 0.72 |
| 601 | Train 10 | Test Hispanic 25 | 0.79 | 0.89 | 0.87 | 1625 | Train 26 | Test Hispanic 25 | 0.79 | 0.88 | 0.87 |
| 602 | Train 10 | Test Hispanic 26 | 0.78 | 0.89 | 0.87 | 1626 | Train 26 | Test Hispanic 26 | 0.78 | 0.89 | 0.87 |
| 603 | Train 10 | Test Hispanic 27 | 0.79 | 0.88 | 0.87 | 1627 | Train 26 | Test Hispanic 27 | 0.79 | 0.88 | 0.86 |
| 604 | Train 10 | Test Hispanic 28 | 0.79 | 0.89 | 0.87 | 1628 | Train 26 | Test Hispanic 28 | 0.79 | 0.89 | 0.87 |
| 605 | Train 10 | Test Hispanic 29 | 0.74 | 0.8 | 0.77 | 1629 | Train 26 | Test Hispanic 29 | 0.74 | 0.8 | 0.77 |
| 606 | Train 10 | Test Hispanic 30 | 0.73 | 0.75 | 0.73 | 1630 | Train 26 | Test Hispanic 30 | 0.73 | 0.75 | 0.72 |
| 607 | Train 10 | Test Hispanic 31 | 0.74 | 0.79 | 0.76 | 1631 | Train 26 | Test Hispanic 31 | 0.74 | 0.79 | 0.76 |
| 608 | Train 10 | Test Hispanic 32 | 0.73 | 0.75 | 0.73 | 1632 | Train 26 | Test Hispanic 32 | 0.73 | 0.75 | 0.72 |
| 609 | Train 10 | Test Hispanic 33 | 0.79 | 0.88 | 0.85 | 1633 | Train 26 | Test Hispanic 33 | 0.79 | 0.88 | 0.85 |
| 610 | Train 10 | Test Hispanic 34 | 0.79 | 0.88 | 0.85 | 1634 | Train 26 | Test Hispanic 34 | 0.79 | 0.88 | 0.85 |
| 611 | Train 10 | Test Hispanic 35 | 0.8 | 0.88 | 0.85 | 1635 | Train 26 | Test Hispanic 35 | 0.8 | 0.88 | 0.85 |
| 612 | Train 10 | Test Hispanic 36 | 0.79 | 0.88 | 0.85 | 1636 | Train 26 | Test Hispanic 36 | 0.79 | 0.88 | 0.85 |
| 613 | Train 10 | Test Hispanic 37 | 0.75 | 0.79 | 0.76 | 1637 | Train 26 | Test Hispanic 37 | 0.75 | 0.79 | 0.76 |
| 614 | Train 10 | Test Hispanic 38 | 0.73 | 0.75 | 0.73 | 1638 | Train 26 | Test Hispanic 38 | 0.73 | 0.75 | 0.72 |
| 615 | Train 10 | Test Hispanic 39 | 0.75 | 0.79 | 0.75 | 1639 | Train 26 | Test Hispanic 39 | 0.75 | 0.79 | 0.76 |
| 616 | Train 10 | Test Hispanic 40 | 0.73 | 0.75 | 0.73 | 1640 | Train 26 | Test Hispanic 40 | 0.73 | 0.75 | 0.72 |
| 617 | Train 10 | Test Hispanic 41 | 0.79 | 0.89 | 0.87 | 1641 | Train 26 | Test Hispanic 41 | 0.79 | 0.89 | 0.87 |
| 618 | Train 10 | Test Hispanic 42 | 0.79 | 0.89 | 0.88 | 1642 | Train 26 | Test Hispanic 42 | 0.79 | 0.89 | 0.87 |
| 619 | Train 10 | Test Hispanic 43 | 0.8 | 0.89 | 0.87 | 1643 | Train 26 | Test Hispanic 43 | 0.8 | 0.89 | 0.87 |
| 620 | Train 10 | Test Hispanic 44 | 0.79 | 0.89 | 0.87 | 1644 | Train 26 | Test Hispanic 44 | 0.8 | 0.89 | 0.87 |
| 621 | Train 10 | Test Hispanic 45 | 0.75 | 0.8 | 0.77 | 1645 | Train 26 | Test Hispanic 45 | 0.75 | 0.8 | 0.77 |
| 622 | Train 10 | Test Hispanic 46 | 0.73 | 0.75 | 0.73 | 1646 | Train 26 | Test Hispanic 46 | 0.73 | 0.75 | 0.72 |
| 623 | Train 10 | Test Hispanic 47 | 0.75 | 0.79 | 0.76 | 1647 | Train 26 | Test Hispanic 47 | 0.75 | 0.79 | 0.77 |
| 624 | Train 10 | Test Hispanic 48 | 0.73 | 0.75 | 0.73 | 1648 | Train 26 | Test Hispanic 48 | 0.73 | 0.75 | 0.73 |
| 625 | Train 10 | Test Hispanic 49 | 0.8 | 0.86 | 0.84 | 1649 | Train 26 | Test Hispanic 49 | 0.8 | 0.86 | 0.84 |
| 626 | Train 10 | Test Hispanic 50 | 0.79 | 0.86 | 0.84 | 1650 | Train 26 | Test Hispanic 50 | 0.79 | 0.86 | 0.84 |
| 627 | Train 10 | Test Hispanic 51 | 0.8 | 0.86 | 0.84 | 1651 | Train 26 | Test Hispanic 51 | 0.8 | 0.86 | 0.84 |
| 628 | Train 10 | Test Hispanic 52 | 0.8 | 0.86 | 0.84 | 1652 | Train 26 | Test Hispanic 52 | 0.8 | 0.86 | 0.84 |
| 629 | Train 10 | Test Hispanic 53 | 0.78 | 0.83 | 0.81 | 1653 | Train 26 | Test Hispanic 53 | 0.78 | 0.83 | 0.81 |
| 630 | Train 10 | Test Hispanic 54 | 0.75 | 0.77 | 0.75 | 1654 | Train 26 | Test Hispanic 54 | 0.75 | 0.77 | 0.74 |
| 631 | Train 10 | Test Hispanic 55 | 0.78 | 0.83 | 0.81 | 1655 | Train 26 | Test Hispanic 55 | 0.78 | 0.83 | 0.81 |
| 632 | Train 10 | Test Hispanic 56 | 0.75 | 0.76 | 0.75 | 1656 | Train 26 | Test Hispanic 56 | 0.75 | 0.76 | 0.74 |
| 633 | Train 10 | Test Hispanic 57 | 0.8 | 0.88 | 0.87 | 1657 | Train 26 | Test Hispanic 57 | 0.8 | 0.88 | 0.87 |
| 634 | Train 10 | Test Hispanic 58 | 0.79 | 0.87 | 0.87 | 1658 | Train 26 | Test Hispanic 58 | 0.79 | 0.87 | 0.87 |
| 635 | Train 10 | Test Hispanic 59 | 0.81 | 0.87 | 0.87 | 1659 | Train 26 | Test Hispanic 59 | 0.81 | 0.87 | 0.87 |
| 636 | Train 10 | Test Hispanic 60 | 0.8 | 0.87 | 0.87 | 1660 | Train 26 | Test Hispanic 60 | 0.8 | 0.87 | 0.86 |
| 637 | Train 10 | Test Hispanic 61 | 0.78 | 0.84 | 0.82 | 1661 | Train 26 | Test Hispanic 61 | 0.78 | 0.84 | 0.82 |
| 638 | Train 10 | Test Hispanic 62 | 0.75 | 0.77 | 0.75 | 1662 | Train 26 | Test Hispanic 62 | 0.75 | 0.77 | 0.75 |
| 639 | Train 10 | Test Hispanic 63 | 0.79 | 0.84 | 0.82 | 1663 | Train 26 | Test Hispanic 63 | 0.79 | 0.84 | 0.82 |
| 640 | Train 10 | Test Hispanic 64 | 0.75 | 0.76 | 0.75 | 1664 | Train 26 | Test Hispanic 64 | 0.75 | 0.76 | 0.75 |
| 641 | Train 11 | Test Hispanic 1 | 0.79 | 0.88 | 0.85 | 1665 | Train 27 | Test Hispanic 1 | 0.79 | 0.88 | 0.85 |
| 642 | Train 11 | Test Hispanic 2 | 0.78 | 0.86 | 0.83 | 1666 | Train 27 | Test Hispanic 2 | 0.78 | 0.86 | 0.83 |
| 643 | Train 11 | Test Hispanic 3 | 0.79 | 0.88 | 0.85 | 1667 | Train 27 | Test Hispanic 3 | 0.79 | 0.88 | 0.85 |
| 644 | Train 11 | Test Hispanic 4 | 0.78 | 0.86 | 0.83 | 1668 | Train 27 | Test Hispanic 4 | 0.78 | 0.86 | 0.84 |
| 645 | Train 11 | Test Hispanic 5 | 0.74 | 0.78 | 0.75 | 1669 | Train 27 | Test Hispanic 5 | 0.74 | 0.78 | 0.75 |
| 646 | Train 11 | Test Hispanic 6 | 0.72 | 0.73 | 0.7 | 1670 | Train 27 | Test Hispanic 6 | 0.72 | 0.73 | 0.7 |
| 647 | Train 11 | Test Hispanic 7 | 0.74 | 0.78 | 0.75 | 1671 | Train 27 | Test Hispanic 7 | 0.74 | 0.78 | 0.75 |
| 648 | Train 11 | Test Hispanic 8 | 0.72 | 0.73 | 0.71 | 1672 | Train 27 | Test Hispanic 8 | 0.72 | 0.73 | 0.71 |
| 649 | Train 11 | Test Hispanic 9 | 0.79 | 0.89 | 0.87 | 1673 | Train 27 | Test Hispanic 9 | 0.79 | 0.89 | 0.88 |
| 650 | Train 11 | Test Hispanic 10 | 0.78 | 0.87 | 0.85 | 1674 | Train 27 | Test Hispanic 10 | 0.78 | 0.87 | 0.86 |
| 651 | Train 11 | Test Hispanic 11 | 0.79 | 0.89 | 0.87 | 1675 | Train 27 | Test Hispanic 11 | 0.79 | 0.89 | 0.88 |
| 652 | Train 11 | Test Hispanic 12 | 0.78 | 0.87 | 0.85 | 1676 | Train 27 | Test Hispanic 12 | 0.78 | 0.87 | 0.86 |
| 653 | Train 11 | Test Hispanic 13 | 0.74 | 0.79 | 0.76 | 1677 | Train 27 | Test Hispanic 13 | 0.74 | 0.79 | 0.76 |
| 654 | Train 11 | Test Hispanic 14 | 0.72 | 0.73 | 0.7 | 1678 | Train 27 | Test Hispanic 14 | 0.72 | 0.73 | 0.7 |
| 655 | Train 11 | Test Hispanic 15 | 0.74 | 0.78 | 0.76 | 1679 | Train 27 | Test Hispanic 15 | 0.74 | 0.78 | 0.76 |
| 656 | Train 11 | Test Hispanic 16 | 0.72 | 0.73 | 0.71 | 1680 | Train 27 | Test Hispanic 16 | 0.72 | 0.73 | 0.71 |
| 657 | Train 11 | Test Hispanic 17 | 0.79 | 0.88 | 0.85 | 1681 | Train 27 | Test Hispanic 17 | 0.79 | 0.88 | 0.85 |
| 658 | Train 11 | Test Hispanic 18 | 0.78 | 0.86 | 0.83 | 1682 | Train 27 | Test Hispanic 18 | 0.78 | 0.86 | 0.83 |
| 659 | Train 11 | Test Hispanic 19 | 0.79 | 0.88 | 0.85 | 1683 | Train 27 | Test Hispanic 19 | 0.79 | 0.88 | 0.85 |
| 660 | Train 11 | Test Hispanic 20 | 0.78 | 0.86 | 0.83 | 1684 | Train 27 | Test Hispanic 20 | 0.78 | 0.86 | 0.84 |
| 661 | Train 11 | Test Hispanic 21 | 0.74 | 0.78 | 0.75 | 1685 | Train 27 | Test Hispanic 21 | 0.74 | 0.78 | 0.75 |
| 662 | Train 11 | Test Hispanic 22 | 0.72 | 0.73 | 0.7 | 1686 | Train 27 | Test Hispanic 22 | 0.72 | 0.73 | 0.7 |
| 663 | Train 11 | Test Hispanic 23 | 0.74 | 0.78 | 0.75 | 1687 | Train 27 | Test Hispanic 23 | 0.74 | 0.78 | 0.75 |
| 664 | Train 11 | Test Hispanic 24 | 0.72 | 0.73 | 0.71 | 1688 | Train 27 | Test Hispanic 24 | 0.72 | 0.73 | 0.71 |
| 665 | Train 11 | Test Hispanic 25 | 0.79 | 0.89 | 0.87 | 1689 | Train 27 | Test Hispanic 25 | 0.79 | 0.89 | 0.88 |
| 666 | Train 11 | Test Hispanic 26 | 0.78 | 0.87 | 0.85 | 1690 | Train 27 | Test Hispanic 26 | 0.78 | 0.87 | 0.86 |
| 667 | Train 11 | Test Hispanic 27 | 0.79 | 0.89 | 0.87 | 1691 | Train 27 | Test Hispanic 27 | 0.79 | 0.89 | 0.88 |
| 668 | Train 11 | Test Hispanic 28 | 0.78 | 0.87 | 0.85 | 1692 | Train 27 | Test Hispanic 28 | 0.78 | 0.87 | 0.86 |
| 669 | Train 11 | Test Hispanic 29 | 0.74 | 0.79 | 0.76 | 1693 | Train 27 | Test Hispanic 29 | 0.74 | 0.79 | 0.76 |
| 670 | Train 11 | Test Hispanic 30 | 0.72 | 0.73 | 0.71 | 1694 | Train 27 | Test Hispanic 30 | 0.72 | 0.73 | 0.7 |
| 671 | Train 11 | Test Hispanic 31 | 0.74 | 0.78 | 0.76 | 1695 | Train 27 | Test Hispanic 31 | 0.74 | 0.78 | 0.76 |
| 672 | Train 11 | Test Hispanic 32 | 0.72 | 0.73 | 0.71 | 1696 | Train 27 | Test Hispanic 32 | 0.72 | 0.73 | 0.71 |
| 673 | Train 11 | Test Hispanic 33 | 0.8 | 0.88 | 0.85 | 1697 | Train 27 | Test Hispanic 33 | 0.8 | 0.88 | 0.86 |
| 674 | Train 11 | Test Hispanic 34 | 0.78 | 0.86 | 0.83 | 1698 | Train 27 | Test Hispanic 34 | 0.78 | 0.86 | 0.84 |
| 675 | Train 11 | Test Hispanic 35 | 0.8 | 0.88 | 0.85 | 1699 | Train 27 | Test Hispanic 35 | 0.8 | 0.88 | 0.86 |
| 676 | Train 11 | Test Hispanic 36 | 0.79 | 0.86 | 0.83 | 1700 | Train 27 | Test Hispanic 36 | 0.79 | 0.86 | 0.84 |
| 677 | Train 11 | Test Hispanic 37 | 0.74 | 0.78 | 0.75 | 1701 | Train 27 | Test Hispanic 37 | 0.74 | 0.78 | 0.75 |
| 678 | Train 11 | Test Hispanic 38 | 0.72 | 0.73 | 0.71 | 1702 | Train 27 | Test Hispanic 38 | 0.72 | 0.73 | 0.71 |
| 679 | Train 11 | Test Hispanic 39 | 0.74 | 0.78 | 0.75 | 1703 | Train 27 | Test Hispanic 39 | 0.74 | 0.78 | 0.76 |
| 680 | Train 11 | Test Hispanic 40 | 0.72 | 0.73 | 0.71 | 1704 | Train 27 | Test Hispanic 40 | 0.72 | 0.73 | 0.71 |
| 681 | Train 11 | Test Hispanic 41 | 0.8 | 0.89 | 0.88 | 1705 | Train 27 | Test Hispanic 41 | 0.8 | 0.89 | 0.88 |
| 682 | Train 11 | Test Hispanic 42 | 0.79 | 0.87 | 0.86 | 1706 | Train 27 | Test Hispanic 42 | 0.79 | 0.87 | 0.86 |
| 683 | Train 11 | Test Hispanic 43 | 0.8 | 0.89 | 0.88 | 1707 | Train 27 | Test Hispanic 43 | 0.8 | 0.89 | 0.88 |
| 684 | Train 11 | Test Hispanic 44 | 0.79 | 0.87 | 0.86 | 1708 | Train 27 | Test Hispanic 44 | 0.79 | 0.87 | 0.86 |
| 685 | Train 11 | Test Hispanic 45 | 0.74 | 0.79 | 0.77 | 1709 | Train 27 | Test Hispanic 45 | 0.74 | 0.79 | 0.77 |
| 686 | Train 11 | Test Hispanic 46 | 0.72 | 0.73 | 0.71 | 1710 | Train 27 | Test Hispanic 46 | 0.72 | 0.73 | 0.71 |
| 687 | Train 11 | Test Hispanic 47 | 0.74 | 0.78 | 0.76 | 1711 | Train 27 | Test Hispanic 47 | 0.74 | 0.79 | 0.77 |
| 688 | Train 11 | Test Hispanic 48 | 0.72 | 0.73 | 0.71 | 1712 | Train 27 | Test Hispanic 48 | 0.72 | 0.73 | 0.71 |
| 689 | Train 11 | Test Hispanic 49 | 0.8 | 0.86 | 0.84 | 1713 | Train 27 | Test Hispanic 49 | 0.8 | 0.86 | 0.85 |
| 690 | Train 11 | Test Hispanic 50 | 0.8 | 0.86 | 0.84 | 1714 | Train 27 | Test Hispanic 50 | 0.8 | 0.86 | 0.84 |
| 691 | Train 11 | Test Hispanic 51 | 0.81 | 0.87 | 0.85 | 1715 | Train 27 | Test Hispanic 51 | 0.81 | 0.87 | 0.85 |
| 692 | Train 11 | Test Hispanic 52 | 0.8 | 0.86 | 0.84 | 1716 | Train 27 | Test Hispanic 52 | 0.8 | 0.86 | 0.85 |
| 693 | Train 11 | Test Hispanic 53 | 0.78 | 0.82 | 0.8 | 1717 | Train 27 | Test Hispanic 53 | 0.78 | 0.82 | 0.8 |
| 694 | Train 11 | Test Hispanic 54 | 0.75 | 0.75 | 0.72 | 1718 | Train 27 | Test Hispanic 54 | 0.75 | 0.74 | 0.71 |
| 695 | Train 11 | Test Hispanic 55 | 0.78 | 0.83 | 0.8 | 1719 | Train 27 | Test Hispanic 55 | 0.78 | 0.83 | 0.81 |
| 696 | Train 11 | Test Hispanic 56 | 0.75 | 0.75 | 0.73 | 1720 | Train 27 | Test Hispanic 56 | 0.75 | 0.74 | 0.72 |
| 697 | Train 11 | Test Hispanic 57 | 0.81 | 0.88 | 0.88 | 1721 | Train 27 | Test Hispanic 57 | 0.81 | 0.88 | 0.88 |
| 698 | Train 11 | Test Hispanic 58 | 0.8 | 0.87 | 0.87 | 1722 | Train 27 | Test Hispanic 58 | 0.8 | 0.87 | 0.87 |
| 699 | Train 11 | Test Hispanic 59 | 0.81 | 0.88 | 0.88 | 1723 | Train 27 | Test Hispanic 59 | 0.81 | 0.88 | 0.88 |
| 700 | Train 11 | Test Hispanic 60 | 0.81 | 0.88 | 0.88 | 1724 | Train 27 | Test Hispanic 60 | 0.81 | 0.88 | 0.88 |
| 701 | Train 11 | Test Hispanic 61 | 0.78 | 0.83 | 0.81 | 1725 | Train 27 | Test Hispanic 61 | 0.78 | 0.83 | 0.82 |
| 702 | Train 11 | Test Hispanic 62 | 0.75 | 0.75 | 0.72 | 1726 | Train 27 | Test Hispanic 62 | 0.75 | 0.75 | 0.71 |
| 703 | Train 11 | Test Hispanic 63 | 0.79 | 0.84 | 0.81 | 1727 | Train 27 | Test Hispanic 63 | 0.79 | 0.84 | 0.82 |
| 704 | Train 11 | Test Hispanic 64 | 0.75 | 0.75 | 0.73 | 1728 | Train 27 | Test Hispanic 64 | 0.75 | 0.75 | 0.72 |
| 705 | Train 12 | Test Hispanic 1 | 0.78 | 0.87 | 0.85 | 1729 | Train 28 | Test Hispanic 1 | 0.78 | 0.87 | 0.85 |
| 706 | Train 12 | Test Hispanic 2 | 0.78 | 0.88 | 0.85 | 1730 | Train 28 | Test Hispanic 2 | 0.78 | 0.88 | 0.85 |
| 707 | Train 12 | Test Hispanic 3 | 0.79 | 0.87 | 0.85 | 1731 | Train 28 | Test Hispanic 3 | 0.79 | 0.87 | 0.85 |
| 708 | Train 12 | Test Hispanic 4 | 0.78 | 0.88 | 0.85 | 1732 | Train 28 | Test Hispanic 4 | 0.78 | 0.88 | 0.85 |
| 709 | Train 12 | Test Hispanic 5 | 0.74 | 0.79 | 0.76 | 1733 | Train 28 | Test Hispanic 5 | 0.74 | 0.79 | 0.76 |
| 710 | Train 12 | Test Hispanic 6 | 0.73 | 0.75 | 0.72 | 1734 | Train 28 | Test Hispanic 6 | 0.72 | 0.75 | 0.72 |
| 711 | Train 12 | Test Hispanic 7 | 0.74 | 0.79 | 0.76 | 1735 | Train 28 | Test Hispanic 7 | 0.74 | 0.79 | 0.75 |
| 712 | Train 12 | Test Hispanic 8 | 0.73 | 0.75 | 0.72 | 1736 | Train 28 | Test Hispanic 8 | 0.73 | 0.75 | 0.72 |
| 713 | Train 12 | Test Hispanic 9 | 0.79 | 0.88 | 0.87 | 1737 | Train 28 | Test Hispanic 9 | 0.79 | 0.88 | 0.87 |
| 714 | Train 12 | Test Hispanic 10 | 0.78 | 0.88 | 0.88 | 1738 | Train 28 | Test Hispanic 10 | 0.78 | 0.89 | 0.87 |
| 715 | Train 12 | Test Hispanic 11 | 0.79 | 0.88 | 0.87 | 1739 | Train 28 | Test Hispanic 11 | 0.79 | 0.88 | 0.87 |
| 716 | Train 12 | Test Hispanic 12 | 0.79 | 0.88 | 0.88 | 1740 | Train 28 | Test Hispanic 12 | 0.79 | 0.89 | 0.87 |
| 717 | Train 12 | Test Hispanic 13 | 0.74 | 0.8 | 0.77 | 1741 | Train 28 | Test Hispanic 13 | 0.74 | 0.8 | 0.77 |
| 718 | Train 12 | Test Hispanic 14 | 0.73 | 0.75 | 0.72 | 1742 | Train 28 | Test Hispanic 14 | 0.73 | 0.75 | 0.72 |
| 719 | Train 12 | Test Hispanic 15 | 0.74 | 0.79 | 0.76 | 1743 | Train 28 | Test Hispanic 15 | 0.74 | 0.79 | 0.76 |
| 720 | Train 12 | Test Hispanic 16 | 0.73 | 0.75 | 0.72 | 1744 | Train 28 | Test Hispanic 16 | 0.73 | 0.75 | 0.72 |
| 721 | Train 12 | Test Hispanic 17 | 0.78 | 0.87 | 0.85 | 1745 | Train 28 | Test Hispanic 17 | 0.78 | 0.87 | 0.85 |
| 722 | Train 12 | Test Hispanic 18 | 0.78 | 0.88 | 0.85 | 1746 | Train 28 | Test Hispanic 18 | 0.78 | 0.88 | 0.85 |
| 723 | Train 12 | Test Hispanic 19 | 0.79 | 0.87 | 0.85 | 1747 | Train 28 | Test Hispanic 19 | 0.79 | 0.87 | 0.85 |
| 724 | Train 12 | Test Hispanic 20 | 0.78 | 0.88 | 0.85 | 1748 | Train 28 | Test Hispanic 20 | 0.78 | 0.88 | 0.85 |
| 725 | Train 12 | Test Hispanic 21 | 0.74 | 0.79 | 0.76 | 1749 | Train 28 | Test Hispanic 21 | 0.74 | 0.79 | 0.76 |
| 726 | Train 12 | Test Hispanic 22 | 0.73 | 0.75 | 0.72 | 1750 | Train 28 | Test Hispanic 22 | 0.73 | 0.75 | 0.72 |
| 727 | Train 12 | Test Hispanic 23 | 0.74 | 0.79 | 0.76 | 1751 | Train 28 | Test Hispanic 23 | 0.74 | 0.79 | 0.75 |
| 728 | Train 12 | Test Hispanic 24 | 0.73 | 0.75 | 0.72 | 1752 | Train 28 | Test Hispanic 24 | 0.73 | 0.75 | 0.72 |
| 729 | Train 12 | Test Hispanic 25 | 0.79 | 0.88 | 0.87 | 1753 | Train 28 | Test Hispanic 25 | 0.79 | 0.88 | 0.87 |
| 730 | Train 12 | Test Hispanic 26 | 0.78 | 0.88 | 0.88 | 1754 | Train 28 | Test Hispanic 26 | 0.78 | 0.89 | 0.87 |
| 731 | Train 12 | Test Hispanic 27 | 0.79 | 0.88 | 0.87 | 1755 | Train 28 | Test Hispanic 27 | 0.79 | 0.88 | 0.87 |
| 732 | Train 12 | Test Hispanic 28 | 0.79 | 0.88 | 0.88 | 1756 | Train 28 | Test Hispanic 28 | 0.79 | 0.89 | 0.87 |
| 733 | Train 12 | Test Hispanic 29 | 0.74 | 0.8 | 0.77 | 1757 | Train 28 | Test Hispanic 29 | 0.74 | 0.8 | 0.77 |
| 734 | Train 12 | Test Hispanic 30 | 0.73 | 0.75 | 0.72 | 1758 | Train 28 | Test Hispanic 30 | 0.73 | 0.75 | 0.72 |
| 735 | Train 12 | Test Hispanic 31 | 0.74 | 0.79 | 0.76 | 1759 | Train 28 | Test Hispanic 31 | 0.74 | 0.79 | 0.76 |
| 736 | Train 12 | Test Hispanic 32 | 0.73 | 0.75 | 0.72 | 1760 | Train 28 | Test Hispanic 32 | 0.73 | 0.75 | 0.72 |
| 737 | Train 12 | Test Hispanic 33 | 0.79 | 0.88 | 0.86 | 1761 | Train 28 | Test Hispanic 33 | 0.79 | 0.88 | 0.85 |
| 738 | Train 12 | Test Hispanic 34 | 0.79 | 0.88 | 0.86 | 1762 | Train 28 | Test Hispanic 34 | 0.79 | 0.88 | 0.85 |
| 739 | Train 12 | Test Hispanic 35 | 0.8 | 0.88 | 0.86 | 1763 | Train 28 | Test Hispanic 35 | 0.8 | 0.88 | 0.85 |
| 740 | Train 12 | Test Hispanic 36 | 0.79 | 0.88 | 0.86 | 1764 | Train 28 | Test Hispanic 36 | 0.79 | 0.88 | 0.85 |
| 741 | Train 12 | Test Hispanic 37 | 0.75 | 0.79 | 0.76 | 1765 | Train 28 | Test Hispanic 37 | 0.75 | 0.79 | 0.76 |
| 742 | Train 12 | Test Hispanic 38 | 0.73 | 0.75 | 0.72 | 1766 | Train 28 | Test Hispanic 38 | 0.73 | 0.75 | 0.72 |
| 743 | Train 12 | Test Hispanic 39 | 0.75 | 0.79 | 0.76 | 1767 | Train 28 | Test Hispanic 39 | 0.75 | 0.79 | 0.76 |
| 744 | Train 12 | Test Hispanic 40 | 0.73 | 0.75 | 0.72 | 1768 | Train 28 | Test Hispanic 40 | 0.73 | 0.75 | 0.72 |
| 745 | Train 12 | Test Hispanic 41 | 0.79 | 0.88 | 0.88 | 1769 | Train 28 | Test Hispanic 41 | 0.79 | 0.88 | 0.87 |
| 746 | Train 12 | Test Hispanic 42 | 0.79 | 0.89 | 0.88 | 1770 | Train 28 | Test Hispanic 42 | 0.79 | 0.89 | 0.87 |
| 747 | Train 12 | Test Hispanic 43 | 0.8 | 0.88 | 0.88 | 1771 | Train 28 | Test Hispanic 43 | 0.8 | 0.88 | 0.87 |
| 748 | Train 12 | Test Hispanic 44 | 0.79 | 0.89 | 0.88 | 1772 | Train 28 | Test Hispanic 44 | 0.79 | 0.89 | 0.87 |
| 749 | Train 12 | Test Hispanic 45 | 0.75 | 0.8 | 0.77 | 1773 | Train 28 | Test Hispanic 45 | 0.75 | 0.8 | 0.77 |
| 750 | Train 12 | Test Hispanic 46 | 0.73 | 0.75 | 0.72 | 1774 | Train 28 | Test Hispanic 46 | 0.73 | 0.75 | 0.72 |
| 751 | Train 12 | Test Hispanic 47 | 0.75 | 0.79 | 0.77 | 1775 | Train 28 | Test Hispanic 47 | 0.75 | 0.79 | 0.77 |
| 752 | Train 12 | Test Hispanic 48 | 0.73 | 0.75 | 0.73 | 1776 | Train 28 | Test Hispanic 48 | 0.73 | 0.75 | 0.73 |
| 753 | Train 12 | Test Hispanic 49 | 0.8 | 0.86 | 0.86 | 1777 | Train 28 | Test Hispanic 49 | 0.8 | 0.86 | 0.85 |
| 754 | Train 12 | Test Hispanic 50 | 0.79 | 0.86 | 0.85 | 1778 | Train 28 | Test Hispanic 50 | 0.79 | 0.86 | 0.84 |
| 755 | Train 12 | Test Hispanic 51 | 0.8 | 0.86 | 0.86 | 1779 | Train 28 | Test Hispanic 51 | 0.8 | 0.86 | 0.85 |
| 756 | Train 12 | Test Hispanic 52 | 0.8 | 0.86 | 0.86 | 1780 | Train 28 | Test Hispanic 52 | 0.8 | 0.86 | 0.85 |
| 757 | Train 12 | Test Hispanic 53 | 0.78 | 0.83 | 0.81 | 1781 | Train 28 | Test Hispanic 53 | 0.78 | 0.82 | 0.8 |
| 758 | Train 12 | Test Hispanic 54 | 0.75 | 0.77 | 0.74 | 1782 | Train 28 | Test Hispanic 54 | 0.75 | 0.76 | 0.74 |
| 759 | Train 12 | Test Hispanic 55 | 0.78 | 0.83 | 0.81 | 1783 | Train 28 | Test Hispanic 55 | 0.78 | 0.83 | 0.8 |
| 760 | Train 12 | Test Hispanic 56 | 0.75 | 0.76 | 0.74 | 1784 | Train 28 | Test Hispanic 56 | 0.75 | 0.76 | 0.74 |
| 761 | Train 12 | Test Hispanic 57 | 0.8 | 0.87 | 0.88 | 1785 | Train 28 | Test Hispanic 57 | 0.8 | 0.87 | 0.88 |
| 762 | Train 12 | Test Hispanic 58 | 0.79 | 0.87 | 0.88 | 1786 | Train 28 | Test Hispanic 58 | 0.79 | 0.87 | 0.87 |
| 763 | Train 12 | Test Hispanic 59 | 0.81 | 0.87 | 0.88 | 1787 | Train 28 | Test Hispanic 59 | 0.81 | 0.87 | 0.88 |
| 764 | Train 12 | Test Hispanic 60 | 0.8 | 0.87 | 0.88 | 1788 | Train 28 | Test Hispanic 60 | 0.8 | 0.87 | 0.88 |
| 765 | Train 12 | Test Hispanic 61 | 0.78 | 0.83 | 0.82 | 1789 | Train 28 | Test Hispanic 61 | 0.78 | 0.83 | 0.81 |
| 766 | Train 12 | Test Hispanic 62 | 0.75 | 0.77 | 0.75 | 1790 | Train 28 | Test Hispanic 62 | 0.75 | 0.77 | 0.74 |
| 767 | Train 12 | Test Hispanic 63 | 0.79 | 0.84 | 0.82 | 1791 | Train 28 | Test Hispanic 63 | 0.79 | 0.84 | 0.82 |
| 768 | Train 12 | Test Hispanic 64 | 0.75 | 0.76 | 0.75 | 1792 | Train 28 | Test Hispanic 64 | 0.75 | 0.76 | 0.74 |
| 769 | Train 13 | Test Hispanic 1 | 0.77 | 0.85 | 0.82 | 1793 | Train 29 | Test Hispanic 1 | 0.77 | 0.85 | 0.82 |
| 770 | Train 13 | Test Hispanic 2 | 0.77 | 0.84 | 0.81 | 1794 | Train 29 | Test Hispanic 2 | 0.77 | 0.84 | 0.81 |
| 771 | Train 13 | Test Hispanic 3 | 0.78 | 0.85 | 0.82 | 1795 | Train 29 | Test Hispanic 3 | 0.78 | 0.85 | 0.82 |
| 772 | Train 13 | Test Hispanic 4 | 0.77 | 0.84 | 0.81 | 1796 | Train 29 | Test Hispanic 4 | 0.77 | 0.84 | 0.81 |
| 773 | Train 13 | Test Hispanic 5 | 0.75 | 0.85 | 0.82 | 1797 | Train 29 | Test Hispanic 5 | 0.75 | 0.85 | 0.82 |
| 774 | Train 13 | Test Hispanic 6 | 0.72 | 0.78 | 0.76 | 1798 | Train 29 | Test Hispanic 6 | 0.72 | 0.77 | 0.77 |
| 775 | Train 13 | Test Hispanic 7 | 0.75 | 0.84 | 0.82 | 1799 | Train 29 | Test Hispanic 7 | 0.75 | 0.84 | 0.81 |
| 776 | Train 13 | Test Hispanic 8 | 0.72 | 0.77 | 0.76 | 1800 | Train 29 | Test Hispanic 8 | 0.72 | 0.77 | 0.77 |
| 777 | Train 13 | Test Hispanic 9 | 0.77 | 0.86 | 0.84 | 1801 | Train 29 | Test Hispanic 9 | 0.77 | 0.86 | 0.84 |
| 778 | Train 13 | Test Hispanic 10 | 0.77 | 0.85 | 0.83 | 1802 | Train 29 | Test Hispanic 10 | 0.77 | 0.85 | 0.83 |
| 779 | Train 13 | Test Hispanic 11 | 0.78 | 0.86 | 0.84 | 1803 | Train 29 | Test Hispanic 11 | 0.78 | 0.86 | 0.84 |
| 780 | Train 13 | Test Hispanic 12 | 0.77 | 0.85 | 0.83 | 1804 | Train 29 | Test Hispanic 12 | 0.77 | 0.85 | 0.83 |
| 781 | Train 13 | Test Hispanic 13 | 0.75 | 0.86 | 0.84 | 1805 | Train 29 | Test Hispanic 13 | 0.75 | 0.86 | 0.84 |
| 782 | Train 13 | Test Hispanic 14 | 0.72 | 0.78 | 0.77 | 1806 | Train 29 | Test Hispanic 14 | 0.72 | 0.77 | 0.78 |
| 783 | Train 13 | Test Hispanic 15 | 0.75 | 0.85 | 0.83 | 1807 | Train 29 | Test Hispanic 15 | 0.75 | 0.85 | 0.83 |
| 784 | Train 13 | Test Hispanic 16 | 0.72 | 0.77 | 0.77 | 1808 | Train 29 | Test Hispanic 16 | 0.72 | 0.77 | 0.77 |
| 785 | Train 13 | Test Hispanic 17 | 0.77 | 0.85 | 0.82 | 1809 | Train 29 | Test Hispanic 17 | 0.77 | 0.85 | 0.82 |
| 786 | Train 13 | Test Hispanic 18 | 0.77 | 0.84 | 0.81 | 1810 | Train 29 | Test Hispanic 18 | 0.77 | 0.84 | 0.81 |
| 787 | Train 13 | Test Hispanic 19 | 0.78 | 0.85 | 0.82 | 1811 | Train 29 | Test Hispanic 19 | 0.78 | 0.85 | 0.82 |
| 788 | Train 13 | Test Hispanic 20 | 0.77 | 0.84 | 0.81 | 1812 | Train 29 | Test Hispanic 20 | 0.77 | 0.84 | 0.81 |
| 789 | Train 13 | Test Hispanic 21 | 0.75 | 0.85 | 0.82 | 1813 | Train 29 | Test Hispanic 21 | 0.75 | 0.85 | 0.82 |
| 790 | Train 13 | Test Hispanic 22 | 0.72 | 0.78 | 0.76 | 1814 | Train 29 | Test Hispanic 22 | 0.72 | 0.77 | 0.77 |
| 791 | Train 13 | Test Hispanic 23 | 0.75 | 0.84 | 0.82 | 1815 | Train 29 | Test Hispanic 23 | 0.75 | 0.84 | 0.81 |
| 792 | Train 13 | Test Hispanic 24 | 0.72 | 0.77 | 0.76 | 1816 | Train 29 | Test Hispanic 24 | 0.72 | 0.77 | 0.77 |
| 793 | Train 13 | Test Hispanic 25 | 0.77 | 0.86 | 0.84 | 1817 | Train 29 | Test Hispanic 25 | 0.77 | 0.86 | 0.84 |
| 794 | Train 13 | Test Hispanic 26 | 0.77 | 0.85 | 0.83 | 1818 | Train 29 | Test Hispanic 26 | 0.77 | 0.85 | 0.83 |
| 795 | Train 13 | Test Hispanic 27 | 0.78 | 0.86 | 0.84 | 1819 | Train 29 | Test Hispanic 27 | 0.78 | 0.86 | 0.84 |
| 796 | Train 13 | Test Hispanic 28 | 0.77 | 0.85 | 0.83 | 1820 | Train 29 | Test Hispanic 28 | 0.77 | 0.85 | 0.83 |
| 797 | Train 13 | Test Hispanic 29 | 0.75 | 0.86 | 0.84 | 1821 | Train 29 | Test Hispanic 29 | 0.75 | 0.86 | 0.84 |
| 798 | Train 13 | Test Hispanic 30 | 0.72 | 0.78 | 0.77 | 1822 | Train 29 | Test Hispanic 30 | 0.72 | 0.78 | 0.78 |
| 799 | Train 13 | Test Hispanic 31 | 0.75 | 0.85 | 0.83 | 1823 | Train 29 | Test Hispanic 31 | 0.75 | 0.85 | 0.83 |
| 800 | Train 13 | Test Hispanic 32 | 0.72 | 0.77 | 0.77 | 1824 | Train 29 | Test Hispanic 32 | 0.72 | 0.77 | 0.77 |
| 801 | Train 13 | Test Hispanic 33 | 0.78 | 0.85 | 0.83 | 1825 | Train 29 | Test Hispanic 33 | 0.78 | 0.85 | 0.83 |
| 802 | Train 13 | Test Hispanic 34 | 0.78 | 0.84 | 0.82 | 1826 | Train 29 | Test Hispanic 34 | 0.78 | 0.84 | 0.81 |
| 803 | Train 13 | Test Hispanic 35 | 0.79 | 0.85 | 0.83 | 1827 | Train 29 | Test Hispanic 35 | 0.79 | 0.85 | 0.83 |
| 804 | Train 13 | Test Hispanic 36 | 0.78 | 0.84 | 0.82 | 1828 | Train 29 | Test Hispanic 36 | 0.78 | 0.84 | 0.81 |
| 805 | Train 13 | Test Hispanic 37 | 0.75 | 0.85 | 0.83 | 1829 | Train 29 | Test Hispanic 37 | 0.75 | 0.85 | 0.82 |
| 806 | Train 13 | Test Hispanic 38 | 0.72 | 0.78 | 0.77 | 1830 | Train 29 | Test Hispanic 38 | 0.72 | 0.77 | 0.78 |
| 807 | Train 13 | Test Hispanic 39 | 0.75 | 0.84 | 0.82 | 1831 | Train 29 | Test Hispanic 39 | 0.75 | 0.84 | 0.82 |
| 808 | Train 13 | Test Hispanic 40 | 0.73 | 0.77 | 0.77 | 1832 | Train 29 | Test Hispanic 40 | 0.73 | 0.77 | 0.77 |
| 809 | Train 13 | Test Hispanic 41 | 0.78 | 0.86 | 0.85 | 1833 | Train 29 | Test Hispanic 41 | 0.78 | 0.86 | 0.85 |
| 810 | Train 13 | Test Hispanic 42 | 0.78 | 0.85 | 0.83 | 1834 | Train 29 | Test Hispanic 42 | 0.78 | 0.85 | 0.83 |
| 811 | Train 13 | Test Hispanic 43 | 0.79 | 0.86 | 0.85 | 1835 | Train 29 | Test Hispanic 43 | 0.79 | 0.86 | 0.85 |
| 812 | Train 13 | Test Hispanic 44 | 0.78 | 0.85 | 0.83 | 1836 | Train 29 | Test Hispanic 44 | 0.78 | 0.85 | 0.83 |
| 813 | Train 13 | Test Hispanic 45 | 0.75 | 0.86 | 0.84 | 1837 | Train 29 | Test Hispanic 45 | 0.75 | 0.86 | 0.84 |
| 814 | Train 13 | Test Hispanic 46 | 0.72 | 0.78 | 0.77 | 1838 | Train 29 | Test Hispanic 46 | 0.72 | 0.77 | 0.78 |
| 815 | Train 13 | Test Hispanic 47 | 0.75 | 0.85 | 0.84 | 1839 | Train 29 | Test Hispanic 47 | 0.75 | 0.85 | 0.84 |
| 816 | Train 13 | Test Hispanic 48 | 0.73 | 0.77 | 0.77 | 1840 | Train 29 | Test Hispanic 48 | 0.73 | 0.77 | 0.78 |
| 817 | Train 13 | Test Hispanic 49 | 0.79 | 0.85 | 0.83 | 1841 | Train 29 | Test Hispanic 49 | 0.79 | 0.85 | 0.83 |
| 818 | Train 13 | Test Hispanic 50 | 0.79 | 0.85 | 0.82 | 1842 | Train 29 | Test Hispanic 50 | 0.79 | 0.85 | 0.82 |
| 819 | Train 13 | Test Hispanic 51 | 0.8 | 0.85 | 0.83 | 1843 | Train 29 | Test Hispanic 51 | 0.8 | 0.85 | 0.83 |
| 820 | Train 13 | Test Hispanic 52 | 0.79 | 0.85 | 0.82 | 1844 | Train 29 | Test Hispanic 52 | 0.79 | 0.85 | 0.82 |
| 821 | Train 13 | Test Hispanic 53 | 0.78 | 0.86 | 0.84 | 1845 | Train 29 | Test Hispanic 53 | 0.78 | 0.86 | 0.84 |
| 822 | Train 13 | Test Hispanic 54 | 0.74 | 0.77 | 0.76 | 1846 | Train 29 | Test Hispanic 54 | 0.74 | 0.76 | 0.78 |
| 823 | Train 13 | Test Hispanic 55 | 0.78 | 0.86 | 0.84 | 1847 | Train 29 | Test Hispanic 55 | 0.78 | 0.86 | 0.84 |
| 824 | Train 13 | Test Hispanic 56 | 0.74 | 0.76 | 0.76 | 1848 | Train 29 | Test Hispanic 56 | 0.74 | 0.75 | 0.77 |
| 825 | Train 13 | Test Hispanic 57 | 0.79 | 0.86 | 0.85 | 1849 | Train 29 | Test Hispanic 57 | 0.79 | 0.87 | 0.86 |
| 826 | Train 13 | Test Hispanic 58 | 0.79 | 0.86 | 0.85 | 1850 | Train 29 | Test Hispanic 58 | 0.79 | 0.86 | 0.85 |
| 827 | Train 13 | Test Hispanic 59 | 0.8 | 0.86 | 0.85 | 1851 | Train 29 | Test Hispanic 59 | 0.8 | 0.86 | 0.86 |
| 828 | Train 13 | Test Hispanic 60 | 0.8 | 0.86 | 0.85 | 1852 | Train 29 | Test Hispanic 60 | 0.8 | 0.86 | 0.85 |
| 829 | Train 13 | Test Hispanic 61 | 0.78 | 0.87 | 0.85 | 1853 | Train 29 | Test Hispanic 61 | 0.78 | 0.87 | 0.86 |
| 830 | Train 13 | Test Hispanic 62 | 0.74 | 0.77 | 0.76 | 1854 | Train 29 | Test Hispanic 62 | 0.74 | 0.77 | 0.78 |
| 831 | Train 13 | Test Hispanic 63 | 0.78 | 0.87 | 0.85 | 1855 | Train 29 | Test Hispanic 63 | 0.78 | 0.87 | 0.86 |
| 832 | Train 13 | Test Hispanic 64 | 0.74 | 0.76 | 0.76 | 1856 | Train 29 | Test Hispanic 64 | 0.74 | 0.76 | 0.78 |
| 833 | Train 14 | Test Hispanic 1 | 0.77 | 0.84 | 0.8 | 1857 | Train 30 | Test Hispanic 1 | 0.77 | 0.84 | 0.77 |
| 834 | Train 14 | Test Hispanic 2 | 0.77 | 0.84 | 0.8 | 1858 | Train 30 | Test Hispanic 2 | 0.77 | 0.84 | 0.78 |
| 835 | Train 14 | Test Hispanic 3 | 0.77 | 0.84 | 0.8 | 1859 | Train 30 | Test Hispanic 3 | 0.77 | 0.84 | 0.78 |
| 836 | Train 14 | Test Hispanic 4 | 0.77 | 0.84 | 0.8 | 1860 | Train 30 | Test Hispanic 4 | 0.77 | 0.84 | 0.78 |
| 837 | Train 14 | Test Hispanic 5 | 0.74 | 0.83 | 0.79 | 1861 | Train 30 | Test Hispanic 5 | 0.74 | 0.83 | 0.77 |
| 838 | Train 14 | Test Hispanic 6 | 0.75 | 0.84 | 0.79 | 1862 | Train 30 | Test Hispanic 6 | 0.75 | 0.84 | 0.78 |
| 839 | Train 14 | Test Hispanic 7 | 0.74 | 0.82 | 0.78 | 1863 | Train 30 | Test Hispanic 7 | 0.74 | 0.82 | 0.77 |
| 840 | Train 14 | Test Hispanic 8 | 0.75 | 0.83 | 0.79 | 1864 | Train 30 | Test Hispanic 8 | 0.75 | 0.83 | 0.77 |
| 841 | Train 14 | Test Hispanic 9 | 0.77 | 0.85 | 0.81 | 1865 | Train 30 | Test Hispanic 9 | 0.77 | 0.84 | 0.78 |
| 842 | Train 14 | Test Hispanic 10 | 0.77 | 0.85 | 0.81 | 1866 | Train 30 | Test Hispanic 10 | 0.77 | 0.85 | 0.79 |
| 843 | Train 14 | Test Hispanic 11 | 0.77 | 0.85 | 0.81 | 1867 | Train 30 | Test Hispanic 11 | 0.77 | 0.85 | 0.79 |
| 844 | Train 14 | Test Hispanic 12 | 0.77 | 0.85 | 0.81 | 1868 | Train 30 | Test Hispanic 12 | 0.77 | 0.85 | 0.79 |
| 845 | Train 14 | Test Hispanic 13 | 0.74 | 0.84 | 0.8 | 1869 | Train 30 | Test Hispanic 13 | 0.74 | 0.84 | 0.78 |
| 846 | Train 14 | Test Hispanic 14 | 0.75 | 0.84 | 0.79 | 1870 | Train 30 | Test Hispanic 14 | 0.75 | 0.84 | 0.78 |
| 847 | Train 14 | Test Hispanic 15 | 0.74 | 0.83 | 0.79 | 1871 | Train 30 | Test Hispanic 15 | 0.74 | 0.83 | 0.77 |
| 848 | Train 14 | Test Hispanic 16 | 0.75 | 0.83 | 0.79 | 1872 | Train 30 | Test Hispanic 16 | 0.75 | 0.83 | 0.77 |
| 849 | Train 14 | Test Hispanic 17 | 0.77 | 0.84 | 0.8 | 1873 | Train 30 | Test Hispanic 17 | 0.77 | 0.84 | 0.77 |
| 850 | Train 14 | Test Hispanic 18 | 0.77 | 0.84 | 0.8 | 1874 | Train 30 | Test Hispanic 18 | 0.77 | 0.84 | 0.78 |
| 851 | Train 14 | Test Hispanic 19 | 0.77 | 0.84 | 0.8 | 1875 | Train 30 | Test Hispanic 19 | 0.77 | 0.84 | 0.78 |
| 852 | Train 14 | Test Hispanic 20 | 0.77 | 0.84 | 0.8 | 1876 | Train 30 | Test Hispanic 20 | 0.77 | 0.84 | 0.78 |
| 853 | Train 14 | Test Hispanic 21 | 0.74 | 0.83 | 0.79 | 1877 | Train 30 | Test Hispanic 21 | 0.74 | 0.83 | 0.77 |
| 854 | Train 14 | Test Hispanic 22 | 0.75 | 0.84 | 0.79 | 1878 | Train 30 | Test Hispanic 22 | 0.75 | 0.84 | 0.78 |
| 855 | Train 14 | Test Hispanic 23 | 0.74 | 0.82 | 0.78 | 1879 | Train 30 | Test Hispanic 23 | 0.74 | 0.82 | 0.77 |
| 856 | Train 14 | Test Hispanic 24 | 0.75 | 0.83 | 0.79 | 1880 | Train 30 | Test Hispanic 24 | 0.75 | 0.83 | 0.77 |
| 857 | Train 14 | Test Hispanic 25 | 0.77 | 0.85 | 0.81 | 1881 | Train 30 | Test Hispanic 25 | 0.77 | 0.84 | 0.78 |
| 858 | Train 14 | Test Hispanic 26 | 0.77 | 0.85 | 0.81 | 1882 | Train 30 | Test Hispanic 26 | 0.77 | 0.85 | 0.79 |
| 859 | Train 14 | Test Hispanic 27 | 0.77 | 0.85 | 0.81 | 1883 | Train 30 | Test Hispanic 27 | 0.77 | 0.85 | 0.79 |
| 860 | Train 14 | Test Hispanic 28 | 0.77 | 0.85 | 0.81 | 1884 | Train 30 | Test Hispanic 28 | 0.77 | 0.85 | 0.79 |
| 861 | Train 14 | Test Hispanic 29 | 0.74 | 0.84 | 0.8 | 1885 | Train 30 | Test Hispanic 29 | 0.74 | 0.84 | 0.78 |
| 862 | Train 14 | Test Hispanic 30 | 0.75 | 0.84 | 0.79 | 1886 | Train 30 | Test Hispanic 30 | 0.75 | 0.84 | 0.78 |
| 863 | Train 14 | Test Hispanic 31 | 0.74 | 0.83 | 0.79 | 1887 | Train 30 | Test Hispanic 31 | 0.74 | 0.83 | 0.77 |
| 864 | Train 14 | Test Hispanic 32 | 0.75 | 0.83 | 0.79 | 1888 | Train 30 | Test Hispanic 32 | 0.75 | 0.83 | 0.77 |
| 865 | Train 14 | Test Hispanic 33 | 0.78 | 0.84 | 0.8 | 1889 | Train 30 | Test Hispanic 33 | 0.78 | 0.84 | 0.78 |
| 866 | Train 14 | Test Hispanic 34 | 0.78 | 0.84 | 0.8 | 1890 | Train 30 | Test Hispanic 34 | 0.78 | 0.84 | 0.78 |
| 867 | Train 14 | Test Hispanic 35 | 0.78 | 0.85 | 0.8 | 1891 | Train 30 | Test Hispanic 35 | 0.78 | 0.84 | 0.78 |
| 868 | Train 14 | Test Hispanic 36 | 0.78 | 0.84 | 0.8 | 1892 | Train 30 | Test Hispanic 36 | 0.78 | 0.84 | 0.79 |
| 869 | Train 14 | Test Hispanic 37 | 0.75 | 0.83 | 0.79 | 1893 | Train 30 | Test Hispanic 37 | 0.75 | 0.83 | 0.78 |
| 870 | Train 14 | Test Hispanic 38 | 0.75 | 0.84 | 0.8 | 1894 | Train 30 | Test Hispanic 38 | 0.75 | 0.84 | 0.78 |
| 871 | Train 14 | Test Hispanic 39 | 0.75 | 0.83 | 0.79 | 1895 | Train 30 | Test Hispanic 39 | 0.75 | 0.83 | 0.77 |
| 872 | Train 14 | Test Hispanic 40 | 0.75 | 0.83 | 0.79 | 1896 | Train 30 | Test Hispanic 40 | 0.75 | 0.83 | 0.78 |
| 873 | Train 14 | Test Hispanic 41 | 0.78 | 0.85 | 0.81 | 1897 | Train 30 | Test Hispanic 41 | 0.78 | 0.85 | 0.79 |
| 874 | Train 14 | Test Hispanic 42 | 0.78 | 0.85 | 0.81 | 1898 | Train 30 | Test Hispanic 42 | 0.78 | 0.85 | 0.79 |
| 875 | Train 14 | Test Hispanic 43 | 0.78 | 0.85 | 0.81 | 1899 | Train 30 | Test Hispanic 43 | 0.78 | 0.85 | 0.79 |
| 876 | Train 14 | Test Hispanic 44 | 0.78 | 0.85 | 0.81 | 1900 | Train 30 | Test Hispanic 44 | 0.78 | 0.85 | 0.79 |
| 877 | Train 14 | Test Hispanic 45 | 0.75 | 0.84 | 0.8 | 1901 | Train 30 | Test Hispanic 45 | 0.75 | 0.84 | 0.78 |
| 878 | Train 14 | Test Hispanic 46 | 0.75 | 0.84 | 0.8 | 1902 | Train 30 | Test Hispanic 46 | 0.75 | 0.84 | 0.78 |
| 879 | Train 14 | Test Hispanic 47 | 0.75 | 0.83 | 0.79 | 1903 | Train 30 | Test Hispanic 47 | 0.75 | 0.83 | 0.78 |
| 880 | Train 14 | Test Hispanic 48 | 0.75 | 0.83 | 0.79 | 1904 | Train 30 | Test Hispanic 48 | 0.75 | 0.83 | 0.78 |
| 881 | Train 14 | Test Hispanic 49 | 0.79 | 0.85 | 0.81 | 1905 | Train 30 | Test Hispanic 49 | 0.79 | 0.85 | 0.8 |
| 882 | Train 14 | Test Hispanic 50 | 0.79 | 0.85 | 0.81 | 1906 | Train 30 | Test Hispanic 50 | 0.79 | 0.85 | 0.8 |
| 883 | Train 14 | Test Hispanic 51 | 0.8 | 0.85 | 0.82 | 1907 | Train 30 | Test Hispanic 51 | 0.8 | 0.85 | 0.8 |
| 884 | Train 14 | Test Hispanic 52 | 0.79 | 0.85 | 0.81 | 1908 | Train 30 | Test Hispanic 52 | 0.79 | 0.85 | 0.8 |
| 885 | Train 14 | Test Hispanic 53 | 0.77 | 0.85 | 0.81 | 1909 | Train 30 | Test Hispanic 53 | 0.77 | 0.85 | 0.79 |
| 886 | Train 14 | Test Hispanic 54 | 0.78 | 0.85 | 0.8 | 1910 | Train 30 | Test Hispanic 54 | 0.78 | 0.85 | 0.79 |
| 887 | Train 14 | Test Hispanic 55 | 0.78 | 0.85 | 0.81 | 1911 | Train 30 | Test Hispanic 55 | 0.78 | 0.85 | 0.79 |
| 888 | Train 14 | Test Hispanic 56 | 0.77 | 0.84 | 0.79 | 1912 | Train 30 | Test Hispanic 56 | 0.77 | 0.84 | 0.78 |
| 889 | Train 14 | Test Hispanic 57 | 0.79 | 0.86 | 0.83 | 1913 | Train 30 | Test Hispanic 57 | 0.79 | 0.86 | 0.83 |
| 890 | Train 14 | Test Hispanic 58 | 0.79 | 0.86 | 0.83 | 1914 | Train 30 | Test Hispanic 58 | 0.79 | 0.86 | 0.82 |
| 891 | Train 14 | Test Hispanic 59 | 0.8 | 0.86 | 0.83 | 1915 | Train 30 | Test Hispanic 59 | 0.8 | 0.86 | 0.83 |
| 892 | Train 14 | Test Hispanic 60 | 0.79 | 0.86 | 0.83 | 1916 | Train 30 | Test Hispanic 60 | 0.79 | 0.86 | 0.82 |
| 893 | Train 14 | Test Hispanic 61 | 0.77 | 0.86 | 0.81 | 1917 | Train 30 | Test Hispanic 61 | 0.77 | 0.86 | 0.8 |
| 894 | Train 14 | Test Hispanic 62 | 0.78 | 0.85 | 0.8 | 1918 | Train 30 | Test Hispanic 62 | 0.78 | 0.85 | 0.79 |
| 895 | Train 14 | Test Hispanic 63 | 0.78 | 0.86 | 0.82 | 1919 | Train 30 | Test Hispanic 63 | 0.78 | 0.86 | 0.8 |
| 896 | Train 14 | Test Hispanic 64 | 0.77 | 0.84 | 0.79 | 1920 | Train 30 | Test Hispanic 64 | 0.77 | 0.84 | 0.78 |
| 897 | Train 15 | Test Hispanic 1 | 0.77 | 0.85 | 0.83 | 1921 | Train 31 | Test Hispanic 1 | 0.77 | 0.85 | 0.83 |
| 898 | Train 15 | Test Hispanic 2 | 0.77 | 0.84 | 0.81 | 1922 | Train 31 | Test Hispanic 2 | 0.77 | 0.84 | 0.82 |
| 899 | Train 15 | Test Hispanic 3 | 0.78 | 0.85 | 0.83 | 1923 | Train 31 | Test Hispanic 3 | 0.78 | 0.85 | 0.83 |
| 900 | Train 15 | Test Hispanic 4 | 0.77 | 0.84 | 0.81 | 1924 | Train 31 | Test Hispanic 4 | 0.77 | 0.84 | 0.82 |
| 901 | Train 15 | Test Hispanic 5 | 0.75 | 0.85 | 0.82 | 1925 | Train 31 | Test Hispanic 5 | 0.75 | 0.85 | 0.82 |
| 902 | Train 15 | Test Hispanic 6 | 0.72 | 0.77 | 0.76 | 1926 | Train 31 | Test Hispanic 6 | 0.72 | 0.77 | 0.76 |
| 903 | Train 15 | Test Hispanic 7 | 0.75 | 0.85 | 0.82 | 1927 | Train 31 | Test Hispanic 7 | 0.75 | 0.85 | 0.82 |
| 904 | Train 15 | Test Hispanic 8 | 0.72 | 0.77 | 0.76 | 1928 | Train 31 | Test Hispanic 8 | 0.72 | 0.77 | 0.77 |
| 905 | Train 15 | Test Hispanic 9 | 0.77 | 0.85 | 0.84 | 1929 | Train 31 | Test Hispanic 9 | 0.77 | 0.85 | 0.84 |
| 906 | Train 15 | Test Hispanic 10 | 0.77 | 0.84 | 0.83 | 1930 | Train 31 | Test Hispanic 10 | 0.77 | 0.84 | 0.83 |
| 907 | Train 15 | Test Hispanic 11 | 0.78 | 0.86 | 0.84 | 1931 | Train 31 | Test Hispanic 11 | 0.78 | 0.86 | 0.85 |
| 908 | Train 15 | Test Hispanic 12 | 0.77 | 0.85 | 0.83 | 1932 | Train 31 | Test Hispanic 12 | 0.77 | 0.85 | 0.84 |
| 909 | Train 15 | Test Hispanic 13 | 0.75 | 0.86 | 0.84 | 1933 | Train 31 | Test Hispanic 13 | 0.75 | 0.86 | 0.84 |
| 910 | Train 15 | Test Hispanic 14 | 0.72 | 0.78 | 0.76 | 1934 | Train 31 | Test Hispanic 14 | 0.72 | 0.77 | 0.76 |
| 911 | Train 15 | Test Hispanic 15 | 0.75 | 0.86 | 0.84 | 1935 | Train 31 | Test Hispanic 15 | 0.75 | 0.86 | 0.84 |
| 912 | Train 15 | Test Hispanic 16 | 0.72 | 0.78 | 0.77 | 1936 | Train 31 | Test Hispanic 16 | 0.72 | 0.77 | 0.77 |
| 913 | Train 15 | Test Hispanic 17 | 0.77 | 0.85 | 0.83 | 1937 | Train 31 | Test Hispanic 17 | 0.77 | 0.85 | 0.83 |
| 914 | Train 15 | Test Hispanic 18 | 0.77 | 0.84 | 0.81 | 1938 | Train 31 | Test Hispanic 18 | 0.77 | 0.84 | 0.82 |
| 915 | Train 15 | Test Hispanic 19 | 0.78 | 0.85 | 0.83 | 1939 | Train 31 | Test Hispanic 19 | 0.78 | 0.85 | 0.83 |
| 916 | Train 15 | Test Hispanic 20 | 0.77 | 0.84 | 0.81 | 1940 | Train 31 | Test Hispanic 20 | 0.77 | 0.84 | 0.82 |
| 917 | Train 15 | Test Hispanic 21 | 0.75 | 0.85 | 0.82 | 1941 | Train 31 | Test Hispanic 21 | 0.75 | 0.85 | 0.82 |
| 918 | Train 15 | Test Hispanic 22 | 0.72 | 0.77 | 0.76 | 1942 | Train 31 | Test Hispanic 22 | 0.72 | 0.77 | 0.76 |
| 919 | Train 15 | Test Hispanic 23 | 0.75 | 0.85 | 0.82 | 1943 | Train 31 | Test Hispanic 23 | 0.75 | 0.85 | 0.82 |
| 920 | Train 15 | Test Hispanic 24 | 0.72 | 0.77 | 0.77 | 1944 | Train 31 | Test Hispanic 24 | 0.72 | 0.77 | 0.77 |
| 921 | Train 15 | Test Hispanic 25 | 0.77 | 0.85 | 0.84 | 1945 | Train 31 | Test Hispanic 25 | 0.77 | 0.85 | 0.84 |
| 922 | Train 15 | Test Hispanic 26 | 0.77 | 0.85 | 0.83 | 1946 | Train 31 | Test Hispanic 26 | 0.77 | 0.84 | 0.83 |
| 923 | Train 15 | Test Hispanic 27 | 0.78 | 0.86 | 0.84 | 1947 | Train 31 | Test Hispanic 27 | 0.78 | 0.86 | 0.85 |
| 924 | Train 15 | Test Hispanic 28 | 0.77 | 0.85 | 0.83 | 1948 | Train 31 | Test Hispanic 28 | 0.77 | 0.85 | 0.84 |
| 925 | Train 15 | Test Hispanic 29 | 0.75 | 0.86 | 0.84 | 1949 | Train 31 | Test Hispanic 29 | 0.75 | 0.86 | 0.84 |
| 926 | Train 15 | Test Hispanic 30 | 0.72 | 0.78 | 0.76 | 1950 | Train 31 | Test Hispanic 30 | 0.72 | 0.78 | 0.76 |
| 927 | Train 15 | Test Hispanic 31 | 0.75 | 0.86 | 0.84 | 1951 | Train 31 | Test Hispanic 31 | 0.75 | 0.86 | 0.84 |
| 928 | Train 15 | Test Hispanic 32 | 0.72 | 0.78 | 0.77 | 1952 | Train 31 | Test Hispanic 32 | 0.72 | 0.77 | 0.77 |
| 929 | Train 15 | Test Hispanic 33 | 0.78 | 0.85 | 0.83 | 1953 | Train 31 | Test Hispanic 33 | 0.78 | 0.85 | 0.84 |
| 930 | Train 15 | Test Hispanic 34 | 0.78 | 0.84 | 0.82 | 1954 | Train 31 | Test Hispanic 34 | 0.78 | 0.84 | 0.82 |
| 931 | Train 15 | Test Hispanic 35 | 0.79 | 0.85 | 0.84 | 1955 | Train 31 | Test Hispanic 35 | 0.79 | 0.85 | 0.84 |
| 932 | Train 15 | Test Hispanic 36 | 0.78 | 0.84 | 0.82 | 1956 | Train 31 | Test Hispanic 36 | 0.78 | 0.84 | 0.83 |
| 933 | Train 15 | Test Hispanic 37 | 0.75 | 0.85 | 0.82 | 1957 | Train 31 | Test Hispanic 37 | 0.75 | 0.85 | 0.83 |
| 934 | Train 15 | Test Hispanic 38 | 0.72 | 0.77 | 0.76 | 1958 | Train 31 | Test Hispanic 38 | 0.72 | 0.77 | 0.77 |
| 935 | Train 15 | Test Hispanic 39 | 0.75 | 0.85 | 0.83 | 1959 | Train 31 | Test Hispanic 39 | 0.75 | 0.85 | 0.83 |
| 936 | Train 15 | Test Hispanic 40 | 0.72 | 0.78 | 0.77 | 1960 | Train 31 | Test Hispanic 40 | 0.72 | 0.77 | 0.77 |
| 937 | Train 15 | Test Hispanic 41 | 0.78 | 0.86 | 0.85 | 1961 | Train 31 | Test Hispanic 41 | 0.78 | 0.86 | 0.85 |
| 938 | Train 15 | Test Hispanic 42 | 0.78 | 0.85 | 0.83 | 1962 | Train 31 | Test Hispanic 42 | 0.78 | 0.85 | 0.84 |
| 939 | Train 15 | Test Hispanic 43 | 0.79 | 0.86 | 0.85 | 1963 | Train 31 | Test Hispanic 43 | 0.79 | 0.86 | 0.85 |
| 940 | Train 15 | Test Hispanic 44 | 0.78 | 0.85 | 0.83 | 1964 | Train 31 | Test Hispanic 44 | 0.78 | 0.85 | 0.84 |
| 941 | Train 15 | Test Hispanic 45 | 0.75 | 0.86 | 0.84 | 1965 | Train 31 | Test Hispanic 45 | 0.75 | 0.86 | 0.84 |
| 942 | Train 15 | Test Hispanic 46 | 0.72 | 0.78 | 0.76 | 1966 | Train 31 | Test Hispanic 46 | 0.72 | 0.78 | 0.77 |
| 943 | Train 15 | Test Hispanic 47 | 0.75 | 0.86 | 0.84 | 1967 | Train 31 | Test Hispanic 47 | 0.75 | 0.86 | 0.84 |
| 944 | Train 15 | Test Hispanic 48 | 0.73 | 0.78 | 0.77 | 1968 | Train 31 | Test Hispanic 48 | 0.73 | 0.77 | 0.77 |
| 945 | Train 15 | Test Hispanic 49 | 0.79 | 0.85 | 0.84 | 1969 | Train 31 | Test Hispanic 49 | 0.79 | 0.85 | 0.84 |
| 946 | Train 15 | Test Hispanic 50 | 0.79 | 0.85 | 0.83 | 1970 | Train 31 | Test Hispanic 50 | 0.79 | 0.85 | 0.83 |
| 947 | Train 15 | Test Hispanic 51 | 0.8 | 0.86 | 0.84 | 1971 | Train 31 | Test Hispanic 51 | 0.8 | 0.85 | 0.84 |
| 948 | Train 15 | Test Hispanic 52 | 0.79 | 0.85 | 0.83 | 1972 | Train 31 | Test Hispanic 52 | 0.79 | 0.85 | 0.84 |
| 949 | Train 15 | Test Hispanic 53 | 0.78 | 0.86 | 0.84 | 1973 | Train 31 | Test Hispanic 53 | 0.78 | 0.86 | 0.84 |
| 950 | Train 15 | Test Hispanic 54 | 0.74 | 0.77 | 0.75 | 1974 | Train 31 | Test Hispanic 54 | 0.74 | 0.77 | 0.74 |
| 951 | Train 15 | Test Hispanic 55 | 0.78 | 0.86 | 0.84 | 1975 | Train 31 | Test Hispanic 55 | 0.78 | 0.86 | 0.84 |
| 952 | Train 15 | Test Hispanic 56 | 0.74 | 0.76 | 0.75 | 1976 | Train 31 | Test Hispanic 56 | 0.74 | 0.76 | 0.75 |
| 953 | Train 15 | Test Hispanic 57 | 0.79 | 0.86 | 0.86 | 1977 | Train 31 | Test Hispanic 57 | 0.79 | 0.86 | 0.86 |
| 954 | Train 15 | Test Hispanic 58 | 0.79 | 0.86 | 0.85 | 1978 | Train 31 | Test Hispanic 58 | 0.79 | 0.86 | 0.86 |
| 955 | Train 15 | Test Hispanic 59 | 0.8 | 0.87 | 0.86 | 1979 | Train 31 | Test Hispanic 59 | 0.8 | 0.86 | 0.86 |
| 956 | Train 15 | Test Hispanic 60 | 0.79 | 0.86 | 0.86 | 1980 | Train 31 | Test Hispanic 60 | 0.79 | 0.86 | 0.86 |
| 957 | Train 15 | Test Hispanic 61 | 0.78 | 0.87 | 0.85 | 1981 | Train 31 | Test Hispanic 61 | 0.78 | 0.87 | 0.86 |
| 958 | Train 15 | Test Hispanic 62 | 0.74 | 0.77 | 0.75 | 1982 | Train 31 | Test Hispanic 62 | 0.74 | 0.77 | 0.74 |
| 959 | Train 15 | Test Hispanic 63 | 0.78 | 0.87 | 0.85 | 1983 | Train 31 | Test Hispanic 63 | 0.78 | 0.87 | 0.86 |
| 960 | Train 15 | Test Hispanic 64 | 0.74 | 0.76 | 0.76 | 1984 | Train 31 | Test Hispanic 64 | 0.74 | 0.76 | 0.75 |
| 961 | Train 16 | Test Hispanic 1 | 0.77 | 0.84 | 0.79 | 1985 | Train 32 | Test Hispanic 1 | 0.77 | 0.84 | 0.79 |
| 962 | Train 16 | Test Hispanic 2 | 0.77 | 0.83 | 0.79 | 1986 | Train 32 | Test Hispanic 2 | 0.77 | 0.84 | 0.79 |
| 963 | Train 16 | Test Hispanic 3 | 0.78 | 0.84 | 0.79 | 1987 | Train 32 | Test Hispanic 3 | 0.78 | 0.84 | 0.79 |
| 964 | Train 16 | Test Hispanic 4 | 0.78 | 0.84 | 0.79 | 1988 | Train 32 | Test Hispanic 4 | 0.77 | 0.84 | 0.8 |
| 965 | Train 16 | Test Hispanic 5 | 0.74 | 0.83 | 0.77 | 1989 | Train 32 | Test Hispanic 5 | 0.74 | 0.83 | 0.78 |
| 966 | Train 16 | Test Hispanic 6 | 0.75 | 0.83 | 0.78 | 1990 | Train 32 | Test Hispanic 6 | 0.75 | 0.83 | 0.78 |
| 967 | Train 16 | Test Hispanic 7 | 0.74 | 0.82 | 0.77 | 1991 | Train 32 | Test Hispanic 7 | 0.74 | 0.82 | 0.78 |
| 968 | Train 16 | Test Hispanic 8 | 0.75 | 0.83 | 0.78 | 1992 | Train 32 | Test Hispanic 8 | 0.75 | 0.83 | 0.78 |
| 969 | Train 16 | Test Hispanic 9 | 0.77 | 0.84 | 0.8 | 1993 | Train 32 | Test Hispanic 9 | 0.77 | 0.84 | 0.8 |
| 970 | Train 16 | Test Hispanic 10 | 0.77 | 0.84 | 0.8 | 1994 | Train 32 | Test Hispanic 10 | 0.77 | 0.84 | 0.8 |
| 971 | Train 16 | Test Hispanic 11 | 0.78 | 0.84 | 0.8 | 1995 | Train 32 | Test Hispanic 11 | 0.78 | 0.84 | 0.8 |
| 972 | Train 16 | Test Hispanic 12 | 0.78 | 0.84 | 0.8 | 1996 | Train 32 | Test Hispanic 12 | 0.78 | 0.84 | 0.81 |
| 973 | Train 16 | Test Hispanic 13 | 0.74 | 0.83 | 0.78 | 1997 | Train 32 | Test Hispanic 13 | 0.74 | 0.83 | 0.79 |
| 974 | Train 16 | Test Hispanic 14 | 0.75 | 0.84 | 0.78 | 1998 | Train 32 | Test Hispanic 14 | 0.75 | 0.84 | 0.78 |
| 975 | Train 16 | Test Hispanic 15 | 0.74 | 0.83 | 0.78 | 1999 | Train 32 | Test Hispanic 15 | 0.74 | 0.83 | 0.78 |
| 976 | Train 16 | Test Hispanic 16 | 0.75 | 0.83 | 0.78 | 2000 | Train 32 | Test Hispanic 16 | 0.75 | 0.83 | 0.78 |
| 977 | Train 16 | Test Hispanic 17 | 0.77 | 0.84 | 0.79 | 2001 | Train 32 | Test Hispanic 17 | 0.77 | 0.84 | 0.79 |
| 978 | Train 16 | Test Hispanic 18 | 0.77 | 0.83 | 0.79 | 2002 | Train 32 | Test Hispanic 18 | 0.77 | 0.84 | 0.79 |
| 979 | Train 16 | Test Hispanic 19 | 0.78 | 0.84 | 0.79 | 2003 | Train 32 | Test Hispanic 19 | 0.78 | 0.84 | 0.79 |
| 980 | Train 16 | Test Hispanic 20 | 0.78 | 0.84 | 0.79 | 2004 | Train 32 | Test Hispanic 20 | 0.77 | 0.84 | 0.8 |
| 981 | Train 16 | Test Hispanic 21 | 0.74 | 0.83 | 0.77 | 2005 | Train 32 | Test Hispanic 21 | 0.74 | 0.83 | 0.78 |
| 982 | Train 16 | Test Hispanic 22 | 0.75 | 0.83 | 0.78 | 2006 | Train 32 | Test Hispanic 22 | 0.75 | 0.83 | 0.78 |
| 983 | Train 16 | Test Hispanic 23 | 0.74 | 0.82 | 0.77 | 2007 | Train 32 | Test Hispanic 23 | 0.74 | 0.82 | 0.78 |
| 984 | Train 16 | Test Hispanic 24 | 0.75 | 0.83 | 0.78 | 2008 | Train 32 | Test Hispanic 24 | 0.75 | 0.83 | 0.78 |
| 985 | Train 16 | Test Hispanic 25 | 0.77 | 0.84 | 0.8 | 2009 | Train 32 | Test Hispanic 25 | 0.77 | 0.84 | 0.8 |
| 986 | Train 16 | Test Hispanic 26 | 0.77 | 0.84 | 0.8 | 2010 | Train 32 | Test Hispanic 26 | 0.77 | 0.84 | 0.8 |
| 987 | Train 16 | Test Hispanic 27 | 0.78 | 0.84 | 0.8 | 2011 | Train 32 | Test Hispanic 27 | 0.78 | 0.84 | 0.8 |
| 988 | Train 16 | Test Hispanic 28 | 0.78 | 0.84 | 0.8 | 2012 | Train 32 | Test Hispanic 28 | 0.78 | 0.84 | 0.81 |
| 989 | Train 16 | Test Hispanic 29 | 0.74 | 0.83 | 0.78 | 2013 | Train 32 | Test Hispanic 29 | 0.74 | 0.83 | 0.79 |
| 990 | Train 16 | Test Hispanic 30 | 0.75 | 0.84 | 0.78 | 2014 | Train 32 | Test Hispanic 30 | 0.75 | 0.84 | 0.78 |
| 991 | Train 16 | Test Hispanic 31 | 0.74 | 0.83 | 0.78 | 2015 | Train 32 | Test Hispanic 31 | 0.74 | 0.83 | 0.78 |
| 992 | Train 16 | Test Hispanic 32 | 0.75 | 0.83 | 0.78 | 2016 | Train 32 | Test Hispanic 32 | 0.75 | 0.83 | 0.78 |
| 993 | Train 16 | Test Hispanic 33 | 0.78 | 0.84 | 0.79 | 2017 | Train 32 | Test Hispanic 33 | 0.78 | 0.84 | 0.8 |
| 994 | Train 16 | Test Hispanic 34 | 0.78 | 0.84 | 0.79 | 2018 | Train 32 | Test Hispanic 34 | 0.78 | 0.84 | 0.8 |
| 995 | Train 16 | Test Hispanic 35 | 0.79 | 0.84 | 0.8 | 2019 | Train 32 | Test Hispanic 35 | 0.79 | 0.84 | 0.8 |
| 996 | Train 16 | Test Hispanic 36 | 0.78 | 0.84 | 0.79 | 2020 | Train 32 | Test Hispanic 36 | 0.78 | 0.84 | 0.8 |
| 997 | Train 16 | Test Hispanic 37 | 0.75 | 0.83 | 0.77 | 2021 | Train 32 | Test Hispanic 37 | 0.75 | 0.83 | 0.78 |
| 998 | Train 16 | Test Hispanic 38 | 0.75 | 0.84 | 0.78 | 2022 | Train 32 | Test Hispanic 38 | 0.75 | 0.84 | 0.78 |
| 999 | Train 16 | Test Hispanic 39 | 0.75 | 0.83 | 0.77 | 2023 | Train 32 | Test Hispanic 39 | 0.75 | 0.83 | 0.78 |
| 1000 | Train 16 | Test Hispanic 40 | 0.76 | 0.83 | 0.78 | 2024 | Train 32 | Test Hispanic 40 | 0.75 | 0.83 | 0.78 |
| 1001 | Train 16 | Test Hispanic 41 | 0.78 | 0.85 | 0.8 | 2025 | Train 32 | Test Hispanic 41 | 0.78 | 0.85 | 0.81 |
| 1002 | Train 16 | Test Hispanic 42 | 0.78 | 0.85 | 0.8 | 2026 | Train 32 | Test Hispanic 42 | 0.78 | 0.85 | 0.81 |
| 1003 | Train 16 | Test Hispanic 43 | 0.79 | 0.85 | 0.8 | 2027 | Train 32 | Test Hispanic 43 | 0.79 | 0.85 | 0.81 |
| 1004 | Train 16 | Test Hispanic 44 | 0.79 | 0.85 | 0.8 | 2028 | Train 32 | Test Hispanic 44 | 0.78 | 0.85 | 0.81 |
| 1005 | Train 16 | Test Hispanic 45 | 0.75 | 0.83 | 0.78 | 2029 | Train 32 | Test Hispanic 45 | 0.75 | 0.83 | 0.79 |
| 1006 | Train 16 | Test Hispanic 46 | 0.75 | 0.84 | 0.78 | 2030 | Train 32 | Test Hispanic 46 | 0.75 | 0.84 | 0.79 |
| 1007 | Train 16 | Test Hispanic 47 | 0.75 | 0.83 | 0.78 | 2031 | Train 32 | Test Hispanic 47 | 0.75 | 0.83 | 0.79 |
| 1008 | Train 16 | Test Hispanic 48 | 0.76 | 0.83 | 0.78 | 2032 | Train 32 | Test Hispanic 48 | 0.75 | 0.83 | 0.78 |
| 1009 | Train 16 | Test Hispanic 49 | 0.8 | 0.85 | 0.8 | 2033 | Train 32 | Test Hispanic 49 | 0.8 | 0.85 | 0.81 |
| 1010 | Train 16 | Test Hispanic 50 | 0.79 | 0.85 | 0.8 | 2034 | Train 32 | Test Hispanic 50 | 0.79 | 0.85 | 0.81 |
| 1011 | Train 16 | Test Hispanic 51 | 0.8 | 0.85 | 0.81 | 2035 | Train 32 | Test Hispanic 51 | 0.8 | 0.85 | 0.82 |
| 1012 | Train 16 | Test Hispanic 52 | 0.8 | 0.85 | 0.8 | 2036 | Train 32 | Test Hispanic 52 | 0.8 | 0.85 | 0.81 |
| 1013 | Train 16 | Test Hispanic 53 | 0.77 | 0.85 | 0.79 | 2037 | Train 32 | Test Hispanic 53 | 0.77 | 0.85 | 0.8 |
| 1014 | Train 16 | Test Hispanic 54 | 0.78 | 0.84 | 0.79 | 2038 | Train 32 | Test Hispanic 54 | 0.78 | 0.84 | 0.79 |
| 1015 | Train 16 | Test Hispanic 55 | 0.78 | 0.85 | 0.8 | 2039 | Train 32 | Test Hispanic 55 | 0.78 | 0.85 | 0.8 |
| 1016 | Train 16 | Test Hispanic 56 | 0.78 | 0.84 | 0.79 | 2040 | Train 32 | Test Hispanic 56 | 0.78 | 0.84 | 0.79 |
| 1017 | Train 16 | Test Hispanic 57 | 0.8 | 0.86 | 0.82 | 2041 | Train 32 | Test Hispanic 57 | 0.8 | 0.86 | 0.83 |
| 1018 | Train 16 | Test Hispanic 58 | 0.79 | 0.86 | 0.81 | 2042 | Train 32 | Test Hispanic 58 | 0.79 | 0.86 | 0.82 |
| 1019 | Train 16 | Test Hispanic 59 | 0.8 | 0.86 | 0.82 | 2043 | Train 32 | Test Hispanic 59 | 0.8 | 0.86 | 0.83 |
| 1020 | Train 16 | Test Hispanic 60 | 0.8 | 0.86 | 0.82 | 2044 | Train 32 | Test Hispanic 60 | 0.8 | 0.86 | 0.83 |
| 1021 | Train 16 | Test Hispanic 61 | 0.78 | 0.86 | 0.8 | 2045 | Train 32 | Test Hispanic 61 | 0.78 | 0.86 | 0.81 |
| 1022 | Train 16 | Test Hispanic 62 | 0.78 | 0.84 | 0.79 | 2046 | Train 32 | Test Hispanic 62 | 0.78 | 0.85 | 0.79 |
| 1023 | Train 16 | Test Hispanic 63 | 0.78 | 0.86 | 0.81 | 2047 | Train 32 | Test Hispanic 63 | 0.78 | 0.86 | 0.81 |
| 1024 | Train 16 | Test Hispanic 64 | 0.78 | 0.84 | 0.79 | 2048 | Train 32 | Test Hispanic 64 | 0.78 | 0.84 | 0.79 |

Table 12. AUC Performance of the ML Models— LR, RF, and GB —for Hispanic Patients Trained on Cohorts 33–64 and Tested Across 64 Cohorts.

| Hispanic | Train  Model | Test  Dataset | AUC (LR) | AUC (RF) | AUC (GB) | Hispanic | Train  Model | Test  Dataset | AUC (LR) | AUC (RF) | AUC (GB) |
| --- | --- | --- | --- | --- | --- | --- | --- | --- | --- | --- | --- |
| 2049 | Train 33 | Test Hispanic 1 | 0.79 | 0.89 | 0.87 | 3073 | Train 49 | Test Hispanic 1 | 0.76 | 0.82 | 0.74 |
| 2050 | Train 33 | Test Hispanic 2 | 0.78 | 0.87 | 0.85 | 3074 | Train 49 | Test Hispanic 2 | 0.75 | 0.8 | 0.72 |
| 2051 | Train 33 | Test Hispanic 3 | 0.79 | 0.89 | 0.87 | 3075 | Train 49 | Test Hispanic 3 | 0.77 | 0.82 | 0.74 |
| 2052 | Train 33 | Test Hispanic 4 | 0.78 | 0.87 | 0.85 | 3076 | Train 49 | Test Hispanic 4 | 0.75 | 0.8 | 0.72 |
| 2053 | Train 33 | Test Hispanic 5 | 0.74 | 0.79 | 0.74 | 3077 | Train 49 | Test Hispanic 5 | 0.73 | 0.75 | 0.66 |
| 2054 | Train 33 | Test Hispanic 6 | 0.72 | 0.73 | 0.68 | 3078 | Train 49 | Test Hispanic 6 | 0.71 | 0.71 | 0.64 |
| 2055 | Train 33 | Test Hispanic 7 | 0.74 | 0.78 | 0.73 | 3079 | Train 49 | Test Hispanic 7 | 0.73 | 0.75 | 0.66 |
| 2056 | Train 33 | Test Hispanic 8 | 0.72 | 0.73 | 0.68 | 3080 | Train 49 | Test Hispanic 8 | 0.72 | 0.71 | 0.64 |
| 2057 | Train 33 | Test Hispanic 9 | 0.79 | 0.88 | 0.84 | 3081 | Train 49 | Test Hispanic 9 | 0.77 | 0.81 | 0.72 |
| 2058 | Train 33 | Test Hispanic 10 | 0.78 | 0.86 | 0.82 | 3082 | Train 49 | Test Hispanic 10 | 0.75 | 0.8 | 0.7 |
| 2059 | Train 33 | Test Hispanic 11 | 0.79 | 0.88 | 0.84 | 3083 | Train 49 | Test Hispanic 11 | 0.77 | 0.81 | 0.72 |
| 2060 | Train 33 | Test Hispanic 12 | 0.78 | 0.86 | 0.82 | 3084 | Train 49 | Test Hispanic 12 | 0.76 | 0.8 | 0.7 |
| 2061 | Train 33 | Test Hispanic 13 | 0.74 | 0.78 | 0.72 | 3085 | Train 49 | Test Hispanic 13 | 0.73 | 0.75 | 0.65 |
| 2062 | Train 33 | Test Hispanic 14 | 0.72 | 0.73 | 0.68 | 3086 | Train 49 | Test Hispanic 14 | 0.71 | 0.71 | 0.64 |
| 2063 | Train 33 | Test Hispanic 15 | 0.74 | 0.77 | 0.72 | 3087 | Train 49 | Test Hispanic 15 | 0.73 | 0.74 | 0.65 |
| 2064 | Train 33 | Test Hispanic 16 | 0.72 | 0.73 | 0.68 | 3088 | Train 49 | Test Hispanic 16 | 0.72 | 0.71 | 0.64 |
| 2065 | Train 33 | Test Hispanic 17 | 0.79 | 0.89 | 0.87 | 3089 | Train 49 | Test Hispanic 17 | 0.76 | 0.82 | 0.74 |
| 2066 | Train 33 | Test Hispanic 18 | 0.78 | 0.87 | 0.85 | 3090 | Train 49 | Test Hispanic 18 | 0.75 | 0.8 | 0.72 |
| 2067 | Train 33 | Test Hispanic 19 | 0.79 | 0.89 | 0.87 | 3091 | Train 49 | Test Hispanic 19 | 0.77 | 0.82 | 0.74 |
| 2068 | Train 33 | Test Hispanic 20 | 0.78 | 0.87 | 0.85 | 3092 | Train 49 | Test Hispanic 20 | 0.75 | 0.8 | 0.72 |
| 2069 | Train 33 | Test Hispanic 21 | 0.74 | 0.79 | 0.74 | 3093 | Train 49 | Test Hispanic 21 | 0.73 | 0.75 | 0.66 |
| 2070 | Train 33 | Test Hispanic 22 | 0.72 | 0.73 | 0.68 | 3094 | Train 49 | Test Hispanic 22 | 0.71 | 0.71 | 0.64 |
| 2071 | Train 33 | Test Hispanic 23 | 0.74 | 0.78 | 0.73 | 3095 | Train 49 | Test Hispanic 23 | 0.73 | 0.75 | 0.66 |
| 2072 | Train 33 | Test Hispanic 24 | 0.72 | 0.73 | 0.68 | 3096 | Train 49 | Test Hispanic 24 | 0.72 | 0.71 | 0.64 |
| 2073 | Train 33 | Test Hispanic 25 | 0.79 | 0.88 | 0.84 | 3097 | Train 49 | Test Hispanic 25 | 0.77 | 0.81 | 0.72 |
| 2074 | Train 33 | Test Hispanic 26 | 0.78 | 0.86 | 0.82 | 3098 | Train 49 | Test Hispanic 26 | 0.75 | 0.8 | 0.7 |
| 2075 | Train 33 | Test Hispanic 27 | 0.79 | 0.88 | 0.84 | 3099 | Train 49 | Test Hispanic 27 | 0.77 | 0.81 | 0.72 |
| 2076 | Train 33 | Test Hispanic 28 | 0.78 | 0.86 | 0.82 | 3100 | Train 49 | Test Hispanic 28 | 0.76 | 0.8 | 0.7 |
| 2077 | Train 33 | Test Hispanic 29 | 0.74 | 0.78 | 0.72 | 3101 | Train 49 | Test Hispanic 29 | 0.73 | 0.75 | 0.65 |
| 2078 | Train 33 | Test Hispanic 30 | 0.72 | 0.73 | 0.68 | 3102 | Train 49 | Test Hispanic 30 | 0.71 | 0.71 | 0.64 |
| 2079 | Train 33 | Test Hispanic 31 | 0.74 | 0.77 | 0.72 | 3103 | Train 49 | Test Hispanic 31 | 0.73 | 0.74 | 0.65 |
| 2080 | Train 33 | Test Hispanic 32 | 0.72 | 0.73 | 0.68 | 3104 | Train 49 | Test Hispanic 32 | 0.72 | 0.71 | 0.64 |
| 2081 | Train 33 | Test Hispanic 33 | 0.8 | 0.9 | 0.88 | 3105 | Train 49 | Test Hispanic 33 | 0.77 | 0.82 | 0.75 |
| 2082 | Train 33 | Test Hispanic 34 | 0.79 | 0.88 | 0.86 | 3106 | Train 49 | Test Hispanic 34 | 0.76 | 0.81 | 0.73 |
| 2083 | Train 33 | Test Hispanic 35 | 0.8 | 0.89 | 0.88 | 3107 | Train 49 | Test Hispanic 35 | 0.78 | 0.83 | 0.76 |
| 2084 | Train 33 | Test Hispanic 36 | 0.79 | 0.88 | 0.85 | 3108 | Train 49 | Test Hispanic 36 | 0.76 | 0.81 | 0.73 |
| 2085 | Train 33 | Test Hispanic 37 | 0.74 | 0.79 | 0.74 | 3109 | Train 49 | Test Hispanic 37 | 0.73 | 0.76 | 0.66 |
| 2086 | Train 33 | Test Hispanic 38 | 0.72 | 0.73 | 0.68 | 3110 | Train 49 | Test Hispanic 38 | 0.72 | 0.71 | 0.64 |
| 2087 | Train 33 | Test Hispanic 39 | 0.74 | 0.79 | 0.74 | 3111 | Train 49 | Test Hispanic 39 | 0.73 | 0.75 | 0.67 |
| 2088 | Train 33 | Test Hispanic 40 | 0.72 | 0.73 | 0.69 | 3112 | Train 49 | Test Hispanic 40 | 0.72 | 0.71 | 0.64 |
| 2089 | Train 33 | Test Hispanic 41 | 0.8 | 0.88 | 0.85 | 3113 | Train 49 | Test Hispanic 41 | 0.78 | 0.82 | 0.73 |
| 2090 | Train 33 | Test Hispanic 42 | 0.79 | 0.86 | 0.83 | 3114 | Train 49 | Test Hispanic 42 | 0.76 | 0.8 | 0.71 |
| 2091 | Train 33 | Test Hispanic 43 | 0.8 | 0.88 | 0.85 | 3115 | Train 49 | Test Hispanic 43 | 0.78 | 0.82 | 0.73 |
| 2092 | Train 33 | Test Hispanic 44 | 0.79 | 0.86 | 0.83 | 3116 | Train 49 | Test Hispanic 44 | 0.77 | 0.81 | 0.71 |
| 2093 | Train 33 | Test Hispanic 45 | 0.74 | 0.78 | 0.73 | 3117 | Train 49 | Test Hispanic 45 | 0.73 | 0.75 | 0.66 |
| 2094 | Train 33 | Test Hispanic 46 | 0.72 | 0.73 | 0.68 | 3118 | Train 49 | Test Hispanic 46 | 0.72 | 0.71 | 0.64 |
| 2095 | Train 33 | Test Hispanic 47 | 0.74 | 0.78 | 0.72 | 3119 | Train 49 | Test Hispanic 47 | 0.73 | 0.75 | 0.66 |
| 2096 | Train 33 | Test Hispanic 48 | 0.73 | 0.73 | 0.69 | 3120 | Train 49 | Test Hispanic 48 | 0.72 | 0.71 | 0.64 |
| 2097 | Train 33 | Test Hispanic 49 | 0.81 | 0.88 | 0.88 | 3121 | Train 49 | Test Hispanic 49 | 0.81 | 0.91 | 0.92 |
| 2098 | Train 33 | Test Hispanic 50 | 0.8 | 0.88 | 0.87 | 3122 | Train 49 | Test Hispanic 50 | 0.8 | 0.91 | 0.92 |
| 2099 | Train 33 | Test Hispanic 51 | 0.81 | 0.88 | 0.87 | 3123 | Train 49 | Test Hispanic 51 | 0.81 | 0.9 | 0.91 |
| 2100 | Train 33 | Test Hispanic 52 | 0.81 | 0.88 | 0.87 | 3124 | Train 49 | Test Hispanic 52 | 0.81 | 0.9 | 0.91 |
| 2101 | Train 33 | Test Hispanic 53 | 0.78 | 0.84 | 0.74 | 3125 | Train 49 | Test Hispanic 53 | 0.77 | 0.84 | 0.78 |
| 2102 | Train 33 | Test Hispanic 54 | 0.75 | 0.75 | 0.68 | 3126 | Train 49 | Test Hispanic 54 | 0.74 | 0.74 | 0.69 |
| 2103 | Train 33 | Test Hispanic 55 | 0.78 | 0.84 | 0.74 | 3127 | Train 49 | Test Hispanic 55 | 0.77 | 0.85 | 0.79 |
| 2104 | Train 33 | Test Hispanic 56 | 0.76 | 0.75 | 0.69 | 3128 | Train 49 | Test Hispanic 56 | 0.75 | 0.75 | 0.69 |
| 2105 | Train 33 | Test Hispanic 57 | 0.81 | 0.87 | 0.85 | 3129 | Train 49 | Test Hispanic 57 | 0.81 | 0.89 | 0.87 |
| 2106 | Train 33 | Test Hispanic 58 | 0.8 | 0.87 | 0.84 | 3130 | Train 49 | Test Hispanic 58 | 0.8 | 0.89 | 0.87 |
| 2107 | Train 33 | Test Hispanic 59 | 0.81 | 0.87 | 0.85 | 3131 | Train 49 | Test Hispanic 59 | 0.81 | 0.89 | 0.86 |
| 2108 | Train 33 | Test Hispanic 60 | 0.81 | 0.87 | 0.84 | 3132 | Train 49 | Test Hispanic 60 | 0.81 | 0.89 | 0.86 |
| 2109 | Train 33 | Test Hispanic 61 | 0.78 | 0.83 | 0.74 | 3133 | Train 49 | Test Hispanic 61 | 0.77 | 0.84 | 0.78 |
| 2110 | Train 33 | Test Hispanic 62 | 0.75 | 0.75 | 0.68 | 3134 | Train 49 | Test Hispanic 62 | 0.75 | 0.74 | 0.7 |
| 2111 | Train 33 | Test Hispanic 63 | 0.79 | 0.84 | 0.74 | 3135 | Train 49 | Test Hispanic 63 | 0.78 | 0.84 | 0.78 |
| 2112 | Train 33 | Test Hispanic 64 | 0.76 | 0.75 | 0.69 | 3136 | Train 49 | Test Hispanic 64 | 0.76 | 0.75 | 0.69 |
| 2113 | Train 34 | Test Hispanic 1 | 0.79 | 0.89 | 0.87 | 3137 | Train 50 | Test Hispanic 1 | 0.76 | 0.79 | 0.76 |
| 2114 | Train 34 | Test Hispanic 2 | 0.78 | 0.89 | 0.87 | 3138 | Train 50 | Test Hispanic 2 | 0.75 | 0.79 | 0.73 |
| 2115 | Train 34 | Test Hispanic 3 | 0.79 | 0.89 | 0.86 | 3139 | Train 50 | Test Hispanic 3 | 0.77 | 0.8 | 0.76 |
| 2116 | Train 34 | Test Hispanic 4 | 0.79 | 0.89 | 0.87 | 3140 | Train 50 | Test Hispanic 4 | 0.75 | 0.79 | 0.73 |
| 2117 | Train 34 | Test Hispanic 5 | 0.74 | 0.8 | 0.77 | 3141 | Train 50 | Test Hispanic 5 | 0.73 | 0.75 | 0.67 |
| 2118 | Train 34 | Test Hispanic 6 | 0.73 | 0.75 | 0.72 | 3142 | Train 50 | Test Hispanic 6 | 0.71 | 0.7 | 0.64 |
| 2119 | Train 34 | Test Hispanic 7 | 0.74 | 0.79 | 0.76 | 3143 | Train 50 | Test Hispanic 7 | 0.73 | 0.74 | 0.67 |
| 2120 | Train 34 | Test Hispanic 8 | 0.73 | 0.75 | 0.72 | 3144 | Train 50 | Test Hispanic 8 | 0.72 | 0.71 | 0.64 |
| 2121 | Train 34 | Test Hispanic 9 | 0.79 | 0.87 | 0.84 | 3145 | Train 50 | Test Hispanic 9 | 0.76 | 0.79 | 0.73 |
| 2122 | Train 34 | Test Hispanic 10 | 0.78 | 0.88 | 0.85 | 3146 | Train 50 | Test Hispanic 10 | 0.75 | 0.78 | 0.71 |
| 2123 | Train 34 | Test Hispanic 11 | 0.79 | 0.87 | 0.84 | 3147 | Train 50 | Test Hispanic 11 | 0.77 | 0.79 | 0.73 |
| 2124 | Train 34 | Test Hispanic 12 | 0.79 | 0.88 | 0.84 | 3148 | Train 50 | Test Hispanic 12 | 0.76 | 0.78 | 0.71 |
| 2125 | Train 34 | Test Hispanic 13 | 0.74 | 0.79 | 0.76 | 3149 | Train 50 | Test Hispanic 13 | 0.73 | 0.75 | 0.66 |
| 2126 | Train 34 | Test Hispanic 14 | 0.73 | 0.75 | 0.72 | 3150 | Train 50 | Test Hispanic 14 | 0.72 | 0.71 | 0.65 |
| 2127 | Train 34 | Test Hispanic 15 | 0.74 | 0.78 | 0.75 | 3151 | Train 50 | Test Hispanic 15 | 0.73 | 0.74 | 0.65 |
| 2128 | Train 34 | Test Hispanic 16 | 0.73 | 0.75 | 0.72 | 3152 | Train 50 | Test Hispanic 16 | 0.72 | 0.71 | 0.64 |
| 2129 | Train 34 | Test Hispanic 17 | 0.79 | 0.89 | 0.87 | 3153 | Train 50 | Test Hispanic 17 | 0.76 | 0.8 | 0.76 |
| 2130 | Train 34 | Test Hispanic 18 | 0.78 | 0.89 | 0.87 | 3154 | Train 50 | Test Hispanic 18 | 0.75 | 0.79 | 0.73 |
| 2131 | Train 34 | Test Hispanic 19 | 0.79 | 0.89 | 0.86 | 3155 | Train 50 | Test Hispanic 19 | 0.77 | 0.8 | 0.76 |
| 2132 | Train 34 | Test Hispanic 20 | 0.79 | 0.89 | 0.87 | 3156 | Train 50 | Test Hispanic 20 | 0.75 | 0.79 | 0.73 |
| 2133 | Train 34 | Test Hispanic 21 | 0.74 | 0.8 | 0.77 | 3157 | Train 50 | Test Hispanic 21 | 0.73 | 0.75 | 0.67 |
| 2134 | Train 34 | Test Hispanic 22 | 0.73 | 0.75 | 0.72 | 3158 | Train 50 | Test Hispanic 22 | 0.71 | 0.71 | 0.64 |
| 2135 | Train 34 | Test Hispanic 23 | 0.74 | 0.79 | 0.76 | 3159 | Train 50 | Test Hispanic 23 | 0.73 | 0.74 | 0.67 |
| 2136 | Train 34 | Test Hispanic 24 | 0.73 | 0.75 | 0.72 | 3160 | Train 50 | Test Hispanic 24 | 0.72 | 0.71 | 0.64 |
| 2137 | Train 34 | Test Hispanic 25 | 0.79 | 0.87 | 0.84 | 3161 | Train 50 | Test Hispanic 25 | 0.76 | 0.79 | 0.73 |
| 2138 | Train 34 | Test Hispanic 26 | 0.79 | 0.88 | 0.85 | 3162 | Train 50 | Test Hispanic 26 | 0.75 | 0.78 | 0.71 |
| 2139 | Train 34 | Test Hispanic 27 | 0.79 | 0.87 | 0.84 | 3163 | Train 50 | Test Hispanic 27 | 0.77 | 0.79 | 0.73 |
| 2140 | Train 34 | Test Hispanic 28 | 0.79 | 0.88 | 0.84 | 3164 | Train 50 | Test Hispanic 28 | 0.76 | 0.78 | 0.71 |
| 2141 | Train 34 | Test Hispanic 29 | 0.74 | 0.79 | 0.76 | 3165 | Train 50 | Test Hispanic 29 | 0.73 | 0.75 | 0.66 |
| 2142 | Train 34 | Test Hispanic 30 | 0.73 | 0.75 | 0.72 | 3166 | Train 50 | Test Hispanic 30 | 0.72 | 0.71 | 0.65 |
| 2143 | Train 34 | Test Hispanic 31 | 0.74 | 0.78 | 0.75 | 3167 | Train 50 | Test Hispanic 31 | 0.73 | 0.74 | 0.65 |
| 2144 | Train 34 | Test Hispanic 32 | 0.73 | 0.75 | 0.72 | 3168 | Train 50 | Test Hispanic 32 | 0.72 | 0.71 | 0.65 |
| 2145 | Train 34 | Test Hispanic 33 | 0.79 | 0.89 | 0.87 | 3169 | Train 50 | Test Hispanic 33 | 0.77 | 0.8 | 0.76 |
| 2146 | Train 34 | Test Hispanic 34 | 0.79 | 0.89 | 0.87 | 3170 | Train 50 | Test Hispanic 34 | 0.76 | 0.79 | 0.74 |
| 2147 | Train 34 | Test Hispanic 35 | 0.8 | 0.89 | 0.87 | 3171 | Train 50 | Test Hispanic 35 | 0.78 | 0.81 | 0.76 |
| 2148 | Train 34 | Test Hispanic 36 | 0.79 | 0.89 | 0.87 | 3172 | Train 50 | Test Hispanic 36 | 0.76 | 0.8 | 0.74 |
| 2149 | Train 34 | Test Hispanic 37 | 0.75 | 0.8 | 0.77 | 3173 | Train 50 | Test Hispanic 37 | 0.74 | 0.75 | 0.68 |
| 2150 | Train 34 | Test Hispanic 38 | 0.73 | 0.75 | 0.72 | 3174 | Train 50 | Test Hispanic 38 | 0.72 | 0.71 | 0.65 |
| 2151 | Train 34 | Test Hispanic 39 | 0.75 | 0.79 | 0.77 | 3175 | Train 50 | Test Hispanic 39 | 0.74 | 0.75 | 0.67 |
| 2152 | Train 34 | Test Hispanic 40 | 0.73 | 0.75 | 0.72 | 3176 | Train 50 | Test Hispanic 40 | 0.72 | 0.71 | 0.65 |
| 2153 | Train 34 | Test Hispanic 41 | 0.79 | 0.88 | 0.85 | 3177 | Train 50 | Test Hispanic 41 | 0.77 | 0.8 | 0.73 |
| 2154 | Train 34 | Test Hispanic 42 | 0.79 | 0.88 | 0.85 | 3178 | Train 50 | Test Hispanic 42 | 0.76 | 0.79 | 0.71 |
| 2155 | Train 34 | Test Hispanic 43 | 0.8 | 0.88 | 0.85 | 3179 | Train 50 | Test Hispanic 43 | 0.78 | 0.8 | 0.74 |
| 2156 | Train 34 | Test Hispanic 44 | 0.8 | 0.88 | 0.85 | 3180 | Train 50 | Test Hispanic 44 | 0.77 | 0.79 | 0.72 |
| 2157 | Train 34 | Test Hispanic 45 | 0.75 | 0.79 | 0.76 | 3181 | Train 50 | Test Hispanic 45 | 0.74 | 0.75 | 0.67 |
| 2158 | Train 34 | Test Hispanic 46 | 0.73 | 0.75 | 0.72 | 3182 | Train 50 | Test Hispanic 46 | 0.72 | 0.71 | 0.65 |
| 2159 | Train 34 | Test Hispanic 47 | 0.75 | 0.79 | 0.76 | 3183 | Train 50 | Test Hispanic 47 | 0.74 | 0.74 | 0.66 |
| 2160 | Train 34 | Test Hispanic 48 | 0.73 | 0.75 | 0.72 | 3184 | Train 50 | Test Hispanic 48 | 0.72 | 0.71 | 0.65 |
| 2161 | Train 34 | Test Hispanic 49 | 0.8 | 0.88 | 0.87 | 3185 | Train 50 | Test Hispanic 49 | 0.81 | 0.91 | 0.93 |
| 2162 | Train 34 | Test Hispanic 50 | 0.79 | 0.88 | 0.86 | 3186 | Train 50 | Test Hispanic 50 | 0.8 | 0.91 | 0.92 |
| 2163 | Train 34 | Test Hispanic 51 | 0.81 | 0.88 | 0.86 | 3187 | Train 50 | Test Hispanic 51 | 0.81 | 0.9 | 0.91 |
| 2164 | Train 34 | Test Hispanic 52 | 0.8 | 0.88 | 0.86 | 3188 | Train 50 | Test Hispanic 52 | 0.81 | 0.9 | 0.91 |
| 2165 | Train 34 | Test Hispanic 53 | 0.78 | 0.83 | 0.8 | 3189 | Train 50 | Test Hispanic 53 | 0.77 | 0.83 | 0.78 |
| 2166 | Train 34 | Test Hispanic 54 | 0.75 | 0.77 | 0.74 | 3190 | Train 50 | Test Hispanic 54 | 0.74 | 0.73 | 0.68 |
| 2167 | Train 34 | Test Hispanic 55 | 0.78 | 0.84 | 0.8 | 3191 | Train 50 | Test Hispanic 55 | 0.78 | 0.83 | 0.78 |
| 2168 | Train 34 | Test Hispanic 56 | 0.75 | 0.77 | 0.74 | 3192 | Train 50 | Test Hispanic 56 | 0.75 | 0.74 | 0.68 |
| 2169 | Train 34 | Test Hispanic 57 | 0.8 | 0.87 | 0.84 | 3193 | Train 50 | Test Hispanic 57 | 0.81 | 0.89 | 0.86 |
| 2170 | Train 34 | Test Hispanic 58 | 0.8 | 0.87 | 0.84 | 3194 | Train 50 | Test Hispanic 58 | 0.8 | 0.89 | 0.86 |
| 2171 | Train 34 | Test Hispanic 59 | 0.81 | 0.87 | 0.84 | 3195 | Train 50 | Test Hispanic 59 | 0.81 | 0.89 | 0.85 |
| 2172 | Train 34 | Test Hispanic 60 | 0.8 | 0.87 | 0.84 | 3196 | Train 50 | Test Hispanic 60 | 0.81 | 0.89 | 0.85 |
| 2173 | Train 34 | Test Hispanic 61 | 0.78 | 0.83 | 0.8 | 3197 | Train 50 | Test Hispanic 61 | 0.77 | 0.82 | 0.77 |
| 2174 | Train 34 | Test Hispanic 62 | 0.75 | 0.77 | 0.74 | 3198 | Train 50 | Test Hispanic 62 | 0.74 | 0.73 | 0.68 |
| 2175 | Train 34 | Test Hispanic 63 | 0.79 | 0.83 | 0.8 | 3199 | Train 50 | Test Hispanic 63 | 0.78 | 0.83 | 0.77 |
| 2176 | Train 34 | Test Hispanic 64 | 0.75 | 0.77 | 0.74 | 3200 | Train 50 | Test Hispanic 64 | 0.75 | 0.74 | 0.68 |
| 2177 | Train 35 | Test Hispanic 1 | 0.79 | 0.89 | 0.87 | 3201 | Train 51 | Test Hispanic 1 | 0.76 | 0.81 | 0.77 |
| 2178 | Train 35 | Test Hispanic 2 | 0.78 | 0.87 | 0.85 | 3202 | Train 51 | Test Hispanic 2 | 0.75 | 0.8 | 0.74 |
| 2179 | Train 35 | Test Hispanic 3 | 0.79 | 0.89 | 0.87 | 3203 | Train 51 | Test Hispanic 3 | 0.77 | 0.82 | 0.78 |
| 2180 | Train 35 | Test Hispanic 4 | 0.78 | 0.87 | 0.85 | 3204 | Train 51 | Test Hispanic 4 | 0.75 | 0.81 | 0.75 |
| 2181 | Train 35 | Test Hispanic 5 | 0.74 | 0.79 | 0.74 | 3205 | Train 51 | Test Hispanic 5 | 0.73 | 0.75 | 0.66 |
| 2182 | Train 35 | Test Hispanic 6 | 0.72 | 0.73 | 0.69 | 3206 | Train 51 | Test Hispanic 6 | 0.71 | 0.71 | 0.62 |
| 2183 | Train 35 | Test Hispanic 7 | 0.74 | 0.78 | 0.74 | 3207 | Train 51 | Test Hispanic 7 | 0.73 | 0.75 | 0.66 |
| 2184 | Train 35 | Test Hispanic 8 | 0.72 | 0.73 | 0.69 | 3208 | Train 51 | Test Hispanic 8 | 0.72 | 0.71 | 0.62 |
| 2185 | Train 35 | Test Hispanic 9 | 0.79 | 0.87 | 0.84 | 3209 | Train 51 | Test Hispanic 9 | 0.76 | 0.81 | 0.74 |
| 2186 | Train 35 | Test Hispanic 10 | 0.78 | 0.86 | 0.82 | 3210 | Train 51 | Test Hispanic 10 | 0.75 | 0.8 | 0.72 |
| 2187 | Train 35 | Test Hispanic 11 | 0.79 | 0.87 | 0.84 | 3211 | Train 51 | Test Hispanic 11 | 0.77 | 0.81 | 0.75 |
| 2188 | Train 35 | Test Hispanic 12 | 0.78 | 0.86 | 0.82 | 3212 | Train 51 | Test Hispanic 12 | 0.76 | 0.8 | 0.72 |
| 2189 | Train 35 | Test Hispanic 13 | 0.74 | 0.78 | 0.73 | 3213 | Train 51 | Test Hispanic 13 | 0.73 | 0.75 | 0.64 |
| 2190 | Train 35 | Test Hispanic 14 | 0.72 | 0.73 | 0.69 | 3214 | Train 51 | Test Hispanic 14 | 0.71 | 0.71 | 0.62 |
| 2191 | Train 35 | Test Hispanic 15 | 0.74 | 0.78 | 0.73 | 3215 | Train 51 | Test Hispanic 15 | 0.73 | 0.74 | 0.64 |
| 2192 | Train 35 | Test Hispanic 16 | 0.72 | 0.73 | 0.69 | 3216 | Train 51 | Test Hispanic 16 | 0.72 | 0.71 | 0.62 |
| 2193 | Train 35 | Test Hispanic 17 | 0.79 | 0.89 | 0.87 | 3217 | Train 51 | Test Hispanic 17 | 0.76 | 0.81 | 0.77 |
| 2194 | Train 35 | Test Hispanic 18 | 0.78 | 0.87 | 0.85 | 3218 | Train 51 | Test Hispanic 18 | 0.75 | 0.8 | 0.74 |
| 2195 | Train 35 | Test Hispanic 19 | 0.79 | 0.89 | 0.87 | 3219 | Train 51 | Test Hispanic 19 | 0.77 | 0.82 | 0.78 |
| 2196 | Train 35 | Test Hispanic 20 | 0.78 | 0.87 | 0.85 | 3220 | Train 51 | Test Hispanic 20 | 0.75 | 0.81 | 0.75 |
| 2197 | Train 35 | Test Hispanic 21 | 0.74 | 0.79 | 0.74 | 3221 | Train 51 | Test Hispanic 21 | 0.73 | 0.75 | 0.66 |
| 2198 | Train 35 | Test Hispanic 22 | 0.72 | 0.73 | 0.69 | 3222 | Train 51 | Test Hispanic 22 | 0.71 | 0.71 | 0.62 |
| 2199 | Train 35 | Test Hispanic 23 | 0.74 | 0.78 | 0.74 | 3223 | Train 51 | Test Hispanic 23 | 0.73 | 0.75 | 0.66 |
| 2200 | Train 35 | Test Hispanic 24 | 0.72 | 0.73 | 0.69 | 3224 | Train 51 | Test Hispanic 24 | 0.72 | 0.71 | 0.62 |
| 2201 | Train 35 | Test Hispanic 25 | 0.79 | 0.87 | 0.84 | 3225 | Train 51 | Test Hispanic 25 | 0.76 | 0.81 | 0.74 |
| 2202 | Train 35 | Test Hispanic 26 | 0.78 | 0.86 | 0.82 | 3226 | Train 51 | Test Hispanic 26 | 0.75 | 0.8 | 0.72 |
| 2203 | Train 35 | Test Hispanic 27 | 0.79 | 0.87 | 0.84 | 3227 | Train 51 | Test Hispanic 27 | 0.77 | 0.81 | 0.75 |
| 2204 | Train 35 | Test Hispanic 28 | 0.78 | 0.86 | 0.82 | 3228 | Train 51 | Test Hispanic 28 | 0.76 | 0.8 | 0.72 |
| 2205 | Train 35 | Test Hispanic 29 | 0.74 | 0.78 | 0.73 | 3229 | Train 51 | Test Hispanic 29 | 0.73 | 0.75 | 0.64 |
| 2206 | Train 35 | Test Hispanic 30 | 0.72 | 0.73 | 0.69 | 3230 | Train 51 | Test Hispanic 30 | 0.71 | 0.71 | 0.62 |
| 2207 | Train 35 | Test Hispanic 31 | 0.74 | 0.78 | 0.73 | 3231 | Train 51 | Test Hispanic 31 | 0.73 | 0.74 | 0.64 |
| 2208 | Train 35 | Test Hispanic 32 | 0.72 | 0.73 | 0.69 | 3232 | Train 51 | Test Hispanic 32 | 0.72 | 0.71 | 0.62 |
| 2209 | Train 35 | Test Hispanic 33 | 0.8 | 0.89 | 0.88 | 3233 | Train 51 | Test Hispanic 33 | 0.77 | 0.82 | 0.78 |
| 2210 | Train 35 | Test Hispanic 34 | 0.78 | 0.87 | 0.85 | 3234 | Train 51 | Test Hispanic 34 | 0.76 | 0.81 | 0.75 |
| 2211 | Train 35 | Test Hispanic 35 | 0.8 | 0.89 | 0.88 | 3235 | Train 51 | Test Hispanic 35 | 0.78 | 0.83 | 0.79 |
| 2212 | Train 35 | Test Hispanic 36 | 0.79 | 0.88 | 0.86 | 3236 | Train 51 | Test Hispanic 36 | 0.76 | 0.81 | 0.76 |
| 2213 | Train 35 | Test Hispanic 37 | 0.74 | 0.79 | 0.75 | 3237 | Train 51 | Test Hispanic 37 | 0.73 | 0.76 | 0.66 |
| 2214 | Train 35 | Test Hispanic 38 | 0.72 | 0.73 | 0.69 | 3238 | Train 51 | Test Hispanic 38 | 0.71 | 0.71 | 0.62 |
| 2215 | Train 35 | Test Hispanic 39 | 0.74 | 0.79 | 0.75 | 3239 | Train 51 | Test Hispanic 39 | 0.73 | 0.75 | 0.66 |
| 2216 | Train 35 | Test Hispanic 40 | 0.72 | 0.73 | 0.7 | 3240 | Train 51 | Test Hispanic 40 | 0.72 | 0.71 | 0.62 |
| 2217 | Train 35 | Test Hispanic 41 | 0.8 | 0.88 | 0.85 | 3241 | Train 51 | Test Hispanic 41 | 0.77 | 0.81 | 0.75 |
| 2218 | Train 35 | Test Hispanic 42 | 0.79 | 0.86 | 0.83 | 3242 | Train 51 | Test Hispanic 42 | 0.76 | 0.8 | 0.73 |
| 2219 | Train 35 | Test Hispanic 43 | 0.8 | 0.88 | 0.85 | 3243 | Train 51 | Test Hispanic 43 | 0.78 | 0.82 | 0.76 |
| 2220 | Train 35 | Test Hispanic 44 | 0.79 | 0.86 | 0.83 | 3244 | Train 51 | Test Hispanic 44 | 0.76 | 0.81 | 0.73 |
| 2221 | Train 35 | Test Hispanic 45 | 0.74 | 0.78 | 0.74 | 3245 | Train 51 | Test Hispanic 45 | 0.73 | 0.75 | 0.65 |
| 2222 | Train 35 | Test Hispanic 46 | 0.72 | 0.73 | 0.69 | 3246 | Train 51 | Test Hispanic 46 | 0.71 | 0.71 | 0.62 |
| 2223 | Train 35 | Test Hispanic 47 | 0.74 | 0.78 | 0.73 | 3247 | Train 51 | Test Hispanic 47 | 0.73 | 0.75 | 0.65 |
| 2224 | Train 35 | Test Hispanic 48 | 0.73 | 0.73 | 0.7 | 3248 | Train 51 | Test Hispanic 48 | 0.72 | 0.72 | 0.62 |
| 2225 | Train 35 | Test Hispanic 49 | 0.81 | 0.88 | 0.87 | 3249 | Train 51 | Test Hispanic 49 | 0.81 | 0.9 | 0.93 |
| 2226 | Train 35 | Test Hispanic 50 | 0.8 | 0.88 | 0.87 | 3250 | Train 51 | Test Hispanic 50 | 0.8 | 0.9 | 0.92 |
| 2227 | Train 35 | Test Hispanic 51 | 0.81 | 0.88 | 0.87 | 3251 | Train 51 | Test Hispanic 51 | 0.81 | 0.91 | 0.93 |
| 2228 | Train 35 | Test Hispanic 52 | 0.81 | 0.88 | 0.87 | 3252 | Train 51 | Test Hispanic 52 | 0.81 | 0.9 | 0.93 |
| 2229 | Train 35 | Test Hispanic 53 | 0.78 | 0.83 | 0.77 | 3253 | Train 51 | Test Hispanic 53 | 0.77 | 0.84 | 0.79 |
| 2230 | Train 35 | Test Hispanic 54 | 0.75 | 0.75 | 0.7 | 3254 | Train 51 | Test Hispanic 54 | 0.74 | 0.75 | 0.66 |
| 2231 | Train 35 | Test Hispanic 55 | 0.78 | 0.84 | 0.78 | 3255 | Train 51 | Test Hispanic 55 | 0.77 | 0.85 | 0.79 |
| 2232 | Train 35 | Test Hispanic 56 | 0.76 | 0.75 | 0.71 | 3256 | Train 51 | Test Hispanic 56 | 0.75 | 0.75 | 0.67 |
| 2233 | Train 35 | Test Hispanic 57 | 0.81 | 0.87 | 0.84 | 3257 | Train 51 | Test Hispanic 57 | 0.81 | 0.89 | 0.86 |
| 2234 | Train 35 | Test Hispanic 58 | 0.8 | 0.87 | 0.84 | 3258 | Train 51 | Test Hispanic 58 | 0.8 | 0.89 | 0.86 |
| 2235 | Train 35 | Test Hispanic 59 | 0.81 | 0.87 | 0.85 | 3259 | Train 51 | Test Hispanic 59 | 0.81 | 0.89 | 0.87 |
| 2236 | Train 35 | Test Hispanic 60 | 0.81 | 0.87 | 0.84 | 3260 | Train 51 | Test Hispanic 60 | 0.81 | 0.89 | 0.87 |
| 2237 | Train 35 | Test Hispanic 61 | 0.78 | 0.83 | 0.77 | 3261 | Train 51 | Test Hispanic 61 | 0.77 | 0.84 | 0.77 |
| 2238 | Train 35 | Test Hispanic 62 | 0.75 | 0.75 | 0.7 | 3262 | Train 51 | Test Hispanic 62 | 0.74 | 0.75 | 0.66 |
| 2239 | Train 35 | Test Hispanic 63 | 0.79 | 0.84 | 0.77 | 3263 | Train 51 | Test Hispanic 63 | 0.78 | 0.84 | 0.78 |
| 2240 | Train 35 | Test Hispanic 64 | 0.76 | 0.75 | 0.71 | 3264 | Train 51 | Test Hispanic 64 | 0.75 | 0.75 | 0.67 |
| 2241 | Train 36 | Test Hispanic 1 | 0.79 | 0.88 | 0.87 | 3265 | Train 52 | Test Hispanic 1 | 0.76 | 0.78 | 0.77 |
| 2242 | Train 36 | Test Hispanic 2 | 0.78 | 0.89 | 0.87 | 3266 | Train 52 | Test Hispanic 2 | 0.75 | 0.78 | 0.75 |
| 2243 | Train 36 | Test Hispanic 3 | 0.79 | 0.88 | 0.87 | 3267 | Train 52 | Test Hispanic 3 | 0.77 | 0.79 | 0.77 |
| 2244 | Train 36 | Test Hispanic 4 | 0.79 | 0.89 | 0.87 | 3268 | Train 52 | Test Hispanic 4 | 0.75 | 0.78 | 0.76 |
| 2245 | Train 36 | Test Hispanic 5 | 0.74 | 0.8 | 0.77 | 3269 | Train 52 | Test Hispanic 5 | 0.73 | 0.75 | 0.68 |
| 2246 | Train 36 | Test Hispanic 6 | 0.72 | 0.75 | 0.71 | 3270 | Train 52 | Test Hispanic 6 | 0.71 | 0.71 | 0.65 |
| 2247 | Train 36 | Test Hispanic 7 | 0.74 | 0.79 | 0.76 | 3271 | Train 52 | Test Hispanic 7 | 0.73 | 0.74 | 0.68 |
| 2248 | Train 36 | Test Hispanic 8 | 0.73 | 0.75 | 0.71 | 3272 | Train 52 | Test Hispanic 8 | 0.72 | 0.71 | 0.65 |
| 2249 | Train 36 | Test Hispanic 9 | 0.79 | 0.87 | 0.84 | 3273 | Train 52 | Test Hispanic 9 | 0.76 | 0.78 | 0.73 |
| 2250 | Train 36 | Test Hispanic 10 | 0.78 | 0.87 | 0.85 | 3274 | Train 52 | Test Hispanic 10 | 0.75 | 0.77 | 0.72 |
| 2251 | Train 36 | Test Hispanic 11 | 0.79 | 0.87 | 0.84 | 3275 | Train 52 | Test Hispanic 11 | 0.77 | 0.78 | 0.74 |
| 2252 | Train 36 | Test Hispanic 12 | 0.79 | 0.87 | 0.85 | 3276 | Train 52 | Test Hispanic 12 | 0.75 | 0.78 | 0.73 |
| 2253 | Train 36 | Test Hispanic 13 | 0.74 | 0.79 | 0.76 | 3277 | Train 52 | Test Hispanic 13 | 0.73 | 0.74 | 0.67 |
| 2254 | Train 36 | Test Hispanic 14 | 0.73 | 0.75 | 0.71 | 3278 | Train 52 | Test Hispanic 14 | 0.71 | 0.71 | 0.66 |
| 2255 | Train 36 | Test Hispanic 15 | 0.74 | 0.78 | 0.75 | 3279 | Train 52 | Test Hispanic 15 | 0.73 | 0.73 | 0.67 |
| 2256 | Train 36 | Test Hispanic 16 | 0.73 | 0.75 | 0.71 | 3280 | Train 52 | Test Hispanic 16 | 0.72 | 0.71 | 0.65 |
| 2257 | Train 36 | Test Hispanic 17 | 0.79 | 0.88 | 0.87 | 3281 | Train 52 | Test Hispanic 17 | 0.76 | 0.78 | 0.77 |
| 2258 | Train 36 | Test Hispanic 18 | 0.78 | 0.89 | 0.87 | 3282 | Train 52 | Test Hispanic 18 | 0.75 | 0.78 | 0.75 |
| 2259 | Train 36 | Test Hispanic 19 | 0.79 | 0.88 | 0.87 | 3283 | Train 52 | Test Hispanic 19 | 0.77 | 0.79 | 0.77 |
| 2260 | Train 36 | Test Hispanic 20 | 0.79 | 0.89 | 0.87 | 3284 | Train 52 | Test Hispanic 20 | 0.75 | 0.78 | 0.76 |
| 2261 | Train 36 | Test Hispanic 21 | 0.74 | 0.8 | 0.77 | 3285 | Train 52 | Test Hispanic 21 | 0.73 | 0.75 | 0.68 |
| 2262 | Train 36 | Test Hispanic 22 | 0.73 | 0.75 | 0.71 | 3286 | Train 52 | Test Hispanic 22 | 0.71 | 0.71 | 0.66 |
| 2263 | Train 36 | Test Hispanic 23 | 0.74 | 0.79 | 0.76 | 3287 | Train 52 | Test Hispanic 23 | 0.73 | 0.74 | 0.68 |
| 2264 | Train 36 | Test Hispanic 24 | 0.73 | 0.75 | 0.71 | 3288 | Train 52 | Test Hispanic 24 | 0.72 | 0.71 | 0.65 |
| 2265 | Train 36 | Test Hispanic 25 | 0.79 | 0.87 | 0.84 | 3289 | Train 52 | Test Hispanic 25 | 0.76 | 0.78 | 0.73 |
| 2266 | Train 36 | Test Hispanic 26 | 0.78 | 0.87 | 0.85 | 3290 | Train 52 | Test Hispanic 26 | 0.75 | 0.77 | 0.72 |
| 2267 | Train 36 | Test Hispanic 27 | 0.79 | 0.87 | 0.84 | 3291 | Train 52 | Test Hispanic 27 | 0.77 | 0.78 | 0.74 |
| 2268 | Train 36 | Test Hispanic 28 | 0.79 | 0.87 | 0.85 | 3292 | Train 52 | Test Hispanic 28 | 0.75 | 0.78 | 0.73 |
| 2269 | Train 36 | Test Hispanic 29 | 0.74 | 0.79 | 0.76 | 3293 | Train 52 | Test Hispanic 29 | 0.73 | 0.74 | 0.67 |
| 2270 | Train 36 | Test Hispanic 30 | 0.73 | 0.75 | 0.71 | 3294 | Train 52 | Test Hispanic 30 | 0.71 | 0.71 | 0.66 |
| 2271 | Train 36 | Test Hispanic 31 | 0.74 | 0.78 | 0.75 | 3295 | Train 52 | Test Hispanic 31 | 0.73 | 0.73 | 0.67 |
| 2272 | Train 36 | Test Hispanic 32 | 0.73 | 0.75 | 0.71 | 3296 | Train 52 | Test Hispanic 32 | 0.72 | 0.71 | 0.65 |
| 2273 | Train 36 | Test Hispanic 33 | 0.79 | 0.89 | 0.87 | 3297 | Train 52 | Test Hispanic 33 | 0.77 | 0.79 | 0.77 |
| 2274 | Train 36 | Test Hispanic 34 | 0.79 | 0.89 | 0.88 | 3298 | Train 52 | Test Hispanic 34 | 0.76 | 0.78 | 0.76 |
| 2275 | Train 36 | Test Hispanic 35 | 0.8 | 0.89 | 0.87 | 3299 | Train 52 | Test Hispanic 35 | 0.78 | 0.8 | 0.78 |
| 2276 | Train 36 | Test Hispanic 36 | 0.79 | 0.89 | 0.88 | 3300 | Train 52 | Test Hispanic 36 | 0.76 | 0.79 | 0.77 |
| 2277 | Train 36 | Test Hispanic 37 | 0.75 | 0.8 | 0.77 | 3301 | Train 52 | Test Hispanic 37 | 0.74 | 0.75 | 0.69 |
| 2278 | Train 36 | Test Hispanic 38 | 0.73 | 0.75 | 0.71 | 3302 | Train 52 | Test Hispanic 38 | 0.72 | 0.71 | 0.66 |
| 2279 | Train 36 | Test Hispanic 39 | 0.75 | 0.8 | 0.77 | 3303 | Train 52 | Test Hispanic 39 | 0.74 | 0.74 | 0.69 |
| 2280 | Train 36 | Test Hispanic 40 | 0.73 | 0.75 | 0.71 | 3304 | Train 52 | Test Hispanic 40 | 0.72 | 0.71 | 0.66 |
| 2281 | Train 36 | Test Hispanic 41 | 0.79 | 0.88 | 0.85 | 3305 | Train 52 | Test Hispanic 41 | 0.77 | 0.79 | 0.74 |
| 2282 | Train 36 | Test Hispanic 42 | 0.79 | 0.88 | 0.85 | 3306 | Train 52 | Test Hispanic 42 | 0.76 | 0.78 | 0.73 |
| 2283 | Train 36 | Test Hispanic 43 | 0.8 | 0.88 | 0.85 | 3307 | Train 52 | Test Hispanic 43 | 0.78 | 0.79 | 0.75 |
| 2284 | Train 36 | Test Hispanic 44 | 0.8 | 0.88 | 0.85 | 3308 | Train 52 | Test Hispanic 44 | 0.76 | 0.79 | 0.74 |
| 2285 | Train 36 | Test Hispanic 45 | 0.75 | 0.79 | 0.76 | 3309 | Train 52 | Test Hispanic 45 | 0.74 | 0.75 | 0.68 |
| 2286 | Train 36 | Test Hispanic 46 | 0.73 | 0.75 | 0.71 | 3310 | Train 52 | Test Hispanic 46 | 0.72 | 0.71 | 0.66 |
| 2287 | Train 36 | Test Hispanic 47 | 0.75 | 0.79 | 0.76 | 3311 | Train 52 | Test Hispanic 47 | 0.74 | 0.74 | 0.67 |
| 2288 | Train 36 | Test Hispanic 48 | 0.73 | 0.75 | 0.71 | 3312 | Train 52 | Test Hispanic 48 | 0.72 | 0.71 | 0.66 |
| 2289 | Train 36 | Test Hispanic 49 | 0.8 | 0.87 | 0.87 | 3313 | Train 52 | Test Hispanic 49 | 0.81 | 0.9 | 0.92 |
| 2290 | Train 36 | Test Hispanic 50 | 0.79 | 0.87 | 0.87 | 3314 | Train 52 | Test Hispanic 50 | 0.8 | 0.9 | 0.92 |
| 2291 | Train 36 | Test Hispanic 51 | 0.8 | 0.88 | 0.87 | 3315 | Train 52 | Test Hispanic 51 | 0.81 | 0.91 | 0.92 |
| 2292 | Train 36 | Test Hispanic 52 | 0.8 | 0.87 | 0.87 | 3316 | Train 52 | Test Hispanic 52 | 0.81 | 0.9 | 0.92 |
| 2293 | Train 36 | Test Hispanic 53 | 0.78 | 0.83 | 0.8 | 3317 | Train 52 | Test Hispanic 53 | 0.77 | 0.82 | 0.79 |
| 2294 | Train 36 | Test Hispanic 54 | 0.75 | 0.77 | 0.72 | 3318 | Train 52 | Test Hispanic 54 | 0.74 | 0.73 | 0.69 |
| 2295 | Train 36 | Test Hispanic 55 | 0.78 | 0.84 | 0.81 | 3319 | Train 52 | Test Hispanic 55 | 0.78 | 0.83 | 0.8 |
| 2296 | Train 36 | Test Hispanic 56 | 0.75 | 0.77 | 0.72 | 3320 | Train 52 | Test Hispanic 56 | 0.75 | 0.73 | 0.69 |
| 2297 | Train 36 | Test Hispanic 57 | 0.8 | 0.86 | 0.84 | 3321 | Train 52 | Test Hispanic 57 | 0.81 | 0.89 | 0.86 |
| 2298 | Train 36 | Test Hispanic 58 | 0.79 | 0.86 | 0.84 | 3322 | Train 52 | Test Hispanic 58 | 0.8 | 0.89 | 0.86 |
| 2299 | Train 36 | Test Hispanic 59 | 0.81 | 0.87 | 0.85 | 3323 | Train 52 | Test Hispanic 59 | 0.81 | 0.89 | 0.86 |
| 2300 | Train 36 | Test Hispanic 60 | 0.8 | 0.87 | 0.84 | 3324 | Train 52 | Test Hispanic 60 | 0.81 | 0.89 | 0.86 |
| 2301 | Train 36 | Test Hispanic 61 | 0.78 | 0.83 | 0.8 | 3325 | Train 52 | Test Hispanic 61 | 0.77 | 0.82 | 0.78 |
| 2302 | Train 36 | Test Hispanic 62 | 0.75 | 0.77 | 0.72 | 3326 | Train 52 | Test Hispanic 62 | 0.74 | 0.73 | 0.69 |
| 2303 | Train 36 | Test Hispanic 63 | 0.79 | 0.83 | 0.8 | 3327 | Train 52 | Test Hispanic 63 | 0.78 | 0.82 | 0.78 |
| 2304 | Train 36 | Test Hispanic 64 | 0.75 | 0.77 | 0.72 | 3328 | Train 52 | Test Hispanic 64 | 0.75 | 0.73 | 0.69 |
| 2305 | Train 37 | Test Hispanic 1 | 0.77 | 0.86 | 0.85 | 3329 | Train 53 | Test Hispanic 1 | 0.76 | 0.82 | 0.71 |
| 2306 | Train 37 | Test Hispanic 2 | 0.77 | 0.85 | 0.83 | 3330 | Train 53 | Test Hispanic 2 | 0.75 | 0.8 | 0.71 |
| 2307 | Train 37 | Test Hispanic 3 | 0.78 | 0.86 | 0.84 | 3331 | Train 53 | Test Hispanic 3 | 0.76 | 0.82 | 0.72 |
| 2308 | Train 37 | Test Hispanic 4 | 0.77 | 0.85 | 0.83 | 3332 | Train 53 | Test Hispanic 4 | 0.75 | 0.8 | 0.71 |
| 2309 | Train 37 | Test Hispanic 5 | 0.75 | 0.86 | 0.84 | 3333 | Train 53 | Test Hispanic 5 | 0.72 | 0.79 | 0.69 |
| 2310 | Train 37 | Test Hispanic 6 | 0.72 | 0.77 | 0.75 | 3334 | Train 53 | Test Hispanic 6 | 0.71 | 0.72 | 0.65 |
| 2311 | Train 37 | Test Hispanic 7 | 0.75 | 0.85 | 0.83 | 3335 | Train 53 | Test Hispanic 7 | 0.72 | 0.78 | 0.69 |
| 2312 | Train 37 | Test Hispanic 8 | 0.72 | 0.77 | 0.75 | 3336 | Train 53 | Test Hispanic 8 | 0.71 | 0.72 | 0.66 |
| 2313 | Train 37 | Test Hispanic 9 | 0.77 | 0.85 | 0.83 | 3337 | Train 53 | Test Hispanic 9 | 0.76 | 0.81 | 0.71 |
| 2314 | Train 37 | Test Hispanic 10 | 0.77 | 0.84 | 0.82 | 3338 | Train 53 | Test Hispanic 10 | 0.76 | 0.8 | 0.71 |
| 2315 | Train 37 | Test Hispanic 11 | 0.78 | 0.85 | 0.83 | 3339 | Train 53 | Test Hispanic 11 | 0.77 | 0.82 | 0.71 |
| 2316 | Train 37 | Test Hispanic 12 | 0.77 | 0.84 | 0.82 | 3340 | Train 53 | Test Hispanic 12 | 0.76 | 0.8 | 0.71 |
| 2317 | Train 37 | Test Hispanic 13 | 0.75 | 0.85 | 0.82 | 3341 | Train 53 | Test Hispanic 13 | 0.72 | 0.79 | 0.69 |
| 2318 | Train 37 | Test Hispanic 14 | 0.72 | 0.77 | 0.75 | 3342 | Train 53 | Test Hispanic 14 | 0.71 | 0.72 | 0.65 |
| 2319 | Train 37 | Test Hispanic 15 | 0.75 | 0.84 | 0.81 | 3343 | Train 53 | Test Hispanic 15 | 0.72 | 0.78 | 0.69 |
| 2320 | Train 37 | Test Hispanic 16 | 0.72 | 0.77 | 0.75 | 3344 | Train 53 | Test Hispanic 16 | 0.71 | 0.72 | 0.66 |
| 2321 | Train 37 | Test Hispanic 17 | 0.77 | 0.86 | 0.84 | 3345 | Train 53 | Test Hispanic 17 | 0.76 | 0.82 | 0.71 |
| 2322 | Train 37 | Test Hispanic 18 | 0.77 | 0.85 | 0.83 | 3346 | Train 53 | Test Hispanic 18 | 0.75 | 0.8 | 0.71 |
| 2323 | Train 37 | Test Hispanic 19 | 0.78 | 0.86 | 0.84 | 3347 | Train 53 | Test Hispanic 19 | 0.76 | 0.82 | 0.72 |
| 2324 | Train 37 | Test Hispanic 20 | 0.77 | 0.85 | 0.83 | 3348 | Train 53 | Test Hispanic 20 | 0.76 | 0.8 | 0.71 |
| 2325 | Train 37 | Test Hispanic 21 | 0.75 | 0.86 | 0.84 | 3349 | Train 53 | Test Hispanic 21 | 0.72 | 0.79 | 0.69 |
| 2326 | Train 37 | Test Hispanic 22 | 0.72 | 0.77 | 0.75 | 3350 | Train 53 | Test Hispanic 22 | 0.71 | 0.72 | 0.65 |
| 2327 | Train 37 | Test Hispanic 23 | 0.75 | 0.85 | 0.83 | 3351 | Train 53 | Test Hispanic 23 | 0.72 | 0.78 | 0.7 |
| 2328 | Train 37 | Test Hispanic 24 | 0.72 | 0.77 | 0.75 | 3352 | Train 53 | Test Hispanic 24 | 0.71 | 0.72 | 0.66 |
| 2329 | Train 37 | Test Hispanic 25 | 0.77 | 0.85 | 0.83 | 3353 | Train 53 | Test Hispanic 25 | 0.76 | 0.81 | 0.71 |
| 2330 | Train 37 | Test Hispanic 26 | 0.77 | 0.84 | 0.82 | 3354 | Train 53 | Test Hispanic 26 | 0.76 | 0.8 | 0.71 |
| 2331 | Train 37 | Test Hispanic 27 | 0.78 | 0.85 | 0.83 | 3355 | Train 53 | Test Hispanic 27 | 0.77 | 0.82 | 0.71 |
| 2332 | Train 37 | Test Hispanic 28 | 0.77 | 0.84 | 0.82 | 3356 | Train 53 | Test Hispanic 28 | 0.76 | 0.8 | 0.71 |
| 2333 | Train 37 | Test Hispanic 29 | 0.75 | 0.85 | 0.82 | 3357 | Train 53 | Test Hispanic 29 | 0.72 | 0.79 | 0.69 |
| 2334 | Train 37 | Test Hispanic 30 | 0.72 | 0.77 | 0.75 | 3358 | Train 53 | Test Hispanic 30 | 0.71 | 0.72 | 0.65 |
| 2335 | Train 37 | Test Hispanic 31 | 0.75 | 0.84 | 0.81 | 3359 | Train 53 | Test Hispanic 31 | 0.72 | 0.78 | 0.69 |
| 2336 | Train 37 | Test Hispanic 32 | 0.72 | 0.77 | 0.75 | 3360 | Train 53 | Test Hispanic 32 | 0.71 | 0.72 | 0.66 |
| 2337 | Train 37 | Test Hispanic 33 | 0.78 | 0.86 | 0.85 | 3361 | Train 53 | Test Hispanic 33 | 0.77 | 0.82 | 0.72 |
| 2338 | Train 37 | Test Hispanic 34 | 0.78 | 0.85 | 0.84 | 3362 | Train 53 | Test Hispanic 34 | 0.76 | 0.81 | 0.72 |
| 2339 | Train 37 | Test Hispanic 35 | 0.79 | 0.86 | 0.85 | 3363 | Train 53 | Test Hispanic 35 | 0.78 | 0.82 | 0.73 |
| 2340 | Train 37 | Test Hispanic 36 | 0.78 | 0.85 | 0.84 | 3364 | Train 53 | Test Hispanic 36 | 0.77 | 0.81 | 0.72 |
| 2341 | Train 37 | Test Hispanic 37 | 0.75 | 0.87 | 0.84 | 3365 | Train 53 | Test Hispanic 37 | 0.73 | 0.79 | 0.7 |
| 2342 | Train 37 | Test Hispanic 38 | 0.72 | 0.77 | 0.76 | 3366 | Train 53 | Test Hispanic 38 | 0.71 | 0.72 | 0.66 |
| 2343 | Train 37 | Test Hispanic 39 | 0.75 | 0.86 | 0.84 | 3367 | Train 53 | Test Hispanic 39 | 0.73 | 0.79 | 0.7 |
| 2344 | Train 37 | Test Hispanic 40 | 0.73 | 0.77 | 0.76 | 3368 | Train 53 | Test Hispanic 40 | 0.72 | 0.72 | 0.66 |
| 2345 | Train 37 | Test Hispanic 41 | 0.78 | 0.85 | 0.83 | 3369 | Train 53 | Test Hispanic 41 | 0.77 | 0.82 | 0.72 |
| 2346 | Train 37 | Test Hispanic 42 | 0.78 | 0.84 | 0.82 | 3370 | Train 53 | Test Hispanic 42 | 0.76 | 0.81 | 0.72 |
| 2347 | Train 37 | Test Hispanic 43 | 0.79 | 0.85 | 0.83 | 3371 | Train 53 | Test Hispanic 43 | 0.78 | 0.82 | 0.72 |
| 2348 | Train 37 | Test Hispanic 44 | 0.78 | 0.84 | 0.82 | 3372 | Train 53 | Test Hispanic 44 | 0.77 | 0.81 | 0.72 |
| 2349 | Train 37 | Test Hispanic 45 | 0.75 | 0.85 | 0.82 | 3373 | Train 53 | Test Hispanic 45 | 0.73 | 0.79 | 0.7 |
| 2350 | Train 37 | Test Hispanic 46 | 0.72 | 0.77 | 0.76 | 3374 | Train 53 | Test Hispanic 46 | 0.71 | 0.72 | 0.66 |
| 2351 | Train 37 | Test Hispanic 47 | 0.75 | 0.84 | 0.82 | 3375 | Train 53 | Test Hispanic 47 | 0.73 | 0.79 | 0.7 |
| 2352 | Train 37 | Test Hispanic 48 | 0.73 | 0.77 | 0.76 | 3376 | Train 53 | Test Hispanic 48 | 0.72 | 0.72 | 0.66 |
| 2353 | Train 37 | Test Hispanic 49 | 0.79 | 0.87 | 0.86 | 3377 | Train 53 | Test Hispanic 49 | 0.8 | 0.88 | 0.86 |
| 2354 | Train 37 | Test Hispanic 50 | 0.79 | 0.86 | 0.85 | 3378 | Train 53 | Test Hispanic 50 | 0.79 | 0.88 | 0.85 |
| 2355 | Train 37 | Test Hispanic 51 | 0.8 | 0.87 | 0.86 | 3379 | Train 53 | Test Hispanic 51 | 0.81 | 0.88 | 0.85 |
| 2356 | Train 37 | Test Hispanic 52 | 0.79 | 0.86 | 0.85 | 3380 | Train 53 | Test Hispanic 52 | 0.8 | 0.88 | 0.85 |
| 2357 | Train 37 | Test Hispanic 53 | 0.78 | 0.87 | 0.84 | 3381 | Train 53 | Test Hispanic 53 | 0.79 | 0.9 | 0.88 |
| 2358 | Train 37 | Test Hispanic 54 | 0.74 | 0.77 | 0.75 | 3382 | Train 53 | Test Hispanic 54 | 0.75 | 0.75 | 0.71 |
| 2359 | Train 37 | Test Hispanic 55 | 0.78 | 0.86 | 0.84 | 3383 | Train 53 | Test Hispanic 55 | 0.79 | 0.89 | 0.87 |
| 2360 | Train 37 | Test Hispanic 56 | 0.74 | 0.76 | 0.75 | 3384 | Train 53 | Test Hispanic 56 | 0.75 | 0.75 | 0.71 |
| 2361 | Train 37 | Test Hispanic 57 | 0.79 | 0.86 | 0.83 | 3385 | Train 53 | Test Hispanic 57 | 0.8 | 0.88 | 0.85 |
| 2362 | Train 37 | Test Hispanic 58 | 0.79 | 0.85 | 0.83 | 3386 | Train 53 | Test Hispanic 58 | 0.8 | 0.88 | 0.85 |
| 2363 | Train 37 | Test Hispanic 59 | 0.8 | 0.86 | 0.83 | 3387 | Train 53 | Test Hispanic 59 | 0.81 | 0.88 | 0.85 |
| 2364 | Train 37 | Test Hispanic 60 | 0.8 | 0.86 | 0.83 | 3388 | Train 53 | Test Hispanic 60 | 0.8 | 0.88 | 0.85 |
| 2365 | Train 37 | Test Hispanic 61 | 0.78 | 0.86 | 0.83 | 3389 | Train 53 | Test Hispanic 61 | 0.79 | 0.89 | 0.85 |
| 2366 | Train 37 | Test Hispanic 62 | 0.74 | 0.77 | 0.75 | 3390 | Train 53 | Test Hispanic 62 | 0.75 | 0.75 | 0.71 |
| 2367 | Train 37 | Test Hispanic 63 | 0.78 | 0.86 | 0.83 | 3391 | Train 53 | Test Hispanic 63 | 0.79 | 0.89 | 0.85 |
| 2368 | Train 37 | Test Hispanic 64 | 0.74 | 0.76 | 0.75 | 3392 | Train 53 | Test Hispanic 64 | 0.75 | 0.75 | 0.71 |
| 2369 | Train 38 | Test Hispanic 1 | 0.77 | 0.84 | 0.78 | 3393 | Train 54 | Test Hispanic 1 | 0.75 | 0.82 | 0.74 |
| 2370 | Train 38 | Test Hispanic 2 | 0.77 | 0.84 | 0.78 | 3394 | Train 54 | Test Hispanic 2 | 0.75 | 0.81 | 0.74 |
| 2371 | Train 38 | Test Hispanic 3 | 0.77 | 0.84 | 0.78 | 3395 | Train 54 | Test Hispanic 3 | 0.76 | 0.82 | 0.74 |
| 2372 | Train 38 | Test Hispanic 4 | 0.77 | 0.84 | 0.78 | 3396 | Train 54 | Test Hispanic 4 | 0.75 | 0.82 | 0.74 |
| 2373 | Train 38 | Test Hispanic 5 | 0.74 | 0.83 | 0.77 | 3397 | Train 54 | Test Hispanic 5 | 0.73 | 0.79 | 0.73 |
| 2374 | Train 38 | Test Hispanic 6 | 0.75 | 0.84 | 0.79 | 3398 | Train 54 | Test Hispanic 6 | 0.74 | 0.8 | 0.74 |
| 2375 | Train 38 | Test Hispanic 7 | 0.74 | 0.82 | 0.77 | 3399 | Train 54 | Test Hispanic 7 | 0.73 | 0.78 | 0.73 |
| 2376 | Train 38 | Test Hispanic 8 | 0.75 | 0.83 | 0.78 | 3400 | Train 54 | Test Hispanic 8 | 0.74 | 0.79 | 0.74 |
| 2377 | Train 38 | Test Hispanic 9 | 0.77 | 0.84 | 0.79 | 3401 | Train 54 | Test Hispanic 9 | 0.75 | 0.82 | 0.75 |
| 2378 | Train 38 | Test Hispanic 10 | 0.77 | 0.85 | 0.79 | 3402 | Train 54 | Test Hispanic 10 | 0.75 | 0.82 | 0.75 |
| 2379 | Train 38 | Test Hispanic 11 | 0.77 | 0.84 | 0.79 | 3403 | Train 54 | Test Hispanic 11 | 0.76 | 0.82 | 0.75 |
| 2380 | Train 38 | Test Hispanic 12 | 0.77 | 0.84 | 0.79 | 3404 | Train 54 | Test Hispanic 12 | 0.76 | 0.82 | 0.75 |
| 2381 | Train 38 | Test Hispanic 13 | 0.74 | 0.83 | 0.78 | 3405 | Train 54 | Test Hispanic 13 | 0.73 | 0.79 | 0.74 |
| 2382 | Train 38 | Test Hispanic 14 | 0.75 | 0.84 | 0.78 | 3406 | Train 54 | Test Hispanic 14 | 0.74 | 0.8 | 0.74 |
| 2383 | Train 38 | Test Hispanic 15 | 0.74 | 0.83 | 0.78 | 3407 | Train 54 | Test Hispanic 15 | 0.73 | 0.78 | 0.73 |
| 2384 | Train 38 | Test Hispanic 16 | 0.75 | 0.83 | 0.78 | 3408 | Train 54 | Test Hispanic 16 | 0.74 | 0.79 | 0.74 |
| 2385 | Train 38 | Test Hispanic 17 | 0.77 | 0.84 | 0.78 | 3409 | Train 54 | Test Hispanic 17 | 0.75 | 0.82 | 0.74 |
| 2386 | Train 38 | Test Hispanic 18 | 0.77 | 0.84 | 0.78 | 3410 | Train 54 | Test Hispanic 18 | 0.75 | 0.81 | 0.74 |
| 2387 | Train 38 | Test Hispanic 19 | 0.77 | 0.84 | 0.78 | 3411 | Train 54 | Test Hispanic 19 | 0.76 | 0.82 | 0.74 |
| 2388 | Train 38 | Test Hispanic 20 | 0.77 | 0.84 | 0.78 | 3412 | Train 54 | Test Hispanic 20 | 0.75 | 0.82 | 0.74 |
| 2389 | Train 38 | Test Hispanic 21 | 0.74 | 0.83 | 0.77 | 3413 | Train 54 | Test Hispanic 21 | 0.73 | 0.79 | 0.73 |
| 2390 | Train 38 | Test Hispanic 22 | 0.75 | 0.84 | 0.79 | 3414 | Train 54 | Test Hispanic 22 | 0.74 | 0.8 | 0.74 |
| 2391 | Train 38 | Test Hispanic 23 | 0.74 | 0.82 | 0.77 | 3415 | Train 54 | Test Hispanic 23 | 0.73 | 0.78 | 0.73 |
| 2392 | Train 38 | Test Hispanic 24 | 0.75 | 0.83 | 0.78 | 3416 | Train 54 | Test Hispanic 24 | 0.74 | 0.79 | 0.74 |
| 2393 | Train 38 | Test Hispanic 25 | 0.77 | 0.84 | 0.79 | 3417 | Train 54 | Test Hispanic 25 | 0.75 | 0.82 | 0.75 |
| 2394 | Train 38 | Test Hispanic 26 | 0.77 | 0.85 | 0.79 | 3418 | Train 54 | Test Hispanic 26 | 0.75 | 0.82 | 0.75 |
| 2395 | Train 38 | Test Hispanic 27 | 0.77 | 0.84 | 0.79 | 3419 | Train 54 | Test Hispanic 27 | 0.76 | 0.82 | 0.75 |
| 2396 | Train 38 | Test Hispanic 28 | 0.77 | 0.84 | 0.79 | 3420 | Train 54 | Test Hispanic 28 | 0.76 | 0.82 | 0.75 |
| 2397 | Train 38 | Test Hispanic 29 | 0.74 | 0.84 | 0.78 | 3421 | Train 54 | Test Hispanic 29 | 0.73 | 0.79 | 0.74 |
| 2398 | Train 38 | Test Hispanic 30 | 0.75 | 0.84 | 0.79 | 3422 | Train 54 | Test Hispanic 30 | 0.74 | 0.8 | 0.74 |
| 2399 | Train 38 | Test Hispanic 31 | 0.74 | 0.83 | 0.78 | 3423 | Train 54 | Test Hispanic 31 | 0.73 | 0.78 | 0.73 |
| 2400 | Train 38 | Test Hispanic 32 | 0.75 | 0.83 | 0.78 | 3424 | Train 54 | Test Hispanic 32 | 0.74 | 0.79 | 0.74 |
| 2401 | Train 38 | Test Hispanic 33 | 0.78 | 0.85 | 0.79 | 3425 | Train 54 | Test Hispanic 33 | 0.76 | 0.83 | 0.75 |
| 2402 | Train 38 | Test Hispanic 34 | 0.78 | 0.85 | 0.79 | 3426 | Train 54 | Test Hispanic 34 | 0.76 | 0.82 | 0.75 |
| 2403 | Train 38 | Test Hispanic 35 | 0.78 | 0.85 | 0.79 | 3427 | Train 54 | Test Hispanic 35 | 0.77 | 0.83 | 0.75 |
| 2404 | Train 38 | Test Hispanic 36 | 0.78 | 0.85 | 0.79 | 3428 | Train 54 | Test Hispanic 36 | 0.76 | 0.82 | 0.75 |
| 2405 | Train 38 | Test Hispanic 37 | 0.75 | 0.84 | 0.78 | 3429 | Train 54 | Test Hispanic 37 | 0.73 | 0.79 | 0.74 |
| 2406 | Train 38 | Test Hispanic 38 | 0.75 | 0.84 | 0.79 | 3430 | Train 54 | Test Hispanic 38 | 0.74 | 0.8 | 0.75 |
| 2407 | Train 38 | Test Hispanic 39 | 0.75 | 0.83 | 0.77 | 3431 | Train 54 | Test Hispanic 39 | 0.74 | 0.79 | 0.73 |
| 2408 | Train 38 | Test Hispanic 40 | 0.75 | 0.83 | 0.78 | 3432 | Train 54 | Test Hispanic 40 | 0.74 | 0.79 | 0.74 |
| 2409 | Train 38 | Test Hispanic 41 | 0.78 | 0.85 | 0.8 | 3433 | Train 54 | Test Hispanic 41 | 0.76 | 0.83 | 0.76 |
| 2410 | Train 38 | Test Hispanic 42 | 0.78 | 0.85 | 0.8 | 3434 | Train 54 | Test Hispanic 42 | 0.76 | 0.82 | 0.76 |
| 2411 | Train 38 | Test Hispanic 43 | 0.78 | 0.85 | 0.8 | 3435 | Train 54 | Test Hispanic 43 | 0.77 | 0.83 | 0.76 |
| 2412 | Train 38 | Test Hispanic 44 | 0.78 | 0.85 | 0.8 | 3436 | Train 54 | Test Hispanic 44 | 0.77 | 0.83 | 0.76 |
| 2413 | Train 38 | Test Hispanic 45 | 0.75 | 0.84 | 0.78 | 3437 | Train 54 | Test Hispanic 45 | 0.74 | 0.79 | 0.74 |
| 2414 | Train 38 | Test Hispanic 46 | 0.75 | 0.84 | 0.79 | 3438 | Train 54 | Test Hispanic 46 | 0.74 | 0.8 | 0.75 |
| 2415 | Train 38 | Test Hispanic 47 | 0.75 | 0.83 | 0.78 | 3439 | Train 54 | Test Hispanic 47 | 0.74 | 0.79 | 0.74 |
| 2416 | Train 38 | Test Hispanic 48 | 0.75 | 0.83 | 0.78 | 3440 | Train 54 | Test Hispanic 48 | 0.74 | 0.79 | 0.74 |
| 2417 | Train 38 | Test Hispanic 49 | 0.79 | 0.86 | 0.8 | 3441 | Train 54 | Test Hispanic 49 | 0.79 | 0.87 | 0.82 |
| 2418 | Train 38 | Test Hispanic 50 | 0.79 | 0.85 | 0.8 | 3442 | Train 54 | Test Hispanic 50 | 0.79 | 0.87 | 0.82 |
| 2419 | Train 38 | Test Hispanic 51 | 0.8 | 0.86 | 0.81 | 3443 | Train 54 | Test Hispanic 51 | 0.8 | 0.87 | 0.82 |
| 2420 | Train 38 | Test Hispanic 52 | 0.79 | 0.85 | 0.8 | 3444 | Train 54 | Test Hispanic 52 | 0.79 | 0.87 | 0.82 |
| 2421 | Train 38 | Test Hispanic 53 | 0.77 | 0.86 | 0.8 | 3445 | Train 54 | Test Hispanic 53 | 0.78 | 0.88 | 0.81 |
| 2422 | Train 38 | Test Hispanic 54 | 0.78 | 0.85 | 0.79 | 3446 | Train 54 | Test Hispanic 54 | 0.78 | 0.88 | 0.81 |
| 2423 | Train 38 | Test Hispanic 55 | 0.78 | 0.86 | 0.8 | 3447 | Train 54 | Test Hispanic 55 | 0.78 | 0.88 | 0.81 |
| 2424 | Train 38 | Test Hispanic 56 | 0.77 | 0.84 | 0.79 | 3448 | Train 54 | Test Hispanic 56 | 0.77 | 0.86 | 0.8 |
| 2425 | Train 38 | Test Hispanic 57 | 0.79 | 0.86 | 0.82 | 3449 | Train 54 | Test Hispanic 57 | 0.79 | 0.88 | 0.84 |
| 2426 | Train 38 | Test Hispanic 58 | 0.79 | 0.86 | 0.82 | 3450 | Train 54 | Test Hispanic 58 | 0.79 | 0.88 | 0.83 |
| 2427 | Train 38 | Test Hispanic 59 | 0.8 | 0.86 | 0.82 | 3451 | Train 54 | Test Hispanic 59 | 0.8 | 0.88 | 0.83 |
| 2428 | Train 38 | Test Hispanic 60 | 0.79 | 0.86 | 0.82 | 3452 | Train 54 | Test Hispanic 60 | 0.79 | 0.87 | 0.83 |
| 2429 | Train 38 | Test Hispanic 61 | 0.77 | 0.86 | 0.81 | 3453 | Train 54 | Test Hispanic 61 | 0.78 | 0.88 | 0.82 |
| 2430 | Train 38 | Test Hispanic 62 | 0.78 | 0.85 | 0.79 | 3454 | Train 54 | Test Hispanic 62 | 0.78 | 0.87 | 0.81 |
| 2431 | Train 38 | Test Hispanic 63 | 0.78 | 0.86 | 0.81 | 3455 | Train 54 | Test Hispanic 63 | 0.78 | 0.88 | 0.82 |
| 2432 | Train 38 | Test Hispanic 64 | 0.77 | 0.84 | 0.79 | 3456 | Train 54 | Test Hispanic 64 | 0.77 | 0.86 | 0.8 |
| 2433 | Train 39 | Test Hispanic 1 | 0.77 | 0.86 | 0.84 | 3457 | Train 55 | Test Hispanic 1 | 0.76 | 0.81 | 0.77 |
| 2434 | Train 39 | Test Hispanic 2 | 0.77 | 0.85 | 0.83 | 3458 | Train 55 | Test Hispanic 2 | 0.75 | 0.8 | 0.76 |
| 2435 | Train 39 | Test Hispanic 3 | 0.78 | 0.86 | 0.84 | 3459 | Train 55 | Test Hispanic 3 | 0.76 | 0.82 | 0.78 |
| 2436 | Train 39 | Test Hispanic 4 | 0.77 | 0.85 | 0.83 | 3460 | Train 55 | Test Hispanic 4 | 0.76 | 0.81 | 0.76 |
| 2437 | Train 39 | Test Hispanic 5 | 0.75 | 0.86 | 0.84 | 3461 | Train 55 | Test Hispanic 5 | 0.73 | 0.79 | 0.71 |
| 2438 | Train 39 | Test Hispanic 6 | 0.72 | 0.77 | 0.75 | 3462 | Train 55 | Test Hispanic 6 | 0.71 | 0.72 | 0.65 |
| 2439 | Train 39 | Test Hispanic 7 | 0.75 | 0.86 | 0.84 | 3463 | Train 55 | Test Hispanic 7 | 0.73 | 0.78 | 0.71 |
| 2440 | Train 39 | Test Hispanic 8 | 0.72 | 0.77 | 0.76 | 3464 | Train 55 | Test Hispanic 8 | 0.71 | 0.73 | 0.65 |
| 2441 | Train 39 | Test Hispanic 9 | 0.77 | 0.84 | 0.82 | 3465 | Train 55 | Test Hispanic 9 | 0.76 | 0.81 | 0.77 |
| 2442 | Train 39 | Test Hispanic 10 | 0.77 | 0.84 | 0.81 | 3466 | Train 55 | Test Hispanic 10 | 0.76 | 0.8 | 0.75 |
| 2443 | Train 39 | Test Hispanic 11 | 0.78 | 0.85 | 0.83 | 3467 | Train 55 | Test Hispanic 11 | 0.77 | 0.82 | 0.77 |
| 2444 | Train 39 | Test Hispanic 12 | 0.77 | 0.84 | 0.82 | 3468 | Train 55 | Test Hispanic 12 | 0.76 | 0.81 | 0.76 |
| 2445 | Train 39 | Test Hispanic 13 | 0.75 | 0.85 | 0.82 | 3469 | Train 55 | Test Hispanic 13 | 0.73 | 0.78 | 0.71 |
| 2446 | Train 39 | Test Hispanic 14 | 0.72 | 0.77 | 0.75 | 3470 | Train 55 | Test Hispanic 14 | 0.71 | 0.72 | 0.65 |
| 2447 | Train 39 | Test Hispanic 15 | 0.75 | 0.84 | 0.82 | 3471 | Train 55 | Test Hispanic 15 | 0.73 | 0.78 | 0.71 |
| 2448 | Train 39 | Test Hispanic 16 | 0.72 | 0.77 | 0.76 | 3472 | Train 55 | Test Hispanic 16 | 0.71 | 0.73 | 0.66 |
| 2449 | Train 39 | Test Hispanic 17 | 0.77 | 0.86 | 0.84 | 3473 | Train 55 | Test Hispanic 17 | 0.76 | 0.82 | 0.77 |
| 2450 | Train 39 | Test Hispanic 18 | 0.77 | 0.85 | 0.83 | 3474 | Train 55 | Test Hispanic 18 | 0.75 | 0.8 | 0.76 |
| 2451 | Train 39 | Test Hispanic 19 | 0.78 | 0.86 | 0.84 | 3475 | Train 55 | Test Hispanic 19 | 0.76 | 0.82 | 0.78 |
| 2452 | Train 39 | Test Hispanic 20 | 0.77 | 0.85 | 0.83 | 3476 | Train 55 | Test Hispanic 20 | 0.76 | 0.81 | 0.76 |
| 2453 | Train 39 | Test Hispanic 21 | 0.75 | 0.86 | 0.84 | 3477 | Train 55 | Test Hispanic 21 | 0.73 | 0.79 | 0.71 |
| 2454 | Train 39 | Test Hispanic 22 | 0.72 | 0.77 | 0.75 | 3478 | Train 55 | Test Hispanic 22 | 0.71 | 0.72 | 0.65 |
| 2455 | Train 39 | Test Hispanic 23 | 0.75 | 0.86 | 0.84 | 3479 | Train 55 | Test Hispanic 23 | 0.73 | 0.78 | 0.71 |
| 2456 | Train 39 | Test Hispanic 24 | 0.72 | 0.77 | 0.76 | 3480 | Train 55 | Test Hispanic 24 | 0.71 | 0.73 | 0.66 |
| 2457 | Train 39 | Test Hispanic 25 | 0.77 | 0.84 | 0.82 | 3481 | Train 55 | Test Hispanic 25 | 0.76 | 0.81 | 0.77 |
| 2458 | Train 39 | Test Hispanic 26 | 0.77 | 0.84 | 0.81 | 3482 | Train 55 | Test Hispanic 26 | 0.76 | 0.8 | 0.75 |
| 2459 | Train 39 | Test Hispanic 27 | 0.78 | 0.85 | 0.83 | 3483 | Train 55 | Test Hispanic 27 | 0.77 | 0.82 | 0.77 |
| 2460 | Train 39 | Test Hispanic 28 | 0.77 | 0.84 | 0.82 | 3484 | Train 55 | Test Hispanic 28 | 0.76 | 0.81 | 0.76 |
| 2461 | Train 39 | Test Hispanic 29 | 0.75 | 0.85 | 0.82 | 3485 | Train 55 | Test Hispanic 29 | 0.73 | 0.78 | 0.71 |
| 2462 | Train 39 | Test Hispanic 30 | 0.72 | 0.77 | 0.75 | 3486 | Train 55 | Test Hispanic 30 | 0.71 | 0.72 | 0.65 |
| 2463 | Train 39 | Test Hispanic 31 | 0.75 | 0.85 | 0.82 | 3487 | Train 55 | Test Hispanic 31 | 0.73 | 0.78 | 0.71 |
| 2464 | Train 39 | Test Hispanic 32 | 0.72 | 0.77 | 0.76 | 3488 | Train 55 | Test Hispanic 32 | 0.71 | 0.73 | 0.66 |
| 2465 | Train 39 | Test Hispanic 33 | 0.78 | 0.86 | 0.85 | 3489 | Train 55 | Test Hispanic 33 | 0.77 | 0.82 | 0.78 |
| 2466 | Train 39 | Test Hispanic 34 | 0.78 | 0.85 | 0.83 | 3490 | Train 55 | Test Hispanic 34 | 0.76 | 0.81 | 0.76 |
| 2467 | Train 39 | Test Hispanic 35 | 0.79 | 0.86 | 0.85 | 3491 | Train 55 | Test Hispanic 35 | 0.78 | 0.83 | 0.79 |
| 2468 | Train 39 | Test Hispanic 36 | 0.78 | 0.85 | 0.84 | 3492 | Train 55 | Test Hispanic 36 | 0.77 | 0.81 | 0.77 |
| 2469 | Train 39 | Test Hispanic 37 | 0.75 | 0.86 | 0.84 | 3493 | Train 55 | Test Hispanic 37 | 0.73 | 0.79 | 0.71 |
| 2470 | Train 39 | Test Hispanic 38 | 0.72 | 0.77 | 0.76 | 3494 | Train 55 | Test Hispanic 38 | 0.71 | 0.72 | 0.65 |
| 2471 | Train 39 | Test Hispanic 39 | 0.75 | 0.86 | 0.84 | 3495 | Train 55 | Test Hispanic 39 | 0.73 | 0.79 | 0.72 |
| 2472 | Train 39 | Test Hispanic 40 | 0.73 | 0.77 | 0.76 | 3496 | Train 55 | Test Hispanic 40 | 0.72 | 0.73 | 0.66 |
| 2473 | Train 39 | Test Hispanic 41 | 0.78 | 0.85 | 0.83 | 3497 | Train 55 | Test Hispanic 41 | 0.77 | 0.82 | 0.78 |
| 2474 | Train 39 | Test Hispanic 42 | 0.78 | 0.84 | 0.82 | 3498 | Train 55 | Test Hispanic 42 | 0.76 | 0.81 | 0.76 |
| 2475 | Train 39 | Test Hispanic 43 | 0.79 | 0.85 | 0.83 | 3499 | Train 55 | Test Hispanic 43 | 0.78 | 0.82 | 0.78 |
| 2476 | Train 39 | Test Hispanic 44 | 0.78 | 0.84 | 0.82 | 3500 | Train 55 | Test Hispanic 44 | 0.77 | 0.81 | 0.76 |
| 2477 | Train 39 | Test Hispanic 45 | 0.75 | 0.85 | 0.82 | 3501 | Train 55 | Test Hispanic 45 | 0.73 | 0.79 | 0.72 |
| 2478 | Train 39 | Test Hispanic 46 | 0.72 | 0.77 | 0.76 | 3502 | Train 55 | Test Hispanic 46 | 0.71 | 0.73 | 0.66 |
| 2479 | Train 39 | Test Hispanic 47 | 0.75 | 0.85 | 0.82 | 3503 | Train 55 | Test Hispanic 47 | 0.73 | 0.79 | 0.72 |
| 2480 | Train 39 | Test Hispanic 48 | 0.73 | 0.77 | 0.76 | 3504 | Train 55 | Test Hispanic 48 | 0.72 | 0.73 | 0.66 |
| 2481 | Train 39 | Test Hispanic 49 | 0.79 | 0.87 | 0.86 | 3505 | Train 55 | Test Hispanic 49 | 0.8 | 0.89 | 0.88 |
| 2482 | Train 39 | Test Hispanic 50 | 0.79 | 0.86 | 0.85 | 3506 | Train 55 | Test Hispanic 50 | 0.8 | 0.88 | 0.87 |
| 2483 | Train 39 | Test Hispanic 51 | 0.8 | 0.87 | 0.86 | 3507 | Train 55 | Test Hispanic 51 | 0.81 | 0.89 | 0.88 |
| 2484 | Train 39 | Test Hispanic 52 | 0.79 | 0.86 | 0.86 | 3508 | Train 55 | Test Hispanic 52 | 0.8 | 0.89 | 0.87 |
| 2485 | Train 39 | Test Hispanic 53 | 0.78 | 0.86 | 0.84 | 3509 | Train 55 | Test Hispanic 53 | 0.79 | 0.9 | 0.9 |
| 2486 | Train 39 | Test Hispanic 54 | 0.74 | 0.77 | 0.75 | 3510 | Train 55 | Test Hispanic 54 | 0.74 | 0.77 | 0.7 |
| 2487 | Train 39 | Test Hispanic 55 | 0.78 | 0.86 | 0.84 | 3511 | Train 55 | Test Hispanic 55 | 0.79 | 0.9 | 0.9 |
| 2488 | Train 39 | Test Hispanic 56 | 0.74 | 0.76 | 0.75 | 3512 | Train 55 | Test Hispanic 56 | 0.75 | 0.77 | 0.71 |
| 2489 | Train 39 | Test Hispanic 57 | 0.79 | 0.86 | 0.84 | 3513 | Train 55 | Test Hispanic 57 | 0.8 | 0.88 | 0.87 |
| 2490 | Train 39 | Test Hispanic 58 | 0.79 | 0.85 | 0.84 | 3514 | Train 55 | Test Hispanic 58 | 0.8 | 0.88 | 0.87 |
| 2491 | Train 39 | Test Hispanic 59 | 0.8 | 0.86 | 0.84 | 3515 | Train 55 | Test Hispanic 59 | 0.81 | 0.89 | 0.87 |
| 2492 | Train 39 | Test Hispanic 60 | 0.8 | 0.86 | 0.84 | 3516 | Train 55 | Test Hispanic 60 | 0.8 | 0.88 | 0.87 |
| 2493 | Train 39 | Test Hispanic 61 | 0.78 | 0.86 | 0.83 | 3517 | Train 55 | Test Hispanic 61 | 0.79 | 0.89 | 0.87 |
| 2494 | Train 39 | Test Hispanic 62 | 0.74 | 0.77 | 0.76 | 3518 | Train 55 | Test Hispanic 62 | 0.74 | 0.77 | 0.71 |
| 2495 | Train 39 | Test Hispanic 63 | 0.78 | 0.86 | 0.83 | 3519 | Train 55 | Test Hispanic 63 | 0.79 | 0.89 | 0.88 |
| 2496 | Train 39 | Test Hispanic 64 | 0.74 | 0.76 | 0.76 | 3520 | Train 55 | Test Hispanic 64 | 0.74 | 0.77 | 0.71 |
| 2497 | Train 40 | Test Hispanic 1 | 0.77 | 0.84 | 0.8 | 3521 | Train 56 | Test Hispanic 1 | 0.75 | 0.81 | 0.76 |
| 2498 | Train 40 | Test Hispanic 2 | 0.77 | 0.84 | 0.8 | 3522 | Train 56 | Test Hispanic 2 | 0.75 | 0.81 | 0.75 |
| 2499 | Train 40 | Test Hispanic 3 | 0.78 | 0.84 | 0.8 | 3523 | Train 56 | Test Hispanic 3 | 0.76 | 0.82 | 0.76 |
| 2500 | Train 40 | Test Hispanic 4 | 0.78 | 0.84 | 0.8 | 3524 | Train 56 | Test Hispanic 4 | 0.75 | 0.81 | 0.76 |
| 2501 | Train 40 | Test Hispanic 5 | 0.74 | 0.83 | 0.79 | 3525 | Train 56 | Test Hispanic 5 | 0.72 | 0.79 | 0.73 |
| 2502 | Train 40 | Test Hispanic 6 | 0.75 | 0.84 | 0.79 | 3526 | Train 56 | Test Hispanic 6 | 0.73 | 0.79 | 0.74 |
| 2503 | Train 40 | Test Hispanic 7 | 0.74 | 0.83 | 0.79 | 3527 | Train 56 | Test Hispanic 7 | 0.72 | 0.78 | 0.73 |
| 2504 | Train 40 | Test Hispanic 8 | 0.75 | 0.83 | 0.79 | 3528 | Train 56 | Test Hispanic 8 | 0.73 | 0.79 | 0.74 |
| 2505 | Train 40 | Test Hispanic 9 | 0.77 | 0.84 | 0.81 | 3529 | Train 56 | Test Hispanic 9 | 0.76 | 0.82 | 0.77 |
| 2506 | Train 40 | Test Hispanic 10 | 0.77 | 0.84 | 0.81 | 3530 | Train 56 | Test Hispanic 10 | 0.75 | 0.81 | 0.76 |
| 2507 | Train 40 | Test Hispanic 11 | 0.78 | 0.84 | 0.81 | 3531 | Train 56 | Test Hispanic 11 | 0.76 | 0.82 | 0.77 |
| 2508 | Train 40 | Test Hispanic 12 | 0.78 | 0.84 | 0.81 | 3532 | Train 56 | Test Hispanic 12 | 0.76 | 0.81 | 0.77 |
| 2509 | Train 40 | Test Hispanic 13 | 0.74 | 0.83 | 0.79 | 3533 | Train 56 | Test Hispanic 13 | 0.72 | 0.79 | 0.74 |
| 2510 | Train 40 | Test Hispanic 14 | 0.75 | 0.83 | 0.79 | 3534 | Train 56 | Test Hispanic 14 | 0.73 | 0.79 | 0.74 |
| 2511 | Train 40 | Test Hispanic 15 | 0.74 | 0.83 | 0.79 | 3535 | Train 56 | Test Hispanic 15 | 0.72 | 0.78 | 0.74 |
| 2512 | Train 40 | Test Hispanic 16 | 0.75 | 0.83 | 0.79 | 3536 | Train 56 | Test Hispanic 16 | 0.73 | 0.79 | 0.74 |
| 2513 | Train 40 | Test Hispanic 17 | 0.77 | 0.84 | 0.8 | 3537 | Train 56 | Test Hispanic 17 | 0.75 | 0.81 | 0.76 |
| 2514 | Train 40 | Test Hispanic 18 | 0.77 | 0.84 | 0.8 | 3538 | Train 56 | Test Hispanic 18 | 0.75 | 0.81 | 0.75 |
| 2515 | Train 40 | Test Hispanic 19 | 0.78 | 0.84 | 0.8 | 3539 | Train 56 | Test Hispanic 19 | 0.76 | 0.82 | 0.76 |
| 2516 | Train 40 | Test Hispanic 20 | 0.78 | 0.84 | 0.8 | 3540 | Train 56 | Test Hispanic 20 | 0.75 | 0.81 | 0.76 |
| 2517 | Train 40 | Test Hispanic 21 | 0.74 | 0.83 | 0.79 | 3541 | Train 56 | Test Hispanic 21 | 0.72 | 0.79 | 0.73 |
| 2518 | Train 40 | Test Hispanic 22 | 0.75 | 0.84 | 0.8 | 3542 | Train 56 | Test Hispanic 22 | 0.73 | 0.79 | 0.74 |
| 2519 | Train 40 | Test Hispanic 23 | 0.74 | 0.83 | 0.79 | 3543 | Train 56 | Test Hispanic 23 | 0.72 | 0.78 | 0.73 |
| 2520 | Train 40 | Test Hispanic 24 | 0.75 | 0.83 | 0.79 | 3544 | Train 56 | Test Hispanic 24 | 0.73 | 0.79 | 0.74 |
| 2521 | Train 40 | Test Hispanic 25 | 0.77 | 0.84 | 0.81 | 3545 | Train 56 | Test Hispanic 25 | 0.76 | 0.82 | 0.77 |
| 2522 | Train 40 | Test Hispanic 26 | 0.77 | 0.84 | 0.81 | 3546 | Train 56 | Test Hispanic 26 | 0.75 | 0.81 | 0.76 |
| 2523 | Train 40 | Test Hispanic 27 | 0.78 | 0.84 | 0.81 | 3547 | Train 56 | Test Hispanic 27 | 0.76 | 0.82 | 0.77 |
| 2524 | Train 40 | Test Hispanic 28 | 0.78 | 0.84 | 0.81 | 3548 | Train 56 | Test Hispanic 28 | 0.76 | 0.81 | 0.77 |
| 2525 | Train 40 | Test Hispanic 29 | 0.74 | 0.83 | 0.79 | 3549 | Train 56 | Test Hispanic 29 | 0.72 | 0.79 | 0.74 |
| 2526 | Train 40 | Test Hispanic 30 | 0.75 | 0.84 | 0.79 | 3550 | Train 56 | Test Hispanic 30 | 0.73 | 0.79 | 0.74 |
| 2527 | Train 40 | Test Hispanic 31 | 0.74 | 0.83 | 0.79 | 3551 | Train 56 | Test Hispanic 31 | 0.72 | 0.78 | 0.74 |
| 2528 | Train 40 | Test Hispanic 32 | 0.75 | 0.83 | 0.79 | 3552 | Train 56 | Test Hispanic 32 | 0.73 | 0.79 | 0.74 |
| 2529 | Train 40 | Test Hispanic 33 | 0.78 | 0.84 | 0.81 | 3553 | Train 56 | Test Hispanic 33 | 0.77 | 0.82 | 0.77 |
| 2530 | Train 40 | Test Hispanic 34 | 0.78 | 0.84 | 0.81 | 3554 | Train 56 | Test Hispanic 34 | 0.76 | 0.81 | 0.76 |
| 2531 | Train 40 | Test Hispanic 35 | 0.79 | 0.85 | 0.81 | 3555 | Train 56 | Test Hispanic 35 | 0.77 | 0.82 | 0.77 |
| 2532 | Train 40 | Test Hispanic 36 | 0.78 | 0.84 | 0.81 | 3556 | Train 56 | Test Hispanic 36 | 0.77 | 0.82 | 0.77 |
| 2533 | Train 40 | Test Hispanic 37 | 0.75 | 0.83 | 0.79 | 3557 | Train 56 | Test Hispanic 37 | 0.73 | 0.79 | 0.74 |
| 2534 | Train 40 | Test Hispanic 38 | 0.75 | 0.84 | 0.8 | 3558 | Train 56 | Test Hispanic 38 | 0.73 | 0.8 | 0.75 |
| 2535 | Train 40 | Test Hispanic 39 | 0.75 | 0.83 | 0.79 | 3559 | Train 56 | Test Hispanic 39 | 0.73 | 0.79 | 0.73 |
| 2536 | Train 40 | Test Hispanic 40 | 0.76 | 0.84 | 0.8 | 3560 | Train 56 | Test Hispanic 40 | 0.74 | 0.79 | 0.75 |
| 2537 | Train 40 | Test Hispanic 41 | 0.78 | 0.85 | 0.81 | 3561 | Train 56 | Test Hispanic 41 | 0.77 | 0.82 | 0.78 |
| 2538 | Train 40 | Test Hispanic 42 | 0.78 | 0.85 | 0.81 | 3562 | Train 56 | Test Hispanic 42 | 0.76 | 0.82 | 0.77 |
| 2539 | Train 40 | Test Hispanic 43 | 0.79 | 0.85 | 0.82 | 3563 | Train 56 | Test Hispanic 43 | 0.77 | 0.83 | 0.79 |
| 2540 | Train 40 | Test Hispanic 44 | 0.79 | 0.85 | 0.82 | 3564 | Train 56 | Test Hispanic 44 | 0.77 | 0.82 | 0.78 |
| 2541 | Train 40 | Test Hispanic 45 | 0.75 | 0.83 | 0.8 | 3565 | Train 56 | Test Hispanic 45 | 0.73 | 0.79 | 0.75 |
| 2542 | Train 40 | Test Hispanic 46 | 0.75 | 0.84 | 0.8 | 3566 | Train 56 | Test Hispanic 46 | 0.73 | 0.79 | 0.75 |
| 2543 | Train 40 | Test Hispanic 47 | 0.75 | 0.83 | 0.8 | 3567 | Train 56 | Test Hispanic 47 | 0.73 | 0.79 | 0.74 |
| 2544 | Train 40 | Test Hispanic 48 | 0.76 | 0.83 | 0.8 | 3568 | Train 56 | Test Hispanic 48 | 0.74 | 0.79 | 0.75 |
| 2545 | Train 40 | Test Hispanic 49 | 0.79 | 0.85 | 0.82 | 3569 | Train 56 | Test Hispanic 49 | 0.8 | 0.87 | 0.81 |
| 2546 | Train 40 | Test Hispanic 50 | 0.79 | 0.85 | 0.81 | 3570 | Train 56 | Test Hispanic 50 | 0.79 | 0.86 | 0.81 |
| 2547 | Train 40 | Test Hispanic 51 | 0.8 | 0.86 | 0.82 | 3571 | Train 56 | Test Hispanic 51 | 0.8 | 0.87 | 0.82 |
| 2548 | Train 40 | Test Hispanic 52 | 0.8 | 0.85 | 0.82 | 3572 | Train 56 | Test Hispanic 52 | 0.8 | 0.87 | 0.82 |
| 2549 | Train 40 | Test Hispanic 53 | 0.77 | 0.85 | 0.81 | 3573 | Train 56 | Test Hispanic 53 | 0.78 | 0.87 | 0.82 |
| 2550 | Train 40 | Test Hispanic 54 | 0.78 | 0.85 | 0.81 | 3574 | Train 56 | Test Hispanic 54 | 0.78 | 0.87 | 0.82 |
| 2551 | Train 40 | Test Hispanic 55 | 0.78 | 0.86 | 0.81 | 3575 | Train 56 | Test Hispanic 55 | 0.78 | 0.87 | 0.82 |
| 2552 | Train 40 | Test Hispanic 56 | 0.78 | 0.84 | 0.81 | 3576 | Train 56 | Test Hispanic 56 | 0.78 | 0.86 | 0.82 |
| 2553 | Train 40 | Test Hispanic 57 | 0.8 | 0.86 | 0.83 | 3577 | Train 56 | Test Hispanic 57 | 0.8 | 0.87 | 0.84 |
| 2554 | Train 40 | Test Hispanic 58 | 0.79 | 0.86 | 0.83 | 3578 | Train 56 | Test Hispanic 58 | 0.79 | 0.87 | 0.84 |
| 2555 | Train 40 | Test Hispanic 59 | 0.8 | 0.86 | 0.84 | 3579 | Train 56 | Test Hispanic 59 | 0.81 | 0.88 | 0.84 |
| 2556 | Train 40 | Test Hispanic 60 | 0.8 | 0.86 | 0.84 | 3580 | Train 56 | Test Hispanic 60 | 0.8 | 0.87 | 0.84 |
| 2557 | Train 40 | Test Hispanic 61 | 0.78 | 0.86 | 0.82 | 3581 | Train 56 | Test Hispanic 61 | 0.78 | 0.88 | 0.83 |
| 2558 | Train 40 | Test Hispanic 62 | 0.78 | 0.85 | 0.81 | 3582 | Train 56 | Test Hispanic 62 | 0.78 | 0.87 | 0.82 |
| 2559 | Train 40 | Test Hispanic 63 | 0.78 | 0.86 | 0.82 | 3583 | Train 56 | Test Hispanic 63 | 0.78 | 0.88 | 0.83 |
| 2560 | Train 40 | Test Hispanic 64 | 0.78 | 0.84 | 0.81 | 3584 | Train 56 | Test Hispanic 64 | 0.78 | 0.86 | 0.82 |
| 2561 | Train 41 | Test Hispanic 1 | 0.79 | 0.88 | 0.84 | 3585 | Train 57 | Test Hispanic 1 | 0.77 | 0.8 | 0.75 |
| 2562 | Train 41 | Test Hispanic 2 | 0.78 | 0.86 | 0.83 | 3586 | Train 57 | Test Hispanic 2 | 0.75 | 0.79 | 0.74 |
| 2563 | Train 41 | Test Hispanic 3 | 0.79 | 0.88 | 0.84 | 3587 | Train 57 | Test Hispanic 3 | 0.77 | 0.81 | 0.76 |
| 2564 | Train 41 | Test Hispanic 4 | 0.78 | 0.86 | 0.82 | 3588 | Train 57 | Test Hispanic 4 | 0.76 | 0.79 | 0.74 |
| 2565 | Train 41 | Test Hispanic 5 | 0.74 | 0.78 | 0.74 | 3589 | Train 57 | Test Hispanic 5 | 0.73 | 0.75 | 0.69 |
| 2566 | Train 41 | Test Hispanic 6 | 0.72 | 0.73 | 0.7 | 3590 | Train 57 | Test Hispanic 6 | 0.71 | 0.7 | 0.67 |
| 2567 | Train 41 | Test Hispanic 7 | 0.74 | 0.78 | 0.74 | 3591 | Train 57 | Test Hispanic 7 | 0.73 | 0.74 | 0.68 |
| 2568 | Train 41 | Test Hispanic 8 | 0.72 | 0.73 | 0.7 | 3592 | Train 57 | Test Hispanic 8 | 0.72 | 0.71 | 0.67 |
| 2569 | Train 41 | Test Hispanic 9 | 0.79 | 0.89 | 0.88 | 3593 | Train 57 | Test Hispanic 9 | 0.77 | 0.81 | 0.78 |
| 2570 | Train 41 | Test Hispanic 10 | 0.78 | 0.87 | 0.85 | 3594 | Train 57 | Test Hispanic 10 | 0.76 | 0.8 | 0.76 |
| 2571 | Train 41 | Test Hispanic 11 | 0.79 | 0.89 | 0.87 | 3595 | Train 57 | Test Hispanic 11 | 0.77 | 0.81 | 0.78 |
| 2572 | Train 41 | Test Hispanic 12 | 0.78 | 0.87 | 0.85 | 3596 | Train 57 | Test Hispanic 12 | 0.76 | 0.8 | 0.76 |
| 2573 | Train 41 | Test Hispanic 13 | 0.74 | 0.79 | 0.76 | 3597 | Train 57 | Test Hispanic 13 | 0.73 | 0.75 | 0.7 |
| 2574 | Train 41 | Test Hispanic 14 | 0.72 | 0.74 | 0.7 | 3598 | Train 57 | Test Hispanic 14 | 0.71 | 0.7 | 0.67 |
| 2575 | Train 41 | Test Hispanic 15 | 0.74 | 0.78 | 0.75 | 3599 | Train 57 | Test Hispanic 15 | 0.73 | 0.74 | 0.69 |
| 2576 | Train 41 | Test Hispanic 16 | 0.72 | 0.73 | 0.71 | 3600 | Train 57 | Test Hispanic 16 | 0.72 | 0.71 | 0.67 |
| 2577 | Train 41 | Test Hispanic 17 | 0.79 | 0.88 | 0.84 | 3601 | Train 57 | Test Hispanic 17 | 0.77 | 0.8 | 0.75 |
| 2578 | Train 41 | Test Hispanic 18 | 0.78 | 0.86 | 0.83 | 3602 | Train 57 | Test Hispanic 18 | 0.75 | 0.79 | 0.74 |
| 2579 | Train 41 | Test Hispanic 19 | 0.79 | 0.88 | 0.84 | 3603 | Train 57 | Test Hispanic 19 | 0.77 | 0.81 | 0.76 |
| 2580 | Train 41 | Test Hispanic 20 | 0.78 | 0.86 | 0.82 | 3604 | Train 57 | Test Hispanic 20 | 0.76 | 0.8 | 0.74 |
| 2581 | Train 41 | Test Hispanic 21 | 0.74 | 0.78 | 0.74 | 3605 | Train 57 | Test Hispanic 21 | 0.73 | 0.75 | 0.69 |
| 2582 | Train 41 | Test Hispanic 22 | 0.72 | 0.73 | 0.7 | 3606 | Train 57 | Test Hispanic 22 | 0.71 | 0.7 | 0.67 |
| 2583 | Train 41 | Test Hispanic 23 | 0.74 | 0.78 | 0.74 | 3607 | Train 57 | Test Hispanic 23 | 0.73 | 0.74 | 0.68 |
| 2584 | Train 41 | Test Hispanic 24 | 0.72 | 0.73 | 0.7 | 3608 | Train 57 | Test Hispanic 24 | 0.72 | 0.71 | 0.67 |
| 2585 | Train 41 | Test Hispanic 25 | 0.79 | 0.89 | 0.88 | 3609 | Train 57 | Test Hispanic 25 | 0.77 | 0.81 | 0.78 |
| 2586 | Train 41 | Test Hispanic 26 | 0.78 | 0.87 | 0.85 | 3610 | Train 57 | Test Hispanic 26 | 0.76 | 0.8 | 0.76 |
| 2587 | Train 41 | Test Hispanic 27 | 0.79 | 0.89 | 0.87 | 3611 | Train 57 | Test Hispanic 27 | 0.77 | 0.81 | 0.78 |
| 2588 | Train 41 | Test Hispanic 28 | 0.78 | 0.87 | 0.85 | 3612 | Train 57 | Test Hispanic 28 | 0.76 | 0.8 | 0.76 |
| 2589 | Train 41 | Test Hispanic 29 | 0.74 | 0.79 | 0.76 | 3613 | Train 57 | Test Hispanic 29 | 0.73 | 0.75 | 0.7 |
| 2590 | Train 41 | Test Hispanic 30 | 0.72 | 0.74 | 0.7 | 3614 | Train 57 | Test Hispanic 30 | 0.71 | 0.71 | 0.67 |
| 2591 | Train 41 | Test Hispanic 31 | 0.74 | 0.78 | 0.75 | 3615 | Train 57 | Test Hispanic 31 | 0.73 | 0.75 | 0.69 |
| 2592 | Train 41 | Test Hispanic 32 | 0.72 | 0.74 | 0.71 | 3616 | Train 57 | Test Hispanic 32 | 0.72 | 0.71 | 0.67 |
| 2593 | Train 41 | Test Hispanic 33 | 0.8 | 0.89 | 0.85 | 3617 | Train 57 | Test Hispanic 33 | 0.78 | 0.81 | 0.76 |
| 2594 | Train 41 | Test Hispanic 34 | 0.79 | 0.87 | 0.83 | 3618 | Train 57 | Test Hispanic 34 | 0.76 | 0.8 | 0.75 |
| 2595 | Train 41 | Test Hispanic 35 | 0.8 | 0.88 | 0.85 | 3619 | Train 57 | Test Hispanic 35 | 0.78 | 0.82 | 0.77 |
| 2596 | Train 41 | Test Hispanic 36 | 0.79 | 0.87 | 0.83 | 3620 | Train 57 | Test Hispanic 36 | 0.77 | 0.8 | 0.75 |
| 2597 | Train 41 | Test Hispanic 37 | 0.74 | 0.79 | 0.74 | 3621 | Train 57 | Test Hispanic 37 | 0.73 | 0.75 | 0.69 |
| 2598 | Train 41 | Test Hispanic 38 | 0.72 | 0.74 | 0.71 | 3622 | Train 57 | Test Hispanic 38 | 0.71 | 0.71 | 0.67 |
| 2599 | Train 41 | Test Hispanic 39 | 0.74 | 0.78 | 0.74 | 3623 | Train 57 | Test Hispanic 39 | 0.73 | 0.75 | 0.69 |
| 2600 | Train 41 | Test Hispanic 40 | 0.73 | 0.74 | 0.71 | 3624 | Train 57 | Test Hispanic 40 | 0.72 | 0.71 | 0.67 |
| 2601 | Train 41 | Test Hispanic 41 | 0.8 | 0.89 | 0.88 | 3625 | Train 57 | Test Hispanic 41 | 0.78 | 0.82 | 0.78 |
| 2602 | Train 41 | Test Hispanic 42 | 0.79 | 0.88 | 0.86 | 3626 | Train 57 | Test Hispanic 42 | 0.76 | 0.81 | 0.77 |
| 2603 | Train 41 | Test Hispanic 43 | 0.8 | 0.89 | 0.88 | 3627 | Train 57 | Test Hispanic 43 | 0.78 | 0.82 | 0.78 |
| 2604 | Train 41 | Test Hispanic 44 | 0.79 | 0.88 | 0.86 | 3628 | Train 57 | Test Hispanic 44 | 0.77 | 0.81 | 0.77 |
| 2605 | Train 41 | Test Hispanic 45 | 0.74 | 0.79 | 0.76 | 3629 | Train 57 | Test Hispanic 45 | 0.73 | 0.75 | 0.7 |
| 2606 | Train 41 | Test Hispanic 46 | 0.72 | 0.74 | 0.71 | 3630 | Train 57 | Test Hispanic 46 | 0.72 | 0.71 | 0.67 |
| 2607 | Train 41 | Test Hispanic 47 | 0.74 | 0.79 | 0.76 | 3631 | Train 57 | Test Hispanic 47 | 0.73 | 0.75 | 0.7 |
| 2608 | Train 41 | Test Hispanic 48 | 0.73 | 0.74 | 0.71 | 3632 | Train 57 | Test Hispanic 48 | 0.72 | 0.71 | 0.67 |
| 2609 | Train 41 | Test Hispanic 49 | 0.81 | 0.87 | 0.84 | 3633 | Train 57 | Test Hispanic 49 | 0.81 | 0.9 | 0.87 |
| 2610 | Train 41 | Test Hispanic 50 | 0.8 | 0.87 | 0.84 | 3634 | Train 57 | Test Hispanic 50 | 0.8 | 0.89 | 0.87 |
| 2611 | Train 41 | Test Hispanic 51 | 0.81 | 0.87 | 0.84 | 3635 | Train 57 | Test Hispanic 51 | 0.81 | 0.89 | 0.87 |
| 2612 | Train 41 | Test Hispanic 52 | 0.8 | 0.87 | 0.83 | 3636 | Train 57 | Test Hispanic 52 | 0.81 | 0.89 | 0.86 |
| 2613 | Train 41 | Test Hispanic 53 | 0.78 | 0.84 | 0.81 | 3637 | Train 57 | Test Hispanic 53 | 0.77 | 0.84 | 0.8 |
| 2614 | Train 41 | Test Hispanic 54 | 0.75 | 0.76 | 0.73 | 3638 | Train 57 | Test Hispanic 54 | 0.74 | 0.74 | 0.71 |
| 2615 | Train 41 | Test Hispanic 55 | 0.79 | 0.84 | 0.81 | 3639 | Train 57 | Test Hispanic 55 | 0.77 | 0.84 | 0.8 |
| 2616 | Train 41 | Test Hispanic 56 | 0.76 | 0.76 | 0.74 | 3640 | Train 57 | Test Hispanic 56 | 0.75 | 0.75 | 0.71 |
| 2617 | Train 41 | Test Hispanic 57 | 0.81 | 0.89 | 0.88 | 3641 | Train 57 | Test Hispanic 57 | 0.81 | 0.91 | 0.93 |
| 2618 | Train 41 | Test Hispanic 58 | 0.8 | 0.88 | 0.88 | 3642 | Train 57 | Test Hispanic 58 | 0.8 | 0.91 | 0.92 |
| 2619 | Train 41 | Test Hispanic 59 | 0.81 | 0.88 | 0.87 | 3643 | Train 57 | Test Hispanic 59 | 0.81 | 0.9 | 0.91 |
| 2620 | Train 41 | Test Hispanic 60 | 0.81 | 0.88 | 0.87 | 3644 | Train 57 | Test Hispanic 60 | 0.81 | 0.9 | 0.91 |
| 2621 | Train 41 | Test Hispanic 61 | 0.78 | 0.84 | 0.83 | 3645 | Train 57 | Test Hispanic 61 | 0.77 | 0.85 | 0.82 |
| 2622 | Train 41 | Test Hispanic 62 | 0.75 | 0.76 | 0.74 | 3646 | Train 57 | Test Hispanic 62 | 0.75 | 0.74 | 0.71 |
| 2623 | Train 41 | Test Hispanic 63 | 0.79 | 0.84 | 0.83 | 3647 | Train 57 | Test Hispanic 63 | 0.78 | 0.85 | 0.82 |
| 2624 | Train 41 | Test Hispanic 64 | 0.76 | 0.76 | 0.74 | 3648 | Train 57 | Test Hispanic 64 | 0.76 | 0.75 | 0.71 |
| 2625 | Train 42 | Test Hispanic 1 | 0.79 | 0.88 | 0.84 | 3649 | Train 58 | Test Hispanic 1 | 0.76 | 0.78 | 0.68 |
| 2626 | Train 42 | Test Hispanic 2 | 0.78 | 0.88 | 0.85 | 3650 | Train 58 | Test Hispanic 2 | 0.75 | 0.77 | 0.68 |
| 2627 | Train 42 | Test Hispanic 3 | 0.79 | 0.88 | 0.84 | 3651 | Train 58 | Test Hispanic 3 | 0.77 | 0.78 | 0.69 |
| 2628 | Train 42 | Test Hispanic 4 | 0.79 | 0.88 | 0.84 | 3652 | Train 58 | Test Hispanic 4 | 0.76 | 0.78 | 0.69 |
| 2629 | Train 42 | Test Hispanic 5 | 0.74 | 0.79 | 0.75 | 3653 | Train 58 | Test Hispanic 5 | 0.73 | 0.74 | 0.65 |
| 2630 | Train 42 | Test Hispanic 6 | 0.73 | 0.75 | 0.71 | 3654 | Train 58 | Test Hispanic 6 | 0.71 | 0.7 | 0.64 |
| 2631 | Train 42 | Test Hispanic 7 | 0.74 | 0.78 | 0.75 | 3655 | Train 58 | Test Hispanic 7 | 0.73 | 0.73 | 0.64 |
| 2632 | Train 42 | Test Hispanic 8 | 0.73 | 0.75 | 0.71 | 3656 | Train 58 | Test Hispanic 8 | 0.72 | 0.71 | 0.64 |
| 2633 | Train 42 | Test Hispanic 9 | 0.79 | 0.88 | 0.87 | 3657 | Train 58 | Test Hispanic 9 | 0.77 | 0.79 | 0.71 |
| 2634 | Train 42 | Test Hispanic 10 | 0.78 | 0.89 | 0.87 | 3658 | Train 58 | Test Hispanic 10 | 0.76 | 0.78 | 0.7 |
| 2635 | Train 42 | Test Hispanic 11 | 0.79 | 0.88 | 0.87 | 3659 | Train 58 | Test Hispanic 11 | 0.77 | 0.79 | 0.71 |
| 2636 | Train 42 | Test Hispanic 12 | 0.79 | 0.89 | 0.87 | 3660 | Train 58 | Test Hispanic 12 | 0.76 | 0.78 | 0.7 |
| 2637 | Train 42 | Test Hispanic 13 | 0.74 | 0.8 | 0.76 | 3661 | Train 58 | Test Hispanic 13 | 0.73 | 0.74 | 0.66 |
| 2638 | Train 42 | Test Hispanic 14 | 0.73 | 0.75 | 0.72 | 3662 | Train 58 | Test Hispanic 14 | 0.72 | 0.71 | 0.64 |
| 2639 | Train 42 | Test Hispanic 15 | 0.74 | 0.79 | 0.76 | 3663 | Train 58 | Test Hispanic 15 | 0.73 | 0.73 | 0.65 |
| 2640 | Train 42 | Test Hispanic 16 | 0.73 | 0.75 | 0.72 | 3664 | Train 58 | Test Hispanic 16 | 0.72 | 0.71 | 0.64 |
| 2641 | Train 42 | Test Hispanic 17 | 0.79 | 0.88 | 0.84 | 3665 | Train 58 | Test Hispanic 17 | 0.76 | 0.78 | 0.68 |
| 2642 | Train 42 | Test Hispanic 18 | 0.78 | 0.88 | 0.85 | 3666 | Train 58 | Test Hispanic 18 | 0.75 | 0.77 | 0.68 |
| 2643 | Train 42 | Test Hispanic 19 | 0.79 | 0.88 | 0.84 | 3667 | Train 58 | Test Hispanic 19 | 0.77 | 0.78 | 0.69 |
| 2644 | Train 42 | Test Hispanic 20 | 0.79 | 0.88 | 0.84 | 3668 | Train 58 | Test Hispanic 20 | 0.76 | 0.78 | 0.69 |
| 2645 | Train 42 | Test Hispanic 21 | 0.74 | 0.79 | 0.75 | 3669 | Train 58 | Test Hispanic 21 | 0.73 | 0.74 | 0.65 |
| 2646 | Train 42 | Test Hispanic 22 | 0.73 | 0.75 | 0.71 | 3670 | Train 58 | Test Hispanic 22 | 0.71 | 0.7 | 0.64 |
| 2647 | Train 42 | Test Hispanic 23 | 0.74 | 0.78 | 0.75 | 3671 | Train 58 | Test Hispanic 23 | 0.73 | 0.73 | 0.64 |
| 2648 | Train 42 | Test Hispanic 24 | 0.73 | 0.75 | 0.71 | 3672 | Train 58 | Test Hispanic 24 | 0.72 | 0.71 | 0.64 |
| 2649 | Train 42 | Test Hispanic 25 | 0.79 | 0.88 | 0.87 | 3673 | Train 58 | Test Hispanic 25 | 0.77 | 0.79 | 0.71 |
| 2650 | Train 42 | Test Hispanic 26 | 0.78 | 0.89 | 0.87 | 3674 | Train 58 | Test Hispanic 26 | 0.76 | 0.78 | 0.7 |
| 2651 | Train 42 | Test Hispanic 27 | 0.79 | 0.88 | 0.87 | 3675 | Train 58 | Test Hispanic 27 | 0.77 | 0.79 | 0.71 |
| 2652 | Train 42 | Test Hispanic 28 | 0.79 | 0.89 | 0.87 | 3676 | Train 58 | Test Hispanic 28 | 0.76 | 0.78 | 0.7 |
| 2653 | Train 42 | Test Hispanic 29 | 0.74 | 0.8 | 0.76 | 3677 | Train 58 | Test Hispanic 29 | 0.73 | 0.74 | 0.66 |
| 2654 | Train 42 | Test Hispanic 30 | 0.73 | 0.76 | 0.72 | 3678 | Train 58 | Test Hispanic 30 | 0.72 | 0.71 | 0.65 |
| 2655 | Train 42 | Test Hispanic 31 | 0.74 | 0.79 | 0.76 | 3679 | Train 58 | Test Hispanic 31 | 0.73 | 0.74 | 0.65 |
| 2656 | Train 42 | Test Hispanic 32 | 0.73 | 0.75 | 0.72 | 3680 | Train 58 | Test Hispanic 32 | 0.72 | 0.71 | 0.64 |
| 2657 | Train 42 | Test Hispanic 33 | 0.79 | 0.88 | 0.85 | 3681 | Train 58 | Test Hispanic 33 | 0.77 | 0.79 | 0.69 |
| 2658 | Train 42 | Test Hispanic 34 | 0.79 | 0.88 | 0.85 | 3682 | Train 58 | Test Hispanic 34 | 0.76 | 0.78 | 0.69 |
| 2659 | Train 42 | Test Hispanic 35 | 0.8 | 0.88 | 0.85 | 3683 | Train 58 | Test Hispanic 35 | 0.78 | 0.79 | 0.7 |
| 2660 | Train 42 | Test Hispanic 36 | 0.79 | 0.88 | 0.85 | 3684 | Train 58 | Test Hispanic 36 | 0.77 | 0.79 | 0.69 |
| 2661 | Train 42 | Test Hispanic 37 | 0.75 | 0.79 | 0.76 | 3685 | Train 58 | Test Hispanic 37 | 0.74 | 0.75 | 0.65 |
| 2662 | Train 42 | Test Hispanic 38 | 0.73 | 0.76 | 0.72 | 3686 | Train 58 | Test Hispanic 38 | 0.72 | 0.71 | 0.65 |
| 2663 | Train 42 | Test Hispanic 39 | 0.75 | 0.79 | 0.75 | 3687 | Train 58 | Test Hispanic 39 | 0.74 | 0.74 | 0.65 |
| 2664 | Train 42 | Test Hispanic 40 | 0.73 | 0.75 | 0.72 | 3688 | Train 58 | Test Hispanic 40 | 0.72 | 0.71 | 0.65 |
| 2665 | Train 42 | Test Hispanic 41 | 0.79 | 0.89 | 0.88 | 3689 | Train 58 | Test Hispanic 41 | 0.78 | 0.79 | 0.71 |
| 2666 | Train 42 | Test Hispanic 42 | 0.79 | 0.89 | 0.88 | 3690 | Train 58 | Test Hispanic 42 | 0.76 | 0.79 | 0.71 |
| 2667 | Train 42 | Test Hispanic 43 | 0.8 | 0.89 | 0.87 | 3691 | Train 58 | Test Hispanic 43 | 0.78 | 0.8 | 0.72 |
| 2668 | Train 42 | Test Hispanic 44 | 0.8 | 0.89 | 0.87 | 3692 | Train 58 | Test Hispanic 44 | 0.77 | 0.79 | 0.71 |
| 2669 | Train 42 | Test Hispanic 45 | 0.75 | 0.8 | 0.77 | 3693 | Train 58 | Test Hispanic 45 | 0.74 | 0.75 | 0.66 |
| 2670 | Train 42 | Test Hispanic 46 | 0.73 | 0.76 | 0.72 | 3694 | Train 58 | Test Hispanic 46 | 0.72 | 0.71 | 0.65 |
| 2671 | Train 42 | Test Hispanic 47 | 0.75 | 0.79 | 0.76 | 3695 | Train 58 | Test Hispanic 47 | 0.74 | 0.74 | 0.66 |
| 2672 | Train 42 | Test Hispanic 48 | 0.73 | 0.76 | 0.72 | 3696 | Train 58 | Test Hispanic 48 | 0.72 | 0.71 | 0.65 |
| 2673 | Train 42 | Test Hispanic 49 | 0.8 | 0.87 | 0.84 | 3697 | Train 58 | Test Hispanic 49 | 0.81 | 0.9 | 0.87 |
| 2674 | Train 42 | Test Hispanic 50 | 0.79 | 0.87 | 0.84 | 3698 | Train 58 | Test Hispanic 50 | 0.8 | 0.9 | 0.87 |
| 2675 | Train 42 | Test Hispanic 51 | 0.8 | 0.87 | 0.84 | 3699 | Train 58 | Test Hispanic 51 | 0.81 | 0.89 | 0.86 |
| 2676 | Train 42 | Test Hispanic 52 | 0.8 | 0.87 | 0.84 | 3700 | Train 58 | Test Hispanic 52 | 0.81 | 0.89 | 0.86 |
| 2677 | Train 42 | Test Hispanic 53 | 0.78 | 0.83 | 0.79 | 3701 | Train 58 | Test Hispanic 53 | 0.77 | 0.82 | 0.78 |
| 2678 | Train 42 | Test Hispanic 54 | 0.75 | 0.78 | 0.72 | 3702 | Train 58 | Test Hispanic 54 | 0.74 | 0.73 | 0.68 |
| 2679 | Train 42 | Test Hispanic 55 | 0.79 | 0.84 | 0.79 | 3703 | Train 58 | Test Hispanic 55 | 0.78 | 0.83 | 0.78 |
| 2680 | Train 42 | Test Hispanic 56 | 0.75 | 0.77 | 0.73 | 3704 | Train 58 | Test Hispanic 56 | 0.75 | 0.74 | 0.68 |
| 2681 | Train 42 | Test Hispanic 57 | 0.8 | 0.88 | 0.88 | 3705 | Train 58 | Test Hispanic 57 | 0.81 | 0.91 | 0.93 |
| 2682 | Train 42 | Test Hispanic 58 | 0.8 | 0.88 | 0.87 | 3706 | Train 58 | Test Hispanic 58 | 0.8 | 0.91 | 0.92 |
| 2683 | Train 42 | Test Hispanic 59 | 0.81 | 0.88 | 0.87 | 3707 | Train 58 | Test Hispanic 59 | 0.81 | 0.9 | 0.91 |
| 2684 | Train 42 | Test Hispanic 60 | 0.8 | 0.88 | 0.87 | 3708 | Train 58 | Test Hispanic 60 | 0.81 | 0.9 | 0.91 |
| 2685 | Train 42 | Test Hispanic 61 | 0.78 | 0.84 | 0.8 | 3709 | Train 58 | Test Hispanic 61 | 0.77 | 0.83 | 0.8 |
| 2686 | Train 42 | Test Hispanic 62 | 0.75 | 0.78 | 0.73 | 3710 | Train 58 | Test Hispanic 62 | 0.74 | 0.73 | 0.69 |
| 2687 | Train 42 | Test Hispanic 63 | 0.79 | 0.84 | 0.8 | 3711 | Train 58 | Test Hispanic 63 | 0.78 | 0.83 | 0.8 |
| 2688 | Train 42 | Test Hispanic 64 | 0.75 | 0.77 | 0.73 | 3712 | Train 58 | Test Hispanic 64 | 0.75 | 0.74 | 0.69 |
| 2689 | Train 43 | Test Hispanic 1 | 0.79 | 0.88 | 0.85 | 3713 | Train 59 | Test Hispanic 1 | 0.76 | 0.81 | 0.73 |
| 2690 | Train 43 | Test Hispanic 2 | 0.78 | 0.86 | 0.83 | 3714 | Train 59 | Test Hispanic 2 | 0.75 | 0.8 | 0.71 |
| 2691 | Train 43 | Test Hispanic 3 | 0.79 | 0.88 | 0.85 | 3715 | Train 59 | Test Hispanic 3 | 0.77 | 0.81 | 0.74 |
| 2692 | Train 43 | Test Hispanic 4 | 0.78 | 0.86 | 0.83 | 3716 | Train 59 | Test Hispanic 4 | 0.75 | 0.8 | 0.72 |
| 2693 | Train 43 | Test Hispanic 5 | 0.74 | 0.78 | 0.74 | 3717 | Train 59 | Test Hispanic 5 | 0.73 | 0.75 | 0.66 |
| 2694 | Train 43 | Test Hispanic 6 | 0.72 | 0.73 | 0.7 | 3718 | Train 59 | Test Hispanic 6 | 0.71 | 0.71 | 0.64 |
| 2695 | Train 43 | Test Hispanic 7 | 0.74 | 0.78 | 0.74 | 3719 | Train 59 | Test Hispanic 7 | 0.73 | 0.75 | 0.66 |
| 2696 | Train 43 | Test Hispanic 8 | 0.72 | 0.73 | 0.7 | 3720 | Train 59 | Test Hispanic 8 | 0.72 | 0.71 | 0.65 |
| 2697 | Train 43 | Test Hispanic 9 | 0.79 | 0.89 | 0.87 | 3721 | Train 59 | Test Hispanic 9 | 0.77 | 0.82 | 0.77 |
| 2698 | Train 43 | Test Hispanic 10 | 0.78 | 0.87 | 0.85 | 3722 | Train 59 | Test Hispanic 10 | 0.75 | 0.8 | 0.75 |
| 2699 | Train 43 | Test Hispanic 11 | 0.79 | 0.89 | 0.87 | 3723 | Train 59 | Test Hispanic 11 | 0.77 | 0.82 | 0.78 |
| 2700 | Train 43 | Test Hispanic 12 | 0.78 | 0.87 | 0.85 | 3724 | Train 59 | Test Hispanic 12 | 0.76 | 0.81 | 0.75 |
| 2701 | Train 43 | Test Hispanic 13 | 0.74 | 0.79 | 0.75 | 3725 | Train 59 | Test Hispanic 13 | 0.73 | 0.75 | 0.68 |
| 2702 | Train 43 | Test Hispanic 14 | 0.72 | 0.74 | 0.7 | 3726 | Train 59 | Test Hispanic 14 | 0.71 | 0.71 | 0.64 |
| 2703 | Train 43 | Test Hispanic 15 | 0.74 | 0.78 | 0.75 | 3727 | Train 59 | Test Hispanic 15 | 0.73 | 0.75 | 0.68 |
| 2704 | Train 43 | Test Hispanic 16 | 0.72 | 0.74 | 0.7 | 3728 | Train 59 | Test Hispanic 16 | 0.72 | 0.71 | 0.65 |
| 2705 | Train 43 | Test Hispanic 17 | 0.79 | 0.88 | 0.85 | 3729 | Train 59 | Test Hispanic 17 | 0.76 | 0.81 | 0.73 |
| 2706 | Train 43 | Test Hispanic 18 | 0.78 | 0.86 | 0.83 | 3730 | Train 59 | Test Hispanic 18 | 0.75 | 0.8 | 0.71 |
| 2707 | Train 43 | Test Hispanic 19 | 0.79 | 0.88 | 0.85 | 3731 | Train 59 | Test Hispanic 19 | 0.77 | 0.81 | 0.74 |
| 2708 | Train 43 | Test Hispanic 20 | 0.78 | 0.86 | 0.83 | 3732 | Train 59 | Test Hispanic 20 | 0.75 | 0.8 | 0.72 |
| 2709 | Train 43 | Test Hispanic 21 | 0.74 | 0.78 | 0.74 | 3733 | Train 59 | Test Hispanic 21 | 0.73 | 0.75 | 0.66 |
| 2710 | Train 43 | Test Hispanic 22 | 0.72 | 0.74 | 0.7 | 3734 | Train 59 | Test Hispanic 22 | 0.71 | 0.71 | 0.64 |
| 2711 | Train 43 | Test Hispanic 23 | 0.74 | 0.78 | 0.74 | 3735 | Train 59 | Test Hispanic 23 | 0.73 | 0.75 | 0.66 |
| 2712 | Train 43 | Test Hispanic 24 | 0.72 | 0.74 | 0.7 | 3736 | Train 59 | Test Hispanic 24 | 0.72 | 0.71 | 0.65 |
| 2713 | Train 43 | Test Hispanic 25 | 0.79 | 0.89 | 0.87 | 3737 | Train 59 | Test Hispanic 25 | 0.77 | 0.82 | 0.77 |
| 2714 | Train 43 | Test Hispanic 26 | 0.78 | 0.87 | 0.85 | 3738 | Train 59 | Test Hispanic 26 | 0.75 | 0.8 | 0.75 |
| 2715 | Train 43 | Test Hispanic 27 | 0.79 | 0.89 | 0.87 | 3739 | Train 59 | Test Hispanic 27 | 0.77 | 0.82 | 0.78 |
| 2716 | Train 43 | Test Hispanic 28 | 0.78 | 0.87 | 0.85 | 3740 | Train 59 | Test Hispanic 28 | 0.76 | 0.81 | 0.75 |
| 2717 | Train 43 | Test Hispanic 29 | 0.74 | 0.79 | 0.75 | 3741 | Train 59 | Test Hispanic 29 | 0.73 | 0.75 | 0.68 |
| 2718 | Train 43 | Test Hispanic 30 | 0.72 | 0.74 | 0.7 | 3742 | Train 59 | Test Hispanic 30 | 0.71 | 0.71 | 0.65 |
| 2719 | Train 43 | Test Hispanic 31 | 0.74 | 0.78 | 0.75 | 3743 | Train 59 | Test Hispanic 31 | 0.73 | 0.75 | 0.68 |
| 2720 | Train 43 | Test Hispanic 32 | 0.72 | 0.74 | 0.7 | 3744 | Train 59 | Test Hispanic 32 | 0.72 | 0.71 | 0.65 |
| 2721 | Train 43 | Test Hispanic 33 | 0.8 | 0.88 | 0.85 | 3745 | Train 59 | Test Hispanic 33 | 0.77 | 0.82 | 0.74 |
| 2722 | Train 43 | Test Hispanic 34 | 0.78 | 0.87 | 0.83 | 3746 | Train 59 | Test Hispanic 34 | 0.76 | 0.8 | 0.72 |
| 2723 | Train 43 | Test Hispanic 35 | 0.8 | 0.88 | 0.86 | 3747 | Train 59 | Test Hispanic 35 | 0.78 | 0.82 | 0.75 |
| 2724 | Train 43 | Test Hispanic 36 | 0.79 | 0.87 | 0.84 | 3748 | Train 59 | Test Hispanic 36 | 0.76 | 0.81 | 0.73 |
| 2725 | Train 43 | Test Hispanic 37 | 0.74 | 0.79 | 0.74 | 3749 | Train 59 | Test Hispanic 37 | 0.73 | 0.76 | 0.67 |
| 2726 | Train 43 | Test Hispanic 38 | 0.72 | 0.74 | 0.7 | 3750 | Train 59 | Test Hispanic 38 | 0.71 | 0.71 | 0.65 |
| 2727 | Train 43 | Test Hispanic 39 | 0.74 | 0.78 | 0.74 | 3751 | Train 59 | Test Hispanic 39 | 0.73 | 0.75 | 0.67 |
| 2728 | Train 43 | Test Hispanic 40 | 0.72 | 0.74 | 0.7 | 3752 | Train 59 | Test Hispanic 40 | 0.72 | 0.72 | 0.65 |
| 2729 | Train 43 | Test Hispanic 41 | 0.8 | 0.89 | 0.87 | 3753 | Train 59 | Test Hispanic 41 | 0.78 | 0.82 | 0.78 |
| 2730 | Train 43 | Test Hispanic 42 | 0.79 | 0.87 | 0.85 | 3754 | Train 59 | Test Hispanic 42 | 0.76 | 0.81 | 0.76 |
| 2731 | Train 43 | Test Hispanic 43 | 0.8 | 0.89 | 0.87 | 3755 | Train 59 | Test Hispanic 43 | 0.78 | 0.83 | 0.79 |
| 2732 | Train 43 | Test Hispanic 44 | 0.79 | 0.87 | 0.85 | 3756 | Train 59 | Test Hispanic 44 | 0.77 | 0.82 | 0.76 |
| 2733 | Train 43 | Test Hispanic 45 | 0.74 | 0.79 | 0.75 | 3757 | Train 59 | Test Hispanic 45 | 0.73 | 0.76 | 0.68 |
| 2734 | Train 43 | Test Hispanic 46 | 0.72 | 0.74 | 0.7 | 3758 | Train 59 | Test Hispanic 46 | 0.71 | 0.71 | 0.65 |
| 2735 | Train 43 | Test Hispanic 47 | 0.74 | 0.79 | 0.75 | 3759 | Train 59 | Test Hispanic 47 | 0.73 | 0.76 | 0.68 |
| 2736 | Train 43 | Test Hispanic 48 | 0.73 | 0.74 | 0.7 | 3760 | Train 59 | Test Hispanic 48 | 0.72 | 0.72 | 0.65 |
| 2737 | Train 43 | Test Hispanic 49 | 0.81 | 0.87 | 0.84 | 3761 | Train 59 | Test Hispanic 49 | 0.81 | 0.89 | 0.87 |
| 2738 | Train 43 | Test Hispanic 50 | 0.8 | 0.87 | 0.84 | 3762 | Train 59 | Test Hispanic 50 | 0.8 | 0.89 | 0.87 |
| 2739 | Train 43 | Test Hispanic 51 | 0.81 | 0.87 | 0.85 | 3763 | Train 59 | Test Hispanic 51 | 0.81 | 0.89 | 0.88 |
| 2740 | Train 43 | Test Hispanic 52 | 0.8 | 0.87 | 0.85 | 3764 | Train 59 | Test Hispanic 52 | 0.81 | 0.89 | 0.88 |
| 2741 | Train 43 | Test Hispanic 53 | 0.78 | 0.83 | 0.8 | 3765 | Train 59 | Test Hispanic 53 | 0.77 | 0.84 | 0.79 |
| 2742 | Train 43 | Test Hispanic 54 | 0.75 | 0.76 | 0.73 | 3766 | Train 59 | Test Hispanic 54 | 0.74 | 0.74 | 0.69 |
| 2743 | Train 43 | Test Hispanic 55 | 0.78 | 0.84 | 0.81 | 3767 | Train 59 | Test Hispanic 55 | 0.77 | 0.85 | 0.8 |
| 2744 | Train 43 | Test Hispanic 56 | 0.75 | 0.76 | 0.73 | 3768 | Train 59 | Test Hispanic 56 | 0.75 | 0.75 | 0.69 |
| 2745 | Train 43 | Test Hispanic 57 | 0.81 | 0.88 | 0.87 | 3769 | Train 59 | Test Hispanic 57 | 0.81 | 0.9 | 0.93 |
| 2746 | Train 43 | Test Hispanic 58 | 0.8 | 0.88 | 0.87 | 3770 | Train 59 | Test Hispanic 58 | 0.8 | 0.9 | 0.93 |
| 2747 | Train 43 | Test Hispanic 59 | 0.81 | 0.88 | 0.88 | 3771 | Train 59 | Test Hispanic 59 | 0.81 | 0.91 | 0.93 |
| 2748 | Train 43 | Test Hispanic 60 | 0.81 | 0.88 | 0.87 | 3772 | Train 59 | Test Hispanic 60 | 0.81 | 0.9 | 0.93 |
| 2749 | Train 43 | Test Hispanic 61 | 0.78 | 0.84 | 0.82 | 3773 | Train 59 | Test Hispanic 61 | 0.77 | 0.85 | 0.82 |
| 2750 | Train 43 | Test Hispanic 62 | 0.75 | 0.76 | 0.73 | 3774 | Train 59 | Test Hispanic 62 | 0.74 | 0.74 | 0.69 |
| 2751 | Train 43 | Test Hispanic 63 | 0.79 | 0.84 | 0.82 | 3775 | Train 59 | Test Hispanic 63 | 0.78 | 0.86 | 0.83 |
| 2752 | Train 43 | Test Hispanic 64 | 0.76 | 0.76 | 0.74 | 3776 | Train 59 | Test Hispanic 64 | 0.75 | 0.75 | 0.69 |
| 2753 | Train 44 | Test Hispanic 1 | 0.79 | 0.87 | 0.84 | 3777 | Train 60 | Test Hispanic 1 | 0.76 | 0.77 | 0.75 |
| 2754 | Train 44 | Test Hispanic 2 | 0.78 | 0.88 | 0.85 | 3778 | Train 60 | Test Hispanic 2 | 0.75 | 0.77 | 0.73 |
| 2755 | Train 44 | Test Hispanic 3 | 0.79 | 0.87 | 0.84 | 3779 | Train 60 | Test Hispanic 3 | 0.77 | 0.78 | 0.76 |
| 2756 | Train 44 | Test Hispanic 4 | 0.78 | 0.88 | 0.85 | 3780 | Train 60 | Test Hispanic 4 | 0.75 | 0.77 | 0.73 |
| 2757 | Train 44 | Test Hispanic 5 | 0.74 | 0.79 | 0.75 | 3781 | Train 60 | Test Hispanic 5 | 0.73 | 0.74 | 0.66 |
| 2758 | Train 44 | Test Hispanic 6 | 0.72 | 0.75 | 0.72 | 3782 | Train 60 | Test Hispanic 6 | 0.71 | 0.71 | 0.65 |
| 2759 | Train 44 | Test Hispanic 7 | 0.74 | 0.79 | 0.75 | 3783 | Train 60 | Test Hispanic 7 | 0.73 | 0.74 | 0.65 |
| 2760 | Train 44 | Test Hispanic 8 | 0.73 | 0.75 | 0.72 | 3784 | Train 60 | Test Hispanic 8 | 0.72 | 0.71 | 0.65 |
| 2761 | Train 44 | Test Hispanic 9 | 0.79 | 0.88 | 0.87 | 3785 | Train 60 | Test Hispanic 9 | 0.76 | 0.78 | 0.78 |
| 2762 | Train 44 | Test Hispanic 10 | 0.78 | 0.89 | 0.87 | 3786 | Train 60 | Test Hispanic 10 | 0.75 | 0.78 | 0.75 |
| 2763 | Train 44 | Test Hispanic 11 | 0.79 | 0.88 | 0.87 | 3787 | Train 60 | Test Hispanic 11 | 0.77 | 0.79 | 0.79 |
| 2764 | Train 44 | Test Hispanic 12 | 0.79 | 0.89 | 0.87 | 3788 | Train 60 | Test Hispanic 12 | 0.76 | 0.78 | 0.76 |
| 2765 | Train 44 | Test Hispanic 13 | 0.74 | 0.8 | 0.76 | 3789 | Train 60 | Test Hispanic 13 | 0.73 | 0.75 | 0.68 |
| 2766 | Train 44 | Test Hispanic 14 | 0.73 | 0.75 | 0.72 | 3790 | Train 60 | Test Hispanic 14 | 0.71 | 0.71 | 0.65 |
| 2767 | Train 44 | Test Hispanic 15 | 0.74 | 0.79 | 0.76 | 3791 | Train 60 | Test Hispanic 15 | 0.73 | 0.74 | 0.67 |
| 2768 | Train 44 | Test Hispanic 16 | 0.73 | 0.75 | 0.73 | 3792 | Train 60 | Test Hispanic 16 | 0.72 | 0.71 | 0.65 |
| 2769 | Train 44 | Test Hispanic 17 | 0.78 | 0.87 | 0.84 | 3793 | Train 60 | Test Hispanic 17 | 0.76 | 0.77 | 0.75 |
| 2770 | Train 44 | Test Hispanic 18 | 0.78 | 0.88 | 0.85 | 3794 | Train 60 | Test Hispanic 18 | 0.75 | 0.77 | 0.73 |
| 2771 | Train 44 | Test Hispanic 19 | 0.79 | 0.87 | 0.84 | 3795 | Train 60 | Test Hispanic 19 | 0.77 | 0.78 | 0.76 |
| 2772 | Train 44 | Test Hispanic 20 | 0.79 | 0.88 | 0.85 | 3796 | Train 60 | Test Hispanic 20 | 0.75 | 0.77 | 0.73 |
| 2773 | Train 44 | Test Hispanic 21 | 0.74 | 0.79 | 0.75 | 3797 | Train 60 | Test Hispanic 21 | 0.73 | 0.74 | 0.66 |
| 2774 | Train 44 | Test Hispanic 22 | 0.73 | 0.75 | 0.72 | 3798 | Train 60 | Test Hispanic 22 | 0.71 | 0.71 | 0.65 |
| 2775 | Train 44 | Test Hispanic 23 | 0.74 | 0.79 | 0.75 | 3799 | Train 60 | Test Hispanic 23 | 0.73 | 0.74 | 0.65 |
| 2776 | Train 44 | Test Hispanic 24 | 0.73 | 0.75 | 0.72 | 3800 | Train 60 | Test Hispanic 24 | 0.72 | 0.71 | 0.65 |
| 2777 | Train 44 | Test Hispanic 25 | 0.79 | 0.88 | 0.87 | 3801 | Train 60 | Test Hispanic 25 | 0.76 | 0.78 | 0.78 |
| 2778 | Train 44 | Test Hispanic 26 | 0.78 | 0.89 | 0.87 | 3802 | Train 60 | Test Hispanic 26 | 0.75 | 0.78 | 0.75 |
| 2779 | Train 44 | Test Hispanic 27 | 0.79 | 0.88 | 0.87 | 3803 | Train 60 | Test Hispanic 27 | 0.77 | 0.79 | 0.79 |
| 2780 | Train 44 | Test Hispanic 28 | 0.79 | 0.89 | 0.87 | 3804 | Train 60 | Test Hispanic 28 | 0.76 | 0.78 | 0.76 |
| 2781 | Train 44 | Test Hispanic 29 | 0.74 | 0.8 | 0.76 | 3805 | Train 60 | Test Hispanic 29 | 0.73 | 0.75 | 0.68 |
| 2782 | Train 44 | Test Hispanic 30 | 0.73 | 0.75 | 0.73 | 3806 | Train 60 | Test Hispanic 30 | 0.71 | 0.71 | 0.65 |
| 2783 | Train 44 | Test Hispanic 31 | 0.74 | 0.79 | 0.76 | 3807 | Train 60 | Test Hispanic 31 | 0.73 | 0.74 | 0.67 |
| 2784 | Train 44 | Test Hispanic 32 | 0.73 | 0.75 | 0.73 | 3808 | Train 60 | Test Hispanic 32 | 0.72 | 0.71 | 0.65 |
| 2785 | Train 44 | Test Hispanic 33 | 0.79 | 0.88 | 0.85 | 3809 | Train 60 | Test Hispanic 33 | 0.77 | 0.78 | 0.76 |
| 2786 | Train 44 | Test Hispanic 34 | 0.79 | 0.88 | 0.85 | 3810 | Train 60 | Test Hispanic 34 | 0.76 | 0.78 | 0.74 |
| 2787 | Train 44 | Test Hispanic 35 | 0.8 | 0.88 | 0.85 | 3811 | Train 60 | Test Hispanic 35 | 0.78 | 0.79 | 0.77 |
| 2788 | Train 44 | Test Hispanic 36 | 0.79 | 0.88 | 0.85 | 3812 | Train 60 | Test Hispanic 36 | 0.76 | 0.78 | 0.74 |
| 2789 | Train 44 | Test Hispanic 37 | 0.75 | 0.79 | 0.76 | 3813 | Train 60 | Test Hispanic 37 | 0.74 | 0.75 | 0.66 |
| 2790 | Train 44 | Test Hispanic 38 | 0.73 | 0.75 | 0.73 | 3814 | Train 60 | Test Hispanic 38 | 0.72 | 0.71 | 0.65 |
| 2791 | Train 44 | Test Hispanic 39 | 0.75 | 0.79 | 0.75 | 3815 | Train 60 | Test Hispanic 39 | 0.74 | 0.74 | 0.66 |
| 2792 | Train 44 | Test Hispanic 40 | 0.73 | 0.75 | 0.73 | 3816 | Train 60 | Test Hispanic 40 | 0.72 | 0.71 | 0.65 |
| 2793 | Train 44 | Test Hispanic 41 | 0.79 | 0.89 | 0.87 | 3817 | Train 60 | Test Hispanic 41 | 0.77 | 0.79 | 0.79 |
| 2794 | Train 44 | Test Hispanic 42 | 0.79 | 0.89 | 0.88 | 3818 | Train 60 | Test Hispanic 42 | 0.76 | 0.78 | 0.76 |
| 2795 | Train 44 | Test Hispanic 43 | 0.8 | 0.89 | 0.88 | 3819 | Train 60 | Test Hispanic 43 | 0.78 | 0.8 | 0.8 |
| 2796 | Train 44 | Test Hispanic 44 | 0.8 | 0.89 | 0.88 | 3820 | Train 60 | Test Hispanic 44 | 0.77 | 0.79 | 0.77 |
| 2797 | Train 44 | Test Hispanic 45 | 0.75 | 0.8 | 0.77 | 3821 | Train 60 | Test Hispanic 45 | 0.74 | 0.75 | 0.68 |
| 2798 | Train 44 | Test Hispanic 46 | 0.73 | 0.76 | 0.73 | 3822 | Train 60 | Test Hispanic 46 | 0.72 | 0.71 | 0.65 |
| 2799 | Train 44 | Test Hispanic 47 | 0.75 | 0.8 | 0.76 | 3823 | Train 60 | Test Hispanic 47 | 0.74 | 0.74 | 0.68 |
| 2800 | Train 44 | Test Hispanic 48 | 0.73 | 0.76 | 0.73 | 3824 | Train 60 | Test Hispanic 48 | 0.72 | 0.71 | 0.65 |
| 2801 | Train 44 | Test Hispanic 49 | 0.8 | 0.86 | 0.84 | 3825 | Train 60 | Test Hispanic 49 | 0.81 | 0.89 | 0.86 |
| 2802 | Train 44 | Test Hispanic 50 | 0.79 | 0.86 | 0.84 | 3826 | Train 60 | Test Hispanic 50 | 0.8 | 0.89 | 0.86 |
| 2803 | Train 44 | Test Hispanic 51 | 0.8 | 0.87 | 0.85 | 3827 | Train 60 | Test Hispanic 51 | 0.81 | 0.9 | 0.87 |
| 2804 | Train 44 | Test Hispanic 52 | 0.8 | 0.86 | 0.84 | 3828 | Train 60 | Test Hispanic 52 | 0.81 | 0.89 | 0.87 |
| 2805 | Train 44 | Test Hispanic 53 | 0.78 | 0.83 | 0.79 | 3829 | Train 60 | Test Hispanic 53 | 0.77 | 0.82 | 0.79 |
| 2806 | Train 44 | Test Hispanic 54 | 0.75 | 0.77 | 0.74 | 3830 | Train 60 | Test Hispanic 54 | 0.74 | 0.73 | 0.7 |
| 2807 | Train 44 | Test Hispanic 55 | 0.78 | 0.84 | 0.8 | 3831 | Train 60 | Test Hispanic 55 | 0.78 | 0.83 | 0.8 |
| 2808 | Train 44 | Test Hispanic 56 | 0.75 | 0.77 | 0.74 | 3832 | Train 60 | Test Hispanic 56 | 0.75 | 0.74 | 0.7 |
| 2809 | Train 44 | Test Hispanic 57 | 0.8 | 0.87 | 0.87 | 3833 | Train 60 | Test Hispanic 57 | 0.81 | 0.91 | 0.92 |
| 2810 | Train 44 | Test Hispanic 58 | 0.8 | 0.87 | 0.87 | 3834 | Train 60 | Test Hispanic 58 | 0.8 | 0.9 | 0.92 |
| 2811 | Train 44 | Test Hispanic 59 | 0.81 | 0.88 | 0.87 | 3835 | Train 60 | Test Hispanic 59 | 0.81 | 0.91 | 0.92 |
| 2812 | Train 44 | Test Hispanic 60 | 0.8 | 0.88 | 0.87 | 3836 | Train 60 | Test Hispanic 60 | 0.81 | 0.91 | 0.92 |
| 2813 | Train 44 | Test Hispanic 61 | 0.78 | 0.84 | 0.8 | 3837 | Train 60 | Test Hispanic 61 | 0.77 | 0.83 | 0.82 |
| 2814 | Train 44 | Test Hispanic 62 | 0.75 | 0.78 | 0.74 | 3838 | Train 60 | Test Hispanic 62 | 0.74 | 0.73 | 0.71 |
| 2815 | Train 44 | Test Hispanic 63 | 0.79 | 0.84 | 0.81 | 3839 | Train 60 | Test Hispanic 63 | 0.78 | 0.83 | 0.83 |
| 2816 | Train 44 | Test Hispanic 64 | 0.75 | 0.77 | 0.74 | 3840 | Train 60 | Test Hispanic 64 | 0.75 | 0.74 | 0.7 |
| 2817 | Train 45 | Test Hispanic 1 | 0.77 | 0.85 | 0.82 | 3841 | Train 61 | Test Hispanic 1 | 0.76 | 0.81 | 0.76 |
| 2818 | Train 45 | Test Hispanic 2 | 0.77 | 0.84 | 0.81 | 3842 | Train 61 | Test Hispanic 2 | 0.75 | 0.8 | 0.75 |
| 2819 | Train 45 | Test Hispanic 3 | 0.78 | 0.85 | 0.83 | 3843 | Train 61 | Test Hispanic 3 | 0.77 | 0.81 | 0.77 |
| 2820 | Train 45 | Test Hispanic 4 | 0.77 | 0.84 | 0.81 | 3844 | Train 61 | Test Hispanic 4 | 0.76 | 0.8 | 0.75 |
| 2821 | Train 45 | Test Hispanic 5 | 0.75 | 0.85 | 0.82 | 3845 | Train 61 | Test Hispanic 5 | 0.72 | 0.78 | 0.71 |
| 2822 | Train 45 | Test Hispanic 6 | 0.72 | 0.78 | 0.75 | 3846 | Train 61 | Test Hispanic 6 | 0.71 | 0.72 | 0.66 |
| 2823 | Train 45 | Test Hispanic 7 | 0.75 | 0.84 | 0.81 | 3847 | Train 61 | Test Hispanic 7 | 0.72 | 0.78 | 0.71 |
| 2824 | Train 45 | Test Hispanic 8 | 0.72 | 0.77 | 0.75 | 3848 | Train 61 | Test Hispanic 8 | 0.71 | 0.72 | 0.66 |
| 2825 | Train 45 | Test Hispanic 9 | 0.77 | 0.86 | 0.84 | 3849 | Train 61 | Test Hispanic 9 | 0.76 | 0.82 | 0.78 |
| 2826 | Train 45 | Test Hispanic 10 | 0.77 | 0.85 | 0.83 | 3850 | Train 61 | Test Hispanic 10 | 0.76 | 0.81 | 0.77 |
| 2827 | Train 45 | Test Hispanic 11 | 0.78 | 0.86 | 0.84 | 3851 | Train 61 | Test Hispanic 11 | 0.77 | 0.82 | 0.78 |
| 2828 | Train 45 | Test Hispanic 12 | 0.77 | 0.85 | 0.83 | 3852 | Train 61 | Test Hispanic 12 | 0.76 | 0.81 | 0.77 |
| 2829 | Train 45 | Test Hispanic 13 | 0.75 | 0.86 | 0.84 | 3853 | Train 61 | Test Hispanic 13 | 0.72 | 0.79 | 0.72 |
| 2830 | Train 45 | Test Hispanic 14 | 0.72 | 0.78 | 0.75 | 3854 | Train 61 | Test Hispanic 14 | 0.71 | 0.72 | 0.66 |
| 2831 | Train 45 | Test Hispanic 15 | 0.75 | 0.85 | 0.83 | 3855 | Train 61 | Test Hispanic 15 | 0.72 | 0.78 | 0.72 |
| 2832 | Train 45 | Test Hispanic 16 | 0.72 | 0.77 | 0.75 | 3856 | Train 61 | Test Hispanic 16 | 0.71 | 0.72 | 0.66 |
| 2833 | Train 45 | Test Hispanic 17 | 0.77 | 0.85 | 0.82 | 3857 | Train 61 | Test Hispanic 17 | 0.76 | 0.81 | 0.76 |
| 2834 | Train 45 | Test Hispanic 18 | 0.77 | 0.84 | 0.81 | 3858 | Train 61 | Test Hispanic 18 | 0.75 | 0.8 | 0.75 |
| 2835 | Train 45 | Test Hispanic 19 | 0.78 | 0.85 | 0.83 | 3859 | Train 61 | Test Hispanic 19 | 0.77 | 0.81 | 0.77 |
| 2836 | Train 45 | Test Hispanic 20 | 0.77 | 0.84 | 0.81 | 3860 | Train 61 | Test Hispanic 20 | 0.76 | 0.8 | 0.75 |
| 2837 | Train 45 | Test Hispanic 21 | 0.75 | 0.85 | 0.82 | 3861 | Train 61 | Test Hispanic 21 | 0.72 | 0.78 | 0.71 |
| 2838 | Train 45 | Test Hispanic 22 | 0.72 | 0.78 | 0.75 | 3862 | Train 61 | Test Hispanic 22 | 0.71 | 0.72 | 0.66 |
| 2839 | Train 45 | Test Hispanic 23 | 0.75 | 0.84 | 0.81 | 3863 | Train 61 | Test Hispanic 23 | 0.72 | 0.78 | 0.71 |
| 2840 | Train 45 | Test Hispanic 24 | 0.72 | 0.77 | 0.75 | 3864 | Train 61 | Test Hispanic 24 | 0.71 | 0.72 | 0.66 |
| 2841 | Train 45 | Test Hispanic 25 | 0.77 | 0.86 | 0.84 | 3865 | Train 61 | Test Hispanic 25 | 0.76 | 0.82 | 0.78 |
| 2842 | Train 45 | Test Hispanic 26 | 0.77 | 0.85 | 0.83 | 3866 | Train 61 | Test Hispanic 26 | 0.76 | 0.81 | 0.77 |
| 2843 | Train 45 | Test Hispanic 27 | 0.78 | 0.86 | 0.84 | 3867 | Train 61 | Test Hispanic 27 | 0.77 | 0.82 | 0.78 |
| 2844 | Train 45 | Test Hispanic 28 | 0.77 | 0.85 | 0.83 | 3868 | Train 61 | Test Hispanic 28 | 0.76 | 0.81 | 0.77 |
| 2845 | Train 45 | Test Hispanic 29 | 0.75 | 0.86 | 0.84 | 3869 | Train 61 | Test Hispanic 29 | 0.72 | 0.79 | 0.72 |
| 2846 | Train 45 | Test Hispanic 30 | 0.72 | 0.78 | 0.75 | 3870 | Train 61 | Test Hispanic 30 | 0.71 | 0.72 | 0.66 |
| 2847 | Train 45 | Test Hispanic 31 | 0.75 | 0.85 | 0.83 | 3871 | Train 61 | Test Hispanic 31 | 0.72 | 0.78 | 0.72 |
| 2848 | Train 45 | Test Hispanic 32 | 0.72 | 0.77 | 0.75 | 3872 | Train 61 | Test Hispanic 32 | 0.71 | 0.72 | 0.67 |
| 2849 | Train 45 | Test Hispanic 33 | 0.78 | 0.86 | 0.83 | 3873 | Train 61 | Test Hispanic 33 | 0.77 | 0.82 | 0.78 |
| 2850 | Train 45 | Test Hispanic 34 | 0.78 | 0.85 | 0.82 | 3874 | Train 61 | Test Hispanic 34 | 0.76 | 0.8 | 0.76 |
| 2851 | Train 45 | Test Hispanic 35 | 0.79 | 0.86 | 0.83 | 3875 | Train 61 | Test Hispanic 35 | 0.78 | 0.82 | 0.78 |
| 2852 | Train 45 | Test Hispanic 36 | 0.78 | 0.85 | 0.82 | 3876 | Train 61 | Test Hispanic 36 | 0.77 | 0.81 | 0.76 |
| 2853 | Train 45 | Test Hispanic 37 | 0.75 | 0.86 | 0.82 | 3877 | Train 61 | Test Hispanic 37 | 0.73 | 0.79 | 0.71 |
| 2854 | Train 45 | Test Hispanic 38 | 0.72 | 0.78 | 0.75 | 3878 | Train 61 | Test Hispanic 38 | 0.71 | 0.72 | 0.67 |
| 2855 | Train 45 | Test Hispanic 39 | 0.75 | 0.85 | 0.82 | 3879 | Train 61 | Test Hispanic 39 | 0.73 | 0.79 | 0.71 |
| 2856 | Train 45 | Test Hispanic 40 | 0.73 | 0.77 | 0.75 | 3880 | Train 61 | Test Hispanic 40 | 0.72 | 0.72 | 0.67 |
| 2857 | Train 45 | Test Hispanic 41 | 0.78 | 0.86 | 0.85 | 3881 | Train 61 | Test Hispanic 41 | 0.77 | 0.82 | 0.79 |
| 2858 | Train 45 | Test Hispanic 42 | 0.78 | 0.85 | 0.84 | 3882 | Train 61 | Test Hispanic 42 | 0.76 | 0.81 | 0.77 |
| 2859 | Train 45 | Test Hispanic 43 | 0.79 | 0.86 | 0.85 | 3883 | Train 61 | Test Hispanic 43 | 0.78 | 0.83 | 0.8 |
| 2860 | Train 45 | Test Hispanic 44 | 0.78 | 0.85 | 0.84 | 3884 | Train 61 | Test Hispanic 44 | 0.77 | 0.81 | 0.78 |
| 2861 | Train 45 | Test Hispanic 45 | 0.75 | 0.86 | 0.84 | 3885 | Train 61 | Test Hispanic 45 | 0.73 | 0.79 | 0.73 |
| 2862 | Train 45 | Test Hispanic 46 | 0.72 | 0.78 | 0.76 | 3886 | Train 61 | Test Hispanic 46 | 0.71 | 0.72 | 0.67 |
| 2863 | Train 45 | Test Hispanic 47 | 0.75 | 0.85 | 0.83 | 3887 | Train 61 | Test Hispanic 47 | 0.73 | 0.79 | 0.73 |
| 2864 | Train 45 | Test Hispanic 48 | 0.73 | 0.77 | 0.75 | 3888 | Train 61 | Test Hispanic 48 | 0.72 | 0.73 | 0.67 |
| 2865 | Train 45 | Test Hispanic 49 | 0.79 | 0.86 | 0.83 | 3889 | Train 61 | Test Hispanic 49 | 0.8 | 0.88 | 0.84 |
| 2866 | Train 45 | Test Hispanic 50 | 0.79 | 0.86 | 0.83 | 3890 | Train 61 | Test Hispanic 50 | 0.79 | 0.88 | 0.83 |
| 2867 | Train 45 | Test Hispanic 51 | 0.8 | 0.86 | 0.83 | 3891 | Train 61 | Test Hispanic 51 | 0.81 | 0.88 | 0.84 |
| 2868 | Train 45 | Test Hispanic 52 | 0.79 | 0.86 | 0.83 | 3892 | Train 61 | Test Hispanic 52 | 0.8 | 0.88 | 0.83 |
| 2869 | Train 45 | Test Hispanic 53 | 0.78 | 0.87 | 0.83 | 3893 | Train 61 | Test Hispanic 53 | 0.79 | 0.89 | 0.85 |
| 2870 | Train 45 | Test Hispanic 54 | 0.74 | 0.77 | 0.74 | 3894 | Train 61 | Test Hispanic 54 | 0.74 | 0.75 | 0.72 |
| 2871 | Train 45 | Test Hispanic 55 | 0.78 | 0.87 | 0.84 | 3895 | Train 61 | Test Hispanic 55 | 0.79 | 0.89 | 0.85 |
| 2872 | Train 45 | Test Hispanic 56 | 0.74 | 0.76 | 0.74 | 3896 | Train 61 | Test Hispanic 56 | 0.75 | 0.75 | 0.72 |
| 2873 | Train 45 | Test Hispanic 57 | 0.79 | 0.87 | 0.86 | 3897 | Train 61 | Test Hispanic 57 | 0.8 | 0.89 | 0.87 |
| 2874 | Train 45 | Test Hispanic 58 | 0.79 | 0.87 | 0.85 | 3898 | Train 61 | Test Hispanic 58 | 0.8 | 0.89 | 0.87 |
| 2875 | Train 45 | Test Hispanic 59 | 0.8 | 0.87 | 0.86 | 3899 | Train 61 | Test Hispanic 59 | 0.81 | 0.89 | 0.87 |
| 2876 | Train 45 | Test Hispanic 60 | 0.8 | 0.87 | 0.85 | 3900 | Train 61 | Test Hispanic 60 | 0.8 | 0.89 | 0.87 |
| 2877 | Train 45 | Test Hispanic 61 | 0.78 | 0.88 | 0.85 | 3901 | Train 61 | Test Hispanic 61 | 0.79 | 0.9 | 0.87 |
| 2878 | Train 45 | Test Hispanic 62 | 0.74 | 0.78 | 0.74 | 3902 | Train 61 | Test Hispanic 62 | 0.74 | 0.76 | 0.73 |
| 2879 | Train 45 | Test Hispanic 63 | 0.78 | 0.87 | 0.85 | 3903 | Train 61 | Test Hispanic 63 | 0.79 | 0.9 | 0.87 |
| 2880 | Train 45 | Test Hispanic 64 | 0.74 | 0.76 | 0.74 | 3904 | Train 61 | Test Hispanic 64 | 0.75 | 0.76 | 0.72 |
| 2881 | Train 46 | Test Hispanic 1 | 0.77 | 0.84 | 0.79 | 3905 | Train 62 | Test Hispanic 1 | 0.75 | 0.82 | 0.75 |
| 2882 | Train 46 | Test Hispanic 2 | 0.77 | 0.84 | 0.79 | 3906 | Train 62 | Test Hispanic 2 | 0.75 | 0.81 | 0.75 |
| 2883 | Train 46 | Test Hispanic 3 | 0.77 | 0.84 | 0.79 | 3907 | Train 62 | Test Hispanic 3 | 0.76 | 0.82 | 0.75 |
| 2884 | Train 46 | Test Hispanic 4 | 0.77 | 0.84 | 0.79 | 3908 | Train 62 | Test Hispanic 4 | 0.76 | 0.81 | 0.75 |
| 2885 | Train 46 | Test Hispanic 5 | 0.74 | 0.83 | 0.78 | 3909 | Train 62 | Test Hispanic 5 | 0.73 | 0.78 | 0.73 |
| 2886 | Train 46 | Test Hispanic 6 | 0.75 | 0.84 | 0.78 | 3910 | Train 62 | Test Hispanic 6 | 0.74 | 0.79 | 0.75 |
| 2887 | Train 46 | Test Hispanic 7 | 0.74 | 0.82 | 0.77 | 3911 | Train 62 | Test Hispanic 7 | 0.73 | 0.78 | 0.73 |
| 2888 | Train 46 | Test Hispanic 8 | 0.75 | 0.83 | 0.77 | 3912 | Train 62 | Test Hispanic 8 | 0.74 | 0.79 | 0.74 |
| 2889 | Train 46 | Test Hispanic 9 | 0.77 | 0.84 | 0.79 | 3913 | Train 62 | Test Hispanic 9 | 0.75 | 0.82 | 0.77 |
| 2890 | Train 46 | Test Hispanic 10 | 0.77 | 0.84 | 0.8 | 3914 | Train 62 | Test Hispanic 10 | 0.76 | 0.82 | 0.77 |
| 2891 | Train 46 | Test Hispanic 11 | 0.77 | 0.84 | 0.79 | 3915 | Train 62 | Test Hispanic 11 | 0.76 | 0.82 | 0.77 |
| 2892 | Train 46 | Test Hispanic 12 | 0.77 | 0.84 | 0.8 | 3916 | Train 62 | Test Hispanic 12 | 0.76 | 0.82 | 0.77 |
| 2893 | Train 46 | Test Hispanic 13 | 0.74 | 0.84 | 0.78 | 3917 | Train 62 | Test Hispanic 13 | 0.73 | 0.79 | 0.74 |
| 2894 | Train 46 | Test Hispanic 14 | 0.75 | 0.84 | 0.78 | 3918 | Train 62 | Test Hispanic 14 | 0.74 | 0.8 | 0.75 |
| 2895 | Train 46 | Test Hispanic 15 | 0.74 | 0.83 | 0.78 | 3919 | Train 62 | Test Hispanic 15 | 0.73 | 0.78 | 0.74 |
| 2896 | Train 46 | Test Hispanic 16 | 0.75 | 0.83 | 0.77 | 3920 | Train 62 | Test Hispanic 16 | 0.74 | 0.79 | 0.74 |
| 2897 | Train 46 | Test Hispanic 17 | 0.77 | 0.84 | 0.79 | 3921 | Train 62 | Test Hispanic 17 | 0.75 | 0.82 | 0.75 |
| 2898 | Train 46 | Test Hispanic 18 | 0.77 | 0.84 | 0.79 | 3922 | Train 62 | Test Hispanic 18 | 0.75 | 0.81 | 0.75 |
| 2899 | Train 46 | Test Hispanic 19 | 0.77 | 0.84 | 0.79 | 3923 | Train 62 | Test Hispanic 19 | 0.76 | 0.82 | 0.75 |
| 2900 | Train 46 | Test Hispanic 20 | 0.77 | 0.84 | 0.79 | 3924 | Train 62 | Test Hispanic 20 | 0.76 | 0.81 | 0.75 |
| 2901 | Train 46 | Test Hispanic 21 | 0.74 | 0.83 | 0.78 | 3925 | Train 62 | Test Hispanic 21 | 0.73 | 0.78 | 0.73 |
| 2902 | Train 46 | Test Hispanic 22 | 0.75 | 0.84 | 0.78 | 3926 | Train 62 | Test Hispanic 22 | 0.74 | 0.8 | 0.75 |
| 2903 | Train 46 | Test Hispanic 23 | 0.74 | 0.82 | 0.77 | 3927 | Train 62 | Test Hispanic 23 | 0.73 | 0.78 | 0.73 |
| 2904 | Train 46 | Test Hispanic 24 | 0.75 | 0.83 | 0.77 | 3928 | Train 62 | Test Hispanic 24 | 0.74 | 0.79 | 0.74 |
| 2905 | Train 46 | Test Hispanic 25 | 0.77 | 0.84 | 0.79 | 3929 | Train 62 | Test Hispanic 25 | 0.75 | 0.82 | 0.77 |
| 2906 | Train 46 | Test Hispanic 26 | 0.77 | 0.84 | 0.8 | 3930 | Train 62 | Test Hispanic 26 | 0.76 | 0.82 | 0.77 |
| 2907 | Train 46 | Test Hispanic 27 | 0.77 | 0.84 | 0.79 | 3931 | Train 62 | Test Hispanic 27 | 0.76 | 0.82 | 0.77 |
| 2908 | Train 46 | Test Hispanic 28 | 0.77 | 0.84 | 0.8 | 3932 | Train 62 | Test Hispanic 28 | 0.76 | 0.82 | 0.77 |
| 2909 | Train 46 | Test Hispanic 29 | 0.74 | 0.84 | 0.78 | 3933 | Train 62 | Test Hispanic 29 | 0.73 | 0.79 | 0.74 |
| 2910 | Train 46 | Test Hispanic 30 | 0.75 | 0.84 | 0.78 | 3934 | Train 62 | Test Hispanic 30 | 0.74 | 0.8 | 0.75 |
| 2911 | Train 46 | Test Hispanic 31 | 0.74 | 0.83 | 0.78 | 3935 | Train 62 | Test Hispanic 31 | 0.73 | 0.78 | 0.74 |
| 2912 | Train 46 | Test Hispanic 32 | 0.75 | 0.83 | 0.77 | 3936 | Train 62 | Test Hispanic 32 | 0.74 | 0.79 | 0.74 |
| 2913 | Train 46 | Test Hispanic 33 | 0.78 | 0.84 | 0.79 | 3937 | Train 62 | Test Hispanic 33 | 0.76 | 0.82 | 0.76 |
| 2914 | Train 46 | Test Hispanic 34 | 0.78 | 0.84 | 0.8 | 3938 | Train 62 | Test Hispanic 34 | 0.76 | 0.82 | 0.76 |
| 2915 | Train 46 | Test Hispanic 35 | 0.78 | 0.84 | 0.8 | 3939 | Train 62 | Test Hispanic 35 | 0.77 | 0.83 | 0.77 |
| 2916 | Train 46 | Test Hispanic 36 | 0.78 | 0.84 | 0.8 | 3940 | Train 62 | Test Hispanic 36 | 0.77 | 0.82 | 0.76 |
| 2917 | Train 46 | Test Hispanic 37 | 0.75 | 0.83 | 0.78 | 3941 | Train 62 | Test Hispanic 37 | 0.74 | 0.79 | 0.74 |
| 2918 | Train 46 | Test Hispanic 38 | 0.75 | 0.84 | 0.78 | 3942 | Train 62 | Test Hispanic 38 | 0.74 | 0.8 | 0.75 |
| 2919 | Train 46 | Test Hispanic 39 | 0.75 | 0.83 | 0.78 | 3943 | Train 62 | Test Hispanic 39 | 0.74 | 0.79 | 0.74 |
| 2920 | Train 46 | Test Hispanic 40 | 0.75 | 0.83 | 0.78 | 3944 | Train 62 | Test Hispanic 40 | 0.74 | 0.79 | 0.75 |
| 2921 | Train 46 | Test Hispanic 41 | 0.78 | 0.85 | 0.8 | 3945 | Train 62 | Test Hispanic 41 | 0.76 | 0.83 | 0.78 |
| 2922 | Train 46 | Test Hispanic 42 | 0.78 | 0.85 | 0.8 | 3946 | Train 62 | Test Hispanic 42 | 0.76 | 0.82 | 0.78 |
| 2923 | Train 46 | Test Hispanic 43 | 0.78 | 0.85 | 0.8 | 3947 | Train 62 | Test Hispanic 43 | 0.77 | 0.83 | 0.78 |
| 2924 | Train 46 | Test Hispanic 44 | 0.78 | 0.85 | 0.8 | 3948 | Train 62 | Test Hispanic 44 | 0.77 | 0.82 | 0.78 |
| 2925 | Train 46 | Test Hispanic 45 | 0.75 | 0.84 | 0.79 | 3949 | Train 62 | Test Hispanic 45 | 0.74 | 0.79 | 0.75 |
| 2926 | Train 46 | Test Hispanic 46 | 0.75 | 0.84 | 0.78 | 3950 | Train 62 | Test Hispanic 46 | 0.74 | 0.8 | 0.75 |
| 2927 | Train 46 | Test Hispanic 47 | 0.75 | 0.83 | 0.78 | 3951 | Train 62 | Test Hispanic 47 | 0.74 | 0.79 | 0.75 |
| 2928 | Train 46 | Test Hispanic 48 | 0.75 | 0.83 | 0.78 | 3952 | Train 62 | Test Hispanic 48 | 0.74 | 0.79 | 0.75 |
| 2929 | Train 46 | Test Hispanic 49 | 0.79 | 0.85 | 0.81 | 3953 | Train 62 | Test Hispanic 49 | 0.79 | 0.87 | 0.83 |
| 2930 | Train 46 | Test Hispanic 50 | 0.79 | 0.85 | 0.81 | 3954 | Train 62 | Test Hispanic 50 | 0.79 | 0.86 | 0.83 |
| 2931 | Train 46 | Test Hispanic 51 | 0.8 | 0.85 | 0.81 | 3955 | Train 62 | Test Hispanic 51 | 0.8 | 0.87 | 0.83 |
| 2932 | Train 46 | Test Hispanic 52 | 0.79 | 0.85 | 0.81 | 3956 | Train 62 | Test Hispanic 52 | 0.79 | 0.86 | 0.83 |
| 2933 | Train 46 | Test Hispanic 53 | 0.77 | 0.85 | 0.79 | 3957 | Train 62 | Test Hispanic 53 | 0.78 | 0.87 | 0.83 |
| 2934 | Train 46 | Test Hispanic 54 | 0.78 | 0.85 | 0.78 | 3958 | Train 62 | Test Hispanic 54 | 0.78 | 0.87 | 0.82 |
| 2935 | Train 46 | Test Hispanic 55 | 0.78 | 0.85 | 0.8 | 3959 | Train 62 | Test Hispanic 55 | 0.78 | 0.87 | 0.83 |
| 2936 | Train 46 | Test Hispanic 56 | 0.77 | 0.84 | 0.78 | 3960 | Train 62 | Test Hispanic 56 | 0.77 | 0.86 | 0.81 |
| 2937 | Train 46 | Test Hispanic 57 | 0.79 | 0.86 | 0.83 | 3961 | Train 62 | Test Hispanic 57 | 0.79 | 0.88 | 0.86 |
| 2938 | Train 46 | Test Hispanic 58 | 0.79 | 0.86 | 0.83 | 3962 | Train 62 | Test Hispanic 58 | 0.79 | 0.88 | 0.85 |
| 2939 | Train 46 | Test Hispanic 59 | 0.8 | 0.86 | 0.83 | 3963 | Train 62 | Test Hispanic 59 | 0.8 | 0.88 | 0.86 |
| 2940 | Train 46 | Test Hispanic 60 | 0.79 | 0.86 | 0.82 | 3964 | Train 62 | Test Hispanic 60 | 0.79 | 0.87 | 0.85 |
| 2941 | Train 46 | Test Hispanic 61 | 0.78 | 0.86 | 0.81 | 3965 | Train 62 | Test Hispanic 61 | 0.78 | 0.88 | 0.84 |
| 2942 | Train 46 | Test Hispanic 62 | 0.78 | 0.85 | 0.78 | 3966 | Train 62 | Test Hispanic 62 | 0.78 | 0.87 | 0.82 |
| 2943 | Train 46 | Test Hispanic 63 | 0.78 | 0.86 | 0.81 | 3967 | Train 62 | Test Hispanic 63 | 0.78 | 0.88 | 0.84 |
| 2944 | Train 46 | Test Hispanic 64 | 0.77 | 0.84 | 0.78 | 3968 | Train 62 | Test Hispanic 64 | 0.77 | 0.86 | 0.81 |
| 2945 | Train 47 | Test Hispanic 1 | 0.77 | 0.85 | 0.82 | 3969 | Train 63 | Test Hispanic 1 | 0.76 | 0.8 | 0.76 |
| 2946 | Train 47 | Test Hispanic 2 | 0.77 | 0.84 | 0.81 | 3970 | Train 63 | Test Hispanic 2 | 0.75 | 0.79 | 0.75 |
| 2947 | Train 47 | Test Hispanic 3 | 0.78 | 0.85 | 0.83 | 3971 | Train 63 | Test Hispanic 3 | 0.77 | 0.81 | 0.77 |
| 2948 | Train 47 | Test Hispanic 4 | 0.77 | 0.84 | 0.82 | 3972 | Train 63 | Test Hispanic 4 | 0.76 | 0.8 | 0.75 |
| 2949 | Train 47 | Test Hispanic 5 | 0.75 | 0.85 | 0.82 | 3973 | Train 63 | Test Hispanic 5 | 0.73 | 0.78 | 0.71 |
| 2950 | Train 47 | Test Hispanic 6 | 0.72 | 0.78 | 0.76 | 3974 | Train 63 | Test Hispanic 6 | 0.71 | 0.72 | 0.68 |
| 2951 | Train 47 | Test Hispanic 7 | 0.75 | 0.85 | 0.82 | 3975 | Train 63 | Test Hispanic 7 | 0.73 | 0.78 | 0.72 |
| 2952 | Train 47 | Test Hispanic 8 | 0.72 | 0.78 | 0.77 | 3976 | Train 63 | Test Hispanic 8 | 0.71 | 0.72 | 0.68 |
| 2953 | Train 47 | Test Hispanic 9 | 0.77 | 0.86 | 0.84 | 3977 | Train 63 | Test Hispanic 9 | 0.76 | 0.81 | 0.78 |
| 2954 | Train 47 | Test Hispanic 10 | 0.77 | 0.85 | 0.83 | 3978 | Train 63 | Test Hispanic 10 | 0.76 | 0.8 | 0.76 |
| 2955 | Train 47 | Test Hispanic 11 | 0.78 | 0.86 | 0.84 | 3979 | Train 63 | Test Hispanic 11 | 0.77 | 0.81 | 0.79 |
| 2956 | Train 47 | Test Hispanic 12 | 0.77 | 0.85 | 0.83 | 3980 | Train 63 | Test Hispanic 12 | 0.76 | 0.8 | 0.77 |
| 2957 | Train 47 | Test Hispanic 13 | 0.75 | 0.86 | 0.84 | 3981 | Train 63 | Test Hispanic 13 | 0.73 | 0.79 | 0.73 |
| 2958 | Train 47 | Test Hispanic 14 | 0.72 | 0.78 | 0.76 | 3982 | Train 63 | Test Hispanic 14 | 0.71 | 0.72 | 0.68 |
| 2959 | Train 47 | Test Hispanic 15 | 0.75 | 0.86 | 0.84 | 3983 | Train 63 | Test Hispanic 15 | 0.73 | 0.79 | 0.73 |
| 2960 | Train 47 | Test Hispanic 16 | 0.72 | 0.78 | 0.77 | 3984 | Train 63 | Test Hispanic 16 | 0.71 | 0.72 | 0.68 |
| 2961 | Train 47 | Test Hispanic 17 | 0.77 | 0.85 | 0.82 | 3985 | Train 63 | Test Hispanic 17 | 0.76 | 0.8 | 0.76 |
| 2962 | Train 47 | Test Hispanic 18 | 0.77 | 0.84 | 0.81 | 3986 | Train 63 | Test Hispanic 18 | 0.75 | 0.79 | 0.75 |
| 2963 | Train 47 | Test Hispanic 19 | 0.78 | 0.85 | 0.83 | 3987 | Train 63 | Test Hispanic 19 | 0.77 | 0.81 | 0.77 |
| 2964 | Train 47 | Test Hispanic 20 | 0.77 | 0.84 | 0.82 | 3988 | Train 63 | Test Hispanic 20 | 0.76 | 0.8 | 0.75 |
| 2965 | Train 47 | Test Hispanic 21 | 0.75 | 0.85 | 0.82 | 3989 | Train 63 | Test Hispanic 21 | 0.73 | 0.78 | 0.71 |
| 2966 | Train 47 | Test Hispanic 22 | 0.72 | 0.78 | 0.76 | 3990 | Train 63 | Test Hispanic 22 | 0.71 | 0.72 | 0.68 |
| 2967 | Train 47 | Test Hispanic 23 | 0.75 | 0.85 | 0.82 | 3991 | Train 63 | Test Hispanic 23 | 0.73 | 0.78 | 0.72 |
| 2968 | Train 47 | Test Hispanic 24 | 0.72 | 0.78 | 0.77 | 3992 | Train 63 | Test Hispanic 24 | 0.71 | 0.72 | 0.68 |
| 2969 | Train 47 | Test Hispanic 25 | 0.77 | 0.86 | 0.84 | 3993 | Train 63 | Test Hispanic 25 | 0.76 | 0.81 | 0.78 |
| 2970 | Train 47 | Test Hispanic 26 | 0.77 | 0.85 | 0.83 | 3994 | Train 63 | Test Hispanic 26 | 0.76 | 0.8 | 0.77 |
| 2971 | Train 47 | Test Hispanic 27 | 0.78 | 0.86 | 0.84 | 3995 | Train 63 | Test Hispanic 27 | 0.77 | 0.81 | 0.79 |
| 2972 | Train 47 | Test Hispanic 28 | 0.77 | 0.85 | 0.83 | 3996 | Train 63 | Test Hispanic 28 | 0.76 | 0.8 | 0.77 |
| 2973 | Train 47 | Test Hispanic 29 | 0.75 | 0.86 | 0.84 | 3997 | Train 63 | Test Hispanic 29 | 0.73 | 0.79 | 0.73 |
| 2974 | Train 47 | Test Hispanic 30 | 0.72 | 0.78 | 0.76 | 3998 | Train 63 | Test Hispanic 30 | 0.71 | 0.72 | 0.68 |
| 2975 | Train 47 | Test Hispanic 31 | 0.75 | 0.86 | 0.84 | 3999 | Train 63 | Test Hispanic 31 | 0.73 | 0.79 | 0.73 |
| 2976 | Train 47 | Test Hispanic 32 | 0.72 | 0.78 | 0.77 | 4000 | Train 63 | Test Hispanic 32 | 0.71 | 0.72 | 0.69 |
| 2977 | Train 47 | Test Hispanic 33 | 0.78 | 0.85 | 0.83 | 4001 | Train 63 | Test Hispanic 33 | 0.77 | 0.81 | 0.77 |
| 2978 | Train 47 | Test Hispanic 34 | 0.78 | 0.84 | 0.82 | 4002 | Train 63 | Test Hispanic 34 | 0.76 | 0.8 | 0.76 |
| 2979 | Train 47 | Test Hispanic 35 | 0.79 | 0.86 | 0.83 | 4003 | Train 63 | Test Hispanic 35 | 0.78 | 0.82 | 0.78 |
| 2980 | Train 47 | Test Hispanic 36 | 0.78 | 0.84 | 0.82 | 4004 | Train 63 | Test Hispanic 36 | 0.77 | 0.8 | 0.76 |
| 2981 | Train 47 | Test Hispanic 37 | 0.75 | 0.85 | 0.82 | 4005 | Train 63 | Test Hispanic 37 | 0.73 | 0.79 | 0.72 |
| 2982 | Train 47 | Test Hispanic 38 | 0.72 | 0.78 | 0.77 | 4006 | Train 63 | Test Hispanic 38 | 0.71 | 0.72 | 0.68 |
| 2983 | Train 47 | Test Hispanic 39 | 0.75 | 0.85 | 0.82 | 4007 | Train 63 | Test Hispanic 39 | 0.73 | 0.79 | 0.72 |
| 2984 | Train 47 | Test Hispanic 40 | 0.72 | 0.78 | 0.77 | 4008 | Train 63 | Test Hispanic 40 | 0.72 | 0.72 | 0.69 |
| 2985 | Train 47 | Test Hispanic 41 | 0.78 | 0.86 | 0.85 | 4009 | Train 63 | Test Hispanic 41 | 0.78 | 0.82 | 0.79 |
| 2986 | Train 47 | Test Hispanic 42 | 0.78 | 0.85 | 0.83 | 4010 | Train 63 | Test Hispanic 42 | 0.77 | 0.8 | 0.77 |
| 2987 | Train 47 | Test Hispanic 43 | 0.79 | 0.86 | 0.85 | 4011 | Train 63 | Test Hispanic 43 | 0.78 | 0.82 | 0.8 |
| 2988 | Train 47 | Test Hispanic 44 | 0.78 | 0.85 | 0.84 | 4012 | Train 63 | Test Hispanic 44 | 0.77 | 0.81 | 0.78 |
| 2989 | Train 47 | Test Hispanic 45 | 0.75 | 0.86 | 0.84 | 4013 | Train 63 | Test Hispanic 45 | 0.73 | 0.79 | 0.74 |
| 2990 | Train 47 | Test Hispanic 46 | 0.72 | 0.78 | 0.77 | 4014 | Train 63 | Test Hispanic 46 | 0.71 | 0.72 | 0.69 |
| 2991 | Train 47 | Test Hispanic 47 | 0.75 | 0.86 | 0.84 | 4015 | Train 63 | Test Hispanic 47 | 0.73 | 0.79 | 0.74 |
| 2992 | Train 47 | Test Hispanic 48 | 0.73 | 0.78 | 0.77 | 4016 | Train 63 | Test Hispanic 48 | 0.72 | 0.72 | 0.69 |
| 2993 | Train 47 | Test Hispanic 49 | 0.79 | 0.86 | 0.83 | 4017 | Train 63 | Test Hispanic 49 | 0.8 | 0.87 | 0.84 |
| 2994 | Train 47 | Test Hispanic 50 | 0.79 | 0.85 | 0.83 | 4018 | Train 63 | Test Hispanic 50 | 0.8 | 0.87 | 0.84 |
| 2995 | Train 47 | Test Hispanic 51 | 0.8 | 0.86 | 0.84 | 4019 | Train 63 | Test Hispanic 51 | 0.81 | 0.88 | 0.85 |
| 2996 | Train 47 | Test Hispanic 52 | 0.79 | 0.86 | 0.84 | 4020 | Train 63 | Test Hispanic 52 | 0.8 | 0.87 | 0.84 |
| 2997 | Train 47 | Test Hispanic 53 | 0.78 | 0.86 | 0.84 | 4021 | Train 63 | Test Hispanic 53 | 0.79 | 0.88 | 0.86 |
| 2998 | Train 47 | Test Hispanic 54 | 0.74 | 0.77 | 0.75 | 4022 | Train 63 | Test Hispanic 54 | 0.74 | 0.75 | 0.73 |
| 2999 | Train 47 | Test Hispanic 55 | 0.78 | 0.87 | 0.84 | 4023 | Train 63 | Test Hispanic 55 | 0.79 | 0.89 | 0.87 |
| 3000 | Train 47 | Test Hispanic 56 | 0.74 | 0.76 | 0.76 | 4024 | Train 63 | Test Hispanic 56 | 0.75 | 0.75 | 0.73 |
| 3001 | Train 47 | Test Hispanic 57 | 0.79 | 0.87 | 0.86 | 4025 | Train 63 | Test Hispanic 57 | 0.8 | 0.89 | 0.88 |
| 3002 | Train 47 | Test Hispanic 58 | 0.79 | 0.86 | 0.86 | 4026 | Train 63 | Test Hispanic 58 | 0.8 | 0.88 | 0.88 |
| 3003 | Train 47 | Test Hispanic 59 | 0.8 | 0.87 | 0.86 | 4027 | Train 63 | Test Hispanic 59 | 0.81 | 0.89 | 0.89 |
| 3004 | Train 47 | Test Hispanic 60 | 0.79 | 0.87 | 0.86 | 4028 | Train 63 | Test Hispanic 60 | 0.8 | 0.88 | 0.88 |
| 3005 | Train 47 | Test Hispanic 61 | 0.78 | 0.87 | 0.86 | 4029 | Train 63 | Test Hispanic 61 | 0.79 | 0.89 | 0.88 |
| 3006 | Train 47 | Test Hispanic 62 | 0.74 | 0.78 | 0.75 | 4030 | Train 63 | Test Hispanic 62 | 0.74 | 0.75 | 0.74 |
| 3007 | Train 47 | Test Hispanic 63 | 0.78 | 0.87 | 0.86 | 4031 | Train 63 | Test Hispanic 63 | 0.79 | 0.89 | 0.89 |
| 3008 | Train 47 | Test Hispanic 64 | 0.74 | 0.77 | 0.76 | 4032 | Train 63 | Test Hispanic 64 | 0.74 | 0.75 | 0.73 |
| 3009 | Train 48 | Test Hispanic 1 | 0.77 | 0.84 | 0.8 | 4033 | Train 64 | Test Hispanic 1 | 0.76 | 0.81 | 0.75 |
| 3010 | Train 48 | Test Hispanic 2 | 0.77 | 0.83 | 0.8 | 4034 | Train 64 | Test Hispanic 2 | 0.75 | 0.81 | 0.74 |
| 3011 | Train 48 | Test Hispanic 3 | 0.78 | 0.84 | 0.8 | 4035 | Train 64 | Test Hispanic 3 | 0.76 | 0.82 | 0.75 |
| 3012 | Train 48 | Test Hispanic 4 | 0.77 | 0.84 | 0.8 | 4036 | Train 64 | Test Hispanic 4 | 0.75 | 0.81 | 0.74 |
| 3013 | Train 48 | Test Hispanic 5 | 0.74 | 0.82 | 0.79 | 4037 | Train 64 | Test Hispanic 5 | 0.72 | 0.79 | 0.72 |
| 3014 | Train 48 | Test Hispanic 6 | 0.75 | 0.83 | 0.79 | 4038 | Train 64 | Test Hispanic 6 | 0.73 | 0.79 | 0.74 |
| 3015 | Train 48 | Test Hispanic 7 | 0.74 | 0.82 | 0.79 | 4039 | Train 64 | Test Hispanic 7 | 0.72 | 0.78 | 0.72 |
| 3016 | Train 48 | Test Hispanic 8 | 0.75 | 0.83 | 0.79 | 4040 | Train 64 | Test Hispanic 8 | 0.73 | 0.79 | 0.74 |
| 3017 | Train 48 | Test Hispanic 9 | 0.77 | 0.84 | 0.81 | 4041 | Train 64 | Test Hispanic 9 | 0.76 | 0.82 | 0.76 |
| 3018 | Train 48 | Test Hispanic 10 | 0.77 | 0.84 | 0.81 | 4042 | Train 64 | Test Hispanic 10 | 0.75 | 0.81 | 0.76 |
| 3019 | Train 48 | Test Hispanic 11 | 0.78 | 0.84 | 0.81 | 4043 | Train 64 | Test Hispanic 11 | 0.76 | 0.82 | 0.77 |
| 3020 | Train 48 | Test Hispanic 12 | 0.78 | 0.84 | 0.81 | 4044 | Train 64 | Test Hispanic 12 | 0.76 | 0.81 | 0.76 |
| 3021 | Train 48 | Test Hispanic 13 | 0.74 | 0.83 | 0.79 | 4045 | Train 64 | Test Hispanic 13 | 0.72 | 0.79 | 0.73 |
| 3022 | Train 48 | Test Hispanic 14 | 0.75 | 0.83 | 0.79 | 4046 | Train 64 | Test Hispanic 14 | 0.73 | 0.79 | 0.74 |
| 3023 | Train 48 | Test Hispanic 15 | 0.74 | 0.83 | 0.79 | 4047 | Train 64 | Test Hispanic 15 | 0.72 | 0.78 | 0.73 |
| 3024 | Train 48 | Test Hispanic 16 | 0.75 | 0.83 | 0.79 | 4048 | Train 64 | Test Hispanic 16 | 0.73 | 0.79 | 0.74 |
| 3025 | Train 48 | Test Hispanic 17 | 0.77 | 0.84 | 0.8 | 4049 | Train 64 | Test Hispanic 17 | 0.76 | 0.81 | 0.75 |
| 3026 | Train 48 | Test Hispanic 18 | 0.77 | 0.83 | 0.8 | 4050 | Train 64 | Test Hispanic 18 | 0.75 | 0.81 | 0.74 |
| 3027 | Train 48 | Test Hispanic 19 | 0.78 | 0.84 | 0.8 | 4051 | Train 64 | Test Hispanic 19 | 0.76 | 0.82 | 0.75 |
| 3028 | Train 48 | Test Hispanic 20 | 0.77 | 0.84 | 0.8 | 4052 | Train 64 | Test Hispanic 20 | 0.75 | 0.81 | 0.74 |
| 3029 | Train 48 | Test Hispanic 21 | 0.74 | 0.82 | 0.79 | 4053 | Train 64 | Test Hispanic 21 | 0.72 | 0.79 | 0.72 |
| 3030 | Train 48 | Test Hispanic 22 | 0.75 | 0.83 | 0.79 | 4054 | Train 64 | Test Hispanic 22 | 0.73 | 0.79 | 0.74 |
| 3031 | Train 48 | Test Hispanic 23 | 0.74 | 0.82 | 0.79 | 4055 | Train 64 | Test Hispanic 23 | 0.72 | 0.78 | 0.72 |
| 3032 | Train 48 | Test Hispanic 24 | 0.75 | 0.83 | 0.79 | 4056 | Train 64 | Test Hispanic 24 | 0.73 | 0.79 | 0.74 |
| 3033 | Train 48 | Test Hispanic 25 | 0.77 | 0.84 | 0.81 | 4057 | Train 64 | Test Hispanic 25 | 0.76 | 0.82 | 0.76 |
| 3034 | Train 48 | Test Hispanic 26 | 0.77 | 0.84 | 0.81 | 4058 | Train 64 | Test Hispanic 26 | 0.76 | 0.81 | 0.76 |
| 3035 | Train 48 | Test Hispanic 27 | 0.78 | 0.84 | 0.81 | 4059 | Train 64 | Test Hispanic 27 | 0.76 | 0.82 | 0.77 |
| 3036 | Train 48 | Test Hispanic 28 | 0.78 | 0.84 | 0.81 | 4060 | Train 64 | Test Hispanic 28 | 0.76 | 0.81 | 0.76 |
| 3037 | Train 48 | Test Hispanic 29 | 0.74 | 0.83 | 0.79 | 4061 | Train 64 | Test Hispanic 29 | 0.72 | 0.79 | 0.73 |
| 3038 | Train 48 | Test Hispanic 30 | 0.75 | 0.83 | 0.79 | 4062 | Train 64 | Test Hispanic 30 | 0.73 | 0.79 | 0.74 |
| 3039 | Train 48 | Test Hispanic 31 | 0.74 | 0.83 | 0.79 | 4063 | Train 64 | Test Hispanic 31 | 0.72 | 0.79 | 0.73 |
| 3040 | Train 48 | Test Hispanic 32 | 0.75 | 0.83 | 0.79 | 4064 | Train 64 | Test Hispanic 32 | 0.73 | 0.79 | 0.74 |
| 3041 | Train 48 | Test Hispanic 33 | 0.78 | 0.84 | 0.81 | 4065 | Train 64 | Test Hispanic 33 | 0.77 | 0.82 | 0.76 |
| 3042 | Train 48 | Test Hispanic 34 | 0.78 | 0.84 | 0.81 | 4066 | Train 64 | Test Hispanic 34 | 0.76 | 0.81 | 0.75 |
| 3043 | Train 48 | Test Hispanic 35 | 0.79 | 0.84 | 0.81 | 4067 | Train 64 | Test Hispanic 35 | 0.77 | 0.82 | 0.76 |
| 3044 | Train 48 | Test Hispanic 36 | 0.78 | 0.84 | 0.81 | 4068 | Train 64 | Test Hispanic 36 | 0.77 | 0.82 | 0.76 |
| 3045 | Train 48 | Test Hispanic 37 | 0.75 | 0.83 | 0.79 | 4069 | Train 64 | Test Hispanic 37 | 0.73 | 0.79 | 0.72 |
| 3046 | Train 48 | Test Hispanic 38 | 0.75 | 0.84 | 0.79 | 4070 | Train 64 | Test Hispanic 38 | 0.73 | 0.79 | 0.74 |
| 3047 | Train 48 | Test Hispanic 39 | 0.75 | 0.83 | 0.79 | 4071 | Train 64 | Test Hispanic 39 | 0.73 | 0.79 | 0.72 |
| 3048 | Train 48 | Test Hispanic 40 | 0.76 | 0.83 | 0.79 | 4072 | Train 64 | Test Hispanic 40 | 0.74 | 0.79 | 0.74 |
| 3049 | Train 48 | Test Hispanic 41 | 0.78 | 0.85 | 0.81 | 4073 | Train 64 | Test Hispanic 41 | 0.77 | 0.83 | 0.78 |
| 3050 | Train 48 | Test Hispanic 42 | 0.78 | 0.85 | 0.81 | 4074 | Train 64 | Test Hispanic 42 | 0.76 | 0.82 | 0.77 |
| 3051 | Train 48 | Test Hispanic 43 | 0.79 | 0.85 | 0.82 | 4075 | Train 64 | Test Hispanic 43 | 0.77 | 0.83 | 0.78 |
| 3052 | Train 48 | Test Hispanic 44 | 0.78 | 0.85 | 0.81 | 4076 | Train 64 | Test Hispanic 44 | 0.77 | 0.82 | 0.77 |
| 3053 | Train 48 | Test Hispanic 45 | 0.75 | 0.83 | 0.8 | 4077 | Train 64 | Test Hispanic 45 | 0.73 | 0.79 | 0.74 |
| 3054 | Train 48 | Test Hispanic 46 | 0.75 | 0.84 | 0.8 | 4078 | Train 64 | Test Hispanic 46 | 0.73 | 0.8 | 0.74 |
| 3055 | Train 48 | Test Hispanic 47 | 0.75 | 0.83 | 0.8 | 4079 | Train 64 | Test Hispanic 47 | 0.73 | 0.79 | 0.74 |
| 3056 | Train 48 | Test Hispanic 48 | 0.75 | 0.83 | 0.79 | 4080 | Train 64 | Test Hispanic 48 | 0.74 | 0.79 | 0.74 |
| 3057 | Train 48 | Test Hispanic 49 | 0.8 | 0.85 | 0.82 | 4081 | Train 64 | Test Hispanic 49 | 0.8 | 0.86 | 0.83 |
| 3058 | Train 48 | Test Hispanic 50 | 0.79 | 0.85 | 0.82 | 4082 | Train 64 | Test Hispanic 50 | 0.79 | 0.86 | 0.83 |
| 3059 | Train 48 | Test Hispanic 51 | 0.8 | 0.85 | 0.83 | 4083 | Train 64 | Test Hispanic 51 | 0.8 | 0.87 | 0.83 |
| 3060 | Train 48 | Test Hispanic 52 | 0.8 | 0.85 | 0.82 | 4084 | Train 64 | Test Hispanic 52 | 0.8 | 0.86 | 0.83 |
| 3061 | Train 48 | Test Hispanic 53 | 0.78 | 0.85 | 0.81 | 4085 | Train 64 | Test Hispanic 53 | 0.78 | 0.87 | 0.83 |
| 3062 | Train 48 | Test Hispanic 54 | 0.78 | 0.84 | 0.8 | 4086 | Train 64 | Test Hispanic 54 | 0.78 | 0.87 | 0.83 |
| 3063 | Train 48 | Test Hispanic 55 | 0.78 | 0.85 | 0.82 | 4087 | Train 64 | Test Hispanic 55 | 0.78 | 0.87 | 0.84 |
| 3064 | Train 48 | Test Hispanic 56 | 0.78 | 0.84 | 0.8 | 4088 | Train 64 | Test Hispanic 56 | 0.78 | 0.86 | 0.83 |
| 3065 | Train 48 | Test Hispanic 57 | 0.8 | 0.86 | 0.83 | 4089 | Train 64 | Test Hispanic 57 | 0.8 | 0.88 | 0.86 |
| 3066 | Train 48 | Test Hispanic 58 | 0.79 | 0.86 | 0.83 | 4090 | Train 64 | Test Hispanic 58 | 0.8 | 0.87 | 0.85 |
| 3067 | Train 48 | Test Hispanic 59 | 0.8 | 0.86 | 0.84 | 4091 | Train 64 | Test Hispanic 59 | 0.81 | 0.88 | 0.86 |
| 3068 | Train 48 | Test Hispanic 60 | 0.8 | 0.86 | 0.83 | 4092 | Train 64 | Test Hispanic 60 | 0.8 | 0.87 | 0.85 |
| 3069 | Train 48 | Test Hispanic 61 | 0.78 | 0.86 | 0.82 | 4093 | Train 64 | Test Hispanic 61 | 0.78 | 0.88 | 0.85 |
| 3070 | Train 48 | Test Hispanic 62 | 0.78 | 0.84 | 0.8 | 4094 | Train 64 | Test Hispanic 62 | 0.78 | 0.87 | 0.83 |
| 3071 | Train 48 | Test Hispanic 63 | 0.78 | 0.86 | 0.82 | 4095 | Train 64 | Test Hispanic 63 | 0.78 | 0.88 | 0.85 |
| 3072 | Train 48 | Test Hispanic 64 | 0.78 | 0.84 | 0.8 | 4096 | Train 64 | Test Hispanic 64 | 0.78 | 0.86 | 0.83 |

Table 13. AUC Performance of the ML Models— LR, RF, and GB —for Non-Hispanic Patients Trained on Cohorts 1–32 and Tested Across 64 Cohorts.

| Not Hispanic | Train  Model | Test  Dataset | AUC (LR) | AUC (RF) | AUC (GB) | Not Hispanic | Train  Model | Test  Dataset | AUC (LR) | AUC (RF) | AUC (GB) |
| --- | --- | --- | --- | --- | --- | --- | --- | --- | --- | --- | --- |
| 1 | Train 1 | Test Not Hispanic 1 | 0.75 | 0.83 | 0.85 | 1025 | Train 17 | Test Not Hispanic 1 | 0.75 | 0.83 | 0.85 |
| 2 | Train 1 | Test Not Hispanic 2 | 0.74 | 0.78 | 0.79 | 1026 | Train 17 | Test Not Hispanic 2 | 0.74 | 0.78 | 0.79 |
| 3 | Train 1 | Test Not Hispanic 3 | 0.75 | 0.83 | 0.85 | 1027 | Train 17 | Test Not Hispanic 3 | 0.75 | 0.83 | 0.85 |
| 4 | Train 1 | Test Not Hispanic 4 | 0.74 | 0.78 | 0.79 | 1028 | Train 17 | Test Not Hispanic 4 | 0.74 | 0.78 | 0.79 |
| 5 | Train 1 | Test Not Hispanic 5 | 0.71 | 0.74 | 0.68 | 1029 | Train 17 | Test Not Hispanic 5 | 0.71 | 0.74 | 0.71 |
| 6 | Train 1 | Test Not Hispanic 6 | 0.69 | 0.7 | 0.64 | 1030 | Train 17 | Test Not Hispanic 6 | 0.69 | 0.7 | 0.65 |
| 7 | Train 1 | Test Not Hispanic 7 | 0.71 | 0.73 | 0.68 | 1031 | Train 17 | Test Not Hispanic 7 | 0.71 | 0.73 | 0.71 |
| 8 | Train 1 | Test Not Hispanic 8 | 0.7 | 0.69 | 0.64 | 1032 | Train 17 | Test Not Hispanic 8 | 0.69 | 0.7 | 0.65 |
| 9 | Train 1 | Test Not Hispanic 9 | 0.75 | 0.82 | 0.81 | 1033 | Train 17 | Test Not Hispanic 9 | 0.75 | 0.82 | 0.81 |
| 10 | Train 1 | Test Not Hispanic 10 | 0.74 | 0.77 | 0.77 | 1034 | Train 17 | Test Not Hispanic 10 | 0.74 | 0.77 | 0.77 |
| 11 | Train 1 | Test Not Hispanic 11 | 0.75 | 0.82 | 0.81 | 1035 | Train 17 | Test Not Hispanic 11 | 0.75 | 0.82 | 0.81 |
| 12 | Train 1 | Test Not Hispanic 12 | 0.74 | 0.77 | 0.77 | 1036 | Train 17 | Test Not Hispanic 12 | 0.74 | 0.77 | 0.77 |
| 13 | Train 1 | Test Not Hispanic 13 | 0.71 | 0.73 | 0.67 | 1037 | Train 17 | Test Not Hispanic 13 | 0.71 | 0.73 | 0.7 |
| 14 | Train 1 | Test Not Hispanic 14 | 0.7 | 0.7 | 0.64 | 1038 | Train 17 | Test Not Hispanic 14 | 0.7 | 0.7 | 0.66 |
| 15 | Train 1 | Test Not Hispanic 15 | 0.71 | 0.73 | 0.66 | 1039 | Train 17 | Test Not Hispanic 15 | 0.71 | 0.73 | 0.7 |
| 16 | Train 1 | Test Not Hispanic 16 | 0.7 | 0.7 | 0.64 | 1040 | Train 17 | Test Not Hispanic 16 | 0.7 | 0.7 | 0.65 |
| 17 | Train 1 | Test Not Hispanic 17 | 0.75 | 0.83 | 0.85 | 1041 | Train 17 | Test Not Hispanic 17 | 0.75 | 0.83 | 0.85 |
| 18 | Train 1 | Test Not Hispanic 18 | 0.74 | 0.78 | 0.79 | 1042 | Train 17 | Test Not Hispanic 18 | 0.74 | 0.78 | 0.79 |
| 19 | Train 1 | Test Not Hispanic 19 | 0.75 | 0.83 | 0.85 | 1043 | Train 17 | Test Not Hispanic 19 | 0.75 | 0.83 | 0.85 |
| 20 | Train 1 | Test Not Hispanic 20 | 0.74 | 0.78 | 0.79 | 1044 | Train 17 | Test Not Hispanic 20 | 0.74 | 0.78 | 0.79 |
| 21 | Train 1 | Test Not Hispanic 21 | 0.71 | 0.74 | 0.68 | 1045 | Train 17 | Test Not Hispanic 21 | 0.71 | 0.74 | 0.71 |
| 22 | Train 1 | Test Not Hispanic 22 | 0.7 | 0.7 | 0.65 | 1046 | Train 17 | Test Not Hispanic 22 | 0.69 | 0.7 | 0.66 |
| 23 | Train 1 | Test Not Hispanic 23 | 0.71 | 0.73 | 0.68 | 1047 | Train 17 | Test Not Hispanic 23 | 0.71 | 0.73 | 0.71 |
| 24 | Train 1 | Test Not Hispanic 24 | 0.7 | 0.69 | 0.64 | 1048 | Train 17 | Test Not Hispanic 24 | 0.69 | 0.7 | 0.65 |
| 25 | Train 1 | Test Not Hispanic 25 | 0.75 | 0.82 | 0.81 | 1049 | Train 17 | Test Not Hispanic 25 | 0.75 | 0.82 | 0.81 |
| 26 | Train 1 | Test Not Hispanic 26 | 0.74 | 0.77 | 0.77 | 1050 | Train 17 | Test Not Hispanic 26 | 0.74 | 0.77 | 0.77 |
| 27 | Train 1 | Test Not Hispanic 27 | 0.75 | 0.82 | 0.81 | 1051 | Train 17 | Test Not Hispanic 27 | 0.75 | 0.82 | 0.81 |
| 28 | Train 1 | Test Not Hispanic 28 | 0.74 | 0.77 | 0.77 | 1052 | Train 17 | Test Not Hispanic 28 | 0.74 | 0.77 | 0.77 |
| 29 | Train 1 | Test Not Hispanic 29 | 0.71 | 0.73 | 0.67 | 1053 | Train 17 | Test Not Hispanic 29 | 0.71 | 0.73 | 0.7 |
| 30 | Train 1 | Test Not Hispanic 30 | 0.7 | 0.7 | 0.65 | 1054 | Train 17 | Test Not Hispanic 30 | 0.7 | 0.7 | 0.66 |
| 31 | Train 1 | Test Not Hispanic 31 | 0.71 | 0.73 | 0.67 | 1055 | Train 17 | Test Not Hispanic 31 | 0.71 | 0.73 | 0.7 |
| 32 | Train 1 | Test Not Hispanic 32 | 0.7 | 0.7 | 0.64 | 1056 | Train 17 | Test Not Hispanic 32 | 0.7 | 0.7 | 0.65 |
| 33 | Train 1 | Test Not Hispanic 33 | 0.76 | 0.83 | 0.85 | 1057 | Train 17 | Test Not Hispanic 33 | 0.76 | 0.83 | 0.85 |
| 34 | Train 1 | Test Not Hispanic 34 | 0.74 | 0.78 | 0.79 | 1058 | Train 17 | Test Not Hispanic 34 | 0.74 | 0.78 | 0.79 |
| 35 | Train 1 | Test Not Hispanic 35 | 0.76 | 0.83 | 0.85 | 1059 | Train 17 | Test Not Hispanic 35 | 0.76 | 0.83 | 0.85 |
| 36 | Train 1 | Test Not Hispanic 36 | 0.74 | 0.78 | 0.79 | 1060 | Train 17 | Test Not Hispanic 36 | 0.74 | 0.78 | 0.79 |
| 37 | Train 1 | Test Not Hispanic 37 | 0.71 | 0.74 | 0.68 | 1061 | Train 17 | Test Not Hispanic 37 | 0.71 | 0.74 | 0.71 |
| 38 | Train 1 | Test Not Hispanic 38 | 0.69 | 0.7 | 0.65 | 1062 | Train 17 | Test Not Hispanic 38 | 0.69 | 0.7 | 0.65 |
| 39 | Train 1 | Test Not Hispanic 39 | 0.71 | 0.73 | 0.68 | 1063 | Train 17 | Test Not Hispanic 39 | 0.71 | 0.74 | 0.71 |
| 40 | Train 1 | Test Not Hispanic 40 | 0.7 | 0.69 | 0.64 | 1064 | Train 17 | Test Not Hispanic 40 | 0.69 | 0.7 | 0.65 |
| 41 | Train 1 | Test Not Hispanic 41 | 0.76 | 0.82 | 0.81 | 1065 | Train 17 | Test Not Hispanic 41 | 0.76 | 0.82 | 0.81 |
| 42 | Train 1 | Test Not Hispanic 42 | 0.74 | 0.77 | 0.77 | 1066 | Train 17 | Test Not Hispanic 42 | 0.74 | 0.77 | 0.77 |
| 43 | Train 1 | Test Not Hispanic 43 | 0.76 | 0.82 | 0.81 | 1067 | Train 17 | Test Not Hispanic 43 | 0.76 | 0.82 | 0.81 |
| 44 | Train 1 | Test Not Hispanic 44 | 0.74 | 0.77 | 0.77 | 1068 | Train 17 | Test Not Hispanic 44 | 0.74 | 0.77 | 0.77 |
| 45 | Train 1 | Test Not Hispanic 45 | 0.71 | 0.73 | 0.67 | 1069 | Train 17 | Test Not Hispanic 45 | 0.71 | 0.73 | 0.7 |
| 46 | Train 1 | Test Not Hispanic 46 | 0.7 | 0.7 | 0.64 | 1070 | Train 17 | Test Not Hispanic 46 | 0.7 | 0.7 | 0.66 |
| 47 | Train 1 | Test Not Hispanic 47 | 0.71 | 0.73 | 0.67 | 1071 | Train 17 | Test Not Hispanic 47 | 0.71 | 0.73 | 0.7 |
| 48 | Train 1 | Test Not Hispanic 48 | 0.7 | 0.7 | 0.64 | 1072 | Train 17 | Test Not Hispanic 48 | 0.7 | 0.7 | 0.65 |
| 49 | Train 1 | Test Not Hispanic 49 | 0.76 | 0.79 | 0.83 | 1073 | Train 17 | Test Not Hispanic 49 | 0.76 | 0.8 | 0.82 |
| 50 | Train 1 | Test Not Hispanic 50 | 0.76 | 0.79 | 0.83 | 1074 | Train 17 | Test Not Hispanic 50 | 0.76 | 0.8 | 0.82 |
| 51 | Train 1 | Test Not Hispanic 51 | 0.76 | 0.79 | 0.83 | 1075 | Train 17 | Test Not Hispanic 51 | 0.76 | 0.79 | 0.82 |
| 52 | Train 1 | Test Not Hispanic 52 | 0.76 | 0.79 | 0.83 | 1076 | Train 17 | Test Not Hispanic 52 | 0.76 | 0.79 | 0.82 |
| 53 | Train 1 | Test Not Hispanic 53 | 0.72 | 0.75 | 0.64 | 1077 | Train 17 | Test Not Hispanic 53 | 0.72 | 0.75 | 0.68 |
| 54 | Train 1 | Test Not Hispanic 54 | 0.71 | 0.71 | 0.65 | 1078 | Train 17 | Test Not Hispanic 54 | 0.71 | 0.71 | 0.67 |
| 55 | Train 1 | Test Not Hispanic 55 | 0.72 | 0.75 | 0.64 | 1079 | Train 17 | Test Not Hispanic 55 | 0.72 | 0.75 | 0.68 |
| 56 | Train 1 | Test Not Hispanic 56 | 0.71 | 0.7 | 0.64 | 1080 | Train 17 | Test Not Hispanic 56 | 0.71 | 0.7 | 0.66 |
| 57 | Train 1 | Test Not Hispanic 57 | 0.76 | 0.79 | 0.8 | 1081 | Train 17 | Test Not Hispanic 57 | 0.76 | 0.79 | 0.79 |
| 58 | Train 1 | Test Not Hispanic 58 | 0.76 | 0.79 | 0.8 | 1082 | Train 17 | Test Not Hispanic 58 | 0.76 | 0.79 | 0.79 |
| 59 | Train 1 | Test Not Hispanic 59 | 0.76 | 0.79 | 0.79 | 1083 | Train 17 | Test Not Hispanic 59 | 0.76 | 0.79 | 0.79 |
| 60 | Train 1 | Test Not Hispanic 60 | 0.76 | 0.79 | 0.79 | 1084 | Train 17 | Test Not Hispanic 60 | 0.76 | 0.79 | 0.79 |
| 61 | Train 1 | Test Not Hispanic 61 | 0.72 | 0.75 | 0.63 | 1085 | Train 17 | Test Not Hispanic 61 | 0.72 | 0.75 | 0.68 |
| 62 | Train 1 | Test Not Hispanic 62 | 0.71 | 0.71 | 0.65 | 1086 | Train 17 | Test Not Hispanic 62 | 0.71 | 0.71 | 0.67 |
| 63 | Train 1 | Test Not Hispanic 63 | 0.72 | 0.75 | 0.63 | 1087 | Train 17 | Test Not Hispanic 63 | 0.72 | 0.75 | 0.68 |
| 64 | Train 1 | Test Not Hispanic 64 | 0.71 | 0.7 | 0.65 | 1088 | Train 17 | Test Not Hispanic 64 | 0.71 | 0.7 | 0.66 |
| 65 | Train 2 | Test Not Hispanic 1 | 0.75 | 0.82 | 0.84 | 1089 | Train 18 | Test Not Hispanic 1 | 0.75 | 0.82 | 0.84 |
| 66 | Train 2 | Test Not Hispanic 2 | 0.76 | 0.83 | 0.85 | 1090 | Train 18 | Test Not Hispanic 2 | 0.76 | 0.83 | 0.85 |
| 67 | Train 2 | Test Not Hispanic 3 | 0.75 | 0.82 | 0.84 | 1091 | Train 18 | Test Not Hispanic 3 | 0.75 | 0.82 | 0.84 |
| 68 | Train 2 | Test Not Hispanic 4 | 0.76 | 0.83 | 0.84 | 1092 | Train 18 | Test Not Hispanic 4 | 0.76 | 0.83 | 0.85 |
| 69 | Train 2 | Test Not Hispanic 5 | 0.71 | 0.74 | 0.69 | 1093 | Train 18 | Test Not Hispanic 5 | 0.71 | 0.73 | 0.7 |
| 70 | Train 2 | Test Not Hispanic 6 | 0.71 | 0.71 | 0.67 | 1094 | Train 18 | Test Not Hispanic 6 | 0.71 | 0.71 | 0.67 |
| 71 | Train 2 | Test Not Hispanic 7 | 0.71 | 0.73 | 0.69 | 1095 | Train 18 | Test Not Hispanic 7 | 0.71 | 0.73 | 0.7 |
| 72 | Train 2 | Test Not Hispanic 8 | 0.71 | 0.71 | 0.66 | 1096 | Train 18 | Test Not Hispanic 8 | 0.71 | 0.71 | 0.67 |
| 73 | Train 2 | Test Not Hispanic 9 | 0.75 | 0.81 | 0.81 | 1097 | Train 18 | Test Not Hispanic 9 | 0.75 | 0.81 | 0.8 |
| 74 | Train 2 | Test Not Hispanic 10 | 0.76 | 0.82 | 0.82 | 1098 | Train 18 | Test Not Hispanic 10 | 0.76 | 0.82 | 0.82 |
| 75 | Train 2 | Test Not Hispanic 11 | 0.75 | 0.81 | 0.81 | 1099 | Train 18 | Test Not Hispanic 11 | 0.75 | 0.81 | 0.8 |
| 76 | Train 2 | Test Not Hispanic 12 | 0.76 | 0.82 | 0.82 | 1100 | Train 18 | Test Not Hispanic 12 | 0.76 | 0.82 | 0.82 |
| 77 | Train 2 | Test Not Hispanic 13 | 0.71 | 0.73 | 0.68 | 1101 | Train 18 | Test Not Hispanic 13 | 0.71 | 0.73 | 0.69 |
| 78 | Train 2 | Test Not Hispanic 14 | 0.71 | 0.71 | 0.67 | 1102 | Train 18 | Test Not Hispanic 14 | 0.71 | 0.71 | 0.67 |
| 79 | Train 2 | Test Not Hispanic 15 | 0.71 | 0.73 | 0.67 | 1103 | Train 18 | Test Not Hispanic 15 | 0.71 | 0.73 | 0.69 |
| 80 | Train 2 | Test Not Hispanic 16 | 0.71 | 0.71 | 0.66 | 1104 | Train 18 | Test Not Hispanic 16 | 0.71 | 0.71 | 0.67 |
| 81 | Train 2 | Test Not Hispanic 17 | 0.75 | 0.82 | 0.84 | 1105 | Train 18 | Test Not Hispanic 17 | 0.75 | 0.82 | 0.84 |
| 82 | Train 2 | Test Not Hispanic 18 | 0.76 | 0.83 | 0.85 | 1106 | Train 18 | Test Not Hispanic 18 | 0.76 | 0.83 | 0.85 |
| 83 | Train 2 | Test Not Hispanic 19 | 0.75 | 0.82 | 0.84 | 1107 | Train 18 | Test Not Hispanic 19 | 0.75 | 0.82 | 0.84 |
| 84 | Train 2 | Test Not Hispanic 20 | 0.76 | 0.83 | 0.84 | 1108 | Train 18 | Test Not Hispanic 20 | 0.76 | 0.83 | 0.85 |
| 85 | Train 2 | Test Not Hispanic 21 | 0.71 | 0.74 | 0.69 | 1109 | Train 18 | Test Not Hispanic 21 | 0.71 | 0.74 | 0.7 |
| 86 | Train 2 | Test Not Hispanic 22 | 0.71 | 0.71 | 0.67 | 1110 | Train 18 | Test Not Hispanic 22 | 0.71 | 0.71 | 0.67 |
| 87 | Train 2 | Test Not Hispanic 23 | 0.71 | 0.73 | 0.69 | 1111 | Train 18 | Test Not Hispanic 23 | 0.71 | 0.73 | 0.7 |
| 88 | Train 2 | Test Not Hispanic 24 | 0.71 | 0.71 | 0.66 | 1112 | Train 18 | Test Not Hispanic 24 | 0.71 | 0.71 | 0.67 |
| 89 | Train 2 | Test Not Hispanic 25 | 0.75 | 0.81 | 0.81 | 1113 | Train 18 | Test Not Hispanic 25 | 0.75 | 0.81 | 0.8 |
| 90 | Train 2 | Test Not Hispanic 26 | 0.76 | 0.82 | 0.82 | 1114 | Train 18 | Test Not Hispanic 26 | 0.76 | 0.82 | 0.82 |
| 91 | Train 2 | Test Not Hispanic 27 | 0.75 | 0.81 | 0.81 | 1115 | Train 18 | Test Not Hispanic 27 | 0.75 | 0.81 | 0.8 |
| 92 | Train 2 | Test Not Hispanic 28 | 0.76 | 0.82 | 0.82 | 1116 | Train 18 | Test Not Hispanic 28 | 0.76 | 0.82 | 0.82 |
| 93 | Train 2 | Test Not Hispanic 29 | 0.71 | 0.73 | 0.68 | 1117 | Train 18 | Test Not Hispanic 29 | 0.71 | 0.73 | 0.69 |
| 94 | Train 2 | Test Not Hispanic 30 | 0.71 | 0.71 | 0.67 | 1118 | Train 18 | Test Not Hispanic 30 | 0.71 | 0.71 | 0.67 |
| 95 | Train 2 | Test Not Hispanic 31 | 0.71 | 0.73 | 0.67 | 1119 | Train 18 | Test Not Hispanic 31 | 0.71 | 0.73 | 0.69 |
| 96 | Train 2 | Test Not Hispanic 32 | 0.71 | 0.71 | 0.66 | 1120 | Train 18 | Test Not Hispanic 32 | 0.71 | 0.71 | 0.67 |
| 97 | Train 2 | Test Not Hispanic 33 | 0.75 | 0.82 | 0.84 | 1121 | Train 18 | Test Not Hispanic 33 | 0.75 | 0.82 | 0.84 |
| 98 | Train 2 | Test Not Hispanic 34 | 0.76 | 0.83 | 0.84 | 1122 | Train 18 | Test Not Hispanic 34 | 0.76 | 0.83 | 0.85 |
| 99 | Train 2 | Test Not Hispanic 35 | 0.75 | 0.82 | 0.84 | 1123 | Train 18 | Test Not Hispanic 35 | 0.75 | 0.82 | 0.83 |
| 100 | Train 2 | Test Not Hispanic 36 | 0.76 | 0.83 | 0.84 | 1124 | Train 18 | Test Not Hispanic 36 | 0.76 | 0.83 | 0.85 |
| 101 | Train 2 | Test Not Hispanic 37 | 0.71 | 0.74 | 0.69 | 1125 | Train 18 | Test Not Hispanic 37 | 0.71 | 0.74 | 0.7 |
| 102 | Train 2 | Test Not Hispanic 38 | 0.71 | 0.71 | 0.67 | 1126 | Train 18 | Test Not Hispanic 38 | 0.71 | 0.71 | 0.67 |
| 103 | Train 2 | Test Not Hispanic 39 | 0.71 | 0.73 | 0.69 | 1127 | Train 18 | Test Not Hispanic 39 | 0.71 | 0.73 | 0.7 |
| 104 | Train 2 | Test Not Hispanic 40 | 0.71 | 0.71 | 0.66 | 1128 | Train 18 | Test Not Hispanic 40 | 0.71 | 0.71 | 0.67 |
| 105 | Train 2 | Test Not Hispanic 41 | 0.75 | 0.81 | 0.81 | 1129 | Train 18 | Test Not Hispanic 41 | 0.75 | 0.81 | 0.8 |
| 106 | Train 2 | Test Not Hispanic 42 | 0.76 | 0.82 | 0.82 | 1130 | Train 18 | Test Not Hispanic 42 | 0.76 | 0.82 | 0.82 |
| 107 | Train 2 | Test Not Hispanic 43 | 0.75 | 0.81 | 0.81 | 1131 | Train 18 | Test Not Hispanic 43 | 0.75 | 0.81 | 0.8 |
| 108 | Train 2 | Test Not Hispanic 44 | 0.76 | 0.82 | 0.82 | 1132 | Train 18 | Test Not Hispanic 44 | 0.76 | 0.82 | 0.82 |
| 109 | Train 2 | Test Not Hispanic 45 | 0.71 | 0.73 | 0.68 | 1133 | Train 18 | Test Not Hispanic 45 | 0.71 | 0.73 | 0.69 |
| 110 | Train 2 | Test Not Hispanic 46 | 0.71 | 0.71 | 0.67 | 1134 | Train 18 | Test Not Hispanic 46 | 0.71 | 0.71 | 0.67 |
| 111 | Train 2 | Test Not Hispanic 47 | 0.71 | 0.73 | 0.67 | 1135 | Train 18 | Test Not Hispanic 47 | 0.71 | 0.73 | 0.69 |
| 112 | Train 2 | Test Not Hispanic 48 | 0.71 | 0.71 | 0.66 | 1136 | Train 18 | Test Not Hispanic 48 | 0.71 | 0.71 | 0.67 |
| 113 | Train 2 | Test Not Hispanic 49 | 0.76 | 0.79 | 0.81 | 1137 | Train 18 | Test Not Hispanic 49 | 0.76 | 0.79 | 0.81 |
| 114 | Train 2 | Test Not Hispanic 50 | 0.76 | 0.79 | 0.81 | 1138 | Train 18 | Test Not Hispanic 50 | 0.76 | 0.79 | 0.81 |
| 115 | Train 2 | Test Not Hispanic 51 | 0.76 | 0.79 | 0.81 | 1139 | Train 18 | Test Not Hispanic 51 | 0.76 | 0.79 | 0.81 |
| 116 | Train 2 | Test Not Hispanic 52 | 0.76 | 0.79 | 0.81 | 1140 | Train 18 | Test Not Hispanic 52 | 0.76 | 0.79 | 0.81 |
| 117 | Train 2 | Test Not Hispanic 53 | 0.72 | 0.76 | 0.66 | 1141 | Train 18 | Test Not Hispanic 53 | 0.72 | 0.76 | 0.64 |
| 118 | Train 2 | Test Not Hispanic 54 | 0.71 | 0.72 | 0.67 | 1142 | Train 18 | Test Not Hispanic 54 | 0.71 | 0.71 | 0.67 |
| 119 | Train 2 | Test Not Hispanic 55 | 0.72 | 0.76 | 0.66 | 1143 | Train 18 | Test Not Hispanic 55 | 0.73 | 0.76 | 0.64 |
| 120 | Train 2 | Test Not Hispanic 56 | 0.71 | 0.71 | 0.66 | 1144 | Train 18 | Test Not Hispanic 56 | 0.71 | 0.71 | 0.66 |
| 121 | Train 2 | Test Not Hispanic 57 | 0.76 | 0.78 | 0.78 | 1145 | Train 18 | Test Not Hispanic 57 | 0.76 | 0.78 | 0.78 |
| 122 | Train 2 | Test Not Hispanic 58 | 0.76 | 0.79 | 0.78 | 1146 | Train 18 | Test Not Hispanic 58 | 0.76 | 0.78 | 0.78 |
| 123 | Train 2 | Test Not Hispanic 59 | 0.76 | 0.78 | 0.78 | 1147 | Train 18 | Test Not Hispanic 59 | 0.76 | 0.78 | 0.78 |
| 124 | Train 2 | Test Not Hispanic 60 | 0.76 | 0.78 | 0.78 | 1148 | Train 18 | Test Not Hispanic 60 | 0.76 | 0.78 | 0.78 |
| 125 | Train 2 | Test Not Hispanic 61 | 0.72 | 0.76 | 0.66 | 1149 | Train 18 | Test Not Hispanic 61 | 0.72 | 0.75 | 0.63 |
| 126 | Train 2 | Test Not Hispanic 62 | 0.71 | 0.72 | 0.67 | 1150 | Train 18 | Test Not Hispanic 62 | 0.72 | 0.71 | 0.67 |
| 127 | Train 2 | Test Not Hispanic 63 | 0.73 | 0.76 | 0.66 | 1151 | Train 18 | Test Not Hispanic 63 | 0.73 | 0.75 | 0.63 |
| 128 | Train 2 | Test Not Hispanic 64 | 0.71 | 0.71 | 0.66 | 1152 | Train 18 | Test Not Hispanic 64 | 0.71 | 0.71 | 0.66 |
| 129 | Train 3 | Test Not Hispanic 1 | 0.75 | 0.83 | 0.85 | 1153 | Train 19 | Test Not Hispanic 1 | 0.75 | 0.83 | 0.85 |
| 130 | Train 3 | Test Not Hispanic 2 | 0.74 | 0.78 | 0.79 | 1154 | Train 19 | Test Not Hispanic 2 | 0.74 | 0.78 | 0.79 |
| 131 | Train 3 | Test Not Hispanic 3 | 0.75 | 0.83 | 0.85 | 1155 | Train 19 | Test Not Hispanic 3 | 0.75 | 0.83 | 0.85 |
| 132 | Train 3 | Test Not Hispanic 4 | 0.74 | 0.78 | 0.79 | 1156 | Train 19 | Test Not Hispanic 4 | 0.74 | 0.78 | 0.79 |
| 133 | Train 3 | Test Not Hispanic 5 | 0.71 | 0.73 | 0.69 | 1157 | Train 19 | Test Not Hispanic 5 | 0.71 | 0.74 | 0.68 |
| 134 | Train 3 | Test Not Hispanic 6 | 0.69 | 0.69 | 0.64 | 1158 | Train 19 | Test Not Hispanic 6 | 0.69 | 0.7 | 0.64 |
| 135 | Train 3 | Test Not Hispanic 7 | 0.71 | 0.73 | 0.69 | 1159 | Train 19 | Test Not Hispanic 7 | 0.71 | 0.73 | 0.68 |
| 136 | Train 3 | Test Not Hispanic 8 | 0.69 | 0.69 | 0.64 | 1160 | Train 19 | Test Not Hispanic 8 | 0.69 | 0.69 | 0.64 |
| 137 | Train 3 | Test Not Hispanic 9 | 0.75 | 0.82 | 0.81 | 1161 | Train 19 | Test Not Hispanic 9 | 0.75 | 0.82 | 0.81 |
| 138 | Train 3 | Test Not Hispanic 10 | 0.74 | 0.77 | 0.76 | 1162 | Train 19 | Test Not Hispanic 10 | 0.74 | 0.77 | 0.77 |
| 139 | Train 3 | Test Not Hispanic 11 | 0.75 | 0.82 | 0.81 | 1163 | Train 19 | Test Not Hispanic 11 | 0.75 | 0.82 | 0.81 |
| 140 | Train 3 | Test Not Hispanic 12 | 0.74 | 0.77 | 0.76 | 1164 | Train 19 | Test Not Hispanic 12 | 0.74 | 0.77 | 0.77 |
| 141 | Train 3 | Test Not Hispanic 13 | 0.71 | 0.73 | 0.68 | 1165 | Train 19 | Test Not Hispanic 13 | 0.71 | 0.73 | 0.67 |
| 142 | Train 3 | Test Not Hispanic 14 | 0.7 | 0.7 | 0.64 | 1166 | Train 19 | Test Not Hispanic 14 | 0.69 | 0.7 | 0.64 |
| 143 | Train 3 | Test Not Hispanic 15 | 0.71 | 0.73 | 0.68 | 1167 | Train 19 | Test Not Hispanic 15 | 0.71 | 0.73 | 0.67 |
| 144 | Train 3 | Test Not Hispanic 16 | 0.7 | 0.69 | 0.64 | 1168 | Train 19 | Test Not Hispanic 16 | 0.7 | 0.7 | 0.64 |
| 145 | Train 3 | Test Not Hispanic 17 | 0.75 | 0.83 | 0.85 | 1169 | Train 19 | Test Not Hispanic 17 | 0.75 | 0.83 | 0.85 |
| 146 | Train 3 | Test Not Hispanic 18 | 0.74 | 0.78 | 0.79 | 1170 | Train 19 | Test Not Hispanic 18 | 0.74 | 0.78 | 0.79 |
| 147 | Train 3 | Test Not Hispanic 19 | 0.75 | 0.83 | 0.85 | 1171 | Train 19 | Test Not Hispanic 19 | 0.75 | 0.83 | 0.85 |
| 148 | Train 3 | Test Not Hispanic 20 | 0.74 | 0.78 | 0.79 | 1172 | Train 19 | Test Not Hispanic 20 | 0.74 | 0.78 | 0.79 |
| 149 | Train 3 | Test Not Hispanic 21 | 0.71 | 0.74 | 0.69 | 1173 | Train 19 | Test Not Hispanic 21 | 0.71 | 0.74 | 0.68 |
| 150 | Train 3 | Test Not Hispanic 22 | 0.69 | 0.7 | 0.64 | 1174 | Train 19 | Test Not Hispanic 22 | 0.69 | 0.7 | 0.64 |
| 151 | Train 3 | Test Not Hispanic 23 | 0.71 | 0.73 | 0.69 | 1175 | Train 19 | Test Not Hispanic 23 | 0.71 | 0.73 | 0.68 |
| 152 | Train 3 | Test Not Hispanic 24 | 0.69 | 0.69 | 0.64 | 1176 | Train 19 | Test Not Hispanic 24 | 0.69 | 0.69 | 0.64 |
| 153 | Train 3 | Test Not Hispanic 25 | 0.75 | 0.82 | 0.81 | 1177 | Train 19 | Test Not Hispanic 25 | 0.75 | 0.82 | 0.81 |
| 154 | Train 3 | Test Not Hispanic 26 | 0.74 | 0.77 | 0.76 | 1178 | Train 19 | Test Not Hispanic 26 | 0.74 | 0.77 | 0.77 |
| 155 | Train 3 | Test Not Hispanic 27 | 0.75 | 0.82 | 0.81 | 1179 | Train 19 | Test Not Hispanic 27 | 0.75 | 0.82 | 0.81 |
| 156 | Train 3 | Test Not Hispanic 28 | 0.74 | 0.77 | 0.76 | 1180 | Train 19 | Test Not Hispanic 28 | 0.74 | 0.77 | 0.77 |
| 157 | Train 3 | Test Not Hispanic 29 | 0.71 | 0.73 | 0.68 | 1181 | Train 19 | Test Not Hispanic 29 | 0.71 | 0.73 | 0.67 |
| 158 | Train 3 | Test Not Hispanic 30 | 0.7 | 0.7 | 0.64 | 1182 | Train 19 | Test Not Hispanic 30 | 0.7 | 0.7 | 0.64 |
| 159 | Train 3 | Test Not Hispanic 31 | 0.71 | 0.73 | 0.68 | 1183 | Train 19 | Test Not Hispanic 31 | 0.71 | 0.73 | 0.67 |
| 160 | Train 3 | Test Not Hispanic 32 | 0.7 | 0.69 | 0.64 | 1184 | Train 19 | Test Not Hispanic 32 | 0.7 | 0.7 | 0.64 |
| 161 | Train 3 | Test Not Hispanic 33 | 0.76 | 0.83 | 0.85 | 1185 | Train 19 | Test Not Hispanic 33 | 0.75 | 0.83 | 0.85 |
| 162 | Train 3 | Test Not Hispanic 34 | 0.74 | 0.78 | 0.79 | 1186 | Train 19 | Test Not Hispanic 34 | 0.74 | 0.78 | 0.79 |
| 163 | Train 3 | Test Not Hispanic 35 | 0.76 | 0.83 | 0.85 | 1187 | Train 19 | Test Not Hispanic 35 | 0.76 | 0.83 | 0.85 |
| 164 | Train 3 | Test Not Hispanic 36 | 0.74 | 0.78 | 0.79 | 1188 | Train 19 | Test Not Hispanic 36 | 0.74 | 0.78 | 0.79 |
| 165 | Train 3 | Test Not Hispanic 37 | 0.71 | 0.74 | 0.69 | 1189 | Train 19 | Test Not Hispanic 37 | 0.71 | 0.74 | 0.68 |
| 166 | Train 3 | Test Not Hispanic 38 | 0.69 | 0.7 | 0.64 | 1190 | Train 19 | Test Not Hispanic 38 | 0.69 | 0.7 | 0.64 |
| 167 | Train 3 | Test Not Hispanic 39 | 0.71 | 0.73 | 0.69 | 1191 | Train 19 | Test Not Hispanic 39 | 0.71 | 0.73 | 0.68 |
| 168 | Train 3 | Test Not Hispanic 40 | 0.69 | 0.69 | 0.64 | 1192 | Train 19 | Test Not Hispanic 40 | 0.69 | 0.69 | 0.64 |
| 169 | Train 3 | Test Not Hispanic 41 | 0.76 | 0.82 | 0.81 | 1193 | Train 19 | Test Not Hispanic 41 | 0.76 | 0.82 | 0.81 |
| 170 | Train 3 | Test Not Hispanic 42 | 0.74 | 0.77 | 0.76 | 1194 | Train 19 | Test Not Hispanic 42 | 0.74 | 0.77 | 0.76 |
| 171 | Train 3 | Test Not Hispanic 43 | 0.76 | 0.82 | 0.81 | 1195 | Train 19 | Test Not Hispanic 43 | 0.76 | 0.82 | 0.81 |
| 172 | Train 3 | Test Not Hispanic 44 | 0.74 | 0.77 | 0.76 | 1196 | Train 19 | Test Not Hispanic 44 | 0.74 | 0.77 | 0.77 |
| 173 | Train 3 | Test Not Hispanic 45 | 0.71 | 0.73 | 0.68 | 1197 | Train 19 | Test Not Hispanic 45 | 0.71 | 0.73 | 0.67 |
| 174 | Train 3 | Test Not Hispanic 46 | 0.7 | 0.7 | 0.64 | 1198 | Train 19 | Test Not Hispanic 46 | 0.69 | 0.7 | 0.64 |
| 175 | Train 3 | Test Not Hispanic 47 | 0.71 | 0.73 | 0.68 | 1199 | Train 19 | Test Not Hispanic 47 | 0.71 | 0.73 | 0.67 |
| 176 | Train 3 | Test Not Hispanic 48 | 0.7 | 0.69 | 0.64 | 1200 | Train 19 | Test Not Hispanic 48 | 0.7 | 0.7 | 0.64 |
| 177 | Train 3 | Test Not Hispanic 49 | 0.76 | 0.8 | 0.83 | 1201 | Train 19 | Test Not Hispanic 49 | 0.76 | 0.8 | 0.82 |
| 178 | Train 3 | Test Not Hispanic 50 | 0.76 | 0.8 | 0.83 | 1202 | Train 19 | Test Not Hispanic 50 | 0.76 | 0.8 | 0.82 |
| 179 | Train 3 | Test Not Hispanic 51 | 0.76 | 0.8 | 0.83 | 1203 | Train 19 | Test Not Hispanic 51 | 0.76 | 0.8 | 0.82 |
| 180 | Train 3 | Test Not Hispanic 52 | 0.76 | 0.8 | 0.83 | 1204 | Train 19 | Test Not Hispanic 52 | 0.76 | 0.8 | 0.82 |
| 181 | Train 3 | Test Not Hispanic 53 | 0.72 | 0.75 | 0.65 | 1205 | Train 19 | Test Not Hispanic 53 | 0.72 | 0.75 | 0.62 |
| 182 | Train 3 | Test Not Hispanic 54 | 0.71 | 0.7 | 0.65 | 1206 | Train 19 | Test Not Hispanic 54 | 0.71 | 0.7 | 0.63 |
| 183 | Train 3 | Test Not Hispanic 55 | 0.72 | 0.75 | 0.65 | 1207 | Train 19 | Test Not Hispanic 55 | 0.72 | 0.75 | 0.63 |
| 184 | Train 3 | Test Not Hispanic 56 | 0.71 | 0.7 | 0.65 | 1208 | Train 19 | Test Not Hispanic 56 | 0.71 | 0.7 | 0.63 |
| 185 | Train 3 | Test Not Hispanic 57 | 0.76 | 0.79 | 0.79 | 1209 | Train 19 | Test Not Hispanic 57 | 0.76 | 0.79 | 0.79 |
| 186 | Train 3 | Test Not Hispanic 58 | 0.76 | 0.79 | 0.79 | 1210 | Train 19 | Test Not Hispanic 58 | 0.76 | 0.79 | 0.79 |
| 187 | Train 3 | Test Not Hispanic 59 | 0.76 | 0.79 | 0.79 | 1211 | Train 19 | Test Not Hispanic 59 | 0.76 | 0.79 | 0.79 |
| 188 | Train 3 | Test Not Hispanic 60 | 0.76 | 0.79 | 0.79 | 1212 | Train 19 | Test Not Hispanic 60 | 0.76 | 0.79 | 0.79 |
| 189 | Train 3 | Test Not Hispanic 61 | 0.72 | 0.75 | 0.64 | 1213 | Train 19 | Test Not Hispanic 61 | 0.72 | 0.75 | 0.62 |
| 190 | Train 3 | Test Not Hispanic 62 | 0.71 | 0.71 | 0.65 | 1214 | Train 19 | Test Not Hispanic 62 | 0.71 | 0.71 | 0.63 |
| 191 | Train 3 | Test Not Hispanic 63 | 0.72 | 0.75 | 0.64 | 1215 | Train 19 | Test Not Hispanic 63 | 0.72 | 0.75 | 0.62 |
| 192 | Train 3 | Test Not Hispanic 64 | 0.71 | 0.7 | 0.65 | 1216 | Train 19 | Test Not Hispanic 64 | 0.71 | 0.7 | 0.63 |
| 193 | Train 4 | Test Not Hispanic 1 | 0.75 | 0.82 | 0.84 | 1217 | Train 20 | Test Not Hispanic 1 | 0.75 | 0.82 | 0.84 |
| 194 | Train 4 | Test Not Hispanic 2 | 0.76 | 0.83 | 0.84 | 1218 | Train 20 | Test Not Hispanic 2 | 0.76 | 0.83 | 0.85 |
| 195 | Train 4 | Test Not Hispanic 3 | 0.75 | 0.82 | 0.84 | 1219 | Train 20 | Test Not Hispanic 3 | 0.75 | 0.82 | 0.84 |
| 196 | Train 4 | Test Not Hispanic 4 | 0.76 | 0.83 | 0.84 | 1220 | Train 20 | Test Not Hispanic 4 | 0.76 | 0.83 | 0.84 |
| 197 | Train 4 | Test Not Hispanic 5 | 0.71 | 0.74 | 0.69 | 1221 | Train 20 | Test Not Hispanic 5 | 0.71 | 0.73 | 0.68 |
| 198 | Train 4 | Test Not Hispanic 6 | 0.71 | 0.71 | 0.67 | 1222 | Train 20 | Test Not Hispanic 6 | 0.71 | 0.71 | 0.66 |
| 199 | Train 4 | Test Not Hispanic 7 | 0.71 | 0.73 | 0.69 | 1223 | Train 20 | Test Not Hispanic 7 | 0.71 | 0.73 | 0.68 |
| 200 | Train 4 | Test Not Hispanic 8 | 0.71 | 0.71 | 0.67 | 1224 | Train 20 | Test Not Hispanic 8 | 0.71 | 0.71 | 0.66 |
| 201 | Train 4 | Test Not Hispanic 9 | 0.75 | 0.81 | 0.81 | 1225 | Train 20 | Test Not Hispanic 9 | 0.75 | 0.81 | 0.81 |
| 202 | Train 4 | Test Not Hispanic 10 | 0.76 | 0.82 | 0.82 | 1226 | Train 20 | Test Not Hispanic 10 | 0.76 | 0.82 | 0.82 |
| 203 | Train 4 | Test Not Hispanic 11 | 0.75 | 0.81 | 0.81 | 1227 | Train 20 | Test Not Hispanic 11 | 0.75 | 0.81 | 0.81 |
| 204 | Train 4 | Test Not Hispanic 12 | 0.76 | 0.82 | 0.82 | 1228 | Train 20 | Test Not Hispanic 12 | 0.76 | 0.82 | 0.82 |
| 205 | Train 4 | Test Not Hispanic 13 | 0.71 | 0.73 | 0.68 | 1229 | Train 20 | Test Not Hispanic 13 | 0.71 | 0.73 | 0.67 |
| 206 | Train 4 | Test Not Hispanic 14 | 0.71 | 0.71 | 0.67 | 1230 | Train 20 | Test Not Hispanic 14 | 0.71 | 0.71 | 0.66 |
| 207 | Train 4 | Test Not Hispanic 15 | 0.71 | 0.73 | 0.68 | 1231 | Train 20 | Test Not Hispanic 15 | 0.71 | 0.73 | 0.67 |
| 208 | Train 4 | Test Not Hispanic 16 | 0.71 | 0.71 | 0.67 | 1232 | Train 20 | Test Not Hispanic 16 | 0.71 | 0.71 | 0.66 |
| 209 | Train 4 | Test Not Hispanic 17 | 0.75 | 0.82 | 0.84 | 1233 | Train 20 | Test Not Hispanic 17 | 0.75 | 0.82 | 0.84 |
| 210 | Train 4 | Test Not Hispanic 18 | 0.76 | 0.83 | 0.84 | 1234 | Train 20 | Test Not Hispanic 18 | 0.76 | 0.83 | 0.85 |
| 211 | Train 4 | Test Not Hispanic 19 | 0.75 | 0.82 | 0.84 | 1235 | Train 20 | Test Not Hispanic 19 | 0.75 | 0.82 | 0.84 |
| 212 | Train 4 | Test Not Hispanic 20 | 0.76 | 0.83 | 0.84 | 1236 | Train 20 | Test Not Hispanic 20 | 0.76 | 0.83 | 0.84 |
| 213 | Train 4 | Test Not Hispanic 21 | 0.71 | 0.74 | 0.69 | 1237 | Train 20 | Test Not Hispanic 21 | 0.71 | 0.73 | 0.68 |
| 214 | Train 4 | Test Not Hispanic 22 | 0.71 | 0.71 | 0.67 | 1238 | Train 20 | Test Not Hispanic 22 | 0.71 | 0.71 | 0.66 |
| 215 | Train 4 | Test Not Hispanic 23 | 0.71 | 0.73 | 0.69 | 1239 | Train 20 | Test Not Hispanic 23 | 0.71 | 0.73 | 0.68 |
| 216 | Train 4 | Test Not Hispanic 24 | 0.71 | 0.71 | 0.67 | 1240 | Train 20 | Test Not Hispanic 24 | 0.71 | 0.71 | 0.66 |
| 217 | Train 4 | Test Not Hispanic 25 | 0.75 | 0.81 | 0.81 | 1241 | Train 20 | Test Not Hispanic 25 | 0.75 | 0.81 | 0.81 |
| 218 | Train 4 | Test Not Hispanic 26 | 0.76 | 0.82 | 0.82 | 1242 | Train 20 | Test Not Hispanic 26 | 0.76 | 0.82 | 0.82 |
| 219 | Train 4 | Test Not Hispanic 27 | 0.75 | 0.81 | 0.81 | 1243 | Train 20 | Test Not Hispanic 27 | 0.75 | 0.81 | 0.81 |
| 220 | Train 4 | Test Not Hispanic 28 | 0.76 | 0.82 | 0.82 | 1244 | Train 20 | Test Not Hispanic 28 | 0.76 | 0.82 | 0.82 |
| 221 | Train 4 | Test Not Hispanic 29 | 0.71 | 0.73 | 0.68 | 1245 | Train 20 | Test Not Hispanic 29 | 0.71 | 0.73 | 0.67 |
| 222 | Train 4 | Test Not Hispanic 30 | 0.71 | 0.71 | 0.67 | 1246 | Train 20 | Test Not Hispanic 30 | 0.71 | 0.71 | 0.66 |
| 223 | Train 4 | Test Not Hispanic 31 | 0.71 | 0.73 | 0.68 | 1247 | Train 20 | Test Not Hispanic 31 | 0.71 | 0.73 | 0.67 |
| 224 | Train 4 | Test Not Hispanic 32 | 0.71 | 0.71 | 0.67 | 1248 | Train 20 | Test Not Hispanic 32 | 0.71 | 0.71 | 0.66 |
| 225 | Train 4 | Test Not Hispanic 33 | 0.75 | 0.82 | 0.84 | 1249 | Train 20 | Test Not Hispanic 33 | 0.75 | 0.82 | 0.84 |
| 226 | Train 4 | Test Not Hispanic 34 | 0.76 | 0.83 | 0.84 | 1250 | Train 20 | Test Not Hispanic 34 | 0.76 | 0.83 | 0.84 |
| 227 | Train 4 | Test Not Hispanic 35 | 0.75 | 0.82 | 0.84 | 1251 | Train 20 | Test Not Hispanic 35 | 0.75 | 0.82 | 0.84 |
| 228 | Train 4 | Test Not Hispanic 36 | 0.76 | 0.83 | 0.84 | 1252 | Train 20 | Test Not Hispanic 36 | 0.76 | 0.83 | 0.84 |
| 229 | Train 4 | Test Not Hispanic 37 | 0.71 | 0.74 | 0.69 | 1253 | Train 20 | Test Not Hispanic 37 | 0.71 | 0.73 | 0.68 |
| 230 | Train 4 | Test Not Hispanic 38 | 0.71 | 0.71 | 0.67 | 1254 | Train 20 | Test Not Hispanic 38 | 0.71 | 0.71 | 0.66 |
| 231 | Train 4 | Test Not Hispanic 39 | 0.71 | 0.73 | 0.69 | 1255 | Train 20 | Test Not Hispanic 39 | 0.71 | 0.73 | 0.68 |
| 232 | Train 4 | Test Not Hispanic 40 | 0.71 | 0.71 | 0.67 | 1256 | Train 20 | Test Not Hispanic 40 | 0.71 | 0.71 | 0.66 |
| 233 | Train 4 | Test Not Hispanic 41 | 0.75 | 0.81 | 0.81 | 1257 | Train 20 | Test Not Hispanic 41 | 0.75 | 0.81 | 0.81 |
| 234 | Train 4 | Test Not Hispanic 42 | 0.76 | 0.82 | 0.82 | 1258 | Train 20 | Test Not Hispanic 42 | 0.76 | 0.82 | 0.82 |
| 235 | Train 4 | Test Not Hispanic 43 | 0.75 | 0.81 | 0.81 | 1259 | Train 20 | Test Not Hispanic 43 | 0.75 | 0.81 | 0.81 |
| 236 | Train 4 | Test Not Hispanic 44 | 0.76 | 0.82 | 0.82 | 1260 | Train 20 | Test Not Hispanic 44 | 0.76 | 0.82 | 0.82 |
| 237 | Train 4 | Test Not Hispanic 45 | 0.71 | 0.73 | 0.68 | 1261 | Train 20 | Test Not Hispanic 45 | 0.71 | 0.73 | 0.67 |
| 238 | Train 4 | Test Not Hispanic 46 | 0.71 | 0.71 | 0.67 | 1262 | Train 20 | Test Not Hispanic 46 | 0.71 | 0.71 | 0.66 |
| 239 | Train 4 | Test Not Hispanic 47 | 0.71 | 0.73 | 0.68 | 1263 | Train 20 | Test Not Hispanic 47 | 0.71 | 0.73 | 0.67 |
| 240 | Train 4 | Test Not Hispanic 48 | 0.71 | 0.71 | 0.67 | 1264 | Train 20 | Test Not Hispanic 48 | 0.71 | 0.71 | 0.66 |
| 241 | Train 4 | Test Not Hispanic 49 | 0.76 | 0.79 | 0.81 | 1265 | Train 20 | Test Not Hispanic 49 | 0.76 | 0.79 | 0.82 |
| 242 | Train 4 | Test Not Hispanic 50 | 0.76 | 0.79 | 0.81 | 1266 | Train 20 | Test Not Hispanic 50 | 0.76 | 0.79 | 0.82 |
| 243 | Train 4 | Test Not Hispanic 51 | 0.76 | 0.79 | 0.81 | 1267 | Train 20 | Test Not Hispanic 51 | 0.76 | 0.79 | 0.82 |
| 244 | Train 4 | Test Not Hispanic 52 | 0.76 | 0.79 | 0.81 | 1268 | Train 20 | Test Not Hispanic 52 | 0.76 | 0.79 | 0.82 |
| 245 | Train 4 | Test Not Hispanic 53 | 0.72 | 0.76 | 0.68 | 1269 | Train 20 | Test Not Hispanic 53 | 0.72 | 0.75 | 0.65 |
| 246 | Train 4 | Test Not Hispanic 54 | 0.71 | 0.72 | 0.67 | 1270 | Train 20 | Test Not Hispanic 54 | 0.71 | 0.71 | 0.66 |
| 247 | Train 4 | Test Not Hispanic 55 | 0.72 | 0.76 | 0.68 | 1271 | Train 20 | Test Not Hispanic 55 | 0.72 | 0.75 | 0.65 |
| 248 | Train 4 | Test Not Hispanic 56 | 0.71 | 0.71 | 0.67 | 1272 | Train 20 | Test Not Hispanic 56 | 0.71 | 0.71 | 0.66 |
| 249 | Train 4 | Test Not Hispanic 57 | 0.76 | 0.78 | 0.78 | 1273 | Train 20 | Test Not Hispanic 57 | 0.76 | 0.78 | 0.79 |
| 250 | Train 4 | Test Not Hispanic 58 | 0.76 | 0.78 | 0.78 | 1274 | Train 20 | Test Not Hispanic 58 | 0.76 | 0.78 | 0.79 |
| 251 | Train 4 | Test Not Hispanic 59 | 0.76 | 0.78 | 0.78 | 1275 | Train 20 | Test Not Hispanic 59 | 0.76 | 0.78 | 0.79 |
| 252 | Train 4 | Test Not Hispanic 60 | 0.76 | 0.78 | 0.78 | 1276 | Train 20 | Test Not Hispanic 60 | 0.76 | 0.78 | 0.79 |
| 253 | Train 4 | Test Not Hispanic 61 | 0.72 | 0.75 | 0.67 | 1277 | Train 20 | Test Not Hispanic 61 | 0.72 | 0.75 | 0.64 |
| 254 | Train 4 | Test Not Hispanic 62 | 0.71 | 0.72 | 0.67 | 1278 | Train 20 | Test Not Hispanic 62 | 0.72 | 0.71 | 0.66 |
| 255 | Train 4 | Test Not Hispanic 63 | 0.72 | 0.75 | 0.67 | 1279 | Train 20 | Test Not Hispanic 63 | 0.73 | 0.75 | 0.64 |
| 256 | Train 4 | Test Not Hispanic 64 | 0.71 | 0.71 | 0.67 | 1280 | Train 20 | Test Not Hispanic 64 | 0.71 | 0.71 | 0.66 |
| 257 | Train 5 | Test Not Hispanic 1 | 0.75 | 0.8 | 0.82 | 1281 | Train 21 | Test Not Hispanic 1 | 0.75 | 0.8 | 0.82 |
| 258 | Train 5 | Test Not Hispanic 2 | 0.75 | 0.78 | 0.78 | 1282 | Train 21 | Test Not Hispanic 2 | 0.75 | 0.78 | 0.78 |
| 259 | Train 5 | Test Not Hispanic 3 | 0.75 | 0.8 | 0.82 | 1283 | Train 21 | Test Not Hispanic 3 | 0.75 | 0.8 | 0.82 |
| 260 | Train 5 | Test Not Hispanic 4 | 0.75 | 0.78 | 0.78 | 1284 | Train 21 | Test Not Hispanic 4 | 0.75 | 0.78 | 0.78 |
| 261 | Train 5 | Test Not Hispanic 5 | 0.73 | 0.78 | 0.77 | 1285 | Train 21 | Test Not Hispanic 5 | 0.73 | 0.78 | 0.77 |
| 262 | Train 5 | Test Not Hispanic 6 | 0.7 | 0.71 | 0.68 | 1286 | Train 21 | Test Not Hispanic 6 | 0.7 | 0.71 | 0.67 |
| 263 | Train 5 | Test Not Hispanic 7 | 0.73 | 0.78 | 0.77 | 1287 | Train 21 | Test Not Hispanic 7 | 0.73 | 0.78 | 0.77 |
| 264 | Train 5 | Test Not Hispanic 8 | 0.7 | 0.71 | 0.68 | 1288 | Train 21 | Test Not Hispanic 8 | 0.7 | 0.71 | 0.67 |
| 265 | Train 5 | Test Not Hispanic 9 | 0.75 | 0.79 | 0.8 | 1289 | Train 21 | Test Not Hispanic 9 | 0.75 | 0.79 | 0.8 |
| 266 | Train 5 | Test Not Hispanic 10 | 0.75 | 0.78 | 0.77 | 1290 | Train 21 | Test Not Hispanic 10 | 0.75 | 0.78 | 0.76 |
| 267 | Train 5 | Test Not Hispanic 11 | 0.75 | 0.79 | 0.79 | 1291 | Train 21 | Test Not Hispanic 11 | 0.75 | 0.79 | 0.79 |
| 268 | Train 5 | Test Not Hispanic 12 | 0.75 | 0.78 | 0.76 | 1292 | Train 21 | Test Not Hispanic 12 | 0.75 | 0.78 | 0.76 |
| 269 | Train 5 | Test Not Hispanic 13 | 0.73 | 0.77 | 0.75 | 1293 | Train 21 | Test Not Hispanic 13 | 0.73 | 0.77 | 0.75 |
| 270 | Train 5 | Test Not Hispanic 14 | 0.7 | 0.71 | 0.68 | 1294 | Train 21 | Test Not Hispanic 14 | 0.7 | 0.71 | 0.67 |
| 271 | Train 5 | Test Not Hispanic 15 | 0.73 | 0.77 | 0.75 | 1295 | Train 21 | Test Not Hispanic 15 | 0.73 | 0.77 | 0.75 |
| 272 | Train 5 | Test Not Hispanic 16 | 0.7 | 0.71 | 0.68 | 1296 | Train 21 | Test Not Hispanic 16 | 0.7 | 0.71 | 0.67 |
| 273 | Train 5 | Test Not Hispanic 17 | 0.75 | 0.8 | 0.82 | 1297 | Train 21 | Test Not Hispanic 17 | 0.75 | 0.8 | 0.82 |
| 274 | Train 5 | Test Not Hispanic 18 | 0.75 | 0.78 | 0.78 | 1298 | Train 21 | Test Not Hispanic 18 | 0.75 | 0.78 | 0.78 |
| 275 | Train 5 | Test Not Hispanic 19 | 0.75 | 0.8 | 0.82 | 1299 | Train 21 | Test Not Hispanic 19 | 0.75 | 0.8 | 0.82 |
| 276 | Train 5 | Test Not Hispanic 20 | 0.75 | 0.78 | 0.78 | 1300 | Train 21 | Test Not Hispanic 20 | 0.75 | 0.78 | 0.78 |
| 277 | Train 5 | Test Not Hispanic 21 | 0.74 | 0.78 | 0.77 | 1301 | Train 21 | Test Not Hispanic 21 | 0.74 | 0.78 | 0.77 |
| 278 | Train 5 | Test Not Hispanic 22 | 0.7 | 0.71 | 0.68 | 1302 | Train 21 | Test Not Hispanic 22 | 0.7 | 0.71 | 0.67 |
| 279 | Train 5 | Test Not Hispanic 23 | 0.73 | 0.78 | 0.77 | 1303 | Train 21 | Test Not Hispanic 23 | 0.73 | 0.78 | 0.77 |
| 280 | Train 5 | Test Not Hispanic 24 | 0.7 | 0.71 | 0.68 | 1304 | Train 21 | Test Not Hispanic 24 | 0.7 | 0.71 | 0.67 |
| 281 | Train 5 | Test Not Hispanic 25 | 0.75 | 0.79 | 0.8 | 1305 | Train 21 | Test Not Hispanic 25 | 0.75 | 0.79 | 0.8 |
| 282 | Train 5 | Test Not Hispanic 26 | 0.75 | 0.78 | 0.77 | 1306 | Train 21 | Test Not Hispanic 26 | 0.75 | 0.78 | 0.76 |
| 283 | Train 5 | Test Not Hispanic 27 | 0.75 | 0.79 | 0.79 | 1307 | Train 21 | Test Not Hispanic 27 | 0.75 | 0.79 | 0.79 |
| 284 | Train 5 | Test Not Hispanic 28 | 0.75 | 0.78 | 0.76 | 1308 | Train 21 | Test Not Hispanic 28 | 0.75 | 0.78 | 0.76 |
| 285 | Train 5 | Test Not Hispanic 29 | 0.74 | 0.77 | 0.75 | 1309 | Train 21 | Test Not Hispanic 29 | 0.74 | 0.77 | 0.75 |
| 286 | Train 5 | Test Not Hispanic 30 | 0.7 | 0.71 | 0.68 | 1310 | Train 21 | Test Not Hispanic 30 | 0.7 | 0.71 | 0.67 |
| 287 | Train 5 | Test Not Hispanic 31 | 0.73 | 0.77 | 0.75 | 1311 | Train 21 | Test Not Hispanic 31 | 0.73 | 0.77 | 0.75 |
| 288 | Train 5 | Test Not Hispanic 32 | 0.7 | 0.71 | 0.68 | 1312 | Train 21 | Test Not Hispanic 32 | 0.7 | 0.71 | 0.68 |
| 289 | Train 5 | Test Not Hispanic 33 | 0.75 | 0.8 | 0.82 | 1313 | Train 21 | Test Not Hispanic 33 | 0.75 | 0.8 | 0.82 |
| 290 | Train 5 | Test Not Hispanic 34 | 0.75 | 0.78 | 0.78 | 1314 | Train 21 | Test Not Hispanic 34 | 0.75 | 0.78 | 0.78 |
| 291 | Train 5 | Test Not Hispanic 35 | 0.75 | 0.8 | 0.82 | 1315 | Train 21 | Test Not Hispanic 35 | 0.75 | 0.8 | 0.82 |
| 292 | Train 5 | Test Not Hispanic 36 | 0.75 | 0.78 | 0.78 | 1316 | Train 21 | Test Not Hispanic 36 | 0.75 | 0.78 | 0.78 |
| 293 | Train 5 | Test Not Hispanic 37 | 0.74 | 0.78 | 0.77 | 1317 | Train 21 | Test Not Hispanic 37 | 0.74 | 0.78 | 0.77 |
| 294 | Train 5 | Test Not Hispanic 38 | 0.7 | 0.71 | 0.68 | 1318 | Train 21 | Test Not Hispanic 38 | 0.7 | 0.71 | 0.67 |
| 295 | Train 5 | Test Not Hispanic 39 | 0.73 | 0.78 | 0.77 | 1319 | Train 21 | Test Not Hispanic 39 | 0.73 | 0.78 | 0.77 |
| 296 | Train 5 | Test Not Hispanic 40 | 0.7 | 0.71 | 0.68 | 1320 | Train 21 | Test Not Hispanic 40 | 0.7 | 0.71 | 0.67 |
| 297 | Train 5 | Test Not Hispanic 41 | 0.75 | 0.8 | 0.8 | 1321 | Train 21 | Test Not Hispanic 41 | 0.75 | 0.8 | 0.8 |
| 298 | Train 5 | Test Not Hispanic 42 | 0.75 | 0.78 | 0.77 | 1322 | Train 21 | Test Not Hispanic 42 | 0.75 | 0.78 | 0.76 |
| 299 | Train 5 | Test Not Hispanic 43 | 0.75 | 0.8 | 0.79 | 1323 | Train 21 | Test Not Hispanic 43 | 0.75 | 0.8 | 0.79 |
| 300 | Train 5 | Test Not Hispanic 44 | 0.75 | 0.78 | 0.76 | 1324 | Train 21 | Test Not Hispanic 44 | 0.75 | 0.78 | 0.76 |
| 301 | Train 5 | Test Not Hispanic 45 | 0.74 | 0.77 | 0.75 | 1325 | Train 21 | Test Not Hispanic 45 | 0.74 | 0.77 | 0.75 |
| 302 | Train 5 | Test Not Hispanic 46 | 0.7 | 0.71 | 0.68 | 1326 | Train 21 | Test Not Hispanic 46 | 0.7 | 0.71 | 0.67 |
| 303 | Train 5 | Test Not Hispanic 47 | 0.73 | 0.77 | 0.75 | 1327 | Train 21 | Test Not Hispanic 47 | 0.73 | 0.77 | 0.75 |
| 304 | Train 5 | Test Not Hispanic 48 | 0.7 | 0.71 | 0.68 | 1328 | Train 21 | Test Not Hispanic 48 | 0.7 | 0.71 | 0.68 |
| 305 | Train 5 | Test Not Hispanic 49 | 0.76 | 0.77 | 0.79 | 1329 | Train 21 | Test Not Hispanic 49 | 0.76 | 0.77 | 0.8 |
| 306 | Train 5 | Test Not Hispanic 50 | 0.76 | 0.77 | 0.79 | 1330 | Train 21 | Test Not Hispanic 50 | 0.76 | 0.77 | 0.8 |
| 307 | Train 5 | Test Not Hispanic 51 | 0.76 | 0.77 | 0.79 | 1331 | Train 21 | Test Not Hispanic 51 | 0.76 | 0.77 | 0.8 |
| 308 | Train 5 | Test Not Hispanic 52 | 0.76 | 0.77 | 0.79 | 1332 | Train 21 | Test Not Hispanic 52 | 0.76 | 0.77 | 0.8 |
| 309 | Train 5 | Test Not Hispanic 53 | 0.75 | 0.78 | 0.74 | 1333 | Train 21 | Test Not Hispanic 53 | 0.75 | 0.78 | 0.76 |
| 310 | Train 5 | Test Not Hispanic 54 | 0.71 | 0.72 | 0.69 | 1334 | Train 21 | Test Not Hispanic 54 | 0.71 | 0.71 | 0.7 |
| 311 | Train 5 | Test Not Hispanic 55 | 0.75 | 0.78 | 0.73 | 1335 | Train 21 | Test Not Hispanic 55 | 0.75 | 0.78 | 0.76 |
| 312 | Train 5 | Test Not Hispanic 56 | 0.71 | 0.71 | 0.69 | 1336 | Train 21 | Test Not Hispanic 56 | 0.71 | 0.71 | 0.7 |
| 313 | Train 5 | Test Not Hispanic 57 | 0.76 | 0.77 | 0.77 | 1337 | Train 21 | Test Not Hispanic 57 | 0.76 | 0.77 | 0.78 |
| 314 | Train 5 | Test Not Hispanic 58 | 0.76 | 0.77 | 0.77 | 1338 | Train 21 | Test Not Hispanic 58 | 0.76 | 0.77 | 0.78 |
| 315 | Train 5 | Test Not Hispanic 59 | 0.76 | 0.77 | 0.77 | 1339 | Train 21 | Test Not Hispanic 59 | 0.76 | 0.77 | 0.78 |
| 316 | Train 5 | Test Not Hispanic 60 | 0.76 | 0.77 | 0.77 | 1340 | Train 21 | Test Not Hispanic 60 | 0.76 | 0.77 | 0.78 |
| 317 | Train 5 | Test Not Hispanic 61 | 0.75 | 0.78 | 0.73 | 1341 | Train 21 | Test Not Hispanic 61 | 0.75 | 0.78 | 0.76 |
| 318 | Train 5 | Test Not Hispanic 62 | 0.71 | 0.72 | 0.69 | 1342 | Train 21 | Test Not Hispanic 62 | 0.72 | 0.72 | 0.7 |
| 319 | Train 5 | Test Not Hispanic 63 | 0.75 | 0.78 | 0.73 | 1343 | Train 21 | Test Not Hispanic 63 | 0.75 | 0.78 | 0.75 |
| 320 | Train 5 | Test Not Hispanic 64 | 0.71 | 0.71 | 0.69 | 1344 | Train 21 | Test Not Hispanic 64 | 0.71 | 0.71 | 0.7 |
| 321 | Train 6 | Test Not Hispanic 1 | 0.73 | 0.77 | 0.75 | 1345 | Train 22 | Test Not Hispanic 1 | 0.73 | 0.77 | 0.75 |
| 322 | Train 6 | Test Not Hispanic 2 | 0.75 | 0.78 | 0.76 | 1346 | Train 22 | Test Not Hispanic 2 | 0.75 | 0.78 | 0.76 |
| 323 | Train 6 | Test Not Hispanic 3 | 0.73 | 0.77 | 0.75 | 1347 | Train 22 | Test Not Hispanic 3 | 0.73 | 0.77 | 0.75 |
| 324 | Train 6 | Test Not Hispanic 4 | 0.75 | 0.78 | 0.76 | 1348 | Train 22 | Test Not Hispanic 4 | 0.75 | 0.78 | 0.76 |
| 325 | Train 6 | Test Not Hispanic 5 | 0.72 | 0.77 | 0.77 | 1349 | Train 22 | Test Not Hispanic 5 | 0.72 | 0.77 | 0.77 |
| 326 | Train 6 | Test Not Hispanic 6 | 0.74 | 0.78 | 0.79 | 1350 | Train 22 | Test Not Hispanic 6 | 0.74 | 0.78 | 0.79 |
| 327 | Train 6 | Test Not Hispanic 7 | 0.72 | 0.77 | 0.77 | 1351 | Train 22 | Test Not Hispanic 7 | 0.72 | 0.77 | 0.77 |
| 328 | Train 6 | Test Not Hispanic 8 | 0.73 | 0.77 | 0.78 | 1352 | Train 22 | Test Not Hispanic 8 | 0.73 | 0.77 | 0.78 |
| 329 | Train 6 | Test Not Hispanic 9 | 0.73 | 0.77 | 0.75 | 1353 | Train 22 | Test Not Hispanic 9 | 0.73 | 0.77 | 0.76 |
| 330 | Train 6 | Test Not Hispanic 10 | 0.75 | 0.78 | 0.76 | 1354 | Train 22 | Test Not Hispanic 10 | 0.75 | 0.78 | 0.77 |
| 331 | Train 6 | Test Not Hispanic 11 | 0.73 | 0.77 | 0.75 | 1355 | Train 22 | Test Not Hispanic 11 | 0.73 | 0.77 | 0.76 |
| 332 | Train 6 | Test Not Hispanic 12 | 0.75 | 0.78 | 0.76 | 1356 | Train 22 | Test Not Hispanic 12 | 0.75 | 0.78 | 0.77 |
| 333 | Train 6 | Test Not Hispanic 13 | 0.72 | 0.77 | 0.78 | 1357 | Train 22 | Test Not Hispanic 13 | 0.72 | 0.77 | 0.78 |
| 334 | Train 6 | Test Not Hispanic 14 | 0.74 | 0.78 | 0.79 | 1358 | Train 22 | Test Not Hispanic 14 | 0.74 | 0.78 | 0.79 |
| 335 | Train 6 | Test Not Hispanic 15 | 0.72 | 0.77 | 0.78 | 1359 | Train 22 | Test Not Hispanic 15 | 0.72 | 0.77 | 0.78 |
| 336 | Train 6 | Test Not Hispanic 16 | 0.74 | 0.78 | 0.78 | 1360 | Train 22 | Test Not Hispanic 16 | 0.74 | 0.78 | 0.78 |
| 337 | Train 6 | Test Not Hispanic 17 | 0.73 | 0.77 | 0.75 | 1361 | Train 22 | Test Not Hispanic 17 | 0.73 | 0.77 | 0.75 |
| 338 | Train 6 | Test Not Hispanic 18 | 0.75 | 0.78 | 0.76 | 1362 | Train 22 | Test Not Hispanic 18 | 0.75 | 0.78 | 0.76 |
| 339 | Train 6 | Test Not Hispanic 19 | 0.73 | 0.77 | 0.75 | 1363 | Train 22 | Test Not Hispanic 19 | 0.73 | 0.77 | 0.75 |
| 340 | Train 6 | Test Not Hispanic 20 | 0.75 | 0.78 | 0.76 | 1364 | Train 22 | Test Not Hispanic 20 | 0.75 | 0.78 | 0.76 |
| 341 | Train 6 | Test Not Hispanic 21 | 0.72 | 0.77 | 0.77 | 1365 | Train 22 | Test Not Hispanic 21 | 0.72 | 0.77 | 0.77 |
| 342 | Train 6 | Test Not Hispanic 22 | 0.74 | 0.78 | 0.79 | 1366 | Train 22 | Test Not Hispanic 22 | 0.74 | 0.78 | 0.79 |
| 343 | Train 6 | Test Not Hispanic 23 | 0.72 | 0.77 | 0.77 | 1367 | Train 22 | Test Not Hispanic 23 | 0.72 | 0.77 | 0.77 |
| 344 | Train 6 | Test Not Hispanic 24 | 0.73 | 0.77 | 0.78 | 1368 | Train 22 | Test Not Hispanic 24 | 0.73 | 0.78 | 0.78 |
| 345 | Train 6 | Test Not Hispanic 25 | 0.73 | 0.77 | 0.75 | 1369 | Train 22 | Test Not Hispanic 25 | 0.73 | 0.77 | 0.76 |
| 346 | Train 6 | Test Not Hispanic 26 | 0.75 | 0.78 | 0.76 | 1370 | Train 22 | Test Not Hispanic 26 | 0.75 | 0.78 | 0.77 |
| 347 | Train 6 | Test Not Hispanic 27 | 0.73 | 0.77 | 0.75 | 1371 | Train 22 | Test Not Hispanic 27 | 0.73 | 0.77 | 0.76 |
| 348 | Train 6 | Test Not Hispanic 28 | 0.75 | 0.78 | 0.76 | 1372 | Train 22 | Test Not Hispanic 28 | 0.75 | 0.78 | 0.77 |
| 349 | Train 6 | Test Not Hispanic 29 | 0.72 | 0.77 | 0.78 | 1373 | Train 22 | Test Not Hispanic 29 | 0.72 | 0.77 | 0.78 |
| 350 | Train 6 | Test Not Hispanic 30 | 0.74 | 0.78 | 0.79 | 1374 | Train 22 | Test Not Hispanic 30 | 0.74 | 0.78 | 0.79 |
| 351 | Train 6 | Test Not Hispanic 31 | 0.72 | 0.77 | 0.78 | 1375 | Train 22 | Test Not Hispanic 31 | 0.72 | 0.77 | 0.78 |
| 352 | Train 6 | Test Not Hispanic 32 | 0.74 | 0.78 | 0.78 | 1376 | Train 22 | Test Not Hispanic 32 | 0.74 | 0.78 | 0.78 |
| 353 | Train 6 | Test Not Hispanic 33 | 0.73 | 0.77 | 0.75 | 1377 | Train 22 | Test Not Hispanic 33 | 0.73 | 0.77 | 0.75 |
| 354 | Train 6 | Test Not Hispanic 34 | 0.75 | 0.78 | 0.76 | 1378 | Train 22 | Test Not Hispanic 34 | 0.75 | 0.78 | 0.77 |
| 355 | Train 6 | Test Not Hispanic 35 | 0.73 | 0.77 | 0.75 | 1379 | Train 22 | Test Not Hispanic 35 | 0.73 | 0.77 | 0.75 |
| 356 | Train 6 | Test Not Hispanic 36 | 0.75 | 0.78 | 0.76 | 1380 | Train 22 | Test Not Hispanic 36 | 0.75 | 0.78 | 0.76 |
| 357 | Train 6 | Test Not Hispanic 37 | 0.72 | 0.77 | 0.77 | 1381 | Train 22 | Test Not Hispanic 37 | 0.72 | 0.77 | 0.77 |
| 358 | Train 6 | Test Not Hispanic 38 | 0.74 | 0.78 | 0.79 | 1382 | Train 22 | Test Not Hispanic 38 | 0.74 | 0.78 | 0.79 |
| 359 | Train 6 | Test Not Hispanic 39 | 0.72 | 0.77 | 0.77 | 1383 | Train 22 | Test Not Hispanic 39 | 0.72 | 0.77 | 0.77 |
| 360 | Train 6 | Test Not Hispanic 40 | 0.73 | 0.77 | 0.78 | 1384 | Train 22 | Test Not Hispanic 40 | 0.73 | 0.77 | 0.78 |
| 361 | Train 6 | Test Not Hispanic 41 | 0.73 | 0.77 | 0.75 | 1385 | Train 22 | Test Not Hispanic 41 | 0.73 | 0.77 | 0.76 |
| 362 | Train 6 | Test Not Hispanic 42 | 0.75 | 0.78 | 0.76 | 1386 | Train 22 | Test Not Hispanic 42 | 0.75 | 0.78 | 0.77 |
| 363 | Train 6 | Test Not Hispanic 43 | 0.73 | 0.77 | 0.75 | 1387 | Train 22 | Test Not Hispanic 43 | 0.73 | 0.77 | 0.76 |
| 364 | Train 6 | Test Not Hispanic 44 | 0.75 | 0.78 | 0.76 | 1388 | Train 22 | Test Not Hispanic 44 | 0.75 | 0.78 | 0.77 |
| 365 | Train 6 | Test Not Hispanic 45 | 0.72 | 0.77 | 0.78 | 1389 | Train 22 | Test Not Hispanic 45 | 0.72 | 0.77 | 0.78 |
| 366 | Train 6 | Test Not Hispanic 46 | 0.74 | 0.78 | 0.79 | 1390 | Train 22 | Test Not Hispanic 46 | 0.74 | 0.78 | 0.79 |
| 367 | Train 6 | Test Not Hispanic 47 | 0.72 | 0.77 | 0.78 | 1391 | Train 22 | Test Not Hispanic 47 | 0.72 | 0.77 | 0.78 |
| 368 | Train 6 | Test Not Hispanic 48 | 0.74 | 0.78 | 0.78 | 1392 | Train 22 | Test Not Hispanic 48 | 0.74 | 0.78 | 0.78 |
| 369 | Train 6 | Test Not Hispanic 49 | 0.75 | 0.78 | 0.76 | 1393 | Train 22 | Test Not Hispanic 49 | 0.75 | 0.78 | 0.77 |
| 370 | Train 6 | Test Not Hispanic 50 | 0.75 | 0.78 | 0.76 | 1394 | Train 22 | Test Not Hispanic 50 | 0.75 | 0.78 | 0.77 |
| 371 | Train 6 | Test Not Hispanic 51 | 0.75 | 0.78 | 0.76 | 1395 | Train 22 | Test Not Hispanic 51 | 0.75 | 0.78 | 0.77 |
| 372 | Train 6 | Test Not Hispanic 52 | 0.75 | 0.78 | 0.76 | 1396 | Train 22 | Test Not Hispanic 52 | 0.75 | 0.78 | 0.77 |
| 373 | Train 6 | Test Not Hispanic 53 | 0.75 | 0.79 | 0.75 | 1397 | Train 22 | Test Not Hispanic 53 | 0.75 | 0.79 | 0.77 |
| 374 | Train 6 | Test Not Hispanic 54 | 0.74 | 0.78 | 0.79 | 1398 | Train 22 | Test Not Hispanic 54 | 0.74 | 0.78 | 0.79 |
| 375 | Train 6 | Test Not Hispanic 55 | 0.74 | 0.79 | 0.75 | 1399 | Train 22 | Test Not Hispanic 55 | 0.75 | 0.79 | 0.76 |
| 376 | Train 6 | Test Not Hispanic 56 | 0.74 | 0.77 | 0.78 | 1400 | Train 22 | Test Not Hispanic 56 | 0.74 | 0.77 | 0.78 |
| 377 | Train 6 | Test Not Hispanic 57 | 0.75 | 0.79 | 0.77 | 1401 | Train 22 | Test Not Hispanic 57 | 0.75 | 0.78 | 0.79 |
| 378 | Train 6 | Test Not Hispanic 58 | 0.75 | 0.79 | 0.77 | 1402 | Train 22 | Test Not Hispanic 58 | 0.75 | 0.79 | 0.79 |
| 379 | Train 6 | Test Not Hispanic 59 | 0.75 | 0.79 | 0.77 | 1403 | Train 22 | Test Not Hispanic 59 | 0.75 | 0.78 | 0.79 |
| 380 | Train 6 | Test Not Hispanic 60 | 0.75 | 0.79 | 0.77 | 1404 | Train 22 | Test Not Hispanic 60 | 0.75 | 0.78 | 0.79 |
| 381 | Train 6 | Test Not Hispanic 61 | 0.75 | 0.79 | 0.76 | 1405 | Train 22 | Test Not Hispanic 61 | 0.75 | 0.79 | 0.77 |
| 382 | Train 6 | Test Not Hispanic 62 | 0.74 | 0.78 | 0.79 | 1406 | Train 22 | Test Not Hispanic 62 | 0.74 | 0.78 | 0.79 |
| 383 | Train 6 | Test Not Hispanic 63 | 0.75 | 0.79 | 0.75 | 1407 | Train 22 | Test Not Hispanic 63 | 0.75 | 0.79 | 0.77 |
| 384 | Train 6 | Test Not Hispanic 64 | 0.74 | 0.77 | 0.78 | 1408 | Train 22 | Test Not Hispanic 64 | 0.74 | 0.77 | 0.78 |
| 385 | Train 7 | Test Not Hispanic 1 | 0.75 | 0.8 | 0.82 | 1409 | Train 23 | Test Not Hispanic 1 | 0.75 | 0.8 | 0.82 |
| 386 | Train 7 | Test Not Hispanic 2 | 0.75 | 0.78 | 0.78 | 1410 | Train 23 | Test Not Hispanic 2 | 0.75 | 0.78 | 0.78 |
| 387 | Train 7 | Test Not Hispanic 3 | 0.75 | 0.8 | 0.82 | 1411 | Train 23 | Test Not Hispanic 3 | 0.75 | 0.8 | 0.82 |
| 388 | Train 7 | Test Not Hispanic 4 | 0.75 | 0.78 | 0.78 | 1412 | Train 23 | Test Not Hispanic 4 | 0.75 | 0.78 | 0.78 |
| 389 | Train 7 | Test Not Hispanic 5 | 0.73 | 0.78 | 0.75 | 1413 | Train 23 | Test Not Hispanic 5 | 0.73 | 0.78 | 0.75 |
| 390 | Train 7 | Test Not Hispanic 6 | 0.7 | 0.71 | 0.68 | 1414 | Train 23 | Test Not Hispanic 6 | 0.7 | 0.7 | 0.67 |
| 391 | Train 7 | Test Not Hispanic 7 | 0.73 | 0.78 | 0.75 | 1415 | Train 23 | Test Not Hispanic 7 | 0.73 | 0.78 | 0.74 |
| 392 | Train 7 | Test Not Hispanic 8 | 0.7 | 0.71 | 0.68 | 1416 | Train 23 | Test Not Hispanic 8 | 0.7 | 0.7 | 0.67 |
| 393 | Train 7 | Test Not Hispanic 9 | 0.75 | 0.79 | 0.79 | 1417 | Train 23 | Test Not Hispanic 9 | 0.75 | 0.79 | 0.79 |
| 394 | Train 7 | Test Not Hispanic 10 | 0.75 | 0.78 | 0.77 | 1418 | Train 23 | Test Not Hispanic 10 | 0.75 | 0.78 | 0.76 |
| 395 | Train 7 | Test Not Hispanic 11 | 0.75 | 0.79 | 0.8 | 1419 | Train 23 | Test Not Hispanic 11 | 0.75 | 0.79 | 0.79 |
| 396 | Train 7 | Test Not Hispanic 12 | 0.75 | 0.78 | 0.77 | 1420 | Train 23 | Test Not Hispanic 12 | 0.75 | 0.78 | 0.76 |
| 397 | Train 7 | Test Not Hispanic 13 | 0.73 | 0.77 | 0.73 | 1421 | Train 23 | Test Not Hispanic 13 | 0.73 | 0.77 | 0.73 |
| 398 | Train 7 | Test Not Hispanic 14 | 0.7 | 0.71 | 0.68 | 1422 | Train 23 | Test Not Hispanic 14 | 0.7 | 0.71 | 0.67 |
| 399 | Train 7 | Test Not Hispanic 15 | 0.73 | 0.77 | 0.73 | 1423 | Train 23 | Test Not Hispanic 15 | 0.73 | 0.77 | 0.73 |
| 400 | Train 7 | Test Not Hispanic 16 | 0.7 | 0.71 | 0.68 | 1424 | Train 23 | Test Not Hispanic 16 | 0.7 | 0.71 | 0.67 |
| 401 | Train 7 | Test Not Hispanic 17 | 0.75 | 0.8 | 0.82 | 1425 | Train 23 | Test Not Hispanic 17 | 0.75 | 0.8 | 0.82 |
| 402 | Train 7 | Test Not Hispanic 18 | 0.75 | 0.78 | 0.78 | 1426 | Train 23 | Test Not Hispanic 18 | 0.75 | 0.78 | 0.78 |
| 403 | Train 7 | Test Not Hispanic 19 | 0.75 | 0.8 | 0.82 | 1427 | Train 23 | Test Not Hispanic 19 | 0.75 | 0.8 | 0.82 |
| 404 | Train 7 | Test Not Hispanic 20 | 0.75 | 0.78 | 0.78 | 1428 | Train 23 | Test Not Hispanic 20 | 0.75 | 0.78 | 0.78 |
| 405 | Train 7 | Test Not Hispanic 21 | 0.74 | 0.78 | 0.75 | 1429 | Train 23 | Test Not Hispanic 21 | 0.74 | 0.78 | 0.75 |
| 406 | Train 7 | Test Not Hispanic 22 | 0.7 | 0.71 | 0.68 | 1430 | Train 23 | Test Not Hispanic 22 | 0.7 | 0.71 | 0.67 |
| 407 | Train 7 | Test Not Hispanic 23 | 0.73 | 0.78 | 0.75 | 1431 | Train 23 | Test Not Hispanic 23 | 0.73 | 0.78 | 0.74 |
| 408 | Train 7 | Test Not Hispanic 24 | 0.7 | 0.71 | 0.68 | 1432 | Train 23 | Test Not Hispanic 24 | 0.7 | 0.7 | 0.67 |
| 409 | Train 7 | Test Not Hispanic 25 | 0.75 | 0.79 | 0.79 | 1433 | Train 23 | Test Not Hispanic 25 | 0.75 | 0.79 | 0.79 |
| 410 | Train 7 | Test Not Hispanic 26 | 0.75 | 0.78 | 0.77 | 1434 | Train 23 | Test Not Hispanic 26 | 0.75 | 0.78 | 0.76 |
| 411 | Train 7 | Test Not Hispanic 27 | 0.75 | 0.79 | 0.8 | 1435 | Train 23 | Test Not Hispanic 27 | 0.75 | 0.79 | 0.79 |
| 412 | Train 7 | Test Not Hispanic 28 | 0.75 | 0.78 | 0.77 | 1436 | Train 23 | Test Not Hispanic 28 | 0.75 | 0.78 | 0.76 |
| 413 | Train 7 | Test Not Hispanic 29 | 0.74 | 0.77 | 0.74 | 1437 | Train 23 | Test Not Hispanic 29 | 0.74 | 0.77 | 0.73 |
| 414 | Train 7 | Test Not Hispanic 30 | 0.7 | 0.71 | 0.68 | 1438 | Train 23 | Test Not Hispanic 30 | 0.7 | 0.71 | 0.67 |
| 415 | Train 7 | Test Not Hispanic 31 | 0.73 | 0.77 | 0.73 | 1439 | Train 23 | Test Not Hispanic 31 | 0.73 | 0.77 | 0.73 |
| 416 | Train 7 | Test Not Hispanic 32 | 0.7 | 0.71 | 0.68 | 1440 | Train 23 | Test Not Hispanic 32 | 0.7 | 0.71 | 0.67 |
| 417 | Train 7 | Test Not Hispanic 33 | 0.75 | 0.8 | 0.82 | 1441 | Train 23 | Test Not Hispanic 33 | 0.75 | 0.8 | 0.82 |
| 418 | Train 7 | Test Not Hispanic 34 | 0.75 | 0.78 | 0.78 | 1442 | Train 23 | Test Not Hispanic 34 | 0.75 | 0.78 | 0.78 |
| 419 | Train 7 | Test Not Hispanic 35 | 0.75 | 0.8 | 0.82 | 1443 | Train 23 | Test Not Hispanic 35 | 0.75 | 0.8 | 0.82 |
| 420 | Train 7 | Test Not Hispanic 36 | 0.75 | 0.78 | 0.78 | 1444 | Train 23 | Test Not Hispanic 36 | 0.75 | 0.78 | 0.78 |
| 421 | Train 7 | Test Not Hispanic 37 | 0.74 | 0.78 | 0.75 | 1445 | Train 23 | Test Not Hispanic 37 | 0.74 | 0.78 | 0.75 |
| 422 | Train 7 | Test Not Hispanic 38 | 0.7 | 0.71 | 0.68 | 1446 | Train 23 | Test Not Hispanic 38 | 0.7 | 0.71 | 0.67 |
| 423 | Train 7 | Test Not Hispanic 39 | 0.73 | 0.78 | 0.75 | 1447 | Train 23 | Test Not Hispanic 39 | 0.73 | 0.78 | 0.74 |
| 424 | Train 7 | Test Not Hispanic 40 | 0.7 | 0.71 | 0.68 | 1448 | Train 23 | Test Not Hispanic 40 | 0.7 | 0.7 | 0.67 |
| 425 | Train 7 | Test Not Hispanic 41 | 0.75 | 0.8 | 0.8 | 1449 | Train 23 | Test Not Hispanic 41 | 0.75 | 0.79 | 0.79 |
| 426 | Train 7 | Test Not Hispanic 42 | 0.75 | 0.78 | 0.77 | 1450 | Train 23 | Test Not Hispanic 42 | 0.75 | 0.78 | 0.76 |
| 427 | Train 7 | Test Not Hispanic 43 | 0.75 | 0.8 | 0.8 | 1451 | Train 23 | Test Not Hispanic 43 | 0.75 | 0.8 | 0.79 |
| 428 | Train 7 | Test Not Hispanic 44 | 0.75 | 0.78 | 0.77 | 1452 | Train 23 | Test Not Hispanic 44 | 0.75 | 0.78 | 0.76 |
| 429 | Train 7 | Test Not Hispanic 45 | 0.74 | 0.77 | 0.74 | 1453 | Train 23 | Test Not Hispanic 45 | 0.74 | 0.77 | 0.73 |
| 430 | Train 7 | Test Not Hispanic 46 | 0.7 | 0.71 | 0.68 | 1454 | Train 23 | Test Not Hispanic 46 | 0.7 | 0.71 | 0.67 |
| 431 | Train 7 | Test Not Hispanic 47 | 0.73 | 0.77 | 0.73 | 1455 | Train 23 | Test Not Hispanic 47 | 0.73 | 0.77 | 0.73 |
| 432 | Train 7 | Test Not Hispanic 48 | 0.7 | 0.71 | 0.68 | 1456 | Train 23 | Test Not Hispanic 48 | 0.7 | 0.71 | 0.67 |
| 433 | Train 7 | Test Not Hispanic 49 | 0.76 | 0.77 | 0.8 | 1457 | Train 23 | Test Not Hispanic 49 | 0.75 | 0.77 | 0.8 |
| 434 | Train 7 | Test Not Hispanic 50 | 0.76 | 0.77 | 0.8 | 1458 | Train 23 | Test Not Hispanic 50 | 0.76 | 0.77 | 0.8 |
| 435 | Train 7 | Test Not Hispanic 51 | 0.76 | 0.77 | 0.8 | 1459 | Train 23 | Test Not Hispanic 51 | 0.76 | 0.77 | 0.8 |
| 436 | Train 7 | Test Not Hispanic 52 | 0.76 | 0.77 | 0.8 | 1460 | Train 23 | Test Not Hispanic 52 | 0.76 | 0.77 | 0.8 |
| 437 | Train 7 | Test Not Hispanic 53 | 0.75 | 0.78 | 0.68 | 1461 | Train 23 | Test Not Hispanic 53 | 0.75 | 0.78 | 0.75 |
| 438 | Train 7 | Test Not Hispanic 54 | 0.71 | 0.71 | 0.68 | 1462 | Train 23 | Test Not Hispanic 54 | 0.71 | 0.71 | 0.67 |
| 439 | Train 7 | Test Not Hispanic 55 | 0.75 | 0.78 | 0.67 | 1463 | Train 23 | Test Not Hispanic 55 | 0.75 | 0.78 | 0.75 |
| 440 | Train 7 | Test Not Hispanic 56 | 0.71 | 0.71 | 0.68 | 1464 | Train 23 | Test Not Hispanic 56 | 0.71 | 0.71 | 0.67 |
| 441 | Train 7 | Test Not Hispanic 57 | 0.76 | 0.77 | 0.78 | 1465 | Train 23 | Test Not Hispanic 57 | 0.76 | 0.77 | 0.78 |
| 442 | Train 7 | Test Not Hispanic 58 | 0.76 | 0.77 | 0.78 | 1466 | Train 23 | Test Not Hispanic 58 | 0.76 | 0.77 | 0.78 |
| 443 | Train 7 | Test Not Hispanic 59 | 0.76 | 0.77 | 0.78 | 1467 | Train 23 | Test Not Hispanic 59 | 0.76 | 0.77 | 0.79 |
| 444 | Train 7 | Test Not Hispanic 60 | 0.76 | 0.77 | 0.78 | 1468 | Train 23 | Test Not Hispanic 60 | 0.76 | 0.77 | 0.79 |
| 445 | Train 7 | Test Not Hispanic 61 | 0.75 | 0.78 | 0.67 | 1469 | Train 23 | Test Not Hispanic 61 | 0.75 | 0.78 | 0.75 |
| 446 | Train 7 | Test Not Hispanic 62 | 0.71 | 0.71 | 0.69 | 1470 | Train 23 | Test Not Hispanic 62 | 0.71 | 0.71 | 0.67 |
| 447 | Train 7 | Test Not Hispanic 63 | 0.75 | 0.78 | 0.67 | 1471 | Train 23 | Test Not Hispanic 63 | 0.75 | 0.78 | 0.74 |
| 448 | Train 7 | Test Not Hispanic 64 | 0.71 | 0.71 | 0.68 | 1472 | Train 23 | Test Not Hispanic 64 | 0.71 | 0.71 | 0.67 |
| 449 | Train 8 | Test Not Hispanic 1 | 0.73 | 0.77 | 0.76 | 1473 | Train 24 | Test Not Hispanic 1 | 0.73 | 0.77 | 0.76 |
| 450 | Train 8 | Test Not Hispanic 2 | 0.75 | 0.78 | 0.77 | 1474 | Train 24 | Test Not Hispanic 2 | 0.75 | 0.78 | 0.77 |
| 451 | Train 8 | Test Not Hispanic 3 | 0.73 | 0.77 | 0.76 | 1475 | Train 24 | Test Not Hispanic 3 | 0.73 | 0.77 | 0.76 |
| 452 | Train 8 | Test Not Hispanic 4 | 0.75 | 0.78 | 0.77 | 1476 | Train 24 | Test Not Hispanic 4 | 0.75 | 0.78 | 0.77 |
| 453 | Train 8 | Test Not Hispanic 5 | 0.72 | 0.77 | 0.77 | 1477 | Train 24 | Test Not Hispanic 5 | 0.72 | 0.77 | 0.77 |
| 454 | Train 8 | Test Not Hispanic 6 | 0.73 | 0.78 | 0.79 | 1478 | Train 24 | Test Not Hispanic 6 | 0.73 | 0.78 | 0.79 |
| 455 | Train 8 | Test Not Hispanic 7 | 0.72 | 0.77 | 0.77 | 1479 | Train 24 | Test Not Hispanic 7 | 0.72 | 0.77 | 0.77 |
| 456 | Train 8 | Test Not Hispanic 8 | 0.73 | 0.78 | 0.79 | 1480 | Train 24 | Test Not Hispanic 8 | 0.73 | 0.78 | 0.79 |
| 457 | Train 8 | Test Not Hispanic 9 | 0.73 | 0.77 | 0.77 | 1481 | Train 24 | Test Not Hispanic 9 | 0.73 | 0.77 | 0.77 |
| 458 | Train 8 | Test Not Hispanic 10 | 0.75 | 0.78 | 0.78 | 1482 | Train 24 | Test Not Hispanic 10 | 0.75 | 0.78 | 0.77 |
| 459 | Train 8 | Test Not Hispanic 11 | 0.73 | 0.77 | 0.77 | 1483 | Train 24 | Test Not Hispanic 11 | 0.73 | 0.77 | 0.77 |
| 460 | Train 8 | Test Not Hispanic 12 | 0.75 | 0.78 | 0.78 | 1484 | Train 24 | Test Not Hispanic 12 | 0.75 | 0.78 | 0.77 |
| 461 | Train 8 | Test Not Hispanic 13 | 0.72 | 0.77 | 0.78 | 1485 | Train 24 | Test Not Hispanic 13 | 0.72 | 0.77 | 0.78 |
| 462 | Train 8 | Test Not Hispanic 14 | 0.74 | 0.78 | 0.79 | 1486 | Train 24 | Test Not Hispanic 14 | 0.74 | 0.78 | 0.79 |
| 463 | Train 8 | Test Not Hispanic 15 | 0.72 | 0.77 | 0.78 | 1487 | Train 24 | Test Not Hispanic 15 | 0.72 | 0.77 | 0.78 |
| 464 | Train 8 | Test Not Hispanic 16 | 0.73 | 0.78 | 0.79 | 1488 | Train 24 | Test Not Hispanic 16 | 0.73 | 0.78 | 0.79 |
| 465 | Train 8 | Test Not Hispanic 17 | 0.73 | 0.77 | 0.76 | 1489 | Train 24 | Test Not Hispanic 17 | 0.73 | 0.77 | 0.76 |
| 466 | Train 8 | Test Not Hispanic 18 | 0.75 | 0.78 | 0.77 | 1490 | Train 24 | Test Not Hispanic 18 | 0.75 | 0.78 | 0.77 |
| 467 | Train 8 | Test Not Hispanic 19 | 0.73 | 0.77 | 0.76 | 1491 | Train 24 | Test Not Hispanic 19 | 0.73 | 0.77 | 0.76 |
| 468 | Train 8 | Test Not Hispanic 20 | 0.75 | 0.78 | 0.77 | 1492 | Train 24 | Test Not Hispanic 20 | 0.75 | 0.78 | 0.77 |
| 469 | Train 8 | Test Not Hispanic 21 | 0.72 | 0.77 | 0.77 | 1493 | Train 24 | Test Not Hispanic 21 | 0.72 | 0.77 | 0.77 |
| 470 | Train 8 | Test Not Hispanic 22 | 0.73 | 0.78 | 0.79 | 1494 | Train 24 | Test Not Hispanic 22 | 0.73 | 0.78 | 0.79 |
| 471 | Train 8 | Test Not Hispanic 23 | 0.72 | 0.77 | 0.77 | 1495 | Train 24 | Test Not Hispanic 23 | 0.72 | 0.77 | 0.77 |
| 472 | Train 8 | Test Not Hispanic 24 | 0.73 | 0.78 | 0.79 | 1496 | Train 24 | Test Not Hispanic 24 | 0.73 | 0.78 | 0.79 |
| 473 | Train 8 | Test Not Hispanic 25 | 0.73 | 0.77 | 0.77 | 1497 | Train 24 | Test Not Hispanic 25 | 0.73 | 0.77 | 0.77 |
| 474 | Train 8 | Test Not Hispanic 26 | 0.75 | 0.78 | 0.78 | 1498 | Train 24 | Test Not Hispanic 26 | 0.75 | 0.78 | 0.77 |
| 475 | Train 8 | Test Not Hispanic 27 | 0.73 | 0.77 | 0.77 | 1499 | Train 24 | Test Not Hispanic 27 | 0.74 | 0.77 | 0.77 |
| 476 | Train 8 | Test Not Hispanic 28 | 0.75 | 0.78 | 0.78 | 1500 | Train 24 | Test Not Hispanic 28 | 0.75 | 0.78 | 0.77 |
| 477 | Train 8 | Test Not Hispanic 29 | 0.72 | 0.77 | 0.78 | 1501 | Train 24 | Test Not Hispanic 29 | 0.72 | 0.77 | 0.78 |
| 478 | Train 8 | Test Not Hispanic 30 | 0.74 | 0.78 | 0.79 | 1502 | Train 24 | Test Not Hispanic 30 | 0.74 | 0.78 | 0.79 |
| 479 | Train 8 | Test Not Hispanic 31 | 0.72 | 0.77 | 0.78 | 1503 | Train 24 | Test Not Hispanic 31 | 0.72 | 0.77 | 0.78 |
| 480 | Train 8 | Test Not Hispanic 32 | 0.74 | 0.78 | 0.79 | 1504 | Train 24 | Test Not Hispanic 32 | 0.74 | 0.78 | 0.79 |
| 481 | Train 8 | Test Not Hispanic 33 | 0.73 | 0.77 | 0.76 | 1505 | Train 24 | Test Not Hispanic 33 | 0.74 | 0.77 | 0.76 |
| 482 | Train 8 | Test Not Hispanic 34 | 0.75 | 0.78 | 0.77 | 1506 | Train 24 | Test Not Hispanic 34 | 0.75 | 0.78 | 0.77 |
| 483 | Train 8 | Test Not Hispanic 35 | 0.73 | 0.77 | 0.76 | 1507 | Train 24 | Test Not Hispanic 35 | 0.74 | 0.77 | 0.76 |
| 484 | Train 8 | Test Not Hispanic 36 | 0.75 | 0.78 | 0.77 | 1508 | Train 24 | Test Not Hispanic 36 | 0.75 | 0.78 | 0.77 |
| 485 | Train 8 | Test Not Hispanic 37 | 0.72 | 0.77 | 0.77 | 1509 | Train 24 | Test Not Hispanic 37 | 0.72 | 0.77 | 0.77 |
| 486 | Train 8 | Test Not Hispanic 38 | 0.73 | 0.78 | 0.79 | 1510 | Train 24 | Test Not Hispanic 38 | 0.73 | 0.78 | 0.79 |
| 487 | Train 8 | Test Not Hispanic 39 | 0.72 | 0.77 | 0.77 | 1511 | Train 24 | Test Not Hispanic 39 | 0.72 | 0.77 | 0.77 |
| 488 | Train 8 | Test Not Hispanic 40 | 0.73 | 0.78 | 0.79 | 1512 | Train 24 | Test Not Hispanic 40 | 0.73 | 0.78 | 0.79 |
| 489 | Train 8 | Test Not Hispanic 41 | 0.74 | 0.77 | 0.77 | 1513 | Train 24 | Test Not Hispanic 41 | 0.74 | 0.77 | 0.77 |
| 490 | Train 8 | Test Not Hispanic 42 | 0.75 | 0.78 | 0.78 | 1514 | Train 24 | Test Not Hispanic 42 | 0.75 | 0.78 | 0.77 |
| 491 | Train 8 | Test Not Hispanic 43 | 0.74 | 0.77 | 0.77 | 1515 | Train 24 | Test Not Hispanic 43 | 0.74 | 0.77 | 0.77 |
| 492 | Train 8 | Test Not Hispanic 44 | 0.75 | 0.78 | 0.78 | 1516 | Train 24 | Test Not Hispanic 44 | 0.75 | 0.78 | 0.77 |
| 493 | Train 8 | Test Not Hispanic 45 | 0.72 | 0.77 | 0.78 | 1517 | Train 24 | Test Not Hispanic 45 | 0.72 | 0.77 | 0.78 |
| 494 | Train 8 | Test Not Hispanic 46 | 0.74 | 0.78 | 0.79 | 1518 | Train 24 | Test Not Hispanic 46 | 0.74 | 0.78 | 0.79 |
| 495 | Train 8 | Test Not Hispanic 47 | 0.72 | 0.77 | 0.78 | 1519 | Train 24 | Test Not Hispanic 47 | 0.72 | 0.77 | 0.78 |
| 496 | Train 8 | Test Not Hispanic 48 | 0.74 | 0.78 | 0.79 | 1520 | Train 24 | Test Not Hispanic 48 | 0.74 | 0.78 | 0.79 |
| 497 | Train 8 | Test Not Hispanic 49 | 0.75 | 0.78 | 0.73 | 1521 | Train 24 | Test Not Hispanic 49 | 0.75 | 0.78 | 0.77 |
| 498 | Train 8 | Test Not Hispanic 50 | 0.75 | 0.78 | 0.73 | 1522 | Train 24 | Test Not Hispanic 50 | 0.75 | 0.78 | 0.77 |
| 499 | Train 8 | Test Not Hispanic 51 | 0.75 | 0.78 | 0.74 | 1523 | Train 24 | Test Not Hispanic 51 | 0.75 | 0.78 | 0.77 |
| 500 | Train 8 | Test Not Hispanic 52 | 0.75 | 0.78 | 0.74 | 1524 | Train 24 | Test Not Hispanic 52 | 0.75 | 0.78 | 0.77 |
| 501 | Train 8 | Test Not Hispanic 53 | 0.74 | 0.79 | 0.73 | 1525 | Train 24 | Test Not Hispanic 53 | 0.74 | 0.79 | 0.76 |
| 502 | Train 8 | Test Not Hispanic 54 | 0.74 | 0.78 | 0.79 | 1526 | Train 24 | Test Not Hispanic 54 | 0.74 | 0.78 | 0.79 |
| 503 | Train 8 | Test Not Hispanic 55 | 0.74 | 0.79 | 0.73 | 1527 | Train 24 | Test Not Hispanic 55 | 0.74 | 0.79 | 0.76 |
| 504 | Train 8 | Test Not Hispanic 56 | 0.74 | 0.78 | 0.79 | 1528 | Train 24 | Test Not Hispanic 56 | 0.74 | 0.78 | 0.79 |
| 505 | Train 8 | Test Not Hispanic 57 | 0.75 | 0.78 | 0.75 | 1529 | Train 24 | Test Not Hispanic 57 | 0.76 | 0.79 | 0.78 |
| 506 | Train 8 | Test Not Hispanic 58 | 0.75 | 0.79 | 0.75 | 1530 | Train 24 | Test Not Hispanic 58 | 0.76 | 0.79 | 0.78 |
| 507 | Train 8 | Test Not Hispanic 59 | 0.75 | 0.78 | 0.75 | 1531 | Train 24 | Test Not Hispanic 59 | 0.76 | 0.79 | 0.78 |
| 508 | Train 8 | Test Not Hispanic 60 | 0.76 | 0.78 | 0.75 | 1532 | Train 24 | Test Not Hispanic 60 | 0.76 | 0.79 | 0.78 |
| 509 | Train 8 | Test Not Hispanic 61 | 0.74 | 0.79 | 0.74 | 1533 | Train 24 | Test Not Hispanic 61 | 0.74 | 0.79 | 0.77 |
| 510 | Train 8 | Test Not Hispanic 62 | 0.74 | 0.78 | 0.79 | 1534 | Train 24 | Test Not Hispanic 62 | 0.74 | 0.78 | 0.79 |
| 511 | Train 8 | Test Not Hispanic 63 | 0.74 | 0.79 | 0.74 | 1535 | Train 24 | Test Not Hispanic 63 | 0.74 | 0.79 | 0.77 |
| 512 | Train 8 | Test Not Hispanic 64 | 0.74 | 0.78 | 0.79 | 1536 | Train 24 | Test Not Hispanic 64 | 0.74 | 0.78 | 0.79 |
| 513 | Train 9 | Test Not Hispanic 1 | 0.75 | 0.82 | 0.81 | 1537 | Train 25 | Test Not Hispanic 1 | 0.75 | 0.82 | 0.81 |
| 514 | Train 9 | Test Not Hispanic 2 | 0.74 | 0.78 | 0.76 | 1538 | Train 25 | Test Not Hispanic 2 | 0.74 | 0.78 | 0.77 |
| 515 | Train 9 | Test Not Hispanic 3 | 0.75 | 0.82 | 0.81 | 1539 | Train 25 | Test Not Hispanic 3 | 0.75 | 0.82 | 0.81 |
| 516 | Train 9 | Test Not Hispanic 4 | 0.74 | 0.78 | 0.76 | 1540 | Train 25 | Test Not Hispanic 4 | 0.74 | 0.78 | 0.77 |
| 517 | Train 9 | Test Not Hispanic 5 | 0.71 | 0.73 | 0.67 | 1541 | Train 25 | Test Not Hispanic 5 | 0.71 | 0.73 | 0.7 |
| 518 | Train 9 | Test Not Hispanic 6 | 0.69 | 0.7 | 0.64 | 1542 | Train 25 | Test Not Hispanic 6 | 0.69 | 0.7 | 0.66 |
| 519 | Train 9 | Test Not Hispanic 7 | 0.71 | 0.73 | 0.67 | 1543 | Train 25 | Test Not Hispanic 7 | 0.71 | 0.73 | 0.7 |
| 520 | Train 9 | Test Not Hispanic 8 | 0.7 | 0.7 | 0.64 | 1544 | Train 25 | Test Not Hispanic 8 | 0.69 | 0.7 | 0.66 |
| 521 | Train 9 | Test Not Hispanic 9 | 0.75 | 0.83 | 0.85 | 1545 | Train 25 | Test Not Hispanic 9 | 0.75 | 0.83 | 0.85 |
| 522 | Train 9 | Test Not Hispanic 10 | 0.74 | 0.78 | 0.79 | 1546 | Train 25 | Test Not Hispanic 10 | 0.74 | 0.78 | 0.8 |
| 523 | Train 9 | Test Not Hispanic 11 | 0.75 | 0.83 | 0.85 | 1547 | Train 25 | Test Not Hispanic 11 | 0.75 | 0.83 | 0.85 |
| 524 | Train 9 | Test Not Hispanic 12 | 0.74 | 0.78 | 0.79 | 1548 | Train 25 | Test Not Hispanic 12 | 0.74 | 0.78 | 0.8 |
| 525 | Train 9 | Test Not Hispanic 13 | 0.71 | 0.74 | 0.68 | 1549 | Train 25 | Test Not Hispanic 13 | 0.71 | 0.73 | 0.71 |
| 526 | Train 9 | Test Not Hispanic 14 | 0.7 | 0.7 | 0.64 | 1550 | Train 25 | Test Not Hispanic 14 | 0.69 | 0.7 | 0.66 |
| 527 | Train 9 | Test Not Hispanic 15 | 0.71 | 0.73 | 0.68 | 1551 | Train 25 | Test Not Hispanic 15 | 0.71 | 0.73 | 0.71 |
| 528 | Train 9 | Test Not Hispanic 16 | 0.7 | 0.7 | 0.64 | 1552 | Train 25 | Test Not Hispanic 16 | 0.7 | 0.7 | 0.66 |
| 529 | Train 9 | Test Not Hispanic 17 | 0.75 | 0.82 | 0.81 | 1553 | Train 25 | Test Not Hispanic 17 | 0.75 | 0.82 | 0.81 |
| 530 | Train 9 | Test Not Hispanic 18 | 0.74 | 0.78 | 0.76 | 1554 | Train 25 | Test Not Hispanic 18 | 0.74 | 0.78 | 0.77 |
| 531 | Train 9 | Test Not Hispanic 19 | 0.75 | 0.82 | 0.81 | 1555 | Train 25 | Test Not Hispanic 19 | 0.75 | 0.82 | 0.81 |
| 532 | Train 9 | Test Not Hispanic 20 | 0.74 | 0.78 | 0.76 | 1556 | Train 25 | Test Not Hispanic 20 | 0.74 | 0.78 | 0.77 |
| 533 | Train 9 | Test Not Hispanic 21 | 0.71 | 0.73 | 0.67 | 1557 | Train 25 | Test Not Hispanic 21 | 0.71 | 0.73 | 0.7 |
| 534 | Train 9 | Test Not Hispanic 22 | 0.7 | 0.7 | 0.64 | 1558 | Train 25 | Test Not Hispanic 22 | 0.69 | 0.7 | 0.66 |
| 535 | Train 9 | Test Not Hispanic 23 | 0.71 | 0.73 | 0.67 | 1559 | Train 25 | Test Not Hispanic 23 | 0.71 | 0.73 | 0.7 |
| 536 | Train 9 | Test Not Hispanic 24 | 0.7 | 0.7 | 0.64 | 1560 | Train 25 | Test Not Hispanic 24 | 0.69 | 0.7 | 0.66 |
| 537 | Train 9 | Test Not Hispanic 25 | 0.75 | 0.83 | 0.85 | 1561 | Train 25 | Test Not Hispanic 25 | 0.75 | 0.83 | 0.85 |
| 538 | Train 9 | Test Not Hispanic 26 | 0.74 | 0.78 | 0.79 | 1562 | Train 25 | Test Not Hispanic 26 | 0.74 | 0.78 | 0.8 |
| 539 | Train 9 | Test Not Hispanic 27 | 0.76 | 0.83 | 0.85 | 1563 | Train 25 | Test Not Hispanic 27 | 0.76 | 0.83 | 0.85 |
| 540 | Train 9 | Test Not Hispanic 28 | 0.74 | 0.78 | 0.79 | 1564 | Train 25 | Test Not Hispanic 28 | 0.74 | 0.78 | 0.8 |
| 541 | Train 9 | Test Not Hispanic 29 | 0.71 | 0.74 | 0.68 | 1565 | Train 25 | Test Not Hispanic 29 | 0.71 | 0.74 | 0.71 |
| 542 | Train 9 | Test Not Hispanic 30 | 0.7 | 0.7 | 0.64 | 1566 | Train 25 | Test Not Hispanic 30 | 0.7 | 0.7 | 0.67 |
| 543 | Train 9 | Test Not Hispanic 31 | 0.71 | 0.73 | 0.68 | 1567 | Train 25 | Test Not Hispanic 31 | 0.71 | 0.73 | 0.71 |
| 544 | Train 9 | Test Not Hispanic 32 | 0.7 | 0.7 | 0.64 | 1568 | Train 25 | Test Not Hispanic 32 | 0.7 | 0.7 | 0.66 |
| 545 | Train 9 | Test Not Hispanic 33 | 0.76 | 0.82 | 0.81 | 1569 | Train 25 | Test Not Hispanic 33 | 0.76 | 0.82 | 0.81 |
| 546 | Train 9 | Test Not Hispanic 34 | 0.74 | 0.78 | 0.76 | 1570 | Train 25 | Test Not Hispanic 34 | 0.74 | 0.78 | 0.77 |
| 547 | Train 9 | Test Not Hispanic 35 | 0.76 | 0.82 | 0.81 | 1571 | Train 25 | Test Not Hispanic 35 | 0.76 | 0.82 | 0.81 |
| 548 | Train 9 | Test Not Hispanic 36 | 0.74 | 0.78 | 0.76 | 1572 | Train 25 | Test Not Hispanic 36 | 0.74 | 0.78 | 0.77 |
| 549 | Train 9 | Test Not Hispanic 37 | 0.71 | 0.73 | 0.67 | 1573 | Train 25 | Test Not Hispanic 37 | 0.71 | 0.73 | 0.7 |
| 550 | Train 9 | Test Not Hispanic 38 | 0.7 | 0.7 | 0.64 | 1574 | Train 25 | Test Not Hispanic 38 | 0.69 | 0.7 | 0.66 |
| 551 | Train 9 | Test Not Hispanic 39 | 0.71 | 0.73 | 0.67 | 1575 | Train 25 | Test Not Hispanic 39 | 0.71 | 0.73 | 0.7 |
| 552 | Train 9 | Test Not Hispanic 40 | 0.7 | 0.7 | 0.64 | 1576 | Train 25 | Test Not Hispanic 40 | 0.69 | 0.7 | 0.66 |
| 553 | Train 9 | Test Not Hispanic 41 | 0.76 | 0.83 | 0.85 | 1577 | Train 25 | Test Not Hispanic 41 | 0.76 | 0.83 | 0.85 |
| 554 | Train 9 | Test Not Hispanic 42 | 0.74 | 0.78 | 0.79 | 1578 | Train 25 | Test Not Hispanic 42 | 0.74 | 0.78 | 0.8 |
| 555 | Train 9 | Test Not Hispanic 43 | 0.76 | 0.83 | 0.85 | 1579 | Train 25 | Test Not Hispanic 43 | 0.76 | 0.83 | 0.85 |
| 556 | Train 9 | Test Not Hispanic 44 | 0.74 | 0.78 | 0.79 | 1580 | Train 25 | Test Not Hispanic 44 | 0.74 | 0.78 | 0.8 |
| 557 | Train 9 | Test Not Hispanic 45 | 0.71 | 0.74 | 0.68 | 1581 | Train 25 | Test Not Hispanic 45 | 0.71 | 0.73 | 0.71 |
| 558 | Train 9 | Test Not Hispanic 46 | 0.7 | 0.7 | 0.64 | 1582 | Train 25 | Test Not Hispanic 46 | 0.69 | 0.7 | 0.67 |
| 559 | Train 9 | Test Not Hispanic 47 | 0.71 | 0.73 | 0.68 | 1583 | Train 25 | Test Not Hispanic 47 | 0.71 | 0.73 | 0.71 |
| 560 | Train 9 | Test Not Hispanic 48 | 0.7 | 0.7 | 0.64 | 1584 | Train 25 | Test Not Hispanic 48 | 0.7 | 0.7 | 0.66 |
| 561 | Train 9 | Test Not Hispanic 49 | 0.76 | 0.79 | 0.79 | 1585 | Train 25 | Test Not Hispanic 49 | 0.76 | 0.79 | 0.79 |
| 562 | Train 9 | Test Not Hispanic 50 | 0.76 | 0.79 | 0.79 | 1586 | Train 25 | Test Not Hispanic 50 | 0.76 | 0.79 | 0.79 |
| 563 | Train 9 | Test Not Hispanic 51 | 0.76 | 0.79 | 0.79 | 1587 | Train 25 | Test Not Hispanic 51 | 0.76 | 0.79 | 0.79 |
| 564 | Train 9 | Test Not Hispanic 52 | 0.76 | 0.79 | 0.79 | 1588 | Train 25 | Test Not Hispanic 52 | 0.76 | 0.79 | 0.79 |
| 565 | Train 9 | Test Not Hispanic 53 | 0.72 | 0.75 | 0.6 | 1589 | Train 25 | Test Not Hispanic 53 | 0.72 | 0.75 | 0.67 |
| 566 | Train 9 | Test Not Hispanic 54 | 0.71 | 0.71 | 0.63 | 1590 | Train 25 | Test Not Hispanic 54 | 0.71 | 0.71 | 0.67 |
| 567 | Train 9 | Test Not Hispanic 55 | 0.72 | 0.75 | 0.6 | 1591 | Train 25 | Test Not Hispanic 55 | 0.72 | 0.75 | 0.67 |
| 568 | Train 9 | Test Not Hispanic 56 | 0.71 | 0.7 | 0.63 | 1592 | Train 25 | Test Not Hispanic 56 | 0.71 | 0.7 | 0.67 |
| 569 | Train 9 | Test Not Hispanic 57 | 0.76 | 0.8 | 0.83 | 1593 | Train 25 | Test Not Hispanic 57 | 0.76 | 0.8 | 0.83 |
| 570 | Train 9 | Test Not Hispanic 58 | 0.76 | 0.8 | 0.83 | 1594 | Train 25 | Test Not Hispanic 58 | 0.76 | 0.8 | 0.83 |
| 571 | Train 9 | Test Not Hispanic 59 | 0.76 | 0.8 | 0.82 | 1595 | Train 25 | Test Not Hispanic 59 | 0.76 | 0.8 | 0.83 |
| 572 | Train 9 | Test Not Hispanic 60 | 0.76 | 0.8 | 0.82 | 1596 | Train 25 | Test Not Hispanic 60 | 0.76 | 0.8 | 0.82 |
| 573 | Train 9 | Test Not Hispanic 61 | 0.72 | 0.75 | 0.6 | 1597 | Train 25 | Test Not Hispanic 61 | 0.72 | 0.75 | 0.68 |
| 574 | Train 9 | Test Not Hispanic 62 | 0.71 | 0.71 | 0.63 | 1598 | Train 25 | Test Not Hispanic 62 | 0.71 | 0.71 | 0.67 |
| 575 | Train 9 | Test Not Hispanic 63 | 0.72 | 0.76 | 0.61 | 1599 | Train 25 | Test Not Hispanic 63 | 0.72 | 0.75 | 0.68 |
| 576 | Train 9 | Test Not Hispanic 64 | 0.71 | 0.7 | 0.63 | 1600 | Train 25 | Test Not Hispanic 64 | 0.71 | 0.7 | 0.67 |
| 577 | Train 10 | Test Not Hispanic 1 | 0.75 | 0.81 | 0.8 | 1601 | Train 26 | Test Not Hispanic 1 | 0.75 | 0.81 | 0.81 |
| 578 | Train 10 | Test Not Hispanic 2 | 0.76 | 0.82 | 0.82 | 1602 | Train 26 | Test Not Hispanic 2 | 0.76 | 0.82 | 0.82 |
| 579 | Train 10 | Test Not Hispanic 3 | 0.75 | 0.81 | 0.8 | 1603 | Train 26 | Test Not Hispanic 3 | 0.75 | 0.81 | 0.8 |
| 580 | Train 10 | Test Not Hispanic 4 | 0.76 | 0.82 | 0.82 | 1604 | Train 26 | Test Not Hispanic 4 | 0.76 | 0.82 | 0.82 |
| 581 | Train 10 | Test Not Hispanic 5 | 0.71 | 0.73 | 0.69 | 1605 | Train 26 | Test Not Hispanic 5 | 0.71 | 0.73 | 0.68 |
| 582 | Train 10 | Test Not Hispanic 6 | 0.71 | 0.71 | 0.68 | 1606 | Train 26 | Test Not Hispanic 6 | 0.71 | 0.72 | 0.68 |
| 583 | Train 10 | Test Not Hispanic 7 | 0.71 | 0.73 | 0.68 | 1607 | Train 26 | Test Not Hispanic 7 | 0.71 | 0.73 | 0.68 |
| 584 | Train 10 | Test Not Hispanic 8 | 0.71 | 0.71 | 0.68 | 1608 | Train 26 | Test Not Hispanic 8 | 0.71 | 0.71 | 0.68 |
| 585 | Train 10 | Test Not Hispanic 9 | 0.75 | 0.82 | 0.83 | 1609 | Train 26 | Test Not Hispanic 9 | 0.75 | 0.82 | 0.83 |
| 586 | Train 10 | Test Not Hispanic 10 | 0.76 | 0.83 | 0.84 | 1610 | Train 26 | Test Not Hispanic 10 | 0.76 | 0.83 | 0.84 |
| 587 | Train 10 | Test Not Hispanic 11 | 0.75 | 0.82 | 0.83 | 1611 | Train 26 | Test Not Hispanic 11 | 0.75 | 0.82 | 0.83 |
| 588 | Train 10 | Test Not Hispanic 12 | 0.76 | 0.83 | 0.84 | 1612 | Train 26 | Test Not Hispanic 12 | 0.76 | 0.83 | 0.84 |
| 589 | Train 10 | Test Not Hispanic 13 | 0.71 | 0.74 | 0.7 | 1613 | Train 26 | Test Not Hispanic 13 | 0.71 | 0.74 | 0.69 |
| 590 | Train 10 | Test Not Hispanic 14 | 0.71 | 0.72 | 0.68 | 1614 | Train 26 | Test Not Hispanic 14 | 0.71 | 0.72 | 0.68 |
| 591 | Train 10 | Test Not Hispanic 15 | 0.71 | 0.74 | 0.7 | 1615 | Train 26 | Test Not Hispanic 15 | 0.71 | 0.73 | 0.69 |
| 592 | Train 10 | Test Not Hispanic 16 | 0.71 | 0.71 | 0.68 | 1616 | Train 26 | Test Not Hispanic 16 | 0.71 | 0.71 | 0.68 |
| 593 | Train 10 | Test Not Hispanic 17 | 0.75 | 0.81 | 0.8 | 1617 | Train 26 | Test Not Hispanic 17 | 0.75 | 0.81 | 0.81 |
| 594 | Train 10 | Test Not Hispanic 18 | 0.76 | 0.82 | 0.82 | 1618 | Train 26 | Test Not Hispanic 18 | 0.76 | 0.82 | 0.82 |
| 595 | Train 10 | Test Not Hispanic 19 | 0.75 | 0.81 | 0.8 | 1619 | Train 26 | Test Not Hispanic 19 | 0.75 | 0.81 | 0.8 |
| 596 | Train 10 | Test Not Hispanic 20 | 0.76 | 0.82 | 0.82 | 1620 | Train 26 | Test Not Hispanic 20 | 0.76 | 0.82 | 0.82 |
| 597 | Train 10 | Test Not Hispanic 21 | 0.71 | 0.73 | 0.69 | 1621 | Train 26 | Test Not Hispanic 21 | 0.71 | 0.73 | 0.68 |
| 598 | Train 10 | Test Not Hispanic 22 | 0.71 | 0.72 | 0.68 | 1622 | Train 26 | Test Not Hispanic 22 | 0.71 | 0.72 | 0.68 |
| 599 | Train 10 | Test Not Hispanic 23 | 0.71 | 0.73 | 0.69 | 1623 | Train 26 | Test Not Hispanic 23 | 0.71 | 0.73 | 0.68 |
| 600 | Train 10 | Test Not Hispanic 24 | 0.71 | 0.71 | 0.68 | 1624 | Train 26 | Test Not Hispanic 24 | 0.71 | 0.71 | 0.68 |
| 601 | Train 10 | Test Not Hispanic 25 | 0.75 | 0.82 | 0.83 | 1625 | Train 26 | Test Not Hispanic 25 | 0.75 | 0.82 | 0.83 |
| 602 | Train 10 | Test Not Hispanic 26 | 0.76 | 0.83 | 0.84 | 1626 | Train 26 | Test Not Hispanic 26 | 0.76 | 0.83 | 0.84 |
| 603 | Train 10 | Test Not Hispanic 27 | 0.75 | 0.82 | 0.83 | 1627 | Train 26 | Test Not Hispanic 27 | 0.75 | 0.82 | 0.83 |
| 604 | Train 10 | Test Not Hispanic 28 | 0.76 | 0.83 | 0.84 | 1628 | Train 26 | Test Not Hispanic 28 | 0.76 | 0.83 | 0.84 |
| 605 | Train 10 | Test Not Hispanic 29 | 0.71 | 0.74 | 0.7 | 1629 | Train 26 | Test Not Hispanic 29 | 0.71 | 0.74 | 0.69 |
| 606 | Train 10 | Test Not Hispanic 30 | 0.71 | 0.72 | 0.68 | 1630 | Train 26 | Test Not Hispanic 30 | 0.71 | 0.72 | 0.68 |
| 607 | Train 10 | Test Not Hispanic 31 | 0.71 | 0.74 | 0.7 | 1631 | Train 26 | Test Not Hispanic 31 | 0.71 | 0.73 | 0.69 |
| 608 | Train 10 | Test Not Hispanic 32 | 0.71 | 0.71 | 0.68 | 1632 | Train 26 | Test Not Hispanic 32 | 0.71 | 0.71 | 0.68 |
| 609 | Train 10 | Test Not Hispanic 33 | 0.75 | 0.81 | 0.8 | 1633 | Train 26 | Test Not Hispanic 33 | 0.75 | 0.81 | 0.81 |
| 610 | Train 10 | Test Not Hispanic 34 | 0.76 | 0.82 | 0.82 | 1634 | Train 26 | Test Not Hispanic 34 | 0.76 | 0.82 | 0.82 |
| 611 | Train 10 | Test Not Hispanic 35 | 0.75 | 0.81 | 0.8 | 1635 | Train 26 | Test Not Hispanic 35 | 0.75 | 0.81 | 0.8 |
| 612 | Train 10 | Test Not Hispanic 36 | 0.76 | 0.82 | 0.82 | 1636 | Train 26 | Test Not Hispanic 36 | 0.76 | 0.82 | 0.82 |
| 613 | Train 10 | Test Not Hispanic 37 | 0.71 | 0.73 | 0.69 | 1637 | Train 26 | Test Not Hispanic 37 | 0.71 | 0.73 | 0.68 |
| 614 | Train 10 | Test Not Hispanic 38 | 0.71 | 0.72 | 0.68 | 1638 | Train 26 | Test Not Hispanic 38 | 0.71 | 0.72 | 0.68 |
| 615 | Train 10 | Test Not Hispanic 39 | 0.71 | 0.73 | 0.69 | 1639 | Train 26 | Test Not Hispanic 39 | 0.71 | 0.73 | 0.68 |
| 616 | Train 10 | Test Not Hispanic 40 | 0.71 | 0.71 | 0.68 | 1640 | Train 26 | Test Not Hispanic 40 | 0.71 | 0.71 | 0.68 |
| 617 | Train 10 | Test Not Hispanic 41 | 0.75 | 0.82 | 0.83 | 1641 | Train 26 | Test Not Hispanic 41 | 0.75 | 0.82 | 0.83 |
| 618 | Train 10 | Test Not Hispanic 42 | 0.76 | 0.83 | 0.84 | 1642 | Train 26 | Test Not Hispanic 42 | 0.76 | 0.83 | 0.84 |
| 619 | Train 10 | Test Not Hispanic 43 | 0.75 | 0.82 | 0.83 | 1643 | Train 26 | Test Not Hispanic 43 | 0.75 | 0.82 | 0.83 |
| 620 | Train 10 | Test Not Hispanic 44 | 0.76 | 0.83 | 0.84 | 1644 | Train 26 | Test Not Hispanic 44 | 0.76 | 0.83 | 0.84 |
| 621 | Train 10 | Test Not Hispanic 45 | 0.71 | 0.74 | 0.7 | 1645 | Train 26 | Test Not Hispanic 45 | 0.71 | 0.74 | 0.69 |
| 622 | Train 10 | Test Not Hispanic 46 | 0.71 | 0.72 | 0.68 | 1646 | Train 26 | Test Not Hispanic 46 | 0.71 | 0.72 | 0.68 |
| 623 | Train 10 | Test Not Hispanic 47 | 0.71 | 0.74 | 0.7 | 1647 | Train 26 | Test Not Hispanic 47 | 0.71 | 0.73 | 0.69 |
| 624 | Train 10 | Test Not Hispanic 48 | 0.71 | 0.71 | 0.68 | 1648 | Train 26 | Test Not Hispanic 48 | 0.71 | 0.71 | 0.68 |
| 625 | Train 10 | Test Not Hispanic 49 | 0.76 | 0.79 | 0.76 | 1649 | Train 26 | Test Not Hispanic 49 | 0.76 | 0.79 | 0.78 |
| 626 | Train 10 | Test Not Hispanic 50 | 0.76 | 0.79 | 0.76 | 1650 | Train 26 | Test Not Hispanic 50 | 0.76 | 0.79 | 0.78 |
| 627 | Train 10 | Test Not Hispanic 51 | 0.76 | 0.79 | 0.76 | 1651 | Train 26 | Test Not Hispanic 51 | 0.76 | 0.79 | 0.78 |
| 628 | Train 10 | Test Not Hispanic 52 | 0.76 | 0.79 | 0.76 | 1652 | Train 26 | Test Not Hispanic 52 | 0.76 | 0.79 | 0.77 |
| 629 | Train 10 | Test Not Hispanic 53 | 0.73 | 0.76 | 0.66 | 1653 | Train 26 | Test Not Hispanic 53 | 0.73 | 0.76 | 0.64 |
| 630 | Train 10 | Test Not Hispanic 54 | 0.71 | 0.72 | 0.68 | 1654 | Train 26 | Test Not Hispanic 54 | 0.71 | 0.72 | 0.67 |
| 631 | Train 10 | Test Not Hispanic 55 | 0.73 | 0.76 | 0.66 | 1655 | Train 26 | Test Not Hispanic 55 | 0.73 | 0.76 | 0.64 |
| 632 | Train 10 | Test Not Hispanic 56 | 0.71 | 0.71 | 0.68 | 1656 | Train 26 | Test Not Hispanic 56 | 0.71 | 0.71 | 0.67 |
| 633 | Train 10 | Test Not Hispanic 57 | 0.76 | 0.79 | 0.8 | 1657 | Train 26 | Test Not Hispanic 57 | 0.76 | 0.79 | 0.81 |
| 634 | Train 10 | Test Not Hispanic 58 | 0.76 | 0.79 | 0.8 | 1658 | Train 26 | Test Not Hispanic 58 | 0.76 | 0.79 | 0.81 |
| 635 | Train 10 | Test Not Hispanic 59 | 0.76 | 0.79 | 0.8 | 1659 | Train 26 | Test Not Hispanic 59 | 0.76 | 0.79 | 0.81 |
| 636 | Train 10 | Test Not Hispanic 60 | 0.76 | 0.79 | 0.8 | 1660 | Train 26 | Test Not Hispanic 60 | 0.76 | 0.79 | 0.81 |
| 637 | Train 10 | Test Not Hispanic 61 | 0.73 | 0.76 | 0.67 | 1661 | Train 26 | Test Not Hispanic 61 | 0.73 | 0.76 | 0.65 |
| 638 | Train 10 | Test Not Hispanic 62 | 0.72 | 0.72 | 0.68 | 1662 | Train 26 | Test Not Hispanic 62 | 0.72 | 0.72 | 0.67 |
| 639 | Train 10 | Test Not Hispanic 63 | 0.73 | 0.76 | 0.67 | 1663 | Train 26 | Test Not Hispanic 63 | 0.73 | 0.76 | 0.65 |
| 640 | Train 10 | Test Not Hispanic 64 | 0.71 | 0.71 | 0.68 | 1664 | Train 26 | Test Not Hispanic 64 | 0.71 | 0.71 | 0.67 |
| 641 | Train 11 | Test Not Hispanic 1 | 0.75 | 0.82 | 0.81 | 1665 | Train 27 | Test Not Hispanic 1 | 0.75 | 0.82 | 0.81 |
| 642 | Train 11 | Test Not Hispanic 2 | 0.74 | 0.78 | 0.76 | 1666 | Train 27 | Test Not Hispanic 2 | 0.74 | 0.78 | 0.76 |
| 643 | Train 11 | Test Not Hispanic 3 | 0.75 | 0.82 | 0.81 | 1667 | Train 27 | Test Not Hispanic 3 | 0.75 | 0.82 | 0.81 |
| 644 | Train 11 | Test Not Hispanic 4 | 0.74 | 0.78 | 0.76 | 1668 | Train 27 | Test Not Hispanic 4 | 0.74 | 0.78 | 0.76 |
| 645 | Train 11 | Test Not Hispanic 5 | 0.71 | 0.73 | 0.68 | 1669 | Train 27 | Test Not Hispanic 5 | 0.71 | 0.73 | 0.68 |
| 646 | Train 11 | Test Not Hispanic 6 | 0.69 | 0.7 | 0.65 | 1670 | Train 27 | Test Not Hispanic 6 | 0.69 | 0.7 | 0.64 |
| 647 | Train 11 | Test Not Hispanic 7 | 0.71 | 0.73 | 0.68 | 1671 | Train 27 | Test Not Hispanic 7 | 0.71 | 0.73 | 0.68 |
| 648 | Train 11 | Test Not Hispanic 8 | 0.69 | 0.7 | 0.65 | 1672 | Train 27 | Test Not Hispanic 8 | 0.69 | 0.7 | 0.64 |
| 649 | Train 11 | Test Not Hispanic 9 | 0.75 | 0.83 | 0.84 | 1673 | Train 27 | Test Not Hispanic 9 | 0.75 | 0.83 | 0.85 |
| 650 | Train 11 | Test Not Hispanic 10 | 0.74 | 0.78 | 0.79 | 1674 | Train 27 | Test Not Hispanic 10 | 0.74 | 0.78 | 0.79 |
| 651 | Train 11 | Test Not Hispanic 11 | 0.75 | 0.83 | 0.84 | 1675 | Train 27 | Test Not Hispanic 11 | 0.75 | 0.83 | 0.85 |
| 652 | Train 11 | Test Not Hispanic 12 | 0.74 | 0.78 | 0.79 | 1676 | Train 27 | Test Not Hispanic 12 | 0.74 | 0.78 | 0.79 |
| 653 | Train 11 | Test Not Hispanic 13 | 0.71 | 0.74 | 0.69 | 1677 | Train 27 | Test Not Hispanic 13 | 0.71 | 0.74 | 0.7 |
| 654 | Train 11 | Test Not Hispanic 14 | 0.7 | 0.7 | 0.65 | 1678 | Train 27 | Test Not Hispanic 14 | 0.7 | 0.7 | 0.64 |
| 655 | Train 11 | Test Not Hispanic 15 | 0.71 | 0.73 | 0.69 | 1679 | Train 27 | Test Not Hispanic 15 | 0.71 | 0.74 | 0.7 |
| 656 | Train 11 | Test Not Hispanic 16 | 0.7 | 0.7 | 0.65 | 1680 | Train 27 | Test Not Hispanic 16 | 0.7 | 0.7 | 0.64 |
| 657 | Train 11 | Test Not Hispanic 17 | 0.75 | 0.82 | 0.81 | 1681 | Train 27 | Test Not Hispanic 17 | 0.75 | 0.82 | 0.81 |
| 658 | Train 11 | Test Not Hispanic 18 | 0.74 | 0.78 | 0.76 | 1682 | Train 27 | Test Not Hispanic 18 | 0.74 | 0.78 | 0.76 |
| 659 | Train 11 | Test Not Hispanic 19 | 0.75 | 0.82 | 0.81 | 1683 | Train 27 | Test Not Hispanic 19 | 0.75 | 0.82 | 0.81 |
| 660 | Train 11 | Test Not Hispanic 20 | 0.74 | 0.78 | 0.76 | 1684 | Train 27 | Test Not Hispanic 20 | 0.74 | 0.78 | 0.76 |
| 661 | Train 11 | Test Not Hispanic 21 | 0.71 | 0.73 | 0.68 | 1685 | Train 27 | Test Not Hispanic 21 | 0.71 | 0.73 | 0.68 |
| 662 | Train 11 | Test Not Hispanic 22 | 0.69 | 0.7 | 0.65 | 1686 | Train 27 | Test Not Hispanic 22 | 0.69 | 0.7 | 0.64 |
| 663 | Train 11 | Test Not Hispanic 23 | 0.71 | 0.73 | 0.68 | 1687 | Train 27 | Test Not Hispanic 23 | 0.71 | 0.73 | 0.68 |
| 664 | Train 11 | Test Not Hispanic 24 | 0.69 | 0.7 | 0.65 | 1688 | Train 27 | Test Not Hispanic 24 | 0.69 | 0.7 | 0.64 |
| 665 | Train 11 | Test Not Hispanic 25 | 0.75 | 0.83 | 0.84 | 1689 | Train 27 | Test Not Hispanic 25 | 0.75 | 0.83 | 0.85 |
| 666 | Train 11 | Test Not Hispanic 26 | 0.74 | 0.78 | 0.79 | 1690 | Train 27 | Test Not Hispanic 26 | 0.74 | 0.78 | 0.79 |
| 667 | Train 11 | Test Not Hispanic 27 | 0.76 | 0.83 | 0.84 | 1691 | Train 27 | Test Not Hispanic 27 | 0.76 | 0.83 | 0.85 |
| 668 | Train 11 | Test Not Hispanic 28 | 0.74 | 0.78 | 0.79 | 1692 | Train 27 | Test Not Hispanic 28 | 0.74 | 0.78 | 0.79 |
| 669 | Train 11 | Test Not Hispanic 29 | 0.71 | 0.74 | 0.69 | 1693 | Train 27 | Test Not Hispanic 29 | 0.71 | 0.74 | 0.7 |
| 670 | Train 11 | Test Not Hispanic 30 | 0.7 | 0.7 | 0.65 | 1694 | Train 27 | Test Not Hispanic 30 | 0.7 | 0.7 | 0.64 |
| 671 | Train 11 | Test Not Hispanic 31 | 0.71 | 0.73 | 0.69 | 1695 | Train 27 | Test Not Hispanic 31 | 0.71 | 0.74 | 0.7 |
| 672 | Train 11 | Test Not Hispanic 32 | 0.7 | 0.7 | 0.65 | 1696 | Train 27 | Test Not Hispanic 32 | 0.7 | 0.7 | 0.64 |
| 673 | Train 11 | Test Not Hispanic 33 | 0.76 | 0.82 | 0.81 | 1697 | Train 27 | Test Not Hispanic 33 | 0.76 | 0.82 | 0.81 |
| 674 | Train 11 | Test Not Hispanic 34 | 0.74 | 0.78 | 0.76 | 1698 | Train 27 | Test Not Hispanic 34 | 0.74 | 0.78 | 0.76 |
| 675 | Train 11 | Test Not Hispanic 35 | 0.76 | 0.82 | 0.81 | 1699 | Train 27 | Test Not Hispanic 35 | 0.76 | 0.82 | 0.81 |
| 676 | Train 11 | Test Not Hispanic 36 | 0.74 | 0.78 | 0.76 | 1700 | Train 27 | Test Not Hispanic 36 | 0.74 | 0.78 | 0.76 |
| 677 | Train 11 | Test Not Hispanic 37 | 0.71 | 0.73 | 0.68 | 1701 | Train 27 | Test Not Hispanic 37 | 0.71 | 0.73 | 0.68 |
| 678 | Train 11 | Test Not Hispanic 38 | 0.69 | 0.7 | 0.65 | 1702 | Train 27 | Test Not Hispanic 38 | 0.69 | 0.7 | 0.64 |
| 679 | Train 11 | Test Not Hispanic 39 | 0.71 | 0.73 | 0.68 | 1703 | Train 27 | Test Not Hispanic 39 | 0.71 | 0.73 | 0.68 |
| 680 | Train 11 | Test Not Hispanic 40 | 0.69 | 0.7 | 0.65 | 1704 | Train 27 | Test Not Hispanic 40 | 0.69 | 0.7 | 0.64 |
| 681 | Train 11 | Test Not Hispanic 41 | 0.76 | 0.83 | 0.84 | 1705 | Train 27 | Test Not Hispanic 41 | 0.76 | 0.83 | 0.85 |
| 682 | Train 11 | Test Not Hispanic 42 | 0.74 | 0.78 | 0.79 | 1706 | Train 27 | Test Not Hispanic 42 | 0.74 | 0.78 | 0.79 |
| 683 | Train 11 | Test Not Hispanic 43 | 0.76 | 0.83 | 0.84 | 1707 | Train 27 | Test Not Hispanic 43 | 0.76 | 0.83 | 0.85 |
| 684 | Train 11 | Test Not Hispanic 44 | 0.74 | 0.78 | 0.79 | 1708 | Train 27 | Test Not Hispanic 44 | 0.74 | 0.78 | 0.79 |
| 685 | Train 11 | Test Not Hispanic 45 | 0.71 | 0.74 | 0.7 | 1709 | Train 27 | Test Not Hispanic 45 | 0.71 | 0.74 | 0.7 |
| 686 | Train 11 | Test Not Hispanic 46 | 0.7 | 0.7 | 0.65 | 1710 | Train 27 | Test Not Hispanic 46 | 0.7 | 0.7 | 0.64 |
| 687 | Train 11 | Test Not Hispanic 47 | 0.71 | 0.73 | 0.69 | 1711 | Train 27 | Test Not Hispanic 47 | 0.71 | 0.74 | 0.7 |
| 688 | Train 11 | Test Not Hispanic 48 | 0.7 | 0.7 | 0.65 | 1712 | Train 27 | Test Not Hispanic 48 | 0.7 | 0.7 | 0.64 |
| 689 | Train 11 | Test Not Hispanic 49 | 0.76 | 0.79 | 0.78 | 1713 | Train 27 | Test Not Hispanic 49 | 0.76 | 0.79 | 0.79 |
| 690 | Train 11 | Test Not Hispanic 50 | 0.76 | 0.79 | 0.78 | 1714 | Train 27 | Test Not Hispanic 50 | 0.76 | 0.79 | 0.79 |
| 691 | Train 11 | Test Not Hispanic 51 | 0.76 | 0.79 | 0.78 | 1715 | Train 27 | Test Not Hispanic 51 | 0.76 | 0.79 | 0.79 |
| 692 | Train 11 | Test Not Hispanic 52 | 0.76 | 0.79 | 0.78 | 1716 | Train 27 | Test Not Hispanic 52 | 0.76 | 0.79 | 0.79 |
| 693 | Train 11 | Test Not Hispanic 53 | 0.72 | 0.75 | 0.64 | 1717 | Train 27 | Test Not Hispanic 53 | 0.72 | 0.75 | 0.66 |
| 694 | Train 11 | Test Not Hispanic 54 | 0.71 | 0.71 | 0.65 | 1718 | Train 27 | Test Not Hispanic 54 | 0.71 | 0.71 | 0.65 |
| 695 | Train 11 | Test Not Hispanic 55 | 0.72 | 0.75 | 0.64 | 1719 | Train 27 | Test Not Hispanic 55 | 0.72 | 0.75 | 0.66 |
| 696 | Train 11 | Test Not Hispanic 56 | 0.71 | 0.7 | 0.65 | 1720 | Train 27 | Test Not Hispanic 56 | 0.71 | 0.7 | 0.65 |
| 697 | Train 11 | Test Not Hispanic 57 | 0.76 | 0.8 | 0.81 | 1721 | Train 27 | Test Not Hispanic 57 | 0.76 | 0.8 | 0.83 |
| 698 | Train 11 | Test Not Hispanic 58 | 0.76 | 0.8 | 0.81 | 1722 | Train 27 | Test Not Hispanic 58 | 0.76 | 0.8 | 0.83 |
| 699 | Train 11 | Test Not Hispanic 59 | 0.76 | 0.8 | 0.81 | 1723 | Train 27 | Test Not Hispanic 59 | 0.76 | 0.8 | 0.83 |
| 700 | Train 11 | Test Not Hispanic 60 | 0.76 | 0.8 | 0.81 | 1724 | Train 27 | Test Not Hispanic 60 | 0.76 | 0.8 | 0.83 |
| 701 | Train 11 | Test Not Hispanic 61 | 0.72 | 0.75 | 0.64 | 1725 | Train 27 | Test Not Hispanic 61 | 0.72 | 0.75 | 0.66 |
| 702 | Train 11 | Test Not Hispanic 62 | 0.71 | 0.71 | 0.66 | 1726 | Train 27 | Test Not Hispanic 62 | 0.71 | 0.71 | 0.65 |
| 703 | Train 11 | Test Not Hispanic 63 | 0.72 | 0.76 | 0.65 | 1727 | Train 27 | Test Not Hispanic 63 | 0.72 | 0.76 | 0.66 |
| 704 | Train 11 | Test Not Hispanic 64 | 0.71 | 0.7 | 0.66 | 1728 | Train 27 | Test Not Hispanic 64 | 0.71 | 0.7 | 0.65 |
| 705 | Train 12 | Test Not Hispanic 1 | 0.75 | 0.81 | 0.81 | 1729 | Train 28 | Test Not Hispanic 1 | 0.75 | 0.81 | 0.81 |
| 706 | Train 12 | Test Not Hispanic 2 | 0.76 | 0.82 | 0.82 | 1730 | Train 28 | Test Not Hispanic 2 | 0.76 | 0.82 | 0.82 |
| 707 | Train 12 | Test Not Hispanic 3 | 0.75 | 0.81 | 0.81 | 1731 | Train 28 | Test Not Hispanic 3 | 0.75 | 0.81 | 0.81 |
| 708 | Train 12 | Test Not Hispanic 4 | 0.76 | 0.82 | 0.82 | 1732 | Train 28 | Test Not Hispanic 4 | 0.76 | 0.82 | 0.82 |
| 709 | Train 12 | Test Not Hispanic 5 | 0.71 | 0.73 | 0.68 | 1733 | Train 28 | Test Not Hispanic 5 | 0.71 | 0.73 | 0.7 |
| 710 | Train 12 | Test Not Hispanic 6 | 0.71 | 0.71 | 0.68 | 1734 | Train 28 | Test Not Hispanic 6 | 0.71 | 0.71 | 0.68 |
| 711 | Train 12 | Test Not Hispanic 7 | 0.71 | 0.73 | 0.68 | 1735 | Train 28 | Test Not Hispanic 7 | 0.71 | 0.73 | 0.7 |
| 712 | Train 12 | Test Not Hispanic 8 | 0.71 | 0.71 | 0.68 | 1736 | Train 28 | Test Not Hispanic 8 | 0.71 | 0.71 | 0.68 |
| 713 | Train 12 | Test Not Hispanic 9 | 0.75 | 0.82 | 0.83 | 1737 | Train 28 | Test Not Hispanic 9 | 0.75 | 0.82 | 0.83 |
| 714 | Train 12 | Test Not Hispanic 10 | 0.76 | 0.83 | 0.84 | 1738 | Train 28 | Test Not Hispanic 10 | 0.76 | 0.83 | 0.84 |
| 715 | Train 12 | Test Not Hispanic 11 | 0.75 | 0.82 | 0.83 | 1739 | Train 28 | Test Not Hispanic 11 | 0.75 | 0.82 | 0.83 |
| 716 | Train 12 | Test Not Hispanic 12 | 0.76 | 0.83 | 0.84 | 1740 | Train 28 | Test Not Hispanic 12 | 0.76 | 0.83 | 0.84 |
| 717 | Train 12 | Test Not Hispanic 13 | 0.71 | 0.74 | 0.69 | 1741 | Train 28 | Test Not Hispanic 13 | 0.71 | 0.74 | 0.71 |
| 718 | Train 12 | Test Not Hispanic 14 | 0.71 | 0.72 | 0.68 | 1742 | Train 28 | Test Not Hispanic 14 | 0.71 | 0.72 | 0.68 |
| 719 | Train 12 | Test Not Hispanic 15 | 0.71 | 0.73 | 0.69 | 1743 | Train 28 | Test Not Hispanic 15 | 0.71 | 0.73 | 0.71 |
| 720 | Train 12 | Test Not Hispanic 16 | 0.71 | 0.71 | 0.68 | 1744 | Train 28 | Test Not Hispanic 16 | 0.71 | 0.71 | 0.68 |
| 721 | Train 12 | Test Not Hispanic 17 | 0.75 | 0.81 | 0.81 | 1745 | Train 28 | Test Not Hispanic 17 | 0.75 | 0.81 | 0.81 |
| 722 | Train 12 | Test Not Hispanic 18 | 0.76 | 0.82 | 0.82 | 1746 | Train 28 | Test Not Hispanic 18 | 0.76 | 0.82 | 0.82 |
| 723 | Train 12 | Test Not Hispanic 19 | 0.75 | 0.81 | 0.81 | 1747 | Train 28 | Test Not Hispanic 19 | 0.75 | 0.81 | 0.81 |
| 724 | Train 12 | Test Not Hispanic 20 | 0.76 | 0.82 | 0.82 | 1748 | Train 28 | Test Not Hispanic 20 | 0.76 | 0.82 | 0.82 |
| 725 | Train 12 | Test Not Hispanic 21 | 0.71 | 0.73 | 0.68 | 1749 | Train 28 | Test Not Hispanic 21 | 0.71 | 0.73 | 0.7 |
| 726 | Train 12 | Test Not Hispanic 22 | 0.71 | 0.71 | 0.68 | 1750 | Train 28 | Test Not Hispanic 22 | 0.71 | 0.72 | 0.68 |
| 727 | Train 12 | Test Not Hispanic 23 | 0.71 | 0.73 | 0.68 | 1751 | Train 28 | Test Not Hispanic 23 | 0.71 | 0.73 | 0.7 |
| 728 | Train 12 | Test Not Hispanic 24 | 0.71 | 0.71 | 0.68 | 1752 | Train 28 | Test Not Hispanic 24 | 0.71 | 0.71 | 0.68 |
| 729 | Train 12 | Test Not Hispanic 25 | 0.75 | 0.82 | 0.83 | 1753 | Train 28 | Test Not Hispanic 25 | 0.75 | 0.82 | 0.83 |
| 730 | Train 12 | Test Not Hispanic 26 | 0.76 | 0.83 | 0.84 | 1754 | Train 28 | Test Not Hispanic 26 | 0.76 | 0.83 | 0.84 |
| 731 | Train 12 | Test Not Hispanic 27 | 0.75 | 0.82 | 0.83 | 1755 | Train 28 | Test Not Hispanic 27 | 0.75 | 0.82 | 0.83 |
| 732 | Train 12 | Test Not Hispanic 28 | 0.76 | 0.83 | 0.84 | 1756 | Train 28 | Test Not Hispanic 28 | 0.76 | 0.83 | 0.84 |
| 733 | Train 12 | Test Not Hispanic 29 | 0.71 | 0.74 | 0.69 | 1757 | Train 28 | Test Not Hispanic 29 | 0.71 | 0.74 | 0.71 |
| 734 | Train 12 | Test Not Hispanic 30 | 0.71 | 0.72 | 0.68 | 1758 | Train 28 | Test Not Hispanic 30 | 0.71 | 0.72 | 0.68 |
| 735 | Train 12 | Test Not Hispanic 31 | 0.71 | 0.73 | 0.69 | 1759 | Train 28 | Test Not Hispanic 31 | 0.71 | 0.73 | 0.71 |
| 736 | Train 12 | Test Not Hispanic 32 | 0.71 | 0.71 | 0.68 | 1760 | Train 28 | Test Not Hispanic 32 | 0.71 | 0.71 | 0.68 |
| 737 | Train 12 | Test Not Hispanic 33 | 0.75 | 0.81 | 0.81 | 1761 | Train 28 | Test Not Hispanic 33 | 0.75 | 0.81 | 0.81 |
| 738 | Train 12 | Test Not Hispanic 34 | 0.76 | 0.82 | 0.82 | 1762 | Train 28 | Test Not Hispanic 34 | 0.76 | 0.82 | 0.82 |
| 739 | Train 12 | Test Not Hispanic 35 | 0.75 | 0.81 | 0.81 | 1763 | Train 28 | Test Not Hispanic 35 | 0.75 | 0.81 | 0.81 |
| 740 | Train 12 | Test Not Hispanic 36 | 0.76 | 0.82 | 0.82 | 1764 | Train 28 | Test Not Hispanic 36 | 0.76 | 0.82 | 0.82 |
| 741 | Train 12 | Test Not Hispanic 37 | 0.71 | 0.73 | 0.68 | 1765 | Train 28 | Test Not Hispanic 37 | 0.71 | 0.73 | 0.7 |
| 742 | Train 12 | Test Not Hispanic 38 | 0.71 | 0.71 | 0.68 | 1766 | Train 28 | Test Not Hispanic 38 | 0.71 | 0.71 | 0.68 |
| 743 | Train 12 | Test Not Hispanic 39 | 0.71 | 0.73 | 0.68 | 1767 | Train 28 | Test Not Hispanic 39 | 0.71 | 0.73 | 0.7 |
| 744 | Train 12 | Test Not Hispanic 40 | 0.71 | 0.71 | 0.68 | 1768 | Train 28 | Test Not Hispanic 40 | 0.71 | 0.71 | 0.68 |
| 745 | Train 12 | Test Not Hispanic 41 | 0.75 | 0.82 | 0.83 | 1769 | Train 28 | Test Not Hispanic 41 | 0.75 | 0.82 | 0.83 |
| 746 | Train 12 | Test Not Hispanic 42 | 0.76 | 0.83 | 0.84 | 1770 | Train 28 | Test Not Hispanic 42 | 0.76 | 0.83 | 0.84 |
| 747 | Train 12 | Test Not Hispanic 43 | 0.75 | 0.82 | 0.83 | 1771 | Train 28 | Test Not Hispanic 43 | 0.75 | 0.82 | 0.83 |
| 748 | Train 12 | Test Not Hispanic 44 | 0.76 | 0.83 | 0.84 | 1772 | Train 28 | Test Not Hispanic 44 | 0.76 | 0.83 | 0.84 |
| 749 | Train 12 | Test Not Hispanic 45 | 0.71 | 0.74 | 0.69 | 1773 | Train 28 | Test Not Hispanic 45 | 0.71 | 0.74 | 0.71 |
| 750 | Train 12 | Test Not Hispanic 46 | 0.71 | 0.72 | 0.68 | 1774 | Train 28 | Test Not Hispanic 46 | 0.71 | 0.72 | 0.68 |
| 751 | Train 12 | Test Not Hispanic 47 | 0.71 | 0.73 | 0.69 | 1775 | Train 28 | Test Not Hispanic 47 | 0.71 | 0.73 | 0.71 |
| 752 | Train 12 | Test Not Hispanic 48 | 0.71 | 0.71 | 0.68 | 1776 | Train 28 | Test Not Hispanic 48 | 0.71 | 0.71 | 0.68 |
| 753 | Train 12 | Test Not Hispanic 49 | 0.76 | 0.79 | 0.78 | 1777 | Train 28 | Test Not Hispanic 49 | 0.76 | 0.78 | 0.79 |
| 754 | Train 12 | Test Not Hispanic 50 | 0.76 | 0.79 | 0.78 | 1778 | Train 28 | Test Not Hispanic 50 | 0.76 | 0.79 | 0.79 |
| 755 | Train 12 | Test Not Hispanic 51 | 0.76 | 0.79 | 0.78 | 1779 | Train 28 | Test Not Hispanic 51 | 0.76 | 0.79 | 0.79 |
| 756 | Train 12 | Test Not Hispanic 52 | 0.76 | 0.79 | 0.78 | 1780 | Train 28 | Test Not Hispanic 52 | 0.76 | 0.79 | 0.79 |
| 757 | Train 12 | Test Not Hispanic 53 | 0.73 | 0.76 | 0.67 | 1781 | Train 28 | Test Not Hispanic 53 | 0.73 | 0.76 | 0.68 |
| 758 | Train 12 | Test Not Hispanic 54 | 0.71 | 0.72 | 0.68 | 1782 | Train 28 | Test Not Hispanic 54 | 0.71 | 0.72 | 0.69 |
| 759 | Train 12 | Test Not Hispanic 55 | 0.73 | 0.76 | 0.67 | 1783 | Train 28 | Test Not Hispanic 55 | 0.73 | 0.76 | 0.68 |
| 760 | Train 12 | Test Not Hispanic 56 | 0.71 | 0.71 | 0.68 | 1784 | Train 28 | Test Not Hispanic 56 | 0.71 | 0.71 | 0.68 |
| 761 | Train 12 | Test Not Hispanic 57 | 0.76 | 0.79 | 0.82 | 1785 | Train 28 | Test Not Hispanic 57 | 0.76 | 0.79 | 0.82 |
| 762 | Train 12 | Test Not Hispanic 58 | 0.76 | 0.79 | 0.82 | 1786 | Train 28 | Test Not Hispanic 58 | 0.76 | 0.79 | 0.82 |
| 763 | Train 12 | Test Not Hispanic 59 | 0.76 | 0.79 | 0.82 | 1787 | Train 28 | Test Not Hispanic 59 | 0.76 | 0.79 | 0.82 |
| 764 | Train 12 | Test Not Hispanic 60 | 0.76 | 0.79 | 0.82 | 1788 | Train 28 | Test Not Hispanic 60 | 0.76 | 0.79 | 0.82 |
| 765 | Train 12 | Test Not Hispanic 61 | 0.73 | 0.76 | 0.68 | 1789 | Train 28 | Test Not Hispanic 61 | 0.73 | 0.76 | 0.68 |
| 766 | Train 12 | Test Not Hispanic 62 | 0.72 | 0.72 | 0.69 | 1790 | Train 28 | Test Not Hispanic 62 | 0.72 | 0.72 | 0.69 |
| 767 | Train 12 | Test Not Hispanic 63 | 0.73 | 0.76 | 0.68 | 1791 | Train 28 | Test Not Hispanic 63 | 0.73 | 0.76 | 0.68 |
| 768 | Train 12 | Test Not Hispanic 64 | 0.71 | 0.71 | 0.68 | 1792 | Train 28 | Test Not Hispanic 64 | 0.71 | 0.71 | 0.68 |
| 769 | Train 13 | Test Not Hispanic 1 | 0.75 | 0.8 | 0.79 | 1793 | Train 29 | Test Not Hispanic 1 | 0.75 | 0.8 | 0.79 |
| 770 | Train 13 | Test Not Hispanic 2 | 0.75 | 0.78 | 0.77 | 1794 | Train 29 | Test Not Hispanic 2 | 0.75 | 0.78 | 0.77 |
| 771 | Train 13 | Test Not Hispanic 3 | 0.75 | 0.8 | 0.79 | 1795 | Train 29 | Test Not Hispanic 3 | 0.75 | 0.8 | 0.79 |
| 772 | Train 13 | Test Not Hispanic 4 | 0.75 | 0.78 | 0.77 | 1796 | Train 29 | Test Not Hispanic 4 | 0.75 | 0.78 | 0.77 |
| 773 | Train 13 | Test Not Hispanic 5 | 0.73 | 0.78 | 0.72 | 1797 | Train 29 | Test Not Hispanic 5 | 0.73 | 0.78 | 0.75 |
| 774 | Train 13 | Test Not Hispanic 6 | 0.7 | 0.71 | 0.68 | 1798 | Train 29 | Test Not Hispanic 6 | 0.7 | 0.71 | 0.7 |
| 775 | Train 13 | Test Not Hispanic 7 | 0.73 | 0.77 | 0.72 | 1799 | Train 29 | Test Not Hispanic 7 | 0.73 | 0.77 | 0.75 |
| 776 | Train 13 | Test Not Hispanic 8 | 0.7 | 0.71 | 0.68 | 1800 | Train 29 | Test Not Hispanic 8 | 0.7 | 0.71 | 0.7 |
| 777 | Train 13 | Test Not Hispanic 9 | 0.75 | 0.8 | 0.81 | 1801 | Train 29 | Test Not Hispanic 9 | 0.75 | 0.8 | 0.82 |
| 778 | Train 13 | Test Not Hispanic 10 | 0.75 | 0.78 | 0.78 | 1802 | Train 29 | Test Not Hispanic 10 | 0.75 | 0.78 | 0.78 |
| 779 | Train 13 | Test Not Hispanic 11 | 0.75 | 0.8 | 0.81 | 1803 | Train 29 | Test Not Hispanic 11 | 0.75 | 0.8 | 0.81 |
| 780 | Train 13 | Test Not Hispanic 12 | 0.75 | 0.78 | 0.78 | 1804 | Train 29 | Test Not Hispanic 12 | 0.75 | 0.78 | 0.78 |
| 781 | Train 13 | Test Not Hispanic 13 | 0.73 | 0.78 | 0.74 | 1805 | Train 29 | Test Not Hispanic 13 | 0.73 | 0.78 | 0.77 |
| 782 | Train 13 | Test Not Hispanic 14 | 0.7 | 0.71 | 0.68 | 1806 | Train 29 | Test Not Hispanic 14 | 0.7 | 0.71 | 0.7 |
| 783 | Train 13 | Test Not Hispanic 15 | 0.73 | 0.78 | 0.73 | 1807 | Train 29 | Test Not Hispanic 15 | 0.73 | 0.78 | 0.77 |
| 784 | Train 13 | Test Not Hispanic 16 | 0.7 | 0.71 | 0.68 | 1808 | Train 29 | Test Not Hispanic 16 | 0.7 | 0.71 | 0.7 |
| 785 | Train 13 | Test Not Hispanic 17 | 0.75 | 0.8 | 0.79 | 1809 | Train 29 | Test Not Hispanic 17 | 0.75 | 0.8 | 0.79 |
| 786 | Train 13 | Test Not Hispanic 18 | 0.75 | 0.78 | 0.77 | 1810 | Train 29 | Test Not Hispanic 18 | 0.75 | 0.78 | 0.77 |
| 787 | Train 13 | Test Not Hispanic 19 | 0.75 | 0.8 | 0.79 | 1811 | Train 29 | Test Not Hispanic 19 | 0.75 | 0.8 | 0.79 |
| 788 | Train 13 | Test Not Hispanic 20 | 0.75 | 0.78 | 0.77 | 1812 | Train 29 | Test Not Hispanic 20 | 0.75 | 0.78 | 0.77 |
| 789 | Train 13 | Test Not Hispanic 21 | 0.74 | 0.78 | 0.72 | 1813 | Train 29 | Test Not Hispanic 21 | 0.74 | 0.78 | 0.75 |
| 790 | Train 13 | Test Not Hispanic 22 | 0.7 | 0.71 | 0.68 | 1814 | Train 29 | Test Not Hispanic 22 | 0.7 | 0.71 | 0.7 |
| 791 | Train 13 | Test Not Hispanic 23 | 0.73 | 0.77 | 0.72 | 1815 | Train 29 | Test Not Hispanic 23 | 0.73 | 0.77 | 0.75 |
| 792 | Train 13 | Test Not Hispanic 24 | 0.7 | 0.71 | 0.68 | 1816 | Train 29 | Test Not Hispanic 24 | 0.7 | 0.71 | 0.7 |
| 793 | Train 13 | Test Not Hispanic 25 | 0.75 | 0.8 | 0.81 | 1817 | Train 29 | Test Not Hispanic 25 | 0.75 | 0.8 | 0.82 |
| 794 | Train 13 | Test Not Hispanic 26 | 0.75 | 0.78 | 0.78 | 1818 | Train 29 | Test Not Hispanic 26 | 0.75 | 0.78 | 0.78 |
| 795 | Train 13 | Test Not Hispanic 27 | 0.75 | 0.8 | 0.81 | 1819 | Train 29 | Test Not Hispanic 27 | 0.75 | 0.8 | 0.81 |
| 796 | Train 13 | Test Not Hispanic 28 | 0.75 | 0.78 | 0.78 | 1820 | Train 29 | Test Not Hispanic 28 | 0.75 | 0.78 | 0.78 |
| 797 | Train 13 | Test Not Hispanic 29 | 0.74 | 0.78 | 0.74 | 1821 | Train 29 | Test Not Hispanic 29 | 0.74 | 0.78 | 0.77 |
| 798 | Train 13 | Test Not Hispanic 30 | 0.7 | 0.71 | 0.68 | 1822 | Train 29 | Test Not Hispanic 30 | 0.7 | 0.71 | 0.7 |
| 799 | Train 13 | Test Not Hispanic 31 | 0.73 | 0.78 | 0.73 | 1823 | Train 29 | Test Not Hispanic 31 | 0.73 | 0.78 | 0.77 |
| 800 | Train 13 | Test Not Hispanic 32 | 0.7 | 0.71 | 0.68 | 1824 | Train 29 | Test Not Hispanic 32 | 0.7 | 0.71 | 0.7 |
| 801 | Train 13 | Test Not Hispanic 33 | 0.75 | 0.8 | 0.79 | 1825 | Train 29 | Test Not Hispanic 33 | 0.75 | 0.8 | 0.79 |
| 802 | Train 13 | Test Not Hispanic 34 | 0.75 | 0.78 | 0.77 | 1826 | Train 29 | Test Not Hispanic 34 | 0.75 | 0.78 | 0.77 |
| 803 | Train 13 | Test Not Hispanic 35 | 0.75 | 0.8 | 0.79 | 1827 | Train 29 | Test Not Hispanic 35 | 0.75 | 0.8 | 0.79 |
| 804 | Train 13 | Test Not Hispanic 36 | 0.75 | 0.78 | 0.77 | 1828 | Train 29 | Test Not Hispanic 36 | 0.75 | 0.78 | 0.77 |
| 805 | Train 13 | Test Not Hispanic 37 | 0.74 | 0.77 | 0.72 | 1829 | Train 29 | Test Not Hispanic 37 | 0.74 | 0.78 | 0.75 |
| 806 | Train 13 | Test Not Hispanic 38 | 0.7 | 0.71 | 0.68 | 1830 | Train 29 | Test Not Hispanic 38 | 0.7 | 0.71 | 0.7 |
| 807 | Train 13 | Test Not Hispanic 39 | 0.73 | 0.77 | 0.72 | 1831 | Train 29 | Test Not Hispanic 39 | 0.73 | 0.77 | 0.75 |
| 808 | Train 13 | Test Not Hispanic 40 | 0.7 | 0.71 | 0.68 | 1832 | Train 29 | Test Not Hispanic 40 | 0.7 | 0.71 | 0.7 |
| 809 | Train 13 | Test Not Hispanic 41 | 0.75 | 0.8 | 0.81 | 1833 | Train 29 | Test Not Hispanic 41 | 0.75 | 0.8 | 0.81 |
| 810 | Train 13 | Test Not Hispanic 42 | 0.75 | 0.78 | 0.78 | 1834 | Train 29 | Test Not Hispanic 42 | 0.75 | 0.78 | 0.78 |
| 811 | Train 13 | Test Not Hispanic 43 | 0.75 | 0.8 | 0.81 | 1835 | Train 29 | Test Not Hispanic 43 | 0.75 | 0.8 | 0.81 |
| 812 | Train 13 | Test Not Hispanic 44 | 0.75 | 0.78 | 0.78 | 1836 | Train 29 | Test Not Hispanic 44 | 0.75 | 0.78 | 0.78 |
| 813 | Train 13 | Test Not Hispanic 45 | 0.74 | 0.78 | 0.74 | 1837 | Train 29 | Test Not Hispanic 45 | 0.74 | 0.78 | 0.77 |
| 814 | Train 13 | Test Not Hispanic 46 | 0.7 | 0.71 | 0.68 | 1838 | Train 29 | Test Not Hispanic 46 | 0.7 | 0.71 | 0.7 |
| 815 | Train 13 | Test Not Hispanic 47 | 0.73 | 0.78 | 0.73 | 1839 | Train 29 | Test Not Hispanic 47 | 0.73 | 0.78 | 0.77 |
| 816 | Train 13 | Test Not Hispanic 48 | 0.7 | 0.71 | 0.68 | 1840 | Train 29 | Test Not Hispanic 48 | 0.7 | 0.71 | 0.7 |
| 817 | Train 13 | Test Not Hispanic 49 | 0.76 | 0.77 | 0.76 | 1841 | Train 29 | Test Not Hispanic 49 | 0.76 | 0.77 | 0.76 |
| 818 | Train 13 | Test Not Hispanic 50 | 0.76 | 0.77 | 0.76 | 1842 | Train 29 | Test Not Hispanic 50 | 0.76 | 0.77 | 0.76 |
| 819 | Train 13 | Test Not Hispanic 51 | 0.76 | 0.77 | 0.76 | 1843 | Train 29 | Test Not Hispanic 51 | 0.76 | 0.77 | 0.76 |
| 820 | Train 13 | Test Not Hispanic 52 | 0.76 | 0.77 | 0.76 | 1844 | Train 29 | Test Not Hispanic 52 | 0.76 | 0.77 | 0.76 |
| 821 | Train 13 | Test Not Hispanic 53 | 0.75 | 0.78 | 0.71 | 1845 | Train 29 | Test Not Hispanic 53 | 0.75 | 0.78 | 0.73 |
| 822 | Train 13 | Test Not Hispanic 54 | 0.71 | 0.72 | 0.68 | 1846 | Train 29 | Test Not Hispanic 54 | 0.71 | 0.72 | 0.71 |
| 823 | Train 13 | Test Not Hispanic 55 | 0.75 | 0.78 | 0.71 | 1847 | Train 29 | Test Not Hispanic 55 | 0.75 | 0.78 | 0.73 |
| 824 | Train 13 | Test Not Hispanic 56 | 0.71 | 0.71 | 0.67 | 1848 | Train 29 | Test Not Hispanic 56 | 0.71 | 0.71 | 0.71 |
| 825 | Train 13 | Test Not Hispanic 57 | 0.76 | 0.77 | 0.78 | 1849 | Train 29 | Test Not Hispanic 57 | 0.76 | 0.77 | 0.78 |
| 826 | Train 13 | Test Not Hispanic 58 | 0.76 | 0.77 | 0.78 | 1850 | Train 29 | Test Not Hispanic 58 | 0.76 | 0.78 | 0.78 |
| 827 | Train 13 | Test Not Hispanic 59 | 0.76 | 0.77 | 0.78 | 1851 | Train 29 | Test Not Hispanic 59 | 0.76 | 0.77 | 0.78 |
| 828 | Train 13 | Test Not Hispanic 60 | 0.76 | 0.77 | 0.78 | 1852 | Train 29 | Test Not Hispanic 60 | 0.76 | 0.77 | 0.78 |
| 829 | Train 13 | Test Not Hispanic 61 | 0.75 | 0.79 | 0.72 | 1853 | Train 29 | Test Not Hispanic 61 | 0.75 | 0.79 | 0.75 |
| 830 | Train 13 | Test Not Hispanic 62 | 0.71 | 0.72 | 0.68 | 1854 | Train 29 | Test Not Hispanic 62 | 0.71 | 0.72 | 0.71 |
| 831 | Train 13 | Test Not Hispanic 63 | 0.75 | 0.79 | 0.72 | 1855 | Train 29 | Test Not Hispanic 63 | 0.75 | 0.79 | 0.75 |
| 832 | Train 13 | Test Not Hispanic 64 | 0.71 | 0.72 | 0.67 | 1856 | Train 29 | Test Not Hispanic 64 | 0.71 | 0.71 | 0.71 |
| 833 | Train 14 | Test Not Hispanic 1 | 0.73 | 0.77 | 0.75 | 1857 | Train 30 | Test Not Hispanic 1 | 0.73 | 0.77 | 0.74 |
| 834 | Train 14 | Test Not Hispanic 2 | 0.75 | 0.78 | 0.76 | 1858 | Train 30 | Test Not Hispanic 2 | 0.75 | 0.78 | 0.75 |
| 835 | Train 14 | Test Not Hispanic 3 | 0.73 | 0.77 | 0.75 | 1859 | Train 30 | Test Not Hispanic 3 | 0.73 | 0.77 | 0.74 |
| 836 | Train 14 | Test Not Hispanic 4 | 0.75 | 0.78 | 0.76 | 1860 | Train 30 | Test Not Hispanic 4 | 0.75 | 0.78 | 0.75 |
| 837 | Train 14 | Test Not Hispanic 5 | 0.72 | 0.77 | 0.77 | 1861 | Train 30 | Test Not Hispanic 5 | 0.72 | 0.77 | 0.77 |
| 838 | Train 14 | Test Not Hispanic 6 | 0.74 | 0.78 | 0.79 | 1862 | Train 30 | Test Not Hispanic 6 | 0.74 | 0.78 | 0.79 |
| 839 | Train 14 | Test Not Hispanic 7 | 0.72 | 0.77 | 0.77 | 1863 | Train 30 | Test Not Hispanic 7 | 0.72 | 0.77 | 0.77 |
| 840 | Train 14 | Test Not Hispanic 8 | 0.73 | 0.77 | 0.78 | 1864 | Train 30 | Test Not Hispanic 8 | 0.73 | 0.77 | 0.78 |
| 841 | Train 14 | Test Not Hispanic 9 | 0.73 | 0.77 | 0.76 | 1865 | Train 30 | Test Not Hispanic 9 | 0.73 | 0.77 | 0.75 |
| 842 | Train 14 | Test Not Hispanic 10 | 0.75 | 0.78 | 0.76 | 1866 | Train 30 | Test Not Hispanic 10 | 0.75 | 0.78 | 0.76 |
| 843 | Train 14 | Test Not Hispanic 11 | 0.73 | 0.77 | 0.76 | 1867 | Train 30 | Test Not Hispanic 11 | 0.73 | 0.77 | 0.75 |
| 844 | Train 14 | Test Not Hispanic 12 | 0.75 | 0.78 | 0.76 | 1868 | Train 30 | Test Not Hispanic 12 | 0.75 | 0.78 | 0.76 |
| 845 | Train 14 | Test Not Hispanic 13 | 0.72 | 0.77 | 0.78 | 1869 | Train 30 | Test Not Hispanic 13 | 0.72 | 0.77 | 0.78 |
| 846 | Train 14 | Test Not Hispanic 14 | 0.74 | 0.78 | 0.79 | 1870 | Train 30 | Test Not Hispanic 14 | 0.74 | 0.78 | 0.79 |
| 847 | Train 14 | Test Not Hispanic 15 | 0.72 | 0.77 | 0.78 | 1871 | Train 30 | Test Not Hispanic 15 | 0.72 | 0.77 | 0.78 |
| 848 | Train 14 | Test Not Hispanic 16 | 0.74 | 0.78 | 0.79 | 1872 | Train 30 | Test Not Hispanic 16 | 0.74 | 0.78 | 0.78 |
| 849 | Train 14 | Test Not Hispanic 17 | 0.73 | 0.77 | 0.75 | 1873 | Train 30 | Test Not Hispanic 17 | 0.73 | 0.77 | 0.74 |
| 850 | Train 14 | Test Not Hispanic 18 | 0.75 | 0.78 | 0.76 | 1874 | Train 30 | Test Not Hispanic 18 | 0.75 | 0.78 | 0.75 |
| 851 | Train 14 | Test Not Hispanic 19 | 0.73 | 0.77 | 0.75 | 1875 | Train 30 | Test Not Hispanic 19 | 0.73 | 0.77 | 0.74 |
| 852 | Train 14 | Test Not Hispanic 20 | 0.75 | 0.78 | 0.76 | 1876 | Train 30 | Test Not Hispanic 20 | 0.75 | 0.78 | 0.75 |
| 853 | Train 14 | Test Not Hispanic 21 | 0.72 | 0.77 | 0.77 | 1877 | Train 30 | Test Not Hispanic 21 | 0.72 | 0.77 | 0.77 |
| 854 | Train 14 | Test Not Hispanic 22 | 0.74 | 0.78 | 0.79 | 1878 | Train 30 | Test Not Hispanic 22 | 0.74 | 0.78 | 0.79 |
| 855 | Train 14 | Test Not Hispanic 23 | 0.72 | 0.77 | 0.77 | 1879 | Train 30 | Test Not Hispanic 23 | 0.72 | 0.77 | 0.77 |
| 856 | Train 14 | Test Not Hispanic 24 | 0.73 | 0.77 | 0.78 | 1880 | Train 30 | Test Not Hispanic 24 | 0.73 | 0.77 | 0.78 |
| 857 | Train 14 | Test Not Hispanic 25 | 0.73 | 0.77 | 0.76 | 1881 | Train 30 | Test Not Hispanic 25 | 0.73 | 0.77 | 0.75 |
| 858 | Train 14 | Test Not Hispanic 26 | 0.75 | 0.78 | 0.76 | 1882 | Train 30 | Test Not Hispanic 26 | 0.75 | 0.78 | 0.76 |
| 859 | Train 14 | Test Not Hispanic 27 | 0.73 | 0.77 | 0.76 | 1883 | Train 30 | Test Not Hispanic 27 | 0.73 | 0.77 | 0.75 |
| 860 | Train 14 | Test Not Hispanic 28 | 0.75 | 0.78 | 0.76 | 1884 | Train 30 | Test Not Hispanic 28 | 0.75 | 0.78 | 0.76 |
| 861 | Train 14 | Test Not Hispanic 29 | 0.72 | 0.77 | 0.78 | 1885 | Train 30 | Test Not Hispanic 29 | 0.72 | 0.77 | 0.78 |
| 862 | Train 14 | Test Not Hispanic 30 | 0.74 | 0.78 | 0.79 | 1886 | Train 30 | Test Not Hispanic 30 | 0.74 | 0.78 | 0.79 |
| 863 | Train 14 | Test Not Hispanic 31 | 0.72 | 0.77 | 0.78 | 1887 | Train 30 | Test Not Hispanic 31 | 0.72 | 0.77 | 0.78 |
| 864 | Train 14 | Test Not Hispanic 32 | 0.74 | 0.78 | 0.79 | 1888 | Train 30 | Test Not Hispanic 32 | 0.74 | 0.78 | 0.79 |
| 865 | Train 14 | Test Not Hispanic 33 | 0.73 | 0.77 | 0.75 | 1889 | Train 30 | Test Not Hispanic 33 | 0.73 | 0.77 | 0.75 |
| 866 | Train 14 | Test Not Hispanic 34 | 0.75 | 0.78 | 0.76 | 1890 | Train 30 | Test Not Hispanic 34 | 0.75 | 0.78 | 0.75 |
| 867 | Train 14 | Test Not Hispanic 35 | 0.73 | 0.77 | 0.75 | 1891 | Train 30 | Test Not Hispanic 35 | 0.73 | 0.77 | 0.74 |
| 868 | Train 14 | Test Not Hispanic 36 | 0.75 | 0.78 | 0.76 | 1892 | Train 30 | Test Not Hispanic 36 | 0.75 | 0.78 | 0.75 |
| 869 | Train 14 | Test Not Hispanic 37 | 0.72 | 0.77 | 0.77 | 1893 | Train 30 | Test Not Hispanic 37 | 0.72 | 0.77 | 0.77 |
| 870 | Train 14 | Test Not Hispanic 38 | 0.74 | 0.78 | 0.79 | 1894 | Train 30 | Test Not Hispanic 38 | 0.74 | 0.78 | 0.79 |
| 871 | Train 14 | Test Not Hispanic 39 | 0.72 | 0.77 | 0.77 | 1895 | Train 30 | Test Not Hispanic 39 | 0.72 | 0.77 | 0.77 |
| 872 | Train 14 | Test Not Hispanic 40 | 0.73 | 0.77 | 0.78 | 1896 | Train 30 | Test Not Hispanic 40 | 0.73 | 0.77 | 0.78 |
| 873 | Train 14 | Test Not Hispanic 41 | 0.73 | 0.77 | 0.76 | 1897 | Train 30 | Test Not Hispanic 41 | 0.73 | 0.77 | 0.75 |
| 874 | Train 14 | Test Not Hispanic 42 | 0.75 | 0.78 | 0.76 | 1898 | Train 30 | Test Not Hispanic 42 | 0.75 | 0.78 | 0.76 |
| 875 | Train 14 | Test Not Hispanic 43 | 0.73 | 0.77 | 0.76 | 1899 | Train 30 | Test Not Hispanic 43 | 0.73 | 0.77 | 0.75 |
| 876 | Train 14 | Test Not Hispanic 44 | 0.75 | 0.78 | 0.76 | 1900 | Train 30 | Test Not Hispanic 44 | 0.75 | 0.78 | 0.76 |
| 877 | Train 14 | Test Not Hispanic 45 | 0.72 | 0.77 | 0.78 | 1901 | Train 30 | Test Not Hispanic 45 | 0.72 | 0.77 | 0.78 |
| 878 | Train 14 | Test Not Hispanic 46 | 0.74 | 0.78 | 0.79 | 1902 | Train 30 | Test Not Hispanic 46 | 0.74 | 0.78 | 0.79 |
| 879 | Train 14 | Test Not Hispanic 47 | 0.72 | 0.77 | 0.78 | 1903 | Train 30 | Test Not Hispanic 47 | 0.72 | 0.77 | 0.78 |
| 880 | Train 14 | Test Not Hispanic 48 | 0.74 | 0.78 | 0.79 | 1904 | Train 30 | Test Not Hispanic 48 | 0.74 | 0.78 | 0.78 |
| 881 | Train 14 | Test Not Hispanic 49 | 0.75 | 0.78 | 0.77 | 1905 | Train 30 | Test Not Hispanic 49 | 0.75 | 0.78 | 0.77 |
| 882 | Train 14 | Test Not Hispanic 50 | 0.75 | 0.78 | 0.77 | 1906 | Train 30 | Test Not Hispanic 50 | 0.75 | 0.78 | 0.77 |
| 883 | Train 14 | Test Not Hispanic 51 | 0.75 | 0.78 | 0.77 | 1907 | Train 30 | Test Not Hispanic 51 | 0.75 | 0.78 | 0.77 |
| 884 | Train 14 | Test Not Hispanic 52 | 0.75 | 0.78 | 0.77 | 1908 | Train 30 | Test Not Hispanic 52 | 0.75 | 0.78 | 0.77 |
| 885 | Train 14 | Test Not Hispanic 53 | 0.75 | 0.79 | 0.74 | 1909 | Train 30 | Test Not Hispanic 53 | 0.75 | 0.79 | 0.75 |
| 886 | Train 14 | Test Not Hispanic 54 | 0.74 | 0.78 | 0.79 | 1910 | Train 30 | Test Not Hispanic 54 | 0.74 | 0.78 | 0.79 |
| 887 | Train 14 | Test Not Hispanic 55 | 0.75 | 0.79 | 0.74 | 1911 | Train 30 | Test Not Hispanic 55 | 0.75 | 0.79 | 0.75 |
| 888 | Train 14 | Test Not Hispanic 56 | 0.74 | 0.77 | 0.78 | 1912 | Train 30 | Test Not Hispanic 56 | 0.74 | 0.77 | 0.78 |
| 889 | Train 14 | Test Not Hispanic 57 | 0.75 | 0.79 | 0.78 | 1913 | Train 30 | Test Not Hispanic 57 | 0.75 | 0.79 | 0.79 |
| 890 | Train 14 | Test Not Hispanic 58 | 0.75 | 0.79 | 0.79 | 1914 | Train 30 | Test Not Hispanic 58 | 0.75 | 0.79 | 0.79 |
| 891 | Train 14 | Test Not Hispanic 59 | 0.75 | 0.79 | 0.78 | 1915 | Train 30 | Test Not Hispanic 59 | 0.75 | 0.79 | 0.79 |
| 892 | Train 14 | Test Not Hispanic 60 | 0.75 | 0.79 | 0.78 | 1916 | Train 30 | Test Not Hispanic 60 | 0.75 | 0.79 | 0.79 |
| 893 | Train 14 | Test Not Hispanic 61 | 0.75 | 0.79 | 0.75 | 1917 | Train 30 | Test Not Hispanic 61 | 0.75 | 0.79 | 0.76 |
| 894 | Train 14 | Test Not Hispanic 62 | 0.74 | 0.78 | 0.79 | 1918 | Train 30 | Test Not Hispanic 62 | 0.74 | 0.78 | 0.79 |
| 895 | Train 14 | Test Not Hispanic 63 | 0.75 | 0.79 | 0.75 | 1919 | Train 30 | Test Not Hispanic 63 | 0.75 | 0.79 | 0.76 |
| 896 | Train 14 | Test Not Hispanic 64 | 0.74 | 0.77 | 0.78 | 1920 | Train 30 | Test Not Hispanic 64 | 0.74 | 0.77 | 0.78 |
| 897 | Train 15 | Test Not Hispanic 1 | 0.75 | 0.8 | 0.79 | 1921 | Train 31 | Test Not Hispanic 1 | 0.75 | 0.8 | 0.8 |
| 898 | Train 15 | Test Not Hispanic 2 | 0.75 | 0.78 | 0.76 | 1922 | Train 31 | Test Not Hispanic 2 | 0.75 | 0.78 | 0.77 |
| 899 | Train 15 | Test Not Hispanic 3 | 0.75 | 0.8 | 0.79 | 1923 | Train 31 | Test Not Hispanic 3 | 0.75 | 0.8 | 0.8 |
| 900 | Train 15 | Test Not Hispanic 4 | 0.75 | 0.78 | 0.76 | 1924 | Train 31 | Test Not Hispanic 4 | 0.75 | 0.78 | 0.77 |
| 901 | Train 15 | Test Not Hispanic 5 | 0.73 | 0.77 | 0.75 | 1925 | Train 31 | Test Not Hispanic 5 | 0.73 | 0.77 | 0.72 |
| 902 | Train 15 | Test Not Hispanic 6 | 0.7 | 0.71 | 0.68 | 1926 | Train 31 | Test Not Hispanic 6 | 0.7 | 0.71 | 0.68 |
| 903 | Train 15 | Test Not Hispanic 7 | 0.73 | 0.77 | 0.75 | 1927 | Train 31 | Test Not Hispanic 7 | 0.73 | 0.77 | 0.71 |
| 904 | Train 15 | Test Not Hispanic 8 | 0.7 | 0.71 | 0.68 | 1928 | Train 31 | Test Not Hispanic 8 | 0.7 | 0.71 | 0.68 |
| 905 | Train 15 | Test Not Hispanic 9 | 0.75 | 0.8 | 0.81 | 1929 | Train 31 | Test Not Hispanic 9 | 0.75 | 0.8 | 0.82 |
| 906 | Train 15 | Test Not Hispanic 10 | 0.75 | 0.78 | 0.78 | 1930 | Train 31 | Test Not Hispanic 10 | 0.75 | 0.78 | 0.79 |
| 907 | Train 15 | Test Not Hispanic 11 | 0.75 | 0.8 | 0.82 | 1931 | Train 31 | Test Not Hispanic 11 | 0.75 | 0.8 | 0.82 |
| 908 | Train 15 | Test Not Hispanic 12 | 0.75 | 0.78 | 0.78 | 1932 | Train 31 | Test Not Hispanic 12 | 0.75 | 0.78 | 0.79 |
| 909 | Train 15 | Test Not Hispanic 13 | 0.73 | 0.78 | 0.77 | 1933 | Train 31 | Test Not Hispanic 13 | 0.73 | 0.78 | 0.73 |
| 910 | Train 15 | Test Not Hispanic 14 | 0.7 | 0.71 | 0.68 | 1934 | Train 31 | Test Not Hispanic 14 | 0.7 | 0.71 | 0.68 |
| 911 | Train 15 | Test Not Hispanic 15 | 0.73 | 0.78 | 0.76 | 1935 | Train 31 | Test Not Hispanic 15 | 0.73 | 0.78 | 0.73 |
| 912 | Train 15 | Test Not Hispanic 16 | 0.7 | 0.71 | 0.68 | 1936 | Train 31 | Test Not Hispanic 16 | 0.7 | 0.71 | 0.68 |
| 913 | Train 15 | Test Not Hispanic 17 | 0.75 | 0.8 | 0.79 | 1937 | Train 31 | Test Not Hispanic 17 | 0.75 | 0.8 | 0.8 |
| 914 | Train 15 | Test Not Hispanic 18 | 0.75 | 0.78 | 0.76 | 1938 | Train 31 | Test Not Hispanic 18 | 0.75 | 0.78 | 0.77 |
| 915 | Train 15 | Test Not Hispanic 19 | 0.75 | 0.8 | 0.79 | 1939 | Train 31 | Test Not Hispanic 19 | 0.75 | 0.8 | 0.8 |
| 916 | Train 15 | Test Not Hispanic 20 | 0.75 | 0.78 | 0.76 | 1940 | Train 31 | Test Not Hispanic 20 | 0.75 | 0.78 | 0.77 |
| 917 | Train 15 | Test Not Hispanic 21 | 0.74 | 0.77 | 0.75 | 1941 | Train 31 | Test Not Hispanic 21 | 0.74 | 0.77 | 0.72 |
| 918 | Train 15 | Test Not Hispanic 22 | 0.7 | 0.71 | 0.68 | 1942 | Train 31 | Test Not Hispanic 22 | 0.7 | 0.71 | 0.68 |
| 919 | Train 15 | Test Not Hispanic 23 | 0.73 | 0.77 | 0.75 | 1943 | Train 31 | Test Not Hispanic 23 | 0.73 | 0.77 | 0.72 |
| 920 | Train 15 | Test Not Hispanic 24 | 0.7 | 0.71 | 0.68 | 1944 | Train 31 | Test Not Hispanic 24 | 0.7 | 0.71 | 0.68 |
| 921 | Train 15 | Test Not Hispanic 25 | 0.75 | 0.8 | 0.81 | 1945 | Train 31 | Test Not Hispanic 25 | 0.75 | 0.8 | 0.82 |
| 922 | Train 15 | Test Not Hispanic 26 | 0.75 | 0.78 | 0.78 | 1946 | Train 31 | Test Not Hispanic 26 | 0.75 | 0.78 | 0.79 |
| 923 | Train 15 | Test Not Hispanic 27 | 0.75 | 0.8 | 0.82 | 1947 | Train 31 | Test Not Hispanic 27 | 0.75 | 0.8 | 0.82 |
| 924 | Train 15 | Test Not Hispanic 28 | 0.75 | 0.78 | 0.78 | 1948 | Train 31 | Test Not Hispanic 28 | 0.75 | 0.78 | 0.79 |
| 925 | Train 15 | Test Not Hispanic 29 | 0.74 | 0.78 | 0.77 | 1949 | Train 31 | Test Not Hispanic 29 | 0.74 | 0.78 | 0.73 |
| 926 | Train 15 | Test Not Hispanic 30 | 0.7 | 0.71 | 0.68 | 1950 | Train 31 | Test Not Hispanic 30 | 0.7 | 0.71 | 0.68 |
| 927 | Train 15 | Test Not Hispanic 31 | 0.73 | 0.78 | 0.76 | 1951 | Train 31 | Test Not Hispanic 31 | 0.73 | 0.78 | 0.73 |
| 928 | Train 15 | Test Not Hispanic 32 | 0.7 | 0.71 | 0.68 | 1952 | Train 31 | Test Not Hispanic 32 | 0.7 | 0.71 | 0.68 |
| 929 | Train 15 | Test Not Hispanic 33 | 0.75 | 0.8 | 0.79 | 1953 | Train 31 | Test Not Hispanic 33 | 0.75 | 0.8 | 0.8 |
| 930 | Train 15 | Test Not Hispanic 34 | 0.75 | 0.78 | 0.76 | 1954 | Train 31 | Test Not Hispanic 34 | 0.75 | 0.78 | 0.77 |
| 931 | Train 15 | Test Not Hispanic 35 | 0.75 | 0.8 | 0.79 | 1955 | Train 31 | Test Not Hispanic 35 | 0.75 | 0.8 | 0.8 |
| 932 | Train 15 | Test Not Hispanic 36 | 0.75 | 0.78 | 0.76 | 1956 | Train 31 | Test Not Hispanic 36 | 0.75 | 0.78 | 0.77 |
| 933 | Train 15 | Test Not Hispanic 37 | 0.74 | 0.77 | 0.75 | 1957 | Train 31 | Test Not Hispanic 37 | 0.74 | 0.77 | 0.72 |
| 934 | Train 15 | Test Not Hispanic 38 | 0.7 | 0.71 | 0.68 | 1958 | Train 31 | Test Not Hispanic 38 | 0.7 | 0.71 | 0.68 |
| 935 | Train 15 | Test Not Hispanic 39 | 0.73 | 0.77 | 0.75 | 1959 | Train 31 | Test Not Hispanic 39 | 0.73 | 0.77 | 0.72 |
| 936 | Train 15 | Test Not Hispanic 40 | 0.7 | 0.71 | 0.68 | 1960 | Train 31 | Test Not Hispanic 40 | 0.7 | 0.71 | 0.68 |
| 937 | Train 15 | Test Not Hispanic 41 | 0.75 | 0.8 | 0.81 | 1961 | Train 31 | Test Not Hispanic 41 | 0.75 | 0.8 | 0.82 |
| 938 | Train 15 | Test Not Hispanic 42 | 0.75 | 0.78 | 0.78 | 1962 | Train 31 | Test Not Hispanic 42 | 0.75 | 0.78 | 0.79 |
| 939 | Train 15 | Test Not Hispanic 43 | 0.75 | 0.8 | 0.81 | 1963 | Train 31 | Test Not Hispanic 43 | 0.75 | 0.8 | 0.82 |
| 940 | Train 15 | Test Not Hispanic 44 | 0.75 | 0.78 | 0.78 | 1964 | Train 31 | Test Not Hispanic 44 | 0.75 | 0.78 | 0.79 |
| 941 | Train 15 | Test Not Hispanic 45 | 0.74 | 0.78 | 0.76 | 1965 | Train 31 | Test Not Hispanic 45 | 0.74 | 0.78 | 0.73 |
| 942 | Train 15 | Test Not Hispanic 46 | 0.7 | 0.71 | 0.68 | 1966 | Train 31 | Test Not Hispanic 46 | 0.7 | 0.71 | 0.68 |
| 943 | Train 15 | Test Not Hispanic 47 | 0.73 | 0.78 | 0.76 | 1967 | Train 31 | Test Not Hispanic 47 | 0.73 | 0.78 | 0.73 |
| 944 | Train 15 | Test Not Hispanic 48 | 0.7 | 0.71 | 0.68 | 1968 | Train 31 | Test Not Hispanic 48 | 0.7 | 0.71 | 0.68 |
| 945 | Train 15 | Test Not Hispanic 49 | 0.76 | 0.77 | 0.78 | 1969 | Train 31 | Test Not Hispanic 49 | 0.76 | 0.77 | 0.78 |
| 946 | Train 15 | Test Not Hispanic 50 | 0.76 | 0.77 | 0.78 | 1970 | Train 31 | Test Not Hispanic 50 | 0.76 | 0.77 | 0.78 |
| 947 | Train 15 | Test Not Hispanic 51 | 0.76 | 0.77 | 0.78 | 1971 | Train 31 | Test Not Hispanic 51 | 0.76 | 0.77 | 0.78 |
| 948 | Train 15 | Test Not Hispanic 52 | 0.76 | 0.77 | 0.78 | 1972 | Train 31 | Test Not Hispanic 52 | 0.76 | 0.77 | 0.78 |
| 949 | Train 15 | Test Not Hispanic 53 | 0.75 | 0.78 | 0.76 | 1973 | Train 31 | Test Not Hispanic 53 | 0.75 | 0.78 | 0.69 |
| 950 | Train 15 | Test Not Hispanic 54 | 0.71 | 0.72 | 0.69 | 1974 | Train 31 | Test Not Hispanic 54 | 0.71 | 0.71 | 0.67 |
| 951 | Train 15 | Test Not Hispanic 55 | 0.75 | 0.78 | 0.75 | 1975 | Train 31 | Test Not Hispanic 55 | 0.75 | 0.78 | 0.69 |
| 952 | Train 15 | Test Not Hispanic 56 | 0.71 | 0.71 | 0.69 | 1976 | Train 31 | Test Not Hispanic 56 | 0.71 | 0.71 | 0.68 |
| 953 | Train 15 | Test Not Hispanic 57 | 0.76 | 0.77 | 0.8 | 1977 | Train 31 | Test Not Hispanic 57 | 0.76 | 0.78 | 0.8 |
| 954 | Train 15 | Test Not Hispanic 58 | 0.76 | 0.77 | 0.8 | 1978 | Train 31 | Test Not Hispanic 58 | 0.76 | 0.78 | 0.8 |
| 955 | Train 15 | Test Not Hispanic 59 | 0.76 | 0.78 | 0.8 | 1979 | Train 31 | Test Not Hispanic 59 | 0.76 | 0.78 | 0.8 |
| 956 | Train 15 | Test Not Hispanic 60 | 0.76 | 0.78 | 0.8 | 1980 | Train 31 | Test Not Hispanic 60 | 0.76 | 0.78 | 0.8 |
| 957 | Train 15 | Test Not Hispanic 61 | 0.75 | 0.79 | 0.77 | 1981 | Train 31 | Test Not Hispanic 61 | 0.75 | 0.79 | 0.7 |
| 958 | Train 15 | Test Not Hispanic 62 | 0.71 | 0.72 | 0.69 | 1982 | Train 31 | Test Not Hispanic 62 | 0.71 | 0.72 | 0.68 |
| 959 | Train 15 | Test Not Hispanic 63 | 0.75 | 0.79 | 0.77 | 1983 | Train 31 | Test Not Hispanic 63 | 0.75 | 0.79 | 0.7 |
| 960 | Train 15 | Test Not Hispanic 64 | 0.71 | 0.71 | 0.69 | 1984 | Train 31 | Test Not Hispanic 64 | 0.71 | 0.71 | 0.68 |
| 961 | Train 16 | Test Not Hispanic 1 | 0.73 | 0.77 | 0.76 | 1985 | Train 32 | Test Not Hispanic 1 | 0.73 | 0.77 | 0.75 |
| 962 | Train 16 | Test Not Hispanic 2 | 0.75 | 0.78 | 0.77 | 1986 | Train 32 | Test Not Hispanic 2 | 0.75 | 0.78 | 0.76 |
| 963 | Train 16 | Test Not Hispanic 3 | 0.73 | 0.77 | 0.76 | 1987 | Train 32 | Test Not Hispanic 3 | 0.73 | 0.77 | 0.76 |
| 964 | Train 16 | Test Not Hispanic 4 | 0.75 | 0.78 | 0.77 | 1988 | Train 32 | Test Not Hispanic 4 | 0.75 | 0.78 | 0.76 |
| 965 | Train 16 | Test Not Hispanic 5 | 0.72 | 0.77 | 0.77 | 1989 | Train 32 | Test Not Hispanic 5 | 0.72 | 0.77 | 0.77 |
| 966 | Train 16 | Test Not Hispanic 6 | 0.73 | 0.78 | 0.79 | 1990 | Train 32 | Test Not Hispanic 6 | 0.73 | 0.78 | 0.79 |
| 967 | Train 16 | Test Not Hispanic 7 | 0.72 | 0.77 | 0.77 | 1991 | Train 32 | Test Not Hispanic 7 | 0.72 | 0.77 | 0.77 |
| 968 | Train 16 | Test Not Hispanic 8 | 0.73 | 0.78 | 0.79 | 1992 | Train 32 | Test Not Hispanic 8 | 0.73 | 0.78 | 0.78 |
| 969 | Train 16 | Test Not Hispanic 9 | 0.73 | 0.77 | 0.77 | 1993 | Train 32 | Test Not Hispanic 9 | 0.73 | 0.77 | 0.76 |
| 970 | Train 16 | Test Not Hispanic 10 | 0.75 | 0.78 | 0.77 | 1994 | Train 32 | Test Not Hispanic 10 | 0.75 | 0.78 | 0.77 |
| 971 | Train 16 | Test Not Hispanic 11 | 0.74 | 0.77 | 0.77 | 1995 | Train 32 | Test Not Hispanic 11 | 0.73 | 0.77 | 0.76 |
| 972 | Train 16 | Test Not Hispanic 12 | 0.75 | 0.78 | 0.77 | 1996 | Train 32 | Test Not Hispanic 12 | 0.75 | 0.78 | 0.77 |
| 973 | Train 16 | Test Not Hispanic 13 | 0.72 | 0.77 | 0.78 | 1997 | Train 32 | Test Not Hispanic 13 | 0.72 | 0.77 | 0.78 |
| 974 | Train 16 | Test Not Hispanic 14 | 0.74 | 0.78 | 0.79 | 1998 | Train 32 | Test Not Hispanic 14 | 0.74 | 0.78 | 0.79 |
| 975 | Train 16 | Test Not Hispanic 15 | 0.72 | 0.77 | 0.78 | 1999 | Train 32 | Test Not Hispanic 15 | 0.72 | 0.77 | 0.78 |
| 976 | Train 16 | Test Not Hispanic 16 | 0.73 | 0.78 | 0.79 | 2000 | Train 32 | Test Not Hispanic 16 | 0.73 | 0.78 | 0.79 |
| 977 | Train 16 | Test Not Hispanic 17 | 0.73 | 0.77 | 0.76 | 2001 | Train 32 | Test Not Hispanic 17 | 0.73 | 0.77 | 0.75 |
| 978 | Train 16 | Test Not Hispanic 18 | 0.75 | 0.78 | 0.77 | 2002 | Train 32 | Test Not Hispanic 18 | 0.75 | 0.78 | 0.76 |
| 979 | Train 16 | Test Not Hispanic 19 | 0.73 | 0.77 | 0.76 | 2003 | Train 32 | Test Not Hispanic 19 | 0.73 | 0.77 | 0.75 |
| 980 | Train 16 | Test Not Hispanic 20 | 0.75 | 0.78 | 0.77 | 2004 | Train 32 | Test Not Hispanic 20 | 0.75 | 0.78 | 0.76 |
| 981 | Train 16 | Test Not Hispanic 21 | 0.72 | 0.77 | 0.77 | 2005 | Train 32 | Test Not Hispanic 21 | 0.72 | 0.77 | 0.77 |
| 982 | Train 16 | Test Not Hispanic 22 | 0.73 | 0.78 | 0.79 | 2006 | Train 32 | Test Not Hispanic 22 | 0.73 | 0.78 | 0.79 |
| 983 | Train 16 | Test Not Hispanic 23 | 0.72 | 0.77 | 0.77 | 2007 | Train 32 | Test Not Hispanic 23 | 0.72 | 0.77 | 0.77 |
| 984 | Train 16 | Test Not Hispanic 24 | 0.73 | 0.78 | 0.79 | 2008 | Train 32 | Test Not Hispanic 24 | 0.73 | 0.78 | 0.78 |
| 985 | Train 16 | Test Not Hispanic 25 | 0.73 | 0.77 | 0.77 | 2009 | Train 32 | Test Not Hispanic 25 | 0.73 | 0.77 | 0.76 |
| 986 | Train 16 | Test Not Hispanic 26 | 0.75 | 0.78 | 0.77 | 2010 | Train 32 | Test Not Hispanic 26 | 0.75 | 0.78 | 0.77 |
| 987 | Train 16 | Test Not Hispanic 27 | 0.74 | 0.77 | 0.77 | 2011 | Train 32 | Test Not Hispanic 27 | 0.73 | 0.77 | 0.76 |
| 988 | Train 16 | Test Not Hispanic 28 | 0.75 | 0.78 | 0.77 | 2012 | Train 32 | Test Not Hispanic 28 | 0.75 | 0.78 | 0.77 |
| 989 | Train 16 | Test Not Hispanic 29 | 0.72 | 0.77 | 0.78 | 2013 | Train 32 | Test Not Hispanic 29 | 0.72 | 0.77 | 0.78 |
| 990 | Train 16 | Test Not Hispanic 30 | 0.74 | 0.78 | 0.79 | 2014 | Train 32 | Test Not Hispanic 30 | 0.74 | 0.78 | 0.79 |
| 991 | Train 16 | Test Not Hispanic 31 | 0.72 | 0.77 | 0.78 | 2015 | Train 32 | Test Not Hispanic 31 | 0.72 | 0.77 | 0.78 |
| 992 | Train 16 | Test Not Hispanic 32 | 0.74 | 0.78 | 0.79 | 2016 | Train 32 | Test Not Hispanic 32 | 0.74 | 0.78 | 0.79 |
| 993 | Train 16 | Test Not Hispanic 33 | 0.74 | 0.77 | 0.76 | 2017 | Train 32 | Test Not Hispanic 33 | 0.74 | 0.77 | 0.75 |
| 994 | Train 16 | Test Not Hispanic 34 | 0.75 | 0.78 | 0.77 | 2018 | Train 32 | Test Not Hispanic 34 | 0.75 | 0.78 | 0.76 |
| 995 | Train 16 | Test Not Hispanic 35 | 0.74 | 0.77 | 0.76 | 2019 | Train 32 | Test Not Hispanic 35 | 0.74 | 0.77 | 0.76 |
| 996 | Train 16 | Test Not Hispanic 36 | 0.75 | 0.78 | 0.77 | 2020 | Train 32 | Test Not Hispanic 36 | 0.75 | 0.78 | 0.76 |
| 997 | Train 16 | Test Not Hispanic 37 | 0.72 | 0.77 | 0.77 | 2021 | Train 32 | Test Not Hispanic 37 | 0.72 | 0.77 | 0.77 |
| 998 | Train 16 | Test Not Hispanic 38 | 0.73 | 0.78 | 0.79 | 2022 | Train 32 | Test Not Hispanic 38 | 0.73 | 0.78 | 0.79 |
| 999 | Train 16 | Test Not Hispanic 39 | 0.72 | 0.77 | 0.77 | 2023 | Train 32 | Test Not Hispanic 39 | 0.72 | 0.77 | 0.77 |
| 1000 | Train 16 | Test Not Hispanic 40 | 0.73 | 0.78 | 0.79 | 2024 | Train 32 | Test Not Hispanic 40 | 0.73 | 0.78 | 0.78 |
| 1001 | Train 16 | Test Not Hispanic 41 | 0.74 | 0.77 | 0.77 | 2025 | Train 32 | Test Not Hispanic 41 | 0.74 | 0.77 | 0.76 |
| 1002 | Train 16 | Test Not Hispanic 42 | 0.75 | 0.78 | 0.77 | 2026 | Train 32 | Test Not Hispanic 42 | 0.75 | 0.78 | 0.77 |
| 1003 | Train 16 | Test Not Hispanic 43 | 0.74 | 0.77 | 0.77 | 2027 | Train 32 | Test Not Hispanic 43 | 0.74 | 0.77 | 0.76 |
| 1004 | Train 16 | Test Not Hispanic 44 | 0.75 | 0.78 | 0.77 | 2028 | Train 32 | Test Not Hispanic 44 | 0.75 | 0.78 | 0.77 |
| 1005 | Train 16 | Test Not Hispanic 45 | 0.72 | 0.77 | 0.78 | 2029 | Train 32 | Test Not Hispanic 45 | 0.72 | 0.77 | 0.78 |
| 1006 | Train 16 | Test Not Hispanic 46 | 0.74 | 0.78 | 0.79 | 2030 | Train 32 | Test Not Hispanic 46 | 0.74 | 0.78 | 0.79 |
| 1007 | Train 16 | Test Not Hispanic 47 | 0.72 | 0.77 | 0.78 | 2031 | Train 32 | Test Not Hispanic 47 | 0.72 | 0.77 | 0.78 |
| 1008 | Train 16 | Test Not Hispanic 48 | 0.74 | 0.78 | 0.79 | 2032 | Train 32 | Test Not Hispanic 48 | 0.74 | 0.78 | 0.79 |
| 1009 | Train 16 | Test Not Hispanic 49 | 0.75 | 0.78 | 0.77 | 2033 | Train 32 | Test Not Hispanic 49 | 0.75 | 0.78 | 0.77 |
| 1010 | Train 16 | Test Not Hispanic 50 | 0.75 | 0.78 | 0.77 | 2034 | Train 32 | Test Not Hispanic 50 | 0.75 | 0.78 | 0.77 |
| 1011 | Train 16 | Test Not Hispanic 51 | 0.75 | 0.78 | 0.77 | 2035 | Train 32 | Test Not Hispanic 51 | 0.75 | 0.78 | 0.77 |
| 1012 | Train 16 | Test Not Hispanic 52 | 0.75 | 0.78 | 0.77 | 2036 | Train 32 | Test Not Hispanic 52 | 0.75 | 0.78 | 0.77 |
| 1013 | Train 16 | Test Not Hispanic 53 | 0.74 | 0.79 | 0.75 | 2037 | Train 32 | Test Not Hispanic 53 | 0.74 | 0.79 | 0.77 |
| 1014 | Train 16 | Test Not Hispanic 54 | 0.74 | 0.78 | 0.78 | 2038 | Train 32 | Test Not Hispanic 54 | 0.74 | 0.78 | 0.78 |
| 1015 | Train 16 | Test Not Hispanic 55 | 0.74 | 0.79 | 0.75 | 2039 | Train 32 | Test Not Hispanic 55 | 0.74 | 0.79 | 0.76 |
| 1016 | Train 16 | Test Not Hispanic 56 | 0.74 | 0.77 | 0.79 | 2040 | Train 32 | Test Not Hispanic 56 | 0.74 | 0.77 | 0.78 |
| 1017 | Train 16 | Test Not Hispanic 57 | 0.76 | 0.79 | 0.78 | 2041 | Train 32 | Test Not Hispanic 57 | 0.76 | 0.79 | 0.79 |
| 1018 | Train 16 | Test Not Hispanic 58 | 0.76 | 0.79 | 0.78 | 2042 | Train 32 | Test Not Hispanic 58 | 0.76 | 0.79 | 0.79 |
| 1019 | Train 16 | Test Not Hispanic 59 | 0.76 | 0.79 | 0.78 | 2043 | Train 32 | Test Not Hispanic 59 | 0.76 | 0.79 | 0.79 |
| 1020 | Train 16 | Test Not Hispanic 60 | 0.76 | 0.79 | 0.78 | 2044 | Train 32 | Test Not Hispanic 60 | 0.76 | 0.79 | 0.79 |
| 1021 | Train 16 | Test Not Hispanic 61 | 0.74 | 0.79 | 0.76 | 2045 | Train 32 | Test Not Hispanic 61 | 0.74 | 0.79 | 0.77 |
| 1022 | Train 16 | Test Not Hispanic 62 | 0.74 | 0.78 | 0.79 | 2046 | Train 32 | Test Not Hispanic 62 | 0.74 | 0.78 | 0.78 |
| 1023 | Train 16 | Test Not Hispanic 63 | 0.74 | 0.79 | 0.76 | 2047 | Train 32 | Test Not Hispanic 63 | 0.74 | 0.79 | 0.77 |
| 1024 | Train 16 | Test Not Hispanic 64 | 0.74 | 0.78 | 0.79 | 2048 | Train 32 | Test Not Hispanic 64 | 0.74 | 0.78 | 0.79 |

Table 14. AUC Performance of the ML Models— LR, RF, and GB —for Non-Hispanic Patients Trained on Cohorts 33–64 and Tested Across 64 Cohorts.

| Not Hispanic | Train  Model | Test  Dataset | AUC (LR) | AUC (RF) | AUC (GB) | Not Hispanic | Train  Model | Test  Dataset | AUC (LR) | AUC (RF) | AUC (GB) |
| --- | --- | --- | --- | --- | --- | --- | --- | --- | --- | --- | --- |
| 2049 | Train 33 | Test Not Hispanic 1 | 0.75 | 0.83 | 0.85 | 3073 | Train 49 | Test Not Hispanic 1 | 0.73 | 0.74 | 0.66 |
| 2050 | Train 33 | Test Not Hispanic 2 | 0.74 | 0.78 | 0.79 | 3074 | Train 49 | Test Not Hispanic 2 | 0.72 | 0.71 | 0.62 |
| 2051 | Train 33 | Test Not Hispanic 3 | 0.75 | 0.83 | 0.85 | 3075 | Train 49 | Test Not Hispanic 3 | 0.73 | 0.74 | 0.66 |
| 2052 | Train 33 | Test Not Hispanic 4 | 0.74 | 0.78 | 0.79 | 3076 | Train 49 | Test Not Hispanic 4 | 0.72 | 0.71 | 0.62 |
| 2053 | Train 33 | Test Not Hispanic 5 | 0.71 | 0.73 | 0.68 | 3077 | Train 49 | Test Not Hispanic 5 | 0.7 | 0.7 | 0.63 |
| 2054 | Train 33 | Test Not Hispanic 6 | 0.69 | 0.7 | 0.64 | 3078 | Train 49 | Test Not Hispanic 6 | 0.69 | 0.68 | 0.61 |
| 2055 | Train 33 | Test Not Hispanic 7 | 0.71 | 0.73 | 0.68 | 3079 | Train 49 | Test Not Hispanic 7 | 0.7 | 0.7 | 0.63 |
| 2056 | Train 33 | Test Not Hispanic 8 | 0.69 | 0.69 | 0.64 | 3080 | Train 49 | Test Not Hispanic 8 | 0.69 | 0.68 | 0.61 |
| 2057 | Train 33 | Test Not Hispanic 9 | 0.75 | 0.82 | 0.81 | 3081 | Train 49 | Test Not Hispanic 9 | 0.73 | 0.73 | 0.65 |
| 2058 | Train 33 | Test Not Hispanic 10 | 0.74 | 0.77 | 0.77 | 3082 | Train 49 | Test Not Hispanic 10 | 0.72 | 0.71 | 0.61 |
| 2059 | Train 33 | Test Not Hispanic 11 | 0.75 | 0.82 | 0.81 | 3083 | Train 49 | Test Not Hispanic 11 | 0.73 | 0.73 | 0.65 |
| 2060 | Train 33 | Test Not Hispanic 12 | 0.74 | 0.77 | 0.77 | 3084 | Train 49 | Test Not Hispanic 12 | 0.72 | 0.71 | 0.61 |
| 2061 | Train 33 | Test Not Hispanic 13 | 0.71 | 0.73 | 0.66 | 3085 | Train 49 | Test Not Hispanic 13 | 0.7 | 0.7 | 0.63 |
| 2062 | Train 33 | Test Not Hispanic 14 | 0.7 | 0.7 | 0.64 | 3086 | Train 49 | Test Not Hispanic 14 | 0.69 | 0.68 | 0.61 |
| 2063 | Train 33 | Test Not Hispanic 15 | 0.71 | 0.73 | 0.66 | 3087 | Train 49 | Test Not Hispanic 15 | 0.7 | 0.7 | 0.63 |
| 2064 | Train 33 | Test Not Hispanic 16 | 0.7 | 0.69 | 0.64 | 3088 | Train 49 | Test Not Hispanic 16 | 0.69 | 0.68 | 0.61 |
| 2065 | Train 33 | Test Not Hispanic 17 | 0.75 | 0.83 | 0.85 | 3089 | Train 49 | Test Not Hispanic 17 | 0.73 | 0.74 | 0.66 |
| 2066 | Train 33 | Test Not Hispanic 18 | 0.74 | 0.78 | 0.79 | 3090 | Train 49 | Test Not Hispanic 18 | 0.72 | 0.71 | 0.62 |
| 2067 | Train 33 | Test Not Hispanic 19 | 0.75 | 0.83 | 0.85 | 3091 | Train 49 | Test Not Hispanic 19 | 0.73 | 0.74 | 0.66 |
| 2068 | Train 33 | Test Not Hispanic 20 | 0.74 | 0.78 | 0.79 | 3092 | Train 49 | Test Not Hispanic 20 | 0.72 | 0.71 | 0.62 |
| 2069 | Train 33 | Test Not Hispanic 21 | 0.71 | 0.73 | 0.68 | 3093 | Train 49 | Test Not Hispanic 21 | 0.7 | 0.7 | 0.63 |
| 2070 | Train 33 | Test Not Hispanic 22 | 0.69 | 0.7 | 0.64 | 3094 | Train 49 | Test Not Hispanic 22 | 0.69 | 0.68 | 0.61 |
| 2071 | Train 33 | Test Not Hispanic 23 | 0.71 | 0.73 | 0.68 | 3095 | Train 49 | Test Not Hispanic 23 | 0.7 | 0.7 | 0.63 |
| 2072 | Train 33 | Test Not Hispanic 24 | 0.69 | 0.69 | 0.64 | 3096 | Train 49 | Test Not Hispanic 24 | 0.69 | 0.68 | 0.61 |
| 2073 | Train 33 | Test Not Hispanic 25 | 0.75 | 0.82 | 0.81 | 3097 | Train 49 | Test Not Hispanic 25 | 0.73 | 0.73 | 0.65 |
| 2074 | Train 33 | Test Not Hispanic 26 | 0.74 | 0.77 | 0.77 | 3098 | Train 49 | Test Not Hispanic 26 | 0.72 | 0.71 | 0.61 |
| 2075 | Train 33 | Test Not Hispanic 27 | 0.75 | 0.82 | 0.81 | 3099 | Train 49 | Test Not Hispanic 27 | 0.73 | 0.73 | 0.65 |
| 2076 | Train 33 | Test Not Hispanic 28 | 0.74 | 0.77 | 0.77 | 3100 | Train 49 | Test Not Hispanic 28 | 0.72 | 0.71 | 0.61 |
| 2077 | Train 33 | Test Not Hispanic 29 | 0.71 | 0.73 | 0.66 | 3101 | Train 49 | Test Not Hispanic 29 | 0.7 | 0.7 | 0.63 |
| 2078 | Train 33 | Test Not Hispanic 30 | 0.7 | 0.7 | 0.64 | 3102 | Train 49 | Test Not Hispanic 30 | 0.69 | 0.68 | 0.61 |
| 2079 | Train 33 | Test Not Hispanic 31 | 0.71 | 0.73 | 0.66 | 3103 | Train 49 | Test Not Hispanic 31 | 0.7 | 0.7 | 0.63 |
| 2080 | Train 33 | Test Not Hispanic 32 | 0.7 | 0.7 | 0.64 | 3104 | Train 49 | Test Not Hispanic 32 | 0.69 | 0.68 | 0.61 |
| 2081 | Train 33 | Test Not Hispanic 33 | 0.75 | 0.83 | 0.85 | 3105 | Train 49 | Test Not Hispanic 33 | 0.73 | 0.74 | 0.67 |
| 2082 | Train 33 | Test Not Hispanic 34 | 0.74 | 0.78 | 0.79 | 3106 | Train 49 | Test Not Hispanic 34 | 0.72 | 0.71 | 0.62 |
| 2083 | Train 33 | Test Not Hispanic 35 | 0.76 | 0.83 | 0.85 | 3107 | Train 49 | Test Not Hispanic 35 | 0.74 | 0.74 | 0.67 |
| 2084 | Train 33 | Test Not Hispanic 36 | 0.74 | 0.78 | 0.79 | 3108 | Train 49 | Test Not Hispanic 36 | 0.72 | 0.71 | 0.62 |
| 2085 | Train 33 | Test Not Hispanic 37 | 0.71 | 0.73 | 0.68 | 3109 | Train 49 | Test Not Hispanic 37 | 0.7 | 0.7 | 0.63 |
| 2086 | Train 33 | Test Not Hispanic 38 | 0.69 | 0.7 | 0.64 | 3110 | Train 49 | Test Not Hispanic 38 | 0.69 | 0.68 | 0.61 |
| 2087 | Train 33 | Test Not Hispanic 39 | 0.71 | 0.73 | 0.68 | 3111 | Train 49 | Test Not Hispanic 39 | 0.7 | 0.7 | 0.63 |
| 2088 | Train 33 | Test Not Hispanic 40 | 0.69 | 0.69 | 0.64 | 3112 | Train 49 | Test Not Hispanic 40 | 0.69 | 0.68 | 0.61 |
| 2089 | Train 33 | Test Not Hispanic 41 | 0.76 | 0.82 | 0.81 | 3113 | Train 49 | Test Not Hispanic 41 | 0.73 | 0.74 | 0.65 |
| 2090 | Train 33 | Test Not Hispanic 42 | 0.74 | 0.77 | 0.77 | 3114 | Train 49 | Test Not Hispanic 42 | 0.72 | 0.71 | 0.61 |
| 2091 | Train 33 | Test Not Hispanic 43 | 0.76 | 0.82 | 0.81 | 3115 | Train 49 | Test Not Hispanic 43 | 0.73 | 0.74 | 0.65 |
| 2092 | Train 33 | Test Not Hispanic 44 | 0.74 | 0.77 | 0.77 | 3116 | Train 49 | Test Not Hispanic 44 | 0.72 | 0.71 | 0.61 |
| 2093 | Train 33 | Test Not Hispanic 45 | 0.71 | 0.73 | 0.66 | 3117 | Train 49 | Test Not Hispanic 45 | 0.7 | 0.7 | 0.63 |
| 2094 | Train 33 | Test Not Hispanic 46 | 0.7 | 0.7 | 0.64 | 3118 | Train 49 | Test Not Hispanic 46 | 0.69 | 0.68 | 0.61 |
| 2095 | Train 33 | Test Not Hispanic 47 | 0.71 | 0.73 | 0.66 | 3119 | Train 49 | Test Not Hispanic 47 | 0.7 | 0.7 | 0.63 |
| 2096 | Train 33 | Test Not Hispanic 48 | 0.7 | 0.7 | 0.64 | 3120 | Train 49 | Test Not Hispanic 48 | 0.69 | 0.68 | 0.61 |
| 2097 | Train 33 | Test Not Hispanic 49 | 0.76 | 0.8 | 0.83 | 3121 | Train 49 | Test Not Hispanic 49 | 0.77 | 0.82 | 0.87 |
| 2098 | Train 33 | Test Not Hispanic 50 | 0.76 | 0.8 | 0.83 | 3122 | Train 49 | Test Not Hispanic 50 | 0.77 | 0.82 | 0.87 |
| 2099 | Train 33 | Test Not Hispanic 51 | 0.76 | 0.79 | 0.82 | 3123 | Train 49 | Test Not Hispanic 51 | 0.77 | 0.82 | 0.87 |
| 2100 | Train 33 | Test Not Hispanic 52 | 0.76 | 0.79 | 0.82 | 3124 | Train 49 | Test Not Hispanic 52 | 0.77 | 0.82 | 0.87 |
| 2101 | Train 33 | Test Not Hispanic 53 | 0.72 | 0.75 | 0.61 | 3125 | Train 49 | Test Not Hispanic 53 | 0.71 | 0.75 | 0.71 |
| 2102 | Train 33 | Test Not Hispanic 54 | 0.71 | 0.71 | 0.63 | 3126 | Train 49 | Test Not Hispanic 54 | 0.7 | 0.69 | 0.65 |
| 2103 | Train 33 | Test Not Hispanic 55 | 0.72 | 0.75 | 0.61 | 3127 | Train 49 | Test Not Hispanic 55 | 0.71 | 0.75 | 0.71 |
| 2104 | Train 33 | Test Not Hispanic 56 | 0.71 | 0.7 | 0.63 | 3128 | Train 49 | Test Not Hispanic 56 | 0.7 | 0.69 | 0.65 |
| 2105 | Train 33 | Test Not Hispanic 57 | 0.76 | 0.79 | 0.8 | 3129 | Train 49 | Test Not Hispanic 57 | 0.77 | 0.81 | 0.8 |
| 2106 | Train 33 | Test Not Hispanic 58 | 0.76 | 0.79 | 0.8 | 3130 | Train 49 | Test Not Hispanic 58 | 0.77 | 0.81 | 0.8 |
| 2107 | Train 33 | Test Not Hispanic 59 | 0.76 | 0.79 | 0.8 | 3131 | Train 49 | Test Not Hispanic 59 | 0.77 | 0.81 | 0.8 |
| 2108 | Train 33 | Test Not Hispanic 60 | 0.76 | 0.79 | 0.8 | 3132 | Train 49 | Test Not Hispanic 60 | 0.77 | 0.81 | 0.8 |
| 2109 | Train 33 | Test Not Hispanic 61 | 0.72 | 0.75 | 0.61 | 3133 | Train 49 | Test Not Hispanic 61 | 0.71 | 0.75 | 0.7 |
| 2110 | Train 33 | Test Not Hispanic 62 | 0.71 | 0.71 | 0.63 | 3134 | Train 49 | Test Not Hispanic 62 | 0.7 | 0.7 | 0.65 |
| 2111 | Train 33 | Test Not Hispanic 63 | 0.72 | 0.75 | 0.61 | 3135 | Train 49 | Test Not Hispanic 63 | 0.71 | 0.75 | 0.7 |
| 2112 | Train 33 | Test Not Hispanic 64 | 0.71 | 0.7 | 0.64 | 3136 | Train 49 | Test Not Hispanic 64 | 0.7 | 0.69 | 0.65 |
| 2113 | Train 34 | Test Not Hispanic 1 | 0.75 | 0.82 | 0.83 | 3137 | Train 50 | Test Not Hispanic 1 | 0.73 | 0.71 | 0.69 |
| 2114 | Train 34 | Test Not Hispanic 2 | 0.76 | 0.83 | 0.84 | 3138 | Train 50 | Test Not Hispanic 2 | 0.72 | 0.7 | 0.66 |
| 2115 | Train 34 | Test Not Hispanic 3 | 0.75 | 0.82 | 0.83 | 3139 | Train 50 | Test Not Hispanic 3 | 0.73 | 0.71 | 0.69 |
| 2116 | Train 34 | Test Not Hispanic 4 | 0.76 | 0.83 | 0.84 | 3140 | Train 50 | Test Not Hispanic 4 | 0.72 | 0.7 | 0.66 |
| 2117 | Train 34 | Test Not Hispanic 5 | 0.71 | 0.73 | 0.68 | 3141 | Train 50 | Test Not Hispanic 5 | 0.7 | 0.68 | 0.66 |
| 2118 | Train 34 | Test Not Hispanic 6 | 0.71 | 0.71 | 0.66 | 3142 | Train 50 | Test Not Hispanic 6 | 0.69 | 0.67 | 0.64 |
| 2119 | Train 34 | Test Not Hispanic 7 | 0.71 | 0.73 | 0.68 | 3143 | Train 50 | Test Not Hispanic 7 | 0.7 | 0.68 | 0.66 |
| 2120 | Train 34 | Test Not Hispanic 8 | 0.71 | 0.71 | 0.66 | 3144 | Train 50 | Test Not Hispanic 8 | 0.69 | 0.67 | 0.64 |
| 2121 | Train 34 | Test Not Hispanic 9 | 0.75 | 0.81 | 0.8 | 3145 | Train 50 | Test Not Hispanic 9 | 0.73 | 0.71 | 0.68 |
| 2122 | Train 34 | Test Not Hispanic 10 | 0.76 | 0.82 | 0.82 | 3146 | Train 50 | Test Not Hispanic 10 | 0.72 | 0.7 | 0.65 |
| 2123 | Train 34 | Test Not Hispanic 11 | 0.75 | 0.81 | 0.8 | 3147 | Train 50 | Test Not Hispanic 11 | 0.73 | 0.71 | 0.68 |
| 2124 | Train 34 | Test Not Hispanic 12 | 0.76 | 0.82 | 0.81 | 3148 | Train 50 | Test Not Hispanic 12 | 0.72 | 0.7 | 0.65 |
| 2125 | Train 34 | Test Not Hispanic 13 | 0.71 | 0.73 | 0.67 | 3149 | Train 50 | Test Not Hispanic 13 | 0.7 | 0.68 | 0.66 |
| 2126 | Train 34 | Test Not Hispanic 14 | 0.71 | 0.71 | 0.66 | 3150 | Train 50 | Test Not Hispanic 14 | 0.69 | 0.67 | 0.64 |
| 2127 | Train 34 | Test Not Hispanic 15 | 0.71 | 0.73 | 0.67 | 3151 | Train 50 | Test Not Hispanic 15 | 0.7 | 0.68 | 0.66 |
| 2128 | Train 34 | Test Not Hispanic 16 | 0.71 | 0.71 | 0.66 | 3152 | Train 50 | Test Not Hispanic 16 | 0.69 | 0.67 | 0.64 |
| 2129 | Train 34 | Test Not Hispanic 17 | 0.75 | 0.82 | 0.83 | 3153 | Train 50 | Test Not Hispanic 17 | 0.73 | 0.71 | 0.69 |
| 2130 | Train 34 | Test Not Hispanic 18 | 0.76 | 0.83 | 0.84 | 3154 | Train 50 | Test Not Hispanic 18 | 0.72 | 0.7 | 0.66 |
| 2131 | Train 34 | Test Not Hispanic 19 | 0.75 | 0.82 | 0.83 | 3155 | Train 50 | Test Not Hispanic 19 | 0.73 | 0.71 | 0.69 |
| 2132 | Train 34 | Test Not Hispanic 20 | 0.76 | 0.83 | 0.84 | 3156 | Train 50 | Test Not Hispanic 20 | 0.72 | 0.7 | 0.66 |
| 2133 | Train 34 | Test Not Hispanic 21 | 0.71 | 0.73 | 0.68 | 3157 | Train 50 | Test Not Hispanic 21 | 0.7 | 0.68 | 0.66 |
| 2134 | Train 34 | Test Not Hispanic 22 | 0.71 | 0.71 | 0.67 | 3158 | Train 50 | Test Not Hispanic 22 | 0.69 | 0.67 | 0.64 |
| 2135 | Train 34 | Test Not Hispanic 23 | 0.71 | 0.73 | 0.68 | 3159 | Train 50 | Test Not Hispanic 23 | 0.7 | 0.68 | 0.66 |
| 2136 | Train 34 | Test Not Hispanic 24 | 0.71 | 0.71 | 0.66 | 3160 | Train 50 | Test Not Hispanic 24 | 0.69 | 0.67 | 0.64 |
| 2137 | Train 34 | Test Not Hispanic 25 | 0.75 | 0.81 | 0.8 | 3161 | Train 50 | Test Not Hispanic 25 | 0.73 | 0.71 | 0.68 |
| 2138 | Train 34 | Test Not Hispanic 26 | 0.76 | 0.82 | 0.82 | 3162 | Train 50 | Test Not Hispanic 26 | 0.72 | 0.7 | 0.65 |
| 2139 | Train 34 | Test Not Hispanic 27 | 0.75 | 0.81 | 0.8 | 3163 | Train 50 | Test Not Hispanic 27 | 0.73 | 0.71 | 0.68 |
| 2140 | Train 34 | Test Not Hispanic 28 | 0.76 | 0.82 | 0.81 | 3164 | Train 50 | Test Not Hispanic 28 | 0.72 | 0.7 | 0.65 |
| 2141 | Train 34 | Test Not Hispanic 29 | 0.71 | 0.73 | 0.67 | 3165 | Train 50 | Test Not Hispanic 29 | 0.7 | 0.68 | 0.66 |
| 2142 | Train 34 | Test Not Hispanic 30 | 0.71 | 0.71 | 0.67 | 3166 | Train 50 | Test Not Hispanic 30 | 0.69 | 0.67 | 0.64 |
| 2143 | Train 34 | Test Not Hispanic 31 | 0.71 | 0.73 | 0.67 | 3167 | Train 50 | Test Not Hispanic 31 | 0.7 | 0.68 | 0.66 |
| 2144 | Train 34 | Test Not Hispanic 32 | 0.71 | 0.71 | 0.66 | 3168 | Train 50 | Test Not Hispanic 32 | 0.69 | 0.67 | 0.64 |
| 2145 | Train 34 | Test Not Hispanic 33 | 0.75 | 0.82 | 0.83 | 3169 | Train 50 | Test Not Hispanic 33 | 0.73 | 0.71 | 0.69 |
| 2146 | Train 34 | Test Not Hispanic 34 | 0.76 | 0.83 | 0.84 | 3170 | Train 50 | Test Not Hispanic 34 | 0.72 | 0.7 | 0.66 |
| 2147 | Train 34 | Test Not Hispanic 35 | 0.75 | 0.82 | 0.83 | 3171 | Train 50 | Test Not Hispanic 35 | 0.73 | 0.71 | 0.69 |
| 2148 | Train 34 | Test Not Hispanic 36 | 0.76 | 0.83 | 0.84 | 3172 | Train 50 | Test Not Hispanic 36 | 0.72 | 0.7 | 0.66 |
| 2149 | Train 34 | Test Not Hispanic 37 | 0.71 | 0.73 | 0.68 | 3173 | Train 50 | Test Not Hispanic 37 | 0.7 | 0.68 | 0.66 |
| 2150 | Train 34 | Test Not Hispanic 38 | 0.71 | 0.71 | 0.66 | 3174 | Train 50 | Test Not Hispanic 38 | 0.69 | 0.67 | 0.64 |
| 2151 | Train 34 | Test Not Hispanic 39 | 0.71 | 0.73 | 0.68 | 3175 | Train 50 | Test Not Hispanic 39 | 0.7 | 0.68 | 0.66 |
| 2152 | Train 34 | Test Not Hispanic 40 | 0.71 | 0.71 | 0.66 | 3176 | Train 50 | Test Not Hispanic 40 | 0.69 | 0.67 | 0.64 |
| 2153 | Train 34 | Test Not Hispanic 41 | 0.75 | 0.81 | 0.81 | 3177 | Train 50 | Test Not Hispanic 41 | 0.73 | 0.71 | 0.68 |
| 2154 | Train 34 | Test Not Hispanic 42 | 0.76 | 0.82 | 0.82 | 3178 | Train 50 | Test Not Hispanic 42 | 0.72 | 0.7 | 0.65 |
| 2155 | Train 34 | Test Not Hispanic 43 | 0.75 | 0.81 | 0.8 | 3179 | Train 50 | Test Not Hispanic 43 | 0.73 | 0.71 | 0.68 |
| 2156 | Train 34 | Test Not Hispanic 44 | 0.76 | 0.82 | 0.82 | 3180 | Train 50 | Test Not Hispanic 44 | 0.72 | 0.7 | 0.65 |
| 2157 | Train 34 | Test Not Hispanic 45 | 0.71 | 0.73 | 0.67 | 3181 | Train 50 | Test Not Hispanic 45 | 0.7 | 0.68 | 0.66 |
| 2158 | Train 34 | Test Not Hispanic 46 | 0.71 | 0.71 | 0.67 | 3182 | Train 50 | Test Not Hispanic 46 | 0.69 | 0.67 | 0.64 |
| 2159 | Train 34 | Test Not Hispanic 47 | 0.71 | 0.73 | 0.67 | 3183 | Train 50 | Test Not Hispanic 47 | 0.7 | 0.68 | 0.66 |
| 2160 | Train 34 | Test Not Hispanic 48 | 0.71 | 0.71 | 0.66 | 3184 | Train 50 | Test Not Hispanic 48 | 0.69 | 0.67 | 0.64 |
| 2161 | Train 34 | Test Not Hispanic 49 | 0.76 | 0.79 | 0.82 | 3185 | Train 50 | Test Not Hispanic 49 | 0.77 | 0.82 | 0.88 |
| 2162 | Train 34 | Test Not Hispanic 50 | 0.76 | 0.79 | 0.82 | 3186 | Train 50 | Test Not Hispanic 50 | 0.77 | 0.82 | 0.88 |
| 2163 | Train 34 | Test Not Hispanic 51 | 0.76 | 0.79 | 0.82 | 3187 | Train 50 | Test Not Hispanic 51 | 0.77 | 0.82 | 0.87 |
| 2164 | Train 34 | Test Not Hispanic 52 | 0.76 | 0.79 | 0.82 | 3188 | Train 50 | Test Not Hispanic 52 | 0.77 | 0.82 | 0.87 |
| 2165 | Train 34 | Test Not Hispanic 53 | 0.72 | 0.75 | 0.67 | 3189 | Train 50 | Test Not Hispanic 53 | 0.71 | 0.73 | 0.73 |
| 2166 | Train 34 | Test Not Hispanic 54 | 0.71 | 0.71 | 0.67 | 3190 | Train 50 | Test Not Hispanic 54 | 0.7 | 0.68 | 0.65 |
| 2167 | Train 34 | Test Not Hispanic 55 | 0.73 | 0.75 | 0.68 | 3191 | Train 50 | Test Not Hispanic 55 | 0.71 | 0.73 | 0.73 |
| 2168 | Train 34 | Test Not Hispanic 56 | 0.71 | 0.7 | 0.66 | 3192 | Train 50 | Test Not Hispanic 56 | 0.7 | 0.68 | 0.65 |
| 2169 | Train 34 | Test Not Hispanic 57 | 0.76 | 0.78 | 0.79 | 3193 | Train 50 | Test Not Hispanic 57 | 0.77 | 0.81 | 0.81 |
| 2170 | Train 34 | Test Not Hispanic 58 | 0.76 | 0.79 | 0.79 | 3194 | Train 50 | Test Not Hispanic 58 | 0.77 | 0.81 | 0.81 |
| 2171 | Train 34 | Test Not Hispanic 59 | 0.76 | 0.78 | 0.79 | 3195 | Train 50 | Test Not Hispanic 59 | 0.77 | 0.81 | 0.8 |
| 2172 | Train 34 | Test Not Hispanic 60 | 0.76 | 0.78 | 0.79 | 3196 | Train 50 | Test Not Hispanic 60 | 0.77 | 0.81 | 0.8 |
| 2173 | Train 34 | Test Not Hispanic 61 | 0.72 | 0.75 | 0.67 | 3197 | Train 50 | Test Not Hispanic 61 | 0.71 | 0.72 | 0.72 |
| 2174 | Train 34 | Test Not Hispanic 62 | 0.72 | 0.71 | 0.67 | 3198 | Train 50 | Test Not Hispanic 62 | 0.7 | 0.69 | 0.66 |
| 2175 | Train 34 | Test Not Hispanic 63 | 0.73 | 0.75 | 0.68 | 3199 | Train 50 | Test Not Hispanic 63 | 0.71 | 0.73 | 0.72 |
| 2176 | Train 34 | Test Not Hispanic 64 | 0.71 | 0.71 | 0.66 | 3200 | Train 50 | Test Not Hispanic 64 | 0.7 | 0.68 | 0.65 |
| 2177 | Train 35 | Test Not Hispanic 1 | 0.75 | 0.83 | 0.84 | 3201 | Train 51 | Test Not Hispanic 1 | 0.73 | 0.74 | 0.7 |
| 2178 | Train 35 | Test Not Hispanic 2 | 0.74 | 0.78 | 0.79 | 3202 | Train 51 | Test Not Hispanic 2 | 0.71 | 0.71 | 0.63 |
| 2179 | Train 35 | Test Not Hispanic 3 | 0.75 | 0.83 | 0.84 | 3203 | Train 51 | Test Not Hispanic 3 | 0.73 | 0.74 | 0.7 |
| 2180 | Train 35 | Test Not Hispanic 4 | 0.74 | 0.78 | 0.79 | 3204 | Train 51 | Test Not Hispanic 4 | 0.71 | 0.71 | 0.63 |
| 2181 | Train 35 | Test Not Hispanic 5 | 0.71 | 0.73 | 0.68 | 3205 | Train 51 | Test Not Hispanic 5 | 0.7 | 0.7 | 0.65 |
| 2182 | Train 35 | Test Not Hispanic 6 | 0.69 | 0.7 | 0.66 | 3206 | Train 51 | Test Not Hispanic 6 | 0.68 | 0.68 | 0.59 |
| 2183 | Train 35 | Test Not Hispanic 7 | 0.71 | 0.73 | 0.68 | 3207 | Train 51 | Test Not Hispanic 7 | 0.7 | 0.7 | 0.65 |
| 2184 | Train 35 | Test Not Hispanic 8 | 0.69 | 0.69 | 0.66 | 3208 | Train 51 | Test Not Hispanic 8 | 0.68 | 0.68 | 0.59 |
| 2185 | Train 35 | Test Not Hispanic 9 | 0.75 | 0.82 | 0.81 | 3209 | Train 51 | Test Not Hispanic 9 | 0.73 | 0.73 | 0.68 |
| 2186 | Train 35 | Test Not Hispanic 10 | 0.74 | 0.77 | 0.77 | 3210 | Train 51 | Test Not Hispanic 10 | 0.71 | 0.71 | 0.62 |
| 2187 | Train 35 | Test Not Hispanic 11 | 0.75 | 0.82 | 0.81 | 3211 | Train 51 | Test Not Hispanic 11 | 0.73 | 0.73 | 0.68 |
| 2188 | Train 35 | Test Not Hispanic 12 | 0.74 | 0.77 | 0.77 | 3212 | Train 51 | Test Not Hispanic 12 | 0.71 | 0.71 | 0.62 |
| 2189 | Train 35 | Test Not Hispanic 13 | 0.71 | 0.73 | 0.66 | 3213 | Train 51 | Test Not Hispanic 13 | 0.7 | 0.7 | 0.64 |
| 2190 | Train 35 | Test Not Hispanic 14 | 0.7 | 0.7 | 0.66 | 3214 | Train 51 | Test Not Hispanic 14 | 0.68 | 0.68 | 0.59 |
| 2191 | Train 35 | Test Not Hispanic 15 | 0.71 | 0.73 | 0.66 | 3215 | Train 51 | Test Not Hispanic 15 | 0.7 | 0.7 | 0.64 |
| 2192 | Train 35 | Test Not Hispanic 16 | 0.7 | 0.69 | 0.66 | 3216 | Train 51 | Test Not Hispanic 16 | 0.69 | 0.68 | 0.59 |
| 2193 | Train 35 | Test Not Hispanic 17 | 0.75 | 0.83 | 0.84 | 3217 | Train 51 | Test Not Hispanic 17 | 0.73 | 0.74 | 0.7 |
| 2194 | Train 35 | Test Not Hispanic 18 | 0.74 | 0.78 | 0.79 | 3218 | Train 51 | Test Not Hispanic 18 | 0.71 | 0.71 | 0.63 |
| 2195 | Train 35 | Test Not Hispanic 19 | 0.75 | 0.83 | 0.84 | 3219 | Train 51 | Test Not Hispanic 19 | 0.73 | 0.74 | 0.7 |
| 2196 | Train 35 | Test Not Hispanic 20 | 0.74 | 0.78 | 0.79 | 3220 | Train 51 | Test Not Hispanic 20 | 0.71 | 0.71 | 0.63 |
| 2197 | Train 35 | Test Not Hispanic 21 | 0.71 | 0.73 | 0.68 | 3221 | Train 51 | Test Not Hispanic 21 | 0.7 | 0.7 | 0.65 |
| 2198 | Train 35 | Test Not Hispanic 22 | 0.69 | 0.7 | 0.66 | 3222 | Train 51 | Test Not Hispanic 22 | 0.68 | 0.68 | 0.59 |
| 2199 | Train 35 | Test Not Hispanic 23 | 0.71 | 0.73 | 0.68 | 3223 | Train 51 | Test Not Hispanic 23 | 0.7 | 0.7 | 0.65 |
| 2200 | Train 35 | Test Not Hispanic 24 | 0.69 | 0.69 | 0.66 | 3224 | Train 51 | Test Not Hispanic 24 | 0.69 | 0.68 | 0.59 |
| 2201 | Train 35 | Test Not Hispanic 25 | 0.75 | 0.82 | 0.81 | 3225 | Train 51 | Test Not Hispanic 25 | 0.73 | 0.73 | 0.68 |
| 2202 | Train 35 | Test Not Hispanic 26 | 0.74 | 0.77 | 0.77 | 3226 | Train 51 | Test Not Hispanic 26 | 0.71 | 0.71 | 0.62 |
| 2203 | Train 35 | Test Not Hispanic 27 | 0.75 | 0.82 | 0.81 | 3227 | Train 51 | Test Not Hispanic 27 | 0.73 | 0.73 | 0.68 |
| 2204 | Train 35 | Test Not Hispanic 28 | 0.74 | 0.77 | 0.77 | 3228 | Train 51 | Test Not Hispanic 28 | 0.71 | 0.71 | 0.62 |
| 2205 | Train 35 | Test Not Hispanic 29 | 0.71 | 0.73 | 0.66 | 3229 | Train 51 | Test Not Hispanic 29 | 0.7 | 0.7 | 0.64 |
| 2206 | Train 35 | Test Not Hispanic 30 | 0.7 | 0.7 | 0.66 | 3230 | Train 51 | Test Not Hispanic 30 | 0.68 | 0.68 | 0.59 |
| 2207 | Train 35 | Test Not Hispanic 31 | 0.71 | 0.73 | 0.66 | 3231 | Train 51 | Test Not Hispanic 31 | 0.7 | 0.7 | 0.64 |
| 2208 | Train 35 | Test Not Hispanic 32 | 0.7 | 0.7 | 0.66 | 3232 | Train 51 | Test Not Hispanic 32 | 0.69 | 0.68 | 0.59 |
| 2209 | Train 35 | Test Not Hispanic 33 | 0.75 | 0.83 | 0.84 | 3233 | Train 51 | Test Not Hispanic 33 | 0.73 | 0.74 | 0.7 |
| 2210 | Train 35 | Test Not Hispanic 34 | 0.74 | 0.78 | 0.79 | 3234 | Train 51 | Test Not Hispanic 34 | 0.71 | 0.71 | 0.63 |
| 2211 | Train 35 | Test Not Hispanic 35 | 0.76 | 0.83 | 0.84 | 3235 | Train 51 | Test Not Hispanic 35 | 0.74 | 0.74 | 0.7 |
| 2212 | Train 35 | Test Not Hispanic 36 | 0.74 | 0.78 | 0.79 | 3236 | Train 51 | Test Not Hispanic 36 | 0.71 | 0.71 | 0.64 |
| 2213 | Train 35 | Test Not Hispanic 37 | 0.71 | 0.74 | 0.68 | 3237 | Train 51 | Test Not Hispanic 37 | 0.7 | 0.7 | 0.65 |
| 2214 | Train 35 | Test Not Hispanic 38 | 0.69 | 0.7 | 0.66 | 3238 | Train 51 | Test Not Hispanic 38 | 0.68 | 0.68 | 0.59 |
| 2215 | Train 35 | Test Not Hispanic 39 | 0.71 | 0.73 | 0.68 | 3239 | Train 51 | Test Not Hispanic 39 | 0.7 | 0.7 | 0.65 |
| 2216 | Train 35 | Test Not Hispanic 40 | 0.69 | 0.69 | 0.66 | 3240 | Train 51 | Test Not Hispanic 40 | 0.69 | 0.68 | 0.59 |
| 2217 | Train 35 | Test Not Hispanic 41 | 0.76 | 0.82 | 0.81 | 3241 | Train 51 | Test Not Hispanic 41 | 0.73 | 0.73 | 0.68 |
| 2218 | Train 35 | Test Not Hispanic 42 | 0.74 | 0.77 | 0.77 | 3242 | Train 51 | Test Not Hispanic 42 | 0.71 | 0.71 | 0.62 |
| 2219 | Train 35 | Test Not Hispanic 43 | 0.76 | 0.82 | 0.81 | 3243 | Train 51 | Test Not Hispanic 43 | 0.74 | 0.74 | 0.68 |
| 2220 | Train 35 | Test Not Hispanic 44 | 0.74 | 0.77 | 0.77 | 3244 | Train 51 | Test Not Hispanic 44 | 0.71 | 0.71 | 0.62 |
| 2221 | Train 35 | Test Not Hispanic 45 | 0.71 | 0.73 | 0.66 | 3245 | Train 51 | Test Not Hispanic 45 | 0.7 | 0.7 | 0.64 |
| 2222 | Train 35 | Test Not Hispanic 46 | 0.7 | 0.7 | 0.66 | 3246 | Train 51 | Test Not Hispanic 46 | 0.68 | 0.68 | 0.59 |
| 2223 | Train 35 | Test Not Hispanic 47 | 0.71 | 0.73 | 0.66 | 3247 | Train 51 | Test Not Hispanic 47 | 0.7 | 0.7 | 0.64 |
| 2224 | Train 35 | Test Not Hispanic 48 | 0.7 | 0.7 | 0.66 | 3248 | Train 51 | Test Not Hispanic 48 | 0.69 | 0.68 | 0.59 |
| 2225 | Train 35 | Test Not Hispanic 49 | 0.76 | 0.79 | 0.83 | 3249 | Train 51 | Test Not Hispanic 49 | 0.77 | 0.82 | 0.86 |
| 2226 | Train 35 | Test Not Hispanic 50 | 0.76 | 0.79 | 0.83 | 3250 | Train 51 | Test Not Hispanic 50 | 0.77 | 0.82 | 0.86 |
| 2227 | Train 35 | Test Not Hispanic 51 | 0.76 | 0.8 | 0.83 | 3251 | Train 51 | Test Not Hispanic 51 | 0.77 | 0.82 | 0.87 |
| 2228 | Train 35 | Test Not Hispanic 52 | 0.76 | 0.8 | 0.83 | 3252 | Train 51 | Test Not Hispanic 52 | 0.77 | 0.82 | 0.86 |
| 2229 | Train 35 | Test Not Hispanic 53 | 0.72 | 0.75 | 0.62 | 3253 | Train 51 | Test Not Hispanic 53 | 0.71 | 0.75 | 0.71 |
| 2230 | Train 35 | Test Not Hispanic 54 | 0.71 | 0.7 | 0.65 | 3254 | Train 51 | Test Not Hispanic 54 | 0.7 | 0.69 | 0.62 |
| 2231 | Train 35 | Test Not Hispanic 55 | 0.72 | 0.75 | 0.62 | 3255 | Train 51 | Test Not Hispanic 55 | 0.71 | 0.75 | 0.71 |
| 2232 | Train 35 | Test Not Hispanic 56 | 0.71 | 0.7 | 0.65 | 3256 | Train 51 | Test Not Hispanic 56 | 0.7 | 0.69 | 0.62 |
| 2233 | Train 35 | Test Not Hispanic 57 | 0.76 | 0.79 | 0.8 | 3257 | Train 51 | Test Not Hispanic 57 | 0.77 | 0.81 | 0.8 |
| 2234 | Train 35 | Test Not Hispanic 58 | 0.76 | 0.79 | 0.8 | 3258 | Train 51 | Test Not Hispanic 58 | 0.77 | 0.81 | 0.8 |
| 2235 | Train 35 | Test Not Hispanic 59 | 0.76 | 0.79 | 0.8 | 3259 | Train 51 | Test Not Hispanic 59 | 0.77 | 0.81 | 0.8 |
| 2236 | Train 35 | Test Not Hispanic 60 | 0.76 | 0.79 | 0.8 | 3260 | Train 51 | Test Not Hispanic 60 | 0.77 | 0.81 | 0.8 |
| 2237 | Train 35 | Test Not Hispanic 61 | 0.72 | 0.75 | 0.62 | 3261 | Train 51 | Test Not Hispanic 61 | 0.71 | 0.75 | 0.7 |
| 2238 | Train 35 | Test Not Hispanic 62 | 0.71 | 0.71 | 0.65 | 3262 | Train 51 | Test Not Hispanic 62 | 0.7 | 0.7 | 0.62 |
| 2239 | Train 35 | Test Not Hispanic 63 | 0.72 | 0.75 | 0.62 | 3263 | Train 51 | Test Not Hispanic 63 | 0.71 | 0.75 | 0.7 |
| 2240 | Train 35 | Test Not Hispanic 64 | 0.71 | 0.7 | 0.65 | 3264 | Train 51 | Test Not Hispanic 64 | 0.7 | 0.69 | 0.62 |
| 2241 | Train 36 | Test Not Hispanic 1 | 0.75 | 0.82 | 0.83 | 3265 | Train 52 | Test Not Hispanic 1 | 0.73 | 0.71 | 0.69 |
| 2242 | Train 36 | Test Not Hispanic 2 | 0.76 | 0.83 | 0.84 | 3266 | Train 52 | Test Not Hispanic 2 | 0.71 | 0.69 | 0.65 |
| 2243 | Train 36 | Test Not Hispanic 3 | 0.75 | 0.82 | 0.83 | 3267 | Train 52 | Test Not Hispanic 3 | 0.73 | 0.71 | 0.69 |
| 2244 | Train 36 | Test Not Hispanic 4 | 0.76 | 0.83 | 0.84 | 3268 | Train 52 | Test Not Hispanic 4 | 0.72 | 0.69 | 0.65 |
| 2245 | Train 36 | Test Not Hispanic 5 | 0.71 | 0.73 | 0.69 | 3269 | Train 52 | Test Not Hispanic 5 | 0.7 | 0.68 | 0.66 |
| 2246 | Train 36 | Test Not Hispanic 6 | 0.71 | 0.71 | 0.67 | 3270 | Train 52 | Test Not Hispanic 6 | 0.68 | 0.67 | 0.63 |
| 2247 | Train 36 | Test Not Hispanic 7 | 0.71 | 0.73 | 0.69 | 3271 | Train 52 | Test Not Hispanic 7 | 0.7 | 0.68 | 0.66 |
| 2248 | Train 36 | Test Not Hispanic 8 | 0.71 | 0.71 | 0.67 | 3272 | Train 52 | Test Not Hispanic 8 | 0.69 | 0.67 | 0.63 |
| 2249 | Train 36 | Test Not Hispanic 9 | 0.75 | 0.81 | 0.81 | 3273 | Train 52 | Test Not Hispanic 9 | 0.73 | 0.71 | 0.67 |
| 2250 | Train 36 | Test Not Hispanic 10 | 0.76 | 0.82 | 0.82 | 3274 | Train 52 | Test Not Hispanic 10 | 0.71 | 0.69 | 0.64 |
| 2251 | Train 36 | Test Not Hispanic 11 | 0.75 | 0.81 | 0.81 | 3275 | Train 52 | Test Not Hispanic 11 | 0.73 | 0.71 | 0.67 |
| 2252 | Train 36 | Test Not Hispanic 12 | 0.76 | 0.82 | 0.82 | 3276 | Train 52 | Test Not Hispanic 12 | 0.71 | 0.69 | 0.64 |
| 2253 | Train 36 | Test Not Hispanic 13 | 0.71 | 0.73 | 0.67 | 3277 | Train 52 | Test Not Hispanic 13 | 0.7 | 0.68 | 0.65 |
| 2254 | Train 36 | Test Not Hispanic 14 | 0.71 | 0.71 | 0.67 | 3278 | Train 52 | Test Not Hispanic 14 | 0.69 | 0.67 | 0.63 |
| 2255 | Train 36 | Test Not Hispanic 15 | 0.71 | 0.73 | 0.67 | 3279 | Train 52 | Test Not Hispanic 15 | 0.7 | 0.68 | 0.65 |
| 2256 | Train 36 | Test Not Hispanic 16 | 0.71 | 0.71 | 0.67 | 3280 | Train 52 | Test Not Hispanic 16 | 0.69 | 0.67 | 0.63 |
| 2257 | Train 36 | Test Not Hispanic 17 | 0.75 | 0.82 | 0.83 | 3281 | Train 52 | Test Not Hispanic 17 | 0.73 | 0.71 | 0.69 |
| 2258 | Train 36 | Test Not Hispanic 18 | 0.76 | 0.83 | 0.84 | 3282 | Train 52 | Test Not Hispanic 18 | 0.72 | 0.69 | 0.65 |
| 2259 | Train 36 | Test Not Hispanic 19 | 0.75 | 0.82 | 0.83 | 3283 | Train 52 | Test Not Hispanic 19 | 0.73 | 0.71 | 0.69 |
| 2260 | Train 36 | Test Not Hispanic 20 | 0.76 | 0.83 | 0.84 | 3284 | Train 52 | Test Not Hispanic 20 | 0.72 | 0.69 | 0.65 |
| 2261 | Train 36 | Test Not Hispanic 21 | 0.71 | 0.74 | 0.69 | 3285 | Train 52 | Test Not Hispanic 21 | 0.7 | 0.68 | 0.66 |
| 2262 | Train 36 | Test Not Hispanic 22 | 0.71 | 0.71 | 0.67 | 3286 | Train 52 | Test Not Hispanic 22 | 0.68 | 0.67 | 0.63 |
| 2263 | Train 36 | Test Not Hispanic 23 | 0.71 | 0.73 | 0.69 | 3287 | Train 52 | Test Not Hispanic 23 | 0.7 | 0.68 | 0.66 |
| 2264 | Train 36 | Test Not Hispanic 24 | 0.71 | 0.71 | 0.67 | 3288 | Train 52 | Test Not Hispanic 24 | 0.69 | 0.67 | 0.63 |
| 2265 | Train 36 | Test Not Hispanic 25 | 0.75 | 0.81 | 0.81 | 3289 | Train 52 | Test Not Hispanic 25 | 0.73 | 0.71 | 0.67 |
| 2266 | Train 36 | Test Not Hispanic 26 | 0.76 | 0.82 | 0.82 | 3290 | Train 52 | Test Not Hispanic 26 | 0.71 | 0.69 | 0.64 |
| 2267 | Train 36 | Test Not Hispanic 27 | 0.75 | 0.81 | 0.81 | 3291 | Train 52 | Test Not Hispanic 27 | 0.73 | 0.71 | 0.67 |
| 2268 | Train 36 | Test Not Hispanic 28 | 0.76 | 0.82 | 0.82 | 3292 | Train 52 | Test Not Hispanic 28 | 0.72 | 0.69 | 0.64 |
| 2269 | Train 36 | Test Not Hispanic 29 | 0.71 | 0.73 | 0.67 | 3293 | Train 52 | Test Not Hispanic 29 | 0.7 | 0.68 | 0.65 |
| 2270 | Train 36 | Test Not Hispanic 30 | 0.71 | 0.71 | 0.67 | 3294 | Train 52 | Test Not Hispanic 30 | 0.69 | 0.67 | 0.63 |
| 2271 | Train 36 | Test Not Hispanic 31 | 0.71 | 0.73 | 0.67 | 3295 | Train 52 | Test Not Hispanic 31 | 0.7 | 0.68 | 0.65 |
| 2272 | Train 36 | Test Not Hispanic 32 | 0.71 | 0.71 | 0.67 | 3296 | Train 52 | Test Not Hispanic 32 | 0.69 | 0.67 | 0.63 |
| 2273 | Train 36 | Test Not Hispanic 33 | 0.75 | 0.82 | 0.83 | 3297 | Train 52 | Test Not Hispanic 33 | 0.73 | 0.71 | 0.69 |
| 2274 | Train 36 | Test Not Hispanic 34 | 0.76 | 0.83 | 0.85 | 3298 | Train 52 | Test Not Hispanic 34 | 0.72 | 0.7 | 0.65 |
| 2275 | Train 36 | Test Not Hispanic 35 | 0.75 | 0.82 | 0.84 | 3299 | Train 52 | Test Not Hispanic 35 | 0.73 | 0.71 | 0.69 |
| 2276 | Train 36 | Test Not Hispanic 36 | 0.76 | 0.83 | 0.85 | 3300 | Train 52 | Test Not Hispanic 36 | 0.72 | 0.7 | 0.66 |
| 2277 | Train 36 | Test Not Hispanic 37 | 0.71 | 0.74 | 0.69 | 3301 | Train 52 | Test Not Hispanic 37 | 0.7 | 0.68 | 0.66 |
| 2278 | Train 36 | Test Not Hispanic 38 | 0.71 | 0.71 | 0.67 | 3302 | Train 52 | Test Not Hispanic 38 | 0.68 | 0.67 | 0.63 |
| 2279 | Train 36 | Test Not Hispanic 39 | 0.71 | 0.73 | 0.69 | 3303 | Train 52 | Test Not Hispanic 39 | 0.7 | 0.68 | 0.66 |
| 2280 | Train 36 | Test Not Hispanic 40 | 0.71 | 0.71 | 0.67 | 3304 | Train 52 | Test Not Hispanic 40 | 0.69 | 0.67 | 0.63 |
| 2281 | Train 36 | Test Not Hispanic 41 | 0.75 | 0.81 | 0.81 | 3305 | Train 52 | Test Not Hispanic 41 | 0.73 | 0.71 | 0.67 |
| 2282 | Train 36 | Test Not Hispanic 42 | 0.76 | 0.82 | 0.82 | 3306 | Train 52 | Test Not Hispanic 42 | 0.72 | 0.69 | 0.64 |
| 2283 | Train 36 | Test Not Hispanic 43 | 0.75 | 0.81 | 0.81 | 3307 | Train 52 | Test Not Hispanic 43 | 0.73 | 0.71 | 0.67 |
| 2284 | Train 36 | Test Not Hispanic 44 | 0.76 | 0.82 | 0.82 | 3308 | Train 52 | Test Not Hispanic 44 | 0.72 | 0.7 | 0.65 |
| 2285 | Train 36 | Test Not Hispanic 45 | 0.71 | 0.73 | 0.68 | 3309 | Train 52 | Test Not Hispanic 45 | 0.7 | 0.68 | 0.65 |
| 2286 | Train 36 | Test Not Hispanic 46 | 0.71 | 0.71 | 0.67 | 3310 | Train 52 | Test Not Hispanic 46 | 0.69 | 0.67 | 0.63 |
| 2287 | Train 36 | Test Not Hispanic 47 | 0.71 | 0.73 | 0.68 | 3311 | Train 52 | Test Not Hispanic 47 | 0.7 | 0.68 | 0.65 |
| 2288 | Train 36 | Test Not Hispanic 48 | 0.71 | 0.71 | 0.67 | 3312 | Train 52 | Test Not Hispanic 48 | 0.69 | 0.67 | 0.63 |
| 2289 | Train 36 | Test Not Hispanic 49 | 0.76 | 0.79 | 0.81 | 3313 | Train 52 | Test Not Hispanic 49 | 0.77 | 0.82 | 0.86 |
| 2290 | Train 36 | Test Not Hispanic 50 | 0.76 | 0.79 | 0.81 | 3314 | Train 52 | Test Not Hispanic 50 | 0.77 | 0.82 | 0.86 |
| 2291 | Train 36 | Test Not Hispanic 51 | 0.76 | 0.79 | 0.81 | 3315 | Train 52 | Test Not Hispanic 51 | 0.77 | 0.82 | 0.86 |
| 2292 | Train 36 | Test Not Hispanic 52 | 0.76 | 0.79 | 0.81 | 3316 | Train 52 | Test Not Hispanic 52 | 0.77 | 0.82 | 0.87 |
| 2293 | Train 36 | Test Not Hispanic 53 | 0.72 | 0.76 | 0.65 | 3317 | Train 52 | Test Not Hispanic 53 | 0.71 | 0.72 | 0.74 |
| 2294 | Train 36 | Test Not Hispanic 54 | 0.71 | 0.71 | 0.67 | 3318 | Train 52 | Test Not Hispanic 54 | 0.7 | 0.68 | 0.66 |
| 2295 | Train 36 | Test Not Hispanic 55 | 0.73 | 0.76 | 0.65 | 3319 | Train 52 | Test Not Hispanic 55 | 0.71 | 0.73 | 0.74 |
| 2296 | Train 36 | Test Not Hispanic 56 | 0.71 | 0.71 | 0.67 | 3320 | Train 52 | Test Not Hispanic 56 | 0.7 | 0.68 | 0.66 |
| 2297 | Train 36 | Test Not Hispanic 57 | 0.76 | 0.78 | 0.78 | 3321 | Train 52 | Test Not Hispanic 57 | 0.77 | 0.81 | 0.8 |
| 2298 | Train 36 | Test Not Hispanic 58 | 0.76 | 0.78 | 0.78 | 3322 | Train 52 | Test Not Hispanic 58 | 0.77 | 0.81 | 0.8 |
| 2299 | Train 36 | Test Not Hispanic 59 | 0.76 | 0.78 | 0.78 | 3323 | Train 52 | Test Not Hispanic 59 | 0.77 | 0.81 | 0.8 |
| 2300 | Train 36 | Test Not Hispanic 60 | 0.76 | 0.79 | 0.78 | 3324 | Train 52 | Test Not Hispanic 60 | 0.77 | 0.81 | 0.8 |
| 2301 | Train 36 | Test Not Hispanic 61 | 0.72 | 0.75 | 0.65 | 3325 | Train 52 | Test Not Hispanic 61 | 0.71 | 0.72 | 0.73 |
| 2302 | Train 36 | Test Not Hispanic 62 | 0.72 | 0.72 | 0.67 | 3326 | Train 52 | Test Not Hispanic 62 | 0.7 | 0.68 | 0.66 |
| 2303 | Train 36 | Test Not Hispanic 63 | 0.73 | 0.76 | 0.65 | 3327 | Train 52 | Test Not Hispanic 63 | 0.71 | 0.72 | 0.73 |
| 2304 | Train 36 | Test Not Hispanic 64 | 0.71 | 0.71 | 0.67 | 3328 | Train 52 | Test Not Hispanic 64 | 0.7 | 0.68 | 0.66 |
| 2305 | Train 37 | Test Not Hispanic 1 | 0.75 | 0.8 | 0.82 | 3329 | Train 53 | Test Not Hispanic 1 | 0.73 | 0.74 | 0.66 |
| 2306 | Train 37 | Test Not Hispanic 2 | 0.75 | 0.78 | 0.79 | 3330 | Train 53 | Test Not Hispanic 2 | 0.73 | 0.72 | 0.65 |
| 2307 | Train 37 | Test Not Hispanic 3 | 0.75 | 0.8 | 0.82 | 3331 | Train 53 | Test Not Hispanic 3 | 0.73 | 0.74 | 0.66 |
| 2308 | Train 37 | Test Not Hispanic 4 | 0.75 | 0.78 | 0.78 | 3332 | Train 53 | Test Not Hispanic 4 | 0.73 | 0.72 | 0.65 |
| 2309 | Train 37 | Test Not Hispanic 5 | 0.73 | 0.78 | 0.77 | 3333 | Train 53 | Test Not Hispanic 5 | 0.72 | 0.74 | 0.69 |
| 2310 | Train 37 | Test Not Hispanic 6 | 0.7 | 0.71 | 0.69 | 3334 | Train 53 | Test Not Hispanic 6 | 0.7 | 0.69 | 0.64 |
| 2311 | Train 37 | Test Not Hispanic 7 | 0.73 | 0.78 | 0.77 | 3335 | Train 53 | Test Not Hispanic 7 | 0.72 | 0.74 | 0.69 |
| 2312 | Train 37 | Test Not Hispanic 8 | 0.7 | 0.71 | 0.69 | 3336 | Train 53 | Test Not Hispanic 8 | 0.7 | 0.69 | 0.64 |
| 2313 | Train 37 | Test Not Hispanic 9 | 0.75 | 0.79 | 0.8 | 3337 | Train 53 | Test Not Hispanic 9 | 0.73 | 0.75 | 0.67 |
| 2314 | Train 37 | Test Not Hispanic 10 | 0.75 | 0.78 | 0.77 | 3338 | Train 53 | Test Not Hispanic 10 | 0.73 | 0.72 | 0.65 |
| 2315 | Train 37 | Test Not Hispanic 11 | 0.75 | 0.79 | 0.79 | 3339 | Train 53 | Test Not Hispanic 11 | 0.73 | 0.75 | 0.67 |
| 2316 | Train 37 | Test Not Hispanic 12 | 0.75 | 0.78 | 0.77 | 3340 | Train 53 | Test Not Hispanic 12 | 0.73 | 0.72 | 0.65 |
| 2317 | Train 37 | Test Not Hispanic 13 | 0.73 | 0.77 | 0.75 | 3341 | Train 53 | Test Not Hispanic 13 | 0.72 | 0.74 | 0.69 |
| 2318 | Train 37 | Test Not Hispanic 14 | 0.7 | 0.71 | 0.69 | 3342 | Train 53 | Test Not Hispanic 14 | 0.7 | 0.69 | 0.64 |
| 2319 | Train 37 | Test Not Hispanic 15 | 0.73 | 0.77 | 0.75 | 3343 | Train 53 | Test Not Hispanic 15 | 0.72 | 0.74 | 0.69 |
| 2320 | Train 37 | Test Not Hispanic 16 | 0.7 | 0.71 | 0.69 | 3344 | Train 53 | Test Not Hispanic 16 | 0.7 | 0.69 | 0.64 |
| 2321 | Train 37 | Test Not Hispanic 17 | 0.75 | 0.8 | 0.82 | 3345 | Train 53 | Test Not Hispanic 17 | 0.73 | 0.74 | 0.66 |
| 2322 | Train 37 | Test Not Hispanic 18 | 0.75 | 0.78 | 0.79 | 3346 | Train 53 | Test Not Hispanic 18 | 0.73 | 0.72 | 0.65 |
| 2323 | Train 37 | Test Not Hispanic 19 | 0.75 | 0.8 | 0.82 | 3347 | Train 53 | Test Not Hispanic 19 | 0.73 | 0.74 | 0.66 |
| 2324 | Train 37 | Test Not Hispanic 20 | 0.75 | 0.78 | 0.78 | 3348 | Train 53 | Test Not Hispanic 20 | 0.73 | 0.72 | 0.65 |
| 2325 | Train 37 | Test Not Hispanic 21 | 0.74 | 0.78 | 0.78 | 3349 | Train 53 | Test Not Hispanic 21 | 0.72 | 0.74 | 0.69 |
| 2326 | Train 37 | Test Not Hispanic 22 | 0.7 | 0.71 | 0.69 | 3350 | Train 53 | Test Not Hispanic 22 | 0.7 | 0.69 | 0.64 |
| 2327 | Train 37 | Test Not Hispanic 23 | 0.73 | 0.78 | 0.77 | 3351 | Train 53 | Test Not Hispanic 23 | 0.72 | 0.74 | 0.69 |
| 2328 | Train 37 | Test Not Hispanic 24 | 0.7 | 0.71 | 0.69 | 3352 | Train 53 | Test Not Hispanic 24 | 0.7 | 0.69 | 0.64 |
| 2329 | Train 37 | Test Not Hispanic 25 | 0.75 | 0.79 | 0.8 | 3353 | Train 53 | Test Not Hispanic 25 | 0.73 | 0.75 | 0.67 |
| 2330 | Train 37 | Test Not Hispanic 26 | 0.75 | 0.78 | 0.77 | 3354 | Train 53 | Test Not Hispanic 26 | 0.73 | 0.72 | 0.65 |
| 2331 | Train 37 | Test Not Hispanic 27 | 0.75 | 0.79 | 0.79 | 3355 | Train 53 | Test Not Hispanic 27 | 0.73 | 0.75 | 0.67 |
| 2332 | Train 37 | Test Not Hispanic 28 | 0.75 | 0.78 | 0.77 | 3356 | Train 53 | Test Not Hispanic 28 | 0.73 | 0.72 | 0.65 |
| 2333 | Train 37 | Test Not Hispanic 29 | 0.73 | 0.77 | 0.75 | 3357 | Train 53 | Test Not Hispanic 29 | 0.72 | 0.74 | 0.69 |
| 2334 | Train 37 | Test Not Hispanic 30 | 0.7 | 0.71 | 0.69 | 3358 | Train 53 | Test Not Hispanic 30 | 0.7 | 0.69 | 0.64 |
| 2335 | Train 37 | Test Not Hispanic 31 | 0.73 | 0.77 | 0.75 | 3359 | Train 53 | Test Not Hispanic 31 | 0.72 | 0.74 | 0.69 |
| 2336 | Train 37 | Test Not Hispanic 32 | 0.7 | 0.71 | 0.69 | 3360 | Train 53 | Test Not Hispanic 32 | 0.7 | 0.69 | 0.64 |
| 2337 | Train 37 | Test Not Hispanic 33 | 0.75 | 0.8 | 0.82 | 3361 | Train 53 | Test Not Hispanic 33 | 0.73 | 0.75 | 0.67 |
| 2338 | Train 37 | Test Not Hispanic 34 | 0.75 | 0.78 | 0.79 | 3362 | Train 53 | Test Not Hispanic 34 | 0.73 | 0.72 | 0.65 |
| 2339 | Train 37 | Test Not Hispanic 35 | 0.75 | 0.8 | 0.82 | 3363 | Train 53 | Test Not Hispanic 35 | 0.73 | 0.75 | 0.67 |
| 2340 | Train 37 | Test Not Hispanic 36 | 0.75 | 0.78 | 0.79 | 3364 | Train 53 | Test Not Hispanic 36 | 0.73 | 0.72 | 0.65 |
| 2341 | Train 37 | Test Not Hispanic 37 | 0.74 | 0.78 | 0.78 | 3365 | Train 53 | Test Not Hispanic 37 | 0.72 | 0.74 | 0.69 |
| 2342 | Train 37 | Test Not Hispanic 38 | 0.7 | 0.71 | 0.69 | 3366 | Train 53 | Test Not Hispanic 38 | 0.7 | 0.69 | 0.64 |
| 2343 | Train 37 | Test Not Hispanic 39 | 0.73 | 0.78 | 0.77 | 3367 | Train 53 | Test Not Hispanic 39 | 0.72 | 0.74 | 0.69 |
| 2344 | Train 37 | Test Not Hispanic 40 | 0.7 | 0.71 | 0.69 | 3368 | Train 53 | Test Not Hispanic 40 | 0.7 | 0.69 | 0.64 |
| 2345 | Train 37 | Test Not Hispanic 41 | 0.75 | 0.79 | 0.8 | 3369 | Train 53 | Test Not Hispanic 41 | 0.73 | 0.75 | 0.67 |
| 2346 | Train 37 | Test Not Hispanic 42 | 0.75 | 0.78 | 0.77 | 3370 | Train 53 | Test Not Hispanic 42 | 0.73 | 0.72 | 0.65 |
| 2347 | Train 37 | Test Not Hispanic 43 | 0.75 | 0.79 | 0.8 | 3371 | Train 53 | Test Not Hispanic 43 | 0.73 | 0.75 | 0.67 |
| 2348 | Train 37 | Test Not Hispanic 44 | 0.75 | 0.78 | 0.77 | 3372 | Train 53 | Test Not Hispanic 44 | 0.73 | 0.72 | 0.65 |
| 2349 | Train 37 | Test Not Hispanic 45 | 0.74 | 0.77 | 0.75 | 3373 | Train 53 | Test Not Hispanic 45 | 0.72 | 0.74 | 0.69 |
| 2350 | Train 37 | Test Not Hispanic 46 | 0.7 | 0.71 | 0.69 | 3374 | Train 53 | Test Not Hispanic 46 | 0.7 | 0.69 | 0.64 |
| 2351 | Train 37 | Test Not Hispanic 47 | 0.73 | 0.77 | 0.75 | 3375 | Train 53 | Test Not Hispanic 47 | 0.72 | 0.74 | 0.69 |
| 2352 | Train 37 | Test Not Hispanic 48 | 0.7 | 0.71 | 0.69 | 3376 | Train 53 | Test Not Hispanic 48 | 0.7 | 0.69 | 0.64 |
| 2353 | Train 37 | Test Not Hispanic 49 | 0.76 | 0.77 | 0.8 | 3377 | Train 53 | Test Not Hispanic 49 | 0.76 | 0.78 | 0.78 |
| 2354 | Train 37 | Test Not Hispanic 50 | 0.76 | 0.77 | 0.8 | 3378 | Train 53 | Test Not Hispanic 50 | 0.76 | 0.78 | 0.78 |
| 2355 | Train 37 | Test Not Hispanic 51 | 0.76 | 0.77 | 0.8 | 3379 | Train 53 | Test Not Hispanic 51 | 0.76 | 0.78 | 0.78 |
| 2356 | Train 37 | Test Not Hispanic 52 | 0.76 | 0.77 | 0.8 | 3380 | Train 53 | Test Not Hispanic 52 | 0.76 | 0.78 | 0.78 |
| 2357 | Train 37 | Test Not Hispanic 53 | 0.75 | 0.79 | 0.73 | 3381 | Train 53 | Test Not Hispanic 53 | 0.76 | 0.83 | 0.77 |
| 2358 | Train 37 | Test Not Hispanic 54 | 0.71 | 0.72 | 0.69 | 3382 | Train 53 | Test Not Hispanic 54 | 0.71 | 0.71 | 0.67 |
| 2359 | Train 37 | Test Not Hispanic 55 | 0.75 | 0.79 | 0.73 | 3383 | Train 53 | Test Not Hispanic 55 | 0.76 | 0.83 | 0.76 |
| 2360 | Train 37 | Test Not Hispanic 56 | 0.71 | 0.71 | 0.7 | 3384 | Train 53 | Test Not Hispanic 56 | 0.71 | 0.71 | 0.66 |
| 2361 | Train 37 | Test Not Hispanic 57 | 0.76 | 0.77 | 0.78 | 3385 | Train 53 | Test Not Hispanic 57 | 0.76 | 0.78 | 0.79 |
| 2362 | Train 37 | Test Not Hispanic 58 | 0.76 | 0.77 | 0.78 | 3386 | Train 53 | Test Not Hispanic 58 | 0.76 | 0.78 | 0.79 |
| 2363 | Train 37 | Test Not Hispanic 59 | 0.76 | 0.77 | 0.78 | 3387 | Train 53 | Test Not Hispanic 59 | 0.76 | 0.78 | 0.78 |
| 2364 | Train 37 | Test Not Hispanic 60 | 0.76 | 0.77 | 0.78 | 3388 | Train 53 | Test Not Hispanic 60 | 0.76 | 0.78 | 0.78 |
| 2365 | Train 37 | Test Not Hispanic 61 | 0.75 | 0.78 | 0.73 | 3389 | Train 53 | Test Not Hispanic 61 | 0.76 | 0.82 | 0.74 |
| 2366 | Train 37 | Test Not Hispanic 62 | 0.72 | 0.72 | 0.69 | 3390 | Train 53 | Test Not Hispanic 62 | 0.72 | 0.71 | 0.67 |
| 2367 | Train 37 | Test Not Hispanic 63 | 0.75 | 0.78 | 0.73 | 3391 | Train 53 | Test Not Hispanic 63 | 0.76 | 0.82 | 0.74 |
| 2368 | Train 37 | Test Not Hispanic 64 | 0.71 | 0.71 | 0.7 | 3392 | Train 53 | Test Not Hispanic 64 | 0.71 | 0.71 | 0.66 |
| 2369 | Train 38 | Test Not Hispanic 1 | 0.73 | 0.77 | 0.76 | 3393 | Train 54 | Test Not Hispanic 1 | 0.72 | 0.73 | 0.65 |
| 2370 | Train 38 | Test Not Hispanic 2 | 0.75 | 0.78 | 0.76 | 3394 | Train 54 | Test Not Hispanic 2 | 0.73 | 0.74 | 0.67 |
| 2371 | Train 38 | Test Not Hispanic 3 | 0.73 | 0.77 | 0.76 | 3395 | Train 54 | Test Not Hispanic 3 | 0.72 | 0.73 | 0.65 |
| 2372 | Train 38 | Test Not Hispanic 4 | 0.75 | 0.78 | 0.76 | 3396 | Train 54 | Test Not Hispanic 4 | 0.73 | 0.74 | 0.67 |
| 2373 | Train 38 | Test Not Hispanic 5 | 0.72 | 0.77 | 0.78 | 3397 | Train 54 | Test Not Hispanic 5 | 0.71 | 0.73 | 0.66 |
| 2374 | Train 38 | Test Not Hispanic 6 | 0.74 | 0.78 | 0.79 | 3398 | Train 54 | Test Not Hispanic 6 | 0.72 | 0.75 | 0.7 |
| 2375 | Train 38 | Test Not Hispanic 7 | 0.72 | 0.77 | 0.77 | 3399 | Train 54 | Test Not Hispanic 7 | 0.71 | 0.73 | 0.66 |
| 2376 | Train 38 | Test Not Hispanic 8 | 0.73 | 0.77 | 0.78 | 3400 | Train 54 | Test Not Hispanic 8 | 0.72 | 0.74 | 0.7 |
| 2377 | Train 38 | Test Not Hispanic 9 | 0.73 | 0.77 | 0.77 | 3401 | Train 54 | Test Not Hispanic 9 | 0.72 | 0.74 | 0.66 |
| 2378 | Train 38 | Test Not Hispanic 10 | 0.75 | 0.78 | 0.77 | 3402 | Train 54 | Test Not Hispanic 10 | 0.73 | 0.74 | 0.68 |
| 2379 | Train 38 | Test Not Hispanic 11 | 0.73 | 0.77 | 0.77 | 3403 | Train 54 | Test Not Hispanic 11 | 0.72 | 0.74 | 0.66 |
| 2380 | Train 38 | Test Not Hispanic 12 | 0.75 | 0.78 | 0.77 | 3404 | Train 54 | Test Not Hispanic 12 | 0.73 | 0.74 | 0.68 |
| 2381 | Train 38 | Test Not Hispanic 13 | 0.72 | 0.77 | 0.78 | 3405 | Train 54 | Test Not Hispanic 13 | 0.71 | 0.73 | 0.67 |
| 2382 | Train 38 | Test Not Hispanic 14 | 0.74 | 0.78 | 0.79 | 3406 | Train 54 | Test Not Hispanic 14 | 0.73 | 0.75 | 0.7 |
| 2383 | Train 38 | Test Not Hispanic 15 | 0.72 | 0.77 | 0.78 | 3407 | Train 54 | Test Not Hispanic 15 | 0.71 | 0.73 | 0.67 |
| 2384 | Train 38 | Test Not Hispanic 16 | 0.74 | 0.77 | 0.78 | 3408 | Train 54 | Test Not Hispanic 16 | 0.72 | 0.75 | 0.7 |
| 2385 | Train 38 | Test Not Hispanic 17 | 0.73 | 0.77 | 0.76 | 3409 | Train 54 | Test Not Hispanic 17 | 0.72 | 0.73 | 0.65 |
| 2386 | Train 38 | Test Not Hispanic 18 | 0.75 | 0.78 | 0.76 | 3410 | Train 54 | Test Not Hispanic 18 | 0.73 | 0.74 | 0.67 |
| 2387 | Train 38 | Test Not Hispanic 19 | 0.73 | 0.77 | 0.76 | 3411 | Train 54 | Test Not Hispanic 19 | 0.72 | 0.73 | 0.65 |
| 2388 | Train 38 | Test Not Hispanic 20 | 0.75 | 0.78 | 0.76 | 3412 | Train 54 | Test Not Hispanic 20 | 0.73 | 0.74 | 0.67 |
| 2389 | Train 38 | Test Not Hispanic 21 | 0.72 | 0.77 | 0.78 | 3413 | Train 54 | Test Not Hispanic 21 | 0.71 | 0.73 | 0.66 |
| 2390 | Train 38 | Test Not Hispanic 22 | 0.74 | 0.78 | 0.79 | 3414 | Train 54 | Test Not Hispanic 22 | 0.72 | 0.75 | 0.7 |
| 2391 | Train 38 | Test Not Hispanic 23 | 0.72 | 0.77 | 0.77 | 3415 | Train 54 | Test Not Hispanic 23 | 0.71 | 0.73 | 0.66 |
| 2392 | Train 38 | Test Not Hispanic 24 | 0.73 | 0.77 | 0.78 | 3416 | Train 54 | Test Not Hispanic 24 | 0.72 | 0.75 | 0.7 |
| 2393 | Train 38 | Test Not Hispanic 25 | 0.73 | 0.77 | 0.77 | 3417 | Train 54 | Test Not Hispanic 25 | 0.72 | 0.74 | 0.66 |
| 2394 | Train 38 | Test Not Hispanic 26 | 0.75 | 0.78 | 0.77 | 3418 | Train 54 | Test Not Hispanic 26 | 0.73 | 0.74 | 0.68 |
| 2395 | Train 38 | Test Not Hispanic 27 | 0.73 | 0.77 | 0.77 | 3419 | Train 54 | Test Not Hispanic 27 | 0.72 | 0.74 | 0.66 |
| 2396 | Train 38 | Test Not Hispanic 28 | 0.75 | 0.78 | 0.77 | 3420 | Train 54 | Test Not Hispanic 28 | 0.73 | 0.74 | 0.68 |
| 2397 | Train 38 | Test Not Hispanic 29 | 0.72 | 0.77 | 0.78 | 3421 | Train 54 | Test Not Hispanic 29 | 0.71 | 0.73 | 0.67 |
| 2398 | Train 38 | Test Not Hispanic 30 | 0.74 | 0.78 | 0.79 | 3422 | Train 54 | Test Not Hispanic 30 | 0.73 | 0.75 | 0.7 |
| 2399 | Train 38 | Test Not Hispanic 31 | 0.72 | 0.77 | 0.78 | 3423 | Train 54 | Test Not Hispanic 31 | 0.71 | 0.73 | 0.67 |
| 2400 | Train 38 | Test Not Hispanic 32 | 0.74 | 0.78 | 0.78 | 3424 | Train 54 | Test Not Hispanic 32 | 0.72 | 0.75 | 0.7 |
| 2401 | Train 38 | Test Not Hispanic 33 | 0.73 | 0.77 | 0.76 | 3425 | Train 54 | Test Not Hispanic 33 | 0.72 | 0.73 | 0.65 |
| 2402 | Train 38 | Test Not Hispanic 34 | 0.75 | 0.78 | 0.77 | 3426 | Train 54 | Test Not Hispanic 34 | 0.73 | 0.74 | 0.67 |
| 2403 | Train 38 | Test Not Hispanic 35 | 0.73 | 0.77 | 0.76 | 3427 | Train 54 | Test Not Hispanic 35 | 0.72 | 0.73 | 0.65 |
| 2404 | Train 38 | Test Not Hispanic 36 | 0.75 | 0.78 | 0.77 | 3428 | Train 54 | Test Not Hispanic 36 | 0.73 | 0.74 | 0.67 |
| 2405 | Train 38 | Test Not Hispanic 37 | 0.72 | 0.77 | 0.78 | 3429 | Train 54 | Test Not Hispanic 37 | 0.71 | 0.73 | 0.66 |
| 2406 | Train 38 | Test Not Hispanic 38 | 0.74 | 0.78 | 0.79 | 3430 | Train 54 | Test Not Hispanic 38 | 0.72 | 0.75 | 0.7 |
| 2407 | Train 38 | Test Not Hispanic 39 | 0.72 | 0.77 | 0.77 | 3431 | Train 54 | Test Not Hispanic 39 | 0.71 | 0.73 | 0.66 |
| 2408 | Train 38 | Test Not Hispanic 40 | 0.73 | 0.78 | 0.78 | 3432 | Train 54 | Test Not Hispanic 40 | 0.72 | 0.75 | 0.7 |
| 2409 | Train 38 | Test Not Hispanic 41 | 0.73 | 0.77 | 0.77 | 3433 | Train 54 | Test Not Hispanic 41 | 0.72 | 0.74 | 0.66 |
| 2410 | Train 38 | Test Not Hispanic 42 | 0.75 | 0.78 | 0.77 | 3434 | Train 54 | Test Not Hispanic 42 | 0.73 | 0.74 | 0.68 |
| 2411 | Train 38 | Test Not Hispanic 43 | 0.73 | 0.77 | 0.77 | 3435 | Train 54 | Test Not Hispanic 43 | 0.72 | 0.74 | 0.66 |
| 2412 | Train 38 | Test Not Hispanic 44 | 0.75 | 0.78 | 0.77 | 3436 | Train 54 | Test Not Hispanic 44 | 0.73 | 0.74 | 0.68 |
| 2413 | Train 38 | Test Not Hispanic 45 | 0.72 | 0.77 | 0.78 | 3437 | Train 54 | Test Not Hispanic 45 | 0.71 | 0.73 | 0.67 |
| 2414 | Train 38 | Test Not Hispanic 46 | 0.74 | 0.78 | 0.79 | 3438 | Train 54 | Test Not Hispanic 46 | 0.73 | 0.75 | 0.7 |
| 2415 | Train 38 | Test Not Hispanic 47 | 0.72 | 0.77 | 0.78 | 3439 | Train 54 | Test Not Hispanic 47 | 0.71 | 0.73 | 0.67 |
| 2416 | Train 38 | Test Not Hispanic 48 | 0.74 | 0.78 | 0.78 | 3440 | Train 54 | Test Not Hispanic 48 | 0.72 | 0.75 | 0.7 |
| 2417 | Train 38 | Test Not Hispanic 49 | 0.75 | 0.78 | 0.77 | 3441 | Train 54 | Test Not Hispanic 49 | 0.76 | 0.79 | 0.76 |
| 2418 | Train 38 | Test Not Hispanic 50 | 0.75 | 0.78 | 0.77 | 3442 | Train 54 | Test Not Hispanic 50 | 0.76 | 0.79 | 0.76 |
| 2419 | Train 38 | Test Not Hispanic 51 | 0.75 | 0.78 | 0.77 | 3443 | Train 54 | Test Not Hispanic 51 | 0.76 | 0.78 | 0.76 |
| 2420 | Train 38 | Test Not Hispanic 52 | 0.75 | 0.78 | 0.77 | 3444 | Train 54 | Test Not Hispanic 52 | 0.76 | 0.79 | 0.76 |
| 2421 | Train 38 | Test Not Hispanic 53 | 0.75 | 0.79 | 0.75 | 3445 | Train 54 | Test Not Hispanic 53 | 0.76 | 0.8 | 0.74 |
| 2422 | Train 38 | Test Not Hispanic 54 | 0.74 | 0.78 | 0.79 | 3446 | Train 54 | Test Not Hispanic 54 | 0.75 | 0.8 | 0.81 |
| 2423 | Train 38 | Test Not Hispanic 55 | 0.75 | 0.79 | 0.74 | 3447 | Train 54 | Test Not Hispanic 55 | 0.75 | 0.8 | 0.73 |
| 2424 | Train 38 | Test Not Hispanic 56 | 0.74 | 0.77 | 0.78 | 3448 | Train 54 | Test Not Hispanic 56 | 0.74 | 0.79 | 0.8 |
| 2425 | Train 38 | Test Not Hispanic 57 | 0.75 | 0.79 | 0.78 | 3449 | Train 54 | Test Not Hispanic 57 | 0.76 | 0.79 | 0.78 |
| 2426 | Train 38 | Test Not Hispanic 58 | 0.75 | 0.79 | 0.78 | 3450 | Train 54 | Test Not Hispanic 58 | 0.76 | 0.79 | 0.78 |
| 2427 | Train 38 | Test Not Hispanic 59 | 0.75 | 0.79 | 0.78 | 3451 | Train 54 | Test Not Hispanic 59 | 0.76 | 0.79 | 0.78 |
| 2428 | Train 38 | Test Not Hispanic 60 | 0.75 | 0.79 | 0.78 | 3452 | Train 54 | Test Not Hispanic 60 | 0.76 | 0.79 | 0.78 |
| 2429 | Train 38 | Test Not Hispanic 61 | 0.75 | 0.79 | 0.75 | 3453 | Train 54 | Test Not Hispanic 61 | 0.76 | 0.81 | 0.74 |
| 2430 | Train 38 | Test Not Hispanic 62 | 0.74 | 0.78 | 0.79 | 3454 | Train 54 | Test Not Hispanic 62 | 0.75 | 0.8 | 0.8 |
| 2431 | Train 38 | Test Not Hispanic 63 | 0.75 | 0.79 | 0.75 | 3455 | Train 54 | Test Not Hispanic 63 | 0.76 | 0.81 | 0.74 |
| 2432 | Train 38 | Test Not Hispanic 64 | 0.74 | 0.77 | 0.78 | 3456 | Train 54 | Test Not Hispanic 64 | 0.74 | 0.79 | 0.8 |
| 2433 | Train 39 | Test Not Hispanic 1 | 0.75 | 0.8 | 0.82 | 3457 | Train 55 | Test Not Hispanic 1 | 0.73 | 0.75 | 0.71 |
| 2434 | Train 39 | Test Not Hispanic 2 | 0.75 | 0.78 | 0.78 | 3458 | Train 55 | Test Not Hispanic 2 | 0.73 | 0.73 | 0.69 |
| 2435 | Train 39 | Test Not Hispanic 3 | 0.75 | 0.8 | 0.82 | 3459 | Train 55 | Test Not Hispanic 3 | 0.73 | 0.75 | 0.71 |
| 2436 | Train 39 | Test Not Hispanic 4 | 0.75 | 0.78 | 0.78 | 3460 | Train 55 | Test Not Hispanic 4 | 0.73 | 0.73 | 0.69 |
| 2437 | Train 39 | Test Not Hispanic 5 | 0.73 | 0.78 | 0.77 | 3461 | Train 55 | Test Not Hispanic 5 | 0.72 | 0.74 | 0.66 |
| 2438 | Train 39 | Test Not Hispanic 6 | 0.7 | 0.71 | 0.69 | 3462 | Train 55 | Test Not Hispanic 6 | 0.7 | 0.69 | 0.63 |
| 2439 | Train 39 | Test Not Hispanic 7 | 0.73 | 0.78 | 0.76 | 3463 | Train 55 | Test Not Hispanic 7 | 0.72 | 0.74 | 0.66 |
| 2440 | Train 39 | Test Not Hispanic 8 | 0.7 | 0.71 | 0.69 | 3464 | Train 55 | Test Not Hispanic 8 | 0.69 | 0.69 | 0.63 |
| 2441 | Train 39 | Test Not Hispanic 9 | 0.75 | 0.79 | 0.79 | 3465 | Train 55 | Test Not Hispanic 9 | 0.73 | 0.75 | 0.71 |
| 2442 | Train 39 | Test Not Hispanic 10 | 0.75 | 0.78 | 0.77 | 3466 | Train 55 | Test Not Hispanic 10 | 0.73 | 0.73 | 0.69 |
| 2443 | Train 39 | Test Not Hispanic 11 | 0.75 | 0.79 | 0.79 | 3467 | Train 55 | Test Not Hispanic 11 | 0.73 | 0.75 | 0.71 |
| 2444 | Train 39 | Test Not Hispanic 12 | 0.75 | 0.78 | 0.77 | 3468 | Train 55 | Test Not Hispanic 12 | 0.73 | 0.73 | 0.69 |
| 2445 | Train 39 | Test Not Hispanic 13 | 0.73 | 0.77 | 0.75 | 3469 | Train 55 | Test Not Hispanic 13 | 0.72 | 0.74 | 0.65 |
| 2446 | Train 39 | Test Not Hispanic 14 | 0.7 | 0.71 | 0.69 | 3470 | Train 55 | Test Not Hispanic 14 | 0.7 | 0.69 | 0.63 |
| 2447 | Train 39 | Test Not Hispanic 15 | 0.73 | 0.77 | 0.74 | 3471 | Train 55 | Test Not Hispanic 15 | 0.72 | 0.74 | 0.65 |
| 2448 | Train 39 | Test Not Hispanic 16 | 0.7 | 0.71 | 0.69 | 3472 | Train 55 | Test Not Hispanic 16 | 0.7 | 0.69 | 0.63 |
| 2449 | Train 39 | Test Not Hispanic 17 | 0.75 | 0.8 | 0.82 | 3473 | Train 55 | Test Not Hispanic 17 | 0.73 | 0.75 | 0.71 |
| 2450 | Train 39 | Test Not Hispanic 18 | 0.75 | 0.78 | 0.78 | 3474 | Train 55 | Test Not Hispanic 18 | 0.73 | 0.73 | 0.69 |
| 2451 | Train 39 | Test Not Hispanic 19 | 0.75 | 0.8 | 0.82 | 3475 | Train 55 | Test Not Hispanic 19 | 0.73 | 0.75 | 0.71 |
| 2452 | Train 39 | Test Not Hispanic 20 | 0.75 | 0.78 | 0.78 | 3476 | Train 55 | Test Not Hispanic 20 | 0.73 | 0.73 | 0.69 |
| 2453 | Train 39 | Test Not Hispanic 21 | 0.74 | 0.78 | 0.77 | 3477 | Train 55 | Test Not Hispanic 21 | 0.72 | 0.74 | 0.66 |
| 2454 | Train 39 | Test Not Hispanic 22 | 0.7 | 0.71 | 0.69 | 3478 | Train 55 | Test Not Hispanic 22 | 0.7 | 0.69 | 0.63 |
| 2455 | Train 39 | Test Not Hispanic 23 | 0.73 | 0.78 | 0.76 | 3479 | Train 55 | Test Not Hispanic 23 | 0.72 | 0.74 | 0.66 |
| 2456 | Train 39 | Test Not Hispanic 24 | 0.7 | 0.71 | 0.69 | 3480 | Train 55 | Test Not Hispanic 24 | 0.7 | 0.69 | 0.63 |
| 2457 | Train 39 | Test Not Hispanic 25 | 0.75 | 0.79 | 0.79 | 3481 | Train 55 | Test Not Hispanic 25 | 0.73 | 0.75 | 0.71 |
| 2458 | Train 39 | Test Not Hispanic 26 | 0.75 | 0.78 | 0.77 | 3482 | Train 55 | Test Not Hispanic 26 | 0.73 | 0.73 | 0.69 |
| 2459 | Train 39 | Test Not Hispanic 27 | 0.75 | 0.79 | 0.79 | 3483 | Train 55 | Test Not Hispanic 27 | 0.73 | 0.75 | 0.71 |
| 2460 | Train 39 | Test Not Hispanic 28 | 0.75 | 0.78 | 0.77 | 3484 | Train 55 | Test Not Hispanic 28 | 0.73 | 0.73 | 0.69 |
| 2461 | Train 39 | Test Not Hispanic 29 | 0.73 | 0.77 | 0.75 | 3485 | Train 55 | Test Not Hispanic 29 | 0.72 | 0.74 | 0.65 |
| 2462 | Train 39 | Test Not Hispanic 30 | 0.7 | 0.71 | 0.69 | 3486 | Train 55 | Test Not Hispanic 30 | 0.7 | 0.69 | 0.63 |
| 2463 | Train 39 | Test Not Hispanic 31 | 0.73 | 0.77 | 0.75 | 3487 | Train 55 | Test Not Hispanic 31 | 0.72 | 0.74 | 0.65 |
| 2464 | Train 39 | Test Not Hispanic 32 | 0.7 | 0.71 | 0.69 | 3488 | Train 55 | Test Not Hispanic 32 | 0.7 | 0.69 | 0.63 |
| 2465 | Train 39 | Test Not Hispanic 33 | 0.75 | 0.8 | 0.82 | 3489 | Train 55 | Test Not Hispanic 33 | 0.73 | 0.75 | 0.71 |
| 2466 | Train 39 | Test Not Hispanic 34 | 0.75 | 0.78 | 0.78 | 3490 | Train 55 | Test Not Hispanic 34 | 0.73 | 0.73 | 0.69 |
| 2467 | Train 39 | Test Not Hispanic 35 | 0.75 | 0.8 | 0.82 | 3491 | Train 55 | Test Not Hispanic 35 | 0.73 | 0.75 | 0.71 |
| 2468 | Train 39 | Test Not Hispanic 36 | 0.75 | 0.78 | 0.78 | 3492 | Train 55 | Test Not Hispanic 36 | 0.73 | 0.73 | 0.69 |
| 2469 | Train 39 | Test Not Hispanic 37 | 0.74 | 0.78 | 0.77 | 3493 | Train 55 | Test Not Hispanic 37 | 0.72 | 0.74 | 0.66 |
| 2470 | Train 39 | Test Not Hispanic 38 | 0.7 | 0.71 | 0.69 | 3494 | Train 55 | Test Not Hispanic 38 | 0.7 | 0.69 | 0.63 |
| 2471 | Train 39 | Test Not Hispanic 39 | 0.73 | 0.78 | 0.77 | 3495 | Train 55 | Test Not Hispanic 39 | 0.72 | 0.74 | 0.66 |
| 2472 | Train 39 | Test Not Hispanic 40 | 0.7 | 0.71 | 0.69 | 3496 | Train 55 | Test Not Hispanic 40 | 0.7 | 0.69 | 0.63 |
| 2473 | Train 39 | Test Not Hispanic 41 | 0.75 | 0.79 | 0.8 | 3497 | Train 55 | Test Not Hispanic 41 | 0.73 | 0.75 | 0.71 |
| 2474 | Train 39 | Test Not Hispanic 42 | 0.75 | 0.78 | 0.77 | 3498 | Train 55 | Test Not Hispanic 42 | 0.73 | 0.73 | 0.69 |
| 2475 | Train 39 | Test Not Hispanic 43 | 0.75 | 0.79 | 0.8 | 3499 | Train 55 | Test Not Hispanic 43 | 0.73 | 0.76 | 0.71 |
| 2476 | Train 39 | Test Not Hispanic 44 | 0.75 | 0.78 | 0.77 | 3500 | Train 55 | Test Not Hispanic 44 | 0.73 | 0.73 | 0.69 |
| 2477 | Train 39 | Test Not Hispanic 45 | 0.74 | 0.77 | 0.75 | 3501 | Train 55 | Test Not Hispanic 45 | 0.72 | 0.74 | 0.65 |
| 2478 | Train 39 | Test Not Hispanic 46 | 0.7 | 0.71 | 0.69 | 3502 | Train 55 | Test Not Hispanic 46 | 0.7 | 0.69 | 0.63 |
| 2479 | Train 39 | Test Not Hispanic 47 | 0.73 | 0.77 | 0.75 | 3503 | Train 55 | Test Not Hispanic 47 | 0.72 | 0.74 | 0.65 |
| 2480 | Train 39 | Test Not Hispanic 48 | 0.7 | 0.71 | 0.69 | 3504 | Train 55 | Test Not Hispanic 48 | 0.7 | 0.69 | 0.63 |
| 2481 | Train 39 | Test Not Hispanic 49 | 0.75 | 0.77 | 0.81 | 3505 | Train 55 | Test Not Hispanic 49 | 0.76 | 0.79 | 0.81 |
| 2482 | Train 39 | Test Not Hispanic 50 | 0.75 | 0.77 | 0.81 | 3506 | Train 55 | Test Not Hispanic 50 | 0.76 | 0.79 | 0.81 |
| 2483 | Train 39 | Test Not Hispanic 51 | 0.76 | 0.77 | 0.81 | 3507 | Train 55 | Test Not Hispanic 51 | 0.76 | 0.8 | 0.81 |
| 2484 | Train 39 | Test Not Hispanic 52 | 0.76 | 0.77 | 0.81 | 3508 | Train 55 | Test Not Hispanic 52 | 0.76 | 0.8 | 0.81 |
| 2485 | Train 39 | Test Not Hispanic 53 | 0.75 | 0.78 | 0.73 | 3509 | Train 55 | Test Not Hispanic 53 | 0.76 | 0.82 | 0.73 |
| 2486 | Train 39 | Test Not Hispanic 54 | 0.71 | 0.71 | 0.7 | 3510 | Train 55 | Test Not Hispanic 54 | 0.71 | 0.71 | 0.66 |
| 2487 | Train 39 | Test Not Hispanic 55 | 0.75 | 0.78 | 0.73 | 3511 | Train 55 | Test Not Hispanic 55 | 0.76 | 0.82 | 0.73 |
| 2488 | Train 39 | Test Not Hispanic 56 | 0.71 | 0.71 | 0.7 | 3512 | Train 55 | Test Not Hispanic 56 | 0.71 | 0.71 | 0.66 |
| 2489 | Train 39 | Test Not Hispanic 57 | 0.76 | 0.77 | 0.79 | 3513 | Train 55 | Test Not Hispanic 57 | 0.76 | 0.79 | 0.8 |
| 2490 | Train 39 | Test Not Hispanic 58 | 0.76 | 0.77 | 0.79 | 3514 | Train 55 | Test Not Hispanic 58 | 0.76 | 0.79 | 0.8 |
| 2491 | Train 39 | Test Not Hispanic 59 | 0.76 | 0.77 | 0.79 | 3515 | Train 55 | Test Not Hispanic 59 | 0.76 | 0.79 | 0.8 |
| 2492 | Train 39 | Test Not Hispanic 60 | 0.76 | 0.77 | 0.79 | 3516 | Train 55 | Test Not Hispanic 60 | 0.76 | 0.79 | 0.8 |
| 2493 | Train 39 | Test Not Hispanic 61 | 0.75 | 0.78 | 0.72 | 3517 | Train 55 | Test Not Hispanic 61 | 0.76 | 0.81 | 0.7 |
| 2494 | Train 39 | Test Not Hispanic 62 | 0.71 | 0.71 | 0.7 | 3518 | Train 55 | Test Not Hispanic 62 | 0.71 | 0.72 | 0.66 |
| 2495 | Train 39 | Test Not Hispanic 63 | 0.75 | 0.78 | 0.72 | 3519 | Train 55 | Test Not Hispanic 63 | 0.76 | 0.82 | 0.7 |
| 2496 | Train 39 | Test Not Hispanic 64 | 0.71 | 0.71 | 0.7 | 3520 | Train 55 | Test Not Hispanic 64 | 0.71 | 0.71 | 0.67 |
| 2497 | Train 40 | Test Not Hispanic 1 | 0.73 | 0.77 | 0.77 | 3521 | Train 56 | Test Not Hispanic 1 | 0.72 | 0.73 | 0.7 |
| 2498 | Train 40 | Test Not Hispanic 2 | 0.75 | 0.78 | 0.77 | 3522 | Train 56 | Test Not Hispanic 2 | 0.72 | 0.74 | 0.71 |
| 2499 | Train 40 | Test Not Hispanic 3 | 0.73 | 0.77 | 0.77 | 3523 | Train 56 | Test Not Hispanic 3 | 0.72 | 0.73 | 0.7 |
| 2500 | Train 40 | Test Not Hispanic 4 | 0.75 | 0.78 | 0.77 | 3524 | Train 56 | Test Not Hispanic 4 | 0.72 | 0.74 | 0.71 |
| 2501 | Train 40 | Test Not Hispanic 5 | 0.72 | 0.77 | 0.77 | 3525 | Train 56 | Test Not Hispanic 5 | 0.7 | 0.73 | 0.71 |
| 2502 | Train 40 | Test Not Hispanic 6 | 0.73 | 0.78 | 0.79 | 3526 | Train 56 | Test Not Hispanic 6 | 0.72 | 0.75 | 0.72 |
| 2503 | Train 40 | Test Not Hispanic 7 | 0.72 | 0.77 | 0.77 | 3527 | Train 56 | Test Not Hispanic 7 | 0.7 | 0.73 | 0.71 |
| 2504 | Train 40 | Test Not Hispanic 8 | 0.73 | 0.78 | 0.79 | 3528 | Train 56 | Test Not Hispanic 8 | 0.72 | 0.75 | 0.72 |
| 2505 | Train 40 | Test Not Hispanic 9 | 0.73 | 0.77 | 0.77 | 3529 | Train 56 | Test Not Hispanic 9 | 0.72 | 0.74 | 0.71 |
| 2506 | Train 40 | Test Not Hispanic 10 | 0.75 | 0.78 | 0.78 | 3530 | Train 56 | Test Not Hispanic 10 | 0.72 | 0.74 | 0.71 |
| 2507 | Train 40 | Test Not Hispanic 11 | 0.73 | 0.77 | 0.77 | 3531 | Train 56 | Test Not Hispanic 11 | 0.72 | 0.74 | 0.71 |
| 2508 | Train 40 | Test Not Hispanic 12 | 0.75 | 0.78 | 0.78 | 3532 | Train 56 | Test Not Hispanic 12 | 0.72 | 0.74 | 0.71 |
| 2509 | Train 40 | Test Not Hispanic 13 | 0.72 | 0.77 | 0.78 | 3533 | Train 56 | Test Not Hispanic 13 | 0.7 | 0.73 | 0.72 |
| 2510 | Train 40 | Test Not Hispanic 14 | 0.74 | 0.78 | 0.79 | 3534 | Train 56 | Test Not Hispanic 14 | 0.72 | 0.75 | 0.72 |
| 2511 | Train 40 | Test Not Hispanic 15 | 0.72 | 0.77 | 0.78 | 3535 | Train 56 | Test Not Hispanic 15 | 0.7 | 0.73 | 0.72 |
| 2512 | Train 40 | Test Not Hispanic 16 | 0.73 | 0.78 | 0.79 | 3536 | Train 56 | Test Not Hispanic 16 | 0.72 | 0.75 | 0.72 |
| 2513 | Train 40 | Test Not Hispanic 17 | 0.73 | 0.77 | 0.77 | 3537 | Train 56 | Test Not Hispanic 17 | 0.72 | 0.73 | 0.7 |
| 2514 | Train 40 | Test Not Hispanic 18 | 0.75 | 0.78 | 0.77 | 3538 | Train 56 | Test Not Hispanic 18 | 0.72 | 0.74 | 0.71 |
| 2515 | Train 40 | Test Not Hispanic 19 | 0.73 | 0.77 | 0.77 | 3539 | Train 56 | Test Not Hispanic 19 | 0.72 | 0.73 | 0.7 |
| 2516 | Train 40 | Test Not Hispanic 20 | 0.75 | 0.78 | 0.77 | 3540 | Train 56 | Test Not Hispanic 20 | 0.72 | 0.74 | 0.71 |
| 2517 | Train 40 | Test Not Hispanic 21 | 0.72 | 0.77 | 0.77 | 3541 | Train 56 | Test Not Hispanic 21 | 0.7 | 0.73 | 0.71 |
| 2518 | Train 40 | Test Not Hispanic 22 | 0.73 | 0.78 | 0.79 | 3542 | Train 56 | Test Not Hispanic 22 | 0.72 | 0.75 | 0.72 |
| 2519 | Train 40 | Test Not Hispanic 23 | 0.72 | 0.77 | 0.77 | 3543 | Train 56 | Test Not Hispanic 23 | 0.7 | 0.73 | 0.71 |
| 2520 | Train 40 | Test Not Hispanic 24 | 0.73 | 0.78 | 0.79 | 3544 | Train 56 | Test Not Hispanic 24 | 0.72 | 0.75 | 0.72 |
| 2521 | Train 40 | Test Not Hispanic 25 | 0.73 | 0.77 | 0.77 | 3545 | Train 56 | Test Not Hispanic 25 | 0.72 | 0.74 | 0.71 |
| 2522 | Train 40 | Test Not Hispanic 26 | 0.75 | 0.78 | 0.78 | 3546 | Train 56 | Test Not Hispanic 26 | 0.72 | 0.74 | 0.71 |
| 2523 | Train 40 | Test Not Hispanic 27 | 0.73 | 0.77 | 0.77 | 3547 | Train 56 | Test Not Hispanic 27 | 0.72 | 0.74 | 0.71 |
| 2524 | Train 40 | Test Not Hispanic 28 | 0.75 | 0.78 | 0.78 | 3548 | Train 56 | Test Not Hispanic 28 | 0.72 | 0.74 | 0.71 |
| 2525 | Train 40 | Test Not Hispanic 29 | 0.72 | 0.77 | 0.78 | 3549 | Train 56 | Test Not Hispanic 29 | 0.7 | 0.73 | 0.72 |
| 2526 | Train 40 | Test Not Hispanic 30 | 0.74 | 0.78 | 0.79 | 3550 | Train 56 | Test Not Hispanic 30 | 0.72 | 0.75 | 0.72 |
| 2527 | Train 40 | Test Not Hispanic 31 | 0.72 | 0.77 | 0.78 | 3551 | Train 56 | Test Not Hispanic 31 | 0.7 | 0.73 | 0.72 |
| 2528 | Train 40 | Test Not Hispanic 32 | 0.74 | 0.78 | 0.79 | 3552 | Train 56 | Test Not Hispanic 32 | 0.72 | 0.75 | 0.72 |
| 2529 | Train 40 | Test Not Hispanic 33 | 0.73 | 0.77 | 0.77 | 3553 | Train 56 | Test Not Hispanic 33 | 0.72 | 0.73 | 0.7 |
| 2530 | Train 40 | Test Not Hispanic 34 | 0.75 | 0.78 | 0.77 | 3554 | Train 56 | Test Not Hispanic 34 | 0.72 | 0.74 | 0.71 |
| 2531 | Train 40 | Test Not Hispanic 35 | 0.74 | 0.77 | 0.77 | 3555 | Train 56 | Test Not Hispanic 35 | 0.72 | 0.73 | 0.7 |
| 2532 | Train 40 | Test Not Hispanic 36 | 0.75 | 0.78 | 0.77 | 3556 | Train 56 | Test Not Hispanic 36 | 0.72 | 0.74 | 0.71 |
| 2533 | Train 40 | Test Not Hispanic 37 | 0.72 | 0.77 | 0.78 | 3557 | Train 56 | Test Not Hispanic 37 | 0.7 | 0.73 | 0.71 |
| 2534 | Train 40 | Test Not Hispanic 38 | 0.73 | 0.78 | 0.79 | 3558 | Train 56 | Test Not Hispanic 38 | 0.72 | 0.75 | 0.72 |
| 2535 | Train 40 | Test Not Hispanic 39 | 0.72 | 0.77 | 0.77 | 3559 | Train 56 | Test Not Hispanic 39 | 0.7 | 0.73 | 0.71 |
| 2536 | Train 40 | Test Not Hispanic 40 | 0.73 | 0.78 | 0.79 | 3560 | Train 56 | Test Not Hispanic 40 | 0.72 | 0.75 | 0.72 |
| 2537 | Train 40 | Test Not Hispanic 41 | 0.74 | 0.77 | 0.77 | 3561 | Train 56 | Test Not Hispanic 41 | 0.72 | 0.74 | 0.71 |
| 2538 | Train 40 | Test Not Hispanic 42 | 0.75 | 0.78 | 0.78 | 3562 | Train 56 | Test Not Hispanic 42 | 0.72 | 0.74 | 0.71 |
| 2539 | Train 40 | Test Not Hispanic 43 | 0.74 | 0.77 | 0.77 | 3563 | Train 56 | Test Not Hispanic 43 | 0.72 | 0.74 | 0.71 |
| 2540 | Train 40 | Test Not Hispanic 44 | 0.75 | 0.78 | 0.78 | 3564 | Train 56 | Test Not Hispanic 44 | 0.72 | 0.74 | 0.71 |
| 2541 | Train 40 | Test Not Hispanic 45 | 0.72 | 0.77 | 0.78 | 3565 | Train 56 | Test Not Hispanic 45 | 0.7 | 0.73 | 0.72 |
| 2542 | Train 40 | Test Not Hispanic 46 | 0.74 | 0.78 | 0.79 | 3566 | Train 56 | Test Not Hispanic 46 | 0.72 | 0.75 | 0.72 |
| 2543 | Train 40 | Test Not Hispanic 47 | 0.72 | 0.77 | 0.78 | 3567 | Train 56 | Test Not Hispanic 47 | 0.7 | 0.73 | 0.72 |
| 2544 | Train 40 | Test Not Hispanic 48 | 0.74 | 0.78 | 0.79 | 3568 | Train 56 | Test Not Hispanic 48 | 0.72 | 0.75 | 0.72 |
| 2545 | Train 40 | Test Not Hispanic 49 | 0.75 | 0.78 | 0.77 | 3569 | Train 56 | Test Not Hispanic 49 | 0.76 | 0.79 | 0.76 |
| 2546 | Train 40 | Test Not Hispanic 50 | 0.75 | 0.78 | 0.77 | 3570 | Train 56 | Test Not Hispanic 50 | 0.76 | 0.79 | 0.76 |
| 2547 | Train 40 | Test Not Hispanic 51 | 0.75 | 0.78 | 0.77 | 3571 | Train 56 | Test Not Hispanic 51 | 0.76 | 0.79 | 0.76 |
| 2548 | Train 40 | Test Not Hispanic 52 | 0.75 | 0.78 | 0.77 | 3572 | Train 56 | Test Not Hispanic 52 | 0.76 | 0.79 | 0.76 |
| 2549 | Train 40 | Test Not Hispanic 53 | 0.74 | 0.79 | 0.75 | 3573 | Train 56 | Test Not Hispanic 53 | 0.75 | 0.8 | 0.74 |
| 2550 | Train 40 | Test Not Hispanic 54 | 0.74 | 0.78 | 0.78 | 3574 | Train 56 | Test Not Hispanic 54 | 0.74 | 0.8 | 0.8 |
| 2551 | Train 40 | Test Not Hispanic 55 | 0.74 | 0.79 | 0.75 | 3575 | Train 56 | Test Not Hispanic 55 | 0.75 | 0.8 | 0.74 |
| 2552 | Train 40 | Test Not Hispanic 56 | 0.74 | 0.78 | 0.78 | 3576 | Train 56 | Test Not Hispanic 56 | 0.74 | 0.8 | 0.8 |
| 2553 | Train 40 | Test Not Hispanic 57 | 0.75 | 0.78 | 0.78 | 3577 | Train 56 | Test Not Hispanic 57 | 0.76 | 0.79 | 0.78 |
| 2554 | Train 40 | Test Not Hispanic 58 | 0.76 | 0.79 | 0.78 | 3578 | Train 56 | Test Not Hispanic 58 | 0.76 | 0.79 | 0.78 |
| 2555 | Train 40 | Test Not Hispanic 59 | 0.75 | 0.78 | 0.78 | 3579 | Train 56 | Test Not Hispanic 59 | 0.76 | 0.79 | 0.78 |
| 2556 | Train 40 | Test Not Hispanic 60 | 0.76 | 0.79 | 0.78 | 3580 | Train 56 | Test Not Hispanic 60 | 0.76 | 0.79 | 0.78 |
| 2557 | Train 40 | Test Not Hispanic 61 | 0.74 | 0.79 | 0.76 | 3581 | Train 56 | Test Not Hispanic 61 | 0.75 | 0.81 | 0.75 |
| 2558 | Train 40 | Test Not Hispanic 62 | 0.74 | 0.78 | 0.78 | 3582 | Train 56 | Test Not Hispanic 62 | 0.74 | 0.8 | 0.8 |
| 2559 | Train 40 | Test Not Hispanic 63 | 0.74 | 0.79 | 0.76 | 3583 | Train 56 | Test Not Hispanic 63 | 0.75 | 0.81 | 0.76 |
| 2560 | Train 40 | Test Not Hispanic 64 | 0.74 | 0.78 | 0.78 | 3584 | Train 56 | Test Not Hispanic 64 | 0.74 | 0.8 | 0.8 |
| 2561 | Train 41 | Test Not Hispanic 1 | 0.75 | 0.82 | 0.81 | 3585 | Train 57 | Test Not Hispanic 1 | 0.73 | 0.73 | 0.7 |
| 2562 | Train 41 | Test Not Hispanic 2 | 0.74 | 0.78 | 0.76 | 3586 | Train 57 | Test Not Hispanic 2 | 0.72 | 0.7 | 0.66 |
| 2563 | Train 41 | Test Not Hispanic 3 | 0.75 | 0.82 | 0.81 | 3587 | Train 57 | Test Not Hispanic 3 | 0.73 | 0.73 | 0.7 |
| 2564 | Train 41 | Test Not Hispanic 4 | 0.74 | 0.78 | 0.76 | 3588 | Train 57 | Test Not Hispanic 4 | 0.72 | 0.7 | 0.66 |
| 2565 | Train 41 | Test Not Hispanic 5 | 0.71 | 0.73 | 0.7 | 3589 | Train 57 | Test Not Hispanic 5 | 0.7 | 0.69 | 0.67 |
| 2566 | Train 41 | Test Not Hispanic 6 | 0.69 | 0.7 | 0.65 | 3590 | Train 57 | Test Not Hispanic 6 | 0.69 | 0.68 | 0.63 |
| 2567 | Train 41 | Test Not Hispanic 7 | 0.71 | 0.73 | 0.69 | 3591 | Train 57 | Test Not Hispanic 7 | 0.7 | 0.69 | 0.67 |
| 2568 | Train 41 | Test Not Hispanic 8 | 0.7 | 0.69 | 0.65 | 3592 | Train 57 | Test Not Hispanic 8 | 0.69 | 0.67 | 0.63 |
| 2569 | Train 41 | Test Not Hispanic 9 | 0.75 | 0.83 | 0.84 | 3593 | Train 57 | Test Not Hispanic 9 | 0.73 | 0.73 | 0.72 |
| 2570 | Train 41 | Test Not Hispanic 10 | 0.74 | 0.78 | 0.78 | 3594 | Train 57 | Test Not Hispanic 10 | 0.72 | 0.71 | 0.67 |
| 2571 | Train 41 | Test Not Hispanic 11 | 0.75 | 0.83 | 0.84 | 3595 | Train 57 | Test Not Hispanic 11 | 0.73 | 0.73 | 0.72 |
| 2572 | Train 41 | Test Not Hispanic 12 | 0.74 | 0.78 | 0.78 | 3596 | Train 57 | Test Not Hispanic 12 | 0.72 | 0.71 | 0.67 |
| 2573 | Train 41 | Test Not Hispanic 13 | 0.71 | 0.73 | 0.71 | 3597 | Train 57 | Test Not Hispanic 13 | 0.7 | 0.7 | 0.67 |
| 2574 | Train 41 | Test Not Hispanic 14 | 0.7 | 0.7 | 0.65 | 3598 | Train 57 | Test Not Hispanic 14 | 0.69 | 0.68 | 0.63 |
| 2575 | Train 41 | Test Not Hispanic 15 | 0.71 | 0.73 | 0.7 | 3599 | Train 57 | Test Not Hispanic 15 | 0.7 | 0.7 | 0.67 |
| 2576 | Train 41 | Test Not Hispanic 16 | 0.7 | 0.7 | 0.65 | 3600 | Train 57 | Test Not Hispanic 16 | 0.69 | 0.67 | 0.63 |
| 2577 | Train 41 | Test Not Hispanic 17 | 0.75 | 0.82 | 0.81 | 3601 | Train 57 | Test Not Hispanic 17 | 0.73 | 0.73 | 0.7 |
| 2578 | Train 41 | Test Not Hispanic 18 | 0.74 | 0.78 | 0.76 | 3602 | Train 57 | Test Not Hispanic 18 | 0.72 | 0.7 | 0.66 |
| 2579 | Train 41 | Test Not Hispanic 19 | 0.75 | 0.82 | 0.81 | 3603 | Train 57 | Test Not Hispanic 19 | 0.73 | 0.73 | 0.7 |
| 2580 | Train 41 | Test Not Hispanic 20 | 0.74 | 0.78 | 0.76 | 3604 | Train 57 | Test Not Hispanic 20 | 0.72 | 0.7 | 0.66 |
| 2581 | Train 41 | Test Not Hispanic 21 | 0.71 | 0.73 | 0.7 | 3605 | Train 57 | Test Not Hispanic 21 | 0.7 | 0.69 | 0.67 |
| 2582 | Train 41 | Test Not Hispanic 22 | 0.7 | 0.7 | 0.65 | 3606 | Train 57 | Test Not Hispanic 22 | 0.69 | 0.68 | 0.63 |
| 2583 | Train 41 | Test Not Hispanic 23 | 0.71 | 0.73 | 0.69 | 3607 | Train 57 | Test Not Hispanic 23 | 0.7 | 0.69 | 0.67 |
| 2584 | Train 41 | Test Not Hispanic 24 | 0.7 | 0.7 | 0.65 | 3608 | Train 57 | Test Not Hispanic 24 | 0.69 | 0.67 | 0.63 |
| 2585 | Train 41 | Test Not Hispanic 25 | 0.75 | 0.83 | 0.84 | 3609 | Train 57 | Test Not Hispanic 25 | 0.73 | 0.73 | 0.72 |
| 2586 | Train 41 | Test Not Hispanic 26 | 0.74 | 0.78 | 0.78 | 3610 | Train 57 | Test Not Hispanic 26 | 0.72 | 0.71 | 0.67 |
| 2587 | Train 41 | Test Not Hispanic 27 | 0.75 | 0.83 | 0.84 | 3611 | Train 57 | Test Not Hispanic 27 | 0.73 | 0.73 | 0.72 |
| 2588 | Train 41 | Test Not Hispanic 28 | 0.74 | 0.78 | 0.78 | 3612 | Train 57 | Test Not Hispanic 28 | 0.72 | 0.71 | 0.67 |
| 2589 | Train 41 | Test Not Hispanic 29 | 0.71 | 0.73 | 0.71 | 3613 | Train 57 | Test Not Hispanic 29 | 0.7 | 0.7 | 0.67 |
| 2590 | Train 41 | Test Not Hispanic 30 | 0.7 | 0.7 | 0.65 | 3614 | Train 57 | Test Not Hispanic 30 | 0.69 | 0.68 | 0.63 |
| 2591 | Train 41 | Test Not Hispanic 31 | 0.71 | 0.73 | 0.7 | 3615 | Train 57 | Test Not Hispanic 31 | 0.7 | 0.7 | 0.67 |
| 2592 | Train 41 | Test Not Hispanic 32 | 0.7 | 0.7 | 0.65 | 3616 | Train 57 | Test Not Hispanic 32 | 0.69 | 0.68 | 0.63 |
| 2593 | Train 41 | Test Not Hispanic 33 | 0.76 | 0.82 | 0.81 | 3617 | Train 57 | Test Not Hispanic 33 | 0.74 | 0.73 | 0.7 |
| 2594 | Train 41 | Test Not Hispanic 34 | 0.74 | 0.78 | 0.76 | 3618 | Train 57 | Test Not Hispanic 34 | 0.72 | 0.71 | 0.66 |
| 2595 | Train 41 | Test Not Hispanic 35 | 0.76 | 0.82 | 0.81 | 3619 | Train 57 | Test Not Hispanic 35 | 0.74 | 0.73 | 0.7 |
| 2596 | Train 41 | Test Not Hispanic 36 | 0.74 | 0.78 | 0.76 | 3620 | Train 57 | Test Not Hispanic 36 | 0.72 | 0.71 | 0.66 |
| 2597 | Train 41 | Test Not Hispanic 37 | 0.71 | 0.73 | 0.7 | 3621 | Train 57 | Test Not Hispanic 37 | 0.7 | 0.7 | 0.67 |
| 2598 | Train 41 | Test Not Hispanic 38 | 0.7 | 0.7 | 0.65 | 3622 | Train 57 | Test Not Hispanic 38 | 0.69 | 0.68 | 0.63 |
| 2599 | Train 41 | Test Not Hispanic 39 | 0.71 | 0.73 | 0.69 | 3623 | Train 57 | Test Not Hispanic 39 | 0.7 | 0.69 | 0.67 |
| 2600 | Train 41 | Test Not Hispanic 40 | 0.7 | 0.7 | 0.65 | 3624 | Train 57 | Test Not Hispanic 40 | 0.69 | 0.67 | 0.63 |
| 2601 | Train 41 | Test Not Hispanic 41 | 0.76 | 0.83 | 0.85 | 3625 | Train 57 | Test Not Hispanic 41 | 0.74 | 0.74 | 0.72 |
| 2602 | Train 41 | Test Not Hispanic 42 | 0.74 | 0.78 | 0.78 | 3626 | Train 57 | Test Not Hispanic 42 | 0.72 | 0.71 | 0.67 |
| 2603 | Train 41 | Test Not Hispanic 43 | 0.76 | 0.83 | 0.84 | 3627 | Train 57 | Test Not Hispanic 43 | 0.74 | 0.74 | 0.72 |
| 2604 | Train 41 | Test Not Hispanic 44 | 0.74 | 0.78 | 0.78 | 3628 | Train 57 | Test Not Hispanic 44 | 0.72 | 0.71 | 0.67 |
| 2605 | Train 41 | Test Not Hispanic 45 | 0.71 | 0.74 | 0.71 | 3629 | Train 57 | Test Not Hispanic 45 | 0.7 | 0.7 | 0.68 |
| 2606 | Train 41 | Test Not Hispanic 46 | 0.7 | 0.7 | 0.65 | 3630 | Train 57 | Test Not Hispanic 46 | 0.69 | 0.68 | 0.63 |
| 2607 | Train 41 | Test Not Hispanic 47 | 0.71 | 0.73 | 0.7 | 3631 | Train 57 | Test Not Hispanic 47 | 0.7 | 0.7 | 0.67 |
| 2608 | Train 41 | Test Not Hispanic 48 | 0.7 | 0.7 | 0.65 | 3632 | Train 57 | Test Not Hispanic 48 | 0.69 | 0.68 | 0.63 |
| 2609 | Train 41 | Test Not Hispanic 49 | 0.76 | 0.79 | 0.79 | 3633 | Train 57 | Test Not Hispanic 49 | 0.77 | 0.81 | 0.81 |
| 2610 | Train 41 | Test Not Hispanic 50 | 0.76 | 0.79 | 0.79 | 3634 | Train 57 | Test Not Hispanic 50 | 0.77 | 0.81 | 0.81 |
| 2611 | Train 41 | Test Not Hispanic 51 | 0.76 | 0.79 | 0.79 | 3635 | Train 57 | Test Not Hispanic 51 | 0.77 | 0.81 | 0.81 |
| 2612 | Train 41 | Test Not Hispanic 52 | 0.76 | 0.79 | 0.79 | 3636 | Train 57 | Test Not Hispanic 52 | 0.77 | 0.81 | 0.81 |
| 2613 | Train 41 | Test Not Hispanic 53 | 0.72 | 0.75 | 0.68 | 3637 | Train 57 | Test Not Hispanic 53 | 0.71 | 0.75 | 0.74 |
| 2614 | Train 41 | Test Not Hispanic 54 | 0.71 | 0.71 | 0.67 | 3638 | Train 57 | Test Not Hispanic 54 | 0.7 | 0.69 | 0.67 |
| 2615 | Train 41 | Test Not Hispanic 55 | 0.72 | 0.75 | 0.68 | 3639 | Train 57 | Test Not Hispanic 55 | 0.71 | 0.75 | 0.74 |
| 2616 | Train 41 | Test Not Hispanic 56 | 0.71 | 0.7 | 0.66 | 3640 | Train 57 | Test Not Hispanic 56 | 0.7 | 0.69 | 0.67 |
| 2617 | Train 41 | Test Not Hispanic 57 | 0.76 | 0.8 | 0.83 | 3641 | Train 57 | Test Not Hispanic 57 | 0.77 | 0.82 | 0.86 |
| 2618 | Train 41 | Test Not Hispanic 58 | 0.76 | 0.8 | 0.83 | 3642 | Train 57 | Test Not Hispanic 58 | 0.77 | 0.82 | 0.86 |
| 2619 | Train 41 | Test Not Hispanic 59 | 0.76 | 0.8 | 0.83 | 3643 | Train 57 | Test Not Hispanic 59 | 0.77 | 0.82 | 0.86 |
| 2620 | Train 41 | Test Not Hispanic 60 | 0.76 | 0.8 | 0.83 | 3644 | Train 57 | Test Not Hispanic 60 | 0.77 | 0.82 | 0.86 |
| 2621 | Train 41 | Test Not Hispanic 61 | 0.72 | 0.75 | 0.69 | 3645 | Train 57 | Test Not Hispanic 61 | 0.71 | 0.75 | 0.75 |
| 2622 | Train 41 | Test Not Hispanic 62 | 0.71 | 0.71 | 0.67 | 3646 | Train 57 | Test Not Hispanic 62 | 0.7 | 0.69 | 0.67 |
| 2623 | Train 41 | Test Not Hispanic 63 | 0.72 | 0.75 | 0.69 | 3647 | Train 57 | Test Not Hispanic 63 | 0.71 | 0.75 | 0.75 |
| 2624 | Train 41 | Test Not Hispanic 64 | 0.71 | 0.7 | 0.66 | 3648 | Train 57 | Test Not Hispanic 64 | 0.7 | 0.69 | 0.67 |
| 2625 | Train 42 | Test Not Hispanic 1 | 0.75 | 0.81 | 0.8 | 3649 | Train 58 | Test Not Hispanic 1 | 0.73 | 0.7 | 0.64 |
| 2626 | Train 42 | Test Not Hispanic 2 | 0.76 | 0.82 | 0.81 | 3650 | Train 58 | Test Not Hispanic 2 | 0.72 | 0.69 | 0.63 |
| 2627 | Train 42 | Test Not Hispanic 3 | 0.75 | 0.81 | 0.8 | 3651 | Train 58 | Test Not Hispanic 3 | 0.73 | 0.7 | 0.64 |
| 2628 | Train 42 | Test Not Hispanic 4 | 0.76 | 0.82 | 0.81 | 3652 | Train 58 | Test Not Hispanic 4 | 0.72 | 0.69 | 0.63 |
| 2629 | Train 42 | Test Not Hispanic 5 | 0.71 | 0.73 | 0.67 | 3653 | Train 58 | Test Not Hispanic 5 | 0.7 | 0.68 | 0.62 |
| 2630 | Train 42 | Test Not Hispanic 6 | 0.71 | 0.71 | 0.67 | 3654 | Train 58 | Test Not Hispanic 6 | 0.69 | 0.67 | 0.61 |
| 2631 | Train 42 | Test Not Hispanic 7 | 0.71 | 0.73 | 0.67 | 3655 | Train 58 | Test Not Hispanic 7 | 0.7 | 0.68 | 0.62 |
| 2632 | Train 42 | Test Not Hispanic 8 | 0.71 | 0.71 | 0.67 | 3656 | Train 58 | Test Not Hispanic 8 | 0.69 | 0.66 | 0.61 |
| 2633 | Train 42 | Test Not Hispanic 9 | 0.75 | 0.82 | 0.83 | 3657 | Train 58 | Test Not Hispanic 9 | 0.73 | 0.71 | 0.66 |
| 2634 | Train 42 | Test Not Hispanic 10 | 0.76 | 0.83 | 0.84 | 3658 | Train 58 | Test Not Hispanic 10 | 0.72 | 0.69 | 0.64 |
| 2635 | Train 42 | Test Not Hispanic 11 | 0.75 | 0.82 | 0.83 | 3659 | Train 58 | Test Not Hispanic 11 | 0.73 | 0.71 | 0.66 |
| 2636 | Train 42 | Test Not Hispanic 12 | 0.76 | 0.83 | 0.84 | 3660 | Train 58 | Test Not Hispanic 12 | 0.72 | 0.69 | 0.64 |
| 2637 | Train 42 | Test Not Hispanic 13 | 0.71 | 0.74 | 0.68 | 3661 | Train 58 | Test Not Hispanic 13 | 0.7 | 0.68 | 0.63 |
| 2638 | Train 42 | Test Not Hispanic 14 | 0.71 | 0.72 | 0.67 | 3662 | Train 58 | Test Not Hispanic 14 | 0.69 | 0.67 | 0.61 |
| 2639 | Train 42 | Test Not Hispanic 15 | 0.71 | 0.73 | 0.68 | 3663 | Train 58 | Test Not Hispanic 15 | 0.7 | 0.68 | 0.63 |
| 2640 | Train 42 | Test Not Hispanic 16 | 0.71 | 0.71 | 0.67 | 3664 | Train 58 | Test Not Hispanic 16 | 0.69 | 0.67 | 0.61 |
| 2641 | Train 42 | Test Not Hispanic 17 | 0.75 | 0.81 | 0.8 | 3665 | Train 58 | Test Not Hispanic 17 | 0.73 | 0.7 | 0.64 |
| 2642 | Train 42 | Test Not Hispanic 18 | 0.76 | 0.82 | 0.81 | 3666 | Train 58 | Test Not Hispanic 18 | 0.72 | 0.69 | 0.63 |
| 2643 | Train 42 | Test Not Hispanic 19 | 0.75 | 0.81 | 0.8 | 3667 | Train 58 | Test Not Hispanic 19 | 0.73 | 0.7 | 0.64 |
| 2644 | Train 42 | Test Not Hispanic 20 | 0.76 | 0.82 | 0.81 | 3668 | Train 58 | Test Not Hispanic 20 | 0.72 | 0.69 | 0.63 |
| 2645 | Train 42 | Test Not Hispanic 21 | 0.71 | 0.73 | 0.67 | 3669 | Train 58 | Test Not Hispanic 21 | 0.7 | 0.68 | 0.62 |
| 2646 | Train 42 | Test Not Hispanic 22 | 0.71 | 0.71 | 0.67 | 3670 | Train 58 | Test Not Hispanic 22 | 0.69 | 0.67 | 0.61 |
| 2647 | Train 42 | Test Not Hispanic 23 | 0.71 | 0.73 | 0.67 | 3671 | Train 58 | Test Not Hispanic 23 | 0.7 | 0.68 | 0.62 |
| 2648 | Train 42 | Test Not Hispanic 24 | 0.71 | 0.71 | 0.67 | 3672 | Train 58 | Test Not Hispanic 24 | 0.69 | 0.67 | 0.61 |
| 2649 | Train 42 | Test Not Hispanic 25 | 0.75 | 0.82 | 0.83 | 3673 | Train 58 | Test Not Hispanic 25 | 0.73 | 0.71 | 0.66 |
| 2650 | Train 42 | Test Not Hispanic 26 | 0.76 | 0.83 | 0.84 | 3674 | Train 58 | Test Not Hispanic 26 | 0.72 | 0.69 | 0.64 |
| 2651 | Train 42 | Test Not Hispanic 27 | 0.75 | 0.82 | 0.83 | 3675 | Train 58 | Test Not Hispanic 27 | 0.73 | 0.71 | 0.66 |
| 2652 | Train 42 | Test Not Hispanic 28 | 0.76 | 0.83 | 0.84 | 3676 | Train 58 | Test Not Hispanic 28 | 0.72 | 0.69 | 0.64 |
| 2653 | Train 42 | Test Not Hispanic 29 | 0.71 | 0.74 | 0.68 | 3677 | Train 58 | Test Not Hispanic 29 | 0.7 | 0.68 | 0.63 |
| 2654 | Train 42 | Test Not Hispanic 30 | 0.71 | 0.72 | 0.67 | 3678 | Train 58 | Test Not Hispanic 30 | 0.69 | 0.67 | 0.61 |
| 2655 | Train 42 | Test Not Hispanic 31 | 0.71 | 0.73 | 0.68 | 3679 | Train 58 | Test Not Hispanic 31 | 0.7 | 0.68 | 0.63 |
| 2656 | Train 42 | Test Not Hispanic 32 | 0.71 | 0.71 | 0.67 | 3680 | Train 58 | Test Not Hispanic 32 | 0.69 | 0.67 | 0.61 |
| 2657 | Train 42 | Test Not Hispanic 33 | 0.75 | 0.81 | 0.8 | 3681 | Train 58 | Test Not Hispanic 33 | 0.73 | 0.7 | 0.64 |
| 2658 | Train 42 | Test Not Hispanic 34 | 0.76 | 0.82 | 0.81 | 3682 | Train 58 | Test Not Hispanic 34 | 0.72 | 0.69 | 0.63 |
| 2659 | Train 42 | Test Not Hispanic 35 | 0.75 | 0.81 | 0.8 | 3683 | Train 58 | Test Not Hispanic 35 | 0.73 | 0.7 | 0.64 |
| 2660 | Train 42 | Test Not Hispanic 36 | 0.76 | 0.82 | 0.81 | 3684 | Train 58 | Test Not Hispanic 36 | 0.72 | 0.69 | 0.63 |
| 2661 | Train 42 | Test Not Hispanic 37 | 0.71 | 0.73 | 0.67 | 3685 | Train 58 | Test Not Hispanic 37 | 0.7 | 0.68 | 0.62 |
| 2662 | Train 42 | Test Not Hispanic 38 | 0.71 | 0.71 | 0.67 | 3686 | Train 58 | Test Not Hispanic 38 | 0.69 | 0.67 | 0.61 |
| 2663 | Train 42 | Test Not Hispanic 39 | 0.71 | 0.73 | 0.67 | 3687 | Train 58 | Test Not Hispanic 39 | 0.7 | 0.68 | 0.62 |
| 2664 | Train 42 | Test Not Hispanic 40 | 0.71 | 0.71 | 0.67 | 3688 | Train 58 | Test Not Hispanic 40 | 0.69 | 0.67 | 0.61 |
| 2665 | Train 42 | Test Not Hispanic 41 | 0.75 | 0.82 | 0.83 | 3689 | Train 58 | Test Not Hispanic 41 | 0.73 | 0.71 | 0.66 |
| 2666 | Train 42 | Test Not Hispanic 42 | 0.76 | 0.83 | 0.84 | 3690 | Train 58 | Test Not Hispanic 42 | 0.72 | 0.7 | 0.64 |
| 2667 | Train 42 | Test Not Hispanic 43 | 0.75 | 0.82 | 0.83 | 3691 | Train 58 | Test Not Hispanic 43 | 0.73 | 0.71 | 0.66 |
| 2668 | Train 42 | Test Not Hispanic 44 | 0.76 | 0.83 | 0.84 | 3692 | Train 58 | Test Not Hispanic 44 | 0.72 | 0.7 | 0.64 |
| 2669 | Train 42 | Test Not Hispanic 45 | 0.71 | 0.74 | 0.68 | 3693 | Train 58 | Test Not Hispanic 45 | 0.7 | 0.68 | 0.63 |
| 2670 | Train 42 | Test Not Hispanic 46 | 0.71 | 0.72 | 0.67 | 3694 | Train 58 | Test Not Hispanic 46 | 0.69 | 0.67 | 0.61 |
| 2671 | Train 42 | Test Not Hispanic 47 | 0.71 | 0.74 | 0.68 | 3695 | Train 58 | Test Not Hispanic 47 | 0.7 | 0.68 | 0.63 |
| 2672 | Train 42 | Test Not Hispanic 48 | 0.71 | 0.71 | 0.67 | 3696 | Train 58 | Test Not Hispanic 48 | 0.69 | 0.67 | 0.61 |
| 2673 | Train 42 | Test Not Hispanic 49 | 0.76 | 0.79 | 0.77 | 3697 | Train 58 | Test Not Hispanic 49 | 0.77 | 0.81 | 0.81 |
| 2674 | Train 42 | Test Not Hispanic 50 | 0.76 | 0.79 | 0.77 | 3698 | Train 58 | Test Not Hispanic 50 | 0.77 | 0.81 | 0.81 |
| 2675 | Train 42 | Test Not Hispanic 51 | 0.76 | 0.79 | 0.77 | 3699 | Train 58 | Test Not Hispanic 51 | 0.77 | 0.81 | 0.81 |
| 2676 | Train 42 | Test Not Hispanic 52 | 0.76 | 0.79 | 0.77 | 3700 | Train 58 | Test Not Hispanic 52 | 0.77 | 0.81 | 0.81 |
| 2677 | Train 42 | Test Not Hispanic 53 | 0.73 | 0.76 | 0.64 | 3701 | Train 58 | Test Not Hispanic 53 | 0.71 | 0.72 | 0.69 |
| 2678 | Train 42 | Test Not Hispanic 54 | 0.71 | 0.72 | 0.66 | 3702 | Train 58 | Test Not Hispanic 54 | 0.7 | 0.68 | 0.63 |
| 2679 | Train 42 | Test Not Hispanic 55 | 0.73 | 0.76 | 0.64 | 3703 | Train 58 | Test Not Hispanic 55 | 0.71 | 0.72 | 0.69 |
| 2680 | Train 42 | Test Not Hispanic 56 | 0.71 | 0.71 | 0.66 | 3704 | Train 58 | Test Not Hispanic 56 | 0.7 | 0.67 | 0.63 |
| 2681 | Train 42 | Test Not Hispanic 57 | 0.76 | 0.79 | 0.81 | 3705 | Train 58 | Test Not Hispanic 57 | 0.77 | 0.82 | 0.87 |
| 2682 | Train 42 | Test Not Hispanic 58 | 0.76 | 0.79 | 0.81 | 3706 | Train 58 | Test Not Hispanic 58 | 0.77 | 0.82 | 0.87 |
| 2683 | Train 42 | Test Not Hispanic 59 | 0.76 | 0.79 | 0.81 | 3707 | Train 58 | Test Not Hispanic 59 | 0.77 | 0.82 | 0.87 |
| 2684 | Train 42 | Test Not Hispanic 60 | 0.76 | 0.79 | 0.81 | 3708 | Train 58 | Test Not Hispanic 60 | 0.77 | 0.82 | 0.87 |
| 2685 | Train 42 | Test Not Hispanic 61 | 0.73 | 0.76 | 0.65 | 3709 | Train 58 | Test Not Hispanic 61 | 0.71 | 0.72 | 0.7 |
| 2686 | Train 42 | Test Not Hispanic 62 | 0.72 | 0.72 | 0.66 | 3710 | Train 58 | Test Not Hispanic 62 | 0.7 | 0.68 | 0.63 |
| 2687 | Train 42 | Test Not Hispanic 63 | 0.73 | 0.76 | 0.65 | 3711 | Train 58 | Test Not Hispanic 63 | 0.71 | 0.72 | 0.7 |
| 2688 | Train 42 | Test Not Hispanic 64 | 0.71 | 0.71 | 0.66 | 3712 | Train 58 | Test Not Hispanic 64 | 0.7 | 0.68 | 0.63 |
| 2689 | Train 43 | Test Not Hispanic 1 | 0.75 | 0.82 | 0.81 | 3713 | Train 59 | Test Not Hispanic 1 | 0.73 | 0.73 | 0.67 |
| 2690 | Train 43 | Test Not Hispanic 2 | 0.74 | 0.78 | 0.76 | 3714 | Train 59 | Test Not Hispanic 2 | 0.72 | 0.71 | 0.64 |
| 2691 | Train 43 | Test Not Hispanic 3 | 0.75 | 0.82 | 0.81 | 3715 | Train 59 | Test Not Hispanic 3 | 0.73 | 0.73 | 0.67 |
| 2692 | Train 43 | Test Not Hispanic 4 | 0.74 | 0.78 | 0.77 | 3716 | Train 59 | Test Not Hispanic 4 | 0.72 | 0.71 | 0.64 |
| 2693 | Train 43 | Test Not Hispanic 5 | 0.71 | 0.73 | 0.69 | 3717 | Train 59 | Test Not Hispanic 5 | 0.7 | 0.7 | 0.64 |
| 2694 | Train 43 | Test Not Hispanic 6 | 0.69 | 0.7 | 0.65 | 3718 | Train 59 | Test Not Hispanic 6 | 0.68 | 0.68 | 0.6 |
| 2695 | Train 43 | Test Not Hispanic 7 | 0.71 | 0.73 | 0.69 | 3719 | Train 59 | Test Not Hispanic 7 | 0.7 | 0.7 | 0.64 |
| 2696 | Train 43 | Test Not Hispanic 8 | 0.69 | 0.69 | 0.65 | 3720 | Train 59 | Test Not Hispanic 8 | 0.69 | 0.68 | 0.6 |
| 2697 | Train 43 | Test Not Hispanic 9 | 0.75 | 0.83 | 0.85 | 3721 | Train 59 | Test Not Hispanic 9 | 0.73 | 0.74 | 0.69 |
| 2698 | Train 43 | Test Not Hispanic 10 | 0.74 | 0.78 | 0.79 | 3722 | Train 59 | Test Not Hispanic 10 | 0.72 | 0.71 | 0.65 |
| 2699 | Train 43 | Test Not Hispanic 11 | 0.75 | 0.83 | 0.85 | 3723 | Train 59 | Test Not Hispanic 11 | 0.73 | 0.74 | 0.69 |
| 2700 | Train 43 | Test Not Hispanic 12 | 0.74 | 0.78 | 0.79 | 3724 | Train 59 | Test Not Hispanic 12 | 0.72 | 0.71 | 0.65 |
| 2701 | Train 43 | Test Not Hispanic 13 | 0.71 | 0.73 | 0.7 | 3725 | Train 59 | Test Not Hispanic 13 | 0.7 | 0.7 | 0.65 |
| 2702 | Train 43 | Test Not Hispanic 14 | 0.7 | 0.7 | 0.65 | 3726 | Train 59 | Test Not Hispanic 14 | 0.69 | 0.68 | 0.6 |
| 2703 | Train 43 | Test Not Hispanic 15 | 0.71 | 0.73 | 0.7 | 3727 | Train 59 | Test Not Hispanic 15 | 0.7 | 0.7 | 0.65 |
| 2704 | Train 43 | Test Not Hispanic 16 | 0.7 | 0.7 | 0.65 | 3728 | Train 59 | Test Not Hispanic 16 | 0.69 | 0.68 | 0.6 |
| 2705 | Train 43 | Test Not Hispanic 17 | 0.75 | 0.82 | 0.81 | 3729 | Train 59 | Test Not Hispanic 17 | 0.73 | 0.73 | 0.67 |
| 2706 | Train 43 | Test Not Hispanic 18 | 0.74 | 0.78 | 0.76 | 3730 | Train 59 | Test Not Hispanic 18 | 0.72 | 0.71 | 0.64 |
| 2707 | Train 43 | Test Not Hispanic 19 | 0.75 | 0.82 | 0.81 | 3731 | Train 59 | Test Not Hispanic 19 | 0.74 | 0.73 | 0.67 |
| 2708 | Train 43 | Test Not Hispanic 20 | 0.74 | 0.78 | 0.77 | 3732 | Train 59 | Test Not Hispanic 20 | 0.72 | 0.71 | 0.64 |
| 2709 | Train 43 | Test Not Hispanic 21 | 0.71 | 0.73 | 0.69 | 3733 | Train 59 | Test Not Hispanic 21 | 0.7 | 0.7 | 0.64 |
| 2710 | Train 43 | Test Not Hispanic 22 | 0.69 | 0.7 | 0.65 | 3734 | Train 59 | Test Not Hispanic 22 | 0.68 | 0.68 | 0.6 |
| 2711 | Train 43 | Test Not Hispanic 23 | 0.71 | 0.73 | 0.69 | 3735 | Train 59 | Test Not Hispanic 23 | 0.7 | 0.7 | 0.64 |
| 2712 | Train 43 | Test Not Hispanic 24 | 0.69 | 0.7 | 0.65 | 3736 | Train 59 | Test Not Hispanic 24 | 0.69 | 0.68 | 0.6 |
| 2713 | Train 43 | Test Not Hispanic 25 | 0.75 | 0.83 | 0.85 | 3737 | Train 59 | Test Not Hispanic 25 | 0.73 | 0.74 | 0.69 |
| 2714 | Train 43 | Test Not Hispanic 26 | 0.74 | 0.78 | 0.79 | 3738 | Train 59 | Test Not Hispanic 26 | 0.72 | 0.71 | 0.65 |
| 2715 | Train 43 | Test Not Hispanic 27 | 0.75 | 0.83 | 0.85 | 3739 | Train 59 | Test Not Hispanic 27 | 0.74 | 0.74 | 0.69 |
| 2716 | Train 43 | Test Not Hispanic 28 | 0.74 | 0.78 | 0.79 | 3740 | Train 59 | Test Not Hispanic 28 | 0.72 | 0.71 | 0.65 |
| 2717 | Train 43 | Test Not Hispanic 29 | 0.71 | 0.73 | 0.7 | 3741 | Train 59 | Test Not Hispanic 29 | 0.7 | 0.7 | 0.65 |
| 2718 | Train 43 | Test Not Hispanic 30 | 0.7 | 0.7 | 0.66 | 3742 | Train 59 | Test Not Hispanic 30 | 0.69 | 0.68 | 0.6 |
| 2719 | Train 43 | Test Not Hispanic 31 | 0.71 | 0.73 | 0.7 | 3743 | Train 59 | Test Not Hispanic 31 | 0.7 | 0.7 | 0.65 |
| 2720 | Train 43 | Test Not Hispanic 32 | 0.7 | 0.7 | 0.65 | 3744 | Train 59 | Test Not Hispanic 32 | 0.69 | 0.68 | 0.6 |
| 2721 | Train 43 | Test Not Hispanic 33 | 0.75 | 0.82 | 0.81 | 3745 | Train 59 | Test Not Hispanic 33 | 0.74 | 0.73 | 0.67 |
| 2722 | Train 43 | Test Not Hispanic 34 | 0.74 | 0.78 | 0.77 | 3746 | Train 59 | Test Not Hispanic 34 | 0.72 | 0.71 | 0.64 |
| 2723 | Train 43 | Test Not Hispanic 35 | 0.76 | 0.82 | 0.81 | 3747 | Train 59 | Test Not Hispanic 35 | 0.74 | 0.74 | 0.67 |
| 2724 | Train 43 | Test Not Hispanic 36 | 0.74 | 0.78 | 0.77 | 3748 | Train 59 | Test Not Hispanic 36 | 0.72 | 0.71 | 0.64 |
| 2725 | Train 43 | Test Not Hispanic 37 | 0.71 | 0.73 | 0.69 | 3749 | Train 59 | Test Not Hispanic 37 | 0.7 | 0.7 | 0.64 |
| 2726 | Train 43 | Test Not Hispanic 38 | 0.69 | 0.7 | 0.65 | 3750 | Train 59 | Test Not Hispanic 38 | 0.68 | 0.68 | 0.6 |
| 2727 | Train 43 | Test Not Hispanic 39 | 0.71 | 0.73 | 0.69 | 3751 | Train 59 | Test Not Hispanic 39 | 0.7 | 0.7 | 0.64 |
| 2728 | Train 43 | Test Not Hispanic 40 | 0.7 | 0.7 | 0.65 | 3752 | Train 59 | Test Not Hispanic 40 | 0.69 | 0.68 | 0.6 |
| 2729 | Train 43 | Test Not Hispanic 41 | 0.76 | 0.83 | 0.85 | 3753 | Train 59 | Test Not Hispanic 41 | 0.74 | 0.74 | 0.69 |
| 2730 | Train 43 | Test Not Hispanic 42 | 0.74 | 0.78 | 0.79 | 3754 | Train 59 | Test Not Hispanic 42 | 0.72 | 0.71 | 0.65 |
| 2731 | Train 43 | Test Not Hispanic 43 | 0.76 | 0.83 | 0.85 | 3755 | Train 59 | Test Not Hispanic 43 | 0.74 | 0.74 | 0.69 |
| 2732 | Train 43 | Test Not Hispanic 44 | 0.74 | 0.78 | 0.79 | 3756 | Train 59 | Test Not Hispanic 44 | 0.72 | 0.71 | 0.65 |
| 2733 | Train 43 | Test Not Hispanic 45 | 0.71 | 0.74 | 0.7 | 3757 | Train 59 | Test Not Hispanic 45 | 0.7 | 0.7 | 0.65 |
| 2734 | Train 43 | Test Not Hispanic 46 | 0.7 | 0.7 | 0.65 | 3758 | Train 59 | Test Not Hispanic 46 | 0.69 | 0.68 | 0.6 |
| 2735 | Train 43 | Test Not Hispanic 47 | 0.71 | 0.73 | 0.7 | 3759 | Train 59 | Test Not Hispanic 47 | 0.7 | 0.7 | 0.65 |
| 2736 | Train 43 | Test Not Hispanic 48 | 0.7 | 0.7 | 0.65 | 3760 | Train 59 | Test Not Hispanic 48 | 0.69 | 0.68 | 0.6 |
| 2737 | Train 43 | Test Not Hispanic 49 | 0.76 | 0.79 | 0.78 | 3761 | Train 59 | Test Not Hispanic 49 | 0.77 | 0.81 | 0.8 |
| 2738 | Train 43 | Test Not Hispanic 50 | 0.76 | 0.79 | 0.78 | 3762 | Train 59 | Test Not Hispanic 50 | 0.77 | 0.81 | 0.8 |
| 2739 | Train 43 | Test Not Hispanic 51 | 0.76 | 0.79 | 0.78 | 3763 | Train 59 | Test Not Hispanic 51 | 0.77 | 0.82 | 0.8 |
| 2740 | Train 43 | Test Not Hispanic 52 | 0.76 | 0.79 | 0.78 | 3764 | Train 59 | Test Not Hispanic 52 | 0.77 | 0.82 | 0.8 |
| 2741 | Train 43 | Test Not Hispanic 53 | 0.72 | 0.75 | 0.66 | 3765 | Train 59 | Test Not Hispanic 53 | 0.71 | 0.75 | 0.72 |
| 2742 | Train 43 | Test Not Hispanic 54 | 0.71 | 0.71 | 0.67 | 3766 | Train 59 | Test Not Hispanic 54 | 0.7 | 0.69 | 0.63 |
| 2743 | Train 43 | Test Not Hispanic 55 | 0.72 | 0.75 | 0.66 | 3767 | Train 59 | Test Not Hispanic 55 | 0.71 | 0.75 | 0.72 |
| 2744 | Train 43 | Test Not Hispanic 56 | 0.71 | 0.7 | 0.67 | 3768 | Train 59 | Test Not Hispanic 56 | 0.7 | 0.69 | 0.63 |
| 2745 | Train 43 | Test Not Hispanic 57 | 0.76 | 0.8 | 0.82 | 3769 | Train 59 | Test Not Hispanic 57 | 0.77 | 0.82 | 0.86 |
| 2746 | Train 43 | Test Not Hispanic 58 | 0.76 | 0.8 | 0.82 | 3770 | Train 59 | Test Not Hispanic 58 | 0.77 | 0.82 | 0.86 |
| 2747 | Train 43 | Test Not Hispanic 59 | 0.76 | 0.8 | 0.82 | 3771 | Train 59 | Test Not Hispanic 59 | 0.77 | 0.83 | 0.86 |
| 2748 | Train 43 | Test Not Hispanic 60 | 0.76 | 0.8 | 0.82 | 3772 | Train 59 | Test Not Hispanic 60 | 0.77 | 0.83 | 0.86 |
| 2749 | Train 43 | Test Not Hispanic 61 | 0.72 | 0.75 | 0.67 | 3773 | Train 59 | Test Not Hispanic 61 | 0.71 | 0.75 | 0.74 |
| 2750 | Train 43 | Test Not Hispanic 62 | 0.71 | 0.71 | 0.67 | 3774 | Train 59 | Test Not Hispanic 62 | 0.7 | 0.7 | 0.63 |
| 2751 | Train 43 | Test Not Hispanic 63 | 0.72 | 0.75 | 0.67 | 3775 | Train 59 | Test Not Hispanic 63 | 0.71 | 0.76 | 0.74 |
| 2752 | Train 43 | Test Not Hispanic 64 | 0.71 | 0.7 | 0.67 | 3776 | Train 59 | Test Not Hispanic 64 | 0.7 | 0.69 | 0.63 |
| 2753 | Train 44 | Test Not Hispanic 1 | 0.75 | 0.81 | 0.8 | 3777 | Train 60 | Test Not Hispanic 1 | 0.73 | 0.7 | 0.7 |
| 2754 | Train 44 | Test Not Hispanic 2 | 0.76 | 0.82 | 0.82 | 3778 | Train 60 | Test Not Hispanic 2 | 0.72 | 0.69 | 0.65 |
| 2755 | Train 44 | Test Not Hispanic 3 | 0.75 | 0.81 | 0.8 | 3779 | Train 60 | Test Not Hispanic 3 | 0.73 | 0.7 | 0.7 |
| 2756 | Train 44 | Test Not Hispanic 4 | 0.76 | 0.82 | 0.82 | 3780 | Train 60 | Test Not Hispanic 4 | 0.72 | 0.69 | 0.65 |
| 2757 | Train 44 | Test Not Hispanic 5 | 0.71 | 0.73 | 0.69 | 3781 | Train 60 | Test Not Hispanic 5 | 0.7 | 0.68 | 0.67 |
| 2758 | Train 44 | Test Not Hispanic 6 | 0.71 | 0.71 | 0.68 | 3782 | Train 60 | Test Not Hispanic 6 | 0.69 | 0.67 | 0.61 |
| 2759 | Train 44 | Test Not Hispanic 7 | 0.71 | 0.73 | 0.69 | 3783 | Train 60 | Test Not Hispanic 7 | 0.7 | 0.68 | 0.67 |
| 2760 | Train 44 | Test Not Hispanic 8 | 0.71 | 0.71 | 0.68 | 3784 | Train 60 | Test Not Hispanic 8 | 0.69 | 0.67 | 0.61 |
| 2761 | Train 44 | Test Not Hispanic 9 | 0.75 | 0.82 | 0.83 | 3785 | Train 60 | Test Not Hispanic 9 | 0.73 | 0.7 | 0.72 |
| 2762 | Train 44 | Test Not Hispanic 10 | 0.76 | 0.83 | 0.84 | 3786 | Train 60 | Test Not Hispanic 10 | 0.72 | 0.69 | 0.67 |
| 2763 | Train 44 | Test Not Hispanic 11 | 0.75 | 0.82 | 0.83 | 3787 | Train 60 | Test Not Hispanic 11 | 0.73 | 0.71 | 0.72 |
| 2764 | Train 44 | Test Not Hispanic 12 | 0.76 | 0.83 | 0.84 | 3788 | Train 60 | Test Not Hispanic 12 | 0.72 | 0.69 | 0.67 |
| 2765 | Train 44 | Test Not Hispanic 13 | 0.71 | 0.73 | 0.7 | 3789 | Train 60 | Test Not Hispanic 13 | 0.7 | 0.68 | 0.68 |
| 2766 | Train 44 | Test Not Hispanic 14 | 0.71 | 0.71 | 0.68 | 3790 | Train 60 | Test Not Hispanic 14 | 0.69 | 0.67 | 0.61 |
| 2767 | Train 44 | Test Not Hispanic 15 | 0.71 | 0.73 | 0.7 | 3791 | Train 60 | Test Not Hispanic 15 | 0.7 | 0.68 | 0.68 |
| 2768 | Train 44 | Test Not Hispanic 16 | 0.71 | 0.71 | 0.68 | 3792 | Train 60 | Test Not Hispanic 16 | 0.69 | 0.67 | 0.62 |
| 2769 | Train 44 | Test Not Hispanic 17 | 0.75 | 0.81 | 0.8 | 3793 | Train 60 | Test Not Hispanic 17 | 0.73 | 0.7 | 0.7 |
| 2770 | Train 44 | Test Not Hispanic 18 | 0.76 | 0.82 | 0.82 | 3794 | Train 60 | Test Not Hispanic 18 | 0.72 | 0.69 | 0.65 |
| 2771 | Train 44 | Test Not Hispanic 19 | 0.75 | 0.81 | 0.8 | 3795 | Train 60 | Test Not Hispanic 19 | 0.73 | 0.7 | 0.7 |
| 2772 | Train 44 | Test Not Hispanic 20 | 0.76 | 0.82 | 0.82 | 3796 | Train 60 | Test Not Hispanic 20 | 0.72 | 0.69 | 0.65 |
| 2773 | Train 44 | Test Not Hispanic 21 | 0.71 | 0.73 | 0.69 | 3797 | Train 60 | Test Not Hispanic 21 | 0.7 | 0.68 | 0.67 |
| 2774 | Train 44 | Test Not Hispanic 22 | 0.71 | 0.71 | 0.68 | 3798 | Train 60 | Test Not Hispanic 22 | 0.69 | 0.67 | 0.61 |
| 2775 | Train 44 | Test Not Hispanic 23 | 0.71 | 0.73 | 0.69 | 3799 | Train 60 | Test Not Hispanic 23 | 0.7 | 0.68 | 0.67 |
| 2776 | Train 44 | Test Not Hispanic 24 | 0.71 | 0.71 | 0.68 | 3800 | Train 60 | Test Not Hispanic 24 | 0.69 | 0.67 | 0.62 |
| 2777 | Train 44 | Test Not Hispanic 25 | 0.75 | 0.82 | 0.83 | 3801 | Train 60 | Test Not Hispanic 25 | 0.73 | 0.7 | 0.72 |
| 2778 | Train 44 | Test Not Hispanic 26 | 0.76 | 0.83 | 0.84 | 3802 | Train 60 | Test Not Hispanic 26 | 0.72 | 0.69 | 0.67 |
| 2779 | Train 44 | Test Not Hispanic 27 | 0.75 | 0.82 | 0.83 | 3803 | Train 60 | Test Not Hispanic 27 | 0.73 | 0.71 | 0.72 |
| 2780 | Train 44 | Test Not Hispanic 28 | 0.76 | 0.83 | 0.84 | 3804 | Train 60 | Test Not Hispanic 28 | 0.72 | 0.69 | 0.67 |
| 2781 | Train 44 | Test Not Hispanic 29 | 0.71 | 0.73 | 0.7 | 3805 | Train 60 | Test Not Hispanic 29 | 0.7 | 0.68 | 0.68 |
| 2782 | Train 44 | Test Not Hispanic 30 | 0.71 | 0.71 | 0.68 | 3806 | Train 60 | Test Not Hispanic 30 | 0.69 | 0.67 | 0.62 |
| 2783 | Train 44 | Test Not Hispanic 31 | 0.71 | 0.73 | 0.7 | 3807 | Train 60 | Test Not Hispanic 31 | 0.7 | 0.68 | 0.68 |
| 2784 | Train 44 | Test Not Hispanic 32 | 0.71 | 0.71 | 0.68 | 3808 | Train 60 | Test Not Hispanic 32 | 0.69 | 0.67 | 0.62 |
| 2785 | Train 44 | Test Not Hispanic 33 | 0.75 | 0.81 | 0.8 | 3809 | Train 60 | Test Not Hispanic 33 | 0.73 | 0.7 | 0.7 |
| 2786 | Train 44 | Test Not Hispanic 34 | 0.76 | 0.82 | 0.82 | 3810 | Train 60 | Test Not Hispanic 34 | 0.72 | 0.69 | 0.65 |
| 2787 | Train 44 | Test Not Hispanic 35 | 0.75 | 0.81 | 0.8 | 3811 | Train 60 | Test Not Hispanic 35 | 0.73 | 0.7 | 0.7 |
| 2788 | Train 44 | Test Not Hispanic 36 | 0.76 | 0.82 | 0.82 | 3812 | Train 60 | Test Not Hispanic 36 | 0.72 | 0.69 | 0.65 |
| 2789 | Train 44 | Test Not Hispanic 37 | 0.71 | 0.73 | 0.69 | 3813 | Train 60 | Test Not Hispanic 37 | 0.7 | 0.68 | 0.67 |
| 2790 | Train 44 | Test Not Hispanic 38 | 0.71 | 0.71 | 0.68 | 3814 | Train 60 | Test Not Hispanic 38 | 0.69 | 0.67 | 0.61 |
| 2791 | Train 44 | Test Not Hispanic 39 | 0.71 | 0.73 | 0.69 | 3815 | Train 60 | Test Not Hispanic 39 | 0.7 | 0.68 | 0.67 |
| 2792 | Train 44 | Test Not Hispanic 40 | 0.71 | 0.71 | 0.68 | 3816 | Train 60 | Test Not Hispanic 40 | 0.69 | 0.67 | 0.62 |
| 2793 | Train 44 | Test Not Hispanic 41 | 0.75 | 0.82 | 0.83 | 3817 | Train 60 | Test Not Hispanic 41 | 0.73 | 0.71 | 0.72 |
| 2794 | Train 44 | Test Not Hispanic 42 | 0.76 | 0.83 | 0.84 | 3818 | Train 60 | Test Not Hispanic 42 | 0.72 | 0.7 | 0.67 |
| 2795 | Train 44 | Test Not Hispanic 43 | 0.75 | 0.82 | 0.83 | 3819 | Train 60 | Test Not Hispanic 43 | 0.73 | 0.71 | 0.72 |
| 2796 | Train 44 | Test Not Hispanic 44 | 0.76 | 0.83 | 0.84 | 3820 | Train 60 | Test Not Hispanic 44 | 0.72 | 0.7 | 0.67 |
| 2797 | Train 44 | Test Not Hispanic 45 | 0.71 | 0.73 | 0.7 | 3821 | Train 60 | Test Not Hispanic 45 | 0.7 | 0.68 | 0.68 |
| 2798 | Train 44 | Test Not Hispanic 46 | 0.71 | 0.71 | 0.68 | 3822 | Train 60 | Test Not Hispanic 46 | 0.69 | 0.67 | 0.62 |
| 2799 | Train 44 | Test Not Hispanic 47 | 0.71 | 0.73 | 0.7 | 3823 | Train 60 | Test Not Hispanic 47 | 0.7 | 0.68 | 0.68 |
| 2800 | Train 44 | Test Not Hispanic 48 | 0.71 | 0.71 | 0.68 | 3824 | Train 60 | Test Not Hispanic 48 | 0.69 | 0.67 | 0.62 |
| 2801 | Train 44 | Test Not Hispanic 49 | 0.76 | 0.79 | 0.79 | 3825 | Train 60 | Test Not Hispanic 49 | 0.77 | 0.81 | 0.8 |
| 2802 | Train 44 | Test Not Hispanic 50 | 0.76 | 0.79 | 0.79 | 3826 | Train 60 | Test Not Hispanic 50 | 0.77 | 0.81 | 0.8 |
| 2803 | Train 44 | Test Not Hispanic 51 | 0.76 | 0.79 | 0.79 | 3827 | Train 60 | Test Not Hispanic 51 | 0.77 | 0.81 | 0.8 |
| 2804 | Train 44 | Test Not Hispanic 52 | 0.76 | 0.79 | 0.79 | 3828 | Train 60 | Test Not Hispanic 52 | 0.77 | 0.81 | 0.8 |
| 2805 | Train 44 | Test Not Hispanic 53 | 0.73 | 0.76 | 0.65 | 3829 | Train 60 | Test Not Hispanic 53 | 0.71 | 0.72 | 0.75 |
| 2806 | Train 44 | Test Not Hispanic 54 | 0.71 | 0.71 | 0.68 | 3830 | Train 60 | Test Not Hispanic 54 | 0.7 | 0.68 | 0.64 |
| 2807 | Train 44 | Test Not Hispanic 55 | 0.73 | 0.76 | 0.66 | 3831 | Train 60 | Test Not Hispanic 55 | 0.71 | 0.72 | 0.75 |
| 2808 | Train 44 | Test Not Hispanic 56 | 0.71 | 0.71 | 0.68 | 3832 | Train 60 | Test Not Hispanic 56 | 0.7 | 0.68 | 0.64 |
| 2809 | Train 44 | Test Not Hispanic 57 | 0.76 | 0.79 | 0.82 | 3833 | Train 60 | Test Not Hispanic 57 | 0.77 | 0.82 | 0.86 |
| 2810 | Train 44 | Test Not Hispanic 58 | 0.76 | 0.79 | 0.82 | 3834 | Train 60 | Test Not Hispanic 58 | 0.77 | 0.82 | 0.86 |
| 2811 | Train 44 | Test Not Hispanic 59 | 0.76 | 0.79 | 0.82 | 3835 | Train 60 | Test Not Hispanic 59 | 0.77 | 0.82 | 0.86 |
| 2812 | Train 44 | Test Not Hispanic 60 | 0.76 | 0.79 | 0.82 | 3836 | Train 60 | Test Not Hispanic 60 | 0.77 | 0.82 | 0.86 |
| 2813 | Train 44 | Test Not Hispanic 61 | 0.73 | 0.76 | 0.65 | 3837 | Train 60 | Test Not Hispanic 61 | 0.71 | 0.72 | 0.77 |
| 2814 | Train 44 | Test Not Hispanic 62 | 0.72 | 0.72 | 0.68 | 3838 | Train 60 | Test Not Hispanic 62 | 0.7 | 0.68 | 0.64 |
| 2815 | Train 44 | Test Not Hispanic 63 | 0.73 | 0.76 | 0.66 | 3839 | Train 60 | Test Not Hispanic 63 | 0.71 | 0.73 | 0.77 |
| 2816 | Train 44 | Test Not Hispanic 64 | 0.71 | 0.71 | 0.68 | 3840 | Train 60 | Test Not Hispanic 64 | 0.7 | 0.68 | 0.64 |
| 2817 | Train 45 | Test Not Hispanic 1 | 0.75 | 0.8 | 0.79 | 3841 | Train 61 | Test Not Hispanic 1 | 0.73 | 0.74 | 0.68 |
| 2818 | Train 45 | Test Not Hispanic 2 | 0.75 | 0.78 | 0.76 | 3842 | Train 61 | Test Not Hispanic 2 | 0.73 | 0.72 | 0.67 |
| 2819 | Train 45 | Test Not Hispanic 3 | 0.75 | 0.8 | 0.79 | 3843 | Train 61 | Test Not Hispanic 3 | 0.73 | 0.74 | 0.68 |
| 2820 | Train 45 | Test Not Hispanic 4 | 0.75 | 0.78 | 0.76 | 3844 | Train 61 | Test Not Hispanic 4 | 0.73 | 0.72 | 0.67 |
| 2821 | Train 45 | Test Not Hispanic 5 | 0.73 | 0.77 | 0.73 | 3845 | Train 61 | Test Not Hispanic 5 | 0.72 | 0.74 | 0.7 |
| 2822 | Train 45 | Test Not Hispanic 6 | 0.7 | 0.71 | 0.68 | 3846 | Train 61 | Test Not Hispanic 6 | 0.7 | 0.68 | 0.63 |
| 2823 | Train 45 | Test Not Hispanic 7 | 0.73 | 0.77 | 0.73 | 3847 | Train 61 | Test Not Hispanic 7 | 0.72 | 0.74 | 0.7 |
| 2824 | Train 45 | Test Not Hispanic 8 | 0.7 | 0.71 | 0.68 | 3848 | Train 61 | Test Not Hispanic 8 | 0.7 | 0.68 | 0.63 |
| 2825 | Train 45 | Test Not Hispanic 9 | 0.75 | 0.8 | 0.82 | 3849 | Train 61 | Test Not Hispanic 9 | 0.73 | 0.75 | 0.7 |
| 2826 | Train 45 | Test Not Hispanic 10 | 0.75 | 0.78 | 0.78 | 3850 | Train 61 | Test Not Hispanic 10 | 0.73 | 0.72 | 0.68 |
| 2827 | Train 45 | Test Not Hispanic 11 | 0.75 | 0.8 | 0.81 | 3851 | Train 61 | Test Not Hispanic 11 | 0.73 | 0.75 | 0.7 |
| 2828 | Train 45 | Test Not Hispanic 12 | 0.75 | 0.78 | 0.78 | 3852 | Train 61 | Test Not Hispanic 12 | 0.73 | 0.72 | 0.68 |
| 2829 | Train 45 | Test Not Hispanic 13 | 0.73 | 0.78 | 0.75 | 3853 | Train 61 | Test Not Hispanic 13 | 0.72 | 0.74 | 0.72 |
| 2830 | Train 45 | Test Not Hispanic 14 | 0.7 | 0.71 | 0.68 | 3854 | Train 61 | Test Not Hispanic 14 | 0.7 | 0.69 | 0.63 |
| 2831 | Train 45 | Test Not Hispanic 15 | 0.73 | 0.78 | 0.74 | 3855 | Train 61 | Test Not Hispanic 15 | 0.72 | 0.74 | 0.72 |
| 2832 | Train 45 | Test Not Hispanic 16 | 0.7 | 0.71 | 0.68 | 3856 | Train 61 | Test Not Hispanic 16 | 0.7 | 0.68 | 0.63 |
| 2833 | Train 45 | Test Not Hispanic 17 | 0.75 | 0.8 | 0.79 | 3857 | Train 61 | Test Not Hispanic 17 | 0.73 | 0.74 | 0.68 |
| 2834 | Train 45 | Test Not Hispanic 18 | 0.75 | 0.78 | 0.76 | 3858 | Train 61 | Test Not Hispanic 18 | 0.73 | 0.72 | 0.67 |
| 2835 | Train 45 | Test Not Hispanic 19 | 0.75 | 0.8 | 0.79 | 3859 | Train 61 | Test Not Hispanic 19 | 0.73 | 0.74 | 0.68 |
| 2836 | Train 45 | Test Not Hispanic 20 | 0.75 | 0.78 | 0.76 | 3860 | Train 61 | Test Not Hispanic 20 | 0.73 | 0.72 | 0.67 |
| 2837 | Train 45 | Test Not Hispanic 21 | 0.74 | 0.78 | 0.73 | 3861 | Train 61 | Test Not Hispanic 21 | 0.72 | 0.74 | 0.7 |
| 2838 | Train 45 | Test Not Hispanic 22 | 0.7 | 0.71 | 0.68 | 3862 | Train 61 | Test Not Hispanic 22 | 0.7 | 0.68 | 0.63 |
| 2839 | Train 45 | Test Not Hispanic 23 | 0.73 | 0.77 | 0.73 | 3863 | Train 61 | Test Not Hispanic 23 | 0.72 | 0.74 | 0.7 |
| 2840 | Train 45 | Test Not Hispanic 24 | 0.7 | 0.71 | 0.68 | 3864 | Train 61 | Test Not Hispanic 24 | 0.7 | 0.68 | 0.63 |
| 2841 | Train 45 | Test Not Hispanic 25 | 0.75 | 0.8 | 0.82 | 3865 | Train 61 | Test Not Hispanic 25 | 0.73 | 0.75 | 0.7 |
| 2842 | Train 45 | Test Not Hispanic 26 | 0.75 | 0.78 | 0.78 | 3866 | Train 61 | Test Not Hispanic 26 | 0.73 | 0.72 | 0.68 |
| 2843 | Train 45 | Test Not Hispanic 27 | 0.75 | 0.8 | 0.81 | 3867 | Train 61 | Test Not Hispanic 27 | 0.73 | 0.75 | 0.7 |
| 2844 | Train 45 | Test Not Hispanic 28 | 0.75 | 0.78 | 0.78 | 3868 | Train 61 | Test Not Hispanic 28 | 0.73 | 0.72 | 0.68 |
| 2845 | Train 45 | Test Not Hispanic 29 | 0.74 | 0.78 | 0.75 | 3869 | Train 61 | Test Not Hispanic 29 | 0.72 | 0.74 | 0.72 |
| 2846 | Train 45 | Test Not Hispanic 30 | 0.7 | 0.71 | 0.68 | 3870 | Train 61 | Test Not Hispanic 30 | 0.7 | 0.69 | 0.63 |
| 2847 | Train 45 | Test Not Hispanic 31 | 0.73 | 0.78 | 0.74 | 3871 | Train 61 | Test Not Hispanic 31 | 0.72 | 0.74 | 0.72 |
| 2848 | Train 45 | Test Not Hispanic 32 | 0.7 | 0.71 | 0.68 | 3872 | Train 61 | Test Not Hispanic 32 | 0.7 | 0.68 | 0.63 |
| 2849 | Train 45 | Test Not Hispanic 33 | 0.75 | 0.8 | 0.8 | 3873 | Train 61 | Test Not Hispanic 33 | 0.73 | 0.74 | 0.68 |
| 2850 | Train 45 | Test Not Hispanic 34 | 0.75 | 0.78 | 0.76 | 3874 | Train 61 | Test Not Hispanic 34 | 0.73 | 0.72 | 0.67 |
| 2851 | Train 45 | Test Not Hispanic 35 | 0.75 | 0.8 | 0.79 | 3875 | Train 61 | Test Not Hispanic 35 | 0.73 | 0.74 | 0.68 |
| 2852 | Train 45 | Test Not Hispanic 36 | 0.75 | 0.78 | 0.76 | 3876 | Train 61 | Test Not Hispanic 36 | 0.73 | 0.72 | 0.67 |
| 2853 | Train 45 | Test Not Hispanic 37 | 0.74 | 0.78 | 0.73 | 3877 | Train 61 | Test Not Hispanic 37 | 0.72 | 0.74 | 0.7 |
| 2854 | Train 45 | Test Not Hispanic 38 | 0.7 | 0.71 | 0.68 | 3878 | Train 61 | Test Not Hispanic 38 | 0.7 | 0.69 | 0.63 |
| 2855 | Train 45 | Test Not Hispanic 39 | 0.73 | 0.77 | 0.73 | 3879 | Train 61 | Test Not Hispanic 39 | 0.72 | 0.74 | 0.7 |
| 2856 | Train 45 | Test Not Hispanic 40 | 0.7 | 0.71 | 0.68 | 3880 | Train 61 | Test Not Hispanic 40 | 0.7 | 0.68 | 0.63 |
| 2857 | Train 45 | Test Not Hispanic 41 | 0.75 | 0.8 | 0.82 | 3881 | Train 61 | Test Not Hispanic 41 | 0.73 | 0.75 | 0.7 |
| 2858 | Train 45 | Test Not Hispanic 42 | 0.75 | 0.78 | 0.78 | 3882 | Train 61 | Test Not Hispanic 42 | 0.73 | 0.72 | 0.68 |
| 2859 | Train 45 | Test Not Hispanic 43 | 0.75 | 0.8 | 0.82 | 3883 | Train 61 | Test Not Hispanic 43 | 0.73 | 0.75 | 0.7 |
| 2860 | Train 45 | Test Not Hispanic 44 | 0.75 | 0.78 | 0.78 | 3884 | Train 61 | Test Not Hispanic 44 | 0.73 | 0.72 | 0.68 |
| 2861 | Train 45 | Test Not Hispanic 45 | 0.74 | 0.78 | 0.75 | 3885 | Train 61 | Test Not Hispanic 45 | 0.72 | 0.74 | 0.72 |
| 2862 | Train 45 | Test Not Hispanic 46 | 0.7 | 0.71 | 0.68 | 3886 | Train 61 | Test Not Hispanic 46 | 0.7 | 0.69 | 0.63 |
| 2863 | Train 45 | Test Not Hispanic 47 | 0.73 | 0.78 | 0.74 | 3887 | Train 61 | Test Not Hispanic 47 | 0.72 | 0.74 | 0.72 |
| 2864 | Train 45 | Test Not Hispanic 48 | 0.7 | 0.71 | 0.68 | 3888 | Train 61 | Test Not Hispanic 48 | 0.7 | 0.69 | 0.63 |
| 2865 | Train 45 | Test Not Hispanic 49 | 0.76 | 0.77 | 0.78 | 3889 | Train 61 | Test Not Hispanic 49 | 0.76 | 0.78 | 0.78 |
| 2866 | Train 45 | Test Not Hispanic 50 | 0.76 | 0.77 | 0.78 | 3890 | Train 61 | Test Not Hispanic 50 | 0.76 | 0.78 | 0.78 |
| 2867 | Train 45 | Test Not Hispanic 51 | 0.76 | 0.77 | 0.77 | 3891 | Train 61 | Test Not Hispanic 51 | 0.76 | 0.78 | 0.78 |
| 2868 | Train 45 | Test Not Hispanic 52 | 0.76 | 0.77 | 0.77 | 3892 | Train 61 | Test Not Hispanic 52 | 0.76 | 0.78 | 0.77 |
| 2869 | Train 45 | Test Not Hispanic 53 | 0.75 | 0.78 | 0.75 | 3893 | Train 61 | Test Not Hispanic 53 | 0.76 | 0.82 | 0.79 |
| 2870 | Train 45 | Test Not Hispanic 54 | 0.71 | 0.72 | 0.69 | 3894 | Train 61 | Test Not Hispanic 54 | 0.71 | 0.71 | 0.66 |
| 2871 | Train 45 | Test Not Hispanic 55 | 0.75 | 0.78 | 0.75 | 3895 | Train 61 | Test Not Hispanic 55 | 0.76 | 0.82 | 0.78 |
| 2872 | Train 45 | Test Not Hispanic 56 | 0.71 | 0.71 | 0.68 | 3896 | Train 61 | Test Not Hispanic 56 | 0.71 | 0.7 | 0.66 |
| 2873 | Train 45 | Test Not Hispanic 57 | 0.76 | 0.78 | 0.8 | 3897 | Train 61 | Test Not Hispanic 57 | 0.76 | 0.79 | 0.81 |
| 2874 | Train 45 | Test Not Hispanic 58 | 0.76 | 0.78 | 0.8 | 3898 | Train 61 | Test Not Hispanic 58 | 0.76 | 0.79 | 0.81 |
| 2875 | Train 45 | Test Not Hispanic 59 | 0.76 | 0.77 | 0.8 | 3899 | Train 61 | Test Not Hispanic 59 | 0.76 | 0.79 | 0.81 |
| 2876 | Train 45 | Test Not Hispanic 60 | 0.76 | 0.78 | 0.8 | 3900 | Train 61 | Test Not Hispanic 60 | 0.76 | 0.79 | 0.81 |
| 2877 | Train 45 | Test Not Hispanic 61 | 0.75 | 0.79 | 0.76 | 3901 | Train 61 | Test Not Hispanic 61 | 0.76 | 0.83 | 0.81 |
| 2878 | Train 45 | Test Not Hispanic 62 | 0.72 | 0.72 | 0.69 | 3902 | Train 61 | Test Not Hispanic 62 | 0.71 | 0.71 | 0.66 |
| 2879 | Train 45 | Test Not Hispanic 63 | 0.75 | 0.79 | 0.76 | 3903 | Train 61 | Test Not Hispanic 63 | 0.76 | 0.83 | 0.81 |
| 2880 | Train 45 | Test Not Hispanic 64 | 0.71 | 0.71 | 0.69 | 3904 | Train 61 | Test Not Hispanic 64 | 0.71 | 0.7 | 0.66 |
| 2881 | Train 46 | Test Not Hispanic 1 | 0.73 | 0.77 | 0.76 | 3905 | Train 62 | Test Not Hispanic 1 | 0.72 | 0.73 | 0.69 |
| 2882 | Train 46 | Test Not Hispanic 2 | 0.75 | 0.78 | 0.77 | 3906 | Train 62 | Test Not Hispanic 2 | 0.73 | 0.74 | 0.69 |
| 2883 | Train 46 | Test Not Hispanic 3 | 0.73 | 0.77 | 0.76 | 3907 | Train 62 | Test Not Hispanic 3 | 0.72 | 0.73 | 0.69 |
| 2884 | Train 46 | Test Not Hispanic 4 | 0.75 | 0.78 | 0.77 | 3908 | Train 62 | Test Not Hispanic 4 | 0.73 | 0.74 | 0.69 |
| 2885 | Train 46 | Test Not Hispanic 5 | 0.72 | 0.77 | 0.77 | 3909 | Train 62 | Test Not Hispanic 5 | 0.71 | 0.73 | 0.69 |
| 2886 | Train 46 | Test Not Hispanic 6 | 0.74 | 0.78 | 0.79 | 3910 | Train 62 | Test Not Hispanic 6 | 0.72 | 0.75 | 0.72 |
| 2887 | Train 46 | Test Not Hispanic 7 | 0.72 | 0.77 | 0.77 | 3911 | Train 62 | Test Not Hispanic 7 | 0.71 | 0.73 | 0.69 |
| 2888 | Train 46 | Test Not Hispanic 8 | 0.73 | 0.77 | 0.78 | 3912 | Train 62 | Test Not Hispanic 8 | 0.72 | 0.74 | 0.72 |
| 2889 | Train 46 | Test Not Hispanic 9 | 0.73 | 0.77 | 0.77 | 3913 | Train 62 | Test Not Hispanic 9 | 0.72 | 0.74 | 0.7 |
| 2890 | Train 46 | Test Not Hispanic 10 | 0.75 | 0.78 | 0.78 | 3914 | Train 62 | Test Not Hispanic 10 | 0.73 | 0.74 | 0.7 |
| 2891 | Train 46 | Test Not Hispanic 11 | 0.73 | 0.77 | 0.77 | 3915 | Train 62 | Test Not Hispanic 11 | 0.72 | 0.74 | 0.7 |
| 2892 | Train 46 | Test Not Hispanic 12 | 0.75 | 0.78 | 0.77 | 3916 | Train 62 | Test Not Hispanic 12 | 0.73 | 0.74 | 0.7 |
| 2893 | Train 46 | Test Not Hispanic 13 | 0.72 | 0.77 | 0.78 | 3917 | Train 62 | Test Not Hispanic 13 | 0.71 | 0.73 | 0.7 |
| 2894 | Train 46 | Test Not Hispanic 14 | 0.74 | 0.78 | 0.79 | 3918 | Train 62 | Test Not Hispanic 14 | 0.73 | 0.75 | 0.72 |
| 2895 | Train 46 | Test Not Hispanic 15 | 0.72 | 0.77 | 0.78 | 3919 | Train 62 | Test Not Hispanic 15 | 0.71 | 0.73 | 0.7 |
| 2896 | Train 46 | Test Not Hispanic 16 | 0.74 | 0.78 | 0.78 | 3920 | Train 62 | Test Not Hispanic 16 | 0.72 | 0.75 | 0.72 |
| 2897 | Train 46 | Test Not Hispanic 17 | 0.73 | 0.77 | 0.76 | 3921 | Train 62 | Test Not Hispanic 17 | 0.72 | 0.73 | 0.69 |
| 2898 | Train 46 | Test Not Hispanic 18 | 0.75 | 0.78 | 0.77 | 3922 | Train 62 | Test Not Hispanic 18 | 0.73 | 0.74 | 0.69 |
| 2899 | Train 46 | Test Not Hispanic 19 | 0.73 | 0.77 | 0.76 | 3923 | Train 62 | Test Not Hispanic 19 | 0.72 | 0.73 | 0.69 |
| 2900 | Train 46 | Test Not Hispanic 20 | 0.75 | 0.78 | 0.77 | 3924 | Train 62 | Test Not Hispanic 20 | 0.73 | 0.74 | 0.69 |
| 2901 | Train 46 | Test Not Hispanic 21 | 0.72 | 0.77 | 0.77 | 3925 | Train 62 | Test Not Hispanic 21 | 0.71 | 0.73 | 0.69 |
| 2902 | Train 46 | Test Not Hispanic 22 | 0.74 | 0.78 | 0.79 | 3926 | Train 62 | Test Not Hispanic 22 | 0.73 | 0.75 | 0.72 |
| 2903 | Train 46 | Test Not Hispanic 23 | 0.72 | 0.77 | 0.77 | 3927 | Train 62 | Test Not Hispanic 23 | 0.71 | 0.73 | 0.69 |
| 2904 | Train 46 | Test Not Hispanic 24 | 0.73 | 0.77 | 0.78 | 3928 | Train 62 | Test Not Hispanic 24 | 0.72 | 0.74 | 0.72 |
| 2905 | Train 46 | Test Not Hispanic 25 | 0.73 | 0.77 | 0.77 | 3929 | Train 62 | Test Not Hispanic 25 | 0.72 | 0.74 | 0.7 |
| 2906 | Train 46 | Test Not Hispanic 26 | 0.75 | 0.78 | 0.77 | 3930 | Train 62 | Test Not Hispanic 26 | 0.73 | 0.74 | 0.7 |
| 2907 | Train 46 | Test Not Hispanic 27 | 0.73 | 0.77 | 0.77 | 3931 | Train 62 | Test Not Hispanic 27 | 0.72 | 0.74 | 0.7 |
| 2908 | Train 46 | Test Not Hispanic 28 | 0.75 | 0.78 | 0.77 | 3932 | Train 62 | Test Not Hispanic 28 | 0.73 | 0.74 | 0.7 |
| 2909 | Train 46 | Test Not Hispanic 29 | 0.72 | 0.77 | 0.78 | 3933 | Train 62 | Test Not Hispanic 29 | 0.71 | 0.73 | 0.7 |
| 2910 | Train 46 | Test Not Hispanic 30 | 0.74 | 0.78 | 0.79 | 3934 | Train 62 | Test Not Hispanic 30 | 0.73 | 0.75 | 0.72 |
| 2911 | Train 46 | Test Not Hispanic 31 | 0.72 | 0.77 | 0.78 | 3935 | Train 62 | Test Not Hispanic 31 | 0.71 | 0.73 | 0.7 |
| 2912 | Train 46 | Test Not Hispanic 32 | 0.74 | 0.78 | 0.78 | 3936 | Train 62 | Test Not Hispanic 32 | 0.73 | 0.75 | 0.72 |
| 2913 | Train 46 | Test Not Hispanic 33 | 0.73 | 0.77 | 0.76 | 3937 | Train 62 | Test Not Hispanic 33 | 0.72 | 0.73 | 0.69 |
| 2914 | Train 46 | Test Not Hispanic 34 | 0.75 | 0.78 | 0.77 | 3938 | Train 62 | Test Not Hispanic 34 | 0.73 | 0.74 | 0.69 |
| 2915 | Train 46 | Test Not Hispanic 35 | 0.73 | 0.77 | 0.76 | 3939 | Train 62 | Test Not Hispanic 35 | 0.72 | 0.73 | 0.69 |
| 2916 | Train 46 | Test Not Hispanic 36 | 0.75 | 0.78 | 0.77 | 3940 | Train 62 | Test Not Hispanic 36 | 0.73 | 0.74 | 0.69 |
| 2917 | Train 46 | Test Not Hispanic 37 | 0.72 | 0.77 | 0.77 | 3941 | Train 62 | Test Not Hispanic 37 | 0.71 | 0.73 | 0.69 |
| 2918 | Train 46 | Test Not Hispanic 38 | 0.74 | 0.78 | 0.79 | 3942 | Train 62 | Test Not Hispanic 38 | 0.72 | 0.75 | 0.72 |
| 2919 | Train 46 | Test Not Hispanic 39 | 0.72 | 0.77 | 0.77 | 3943 | Train 62 | Test Not Hispanic 39 | 0.71 | 0.73 | 0.69 |
| 2920 | Train 46 | Test Not Hispanic 40 | 0.73 | 0.77 | 0.78 | 3944 | Train 62 | Test Not Hispanic 40 | 0.72 | 0.74 | 0.72 |
| 2921 | Train 46 | Test Not Hispanic 41 | 0.73 | 0.77 | 0.77 | 3945 | Train 62 | Test Not Hispanic 41 | 0.72 | 0.74 | 0.7 |
| 2922 | Train 46 | Test Not Hispanic 42 | 0.75 | 0.78 | 0.78 | 3946 | Train 62 | Test Not Hispanic 42 | 0.73 | 0.74 | 0.7 |
| 2923 | Train 46 | Test Not Hispanic 43 | 0.73 | 0.77 | 0.77 | 3947 | Train 62 | Test Not Hispanic 43 | 0.72 | 0.74 | 0.7 |
| 2924 | Train 46 | Test Not Hispanic 44 | 0.75 | 0.78 | 0.78 | 3948 | Train 62 | Test Not Hispanic 44 | 0.73 | 0.74 | 0.7 |
| 2925 | Train 46 | Test Not Hispanic 45 | 0.72 | 0.77 | 0.78 | 3949 | Train 62 | Test Not Hispanic 45 | 0.71 | 0.73 | 0.7 |
| 2926 | Train 46 | Test Not Hispanic 46 | 0.74 | 0.78 | 0.79 | 3950 | Train 62 | Test Not Hispanic 46 | 0.73 | 0.75 | 0.72 |
| 2927 | Train 46 | Test Not Hispanic 47 | 0.72 | 0.77 | 0.78 | 3951 | Train 62 | Test Not Hispanic 47 | 0.71 | 0.73 | 0.7 |
| 2928 | Train 46 | Test Not Hispanic 48 | 0.74 | 0.78 | 0.78 | 3952 | Train 62 | Test Not Hispanic 48 | 0.73 | 0.75 | 0.72 |
| 2929 | Train 46 | Test Not Hispanic 49 | 0.75 | 0.78 | 0.77 | 3953 | Train 62 | Test Not Hispanic 49 | 0.76 | 0.78 | 0.77 |
| 2930 | Train 46 | Test Not Hispanic 50 | 0.75 | 0.78 | 0.77 | 3954 | Train 62 | Test Not Hispanic 50 | 0.76 | 0.79 | 0.77 |
| 2931 | Train 46 | Test Not Hispanic 51 | 0.75 | 0.78 | 0.77 | 3955 | Train 62 | Test Not Hispanic 51 | 0.76 | 0.78 | 0.76 |
| 2932 | Train 46 | Test Not Hispanic 52 | 0.75 | 0.78 | 0.77 | 3956 | Train 62 | Test Not Hispanic 52 | 0.76 | 0.78 | 0.76 |
| 2933 | Train 46 | Test Not Hispanic 53 | 0.75 | 0.79 | 0.77 | 3957 | Train 62 | Test Not Hispanic 53 | 0.76 | 0.8 | 0.74 |
| 2934 | Train 46 | Test Not Hispanic 54 | 0.74 | 0.78 | 0.78 | 3958 | Train 62 | Test Not Hispanic 54 | 0.75 | 0.8 | 0.81 |
| 2935 | Train 46 | Test Not Hispanic 55 | 0.75 | 0.79 | 0.76 | 3959 | Train 62 | Test Not Hispanic 55 | 0.75 | 0.8 | 0.74 |
| 2936 | Train 46 | Test Not Hispanic 56 | 0.74 | 0.77 | 0.78 | 3960 | Train 62 | Test Not Hispanic 56 | 0.74 | 0.79 | 0.8 |
| 2937 | Train 46 | Test Not Hispanic 57 | 0.75 | 0.79 | 0.79 | 3961 | Train 62 | Test Not Hispanic 57 | 0.76 | 0.79 | 0.78 |
| 2938 | Train 46 | Test Not Hispanic 58 | 0.75 | 0.79 | 0.79 | 3962 | Train 62 | Test Not Hispanic 58 | 0.76 | 0.79 | 0.78 |
| 2939 | Train 46 | Test Not Hispanic 59 | 0.75 | 0.79 | 0.79 | 3963 | Train 62 | Test Not Hispanic 59 | 0.76 | 0.79 | 0.78 |
| 2940 | Train 46 | Test Not Hispanic 60 | 0.75 | 0.79 | 0.79 | 3964 | Train 62 | Test Not Hispanic 60 | 0.76 | 0.79 | 0.78 |
| 2941 | Train 46 | Test Not Hispanic 61 | 0.75 | 0.79 | 0.78 | 3965 | Train 62 | Test Not Hispanic 61 | 0.76 | 0.81 | 0.75 |
| 2942 | Train 46 | Test Not Hispanic 62 | 0.74 | 0.78 | 0.79 | 3966 | Train 62 | Test Not Hispanic 62 | 0.75 | 0.8 | 0.81 |
| 2943 | Train 46 | Test Not Hispanic 63 | 0.75 | 0.79 | 0.77 | 3967 | Train 62 | Test Not Hispanic 63 | 0.76 | 0.81 | 0.75 |
| 2944 | Train 46 | Test Not Hispanic 64 | 0.74 | 0.77 | 0.78 | 3968 | Train 62 | Test Not Hispanic 64 | 0.74 | 0.79 | 0.8 |
| 2945 | Train 47 | Test Not Hispanic 1 | 0.75 | 0.79 | 0.79 | 3969 | Train 63 | Test Not Hispanic 1 | 0.73 | 0.74 | 0.7 |
| 2946 | Train 47 | Test Not Hispanic 2 | 0.75 | 0.78 | 0.77 | 3970 | Train 63 | Test Not Hispanic 2 | 0.73 | 0.72 | 0.67 |
| 2947 | Train 47 | Test Not Hispanic 3 | 0.75 | 0.8 | 0.79 | 3971 | Train 63 | Test Not Hispanic 3 | 0.73 | 0.74 | 0.7 |
| 2948 | Train 47 | Test Not Hispanic 4 | 0.75 | 0.78 | 0.77 | 3972 | Train 63 | Test Not Hispanic 4 | 0.73 | 0.72 | 0.67 |
| 2949 | Train 47 | Test Not Hispanic 5 | 0.73 | 0.77 | 0.72 | 3973 | Train 63 | Test Not Hispanic 5 | 0.72 | 0.74 | 0.71 |
| 2950 | Train 47 | Test Not Hispanic 6 | 0.7 | 0.71 | 0.67 | 3974 | Train 63 | Test Not Hispanic 6 | 0.7 | 0.68 | 0.64 |
| 2951 | Train 47 | Test Not Hispanic 7 | 0.73 | 0.77 | 0.72 | 3975 | Train 63 | Test Not Hispanic 7 | 0.72 | 0.74 | 0.71 |
| 2952 | Train 47 | Test Not Hispanic 8 | 0.7 | 0.71 | 0.67 | 3976 | Train 63 | Test Not Hispanic 8 | 0.7 | 0.68 | 0.65 |
| 2953 | Train 47 | Test Not Hispanic 9 | 0.75 | 0.8 | 0.81 | 3977 | Train 63 | Test Not Hispanic 9 | 0.73 | 0.74 | 0.71 |
| 2954 | Train 47 | Test Not Hispanic 10 | 0.75 | 0.78 | 0.78 | 3978 | Train 63 | Test Not Hispanic 10 | 0.73 | 0.72 | 0.68 |
| 2955 | Train 47 | Test Not Hispanic 11 | 0.75 | 0.8 | 0.81 | 3979 | Train 63 | Test Not Hispanic 11 | 0.73 | 0.74 | 0.71 |
| 2956 | Train 47 | Test Not Hispanic 12 | 0.75 | 0.78 | 0.78 | 3980 | Train 63 | Test Not Hispanic 12 | 0.73 | 0.72 | 0.68 |
| 2957 | Train 47 | Test Not Hispanic 13 | 0.73 | 0.78 | 0.74 | 3981 | Train 63 | Test Not Hispanic 13 | 0.72 | 0.74 | 0.73 |
| 2958 | Train 47 | Test Not Hispanic 14 | 0.7 | 0.71 | 0.67 | 3982 | Train 63 | Test Not Hispanic 14 | 0.7 | 0.69 | 0.64 |
| 2959 | Train 47 | Test Not Hispanic 15 | 0.73 | 0.78 | 0.73 | 3983 | Train 63 | Test Not Hispanic 15 | 0.72 | 0.74 | 0.73 |
| 2960 | Train 47 | Test Not Hispanic 16 | 0.7 | 0.71 | 0.67 | 3984 | Train 63 | Test Not Hispanic 16 | 0.7 | 0.69 | 0.65 |
| 2961 | Train 47 | Test Not Hispanic 17 | 0.75 | 0.79 | 0.79 | 3985 | Train 63 | Test Not Hispanic 17 | 0.73 | 0.74 | 0.7 |
| 2962 | Train 47 | Test Not Hispanic 18 | 0.75 | 0.78 | 0.77 | 3986 | Train 63 | Test Not Hispanic 18 | 0.73 | 0.72 | 0.67 |
| 2963 | Train 47 | Test Not Hispanic 19 | 0.75 | 0.8 | 0.79 | 3987 | Train 63 | Test Not Hispanic 19 | 0.73 | 0.74 | 0.7 |
| 2964 | Train 47 | Test Not Hispanic 20 | 0.75 | 0.78 | 0.77 | 3988 | Train 63 | Test Not Hispanic 20 | 0.73 | 0.72 | 0.67 |
| 2965 | Train 47 | Test Not Hispanic 21 | 0.74 | 0.77 | 0.72 | 3989 | Train 63 | Test Not Hispanic 21 | 0.72 | 0.74 | 0.71 |
| 2966 | Train 47 | Test Not Hispanic 22 | 0.7 | 0.71 | 0.67 | 3990 | Train 63 | Test Not Hispanic 22 | 0.7 | 0.69 | 0.64 |
| 2967 | Train 47 | Test Not Hispanic 23 | 0.73 | 0.77 | 0.72 | 3991 | Train 63 | Test Not Hispanic 23 | 0.72 | 0.74 | 0.71 |
| 2968 | Train 47 | Test Not Hispanic 24 | 0.7 | 0.71 | 0.67 | 3992 | Train 63 | Test Not Hispanic 24 | 0.7 | 0.68 | 0.65 |
| 2969 | Train 47 | Test Not Hispanic 25 | 0.75 | 0.8 | 0.81 | 3993 | Train 63 | Test Not Hispanic 25 | 0.73 | 0.74 | 0.71 |
| 2970 | Train 47 | Test Not Hispanic 26 | 0.75 | 0.78 | 0.78 | 3994 | Train 63 | Test Not Hispanic 26 | 0.73 | 0.72 | 0.68 |
| 2971 | Train 47 | Test Not Hispanic 27 | 0.75 | 0.8 | 0.81 | 3995 | Train 63 | Test Not Hispanic 27 | 0.73 | 0.74 | 0.71 |
| 2972 | Train 47 | Test Not Hispanic 28 | 0.75 | 0.78 | 0.78 | 3996 | Train 63 | Test Not Hispanic 28 | 0.73 | 0.72 | 0.68 |
| 2973 | Train 47 | Test Not Hispanic 29 | 0.74 | 0.78 | 0.74 | 3997 | Train 63 | Test Not Hispanic 29 | 0.72 | 0.74 | 0.73 |
| 2974 | Train 47 | Test Not Hispanic 30 | 0.7 | 0.71 | 0.67 | 3998 | Train 63 | Test Not Hispanic 30 | 0.7 | 0.69 | 0.65 |
| 2975 | Train 47 | Test Not Hispanic 31 | 0.73 | 0.78 | 0.73 | 3999 | Train 63 | Test Not Hispanic 31 | 0.72 | 0.74 | 0.73 |
| 2976 | Train 47 | Test Not Hispanic 32 | 0.7 | 0.71 | 0.67 | 4000 | Train 63 | Test Not Hispanic 32 | 0.7 | 0.69 | 0.65 |
| 2977 | Train 47 | Test Not Hispanic 33 | 0.75 | 0.8 | 0.79 | 4001 | Train 63 | Test Not Hispanic 33 | 0.73 | 0.74 | 0.7 |
| 2978 | Train 47 | Test Not Hispanic 34 | 0.75 | 0.78 | 0.77 | 4002 | Train 63 | Test Not Hispanic 34 | 0.73 | 0.72 | 0.67 |
| 2979 | Train 47 | Test Not Hispanic 35 | 0.75 | 0.8 | 0.8 | 4003 | Train 63 | Test Not Hispanic 35 | 0.73 | 0.74 | 0.7 |
| 2980 | Train 47 | Test Not Hispanic 36 | 0.75 | 0.78 | 0.77 | 4004 | Train 63 | Test Not Hispanic 36 | 0.73 | 0.72 | 0.67 |
| 2981 | Train 47 | Test Not Hispanic 37 | 0.74 | 0.78 | 0.72 | 4005 | Train 63 | Test Not Hispanic 37 | 0.72 | 0.74 | 0.71 |
| 2982 | Train 47 | Test Not Hispanic 38 | 0.7 | 0.71 | 0.67 | 4006 | Train 63 | Test Not Hispanic 38 | 0.7 | 0.69 | 0.64 |
| 2983 | Train 47 | Test Not Hispanic 39 | 0.73 | 0.77 | 0.72 | 4007 | Train 63 | Test Not Hispanic 39 | 0.72 | 0.74 | 0.71 |
| 2984 | Train 47 | Test Not Hispanic 40 | 0.7 | 0.71 | 0.67 | 4008 | Train 63 | Test Not Hispanic 40 | 0.7 | 0.68 | 0.65 |
| 2985 | Train 47 | Test Not Hispanic 41 | 0.75 | 0.8 | 0.81 | 4009 | Train 63 | Test Not Hispanic 41 | 0.73 | 0.74 | 0.71 |
| 2986 | Train 47 | Test Not Hispanic 42 | 0.75 | 0.78 | 0.78 | 4010 | Train 63 | Test Not Hispanic 42 | 0.73 | 0.73 | 0.68 |
| 2987 | Train 47 | Test Not Hispanic 43 | 0.75 | 0.8 | 0.82 | 4011 | Train 63 | Test Not Hispanic 43 | 0.73 | 0.75 | 0.71 |
| 2988 | Train 47 | Test Not Hispanic 44 | 0.75 | 0.78 | 0.78 | 4012 | Train 63 | Test Not Hispanic 44 | 0.73 | 0.73 | 0.68 |
| 2989 | Train 47 | Test Not Hispanic 45 | 0.74 | 0.78 | 0.74 | 4013 | Train 63 | Test Not Hispanic 45 | 0.72 | 0.74 | 0.73 |
| 2990 | Train 47 | Test Not Hispanic 46 | 0.7 | 0.71 | 0.67 | 4014 | Train 63 | Test Not Hispanic 46 | 0.7 | 0.69 | 0.65 |
| 2991 | Train 47 | Test Not Hispanic 47 | 0.73 | 0.78 | 0.74 | 4015 | Train 63 | Test Not Hispanic 47 | 0.72 | 0.74 | 0.73 |
| 2992 | Train 47 | Test Not Hispanic 48 | 0.7 | 0.71 | 0.67 | 4016 | Train 63 | Test Not Hispanic 48 | 0.7 | 0.69 | 0.65 |
| 2993 | Train 47 | Test Not Hispanic 49 | 0.75 | 0.77 | 0.78 | 4017 | Train 63 | Test Not Hispanic 49 | 0.76 | 0.78 | 0.78 |
| 2994 | Train 47 | Test Not Hispanic 50 | 0.76 | 0.77 | 0.78 | 4018 | Train 63 | Test Not Hispanic 50 | 0.76 | 0.78 | 0.77 |
| 2995 | Train 47 | Test Not Hispanic 51 | 0.76 | 0.77 | 0.78 | 4019 | Train 63 | Test Not Hispanic 51 | 0.76 | 0.78 | 0.78 |
| 2996 | Train 47 | Test Not Hispanic 52 | 0.76 | 0.77 | 0.78 | 4020 | Train 63 | Test Not Hispanic 52 | 0.76 | 0.78 | 0.78 |
| 2997 | Train 47 | Test Not Hispanic 53 | 0.75 | 0.78 | 0.67 | 4021 | Train 63 | Test Not Hispanic 53 | 0.76 | 0.82 | 0.78 |
| 2998 | Train 47 | Test Not Hispanic 54 | 0.71 | 0.71 | 0.67 | 4022 | Train 63 | Test Not Hispanic 54 | 0.71 | 0.71 | 0.67 |
| 2999 | Train 47 | Test Not Hispanic 55 | 0.75 | 0.78 | 0.67 | 4023 | Train 63 | Test Not Hispanic 55 | 0.76 | 0.82 | 0.78 |
| 3000 | Train 47 | Test Not Hispanic 56 | 0.71 | 0.71 | 0.66 | 4024 | Train 63 | Test Not Hispanic 56 | 0.71 | 0.7 | 0.67 |
| 3001 | Train 47 | Test Not Hispanic 57 | 0.76 | 0.78 | 0.8 | 4025 | Train 63 | Test Not Hispanic 57 | 0.76 | 0.79 | 0.81 |
| 3002 | Train 47 | Test Not Hispanic 58 | 0.76 | 0.78 | 0.8 | 4026 | Train 63 | Test Not Hispanic 58 | 0.76 | 0.79 | 0.81 |
| 3003 | Train 47 | Test Not Hispanic 59 | 0.76 | 0.78 | 0.8 | 4027 | Train 63 | Test Not Hispanic 59 | 0.76 | 0.79 | 0.81 |
| 3004 | Train 47 | Test Not Hispanic 60 | 0.76 | 0.78 | 0.8 | 4028 | Train 63 | Test Not Hispanic 60 | 0.76 | 0.79 | 0.81 |
| 3005 | Train 47 | Test Not Hispanic 61 | 0.75 | 0.79 | 0.68 | 4029 | Train 63 | Test Not Hispanic 61 | 0.76 | 0.83 | 0.8 |
| 3006 | Train 47 | Test Not Hispanic 62 | 0.71 | 0.72 | 0.67 | 4030 | Train 63 | Test Not Hispanic 62 | 0.71 | 0.71 | 0.67 |
| 3007 | Train 47 | Test Not Hispanic 63 | 0.75 | 0.79 | 0.68 | 4031 | Train 63 | Test Not Hispanic 63 | 0.76 | 0.83 | 0.8 |
| 3008 | Train 47 | Test Not Hispanic 64 | 0.71 | 0.71 | 0.67 | 4032 | Train 63 | Test Not Hispanic 64 | 0.71 | 0.7 | 0.67 |
| 3009 | Train 48 | Test Not Hispanic 1 | 0.73 | 0.77 | 0.76 | 4033 | Train 64 | Test Not Hispanic 1 | 0.72 | 0.73 | 0.7 |
| 3010 | Train 48 | Test Not Hispanic 2 | 0.75 | 0.78 | 0.77 | 4034 | Train 64 | Test Not Hispanic 2 | 0.72 | 0.74 | 0.69 |
| 3011 | Train 48 | Test Not Hispanic 3 | 0.73 | 0.77 | 0.76 | 4035 | Train 64 | Test Not Hispanic 3 | 0.72 | 0.74 | 0.7 |
| 3012 | Train 48 | Test Not Hispanic 4 | 0.75 | 0.78 | 0.77 | 4036 | Train 64 | Test Not Hispanic 4 | 0.72 | 0.74 | 0.69 |
| 3013 | Train 48 | Test Not Hispanic 5 | 0.72 | 0.77 | 0.77 | 4037 | Train 64 | Test Not Hispanic 5 | 0.7 | 0.73 | 0.7 |
| 3014 | Train 48 | Test Not Hispanic 6 | 0.73 | 0.78 | 0.79 | 4038 | Train 64 | Test Not Hispanic 6 | 0.72 | 0.75 | 0.72 |
| 3015 | Train 48 | Test Not Hispanic 7 | 0.72 | 0.77 | 0.77 | 4039 | Train 64 | Test Not Hispanic 7 | 0.7 | 0.73 | 0.7 |
| 3016 | Train 48 | Test Not Hispanic 8 | 0.73 | 0.78 | 0.78 | 4040 | Train 64 | Test Not Hispanic 8 | 0.72 | 0.75 | 0.72 |
| 3017 | Train 48 | Test Not Hispanic 9 | 0.73 | 0.77 | 0.77 | 4041 | Train 64 | Test Not Hispanic 9 | 0.72 | 0.74 | 0.71 |
| 3018 | Train 48 | Test Not Hispanic 10 | 0.75 | 0.78 | 0.78 | 4042 | Train 64 | Test Not Hispanic 10 | 0.72 | 0.74 | 0.7 |
| 3019 | Train 48 | Test Not Hispanic 11 | 0.73 | 0.77 | 0.77 | 4043 | Train 64 | Test Not Hispanic 11 | 0.72 | 0.74 | 0.71 |
| 3020 | Train 48 | Test Not Hispanic 12 | 0.75 | 0.78 | 0.78 | 4044 | Train 64 | Test Not Hispanic 12 | 0.72 | 0.74 | 0.7 |
| 3021 | Train 48 | Test Not Hispanic 13 | 0.72 | 0.77 | 0.78 | 4045 | Train 64 | Test Not Hispanic 13 | 0.7 | 0.74 | 0.71 |
| 3022 | Train 48 | Test Not Hispanic 14 | 0.74 | 0.78 | 0.79 | 4046 | Train 64 | Test Not Hispanic 14 | 0.72 | 0.75 | 0.72 |
| 3023 | Train 48 | Test Not Hispanic 15 | 0.72 | 0.77 | 0.78 | 4047 | Train 64 | Test Not Hispanic 15 | 0.7 | 0.73 | 0.71 |
| 3024 | Train 48 | Test Not Hispanic 16 | 0.73 | 0.78 | 0.79 | 4048 | Train 64 | Test Not Hispanic 16 | 0.72 | 0.75 | 0.72 |
| 3025 | Train 48 | Test Not Hispanic 17 | 0.73 | 0.77 | 0.76 | 4049 | Train 64 | Test Not Hispanic 17 | 0.72 | 0.73 | 0.7 |
| 3026 | Train 48 | Test Not Hispanic 18 | 0.75 | 0.78 | 0.77 | 4050 | Train 64 | Test Not Hispanic 18 | 0.72 | 0.74 | 0.69 |
| 3027 | Train 48 | Test Not Hispanic 19 | 0.73 | 0.77 | 0.76 | 4051 | Train 64 | Test Not Hispanic 19 | 0.72 | 0.74 | 0.7 |
| 3028 | Train 48 | Test Not Hispanic 20 | 0.75 | 0.78 | 0.77 | 4052 | Train 64 | Test Not Hispanic 20 | 0.72 | 0.74 | 0.69 |
| 3029 | Train 48 | Test Not Hispanic 21 | 0.72 | 0.77 | 0.77 | 4053 | Train 64 | Test Not Hispanic 21 | 0.7 | 0.73 | 0.7 |
| 3030 | Train 48 | Test Not Hispanic 22 | 0.73 | 0.78 | 0.79 | 4054 | Train 64 | Test Not Hispanic 22 | 0.72 | 0.75 | 0.72 |
| 3031 | Train 48 | Test Not Hispanic 23 | 0.72 | 0.77 | 0.77 | 4055 | Train 64 | Test Not Hispanic 23 | 0.7 | 0.73 | 0.7 |
| 3032 | Train 48 | Test Not Hispanic 24 | 0.73 | 0.78 | 0.78 | 4056 | Train 64 | Test Not Hispanic 24 | 0.72 | 0.75 | 0.72 |
| 3033 | Train 48 | Test Not Hispanic 25 | 0.73 | 0.77 | 0.77 | 4057 | Train 64 | Test Not Hispanic 25 | 0.72 | 0.74 | 0.71 |
| 3034 | Train 48 | Test Not Hispanic 26 | 0.75 | 0.78 | 0.78 | 4058 | Train 64 | Test Not Hispanic 26 | 0.72 | 0.74 | 0.7 |
| 3035 | Train 48 | Test Not Hispanic 27 | 0.73 | 0.77 | 0.77 | 4059 | Train 64 | Test Not Hispanic 27 | 0.72 | 0.74 | 0.71 |
| 3036 | Train 48 | Test Not Hispanic 28 | 0.75 | 0.78 | 0.78 | 4060 | Train 64 | Test Not Hispanic 28 | 0.72 | 0.74 | 0.7 |
| 3037 | Train 48 | Test Not Hispanic 29 | 0.72 | 0.77 | 0.78 | 4061 | Train 64 | Test Not Hispanic 29 | 0.7 | 0.74 | 0.71 |
| 3038 | Train 48 | Test Not Hispanic 30 | 0.74 | 0.78 | 0.79 | 4062 | Train 64 | Test Not Hispanic 30 | 0.72 | 0.75 | 0.72 |
| 3039 | Train 48 | Test Not Hispanic 31 | 0.72 | 0.77 | 0.78 | 4063 | Train 64 | Test Not Hispanic 31 | 0.7 | 0.73 | 0.71 |
| 3040 | Train 48 | Test Not Hispanic 32 | 0.74 | 0.78 | 0.79 | 4064 | Train 64 | Test Not Hispanic 32 | 0.72 | 0.75 | 0.72 |
| 3041 | Train 48 | Test Not Hispanic 33 | 0.74 | 0.77 | 0.76 | 4065 | Train 64 | Test Not Hispanic 33 | 0.72 | 0.74 | 0.7 |
| 3042 | Train 48 | Test Not Hispanic 34 | 0.75 | 0.78 | 0.77 | 4066 | Train 64 | Test Not Hispanic 34 | 0.72 | 0.74 | 0.7 |
| 3043 | Train 48 | Test Not Hispanic 35 | 0.74 | 0.77 | 0.76 | 4067 | Train 64 | Test Not Hispanic 35 | 0.72 | 0.74 | 0.7 |
| 3044 | Train 48 | Test Not Hispanic 36 | 0.75 | 0.78 | 0.77 | 4068 | Train 64 | Test Not Hispanic 36 | 0.72 | 0.74 | 0.7 |
| 3045 | Train 48 | Test Not Hispanic 37 | 0.72 | 0.77 | 0.77 | 4069 | Train 64 | Test Not Hispanic 37 | 0.7 | 0.73 | 0.7 |
| 3046 | Train 48 | Test Not Hispanic 38 | 0.73 | 0.78 | 0.79 | 4070 | Train 64 | Test Not Hispanic 38 | 0.72 | 0.75 | 0.72 |
| 3047 | Train 48 | Test Not Hispanic 39 | 0.72 | 0.77 | 0.77 | 4071 | Train 64 | Test Not Hispanic 39 | 0.7 | 0.73 | 0.7 |
| 3048 | Train 48 | Test Not Hispanic 40 | 0.73 | 0.78 | 0.78 | 4072 | Train 64 | Test Not Hispanic 40 | 0.72 | 0.75 | 0.72 |
| 3049 | Train 48 | Test Not Hispanic 41 | 0.74 | 0.77 | 0.77 | 4073 | Train 64 | Test Not Hispanic 41 | 0.72 | 0.74 | 0.71 |
| 3050 | Train 48 | Test Not Hispanic 42 | 0.75 | 0.78 | 0.78 | 4074 | Train 64 | Test Not Hispanic 42 | 0.72 | 0.75 | 0.7 |
| 3051 | Train 48 | Test Not Hispanic 43 | 0.74 | 0.77 | 0.77 | 4075 | Train 64 | Test Not Hispanic 43 | 0.72 | 0.74 | 0.71 |
| 3052 | Train 48 | Test Not Hispanic 44 | 0.75 | 0.78 | 0.78 | 4076 | Train 64 | Test Not Hispanic 44 | 0.72 | 0.75 | 0.7 |
| 3053 | Train 48 | Test Not Hispanic 45 | 0.72 | 0.77 | 0.78 | 4077 | Train 64 | Test Not Hispanic 45 | 0.7 | 0.74 | 0.71 |
| 3054 | Train 48 | Test Not Hispanic 46 | 0.74 | 0.78 | 0.79 | 4078 | Train 64 | Test Not Hispanic 46 | 0.72 | 0.75 | 0.72 |
| 3055 | Train 48 | Test Not Hispanic 47 | 0.72 | 0.77 | 0.78 | 4079 | Train 64 | Test Not Hispanic 47 | 0.7 | 0.73 | 0.71 |
| 3056 | Train 48 | Test Not Hispanic 48 | 0.74 | 0.78 | 0.79 | 4080 | Train 64 | Test Not Hispanic 48 | 0.72 | 0.75 | 0.72 |
| 3057 | Train 48 | Test Not Hispanic 49 | 0.75 | 0.78 | 0.78 | 4081 | Train 64 | Test Not Hispanic 49 | 0.76 | 0.78 | 0.76 |
| 3058 | Train 48 | Test Not Hispanic 50 | 0.75 | 0.78 | 0.78 | 4082 | Train 64 | Test Not Hispanic 50 | 0.76 | 0.78 | 0.76 |
| 3059 | Train 48 | Test Not Hispanic 51 | 0.75 | 0.78 | 0.78 | 4083 | Train 64 | Test Not Hispanic 51 | 0.76 | 0.78 | 0.76 |
| 3060 | Train 48 | Test Not Hispanic 52 | 0.75 | 0.78 | 0.78 | 4084 | Train 64 | Test Not Hispanic 52 | 0.76 | 0.79 | 0.76 |
| 3061 | Train 48 | Test Not Hispanic 53 | 0.74 | 0.79 | 0.76 | 4085 | Train 64 | Test Not Hispanic 53 | 0.75 | 0.8 | 0.73 |
| 3062 | Train 48 | Test Not Hispanic 54 | 0.74 | 0.78 | 0.78 | 4086 | Train 64 | Test Not Hispanic 54 | 0.74 | 0.8 | 0.8 |
| 3063 | Train 48 | Test Not Hispanic 55 | 0.74 | 0.79 | 0.76 | 4087 | Train 64 | Test Not Hispanic 55 | 0.75 | 0.8 | 0.73 |
| 3064 | Train 48 | Test Not Hispanic 56 | 0.74 | 0.77 | 0.78 | 4088 | Train 64 | Test Not Hispanic 56 | 0.74 | 0.8 | 0.8 |
| 3065 | Train 48 | Test Not Hispanic 57 | 0.76 | 0.79 | 0.78 | 4089 | Train 64 | Test Not Hispanic 57 | 0.76 | 0.79 | 0.78 |
| 3066 | Train 48 | Test Not Hispanic 58 | 0.76 | 0.79 | 0.79 | 4090 | Train 64 | Test Not Hispanic 58 | 0.76 | 0.79 | 0.78 |
| 3067 | Train 48 | Test Not Hispanic 59 | 0.76 | 0.78 | 0.79 | 4091 | Train 64 | Test Not Hispanic 59 | 0.76 | 0.79 | 0.78 |
| 3068 | Train 48 | Test Not Hispanic 60 | 0.76 | 0.79 | 0.79 | 4092 | Train 64 | Test Not Hispanic 60 | 0.76 | 0.79 | 0.78 |
| 3069 | Train 48 | Test Not Hispanic 61 | 0.74 | 0.79 | 0.77 | 4093 | Train 64 | Test Not Hispanic 61 | 0.75 | 0.81 | 0.74 |
| 3070 | Train 48 | Test Not Hispanic 62 | 0.74 | 0.78 | 0.78 | 4094 | Train 64 | Test Not Hispanic 62 | 0.74 | 0.8 | 0.8 |
| 3071 | Train 48 | Test Not Hispanic 63 | 0.74 | 0.79 | 0.77 | 4095 | Train 64 | Test Not Hispanic 63 | 0.75 | 0.81 | 0.74 |
| 3072 | Train 48 | Test Not Hispanic 64 | 0.74 | 0.78 | 0.79 | 4096 | Train 64 | Test Not Hispanic 64 | 0.74 | 0.8 | 0.8 |
