# Supplementary material for: A Systematic Evaluation of Cohort Selection Criteria and Their Impact on Machine Learning Model Performance and Demographic Disparities in COVID-19 Outcomes: Cohort Study
Source: JMIR Form Res. 2026 Aug 20;10:e88406. doi: 10.2196/88406 (PMC13492188; doi:10.2196/88406)
Supplement: Supplementary file 5 [file formative-v10-e88406-s005.docx]

Appendix 7

This multimedia appendix presents supplementary analyses evaluating the effects of cohort construction decisions on model performance, fairness, and calibration. It includes sex-specific feature-importance comparisons, demographic parity and equality of opportunity across demographic subgroups, and representative calibration curves and predicted probability distributions for the 16- and 64-cohort analyses.

Figure S1. Feature importance comparing sex groups, female and male, for Approach 1 using the RF model. RF: random forest.

Figure S2. Demographic parity across demographic subgroups within cohorts 1 to 16 using the RF model. RF: random forest.

Figure S3. Equality of opportunity across demographic subgroups within cohorts 1 to 16 using the RF model. RF: random forest.

Figure S4. Calibration curves and distributions of predicted probabilities for 16 random forest models evaluated using test cohort 14.

Figure S5. Calibration curves and distributions of predicted probabilities for 64 logistic regression models evaluated using test cohort 38.
