# Supplementary material for: A Systematic Evaluation of Cohort Selection Criteria and Their Impact on Machine Learning Model Performance and Demographic Disparities in COVID-19 Outcomes: Cohort Study
Source: JMIR Form Res. 2026 Aug 20;10:e88406. doi: 10.2196/88406 (PMC13492188; doi:10.2196/88406)
Supplement: Supplementary file 6 [file formative-v10-e88406-s006.docx]

Appendix 5

Appendix 5 presents calibration analyses across all 16 cohorts. The series of plots illustrates the calibration results, evaluating how well predicted probabilities align with observed outcomes. For each test cohort (1 through 16), models were trained on each of the 16 training cohorts, and calibration plots were generated to assess the reliability and generalizability of predictions across all test-training cohort combinations. These analyses were performed separately for LR, RF, and GB models.

| ***Calibration (LR) – Original Decisions*** | |
| --- | --- |
| ***Cohorts 1***   | ***Cohorts 2***   |
| ***Cohorts 3***   | ***Cohorts 4***   |

| ***Calibration (LR) – Original Decisions*** | |
| --- | --- |
| ***Cohorts 5***   | ***Cohorts 6***   |
| ***Cohorts 7***   | ***Cohorts 8***   |

| ***Calibration (LR) – Original Decisions*** | |
| --- | --- |
| ***Cohorts 9***   | ***Cohorts 10***   |
| ***Cohorts 11***   | ***Cohorts 12***   |

| ***Calibration (LR) – Original Decisions*** | |
| --- | --- |
| ***Cohorts 13***   | ***Cohorts 14***   |
| ***Cohorts 15***   | ***Cohorts 16***   |

| ***Calibration (RF) – Original Decisions*** | |
| --- | --- |
| ***Cohorts 1***   | ***Cohorts 2***   |
| ***Cohorts 3***   | ***Cohorts 4***   |

| ***Calibration (RF) – Original Decisions*** | |
| --- | --- |
| ***Cohorts 5***   | ***Cohorts 6***   |
| ***Cohorts 7***   | ***Cohorts 8***   |

| ***Calibration (RF) – Original Decisions*** | |
| --- | --- |
| ***Cohorts 9***   | ***Cohorts 10***   |
| ***Cohorts 11***   | ***Cohorts 12***   |

| ***Calibration (RF) – Original Decisions*** | |
| --- | --- |
| ***Cohorts 13***   | ***Cohorts 14***   |
| ***Cohorts 15***   | ***Cohorts 16***   |

| ***Calibration (GB) – Original Decisions*** | |
| --- | --- |
| ***Cohorts 1***   | ***Cohorts 2***   |
| ***Cohorts 3***   | ***Cohorts 4***   |

| ***Calibration (GB) – Original Decisions*** | |
| --- | --- |
| ***Cohorts 5***   | ***Cohorts 6***   |
| ***Cohorts 7***   | ***Cohorts 8***   |

| ***Calibration (GB) – Original Decisions*** | |
| --- | --- |
| ***Cohorts 9***   | ***Cohorts 10***   |
| ***Cohorts 11***   | ***Cohorts 12***   |

| ***Calibration (GB) – Original Decisions*** | |
| --- | --- |
| ***Cohorts 13***   | ***Cohorts 14***   |
| ***Cohorts 15***   | ***Cohorts 16***   |
