# Supplementary material for: A Systematic Evaluation of Cohort Selection Criteria and Their Impact on Machine Learning Model Performance and Demographic Disparities in COVID-19 Outcomes: Cohort Study
Source: JMIR Form Res. 2026 Aug 20;10:e88406. doi: 10.2196/88406 (PMC13492188; doi:10.2196/88406)
Supplement: Supplementary file 7 [file formative-v10-e88406-s007.docx]

Appendix 6

Appendix 6 presents calibration analyses across all 64 cohorts, illustrating how predicted probabilities align with observed outcomes. For each test cohort (1 through 64), models were trained on all corresponding training cohorts, and calibration plots were generated for each training–testing combination to assess the reliability and generalizability of predictions. Analyses were conducted separately for Logistic Regression (LR), Random Forest (RF), and Gradient Boosting (GB).

| ***Calibration (LR) – Add Additional Decisions*** | |
| --- | --- |
| ***Cohorts 1***   | ***Cohorts 2***   |
| ***Cohorts 3***   | ***Cohorts 4***   |

| ***Calibration (LR) – Add Additional Decisions*** | |
| --- | --- |
| ***Cohorts 5***   | ***Cohorts 6***   |
| ***Cohorts 7***   | ***Cohorts 8***   |

| ***Calibration (LR) – Add Additional Decisions*** | |
| --- | --- |
| ***Cohorts 9***   | ***Cohorts 10***   |
| ***Cohorts 11***   | ***Cohorts 12***   |

| ***Calibration (LR) – Add Additional Decisions*** | |
| --- | --- |
| ***Cohorts 13***   | ***Cohorts 14***   |
| ***Cohorts 15***   | ***Cohorts 16***   |

| ***Calibration (LR) – Add Additional Decisions*** | |
| --- | --- |
| ***Cohorts 17***   | ***Cohorts 18***   |
| ***Cohorts 19***   | ***Cohorts 20***   |

| ***Calibration (LR) – Add Additional Decisions*** | |
| --- | --- |
| ***Cohorts 21***   | ***Cohorts 22***   |
| ***Cohorts 23***   | ***Cohorts 24***   |

| ***Calibration (LR) – Add Additional Decisions*** | |
| --- | --- |
| ***Cohorts 25***   | ***Cohorts 26***   |
| ***Cohorts 27***   | ***Cohorts 28***   |

| ***Calibration (LR) – Add Additional Decisions*** | |
| --- | --- |
| ***Cohorts 29***   | ***Cohorts 30***   |
| ***Cohorts 31***   | ***Cohorts 32***   |

| ***Calibration (LR) – Add Additional Decisions*** | |
| --- | --- |
| ***Cohorts 33***   | ***Cohorts 34***   |
| ***Cohorts 35***   | ***Cohorts 36***   |

| ***Calibration (LR) – Add Additional Decisions*** | |
| --- | --- |
| ***Cohorts 37***   | ***Cohorts 38***   |
| ***Cohorts 39***   | ***Cohorts 40***   |

| ***Calibration (LR) – Add Additional Decisions*** | |
| --- | --- |
| ***Cohorts 41***   | ***Cohorts 42***   |
| ***Cohorts 43***   | ***Cohorts 44***   |

| ***Calibration (LR) – Add Additional Decisions*** | |
| --- | --- |
| ***Cohorts 45***   | ***Cohorts 46***   |
| ***Cohorts 47***   | ***Cohorts 48***   |

| ***Calibration (LR) – Add Additional Decisions*** | |
| --- | --- |
| ***Cohorts 49***   | ***Cohorts 50***   |
| ***Cohorts 51***   | ***Cohorts 52***   |

| ***Calibration (LR) – Add Additional Decisions*** | |
| --- | --- |
| ***Cohorts 53***   | ***Cohorts 54***   |
| ***Cohorts 55***   | ***Cohorts 56***   |

| ***Calibration (LR) – Add Additional Decisions*** | |
| --- | --- |
| ***Cohorts 57***   | ***Cohorts 58***   |
| ***Cohorts 59***   | ***Cohorts 60***   |

| ***Calibration (LR) – Add Additional Decisions*** | |
| --- | --- |
| ***Cohorts 61***   | ***Cohorts 62***   |
| ***Cohorts 63***   | ***Cohorts 64***   |

| ***Calibration (RF) – Add Additional Decisions*** | |
| --- | --- |
| ***Cohorts 1***   | ***Cohorts 2***   |
| ***Cohorts 3***   | ***Cohorts 4***   |

| ***Calibration (RF) – Add Additional Decisions*** | |
| --- | --- |
| ***Cohorts 5***   | ***Cohorts 6***   |
| ***Cohorts 7***   | ***Cohorts 8***   |

| ***Calibration (RF) – Add Additional Decisions*** | |
| --- | --- |
| ***Cohorts 9***   | ***Cohorts 10***   |
| ***Cohorts 11***   | ***Cohorts 12***   |

| ***Calibration (RF) – Add Additional Decisions*** | |
| --- | --- |
| ***Cohorts 13***   | ***Cohorts 14***   |
| ***Cohorts 15***   | ***Cohorts 16***   |

| ***Calibration (RF) – Add Additional Decisions*** | |
| --- | --- |
| ***Cohorts 17***   | ***Cohorts 18***   |
| ***Cohorts 19***   | ***Cohorts 20***   |

| ***Calibration (RF) – Add Additional Decisions*** | |
| --- | --- |
| ***Cohorts 21***   | ***Cohorts 22***   |
| ***Cohorts 23***   | ***Cohorts 24***   |

| ***Calibration (RF) – Add Additional Decisions*** | |
| --- | --- |
| ***Cohorts 25***   | ***Cohorts 26***   |
| ***Cohorts 27***   | ***Cohorts 28***   |

| ***Calibration (RF) – Add Additional Decisions*** | |
| --- | --- |
| ***Cohorts 29***   | ***Cohorts 30***   |
| ***Cohorts 31***   | ***Cohorts 32***   |

| ***Calibration (RF) – Add Additional Decisions*** | |
| --- | --- |
| ***Cohorts 33***   | ***Cohorts 34***   |
| ***Cohorts 35***   | ***Cohorts 36***   |

| ***Calibration (RF) – Add Additional Decisions*** | |
| --- | --- |
| ***Cohorts 37*** | ***Cohorts 38*** |
| ***Cohorts 39*** | ***Cohorts 40*** |

| ***Calibration (RF) – Add Additional Decisions*** | |
| --- | --- |
| ***Cohorts 41*** | ***Cohorts 42*** |
| ***Cohorts 43*** | ***Cohorts 44*** |

| ***Calibration (RF) – Add Additional Decisions*** | |
| --- | --- |
| ***Cohorts 45*** | ***Cohorts 46*** |
| ***Cohorts 47*** | ***Cohorts 48*** |

| ***Calibration (RF) – Add Additional Decisions*** | |
| --- | --- |
| ***Cohorts 49*** | ***Cohorts 50*** |
| ***Cohorts 51*** | ***Cohorts 52*** |

| ***Calibration (RF) – Add Additional Decisions*** | |
| --- | --- |
| ***Cohorts 53*** | ***Cohorts 54*** |
| ***Cohorts 55*** | ***Cohorts 56*** |

| ***Calibration (RF) – Add Additional Decisions*** | |
| --- | --- |
| ***Cohorts 57*** | ***Cohorts 58*** |
| ***Cohorts 59*** | ***Cohorts 60*** |

| ***Calibration (RF) – Add Additional Decisions*** | |
| --- | --- |
| ***Cohorts 61*** | ***Cohorts 62*** |
| ***Cohorts 63*** | ***Cohorts 64*** |

| ***Calibration (GB) – Add Additional Decisions*** | |
| --- | --- |
| ***Cohorts 1*** | ***Cohorts 2*** |
| ***Cohorts 3*** | ***Cohorts 4*** |

| ***Calibration (GB) – Add Additional Decisions*** | |
| --- | --- |
| ***Cohorts 5*** | ***Cohorts 6*** |
| ***Cohorts 7*** | ***Cohorts 8*** |

| ***Calibration (GB) – Add Additional Decisions*** | |
| --- | --- |
| ***Cohorts 9*** | ***Cohorts 10*** |
| ***Cohorts 11*** | ***Cohorts 12*** |

| ***Calibration (GB) – Add Additional Decisions*** | |
| --- | --- |
| ***Cohorts 13*** | ***Cohorts 14*** |
| ***Cohorts 15*** | ***Cohorts 16*** |

| ***Calibration (GB) – Add Additional Decisions*** | |
| --- | --- |
| ***Cohorts 17*** | ***Cohorts 18*** |
| ***Cohorts 19*** | ***Cohorts 20*** |

| ***Calibration (GB) – Add Additional Decisions*** | |
| --- | --- |
| ***Cohorts 21*** | ***Cohorts 22*** |
| ***Cohorts 23*** | ***Cohorts 24*** |

| ***Calibration (GB) – Add Additional Decisions*** | |
| --- | --- |
| ***Cohorts 25*** | ***Cohorts 26*** |
| ***Cohorts 27*** | ***Cohorts 28*** |

| ***Calibration (GB) – Add Additional Decisions*** | |
| --- | --- |
| ***Cohorts 29*** | ***Cohorts 30*** |
| ***Cohorts 31*** | ***Cohorts 32*** |

| ***Calibration (GB) – Add Additional Decisions*** | |
| --- | --- |
| ***Cohorts 33*** | ***Cohorts 34*** |
| ***Cohorts 35*** | ***Cohorts 36*** |

| ***Calibration (GB) – Add Additional Decisions*** | |
| --- | --- |
| ***Cohorts 37*** | ***Cohorts 38*** |
| ***Cohorts 39*** | ***Cohorts 40*** |

| ***Calibration (GB) – Add Additional Decisions*** | |
| --- | --- |
| ***Cohorts 41*** | ***Cohorts 42*** |
| ***Cohorts 43*** | ***Cohorts 44*** |

| ***Calibration (GB) – Add Additional Decisions*** | |
| --- | --- |
| ***Cohorts 45*** | ***Cohorts 46*** |
| ***Cohorts 47*** | ***Cohorts 48*** |

| ***Calibration (GB) – Add Additional Decisions*** | |
| --- | --- |
| ***Cohorts 49*** | ***Cohorts 50*** |
| ***Cohorts 51*** | ***Cohorts 52*** |

| ***Calibration (GB) – Add Additional Decisions*** | |
| --- | --- |
| ***Cohorts 53*** | ***Cohorts 54*** |
| ***Cohorts 55*** | ***Cohorts 56*** |

| ***Calibration (GB) – Add Additional Decisions*** | |
| --- | --- |
| ***Cohorts 57*** | ***Cohorts 58*** |
| ***Cohorts 59*** | ***Cohorts 60*** |

| ***Calibration (GB) – Add Additional Decisions*** | |
| --- | --- |
| ***Cohorts 61*** | ***Cohorts 62*** |
| ***Cohorts 63*** | ***Cohorts 64*** |
